# Transcriptome-Wide Analyses of 5′-Ends in RNase J Mutants of a Gram-Positive Pathogen Reveal a Role in RNA Maturation, Regulation and Degradation

**DOI:** 10.1371/journal.pgen.1004207

**Published:** 2014-02-27

**Authors:** Patrick Linder, Sylvain Lemeille, Peter Redder

**Affiliations:** Department of Microbiology and Molecular Medicine, Faculty of Medicine, University of Geneva, Geneva, Switzerland; Institut de Biologie Physico-Chimique, France

## Abstract

RNA decay and maturation have in recent years been recognised as major regulatory mechanisms in bacteria. In contrast to *Escherichia coli*, the Firmicute (Gram-positive) bacteria often do not encode the well-studied endonuclease RNase E, but instead rely on the endonucleases RNase Y, RNase J1 and RNase J2, of which the latter two have additionally been shown to have 5′ to 3′ exonucleolytic activity. We have previously demonstrated that these RNases could be deleted individually in the pathogenic Firmicute *Staphylococcus aureus*; however, we here present that, outside a narrow permissive window of growth conditions, deleting one or both of the RNase J genes presents serious difficulties for the cell. Moreover, an active site mutant of RNase J1 behaved like a deletion, whereas no phenotypes were detected for the RNase J2 active site mutant. Furthermore, in order to study the *in vivo* enzymatic activity of RNase J1 and J2, a method was developed to map the exact 5′-ends of mature and processed RNA, on a global scale. An enrichment of 5′ RNA ends could be seen in the RNase J mutants, suggesting that their exonucleolytic activity is crucial for normal degradation of bulk RNA. Using the data to examine specific RNAs, we demonstrated that RNase J activity is needed for correct 5′ maturation of both the 16S rRNA and the RNase P ribozyme, and can also inactivate the latter, possibly as quality control. Additional examples show that RNase J perform initial cleavages, apparently competing with ribosomes for access to mRNAs. The novel 5′ mapping assay offers an exceptionally detailed view of RNase activity, and reveals that the roles of RNase J proteins are diverse, ranging from maturation and post-transcriptional regulation to degradation.

## Author Summary

The long RNA (ribonucleic acid) chains are key intermediates in the transfer of information from the genes on chromosomes to the production of protein. An RNA copy (mRNA) is transcribed from the gene, and this copy is then translated into protein by a complex molecular machine called the ribosome. The amount of mRNA copies of a given gene is therefore important for how much of the corresponding protein can be generated. This “pool” of mRNA depends on how many copies are made per second, but also on how many copies disappear due to degradation. In this study, we examine mutants of the bacterial pathogen *Staphylococcus aureus*, which have lost one or both of their RNase J genes (ribonuclease J). These mutants grow relatively fine under standard laboratory conditions, but will stop growing if they are stressed by alternative food sources or temperatures equivalent to a high fever (42°C). We show that the RNase J enzymes are major players in the degradation of RNA by removing one link at a time in the RNA-chains, thus influencing the pool of mRNAs in the cell. Furthermore, certain RNAs are processed to stable and active forms by RNase J, instead of being degraded.

## Introduction

RNA decay is an important regulatory process in bacteria and is thought to be a major factor in post-transcriptional control [1]. Some bacterial mRNAs have halflives of only seconds, most mRNAs have halflives of minutes, and a few especially stable RNAs can last for hours [2], however the signals that distinguish a short-lived from a long-lived RNA are only poorly understood. The standard RNA decay paradigm, based on the model organism *Escherichia coli*, describes an initial endonucleolytic cut made by RNase E and regulated by largely unknown mechanism(s). Then 3′ to 5′ exonucleolytic degradation follows, carried out by polynucleotide phosphorylase (PNPase), which is normally inhibited by hairpin structures at the 3′-end of the RNA [3]–[5]. However, this model can rarely be applied to bacteria outside the beta and gamma-proteobacteria, since large families such as the Firmicutes often do not encode RNase E homologs, but instead encode RNase Y [6] and one or more RNase J paralog(s) [7]. A role for RNase J1 in general RNA decay was shown by depleting it in *Bacillus subtilis* and *Streptococcus pyogenes*, which greatly increases the half-lives of many mRNAs [8]–[10]. Moreover, *B. subtilis* and *S. aureus* RNase Y, RNase J1, and RNase J2 have been proposed to be a part of a larger degradosome-complex, identified via bacterial two-hybrid interactions [11], [12], which might coordinate and/or regulate the RNA decay.

The characterisation of the *in vivo* enzyme activity of *B. subtilis* RNase J1 revealed a 5′ to 3′ exoribonucleolytic activity, previously unheard of in bacteria [13], and structural studies have provided important clues to the molecular basis for this activity [14]–[16]. Removal of the final 38 nucleotides from the 5′-end of pre-16S rRNA in *B. subtilis* is dependent on this exonucleolytic activity, underlining its importance [13], [17].

In contrast, RNase J2 exhibits little to no exonucleolytic activity [18], which is accompanied by a virtual lack of phenotypes in a *B. subtilis* RNase J2 mutant [7], [8]. A hint to the role of RNase J2 may be found in bacterial two-hybrid experiments, where RNase J1 and J2 interact strongly with each other [11], [12], and several lines of evidence from both *B. subtilis* and *S. aureus* suggest that RNase J1 and J2 can form both homo- and hetero-dimers as well as hetero-tetramers, although reports do not agree on which form is predominant [12], [16], [18].

Moreover, individually both *B. subtilis* RNase J1 and J2 have been shown *in vitro* to endonucleolytically cut a number of RNAs, and that the interplay between the two proteins changes the specificity of this activity [7], [18]. However, the physiological role of this, and to what extent it complements the endonucleolytic activity of RNase Y, is not clear [9].

RNase J1 was originally identified as an essential gene in *B. subtilis* [19], and both RNase J1 and J2 were reported to be essential in *S. pyogenes* [10]. In *S. aureus*, a saturated transposon insertion screen of the genome failed to find insertions in either RNase J1 or RNase J2, suggesting that both were essential in this organism [20]. In contrast, using our recently developed system for generating allelic replacements in *S. aureus*, we were able to isolate deletion mutants of both RNase J1 and J2 [21].

In this work we examine the range of severe phenotypes caused by the loss of RNase J1 and RNase J2, however at the same time we show that a deletion of both is viable in *S. aureus*. This is in concert with results published during the preparation of this manuscript by Figaro and coworkers [22], where, in contrast to previous data, the two RNase J genes were shown to be non-essential in *B. subtilis*. In addition, we show that mutations in the RNase J1 active site are virtually equivalent to a complete deletion of the RNase J1 gene, whereas the RNase J2 active site appears to have no function under the tested conditions, even though a complete deletion of RNase J2 results in growth defects that approach the RNase J1 deletion. To study the details of RNase J1 and J2 mutations, we furthermore developed a new method for examining the 5′-ends of the entire mono-phosphorylated transcriptome. Using this assay, we show that the major role of RNase J (RNase J will be used as generic term, where we discuss J1 and J2, and not a specific one of them) in RNA decay is the 5′ to 3′ exonucleolytic activity, but examples are also given where the RNase J endonucleolytic activity might play an important role. Finally, we show that RNase J activity is responsible for the normal pathway of RNase P RNA and 16S rRNA 5′-maturation.

## Results

### RNase J1 and J2 are only essential under specific growth conditions in *S. aureus*

In order to consolidate the apparent contradiction between the findings of Chaudhuri and coworkers (2009), which indicated essentiality of RNase J1 and J2, and our own previous findings, in which the two genes are non-essential [21], we used total genome sequencing to confirm the deletions of the two RNase J genes (S0940 and SA1118, using the *S. aureus* N315 nomenclature, which will be used throughout this manuscript). Out of more than three million 100 bp reads from the Illumina machine, none mapped to the deleted regions, in contrast to the sequence of unrelated strains, where at least 1100 reads would map to each of the two RNase J genes (Table 1). This confirmed that RNase J1 and RNase J2 regions had indeed been deleted in our mutants, and additionally ruled out that the rnase genes had been moved to an alternative genomic location by an unfortunate recombination event. The sequencing data was also used to perform a search for single nucleotide polymorphisms (SNPs) and small inserts and deletions (indels) in our mutant strains, localising any potential second-site mutations which might aid the cell in surviving the loss of the RNase J genes. However, the only secondary mutation identified in the RNase J1 deletion strain (strain ΔJ1) was a silent valine to valine in the gluconate operon repressor (C to A at position 2578631 in SA2295), and no secondary mutations were detected in the RNase J2 deletion strain (strain ΔJ2).

**Table 1:** Reads mapping to the RNase J1 and RNase J2 genes in the RNase J deletion mutants and in unrelated strains (GHU-12 to 26).

Strain	J1 gene (SA0940)	J2 gene (SA1118)	Total # of reads from strain that map to *S. aureus*
ΔJ1	0	1509	3735419
ΔJ2	1650	0	3334625
ΔJ1ΔJ2	0	0	3549198
GHU-12*	1311	1314	3858237
GHU-13	1121	1180	3043123
GHU-14	1226	1359	3578899
GHU-15	1341	1383	3282388
GHU-16	1385	1358	3409154
GHU-17	1203	1236	3846389
GHU-18	1157	1159	3094900
GHU-22	2862	2980	5969805
GHU-23	1661	1559	3766296
GHU-24	1974	1783	4377388
GHU-25	2158	2011	4682005
GHU-26	1976	1905	4267860

*) The GHU strains have wild-type RNase J loci and are unrelated to this study, except that they originate from the same lineage as strains ΔJ1, ΔJ1 and ΔJ1ΔJ2.

Furthermore, to ensure that the viability of the RNase J1 deletion mutants was not a peculiarity of the SA564-strain used [21], the RNase J1 deletion was generated twice, independently, in an RN4220 background (strains PR02-03 and 06, Table 2).

**Table 2:** Strains used.

Name	Strain	Mutation	Reference
SA564-based strains			
WT	PR01	Δ*pyrFE*	[21]
ΔJ1	PR01-01	Δ*rnjA* (SA0940)	[21]
ΔJ2	PR01-04	Δ*rnjB* (SA1118)	[21]
ΔJ1ΔJ2	PR01-17	Δ*rnjA::ermC, ΔrnjB*	This work
J1^AGA^	PR01-27	GTGACCATGT to GGCGCCTGCT at position 1068202*	This work
J2^AGA^	PR01-37	ACACGGACATG to AGCAGGCGCCG at position 1268999*	This work
ΔY	PR01-02	Δ*rny*	[21]
ΔcshA	PR01-15	Δ*cshA*::*kanA* Chromosomal region between 2134254 and 2137592* substituted with the *kanA* gene	This work
RN4220-based strains			
RN4220ΔpyrFE	PR02		[21]
RN4220ΔJ1C	PR02-03	ΔJ1::CAT194	This work
RN4220ΔJ1E	PR02-06	ΔJ1::*ermC*	This work

*) *S. aureus* N315 nomenclature and chromosomal positions are used.

**Figure 1:**
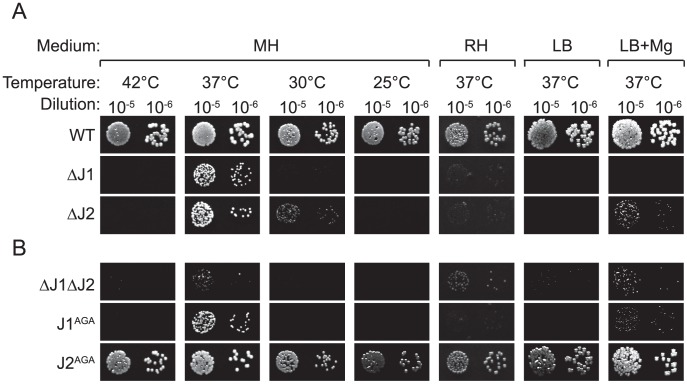
RNase J mutant phenotypes. Cultures grown over night, were diluted 10^5^ and 10^6^ times, whereupon 5 µl were spotted on either MH, RH, LB, or LB+Mg agar plates with 10 mg/l uracil, and incubated at the indicated temperatures until the WT colonies reached an approximately similar size. LB+Mg denotes LB plates supplemented with 5 mM MgCl_2_. Growth of a strain was then scored by the size (or absence) of visible colonies compared to the WT colonies. A) Previously published strains. B) RNase J mutants generated in this study. A and B were spotted on the same plates.

The main difference between our method for generating mutants and that of Chaudhuri and coworkers, was the passages at 44°C used by the latter to eliminate their thermosensitive transposon-carrying plasmid. Indeed, the authors warn that their list of essential genes reflect conditions at 44°C in the specific medium used, and state: “Consequently, genes required for high temperature survival will be scored as putatively essential” [20]. Therefore, we tested whether high temperature was limiting growth of the RNase J mutants, by spotting a dilution series of RNase J1 and J2 mutants on Mueller-Hinton (MH) plates, incubated at a range of temperatures. Corresponding with the warning from Chaudhuri and coworkers, the RNase J deletion mutants did indeed grow extremely poorly at 42°C, but, surprisingly, also at 30°C and below, and it is therefore only at 37°C that the mutants will grow relatively unaffected (Figure 1).

The requirements for the medium were examined by using a defined medium (RH-medium, containing only glucose, amino acids, vitamins, buffer, and salts), along with LB-medium and MH-medium. The spot-tests revealed that RNase J deletion mutants require a complex medium for growth, and will not grow on RH-medium. Furthermore, even though LB medium contains a complex range of various salts and nutrients, it has a low Magnesium concentration (30 to 50 µM; [23]), and this deficiency apparently also inhibits growth of the RNase J mutants, unless additional MgCl_2_ is added, in which case the ΔJ2 mutant will grow (Figure 1A). Thus, RNase J mutants are only viable at a very restrictive temperature range and are highly susceptible to medium changes.

### A double RNase J1/J2 mutant is viable

In the single RNase J1 and J2 mutants, it is still a possibility that the activity of the remaining RNase J partially compensates for the loss of the other RNase J. To examine the consequences of a complete absence of RNase J activity, a double mutant (ΔJ1ΔJ2) was generated, where the RNase J2 mutant was used as parent for a second round of mutagenesis, to substitute the RNase J1 gene with an erythromycin resistance cassette. The full genome of the double mutant was also sequenced, to confirm the deletions (Table 1). The double mutant does grow at 37°C, but even slower than the single mutants (Figure 1B). Both strains ΔJ1 and ΔJ1ΔJ2 generate visible aggregates when grown in liquid culture, making it difficult to obtain reproducible growth data by measuring OD_600_ or CFU-counts. We therefore used a semi-quantitative spot-assay to evaluate the growth of the various mutant strains, by comparing the size of individual colonies on the same petri-dish.

### RNase J1 and RNase J2 are both needed for full functionality, but the active site of RNase J2 is dispensable

RNase J2 is not essential in *B. subtilis*, and a deletion mutant exhibits only very mild phenotypes [8]. In *S. aureus* the RNase J2 deletion does result in strong growth defects, however these defects are less severe than those observed for the ΔJ1 strain (Figure 1). This raised the possibility that the effects observed from deleting RNase J2 were in fact due to a partially malfunctioning RNase J1, in the absence of RNase J2. Alternatively, since the interaction of RNase J2 with the degradosome appears to go through RNase J1 [11], [12] it is possible that the stronger phenotypes observed for the ΔJ1 strain are due to removing RNase J2 from the degradosome in absence of RNase J1.

Active-site mutants were therefore generated, in order to maintain the possibility of correctly forming a J1+J2 complex, but eliminating the enzymatic activity of either one or the other of the ribonucleases. The active site of *S. aureus* RNase J1 has a 74-HGHEDH-motif, which matches the signature motif (HxHxDH) of the metallo-β-lactamase family [18], while the *S. aureus* RNase J2 active site sequence 76-HGHEHA exhibits several differences from the motif (Figure S1). A H76A mutation disrupts the unique active site of *B. subtilis* RNase J1 [13], [16], [17], and since both RNase J1 and J2 of *S. aureus* have the first two Zinc-coordinating histidines of the HxHxDH motif, these were chosen for substitution with alanines, generating the active site mutants RNase J1 H74A-G-H76A (J1^AGA^) and RNase J2 H76A-G-H78A (J2^AGA^) in place of the wild-type genes on the chromosome (Table 2; Figure S1).

The growth of J1^AGA^ and J2^AGA^ mutants were then examined, under the conditions where ΔJ1 and ΔJ2 are inhibited. The J2^AGA^ mutant grew like wild-type under all tested conditions consistent with a proposed structural role of RNase J2, whereas the J1^AGA^ strain exhibited defects that were very similar to the ΔJ1 mutant (Figure 1B), and it is therefore not dissociation of the degradosome that produces the phenotypes.

#### Overexpressing RNase J1 partially complements the loss of RNase J2

**Figure 2:**
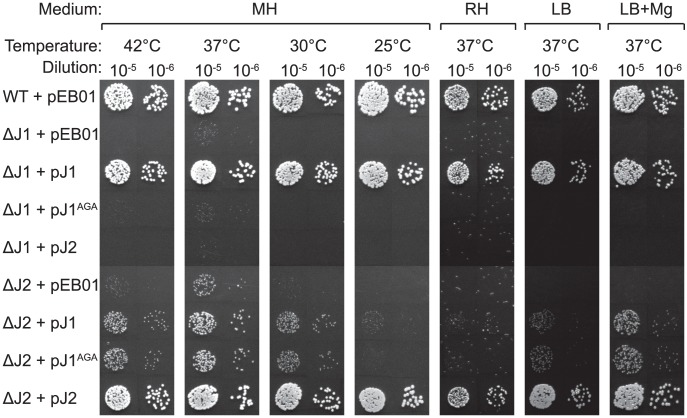
Complementation of the RNase J deletions. Spotting and interpretation was carried out as in Figure 1, but with 10 µg/ml chloramphenicol added to both the overnight culture and in the agar plates, to ensure against loss of the complementing plasmids. pEB01 is the empty plasmid. The faint white rings seen randomly distributed on the RH medium are not colonies, but are air bubbles that often form inside the agar matrix during incubation of this medium.

Using the growth-inhibitory conditions established above, it was tested whether overexpression of one RNase J could rescue the loss of the other. The genes for RNase J1 and RNase J2 were cloned into the multicopy plasmid pEB01 including upstream regions containing ribosome binding sites (RBSs) and putative promoters. In the case of RNase J1, this meant inclusion of a small upstream ORF (SA0941) (Table 3). The ΔJ1 mutant was completely rescued by introduction of the pJ1 plasmid, but could not be complemented by pJ2 or by pJ1^AGA^. In contrast, the ΔJ2 strain could be partially rescued by the pJ1 plasmid, and fully complemented by pJ2 (Figure 2). Interestingly, pJ1^AGA^ was also able to partially complement the loss of RNase J2, supporting the hypothesis that the role of RNase J2 is mainly structural, and does not require enzymatic activity.

**Table 3:** Plasmids and vectors.

Name	Comment	Reference
Vectors for generating allelic replacements		
pRLY-vector series	Range of vectors for allelic replacements	[21]
pRLYC9-J1ERYS-1	Used for generating the RNase J1 deletion in strain PR01-17	This work
pRLYT9-J1ERYS-1	Used for generating the RNase J1 deletion in strains PR02-06	This work
pRLYE9-J1CATS-1	Used for generating the RNase J1 deletion in strain PR02-03	This work
pRLYT-J1AGA-1	Used for generating the J1^AGA^ mutant PR01-27	This work
pRLYT-J2AGA-3	Used for generating the J2^AGA^ mutant PR01-37	This work
pRLYE-85RL-Kan-1	Used for generating the cshA deletion mutant PR01-15	This work
Vectors for complementation		
pEB01	Empty vector Multi-copy shuttle vector for *E. coli* and *S. aureus*	[32]
pJ1	Complementation plasmid with RNase J1 pEB01 with chromosomal region 1069181–1066588*	This work
pJ2	Complementation plasmid with RNase J2 pEB01 with chromosomal region 1268514–1270625*	This work
pJ1^AGA^	Complementation plasmid with RNase J1^AGA^Same as pJ1, but with the AGA active site mutation.	This work

*) Chromosomal positions from *S. aureus* N315 are used. The RNase J1 gene is in a putative operon with an upstream transcriptional regulator (SA0941), and both genes were included in the pJ1 plasmid, in order to include the promoter and to avoid the problems with RNase J1 expression described by Mäder and coworkers [8].

### Mapping the 5′-ends of mono-phosphorylated RNAs

**Figure 3:**
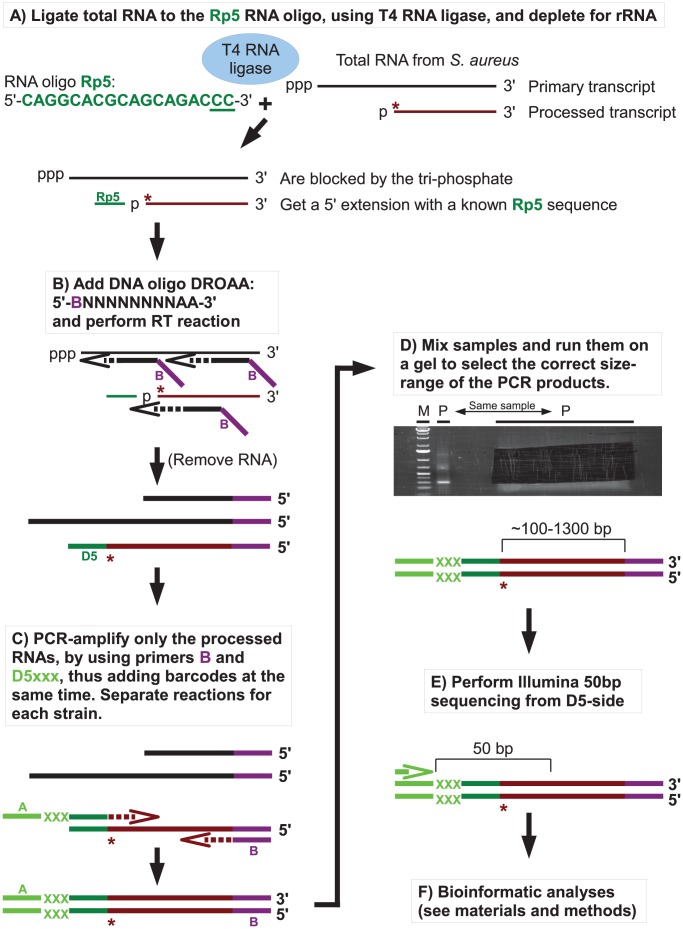
Flowchart showing the mapping of 5′ RNA ends with the EMOTE assay. The 5′-bases of mono-phosphorylated RNA (asterisks) were identified using the following technique. Thin and thick lines denote RNA and DNA, respectively. Open arrows indicate primer elongation. A) The RNA oligo Rp5 (green) is ligated to total RNA from each strain, adding a known sequence to all mono-phosphorylated RNAs (brown), and excluding tri-phosphorylated RNAs (black). The majority of 16S and 23S rRNA are removed by hybridisation to magnetic beads. The two underlined cytidine nucleotides at the 3′-end of Rp5 will be explained below. B) Reverse transcription is performed with a semi-random primer (DROAA), which cannot anneal to the Rp5 sequence. The 5′-ends of the cDNA will all have the sequence of Illumina adaptor B (purple), but only cDNA made from Rp5-ligated RNA will end in a known sequence (D5), which is complementary to Rp5 (green). C) The cDNA from each strain is amplified using one primer, B, that anneals to the Illumina adaptor B (purple), and a second primer, D5xxx, that anneals to D5 (green) and adds the Illumina adaptor A sequence (light green) as well as a short bar-code (light green “XXX”) which is unique to each strain. The D5xxx primers only have the first 16 nt of Rp5, so any PCR product where the D5xxx primers have annealed to non-Rp5 sequence, will not include base 17 and 18 of Rp5. D) The PCR products from all the strains are mixed together and run on an agarose gel, whereupon the appropriate size-range of the smear (P-lanes) is extracted. E) Illumina sequencing of 50 bases from the D5-side then reveals the bar-code (light green, identifying from which strain the original RNA came), the Rp5 sequence (green), the first base in the original mono-phosphorylated RNA (asterisk), and the subsequent 23 bases (brown), allowing an unambiguous mapping of the 5′-end of each detected RNA. F) Bioinformatic analyses to (I) use the bar-code sequence to assign each read to the correct strain. (II) Verify that the two cytosine bases at the 3′-end of the Rp5 sequence are present to remove reads that originate from a mis-priming of the D5xxx primer. (III) align the 24 bases to the genome, to determine the exact position and orientation of each 5′-base. (IV) Tabulate and analyse the data (see materials and methods).

RNase J1, and to a lesser degree RNase J2, from *B. subtilis* were shown in several studies to have 5′ to 3′ exoribonucleolytic activity, and that mono-phosphorylated 5′ ends were strongly preferred over tri-phosphorylated ends. To examine the effects of RNase J1 and J2 on 5′-ends of RNAs in *S. aureus*, the Exact Mapping Of Transcriptome Ends (EMOTE) assay was developed to map the exact 5′ base of a large number of mono-phophorylated RNAs in the cell (see flowchart in Figure 3). Briefly, large excess of a custom-designed 18 nt RNA oligo (Rp5) was ligated to total RNA preparations from WT, ΔJ1, ΔJ2, ΔJ1ΔJ2, J1^AGA^, ΔY, and ΔcshA strains (Table 2). The enzyme used, T4 RNA ligase 1, will not ligate tri-phosphorylated ends to Rp5, and the assay is therefore specific for the 5′ mono-phosphorylated ends that are preferred and generated by RNase J. The cDNA was generated using a semi-random primer (DROAA), which also adds one of the two linkers needed for sequencing with Illumina HiSeq technology. In order to avoid cDNA generated from the excess of Rp5, the DROAA primer terminated in two deoxyadenosines, a combination which is extremely common in the *S. aureus* genome, but is unable to basepair with the uridine-lacking Rp5 oligo. Second strand synthesis and PCR amplification was carried out using a primer specific for the Illumina linker in DROAA, paired with a Rp5-specific primer which also added the second Illumina linker sequence to the DNA. 50 bp Illumina sequencing was then performed from the Rp5-end of the DNA, revealing the 24 first bases of each RNA, more than enough to map the reads precisely in a bacterial genome (Figure 3). The first of these 24 bases thus represents the 5′-end of an RNA molecule in the original sample, and the number of Illumina reads that correspond to each original 5′ position, are then added up and tabularised (see Tables S1, S2 and S3 for examples). In the tables, the position given is the number of the first (5′) base in the detected RNA, and a cleavage at position X is therefore defined as a cleavage between base X-1 and base X. Each column shows the number of reads that detect a specific 5′-end from a given strain.

Table 4 gives a general overview of the output from the EMOTE assay. The first step was to remove reads that do not have the Rp5 sequence (about 6%), and therefore cannot be used to determine 5′-ends. Next the barcodes were identified to assign each read to a specific bacterial strain. These pools of reads were then examined for the presence of the CC motif, which ensures that the read reflects a genuine RNA 5′-end. Aligning the 24 bases after the CC motif to the *S. aureus* N315 genome sequence resulted in either uniquely mapping reads, or reads that mapped to multiple locations on the genome (such as rRNA genes, IS elements, etc.). Uniquely mapping and multiply mapping reads were organised in separate tables for further analyses, as shown in Table S1 and S2.

**Table 4:** Overview of the EMOTE data.

Bioreplicate:	#1	Total reads:	43619821	Reads with Rp5 motif:		41423045			
Primer	Strain	# of reads with barcode	# of reads with CC ( = reads included in the analyses)	# of reads mapping to unique positions	# of reads mapping to multiple positions	% of all reads mapping to tRNA	% of all reads mapping to rRNA	% of all reads mapping within ORFs+200 nt upstream	Number of genes with more than 10 mapped reads
D5a	WT	11385677	8231917	122318	5477051	0.86	96.97	2.17	1565
D5b	ΔY	5361790	4625509	63075	2853357	0.49	97.35	2.16	1074
D5c	ΔJ1	5947519	5040905	193442	2471613	0.21	92.51	7.28	1659
D5d	ΔJ2	6415079	5351695	366785	2937292	0.64	88.24	11.12	1976
D5e	ΔJ1ΔJ2	5880549	3956054	191270	2066206	0.39	91.11	8.49	1703
D5f	ΔcshA	6278524	4793096	42131	2694536	0.63	97.84	1.54	1005

Bioreplicate:	#2	Total reads:	38862898	Reads with Rp5 motif:		36696917			
D5La	WT	6471950	5917900	46537	5477653	0.11	99.05	0.84	1068
D5Lb	ΔY	3514379	3207334	38367	2921682	0.1	98.6	1.3	700
D5Lc	ΔJ1	4435717	4210978	174912	3678502	0.17	95.26	4.57	1584
D5Ld	ΔJ2	3767796	3491261	117818	3080977	0.14	96.16	3.7	1395
D5Le	ΔJ1ΔJ2	2999814	2690302	111099	2285019	0.11	95.25	4.64	1356
D5Lf	ΔcshA	4488874	4170088	41016	3744046	0.15	98.77	1.09	1008
D5Lh	J1^AGA^	4429992	4122024	166087	3620870	0.13	95.46	4.41	1564

#### Limitations in the EMOTE method

It is important to note that even though the RNA ligation step confers specificity to mono-phophorylated RNAs, the EMOTE data does not distinguish between mono-phosphorylated 5′-ends generated by endonucleases, exonucleases, or pyrophosphohydrolases. It is therefore often possible to detect transcription start-sites, because tri-phosphorylated ends are converted to mono-phosphorylated ends by one of the *S. aureus* homologues of the Nudix family of pyrophosphohydrolases. In *B. subtilis*, only one of several has so far been identified [24] (the homolog in *S. aureus* is SA1612). Furthermore, the RNA ligase exhibits some sequence specific bias in its activity, and although the ligation step is carried out with large excess of Rp5, it is still possible that some 5′-ends are under- or over-represented. This bias is however identical in all strains for 5′-ends at a given position, and the bias is therefore removed when the data from two strains are compared.

If an RNA fragment is shorter than about 100 nucleotides, then it will be under-represented in our EMOTE assay, since the chosen RNA purification method depletes small RNA from the sample. This is further accentuated by the paucity of potential priming sites for the DROAA oligo on very short RNAs, however, we are still able to detect 5S rRNA and some tRNAs in our EMOTE data (Table S1 and S2).

In addition to the potential biases and interpretation problems mentioned above, an additional problem presented itself, which was not directly related to the EMOTE method. We had great difficulty removing ribosomal RNA from our samples, and in our hands, the Ambion MicrobeExpress kit works very poorly for *S. aureus* rRNA, and we suspect that the pull-down oligos do not hybridise fully to the rRNA of this organism. This led to high percentage of reads mapping to the 5′-ends of 16S and 23S rRNA (Table 4), so it is certain that an improved rRNA depletion method/protocol would improve the depth of the EMOTE data. The data presented here are therefore limited to relatively high abundance RNAs, however we fully expect that a deeper sequencing would allow detection of even poorly expressed RNAs. A rough estimate of how many genes we are able to detect is illustrated in the final column of Table 4, where the number of genes with more than 10 reads are listed.

### Validation of the EMOTE assay

The EMOTE method was evaluated by comparing our data to the results from the recent landmark paper on RNase III function in *S. aureus* [25]. The described RNase III cleavage of the 16 rRNA processing stem, the cut inside the *rnc* gene, and the double-stranded cut of a hairpin in the *cspA* gene were all detected by our method (Tables S3, S4 and S5). The cleavage of the 16S rRNA processing stem by RNase III was detected at the exact previously published site, and served as a basis for further analyses of the 16S rRNA gene (see below). In the *rnc* gene we detect a large number of RNA with a 5′ base at position 1216563 and a smaller sub-population at the adjacent base 1216564, and in addition, a weaker signal is seen for 5′-bases 10–16 bases downstream, corresponding to the cleavage detected *in vivo* by primer extension. In the *cspA* gene, we could detect a high number of 5′-ends at positions 1409120 and 1409083, which not only confirm the previously published results, but additionally our exact method serves to refine the positions of the RNase III cleavages to one base further towards the base of the *cspA* hairpin [25].

### Lack of RNase J activity results in a transcriptome-wide accumulation of mono-phosphorylated 5′ ends

RNase J1 has been proposed to be a major player in bulk RNA decay [7], by processively digesting the RNAs from the 5′-end once they become mono-phosphorylated, either by an endonucleolytic cut, or by the action of a pyrophosphohydrolase. This hypothesis has been supported by the observed stabilisation of individual RNAs during depletion of RNase J1 in *B. subtilis* and *S. pyogenes* [8], [10]. However, until now it has been unclear to what extent it is a general mechanism that affects a majority of the transcriptome, or whether only a small subset of RNAs are dependent on RNase J1 for correct degradation.

To resolve this, the number of reads for a given position in the WT data-set was compared to the number of reads in the RNase J mutants. Only positions where at least two reads mapped in both the WT and mutant data-sets were included, reducing background noise, and potential bias caused by lack of sequencing depth. Table 5 shows the number of positions that fulfilled the criteria, and the percentage of these that are either 4-fold more abundant or 4-fold less abundant in the mutants. The ΔY and ΔcshA data are included as controls, and show neither strong enrichment nor strong reduction in the 5′-ends. The strong enrichment of 5′-ends in the RNase J mutants, signifies that under wild-type conditions, the bulk of the RNA is indeed degraded by the 5′ exonucleolytic activity of the RNase J complex. The assay does not reveal what happens to the 3′-ends of the RNA, however, the strains are viable, and it must therefore be presumed that the RNA is degraded via the 3′-ends in the RNase J mutants.

**Table 5:** Enrichment of 5′-ends in the RNase J mutants.

Strain	Total # of positions^a^	Enriched^b^ %	Similar^c^ %	Reduced^d^ %
**ΔJ1**	831	20.0	73.4	6.6
**ΔJ2**	905	15.8	80.0	4.2
**ΔJ1ΔJ2**	918	17.9	75.9	6.2
**J1^AGA^**	830	20.6	73.4	6.0
**ΔY**	981	2.1	95.7	2.1
**ΔcshA**	1172	4.3	91.6	4.1

a ) Only positions where both WT and mutant strain exhibited 2 reads or more (normalised with total number reads from the strain) were included.

b ) Enriched: Positions where the normalised mutant strain exhibits more than 4× the number of reads in the WT (Mutant>4×WT).

c ) Similar: (WT/4<Mutant<4×WT).

d ) Reduced: Positions where the normalised mutant strain exhibits less than 4× the number of reads in the WT (WT/4>Mutant).

### Possible outcomes caused by the loss of RNase J activity

When an RNase is analysed in an isolated *in vitro* system, the interpretation is often straight-forward since the presence/absence of the RNase will generate certain specific patterns of RNA products. *In vivo* however, many RNases function together, often with overlapping functions, to degrade or mature RNA, and it is therefore important to take this into account when interpreting the data. Several scenarios can be imagined when the RNase J genes are deleted, due to the potential combination of exo- and endo-nucleolytic activities.

- If an RNA is degraded (or matured) directly from the original 5′-end, after a pyrophosphohydrolase has generated a mono-phosphate, then a loss of 5′ exonucleolytic activity will result in an accumulation of 5′-ends, which can be detected by the EMOTE assay.
- Endonucleolytic cuts performed by a non-RNase J, which are then 5′ degraded by the RNase J1+J2 will be poorly detectable in the EMOTE data from the WT strain, since these RNA species are quickly removed, as soon as they are generated. However in the RNase J mutants, where the 5′ exonuclease activity is absent, such a cleavage site will be visible in the EMOTE data, since the generated 5′-ends will accumulate (for example at position +452 to +477 in the SA1279-*rnpB* transcript). Sites from i and ii will contribute to the accumulation observed for all the examined RNase J mutants (Table 5), which shows that RNase J2 is needed for exonucleolytic activity, but also that mutating the active site of RNase J1 is sufficient to see the same level of 5′-end accumulation as in the ΔJ1ΔJ2 strain (Table 5), strongly suggesting that *in vivo*, it is primarily the RNase J1+J2 complex which is the 5′ exonuclease.
- A more complex situation arises when, for example, an endonucleolytic cut is made by RNase J1, and then 5′ exonucleolytically degraded. Then we would expect to observe an accumulation in the ΔJ2 strain, since the endonucleolytic cut can be made by RNase J1, and the exonucleolytic degradation is inhibited by the loss of RNase J2 from the RNase J1+J2 complex. Both the WT and the ΔJ1ΔJ2 strains will exhibit low signals in the EMOTE data, but for different reasons: In the WT, the 5′-end generated by the endonucleolytic activity of RNase J1 is removed by the exonucleolytic activity, whereas in the ΔJ1ΔJ2 strain, there is no RNase J to perform the endonucleolytic cleavage in first place. The latter can additionally be used to distinguish between scenario ii and iii, because a 5′-end generated by a non-RNase J will accumulate in the ΔJ1ΔJ2 strain, whereas it will be absent if the cut is made by an RNase J.
- Finally, to complicate matters even further, it is almost certain that the *in vivo* 5′-exonucleolytic activity of RNase J can be blocked or paused by external factors such as ribosome binding or secondary structures. It is therefore not immediately possible to determine whether a 5′-end was generated by exo- or endo-nucleolytic cleavage, and consequently the interpretation of both EMOTE and other types of data is often not straight-forward.

**Figure 4:**
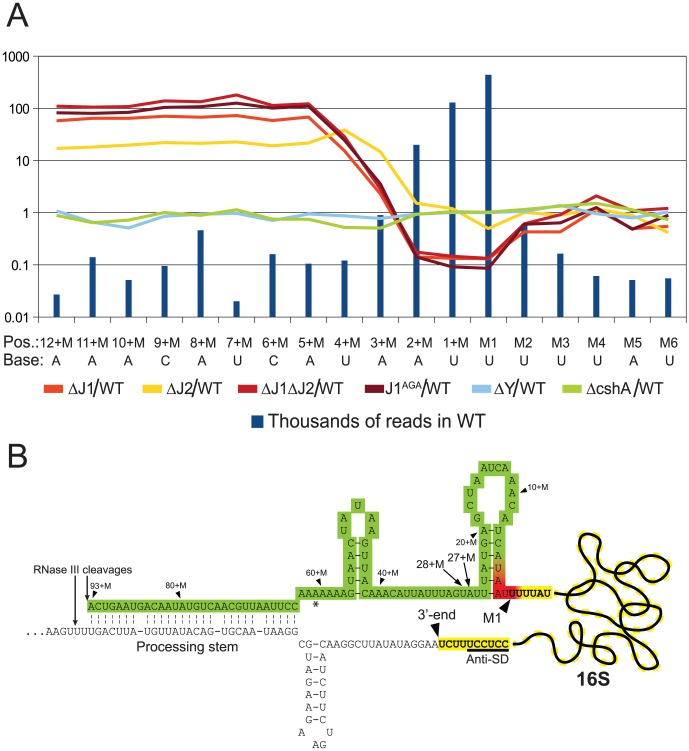
Enrichment of immature 16S rRNA in RNase J mutants. A) The curves show the number of reads mapping to each position of the immature 16S rRNA (mature 16S starts at position M1) in the mutant strains, divided by the number of reads mapping to the same position in the WT data. Data from RNase Y and cshA deletion mutants [21] have been included as controls. The number of reads for each strain has been normalised to the sum of reads mapping to positions between the 16S rRNA RNase III processing site [25] and the 3′-end of the mature 16S rRNA. Blue bars indicate how many thousands of reads mapped to each position in the WT data-set. B) Overview of the region important for the maturation steps after RNase III has cleaved the processing stem. Positions of 5′-ends that accumulate in the RNase J mutants are indicated in green, and red positions are much less abundant in the RNase J mutants compared to the WT strain. No significant changes are observed at the yellow positions. M1 denotes the first nucleotide of the mature 16S rRNA. The Anti-Shine-Dalgarno (Anti-SD) and the 3′-end of the mature 16S rRNA are indicated. 5′-ends at positions 28+M and 27+M are relatively frequently observed (Table S3), and might be a hotspot for the ribonuclease that cleaves between 93+M and M1, prior to trimming by RNase J (see discussion for details). The asterisk indicates where two of the five 16S rRNA genes in *S. aureus* have one base less.

**Figure 5:**
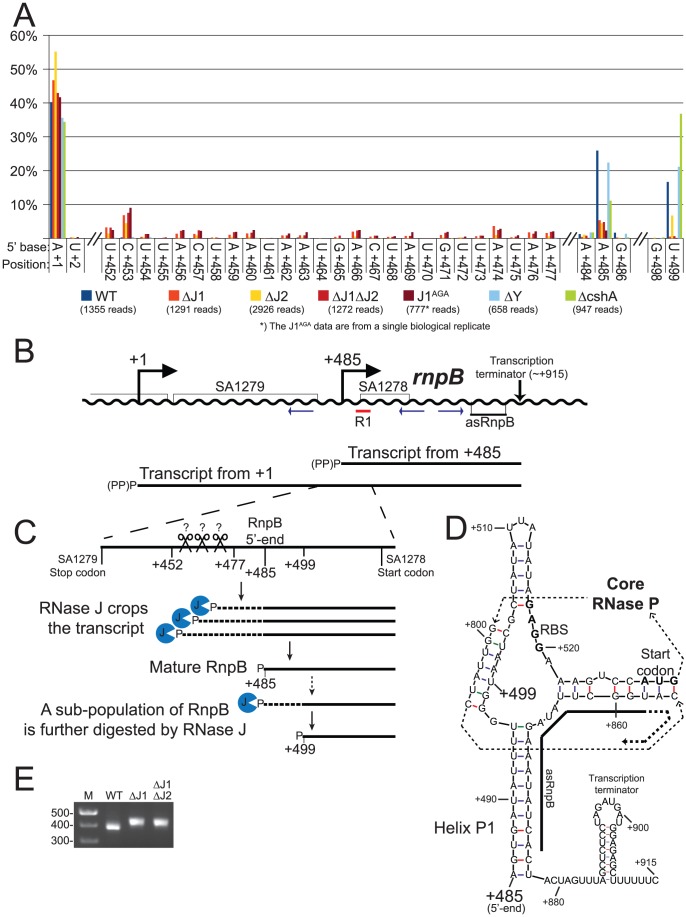
Both maturation and inactivation of RNase P RNA is carried out by RNase J. A) Histogram showing the percentage of reads mapping to a given position, out of the total number of reads mapping to the putative SA1279-*rnpB* operon in each strain (shown in parentheses). Only positions of interest are included, but the full data-set can be found in Table S6. +1: The putative transcription start site of SA1279. +452 to +477: The RNase J mutants accumulate RNA with 5′-ends in this region. +485: The putative transcription start site of *rnpB*, a major detected RNA species in the WT, ΔY and ΔcshA, but very reduced in the RNase J mutants. +499: A major detected RNA species in the WT, ΔY and ΔcshA, however it is absent from the RNase J1 mutants and reduced in the ΔJ2 strain. B) The layout of the region around SA1279 and *rnpB*. DNA is represented as a wavy line, and RNA transcripts as straight black lines. (PP)P indicates a mix of tri- and mono-phosphorylated RNA, generated by pyrophosphohydrolases. Small blue arrows indicate the PCR-primers used to amplify circularised RnpB and SA1279-RnpB for mapping the 5′ and 3′-ends. R1 indicates the probe used for the Northern blot shown in Figure S2. C) A blow-up of the region from +420 to +540, showing the proposed model for converting the +1 transcript into mature RnpB. P indicates mono-phosphorylation. D) Predicted secondary structures of RnpB, generated using mfold with default settings [30], and based on the crystal structures of RNase P RNA [27], [31]. Within the RNase P structure, the thin dotted arrows indicate the path of the RNA through the secondary and tertiary structure of RNase P, the RBS and start codon of SA1278 are in bold, and the region where the anti-sense RNA can hybridise is indicated with a thick black line. E) The difference in average length of RnpB in WT, ΔJ1, and ΔJ1ΔJ2 strains, revealed by the length of the PCR-product generated across the 5′/3′ junction. Results of the cloned and sequenced PCR-products are shown in Table 6. M: Marker.

**Figure 6:**
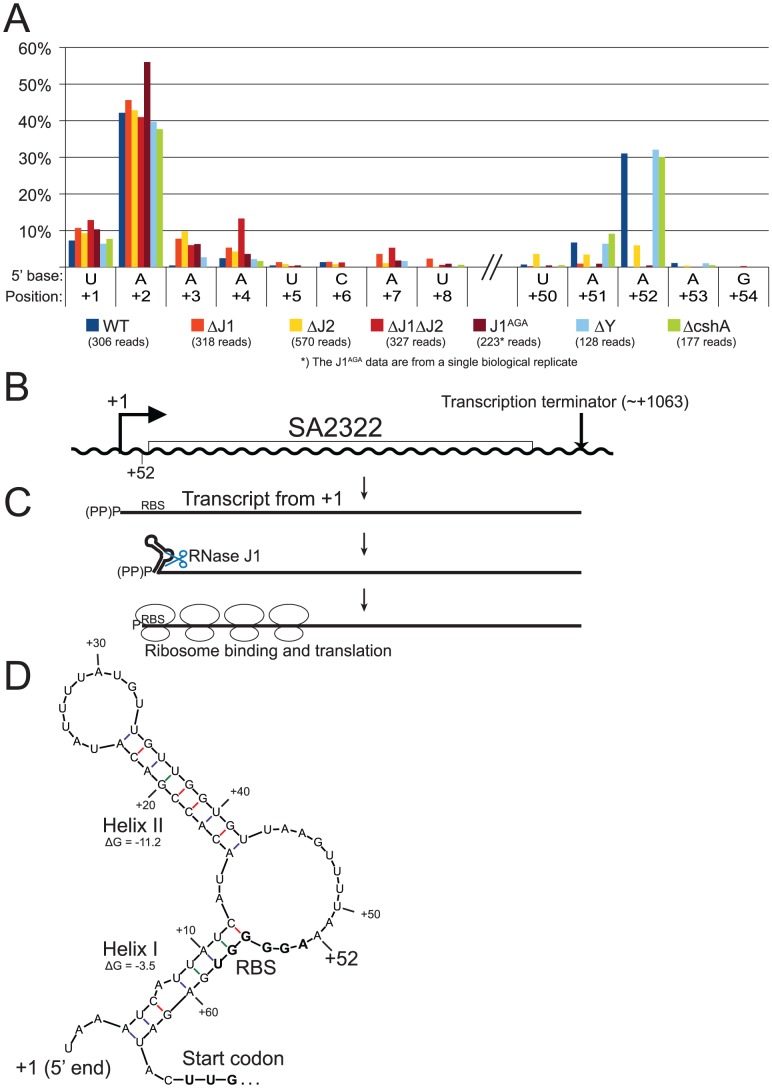
mRNA maturation by RNase J reveals a potential regulation of translation. A) Histogram showing the percentage of reads mapping to a given position, out of the total number of reads mapping to the SA2322 transcript in each strain (shown in parentheses). Only positions of interest are included, but the full data-set can be found in Table S7. +1 and +2: The putative transcription start sites. +52: A major detected RNA species in the WT, ΔY and ΔcshA, however it is absent from the RNase J1 mutants and strongly reduced in the ΔJ2 strain. B) The SA2322 locus with important positions indicated. C) A schematic view of the fate of SA2322 transcripts. A newly formed transcript can form a secondary structure, shown in panel D, which partially sequesters the ribosome binding site (RBS). RNase J can shorten the transcript by 51 nt, and is presumably blocked from further exonucleolytic digestion by ribosomes binding to the RBS. (PP)P indicates a mix of tri- and mono-phosphorylated RNA, generated by pyrophosphohydrolases. D) Predicted secondary structure at the 5′-end of the SA2322 transcript. ΔG values predicted by the mfold algorithm are in kcal/mol. RBS and start codon are indicated in bold.

**Figure 7:**
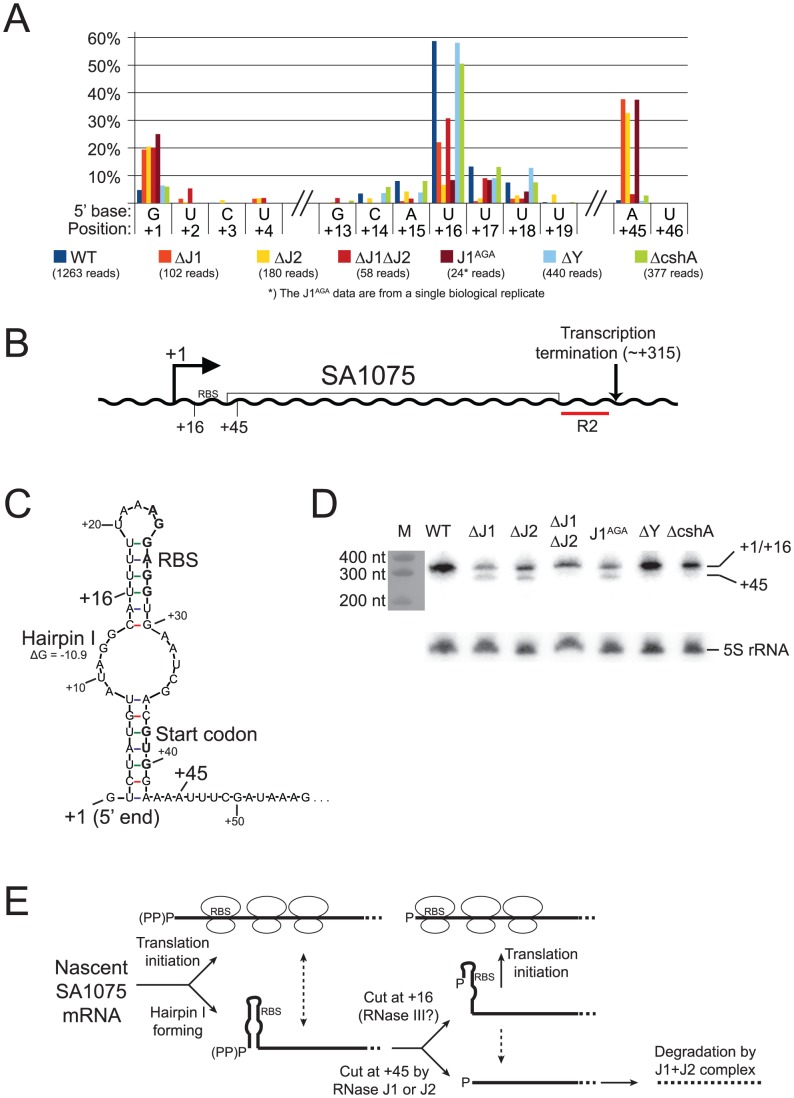
SA1075 mRNA inactivation by RNase J competes with translation initiation. A) Histogram showing the percentage of reads mapping to a given position, out of the total number of reads mapping to the SA1075 transcript in each strain (shown in parentheses). Only positions of interest are included, but the full data-set can be found in Table S8. +1: The putative transcription start site. +16: The major detected RNA species in the WT, ΔY and ΔcshA. +45: Position where ΔJ1ΔJ2 differs from ΔJ1, ΔJ2 and J1^AGA^, and appears similar to WT. B) Important positions indicated on the SA1075 gene. +1: Transcription Start Site. RBS: Ribosome Binding Site. R2 indicates the probe used for the Northern blot in panel D. C) Hairpin structure predicted by the mfold algorithm, which sequesters the RBS and start-codon (shown in bold). No secondary structure was predicted for the 50 nucleotides downstream of position +45. D) Northern blot of the SA1075 transcript, using probe R2. The +1 and +16 RNA species are not resolved, and can be seen as a single band, however the +45 species is clearly visible in the ΔJ1, ΔJ2, and J1^AGA^ strains. The marker was stained with methylene blue and photographed. As a loading control, the Northern blot was stripped and re-probed to detect the 5S rRNA. E) The proposed model for determining the fate of SA1075 mRNA via competition between RNase J, ribosomes and the nuclease that cleaves at position +16. (PP)P indicates a mix of tri- and mono-phosphorylated RNA, generated by pyrophosphohydrolases. Nascent SA1075 mRNA can either be occupied by ribosomes, binding to the RBS, or form Hairpin I which sequesters the RBS. Ribosomes will shield position +45 from RNase J, but the hairpin will not. If cleavage at position +16 occurs before RNase J has cleaved at position +45, then the RBS will be liberated from Hairpin I, and ribosomes can initiate translation. If ever the +45 cut is made by RNase J, then the mRNA, which no longer has RBS or start-codon, is immediately degraded (possibly by the RNase J1+J2 complex). Either RNase J1 or RNase J2 can perform a cleavage at position +45. The loss of both RNases prevents the +45 RNA species from being generated, thus explaining why the WT and the ΔJ1ΔJ2 strains appear similar in panel A (see discussion for details and other potential explanations).

Below, we present four examples where RNase J activity impacts on RNAs (Figures 4, 5, 6 and 7) and although they are certainly not exhaustive for all situations, they illustrate different outcomes of the absence of RNase J activity.

#### Example 1: The final step in 5′ maturation of 16S rRNA is carried out by RNase J1, aided by RNase J2

Not surprisingly, and despite the ribosome depletion step, the vast majority of reads from the EMOTE mapped to the 16S and 23S rRNAs (small RNAs like 5S rRNA and tRNAs were depleted during the RNA purification procedure). The rRNAs represent a large and stable population of mono-phosphorylated RNAs in the cell, whereas mono-phosphorylated mRNAs are often undergoing degradation and therefore have short half-lives and much lower steady-state levels. When comparing the number of reads for each position at the 5′-end of 16S rRNA, it became clear that the RNase J mutants were unable to properly finalise the 5′ maturation of this RNA. Instead, there was an accumulation of immature 16S rRNA (Figure 4, Table S5), showing that RNase J1 and J2 are responsible for cropping the final 4 nucleotides to form mature 16S rRNA. The J1^AGA^ mutant exhibits identical deficiencies to ΔJ1 and ΔJ1ΔJ2, indicating that the active site of RNase J1 is required for 16S maturation in *S. aureus*. A similar analysis of the 5′-end of 23S rRNA revealed no enrichment in the +M region of any of our examined mutants (Table S2), but this is not surprising, since 5′ maturation of *B. subtilis* 23S rRNA was shown to be carried out by the Mini-III RNase, and RNase J activity is only important when Mini-III is missing [26].

#### Example 2: RNase J matures and degrades the 5′-end of RNase P RNA

The second example is the region containing SA1279, SA1278 and *rnpB*, the RNA component of the ribozyme RNase P. SA1279 encodes cell division protein GpsB, whereas the SA1278 ORF is entirely inside the *rnpB* gene, and might not be translated.

RNA species with three different 5′-ends are abundant in this region (Figure 5A, Table S6). Position +1 is within the SA1280 ORF, and has upstream −10 (TATA) and −35 (TTGAtt) motifs. It is probably the major transcription start site for the SA1279 gene, but there are no obvious upstream transcription termination sites, so transcription start sites even further upstream could also contribute to SA1279 expression. Furthermore, a strong hairpin can form at +1, protecting the RNA from 5′ degradation, without interfering with translation of SA1279. The +485 site corresponds to the 5′-end of the RNase P RNA (RnpB), and is also a possible transcription start site, although the −35 motif is very weak (cTGtCt). The +485 5′-end is protected by Helix P1, which forms between the beginning and the end of RnpB (Figure 5D and [27]). No obvious transcription termination site could be identified between +1 and +485, and it is likely that both transcription start sites contribute to *rnpB* expression. The +499 5′-end is more difficult to explain, since no convincing transcription start motifs are present, and the +499 RNA would be unable to form the conserved Helix P1 in RNase P. The +499 RNA species are much less abundant in the RNase J mutants, although the ΔJ2 strain still has a small population of this RNA (Figure 5A). Similarly, a smaller proportion of the +485 RNA was detected in the RNase J mutants, but this was counterbalanced by the appearance of several small populations of RNAs starting at positions +452 to +477 (Figure 5A and 5C).

RnpB molecules with a 5′-end at +499, where the P1 Helix cannot form a protecting secondary structure with the 3′-end, could potentially provide an entry-point for 3′ to 5′ exonucleases. To determine whether this was the case, total RNA was circularised with T4 RNA ligase 1, an *rnpB*-specific oligo was used to generate cDNA, and PCR products across the ligated 5′-3′ junction of RnpB were obtained using divergent *rnpB*-specific primers (Figure 5B). The average length of PCR-products amplified from the WT strain were visibly shorter than those from the ΔJ1 and ΔJ1ΔJ2 strains (Figure 5E). To obtain the exact 5′ and 3′-ends of individual RnpB molecules, the bands from WT and ΔJ1ΔJ2 were cut from the gel and ligated to a cloning vector, whereupon twelve clones from each strain were sequenced. The pairs of 5′-ends and 3′-ends shown in Table 6 qualitatively proves the existence of both the +485 and +499 RNA species in the WT strain, and reveal that 3′ heterogeneity of RnpB is limited, such that the size differences of the PCR-products in Figure 5E are due to lack of 5′ processing in the ΔJ1ΔJ2 strain.

**Table 6:** In ΔJ1ΔJ2, RnpB begins upstream of the +485 putative transcription start site, but 3′-ends are unaffected.

RnpB Clone	5′ base*	3′ base*
WT clone 1	+499	+904
WT clone 2	+536	+904
WT clone 3	+499 to +500	+908 to +909
WT clone 4	+499 to +500	+910 to +911
WT clone 5	+484 to +485	+805 to +806
WT clone 6	+499 to +500	+911 to +912
WT clone 7	+499	+904
WT clone 8	+499 to +501	+884 to +886
WT clone 9	+499 to +500	+910 to +911
WT clone 10	+497 to +499	+901 to +903
WT clone 11	+498 to +499	+906 to +907
WT clone 12	+499 to +500	+909 to +910
ΔJ1ΔJ2 clone 1	+463	+908
ΔJ1ΔJ2 clone 2	+478	+907
ΔJ1ΔJ2 clone 3	+475 to +476	+883 to +884
ΔJ1ΔJ2 clone 4	+470 to +471	+909 to +910
ΔJ1ΔJ2 clone 5	+453 to +456	+906 to +910
ΔJ1ΔJ2 clone 6	+459	+904
ΔJ1ΔJ2 clone 7	+472 to +474	+910 to +912
ΔJ1ΔJ2 clone 8	+464 to +465	+913 to +914
ΔJ1ΔJ2 clone 9	+471 to +474	+881 to +884
ΔJ1ΔJ2 clone 10	+452 to +453	+911 to +912
ΔJ1ΔJ2 clone 11	+473 to +474	+908 to +909
ΔJ1ΔJ2 clone 12	+467 to +469	+887 to +889

*) If the 5′ base and the 3′ base are identical, thus preventing exact mapping of the ends, then a range of possible 5′ and 3′ positions are given.

To show that an RNA species really does start at +1 and extends into the *rnpB* gene, we first performed a Northern blot, were we could detect a faint band corresponding in size to this longer RNA (Figure S2A). This was then confirmed by using the cDNA generated from circularised RNA (described above) as template for a PCR where the reverse rnpB-primer was substituted with a reverse primer annealing inside the upstream SA1279 ORF (Figure 5B). The extremely faint bands (Figure S2B) were again cut from the gel, and six clones from each strain were sequenced. Table 7 shows the existence of RNA molecules with a 5′-end at +1, but also that some slightly (7 to 14 nt) longer RNAs can be found when no RNase J is present to trim the 5′-ends.

**Table 7:** Sequencing circularised SA1279-RnpB.

SA1279-RnpB Clone	5′ base*	3′ base*
WT clone 1	+1 to +3	+915 to +917
WT clone 2	+351	+915
WT clone 3	+1 to +3	+915 to +917
WT clone 4	+63	+915
WT clone 5	+173	+915
WT clone 6	+1 to +3	+915 to +917
ΔJ1ΔJ2 clone 1	−8 to to7	+914 to +915
ΔJ1ΔJ2 clone 2	+1 to +3	+915 to +917
ΔJ1ΔJ2 clone 3	+1 to +2	+903 to +904
ΔJ1ΔJ2 clone 4	−1 to +1	+913 to +914
ΔJ1ΔJ2 clone 5	−8	+913
ΔJ1ΔJ2 clone 6	−14	+912

*) If the 5′ base and the 3′ base are identical, thus preventing exact mapping of the ends, then a range of possible 5′ and 3′ positions are given.

#### Example 3: RNase J removes the first 51 nucleotides from SA2322 mRNA

Newly transcribed full-length SA2322 mRNA, encoding a hypothetical membrane protein, starting at position +1 or +2, can form a predicted energetically favourable 9 bp hairpin structure (Helix II in Figure 6) between nucleotide +15 and +42, and this is possibly further stabilised by a secondary helix between nt +4 to +12 and +56 to +63 (Helix I, in Figure 6). However, a significant proportion of RNA is detected which starts at position +52 (Figure 6A, Table S7), a position that lack recognisable transcription initiation signals, and is not caused by cleavage by RNase Y, since the +52 position is easily detected in the ΔY strain. The RNase J mutants however, either lack the cut (ΔJ1, ΔJ1ΔJ2 and J1^AGA^) or have a severe paucity of it, compared to the WT strain (ΔJ2) (Figure 6A), and it is therefore likely that RNase J activity is responsible for the +52 cleavage (Figure 6, see discussion for details).

#### Example 4: Strain ΔJ1ΔJ2 has different RNA sub-populations than WT, ΔJ1 and ΔJ2

The fourth, and perhaps most intriguing, example is the SA1075 mRNA, encoding the acyl carrier protein *hmrB*, in which the RNase J single mutants behave different from the double RNase J deletion (Figure 7), as opposed to previous examples, where the four RNase J mutants follow each other closely (Figures 4, 5, and 6).

Newly formed SA1075 mRNA, is predicted to form a secondary structure that seems to block translation by sequestering the RBS and the start codon (Figure 7B and C). However, cleavage at +16, generating a new 5′-end, renders the RBS accessible, presumably allowing translation to occur. In the WT, ΔJ1, ΔJ1ΔJ2, and ΔY strains, a major population starting at +16 can be observed, however, in strains ΔJ2 and J1^AGA^, this RNA species is much less visible. Even though the overall abundance of detected SA1075 RNA is reduced in the RNase J mutants, the percentage with 5′-ends at +1 goes up in the RNase J mutants, (Figure 7A, Table S8). The cleavage at position +45 of the SA1075 is very noticeable in strains ΔJ1, ΔJ2 and J1^AGA^, but is only at background level in ΔJ1ΔJ2, WT, ΔY, and ΔcshA (Figure 7A). A Northern blot was used to confirm the observed data, using probe R2, which hybridises near the 3′-end (Figure 7B). The +1 and +16 bands are not separated, however the +45 band is visible in all RNase J mutant strains except ΔJ1ΔJ2 (Figure 7D). Either RNase J1 or RNase J2 must therefore be needed for this cleavage to occur, whether by exo- or endonucleolysis, and since the +45 position is also detected in the J1^AGA^ strain, which encodes the entire RNase J1 protein, but only lacks an active site, it seems highly unlikely (albeit possible) that the cut is made by another RNase, requiring the presence of one of the RNase J proteins for its activity (Figure 7, see discussion for details).

## Discussion

We have previously shown that RNase J1 and RNase J2 individually are not needed for *S. aureus* viability [21], and we have here shown that the double mutant is also viable, proving that it is not a case of one RNase J taking over the function of the other in the single mutants. Several growth conditions were examined, and it was found that growth of the RNase J mutants was strongly inhibited outside a narrow range of laboratory growth conditions. These pronounced growth defects reflect the importance of RNA degradation and are in accordance with the major defects in cell morphology observed in *B. subtilis* [22]. We believe that the previous failure to isolate RNase J mutants could at least partially be attributed to their reduced growth under various conditions and that our recently developed selection/counterselection system was a key element in overcoming the difficulties of isolating RNase J mutants [21]. Moreover, due to the controversy of our findings when comparing with earlier publications from both *S. aureus* and other Firmicutes [7], [10], [19], [20], we confirmed the deletion of the RNase J genes by full genome sequencing (Table 1), and only identified a single valine to valine point mutation on the ΔJ1 chromosome, which appears to be unrelated to the viability of ΔJ1.

### RNase J1 and J2 are working together to correctly cleave and degrade RNA

RNase J1 and RNase J2 active-site mutants were examined, and surprisingly, the J2^AGA^ mutant exhibited no growth defects, whereas the J1^AGA^ mutant behaved almost exactly like the full RNase J1 deletion mutant, both in the 5′-mapping experiments and in terms of growth, with the only major difference being an ability to grow on magnesium complemented LB-medium (Figure 1). This seems to indicate that the only active RNase J in *S. aureus* is RNase J1, but that the RNase J2 protein is needed for RNase J1 to function correctly.

Taking into account data mainly from *B. subtilis* [11], , but also from *S. aureus* [12], RNase J1 and RNase J2 strongly interact, and will generate a population of hetero-dimers or tetramers. We therefore propose a model where RNase J1 provides the active site of the complex, accounting for the strong phenotype of the J1^AGA^ mutant, and the lack of phenotypes for the J2^AGA^ mutant. In contrast, RNase J2 serves mainly as a structural protein, ensuring the proper function of the RNase J1+J2 complex. The phenotypes seen for the RNase J2 deletion mutants are therefore caused by a disruption of the RNase J1+J2 complex, which can be partially overcome by over-expressing RNase J1 from a plasmid, however, the over-expressed RNase J1 does not need to be enzymatically active (Figure 2). This hypothesis is strengthened by the almost identical molecular data from ΔJ1 and J1^AGA^, and ΔJ2, found throughout our EMOTE and Northern blot data (Figures 4, 5, 6 and 7; Table S1 and S2). Nevertheless, the model does not completely exclude a minor enzymatic activity of RNase J2, perhaps when it is not complexed to RNase J1, an activity that would account for the +45 cut of SA1075 in ΔJ1 (Figure 7 and see below). Moreover, the interplay between RNase J1 and J2 may be different in organisms where RNase J2 was shown to be either essential or virtually dispensable, such as *S. pyogenes* and *B. subtilis*, respectively [10], [7].

### 5′ EMOTE analyses of RNase J mutants

RNase J enzymes were reported to have endonucleolytic and 5′ to 3′ exonucleolytic activities. To obtain a better picture of these enzymatic activities within the cell, it was necessary to develop an assay that allowed us to examine the 5′-ends of all cellular RNA in the mutants, and compare it to the wild-type (Figure 3). Two additional mutants of the RNA decay machinery (ΔY and ΔcshA) were included as controls, to ensure that observed effects were RNase J specific. Processed RNA and degradation intermediates possess a mono-phosphate 5′-end, and are readily detected in our assay, whereas primary 5′-ends are generally protected by their triphosphate ends, and are only detected due to the pyrophosphohydrolase activity of enzymes such as RppH [24]. The assay is therefore useful for studying enzymes like RNase J, that endo- or exonucleolytically cleave RNA and produce molecules with monophosphorylated 5′-ends.

### RNase J1 and J2 are responsible for 5′ maturation of 16S rRNA

The EMOTE analysis shows a clear enrichment in the RNase J1 mutants at all positions upstream of the 4+M position (defined here as the mature rRNA with additional 4 nucleotides at the 5′-end), whereupon the abundance rapidly decreases to an extreme under-representation at position M1 (the first nucleotide of the mature rRNA). The proportion between the RNase J1 mutants and the WT stays at the same high plateau from position 13+M to 4+M (Figure 4), and our data indicate that this plateau extends all the way to the RNase III cleavage site (93+M) (Table S3).

Downstream of position 5+M, in the wild-type, 5′ ends are strongly enriched, presumably due to rapid exonucleolytic activity of RNase J trimming the 16S rRNA down to position 4+M, and then a somewhat slower final trimming to obtain the fully mature 16S rRNA at position M1. It is furthermore possible that the difference between the severity of phenotypes seen for ΔJ2 and ΔJ1 (Figure 1) can be explained by the higher proportion of mature 16S rRNA observed in ΔJ2 (Figure 4).

Our interpretation is that an unidentified endonuclease cleaves semi-randomly between 93+M and 5+M, whereupon the RNase J1+J2 complex exonucleolytically degrades the resulting 5′-end until it reaches the M1 position. The endonuclease responsible for the random cuts in *S. aureus* is not likely to be RNase Y, since the ΔY data follow the WT data closely (Figure 4). Even in the total absence of RNase J, there is however still a small but significant amount of 16S rRNA with a correctly matured 5′-end, but it is unclear whether this is caused by occasional cleavage at the M1 position by the above mentioned unidentified endonuclease, or by a backup-system for RNase J (Figure 4).

In *B. subtilis*, the 16S rRNA is specifically cut by the unidentified endonuclease at position 38+M, whereupon it is trimmed to maturity by RNase J1 [13], [17]. In *S. aureus*, a peak in the reads at positions 27+M and 28+M in both WT and all mutant strains (Figure 4, Table S3) suggests that these two positions are equivalent to 38+M in *B. subtilis*, but that the *S. aureus* enzyme is less specific.

Since RNase J is responsible for maturing the 5′ end of 16S rRNA, they must somehow limit their processive exonuclease activity, in order to avoid degrading the entire rRNA. Mathy and coworkers [13] observed that *in vitro*, the *B. subtilis* RNase J1 will digest the entire 16S rRNA molecule, but this is obviously not the case *in vivo*, where RNase J processivity is arrested at the M1 site, presumably by ribosomal proteins.

### RnpB-maturation and possibly quality control by RNase J

We have shown that the +1 transcript of SA1279 continues into *rnpB* (Figure 5) and that this long RNA is processed to obtain a mature RNase P RNA (Table 7, Figure S2). We propose that the accumulation of RNA species with 5′-ends in the region downstream of the SA1279 ORF (+452 to +477), detected in the RNase J mutants, represent cleavage products by an unidentified endonuclease, which in the WT strain are trimmed by RNase J to form the mature RNase P RNA at position +485 (Figure 5C). This model thus explains the reduction in observed +485 RNA in the RNase J mutant strains, and the remaining, lower, level of mono-phosphorylated mature RNase P RNA is generated directly by transcription initiation and pyrophosphohydrolysis at this site (Figure 5B), despite the poor −35 motif. It is possible that it is RNase Y that performs the endonucleolytic cleavage of the +452 to +477 region. However, our data can neither confirm nor reject this, since the resulting 5′-ends are removed so quickly by RNase J in the WT strain that they do not appear in our data, and thus the absence of these species in the ΔY mutant is not conclusive (Figure 5).

The formation of +499 RNA population is less obvious, however our data clearly show that it is caused by RNase J. The evolutionary conserved P1 Helix, which presumably protects the 5′-end of the RnpB RNA from 5′ degradation cannot form during transcription, but only once the entire RNA has been made. One possibility is therefore that a 5′ exonucleolytic attack is made by RNase J on nascent RNA, which has not yet formed the P1 Helix (Figure 5D). This exonucleolytic digestion would then continue until blocked by one of the many secondary structures in RNase P RNA, or by the protein component (RnpA) of the ribozyme. The +499 end would in this model represent a degradation intermediate, where RNase J either is blocked or pauses long enough to accumulate a detectable population of +499 RNA. The EMOTE data, due to potential ligation and PCR biases, cannot be used quantitatively to compare the relative abundance of two different 5′-ends such as +485 and +499, and the same is the case for the circularised and cloned junctions shown in Table 6, which seem to indicate that the +499 5′-end is more abundant in the WT strain than the +485 end. Instead, Table 6 confirms qualitatively that the 5′-end of the RNA can be both +485 and +499 in WT.

Adding further complexity to the maturation of RNase P is the existence of a small anti-sense RNA, which can base-pair to the 3′-end of RnpB [2], [28]. One could imagine that this anti-sense RNA is in competition with the 5′-end of the RnpB to hybridise to the 3′-end, and if the competition is successful, then the P1 Helix will fail to form, and the 5′-end is free to be degraded by RNase J (Figure 5D).

### SA2322 mRNA maturation by RNase J

Our data cannot directly determine whether the +52 cut of SA2322 is due exonucleolytic digestion of the primary transcript, or an endonucleolytic cut directly at +52. In either scenario, 5′ to 3′ exonucleolytic digestion beyond +52 is very likely inhibited by ribosomes binding the RBS, as seen for the *hbs* gene in *B. subtilis* [29]. Importantly, if Helix I, helped by Helix II, forms *in vivo* in full-length mRNA, the +52 cut may serve a regulatory function, since Helix I partially sequesters the RBS of SA2322. It is also worth noting that the transcription start site of SA2322 appears to be less precise in all the RNase J mutants (Figure 6A). This might be due to lessened fidelity of the transcription initiation complex, altered activity of pyrophosphohydrolases, which would reveal new products to our assay, or perhaps that RNAs starting at position +3 or +4 are so rapidly degraded by RNases J in the WT strain, that these RNA species simply are not observed.

### RNase J1 and J2 are individually responsible for cleavage at position +45 of SA1075 mRNA

The essential [20] SA1075 gene, where a cut at position +16 liberates the RBS, serves as a second example of an RNase-mediated regulatory mechanism. This cleavage however, is not mediated by RNase J. Instead, a second cleavage at position +45, inside the SA1075 ORF, is performed by either RNase J1 or J2 individually, and reveals an intriguing difference in activity between the RNase J1+J2 complex, and RNase J1 or J2 alone.

It appears that three scenarios are possible for a newly transcribed SA1075 mRNA: i) Translation is initiated before Hairpin I can form, and successively loading ribosomes prevent the formation of Hairpin I. If ever a pause in translation initiation occurs, then Hairpin I will form, blocking further initiation, unless ii) an RNase cleaves at position +16, to destroy Hairpin I and allow translation initiation. However, if an SA1075 mRNA molecule is not shielded by ribosomes, then it is a potential target for iii) an RNase J cleavage at position +45, which removes the RBS and start codon, and initiates degradation of the mRNA (Figures 7C and E).

While our data cannot identify the enzyme that cleaves SA1075 mRNA at position +16, it is possible that it is RNase III, because the 5′ fragment of the SA1075 mRNA was recovered in a pull-down experiment, using an inactive RNase III mutant as bait, whereas a fragment starting at +16 was obtained when wild-type RNase III was used ([25]; P. Romby, personal communication). If so, then the fate of an SA1075 mRNA is decided by a competition between RNase J, RNase III and the ribosome (which itself is matured by RNase J and RNase III).

Several hypotheses can explain difference at position +45, between the ΔJ1, ΔJ2 and J1^AGA^ mutants and ΔJ1ΔJ2, observed in both the Northern blot and EMOTE data (Figure 7). It is possible that the +45 cleavage is caused by an unrelated RNase, which is over-expressed in the ΔJ1, ΔJ2 and J1^AGA^ mutants, but not in the ΔJ1ΔJ2 strain. It is also possible that RNase J1 or J2 individually still retain a small amount of exonucleolytic activity, even though no such activity has been shown for RNase J2 [18], and that this activity is able to digest until position +45, where it is blocked by an unknown mechanism. What this mechanism might be is uncertain, since the +45 RNA does not contain a ribosomal binding site, nor is any secondary structure predicted which could block RNase J exonucleolysis.

Our preferred hypothesis, is that the +45 is an endonucleolytic cleavage which can be performed by either of the two RNase J proteins individually, but that any subsequent 5′ to 3′ exonucleolytic degradation can only be carried out by a functional RNase J1+J2 complex. This would explain why 5′-ends at position +45 are not seen in the WT strain, since the RNAs are rapidly 5′ to 3′ digested, neither seen in the ΔJ1ΔJ2 strain, because the +45 cleavage is never made. In contrast, the +45 RNA is highly enriched in the ΔJ1, ΔJ2 and J1^AGA^ mutants, because they are able to perform the +45 cut, but unable to exonucleolytically degrade the resulting product. This hypothesis would also correspond with the observation by Mäder and coworkers [8] that several genes had significantly altered abundance in their *B. subtilis* RNase J1 and/or RNase J2 mutants, but did not exhibit any change in the double mutant.

Supporting this hypothesis are the data presented in Table 5, where the severely inhibited or lacking *in vivo* exonuclease activity in strains ΔJ1, ΔJ2 and J1^AGA^ results in a similar accumulation of 5′-ends to that found in strain ΔJ1ΔJ2.

### Concluding remarks

As seen in Figure 1, the RNase J mutants exhibit severe growth defects when taken outside the narrow ‘comfort zone’ of 37°C and Mueller-Hinton medium. The exact cause(s) of these problems have not been identified, but since our 5′ data shows that RNase J exerts a global influence on mRNAs (as well as on maturation of stable RNAs), the growth defects are most likely due to mis-regulation of a number of genes, each of which probably not causing major phenotypes, but generating severe effects when combined. Possible candidate genes could be SA1075 (*hmrB*) and RNase P, which are both essential [20]. In all mutant studies, especially those where the growth of the cell is severely affected, there is always a risk of detecting secondary effects of the mutation. This is important to keep in mind when interpreting the RNase J mutant data, even though we have attempted to strengthen the observations by using a range of different RNase J mutants and including the ΔY and ΔcshA strains as controls.

It appears that the 5′ to 3′ exonucleolytic activity is crucial for normal growth, whereas the endonucleolytic activity only serves a secondary function. The normal growth of strain J2^AGA^ supports this, if the *S. aureus* RNase J2 is similar to its *B. subtilis* counterpart, which almost exclusively exhibits endonucleolytic activity *in vitro* [18].

While we do not assume that our four presented examples are exhaustive for all the functions of RNase J, they reveal how RNase J activity remodels sub-populations of RNA (Figures 4, 5, 6 and 7), and show that the roles of RNase J are diverse, ranging from maturation and post-transcriptional regulation to degradation. Furthermore, our data clearly show that RNase J1 is the work-horse, while RNase J2 plays a supporting role, with no detected phenotypes of the J2^AGA^ mutant. However, we also detect that an important RNase J-mediated cleavage takes place in the SA1075 gene in ΔJ1, which demonstrates that RNase J2 is capable of performing some tasks by itself. The implied interplay between RNase J1, RNase J2 and the RNase J1+J2 complex *in vivo*, which might correlate with switching between endo- and exo-nucleolytic activity, is likely to have fine-tuned regulatory functions, arising from the evolutionary event that duplicated the RNase J gene in an early Firmicute ancestor.

## Materials and Methods

### Growth mediums

Mueller-Hinton (Beckton-Dickinson, Sparks, MD, USA) and LB-plates (Merck, Darmstadt, Germany) were prepared with 13 g/l agar (Merck), and RH-plates were made by autoclaving 100 ml milliQ water with 7.5 g agar, cooling it to 55°C and mixing with 500 ml modified RPMI-medium (Sigma, R7388) prewarmed to 55°C. +U, +C, and +Mg, indicates addition of 20 mg/l uracil, 10 mg/l chloramphenicol, and 5 mM MgCl_2_, respectively.

### Constructing allelic replacement mutants

Mutants were generated using the pRLY-vector series (Table 3), and following the protocol described in Redder and Linder, 2012. However, since the RNase J mutants are sensitive to both heat and cold, strains PR01-20, PR01-25, PR02-03, and PR02-06 were kept between 32°C and 39°C throughout their construction, and strain PR01-27 and PR01-37 were grown at 37°C at all times, since the lack of origin of replication in the pRLY-vectors used for these strains precluded the need for plasmid establishment and plasmid elimination phases (see also Redder and Linder, 2012).

### Spotting dilutions to determine growth defects

Strains were cultured overnight in liquid MH+U with agitation. Dilution series were then made in MH medium, to obtain 10^−5^ and 10^−6^ dilutions, 5 µl of which were then spotted on MH and RH plates with added uracil (10 mg/l). The plates were then incubated at the indicated temperature(s) until the WT colonies reached appropriate size, typically 18 hours for 42°C and 37°C, 24 hours for 30°C and 48 hours for 25°. All strains compared in Figure 1 were spotted on the same plate, to eliminate variations between batches. The same was the case for the strains in Figure 2.

### EMOTE assay of mono-phosphorylated 5′-ends

2 ml of mid-exponential phase cultures (OD_600_ between 0.40 and 0.45) were harvested by centrifugation, the supernatant removed, and 1 ml cold 1∶1 ethanol/acetone was immediately added to protect the RNA. After washing the bacterials pellets in 1×TE buffer (10 mM Tris, 1 mM EDTA, pH 8), the cells were lysed with 100 µg lysostaphin (AMBI Products LLC, Lawrence, NY, USA) in 200 µl TE with 1 U/µl Murine RNase Inhibitor (M0314S, New England Biolabs, Ipswich, MA, USA), and homogenised using RNeasy Shredder columns (Qiagen, Hombrechtikon, Switzerland), whereupon total RNA was prepared using the RNeasy mini kit (Qiagen) with an on-column DNase I treatment (Cat: 79254; Qiagen).

For each strain, 0.5 nmol of Rp5 oligo (Table 8), 5 µg of total RNA, and TE buffer to a total volume of 55 µl was heated for 120 seconds at 95°C and then flash-cooled in ice/water. A premix of 10 µl 10× T4 RNA ligase buffer, 10 µl 10 mM ATP, 30 U T4 RNA ligase 1, 1 µl Murine RNase inhibitor (all from New England Biolabs), and 21 µl water was added to each tube of denatured RNA, and incubated for 3 hours at 37°C. After ethanol precipitation and re-suspension in 20 µl TE, the protocol for the MicrobeExpress kit (Ambion, Life Technologies, Zug, Switzerland) was followed to deplete the ribosomal RNA, followed by re-suspension in 25 µl water.

**Table 8:** Oligos for 5′ EMOTE.

Name	Sequence
Rp5	CAGGCACGCAGCAGACCC (RNA)
DROAA	GGCATTCCTGCTGAACCGCTCTTCCGATCTNNNNNNNNAA
B-oligo	GGCATTCCTGCTGAACCGC
	D5-oligos for biological replicate #1 (barcode is underlined)
D5a	CTCTTTCCCTACACGACGCTCTTCCGATCTTACACAGGCACGCAGCAGAC
D5b	CTCTTTCCCTACACGACGCTCTTCCGATCTAAGCCAGGCACGCAGCAGAC
D5c	CTCTTTCCCTACACGACGCTCTTCCGATCTCATGCAGGCACGCAGCAGAC
D5d	CTCTTTCCCTACACGACGCTCTTCCGATCTGAATCAGGCACGCAGCAGAC
D5e	CTCTTTCCCTACACGACGCTCTTCCGATCTTCGACAGGCACGCAGCAGAC
D5f	CTCTTTCCCTACACGACGCTCTTCCGATCTACTCCAGGCACGCAGCAGAC
	D5-oligos for biological replicate #2 (barcode is underlined)
D5La	CTCTTTCCCTACACGACGCTCTTCCGATCTNNAAGTCGCAGGCACGCAGCAGAC
D5Lb	CTCTTTCCCTACACGACGCTCTTCCGATCTNNCATACCCAGGCACGCAGCAGAC
D5Lc	CTCTTTCCCTACACGACGCTCTTCCGATCTNNGCTGCACAGGCACGCAGCAGAC
D5Ld	CTCTTTCCCTACACGACGCTCTTCCGATCTNNTCACGTCAGGCACGCAGCAGAC
D5Le	CTCTTTCCCTACACGACGCTCTTCCGATCTNNAGATGGCAGGCACGCAGCAGAC
D5Lf	CTCTTTCCCTACACGACGCTCTTCCGATCTNNCGCAGCCAGGCACGCAGCAGAC
D5Lh	CTCTTTCCCTACACGACGCTCTTCCGATCTNNTTGCATCAGGCACGCAGCAGAC

cDNA with one Illumina-sequencing adaptor was generated from 4.5 µl Rp5-ligated and rRNA-depleted total RNA, with 100 pmol DROAA oligo, 6 µl water, 4 µl 5× Reverse Transcriptase buffer, 2 µl 100 mM DTT, 1 µl 10 mM dNTP, 0.5 µl Murine RNase inhibitor, and 1 µl M-MLV (-H) Reverse Transcriptase (New England Biolabs), which was incubated for 10 minutes at room temperature, 50 minutes at 42°C, and 30 minutes at 65°C to inactivate the enzymes. 180 µl water with 1 mg RNase I was added and the cDNA was purified using the Promega SV PCR-purification kit (Promega, Madison, WI, USA).

The second Illumina sequencing adaptor, together with barcodes for identifying from which strain the sequence originated, was added in a second-strand PCR: 32 µl H_2_O, 1.5 µl 10 µM oligo D5La (D5Lb, D5Lc, etc.), 1.5 µl 10 µM B-oligo (Table 8), 10 µl 5× Phusion HF buffer, 1.5 µl 10 mM dNTP, 0.5 µl Phusion enzyme, 3 µl cDNA. Program: 2 min @ 98°C, 20 sec @ 98°C, 15 sec @ 55°C, 30 sec @ 72°C (30 cycles), 3 min @ 72°C. 6 µl of each PCR product was loaded on an agarose gel, to verify that the yields were similar. 40 µl of the WT PCR product was mixed with 20 µl from each of the other strains, and the mixture was purified using the Promega SV PCR-purification kit, and eluted twice with 50 µl water. The purified mixture was loaded on an agarose gel, and the smear between 300 bp and 1400 bp was extracted using the Promega SV Gel extraction kit (Promega), according to protocol (Figure 3), and the DNA was sent for 50 bp Illumina sequencing (Fasteris, Plan-des-Ouates, Switzerland).

Two biological replicates were carried out for strains WT, ΔJ1, ΔJ2, ΔJ1ΔJ2, ΔY and ΔcshA, but only one for strain J1^AGA^. Data presented for J1^AGA^ and in Figure 4 are from the second biological replicate, and the latter exhibit the same patterns as the first biological replicate.

### Analysing the EMOTE data

Before mapping on the reference genome, reads were split into groups looking for the exact match with the corresponding barcode sequence. After sorting, only reads having the control sequence (CC) at the proper position were selected. Then, reads were trimmed in order to only keep the sequence corresponding to the RNA. Reads were mapped on the reference genome (*Staphylococcus aureus* N315, accession number: NC_002745.2) using Bowtie 0.12.7 with the standard parameters. A first mapping was performed, keeping reads that mapped to unique positions on the chromosome (Table S1). Reads mapping at multiple positions on the chromosome were used on a second step (Table S2). Both mappings were analysed separately.

Analysing the 5′-ends of rRNAs used data from the group of reads that mapped to multiple locations (the five rRNA operons for example). The percentage of detected RNAs with 5′-ends at a given position was determined by dividing the number of reads mapping to that position by the total number of reads with a 5′-end within the region from the 16S rRNA processing stem cleavage by RNase III (93+M) to the 3′-end of the mature 16S rRNA. The proportion of 5′-ends compared to the WT strain (shown in Figure 4) was then obtained by dividing the percentage from the mutant strain with the percentage from the WT strain. The full data-set for 16S rRNA is shown in Table S3.

5′-ends of mRNAs and RNase P RNA used data from the group of reads that mapped to unique positions on the chromosome. The percentages for a given position was calculated as described above, however, the proportion between mutant and WT percentages was not calculated, since several positions had a WT percentage of zero, which would lead to division by zero. The full data-sets for the examples given in the text are shown in Tables S6, S7, and S8.

### Northern blots

Total RNA was prepared in the same way as for the EMOTE assay, but with an additional step of ethanol precipitation to concentrate the RNA. 4 µg total RNA from each strain was loaded on a 5% polyacryl amide gel with 8M urea, and afterwards transferred to a Hybond-N nitrocellulose membrane (Amersham) using a Biorad Protean Tetra-cell blotting system. The RNA was crosslinked to the membrane with a UV Stratalinker 2400 (Stratagene), and the marker was revealed using Methylene blue. Probes were hybridised over night at 37°C in ExpressHyb hybridization solution (Clontech, Mountain View, CA, USA), excess probe was washed away, and the signal was detected using a Typhoon FLA 7000 phosphorimager (General Electric). The membrane was stripped for 2 hours at 75°C with 0.2% SDS and 10 mM Tris pH 7.5, whereupon a new probe was hybridised. Loading control was done with a 5S rRNA probe, used last to ensure that the strong 5S signal did not interfere with the other experiments. Probes used for Northern blotting (Table S9) were 5′ labelled using ^32^P γ-ATP and T4 polynucleotide kinase (New England Biolabs).

## Supporting Information

Figure S1 Alignment of RNase J1 and J2 from *S. aureus* and *B. subtilis*. SaJ1 and SaJ2: RNase J1 and J2 from *S. aureus*, respectively. BsJ1 and BsJ2: RNase J1 and J2 from *B. subtilis*, respectively. Asterisks indicate the two histidines mutated to alanines in the J1^AGA^ and J2^AGA^ mutants.
(EPS)

Figure S2 Confirming the existence of a +1 to +915 transcript at the *rnpB* locus. A) Northern blot with a probe that anneals to RnpB, shows the major band with a size around 400 nt, representing RNA species that begin between +452 and +499, and a minor band of approximately 900 nt, which corresponds to the +1 to +915 transcript. 5S rRNA is shown below as loading control. B) The extremely faint bands generated by a PCR reaction across the ligated +1/+915 junction. The bands were cloned, and the 5′/3′ junctions are presented in Table 7.
(EPS)

Table S1 A full EMOTE data-set of WT and RNase J mutant reads, mapping to unique locations on the *S. aureus* N315 chromosome.
(PDF)

Table S2 A full EMOTE data-set of WT and RNase J mutant reads, mapping to multiple locations on the *S. aureus* N315 chromosome.
(PDF)

Table S3 The full data-set of 16S rRNA, which is analysed in Figure 4.
(XLS)

Table S4 The full data-set of *rnc.*
(XLS)

Table S5 The full data-set of *cspA.*
(XLS)

Table S6 The full data-set of the SA1279-*rnpB* operon, from which the highlights are shown in Figure 5.
(XLS)

Table S7 The full data-set of the SA2322 operon, from which the highlights are shown in Figure 6.
(XLS)

Table S8 The full data-set of the SA1075 operon, from which the highlights are shown in Figure 7.
(XLS)

Table S9 List of oligos used in addition to the oligos shown in Table 8.
(PDF)
