# Supplementary material for: Transcriptome-Wide Analyses of 5′-Ends in RNase J Mutants of a Gram-Positive Pathogen Reveal a Role in RNA Maturation, Regulation and Degradation
Source: PLoS Genet. 2014 Feb 27;10(2):e1004207. doi: 10.1371/journal.pgen.1004207 (PMC3937233; doi:10.1371/journal.pgen.1004207)
Supplement: Table S2 — A full EMOTE data-set of WT and RNase J mutant reads, mapping to multiple locations on the S. aureus N315 chromosome. (PDF) [file pgen.1004207.s004.pdf]

N315 chromosome assembly	position	strand	5'nt	WT	dJ1	dJ2	dJ1dJ2	J1AGA	ORF
gi|29165615|ref|NC_002745.2|	6801	+	C	0	0	1	0	0	SA0005
gi|29165615|ref|NC_002745.2|	6802	+	A	0	1	0	0	0	SA0005
gi|29165615|ref|NC_002745.2|	6803	+	A	0	1	0	0	0	SA0005
gi|29165615|ref|NC_002745.2|	7100	+	U	2	1	0	0	0	SA0006
gi|29165615|ref|NC_002745.2|	7408	+	U	1	0	0	0	0	SA0006
gi|29165615|ref|NC_002745.2|	7640	+	U	0	2	0	0	1	SA0006
gi|29165615|ref|NC_002745.2|	9144	+	G	0	0	1	0	0	SA0006
gi|29165615|ref|NC_002745.2|	9145	+	A	1	0	0	0	0	SA0006
gi|29165615|ref|NC_002745.2|	24160	+	G	0	2	0	0	0	SAtRNA01
gi|29165615|ref|NC_002745.2|	24175	+	C	0	0	1	0	0	SAtRNA01
gi|29165615|ref|NC_002745.2|	24237	+	U	1	0	0	0	0	-
gi|29165615|ref|NC_002745.2|	24255	+	A	0	0	0	0	1	SAtRNA02
gi|29165615|ref|NC_002745.2|	24263	+	A	0	0	0	1	0	SAtRNA02
gi|29165615|ref|NC_002745.2|	24277	+	C	1	1	0	0	0	SAtRNA02
gi|29165615|ref|NC_002745.2|	24278	+	A	0	0	0	1	0	SAtRNA02
gi|29165615|ref|NC_002745.2|	24279	+	C	0	0	0	0	1	SAtRNA02
gi|29165615|ref|NC_002745.2|	24280	+	G	0	0	0	0	1	SAtRNA02
gi|29165615|ref|NC_002745.2|	24893	+	A	0	1	0	1	3	-
gi|29165615|ref|NC_002745.2|	25207	+	U	0	0	1	0	1	SA0017
gi|29165615|ref|NC_002745.2|	34624	+	A	0	0	2	0	0	SA0024
gi|29165615|ref|NC_002745.2|	34625	+	A	5	4	2	3	1	SA0024
gi|29165615|ref|NC_002745.2|	47100	+	A	0	0	0	0	1	-
gi|29165615|ref|NC_002745.2|	53574	+	U	0	0	0	0	1	SA0045
gi|29165615|ref|NC_002745.2|	53575	+	A	0	0	0	0	1	SA0045
gi|29165615|ref|NC_002745.2|	53576	+	A	0	1	0	0	0	SA0045
gi|29165615|ref|NC_002745.2|	53579	+	A	0	0	1	0	0	SA0045
gi|29165615|ref|NC_002745.2|	77533	+	U	0	1	0	0	0	SA0068
gi|29165615|ref|NC_002745.2|	77534	+	C	0	0	1	0	0	SA0068
gi|29165615|ref|NC_002745.2|	77588	+	U	0	0	1	0	1	SA0068
gi|29165615|ref|NC_002745.2|	80117	+	U	0	0	1	0	0	SA0070
gi|29165615|ref|NC_002745.2|	80119	+	U	0	0	1	0	0	SA0070
gi|29165615|ref|NC_002745.2|	80120	+	A	0	0	0	0	1	SA0070
gi|29165615|ref|NC_002745.2|	80121	+	U	0	0	1	0	0	SA0070
gi|29165615|ref|NC_002745.2|	80261	+	U	2	0	0	0	0	SA0070
gi|29165615|ref|NC_002745.2|	80315	+	A	0	0	0	1	0	SA0070
gi|29165615|ref|NC_002745.2|	86607	+	A	0	1	0	0	0	SA0077
gi|29165615|ref|NC_002745.2|	89513	+	A	1	0	0	0	0	-
gi|29165615|ref|NC_002745.2|	89519	+	C	0	0	0	1	0	-
gi|29165615|ref|NC_002745.2|	89522	+	A	0	0	0	1	0	-
gi|29165615|ref|NC_002745.2|	89523	+	C	0	0	2	0	0	-
gi|29165615|ref|NC_002745.2|	89531	+	C	0	0	0	1	0	-
gi|29165615|ref|NC_002745.2|	89542	+	U	0	0	0	1	0	-
gi|29165615|ref|NC_002745.2|	89707	+	A	0	0	0	1	0	-
gi|29165615|ref|NC_002745.2|	89723	+	U	0	1	0	0	0	-
gi|29165615|ref|NC_002745.2|	89725	+	A	0	0	0	1	1	-
gi|29165615|ref|NC_002745.2|	89736	+	A	0	0	0	1	0	-
gi|29165615|ref|NC_002745.2|	89746	+	A	0	2	0	0	0	-
gi|29165615|ref|NC_002745.2|	89895	+	A	0	0	0	1	0	-
gi|29165615|ref|NC_002745.2|	89915	+	U	1	0	0	2	0	-
gi|29165615|ref|NC_002745.2|	89933	+	C	0	0	0	0	1	-
gi|29165615|ref|NC_002745.2|	89934	+	A	0	0	0	1	0	-
gi|29165615|ref|NC_002745.2|	89942	+	C	0	0	1	0	3	-
gi|29165615|ref|NC_002745.2|	89950	+	U	0	0	0	0	1	-
gi|29165615|ref|NC_002745.2|	89959	+	U	0	0	1	0	0	-
gi|29165615|ref|NC_002745.2|	89969	+	C	1	0	0	0	0	-
gi|29165615|ref|NC_002745.2|	89971	+	A	0	2	0	0	0	-
gi|29165615|ref|NC_002745.2|	89975	+	U	0	1	0	1	1	-
gi|29165615|ref|NC_002745.2|	89977	+	U	1	1	0	0	0	-
gi|29165615|ref|NC_002745.2|	89981	+	G	0	0	1	0	0	-
gi|29165615|ref|NC_002745.2|	90009	+	U	0	0	0	0	1	-
gi|29165615|ref|NC_002745.2|	90012	+	C	0	0	1	0	0	-
gi|29165615|ref|NC_002745.2|	90014	+	A	0	0	0	0	6	-
gi|29165615|ref|NC_002745.2|	90019	+	A	0	0	1	0	0	-
gi|29165615|ref|NC_002745.2|	90024	+	C	1	3	4	2	1	-
gi|29165615|ref|NC_002745.2|	90026	+	U	0	1	0	0	0	-
gi|29165615|ref|NC_002745.2|	90031	+	U	0	0	0	0	1	-
gi|29165615|ref|NC_002745.2|	90032	+	C	0	1	0	2	1	-
gi|29165615|ref|NC_002745.2|	90036	+	U	3	2	0	0	1	-
gi|29165615|ref|NC_002745.2|	90037	+	A	0	0	1	0	1	-
gi|29165615|ref|NC_002745.2|	90039	+	U	0	0	1	0	1	-
gi|29165615|ref|NC_002745.2|	90041	+	C	0	0	1	1	1	-
gi|29165615|ref|NC_002745.2|	90042	+	C	1	0	0	0	0	-
gi|29165615|ref|NC_002745.2|	90043	+	A	0	1	1	0	0	-
gi|29165615|ref|NC_002745.2|	90044	+	A	1	1	2	0	0	-
gi|29165615|ref|NC_002745.2|	90046	+	A	0	0	0	0	1	-
gi|29165615|ref|NC_002745.2|	90047	+	U	2	0	0	0	0	-
gi|29165615|ref|NC_002745.2|	90051	+	U	6	2	0	0	3	-
gi|29165615|ref|NC_002745.2|	90054	+	G	0	0	0	0	1	-
gi|29165615|ref|NC_002745.2|	90056	+	A	0	0	1	0	1	-
gi|29165615|ref|NC_002745.2|	90057	+	G	0	1	0	0	0	-

gi|29165615|ref|NC_002745.2|	90060	+	C	0	1	1	0	0	-
gi|29165615|ref|NC_002745.2|	90061	+	A	0	1	0	1	0	-
gi|29165615|ref|NC_002745.2|	90066	+	A	0	1	0	2	0	-
gi|29165615|ref|NC_002745.2|	91749	+	A	0	0	0	1	0	SA0082
gi|29165615|ref|NC_002745.2|	92079	+	A	0	0	1	0	0	SA0082
gi|29165615|ref|NC_002745.2|	92775	+	G	0	0	0	0	1	SA0083
gi|29165615|ref|NC_002745.2|	93825	+	A	0	0	0	0	1	SA0083
gi|29165615|ref|NC_002745.2|	93832	+	U	0	0	0	1	0	SA0083
gi|29165615|ref|NC_002745.2|	95375	+	A	0	0	1	1	0	-
gi|29165615|ref|NC_002745.2|	95819	+	A	1	0	0	0	0	-
gi|29165615|ref|NC_002745.2|	95825	+	C	0	0	0	1	0	-
gi|29165615|ref|NC_002745.2|	95828	+	A	0	0	0	1	0	-
gi|29165615|ref|NC_002745.2|	95829	+	C	0	0	2	0	0	-
gi|29165615|ref|NC_002745.2|	95837	+	C	0	0	0	1	0	-
gi|29165615|ref|NC_002745.2|	95848	+	U	0	0	0	1	0	-
gi|29165615|ref|NC_002745.2|	96014	+	A	0	0	0	1	0	-
gi|29165615|ref|NC_002745.2|	96030	+	U	0	1	0	0	0	-
gi|29165615|ref|NC_002745.2|	96032	+	A	0	0	0	1	1	-
gi|29165615|ref|NC_002745.2|	96043	+	A	0	0	0	1	0	-
gi|29165615|ref|NC_002745.2|	96053	+	A	0	2	0	0	0	-
gi|29165615|ref|NC_002745.2|	103839	+	A	0	0	1	0	0	-
gi|29165615|ref|NC_002745.2|	103853	+	A	0	1	0	0	0	SA0092
gi|29165615|ref|NC_002745.2|	103854	+	U	0	1	0	0	0	SA0092
gi|29165615|ref|NC_002745.2|	104009	+	U	0	1	0	0	0	SA0092
gi|29165615|ref|NC_002745.2|	104373	+	C	0	1	0	0	0	SA0092
gi|29165615|ref|NC_002745.2|	104831	+	U	0	1	0	0	0	SA0093
gi|29165615|ref|NC_002745.2|	105195	+	C	0	1	0	0	0	SA0093
gi|29165615|ref|NC_002745.2|	105498	+	U	1	0	0	0	0	-
gi|29165615|ref|NC_002745.2|	105499	+	A	0	1	0	0	0	-
gi|29165615|ref|NC_002745.2|	105511	+	U	0	1	0	0	0	-
gi|29165615|ref|NC_002745.2|	105530	+	A	0	2	0	0	0	SA0094
gi|29165615|ref|NC_002745.2|	105666	+	U	0	1	0	0	0	SA0094
gi|29165615|ref|NC_002745.2|	105676	+	A	0	0	2	0	0	SA0094
gi|29165615|ref|NC_002745.2|	106331	+	A	0	0	1	0	0	-
gi|29165615|ref|NC_002745.2|	106594	+	G	0	0	1	0	0	SA0095
gi|29165615|ref|NC_002745.2|	106844	+	A	0	0	0	1	0	SA0095
gi|29165615|ref|NC_002745.2|	106865	+	C	0	1	0	0	0	SA0095
gi|29165615|ref|NC_002745.2|	107167	+	U	1	0	0	0	0	-
gi|29165615|ref|NC_002745.2|	107168	+	A	0	1	0	0	0	-
gi|29165615|ref|NC_002745.2|	107179	+	A	0	1	0	0	0	-
gi|29165615|ref|NC_002745.2|	107180	+	U	0	1	0	0	0	-
gi|29165615|ref|NC_002745.2|	107199	+	A	0	2	0	0	0	SA0096
gi|29165615|ref|NC_002745.2|	107335	+	U	0	1	0	0	0	SA0096
gi|29165615|ref|NC_002745.2|	107345	+	A	0	0	1	0	0	SA0096
gi|29165615|ref|NC_002745.2|	107428	+	G	0	0	1	0	0	SA0096
gi|29165615|ref|NC_002745.2|	107699	+	C	0	1	0	0	0	SA0096
gi|29165615|ref|NC_002745.2|	112012	+	G	0	1	0	0	0	SA0099
gi|29165615|ref|NC_002745.2|	112018	+	C	0	0	0	0	1	SA0099
gi|29165615|ref|NC_002745.2|	112024	+	U	1	0	0	0	0	SA0099
gi|29165615|ref|NC_002745.2|	112025	+	U	0	1	0	0	0	SA0099
gi|29165615|ref|NC_002745.2|	112026	+	A	0	0	0	0	1	SA0099
gi|29165615|ref|NC_002745.2|	112032	+	C	0	0	1	0	0	SA0099
gi|29165615|ref|NC_002745.2|	112035	+	U	0	0	1	0	0	SA0099
gi|29165615|ref|NC_002745.2|	112380	+	A	0	1	0	0	0	SA0099
gi|29165615|ref|NC_002745.2|	115406	+	A	0	0	0	0	1	-
gi|29165615|ref|NC_002745.2|	120810	+	U	0	0	1	0	0	SA0106
gi|29165615|ref|NC_002745.2|	120811	+	A	0	4	0	1	0	SA0106
gi|29165615|ref|NC_002745.2|	120812	+	U	0	1	1	0	0	SA0106
gi|29165615|ref|NC_002745.2|	120813	+	G	0	0	1	0	0	SA0106
gi|29165615|ref|NC_002745.2|	120814	+	C	0	9	12	19	7	SA0106
gi|29165615|ref|NC_002745.2|	120815	+	U	0	2	1	2	2	SA0106
gi|29165615|ref|NC_002745.2|	120816	+	U	0	0	1	0	1	SA0106
gi|29165615|ref|NC_002745.2|	120817	+	A	0	1	3	0	1	SA0106
gi|29165615|ref|NC_002745.2|	120818	+	A	0	3	1	0	1	SA0106
gi|29165615|ref|NC_002745.2|	120819	+	C	0	0	1	3	0	SA0106
gi|29165615|ref|NC_002745.2|	120822	+	U	0	10	10	11	11	SA0106
gi|29165615|ref|NC_002745.2|	120823	+	U	1	0	5	2	4	SA0106
gi|29165615|ref|NC_002745.2|	120824	+	U	0	3	1	2	2	SA0106
gi|29165615|ref|NC_002745.2|	120826	+	U	1	0	2	4	1	SA0106
gi|29165615|ref|NC_002745.2|	120827	+	A	1	18	23	10	19	SA0106
gi|29165615|ref|NC_002745.2|	121028	+	A	0	0	0	0	2	SA0106
gi|29165615|ref|NC_002745.2|	121031	+	C	0	4	3	2	1	SA0106
gi|29165615|ref|NC_002745.2|	121093	+	U	0	1	0	0	0	SA0106
gi|29165615|ref|NC_002745.2|	121099	+	C	0	0	2	0	0	SA0106
gi|29165615|ref|NC_002745.2|	121101	+	C	0	3	2	0	0	SA0106
gi|29165615|ref|NC_002745.2|	121105	+	U	0	0	0	1	0	SA0106
gi|29165615|ref|NC_002745.2|	121106	+	U	0	1	0	0	0	SA0106
gi|29165615|ref|NC_002745.2|	121116	+	C	0	2	0	0	0	SA0106
gi|29165615|ref|NC_002745.2|	121123	+	A	0	0	1	0	0	SA0106
gi|29165615|ref|NC_002745.2|	121176	+	A	0	1	0	0	0	SA0106
gi|29165615|ref|NC_002745.2|	122046	+	U	1	0	0	0	0	SA0106

gi|29165615|ref|NC_002745.2|	122170	+	A	0	0	0	0	1	SA0106
gi|29165615|ref|NC_002745.2|	122174	+	A	1	0	0	0	0	SA0106
gi|29165615|ref|NC_002745.2|	122940	+	G	0	0	1	0	0	-
gi|29165615|ref|NC_002745.2|	122943	+	G	0	0	0	0	1	-
gi|29165615|ref|NC_002745.2|	122947	+	U	0	4	0	0	0	-
gi|29165615|ref|NC_002745.2|	122971	+	U	0	1	0	0	0	-
gi|29165615|ref|NC_002745.2|	123000	+	U	0	0	0	1	0	-
gi|29165615|ref|NC_002745.2|	123019	+	U	0	1	0	0	0	-
gi|29165615|ref|NC_002745.2|	123048	+	U	0	0	0	1	0	-
gi|29165615|ref|NC_002745.2|	123067	+	U	0	1	0	0	0	-
gi|29165615|ref|NC_002745.2|	123084	+	G	0	0	1	0	0	-
gi|29165615|ref|NC_002745.2|	123086	+	U	0	0	0	0	1	-
gi|29165615|ref|NC_002745.2|	123087	+	G	0	0	0	0	1	-
gi|29165615|ref|NC_002745.2|	123096	+	U	0	0	0	1	0	-
gi|29165615|ref|NC_002745.2|	123115	+	U	0	1	0	0	0	-
gi|29165615|ref|NC_002745.2|	123139	+	U	0	3	0	0	0	-
gi|29165615|ref|NC_002745.2|	123144	+	U	0	0	0	1	0	-
gi|29165615|ref|NC_002745.2|	123156	+	G	0	0	1	0	0	-
gi|29165615|ref|NC_002745.2|	123158	+	U	0	0	0	0	1	-
gi|29165615|ref|NC_002745.2|	123159	+	G	0	0	0	0	1	-
gi|29165615|ref|NC_002745.2|	123168	+	U	0	0	0	1	0	-
gi|29165615|ref|NC_002745.2|	128934	+	A	0	2	0	0	1	SA0112
gi|29165615|ref|NC_002745.2|	143076	+	U	0	1	0	0	0	SA0123
gi|29165615|ref|NC_002745.2|	148063	+	A	0	3	0	1	3	SA0128
gi|29165615|ref|NC_002745.2|	149827	+	G	0	0	0	0	1	-
gi|29165615|ref|NC_002745.2|	149833	+	U	0	0	1	0	0	-
gi|29165615|ref|NC_002745.2|	151087	+	A	0	0	0	0	1	SA0131
gi|29165615|ref|NC_002745.2|	151091	+	A	0	1	0	0	0	SA0131
gi|29165615|ref|NC_002745.2|	151092	+	A	0	1	0	0	0	SA0131
gi|29165615|ref|NC_002745.2|	151101	+	A	0	1	0	1	0	SA0131
gi|29165615|ref|NC_002745.2|	151105	+	U	0	0	1	0	0	SA0131
gi|29165615|ref|NC_002745.2|	151106	+	U	0	0	0	1	0	SA0131
gi|29165615|ref|NC_002745.2|	151233	+	U	0	0	1	0	0	SA0131
gi|29165615|ref|NC_002745.2|	151451	+	A	0	0	0	0	1	SA0131
gi|29165615|ref|NC_002745.2|	153091	+	A	0	0	0	0	2	SA0133
gi|29165615|ref|NC_002745.2|	153092	+	U	0	1	0	0	1	SA0133
gi|29165615|ref|NC_002745.2|	153095	+	A	1	2	0	0	0	SA0133
gi|29165615|ref|NC_002745.2|	153099	+	C	0	0	1	0	1	SA0133
gi|29165615|ref|NC_002745.2|	153115	+	G	0	0	1	0	0	SA0133
gi|29165615|ref|NC_002745.2|	153126	+	U	0	0	0	1	0	SA0133
gi|29165615|ref|NC_002745.2|	153172	+	A	0	0	0	0	1	SA0133
gi|29165615|ref|NC_002745.2|	153174	+	A	0	1	0	0	0	SA0133
gi|29165615|ref|NC_002745.2|	153202	+	A	0	1	0	0	0	SA0133
gi|29165615|ref|NC_002745.2|	153204	+	A	0	0	0	0	1	SA0133
gi|29165615|ref|NC_002745.2|	153206	+	A	0	2	0	0	0	SA0133
gi|29165615|ref|NC_002745.2|	153217	+	C	0	0	0	0	1	SA0133
gi|29165615|ref|NC_002745.2|	153219	+	A	0	0	0	1	0	SA0133
gi|29165615|ref|NC_002745.2|	153220	+	C	0	0	0	1	0	SA0133
gi|29165615|ref|NC_002745.2|	153221	+	U	1	0	0	0	0	SA0133
gi|29165615|ref|NC_002745.2|	153251	+	U	1	0	0	0	0	SA0133
gi|29165615|ref|NC_002745.2|	153263	+	C	0	0	0	1	0	SA0133
gi|29165615|ref|NC_002745.2|	153302	+	C	0	1	0	0	2	SA0133
gi|29165615|ref|NC_002745.2|	153320	+	A	0	2	0	0	0	SA0133
gi|29165615|ref|NC_002745.2|	153329	+	A	0	0	0	0	1	SA0133
gi|29165615|ref|NC_002745.2|	153332	+	A	0	1	0	0	0	SA0133
gi|29165615|ref|NC_002745.2|	153357	+	C	0	0	0	1	0	SA0133
gi|29165615|ref|NC_002745.2|	153359	+	C	0	0	1	0	0	SA0133
gi|29165615|ref|NC_002745.2|	153376	+	U	0	0	1	0	0	SA0133
gi|29165615|ref|NC_002745.2|	153389	+	A	0	1	0	0	0	SA0133
gi|29165615|ref|NC_002745.2|	153395	+	A	0	1	0	0	0	SA0133
gi|29165615|ref|NC_002745.2|	153407	+	U	2	0	0	0	0	SA0133
gi|29165615|ref|NC_002745.2|	153412	+	A	0	1	0	0	0	SA0133
gi|29165615|ref|NC_002745.2|	153423	+	A	0	1	0	0	0	SA0133
gi|29165615|ref|NC_002745.2|	153424	+	G	0	2	0	0	1	SA0133
gi|29165615|ref|NC_002745.2|	153444	+	U	0	0	0	0	1	SA0133
gi|29165615|ref|NC_002745.2|	153458	+	U	1	0	0	0	0	SA0133
gi|29165615|ref|NC_002745.2|	153475	+	A	0	0	1	0	0	SA0133
gi|29165615|ref|NC_002745.2|	153476	+	U	0	1	0	0	0	SA0133
gi|29165615|ref|NC_002745.2|	153478	+	G	0	0	0	0	1	SA0133
gi|29165615|ref|NC_002745.2|	153487	+	A	0	0	0	0	1	SA0133
gi|29165615|ref|NC_002745.2|	153489	+	U	0	0	0	1	0	SA0133
gi|29165615|ref|NC_002745.2|	153491	+	A	0	0	1	0	0	SA0133
gi|29165615|ref|NC_002745.2|	153500	+	A	0	0	1	1	0	SA0133
gi|29165615|ref|NC_002745.2|	153502	+	G	0	0	0	0	1	SA0133
gi|29165615|ref|NC_002745.2|	153530	+	C	0	0	1	0	0	SA0133
gi|29165615|ref|NC_002745.2|	153572	+	A	0	0	1	1	0	SA0133
gi|29165615|ref|NC_002745.2|	153573	+	A	0	1	0	0	0	SA0133
gi|29165615|ref|NC_002745.2|	153574	+	U	0	0	1	0	0	SA0133
gi|29165615|ref|NC_002745.2|	153576	+	A	0	0	0	0	2	SA0133
gi|29165615|ref|NC_002745.2|	153578	+	U	0	0	0	0	1	SA0133
gi|29165615|ref|NC_002745.2|	153580	+	A	0	2	0	0	2	SA0133

gi|29165615|ref|NC_002745.2|	153583	+	G	0	1	1	0	0	SA0133
gi|29165615|ref|NC_002745.2|	153586	+	A	0	0	0	1	1	SA0133
gi|29165615|ref|NC_002745.2|	153587	+	C	0	1	0	0	1	SA0133
gi|29165615|ref|NC_002745.2|	153609	+	A	1	0	0	0	0	SA0133
gi|29165615|ref|NC_002745.2|	153616	+	U	0	1	0	0	0	SA0133
gi|29165615|ref|NC_002745.2|	153618	+	A	0	0	2	0	1	SA0133
gi|29165615|ref|NC_002745.2|	153630	+	U	0	0	0	1	0	SA0133
gi|29165615|ref|NC_002745.2|	153631	+	A	0	1	0	0	0	SA0133
gi|29165615|ref|NC_002745.2|	153637	+	G	2	0	0	0	0	SA0133
gi|29165615|ref|NC_002745.2|	153638	+	A	0	1	0	0	0	SA0133
gi|29165615|ref|NC_002745.2|	153643	+	U	0	0	0	0	1	SA0133
gi|29165615|ref|NC_002745.2|	153646	+	A	0	0	0	0	1	SA0133
gi|29165615|ref|NC_002745.2|	153648	+	U	0	1	0	1	0	SA0133
gi|29165615|ref|NC_002745.2|	153650	+	A	0	1	0	0	0	SA0133
gi|29165615|ref|NC_002745.2|	153652	+	A	0	4	0	1	2	SA0133
gi|29165615|ref|NC_002745.2|	153656	+	U	4	0	0	0	0	SA0133
gi|29165615|ref|NC_002745.2|	153660	+	A	2	0	0	0	0	SA0133
gi|29165615|ref|NC_002745.2|	153668	+	C	0	0	1	0	0	SA0133
gi|29165615|ref|NC_002745.2|	153672	+	A	0	0	0	0	1	SA0133
gi|29165615|ref|NC_002745.2|	153673	+	C	0	0	0	0	2	SA0133
gi|29165615|ref|NC_002745.2|	153675	+	U	1	0	0	0	0	SA0133
gi|29165615|ref|NC_002745.2|	153677	+	U	0	0	1	0	1	SA0133
gi|29165615|ref|NC_002745.2|	153693	+	C	0	0	0	0	1	SA0133
gi|29165615|ref|NC_002745.2|	153706	+	C	0	0	0	1	0	SA0133
gi|29165615|ref|NC_002745.2|	153716	+	A	0	0	0	0	1	SA0133
gi|29165615|ref|NC_002745.2|	183508	+	A	0	1	0	1	1	SA0159
gi|29165615|ref|NC_002745.2|	186344	+	U	0	0	1	0	0	SA0162
gi|29165615|ref|NC_002745.2|	186831	+	A	0	0	1	1	0	-
gi|29165615|ref|NC_002745.2|	188302	+	C	0	0	0	1	0	-
gi|29165615|ref|NC_002745.2|	188315	+	U	0	1	0	0	0	-
gi|29165615|ref|NC_002745.2|	188330	+	G	0	0	0	0	1	-
gi|29165615|ref|NC_002745.2|	188336	+	U	0	0	1	0	0	-
gi|29165615|ref|NC_002745.2|	188353	+	A	0	0	0	0	1	-
gi|29165615|ref|NC_002745.2|	188360	+	A	0	0	0	0	1	-
gi|29165615|ref|NC_002745.2|	188363	+	C	0	0	0	1	0	-
gi|29165615|ref|NC_002745.2|	188403	+	A	0	0	0	0	1	-
gi|29165615|ref|NC_002745.2|	188405	+	C	0	0	1	0	0	-
gi|29165615|ref|NC_002745.2|	188406	+	A	0	0	0	1	0	-
gi|29165615|ref|NC_002745.2|	188411	+	G	0	0	0	1	0	-
gi|29165615|ref|NC_002745.2|	188419	+	A	0	0	0	5	0	-
gi|29165615|ref|NC_002745.2|	188420	+	A	0	0	0	1	0	-
gi|29165615|ref|NC_002745.2|	188421	+	A	0	0	0	0	1	-
gi|29165615|ref|NC_002745.2|	188424	+	A	0	1	0	3	2	-
gi|29165615|ref|NC_002745.2|	188425	+	A	0	0	0	1	0	-
gi|29165615|ref|NC_002745.2|	188431	+	A	1	0	0	0	0	-
gi|29165615|ref|NC_002745.2|	188465	+	A	0	0	0	3	0	-
gi|29165615|ref|NC_002745.2|	188471	+	A	0	0	1	0	0	-
gi|29165615|ref|NC_002745.2|	188483	+	A	0	0	0	2	1	-
gi|29165615|ref|NC_002745.2|	188484	+	A	0	0	0	1	0	-
gi|29165615|ref|NC_002745.2|	188487	+	U	0	1	0	0	0	-
gi|29165615|ref|NC_002745.2|	188490	+	A	1	0	0	0	0	-
gi|29165615|ref|NC_002745.2|	188492	+	A	0	1	0	0	1	-
gi|29165615|ref|NC_002745.2|	188519	+	G	0	2	0	0	0	-
gi|29165615|ref|NC_002745.2|	188534	+	A	0	0	0	0	1	-
gi|29165615|ref|NC_002745.2|	188535	+	A	0	2	0	1	0	-
gi|29165615|ref|NC_002745.2|	227619	+	A	0	1	0	0	1	SA0192
gi|29165615|ref|NC_002745.2|	227624	+	C	1	0	0	0	1	SA0192
gi|29165615|ref|NC_002745.2|	227628	+	C	0	0	0	1	0	SA0192
gi|29165615|ref|NC_002745.2|	248818	+	A	0	1	0	0	0	SA0209
gi|29165615|ref|NC_002745.2|	248964	+	G	0	0	1	0	0	-
gi|29165615|ref|NC_002745.2|	260554	+	C	0	0	0	0	1	SA0218
gi|29165615|ref|NC_002745.2|	279322	+	C	0	2	0	0	0	-
gi|29165615|ref|NC_002745.2|	279337	+	A	0	0	0	0	1	-
gi|29165615|ref|NC_002745.2|	279338	+	A	0	2	0	1	0	-
gi|29165615|ref|NC_002745.2|	293573	+	C	0	1	0	0	0	SA0241
gi|29165615|ref|NC_002745.2|	293606	+	U	1	0	0	0	0	SA0241
gi|29165615|ref|NC_002745.2|	293609	+	U	0	0	1	0	0	SA0241
gi|29165615|ref|NC_002745.2|	293613	+	A	2	0	0	0	0	SA0241
gi|29165615|ref|NC_002745.2|	293620	+	A	0	1	0	0	0	SA0241
gi|29165615|ref|NC_002745.2|	293797	+	A	0	4	0	0	0	SA0242
gi|29165615|ref|NC_002745.2|	294124	+	C	0	1	0	0	0	SA0242
gi|29165615|ref|NC_002745.2|	294142	+	U	0	0	0	0	1	SA0242
gi|29165615|ref|NC_002745.2|	294439	+	A	0	1	0	0	0	SA0242
gi|29165615|ref|NC_002745.2|	294613	+	G	0	1	0	0	0	SA0242
gi|29165615|ref|NC_002745.2|	295458	+	G	1	0	0	0	0	SA0243
gi|29165615|ref|NC_002745.2|	295472	+	U	1	0	0	0	0	SA0243
gi|29165615|ref|NC_002745.2|	295563	+	U	0	0	0	0	1	SA0243
gi|29165615|ref|NC_002745.2|	295567	+	U	0	0	1	0	0	SA0243
gi|29165615|ref|NC_002745.2|	295568	+	C	0	0	2	1	1	SA0243
gi|29165615|ref|NC_002745.2|	295575	+	G	1	0	0	0	0	SA0243
gi|29165615|ref|NC_002745.2|	295594	+	U	0	0	0	0	1	SA0243

gi|29165615|ref|NC_002745.2|	295602	+	U	0	0	1	0	0	SA0243
gi|29165615|ref|NC_002745.2|	295676	+	U	0	2	1	0	0	SA0243
gi|29165615|ref|NC_002745.2|	295677	+	A	1	0	0	0	0	SA0243
gi|29165615|ref|NC_002745.2|	295678	+	U	0	0	0	3	0	SA0243
gi|29165615|ref|NC_002745.2|	295680	+	U	1	0	0	0	0	SA0243
gi|29165615|ref|NC_002745.2|	295689	+	A	0	4	1	0	2	SA0243
gi|29165615|ref|NC_002745.2|	295693	+	C	0	0	0	0	1	SA0243
gi|29165615|ref|NC_002745.2|	295695	+	A	4	15	8	13	8	SA0243
gi|29165615|ref|NC_002745.2|	295696	+	U	0	0	0	1	0	SA0243
gi|29165615|ref|NC_002745.2|	295699	+	U	0	0	0	1	0	SA0243
gi|29165615|ref|NC_002745.2|	295756	+	A	0	0	1	0	0	SA0243
gi|29165615|ref|NC_002745.2|	295761	+	G	1	0	0	0	0	SA0243
gi|29165615|ref|NC_002745.2|	295806	+	A	0	1	0	0	0	SA0243
gi|29165615|ref|NC_002745.2|	295836	+	A	0	1	0	0	0	SA0243
gi|29165615|ref|NC_002745.2|	295854	+	U	1	0	0	0	0	SA0243
gi|29165615|ref|NC_002745.2|	295913	+	A	0	1	0	0	0	SA0243
gi|29165615|ref|NC_002745.2|	296084	+	U	0	0	0	1	0	SA0243
gi|29165615|ref|NC_002745.2|	296087	+	A	0	0	0	0	1	SA0243
gi|29165615|ref|NC_002745.2|	296091	+	G	0	0	1	0	0	SA0243
gi|29165615|ref|NC_002745.2|	296093	+	G	0	1	0	0	1	SA0243
gi|29165615|ref|NC_002745.2|	296094	+	A	0	0	0	0	1	SA0243
gi|29165615|ref|NC_002745.2|	296107	+	A	0	3	0	0	0	SA0243
gi|29165615|ref|NC_002745.2|	296113	+	U	0	1	0	0	0	SA0243
gi|29165615|ref|NC_002745.2|	296114	+	G	0	0	0	0	1	SA0243
gi|29165615|ref|NC_002745.2|	296115	+	A	0	1	0	0	1	SA0243
gi|29165615|ref|NC_002745.2|	296116	+	A	0	0	0	0	1	SA0243
gi|29165615|ref|NC_002745.2|	296118	+	A	0	0	0	0	1	SA0243
gi|29165615|ref|NC_002745.2|	296124	+	U	0	0	0	1	0	SA0243
gi|29165615|ref|NC_002745.2|	296125	+	A	0	1	0	0	0	SA0243
gi|29165615|ref|NC_002745.2|	296126	+	U	0	0	0	0	1	SA0243
gi|29165615|ref|NC_002745.2|	296131	+	A	0	1	1	0	1	SA0243
gi|29165615|ref|NC_002745.2|	296132	+	A	0	0	0	1	0	SA0243
gi|29165615|ref|NC_002745.2|	296135	+	C	0	1	1	0	0	SA0243
gi|29165615|ref|NC_002745.2|	296138	+	A	0	2	0	0	0	SA0243
gi|29165615|ref|NC_002745.2|	296146	+	C	0	1	0	0	0	SA0243
gi|29165615|ref|NC_002745.2|	296151	+	C	0	0	0	1	0	SA0243
gi|29165615|ref|NC_002745.2|	296163	+	A	0	1	1	0	0	SA0243
gi|29165615|ref|NC_002745.2|	296165	+	G	0	0	0	0	1	SA0243
gi|29165615|ref|NC_002745.2|	296169	+	A	0	0	1	0	0	SA0243
gi|29165615|ref|NC_002745.2|	296170	+	C	0	3	0	0	0	SA0243
gi|29165615|ref|NC_002745.2|	296171	+	A	0	0	0	1	0	SA0243
gi|29165615|ref|NC_002745.2|	296173	+	U	0	1	0	0	0	SA0243
gi|29165615|ref|NC_002745.2|	296174	+	A	0	0	0	0	1	SA0243
gi|29165615|ref|NC_002745.2|	296177	+	A	0	0	1	0	0	SA0243
gi|29165615|ref|NC_002745.2|	296184	+	A	0	2	0	0	0	SA0243
gi|29165615|ref|NC_002745.2|	296198	+	U	0	0	0	0	1	SA0243
gi|29165615|ref|NC_002745.2|	296200	+	U	0	1	0	0	0	SA0243
gi|29165615|ref|NC_002745.2|	296211	+	U	1	0	0	0	0	SA0243
gi|29165615|ref|NC_002745.2|	296214	+	C	0	0	2	2	0	SA0243
gi|29165615|ref|NC_002745.2|	296292	+	C	0	0	2	0	0	SA0243
gi|29165615|ref|NC_002745.2|	296293	+	A	0	0	0	1	0	SA0243
gi|29165615|ref|NC_002745.2|	296295	+	U	0	0	0	1	0	SA0243
gi|29165615|ref|NC_002745.2|	296299	+	A	0	0	0	0	1	SA0243
gi|29165615|ref|NC_002745.2|	296306	+	C	0	0	0	0	1	SA0243
gi|29165615|ref|NC_002745.2|	296308	+	U	0	0	1	0	0	SA0243
gi|29165615|ref|NC_002745.2|	296310	+	U	0	0	0	0	1	SA0243
gi|29165615|ref|NC_002745.2|	296312	+	A	0	0	2	0	2	SA0243
gi|29165615|ref|NC_002745.2|	296313	+	A	0	1	0	0	0	SA0243
gi|29165615|ref|NC_002745.2|	296314	+	A	0	3	1	0	6	SA0243
gi|29165615|ref|NC_002745.2|	298896	+	C	0	1	0	0	0	SA0245
gi|29165615|ref|NC_002745.2|	298929	+	U	1	0	0	0	0	SA0245
gi|29165615|ref|NC_002745.2|	298932	+	U	0	0	1	0	0	SA0245
gi|29165615|ref|NC_002745.2|	298936	+	A	2	0	0	0	0	SA0245
gi|29165615|ref|NC_002745.2|	298943	+	A	0	1	0	0	0	SA0245
gi|29165615|ref|NC_002745.2|	299120	+	A	0	4	0	0	0	SA0246
gi|29165615|ref|NC_002745.2|	299447	+	A	0	1	0	0	0	SA0246
gi|29165615|ref|NC_002745.2|	299465	+	U	0	0	0	0	1	SA0246
gi|29165615|ref|NC_002745.2|	299762	+	A	0	1	0	0	0	SA0246
gi|29165615|ref|NC_002745.2|	299936	+	U	0	1	0	0	0	SA0246
gi|29165615|ref|NC_002745.2|	300275	+	A	0	0	0	1	0	SA0247
gi|29165615|ref|NC_002745.2|	300775	+	U	1	0	0	0	0	SA0247
gi|29165615|ref|NC_002745.2|	300789	+	U	1	0	0	0	0	SA0247
gi|29165615|ref|NC_002745.2|	300880	+	U	0	0	0	0	1	SA0247
gi|29165615|ref|NC_002745.2|	300884	+	U	0	0	1	0	0	SA0247
gi|29165615|ref|NC_002745.2|	300885	+	C	0	0	2	1	1	SA0247
gi|29165615|ref|NC_002745.2|	300892	+	G	1	0	0	0	0	SA0247
gi|29165615|ref|NC_002745.2|	300911	+	U	0	0	0	0	1	SA0247
gi|29165615|ref|NC_002745.2|	300919	+	U	0	0	1	0	0	SA0247
gi|29165615|ref|NC_002745.2|	300993	+	U	0	2	1	0	0	SA0247
gi|29165615|ref|NC_002745.2|	300994	+	A	1	0	0	0	0	SA0247
gi|29165615|ref|NC_002745.2|	300995	+	U	0	0	0	3	0	SA0247

gi|29165615|ref|NC_002745.2|	300997	+	U	1	0	0	0	0	SA0247
gi|29165615|ref|NC_002745.2|	301006	+	A	0	4	1	0	2	SA0247
gi|29165615|ref|NC_002745.2|	301010	+	C	0	0	0	0	1	SA0247
gi|29165615|ref|NC_002745.2|	301012	+	A	4	15	8	13	8	SA0247
gi|29165615|ref|NC_002745.2|	301013	+	U	0	0	0	1	0	SA0247
gi|29165615|ref|NC_002745.2|	301016	+	U	0	0	0	1	0	SA0247
gi|29165615|ref|NC_002745.2|	301073	+	U	0	0	1	0	0	SA0247
gi|29165615|ref|NC_002745.2|	301078	+	G	1	0	0	0	0	SA0247
gi|29165615|ref|NC_002745.2|	301123	+	A	0	1	0	0	0	SA0247
gi|29165615|ref|NC_002745.2|	301153	+	A	0	1	0	0	0	SA0247
gi|29165615|ref|NC_002745.2|	301171	+	U	1	0	0	0	0	SA0247
gi|29165615|ref|NC_002745.2|	301230	+	A	0	1	0	0	0	SA0247
gi|29165615|ref|NC_002745.2|	301401	+	U	0	0	0	1	0	SA0247
gi|29165615|ref|NC_002745.2|	301404	+	A	0	0	0	0	1	SA0247
gi|29165615|ref|NC_002745.2|	301408	+	G	0	0	1	0	0	SA0247
gi|29165615|ref|NC_002745.2|	301410	+	G	0	1	0	0	1	SA0247
gi|29165615|ref|NC_002745.2|	301411	+	A	0	0	0	0	1	SA0247
gi|29165615|ref|NC_002745.2|	301424	+	A	0	3	0	0	0	SA0247
gi|29165615|ref|NC_002745.2|	301430	+	U	0	1	0	0	0	SA0247
gi|29165615|ref|NC_002745.2|	301431	+	G	0	0	0	0	1	SA0247
gi|29165615|ref|NC_002745.2|	301432	+	A	0	1	0	0	1	SA0247
gi|29165615|ref|NC_002745.2|	301433	+	A	0	0	0	0	1	SA0247
gi|29165615|ref|NC_002745.2|	301435	+	A	0	0	0	0	1	SA0247
gi|29165615|ref|NC_002745.2|	301441	+	U	0	0	0	1	0	SA0247
gi|29165615|ref|NC_002745.2|	301442	+	A	0	1	0	0	0	SA0247
gi|29165615|ref|NC_002745.2|	301443	+	U	0	0	0	0	1	SA0247
gi|29165615|ref|NC_002745.2|	301448	+	A	0	1	1	0	1	SA0247
gi|29165615|ref|NC_002745.2|	301449	+	A	0	0	0	1	0	SA0247
gi|29165615|ref|NC_002745.2|	301452	+	C	0	1	1	0	0	SA0247
gi|29165615|ref|NC_002745.2|	301455	+	A	0	2	0	0	0	SA0247
gi|29165615|ref|NC_002745.2|	301463	+	C	0	1	0	0	0	SA0247
gi|29165615|ref|NC_002745.2|	301468	+	C	0	0	0	1	0	SA0247
gi|29165615|ref|NC_002745.2|	301480	+	A	0	1	1	0	0	SA0247
gi|29165615|ref|NC_002745.2|	301482	+	G	0	0	0	0	1	SA0247
gi|29165615|ref|NC_002745.2|	301486	+	A	0	0	1	0	0	SA0247
gi|29165615|ref|NC_002745.2|	301487	+	U	0	3	0	0	0	SA0247
gi|29165615|ref|NC_002745.2|	301488	+	A	0	0	0	1	0	SA0247
gi|29165615|ref|NC_002745.2|	301490	+	U	0	1	0	0	0	SA0247
gi|29165615|ref|NC_002745.2|	301491	+	A	0	0	0	0	1	SA0247
gi|29165615|ref|NC_002745.2|	301494	+	A	0	0	1	0	0	SA0247
gi|29165615|ref|NC_002745.2|	301501	+	A	0	2	0	0	0	SA0247
gi|29165615|ref|NC_002745.2|	301515	+	U	0	0	0	0	1	SA0247
gi|29165615|ref|NC_002745.2|	301517	+	U	0	1	0	0	0	SA0247
gi|29165615|ref|NC_002745.2|	301528	+	U	1	0	0	0	0	SA0247
gi|29165615|ref|NC_002745.2|	301531	+	C	0	0	2	2	0	SA0247
gi|29165615|ref|NC_002745.2|	301609	+	C	0	0	2	0	0	SA0247
gi|29165615|ref|NC_002745.2|	301610	+	A	0	0	0	1	0	SA0247
gi|29165615|ref|NC_002745.2|	301612	+	U	0	0	0	1	0	SA0247
gi|29165615|ref|NC_002745.2|	301616	+	A	0	0	0	0	1	SA0247
gi|29165615|ref|NC_002745.2|	301623	+	C	0	0	0	0	1	SA0247
gi|29165615|ref|NC_002745.2|	301625	+	U	0	0	1	0	0	SA0247
gi|29165615|ref|NC_002745.2|	301627	+	U	0	0	0	0	1	SA0247
gi|29165615|ref|NC_002745.2|	301629	+	A	0	0	2	0	2	SA0247
gi|29165615|ref|NC_002745.2|	301630	+	A	0	1	0	0	0	SA0247
gi|29165615|ref|NC_002745.2|	301631	+	A	0	3	1	0	6	SA0247
gi|29165615|ref|NC_002745.2|	302996	+	G	1	0	0	0	0	SA0248
gi|29165615|ref|NC_002745.2|	302997	+	A	0	0	0	2	7	SA0248
gi|29165615|ref|NC_002745.2|	302998	+	A	0	1	0	1	1	SA0248
gi|29165615|ref|NC_002745.2|	310231	+	A	0	1	0	0	2	SA0256
gi|29165615|ref|NC_002745.2|	311538	+	A	0	0	0	0	1	-
gi|29165615|ref|NC_002745.2|	311540	+	C	0	0	1	0	0	-
gi|29165615|ref|NC_002745.2|	311541	+	A	0	0	0	1	0	-
gi|29165615|ref|NC_002745.2|	311546	+	G	0	0	0	1	0	-
gi|29165615|ref|NC_002745.2|	311554	+	A	0	0	0	5	0	-
gi|29165615|ref|NC_002745.2|	311555	+	A	0	0	0	1	0	-
gi|29165615|ref|NC_002745.2|	311556	+	A	0	0	0	0	1	-
gi|29165615|ref|NC_002745.2|	311559	+	A	0	1	0	1	1	-
gi|29165615|ref|NC_002745.2|	311560	+	A	0	0	0	1	0	-
gi|29165615|ref|NC_002745.2|	311563	+	U	0	0	0	1	0	-
gi|29165615|ref|NC_002745.2|	311564	+	C	0	1	0	0	0	-
gi|29165615|ref|NC_002745.2|	311565	+	U	0	0	0	1	0	-
gi|29165615|ref|NC_002745.2|	311566	+	A	0	0	1	0	0	-
gi|29165615|ref|NC_002745.2|	311572	+	A	0	0	0	0	1	-
gi|29165615|ref|NC_002745.2|	311577	+	A	0	1	0	0	0	-
gi|29165615|ref|NC_002745.2|	311593	+	A	0	0	0	0	1	-
gi|29165615|ref|NC_002745.2|	311595	+	C	0	0	1	0	0	-
gi|29165615|ref|NC_002745.2|	311596	+	A	0	0	0	1	0	-
gi|29165615|ref|NC_002745.2|	311601	+	G	0	0	0	1	0	-
gi|29165615|ref|NC_002745.2|	311609	+	A	0	0	0	5	0	-
gi|29165615|ref|NC_002745.2|	311610	+	A	0	0	0	1	0	-
gi|29165615|ref|NC_002745.2|	311611	+	A	0	0	0	0	1	-

gi|29165615|ref|NC_002745.2|	311614	+	A	0	1	0	3	2	-
gi|29165615|ref|NC_002745.2|	311615	+	A	0	0	0	1	0	-
gi|29165615|ref|NC_002745.2|	311618	+	U	0	0	0	1	0	-
gi|29165615|ref|NC_002745.2|	311619	+	C	0	1	0	0	0	-
gi|29165615|ref|NC_002745.2|	311620	+	U	0	0	0	1	0	-
gi|29165615|ref|NC_002745.2|	311621	+	A	0	0	1	0	0	-
gi|29165615|ref|NC_002745.2|	311627	+	A	0	0	0	0	1	-
gi|29165615|ref|NC_002745.2|	311632	+	A	0	1	0	0	0	-
gi|29165615|ref|NC_002745.2|	311648	+	A	0	0	0	0	1	-
gi|29165615|ref|NC_002745.2|	311650	+	C	0	0	1	0	0	-
gi|29165615|ref|NC_002745.2|	311651	+	A	0	0	0	1	0	-
gi|29165615|ref|NC_002745.2|	311656	+	G	0	0	0	1	0	-
gi|29165615|ref|NC_002745.2|	311664	+	A	0	0	0	5	0	-
gi|29165615|ref|NC_002745.2|	311665	+	A	0	0	0	1	0	-
gi|29165615|ref|NC_002745.2|	311666	+	A	0	0	0	0	1	-
gi|29165615|ref|NC_002745.2|	311669	+	A	0	1	0	1	1	-
gi|29165615|ref|NC_002745.2|	311670	+	A	0	0	0	1	0	-
gi|29165615|ref|NC_002745.2|	311673	+	U	0	0	0	1	0	-
gi|29165615|ref|NC_002745.2|	311703	+	G	0	2	0	0	0	-
gi|29165615|ref|NC_002745.2|	311718	+	A	0	0	0	0	1	-
gi|29165615|ref|NC_002745.2|	311719	+	A	0	2	0	1	0	-
gi|29165615|ref|NC_002745.2|	316871	+	U	0	0	0	0	1	SA0262
gi|29165615|ref|NC_002745.2|	321925	+	C	0	1	0	0	0	-
gi|29165615|ref|NC_002745.2|	340594	+	U	0	0	1	0	0	SA0282
gi|29165615|ref|NC_002745.2|	340602	+	G	0	0	0	1	0	SA0282
gi|29165615|ref|NC_002745.2|	340605	+	A	0	0	0	1	0	SA0282
gi|29165615|ref|NC_002745.2|	340608	+	G	0	1	0	1	0	SA0282
gi|29165615|ref|NC_002745.2|	340609	+	C	0	0	0	1	0	SA0282
gi|29165615|ref|NC_002745.2|	340615	+	A	0	0	0	0	1	SA0282
gi|29165615|ref|NC_002745.2|	340618	+	U	0	1	0	0	0	SA0282
gi|29165615|ref|NC_002745.2|	340619	+	U	0	0	0	0	1	SA0282
gi|29165615|ref|NC_002745.2|	340623	+	A	0	1	0	0	0	SA0282
gi|29165615|ref|NC_002745.2|	340634	+	G	0	0	0	0	1	SA0282
gi|29165615|ref|NC_002745.2|	340635	+	G	0	0	0	0	1	SA0282
gi|29165615|ref|NC_002745.2|	340636	+	U	0	0	0	1	0	SA0282
gi|29165615|ref|NC_002745.2|	340638	+	G	0	0	0	1	0	SA0282
gi|29165615|ref|NC_002745.2|	340651	+	U	0	0	0	0	1	SA0282
gi|29165615|ref|NC_002745.2|	340659	+	A	1	0	0	0	0	SA0282
gi|29165615|ref|NC_002745.2|	340676	+	U	0	1	0	0	0	SA0282
gi|29165615|ref|NC_002745.2|	340698	+	A	0	0	1	0	0	SA0282
gi|29165615|ref|NC_002745.2|	340711	+	C	1	0	0	0	0	SA0282
gi|29165615|ref|NC_002745.2|	340725	+	U	0	0	1	0	0	SA0282
gi|29165615|ref|NC_002745.2|	340729	+	A	0	0	0	0	1	SA0282
gi|29165615|ref|NC_002745.2|	340742	+	U	2	0	0	0	0	SA0282
gi|29165615|ref|NC_002745.2|	340759	+	A	0	0	0	0	1	SA0282
gi|29165615|ref|NC_002745.2|	340760	+	C	0	0	1	0	0	SA0282
gi|29165615|ref|NC_002745.2|	340762	+	U	1	0	0	0	0	SA0282
gi|29165615|ref|NC_002745.2|	340775	+	A	0	0	1	0	0	SA0282
gi|29165615|ref|NC_002745.2|	340804	+	A	0	1	0	0	0	SA0282
gi|29165615|ref|NC_002745.2|	340806	+	U	0	0	0	0	2	SA0282
gi|29165615|ref|NC_002745.2|	340839	+	G	0	0	0	0	1	SA0282
gi|29165615|ref|NC_002745.2|	340844	+	U	0	0	0	0	1	SA0282
gi|29165615|ref|NC_002745.2|	340849	+	A	1	0	0	0	0	SA0282
gi|29165615|ref|NC_002745.2|	340852	+	C	0	0	0	0	1	SA0282
gi|29165615|ref|NC_002745.2|	340853	+	A	0	0	1	0	0	SA0282
gi|29165615|ref|NC_002745.2|	340861	+	C	1	0	0	0	0	SA0282
gi|29165615|ref|NC_002745.2|	340868	+	A	0	0	1	0	0	SA0282
gi|29165615|ref|NC_002745.2|	340879	+	C	0	0	0	1	0	SA0282
gi|29165615|ref|NC_002745.2|	340880	+	A	3	0	0	0	0	SA0282
gi|29165615|ref|NC_002745.2|	340885	+	A	0	0	0	1	0	SA0282
gi|29165615|ref|NC_002745.2|	340891	+	A	0	1	0	0	0	SA0282
gi|29165615|ref|NC_002745.2|	340901	+	A	0	0	0	1	0	SA0282
gi|29165615|ref|NC_002745.2|	340909	+	A	0	0	0	1	0	SA0282
gi|29165615|ref|NC_002745.2|	340918	+	A	0	0	0	1	0	SA0282
gi|29165615|ref|NC_002745.2|	340927	+	A	0	3	0	0	0	SA0282
gi|29165615|ref|NC_002745.2|	340933	+	A	0	0	1	0	0	SA0282
gi|29165615|ref|NC_002745.2|	340940	+	U	1	0	0	0	0	SA0282
gi|29165615|ref|NC_002745.2|	340960	+	A	0	0	0	2	0	SA0282
gi|29165615|ref|NC_002745.2|	340969	+	A	0	0	0	1	0	SA0282
gi|29165615|ref|NC_002745.2|	340976	+	A	0	1	0	0	0	SA0282
gi|29165615|ref|NC_002745.2|	340977	+	U	1	0	0	0	0	SA0282
gi|29165615|ref|NC_002745.2|	340978	+	U	1	0	0	0	0	SA0282
gi|29165615|ref|NC_002745.2|	340995	+	A	1	0	0	0	0	SA0282
gi|29165615|ref|NC_002745.2|	343253	+	A	0	0	0	0	1	SA0286
gi|29165615|ref|NC_002745.2|	343254	+	G	0	0	0	0	1	SA0286
gi|29165615|ref|NC_002745.2|	343255	+	U	0	0	0	1	0	SA0286
gi|29165615|ref|NC_002745.2|	343257	+	G	0	0	0	1	0	SA0286
gi|29165615|ref|NC_002745.2|	343270	+	U	0	0	0	0	1	SA0286
gi|29165615|ref|NC_002745.2|	343278	+	A	1	0	0	0	0	SA0286
gi|29165615|ref|NC_002745.2|	343468	+	A	1	0	0	0	0	SA0286
gi|29165615|ref|NC_002745.2|	343471	+	C	0	0	0	0	1	SA0286

gi|29165615|ref|NC_002745.2|	343472	+	A	0	0	1	0	0	SA0286
gi|29165615|ref|NC_002745.2|	343508	+	U	0	2	0	0	0	SA0286
gi|29165615|ref|NC_002745.2|	343510	+	A	0	1	0	0	0	SA0286
gi|29165615|ref|NC_002745.2|	343520	+	U	0	0	0	1	0	SA0286
gi|29165615|ref|NC_002745.2|	343528	+	A	0	0	0	1	0	SA0286
gi|29165615|ref|NC_002745.2|	343537	+	A	0	0	0	1	0	SA0286
gi|29165615|ref|NC_002745.2|	343546	+	A	0	3	0	0	0	SA0286
gi|29165615|ref|NC_002745.2|	343552	+	A	0	0	1	0	0	SA0286
gi|29165615|ref|NC_002745.2|	343559	+	U	1	0	0	0	0	SA0286
gi|29165615|ref|NC_002745.2|	343579	+	A	0	0	0	2	0	SA0286
gi|29165615|ref|NC_002745.2|	343588	+	A	0	0	0	1	0	SA0286
gi|29165615|ref|NC_002745.2|	343595	+	A	0	1	0	0	0	SA0286
gi|29165615|ref|NC_002745.2|	343596	+	U	1	0	0	0	0	SA0286
gi|29165615|ref|NC_002745.2|	343597	+	U	1	0	0	0	0	SA0286
gi|29165615|ref|NC_002745.2|	343614	+	A	1	0	0	0	0	SA0286
gi|29165615|ref|NC_002745.2|	343724	+	U	0	0	1	0	0	SA0287
gi|29165615|ref|NC_002745.2|	343732	+	G	0	0	0	1	0	SA0287
gi|29165615|ref|NC_002745.2|	343735	+	A	0	0	0	1	0	SA0287
gi|29165615|ref|NC_002745.2|	343738	+	G	0	1	0	1	0	SA0287
gi|29165615|ref|NC_002745.2|	343739	+	C	0	0	0	1	0	SA0287
gi|29165615|ref|NC_002745.2|	343745	+	A	0	0	0	0	1	SA0287
gi|29165615|ref|NC_002745.2|	343748	+	U	0	1	0	0	0	SA0287
gi|29165615|ref|NC_002745.2|	343749	+	U	0	0	0	0	1	SA0287
gi|29165615|ref|NC_002745.2|	343753	+	A	0	1	0	0	0	SA0287
gi|29165615|ref|NC_002745.2|	343764	+	G	0	0	0	0	1	SA0287
gi|29165615|ref|NC_002745.2|	343765	+	G	0	0	0	0	1	SA0287
gi|29165615|ref|NC_002745.2|	343766	+	U	0	0	0	1	0	SA0287
gi|29165615|ref|NC_002745.2|	343768	+	G	0	0	0	1	0	SA0287
gi|29165615|ref|NC_002745.2|	343781	+	U	0	0	0	0	1	SA0287
gi|29165615|ref|NC_002745.2|	343789	+	A	1	0	0	0	0	SA0287
gi|29165615|ref|NC_002745.2|	343806	+	U	0	1	0	0	0	SA0287
gi|29165615|ref|NC_002745.2|	343828	+	A	0	0	1	0	0	SA0287
gi|29165615|ref|NC_002745.2|	343841	+	C	1	0	0	0	0	SA0287
gi|29165615|ref|NC_002745.2|	343855	+	U	0	0	1	0	0	SA0287
gi|29165615|ref|NC_002745.2|	343859	+	A	0	0	0	0	1	SA0287
gi|29165615|ref|NC_002745.2|	343872	+	U	2	0	0	0	0	SA0287
gi|29165615|ref|NC_002745.2|	343889	+	A	0	0	0	0	1	SA0287
gi|29165615|ref|NC_002745.2|	343890	+	C	0	0	1	0	0	SA0287
gi|29165615|ref|NC_002745.2|	343892	+	U	1	0	0	0	0	SA0287
gi|29165615|ref|NC_002745.2|	343905	+	A	0	0	1	0	0	SA0287
gi|29165615|ref|NC_002745.2|	343934	+	A	0	1	0	0	0	SA0287
gi|29165615|ref|NC_002745.2|	343936	+	U	0	0	0	0	2	SA0287
gi|29165615|ref|NC_002745.2|	343969	+	G	0	0	0	0	1	SA0287
gi|29165615|ref|NC_002745.2|	343974	+	U	0	0	0	0	1	SA0287
gi|29165615|ref|NC_002745.2|	343979	+	A	1	0	0	0	0	SA0287
gi|29165615|ref|NC_002745.2|	343982	+	C	0	0	0	0	1	SA0287
gi|29165615|ref|NC_002745.2|	343983	+	A	0	0	1	0	0	SA0287
gi|29165615|ref|NC_002745.2|	343991	+	C	1	0	0	0	0	SA0287
gi|29165615|ref|NC_002745.2|	344019	+	U	0	2	0	0	0	SA0287
gi|29165615|ref|NC_002745.2|	344021	+	A	0	1	0	0	0	SA0287
gi|29165615|ref|NC_002745.2|	344039	+	A	0	0	0	1	0	SA0287
gi|29165615|ref|NC_002745.2|	344048	+	A	0	0	0	1	0	SA0287
gi|29165615|ref|NC_002745.2|	344057	+	A	0	3	0	0	0	SA0287
gi|29165615|ref|NC_002745.2|	344063	+	A	0	0	1	0	0	SA0287
gi|29165615|ref|NC_002745.2|	344070	+	U	1	0	0	0	0	SA0287
gi|29165615|ref|NC_002745.2|	344090	+	A	0	0	0	2	0	SA0287
gi|29165615|ref|NC_002745.2|	344099	+	A	0	0	0	1	0	SA0287
gi|29165615|ref|NC_002745.2|	344106	+	A	0	1	0	0	0	SA0287
gi|29165615|ref|NC_002745.2|	344107	+	U	1	0	0	0	0	SA0287
gi|29165615|ref|NC_002745.2|	344108	+	U	1	0	0	0	0	SA0287
gi|29165615|ref|NC_002745.2|	344256	+	A	0	0	0	0	1	SA0288
gi|29165615|ref|NC_002745.2|	344259	+	U	0	1	0	0	0	SA0288
gi|29165615|ref|NC_002745.2|	344260	+	U	0	0	0	0	1	SA0288
gi|29165615|ref|NC_002745.2|	344264	+	A	0	1	0	0	0	SA0288
gi|29165615|ref|NC_002745.2|	344490	+	A	1	0	0	0	0	SA0288
gi|29165615|ref|NC_002745.2|	344493	+	C	0	0	0	0	1	SA0288
gi|29165615|ref|NC_002745.2|	344494	+	A	0	0	1	0	0	SA0288
gi|29165615|ref|NC_002745.2|	344502	+	C	1	0	0	0	0	SA0288
gi|29165615|ref|NC_002745.2|	344509	+	A	0	0	1	0	0	SA0288
gi|29165615|ref|NC_002745.2|	344532	+	A	0	1	0	0	0	SA0288
gi|29165615|ref|NC_002745.2|	344849	+	A	0	0	1	0	0	SA0289
gi|29165615|ref|NC_002745.2|	344862	+	C	1	0	0	0	0	SA0289
gi|29165615|ref|NC_002745.2|	344990	+	G	0	0	0	0	1	SA0289
gi|29165615|ref|NC_002745.2|	344995	+	U	0	0	0	0	1	SA0289
gi|29165615|ref|NC_002745.2|	345000	+	A	1	0	0	0	0	SA0289
gi|29165615|ref|NC_002745.2|	345003	+	C	0	0	0	0	1	SA0289
gi|29165615|ref|NC_002745.2|	345004	+	A	0	0	1	0	0	SA0289
gi|29165615|ref|NC_002745.2|	345012	+	C	1	0	0	0	0	SA0289
gi|29165615|ref|NC_002745.2|	345019	+	A	0	0	1	0	0	SA0289
gi|29165615|ref|NC_002745.2|	345040	+	U	0	2	0	0	0	SA0289
gi|29165615|ref|NC_002745.2|	345042	+	A	0	1	0	0	0	SA0289

gi|29165615|ref|NC_002745.2|	345052	+	U	0	0	0	1	0	SA0289
gi|29165615|ref|NC_002745.2|	345060	+	A	0	0	0	1	0	SA0289
gi|29165615|ref|NC_002745.2|	345069	+	A	0	0	0	1	0	SA0289
gi|29165615|ref|NC_002745.2|	345078	+	A	0	3	0	0	0	SA0289
gi|29165615|ref|NC_002745.2|	345084	+	A	0	0	1	0	0	SA0289
gi|29165615|ref|NC_002745.2|	345091	+	U	1	0	0	0	0	SA0289
gi|29165615|ref|NC_002745.2|	345111	+	A	0	0	0	2	0	SA0289
gi|29165615|ref|NC_002745.2|	345120	+	A	0	0	0	1	0	SA0289
gi|29165615|ref|NC_002745.2|	345127	+	A	0	1	0	0	0	SA0289
gi|29165615|ref|NC_002745.2|	345128	+	U	1	0	0	0	0	SA0289
gi|29165615|ref|NC_002745.2|	345129	+	U	1	0	0	0	0	SA0289
gi|29165615|ref|NC_002745.2|	345296	+	A	0	0	0	0	1	SA0290
gi|29165615|ref|NC_002745.2|	345297	+	G	0	0	0	0	1	SA0290
gi|29165615|ref|NC_002745.2|	345298	+	U	0	0	0	1	0	SA0290
gi|29165615|ref|NC_002745.2|	345300	+	G	0	0	0	1	0	SA0290
gi|29165615|ref|NC_002745.2|	345313	+	U	0	0	0	0	1	SA0290
gi|29165615|ref|NC_002745.2|	345360	+	A	0	0	1	0	0	SA0290
gi|29165615|ref|NC_002745.2|	345373	+	C	1	0	0	0	0	SA0290
gi|29165615|ref|NC_002745.2|	345498	+	G	0	0	0	0	1	SA0290
gi|29165615|ref|NC_002745.2|	345503	+	U	0	0	0	0	1	SA0290
gi|29165615|ref|NC_002745.2|	345508	+	A	1	0	0	0	0	SA0290
gi|29165615|ref|NC_002745.2|	345511	+	C	0	0	0	0	1	SA0290
gi|29165615|ref|NC_002745.2|	345512	+	A	0	0	1	0	0	SA0290
gi|29165615|ref|NC_002745.2|	345520	+	C	1	0	0	0	0	SA0290
gi|29165615|ref|NC_002745.2|	345527	+	A	0	0	1	0	0	SA0290
gi|29165615|ref|NC_002745.2|	345538	+	C	0	0	0	1	0	SA0290
gi|29165615|ref|NC_002745.2|	345539	+	A	3	0	0	0	0	SA0290
gi|29165615|ref|NC_002745.2|	345544	+	A	0	0	0	1	0	SA0290
gi|29165615|ref|NC_002745.2|	345560	+	A	0	0	0	1	0	SA0290
gi|29165615|ref|NC_002745.2|	352950	+	A	0	6	2	8	7	SA0297
gi|29165615|ref|NC_002745.2|	356177	+	A	0	1	0	1	0	SA0301
gi|29165615|ref|NC_002745.2|	366516	+	A	0	1	0	0	0	SA0309
gi|29165615|ref|NC_002745.2|	366576	+	U	1	0	0	0	0	SA0309
gi|29165615|ref|NC_002745.2|	366581	+	U	0	0	1	0	0	SA0309
gi|29165615|ref|NC_002745.2|	366588	+	U	0	0	0	0	1	SA0309
gi|29165615|ref|NC_002745.2|	366596	+	C	0	0	1	0	0	SA0309
gi|29165615|ref|NC_002745.2|	366838	+	A	0	1	0	0	1	SA0309
gi|29165615|ref|NC_002745.2|	366841	+	U	0	1	1	0	0	SA0309
gi|29165615|ref|NC_002745.2|	366842	+	U	0	0	0	0	1	SA0309
gi|29165615|ref|NC_002745.2|	366846	+	A	0	0	0	1	0	SA0309
gi|29165615|ref|NC_002745.2|	373933	+	U	0	0	0	0	1	SA0316
gi|29165615|ref|NC_002745.2|	373934	+	G	0	1	0	0	0	SA0316
gi|29165615|ref|NC_002745.2|	383948	+	A	0	1	0	0	0	-
gi|29165615|ref|NC_002745.2|	387960	+	G	0	0	1	0	0	-
gi|29165615|ref|NC_002745.2|	387964	+	A	0	0	0	0	1	-
gi|29165615|ref|NC_002745.2|	387973	+	C	1	2	0	0	0	-
gi|29165615|ref|NC_002745.2|	387979	+	A	0	0	0	0	1	-
gi|29165615|ref|NC_002745.2|	387981	+	A	0	0	1	0	0	-
gi|29165615|ref|NC_002745.2|	388028	+	A	0	0	0	2	1	-
gi|29165615|ref|NC_002745.2|	388029	+	A	0	0	0	1	0	-
gi|29165615|ref|NC_002745.2|	388032	+	U	0	0	0	1	0	-
gi|29165615|ref|NC_002745.2|	388033	+	C	0	1	0	0	0	-
gi|29165615|ref|NC_002745.2|	388034	+	U	0	0	0	1	0	-
gi|29165615|ref|NC_002745.2|	388041	+	A	0	0	0	0	1	-
gi|29165615|ref|NC_002745.2|	389673	+	U	0	0	0	1	0	SA0331
gi|29165615|ref|NC_002745.2|	389674	+	G	0	0	2	0	1	SA0331
gi|29165615|ref|NC_002745.2|	389675	+	A	0	1	1	0	0	SA0331
gi|29165615|ref|NC_002745.2|	395864	+	G	0	0	0	1	0	SA0339
gi|29165615|ref|NC_002745.2|	399900	+	A	0	0	0	0	1	SA0342
gi|29165615|ref|NC_002745.2|	406654	+	U	0	0	2	0	1	-
gi|29165615|ref|NC_002745.2|	411028	+	U	0	1	0	0	0	SA0351
gi|29165615|ref|NC_002745.2|	412720	+	A	0	0	1	1	1	-
gi|29165615|ref|NC_002745.2|	412721	+	A	0	5	2	3	2	-
gi|29165615|ref|NC_002745.2|	412722	+	A	0	6	1	0	3	-
gi|29165615|ref|NC_002745.2|	412724	+	A	0	14	0	0	3	-
gi|29165615|ref|NC_002745.2|	417186	+	A	0	0	0	0	3	SA0359
gi|29165615|ref|NC_002745.2|	417187	+	A	0	2	0	0	1	SA0359
gi|29165615|ref|NC_002745.2|	417188	+	A	0	2	2	0	0	SA0359
gi|29165615|ref|NC_002745.2|	426656	+	A	0	0	0	1	0	-
gi|29165615|ref|NC_002745.2|	426661	+	A	15	15	14	7	8	-
gi|29165615|ref|NC_002745.2|	426666	+	A	1	1	0	0	1	-
gi|29165615|ref|NC_002745.2|	426669	+	C	0	2	1	0	1	-
gi|29165615|ref|NC_002745.2|	426709	+	A	0	0	0	2	1	SA0369
gi|29165615|ref|NC_002745.2|	426714	+	A	0	0	0	1	1	SA0369
gi|29165615|ref|NC_002745.2|	426720	+	A	0	0	0	0	1	SA0369
gi|29165615|ref|NC_002745.2|	426722	+	A	0	0	1	0	0	SA0369
gi|29165615|ref|NC_002745.2|	426723	+	A	0	0	0	1	0	SA0369
gi|29165615|ref|NC_002745.2|	426726	+	U	0	0	1	0	0	SA0369
gi|29165615|ref|NC_002745.2|	426728	+	A	0	0	0	0	2	SA0369
gi|29165615|ref|NC_002745.2|	426729	+	A	0	2	0	0	0	SA0369
gi|29165615|ref|NC_002745.2|	426731	+	U	0	0	1	0	0	SA0369

gi|29165615|ref|NC_002745.2|	426732	+	A	0	0	1	0	0	SA0369
gi|29165615|ref|NC_002745.2|	426734	+	A	0	0	2	0	0	SA0369
gi|29165615|ref|NC_002745.2|	426735	+	U	0	1	2	0	2	SA0369
gi|29165615|ref|NC_002745.2|	426736	+	A	0	1	0	0	2	SA0369
gi|29165615|ref|NC_002745.2|	426737	+	A	0	0	0	0	1	SA0369
gi|29165615|ref|NC_002745.2|	426738	+	A	0	1	0	0	0	SA0369
gi|29165615|ref|NC_002745.2|	426739	+	C	0	3	0	0	0	SA0369
gi|29165615|ref|NC_002745.2|	426741	+	A	0	0	0	0	2	SA0369
gi|29165615|ref|NC_002745.2|	426743	+	G	0	0	0	0	1	SA0369
gi|29165615|ref|NC_002745.2|	426744	+	A	2	6	1	1	6	SA0369
gi|29165615|ref|NC_002745.2|	426745	+	A	0	0	1	0	0	SA0369
gi|29165615|ref|NC_002745.2|	426750	+	A	0	1	3	0	2	SA0369
gi|29165615|ref|NC_002745.2|	426753	+	U	0	0	2	0	0	SA0369
gi|29165615|ref|NC_002745.2|	426756	+	U	0	1	0	0	1	SA0369
gi|29165615|ref|NC_002745.2|	426757	+	U	0	0	1	0	0	SA0369
gi|29165615|ref|NC_002745.2|	426761	+	A	0	2	1	0	2	SA0369
gi|29165615|ref|NC_002745.2|	426762	+	A	0	0	4	1	0	SA0369
gi|29165615|ref|NC_002745.2|	426765	+	G	0	0	0	0	1	SA0369
gi|29165615|ref|NC_002745.2|	426766	+	A	0	0	0	1	0	SA0369
gi|29165615|ref|NC_002745.2|	426767	+	A	0	1	0	0	0	SA0369
gi|29165615|ref|NC_002745.2|	426769	+	A	0	0	1	0	1	SA0369
gi|29165615|ref|NC_002745.2|	426770	+	A	1	1	2	1	3	SA0369
gi|29165615|ref|NC_002745.2|	426775	+	A	0	0	1	0	0	SA0369
gi|29165615|ref|NC_002745.2|	426777	+	C	1	0	0	0	1	SA0369
gi|29165615|ref|NC_002745.2|	426785	+	A	0	0	2	0	0	SA0369
gi|29165615|ref|NC_002745.2|	426791	+	G	0	1	0	0	0	SA0369
gi|29165615|ref|NC_002745.2|	426796	+	A	0	0	1	0	0	SA0369
gi|29165615|ref|NC_002745.2|	426798	+	C	0	0	0	0	1	SA0369
gi|29165615|ref|NC_002745.2|	426800	+	U	0	1	0	0	0	SA0369
gi|29165615|ref|NC_002745.2|	426809	+	U	0	0	1	0	0	SA0369
gi|29165615|ref|NC_002745.2|	426830	+	G	0	1	0	0	0	SA0369
gi|29165615|ref|NC_002745.2|	426847	+	A	0	0	0	1	0	SA0369
gi|29165615|ref|NC_002745.2|	426848	+	A	0	1	0	0	0	SA0369
gi|29165615|ref|NC_002745.2|	426892	+	A	0	1	0	0	0	SA0369
gi|29165615|ref|NC_002745.2|	426893	+	A	0	1	0	0	0	SA0369
gi|29165615|ref|NC_002745.2|	426924	+	C	0	1	0	0	0	SA0369
gi|29165615|ref|NC_002745.2|	426934	+	U	0	1	0	0	0	SA0369
gi|29165615|ref|NC_002745.2|	427029	+	U	0	0	0	0	1	SA0369
gi|29165615|ref|NC_002745.2|	427089	+	C	1	0	0	0	0	SA0369
gi|29165615|ref|NC_002745.2|	427200	+	U	0	1	0	0	0	SA0369
gi|29165615|ref|NC_002745.2|	427201	+	A	0	0	1	0	0	SA0369
gi|29165615|ref|NC_002745.2|	427236	+	A	0	1	0	0	0	SA0369
gi|29165615|ref|NC_002745.2|	427255	+	U	0	2	0	0	0	SA0369
gi|29165615|ref|NC_002745.2|	427263	+	A	0	1	0	0	0	SA0369
gi|29165615|ref|NC_002745.2|	427266	+	A	0	0	1	0	0	SA0369
gi|29165615|ref|NC_002745.2|	427358	+	C	0	0	0	0	1	SA0369
gi|29165615|ref|NC_002745.2|	427434	+	U	0	0	2	0	0	SA0369
gi|29165615|ref|NC_002745.2|	427435	+	A	0	0	0	0	1	SA0369
gi|29165615|ref|NC_002745.2|	427457	+	C	1	0	0	0	0	SA0369
gi|29165615|ref|NC_002745.2|	427520	+	A	0	0	1	0	0	SA0369
gi|29165615|ref|NC_002745.2|	427547	+	A	0	0	1	0	0	SA0369
gi|29165615|ref|NC_002745.2|	427548	+	G	0	0	1	0	0	SA0369
gi|29165615|ref|NC_002745.2|	427682	+	A	0	0	0	0	1	SA0369
gi|29165615|ref|NC_002745.2|	427699	+	U	0	0	0	0	1	SA0369
gi|29165615|ref|NC_002745.2|	427704	+	G	0	0	0	0	2	SA0369
gi|29165615|ref|NC_002745.2|	427734	+	U	0	0	0	0	1	SA0369
gi|29165615|ref|NC_002745.2|	427737	+	G	0	0	0	0	1	SA0369
gi|29165615|ref|NC_002745.2|	427761	+	U	0	0	1	0	0	SA0369
gi|29165615|ref|NC_002745.2|	427779	+	A	0	0	1	0	0	SA0369
gi|29165615|ref|NC_002745.2|	427812	+	C	0	0	0	1	0	SA0369
gi|29165615|ref|NC_002745.2|	427862	+	A	0	1	0	0	0	SA0369
gi|29165615|ref|NC_002745.2|	427873	+	A	0	1	0	1	0	SA0369
gi|29165615|ref|NC_002745.2|	427877	+	A	0	0	1	0	0	SA0369
gi|29165615|ref|NC_002745.2|	427908	+	A	0	0	1	0	0	SA0369
gi|29165615|ref|NC_002745.2|	427922	+	C	0	0	0	1	0	SA0369
gi|29165615|ref|NC_002745.2|	427929	+	U	0	0	0	1	0	SA0369
gi|29165615|ref|NC_002745.2|	427989	+	U	0	0	1	0	0	SA0369
gi|29165615|ref|NC_002745.2|	427997	+	U	0	0	0	1	0	SA0369
gi|29165615|ref|NC_002745.2|	427998	+	A	0	0	0	0	1	SA0369
gi|29165615|ref|NC_002745.2|	428013	+	A	0	0	0	0	1	-
gi|29165615|ref|NC_002745.2|	428019	+	U	0	0	0	0	1	-
gi|29165615|ref|NC_002745.2|	428021	+	U	0	0	0	1	0	-
gi|29165615|ref|NC_002745.2|	428024	+	U	0	0	0	0	1	-
gi|29165615|ref|NC_002745.2|	428027	+	A	0	0	0	1	2	-
gi|29165615|ref|NC_002745.2|	428028	+	A	0	1	0	1	0	-
gi|29165615|ref|NC_002745.2|	428029	+	A	0	0	0	1	0	-
gi|29165615|ref|NC_002745.2|	428030	+	A	1	0	0	0	0	-
gi|29165615|ref|NC_002745.2|	428034	+	U	0	1	0	0	0	-
gi|29165615|ref|NC_002745.2|	428041	+	A	0	0	0	0	1	-
gi|29165615|ref|NC_002745.2|	428043	+	U	0	1	2	3	0	-
gi|29165615|ref|NC_002745.2|	428044	+	U	0	0	1	0	2	-

gi|29165615|ref|NC_002745.2|	428052	+	C	0	0	0	0	1	-
gi|29165615|ref|NC_002745.2|	432844	+	A	0	0	0	1	0	SA0374
gi|29165615|ref|NC_002745.2|	433607	+	U	0	0	0	1	0	SA0375
gi|29165615|ref|NC_002745.2|	438472	+	G	0	0	0	0	1	-
gi|29165615|ref|NC_002745.2|	438491	+	C	0	0	0	0	1	-
gi|29165615|ref|NC_002745.2|	442010	+	G	0	0	0	1	1	-
gi|29165615|ref|NC_002745.2|	443063	+	A	0	0	0	0	1	SA0384
gi|29165615|ref|NC_002745.2|	444001	+	C	0	0	0	1	0	SA0384
gi|29165615|ref|NC_002745.2|	444011	+	A	0	0	0	0	1	SA0384
gi|29165615|ref|NC_002745.2|	444021	+	G	0	0	0	0	1	SA0384
gi|29165615|ref|NC_002745.2|	444497	+	A	0	0	0	0	1	SA0385
gi|29165615|ref|NC_002745.2|	445243	+	U	0	0	0	1	0	SA0385
gi|29165615|ref|NC_002745.2|	445253	+	A	0	0	0	0	1	SA0385
gi|29165615|ref|NC_002745.2|	445263	+	G	0	0	0	0	1	SA0385
gi|29165615|ref|NC_002745.2|	446798	+	G	12	1	1	3	2	-
gi|29165615|ref|NC_002745.2|	447855	+	G	0	0	0	1	1	-
gi|29165615|ref|NC_002745.2|	448989	+	A	0	0	1	0	0	SA0389
gi|29165615|ref|NC_002745.2|	449994	+	G	0	0	0	1	1	-
gi|29165615|ref|NC_002745.2|	450053	+	A	0	0	1	0	0	SA0390
gi|29165615|ref|NC_002745.2|	450952	+	U	0	2	3	0	2	-
gi|29165615|ref|NC_002745.2|	450954	+	U	0	0	3	0	1	-
gi|29165615|ref|NC_002745.2|	450955	+	A	15	66	45	42	56	-
gi|29165615|ref|NC_002745.2|	450956	+	U	0	0	0	2	1	-
gi|29165615|ref|NC_002745.2|	450957	+	U	0	1	3	0	2	-
gi|29165615|ref|NC_002745.2|	450958	+	A	1	0	0	0	1	-
gi|29165615|ref|NC_002745.2|	450959	+	C	0	0	0	0	1	-
gi|29165615|ref|NC_002745.2|	450960	+	A	0	0	1	0	0	-
gi|29165615|ref|NC_002745.2|	450961	+	C	0	0	1	0	0	-
gi|29165615|ref|NC_002745.2|	450965	+	G	0	1	0	0	0	-
gi|29165615|ref|NC_002745.2|	450966	+	A	0	0	0	1	0	-
gi|29165615|ref|NC_002745.2|	450967	+	G	0	0	0	1	0	-
gi|29165615|ref|NC_002745.2|	450968	+	A	0	1	2	1	0	-
gi|29165615|ref|NC_002745.2|	450969	+	U	0	1	0	1	1	-
gi|29165615|ref|NC_002745.2|	450970	+	A	0	0	1	0	2	-
gi|29165615|ref|NC_002745.2|	450971	+	A	0	0	0	1	0	-
gi|29165615|ref|NC_002745.2|	450978	+	U	0	1	4	0	2	-
gi|29165615|ref|NC_002745.2|	450981	+	U	0	1	0	0	0	-
gi|29165615|ref|NC_002745.2|	450982	+	C	0	0	1	0	0	-
gi|29165615|ref|NC_002745.2|	450990	+	G	0	0	2	0	0	-
gi|29165615|ref|NC_002745.2|	450993	+	G	1	1	0	0	0	-
gi|29165615|ref|NC_002745.2|	450994	+	A	0	0	0	0	1	-
gi|29165615|ref|NC_002745.2|	450998	+	A	0	0	0	0	2	-
gi|29165615|ref|NC_002745.2|	450999	+	C	0	0	0	1	0	-
gi|29165615|ref|NC_002745.2|	451000	+	A	0	0	1	0	0	SA0391
gi|29165615|ref|NC_002745.2|	451002	+	G	0	1	0	0	0	SA0391
gi|29165615|ref|NC_002745.2|	451005	+	U	0	1	1	1	3	SA0391
gi|29165615|ref|NC_002745.2|	451006	+	A	0	1	0	0	1	SA0391
gi|29165615|ref|NC_002745.2|	451007	+	U	0	0	0	1	0	SA0391
gi|29165615|ref|NC_002745.2|	451008	+	U	0	3	2	1	1	SA0391
gi|29165615|ref|NC_002745.2|	451009	+	A	1	0	2	0	2	SA0391
gi|29165615|ref|NC_002745.2|	451011	+	U	0	0	1	0	0	SA0391
gi|29165615|ref|NC_002745.2|	451013	+	A	0	23	6	7	19	SA0391
gi|29165615|ref|NC_002745.2|	451014	+	A	1	20	19	23	35	SA0391
gi|29165615|ref|NC_002745.2|	451015	+	A	2	12	3	10	14	SA0391
gi|29165615|ref|NC_002745.2|	451016	+	A	0	22	12	9	15	SA0391
gi|29165615|ref|NC_002745.2|	451017	+	A	0	4	2	0	1	SA0391
gi|29165615|ref|NC_002745.2|	451026	+	G	0	1	0	0	0	SA0391
gi|29165615|ref|NC_002745.2|	451031	+	A	1	0	0	0	0	SA0391
gi|29165615|ref|NC_002745.2|	451040	+	U	0	1	1	0	0	SA0391
gi|29165615|ref|NC_002745.2|	451041	+	A	0	1	0	0	0	SA0391
gi|29165615|ref|NC_002745.2|	451043	+	A	1	6	3	0	3	SA0391
gi|29165615|ref|NC_002745.2|	451044	+	U	0	1	0	1	0	SA0391
gi|29165615|ref|NC_002745.2|	451045	+	A	0	4	5	1	5	SA0391
gi|29165615|ref|NC_002745.2|	451046	+	A	1	2	1	2	5	SA0391
gi|29165615|ref|NC_002745.2|	451047	+	A	1	16	11	6	17	SA0391
gi|29165615|ref|NC_002745.2|	451048	+	A	0	10	3	2	15	SA0391
gi|29165615|ref|NC_002745.2|	451049	+	A	0	1	0	1	1	SA0391
gi|29165615|ref|NC_002745.2|	451050	+	A	0	4	1	1	5	SA0391
gi|29165615|ref|NC_002745.2|	451051	+	U	0	1	1	0	0	SA0391
gi|29165615|ref|NC_002745.2|	451052	+	U	0	1	1	0	1	SA0391
gi|29165615|ref|NC_002745.2|	451057	+	U	0	1	1	0	0	SA0391
gi|29165615|ref|NC_002745.2|	451059	+	G	0	1	0	0	0	SA0391
gi|29165615|ref|NC_002745.2|	451060	+	A	0	5	0	1	1	SA0391
gi|29165615|ref|NC_002745.2|	451064	+	C	0	1	0	0	2	SA0391
gi|29165615|ref|NC_002745.2|	451065	+	G	0	1	0	0	0	SA0391
gi|29165615|ref|NC_002745.2|	451066	+	A	0	1	0	0	0	SA0391
gi|29165615|ref|NC_002745.2|	451069	+	G	0	0	0	0	1	SA0391
gi|29165615|ref|NC_002745.2|	451070	+	A	0	2	1	0	2	SA0391
gi|29165615|ref|NC_002745.2|	451074	+	A	0	0	1	1	1	SA0391
gi|29165615|ref|NC_002745.2|	451075	+	A	0	0	1	0	0	SA0391
gi|29165615|ref|NC_002745.2|	451081	+	A	0	0	3	0	2	SA0391

gi|29165615|ref|NC_002745.2|	451082	+	A	2	0	1	0	0	SA0391
gi|29165615|ref|NC_002745.2|	451083	+	U	0	0	0	0	1	SA0391
gi|29165615|ref|NC_002745.2|	451084	+	A	0	0	1	0	0	SA0391
gi|29165615|ref|NC_002745.2|	451091	+	C	0	0	0	0	1	SA0391
gi|29165615|ref|NC_002745.2|	451104	+	U	0	0	0	0	1	SA0391
gi|29165615|ref|NC_002745.2|	451106	+	A	0	1	0	0	0	SA0391
gi|29165615|ref|NC_002745.2|	451115	+	U	0	0	1	0	0	SA0391
gi|29165615|ref|NC_002745.2|	451123	+	A	0	0	1	0	0	SA0391
gi|29165615|ref|NC_002745.2|	451125	+	U	0	0	1	0	0	SA0391
gi|29165615|ref|NC_002745.2|	451151	+	C	0	0	0	1	0	SA0391
gi|29165615|ref|NC_002745.2|	451155	+	A	0	1	0	0	0	SA0391
gi|29165615|ref|NC_002745.2|	451158	+	A	0	0	0	1	0	SA0391
gi|29165615|ref|NC_002745.2|	451160	+	A	0	1	2	0	0	SA0391
gi|29165615|ref|NC_002745.2|	451161	+	A	0	0	0	1	1	SA0391
gi|29165615|ref|NC_002745.2|	451274	+	A	0	0	0	0	1	SA0391
gi|29165615|ref|NC_002745.2|	451358	+	A	0	0	0	0	1	SA0391
gi|29165615|ref|NC_002745.2|	451371	+	A	0	0	0	1	0	SA0391
gi|29165615|ref|NC_002745.2|	451379	+	G	0	0	0	1	0	SA0391
gi|29165615|ref|NC_002745.2|	451391	+	A	0	0	1	1	0	SA0391
gi|29165615|ref|NC_002745.2|	451397	+	A	0	1	0	2	1	SA0391
gi|29165615|ref|NC_002745.2|	451399	+	U	1	0	0	0	0	SA0391
gi|29165615|ref|NC_002745.2|	451401	+	U	0	0	1	0	0	SA0391
gi|29165615|ref|NC_002745.2|	451429	+	A	0	1	0	0	0	SA0391
gi|29165615|ref|NC_002745.2|	451449	+	C	0	1	0	0	0	SA0391
gi|29165615|ref|NC_002745.2|	451450	+	A	0	1	0	0	0	SA0391
gi|29165615|ref|NC_002745.2|	451451	+	A	0	0	0	0	1	SA0391
gi|29165615|ref|NC_002745.2|	451487	+	U	0	1	0	0	0	SA0391
gi|29165615|ref|NC_002745.2|	451505	+	U	1	0	0	0	0	SA0391
gi|29165615|ref|NC_002745.2|	451516	+	C	0	0	0	0	1	SA0391
gi|29165615|ref|NC_002745.2|	451660	+	A	0	0	0	0	1	SA0391
gi|29165615|ref|NC_002745.2|	451674	+	U	1	0	0	0	0	SA0391
gi|29165615|ref|NC_002745.2|	451686	+	C	0	0	0	0	1	SA0391
gi|29165615|ref|NC_002745.2|	451719	+	U	0	0	0	0	1	SA0391
gi|29165615|ref|NC_002745.2|	451756	+	C	0	0	0	1	0	SA0391
gi|29165615|ref|NC_002745.2|	451778	+	A	0	0	0	0	1	SA0391
gi|29165615|ref|NC_002745.2|	451802	+	U	1	0	0	0	0	SA0391
gi|29165615|ref|NC_002745.2|	451880	+	C	0	2	0	0	0	SA0391
gi|29165615|ref|NC_002745.2|	451926	+	U	1	0	0	0	0	SA0391
gi|29165615|ref|NC_002745.2|	451928	+	C	0	1	0	0	0	SA0391
gi|29165615|ref|NC_002745.2|	451930	+	G	0	0	1	0	0	SA0391
gi|29165615|ref|NC_002745.2|	451938	+	A	0	1	0	0	0	SA0391
gi|29165615|ref|NC_002745.2|	451939	+	U	0	0	0	1	0	SA0391
gi|29165615|ref|NC_002745.2|	451949	+	A	0	1	0	0	0	SA0391
gi|29165615|ref|NC_002745.2|	451989	+	C	3	0	0	0	0	SA0391
gi|29165615|ref|NC_002745.2|	452029	+	C	2	0	0	0	0	SA0391
gi|29165615|ref|NC_002745.2|	452221	+	C	0	0	0	0	1	SA0391
gi|29165615|ref|NC_002745.2|	452233	+	G	0	0	0	0	1	SA0391
gi|29165615|ref|NC_002745.2|	452274	+	A	0	1	0	0	0	SA0391
gi|29165615|ref|NC_002745.2|	452282	+	A	0	0	1	0	0	SA0391
gi|29165615|ref|NC_002745.2|	452327	+	A	0	0	0	1	1	SA0391
gi|29165615|ref|NC_002745.2|	452357	+	A	0	1	0	0	0	SA0391
gi|29165615|ref|NC_002745.2|	452368	+	U	1	1	0	0	0	SA0391
gi|29165615|ref|NC_002745.2|	452373	+	A	0	0	1	0	0	SA0391
gi|29165615|ref|NC_002745.2|	452376	+	G	0	0	1	0	0	SA0391
gi|29165615|ref|NC_002745.2|	452381	+	C	0	0	0	1	1	SA0391
gi|29165615|ref|NC_002745.2|	452384	+	A	0	1	0	0	0	SA0391
gi|29165615|ref|NC_002745.2|	452387	+	A	0	0	1	0	0	SA0391
gi|29165615|ref|NC_002745.2|	452417	+	U	0	0	0	0	1	SA0391
gi|29165615|ref|NC_002745.2|	452423	+	C	0	0	1	0	0	SA0391
gi|29165615|ref|NC_002745.2|	452450	+	U	0	0	0	1	0	SA0391
gi|29165615|ref|NC_002745.2|	452456	+	A	0	0	0	1	0	SA0391
gi|29165615|ref|NC_002745.2|	452462	+	A	1	0	0	0	0	SA0391
gi|29165615|ref|NC_002745.2|	452465	+	A	0	0	0	0	1	SA0391
gi|29165615|ref|NC_002745.2|	452474	+	A	0	0	0	0	1	SA0391
gi|29165615|ref|NC_002745.2|	452477	+	A	0	1	0	0	0	SA0391
gi|29165615|ref|NC_002745.2|	452481	+	C	1	1	0	0	0	SA0391
gi|29165615|ref|NC_002745.2|	452482	+	G	0	0	1	0	0	SA0391
gi|29165615|ref|NC_002745.2|	452483	+	A	0	1	0	0	0	SA0391
gi|29165615|ref|NC_002745.2|	452488	+	G	0	0	0	0	1	SA0391
gi|29165615|ref|NC_002745.2|	452489	+	A	0	0	0	1	1	SA0391
gi|29165615|ref|NC_002745.2|	452491	+	A	0	1	0	0	2	SA0391
gi|29165615|ref|NC_002745.2|	452493	+	C	1	0	0	0	0	SA0391
gi|29165615|ref|NC_002745.2|	452498	+	A	0	0	2	1	0	SA0391
gi|29165615|ref|NC_002745.2|	452501	+	U	0	0	0	0	1	SA0391
gi|29165615|ref|NC_002745.2|	452502	+	U	1	0	0	0	0	SA0391
gi|29165615|ref|NC_002745.2|	452503	+	G	0	1	0	0	0	SA0391
gi|29165615|ref|NC_002745.2|	452504	+	A	0	0	2	1	0	SA0391
gi|29165615|ref|NC_002745.2|	452505	+	A	0	2	1	1	1	SA0391
gi|29165615|ref|NC_002745.2|	452506	+	C	1	1	3	2	1	SA0391
gi|29165615|ref|NC_002745.2|	452509	+	G	0	1	1	0	0	SA0391
gi|29165615|ref|NC_002745.2|	452510	+	A	1	16	8	8	7	SA0391

gi|29165615|ref|NC_002745.2|	452511	+	A	0	3	1	0	0	SA0391
gi|29165615|ref|NC_002745.2|	452513	+	U	2	3	1	0	1	SA0391
gi|29165615|ref|NC_002745.2|	452514	+	C	0	1	2	1	0	SA0391
gi|29165615|ref|NC_002745.2|	452515	+	A	0	2	0	0	5	SA0391
gi|29165615|ref|NC_002745.2|	452516	+	A	2	0	0	0	0	SA0391
gi|29165615|ref|NC_002745.2|	452518	+	G	2	0	0	0	0	SA0391
gi|29165615|ref|NC_002745.2|	452519	+	C	2	0	0	0	0	SA0391
gi|29165615|ref|NC_002745.2|	452520	+	A	0	0	0	1	0	SA0391
gi|29165615|ref|NC_002745.2|	452525	+	U	1	0	0	0	0	SA0391
gi|29165615|ref|NC_002745.2|	452526	+	G	1	0	0	0	0	SA0391
gi|29165615|ref|NC_002745.2|	452527	+	A	0	2	1	3	1	SA0391
gi|29165615|ref|NC_002745.2|	452530	+	G	0	0	0	0	1	SA0391
gi|29165615|ref|NC_002745.2|	452531	+	A	1	0	0	0	0	SA0391
gi|29165615|ref|NC_002745.2|	452532	+	A	2	0	1	0	0	SA0391
gi|29165615|ref|NC_002745.2|	452533	+	C	2	0	3	0	0	SA0391
gi|29165615|ref|NC_002745.2|	452534	+	U	3	0	0	0	0	SA0391
gi|29165615|ref|NC_002745.2|	452535	+	U	1	0	0	0	0	SA0391
gi|29165615|ref|NC_002745.2|	452536	+	G	1	0	0	0	0	SA0391
gi|29165615|ref|NC_002745.2|	452537	+	G	4	0	0	0	0	SA0391
gi|29165615|ref|NC_002745.2|	452538	+	G	5	0	1	1	0	SA0391
gi|29165615|ref|NC_002745.2|	452540	+	U	5	0	0	0	0	SA0391
gi|29165615|ref|NC_002745.2|	452551	+	G	0	1	0	0	0	SA0391_SA0392
gi|29165615|ref|NC_002745.2|	452552	+	A	0	0	2	0	2	SA0391_SA0392
gi|29165615|ref|NC_002745.2|	452553	+	G	1	0	0	0	0	SA0391_SA0392
gi|29165615|ref|NC_002745.2|	452555	+	A	0	1	0	0	0	SA0391_SA0392
gi|29165615|ref|NC_002745.2|	452556	+	A	1	0	0	0	1	SA0391_SA0392
gi|29165615|ref|NC_002745.2|	452557	+	U	0	2	0	0	2	SA0392
gi|29165615|ref|NC_002745.2|	452558	+	A	0	0	1	0	0	SA0392
gi|29165615|ref|NC_002745.2|	452559	+	C	0	1	1	0	1	SA0392
gi|29165615|ref|NC_002745.2|	452560	+	A	0	1	2	4	1	SA0392
gi|29165615|ref|NC_002745.2|	452561	+	C	0	0	0	0	1	SA0392
gi|29165615|ref|NC_002745.2|	452562	+	A	0	1	0	0	0	SA0392
gi|29165615|ref|NC_002745.2|	452563	+	A	0	2	0	0	1	SA0392
gi|29165615|ref|NC_002745.2|	452564	+	A	0	0	0	0	1	SA0392
gi|29165615|ref|NC_002745.2|	452565	+	A	0	0	0	0	2	SA0392
gi|29165615|ref|NC_002745.2|	452566	+	G	0	1	0	0	0	SA0392
gi|29165615|ref|NC_002745.2|	452567	+	A	1	1	0	0	1	SA0392
gi|29165615|ref|NC_002745.2|	452569	+	A	0	0	0	0	1	SA0392
gi|29165615|ref|NC_002745.2|	452570	+	A	0	0	2	0	1	SA0392
gi|29165615|ref|NC_002745.2|	452571	+	A	1	0	0	0	2	SA0392
gi|29165615|ref|NC_002745.2|	452576	+	C	0	0	1	0	0	SA0392
gi|29165615|ref|NC_002745.2|	452589	+	U	4	0	0	0	0	SA0392
gi|29165615|ref|NC_002745.2|	452591	+	C	1	0	0	0	0	SA0392
gi|29165615|ref|NC_002745.2|	452592	+	C	1	0	0	0	0	SA0392
gi|29165615|ref|NC_002745.2|	454083	+	G	12	1	1	3	2	-
gi|29165615|ref|NC_002745.2|	457218	+	A	0	0	0	0	1	SA0396
gi|29165615|ref|NC_002745.2|	457290	+	U	0	1	0	0	0	SA0396
gi|29165615|ref|NC_002745.2|	458034	+	A	0	0	0	0	1	SA0397
gi|29165615|ref|NC_002745.2|	458106	+	U	0	1	0	0	0	SA0397
gi|29165615|ref|NC_002745.2|	458743	+	U	0	0	0	0	1	-
gi|29165615|ref|NC_002745.2|	458745	+	A	0	1	0	0	0	-
gi|29165615|ref|NC_002745.2|	458864	+	A	0	0	0	0	1	SA0398
gi|29165615|ref|NC_002745.2|	458936	+	U	0	1	0	0	0	SA0398
gi|29165615|ref|NC_002745.2|	458947	+	A	0	1	0	0	0	SA0398
gi|29165615|ref|NC_002745.2|	458965	+	A	0	1	0	0	0	SA0398
gi|29165615|ref|NC_002745.2|	459748	+	U	0	1	0	0	0	-
gi|29165615|ref|NC_002745.2|	460022	+	A	0	0	0	0	1	SA0399
gi|29165615|ref|NC_002745.2|	460516	+	A	0	0	0	0	1	SA0400
gi|29165615|ref|NC_002745.2|	460588	+	U	0	1	0	0	0	SA0400
gi|29165615|ref|NC_002745.2|	460599	+	A	0	1	0	0	0	SA0400
gi|29165615|ref|NC_002745.2|	461320	+	A	0	0	0	0	1	SA0401
gi|29165615|ref|NC_002745.2|	461392	+	U	0	1	0	0	0	SA0401
gi|29165615|ref|NC_002745.2|	461403	+	A	0	1	0	0	0	SA0401
gi|29165615|ref|NC_002745.2|	461421	+	A	0	1	0	0	0	SA0401
gi|29165615|ref|NC_002745.2|	462174	+	A	0	0	0	0	1	SA0402
gi|29165615|ref|NC_002745.2|	462256	+	A	0	0	1	0	0	SA0402
gi|29165615|ref|NC_002745.2|	462257	+	A	0	1	0	0	0	SA0402
gi|29165615|ref|NC_002745.2|	462275	+	A	0	1	0	0	0	SA0402
gi|29165615|ref|NC_002745.2|	463100	+	A	0	1	0	0	0	SA0403
gi|29165615|ref|NC_002745.2|	463821	+	A	0	0	0	0	1	SA0404
gi|29165615|ref|NC_002745.2|	463878	+	A	0	0	0	0	1	SA0404
gi|29165615|ref|NC_002745.2|	463893	+	U	0	1	0	0	0	SA0404
gi|29165615|ref|NC_002745.2|	464667	+	U	0	0	0	0	1	-
gi|29165615|ref|NC_002745.2|	464669	+	A	0	1	0	0	0	-
gi|29165615|ref|NC_002745.2|	464894	+	A	0	0	1	0	0	SA0405
gi|29165615|ref|NC_002745.2|	472239	+	G	0	0	1	0	1	SA0411
gi|29165615|ref|NC_002745.2|	484436	+	A	0	0	0	1	0	SA0422
gi|29165615|ref|NC_002745.2|	485205	+	G	0	0	1	0	0	SA0423
gi|29165615|ref|NC_002745.2|	485250	+	A	1	0	0	0	0	SA0423
gi|29165615|ref|NC_002745.2|	485406	+	G	0	0	1	0	0	SA0423
gi|29165615|ref|NC_002745.2|	485451	+	A	1	0	0	0	0	SA0423

gi|29165615|ref|NC_002745.2|	496913	+	G	1	3	0	0	0	SAtRNA03
gi|29165615|ref|NC_002745.2|	496929	+	C	1	0	0	0	0	SAtRNA03
gi|29165615|ref|NC_002745.2|	496944	+	G	0	1	0	0	0	SAtRNA03
gi|29165615|ref|NC_002745.2|	496954	+	A	0	0	0	0	1	SAtRNA03
gi|29165615|ref|NC_002745.2|	496970	+	C	0	0	0	0	2	SAtRNA03
gi|29165615|ref|NC_002745.2|	496971	+	G	0	0	0	0	1	SAtRNA03
gi|29165615|ref|NC_002745.2|	498627	+	U	0	1	0	0	0	SA0432
gi|29165615|ref|NC_002745.2|	498628	+	U	0	0	2	0	0	SA0432
gi|29165615|ref|NC_002745.2|	498632	+	G	0	0	1	1	3	SA0432
gi|29165615|ref|NC_002745.2|	498633	+	A	1	0	1	0	0	SA0432
gi|29165615|ref|NC_002745.2|	498635	+	C	0	0	0	0	2	SA0432
gi|29165615|ref|NC_002745.2|	504333	+	A	0	0	0	1	0	-
gi|29165615|ref|NC_002745.2|	504334	+	U	0	1	0	0	0	-
gi|29165615|ref|NC_002745.2|	504340	+	U	0	1	2	0	1	-
gi|29165615|ref|NC_002745.2|	506051	+	A	0	0	0	0	1	-
gi|29165615|ref|NC_002745.2|	506052	+	A	0	1	1	0	1	-
gi|29165615|ref|NC_002745.2|	506054	+	A	0	5	3	1	1	-
gi|29165615|ref|NC_002745.2|	506055	+	A	0	5	2	1	8	-
gi|29165615|ref|NC_002745.2|	506056	+	U	0	0	1	0	0	-
gi|29165615|ref|NC_002745.2|	506057	+	G	0	0	0	0	1	-
gi|29165615|ref|NC_002745.2|	506058	+	A	0	4	3	3	6	-
gi|29165615|ref|NC_002745.2|	506059	+	A	0	3	3	2	2	-
gi|29165615|ref|NC_002745.2|	506060	+	C	0	2	4	2	2	-
gi|29165615|ref|NC_002745.2|	506061	+	A	0	6	0	5	2	-
gi|29165615|ref|NC_002745.2|	506062	+	U	1	1	0	0	0	-
gi|29165615|ref|NC_002745.2|	506063	+	U	0	0	0	0	2	-
gi|29165615|ref|NC_002745.2|	506064	+	G	0	3	0	0	1	-
gi|29165615|ref|NC_002745.2|	506065	+	A	0	13	9	10	10	-
gi|29165615|ref|NC_002745.2|	506066	+	A	0	12	8	6	14	-
gi|29165615|ref|NC_002745.2|	506067	+	A	10	63	29	41	79	-
gi|29165615|ref|NC_002745.2|	506068	+	A	6395	28137	20043	16082	34402	-
gi|29165615|ref|NC_002745.2|	506069	+	C	70	1473	732	776	2100	-
gi|29165615|ref|NC_002745.2|	506070	+	U	27	357	170	239	520	-
gi|29165615|ref|NC_002745.2|	506071	+	G	25	349	192	268	521	-
gi|29165615|ref|NC_002745.2|	506072	+	A	18	331	187	337	562	-
gi|29165615|ref|NC_002745.2|	506073	+	A	9	120	68	95	204	-
gi|29165615|ref|NC_002745.2|	506074	+	U	1	16	8	15	25	-
gi|29165615|ref|NC_002745.2|	506075	+	G	15	351	143	245	509	-
gi|29165615|ref|NC_002745.2|	506076	+	A	8	107	76	100	166	-
gi|29165615|ref|NC_002745.2|	506077	+	C	4	51	34	46	58	-
gi|29165615|ref|NC_002745.2|	506078	+	A	12	117	58	86	160	-
gi|29165615|ref|NC_002745.2|	506079	+	A	30	694	296	468	885	-
gi|29165615|ref|NC_002745.2|	506080	+	U	9	120	57	95	175	-
gi|29165615|ref|NC_002745.2|	506081	+	A	15	310	134	245	433	-
gi|29165615|ref|NC_002745.2|	506082	+	U	0	2	4	4	4	-
gi|29165615|ref|NC_002745.2|	506083	+	G	1	30	8	27	40	-
gi|29165615|ref|NC_002745.2|	506084	+	U	22	253	74	209	319	-
gi|29165615|ref|NC_002745.2|	506085	+	C	6	70	35	64	81	-
gi|29165615|ref|NC_002745.2|	506086	+	A	21	383	120	291	509	-
gi|29165615|ref|NC_002745.2|	506087	+	A	7	139	61	103	195	-
gi|29165615|ref|NC_002745.2|	506088	+	C	2	19	5	14	25	-
gi|29165615|ref|NC_002745.2|	506089	+	G	2	37	22	28	83	-
gi|29165615|ref|NC_002745.2|	506090	+	U	3	82	24	42	118	-
gi|29165615|ref|NC_002745.2|	506091	+	U	0	18	7	21	34	-
gi|29165615|ref|NC_002745.2|	506092	+	A	28	438	141	321	656	-
gi|29165615|ref|NC_002745.2|	506093	+	A	13	311	93	221	465	-
gi|29165615|ref|NC_002745.2|	506094	+	U	1	6	4	15	21	-
gi|29165615|ref|NC_002745.2|	506095	+	U	0	13	3	16	24	-
gi|29165615|ref|NC_002745.2|	506096	+	C	6	94	32	91	120	-
gi|29165615|ref|NC_002745.2|	506097	+	C	16	202	87	229	387	-
gi|29165615|ref|NC_002745.2|	506098	+	A	31	557	172	494	1037	-
gi|29165615|ref|NC_002745.2|	506099	+	A	18	283	63	293	593	-
gi|29165615|ref|NC_002745.2|	506100	+	A	3	92	22	117	219	-
gi|29165615|ref|NC_002745.2|	506101	+	A	0	48	10	48	91	-
gi|29165615|ref|NC_002745.2|	506102	+	A	3	83	11	26	98	-
gi|29165615|ref|NC_002745.2|	506103	+	A	2	28	6	5	25	-
gi|29165615|ref|NC_002745.2|	506104	+	C	1	8	5	7	6	-
gi|29165615|ref|NC_002745.2|	506105	+	G	0	2	1	1	2	-
gi|29165615|ref|NC_002745.2|	506106	+	U	0	3	1	2	3	-
gi|29165615|ref|NC_002745.2|	506107	+	A	0	31	17	22	43	-
gi|29165615|ref|NC_002745.2|	506108	+	A	0	41	14	50	59	-
gi|29165615|ref|NC_002745.2|	506109	+	C	0	20	10	27	39	-
gi|29165615|ref|NC_002745.2|	506110	+	U	4	10	1	5	10	-
gi|29165615|ref|NC_002745.2|	506111	+	A	1	18	6	15	38	-
gi|29165615|ref|NC_002745.2|	506112	+	U	1	16	6	24	22	-
gi|29165615|ref|NC_002745.2|	506113	+	A	0	43	16	34	61	-
gi|29165615|ref|NC_002745.2|	506114	+	A	3	54	15	32	45	-
gi|29165615|ref|NC_002745.2|	506115	+	G	4	32	14	36	48	-
gi|29165615|ref|NC_002745.2|	506116	+	U	0	2	2	3	6	-
gi|29165615|ref|NC_002745.2|	506117	+	U	2	5	1	3	9	-
gi|29165615|ref|NC_002745.2|	506118	+	A	1	61	30	55	105	-

gi|29165615|ref|NC_002745.2|	506119	+	C	2	15	7	27	48	-
gi|29165615|ref|NC_002745.2|	506120	+	A	3	135	42	149	204	-
gi|29165615|ref|NC_002745.2|	506121	+	A	50	1002	283	991	1965	-
gi|29165615|ref|NC_002745.2|	506122	+	A	23	505	145	427	892	-
gi|29165615|ref|NC_002745.2|	506123	+	C	5	131	52	124	235	-
gi|29165615|ref|NC_002745.2|	506124	+	A	10	100	54	141	231	-
gi|29165615|ref|NC_002745.2|	506125	+	U	13	141	50	154	228	-
gi|29165615|ref|NC_002745.2|	506126	+	U	17	205	116	180	298	-
gi|29165615|ref|NC_002745.2|	506127	+	A	16	189	116	223	392	-
gi|29165615|ref|NC_002745.2|	506128	+	U	12	246	145	246	506	-
gi|29165615|ref|NC_002745.2|	506129	+	U	13	235	108	248	456	-
gi|29165615|ref|NC_002745.2|	506130	+	U	4	145	62	120	262	-
gi|29165615|ref|NC_002745.2|	506131	+	A	41	743	319	699	1579	-
gi|29165615|ref|NC_002745.2|	506132	+	G	126	1881	925	1908	4827	-
gi|29165615|ref|NC_002745.2|	506133	+	U	581	9005	4237	9137	18497	-
gi|29165615|ref|NC_002745.2|	506134	+	A	652	9769	4105	11799	22778	-
gi|29165615|ref|NC_002745.2|	506135	+	U	177	2450	1176	2819	5923	-
gi|29165615|ref|NC_002745.2|	506136	+	U	71	663	306	1025	1803	-
gi|29165615|ref|NC_002745.2|	506137	+	U	29	455	241	567	1206	-
gi|29165615|ref|NC_002745.2|	506138	+	A	150	1778	944	2409	5056	-
gi|29165615|ref|NC_002745.2|	506139	+	U	6	62	29	68	359	-
gi|29165615|ref|NC_002745.2|	506140	+	G	14	104	37	134	273	-
gi|29165615|ref|NC_002745.2|	506141	+	A	39	588	266	680	1544	-
gi|29165615|ref|NC_002745.2|	506142	+	G	72	1167	427	1302	2372	-
gi|29165615|ref|NC_002745.2|	506143	+	C	62	864	353	1152	2058	-
gi|29165615|ref|NC_002745.2|	506144	+	U	17	159	68	211	348	-
gi|29165615|ref|NC_002745.2|	506145	+	A	27	483	191	605	1085	-
gi|29165615|ref|NC_002745.2|	506146	+	A	15	194	77	253	472	-
gi|29165615|ref|NC_002745.2|	506147	+	U	1	12	2	16	26	-
gi|29165615|ref|NC_002745.2|	506148	+	C	5	77	23	138	156	-
gi|29165615|ref|NC_002745.2|	506149	+	A	27	311	122	535	723	-
gi|29165615|ref|NC_002745.2|	506150	+	A	140	1794	671	2660	3656	-
gi|29165615|ref|NC_002745.2|	506151	+	A	51	654	267	992	1393	-
gi|29165615|ref|NC_002745.2|	506152	+	C	95	1334	554	2375	3232	-
gi|29165615|ref|NC_002745.2|	506153	+	A	459	6162	2598	11041	16052	-
gi|29165615|ref|NC_002745.2|	506154	+	U	20	290	120	650	821	-
gi|29165615|ref|NC_002745.2|	506155	+	C	160	1860	812	3271	5259	-
gi|29165615|ref|NC_002745.2|	506156	+	A	105	1422	601	2312	3778	-
gi|29165615|ref|NC_002745.2|	506157	+	U	121	370	1228	630	972	-
gi|29165615|ref|NC_002745.2|	506158	+	A	895	406	3481	459	1038	-
gi|29165615|ref|NC_002745.2|	506159	+	A	19984	566	8000	630	919	-
gi|29165615|ref|NC_002745.2|	506160	+	U	128816	3410	40795	3380	3824	-
gi|29165615|ref|NC_002745.2|	506161	+	U	440756	11429	58051	10561	12323	SArRNA01
gi|29165615|ref|NC_002745.2|	506162	+	U	610	52	163	67	119	SArRNA01
gi|29165615|ref|NC_002745.2|	506163	+	U	164	14	39	27	34	SArRNA01
gi|29165615|ref|NC_002745.2|	506164	+	U	61	14	19	23	25	SArRNA01
gi|29165615|ref|NC_002745.2|	506165	+	A	51	5	12	10	8	SArRNA01
gi|29165615|ref|NC_002745.2|	506166	+	U	55	6	6	12	16	SArRNA01
gi|29165615|ref|NC_002745.2|	506167	+	G	13	6	3	4	11	SArRNA01
gi|29165615|ref|NC_002745.2|	506168	+	G	20	2	6	12	12	SArRNA01
gi|29165615|ref|NC_002745.2|	506169	+	A	13	0	4	6	6	SArRNA01
gi|29165615|ref|NC_002745.2|	506170	+	G	10	5	5	5	12	SArRNA01
gi|29165615|ref|NC_002745.2|	506171	+	A	15	3	3	3	8	SArRNA01
gi|29165615|ref|NC_002745.2|	506172	+	G	5	4	2	3	3	SArRNA01
gi|29165615|ref|NC_002745.2|	506173	+	U	37	6	3	1	15	SArRNA01
gi|29165615|ref|NC_002745.2|	506174	+	U	8	0	0	3	5	SArRNA01
gi|29165615|ref|NC_002745.2|	506175	+	U	12	2	0	1	4	SArRNA01
gi|29165615|ref|NC_002745.2|	506176	+	G	14	2	2	1	6	SArRNA01
gi|29165615|ref|NC_002745.2|	506177	+	A	5	1	0	2	3	SArRNA01
gi|29165615|ref|NC_002745.2|	506178	+	U	4	0	1	0	4	SArRNA01
gi|29165615|ref|NC_002745.2|	506179	+	C	8	0	2	0	2	SArRNA01
gi|29165615|ref|NC_002745.2|	506180	+	C	37	17	10	8	20	SArRNA01
gi|29165615|ref|NC_002745.2|	506181	+	U	10	5	4	2	8	SArRNA01
gi|29165615|ref|NC_002745.2|	506182	+	G	13	2	0	3	1	SArRNA01
gi|29165615|ref|NC_002745.2|	506183	+	G	6	0	0	0	1	SArRNA01
gi|29165615|ref|NC_002745.2|	506184	+	C	26	1	3	1	2	SArRNA01
gi|29165615|ref|NC_002745.2|	506185	+	U	9	0	2	2	4	SArRNA01
gi|29165615|ref|NC_002745.2|	506186	+	C	29	2	5	2	6	SArRNA01
gi|29165615|ref|NC_002745.2|	506187	+	A	8	3	4	0	4	SArRNA01
gi|29165615|ref|NC_002745.2|	506188	+	G	5	2	0	1	2	SArRNA01
gi|29165615|ref|NC_002745.2|	506189	+	G	5	5	2	2	4	SArRNA01
gi|29165615|ref|NC_002745.2|	506190	+	A	13	0	5	3	2	SArRNA01
gi|29165615|ref|NC_002745.2|	506191	+	U	11	3	0	4	2	SArRNA01
gi|29165615|ref|NC_002745.2|	506192	+	G	14	0	1	5	2	SArRNA01
gi|29165615|ref|NC_002745.2|	506193	+	A	11	2	2	1	0	SArRNA01
gi|29165615|ref|NC_002745.2|	506194	+	A	7	0	5	2	1	SArRNA01
gi|29165615|ref|NC_002745.2|	506195	+	C	101	4	6	0	9	SArRNA01
gi|29165615|ref|NC_002745.2|	506196	+	G	42	5	5	0	3	SArRNA01
gi|29165615|ref|NC_002745.2|	506197	+	C	13	1	0	1	2	SArRNA01
gi|29165615|ref|NC_002745.2|	506198	+	U	18	2	2	0	2	SArRNA01
gi|29165615|ref|NC_002745.2|	506199	+	G	20	0	1	1	2	SArRNA01

gi|29165615|ref|NC_002745.2|	506201	+	C	3	0	2	1	0	SArRNA01
gi|29165615|ref|NC_002745.2|	506202	+	G	0	2	0	0	1	SArRNA01
gi|29165615|ref|NC_002745.2|	506204	+	C	3	1	0	0	2	SArRNA01
gi|29165615|ref|NC_002745.2|	506205	+	G	1	0	0	0	1	SArRNA01
gi|29165615|ref|NC_002745.2|	506206	+	U	1	0	0	0	1	SArRNA01
gi|29165615|ref|NC_002745.2|	506207	+	G	1	0	1	0	1	SArRNA01
gi|29165615|ref|NC_002745.2|	506208	+	C	1	0	0	2	0	SArRNA01
gi|29165615|ref|NC_002745.2|	506209	+	C	6	9	3	3	16	SArRNA01
gi|29165615|ref|NC_002745.2|	506210	+	U	7	14	1	1	7	SArRNA01
gi|29165615|ref|NC_002745.2|	506211	+	A	11	15	6	7	6	SArRNA01
gi|29165615|ref|NC_002745.2|	506212	+	A	15	31	5	2	17	SArRNA01
gi|29165615|ref|NC_002745.2|	506213	+	U	18	34	4	7	20	SArRNA01
gi|29165615|ref|NC_002745.2|	506214	+	A	66	56	12	12	37	SArRNA01
gi|29165615|ref|NC_002745.2|	506215	+	C	229	156	58	98	147	SArRNA01
gi|29165615|ref|NC_002745.2|	506216	+	A	59	44	19	17	47	SArRNA01
gi|29165615|ref|NC_002745.2|	506217	+	U	5	2	3	3	3	SArRNA01
gi|29165615|ref|NC_002745.2|	506218	+	G	6	7	1	7	5	SArRNA01
gi|29165615|ref|NC_002745.2|	506219	+	C	57	45	17	24	33	SArRNA01
gi|29165615|ref|NC_002745.2|	506220	+	A	9	18	4	7	12	SArRNA01
gi|29165615|ref|NC_002745.2|	506221	+	A	4	8	3	1	7	SArRNA01
gi|29165615|ref|NC_002745.2|	506222	+	G	5	11	4	2	2	SArRNA01
gi|29165615|ref|NC_002745.2|	506223	+	U	28	12	4	7	19	SArRNA01
gi|29165615|ref|NC_002745.2|	506224	+	C	64	37	15	26	42	SArRNA01
gi|29165615|ref|NC_002745.2|	506225	+	G	23	21	8	9	17	SArRNA01
gi|29165615|ref|NC_002745.2|	506226	+	A	7	3	3	3	4	SArRNA01
gi|29165615|ref|NC_002745.2|	506227	+	G	6	1	3	2	2	SArRNA01
gi|29165615|ref|NC_002745.2|	506228	+	C	33	19	8	6	15	SArRNA01
gi|29165615|ref|NC_002745.2|	506229	+	G	9	4	3	3	4	SArRNA01
gi|29165615|ref|NC_002745.2|	506230	+	A	15	5	5	2	8	SArRNA01
gi|29165615|ref|NC_002745.2|	506231	+	A	13	29	7	6	17	SArRNA01
gi|29165615|ref|NC_002745.2|	506232	+	C	21	16	7	5	28	SArRNA01
gi|29165615|ref|NC_002745.2|	506233	+	G	10	10	8	4	11	SArRNA01
gi|29165615|ref|NC_002745.2|	506234	+	G	9	14	6	8	8	SArRNA01
gi|29165615|ref|NC_002745.2|	506235	+	A	13	4	3	4	8	SArRNA01
gi|29165615|ref|NC_002745.2|	506236	+	C	54	16	5	7	18	SArRNA01
gi|29165615|ref|NC_002745.2|	506237	+	G	3	1	1	0	2	SArRNA01
gi|29165615|ref|NC_002745.2|	506238	+	A	1	2	0	4	8	SArRNA01
gi|29165615|ref|NC_002745.2|	506239	+	G	2	4	1	0	6	SArRNA01
gi|29165615|ref|NC_002745.2|	506240	+	A	6	6	2	3	9	SArRNA01
gi|29165615|ref|NC_002745.2|	506241	+	A	5	1	3	3	2	SArRNA01
gi|29165615|ref|NC_002745.2|	506242	+	G	0	1	0	0	1	SArRNA01
gi|29165615|ref|NC_002745.2|	506243	+	C	15	18	3	8	9	SArRNA01
gi|29165615|ref|NC_002745.2|	506244	+	U	9	1	1	3	7	SArRNA01
gi|29165615|ref|NC_002745.2|	506245	+	U	7	2	0	1	1	SArRNA01
gi|29165615|ref|NC_002745.2|	506246	+	G	0	2	0	1	1	SArRNA01
gi|29165615|ref|NC_002745.2|	506247	+	C	2	0	2	2	8	SArRNA01
gi|29165615|ref|NC_002745.2|	506248	+	U	4	2	1	3	3	SArRNA01
gi|29165615|ref|NC_002745.2|	506249	+	U	0	1	0	0	0	SArRNA01
gi|29165615|ref|NC_002745.2|	506250	+	C	5	3	2	2	4	SArRNA01
gi|29165615|ref|NC_002745.2|	506251	+	U	0	2	2	1	4	SArRNA01
gi|29165615|ref|NC_002745.2|	506252	+	C	8	8	1	4	2	SArRNA01
gi|29165615|ref|NC_002745.2|	506253	+	U	18	6	6	3	6	SArRNA01
gi|29165615|ref|NC_002745.2|	506254	+	G	13	3	3	2	11	SArRNA01
gi|29165615|ref|NC_002745.2|	506255	+	A	15	7	6	10	10	SArRNA01
gi|29165615|ref|NC_002745.2|	506256	+	U	39	8	2	4	7	SArRNA01
gi|29165615|ref|NC_002745.2|	506257	+	G	14	3	1	4	3	SArRNA01
gi|29165615|ref|NC_002745.2|	506258	+	U	13	7	4	2	4	SArRNA01
gi|29165615|ref|NC_002745.2|	506259	+	U	27	16	11	5	13	SArRNA01
gi|29165615|ref|NC_002745.2|	506260	+	A	17	20	2	5	4	SArRNA01
gi|29165615|ref|NC_002745.2|	506261	+	G	3	2	1	5	1	SArRNA01
gi|29165615|ref|NC_002745.2|	506262	+	C	47	10	12	10	28	SArRNA01
gi|29165615|ref|NC_002745.2|	506263	+	G	20	5	4	1	11	SArRNA01
gi|29165615|ref|NC_002745.2|	506264	+	G	10	0	4	1	5	SArRNA01
gi|29165615|ref|NC_002745.2|	506265	+	C	26	4	9	14	13	SArRNA01
gi|29165615|ref|NC_002745.2|	506266	+	G	15	6	3	5	10	SArRNA01
gi|29165615|ref|NC_002745.2|	506267	+	G	23	4	6	5	8	SArRNA01
gi|29165615|ref|NC_002745.2|	506268	+	A	6	1	0	1	4	SArRNA01
gi|29165615|ref|NC_002745.2|	506269	+	C	89	46	41	42	59	SArRNA01
gi|29165615|ref|NC_002745.2|	506270	+	G	8	13	7	9	5	SArRNA01
gi|29165615|ref|NC_002745.2|	506271	+	G	2	0	1	0	1	SArRNA01
gi|29165615|ref|NC_002745.2|	506272	+	G	1	2	0	0	1	SArRNA01
gi|29165615|ref|NC_002745.2|	506273	+	U	22	8	9	8	5	SArRNA01
gi|29165615|ref|NC_002745.2|	506274	+	G	5	1	2	1	3	SArRNA01
gi|29165615|ref|NC_002745.2|	506275	+	A	0	1	1	2	0	SArRNA01
gi|29165615|ref|NC_002745.2|	506276	+	G	1	2	0	0	2	SArRNA01
gi|29165615|ref|NC_002745.2|	506277	+	U	2	1	1	3	1	SArRNA01
gi|29165615|ref|NC_002745.2|	506278	+	A	5	8	2	5	4	SArRNA01
gi|29165615|ref|NC_002745.2|	506279	+	A	12	50	6	2	24	SArRNA01
gi|29165615|ref|NC_002745.2|	506280	+	C	55	28	10	27	29	SArRNA01
gi|29165615|ref|NC_002745.2|	506281	+	A	12	6	1	4	6	SArRNA01
gi|29165615|ref|NC_002745.2|	506282	+	C	9	1	3	2	5	SArRNA01

gi|29165615|ref|NC_002745.2|	506283	+	G	4	1	2	3	4	SArRNA01
gi|29165615|ref|NC_002745.2|	506284	+	U	7	4	2	2	2	SArRNA01
gi|29165615|ref|NC_002745.2|	506285	+	G	0	0	0	0	1	SArRNA01
gi|29165615|ref|NC_002745.2|	506286	+	G	0	0	1	0	1	SArRNA01
gi|29165615|ref|NC_002745.2|	506287	+	A	2	2	0	0	6	SArRNA01
gi|29165615|ref|NC_002745.2|	506288	+	U	5	2	1	0	1	SArRNA01
gi|29165615|ref|NC_002745.2|	506289	+	A	1	2	0	0	4	SArRNA01
gi|29165615|ref|NC_002745.2|	506290	+	A	0	4	0	2	3	SArRNA01
gi|29165615|ref|NC_002745.2|	506291	+	C	5	5	2	1	6	SArRNA01
gi|29165615|ref|NC_002745.2|	506292	+	C	13	16	5	3	13	SArRNA01
gi|29165615|ref|NC_002745.2|	506293	+	U	13	7	4	4	6	SArRNA01
gi|29165615|ref|NC_002745.2|	506294	+	A	9	5	2	1	8	SArRNA01
gi|29165615|ref|NC_002745.2|	506295	+	C	13	6	4	2	5	SArRNA01
gi|29165615|ref|NC_002745.2|	506296	+	C	49	29	18	17	18	SArRNA01
gi|29165615|ref|NC_002745.2|	506297	+	U	8	12	1	3	10	SArRNA01
gi|29165615|ref|NC_002745.2|	506298	+	A	11	3	4	2	9	SArRNA01
gi|29165615|ref|NC_002745.2|	506299	+	U	14	4	2	8	15	SArRNA01
gi|29165615|ref|NC_002745.2|	506300	+	A	10	9	5	3	12	SArRNA01
gi|29165615|ref|NC_002745.2|	506301	+	A	4	6	1	2	7	SArRNA01
gi|29165615|ref|NC_002745.2|	506302	+	G	18	10	1	7	5	SArRNA01
gi|29165615|ref|NC_002745.2|	506303	+	A	1	3	0	4	3	SArRNA01
gi|29165615|ref|NC_002745.2|	506304	+	C	17	22	8	14	25	SArRNA01
gi|29165615|ref|NC_002745.2|	506305	+	U	10	9	2	7	7	SArRNA01
gi|29165615|ref|NC_002745.2|	506306	+	G	3	5	1	0	6	SArRNA01
gi|29165615|ref|NC_002745.2|	506307	+	G	3	2	0	0	3	SArRNA01
gi|29165615|ref|NC_002745.2|	506308	+	G	6	1	4	1	4	SArRNA01
gi|29165615|ref|NC_002745.2|	506309	+	A	8	1	2	2	3	SArRNA01
gi|29165615|ref|NC_002745.2|	506310	+	U	5	2	0	1	2	SArRNA01
gi|29165615|ref|NC_002745.2|	506311	+	A	6	8	1	2	7	SArRNA01
gi|29165615|ref|NC_002745.2|	506312	+	A	4	4	1	2	4	SArRNA01
gi|29165615|ref|NC_002745.2|	506313	+	C	7	3	2	1	6	SArRNA01
gi|29165615|ref|NC_002745.2|	506314	+	U	10	1	2	2	6	SArRNA01
gi|29165615|ref|NC_002745.2|	506315	+	U	12	2	5	6	7	SArRNA01
gi|29165615|ref|NC_002745.2|	506316	+	C	25	9	12	5	19	SArRNA01
gi|29165615|ref|NC_002745.2|	506317	+	G	26	14	8	6	17	SArRNA01
gi|29165615|ref|NC_002745.2|	506318	+	G	19	5	3	5	14	SArRNA01
gi|29165615|ref|NC_002745.2|	506319	+	G	9	4	5	5	13	SArRNA01
gi|29165615|ref|NC_002745.2|	506320	+	A	7	3	3	2	8	SArRNA01
gi|29165615|ref|NC_002745.2|	506321	+	A	11	6	6	2	4	SArRNA01
gi|29165615|ref|NC_002745.2|	506322	+	A	4	0	3	0	6	SArRNA01
gi|29165615|ref|NC_002745.2|	506323	+	C	2	4	0	0	0	SArRNA01
gi|29165615|ref|NC_002745.2|	506324	+	C	4	4	1	2	4	SArRNA01
gi|29165615|ref|NC_002745.2|	506325	+	G	0	3	2	2	5	SArRNA01
gi|29165615|ref|NC_002745.2|	506326	+	G	4	1	0	1	2	SArRNA01
gi|29165615|ref|NC_002745.2|	506327	+	A	2	2	2	1	2	SArRNA01
gi|29165615|ref|NC_002745.2|	506328	+	G	2	1	0	0	0	SArRNA01
gi|29165615|ref|NC_002745.2|	506329	+	C	5	10	5	3	10	SArRNA01
gi|29165615|ref|NC_002745.2|	506330	+	U	7	3	1	2	8	SArRNA01
gi|29165615|ref|NC_002745.2|	506331	+	A	4	6	2	3	4	SArRNA01
gi|29165615|ref|NC_002745.2|	506332	+	A	21	12	3	5	31	SArRNA01
gi|29165615|ref|NC_002745.2|	506333	+	U	49	32	18	15	52	SArRNA01
gi|29165615|ref|NC_002745.2|	506334	+	A	11	3	5	5	20	SArRNA01
gi|29165615|ref|NC_002745.2|	506335	+	C	29	8	10	9	14	SArRNA01
gi|29165615|ref|NC_002745.2|	506336	+	C	54	19	10	17	61	SArRNA01
gi|29165615|ref|NC_002745.2|	506337	+	G	13	12	4	5	25	SArRNA01
gi|29165615|ref|NC_002745.2|	506338	+	G	8	6	2	2	14	SArRNA01
gi|29165615|ref|NC_002745.2|	506339	+	A	10	5	3	2	23	SArRNA01
gi|29165615|ref|NC_002745.2|	506340	+	U	8	16	6	4	23	SArRNA01
gi|29165615|ref|NC_002745.2|	506341	+	A	12	3	1	4	18	SArRNA01
gi|29165615|ref|NC_002745.2|	506342	+	A	13	26	6	7	35	SArRNA01
gi|29165615|ref|NC_002745.2|	506343	+	U	0	6	8	2	8	SArRNA01
gi|29165615|ref|NC_002745.2|	506344	+	A	1	0	0	1	0	SArRNA01
gi|29165615|ref|NC_002745.2|	506345	+	U	0	1	0	0	0	SArRNA01
gi|29165615|ref|NC_002745.2|	506346	+	U	3	0	1	2	4	SArRNA01
gi|29165615|ref|NC_002745.2|	506347	+	U	2	1	0	0	0	SArRNA01
gi|29165615|ref|NC_002745.2|	506348	+	U	4	3	0	0	4	SArRNA01
gi|29165615|ref|NC_002745.2|	506349	+	G	1	0	0	1	1	SArRNA01
gi|29165615|ref|NC_002745.2|	506350	+	A	7	6	1	2	5	SArRNA01
gi|29165615|ref|NC_002745.2|	506351	+	A	9	13	8	13	14	SArRNA01
gi|29165615|ref|NC_002745.2|	506352	+	C	6	4	2	4	7	SArRNA01
gi|29165615|ref|NC_002745.2|	506353	+	C	12	6	2	5	15	SArRNA01
gi|29165615|ref|NC_002745.2|	506354	+	G	12	9	7	2	19	SArRNA01
gi|29165615|ref|NC_002745.2|	506355	+	C	40	20	17	19	54	SArRNA01
gi|29165615|ref|NC_002745.2|	506356	+	A	83	47	17	22	58	SArRNA01
gi|29165615|ref|NC_002745.2|	506357	+	U	26	18	10	16	25	SArRNA01
gi|29165615|ref|NC_002745.2|	506358	+	G	20	10	2	2	26	SArRNA01
gi|29165615|ref|NC_002745.2|	506359	+	G	5	5	3	2	15	SArRNA01
gi|29165615|ref|NC_002745.2|	506360	+	U	4	3	2	3	16	SArRNA01
gi|29165615|ref|NC_002745.2|	506361	+	U	2	6	0	2	5	SArRNA01
gi|29165615|ref|NC_002745.2|	506362	+	C	12	14	3	7	16	SArRNA01
gi|29165615|ref|NC_002745.2|	506363	+	A	15	12	11	13	13	SArRNA01

gi|29165615|ref|NC_002745.2|	506364	+	A	18	21	8	8	22 SArRNA01
gi|29165615|ref|NC_002745.2|	506365	+	A	26	16	12	10	31 SArRNA01
gi|29165615|ref|NC_002745.2|	506366	+	A	20	17	12	13	35 SArRNA01
gi|29165615|ref|NC_002745.2|	506367	+	G	76	75	26	49	76 SArRNA01
gi|29165615|ref|NC_002745.2|	506368	+	U	6	10	4	9	36 SArRNA01
gi|29165615|ref|NC_002745.2|	506369	+	G	21	18	15	12	23 SArRNA01
gi|29165615|ref|NC_002745.2|	506370	+	A	24	16	5	13	25 SArRNA01
gi|29165615|ref|NC_002745.2|	506371	+	A	227	105	66	139	324 SArRNA01
gi|29165615|ref|NC_002745.2|	506372	+	A	29	15	15	14	27 SArRNA01
gi|29165615|ref|NC_002745.2|	506373	+	G	14	17	19	15	19 SArRNA01
gi|29165615|ref|NC_002745.2|	506374	+	A	2	0	0	0	4 SArRNA01
gi|29165615|ref|NC_002745.2|	506375	+	C	10	11	5	2	7 SArRNA01
gi|29165615|ref|NC_002745.2|	506376	+	G	1	13	2	1	2 SArRNA01
gi|29165615|ref|NC_002745.2|	506377	+	G	2	2	3	3	5 SArRNA01
gi|29165615|ref|NC_002745.2|	506378	+	U	40	11	9	6	15 SArRNA01
gi|29165615|ref|NC_002745.2|	506379	+	C	103	101	20	39	60 SArRNA01
gi|29165615|ref|NC_002745.2|	506380	+	U	22	15	17	10	26 SArRNA01
gi|29165615|ref|NC_002745.2|	506381	+	U	8	6	2	0	14 SArRNA01
gi|29165615|ref|NC_002745.2|	506382	+	G	3	5	2	6	2 SArRNA01
gi|29165615|ref|NC_002745.2|	506383	+	C	16	22	5	4	10 SArRNA01
gi|29165615|ref|NC_002745.2|	506384	+	U	10	5	2	5	10 SArRNA01
gi|29165615|ref|NC_002745.2|	506385	+	G	1	1	1	1	3 SArRNA01
gi|29165615|ref|NC_002745.2|	506386	+	U	3	4	2	2	5 SArRNA01
gi|29165615|ref|NC_002745.2|	506387	+	C	40	43	23	15	25 SArRNA01
gi|29165615|ref|NC_002745.2|	506388	+	A	12	13	9	2	15 SArRNA01
gi|29165615|ref|NC_002745.2|	506389	+	C	14	3	2	4	22 SArRNA01
gi|29165615|ref|NC_002745.2|	506390	+	U	15	8	5	4	13 SArRNA01
gi|29165615|ref|NC_002745.2|	506391	+	U	17	9	3	8	15 SArRNA01
gi|29165615|ref|NC_002745.2|	506392	+	A	16	6	10	2	18 SArRNA01
gi|29165615|ref|NC_002745.2|	506393	+	U	23	17	10	8	15 SArRNA01
gi|29165615|ref|NC_002745.2|	506394	+	A	20	13	4	2	11 SArRNA01
gi|29165615|ref|NC_002745.2|	506395	+	G	20	13	4	7	18 SArRNA01
gi|29165615|ref|NC_002745.2|	506396	+	A	13	23	8	9	16 SArRNA01
gi|29165615|ref|NC_002745.2|	506397	+	U	10	6	3	14	11 SArRNA01
gi|29165615|ref|NC_002745.2|	506398	+	G	2	1	0	6	2 SArRNA01
gi|29165615|ref|NC_002745.2|	506399	+	G	8	15	2	5	5 SArRNA01
gi|29165615|ref|NC_002745.2|	506400	+	A	5	11	4	3	6 SArRNA01
gi|29165615|ref|NC_002745.2|	506401	+	U	7	5	1	1	4 SArRNA01
gi|29165615|ref|NC_002745.2|	506402	+	C	20	8	0	2	12 SArRNA01
gi|29165615|ref|NC_002745.2|	506403	+	C	42	27	12	7	32 SArRNA01
gi|29165615|ref|NC_002745.2|	506404	+	G	10	8	2	9	9 SArRNA01
gi|29165615|ref|NC_002745.2|	506405	+	C	34	11	3	3	13 SArRNA01
gi|29165615|ref|NC_002745.2|	506406	+	G	3	3	0	0	2 SArRNA01
gi|29165615|ref|NC_002745.2|	506407	+	C	11	18	9	5	9 SArRNA01
gi|29165615|ref|NC_002745.2|	506408	+	U	6	11	3	3	6 SArRNA01
gi|29165615|ref|NC_002745.2|	506409	+	G	7	3	0	0	0 SArRNA01
gi|29165615|ref|NC_002745.2|	506410	+	C	34	19	7	12	9 SArRNA01
gi|29165615|ref|NC_002745.2|	506411	+	A	17	27	8	15	18 SArRNA01
gi|29165615|ref|NC_002745.2|	506412	+	U	33	18	14	10	15 SArRNA01
gi|29165615|ref|NC_002745.2|	506413	+	U	24	8	7	17	20 SArRNA01
gi|29165615|ref|NC_002745.2|	506414	+	A	8	3	7	6	8 SArRNA01
gi|29165615|ref|NC_002745.2|	506415	+	G	1	1	3	0	3 SArRNA01
gi|29165615|ref|NC_002745.2|	506416	+	C	108	116	34	56	51 SArRNA01
gi|29165615|ref|NC_002745.2|	506417	+	U	39	14	11	8	19 SArRNA01
gi|29165615|ref|NC_002745.2|	506418	+	A	18	13	4	4	8 SArRNA01
gi|29165615|ref|NC_002745.2|	506419	+	G	11	3	6	4	3 SArRNA01
gi|29165615|ref|NC_002745.2|	506420	+	U	2	5	0	2	1 SArRNA01
gi|29165615|ref|NC_002745.2|	506421	+	U	3	3	0	5	0 SArRNA01
gi|29165615|ref|NC_002745.2|	506422	+	G	0	3	0	2	0 SArRNA01
gi|29165615|ref|NC_002745.2|	506423	+	G	20	6	4	4	14 SArRNA01
gi|29165615|ref|NC_002745.2|	506424	+	U	18	10	6	6	19 SArRNA01
gi|29165615|ref|NC_002745.2|	506425	+	A	18	12	8	8	20 SArRNA01
gi|29165615|ref|NC_002745.2|	506426	+	A	16	15	5	4	8 SArRNA01
gi|29165615|ref|NC_002745.2|	506427	+	G	0	0	1	4	3 SArRNA01
gi|29165615|ref|NC_002745.2|	506428	+	G	11	6	2	1	10 SArRNA01
gi|29165615|ref|NC_002745.2|	506429	+	U	16	6	6	4	13 SArRNA01
gi|29165615|ref|NC_002745.2|	506430	+	A	18	11	6	4	11 SArRNA01
gi|29165615|ref|NC_002745.2|	506431	+	A	47	14	6	5	7 SArRNA01
gi|29165615|ref|NC_002745.2|	506432	+	C	94	98	31	57	42 SArRNA01
gi|29165615|ref|NC_002745.2|	506433	+	G	18	21	5	6	12 SArRNA01
gi|29165615|ref|NC_002745.2|	506434	+	G	3	2	0	0	3 SArRNA01
gi|29165615|ref|NC_002745.2|	506435	+	C	33	17	15	5	20 SArRNA01
gi|29165615|ref|NC_002745.2|	506436	+	U	27	28	10	12	18 SArRNA01
gi|29165615|ref|NC_002745.2|	506437	+	U	60	50	31	39	34 SArRNA01
gi|29165615|ref|NC_002745.2|	506438	+	A	8	5	5	8	12 SArRNA01
gi|29165615|ref|NC_002745.2|	506439	+	C	19	18	7	9	10 SArRNA01
gi|29165615|ref|NC_002745.2|	506440	+	C	148	80	49	51	91 SArRNA01
gi|29165615|ref|NC_002745.2|	506441	+	A	95	69	30	46	51 SArRNA01
gi|29165615|ref|NC_002745.2|	506442	+	A	95	46	31	26	33 SArRNA01
gi|29165615|ref|NC_002745.2|	506443	+	G	45	10	4	6	19 SArRNA01
gi|29165615|ref|NC_002745.2|	506444	+	G	27	6	5	10	23 SArRNA01

gi|29165615|ref|NC_002745.2|	506445 +	C	78	35	24	16	36	SArRNA01
gi|29165615|ref|NC_002745.2|	506446 +	A	46	24	17	14	39	SArRNA01
gi|29165615|ref|NC_002745.2|	506447 +	A	57	23	12	26	44	SArRNA01
gi|29165615|ref|NC_002745.2|	506448 +	C	125	111	39	43	98	SArRNA01
gi|29165615|ref|NC_002745.2|	506449 +	G	162	159	80	79	93	SArRNA01
gi|29165615|ref|NC_002745.2|	506450 +	A	52	31	16	16	34	SArRNA01
gi|29165615|ref|NC_002745.2|	506451 +	U	35	17	10	8	30	SArRNA01
gi|29165615|ref|NC_002745.2|	506452 +	G	16	5	2	5	8	SArRNA01
gi|29165615|ref|NC_002745.2|	506453 +	C	76	28	13	16	11	SArRNA01
gi|29165615|ref|NC_002745.2|	506454 +	A	33	32	8	8	19	SArRNA01
gi|29165615|ref|NC_002745.2|	506455 +	U	49	50	28	52	39	SArRNA01
gi|29165615|ref|NC_002745.2|	506456 +	A	13	13	1	3	5	SArRNA01
gi|29165615|ref|NC_002745.2|	506457 +	G	12	8	1	0	4	SArRNA01
gi|29165615|ref|NC_002745.2|	506458 +	C	10	8	3	2	4	SArRNA01
gi|29165615|ref|NC_002745.2|	506459 +	C	35	60	18	14	31	SArRNA01
gi|29165615|ref|NC_002745.2|	506460 +	G	26	34	5	10	22	SArRNA01
gi|29165615|ref|NC_002745.2|	506461 +	A	10	9	5	5	11	SArRNA01
gi|29165615|ref|NC_002745.2|	506462 +	C	47	65	19	51	46	SArRNA01
gi|29165615|ref|NC_002745.2|	506463 +	C	93	53	28	39	72	SArRNA01
gi|29165615|ref|NC_002745.2|	506464 +	U	71	51	33	45	58	SArRNA01
gi|29165615|ref|NC_002745.2|	506465 +	G	56	36	22	15	44	SArRNA01
gi|29165615|ref|NC_002745.2|	506466 +	A	91	25	15	26	68	SArRNA01
gi|29165615|ref|NC_002745.2|	506467 +	G	53	28	13	16	32	SArRNA01
gi|29165615|ref|NC_002745.2|	506468 +	A	29	15	16	10	29	SArRNA01
gi|29165615|ref|NC_002745.2|	506469 +	G	19	14	8	7	16	SArRNA01
gi|29165615|ref|NC_002745.2|	506470 +	G	16	12	2	5	11	SArRNA01
gi|29165615|ref|NC_002745.2|	506471 +	G	19	7	2	4	22	SArRNA01
gi|29165615|ref|NC_002745.2|	506472 +	U	34	11	8	13	17	SArRNA01
gi|29165615|ref|NC_002745.2|	506473 +	G	22	6	2	11	19	SArRNA01
gi|29165615|ref|NC_002745.2|	506474 +	A	49	22	13	16	21	SArRNA01
gi|29165615|ref|NC_002745.2|	506475 +	U	71	31	10	9	34	SArRNA01
gi|29165615|ref|NC_002745.2|	506476 +	C	95	57	34	34	59	SArRNA01
gi|29165615|ref|NC_002745.2|	506477 +	G	24	21	10	12	6	SArRNA01
gi|29165615|ref|NC_002745.2|	506478 +	G	6	2	1	1	6	SArRNA01
gi|29165615|ref|NC_002745.2|	506479 +	C	29	22	7	10	16	SArRNA01
gi|29165615|ref|NC_002745.2|	506480 +	C	71	61	15	19	23	SArRNA01
gi|29165615|ref|NC_002745.2|	506481 +	A	16	26	10	9	15	SArRNA01
gi|29165615|ref|NC_002745.2|	506482 +	C	145	96	31	49	57	SArRNA01
gi|29165615|ref|NC_002745.2|	506483 +	A	18	35	7	10	19	SArRNA01
gi|29165615|ref|NC_002745.2|	506484 +	C	57	35	19	18	24	SArRNA01
gi|29165615|ref|NC_002745.2|	506485 +	U	54	19	9	15	39	SArRNA01
gi|29165615|ref|NC_002745.2|	506486 +	G	7	9	8	11	29	SArRNA01
gi|29165615|ref|NC_002745.2|	506487 +	G	23	18	6	8	24	SArRNA01
gi|29165615|ref|NC_002745.2|	506488 +	A	65	65	15	25	33	SArRNA01
gi|29165615|ref|NC_002745.2|	506489 +	A	29	18	12	12	9	SArRNA01
gi|29165615|ref|NC_002745.2|	506490 +	C	63	52	23	26	35	SArRNA01
gi|29165615|ref|NC_002745.2|	506491 +	U	50	39	18	31	21	SArRNA01
gi|29165615|ref|NC_002745.2|	506492 +	G	26	17	12	13	20	SArRNA01
gi|29165615|ref|NC_002745.2|	506493 +	A	76	39	26	39	38	SArRNA01
gi|29165615|ref|NC_002745.2|	506494 +	G	55	43	19	32	40	SArRNA01
gi|29165615|ref|NC_002745.2|	506495 +	A	68	24	10	13	27	SArRNA01
gi|29165615|ref|NC_002745.2|	506496 +	C	199	144	42	75	110	SArRNA01
gi|29165615|ref|NC_002745.2|	506497 +	A	26	28	18	16	29	SArRNA01
gi|29165615|ref|NC_002745.2|	506498 +	C	67	44	19	14	33	SArRNA01
gi|29165615|ref|NC_002745.2|	506499 +	G	16	20	15	13	24	SArRNA01
gi|29165615|ref|NC_002745.2|	506500 +	G	24	6	6	16	27	SArRNA01
gi|29165615|ref|NC_002745.2|	506501 +	U	22	15	7	12	20	SArRNA01
gi|29165615|ref|NC_002745.2|	506502 +	C	24	20	11	4	20	SArRNA01
gi|29165615|ref|NC_002745.2|	506503 +	C	129	50	16	21	38	SArRNA01
gi|29165615|ref|NC_002745.2|	506504 +	A	26	14	10	16	17	SArRNA01
gi|29165615|ref|NC_002745.2|	506505 +	G	27	24	9	3	12	SArRNA01
gi|29165615|ref|NC_002745.2|	506506 +	A	17	12	4	11	13	SArRNA01
gi|29165615|ref|NC_002745.2|	506507 +	C	95	56	20	32	37	SArRNA01
gi|29165615|ref|NC_002745.2|	506508 +	U	134	72	21	26	53	SArRNA01
gi|29165615|ref|NC_002745.2|	506509 +	C	76	34	9	10	32	SArRNA01
gi|29165615|ref|NC_002745.2|	506510 +	C	163	71	46	50	116	SArRNA01
gi|29165615|ref|NC_002745.2|	506511 +	U	140	88	39	44	100	SArRNA01
gi|29165615|ref|NC_002745.2|	506512 +	A	50	41	20	31	56	SArRNA01
gi|29165615|ref|NC_002745.2|	506513 +	C	281	173	90	141	129	SArRNA01
gi|29165615|ref|NC_002745.2|	506514 +	G	49	46	13	21	35	SArRNA01
gi|29165615|ref|NC_002745.2|	506515 +	G	68	20	9	28	43	SArRNA01
gi|29165615|ref|NC_002745.2|	506516 +	G	143	89	38	81	95	SArRNA01
gi|29165615|ref|NC_002745.2|	506517 +	A	414	178	120	145	174	SArRNA01
gi|29165615|ref|NC_002745.2|	506518 +	G	62	34	22	29	45	SArRNA01
gi|29165615|ref|NC_002745.2|	506519 +	G	72	52	19	26	45	SArRNA01
gi|29165615|ref|NC_002745.2|	506520 +	C	241	82	50	60	97	SArRNA01
gi|29165615|ref|NC_002745.2|	506521 +	A	187	114	59	66	108	SArRNA01
gi|29165615|ref|NC_002745.2|	506522 +	G	110	63	30	40	71	SArRNA01
gi|29165615|ref|NC_002745.2|	506523 +	C	193	122	46	76	129	SArRNA01
gi|29165615|ref|NC_002745.2|	506524 +	A	194	120	53	91	176	SArRNA01
gi|29165615|ref|NC_002745.2|	506525 +	G	169	103	51	99	185	SArRNA01

gi|29165615|ref|NC_002745.2|	506526 +	U	106	106	40	59	85	SArRNA01
gi|29165615|ref|NC_002745.2|	506527 +	A	77	61	32	35	60	SArRNA01
gi|29165615|ref|NC_002745.2|	506528 +	G	43	29	19	27	44	SArRNA01
gi|29165615|ref|NC_002745.2|	506529 +	G	59	27	19	16	32	SArRNA01
gi|29165615|ref|NC_002745.2|	506530 +	G	37	16	12	17	33	SArRNA01
gi|29165615|ref|NC_002745.2|	506531 +	A	37	26	18	12	25	SArRNA01
gi|29165615|ref|NC_002745.2|	506532 +	A	125	419	50	54	177	SArRNA01
gi|29165615|ref|NC_002745.2|	506533 +	U	75	57	18	44	56	SArRNA01
gi|29165615|ref|NC_002745.2|	506534 +	C	1125	685	259	374	472	SArRNA01
gi|29165615|ref|NC_002745.2|	506535 +	U	189	144	74	109	137	SArRNA01
gi|29165615|ref|NC_002745.2|	506536 +	U	34	16	13	17	25	SArRNA01
gi|29165615|ref|NC_002745.2|	506537 +	C	80	29	14	39	59	SArRNA01
gi|29165615|ref|NC_002745.2|	506538 +	C	182	67	35	53	111	SArRNA01
gi|29165615|ref|NC_002745.2|	506539 +	G	134	45	35	45	90	SArRNA01
gi|29165615|ref|NC_002745.2|	506540 +	C	218	121	48	68	114	SArRNA01
gi|29165615|ref|NC_002745.2|	506541 +	A	139	101	40	66	81	SArRNA01
gi|29165615|ref|NC_002745.2|	506542 +	A	186	241	52	53	210	SArRNA01
gi|29165615|ref|NC_002745.2|	506543 +	U	351	182	56	126	157	SArRNA01
gi|29165615|ref|NC_002745.2|	506544 +	G	147	76	40	71	157	SArRNA01
gi|29165615|ref|NC_002745.2|	506545 +	G	114	66	26	43	102	SArRNA01
gi|29165615|ref|NC_002745.2|	506546 +	G	27	20	4	14	41	SArRNA01
gi|29165615|ref|NC_002745.2|	506547 +	C	66	30	20	26	52	SArRNA01
gi|29165615|ref|NC_002745.2|	506548 +	G	43	31	14	25	58	SArRNA01
gi|29165615|ref|NC_002745.2|	506549 +	A	67	22	17	18	45	SArRNA01
gi|29165615|ref|NC_002745.2|	506550 +	A	28	16	10	18	36	SArRNA01
gi|29165615|ref|NC_002745.2|	506551 +	A	22	9	12	6	16	SArRNA01
gi|29165615|ref|NC_002745.2|	506552 +	G	1	0	0	4	2	SArRNA01
gi|29165615|ref|NC_002745.2|	506553 +	C	35	9	6	17	16	SArRNA01
gi|29165615|ref|NC_002745.2|	506554 +	C	46	21	14	17	31	SArRNA01
gi|29165615|ref|NC_002745.2|	506555 +	U	60	31	18	15	37	SArRNA01
gi|29165615|ref|NC_002745.2|	506556 +	G	46	45	18	23	45	SArRNA01
gi|29165615|ref|NC_002745.2|	506557 +	A	69	23	25	26	50	SArRNA01
gi|29165615|ref|NC_002745.2|	506558 +	C	106	68	28	25	92	SArRNA01
gi|29165615|ref|NC_002745.2|	506559 +	G	90	63	29	38	76	SArRNA01
gi|29165615|ref|NC_002745.2|	506560 +	G	47	36	18	26	37	SArRNA01
gi|29165615|ref|NC_002745.2|	506561 +	A	45	39	13	20	40	SArRNA01
gi|29165615|ref|NC_002745.2|	506562 +	G	33	27	17	22	38	SArRNA01
gi|29165615|ref|NC_002745.2|	506563 +	C	226	107	51	72	121	SArRNA01
gi|29165615|ref|NC_002745.2|	506564 +	A	77	64	31	30	80	SArRNA01
gi|29165615|ref|NC_002745.2|	506565 +	A	39	25	12	27	46	SArRNA01
gi|29165615|ref|NC_002745.2|	506566 +	C	87	53	27	43	83	SArRNA01
gi|29165615|ref|NC_002745.2|	506567 +	G	27	7	8	11	19	SArRNA01
gi|29165615|ref|NC_002745.2|	506568 +	C	50	29	19	38	52	SArRNA01
gi|29165615|ref|NC_002745.2|	506569 +	C	90	58	30	27	78	SArRNA01
gi|29165615|ref|NC_002745.2|	506570 +	G	12	18	8	6	13	SArRNA01
gi|29165615|ref|NC_002745.2|	506571 +	C	34	29	18	24	35	SArRNA01
gi|29165615|ref|NC_002745.2|	506572 +	G	22	21	15	14	38	SArRNA01
gi|29165615|ref|NC_002745.2|	506573 +	U	59	50	17	22	54	SArRNA01
gi|29165615|ref|NC_002745.2|	506574 +	G	63	53	41	39	48	SArRNA01
gi|29165615|ref|NC_002745.2|	506575 +	A	78	65	40	51	84	SArRNA01
gi|29165615|ref|NC_002745.2|	506576 +	G	131	119	58	72	112	SArRNA01
gi|29165615|ref|NC_002745.2|	506577 +	U	94	67	48	45	115	SArRNA01
gi|29165615|ref|NC_002745.2|	506578 +	G	100	78	51	66	84	SArRNA01
gi|29165615|ref|NC_002745.2|	506579 +	A	104	71	44	50	93	SArRNA01
gi|29165615|ref|NC_002745.2|	506580 +	U	88	58	31	48	78	SArRNA01
gi|29165615|ref|NC_002745.2|	506581 +	G	143	79	41	52	67	SArRNA01
gi|29165615|ref|NC_002745.2|	506582 +	A	116	76	33	61	101	SArRNA01
gi|29165615|ref|NC_002745.2|	506583 +	A	153	111	61	88	114	SArRNA01
gi|29165615|ref|NC_002745.2|	506584 +	G	74	58	56	127	81	SArRNA01
gi|29165615|ref|NC_002745.2|	506585 +	G	237	86	56	56	159	SArRNA01
gi|29165615|ref|NC_002745.2|	506586 +	U	117	60	25	32	76	SArRNA01
gi|29165615|ref|NC_002745.2|	506587 +	C	77	27	24	13	37	SArRNA01
gi|29165615|ref|NC_002745.2|	506588 +	U	16	7	6	10	29	SArRNA01
gi|29165615|ref|NC_002745.2|	506589 +	U	15	12	13	11	9	SArRNA01
gi|29165615|ref|NC_002745.2|	506590 +	C	189	108	69	80	107	SArRNA01
gi|29165615|ref|NC_002745.2|	506591 +	G	56	40	23	26	39	SArRNA01
gi|29165615|ref|NC_002745.2|	506592 +	G	100	64	50	53	105	SArRNA01
gi|29165615|ref|NC_002745.2|	506593 +	A	123	74	52	53	158	SArRNA01
gi|29165615|ref|NC_002745.2|	506594 +	U	89	58	45	51	99	SArRNA01
gi|29165615|ref|NC_002745.2|	506595 +	C	55	50	20	29	76	SArRNA01
gi|29165615|ref|NC_002745.2|	506596 +	G	49	47	39	43	88	SArRNA01
gi|29165615|ref|NC_002745.2|	506597 +	U	87	78	79	56	132	SArRNA01
gi|29165615|ref|NC_002745.2|	506598 +	A	29	19	13	13	28	SArRNA01
gi|29165615|ref|NC_002745.2|	506599 +	A	23	18	8	24	35	SArRNA01
gi|29165615|ref|NC_002745.2|	506600 +	A	32	44	12	28	46	SArRNA01
gi|29165615|ref|NC_002745.2|	506601 +	A	112	955	115	29	409	SArRNA01
gi|29165615|ref|NC_002745.2|	506602 +	C	402	305	150	152	272	SArRNA01
gi|29165615|ref|NC_002745.2|	506603 +	U	91	67	25	35	61	SArRNA01
gi|29165615|ref|NC_002745.2|	506604 +	C	55	43	36	22	46	SArRNA01
gi|29165615|ref|NC_002745.2|	506605 +	U	46	22	19	15	40	SArRNA01
gi|29165615|ref|NC_002745.2|	506606 +	G	16	16	7	17	19	SArRNA01

gi|29165615|ref|NC_002745.2|	506607 +	U	25	29	12	16	55	SArRNA01
gi|29165615|ref|NC_002745.2|	506608 +	U	25	36	22	18	48	SArRNA01
gi|29165615|ref|NC_002745.2|	506609 +	A	15	11	6	6	15	SArRNA01
gi|29165615|ref|NC_002745.2|	506610 +	U	46	33	19	44	76	SArRNA01
gi|29165615|ref|NC_002745.2|	506611 +	U	140	101	42	55	121	SArRNA01
gi|29165615|ref|NC_002745.2|	506612 +	A	469	260	191	250	272	SArRNA01
gi|29165615|ref|NC_002745.2|	506613 +	G	18	33	13	10	21	SArRNA01
gi|29165615|ref|NC_002745.2|	506614 +	G	15	18	11	5	13	SArRNA01
gi|29165615|ref|NC_002745.2|	506615 +	G	111	83	47	52	84	SArRNA01
gi|29165615|ref|NC_002745.2|	506616 +	A	32	28	21	15	52	SArRNA01
gi|29165615|ref|NC_002745.2|	506617 +	A	30	30	14	17	40	SArRNA01
gi|29165615|ref|NC_002745.2|	506618 +	G	57	70	21	32	67	SArRNA01
gi|29165615|ref|NC_002745.2|	506619 +	A	55	39	24	31	72	SArRNA01
gi|29165615|ref|NC_002745.2|	506620 +	A	45	44	17	18	50	SArRNA01
gi|29165615|ref|NC_002745.2|	506621 +	C	260	250	65	131	213	SArRNA01
gi|29165615|ref|NC_002745.2|	506622 +	A	103	133	41	56	135	SArRNA01
gi|29165615|ref|NC_002745.2|	506623 +	U	169	173	71	88	154	SArRNA01
gi|29165615|ref|NC_002745.2|	506624 +	A	100	120	34	61	95	SArRNA01
gi|29165615|ref|NC_002745.2|	506625 +	U	91	64	50	46	79	SArRNA01
gi|29165615|ref|NC_002745.2|	506626 +	G	12	19	13	15	24	SArRNA01
gi|29165615|ref|NC_002745.2|	506627 +	U	75	44	28	40	82	SArRNA01
gi|29165615|ref|NC_002745.2|	506628 +	G	57	39	27	49	80	SArRNA01
gi|29165615|ref|NC_002745.2|	506629 +	U	80	57	31	34	115	SArRNA01
gi|29165615|ref|NC_002745.2|	506630 +	A	30	22	14	26	51	SArRNA01
gi|29165615|ref|NC_002745.2|	506631 +	A	42	26	24	26	59	SArRNA01
gi|29165615|ref|NC_002745.2|	506632 +	G	38	39	25	24	53	SArRNA01
gi|29165615|ref|NC_002745.2|	506633 +	U	80	44	43	41	84	SArRNA01
gi|29165615|ref|NC_002745.2|	506634 +	A	41	61	21	24	63	SArRNA01
gi|29165615|ref|NC_002745.2|	506635 +	A	50	51	28	21	70	SArRNA01
gi|29165615|ref|NC_002745.2|	506636 +	C	167	163	24	36	213	SArRNA01
gi|29165615|ref|NC_002745.2|	506637 +	U	88	81	28	38	117	SArRNA01
gi|29165615|ref|NC_002745.2|	506638 +	G	31	38	7	8	36	SArRNA01
gi|29165615|ref|NC_002745.2|	506639 +	U	18	15	6	6	21	SArRNA01
gi|29165615|ref|NC_002745.2|	506640 +	G	11	7	5	2	13	SArRNA01
gi|29165615|ref|NC_002745.2|	506641 +	C	94	53	39	39	70	SArRNA01
gi|29165615|ref|NC_002745.2|	506642 +	A	53	37	25	22	59	SArRNA01
gi|29165615|ref|NC_002745.2|	506643 +	C	224	161	102	98	176	SArRNA01
gi|29165615|ref|NC_002745.2|	506644 +	A	85	69	46	46	120	SArRNA01
gi|29165615|ref|NC_002745.2|	506645 +	U	75	28	25	38	56	SArRNA01
gi|29165615|ref|NC_002745.2|	506646 +	C	117	97	36	49	98	SArRNA01
gi|29165615|ref|NC_002745.2|	506647 +	U	85	62	37	49	121	SArRNA01
gi|29165615|ref|NC_002745.2|	506648 +	U	73	43	14	25	58	SArRNA01
gi|29165615|ref|NC_002745.2|	506649 +	G	74	62	28	50	72	SArRNA01
gi|29165615|ref|NC_002745.2|	506650 +	A	43	34	17	24	49	SArRNA01
gi|29165615|ref|NC_002745.2|	506651 +	C	137	85	44	56	102	SArRNA01
gi|29165615|ref|NC_002745.2|	506652 +	G	24	44	18	19	40	SArRNA01
gi|29165615|ref|NC_002745.2|	506653 +	G	10	18	7	4	19	SArRNA01
gi|29165615|ref|NC_002745.2|	506654 +	U	28	42	17	15	56	SArRNA01
gi|29165615|ref|NC_002745.2|	506655 +	A	16	19	8	5	35	SArRNA01
gi|29165615|ref|NC_002745.2|	506656 +	C	45	46	27	19	72	SArRNA01
gi|29165615|ref|NC_002745.2|	506657 +	C	386	342	203	208	362	SArRNA01
gi|29165615|ref|NC_002745.2|	506658 +	U	73	52	35	36	87	SArRNA01
gi|29165615|ref|NC_002745.2|	506659 +	A	30	20	17	20	38	SArRNA01
gi|29165615|ref|NC_002745.2|	506660 +	A	83	96	42	46	137	SArRNA01
gi|29165615|ref|NC_002745.2|	506661 +	U	108	45	30	40	91	SArRNA01
gi|29165615|ref|NC_002745.2|	506662 +	C	114	63	42	58	121	SArRNA01
gi|29165615|ref|NC_002745.2|	506663 +	A	31	37	26	16	68	SArRNA01
gi|29165615|ref|NC_002745.2|	506664 +	G	11	5	10	4	18	SArRNA01
gi|29165615|ref|NC_002745.2|	506665 +	A	24	12	14	14	31	SArRNA01
gi|29165615|ref|NC_002745.2|	506666 +	A	17	8	4	13	23	SArRNA01
gi|29165615|ref|NC_002745.2|	506667 +	A	8	7	0	6	14	SArRNA01
gi|29165615|ref|NC_002745.2|	506668 +	G	1	0	3	1	5	SArRNA01
gi|29165615|ref|NC_002745.2|	506669 +	C	23	15	10	11	42	SArRNA01
gi|29165615|ref|NC_002745.2|	506670 +	C	81	51	32	38	96	SArRNA01
gi|29165615|ref|NC_002745.2|	506671 +	A	29	10	16	14	51	SArRNA01
gi|29165615|ref|NC_002745.2|	506672 +	C	89	62	45	55	128	SArRNA01
gi|29165615|ref|NC_002745.2|	506673 +	G	11	15	8	8	28	SArRNA01
gi|29165615|ref|NC_002745.2|	506674 +	G	6	3	4	1	16	SArRNA01
gi|29165615|ref|NC_002745.2|	506675 +	C	39	18	17	16	91	SArRNA01
gi|29165615|ref|NC_002745.2|	506676 +	U	23	15	14	15	70	SArRNA01
gi|29165615|ref|NC_002745.2|	506677 +	A	21	13	6	6	30	SArRNA01
gi|29165615|ref|NC_002745.2|	506678 +	A	4	5	3	2	7	SArRNA01
gi|29165615|ref|NC_002745.2|	506679 +	C	6	5	3	8	21	SArRNA01
gi|29165615|ref|NC_002745.2|	506680 +	U	11	7	9	12	37	SArRNA01
gi|29165615|ref|NC_002745.2|	506681 +	A	9	9	3	7	32	SArRNA01
gi|29165615|ref|NC_002745.2|	506682 +	C	19	10	9	12	28	SArRNA01
gi|29165615|ref|NC_002745.2|	506683 +	G	6	2	7	2	9	SArRNA01
gi|29165615|ref|NC_002745.2|	506684 +	U	35	11	16	11	38	SArRNA01
gi|29165615|ref|NC_002745.2|	506685 +	G	46	31	29	17	59	SArRNA01
gi|29165615|ref|NC_002745.2|	506686 +	C	87	66	36	46	98	SArRNA01
gi|29165615|ref|NC_002745.2|	506687 +	C	136	95	49	52	140	SArRNA01

gi|29165615|ref|NC_002745.2|	506688	+	A	75	52	31	34	95	SArRNA01
gi|29165615|ref|NC_002745.2|	506689	+	G	62	32	26	30	55	SArRNA01
gi|29165615|ref|NC_002745.2|	506690	+	C	119	59	50	33	125	SArRNA01
gi|29165615|ref|NC_002745.2|	506691	+	A	79	49	34	36	80	SArRNA01
gi|29165615|ref|NC_002745.2|	506692	+	G	19	13	7	7	10	SArRNA01
gi|29165615|ref|NC_002745.2|	506693	+	C	104	43	20	26	73	SArRNA01
gi|29165615|ref|NC_002745.2|	506694	+	C	122	79	42	40	99	SArRNA01
gi|29165615|ref|NC_002745.2|	506695	+	G	60	70	26	22	83	SArRNA01
gi|29165615|ref|NC_002745.2|	506696	+	C	45	31	17	26	43	SArRNA01
gi|29165615|ref|NC_002745.2|	506697	+	G	61	45	23	27	67	SArRNA01
gi|29165615|ref|NC_002745.2|	506698	+	G	67	56	32	33	94	SArRNA01
gi|29165615|ref|NC_002745.2|	506699	+	U	124	92	61	69	143	SArRNA01
gi|29165615|ref|NC_002745.2|	506700	+	A	31	37	17	18	26	SArRNA01
gi|29165615|ref|NC_002745.2|	506701	+	A	112	109	52	48	161	SArRNA01
gi|29165615|ref|NC_002745.2|	506702	+	U	204	222	115	101	261	SArRNA01
gi|29165615|ref|NC_002745.2|	506703	+	A	129	89	51	55	137	SArRNA01
gi|29165615|ref|NC_002745.2|	506704	+	C	156	87	49	72	151	SArRNA01
gi|29165615|ref|NC_002745.2|	506705	+	G	152	98	51	57	149	SArRNA01
gi|29165615|ref|NC_002745.2|	506706	+	U	83	99	54	55	136	SArRNA01
gi|29165615|ref|NC_002745.2|	506707	+	A	44	23	22	27	69	SArRNA01
gi|29165615|ref|NC_002745.2|	506708	+	G	29	23	20	17	44	SArRNA01
gi|29165615|ref|NC_002745.2|	506709	+	G	113	107	48	53	89	SArRNA01
gi|29165615|ref|NC_002745.2|	506710	+	U	212	296	73	95	211	SArRNA01
gi|29165615|ref|NC_002745.2|	506711	+	G	35	34	10	20	35	SArRNA01
gi|29165615|ref|NC_002745.2|	506712	+	G	27	24	10	12	34	SArRNA01
gi|29165615|ref|NC_002745.2|	506713	+	C	62	59	34	27	85	SArRNA01
gi|29165615|ref|NC_002745.2|	506714	+	A	51	57	29	23	86	SArRNA01
gi|29165615|ref|NC_002745.2|	506715	+	A	50	27	20	15	45	SArRNA01
gi|29165615|ref|NC_002745.2|	506716	+	G	15	7	5	6	10	SArRNA01
gi|29165615|ref|NC_002745.2|	506717	+	C	82	37	35	37	80	SArRNA01
gi|29165615|ref|NC_002745.2|	506718	+	G	92	50	42	32	99	SArRNA01
gi|29165615|ref|NC_002745.2|	506719	+	U	89	67	30	43	111	SArRNA01
gi|29165615|ref|NC_002745.2|	506720	+	U	106	63	41	54	109	SArRNA01
gi|29165615|ref|NC_002745.2|	506721	+	A	71	38	37	37	99	SArRNA01
gi|29165615|ref|NC_002745.2|	506722	+	U	80	68	40	47	96	SArRNA01
gi|29165615|ref|NC_002745.2|	506723	+	C	229	190	66	77	182	SArRNA01
gi|29165615|ref|NC_002745.2|	506724	+	C	615	390	182	243	499	SArRNA01
gi|29165615|ref|NC_002745.2|	506725	+	G	257	170	70	78	234	SArRNA01
gi|29165615|ref|NC_002745.2|	506726	+	G	127	85	69	57	177	SArRNA01
gi|29165615|ref|NC_002745.2|	506727	+	A	130	70	76	57	163	SArRNA01
gi|29165615|ref|NC_002745.2|	506728	+	A	191	149	87	89	209	SArRNA01
gi|29165615|ref|NC_002745.2|	506729	+	U	148	142	83	95	245	SArRNA01
gi|29165615|ref|NC_002745.2|	506730	+	U	277	152	136	125	202	SArRNA01
gi|29165615|ref|NC_002745.2|	506731	+	A	200	89	61	65	152	SArRNA01
gi|29165615|ref|NC_002745.2|	506732	+	U	130	58	43	34	112	SArRNA01
gi|29165615|ref|NC_002745.2|	506733	+	U	86	47	12	15	80	SArRNA01
gi|29165615|ref|NC_002745.2|	506734	+	G	24	11	9	6	17	SArRNA01
gi|29165615|ref|NC_002745.2|	506735	+	G	24	19	16	16	30	SArRNA01
gi|29165615|ref|NC_002745.2|	506736	+	G	11	15	4	4	10	SArRNA01
gi|29165615|ref|NC_002745.2|	506737	+	C	95	43	39	39	120	SArRNA01
gi|29165615|ref|NC_002745.2|	506738	+	G	50	50	27	28	93	SArRNA01
gi|29165615|ref|NC_002745.2|	506739	+	U	118	45	35	36	95	SArRNA01
gi|29165615|ref|NC_002745.2|	506740	+	A	123	48	24	33	45	SArRNA01
gi|29165615|ref|NC_002745.2|	506741	+	A	130	27	26	25	61	SArRNA01
gi|29165615|ref|NC_002745.2|	506742	+	A	53	36	15	17	52	SArRNA01
gi|29165615|ref|NC_002745.2|	506743	+	G	29	9	6	13	16	SArRNA01
gi|29165615|ref|NC_002745.2|	506744	+	C	132	53	37	34	71	SArRNA01
gi|29165615|ref|NC_002745.2|	506745	+	G	19	18	8	19	28	SArRNA01
gi|29165615|ref|NC_002745.2|	506746	+	C	76	32	32	30	40	SArRNA01
gi|29165615|ref|NC_002745.2|	506747	+	G	36	16	8	8	19	SArRNA01
gi|29165615|ref|NC_002745.2|	506748	+	C	113	13	17	16	37	SArRNA01
gi|29165615|ref|NC_002745.2|	506749	+	G	38	14	11	16	32	SArRNA01
gi|29165615|ref|NC_002745.2|	506750	+	U	61	29	18	12	40	SArRNA01
gi|29165615|ref|NC_002745.2|	506751	+	A	26	8	7	5	17	SArRNA01
gi|29165615|ref|NC_002745.2|	506752	+	G	5	7	1	6	6	SArRNA01
gi|29165615|ref|NC_002745.2|	506753	+	G	5	5	2	4	5	SArRNA01
gi|29165615|ref|NC_002745.2|	506754	+	C	29	24	15	14	20	SArRNA01
gi|29165615|ref|NC_002745.2|	506755	+	G	22	15	10	15	18	SArRNA01
gi|29165615|ref|NC_002745.2|	506756	+	G	39	44	21	28	63	SArRNA01
gi|29165615|ref|NC_002745.2|	506757	+	U	139	97	67	65	162	SArRNA01
gi|29165615|ref|NC_002745.2|	506758	+	U	125	105	93	48	143	SArRNA01
gi|29165615|ref|NC_002745.2|	506759	+	U	88	67	58	59	132	SArRNA01
gi|29165615|ref|NC_002745.2|	506760	+	U	70	88	36	41	99	SArRNA01
gi|29165615|ref|NC_002745.2|	506761	+	U	36	29	15	20	60	SArRNA01
gi|29165615|ref|NC_002745.2|	506762	+	U	27	53	19	21	40	SArRNA01
gi|29165615|ref|NC_002745.2|	506763	+	A	10	13	6	8	12	SArRNA01
gi|29165615|ref|NC_002745.2|	506764	+	A	3	3	1	5	10	SArRNA01
gi|29165615|ref|NC_002745.2|	506765	+	G	3	3	6	2	3	SArRNA01
gi|29165615|ref|NC_002745.2|	506766	+	U	28	25	13	19	33	SArRNA01
gi|29165615|ref|NC_002745.2|	506767	+	C	170	187	80	66	137	SArRNA01
gi|29165615|ref|NC_002745.2|	506768	+	U	90	48	26	26	80	SArRNA01

gi|29165615|ref|NC_002745.2|	506769	+	G	25	39	28	26	47	SArRNA01
gi|29165615|ref|NC_002745.2|	506770	+	A	55	44	38	33	82	SArRNA01
gi|29165615|ref|NC_002745.2|	506771	+	U	44	45	22	16	58	SArRNA01
gi|29165615|ref|NC_002745.2|	506772	+	G	34	40	21	18	45	SArRNA01
gi|29165615|ref|NC_002745.2|	506773	+	U	55	34	30	21	46	SArRNA01
gi|29165615|ref|NC_002745.2|	506774	+	G	41	38	15	33	52	SArRNA01
gi|29165615|ref|NC_002745.2|	506775	+	A	52	48	35	16	59	SArRNA01
gi|29165615|ref|NC_002745.2|	506776	+	A	43	44	23	20	52	SArRNA01
gi|29165615|ref|NC_002745.2|	506777	+	A	54	31	19	20	43	SArRNA01
gi|29165615|ref|NC_002745.2|	506778	+	G	38	21	15	13	29	SArRNA01
gi|29165615|ref|NC_002745.2|	506779	+	C	24	18	5	5	14	SArRNA01
gi|29165615|ref|NC_002745.2|	506780	+	C	8	5	3	8	17	SArRNA01
gi|29165615|ref|NC_002745.2|	506781	+	C	43	14	16	26	19	SArRNA01
gi|29165615|ref|NC_002745.2|	506782	+	A	19	22	12	9	29	SArRNA01
gi|29165615|ref|NC_002745.2|	506783	+	C	31	17	9	13	19	SArRNA01
gi|29165615|ref|NC_002745.2|	506784	+	G	10	23	5	8	17	SArRNA01
gi|29165615|ref|NC_002745.2|	506785	+	G	8	9	3	3	13	SArRNA01
gi|29165615|ref|NC_002745.2|	506786	+	C	23	19	14	13	15	SArRNA01
gi|29165615|ref|NC_002745.2|	506787	+	U	31	16	15	8	39	SArRNA01
gi|29165615|ref|NC_002745.2|	506788	+	C	85	77	33	38	62	SArRNA01
gi|29165615|ref|NC_002745.2|	506789	+	A	38	38	16	19	42	SArRNA01
gi|29165615|ref|NC_002745.2|	506790	+	A	15	16	21	8	24	SArRNA01
gi|29165615|ref|NC_002745.2|	506791	+	C	23	25	13	10	28	SArRNA01
gi|29165615|ref|NC_002745.2|	506792	+	C	40	22	21	20	31	SArRNA01
gi|29165615|ref|NC_002745.2|	506793	+	G	22	21	14	8	23	SArRNA01
gi|29165615|ref|NC_002745.2|	506794	+	U	12	13	5	7	23	SArRNA01
gi|29165615|ref|NC_002745.2|	506795	+	G	15	9	12	3	18	SArRNA01
gi|29165615|ref|NC_002745.2|	506796	+	G	7	3	2	5	12	SArRNA01
gi|29165615|ref|NC_002745.2|	506797	+	A	2	4	0	1	3	SArRNA01
gi|29165615|ref|NC_002745.2|	506798	+	G	6	6	1	2	6	SArRNA01
gi|29165615|ref|NC_002745.2|	506799	+	G	11	12	2	6	6	SArRNA01
gi|29165615|ref|NC_002745.2|	506800	+	G	17	11	2	10	15	SArRNA01
gi|29165615|ref|NC_002745.2|	506801	+	U	3	4	8	5	10	SArRNA01
gi|29165615|ref|NC_002745.2|	506802	+	C	133	94	41	36	76	SArRNA01
gi|29165615|ref|NC_002745.2|	506803	+	A	22	31	13	11	29	SArRNA01
gi|29165615|ref|NC_002745.2|	506804	+	U	0	5	3	5	8	SArRNA01
gi|29165615|ref|NC_002745.2|	506805	+	U	6	7	3	1	4	SArRNA01
gi|29165615|ref|NC_002745.2|	506806	+	G	0	1	1	0	2	SArRNA01
gi|29165615|ref|NC_002745.2|	506807	+	G	0	4	1	3	6	SArRNA01
gi|29165615|ref|NC_002745.2|	506808	+	A	5	0	3	0	1	SArRNA01
gi|29165615|ref|NC_002745.2|	506809	+	A	0	3	1	0	2	SArRNA01
gi|29165615|ref|NC_002745.2|	506810	+	A	2	3	1	0	3	SArRNA01
gi|29165615|ref|NC_002745.2|	506811	+	C	12	11	6	3	13	SArRNA01
gi|29165615|ref|NC_002745.2|	506812	+	U	7	5	4	2	3	SArRNA01
gi|29165615|ref|NC_002745.2|	506813	+	G	0	0	0	0	3	SArRNA01
gi|29165615|ref|NC_002745.2|	506814	+	G	0	0	0	0	1	SArRNA01
gi|29165615|ref|NC_002745.2|	506815	+	A	0	0	2	0	3	SArRNA01
gi|29165615|ref|NC_002745.2|	506816	+	A	2	1	0	2	5	SArRNA01
gi|29165615|ref|NC_002745.2|	506817	+	A	4	1	2	1	3	SArRNA01
gi|29165615|ref|NC_002745.2|	506818	+	A	24	8	0	3	7	SArRNA01
gi|29165615|ref|NC_002745.2|	506819	+	C	41	6	3	1	8	SArRNA01
gi|29165615|ref|NC_002745.2|	506820	+	U	10	6	1	2	3	SArRNA01
gi|29165615|ref|NC_002745.2|	506821	+	U	2	3	1	1	3	SArRNA01
gi|29165615|ref|NC_002745.2|	506822	+	G	10	2	0	0	1	SArRNA01
gi|29165615|ref|NC_002745.2|	506823	+	A	6	2	1	2	1	SArRNA01
gi|29165615|ref|NC_002745.2|	506824	+	G	3	0	0	0	0	SArRNA01
gi|29165615|ref|NC_002745.2|	506825	+	U	3	2	0	0	0	SArRNA01
gi|29165615|ref|NC_002745.2|	506826	+	G	1	0	1	0	1	SArRNA01
gi|29165615|ref|NC_002745.2|	506827	+	C	5	4	5	2	2	SArRNA01
gi|29165615|ref|NC_002745.2|	506828	+	A	11	12	2	3	5	SArRNA01
gi|29165615|ref|NC_002745.2|	506829	+	G	1	3	0	1	1	SArRNA01
gi|29165615|ref|NC_002745.2|	506830	+	A	4	2	2	1	1	SArRNA01
gi|29165615|ref|NC_002745.2|	506831	+	A	1	4	1	2	1	SArRNA01
gi|29165615|ref|NC_002745.2|	506832	+	G	11	1	2	1	2	SArRNA01
gi|29165615|ref|NC_002745.2|	506833	+	A	1	1	1	0	1	SArRNA01
gi|29165615|ref|NC_002745.2|	506834	+	G	3	0	0	0	1	SArRNA01
gi|29165615|ref|NC_002745.2|	506835	+	G	0	0	0	1	1	SArRNA01
gi|29165615|ref|NC_002745.2|	506836	+	A	0	0	0	0	1	SArRNA01
gi|29165615|ref|NC_002745.2|	506837	+	A	0	2	0	1	0	SArRNA01
gi|29165615|ref|NC_002745.2|	506838	+	A	1	0	1	0	0	SArRNA01
gi|29165615|ref|NC_002745.2|	506839	+	G	1	1	0	0	0	SArRNA01
gi|29165615|ref|NC_002745.2|	506840	+	U	2	0	0	0	2	SArRNA01
gi|29165615|ref|NC_002745.2|	506842	+	G	0	1	0	1	0	SArRNA01
gi|29165615|ref|NC_002745.2|	506843	+	A	2	0	0	0	0	SArRNA01
gi|29165615|ref|NC_002745.2|	506844	+	A	0	1	0	0	1	SArRNA01
gi|29165615|ref|NC_002745.2|	506847	+	C	1	3	0	4	1	SArRNA01
gi|29165615|ref|NC_002745.2|	506848	+	C	4	2	3	3	6	SArRNA01
gi|29165615|ref|NC_002745.2|	506849	+	A	1	5	2	4	3	SArRNA01
gi|29165615|ref|NC_002745.2|	506850	+	U	0	1	0	1	2	SArRNA01
gi|29165615|ref|NC_002745.2|	506851	+	G	3	2	0	1	2	SArRNA01
gi|29165615|ref|NC_002745.2|	506852	+	U	2	4	0	1	2	SArRNA01

gi|29165615|ref|NC_002745.2|	506853	+	G	3	4	1	2	4	SArRNA01
gi|29165615|ref|NC_002745.2|	506854	+	U	2	3	1	1	3	SArRNA01
gi|29165615|ref|NC_002745.2|	506855	+	A	3	4	2	1	3	SArRNA01
gi|29165615|ref|NC_002745.2|	506856	+	G	0	1	0	0	1	SArRNA01
gi|29165615|ref|NC_002745.2|	506857	+	C	2	0	0	1	1	SArRNA01
gi|29165615|ref|NC_002745.2|	506858	+	G	0	4	0	1	7	SArRNA01
gi|29165615|ref|NC_002745.2|	506859	+	G	1	3	0	2	1	SArRNA01
gi|29165615|ref|NC_002745.2|	506860	+	U	5	1	0	0	0	SArRNA01
gi|29165615|ref|NC_002745.2|	506862	+	A	3	0	0	0	1	SArRNA01
gi|29165615|ref|NC_002745.2|	506863	+	A	0	0	0	0	1	SArRNA01
gi|29165615|ref|NC_002745.2|	506864	+	A	0	6	0	1	1	SArRNA01
gi|29165615|ref|NC_002745.2|	506865	+	U	5	2	1	2	0	SArRNA01
gi|29165615|ref|NC_002745.2|	506867	+	C	3	2	0	0	0	SArRNA01
gi|29165615|ref|NC_002745.2|	506868	+	G	0	0	0	0	2	SArRNA01
gi|29165615|ref|NC_002745.2|	506869	+	C	0	0	1	0	1	SArRNA01
gi|29165615|ref|NC_002745.2|	506870	+	A	1	2	0	0	2	SArRNA01
gi|29165615|ref|NC_002745.2|	506871	+	G	0	3	0	0	2	SArRNA01
gi|29165615|ref|NC_002745.2|	506872	+	A	1	0	0	0	0	SArRNA01
gi|29165615|ref|NC_002745.2|	506873	+	G	2	2	0	0	0	SArRNA01
gi|29165615|ref|NC_002745.2|	506874	+	A	2	0	0	0	1	SArRNA01
gi|29165615|ref|NC_002745.2|	506875	+	U	1	1	1	1	2	SArRNA01
gi|29165615|ref|NC_002745.2|	506876	+	A	1	1	1	1	3	SArRNA01
gi|29165615|ref|NC_002745.2|	506877	+	U	3	1	0	2	1	SArRNA01
gi|29165615|ref|NC_002745.2|	506878	+	G	0	0	0	1	2	SArRNA01
gi|29165615|ref|NC_002745.2|	506879	+	G	2	2	0	1	1	SArRNA01
gi|29165615|ref|NC_002745.2|	506880	+	A	2	2	1	0	1	SArRNA01
gi|29165615|ref|NC_002745.2|	506881	+	G	0	0	0	1	1	SArRNA01
gi|29165615|ref|NC_002745.2|	506882	+	G	0	1	0	0	1	SArRNA01
gi|29165615|ref|NC_002745.2|	506883	+	A	0	2	0	0	1	SArRNA01
gi|29165615|ref|NC_002745.2|	506884	+	A	2	3	1	0	0	SArRNA01
gi|29165615|ref|NC_002745.2|	506885	+	C	0	3	0	1	1	SArRNA01
gi|29165615|ref|NC_002745.2|	506886	+	A	2	2	0	0	1	SArRNA01
gi|29165615|ref|NC_002745.2|	506887	+	C	3	1	0	0	1	SArRNA01
gi|29165615|ref|NC_002745.2|	506888	+	C	1	7	0	0	2	SArRNA01
gi|29165615|ref|NC_002745.2|	506889	+	A	0	0	0	0	1	SArRNA01
gi|29165615|ref|NC_002745.2|	506890	+	G	0	5	2	1	4	SArRNA01
gi|29165615|ref|NC_002745.2|	506891	+	U	0	0	1	1	1	SArRNA01
gi|29165615|ref|NC_002745.2|	506892	+	G	0	0	0	0	1	SArRNA01
gi|29165615|ref|NC_002745.2|	506893	+	G	3	0	0	2	1	SArRNA01
gi|29165615|ref|NC_002745.2|	506894	+	C	0	1	0	1	0	SArRNA01
gi|29165615|ref|NC_002745.2|	506895	+	G	3	0	0	1	2	SArRNA01
gi|29165615|ref|NC_002745.2|	506896	+	A	0	1	0	1	2	SArRNA01
gi|29165615|ref|NC_002745.2|	506897	+	A	1	0	1	0	0	SArRNA01
gi|29165615|ref|NC_002745.2|	506899	+	G	0	0	0	1	1	SArRNA01
gi|29165615|ref|NC_002745.2|	506900	+	C	3	0	1	1	2	SArRNA01
gi|29165615|ref|NC_002745.2|	506901	+	G	2	2	0	1	1	SArRNA01
gi|29165615|ref|NC_002745.2|	506902	+	A	5	2	0	1	2	SArRNA01
gi|29165615|ref|NC_002745.2|	506903	+	C	6	1	1	0	1	SArRNA01
gi|29165615|ref|NC_002745.2|	506904	+	U	1	0	0	0	0	SArRNA01
gi|29165615|ref|NC_002745.2|	506905	+	U	0	0	3	0	1	SArRNA01
gi|29165615|ref|NC_002745.2|	506906	+	U	1	0	0	0	1	SArRNA01
gi|29165615|ref|NC_002745.2|	506907	+	C	1	1	0	0	0	SArRNA01
gi|29165615|ref|NC_002745.2|	506908	+	U	0	0	0	1	3	SArRNA01
gi|29165615|ref|NC_002745.2|	506910	+	G	0	0	0	1	0	SArRNA01
gi|29165615|ref|NC_002745.2|	506911	+	U	0	3	1	0	1	SArRNA01
gi|29165615|ref|NC_002745.2|	506912	+	C	0	3	0	1	2	SArRNA01
gi|29165615|ref|NC_002745.2|	506913	+	U	2	1	0	1	2	SArRNA01
gi|29165615|ref|NC_002745.2|	506914	+	G	0	0	1	1	1	SArRNA01
gi|29165615|ref|NC_002745.2|	506915	+	U	0	2	1	2	1	SArRNA01
gi|29165615|ref|NC_002745.2|	506916	+	A	2	1	1	0	1	SArRNA01
gi|29165615|ref|NC_002745.2|	506917	+	A	5	0	2	0	3	SArRNA01
gi|29165615|ref|NC_002745.2|	506918	+	C	9	3	2	2	6	SArRNA01
gi|29165615|ref|NC_002745.2|	506919	+	U	3	5	0	0	1	SArRNA01
gi|29165615|ref|NC_002745.2|	506920	+	G	0	1	1	1	0	SArRNA01
gi|29165615|ref|NC_002745.2|	506921	+	A	0	0	1	0	1	SArRNA01
gi|29165615|ref|NC_002745.2|	506922	+	C	0	0	2	1	0	SArRNA01
gi|29165615|ref|NC_002745.2|	506924	+	C	0	2	1	1	1	SArRNA01
gi|29165615|ref|NC_002745.2|	506925	+	U	3	0	0	0	0	SArRNA01
gi|29165615|ref|NC_002745.2|	506926	+	G	4	2	0	0	2	SArRNA01
gi|29165615|ref|NC_002745.2|	506927	+	A	1	2	0	1	0	SArRNA01
gi|29165615|ref|NC_002745.2|	506928	+	U	3	2	1	2	4	SArRNA01
gi|29165615|ref|NC_002745.2|	506930	+	U	0	1	0	1	0	SArRNA01
gi|29165615|ref|NC_002745.2|	506932	+	C	0	4	0	0	0	SArRNA01
gi|29165615|ref|NC_002745.2|	506933	+	G	2	1	1	1	4	SArRNA01
gi|29165615|ref|NC_002745.2|	506934	+	A	1	0	0	1	0	SArRNA01
gi|29165615|ref|NC_002745.2|	506936	+	A	1	0	0	0	0	SArRNA01
gi|29165615|ref|NC_002745.2|	506944	+	G	0	0	0	0	1	SArRNA01
gi|29165615|ref|NC_002745.2|	506945	+	A	0	0	0	1	0	SArRNA01
gi|29165615|ref|NC_002745.2|	506950	+	A	0	0	1	0	0	SArRNA01
gi|29165615|ref|NC_002745.2|	506951	+	C	0	2	0	0	1	SArRNA01
gi|29165615|ref|NC_002745.2|	506952	+	A	1	2	0	0	0	SArRNA01

gi|29165615|ref|NC_002745.2|	506954	+	G	1	0	0	0	0	SArRNA01
gi|29165615|ref|NC_002745.2|	506956	+	U	0	0	0	0	1	SArRNA01
gi|29165615|ref|NC_002745.2|	506958	+	A	0	0	1	0	1	SArRNA01
gi|29165615|ref|NC_002745.2|	506960	+	A	3	0	1	0	0	SArRNA01
gi|29165615|ref|NC_002745.2|	506961	+	U	7	3	0	1	7	SArRNA01
gi|29165615|ref|NC_002745.2|	506962	+	A	0	2	0	1	3	SArRNA01
gi|29165615|ref|NC_002745.2|	506963	+	C	6	22	0	0	8	SArRNA01
gi|29165615|ref|NC_002745.2|	506964	+	C	5	9	3	0	7	SArRNA01
gi|29165615|ref|NC_002745.2|	506965	+	C	11	17	2	4	13	SArRNA01
gi|29165615|ref|NC_002745.2|	506966	+	U	7	7	0	0	5	SArRNA01
gi|29165615|ref|NC_002745.2|	506967	+	G	2	1	0	0	2	SArRNA01
gi|29165615|ref|NC_002745.2|	506968	+	G	0	3	0	0	2	SArRNA01
gi|29165615|ref|NC_002745.2|	506969	+	U	3	0	3	1	2	SArRNA01
gi|29165615|ref|NC_002745.2|	506970	+	A	6	5	1	2	2	SArRNA01
gi|29165615|ref|NC_002745.2|	506971	+	G	0	1	0	2	2	SArRNA01
gi|29165615|ref|NC_002745.2|	506972	+	U	7	0	1	2	2	SArRNA01
gi|29165615|ref|NC_002745.2|	506973	+	C	6	5	2	1	0	SArRNA01
gi|29165615|ref|NC_002745.2|	506974	+	C	18	13	3	10	7	SArRNA01
gi|29165615|ref|NC_002745.2|	506975	+	A	6	7	2	3	9	SArRNA01
gi|29165615|ref|NC_002745.2|	506976	+	C	6	6	3	4	10	SArRNA01
gi|29165615|ref|NC_002745.2|	506977	+	G	4	1	0	2	2	SArRNA01
gi|29165615|ref|NC_002745.2|	506978	+	C	0	3	0	1	5	SArRNA01
gi|29165615|ref|NC_002745.2|	506979	+	C	18	15	6	4	5	SArRNA01
gi|29165615|ref|NC_002745.2|	506980	+	G	11	7	9	14	16	SArRNA01
gi|29165615|ref|NC_002745.2|	506981	+	U	10	27	13	18	36	SArRNA01
gi|29165615|ref|NC_002745.2|	506982	+	A	5	5	5	3	5	SArRNA01
gi|29165615|ref|NC_002745.2|	506983	+	A	38	30	13	13	15	SArRNA01
gi|29165615|ref|NC_002745.2|	506984	+	A	9	50	15	4	33	SArRNA01
gi|29165615|ref|NC_002745.2|	506985	+	C	46	62	14	20	40	SArRNA01
gi|29165615|ref|NC_002745.2|	506986	+	G	9	22	5	12	20	SArRNA01
gi|29165615|ref|NC_002745.2|	506987	+	A	8	1	4	9	6	SArRNA01
gi|29165615|ref|NC_002745.2|	506988	+	U	11	12	3	4	9	SArRNA01
gi|29165615|ref|NC_002745.2|	506989	+	G	2	7	0	2	3	SArRNA01
gi|29165615|ref|NC_002745.2|	506990	+	A	5	2	1	0	2	SArRNA01
gi|29165615|ref|NC_002745.2|	506991	+	G	2	0	0	0	1	SArRNA01
gi|29165615|ref|NC_002745.2|	506992	+	U	4	4	2	1	4	SArRNA01
gi|29165615|ref|NC_002745.2|	506993	+	G	1	0	1	1	2	SArRNA01
gi|29165615|ref|NC_002745.2|	506994	+	C	9	9	4	7	8	SArRNA01
gi|29165615|ref|NC_002745.2|	506995	+	U	8	3	0	6	9	SArRNA01
gi|29165615|ref|NC_002745.2|	506996	+	A	6	6	1	0	4	SArRNA01
gi|29165615|ref|NC_002745.2|	506997	+	A	13	10	4	1	9	SArRNA01
gi|29165615|ref|NC_002745.2|	506998	+	G	8	5	5	2	11	SArRNA01
gi|29165615|ref|NC_002745.2|	506999	+	U	8	5	5	3	8	SArRNA01
gi|29165615|ref|NC_002745.2|	507000	+	G	5	0	3	4	11	SArRNA01
gi|29165615|ref|NC_002745.2|	507001	+	U	2	3	3	1	12	SArRNA01
gi|29165615|ref|NC_002745.2|	507002	+	U	7	2	0	0	7	SArRNA01
gi|29165615|ref|NC_002745.2|	507003	+	A	3	0	1	0	2	SArRNA01
gi|29165615|ref|NC_002745.2|	507004	+	G	0	1	0	0	0	SArRNA01
gi|29165615|ref|NC_002745.2|	507008	+	G	0	1	0	0	1	SArRNA01
gi|29165615|ref|NC_002745.2|	507009	+	U	0	0	0	0	3	SArRNA01
gi|29165615|ref|NC_002745.2|	507010	+	U	0	3	0	1	1	SArRNA01
gi|29165615|ref|NC_002745.2|	507011	+	U	3	1	2	1	3	SArRNA01
gi|29165615|ref|NC_002745.2|	507012	+	C	2	5	0	1	3	SArRNA01
gi|29165615|ref|NC_002745.2|	507013	+	C	18	35	5	10	27	SArRNA01
gi|29165615|ref|NC_002745.2|	507014	+	G	4	10	2	4	7	SArRNA01
gi|29165615|ref|NC_002745.2|	507015	+	C	11	8	5	7	22	SArRNA01
gi|29165615|ref|NC_002745.2|	507016	+	C	15	5	6	2	12	SArRNA01
gi|29165615|ref|NC_002745.2|	507017	+	C	15	3	3	3	9	SArRNA01
gi|29165615|ref|NC_002745.2|	507018	+	C	11	4	4	3	10	SArRNA01
gi|29165615|ref|NC_002745.2|	507019	+	U	22	12	3	3	10	SArRNA01
gi|29165615|ref|NC_002745.2|	507020	+	U	12	6	3	5	11	SArRNA01
gi|29165615|ref|NC_002745.2|	507021	+	A	16	10	7	3	16	SArRNA01
gi|29165615|ref|NC_002745.2|	507022	+	G	5	4	2	4	11	SArRNA01
gi|29165615|ref|NC_002745.2|	507023	+	U	6	0	1	2	5	SArRNA01
gi|29165615|ref|NC_002745.2|	507024	+	G	1	0	0	0	0	SArRNA01
gi|29165615|ref|NC_002745.2|	507025	+	C	10	4	6	4	10	SArRNA01
gi|29165615|ref|NC_002745.2|	507026	+	U	0	5	2	1	5	SArRNA01
gi|29165615|ref|NC_002745.2|	507028	+	C	7	16	1	6	10	SArRNA01
gi|29165615|ref|NC_002745.2|	507029	+	A	3	5	2	1	6	SArRNA01
gi|29165615|ref|NC_002745.2|	507030	+	G	2	0	0	1	1	SArRNA01
gi|29165615|ref|NC_002745.2|	507031	+	C	2	4	0	3	7	SArRNA01
gi|29165615|ref|NC_002745.2|	507032	+	U	3	5	0	2	1	SArRNA01
gi|29165615|ref|NC_002745.2|	507033	+	A	3	6	0	1	5	SArRNA01
gi|29165615|ref|NC_002745.2|	507034	+	A	3	2	0	0	0	SArRNA01
gi|29165615|ref|NC_002745.2|	507035	+	C	5	4	1	0	3	SArRNA01
gi|29165615|ref|NC_002745.2|	507036	+	G	0	0	0	0	4	SArRNA01
gi|29165615|ref|NC_002745.2|	507037	+	C	3	4	1	2	1	SArRNA01
gi|29165615|ref|NC_002745.2|	507038	+	A	5	0	0	0	4	SArRNA01
gi|29165615|ref|NC_002745.2|	507039	+	U	3	3	0	0	2	SArRNA01
gi|29165615|ref|NC_002745.2|	507040	+	U	2	5	1	1	3	SArRNA01
gi|29165615|ref|NC_002745.2|	507041	+	A	3	1	1	3	4	SArRNA01

gi|29165615|ref|NC_002745.2|	507042	+	A	0	5	0	2	5	SArRNA01
gi|29165615|ref|NC_002745.2|	507043	+	G	0	0	0	1	0	SArRNA01
gi|29165615|ref|NC_002745.2|	507044	+	C	5	6	0	0	2	SArRNA01
gi|29165615|ref|NC_002745.2|	507045	+	A	3	2	0	1	0	SArRNA01
gi|29165615|ref|NC_002745.2|	507046	+	C	4	4	0	1	2	SArRNA01
gi|29165615|ref|NC_002745.2|	507047	+	U	2	0	2	0	0	SArRNA01
gi|29165615|ref|NC_002745.2|	507048	+	C	4	1	1	0	2	SArRNA01
gi|29165615|ref|NC_002745.2|	507049	+	C	5	4	6	2	10	SArRNA01
gi|29165615|ref|NC_002745.2|	507050	+	G	1	2	0	0	2	SArRNA01
gi|29165615|ref|NC_002745.2|	507051	+	C	4	1	0	0	0	SArRNA01
gi|29165615|ref|NC_002745.2|	507052	+	C	10	35	7	11	26	SArRNA01
gi|29165615|ref|NC_002745.2|	507053	+	U	3	10	1	1	2	SArRNA01
gi|29165615|ref|NC_002745.2|	507054	+	G	0	0	0	0	1	SArRNA01
gi|29165615|ref|NC_002745.2|	507055	+	G	1	0	0	0	1	SArRNA01
gi|29165615|ref|NC_002745.2|	507056	+	G	3	0	0	1	1	SArRNA01
gi|29165615|ref|NC_002745.2|	507058	+	A	1	0	0	0	0	SArRNA01
gi|29165615|ref|NC_002745.2|	507059	+	G	1	0	0	1	1	SArRNA01
gi|29165615|ref|NC_002745.2|	507060	+	U	2	6	3	3	7	SArRNA01
gi|29165615|ref|NC_002745.2|	507061	+	A	0	4	0	0	0	SArRNA01
gi|29165615|ref|NC_002745.2|	507062	+	C	7	2	1	1	4	SArRNA01
gi|29165615|ref|NC_002745.2|	507063	+	G	0	9	0	0	2	SArRNA01
gi|29165615|ref|NC_002745.2|	507064	+	A	1	1	1	0	2	SArRNA01
gi|29165615|ref|NC_002745.2|	507065	+	C	1	1	1	0	0	SArRNA01
gi|29165615|ref|NC_002745.2|	507066	+	C	0	2	0	3	1	SArRNA01
gi|29165615|ref|NC_002745.2|	507067	+	G	0	2	0	0	2	SArRNA01
gi|29165615|ref|NC_002745.2|	507068	+	C	4	5	0	0	1	SArRNA01
gi|29165615|ref|NC_002745.2|	507069	+	A	3	4	1	2	3	SArRNA01
gi|29165615|ref|NC_002745.2|	507070	+	A	1	0	0	0	1	SArRNA01
gi|29165615|ref|NC_002745.2|	507071	+	G	0	0	0	0	1	SArRNA01
gi|29165615|ref|NC_002745.2|	507072	+	G	0	2	0	1	1	SArRNA01
gi|29165615|ref|NC_002745.2|	507073	+	U	0	1	0	0	0	SArRNA01
gi|29165615|ref|NC_002745.2|	507074	+	U	2	0	0	1	1	SArRNA01
gi|29165615|ref|NC_002745.2|	507075	+	G	1	2	0	2	4	SArRNA01
gi|29165615|ref|NC_002745.2|	507076	+	A	1	0	1	0	0	SArRNA01
gi|29165615|ref|NC_002745.2|	507077	+	A	0	9	1	0	5	SArRNA01
gi|29165615|ref|NC_002745.2|	507078	+	A	1	4	0	1	6	SArRNA01
gi|29165615|ref|NC_002745.2|	507079	+	C	1	0	1	1	0	SArRNA01
gi|29165615|ref|NC_002745.2|	507080	+	U	1	6	0	1	0	SArRNA01
gi|29165615|ref|NC_002745.2|	507081	+	C	0	3	0	0	1	SArRNA01
gi|29165615|ref|NC_002745.2|	507082	+	A	0	1	0	0	3	SArRNA01
gi|29165615|ref|NC_002745.2|	507083	+	A	0	5	0	0	1	SArRNA01
gi|29165615|ref|NC_002745.2|	507084	+	A	0	0	2	1	3	SArRNA01
gi|29165615|ref|NC_002745.2|	507085	+	G	1	1	1	2	0	SArRNA01
gi|29165615|ref|NC_002745.2|	507086	+	G	1	1	0	1	2	SArRNA01
gi|29165615|ref|NC_002745.2|	507087	+	A	4	4	4	3	2	SArRNA01
gi|29165615|ref|NC_002745.2|	507088	+	A	3	20	3	1	11	SArRNA01
gi|29165615|ref|NC_002745.2|	507089	+	U	2	6	1	0	2	SArRNA01
gi|29165615|ref|NC_002745.2|	507090	+	U	3	3	0	0	2	SArRNA01
gi|29165615|ref|NC_002745.2|	507091	+	G	5	6	2	2	2	SArRNA01
gi|29165615|ref|NC_002745.2|	507092	+	A	0	0	0	0	1	SArRNA01
gi|29165615|ref|NC_002745.2|	507093	+	C	5	7	0	0	0	SArRNA01
gi|29165615|ref|NC_002745.2|	507094	+	G	0	0	0	0	2	SArRNA01
gi|29165615|ref|NC_002745.2|	507096	+	G	0	0	2	0	0	SArRNA01
gi|29165615|ref|NC_002745.2|	507097	+	G	2	2	0	0	1	SArRNA01
gi|29165615|ref|NC_002745.2|	507098	+	A	0	2	0	0	1	SArRNA01
gi|29165615|ref|NC_002745.2|	507099	+	C	21	10	6	2	21	SArRNA01
gi|29165615|ref|NC_002745.2|	507100	+	C	8	5	2	2	4	SArRNA01
gi|29165615|ref|NC_002745.2|	507101	+	C	8	6	1	1	5	SArRNA01
gi|29165615|ref|NC_002745.2|	507102	+	G	1	1	5	0	8	SArRNA01
gi|29165615|ref|NC_002745.2|	507103	+	C	6	8	2	4	6	SArRNA01
gi|29165615|ref|NC_002745.2|	507104	+	A	8	6	1	3	2	SArRNA01
gi|29165615|ref|NC_002745.2|	507105	+	C	19	9	6	5	9	SArRNA01
gi|29165615|ref|NC_002745.2|	507106	+	A	4	9	3	3	8	SArRNA01
gi|29165615|ref|NC_002745.2|	507107	+	A	2	7	0	1	3	SArRNA01
gi|29165615|ref|NC_002745.2|	507108	+	G	5	0	1	0	0	SArRNA01
gi|29165615|ref|NC_002745.2|	507109	+	C	9	3	2	4	4	SArRNA01
gi|29165615|ref|NC_002745.2|	507110	+	G	3	1	0	0	1	SArRNA01
gi|29165615|ref|NC_002745.2|	507111	+	G	0	1	1	0	3	SArRNA01
gi|29165615|ref|NC_002745.2|	507112	+	U	10	0	1	5	2	SArRNA01
gi|29165615|ref|NC_002745.2|	507113	+	G	0	2	0	0	2	SArRNA01
gi|29165615|ref|NC_002745.2|	507114	+	G	2	1	1	1	1	SArRNA01
gi|29165615|ref|NC_002745.2|	507115	+	A	2	1	0	2	4	SArRNA01
gi|29165615|ref|NC_002745.2|	507116	+	G	4	1	1	3	3	SArRNA01
gi|29165615|ref|NC_002745.2|	507117	+	C	11	4	4	4	3	SArRNA01
gi|29165615|ref|NC_002745.2|	507118	+	A	4	2	3	3	1	SArRNA01
gi|29165615|ref|NC_002745.2|	507120	+	G	1	0	0	1	0	SArRNA01
gi|29165615|ref|NC_002745.2|	507121	+	U	0	1	0	0	0	SArRNA01
gi|29165615|ref|NC_002745.2|	507122	+	G	1	1	0	1	0	SArRNA01
gi|29165615|ref|NC_002745.2|	507123	+	G	0	0	0	1	0	SArRNA01
gi|29165615|ref|NC_002745.2|	507124	+	U	0	2	0	0	1	SArRNA01
gi|29165615|ref|NC_002745.2|	507125	+	U	0	1	1	0	1	SArRNA01

gi|29165615|ref|NC_002745.2|	507126	+	U	1	2	0	0	0	SArRNA01
gi|29165615|ref|NC_002745.2|	507127	+	A	3	6	0	0	2	SArRNA01
gi|29165615|ref|NC_002745.2|	507128	+	A	6	14	3	2	7	SArRNA01
gi|29165615|ref|NC_002745.2|	507129	+	U	0	2	0	0	2	SArRNA01
gi|29165615|ref|NC_002745.2|	507130	+	U	1	0	1	1	3	SArRNA01
gi|29165615|ref|NC_002745.2|	507131	+	C	1	7	0	1	1	SArRNA01
gi|29165615|ref|NC_002745.2|	507132	+	G	1	2	0	0	1	SArRNA01
gi|29165615|ref|NC_002745.2|	507133	+	A	1	1	1	1	2	SArRNA01
gi|29165615|ref|NC_002745.2|	507134	+	A	1	0	0	2	0	SArRNA01
gi|29165615|ref|NC_002745.2|	507135	+	G	2	3	0	1	0	SArRNA01
gi|29165615|ref|NC_002745.2|	507136	+	C	1	2	0	1	0	SArRNA01
gi|29165615|ref|NC_002745.2|	507137	+	A	15	5	1	4	5	SArRNA01
gi|29165615|ref|NC_002745.2|	507138	+	A	0	2	0	0	0	SArRNA01
gi|29165615|ref|NC_002745.2|	507139	+	C	9	7	1	2	4	SArRNA01
gi|29165615|ref|NC_002745.2|	507140	+	G	4	1	0	0	1	SArRNA01
gi|29165615|ref|NC_002745.2|	507141	+	C	2	0	2	0	1	SArRNA01
gi|29165615|ref|NC_002745.2|	507142	+	G	8	2	1	3	4	SArRNA01
gi|29165615|ref|NC_002745.2|	507143	+	A	3	0	1	0	2	SArRNA01
gi|29165615|ref|NC_002745.2|	507144	+	A	1	2	1	2	1	SArRNA01
gi|29165615|ref|NC_002745.2|	507145	+	G	2	0	0	0	1	SArRNA01
gi|29165615|ref|NC_002745.2|	507146	+	A	1	3	2	1	2	SArRNA01
gi|29165615|ref|NC_002745.2|	507147	+	A	0	2	1	0	1	SArRNA01
gi|29165615|ref|NC_002745.2|	507148	+	C	7	3	0	1	1	SArRNA01
gi|29165615|ref|NC_002745.2|	507149	+	C	7	2	1	5	6	SArRNA01
gi|29165615|ref|NC_002745.2|	507150	+	U	1	4	0	1	2	SArRNA01
gi|29165615|ref|NC_002745.2|	507151	+	U	2	3	2	3	1	SArRNA01
gi|29165615|ref|NC_002745.2|	507152	+	A	0	0	1	0	0	SArRNA01
gi|29165615|ref|NC_002745.2|	507153	+	C	1	0	1	0	0	SArRNA01
gi|29165615|ref|NC_002745.2|	507154	+	C	2	2	3	11	3	SArRNA01
gi|29165615|ref|NC_002745.2|	507155	+	A	5	2	1	3	2	SArRNA01
gi|29165615|ref|NC_002745.2|	507156	+	A	1	0	0	1	1	SArRNA01
gi|29165615|ref|NC_002745.2|	507157	+	A	0	0	0	0	1	SArRNA01
gi|29165615|ref|NC_002745.2|	507158	+	U	0	2	2	0	0	SArRNA01
gi|29165615|ref|NC_002745.2|	507159	+	C	2	6	1	1	1	SArRNA01
gi|29165615|ref|NC_002745.2|	507160	+	U	4	4	1	4	0	SArRNA01
gi|29165615|ref|NC_002745.2|	507161	+	U	1	1	1	0	0	SArRNA01
gi|29165615|ref|NC_002745.2|	507163	+	A	0	0	0	0	1	SArRNA01
gi|29165615|ref|NC_002745.2|	507164	+	C	7	5	5	6	8	SArRNA01
gi|29165615|ref|NC_002745.2|	507165	+	A	0	8	2	2	0	SArRNA01
gi|29165615|ref|NC_002745.2|	507166	+	U	0	0	0	2	0	SArRNA01
gi|29165615|ref|NC_002745.2|	507167	+	C	1	2	0	0	0	SArRNA01
gi|29165615|ref|NC_002745.2|	507168	+	C	1	4	0	1	1	SArRNA01
gi|29165615|ref|NC_002745.2|	507169	+	U	2	0	2	1	2	SArRNA01
gi|29165615|ref|NC_002745.2|	507170	+	U	0	3	1	0	1	SArRNA01
gi|29165615|ref|NC_002745.2|	507171	+	U	0	3	0	0	1	SArRNA01
gi|29165615|ref|NC_002745.2|	507174	+	C	3	2	1	0	2	SArRNA01
gi|29165615|ref|NC_002745.2|	507175	+	A	0	1	0	1	1	SArRNA01
gi|29165615|ref|NC_002745.2|	507176	+	A	5	2	2	0	0	SArRNA01
gi|29165615|ref|NC_002745.2|	507177	+	C	1	0	0	3	2	SArRNA01
gi|29165615|ref|NC_002745.2|	507178	+	U	0	0	0	0	4	SArRNA01
gi|29165615|ref|NC_002745.2|	507179	+	C	5	1	0	1	1	SArRNA01
gi|29165615|ref|NC_002745.2|	507180	+	U	1	2	0	0	2	SArRNA01
gi|29165615|ref|NC_002745.2|	507181	+	A	2	1	0	0	0	SArRNA01
gi|29165615|ref|NC_002745.2|	507182	+	G	2	1	1	1	1	SArRNA01
gi|29165615|ref|NC_002745.2|	507183	+	A	0	0	1	1	1	SArRNA01
gi|29165615|ref|NC_002745.2|	507184	+	G	2	1	0	0	3	SArRNA01
gi|29165615|ref|NC_002745.2|	507185	+	A	0	2	2	1	1	SArRNA01
gi|29165615|ref|NC_002745.2|	507186	+	U	3	0	1	1	0	SArRNA01
gi|29165615|ref|NC_002745.2|	507187	+	A	1	0	0	1	1	SArRNA01
gi|29165615|ref|NC_002745.2|	507188	+	G	0	0	1	0	0	SArRNA01
gi|29165615|ref|NC_002745.2|	507190	+	G	0	0	0	1	0	SArRNA01
gi|29165615|ref|NC_002745.2|	507191	+	C	2	0	0	1	0	SArRNA01
gi|29165615|ref|NC_002745.2|	507192	+	U	32	7	3	6	4	SArRNA01
gi|29165615|ref|NC_002745.2|	507193	+	U	1	1	0	0	0	SArRNA01
gi|29165615|ref|NC_002745.2|	507194	+	U	2	1	1	0	0	SArRNA01
gi|29165615|ref|NC_002745.2|	507195	+	C	14	4	1	5	3	SArRNA01
gi|29165615|ref|NC_002745.2|	507196	+	C	4	6	1	1	2	SArRNA01
gi|29165615|ref|NC_002745.2|	507197	+	C	0	0	0	1	2	SArRNA01
gi|29165615|ref|NC_002745.2|	507198	+	C	0	0	1	0	3	SArRNA01
gi|29165615|ref|NC_002745.2|	507199	+	U	0	1	0	0	0	SArRNA01
gi|29165615|ref|NC_002745.2|	507201	+	C	3	1	0	0	1	SArRNA01
gi|29165615|ref|NC_002745.2|	507203	+	G	0	1	0	0	0	SArRNA01
gi|29165615|ref|NC_002745.2|	507205	+	G	0	0	0	0	2	SArRNA01
gi|29165615|ref|NC_002745.2|	507206	+	G	0	1	0	0	0	SArRNA01
gi|29165615|ref|NC_002745.2|	507207	+	A	0	0	1	0	1	SArRNA01
gi|29165615|ref|NC_002745.2|	507208	+	C	9	0	1	0	0	SArRNA01
gi|29165615|ref|NC_002745.2|	507209	+	A	3	1	0	0	1	SArRNA01
gi|29165615|ref|NC_002745.2|	507210	+	A	0	1	0	0	0	SArRNA01
gi|29165615|ref|NC_002745.2|	507211	+	A	0	1	0	0	3	SArRNA01
gi|29165615|ref|NC_002745.2|	507212	+	G	0	1	0	0	3	SArRNA01
gi|29165615|ref|NC_002745.2|	507213	+	U	0	0	0	0	1	SArRNA01

gi|29165615|ref|NC_002745.2|	507214	+	G	1	1	1	0	1	SArRNA01
gi|29165615|ref|NC_002745.2|	507215	+	A	0	1	1	0	0	SArRNA01
gi|29165615|ref|NC_002745.2|	507216	+	C	21	35	8	18	16	SArRNA01
gi|29165615|ref|NC_002745.2|	507217	+	A	4	2	0	0	1	SArRNA01
gi|29165615|ref|NC_002745.2|	507218	+	G	0	1	0	0	2	SArRNA01
gi|29165615|ref|NC_002745.2|	507219	+	G	0	1	0	0	2	SArRNA01
gi|29165615|ref|NC_002745.2|	507220	+	U	4	1	0	0	1	SArRNA01
gi|29165615|ref|NC_002745.2|	507222	+	G	2	1	0	1	1	SArRNA01
gi|29165615|ref|NC_002745.2|	507223	+	U	1	0	2	1	0	SArRNA01
gi|29165615|ref|NC_002745.2|	507224	+	G	2	0	0	0	0	SArRNA01
gi|29165615|ref|NC_002745.2|	507225	+	C	0	0	0	0	1	SArRNA01
gi|29165615|ref|NC_002745.2|	507226	+	A	2	0	0	0	2	SArRNA01
gi|29165615|ref|NC_002745.2|	507227	+	U	1	1	0	0	0	SArRNA01
gi|29165615|ref|NC_002745.2|	507228	+	G	3	1	2	0	0	SArRNA01
gi|29165615|ref|NC_002745.2|	507230	+	U	0	0	0	0	3	SArRNA01
gi|29165615|ref|NC_002745.2|	507231	+	U	3	0	0	0	0	SArRNA01
gi|29165615|ref|NC_002745.2|	507233	+	U	1	0	0	0	0	SArRNA01
gi|29165615|ref|NC_002745.2|	507234	+	C	2	0	0	0	0	SArRNA01
gi|29165615|ref|NC_002745.2|	507235	+	G	0	0	0	0	1	SArRNA01
gi|29165615|ref|NC_002745.2|	507236	+	U	0	0	0	0	2	SArRNA01
gi|29165615|ref|NC_002745.2|	507237	+	C	1	0	0	0	0	SArRNA01
gi|29165615|ref|NC_002745.2|	507240	+	C	0	1	0	1	1	SArRNA01
gi|29165615|ref|NC_002745.2|	507242	+	C	1	1	0	0	0	SArRNA01
gi|29165615|ref|NC_002745.2|	507249	+	U	2	0	0	0	0	SArRNA01
gi|29165615|ref|NC_002745.2|	507251	+	A	1	0	0	0	0	SArRNA01
gi|29165615|ref|NC_002745.2|	507252	+	G	1	0	0	0	2	SArRNA01
gi|29165615|ref|NC_002745.2|	507254	+	U	1	2	0	0	0	SArRNA01
gi|29165615|ref|NC_002745.2|	507259	+	G	0	0	1	5	1	SArRNA01
gi|29165615|ref|NC_002745.2|	507262	+	U	0	0	1	0	0	SArRNA01
gi|29165615|ref|NC_002745.2|	507263	+	A	0	0	1	0	0	SArRNA01
gi|29165615|ref|NC_002745.2|	507264	+	A	1	1	0	0	3	SArRNA01
gi|29165615|ref|NC_002745.2|	507265	+	G	0	0	0	0	1	SArRNA01
gi|29165615|ref|NC_002745.2|	507266	+	U	2	0	0	0	0	SArRNA01
gi|29165615|ref|NC_002745.2|	507267	+	C	2	4	0	0	2	SArRNA01
gi|29165615|ref|NC_002745.2|	507268	+	C	1	4	1	1	1	SArRNA01
gi|29165615|ref|NC_002745.2|	507269	+	C	3	3	0	0	0	SArRNA01
gi|29165615|ref|NC_002745.2|	507270	+	G	2	6	0	0	1	SArRNA01
gi|29165615|ref|NC_002745.2|	507271	+	C	0	2	0	0	0	SArRNA01
gi|29165615|ref|NC_002745.2|	507272	+	A	4	0	0	0	0	SArRNA01
gi|29165615|ref|NC_002745.2|	507273	+	A	16	0	1	0	2	SArRNA01
gi|29165615|ref|NC_002745.2|	507274	+	C	34	4	2	5	4	SArRNA01
gi|29165615|ref|NC_002745.2|	507275	+	G	13	2	1	1	1	SArRNA01
gi|29165615|ref|NC_002745.2|	507276	+	A	8	2	1	1	2	SArRNA01
gi|29165615|ref|NC_002745.2|	507277	+	G	2	0	0	0	1	SArRNA01
gi|29165615|ref|NC_002745.2|	507278	+	C	12	4	2	1	1	SArRNA01
gi|29165615|ref|NC_002745.2|	507279	+	G	5	4	0	1	1	SArRNA01
gi|29165615|ref|NC_002745.2|	507280	+	C	23	2	2	3	2	SArRNA01
gi|29165615|ref|NC_002745.2|	507281	+	A	14	11	0	3	2	SArRNA01
gi|29165615|ref|NC_002745.2|	507282	+	A	6	5	2	3	8	SArRNA01
gi|29165615|ref|NC_002745.2|	507283	+	C	34	3	8	4	7	SArRNA01
gi|29165615|ref|NC_002745.2|	507284	+	C	24	14	2	4	4	SArRNA01
gi|29165615|ref|NC_002745.2|	507285	+	C	13	2	1	2	3	SArRNA01
gi|29165615|ref|NC_002745.2|	507286	+	U	4	8	2	4	6	SArRNA01
gi|29165615|ref|NC_002745.2|	507287	+	U	5	7	0	0	2	SArRNA01
gi|29165615|ref|NC_002745.2|	507288	+	A	2	8	0	1	1	SArRNA01
gi|29165615|ref|NC_002745.2|	507289	+	A	1	0	0	0	0	SArRNA01
gi|29165615|ref|NC_002745.2|	507291	+	C	0	0	0	0	2	SArRNA01
gi|29165615|ref|NC_002745.2|	507292	+	U	0	0	0	1	0	SArRNA01
gi|29165615|ref|NC_002745.2|	507293	+	U	0	2	0	1	3	SArRNA01
gi|29165615|ref|NC_002745.2|	507294	+	A	0	6	0	1	2	SArRNA01
gi|29165615|ref|NC_002745.2|	507295	+	G	1	2	0	0	3	SArRNA01
gi|29165615|ref|NC_002745.2|	507296	+	U	6	8	0	5	1	SArRNA01
gi|29165615|ref|NC_002745.2|	507297	+	U	5	4	2	0	2	SArRNA01
gi|29165615|ref|NC_002745.2|	507298	+	G	5	6	3	1	2	SArRNA01
gi|29165615|ref|NC_002745.2|	507299	+	C	4	9	0	1	3	SArRNA01
gi|29165615|ref|NC_002745.2|	507300	+	C	13	7	9	4	11	SArRNA01
gi|29165615|ref|NC_002745.2|	507301	+	A	10	15	4	6	7	SArRNA01
gi|29165615|ref|NC_002745.2|	507302	+	U	21	28	4	8	7	SArRNA01
gi|29165615|ref|NC_002745.2|	507303	+	C	11	5	2	8	9	SArRNA01
gi|29165615|ref|NC_002745.2|	507304	+	A	7	3	1	5	11	SArRNA01
gi|29165615|ref|NC_002745.2|	507305	+	U	8	5	3	0	5	SArRNA01
gi|29165615|ref|NC_002745.2|	507306	+	U	4	13	0	3	9	SArRNA01
gi|29165615|ref|NC_002745.2|	507307	+	A	11	3	0	3	3	SArRNA01
gi|29165615|ref|NC_002745.2|	507308	+	A	8	5	4	4	4	SArRNA01
gi|29165615|ref|NC_002745.2|	507309	+	G	5	5	1	2	4	SArRNA01
gi|29165615|ref|NC_002745.2|	507310	+	U	2	1	1	1	1	SArRNA01
gi|29165615|ref|NC_002745.2|	507311	+	U	2	12	1	1	4	SArRNA01
gi|29165615|ref|NC_002745.2|	507312	+	G	0	3	2	1	2	SArRNA01
gi|29165615|ref|NC_002745.2|	507313	+	G	1	2	0	1	1	SArRNA01
gi|29165615|ref|NC_002745.2|	507314	+	G	1	3	1	0	3	SArRNA01
gi|29165615|ref|NC_002745.2|	507315	+	C	12	14	8	4	10	SArRNA01

gi|29165615|ref|NC_002745.2|	507316	+	A	3	1	2	4	2	SArRNA01
gi|29165615|ref|NC_002745.2|	507317	+	C	2	7	0	5	5	SArRNA01
gi|29165615|ref|NC_002745.2|	507318	+	U	1	5	2	0	2	SArRNA01
gi|29165615|ref|NC_002745.2|	507319	+	C	4	8	2	1	1	SArRNA01
gi|29165615|ref|NC_002745.2|	507320	+	U	2	4	0	0	5	SArRNA01
gi|29165615|ref|NC_002745.2|	507321	+	A	3	4	0	3	1	SArRNA01
gi|29165615|ref|NC_002745.2|	507322	+	A	4	4	1	0	2	SArRNA01
gi|29165615|ref|NC_002745.2|	507323	+	G	9	14	1	1	7	SArRNA01
gi|29165615|ref|NC_002745.2|	507324	+	U	1	3	0	0	5	SArRNA01
gi|29165615|ref|NC_002745.2|	507325	+	U	0	0	3	2	1	SArRNA01
gi|29165615|ref|NC_002745.2|	507326	+	G	0	3	0	1	1	SArRNA01
gi|29165615|ref|NC_002745.2|	507327	+	A	1	3	0	0	1	SArRNA01
gi|29165615|ref|NC_002745.2|	507328	+	C	3	7	1	2	3	SArRNA01
gi|29165615|ref|NC_002745.2|	507329	+	U	6	1	1	1	1	SArRNA01
gi|29165615|ref|NC_002745.2|	507330	+	G	2	0	0	0	0	SArRNA01
gi|29165615|ref|NC_002745.2|	507331	+	C	1	0	0	0	0	SArRNA01
gi|29165615|ref|NC_002745.2|	507332	+	C	3	5	0	0	4	SArRNA01
gi|29165615|ref|NC_002745.2|	507333	+	G	7	3	0	0	1	SArRNA01
gi|29165615|ref|NC_002745.2|	507334	+	G	1	4	0	1	1	SArRNA01
gi|29165615|ref|NC_002745.2|	507335	+	U	1	3	3	2	4	SArRNA01
gi|29165615|ref|NC_002745.2|	507336	+	G	6	3	1	1	2	SArRNA01
gi|29165615|ref|NC_002745.2|	507337	+	A	1	4	2	1	1	SArRNA01
gi|29165615|ref|NC_002745.2|	507338	+	C	8	19	6	3	15	SArRNA01
gi|29165615|ref|NC_002745.2|	507339	+	A	5	6	0	3	6	SArRNA01
gi|29165615|ref|NC_002745.2|	507340	+	A	15	50	2	3	17	SArRNA01
gi|29165615|ref|NC_002745.2|	507341	+	A	2	8	1	0	6	SArRNA01
gi|29165615|ref|NC_002745.2|	507342	+	C	7	3	5	4	4	SArRNA01
gi|29165615|ref|NC_002745.2|	507343	+	C	11	13	4	5	3	SArRNA01
gi|29165615|ref|NC_002745.2|	507344	+	G	5	10	1	0	4	SArRNA01
gi|29165615|ref|NC_002745.2|	507345	+	G	11	3	3	6	2	SArRNA01
gi|29165615|ref|NC_002745.2|	507346	+	A	4	3	0	5	2	SArRNA01
gi|29165615|ref|NC_002745.2|	507347	+	G	4	8	1	3	2	SArRNA01
gi|29165615|ref|NC_002745.2|	507348	+	G	1	6	3	2	5	SArRNA01
gi|29165615|ref|NC_002745.2|	507349	+	A	16	14	13	15	20	SArRNA01
gi|29165615|ref|NC_002745.2|	507350	+	A	10	16	3	7	7	SArRNA01
gi|29165615|ref|NC_002745.2|	507351	+	G	2	1	0	1	1	SArRNA01
gi|29165615|ref|NC_002745.2|	507352	+	G	8	5	3	3	6	SArRNA01
gi|29165615|ref|NC_002745.2|	507353	+	U	3	3	1	0	0	SArRNA01
gi|29165615|ref|NC_002745.2|	507354	+	G	0	0	0	2	0	SArRNA01
gi|29165615|ref|NC_002745.2|	507355	+	G	0	1	0	0	0	SArRNA01
gi|29165615|ref|NC_002745.2|	507357	+	G	2	3	0	3	2	SArRNA01
gi|29165615|ref|NC_002745.2|	507358	+	A	1	4	2	3	1	SArRNA01
gi|29165615|ref|NC_002745.2|	507359	+	U	3	7	1	1	3	SArRNA01
gi|29165615|ref|NC_002745.2|	507360	+	G	3	2	0	1	8	SArRNA01
gi|29165615|ref|NC_002745.2|	507361	+	A	10	4	2	2	8	SArRNA01
gi|29165615|ref|NC_002745.2|	507362	+	C	3	3	2	2	7	SArRNA01
gi|29165615|ref|NC_002745.2|	507363	+	G	2	2	5	2	2	SArRNA01
gi|29165615|ref|NC_002745.2|	507364	+	U	0	3	1	3	1	SArRNA01
gi|29165615|ref|NC_002745.2|	507365	+	C	4	1	0	1	4	SArRNA01
gi|29165615|ref|NC_002745.2|	507366	+	A	0	0	0	2	4	SArRNA01
gi|29165615|ref|NC_002745.2|	507367	+	A	7	20	2	3	10	SArRNA01
gi|29165615|ref|NC_002745.2|	507368	+	A	7	14	2	6	10	SArRNA01
gi|29165615|ref|NC_002745.2|	507369	+	U	9	8	6	6	10	SArRNA01
gi|29165615|ref|NC_002745.2|	507370	+	C	11	20	4	8	10	SArRNA01
gi|29165615|ref|NC_002745.2|	507371	+	A	7	11	3	8	3	SArRNA01
gi|29165615|ref|NC_002745.2|	507372	+	U	5	2	0	1	5	SArRNA01
gi|29165615|ref|NC_002745.2|	507373	+	C	6	15	0	6	4	SArRNA01
gi|29165615|ref|NC_002745.2|	507374	+	A	4	4	0	1	4	SArRNA01
gi|29165615|ref|NC_002745.2|	507375	+	U	4	4	0	0	1	SArRNA01
gi|29165615|ref|NC_002745.2|	507376	+	G	0	1	0	1	1	SArRNA01
gi|29165615|ref|NC_002745.2|	507377	+	C	22	17	5	11	13	SArRNA01
gi|29165615|ref|NC_002745.2|	507378	+	C	7	13	3	5	3	SArRNA01
gi|29165615|ref|NC_002745.2|	507379	+	C	4	4	0	1	5	SArRNA01
gi|29165615|ref|NC_002745.2|	507380	+	C	2	4	4	1	7	SArRNA01
gi|29165615|ref|NC_002745.2|	507381	+	U	5	3	2	0	2	SArRNA01
gi|29165615|ref|NC_002745.2|	507382	+	U	7	7	3	1	4	SArRNA01
gi|29165615|ref|NC_002745.2|	507383	+	A	7	4	2	3	4	SArRNA01
gi|29165615|ref|NC_002745.2|	507384	+	U	11	7	6	6	11	SArRNA01
gi|29165615|ref|NC_002745.2|	507385	+	G	16	10	6	10	11	SArRNA01
gi|29165615|ref|NC_002745.2|	507386	+	A	9	5	1	8	13	SArRNA01
gi|29165615|ref|NC_002745.2|	507387	+	U	5	7	3	7	13	SArRNA01
gi|29165615|ref|NC_002745.2|	507388	+	U	6	1	0	0	5	SArRNA01
gi|29165615|ref|NC_002745.2|	507389	+	U	1	4	0	0	0	SArRNA01
gi|29165615|ref|NC_002745.2|	507390	+	G	1	1	1	1	0	SArRNA01
gi|29165615|ref|NC_002745.2|	507391	+	G	0	1	0	1	2	SArRNA01
gi|29165615|ref|NC_002745.2|	507392	+	G	2	1	0	1	1	SArRNA01
gi|29165615|ref|NC_002745.2|	507393	+	C	8	9	1	2	2	SArRNA01
gi|29165615|ref|NC_002745.2|	507394	+	U	8	9	3	5	6	SArRNA01
gi|29165615|ref|NC_002745.2|	507395	+	A	6	2	0	3	4	SArRNA01
gi|29165615|ref|NC_002745.2|	507396	+	C	6	7	0	2	3	SArRNA01
gi|29165615|ref|NC_002745.2|	507397	+	A	6	7	2	0	8	SArRNA01

gi|29165615|ref|NC_002745.2|	507398	+	C	46	29	17	14	30	SArRNA01
gi|29165615|ref|NC_002745.2|	507399	+	A	4	12	0	0	6	SArRNA01
gi|29165615|ref|NC_002745.2|	507400	+	C	0	5	1	3	0	SArRNA01
gi|29165615|ref|NC_002745.2|	507401	+	G	4	2	3	2	2	SArRNA01
gi|29165615|ref|NC_002745.2|	507402	+	U	0	0	1	0	0	SArRNA01
gi|29165615|ref|NC_002745.2|	507403	+	G	1	0	0	0	0	SArRNA01
gi|29165615|ref|NC_002745.2|	507404	+	C	5	3	4	0	2	SArRNA01
gi|29165615|ref|NC_002745.2|	507405	+	U	1	5	3	5	3	SArRNA01
gi|29165615|ref|NC_002745.2|	507406	+	A	0	2	0	0	0	SArRNA01
gi|29165615|ref|NC_002745.2|	507407	+	C	14	14	4	8	16	SArRNA01
gi|29165615|ref|NC_002745.2|	507408	+	A	1	7	4	4	5	SArRNA01
gi|29165615|ref|NC_002745.2|	507409	+	A	0	7	0	0	1	SArRNA01
gi|29165615|ref|NC_002745.2|	507410	+	U	0	5	1	1	1	SArRNA01
gi|29165615|ref|NC_002745.2|	507411	+	G	6	6	0	1	3	SArRNA01
gi|29165615|ref|NC_002745.2|	507412	+	G	15	11	6	7	2	SArRNA01
gi|29165615|ref|NC_002745.2|	507413	+	A	1	6	0	0	1	SArRNA01
gi|29165615|ref|NC_002745.2|	507414	+	C	51	40	21	15	29	SArRNA01
gi|29165615|ref|NC_002745.2|	507415	+	A	18	17	8	5	10	SArRNA01
gi|29165615|ref|NC_002745.2|	507416	+	A	24	213	56	12	114	SArRNA01
gi|29165615|ref|NC_002745.2|	507417	+	U	4	3	2	2	5	SArRNA01
gi|29165615|ref|NC_002745.2|	507418	+	A	2	9	3	6	6	SArRNA01
gi|29165615|ref|NC_002745.2|	507419	+	C	3	11	2	1	3	SArRNA01
gi|29165615|ref|NC_002745.2|	507420	+	A	1	1	0	2	3	SArRNA01
gi|29165615|ref|NC_002745.2|	507421	+	A	3	17	0	1	6	SArRNA01
gi|29165615|ref|NC_002745.2|	507422	+	A	4	20	0	2	9	SArRNA01
gi|29165615|ref|NC_002745.2|	507423	+	G	0	0	0	2	0	SArRNA01
gi|29165615|ref|NC_002745.2|	507424	+	G	0	2	0	0	0	SArRNA01
gi|29165615|ref|NC_002745.2|	507425	+	G	1	1	0	1	0	SArRNA01
gi|29165615|ref|NC_002745.2|	507426	+	C	10	5	3	4	1	SArRNA01
gi|29165615|ref|NC_002745.2|	507427	+	A	1	8	1	0	3	SArRNA01
gi|29165615|ref|NC_002745.2|	507428	+	G	0	2	0	0	0	SArRNA01
gi|29165615|ref|NC_002745.2|	507429	+	C	2	2	3	3	3	SArRNA01
gi|29165615|ref|NC_002745.2|	507430	+	G	0	0	0	0	2	SArRNA01
gi|29165615|ref|NC_002745.2|	507431	+	A	0	0	0	0	2	SArRNA01
gi|29165615|ref|NC_002745.2|	507432	+	A	6	7	3	3	6	SArRNA01
gi|29165615|ref|NC_002745.2|	507433	+	A	3	5	2	3	2	SArRNA01
gi|29165615|ref|NC_002745.2|	507434	+	C	2	1	0	0	2	SArRNA01
gi|29165615|ref|NC_002745.2|	507435	+	C	3	4	2	2	6	SArRNA01
gi|29165615|ref|NC_002745.2|	507436	+	G	0	0	0	0	4	SArRNA01
gi|29165615|ref|NC_002745.2|	507437	+	U	0	3	0	0	0	SArRNA01
gi|29165615|ref|NC_002745.2|	507439	+	A	1	1	0	0	1	SArRNA01
gi|29165615|ref|NC_002745.2|	507440	+	G	0	1	0	0	0	SArRNA01
gi|29165615|ref|NC_002745.2|	507441	+	G	1	0	0	1	2	SArRNA01
gi|29165615|ref|NC_002745.2|	507442	+	U	0	1	0	0	1	SArRNA01
gi|29165615|ref|NC_002745.2|	507443	+	C	11	9	6	5	3	SArRNA01
gi|29165615|ref|NC_002745.2|	507444	+	A	2	1	1	1	4	SArRNA01
gi|29165615|ref|NC_002745.2|	507445	+	A	1	0	2	1	1	SArRNA01
gi|29165615|ref|NC_002745.2|	507446	+	G	2	0	0	1	0	SArRNA01
gi|29165615|ref|NC_002745.2|	507447	+	C	0	0	0	0	1	SArRNA01
gi|29165615|ref|NC_002745.2|	507448	+	A	2	2	0	1	1	SArRNA01
gi|29165615|ref|NC_002745.2|	507449	+	A	0	2	0	0	3	SArRNA01
gi|29165615|ref|NC_002745.2|	507450	+	A	9	16	1	3	11	SArRNA01
gi|29165615|ref|NC_002745.2|	507451	+	U	14	20	3	13	13	SArRNA01
gi|29165615|ref|NC_002745.2|	507452	+	C	14	29	12	13	14	SArRNA01
gi|29165615|ref|NC_002745.2|	507453	+	C	14	11	7	5	8	SArRNA01
gi|29165615|ref|NC_002745.2|	507454	+	C	2	3	1	1	1	SArRNA01
gi|29165615|ref|NC_002745.2|	507455	+	A	0	3	1	1	1	SArRNA01
gi|29165615|ref|NC_002745.2|	507456	+	U	1	3	1	0	2	SArRNA01
gi|29165615|ref|NC_002745.2|	507457	+	A	2	3	0	0	1	SArRNA01
gi|29165615|ref|NC_002745.2|	507458	+	A	0	1	0	0	3	SArRNA01
gi|29165615|ref|NC_002745.2|	507459	+	A	1	3	1	0	2	SArRNA01
gi|29165615|ref|NC_002745.2|	507460	+	G	2	0	0	0	0	SArRNA01
gi|29165615|ref|NC_002745.2|	507461	+	U	0	1	1	1	2	SArRNA01
gi|29165615|ref|NC_002745.2|	507462	+	U	2	4	0	1	0	SArRNA01
gi|29165615|ref|NC_002745.2|	507463	+	G	4	6	1	0	5	SArRNA01
gi|29165615|ref|NC_002745.2|	507464	+	U	0	0	1	0	1	SArRNA01
gi|29165615|ref|NC_002745.2|	507465	+	U	3	1	0	0	1	SArRNA01
gi|29165615|ref|NC_002745.2|	507466	+	C	3	6	3	1	5	SArRNA01
gi|29165615|ref|NC_002745.2|	507467	+	U	2	1	3	0	1	SArRNA01
gi|29165615|ref|NC_002745.2|	507468	+	C	1	8	0	0	1	SArRNA01
gi|29165615|ref|NC_002745.2|	507469	+	A	2	1	3	0	3	SArRNA01
gi|29165615|ref|NC_002745.2|	507470	+	G	0	1	0	2	1	SArRNA01
gi|29165615|ref|NC_002745.2|	507471	+	U	2	3	2	0	1	SArRNA01
gi|29165615|ref|NC_002745.2|	507472	+	U	0	3	2	2	1	SArRNA01
gi|29165615|ref|NC_002745.2|	507473	+	C	3	5	4	2	6	SArRNA01
gi|29165615|ref|NC_002745.2|	507474	+	G	0	0	0	0	1	SArRNA01
gi|29165615|ref|NC_002745.2|	507475	+	G	0	0	1	0	0	SArRNA01
gi|29165615|ref|NC_002745.2|	507476	+	A	0	1	0	0	0	SArRNA01
gi|29165615|ref|NC_002745.2|	507478	+	U	0	2	0	0	0	SArRNA01
gi|29165615|ref|NC_002745.2|	507481	+	A	0	0	0	0	2	SArRNA01
gi|29165615|ref|NC_002745.2|	507483	+	U	1	0	0	1	0	SArRNA01

gi|29165615|ref|NC_002745.2|	507485	+	U	0	1	0	0	0	SArRNA01
gi|29165615|ref|NC_002745.2|	507487	+	C	0	7	1	1	2	SArRNA01
gi|29165615|ref|NC_002745.2|	507488	+	A	0	2	0	0	1	SArRNA01
gi|29165615|ref|NC_002745.2|	507489	+	A	0	1	0	0	1	SArRNA01
gi|29165615|ref|NC_002745.2|	507490	+	C	2	2	0	0	3	SArRNA01
gi|29165615|ref|NC_002745.2|	507491	+	U	1	3	0	0	1	SArRNA01
gi|29165615|ref|NC_002745.2|	507492	+	C	32	30	9	13	26	SArRNA01
gi|29165615|ref|NC_002745.2|	507493	+	G	2	7	0	4	2	SArRNA01
gi|29165615|ref|NC_002745.2|	507495	+	C	3	2	0	0	0	SArRNA01
gi|29165615|ref|NC_002745.2|	507496	+	U	0	2	0	0	0	SArRNA01
gi|29165615|ref|NC_002745.2|	507497	+	A	0	2	2	1	1	SArRNA01
gi|29165615|ref|NC_002745.2|	507498	+	C	1	0	0	1	0	SArRNA01
gi|29165615|ref|NC_002745.2|	507499	+	A	2	2	0	1	0	SArRNA01
gi|29165615|ref|NC_002745.2|	507500	+	U	0	0	2	0	1	SArRNA01
gi|29165615|ref|NC_002745.2|	507501	+	G	4	8	1	3	4	SArRNA01
gi|29165615|ref|NC_002745.2|	507502	+	A	0	0	0	1	3	SArRNA01
gi|29165615|ref|NC_002745.2|	507503	+	A	3	3	0	2	2	SArRNA01
gi|29165615|ref|NC_002745.2|	507504	+	G	3	1	0	1	1	SArRNA01
gi|29165615|ref|NC_002745.2|	507505	+	C	2	2	0	0	2	SArRNA01
gi|29165615|ref|NC_002745.2|	507506	+	U	0	1	0	0	2	SArRNA01
gi|29165615|ref|NC_002745.2|	507507	+	G	0	0	0	0	1	SArRNA01
gi|29165615|ref|NC_002745.2|	507508	+	G	0	3	1	1	1	SArRNA01
gi|29165615|ref|NC_002745.2|	507509	+	A	0	3	0	2	7	SArRNA01
gi|29165615|ref|NC_002745.2|	507510	+	A	0	5	0	1	8	SArRNA01
gi|29165615|ref|NC_002745.2|	507511	+	U	1	1	1	0	2	SArRNA01
gi|29165615|ref|NC_002745.2|	507512	+	C	0	1	0	0	2	SArRNA01
gi|29165615|ref|NC_002745.2|	507513	+	G	3	5	0	1	0	SArRNA01
gi|29165615|ref|NC_002745.2|	507514	+	C	4	6	3	4	4	SArRNA01
gi|29165615|ref|NC_002745.2|	507515	+	U	0	1	0	0	3	SArRNA01
gi|29165615|ref|NC_002745.2|	507516	+	A	0	1	0	1	0	SArRNA01
gi|29165615|ref|NC_002745.2|	507517	+	G	0	1	1	0	2	SArRNA01
gi|29165615|ref|NC_002745.2|	507518	+	U	0	1	0	0	0	SArRNA01
gi|29165615|ref|NC_002745.2|	507519	+	A	0	1	0	0	1	SArRNA01
gi|29165615|ref|NC_002745.2|	507520	+	A	1	11	1	0	5	SArRNA01
gi|29165615|ref|NC_002745.2|	507523	+	G	0	1	0	0	1	SArRNA01
gi|29165615|ref|NC_002745.2|	507524	+	U	0	4	0	1	3	SArRNA01
gi|29165615|ref|NC_002745.2|	507525	+	A	0	0	0	0	1	SArRNA01
gi|29165615|ref|NC_002745.2|	507526	+	G	0	0	0	0	1	SArRNA01
gi|29165615|ref|NC_002745.2|	507527	+	A	2	1	0	0	2	SArRNA01
gi|29165615|ref|NC_002745.2|	507528	+	U	1	3	0	2	2	SArRNA01
gi|29165615|ref|NC_002745.2|	507529	+	C	1	1	0	0	3	SArRNA01
gi|29165615|ref|NC_002745.2|	507530	+	A	0	3	0	1	1	SArRNA01
gi|29165615|ref|NC_002745.2|	507532	+	C	4	2	0	2	2	SArRNA01
gi|29165615|ref|NC_002745.2|	507533	+	A	2	3	0	0	1	SArRNA01
gi|29165615|ref|NC_002745.2|	507534	+	U	2	1	0	0	2	SArRNA01
gi|29165615|ref|NC_002745.2|	507535	+	G	0	2	0	0	0	SArRNA01
gi|29165615|ref|NC_002745.2|	507536	+	C	3	2	0	0	2	SArRNA01
gi|29165615|ref|NC_002745.2|	507537	+	U	0	0	0	0	1	SArRNA01
gi|29165615|ref|NC_002745.2|	507538	+	A	0	0	0	1	0	SArRNA01
gi|29165615|ref|NC_002745.2|	507539	+	C	0	0	0	2	1	SArRNA01
gi|29165615|ref|NC_002745.2|	507540	+	G	0	2	0	0	1	SArRNA01
gi|29165615|ref|NC_002745.2|	507541	+	G	1	3	1	0	0	SArRNA01
gi|29165615|ref|NC_002745.2|	507542	+	U	0	0	0	0	1	SArRNA01
gi|29165615|ref|NC_002745.2|	507544	+	A	0	1	0	0	0	SArRNA01
gi|29165615|ref|NC_002745.2|	507545	+	A	1	5	0	3	0	SArRNA01
gi|29165615|ref|NC_002745.2|	507546	+	U	1	5	0	1	1	SArRNA01
gi|29165615|ref|NC_002745.2|	507547	+	A	0	3	0	0	0	SArRNA01
gi|29165615|ref|NC_002745.2|	507548	+	C	1	0	0	2	0	SArRNA01
gi|29165615|ref|NC_002745.2|	507549	+	G	2	0	0	0	1	SArRNA01
gi|29165615|ref|NC_002745.2|	507551	+	U	2	1	0	0	2	SArRNA01
gi|29165615|ref|NC_002745.2|	507552	+	C	0	0	1	0	0	SArRNA01
gi|29165615|ref|NC_002745.2|	507553	+	C	0	0	0	0	1	SArRNA01
gi|29165615|ref|NC_002745.2|	507558	+	U	1	0	0	0	0	SArRNA01
gi|29165615|ref|NC_002745.2|	507559	+	C	2	3	0	0	0	SArRNA01
gi|29165615|ref|NC_002745.2|	507560	+	U	0	0	0	0	3	SArRNA01
gi|29165615|ref|NC_002745.2|	507565	+	C	0	3	0	0	0	SArRNA01
gi|29165615|ref|NC_002745.2|	507567	+	C	1	3	1	0	4	SArRNA01
gi|29165615|ref|NC_002745.2|	507568	+	A	0	1	0	0	0	SArRNA01
gi|29165615|ref|NC_002745.2|	507569	+	C	0	3	0	0	0	SArRNA01
gi|29165615|ref|NC_002745.2|	507570	+	C	0	1	0	0	1	SArRNA01
gi|29165615|ref|NC_002745.2|	507571	+	G	0	3	0	0	0	SArRNA01
gi|29165615|ref|NC_002745.2|	507574	+	C	0	1	0	0	0	SArRNA01
gi|29165615|ref|NC_002745.2|	507577	+	C	4	2	2	1	1	SArRNA01
gi|29165615|ref|NC_002745.2|	507579	+	C	5	1	0	2	0	SArRNA01
gi|29165615|ref|NC_002745.2|	507580	+	A	2	3	1	0	0	SArRNA01
gi|29165615|ref|NC_002745.2|	507581	+	C	0	3	1	0	2	SArRNA01
gi|29165615|ref|NC_002745.2|	507582	+	C	4	13	4	9	15	SArRNA01
gi|29165615|ref|NC_002745.2|	507583	+	A	0	1	3	0	1	SArRNA01
gi|29165615|ref|NC_002745.2|	507586	+	A	2	0	0	0	0	SArRNA01
gi|29165615|ref|NC_002745.2|	507587	+	G	1	0	0	0	0	SArRNA01
gi|29165615|ref|NC_002745.2|	507590	+	U	0	0	0	1	0	SArRNA01

gi|29165615|ref|NC_002745.2|	507592	+	U	0	0	0	0	1	SArRNA01
gi|29165615|ref|NC_002745.2|	507593	+	G	0	0	0	1	0	SArRNA01
gi|29165615|ref|NC_002745.2|	507594	+	U	0	0	0	0	3	SArRNA01
gi|29165615|ref|NC_002745.2|	507595	+	A	1	0	1	1	1	SArRNA01
gi|29165615|ref|NC_002745.2|	507596	+	A	1	3	0	0	1	SArRNA01
gi|29165615|ref|NC_002745.2|	507597	+	C	37	43	21	14	45	SArRNA01
gi|29165615|ref|NC_002745.2|	507598	+	A	11	18	2	4	6	SArRNA01
gi|29165615|ref|NC_002745.2|	507599	+	C	41	30	16	8	19	SArRNA01
gi|29165615|ref|NC_002745.2|	507600	+	C	3	6	3	2	11	SArRNA01
gi|29165615|ref|NC_002745.2|	507601	+	C	18	20	4	7	16	SArRNA01
gi|29165615|ref|NC_002745.2|	507602	+	G	21	19	10	6	9	SArRNA01
gi|29165615|ref|NC_002745.2|	507603	+	A	3	6	2	2	3	SArRNA01
gi|29165615|ref|NC_002745.2|	507604	+	A	0	1	0	0	0	SArRNA01
gi|29165615|ref|NC_002745.2|	507605	+	G	0	1	1	0	0	SArRNA01
gi|29165615|ref|NC_002745.2|	507606	+	C	7	14	1	5	5	SArRNA01
gi|29165615|ref|NC_002745.2|	507607	+	C	10	15	4	5	4	SArRNA01
gi|29165615|ref|NC_002745.2|	507608	+	G	5	6	1	1	2	SArRNA01
gi|29165615|ref|NC_002745.2|	507610	+	U	1	4	0	0	0	SArRNA01
gi|29165615|ref|NC_002745.2|	507611	+	G	0	0	0	0	1	SArRNA01
gi|29165615|ref|NC_002745.2|	507612	+	G	2	0	0	1	0	SArRNA01
gi|29165615|ref|NC_002745.2|	507613	+	A	1	0	0	0	4	SArRNA01
gi|29165615|ref|NC_002745.2|	507614	+	G	3	4	2	0	2	SArRNA01
gi|29165615|ref|NC_002745.2|	507615	+	U	1	4	0	1	3	SArRNA01
gi|29165615|ref|NC_002745.2|	507616	+	A	13	30	3	5	11	SArRNA01
gi|29165615|ref|NC_002745.2|	507617	+	A	5	7	0	2	8	SArRNA01
gi|29165615|ref|NC_002745.2|	507618	+	C	17	31	6	11	22	SArRNA01
gi|29165615|ref|NC_002745.2|	507619	+	C	3	7	2	1	6	SArRNA01
gi|29165615|ref|NC_002745.2|	507620	+	U	10	2	4	4	4	SArRNA01
gi|29165615|ref|NC_002745.2|	507621	+	U	6	3	3	0	8	SArRNA01
gi|29165615|ref|NC_002745.2|	507622	+	U	0	1	0	0	4	SArRNA01
gi|29165615|ref|NC_002745.2|	507623	+	U	6	8	0	3	5	SArRNA01
gi|29165615|ref|NC_002745.2|	507624	+	A	1	11	0	1	4	SArRNA01
gi|29165615|ref|NC_002745.2|	507625	+	G	5	5	2	0	0	SArRNA01
gi|29165615|ref|NC_002745.2|	507626	+	G	3	0	1	1	4	SArRNA01
gi|29165615|ref|NC_002745.2|	507627	+	A	3	1	2	1	1	SArRNA01
gi|29165615|ref|NC_002745.2|	507628	+	G	1	0	3	1	1	SArRNA01
gi|29165615|ref|NC_002745.2|	507629	+	C	203	158	56	42	112	SArRNA01
gi|29165615|ref|NC_002745.2|	507630	+	U	16	26	3	3	23	SArRNA01
gi|29165615|ref|NC_002745.2|	507631	+	A	4	7	0	0	9	SArRNA01
gi|29165615|ref|NC_002745.2|	507632	+	G	8	6	3	2	7	SArRNA01
gi|29165615|ref|NC_002745.2|	507633	+	C	4	5	3	4	4	SArRNA01
gi|29165615|ref|NC_002745.2|	507634	+	C	62	37	21	17	22	SArRNA01
gi|29165615|ref|NC_002745.2|	507635	+	G	19	16	8	4	7	SArRNA01
gi|29165615|ref|NC_002745.2|	507636	+	U	0	0	2	1	0	SArRNA01
gi|29165615|ref|NC_002745.2|	507637	+	C	8	11	0	3	4	SArRNA01
gi|29165615|ref|NC_002745.2|	507638	+	G	6	3	2	2	6	SArRNA01
gi|29165615|ref|NC_002745.2|	507639	+	A	0	2	0	0	0	SArRNA01
gi|29165615|ref|NC_002745.2|	507641	+	G	1	0	0	0	0	SArRNA01
gi|29165615|ref|NC_002745.2|	507642	+	G	0	0	1	0	0	SArRNA01
gi|29165615|ref|NC_002745.2|	507643	+	U	1	0	0	0	0	SArRNA01
gi|29165615|ref|NC_002745.2|	507645	+	G	0	1	0	0	0	SArRNA01
gi|29165615|ref|NC_002745.2|	507672	+	C	1	0	0	0	0	SArRNA01
gi|29165615|ref|NC_002745.2|	507691	+	G	0	1	0	0	0	SArRNA01
gi|29165615|ref|NC_002745.2|	507706	+	C	2	0	0	0	0	SArRNA01
gi|29165615|ref|NC_002745.2|	507707	+	C	8	2	1	2	0	SArRNA01
gi|29165615|ref|NC_002745.2|	507708	+	U	0	0	0	0	1	SArRNA01
gi|29165615|ref|NC_002745.2|	507709	+	C	0	0	0	0	2	SArRNA01
gi|29165615|ref|NC_002745.2|	507710	+	C	0	1	0	0	0	SArRNA01
gi|29165615|ref|NC_002745.2|	507718	+	G	7	42	52	13	17	-
gi|29165615|ref|NC_002745.2|	507719	+	G	1	0	3	3	5	-
gi|29165615|ref|NC_002745.2|	507720	+	A	0	0	3	2	2	-
gi|29165615|ref|NC_002745.2|	507722	+	A	0	1	0	0	1	-
gi|29165615|ref|NC_002745.2|	507724	+	A	0	0	0	0	1	-
gi|29165615|ref|NC_002745.2|	507732	+	C	2	0	0	0	0	-
gi|29165615|ref|NC_002745.2|	507736	+	U	0	0	1	0	0	-
gi|29165615|ref|NC_002745.2|	507737	+	U	0	0	1	0	0	-
gi|29165615|ref|NC_002745.2|	507740	+	G	0	1	1	0	3	-
gi|29165615|ref|NC_002745.2|	507741	+	A	0	1	2	0	1	-
gi|29165615|ref|NC_002745.2|	507743	+	G	0	0	2	0	0	-
gi|29165615|ref|NC_002745.2|	507744	+	A	0	1	0	0	0	-
gi|29165615|ref|NC_002745.2|	507745	+	A	0	1	0	0	0	-
gi|29165615|ref|NC_002745.2|	507746	+	G	0	0	1	0	0	-
gi|29165615|ref|NC_002745.2|	507779	+	U	0	0	0	0	1	-
gi|29165615|ref|NC_002745.2|	507780	+	U	8	1	3	3	1	-
gi|29165615|ref|NC_002745.2|	507801	+	U	0	0	1	0	0	-
gi|29165615|ref|NC_002745.2|	507805	+	G	9	2	1	1	2	SAtRNA04
gi|29165615|ref|NC_002745.2|	507812	+	U	1	0	0	0	0	SAtRNA04
gi|29165615|ref|NC_002745.2|	507824	+	U	0	0	0	0	1	SAtRNA04
gi|29165615|ref|NC_002745.2|	507840	+	A	1	0	0	0	0	SAtRNA04
gi|29165615|ref|NC_002745.2|	507890	+	A	1	0	0	0	0	-
gi|29165615|ref|NC_002745.2|	507899	+	U	9	5	4	2	4	-

gi|29165615|ref|NC_002745.2|	507900	+	G	1287	1680	1600	664	1174	SAtRNA05
gi|29165615|ref|NC_002745.2|	507901	+	G	1	1	0	1	1	SAtRNA05
gi|29165615|ref|NC_002745.2|	507902	+	G	0	1	0	0	0	SAtRNA05
gi|29165615|ref|NC_002745.2|	507903	+	G	0	0	1	0	0	SAtRNA05
gi|29165615|ref|NC_002745.2|	507910	+	C	1	0	0	0	0	SAtRNA05
gi|29165615|ref|NC_002745.2|	507918	+	G	0	0	0	0	1	SAtRNA05
gi|29165615|ref|NC_002745.2|	507924	+	C	0	0	0	0	2	SAtRNA05
gi|29165615|ref|NC_002745.2|	507931	+	U	2	0	0	0	0	SAtRNA05
gi|29165615|ref|NC_002745.2|	507932	+	U	0	0	0	2	1	SAtRNA05
gi|29165615|ref|NC_002745.2|	507933	+	U	0	0	1	0	0	SAtRNA05
gi|29165615|ref|NC_002745.2|	507936	+	A	1	0	0	0	0	SAtRNA05
gi|29165615|ref|NC_002745.2|	507941	+	G	3	0	0	0	0	SAtRNA05
gi|29165615|ref|NC_002745.2|	507955	+	C	1	0	0	0	0	SAtRNA05
gi|29165615|ref|NC_002745.2|	507959	+	C	2	0	0	0	0	SAtRNA05
gi|29165615|ref|NC_002745.2|	507969	+	U	1	0	0	0	0	SAtRNA05
gi|29165615|ref|NC_002745.2|	507971	+	C	1	0	0	0	2	SAtRNA05
gi|29165615|ref|NC_002745.2|	507972	+	A	0	1	0	0	0	SAtRNA05
gi|29165615|ref|NC_002745.2|	507973	+	C	0	0	0	1	0	SAtRNA05
gi|29165615|ref|NC_002745.2|	507974	+	C	0	1	0	0	0	SAtRNA05
gi|29165615|ref|NC_002745.2|	507976	+	U	0	2	1	5	1	-
gi|29165615|ref|NC_002745.2|	507977	+	U	0	0	0	2	2	-
gi|29165615|ref|NC_002745.2|	507979	+	U	0	0	0	0	1	-
gi|29165615|ref|NC_002745.2|	507980	+	U	0	0	1	0	0	-
gi|29165615|ref|NC_002745.2|	507982	+	G	1	0	0	0	0	-
gi|29165615|ref|NC_002745.2|	507983	+	U	0	1	0	0	0	-
gi|29165615|ref|NC_002745.2|	507984	+	A	0	0	1	0	0	-
gi|29165615|ref|NC_002745.2|	507985	+	C	1	0	2	2	0	-
gi|29165615|ref|NC_002745.2|	507989	+	G	0	1	1	0	0	-
gi|29165615|ref|NC_002745.2|	507990	+	A	0	2	1	0	0	-
gi|29165615|ref|NC_002745.2|	507991	+	A	2	1	0	1	0	-
gi|29165615|ref|NC_002745.2|	507992	+	A	7	2	5	4	2	-
gi|29165615|ref|NC_002745.2|	507993	+	A	1136	220	223	625	436	-
gi|29165615|ref|NC_002745.2|	507994	+	C	2	2	2	9	6	-
gi|29165615|ref|NC_002745.2|	507995	+	U	1	0	1	2	1	-
gi|29165615|ref|NC_002745.2|	507996	+	A	0	2	0	3	2	-
gi|29165615|ref|NC_002745.2|	507997	+	G	1	1	2	2	1	-
gi|29165615|ref|NC_002745.2|	507998	+	A	1	1	1	4	3	-
gi|29165615|ref|NC_002745.2|	507999	+	U	0	0	0	0	1	-
gi|29165615|ref|NC_002745.2|	508000	+	A	0	1	1	1	0	-
gi|29165615|ref|NC_002745.2|	508002	+	G	0	0	0	1	0	-
gi|29165615|ref|NC_002745.2|	508003	+	U	2	0	0	1	0	-
gi|29165615|ref|NC_002745.2|	508004	+	A	0	0	1	2	0	-
gi|29165615|ref|NC_002745.2|	508005	+	A	0	0	0	1	0	-
gi|29165615|ref|NC_002745.2|	508006	+	G	0	0	0	1	0	-
gi|29165615|ref|NC_002745.2|	508008	+	A	0	0	1	7	0	-
gi|29165615|ref|NC_002745.2|	508009	+	A	0	0	0	2	0	-
gi|29165615|ref|NC_002745.2|	508010	+	A	1	3	0	2	0	-
gi|29165615|ref|NC_002745.2|	508011	+	A	0	1	0	4	0	-
gi|29165615|ref|NC_002745.2|	508013	+	A	0	0	0	3	0	-
gi|29165615|ref|NC_002745.2|	508014	+	U	0	0	0	2	0	-
gi|29165615|ref|NC_002745.2|	508015	+	A	0	0	1	1	0	-
gi|29165615|ref|NC_002745.2|	508016	+	G	0	4	3	5	3	-
gi|29165615|ref|NC_002745.2|	508017	+	A	0	2	2	7	1	-
gi|29165615|ref|NC_002745.2|	508019	+	U	0	1	1	0	0	-
gi|29165615|ref|NC_002745.2|	508021	+	U	0	0	1	0	0	-
gi|29165615|ref|NC_002745.2|	508022	+	A	0	1	0	1	0	-
gi|29165615|ref|NC_002745.2|	508023	+	C	1	2	0	1	0	-
gi|29165615|ref|NC_002745.2|	508024	+	C	4	1	2	9	3	-
gi|29165615|ref|NC_002745.2|	508025	+	A	1	0	0	3	2	-
gi|29165615|ref|NC_002745.2|	508026	+	A	0	0	0	1	1	-
gi|29165615|ref|NC_002745.2|	508027	+	G	1	0	0	1	0	-
gi|29165615|ref|NC_002745.2|	508028	+	C	2	0	0	5	1	-
gi|29165615|ref|NC_002745.2|	508029	+	A	1	1	1	8	1	-
gi|29165615|ref|NC_002745.2|	508030	+	A	0	5	2	13	1	-
gi|29165615|ref|NC_002745.2|	508031	+	A	1	1	0	4	1	-
gi|29165615|ref|NC_002745.2|	508032	+	A	0	0	1	0	0	-
gi|29165615|ref|NC_002745.2|	508033	+	C	0	0	0	0	2	-
gi|29165615|ref|NC_002745.2|	508034	+	C	3	1	1	1	3	-
gi|29165615|ref|NC_002745.2|	508035	+	G	1	2	0	4	1	-
gi|29165615|ref|NC_002745.2|	508036	+	A	0	0	0	2	0	-
gi|29165615|ref|NC_002745.2|	508037	+	G	0	0	0	0	1	-
gi|29165615|ref|NC_002745.2|	508038	+	U	0	0	0	1	0	-
gi|29165615|ref|NC_002745.2|	508040	+	A	0	1	0	8	0	-
gi|29165615|ref|NC_002745.2|	508041	+	A	0	0	0	0	1	-
gi|29165615|ref|NC_002745.2|	508043	+	A	1	0	1	2	1	-
gi|29165615|ref|NC_002745.2|	508044	+	A	0	1	0	2	0	-
gi|29165615|ref|NC_002745.2|	508046	+	G	0	0	0	2	0	-
gi|29165615|ref|NC_002745.2|	508047	+	A	0	1	1	11	2	-
gi|29165615|ref|NC_002745.2|	508048	+	G	0	0	0	2	1	-
gi|29165615|ref|NC_002745.2|	508049	+	U	0	0	0	1	0	-
gi|29165615|ref|NC_002745.2|	508051	+	U	0	0	1	0	0	-

gi|29165615|ref|NC_002745.2|	508052	+	U	2	0	1	5	0	-
gi|29165615|ref|NC_002745.2|	508053	+	A	1	4	0	36	3	-
gi|29165615|ref|NC_002745.2|	508054	+	A	0	9	1	30	5	-
gi|29165615|ref|NC_002745.2|	508055	+	A	0	4	2	3	4	-
gi|29165615|ref|NC_002745.2|	508056	+	U	2	1	1	3	0	-
gi|29165615|ref|NC_002745.2|	508057	+	A	1	1	1	11	0	-
gi|29165615|ref|NC_002745.2|	508058	+	A	0	0	1	3	0	-
gi|29165615|ref|NC_002745.2|	508059	+	G	0	1	0	2	0	-
gi|29165615|ref|NC_002745.2|	508060	+	C	0	0	0	1	0	-
gi|29165615|ref|NC_002745.2|	508061	+	U	1	0	0	0	0	-
gi|29165615|ref|NC_002745.2|	508062	+	U	1	0	0	0	0	-
gi|29165615|ref|NC_002745.2|	508063	+	G	4	0	1	8	1	-
gi|29165615|ref|NC_002745.2|	508064	+	A	1	4	3	31	2	-
gi|29165615|ref|NC_002745.2|	508065	+	A	1	2	3	14	3	-
gi|29165615|ref|NC_002745.2|	508066	+	U	0	3	0	4	0	-
gi|29165615|ref|NC_002745.2|	508067	+	U	1	0	0	1	0	-
gi|29165615|ref|NC_002745.2|	508068	+	C	1	1	0	2	0	-
gi|29165615|ref|NC_002745.2|	508069	+	A	3	2	0	11	0	-
gi|29165615|ref|NC_002745.2|	508070	+	U	9	23	16	165	27	-
gi|29165615|ref|NC_002745.2|	508071	+	A	8	48	14	417	49	-
gi|29165615|ref|NC_002745.2|	508072	+	A	4	26	10	263	28	-
gi|29165615|ref|NC_002745.2|	508073	+	G	8	22	10	187	24	-
gi|29165615|ref|NC_002745.2|	508074	+	A	3	17	3	135	9	-
gi|29165615|ref|NC_002745.2|	508075	+	A	9	23	6	185	16	-
gi|29165615|ref|NC_002745.2|	508076	+	A	3	11	4	89	10	-
gi|29165615|ref|NC_002745.2|	508077	+	U	2	4	1	19	1	-
gi|29165615|ref|NC_002745.2|	508078	+	A	8	4	3	112	9	-
gi|29165615|ref|NC_002745.2|	508079	+	A	5	14	2	56	12	-
gi|29165615|ref|NC_002745.2|	508080	+	U	0	1	0	9	2	-
gi|29165615|ref|NC_002745.2|	508081	+	C	1	1	0	2	1	-
gi|29165615|ref|NC_002745.2|	508083	+	C	0	0	0	1	0	-
gi|29165615|ref|NC_002745.2|	508085	+	A	1	0	0	1	0	-
gi|29165615|ref|NC_002745.2|	508089	+	U	3	1	0	0	0	-
gi|29165615|ref|NC_002745.2|	508090	+	U	1	0	0	0	0	-
gi|29165615|ref|NC_002745.2|	508091	+	C	0	0	0	2	1	-
gi|29165615|ref|NC_002745.2|	508092	+	G	1	0	0	5	0	-
gi|29165615|ref|NC_002745.2|	508093	+	A	0	1	2	2	0	-
gi|29165615|ref|NC_002745.2|	508094	+	A	0	0	0	3	0	-
gi|29165615|ref|NC_002745.2|	508095	+	A	0	0	0	1	0	-
gi|29165615|ref|NC_002745.2|	508097	+	A	6	0	0	1	1	-
gi|29165615|ref|NC_002745.2|	508098	+	A	4	0	0	0	0	-
gi|29165615|ref|NC_002745.2|	508099	+	C	2	2	1	1	3	-
gi|29165615|ref|NC_002745.2|	508100	+	A	7	1	2	1	4	-
gi|29165615|ref|NC_002745.2|	508101	+	C	2	0	0	1	0	-
gi|29165615|ref|NC_002745.2|	508102	+	U	0	0	1	0	0	-
gi|29165615|ref|NC_002745.2|	508104	+	A	1	0	0	0	0	-
gi|29165615|ref|NC_002745.2|	508105	+	C	3	0	0	0	0	-
gi|29165615|ref|NC_002745.2|	508106	+	A	2	0	1	1	0	-
gi|29165615|ref|NC_002745.2|	508107	+	A	0	0	1	0	0	-
gi|29165615|ref|NC_002745.2|	508108	+	G	1	0	0	0	0	-
gi|29165615|ref|NC_002745.2|	508109	+	A	0	1	0	1	0	-
gi|29165615|ref|NC_002745.2|	508110	+	U	0	0	2	0	0	-
gi|29165615|ref|NC_002745.2|	508111	+	U	1	0	1	0	0	-
gi|29165615|ref|NC_002745.2|	508112	+	A	0	0	0	0	1	-
gi|29165615|ref|NC_002745.2|	508113	+	A	0	3	1	20	2	-
gi|29165615|ref|NC_002745.2|	508114	+	U	1	0	1	0	1	-
gi|29165615|ref|NC_002745.2|	508115	+	A	2	0	1	19	2	-
gi|29165615|ref|NC_002745.2|	508116	+	A	1	0	3	10	0	-
gi|29165615|ref|NC_002745.2|	508117	+	C	2	0	1	0	1	-
gi|29165615|ref|NC_002745.2|	508118	+	G	0	0	0	2	0	-
gi|29165615|ref|NC_002745.2|	508119	+	C	14	62	29	374	44	-
gi|29165615|ref|NC_002745.2|	508120	+	G	2	1	3	39	3	-
gi|29165615|ref|NC_002745.2|	508121	+	U	0	1	2	8	0	-
gi|29165615|ref|NC_002745.2|	508122	+	U	0	2	2	1	0	-
gi|29165615|ref|NC_002745.2|	508123	+	U	0	1	0	3	0	-
gi|29165615|ref|NC_002745.2|	508124	+	A	0	0	1	2	0	-
gi|29165615|ref|NC_002745.2|	508125	+	A	1	0	0	5	0	-
gi|29165615|ref|NC_002745.2|	508126	+	A	0	3	3	5	1	-
gi|29165615|ref|NC_002745.2|	508128	+	C	2	1	1	7	0	-
gi|29165615|ref|NC_002745.2|	508129	+	U	0	0	0	1	0	-
gi|29165615|ref|NC_002745.2|	508132	+	U	0	0	0	0	1	-
gi|29165615|ref|NC_002745.2|	508133	+	U	1	0	0	1	1	-
gi|29165615|ref|NC_002745.2|	508134	+	A	1	2	1	37	2	-
gi|29165615|ref|NC_002745.2|	508135	+	U	0	1	1	6	0	-
gi|29165615|ref|NC_002745.2|	508136	+	A	1	0	0	44	0	-
gi|29165615|ref|NC_002745.2|	508137	+	A	0	0	1	20	0	-
gi|29165615|ref|NC_002745.2|	508138	+	A	0	0	0	5	0	-
gi|29165615|ref|NC_002745.2|	508139	+	A	0	0	0	6	0	-
gi|29165615|ref|NC_002745.2|	508140	+	G	3	0	0	3	0	-
gi|29165615|ref|NC_002745.2|	508141	+	A	3	1	2	28	0	-
gi|29165615|ref|NC_002745.2|	508142	+	A	0	0	0	2	1	-

gi|29165615|ref|NC_002745.2|	508143	+	C	0	0	0	2	1	-
gi|29165615|ref|NC_002745.2|	508144	+	G	0	0	0	4	0	-
gi|29165615|ref|NC_002745.2|	508145	+	U	0	0	0	11	0	-
gi|29165615|ref|NC_002745.2|	508146	+	A	3	0	0	86	1	-
gi|29165615|ref|NC_002745.2|	508147	+	A	2	2	0	36	1	-
gi|29165615|ref|NC_002745.2|	508148	+	U	2	0	0	7	1	-
gi|29165615|ref|NC_002745.2|	508149	+	U	0	0	1	12	1	-
gi|29165615|ref|NC_002745.2|	508150	+	U	1	1	0	7	0	-
gi|29165615|ref|NC_002745.2|	508151	+	C	3	0	0	29	1	-
gi|29165615|ref|NC_002745.2|	508152	+	A	0	0	0	7	0	-
gi|29165615|ref|NC_002745.2|	508153	+	U	0	0	0	4	0	-
gi|29165615|ref|NC_002745.2|	508154	+	G	0	1	0	4	0	-
gi|29165615|ref|NC_002745.2|	508155	+	U	1	0	0	3	1	-
gi|29165615|ref|NC_002745.2|	508156	+	U	1	1	0	4	0	-
gi|29165615|ref|NC_002745.2|	508157	+	A	1	2	1	6	3	-
gi|29165615|ref|NC_002745.2|	508158	+	A	5	0	1	11	2	-
gi|29165615|ref|NC_002745.2|	508159	+	C	3	0	0	2	1	-
gi|29165615|ref|NC_002745.2|	508160	+	G	1	0	0	1	0	-
gi|29165615|ref|NC_002745.2|	508161	+	U	1	0	1	0	0	-
gi|29165615|ref|NC_002745.2|	508162	+	U	1	0	0	0	0	-
gi|29165615|ref|NC_002745.2|	508164	+	G	0	0	0	1	0	-
gi|29165615|ref|NC_002745.2|	508165	+	A	2	4	2	66	1	-
gi|29165615|ref|NC_002745.2|	508166	+	C	5	2	0	6	0	-
gi|29165615|ref|NC_002745.2|	508167	+	U	1	2	0	0	1	-
gi|29165615|ref|NC_002745.2|	508168	+	U	0	0	0	1	0	-
gi|29165615|ref|NC_002745.2|	508169	+	A	4	0	1	13	3	-
gi|29165615|ref|NC_002745.2|	508170	+	U	2	0	0	3	0	-
gi|29165615|ref|NC_002745.2|	508171	+	A	0	2	0	13	3	-
gi|29165615|ref|NC_002745.2|	508172	+	A	1	1	1	17	2	-
gi|29165615|ref|NC_002745.2|	508173	+	A	3	2	0	17	2	-
gi|29165615|ref|NC_002745.2|	508174	+	A	2	5	1	34	0	-
gi|29165615|ref|NC_002745.2|	508175	+	A	4	5	0	14	14	-
gi|29165615|ref|NC_002745.2|	508176	+	U	1	0	0	5	0	-
gi|29165615|ref|NC_002745.2|	508177	+	G	5	1	2	23	4	-
gi|29165615|ref|NC_002745.2|	508178	+	G	2	2	5	33	4	-
gi|29165615|ref|NC_002745.2|	508179	+	U	4	0	2	0	1	-
gi|29165615|ref|NC_002745.2|	508180	+	G	0	0	0	10	1	-
gi|29165615|ref|NC_002745.2|	508181	+	G	4	0	2	27	4	-
gi|29165615|ref|NC_002745.2|	508182	+	A	7	5	3	33	4	-
gi|29165615|ref|NC_002745.2|	508183	+	A	12	2	3	55	7	-
gi|29165615|ref|NC_002745.2|	508184	+	A	5	3	3	32	0	-
gi|29165615|ref|NC_002745.2|	508185	+	C	22	4	3	15	6	-
gi|29165615|ref|NC_002745.2|	508186	+	A	19	10	4	26	14	-
gi|29165615|ref|NC_002745.2|	508187	+	U	56	21	23	51	27	-
gi|29165615|ref|NC_002745.2|	508188	+	A	77	25	17	21	19	-
gi|29165615|ref|NC_002745.2|	508189	+	G	236	167	138	146	174	SArRNA02
gi|29165615|ref|NC_002745.2|	508190	+	A	13289	9658	6342	5268	7428	SArRNA02
gi|29165615|ref|NC_002745.2|	508191	+	U	4688426	3458875	2860835	2114435	3322169	SArRNA02
gi|29165615|ref|NC_002745.2|	508192	+	U	2404	4642	3181	3137	4527	SArRNA02
gi|29165615|ref|NC_002745.2|	508193	+	A	8496	3514	2335	2876	3068	SArRNA02
gi|29165615|ref|NC_002745.2|	508194	+	A	18974	1553	1313	1189	1437	SArRNA02
gi|29165615|ref|NC_002745.2|	508195	+	G	3105	1040	814	698	995	SArRNA02
gi|29165615|ref|NC_002745.2|	508196	+	U	3050	1134	903	873	1058	SArRNA02
gi|29165615|ref|NC_002745.2|	508197	+	U	2637	1112	902	775	1163	SArRNA02
gi|29165615|ref|NC_002745.2|	508198	+	A	835	841	506	578	829	SArRNA02
gi|29165615|ref|NC_002745.2|	508199	+	U	748	265	365	187	307	SArRNA02
gi|29165615|ref|NC_002745.2|	508200	+	U	3981	458	739	280	397	SArRNA02
gi|29165615|ref|NC_002745.2|	508201	+	A	422	149	104	47	98	SArRNA02
gi|29165615|ref|NC_002745.2|	508202	+	A	230	64	26	13	38	SArRNA02
gi|29165615|ref|NC_002745.2|	508203	+	G	119	23	14	13	16	SArRNA02
gi|29165615|ref|NC_002745.2|	508204	+	G	412	34	33	21	36	SArRNA02
gi|29165615|ref|NC_002745.2|	508205	+	G	839	183	146	59	118	SArRNA02
gi|29165615|ref|NC_002745.2|	508206	+	C	1179	89	111	44	96	SArRNA02
gi|29165615|ref|NC_002745.2|	508207	+	G	137	34	26	16	29	SArRNA02
gi|29165615|ref|NC_002745.2|	508208	+	C	305	178	109	69	115	SArRNA02
gi|29165615|ref|NC_002745.2|	508209	+	A	55	41	28	20	48	SArRNA02
gi|29165615|ref|NC_002745.2|	508210	+	C	199	85	55	40	80	SArRNA02
gi|29165615|ref|NC_002745.2|	508211	+	G	44	47	28	20	39	SArRNA02
gi|29165615|ref|NC_002745.2|	508212	+	G	23	23	13	12	27	SArRNA02
gi|29165615|ref|NC_002745.2|	508213	+	U	47	16	9	7	22	SArRNA02
gi|29165615|ref|NC_002745.2|	508214	+	G	29	18	6	9	13	SArRNA02
gi|29165615|ref|NC_002745.2|	508215	+	G	78	30	17	12	26	SArRNA02
gi|29165615|ref|NC_002745.2|	508216	+	A	108	14	30	12	27	SArRNA02
gi|29165615|ref|NC_002745.2|	508217	+	U	4	1	3	1	8	SArRNA02
gi|29165615|ref|NC_002745.2|	508218	+	G	0	0	2	0	0	SArRNA02
gi|29165615|ref|NC_002745.2|	508219	+	C	37	16	7	2	12	SArRNA02
gi|29165615|ref|NC_002745.2|	508220	+	C	39	84	13	10	50	SArRNA02
gi|29165615|ref|NC_002745.2|	508221	+	U	19	19	6	0	12	SArRNA02
gi|29165615|ref|NC_002745.2|	508222	+	U	25	26	8	9	24	SArRNA02
gi|29165615|ref|NC_002745.2|	508223	+	G	1	8	3	1	4	SArRNA02
gi|29165615|ref|NC_002745.2|	508224	+	G	7	10	4	3	13	SArRNA02

gi|29165615|ref|NC_002745.2|	508225 +	C	57	52	31	20	45	SArRNA02
gi|29165615|ref|NC_002745.2|	508226 +	A	30	41	13	11	20	SArRNA02
gi|29165615|ref|NC_002745.2|	508227 +	C	46	35	14	21	35	SArRNA02
gi|29165615|ref|NC_002745.2|	508228 +	U	41	27	11	11	31	SArRNA02
gi|29165615|ref|NC_002745.2|	508229 +	A	14	17	3	3	4	SArRNA02
gi|29165615|ref|NC_002745.2|	508230 +	G	57	34	20	12	33	SArRNA02
gi|29165615|ref|NC_002745.2|	508231 +	A	90	18	12	7	23	SArRNA02
gi|29165615|ref|NC_002745.2|	508232 +	A	130	32	11	5	15	SArRNA02
gi|29165615|ref|NC_002745.2|	508233 +	G	11	21	11	6	11	SArRNA02
gi|29165615|ref|NC_002745.2|	508234 +	C	8	6	5	2	12	SArRNA02
gi|29165615|ref|NC_002745.2|	508235 +	C	51	83	17	12	80	SArRNA02
gi|29165615|ref|NC_002745.2|	508236 +	G	94	77	31	42	70	SArRNA02
gi|29165615|ref|NC_002745.2|	508237 +	A	10	10	3	3	16	SArRNA02
gi|29165615|ref|NC_002745.2|	508238 +	U	9	6	12	13	31	SArRNA02
gi|29165615|ref|NC_002745.2|	508239 +	G	16	9	11	11	23	SArRNA02
gi|29165615|ref|NC_002745.2|	508240 +	A	18	14	4	12	11	SArRNA02
gi|29165615|ref|NC_002745.2|	508241 +	A	13	17	4	2	14	SArRNA02
gi|29165615|ref|NC_002745.2|	508242 +	G	4	6	4	4	9	SArRNA02
gi|29165615|ref|NC_002745.2|	508243 +	G	18	22	11	9	22	SArRNA02
gi|29165615|ref|NC_002745.2|	508244 +	A	15	6	3	5	12	SArRNA02
gi|29165615|ref|NC_002745.2|	508245 +	C	29	27	18	16	22	SArRNA02
gi|29165615|ref|NC_002745.2|	508246 +	G	35	19	9	13	20	SArRNA02
gi|29165615|ref|NC_002745.2|	508247 +	U	27	32	19	8	37	SArRNA02
gi|29165615|ref|NC_002745.2|	508248 +	U	23	51	23	21	33	SArRNA02
gi|29165615|ref|NC_002745.2|	508249 +	A	20	12	8	14	22	SArRNA02
gi|29165615|ref|NC_002745.2|	508250 +	C	24	34	13	24	42	SArRNA02
gi|29165615|ref|NC_002745.2|	508251 +	U	41	41	12	23	47	SArRNA02
gi|29165615|ref|NC_002745.2|	508252 +	A	34	48	14	28	45	SArRNA02
gi|29165615|ref|NC_002745.2|	508253 +	A	1007	878	350	1165	819	SArRNA02
gi|29165615|ref|NC_002745.2|	508254 +	C	50	42	22	36	46	SArRNA02
gi|29165615|ref|NC_002745.2|	508255 +	G	29	15	7	13	22	SArRNA02
gi|29165615|ref|NC_002745.2|	508256 +	A	26	8	7	13	17	SArRNA02
gi|29165615|ref|NC_002745.2|	508257 +	C	34	36	18	22	23	SArRNA02
gi|29165615|ref|NC_002745.2|	508258 +	G	34	19	4	8	22	SArRNA02
gi|29165615|ref|NC_002745.2|	508259 +	A	31	32	16	16	39	SArRNA02
gi|29165615|ref|NC_002745.2|	508260 +	U	113	122	65	65	114	SArRNA02
gi|29165615|ref|NC_002745.2|	508261 +	A	56	37	8	15	44	SArRNA02
gi|29165615|ref|NC_002745.2|	508262 +	U	57	37	21	30	59	SArRNA02
gi|29165615|ref|NC_002745.2|	508263 +	G	44	24	14	21	23	SArRNA02
gi|29165615|ref|NC_002745.2|	508264 +	C	65	29	16	11	32	SArRNA02
gi|29165615|ref|NC_002745.2|	508265 +	U	42	34	23	22	55	SArRNA02
gi|29165615|ref|NC_002745.2|	508266 +	U	53	20	20	21	44	SArRNA02
gi|29165615|ref|NC_002745.2|	508267 +	U	21	14	4	15	19	SArRNA02
gi|29165615|ref|NC_002745.2|	508268 +	G	4	12	5	4	10	SArRNA02
gi|29165615|ref|NC_002745.2|	508269 +	G	11	11	4	2	8	SArRNA02
gi|29165615|ref|NC_002745.2|	508270 +	G	15	13	8	10	14	SArRNA02
gi|29165615|ref|NC_002745.2|	508271 +	G	70	35	27	24	76	SArRNA02
gi|29165615|ref|NC_002745.2|	508272 +	A	54	35	34	21	76	SArRNA02
gi|29165615|ref|NC_002745.2|	508273 +	G	13	12	13	12	14	SArRNA02
gi|29165615|ref|NC_002745.2|	508274 +	C	26	13	5	4	5	SArRNA02
gi|29165615|ref|NC_002745.2|	508275 +	U	19	8	3	7	14	SArRNA02
gi|29165615|ref|NC_002745.2|	508276 +	G	9	8	1	5	9	SArRNA02
gi|29165615|ref|NC_002745.2|	508277 +	U	36	27	10	11	25	SArRNA02
gi|29165615|ref|NC_002745.2|	508278 +	A	24	24	4	6	24	SArRNA02
gi|29165615|ref|NC_002745.2|	508279 +	A	29	8	14	14	32	SArRNA02
gi|29165615|ref|NC_002745.2|	508280 +	G	33	25	10	18	30	SArRNA02
gi|29165615|ref|NC_002745.2|	508281 +	U	26	42	20	42	43	SArRNA02
gi|29165615|ref|NC_002745.2|	508282 +	A	12	15	11	13	18	SArRNA02
gi|29165615|ref|NC_002745.2|	508283 +	A	13	11	4	6	8	SArRNA02
gi|29165615|ref|NC_002745.2|	508284 +	G	1	3	3	0	3	SArRNA02
gi|29165615|ref|NC_002745.2|	508285 +	C	22	7	10	6	18	SArRNA02
gi|29165615|ref|NC_002745.2|	508286 +	U	5	4	1	0	9	SArRNA02
gi|29165615|ref|NC_002745.2|	508287 +	U	5	3	5	4	10	SArRNA02
gi|29165615|ref|NC_002745.2|	508288 +	U	13	28	14	7	12	SArRNA02
gi|29165615|ref|NC_002745.2|	508289 +	G	27	43	19	18	30	SArRNA02
gi|29165615|ref|NC_002745.2|	508290 +	A	6	7	1	5	16	SArRNA02
gi|29165615|ref|NC_002745.2|	508291 +	U	3	5	1	4	3	SArRNA02
gi|29165615|ref|NC_002745.2|	508292 +	C	17	36	3	7	19	SArRNA02
gi|29165615|ref|NC_002745.2|	508293 +	C	30	28	14	23	28	SArRNA02
gi|29165615|ref|NC_002745.2|	508294 +	A	15	19	2	4	19	SArRNA02
gi|29165615|ref|NC_002745.2|	508295 +	G	21	35	12	20	27	SArRNA02
gi|29165615|ref|NC_002745.2|	508296 +	A	21	15	1	7	10	SArRNA02
gi|29165615|ref|NC_002745.2|	508297 +	G	53	58	20	16	25	SArRNA02
gi|29165615|ref|NC_002745.2|	508298 +	A	23	32	19	12	11	SArRNA02
gi|29165615|ref|NC_002745.2|	508299 +	U	5	4	3	2	6	SArRNA02
gi|29165615|ref|NC_002745.2|	508300 +	U	4	3	3	4	3	SArRNA02
gi|29165615|ref|NC_002745.2|	508301 +	U	3	3	2	5	3	SArRNA02
gi|29165615|ref|NC_002745.2|	508302 +	C	5	10	4	6	11	SArRNA02
gi|29165615|ref|NC_002745.2|	508303 +	C	54	64	16	32	28	SArRNA02
gi|29165615|ref|NC_002745.2|	508304 +	G	10	23	5	4	14	SArRNA02
gi|29165615|ref|NC_002745.2|	508305 +	A	5	7	1	4	4	SArRNA02

gi|29165615|ref|NC_002745.2|	508306	+	A	5	15	0	2	4	SArRNA02
gi|29165615|ref|NC_002745.2|	508307	+	U	6	9	0	2	3	SArRNA02
gi|29165615|ref|NC_002745.2|	508308	+	G	3	4	1	1	4	SArRNA02
gi|29165615|ref|NC_002745.2|	508309	+	G	0	2	1	0	4	SArRNA02
gi|29165615|ref|NC_002745.2|	508310	+	G	0	0	5	0	2	SArRNA02
gi|29165615|ref|NC_002745.2|	508311	+	G	3	1	0	2	2	SArRNA02
gi|29165615|ref|NC_002745.2|	508312	+	A	0	6	0	0	3	SArRNA02
gi|29165615|ref|NC_002745.2|	508313	+	A	31	58	10	9	28	SArRNA02
gi|29165615|ref|NC_002745.2|	508314	+	A	3	7	1	0	5	SArRNA02
gi|29165615|ref|NC_002745.2|	508315	+	C	21	22	3	1	31	SArRNA02
gi|29165615|ref|NC_002745.2|	508316	+	C	30	32	9	13	32	SArRNA02
gi|29165615|ref|NC_002745.2|	508317	+	C	14	21	10	10	14	SArRNA02
gi|29165615|ref|NC_002745.2|	508318	+	A	23	47	20	25	24	SArRNA02
gi|29165615|ref|NC_002745.2|	508319	+	G	3	1	0	2	1	SArRNA02
gi|29165615|ref|NC_002745.2|	508320	+	C	2	1	3	2	9	SArRNA02
gi|29165615|ref|NC_002745.2|	508321	+	A	4	11	5	4	11	SArRNA02
gi|29165615|ref|NC_002745.2|	508322	+	U	10	19	7	4	16	SArRNA02
gi|29165615|ref|NC_002745.2|	508323	+	G	24	18	11	13	11	SArRNA02
gi|29165615|ref|NC_002745.2|	508324	+	A	12	9	2	9	8	SArRNA02
gi|29165615|ref|NC_002745.2|	508325	+	G	8	6	7	21	17	SArRNA02
gi|29165615|ref|NC_002745.2|	508326	+	U	17	5	4	2	6	SArRNA02
gi|29165615|ref|NC_002745.2|	508327	+	U	42	29	13	18	31	SArRNA02
gi|29165615|ref|NC_002745.2|	508328	+	A	16	14	6	3	5	SArRNA02
gi|29165615|ref|NC_002745.2|	508329	+	U	7	6	4	2	3	SArRNA02
gi|29165615|ref|NC_002745.2|	508330	+	G	2	1	2	0	7	SArRNA02
gi|29165615|ref|NC_002745.2|	508331	+	U	5	13	2	2	2	SArRNA02
gi|29165615|ref|NC_002745.2|	508332	+	C	132	85	45	52	59	SArRNA02
gi|29165615|ref|NC_002745.2|	508333	+	A	27	24	7	10	9	SArRNA02
gi|29165615|ref|NC_002745.2|	508334	+	U	4	7	1	4	8	SArRNA02
gi|29165615|ref|NC_002745.2|	508335	+	G	2	4	0	1	5	SArRNA02
gi|29165615|ref|NC_002745.2|	508336	+	U	9	7	3	2	5	SArRNA02
gi|29165615|ref|NC_002745.2|	508337	+	U	25	23	13	11	16	SArRNA02
gi|29165615|ref|NC_002745.2|	508338	+	A	0	8	1	1	6	SArRNA02
gi|29165615|ref|NC_002745.2|	508339	+	U	0	7	0	1	2	SArRNA02
gi|29165615|ref|NC_002745.2|	508340	+	C	33	44	19	20	22	SArRNA02
gi|29165615|ref|NC_002745.2|	508341	+	G	9	19	5	6	8	SArRNA02
gi|29165615|ref|NC_002745.2|	508342	+	A	2	7	1	0	4	SArRNA02
gi|29165615|ref|NC_002745.2|	508343	+	U	28	30	8	11	22	SArRNA02
gi|29165615|ref|NC_002745.2|	508344	+	A	7	7	3	0	4	SArRNA02
gi|29165615|ref|NC_002745.2|	508345	+	U	13	3	6	4	10	SArRNA02
gi|29165615|ref|NC_002745.2|	508346	+	G	6	0	6	6	4	SArRNA02
gi|29165615|ref|NC_002745.2|	508347	+	U	5	3	2	3	5	SArRNA02
gi|29165615|ref|NC_002745.2|	508348	+	G	10	6	2	9	8	SArRNA02
gi|29165615|ref|NC_002745.2|	508349	+	A	16	6	4	5	8	SArRNA02
gi|29165615|ref|NC_002745.2|	508350	+	A	7	17	2	7	12	SArRNA02
gi|29165615|ref|NC_002745.2|	508351	+	U	8	16	3	1	17	SArRNA02
gi|29165615|ref|NC_002745.2|	508352	+	A	38	31	4	7	30	SArRNA02
gi|29165615|ref|NC_002745.2|	508353	+	C	31	48	4	10	36	SArRNA02
gi|29165615|ref|NC_002745.2|	508354	+	A	20	33	6	11	22	SArRNA02
gi|29165615|ref|NC_002745.2|	508355	+	U	22	18	3	5	17	SArRNA02
gi|29165615|ref|NC_002745.2|	508356	+	A	3	9	1	1	2	SArRNA02
gi|29165615|ref|NC_002745.2|	508357	+	G	6	1	1	3	4	SArRNA02
gi|29165615|ref|NC_002745.2|	508358	+	C	28	25	9	10	15	SArRNA02
gi|29165615|ref|NC_002745.2|	508359	+	A	14	9	1	5	7	SArRNA02
gi|29165615|ref|NC_002745.2|	508360	+	U	5	3	1	0	7	SArRNA02
gi|29165615|ref|NC_002745.2|	508361	+	A	3	6	2	0	5	SArRNA02
gi|29165615|ref|NC_002745.2|	508362	+	U	11	7	3	5	7	SArRNA02
gi|29165615|ref|NC_002745.2|	508363	+	C	18	9	6	2	7	SArRNA02
gi|29165615|ref|NC_002745.2|	508364	+	A	13	8	5	3	5	SArRNA02
gi|29165615|ref|NC_002745.2|	508365	+	G	8	7	0	6	2	SArRNA02
gi|29165615|ref|NC_002745.2|	508366	+	A	25	60	18	8	6	SArRNA02
gi|29165615|ref|NC_002745.2|	508367	+	A	131	231	47	58	77	SArRNA02
gi|29165615|ref|NC_002745.2|	508368	+	G	2	2	0	0	0	SArRNA02
gi|29165615|ref|NC_002745.2|	508369	+	G	0	2	0	1	0	SArRNA02
gi|29165615|ref|NC_002745.2|	508370	+	C	24	17	4	12	13	SArRNA02
gi|29165615|ref|NC_002745.2|	508371	+	A	14	22	2	2	7	SArRNA02
gi|29165615|ref|NC_002745.2|	508372	+	C	44	27	21	16	18	SArRNA02
gi|29165615|ref|NC_002745.2|	508373	+	A	14	20	4	5	9	SArRNA02
gi|29165615|ref|NC_002745.2|	508374	+	C	27	23	6	9	7	SArRNA02
gi|29165615|ref|NC_002745.2|	508375	+	C	8	10	2	3	7	SArRNA02
gi|29165615|ref|NC_002745.2|	508376	+	C	7	18	5	8	2	SArRNA02
gi|29165615|ref|NC_002745.2|	508377	+	G	67	174	20	22	67	SArRNA02
gi|29165615|ref|NC_002745.2|	508378	+	G	6	11	3	1	5	SArRNA02
gi|29165615|ref|NC_002745.2|	508379	+	A	7	9	1	1	5	SArRNA02
gi|29165615|ref|NC_002745.2|	508380	+	G	14	6	2	6	6	SArRNA02
gi|29165615|ref|NC_002745.2|	508381	+	A	7	8	3	1	8	SArRNA02
gi|29165615|ref|NC_002745.2|	508382	+	A	1	0	1	0	1	SArRNA02
gi|29165615|ref|NC_002745.2|	508383	+	C	7	4	0	1	2	SArRNA02
gi|29165615|ref|NC_002745.2|	508384	+	U	0	1	1	1	1	SArRNA02
gi|29165615|ref|NC_002745.2|	508385	+	G	1	1	0	0	0	SArRNA02
gi|29165615|ref|NC_002745.2|	508386	+	A	3	1	0	0	1	SArRNA02

gi|29165615|ref|NC_002745.2|	508387	+	A	1	0	0	1	0	SArRNA02
gi|29165615|ref|NC_002745.2|	508388	+	A	0	2	3	0	0	SArRNA02
gi|29165615|ref|NC_002745.2|	508389	+	C	11	12	4	9	11	SArRNA02
gi|29165615|ref|NC_002745.2|	508390	+	A	4	13	2	0	7	SArRNA02
gi|29165615|ref|NC_002745.2|	508391	+	U	3	3	5	3	2	SArRNA02
gi|29165615|ref|NC_002745.2|	508392	+	C	2	9	1	2	0	SArRNA02
gi|29165615|ref|NC_002745.2|	508393	+	U	0	0	0	0	1	SArRNA02
gi|29165615|ref|NC_002745.2|	508394	+	U	0	1	0	1	2	SArRNA02
gi|29165615|ref|NC_002745.2|	508395	+	A	0	1	0	2	2	SArRNA02
gi|29165615|ref|NC_002745.2|	508396	+	G	1	3	0	0	1	SArRNA02
gi|29165615|ref|NC_002745.2|	508397	+	U	2	5	1	0	6	SArRNA02
gi|29165615|ref|NC_002745.2|	508398	+	A	0	2	1	0	1	SArRNA02
gi|29165615|ref|NC_002745.2|	508399	+	C	12	8	4	2	5	SArRNA02
gi|29165615|ref|NC_002745.2|	508400	+	C	10	14	3	6	8	SArRNA02
gi|29165615|ref|NC_002745.2|	508401	+	C	11	8	8	3	12	SArRNA02
gi|29165615|ref|NC_002745.2|	508402	+	G	0	4	1	1	3	SArRNA02
gi|29165615|ref|NC_002745.2|	508403	+	G	5	6	4	6	4	SArRNA02
gi|29165615|ref|NC_002745.2|	508404	+	A	0	5	2	1	1	SArRNA02
gi|29165615|ref|NC_002745.2|	508405	+	G	1	0	1	0	0	SArRNA02
gi|29165615|ref|NC_002745.2|	508406	+	G	0	1	0	0	2	SArRNA02
gi|29165615|ref|NC_002745.2|	508407	+	A	4	10	0	0	4	SArRNA02
gi|29165615|ref|NC_002745.2|	508408	+	A	38	45	11	18	15	SArRNA02
gi|29165615|ref|NC_002745.2|	508409	+	G	2	2	2	3	2	SArRNA02
gi|29165615|ref|NC_002745.2|	508410	+	A	1	0	0	1	1	SArRNA02
gi|29165615|ref|NC_002745.2|	508411	+	G	0	2	0	3	5	SArRNA02
gi|29165615|ref|NC_002745.2|	508412	+	A	1	1	0	0	1	SArRNA02
gi|29165615|ref|NC_002745.2|	508413	+	A	0	2	0	2	1	SArRNA02
gi|29165615|ref|NC_002745.2|	508414	+	A	1	1	0	3	0	SArRNA02
gi|29165615|ref|NC_002745.2|	508415	+	G	0	4	1	1	0	SArRNA02
gi|29165615|ref|NC_002745.2|	508416	+	A	0	2	0	0	0	SArRNA02
gi|29165615|ref|NC_002745.2|	508417	+	A	2	3	0	0	0	SArRNA02
gi|29165615|ref|NC_002745.2|	508418	+	A	1	1	1	1	2	SArRNA02
gi|29165615|ref|NC_002745.2|	508419	+	A	9	124	10	3	21	SArRNA02
gi|29165615|ref|NC_002745.2|	508420	+	U	2	6	4	1	4	SArRNA02
gi|29165615|ref|NC_002745.2|	508421	+	U	2	0	2	0	4	SArRNA02
gi|29165615|ref|NC_002745.2|	508422	+	C	2	6	1	0	4	SArRNA02
gi|29165615|ref|NC_002745.2|	508423	+	G	0	3	1	0	0	SArRNA02
gi|29165615|ref|NC_002745.2|	508424	+	A	1	3	1	1	0	SArRNA02
gi|29165615|ref|NC_002745.2|	508426	+	U	1	8	0	1	1	SArRNA02
gi|29165615|ref|NC_002745.2|	508427	+	C	27	20	9	13	8	SArRNA02
gi|29165615|ref|NC_002745.2|	508428	+	C	16	5	5	2	3	SArRNA02
gi|29165615|ref|NC_002745.2|	508429	+	C	5	2	2	4	7	SArRNA02
gi|29165615|ref|NC_002745.2|	508430	+	U	7	5	3	5	7	SArRNA02
gi|29165615|ref|NC_002745.2|	508431	+	U	5	3	4	5	5	SArRNA02
gi|29165615|ref|NC_002745.2|	508432	+	A	6	2	1	1	4	SArRNA02
gi|29165615|ref|NC_002745.2|	508433	+	G	3	6	2	2	4	SArRNA02
gi|29165615|ref|NC_002745.2|	508434	+	U	9	5	2	2	7	SArRNA02
gi|29165615|ref|NC_002745.2|	508435	+	A	7	3	3	1	5	SArRNA02
gi|29165615|ref|NC_002745.2|	508436	+	G	1	0	1	0	0	SArRNA02
gi|29165615|ref|NC_002745.2|	508437	+	C	7	4	4	2	2	SArRNA02
gi|29165615|ref|NC_002745.2|	508438	+	G	2	0	1	0	1	SArRNA02
gi|29165615|ref|NC_002745.2|	508439	+	G	1	1	0	0	1	SArRNA02
gi|29165615|ref|NC_002745.2|	508440	+	C	16	15	4	8	19	SArRNA02
gi|29165615|ref|NC_002745.2|	508441	+	G	6	5	1	1	8	SArRNA02
gi|29165615|ref|NC_002745.2|	508442	+	A	1	5	0	1	5	SArRNA02
gi|29165615|ref|NC_002745.2|	508443	+	G	1	2	1	2	0	SArRNA02
gi|29165615|ref|NC_002745.2|	508444	+	C	5	6	1	2	3	SArRNA02
gi|29165615|ref|NC_002745.2|	508445	+	G	5	5	0	1	3	SArRNA02
gi|29165615|ref|NC_002745.2|	508446	+	A	1	1	1	0	0	SArRNA02
gi|29165615|ref|NC_002745.2|	508447	+	A	3	1	0	3	4	SArRNA02
gi|29165615|ref|NC_002745.2|	508448	+	A	1	2	0	0	3	SArRNA02
gi|29165615|ref|NC_002745.2|	508449	+	C	7	3	1	1	3	SArRNA02
gi|29165615|ref|NC_002745.2|	508450	+	G	0	0	0	1	2	SArRNA02
gi|29165615|ref|NC_002745.2|	508451	+	G	2	0	0	0	0	SArRNA02
gi|29165615|ref|NC_002745.2|	508452	+	G	1	0	0	0	0	SArRNA02
gi|29165615|ref|NC_002745.2|	508453	+	A	0	1	1	0	1	SArRNA02
gi|29165615|ref|NC_002745.2|	508454	+	A	5	1	2	0	0	SArRNA02
gi|29165615|ref|NC_002745.2|	508455	+	G	0	0	1	0	0	SArRNA02
gi|29165615|ref|NC_002745.2|	508456	+	A	1	0	0	1	0	SArRNA02
gi|29165615|ref|NC_002745.2|	508457	+	G	0	1	0	0	0	SArRNA02
gi|29165615|ref|NC_002745.2|	508458	+	C	10	8	1	2	2	SArRNA02
gi|29165615|ref|NC_002745.2|	508459	+	C	3	4	0	3	5	SArRNA02
gi|29165615|ref|NC_002745.2|	508460	+	C	15	11	3	7	7	SArRNA02
gi|29165615|ref|NC_002745.2|	508461	+	A	2	2	1	3	1	SArRNA02
gi|29165615|ref|NC_002745.2|	508462	+	A	2	7	3	1	3	SArRNA02
gi|29165615|ref|NC_002745.2|	508463	+	A	3	2	3	2	2	SArRNA02
gi|29165615|ref|NC_002745.2|	508464	+	C	1	2	1	2	4	SArRNA02
gi|29165615|ref|NC_002745.2|	508465	+	C	16	2	2	7	4	SArRNA02
gi|29165615|ref|NC_002745.2|	508466	+	A	7	4	4	4	6	SArRNA02
gi|29165615|ref|NC_002745.2|	508467	+	A	5	3	1	1	5	SArRNA02
gi|29165615|ref|NC_002745.2|	508468	+	C	6	1	3	1	4	SArRNA02

gi|29165615|ref|NC_002745.2|	508469	+	A	7	4	1	1	3	SArRNA02
gi|29165615|ref|NC_002745.2|	508470	+	A	3	2	1	1	0	SArRNA02
gi|29165615|ref|NC_002745.2|	508471	+	G	1	2	0	0	0	SArRNA02
gi|29165615|ref|NC_002745.2|	508472	+	C	10	6	3	2	5	SArRNA02
gi|29165615|ref|NC_002745.2|	508473	+	U	5	3	0	2	5	SArRNA02
gi|29165615|ref|NC_002745.2|	508474	+	U	0	1	1	0	1	SArRNA02
gi|29165615|ref|NC_002745.2|	508475	+	G	1	1	1	1	1	SArRNA02
gi|29165615|ref|NC_002745.2|	508476	+	C	3	6	2	1	3	SArRNA02
gi|29165615|ref|NC_002745.2|	508477	+	U	0	0	0	1	3	SArRNA02
gi|29165615|ref|NC_002745.2|	508478	+	U	2	1	3	0	1	SArRNA02
gi|29165615|ref|NC_002745.2|	508479	+	G	3	2	1	0	0	SArRNA02
gi|29165615|ref|NC_002745.2|	508480	+	U	5	0	2	1	2	SArRNA02
gi|29165615|ref|NC_002745.2|	508481	+	U	6	4	2	2	0	SArRNA02
gi|29165615|ref|NC_002745.2|	508482	+	G	0	0	1	0	1	SArRNA02
gi|29165615|ref|NC_002745.2|	508484	+	G	1	1	0	2	2	SArRNA02
gi|29165615|ref|NC_002745.2|	508485	+	G	1	0	0	0	3	SArRNA02
gi|29165615|ref|NC_002745.2|	508486	+	U	0	2	2	0	1	SArRNA02
gi|29165615|ref|NC_002745.2|	508487	+	U	1	0	1	0	0	SArRNA02
gi|29165615|ref|NC_002745.2|	508488	+	G	1	1	0	1	0	SArRNA02
gi|29165615|ref|NC_002745.2|	508489	+	U	2	4	0	3	0	SArRNA02
gi|29165615|ref|NC_002745.2|	508490	+	A	0	5	0	1	0	SArRNA02
gi|29165615|ref|NC_002745.2|	508491	+	G	2	1	1	0	0	SArRNA02
gi|29165615|ref|NC_002745.2|	508494	+	C	22	22	7	5	10	SArRNA02
gi|29165615|ref|NC_002745.2|	508495	+	A	8	5	3	4	4	SArRNA02
gi|29165615|ref|NC_002745.2|	508496	+	C	7	4	3	4	5	SArRNA02
gi|29165615|ref|NC_002745.2|	508497	+	U	0	2	0	0	2	SArRNA02
gi|29165615|ref|NC_002745.2|	508498	+	C	1	3	1	3	4	SArRNA02
gi|29165615|ref|NC_002745.2|	508499	+	U	2	2	0	0	1	SArRNA02
gi|29165615|ref|NC_002745.2|	508500	+	G	0	2	0	0	3	SArRNA02
gi|29165615|ref|NC_002745.2|	508501	+	U	6	2	1	0	6	SArRNA02
gi|29165615|ref|NC_002745.2|	508502	+	A	2	0	1	2	0	SArRNA02
gi|29165615|ref|NC_002745.2|	508503	+	C	3	12	1	1	2	SArRNA02
gi|29165615|ref|NC_002745.2|	508504	+	G	1	9	2	2	6	SArRNA02
gi|29165615|ref|NC_002745.2|	508505	+	G	1	5	1	4	0	SArRNA02
gi|29165615|ref|NC_002745.2|	508506	+	A	1	1	0	2	2	SArRNA02
gi|29165615|ref|NC_002745.2|	508507	+	G	1	5	0	0	2	SArRNA02
gi|29165615|ref|NC_002745.2|	508508	+	U	1	2	0	1	0	SArRNA02
gi|29165615|ref|NC_002745.2|	508509	+	U	10	14	5	4	7	SArRNA02
gi|29165615|ref|NC_002745.2|	508510	+	A	1	0	0	1	1	SArRNA02
gi|29165615|ref|NC_002745.2|	508511	+	C	8	16	4	6	8	SArRNA02
gi|29165615|ref|NC_002745.2|	508512	+	A	1	10	0	2	3	SArRNA02
gi|29165615|ref|NC_002745.2|	508513	+	A	2	7	0	0	0	SArRNA02
gi|29165615|ref|NC_002745.2|	508514	+	A	2	2	0	0	0	SArRNA02
gi|29165615|ref|NC_002745.2|	508515	+	G	3	2	3	2	0	SArRNA02
gi|29165615|ref|NC_002745.2|	508516	+	G	0	2	1	0	0	SArRNA02
gi|29165615|ref|NC_002745.2|	508517	+	A	1	1	1	0	2	SArRNA02
gi|29165615|ref|NC_002745.2|	508518	+	C	7	4	1	3	4	SArRNA02
gi|29165615|ref|NC_002745.2|	508519	+	G	0	1	0	1	2	SArRNA02
gi|29165615|ref|NC_002745.2|	508520	+	A	5	0	1	1	0	SArRNA02
gi|29165615|ref|NC_002745.2|	508521	+	C	6	1	1	1	2	SArRNA02
gi|29165615|ref|NC_002745.2|	508522	+	A	2	3	0	1	0	SArRNA02
gi|29165615|ref|NC_002745.2|	508523	+	U	7	3	0	1	3	SArRNA02
gi|29165615|ref|NC_002745.2|	508524	+	U	2	6	1	2	0	SArRNA02
gi|29165615|ref|NC_002745.2|	508525	+	A	0	1	0	0	2	SArRNA02
gi|29165615|ref|NC_002745.2|	508526	+	G	0	1	0	2	1	SArRNA02
gi|29165615|ref|NC_002745.2|	508527	+	A	0	0	0	0	1	SArRNA02
gi|29165615|ref|NC_002745.2|	508528	+	C	1	3	1	1	0	SArRNA02
gi|29165615|ref|NC_002745.2|	508529	+	G	0	1	1	0	0	SArRNA02
gi|29165615|ref|NC_002745.2|	508530	+	A	0	1	0	0	2	SArRNA02
gi|29165615|ref|NC_002745.2|	508531	+	A	1	9	0	0	8	SArRNA02
gi|29165615|ref|NC_002745.2|	508532	+	U	6	1	1	3	0	SArRNA02
gi|29165615|ref|NC_002745.2|	508533	+	C	9	4	3	4	2	SArRNA02
gi|29165615|ref|NC_002745.2|	508534	+	A	3	5	1	1	1	SArRNA02
gi|29165615|ref|NC_002745.2|	508535	+	U	2	3	1	0	0	SArRNA02
gi|29165615|ref|NC_002745.2|	508536	+	C	7	9	7	5	5	SArRNA02
gi|29165615|ref|NC_002745.2|	508537	+	U	3	4	2	1	1	SArRNA02
gi|29165615|ref|NC_002745.2|	508538	+	G	0	0	1	1	0	SArRNA02
gi|29165615|ref|NC_002745.2|	508539	+	G	7	1	1	0	5	SArRNA02
gi|29165615|ref|NC_002745.2|	508540	+	A	1	5	2	2	2	SArRNA02
gi|29165615|ref|NC_002745.2|	508541	+	A	8	9	0	0	0	SArRNA02
gi|29165615|ref|NC_002745.2|	508542	+	A	3	5	2	1	3	SArRNA02
gi|29165615|ref|NC_002745.2|	508543	+	G	2	3	1	3	2	SArRNA02
gi|29165615|ref|NC_002745.2|	508544	+	A	2	3	0	0	3	SArRNA02
gi|29165615|ref|NC_002745.2|	508545	+	U	4	4	2	2	2	SArRNA02
gi|29165615|ref|NC_002745.2|	508546	+	G	15	4	2	6	13	SArRNA02
gi|29165615|ref|NC_002745.2|	508547	+	A	9	14	6	4	3	SArRNA02
gi|29165615|ref|NC_002745.2|	508548	+	A	25	31	10	22	25	SArRNA02
gi|29165615|ref|NC_002745.2|	508549	+	U	0	1	0	0	1	SArRNA02
gi|29165615|ref|NC_002745.2|	508550	+	C	7	18	0	4	7	SArRNA02
gi|29165615|ref|NC_002745.2|	508551	+	A	3	1	1	2	6	SArRNA02
gi|29165615|ref|NC_002745.2|	508552	+	A	3	0	1	1	3	SArRNA02

gi|29165615|ref|NC_002745.2|	508553	+	A	3	9	4	0	1	SArRNA02
gi|29165615|ref|NC_002745.2|	508554	+	G	7	7	1	6	4	SArRNA02
gi|29165615|ref|NC_002745.2|	508555	+	A	3	7	3	3	1	SArRNA02
gi|29165615|ref|NC_002745.2|	508556	+	A	3	0	1	1	3	SArRNA02
gi|29165615|ref|NC_002745.2|	508557	+	G	0	2	0	1	3	SArRNA02
gi|29165615|ref|NC_002745.2|	508558	+	G	2	2	2	2	2	SArRNA02
gi|29165615|ref|NC_002745.2|	508559	+	U	7	2	0	0	5	SArRNA02
gi|29165615|ref|NC_002745.2|	508560	+	A	7	4	1	4	9	SArRNA02
gi|29165615|ref|NC_002745.2|	508561	+	A	10	10	0	2	6	SArRNA02
gi|29165615|ref|NC_002745.2|	508562	+	U	11	3	6	5	7	SArRNA02
gi|29165615|ref|NC_002745.2|	508563	+	A	6	4	0	2	4	SArRNA02
gi|29165615|ref|NC_002745.2|	508564	+	A	1	14	2	5	8	SArRNA02
gi|29165615|ref|NC_002745.2|	508565	+	U	4	10	1	4	7	SArRNA02
gi|29165615|ref|NC_002745.2|	508566	+	C	5	7	4	7	6	SArRNA02
gi|29165615|ref|NC_002745.2|	508567	+	C	9	7	3	1	4	SArRNA02
gi|29165615|ref|NC_002745.2|	508568	+	U	3	8	0	0	9	SArRNA02
gi|29165615|ref|NC_002745.2|	508569	+	G	13	13	1	7	7	SArRNA02
gi|29165615|ref|NC_002745.2|	508570	+	U	3	4	1	2	1	SArRNA02
gi|29165615|ref|NC_002745.2|	508571	+	A	5	3	0	4	2	SArRNA02
gi|29165615|ref|NC_002745.2|	508572	+	G	4	1	1	1	1	SArRNA02
gi|29165615|ref|NC_002745.2|	508573	+	U	4	4	1	2	1	SArRNA02
gi|29165615|ref|NC_002745.2|	508574	+	C	4	3	1	1	1	SArRNA02
gi|29165615|ref|NC_002745.2|	508575	+	G	2	0	1	0	6	SArRNA02
gi|29165615|ref|NC_002745.2|	508576	+	A	2	2	1	0	0	SArRNA02
gi|29165615|ref|NC_002745.2|	508577	+	A	12	9	0	1	2	SArRNA02
gi|29165615|ref|NC_002745.2|	508578	+	A	9	5	1	4	6	SArRNA02
gi|29165615|ref|NC_002745.2|	508579	+	A	9	0	1	2	2	SArRNA02
gi|29165615|ref|NC_002745.2|	508580	+	U	4	4	1	1	2	SArRNA02
gi|29165615|ref|NC_002745.2|	508581	+	G	1	1	0	1	0	SArRNA02
gi|29165615|ref|NC_002745.2|	508582	+	U	2	2	0	0	1	SArRNA02
gi|29165615|ref|NC_002745.2|	508583	+	U	2	1	0	1	1	SArRNA02
gi|29165615|ref|NC_002745.2|	508584	+	G	2	0	0	3	0	SArRNA02
gi|29165615|ref|NC_002745.2|	508585	+	U	7	2	1	0	0	SArRNA02
gi|29165615|ref|NC_002745.2|	508586	+	C	6	8	2	1	3	SArRNA02
gi|29165615|ref|NC_002745.2|	508587	+	U	2	5	1	4	2	SArRNA02
gi|29165615|ref|NC_002745.2|	508588	+	C	5	9	3	3	5	SArRNA02
gi|29165615|ref|NC_002745.2|	508589	+	U	13	0	1	7	4	SArRNA02
gi|29165615|ref|NC_002745.2|	508590	+	C	1	2	2	2	0	SArRNA02
gi|29165615|ref|NC_002745.2|	508591	+	U	4	2	1	0	1	SArRNA02
gi|29165615|ref|NC_002745.2|	508592	+	U	2	6	0	1	4	SArRNA02
gi|29165615|ref|NC_002745.2|	508593	+	G	2	1	0	1	1	SArRNA02
gi|29165615|ref|NC_002745.2|	508594	+	A	3	1	0	1	3	SArRNA02
gi|29165615|ref|NC_002745.2|	508595	+	G	2	2	0	5	0	SArRNA02
gi|29165615|ref|NC_002745.2|	508596	+	U	1	2	0	3	3	SArRNA02
gi|29165615|ref|NC_002745.2|	508597	+	G	0	1	0	0	0	SArRNA02
gi|29165615|ref|NC_002745.2|	508598	+	G	4	5	7	3	7	SArRNA02
gi|29165615|ref|NC_002745.2|	508599	+	A	5	11	6	9	4	SArRNA02
gi|29165615|ref|NC_002745.2|	508600	+	U	4	3	0	1	3	SArRNA02
gi|29165615|ref|NC_002745.2|	508601	+	C	8	7	1	1	6	SArRNA02
gi|29165615|ref|NC_002745.2|	508602	+	C	24	9	2	7	7	SArRNA02
gi|29165615|ref|NC_002745.2|	508603	+	U	11	6	2	2	4	SArRNA02
gi|29165615|ref|NC_002745.2|	508604	+	G	2	1	0	0	2	SArRNA02
gi|29165615|ref|NC_002745.2|	508605	+	A	1	1	0	0	3	SArRNA02
gi|29165615|ref|NC_002745.2|	508606	+	G	11	10	3	3	0	SArRNA02
gi|29165615|ref|NC_002745.2|	508607	+	U	40	12	2	12	13	SArRNA02
gi|29165615|ref|NC_002745.2|	508608	+	A	5	5	3	6	4	SArRNA02
gi|29165615|ref|NC_002745.2|	508609	+	C	7	3	0	2	6	SArRNA02
gi|29165615|ref|NC_002745.2|	508610	+	G	8	6	0	2	12	SArRNA02
gi|29165615|ref|NC_002745.2|	508611	+	A	10	5	1	6	4	SArRNA02
gi|29165615|ref|NC_002745.2|	508612	+	C	5	8	3	3	8	SArRNA02
gi|29165615|ref|NC_002745.2|	508613	+	G	4	4	1	0	6	SArRNA02
gi|29165615|ref|NC_002745.2|	508614	+	G	12	6	5	9	6	SArRNA02
gi|29165615|ref|NC_002745.2|	508615	+	A	6	3	0	3	7	SArRNA02
gi|29165615|ref|NC_002745.2|	508616	+	G	4	2	0	1	0	SArRNA02
gi|29165615|ref|NC_002745.2|	508617	+	C	21	25	9	12	15	SArRNA02
gi|29165615|ref|NC_002745.2|	508618	+	A	14	10	2	4	6	SArRNA02
gi|29165615|ref|NC_002745.2|	508619	+	C	22	13	6	12	16	SArRNA02
gi|29165615|ref|NC_002745.2|	508620	+	G	2	3	2	5	4	SArRNA02
gi|29165615|ref|NC_002745.2|	508621	+	U	6	1	0	3	3	SArRNA02
gi|29165615|ref|NC_002745.2|	508622	+	G	2	2	0	3	2	SArRNA02
gi|29165615|ref|NC_002745.2|	508623	+	A	3	1	2	0	0	SArRNA02
gi|29165615|ref|NC_002745.2|	508624	+	A	0	4	0	1	3	SArRNA02
gi|29165615|ref|NC_002745.2|	508625	+	A	2	7	0	1	6	SArRNA02
gi|29165615|ref|NC_002745.2|	508626	+	U	3	1	0	1	1	SArRNA02
gi|29165615|ref|NC_002745.2|	508627	+	U	0	2	2	0	2	SArRNA02
gi|29165615|ref|NC_002745.2|	508628	+	C	6	1	2	0	1	SArRNA02
gi|29165615|ref|NC_002745.2|	508629	+	C	3	0	3	1	3	SArRNA02
gi|29165615|ref|NC_002745.2|	508630	+	G	0	4	1	0	2	SArRNA02
gi|29165615|ref|NC_002745.2|	508631	+	U	5	2	0	1	0	SArRNA02
gi|29165615|ref|NC_002745.2|	508632	+	C	6	1	0	1	1	SArRNA02
gi|29165615|ref|NC_002745.2|	508633	+	G	0	1	1	0	0	SArRNA02

gi|29165615|ref|NC_002745.2|	508634	+	G	3	5	1	2	6	SArRNA02
gi|29165615|ref|NC_002745.2|	508635	+	A	6	3	0	1	4	SArRNA02
gi|29165615|ref|NC_002745.2|	508636	+	A	3	6	0	1	6	SArRNA02
gi|29165615|ref|NC_002745.2|	508637	+	U	6	4	1	3	7	SArRNA02
gi|29165615|ref|NC_002745.2|	508638	+	C	5	20	3	3	4	SArRNA02
gi|29165615|ref|NC_002745.2|	508639	+	U	4	5	1	6	5	SArRNA02
gi|29165615|ref|NC_002745.2|	508640	+	G	0	0	0	0	2	SArRNA02
gi|29165615|ref|NC_002745.2|	508641	+	G	0	2	0	0	1	SArRNA02
gi|29165615|ref|NC_002745.2|	508642	+	G	0	1	1	0	3	SArRNA02
gi|29165615|ref|NC_002745.2|	508643	+	A	0	1	0	0	1	SArRNA02
gi|29165615|ref|NC_002745.2|	508644	+	G	0	1	0	1	0	SArRNA02
gi|29165615|ref|NC_002745.2|	508645	+	G	6	0	1	2	8	SArRNA02
gi|29165615|ref|NC_002745.2|	508646	+	A	3	2	0	1	5	SArRNA02
gi|29165615|ref|NC_002745.2|	508647	+	C	18	28	9	11	21	SArRNA02
gi|29165615|ref|NC_002745.2|	508648	+	C	42	28	13	22	17	SArRNA02
gi|29165615|ref|NC_002745.2|	508649	+	A	8	9	4	3	7	SArRNA02
gi|29165615|ref|NC_002745.2|	508650	+	U	1	3	4	5	7	SArRNA02
gi|29165615|ref|NC_002745.2|	508651	+	C	15	24	11	24	21	SArRNA02
gi|29165615|ref|NC_002745.2|	508652	+	U	5	9	2	1	8	SArRNA02
gi|29165615|ref|NC_002745.2|	508653	+	C	1	1	1	4	2	SArRNA02
gi|29165615|ref|NC_002745.2|	508654	+	C	5	2	4	3	7	SArRNA02
gi|29165615|ref|NC_002745.2|	508655	+	U	5	8	3	4	6	SArRNA02
gi|29165615|ref|NC_002745.2|	508656	+	A	3	2	3	1	4	SArRNA02
gi|29165615|ref|NC_002745.2|	508657	+	A	1	4	3	3	6	SArRNA02
gi|29165615|ref|NC_002745.2|	508658	+	G	2	3	0	1	3	SArRNA02
gi|29165615|ref|NC_002745.2|	508659	+	G	1	1	2	1	3	SArRNA02
gi|29165615|ref|NC_002745.2|	508660	+	C	10	3	1	7	9	SArRNA02
gi|29165615|ref|NC_002745.2|	508661	+	U	7	12	2	1	17	SArRNA02
gi|29165615|ref|NC_002745.2|	508662	+	A	6	2	2	1	5	SArRNA02
gi|29165615|ref|NC_002745.2|	508663	+	A	3	4	2	4	4	SArRNA02
gi|29165615|ref|NC_002745.2|	508664	+	A	15	153	14	8	47	SArRNA02
gi|29165615|ref|NC_002745.2|	508665	+	U	10	27	6	8	16	SArRNA02
gi|29165615|ref|NC_002745.2|	508666	+	A	4	3	2	1	5	SArRNA02
gi|29165615|ref|NC_002745.2|	508667	+	C	31	27	10	10	32	SArRNA02
gi|29165615|ref|NC_002745.2|	508668	+	U	18	19	2	13	11	SArRNA02
gi|29165615|ref|NC_002745.2|	508669	+	C	7	8	2	6	7	SArRNA02
gi|29165615|ref|NC_002745.2|	508670	+	U	9	8	4	4	18	SArRNA02
gi|29165615|ref|NC_002745.2|	508671	+	C	46	37	7	11	16	SArRNA02
gi|29165615|ref|NC_002745.2|	508672	+	U	20	11	4	6	11	SArRNA02
gi|29165615|ref|NC_002745.2|	508673	+	A	7	6	3	1	6	SArRNA02
gi|29165615|ref|NC_002745.2|	508674	+	G	7	1	0	0	1	SArRNA02
gi|29165615|ref|NC_002745.2|	508675	+	U	9	1	0	3	6	SArRNA02
gi|29165615|ref|NC_002745.2|	508676	+	G	7	2	4	4	2	SArRNA02
gi|29165615|ref|NC_002745.2|	508677	+	A	5	6	1	1	2	SArRNA02
gi|29165615|ref|NC_002745.2|	508678	+	C	8	14	3	1	4	SArRNA02
gi|29165615|ref|NC_002745.2|	508679	+	C	18	17	6	3	11	SArRNA02
gi|29165615|ref|NC_002745.2|	508680	+	G	11	7	2	5	9	SArRNA02
gi|29165615|ref|NC_002745.2|	508681	+	A	16	14	5	6	14	SArRNA02
gi|29165615|ref|NC_002745.2|	508682	+	U	17	17	6	13	23	SArRNA02
gi|29165615|ref|NC_002745.2|	508683	+	A	8	6	5	3	8	SArRNA02
gi|29165615|ref|NC_002745.2|	508684	+	G	6	4	0	1	10	SArRNA02
gi|29165615|ref|NC_002745.2|	508685	+	U	9	8	2	5	11	SArRNA02
gi|29165615|ref|NC_002745.2|	508686	+	G	9	7	3	4	5	SArRNA02
gi|29165615|ref|NC_002745.2|	508687	+	A	6	4	0	7	6	SArRNA02
gi|29165615|ref|NC_002745.2|	508688	+	A	4	3	2	3	7	SArRNA02
gi|29165615|ref|NC_002745.2|	508689	+	C	43	27	6	13	20	SArRNA02
gi|29165615|ref|NC_002745.2|	508690	+	C	33	26	11	7	17	SArRNA02
gi|29165615|ref|NC_002745.2|	508691	+	A	6	10	5	3	11	SArRNA02
gi|29165615|ref|NC_002745.2|	508692	+	G	7	2	3	2	5	SArRNA02
gi|29165615|ref|NC_002745.2|	508693	+	U	11	14	3	9	6	SArRNA02
gi|29165615|ref|NC_002745.2|	508694	+	A	15	17	1	4	24	SArRNA02
gi|29165615|ref|NC_002745.2|	508695	+	C	30	14	7	2	19	SArRNA02
gi|29165615|ref|NC_002745.2|	508696	+	C	113	46	16	20	74	SArRNA02
gi|29165615|ref|NC_002745.2|	508697	+	G	35	23	11	8	27	SArRNA02
gi|29165615|ref|NC_002745.2|	508698	+	U	24	12	9	8	20	SArRNA02
gi|29165615|ref|NC_002745.2|	508699	+	G	15	9	7	10	12	SArRNA02
gi|29165615|ref|NC_002745.2|	508700	+	A	11	6	2	2	6	SArRNA02
gi|29165615|ref|NC_002745.2|	508701	+	G	1	2	0	0	4	SArRNA02
gi|29165615|ref|NC_002745.2|	508702	+	G	2	2	1	0	5	SArRNA02
gi|29165615|ref|NC_002745.2|	508703	+	G	4	2	4	2	7	SArRNA02
gi|29165615|ref|NC_002745.2|	508704	+	A	8	10	2	6	10	SArRNA02
gi|29165615|ref|NC_002745.2|	508705	+	A	11	4	4	5	15	SArRNA02
gi|29165615|ref|NC_002745.2|	508706	+	A	6	8	2	3	11	SArRNA02
gi|29165615|ref|NC_002745.2|	508707	+	G	1	2	2	1	2	SArRNA02
gi|29165615|ref|NC_002745.2|	508708	+	G	9	9	5	10	10	SArRNA02
gi|29165615|ref|NC_002745.2|	508709	+	U	13	7	9	0	14	SArRNA02
gi|29165615|ref|NC_002745.2|	508710	+	G	7	2	1	1	5	SArRNA02
gi|29165615|ref|NC_002745.2|	508711	+	A	12	10	5	3	16	SArRNA02
gi|29165615|ref|NC_002745.2|	508712	+	A	5	2	0	0	6	SArRNA02
gi|29165615|ref|NC_002745.2|	508713	+	A	5	0	0	0	2	SArRNA02
gi|29165615|ref|NC_002745.2|	508714	+	A	3	3	1	0	3	SArRNA02

gi|29165615|ref|NC_002745.2|	508715	+	G	3	1	1	1	2	SArRNA02
gi|29165615|ref|NC_002745.2|	508716	+	C	41	37	12	11	25	SArRNA02
gi|29165615|ref|NC_002745.2|	508717	+	A	9	12	3	0	6	SArRNA02
gi|29165615|ref|NC_002745.2|	508718	+	C	53	28	14	27	30	SArRNA02
gi|29165615|ref|NC_002745.2|	508719	+	C	28	13	7	9	15	SArRNA02
gi|29165615|ref|NC_002745.2|	508720	+	C	14	10	4	7	7	SArRNA02
gi|29165615|ref|NC_002745.2|	508721	+	C	15	1	7	4	13	SArRNA02
gi|29165615|ref|NC_002745.2|	508722	+	G	9	6	5	2	13	SArRNA02
gi|29165615|ref|NC_002745.2|	508723	+	G	9	7	6	8	15	SArRNA02
gi|29165615|ref|NC_002745.2|	508724	+	A	10	7	7	4	3	SArRNA02
gi|29165615|ref|NC_002745.2|	508725	+	A	16	2	2	3	8	SArRNA02
gi|29165615|ref|NC_002745.2|	508726	+	G	1	2	2	3	8	SArRNA02
gi|29165615|ref|NC_002745.2|	508727	+	G	1	0	0	0	4	SArRNA02
gi|29165615|ref|NC_002745.2|	508728	+	G	1	4	2	1	4	SArRNA02
gi|29165615|ref|NC_002745.2|	508729	+	G	4	7	4	8	10	SArRNA02
gi|29165615|ref|NC_002745.2|	508730	+	A	10	9	4	5	12	SArRNA02
gi|29165615|ref|NC_002745.2|	508731	+	G	9	2	1	8	9	SArRNA02
gi|29165615|ref|NC_002745.2|	508732	+	U	8	11	3	3	13	SArRNA02
gi|29165615|ref|NC_002745.2|	508733	+	G	4	5	4	5	7	SArRNA02
gi|29165615|ref|NC_002745.2|	508734	+	A	3	4	3	3	17	SArRNA02
gi|29165615|ref|NC_002745.2|	508735	+	A	10	3	6	5	21	SArRNA02
gi|29165615|ref|NC_002745.2|	508736	+	A	7	53	4	9	31	SArRNA02
gi|29165615|ref|NC_002745.2|	508737	+	U	12	37	21	18	43	SArRNA02
gi|29165615|ref|NC_002745.2|	508738	+	A	8	22	2	1	16	SArRNA02
gi|29165615|ref|NC_002745.2|	508739	+	G	17	13	7	5	9	SArRNA02
gi|29165615|ref|NC_002745.2|	508740	+	A	20	23	8	10	21	SArRNA02
gi|29165615|ref|NC_002745.2|	508741	+	A	12	13	1	4	12	SArRNA02
gi|29165615|ref|NC_002745.2|	508742	+	C	39	23	12	15	28	SArRNA02
gi|29165615|ref|NC_002745.2|	508743	+	C	27	34	8	15	25	SArRNA02
gi|29165615|ref|NC_002745.2|	508744	+	U	10	12	2	7	17	SArRNA02
gi|29165615|ref|NC_002745.2|	508745	+	G	8	6	6	3	7	SArRNA02
gi|29165615|ref|NC_002745.2|	508746	+	A	4	3	0	4	11	SArRNA02
gi|29165615|ref|NC_002745.2|	508747	+	A	12	2	3	2	6	SArRNA02
gi|29165615|ref|NC_002745.2|	508748	+	A	9	6	3	1	10	SArRNA02
gi|29165615|ref|NC_002745.2|	508749	+	C	8	4	3	1	13	SArRNA02
gi|29165615|ref|NC_002745.2|	508750	+	C	6	10	5	1	11	SArRNA02
gi|29165615|ref|NC_002745.2|	508751	+	G	4	4	2	4	7	SArRNA02
gi|29165615|ref|NC_002745.2|	508752	+	U	17	8	4	7	11	SArRNA02
gi|29165615|ref|NC_002745.2|	508753	+	G	4	6	2	0	6	SArRNA02
gi|29165615|ref|NC_002745.2|	508754	+	U	5	5	2	3	6	SArRNA02
gi|29165615|ref|NC_002745.2|	508755	+	G	7	3	1	0	6	SArRNA02
gi|29165615|ref|NC_002745.2|	508756	+	C	7	8	6	7	7	SArRNA02
gi|29165615|ref|NC_002745.2|	508757	+	U	4	1	5	7	6	SArRNA02
gi|29165615|ref|NC_002745.2|	508758	+	U	16	35	17	24	44	SArRNA02
gi|29165615|ref|NC_002745.2|	508759	+	A	7	7	4	2	15	SArRNA02
gi|29165615|ref|NC_002745.2|	508760	+	C	28	28	6	17	29	SArRNA02
gi|29165615|ref|NC_002745.2|	508761	+	A	8	13	7	13	25	SArRNA02
gi|29165615|ref|NC_002745.2|	508762	+	A	8	11	4	6	10	SArRNA02
gi|29165615|ref|NC_002745.2|	508763	+	G	16	10	7	4	12	SArRNA02
gi|29165615|ref|NC_002745.2|	508764	+	U	19	18	16	15	19	SArRNA02
gi|29165615|ref|NC_002745.2|	508765	+	A	11	3	2	4	6	SArRNA02
gi|29165615|ref|NC_002745.2|	508766	+	G	4	5	2	2	5	SArRNA02
gi|29165615|ref|NC_002745.2|	508767	+	U	30	16	13	7	26	SArRNA02
gi|29165615|ref|NC_002745.2|	508768	+	C	10	6	5	3	7	SArRNA02
gi|29165615|ref|NC_002745.2|	508769	+	A	7	11	3	6	3	SArRNA02
gi|29165615|ref|NC_002745.2|	508770	+	G	9	3	1	2	6	SArRNA02
gi|29165615|ref|NC_002745.2|	508771	+	A	2	3	1	1	2	SArRNA02
gi|29165615|ref|NC_002745.2|	508772	+	G	3	3	0	0	4	SArRNA02
gi|29165615|ref|NC_002745.2|	508773	+	C	6	5	1	3	12	SArRNA02
gi|29165615|ref|NC_002745.2|	508774	+	C	4	3	5	5	4	SArRNA02
gi|29165615|ref|NC_002745.2|	508775	+	C	35	18	9	16	24	SArRNA02
gi|29165615|ref|NC_002745.2|	508776	+	G	7	2	5	2	7	SArRNA02
gi|29165615|ref|NC_002745.2|	508777	+	U	4	2	0	3	2	SArRNA02
gi|29165615|ref|NC_002745.2|	508778	+	U	0	2	3	1	5	SArRNA02
gi|29165615|ref|NC_002745.2|	508779	+	A	2	5	1	3	6	SArRNA02
gi|29165615|ref|NC_002745.2|	508780	+	A	2	3	2	2	6	SArRNA02
gi|29165615|ref|NC_002745.2|	508781	+	U	0	3	1	0	1	SArRNA02
gi|29165615|ref|NC_002745.2|	508782	+	G	1	0	1	1	0	SArRNA02
gi|29165615|ref|NC_002745.2|	508783	+	G	5	10	5	0	4	SArRNA02
gi|29165615|ref|NC_002745.2|	508784	+	G	10	2	1	2	7	SArRNA02
gi|29165615|ref|NC_002745.2|	508785	+	U	8	2	4	4	7	SArRNA02
gi|29165615|ref|NC_002745.2|	508786	+	G	1	0	0	1	0	SArRNA02
gi|29165615|ref|NC_002745.2|	508787	+	A	6	3	2	1	2	SArRNA02
gi|29165615|ref|NC_002745.2|	508788	+	U	2	0	0	1	0	SArRNA02
gi|29165615|ref|NC_002745.2|	508789	+	G	1	0	0	0	0	SArRNA02
gi|29165615|ref|NC_002745.2|	508791	+	C	7	3	2	0	0	SArRNA02
gi|29165615|ref|NC_002745.2|	508792	+	G	2	0	2	0	0	SArRNA02
gi|29165615|ref|NC_002745.2|	508793	+	U	4	5	2	2	1	SArRNA02
gi|29165615|ref|NC_002745.2|	508794	+	G	1	1	0	1	0	SArRNA02
gi|29165615|ref|NC_002745.2|	508795	+	C	10	13	6	8	10	SArRNA02
gi|29165615|ref|NC_002745.2|	508796	+	C	20	16	4	7	9	SArRNA02

gi|29165615|ref|NC_002745.2|	508797	+	U	19	117	92	100	108	SArRNA02
gi|29165615|ref|NC_002745.2|	508798	+	U	44	145	94	150	124	SArRNA02
gi|29165615|ref|NC_002745.2|	508799	+	U	16	72	36	73	53	SArRNA02
gi|29165615|ref|NC_002745.2|	508800	+	U	4	8	1	1	7	SArRNA02
gi|29165615|ref|NC_002745.2|	508801	+	G	16	17	6	7	8	SArRNA02
gi|29165615|ref|NC_002745.2|	508802	+	U	8	6	2	8	4	SArRNA02
gi|29165615|ref|NC_002745.2|	508803	+	A	2	8	1	4	2	SArRNA02
gi|29165615|ref|NC_002745.2|	508804	+	G	8	8	2	1	6	SArRNA02
gi|29165615|ref|NC_002745.2|	508805	+	A	22	12	8	13	6	SArRNA02
gi|29165615|ref|NC_002745.2|	508806	+	A	39	304	57	12	81	SArRNA02
gi|29165615|ref|NC_002745.2|	508807	+	U	8	9	1	1	5	SArRNA02
gi|29165615|ref|NC_002745.2|	508808	+	G	2	4	0	3	2	SArRNA02
gi|29165615|ref|NC_002745.2|	508809	+	A	3	4	2	2	3	SArRNA02
gi|29165615|ref|NC_002745.2|	508810	+	A	3	5	0	2	4	SArRNA02
gi|29165615|ref|NC_002745.2|	508811	+	C	9	15	4	5	10	SArRNA02
gi|29165615|ref|NC_002745.2|	508812	+	C	26	15	5	6	9	SArRNA02
gi|29165615|ref|NC_002745.2|	508813	+	G	4	12	2	4	1	SArRNA02
gi|29165615|ref|NC_002745.2|	508814	+	G	4	1	2	1	3	SArRNA02
gi|29165615|ref|NC_002745.2|	508815	+	C	3	6	4	3	3	SArRNA02
gi|29165615|ref|NC_002745.2|	508816	+	G	8	9	2	4	4	SArRNA02
gi|29165615|ref|NC_002745.2|	508817	+	A	5	4	2	4	5	SArRNA02
gi|29165615|ref|NC_002745.2|	508818	+	G	17	2	1	6	5	SArRNA02
gi|29165615|ref|NC_002745.2|	508819	+	U	1	2	3	2	10	SArRNA02
gi|29165615|ref|NC_002745.2|	508820	+	U	3	5	1	5	15	SArRNA02
gi|29165615|ref|NC_002745.2|	508821	+	A	18	6	7	2	7	SArRNA02
gi|29165615|ref|NC_002745.2|	508822	+	C	19	13	5	10	15	SArRNA02
gi|29165615|ref|NC_002745.2|	508823	+	G	26	27	14	25	30	SArRNA02
gi|29165615|ref|NC_002745.2|	508824	+	A	36	19	9	11	22	SArRNA02
gi|29165615|ref|NC_002745.2|	508825	+	U	15	17	9	8	19	SArRNA02
gi|29165615|ref|NC_002745.2|	508826	+	U	12	16	9	6	18	SArRNA02
gi|29165615|ref|NC_002745.2|	508827	+	U	29	17	7	9	12	SArRNA02
gi|29165615|ref|NC_002745.2|	508828	+	G	52	48	20	34	32	SArRNA02
gi|29165615|ref|NC_002745.2|	508829	+	A	26	18	3	4	15	SArRNA02
gi|29165615|ref|NC_002745.2|	508830	+	U	20	10	3	9	9	SArRNA02
gi|29165615|ref|NC_002745.2|	508831	+	G	5	3	3	5	4	SArRNA02
gi|29165615|ref|NC_002745.2|	508832	+	C	35	15	13	13	27	SArRNA02
gi|29165615|ref|NC_002745.2|	508833	+	A	24	31	8	9	21	SArRNA02
gi|29165615|ref|NC_002745.2|	508834	+	A	16	4	7	4	9	SArRNA02
gi|29165615|ref|NC_002745.2|	508835	+	G	7	4	4	3	0	SArRNA02
gi|29165615|ref|NC_002745.2|	508836	+	G	20	13	11	10	21	SArRNA02
gi|29165615|ref|NC_002745.2|	508837	+	U	18	17	9	10	23	SArRNA02
gi|29165615|ref|NC_002745.2|	508838	+	U	30	26	9	8	17	SArRNA02
gi|29165615|ref|NC_002745.2|	508839	+	A	11	7	5	12	13	SArRNA02
gi|29165615|ref|NC_002745.2|	508840	+	A	23	11	7	8	22	SArRNA02
gi|29165615|ref|NC_002745.2|	508841	+	G	19	21	5	6	9	SArRNA02
gi|29165615|ref|NC_002745.2|	508842	+	C	29	32	10	10	19	SArRNA02
gi|29165615|ref|NC_002745.2|	508843	+	A	22	18	9	3	14	SArRNA02
gi|29165615|ref|NC_002745.2|	508844	+	G	17	18	7	15	24	SArRNA02
gi|29165615|ref|NC_002745.2|	508845	+	U	16	10	7	10	24	SArRNA02
gi|29165615|ref|NC_002745.2|	508846	+	A	35	17	8	7	18	SArRNA02
gi|29165615|ref|NC_002745.2|	508847	+	A	39	24	9	6	26	SArRNA02
gi|29165615|ref|NC_002745.2|	508848	+	A	23	48	6	4	32	SArRNA02
gi|29165615|ref|NC_002745.2|	508849	+	U	8	5	2	10	6	SArRNA02
gi|29165615|ref|NC_002745.2|	508850	+	G	5	3	7	4	7	SArRNA02
gi|29165615|ref|NC_002745.2|	508851	+	U	6	3	4	1	5	SArRNA02
gi|29165615|ref|NC_002745.2|	508852	+	G	3	1	0	1	2	SArRNA02
gi|29165615|ref|NC_002745.2|	508853	+	G	1	6	2	2	6	SArRNA02
gi|29165615|ref|NC_002745.2|	508854	+	A	14	10	3	0	12	SArRNA02
gi|29165615|ref|NC_002745.2|	508855	+	G	34	8	2	2	1	SArRNA02
gi|29165615|ref|NC_002745.2|	508856	+	C	1122	81	54	59	67	SArRNA02
gi|29165615|ref|NC_002745.2|	508857	+	C	169	21	11	18	21	SArRNA02
gi|29165615|ref|NC_002745.2|	508858	+	G	33	20	6	9	18	SArRNA02
gi|29165615|ref|NC_002745.2|	508859	+	U	38	42	22	23	29	SArRNA02
gi|29165615|ref|NC_002745.2|	508860	+	A	23	20	8	13	15	SArRNA02
gi|29165615|ref|NC_002745.2|	508861	+	G	7	7	2	7	9	SArRNA02
gi|29165615|ref|NC_002745.2|	508862	+	C	51	26	7	11	28	SArRNA02
gi|29165615|ref|NC_002745.2|	508863	+	G	28	10	4	6	12	SArRNA02
gi|29165615|ref|NC_002745.2|	508864	+	A	27	12	5	7	17	SArRNA02
gi|29165615|ref|NC_002745.2|	508865	+	A	10	9	7	14	14	SArRNA02
gi|29165615|ref|NC_002745.2|	508866	+	A	21	14	5	15	12	SArRNA02
gi|29165615|ref|NC_002745.2|	508867	+	G	0	2	0	0	1	SArRNA02
gi|29165615|ref|NC_002745.2|	508868	+	C	40	21	21	18	13	SArRNA02
gi|29165615|ref|NC_002745.2|	508869	+	G	3	10	4	4	1	SArRNA02
gi|29165615|ref|NC_002745.2|	508870	+	A	1	2	1	1	1	SArRNA02
gi|29165615|ref|NC_002745.2|	508871	+	G	4	0	1	2	0	SArRNA02
gi|29165615|ref|NC_002745.2|	508872	+	U	31	16	4	9	8	SArRNA02
gi|29165615|ref|NC_002745.2|	508873	+	C	48	35	13	15	17	SArRNA02
gi|29165615|ref|NC_002745.2|	508874	+	U	9	11	7	3	4	SArRNA02
gi|29165615|ref|NC_002745.2|	508875	+	G	2	2	0	4	1	SArRNA02
gi|29165615|ref|NC_002745.2|	508876	+	A	4	9	3	3	4	SArRNA02
gi|29165615|ref|NC_002745.2|	508877	+	A	8	22	2	4	7	SArRNA02

gi|29165615|ref|NC_002745.2|	508878	+	U	14	16	11	17	20	SArRNA02
gi|29165615|ref|NC_002745.2|	508879	+	A	9	16	3	9	8	SArRNA02
gi|29165615|ref|NC_002745.2|	508880	+	G	5	2	0	5	1	SArRNA02
gi|29165615|ref|NC_002745.2|	508881	+	G	3	4	1	2	2	SArRNA02
gi|29165615|ref|NC_002745.2|	508882	+	G	4	2	0	0	2	SArRNA02
gi|29165615|ref|NC_002745.2|	508883	+	C	9	5	1	2	5	SArRNA02
gi|29165615|ref|NC_002745.2|	508884	+	G	8	1	3	6	9	SArRNA02
gi|29165615|ref|NC_002745.2|	508885	+	U	8	5	7	2	15	SArRNA02
gi|29165615|ref|NC_002745.2|	508886	+	U	13	6	3	1	8	SArRNA02
gi|29165615|ref|NC_002745.2|	508887	+	U	9	10	11	5	9	SArRNA02
gi|29165615|ref|NC_002745.2|	508888	+	A	9	10	7	7	9	SArRNA02
gi|29165615|ref|NC_002745.2|	508889	+	G	9	5	3	1	5	SArRNA02
gi|29165615|ref|NC_002745.2|	508890	+	U	13	4	7	3	7	SArRNA02
gi|29165615|ref|NC_002745.2|	508891	+	A	13	11	3	6	9	SArRNA02
gi|29165615|ref|NC_002745.2|	508892	+	U	2	2	1	3	3	SArRNA02
gi|29165615|ref|NC_002745.2|	508893	+	U	4	1	0	0	5	SArRNA02
gi|29165615|ref|NC_002745.2|	508894	+	U	0	1	0	1	2	SArRNA02
gi|29165615|ref|NC_002745.2|	508895	+	G	0	1	0	0	0	SArRNA02
gi|29165615|ref|NC_002745.2|	508896	+	G	0	0	0	0	1	SArRNA02
gi|29165615|ref|NC_002745.2|	508898	+	C	1	3	1	0	1	SArRNA02
gi|29165615|ref|NC_002745.2|	508899	+	G	6	3	1	2	2	SArRNA02
gi|29165615|ref|NC_002745.2|	508900	+	U	1	1	1	0	1	SArRNA02
gi|29165615|ref|NC_002745.2|	508901	+	A	3	3	0	0	0	SArRNA02
gi|29165615|ref|NC_002745.2|	508902	+	G	12	16	2	7	5	SArRNA02
gi|29165615|ref|NC_002745.2|	508903	+	A	1	2	0	2	1	SArRNA02
gi|29165615|ref|NC_002745.2|	508904	+	C	47	44	14	21	26	SArRNA02
gi|29165615|ref|NC_002745.2|	508905	+	C	11	6	4	1	7	SArRNA02
gi|29165615|ref|NC_002745.2|	508906	+	C	21	12	6	2	3	SArRNA02
gi|29165615|ref|NC_002745.2|	508907	+	G	182	102	70	74	75	SArRNA02
gi|29165615|ref|NC_002745.2|	508908	+	A	2	1	0	0	2	SArRNA02
gi|29165615|ref|NC_002745.2|	508909	+	A	6	1	0	0	4	SArRNA02
gi|29165615|ref|NC_002745.2|	508910	+	A	2	1	0	0	0	SArRNA02
gi|29165615|ref|NC_002745.2|	508911	+	C	0	1	0	0	1	SArRNA02
gi|29165615|ref|NC_002745.2|	508912	+	C	4	6	0	0	3	SArRNA02
gi|29165615|ref|NC_002745.2|	508914	+	G	0	0	0	0	1	SArRNA02
gi|29165615|ref|NC_002745.2|	508915	+	G	1	1	0	0	0	SArRNA02
gi|29165615|ref|NC_002745.2|	508916	+	U	2	1	0	1	0	SArRNA02
gi|29165615|ref|NC_002745.2|	508917	+	G	2	3	0	0	1	SArRNA02
gi|29165615|ref|NC_002745.2|	508918	+	A	1	0	0	2	1	SArRNA02
gi|29165615|ref|NC_002745.2|	508920	+	C	2	1	0	1	0	SArRNA02
gi|29165615|ref|NC_002745.2|	508921	+	U	1	3	1	0	1	SArRNA02
gi|29165615|ref|NC_002745.2|	508922	+	A	5	1	0	1	0	SArRNA02
gi|29165615|ref|NC_002745.2|	508923	+	C	26	31	9	14	13	SArRNA02
gi|29165615|ref|NC_002745.2|	508924	+	C	4	14	3	3	0	SArRNA02
gi|29165615|ref|NC_002745.2|	508925	+	C	9	4	0	1	1	SArRNA02
gi|29165615|ref|NC_002745.2|	508926	+	U	3	0	1	2	3	SArRNA02
gi|29165615|ref|NC_002745.2|	508927	+	U	13	6	3	6	3	SArRNA02
gi|29165615|ref|NC_002745.2|	508928	+	G	1	1	1	3	1	SArRNA02
gi|29165615|ref|NC_002745.2|	508929	+	G	2	0	0	2	4	SArRNA02
gi|29165615|ref|NC_002745.2|	508930	+	U	9	6	2	2	5	SArRNA02
gi|29165615|ref|NC_002745.2|	508931	+	C	26	37	8	11	18	SArRNA02
gi|29165615|ref|NC_002745.2|	508932	+	A	17	25	3	4	16	SArRNA02
gi|29165615|ref|NC_002745.2|	508933	+	G	1	6	1	1	2	SArRNA02
gi|29165615|ref|NC_002745.2|	508934	+	G	4	0	5	6	5	SArRNA02
gi|29165615|ref|NC_002745.2|	508935	+	U	3	1	5	0	6	SArRNA02
gi|29165615|ref|NC_002745.2|	508936	+	U	2	1	1	0	1	SArRNA02
gi|29165615|ref|NC_002745.2|	508937	+	G	0	3	0	1	0	SArRNA02
gi|29165615|ref|NC_002745.2|	508938	+	A	0	2	1	0	2	SArRNA02
gi|29165615|ref|NC_002745.2|	508939	+	A	3	3	0	0	4	SArRNA02
gi|29165615|ref|NC_002745.2|	508940	+	G	1	1	0	0	0	SArRNA02
gi|29165615|ref|NC_002745.2|	508941	+	U	0	0	0	0	2	SArRNA02
gi|29165615|ref|NC_002745.2|	508942	+	U	0	2	0	1	2	SArRNA02
gi|29165615|ref|NC_002745.2|	508943	+	C	21	21	7	7	14	SArRNA02
gi|29165615|ref|NC_002745.2|	508944	+	A	18	5	0	5	8	SArRNA02
gi|29165615|ref|NC_002745.2|	508945	+	G	8	9	4	4	14	SArRNA02
gi|29165615|ref|NC_002745.2|	508946	+	G	14	6	5	3	11	SArRNA02
gi|29165615|ref|NC_002745.2|	508947	+	U	18	13	7	2	18	SArRNA02
gi|29165615|ref|NC_002745.2|	508948	+	A	55	53	17	19	38	SArRNA02
gi|29165615|ref|NC_002745.2|	508949	+	A	4	3	1	1	4	SArRNA02
gi|29165615|ref|NC_002745.2|	508950	+	C	99	96	36	43	59	SArRNA02
gi|29165615|ref|NC_002745.2|	508951	+	A	38	35	9	18	19	SArRNA02
gi|29165615|ref|NC_002745.2|	508952	+	C	6	8	0	1	5	SArRNA02
gi|29165615|ref|NC_002745.2|	508953	+	U	4	2	1	2	4	SArRNA02
gi|29165615|ref|NC_002745.2|	508954	+	G	2	4	0	1	2	SArRNA02
gi|29165615|ref|NC_002745.2|	508955	+	A	6	4	2	0	4	SArRNA02
gi|29165615|ref|NC_002745.2|	508956	+	A	3	2	1	0	0	SArRNA02
gi|29165615|ref|NC_002745.2|	508957	+	U	3	3	0	1	5	SArRNA02
gi|29165615|ref|NC_002745.2|	508958	+	G	0	0	0	0	1	SArRNA02
gi|29165615|ref|NC_002745.2|	508959	+	G	5	5	2	0	5	SArRNA02
gi|29165615|ref|NC_002745.2|	508960	+	A	8	10	0	1	5	SArRNA02
gi|29165615|ref|NC_002745.2|	508961	+	G	4	3	1	4	2	SArRNA02

gi|29165615|ref|NC_002745.2|	508962	+	G	7	7	3	5	5	SArRNA02
gi|29165615|ref|NC_002745.2|	508963	+	A	2	2	1	0	0	SArRNA02
gi|29165615|ref|NC_002745.2|	508964	+	C	7	14	6	3	5	SArRNA02
gi|29165615|ref|NC_002745.2|	508965	+	C	31	53	22	9	31	SArRNA02
gi|29165615|ref|NC_002745.2|	508966	+	G	23	23	12	12	19	SArRNA02
gi|29165615|ref|NC_002745.2|	508967	+	A	14	14	9	15	9	SArRNA02
gi|29165615|ref|NC_002745.2|	508968	+	A	2	4	0	1	2	SArRNA02
gi|29165615|ref|NC_002745.2|	508969	+	C	5	8	0	0	3	SArRNA02
gi|29165615|ref|NC_002745.2|	508970	+	C	27	21	8	5	8	SArRNA02
gi|29165615|ref|NC_002745.2|	508971	+	G	2	4	2	2	6	SArRNA02
gi|29165615|ref|NC_002745.2|	508972	+	A	2	4	2	4	5	SArRNA02
gi|29165615|ref|NC_002745.2|	508973	+	C	7	7	5	0	8	SArRNA02
gi|29165615|ref|NC_002745.2|	508974	+	U	7	9	4	6	5	SArRNA02
gi|29165615|ref|NC_002745.2|	508975	+	U	28	25	11	14	25	SArRNA02
gi|29165615|ref|NC_002745.2|	508976	+	A	12	14	0	4	8	SArRNA02
gi|29165615|ref|NC_002745.2|	508977	+	C	45	34	24	29	37	SArRNA02
gi|29165615|ref|NC_002745.2|	508978	+	G	13	18	7	13	17	SArRNA02
gi|29165615|ref|NC_002745.2|	508979	+	U	16	7	3	3	15	SArRNA02
gi|29165615|ref|NC_002745.2|	508980	+	U	30	28	5	20	24	SArRNA02
gi|29165615|ref|NC_002745.2|	508981	+	G	10	8	2	9	12	SArRNA02
gi|29165615|ref|NC_002745.2|	508982	+	A	10	8	4	3	10	SArRNA02
gi|29165615|ref|NC_002745.2|	508983	+	A	7	6	5	5	7	SArRNA02
gi|29165615|ref|NC_002745.2|	508984	+	A	19	31	14	16	29	SArRNA02
gi|29165615|ref|NC_002745.2|	508985	+	A	14	15	8	6	12	SArRNA02
gi|29165615|ref|NC_002745.2|	508986	+	G	7	14	2	9	14	SArRNA02
gi|29165615|ref|NC_002745.2|	508987	+	U	36	68	27	57	43	SArRNA02
gi|29165615|ref|NC_002745.2|	508988	+	G	9	20	18	22	17	SArRNA02
gi|29165615|ref|NC_002745.2|	508989	+	A	12	16	9	13	18	SArRNA02
gi|29165615|ref|NC_002745.2|	508990	+	G	5	8	2	0	4	SArRNA02
gi|29165615|ref|NC_002745.2|	508991	+	C	147	96	45	43	57	SArRNA02
gi|29165615|ref|NC_002745.2|	508992	+	G	54	48	14	27	33	SArRNA02
gi|29165615|ref|NC_002745.2|	508993	+	G	11	15	6	4	7	SArRNA02
gi|29165615|ref|NC_002745.2|	508994	+	A	16	16	8	7	16	SArRNA02
gi|29165615|ref|NC_002745.2|	508995	+	U	23	16	9	8	13	SArRNA02
gi|29165615|ref|NC_002745.2|	508996	+	G	46	49	16	20	30	SArRNA02
gi|29165615|ref|NC_002745.2|	508997	+	A	108	108	34	42	62	SArRNA02
gi|29165615|ref|NC_002745.2|	508998	+	A	31	34	14	23	17	SArRNA02
gi|29165615|ref|NC_002745.2|	508999	+	C	41	30	8	12	27	SArRNA02
gi|29165615|ref|NC_002745.2|	509000	+	U	32	43	7	10	12	SArRNA02
gi|29165615|ref|NC_002745.2|	509001	+	G	1	5	1	0	3	SArRNA02
gi|29165615|ref|NC_002745.2|	509002	+	A	6	3	1	1	5	SArRNA02
gi|29165615|ref|NC_002745.2|	509004	+	G	1	0	0	1	1	SArRNA02
gi|29165615|ref|NC_002745.2|	509005	+	G	10	7	4	3	8	SArRNA02
gi|29165615|ref|NC_002745.2|	509006	+	U	4	6	3	2	3	SArRNA02
gi|29165615|ref|NC_002745.2|	509007	+	A	1	1	0	0	4	SArRNA02
gi|29165615|ref|NC_002745.2|	509008	+	G	0	0	0	1	1	SArRNA02
gi|29165615|ref|NC_002745.2|	509009	+	C	13	10	7	2	5	SArRNA02
gi|29165615|ref|NC_002745.2|	509010	+	G	1	12	3	3	4	SArRNA02
gi|29165615|ref|NC_002745.2|	509011	+	G	4	1	1	4	4	SArRNA02
gi|29165615|ref|NC_002745.2|	509012	+	A	6	1	1	0	5	SArRNA02
gi|29165615|ref|NC_002745.2|	509013	+	G	3	6	2	2	1	SArRNA02
gi|29165615|ref|NC_002745.2|	509014	+	A	7	4	2	3	9	SArRNA02
gi|29165615|ref|NC_002745.2|	509015	+	A	16	17	5	1	8	SArRNA02
gi|29165615|ref|NC_002745.2|	509016	+	A	25	125	24	11	35	SArRNA02
gi|29165615|ref|NC_002745.2|	509017	+	U	7	10	5	10	4	SArRNA02
gi|29165615|ref|NC_002745.2|	509018	+	U	7	4	0	1	5	SArRNA02
gi|29165615|ref|NC_002745.2|	509019	+	C	18	26	4	1	10	SArRNA02
gi|29165615|ref|NC_002745.2|	509020	+	C	127	100	35	49	54	SArRNA02
gi|29165615|ref|NC_002745.2|	509021	+	A	31	40	15	8	14	SArRNA02
gi|29165615|ref|NC_002745.2|	509022	+	A	8	54	6	5	10	SArRNA02
gi|29165615|ref|NC_002745.2|	509023	+	U	3	3	0	0	1	SArRNA02
gi|29165615|ref|NC_002745.2|	509024	+	C	7	15	2	3	4	SArRNA02
gi|29165615|ref|NC_002745.2|	509025	+	G	11	11	3	10	6	SArRNA02
gi|29165615|ref|NC_002745.2|	509026	+	A	18	22	3	5	3	SArRNA02
gi|29165615|ref|NC_002745.2|	509027	+	A	4	6	2	3	9	SArRNA02
gi|29165615|ref|NC_002745.2|	509028	+	C	11	6	6	3	3	SArRNA02
gi|29165615|ref|NC_002745.2|	509029	+	C	15	10	6	3	7	SArRNA02
gi|29165615|ref|NC_002745.2|	509030	+	U	10	9	1	4	3	SArRNA02
gi|29165615|ref|NC_002745.2|	509031	+	G	6	6	5	7	0	SArRNA02
gi|29165615|ref|NC_002745.2|	509032	+	G	8	3	0	1	7	SArRNA02
gi|29165615|ref|NC_002745.2|	509033	+	A	10	3	4	2	3	SArRNA02
gi|29165615|ref|NC_002745.2|	509034	+	G	11	13	5	3	9	SArRNA02
gi|29165615|ref|NC_002745.2|	509035	+	A	23	20	4	7	14	SArRNA02
gi|29165615|ref|NC_002745.2|	509036	+	U	12	19	13	13	18	SArRNA02
gi|29165615|ref|NC_002745.2|	509037	+	A	5	0	1	2	3	SArRNA02
gi|29165615|ref|NC_002745.2|	509038	+	G	2	0	1	1	1	SArRNA02
gi|29165615|ref|NC_002745.2|	509039	+	C	7	14	2	1	8	SArRNA02
gi|29165615|ref|NC_002745.2|	509040	+	U	4	6	1	1	3	SArRNA02
gi|29165615|ref|NC_002745.2|	509041	+	G	3	1	0	0	1	SArRNA02
gi|29165615|ref|NC_002745.2|	509042	+	G	7	1	1	1	1	SArRNA02
gi|29165615|ref|NC_002745.2|	509043	+	U	7	0	0	2	2	SArRNA02

gi|29165615|ref|NC_002745.2|	509044	+	U	2	4	1	0	2	SArRNA02
gi|29165615|ref|NC_002745.2|	509045	+	C	6	9	6	3	9	SArRNA02
gi|29165615|ref|NC_002745.2|	509046	+	U	3	5	1	5	1	SArRNA02
gi|29165615|ref|NC_002745.2|	509047	+	C	35	39	14	22	20	SArRNA02
gi|29165615|ref|NC_002745.2|	509048	+	U	13	9	2	4	6	SArRNA02
gi|29165615|ref|NC_002745.2|	509049	+	C	4	5	0	0	2	SArRNA02
gi|29165615|ref|NC_002745.2|	509050	+	C	8	9	3	4	6	SArRNA02
gi|29165615|ref|NC_002745.2|	509051	+	G	1499	1071	669	908	645	SArRNA02
gi|29165615|ref|NC_002745.2|	509052	+	A	6	7	1	2	3	SArRNA02
gi|29165615|ref|NC_002745.2|	509053	+	A	6	7	1	3	7	SArRNA02
gi|29165615|ref|NC_002745.2|	509054	+	A	23	204	51	17	63	SArRNA02
gi|29165615|ref|NC_002745.2|	509055	+	U	9	9	4	6	9	SArRNA02
gi|29165615|ref|NC_002745.2|	509056	+	A	7	7	6	3	2	SArRNA02
gi|29165615|ref|NC_002745.2|	509057	+	G	7	6	4	4	3	SArRNA02
gi|29165615|ref|NC_002745.2|	509058	+	C	28	19	4	11	14	SArRNA02
gi|29165615|ref|NC_002745.2|	509059	+	U	13	7	5	6	10	SArRNA02
gi|29165615|ref|NC_002745.2|	509060	+	U	7	9	5	4	4	SArRNA02
gi|29165615|ref|NC_002745.2|	509061	+	U	9	14	3	5	11	SArRNA02
gi|29165615|ref|NC_002745.2|	509062	+	A	7	12	1	0	7	SArRNA02
gi|29165615|ref|NC_002745.2|	509063	+	G	2	1	5	1	2	SArRNA02
gi|29165615|ref|NC_002745.2|	509064	+	G	1	1	1	1	4	SArRNA02
gi|29165615|ref|NC_002745.2|	509065	+	G	6	8	0	3	4	SArRNA02
gi|29165615|ref|NC_002745.2|	509066	+	C	73	29	10	10	17	SArRNA02
gi|29165615|ref|NC_002745.2|	509067	+	U	7	18	1	4	3	SArRNA02
gi|29165615|ref|NC_002745.2|	509068	+	A	6	8	1	1	4	SArRNA02
gi|29165615|ref|NC_002745.2|	509069	+	G	11	10	2	3	5	SArRNA02
gi|29165615|ref|NC_002745.2|	509070	+	C	17	19	11	10	7	SArRNA02
gi|29165615|ref|NC_002745.2|	509071	+	C	5	7	3	2	2	SArRNA02
gi|29165615|ref|NC_002745.2|	509072	+	U	5	3	1	0	1	SArRNA02
gi|29165615|ref|NC_002745.2|	509073	+	C	65	72	36	25	22	SArRNA02
gi|29165615|ref|NC_002745.2|	509074	+	A	16	17	5	5	9	SArRNA02
gi|29165615|ref|NC_002745.2|	509075	+	A	9	15	3	4	6	SArRNA02
gi|29165615|ref|NC_002745.2|	509076	+	G	5	1	2	5	8	SArRNA02
gi|29165615|ref|NC_002745.2|	509077	+	U	6	5	3	2	7	SArRNA02
gi|29165615|ref|NC_002745.2|	509078	+	G	87	90	43	38	48	SArRNA02
gi|29165615|ref|NC_002745.2|	509079	+	A	19	8	5	4	8	SArRNA02
gi|29165615|ref|NC_002745.2|	509080	+	U	10	18	7	12	15	SArRNA02
gi|29165615|ref|NC_002745.2|	509081	+	G	12	20	6	2	6	SArRNA02
gi|29165615|ref|NC_002745.2|	509082	+	A	3	8	2	2	7	SArRNA02
gi|29165615|ref|NC_002745.2|	509083	+	U	10	10	1	10	8	SArRNA02
gi|29165615|ref|NC_002745.2|	509084	+	U	32	67	24	21	19	SArRNA02
gi|29165615|ref|NC_002745.2|	509085	+	A	9	8	6	2	4	SArRNA02
gi|29165615|ref|NC_002745.2|	509086	+	U	19	5	3	5	8	SArRNA02
gi|29165615|ref|NC_002745.2|	509087	+	U	17	6	5	6	9	SArRNA02
gi|29165615|ref|NC_002745.2|	509088	+	G	1	3	1	3	6	SArRNA02
gi|29165615|ref|NC_002745.2|	509089	+	G	11	11	4	8	4	SArRNA02
gi|29165615|ref|NC_002745.2|	509090	+	A	21	12	3	10	10	SArRNA02
gi|29165615|ref|NC_002745.2|	509091	+	G	5	2	0	1	3	SArRNA02
gi|29165615|ref|NC_002745.2|	509092	+	G	14	8	5	5	6	SArRNA02
gi|29165615|ref|NC_002745.2|	509093	+	U	18	16	1	6	15	SArRNA02
gi|29165615|ref|NC_002745.2|	509094	+	A	3	24	2	7	11	SArRNA02
gi|29165615|ref|NC_002745.2|	509095	+	G	23	27	9	9	8	SArRNA02
gi|29165615|ref|NC_002745.2|	509096	+	A	10	11	3	11	5	SArRNA02
gi|29165615|ref|NC_002745.2|	509097	+	G	6	4	4	4	11	SArRNA02
gi|29165615|ref|NC_002745.2|	509098	+	C	76	36	13	13	20	SArRNA02
gi|29165615|ref|NC_002745.2|	509099	+	A	31	11	2	13	14	SArRNA02
gi|29165615|ref|NC_002745.2|	509100	+	C	35	38	14	10	16	SArRNA02
gi|29165615|ref|NC_002745.2|	509101	+	U	5	5	1	1	2	SArRNA02
gi|29165615|ref|NC_002745.2|	509102	+	G	1	0	0	0	0	SArRNA02
gi|29165615|ref|NC_002745.2|	509103	+	U	8	9	3	2	8	SArRNA02
gi|29165615|ref|NC_002745.2|	509104	+	U	1	4	0	1	2	SArRNA02
gi|29165615|ref|NC_002745.2|	509105	+	U	4	2	1	3	3	SArRNA02
gi|29165615|ref|NC_002745.2|	509106	+	G	1	0	2	3	0	SArRNA02
gi|29165615|ref|NC_002745.2|	509107	+	G	1	2	0	0	0	SArRNA02
gi|29165615|ref|NC_002745.2|	509108	+	A	7	1	0	4	2	SArRNA02
gi|29165615|ref|NC_002745.2|	509109	+	C	6	2	3	3	4	SArRNA02
gi|29165615|ref|NC_002745.2|	509110	+	G	2	1	0	2	2	SArRNA02
gi|29165615|ref|NC_002745.2|	509111	+	A	5	3	1	0	3	SArRNA02
gi|29165615|ref|NC_002745.2|	509112	+	G	3	0	0	0	1	SArRNA02
gi|29165615|ref|NC_002745.2|	509113	+	G	2	2	0	0	1	SArRNA02
gi|29165615|ref|NC_002745.2|	509114	+	G	1	3	0	0	1	SArRNA02
gi|29165615|ref|NC_002745.2|	509115	+	G	4	2	0	1	3	SArRNA02
gi|29165615|ref|NC_002745.2|	509116	+	C	636	397	286	203	277	SArRNA02
gi|29165615|ref|NC_002745.2|	509117	+	C	216	140	65	85	93	SArRNA02
gi|29165615|ref|NC_002745.2|	509118	+	C	93	50	18	30	30	SArRNA02
gi|29165615|ref|NC_002745.2|	509119	+	C	52	47	25	18	37	SArRNA02
gi|29165615|ref|NC_002745.2|	509120	+	U	35	29	16	10	37	SArRNA02
gi|29165615|ref|NC_002745.2|	509121	+	C	39	29	11	20	19	SArRNA02
gi|29165615|ref|NC_002745.2|	509122	+	U	10	8	3	10	14	SArRNA02
gi|29165615|ref|NC_002745.2|	509123	+	C	558	587	222	330	303	SArRNA02
gi|29165615|ref|NC_002745.2|	509124	+	G	88	113	52	47	56	SArRNA02

gi|29165615|ref|NC_002745.2|	509125	+	G	4	2	0	2	4	SArRNA02
gi|29165615|ref|NC_002745.2|	509126	+	G	4	3	1	2	4	SArRNA02
gi|29165615|ref|NC_002745.2|	509127	+	U	10	12	4	6	4	SArRNA02
gi|29165615|ref|NC_002745.2|	509128	+	U	33	42	13	18	25	SArRNA02
gi|29165615|ref|NC_002745.2|	509129	+	A	14	6	1	6	8	SArRNA02
gi|29165615|ref|NC_002745.2|	509130	+	C	23	8	1	8	12	SArRNA02
gi|29165615|ref|NC_002745.2|	509131	+	C	45	17	10	7	32	SArRNA02
gi|29165615|ref|NC_002745.2|	509132	+	G	21	16	0	11	17	SArRNA02
gi|29165615|ref|NC_002745.2|	509133	+	A	62	57	19	19	36	SArRNA02
gi|29165615|ref|NC_002745.2|	509134	+	A	23	31	13	13	18	SArRNA02
gi|29165615|ref|NC_002745.2|	509135	+	U	17	19	6	10	10	SArRNA02
gi|29165615|ref|NC_002745.2|	509136	+	U	15	21	9	12	13	SArRNA02
gi|29165615|ref|NC_002745.2|	509137	+	C	131	96	60	51	59	SArRNA02
gi|29165615|ref|NC_002745.2|	509138	+	A	54	37	15	19	29	SArRNA02
gi|29165615|ref|NC_002745.2|	509139	+	G	9	14	6	6	6	SArRNA02
gi|29165615|ref|NC_002745.2|	509140	+	A	5	11	7	8	16	SArRNA02
gi|29165615|ref|NC_002745.2|	509141	+	C	113	113	69	57	51	SArRNA02
gi|29165615|ref|NC_002745.2|	509142	+	A	30	36	8	18	22	SArRNA02
gi|29165615|ref|NC_002745.2|	509143	+	A	36	43	13	20	35	SArRNA02
gi|29165615|ref|NC_002745.2|	509144	+	A	69	59	23	43	50	SArRNA02
gi|29165615|ref|NC_002745.2|	509145	+	C	219	234	101	139	100	SArRNA02
gi|29165615|ref|NC_002745.2|	509146	+	U	64	59	26	42	55	SArRNA02
gi|29165615|ref|NC_002745.2|	509147	+	C	100	47	18	34	41	SArRNA02
gi|29165615|ref|NC_002745.2|	509148	+	C	211	145	53	72	109	SArRNA02
gi|29165615|ref|NC_002745.2|	509149	+	G	178	149	57	78	119	SArRNA02
gi|29165615|ref|NC_002745.2|	509150	+	A	569	466	239	312	316	SArRNA02
gi|29165615|ref|NC_002745.2|	509151	+	A	236	237	84	150	159	SArRNA02
gi|29165615|ref|NC_002745.2|	509152	+	U	47	47	21	22	39	SArRNA02
gi|29165615|ref|NC_002745.2|	509153	+	G	20	11	2	4	10	SArRNA02
gi|29165615|ref|NC_002745.2|	509154	+	C	52	38	11	19	26	SArRNA02
gi|29165615|ref|NC_002745.2|	509155	+	C	409	436	175	254	197	SArRNA02
gi|29165615|ref|NC_002745.2|	509156	+	A	83	78	34	37	67	SArRNA02
gi|29165615|ref|NC_002745.2|	509157	+	A	52	24	13	20	35	SArRNA02
gi|29165615|ref|NC_002745.2|	509158	+	U	40	30	25	38	53	SArRNA02
gi|29165615|ref|NC_002745.2|	509159	+	U	81	62	27	30	69	SArRNA02
gi|29165615|ref|NC_002745.2|	509160	+	A	72	55	20	31	55	SArRNA02
gi|29165615|ref|NC_002745.2|	509161	+	A	115	103	43	48	94	SArRNA02
gi|29165615|ref|NC_002745.2|	509162	+	U	60	38	16	34	49	SArRNA02
gi|29165615|ref|NC_002745.2|	509163	+	U	30	24	21	19	45	SArRNA02
gi|29165615|ref|NC_002745.2|	509164	+	U	59	53	17	22	37	SArRNA02
gi|29165615|ref|NC_002745.2|	509165	+	A	20	19	11	12	19	SArRNA02
gi|29165615|ref|NC_002745.2|	509166	+	A	29	28	11	16	24	SArRNA02
gi|29165615|ref|NC_002745.2|	509167	+	C	125	113	39	59	55	SArRNA02
gi|29165615|ref|NC_002745.2|	509168	+	U	33	26	10	16	38	SArRNA02
gi|29165615|ref|NC_002745.2|	509169	+	U	12	11	5	10	18	SArRNA02
gi|29165615|ref|NC_002745.2|	509170	+	G	13	9	5	13	12	SArRNA02
gi|29165615|ref|NC_002745.2|	509171	+	G	104	64	38	46	50	SArRNA02
gi|29165615|ref|NC_002745.2|	509172	+	G	170	112	69	63	74	SArRNA02
gi|29165615|ref|NC_002745.2|	509173	+	A	54	36	13	18	53	SArRNA02
gi|29165615|ref|NC_002745.2|	509174	+	G	60	34	22	22	47	SArRNA02
gi|29165615|ref|NC_002745.2|	509175	+	U	287	205	80	83	128	SArRNA02
gi|29165615|ref|NC_002745.2|	509176	+	C	1680	1188	549	620	777	SArRNA02
gi|29165615|ref|NC_002745.2|	509177	+	A	367	270	111	162	180	SArRNA02
gi|29165615|ref|NC_002745.2|	509178	+	G	14	14	7	1	12	SArRNA02
gi|29165615|ref|NC_002745.2|	509179	+	A	173	70	32	65	104	SArRNA02
gi|29165615|ref|NC_002745.2|	509180	+	A	104	63	21	36	56	SArRNA02
gi|29165615|ref|NC_002745.2|	509181	+	C	70	73	44	31	41	SArRNA02
gi|29165615|ref|NC_002745.2|	509182	+	A	26	44	9	16	19	SArRNA02
gi|29165615|ref|NC_002745.2|	509183	+	U	48	50	13	16	17	SArRNA02
gi|29165615|ref|NC_002745.2|	509184	+	G	23	15	6	14	13	SArRNA02
gi|29165615|ref|NC_002745.2|	509185	+	G	148	91	46	69	139	SArRNA02
gi|29165615|ref|NC_002745.2|	509186	+	G	144	111	57	53	139	SArRNA02
gi|29165615|ref|NC_002745.2|	509187	+	U	253	139	73	96	190	SArRNA02
gi|29165615|ref|NC_002745.2|	509188	+	G	143	87	53	61	112	SArRNA02
gi|29165615|ref|NC_002745.2|	509189	+	A	190	128	85	78	198	SArRNA02
gi|29165615|ref|NC_002745.2|	509190	+	U	76	85	26	43	84	SArRNA02
gi|29165615|ref|NC_002745.2|	509191	+	A	49	56	18	26	54	SArRNA02
gi|29165615|ref|NC_002745.2|	509192	+	A	38	36	16	28	43	SArRNA02
gi|29165615|ref|NC_002745.2|	509193	+	G	26	25	12	13	24	SArRNA02
gi|29165615|ref|NC_002745.2|	509194	+	G	18	16	1	11	16	SArRNA02
gi|29165615|ref|NC_002745.2|	509195	+	U	9	13	4	2	12	SArRNA02
gi|29165615|ref|NC_002745.2|	509196	+	C	57	39	9	5	36	SArRNA02
gi|29165615|ref|NC_002745.2|	509197	+	C	171	102	67	60	90	SArRNA02
gi|29165615|ref|NC_002745.2|	509198	+	G	29	43	17	17	13	SArRNA02
gi|29165615|ref|NC_002745.2|	509199	+	U	28	28	15	25	35	SArRNA02
gi|29165615|ref|NC_002745.2|	509200	+	G	12	13	6	10	10	SArRNA02
gi|29165615|ref|NC_002745.2|	509201	+	U	50	43	19	29	43	SArRNA02
gi|29165615|ref|NC_002745.2|	509202	+	U	69	56	32	55	44	SArRNA02
gi|29165615|ref|NC_002745.2|	509203	+	C	62	34	15	19	38	SArRNA02
gi|29165615|ref|NC_002745.2|	509204	+	G	44	43	24	30	41	SArRNA02
gi|29165615|ref|NC_002745.2|	509205	+	A	54	36	14	24	27	SArRNA02

gi|29165615|ref|NC_002745.2|	509206	+	A	49	72	19	22	37	SArRNA02
gi|29165615|ref|NC_002745.2|	509207	+	A	51	46	14	17	29	SArRNA02
gi|29165615|ref|NC_002745.2|	509208	+	G	14	9	3	10	9	SArRNA02
gi|29165615|ref|NC_002745.2|	509209	+	G	17	26	4	7	7	SArRNA02
gi|29165615|ref|NC_002745.2|	509210	+	G	11	27	7	8	3	SArRNA02
gi|29165615|ref|NC_002745.2|	509211	+	A	11	4	0	2	7	SArRNA02
gi|29165615|ref|NC_002745.2|	509212	+	A	10	13	6	8	12	SArRNA02
gi|29165615|ref|NC_002745.2|	509213	+	A	23	20	3	7	15	SArRNA02
gi|29165615|ref|NC_002745.2|	509214	+	C	164	171	49	80	110	SArRNA02
gi|29165615|ref|NC_002745.2|	509215	+	A	22	33	9	5	21	SArRNA02
gi|29165615|ref|NC_002745.2|	509216	+	G	4	4	3	2	1	SArRNA02
gi|29165615|ref|NC_002745.2|	509217	+	C	145	145	51	48	65	SArRNA02
gi|29165615|ref|NC_002745.2|	509218	+	C	132	153	48	46	66	SArRNA02
gi|29165615|ref|NC_002745.2|	509219	+	C	374	180	91	89	113	SArRNA02
gi|29165615|ref|NC_002745.2|	509220	+	A	189	80	50	34	62	SArRNA02
gi|29165615|ref|NC_002745.2|	509221	+	G	19	6	5	9	13	SArRNA02
gi|29165615|ref|NC_002745.2|	509222	+	A	15	20	5	8	15	SArRNA02
gi|29165615|ref|NC_002745.2|	509223	+	C	353	220	136	125	196	SArRNA02
gi|29165615|ref|NC_002745.2|	509224	+	C	915	512	368	299	530	SArRNA02
gi|29165615|ref|NC_002745.2|	509225	+	A	238	163	81	69	107	SArRNA02
gi|29165615|ref|NC_002745.2|	509226	+	C	189	123	52	46	97	SArRNA02
gi|29165615|ref|NC_002745.2|	509227	+	C	417	292	162	134	193	SArRNA02
gi|29165615|ref|NC_002745.2|	509228	+	A	126	104	37	53	62	SArRNA02
gi|29165615|ref|NC_002745.2|	509229	+	G	108	56	11	31	74	SArRNA02
gi|29165615|ref|NC_002745.2|	509230	+	C	805	166	161	130	350	SArRNA02
gi|29165615|ref|NC_002745.2|	509231	+	U	1706	176	135	143	249	SArRNA02
gi|29165615|ref|NC_002745.2|	509232	+	A	451	230	97	108	264	SArRNA02
gi|29165615|ref|NC_002745.2|	509233	+	A	316	214	114	147	300	SArRNA02
gi|29165615|ref|NC_002745.2|	509234	+	G	316	222	99	123	330	SArRNA02
gi|29165615|ref|NC_002745.2|	509235	+	G	301	266	136	154	397	SArRNA02
gi|29165615|ref|NC_002745.2|	509236	+	U	284	209	117	109	284	SArRNA02
gi|29165615|ref|NC_002745.2|	509237	+	C	531	407	230	198	374	SArRNA02
gi|29165615|ref|NC_002745.2|	509238	+	C	354	282	165	135	263	SArRNA02
gi|29165615|ref|NC_002745.2|	509239	+	C	235	174	87	84	271	SArRNA02
gi|29165615|ref|NC_002745.2|	509240	+	A	254	167	90	111	256	SArRNA02
gi|29165615|ref|NC_002745.2|	509241	+	A	413	257	132	115	304	SArRNA02
gi|29165615|ref|NC_002745.2|	509242	+	A	351	227	130	125	338	SArRNA02
gi|29165615|ref|NC_002745.2|	509243	+	A	455	1097	359	183	825	SArRNA02
gi|29165615|ref|NC_002745.2|	509244	+	U	446	805	517	339	851	SArRNA02
gi|29165615|ref|NC_002745.2|	509245	+	A	416	426	186	206	426	SArRNA02
gi|29165615|ref|NC_002745.2|	509246	+	U	817	637	329	313	600	SArRNA02
gi|29165615|ref|NC_002745.2|	509247	+	A	441	287	188	192	435	SArRNA02
gi|29165615|ref|NC_002745.2|	509248	+	U	565	283	201	177	472	SArRNA02
gi|29165615|ref|NC_002745.2|	509249	+	G	452	318	196	200	533	SArRNA02
gi|29165615|ref|NC_002745.2|	509250	+	U	388	283	211	187	495	SArRNA02
gi|29165615|ref|NC_002745.2|	509251	+	U	671	528	253	331	640	SArRNA02
gi|29165615|ref|NC_002745.2|	509252	+	A	552	445	228	247	466	SArRNA02
gi|29165615|ref|NC_002745.2|	509253	+	A	490	350	197	251	525	SArRNA02
gi|29165615|ref|NC_002745.2|	509254	+	G	1404	969	490	728	1118	SArRNA02
gi|29165615|ref|NC_002745.2|	509255	+	U	751	572	298	316	655	SArRNA02
gi|29165615|ref|NC_002745.2|	509256	+	G	800	708	397	544	894	SArRNA02
gi|29165615|ref|NC_002745.2|	509257	+	G	454	345	188	206	505	SArRNA02
gi|29165615|ref|NC_002745.2|	509258	+	A	400	341	169	198	418	SArRNA02
gi|29165615|ref|NC_002745.2|	509259	+	A	245	213	109	125	263	SArRNA02
gi|29165615|ref|NC_002745.2|	509260	+	A	265	242	134	141	310	SArRNA02
gi|29165615|ref|NC_002745.2|	509261	+	A	320	188	124	117	334	SArRNA02
gi|29165615|ref|NC_002745.2|	509262	+	G	177	115	76	93	186	SArRNA02
gi|29165615|ref|NC_002745.2|	509263	+	G	395	246	145	169	495	SArRNA02
gi|29165615|ref|NC_002745.2|	509264	+	A	538	210	182	157	427	SArRNA02
gi|29165615|ref|NC_002745.2|	509265	+	U	717	191	136	111	314	SArRNA02
gi|29165615|ref|NC_002745.2|	509266	+	G	245	153	120	106	285	SArRNA02
gi|29165615|ref|NC_002745.2|	509267	+	U	1319	440	275	257	685	SArRNA02
gi|29165615|ref|NC_002745.2|	509268	+	G	63	17	14	22	36	SArRNA02
gi|29165615|ref|NC_002745.2|	509269	+	G	144	12	8	5	19	SArRNA02
gi|29165615|ref|NC_002745.2|	509270	+	C	9042	427	333	314	477	SArRNA02
gi|29165615|ref|NC_002745.2|	509271	+	G	514	62	46	36	78	SArRNA02
gi|29165615|ref|NC_002745.2|	509272	+	U	123	87	49	46	98	SArRNA02
gi|29165615|ref|NC_002745.2|	509273	+	U	40	55	18	15	60	SArRNA02
gi|29165615|ref|NC_002745.2|	509274	+	G	28	17	5	17	31	SArRNA02
gi|29165615|ref|NC_002745.2|	509275	+	C	83	86	40	48	127	SArRNA02
gi|29165615|ref|NC_002745.2|	509276	+	C	291	175	105	101	338	SArRNA02
gi|29165615|ref|NC_002745.2|	509277	+	C	478	302	227	232	493	SArRNA02
gi|29165615|ref|NC_002745.2|	509278	+	A	207	145	108	111	287	SArRNA02
gi|29165615|ref|NC_002745.2|	509279	+	G	128	91	65	62	160	SArRNA02
gi|29165615|ref|NC_002745.2|	509280	+	A	93	50	55	45	161	SArRNA02
gi|29165615|ref|NC_002745.2|	509281	+	C	1002	196	135	151	410	SArRNA02
gi|29165615|ref|NC_002745.2|	509282	+	A	259	111	62	77	176	SArRNA02
gi|29165615|ref|NC_002745.2|	509283	+	A	250	159	42	34	140	SArRNA02
gi|29165615|ref|NC_002745.2|	509284	+	C	1250	580	338	329	760	SArRNA02
gi|29165615|ref|NC_002745.2|	509285	+	U	1890	212	137	143	279	SArRNA02
gi|29165615|ref|NC_002745.2|	509286	+	A	323	56	22	36	74	SArRNA02

gi|29165615|ref|NC_002745.2|	509287	+	G	5	7	8	8	20	SArRNA02
gi|29165615|ref|NC_002745.2|	509288	+	G	6	19	8	9	20	SArRNA02
gi|29165615|ref|NC_002745.2|	509289	+	A	34	19	11	11	32	SArRNA02
gi|29165615|ref|NC_002745.2|	509290	+	U	51	24	23	24	33	SArRNA02
gi|29165615|ref|NC_002745.2|	509291	+	G	45	7	14	8	30	SArRNA02
gi|29165615|ref|NC_002745.2|	509292	+	U	51	34	32	28	91	SArRNA02
gi|29165615|ref|NC_002745.2|	509293	+	U	34	35	25	39	50	SArRNA02
gi|29165615|ref|NC_002745.2|	509294	+	G	6	9	5	3	6	SArRNA02
gi|29165615|ref|NC_002745.2|	509295	+	G	41	26	12	27	46	SArRNA02
gi|29165615|ref|NC_002745.2|	509296	+	C	91	83	47	52	184	SArRNA02
gi|29165615|ref|NC_002745.2|	509297	+	U	80	39	34	25	119	SArRNA02
gi|29165615|ref|NC_002745.2|	509298	+	U	67	75	49	40	144	SArRNA02
gi|29165615|ref|NC_002745.2|	509299	+	A	66	55	26	38	118	SArRNA02
gi|29165615|ref|NC_002745.2|	509300	+	G	312	403	146	169	330	SArRNA02
gi|29165615|ref|NC_002745.2|	509301	+	A	13	34	18	6	27	SArRNA02
gi|29165615|ref|NC_002745.2|	509302	+	A	58	64	19	22	68	SArRNA02
gi|29165615|ref|NC_002745.2|	509303	+	G	55	48	18	15	45	SArRNA02
gi|29165615|ref|NC_002745.2|	509304	+	C	400	533	245	246	467	SArRNA02
gi|29165615|ref|NC_002745.2|	509305	+	A	144	176	47	57	134	SArRNA02
gi|29165615|ref|NC_002745.2|	509306	+	G	57	35	27	13	65	SArRNA02
gi|29165615|ref|NC_002745.2|	509307	+	C	131	123	68	94	186	SArRNA02
gi|29165615|ref|NC_002745.2|	509308	+	C	288	240	164	158	315	SArRNA02
gi|29165615|ref|NC_002745.2|	509309	+	A	106	121	66	55	205	SArRNA02
gi|29165615|ref|NC_002745.2|	509310	+	U	89	54	26	31	123	SArRNA02
gi|29165615|ref|NC_002745.2|	509311	+	C	484	386	226	169	424	SArRNA02
gi|29165615|ref|NC_002745.2|	509312	+	A	223	204	132	106	364	SArRNA02
gi|29165615|ref|NC_002745.2|	509313	+	U	123	111	62	62	227	SArRNA02
gi|29165615|ref|NC_002745.2|	509314	+	U	109	76	58	42	179	SArRNA02
gi|29165615|ref|NC_002745.2|	509315	+	U	128	100	60	49	194	SArRNA02
gi|29165615|ref|NC_002745.2|	509316	+	A	132	84	45	53	155	SArRNA02
gi|29165615|ref|NC_002745.2|	509317	+	A	119	89	59	47	171	SArRNA02
gi|29165615|ref|NC_002745.2|	509318	+	A	108	82	64	88	131	SArRNA02
gi|29165615|ref|NC_002745.2|	509319	+	G	958	595	533	1258	2364	SArRNA02
gi|29165615|ref|NC_002745.2|	509320	+	A	89	69	48	69	132	SArRNA02
gi|29165615|ref|NC_002745.2|	509321	+	G	30	19	14	19	60	SArRNA02
gi|29165615|ref|NC_002745.2|	509322	+	U	33	25	11	16	63	SArRNA02
gi|29165615|ref|NC_002745.2|	509323	+	G	40	29	13	21	40	SArRNA02
gi|29165615|ref|NC_002745.2|	509324	+	C	164	110	73	88	190	SArRNA02
gi|29165615|ref|NC_002745.2|	509325	+	G	57	74	31	28	120	SArRNA02
gi|29165615|ref|NC_002745.2|	509326	+	U	90	91	54	36	120	SArRNA02
gi|29165615|ref|NC_002745.2|	509327	+	A	38	71	30	22	65	SArRNA02
gi|29165615|ref|NC_002745.2|	509328	+	A	114	368	68	46	279	SArRNA02
gi|29165615|ref|NC_002745.2|	509329	+	U	65	140	87	69	205	SArRNA02
gi|29165615|ref|NC_002745.2|	509330	+	A	19	54	12	10	38	SArRNA02
gi|29165615|ref|NC_002745.2|	509331	+	G	11	15	4	3	13	SArRNA02
gi|29165615|ref|NC_002745.2|	509332	+	C	71	59	30	18	101	SArRNA02
gi|29165615|ref|NC_002745.2|	509333	+	U	40	38	23	24	66	SArRNA02
gi|29165615|ref|NC_002745.2|	509334	+	C	87	84	32	26	85	SArRNA02
gi|29165615|ref|NC_002745.2|	509335	+	A	48	43	29	28	87	SArRNA02
gi|29165615|ref|NC_002745.2|	509336	+	C	183	242	98	89	164	SArRNA02
gi|29165615|ref|NC_002745.2|	509337	+	U	183	142	99	218	363	SArRNA02
gi|29165615|ref|NC_002745.2|	509338	+	A	43	49	22	25	52	SArRNA02
gi|29165615|ref|NC_002745.2|	509339	+	G	45	43	23	22	59	SArRNA02
gi|29165615|ref|NC_002745.2|	509340	+	U	98	58	35	23	99	SArRNA02
gi|29165615|ref|NC_002745.2|	509341	+	C	714	707	287	514	634	SArRNA02
gi|29165615|ref|NC_002745.2|	509342	+	G	152	167	88	92	208	SArRNA02
gi|29165615|ref|NC_002745.2|	509343	+	A	104	102	61	36	146	SArRNA02
gi|29165615|ref|NC_002745.2|	509344	+	G	67	93	42	36	113	SArRNA02
gi|29165615|ref|NC_002745.2|	509345	+	U	68	65	38	22	72	SArRNA02
gi|29165615|ref|NC_002745.2|	509346	+	G	27	34	24	22	46	SArRNA02
gi|29165615|ref|NC_002745.2|	509347	+	A	71	47	15	30	41	SArRNA02
gi|29165615|ref|NC_002745.2|	509348	+	C	125	92	45	43	112	SArRNA02
gi|29165615|ref|NC_002745.2|	509349	+	A	22	25	22	13	42	SArRNA02
gi|29165615|ref|NC_002745.2|	509350	+	C	47	58	26	20	82	SArRNA02
gi|29165615|ref|NC_002745.2|	509351	+	U	31	50	17	11	42	SArRNA02
gi|29165615|ref|NC_002745.2|	509352	+	G	13	11	1	0	3	SArRNA02
gi|29165615|ref|NC_002745.2|	509353	+	C	44	42	28	23	24	SArRNA02
gi|29165615|ref|NC_002745.2|	509354	+	G	13	9	8	8	14	SArRNA02
gi|29165615|ref|NC_002745.2|	509355	+	C	22	29	20	11	44	SArRNA02
gi|29165615|ref|NC_002745.2|	509356	+	C	49	32	22	17	66	SArRNA02
gi|29165615|ref|NC_002745.2|	509357	+	G	34	51	23	27	49	SArRNA02
gi|29165615|ref|NC_002745.2|	509358	+	A	30	45	26	16	52	SArRNA02
gi|29165615|ref|NC_002745.2|	509359	+	A	59	57	29	25	81	SArRNA02
gi|29165615|ref|NC_002745.2|	509360	+	A	108	87	66	48	89	SArRNA02
gi|29165615|ref|NC_002745.2|	509361	+	A	109	188	71	53	193	SArRNA02
gi|29165615|ref|NC_002745.2|	509362	+	U	120	152	77	66	164	SArRNA02
gi|29165615|ref|NC_002745.2|	509363	+	G	81	138	62	54	122	SArRNA02
gi|29165615|ref|NC_002745.2|	509364	+	U	39	69	45	57	100	SArRNA02
gi|29165615|ref|NC_002745.2|	509365	+	A	50	43	21	13	40	SArRNA02
gi|29165615|ref|NC_002745.2|	509366	+	C	74	75	19	24	94	SArRNA02
gi|29165615|ref|NC_002745.2|	509367	+	C	227	264	51	55	276	SArRNA02

gi|29165615|ref|NC_002745.2|	509368	+	G	92	142	34	28	133	SArRNA02
gi|29165615|ref|NC_002745.2|	509369	+	G	10	15	4	4	9	SArRNA02
gi|29165615|ref|NC_002745.2|	509370	+	G	11	4	4	3	6	SArRNA02
gi|29165615|ref|NC_002745.2|	509371	+	G	5	13	6	2	17	SArRNA02
gi|29165615|ref|NC_002745.2|	509372	+	C	71	65	39	32	107	SArRNA02
gi|29165615|ref|NC_002745.2|	509373	+	U	51	60	37	19	78	SArRNA02
gi|29165615|ref|NC_002745.2|	509374	+	A	38	44	30	19	55	SArRNA02
gi|29165615|ref|NC_002745.2|	509375	+	A	49	65	40	20	72	SArRNA02
gi|29165615|ref|NC_002745.2|	509376	+	A	69	59	47	26	106	SArRNA02
gi|29165615|ref|NC_002745.2|	509377	+	C	157	127	50	37	137	SArRNA02
gi|29165615|ref|NC_002745.2|	509378	+	A	128	116	74	50	179	SArRNA02
gi|29165615|ref|NC_002745.2|	509379	+	U	451	92	51	40	143	SArRNA02
gi|29165615|ref|NC_002745.2|	509380	+	A	165	81	41	33	108	SArRNA02
gi|29165615|ref|NC_002745.2|	509381	+	U	72	54	42	27	87	SArRNA02
gi|29165615|ref|NC_002745.2|	509382	+	U	50	61	20	20	44	SArRNA02
gi|29165615|ref|NC_002745.2|	509383	+	A	51	46	28	20	53	SArRNA02
gi|29165615|ref|NC_002745.2|	509384	+	C	72	67	34	25	60	SArRNA02
gi|29165615|ref|NC_002745.2|	509385	+	C	108	115	57	58	139	SArRNA02
gi|29165615|ref|NC_002745.2|	509386	+	G	98	83	32	40	98	SArRNA02
gi|29165615|ref|NC_002745.2|	509387	+	A	65	83	39	25	103	SArRNA02
gi|29165615|ref|NC_002745.2|	509388	+	A	50	62	23	20	59	SArRNA02
gi|29165615|ref|NC_002745.2|	509389	+	G	1	0	0	1	3	SArRNA02
gi|29165615|ref|NC_002745.2|	509390	+	C	3	7	1	3	3	SArRNA02
gi|29165615|ref|NC_002745.2|	509391	+	U	8	8	2	3	14	SArRNA02
gi|29165615|ref|NC_002745.2|	509392	+	G	6	2	1	1	4	SArRNA02
gi|29165615|ref|NC_002745.2|	509393	+	U	4	2	3	5	3	SArRNA02
gi|29165615|ref|NC_002745.2|	509394	+	G	2	1	1	1	0	SArRNA02
gi|29165615|ref|NC_002745.2|	509395	+	G	3	2	1	0	3	SArRNA02
gi|29165615|ref|NC_002745.2|	509396	+	A	1	2	0	0	1	SArRNA02
gi|29165615|ref|NC_002745.2|	509397	+	U	2	3	2	2	2	SArRNA02
gi|29165615|ref|NC_002745.2|	509398	+	U	5	2	0	2	1	SArRNA02
gi|29165615|ref|NC_002745.2|	509399	+	G	2	1	0	0	0	SArRNA02
gi|29165615|ref|NC_002745.2|	509400	+	U	0	1	1	2	1	SArRNA02
gi|29165615|ref|NC_002745.2|	509401	+	C	8	6	4	6	7	SArRNA02
gi|29165615|ref|NC_002745.2|	509402	+	C	2	4	2	3	2	SArRNA02
gi|29165615|ref|NC_002745.2|	509403	+	U	2	3	1	5	6	SArRNA02
gi|29165615|ref|NC_002745.2|	509404	+	U	4	4	1	3	8	SArRNA02
gi|29165615|ref|NC_002745.2|	509405	+	U	8	2	2	0	2	SArRNA02
gi|29165615|ref|NC_002745.2|	509406	+	G	7	3	2	1	2	SArRNA02
gi|29165615|ref|NC_002745.2|	509407	+	G	5	5	7	4	15	SArRNA02
gi|29165615|ref|NC_002745.2|	509408	+	A	5	2	3	2	3	SArRNA02
gi|29165615|ref|NC_002745.2|	509409	+	C	13	7	4	4	4	SArRNA02
gi|29165615|ref|NC_002745.2|	509410	+	A	9	7	2	2	7	SArRNA02
gi|29165615|ref|NC_002745.2|	509411	+	A	6	11	6	3	19	SArRNA02
gi|29165615|ref|NC_002745.2|	509412	+	U	4	6	1	2	2	SArRNA02
gi|29165615|ref|NC_002745.2|	509413	+	G	1	1	1	3	1	SArRNA02
gi|29165615|ref|NC_002745.2|	509414	+	G	12	12	4	10	9	SArRNA02
gi|29165615|ref|NC_002745.2|	509415	+	U	6	7	0	4	3	SArRNA02
gi|29165615|ref|NC_002745.2|	509416	+	A	2	2	1	0	6	SArRNA02
gi|29165615|ref|NC_002745.2|	509417	+	G	1	1	0	1	0	SArRNA02
gi|29165615|ref|NC_002745.2|	509418	+	G	3	7	6	2	3	SArRNA02
gi|29165615|ref|NC_002745.2|	509419	+	A	8	3	8	3	4	SArRNA02
gi|29165615|ref|NC_002745.2|	509420	+	G	2	4	2	2	4	SArRNA02
gi|29165615|ref|NC_002745.2|	509421	+	A	9	2	0	1	1	SArRNA02
gi|29165615|ref|NC_002745.2|	509422	+	G	3	2	0	0	2	SArRNA02
gi|29165615|ref|NC_002745.2|	509423	+	C	11	9	2	2	8	SArRNA02
gi|29165615|ref|NC_002745.2|	509424	+	G	6	5	5	3	8	SArRNA02
gi|29165615|ref|NC_002745.2|	509425	+	U	0	1	0	0	1	SArRNA02
gi|29165615|ref|NC_002745.2|	509426	+	U	1	0	1	0	1	SArRNA02
gi|29165615|ref|NC_002745.2|	509427	+	C	2	2	2	1	3	SArRNA02
gi|29165615|ref|NC_002745.2|	509428	+	U	2	6	0	0	7	SArRNA02
gi|29165615|ref|NC_002745.2|	509429	+	A	9	9	2	2	9	SArRNA02
gi|29165615|ref|NC_002745.2|	509430	+	A	6	4	3	2	4	SArRNA02
gi|29165615|ref|NC_002745.2|	509431	+	G	1	1	0	0	0	SArRNA02
gi|29165615|ref|NC_002745.2|	509432	+	G	1	1	0	0	0	SArRNA02
gi|29165615|ref|NC_002745.2|	509433	+	G	0	2	0	2	0	SArRNA02
gi|29165615|ref|NC_002745.2|	509434	+	C	9	2	1	1	2	SArRNA02
gi|29165615|ref|NC_002745.2|	509435	+	G	4	2	3	2	2	SArRNA02
gi|29165615|ref|NC_002745.2|	509436	+	U	3	7	6	5	4	SArRNA02
gi|29165615|ref|NC_002745.2|	509437	+	U	7	3	1	2	7	SArRNA02
gi|29165615|ref|NC_002745.2|	509438	+	G	8	26	4	6	10	SArRNA02
gi|29165615|ref|NC_002745.2|	509439	+	A	0	0	1	0	2	SArRNA02
gi|29165615|ref|NC_002745.2|	509440	+	A	2	0	1	0	3	SArRNA02
gi|29165615|ref|NC_002745.2|	509441	+	G	0	1	0	0	1	SArRNA02
gi|29165615|ref|NC_002745.2|	509442	+	C	12	15	3	13	6	SArRNA02
gi|29165615|ref|NC_002745.2|	509443	+	A	4	8	3	2	4	SArRNA02
gi|29165615|ref|NC_002745.2|	509444	+	U	3	4	0	4	3	SArRNA02
gi|29165615|ref|NC_002745.2|	509445	+	G	4	14	1	1	4	SArRNA02
gi|29165615|ref|NC_002745.2|	509446	+	A	2	12	4	3	11	SArRNA02
gi|29165615|ref|NC_002745.2|	509447	+	U	12	11	6	9	12	SArRNA02
gi|29165615|ref|NC_002745.2|	509448	+	C	8	7	6	4	12	SArRNA02

gi|29165615|ref|NC_002745.2|	509449	+	G	24	13	6	7	24	SArRNA02
gi|29165615|ref|NC_002745.2|	509450	+	U	4	20	5	6	10	SArRNA02
gi|29165615|ref|NC_002745.2|	509451	+	A	9	22	2	2	7	SArRNA02
gi|29165615|ref|NC_002745.2|	509452	+	A	9	7	5	3	8	SArRNA02
gi|29165615|ref|NC_002745.2|	509453	+	G	4	3	5	4	6	SArRNA02
gi|29165615|ref|NC_002745.2|	509454	+	G	20	20	5	5	19	SArRNA02
gi|29165615|ref|NC_002745.2|	509455	+	A	6	15	6	4	7	SArRNA02
gi|29165615|ref|NC_002745.2|	509456	+	C	10	6	4	3	7	SArRNA02
gi|29165615|ref|NC_002745.2|	509457	+	A	9	8	6	9	11	SArRNA02
gi|29165615|ref|NC_002745.2|	509458	+	U	2	6	3	4	6	SArRNA02
gi|29165615|ref|NC_002745.2|	509459	+	G	4	1	2	2	2	SArRNA02
gi|29165615|ref|NC_002745.2|	509460	+	U	4	1	3	2	1	SArRNA02
gi|29165615|ref|NC_002745.2|	509461	+	G	7	1	1	3	1	SArRNA02
gi|29165615|ref|NC_002745.2|	509462	+	G	9	8	2	0	3	SArRNA02
gi|29165615|ref|NC_002745.2|	509463	+	A	6	4	1	2	4	SArRNA02
gi|29165615|ref|NC_002745.2|	509464	+	G	1	3	0	2	3	SArRNA02
gi|29165615|ref|NC_002745.2|	509465	+	C	5	4	4	4	10	SArRNA02
gi|29165615|ref|NC_002745.2|	509466	+	G	0	1	1	1	4	SArRNA02
gi|29165615|ref|NC_002745.2|	509467	+	C	4	2	1	2	8	SArRNA02
gi|29165615|ref|NC_002745.2|	509468	+	U	6	6	5	2	11	SArRNA02
gi|29165615|ref|NC_002745.2|	509469	+	U	21	21	8	12	19	SArRNA02
gi|29165615|ref|NC_002745.2|	509470	+	A	8	10	2	7	8	SArRNA02
gi|29165615|ref|NC_002745.2|	509471	+	G	29	26	10	9	11	SArRNA02
gi|29165615|ref|NC_002745.2|	509472	+	A	20	9	11	9	22	SArRNA02
gi|29165615|ref|NC_002745.2|	509473	+	A	10	21	7	6	25	SArRNA02
gi|29165615|ref|NC_002745.2|	509474	+	G	16	13	7	9	19	SArRNA02
gi|29165615|ref|NC_002745.2|	509475	+	U	16	14	5	8	15	SArRNA02
gi|29165615|ref|NC_002745.2|	509476	+	G	17	13	10	5	18	SArRNA02
gi|29165615|ref|NC_002745.2|	509477	+	A	11	6	5	4	8	SArRNA02
gi|29165615|ref|NC_002745.2|	509478	+	G	8	9	7	3	17	SArRNA02
gi|29165615|ref|NC_002745.2|	509479	+	A	25	18	12	17	15	SArRNA02
gi|29165615|ref|NC_002745.2|	509480	+	A	24	20	8	6	10	SArRNA02
gi|29165615|ref|NC_002745.2|	509481	+	U	10	12	3	4	9	SArRNA02
gi|29165615|ref|NC_002745.2|	509482	+	G	2	4	0	0	0	SArRNA02
gi|29165615|ref|NC_002745.2|	509483	+	C	10	6	5	5	1	SArRNA02
gi|29165615|ref|NC_002745.2|	509484	+	C	43	37	8	14	20	SArRNA02
gi|29165615|ref|NC_002745.2|	509485	+	G	9	5	5	2	5	SArRNA02
gi|29165615|ref|NC_002745.2|	509486	+	G	5	5	4	1	8	SArRNA02
gi|29165615|ref|NC_002745.2|	509487	+	U	10	11	2	7	9	SArRNA02
gi|29165615|ref|NC_002745.2|	509488	+	G	9	7	2	4	8	SArRNA02
gi|29165615|ref|NC_002745.2|	509489	+	U	10	5	7	2	10	SArRNA02
gi|29165615|ref|NC_002745.2|	509490	+	G	11	8	6	5	5	SArRNA02
gi|29165615|ref|NC_002745.2|	509491	+	A	7	11	4	3	11	SArRNA02
gi|29165615|ref|NC_002745.2|	509492	+	G	35	26	10	12	15	SArRNA02
gi|29165615|ref|NC_002745.2|	509493	+	U	30	30	15	13	17	SArRNA02
gi|29165615|ref|NC_002745.2|	509494	+	A	35	35	8	11	16	SArRNA02
gi|29165615|ref|NC_002745.2|	509495	+	G	9	5	1	0	2	SArRNA02
gi|29165615|ref|NC_002745.2|	509496	+	C	8	9	0	6	4	SArRNA02
gi|29165615|ref|NC_002745.2|	509497	+	G	4	6	3	1	2	SArRNA02
gi|29165615|ref|NC_002745.2|	509498	+	A	2	1	1	1	3	SArRNA02
gi|29165615|ref|NC_002745.2|	509499	+	A	1	3	0	0	2	SArRNA02
gi|29165615|ref|NC_002745.2|	509500	+	A	0	1	0	0	1	SArRNA02
gi|29165615|ref|NC_002745.2|	509501	+	G	0	2	0	2	2	SArRNA02
gi|29165615|ref|NC_002745.2|	509503	+	C	0	1	0	0	0	SArRNA02
gi|29165615|ref|NC_002745.2|	509505	+	G	0	0	1	0	1	SArRNA02
gi|29165615|ref|NC_002745.2|	509506	+	G	6	2	1	1	0	SArRNA02
gi|29165615|ref|NC_002745.2|	509507	+	U	0	0	1	2	1	SArRNA02
gi|29165615|ref|NC_002745.2|	509508	+	G	0	0	0	0	2	SArRNA02
gi|29165615|ref|NC_002745.2|	509509	+	A	0	0	0	1	1	SArRNA02
gi|29165615|ref|NC_002745.2|	509510	+	G	0	0	0	0	1	SArRNA02
gi|29165615|ref|NC_002745.2|	509511	+	A	0	3	0	0	3	SArRNA02
gi|29165615|ref|NC_002745.2|	509512	+	A	0	2	1	0	1	SArRNA02
gi|29165615|ref|NC_002745.2|	509513	+	U	0	1	0	0	1	SArRNA02
gi|29165615|ref|NC_002745.2|	509514	+	C	26	10	5	4	9	SArRNA02
gi|29165615|ref|NC_002745.2|	509515	+	C	3	5	0	1	6	SArRNA02
gi|29165615|ref|NC_002745.2|	509516	+	C	3	1	2	0	0	SArRNA02
gi|29165615|ref|NC_002745.2|	509517	+	G	1	3	1	2	1	SArRNA02
gi|29165615|ref|NC_002745.2|	509518	+	U	0	1	0	1	0	SArRNA02
gi|29165615|ref|NC_002745.2|	509519	+	C	0	1	0	0	1	SArRNA02
gi|29165615|ref|NC_002745.2|	509520	+	C	2	3	2	0	0	SArRNA02
gi|29165615|ref|NC_002745.2|	509521	+	A	1	2	1	1	1	SArRNA02
gi|29165615|ref|NC_002745.2|	509522	+	C	1	4	0	0	1	SArRNA02
gi|29165615|ref|NC_002745.2|	509523	+	C	60	40	14	15	27	SArRNA02
gi|29165615|ref|NC_002745.2|	509524	+	G	26	20	10	18	25	SArRNA02
gi|29165615|ref|NC_002745.2|	509525	+	A	28	31	15	10	23	SArRNA02
gi|29165615|ref|NC_002745.2|	509526	+	U	38	31	16	22	35	SArRNA02
gi|29165615|ref|NC_002745.2|	509527	+	U	25	16	11	7	23	SArRNA02
gi|29165615|ref|NC_002745.2|	509528	+	G	0	2	0	2	0	SArRNA02
gi|29165615|ref|NC_002745.2|	509529	+	A	7	13	6	7	18	SArRNA02
gi|29165615|ref|NC_002745.2|	509530	+	C	12	17	4	9	21	SArRNA02
gi|29165615|ref|NC_002745.2|	509531	+	U	34	38	15	23	27	SArRNA02

gi|29165615|ref|NC_002745.2|	509532	+	A	16	19	9	7	19	SArRNA02
gi|29165615|ref|NC_002745.2|	509533	+	A	37	36	14	11	39	SArRNA02
gi|29165615|ref|NC_002745.2|	509534	+	G	36	22	13	7	23	SArRNA02
gi|29165615|ref|NC_002745.2|	509535	+	G	22	16	15	11	35	SArRNA02
gi|29165615|ref|NC_002745.2|	509536	+	U	10	11	6	9	19	SArRNA02
gi|29165615|ref|NC_002745.2|	509537	+	U	5	3	0	1	7	SArRNA02
gi|29165615|ref|NC_002745.2|	509538	+	U	2	2	0	0	1	SArRNA02
gi|29165615|ref|NC_002745.2|	509539	+	C	2	2	0	0	2	SArRNA02
gi|29165615|ref|NC_002745.2|	509540	+	C	3	1	1	3	7	SArRNA02
gi|29165615|ref|NC_002745.2|	509541	+	A	4	5	3	2	10	SArRNA02
gi|29165615|ref|NC_002745.2|	509542	+	G	3	1	5	3	9	SArRNA02
gi|29165615|ref|NC_002745.2|	509543	+	A	11	1	2	4	13	SArRNA02
gi|29165615|ref|NC_002745.2|	509544	+	G	6	6	5	5	11	SArRNA02
gi|29165615|ref|NC_002745.2|	509545	+	G	15	13	3	4	13	SArRNA02
gi|29165615|ref|NC_002745.2|	509546	+	A	9	20	2	7	10	SArRNA02
gi|29165615|ref|NC_002745.2|	509547	+	A	11	9	5	5	13	SArRNA02
gi|29165615|ref|NC_002745.2|	509548	+	G	1	3	0	3	3	SArRNA02
gi|29165615|ref|NC_002745.2|	509549	+	G	2	1	1	2	2	SArRNA02
gi|29165615|ref|NC_002745.2|	509550	+	C	0	0	1	0	2	SArRNA02
gi|29165615|ref|NC_002745.2|	509551	+	U	3	8	2	0	2	SArRNA02
gi|29165615|ref|NC_002745.2|	509552	+	C	4	2	2	0	4	SArRNA02
gi|29165615|ref|NC_002745.2|	509553	+	G	2	3	0	0	7	SArRNA02
gi|29165615|ref|NC_002745.2|	509554	+	U	2	2	0	1	5	SArRNA02
gi|29165615|ref|NC_002745.2|	509555	+	C	4	5	0	1	5	SArRNA02
gi|29165615|ref|NC_002745.2|	509556	+	C	17	24	2	9	20	SArRNA02
gi|29165615|ref|NC_002745.2|	509557	+	G	7	12	7	7	16	SArRNA02
gi|29165615|ref|NC_002745.2|	509558	+	C	10	12	3	5	12	SArRNA02
gi|29165615|ref|NC_002745.2|	509559	+	U	8	11	3	5	5	SArRNA02
gi|29165615|ref|NC_002745.2|	509560	+	C	2	6	2	1	4	SArRNA02
gi|29165615|ref|NC_002745.2|	509561	+	U	15	13	7	4	17	SArRNA02
gi|29165615|ref|NC_002745.2|	509562	+	G	6	3	3	3	3	SArRNA02
gi|29165615|ref|NC_002745.2|	509563	+	G	10	8	1	5	7	SArRNA02
gi|29165615|ref|NC_002745.2|	509564	+	G	12	7	4	5	13	SArRNA02
gi|29165615|ref|NC_002745.2|	509565	+	U	11	12	8	1	16	SArRNA02
gi|29165615|ref|NC_002745.2|	509566	+	U	6	12	8	8	13	SArRNA02
gi|29165615|ref|NC_002745.2|	509567	+	A	12	10	4	6	13	SArRNA02
gi|29165615|ref|NC_002745.2|	509568	+	G	15	5	4	6	6	SArRNA02
gi|29165615|ref|NC_002745.2|	509569	+	U	4	0	1	0	4	SArRNA02
gi|29165615|ref|NC_002745.2|	509570	+	C	1	8	0	2	2	SArRNA02
gi|29165615|ref|NC_002745.2|	509571	+	G	2	4	1	1	0	SArRNA02
gi|29165615|ref|NC_002745.2|	509572	+	G	4	2	1	0	1	SArRNA02
gi|29165615|ref|NC_002745.2|	509573	+	G	0	3	0	1	0	SArRNA02
gi|29165615|ref|NC_002745.2|	509574	+	U	0	3	0	0	1	SArRNA02
gi|29165615|ref|NC_002745.2|	509575	+	C	1	2	0	2	0	SArRNA02
gi|29165615|ref|NC_002745.2|	509576	+	C	5	14	0	5	8	SArRNA02
gi|29165615|ref|NC_002745.2|	509577	+	U	2	11	3	1	11	SArRNA02
gi|29165615|ref|NC_002745.2|	509578	+	A	6	5	1	1	5	SArRNA02
gi|29165615|ref|NC_002745.2|	509579	+	A	10	4	1	3	3	SArRNA02
gi|29165615|ref|NC_002745.2|	509580	+	G	0	0	0	0	1	SArRNA02
gi|29165615|ref|NC_002745.2|	509581	+	C	4	2	2	4	1	SArRNA02
gi|29165615|ref|NC_002745.2|	509582	+	U	5	6	4	0	3	SArRNA02
gi|29165615|ref|NC_002745.2|	509583	+	G	6	14	7	1	9	SArRNA02
gi|29165615|ref|NC_002745.2|	509584	+	A	3	1	2	0	0	SArRNA02
gi|29165615|ref|NC_002745.2|	509585	+	G	0	1	0	1	1	SArRNA02
gi|29165615|ref|NC_002745.2|	509586	+	G	3	1	0	1	2	SArRNA02
gi|29165615|ref|NC_002745.2|	509587	+	C	0	3	0	1	2	SArRNA02
gi|29165615|ref|NC_002745.2|	509588	+	C	11	7	2	5	10	SArRNA02
gi|29165615|ref|NC_002745.2|	509589	+	G	7	5	3	1	6	SArRNA02
gi|29165615|ref|NC_002745.2|	509590	+	A	1	5	0	0	8	SArRNA02
gi|29165615|ref|NC_002745.2|	509591	+	C	29	46	11	9	29	SArRNA02
gi|29165615|ref|NC_002745.2|	509592	+	A	6	14	2	3	6	SArRNA02
gi|29165615|ref|NC_002745.2|	509593	+	G	7	16	1	3	7	SArRNA02
gi|29165615|ref|NC_002745.2|	509594	+	G	2	1	1	0	2	SArRNA02
gi|29165615|ref|NC_002745.2|	509595	+	C	4	1	2	1	7	SArRNA02
gi|29165615|ref|NC_002745.2|	509596	+	G	5	6	2	1	2	SArRNA02
gi|29165615|ref|NC_002745.2|	509597	+	U	15	13	3	11	8	SArRNA02
gi|29165615|ref|NC_002745.2|	509598	+	A	1	2	3	0	3	SArRNA02
gi|29165615|ref|NC_002745.2|	509599	+	G	0	2	1	0	1	SArRNA02
gi|29165615|ref|NC_002745.2|	509600	+	G	0	1	0	0	0	SArRNA02
gi|29165615|ref|NC_002745.2|	509601	+	C	5	6	4	3	12	SArRNA02
gi|29165615|ref|NC_002745.2|	509602	+	G	17	21	12	8	17	SArRNA02
gi|29165615|ref|NC_002745.2|	509603	+	A	4	6	1	1	6	SArRNA02
gi|29165615|ref|NC_002745.2|	509604	+	U	5	6	4	1	1	SArRNA02
gi|29165615|ref|NC_002745.2|	509605	+	G	2	3	1	1	2	SArRNA02
gi|29165615|ref|NC_002745.2|	509606	+	G	0	1	1	2	3	SArRNA02
gi|29165615|ref|NC_002745.2|	509607	+	A	1	1	1	5	5	SArRNA02
gi|29165615|ref|NC_002745.2|	509608	+	U	1	1	1	0	0	SArRNA02
gi|29165615|ref|NC_002745.2|	509609	+	A	1	0	0	1	5	SArRNA02
gi|29165615|ref|NC_002745.2|	509610	+	A	0	1	0	0	2	SArRNA02
gi|29165615|ref|NC_002745.2|	509611	+	C	7	7	4	1	1	SArRNA02
gi|29165615|ref|NC_002745.2|	509612	+	A	1	3	0	0	2	SArRNA02

gi|29165615|ref|NC_002745.2|	509613	+	G	1	0	1	0	1	SArRNA02
gi|29165615|ref|NC_002745.2|	509614	+	G	0	1	0	1	0	SArRNA02
gi|29165615|ref|NC_002745.2|	509615	+	U	1	0	0	1	3	SArRNA02
gi|29165615|ref|NC_002745.2|	509616	+	U	0	1	1	1	2	SArRNA02
gi|29165615|ref|NC_002745.2|	509617	+	G	3	0	2	0	2	SArRNA02
gi|29165615|ref|NC_002745.2|	509618	+	A	2	5	0	2	2	SArRNA02
gi|29165615|ref|NC_002745.2|	509619	+	U	0	4	0	3	4	SArRNA02
gi|29165615|ref|NC_002745.2|	509620	+	A	1	1	2	0	4	SArRNA02
gi|29165615|ref|NC_002745.2|	509621	+	U	3	0	3	0	2	SArRNA02
gi|29165615|ref|NC_002745.2|	509622	+	U	4	2	0	0	0	SArRNA02
gi|29165615|ref|NC_002745.2|	509623	+	C	2	1	0	2	1	SArRNA02
gi|29165615|ref|NC_002745.2|	509624	+	C	2	0	0	1	0	SArRNA02
gi|29165615|ref|NC_002745.2|	509625	+	U	7	3	0	1	1	SArRNA02
gi|29165615|ref|NC_002745.2|	509626	+	G	0	1	0	0	1	SArRNA02
gi|29165615|ref|NC_002745.2|	509627	+	U	1	2	0	4	2	SArRNA02
gi|29165615|ref|NC_002745.2|	509628	+	A	0	1	0	0	0	SArRNA02
gi|29165615|ref|NC_002745.2|	509629	+	C	0	4	1	2	6	SArRNA02
gi|29165615|ref|NC_002745.2|	509630	+	C	15	13	3	2	13	SArRNA02
gi|29165615|ref|NC_002745.2|	509631	+	A	1	5	0	2	3	SArRNA02
gi|29165615|ref|NC_002745.2|	509632	+	C	1	3	1	2	2	SArRNA02
gi|29165615|ref|NC_002745.2|	509633	+	C	6	4	1	2	6	SArRNA02
gi|29165615|ref|NC_002745.2|	509634	+	U	4	5	2	3	6	SArRNA02
gi|29165615|ref|NC_002745.2|	509635	+	A	3	1	1	1	0	SArRNA02
gi|29165615|ref|NC_002745.2|	509636	+	U	6	8	1	2	6	SArRNA02
gi|29165615|ref|NC_002745.2|	509637	+	A	4	1	1	2	5	SArRNA02
gi|29165615|ref|NC_002745.2|	509638	+	A	3	11	1	2	13	SArRNA02
gi|29165615|ref|NC_002745.2|	509639	+	U	6	1	2	2	1	SArRNA02
gi|29165615|ref|NC_002745.2|	509640	+	C	4	3	2	1	5	SArRNA02
gi|29165615|ref|NC_002745.2|	509641	+	G	1	2	2	3	4	SArRNA02
gi|29165615|ref|NC_002745.2|	509642	+	U	0	4	4	2	2	SArRNA02
gi|29165615|ref|NC_002745.2|	509643	+	U	8	4	1	0	3	SArRNA02
gi|29165615|ref|NC_002745.2|	509644	+	U	6	8	3	0	1	SArRNA02
gi|29165615|ref|NC_002745.2|	509645	+	U	5	7	3	5	7	SArRNA02
gi|29165615|ref|NC_002745.2|	509646	+	A	2	3	1	1	4	SArRNA02
gi|29165615|ref|NC_002745.2|	509647	+	A	9	75	22	3	28	SArRNA02
gi|29165615|ref|NC_002745.2|	509648	+	U	4	3	1	2	5	SArRNA02
gi|29165615|ref|NC_002745.2|	509649	+	C	8	5	0	3	2	SArRNA02
gi|29165615|ref|NC_002745.2|	509650	+	G	6	10	3	2	6	SArRNA02
gi|29165615|ref|NC_002745.2|	509651	+	A	3	2	1	2	2	SArRNA02
gi|29165615|ref|NC_002745.2|	509652	+	U	7	12	0	2	1	SArRNA02
gi|29165615|ref|NC_002745.2|	509653	+	G	0	1	1	0	2	SArRNA02
gi|29165615|ref|NC_002745.2|	509654	+	G	0	2	0	1	0	SArRNA02
gi|29165615|ref|NC_002745.2|	509655	+	G	1	3	3	0	2	SArRNA02
gi|29165615|ref|NC_002745.2|	509656	+	G	0	0	0	0	1	SArRNA02
gi|29165615|ref|NC_002745.2|	509658	+	G	0	0	1	0	1	SArRNA02
gi|29165615|ref|NC_002745.2|	509660	+	C	1	12	0	0	4	SArRNA02
gi|29165615|ref|NC_002745.2|	509661	+	G	1	4	4	1	1	SArRNA02
gi|29165615|ref|NC_002745.2|	509662	+	C	3	0	0	1	0	SArRNA02
gi|29165615|ref|NC_002745.2|	509663	+	A	0	1	1	0	1	SArRNA02
gi|29165615|ref|NC_002745.2|	509664	+	G	2	1	0	0	3	SArRNA02
gi|29165615|ref|NC_002745.2|	509665	+	U	7	2	5	0	1	SArRNA02
gi|29165615|ref|NC_002745.2|	509666	+	A	1	3	2	0	1	SArRNA02
gi|29165615|ref|NC_002745.2|	509667	+	G	0	0	0	1	0	SArRNA02
gi|29165615|ref|NC_002745.2|	509668	+	G	0	1	0	1	1	SArRNA02
gi|29165615|ref|NC_002745.2|	509669	+	A	2	5	0	1	5	SArRNA02
gi|29165615|ref|NC_002745.2|	509670	+	U	5	5	0	3	3	SArRNA02
gi|29165615|ref|NC_002745.2|	509671	+	A	2	0	2	1	1	SArRNA02
gi|29165615|ref|NC_002745.2|	509672	+	G	0	1	0	0	0	SArRNA02
gi|29165615|ref|NC_002745.2|	509674	+	C	3	0	1	2	3	SArRNA02
gi|29165615|ref|NC_002745.2|	509675	+	G	1	4	0	0	1	SArRNA02
gi|29165615|ref|NC_002745.2|	509676	+	A	0	1	0	0	0	SArRNA02
gi|29165615|ref|NC_002745.2|	509681	+	U	0	1	0	0	0	SArRNA02
gi|29165615|ref|NC_002745.2|	509682	+	G	0	1	0	0	0	SArRNA02
gi|29165615|ref|NC_002745.2|	509683	+	C	4	1	3	0	4	SArRNA02
gi|29165615|ref|NC_002745.2|	509684	+	G	1	3	0	0	3	SArRNA02
gi|29165615|ref|NC_002745.2|	509685	+	A	0	2	3	0	2	SArRNA02
gi|29165615|ref|NC_002745.2|	509686	+	U	0	0	2	3	3	SArRNA02
gi|29165615|ref|NC_002745.2|	509687	+	U	1	1	0	0	1	SArRNA02
gi|29165615|ref|NC_002745.2|	509688	+	G	2	1	0	0	1	SArRNA02
gi|29165615|ref|NC_002745.2|	509689	+	G	1	3	0	0	1	SArRNA02
gi|29165615|ref|NC_002745.2|	509690	+	A	1	3	3	0	5	SArRNA02
gi|29165615|ref|NC_002745.2|	509691	+	U	3	2	0	0	1	SArRNA02
gi|29165615|ref|NC_002745.2|	509692	+	U	0	0	0	0	2	SArRNA02
gi|29165615|ref|NC_002745.2|	509693	+	G	0	1	0	0	1	SArRNA02
gi|29165615|ref|NC_002745.2|	509694	+	C	0	7	2	0	3	SArRNA02
gi|29165615|ref|NC_002745.2|	509695	+	A	0	2	0	0	1	SArRNA02
gi|29165615|ref|NC_002745.2|	509696	+	C	1	0	0	0	2	SArRNA02
gi|29165615|ref|NC_002745.2|	509697	+	G	1	2	0	1	0	SArRNA02
gi|29165615|ref|NC_002745.2|	509698	+	U	1	0	0	0	0	SArRNA02
gi|29165615|ref|NC_002745.2|	509699	+	C	1	6	1	2	12	SArRNA02
gi|29165615|ref|NC_002745.2|	509700	+	U	2	6	1	0	2	SArRNA02

gi|29165615|ref|NC_002745.2|	509701 +	A	0	2	0	0	1	SArRNA02
gi|29165615|ref|NC_002745.2|	509702 +	A	0	0	0	0	1	SArRNA02
gi|29165615|ref|NC_002745.2|	509704 +	C	4	3	0	1	0	SArRNA02
gi|29165615|ref|NC_002745.2|	509705 +	A	3	0	0	0	2	SArRNA02
gi|29165615|ref|NC_002745.2|	509706 +	G	0	0	0	3	1	SArRNA02
gi|29165615|ref|NC_002745.2|	509707 +	U	2	0	0	0	2	SArRNA02
gi|29165615|ref|NC_002745.2|	509708 +	A	2	4	0	0	3	SArRNA02
gi|29165615|ref|NC_002745.2|	509709 +	A	1	1	0	0	1	SArRNA02
gi|29165615|ref|NC_002745.2|	509711 +	G	1	1	0	0	1	SArRNA02
gi|29165615|ref|NC_002745.2|	509712 +	C	4	0	0	1	1	SArRNA02
gi|29165615|ref|NC_002745.2|	509713 +	U	0	1	0	1	0	SArRNA02
gi|29165615|ref|NC_002745.2|	509714 +	G	0	0	0	0	1	SArRNA02
gi|29165615|ref|NC_002745.2|	509715 +	A	0	1	0	0	3	SArRNA02
gi|29165615|ref|NC_002745.2|	509717 +	U	0	1	0	0	0	SArRNA02
gi|29165615|ref|NC_002745.2|	509718 +	A	0	1	1	0	2	SArRNA02
gi|29165615|ref|NC_002745.2|	509720 +	U	2	3	0	1	1	SArRNA02
gi|29165615|ref|NC_002745.2|	509721 +	A	2	1	0	1	1	SArRNA02
gi|29165615|ref|NC_002745.2|	509722 +	G	2	0	0	0	1	SArRNA02
gi|29165615|ref|NC_002745.2|	509725 +	A	1	1	0	0	1	SArRNA02
gi|29165615|ref|NC_002745.2|	509726 +	A	0	0	0	0	1	SArRNA02
gi|29165615|ref|NC_002745.2|	509727 +	A	4	3	1	1	1	SArRNA02
gi|29165615|ref|NC_002745.2|	509728 +	U	1	1	1	0	1	SArRNA02
gi|29165615|ref|NC_002745.2|	509729 +	C	1	2	0	0	2	SArRNA02
gi|29165615|ref|NC_002745.2|	509730 +	C	1	4	0	0	0	SArRNA02
gi|29165615|ref|NC_002745.2|	509731 +	G	0	0	0	1	0	SArRNA02
gi|29165615|ref|NC_002745.2|	509732 +	G	2	0	0	1	0	SArRNA02
gi|29165615|ref|NC_002745.2|	509734 +	A	0	1	1	0	0	SArRNA02
gi|29165615|ref|NC_002745.2|	509735 +	C	0	2	0	0	1	SArRNA02
gi|29165615|ref|NC_002745.2|	509736 +	U	0	1	1	0	1	SArRNA02
gi|29165615|ref|NC_002745.2|	509737 +	C	0	0	0	0	1	SArRNA02
gi|29165615|ref|NC_002745.2|	509738 +	G	1	0	0	0	5	SArRNA02
gi|29165615|ref|NC_002745.2|	509739 +	U	1	0	0	0	1	SArRNA02
gi|29165615|ref|NC_002745.2|	509740 +	U	3	2	0	0	6	SArRNA02
gi|29165615|ref|NC_002745.2|	509741 +	A	0	6	0	0	1	SArRNA02
gi|29165615|ref|NC_002745.2|	509742 +	A	3	2	0	0	2	SArRNA02
gi|29165615|ref|NC_002745.2|	509743 +	G	0	2	0	1	0	SArRNA02
gi|29165615|ref|NC_002745.2|	509744 +	G	1	1	0	0	0	SArRNA02
gi|29165615|ref|NC_002745.2|	509745 +	C	1	1	1	0	5	SArRNA02
gi|29165615|ref|NC_002745.2|	509746 +	U	1	2	0	1	2	SArRNA02
gi|29165615|ref|NC_002745.2|	509747 +	G	0	0	1	0	1	SArRNA02
gi|29165615|ref|NC_002745.2|	509748 +	A	4	0	0	1	2	SArRNA02
gi|29165615|ref|NC_002745.2|	509750 +	C	1	0	1	0	0	SArRNA02
gi|29165615|ref|NC_002745.2|	509751 +	U	2	0	3	0	0	SArRNA02
gi|29165615|ref|NC_002745.2|	509752 +	G	0	0	0	0	1	SArRNA02
gi|29165615|ref|NC_002745.2|	509753 +	U	0	0	0	0	1	SArRNA02
gi|29165615|ref|NC_002745.2|	509754 +	G	1	3	1	0	0	SArRNA02
gi|29165615|ref|NC_002745.2|	509755 +	A	1	0	0	0	0	SArRNA02
gi|29165615|ref|NC_002745.2|	509756 +	U	1	8	2	2	6	SArRNA02
gi|29165615|ref|NC_002745.2|	509757 +	G	1	1	1	1	0	SArRNA02
gi|29165615|ref|NC_002745.2|	509758 +	G	1	1	1	0	0	SArRNA02
gi|29165615|ref|NC_002745.2|	509763 +	A	1	0	0	0	0	SArRNA02
gi|29165615|ref|NC_002745.2|	509764 +	A	2	0	0	0	0	SArRNA02
gi|29165615|ref|NC_002745.2|	509765 +	G	0	1	0	0	0	SArRNA02
gi|29165615|ref|NC_002745.2|	509766 +	A	1	0	0	0	3	SArRNA02
gi|29165615|ref|NC_002745.2|	509767 +	C	1	0	1	2	2	SArRNA02
gi|29165615|ref|NC_002745.2|	509768 +	A	3	1	0	0	2	SArRNA02
gi|29165615|ref|NC_002745.2|	509769 +	U	0	0	1	1	1	SArRNA02
gi|29165615|ref|NC_002745.2|	509770 +	U	1	0	0	0	0	SArRNA02
gi|29165615|ref|NC_002745.2|	509772 +	A	0	0	0	0	3	SArRNA02
gi|29165615|ref|NC_002745.2|	509773 +	G	1	0	0	0	1	SArRNA02
gi|29165615|ref|NC_002745.2|	509774 +	U	0	0	0	0	1	SArRNA02
gi|29165615|ref|NC_002745.2|	509775 +	C	0	1	0	0	0	SArRNA02
gi|29165615|ref|NC_002745.2|	509776 +	U	1	1	1	0	0	SArRNA02
gi|29165615|ref|NC_002745.2|	509777 +	U	0	1	0	0	1	SArRNA02
gi|29165615|ref|NC_002745.2|	509778 +	C	0	1	0	0	1	SArRNA02
gi|29165615|ref|NC_002745.2|	509779 +	G	0	1	1	0	0	SArRNA02
gi|29165615|ref|NC_002745.2|	509781 +	G	0	0	0	0	1	SArRNA02
gi|29165615|ref|NC_002745.2|	509782 +	U	2	0	1	0	1	SArRNA02
gi|29165615|ref|NC_002745.2|	509783 +	C	2	0	0	0	0	SArRNA02
gi|29165615|ref|NC_002745.2|	509784 +	G	1	1	0	0	1	SArRNA02
gi|29165615|ref|NC_002745.2|	509785 +	U	0	0	1	0	0	SArRNA02
gi|29165615|ref|NC_002745.2|	509788 +	A	0	1	0	0	1	SArRNA02
gi|29165615|ref|NC_002745.2|	509789 +	U	3	0	0	0	0	SArRNA02
gi|29165615|ref|NC_002745.2|	509790 +	U	0	0	1	1	0	SArRNA02
gi|29165615|ref|NC_002745.2|	509791 +	U	0	1	0	0	1	SArRNA02
gi|29165615|ref|NC_002745.2|	509792 +	C	2	2	2	1	0	SArRNA02
gi|29165615|ref|NC_002745.2|	509793 +	A	2	0	0	0	1	SArRNA02
gi|29165615|ref|NC_002745.2|	509794 +	C	3	5	2	0	2	SArRNA02
gi|29165615|ref|NC_002745.2|	509795 +	A	2	1	0	1	0	SArRNA02
gi|29165615|ref|NC_002745.2|	509796 +	C	2	1	0	2	1	SArRNA02
gi|29165615|ref|NC_002745.2|	509797 +	U	0	0	1	0	1	SArRNA02

gi|29165615|ref|NC_002745.2|	509798	+	G	0	0	0	1	0	SArRNA02
gi|29165615|ref|NC_002745.2|	509800	+	C	2	3	1	0	0	SArRNA02
gi|29165615|ref|NC_002745.2|	509801	+	G	0	0	0	1	0	SArRNA02
gi|29165615|ref|NC_002745.2|	509802	+	A	0	0	0	0	1	SArRNA02
gi|29165615|ref|NC_002745.2|	509803	+	G	1	0	1	0	1	SArRNA02
gi|29165615|ref|NC_002745.2|	509804	+	A	1	0	1	0	0	SArRNA02
gi|29165615|ref|NC_002745.2|	509805	+	A	1	0	0	0	1	SArRNA02
gi|29165615|ref|NC_002745.2|	509806	+	A	1	1	0	0	0	SArRNA02
gi|29165615|ref|NC_002745.2|	509807	+	A	1	1	0	0	1	SArRNA02
gi|29165615|ref|NC_002745.2|	509809	+	C	2	0	1	0	0	SArRNA02
gi|29165615|ref|NC_002745.2|	509810	+	C	3	1	3	1	2	SArRNA02
gi|29165615|ref|NC_002745.2|	509811	+	U	2	1	0	1	2	SArRNA02
gi|29165615|ref|NC_002745.2|	509812	+	C	1	4	0	1	5	SArRNA02
gi|29165615|ref|NC_002745.2|	509813	+	U	1	9	2	0	3	SArRNA02
gi|29165615|ref|NC_002745.2|	509814	+	A	2	4	1	0	5	SArRNA02
gi|29165615|ref|NC_002745.2|	509815	+	G	0	3	1	4	1	SArRNA02
gi|29165615|ref|NC_002745.2|	509816	+	A	4	8	1	2	5	SArRNA02
gi|29165615|ref|NC_002745.2|	509817	+	U	4	5	1	3	6	SArRNA02
gi|29165615|ref|NC_002745.2|	509818	+	A	0	1	3	1	3	SArRNA02
gi|29165615|ref|NC_002745.2|	509819	+	G	1	1	4	3	0	SArRNA02
gi|29165615|ref|NC_002745.2|	509820	+	A	0	3	2	2	7	SArRNA02
gi|29165615|ref|NC_002745.2|	509821	+	A	4	3	0	3	3	SArRNA02
gi|29165615|ref|NC_002745.2|	509822	+	A	3	0	3	1	4	SArRNA02
gi|29165615|ref|NC_002745.2|	509823	+	A	13	72	1	2	15	SArRNA02
gi|29165615|ref|NC_002745.2|	509824	+	U	0	3	1	1	1	SArRNA02
gi|29165615|ref|NC_002745.2|	509825	+	A	3	1	1	1	2	SArRNA02
gi|29165615|ref|NC_002745.2|	509830	+	C	0	0	1	0	1	SArRNA02
gi|29165615|ref|NC_002745.2|	509831	+	C	0	6	0	0	0	SArRNA02
gi|29165615|ref|NC_002745.2|	509832	+	C	2	1	0	0	1	SArRNA02
gi|29165615|ref|NC_002745.2|	509833	+	G	0	4	0	0	0	SArRNA02
gi|29165615|ref|NC_002745.2|	509834	+	U	1	7	1	0	2	SArRNA02
gi|29165615|ref|NC_002745.2|	509836	+	C	1	0	0	0	1	SArRNA02
gi|29165615|ref|NC_002745.2|	509837	+	C	0	1	0	0	0	SArRNA02
gi|29165615|ref|NC_002745.2|	509838	+	G	1	1	0	1	2	SArRNA02
gi|29165615|ref|NC_002745.2|	509839	+	C	5	6	2	1	4	SArRNA02
gi|29165615|ref|NC_002745.2|	509840	+	A	0	1	0	1	4	SArRNA02
gi|29165615|ref|NC_002745.2|	509841	+	A	1	7	1	0	5	SArRNA02
gi|29165615|ref|NC_002745.2|	509842	+	A	1	16	1	0	6	SArRNA02
gi|29165615|ref|NC_002745.2|	509843	+	C	1	1	1	0	4	SArRNA02
gi|29165615|ref|NC_002745.2|	509844	+	C	6	21	2	2	6	SArRNA02
gi|29165615|ref|NC_002745.2|	509845	+	G	1	8	0	2	4	SArRNA02
gi|29165615|ref|NC_002745.2|	509847	+	C	4	8	0	0	6	SArRNA02
gi|29165615|ref|NC_002745.2|	509848	+	A	0	13	1	0	3	SArRNA02
gi|29165615|ref|NC_002745.2|	509849	+	C	15	23	5	8	10	SArRNA02
gi|29165615|ref|NC_002745.2|	509850	+	A	2	12	1	1	3	SArRNA02
gi|29165615|ref|NC_002745.2|	509851	+	G	0	1	0	1	0	SArRNA02
gi|29165615|ref|NC_002745.2|	509852	+	G	1	2	0	2	1	SArRNA02
gi|29165615|ref|NC_002745.2|	509853	+	U	3	7	0	0	2	SArRNA02
gi|29165615|ref|NC_002745.2|	509855	+	G	1	0	0	0	0	SArRNA02
gi|29165615|ref|NC_002745.2|	509856	+	U	0	0	0	1	1	SArRNA02
gi|29165615|ref|NC_002745.2|	509857	+	C	18	18	7	11	13	SArRNA02
gi|29165615|ref|NC_002745.2|	509858	+	A	1	5	2	0	1	SArRNA02
gi|29165615|ref|NC_002745.2|	509859	+	A	0	3	1	0	3	SArRNA02
gi|29165615|ref|NC_002745.2|	509860	+	G	1	1	0	0	0	SArRNA02
gi|29165615|ref|NC_002745.2|	509861	+	A	0	1	0	0	0	SArRNA02
gi|29165615|ref|NC_002745.2|	509862	+	U	0	0	0	1	3	SArRNA02
gi|29165615|ref|NC_002745.2|	509863	+	G	1	1	0	0	1	SArRNA02
gi|29165615|ref|NC_002745.2|	509864	+	A	1	2	0	1	3	SArRNA02
gi|29165615|ref|NC_002745.2|	509865	+	G	4	3	0	0	3	SArRNA02
gi|29165615|ref|NC_002745.2|	509866	+	A	8	3	4	3	5	SArRNA02
gi|29165615|ref|NC_002745.2|	509867	+	A	15	54	8	5	19	SArRNA02
gi|29165615|ref|NC_002745.2|	509868	+	U	1	1	0	2	5	SArRNA02
gi|29165615|ref|NC_002745.2|	509869	+	U	1	3	0	0	0	SArRNA02
gi|29165615|ref|NC_002745.2|	509870	+	C	9	11	1	3	9	SArRNA02
gi|29165615|ref|NC_002745.2|	509871	+	U	3	9	1	2	5	SArRNA02
gi|29165615|ref|NC_002745.2|	509872	+	A	2	15	3	13	8	SArRNA02
gi|29165615|ref|NC_002745.2|	509873	+	A	3	4	1	4	2	SArRNA02
gi|29165615|ref|NC_002745.2|	509874	+	G	0	0	0	1	1	SArRNA02
gi|29165615|ref|NC_002745.2|	509875	+	G	0	1	0	0	1	SArRNA02
gi|29165615|ref|NC_002745.2|	509876	+	U	5	2	4	6	3	SArRNA02
gi|29165615|ref|NC_002745.2|	509877	+	G	1	2	0	1	1	SArRNA02
gi|29165615|ref|NC_002745.2|	509878	+	A	0	2	0	2	0	SArRNA02
gi|29165615|ref|NC_002745.2|	509879	+	G	0	0	0	0	1	SArRNA02
gi|29165615|ref|NC_002745.2|	509880	+	C	4	3	0	1	4	SArRNA02
gi|29165615|ref|NC_002745.2|	509881	+	G	1	2	0	1	0	SArRNA02
gi|29165615|ref|NC_002745.2|	509883	+	G	1	0	0	1	0	SArRNA02
gi|29165615|ref|NC_002745.2|	509884	+	C	3	1	1	1	0	SArRNA02
gi|29165615|ref|NC_002745.2|	509885	+	G	0	4	0	2	0	SArRNA02
gi|29165615|ref|NC_002745.2|	509886	+	A	3	2	0	0	0	SArRNA02
gi|29165615|ref|NC_002745.2|	509888	+	C	1	2	1	1	1	SArRNA02
gi|29165615|ref|NC_002745.2|	509890	+	C	1	0	0	2	1	SArRNA02

gi|29165615|ref|NC_002745.2|	509891	+	U	1	0	2	0	2	SArRNA02
gi|29165615|ref|NC_002745.2|	509892	+	C	3	1	0	1	2	SArRNA02
gi|29165615|ref|NC_002745.2|	509893	+	G	1	1	0	0	1	SArRNA02
gi|29165615|ref|NC_002745.2|	509894	+	U	0	0	0	1	0	SArRNA02
gi|29165615|ref|NC_002745.2|	509895	+	U	0	0	0	1	4	SArRNA02
gi|29165615|ref|NC_002745.2|	509896	+	A	0	1	3	0	0	SArRNA02
gi|29165615|ref|NC_002745.2|	509897	+	A	7	2	0	2	2	SArRNA02
gi|29165615|ref|NC_002745.2|	509898	+	G	0	0	0	0	1	SArRNA02
gi|29165615|ref|NC_002745.2|	509899	+	G	0	1	0	0	0	SArRNA02
gi|29165615|ref|NC_002745.2|	509900	+	A	1	1	0	0	0	SArRNA02
gi|29165615|ref|NC_002745.2|	509902	+	C	1	1	1	2	0	SArRNA02
gi|29165615|ref|NC_002745.2|	509903	+	U	0	1	2	4	4	SArRNA02
gi|29165615|ref|NC_002745.2|	509904	+	C	2	1	0	2	1	SArRNA02
gi|29165615|ref|NC_002745.2|	509906	+	G	0	0	0	0	1	SArRNA02
gi|29165615|ref|NC_002745.2|	509908	+	A	2	1	0	0	1	SArRNA02
gi|29165615|ref|NC_002745.2|	509909	+	A	0	0	0	0	1	SArRNA02
gi|29165615|ref|NC_002745.2|	509910	+	A	4	1	0	0	4	SArRNA02
gi|29165615|ref|NC_002745.2|	509911	+	A	0	1	0	0	1	SArRNA02
gi|29165615|ref|NC_002745.2|	509913	+	G	0	0	1	0	1	SArRNA02
gi|29165615|ref|NC_002745.2|	509915	+	C	4	8	1	1	6	SArRNA02
gi|29165615|ref|NC_002745.2|	509916	+	C	0	2	0	1	1	SArRNA02
gi|29165615|ref|NC_002745.2|	509917	+	C	1	0	0	0	0	SArRNA02
gi|29165615|ref|NC_002745.2|	509918	+	C	1	1	0	0	0	SArRNA02
gi|29165615|ref|NC_002745.2|	509919	+	G	0	1	1	0	1	SArRNA02
gi|29165615|ref|NC_002745.2|	509920	+	U	1	0	0	0	2	SArRNA02
gi|29165615|ref|NC_002745.2|	509921	+	A	1	1	0	0	0	SArRNA02
gi|29165615|ref|NC_002745.2|	509922	+	A	0	1	0	1	1	SArRNA02
gi|29165615|ref|NC_002745.2|	509923	+	C	6	3	2	1	0	SArRNA02
gi|29165615|ref|NC_002745.2|	509924	+	U	2	2	0	4	2	SArRNA02
gi|29165615|ref|NC_002745.2|	509925	+	U	1	2	2	1	2	SArRNA02
gi|29165615|ref|NC_002745.2|	509926	+	C	2	1	0	3	3	SArRNA02
gi|29165615|ref|NC_002745.2|	509927	+	G	1	1	1	0	0	SArRNA02
gi|29165615|ref|NC_002745.2|	509928	+	G	2	0	0	2	1	SArRNA02
gi|29165615|ref|NC_002745.2|	509930	+	A	1	0	2	1	1	SArRNA02
gi|29165615|ref|NC_002745.2|	509931	+	G	1	1	0	0	3	SArRNA02
gi|29165615|ref|NC_002745.2|	509932	+	A	1	0	1	1	3	SArRNA02
gi|29165615|ref|NC_002745.2|	509934	+	G	0	0	0	0	1	SArRNA02
gi|29165615|ref|NC_002745.2|	509941	+	U	0	1	0	0	3	SArRNA02
gi|29165615|ref|NC_002745.2|	509942	+	C	2	0	0	0	2	SArRNA02
gi|29165615|ref|NC_002745.2|	509943	+	U	2	0	1	0	0	SArRNA02
gi|29165615|ref|NC_002745.2|	509944	+	U	1	0	1	0	1	SArRNA02
gi|29165615|ref|NC_002745.2|	509948	+	G	0	1	0	0	0	SArRNA02
gi|29165615|ref|NC_002745.2|	509949	+	G	0	0	0	0	1	SArRNA02
gi|29165615|ref|NC_002745.2|	509951	+	U	1	0	0	0	0	SArRNA02
gi|29165615|ref|NC_002745.2|	509953	+	A	1	1	2	0	2	SArRNA02
gi|29165615|ref|NC_002745.2|	509954	+	C	30	21	11	17	19	SArRNA02
gi|29165615|ref|NC_002745.2|	509955	+	G	8	3	0	2	6	SArRNA02
gi|29165615|ref|NC_002745.2|	509957	+	C	6	1	0	1	3	SArRNA02
gi|29165615|ref|NC_002745.2|	509958	+	C	2	2	1	2	0	SArRNA02
gi|29165615|ref|NC_002745.2|	509959	+	A	0	0	1	1	0	SArRNA02
gi|29165615|ref|NC_002745.2|	509960	+	G	1	0	0	0	2	SArRNA02
gi|29165615|ref|NC_002745.2|	509961	+	A	1	1	0	3	0	SArRNA02
gi|29165615|ref|NC_002745.2|	509962	+	A	0	2	2	0	1	SArRNA02
gi|29165615|ref|NC_002745.2|	509963	+	G	1	3	0	0	1	SArRNA02
gi|29165615|ref|NC_002745.2|	509964	+	A	1	0	0	0	1	SArRNA02
gi|29165615|ref|NC_002745.2|	509965	+	G	0	0	0	1	1	SArRNA02
gi|29165615|ref|NC_002745.2|	509966	+	C	3	2	0	2	1	SArRNA02
gi|29165615|ref|NC_002745.2|	509967	+	C	1	0	0	0	1	SArRNA02
gi|29165615|ref|NC_002745.2|	509968	+	G	2	0	1	0	2	SArRNA02
gi|29165615|ref|NC_002745.2|	509969	+	C	1	1	2	1	3	SArRNA02
gi|29165615|ref|NC_002745.2|	509970	+	A	5	1	0	2	2	SArRNA02
gi|29165615|ref|NC_002745.2|	509971	+	G	1	0	2	0	2	SArRNA02
gi|29165615|ref|NC_002745.2|	509972	+	U	2	1	2	2	0	SArRNA02
gi|29165615|ref|NC_002745.2|	509973	+	G	2	2	1	8	1	SArRNA02
gi|29165615|ref|NC_002745.2|	509974	+	A	3	2	0	1	1	SArRNA02
gi|29165615|ref|NC_002745.2|	509975	+	A	4	7	3	1	3	SArRNA02
gi|29165615|ref|NC_002745.2|	509976	+	U	1	3	0	0	2	SArRNA02
gi|29165615|ref|NC_002745.2|	509977	+	A	3	4	0	0	0	SArRNA02
gi|29165615|ref|NC_002745.2|	509978	+	G	0	1	0	0	2	SArRNA02
gi|29165615|ref|NC_002745.2|	509979	+	G	0	0	1	2	0	SArRNA02
gi|29165615|ref|NC_002745.2|	509980	+	C	3	3	2	3	6	SArRNA02
gi|29165615|ref|NC_002745.2|	509981	+	C	0	1	1	1	5	SArRNA02
gi|29165615|ref|NC_002745.2|	509982	+	C	3	1	3	2	9	SArRNA02
gi|29165615|ref|NC_002745.2|	509983	+	A	6	5	5	1	2	SArRNA02
gi|29165615|ref|NC_002745.2|	509984	+	A	2	1	0	1	3	SArRNA02
gi|29165615|ref|NC_002745.2|	509985	+	G	0	1	0	0	2	SArRNA02
gi|29165615|ref|NC_002745.2|	509986	+	C	2	0	1	0	1	SArRNA02
gi|29165615|ref|NC_002745.2|	509987	+	G	0	2	3	2	0	SArRNA02
gi|29165615|ref|NC_002745.2|	509988	+	A	0	0	1	0	1	SArRNA02
gi|29165615|ref|NC_002745.2|	509989	+	C	1	0	2	0	0	SArRNA02
gi|29165615|ref|NC_002745.2|	509990	+	U	0	0	1	1	2	SArRNA02

gi|29165615|ref|NC_002745.2|	509992	+	U	2	0	0	1	1 SArRNA02
gi|29165615|ref|NC_002745.2|	509993	+	U	0	0	0	0	3 SArRNA02
gi|29165615|ref|NC_002745.2|	509994	+	U	0	1	2	2	3 SArRNA02
gi|29165615|ref|NC_002745.2|	509995	+	A	1	2	1	0	0 SArRNA02
gi|29165615|ref|NC_002745.2|	509996	+	U	4	4	0	0	2 SArRNA02
gi|29165615|ref|NC_002745.2|	509997	+	C	2	0	1	1	4 SArRNA02
gi|29165615|ref|NC_002745.2|	509998	+	A	1	0	1	0	1 SArRNA02
gi|29165615|ref|NC_002745.2|	509999	+	A	4	0	0	0	3 SArRNA02
gi|29165615|ref|NC_002745.2|	510000	+	A	0	1	0	2	1 SArRNA02
gi|29165615|ref|NC_002745.2|	510001	+	A	2	1	3	0	3 SArRNA02
gi|29165615|ref|NC_002745.2|	510002	+	A	5	2	1	0	3 SArRNA02
gi|29165615|ref|NC_002745.2|	510003	+	C	11	2	1	4	1 SArRNA02
gi|29165615|ref|NC_002745.2|	510004	+	A	4	0	2	1	1 SArRNA02
gi|29165615|ref|NC_002745.2|	510007	+	G	0	0	0	0	1 SArRNA02
gi|29165615|ref|NC_002745.2|	510008	+	G	1	0	0	0	1 SArRNA02
gi|29165615|ref|NC_002745.2|	510009	+	U	167	6	10	2	7 SArRNA02
gi|29165615|ref|NC_002745.2|	510010	+	C	30	0	1	0	2 SArRNA02
gi|29165615|ref|NC_002745.2|	510011	+	U	5	1	1	2	2 SArRNA02
gi|29165615|ref|NC_002745.2|	510012	+	C	2	0	0	0	3 SArRNA02
gi|29165615|ref|NC_002745.2|	510013	+	U	0	0	2	0	1 SArRNA02
gi|29165615|ref|NC_002745.2|	510014	+	G	3	1	2	1	0 SArRNA02
gi|29165615|ref|NC_002745.2|	510015	+	C	3	3	0	2	5 SArRNA02
gi|29165615|ref|NC_002745.2|	510016	+	U	3	0	3	0	3 SArRNA02
gi|29165615|ref|NC_002745.2|	510017	+	A	1	1	0	0	2 SArRNA02
gi|29165615|ref|NC_002745.2|	510018	+	A	3	3	0	0	3 SArRNA02
gi|29165615|ref|NC_002745.2|	510019	+	A	1	0	0	0	0 SArRNA02
gi|29165615|ref|NC_002745.2|	510020	+	C	2	1	1	0	4 SArRNA02
gi|29165615|ref|NC_002745.2|	510021	+	C	2	1	2	0	3 SArRNA02
gi|29165615|ref|NC_002745.2|	510022	+	G	0	0	1	0	2 SArRNA02
gi|29165615|ref|NC_002745.2|	510023	+	U	2	1	0	0	1 SArRNA02
gi|29165615|ref|NC_002745.2|	510024	+	A	1	2	1	0	1 SArRNA02
gi|29165615|ref|NC_002745.2|	510025	+	A	0	0	2	0	1 SArRNA02
gi|29165615|ref|NC_002745.2|	510026	+	G	2	3	1	0	1 SArRNA02
gi|29165615|ref|NC_002745.2|	510027	+	G	0	0	0	0	1 SArRNA02
gi|29165615|ref|NC_002745.2|	510028	+	U	0	1	2	0	0 SArRNA02
gi|29165615|ref|NC_002745.2|	510029	+	G	0	2	0	1	1 SArRNA02
gi|29165615|ref|NC_002745.2|	510030	+	A	1	0	1	1	0 SArRNA02
gi|29165615|ref|NC_002745.2|	510032	+	G	1	0	0	0	0 SArRNA02
gi|29165615|ref|NC_002745.2|	510033	+	U	0	1	0	0	0 SArRNA02
gi|29165615|ref|NC_002745.2|	510034	+	A	1	1	1	0	0 SArRNA02
gi|29165615|ref|NC_002745.2|	510035	+	U	2	0	2	0	1 SArRNA02
gi|29165615|ref|NC_002745.2|	510036	+	A	2	1	0	0	0 SArRNA02
gi|29165615|ref|NC_002745.2|	510038	+	G	0	1	1	0	1 SArRNA02
gi|29165615|ref|NC_002745.2|	510039	+	G	1	0	0	0	0 SArRNA02
gi|29165615|ref|NC_002745.2|	510041	+	C	0	0	1	0	1 SArRNA02
gi|29165615|ref|NC_002745.2|	510042	+	U	0	1	0	0	0 SArRNA02
gi|29165615|ref|NC_002745.2|	510046	+	G	1	0	0	0	0 SArRNA02
gi|29165615|ref|NC_002745.2|	510047	+	C	0	1	0	0	0 SArRNA02
gi|29165615|ref|NC_002745.2|	510048	+	C	1	1	0	0	0 SArRNA02
gi|29165615|ref|NC_002745.2|	510049	+	U	0	0	1	0	0 SArRNA02
gi|29165615|ref|NC_002745.2|	510051	+	C	2	1	0	0	1 SArRNA02
gi|29165615|ref|NC_002745.2|	510052	+	C	1	1	0	0	0 SArRNA02
gi|29165615|ref|NC_002745.2|	510053	+	C	0	1	0	0	0 SArRNA02
gi|29165615|ref|NC_002745.2|	510054	+	G	0	0	0	1	0 SArRNA02
gi|29165615|ref|NC_002745.2|	510061	+	G	2	8	5	1	5 SArRNA02
gi|29165615|ref|NC_002745.2|	510063	+	A	0	0	0	1	2 SArRNA02
gi|29165615|ref|NC_002745.2|	510065	+	G	0	1	0	0	0 SArRNA02
gi|29165615|ref|NC_002745.2|	510067	+	U	0	1	0	0	0 SArRNA02
gi|29165615|ref|NC_002745.2|	510068	+	A	0	1	0	0	0 SArRNA02
gi|29165615|ref|NC_002745.2|	510070	+	G	0	0	0	1	0 SArRNA02
gi|29165615|ref|NC_002745.2|	510071	+	A	1	2	0	0	0 SArRNA02
gi|29165615|ref|NC_002745.2|	510072	+	G	0	1	0	0	0 SArRNA02
gi|29165615|ref|NC_002745.2|	510073	+	G	1	1	0	0	1 SArRNA02
gi|29165615|ref|NC_002745.2|	510074	+	A	0	0	1	0	0 SArRNA02
gi|29165615|ref|NC_002745.2|	510075	+	G	0	1	0	0	0 SArRNA02
gi|29165615|ref|NC_002745.2|	510076	+	U	0	1	1	0	0 SArRNA02
gi|29165615|ref|NC_002745.2|	510077	+	G	0	0	0	0	2 SArRNA02
gi|29165615|ref|NC_002745.2|	510079	+	U	0	0	0	1	1 SArRNA02
gi|29165615|ref|NC_002745.2|	510080	+	U	0	0	0	0	1 SArRNA02
gi|29165615|ref|NC_002745.2|	510081	+	A	0	0	1	0	0 SArRNA02
gi|29165615|ref|NC_002745.2|	510082	+	G	0	1	0	0	0 SArRNA02
gi|29165615|ref|NC_002745.2|	510083	+	C	0	1	0	0	0 SArRNA02
gi|29165615|ref|NC_002745.2|	510086	+	C	0	0	0	0	2 SArRNA02
gi|29165615|ref|NC_002745.2|	510088	+	G	0	0	0	1	0 SArRNA02
gi|29165615|ref|NC_002745.2|	510090	+	G	0	1	0	0	0 SArRNA02
gi|29165615|ref|NC_002745.2|	510092	+	A	0	0	1	0	0 SArRNA02
gi|29165615|ref|NC_002745.2|	510097	+	C	0	0	0	0	2 SArRNA02
gi|29165615|ref|NC_002745.2|	510107	+	C	4	3	0	0	1 SArRNA02
gi|29165615|ref|NC_002745.2|	510109	+	C	0	1	0	0	1 SArRNA02
gi|29165615|ref|NC_002745.2|	510110	+	C	5	2	0	1	1 SArRNA02
gi|29165615|ref|NC_002745.2|	510111	+	A	0	1	1	0	0 SArRNA02

gi|29165615|ref|NC_002745.2|	510114	+	A	0	1	0	0	0	SArRNA02
gi|29165615|ref|NC_002745.2|	510115	+	A	0	1	0	0	0	SArRNA02
gi|29165615|ref|NC_002745.2|	510117	+	C	1	1	0	0	2	SArRNA02
gi|29165615|ref|NC_002745.2|	510119	+	G	0	2	0	0	0	SArRNA02
gi|29165615|ref|NC_002745.2|	510121	+	G	0	1	0	0	0	SArRNA02
gi|29165615|ref|NC_002745.2|	510122	+	G	0	1	0	0	0	SArRNA02
gi|29165615|ref|NC_002745.2|	510125	+	G	1	0	0	0	0	SArRNA02
gi|29165615|ref|NC_002745.2|	510126	+	U	0	0	0	0	1	SArRNA02
gi|29165615|ref|NC_002745.2|	510127	+	A	0	0	0	1	0	SArRNA02
gi|29165615|ref|NC_002745.2|	510128	+	A	0	0	0	0	3	SArRNA02
gi|29165615|ref|NC_002745.2|	510130	+	U	0	0	0	0	2	SArRNA02
gi|29165615|ref|NC_002745.2|	510131	+	A	1	1	2	2	1	SArRNA02
gi|29165615|ref|NC_002745.2|	510133	+	A	0	0	1	0	2	SArRNA02
gi|29165615|ref|NC_002745.2|	510134	+	A	1	2	0	0	0	SArRNA02
gi|29165615|ref|NC_002745.2|	510135	+	C	0	0	1	0	0	SArRNA02
gi|29165615|ref|NC_002745.2|	510140	+	C	3	0	0	0	1	SArRNA02
gi|29165615|ref|NC_002745.2|	510141	+	U	0	2	0	0	0	SArRNA02
gi|29165615|ref|NC_002745.2|	510143	+	A	0	0	0	1	0	SArRNA02
gi|29165615|ref|NC_002745.2|	510145	+	G	0	0	0	0	1	SArRNA02
gi|29165615|ref|NC_002745.2|	510146	+	U	0	1	0	0	1	SArRNA02
gi|29165615|ref|NC_002745.2|	510147	+	A	0	1	0	0	0	SArRNA02
gi|29165615|ref|NC_002745.2|	510149	+	C	2	0	0	0	1	SArRNA02
gi|29165615|ref|NC_002745.2|	510150	+	G	0	0	1	1	1	SArRNA02
gi|29165615|ref|NC_002745.2|	510151	+	A	1	8	1	4	4	SArRNA02
gi|29165615|ref|NC_002745.2|	510152	+	A	2	4	3	4	7	SArRNA02
gi|29165615|ref|NC_002745.2|	510153	+	A	11	67	7	3	37	SArRNA02
gi|29165615|ref|NC_002745.2|	510154	+	U	0	4	3	0	2	SArRNA02
gi|29165615|ref|NC_002745.2|	510155	+	U	1	1	0	1	2	SArRNA02
gi|29165615|ref|NC_002745.2|	510156	+	C	5	17	1	3	10	SArRNA02
gi|29165615|ref|NC_002745.2|	510157	+	C	75	135	30	39	92	SArRNA02
gi|29165615|ref|NC_002745.2|	510158	+	U	9	12	3	4	12	SArRNA02
gi|29165615|ref|NC_002745.2|	510159	+	U	8	4	3	1	11	SArRNA02
gi|29165615|ref|NC_002745.2|	510160	+	G	12	4	1	0	6	SArRNA02
gi|29165615|ref|NC_002745.2|	510161	+	U	10	9	2	1	9	SArRNA02
gi|29165615|ref|NC_002745.2|	510162	+	C	21	18	3	6	13	SArRNA02
gi|29165615|ref|NC_002745.2|	510163	+	G	5	10	0	0	4	SArRNA02
gi|29165615|ref|NC_002745.2|	510164	+	G	1	1	0	0	2	SArRNA02
gi|29165615|ref|NC_002745.2|	510165	+	G	1	1	4	0	3	SArRNA02
gi|29165615|ref|NC_002745.2|	510166	+	U	3	0	2	1	3	SArRNA02
gi|29165615|ref|NC_002745.2|	510167	+	A	2	4	0	1	3	SArRNA02
gi|29165615|ref|NC_002745.2|	510168	+	A	3	1	0	2	0	SArRNA02
gi|29165615|ref|NC_002745.2|	510169	+	G	0	0	0	0	1	SArRNA02
gi|29165615|ref|NC_002745.2|	510170	+	U	0	2	1	0	0	SArRNA02
gi|29165615|ref|NC_002745.2|	510171	+	U	1	3	0	0	0	SArRNA02
gi|29165615|ref|NC_002745.2|	510172	+	C	3	6	0	0	2	SArRNA02
gi|29165615|ref|NC_002745.2|	510173	+	C	1	4	2	1	1	SArRNA02
gi|29165615|ref|NC_002745.2|	510174	+	G	4	6	0	1	2	SArRNA02
gi|29165615|ref|NC_002745.2|	510175	+	A	0	8	0	0	6	SArRNA02
gi|29165615|ref|NC_002745.2|	510176	+	C	65	32	10	9	35	SArRNA02
gi|29165615|ref|NC_002745.2|	510177	+	C	20	35	18	5	28	SArRNA02
gi|29165615|ref|NC_002745.2|	510178	+	C	11	15	1	1	8	SArRNA02
gi|29165615|ref|NC_002745.2|	510179	+	G	14	17	3	1	19	SArRNA02
gi|29165615|ref|NC_002745.2|	510180	+	C	41	46	19	9	37	SArRNA02
gi|29165615|ref|NC_002745.2|	510181	+	A	7	14	6	4	11	SArRNA02
gi|29165615|ref|NC_002745.2|	510182	+	C	4	5	3	1	6	SArRNA02
gi|29165615|ref|NC_002745.2|	510183	+	G	10	2	7	1	15	SArRNA02
gi|29165615|ref|NC_002745.2|	510184	+	A	3	5	3	3	8	SArRNA02
gi|29165615|ref|NC_002745.2|	510185	+	A	20	25	10	5	18	SArRNA02
gi|29165615|ref|NC_002745.2|	510186	+	A	15	6	4	1	6	SArRNA02
gi|29165615|ref|NC_002745.2|	510187	+	G	1	0	0	0	1	SArRNA02
gi|29165615|ref|NC_002745.2|	510188	+	G	10	0	0	0	5	SArRNA02
gi|29165615|ref|NC_002745.2|	510189	+	C	6	4	1	2	4	SArRNA02
gi|29165615|ref|NC_002745.2|	510190	+	G	7	9	2	0	2	SArRNA02
gi|29165615|ref|NC_002745.2|	510191	+	U	3	6	6	3	7	SArRNA02
gi|29165615|ref|NC_002745.2|	510192	+	A	0	17	10	9	11	SArRNA02
gi|29165615|ref|NC_002745.2|	510193	+	A	4	12	12	7	19	SArRNA02
gi|29165615|ref|NC_002745.2|	510194	+	C	10	33	12	15	24	SArRNA02
gi|29165615|ref|NC_002745.2|	510195	+	G	14	17	4	7	18	SArRNA02
gi|29165615|ref|NC_002745.2|	510196	+	A	36	228	142	235	273	SArRNA02
gi|29165615|ref|NC_002745.2|	510197	+	U	10	34	17	11	18	SArRNA02
gi|29165615|ref|NC_002745.2|	510198	+	U	3	10	4	7	6	SArRNA02
gi|29165615|ref|NC_002745.2|	510199	+	U	3	10	4	1	6	SArRNA02
gi|29165615|ref|NC_002745.2|	510200	+	G	6	26	0	0	13	SArRNA02
gi|29165615|ref|NC_002745.2|	510201	+	G	0	2	0	0	0	SArRNA02
gi|29165615|ref|NC_002745.2|	510202	+	G	0	2	0	0	1	SArRNA02
gi|29165615|ref|NC_002745.2|	510203	+	C	8	8	6	7	6	SArRNA02
gi|29165615|ref|NC_002745.2|	510204	+	A	1	5	3	1	2	SArRNA02
gi|29165615|ref|NC_002745.2|	510205	+	C	12	11	3	3	5	SArRNA02
gi|29165615|ref|NC_002745.2|	510206	+	U	2	3	0	1	1	SArRNA02
gi|29165615|ref|NC_002745.2|	510207	+	G	0	0	0	0	1	SArRNA02
gi|29165615|ref|NC_002745.2|	510208	+	U	0	2	0	1	2	SArRNA02

gi|29165615|ref|NC_002745.2|	510209 +	C	6	13	2	2	2	SArRNA02
gi|29165615|ref|NC_002745.2|	510210 +	U	1	4	2	1	6	SArRNA02
gi|29165615|ref|NC_002745.2|	510211 +	C	49	48	13	17	34	SArRNA02
gi|29165615|ref|NC_002745.2|	510212 +	A	42	48	24	10	26	SArRNA02
gi|29165615|ref|NC_002745.2|	510213 +	A	1	6	5	1	6	SArRNA02
gi|29165615|ref|NC_002745.2|	510214 +	C	52	42	16	10	27	SArRNA02
gi|29165615|ref|NC_002745.2|	510215 +	G	8	12	3	0	9	SArRNA02
gi|29165615|ref|NC_002745.2|	510216 +	A	2	3	0	2	1	SArRNA02
gi|29165615|ref|NC_002745.2|	510217 +	G	4	0	3	1	0	SArRNA02
gi|29165615|ref|NC_002745.2|	510218 +	A	1	5	1	0	4	SArRNA02
gi|29165615|ref|NC_002745.2|	510219 +	G	6	3	4	4	7	SArRNA02
gi|29165615|ref|NC_002745.2|	510220 +	A	5	7	4	1	6	SArRNA02
gi|29165615|ref|NC_002745.2|	510221 +	C	11	9	3	1	9	SArRNA02
gi|29165615|ref|NC_002745.2|	510222 +	U	5	13	1	3	8	SArRNA02
gi|29165615|ref|NC_002745.2|	510223 +	C	19	22	8	5	12	SArRNA02
gi|29165615|ref|NC_002745.2|	510224 +	G	1	6	1	0	2	SArRNA02
gi|29165615|ref|NC_002745.2|	510225 +	G	1	2	0	0	1	SArRNA02
gi|29165615|ref|NC_002745.2|	510226 +	U	4	1	0	1	2	SArRNA02
gi|29165615|ref|NC_002745.2|	510227 +	G	0	3	1	0	0	SArRNA02
gi|29165615|ref|NC_002745.2|	510228 +	A	0	0	1	0	2	SArRNA02
gi|29165615|ref|NC_002745.2|	510229 +	A	5	6	3	6	7	SArRNA02
gi|29165615|ref|NC_002745.2|	510230 +	A	15	147	24	3	35	SArRNA02
gi|29165615|ref|NC_002745.2|	510231 +	U	2	2	0	0	4	SArRNA02
gi|29165615|ref|NC_002745.2|	510232 +	C	14	37	3	3	25	SArRNA02
gi|29165615|ref|NC_002745.2|	510233 +	A	20	10	6	2	10	SArRNA02
gi|29165615|ref|NC_002745.2|	510234 +	U	8	7	2	7	11	SArRNA02
gi|29165615|ref|NC_002745.2|	510235 +	A	0	2	0	0	1	SArRNA02
gi|29165615|ref|NC_002745.2|	510236 +	G	0	2	1	0	0	SArRNA02
gi|29165615|ref|NC_002745.2|	510237 +	U	5	9	2	0	5	SArRNA02
gi|29165615|ref|NC_002745.2|	510238 +	A	1	2	2	1	0	SArRNA02
gi|29165615|ref|NC_002745.2|	510239 +	C	1	1	3	1	3	SArRNA02
gi|29165615|ref|NC_002745.2|	510240 +	C	3	3	0	3	1	SArRNA02
gi|29165615|ref|NC_002745.2|	510241 +	U	6	6	0	1	2	SArRNA02
gi|29165615|ref|NC_002745.2|	510242 +	G	2	1	0	1	1	SArRNA02
gi|29165615|ref|NC_002745.2|	510243 +	U	6	3	4	2	2	SArRNA02
gi|29165615|ref|NC_002745.2|	510244 +	G	1	0	2	5	3	SArRNA02
gi|29165615|ref|NC_002745.2|	510245 +	A	4	3	2	2	4	SArRNA02
gi|29165615|ref|NC_002745.2|	510246 +	A	10	14	0	2	9	SArRNA02
gi|29165615|ref|NC_002745.2|	510247 +	G	10	12	3	4	5	SArRNA02
gi|29165615|ref|NC_002745.2|	510248 +	A	6	9	6	4	7	SArRNA02
gi|29165615|ref|NC_002745.2|	510249 +	U	4	5	3	3	5	SArRNA02
gi|29165615|ref|NC_002745.2|	510250 +	G	2	4	2	0	7	SArRNA02
gi|29165615|ref|NC_002745.2|	510251 +	C	13	13	1	3	0	SArRNA02
gi|29165615|ref|NC_002745.2|	510252 +	A	7	4	1	4	7	SArRNA02
gi|29165615|ref|NC_002745.2|	510253 +	G	0	1	0	0	1	SArRNA02
gi|29165615|ref|NC_002745.2|	510254 +	G	1	1	1	1	0	SArRNA02
gi|29165615|ref|NC_002745.2|	510255 +	U	6	2	2	0	6	SArRNA02
gi|29165615|ref|NC_002745.2|	510256 +	U	7	5	0	1	6	SArRNA02
gi|29165615|ref|NC_002745.2|	510257 +	A	6	3	1	0	3	SArRNA02
gi|29165615|ref|NC_002745.2|	510258 +	C	10	10	5	2	10	SArRNA02
gi|29165615|ref|NC_002745.2|	510259 +	C	5	7	4	2	4	SArRNA02
gi|29165615|ref|NC_002745.2|	510260 +	C	9	1	3	5	7	SArRNA02
gi|29165615|ref|NC_002745.2|	510261 +	G	0	2	0	0	1	SArRNA02
gi|29165615|ref|NC_002745.2|	510262 +	C	9	2	2	5	2	SArRNA02
gi|29165615|ref|NC_002745.2|	510263 +	G	1	3	0	2	1	SArRNA02
gi|29165615|ref|NC_002745.2|	510264 +	A	6	3	2	1	2	SArRNA02
gi|29165615|ref|NC_002745.2|	510265 +	C	20	23	3	1	11	SArRNA02
gi|29165615|ref|NC_002745.2|	510266 +	A	5	5	2	0	1	SArRNA02
gi|29165615|ref|NC_002745.2|	510267 +	G	5	4	0	1	12	SArRNA02
gi|29165615|ref|NC_002745.2|	510268 +	G	2	4	4	1	4	SArRNA02
gi|29165615|ref|NC_002745.2|	510269 +	A	5	1	2	0	2	SArRNA02
gi|29165615|ref|NC_002745.2|	510270 +	C	7	2	1	0	4	SArRNA02
gi|29165615|ref|NC_002745.2|	510271 +	G	1	5	0	1	2	SArRNA02
gi|29165615|ref|NC_002745.2|	510272 +	G	0	4	1	0	1	SArRNA02
gi|29165615|ref|NC_002745.2|	510273 +	A	1	0	1	2	3	SArRNA02
gi|29165615|ref|NC_002745.2|	510274 +	A	5	3	2	2	3	SArRNA02
gi|29165615|ref|NC_002745.2|	510275 +	A	3	3	1	0	5	SArRNA02
gi|29165615|ref|NC_002745.2|	510276 +	G	10	7	2	0	4	SArRNA02
gi|29165615|ref|NC_002745.2|	510277 +	A	5	3	3	1	6	SArRNA02
gi|29165615|ref|NC_002745.2|	510278 +	C	7	9	2	3	17	SArRNA02
gi|29165615|ref|NC_002745.2|	510279 +	C	19	5	2	3	4	SArRNA02
gi|29165615|ref|NC_002745.2|	510280 +	C	6	1	3	1	6	SArRNA02
gi|29165615|ref|NC_002745.2|	510281 +	C	17	22	4	24	10	SArRNA02
gi|29165615|ref|NC_002745.2|	510282 +	G	2	4	2	1	4	SArRNA02
gi|29165615|ref|NC_002745.2|	510283 +	U	6	3	1	1	5	SArRNA02
gi|29165615|ref|NC_002745.2|	510284 +	G	1	1	0	0	1	SArRNA02
gi|29165615|ref|NC_002745.2|	510285 +	G	3	2	1	2	2	SArRNA02
gi|29165615|ref|NC_002745.2|	510286 +	A	3	0	2	0	4	SArRNA02
gi|29165615|ref|NC_002745.2|	510287 +	G	7	4	0	0	4	SArRNA02
gi|29165615|ref|NC_002745.2|	510288 +	C	8	6	3	1	7	SArRNA02
gi|29165615|ref|NC_002745.2|	510289 +	U	6	2	1	2	3	SArRNA02

gi|29165615|ref|NC_002745.2|	510290	+	U	2	1	0	0	0	SArRNA02
gi|29165615|ref|NC_002745.2|	510291	+	U	6	4	0	0	4	SArRNA02
gi|29165615|ref|NC_002745.2|	510292	+	A	0	4	0	0	3	SArRNA02
gi|29165615|ref|NC_002745.2|	510293	+	C	2	3	4	5	2	SArRNA02
gi|29165615|ref|NC_002745.2|	510294	+	U	2	5	0	2	5	SArRNA02
gi|29165615|ref|NC_002745.2|	510295	+	G	1	1	1	0	0	SArRNA02
gi|29165615|ref|NC_002745.2|	510296	+	U	2	0	0	2	1	SArRNA02
gi|29165615|ref|NC_002745.2|	510297	+	A	3	1	0	0	0	SArRNA02
gi|29165615|ref|NC_002745.2|	510299	+	C	1	2	0	0	1	SArRNA02
gi|29165615|ref|NC_002745.2|	510300	+	C	16	13	10	6	6	SArRNA02
gi|29165615|ref|NC_002745.2|	510301	+	U	0	5	0	1	4	SArRNA02
gi|29165615|ref|NC_002745.2|	510302	+	G	0	1	0	0	1	SArRNA02
gi|29165615|ref|NC_002745.2|	510303	+	A	5	2	1	0	8	SArRNA02
gi|29165615|ref|NC_002745.2|	510304	+	U	10	3	1	1	6	SArRNA02
gi|29165615|ref|NC_002745.2|	510305	+	A	1	2	2	2	4	SArRNA02
gi|29165615|ref|NC_002745.2|	510306	+	U	0	0	0	0	3	SArRNA02
gi|29165615|ref|NC_002745.2|	510308	+	G	0	0	0	1	1	SArRNA02
gi|29165615|ref|NC_002745.2|	510309	+	A	1	4	1	0	3	SArRNA02
gi|29165615|ref|NC_002745.2|	510310	+	A	0	0	1	1	0	SArRNA02
gi|29165615|ref|NC_002745.2|	510311	+	A	2	15	1	0	7	SArRNA02
gi|29165615|ref|NC_002745.2|	510312	+	U	6	6	2	0	1	SArRNA02
gi|29165615|ref|NC_002745.2|	510313	+	U	2	5	2	1	2	SArRNA02
gi|29165615|ref|NC_002745.2|	510314	+	C	11	22	6	5	10	SArRNA02
gi|29165615|ref|NC_002745.2|	510315	+	G	1	1	0	0	2	SArRNA02
gi|29165615|ref|NC_002745.2|	510317	+	C	0	1	0	2	0	SArRNA02
gi|29165615|ref|NC_002745.2|	510318	+	A	0	2	0	0	1	SArRNA02
gi|29165615|ref|NC_002745.2|	510319	+	C	1	1	0	0	3	SArRNA02
gi|29165615|ref|NC_002745.2|	510320	+	A	0	2	0	0	0	SArRNA02
gi|29165615|ref|NC_002745.2|	510321	+	G	1	2	0	0	0	SArRNA02
gi|29165615|ref|NC_002745.2|	510322	+	C	11	8	3	1	4	SArRNA02
gi|29165615|ref|NC_002745.2|	510323	+	U	3	8	3	5	7	SArRNA02
gi|29165615|ref|NC_002745.2|	510324	+	U	6	2	0	4	4	SArRNA02
gi|29165615|ref|NC_002745.2|	510325	+	G	6	4	0	1	10	SArRNA02
gi|29165615|ref|NC_002745.2|	510326	+	U	5	13	3	2	3	SArRNA02
gi|29165615|ref|NC_002745.2|	510327	+	A	3	2	0	0	3	SArRNA02
gi|29165615|ref|NC_002745.2|	510328	+	C	35	76	12	23	51	SArRNA02
gi|29165615|ref|NC_002745.2|	510329	+	A	14	28	4	11	23	SArRNA02
gi|29165615|ref|NC_002745.2|	510330	+	G	4	4	3	3	6	SArRNA02
gi|29165615|ref|NC_002745.2|	510331	+	G	141	9	56	5	15	SArRNA02
gi|29165615|ref|NC_002745.2|	510332	+	A	162	13	47	10	13	SArRNA02
gi|29165615|ref|NC_002745.2|	510333	+	U	15	24	5	14	11	SArRNA02
gi|29165615|ref|NC_002745.2|	510334	+	A	2	3	1	2	4	SArRNA02
gi|29165615|ref|NC_002745.2|	510335	+	G	3	3	2	1	1	SArRNA02
gi|29165615|ref|NC_002745.2|	510336	+	G	6	1	2	5	5	SArRNA02
gi|29165615|ref|NC_002745.2|	510337	+	U	17	8	3	5	8	SArRNA02
gi|29165615|ref|NC_002745.2|	510338	+	A	7	8	4	2	5	SArRNA02
gi|29165615|ref|NC_002745.2|	510339	+	G	4	2	0	3	5	SArRNA02
gi|29165615|ref|NC_002745.2|	510340	+	G	10	4	5	6	8	SArRNA02
gi|29165615|ref|NC_002745.2|	510341	+	A	5	6	2	1	4	SArRNA02
gi|29165615|ref|NC_002745.2|	510343	+	C	13	6	3	8	11	SArRNA02
gi|29165615|ref|NC_002745.2|	510344	+	C	6	4	1	1	6	SArRNA02
gi|29165615|ref|NC_002745.2|	510345	+	U	1	4	3	1	5	SArRNA02
gi|29165615|ref|NC_002745.2|	510346	+	U	11	3	0	1	7	SArRNA02
gi|29165615|ref|NC_002745.2|	510347	+	U	5	1	1	0	2	SArRNA02
gi|29165615|ref|NC_002745.2|	510348	+	G	0	0	0	1	0	SArRNA02
gi|29165615|ref|NC_002745.2|	510349	+	A	1	1	0	1	4	SArRNA02
gi|29165615|ref|NC_002745.2|	510350	+	A	1	0	0	2	2	SArRNA02
gi|29165615|ref|NC_002745.2|	510352	+	C	4	0	0	1	2	SArRNA02
gi|29165615|ref|NC_002745.2|	510353	+	G	0	0	0	1	1	SArRNA02
gi|29165615|ref|NC_002745.2|	510354	+	U	1	0	2	1	3	SArRNA02
gi|29165615|ref|NC_002745.2|	510355	+	G	1	0	0	0	0	SArRNA02
gi|29165615|ref|NC_002745.2|	510356	+	A	0	0	1	1	1	SArRNA02
gi|29165615|ref|NC_002745.2|	510357	+	G	0	0	0	0	2	SArRNA02
gi|29165615|ref|NC_002745.2|	510358	+	C	1	3	0	1	5	SArRNA02
gi|29165615|ref|NC_002745.2|	510359	+	G	0	1	0	0	2	SArRNA02
gi|29165615|ref|NC_002745.2|	510360	+	C	2	7	3	1	6	SArRNA02
gi|29165615|ref|NC_002745.2|	510361	+	U	5	1	3	5	5	SArRNA02
gi|29165615|ref|NC_002745.2|	510362	+	A	3	4	0	0	2	SArRNA02
gi|29165615|ref|NC_002745.2|	510363	+	G	2	0	1	1	2	SArRNA02
gi|29165615|ref|NC_002745.2|	510364	+	C	6	6	1	1	2	SArRNA02
gi|29165615|ref|NC_002745.2|	510365	+	U	0	5	2	1	5	SArRNA02
gi|29165615|ref|NC_002745.2|	510366	+	U	1	1	0	0	2	SArRNA02
gi|29165615|ref|NC_002745.2|	510367	+	A	1	5	0	0	2	SArRNA02
gi|29165615|ref|NC_002745.2|	510368	+	C	0	1	1	0	1	SArRNA02
gi|29165615|ref|NC_002745.2|	510369	+	G	1	0	0	0	2	SArRNA02
gi|29165615|ref|NC_002745.2|	510370	+	U	0	2	0	2	1	SArRNA02
gi|29165615|ref|NC_002745.2|	510371	+	G	1	2	0	0	1	SArRNA02
gi|29165615|ref|NC_002745.2|	510372	+	G	1	1	1	0	2	SArRNA02
gi|29165615|ref|NC_002745.2|	510373	+	A	0	2	2	2	2	SArRNA02
gi|29165615|ref|NC_002745.2|	510374	+	G	2	1	1	1	6	SArRNA02
gi|29165615|ref|NC_002745.2|	510375	+	G	2	0	2	2	1	SArRNA02

gi|29165615|ref|NC_002745.2|	510376	+	C	1	1	1	1	5	SArRNA02
gi|29165615|ref|NC_002745.2|	510377	+	G	0	1	0	1	1	SArRNA02
gi|29165615|ref|NC_002745.2|	510378	+	C	4	7	2	2	7	SArRNA02
gi|29165615|ref|NC_002745.2|	510379	+	U	17	9	2	1	12	SArRNA02
gi|29165615|ref|NC_002745.2|	510380	+	G	1	3	1	0	4	SArRNA02
gi|29165615|ref|NC_002745.2|	510381	+	G	5	6	0	1	4	SArRNA02
gi|29165615|ref|NC_002745.2|	510382	+	U	3	8	0	1	4	SArRNA02
gi|29165615|ref|NC_002745.2|	510383	+	G	1	2	0	0	1	SArRNA02
gi|29165615|ref|NC_002745.2|	510384	+	G	0	2	1	0	1	SArRNA02
gi|29165615|ref|NC_002745.2|	510385	+	G	6	2	0	3	5	SArRNA02
gi|29165615|ref|NC_002745.2|	510386	+	A	3	6	2	0	3	SArRNA02
gi|29165615|ref|NC_002745.2|	510387	+	U	7	6	4	1	5	SArRNA02
gi|29165615|ref|NC_002745.2|	510388	+	A	0	2	1	0	3	SArRNA02
gi|29165615|ref|NC_002745.2|	510389	+	C	14	14	4	9	15	SArRNA02
gi|29165615|ref|NC_002745.2|	510390	+	U	53	28	14	13	40	SArRNA02
gi|29165615|ref|NC_002745.2|	510391	+	A	15	11	3	3	9	SArRNA02
gi|29165615|ref|NC_002745.2|	510392	+	C	10	12	6	2	3	SArRNA02
gi|29165615|ref|NC_002745.2|	510393	+	C	1	9	2	2	3	SArRNA02
gi|29165615|ref|NC_002745.2|	510394	+	C	5	18	9	5	11	SArRNA02
gi|29165615|ref|NC_002745.2|	510395	+	U	3	2	0	3	3	SArRNA02
gi|29165615|ref|NC_002745.2|	510396	+	A	2	5	0	2	2	SArRNA02
gi|29165615|ref|NC_002745.2|	510398	+	C	1	1	0	1	2	SArRNA02
gi|29165615|ref|NC_002745.2|	510399	+	U	1	0	0	0	0	SArRNA02
gi|29165615|ref|NC_002745.2|	510400	+	G	2	0	0	0	0	SArRNA02
gi|29165615|ref|NC_002745.2|	510401	+	U	0	1	1	0	5	SArRNA02
gi|29165615|ref|NC_002745.2|	510402	+	G	2	1	0	0	0	SArRNA02
gi|29165615|ref|NC_002745.2|	510403	+	U	6	3	0	1	3	SArRNA02
gi|29165615|ref|NC_002745.2|	510404	+	U	0	0	0	2	3	SArRNA02
gi|29165615|ref|NC_002745.2|	510405	+	G	1	0	0	0	0	SArRNA02
gi|29165615|ref|NC_002745.2|	510407	+	C	5	1	0	0	2	SArRNA02
gi|29165615|ref|NC_002745.2|	510408	+	U	5	1	3	1	0	SArRNA02
gi|29165615|ref|NC_002745.2|	510409	+	U	3	0	0	0	3	SArRNA02
gi|29165615|ref|NC_002745.2|	510410	+	U	3	0	1	1	4	SArRNA02
gi|29165615|ref|NC_002745.2|	510411	+	C	4	5	2	0	8	SArRNA02
gi|29165615|ref|NC_002745.2|	510412	+	U	0	2	0	1	5	SArRNA02
gi|29165615|ref|NC_002745.2|	510413	+	A	0	0	1	1	2	SArRNA02
gi|29165615|ref|NC_002745.2|	510414	+	A	0	1	0	1	2	SArRNA02
gi|29165615|ref|NC_002745.2|	510415	+	C	7	11	4	1	7	SArRNA02
gi|29165615|ref|NC_002745.2|	510416	+	C	3	1	1	1	1	SArRNA02
gi|29165615|ref|NC_002745.2|	510417	+	C	2	2	0	1	1	SArRNA02
gi|29165615|ref|NC_002745.2|	510418	+	G	0	2	0	0	1	SArRNA02
gi|29165615|ref|NC_002745.2|	510419	+	C	1	1	0	0	0	SArRNA02
gi|29165615|ref|NC_002745.2|	510420	+	A	1	0	0	0	0	SArRNA02
gi|29165615|ref|NC_002745.2|	510421	+	C	0	1	0	0	0	SArRNA02
gi|29165615|ref|NC_002745.2|	510422	+	C	5	3	0	0	1	SArRNA02
gi|29165615|ref|NC_002745.2|	510423	+	A	1	4	2	0	3	SArRNA02
gi|29165615|ref|NC_002745.2|	510424	+	C	6	2	0	3	1	SArRNA02
gi|29165615|ref|NC_002745.2|	510425	+	U	2	1	2	1	5	SArRNA02
gi|29165615|ref|NC_002745.2|	510426	+	U	2	6	0	0	4	SArRNA02
gi|29165615|ref|NC_002745.2|	510427	+	A	3	1	0	1	3	SArRNA02
gi|29165615|ref|NC_002745.2|	510428	+	U	1	2	2	0	3	SArRNA02
gi|29165615|ref|NC_002745.2|	510429	+	C	1	2	4	1	2	SArRNA02
gi|29165615|ref|NC_002745.2|	510430	+	G	0	2	1	1	3	SArRNA02
gi|29165615|ref|NC_002745.2|	510433	+	G	0	0	0	0	1	SArRNA02
gi|29165615|ref|NC_002745.2|	510434	+	U	0	0	0	0	1	SArRNA02
gi|29165615|ref|NC_002745.2|	510435	+	G	0	0	0	0	1	SArRNA02
gi|29165615|ref|NC_002745.2|	510436	+	G	0	0	1	1	0	SArRNA02
gi|29165615|ref|NC_002745.2|	510437	+	G	5	3	0	0	2	SArRNA02
gi|29165615|ref|NC_002745.2|	510438	+	A	3	5	4	1	2	SArRNA02
gi|29165615|ref|NC_002745.2|	510439	+	G	1	3	3	0	0	SArRNA02
gi|29165615|ref|NC_002745.2|	510440	+	A	4	5	2	2	3	SArRNA02
gi|29165615|ref|NC_002745.2|	510441	+	C	16	22	5	9	20	SArRNA02
gi|29165615|ref|NC_002745.2|	510442	+	A	5	9	2	5	1	SArRNA02
gi|29165615|ref|NC_002745.2|	510443	+	G	3	1	0	3	5	SArRNA02
gi|29165615|ref|NC_002745.2|	510444	+	U	0	0	0	4	2	SArRNA02
gi|29165615|ref|NC_002745.2|	510445	+	G	1	0	0	1	1	SArRNA02
gi|29165615|ref|NC_002745.2|	510446	+	U	4	1	0	1	6	SArRNA02
gi|29165615|ref|NC_002745.2|	510447	+	C	5	0	1	0	2	SArRNA02
gi|29165615|ref|NC_002745.2|	510448	+	A	0	1	1	1	1	SArRNA02
gi|29165615|ref|NC_002745.2|	510449	+	A	0	0	0	2	3	SArRNA02
gi|29165615|ref|NC_002745.2|	510450	+	G	0	2	0	1	0	SArRNA02
gi|29165615|ref|NC_002745.2|	510451	+	C	1	5	2	2	1	SArRNA02
gi|29165615|ref|NC_002745.2|	510452	+	G	4	1	0	0	3	SArRNA02
gi|29165615|ref|NC_002745.2|	510453	+	G	3	3	0	0	1	SArRNA02
gi|29165615|ref|NC_002745.2|	510454	+	G	2	1	0	0	3	SArRNA02
gi|29165615|ref|NC_002745.2|	510455	+	C	9	3	2	2	1	SArRNA02
gi|29165615|ref|NC_002745.2|	510456	+	A	0	3	1	1	2	SArRNA02
gi|29165615|ref|NC_002745.2|	510457	+	G	0	0	1	0	0	SArRNA02
gi|29165615|ref|NC_002745.2|	510458	+	U	3	1	0	3	3	SArRNA02
gi|29165615|ref|NC_002745.2|	510459	+	U	4	5	0	1	3	SArRNA02
gi|29165615|ref|NC_002745.2|	510460	+	U	1	3	1	0	4	SArRNA02

gi|29165615|ref|NC_002745.2|	510461 +	G	0	2	3	0	1	SArRNA02
gi|29165615|ref|NC_002745.2|	510462 +	A	0	2	0	1	0	SArRNA02
gi|29165615|ref|NC_002745.2|	510463 +	C	6	7	1	0	0	SArRNA02
gi|29165615|ref|NC_002745.2|	510464 +	U	5	1	2	1	2	SArRNA02
gi|29165615|ref|NC_002745.2|	510465 +	G	0	2	0	0	1	SArRNA02
gi|29165615|ref|NC_002745.2|	510466 +	G	1	0	0	0	0	SArRNA02
gi|29165615|ref|NC_002745.2|	510469 +	C	0	0	0	0	1	SArRNA02
gi|29165615|ref|NC_002745.2|	510472 +	U	1	0	0	0	0	SArRNA02
gi|29165615|ref|NC_002745.2|	510473 +	C	0	0	1	0	2	SArRNA02
gi|29165615|ref|NC_002745.2|	510475 +	C	1	1	0	0	0	SArRNA02
gi|29165615|ref|NC_002745.2|	510476 +	C	0	3	0	0	0	SArRNA02
gi|29165615|ref|NC_002745.2|	510477 +	U	0	1	0	1	0	SArRNA02
gi|29165615|ref|NC_002745.2|	510478 +	C	0	4	2	0	0	SArRNA02
gi|29165615|ref|NC_002745.2|	510479 +	C	4	0	1	1	3	SArRNA02
gi|29165615|ref|NC_002745.2|	510481 +	A	0	1	0	1	2	SArRNA02
gi|29165615|ref|NC_002745.2|	510482 +	A	0	0	1	0	0	SArRNA02
gi|29165615|ref|NC_002745.2|	510483 +	A	17	29	0	5	14	SArRNA02
gi|29165615|ref|NC_002745.2|	510484 +	A	4	7	1	1	7	SArRNA02
gi|29165615|ref|NC_002745.2|	510485 +	G	0	1	0	0	0	SArRNA02
gi|29165615|ref|NC_002745.2|	510486 +	G	0	1	0	0	1	SArRNA02
gi|29165615|ref|NC_002745.2|	510487 +	U	2	1	2	2	1	SArRNA02
gi|29165615|ref|NC_002745.2|	510488 +	A	0	0	0	0	1	SArRNA02
gi|29165615|ref|NC_002745.2|	510490 +	C	1	5	0	1	4	SArRNA02
gi|29165615|ref|NC_002745.2|	510491 +	G	0	1	2	0	0	SArRNA02
gi|29165615|ref|NC_002745.2|	510492 +	G	5	1	0	0	0	SArRNA02
gi|29165615|ref|NC_002745.2|	510493 +	A	1	1	1	0	1	SArRNA02
gi|29165615|ref|NC_002745.2|	510494 +	G	0	0	0	1	0	SArRNA02
gi|29165615|ref|NC_002745.2|	510495 +	G	5	1	2	0	0	SArRNA02
gi|29165615|ref|NC_002745.2|	510496 +	C	1	0	0	0	0	SArRNA02
gi|29165615|ref|NC_002745.2|	510498 +	C	3	1	0	0	2	SArRNA02
gi|29165615|ref|NC_002745.2|	510499 +	U	3	1	1	0	0	SArRNA02
gi|29165615|ref|NC_002745.2|	510500 +	C	0	2	0	2	0	SArRNA02
gi|29165615|ref|NC_002745.2|	510501 +	A	0	0	0	0	1	SArRNA02
gi|29165615|ref|NC_002745.2|	510502 +	A	0	0	0	0	1	SArRNA02
gi|29165615|ref|NC_002745.2|	510503 +	A	2	0	0	0	0	SArRNA02
gi|29165615|ref|NC_002745.2|	510504 +	G	0	1	0	0	0	SArRNA02
gi|29165615|ref|NC_002745.2|	510505 +	G	0	1	0	0	1	SArRNA02
gi|29165615|ref|NC_002745.2|	510506 +	U	0	3	0	1	0	SArRNA02
gi|29165615|ref|NC_002745.2|	510507 +	U	1	2	0	0	9	SArRNA02
gi|29165615|ref|NC_002745.2|	510508 +	C	6	2	2	1	1	SArRNA02
gi|29165615|ref|NC_002745.2|	510509 +	C	1	6	2	0	1	SArRNA02
gi|29165615|ref|NC_002745.2|	510510 +	C	3	1	0	2	2	SArRNA02
gi|29165615|ref|NC_002745.2|	510511 +	U	0	0	1	0	0	SArRNA02
gi|29165615|ref|NC_002745.2|	510512 +	C	13	2	5	3	6	SArRNA02
gi|29165615|ref|NC_002745.2|	510513 +	A	1	1	0	0	1	SArRNA02
gi|29165615|ref|NC_002745.2|	510515 +	A	3	3	0	1	3	SArRNA02
gi|29165615|ref|NC_002745.2|	510516 +	A	3	7	4	3	5	SArRNA02
gi|29165615|ref|NC_002745.2|	510517 +	U	3	2	2	1	6	SArRNA02
gi|29165615|ref|NC_002745.2|	510518 +	G	4	6	0	2	5	SArRNA02
gi|29165615|ref|NC_002745.2|	510519 +	G	3	13	2	0	8	SArRNA02
gi|29165615|ref|NC_002745.2|	510520 +	U	3	2	2	4	7	SArRNA02
gi|29165615|ref|NC_002745.2|	510521 +	U	0	3	1	1	3	SArRNA02
gi|29165615|ref|NC_002745.2|	510522 +	G	6	4	4	2	6	SArRNA02
gi|29165615|ref|NC_002745.2|	510523 +	G	0	2	1	0	1	SArRNA02
gi|29165615|ref|NC_002745.2|	510524 +	A	0	2	0	0	1	SArRNA02
gi|29165615|ref|NC_002745.2|	510525 +	A	1	4	1	0	1	SArRNA02
gi|29165615|ref|NC_002745.2|	510526 +	A	9	70	8	4	20	SArRNA02
gi|29165615|ref|NC_002745.2|	510527 +	U	0	2	1	0	1	SArRNA02
gi|29165615|ref|NC_002745.2|	510528 +	C	2	4	1	4	3	SArRNA02
gi|29165615|ref|NC_002745.2|	510529 +	A	0	4	4	1	4	SArRNA02
gi|29165615|ref|NC_002745.2|	510530 +	U	0	1	1	0	4	SArRNA02
gi|29165615|ref|NC_002745.2|	510531 +	U	1	3	4	3	3	SArRNA02
gi|29165615|ref|NC_002745.2|	510532 +	C	5	5	2	0	4	SArRNA02
gi|29165615|ref|NC_002745.2|	510533 +	A	2	5	5	1	3	SArRNA02
gi|29165615|ref|NC_002745.2|	510534 +	U	3	4	2	2	7	SArRNA02
gi|29165615|ref|NC_002745.2|	510535 +	A	7	4	3	2	6	SArRNA02
gi|29165615|ref|NC_002745.2|	510536 +	G	1	2	5	2	5	SArRNA02
gi|29165615|ref|NC_002745.2|	510537 +	A	1	3	0	0	2	SArRNA02
gi|29165615|ref|NC_002745.2|	510538 +	G	3	2	1	2	7	SArRNA02
gi|29165615|ref|NC_002745.2|	510540 +	G	4	3	0	1	3	SArRNA02
gi|29165615|ref|NC_002745.2|	510541 +	U	0	4	0	0	3	SArRNA02
gi|29165615|ref|NC_002745.2|	510542 +	A	1	1	3	0	1	SArRNA02
gi|29165615|ref|NC_002745.2|	510543 +	A	4	6	0	3	4	SArRNA02
gi|29165615|ref|NC_002745.2|	510544 +	A	5	0	1	0	1	SArRNA02
gi|29165615|ref|NC_002745.2|	510545 +	G	1	5	2	0	1	SArRNA02
gi|29165615|ref|NC_002745.2|	510546 +	G	2	3	1	2	6	SArRNA02
gi|29165615|ref|NC_002745.2|	510547 +	C	1	2	0	0	1	SArRNA02
gi|29165615|ref|NC_002745.2|	510548 +	A	4	1	4	3	5	SArRNA02
gi|29165615|ref|NC_002745.2|	510549 +	U	6	7	4	4	12	SArRNA02
gi|29165615|ref|NC_002745.2|	510550 +	A	0	1	1	0	4	SArRNA02
gi|29165615|ref|NC_002745.2|	510551 +	A	0	1	0	0	0	SArRNA02

gi|29165615|ref|NC_002745.2|	510553	+	G	0	0	0	0	1 SArRNA02
gi|29165615|ref|NC_002745.2|	510554	+	G	0	1	1	0	0 SArRNA02
gi|29165615|ref|NC_002745.2|	510557	+	C	1	1	0	0	0 SArRNA02
gi|29165615|ref|NC_002745.2|	510563	+	U	6	0	2	2	4 SArRNA02
gi|29165615|ref|NC_002745.2|	510564	+	G	0	1	0	0	0 SArRNA02
gi|29165615|ref|NC_002745.2|	510565	+	C	0	3	0	1	0 SArRNA02
gi|29165615|ref|NC_002745.2|	510566	+	G	0	1	0	1	0 SArRNA02
gi|29165615|ref|NC_002745.2|	510567	+	A	0	2	0	0	0 SArRNA02
gi|29165615|ref|NC_002745.2|	510568	+	G	0	0	0	0	2 SArRNA02
gi|29165615|ref|NC_002745.2|	510570	+	C	1	0	0	0	0 SArRNA02
gi|29165615|ref|NC_002745.2|	510572	+	U	0	1	0	0	1 SArRNA02
gi|29165615|ref|NC_002745.2|	510573	+	A	1	0	0	0	0 SArRNA02
gi|29165615|ref|NC_002745.2|	510574	+	C	7	9	0	6	5 SArRNA02
gi|29165615|ref|NC_002745.2|	510575	+	A	3	4	3	0	1 SArRNA02
gi|29165615|ref|NC_002745.2|	510576	+	A	1	0	0	0	0 SArRNA02
gi|29165615|ref|NC_002745.2|	510577	+	G	1	0	0	0	1 SArRNA02
gi|29165615|ref|NC_002745.2|	510578	+	U	0	0	0	0	2 SArRNA02
gi|29165615|ref|NC_002745.2|	510579	+	C	3	2	1	0	5 SArRNA02
gi|29165615|ref|NC_002745.2|	510581	+	A	0	0	1	0	1 SArRNA02
gi|29165615|ref|NC_002745.2|	510582	+	G	0	0	0	0	2 SArRNA02
gi|29165615|ref|NC_002745.2|	510584	+	A	0	0	1	0	2 SArRNA02
gi|29165615|ref|NC_002745.2|	510591	+	A	0	0	0	0	1 SArRNA02
gi|29165615|ref|NC_002745.2|	510592	+	A	0	2	0	0	1 SArRNA02
gi|29165615|ref|NC_002745.2|	510595	+	A	1	0	0	0	0 SArRNA02
gi|29165615|ref|NC_002745.2|	510596	+	C	1	0	0	0	0 SArRNA02
gi|29165615|ref|NC_002745.2|	510600	+	C	1	2	0	0	0 SArRNA02
gi|29165615|ref|NC_002745.2|	510603	+	A	1	0	0	0	0 SArRNA02
gi|29165615|ref|NC_002745.2|	510606	+	G	1	1	0	0	0 SArRNA02
gi|29165615|ref|NC_002745.2|	510609	+	C	0	1	0	2	1 SArRNA02
gi|29165615|ref|NC_002745.2|	510610	+	C	0	0	1	0	0 SArRNA02
gi|29165615|ref|NC_002745.2|	510622	+	A	0	0	0	0	2 SArRNA02
gi|29165615|ref|NC_002745.2|	510626	+	A	0	0	0	0	1 SArRNA02
gi|29165615|ref|NC_002745.2|	510627	+	A	0	0	0	0	1 SArRNA02
gi|29165615|ref|NC_002745.2|	510633	+	A	0	0	0	0	1 SArRNA02
gi|29165615|ref|NC_002745.2|	510635	+	C	1	1	0	0	0 SArRNA02
gi|29165615|ref|NC_002745.2|	510637	+	C	0	0	0	0	1 SArRNA02
gi|29165615|ref|NC_002745.2|	510639	+	C	0	1	0	0	0 SArRNA02
gi|29165615|ref|NC_002745.2|	510640	+	A	2	1	0	0	0 SArRNA02
gi|29165615|ref|NC_002745.2|	510641	+	A	0	1	0	0	0 SArRNA02
gi|29165615|ref|NC_002745.2|	510642	+	C	0	1	0	0	0 SArRNA02
gi|29165615|ref|NC_002745.2|	510644	+	G	0	0	0	1	0 SArRNA02
gi|29165615|ref|NC_002745.2|	510646	+	U	1	0	0	1	0 SArRNA02
gi|29165615|ref|NC_002745.2|	510652	+	C	0	1	0	0	0 SArRNA02
gi|29165615|ref|NC_002745.2|	510654	+	A	0	0	0	0	1 SArRNA02
gi|29165615|ref|NC_002745.2|	510655	+	C	1	2	0	0	0 SArRNA02
gi|29165615|ref|NC_002745.2|	510656	+	C	0	1	1	0	0 SArRNA02
gi|29165615|ref|NC_002745.2|	510657	+	C	1	0	0	0	0 SArRNA02
gi|29165615|ref|NC_002745.2|	510658	+	C	0	1	1	0	0 SArRNA02
gi|29165615|ref|NC_002745.2|	510659	+	G	2	1	0	0	0 SArRNA02
gi|29165615|ref|NC_002745.2|	510663	+	A	0	0	0	1	0 SArRNA02
gi|29165615|ref|NC_002745.2|	510666	+	A	1	0	0	0	0 SArRNA02
gi|29165615|ref|NC_002745.2|	510667	+	C	0	1	0	2	2 SArRNA02
gi|29165615|ref|NC_002745.2|	510668	+	A	0	1	0	0	0 SArRNA02
gi|29165615|ref|NC_002745.2|	510675	+	U	0	1	0	0	0 SArRNA02
gi|29165615|ref|NC_002745.2|	510676	+	C	5	1	0	0	0 SArRNA02
gi|29165615|ref|NC_002745.2|	510677	+	U	0	1	1	0	0 SArRNA02
gi|29165615|ref|NC_002745.2|	510678	+	C	0	3	2	1	2 SArRNA02
gi|29165615|ref|NC_002745.2|	510679	+	C	12	4	4	2	4 SArRNA02
gi|29165615|ref|NC_002745.2|	510680	+	C	3	1	1	0	0 SArRNA02
gi|29165615|ref|NC_002745.2|	510681	+	C	0	1	1	0	0 SArRNA02
gi|29165615|ref|NC_002745.2|	510682	+	C	0	0	0	1	0 SArRNA02
gi|29165615|ref|NC_002745.2|	510684	+	A	0	0	0	1	1 SArRNA02
gi|29165615|ref|NC_002745.2|	510686	+	A	0	2	0	0	2 SArRNA02
gi|29165615|ref|NC_002745.2|	510689	+	U	0	1	0	0	0 SArRNA02
gi|29165615|ref|NC_002745.2|	510690	+	C	8	17	4	1	14 SArRNA02
gi|29165615|ref|NC_002745.2|	510691	+	A	5	6	0	4	1 SArRNA02
gi|29165615|ref|NC_002745.2|	510692	+	C	11	17	8	7	9 SArRNA02
gi|29165615|ref|NC_002745.2|	510693	+	A	8	12	0	1	6 SArRNA02
gi|29165615|ref|NC_002745.2|	510694	+	U	0	0	0	1	0 SArRNA02
gi|29165615|ref|NC_002745.2|	510695	+	C	0	1	1	2	2 SArRNA02
gi|29165615|ref|NC_002745.2|	510696	+	G	1	0	0	0	0 SArRNA02
gi|29165615|ref|NC_002745.2|	510697	+	A	0	0	0	1	0 SArRNA02
gi|29165615|ref|NC_002745.2|	510698	+	C	3	2	0	0	1 SArRNA02
gi|29165615|ref|NC_002745.2|	510699	+	G	0	1	0	0	0 SArRNA02
gi|29165615|ref|NC_002745.2|	510700	+	G	0	0	1	0	0 SArRNA02
gi|29165615|ref|NC_002745.2|	510704	+	G	1	0	0	0	0 SArRNA02
gi|29165615|ref|NC_002745.2|	510705	+	G	1	0	0	0	1 SArRNA02
gi|29165615|ref|NC_002745.2|	510706	+	U	0	0	0	0	1 SArRNA02
gi|29165615|ref|NC_002745.2|	510707	+	U	2	16	1	2	5 SArRNA02
gi|29165615|ref|NC_002745.2|	510708	+	U	56	109	5	2	58 SArRNA02
gi|29165615|ref|NC_002745.2|	510711	+	C	12	22	2	7	28 SArRNA02

gi|29165615|ref|NC_002745.2|	510712	+	A	6	5	0	1	3	SArRNA02
gi|29165615|ref|NC_002745.2|	510713	+	C	3	4	2	1	3	SArRNA02
gi|29165615|ref|NC_002745.2|	510714	+	C	2	7	2	3	4	SArRNA02
gi|29165615|ref|NC_002745.2|	510715	+	U	3	2	1	0	4	SArRNA02
gi|29165615|ref|NC_002745.2|	510716	+	C	5	18	1	6	18	SArRNA02
gi|29165615|ref|NC_002745.2|	510717	+	G	6	30	5	4	12	SArRNA02
gi|29165615|ref|NC_002745.2|	510718	+	A	1	0	0	1	0	SArRNA02
gi|29165615|ref|NC_002745.2|	510719	+	U	0	0	0	0	1	SArRNA02
gi|29165615|ref|NC_002745.2|	510720	+	G	2	0	0	0	0	SArRNA02
gi|29165615|ref|NC_002745.2|	510721	+	U	0	1	0	0	3	SArRNA02
gi|29165615|ref|NC_002745.2|	510722	+	C	5	9	5	4	2	SArRNA02
gi|29165615|ref|NC_002745.2|	510723	+	G	1	2	0	0	1	SArRNA02
gi|29165615|ref|NC_002745.2|	510724	+	G	0	0	0	1	1	SArRNA02
gi|29165615|ref|NC_002745.2|	510725	+	C	0	4	3	1	1	SArRNA02
gi|29165615|ref|NC_002745.2|	510726	+	U	2	3	1	1	7	SArRNA02
gi|29165615|ref|NC_002745.2|	510727	+	C	39	40	4	15	40	SArRNA02
gi|29165615|ref|NC_002745.2|	510728	+	A	21	29	4	7	14	SArRNA02
gi|29165615|ref|NC_002745.2|	510729	+	U	8	2	3	3	9	SArRNA02
gi|29165615|ref|NC_002745.2|	510730	+	C	10	16	6	3	9	SArRNA02
gi|29165615|ref|NC_002745.2|	510731	+	G	5	5	2	2	9	SArRNA02
gi|29165615|ref|NC_002745.2|	510732	+	C	23	25	12	8	15	SArRNA02
gi|29165615|ref|NC_002745.2|	510733	+	A	8	16	7	5	17	SArRNA02
gi|29165615|ref|NC_002745.2|	510734	+	U	27	9	6	8	14	SArRNA02
gi|29165615|ref|NC_002745.2|	510735	+	C	49	12	8	7	26	SArRNA02
gi|29165615|ref|NC_002745.2|	510736	+	C	59	19	14	10	20	SArRNA02
gi|29165615|ref|NC_002745.2|	510737	+	U	26	16	9	10	13	SArRNA02
gi|29165615|ref|NC_002745.2|	510738	+	G	0	1	0	0	1	SArRNA02
gi|29165615|ref|NC_002745.2|	510739	+	G	4	5	4	3	3	SArRNA02
gi|29165615|ref|NC_002745.2|	510740	+	G	32	10	5	11	15	SArRNA02
gi|29165615|ref|NC_002745.2|	510741	+	G	3	4	1	1	1	SArRNA02
gi|29165615|ref|NC_002745.2|	510742	+	C	3	8	2	4	5	SArRNA02
gi|29165615|ref|NC_002745.2|	510743	+	U	8	3	4	4	17	SArRNA02
gi|29165615|ref|NC_002745.2|	510744	+	G	10	13	3	3	10	SArRNA02
gi|29165615|ref|NC_002745.2|	510745	+	U	6	4	5	4	12	SArRNA02
gi|29165615|ref|NC_002745.2|	510746	+	A	6	1	1	3	3	SArRNA02
gi|29165615|ref|NC_002745.2|	510747	+	G	3	8	1	2	6	SArRNA02
gi|29165615|ref|NC_002745.2|	510748	+	U	31	50	6	14	34	SArRNA02
gi|29165615|ref|NC_002745.2|	510749	+	C	13	22	6	5	19	SArRNA02
gi|29165615|ref|NC_002745.2|	510750	+	G	21	19	14	6	9	SArRNA02
gi|29165615|ref|NC_002745.2|	510751	+	G	21	21	8	11	17	SArRNA02
gi|29165615|ref|NC_002745.2|	510752	+	U	26	17	10	5	9	SArRNA02
gi|29165615|ref|NC_002745.2|	510753	+	C	21	28	5	9	17	SArRNA02
gi|29165615|ref|NC_002745.2|	510754	+	C	28	26	10	9	23	SArRNA02
gi|29165615|ref|NC_002745.2|	510755	+	C	24	15	10	8	17	SArRNA02
gi|29165615|ref|NC_002745.2|	510756	+	A	6	7	1	1	6	SArRNA02
gi|29165615|ref|NC_002745.2|	510757	+	A	6	6	0	6	10	SArRNA02
gi|29165615|ref|NC_002745.2|	510758	+	G	4	3	1	2	0	SArRNA02
gi|29165615|ref|NC_002745.2|	510759	+	G	12	3	2	6	13	SArRNA02
gi|29165615|ref|NC_002745.2|	510760	+	G	10	10	5	3	9	SArRNA02
gi|29165615|ref|NC_002745.2|	510761	+	U	13	3	2	3	3	SArRNA02
gi|29165615|ref|NC_002745.2|	510762	+	U	2	0	1	1	5	SArRNA02
gi|29165615|ref|NC_002745.2|	510763	+	G	2	3	3	0	7	SArRNA02
gi|29165615|ref|NC_002745.2|	510764	+	G	1	2	0	1	2	SArRNA02
gi|29165615|ref|NC_002745.2|	510765	+	G	1	2	0	0	3	SArRNA02
gi|29165615|ref|NC_002745.2|	510766	+	C	7	0	0	1	3	SArRNA02
gi|29165615|ref|NC_002745.2|	510767	+	U	12	2	1	0	0	SArRNA02
gi|29165615|ref|NC_002745.2|	510768	+	G	24	0	7	1	3	SArRNA02
gi|29165615|ref|NC_002745.2|	510769	+	U	1	0	0	0	0	SArRNA02
gi|29165615|ref|NC_002745.2|	510770	+	U	0	3	0	0	2	SArRNA02
gi|29165615|ref|NC_002745.2|	510771	+	C	106	105	44	53	73	SArRNA02
gi|29165615|ref|NC_002745.2|	510772	+	G	14	28	3	11	28	SArRNA02
gi|29165615|ref|NC_002745.2|	510773	+	C	18	13	2	4	13	SArRNA02
gi|29165615|ref|NC_002745.2|	510774	+	C	5	10	2	1	7	SArRNA02
gi|29165615|ref|NC_002745.2|	510775	+	C	1	8	4	0	9	SArRNA02
gi|29165615|ref|NC_002745.2|	510776	+	A	5	3	2	1	9	SArRNA02
gi|29165615|ref|NC_002745.2|	510777	+	U	9	2	5	4	11	SArRNA02
gi|29165615|ref|NC_002745.2|	510778	+	U	3	6	0	3	7	SArRNA02
gi|29165615|ref|NC_002745.2|	510779	+	A	9	6	2	3	4	SArRNA02
gi|29165615|ref|NC_002745.2|	510780	+	A	6	6	2	2	9	SArRNA02
gi|29165615|ref|NC_002745.2|	510781	+	A	4	15	0	2	10	SArRNA02
gi|29165615|ref|NC_002745.2|	510782	+	G	7	8	3	0	3	SArRNA02
gi|29165615|ref|NC_002745.2|	510783	+	C	16	7	3	2	10	SArRNA02
gi|29165615|ref|NC_002745.2|	510784	+	G	7	4	2	3	1	SArRNA02
gi|29165615|ref|NC_002745.2|	510785	+	G	3	10	0	1	4	SArRNA02
gi|29165615|ref|NC_002745.2|	510786	+	U	29	38	14	9	21	SArRNA02
gi|29165615|ref|NC_002745.2|	510787	+	A	2	4	1	1	1	SArRNA02
gi|29165615|ref|NC_002745.2|	510788	+	C	4	4	1	3	2	SArRNA02
gi|29165615|ref|NC_002745.2|	510789	+	G	2	5	2	2	3	SArRNA02
gi|29165615|ref|NC_002745.2|	510790	+	C	17	18	6	3	11	SArRNA02
gi|29165615|ref|NC_002745.2|	510791	+	G	8	12	4	1	7	SArRNA02
gi|29165615|ref|NC_002745.2|	510792	+	A	7	3	2	2	11	SArRNA02

gi|29165615|ref|NC_002745.2|	510793	+	G	1	1	2	1	3	SArRNA02
gi|29165615|ref|NC_002745.2|	510794	+	C	7	13	0	3	10	SArRNA02
gi|29165615|ref|NC_002745.2|	510795	+	U	2	16	3	1	12	SArRNA02
gi|29165615|ref|NC_002745.2|	510796	+	G	1	0	0	1	2	SArRNA02
gi|29165615|ref|NC_002745.2|	510797	+	G	1	1	0	0	0	SArRNA02
gi|29165615|ref|NC_002745.2|	510798	+	G	0	1	0	1	1	SArRNA02
gi|29165615|ref|NC_002745.2|	510799	+	U	2	0	0	0	2	SArRNA02
gi|29165615|ref|NC_002745.2|	510800	+	U	0	2	0	0	6	SArRNA02
gi|29165615|ref|NC_002745.2|	510801	+	C	15	29	8	12	22	SArRNA02
gi|29165615|ref|NC_002745.2|	510802	+	A	1	8	2	0	6	SArRNA02
gi|29165615|ref|NC_002745.2|	510804	+	A	1	3	0	0	0	SArRNA02
gi|29165615|ref|NC_002745.2|	510805	+	A	2	1	0	0	0	SArRNA02
gi|29165615|ref|NC_002745.2|	510806	+	C	6	17	1	4	16	SArRNA02
gi|29165615|ref|NC_002745.2|	510807	+	G	6	8	3	2	3	SArRNA02
gi|29165615|ref|NC_002745.2|	510808	+	U	1	2	2	1	1	SArRNA02
gi|29165615|ref|NC_002745.2|	510809	+	C	1	9	4	1	4	SArRNA02
gi|29165615|ref|NC_002745.2|	510810	+	G	6	1	2	3	1	SArRNA02
gi|29165615|ref|NC_002745.2|	510811	+	U	3	6	2	2	10	SArRNA02
gi|29165615|ref|NC_002745.2|	510812	+	G	1	3	1	2	0	SArRNA02
gi|29165615|ref|NC_002745.2|	510813	+	A	1	2	1	0	2	SArRNA02
gi|29165615|ref|NC_002745.2|	510815	+	A	4	2	1	2	3	SArRNA02
gi|29165615|ref|NC_002745.2|	510816	+	C	4	4	0	4	0	SArRNA02
gi|29165615|ref|NC_002745.2|	510817	+	A	2	7	2	3	1	SArRNA02
gi|29165615|ref|NC_002745.2|	510818	+	G	0	3	0	0	2	SArRNA02
gi|29165615|ref|NC_002745.2|	510819	+	U	2	0	0	0	0	SArRNA02
gi|29165615|ref|NC_002745.2|	510820	+	U	0	1	0	0	3	SArRNA02
gi|29165615|ref|NC_002745.2|	510821	+	C	4	3	0	0	7	SArRNA02
gi|29165615|ref|NC_002745.2|	510822	+	G	1	2	0	0	1	SArRNA02
gi|29165615|ref|NC_002745.2|	510823	+	G	2	1	0	0	0	SArRNA02
gi|29165615|ref|NC_002745.2|	510824	+	U	0	0	0	0	1	SArRNA02
gi|29165615|ref|NC_002745.2|	510825	+	C	3	9	3	2	6	SArRNA02
gi|29165615|ref|NC_002745.2|	510826	+	C	1	11	0	1	6	SArRNA02
gi|29165615|ref|NC_002745.2|	510827	+	C	6	17	0	1	4	SArRNA02
gi|29165615|ref|NC_002745.2|	510828	+	U	8	25	1	0	5	SArRNA02
gi|29165615|ref|NC_002745.2|	510829	+	A	4	17	2	2	7	SArRNA02
gi|29165615|ref|NC_002745.2|	510830	+	U	1	0	2	1	2	SArRNA02
gi|29165615|ref|NC_002745.2|	510831	+	C	2	8	1	0	5	SArRNA02
gi|29165615|ref|NC_002745.2|	510832	+	C	11	10	2	8	11	SArRNA02
gi|29165615|ref|NC_002745.2|	510833	+	G	2	15	3	4	5	SArRNA02
gi|29165615|ref|NC_002745.2|	510834	+	U	8	1	0	0	4	SArRNA02
gi|29165615|ref|NC_002745.2|	510835	+	C	3	3	2	1	3	SArRNA02
gi|29165615|ref|NC_002745.2|	510836	+	G	0	2	1	0	0	SArRNA02
gi|29165615|ref|NC_002745.2|	510837	+	U	6	2	2	0	1	SArRNA02
gi|29165615|ref|NC_002745.2|	510838	+	G	0	1	0	0	2	SArRNA02
gi|29165615|ref|NC_002745.2|	510839	+	G	1	2	0	0	1	SArRNA02
gi|29165615|ref|NC_002745.2|	510840	+	G	0	1	0	0	0	SArRNA02
gi|29165615|ref|NC_002745.2|	510841	+	C	6	4	0	1	2	SArRNA02
gi|29165615|ref|NC_002745.2|	510842	+	G	6	5	2	1	3	SArRNA02
gi|29165615|ref|NC_002745.2|	510843	+	U	1	11	2	6	6	SArRNA02
gi|29165615|ref|NC_002745.2|	510844	+	A	1	1	0	0	2	SArRNA02
gi|29165615|ref|NC_002745.2|	510845	+	G	0	2	0	0	1	SArRNA02
gi|29165615|ref|NC_002745.2|	510846	+	G	1	1	1	0	1	SArRNA02
gi|29165615|ref|NC_002745.2|	510847	+	A	0	2	0	3	1	SArRNA02
gi|29165615|ref|NC_002745.2|	510848	+	A	10	6	2	2	6	SArRNA02
gi|29165615|ref|NC_002745.2|	510849	+	A	13	64	3	6	24	SArRNA02
gi|29165615|ref|NC_002745.2|	510850	+	U	10	9	3	3	6	SArRNA02
gi|29165615|ref|NC_002745.2|	510851	+	U	10	5	1	3	12	SArRNA02
gi|29165615|ref|NC_002745.2|	510852	+	U	7	8	3	3	7	SArRNA02
gi|29165615|ref|NC_002745.2|	510853	+	G	12	7	2	4	9	SArRNA02
gi|29165615|ref|NC_002745.2|	510854	+	A	0	2	1	0	1	SArRNA02
gi|29165615|ref|NC_002745.2|	510855	+	G	2	3	2	2	0	SArRNA02
gi|29165615|ref|NC_002745.2|	510856	+	A	3	2	1	3	10	SArRNA02
gi|29165615|ref|NC_002745.2|	510857	+	G	1	2	1	0	1	SArRNA02
gi|29165615|ref|NC_002745.2|	510858	+	G	5	1	2	4	6	SArRNA02
gi|29165615|ref|NC_002745.2|	510859	+	A	6	3	1	5	7	SArRNA02
gi|29165615|ref|NC_002745.2|	510860	+	G	5	4	1	0	1	SArRNA02
gi|29165615|ref|NC_002745.2|	510861	+	C	1	1	2	2	3	SArRNA02
gi|29165615|ref|NC_002745.2|	510862	+	U	2	1	1	0	4	SArRNA02
gi|29165615|ref|NC_002745.2|	510863	+	G	0	2	0	0	1	SArRNA02
gi|29165615|ref|NC_002745.2|	510864	+	U	3	3	0	1	2	SArRNA02
gi|29165615|ref|NC_002745.2|	510865	+	C	6	1	4	0	4	SArRNA02
gi|29165615|ref|NC_002745.2|	510866	+	C	4	4	0	1	6	SArRNA02
gi|29165615|ref|NC_002745.2|	510867	+	U	4	4	4	1	3	SArRNA02
gi|29165615|ref|NC_002745.2|	510868	+	U	7	8	3	4	0	SArRNA02
gi|29165615|ref|NC_002745.2|	510869	+	A	2	4	1	1	3	SArRNA02
gi|29165615|ref|NC_002745.2|	510870	+	G	2	3	1	0	4	SArRNA02
gi|29165615|ref|NC_002745.2|	510871	+	U	1	14	3	1	3	SArRNA02
gi|29165615|ref|NC_002745.2|	510872	+	A	8	9	1	0	1	SArRNA02
gi|29165615|ref|NC_002745.2|	510873	+	C	7	11	2	2	3	SArRNA02
gi|29165615|ref|NC_002745.2|	510874	+	G	2	9	2	2	8	SArRNA02
gi|29165615|ref|NC_002745.2|	510875	+	A	2	1	1	1	1	SArRNA02

gi|29165615|ref|NC_002745.2|	510876	+	G	0	0	0	0	2	SArRNA02
gi|29165615|ref|NC_002745.2|	510877	+	A	2	4	0	2	1	SArRNA02
gi|29165615|ref|NC_002745.2|	510878	+	G	4	2	0	0	1	SArRNA02
gi|29165615|ref|NC_002745.2|	510879	+	G	0	0	0	1	2	SArRNA02
gi|29165615|ref|NC_002745.2|	510880	+	A	3	1	0	1	0	SArRNA02
gi|29165615|ref|NC_002745.2|	510881	+	C	3	9	0	0	3	SArRNA02
gi|29165615|ref|NC_002745.2|	510882	+	C	9	10	3	6	15	SArRNA02
gi|29165615|ref|NC_002745.2|	510883	+	G	2	5	1	0	4	SArRNA02
gi|29165615|ref|NC_002745.2|	510884	+	G	1	2	0	2	1	SArRNA02
gi|29165615|ref|NC_002745.2|	510885	+	G	6	0	0	4	8	SArRNA02
gi|29165615|ref|NC_002745.2|	510886	+	A	14	9	2	3	5	SArRNA02
gi|29165615|ref|NC_002745.2|	510887	+	U	12	11	1	0	4	SArRNA02
gi|29165615|ref|NC_002745.2|	510888	+	G	4	0	0	2	2	SArRNA02
gi|29165615|ref|NC_002745.2|	510889	+	G	14	1	0	0	3	SArRNA02
gi|29165615|ref|NC_002745.2|	510890	+	A	23	2	3	1	7	SArRNA02
gi|29165615|ref|NC_002745.2|	510891	+	C	3	9	4	3	6	SArRNA02
gi|29165615|ref|NC_002745.2|	510892	+	A	4	21	3	7	12	SArRNA02
gi|29165615|ref|NC_002745.2|	510893	+	U	9	19	8	7	25	SArRNA02
gi|29165615|ref|NC_002745.2|	510894	+	A	13	10	1	4	10	SArRNA02
gi|29165615|ref|NC_002745.2|	510895	+	C	11	6	4	2	8	SArRNA02
gi|29165615|ref|NC_002745.2|	510896	+	C	8	5	5	1	7	SArRNA02
gi|29165615|ref|NC_002745.2|	510897	+	U	4	1	5	4	6	SArRNA02
gi|29165615|ref|NC_002745.2|	510898	+	C	8	1	2	4	6	SArRNA02
gi|29165615|ref|NC_002745.2|	510899	+	U	3	3	0	1	7	SArRNA02
gi|29165615|ref|NC_002745.2|	510900	+	G	1	5	3	2	2	SArRNA02
gi|29165615|ref|NC_002745.2|	510901	+	G	2	3	3	6	6	SArRNA02
gi|29165615|ref|NC_002745.2|	510902	+	U	8	9	3	1	12	SArRNA02
gi|29165615|ref|NC_002745.2|	510903	+	G	7	9	2	5	5	SArRNA02
gi|29165615|ref|NC_002745.2|	510904	+	U	5	7	3	3	12	SArRNA02
gi|29165615|ref|NC_002745.2|	510905	+	A	8	7	5	3	10	SArRNA02
gi|29165615|ref|NC_002745.2|	510906	+	C	12	12	4	4	11	SArRNA02
gi|29165615|ref|NC_002745.2|	510907	+	C	312	108	47	55	199	SArRNA02
gi|29165615|ref|NC_002745.2|	510908	+	A	26	5	4	9	33	SArRNA02
gi|29165615|ref|NC_002745.2|	510909	+	G	4	1	0	3	11	SArRNA02
gi|29165615|ref|NC_002745.2|	510910	+	U	6	0	3	1	9	SArRNA02
gi|29165615|ref|NC_002745.2|	510911	+	U	4	5	1	0	4	SArRNA02
gi|29165615|ref|NC_002745.2|	510912	+	G	1	3	1	0	8	SArRNA02
gi|29165615|ref|NC_002745.2|	510913	+	U	1	4	0	0	7	SArRNA02
gi|29165615|ref|NC_002745.2|	510914	+	C	4	2	1	1	0	SArRNA02
gi|29165615|ref|NC_002745.2|	510915	+	G	0	2	0	1	1	SArRNA02
gi|29165615|ref|NC_002745.2|	510916	+	U	0	0	0	0	1	SArRNA02
gi|29165615|ref|NC_002745.2|	510918	+	C	1	6	0	0	2	SArRNA02
gi|29165615|ref|NC_002745.2|	510919	+	C	5	10	0	3	2	SArRNA02
gi|29165615|ref|NC_002745.2|	510920	+	A	3	18	0	0	8	SArRNA02
gi|29165615|ref|NC_002745.2|	510921	+	A	1	25	1	0	12	SArRNA02
gi|29165615|ref|NC_002745.2|	510922	+	C	2	7	4	1	1	SArRNA02
gi|29165615|ref|NC_002745.2|	510923	+	G	1	8	0	1	3	SArRNA02
gi|29165615|ref|NC_002745.2|	510925	+	C	0	4	0	3	1	SArRNA02
gi|29165615|ref|NC_002745.2|	510926	+	A	1	5	0	1	3	SArRNA02
gi|29165615|ref|NC_002745.2|	510927	+	U	1	1	0	0	3	SArRNA02
gi|29165615|ref|NC_002745.2|	510928	+	A	4	1	0	0	2	SArRNA02
gi|29165615|ref|NC_002745.2|	510929	+	G	1	2	1	0	1	SArRNA02
gi|29165615|ref|NC_002745.2|	510930	+	C	2	4	1	0	1	SArRNA02
gi|29165615|ref|NC_002745.2|	510931	+	U	3	3	3	0	1	SArRNA02
gi|29165615|ref|NC_002745.2|	510932	+	G	0	0	0	1	0	SArRNA02
gi|29165615|ref|NC_002745.2|	510934	+	G	0	1	0	0	3	SArRNA02
gi|29165615|ref|NC_002745.2|	510935	+	U	6	4	0	0	4	SArRNA02
gi|29165615|ref|NC_002745.2|	510936	+	A	0	1	1	0	0	SArRNA02
gi|29165615|ref|NC_002745.2|	510938	+	C	2	9	0	0	3	SArRNA02
gi|29165615|ref|NC_002745.2|	510939	+	U	2	17	0	1	8	SArRNA02
gi|29165615|ref|NC_002745.2|	510940	+	A	3	8	2	0	0	SArRNA02
gi|29165615|ref|NC_002745.2|	510942	+	G	0	1	0	0	0	SArRNA02
gi|29165615|ref|NC_002745.2|	510943	+	U	0	1	0	0	0	SArRNA02
gi|29165615|ref|NC_002745.2|	510945	+	U	0	1	0	1	1	SArRNA02
gi|29165615|ref|NC_002745.2|	510946	+	G	1	1	0	1	3	SArRNA02
gi|29165615|ref|NC_002745.2|	510947	+	G	3	7	0	1	0	SArRNA02
gi|29165615|ref|NC_002745.2|	510948	+	A	2	0	0	0	0	SArRNA02
gi|29165615|ref|NC_002745.2|	510949	+	C	17	20	1	5	4	SArRNA02
gi|29165615|ref|NC_002745.2|	510950	+	G	3	11	3	4	7	SArRNA02
gi|29165615|ref|NC_002745.2|	510952	+	G	0	0	0	0	2	SArRNA02
gi|29165615|ref|NC_002745.2|	510953	+	A	1	2	1	0	0	SArRNA02
gi|29165615|ref|NC_002745.2|	510954	+	U	1	17	1	1	3	SArRNA02
gi|29165615|ref|NC_002745.2|	510955	+	A	4	23	0	1	10	SArRNA02
gi|29165615|ref|NC_002745.2|	510956	+	A	0	9	1	0	3	SArRNA02
gi|29165615|ref|NC_002745.2|	510957	+	G	1	7	0	0	1	SArRNA02
gi|29165615|ref|NC_002745.2|	510958	+	U	1	0	0	0	6	SArRNA02
gi|29165615|ref|NC_002745.2|	510960	+	C	0	1	0	0	0	SArRNA02
gi|29165615|ref|NC_002745.2|	510961	+	U	0	1	0	0	0	SArRNA02
gi|29165615|ref|NC_002745.2|	510962	+	G	0	1	0	0	0	SArRNA02
gi|29165615|ref|NC_002745.2|	510963	+	A	1	3	0	0	1	SArRNA02
gi|29165615|ref|NC_002745.2|	510964	+	A	0	0	0	0	1	SArRNA02

gi|29165615|ref|NC_002745.2|	510965	+	A	0	2	0	0	0	SArRNA02
gi|29165615|ref|NC_002745.2|	510966	+	G	1	1	0	0	0	SArRNA02
gi|29165615|ref|NC_002745.2|	510967	+	C	1	5	1	1	4	SArRNA02
gi|29165615|ref|NC_002745.2|	510968	+	A	2	3	2	0	1	SArRNA02
gi|29165615|ref|NC_002745.2|	510969	+	U	0	7	1	1	1	SArRNA02
gi|29165615|ref|NC_002745.2|	510970	+	C	3	19	0	1	12	SArRNA02
gi|29165615|ref|NC_002745.2|	510971	+	U	0	11	1	3	5	SArRNA02
gi|29165615|ref|NC_002745.2|	510972	+	A	2	3	0	0	0	SArRNA02
gi|29165615|ref|NC_002745.2|	510973	+	A	0	1	1	0	2	SArRNA02
gi|29165615|ref|NC_002745.2|	510975	+	C	0	1	0	2	1	SArRNA02
gi|29165615|ref|NC_002745.2|	510976	+	A	1	1	0	0	1	SArRNA02
gi|29165615|ref|NC_002745.2|	510978	+	G	0	0	0	1	0	SArRNA02
gi|29165615|ref|NC_002745.2|	510979	+	A	0	0	1	0	0	SArRNA02
gi|29165615|ref|NC_002745.2|	510980	+	A	0	1	0	0	1	SArRNA02
gi|29165615|ref|NC_002745.2|	510981	+	G	1	0	0	0	0	SArRNA02
gi|29165615|ref|NC_002745.2|	510982	+	C	16	21	14	11	19	SArRNA02
gi|29165615|ref|NC_002745.2|	510983	+	C	23	26	14	13	25	SArRNA02
gi|29165615|ref|NC_002745.2|	510984	+	C	32	29	17	8	26	SArRNA02
gi|29165615|ref|NC_002745.2|	510985	+	C	6	7	4	6	11	SArRNA02
gi|29165615|ref|NC_002745.2|	510986	+	C	1	9	1	1	3	SArRNA02
gi|29165615|ref|NC_002745.2|	510987	+	C	0	2	1	0	2	SArRNA02
gi|29165615|ref|NC_002745.2|	510988	+	U	0	2	0	0	0	SArRNA02
gi|29165615|ref|NC_002745.2|	510989	+	C	2	6	1	2	3	SArRNA02
gi|29165615|ref|NC_002745.2|	510990	+	A	1	5	1	0	1	SArRNA02
gi|29165615|ref|NC_002745.2|	510998	+	A	0	0	0	1	0	SArRNA02
gi|29165615|ref|NC_002745.2|	510999	+	U	0	3	0	0	0	SArRNA02
gi|29165615|ref|NC_002745.2|	511000	+	U	0	1	0	0	0	SArRNA02
gi|29165615|ref|NC_002745.2|	511001	+	U	1	0	1	0	1	SArRNA02
gi|29165615|ref|NC_002745.2|	511002	+	C	1	2	0	1	1	SArRNA02
gi|29165615|ref|NC_002745.2|	511003	+	C	0	2	0	0	1	SArRNA02
gi|29165615|ref|NC_002745.2|	511004	+	C	1	3	1	0	1	SArRNA02
gi|29165615|ref|NC_002745.2|	511006	+	A	0	0	2	1	0	SArRNA02
gi|29165615|ref|NC_002745.2|	511007	+	C	1	0	0	0	1	SArRNA02
gi|29165615|ref|NC_002745.2|	511008	+	U	0	2	0	0	0	SArRNA02
gi|29165615|ref|NC_002745.2|	511009	+	U	1	0	0	0	0	SArRNA02
gi|29165615|ref|NC_002745.2|	511010	+	C	14	5	0	4	1	SArRNA02
gi|29165615|ref|NC_002745.2|	511011	+	G	4	0	0	0	0	SArRNA02
gi|29165615|ref|NC_002745.2|	511013	+	U	0	0	0	1	0	SArRNA02
gi|29165615|ref|NC_002745.2|	511014	+	U	0	0	2	0	0	SArRNA02
gi|29165615|ref|NC_002745.2|	511015	+	A	1	0	0	0	2	SArRNA02
gi|29165615|ref|NC_002745.2|	511017	+	A	0	0	0	1	0	SArRNA02
gi|29165615|ref|NC_002745.2|	511018	+	A	0	0	0	0	1	SArRNA02
gi|29165615|ref|NC_002745.2|	511019	+	G	2	1	5	3	0	SArRNA02
gi|29165615|ref|NC_002745.2|	511020	+	A	3	11	0	1	4	SArRNA02
gi|29165615|ref|NC_002745.2|	511021	+	U	6	7	4	3	5	SArRNA02
gi|29165615|ref|NC_002745.2|	511022	+	C	22	19	8	8	13	SArRNA02
gi|29165615|ref|NC_002745.2|	511023	+	C	27	28	12	10	13	-
gi|29165615|ref|NC_002745.2|	511024	+	C	3	6	3	2	2	-
gi|29165615|ref|NC_002745.2|	511025	+	U	4	2	0	0	1	-
gi|29165615|ref|NC_002745.2|	511026	+	C	1	3	0	0	1	-
gi|29165615|ref|NC_002745.2|	511027	+	A	0	3	1	0	1	-
gi|29165615|ref|NC_002745.2|	511028	+	A	0	0	2	1	2	-
gi|29165615|ref|NC_002745.2|	511029	+	A	0	2	0	0	0	-
gi|29165615|ref|NC_002745.2|	511030	+	G	2	0	1	4	1	-
gi|29165615|ref|NC_002745.2|	511031	+	A	2	1	0	1	0	-
gi|29165615|ref|NC_002745.2|	511032	+	U	2	0	1	0	0	-
gi|29165615|ref|NC_002745.2|	511033	+	G	3	1	0	1	1	-
gi|29165615|ref|NC_002745.2|	511034	+	A	4	0	1	1	0	-
gi|29165615|ref|NC_002745.2|	511036	+	G	0	0	0	1	2	-
gi|29165615|ref|NC_002745.2|	511040	+	U	0	4	0	0	0	-
gi|29165615|ref|NC_002745.2|	511041	+	U	0	0	1	0	0	-
gi|29165615|ref|NC_002745.2|	511043	+	A	0	7	0	0	6	-
gi|29165615|ref|NC_002745.2|	511044	+	U	2	1	1	0	0	-
gi|29165615|ref|NC_002745.2|	511045	+	A	0	2	0	0	0	-
gi|29165615|ref|NC_002745.2|	511048	+	U	0	0	0	0	1	-
gi|29165615|ref|NC_002745.2|	511050	+	C	0	3	0	0	1	-
gi|29165615|ref|NC_002745.2|	511051	+	G	3	1	0	1	4	-
gi|29165615|ref|NC_002745.2|	511052	+	A	2	1	0	0	0	-
gi|29165615|ref|NC_002745.2|	511056	+	G	0	0	0	0	1	-
gi|29165615|ref|NC_002745.2|	511057	+	G	1	3	1	2	1	-
gi|29165615|ref|NC_002745.2|	511058	+	A	1	0	0	0	0	-
gi|29165615|ref|NC_002745.2|	511060	+	G	0	0	0	0	1	-
gi|29165615|ref|NC_002745.2|	511061	+	C	0	1	1	2	0	-
gi|29165615|ref|NC_002745.2|	511066	+	U	1	0	0	0	1	-
gi|29165615|ref|NC_002745.2|	511068	+	A	0	1	1	0	0	-
gi|29165615|ref|NC_002745.2|	511069	+	C	2	0	0	0	0	-
gi|29165615|ref|NC_002745.2|	511071	+	U	0	0	0	0	1	-
gi|29165615|ref|NC_002745.2|	511072	+	G	2	0	0	0	0	-
gi|29165615|ref|NC_002745.2|	511073	+	U	1	0	0	1	0	-
gi|29165615|ref|NC_002745.2|	511074	+	G	0	0	1	0	0	-
gi|29165615|ref|NC_002745.2|	511076	+	A	0	0	1	0	0	-

gi|29165615|ref|NC_002745.2|	511083	+	G	0	0	0	1	0	-
gi|29165615|ref|NC_002745.2|	511084	+	A	0	0	1	0	0	-
gi|29165615|ref|NC_002745.2|	511086	+	U	1	0	0	0	0	-
gi|29165615|ref|NC_002745.2|	511087	+	A	1	0	0	0	1	-
gi|29165615|ref|NC_002745.2|	511091	+	A	0	0	0	0	2	-
gi|29165615|ref|NC_002745.2|	511103	+	C	1	0	0	0	0	-
gi|29165615|ref|NC_002745.2|	511109	+	C	3	0	0	0	0	-
gi|29165615|ref|NC_002745.2|	511110	+	A	0	1	0	1	1	-
gi|29165615|ref|NC_002745.2|	511146	+	U	0	1	0	0	0	-
gi|29165615|ref|NC_002745.2|	511159	+	U	40	8	11	6	12	-
gi|29165615|ref|NC_002745.2|	511182	+	U	2	2	2	1	0	-
gi|29165615|ref|NC_002745.2|	511183	+	G	4	7	6	5	11	-
gi|29165615|ref|NC_002745.2|	511184	+	U	2502	2241	1275	977	1934	SArRNA03
gi|29165615|ref|NC_002745.2|	511185	+	C	1	15	3	1	9	SArRNA03
gi|29165615|ref|NC_002745.2|	511186	+	U	7	6	2	4	4	SArRNA03
gi|29165615|ref|NC_002745.2|	511187	+	G	1	0	1	0	1	SArRNA03
gi|29165615|ref|NC_002745.2|	511188	+	G	3	0	0	0	2	SArRNA03
gi|29165615|ref|NC_002745.2|	511189	+	U	4	5	3	0	0	SArRNA03
gi|29165615|ref|NC_002745.2|	511190	+	G	5	10	4	2	2	SArRNA03
gi|29165615|ref|NC_002745.2|	511191	+	A	0	0	0	1	0	SArRNA03
gi|29165615|ref|NC_002745.2|	511192	+	C	34	29	12	6	21	SArRNA03
gi|29165615|ref|NC_002745.2|	511193	+	U	8	22	3	2	25	SArRNA03
gi|29165615|ref|NC_002745.2|	511194	+	A	30	44	31	10	27	SArRNA03
gi|29165615|ref|NC_002745.2|	511195	+	U	1	1	0	2	1	SArRNA03
gi|29165615|ref|NC_002745.2|	511196	+	A	1	0	0	1	0	SArRNA03
gi|29165615|ref|NC_002745.2|	511197	+	G	0	0	0	1	0	SArRNA03
gi|29165615|ref|NC_002745.2|	511198	+	C	19	24	16	8	21	SArRNA03
gi|29165615|ref|NC_002745.2|	511199	+	A	4	2	1	2	4	SArRNA03
gi|29165615|ref|NC_002745.2|	511200	+	A	9	4	2	1	4	SArRNA03
gi|29165615|ref|NC_002745.2|	511201	+	G	0	2	0	0	1	SArRNA03
gi|29165615|ref|NC_002745.2|	511202	+	G	1	6	1	1	2	SArRNA03
gi|29165615|ref|NC_002745.2|	511203	+	A	0	0	1	1	1	SArRNA03
gi|29165615|ref|NC_002745.2|	511204	+	G	0	1	1	0	1	SArRNA03
gi|29165615|ref|NC_002745.2|	511206	+	U	0	1	0	0	1	SArRNA03
gi|29165615|ref|NC_002745.2|	511207	+	C	4	16	4	0	13	SArRNA03
gi|29165615|ref|NC_002745.2|	511208	+	A	2	6	0	1	6	SArRNA03
gi|29165615|ref|NC_002745.2|	511209	+	C	68	107	63	56	92	SArRNA03
gi|29165615|ref|NC_002745.2|	511210	+	A	7	25	5	7	11	SArRNA03
gi|29165615|ref|NC_002745.2|	511211	+	C	28	65	26	32	33	SArRNA03
gi|29165615|ref|NC_002745.2|	511212	+	C	8	31	9	15	7	SArRNA03
gi|29165615|ref|NC_002745.2|	511213	+	U	6	3	0	0	4	SArRNA03
gi|29165615|ref|NC_002745.2|	511214	+	G	0	1	0	4	0	SArRNA03
gi|29165615|ref|NC_002745.2|	511215	+	U	0	0	0	0	1	SArRNA03
gi|29165615|ref|NC_002745.2|	511216	+	U	0	2	1	1	1	SArRNA03
gi|29165615|ref|NC_002745.2|	511217	+	C	7	6	4	1	3	SArRNA03
gi|29165615|ref|NC_002745.2|	511218	+	C	1	23	1	0	9	SArRNA03
gi|29165615|ref|NC_002745.2|	511219	+	C	11	31	9	5	24	SArRNA03
gi|29165615|ref|NC_002745.2|	511220	+	A	3	7	1	0	6	SArRNA03
gi|29165615|ref|NC_002745.2|	511221	+	U	0	5	0	0	0	SArRNA03
gi|29165615|ref|NC_002745.2|	511222	+	G	2	2	0	0	0	SArRNA03
gi|29165615|ref|NC_002745.2|	511223	+	C	23	46	4	5	37	SArRNA03
gi|29165615|ref|NC_002745.2|	511224	+	C	70	92	45	26	54	SArRNA03
gi|29165615|ref|NC_002745.2|	511225	+	G	19	17	10	4	16	SArRNA03
gi|29165615|ref|NC_002745.2|	511226	+	A	2	2	1	1	1	SArRNA03
gi|29165615|ref|NC_002745.2|	511228	+	C	24	41	18	9	19	SArRNA03
gi|29165615|ref|NC_002745.2|	511229	+	A	2	9	0	2	6	SArRNA03
gi|29165615|ref|NC_002745.2|	511230	+	C	3	9	1	0	5	SArRNA03
gi|29165615|ref|NC_002745.2|	511231	+	A	1	7	0	1	3	SArRNA03
gi|29165615|ref|NC_002745.2|	511232	+	G	2	11	0	1	3	SArRNA03
gi|29165615|ref|NC_002745.2|	511233	+	A	0	4	1	1	1	SArRNA03
gi|29165615|ref|NC_002745.2|	511234	+	A	1	1	0	1	0	SArRNA03
gi|29165615|ref|NC_002745.2|	511235	+	G	0	0	0	0	2	SArRNA03
gi|29165615|ref|NC_002745.2|	511236	+	U	1	0	0	0	2	SArRNA03
gi|29165615|ref|NC_002745.2|	511237	+	U	2	12	3	0	2	SArRNA03
gi|29165615|ref|NC_002745.2|	511238	+	A	3	0	1	0	2	SArRNA03
gi|29165615|ref|NC_002745.2|	511239	+	A	1	0	0	1	1	SArRNA03
gi|29165615|ref|NC_002745.2|	511241	+	C	2	7	4	5	5	SArRNA03
gi|29165615|ref|NC_002745.2|	511242	+	U	1	2	1	2	0	SArRNA03
gi|29165615|ref|NC_002745.2|	511243	+	C	2	0	0	1	0	SArRNA03
gi|29165615|ref|NC_002745.2|	511244	+	C	2	2	1	0	3	SArRNA03
gi|29165615|ref|NC_002745.2|	511245	+	U	0	2	0	0	0	SArRNA03
gi|29165615|ref|NC_002745.2|	511246	+	U	1	1	0	0	0	SArRNA03
gi|29165615|ref|NC_002745.2|	511247	+	A	0	2	0	0	0	SArRNA03
gi|29165615|ref|NC_002745.2|	511249	+	C	1	3	2	0	1	SArRNA03
gi|29165615|ref|NC_002745.2|	511250	+	G	0	0	0	0	3	SArRNA03
gi|29165615|ref|NC_002745.2|	511251	+	U	1	0	0	0	1	SArRNA03
gi|29165615|ref|NC_002745.2|	511252	+	C	0	7	0	0	4	SArRNA03
gi|29165615|ref|NC_002745.2|	511253	+	G	0	7	0	3	2	SArRNA03
gi|29165615|ref|NC_002745.2|	511254	+	A	0	3	0	0	1	SArRNA03
gi|29165615|ref|NC_002745.2|	511256	+	G	0	0	0	0	1	SArRNA03
gi|29165615|ref|NC_002745.2|	511257	+	G	0	2	1	0	0	SArRNA03

gi|29165615|ref|NC_002745.2|	511258	+	U	0	2	0	0	0	SArRNA03
gi|29165615|ref|NC_002745.2|	511260	+	G	0	0	0	0	1	SArRNA03
gi|29165615|ref|NC_002745.2|	511268	+	U	1	0	0	0	0	SArRNA03
gi|29165615|ref|NC_002745.2|	511269	+	A	0	0	0	0	1	SArRNA03
gi|29165615|ref|NC_002745.2|	511270	+	C	2	2	2	0	4	SArRNA03
gi|29165615|ref|NC_002745.2|	511271	+	G	1	6	1	2	1	SArRNA03
gi|29165615|ref|NC_002745.2|	511272	+	U	0	0	0	0	1	SArRNA03
gi|29165615|ref|NC_002745.2|	511273	+	U	1	5	0	0	2	SArRNA03
gi|29165615|ref|NC_002745.2|	511274	+	C	6	2	3	3	3	SArRNA03
gi|29165615|ref|NC_002745.2|	511275	+	C	0	5	1	0	0	SArRNA03
gi|29165615|ref|NC_002745.2|	511276	+	G	0	1	0	0	2	SArRNA03
gi|29165615|ref|NC_002745.2|	511277	+	C	2	4	2	0	2	SArRNA03
gi|29165615|ref|NC_002745.2|	511278	+	U	0	1	0	1	0	SArRNA03
gi|29165615|ref|NC_002745.2|	511279	+	A	0	0	1	0	0	SArRNA03
gi|29165615|ref|NC_002745.2|	511299	+	A	1	30	8	8	12	-
gi|29165615|ref|NC_002745.2|	511300	+	A	2	69	35	21	48	-
gi|29165615|ref|NC_002745.2|	511301	+	A	3	79	17	19	58	-
gi|29165615|ref|NC_002745.2|	511302	+	A	1	58	11	14	39	-
gi|29165615|ref|NC_002745.2|	511303	+	A	1	10	7	3	13	-
gi|29165615|ref|NC_002745.2|	511304	+	A	0	2	1	0	0	-
gi|29165615|ref|NC_002745.2|	511370	+	A	0	0	1	0	0	-
gi|29165615|ref|NC_002745.2|	511376	+	A	0	0	0	0	2	-
gi|29165615|ref|NC_002745.2|	511377	+	A	0	0	0	0	2	-
gi|29165615|ref|NC_002745.2|	511383	+	A	0	0	0	0	1	-
gi|29165615|ref|NC_002745.2|	511547	+	U	1	0	0	0	0	-
gi|29165615|ref|NC_002745.2|	511548	+	A	0	0	0	0	1	-
gi|29165615|ref|NC_002745.2|	511549	+	C	2	2	2	0	4	-
gi|29165615|ref|NC_002745.2|	511550	+	G	1	6	1	2	1	-
gi|29165615|ref|NC_002745.2|	511551	+	U	0	0	0	0	1	-
gi|29165615|ref|NC_002745.2|	511552	+	U	1	5	0	0	2	-
gi|29165615|ref|NC_002745.2|	511553	+	C	6	2	3	3	3	-
gi|29165615|ref|NC_002745.2|	511554	+	C	0	5	1	0	0	-
gi|29165615|ref|NC_002745.2|	511555	+	G	0	1	0	0	2	-
gi|29165615|ref|NC_002745.2|	511556	+	C	2	4	2	0	2	-
gi|29165615|ref|NC_002745.2|	511557	+	U	0	1	0	1	0	-
gi|29165615|ref|NC_002745.2|	511558	+	A	0	0	1	0	0	-
gi|29165615|ref|NC_002745.2|	511577	+	C	1	30	8	8	12	-
gi|29165615|ref|NC_002745.2|	511578	+	A	2	69	35	21	48	-
gi|29165615|ref|NC_002745.2|	511579	+	A	3	79	17	19	58	-
gi|29165615|ref|NC_002745.2|	511580	+	A	1	58	11	14	39	-
gi|29165615|ref|NC_002745.2|	511581	+	A	1	10	7	3	13	-
gi|29165615|ref|NC_002745.2|	511582	+	A	0	2	1	0	0	-
gi|29165615|ref|NC_002745.2|	511647	+	A	0	0	1	0	0	-
gi|29165615|ref|NC_002745.2|	511653	+	A	0	0	0	0	2	-
gi|29165615|ref|NC_002745.2|	511654	+	A	0	0	0	0	2	-
gi|29165615|ref|NC_002745.2|	511660	+	A	0	0	0	0	1	-
gi|29165615|ref|NC_002745.2|	511824	+	U	1	0	0	0	0	-
gi|29165615|ref|NC_002745.2|	511825	+	A	0	0	0	0	1	-
gi|29165615|ref|NC_002745.2|	511826	+	C	2	2	2	0	4	-
gi|29165615|ref|NC_002745.2|	511827	+	G	1	6	1	2	1	-
gi|29165615|ref|NC_002745.2|	511828	+	U	0	0	0	0	1	-
gi|29165615|ref|NC_002745.2|	511829	+	U	1	5	0	0	2	-
gi|29165615|ref|NC_002745.2|	511830	+	C	6	2	2	2	3	-
gi|29165615|ref|NC_002745.2|	511831	+	C	0	0	1	0	0	-
gi|29165615|ref|NC_002745.2|	511832	+	G	0	0	0	0	1	-
gi|29165615|ref|NC_002745.2|	511834	+	U	0	0	0	1	0	-
gi|29165615|ref|NC_002745.2|	511855	+	A	1	28	8	7	12	-
gi|29165615|ref|NC_002745.2|	511856	+	A	2	67	35	20	48	-
gi|29165615|ref|NC_002745.2|	511857	+	G	3	79	15	19	57	-
gi|29165615|ref|NC_002745.2|	511858	+	A	1	58	11	14	39	-
gi|29165615|ref|NC_002745.2|	511859	+	A	1	10	6	3	13	-
gi|29165615|ref|NC_002745.2|	511860	+	A	0	2	1	0	0	-
gi|29165615|ref|NC_002745.2|	511975	+	G	1	0	0	0	0	-
gi|29165615|ref|NC_002745.2|	514328	+	C	0	0	0	0	1	SA0441
gi|29165615|ref|NC_002745.2|	520109	+	A	0	0	0	1	0	SA0448
gi|29165615|ref|NC_002745.2|	520112	+	A	0	1	1	0	0	SA0448
gi|29165615|ref|NC_002745.2|	522913	+	G	0	1	0	0	0	SA0451
gi|29165615|ref|NC_002745.2|	522914	+	A	0	0	0	0	2	SA0451
gi|29165615|ref|NC_002745.2|	524928	+	U	0	1	0	1	1	SA0453
gi|29165615|ref|NC_002745.2|	534200	+	A	0	2	0	0	1	SA0461
gi|29165615|ref|NC_002745.2|	540110	+	U	0	0	0	0	1	SA0468
gi|29165615|ref|NC_002745.2|	541410	+	A	0	1	2	0	1	SA0469
gi|29165615|ref|NC_002745.2|	541411	+	A	0	1	1	1	3	SA0469
gi|29165615|ref|NC_002745.2|	542354	+	A	1	0	0	0	0	SA0469
gi|29165615|ref|NC_002745.2|	543815	+	C	0	1	1	0	0	SA0470
gi|29165615|ref|NC_002745.2|	548529	+	A	0	1	0	0	0	SA0475
gi|29165615|ref|NC_002745.2|	549615	+	G	3	6	6	5	11	-
gi|29165615|ref|NC_002745.2|	549616	+	U	2460	2216	1260	961	1917	SArRNA04
gi|29165615|ref|NC_002745.2|	549617	+	C	1	15	3	1	9	SArRNA04
gi|29165615|ref|NC_002745.2|	549618	+	U	0	6	1	0	1	SArRNA04
gi|29165615|ref|NC_002745.2|	549619	+	G	1	0	0	0	1	SArRNA04

gi|29165615|ref|NC_002745.2|	549620 +	G	3	0	0	0	2	SArRNA04
gi|29165615|ref|NC_002745.2|	549621 +	U	4	5	3	0	0	SArRNA04
gi|29165615|ref|NC_002745.2|	549622 +	G	5	10	4	2	2	SArRNA04
gi|29165615|ref|NC_002745.2|	549623 +	G	0	0	0	1	0	SArRNA04
gi|29165615|ref|NC_002745.2|	549624 +	C	34	29	12	6	21	SArRNA04
gi|29165615|ref|NC_002745.2|	549625 +	U	8	22	3	2	25	SArRNA04
gi|29165615|ref|NC_002745.2|	549626 +	A	30	44	31	10	27	SArRNA04
gi|29165615|ref|NC_002745.2|	549627 +	U	1	1	0	2	1	SArRNA04
gi|29165615|ref|NC_002745.2|	549628 +	A	1	0	0	1	0	SArRNA04
gi|29165615|ref|NC_002745.2|	549629 +	G	0	0	0	1	0	SArRNA04
gi|29165615|ref|NC_002745.2|	549630 +	C	19	24	16	8	21	SArRNA04
gi|29165615|ref|NC_002745.2|	549631 +	A	4	2	1	2	4	SArRNA04
gi|29165615|ref|NC_002745.2|	549632 +	A	9	4	2	1	4	SArRNA04
gi|29165615|ref|NC_002745.2|	549633 +	G	0	2	0	0	1	SArRNA04
gi|29165615|ref|NC_002745.2|	549634 +	G	1	6	1	1	2	SArRNA04
gi|29165615|ref|NC_002745.2|	549635 +	A	0	0	1	1	1	SArRNA04
gi|29165615|ref|NC_002745.2|	549636 +	G	0	1	1	0	1	SArRNA04
gi|29165615|ref|NC_002745.2|	549638 +	U	0	1	0	0	1	SArRNA04
gi|29165615|ref|NC_002745.2|	549639 +	C	4	16	4	0	13	SArRNA04
gi|29165615|ref|NC_002745.2|	549640 +	A	2	6	0	1	6	SArRNA04
gi|29165615|ref|NC_002745.2|	549641 +	C	68	107	63	56	92	SArRNA04
gi|29165615|ref|NC_002745.2|	549642 +	A	7	25	5	7	11	SArRNA04
gi|29165615|ref|NC_002745.2|	549643 +	C	28	65	26	32	33	SArRNA04
gi|29165615|ref|NC_002745.2|	549644 +	C	8	31	9	15	7	SArRNA04
gi|29165615|ref|NC_002745.2|	549645 +	U	6	3	0	0	4	SArRNA04
gi|29165615|ref|NC_002745.2|	549646 +	G	0	1	0	4	0	SArRNA04
gi|29165615|ref|NC_002745.2|	549647 +	U	0	0	0	0	1	SArRNA04
gi|29165615|ref|NC_002745.2|	549648 +	U	0	2	1	1	1	SArRNA04
gi|29165615|ref|NC_002745.2|	549649 +	C	7	6	4	1	3	SArRNA04
gi|29165615|ref|NC_002745.2|	549650 +	C	1	23	1	0	9	SArRNA04
gi|29165615|ref|NC_002745.2|	549651 +	C	11	31	9	5	24	SArRNA04
gi|29165615|ref|NC_002745.2|	549652 +	A	3	7	1	0	6	SArRNA04
gi|29165615|ref|NC_002745.2|	549653 +	U	0	5	0	0	0	SArRNA04
gi|29165615|ref|NC_002745.2|	549654 +	G	2	2	0	0	0	SArRNA04
gi|29165615|ref|NC_002745.2|	549655 +	C	23	46	4	5	37	SArRNA04
gi|29165615|ref|NC_002745.2|	549656 +	C	70	92	45	26	54	SArRNA04
gi|29165615|ref|NC_002745.2|	549657 +	G	19	17	10	4	16	SArRNA04
gi|29165615|ref|NC_002745.2|	549658 +	A	2	2	1	1	1	SArRNA04
gi|29165615|ref|NC_002745.2|	549660 +	C	24	41	18	9	19	SArRNA04
gi|29165615|ref|NC_002745.2|	549661 +	A	2	9	0	2	6	SArRNA04
gi|29165615|ref|NC_002745.2|	549662 +	C	3	9	1	0	5	SArRNA04
gi|29165615|ref|NC_002745.2|	549663 +	A	1	7	0	1	3	SArRNA04
gi|29165615|ref|NC_002745.2|	549664 +	G	2	11	0	1	3	SArRNA04
gi|29165615|ref|NC_002745.2|	549665 +	A	0	4	1	1	1	SArRNA04
gi|29165615|ref|NC_002745.2|	549666 +	A	1	1	0	1	0	SArRNA04
gi|29165615|ref|NC_002745.2|	549667 +	G	0	0	0	0	2	SArRNA04
gi|29165615|ref|NC_002745.2|	549668 +	U	1	0	0	0	2	SArRNA04
gi|29165615|ref|NC_002745.2|	549669 +	U	2	12	3	0	2	SArRNA04
gi|29165615|ref|NC_002745.2|	549670 +	A	3	0	1	0	2	SArRNA04
gi|29165615|ref|NC_002745.2|	549671 +	A	1	0	0	1	1	SArRNA04
gi|29165615|ref|NC_002745.2|	549673 +	C	2	7	4	5	5	SArRNA04
gi|29165615|ref|NC_002745.2|	549674 +	U	1	2	1	2	0	SArRNA04
gi|29165615|ref|NC_002745.2|	549675 +	C	2	0	0	1	0	SArRNA04
gi|29165615|ref|NC_002745.2|	549676 +	C	2	2	1	0	3	SArRNA04
gi|29165615|ref|NC_002745.2|	549677 +	U	0	2	0	0	0	SArRNA04
gi|29165615|ref|NC_002745.2|	549678 +	U	1	1	0	0	0	SArRNA04
gi|29165615|ref|NC_002745.2|	549679 +	A	0	2	0	0	0	SArRNA04
gi|29165615|ref|NC_002745.2|	549681 +	C	1	3	2	0	1	SArRNA04
gi|29165615|ref|NC_002745.2|	549682 +	G	0	0	0	0	3	SArRNA04
gi|29165615|ref|NC_002745.2|	549683 +	U	1	0	0	0	1	SArRNA04
gi|29165615|ref|NC_002745.2|	549684 +	C	0	7	0	0	4	SArRNA04
gi|29165615|ref|NC_002745.2|	549685 +	G	0	7	0	3	2	SArRNA04
gi|29165615|ref|NC_002745.2|	549686 +	A	0	3	0	0	1	SArRNA04
gi|29165615|ref|NC_002745.2|	549688 +	G	0	0	0	0	1	SArRNA04
gi|29165615|ref|NC_002745.2|	549689 +	G	0	2	1	0	0	SArRNA04
gi|29165615|ref|NC_002745.2|	549690 +	U	0	2	0	0	0	SArRNA04
gi|29165615|ref|NC_002745.2|	549692 +	G	0	0	0	0	1	SArRNA04
gi|29165615|ref|NC_002745.2|	549700 +	U	1	0	0	0	0	SArRNA04
gi|29165615|ref|NC_002745.2|	549701 +	A	0	0	0	0	1	SArRNA04
gi|29165615|ref|NC_002745.2|	549702 +	C	2	2	2	0	4	SArRNA04
gi|29165615|ref|NC_002745.2|	549703 +	G	1	6	1	2	1	SArRNA04
gi|29165615|ref|NC_002745.2|	549704 +	U	0	0	0	0	1	SArRNA04
gi|29165615|ref|NC_002745.2|	549705 +	U	1	5	0	0	2	SArRNA04
gi|29165615|ref|NC_002745.2|	549706 +	C	6	2	3	3	3	SArRNA04
gi|29165615|ref|NC_002745.2|	549707 +	C	0	5	1	0	0	SArRNA04
gi|29165615|ref|NC_002745.2|	549708 +	G	0	1	0	0	2	SArRNA04
gi|29165615|ref|NC_002745.2|	549709 +	C	2	4	2	0	2	SArRNA04
gi|29165615|ref|NC_002745.2|	549710 +	U	0	1	0	1	0	SArRNA04
gi|29165615|ref|NC_002745.2|	549743 +	G	14	12	14	5	8	SAtRNA06
gi|29165615|ref|NC_002745.2|	549753 +	C	0	1	0	0	0	SAtRNA06
gi|29165615|ref|NC_002745.2|	549756 +	A	0	0	1	0	0	SAtRNA06

gi|29165615|ref|NC_002745.2|	549758	+	C	0	0	0	0	1	SAtRNA06
gi|29165615|ref|NC_002745.2|	549772	+	G	0	0	0	0	1	SAtRNA06
gi|29165615|ref|NC_002745.2|	549774	+	C	1	0	0	0	0	SAtRNA06
gi|29165615|ref|NC_002745.2|	549778	+	C	0	0	0	0	2	SAtRNA06
gi|29165615|ref|NC_002745.2|	549793	+	C	1	0	0	0	0	SAtRNA06
gi|29165615|ref|NC_002745.2|	549866	+	C	0	0	1	1	0	SAtRNA07
gi|29165615|ref|NC_002745.2|	549867	+	U	1	0	0	0	0	SAtRNA07
gi|29165615|ref|NC_002745.2|	549927	+	C	0	1	0	0	0	SAtRNA08
gi|29165615|ref|NC_002745.2|	549954	+	A	1	0	0	0	0	SAtRNA08
gi|29165615|ref|NC_002745.2|	549955	+	U	0	1	0	0	0	SAtRNA08
gi|29165615|ref|NC_002745.2|	549956	+	C	1	0	0	0	0	SAtRNA08
gi|29165615|ref|NC_002745.2|	550023	+	G	11	6	10	6	3	SAtRNA09
gi|29165615|ref|NC_002745.2|	550026	+	G	0	0	0	0	1	SAtRNA09
gi|29165615|ref|NC_002745.2|	550028	+	A	0	2	0	0	0	SAtRNA09
gi|29165615|ref|NC_002745.2|	550036	+	A	0	2	0	0	0	SAtRNA09
gi|29165615|ref|NC_002745.2|	550160	+	C	0	0	1	0	0	SAtRNA10
gi|29165615|ref|NC_002745.2|	550196	+	U	0	2	0	0	0	-
gi|29165615|ref|NC_002745.2|	550199	+	G	18	9	7	3	13	SAtRNA11
gi|29165615|ref|NC_002745.2|	550211	+	C	0	0	0	1	0	SAtRNA11
gi|29165615|ref|NC_002745.2|	550224	+	C	0	1	0	0	0	SAtRNA11
gi|29165615|ref|NC_002745.2|	550238	+	U	0	1	0	0	0	SAtRNA11
gi|29165615|ref|NC_002745.2|	550239	+	C	0	0	0	0	1	SAtRNA11
gi|29165615|ref|NC_002745.2|	550240	+	A	0	1	0	0	1	SAtRNA11
gi|29165615|ref|NC_002745.2|	550241	+	A	1	0	0	0	0	SAtRNA11
gi|29165615|ref|NC_002745.2|	550246	+	U	0	0	1	0	0	SAtRNA11
gi|29165615|ref|NC_002745.2|	550247	+	U	1	0	0	0	0	SAtRNA11
gi|29165615|ref|NC_002745.2|	550296	+	C	2	2	1	1	2	SAtRNA12
gi|29165615|ref|NC_002745.2|	550337	+	A	0	0	2	0	0	SAtRNA12
gi|29165615|ref|NC_002745.2|	550392	+	U	9	5	4	2	4	-
gi|29165615|ref|NC_002745.2|	550393	+	G	1287	1680	1600	664	1174	SAtRNA13
gi|29165615|ref|NC_002745.2|	550394	+	G	1	1	0	1	1	SAtRNA13
gi|29165615|ref|NC_002745.2|	550395	+	G	0	1	0	0	0	SAtRNA13
gi|29165615|ref|NC_002745.2|	550396	+	G	0	0	1	0	0	SAtRNA13
gi|29165615|ref|NC_002745.2|	550403	+	C	1	0	0	0	0	SAtRNA13
gi|29165615|ref|NC_002745.2|	550411	+	G	0	0	0	0	1	SAtRNA13
gi|29165615|ref|NC_002745.2|	550417	+	C	0	0	0	0	2	SAtRNA13
gi|29165615|ref|NC_002745.2|	550424	+	U	2	0	0	0	0	SAtRNA13
gi|29165615|ref|NC_002745.2|	550425	+	U	0	0	0	2	1	SAtRNA13
gi|29165615|ref|NC_002745.2|	550426	+	U	0	0	1	0	0	SAtRNA13
gi|29165615|ref|NC_002745.2|	550429	+	A	1	0	0	0	0	SAtRNA13
gi|29165615|ref|NC_002745.2|	550434	+	G	3	0	0	0	0	SAtRNA13
gi|29165615|ref|NC_002745.2|	550448	+	C	1	0	0	0	0	SAtRNA13
gi|29165615|ref|NC_002745.2|	550480	+	A	0	0	1	0	1	-
gi|29165615|ref|NC_002745.2|	550482	+	G	0	5	3	1	1	-
gi|29165615|ref|NC_002745.2|	550483	+	A	0	5	2	1	8	-
gi|29165615|ref|NC_002745.2|	550484	+	U	0	0	1	0	0	-
gi|29165615|ref|NC_002745.2|	550485	+	G	0	0	0	0	1	-
gi|29165615|ref|NC_002745.2|	550486	+	A	0	4	3	3	6	-
gi|29165615|ref|NC_002745.2|	550487	+	A	0	3	3	2	2	-
gi|29165615|ref|NC_002745.2|	550488	+	C	0	2	4	2	2	-
gi|29165615|ref|NC_002745.2|	550489	+	A	0	6	0	5	2	-
gi|29165615|ref|NC_002745.2|	550490	+	U	1	1	0	0	0	-
gi|29165615|ref|NC_002745.2|	550491	+	U	0	0	0	0	2	-
gi|29165615|ref|NC_002745.2|	550492	+	G	0	3	0	0	1	-
gi|29165615|ref|NC_002745.2|	550493	+	A	0	13	9	10	10	-
gi|29165615|ref|NC_002745.2|	550494	+	A	0	12	8	6	14	-
gi|29165615|ref|NC_002745.2|	550495	+	A	10	63	29	41	79	-
gi|29165615|ref|NC_002745.2|	550496	+	A	6395	28137	20043	16082	34402	-
gi|29165615|ref|NC_002745.2|	550497	+	C	70	1473	732	776	2100	-
gi|29165615|ref|NC_002745.2|	550498	+	U	27	357	170	239	520	-
gi|29165615|ref|NC_002745.2|	550499	+	G	25	349	192	268	521	-
gi|29165615|ref|NC_002745.2|	550500	+	A	18	331	187	337	562	-
gi|29165615|ref|NC_002745.2|	550501	+	A	9	120	68	95	204	-
gi|29165615|ref|NC_002745.2|	550502	+	U	1	16	8	15	25	-
gi|29165615|ref|NC_002745.2|	550503	+	G	15	351	143	245	509	-
gi|29165615|ref|NC_002745.2|	550504	+	A	8	107	76	100	166	-
gi|29165615|ref|NC_002745.2|	550505	+	C	4	51	34	46	58	-
gi|29165615|ref|NC_002745.2|	550506	+	A	12	117	58	86	160	-
gi|29165615|ref|NC_002745.2|	550507	+	A	30	694	296	468	885	-
gi|29165615|ref|NC_002745.2|	550508	+	U	9	120	57	95	175	-
gi|29165615|ref|NC_002745.2|	550509	+	A	15	310	134	245	433	-
gi|29165615|ref|NC_002745.2|	550510	+	U	0	2	4	4	4	-
gi|29165615|ref|NC_002745.2|	550511	+	G	1	30	8	27	40	-
gi|29165615|ref|NC_002745.2|	550512	+	U	22	253	74	209	319	-
gi|29165615|ref|NC_002745.2|	550513	+	C	6	70	35	64	81	-
gi|29165615|ref|NC_002745.2|	550514	+	A	21	383	120	291	509	-
gi|29165615|ref|NC_002745.2|	550515	+	A	7	139	61	103	195	-
gi|29165615|ref|NC_002745.2|	550516	+	C	2	19	5	14	25	-
gi|29165615|ref|NC_002745.2|	550517	+	G	2	37	22	28	83	-
gi|29165615|ref|NC_002745.2|	550518	+	U	3	82	24	42	118	-
gi|29165615|ref|NC_002745.2|	550519	+	U	0	18	7	21	34	-

gi|29165615|ref|NC_002745.2|	550520	+	A	28	438	141	321	656	-
gi|29165615|ref|NC_002745.2|	550521	+	A	13	311	93	221	465	-
gi|29165615|ref|NC_002745.2|	550522	+	U	1	6	4	15	21	-
gi|29165615|ref|NC_002745.2|	550523	+	U	0	13	3	16	24	-
gi|29165615|ref|NC_002745.2|	550524	+	C	6	94	32	91	120	-
gi|29165615|ref|NC_002745.2|	550525	+	C	16	202	87	229	387	-
gi|29165615|ref|NC_002745.2|	550526	+	A	31	557	172	494	1037	-
gi|29165615|ref|NC_002745.2|	550527	+	A	18	283	63	293	593	-
gi|29165615|ref|NC_002745.2|	550528	+	A	3	92	22	117	219	-
gi|29165615|ref|NC_002745.2|	550529	+	A	0	48	10	48	91	-
gi|29165615|ref|NC_002745.2|	550530	+	A	3	83	11	26	98	-
gi|29165615|ref|NC_002745.2|	550531	+	A	2	28	6	5	25	-
gi|29165615|ref|NC_002745.2|	550532	+	C	1	8	5	7	6	-
gi|29165615|ref|NC_002745.2|	550533	+	G	0	2	1	1	2	-
gi|29165615|ref|NC_002745.2|	550534	+	U	0	3	1	2	3	-
gi|29165615|ref|NC_002745.2|	550535	+	A	0	31	17	22	43	-
gi|29165615|ref|NC_002745.2|	550536	+	A	0	41	14	50	59	-
gi|29165615|ref|NC_002745.2|	550537	+	C	0	20	10	27	39	-
gi|29165615|ref|NC_002745.2|	550538	+	U	4	10	1	5	10	-
gi|29165615|ref|NC_002745.2|	550539	+	A	1	18	6	15	38	-
gi|29165615|ref|NC_002745.2|	550540	+	U	1	16	6	24	22	-
gi|29165615|ref|NC_002745.2|	550541	+	A	0	43	16	34	61	-
gi|29165615|ref|NC_002745.2|	550542	+	A	3	54	15	32	45	-
gi|29165615|ref|NC_002745.2|	550543	+	G	4	32	14	36	48	-
gi|29165615|ref|NC_002745.2|	550544	+	U	0	2	2	3	6	-
gi|29165615|ref|NC_002745.2|	550545	+	U	2	5	1	3	9	-
gi|29165615|ref|NC_002745.2|	550546	+	A	1	61	30	55	105	-
gi|29165615|ref|NC_002745.2|	550547	+	C	2	15	7	27	48	-
gi|29165615|ref|NC_002745.2|	550548	+	A	3	135	42	149	204	-
gi|29165615|ref|NC_002745.2|	550549	+	A	50	1002	283	991	1965	-
gi|29165615|ref|NC_002745.2|	550550	+	A	23	505	145	427	892	-
gi|29165615|ref|NC_002745.2|	550551	+	C	5	131	52	124	235	-
gi|29165615|ref|NC_002745.2|	550552	+	A	10	100	54	141	231	-
gi|29165615|ref|NC_002745.2|	550553	+	U	13	141	50	154	228	-
gi|29165615|ref|NC_002745.2|	550554	+	U	17	205	116	180	298	-
gi|29165615|ref|NC_002745.2|	550555	+	A	16	189	116	223	392	-
gi|29165615|ref|NC_002745.2|	550556	+	U	12	246	145	246	506	-
gi|29165615|ref|NC_002745.2|	550557	+	U	13	235	108	248	456	-
gi|29165615|ref|NC_002745.2|	550558	+	U	4	145	62	120	262	-
gi|29165615|ref|NC_002745.2|	550559	+	A	41	743	319	699	1579	-
gi|29165615|ref|NC_002745.2|	550560	+	G	126	1881	925	1908	4827	-
gi|29165615|ref|NC_002745.2|	550561	+	U	581	9005	4237	9137	18497	-
gi|29165615|ref|NC_002745.2|	550562	+	A	652	9769	4105	11799	22778	-
gi|29165615|ref|NC_002745.2|	550563	+	U	177	2450	1176	2819	5923	-
gi|29165615|ref|NC_002745.2|	550564	+	U	71	663	306	1025	1803	-
gi|29165615|ref|NC_002745.2|	550565	+	U	29	455	241	567	1206	-
gi|29165615|ref|NC_002745.2|	550566	+	A	150	1778	944	2409	5056	-
gi|29165615|ref|NC_002745.2|	550567	+	U	6	62	29	68	359	-
gi|29165615|ref|NC_002745.2|	550568	+	G	14	104	37	134	273	-
gi|29165615|ref|NC_002745.2|	550569	+	A	39	588	266	680	1544	-
gi|29165615|ref|NC_002745.2|	550570	+	G	72	1167	427	1302	2372	-
gi|29165615|ref|NC_002745.2|	550571	+	C	62	864	353	1152	2058	-
gi|29165615|ref|NC_002745.2|	550572	+	U	17	159	68	211	348	-
gi|29165615|ref|NC_002745.2|	550573	+	A	27	483	191	605	1085	-
gi|29165615|ref|NC_002745.2|	550574	+	A	15	194	77	253	472	-
gi|29165615|ref|NC_002745.2|	550575	+	U	1	12	2	16	26	-
gi|29165615|ref|NC_002745.2|	550576	+	C	5	77	23	138	156	-
gi|29165615|ref|NC_002745.2|	550577	+	A	27	311	122	535	723	-
gi|29165615|ref|NC_002745.2|	550578	+	A	140	1794	671	2660	3656	-
gi|29165615|ref|NC_002745.2|	550579	+	A	51	654	267	992	1393	-
gi|29165615|ref|NC_002745.2|	550580	+	C	95	1334	554	2375	3232	-
gi|29165615|ref|NC_002745.2|	550581	+	A	459	6162	2598	11041	16052	-
gi|29165615|ref|NC_002745.2|	550582	+	U	20	290	120	650	821	-
gi|29165615|ref|NC_002745.2|	550583	+	C	160	1860	812	3271	5259	-
gi|29165615|ref|NC_002745.2|	550584	+	A	105	1422	601	2312	3778	-
gi|29165615|ref|NC_002745.2|	550585	+	U	121	370	1228	630	972	-
gi|29165615|ref|NC_002745.2|	550586	+	A	895	406	3481	459	1038	-
gi|29165615|ref|NC_002745.2|	550587	+	A	19984	566	8000	630	919	-
gi|29165615|ref|NC_002745.2|	550588	+	U	128816	3410	40795	3380	3824	-
gi|29165615|ref|NC_002745.2|	550589	+	U	440756	11429	58051	10561	12323	SArRNA05
gi|29165615|ref|NC_002745.2|	550590	+	U	610	52	163	67	119	SArRNA05
gi|29165615|ref|NC_002745.2|	550591	+	U	164	14	39	27	34	SArRNA05
gi|29165615|ref|NC_002745.2|	550592	+	U	61	14	19	23	25	SArRNA05
gi|29165615|ref|NC_002745.2|	550593	+	A	51	5	12	10	8	SArRNA05
gi|29165615|ref|NC_002745.2|	550594	+	U	55	6	6	12	16	SArRNA05
gi|29165615|ref|NC_002745.2|	550595	+	G	13	6	3	4	11	SArRNA05
gi|29165615|ref|NC_002745.2|	550596	+	G	20	2	6	12	12	SArRNA05
gi|29165615|ref|NC_002745.2|	550597	+	A	13	0	4	6	6	SArRNA05
gi|29165615|ref|NC_002745.2|	550598	+	G	10	5	5	5	12	SArRNA05
gi|29165615|ref|NC_002745.2|	550599	+	A	15	3	3	3	8	SArRNA05
gi|29165615|ref|NC_002745.2|	550600	+	G	5	4	2	3	3	SArRNA05

gi|29165615|ref|NC_002745.2|	550601	+	U	37	6	3	1	15	SArRNA05
gi|29165615|ref|NC_002745.2|	550602	+	U	8	0	0	3	5	SArRNA05
gi|29165615|ref|NC_002745.2|	550603	+	U	12	2	0	1	4	SArRNA05
gi|29165615|ref|NC_002745.2|	550604	+	G	14	2	2	1	6	SArRNA05
gi|29165615|ref|NC_002745.2|	550605	+	A	5	1	0	2	3	SArRNA05
gi|29165615|ref|NC_002745.2|	550606	+	U	4	0	1	0	4	SArRNA05
gi|29165615|ref|NC_002745.2|	550607	+	C	8	0	2	0	2	SArRNA05
gi|29165615|ref|NC_002745.2|	550608	+	C	37	17	10	8	20	SArRNA05
gi|29165615|ref|NC_002745.2|	550609	+	U	10	5	4	2	8	SArRNA05
gi|29165615|ref|NC_002745.2|	550610	+	G	13	2	0	3	1	SArRNA05
gi|29165615|ref|NC_002745.2|	550611	+	G	6	0	0	0	1	SArRNA05
gi|29165615|ref|NC_002745.2|	550612	+	C	26	1	3	1	2	SArRNA05
gi|29165615|ref|NC_002745.2|	550613	+	U	9	0	2	2	4	SArRNA05
gi|29165615|ref|NC_002745.2|	550614	+	C	29	2	5	2	6	SArRNA05
gi|29165615|ref|NC_002745.2|	550615	+	A	8	3	4	0	4	SArRNA05
gi|29165615|ref|NC_002745.2|	550616	+	G	5	2	0	1	2	SArRNA05
gi|29165615|ref|NC_002745.2|	550617	+	G	5	5	2	2	4	SArRNA05
gi|29165615|ref|NC_002745.2|	550618	+	A	13	0	5	3	2	SArRNA05
gi|29165615|ref|NC_002745.2|	550619	+	U	11	3	0	4	2	SArRNA05
gi|29165615|ref|NC_002745.2|	550620	+	G	14	0	1	5	2	SArRNA05
gi|29165615|ref|NC_002745.2|	550621	+	A	11	2	2	1	0	SArRNA05
gi|29165615|ref|NC_002745.2|	550622	+	A	7	0	5	2	1	SArRNA05
gi|29165615|ref|NC_002745.2|	550623	+	C	101	4	6	0	9	SArRNA05
gi|29165615|ref|NC_002745.2|	550624	+	G	42	5	5	0	3	SArRNA05
gi|29165615|ref|NC_002745.2|	550625	+	C	13	1	0	1	2	SArRNA05
gi|29165615|ref|NC_002745.2|	550626	+	U	18	2	2	0	2	SArRNA05
gi|29165615|ref|NC_002745.2|	550627	+	G	20	0	1	1	2	SArRNA05
gi|29165615|ref|NC_002745.2|	550629	+	C	3	0	2	1	0	SArRNA05
gi|29165615|ref|NC_002745.2|	550630	+	G	0	2	0	0	1	SArRNA05
gi|29165615|ref|NC_002745.2|	550632	+	C	3	1	0	0	2	SArRNA05
gi|29165615|ref|NC_002745.2|	550633	+	G	1	0	0	0	1	SArRNA05
gi|29165615|ref|NC_002745.2|	550634	+	U	1	0	0	0	1	SArRNA05
gi|29165615|ref|NC_002745.2|	550635	+	G	1	0	1	0	1	SArRNA05
gi|29165615|ref|NC_002745.2|	550636	+	C	1	0	0	2	0	SArRNA05
gi|29165615|ref|NC_002745.2|	550637	+	C	6	9	3	3	16	SArRNA05
gi|29165615|ref|NC_002745.2|	550638	+	U	7	14	1	1	7	SArRNA05
gi|29165615|ref|NC_002745.2|	550639	+	A	11	15	6	7	6	SArRNA05
gi|29165615|ref|NC_002745.2|	550640	+	A	15	31	5	2	17	SArRNA05
gi|29165615|ref|NC_002745.2|	550641	+	U	18	34	4	7	20	SArRNA05
gi|29165615|ref|NC_002745.2|	550642	+	A	66	56	12	12	37	SArRNA05
gi|29165615|ref|NC_002745.2|	550643	+	C	228	153	58	98	146	SArRNA05
gi|29165615|ref|NC_002745.2|	550644	+	A	59	44	19	17	47	SArRNA05
gi|29165615|ref|NC_002745.2|	550645	+	U	5	2	3	3	3	SArRNA05
gi|29165615|ref|NC_002745.2|	550646	+	G	6	7	1	7	5	SArRNA05
gi|29165615|ref|NC_002745.2|	550647	+	C	56	45	17	24	33	SArRNA05
gi|29165615|ref|NC_002745.2|	550648	+	A	9	18	4	7	12	SArRNA05
gi|29165615|ref|NC_002745.2|	550649	+	A	4	8	3	1	7	SArRNA05
gi|29165615|ref|NC_002745.2|	550650	+	G	5	11	4	2	2	SArRNA05
gi|29165615|ref|NC_002745.2|	550651	+	U	28	12	4	7	19	SArRNA05
gi|29165615|ref|NC_002745.2|	550652	+	C	64	37	15	25	42	SArRNA05
gi|29165615|ref|NC_002745.2|	550653	+	G	23	21	8	9	17	SArRNA05
gi|29165615|ref|NC_002745.2|	550654	+	A	7	3	3	3	4	SArRNA05
gi|29165615|ref|NC_002745.2|	550655	+	G	6	1	3	2	2	SArRNA05
gi|29165615|ref|NC_002745.2|	550656	+	C	33	19	8	6	15	SArRNA05
gi|29165615|ref|NC_002745.2|	550657	+	G	9	4	3	3	4	SArRNA05
gi|29165615|ref|NC_002745.2|	550658	+	A	15	5	5	2	8	SArRNA05
gi|29165615|ref|NC_002745.2|	550659	+	A	13	29	7	5	17	SArRNA05
gi|29165615|ref|NC_002745.2|	550660	+	C	21	16	7	5	28	SArRNA05
gi|29165615|ref|NC_002745.2|	550661	+	G	10	10	8	4	11	SArRNA05
gi|29165615|ref|NC_002745.2|	550662	+	G	9	13	6	8	8	SArRNA05
gi|29165615|ref|NC_002745.2|	550663	+	A	13	4	3	4	8	SArRNA05
gi|29165615|ref|NC_002745.2|	550664	+	U	52	16	5	7	18	SArRNA05
gi|29165615|ref|NC_002745.2|	550665	+	G	3	1	1	0	2	SArRNA05
gi|29165615|ref|NC_002745.2|	550666	+	A	1	2	0	4	8	SArRNA05
gi|29165615|ref|NC_002745.2|	550667	+	G	2	4	1	0	6	SArRNA05
gi|29165615|ref|NC_002745.2|	550668	+	A	6	6	2	3	9	SArRNA05
gi|29165615|ref|NC_002745.2|	550669	+	A	5	1	3	3	2	SArRNA05
gi|29165615|ref|NC_002745.2|	550670	+	G	0	1	0	0	1	SArRNA05
gi|29165615|ref|NC_002745.2|	550671	+	C	15	18	3	8	9	SArRNA05
gi|29165615|ref|NC_002745.2|	550672	+	U	9	1	1	3	7	SArRNA05
gi|29165615|ref|NC_002745.2|	550673	+	U	7	2	0	1	1	SArRNA05
gi|29165615|ref|NC_002745.2|	550674	+	G	0	2	0	1	1	SArRNA05
gi|29165615|ref|NC_002745.2|	550675	+	C	2	0	2	2	8	SArRNA05
gi|29165615|ref|NC_002745.2|	550676	+	U	4	2	1	3	3	SArRNA05
gi|29165615|ref|NC_002745.2|	550677	+	U	0	1	0	0	0	SArRNA05
gi|29165615|ref|NC_002745.2|	550678	+	C	5	3	2	2	4	SArRNA05
gi|29165615|ref|NC_002745.2|	550679	+	U	0	2	2	1	4	SArRNA05
gi|29165615|ref|NC_002745.2|	550680	+	C	8	8	1	4	2	SArRNA05
gi|29165615|ref|NC_002745.2|	550681	+	U	18	6	6	3	6	SArRNA05
gi|29165615|ref|NC_002745.2|	550682	+	G	13	3	3	2	11	SArRNA05
gi|29165615|ref|NC_002745.2|	550683	+	A	15	7	6	10	10	SArRNA05

gi|29165615|ref|NC_002745.2|	550684	+	U	39	8	2	4	7	SArRNA05
gi|29165615|ref|NC_002745.2|	550685	+	G	14	3	1	4	3	SArRNA05
gi|29165615|ref|NC_002745.2|	550686	+	U	13	7	4	2	4	SArRNA05
gi|29165615|ref|NC_002745.2|	550687	+	U	27	16	11	5	13	SArRNA05
gi|29165615|ref|NC_002745.2|	550688	+	A	17	20	2	5	4	SArRNA05
gi|29165615|ref|NC_002745.2|	550689	+	G	3	2	1	5	1	SArRNA05
gi|29165615|ref|NC_002745.2|	550690	+	C	47	10	12	10	28	SArRNA05
gi|29165615|ref|NC_002745.2|	550691	+	G	20	5	4	1	11	SArRNA05
gi|29165615|ref|NC_002745.2|	550692	+	G	10	0	4	1	5	SArRNA05
gi|29165615|ref|NC_002745.2|	550693	+	C	26	4	9	14	13	SArRNA05
gi|29165615|ref|NC_002745.2|	550694	+	G	15	6	3	5	10	SArRNA05
gi|29165615|ref|NC_002745.2|	550695	+	G	23	4	6	5	8	SArRNA05
gi|29165615|ref|NC_002745.2|	550696	+	A	6	1	0	1	4	SArRNA05
gi|29165615|ref|NC_002745.2|	550697	+	C	89	46	41	42	59	SArRNA05
gi|29165615|ref|NC_002745.2|	550698	+	G	8	13	7	9	5	SArRNA05
gi|29165615|ref|NC_002745.2|	550699	+	G	2	0	1	0	1	SArRNA05
gi|29165615|ref|NC_002745.2|	550700	+	G	1	2	0	0	1	SArRNA05
gi|29165615|ref|NC_002745.2|	550701	+	U	22	8	9	8	5	SArRNA05
gi|29165615|ref|NC_002745.2|	550702	+	G	5	1	2	1	3	SArRNA05
gi|29165615|ref|NC_002745.2|	550703	+	A	0	1	1	2	0	SArRNA05
gi|29165615|ref|NC_002745.2|	550704	+	G	1	2	0	0	2	SArRNA05
gi|29165615|ref|NC_002745.2|	550705	+	U	2	1	1	3	1	SArRNA05
gi|29165615|ref|NC_002745.2|	550706	+	A	5	8	2	5	4	SArRNA05
gi|29165615|ref|NC_002745.2|	550707	+	A	12	50	6	2	24	SArRNA05
gi|29165615|ref|NC_002745.2|	550708	+	C	55	28	10	27	29	SArRNA05
gi|29165615|ref|NC_002745.2|	550709	+	A	12	6	1	4	6	SArRNA05
gi|29165615|ref|NC_002745.2|	550710	+	C	9	1	3	2	5	SArRNA05
gi|29165615|ref|NC_002745.2|	550711	+	G	4	1	2	3	4	SArRNA05
gi|29165615|ref|NC_002745.2|	550712	+	U	7	4	2	2	2	SArRNA05
gi|29165615|ref|NC_002745.2|	550713	+	G	0	0	0	0	1	SArRNA05
gi|29165615|ref|NC_002745.2|	550714	+	G	0	0	1	0	1	SArRNA05
gi|29165615|ref|NC_002745.2|	550715	+	A	2	2	0	0	6	SArRNA05
gi|29165615|ref|NC_002745.2|	550716	+	U	5	2	1	0	1	SArRNA05
gi|29165615|ref|NC_002745.2|	550717	+	A	1	2	0	0	4	SArRNA05
gi|29165615|ref|NC_002745.2|	550718	+	A	0	4	0	2	3	SArRNA05
gi|29165615|ref|NC_002745.2|	550719	+	C	5	5	2	1	6	SArRNA05
gi|29165615|ref|NC_002745.2|	550720	+	C	13	16	5	3	13	SArRNA05
gi|29165615|ref|NC_002745.2|	550721	+	U	13	7	4	4	6	SArRNA05
gi|29165615|ref|NC_002745.2|	550722	+	A	9	5	2	1	8	SArRNA05
gi|29165615|ref|NC_002745.2|	550723	+	C	13	6	4	2	5	SArRNA05
gi|29165615|ref|NC_002745.2|	550724	+	C	49	29	18	17	18	SArRNA05
gi|29165615|ref|NC_002745.2|	550725	+	U	8	12	1	3	10	SArRNA05
gi|29165615|ref|NC_002745.2|	550726	+	A	11	3	4	2	9	SArRNA05
gi|29165615|ref|NC_002745.2|	550727	+	U	14	4	2	8	15	SArRNA05
gi|29165615|ref|NC_002745.2|	550728	+	A	10	9	5	3	12	SArRNA05
gi|29165615|ref|NC_002745.2|	550729	+	A	4	6	1	2	7	SArRNA05
gi|29165615|ref|NC_002745.2|	550730	+	G	18	10	1	7	5	SArRNA05
gi|29165615|ref|NC_002745.2|	550731	+	A	1	3	0	4	3	SArRNA05
gi|29165615|ref|NC_002745.2|	550732	+	C	17	22	8	14	25	SArRNA05
gi|29165615|ref|NC_002745.2|	550733	+	U	10	9	2	7	7	SArRNA05
gi|29165615|ref|NC_002745.2|	550734	+	G	3	5	1	0	6	SArRNA05
gi|29165615|ref|NC_002745.2|	550735	+	G	3	2	0	0	3	SArRNA05
gi|29165615|ref|NC_002745.2|	550736	+	G	6	1	4	1	4	SArRNA05
gi|29165615|ref|NC_002745.2|	550737	+	A	8	1	2	2	3	SArRNA05
gi|29165615|ref|NC_002745.2|	550738	+	U	5	2	0	1	2	SArRNA05
gi|29165615|ref|NC_002745.2|	550739	+	A	6	8	1	2	7	SArRNA05
gi|29165615|ref|NC_002745.2|	550740	+	A	4	4	1	2	4	SArRNA05
gi|29165615|ref|NC_002745.2|	550741	+	C	7	3	2	1	6	SArRNA05
gi|29165615|ref|NC_002745.2|	550742	+	U	10	1	2	2	6	SArRNA05
gi|29165615|ref|NC_002745.2|	550743	+	U	12	2	5	6	7	SArRNA05
gi|29165615|ref|NC_002745.2|	550744	+	C	25	9	12	5	19	SArRNA05
gi|29165615|ref|NC_002745.2|	550745	+	G	26	14	8	6	17	SArRNA05
gi|29165615|ref|NC_002745.2|	550746	+	G	19	5	3	5	14	SArRNA05
gi|29165615|ref|NC_002745.2|	550747	+	G	9	4	5	5	13	SArRNA05
gi|29165615|ref|NC_002745.2|	550748	+	A	7	3	3	2	8	SArRNA05
gi|29165615|ref|NC_002745.2|	550749	+	A	11	6	6	2	4	SArRNA05
gi|29165615|ref|NC_002745.2|	550750	+	A	4	0	3	0	6	SArRNA05
gi|29165615|ref|NC_002745.2|	550751	+	C	2	4	0	0	0	SArRNA05
gi|29165615|ref|NC_002745.2|	550752	+	C	4	4	1	2	4	SArRNA05
gi|29165615|ref|NC_002745.2|	550753	+	G	0	3	2	2	5	SArRNA05
gi|29165615|ref|NC_002745.2|	550754	+	G	4	1	0	1	2	SArRNA05
gi|29165615|ref|NC_002745.2|	550755	+	A	2	2	2	1	2	SArRNA05
gi|29165615|ref|NC_002745.2|	550756	+	G	2	1	0	0	0	SArRNA05
gi|29165615|ref|NC_002745.2|	550757	+	C	5	10	5	3	10	SArRNA05
gi|29165615|ref|NC_002745.2|	550758	+	U	7	3	1	2	8	SArRNA05
gi|29165615|ref|NC_002745.2|	550759	+	A	4	6	2	3	4	SArRNA05
gi|29165615|ref|NC_002745.2|	550760	+	A	21	12	3	5	31	SArRNA05
gi|29165615|ref|NC_002745.2|	550761	+	U	49	32	18	15	52	SArRNA05
gi|29165615|ref|NC_002745.2|	550762	+	A	11	3	5	5	20	SArRNA05
gi|29165615|ref|NC_002745.2|	550763	+	C	29	8	10	9	14	SArRNA05
gi|29165615|ref|NC_002745.2|	550764	+	C	54	19	10	17	61	SArRNA05

gi|29165615|ref|NC_002745.2|	550765	+	G	13	12	4	5	25 SArRNA05
gi|29165615|ref|NC_002745.2|	550766	+	G	8	6	2	2	14 SArRNA05
gi|29165615|ref|NC_002745.2|	550767	+	A	10	5	3	2	23 SArRNA05
gi|29165615|ref|NC_002745.2|	550768	+	U	8	16	6	4	23 SArRNA05
gi|29165615|ref|NC_002745.2|	550769	+	A	12	3	1	4	18 SArRNA05
gi|29165615|ref|NC_002745.2|	550770	+	A	13	26	6	7	35 SArRNA05
gi|29165615|ref|NC_002745.2|	550771	+	U	0	6	8	2	8 SArRNA05
gi|29165615|ref|NC_002745.2|	550772	+	A	1	0	0	1	0 SArRNA05
gi|29165615|ref|NC_002745.2|	550773	+	U	0	1	0	0	0 SArRNA05
gi|29165615|ref|NC_002745.2|	550774	+	U	3	0	1	2	4 SArRNA05
gi|29165615|ref|NC_002745.2|	550775	+	U	2	1	0	0	0 SArRNA05
gi|29165615|ref|NC_002745.2|	550776	+	U	4	3	0	0	4 SArRNA05
gi|29165615|ref|NC_002745.2|	550777	+	G	1	0	0	1	1 SArRNA05
gi|29165615|ref|NC_002745.2|	550778	+	A	7	6	1	2	5 SArRNA05
gi|29165615|ref|NC_002745.2|	550779	+	A	9	13	8	13	14 SArRNA05
gi|29165615|ref|NC_002745.2|	550780	+	C	6	4	2	4	7 SArRNA05
gi|29165615|ref|NC_002745.2|	550781	+	C	12	6	2	5	15 SArRNA05
gi|29165615|ref|NC_002745.2|	550782	+	G	12	9	7	2	19 SArRNA05
gi|29165615|ref|NC_002745.2|	550783	+	C	40	20	17	19	54 SArRNA05
gi|29165615|ref|NC_002745.2|	550784	+	A	83	47	17	22	58 SArRNA05
gi|29165615|ref|NC_002745.2|	550785	+	U	26	18	10	16	25 SArRNA05
gi|29165615|ref|NC_002745.2|	550786	+	G	20	10	2	2	26 SArRNA05
gi|29165615|ref|NC_002745.2|	550787	+	G	5	5	3	2	15 SArRNA05
gi|29165615|ref|NC_002745.2|	550788	+	U	4	3	2	3	16 SArRNA05
gi|29165615|ref|NC_002745.2|	550789	+	U	2	6	0	2	5 SArRNA05
gi|29165615|ref|NC_002745.2|	550790	+	C	12	14	3	7	16 SArRNA05
gi|29165615|ref|NC_002745.2|	550791	+	A	15	12	11	13	13 SArRNA05
gi|29165615|ref|NC_002745.2|	550792	+	A	18	21	8	8	22 SArRNA05
gi|29165615|ref|NC_002745.2|	550793	+	A	26	16	12	10	31 SArRNA05
gi|29165615|ref|NC_002745.2|	550794	+	A	20	17	12	13	35 SArRNA05
gi|29165615|ref|NC_002745.2|	550795	+	G	76	75	26	49	76 SArRNA05
gi|29165615|ref|NC_002745.2|	550796	+	U	6	10	4	9	36 SArRNA05
gi|29165615|ref|NC_002745.2|	550797	+	G	21	18	15	12	23 SArRNA05
gi|29165615|ref|NC_002745.2|	550798	+	A	24	16	5	13	25 SArRNA05
gi|29165615|ref|NC_002745.2|	550799	+	A	227	105	66	139	324 SArRNA05
gi|29165615|ref|NC_002745.2|	550800	+	A	29	15	15	14	27 SArRNA05
gi|29165615|ref|NC_002745.2|	550801	+	G	14	17	19	15	19 SArRNA05
gi|29165615|ref|NC_002745.2|	550802	+	A	2	0	0	0	4 SArRNA05
gi|29165615|ref|NC_002745.2|	550803	+	C	10	11	5	2	7 SArRNA05
gi|29165615|ref|NC_002745.2|	550804	+	G	1	13	2	1	2 SArRNA05
gi|29165615|ref|NC_002745.2|	550805	+	G	2	2	3	3	5 SArRNA05
gi|29165615|ref|NC_002745.2|	550806	+	U	40	11	9	6	15 SArRNA05
gi|29165615|ref|NC_002745.2|	550807	+	C	103	101	20	39	60 SArRNA05
gi|29165615|ref|NC_002745.2|	550808	+	U	22	15	17	10	26 SArRNA05
gi|29165615|ref|NC_002745.2|	550809	+	U	8	6	2	0	14 SArRNA05
gi|29165615|ref|NC_002745.2|	550810	+	G	3	5	2	6	2 SArRNA05
gi|29165615|ref|NC_002745.2|	550811	+	C	16	22	5	4	10 SArRNA05
gi|29165615|ref|NC_002745.2|	550812	+	U	10	5	2	5	10 SArRNA05
gi|29165615|ref|NC_002745.2|	550813	+	G	1	1	1	1	3 SArRNA05
gi|29165615|ref|NC_002745.2|	550814	+	U	3	4	2	2	5 SArRNA05
gi|29165615|ref|NC_002745.2|	550815	+	C	40	43	23	15	25 SArRNA05
gi|29165615|ref|NC_002745.2|	550816	+	A	12	13	9	2	15 SArRNA05
gi|29165615|ref|NC_002745.2|	550817	+	C	14	3	2	4	22 SArRNA05
gi|29165615|ref|NC_002745.2|	550818	+	U	15	8	5	4	13 SArRNA05
gi|29165615|ref|NC_002745.2|	550819	+	U	17	9	3	8	15 SArRNA05
gi|29165615|ref|NC_002745.2|	550820	+	A	16	6	10	2	18 SArRNA05
gi|29165615|ref|NC_002745.2|	550821	+	U	23	17	10	8	15 SArRNA05
gi|29165615|ref|NC_002745.2|	550822	+	A	20	13	4	2	11 SArRNA05
gi|29165615|ref|NC_002745.2|	550823	+	G	20	13	4	7	18 SArRNA05
gi|29165615|ref|NC_002745.2|	550824	+	A	13	23	8	9	16 SArRNA05
gi|29165615|ref|NC_002745.2|	550825	+	U	10	6	3	14	11 SArRNA05
gi|29165615|ref|NC_002745.2|	550826	+	G	2	1	0	6	2 SArRNA05
gi|29165615|ref|NC_002745.2|	550827	+	G	8	15	2	5	5 SArRNA05
gi|29165615|ref|NC_002745.2|	550828	+	A	5	11	4	3	6 SArRNA05
gi|29165615|ref|NC_002745.2|	550829	+	U	7	5	1	1	4 SArRNA05
gi|29165615|ref|NC_002745.2|	550830	+	C	20	8	0	2	12 SArRNA05
gi|29165615|ref|NC_002745.2|	550831	+	C	42	27	12	7	32 SArRNA05
gi|29165615|ref|NC_002745.2|	550832	+	G	10	8	2	9	9 SArRNA05
gi|29165615|ref|NC_002745.2|	550833	+	C	34	11	3	3	13 SArRNA05
gi|29165615|ref|NC_002745.2|	550834	+	G	3	3	0	0	2 SArRNA05
gi|29165615|ref|NC_002745.2|	550835	+	C	11	18	9	5	9 SArRNA05
gi|29165615|ref|NC_002745.2|	550836	+	U	6	11	3	3	6 SArRNA05
gi|29165615|ref|NC_002745.2|	550837	+	G	7	3	0	0	0 SArRNA05
gi|29165615|ref|NC_002745.2|	550838	+	C	34	19	7	12	9 SArRNA05
gi|29165615|ref|NC_002745.2|	550839	+	A	17	27	8	15	18 SArRNA05
gi|29165615|ref|NC_002745.2|	550840	+	U	33	18	14	10	15 SArRNA05
gi|29165615|ref|NC_002745.2|	550841	+	U	24	8	7	17	20 SArRNA05
gi|29165615|ref|NC_002745.2|	550842	+	A	8	3	7	6	8 SArRNA05
gi|29165615|ref|NC_002745.2|	550843	+	G	1	1	3	0	3 SArRNA05
gi|29165615|ref|NC_002745.2|	550844	+	C	108	116	34	56	51 SArRNA05
gi|29165615|ref|NC_002745.2|	550845	+	U	39	14	11	8	19 SArRNA05

gi|29165615|ref|NC_002745.2|	550846	+	A	18	13	4	4	8	SArRNA05
gi|29165615|ref|NC_002745.2|	550847	+	G	11	3	6	4	3	SArRNA05
gi|29165615|ref|NC_002745.2|	550848	+	U	2	5	0	2	1	SArRNA05
gi|29165615|ref|NC_002745.2|	550849	+	U	3	3	0	5	0	SArRNA05
gi|29165615|ref|NC_002745.2|	550850	+	G	0	3	0	2	0	SArRNA05
gi|29165615|ref|NC_002745.2|	550851	+	G	20	6	4	4	14	SArRNA05
gi|29165615|ref|NC_002745.2|	550852	+	U	18	10	6	6	19	SArRNA05
gi|29165615|ref|NC_002745.2|	550853	+	A	18	12	8	8	20	SArRNA05
gi|29165615|ref|NC_002745.2|	550854	+	A	16	15	5	4	8	SArRNA05
gi|29165615|ref|NC_002745.2|	550855	+	G	0	0	1	4	3	SArRNA05
gi|29165615|ref|NC_002745.2|	550856	+	G	11	6	2	1	10	SArRNA05
gi|29165615|ref|NC_002745.2|	550857	+	U	16	6	6	4	13	SArRNA05
gi|29165615|ref|NC_002745.2|	550858	+	A	18	11	6	4	11	SArRNA05
gi|29165615|ref|NC_002745.2|	550859	+	A	47	14	6	5	7	SArRNA05
gi|29165615|ref|NC_002745.2|	550860	+	C	94	98	31	57	42	SArRNA05
gi|29165615|ref|NC_002745.2|	550861	+	G	18	21	5	6	12	SArRNA05
gi|29165615|ref|NC_002745.2|	550862	+	G	3	2	0	0	3	SArRNA05
gi|29165615|ref|NC_002745.2|	550863	+	C	33	17	15	5	20	SArRNA05
gi|29165615|ref|NC_002745.2|	550864	+	U	27	28	10	12	18	SArRNA05
gi|29165615|ref|NC_002745.2|	550865	+	U	60	50	31	39	34	SArRNA05
gi|29165615|ref|NC_002745.2|	550866	+	A	8	5	5	8	12	SArRNA05
gi|29165615|ref|NC_002745.2|	550867	+	C	19	18	7	9	10	SArRNA05
gi|29165615|ref|NC_002745.2|	550868	+	C	148	80	49	51	91	SArRNA05
gi|29165615|ref|NC_002745.2|	550869	+	A	95	69	30	46	51	SArRNA05
gi|29165615|ref|NC_002745.2|	550870	+	A	95	46	31	26	33	SArRNA05
gi|29165615|ref|NC_002745.2|	550871	+	G	45	10	4	6	19	SArRNA05
gi|29165615|ref|NC_002745.2|	550872	+	G	27	6	5	10	23	SArRNA05
gi|29165615|ref|NC_002745.2|	550873	+	C	78	35	24	16	36	SArRNA05
gi|29165615|ref|NC_002745.2|	550874	+	A	46	24	17	14	39	SArRNA05
gi|29165615|ref|NC_002745.2|	550875	+	A	57	23	12	26	44	SArRNA05
gi|29165615|ref|NC_002745.2|	550876	+	C	125	111	39	43	98	SArRNA05
gi|29165615|ref|NC_002745.2|	550877	+	G	162	159	80	79	93	SArRNA05
gi|29165615|ref|NC_002745.2|	550878	+	A	52	31	16	16	34	SArRNA05
gi|29165615|ref|NC_002745.2|	550879	+	U	35	17	10	8	30	SArRNA05
gi|29165615|ref|NC_002745.2|	550880	+	G	16	5	2	5	8	SArRNA05
gi|29165615|ref|NC_002745.2|	550881	+	C	76	28	13	16	11	SArRNA05
gi|29165615|ref|NC_002745.2|	550882	+	A	33	32	8	8	19	SArRNA05
gi|29165615|ref|NC_002745.2|	550883	+	U	49	50	28	52	39	SArRNA05
gi|29165615|ref|NC_002745.2|	550884	+	A	13	13	1	3	5	SArRNA05
gi|29165615|ref|NC_002745.2|	550885	+	G	12	8	1	0	4	SArRNA05
gi|29165615|ref|NC_002745.2|	550886	+	C	10	8	3	2	4	SArRNA05
gi|29165615|ref|NC_002745.2|	550887	+	C	35	60	18	14	31	SArRNA05
gi|29165615|ref|NC_002745.2|	550888	+	G	26	34	5	10	22	SArRNA05
gi|29165615|ref|NC_002745.2|	550889	+	A	10	9	5	5	11	SArRNA05
gi|29165615|ref|NC_002745.2|	550890	+	C	47	65	19	51	46	SArRNA05
gi|29165615|ref|NC_002745.2|	550891	+	C	93	53	28	39	72	SArRNA05
gi|29165615|ref|NC_002745.2|	550892	+	U	71	51	33	45	58	SArRNA05
gi|29165615|ref|NC_002745.2|	550893	+	G	56	36	22	15	44	SArRNA05
gi|29165615|ref|NC_002745.2|	550894	+	A	91	25	15	26	68	SArRNA05
gi|29165615|ref|NC_002745.2|	550895	+	G	53	28	13	16	32	SArRNA05
gi|29165615|ref|NC_002745.2|	550896	+	A	29	15	16	10	29	SArRNA05
gi|29165615|ref|NC_002745.2|	550897	+	G	19	14	8	7	16	SArRNA05
gi|29165615|ref|NC_002745.2|	550898	+	G	16	12	2	5	11	SArRNA05
gi|29165615|ref|NC_002745.2|	550899	+	G	19	7	2	4	22	SArRNA05
gi|29165615|ref|NC_002745.2|	550900	+	U	34	11	8	13	17	SArRNA05
gi|29165615|ref|NC_002745.2|	550901	+	G	22	6	2	11	19	SArRNA05
gi|29165615|ref|NC_002745.2|	550902	+	A	49	22	13	16	21	SArRNA05
gi|29165615|ref|NC_002745.2|	550903	+	U	71	31	10	9	34	SArRNA05
gi|29165615|ref|NC_002745.2|	550904	+	C	95	57	34	34	59	SArRNA05
gi|29165615|ref|NC_002745.2|	550905	+	G	24	21	10	12	6	SArRNA05
gi|29165615|ref|NC_002745.2|	550906	+	G	6	2	1	1	6	SArRNA05
gi|29165615|ref|NC_002745.2|	550907	+	C	29	22	7	10	16	SArRNA05
gi|29165615|ref|NC_002745.2|	550908	+	C	71	61	15	19	23	SArRNA05
gi|29165615|ref|NC_002745.2|	550909	+	A	16	26	10	9	15	SArRNA05
gi|29165615|ref|NC_002745.2|	550910	+	C	145	96	31	49	57	SArRNA05
gi|29165615|ref|NC_002745.2|	550911	+	A	18	35	7	10	19	SArRNA05
gi|29165615|ref|NC_002745.2|	550912	+	C	57	35	19	18	24	SArRNA05
gi|29165615|ref|NC_002745.2|	550913	+	U	54	19	9	15	39	SArRNA05
gi|29165615|ref|NC_002745.2|	550914	+	G	7	9	8	11	29	SArRNA05
gi|29165615|ref|NC_002745.2|	550915	+	G	23	18	6	8	24	SArRNA05
gi|29165615|ref|NC_002745.2|	550916	+	A	65	65	15	25	33	SArRNA05
gi|29165615|ref|NC_002745.2|	550917	+	A	29	18	12	12	9	SArRNA05
gi|29165615|ref|NC_002745.2|	550918	+	C	63	52	23	26	35	SArRNA05
gi|29165615|ref|NC_002745.2|	550919	+	U	50	39	18	31	21	SArRNA05
gi|29165615|ref|NC_002745.2|	550920	+	G	26	17	12	13	20	SArRNA05
gi|29165615|ref|NC_002745.2|	550921	+	A	76	39	26	39	38	SArRNA05
gi|29165615|ref|NC_002745.2|	550922	+	G	55	43	19	32	40	SArRNA05
gi|29165615|ref|NC_002745.2|	550923	+	A	68	24	10	13	27	SArRNA05
gi|29165615|ref|NC_002745.2|	550924	+	C	199	144	42	75	110	SArRNA05
gi|29165615|ref|NC_002745.2|	550925	+	A	26	28	18	16	29	SArRNA05
gi|29165615|ref|NC_002745.2|	550926	+	C	67	44	19	14	33	SArRNA05

gi|29165615|ref|NC_002745.2|	550927	+	G	16	20	15	13	24	SArRNA05
gi|29165615|ref|NC_002745.2|	550928	+	G	24	6	6	16	27	SArRNA05
gi|29165615|ref|NC_002745.2|	550929	+	U	22	15	7	12	20	SArRNA05
gi|29165615|ref|NC_002745.2|	550930	+	C	24	20	11	4	20	SArRNA05
gi|29165615|ref|NC_002745.2|	550931	+	C	129	50	16	21	38	SArRNA05
gi|29165615|ref|NC_002745.2|	550932	+	A	26	14	10	16	17	SArRNA05
gi|29165615|ref|NC_002745.2|	550933	+	G	27	24	9	3	12	SArRNA05
gi|29165615|ref|NC_002745.2|	550934	+	A	17	12	4	11	13	SArRNA05
gi|29165615|ref|NC_002745.2|	550935	+	C	95	56	20	32	37	SArRNA05
gi|29165615|ref|NC_002745.2|	550936	+	U	134	72	21	26	53	SArRNA05
gi|29165615|ref|NC_002745.2|	550937	+	C	76	34	9	10	32	SArRNA05
gi|29165615|ref|NC_002745.2|	550938	+	C	163	71	46	50	116	SArRNA05
gi|29165615|ref|NC_002745.2|	550939	+	U	140	88	39	44	100	SArRNA05
gi|29165615|ref|NC_002745.2|	550940	+	A	50	41	20	31	56	SArRNA05
gi|29165615|ref|NC_002745.2|	550941	+	C	281	173	90	141	129	SArRNA05
gi|29165615|ref|NC_002745.2|	550942	+	G	49	46	13	21	35	SArRNA05
gi|29165615|ref|NC_002745.2|	550943	+	G	68	20	9	28	43	SArRNA05
gi|29165615|ref|NC_002745.2|	550944	+	G	143	89	38	81	95	SArRNA05
gi|29165615|ref|NC_002745.2|	550945	+	A	414	178	120	145	174	SArRNA05
gi|29165615|ref|NC_002745.2|	550946	+	G	62	34	22	29	45	SArRNA05
gi|29165615|ref|NC_002745.2|	550947	+	G	72	52	19	26	45	SArRNA05
gi|29165615|ref|NC_002745.2|	550948	+	C	241	82	50	60	97	SArRNA05
gi|29165615|ref|NC_002745.2|	550949	+	A	187	114	59	66	108	SArRNA05
gi|29165615|ref|NC_002745.2|	550950	+	G	110	63	30	40	71	SArRNA05
gi|29165615|ref|NC_002745.2|	550951	+	C	193	122	46	76	129	SArRNA05
gi|29165615|ref|NC_002745.2|	550952	+	A	194	120	53	91	176	SArRNA05
gi|29165615|ref|NC_002745.2|	550953	+	G	169	103	51	99	185	SArRNA05
gi|29165615|ref|NC_002745.2|	550954	+	U	106	106	40	59	85	SArRNA05
gi|29165615|ref|NC_002745.2|	550955	+	A	77	61	32	35	60	SArRNA05
gi|29165615|ref|NC_002745.2|	550956	+	G	43	29	19	27	44	SArRNA05
gi|29165615|ref|NC_002745.2|	550957	+	G	59	27	19	16	32	SArRNA05
gi|29165615|ref|NC_002745.2|	550958	+	G	37	16	12	17	33	SArRNA05
gi|29165615|ref|NC_002745.2|	550959	+	A	37	26	18	12	25	SArRNA05
gi|29165615|ref|NC_002745.2|	550960	+	A	125	419	50	54	177	SArRNA05
gi|29165615|ref|NC_002745.2|	550961	+	U	75	57	18	44	56	SArRNA05
gi|29165615|ref|NC_002745.2|	550962	+	C	1125	685	259	374	472	SArRNA05
gi|29165615|ref|NC_002745.2|	550963	+	U	189	144	74	109	137	SArRNA05
gi|29165615|ref|NC_002745.2|	550964	+	U	34	16	13	17	25	SArRNA05
gi|29165615|ref|NC_002745.2|	550965	+	C	80	29	14	39	59	SArRNA05
gi|29165615|ref|NC_002745.2|	550966	+	C	182	67	35	53	111	SArRNA05
gi|29165615|ref|NC_002745.2|	550967	+	G	134	45	35	45	90	SArRNA05
gi|29165615|ref|NC_002745.2|	550968	+	C	218	121	48	68	114	SArRNA05
gi|29165615|ref|NC_002745.2|	550969	+	A	139	101	40	66	81	SArRNA05
gi|29165615|ref|NC_002745.2|	550970	+	A	186	241	52	53	210	SArRNA05
gi|29165615|ref|NC_002745.2|	550971	+	U	351	182	56	126	157	SArRNA05
gi|29165615|ref|NC_002745.2|	550972	+	G	147	76	40	71	157	SArRNA05
gi|29165615|ref|NC_002745.2|	550973	+	G	114	66	26	43	102	SArRNA05
gi|29165615|ref|NC_002745.2|	550974	+	G	27	20	4	14	41	SArRNA05
gi|29165615|ref|NC_002745.2|	550975	+	C	66	30	20	26	52	SArRNA05
gi|29165615|ref|NC_002745.2|	550976	+	G	43	31	14	25	58	SArRNA05
gi|29165615|ref|NC_002745.2|	550977	+	A	67	22	17	18	45	SArRNA05
gi|29165615|ref|NC_002745.2|	550978	+	A	28	16	10	18	36	SArRNA05
gi|29165615|ref|NC_002745.2|	550979	+	A	22	9	12	6	16	SArRNA05
gi|29165615|ref|NC_002745.2|	550980	+	G	1	0	0	4	2	SArRNA05
gi|29165615|ref|NC_002745.2|	550981	+	C	35	9	6	17	16	SArRNA05
gi|29165615|ref|NC_002745.2|	550982	+	C	46	21	14	17	31	SArRNA05
gi|29165615|ref|NC_002745.2|	550983	+	U	60	31	18	15	37	SArRNA05
gi|29165615|ref|NC_002745.2|	550984	+	G	46	45	18	23	45	SArRNA05
gi|29165615|ref|NC_002745.2|	550985	+	A	69	23	25	26	50	SArRNA05
gi|29165615|ref|NC_002745.2|	550986	+	C	106	68	28	25	92	SArRNA05
gi|29165615|ref|NC_002745.2|	550987	+	G	90	63	29	38	76	SArRNA05
gi|29165615|ref|NC_002745.2|	550988	+	G	47	36	18	26	37	SArRNA05
gi|29165615|ref|NC_002745.2|	550989	+	A	45	39	13	20	40	SArRNA05
gi|29165615|ref|NC_002745.2|	550990	+	G	33	27	17	22	38	SArRNA05
gi|29165615|ref|NC_002745.2|	550991	+	C	226	107	51	72	121	SArRNA05
gi|29165615|ref|NC_002745.2|	550992	+	A	77	64	31	30	80	SArRNA05
gi|29165615|ref|NC_002745.2|	550993	+	A	39	25	12	27	46	SArRNA05
gi|29165615|ref|NC_002745.2|	550994	+	C	87	53	27	43	83	SArRNA05
gi|29165615|ref|NC_002745.2|	550995	+	G	27	7	8	11	19	SArRNA05
gi|29165615|ref|NC_002745.2|	550996	+	C	50	29	19	38	52	SArRNA05
gi|29165615|ref|NC_002745.2|	550997	+	C	90	58	30	27	78	SArRNA05
gi|29165615|ref|NC_002745.2|	550998	+	G	12	18	8	6	13	SArRNA05
gi|29165615|ref|NC_002745.2|	550999	+	C	34	29	18	24	35	SArRNA05
gi|29165615|ref|NC_002745.2|	551000	+	G	22	21	15	14	38	SArRNA05
gi|29165615|ref|NC_002745.2|	551001	+	U	59	50	17	22	54	SArRNA05
gi|29165615|ref|NC_002745.2|	551002	+	G	63	53	41	39	48	SArRNA05
gi|29165615|ref|NC_002745.2|	551003	+	A	78	65	40	51	84	SArRNA05
gi|29165615|ref|NC_002745.2|	551004	+	G	131	119	58	72	112	SArRNA05
gi|29165615|ref|NC_002745.2|	551005	+	U	94	67	48	45	115	SArRNA05
gi|29165615|ref|NC_002745.2|	551006	+	G	100	78	51	66	84	SArRNA05
gi|29165615|ref|NC_002745.2|	551007	+	A	104	71	44	50	93	SArRNA05

gi|29165615|ref|NC_002745.2|	551008	+	U	88	58	31	48	78	SArRNA05
gi|29165615|ref|NC_002745.2|	551009	+	G	143	79	41	52	67	SArRNA05
gi|29165615|ref|NC_002745.2|	551010	+	A	116	76	33	61	101	SArRNA05
gi|29165615|ref|NC_002745.2|	551011	+	A	153	111	61	88	114	SArRNA05
gi|29165615|ref|NC_002745.2|	551012	+	G	74	58	56	127	81	SArRNA05
gi|29165615|ref|NC_002745.2|	551013	+	G	237	86	56	56	159	SArRNA05
gi|29165615|ref|NC_002745.2|	551014	+	U	117	60	25	32	76	SArRNA05
gi|29165615|ref|NC_002745.2|	551015	+	C	77	27	24	13	37	SArRNA05
gi|29165615|ref|NC_002745.2|	551016	+	U	16	7	6	10	29	SArRNA05
gi|29165615|ref|NC_002745.2|	551017	+	U	15	12	13	11	9	SArRNA05
gi|29165615|ref|NC_002745.2|	551018	+	C	189	108	69	80	107	SArRNA05
gi|29165615|ref|NC_002745.2|	551019	+	G	56	40	23	26	39	SArRNA05
gi|29165615|ref|NC_002745.2|	551020	+	G	100	64	50	53	105	SArRNA05
gi|29165615|ref|NC_002745.2|	551021	+	A	123	74	52	53	158	SArRNA05
gi|29165615|ref|NC_002745.2|	551022	+	U	89	58	45	51	99	SArRNA05
gi|29165615|ref|NC_002745.2|	551023	+	C	55	50	20	29	76	SArRNA05
gi|29165615|ref|NC_002745.2|	551024	+	G	49	47	39	43	88	SArRNA05
gi|29165615|ref|NC_002745.2|	551025	+	U	87	78	79	56	132	SArRNA05
gi|29165615|ref|NC_002745.2|	551026	+	A	29	19	13	13	28	SArRNA05
gi|29165615|ref|NC_002745.2|	551027	+	A	23	18	8	24	35	SArRNA05
gi|29165615|ref|NC_002745.2|	551028	+	A	32	44	12	28	46	SArRNA05
gi|29165615|ref|NC_002745.2|	551029	+	A	112	955	115	29	409	SArRNA05
gi|29165615|ref|NC_002745.2|	551030	+	C	402	305	150	152	272	SArRNA05
gi|29165615|ref|NC_002745.2|	551031	+	U	91	67	25	35	61	SArRNA05
gi|29165615|ref|NC_002745.2|	551032	+	C	55	43	36	22	46	SArRNA05
gi|29165615|ref|NC_002745.2|	551033	+	U	46	22	19	15	40	SArRNA05
gi|29165615|ref|NC_002745.2|	551034	+	G	16	16	7	17	19	SArRNA05
gi|29165615|ref|NC_002745.2|	551035	+	U	25	29	12	16	55	SArRNA05
gi|29165615|ref|NC_002745.2|	551036	+	U	25	36	22	18	48	SArRNA05
gi|29165615|ref|NC_002745.2|	551037	+	A	15	11	6	6	15	SArRNA05
gi|29165615|ref|NC_002745.2|	551038	+	U	46	33	19	44	76	SArRNA05
gi|29165615|ref|NC_002745.2|	551039	+	U	140	101	42	55	121	SArRNA05
gi|29165615|ref|NC_002745.2|	551040	+	A	469	260	191	250	272	SArRNA05
gi|29165615|ref|NC_002745.2|	551041	+	G	18	33	13	10	21	SArRNA05
gi|29165615|ref|NC_002745.2|	551042	+	G	15	18	11	5	13	SArRNA05
gi|29165615|ref|NC_002745.2|	551043	+	G	111	83	47	52	84	SArRNA05
gi|29165615|ref|NC_002745.2|	551044	+	A	32	28	21	15	52	SArRNA05
gi|29165615|ref|NC_002745.2|	551045	+	A	30	30	14	17	40	SArRNA05
gi|29165615|ref|NC_002745.2|	551046	+	G	57	70	21	32	67	SArRNA05
gi|29165615|ref|NC_002745.2|	551047	+	A	55	39	24	31	72	SArRNA05
gi|29165615|ref|NC_002745.2|	551048	+	A	45	44	17	18	50	SArRNA05
gi|29165615|ref|NC_002745.2|	551049	+	C	260	250	65	131	213	SArRNA05
gi|29165615|ref|NC_002745.2|	551050	+	A	103	133	41	56	135	SArRNA05
gi|29165615|ref|NC_002745.2|	551051	+	U	169	173	71	88	154	SArRNA05
gi|29165615|ref|NC_002745.2|	551052	+	A	100	120	34	61	95	SArRNA05
gi|29165615|ref|NC_002745.2|	551053	+	U	91	64	50	46	79	SArRNA05
gi|29165615|ref|NC_002745.2|	551054	+	G	12	19	13	15	24	SArRNA05
gi|29165615|ref|NC_002745.2|	551055	+	U	75	44	28	40	82	SArRNA05
gi|29165615|ref|NC_002745.2|	551056	+	G	57	39	27	49	80	SArRNA05
gi|29165615|ref|NC_002745.2|	551057	+	U	80	57	31	34	115	SArRNA05
gi|29165615|ref|NC_002745.2|	551058	+	A	30	22	14	26	51	SArRNA05
gi|29165615|ref|NC_002745.2|	551059	+	A	42	26	24	26	59	SArRNA05
gi|29165615|ref|NC_002745.2|	551060	+	G	38	39	25	24	53	SArRNA05
gi|29165615|ref|NC_002745.2|	551061	+	U	80	44	43	41	84	SArRNA05
gi|29165615|ref|NC_002745.2|	551062	+	A	41	61	21	24	63	SArRNA05
gi|29165615|ref|NC_002745.2|	551063	+	A	50	51	28	21	70	SArRNA05
gi|29165615|ref|NC_002745.2|	551064	+	C	167	163	24	36	213	SArRNA05
gi|29165615|ref|NC_002745.2|	551065	+	U	88	81	28	38	117	SArRNA05
gi|29165615|ref|NC_002745.2|	551066	+	G	31	38	7	8	36	SArRNA05
gi|29165615|ref|NC_002745.2|	551067	+	U	18	15	6	6	21	SArRNA05
gi|29165615|ref|NC_002745.2|	551068	+	G	11	7	5	2	13	SArRNA05
gi|29165615|ref|NC_002745.2|	551069	+	C	94	53	39	39	70	SArRNA05
gi|29165615|ref|NC_002745.2|	551070	+	A	53	37	25	22	59	SArRNA05
gi|29165615|ref|NC_002745.2|	551071	+	C	224	161	102	98	176	SArRNA05
gi|29165615|ref|NC_002745.2|	551072	+	A	85	69	46	46	120	SArRNA05
gi|29165615|ref|NC_002745.2|	551073	+	U	75	28	25	38	56	SArRNA05
gi|29165615|ref|NC_002745.2|	551074	+	C	117	97	36	49	98	SArRNA05
gi|29165615|ref|NC_002745.2|	551075	+	U	85	62	37	49	121	SArRNA05
gi|29165615|ref|NC_002745.2|	551076	+	U	73	43	14	25	58	SArRNA05
gi|29165615|ref|NC_002745.2|	551077	+	G	74	62	28	50	72	SArRNA05
gi|29165615|ref|NC_002745.2|	551078	+	A	43	34	17	24	49	SArRNA05
gi|29165615|ref|NC_002745.2|	551079	+	C	137	85	44	56	102	SArRNA05
gi|29165615|ref|NC_002745.2|	551080	+	G	24	44	18	19	40	SArRNA05
gi|29165615|ref|NC_002745.2|	551081	+	G	10	18	7	4	19	SArRNA05
gi|29165615|ref|NC_002745.2|	551082	+	U	28	42	17	15	56	SArRNA05
gi|29165615|ref|NC_002745.2|	551083	+	A	16	19	8	5	35	SArRNA05
gi|29165615|ref|NC_002745.2|	551084	+	C	45	46	27	19	72	SArRNA05
gi|29165615|ref|NC_002745.2|	551085	+	C	386	342	203	208	362	SArRNA05
gi|29165615|ref|NC_002745.2|	551086	+	U	73	52	35	36	87	SArRNA05
gi|29165615|ref|NC_002745.2|	551087	+	A	30	20	17	20	38	SArRNA05
gi|29165615|ref|NC_002745.2|	551088	+	A	83	96	42	46	137	SArRNA05

gi|29165615|ref|NC_002745.2|	551089	+	U	108	45	30	40	91	SArRNA05
gi|29165615|ref|NC_002745.2|	551090	+	C	114	63	42	58	121	SArRNA05
gi|29165615|ref|NC_002745.2|	551091	+	A	31	37	26	16	68	SArRNA05
gi|29165615|ref|NC_002745.2|	551092	+	G	11	5	10	4	18	SArRNA05
gi|29165615|ref|NC_002745.2|	551093	+	A	24	12	14	14	31	SArRNA05
gi|29165615|ref|NC_002745.2|	551094	+	A	17	8	4	13	23	SArRNA05
gi|29165615|ref|NC_002745.2|	551095	+	A	8	7	0	6	14	SArRNA05
gi|29165615|ref|NC_002745.2|	551096	+	G	1	0	3	1	5	SArRNA05
gi|29165615|ref|NC_002745.2|	551097	+	C	23	15	10	11	42	SArRNA05
gi|29165615|ref|NC_002745.2|	551098	+	C	81	51	32	38	96	SArRNA05
gi|29165615|ref|NC_002745.2|	551099	+	A	29	10	16	14	51	SArRNA05
gi|29165615|ref|NC_002745.2|	551100	+	C	89	62	45	55	128	SArRNA05
gi|29165615|ref|NC_002745.2|	551101	+	G	11	15	8	8	28	SArRNA05
gi|29165615|ref|NC_002745.2|	551102	+	G	6	3	4	1	16	SArRNA05
gi|29165615|ref|NC_002745.2|	551103	+	C	39	18	17	16	91	SArRNA05
gi|29165615|ref|NC_002745.2|	551104	+	U	23	15	14	15	70	SArRNA05
gi|29165615|ref|NC_002745.2|	551105	+	A	21	13	6	6	30	SArRNA05
gi|29165615|ref|NC_002745.2|	551106	+	A	4	5	3	2	7	SArRNA05
gi|29165615|ref|NC_002745.2|	551107	+	C	6	5	3	8	21	SArRNA05
gi|29165615|ref|NC_002745.2|	551108	+	U	11	7	9	12	37	SArRNA05
gi|29165615|ref|NC_002745.2|	551109	+	A	9	9	3	7	32	SArRNA05
gi|29165615|ref|NC_002745.2|	551110	+	C	19	10	9	12	28	SArRNA05
gi|29165615|ref|NC_002745.2|	551111	+	G	6	2	7	2	9	SArRNA05
gi|29165615|ref|NC_002745.2|	551112	+	U	35	11	16	11	38	SArRNA05
gi|29165615|ref|NC_002745.2|	551113	+	G	46	31	29	17	59	SArRNA05
gi|29165615|ref|NC_002745.2|	551114	+	C	87	66	36	46	98	SArRNA05
gi|29165615|ref|NC_002745.2|	551115	+	C	136	95	49	52	140	SArRNA05
gi|29165615|ref|NC_002745.2|	551116	+	A	75	52	31	34	95	SArRNA05
gi|29165615|ref|NC_002745.2|	551117	+	G	62	32	26	30	55	SArRNA05
gi|29165615|ref|NC_002745.2|	551118	+	C	119	59	50	33	125	SArRNA05
gi|29165615|ref|NC_002745.2|	551119	+	A	79	49	34	36	80	SArRNA05
gi|29165615|ref|NC_002745.2|	551120	+	G	19	13	7	7	10	SArRNA05
gi|29165615|ref|NC_002745.2|	551121	+	C	104	43	20	26	73	SArRNA05
gi|29165615|ref|NC_002745.2|	551122	+	C	122	79	42	40	99	SArRNA05
gi|29165615|ref|NC_002745.2|	551123	+	G	60	70	26	22	83	SArRNA05
gi|29165615|ref|NC_002745.2|	551124	+	C	45	31	17	26	43	SArRNA05
gi|29165615|ref|NC_002745.2|	551125	+	G	61	45	23	27	67	SArRNA05
gi|29165615|ref|NC_002745.2|	551126	+	G	67	56	32	33	94	SArRNA05
gi|29165615|ref|NC_002745.2|	551127	+	U	124	92	61	69	143	SArRNA05
gi|29165615|ref|NC_002745.2|	551128	+	A	31	37	17	18	26	SArRNA05
gi|29165615|ref|NC_002745.2|	551129	+	A	112	109	52	48	161	SArRNA05
gi|29165615|ref|NC_002745.2|	551130	+	U	204	222	115	101	261	SArRNA05
gi|29165615|ref|NC_002745.2|	551131	+	A	129	89	51	55	137	SArRNA05
gi|29165615|ref|NC_002745.2|	551132	+	C	156	87	49	72	151	SArRNA05
gi|29165615|ref|NC_002745.2|	551133	+	G	152	98	51	57	149	SArRNA05
gi|29165615|ref|NC_002745.2|	551134	+	U	83	99	54	55	136	SArRNA05
gi|29165615|ref|NC_002745.2|	551135	+	A	44	23	22	27	69	SArRNA05
gi|29165615|ref|NC_002745.2|	551136	+	G	29	23	20	17	44	SArRNA05
gi|29165615|ref|NC_002745.2|	551137	+	G	113	107	48	53	89	SArRNA05
gi|29165615|ref|NC_002745.2|	551138	+	U	212	296	73	95	211	SArRNA05
gi|29165615|ref|NC_002745.2|	551139	+	G	35	34	10	20	35	SArRNA05
gi|29165615|ref|NC_002745.2|	551140	+	G	27	24	10	12	34	SArRNA05
gi|29165615|ref|NC_002745.2|	551141	+	C	62	59	34	27	85	SArRNA05
gi|29165615|ref|NC_002745.2|	551142	+	A	51	57	29	23	86	SArRNA05
gi|29165615|ref|NC_002745.2|	551143	+	A	50	27	20	15	45	SArRNA05
gi|29165615|ref|NC_002745.2|	551144	+	G	15	7	5	6	10	SArRNA05
gi|29165615|ref|NC_002745.2|	551145	+	C	82	37	35	37	80	SArRNA05
gi|29165615|ref|NC_002745.2|	551146	+	G	92	50	42	32	99	SArRNA05
gi|29165615|ref|NC_002745.2|	551147	+	U	89	67	30	43	111	SArRNA05
gi|29165615|ref|NC_002745.2|	551148	+	U	106	63	41	54	109	SArRNA05
gi|29165615|ref|NC_002745.2|	551149	+	A	71	38	37	37	99	SArRNA05
gi|29165615|ref|NC_002745.2|	551150	+	U	80	68	40	47	96	SArRNA05
gi|29165615|ref|NC_002745.2|	551151	+	C	229	190	66	77	182	SArRNA05
gi|29165615|ref|NC_002745.2|	551152	+	C	615	390	182	243	499	SArRNA05
gi|29165615|ref|NC_002745.2|	551153	+	G	257	170	70	78	234	SArRNA05
gi|29165615|ref|NC_002745.2|	551154	+	G	127	85	69	57	177	SArRNA05
gi|29165615|ref|NC_002745.2|	551155	+	A	130	70	76	57	163	SArRNA05
gi|29165615|ref|NC_002745.2|	551156	+	A	191	149	87	89	209	SArRNA05
gi|29165615|ref|NC_002745.2|	551157	+	U	148	142	83	95	245	SArRNA05
gi|29165615|ref|NC_002745.2|	551158	+	U	277	152	136	125	202	SArRNA05
gi|29165615|ref|NC_002745.2|	551159	+	A	200	89	61	65	152	SArRNA05
gi|29165615|ref|NC_002745.2|	551160	+	U	130	58	43	34	112	SArRNA05
gi|29165615|ref|NC_002745.2|	551161	+	U	86	47	12	15	80	SArRNA05
gi|29165615|ref|NC_002745.2|	551162	+	G	24	11	9	6	17	SArRNA05
gi|29165615|ref|NC_002745.2|	551163	+	G	24	19	16	16	30	SArRNA05
gi|29165615|ref|NC_002745.2|	551164	+	G	11	15	4	4	10	SArRNA05
gi|29165615|ref|NC_002745.2|	551165	+	C	95	43	39	39	120	SArRNA05
gi|29165615|ref|NC_002745.2|	551166	+	G	50	50	27	28	93	SArRNA05
gi|29165615|ref|NC_002745.2|	551167	+	U	118	45	35	36	95	SArRNA05
gi|29165615|ref|NC_002745.2|	551168	+	A	123	48	24	33	45	SArRNA05
gi|29165615|ref|NC_002745.2|	551169	+	A	130	27	26	25	61	SArRNA05

gi|29165615|ref|NC_002745.2|	551170	+	A	53	36	15	17	52	SArRNA05
gi|29165615|ref|NC_002745.2|	551171	+	G	29	9	6	13	16	SArRNA05
gi|29165615|ref|NC_002745.2|	551172	+	C	132	53	37	34	71	SArRNA05
gi|29165615|ref|NC_002745.2|	551173	+	G	19	18	8	19	28	SArRNA05
gi|29165615|ref|NC_002745.2|	551174	+	C	76	32	32	30	40	SArRNA05
gi|29165615|ref|NC_002745.2|	551175	+	G	36	16	8	8	19	SArRNA05
gi|29165615|ref|NC_002745.2|	551176	+	C	113	13	17	16	37	SArRNA05
gi|29165615|ref|NC_002745.2|	551177	+	G	38	14	11	16	32	SArRNA05
gi|29165615|ref|NC_002745.2|	551178	+	U	61	29	18	12	40	SArRNA05
gi|29165615|ref|NC_002745.2|	551179	+	A	26	8	7	5	17	SArRNA05
gi|29165615|ref|NC_002745.2|	551180	+	G	5	7	1	6	6	SArRNA05
gi|29165615|ref|NC_002745.2|	551181	+	G	5	5	2	4	5	SArRNA05
gi|29165615|ref|NC_002745.2|	551182	+	C	29	24	15	14	20	SArRNA05
gi|29165615|ref|NC_002745.2|	551183	+	G	22	15	10	15	18	SArRNA05
gi|29165615|ref|NC_002745.2|	551184	+	G	39	44	21	28	63	SArRNA05
gi|29165615|ref|NC_002745.2|	551185	+	U	139	97	67	65	162	SArRNA05
gi|29165615|ref|NC_002745.2|	551186	+	U	125	105	93	48	143	SArRNA05
gi|29165615|ref|NC_002745.2|	551187	+	U	88	67	58	59	132	SArRNA05
gi|29165615|ref|NC_002745.2|	551188	+	U	70	88	36	41	99	SArRNA05
gi|29165615|ref|NC_002745.2|	551189	+	U	36	29	15	20	60	SArRNA05
gi|29165615|ref|NC_002745.2|	551190	+	U	27	53	19	21	40	SArRNA05
gi|29165615|ref|NC_002745.2|	551191	+	A	10	13	6	8	12	SArRNA05
gi|29165615|ref|NC_002745.2|	551192	+	A	3	3	1	5	10	SArRNA05
gi|29165615|ref|NC_002745.2|	551193	+	G	3	3	6	2	3	SArRNA05
gi|29165615|ref|NC_002745.2|	551194	+	U	28	25	13	19	33	SArRNA05
gi|29165615|ref|NC_002745.2|	551195	+	C	170	187	80	66	137	SArRNA05
gi|29165615|ref|NC_002745.2|	551196	+	U	90	48	26	26	80	SArRNA05
gi|29165615|ref|NC_002745.2|	551197	+	G	25	39	28	26	47	SArRNA05
gi|29165615|ref|NC_002745.2|	551198	+	A	55	44	38	33	82	SArRNA05
gi|29165615|ref|NC_002745.2|	551199	+	U	44	45	22	16	58	SArRNA05
gi|29165615|ref|NC_002745.2|	551200	+	G	34	40	21	18	45	SArRNA05
gi|29165615|ref|NC_002745.2|	551201	+	U	55	34	30	21	46	SArRNA05
gi|29165615|ref|NC_002745.2|	551202	+	G	41	38	15	33	52	SArRNA05
gi|29165615|ref|NC_002745.2|	551203	+	A	52	48	35	16	59	SArRNA05
gi|29165615|ref|NC_002745.2|	551204	+	A	43	44	23	20	52	SArRNA05
gi|29165615|ref|NC_002745.2|	551205	+	A	54	31	19	20	43	SArRNA05
gi|29165615|ref|NC_002745.2|	551206	+	G	38	21	15	13	29	SArRNA05
gi|29165615|ref|NC_002745.2|	551207	+	C	24	18	5	5	14	SArRNA05
gi|29165615|ref|NC_002745.2|	551208	+	C	8	5	3	8	17	SArRNA05
gi|29165615|ref|NC_002745.2|	551209	+	C	43	14	16	26	19	SArRNA05
gi|29165615|ref|NC_002745.2|	551210	+	A	19	22	12	9	29	SArRNA05
gi|29165615|ref|NC_002745.2|	551211	+	C	31	17	9	13	19	SArRNA05
gi|29165615|ref|NC_002745.2|	551212	+	G	10	23	5	8	17	SArRNA05
gi|29165615|ref|NC_002745.2|	551213	+	G	8	9	3	3	13	SArRNA05
gi|29165615|ref|NC_002745.2|	551214	+	C	23	19	14	13	15	SArRNA05
gi|29165615|ref|NC_002745.2|	551215	+	U	31	16	15	8	39	SArRNA05
gi|29165615|ref|NC_002745.2|	551216	+	C	85	77	33	38	62	SArRNA05
gi|29165615|ref|NC_002745.2|	551217	+	A	38	38	16	19	42	SArRNA05
gi|29165615|ref|NC_002745.2|	551218	+	A	15	16	21	8	24	SArRNA05
gi|29165615|ref|NC_002745.2|	551219	+	C	23	25	13	10	28	SArRNA05
gi|29165615|ref|NC_002745.2|	551220	+	C	40	22	21	20	31	SArRNA05
gi|29165615|ref|NC_002745.2|	551221	+	G	22	21	14	8	23	SArRNA05
gi|29165615|ref|NC_002745.2|	551222	+	U	12	13	5	7	23	SArRNA05
gi|29165615|ref|NC_002745.2|	551223	+	G	15	9	12	3	18	SArRNA05
gi|29165615|ref|NC_002745.2|	551224	+	G	7	3	2	5	12	SArRNA05
gi|29165615|ref|NC_002745.2|	551225	+	A	2	4	0	1	3	SArRNA05
gi|29165615|ref|NC_002745.2|	551226	+	G	6	6	1	2	6	SArRNA05
gi|29165615|ref|NC_002745.2|	551227	+	G	11	12	2	6	6	SArRNA05
gi|29165615|ref|NC_002745.2|	551228	+	G	17	11	2	10	15	SArRNA05
gi|29165615|ref|NC_002745.2|	551229	+	U	3	4	8	5	10	SArRNA05
gi|29165615|ref|NC_002745.2|	551230	+	C	133	94	41	36	76	SArRNA05
gi|29165615|ref|NC_002745.2|	551231	+	A	22	31	13	11	29	SArRNA05
gi|29165615|ref|NC_002745.2|	551232	+	U	0	5	3	5	8	SArRNA05
gi|29165615|ref|NC_002745.2|	551233	+	U	6	7	3	1	4	SArRNA05
gi|29165615|ref|NC_002745.2|	551234	+	G	0	1	1	0	2	SArRNA05
gi|29165615|ref|NC_002745.2|	551235	+	G	0	4	1	3	6	SArRNA05
gi|29165615|ref|NC_002745.2|	551236	+	A	5	0	3	0	1	SArRNA05
gi|29165615|ref|NC_002745.2|	551237	+	A	0	3	1	0	2	SArRNA05
gi|29165615|ref|NC_002745.2|	551238	+	A	2	3	1	0	3	SArRNA05
gi|29165615|ref|NC_002745.2|	551239	+	C	12	11	6	3	13	SArRNA05
gi|29165615|ref|NC_002745.2|	551240	+	U	7	5	4	2	3	SArRNA05
gi|29165615|ref|NC_002745.2|	551241	+	G	0	0	0	0	3	SArRNA05
gi|29165615|ref|NC_002745.2|	551242	+	G	0	0	0	0	1	SArRNA05
gi|29165615|ref|NC_002745.2|	551243	+	A	0	0	2	0	3	SArRNA05
gi|29165615|ref|NC_002745.2|	551244	+	A	2	1	0	2	5	SArRNA05
gi|29165615|ref|NC_002745.2|	551245	+	A	4	1	2	1	3	SArRNA05
gi|29165615|ref|NC_002745.2|	551246	+	A	24	8	0	3	7	SArRNA05
gi|29165615|ref|NC_002745.2|	551247	+	C	41	6	3	1	8	SArRNA05
gi|29165615|ref|NC_002745.2|	551248	+	U	10	6	1	2	3	SArRNA05
gi|29165615|ref|NC_002745.2|	551249	+	U	2	3	1	1	3	SArRNA05
gi|29165615|ref|NC_002745.2|	551250	+	G	10	2	0	0	1	SArRNA05

gi|29165615|ref|NC_002745.2|	551251	+	A	6	2	1	2	1	SArRNA05
gi|29165615|ref|NC_002745.2|	551252	+	G	3	0	0	0	0	SArRNA05
gi|29165615|ref|NC_002745.2|	551253	+	U	3	2	0	0	0	SArRNA05
gi|29165615|ref|NC_002745.2|	551254	+	G	1	0	1	0	1	SArRNA05
gi|29165615|ref|NC_002745.2|	551255	+	C	5	4	5	2	2	SArRNA05
gi|29165615|ref|NC_002745.2|	551256	+	A	11	12	2	3	5	SArRNA05
gi|29165615|ref|NC_002745.2|	551257	+	G	1	3	0	1	1	SArRNA05
gi|29165615|ref|NC_002745.2|	551258	+	A	4	2	2	1	1	SArRNA05
gi|29165615|ref|NC_002745.2|	551259	+	A	1	4	1	2	1	SArRNA05
gi|29165615|ref|NC_002745.2|	551260	+	G	11	1	2	1	2	SArRNA05
gi|29165615|ref|NC_002745.2|	551261	+	A	1	1	1	0	1	SArRNA05
gi|29165615|ref|NC_002745.2|	551262	+	G	3	0	0	0	1	SArRNA05
gi|29165615|ref|NC_002745.2|	551263	+	G	0	0	0	1	1	SArRNA05
gi|29165615|ref|NC_002745.2|	551264	+	A	0	0	0	0	1	SArRNA05
gi|29165615|ref|NC_002745.2|	551265	+	A	0	2	0	1	0	SArRNA05
gi|29165615|ref|NC_002745.2|	551266	+	A	1	0	1	0	0	SArRNA05
gi|29165615|ref|NC_002745.2|	551267	+	G	1	1	0	0	0	SArRNA05
gi|29165615|ref|NC_002745.2|	551268	+	U	2	0	0	0	2	SArRNA05
gi|29165615|ref|NC_002745.2|	551270	+	G	0	1	0	1	0	SArRNA05
gi|29165615|ref|NC_002745.2|	551271	+	A	2	0	0	0	0	SArRNA05
gi|29165615|ref|NC_002745.2|	551272	+	A	0	1	0	0	1	SArRNA05
gi|29165615|ref|NC_002745.2|	551275	+	C	1	3	0	4	1	SArRNA05
gi|29165615|ref|NC_002745.2|	551276	+	C	4	2	3	3	6	SArRNA05
gi|29165615|ref|NC_002745.2|	551277	+	A	1	5	2	4	3	SArRNA05
gi|29165615|ref|NC_002745.2|	551278	+	U	0	1	0	1	2	SArRNA05
gi|29165615|ref|NC_002745.2|	551279	+	G	3	2	0	1	2	SArRNA05
gi|29165615|ref|NC_002745.2|	551280	+	U	2	4	0	1	2	SArRNA05
gi|29165615|ref|NC_002745.2|	551281	+	G	3	4	1	2	4	SArRNA05
gi|29165615|ref|NC_002745.2|	551282	+	U	2	3	1	1	3	SArRNA05
gi|29165615|ref|NC_002745.2|	551283	+	A	3	4	2	1	3	SArRNA05
gi|29165615|ref|NC_002745.2|	551284	+	G	0	1	0	0	1	SArRNA05
gi|29165615|ref|NC_002745.2|	551285	+	C	2	0	0	1	1	SArRNA05
gi|29165615|ref|NC_002745.2|	551286	+	G	0	4	0	1	7	SArRNA05
gi|29165615|ref|NC_002745.2|	551287	+	G	1	3	0	2	1	SArRNA05
gi|29165615|ref|NC_002745.2|	551288	+	U	5	1	0	0	0	SArRNA05
gi|29165615|ref|NC_002745.2|	551290	+	A	3	0	0	0	1	SArRNA05
gi|29165615|ref|NC_002745.2|	551291	+	A	0	0	0	0	1	SArRNA05
gi|29165615|ref|NC_002745.2|	551292	+	A	0	6	0	1	1	SArRNA05
gi|29165615|ref|NC_002745.2|	551293	+	U	5	2	1	2	0	SArRNA05
gi|29165615|ref|NC_002745.2|	551295	+	C	3	2	0	0	0	SArRNA05
gi|29165615|ref|NC_002745.2|	551296	+	G	0	0	0	0	2	SArRNA05
gi|29165615|ref|NC_002745.2|	551297	+	C	0	0	1	0	1	SArRNA05
gi|29165615|ref|NC_002745.2|	551298	+	A	1	2	0	0	2	SArRNA05
gi|29165615|ref|NC_002745.2|	551299	+	G	0	3	0	0	2	SArRNA05
gi|29165615|ref|NC_002745.2|	551300	+	A	1	0	0	0	0	SArRNA05
gi|29165615|ref|NC_002745.2|	551301	+	G	2	2	0	0	0	SArRNA05
gi|29165615|ref|NC_002745.2|	551302	+	A	2	0	0	0	1	SArRNA05
gi|29165615|ref|NC_002745.2|	551303	+	U	1	1	1	1	2	SArRNA05
gi|29165615|ref|NC_002745.2|	551304	+	A	1	1	1	1	3	SArRNA05
gi|29165615|ref|NC_002745.2|	551305	+	U	3	1	0	2	1	SArRNA05
gi|29165615|ref|NC_002745.2|	551306	+	G	0	0	0	1	2	SArRNA05
gi|29165615|ref|NC_002745.2|	551307	+	G	2	2	0	1	1	SArRNA05
gi|29165615|ref|NC_002745.2|	551308	+	A	2	2	1	0	1	SArRNA05
gi|29165615|ref|NC_002745.2|	551309	+	G	0	0	0	1	1	SArRNA05
gi|29165615|ref|NC_002745.2|	551310	+	G	0	1	0	0	1	SArRNA05
gi|29165615|ref|NC_002745.2|	551311	+	A	0	2	0	0	1	SArRNA05
gi|29165615|ref|NC_002745.2|	551312	+	A	2	3	1	0	0	SArRNA05
gi|29165615|ref|NC_002745.2|	551313	+	C	0	3	0	1	1	SArRNA05
gi|29165615|ref|NC_002745.2|	551314	+	A	2	2	0	0	1	SArRNA05
gi|29165615|ref|NC_002745.2|	551315	+	C	3	1	0	0	1	SArRNA05
gi|29165615|ref|NC_002745.2|	551316	+	C	1	7	0	0	2	SArRNA05
gi|29165615|ref|NC_002745.2|	551317	+	A	0	0	0	0	1	SArRNA05
gi|29165615|ref|NC_002745.2|	551318	+	G	0	5	2	1	4	SArRNA05
gi|29165615|ref|NC_002745.2|	551319	+	U	0	0	1	1	1	SArRNA05
gi|29165615|ref|NC_002745.2|	551320	+	G	0	0	0	0	1	SArRNA05
gi|29165615|ref|NC_002745.2|	551321	+	G	3	0	0	2	1	SArRNA05
gi|29165615|ref|NC_002745.2|	551322	+	C	0	1	0	1	0	SArRNA05
gi|29165615|ref|NC_002745.2|	551323	+	G	3	0	0	1	2	SArRNA05
gi|29165615|ref|NC_002745.2|	551324	+	A	0	1	0	1	2	SArRNA05
gi|29165615|ref|NC_002745.2|	551325	+	A	1	0	1	0	0	SArRNA05
gi|29165615|ref|NC_002745.2|	551327	+	G	0	0	0	1	1	SArRNA05
gi|29165615|ref|NC_002745.2|	551328	+	C	3	0	1	1	2	SArRNA05
gi|29165615|ref|NC_002745.2|	551329	+	G	2	2	0	1	1	SArRNA05
gi|29165615|ref|NC_002745.2|	551330	+	A	5	2	0	1	2	SArRNA05
gi|29165615|ref|NC_002745.2|	551331	+	C	6	1	1	0	1	SArRNA05
gi|29165615|ref|NC_002745.2|	551332	+	U	1	0	0	0	0	SArRNA05
gi|29165615|ref|NC_002745.2|	551333	+	U	0	0	3	0	1	SArRNA05
gi|29165615|ref|NC_002745.2|	551334	+	U	1	0	0	0	1	SArRNA05
gi|29165615|ref|NC_002745.2|	551335	+	C	1	1	0	0	0	SArRNA05
gi|29165615|ref|NC_002745.2|	551336	+	U	0	0	0	1	3	SArRNA05
gi|29165615|ref|NC_002745.2|	551338	+	G	0	0	0	1	0	SArRNA05

gi|29165615|ref|NC_002745.2|	551339	+	U	0	3	1	0	1	SArRNA05
gi|29165615|ref|NC_002745.2|	551340	+	C	0	3	0	1	2	SArRNA05
gi|29165615|ref|NC_002745.2|	551341	+	U	2	1	0	1	2	SArRNA05
gi|29165615|ref|NC_002745.2|	551342	+	G	0	0	1	1	1	SArRNA05
gi|29165615|ref|NC_002745.2|	551343	+	U	0	2	1	2	1	SArRNA05
gi|29165615|ref|NC_002745.2|	551344	+	A	2	1	1	0	1	SArRNA05
gi|29165615|ref|NC_002745.2|	551345	+	A	5	0	2	0	3	SArRNA05
gi|29165615|ref|NC_002745.2|	551346	+	C	9	3	2	2	6	SArRNA05
gi|29165615|ref|NC_002745.2|	551347	+	U	3	5	0	0	1	SArRNA05
gi|29165615|ref|NC_002745.2|	551348	+	G	0	1	1	1	0	SArRNA05
gi|29165615|ref|NC_002745.2|	551349	+	A	0	0	1	0	1	SArRNA05
gi|29165615|ref|NC_002745.2|	551350	+	C	0	0	2	1	0	SArRNA05
gi|29165615|ref|NC_002745.2|	551352	+	C	0	2	1	1	1	SArRNA05
gi|29165615|ref|NC_002745.2|	551353	+	U	3	0	0	0	0	SArRNA05
gi|29165615|ref|NC_002745.2|	551354	+	G	4	2	0	0	2	SArRNA05
gi|29165615|ref|NC_002745.2|	551355	+	A	1	2	0	1	0	SArRNA05
gi|29165615|ref|NC_002745.2|	551356	+	U	3	2	1	2	4	SArRNA05
gi|29165615|ref|NC_002745.2|	551358	+	U	0	1	0	1	0	SArRNA05
gi|29165615|ref|NC_002745.2|	551360	+	C	0	4	0	0	0	SArRNA05
gi|29165615|ref|NC_002745.2|	551361	+	G	2	1	1	1	4	SArRNA05
gi|29165615|ref|NC_002745.2|	551362	+	A	1	0	0	1	0	SArRNA05
gi|29165615|ref|NC_002745.2|	551364	+	A	1	0	0	0	0	SArRNA05
gi|29165615|ref|NC_002745.2|	551372	+	G	0	0	0	0	1	SArRNA05
gi|29165615|ref|NC_002745.2|	551373	+	A	0	0	0	1	0	SArRNA05
gi|29165615|ref|NC_002745.2|	551378	+	A	0	0	1	0	0	SArRNA05
gi|29165615|ref|NC_002745.2|	551379	+	C	0	2	0	0	1	SArRNA05
gi|29165615|ref|NC_002745.2|	551380	+	A	1	2	0	0	0	SArRNA05
gi|29165615|ref|NC_002745.2|	551382	+	G	1	0	0	0	0	SArRNA05
gi|29165615|ref|NC_002745.2|	551384	+	U	0	0	0	0	1	SArRNA05
gi|29165615|ref|NC_002745.2|	551386	+	A	0	0	1	0	1	SArRNA05
gi|29165615|ref|NC_002745.2|	551388	+	A	3	0	1	0	0	SArRNA05
gi|29165615|ref|NC_002745.2|	551389	+	U	7	3	0	1	7	SArRNA05
gi|29165615|ref|NC_002745.2|	551390	+	A	0	2	0	1	3	SArRNA05
gi|29165615|ref|NC_002745.2|	551391	+	C	6	22	0	0	8	SArRNA05
gi|29165615|ref|NC_002745.2|	551392	+	C	5	9	3	0	7	SArRNA05
gi|29165615|ref|NC_002745.2|	551393	+	C	11	17	2	4	13	SArRNA05
gi|29165615|ref|NC_002745.2|	551394	+	U	7	7	0	0	5	SArRNA05
gi|29165615|ref|NC_002745.2|	551395	+	G	2	1	0	0	2	SArRNA05
gi|29165615|ref|NC_002745.2|	551396	+	G	0	3	0	0	2	SArRNA05
gi|29165615|ref|NC_002745.2|	551397	+	U	3	0	3	1	2	SArRNA05
gi|29165615|ref|NC_002745.2|	551398	+	A	6	5	1	2	2	SArRNA05
gi|29165615|ref|NC_002745.2|	551399	+	G	0	1	0	2	2	SArRNA05
gi|29165615|ref|NC_002745.2|	551400	+	U	7	0	1	2	2	SArRNA05
gi|29165615|ref|NC_002745.2|	551401	+	C	6	5	2	1	0	SArRNA05
gi|29165615|ref|NC_002745.2|	551402	+	C	18	13	3	10	7	SArRNA05
gi|29165615|ref|NC_002745.2|	551403	+	A	6	7	2	3	9	SArRNA05
gi|29165615|ref|NC_002745.2|	551404	+	C	6	6	3	4	10	SArRNA05
gi|29165615|ref|NC_002745.2|	551405	+	G	4	1	0	2	2	SArRNA05
gi|29165615|ref|NC_002745.2|	551406	+	C	0	3	0	1	5	SArRNA05
gi|29165615|ref|NC_002745.2|	551407	+	C	18	15	6	4	5	SArRNA05
gi|29165615|ref|NC_002745.2|	551408	+	G	11	7	9	14	16	SArRNA05
gi|29165615|ref|NC_002745.2|	551409	+	U	10	27	13	18	36	SArRNA05
gi|29165615|ref|NC_002745.2|	551410	+	A	5	5	5	3	5	SArRNA05
gi|29165615|ref|NC_002745.2|	551411	+	A	38	30	13	13	15	SArRNA05
gi|29165615|ref|NC_002745.2|	551412	+	A	9	50	15	4	33	SArRNA05
gi|29165615|ref|NC_002745.2|	551413	+	C	46	62	14	20	40	SArRNA05
gi|29165615|ref|NC_002745.2|	551414	+	G	9	22	5	12	20	SArRNA05
gi|29165615|ref|NC_002745.2|	551415	+	A	8	1	4	9	6	SArRNA05
gi|29165615|ref|NC_002745.2|	551416	+	U	11	12	3	4	9	SArRNA05
gi|29165615|ref|NC_002745.2|	551417	+	G	2	7	0	2	3	SArRNA05
gi|29165615|ref|NC_002745.2|	551418	+	A	5	2	1	0	2	SArRNA05
gi|29165615|ref|NC_002745.2|	551419	+	G	2	0	0	0	1	SArRNA05
gi|29165615|ref|NC_002745.2|	551420	+	U	4	4	2	1	4	SArRNA05
gi|29165615|ref|NC_002745.2|	551421	+	G	1	0	1	1	2	SArRNA05
gi|29165615|ref|NC_002745.2|	551422	+	C	9	9	4	7	8	SArRNA05
gi|29165615|ref|NC_002745.2|	551423	+	U	8	3	0	6	9	SArRNA05
gi|29165615|ref|NC_002745.2|	551424	+	A	6	6	1	0	4	SArRNA05
gi|29165615|ref|NC_002745.2|	551425	+	A	13	10	4	1	9	SArRNA05
gi|29165615|ref|NC_002745.2|	551426	+	G	8	5	5	2	11	SArRNA05
gi|29165615|ref|NC_002745.2|	551427	+	U	8	5	5	3	8	SArRNA05
gi|29165615|ref|NC_002745.2|	551428	+	G	5	0	3	4	11	SArRNA05
gi|29165615|ref|NC_002745.2|	551429	+	U	2	3	3	1	12	SArRNA05
gi|29165615|ref|NC_002745.2|	551430	+	U	7	2	0	0	7	SArRNA05
gi|29165615|ref|NC_002745.2|	551431	+	A	3	0	1	0	2	SArRNA05
gi|29165615|ref|NC_002745.2|	551432	+	G	0	1	0	0	0	SArRNA05
gi|29165615|ref|NC_002745.2|	551436	+	G	0	1	0	0	1	SArRNA05
gi|29165615|ref|NC_002745.2|	551437	+	U	0	0	0	0	3	SArRNA05
gi|29165615|ref|NC_002745.2|	551438	+	U	0	3	0	1	1	SArRNA05
gi|29165615|ref|NC_002745.2|	551439	+	U	3	1	2	1	3	SArRNA05
gi|29165615|ref|NC_002745.2|	551440	+	C	2	5	0	1	3	SArRNA05
gi|29165615|ref|NC_002745.2|	551441	+	C	18	35	5	10	27	SArRNA05

gi|29165615|ref|NC_002745.2|	551442	+	G	4	10	2	4	7	SArRNA05
gi|29165615|ref|NC_002745.2|	551443	+	C	11	8	5	7	22	SArRNA05
gi|29165615|ref|NC_002745.2|	551444	+	C	15	5	6	2	12	SArRNA05
gi|29165615|ref|NC_002745.2|	551445	+	C	15	3	3	3	9	SArRNA05
gi|29165615|ref|NC_002745.2|	551446	+	C	11	4	4	3	10	SArRNA05
gi|29165615|ref|NC_002745.2|	551447	+	U	22	12	3	3	10	SArRNA05
gi|29165615|ref|NC_002745.2|	551448	+	U	12	6	3	5	11	SArRNA05
gi|29165615|ref|NC_002745.2|	551449	+	A	16	10	7	3	16	SArRNA05
gi|29165615|ref|NC_002745.2|	551450	+	G	5	4	2	4	11	SArRNA05
gi|29165615|ref|NC_002745.2|	551451	+	U	6	0	1	2	5	SArRNA05
gi|29165615|ref|NC_002745.2|	551452	+	G	1	0	0	0	0	SArRNA05
gi|29165615|ref|NC_002745.2|	551453	+	C	10	4	6	4	10	SArRNA05
gi|29165615|ref|NC_002745.2|	551454	+	U	0	5	2	1	5	SArRNA05
gi|29165615|ref|NC_002745.2|	551456	+	C	7	16	1	6	10	SArRNA05
gi|29165615|ref|NC_002745.2|	551457	+	A	3	5	2	1	6	SArRNA05
gi|29165615|ref|NC_002745.2|	551458	+	G	2	0	0	1	1	SArRNA05
gi|29165615|ref|NC_002745.2|	551459	+	C	2	4	0	3	7	SArRNA05
gi|29165615|ref|NC_002745.2|	551460	+	U	3	5	0	2	1	SArRNA05
gi|29165615|ref|NC_002745.2|	551461	+	A	3	6	0	1	5	SArRNA05
gi|29165615|ref|NC_002745.2|	551462	+	A	3	2	0	0	0	SArRNA05
gi|29165615|ref|NC_002745.2|	551463	+	C	5	4	1	0	3	SArRNA05
gi|29165615|ref|NC_002745.2|	551464	+	G	0	0	0	0	4	SArRNA05
gi|29165615|ref|NC_002745.2|	551465	+	C	3	4	1	2	1	SArRNA05
gi|29165615|ref|NC_002745.2|	551466	+	A	5	0	0	0	4	SArRNA05
gi|29165615|ref|NC_002745.2|	551467	+	U	3	3	0	0	2	SArRNA05
gi|29165615|ref|NC_002745.2|	551468	+	U	2	5	1	1	3	SArRNA05
gi|29165615|ref|NC_002745.2|	551469	+	A	3	1	1	3	4	SArRNA05
gi|29165615|ref|NC_002745.2|	551470	+	A	0	5	0	2	5	SArRNA05
gi|29165615|ref|NC_002745.2|	551471	+	G	0	0	0	1	0	SArRNA05
gi|29165615|ref|NC_002745.2|	551472	+	C	5	6	0	0	2	SArRNA05
gi|29165615|ref|NC_002745.2|	551473	+	A	3	2	0	1	0	SArRNA05
gi|29165615|ref|NC_002745.2|	551474	+	C	4	4	0	1	2	SArRNA05
gi|29165615|ref|NC_002745.2|	551475	+	U	2	0	2	0	0	SArRNA05
gi|29165615|ref|NC_002745.2|	551476	+	C	4	1	1	0	2	SArRNA05
gi|29165615|ref|NC_002745.2|	551477	+	C	5	4	6	2	10	SArRNA05
gi|29165615|ref|NC_002745.2|	551478	+	G	1	2	0	0	2	SArRNA05
gi|29165615|ref|NC_002745.2|	551479	+	C	4	1	0	0	0	SArRNA05
gi|29165615|ref|NC_002745.2|	551480	+	C	10	35	7	11	26	SArRNA05
gi|29165615|ref|NC_002745.2|	551481	+	U	3	10	1	1	2	SArRNA05
gi|29165615|ref|NC_002745.2|	551482	+	G	0	0	0	0	1	SArRNA05
gi|29165615|ref|NC_002745.2|	551483	+	G	1	0	0	0	1	SArRNA05
gi|29165615|ref|NC_002745.2|	551484	+	G	3	0	0	1	1	SArRNA05
gi|29165615|ref|NC_002745.2|	551486	+	A	1	0	0	0	0	SArRNA05
gi|29165615|ref|NC_002745.2|	551487	+	G	1	0	0	1	1	SArRNA05
gi|29165615|ref|NC_002745.2|	551488	+	U	2	6	3	3	7	SArRNA05
gi|29165615|ref|NC_002745.2|	551489	+	A	0	4	0	0	0	SArRNA05
gi|29165615|ref|NC_002745.2|	551490	+	C	7	2	1	1	4	SArRNA05
gi|29165615|ref|NC_002745.2|	551491	+	G	0	9	0	0	2	SArRNA05
gi|29165615|ref|NC_002745.2|	551492	+	A	1	1	1	0	2	SArRNA05
gi|29165615|ref|NC_002745.2|	551493	+	C	1	1	1	0	0	SArRNA05
gi|29165615|ref|NC_002745.2|	551494	+	C	0	2	0	3	1	SArRNA05
gi|29165615|ref|NC_002745.2|	551495	+	G	0	2	0	0	2	SArRNA05
gi|29165615|ref|NC_002745.2|	551496	+	C	4	5	0	0	1	SArRNA05
gi|29165615|ref|NC_002745.2|	551497	+	A	3	4	1	2	3	SArRNA05
gi|29165615|ref|NC_002745.2|	551498	+	A	1	0	0	0	1	SArRNA05
gi|29165615|ref|NC_002745.2|	551499	+	G	0	0	0	0	1	SArRNA05
gi|29165615|ref|NC_002745.2|	551500	+	G	0	2	0	1	1	SArRNA05
gi|29165615|ref|NC_002745.2|	551501	+	U	0	1	0	0	0	SArRNA05
gi|29165615|ref|NC_002745.2|	551502	+	U	2	0	0	1	1	SArRNA05
gi|29165615|ref|NC_002745.2|	551503	+	G	1	2	0	2	4	SArRNA05
gi|29165615|ref|NC_002745.2|	551504	+	A	1	0	1	0	0	SArRNA05
gi|29165615|ref|NC_002745.2|	551505	+	A	0	9	1	0	5	SArRNA05
gi|29165615|ref|NC_002745.2|	551506	+	A	1	4	0	1	6	SArRNA05
gi|29165615|ref|NC_002745.2|	551507	+	C	1	0	1	1	0	SArRNA05
gi|29165615|ref|NC_002745.2|	551508	+	U	1	6	0	1	0	SArRNA05
gi|29165615|ref|NC_002745.2|	551509	+	C	0	3	0	0	1	SArRNA05
gi|29165615|ref|NC_002745.2|	551510	+	A	0	1	0	0	3	SArRNA05
gi|29165615|ref|NC_002745.2|	551511	+	A	0	5	0	0	1	SArRNA05
gi|29165615|ref|NC_002745.2|	551512	+	A	0	0	2	1	3	SArRNA05
gi|29165615|ref|NC_002745.2|	551513	+	G	1	1	1	2	0	SArRNA05
gi|29165615|ref|NC_002745.2|	551514	+	G	1	1	0	1	2	SArRNA05
gi|29165615|ref|NC_002745.2|	551515	+	A	4	4	4	3	2	SArRNA05
gi|29165615|ref|NC_002745.2|	551516	+	A	3	20	3	1	11	SArRNA05
gi|29165615|ref|NC_002745.2|	551517	+	U	2	6	1	0	2	SArRNA05
gi|29165615|ref|NC_002745.2|	551518	+	U	3	3	0	0	2	SArRNA05
gi|29165615|ref|NC_002745.2|	551519	+	G	5	6	2	2	2	SArRNA05
gi|29165615|ref|NC_002745.2|	551520	+	A	0	0	0	0	1	SArRNA05
gi|29165615|ref|NC_002745.2|	551521	+	C	5	7	0	0	0	SArRNA05
gi|29165615|ref|NC_002745.2|	551522	+	G	0	0	0	0	2	SArRNA05
gi|29165615|ref|NC_002745.2|	551524	+	G	0	0	2	0	0	SArRNA05
gi|29165615|ref|NC_002745.2|	551525	+	G	2	2	0	0	1	SArRNA05

gi|29165615|ref|NC_002745.2|	551526	+	A	0	2	0	0	1	SArRNA05
gi|29165615|ref|NC_002745.2|	551527	+	C	21	10	6	2	21	SArRNA05
gi|29165615|ref|NC_002745.2|	551528	+	C	8	5	2	2	4	SArRNA05
gi|29165615|ref|NC_002745.2|	551529	+	C	8	6	1	1	5	SArRNA05
gi|29165615|ref|NC_002745.2|	551530	+	G	1	1	5	0	8	SArRNA05
gi|29165615|ref|NC_002745.2|	551531	+	C	6	8	2	4	6	SArRNA05
gi|29165615|ref|NC_002745.2|	551532	+	A	8	6	1	3	2	SArRNA05
gi|29165615|ref|NC_002745.2|	551533	+	C	19	9	6	5	9	SArRNA05
gi|29165615|ref|NC_002745.2|	551534	+	A	4	9	3	3	8	SArRNA05
gi|29165615|ref|NC_002745.2|	551535	+	A	2	7	0	1	3	SArRNA05
gi|29165615|ref|NC_002745.2|	551536	+	G	5	0	1	0	0	SArRNA05
gi|29165615|ref|NC_002745.2|	551537	+	C	9	3	2	4	4	SArRNA05
gi|29165615|ref|NC_002745.2|	551538	+	G	3	1	0	0	1	SArRNA05
gi|29165615|ref|NC_002745.2|	551539	+	G	0	1	1	0	3	SArRNA05
gi|29165615|ref|NC_002745.2|	551540	+	U	10	0	1	5	2	SArRNA05
gi|29165615|ref|NC_002745.2|	551541	+	G	0	2	0	0	2	SArRNA05
gi|29165615|ref|NC_002745.2|	551542	+	G	2	1	1	1	1	SArRNA05
gi|29165615|ref|NC_002745.2|	551543	+	A	2	1	0	2	4	SArRNA05
gi|29165615|ref|NC_002745.2|	551544	+	G	4	1	1	3	3	SArRNA05
gi|29165615|ref|NC_002745.2|	551545	+	C	11	4	4	4	3	SArRNA05
gi|29165615|ref|NC_002745.2|	551546	+	A	4	2	3	3	1	SArRNA05
gi|29165615|ref|NC_002745.2|	551548	+	G	1	0	0	1	0	SArRNA05
gi|29165615|ref|NC_002745.2|	551549	+	U	0	1	0	0	0	SArRNA05
gi|29165615|ref|NC_002745.2|	551550	+	G	1	1	0	1	0	SArRNA05
gi|29165615|ref|NC_002745.2|	551551	+	G	0	0	0	1	0	SArRNA05
gi|29165615|ref|NC_002745.2|	551552	+	U	0	2	0	0	1	SArRNA05
gi|29165615|ref|NC_002745.2|	551553	+	U	0	1	1	0	1	SArRNA05
gi|29165615|ref|NC_002745.2|	551554	+	U	1	2	0	0	0	SArRNA05
gi|29165615|ref|NC_002745.2|	551555	+	A	3	6	0	0	2	SArRNA05
gi|29165615|ref|NC_002745.2|	551556	+	A	6	14	3	2	7	SArRNA05
gi|29165615|ref|NC_002745.2|	551557	+	U	0	2	0	0	2	SArRNA05
gi|29165615|ref|NC_002745.2|	551558	+	U	1	0	1	1	3	SArRNA05
gi|29165615|ref|NC_002745.2|	551559	+	C	1	7	0	1	1	SArRNA05
gi|29165615|ref|NC_002745.2|	551560	+	G	1	2	0	0	1	SArRNA05
gi|29165615|ref|NC_002745.2|	551561	+	A	1	1	1	1	2	SArRNA05
gi|29165615|ref|NC_002745.2|	551562	+	A	1	0	0	2	0	SArRNA05
gi|29165615|ref|NC_002745.2|	551563	+	G	2	3	0	1	0	SArRNA05
gi|29165615|ref|NC_002745.2|	551564	+	C	1	2	0	1	0	SArRNA05
gi|29165615|ref|NC_002745.2|	551565	+	A	15	5	1	4	5	SArRNA05
gi|29165615|ref|NC_002745.2|	551566	+	A	0	2	0	0	0	SArRNA05
gi|29165615|ref|NC_002745.2|	551567	+	C	9	7	1	2	4	SArRNA05
gi|29165615|ref|NC_002745.2|	551568	+	G	4	1	0	0	1	SArRNA05
gi|29165615|ref|NC_002745.2|	551569	+	C	2	0	2	0	1	SArRNA05
gi|29165615|ref|NC_002745.2|	551570	+	G	8	2	1	3	4	SArRNA05
gi|29165615|ref|NC_002745.2|	551571	+	A	3	0	1	0	2	SArRNA05
gi|29165615|ref|NC_002745.2|	551572	+	A	1	2	1	2	1	SArRNA05
gi|29165615|ref|NC_002745.2|	551573	+	G	2	0	0	0	1	SArRNA05
gi|29165615|ref|NC_002745.2|	551574	+	A	1	3	2	1	2	SArRNA05
gi|29165615|ref|NC_002745.2|	551575	+	A	0	2	1	0	1	SArRNA05
gi|29165615|ref|NC_002745.2|	551576	+	C	7	3	0	1	1	SArRNA05
gi|29165615|ref|NC_002745.2|	551577	+	C	7	2	1	5	6	SArRNA05
gi|29165615|ref|NC_002745.2|	551578	+	U	1	4	0	1	2	SArRNA05
gi|29165615|ref|NC_002745.2|	551579	+	U	2	3	2	3	1	SArRNA05
gi|29165615|ref|NC_002745.2|	551580	+	A	0	0	1	0	0	SArRNA05
gi|29165615|ref|NC_002745.2|	551581	+	C	1	0	1	0	0	SArRNA05
gi|29165615|ref|NC_002745.2|	551582	+	C	2	2	3	11	3	SArRNA05
gi|29165615|ref|NC_002745.2|	551583	+	A	5	2	1	3	2	SArRNA05
gi|29165615|ref|NC_002745.2|	551584	+	A	1	0	0	1	1	SArRNA05
gi|29165615|ref|NC_002745.2|	551585	+	A	0	0	0	0	1	SArRNA05
gi|29165615|ref|NC_002745.2|	551586	+	U	0	2	2	0	0	SArRNA05
gi|29165615|ref|NC_002745.2|	551587	+	C	2	6	1	1	1	SArRNA05
gi|29165615|ref|NC_002745.2|	551588	+	U	4	4	1	4	0	SArRNA05
gi|29165615|ref|NC_002745.2|	551589	+	U	1	1	1	0	0	SArRNA05
gi|29165615|ref|NC_002745.2|	551591	+	A	0	0	0	0	1	SArRNA05
gi|29165615|ref|NC_002745.2|	551592	+	C	7	5	5	6	8	SArRNA05
gi|29165615|ref|NC_002745.2|	551593	+	A	0	8	2	2	0	SArRNA05
gi|29165615|ref|NC_002745.2|	551594	+	U	0	0	0	2	0	SArRNA05
gi|29165615|ref|NC_002745.2|	551595	+	C	1	2	0	0	0	SArRNA05
gi|29165615|ref|NC_002745.2|	551596	+	C	1	4	0	1	1	SArRNA05
gi|29165615|ref|NC_002745.2|	551597	+	U	2	0	2	1	2	SArRNA05
gi|29165615|ref|NC_002745.2|	551598	+	U	0	3	1	0	1	SArRNA05
gi|29165615|ref|NC_002745.2|	551599	+	U	0	3	0	0	1	SArRNA05
gi|29165615|ref|NC_002745.2|	551602	+	C	3	2	1	0	2	SArRNA05
gi|29165615|ref|NC_002745.2|	551603	+	A	0	1	0	1	1	SArRNA05
gi|29165615|ref|NC_002745.2|	551604	+	A	5	2	2	0	0	SArRNA05
gi|29165615|ref|NC_002745.2|	551605	+	C	1	0	0	3	2	SArRNA05
gi|29165615|ref|NC_002745.2|	551606	+	U	0	0	0	0	4	SArRNA05
gi|29165615|ref|NC_002745.2|	551607	+	C	5	1	0	1	1	SArRNA05
gi|29165615|ref|NC_002745.2|	551608	+	U	1	2	0	0	2	SArRNA05
gi|29165615|ref|NC_002745.2|	551609	+	A	2	1	0	0	0	SArRNA05
gi|29165615|ref|NC_002745.2|	551610	+	G	2	1	1	1	1	SArRNA05

gi|29165615|ref|NC_002745.2|	551611	+	A	0	0	1	1	1	SArRNA05
gi|29165615|ref|NC_002745.2|	551612	+	G	2	1	0	0	3	SArRNA05
gi|29165615|ref|NC_002745.2|	551613	+	A	0	2	2	1	1	SArRNA05
gi|29165615|ref|NC_002745.2|	551614	+	U	3	0	1	1	0	SArRNA05
gi|29165615|ref|NC_002745.2|	551615	+	A	1	0	0	1	1	SArRNA05
gi|29165615|ref|NC_002745.2|	551616	+	G	0	0	1	0	0	SArRNA05
gi|29165615|ref|NC_002745.2|	551618	+	G	0	0	0	1	0	SArRNA05
gi|29165615|ref|NC_002745.2|	551619	+	C	2	0	0	1	0	SArRNA05
gi|29165615|ref|NC_002745.2|	551620	+	C	33	7	4	6	4	SArRNA05
gi|29165615|ref|NC_002745.2|	551621	+	U	1	1	0	0	0	SArRNA05
gi|29165615|ref|NC_002745.2|	551622	+	U	2	1	1	0	0	SArRNA05
gi|29165615|ref|NC_002745.2|	551623	+	C	14	4	1	5	3	SArRNA05
gi|29165615|ref|NC_002745.2|	551624	+	C	4	6	1	1	2	SArRNA05
gi|29165615|ref|NC_002745.2|	551625	+	C	0	0	0	1	2	SArRNA05
gi|29165615|ref|NC_002745.2|	551626	+	C	0	0	1	0	3	SArRNA05
gi|29165615|ref|NC_002745.2|	551627	+	U	0	1	0	0	0	SArRNA05
gi|29165615|ref|NC_002745.2|	551629	+	C	3	1	0	0	1	SArRNA05
gi|29165615|ref|NC_002745.2|	551631	+	G	0	1	0	0	0	SArRNA05
gi|29165615|ref|NC_002745.2|	551633	+	G	0	0	0	0	2	SArRNA05
gi|29165615|ref|NC_002745.2|	551634	+	G	0	1	0	0	0	SArRNA05
gi|29165615|ref|NC_002745.2|	551635	+	A	0	0	1	0	1	SArRNA05
gi|29165615|ref|NC_002745.2|	551636	+	C	9	0	1	0	0	SArRNA05
gi|29165615|ref|NC_002745.2|	551637	+	A	3	1	0	0	1	SArRNA05
gi|29165615|ref|NC_002745.2|	551638	+	A	0	1	0	0	0	SArRNA05
gi|29165615|ref|NC_002745.2|	551639	+	A	0	1	0	0	3	SArRNA05
gi|29165615|ref|NC_002745.2|	551640	+	G	0	1	0	0	3	SArRNA05
gi|29165615|ref|NC_002745.2|	551641	+	U	0	0	0	0	1	SArRNA05
gi|29165615|ref|NC_002745.2|	551642	+	G	1	1	1	0	1	SArRNA05
gi|29165615|ref|NC_002745.2|	551643	+	A	0	1	1	0	0	SArRNA05
gi|29165615|ref|NC_002745.2|	551644	+	C	21	35	8	18	16	SArRNA05
gi|29165615|ref|NC_002745.2|	551645	+	A	4	2	0	0	1	SArRNA05
gi|29165615|ref|NC_002745.2|	551646	+	G	0	1	0	0	2	SArRNA05
gi|29165615|ref|NC_002745.2|	551647	+	G	0	1	0	0	2	SArRNA05
gi|29165615|ref|NC_002745.2|	551648	+	U	4	1	0	0	1	SArRNA05
gi|29165615|ref|NC_002745.2|	551650	+	G	2	1	0	1	1	SArRNA05
gi|29165615|ref|NC_002745.2|	551651	+	U	1	0	2	1	0	SArRNA05
gi|29165615|ref|NC_002745.2|	551652	+	G	2	0	0	0	0	SArRNA05
gi|29165615|ref|NC_002745.2|	551653	+	C	0	0	0	0	1	SArRNA05
gi|29165615|ref|NC_002745.2|	551654	+	A	2	0	0	0	2	SArRNA05
gi|29165615|ref|NC_002745.2|	551655	+	U	1	1	0	0	0	SArRNA05
gi|29165615|ref|NC_002745.2|	551656	+	G	3	1	2	0	0	SArRNA05
gi|29165615|ref|NC_002745.2|	551658	+	U	0	0	0	0	3	SArRNA05
gi|29165615|ref|NC_002745.2|	551659	+	U	3	0	0	0	0	SArRNA05
gi|29165615|ref|NC_002745.2|	551661	+	U	1	0	0	0	0	SArRNA05
gi|29165615|ref|NC_002745.2|	551662	+	C	2	0	0	0	0	SArRNA05
gi|29165615|ref|NC_002745.2|	551663	+	G	0	0	0	0	1	SArRNA05
gi|29165615|ref|NC_002745.2|	551664	+	U	0	0	0	0	2	SArRNA05
gi|29165615|ref|NC_002745.2|	551665	+	C	1	0	0	0	0	SArRNA05
gi|29165615|ref|NC_002745.2|	551668	+	C	0	1	0	1	1	SArRNA05
gi|29165615|ref|NC_002745.2|	551670	+	C	1	1	0	0	0	SArRNA05
gi|29165615|ref|NC_002745.2|	551677	+	U	2	0	0	0	0	SArRNA05
gi|29165615|ref|NC_002745.2|	551679	+	A	1	0	0	0	0	SArRNA05
gi|29165615|ref|NC_002745.2|	551680	+	G	1	0	0	0	2	SArRNA05
gi|29165615|ref|NC_002745.2|	551682	+	U	1	2	0	0	0	SArRNA05
gi|29165615|ref|NC_002745.2|	551687	+	G	0	0	1	5	1	SArRNA05
gi|29165615|ref|NC_002745.2|	551690	+	U	0	0	1	0	0	SArRNA05
gi|29165615|ref|NC_002745.2|	551691	+	A	0	0	1	0	0	SArRNA05
gi|29165615|ref|NC_002745.2|	551692	+	A	1	1	0	0	3	SArRNA05
gi|29165615|ref|NC_002745.2|	551693	+	G	0	0	0	0	1	SArRNA05
gi|29165615|ref|NC_002745.2|	551694	+	U	2	0	0	0	0	SArRNA05
gi|29165615|ref|NC_002745.2|	551695	+	C	2	4	0	0	2	SArRNA05
gi|29165615|ref|NC_002745.2|	551696	+	C	1	4	1	1	1	SArRNA05
gi|29165615|ref|NC_002745.2|	551697	+	C	3	3	0	0	0	SArRNA05
gi|29165615|ref|NC_002745.2|	551698	+	G	2	6	0	0	1	SArRNA05
gi|29165615|ref|NC_002745.2|	551699	+	C	0	2	0	0	0	SArRNA05
gi|29165615|ref|NC_002745.2|	551700	+	A	4	0	0	0	0	SArRNA05
gi|29165615|ref|NC_002745.2|	551701	+	A	16	0	1	0	2	SArRNA05
gi|29165615|ref|NC_002745.2|	551702	+	C	34	4	2	5	4	SArRNA05
gi|29165615|ref|NC_002745.2|	551703	+	G	13	2	1	1	1	SArRNA05
gi|29165615|ref|NC_002745.2|	551704	+	A	8	2	1	1	2	SArRNA05
gi|29165615|ref|NC_002745.2|	551705	+	G	2	0	0	0	1	SArRNA05
gi|29165615|ref|NC_002745.2|	551706	+	C	12	4	2	1	1	SArRNA05
gi|29165615|ref|NC_002745.2|	551707	+	G	5	4	0	1	1	SArRNA05
gi|29165615|ref|NC_002745.2|	551708	+	C	23	2	2	3	2	SArRNA05
gi|29165615|ref|NC_002745.2|	551709	+	A	14	11	0	3	2	SArRNA05
gi|29165615|ref|NC_002745.2|	551710	+	A	6	5	2	3	8	SArRNA05
gi|29165615|ref|NC_002745.2|	551711	+	C	34	3	8	4	7	SArRNA05
gi|29165615|ref|NC_002745.2|	551712	+	C	24	14	2	4	4	SArRNA05
gi|29165615|ref|NC_002745.2|	551713	+	C	13	2	1	2	3	SArRNA05
gi|29165615|ref|NC_002745.2|	551714	+	U	4	8	2	4	6	SArRNA05
gi|29165615|ref|NC_002745.2|	551715	+	U	5	7	0	0	2	SArRNA05

gi|29165615|ref|NC_002745.2|	551716	+	A	2	8	0	1	1	SArRNA05
gi|29165615|ref|NC_002745.2|	551717	+	A	1	0	0	0	0	SArRNA05
gi|29165615|ref|NC_002745.2|	551719	+	C	0	0	0	0	2	SArRNA05
gi|29165615|ref|NC_002745.2|	551720	+	U	0	0	0	1	0	SArRNA05
gi|29165615|ref|NC_002745.2|	551721	+	U	0	2	0	1	3	SArRNA05
gi|29165615|ref|NC_002745.2|	551722	+	A	0	6	0	1	2	SArRNA05
gi|29165615|ref|NC_002745.2|	551723	+	G	1	2	0	0	3	SArRNA05
gi|29165615|ref|NC_002745.2|	551724	+	U	6	8	0	5	1	SArRNA05
gi|29165615|ref|NC_002745.2|	551725	+	U	5	4	2	0	2	SArRNA05
gi|29165615|ref|NC_002745.2|	551726	+	G	5	6	3	1	2	SArRNA05
gi|29165615|ref|NC_002745.2|	551727	+	C	4	9	0	1	3	SArRNA05
gi|29165615|ref|NC_002745.2|	551728	+	C	13	7	9	4	11	SArRNA05
gi|29165615|ref|NC_002745.2|	551729	+	A	10	15	4	6	7	SArRNA05
gi|29165615|ref|NC_002745.2|	551730	+	U	21	28	4	8	7	SArRNA05
gi|29165615|ref|NC_002745.2|	551731	+	C	11	5	2	8	9	SArRNA05
gi|29165615|ref|NC_002745.2|	551732	+	A	7	3	1	5	11	SArRNA05
gi|29165615|ref|NC_002745.2|	551733	+	U	8	5	3	0	5	SArRNA05
gi|29165615|ref|NC_002745.2|	551734	+	U	4	13	0	3	9	SArRNA05
gi|29165615|ref|NC_002745.2|	551735	+	A	11	3	0	3	3	SArRNA05
gi|29165615|ref|NC_002745.2|	551736	+	A	8	5	4	4	4	SArRNA05
gi|29165615|ref|NC_002745.2|	551737	+	G	5	5	1	2	4	SArRNA05
gi|29165615|ref|NC_002745.2|	551738	+	U	2	1	1	1	1	SArRNA05
gi|29165615|ref|NC_002745.2|	551739	+	U	2	12	1	1	4	SArRNA05
gi|29165615|ref|NC_002745.2|	551740	+	G	0	3	2	1	2	SArRNA05
gi|29165615|ref|NC_002745.2|	551741	+	G	1	2	0	1	1	SArRNA05
gi|29165615|ref|NC_002745.2|	551742	+	G	1	3	1	0	3	SArRNA05
gi|29165615|ref|NC_002745.2|	551743	+	C	12	14	8	4	10	SArRNA05
gi|29165615|ref|NC_002745.2|	551744	+	A	3	1	2	4	2	SArRNA05
gi|29165615|ref|NC_002745.2|	551745	+	C	2	7	0	5	5	SArRNA05
gi|29165615|ref|NC_002745.2|	551746	+	U	1	5	2	0	2	SArRNA05
gi|29165615|ref|NC_002745.2|	551747	+	C	4	8	2	1	1	SArRNA05
gi|29165615|ref|NC_002745.2|	551748	+	U	2	4	0	0	5	SArRNA05
gi|29165615|ref|NC_002745.2|	551749	+	A	3	4	0	3	1	SArRNA05
gi|29165615|ref|NC_002745.2|	551750	+	A	4	4	1	0	2	SArRNA05
gi|29165615|ref|NC_002745.2|	551751	+	G	9	14	1	1	7	SArRNA05
gi|29165615|ref|NC_002745.2|	551752	+	U	1	3	0	0	5	SArRNA05
gi|29165615|ref|NC_002745.2|	551753	+	U	0	0	3	2	1	SArRNA05
gi|29165615|ref|NC_002745.2|	551754	+	G	0	3	0	1	1	SArRNA05
gi|29165615|ref|NC_002745.2|	551755	+	A	1	3	0	0	1	SArRNA05
gi|29165615|ref|NC_002745.2|	551756	+	C	3	7	1	2	3	SArRNA05
gi|29165615|ref|NC_002745.2|	551757	+	U	6	1	1	1	1	SArRNA05
gi|29165615|ref|NC_002745.2|	551758	+	G	2	0	0	0	0	SArRNA05
gi|29165615|ref|NC_002745.2|	551759	+	C	1	0	0	0	0	SArRNA05
gi|29165615|ref|NC_002745.2|	551760	+	C	3	5	0	0	4	SArRNA05
gi|29165615|ref|NC_002745.2|	551761	+	G	7	3	0	0	1	SArRNA05
gi|29165615|ref|NC_002745.2|	551762	+	G	1	4	0	1	1	SArRNA05
gi|29165615|ref|NC_002745.2|	551763	+	U	1	3	3	2	4	SArRNA05
gi|29165615|ref|NC_002745.2|	551764	+	G	6	3	1	1	2	SArRNA05
gi|29165615|ref|NC_002745.2|	551765	+	A	1	4	2	1	1	SArRNA05
gi|29165615|ref|NC_002745.2|	551766	+	C	8	19	6	3	15	SArRNA05
gi|29165615|ref|NC_002745.2|	551767	+	A	5	6	0	3	6	SArRNA05
gi|29165615|ref|NC_002745.2|	551768	+	A	15	50	2	3	17	SArRNA05
gi|29165615|ref|NC_002745.2|	551769	+	A	2	8	1	0	6	SArRNA05
gi|29165615|ref|NC_002745.2|	551770	+	C	7	3	5	4	4	SArRNA05
gi|29165615|ref|NC_002745.2|	551771	+	C	11	13	4	5	3	SArRNA05
gi|29165615|ref|NC_002745.2|	551772	+	G	5	10	1	0	4	SArRNA05
gi|29165615|ref|NC_002745.2|	551773	+	G	11	3	3	6	2	SArRNA05
gi|29165615|ref|NC_002745.2|	551774	+	A	4	3	0	5	2	SArRNA05
gi|29165615|ref|NC_002745.2|	551775	+	G	4	8	1	3	2	SArRNA05
gi|29165615|ref|NC_002745.2|	551776	+	G	1	6	3	2	5	SArRNA05
gi|29165615|ref|NC_002745.2|	551777	+	A	16	14	13	15	20	SArRNA05
gi|29165615|ref|NC_002745.2|	551778	+	A	10	16	3	7	7	SArRNA05
gi|29165615|ref|NC_002745.2|	551779	+	G	2	1	0	1	1	SArRNA05
gi|29165615|ref|NC_002745.2|	551780	+	G	8	5	3	3	6	SArRNA05
gi|29165615|ref|NC_002745.2|	551781	+	U	3	3	1	0	0	SArRNA05
gi|29165615|ref|NC_002745.2|	551782	+	G	0	0	0	2	0	SArRNA05
gi|29165615|ref|NC_002745.2|	551783	+	G	0	1	0	0	0	SArRNA05
gi|29165615|ref|NC_002745.2|	551785	+	G	2	3	0	3	2	SArRNA05
gi|29165615|ref|NC_002745.2|	551786	+	A	1	4	2	3	1	SArRNA05
gi|29165615|ref|NC_002745.2|	551787	+	U	3	7	1	1	3	SArRNA05
gi|29165615|ref|NC_002745.2|	551788	+	G	3	2	0	1	8	SArRNA05
gi|29165615|ref|NC_002745.2|	551789	+	A	10	4	2	2	8	SArRNA05
gi|29165615|ref|NC_002745.2|	551790	+	C	3	3	2	2	7	SArRNA05
gi|29165615|ref|NC_002745.2|	551791	+	G	2	2	5	2	2	SArRNA05
gi|29165615|ref|NC_002745.2|	551792	+	U	0	3	1	3	1	SArRNA05
gi|29165615|ref|NC_002745.2|	551793	+	C	4	1	0	1	4	SArRNA05
gi|29165615|ref|NC_002745.2|	551794	+	A	0	0	0	2	4	SArRNA05
gi|29165615|ref|NC_002745.2|	551795	+	A	7	20	2	3	10	SArRNA05
gi|29165615|ref|NC_002745.2|	551796	+	A	7	14	2	6	10	SArRNA05
gi|29165615|ref|NC_002745.2|	551797	+	U	9	8	6	6	10	SArRNA05
gi|29165615|ref|NC_002745.2|	551798	+	C	11	20	4	8	10	SArRNA05

gi|29165615|ref|NC_002745.2|	551799	+	A	7	11	3	8	3	SArRNA05
gi|29165615|ref|NC_002745.2|	551800	+	U	5	2	0	1	5	SArRNA05
gi|29165615|ref|NC_002745.2|	551801	+	C	6	15	0	6	4	SArRNA05
gi|29165615|ref|NC_002745.2|	551802	+	A	4	4	0	1	4	SArRNA05
gi|29165615|ref|NC_002745.2|	551803	+	U	4	4	0	0	1	SArRNA05
gi|29165615|ref|NC_002745.2|	551804	+	G	0	1	0	1	1	SArRNA05
gi|29165615|ref|NC_002745.2|	551805	+	C	22	17	5	11	13	SArRNA05
gi|29165615|ref|NC_002745.2|	551806	+	C	7	13	3	5	3	SArRNA05
gi|29165615|ref|NC_002745.2|	551807	+	C	4	4	0	1	5	SArRNA05
gi|29165615|ref|NC_002745.2|	551808	+	C	2	4	4	1	7	SArRNA05
gi|29165615|ref|NC_002745.2|	551809	+	U	5	3	2	0	2	SArRNA05
gi|29165615|ref|NC_002745.2|	551810	+	U	7	7	3	1	4	SArRNA05
gi|29165615|ref|NC_002745.2|	551811	+	A	7	4	2	3	4	SArRNA05
gi|29165615|ref|NC_002745.2|	551812	+	U	11	7	6	6	11	SArRNA05
gi|29165615|ref|NC_002745.2|	551813	+	G	16	10	6	10	11	SArRNA05
gi|29165615|ref|NC_002745.2|	551814	+	A	9	5	1	8	13	SArRNA05
gi|29165615|ref|NC_002745.2|	551815	+	U	5	7	3	7	13	SArRNA05
gi|29165615|ref|NC_002745.2|	551816	+	U	6	1	0	0	5	SArRNA05
gi|29165615|ref|NC_002745.2|	551817	+	U	1	4	0	0	0	SArRNA05
gi|29165615|ref|NC_002745.2|	551818	+	G	1	1	1	1	0	SArRNA05
gi|29165615|ref|NC_002745.2|	551819	+	G	0	1	0	1	2	SArRNA05
gi|29165615|ref|NC_002745.2|	551820	+	G	2	1	0	1	1	SArRNA05
gi|29165615|ref|NC_002745.2|	551821	+	C	8	9	1	2	2	SArRNA05
gi|29165615|ref|NC_002745.2|	551822	+	U	8	9	3	5	6	SArRNA05
gi|29165615|ref|NC_002745.2|	551823	+	A	6	2	0	3	4	SArRNA05
gi|29165615|ref|NC_002745.2|	551824	+	C	6	7	0	2	3	SArRNA05
gi|29165615|ref|NC_002745.2|	551825	+	A	6	7	2	0	8	SArRNA05
gi|29165615|ref|NC_002745.2|	551826	+	C	46	29	17	14	30	SArRNA05
gi|29165615|ref|NC_002745.2|	551827	+	A	4	12	0	0	6	SArRNA05
gi|29165615|ref|NC_002745.2|	551828	+	C	0	5	1	3	0	SArRNA05
gi|29165615|ref|NC_002745.2|	551829	+	G	4	2	3	2	2	SArRNA05
gi|29165615|ref|NC_002745.2|	551830	+	U	0	0	1	0	0	SArRNA05
gi|29165615|ref|NC_002745.2|	551831	+	G	1	0	0	0	0	SArRNA05
gi|29165615|ref|NC_002745.2|	551832	+	C	5	3	4	0	2	SArRNA05
gi|29165615|ref|NC_002745.2|	551833	+	U	1	5	3	5	3	SArRNA05
gi|29165615|ref|NC_002745.2|	551834	+	A	0	2	0	0	0	SArRNA05
gi|29165615|ref|NC_002745.2|	551835	+	C	14	14	4	8	16	SArRNA05
gi|29165615|ref|NC_002745.2|	551836	+	A	1	7	4	4	5	SArRNA05
gi|29165615|ref|NC_002745.2|	551837	+	A	0	7	0	0	1	SArRNA05
gi|29165615|ref|NC_002745.2|	551838	+	U	0	5	1	1	1	SArRNA05
gi|29165615|ref|NC_002745.2|	551839	+	G	6	6	0	1	3	SArRNA05
gi|29165615|ref|NC_002745.2|	551840	+	G	15	11	6	7	2	SArRNA05
gi|29165615|ref|NC_002745.2|	551841	+	A	1	6	0	0	1	SArRNA05
gi|29165615|ref|NC_002745.2|	551842	+	C	51	41	21	15	29	SArRNA05
gi|29165615|ref|NC_002745.2|	551843	+	A	18	17	8	5	10	SArRNA05
gi|29165615|ref|NC_002745.2|	551844	+	A	25	213	56	12	114	SArRNA05
gi|29165615|ref|NC_002745.2|	551845	+	U	4	3	2	2	6	SArRNA05
gi|29165615|ref|NC_002745.2|	551846	+	A	2	9	3	6	6	SArRNA05
gi|29165615|ref|NC_002745.2|	551847	+	C	3	11	2	1	3	SArRNA05
gi|29165615|ref|NC_002745.2|	551848	+	A	1	1	0	2	3	SArRNA05
gi|29165615|ref|NC_002745.2|	551849	+	A	3	17	0	1	6	SArRNA05
gi|29165615|ref|NC_002745.2|	551850	+	A	4	20	0	2	10	SArRNA05
gi|29165615|ref|NC_002745.2|	551851	+	G	0	0	0	2	0	SArRNA05
gi|29165615|ref|NC_002745.2|	551852	+	G	0	2	0	0	0	SArRNA05
gi|29165615|ref|NC_002745.2|	551853	+	G	1	1	0	1	0	SArRNA05
gi|29165615|ref|NC_002745.2|	551854	+	C	10	5	3	4	1	SArRNA05
gi|29165615|ref|NC_002745.2|	551855	+	A	1	8	1	0	3	SArRNA05
gi|29165615|ref|NC_002745.2|	551856	+	G	0	2	0	0	0	SArRNA05
gi|29165615|ref|NC_002745.2|	551857	+	C	2	2	3	3	3	SArRNA05
gi|29165615|ref|NC_002745.2|	551858	+	G	0	0	0	0	2	SArRNA05
gi|29165615|ref|NC_002745.2|	551859	+	A	0	0	0	0	2	SArRNA05
gi|29165615|ref|NC_002745.2|	551860	+	A	6	7	3	3	6	SArRNA05
gi|29165615|ref|NC_002745.2|	551861	+	A	3	5	2	3	2	SArRNA05
gi|29165615|ref|NC_002745.2|	551862	+	C	2	1	0	0	2	SArRNA05
gi|29165615|ref|NC_002745.2|	551863	+	C	3	4	2	2	6	SArRNA05
gi|29165615|ref|NC_002745.2|	551864	+	G	0	0	0	0	4	SArRNA05
gi|29165615|ref|NC_002745.2|	551865	+	C	0	3	0	0	0	SArRNA05
gi|29165615|ref|NC_002745.2|	551867	+	A	1	1	0	0	1	SArRNA05
gi|29165615|ref|NC_002745.2|	551868	+	G	0	1	0	0	0	SArRNA05
gi|29165615|ref|NC_002745.2|	551869	+	G	1	0	0	1	2	SArRNA05
gi|29165615|ref|NC_002745.2|	551870	+	U	0	1	0	0	1	SArRNA05
gi|29165615|ref|NC_002745.2|	551871	+	C	11	9	6	5	3	SArRNA05
gi|29165615|ref|NC_002745.2|	551872	+	A	2	1	1	1	4	SArRNA05
gi|29165615|ref|NC_002745.2|	551873	+	A	1	0	2	1	1	SArRNA05
gi|29165615|ref|NC_002745.2|	551874	+	G	2	0	0	1	0	SArRNA05
gi|29165615|ref|NC_002745.2|	551875	+	C	0	0	0	0	1	SArRNA05
gi|29165615|ref|NC_002745.2|	551876	+	A	2	2	0	1	1	SArRNA05
gi|29165615|ref|NC_002745.2|	551877	+	A	0	2	0	0	3	SArRNA05
gi|29165615|ref|NC_002745.2|	551878	+	A	9	16	1	3	11	SArRNA05
gi|29165615|ref|NC_002745.2|	551879	+	U	14	20	3	13	13	SArRNA05
gi|29165615|ref|NC_002745.2|	551880	+	C	14	29	12	13	14	SArRNA05

gi|29165615|ref|NC_002745.2|	551881	+	C	14	11	7	5	8	SArRNA05
gi|29165615|ref|NC_002745.2|	551882	+	C	2	3	1	1	1	SArRNA05
gi|29165615|ref|NC_002745.2|	551883	+	A	0	3	1	1	1	SArRNA05
gi|29165615|ref|NC_002745.2|	551884	+	U	1	3	1	0	2	SArRNA05
gi|29165615|ref|NC_002745.2|	551885	+	A	2	3	0	0	1	SArRNA05
gi|29165615|ref|NC_002745.2|	551886	+	A	0	1	0	0	3	SArRNA05
gi|29165615|ref|NC_002745.2|	551887	+	A	1	3	1	0	2	SArRNA05
gi|29165615|ref|NC_002745.2|	551888	+	G	2	0	0	0	0	SArRNA05
gi|29165615|ref|NC_002745.2|	551889	+	U	0	1	1	1	2	SArRNA05
gi|29165615|ref|NC_002745.2|	551890	+	U	2	4	0	1	0	SArRNA05
gi|29165615|ref|NC_002745.2|	551891	+	G	4	6	1	0	5	SArRNA05
gi|29165615|ref|NC_002745.2|	551892	+	U	0	0	1	0	1	SArRNA05
gi|29165615|ref|NC_002745.2|	551893	+	U	3	1	0	0	1	SArRNA05
gi|29165615|ref|NC_002745.2|	551894	+	C	3	6	3	1	5	SArRNA05
gi|29165615|ref|NC_002745.2|	551895	+	U	2	1	3	0	1	SArRNA05
gi|29165615|ref|NC_002745.2|	551896	+	C	1	8	0	0	1	SArRNA05
gi|29165615|ref|NC_002745.2|	551897	+	A	2	1	3	0	3	SArRNA05
gi|29165615|ref|NC_002745.2|	551898	+	G	0	1	0	2	1	SArRNA05
gi|29165615|ref|NC_002745.2|	551899	+	U	2	3	2	0	1	SArRNA05
gi|29165615|ref|NC_002745.2|	551900	+	U	0	3	2	2	1	SArRNA05
gi|29165615|ref|NC_002745.2|	551901	+	C	3	5	4	2	6	SArRNA05
gi|29165615|ref|NC_002745.2|	551902	+	G	0	0	0	0	1	SArRNA05
gi|29165615|ref|NC_002745.2|	551903	+	G	0	0	1	0	0	SArRNA05
gi|29165615|ref|NC_002745.2|	551904	+	A	0	1	0	0	0	SArRNA05
gi|29165615|ref|NC_002745.2|	551906	+	U	0	2	0	0	0	SArRNA05
gi|29165615|ref|NC_002745.2|	551909	+	A	0	0	0	0	2	SArRNA05
gi|29165615|ref|NC_002745.2|	551911	+	U	1	0	0	1	0	SArRNA05
gi|29165615|ref|NC_002745.2|	551913	+	U	0	1	0	0	0	SArRNA05
gi|29165615|ref|NC_002745.2|	551915	+	C	0	7	1	1	2	SArRNA05
gi|29165615|ref|NC_002745.2|	551916	+	A	0	2	0	0	1	SArRNA05
gi|29165615|ref|NC_002745.2|	551917	+	A	0	1	0	0	1	SArRNA05
gi|29165615|ref|NC_002745.2|	551918	+	C	2	2	0	0	3	SArRNA05
gi|29165615|ref|NC_002745.2|	551919	+	U	1	3	0	0	1	SArRNA05
gi|29165615|ref|NC_002745.2|	551920	+	C	32	30	9	13	26	SArRNA05
gi|29165615|ref|NC_002745.2|	551921	+	G	2	7	0	4	2	SArRNA05
gi|29165615|ref|NC_002745.2|	551923	+	C	3	2	0	0	0	SArRNA05
gi|29165615|ref|NC_002745.2|	551924	+	U	0	2	0	0	0	SArRNA05
gi|29165615|ref|NC_002745.2|	551925	+	A	0	2	2	1	1	SArRNA05
gi|29165615|ref|NC_002745.2|	551926	+	C	1	0	0	1	0	SArRNA05
gi|29165615|ref|NC_002745.2|	551927	+	A	2	2	0	1	0	SArRNA05
gi|29165615|ref|NC_002745.2|	551928	+	U	0	0	2	0	1	SArRNA05
gi|29165615|ref|NC_002745.2|	551929	+	G	4	8	1	3	4	SArRNA05
gi|29165615|ref|NC_002745.2|	551930	+	A	0	0	0	1	3	SArRNA05
gi|29165615|ref|NC_002745.2|	551931	+	A	3	3	0	2	2	SArRNA05
gi|29165615|ref|NC_002745.2|	551932	+	G	3	1	0	1	1	SArRNA05
gi|29165615|ref|NC_002745.2|	551933	+	C	2	2	0	0	2	SArRNA05
gi|29165615|ref|NC_002745.2|	551934	+	U	0	1	0	0	2	SArRNA05
gi|29165615|ref|NC_002745.2|	551935	+	G	0	0	0	0	1	SArRNA05
gi|29165615|ref|NC_002745.2|	551936	+	G	0	3	1	1	1	SArRNA05
gi|29165615|ref|NC_002745.2|	551937	+	A	0	3	0	2	7	SArRNA05
gi|29165615|ref|NC_002745.2|	551938	+	A	0	5	0	1	8	SArRNA05
gi|29165615|ref|NC_002745.2|	551939	+	U	1	1	1	0	2	SArRNA05
gi|29165615|ref|NC_002745.2|	551940	+	C	0	1	0	0	2	SArRNA05
gi|29165615|ref|NC_002745.2|	551941	+	G	3	5	0	1	0	SArRNA05
gi|29165615|ref|NC_002745.2|	551942	+	C	4	6	3	4	4	SArRNA05
gi|29165615|ref|NC_002745.2|	551943	+	U	0	1	0	0	3	SArRNA05
gi|29165615|ref|NC_002745.2|	551944	+	A	0	1	0	1	0	SArRNA05
gi|29165615|ref|NC_002745.2|	551945	+	G	0	1	1	0	2	SArRNA05
gi|29165615|ref|NC_002745.2|	551946	+	U	0	1	0	0	0	SArRNA05
gi|29165615|ref|NC_002745.2|	551947	+	A	0	1	0	0	1	SArRNA05
gi|29165615|ref|NC_002745.2|	551948	+	A	1	11	1	0	5	SArRNA05
gi|29165615|ref|NC_002745.2|	551951	+	G	0	1	0	0	1	SArRNA05
gi|29165615|ref|NC_002745.2|	551952	+	U	0	4	0	1	3	SArRNA05
gi|29165615|ref|NC_002745.2|	551953	+	A	0	0	0	0	1	SArRNA05
gi|29165615|ref|NC_002745.2|	551954	+	G	0	0	0	0	1	SArRNA05
gi|29165615|ref|NC_002745.2|	551955	+	A	2	1	0	0	2	SArRNA05
gi|29165615|ref|NC_002745.2|	551956	+	U	1	3	0	2	2	SArRNA05
gi|29165615|ref|NC_002745.2|	551957	+	C	1	1	0	0	3	SArRNA05
gi|29165615|ref|NC_002745.2|	551958	+	A	0	3	0	1	1	SArRNA05
gi|29165615|ref|NC_002745.2|	551960	+	C	4	2	0	2	2	SArRNA05
gi|29165615|ref|NC_002745.2|	551961	+	A	2	3	0	0	1	SArRNA05
gi|29165615|ref|NC_002745.2|	551962	+	U	2	1	0	0	2	SArRNA05
gi|29165615|ref|NC_002745.2|	551963	+	G	0	2	0	0	0	SArRNA05
gi|29165615|ref|NC_002745.2|	551964	+	C	3	2	0	0	2	SArRNA05
gi|29165615|ref|NC_002745.2|	551965	+	U	0	0	0	0	1	SArRNA05
gi|29165615|ref|NC_002745.2|	551966	+	A	0	0	0	1	0	SArRNA05
gi|29165615|ref|NC_002745.2|	551967	+	C	0	0	0	2	1	SArRNA05
gi|29165615|ref|NC_002745.2|	551968	+	G	0	2	0	0	1	SArRNA05
gi|29165615|ref|NC_002745.2|	551969	+	G	1	3	1	0	0	SArRNA05
gi|29165615|ref|NC_002745.2|	551970	+	U	0	0	0	0	1	SArRNA05
gi|29165615|ref|NC_002745.2|	551972	+	A	0	1	0	0	0	SArRNA05

gi|29165615|ref|NC_002745.2|	551973	+	A	1	5	0	3	0	SArRNA05
gi|29165615|ref|NC_002745.2|	551974	+	U	1	5	0	1	1	SArRNA05
gi|29165615|ref|NC_002745.2|	551975	+	A	0	3	0	0	0	SArRNA05
gi|29165615|ref|NC_002745.2|	551976	+	C	1	0	0	2	0	SArRNA05
gi|29165615|ref|NC_002745.2|	551977	+	G	2	0	0	0	1	SArRNA05
gi|29165615|ref|NC_002745.2|	551979	+	U	2	1	0	0	2	SArRNA05
gi|29165615|ref|NC_002745.2|	551980	+	C	0	0	1	0	0	SArRNA05
gi|29165615|ref|NC_002745.2|	551981	+	C	0	0	0	0	1	SArRNA05
gi|29165615|ref|NC_002745.2|	551986	+	U	1	0	0	0	0	SArRNA05
gi|29165615|ref|NC_002745.2|	551987	+	C	2	3	0	0	0	SArRNA05
gi|29165615|ref|NC_002745.2|	551988	+	U	0	0	0	0	3	SArRNA05
gi|29165615|ref|NC_002745.2|	551993	+	C	0	3	0	0	0	SArRNA05
gi|29165615|ref|NC_002745.2|	551995	+	C	1	3	1	0	4	SArRNA05
gi|29165615|ref|NC_002745.2|	551996	+	A	0	1	0	0	0	SArRNA05
gi|29165615|ref|NC_002745.2|	551997	+	C	0	3	0	0	0	SArRNA05
gi|29165615|ref|NC_002745.2|	551998	+	C	0	1	0	0	1	SArRNA05
gi|29165615|ref|NC_002745.2|	551999	+	G	0	3	0	0	0	SArRNA05
gi|29165615|ref|NC_002745.2|	552002	+	C	0	1	0	0	0	SArRNA05
gi|29165615|ref|NC_002745.2|	552005	+	C	4	2	2	1	1	SArRNA05
gi|29165615|ref|NC_002745.2|	552007	+	C	5	1	0	2	0	SArRNA05
gi|29165615|ref|NC_002745.2|	552008	+	A	2	3	1	0	0	SArRNA05
gi|29165615|ref|NC_002745.2|	552009	+	C	0	3	1	0	2	SArRNA05
gi|29165615|ref|NC_002745.2|	552010	+	C	4	13	4	9	15	SArRNA05
gi|29165615|ref|NC_002745.2|	552011	+	A	0	1	3	0	1	SArRNA05
gi|29165615|ref|NC_002745.2|	552014	+	A	2	0	0	0	0	SArRNA05
gi|29165615|ref|NC_002745.2|	552015	+	G	1	0	0	0	0	SArRNA05
gi|29165615|ref|NC_002745.2|	552018	+	U	0	0	0	1	0	SArRNA05
gi|29165615|ref|NC_002745.2|	552020	+	U	0	0	0	0	1	SArRNA05
gi|29165615|ref|NC_002745.2|	552021	+	G	0	0	0	1	0	SArRNA05
gi|29165615|ref|NC_002745.2|	552022	+	U	0	0	0	0	3	SArRNA05
gi|29165615|ref|NC_002745.2|	552023	+	A	1	0	1	1	1	SArRNA05
gi|29165615|ref|NC_002745.2|	552024	+	A	1	3	0	0	1	SArRNA05
gi|29165615|ref|NC_002745.2|	552025	+	C	37	43	21	14	45	SArRNA05
gi|29165615|ref|NC_002745.2|	552026	+	A	11	18	2	4	6	SArRNA05
gi|29165615|ref|NC_002745.2|	552027	+	C	41	30	16	8	19	SArRNA05
gi|29165615|ref|NC_002745.2|	552028	+	C	3	6	3	2	11	SArRNA05
gi|29165615|ref|NC_002745.2|	552029	+	C	18	20	4	7	16	SArRNA05
gi|29165615|ref|NC_002745.2|	552030	+	G	21	19	10	6	9	SArRNA05
gi|29165615|ref|NC_002745.2|	552031	+	A	3	6	2	2	3	SArRNA05
gi|29165615|ref|NC_002745.2|	552032	+	A	0	1	0	0	0	SArRNA05
gi|29165615|ref|NC_002745.2|	552033	+	G	0	1	1	0	0	SArRNA05
gi|29165615|ref|NC_002745.2|	552034	+	C	7	14	1	5	5	SArRNA05
gi|29165615|ref|NC_002745.2|	552035	+	C	10	15	4	5	4	SArRNA05
gi|29165615|ref|NC_002745.2|	552036	+	G	5	6	1	1	2	SArRNA05
gi|29165615|ref|NC_002745.2|	552038	+	U	1	4	0	0	0	SArRNA05
gi|29165615|ref|NC_002745.2|	552039	+	G	0	0	0	0	1	SArRNA05
gi|29165615|ref|NC_002745.2|	552040	+	G	2	0	0	1	0	SArRNA05
gi|29165615|ref|NC_002745.2|	552041	+	A	1	0	0	0	4	SArRNA05
gi|29165615|ref|NC_002745.2|	552042	+	G	3	4	2	0	2	SArRNA05
gi|29165615|ref|NC_002745.2|	552043	+	U	1	4	0	1	3	SArRNA05
gi|29165615|ref|NC_002745.2|	552044	+	A	13	30	3	5	11	SArRNA05
gi|29165615|ref|NC_002745.2|	552045	+	A	5	7	0	2	8	SArRNA05
gi|29165615|ref|NC_002745.2|	552046	+	C	17	31	6	11	22	SArRNA05
gi|29165615|ref|NC_002745.2|	552047	+	C	3	7	2	1	6	SArRNA05
gi|29165615|ref|NC_002745.2|	552048	+	U	10	2	4	4	4	SArRNA05
gi|29165615|ref|NC_002745.2|	552049	+	U	6	3	3	0	8	SArRNA05
gi|29165615|ref|NC_002745.2|	552050	+	U	0	1	0	0	4	SArRNA05
gi|29165615|ref|NC_002745.2|	552051	+	U	6	8	0	3	5	SArRNA05
gi|29165615|ref|NC_002745.2|	552052	+	A	1	11	0	1	4	SArRNA05
gi|29165615|ref|NC_002745.2|	552053	+	G	5	5	2	0	0	SArRNA05
gi|29165615|ref|NC_002745.2|	552054	+	G	3	0	1	1	4	SArRNA05
gi|29165615|ref|NC_002745.2|	552055	+	A	3	1	2	1	1	SArRNA05
gi|29165615|ref|NC_002745.2|	552056	+	G	1	0	3	1	1	SArRNA05
gi|29165615|ref|NC_002745.2|	552057	+	C	203	158	56	42	112	SArRNA05
gi|29165615|ref|NC_002745.2|	552058	+	U	16	26	3	3	23	SArRNA05
gi|29165615|ref|NC_002745.2|	552059	+	A	4	7	0	0	9	SArRNA05
gi|29165615|ref|NC_002745.2|	552060	+	G	8	6	3	2	7	SArRNA05
gi|29165615|ref|NC_002745.2|	552061	+	C	4	5	3	4	4	SArRNA05
gi|29165615|ref|NC_002745.2|	552062	+	C	62	37	21	17	22	SArRNA05
gi|29165615|ref|NC_002745.2|	552063	+	G	19	16	8	4	7	SArRNA05
gi|29165615|ref|NC_002745.2|	552064	+	U	0	0	2	1	0	SArRNA05
gi|29165615|ref|NC_002745.2|	552065	+	C	8	11	0	3	4	SArRNA05
gi|29165615|ref|NC_002745.2|	552066	+	G	6	3	2	2	6	SArRNA05
gi|29165615|ref|NC_002745.2|	552067	+	A	0	2	0	0	0	SArRNA05
gi|29165615|ref|NC_002745.2|	552069	+	G	1	0	0	0	0	SArRNA05
gi|29165615|ref|NC_002745.2|	552070	+	G	0	0	1	0	0	SArRNA05
gi|29165615|ref|NC_002745.2|	552071	+	U	1	0	0	0	0	SArRNA05
gi|29165615|ref|NC_002745.2|	552073	+	G	0	1	0	0	0	SArRNA05
gi|29165615|ref|NC_002745.2|	552100	+	C	1	0	0	0	0	SArRNA05
gi|29165615|ref|NC_002745.2|	552119	+	G	0	1	0	0	0	SArRNA05
gi|29165615|ref|NC_002745.2|	552134	+	C	2	0	0	0	0	SArRNA05

gi|29165615|ref|NC_002745.2|	552135	+	C	8	2	1	2	0	SArRNA05
gi|29165615|ref|NC_002745.2|	552136	+	U	0	0	0	0	1	SArRNA05
gi|29165615|ref|NC_002745.2|	552137	+	C	0	0	0	0	2	SArRNA05
gi|29165615|ref|NC_002745.2|	552138	+	C	0	1	0	0	0	SArRNA05
gi|29165615|ref|NC_002745.2|	552146	+	G	7	41	52	14	17	-
gi|29165615|ref|NC_002745.2|	552147	+	G	1	0	3	3	5	-
gi|29165615|ref|NC_002745.2|	552148	+	A	0	0	3	2	2	-
gi|29165615|ref|NC_002745.2|	552150	+	A	0	1	0	0	1	-
gi|29165615|ref|NC_002745.2|	552152	+	A	0	0	0	0	1	-
gi|29165615|ref|NC_002745.2|	552160	+	C	2	0	0	0	0	-
gi|29165615|ref|NC_002745.2|	552164	+	U	0	0	1	0	0	-
gi|29165615|ref|NC_002745.2|	552165	+	U	0	0	1	0	0	-
gi|29165615|ref|NC_002745.2|	552168	+	U	0	1	1	0	3	-
gi|29165615|ref|NC_002745.2|	552169	+	C	0	1	2	0	1	-
gi|29165615|ref|NC_002745.2|	552171	+	G	0	0	2	0	0	-
gi|29165615|ref|NC_002745.2|	552172	+	A	0	1	0	0	0	-
gi|29165615|ref|NC_002745.2|	552173	+	A	0	1	0	0	0	-
gi|29165615|ref|NC_002745.2|	552174	+	G	0	0	1	0	0	-
gi|29165615|ref|NC_002745.2|	552207	+	U	1	0	0	0	0	-
gi|29165615|ref|NC_002745.2|	552208	+	U	23	6	2	7	13	-
gi|29165615|ref|NC_002745.2|	552232	+	A	0	0	1	0	0	-
gi|29165615|ref|NC_002745.2|	552236	+	G	9	2	1	1	2	SAtRNA14
gi|29165615|ref|NC_002745.2|	552243	+	U	1	0	0	0	0	SAtRNA14
gi|29165615|ref|NC_002745.2|	552255	+	U	0	0	0	0	1	SAtRNA14
gi|29165615|ref|NC_002745.2|	552271	+	A	1	0	0	0	0	SAtRNA14
gi|29165615|ref|NC_002745.2|	552306	+	C	1	0	0	0	0	SAtRNA14
gi|29165615|ref|NC_002745.2|	552308	+	C	1	0	0	0	2	SAtRNA14
gi|29165615|ref|NC_002745.2|	552309	+	A	0	1	0	0	0	SAtRNA14
gi|29165615|ref|NC_002745.2|	552310	+	C	0	0	0	1	0	SAtRNA14
gi|29165615|ref|NC_002745.2|	552311	+	C	0	1	0	0	0	SAtRNA14
gi|29165615|ref|NC_002745.2|	552313	+	U	0	2	1	5	1	-
gi|29165615|ref|NC_002745.2|	552314	+	U	0	0	0	2	2	-
gi|29165615|ref|NC_002745.2|	552316	+	U	0	0	0	0	1	-
gi|29165615|ref|NC_002745.2|	552317	+	U	0	0	1	0	0	-
gi|29165615|ref|NC_002745.2|	552319	+	G	1	0	0	0	0	-
gi|29165615|ref|NC_002745.2|	552320	+	U	0	1	0	0	0	-
gi|29165615|ref|NC_002745.2|	552321	+	A	0	0	1	0	0	-
gi|29165615|ref|NC_002745.2|	552322	+	C	1	0	2	2	0	-
gi|29165615|ref|NC_002745.2|	552326	+	G	0	1	1	0	0	-
gi|29165615|ref|NC_002745.2|	552327	+	A	0	2	1	0	0	-
gi|29165615|ref|NC_002745.2|	552328	+	A	2	1	0	1	0	-
gi|29165615|ref|NC_002745.2|	552329	+	A	7	2	5	4	2	-
gi|29165615|ref|NC_002745.2|	552330	+	A	1136	220	223	625	436	-
gi|29165615|ref|NC_002745.2|	552331	+	C	2	2	2	9	6	-
gi|29165615|ref|NC_002745.2|	552332	+	U	1	0	1	2	1	-
gi|29165615|ref|NC_002745.2|	552333	+	A	0	2	0	3	2	-
gi|29165615|ref|NC_002745.2|	552334	+	G	1	1	2	2	1	-
gi|29165615|ref|NC_002745.2|	552335	+	A	1	1	1	4	3	-
gi|29165615|ref|NC_002745.2|	552336	+	U	0	0	0	0	1	-
gi|29165615|ref|NC_002745.2|	552337	+	A	0	1	1	1	0	-
gi|29165615|ref|NC_002745.2|	552339	+	G	0	0	0	1	0	-
gi|29165615|ref|NC_002745.2|	552340	+	U	2	0	0	1	0	-
gi|29165615|ref|NC_002745.2|	552341	+	A	0	0	1	2	0	-
gi|29165615|ref|NC_002745.2|	552342	+	A	0	0	0	1	0	-
gi|29165615|ref|NC_002745.2|	552343	+	G	0	0	0	1	0	-
gi|29165615|ref|NC_002745.2|	552345	+	A	0	0	1	7	0	-
gi|29165615|ref|NC_002745.2|	552346	+	A	0	0	0	2	0	-
gi|29165615|ref|NC_002745.2|	552347	+	A	1	3	0	2	0	-
gi|29165615|ref|NC_002745.2|	552348	+	A	0	1	0	4	0	-
gi|29165615|ref|NC_002745.2|	552350	+	A	0	0	0	3	0	-
gi|29165615|ref|NC_002745.2|	552351	+	U	0	0	0	2	0	-
gi|29165615|ref|NC_002745.2|	552352	+	A	0	0	1	1	0	-
gi|29165615|ref|NC_002745.2|	552353	+	G	0	4	3	5	3	-
gi|29165615|ref|NC_002745.2|	552354	+	A	0	2	2	7	1	-
gi|29165615|ref|NC_002745.2|	552356	+	U	0	1	1	0	0	-
gi|29165615|ref|NC_002745.2|	552358	+	U	0	0	1	0	0	-
gi|29165615|ref|NC_002745.2|	552359	+	A	0	1	0	1	0	-
gi|29165615|ref|NC_002745.2|	552360	+	C	1	2	0	1	0	-
gi|29165615|ref|NC_002745.2|	552361	+	C	4	1	2	9	3	-
gi|29165615|ref|NC_002745.2|	552362	+	A	1	0	0	3	2	-
gi|29165615|ref|NC_002745.2|	552363	+	A	0	0	0	1	1	-
gi|29165615|ref|NC_002745.2|	552364	+	G	1	0	0	1	0	-
gi|29165615|ref|NC_002745.2|	552365	+	C	2	0	0	5	1	-
gi|29165615|ref|NC_002745.2|	552366	+	A	1	1	1	8	1	-
gi|29165615|ref|NC_002745.2|	552367	+	A	0	5	2	13	1	-
gi|29165615|ref|NC_002745.2|	552368	+	A	1	1	0	4	1	-
gi|29165615|ref|NC_002745.2|	552369	+	A	0	0	1	0	0	-
gi|29165615|ref|NC_002745.2|	552370	+	C	0	0	0	0	2	-
gi|29165615|ref|NC_002745.2|	552371	+	C	3	1	1	1	3	-
gi|29165615|ref|NC_002745.2|	552372	+	G	1	2	0	4	1	-
gi|29165615|ref|NC_002745.2|	552373	+	A	0	0	0	2	0	-

gi|29165615|ref|NC_002745.2|	552374	+	G	0	0	0	0	1	-
gi|29165615|ref|NC_002745.2|	552375	+	U	0	0	0	1	0	-
gi|29165615|ref|NC_002745.2|	552377	+	A	0	1	0	8	0	-
gi|29165615|ref|NC_002745.2|	552378	+	A	0	0	0	0	1	-
gi|29165615|ref|NC_002745.2|	552380	+	A	1	0	1	2	1	-
gi|29165615|ref|NC_002745.2|	552381	+	A	0	1	0	2	0	-
gi|29165615|ref|NC_002745.2|	552383	+	G	0	0	0	2	0	-
gi|29165615|ref|NC_002745.2|	552384	+	A	0	1	1	11	2	-
gi|29165615|ref|NC_002745.2|	552385	+	G	0	0	0	2	1	-
gi|29165615|ref|NC_002745.2|	552386	+	U	0	0	0	1	0	-
gi|29165615|ref|NC_002745.2|	552388	+	U	0	0	1	0	0	-
gi|29165615|ref|NC_002745.2|	552389	+	U	2	0	1	5	0	-
gi|29165615|ref|NC_002745.2|	552390	+	A	1	4	0	36	3	-
gi|29165615|ref|NC_002745.2|	552391	+	A	0	9	1	30	5	-
gi|29165615|ref|NC_002745.2|	552392	+	A	0	4	2	3	4	-
gi|29165615|ref|NC_002745.2|	552393	+	U	2	1	1	3	0	-
gi|29165615|ref|NC_002745.2|	552394	+	A	1	1	1	11	0	-
gi|29165615|ref|NC_002745.2|	552395	+	A	0	0	1	3	0	-
gi|29165615|ref|NC_002745.2|	552396	+	G	0	1	0	2	0	-
gi|29165615|ref|NC_002745.2|	552397	+	C	0	0	0	1	0	-
gi|29165615|ref|NC_002745.2|	552398	+	U	1	0	0	0	0	-
gi|29165615|ref|NC_002745.2|	552399	+	U	1	0	0	0	0	-
gi|29165615|ref|NC_002745.2|	552400	+	G	4	0	1	8	1	-
gi|29165615|ref|NC_002745.2|	552401	+	A	1	4	3	31	2	-
gi|29165615|ref|NC_002745.2|	552402	+	A	1	2	3	14	3	-
gi|29165615|ref|NC_002745.2|	552403	+	U	0	3	0	4	0	-
gi|29165615|ref|NC_002745.2|	552404	+	U	1	0	0	1	0	-
gi|29165615|ref|NC_002745.2|	552405	+	C	1	1	0	2	0	-
gi|29165615|ref|NC_002745.2|	552406	+	A	3	2	0	11	0	-
gi|29165615|ref|NC_002745.2|	552407	+	U	9	23	16	165	27	-
gi|29165615|ref|NC_002745.2|	552408	+	A	8	48	14	417	49	-
gi|29165615|ref|NC_002745.2|	552409	+	A	4	26	10	263	28	-
gi|29165615|ref|NC_002745.2|	552410	+	G	8	22	10	187	24	-
gi|29165615|ref|NC_002745.2|	552411	+	A	3	17	3	135	9	-
gi|29165615|ref|NC_002745.2|	552412	+	A	9	23	6	185	16	-
gi|29165615|ref|NC_002745.2|	552413	+	A	3	11	4	89	10	-
gi|29165615|ref|NC_002745.2|	552414	+	U	2	4	1	19	1	-
gi|29165615|ref|NC_002745.2|	552415	+	A	8	4	3	112	9	-
gi|29165615|ref|NC_002745.2|	552416	+	A	5	14	2	56	12	-
gi|29165615|ref|NC_002745.2|	552417	+	U	0	1	0	9	2	-
gi|29165615|ref|NC_002745.2|	552418	+	C	1	1	0	2	1	-
gi|29165615|ref|NC_002745.2|	552420	+	C	0	0	0	1	0	-
gi|29165615|ref|NC_002745.2|	552422	+	A	1	0	0	1	0	-
gi|29165615|ref|NC_002745.2|	552426	+	U	3	1	0	0	0	-
gi|29165615|ref|NC_002745.2|	552427	+	U	1	0	0	0	0	-
gi|29165615|ref|NC_002745.2|	552428	+	C	0	0	0	2	1	-
gi|29165615|ref|NC_002745.2|	552429	+	G	1	0	0	5	0	-
gi|29165615|ref|NC_002745.2|	552430	+	A	0	1	2	2	0	-
gi|29165615|ref|NC_002745.2|	552431	+	A	0	0	0	3	0	-
gi|29165615|ref|NC_002745.2|	552432	+	A	0	0	0	1	0	-
gi|29165615|ref|NC_002745.2|	552434	+	A	6	0	0	1	1	-
gi|29165615|ref|NC_002745.2|	552435	+	A	4	0	0	0	0	-
gi|29165615|ref|NC_002745.2|	552436	+	C	2	2	1	1	3	-
gi|29165615|ref|NC_002745.2|	552437	+	A	7	1	2	1	4	-
gi|29165615|ref|NC_002745.2|	552438	+	C	2	0	0	1	0	-
gi|29165615|ref|NC_002745.2|	552439	+	U	0	0	1	0	0	-
gi|29165615|ref|NC_002745.2|	552466	+	A	4	3	0	14	13	-
gi|29165615|ref|NC_002745.2|	552467	+	G	1	0	0	5	0	-
gi|29165615|ref|NC_002745.2|	552468	+	G	5	1	2	23	4	-
gi|29165615|ref|NC_002745.2|	552469	+	A	2	2	5	33	4	-
gi|29165615|ref|NC_002745.2|	552470	+	U	4	0	2	0	1	-
gi|29165615|ref|NC_002745.2|	552471	+	G	0	0	0	10	1	-
gi|29165615|ref|NC_002745.2|	552472	+	G	4	0	2	27	4	-
gi|29165615|ref|NC_002745.2|	552473	+	A	7	5	3	33	4	-
gi|29165615|ref|NC_002745.2|	552474	+	A	12	2	3	55	7	-
gi|29165615|ref|NC_002745.2|	552475	+	A	5	3	3	32	0	-
gi|29165615|ref|NC_002745.2|	552476	+	C	22	4	3	15	6	-
gi|29165615|ref|NC_002745.2|	552477	+	A	19	10	4	26	14	-
gi|29165615|ref|NC_002745.2|	552478	+	U	56	21	23	51	27	-
gi|29165615|ref|NC_002745.2|	552479	+	A	77	25	17	21	19	-
gi|29165615|ref|NC_002745.2|	552480	+	G	236	167	138	146	174	SArRNA06
gi|29165615|ref|NC_002745.2|	552481	+	A	13289	9658	6342	5268	7428	SArRNA06
gi|29165615|ref|NC_002745.2|	552482	+	U	4688426	3458875	2860835	2114435	3322169	SArRNA06
gi|29165615|ref|NC_002745.2|	552483	+	U	2404	4642	3181	3137	4527	SArRNA06
gi|29165615|ref|NC_002745.2|	552484	+	A	8496	3514	2335	2876	3068	SArRNA06
gi|29165615|ref|NC_002745.2|	552485	+	A	18974	1553	1313	1189	1437	SArRNA06
gi|29165615|ref|NC_002745.2|	552486	+	G	3105	1040	814	698	995	SArRNA06
gi|29165615|ref|NC_002745.2|	552487	+	U	3050	1134	903	873	1058	SArRNA06
gi|29165615|ref|NC_002745.2|	552488	+	U	2637	1112	902	775	1163	SArRNA06
gi|29165615|ref|NC_002745.2|	552489	+	A	835	841	506	578	829	SArRNA06
gi|29165615|ref|NC_002745.2|	552490	+	U	748	265	365	187	307	SArRNA06

gi|29165615|ref|NC_002745.2|	552491 +	U	3981	458	739	280	397	SArRNA06
gi|29165615|ref|NC_002745.2|	552492 +	A	422	149	104	47	98	SArRNA06
gi|29165615|ref|NC_002745.2|	552493 +	A	230	64	26	13	38	SArRNA06
gi|29165615|ref|NC_002745.2|	552494 +	G	119	23	14	13	16	SArRNA06
gi|29165615|ref|NC_002745.2|	552495 +	G	412	34	33	21	36	SArRNA06
gi|29165615|ref|NC_002745.2|	552496 +	G	839	183	146	59	118	SArRNA06
gi|29165615|ref|NC_002745.2|	552497 +	C	1179	89	111	44	96	SArRNA06
gi|29165615|ref|NC_002745.2|	552498 +	G	137	34	26	16	29	SArRNA06
gi|29165615|ref|NC_002745.2|	552499 +	C	305	178	109	69	115	SArRNA06
gi|29165615|ref|NC_002745.2|	552500 +	A	55	41	28	20	48	SArRNA06
gi|29165615|ref|NC_002745.2|	552501 +	C	199	85	55	40	80	SArRNA06
gi|29165615|ref|NC_002745.2|	552502 +	G	44	47	28	20	39	SArRNA06
gi|29165615|ref|NC_002745.2|	552503 +	G	23	23	13	12	27	SArRNA06
gi|29165615|ref|NC_002745.2|	552504 +	U	47	16	9	7	22	SArRNA06
gi|29165615|ref|NC_002745.2|	552505 +	G	29	18	6	9	13	SArRNA06
gi|29165615|ref|NC_002745.2|	552506 +	G	78	30	17	12	26	SArRNA06
gi|29165615|ref|NC_002745.2|	552507 +	A	108	14	30	12	27	SArRNA06
gi|29165615|ref|NC_002745.2|	552508 +	U	4	1	3	1	8	SArRNA06
gi|29165615|ref|NC_002745.2|	552509 +	G	0	0	2	0	0	SArRNA06
gi|29165615|ref|NC_002745.2|	552510 +	C	37	16	7	2	12	SArRNA06
gi|29165615|ref|NC_002745.2|	552511 +	C	39	84	13	10	50	SArRNA06
gi|29165615|ref|NC_002745.2|	552512 +	U	19	19	6	0	12	SArRNA06
gi|29165615|ref|NC_002745.2|	552513 +	U	25	26	8	9	24	SArRNA06
gi|29165615|ref|NC_002745.2|	552514 +	G	1	8	3	1	4	SArRNA06
gi|29165615|ref|NC_002745.2|	552515 +	G	7	10	4	3	13	SArRNA06
gi|29165615|ref|NC_002745.2|	552516 +	C	57	52	31	20	45	SArRNA06
gi|29165615|ref|NC_002745.2|	552517 +	A	30	41	13	11	20	SArRNA06
gi|29165615|ref|NC_002745.2|	552518 +	C	46	35	14	21	35	SArRNA06
gi|29165615|ref|NC_002745.2|	552519 +	U	41	27	11	11	31	SArRNA06
gi|29165615|ref|NC_002745.2|	552520 +	A	14	17	3	3	4	SArRNA06
gi|29165615|ref|NC_002745.2|	552521 +	G	57	34	20	12	33	SArRNA06
gi|29165615|ref|NC_002745.2|	552522 +	A	90	18	12	7	23	SArRNA06
gi|29165615|ref|NC_002745.2|	552523 +	A	130	32	11	5	15	SArRNA06
gi|29165615|ref|NC_002745.2|	552524 +	G	11	21	11	6	11	SArRNA06
gi|29165615|ref|NC_002745.2|	552525 +	C	8	6	5	2	12	SArRNA06
gi|29165615|ref|NC_002745.2|	552526 +	C	51	83	17	12	80	SArRNA06
gi|29165615|ref|NC_002745.2|	552527 +	G	94	77	31	42	70	SArRNA06
gi|29165615|ref|NC_002745.2|	552528 +	A	10	10	3	3	16	SArRNA06
gi|29165615|ref|NC_002745.2|	552529 +	U	9	6	12	13	31	SArRNA06
gi|29165615|ref|NC_002745.2|	552530 +	G	16	9	11	11	23	SArRNA06
gi|29165615|ref|NC_002745.2|	552531 +	A	18	14	4	12	11	SArRNA06
gi|29165615|ref|NC_002745.2|	552532 +	A	13	17	4	2	14	SArRNA06
gi|29165615|ref|NC_002745.2|	552533 +	G	4	6	4	4	9	SArRNA06
gi|29165615|ref|NC_002745.2|	552534 +	G	18	22	11	9	22	SArRNA06
gi|29165615|ref|NC_002745.2|	552535 +	A	15	6	3	5	12	SArRNA06
gi|29165615|ref|NC_002745.2|	552536 +	C	29	27	18	16	22	SArRNA06
gi|29165615|ref|NC_002745.2|	552537 +	G	35	19	9	13	20	SArRNA06
gi|29165615|ref|NC_002745.2|	552538 +	U	27	32	19	8	37	SArRNA06
gi|29165615|ref|NC_002745.2|	552539 +	U	23	51	23	21	33	SArRNA06
gi|29165615|ref|NC_002745.2|	552540 +	A	20	12	8	14	22	SArRNA06
gi|29165615|ref|NC_002745.2|	552541 +	C	24	34	13	24	42	SArRNA06
gi|29165615|ref|NC_002745.2|	552542 +	U	41	41	12	23	47	SArRNA06
gi|29165615|ref|NC_002745.2|	552543 +	A	34	48	14	28	45	SArRNA06
gi|29165615|ref|NC_002745.2|	552544 +	A	1007	878	350	1165	819	SArRNA06
gi|29165615|ref|NC_002745.2|	552545 +	C	50	42	22	36	46	SArRNA06
gi|29165615|ref|NC_002745.2|	552546 +	G	29	15	7	13	22	SArRNA06
gi|29165615|ref|NC_002745.2|	552547 +	A	26	8	7	13	17	SArRNA06
gi|29165615|ref|NC_002745.2|	552548 +	C	34	36	18	22	23	SArRNA06
gi|29165615|ref|NC_002745.2|	552549 +	G	34	19	4	8	22	SArRNA06
gi|29165615|ref|NC_002745.2|	552550 +	A	31	32	16	16	39	SArRNA06
gi|29165615|ref|NC_002745.2|	552551 +	U	113	122	65	65	114	SArRNA06
gi|29165615|ref|NC_002745.2|	552552 +	A	56	37	8	15	44	SArRNA06
gi|29165615|ref|NC_002745.2|	552553 +	U	57	37	21	30	59	SArRNA06
gi|29165615|ref|NC_002745.2|	552554 +	G	44	24	14	21	23	SArRNA06
gi|29165615|ref|NC_002745.2|	552555 +	C	65	29	16	11	32	SArRNA06
gi|29165615|ref|NC_002745.2|	552556 +	U	42	34	23	22	55	SArRNA06
gi|29165615|ref|NC_002745.2|	552557 +	U	53	20	20	21	44	SArRNA06
gi|29165615|ref|NC_002745.2|	552558 +	U	21	14	4	15	19	SArRNA06
gi|29165615|ref|NC_002745.2|	552559 +	G	4	12	5	4	10	SArRNA06
gi|29165615|ref|NC_002745.2|	552560 +	G	11	11	4	2	8	SArRNA06
gi|29165615|ref|NC_002745.2|	552561 +	G	15	13	8	10	14	SArRNA06
gi|29165615|ref|NC_002745.2|	552562 +	G	70	35	27	24	76	SArRNA06
gi|29165615|ref|NC_002745.2|	552563 +	A	54	35	34	21	76	SArRNA06
gi|29165615|ref|NC_002745.2|	552564 +	G	13	12	13	12	14	SArRNA06
gi|29165615|ref|NC_002745.2|	552565 +	C	26	13	5	4	5	SArRNA06
gi|29165615|ref|NC_002745.2|	552566 +	U	19	8	3	7	14	SArRNA06
gi|29165615|ref|NC_002745.2|	552567 +	G	9	8	1	5	9	SArRNA06
gi|29165615|ref|NC_002745.2|	552568 +	U	36	27	10	11	25	SArRNA06
gi|29165615|ref|NC_002745.2|	552569 +	A	24	24	4	6	24	SArRNA06
gi|29165615|ref|NC_002745.2|	552570 +	A	29	8	14	14	32	SArRNA06
gi|29165615|ref|NC_002745.2|	552571 +	G	33	25	10	18	30	SArRNA06

gi|29165615|ref|NC_002745.2|	552572 +	U	26	42	20	42	43	SArRNA06
gi|29165615|ref|NC_002745.2|	552573 +	A	12	15	11	13	18	SArRNA06
gi|29165615|ref|NC_002745.2|	552574 +	A	13	11	4	6	8	SArRNA06
gi|29165615|ref|NC_002745.2|	552575 +	G	1	3	3	0	3	SArRNA06
gi|29165615|ref|NC_002745.2|	552576 +	C	22	7	10	6	18	SArRNA06
gi|29165615|ref|NC_002745.2|	552577 +	U	5	4	1	0	9	SArRNA06
gi|29165615|ref|NC_002745.2|	552578 +	U	5	3	5	4	10	SArRNA06
gi|29165615|ref|NC_002745.2|	552579 +	U	13	28	14	7	12	SArRNA06
gi|29165615|ref|NC_002745.2|	552580 +	G	27	43	19	18	30	SArRNA06
gi|29165615|ref|NC_002745.2|	552581 +	A	6	7	1	5	16	SArRNA06
gi|29165615|ref|NC_002745.2|	552582 +	U	3	5	1	4	3	SArRNA06
gi|29165615|ref|NC_002745.2|	552583 +	C	17	36	3	7	19	SArRNA06
gi|29165615|ref|NC_002745.2|	552584 +	C	30	28	14	23	28	SArRNA06
gi|29165615|ref|NC_002745.2|	552585 +	A	15	19	2	4	19	SArRNA06
gi|29165615|ref|NC_002745.2|	552586 +	G	21	35	12	20	27	SArRNA06
gi|29165615|ref|NC_002745.2|	552587 +	A	21	15	1	7	10	SArRNA06
gi|29165615|ref|NC_002745.2|	552588 +	G	53	58	20	16	25	SArRNA06
gi|29165615|ref|NC_002745.2|	552589 +	A	23	32	19	12	11	SArRNA06
gi|29165615|ref|NC_002745.2|	552590 +	U	5	4	3	2	6	SArRNA06
gi|29165615|ref|NC_002745.2|	552591 +	U	4	3	3	4	3	SArRNA06
gi|29165615|ref|NC_002745.2|	552592 +	U	3	3	2	5	3	SArRNA06
gi|29165615|ref|NC_002745.2|	552593 +	C	5	10	4	6	11	SArRNA06
gi|29165615|ref|NC_002745.2|	552594 +	C	54	64	16	32	28	SArRNA06
gi|29165615|ref|NC_002745.2|	552595 +	G	10	23	5	4	14	SArRNA06
gi|29165615|ref|NC_002745.2|	552596 +	A	5	7	1	4	4	SArRNA06
gi|29165615|ref|NC_002745.2|	552597 +	A	5	15	0	2	4	SArRNA06
gi|29165615|ref|NC_002745.2|	552598 +	U	6	9	0	2	3	SArRNA06
gi|29165615|ref|NC_002745.2|	552599 +	G	3	4	1	1	4	SArRNA06
gi|29165615|ref|NC_002745.2|	552600 +	G	0	2	1	0	4	SArRNA06
gi|29165615|ref|NC_002745.2|	552601 +	G	0	0	5	0	2	SArRNA06
gi|29165615|ref|NC_002745.2|	552602 +	G	3	1	0	2	2	SArRNA06
gi|29165615|ref|NC_002745.2|	552603 +	A	0	6	0	0	3	SArRNA06
gi|29165615|ref|NC_002745.2|	552604 +	A	31	58	10	9	28	SArRNA06
gi|29165615|ref|NC_002745.2|	552605 +	A	3	7	1	0	5	SArRNA06
gi|29165615|ref|NC_002745.2|	552606 +	C	21	22	3	1	31	SArRNA06
gi|29165615|ref|NC_002745.2|	552607 +	C	30	32	9	13	32	SArRNA06
gi|29165615|ref|NC_002745.2|	552608 +	C	14	21	10	10	14	SArRNA06
gi|29165615|ref|NC_002745.2|	552609 +	A	23	47	20	25	24	SArRNA06
gi|29165615|ref|NC_002745.2|	552610 +	G	3	1	0	2	1	SArRNA06
gi|29165615|ref|NC_002745.2|	552611 +	C	2	1	3	2	9	SArRNA06
gi|29165615|ref|NC_002745.2|	552612 +	A	4	11	5	4	11	SArRNA06
gi|29165615|ref|NC_002745.2|	552613 +	U	10	19	7	4	16	SArRNA06
gi|29165615|ref|NC_002745.2|	552614 +	G	24	18	11	13	11	SArRNA06
gi|29165615|ref|NC_002745.2|	552615 +	A	12	9	2	9	8	SArRNA06
gi|29165615|ref|NC_002745.2|	552616 +	G	8	6	7	21	17	SArRNA06
gi|29165615|ref|NC_002745.2|	552617 +	U	17	5	4	2	6	SArRNA06
gi|29165615|ref|NC_002745.2|	552618 +	U	42	29	13	18	31	SArRNA06
gi|29165615|ref|NC_002745.2|	552619 +	A	16	14	6	3	5	SArRNA06
gi|29165615|ref|NC_002745.2|	552620 +	U	7	6	4	2	3	SArRNA06
gi|29165615|ref|NC_002745.2|	552621 +	G	2	1	2	0	7	SArRNA06
gi|29165615|ref|NC_002745.2|	552622 +	U	5	13	2	2	2	SArRNA06
gi|29165615|ref|NC_002745.2|	552623 +	C	132	85	45	52	59	SArRNA06
gi|29165615|ref|NC_002745.2|	552624 +	A	27	24	7	10	9	SArRNA06
gi|29165615|ref|NC_002745.2|	552625 +	U	4	7	1	4	8	SArRNA06
gi|29165615|ref|NC_002745.2|	552626 +	G	2	4	0	1	5	SArRNA06
gi|29165615|ref|NC_002745.2|	552627 +	U	9	7	3	2	5	SArRNA06
gi|29165615|ref|NC_002745.2|	552628 +	U	25	23	13	11	16	SArRNA06
gi|29165615|ref|NC_002745.2|	552629 +	A	0	8	1	1	6	SArRNA06
gi|29165615|ref|NC_002745.2|	552630 +	U	0	7	0	1	2	SArRNA06
gi|29165615|ref|NC_002745.2|	552631 +	C	33	44	19	20	22	SArRNA06
gi|29165615|ref|NC_002745.2|	552632 +	G	9	19	5	6	8	SArRNA06
gi|29165615|ref|NC_002745.2|	552633 +	A	2	7	1	0	4	SArRNA06
gi|29165615|ref|NC_002745.2|	552634 +	U	28	30	8	11	22	SArRNA06
gi|29165615|ref|NC_002745.2|	552635 +	A	7	7	3	0	4	SArRNA06
gi|29165615|ref|NC_002745.2|	552636 +	U	13	3	6	4	10	SArRNA06
gi|29165615|ref|NC_002745.2|	552637 +	G	6	0	6	6	4	SArRNA06
gi|29165615|ref|NC_002745.2|	552638 +	U	5	3	2	3	5	SArRNA06
gi|29165615|ref|NC_002745.2|	552639 +	G	10	6	2	9	8	SArRNA06
gi|29165615|ref|NC_002745.2|	552640 +	A	16	6	4	5	8	SArRNA06
gi|29165615|ref|NC_002745.2|	552641 +	A	7	17	2	7	12	SArRNA06
gi|29165615|ref|NC_002745.2|	552642 +	U	8	16	3	1	17	SArRNA06
gi|29165615|ref|NC_002745.2|	552643 +	A	38	31	4	7	30	SArRNA06
gi|29165615|ref|NC_002745.2|	552644 +	C	31	48	4	10	36	SArRNA06
gi|29165615|ref|NC_002745.2|	552645 +	A	20	33	6	11	22	SArRNA06
gi|29165615|ref|NC_002745.2|	552646 +	U	22	18	3	5	17	SArRNA06
gi|29165615|ref|NC_002745.2|	552647 +	A	3	9	1	1	2	SArRNA06
gi|29165615|ref|NC_002745.2|	552648 +	G	6	1	1	3	4	SArRNA06
gi|29165615|ref|NC_002745.2|	552649 +	C	28	25	9	10	15	SArRNA06
gi|29165615|ref|NC_002745.2|	552650 +	A	14	9	1	5	7	SArRNA06
gi|29165615|ref|NC_002745.2|	552651 +	U	5	3	1	0	7	SArRNA06
gi|29165615|ref|NC_002745.2|	552652 +	A	3	6	2	0	5	SArRNA06

gi|29165615|ref|NC_002745.2|	552653 +	U	11	7	3	5	7	SArRNA06
gi|29165615|ref|NC_002745.2|	552654 +	C	18	9	6	2	7	SArRNA06
gi|29165615|ref|NC_002745.2|	552655 +	A	13	8	5	3	5	SArRNA06
gi|29165615|ref|NC_002745.2|	552656 +	G	8	7	0	6	2	SArRNA06
gi|29165615|ref|NC_002745.2|	552657 +	A	25	60	18	8	6	SArRNA06
gi|29165615|ref|NC_002745.2|	552658 +	A	131	231	47	58	77	SArRNA06
gi|29165615|ref|NC_002745.2|	552659 +	G	2	2	0	0	0	SArRNA06
gi|29165615|ref|NC_002745.2|	552660 +	G	0	2	0	1	0	SArRNA06
gi|29165615|ref|NC_002745.2|	552661 +	C	24	17	4	12	13	SArRNA06
gi|29165615|ref|NC_002745.2|	552662 +	A	14	22	2	2	7	SArRNA06
gi|29165615|ref|NC_002745.2|	552663 +	C	44	27	21	16	18	SArRNA06
gi|29165615|ref|NC_002745.2|	552664 +	A	14	20	4	5	9	SArRNA06
gi|29165615|ref|NC_002745.2|	552665 +	C	27	23	6	9	7	SArRNA06
gi|29165615|ref|NC_002745.2|	552666 +	C	8	10	2	3	7	SArRNA06
gi|29165615|ref|NC_002745.2|	552667 +	C	7	18	5	8	2	SArRNA06
gi|29165615|ref|NC_002745.2|	552668 +	G	67	174	20	22	67	SArRNA06
gi|29165615|ref|NC_002745.2|	552669 +	G	6	11	3	1	5	SArRNA06
gi|29165615|ref|NC_002745.2|	552670 +	A	7	9	1	1	5	SArRNA06
gi|29165615|ref|NC_002745.2|	552671 +	G	14	6	2	6	6	SArRNA06
gi|29165615|ref|NC_002745.2|	552672 +	A	7	8	3	1	8	SArRNA06
gi|29165615|ref|NC_002745.2|	552673 +	A	1	0	1	0	1	SArRNA06
gi|29165615|ref|NC_002745.2|	552674 +	C	7	4	0	1	2	SArRNA06
gi|29165615|ref|NC_002745.2|	552675 +	U	0	1	1	1	1	SArRNA06
gi|29165615|ref|NC_002745.2|	552676 +	G	1	1	0	0	0	SArRNA06
gi|29165615|ref|NC_002745.2|	552677 +	A	3	1	0	0	1	SArRNA06
gi|29165615|ref|NC_002745.2|	552678 +	A	1	0	0	1	0	SArRNA06
gi|29165615|ref|NC_002745.2|	552679 +	A	0	2	3	0	0	SArRNA06
gi|29165615|ref|NC_002745.2|	552680 +	C	11	12	4	9	11	SArRNA06
gi|29165615|ref|NC_002745.2|	552681 +	A	4	13	2	0	7	SArRNA06
gi|29165615|ref|NC_002745.2|	552682 +	U	3	3	5	3	2	SArRNA06
gi|29165615|ref|NC_002745.2|	552683 +	C	2	9	1	2	0	SArRNA06
gi|29165615|ref|NC_002745.2|	552684 +	U	0	0	0	0	1	SArRNA06
gi|29165615|ref|NC_002745.2|	552685 +	U	0	1	0	1	2	SArRNA06
gi|29165615|ref|NC_002745.2|	552686 +	A	0	1	0	2	2	SArRNA06
gi|29165615|ref|NC_002745.2|	552687 +	G	1	3	0	0	1	SArRNA06
gi|29165615|ref|NC_002745.2|	552688 +	U	2	5	1	0	6	SArRNA06
gi|29165615|ref|NC_002745.2|	552689 +	A	0	2	1	0	1	SArRNA06
gi|29165615|ref|NC_002745.2|	552690 +	C	12	8	4	2	5	SArRNA06
gi|29165615|ref|NC_002745.2|	552691 +	C	10	14	3	6	8	SArRNA06
gi|29165615|ref|NC_002745.2|	552692 +	C	11	8	8	3	12	SArRNA06
gi|29165615|ref|NC_002745.2|	552693 +	G	0	4	1	1	3	SArRNA06
gi|29165615|ref|NC_002745.2|	552694 +	G	5	6	4	6	4	SArRNA06
gi|29165615|ref|NC_002745.2|	552695 +	A	0	5	2	1	1	SArRNA06
gi|29165615|ref|NC_002745.2|	552696 +	G	1	0	1	0	0	SArRNA06
gi|29165615|ref|NC_002745.2|	552697 +	G	0	1	0	0	2	SArRNA06
gi|29165615|ref|NC_002745.2|	552698 +	A	4	10	0	0	4	SArRNA06
gi|29165615|ref|NC_002745.2|	552699 +	A	38	45	11	18	15	SArRNA06
gi|29165615|ref|NC_002745.2|	552700 +	G	2	2	2	3	2	SArRNA06
gi|29165615|ref|NC_002745.2|	552701 +	A	1	0	0	1	1	SArRNA06
gi|29165615|ref|NC_002745.2|	552702 +	G	0	2	0	3	5	SArRNA06
gi|29165615|ref|NC_002745.2|	552703 +	A	1	1	0	0	1	SArRNA06
gi|29165615|ref|NC_002745.2|	552704 +	A	0	2	0	2	1	SArRNA06
gi|29165615|ref|NC_002745.2|	552705 +	A	1	1	0	3	0	SArRNA06
gi|29165615|ref|NC_002745.2|	552706 +	G	0	4	1	1	0	SArRNA06
gi|29165615|ref|NC_002745.2|	552707 +	A	0	2	0	0	0	SArRNA06
gi|29165615|ref|NC_002745.2|	552708 +	A	2	3	0	0	0	SArRNA06
gi|29165615|ref|NC_002745.2|	552709 +	A	1	1	1	1	2	SArRNA06
gi|29165615|ref|NC_002745.2|	552710 +	A	9	124	10	3	21	SArRNA06
gi|29165615|ref|NC_002745.2|	552711 +	U	2	6	4	1	4	SArRNA06
gi|29165615|ref|NC_002745.2|	552712 +	U	2	0	2	0	4	SArRNA06
gi|29165615|ref|NC_002745.2|	552713 +	C	2	6	1	0	4	SArRNA06
gi|29165615|ref|NC_002745.2|	552714 +	G	0	3	1	0	0	SArRNA06
gi|29165615|ref|NC_002745.2|	552715 +	A	1	3	1	1	0	SArRNA06
gi|29165615|ref|NC_002745.2|	552717 +	U	1	8	0	1	1	SArRNA06
gi|29165615|ref|NC_002745.2|	552718 +	C	27	20	9	13	8	SArRNA06
gi|29165615|ref|NC_002745.2|	552719 +	C	16	5	5	2	3	SArRNA06
gi|29165615|ref|NC_002745.2|	552720 +	C	5	2	2	4	7	SArRNA06
gi|29165615|ref|NC_002745.2|	552721 +	U	7	5	3	5	7	SArRNA06
gi|29165615|ref|NC_002745.2|	552722 +	U	5	3	4	5	5	SArRNA06
gi|29165615|ref|NC_002745.2|	552723 +	A	6	2	1	1	4	SArRNA06
gi|29165615|ref|NC_002745.2|	552724 +	G	3	6	2	2	4	SArRNA06
gi|29165615|ref|NC_002745.2|	552725 +	U	9	5	2	2	7	SArRNA06
gi|29165615|ref|NC_002745.2|	552726 +	A	7	3	3	1	5	SArRNA06
gi|29165615|ref|NC_002745.2|	552727 +	G	1	0	1	0	0	SArRNA06
gi|29165615|ref|NC_002745.2|	552728 +	C	7	4	4	2	2	SArRNA06
gi|29165615|ref|NC_002745.2|	552729 +	G	2	0	1	0	1	SArRNA06
gi|29165615|ref|NC_002745.2|	552730 +	G	1	1	0	0	1	SArRNA06
gi|29165615|ref|NC_002745.2|	552731 +	C	16	15	4	8	19	SArRNA06
gi|29165615|ref|NC_002745.2|	552732 +	G	6	5	1	1	8	SArRNA06
gi|29165615|ref|NC_002745.2|	552733 +	A	1	5	0	1	5	SArRNA06
gi|29165615|ref|NC_002745.2|	552734 +	G	1	2	1	2	0	SArRNA06

gi|29165615|ref|NC_002745.2|	552735	+	C	5	6	1	2	3	SArRNA06
gi|29165615|ref|NC_002745.2|	552736	+	G	5	5	0	1	3	SArRNA06
gi|29165615|ref|NC_002745.2|	552737	+	A	1	1	1	0	0	SArRNA06
gi|29165615|ref|NC_002745.2|	552738	+	A	3	1	0	3	4	SArRNA06
gi|29165615|ref|NC_002745.2|	552739	+	A	1	2	0	0	3	SArRNA06
gi|29165615|ref|NC_002745.2|	552740	+	C	7	3	1	1	3	SArRNA06
gi|29165615|ref|NC_002745.2|	552741	+	G	0	0	0	1	2	SArRNA06
gi|29165615|ref|NC_002745.2|	552742	+	G	2	0	0	0	0	SArRNA06
gi|29165615|ref|NC_002745.2|	552743	+	G	1	0	0	0	0	SArRNA06
gi|29165615|ref|NC_002745.2|	552744	+	A	0	1	1	0	1	SArRNA06
gi|29165615|ref|NC_002745.2|	552745	+	A	5	1	2	0	0	SArRNA06
gi|29165615|ref|NC_002745.2|	552746	+	G	0	0	1	0	0	SArRNA06
gi|29165615|ref|NC_002745.2|	552747	+	A	1	0	0	1	0	SArRNA06
gi|29165615|ref|NC_002745.2|	552748	+	G	0	1	0	0	0	SArRNA06
gi|29165615|ref|NC_002745.2|	552749	+	C	10	8	1	2	2	SArRNA06
gi|29165615|ref|NC_002745.2|	552750	+	C	3	4	0	3	5	SArRNA06
gi|29165615|ref|NC_002745.2|	552751	+	C	15	11	3	7	7	SArRNA06
gi|29165615|ref|NC_002745.2|	552752	+	A	2	2	1	3	1	SArRNA06
gi|29165615|ref|NC_002745.2|	552753	+	A	2	7	3	1	3	SArRNA06
gi|29165615|ref|NC_002745.2|	552754	+	A	3	2	3	2	2	SArRNA06
gi|29165615|ref|NC_002745.2|	552755	+	C	1	2	1	2	4	SArRNA06
gi|29165615|ref|NC_002745.2|	552756	+	C	16	2	2	7	4	SArRNA06
gi|29165615|ref|NC_002745.2|	552757	+	A	7	4	4	4	6	SArRNA06
gi|29165615|ref|NC_002745.2|	552758	+	A	5	3	1	1	5	SArRNA06
gi|29165615|ref|NC_002745.2|	552759	+	A	6	1	3	1	4	SArRNA06
gi|29165615|ref|NC_002745.2|	552760	+	A	7	4	1	1	3	SArRNA06
gi|29165615|ref|NC_002745.2|	552761	+	A	3	2	1	1	0	SArRNA06
gi|29165615|ref|NC_002745.2|	552762	+	G	1	2	0	0	0	SArRNA06
gi|29165615|ref|NC_002745.2|	552763	+	C	10	6	3	2	5	SArRNA06
gi|29165615|ref|NC_002745.2|	552764	+	U	5	3	0	2	5	SArRNA06
gi|29165615|ref|NC_002745.2|	552765	+	U	0	1	1	0	1	SArRNA06
gi|29165615|ref|NC_002745.2|	552766	+	G	1	1	1	1	1	SArRNA06
gi|29165615|ref|NC_002745.2|	552767	+	C	3	6	2	1	3	SArRNA06
gi|29165615|ref|NC_002745.2|	552768	+	U	0	0	0	1	3	SArRNA06
gi|29165615|ref|NC_002745.2|	552769	+	U	2	1	3	0	1	SArRNA06
gi|29165615|ref|NC_002745.2|	552770	+	G	3	2	1	0	0	SArRNA06
gi|29165615|ref|NC_002745.2|	552771	+	U	5	0	2	1	2	SArRNA06
gi|29165615|ref|NC_002745.2|	552772	+	U	6	4	2	2	0	SArRNA06
gi|29165615|ref|NC_002745.2|	552773	+	G	0	0	1	0	1	SArRNA06
gi|29165615|ref|NC_002745.2|	552775	+	G	1	1	0	2	2	SArRNA06
gi|29165615|ref|NC_002745.2|	552776	+	G	1	0	0	0	3	SArRNA06
gi|29165615|ref|NC_002745.2|	552777	+	U	0	2	2	0	1	SArRNA06
gi|29165615|ref|NC_002745.2|	552778	+	U	1	0	1	0	0	SArRNA06
gi|29165615|ref|NC_002745.2|	552779	+	G	1	1	0	1	0	SArRNA06
gi|29165615|ref|NC_002745.2|	552780	+	U	2	5	0	3	0	SArRNA06
gi|29165615|ref|NC_002745.2|	552781	+	A	0	5	0	1	0	SArRNA06
gi|29165615|ref|NC_002745.2|	552782	+	G	2	1	1	0	0	SArRNA06
gi|29165615|ref|NC_002745.2|	552785	+	C	22	22	7	5	10	SArRNA06
gi|29165615|ref|NC_002745.2|	552786	+	A	8	5	3	5	4	SArRNA06
gi|29165615|ref|NC_002745.2|	552787	+	C	7	5	3	4	5	SArRNA06
gi|29165615|ref|NC_002745.2|	552788	+	U	0	2	0	0	2	SArRNA06
gi|29165615|ref|NC_002745.2|	552789	+	C	2	3	1	3	4	SArRNA06
gi|29165615|ref|NC_002745.2|	552790	+	U	2	2	0	0	1	SArRNA06
gi|29165615|ref|NC_002745.2|	552791	+	A	0	2	0	0	3	SArRNA06
gi|29165615|ref|NC_002745.2|	552792	+	U	6	2	1	0	6	SArRNA06
gi|29165615|ref|NC_002745.2|	552793	+	A	2	0	1	2	0	SArRNA06
gi|29165615|ref|NC_002745.2|	552794	+	C	3	12	1	1	2	SArRNA06
gi|29165615|ref|NC_002745.2|	552795	+	G	1	9	2	2	6	SArRNA06
gi|29165615|ref|NC_002745.2|	552796	+	G	1	5	1	4	0	SArRNA06
gi|29165615|ref|NC_002745.2|	552797	+	A	1	1	0	2	2	SArRNA06
gi|29165615|ref|NC_002745.2|	552798	+	G	1	5	0	0	2	SArRNA06
gi|29165615|ref|NC_002745.2|	552799	+	U	1	2	0	1	0	SArRNA06
gi|29165615|ref|NC_002745.2|	552800	+	U	10	14	5	4	7	SArRNA06
gi|29165615|ref|NC_002745.2|	552801	+	A	1	0	0	1	1	SArRNA06
gi|29165615|ref|NC_002745.2|	552802	+	C	8	16	4	6	8	SArRNA06
gi|29165615|ref|NC_002745.2|	552803	+	A	1	10	0	2	3	SArRNA06
gi|29165615|ref|NC_002745.2|	552804	+	A	2	7	0	0	0	SArRNA06
gi|29165615|ref|NC_002745.2|	552805	+	A	2	2	0	0	0	SArRNA06
gi|29165615|ref|NC_002745.2|	552806	+	G	3	2	3	2	0	SArRNA06
gi|29165615|ref|NC_002745.2|	552807	+	G	0	2	1	0	0	SArRNA06
gi|29165615|ref|NC_002745.2|	552808	+	A	1	1	1	0	2	SArRNA06
gi|29165615|ref|NC_002745.2|	552809	+	C	7	4	1	3	4	SArRNA06
gi|29165615|ref|NC_002745.2|	552810	+	G	0	1	0	1	2	SArRNA06
gi|29165615|ref|NC_002745.2|	552811	+	A	5	0	1	1	0	SArRNA06
gi|29165615|ref|NC_002745.2|	552812	+	C	6	1	1	1	2	SArRNA06
gi|29165615|ref|NC_002745.2|	552813	+	A	2	3	0	1	0	SArRNA06
gi|29165615|ref|NC_002745.2|	552814	+	U	7	3	0	1	3	SArRNA06
gi|29165615|ref|NC_002745.2|	552815	+	U	2	6	1	2	0	SArRNA06
gi|29165615|ref|NC_002745.2|	552816	+	A	0	1	0	0	2	SArRNA06
gi|29165615|ref|NC_002745.2|	552817	+	G	0	1	0	2	1	SArRNA06
gi|29165615|ref|NC_002745.2|	552818	+	A	0	0	0	0	1	SArRNA06

gi|29165615|ref|NC_002745.2|	552819	+	C	1	3	1	1	0	SArRNA06
gi|29165615|ref|NC_002745.2|	552820	+	G	0	1	1	0	0	SArRNA06
gi|29165615|ref|NC_002745.2|	552821	+	A	0	1	0	0	2	SArRNA06
gi|29165615|ref|NC_002745.2|	552822	+	A	1	9	0	0	8	SArRNA06
gi|29165615|ref|NC_002745.2|	552823	+	U	6	1	1	3	0	SArRNA06
gi|29165615|ref|NC_002745.2|	552824	+	C	9	4	3	4	2	SArRNA06
gi|29165615|ref|NC_002745.2|	552825	+	A	3	5	1	1	1	SArRNA06
gi|29165615|ref|NC_002745.2|	552826	+	U	2	3	1	0	0	SArRNA06
gi|29165615|ref|NC_002745.2|	552827	+	C	7	9	7	5	5	SArRNA06
gi|29165615|ref|NC_002745.2|	552828	+	U	3	4	2	1	1	SArRNA06
gi|29165615|ref|NC_002745.2|	552829	+	G	0	0	1	1	0	SArRNA06
gi|29165615|ref|NC_002745.2|	552830	+	G	7	1	1	0	5	SArRNA06
gi|29165615|ref|NC_002745.2|	552831	+	A	1	5	2	2	2	SArRNA06
gi|29165615|ref|NC_002745.2|	552832	+	A	8	9	0	0	0	SArRNA06
gi|29165615|ref|NC_002745.2|	552833	+	A	3	5	2	1	3	SArRNA06
gi|29165615|ref|NC_002745.2|	552834	+	G	2	3	1	3	2	SArRNA06
gi|29165615|ref|NC_002745.2|	552835	+	A	2	3	0	0	3	SArRNA06
gi|29165615|ref|NC_002745.2|	552836	+	U	4	4	2	2	2	SArRNA06
gi|29165615|ref|NC_002745.2|	552837	+	G	15	4	2	6	13	SArRNA06
gi|29165615|ref|NC_002745.2|	552838	+	A	9	14	6	4	3	SArRNA06
gi|29165615|ref|NC_002745.2|	552839	+	A	25	31	10	22	25	SArRNA06
gi|29165615|ref|NC_002745.2|	552840	+	U	0	1	0	0	1	SArRNA06
gi|29165615|ref|NC_002745.2|	552841	+	C	7	18	0	4	7	SArRNA06
gi|29165615|ref|NC_002745.2|	552842	+	A	3	1	1	2	6	SArRNA06
gi|29165615|ref|NC_002745.2|	552843	+	A	3	0	1	1	3	SArRNA06
gi|29165615|ref|NC_002745.2|	552844	+	A	3	9	4	0	1	SArRNA06
gi|29165615|ref|NC_002745.2|	552845	+	G	7	7	1	6	4	SArRNA06
gi|29165615|ref|NC_002745.2|	552846	+	A	3	7	3	3	1	SArRNA06
gi|29165615|ref|NC_002745.2|	552847	+	A	3	0	1	1	3	SArRNA06
gi|29165615|ref|NC_002745.2|	552848	+	G	0	2	0	1	3	SArRNA06
gi|29165615|ref|NC_002745.2|	552849	+	G	2	2	2	2	2	SArRNA06
gi|29165615|ref|NC_002745.2|	552850	+	U	7	2	0	0	5	SArRNA06
gi|29165615|ref|NC_002745.2|	552851	+	A	7	4	1	4	9	SArRNA06
gi|29165615|ref|NC_002745.2|	552852	+	A	10	10	0	2	6	SArRNA06
gi|29165615|ref|NC_002745.2|	552853	+	U	11	3	6	5	7	SArRNA06
gi|29165615|ref|NC_002745.2|	552854	+	A	6	4	0	2	4	SArRNA06
gi|29165615|ref|NC_002745.2|	552855	+	A	1	14	2	5	8	SArRNA06
gi|29165615|ref|NC_002745.2|	552856	+	U	4	10	1	4	7	SArRNA06
gi|29165615|ref|NC_002745.2|	552857	+	C	5	7	4	7	6	SArRNA06
gi|29165615|ref|NC_002745.2|	552858	+	C	9	7	3	1	4	SArRNA06
gi|29165615|ref|NC_002745.2|	552859	+	U	3	8	0	0	9	SArRNA06
gi|29165615|ref|NC_002745.2|	552860	+	G	13	13	1	7	7	SArRNA06
gi|29165615|ref|NC_002745.2|	552861	+	U	3	4	1	2	1	SArRNA06
gi|29165615|ref|NC_002745.2|	552862	+	A	5	3	0	4	2	SArRNA06
gi|29165615|ref|NC_002745.2|	552863	+	G	4	1	1	1	1	SArRNA06
gi|29165615|ref|NC_002745.2|	552864	+	U	4	4	1	2	1	SArRNA06
gi|29165615|ref|NC_002745.2|	552865	+	C	4	3	1	1	1	SArRNA06
gi|29165615|ref|NC_002745.2|	552866	+	G	2	0	1	0	6	SArRNA06
gi|29165615|ref|NC_002745.2|	552867	+	A	2	2	1	0	0	SArRNA06
gi|29165615|ref|NC_002745.2|	552868	+	A	12	9	0	1	2	SArRNA06
gi|29165615|ref|NC_002745.2|	552869	+	A	9	5	1	4	6	SArRNA06
gi|29165615|ref|NC_002745.2|	552870	+	A	9	0	1	2	2	SArRNA06
gi|29165615|ref|NC_002745.2|	552871	+	U	4	4	1	1	2	SArRNA06
gi|29165615|ref|NC_002745.2|	552872	+	G	1	1	0	1	0	SArRNA06
gi|29165615|ref|NC_002745.2|	552873	+	U	2	2	0	0	1	SArRNA06
gi|29165615|ref|NC_002745.2|	552874	+	U	2	1	0	1	1	SArRNA06
gi|29165615|ref|NC_002745.2|	552875	+	G	2	0	0	3	0	SArRNA06
gi|29165615|ref|NC_002745.2|	552876	+	U	7	2	1	0	0	SArRNA06
gi|29165615|ref|NC_002745.2|	552877	+	C	6	8	2	1	3	SArRNA06
gi|29165615|ref|NC_002745.2|	552878	+	U	2	5	1	4	2	SArRNA06
gi|29165615|ref|NC_002745.2|	552879	+	C	5	9	3	3	5	SArRNA06
gi|29165615|ref|NC_002745.2|	552880	+	U	13	0	1	7	4	SArRNA06
gi|29165615|ref|NC_002745.2|	552881	+	C	1	2	2	2	0	SArRNA06
gi|29165615|ref|NC_002745.2|	552882	+	U	4	2	1	0	1	SArRNA06
gi|29165615|ref|NC_002745.2|	552883	+	U	2	6	0	1	4	SArRNA06
gi|29165615|ref|NC_002745.2|	552884	+	G	2	1	0	1	1	SArRNA06
gi|29165615|ref|NC_002745.2|	552885	+	A	3	1	0	1	3	SArRNA06
gi|29165615|ref|NC_002745.2|	552886	+	G	2	2	0	5	0	SArRNA06
gi|29165615|ref|NC_002745.2|	552887	+	U	1	2	0	3	3	SArRNA06
gi|29165615|ref|NC_002745.2|	552888	+	G	0	1	0	0	0	SArRNA06
gi|29165615|ref|NC_002745.2|	552889	+	G	4	5	7	3	7	SArRNA06
gi|29165615|ref|NC_002745.2|	552890	+	A	5	11	6	9	4	SArRNA06
gi|29165615|ref|NC_002745.2|	552891	+	U	4	3	0	1	3	SArRNA06
gi|29165615|ref|NC_002745.2|	552892	+	C	8	7	1	1	6	SArRNA06
gi|29165615|ref|NC_002745.2|	552893	+	C	24	9	2	7	7	SArRNA06
gi|29165615|ref|NC_002745.2|	552894	+	U	11	6	2	2	4	SArRNA06
gi|29165615|ref|NC_002745.2|	552895	+	G	2	1	0	0	2	SArRNA06
gi|29165615|ref|NC_002745.2|	552896	+	A	1	1	0	0	3	SArRNA06
gi|29165615|ref|NC_002745.2|	552897	+	G	11	10	3	3	0	SArRNA06
gi|29165615|ref|NC_002745.2|	552898	+	U	40	12	2	12	13	SArRNA06
gi|29165615|ref|NC_002745.2|	552899	+	A	5	5	3	6	4	SArRNA06

gi|29165615|ref|NC_002745.2|	552900	+	C	7	3	0	2	6	SArRNA06
gi|29165615|ref|NC_002745.2|	552901	+	G	8	6	0	2	12	SArRNA06
gi|29165615|ref|NC_002745.2|	552902	+	A	10	5	1	6	4	SArRNA06
gi|29165615|ref|NC_002745.2|	552903	+	C	5	8	3	3	8	SArRNA06
gi|29165615|ref|NC_002745.2|	552904	+	G	4	4	1	0	6	SArRNA06
gi|29165615|ref|NC_002745.2|	552905	+	G	12	6	5	9	6	SArRNA06
gi|29165615|ref|NC_002745.2|	552906	+	A	6	3	0	3	7	SArRNA06
gi|29165615|ref|NC_002745.2|	552907	+	G	4	2	0	1	0	SArRNA06
gi|29165615|ref|NC_002745.2|	552908	+	C	21	25	9	12	15	SArRNA06
gi|29165615|ref|NC_002745.2|	552909	+	A	14	10	2	4	6	SArRNA06
gi|29165615|ref|NC_002745.2|	552910	+	C	22	13	6	12	16	SArRNA06
gi|29165615|ref|NC_002745.2|	552911	+	G	2	3	2	5	4	SArRNA06
gi|29165615|ref|NC_002745.2|	552912	+	U	6	1	0	3	3	SArRNA06
gi|29165615|ref|NC_002745.2|	552913	+	G	2	2	0	3	2	SArRNA06
gi|29165615|ref|NC_002745.2|	552914	+	A	3	1	2	0	0	SArRNA06
gi|29165615|ref|NC_002745.2|	552915	+	A	0	4	0	1	3	SArRNA06
gi|29165615|ref|NC_002745.2|	552916	+	A	2	7	0	1	6	SArRNA06
gi|29165615|ref|NC_002745.2|	552917	+	U	3	1	0	1	1	SArRNA06
gi|29165615|ref|NC_002745.2|	552918	+	U	0	2	2	0	2	SArRNA06
gi|29165615|ref|NC_002745.2|	552919	+	C	6	1	2	0	1	SArRNA06
gi|29165615|ref|NC_002745.2|	552920	+	C	3	0	3	1	3	SArRNA06
gi|29165615|ref|NC_002745.2|	552921	+	G	0	4	1	0	2	SArRNA06
gi|29165615|ref|NC_002745.2|	552922	+	U	5	2	0	1	0	SArRNA06
gi|29165615|ref|NC_002745.2|	552923	+	C	6	1	0	1	1	SArRNA06
gi|29165615|ref|NC_002745.2|	552924	+	G	0	1	1	0	0	SArRNA06
gi|29165615|ref|NC_002745.2|	552925	+	G	3	5	1	2	6	SArRNA06
gi|29165615|ref|NC_002745.2|	552926	+	A	6	3	0	1	4	SArRNA06
gi|29165615|ref|NC_002745.2|	552927	+	A	3	6	0	1	6	SArRNA06
gi|29165615|ref|NC_002745.2|	552928	+	U	6	4	1	3	7	SArRNA06
gi|29165615|ref|NC_002745.2|	552929	+	C	5	20	3	3	4	SArRNA06
gi|29165615|ref|NC_002745.2|	552930	+	U	4	5	1	6	5	SArRNA06
gi|29165615|ref|NC_002745.2|	552931	+	G	0	0	0	0	2	SArRNA06
gi|29165615|ref|NC_002745.2|	552932	+	G	0	2	0	0	1	SArRNA06
gi|29165615|ref|NC_002745.2|	552933	+	G	0	1	1	0	3	SArRNA06
gi|29165615|ref|NC_002745.2|	552934	+	A	0	1	0	0	1	SArRNA06
gi|29165615|ref|NC_002745.2|	552935	+	G	0	1	0	1	0	SArRNA06
gi|29165615|ref|NC_002745.2|	552936	+	G	6	0	1	2	8	SArRNA06
gi|29165615|ref|NC_002745.2|	552937	+	A	3	2	0	1	5	SArRNA06
gi|29165615|ref|NC_002745.2|	552938	+	C	18	28	9	11	21	SArRNA06
gi|29165615|ref|NC_002745.2|	552939	+	C	42	28	13	22	17	SArRNA06
gi|29165615|ref|NC_002745.2|	552940	+	A	8	9	4	3	7	SArRNA06
gi|29165615|ref|NC_002745.2|	552941	+	U	1	3	4	5	7	SArRNA06
gi|29165615|ref|NC_002745.2|	552942	+	C	15	24	11	24	21	SArRNA06
gi|29165615|ref|NC_002745.2|	552943	+	U	5	9	2	1	8	SArRNA06
gi|29165615|ref|NC_002745.2|	552944	+	C	1	1	1	4	2	SArRNA06
gi|29165615|ref|NC_002745.2|	552945	+	C	5	2	4	3	7	SArRNA06
gi|29165615|ref|NC_002745.2|	552946	+	U	5	8	3	4	6	SArRNA06
gi|29165615|ref|NC_002745.2|	552947	+	A	3	2	3	1	4	SArRNA06
gi|29165615|ref|NC_002745.2|	552948	+	A	1	4	3	3	6	SArRNA06
gi|29165615|ref|NC_002745.2|	552949	+	G	2	3	0	1	3	SArRNA06
gi|29165615|ref|NC_002745.2|	552950	+	G	1	1	2	1	3	SArRNA06
gi|29165615|ref|NC_002745.2|	552951	+	C	10	3	1	7	9	SArRNA06
gi|29165615|ref|NC_002745.2|	552952	+	U	7	12	2	1	17	SArRNA06
gi|29165615|ref|NC_002745.2|	552953	+	A	6	2	2	1	5	SArRNA06
gi|29165615|ref|NC_002745.2|	552954	+	A	3	4	2	4	4	SArRNA06
gi|29165615|ref|NC_002745.2|	552955	+	A	15	153	14	8	47	SArRNA06
gi|29165615|ref|NC_002745.2|	552956	+	U	10	27	6	8	16	SArRNA06
gi|29165615|ref|NC_002745.2|	552957	+	A	4	3	2	1	5	SArRNA06
gi|29165615|ref|NC_002745.2|	552958	+	C	31	27	10	10	32	SArRNA06
gi|29165615|ref|NC_002745.2|	552959	+	U	18	19	2	13	11	SArRNA06
gi|29165615|ref|NC_002745.2|	552960	+	C	7	8	2	6	7	SArRNA06
gi|29165615|ref|NC_002745.2|	552961	+	U	9	8	4	4	18	SArRNA06
gi|29165615|ref|NC_002745.2|	552962	+	C	46	37	7	11	16	SArRNA06
gi|29165615|ref|NC_002745.2|	552963	+	U	20	11	4	6	11	SArRNA06
gi|29165615|ref|NC_002745.2|	552964	+	A	7	6	3	1	6	SArRNA06
gi|29165615|ref|NC_002745.2|	552965	+	G	7	1	0	0	1	SArRNA06
gi|29165615|ref|NC_002745.2|	552966	+	U	9	1	0	3	6	SArRNA06
gi|29165615|ref|NC_002745.2|	552967	+	G	7	2	4	4	2	SArRNA06
gi|29165615|ref|NC_002745.2|	552968	+	A	5	6	1	1	2	SArRNA06
gi|29165615|ref|NC_002745.2|	552969	+	C	8	14	3	1	4	SArRNA06
gi|29165615|ref|NC_002745.2|	552970	+	C	18	17	6	3	11	SArRNA06
gi|29165615|ref|NC_002745.2|	552971	+	G	11	7	2	5	9	SArRNA06
gi|29165615|ref|NC_002745.2|	552972	+	A	16	14	5	6	14	SArRNA06
gi|29165615|ref|NC_002745.2|	552973	+	U	17	17	6	13	23	SArRNA06
gi|29165615|ref|NC_002745.2|	552974	+	A	8	6	5	3	8	SArRNA06
gi|29165615|ref|NC_002745.2|	552975	+	G	6	4	0	1	10	SArRNA06
gi|29165615|ref|NC_002745.2|	552976	+	U	9	8	2	5	11	SArRNA06
gi|29165615|ref|NC_002745.2|	552977	+	G	9	7	3	4	5	SArRNA06
gi|29165615|ref|NC_002745.2|	552978	+	A	6	4	0	7	6	SArRNA06
gi|29165615|ref|NC_002745.2|	552979	+	A	4	3	2	3	7	SArRNA06
gi|29165615|ref|NC_002745.2|	552980	+	C	43	27	6	13	20	SArRNA06

gi|29165615|ref|NC_002745.2|	552981 +	C	33	26	11	7	17	SArRNA06
gi|29165615|ref|NC_002745.2|	552982 +	A	6	10	5	3	11	SArRNA06
gi|29165615|ref|NC_002745.2|	552983 +	G	7	2	3	2	5	SArRNA06
gi|29165615|ref|NC_002745.2|	552984 +	U	11	14	3	9	6	SArRNA06
gi|29165615|ref|NC_002745.2|	552985 +	A	15	17	1	4	24	SArRNA06
gi|29165615|ref|NC_002745.2|	552986 +	C	30	14	7	2	19	SArRNA06
gi|29165615|ref|NC_002745.2|	552987 +	C	113	46	16	20	74	SArRNA06
gi|29165615|ref|NC_002745.2|	552988 +	G	35	23	11	8	27	SArRNA06
gi|29165615|ref|NC_002745.2|	552989 +	U	24	12	9	8	20	SArRNA06
gi|29165615|ref|NC_002745.2|	552990 +	G	15	9	7	10	12	SArRNA06
gi|29165615|ref|NC_002745.2|	552991 +	A	11	6	2	2	6	SArRNA06
gi|29165615|ref|NC_002745.2|	552992 +	G	1	2	0	0	4	SArRNA06
gi|29165615|ref|NC_002745.2|	552993 +	G	2	2	1	0	5	SArRNA06
gi|29165615|ref|NC_002745.2|	552994 +	G	4	2	4	2	7	SArRNA06
gi|29165615|ref|NC_002745.2|	552995 +	A	8	10	2	6	10	SArRNA06
gi|29165615|ref|NC_002745.2|	552996 +	A	11	4	4	5	15	SArRNA06
gi|29165615|ref|NC_002745.2|	552997 +	A	6	8	2	3	11	SArRNA06
gi|29165615|ref|NC_002745.2|	552998 +	G	1	2	2	1	2	SArRNA06
gi|29165615|ref|NC_002745.2|	552999 +	G	9	9	5	10	10	SArRNA06
gi|29165615|ref|NC_002745.2|	553000 +	U	13	7	9	0	14	SArRNA06
gi|29165615|ref|NC_002745.2|	553001 +	G	7	2	1	1	5	SArRNA06
gi|29165615|ref|NC_002745.2|	553002 +	A	12	10	5	3	16	SArRNA06
gi|29165615|ref|NC_002745.2|	553003 +	A	5	2	0	0	6	SArRNA06
gi|29165615|ref|NC_002745.2|	553004 +	A	5	0	0	0	2	SArRNA06
gi|29165615|ref|NC_002745.2|	553005 +	A	3	3	1	0	3	SArRNA06
gi|29165615|ref|NC_002745.2|	553006 +	G	3	1	1	1	2	SArRNA06
gi|29165615|ref|NC_002745.2|	553007 +	C	41	37	12	11	25	SArRNA06
gi|29165615|ref|NC_002745.2|	553008 +	A	9	12	3	0	6	SArRNA06
gi|29165615|ref|NC_002745.2|	553009 +	C	53	28	14	27	30	SArRNA06
gi|29165615|ref|NC_002745.2|	553010 +	C	28	13	7	9	15	SArRNA06
gi|29165615|ref|NC_002745.2|	553011 +	C	14	10	4	7	7	SArRNA06
gi|29165615|ref|NC_002745.2|	553012 +	C	15	1	7	4	13	SArRNA06
gi|29165615|ref|NC_002745.2|	553013 +	G	9	6	5	2	13	SArRNA06
gi|29165615|ref|NC_002745.2|	553014 +	G	9	7	6	8	15	SArRNA06
gi|29165615|ref|NC_002745.2|	553015 +	A	10	7	7	4	3	SArRNA06
gi|29165615|ref|NC_002745.2|	553016 +	A	16	2	2	3	8	SArRNA06
gi|29165615|ref|NC_002745.2|	553017 +	G	1	2	2	3	8	SArRNA06
gi|29165615|ref|NC_002745.2|	553018 +	G	1	0	0	0	4	SArRNA06
gi|29165615|ref|NC_002745.2|	553019 +	G	1	4	2	1	4	SArRNA06
gi|29165615|ref|NC_002745.2|	553020 +	G	4	7	4	8	10	SArRNA06
gi|29165615|ref|NC_002745.2|	553021 +	A	10	9	4	5	12	SArRNA06
gi|29165615|ref|NC_002745.2|	553022 +	G	9	2	1	8	9	SArRNA06
gi|29165615|ref|NC_002745.2|	553023 +	U	8	11	3	3	13	SArRNA06
gi|29165615|ref|NC_002745.2|	553024 +	G	4	5	4	5	7	SArRNA06
gi|29165615|ref|NC_002745.2|	553025 +	A	3	4	3	3	17	SArRNA06
gi|29165615|ref|NC_002745.2|	553026 +	A	10	3	6	5	21	SArRNA06
gi|29165615|ref|NC_002745.2|	553027 +	A	7	53	4	9	31	SArRNA06
gi|29165615|ref|NC_002745.2|	553028 +	U	12	37	21	18	43	SArRNA06
gi|29165615|ref|NC_002745.2|	553029 +	A	8	22	2	1	16	SArRNA06
gi|29165615|ref|NC_002745.2|	553030 +	G	17	13	7	5	9	SArRNA06
gi|29165615|ref|NC_002745.2|	553031 +	A	20	23	8	10	21	SArRNA06
gi|29165615|ref|NC_002745.2|	553032 +	A	12	13	1	4	12	SArRNA06
gi|29165615|ref|NC_002745.2|	553033 +	C	39	23	12	15	28	SArRNA06
gi|29165615|ref|NC_002745.2|	553034 +	C	27	34	8	15	25	SArRNA06
gi|29165615|ref|NC_002745.2|	553035 +	U	10	12	2	7	17	SArRNA06
gi|29165615|ref|NC_002745.2|	553036 +	G	8	6	6	3	7	SArRNA06
gi|29165615|ref|NC_002745.2|	553037 +	A	4	3	0	4	11	SArRNA06
gi|29165615|ref|NC_002745.2|	553038 +	A	12	2	3	2	6	SArRNA06
gi|29165615|ref|NC_002745.2|	553039 +	A	9	6	3	1	10	SArRNA06
gi|29165615|ref|NC_002745.2|	553040 +	C	8	4	3	1	13	SArRNA06
gi|29165615|ref|NC_002745.2|	553041 +	C	6	10	5	1	11	SArRNA06
gi|29165615|ref|NC_002745.2|	553042 +	G	4	4	2	4	7	SArRNA06
gi|29165615|ref|NC_002745.2|	553043 +	U	17	8	4	7	11	SArRNA06
gi|29165615|ref|NC_002745.2|	553044 +	G	4	6	2	0	6	SArRNA06
gi|29165615|ref|NC_002745.2|	553045 +	U	5	5	2	3	6	SArRNA06
gi|29165615|ref|NC_002745.2|	553046 +	G	7	3	1	0	6	SArRNA06
gi|29165615|ref|NC_002745.2|	553047 +	C	7	8	6	7	7	SArRNA06
gi|29165615|ref|NC_002745.2|	553048 +	U	4	1	5	7	6	SArRNA06
gi|29165615|ref|NC_002745.2|	553049 +	U	16	35	17	24	44	SArRNA06
gi|29165615|ref|NC_002745.2|	553050 +	A	7	7	4	2	15	SArRNA06
gi|29165615|ref|NC_002745.2|	553051 +	C	28	28	6	17	29	SArRNA06
gi|29165615|ref|NC_002745.2|	553052 +	A	8	13	7	13	25	SArRNA06
gi|29165615|ref|NC_002745.2|	553053 +	A	8	11	4	6	10	SArRNA06
gi|29165615|ref|NC_002745.2|	553054 +	G	16	10	7	4	12	SArRNA06
gi|29165615|ref|NC_002745.2|	553055 +	U	19	18	16	15	19	SArRNA06
gi|29165615|ref|NC_002745.2|	553056 +	A	11	3	2	4	6	SArRNA06
gi|29165615|ref|NC_002745.2|	553057 +	G	4	5	2	2	5	SArRNA06
gi|29165615|ref|NC_002745.2|	553058 +	U	30	16	13	7	26	SArRNA06
gi|29165615|ref|NC_002745.2|	553059 +	C	10	6	5	3	7	SArRNA06
gi|29165615|ref|NC_002745.2|	553060 +	A	7	11	3	6	3	SArRNA06
gi|29165615|ref|NC_002745.2|	553061 +	G	9	3	1	2	6	SArRNA06

gi|29165615|ref|NC_002745.2|	553062	+	A	2	3	1	1	2	SArRNA06
gi|29165615|ref|NC_002745.2|	553063	+	G	3	3	0	0	4	SArRNA06
gi|29165615|ref|NC_002745.2|	553064	+	C	6	5	1	3	12	SArRNA06
gi|29165615|ref|NC_002745.2|	553065	+	C	4	3	5	5	4	SArRNA06
gi|29165615|ref|NC_002745.2|	553066	+	C	35	18	9	16	24	SArRNA06
gi|29165615|ref|NC_002745.2|	553067	+	G	7	2	5	2	7	SArRNA06
gi|29165615|ref|NC_002745.2|	553068	+	U	4	2	0	3	2	SArRNA06
gi|29165615|ref|NC_002745.2|	553069	+	U	0	2	3	1	5	SArRNA06
gi|29165615|ref|NC_002745.2|	553070	+	A	2	5	1	3	6	SArRNA06
gi|29165615|ref|NC_002745.2|	553071	+	A	2	3	2	2	6	SArRNA06
gi|29165615|ref|NC_002745.2|	553072	+	U	0	3	1	0	1	SArRNA06
gi|29165615|ref|NC_002745.2|	553073	+	G	1	0	1	1	0	SArRNA06
gi|29165615|ref|NC_002745.2|	553074	+	G	5	10	5	0	4	SArRNA06
gi|29165615|ref|NC_002745.2|	553075	+	G	10	2	1	2	7	SArRNA06
gi|29165615|ref|NC_002745.2|	553076	+	U	8	2	4	4	7	SArRNA06
gi|29165615|ref|NC_002745.2|	553077	+	G	1	0	0	1	0	SArRNA06
gi|29165615|ref|NC_002745.2|	553078	+	A	6	3	2	1	2	SArRNA06
gi|29165615|ref|NC_002745.2|	553079	+	U	2	0	0	1	0	SArRNA06
gi|29165615|ref|NC_002745.2|	553080	+	G	1	0	0	0	0	SArRNA06
gi|29165615|ref|NC_002745.2|	553082	+	C	7	3	2	0	0	SArRNA06
gi|29165615|ref|NC_002745.2|	553083	+	G	2	0	2	0	0	SArRNA06
gi|29165615|ref|NC_002745.2|	553084	+	U	4	5	2	2	1	SArRNA06
gi|29165615|ref|NC_002745.2|	553085	+	G	1	1	0	1	0	SArRNA06
gi|29165615|ref|NC_002745.2|	553086	+	C	10	13	6	8	10	SArRNA06
gi|29165615|ref|NC_002745.2|	553087	+	C	20	16	4	7	9	SArRNA06
gi|29165615|ref|NC_002745.2|	553088	+	U	19	117	92	100	108	SArRNA06
gi|29165615|ref|NC_002745.2|	553089	+	U	44	145	94	150	124	SArRNA06
gi|29165615|ref|NC_002745.2|	553090	+	U	16	72	36	73	53	SArRNA06
gi|29165615|ref|NC_002745.2|	553091	+	U	4	8	1	1	7	SArRNA06
gi|29165615|ref|NC_002745.2|	553092	+	G	16	17	6	7	8	SArRNA06
gi|29165615|ref|NC_002745.2|	553093	+	U	8	6	2	8	4	SArRNA06
gi|29165615|ref|NC_002745.2|	553094	+	A	2	8	1	4	2	SArRNA06
gi|29165615|ref|NC_002745.2|	553095	+	G	8	8	2	1	6	SArRNA06
gi|29165615|ref|NC_002745.2|	553096	+	A	22	12	8	13	6	SArRNA06
gi|29165615|ref|NC_002745.2|	553097	+	A	39	304	57	12	81	SArRNA06
gi|29165615|ref|NC_002745.2|	553098	+	U	8	9	1	1	5	SArRNA06
gi|29165615|ref|NC_002745.2|	553099	+	G	2	4	0	3	2	SArRNA06
gi|29165615|ref|NC_002745.2|	553100	+	A	3	4	2	2	3	SArRNA06
gi|29165615|ref|NC_002745.2|	553101	+	A	3	5	0	2	4	SArRNA06
gi|29165615|ref|NC_002745.2|	553102	+	C	9	15	4	5	10	SArRNA06
gi|29165615|ref|NC_002745.2|	553103	+	C	26	15	5	6	9	SArRNA06
gi|29165615|ref|NC_002745.2|	553104	+	G	4	12	2	4	1	SArRNA06
gi|29165615|ref|NC_002745.2|	553105	+	G	4	1	2	1	3	SArRNA06
gi|29165615|ref|NC_002745.2|	553106	+	C	3	6	4	3	3	SArRNA06
gi|29165615|ref|NC_002745.2|	553107	+	G	8	9	2	4	4	SArRNA06
gi|29165615|ref|NC_002745.2|	553108	+	A	5	4	2	4	5	SArRNA06
gi|29165615|ref|NC_002745.2|	553109	+	G	17	2	1	6	5	SArRNA06
gi|29165615|ref|NC_002745.2|	553110	+	U	1	2	3	2	10	SArRNA06
gi|29165615|ref|NC_002745.2|	553111	+	U	3	5	1	5	15	SArRNA06
gi|29165615|ref|NC_002745.2|	553112	+	A	18	6	7	2	7	SArRNA06
gi|29165615|ref|NC_002745.2|	553113	+	C	19	13	5	10	15	SArRNA06
gi|29165615|ref|NC_002745.2|	553114	+	G	26	27	14	25	30	SArRNA06
gi|29165615|ref|NC_002745.2|	553115	+	A	36	19	9	11	22	SArRNA06
gi|29165615|ref|NC_002745.2|	553116	+	U	15	17	9	8	19	SArRNA06
gi|29165615|ref|NC_002745.2|	553117	+	U	12	16	9	6	18	SArRNA06
gi|29165615|ref|NC_002745.2|	553118	+	U	29	17	7	9	12	SArRNA06
gi|29165615|ref|NC_002745.2|	553119	+	G	52	48	20	34	32	SArRNA06
gi|29165615|ref|NC_002745.2|	553120	+	A	26	18	3	4	15	SArRNA06
gi|29165615|ref|NC_002745.2|	553121	+	U	20	10	3	9	9	SArRNA06
gi|29165615|ref|NC_002745.2|	553122	+	G	5	3	3	5	4	SArRNA06
gi|29165615|ref|NC_002745.2|	553123	+	C	35	15	13	13	27	SArRNA06
gi|29165615|ref|NC_002745.2|	553124	+	A	24	31	8	9	21	SArRNA06
gi|29165615|ref|NC_002745.2|	553125	+	A	16	4	7	4	9	SArRNA06
gi|29165615|ref|NC_002745.2|	553126	+	G	7	4	4	3	0	SArRNA06
gi|29165615|ref|NC_002745.2|	553127	+	G	20	13	11	10	21	SArRNA06
gi|29165615|ref|NC_002745.2|	553128	+	U	18	17	9	10	23	SArRNA06
gi|29165615|ref|NC_002745.2|	553129	+	U	30	26	9	8	17	SArRNA06
gi|29165615|ref|NC_002745.2|	553130	+	A	11	7	5	12	13	SArRNA06
gi|29165615|ref|NC_002745.2|	553131	+	A	23	11	7	8	22	SArRNA06
gi|29165615|ref|NC_002745.2|	553132	+	G	19	21	5	6	9	SArRNA06
gi|29165615|ref|NC_002745.2|	553133	+	C	29	32	10	10	19	SArRNA06
gi|29165615|ref|NC_002745.2|	553134	+	A	22	18	9	3	14	SArRNA06
gi|29165615|ref|NC_002745.2|	553135	+	G	17	18	7	15	24	SArRNA06
gi|29165615|ref|NC_002745.2|	553136	+	U	16	10	7	10	24	SArRNA06
gi|29165615|ref|NC_002745.2|	553137	+	A	35	17	8	7	18	SArRNA06
gi|29165615|ref|NC_002745.2|	553138	+	A	39	24	9	6	26	SArRNA06
gi|29165615|ref|NC_002745.2|	553139	+	A	23	48	6	4	32	SArRNA06
gi|29165615|ref|NC_002745.2|	553140	+	U	8	5	2	10	6	SArRNA06
gi|29165615|ref|NC_002745.2|	553141	+	G	5	3	7	4	7	SArRNA06
gi|29165615|ref|NC_002745.2|	553142	+	U	6	3	4	1	5	SArRNA06
gi|29165615|ref|NC_002745.2|	553143	+	G	3	1	0	1	2	SArRNA06

gi|29165615|ref|NC_002745.2|	553144	+	G	1	6	2	2	6	SArRNA06
gi|29165615|ref|NC_002745.2|	553145	+	A	14	10	3	0	12	SArRNA06
gi|29165615|ref|NC_002745.2|	553146	+	G	34	8	2	2	1	SArRNA06
gi|29165615|ref|NC_002745.2|	553147	+	C	1122	81	54	59	67	SArRNA06
gi|29165615|ref|NC_002745.2|	553148	+	C	169	21	11	18	21	SArRNA06
gi|29165615|ref|NC_002745.2|	553149	+	G	33	20	6	9	18	SArRNA06
gi|29165615|ref|NC_002745.2|	553150	+	U	38	42	22	23	29	SArRNA06
gi|29165615|ref|NC_002745.2|	553151	+	A	23	20	8	13	15	SArRNA06
gi|29165615|ref|NC_002745.2|	553152	+	G	7	7	2	7	9	SArRNA06
gi|29165615|ref|NC_002745.2|	553153	+	C	51	26	7	11	28	SArRNA06
gi|29165615|ref|NC_002745.2|	553154	+	G	28	10	4	6	12	SArRNA06
gi|29165615|ref|NC_002745.2|	553155	+	A	27	12	5	7	17	SArRNA06
gi|29165615|ref|NC_002745.2|	553156	+	A	10	9	7	14	14	SArRNA06
gi|29165615|ref|NC_002745.2|	553157	+	A	21	14	5	15	12	SArRNA06
gi|29165615|ref|NC_002745.2|	553158	+	G	0	2	0	0	1	SArRNA06
gi|29165615|ref|NC_002745.2|	553159	+	C	40	21	21	18	13	SArRNA06
gi|29165615|ref|NC_002745.2|	553160	+	G	3	10	4	4	1	SArRNA06
gi|29165615|ref|NC_002745.2|	553161	+	A	1	2	1	1	1	SArRNA06
gi|29165615|ref|NC_002745.2|	553162	+	G	4	0	1	2	0	SArRNA06
gi|29165615|ref|NC_002745.2|	553163	+	U	31	16	4	9	8	SArRNA06
gi|29165615|ref|NC_002745.2|	553164	+	C	48	35	13	15	17	SArRNA06
gi|29165615|ref|NC_002745.2|	553165	+	U	9	11	7	3	4	SArRNA06
gi|29165615|ref|NC_002745.2|	553166	+	G	2	2	0	4	1	SArRNA06
gi|29165615|ref|NC_002745.2|	553167	+	A	4	9	3	3	4	SArRNA06
gi|29165615|ref|NC_002745.2|	553168	+	A	8	22	2	4	7	SArRNA06
gi|29165615|ref|NC_002745.2|	553169	+	U	14	16	11	17	20	SArRNA06
gi|29165615|ref|NC_002745.2|	553170	+	A	9	16	3	9	8	SArRNA06
gi|29165615|ref|NC_002745.2|	553171	+	G	5	2	0	5	1	SArRNA06
gi|29165615|ref|NC_002745.2|	553172	+	G	3	4	1	2	2	SArRNA06
gi|29165615|ref|NC_002745.2|	553173	+	G	4	2	0	0	2	SArRNA06
gi|29165615|ref|NC_002745.2|	553174	+	C	9	5	1	2	5	SArRNA06
gi|29165615|ref|NC_002745.2|	553175	+	G	8	1	3	6	9	SArRNA06
gi|29165615|ref|NC_002745.2|	553176	+	U	8	5	7	2	15	SArRNA06
gi|29165615|ref|NC_002745.2|	553177	+	U	13	6	3	1	8	SArRNA06
gi|29165615|ref|NC_002745.2|	553178	+	U	9	10	11	5	9	SArRNA06
gi|29165615|ref|NC_002745.2|	553179	+	A	9	10	7	7	9	SArRNA06
gi|29165615|ref|NC_002745.2|	553180	+	G	9	5	3	1	5	SArRNA06
gi|29165615|ref|NC_002745.2|	553181	+	U	13	4	7	3	7	SArRNA06
gi|29165615|ref|NC_002745.2|	553182	+	A	13	11	3	6	9	SArRNA06
gi|29165615|ref|NC_002745.2|	553183	+	U	2	2	1	3	3	SArRNA06
gi|29165615|ref|NC_002745.2|	553184	+	U	4	1	0	0	5	SArRNA06
gi|29165615|ref|NC_002745.2|	553185	+	U	0	1	0	1	2	SArRNA06
gi|29165615|ref|NC_002745.2|	553186	+	G	0	1	0	0	0	SArRNA06
gi|29165615|ref|NC_002745.2|	553187	+	G	0	0	0	0	1	SArRNA06
gi|29165615|ref|NC_002745.2|	553189	+	C	1	3	1	0	1	SArRNA06
gi|29165615|ref|NC_002745.2|	553190	+	G	6	3	1	2	2	SArRNA06
gi|29165615|ref|NC_002745.2|	553191	+	U	1	1	1	0	1	SArRNA06
gi|29165615|ref|NC_002745.2|	553192	+	A	3	3	0	0	0	SArRNA06
gi|29165615|ref|NC_002745.2|	553193	+	G	12	16	2	7	5	SArRNA06
gi|29165615|ref|NC_002745.2|	553194	+	A	1	2	0	2	1	SArRNA06
gi|29165615|ref|NC_002745.2|	553195	+	C	47	44	14	21	26	SArRNA06
gi|29165615|ref|NC_002745.2|	553196	+	C	11	6	4	1	7	SArRNA06
gi|29165615|ref|NC_002745.2|	553197	+	C	21	12	6	2	3	SArRNA06
gi|29165615|ref|NC_002745.2|	553198	+	G	182	102	70	74	75	SArRNA06
gi|29165615|ref|NC_002745.2|	553199	+	A	2	1	0	0	2	SArRNA06
gi|29165615|ref|NC_002745.2|	553200	+	A	6	1	0	0	4	SArRNA06
gi|29165615|ref|NC_002745.2|	553201	+	A	2	1	0	0	0	SArRNA06
gi|29165615|ref|NC_002745.2|	553202	+	C	0	1	0	0	1	SArRNA06
gi|29165615|ref|NC_002745.2|	553203	+	C	4	6	0	0	3	SArRNA06
gi|29165615|ref|NC_002745.2|	553205	+	G	0	0	0	0	1	SArRNA06
gi|29165615|ref|NC_002745.2|	553206	+	G	1	1	0	0	0	SArRNA06
gi|29165615|ref|NC_002745.2|	553207	+	U	2	1	0	1	0	SArRNA06
gi|29165615|ref|NC_002745.2|	553208	+	G	2	3	0	0	1	SArRNA06
gi|29165615|ref|NC_002745.2|	553209	+	A	1	0	0	2	1	SArRNA06
gi|29165615|ref|NC_002745.2|	553211	+	C	2	1	0	1	0	SArRNA06
gi|29165615|ref|NC_002745.2|	553212	+	U	1	3	1	0	1	SArRNA06
gi|29165615|ref|NC_002745.2|	553213	+	A	5	1	0	1	0	SArRNA06
gi|29165615|ref|NC_002745.2|	553214	+	C	26	31	9	14	13	SArRNA06
gi|29165615|ref|NC_002745.2|	553215	+	C	4	14	3	3	0	SArRNA06
gi|29165615|ref|NC_002745.2|	553216	+	C	9	4	0	1	1	SArRNA06
gi|29165615|ref|NC_002745.2|	553217	+	U	3	0	1	2	3	SArRNA06
gi|29165615|ref|NC_002745.2|	553218	+	U	13	6	3	6	3	SArRNA06
gi|29165615|ref|NC_002745.2|	553219	+	G	1	1	1	3	1	SArRNA06
gi|29165615|ref|NC_002745.2|	553220	+	G	2	0	0	2	4	SArRNA06
gi|29165615|ref|NC_002745.2|	553221	+	U	9	6	2	2	5	SArRNA06
gi|29165615|ref|NC_002745.2|	553222	+	C	26	37	8	11	18	SArRNA06
gi|29165615|ref|NC_002745.2|	553223	+	A	17	25	3	4	16	SArRNA06
gi|29165615|ref|NC_002745.2|	553224	+	G	1	6	1	1	2	SArRNA06
gi|29165615|ref|NC_002745.2|	553225	+	G	4	0	5	6	5	SArRNA06
gi|29165615|ref|NC_002745.2|	553226	+	U	3	1	5	0	6	SArRNA06
gi|29165615|ref|NC_002745.2|	553227	+	U	2	1	1	0	1	SArRNA06

gi|29165615|ref|NC_002745.2|	553228	+	G	0	3	0	1	0	SArRNA06
gi|29165615|ref|NC_002745.2|	553229	+	A	0	2	1	0	2	SArRNA06
gi|29165615|ref|NC_002745.2|	553230	+	A	3	3	0	0	4	SArRNA06
gi|29165615|ref|NC_002745.2|	553231	+	G	1	1	0	0	0	SArRNA06
gi|29165615|ref|NC_002745.2|	553232	+	U	0	0	0	0	2	SArRNA06
gi|29165615|ref|NC_002745.2|	553233	+	U	0	2	0	1	2	SArRNA06
gi|29165615|ref|NC_002745.2|	553234	+	C	21	21	7	7	14	SArRNA06
gi|29165615|ref|NC_002745.2|	553235	+	A	18	5	0	5	8	SArRNA06
gi|29165615|ref|NC_002745.2|	553236	+	G	8	9	4	4	14	SArRNA06
gi|29165615|ref|NC_002745.2|	553237	+	G	14	6	5	3	11	SArRNA06
gi|29165615|ref|NC_002745.2|	553238	+	U	18	13	7	2	18	SArRNA06
gi|29165615|ref|NC_002745.2|	553239	+	A	55	53	17	19	38	SArRNA06
gi|29165615|ref|NC_002745.2|	553240	+	A	4	3	1	1	4	SArRNA06
gi|29165615|ref|NC_002745.2|	553241	+	C	99	96	36	43	59	SArRNA06
gi|29165615|ref|NC_002745.2|	553242	+	A	38	35	9	18	19	SArRNA06
gi|29165615|ref|NC_002745.2|	553243	+	C	6	8	0	1	5	SArRNA06
gi|29165615|ref|NC_002745.2|	553244	+	U	4	2	1	2	4	SArRNA06
gi|29165615|ref|NC_002745.2|	553245	+	G	2	4	0	1	2	SArRNA06
gi|29165615|ref|NC_002745.2|	553246	+	A	6	4	2	0	4	SArRNA06
gi|29165615|ref|NC_002745.2|	553247	+	A	3	2	1	0	0	SArRNA06
gi|29165615|ref|NC_002745.2|	553248	+	U	3	3	0	1	5	SArRNA06
gi|29165615|ref|NC_002745.2|	553249	+	G	0	0	0	0	1	SArRNA06
gi|29165615|ref|NC_002745.2|	553250	+	G	5	5	2	0	5	SArRNA06
gi|29165615|ref|NC_002745.2|	553251	+	A	8	10	0	1	5	SArRNA06
gi|29165615|ref|NC_002745.2|	553252	+	G	4	3	1	4	2	SArRNA06
gi|29165615|ref|NC_002745.2|	553253	+	G	7	7	3	5	5	SArRNA06
gi|29165615|ref|NC_002745.2|	553254	+	A	2	2	1	0	0	SArRNA06
gi|29165615|ref|NC_002745.2|	553255	+	C	7	14	6	3	5	SArRNA06
gi|29165615|ref|NC_002745.2|	553256	+	C	31	53	22	9	31	SArRNA06
gi|29165615|ref|NC_002745.2|	553257	+	G	23	23	12	12	19	SArRNA06
gi|29165615|ref|NC_002745.2|	553258	+	A	14	14	9	15	9	SArRNA06
gi|29165615|ref|NC_002745.2|	553259	+	A	2	4	0	1	2	SArRNA06
gi|29165615|ref|NC_002745.2|	553260	+	C	5	8	0	0	3	SArRNA06
gi|29165615|ref|NC_002745.2|	553261	+	C	27	21	8	5	8	SArRNA06
gi|29165615|ref|NC_002745.2|	553262	+	G	2	4	2	2	6	SArRNA06
gi|29165615|ref|NC_002745.2|	553263	+	A	2	4	2	4	5	SArRNA06
gi|29165615|ref|NC_002745.2|	553264	+	C	7	7	5	0	8	SArRNA06
gi|29165615|ref|NC_002745.2|	553265	+	U	7	9	4	6	5	SArRNA06
gi|29165615|ref|NC_002745.2|	553266	+	U	28	25	11	14	25	SArRNA06
gi|29165615|ref|NC_002745.2|	553267	+	A	12	14	0	4	8	SArRNA06
gi|29165615|ref|NC_002745.2|	553268	+	C	45	34	24	29	37	SArRNA06
gi|29165615|ref|NC_002745.2|	553269	+	G	13	18	7	13	17	SArRNA06
gi|29165615|ref|NC_002745.2|	553270	+	U	16	7	3	3	15	SArRNA06
gi|29165615|ref|NC_002745.2|	553271	+	U	30	28	5	20	24	SArRNA06
gi|29165615|ref|NC_002745.2|	553272	+	G	10	8	2	9	12	SArRNA06
gi|29165615|ref|NC_002745.2|	553273	+	A	10	8	4	3	10	SArRNA06
gi|29165615|ref|NC_002745.2|	553274	+	A	7	6	5	5	7	SArRNA06
gi|29165615|ref|NC_002745.2|	553275	+	A	19	31	14	16	29	SArRNA06
gi|29165615|ref|NC_002745.2|	553276	+	A	14	15	8	6	12	SArRNA06
gi|29165615|ref|NC_002745.2|	553277	+	G	7	14	2	9	14	SArRNA06
gi|29165615|ref|NC_002745.2|	553278	+	U	36	68	27	57	43	SArRNA06
gi|29165615|ref|NC_002745.2|	553279	+	G	9	20	18	22	17	SArRNA06
gi|29165615|ref|NC_002745.2|	553280	+	A	12	16	9	13	18	SArRNA06
gi|29165615|ref|NC_002745.2|	553281	+	G	5	8	2	0	4	SArRNA06
gi|29165615|ref|NC_002745.2|	553282	+	C	147	96	45	43	57	SArRNA06
gi|29165615|ref|NC_002745.2|	553283	+	G	54	48	14	27	33	SArRNA06
gi|29165615|ref|NC_002745.2|	553284	+	G	11	15	6	4	7	SArRNA06
gi|29165615|ref|NC_002745.2|	553285	+	A	16	16	8	7	16	SArRNA06
gi|29165615|ref|NC_002745.2|	553286	+	U	23	16	9	8	13	SArRNA06
gi|29165615|ref|NC_002745.2|	553287	+	G	46	49	16	20	30	SArRNA06
gi|29165615|ref|NC_002745.2|	553288	+	A	108	108	34	42	62	SArRNA06
gi|29165615|ref|NC_002745.2|	553289	+	A	31	34	14	23	17	SArRNA06
gi|29165615|ref|NC_002745.2|	553290	+	C	41	30	8	12	27	SArRNA06
gi|29165615|ref|NC_002745.2|	553291	+	U	32	43	7	10	12	SArRNA06
gi|29165615|ref|NC_002745.2|	553292	+	G	1	5	1	0	3	SArRNA06
gi|29165615|ref|NC_002745.2|	553293	+	A	6	3	1	1	5	SArRNA06
gi|29165615|ref|NC_002745.2|	553295	+	G	1	0	0	1	1	SArRNA06
gi|29165615|ref|NC_002745.2|	553296	+	G	10	7	4	3	8	SArRNA06
gi|29165615|ref|NC_002745.2|	553297	+	U	4	6	3	2	3	SArRNA06
gi|29165615|ref|NC_002745.2|	553298	+	A	1	1	0	0	4	SArRNA06
gi|29165615|ref|NC_002745.2|	553299	+	G	0	0	0	1	1	SArRNA06
gi|29165615|ref|NC_002745.2|	553300	+	C	13	10	7	2	5	SArRNA06
gi|29165615|ref|NC_002745.2|	553301	+	G	1	12	3	3	4	SArRNA06
gi|29165615|ref|NC_002745.2|	553302	+	G	4	1	1	4	4	SArRNA06
gi|29165615|ref|NC_002745.2|	553303	+	A	6	1	1	0	5	SArRNA06
gi|29165615|ref|NC_002745.2|	553304	+	G	3	6	2	2	1	SArRNA06
gi|29165615|ref|NC_002745.2|	553305	+	A	7	4	2	3	9	SArRNA06
gi|29165615|ref|NC_002745.2|	553306	+	A	16	17	5	1	8	SArRNA06
gi|29165615|ref|NC_002745.2|	553307	+	A	25	125	24	11	35	SArRNA06
gi|29165615|ref|NC_002745.2|	553308	+	U	7	10	5	10	4	SArRNA06
gi|29165615|ref|NC_002745.2|	553309	+	U	7	4	0	1	5	SArRNA06

gi|29165615|ref|NC_002745.2|	553310	+	C	18	26	4	1	10	SArRNA06
gi|29165615|ref|NC_002745.2|	553311	+	C	127	100	35	49	54	SArRNA06
gi|29165615|ref|NC_002745.2|	553312	+	A	31	40	15	8	14	SArRNA06
gi|29165615|ref|NC_002745.2|	553313	+	A	8	54	6	5	10	SArRNA06
gi|29165615|ref|NC_002745.2|	553314	+	U	3	3	0	0	1	SArRNA06
gi|29165615|ref|NC_002745.2|	553315	+	C	7	15	2	3	4	SArRNA06
gi|29165615|ref|NC_002745.2|	553316	+	G	11	11	3	10	6	SArRNA06
gi|29165615|ref|NC_002745.2|	553317	+	A	18	22	3	5	3	SArRNA06
gi|29165615|ref|NC_002745.2|	553318	+	A	4	6	2	3	9	SArRNA06
gi|29165615|ref|NC_002745.2|	553319	+	C	11	6	6	3	3	SArRNA06
gi|29165615|ref|NC_002745.2|	553320	+	C	15	10	6	3	7	SArRNA06
gi|29165615|ref|NC_002745.2|	553321	+	U	10	9	1	4	3	SArRNA06
gi|29165615|ref|NC_002745.2|	553322	+	G	6	6	5	7	0	SArRNA06
gi|29165615|ref|NC_002745.2|	553323	+	G	8	3	0	1	7	SArRNA06
gi|29165615|ref|NC_002745.2|	553324	+	A	10	3	4	2	3	SArRNA06
gi|29165615|ref|NC_002745.2|	553325	+	G	11	13	5	3	9	SArRNA06
gi|29165615|ref|NC_002745.2|	553326	+	A	23	20	4	7	14	SArRNA06
gi|29165615|ref|NC_002745.2|	553327	+	U	12	19	13	13	18	SArRNA06
gi|29165615|ref|NC_002745.2|	553328	+	A	5	0	1	2	3	SArRNA06
gi|29165615|ref|NC_002745.2|	553329	+	G	2	0	1	1	1	SArRNA06
gi|29165615|ref|NC_002745.2|	553330	+	C	7	14	2	1	8	SArRNA06
gi|29165615|ref|NC_002745.2|	553331	+	U	4	6	1	1	3	SArRNA06
gi|29165615|ref|NC_002745.2|	553332	+	G	3	1	0	0	1	SArRNA06
gi|29165615|ref|NC_002745.2|	553333	+	G	7	1	1	1	1	SArRNA06
gi|29165615|ref|NC_002745.2|	553334	+	U	7	0	0	2	2	SArRNA06
gi|29165615|ref|NC_002745.2|	553335	+	U	2	4	1	0	2	SArRNA06
gi|29165615|ref|NC_002745.2|	553336	+	C	6	9	6	3	9	SArRNA06
gi|29165615|ref|NC_002745.2|	553337	+	U	3	5	1	5	1	SArRNA06
gi|29165615|ref|NC_002745.2|	553338	+	C	35	39	14	22	20	SArRNA06
gi|29165615|ref|NC_002745.2|	553339	+	U	13	9	2	4	6	SArRNA06
gi|29165615|ref|NC_002745.2|	553340	+	C	4	5	0	0	2	SArRNA06
gi|29165615|ref|NC_002745.2|	553341	+	C	8	9	3	4	6	SArRNA06
gi|29165615|ref|NC_002745.2|	553342	+	G	1499	1071	669	908	645	SArRNA06
gi|29165615|ref|NC_002745.2|	553343	+	A	6	7	1	2	3	SArRNA06
gi|29165615|ref|NC_002745.2|	553344	+	A	6	7	1	3	7	SArRNA06
gi|29165615|ref|NC_002745.2|	553345	+	A	23	204	51	17	63	SArRNA06
gi|29165615|ref|NC_002745.2|	553346	+	U	9	9	4	6	9	SArRNA06
gi|29165615|ref|NC_002745.2|	553347	+	A	7	7	6	3	2	SArRNA06
gi|29165615|ref|NC_002745.2|	553348	+	G	7	6	4	4	3	SArRNA06
gi|29165615|ref|NC_002745.2|	553349	+	C	28	19	4	11	14	SArRNA06
gi|29165615|ref|NC_002745.2|	553350	+	U	13	7	5	6	10	SArRNA06
gi|29165615|ref|NC_002745.2|	553351	+	U	7	9	5	4	4	SArRNA06
gi|29165615|ref|NC_002745.2|	553352	+	U	9	14	3	5	11	SArRNA06
gi|29165615|ref|NC_002745.2|	553353	+	A	7	12	1	0	7	SArRNA06
gi|29165615|ref|NC_002745.2|	553354	+	G	2	1	5	1	2	SArRNA06
gi|29165615|ref|NC_002745.2|	553355	+	G	1	1	1	1	4	SArRNA06
gi|29165615|ref|NC_002745.2|	553356	+	G	6	8	0	3	4	SArRNA06
gi|29165615|ref|NC_002745.2|	553357	+	C	73	29	10	10	17	SArRNA06
gi|29165615|ref|NC_002745.2|	553358	+	U	7	18	1	4	3	SArRNA06
gi|29165615|ref|NC_002745.2|	553359	+	A	6	8	1	1	4	SArRNA06
gi|29165615|ref|NC_002745.2|	553360	+	G	11	10	2	3	5	SArRNA06
gi|29165615|ref|NC_002745.2|	553361	+	C	17	19	11	10	7	SArRNA06
gi|29165615|ref|NC_002745.2|	553362	+	C	5	7	3	2	2	SArRNA06
gi|29165615|ref|NC_002745.2|	553363	+	U	5	3	1	0	1	SArRNA06
gi|29165615|ref|NC_002745.2|	553364	+	C	65	72	36	25	22	SArRNA06
gi|29165615|ref|NC_002745.2|	553365	+	A	16	17	5	5	9	SArRNA06
gi|29165615|ref|NC_002745.2|	553366	+	A	9	15	3	4	6	SArRNA06
gi|29165615|ref|NC_002745.2|	553367	+	G	5	1	2	5	8	SArRNA06
gi|29165615|ref|NC_002745.2|	553368	+	U	6	5	3	2	7	SArRNA06
gi|29165615|ref|NC_002745.2|	553369	+	G	87	90	43	38	48	SArRNA06
gi|29165615|ref|NC_002745.2|	553370	+	A	19	8	5	4	8	SArRNA06
gi|29165615|ref|NC_002745.2|	553371	+	U	10	18	7	12	15	SArRNA06
gi|29165615|ref|NC_002745.2|	553372	+	G	12	20	6	2	6	SArRNA06
gi|29165615|ref|NC_002745.2|	553373	+	A	3	8	2	2	7	SArRNA06
gi|29165615|ref|NC_002745.2|	553374	+	U	10	10	1	10	8	SArRNA06
gi|29165615|ref|NC_002745.2|	553375	+	U	32	67	24	21	19	SArRNA06
gi|29165615|ref|NC_002745.2|	553376	+	A	9	8	6	2	4	SArRNA06
gi|29165615|ref|NC_002745.2|	553377	+	U	19	5	3	5	8	SArRNA06
gi|29165615|ref|NC_002745.2|	553378	+	U	17	6	5	6	9	SArRNA06
gi|29165615|ref|NC_002745.2|	553379	+	G	1	3	1	3	6	SArRNA06
gi|29165615|ref|NC_002745.2|	553380	+	G	11	11	4	8	4	SArRNA06
gi|29165615|ref|NC_002745.2|	553381	+	A	21	12	3	10	10	SArRNA06
gi|29165615|ref|NC_002745.2|	553382	+	G	5	2	0	1	3	SArRNA06
gi|29165615|ref|NC_002745.2|	553383	+	G	14	8	5	5	6	SArRNA06
gi|29165615|ref|NC_002745.2|	553384	+	U	18	16	1	6	15	SArRNA06
gi|29165615|ref|NC_002745.2|	553385	+	A	3	24	2	7	11	SArRNA06
gi|29165615|ref|NC_002745.2|	553386	+	G	23	27	9	9	8	SArRNA06
gi|29165615|ref|NC_002745.2|	553387	+	A	10	11	3	11	5	SArRNA06
gi|29165615|ref|NC_002745.2|	553388	+	G	6	4	4	4	11	SArRNA06
gi|29165615|ref|NC_002745.2|	553389	+	C	76	36	13	13	20	SArRNA06
gi|29165615|ref|NC_002745.2|	553390	+	A	31	11	2	13	14	SArRNA06

gi|29165615|ref|NC_002745.2|	553391 +	C	35	38	14	10	16	SArRNA06
gi|29165615|ref|NC_002745.2|	553392 +	U	5	5	1	1	2	SArRNA06
gi|29165615|ref|NC_002745.2|	553393 +	G	1	0	0	0	0	SArRNA06
gi|29165615|ref|NC_002745.2|	553394 +	U	8	9	3	2	8	SArRNA06
gi|29165615|ref|NC_002745.2|	553395 +	U	1	4	0	1	2	SArRNA06
gi|29165615|ref|NC_002745.2|	553396 +	U	4	2	1	3	3	SArRNA06
gi|29165615|ref|NC_002745.2|	553397 +	G	1	0	2	3	0	SArRNA06
gi|29165615|ref|NC_002745.2|	553398 +	G	1	2	0	0	0	SArRNA06
gi|29165615|ref|NC_002745.2|	553399 +	A	7	1	0	4	2	SArRNA06
gi|29165615|ref|NC_002745.2|	553400 +	C	6	2	3	3	4	SArRNA06
gi|29165615|ref|NC_002745.2|	553401 +	G	2	1	0	2	2	SArRNA06
gi|29165615|ref|NC_002745.2|	553402 +	A	5	3	1	0	3	SArRNA06
gi|29165615|ref|NC_002745.2|	553403 +	G	3	0	0	0	1	SArRNA06
gi|29165615|ref|NC_002745.2|	553404 +	G	2	2	0	0	1	SArRNA06
gi|29165615|ref|NC_002745.2|	553405 +	G	1	3	0	0	1	SArRNA06
gi|29165615|ref|NC_002745.2|	553406 +	G	4	2	0	1	3	SArRNA06
gi|29165615|ref|NC_002745.2|	553407 +	C	636	397	286	203	277	SArRNA06
gi|29165615|ref|NC_002745.2|	553408 +	C	216	140	65	85	93	SArRNA06
gi|29165615|ref|NC_002745.2|	553409 +	C	93	50	18	30	30	SArRNA06
gi|29165615|ref|NC_002745.2|	553410 +	C	52	47	25	18	37	SArRNA06
gi|29165615|ref|NC_002745.2|	553411 +	U	35	29	16	10	37	SArRNA06
gi|29165615|ref|NC_002745.2|	553412 +	C	39	29	11	20	19	SArRNA06
gi|29165615|ref|NC_002745.2|	553413 +	U	10	8	3	10	14	SArRNA06
gi|29165615|ref|NC_002745.2|	553414 +	C	558	587	222	330	303	SArRNA06
gi|29165615|ref|NC_002745.2|	553415 +	G	88	113	52	47	56	SArRNA06
gi|29165615|ref|NC_002745.2|	553416 +	G	4	2	0	2	4	SArRNA06
gi|29165615|ref|NC_002745.2|	553417 +	G	4	3	1	2	4	SArRNA06
gi|29165615|ref|NC_002745.2|	553418 +	U	10	12	4	6	4	SArRNA06
gi|29165615|ref|NC_002745.2|	553419 +	U	33	42	13	18	25	SArRNA06
gi|29165615|ref|NC_002745.2|	553420 +	A	14	6	1	6	8	SArRNA06
gi|29165615|ref|NC_002745.2|	553421 +	C	23	8	1	8	12	SArRNA06
gi|29165615|ref|NC_002745.2|	553422 +	C	45	17	10	7	32	SArRNA06
gi|29165615|ref|NC_002745.2|	553423 +	G	21	16	0	11	17	SArRNA06
gi|29165615|ref|NC_002745.2|	553424 +	A	62	57	19	19	36	SArRNA06
gi|29165615|ref|NC_002745.2|	553425 +	A	23	31	13	13	18	SArRNA06
gi|29165615|ref|NC_002745.2|	553426 +	U	17	19	6	10	10	SArRNA06
gi|29165615|ref|NC_002745.2|	553427 +	U	15	21	9	12	13	SArRNA06
gi|29165615|ref|NC_002745.2|	553428 +	C	131	96	60	51	59	SArRNA06
gi|29165615|ref|NC_002745.2|	553429 +	A	54	37	15	19	29	SArRNA06
gi|29165615|ref|NC_002745.2|	553430 +	G	9	14	6	6	6	SArRNA06
gi|29165615|ref|NC_002745.2|	553431 +	A	5	11	7	8	16	SArRNA06
gi|29165615|ref|NC_002745.2|	553432 +	C	113	113	69	57	51	SArRNA06
gi|29165615|ref|NC_002745.2|	553433 +	A	30	36	8	18	22	SArRNA06
gi|29165615|ref|NC_002745.2|	553434 +	A	36	43	13	20	35	SArRNA06
gi|29165615|ref|NC_002745.2|	553435 +	A	69	59	23	43	50	SArRNA06
gi|29165615|ref|NC_002745.2|	553436 +	C	219	234	101	139	100	SArRNA06
gi|29165615|ref|NC_002745.2|	553437 +	U	64	59	26	42	55	SArRNA06
gi|29165615|ref|NC_002745.2|	553438 +	C	100	47	18	34	41	SArRNA06
gi|29165615|ref|NC_002745.2|	553439 +	C	211	145	53	72	109	SArRNA06
gi|29165615|ref|NC_002745.2|	553440 +	G	178	149	57	78	119	SArRNA06
gi|29165615|ref|NC_002745.2|	553441 +	A	569	466	239	312	316	SArRNA06
gi|29165615|ref|NC_002745.2|	553442 +	A	236	237	84	150	159	SArRNA06
gi|29165615|ref|NC_002745.2|	553443 +	U	47	47	21	22	39	SArRNA06
gi|29165615|ref|NC_002745.2|	553444 +	G	20	11	2	4	10	SArRNA06
gi|29165615|ref|NC_002745.2|	553445 +	C	52	38	11	19	26	SArRNA06
gi|29165615|ref|NC_002745.2|	553446 +	C	409	436	175	254	197	SArRNA06
gi|29165615|ref|NC_002745.2|	553447 +	A	83	78	34	37	67	SArRNA06
gi|29165615|ref|NC_002745.2|	553448 +	A	52	24	13	20	35	SArRNA06
gi|29165615|ref|NC_002745.2|	553449 +	U	40	30	25	38	53	SArRNA06
gi|29165615|ref|NC_002745.2|	553450 +	U	81	62	27	30	69	SArRNA06
gi|29165615|ref|NC_002745.2|	553451 +	A	72	55	20	31	55	SArRNA06
gi|29165615|ref|NC_002745.2|	553452 +	A	115	103	43	48	94	SArRNA06
gi|29165615|ref|NC_002745.2|	553453 +	U	60	38	16	34	49	SArRNA06
gi|29165615|ref|NC_002745.2|	553454 +	U	30	24	21	19	45	SArRNA06
gi|29165615|ref|NC_002745.2|	553455 +	U	59	53	17	22	37	SArRNA06
gi|29165615|ref|NC_002745.2|	553456 +	A	20	19	11	12	19	SArRNA06
gi|29165615|ref|NC_002745.2|	553457 +	A	29	28	11	16	24	SArRNA06
gi|29165615|ref|NC_002745.2|	553458 +	C	125	113	39	59	55	SArRNA06
gi|29165615|ref|NC_002745.2|	553459 +	U	33	26	10	16	38	SArRNA06
gi|29165615|ref|NC_002745.2|	553460 +	U	12	11	5	10	18	SArRNA06
gi|29165615|ref|NC_002745.2|	553461 +	G	13	9	5	13	12	SArRNA06
gi|29165615|ref|NC_002745.2|	553462 +	G	104	64	38	46	50	SArRNA06
gi|29165615|ref|NC_002745.2|	553463 +	G	170	112	69	63	74	SArRNA06
gi|29165615|ref|NC_002745.2|	553464 +	A	54	36	13	18	53	SArRNA06
gi|29165615|ref|NC_002745.2|	553465 +	G	60	34	22	22	47	SArRNA06
gi|29165615|ref|NC_002745.2|	553466 +	U	287	205	80	83	128	SArRNA06
gi|29165615|ref|NC_002745.2|	553467 +	C	1680	1188	549	620	777	SArRNA06
gi|29165615|ref|NC_002745.2|	553468 +	A	367	270	111	162	180	SArRNA06
gi|29165615|ref|NC_002745.2|	553469 +	G	14	14	7	1	12	SArRNA06
gi|29165615|ref|NC_002745.2|	553470 +	A	173	70	32	65	104	SArRNA06
gi|29165615|ref|NC_002745.2|	553471 +	A	104	63	21	36	56	SArRNA06

gi|29165615|ref|NC_002745.2|	553472 +	C	70	73	44	31	41	SArRNA06
gi|29165615|ref|NC_002745.2|	553473 +	A	26	44	9	16	19	SArRNA06
gi|29165615|ref|NC_002745.2|	553474 +	U	48	50	13	16	17	SArRNA06
gi|29165615|ref|NC_002745.2|	553475 +	G	23	15	6	14	13	SArRNA06
gi|29165615|ref|NC_002745.2|	553476 +	G	148	91	46	69	139	SArRNA06
gi|29165615|ref|NC_002745.2|	553477 +	G	144	111	57	53	139	SArRNA06
gi|29165615|ref|NC_002745.2|	553478 +	U	253	139	73	96	190	SArRNA06
gi|29165615|ref|NC_002745.2|	553479 +	G	143	87	53	61	112	SArRNA06
gi|29165615|ref|NC_002745.2|	553480 +	A	190	128	85	78	198	SArRNA06
gi|29165615|ref|NC_002745.2|	553481 +	U	76	85	26	43	84	SArRNA06
gi|29165615|ref|NC_002745.2|	553482 +	A	49	56	18	26	54	SArRNA06
gi|29165615|ref|NC_002745.2|	553483 +	A	38	36	16	28	43	SArRNA06
gi|29165615|ref|NC_002745.2|	553484 +	G	26	25	12	13	24	SArRNA06
gi|29165615|ref|NC_002745.2|	553485 +	G	18	16	1	11	16	SArRNA06
gi|29165615|ref|NC_002745.2|	553486 +	U	9	13	4	2	12	SArRNA06
gi|29165615|ref|NC_002745.2|	553487 +	C	57	39	9	5	36	SArRNA06
gi|29165615|ref|NC_002745.2|	553488 +	C	171	102	67	60	90	SArRNA06
gi|29165615|ref|NC_002745.2|	553489 +	G	29	43	17	17	13	SArRNA06
gi|29165615|ref|NC_002745.2|	553490 +	U	28	28	15	25	35	SArRNA06
gi|29165615|ref|NC_002745.2|	553491 +	G	12	13	6	10	10	SArRNA06
gi|29165615|ref|NC_002745.2|	553492 +	U	50	43	19	29	43	SArRNA06
gi|29165615|ref|NC_002745.2|	553493 +	U	69	56	32	55	44	SArRNA06
gi|29165615|ref|NC_002745.2|	553494 +	C	62	34	15	19	38	SArRNA06
gi|29165615|ref|NC_002745.2|	553495 +	G	44	43	24	30	41	SArRNA06
gi|29165615|ref|NC_002745.2|	553496 +	A	54	36	14	24	27	SArRNA06
gi|29165615|ref|NC_002745.2|	553497 +	A	49	72	19	22	37	SArRNA06
gi|29165615|ref|NC_002745.2|	553498 +	A	51	46	14	17	29	SArRNA06
gi|29165615|ref|NC_002745.2|	553499 +	G	14	9	3	10	9	SArRNA06
gi|29165615|ref|NC_002745.2|	553500 +	G	17	26	4	7	7	SArRNA06
gi|29165615|ref|NC_002745.2|	553501 +	G	11	27	7	8	3	SArRNA06
gi|29165615|ref|NC_002745.2|	553502 +	A	11	4	0	2	7	SArRNA06
gi|29165615|ref|NC_002745.2|	553503 +	A	10	13	6	8	12	SArRNA06
gi|29165615|ref|NC_002745.2|	553504 +	A	23	20	3	7	15	SArRNA06
gi|29165615|ref|NC_002745.2|	553505 +	C	164	171	49	80	110	SArRNA06
gi|29165615|ref|NC_002745.2|	553506 +	A	22	33	9	5	21	SArRNA06
gi|29165615|ref|NC_002745.2|	553507 +	G	4	4	3	2	1	SArRNA06
gi|29165615|ref|NC_002745.2|	553508 +	C	145	145	51	48	65	SArRNA06
gi|29165615|ref|NC_002745.2|	553509 +	C	132	153	48	46	66	SArRNA06
gi|29165615|ref|NC_002745.2|	553510 +	C	374	180	91	89	113	SArRNA06
gi|29165615|ref|NC_002745.2|	553511 +	A	189	80	50	34	62	SArRNA06
gi|29165615|ref|NC_002745.2|	553512 +	G	19	6	5	9	13	SArRNA06
gi|29165615|ref|NC_002745.2|	553513 +	A	15	20	5	8	15	SArRNA06
gi|29165615|ref|NC_002745.2|	553514 +	C	353	220	136	125	196	SArRNA06
gi|29165615|ref|NC_002745.2|	553515 +	C	915	512	368	299	530	SArRNA06
gi|29165615|ref|NC_002745.2|	553516 +	A	238	163	81	69	107	SArRNA06
gi|29165615|ref|NC_002745.2|	553517 +	C	189	123	52	46	97	SArRNA06
gi|29165615|ref|NC_002745.2|	553518 +	C	417	292	162	134	193	SArRNA06
gi|29165615|ref|NC_002745.2|	553519 +	A	126	104	37	53	62	SArRNA06
gi|29165615|ref|NC_002745.2|	553520 +	G	108	56	11	31	74	SArRNA06
gi|29165615|ref|NC_002745.2|	553521 +	C	805	166	161	130	350	SArRNA06
gi|29165615|ref|NC_002745.2|	553522 +	U	1706	176	135	143	249	SArRNA06
gi|29165615|ref|NC_002745.2|	553523 +	A	451	230	97	108	264	SArRNA06
gi|29165615|ref|NC_002745.2|	553524 +	A	316	214	114	147	300	SArRNA06
gi|29165615|ref|NC_002745.2|	553525 +	G	316	222	99	123	330	SArRNA06
gi|29165615|ref|NC_002745.2|	553526 +	G	301	266	136	154	397	SArRNA06
gi|29165615|ref|NC_002745.2|	553527 +	U	284	209	117	109	284	SArRNA06
gi|29165615|ref|NC_002745.2|	553528 +	C	531	407	230	198	374	SArRNA06
gi|29165615|ref|NC_002745.2|	553529 +	C	354	282	165	135	263	SArRNA06
gi|29165615|ref|NC_002745.2|	553530 +	C	235	174	87	84	271	SArRNA06
gi|29165615|ref|NC_002745.2|	553531 +	A	254	167	90	111	256	SArRNA06
gi|29165615|ref|NC_002745.2|	553532 +	A	413	257	132	115	304	SArRNA06
gi|29165615|ref|NC_002745.2|	553533 +	A	351	227	130	125	338	SArRNA06
gi|29165615|ref|NC_002745.2|	553534 +	A	455	1097	359	183	825	SArRNA06
gi|29165615|ref|NC_002745.2|	553535 +	U	446	805	517	339	851	SArRNA06
gi|29165615|ref|NC_002745.2|	553536 +	A	416	426	186	206	426	SArRNA06
gi|29165615|ref|NC_002745.2|	553537 +	U	817	637	329	313	600	SArRNA06
gi|29165615|ref|NC_002745.2|	553538 +	A	441	287	188	192	435	SArRNA06
gi|29165615|ref|NC_002745.2|	553539 +	U	565	283	201	177	472	SArRNA06
gi|29165615|ref|NC_002745.2|	553540 +	G	452	318	196	200	533	SArRNA06
gi|29165615|ref|NC_002745.2|	553541 +	U	388	283	211	187	495	SArRNA06
gi|29165615|ref|NC_002745.2|	553542 +	U	671	528	253	331	640	SArRNA06
gi|29165615|ref|NC_002745.2|	553543 +	A	552	445	228	247	466	SArRNA06
gi|29165615|ref|NC_002745.2|	553544 +	A	490	350	197	251	525	SArRNA06
gi|29165615|ref|NC_002745.2|	553545 +	G	1404	969	490	728	1118	SArRNA06
gi|29165615|ref|NC_002745.2|	553546 +	U	751	572	298	316	655	SArRNA06
gi|29165615|ref|NC_002745.2|	553547 +	G	800	708	397	544	894	SArRNA06
gi|29165615|ref|NC_002745.2|	553548 +	G	454	345	188	206	505	SArRNA06
gi|29165615|ref|NC_002745.2|	553549 +	A	400	341	169	198	418	SArRNA06
gi|29165615|ref|NC_002745.2|	553550 +	A	245	213	109	125	263	SArRNA06
gi|29165615|ref|NC_002745.2|	553551 +	A	265	242	134	141	310	SArRNA06
gi|29165615|ref|NC_002745.2|	553552 +	A	320	188	124	117	334	SArRNA06

gi|29165615|ref|NC_002745.2|	553553 +	G	177	115	76	93	186	SArRNA06
gi|29165615|ref|NC_002745.2|	553554 +	G	395	246	145	169	495	SArRNA06
gi|29165615|ref|NC_002745.2|	553555 +	A	538	210	182	157	427	SArRNA06
gi|29165615|ref|NC_002745.2|	553556 +	U	717	191	136	111	314	SArRNA06
gi|29165615|ref|NC_002745.2|	553557 +	G	245	153	120	106	285	SArRNA06
gi|29165615|ref|NC_002745.2|	553558 +	U	1319	440	275	257	685	SArRNA06
gi|29165615|ref|NC_002745.2|	553559 +	G	63	17	14	22	36	SArRNA06
gi|29165615|ref|NC_002745.2|	553560 +	G	144	12	8	5	19	SArRNA06
gi|29165615|ref|NC_002745.2|	553561 +	C	9042	427	333	314	477	SArRNA06
gi|29165615|ref|NC_002745.2|	553562 +	G	514	62	46	36	78	SArRNA06
gi|29165615|ref|NC_002745.2|	553563 +	U	123	87	49	46	98	SArRNA06
gi|29165615|ref|NC_002745.2|	553564 +	U	40	55	18	15	60	SArRNA06
gi|29165615|ref|NC_002745.2|	553565 +	G	28	17	5	17	31	SArRNA06
gi|29165615|ref|NC_002745.2|	553566 +	C	83	86	40	48	127	SArRNA06
gi|29165615|ref|NC_002745.2|	553567 +	C	291	175	105	101	338	SArRNA06
gi|29165615|ref|NC_002745.2|	553568 +	C	478	302	227	232	493	SArRNA06
gi|29165615|ref|NC_002745.2|	553569 +	A	207	145	108	111	287	SArRNA06
gi|29165615|ref|NC_002745.2|	553570 +	G	128	91	65	62	160	SArRNA06
gi|29165615|ref|NC_002745.2|	553571 +	A	93	50	55	45	161	SArRNA06
gi|29165615|ref|NC_002745.2|	553572 +	C	1002	196	135	151	410	SArRNA06
gi|29165615|ref|NC_002745.2|	553573 +	A	259	111	62	77	176	SArRNA06
gi|29165615|ref|NC_002745.2|	553574 +	A	250	159	42	34	140	SArRNA06
gi|29165615|ref|NC_002745.2|	553575 +	C	1250	580	338	329	760	SArRNA06
gi|29165615|ref|NC_002745.2|	553576 +	U	1890	212	137	143	279	SArRNA06
gi|29165615|ref|NC_002745.2|	553577 +	A	323	56	22	36	74	SArRNA06
gi|29165615|ref|NC_002745.2|	553578 +	G	5	7	8	8	20	SArRNA06
gi|29165615|ref|NC_002745.2|	553579 +	G	6	19	8	9	20	SArRNA06
gi|29165615|ref|NC_002745.2|	553580 +	A	34	19	11	11	32	SArRNA06
gi|29165615|ref|NC_002745.2|	553581 +	U	51	24	23	24	33	SArRNA06
gi|29165615|ref|NC_002745.2|	553582 +	G	45	7	14	8	30	SArRNA06
gi|29165615|ref|NC_002745.2|	553583 +	U	51	34	32	28	91	SArRNA06
gi|29165615|ref|NC_002745.2|	553584 +	U	34	35	25	39	50	SArRNA06
gi|29165615|ref|NC_002745.2|	553585 +	G	6	9	5	3	6	SArRNA06
gi|29165615|ref|NC_002745.2|	553586 +	G	41	26	12	27	46	SArRNA06
gi|29165615|ref|NC_002745.2|	553587 +	C	91	83	47	52	184	SArRNA06
gi|29165615|ref|NC_002745.2|	553588 +	U	80	39	34	25	119	SArRNA06
gi|29165615|ref|NC_002745.2|	553589 +	U	67	75	49	40	144	SArRNA06
gi|29165615|ref|NC_002745.2|	553590 +	A	66	55	26	38	118	SArRNA06
gi|29165615|ref|NC_002745.2|	553591 +	G	312	403	146	169	330	SArRNA06
gi|29165615|ref|NC_002745.2|	553592 +	A	13	34	18	6	27	SArRNA06
gi|29165615|ref|NC_002745.2|	553593 +	A	58	64	19	22	68	SArRNA06
gi|29165615|ref|NC_002745.2|	553594 +	G	55	48	18	15	45	SArRNA06
gi|29165615|ref|NC_002745.2|	553595 +	C	400	533	245	246	467	SArRNA06
gi|29165615|ref|NC_002745.2|	553596 +	A	144	176	47	57	134	SArRNA06
gi|29165615|ref|NC_002745.2|	553597 +	G	57	35	27	13	65	SArRNA06
gi|29165615|ref|NC_002745.2|	553598 +	C	131	123	68	94	186	SArRNA06
gi|29165615|ref|NC_002745.2|	553599 +	C	288	240	164	158	315	SArRNA06
gi|29165615|ref|NC_002745.2|	553600 +	A	106	121	66	55	205	SArRNA06
gi|29165615|ref|NC_002745.2|	553601 +	U	89	54	26	31	123	SArRNA06
gi|29165615|ref|NC_002745.2|	553602 +	C	484	386	226	169	424	SArRNA06
gi|29165615|ref|NC_002745.2|	553603 +	A	223	204	132	106	364	SArRNA06
gi|29165615|ref|NC_002745.2|	553604 +	U	123	111	62	62	227	SArRNA06
gi|29165615|ref|NC_002745.2|	553605 +	U	109	76	58	42	179	SArRNA06
gi|29165615|ref|NC_002745.2|	553606 +	U	128	100	60	49	194	SArRNA06
gi|29165615|ref|NC_002745.2|	553607 +	A	132	84	45	53	155	SArRNA06
gi|29165615|ref|NC_002745.2|	553608 +	A	119	89	59	47	171	SArRNA06
gi|29165615|ref|NC_002745.2|	553609 +	A	108	82	64	88	131	SArRNA06
gi|29165615|ref|NC_002745.2|	553610 +	G	958	595	533	1258	2364	SArRNA06
gi|29165615|ref|NC_002745.2|	553611 +	A	89	69	48	69	132	SArRNA06
gi|29165615|ref|NC_002745.2|	553612 +	G	30	19	14	19	60	SArRNA06
gi|29165615|ref|NC_002745.2|	553613 +	U	33	25	11	16	63	SArRNA06
gi|29165615|ref|NC_002745.2|	553614 +	G	40	29	13	21	40	SArRNA06
gi|29165615|ref|NC_002745.2|	553615 +	C	164	110	73	88	190	SArRNA06
gi|29165615|ref|NC_002745.2|	553616 +	G	57	74	31	28	120	SArRNA06
gi|29165615|ref|NC_002745.2|	553617 +	U	90	91	54	36	120	SArRNA06
gi|29165615|ref|NC_002745.2|	553618 +	A	38	71	30	22	65	SArRNA06
gi|29165615|ref|NC_002745.2|	553619 +	A	114	368	68	46	279	SArRNA06
gi|29165615|ref|NC_002745.2|	553620 +	U	65	140	87	69	205	SArRNA06
gi|29165615|ref|NC_002745.2|	553621 +	A	19	54	12	10	38	SArRNA06
gi|29165615|ref|NC_002745.2|	553622 +	G	11	15	4	3	13	SArRNA06
gi|29165615|ref|NC_002745.2|	553623 +	C	71	59	30	18	101	SArRNA06
gi|29165615|ref|NC_002745.2|	553624 +	U	40	38	23	24	66	SArRNA06
gi|29165615|ref|NC_002745.2|	553625 +	C	87	84	32	26	85	SArRNA06
gi|29165615|ref|NC_002745.2|	553626 +	A	48	43	29	28	87	SArRNA06
gi|29165615|ref|NC_002745.2|	553627 +	C	183	242	98	89	164	SArRNA06
gi|29165615|ref|NC_002745.2|	553628 +	U	183	142	99	218	363	SArRNA06
gi|29165615|ref|NC_002745.2|	553629 +	A	43	49	22	25	52	SArRNA06
gi|29165615|ref|NC_002745.2|	553630 +	G	45	43	23	22	59	SArRNA06
gi|29165615|ref|NC_002745.2|	553631 +	U	98	58	35	23	99	SArRNA06
gi|29165615|ref|NC_002745.2|	553632 +	C	714	707	287	514	634	SArRNA06
gi|29165615|ref|NC_002745.2|	553633 +	G	152	167	88	92	208	SArRNA06

gi|29165615|ref|NC_002745.2|	553634	+	A	104	102	61	36	146	SArRNA06
gi|29165615|ref|NC_002745.2|	553635	+	G	67	93	42	36	113	SArRNA06
gi|29165615|ref|NC_002745.2|	553636	+	U	68	65	38	22	72	SArRNA06
gi|29165615|ref|NC_002745.2|	553637	+	G	27	34	24	22	46	SArRNA06
gi|29165615|ref|NC_002745.2|	553638	+	A	71	47	15	30	41	SArRNA06
gi|29165615|ref|NC_002745.2|	553639	+	C	125	92	45	43	112	SArRNA06
gi|29165615|ref|NC_002745.2|	553640	+	A	22	25	22	13	42	SArRNA06
gi|29165615|ref|NC_002745.2|	553641	+	C	47	58	26	20	82	SArRNA06
gi|29165615|ref|NC_002745.2|	553642	+	U	31	50	17	11	42	SArRNA06
gi|29165615|ref|NC_002745.2|	553643	+	G	13	11	1	0	3	SArRNA06
gi|29165615|ref|NC_002745.2|	553644	+	C	44	42	28	23	24	SArRNA06
gi|29165615|ref|NC_002745.2|	553645	+	G	13	9	8	8	14	SArRNA06
gi|29165615|ref|NC_002745.2|	553646	+	C	22	29	20	11	44	SArRNA06
gi|29165615|ref|NC_002745.2|	553647	+	C	49	32	22	17	66	SArRNA06
gi|29165615|ref|NC_002745.2|	553648	+	G	34	51	23	27	49	SArRNA06
gi|29165615|ref|NC_002745.2|	553649	+	A	30	45	26	16	52	SArRNA06
gi|29165615|ref|NC_002745.2|	553650	+	A	59	57	29	25	81	SArRNA06
gi|29165615|ref|NC_002745.2|	553651	+	A	108	87	66	48	89	SArRNA06
gi|29165615|ref|NC_002745.2|	553652	+	A	109	188	71	53	193	SArRNA06
gi|29165615|ref|NC_002745.2|	553653	+	U	120	152	77	66	164	SArRNA06
gi|29165615|ref|NC_002745.2|	553654	+	G	81	138	62	54	122	SArRNA06
gi|29165615|ref|NC_002745.2|	553655	+	U	39	69	45	57	100	SArRNA06
gi|29165615|ref|NC_002745.2|	553656	+	A	50	43	21	13	40	SArRNA06
gi|29165615|ref|NC_002745.2|	553657	+	C	74	75	19	24	94	SArRNA06
gi|29165615|ref|NC_002745.2|	553658	+	C	227	264	51	55	276	SArRNA06
gi|29165615|ref|NC_002745.2|	553659	+	G	92	142	34	28	133	SArRNA06
gi|29165615|ref|NC_002745.2|	553660	+	G	10	15	4	4	9	SArRNA06
gi|29165615|ref|NC_002745.2|	553661	+	G	11	4	4	3	6	SArRNA06
gi|29165615|ref|NC_002745.2|	553662	+	G	5	13	6	2	17	SArRNA06
gi|29165615|ref|NC_002745.2|	553663	+	C	71	65	39	32	107	SArRNA06
gi|29165615|ref|NC_002745.2|	553664	+	U	51	60	37	19	78	SArRNA06
gi|29165615|ref|NC_002745.2|	553665	+	A	38	44	30	19	55	SArRNA06
gi|29165615|ref|NC_002745.2|	553666	+	A	49	65	40	20	72	SArRNA06
gi|29165615|ref|NC_002745.2|	553667	+	A	69	59	47	26	106	SArRNA06
gi|29165615|ref|NC_002745.2|	553668	+	C	157	127	50	37	137	SArRNA06
gi|29165615|ref|NC_002745.2|	553669	+	A	128	116	74	50	179	SArRNA06
gi|29165615|ref|NC_002745.2|	553670	+	U	451	92	51	40	143	SArRNA06
gi|29165615|ref|NC_002745.2|	553671	+	A	165	81	41	33	108	SArRNA06
gi|29165615|ref|NC_002745.2|	553672	+	U	72	54	42	27	87	SArRNA06
gi|29165615|ref|NC_002745.2|	553673	+	U	50	61	20	20	44	SArRNA06
gi|29165615|ref|NC_002745.2|	553674	+	A	51	46	28	20	53	SArRNA06
gi|29165615|ref|NC_002745.2|	553675	+	C	72	67	34	25	60	SArRNA06
gi|29165615|ref|NC_002745.2|	553676	+	C	108	115	57	58	139	SArRNA06
gi|29165615|ref|NC_002745.2|	553677	+	G	98	83	32	40	98	SArRNA06
gi|29165615|ref|NC_002745.2|	553678	+	A	65	83	39	25	103	SArRNA06
gi|29165615|ref|NC_002745.2|	553679	+	A	50	62	23	20	59	SArRNA06
gi|29165615|ref|NC_002745.2|	553680	+	G	1	0	0	1	3	SArRNA06
gi|29165615|ref|NC_002745.2|	553681	+	C	3	7	1	3	3	SArRNA06
gi|29165615|ref|NC_002745.2|	553682	+	U	8	8	2	3	14	SArRNA06
gi|29165615|ref|NC_002745.2|	553683	+	G	6	2	1	1	4	SArRNA06
gi|29165615|ref|NC_002745.2|	553684	+	U	4	2	3	5	3	SArRNA06
gi|29165615|ref|NC_002745.2|	553685	+	G	2	1	1	1	0	SArRNA06
gi|29165615|ref|NC_002745.2|	553686	+	G	3	2	1	0	3	SArRNA06
gi|29165615|ref|NC_002745.2|	553687	+	A	1	2	0	0	1	SArRNA06
gi|29165615|ref|NC_002745.2|	553688	+	U	2	3	2	2	2	SArRNA06
gi|29165615|ref|NC_002745.2|	553689	+	U	5	2	0	2	1	SArRNA06
gi|29165615|ref|NC_002745.2|	553690	+	G	2	1	0	0	0	SArRNA06
gi|29165615|ref|NC_002745.2|	553691	+	U	0	1	1	2	1	SArRNA06
gi|29165615|ref|NC_002745.2|	553692	+	C	8	6	4	6	7	SArRNA06
gi|29165615|ref|NC_002745.2|	553693	+	C	2	4	2	3	2	SArRNA06
gi|29165615|ref|NC_002745.2|	553694	+	U	2	3	1	5	6	SArRNA06
gi|29165615|ref|NC_002745.2|	553695	+	U	4	4	1	3	8	SArRNA06
gi|29165615|ref|NC_002745.2|	553696	+	U	8	2	2	0	2	SArRNA06
gi|29165615|ref|NC_002745.2|	553697	+	G	7	3	2	1	2	SArRNA06
gi|29165615|ref|NC_002745.2|	553698	+	G	5	5	7	4	15	SArRNA06
gi|29165615|ref|NC_002745.2|	553699	+	A	5	2	3	2	3	SArRNA06
gi|29165615|ref|NC_002745.2|	553700	+	C	13	7	4	4	4	SArRNA06
gi|29165615|ref|NC_002745.2|	553701	+	A	9	7	2	2	7	SArRNA06
gi|29165615|ref|NC_002745.2|	553702	+	A	6	11	6	3	19	SArRNA06
gi|29165615|ref|NC_002745.2|	553703	+	U	4	6	1	2	2	SArRNA06
gi|29165615|ref|NC_002745.2|	553704	+	G	1	1	1	3	1	SArRNA06
gi|29165615|ref|NC_002745.2|	553705	+	G	12	12	4	10	9	SArRNA06
gi|29165615|ref|NC_002745.2|	553706	+	U	6	7	0	4	3	SArRNA06
gi|29165615|ref|NC_002745.2|	553707	+	A	2	2	1	0	6	SArRNA06
gi|29165615|ref|NC_002745.2|	553708	+	G	1	1	0	1	0	SArRNA06
gi|29165615|ref|NC_002745.2|	553709	+	G	3	7	6	2	3	SArRNA06
gi|29165615|ref|NC_002745.2|	553710	+	A	8	3	8	3	4	SArRNA06
gi|29165615|ref|NC_002745.2|	553711	+	G	2	4	2	2	4	SArRNA06
gi|29165615|ref|NC_002745.2|	553712	+	A	9	2	0	1	1	SArRNA06
gi|29165615|ref|NC_002745.2|	553713	+	G	3	2	0	0	2	SArRNA06
gi|29165615|ref|NC_002745.2|	553714	+	C	11	9	2	2	8	SArRNA06

gi|29165615|ref|NC_002745.2|	553715 +	G	6	5	5	3	8	SArRNA06
gi|29165615|ref|NC_002745.2|	553716 +	U	0	1	0	0	1	SArRNA06
gi|29165615|ref|NC_002745.2|	553717 +	U	1	0	1	0	1	SArRNA06
gi|29165615|ref|NC_002745.2|	553718 +	C	2	2	2	1	3	SArRNA06
gi|29165615|ref|NC_002745.2|	553719 +	U	2	6	0	0	7	SArRNA06
gi|29165615|ref|NC_002745.2|	553720 +	A	9	9	2	2	9	SArRNA06
gi|29165615|ref|NC_002745.2|	553721 +	A	6	4	3	2	4	SArRNA06
gi|29165615|ref|NC_002745.2|	553722 +	G	1	1	0	0	0	SArRNA06
gi|29165615|ref|NC_002745.2|	553723 +	G	1	1	0	0	0	SArRNA06
gi|29165615|ref|NC_002745.2|	553724 +	G	0	2	0	2	0	SArRNA06
gi|29165615|ref|NC_002745.2|	553725 +	C	9	2	1	1	2	SArRNA06
gi|29165615|ref|NC_002745.2|	553726 +	G	4	2	3	2	2	SArRNA06
gi|29165615|ref|NC_002745.2|	553727 +	U	3	7	6	5	4	SArRNA06
gi|29165615|ref|NC_002745.2|	553728 +	U	7	3	1	2	7	SArRNA06
gi|29165615|ref|NC_002745.2|	553729 +	G	8	26	4	6	10	SArRNA06
gi|29165615|ref|NC_002745.2|	553730 +	A	0	0	1	0	2	SArRNA06
gi|29165615|ref|NC_002745.2|	553731 +	A	2	0	1	0	3	SArRNA06
gi|29165615|ref|NC_002745.2|	553732 +	G	0	1	0	0	1	SArRNA06
gi|29165615|ref|NC_002745.2|	553733 +	C	12	15	3	13	6	SArRNA06
gi|29165615|ref|NC_002745.2|	553734 +	A	4	8	3	2	4	SArRNA06
gi|29165615|ref|NC_002745.2|	553735 +	U	3	4	0	4	3	SArRNA06
gi|29165615|ref|NC_002745.2|	553736 +	G	4	14	1	1	4	SArRNA06
gi|29165615|ref|NC_002745.2|	553737 +	A	2	12	4	3	11	SArRNA06
gi|29165615|ref|NC_002745.2|	553738 +	U	12	11	6	9	12	SArRNA06
gi|29165615|ref|NC_002745.2|	553739 +	C	8	7	6	4	12	SArRNA06
gi|29165615|ref|NC_002745.2|	553740 +	G	24	13	6	7	24	SArRNA06
gi|29165615|ref|NC_002745.2|	553741 +	U	4	20	5	6	10	SArRNA06
gi|29165615|ref|NC_002745.2|	553742 +	A	9	22	2	2	7	SArRNA06
gi|29165615|ref|NC_002745.2|	553743 +	A	9	7	5	3	8	SArRNA06
gi|29165615|ref|NC_002745.2|	553744 +	G	4	3	5	4	6	SArRNA06
gi|29165615|ref|NC_002745.2|	553745 +	G	20	20	5	5	19	SArRNA06
gi|29165615|ref|NC_002745.2|	553746 +	A	6	15	6	4	7	SArRNA06
gi|29165615|ref|NC_002745.2|	553747 +	C	10	6	4	3	7	SArRNA06
gi|29165615|ref|NC_002745.2|	553748 +	A	9	8	6	9	11	SArRNA06
gi|29165615|ref|NC_002745.2|	553749 +	U	2	6	3	4	6	SArRNA06
gi|29165615|ref|NC_002745.2|	553750 +	G	4	1	2	2	2	SArRNA06
gi|29165615|ref|NC_002745.2|	553751 +	U	4	1	3	2	1	SArRNA06
gi|29165615|ref|NC_002745.2|	553752 +	G	7	1	1	3	1	SArRNA06
gi|29165615|ref|NC_002745.2|	553753 +	G	9	8	2	0	3	SArRNA06
gi|29165615|ref|NC_002745.2|	553754 +	A	6	4	1	2	4	SArRNA06
gi|29165615|ref|NC_002745.2|	553755 +	G	1	3	0	2	3	SArRNA06
gi|29165615|ref|NC_002745.2|	553756 +	C	5	4	4	4	10	SArRNA06
gi|29165615|ref|NC_002745.2|	553757 +	G	0	1	1	1	4	SArRNA06
gi|29165615|ref|NC_002745.2|	553758 +	C	4	2	1	2	8	SArRNA06
gi|29165615|ref|NC_002745.2|	553759 +	U	6	6	5	2	11	SArRNA06
gi|29165615|ref|NC_002745.2|	553760 +	U	21	21	8	12	19	SArRNA06
gi|29165615|ref|NC_002745.2|	553761 +	A	8	10	2	7	8	SArRNA06
gi|29165615|ref|NC_002745.2|	553762 +	G	29	26	10	9	11	SArRNA06
gi|29165615|ref|NC_002745.2|	553763 +	A	20	9	11	9	22	SArRNA06
gi|29165615|ref|NC_002745.2|	553764 +	A	10	21	7	6	25	SArRNA06
gi|29165615|ref|NC_002745.2|	553765 +	G	16	13	7	9	19	SArRNA06
gi|29165615|ref|NC_002745.2|	553766 +	U	16	14	5	8	15	SArRNA06
gi|29165615|ref|NC_002745.2|	553767 +	G	17	13	10	5	18	SArRNA06
gi|29165615|ref|NC_002745.2|	553768 +	A	11	6	5	4	8	SArRNA06
gi|29165615|ref|NC_002745.2|	553769 +	G	8	9	7	3	17	SArRNA06
gi|29165615|ref|NC_002745.2|	553770 +	A	25	18	12	17	15	SArRNA06
gi|29165615|ref|NC_002745.2|	553771 +	A	24	20	8	6	10	SArRNA06
gi|29165615|ref|NC_002745.2|	553772 +	U	10	12	3	4	9	SArRNA06
gi|29165615|ref|NC_002745.2|	553773 +	G	2	4	0	0	0	SArRNA06
gi|29165615|ref|NC_002745.2|	553774 +	C	10	6	5	5	1	SArRNA06
gi|29165615|ref|NC_002745.2|	553775 +	C	43	37	8	14	20	SArRNA06
gi|29165615|ref|NC_002745.2|	553776 +	G	9	5	5	2	5	SArRNA06
gi|29165615|ref|NC_002745.2|	553777 +	G	5	5	4	1	8	SArRNA06
gi|29165615|ref|NC_002745.2|	553778 +	U	10	11	2	7	9	SArRNA06
gi|29165615|ref|NC_002745.2|	553779 +	G	9	7	2	4	8	SArRNA06
gi|29165615|ref|NC_002745.2|	553780 +	U	10	5	7	2	10	SArRNA06
gi|29165615|ref|NC_002745.2|	553781 +	G	11	8	6	5	5	SArRNA06
gi|29165615|ref|NC_002745.2|	553782 +	A	7	11	4	3	11	SArRNA06
gi|29165615|ref|NC_002745.2|	553783 +	G	35	26	10	12	15	SArRNA06
gi|29165615|ref|NC_002745.2|	553784 +	U	30	30	15	13	17	SArRNA06
gi|29165615|ref|NC_002745.2|	553785 +	A	35	35	8	11	16	SArRNA06
gi|29165615|ref|NC_002745.2|	553786 +	G	9	5	1	0	2	SArRNA06
gi|29165615|ref|NC_002745.2|	553787 +	C	8	9	0	6	4	SArRNA06
gi|29165615|ref|NC_002745.2|	553788 +	G	4	6	3	1	2	SArRNA06
gi|29165615|ref|NC_002745.2|	553789 +	A	2	1	1	1	3	SArRNA06
gi|29165615|ref|NC_002745.2|	553790 +	A	1	3	0	0	2	SArRNA06
gi|29165615|ref|NC_002745.2|	553791 +	A	0	1	0	0	1	SArRNA06
gi|29165615|ref|NC_002745.2|	553792 +	G	0	2	0	2	2	SArRNA06
gi|29165615|ref|NC_002745.2|	553794 +	C	0	1	0	0	0	SArRNA06
gi|29165615|ref|NC_002745.2|	553796 +	G	0	0	1	0	1	SArRNA06
gi|29165615|ref|NC_002745.2|	553797 +	G	6	2	1	1	0	SArRNA06

gi|29165615|ref|NC_002745.2|	553798	+	U	0	0	1	2	1	SArRNA06
gi|29165615|ref|NC_002745.2|	553799	+	G	0	0	0	0	2	SArRNA06
gi|29165615|ref|NC_002745.2|	553800	+	A	0	0	0	1	1	SArRNA06
gi|29165615|ref|NC_002745.2|	553801	+	G	0	0	0	0	1	SArRNA06
gi|29165615|ref|NC_002745.2|	553802	+	A	0	3	0	0	3	SArRNA06
gi|29165615|ref|NC_002745.2|	553803	+	A	0	2	1	0	1	SArRNA06
gi|29165615|ref|NC_002745.2|	553804	+	U	0	1	0	0	1	SArRNA06
gi|29165615|ref|NC_002745.2|	553805	+	C	26	10	5	4	9	SArRNA06
gi|29165615|ref|NC_002745.2|	553806	+	C	3	5	0	1	6	SArRNA06
gi|29165615|ref|NC_002745.2|	553807	+	C	3	1	2	0	0	SArRNA06
gi|29165615|ref|NC_002745.2|	553808	+	G	1	3	1	2	1	SArRNA06
gi|29165615|ref|NC_002745.2|	553809	+	U	0	1	0	1	0	SArRNA06
gi|29165615|ref|NC_002745.2|	553810	+	C	0	1	0	0	1	SArRNA06
gi|29165615|ref|NC_002745.2|	553811	+	C	2	3	2	0	0	SArRNA06
gi|29165615|ref|NC_002745.2|	553812	+	A	1	2	1	1	1	SArRNA06
gi|29165615|ref|NC_002745.2|	553813	+	C	1	4	0	0	1	SArRNA06
gi|29165615|ref|NC_002745.2|	553814	+	C	60	40	14	15	27	SArRNA06
gi|29165615|ref|NC_002745.2|	553815	+	G	26	20	10	18	25	SArRNA06
gi|29165615|ref|NC_002745.2|	553816	+	A	28	31	15	10	23	SArRNA06
gi|29165615|ref|NC_002745.2|	553817	+	U	38	31	16	22	35	SArRNA06
gi|29165615|ref|NC_002745.2|	553818	+	U	25	16	11	7	23	SArRNA06
gi|29165615|ref|NC_002745.2|	553819	+	G	0	2	0	2	0	SArRNA06
gi|29165615|ref|NC_002745.2|	553820	+	A	7	13	6	7	18	SArRNA06
gi|29165615|ref|NC_002745.2|	553821	+	C	12	17	4	9	21	SArRNA06
gi|29165615|ref|NC_002745.2|	553822	+	U	34	38	15	23	27	SArRNA06
gi|29165615|ref|NC_002745.2|	553823	+	A	16	19	9	7	19	SArRNA06
gi|29165615|ref|NC_002745.2|	553824	+	A	37	36	14	11	39	SArRNA06
gi|29165615|ref|NC_002745.2|	553825	+	G	36	22	13	7	23	SArRNA06
gi|29165615|ref|NC_002745.2|	553826	+	G	22	16	15	11	35	SArRNA06
gi|29165615|ref|NC_002745.2|	553827	+	U	10	11	6	9	19	SArRNA06
gi|29165615|ref|NC_002745.2|	553828	+	U	5	3	0	1	7	SArRNA06
gi|29165615|ref|NC_002745.2|	553829	+	U	2	2	0	0	1	SArRNA06
gi|29165615|ref|NC_002745.2|	553830	+	C	2	2	0	0	2	SArRNA06
gi|29165615|ref|NC_002745.2|	553831	+	C	3	1	1	3	7	SArRNA06
gi|29165615|ref|NC_002745.2|	553832	+	A	4	5	3	2	10	SArRNA06
gi|29165615|ref|NC_002745.2|	553833	+	G	3	1	5	3	9	SArRNA06
gi|29165615|ref|NC_002745.2|	553834	+	A	11	1	2	4	13	SArRNA06
gi|29165615|ref|NC_002745.2|	553835	+	G	6	6	5	5	11	SArRNA06
gi|29165615|ref|NC_002745.2|	553836	+	G	15	13	3	4	13	SArRNA06
gi|29165615|ref|NC_002745.2|	553837	+	A	9	20	2	7	10	SArRNA06
gi|29165615|ref|NC_002745.2|	553838	+	A	11	9	5	5	13	SArRNA06
gi|29165615|ref|NC_002745.2|	553839	+	G	1	3	0	3	3	SArRNA06
gi|29165615|ref|NC_002745.2|	553840	+	G	2	1	1	2	2	SArRNA06
gi|29165615|ref|NC_002745.2|	553841	+	C	0	0	1	0	2	SArRNA06
gi|29165615|ref|NC_002745.2|	553842	+	U	3	8	2	0	2	SArRNA06
gi|29165615|ref|NC_002745.2|	553843	+	C	4	2	2	0	4	SArRNA06
gi|29165615|ref|NC_002745.2|	553844	+	G	2	3	0	0	7	SArRNA06
gi|29165615|ref|NC_002745.2|	553845	+	U	2	2	0	1	5	SArRNA06
gi|29165615|ref|NC_002745.2|	553846	+	C	4	5	0	1	5	SArRNA06
gi|29165615|ref|NC_002745.2|	553847	+	C	17	24	2	9	20	SArRNA06
gi|29165615|ref|NC_002745.2|	553848	+	G	7	12	7	7	16	SArRNA06
gi|29165615|ref|NC_002745.2|	553849	+	C	10	12	3	5	12	SArRNA06
gi|29165615|ref|NC_002745.2|	553850	+	U	8	11	3	5	5	SArRNA06
gi|29165615|ref|NC_002745.2|	553851	+	C	2	6	2	1	4	SArRNA06
gi|29165615|ref|NC_002745.2|	553852	+	U	15	13	7	4	17	SArRNA06
gi|29165615|ref|NC_002745.2|	553853	+	G	6	3	3	3	3	SArRNA06
gi|29165615|ref|NC_002745.2|	553854	+	G	10	8	1	5	7	SArRNA06
gi|29165615|ref|NC_002745.2|	553855	+	G	12	7	4	5	13	SArRNA06
gi|29165615|ref|NC_002745.2|	553856	+	U	11	12	8	1	16	SArRNA06
gi|29165615|ref|NC_002745.2|	553857	+	U	6	12	8	8	13	SArRNA06
gi|29165615|ref|NC_002745.2|	553858	+	A	12	10	4	6	13	SArRNA06
gi|29165615|ref|NC_002745.2|	553859	+	G	15	5	4	6	6	SArRNA06
gi|29165615|ref|NC_002745.2|	553860	+	U	4	0	1	0	4	SArRNA06
gi|29165615|ref|NC_002745.2|	553861	+	C	1	8	0	2	2	SArRNA06
gi|29165615|ref|NC_002745.2|	553862	+	G	2	4	1	1	0	SArRNA06
gi|29165615|ref|NC_002745.2|	553863	+	G	4	2	1	0	1	SArRNA06
gi|29165615|ref|NC_002745.2|	553864	+	G	0	3	0	1	0	SArRNA06
gi|29165615|ref|NC_002745.2|	553865	+	U	0	3	0	0	1	SArRNA06
gi|29165615|ref|NC_002745.2|	553866	+	C	1	2	0	2	0	SArRNA06
gi|29165615|ref|NC_002745.2|	553867	+	C	5	14	0	5	8	SArRNA06
gi|29165615|ref|NC_002745.2|	553868	+	U	2	11	3	1	11	SArRNA06
gi|29165615|ref|NC_002745.2|	553869	+	A	6	5	1	1	5	SArRNA06
gi|29165615|ref|NC_002745.2|	553870	+	A	10	4	1	3	3	SArRNA06
gi|29165615|ref|NC_002745.2|	553871	+	G	0	0	0	0	1	SArRNA06
gi|29165615|ref|NC_002745.2|	553872	+	C	4	2	2	4	1	SArRNA06
gi|29165615|ref|NC_002745.2|	553873	+	U	5	6	4	0	3	SArRNA06
gi|29165615|ref|NC_002745.2|	553874	+	G	6	14	7	1	9	SArRNA06
gi|29165615|ref|NC_002745.2|	553875	+	A	3	1	2	0	0	SArRNA06
gi|29165615|ref|NC_002745.2|	553876	+	G	0	1	0	1	1	SArRNA06
gi|29165615|ref|NC_002745.2|	553877	+	G	3	1	0	1	2	SArRNA06
gi|29165615|ref|NC_002745.2|	553878	+	C	0	3	0	1	2	SArRNA06

gi|29165615|ref|NC_002745.2|	553879	+	C	11	7	2	5	10 SArRNA06
gi|29165615|ref|NC_002745.2|	553880	+	G	7	5	3	1	6 SArRNA06
gi|29165615|ref|NC_002745.2|	553881	+	A	1	5	0	0	8 SArRNA06
gi|29165615|ref|NC_002745.2|	553882	+	C	29	46	11	9	29 SArRNA06
gi|29165615|ref|NC_002745.2|	553883	+	A	6	14	2	3	6 SArRNA06
gi|29165615|ref|NC_002745.2|	553884	+	G	7	16	1	3	7 SArRNA06
gi|29165615|ref|NC_002745.2|	553885	+	G	2	1	1	0	2 SArRNA06
gi|29165615|ref|NC_002745.2|	553886	+	C	4	1	2	1	7 SArRNA06
gi|29165615|ref|NC_002745.2|	553887	+	G	5	6	2	1	2 SArRNA06
gi|29165615|ref|NC_002745.2|	553888	+	U	15	13	3	11	8 SArRNA06
gi|29165615|ref|NC_002745.2|	553889	+	A	1	2	3	0	3 SArRNA06
gi|29165615|ref|NC_002745.2|	553890	+	G	0	2	1	0	1 SArRNA06
gi|29165615|ref|NC_002745.2|	553891	+	G	0	1	0	0	0 SArRNA06
gi|29165615|ref|NC_002745.2|	553892	+	C	5	6	4	3	12 SArRNA06
gi|29165615|ref|NC_002745.2|	553893	+	G	17	21	12	8	17 SArRNA06
gi|29165615|ref|NC_002745.2|	553894	+	A	4	6	1	1	6 SArRNA06
gi|29165615|ref|NC_002745.2|	553895	+	U	5	6	4	1	1 SArRNA06
gi|29165615|ref|NC_002745.2|	553896	+	G	2	3	1	1	2 SArRNA06
gi|29165615|ref|NC_002745.2|	553897	+	G	0	1	1	2	3 SArRNA06
gi|29165615|ref|NC_002745.2|	553898	+	A	1	1	1	5	5 SArRNA06
gi|29165615|ref|NC_002745.2|	553899	+	U	1	1	1	0	0 SArRNA06
gi|29165615|ref|NC_002745.2|	553900	+	A	1	0	0	1	5 SArRNA06
gi|29165615|ref|NC_002745.2|	553901	+	A	0	1	0	0	2 SArRNA06
gi|29165615|ref|NC_002745.2|	553902	+	C	7	7	4	1	1 SArRNA06
gi|29165615|ref|NC_002745.2|	553903	+	A	1	3	0	0	2 SArRNA06
gi|29165615|ref|NC_002745.2|	553904	+	G	1	0	1	0	1 SArRNA06
gi|29165615|ref|NC_002745.2|	553905	+	G	0	1	0	1	0 SArRNA06
gi|29165615|ref|NC_002745.2|	553906	+	U	1	0	0	1	3 SArRNA06
gi|29165615|ref|NC_002745.2|	553907	+	U	0	1	1	1	2 SArRNA06
gi|29165615|ref|NC_002745.2|	553908	+	G	3	0	2	0	2 SArRNA06
gi|29165615|ref|NC_002745.2|	553909	+	A	2	5	0	2	2 SArRNA06
gi|29165615|ref|NC_002745.2|	553910	+	U	0	4	0	3	4 SArRNA06
gi|29165615|ref|NC_002745.2|	553911	+	A	1	1	2	0	4 SArRNA06
gi|29165615|ref|NC_002745.2|	553912	+	U	3	0	3	0	2 SArRNA06
gi|29165615|ref|NC_002745.2|	553913	+	U	4	2	0	0	0 SArRNA06
gi|29165615|ref|NC_002745.2|	553914	+	C	2	1	0	2	1 SArRNA06
gi|29165615|ref|NC_002745.2|	553915	+	C	2	0	0	1	0 SArRNA06
gi|29165615|ref|NC_002745.2|	553916	+	U	7	3	0	1	1 SArRNA06
gi|29165615|ref|NC_002745.2|	553917	+	G	0	1	0	0	1 SArRNA06
gi|29165615|ref|NC_002745.2|	553918	+	U	1	2	0	4	2 SArRNA06
gi|29165615|ref|NC_002745.2|	553919	+	A	0	1	0	0	0 SArRNA06
gi|29165615|ref|NC_002745.2|	553920	+	C	0	4	1	2	6 SArRNA06
gi|29165615|ref|NC_002745.2|	553921	+	C	15	13	3	2	13 SArRNA06
gi|29165615|ref|NC_002745.2|	553922	+	A	1	5	0	2	3 SArRNA06
gi|29165615|ref|NC_002745.2|	553923	+	C	1	3	1	2	2 SArRNA06
gi|29165615|ref|NC_002745.2|	553924	+	C	6	4	1	2	6 SArRNA06
gi|29165615|ref|NC_002745.2|	553925	+	U	4	5	2	3	6 SArRNA06
gi|29165615|ref|NC_002745.2|	553926	+	A	3	1	1	1	0 SArRNA06
gi|29165615|ref|NC_002745.2|	553927	+	U	6	8	1	2	6 SArRNA06
gi|29165615|ref|NC_002745.2|	553928	+	A	4	1	1	2	5 SArRNA06
gi|29165615|ref|NC_002745.2|	553929	+	A	3	11	1	2	13 SArRNA06
gi|29165615|ref|NC_002745.2|	553930	+	U	6	1	2	2	1 SArRNA06
gi|29165615|ref|NC_002745.2|	553931	+	C	4	3	2	1	5 SArRNA06
gi|29165615|ref|NC_002745.2|	553932	+	G	1	2	2	3	4 SArRNA06
gi|29165615|ref|NC_002745.2|	553933	+	U	0	4	4	2	2 SArRNA06
gi|29165615|ref|NC_002745.2|	553934	+	U	8	4	1	0	3 SArRNA06
gi|29165615|ref|NC_002745.2|	553935	+	U	6	8	3	0	1 SArRNA06
gi|29165615|ref|NC_002745.2|	553936	+	U	5	7	3	5	7 SArRNA06
gi|29165615|ref|NC_002745.2|	553937	+	A	2	3	1	1	4 SArRNA06
gi|29165615|ref|NC_002745.2|	553938	+	A	9	75	22	3	28 SArRNA06
gi|29165615|ref|NC_002745.2|	553939	+	U	4	3	1	2	5 SArRNA06
gi|29165615|ref|NC_002745.2|	553940	+	C	8	5	0	3	2 SArRNA06
gi|29165615|ref|NC_002745.2|	553941	+	G	6	10	3	2	6 SArRNA06
gi|29165615|ref|NC_002745.2|	553942	+	A	3	2	1	2	2 SArRNA06
gi|29165615|ref|NC_002745.2|	553943	+	U	7	12	0	2	1 SArRNA06
gi|29165615|ref|NC_002745.2|	553944	+	G	0	1	1	0	2 SArRNA06
gi|29165615|ref|NC_002745.2|	553945	+	G	0	2	0	1	0 SArRNA06
gi|29165615|ref|NC_002745.2|	553946	+	G	1	3	3	0	2 SArRNA06
gi|29165615|ref|NC_002745.2|	553947	+	G	0	0	0	0	1 SArRNA06
gi|29165615|ref|NC_002745.2|	553949	+	G	0	0	1	0	1 SArRNA06
gi|29165615|ref|NC_002745.2|	553951	+	C	1	12	0	0	4 SArRNA06
gi|29165615|ref|NC_002745.2|	553952	+	G	1	4	4	1	1 SArRNA06
gi|29165615|ref|NC_002745.2|	553953	+	C	3	0	0	1	0 SArRNA06
gi|29165615|ref|NC_002745.2|	553954	+	A	0	1	1	0	1 SArRNA06
gi|29165615|ref|NC_002745.2|	553955	+	G	2	1	0	0	3 SArRNA06
gi|29165615|ref|NC_002745.2|	553956	+	U	7	2	5	0	1 SArRNA06
gi|29165615|ref|NC_002745.2|	553957	+	A	1	3	2	0	1 SArRNA06
gi|29165615|ref|NC_002745.2|	553958	+	G	0	0	0	1	0 SArRNA06
gi|29165615|ref|NC_002745.2|	553959	+	G	0	1	0	1	1 SArRNA06
gi|29165615|ref|NC_002745.2|	553960	+	A	2	5	0	1	5 SArRNA06
gi|29165615|ref|NC_002745.2|	553961	+	U	5	5	0	3	3 SArRNA06

gi|29165615|ref|NC_002745.2|	553962	+	A	2	0	2	1	1	SArRNA06
gi|29165615|ref|NC_002745.2|	553963	+	G	0	1	0	0	0	SArRNA06
gi|29165615|ref|NC_002745.2|	553965	+	C	3	0	1	2	3	SArRNA06
gi|29165615|ref|NC_002745.2|	553966	+	G	1	4	0	0	1	SArRNA06
gi|29165615|ref|NC_002745.2|	553967	+	A	0	1	0	0	0	SArRNA06
gi|29165615|ref|NC_002745.2|	553972	+	U	0	1	0	0	0	SArRNA06
gi|29165615|ref|NC_002745.2|	553973	+	G	0	1	0	0	0	SArRNA06
gi|29165615|ref|NC_002745.2|	553974	+	C	4	1	3	0	4	SArRNA06
gi|29165615|ref|NC_002745.2|	553975	+	G	1	3	0	0	3	SArRNA06
gi|29165615|ref|NC_002745.2|	553976	+	A	0	2	3	0	2	SArRNA06
gi|29165615|ref|NC_002745.2|	553977	+	U	0	0	2	3	3	SArRNA06
gi|29165615|ref|NC_002745.2|	553978	+	U	1	1	0	0	1	SArRNA06
gi|29165615|ref|NC_002745.2|	553979	+	G	2	1	0	0	1	SArRNA06
gi|29165615|ref|NC_002745.2|	553980	+	G	1	3	0	0	1	SArRNA06
gi|29165615|ref|NC_002745.2|	553981	+	A	1	3	3	0	5	SArRNA06
gi|29165615|ref|NC_002745.2|	553982	+	U	3	2	0	0	1	SArRNA06
gi|29165615|ref|NC_002745.2|	553983	+	U	0	0	0	0	2	SArRNA06
gi|29165615|ref|NC_002745.2|	553984	+	G	0	1	0	0	1	SArRNA06
gi|29165615|ref|NC_002745.2|	553985	+	C	0	7	2	0	3	SArRNA06
gi|29165615|ref|NC_002745.2|	553986	+	A	0	2	0	0	1	SArRNA06
gi|29165615|ref|NC_002745.2|	553987	+	C	1	0	0	0	2	SArRNA06
gi|29165615|ref|NC_002745.2|	553988	+	G	1	2	0	1	0	SArRNA06
gi|29165615|ref|NC_002745.2|	553989	+	U	1	0	0	0	0	SArRNA06
gi|29165615|ref|NC_002745.2|	553990	+	C	1	6	1	2	12	SArRNA06
gi|29165615|ref|NC_002745.2|	553991	+	U	2	6	1	0	2	SArRNA06
gi|29165615|ref|NC_002745.2|	553992	+	A	0	2	0	0	1	SArRNA06
gi|29165615|ref|NC_002745.2|	553993	+	A	0	0	0	0	1	SArRNA06
gi|29165615|ref|NC_002745.2|	553995	+	C	4	3	0	1	0	SArRNA06
gi|29165615|ref|NC_002745.2|	553996	+	A	3	0	0	0	2	SArRNA06
gi|29165615|ref|NC_002745.2|	553997	+	G	0	0	0	3	1	SArRNA06
gi|29165615|ref|NC_002745.2|	553998	+	U	2	0	0	0	2	SArRNA06
gi|29165615|ref|NC_002745.2|	553999	+	A	2	4	0	0	3	SArRNA06
gi|29165615|ref|NC_002745.2|	554000	+	A	1	1	0	0	1	SArRNA06
gi|29165615|ref|NC_002745.2|	554002	+	G	1	1	0	0	1	SArRNA06
gi|29165615|ref|NC_002745.2|	554003	+	C	4	0	0	1	1	SArRNA06
gi|29165615|ref|NC_002745.2|	554004	+	U	0	1	0	1	0	SArRNA06
gi|29165615|ref|NC_002745.2|	554005	+	G	0	0	0	0	1	SArRNA06
gi|29165615|ref|NC_002745.2|	554006	+	A	0	1	0	0	3	SArRNA06
gi|29165615|ref|NC_002745.2|	554008	+	U	0	1	0	0	0	SArRNA06
gi|29165615|ref|NC_002745.2|	554009	+	A	0	1	1	0	2	SArRNA06
gi|29165615|ref|NC_002745.2|	554011	+	U	2	3	0	1	1	SArRNA06
gi|29165615|ref|NC_002745.2|	554012	+	A	2	1	0	1	1	SArRNA06
gi|29165615|ref|NC_002745.2|	554013	+	G	2	0	0	0	1	SArRNA06
gi|29165615|ref|NC_002745.2|	554016	+	A	1	1	0	0	1	SArRNA06
gi|29165615|ref|NC_002745.2|	554017	+	A	0	0	0	0	1	SArRNA06
gi|29165615|ref|NC_002745.2|	554018	+	A	4	3	1	1	1	SArRNA06
gi|29165615|ref|NC_002745.2|	554019	+	U	1	1	1	0	1	SArRNA06
gi|29165615|ref|NC_002745.2|	554020	+	C	1	2	0	0	2	SArRNA06
gi|29165615|ref|NC_002745.2|	554021	+	C	1	4	0	0	0	SArRNA06
gi|29165615|ref|NC_002745.2|	554022	+	G	0	0	0	1	0	SArRNA06
gi|29165615|ref|NC_002745.2|	554023	+	G	2	0	0	1	0	SArRNA06
gi|29165615|ref|NC_002745.2|	554025	+	A	0	1	1	0	0	SArRNA06
gi|29165615|ref|NC_002745.2|	554026	+	C	0	2	0	0	1	SArRNA06
gi|29165615|ref|NC_002745.2|	554027	+	U	0	1	1	0	1	SArRNA06
gi|29165615|ref|NC_002745.2|	554028	+	C	0	0	0	0	1	SArRNA06
gi|29165615|ref|NC_002745.2|	554029	+	G	1	0	0	0	5	SArRNA06
gi|29165615|ref|NC_002745.2|	554030	+	U	1	0	0	0	1	SArRNA06
gi|29165615|ref|NC_002745.2|	554031	+	U	3	2	0	0	6	SArRNA06
gi|29165615|ref|NC_002745.2|	554032	+	A	0	6	0	0	1	SArRNA06
gi|29165615|ref|NC_002745.2|	554033	+	A	3	2	0	0	2	SArRNA06
gi|29165615|ref|NC_002745.2|	554034	+	G	0	2	0	1	0	SArRNA06
gi|29165615|ref|NC_002745.2|	554035	+	G	1	1	0	0	0	SArRNA06
gi|29165615|ref|NC_002745.2|	554036	+	C	1	1	1	0	5	SArRNA06
gi|29165615|ref|NC_002745.2|	554037	+	U	1	2	0	1	2	SArRNA06
gi|29165615|ref|NC_002745.2|	554038	+	G	0	0	1	0	1	SArRNA06
gi|29165615|ref|NC_002745.2|	554039	+	A	4	0	0	1	2	SArRNA06
gi|29165615|ref|NC_002745.2|	554041	+	C	1	0	1	0	0	SArRNA06
gi|29165615|ref|NC_002745.2|	554042	+	U	2	0	3	0	0	SArRNA06
gi|29165615|ref|NC_002745.2|	554043	+	G	0	0	0	0	1	SArRNA06
gi|29165615|ref|NC_002745.2|	554044	+	U	0	0	0	0	1	SArRNA06
gi|29165615|ref|NC_002745.2|	554045	+	G	1	3	1	0	0	SArRNA06
gi|29165615|ref|NC_002745.2|	554046	+	A	1	0	0	0	0	SArRNA06
gi|29165615|ref|NC_002745.2|	554047	+	U	1	8	2	2	6	SArRNA06
gi|29165615|ref|NC_002745.2|	554048	+	G	1	1	1	1	0	SArRNA06
gi|29165615|ref|NC_002745.2|	554049	+	G	1	1	1	0	0	SArRNA06
gi|29165615|ref|NC_002745.2|	554054	+	A	1	0	0	0	0	SArRNA06
gi|29165615|ref|NC_002745.2|	554055	+	A	2	0	0	0	0	SArRNA06
gi|29165615|ref|NC_002745.2|	554056	+	G	0	1	0	0	0	SArRNA06
gi|29165615|ref|NC_002745.2|	554057	+	A	1	0	0	0	3	SArRNA06
gi|29165615|ref|NC_002745.2|	554058	+	C	1	0	1	2	2	SArRNA06
gi|29165615|ref|NC_002745.2|	554059	+	A	3	1	0	0	2	SArRNA06

gi|29165615|ref|NC_002745.2|	554060	+	U	0	0	1	1	1	SArRNA06
gi|29165615|ref|NC_002745.2|	554061	+	U	1	0	0	0	0	SArRNA06
gi|29165615|ref|NC_002745.2|	554063	+	A	0	0	0	0	3	SArRNA06
gi|29165615|ref|NC_002745.2|	554064	+	G	1	0	0	0	1	SArRNA06
gi|29165615|ref|NC_002745.2|	554065	+	U	0	0	0	0	1	SArRNA06
gi|29165615|ref|NC_002745.2|	554066	+	C	0	1	0	0	0	SArRNA06
gi|29165615|ref|NC_002745.2|	554067	+	U	1	1	1	0	0	SArRNA06
gi|29165615|ref|NC_002745.2|	554068	+	U	0	1	0	0	1	SArRNA06
gi|29165615|ref|NC_002745.2|	554069	+	C	0	1	0	0	1	SArRNA06
gi|29165615|ref|NC_002745.2|	554070	+	G	0	1	1	0	0	SArRNA06
gi|29165615|ref|NC_002745.2|	554072	+	G	0	0	0	0	1	SArRNA06
gi|29165615|ref|NC_002745.2|	554073	+	U	2	0	1	0	1	SArRNA06
gi|29165615|ref|NC_002745.2|	554074	+	C	2	0	0	0	0	SArRNA06
gi|29165615|ref|NC_002745.2|	554075	+	G	1	1	0	0	1	SArRNA06
gi|29165615|ref|NC_002745.2|	554076	+	U	0	0	1	0	0	SArRNA06
gi|29165615|ref|NC_002745.2|	554079	+	A	0	1	0	0	1	SArRNA06
gi|29165615|ref|NC_002745.2|	554080	+	U	3	0	0	0	0	SArRNA06
gi|29165615|ref|NC_002745.2|	554081	+	U	0	0	1	1	0	SArRNA06
gi|29165615|ref|NC_002745.2|	554082	+	U	0	1	0	0	1	SArRNA06
gi|29165615|ref|NC_002745.2|	554083	+	C	2	2	2	1	0	SArRNA06
gi|29165615|ref|NC_002745.2|	554084	+	A	2	0	0	0	1	SArRNA06
gi|29165615|ref|NC_002745.2|	554085	+	C	3	5	2	0	2	SArRNA06
gi|29165615|ref|NC_002745.2|	554086	+	A	2	1	0	1	0	SArRNA06
gi|29165615|ref|NC_002745.2|	554087	+	C	2	1	0	2	1	SArRNA06
gi|29165615|ref|NC_002745.2|	554088	+	U	0	0	1	0	1	SArRNA06
gi|29165615|ref|NC_002745.2|	554089	+	G	0	0	0	1	0	SArRNA06
gi|29165615|ref|NC_002745.2|	554091	+	C	2	3	1	0	0	SArRNA06
gi|29165615|ref|NC_002745.2|	554092	+	G	0	0	0	1	0	SArRNA06
gi|29165615|ref|NC_002745.2|	554093	+	A	0	0	0	0	1	SArRNA06
gi|29165615|ref|NC_002745.2|	554094	+	G	1	0	1	0	1	SArRNA06
gi|29165615|ref|NC_002745.2|	554095	+	A	1	0	1	0	0	SArRNA06
gi|29165615|ref|NC_002745.2|	554096	+	A	1	0	0	0	1	SArRNA06
gi|29165615|ref|NC_002745.2|	554097	+	A	1	1	0	0	0	SArRNA06
gi|29165615|ref|NC_002745.2|	554098	+	A	1	1	0	0	1	SArRNA06
gi|29165615|ref|NC_002745.2|	554100	+	C	2	0	1	0	0	SArRNA06
gi|29165615|ref|NC_002745.2|	554101	+	C	3	1	3	1	2	SArRNA06
gi|29165615|ref|NC_002745.2|	554102	+	U	2	1	0	1	2	SArRNA06
gi|29165615|ref|NC_002745.2|	554103	+	C	1	4	0	1	5	SArRNA06
gi|29165615|ref|NC_002745.2|	554104	+	U	1	9	2	0	3	SArRNA06
gi|29165615|ref|NC_002745.2|	554105	+	A	2	4	1	0	5	SArRNA06
gi|29165615|ref|NC_002745.2|	554106	+	G	0	3	1	4	1	SArRNA06
gi|29165615|ref|NC_002745.2|	554107	+	A	4	8	1	2	5	SArRNA06
gi|29165615|ref|NC_002745.2|	554108	+	U	4	5	1	3	6	SArRNA06
gi|29165615|ref|NC_002745.2|	554109	+	A	0	1	3	1	3	SArRNA06
gi|29165615|ref|NC_002745.2|	554110	+	G	1	1	4	3	0	SArRNA06
gi|29165615|ref|NC_002745.2|	554111	+	A	0	3	2	2	7	SArRNA06
gi|29165615|ref|NC_002745.2|	554112	+	A	4	3	0	3	3	SArRNA06
gi|29165615|ref|NC_002745.2|	554113	+	A	3	0	3	1	4	SArRNA06
gi|29165615|ref|NC_002745.2|	554114	+	A	13	72	1	2	15	SArRNA06
gi|29165615|ref|NC_002745.2|	554115	+	U	0	3	1	1	1	SArRNA06
gi|29165615|ref|NC_002745.2|	554116	+	A	3	1	1	1	2	SArRNA06
gi|29165615|ref|NC_002745.2|	554121	+	C	0	0	1	0	1	SArRNA06
gi|29165615|ref|NC_002745.2|	554122	+	C	0	6	0	0	0	SArRNA06
gi|29165615|ref|NC_002745.2|	554123	+	C	2	1	0	0	1	SArRNA06
gi|29165615|ref|NC_002745.2|	554124	+	G	0	4	0	0	0	SArRNA06
gi|29165615|ref|NC_002745.2|	554125	+	U	1	7	1	0	2	SArRNA06
gi|29165615|ref|NC_002745.2|	554127	+	C	1	0	0	0	1	SArRNA06
gi|29165615|ref|NC_002745.2|	554128	+	C	0	1	0	0	0	SArRNA06
gi|29165615|ref|NC_002745.2|	554129	+	G	1	1	0	1	2	SArRNA06
gi|29165615|ref|NC_002745.2|	554130	+	C	5	6	2	1	4	SArRNA06
gi|29165615|ref|NC_002745.2|	554131	+	A	0	1	0	1	4	SArRNA06
gi|29165615|ref|NC_002745.2|	554132	+	A	1	7	1	0	5	SArRNA06
gi|29165615|ref|NC_002745.2|	554133	+	A	1	16	1	0	6	SArRNA06
gi|29165615|ref|NC_002745.2|	554134	+	C	1	1	1	0	4	SArRNA06
gi|29165615|ref|NC_002745.2|	554135	+	C	6	21	2	2	6	SArRNA06
gi|29165615|ref|NC_002745.2|	554136	+	G	1	8	0	2	4	SArRNA06
gi|29165615|ref|NC_002745.2|	554138	+	C	4	8	0	0	6	SArRNA06
gi|29165615|ref|NC_002745.2|	554139	+	A	0	13	1	0	3	SArRNA06
gi|29165615|ref|NC_002745.2|	554140	+	C	15	23	5	8	10	SArRNA06
gi|29165615|ref|NC_002745.2|	554141	+	A	2	12	1	1	3	SArRNA06
gi|29165615|ref|NC_002745.2|	554142	+	G	0	1	0	1	0	SArRNA06
gi|29165615|ref|NC_002745.2|	554143	+	G	1	2	0	2	1	SArRNA06
gi|29165615|ref|NC_002745.2|	554144	+	U	3	7	0	0	2	SArRNA06
gi|29165615|ref|NC_002745.2|	554146	+	G	1	0	0	0	0	SArRNA06
gi|29165615|ref|NC_002745.2|	554147	+	U	0	0	0	1	1	SArRNA06
gi|29165615|ref|NC_002745.2|	554148	+	C	18	18	7	11	13	SArRNA06
gi|29165615|ref|NC_002745.2|	554149	+	A	1	5	2	0	1	SArRNA06
gi|29165615|ref|NC_002745.2|	554150	+	A	0	3	1	0	3	SArRNA06
gi|29165615|ref|NC_002745.2|	554151	+	G	1	1	0	0	0	SArRNA06
gi|29165615|ref|NC_002745.2|	554152	+	A	0	1	0	0	0	SArRNA06
gi|29165615|ref|NC_002745.2|	554153	+	U	0	0	0	1	3	SArRNA06

gi|29165615|ref|NC_002745.2|	554154	+	G	1	1	0	0	1	SArRNA06
gi|29165615|ref|NC_002745.2|	554155	+	A	1	2	0	1	3	SArRNA06
gi|29165615|ref|NC_002745.2|	554156	+	G	4	3	0	0	3	SArRNA06
gi|29165615|ref|NC_002745.2|	554157	+	A	8	3	4	3	5	SArRNA06
gi|29165615|ref|NC_002745.2|	554158	+	A	15	54	8	5	19	SArRNA06
gi|29165615|ref|NC_002745.2|	554159	+	U	1	1	0	2	5	SArRNA06
gi|29165615|ref|NC_002745.2|	554160	+	U	1	3	0	0	0	SArRNA06
gi|29165615|ref|NC_002745.2|	554161	+	C	9	11	1	3	9	SArRNA06
gi|29165615|ref|NC_002745.2|	554162	+	U	3	9	1	2	5	SArRNA06
gi|29165615|ref|NC_002745.2|	554163	+	A	2	15	3	13	8	SArRNA06
gi|29165615|ref|NC_002745.2|	554164	+	A	3	4	1	4	2	SArRNA06
gi|29165615|ref|NC_002745.2|	554165	+	G	0	0	0	1	1	SArRNA06
gi|29165615|ref|NC_002745.2|	554166	+	G	0	1	0	0	1	SArRNA06
gi|29165615|ref|NC_002745.2|	554167	+	U	5	2	4	6	3	SArRNA06
gi|29165615|ref|NC_002745.2|	554168	+	G	1	2	0	1	1	SArRNA06
gi|29165615|ref|NC_002745.2|	554169	+	A	0	2	0	2	0	SArRNA06
gi|29165615|ref|NC_002745.2|	554170	+	G	0	0	0	0	1	SArRNA06
gi|29165615|ref|NC_002745.2|	554171	+	C	4	3	0	1	4	SArRNA06
gi|29165615|ref|NC_002745.2|	554172	+	G	1	2	0	1	0	SArRNA06
gi|29165615|ref|NC_002745.2|	554174	+	G	1	0	0	1	0	SArRNA06
gi|29165615|ref|NC_002745.2|	554175	+	C	3	1	1	1	0	SArRNA06
gi|29165615|ref|NC_002745.2|	554176	+	G	0	4	0	2	0	SArRNA06
gi|29165615|ref|NC_002745.2|	554177	+	A	3	2	0	0	0	SArRNA06
gi|29165615|ref|NC_002745.2|	554179	+	C	1	2	1	1	1	SArRNA06
gi|29165615|ref|NC_002745.2|	554181	+	C	1	0	0	2	1	SArRNA06
gi|29165615|ref|NC_002745.2|	554182	+	U	1	0	2	0	2	SArRNA06
gi|29165615|ref|NC_002745.2|	554183	+	C	3	1	0	1	2	SArRNA06
gi|29165615|ref|NC_002745.2|	554184	+	G	1	1	0	0	1	SArRNA06
gi|29165615|ref|NC_002745.2|	554185	+	U	0	0	0	1	0	SArRNA06
gi|29165615|ref|NC_002745.2|	554186	+	U	0	0	0	1	4	SArRNA06
gi|29165615|ref|NC_002745.2|	554187	+	A	0	1	3	0	0	SArRNA06
gi|29165615|ref|NC_002745.2|	554188	+	A	7	2	0	2	2	SArRNA06
gi|29165615|ref|NC_002745.2|	554189	+	G	0	0	0	0	1	SArRNA06
gi|29165615|ref|NC_002745.2|	554190	+	G	0	1	0	0	0	SArRNA06
gi|29165615|ref|NC_002745.2|	554191	+	A	1	1	0	0	0	SArRNA06
gi|29165615|ref|NC_002745.2|	554193	+	C	1	1	1	2	0	SArRNA06
gi|29165615|ref|NC_002745.2|	554194	+	U	0	1	2	4	4	SArRNA06
gi|29165615|ref|NC_002745.2|	554195	+	C	2	1	0	2	1	SArRNA06
gi|29165615|ref|NC_002745.2|	554197	+	G	0	0	0	0	1	SArRNA06
gi|29165615|ref|NC_002745.2|	554199	+	A	2	1	0	0	1	SArRNA06
gi|29165615|ref|NC_002745.2|	554200	+	A	0	0	0	0	1	SArRNA06
gi|29165615|ref|NC_002745.2|	554201	+	A	4	1	0	0	4	SArRNA06
gi|29165615|ref|NC_002745.2|	554202	+	A	0	1	0	0	1	SArRNA06
gi|29165615|ref|NC_002745.2|	554204	+	G	0	0	1	0	1	SArRNA06
gi|29165615|ref|NC_002745.2|	554206	+	C	4	8	1	1	6	SArRNA06
gi|29165615|ref|NC_002745.2|	554207	+	C	0	2	0	1	1	SArRNA06
gi|29165615|ref|NC_002745.2|	554208	+	C	1	0	0	0	0	SArRNA06
gi|29165615|ref|NC_002745.2|	554209	+	C	1	1	0	0	0	SArRNA06
gi|29165615|ref|NC_002745.2|	554210	+	G	0	1	1	0	1	SArRNA06
gi|29165615|ref|NC_002745.2|	554211	+	U	1	0	0	0	2	SArRNA06
gi|29165615|ref|NC_002745.2|	554212	+	A	1	1	0	0	0	SArRNA06
gi|29165615|ref|NC_002745.2|	554213	+	A	0	1	0	1	1	SArRNA06
gi|29165615|ref|NC_002745.2|	554214	+	C	6	3	2	1	0	SArRNA06
gi|29165615|ref|NC_002745.2|	554215	+	U	2	2	0	4	2	SArRNA06
gi|29165615|ref|NC_002745.2|	554216	+	U	1	2	2	1	2	SArRNA06
gi|29165615|ref|NC_002745.2|	554217	+	C	2	1	0	3	3	SArRNA06
gi|29165615|ref|NC_002745.2|	554218	+	G	1	1	1	0	0	SArRNA06
gi|29165615|ref|NC_002745.2|	554219	+	G	2	0	0	2	1	SArRNA06
gi|29165615|ref|NC_002745.2|	554221	+	A	1	0	2	1	1	SArRNA06
gi|29165615|ref|NC_002745.2|	554222	+	G	1	1	0	0	3	SArRNA06
gi|29165615|ref|NC_002745.2|	554223	+	A	1	0	1	1	3	SArRNA06
gi|29165615|ref|NC_002745.2|	554225	+	G	0	0	0	0	1	SArRNA06
gi|29165615|ref|NC_002745.2|	554232	+	U	0	1	0	0	3	SArRNA06
gi|29165615|ref|NC_002745.2|	554233	+	C	2	0	0	0	2	SArRNA06
gi|29165615|ref|NC_002745.2|	554234	+	U	2	0	1	0	0	SArRNA06
gi|29165615|ref|NC_002745.2|	554235	+	U	1	0	1	0	1	SArRNA06
gi|29165615|ref|NC_002745.2|	554239	+	G	0	1	0	0	0	SArRNA06
gi|29165615|ref|NC_002745.2|	554240	+	G	0	0	0	0	1	SArRNA06
gi|29165615|ref|NC_002745.2|	554242	+	U	1	0	0	0	0	SArRNA06
gi|29165615|ref|NC_002745.2|	554244	+	A	1	1	2	0	2	SArRNA06
gi|29165615|ref|NC_002745.2|	554245	+	C	30	21	11	17	19	SArRNA06
gi|29165615|ref|NC_002745.2|	554246	+	G	8	3	0	2	6	SArRNA06
gi|29165615|ref|NC_002745.2|	554248	+	C	6	1	0	1	3	SArRNA06
gi|29165615|ref|NC_002745.2|	554249	+	C	2	2	1	2	0	SArRNA06
gi|29165615|ref|NC_002745.2|	554250	+	A	0	0	1	1	0	SArRNA06
gi|29165615|ref|NC_002745.2|	554251	+	G	1	0	0	0	2	SArRNA06
gi|29165615|ref|NC_002745.2|	554252	+	A	1	1	0	3	0	SArRNA06
gi|29165615|ref|NC_002745.2|	554253	+	A	0	2	2	0	1	SArRNA06
gi|29165615|ref|NC_002745.2|	554254	+	G	1	3	0	0	1	SArRNA06
gi|29165615|ref|NC_002745.2|	554255	+	A	1	0	0	0	1	SArRNA06
gi|29165615|ref|NC_002745.2|	554256	+	G	0	0	0	1	1	SArRNA06

gi|29165615|ref|NC_002745.2|	554257	+	C	3	2	0	2	1	SArRNA06
gi|29165615|ref|NC_002745.2|	554258	+	C	1	0	0	0	1	SArRNA06
gi|29165615|ref|NC_002745.2|	554259	+	G	2	0	1	0	2	SArRNA06
gi|29165615|ref|NC_002745.2|	554260	+	C	1	1	2	1	3	SArRNA06
gi|29165615|ref|NC_002745.2|	554261	+	A	5	1	0	2	2	SArRNA06
gi|29165615|ref|NC_002745.2|	554262	+	G	1	0	2	0	2	SArRNA06
gi|29165615|ref|NC_002745.2|	554263	+	U	2	1	2	2	0	SArRNA06
gi|29165615|ref|NC_002745.2|	554264	+	G	2	2	1	8	1	SArRNA06
gi|29165615|ref|NC_002745.2|	554265	+	A	3	2	0	1	1	SArRNA06
gi|29165615|ref|NC_002745.2|	554266	+	A	4	7	3	1	3	SArRNA06
gi|29165615|ref|NC_002745.2|	554267	+	U	1	3	0	0	2	SArRNA06
gi|29165615|ref|NC_002745.2|	554268	+	A	3	4	0	0	0	SArRNA06
gi|29165615|ref|NC_002745.2|	554269	+	G	0	1	0	0	2	SArRNA06
gi|29165615|ref|NC_002745.2|	554270	+	G	0	0	1	2	0	SArRNA06
gi|29165615|ref|NC_002745.2|	554271	+	C	3	3	2	3	6	SArRNA06
gi|29165615|ref|NC_002745.2|	554272	+	C	0	1	1	1	5	SArRNA06
gi|29165615|ref|NC_002745.2|	554273	+	C	3	1	3	2	9	SArRNA06
gi|29165615|ref|NC_002745.2|	554274	+	A	6	5	5	1	2	SArRNA06
gi|29165615|ref|NC_002745.2|	554275	+	A	2	1	0	1	3	SArRNA06
gi|29165615|ref|NC_002745.2|	554276	+	G	0	1	0	0	2	SArRNA06
gi|29165615|ref|NC_002745.2|	554277	+	C	2	0	1	0	1	SArRNA06
gi|29165615|ref|NC_002745.2|	554278	+	G	0	2	3	2	0	SArRNA06
gi|29165615|ref|NC_002745.2|	554279	+	A	0	0	1	0	1	SArRNA06
gi|29165615|ref|NC_002745.2|	554280	+	C	1	0	2	0	0	SArRNA06
gi|29165615|ref|NC_002745.2|	554281	+	U	0	0	1	1	2	SArRNA06
gi|29165615|ref|NC_002745.2|	554283	+	U	2	0	0	1	1	SArRNA06
gi|29165615|ref|NC_002745.2|	554284	+	U	0	0	0	0	3	SArRNA06
gi|29165615|ref|NC_002745.2|	554285	+	U	0	1	2	2	3	SArRNA06
gi|29165615|ref|NC_002745.2|	554286	+	A	1	2	1	0	0	SArRNA06
gi|29165615|ref|NC_002745.2|	554287	+	U	4	4	0	0	2	SArRNA06
gi|29165615|ref|NC_002745.2|	554288	+	C	2	0	1	1	4	SArRNA06
gi|29165615|ref|NC_002745.2|	554289	+	A	1	0	1	0	1	SArRNA06
gi|29165615|ref|NC_002745.2|	554290	+	A	4	0	0	0	3	SArRNA06
gi|29165615|ref|NC_002745.2|	554291	+	A	0	1	0	2	1	SArRNA06
gi|29165615|ref|NC_002745.2|	554292	+	A	2	1	3	0	3	SArRNA06
gi|29165615|ref|NC_002745.2|	554293	+	A	5	2	1	0	3	SArRNA06
gi|29165615|ref|NC_002745.2|	554294	+	C	11	2	1	4	1	SArRNA06
gi|29165615|ref|NC_002745.2|	554295	+	A	4	0	2	1	1	SArRNA06
gi|29165615|ref|NC_002745.2|	554298	+	G	0	0	0	0	1	SArRNA06
gi|29165615|ref|NC_002745.2|	554299	+	G	1	0	0	0	1	SArRNA06
gi|29165615|ref|NC_002745.2|	554300	+	U	167	6	10	2	7	SArRNA06
gi|29165615|ref|NC_002745.2|	554301	+	C	30	0	1	0	2	SArRNA06
gi|29165615|ref|NC_002745.2|	554302	+	U	5	1	1	2	2	SArRNA06
gi|29165615|ref|NC_002745.2|	554303	+	C	2	0	0	0	3	SArRNA06
gi|29165615|ref|NC_002745.2|	554304	+	U	0	0	2	0	1	SArRNA06
gi|29165615|ref|NC_002745.2|	554305	+	G	3	1	2	1	0	SArRNA06
gi|29165615|ref|NC_002745.2|	554306	+	C	3	3	0	2	5	SArRNA06
gi|29165615|ref|NC_002745.2|	554307	+	U	3	0	3	0	3	SArRNA06
gi|29165615|ref|NC_002745.2|	554308	+	A	1	1	0	0	2	SArRNA06
gi|29165615|ref|NC_002745.2|	554309	+	A	3	3	0	0	3	SArRNA06
gi|29165615|ref|NC_002745.2|	554310	+	A	1	0	0	0	0	SArRNA06
gi|29165615|ref|NC_002745.2|	554311	+	C	2	1	1	0	4	SArRNA06
gi|29165615|ref|NC_002745.2|	554312	+	C	2	1	2	0	3	SArRNA06
gi|29165615|ref|NC_002745.2|	554313	+	G	0	0	1	0	2	SArRNA06
gi|29165615|ref|NC_002745.2|	554314	+	U	2	1	0	0	1	SArRNA06
gi|29165615|ref|NC_002745.2|	554315	+	A	1	2	1	0	1	SArRNA06
gi|29165615|ref|NC_002745.2|	554316	+	A	0	0	2	0	1	SArRNA06
gi|29165615|ref|NC_002745.2|	554317	+	G	2	3	1	0	1	SArRNA06
gi|29165615|ref|NC_002745.2|	554318	+	G	0	0	0	0	1	SArRNA06
gi|29165615|ref|NC_002745.2|	554319	+	U	0	1	2	0	0	SArRNA06
gi|29165615|ref|NC_002745.2|	554320	+	G	0	2	0	1	1	SArRNA06
gi|29165615|ref|NC_002745.2|	554321	+	A	1	0	1	1	0	SArRNA06
gi|29165615|ref|NC_002745.2|	554323	+	G	1	0	0	0	0	SArRNA06
gi|29165615|ref|NC_002745.2|	554324	+	U	0	1	0	0	0	SArRNA06
gi|29165615|ref|NC_002745.2|	554325	+	A	1	1	1	0	0	SArRNA06
gi|29165615|ref|NC_002745.2|	554326	+	U	2	0	2	0	1	SArRNA06
gi|29165615|ref|NC_002745.2|	554327	+	A	2	1	0	0	0	SArRNA06
gi|29165615|ref|NC_002745.2|	554329	+	G	0	1	1	0	1	SArRNA06
gi|29165615|ref|NC_002745.2|	554330	+	G	1	0	0	0	0	SArRNA06
gi|29165615|ref|NC_002745.2|	554332	+	C	0	0	1	0	1	SArRNA06
gi|29165615|ref|NC_002745.2|	554333	+	U	0	1	0	0	0	SArRNA06
gi|29165615|ref|NC_002745.2|	554337	+	G	1	0	0	0	0	SArRNA06
gi|29165615|ref|NC_002745.2|	554338	+	C	0	1	0	0	0	SArRNA06
gi|29165615|ref|NC_002745.2|	554339	+	C	1	1	0	0	0	SArRNA06
gi|29165615|ref|NC_002745.2|	554340	+	U	0	0	1	0	0	SArRNA06
gi|29165615|ref|NC_002745.2|	554342	+	C	2	1	0	0	1	SArRNA06
gi|29165615|ref|NC_002745.2|	554343	+	C	1	1	0	0	0	SArRNA06
gi|29165615|ref|NC_002745.2|	554344	+	C	0	1	0	0	0	SArRNA06
gi|29165615|ref|NC_002745.2|	554345	+	G	0	0	0	1	0	SArRNA06
gi|29165615|ref|NC_002745.2|	554352	+	G	2	8	5	1	5	SArRNA06
gi|29165615|ref|NC_002745.2|	554354	+	A	0	0	0	1	2	SArRNA06

gi|29165615|ref|NC_002745.2|	554356	+	G	0	1	0	0	0	SArRNA06
gi|29165615|ref|NC_002745.2|	554358	+	U	0	1	0	0	0	SArRNA06
gi|29165615|ref|NC_002745.2|	554359	+	A	0	1	0	0	0	SArRNA06
gi|29165615|ref|NC_002745.2|	554361	+	G	0	0	0	1	0	SArRNA06
gi|29165615|ref|NC_002745.2|	554362	+	A	1	2	0	0	0	SArRNA06
gi|29165615|ref|NC_002745.2|	554363	+	G	0	1	0	0	0	SArRNA06
gi|29165615|ref|NC_002745.2|	554364	+	G	1	1	0	0	1	SArRNA06
gi|29165615|ref|NC_002745.2|	554365	+	A	0	0	1	0	0	SArRNA06
gi|29165615|ref|NC_002745.2|	554366	+	G	0	1	0	0	0	SArRNA06
gi|29165615|ref|NC_002745.2|	554367	+	U	0	1	1	0	0	SArRNA06
gi|29165615|ref|NC_002745.2|	554368	+	G	0	0	0	0	2	SArRNA06
gi|29165615|ref|NC_002745.2|	554370	+	U	0	0	0	1	1	SArRNA06
gi|29165615|ref|NC_002745.2|	554371	+	U	0	0	0	0	1	SArRNA06
gi|29165615|ref|NC_002745.2|	554372	+	A	0	0	1	0	0	SArRNA06
gi|29165615|ref|NC_002745.2|	554373	+	G	0	1	0	0	0	SArRNA06
gi|29165615|ref|NC_002745.2|	554374	+	C	0	1	0	0	0	SArRNA06
gi|29165615|ref|NC_002745.2|	554377	+	C	0	0	0	0	2	SArRNA06
gi|29165615|ref|NC_002745.2|	554379	+	G	0	0	0	1	0	SArRNA06
gi|29165615|ref|NC_002745.2|	554381	+	G	0	1	0	0	0	SArRNA06
gi|29165615|ref|NC_002745.2|	554383	+	A	0	0	1	0	0	SArRNA06
gi|29165615|ref|NC_002745.2|	554388	+	C	0	0	0	0	2	SArRNA06
gi|29165615|ref|NC_002745.2|	554398	+	C	4	3	0	0	1	SArRNA06
gi|29165615|ref|NC_002745.2|	554400	+	C	0	1	0	0	1	SArRNA06
gi|29165615|ref|NC_002745.2|	554401	+	C	5	2	0	1	1	SArRNA06
gi|29165615|ref|NC_002745.2|	554402	+	A	0	1	1	0	0	SArRNA06
gi|29165615|ref|NC_002745.2|	554405	+	A	0	1	0	0	0	SArRNA06
gi|29165615|ref|NC_002745.2|	554406	+	A	0	1	0	0	0	SArRNA06
gi|29165615|ref|NC_002745.2|	554408	+	C	1	1	0	0	2	SArRNA06
gi|29165615|ref|NC_002745.2|	554410	+	G	0	2	0	0	0	SArRNA06
gi|29165615|ref|NC_002745.2|	554412	+	G	0	1	0	0	0	SArRNA06
gi|29165615|ref|NC_002745.2|	554413	+	G	0	1	0	0	0	SArRNA06
gi|29165615|ref|NC_002745.2|	554416	+	G	1	0	0	0	0	SArRNA06
gi|29165615|ref|NC_002745.2|	554417	+	U	0	0	0	0	1	SArRNA06
gi|29165615|ref|NC_002745.2|	554418	+	A	0	0	0	1	0	SArRNA06
gi|29165615|ref|NC_002745.2|	554419	+	A	0	0	0	0	3	SArRNA06
gi|29165615|ref|NC_002745.2|	554421	+	U	0	0	0	0	2	SArRNA06
gi|29165615|ref|NC_002745.2|	554422	+	A	1	1	2	2	1	SArRNA06
gi|29165615|ref|NC_002745.2|	554424	+	A	0	0	1	0	2	SArRNA06
gi|29165615|ref|NC_002745.2|	554425	+	A	1	2	0	0	0	SArRNA06
gi|29165615|ref|NC_002745.2|	554426	+	C	0	0	1	0	0	SArRNA06
gi|29165615|ref|NC_002745.2|	554431	+	C	3	0	0	0	1	SArRNA06
gi|29165615|ref|NC_002745.2|	554432	+	U	0	2	0	0	0	SArRNA06
gi|29165615|ref|NC_002745.2|	554434	+	A	0	0	0	1	0	SArRNA06
gi|29165615|ref|NC_002745.2|	554436	+	G	0	0	0	0	1	SArRNA06
gi|29165615|ref|NC_002745.2|	554437	+	U	0	1	0	0	1	SArRNA06
gi|29165615|ref|NC_002745.2|	554438	+	A	0	1	0	0	0	SArRNA06
gi|29165615|ref|NC_002745.2|	554440	+	C	2	0	0	0	1	SArRNA06
gi|29165615|ref|NC_002745.2|	554441	+	G	0	0	1	1	1	SArRNA06
gi|29165615|ref|NC_002745.2|	554442	+	A	1	8	1	4	4	SArRNA06
gi|29165615|ref|NC_002745.2|	554443	+	A	2	4	3	4	7	SArRNA06
gi|29165615|ref|NC_002745.2|	554444	+	A	11	67	7	3	37	SArRNA06
gi|29165615|ref|NC_002745.2|	554445	+	U	0	4	3	0	2	SArRNA06
gi|29165615|ref|NC_002745.2|	554446	+	U	1	1	0	1	2	SArRNA06
gi|29165615|ref|NC_002745.2|	554447	+	C	5	17	1	3	10	SArRNA06
gi|29165615|ref|NC_002745.2|	554448	+	C	75	135	30	39	92	SArRNA06
gi|29165615|ref|NC_002745.2|	554449	+	U	9	12	3	4	12	SArRNA06
gi|29165615|ref|NC_002745.2|	554450	+	U	8	4	3	1	11	SArRNA06
gi|29165615|ref|NC_002745.2|	554451	+	G	12	4	1	0	6	SArRNA06
gi|29165615|ref|NC_002745.2|	554452	+	U	10	9	2	1	9	SArRNA06
gi|29165615|ref|NC_002745.2|	554453	+	C	21	18	3	6	13	SArRNA06
gi|29165615|ref|NC_002745.2|	554454	+	G	5	10	0	0	4	SArRNA06
gi|29165615|ref|NC_002745.2|	554455	+	G	1	1	0	0	2	SArRNA06
gi|29165615|ref|NC_002745.2|	554456	+	G	1	1	4	0	3	SArRNA06
gi|29165615|ref|NC_002745.2|	554457	+	U	3	0	2	1	3	SArRNA06
gi|29165615|ref|NC_002745.2|	554458	+	A	2	4	0	1	3	SArRNA06
gi|29165615|ref|NC_002745.2|	554459	+	A	3	1	0	2	0	SArRNA06
gi|29165615|ref|NC_002745.2|	554460	+	G	0	0	0	0	1	SArRNA06
gi|29165615|ref|NC_002745.2|	554461	+	U	0	2	1	0	0	SArRNA06
gi|29165615|ref|NC_002745.2|	554462	+	U	1	3	0	0	0	SArRNA06
gi|29165615|ref|NC_002745.2|	554463	+	C	3	6	0	0	2	SArRNA06
gi|29165615|ref|NC_002745.2|	554464	+	C	1	4	2	1	1	SArRNA06
gi|29165615|ref|NC_002745.2|	554465	+	G	4	6	0	1	2	SArRNA06
gi|29165615|ref|NC_002745.2|	554466	+	A	0	8	0	0	6	SArRNA06
gi|29165615|ref|NC_002745.2|	554467	+	C	65	32	10	9	35	SArRNA06
gi|29165615|ref|NC_002745.2|	554468	+	C	20	35	18	5	28	SArRNA06
gi|29165615|ref|NC_002745.2|	554469	+	C	11	15	1	1	8	SArRNA06
gi|29165615|ref|NC_002745.2|	554470	+	G	14	17	3	1	19	SArRNA06
gi|29165615|ref|NC_002745.2|	554471	+	C	41	46	19	9	37	SArRNA06
gi|29165615|ref|NC_002745.2|	554472	+	A	7	14	6	4	11	SArRNA06
gi|29165615|ref|NC_002745.2|	554473	+	C	4	5	3	1	6	SArRNA06
gi|29165615|ref|NC_002745.2|	554474	+	G	10	2	7	1	15	SArRNA06

gi|29165615|ref|NC_002745.2|	554475	+	A	3	5	3	3	8	SArRNA06
gi|29165615|ref|NC_002745.2|	554476	+	A	20	25	10	5	18	SArRNA06
gi|29165615|ref|NC_002745.2|	554477	+	A	15	6	4	1	6	SArRNA06
gi|29165615|ref|NC_002745.2|	554478	+	G	1	0	0	0	1	SArRNA06
gi|29165615|ref|NC_002745.2|	554479	+	G	10	0	0	0	5	SArRNA06
gi|29165615|ref|NC_002745.2|	554480	+	C	6	4	1	2	4	SArRNA06
gi|29165615|ref|NC_002745.2|	554481	+	G	7	9	2	0	2	SArRNA06
gi|29165615|ref|NC_002745.2|	554482	+	U	3	6	6	3	7	SArRNA06
gi|29165615|ref|NC_002745.2|	554483	+	A	0	17	10	9	11	SArRNA06
gi|29165615|ref|NC_002745.2|	554484	+	A	4	12	12	7	19	SArRNA06
gi|29165615|ref|NC_002745.2|	554485	+	C	10	33	12	15	24	SArRNA06
gi|29165615|ref|NC_002745.2|	554486	+	G	14	17	4	7	18	SArRNA06
gi|29165615|ref|NC_002745.2|	554487	+	A	36	228	142	235	273	SArRNA06
gi|29165615|ref|NC_002745.2|	554488	+	U	10	34	17	11	18	SArRNA06
gi|29165615|ref|NC_002745.2|	554489	+	U	3	10	4	7	6	SArRNA06
gi|29165615|ref|NC_002745.2|	554490	+	U	3	10	4	1	6	SArRNA06
gi|29165615|ref|NC_002745.2|	554491	+	G	6	26	0	0	13	SArRNA06
gi|29165615|ref|NC_002745.2|	554492	+	G	0	2	0	0	0	SArRNA06
gi|29165615|ref|NC_002745.2|	554493	+	G	0	2	0	0	1	SArRNA06
gi|29165615|ref|NC_002745.2|	554494	+	C	8	8	6	7	6	SArRNA06
gi|29165615|ref|NC_002745.2|	554495	+	A	1	5	3	1	2	SArRNA06
gi|29165615|ref|NC_002745.2|	554496	+	C	12	11	3	3	5	SArRNA06
gi|29165615|ref|NC_002745.2|	554497	+	U	2	3	0	1	1	SArRNA06
gi|29165615|ref|NC_002745.2|	554498	+	G	0	0	0	0	1	SArRNA06
gi|29165615|ref|NC_002745.2|	554499	+	U	0	2	0	1	2	SArRNA06
gi|29165615|ref|NC_002745.2|	554500	+	C	6	13	2	2	2	SArRNA06
gi|29165615|ref|NC_002745.2|	554501	+	U	1	4	2	1	6	SArRNA06
gi|29165615|ref|NC_002745.2|	554502	+	C	49	48	13	17	34	SArRNA06
gi|29165615|ref|NC_002745.2|	554503	+	A	42	48	24	10	26	SArRNA06
gi|29165615|ref|NC_002745.2|	554504	+	A	1	6	5	1	6	SArRNA06
gi|29165615|ref|NC_002745.2|	554505	+	C	52	42	16	10	27	SArRNA06
gi|29165615|ref|NC_002745.2|	554506	+	G	8	12	3	0	9	SArRNA06
gi|29165615|ref|NC_002745.2|	554507	+	A	2	3	0	2	1	SArRNA06
gi|29165615|ref|NC_002745.2|	554508	+	G	4	0	3	1	0	SArRNA06
gi|29165615|ref|NC_002745.2|	554509	+	A	1	5	1	0	4	SArRNA06
gi|29165615|ref|NC_002745.2|	554510	+	G	6	3	4	4	7	SArRNA06
gi|29165615|ref|NC_002745.2|	554511	+	A	5	7	4	1	6	SArRNA06
gi|29165615|ref|NC_002745.2|	554512	+	C	11	9	3	1	9	SArRNA06
gi|29165615|ref|NC_002745.2|	554513	+	U	5	13	1	3	8	SArRNA06
gi|29165615|ref|NC_002745.2|	554514	+	C	19	22	8	5	12	SArRNA06
gi|29165615|ref|NC_002745.2|	554515	+	G	1	6	1	0	2	SArRNA06
gi|29165615|ref|NC_002745.2|	554516	+	G	1	2	0	0	1	SArRNA06
gi|29165615|ref|NC_002745.2|	554517	+	U	4	1	0	1	2	SArRNA06
gi|29165615|ref|NC_002745.2|	554518	+	G	0	3	1	0	0	SArRNA06
gi|29165615|ref|NC_002745.2|	554519	+	A	0	0	1	0	2	SArRNA06
gi|29165615|ref|NC_002745.2|	554520	+	A	5	6	3	6	7	SArRNA06
gi|29165615|ref|NC_002745.2|	554521	+	A	15	147	24	3	35	SArRNA06
gi|29165615|ref|NC_002745.2|	554522	+	U	2	2	0	0	4	SArRNA06
gi|29165615|ref|NC_002745.2|	554523	+	C	14	37	3	3	25	SArRNA06
gi|29165615|ref|NC_002745.2|	554524	+	A	20	10	6	2	10	SArRNA06
gi|29165615|ref|NC_002745.2|	554525	+	U	8	7	2	7	11	SArRNA06
gi|29165615|ref|NC_002745.2|	554526	+	A	0	2	0	0	1	SArRNA06
gi|29165615|ref|NC_002745.2|	554527	+	G	0	2	1	0	0	SArRNA06
gi|29165615|ref|NC_002745.2|	554528	+	U	5	9	2	0	5	SArRNA06
gi|29165615|ref|NC_002745.2|	554529	+	A	1	2	2	1	0	SArRNA06
gi|29165615|ref|NC_002745.2|	554530	+	C	1	1	3	1	3	SArRNA06
gi|29165615|ref|NC_002745.2|	554531	+	C	3	3	0	3	1	SArRNA06
gi|29165615|ref|NC_002745.2|	554532	+	U	6	6	0	1	2	SArRNA06
gi|29165615|ref|NC_002745.2|	554533	+	G	2	1	0	1	1	SArRNA06
gi|29165615|ref|NC_002745.2|	554534	+	U	6	3	4	2	2	SArRNA06
gi|29165615|ref|NC_002745.2|	554535	+	G	1	0	2	5	3	SArRNA06
gi|29165615|ref|NC_002745.2|	554536	+	A	4	3	2	2	4	SArRNA06
gi|29165615|ref|NC_002745.2|	554537	+	A	10	14	0	2	9	SArRNA06
gi|29165615|ref|NC_002745.2|	554538	+	G	10	12	3	4	5	SArRNA06
gi|29165615|ref|NC_002745.2|	554539	+	A	6	9	6	4	7	SArRNA06
gi|29165615|ref|NC_002745.2|	554540	+	U	4	5	3	3	5	SArRNA06
gi|29165615|ref|NC_002745.2|	554541	+	G	2	4	2	0	7	SArRNA06
gi|29165615|ref|NC_002745.2|	554542	+	C	13	13	1	3	0	SArRNA06
gi|29165615|ref|NC_002745.2|	554543	+	A	7	4	1	4	7	SArRNA06
gi|29165615|ref|NC_002745.2|	554544	+	G	0	1	0	0	1	SArRNA06
gi|29165615|ref|NC_002745.2|	554545	+	G	1	1	1	1	0	SArRNA06
gi|29165615|ref|NC_002745.2|	554546	+	U	6	2	2	0	6	SArRNA06
gi|29165615|ref|NC_002745.2|	554547	+	U	7	5	0	1	6	SArRNA06
gi|29165615|ref|NC_002745.2|	554548	+	A	6	3	1	0	3	SArRNA06
gi|29165615|ref|NC_002745.2|	554549	+	C	10	10	5	2	10	SArRNA06
gi|29165615|ref|NC_002745.2|	554550	+	C	5	7	4	2	4	SArRNA06
gi|29165615|ref|NC_002745.2|	554551	+	C	9	1	3	5	7	SArRNA06
gi|29165615|ref|NC_002745.2|	554552	+	G	0	2	0	0	1	SArRNA06
gi|29165615|ref|NC_002745.2|	554553	+	C	9	2	2	5	2	SArRNA06
gi|29165615|ref|NC_002745.2|	554554	+	G	1	3	0	2	1	SArRNA06
gi|29165615|ref|NC_002745.2|	554555	+	A	6	3	2	1	2	SArRNA06

gi|29165615|ref|NC_002745.2|	554556	+	C	20	23	3	1	11	SArRNA06
gi|29165615|ref|NC_002745.2|	554557	+	A	5	5	2	0	1	SArRNA06
gi|29165615|ref|NC_002745.2|	554558	+	G	5	4	0	1	12	SArRNA06
gi|29165615|ref|NC_002745.2|	554559	+	G	2	4	4	1	4	SArRNA06
gi|29165615|ref|NC_002745.2|	554560	+	A	5	1	2	0	2	SArRNA06
gi|29165615|ref|NC_002745.2|	554561	+	C	7	2	1	0	4	SArRNA06
gi|29165615|ref|NC_002745.2|	554562	+	G	1	5	0	1	2	SArRNA06
gi|29165615|ref|NC_002745.2|	554563	+	G	0	4	1	0	1	SArRNA06
gi|29165615|ref|NC_002745.2|	554564	+	A	1	0	1	2	3	SArRNA06
gi|29165615|ref|NC_002745.2|	554565	+	A	5	3	2	2	3	SArRNA06
gi|29165615|ref|NC_002745.2|	554566	+	A	3	3	1	0	5	SArRNA06
gi|29165615|ref|NC_002745.2|	554567	+	G	10	7	2	0	4	SArRNA06
gi|29165615|ref|NC_002745.2|	554568	+	A	5	3	3	1	6	SArRNA06
gi|29165615|ref|NC_002745.2|	554569	+	C	7	9	2	3	17	SArRNA06
gi|29165615|ref|NC_002745.2|	554570	+	C	19	5	2	3	4	SArRNA06
gi|29165615|ref|NC_002745.2|	554571	+	C	6	1	3	1	6	SArRNA06
gi|29165615|ref|NC_002745.2|	554572	+	C	17	22	4	24	10	SArRNA06
gi|29165615|ref|NC_002745.2|	554573	+	G	2	4	2	1	4	SArRNA06
gi|29165615|ref|NC_002745.2|	554574	+	U	6	3	1	1	5	SArRNA06
gi|29165615|ref|NC_002745.2|	554575	+	G	1	1	0	0	1	SArRNA06
gi|29165615|ref|NC_002745.2|	554576	+	G	3	2	1	2	2	SArRNA06
gi|29165615|ref|NC_002745.2|	554577	+	A	3	0	2	0	4	SArRNA06
gi|29165615|ref|NC_002745.2|	554578	+	G	7	4	0	0	4	SArRNA06
gi|29165615|ref|NC_002745.2|	554579	+	C	8	6	3	1	7	SArRNA06
gi|29165615|ref|NC_002745.2|	554580	+	U	6	2	1	2	3	SArRNA06
gi|29165615|ref|NC_002745.2|	554581	+	U	2	1	0	0	0	SArRNA06
gi|29165615|ref|NC_002745.2|	554582	+	U	6	4	0	0	4	SArRNA06
gi|29165615|ref|NC_002745.2|	554583	+	A	0	4	0	0	3	SArRNA06
gi|29165615|ref|NC_002745.2|	554584	+	C	2	3	4	5	2	SArRNA06
gi|29165615|ref|NC_002745.2|	554585	+	U	2	5	0	2	5	SArRNA06
gi|29165615|ref|NC_002745.2|	554586	+	G	1	1	1	0	0	SArRNA06
gi|29165615|ref|NC_002745.2|	554587	+	U	2	0	0	2	1	SArRNA06
gi|29165615|ref|NC_002745.2|	554588	+	A	3	1	0	0	0	SArRNA06
gi|29165615|ref|NC_002745.2|	554590	+	C	1	2	0	0	1	SArRNA06
gi|29165615|ref|NC_002745.2|	554591	+	C	16	13	10	6	6	SArRNA06
gi|29165615|ref|NC_002745.2|	554592	+	U	0	5	0	1	4	SArRNA06
gi|29165615|ref|NC_002745.2|	554593	+	G	0	1	0	0	1	SArRNA06
gi|29165615|ref|NC_002745.2|	554594	+	A	5	2	1	0	8	SArRNA06
gi|29165615|ref|NC_002745.2|	554595	+	U	10	3	1	1	6	SArRNA06
gi|29165615|ref|NC_002745.2|	554596	+	A	1	2	2	2	4	SArRNA06
gi|29165615|ref|NC_002745.2|	554597	+	U	0	0	0	0	3	SArRNA06
gi|29165615|ref|NC_002745.2|	554599	+	G	0	0	0	1	1	SArRNA06
gi|29165615|ref|NC_002745.2|	554600	+	A	1	4	1	0	3	SArRNA06
gi|29165615|ref|NC_002745.2|	554601	+	A	0	0	1	1	0	SArRNA06
gi|29165615|ref|NC_002745.2|	554602	+	A	2	15	1	0	7	SArRNA06
gi|29165615|ref|NC_002745.2|	554603	+	U	6	6	2	0	1	SArRNA06
gi|29165615|ref|NC_002745.2|	554604	+	U	2	5	2	1	2	SArRNA06
gi|29165615|ref|NC_002745.2|	554605	+	C	11	22	6	5	10	SArRNA06
gi|29165615|ref|NC_002745.2|	554606	+	G	1	1	0	0	2	SArRNA06
gi|29165615|ref|NC_002745.2|	554608	+	C	0	1	0	2	0	SArRNA06
gi|29165615|ref|NC_002745.2|	554609	+	A	0	2	0	0	1	SArRNA06
gi|29165615|ref|NC_002745.2|	554610	+	C	1	1	0	0	3	SArRNA06
gi|29165615|ref|NC_002745.2|	554611	+	A	0	2	0	0	0	SArRNA06
gi|29165615|ref|NC_002745.2|	554612	+	G	1	2	0	0	0	SArRNA06
gi|29165615|ref|NC_002745.2|	554613	+	C	11	8	3	1	4	SArRNA06
gi|29165615|ref|NC_002745.2|	554614	+	U	3	8	3	5	7	SArRNA06
gi|29165615|ref|NC_002745.2|	554615	+	U	6	2	0	4	4	SArRNA06
gi|29165615|ref|NC_002745.2|	554616	+	G	6	4	0	1	10	SArRNA06
gi|29165615|ref|NC_002745.2|	554617	+	U	5	13	3	2	3	SArRNA06
gi|29165615|ref|NC_002745.2|	554618	+	A	3	2	0	0	3	SArRNA06
gi|29165615|ref|NC_002745.2|	554619	+	C	35	76	12	23	51	SArRNA06
gi|29165615|ref|NC_002745.2|	554620	+	A	14	28	4	11	23	SArRNA06
gi|29165615|ref|NC_002745.2|	554621	+	G	4	4	3	3	6	SArRNA06
gi|29165615|ref|NC_002745.2|	554622	+	G	141	9	56	5	15	SArRNA06
gi|29165615|ref|NC_002745.2|	554623	+	A	162	13	47	10	13	SArRNA06
gi|29165615|ref|NC_002745.2|	554624	+	U	15	24	5	14	11	SArRNA06
gi|29165615|ref|NC_002745.2|	554625	+	A	2	3	1	2	4	SArRNA06
gi|29165615|ref|NC_002745.2|	554626	+	G	3	3	2	1	1	SArRNA06
gi|29165615|ref|NC_002745.2|	554627	+	G	6	1	2	5	5	SArRNA06
gi|29165615|ref|NC_002745.2|	554628	+	U	17	8	3	5	8	SArRNA06
gi|29165615|ref|NC_002745.2|	554629	+	A	7	8	4	2	5	SArRNA06
gi|29165615|ref|NC_002745.2|	554630	+	G	4	2	0	3	5	SArRNA06
gi|29165615|ref|NC_002745.2|	554631	+	G	10	4	5	6	8	SArRNA06
gi|29165615|ref|NC_002745.2|	554632	+	A	5	6	2	1	4	SArRNA06
gi|29165615|ref|NC_002745.2|	554634	+	C	13	6	3	8	11	SArRNA06
gi|29165615|ref|NC_002745.2|	554635	+	C	6	4	1	1	6	SArRNA06
gi|29165615|ref|NC_002745.2|	554636	+	U	1	4	3	1	5	SArRNA06
gi|29165615|ref|NC_002745.2|	554637	+	U	11	3	0	1	7	SArRNA06
gi|29165615|ref|NC_002745.2|	554638	+	U	5	1	1	0	2	SArRNA06
gi|29165615|ref|NC_002745.2|	554639	+	G	0	0	0	1	0	SArRNA06
gi|29165615|ref|NC_002745.2|	554640	+	A	1	1	0	1	4	SArRNA06

gi|29165615|ref|NC_002745.2|	554641	+	A	1	0	0	2	2	SArRNA06
gi|29165615|ref|NC_002745.2|	554643	+	C	4	0	0	1	2	SArRNA06
gi|29165615|ref|NC_002745.2|	554644	+	G	0	0	0	1	1	SArRNA06
gi|29165615|ref|NC_002745.2|	554645	+	U	1	0	2	1	3	SArRNA06
gi|29165615|ref|NC_002745.2|	554646	+	G	1	0	0	0	0	SArRNA06
gi|29165615|ref|NC_002745.2|	554647	+	A	0	0	1	1	1	SArRNA06
gi|29165615|ref|NC_002745.2|	554648	+	G	0	0	0	0	2	SArRNA06
gi|29165615|ref|NC_002745.2|	554649	+	C	1	3	0	1	5	SArRNA06
gi|29165615|ref|NC_002745.2|	554650	+	G	0	1	0	0	2	SArRNA06
gi|29165615|ref|NC_002745.2|	554651	+	C	2	7	3	1	6	SArRNA06
gi|29165615|ref|NC_002745.2|	554652	+	U	5	1	3	5	5	SArRNA06
gi|29165615|ref|NC_002745.2|	554653	+	A	3	4	0	0	2	SArRNA06
gi|29165615|ref|NC_002745.2|	554654	+	G	2	0	1	1	2	SArRNA06
gi|29165615|ref|NC_002745.2|	554655	+	C	6	6	1	1	2	SArRNA06
gi|29165615|ref|NC_002745.2|	554656	+	U	0	5	2	1	5	SArRNA06
gi|29165615|ref|NC_002745.2|	554657	+	U	1	1	0	0	2	SArRNA06
gi|29165615|ref|NC_002745.2|	554658	+	A	1	5	0	0	2	SArRNA06
gi|29165615|ref|NC_002745.2|	554659	+	C	0	1	1	0	1	SArRNA06
gi|29165615|ref|NC_002745.2|	554660	+	G	1	0	0	0	2	SArRNA06
gi|29165615|ref|NC_002745.2|	554661	+	U	0	2	0	2	1	SArRNA06
gi|29165615|ref|NC_002745.2|	554662	+	G	1	2	0	0	1	SArRNA06
gi|29165615|ref|NC_002745.2|	554663	+	G	1	1	1	0	2	SArRNA06
gi|29165615|ref|NC_002745.2|	554664	+	A	0	2	2	2	2	SArRNA06
gi|29165615|ref|NC_002745.2|	554665	+	G	2	1	1	1	6	SArRNA06
gi|29165615|ref|NC_002745.2|	554666	+	G	2	0	2	2	1	SArRNA06
gi|29165615|ref|NC_002745.2|	554667	+	C	1	1	1	1	5	SArRNA06
gi|29165615|ref|NC_002745.2|	554668	+	G	0	1	0	1	1	SArRNA06
gi|29165615|ref|NC_002745.2|	554669	+	C	4	7	2	2	7	SArRNA06
gi|29165615|ref|NC_002745.2|	554670	+	U	17	9	2	1	12	SArRNA06
gi|29165615|ref|NC_002745.2|	554671	+	G	1	3	1	0	4	SArRNA06
gi|29165615|ref|NC_002745.2|	554672	+	G	5	6	0	1	4	SArRNA06
gi|29165615|ref|NC_002745.2|	554673	+	U	3	8	0	1	4	SArRNA06
gi|29165615|ref|NC_002745.2|	554674	+	G	1	2	0	0	1	SArRNA06
gi|29165615|ref|NC_002745.2|	554675	+	G	0	2	1	0	1	SArRNA06
gi|29165615|ref|NC_002745.2|	554676	+	G	6	2	0	3	5	SArRNA06
gi|29165615|ref|NC_002745.2|	554677	+	A	3	6	2	0	3	SArRNA06
gi|29165615|ref|NC_002745.2|	554678	+	U	7	6	4	1	5	SArRNA06
gi|29165615|ref|NC_002745.2|	554679	+	A	0	2	1	0	3	SArRNA06
gi|29165615|ref|NC_002745.2|	554680	+	C	14	14	4	9	15	SArRNA06
gi|29165615|ref|NC_002745.2|	554681	+	U	53	28	14	13	40	SArRNA06
gi|29165615|ref|NC_002745.2|	554682	+	A	15	11	3	3	9	SArRNA06
gi|29165615|ref|NC_002745.2|	554683	+	C	10	12	6	2	3	SArRNA06
gi|29165615|ref|NC_002745.2|	554684	+	C	1	9	2	2	3	SArRNA06
gi|29165615|ref|NC_002745.2|	554685	+	C	5	18	9	5	11	SArRNA06
gi|29165615|ref|NC_002745.2|	554686	+	U	3	2	0	3	3	SArRNA06
gi|29165615|ref|NC_002745.2|	554687	+	A	2	5	0	2	2	SArRNA06
gi|29165615|ref|NC_002745.2|	554689	+	C	1	1	0	1	2	SArRNA06
gi|29165615|ref|NC_002745.2|	554690	+	U	1	0	0	0	0	SArRNA06
gi|29165615|ref|NC_002745.2|	554691	+	G	2	0	0	0	0	SArRNA06
gi|29165615|ref|NC_002745.2|	554692	+	U	0	1	1	0	5	SArRNA06
gi|29165615|ref|NC_002745.2|	554693	+	G	2	1	0	0	0	SArRNA06
gi|29165615|ref|NC_002745.2|	554694	+	U	6	3	0	1	3	SArRNA06
gi|29165615|ref|NC_002745.2|	554695	+	U	0	0	0	2	3	SArRNA06
gi|29165615|ref|NC_002745.2|	554696	+	G	1	0	0	0	0	SArRNA06
gi|29165615|ref|NC_002745.2|	554698	+	C	5	1	0	0	2	SArRNA06
gi|29165615|ref|NC_002745.2|	554699	+	U	5	1	3	1	0	SArRNA06
gi|29165615|ref|NC_002745.2|	554700	+	U	3	0	0	0	3	SArRNA06
gi|29165615|ref|NC_002745.2|	554701	+	U	3	0	1	1	4	SArRNA06
gi|29165615|ref|NC_002745.2|	554702	+	C	4	5	2	0	8	SArRNA06
gi|29165615|ref|NC_002745.2|	554703	+	U	0	2	0	1	5	SArRNA06
gi|29165615|ref|NC_002745.2|	554704	+	A	0	0	1	1	2	SArRNA06
gi|29165615|ref|NC_002745.2|	554705	+	A	0	1	0	1	2	SArRNA06
gi|29165615|ref|NC_002745.2|	554706	+	C	7	11	4	1	7	SArRNA06
gi|29165615|ref|NC_002745.2|	554707	+	C	3	1	1	1	1	SArRNA06
gi|29165615|ref|NC_002745.2|	554708	+	C	2	2	0	1	1	SArRNA06
gi|29165615|ref|NC_002745.2|	554709	+	G	0	2	0	0	1	SArRNA06
gi|29165615|ref|NC_002745.2|	554710	+	C	1	1	0	0	0	SArRNA06
gi|29165615|ref|NC_002745.2|	554711	+	A	1	0	0	0	0	SArRNA06
gi|29165615|ref|NC_002745.2|	554712	+	C	0	1	0	0	0	SArRNA06
gi|29165615|ref|NC_002745.2|	554713	+	C	5	3	0	0	1	SArRNA06
gi|29165615|ref|NC_002745.2|	554714	+	A	1	4	2	0	3	SArRNA06
gi|29165615|ref|NC_002745.2|	554715	+	C	6	2	0	3	1	SArRNA06
gi|29165615|ref|NC_002745.2|	554716	+	U	2	1	2	1	5	SArRNA06
gi|29165615|ref|NC_002745.2|	554717	+	U	2	6	0	0	4	SArRNA06
gi|29165615|ref|NC_002745.2|	554718	+	A	3	1	0	1	3	SArRNA06
gi|29165615|ref|NC_002745.2|	554719	+	U	2	2	2	0	3	SArRNA06
gi|29165615|ref|NC_002745.2|	554720	+	C	1	2	4	1	2	SArRNA06
gi|29165615|ref|NC_002745.2|	554721	+	G	0	2	1	1	3	SArRNA06
gi|29165615|ref|NC_002745.2|	554724	+	G	0	0	0	0	1	SArRNA06
gi|29165615|ref|NC_002745.2|	554725	+	U	0	0	0	0	1	SArRNA06
gi|29165615|ref|NC_002745.2|	554726	+	G	0	0	0	0	1	SArRNA06

gi|29165615|ref|NC_002745.2|	554727	+	G	0	0	1	1	0	SArRNA06
gi|29165615|ref|NC_002745.2|	554728	+	G	5	3	0	0	2	SArRNA06
gi|29165615|ref|NC_002745.2|	554729	+	A	3	5	4	1	2	SArRNA06
gi|29165615|ref|NC_002745.2|	554730	+	G	1	3	3	0	0	SArRNA06
gi|29165615|ref|NC_002745.2|	554731	+	A	4	5	2	2	3	SArRNA06
gi|29165615|ref|NC_002745.2|	554732	+	C	16	22	5	10	20	SArRNA06
gi|29165615|ref|NC_002745.2|	554733	+	A	5	9	2	5	1	SArRNA06
gi|29165615|ref|NC_002745.2|	554734	+	G	3	1	0	3	5	SArRNA06
gi|29165615|ref|NC_002745.2|	554735	+	U	0	0	0	4	2	SArRNA06
gi|29165615|ref|NC_002745.2|	554736	+	G	1	0	0	1	1	SArRNA06
gi|29165615|ref|NC_002745.2|	554737	+	U	4	1	0	1	6	SArRNA06
gi|29165615|ref|NC_002745.2|	554738	+	C	5	0	1	0	2	SArRNA06
gi|29165615|ref|NC_002745.2|	554739	+	A	0	1	1	1	1	SArRNA06
gi|29165615|ref|NC_002745.2|	554740	+	G	0	0	0	2	3	SArRNA06
gi|29165615|ref|NC_002745.2|	554741	+	G	0	2	0	1	0	SArRNA06
gi|29165615|ref|NC_002745.2|	554742	+	C	1	5	2	2	1	SArRNA06
gi|29165615|ref|NC_002745.2|	554743	+	G	4	1	0	0	3	SArRNA06
gi|29165615|ref|NC_002745.2|	554744	+	G	3	3	0	0	1	SArRNA06
gi|29165615|ref|NC_002745.2|	554745	+	G	2	1	0	0	3	SArRNA06
gi|29165615|ref|NC_002745.2|	554746	+	C	9	3	2	2	1	SArRNA06
gi|29165615|ref|NC_002745.2|	554747	+	A	0	3	1	1	2	SArRNA06
gi|29165615|ref|NC_002745.2|	554748	+	G	0	0	1	0	0	SArRNA06
gi|29165615|ref|NC_002745.2|	554749	+	U	3	1	0	3	3	SArRNA06
gi|29165615|ref|NC_002745.2|	554750	+	U	4	5	0	1	3	SArRNA06
gi|29165615|ref|NC_002745.2|	554751	+	U	1	3	1	0	4	SArRNA06
gi|29165615|ref|NC_002745.2|	554752	+	G	0	2	3	0	1	SArRNA06
gi|29165615|ref|NC_002745.2|	554753	+	A	0	2	0	1	0	SArRNA06
gi|29165615|ref|NC_002745.2|	554754	+	C	6	7	1	0	0	SArRNA06
gi|29165615|ref|NC_002745.2|	554755	+	U	5	1	2	1	2	SArRNA06
gi|29165615|ref|NC_002745.2|	554756	+	G	0	2	0	0	1	SArRNA06
gi|29165615|ref|NC_002745.2|	554757	+	G	1	0	0	0	0	SArRNA06
gi|29165615|ref|NC_002745.2|	554760	+	C	0	0	0	0	1	SArRNA06
gi|29165615|ref|NC_002745.2|	554763	+	U	1	0	0	0	0	SArRNA06
gi|29165615|ref|NC_002745.2|	554764	+	C	0	0	1	0	2	SArRNA06
gi|29165615|ref|NC_002745.2|	554766	+	C	1	1	0	0	0	SArRNA06
gi|29165615|ref|NC_002745.2|	554767	+	C	0	3	0	0	0	SArRNA06
gi|29165615|ref|NC_002745.2|	554768	+	U	0	1	0	1	0	SArRNA06
gi|29165615|ref|NC_002745.2|	554769	+	C	0	4	2	0	0	SArRNA06
gi|29165615|ref|NC_002745.2|	554770	+	C	4	0	1	1	3	SArRNA06
gi|29165615|ref|NC_002745.2|	554772	+	A	0	1	0	1	2	SArRNA06
gi|29165615|ref|NC_002745.2|	554773	+	A	0	0	1	0	0	SArRNA06
gi|29165615|ref|NC_002745.2|	554774	+	A	17	29	0	5	14	SArRNA06
gi|29165615|ref|NC_002745.2|	554775	+	A	4	7	1	1	7	SArRNA06
gi|29165615|ref|NC_002745.2|	554776	+	G	0	1	0	0	0	SArRNA06
gi|29165615|ref|NC_002745.2|	554777	+	G	0	1	0	0	1	SArRNA06
gi|29165615|ref|NC_002745.2|	554778	+	U	2	1	2	2	1	SArRNA06
gi|29165615|ref|NC_002745.2|	554779	+	A	0	0	0	0	1	SArRNA06
gi|29165615|ref|NC_002745.2|	554781	+	C	1	5	0	1	4	SArRNA06
gi|29165615|ref|NC_002745.2|	554782	+	G	0	1	2	0	0	SArRNA06
gi|29165615|ref|NC_002745.2|	554783	+	G	5	1	0	0	0	SArRNA06
gi|29165615|ref|NC_002745.2|	554784	+	A	1	1	1	0	1	SArRNA06
gi|29165615|ref|NC_002745.2|	554785	+	G	0	0	0	1	0	SArRNA06
gi|29165615|ref|NC_002745.2|	554786	+	G	5	1	2	0	0	SArRNA06
gi|29165615|ref|NC_002745.2|	554787	+	C	1	0	0	0	0	SArRNA06
gi|29165615|ref|NC_002745.2|	554789	+	C	3	1	0	0	2	SArRNA06
gi|29165615|ref|NC_002745.2|	554790	+	U	3	1	1	0	0	SArRNA06
gi|29165615|ref|NC_002745.2|	554791	+	C	0	2	0	2	0	SArRNA06
gi|29165615|ref|NC_002745.2|	554792	+	A	0	0	0	0	1	SArRNA06
gi|29165615|ref|NC_002745.2|	554793	+	A	0	0	0	0	1	SArRNA06
gi|29165615|ref|NC_002745.2|	554794	+	A	2	0	0	0	0	SArRNA06
gi|29165615|ref|NC_002745.2|	554795	+	G	0	1	0	0	0	SArRNA06
gi|29165615|ref|NC_002745.2|	554796	+	G	0	1	0	0	1	SArRNA06
gi|29165615|ref|NC_002745.2|	554797	+	U	0	3	0	1	0	SArRNA06
gi|29165615|ref|NC_002745.2|	554798	+	U	1	2	0	0	9	SArRNA06
gi|29165615|ref|NC_002745.2|	554799	+	C	6	2	2	1	1	SArRNA06
gi|29165615|ref|NC_002745.2|	554800	+	C	1	6	2	0	1	SArRNA06
gi|29165615|ref|NC_002745.2|	554801	+	C	3	1	0	2	2	SArRNA06
gi|29165615|ref|NC_002745.2|	554802	+	U	0	0	1	0	0	SArRNA06
gi|29165615|ref|NC_002745.2|	554803	+	C	13	2	5	3	6	SArRNA06
gi|29165615|ref|NC_002745.2|	554804	+	A	1	1	0	0	1	SArRNA06
gi|29165615|ref|NC_002745.2|	554806	+	A	3	3	0	1	3	SArRNA06
gi|29165615|ref|NC_002745.2|	554807	+	A	3	7	4	3	5	SArRNA06
gi|29165615|ref|NC_002745.2|	554808	+	U	3	2	2	1	6	SArRNA06
gi|29165615|ref|NC_002745.2|	554809	+	G	4	6	0	2	5	SArRNA06
gi|29165615|ref|NC_002745.2|	554810	+	G	3	13	2	0	8	SArRNA06
gi|29165615|ref|NC_002745.2|	554811	+	U	3	2	2	4	7	SArRNA06
gi|29165615|ref|NC_002745.2|	554812	+	U	0	3	1	1	3	SArRNA06
gi|29165615|ref|NC_002745.2|	554813	+	G	6	4	4	2	6	SArRNA06
gi|29165615|ref|NC_002745.2|	554814	+	G	0	2	1	0	1	SArRNA06
gi|29165615|ref|NC_002745.2|	554815	+	A	0	2	0	0	1	SArRNA06
gi|29165615|ref|NC_002745.2|	554816	+	A	1	4	1	0	1	SArRNA06

gi|29165615|ref|NC_002745.2|	554817	+	A	9	70	8	4	20	SArRNA06
gi|29165615|ref|NC_002745.2|	554818	+	U	0	2	1	0	1	SArRNA06
gi|29165615|ref|NC_002745.2|	554819	+	C	2	4	1	4	3	SArRNA06
gi|29165615|ref|NC_002745.2|	554820	+	A	0	4	4	1	4	SArRNA06
gi|29165615|ref|NC_002745.2|	554821	+	U	0	1	1	0	4	SArRNA06
gi|29165615|ref|NC_002745.2|	554822	+	U	1	3	4	3	3	SArRNA06
gi|29165615|ref|NC_002745.2|	554823	+	C	5	5	2	0	4	SArRNA06
gi|29165615|ref|NC_002745.2|	554824	+	A	2	5	5	1	3	SArRNA06
gi|29165615|ref|NC_002745.2|	554825	+	U	3	4	2	2	7	SArRNA06
gi|29165615|ref|NC_002745.2|	554826	+	A	7	4	3	2	6	SArRNA06
gi|29165615|ref|NC_002745.2|	554827	+	G	1	2	5	2	5	SArRNA06
gi|29165615|ref|NC_002745.2|	554828	+	A	1	3	0	0	2	SArRNA06
gi|29165615|ref|NC_002745.2|	554829	+	G	3	2	1	2	7	SArRNA06
gi|29165615|ref|NC_002745.2|	554831	+	G	4	3	0	1	3	SArRNA06
gi|29165615|ref|NC_002745.2|	554832	+	U	0	4	0	0	3	SArRNA06
gi|29165615|ref|NC_002745.2|	554833	+	A	1	1	3	0	1	SArRNA06
gi|29165615|ref|NC_002745.2|	554834	+	A	4	6	0	3	4	SArRNA06
gi|29165615|ref|NC_002745.2|	554835	+	A	5	0	1	0	1	SArRNA06
gi|29165615|ref|NC_002745.2|	554836	+	G	1	5	2	0	1	SArRNA06
gi|29165615|ref|NC_002745.2|	554837	+	G	2	3	1	2	6	SArRNA06
gi|29165615|ref|NC_002745.2|	554838	+	C	1	2	0	0	1	SArRNA06
gi|29165615|ref|NC_002745.2|	554839	+	A	4	1	4	3	5	SArRNA06
gi|29165615|ref|NC_002745.2|	554840	+	U	6	7	4	4	12	SArRNA06
gi|29165615|ref|NC_002745.2|	554841	+	A	0	1	1	0	4	SArRNA06
gi|29165615|ref|NC_002745.2|	554842	+	A	0	1	0	0	0	SArRNA06
gi|29165615|ref|NC_002745.2|	554844	+	G	0	0	0	0	1	SArRNA06
gi|29165615|ref|NC_002745.2|	554845	+	G	0	1	1	0	0	SArRNA06
gi|29165615|ref|NC_002745.2|	554848	+	C	1	1	0	0	0	SArRNA06
gi|29165615|ref|NC_002745.2|	554854	+	U	6	0	2	2	4	SArRNA06
gi|29165615|ref|NC_002745.2|	554855	+	G	0	1	0	0	0	SArRNA06
gi|29165615|ref|NC_002745.2|	554856	+	C	0	3	0	1	0	SArRNA06
gi|29165615|ref|NC_002745.2|	554857	+	G	0	1	0	1	0	SArRNA06
gi|29165615|ref|NC_002745.2|	554858	+	A	0	2	0	0	0	SArRNA06
gi|29165615|ref|NC_002745.2|	554859	+	G	0	0	0	0	2	SArRNA06
gi|29165615|ref|NC_002745.2|	554861	+	C	1	0	0	0	0	SArRNA06
gi|29165615|ref|NC_002745.2|	554863	+	U	0	1	0	0	1	SArRNA06
gi|29165615|ref|NC_002745.2|	554864	+	A	1	0	0	0	0	SArRNA06
gi|29165615|ref|NC_002745.2|	554865	+	C	7	9	0	6	5	SArRNA06
gi|29165615|ref|NC_002745.2|	554866	+	A	3	4	3	0	1	SArRNA06
gi|29165615|ref|NC_002745.2|	554867	+	A	1	0	0	0	0	SArRNA06
gi|29165615|ref|NC_002745.2|	554868	+	G	1	0	0	0	1	SArRNA06
gi|29165615|ref|NC_002745.2|	554869	+	U	0	0	0	0	2	SArRNA06
gi|29165615|ref|NC_002745.2|	554870	+	C	3	2	1	0	5	SArRNA06
gi|29165615|ref|NC_002745.2|	554872	+	A	0	0	1	0	1	SArRNA06
gi|29165615|ref|NC_002745.2|	554873	+	G	0	0	0	0	2	SArRNA06
gi|29165615|ref|NC_002745.2|	554875	+	A	0	0	1	0	2	SArRNA06
gi|29165615|ref|NC_002745.2|	554882	+	A	0	0	0	0	1	SArRNA06
gi|29165615|ref|NC_002745.2|	554883	+	A	0	2	0	0	1	SArRNA06
gi|29165615|ref|NC_002745.2|	554886	+	A	1	0	0	0	0	SArRNA06
gi|29165615|ref|NC_002745.2|	554887	+	C	1	0	0	0	0	SArRNA06
gi|29165615|ref|NC_002745.2|	554891	+	C	1	2	0	0	0	SArRNA06
gi|29165615|ref|NC_002745.2|	554894	+	A	1	0	0	0	0	SArRNA06
gi|29165615|ref|NC_002745.2|	554897	+	G	1	1	0	0	0	SArRNA06
gi|29165615|ref|NC_002745.2|	554900	+	C	0	1	0	2	1	SArRNA06
gi|29165615|ref|NC_002745.2|	554901	+	C	0	0	1	0	0	SArRNA06
gi|29165615|ref|NC_002745.2|	554913	+	A	0	0	0	0	2	SArRNA06
gi|29165615|ref|NC_002745.2|	554917	+	A	0	0	0	0	1	SArRNA06
gi|29165615|ref|NC_002745.2|	554918	+	A	0	0	0	0	1	SArRNA06
gi|29165615|ref|NC_002745.2|	554924	+	A	0	0	0	0	1	SArRNA06
gi|29165615|ref|NC_002745.2|	554926	+	C	1	1	0	0	0	SArRNA06
gi|29165615|ref|NC_002745.2|	554928	+	C	0	0	0	0	1	SArRNA06
gi|29165615|ref|NC_002745.2|	554930	+	C	0	1	0	0	0	SArRNA06
gi|29165615|ref|NC_002745.2|	554931	+	A	2	1	0	0	0	SArRNA06
gi|29165615|ref|NC_002745.2|	554932	+	A	0	1	0	0	0	SArRNA06
gi|29165615|ref|NC_002745.2|	554933	+	C	0	1	0	0	0	SArRNA06
gi|29165615|ref|NC_002745.2|	554935	+	G	0	0	0	1	0	SArRNA06
gi|29165615|ref|NC_002745.2|	554937	+	U	1	0	0	1	0	SArRNA06
gi|29165615|ref|NC_002745.2|	554943	+	C	0	1	0	0	0	SArRNA06
gi|29165615|ref|NC_002745.2|	554945	+	A	0	0	0	0	1	SArRNA06
gi|29165615|ref|NC_002745.2|	554946	+	C	1	2	0	0	0	SArRNA06
gi|29165615|ref|NC_002745.2|	554947	+	C	0	1	1	0	0	SArRNA06
gi|29165615|ref|NC_002745.2|	554948	+	C	1	0	0	0	0	SArRNA06
gi|29165615|ref|NC_002745.2|	554949	+	C	0	1	1	0	0	SArRNA06
gi|29165615|ref|NC_002745.2|	554950	+	G	2	1	0	0	0	SArRNA06
gi|29165615|ref|NC_002745.2|	554954	+	A	0	0	0	1	0	SArRNA06
gi|29165615|ref|NC_002745.2|	554957	+	A	1	0	0	0	0	SArRNA06
gi|29165615|ref|NC_002745.2|	554958	+	C	0	1	0	2	2	SArRNA06
gi|29165615|ref|NC_002745.2|	554959	+	A	0	1	0	0	0	SArRNA06
gi|29165615|ref|NC_002745.2|	554966	+	U	0	1	0	0	0	SArRNA06
gi|29165615|ref|NC_002745.2|	554967	+	C	5	1	0	0	0	SArRNA06
gi|29165615|ref|NC_002745.2|	554968	+	U	0	1	1	0	0	SArRNA06

gi|29165615|ref|NC_002745.2|	554969	+	C	0	3	2	1	2	SArRNA06
gi|29165615|ref|NC_002745.2|	554970	+	C	12	4	4	2	4	SArRNA06
gi|29165615|ref|NC_002745.2|	554971	+	C	3	1	1	0	0	SArRNA06
gi|29165615|ref|NC_002745.2|	554972	+	C	0	1	1	0	0	SArRNA06
gi|29165615|ref|NC_002745.2|	554973	+	C	0	0	0	1	0	SArRNA06
gi|29165615|ref|NC_002745.2|	554975	+	A	0	0	0	1	1	SArRNA06
gi|29165615|ref|NC_002745.2|	554977	+	A	0	2	0	0	2	SArRNA06
gi|29165615|ref|NC_002745.2|	554980	+	U	0	1	0	0	0	SArRNA06
gi|29165615|ref|NC_002745.2|	554981	+	C	8	17	4	1	14	SArRNA06
gi|29165615|ref|NC_002745.2|	554982	+	A	5	6	0	4	1	SArRNA06
gi|29165615|ref|NC_002745.2|	554983	+	C	11	17	8	7	9	SArRNA06
gi|29165615|ref|NC_002745.2|	554984	+	A	8	12	0	1	6	SArRNA06
gi|29165615|ref|NC_002745.2|	554985	+	U	0	0	0	1	0	SArRNA06
gi|29165615|ref|NC_002745.2|	554986	+	C	0	1	1	2	2	SArRNA06
gi|29165615|ref|NC_002745.2|	554987	+	G	1	0	0	0	0	SArRNA06
gi|29165615|ref|NC_002745.2|	554988	+	A	0	0	0	1	0	SArRNA06
gi|29165615|ref|NC_002745.2|	554989	+	C	3	2	0	0	1	SArRNA06
gi|29165615|ref|NC_002745.2|	554990	+	G	0	1	0	0	0	SArRNA06
gi|29165615|ref|NC_002745.2|	554991	+	G	0	0	1	0	0	SArRNA06
gi|29165615|ref|NC_002745.2|	554995	+	G	1	0	0	0	0	SArRNA06
gi|29165615|ref|NC_002745.2|	554996	+	G	1	0	0	0	1	SArRNA06
gi|29165615|ref|NC_002745.2|	554997	+	U	0	0	0	0	1	SArRNA06
gi|29165615|ref|NC_002745.2|	554998	+	U	2	16	1	2	5	SArRNA06
gi|29165615|ref|NC_002745.2|	554999	+	U	56	109	5	2	58	SArRNA06
gi|29165615|ref|NC_002745.2|	555002	+	C	12	22	2	7	28	SArRNA06
gi|29165615|ref|NC_002745.2|	555003	+	A	6	5	0	1	3	SArRNA06
gi|29165615|ref|NC_002745.2|	555004	+	C	3	4	2	1	3	SArRNA06
gi|29165615|ref|NC_002745.2|	555005	+	C	2	7	2	3	4	SArRNA06
gi|29165615|ref|NC_002745.2|	555006	+	U	3	2	1	0	4	SArRNA06
gi|29165615|ref|NC_002745.2|	555007	+	C	5	18	1	6	18	SArRNA06
gi|29165615|ref|NC_002745.2|	555008	+	G	6	30	5	4	12	SArRNA06
gi|29165615|ref|NC_002745.2|	555009	+	A	1	0	0	1	0	SArRNA06
gi|29165615|ref|NC_002745.2|	555010	+	U	0	0	0	0	1	SArRNA06
gi|29165615|ref|NC_002745.2|	555011	+	G	2	0	0	0	0	SArRNA06
gi|29165615|ref|NC_002745.2|	555012	+	U	0	1	0	0	3	SArRNA06
gi|29165615|ref|NC_002745.2|	555013	+	C	5	9	5	4	2	SArRNA06
gi|29165615|ref|NC_002745.2|	555014	+	G	1	2	0	0	1	SArRNA06
gi|29165615|ref|NC_002745.2|	555015	+	G	0	0	0	1	1	SArRNA06
gi|29165615|ref|NC_002745.2|	555016	+	C	0	4	3	1	1	SArRNA06
gi|29165615|ref|NC_002745.2|	555017	+	U	2	3	1	1	7	SArRNA06
gi|29165615|ref|NC_002745.2|	555018	+	C	39	40	4	15	40	SArRNA06
gi|29165615|ref|NC_002745.2|	555019	+	A	21	29	4	7	14	SArRNA06
gi|29165615|ref|NC_002745.2|	555020	+	U	8	2	3	3	9	SArRNA06
gi|29165615|ref|NC_002745.2|	555021	+	C	10	16	6	3	9	SArRNA06
gi|29165615|ref|NC_002745.2|	555022	+	G	5	5	2	2	9	SArRNA06
gi|29165615|ref|NC_002745.2|	555023	+	C	23	25	12	8	15	SArRNA06
gi|29165615|ref|NC_002745.2|	555024	+	A	8	16	7	5	17	SArRNA06
gi|29165615|ref|NC_002745.2|	555025	+	U	27	9	6	8	14	SArRNA06
gi|29165615|ref|NC_002745.2|	555026	+	C	49	12	8	7	26	SArRNA06
gi|29165615|ref|NC_002745.2|	555027	+	C	59	19	14	10	20	SArRNA06
gi|29165615|ref|NC_002745.2|	555028	+	U	26	16	9	10	13	SArRNA06
gi|29165615|ref|NC_002745.2|	555029	+	G	0	1	0	0	1	SArRNA06
gi|29165615|ref|NC_002745.2|	555030	+	G	4	5	4	3	3	SArRNA06
gi|29165615|ref|NC_002745.2|	555031	+	G	32	10	5	11	15	SArRNA06
gi|29165615|ref|NC_002745.2|	555032	+	G	3	4	1	1	1	SArRNA06
gi|29165615|ref|NC_002745.2|	555033	+	C	3	8	2	4	5	SArRNA06
gi|29165615|ref|NC_002745.2|	555034	+	U	8	3	4	4	17	SArRNA06
gi|29165615|ref|NC_002745.2|	555035	+	G	10	13	3	3	10	SArRNA06
gi|29165615|ref|NC_002745.2|	555036	+	U	6	4	5	4	12	SArRNA06
gi|29165615|ref|NC_002745.2|	555037	+	A	6	1	1	3	3	SArRNA06
gi|29165615|ref|NC_002745.2|	555038	+	G	3	8	1	2	6	SArRNA06
gi|29165615|ref|NC_002745.2|	555039	+	U	31	50	6	14	34	SArRNA06
gi|29165615|ref|NC_002745.2|	555040	+	C	13	22	6	5	19	SArRNA06
gi|29165615|ref|NC_002745.2|	555041	+	G	21	19	14	6	9	SArRNA06
gi|29165615|ref|NC_002745.2|	555042	+	G	21	21	8	11	17	SArRNA06
gi|29165615|ref|NC_002745.2|	555043	+	U	26	17	10	5	9	SArRNA06
gi|29165615|ref|NC_002745.2|	555044	+	C	21	28	5	9	17	SArRNA06
gi|29165615|ref|NC_002745.2|	555045	+	C	28	26	10	9	23	SArRNA06
gi|29165615|ref|NC_002745.2|	555046	+	C	24	15	10	8	17	SArRNA06
gi|29165615|ref|NC_002745.2|	555047	+	A	6	7	1	1	6	SArRNA06
gi|29165615|ref|NC_002745.2|	555048	+	A	6	6	0	6	10	SArRNA06
gi|29165615|ref|NC_002745.2|	555049	+	G	4	3	1	2	0	SArRNA06
gi|29165615|ref|NC_002745.2|	555050	+	G	12	3	2	6	13	SArRNA06
gi|29165615|ref|NC_002745.2|	555051	+	G	10	10	5	3	9	SArRNA06
gi|29165615|ref|NC_002745.2|	555052	+	U	13	3	2	3	3	SArRNA06
gi|29165615|ref|NC_002745.2|	555053	+	U	2	0	1	1	5	SArRNA06
gi|29165615|ref|NC_002745.2|	555054	+	G	2	3	3	0	7	SArRNA06
gi|29165615|ref|NC_002745.2|	555055	+	G	1	2	0	1	2	SArRNA06
gi|29165615|ref|NC_002745.2|	555056	+	G	1	2	0	0	3	SArRNA06
gi|29165615|ref|NC_002745.2|	555057	+	C	7	0	0	1	3	SArRNA06
gi|29165615|ref|NC_002745.2|	555058	+	U	12	2	1	0	0	SArRNA06

gi|29165615|ref|NC_002745.2|	555059	+	G	24	0	7	1	3	SArRNA06
gi|29165615|ref|NC_002745.2|	555060	+	U	1	0	0	0	0	SArRNA06
gi|29165615|ref|NC_002745.2|	555061	+	U	0	3	0	0	2	SArRNA06
gi|29165615|ref|NC_002745.2|	555062	+	C	106	105	44	53	73	SArRNA06
gi|29165615|ref|NC_002745.2|	555063	+	G	14	28	3	11	28	SArRNA06
gi|29165615|ref|NC_002745.2|	555064	+	C	18	13	2	4	13	SArRNA06
gi|29165615|ref|NC_002745.2|	555065	+	C	5	10	2	1	7	SArRNA06
gi|29165615|ref|NC_002745.2|	555066	+	C	1	8	4	0	9	SArRNA06
gi|29165615|ref|NC_002745.2|	555067	+	A	5	3	2	1	9	SArRNA06
gi|29165615|ref|NC_002745.2|	555068	+	U	9	2	5	4	11	SArRNA06
gi|29165615|ref|NC_002745.2|	555069	+	U	3	6	0	3	7	SArRNA06
gi|29165615|ref|NC_002745.2|	555070	+	A	9	6	2	3	4	SArRNA06
gi|29165615|ref|NC_002745.2|	555071	+	A	6	6	2	2	9	SArRNA06
gi|29165615|ref|NC_002745.2|	555072	+	A	4	15	0	2	10	SArRNA06
gi|29165615|ref|NC_002745.2|	555073	+	G	7	8	3	0	3	SArRNA06
gi|29165615|ref|NC_002745.2|	555074	+	C	16	7	3	2	10	SArRNA06
gi|29165615|ref|NC_002745.2|	555075	+	G	7	4	2	3	1	SArRNA06
gi|29165615|ref|NC_002745.2|	555076	+	G	3	10	0	1	4	SArRNA06
gi|29165615|ref|NC_002745.2|	555077	+	U	29	38	14	9	21	SArRNA06
gi|29165615|ref|NC_002745.2|	555078	+	A	2	4	1	1	1	SArRNA06
gi|29165615|ref|NC_002745.2|	555079	+	C	4	4	1	3	2	SArRNA06
gi|29165615|ref|NC_002745.2|	555080	+	G	2	5	2	2	3	SArRNA06
gi|29165615|ref|NC_002745.2|	555081	+	C	17	18	6	3	11	SArRNA06
gi|29165615|ref|NC_002745.2|	555082	+	G	8	12	4	1	7	SArRNA06
gi|29165615|ref|NC_002745.2|	555083	+	A	7	3	2	2	11	SArRNA06
gi|29165615|ref|NC_002745.2|	555084	+	G	1	1	2	1	3	SArRNA06
gi|29165615|ref|NC_002745.2|	555085	+	C	7	13	0	3	10	SArRNA06
gi|29165615|ref|NC_002745.2|	555086	+	U	2	16	3	1	12	SArRNA06
gi|29165615|ref|NC_002745.2|	555087	+	G	1	0	0	1	2	SArRNA06
gi|29165615|ref|NC_002745.2|	555088	+	G	1	1	0	0	0	SArRNA06
gi|29165615|ref|NC_002745.2|	555089	+	G	0	1	0	1	1	SArRNA06
gi|29165615|ref|NC_002745.2|	555090	+	U	2	0	0	0	2	SArRNA06
gi|29165615|ref|NC_002745.2|	555091	+	U	0	2	0	0	6	SArRNA06
gi|29165615|ref|NC_002745.2|	555092	+	C	15	29	8	12	22	SArRNA06
gi|29165615|ref|NC_002745.2|	555093	+	A	1	8	2	0	6	SArRNA06
gi|29165615|ref|NC_002745.2|	555095	+	A	1	3	0	0	0	SArRNA06
gi|29165615|ref|NC_002745.2|	555096	+	A	2	1	0	0	0	SArRNA06
gi|29165615|ref|NC_002745.2|	555097	+	C	6	17	1	4	16	SArRNA06
gi|29165615|ref|NC_002745.2|	555098	+	G	6	8	3	2	3	SArRNA06
gi|29165615|ref|NC_002745.2|	555099	+	U	1	2	2	1	1	SArRNA06
gi|29165615|ref|NC_002745.2|	555100	+	C	1	9	4	1	4	SArRNA06
gi|29165615|ref|NC_002745.2|	555101	+	G	6	1	2	3	1	SArRNA06
gi|29165615|ref|NC_002745.2|	555102	+	U	3	6	2	2	10	SArRNA06
gi|29165615|ref|NC_002745.2|	555103	+	G	1	3	1	2	0	SArRNA06
gi|29165615|ref|NC_002745.2|	555104	+	A	1	2	1	0	2	SArRNA06
gi|29165615|ref|NC_002745.2|	555106	+	A	4	2	1	2	3	SArRNA06
gi|29165615|ref|NC_002745.2|	555107	+	C	4	4	0	4	0	SArRNA06
gi|29165615|ref|NC_002745.2|	555108	+	A	2	7	2	3	1	SArRNA06
gi|29165615|ref|NC_002745.2|	555109	+	G	0	3	0	0	2	SArRNA06
gi|29165615|ref|NC_002745.2|	555110	+	U	2	0	0	0	0	SArRNA06
gi|29165615|ref|NC_002745.2|	555111	+	U	0	1	0	0	3	SArRNA06
gi|29165615|ref|NC_002745.2|	555112	+	C	4	3	0	0	7	SArRNA06
gi|29165615|ref|NC_002745.2|	555113	+	G	1	2	0	0	1	SArRNA06
gi|29165615|ref|NC_002745.2|	555114	+	G	2	1	0	0	0	SArRNA06
gi|29165615|ref|NC_002745.2|	555115	+	U	0	0	0	0	1	SArRNA06
gi|29165615|ref|NC_002745.2|	555116	+	C	3	9	3	2	6	SArRNA06
gi|29165615|ref|NC_002745.2|	555117	+	C	1	11	0	1	6	SArRNA06
gi|29165615|ref|NC_002745.2|	555118	+	C	6	17	0	1	4	SArRNA06
gi|29165615|ref|NC_002745.2|	555119	+	U	8	25	1	0	5	SArRNA06
gi|29165615|ref|NC_002745.2|	555120	+	A	4	17	2	2	7	SArRNA06
gi|29165615|ref|NC_002745.2|	555121	+	U	1	0	2	1	2	SArRNA06
gi|29165615|ref|NC_002745.2|	555122	+	C	2	8	1	0	5	SArRNA06
gi|29165615|ref|NC_002745.2|	555123	+	C	11	10	2	8	11	SArRNA06
gi|29165615|ref|NC_002745.2|	555124	+	G	2	15	3	4	5	SArRNA06
gi|29165615|ref|NC_002745.2|	555125	+	U	8	1	0	0	4	SArRNA06
gi|29165615|ref|NC_002745.2|	555126	+	C	3	3	2	1	3	SArRNA06
gi|29165615|ref|NC_002745.2|	555127	+	G	0	2	1	0	0	SArRNA06
gi|29165615|ref|NC_002745.2|	555128	+	U	6	2	2	0	1	SArRNA06
gi|29165615|ref|NC_002745.2|	555129	+	G	0	1	0	0	2	SArRNA06
gi|29165615|ref|NC_002745.2|	555130	+	G	1	2	0	0	1	SArRNA06
gi|29165615|ref|NC_002745.2|	555131	+	G	0	1	0	0	0	SArRNA06
gi|29165615|ref|NC_002745.2|	555132	+	C	6	4	0	1	2	SArRNA06
gi|29165615|ref|NC_002745.2|	555133	+	G	6	5	2	1	3	SArRNA06
gi|29165615|ref|NC_002745.2|	555134	+	U	1	11	2	6	6	SArRNA06
gi|29165615|ref|NC_002745.2|	555135	+	A	1	1	0	0	2	SArRNA06
gi|29165615|ref|NC_002745.2|	555136	+	G	0	2	0	0	1	SArRNA06
gi|29165615|ref|NC_002745.2|	555137	+	G	1	1	1	0	1	SArRNA06
gi|29165615|ref|NC_002745.2|	555138	+	A	0	2	0	3	1	SArRNA06
gi|29165615|ref|NC_002745.2|	555139	+	A	10	6	2	2	6	SArRNA06
gi|29165615|ref|NC_002745.2|	555140	+	A	13	64	3	6	24	SArRNA06
gi|29165615|ref|NC_002745.2|	555141	+	U	10	9	3	3	6	SArRNA06

gi|29165615|ref|NC_002745.2|	555142	+	U	10	5	1	3	12	SArRNA06
gi|29165615|ref|NC_002745.2|	555143	+	U	7	8	3	3	7	SArRNA06
gi|29165615|ref|NC_002745.2|	555144	+	G	12	7	2	4	9	SArRNA06
gi|29165615|ref|NC_002745.2|	555145	+	A	0	2	1	0	1	SArRNA06
gi|29165615|ref|NC_002745.2|	555146	+	G	2	3	2	2	0	SArRNA06
gi|29165615|ref|NC_002745.2|	555147	+	A	3	2	1	3	10	SArRNA06
gi|29165615|ref|NC_002745.2|	555148	+	G	1	2	1	0	1	SArRNA06
gi|29165615|ref|NC_002745.2|	555149	+	G	5	1	2	4	6	SArRNA06
gi|29165615|ref|NC_002745.2|	555150	+	A	6	3	1	5	7	SArRNA06
gi|29165615|ref|NC_002745.2|	555151	+	G	5	4	1	0	1	SArRNA06
gi|29165615|ref|NC_002745.2|	555152	+	C	1	1	2	2	3	SArRNA06
gi|29165615|ref|NC_002745.2|	555153	+	U	2	1	1	0	4	SArRNA06
gi|29165615|ref|NC_002745.2|	555154	+	G	0	2	0	0	1	SArRNA06
gi|29165615|ref|NC_002745.2|	555155	+	U	3	3	0	1	2	SArRNA06
gi|29165615|ref|NC_002745.2|	555156	+	C	6	1	4	0	4	SArRNA06
gi|29165615|ref|NC_002745.2|	555157	+	C	4	4	0	1	6	SArRNA06
gi|29165615|ref|NC_002745.2|	555158	+	U	4	4	4	1	3	SArRNA06
gi|29165615|ref|NC_002745.2|	555159	+	U	7	8	3	4	0	SArRNA06
gi|29165615|ref|NC_002745.2|	555160	+	A	2	4	1	1	3	SArRNA06
gi|29165615|ref|NC_002745.2|	555161	+	G	2	3	1	0	4	SArRNA06
gi|29165615|ref|NC_002745.2|	555162	+	U	1	14	3	1	3	SArRNA06
gi|29165615|ref|NC_002745.2|	555163	+	A	8	9	1	0	1	SArRNA06
gi|29165615|ref|NC_002745.2|	555164	+	C	7	11	2	2	3	SArRNA06
gi|29165615|ref|NC_002745.2|	555165	+	G	2	9	2	2	8	SArRNA06
gi|29165615|ref|NC_002745.2|	555166	+	A	2	1	1	1	1	SArRNA06
gi|29165615|ref|NC_002745.2|	555167	+	G	0	0	0	0	2	SArRNA06
gi|29165615|ref|NC_002745.2|	555168	+	A	2	4	0	2	1	SArRNA06
gi|29165615|ref|NC_002745.2|	555169	+	G	4	2	0	0	1	SArRNA06
gi|29165615|ref|NC_002745.2|	555170	+	G	0	0	0	1	2	SArRNA06
gi|29165615|ref|NC_002745.2|	555171	+	A	3	1	0	1	0	SArRNA06
gi|29165615|ref|NC_002745.2|	555172	+	C	3	9	0	0	3	SArRNA06
gi|29165615|ref|NC_002745.2|	555173	+	C	9	10	3	6	15	SArRNA06
gi|29165615|ref|NC_002745.2|	555174	+	G	2	5	1	0	4	SArRNA06
gi|29165615|ref|NC_002745.2|	555175	+	G	1	2	0	2	1	SArRNA06
gi|29165615|ref|NC_002745.2|	555176	+	G	6	0	0	4	8	SArRNA06
gi|29165615|ref|NC_002745.2|	555177	+	A	14	9	2	3	5	SArRNA06
gi|29165615|ref|NC_002745.2|	555178	+	U	12	11	1	0	4	SArRNA06
gi|29165615|ref|NC_002745.2|	555179	+	G	4	0	0	2	2	SArRNA06
gi|29165615|ref|NC_002745.2|	555180	+	G	14	1	0	0	3	SArRNA06
gi|29165615|ref|NC_002745.2|	555181	+	A	23	2	3	1	7	SArRNA06
gi|29165615|ref|NC_002745.2|	555182	+	C	3	9	4	3	6	SArRNA06
gi|29165615|ref|NC_002745.2|	555183	+	A	4	21	3	7	12	SArRNA06
gi|29165615|ref|NC_002745.2|	555184	+	U	9	19	8	7	25	SArRNA06
gi|29165615|ref|NC_002745.2|	555185	+	A	13	10	1	4	10	SArRNA06
gi|29165615|ref|NC_002745.2|	555186	+	C	11	6	4	2	8	SArRNA06
gi|29165615|ref|NC_002745.2|	555187	+	C	8	5	5	1	7	SArRNA06
gi|29165615|ref|NC_002745.2|	555188	+	U	4	1	5	4	6	SArRNA06
gi|29165615|ref|NC_002745.2|	555189	+	C	8	1	2	4	6	SArRNA06
gi|29165615|ref|NC_002745.2|	555190	+	U	3	3	0	1	7	SArRNA06
gi|29165615|ref|NC_002745.2|	555191	+	G	1	5	3	2	2	SArRNA06
gi|29165615|ref|NC_002745.2|	555192	+	G	2	3	3	6	6	SArRNA06
gi|29165615|ref|NC_002745.2|	555193	+	U	8	9	3	1	12	SArRNA06
gi|29165615|ref|NC_002745.2|	555194	+	G	7	9	2	5	5	SArRNA06
gi|29165615|ref|NC_002745.2|	555195	+	U	5	7	3	3	12	SArRNA06
gi|29165615|ref|NC_002745.2|	555196	+	A	8	7	5	3	10	SArRNA06
gi|29165615|ref|NC_002745.2|	555197	+	C	12	12	4	4	11	SArRNA06
gi|29165615|ref|NC_002745.2|	555198	+	C	312	108	47	55	199	SArRNA06
gi|29165615|ref|NC_002745.2|	555199	+	A	26	5	4	9	33	SArRNA06
gi|29165615|ref|NC_002745.2|	555200	+	G	4	1	0	3	11	SArRNA06
gi|29165615|ref|NC_002745.2|	555201	+	U	6	0	3	1	9	SArRNA06
gi|29165615|ref|NC_002745.2|	555202	+	U	4	5	1	0	4	SArRNA06
gi|29165615|ref|NC_002745.2|	555203	+	G	1	3	1	0	8	SArRNA06
gi|29165615|ref|NC_002745.2|	555204	+	U	1	4	0	0	7	SArRNA06
gi|29165615|ref|NC_002745.2|	555205	+	C	4	2	1	1	0	SArRNA06
gi|29165615|ref|NC_002745.2|	555206	+	G	0	2	0	1	1	SArRNA06
gi|29165615|ref|NC_002745.2|	555207	+	U	0	0	0	0	1	SArRNA06
gi|29165615|ref|NC_002745.2|	555209	+	C	1	6	0	0	2	SArRNA06
gi|29165615|ref|NC_002745.2|	555210	+	C	5	10	0	3	2	SArRNA06
gi|29165615|ref|NC_002745.2|	555211	+	A	3	18	0	0	8	SArRNA06
gi|29165615|ref|NC_002745.2|	555212	+	A	1	25	1	0	12	SArRNA06
gi|29165615|ref|NC_002745.2|	555213	+	C	2	7	4	1	1	SArRNA06
gi|29165615|ref|NC_002745.2|	555214	+	G	1	8	0	1	3	SArRNA06
gi|29165615|ref|NC_002745.2|	555216	+	C	0	4	0	3	1	SArRNA06
gi|29165615|ref|NC_002745.2|	555217	+	A	1	5	0	1	3	SArRNA06
gi|29165615|ref|NC_002745.2|	555218	+	U	1	1	0	0	3	SArRNA06
gi|29165615|ref|NC_002745.2|	555219	+	A	4	1	0	0	2	SArRNA06
gi|29165615|ref|NC_002745.2|	555220	+	G	1	2	1	0	1	SArRNA06
gi|29165615|ref|NC_002745.2|	555221	+	C	2	4	1	0	1	SArRNA06
gi|29165615|ref|NC_002745.2|	555222	+	U	3	3	3	0	1	SArRNA06
gi|29165615|ref|NC_002745.2|	555223	+	G	0	0	0	1	0	SArRNA06
gi|29165615|ref|NC_002745.2|	555225	+	G	0	1	0	0	3	SArRNA06

gi|29165615|ref|NC_002745.2|	555226	+	U	6	4	0	0	4	SArRNA06
gi|29165615|ref|NC_002745.2|	555227	+	A	0	1	1	0	0	SArRNA06
gi|29165615|ref|NC_002745.2|	555229	+	C	2	9	0	0	3	SArRNA06
gi|29165615|ref|NC_002745.2|	555230	+	U	2	17	0	1	8	SArRNA06
gi|29165615|ref|NC_002745.2|	555231	+	A	3	8	2	0	0	SArRNA06
gi|29165615|ref|NC_002745.2|	555233	+	G	0	1	0	0	0	SArRNA06
gi|29165615|ref|NC_002745.2|	555234	+	U	0	1	0	0	0	SArRNA06
gi|29165615|ref|NC_002745.2|	555236	+	U	0	1	0	1	1	SArRNA06
gi|29165615|ref|NC_002745.2|	555237	+	G	1	1	0	1	3	SArRNA06
gi|29165615|ref|NC_002745.2|	555238	+	G	3	7	0	1	0	SArRNA06
gi|29165615|ref|NC_002745.2|	555239	+	A	2	0	0	0	0	SArRNA06
gi|29165615|ref|NC_002745.2|	555240	+	C	17	20	1	5	4	SArRNA06
gi|29165615|ref|NC_002745.2|	555241	+	G	3	11	3	4	7	SArRNA06
gi|29165615|ref|NC_002745.2|	555243	+	G	0	0	0	0	2	SArRNA06
gi|29165615|ref|NC_002745.2|	555244	+	A	1	2	1	0	0	SArRNA06
gi|29165615|ref|NC_002745.2|	555245	+	U	1	17	1	1	3	SArRNA06
gi|29165615|ref|NC_002745.2|	555246	+	A	4	23	0	1	10	SArRNA06
gi|29165615|ref|NC_002745.2|	555247	+	A	0	9	1	0	3	SArRNA06
gi|29165615|ref|NC_002745.2|	555248	+	G	1	7	0	0	1	SArRNA06
gi|29165615|ref|NC_002745.2|	555249	+	U	1	0	0	0	6	SArRNA06
gi|29165615|ref|NC_002745.2|	555251	+	C	0	1	0	0	0	SArRNA06
gi|29165615|ref|NC_002745.2|	555252	+	U	0	1	0	0	0	SArRNA06
gi|29165615|ref|NC_002745.2|	555253	+	G	0	1	0	0	0	SArRNA06
gi|29165615|ref|NC_002745.2|	555254	+	A	1	3	0	0	1	SArRNA06
gi|29165615|ref|NC_002745.2|	555255	+	A	0	0	0	0	1	SArRNA06
gi|29165615|ref|NC_002745.2|	555256	+	A	0	2	0	0	0	SArRNA06
gi|29165615|ref|NC_002745.2|	555257	+	G	1	1	0	0	0	SArRNA06
gi|29165615|ref|NC_002745.2|	555258	+	C	1	5	1	1	4	SArRNA06
gi|29165615|ref|NC_002745.2|	555259	+	A	2	3	2	0	1	SArRNA06
gi|29165615|ref|NC_002745.2|	555260	+	U	0	7	1	1	1	SArRNA06
gi|29165615|ref|NC_002745.2|	555261	+	C	3	19	0	1	12	SArRNA06
gi|29165615|ref|NC_002745.2|	555262	+	U	0	11	1	3	5	SArRNA06
gi|29165615|ref|NC_002745.2|	555263	+	A	2	3	0	0	0	SArRNA06
gi|29165615|ref|NC_002745.2|	555264	+	A	0	1	1	0	2	SArRNA06
gi|29165615|ref|NC_002745.2|	555266	+	C	0	1	0	2	1	SArRNA06
gi|29165615|ref|NC_002745.2|	555267	+	A	1	1	0	0	1	SArRNA06
gi|29165615|ref|NC_002745.2|	555269	+	G	0	0	0	1	0	SArRNA06
gi|29165615|ref|NC_002745.2|	555270	+	A	0	0	1	0	0	SArRNA06
gi|29165615|ref|NC_002745.2|	555271	+	A	0	1	0	0	1	SArRNA06
gi|29165615|ref|NC_002745.2|	555272	+	G	1	0	0	0	0	SArRNA06
gi|29165615|ref|NC_002745.2|	555273	+	C	16	21	14	11	19	SArRNA06
gi|29165615|ref|NC_002745.2|	555274	+	C	23	26	14	13	25	SArRNA06
gi|29165615|ref|NC_002745.2|	555275	+	C	32	29	17	8	26	SArRNA06
gi|29165615|ref|NC_002745.2|	555276	+	C	6	7	4	6	11	SArRNA06
gi|29165615|ref|NC_002745.2|	555277	+	C	1	9	1	1	3	SArRNA06
gi|29165615|ref|NC_002745.2|	555278	+	C	0	2	1	0	2	SArRNA06
gi|29165615|ref|NC_002745.2|	555279	+	U	0	2	0	0	0	SArRNA06
gi|29165615|ref|NC_002745.2|	555280	+	C	2	6	1	2	3	SArRNA06
gi|29165615|ref|NC_002745.2|	555281	+	A	1	5	1	0	1	SArRNA06
gi|29165615|ref|NC_002745.2|	555289	+	A	0	0	0	1	0	SArRNA06
gi|29165615|ref|NC_002745.2|	555290	+	U	0	3	0	0	0	SArRNA06
gi|29165615|ref|NC_002745.2|	555291	+	U	0	1	0	0	0	SArRNA06
gi|29165615|ref|NC_002745.2|	555292	+	U	1	0	1	0	1	SArRNA06
gi|29165615|ref|NC_002745.2|	555293	+	C	1	2	0	1	1	SArRNA06
gi|29165615|ref|NC_002745.2|	555294	+	C	0	2	0	0	1	SArRNA06
gi|29165615|ref|NC_002745.2|	555295	+	C	1	3	1	0	1	SArRNA06
gi|29165615|ref|NC_002745.2|	555297	+	A	0	0	2	1	0	SArRNA06
gi|29165615|ref|NC_002745.2|	555298	+	C	1	0	0	0	1	SArRNA06
gi|29165615|ref|NC_002745.2|	555299	+	U	0	2	0	0	0	SArRNA06
gi|29165615|ref|NC_002745.2|	555300	+	U	1	0	0	0	0	SArRNA06
gi|29165615|ref|NC_002745.2|	555301	+	C	14	5	0	4	1	SArRNA06
gi|29165615|ref|NC_002745.2|	555302	+	G	4	0	0	0	0	SArRNA06
gi|29165615|ref|NC_002745.2|	555304	+	U	0	0	0	1	0	SArRNA06
gi|29165615|ref|NC_002745.2|	555305	+	U	0	0	2	0	0	SArRNA06
gi|29165615|ref|NC_002745.2|	555306	+	A	1	0	0	0	2	SArRNA06
gi|29165615|ref|NC_002745.2|	555308	+	A	0	0	0	1	0	SArRNA06
gi|29165615|ref|NC_002745.2|	555309	+	A	0	0	0	0	1	SArRNA06
gi|29165615|ref|NC_002745.2|	555310	+	G	2	1	5	3	0	SArRNA06
gi|29165615|ref|NC_002745.2|	555311	+	A	3	11	0	1	4	SArRNA06
gi|29165615|ref|NC_002745.2|	555312	+	U	6	7	4	3	5	SArRNA06
gi|29165615|ref|NC_002745.2|	555313	+	C	22	19	8	8	13	SArRNA06
gi|29165615|ref|NC_002745.2|	555314	+	C	27	28	12	10	13	-
gi|29165615|ref|NC_002745.2|	555315	+	C	3	6	3	2	2	-
gi|29165615|ref|NC_002745.2|	555316	+	U	4	2	0	0	1	-
gi|29165615|ref|NC_002745.2|	555317	+	C	1	3	0	0	1	-
gi|29165615|ref|NC_002745.2|	555318	+	A	0	3	1	0	1	-
gi|29165615|ref|NC_002745.2|	555319	+	A	0	0	2	1	2	-
gi|29165615|ref|NC_002745.2|	555320	+	A	0	2	0	0	0	-
gi|29165615|ref|NC_002745.2|	555321	+	G	2	0	1	4	1	-
gi|29165615|ref|NC_002745.2|	555322	+	A	2	1	0	1	0	-
gi|29165615|ref|NC_002745.2|	555323	+	U	2	0	1	0	0	-

gi|29165615|ref|NC_002745.2|	555324	+	G	3	1	0	1	1	-
gi|29165615|ref|NC_002745.2|	555325	+	A	4	0	1	1	0	-
gi|29165615|ref|NC_002745.2|	555327	+	G	0	0	0	1	2	-
gi|29165615|ref|NC_002745.2|	555331	+	U	0	4	0	0	0	-
gi|29165615|ref|NC_002745.2|	555332	+	U	0	0	1	0	0	-
gi|29165615|ref|NC_002745.2|	555334	+	A	0	7	0	0	6	-
gi|29165615|ref|NC_002745.2|	555335	+	U	2	1	1	0	0	-
gi|29165615|ref|NC_002745.2|	555336	+	A	0	2	0	0	0	-
gi|29165615|ref|NC_002745.2|	555339	+	U	0	0	0	0	1	-
gi|29165615|ref|NC_002745.2|	555341	+	C	0	3	0	0	1	-
gi|29165615|ref|NC_002745.2|	555342	+	G	3	1	0	1	4	-
gi|29165615|ref|NC_002745.2|	555343	+	A	2	1	0	0	0	-
gi|29165615|ref|NC_002745.2|	555347	+	G	0	0	0	0	1	-
gi|29165615|ref|NC_002745.2|	555348	+	G	1	3	1	2	1	-
gi|29165615|ref|NC_002745.2|	555349	+	A	1	0	0	0	0	-
gi|29165615|ref|NC_002745.2|	555351	+	G	0	0	0	0	1	-
gi|29165615|ref|NC_002745.2|	555352	+	C	0	1	1	2	0	-
gi|29165615|ref|NC_002745.2|	555357	+	U	1	0	0	0	1	-
gi|29165615|ref|NC_002745.2|	555359	+	A	0	1	1	0	0	-
gi|29165615|ref|NC_002745.2|	555360	+	C	2	0	0	0	0	-
gi|29165615|ref|NC_002745.2|	555362	+	U	0	0	0	0	1	-
gi|29165615|ref|NC_002745.2|	555363	+	G	2	0	0	0	0	-
gi|29165615|ref|NC_002745.2|	555364	+	U	1	0	0	1	0	-
gi|29165615|ref|NC_002745.2|	555365	+	G	0	0	1	0	0	-
gi|29165615|ref|NC_002745.2|	555367	+	A	0	0	1	0	0	-
gi|29165615|ref|NC_002745.2|	555374	+	G	0	0	0	1	0	-
gi|29165615|ref|NC_002745.2|	555375	+	A	0	0	1	0	0	-
gi|29165615|ref|NC_002745.2|	555377	+	U	1	0	0	0	0	-
gi|29165615|ref|NC_002745.2|	555378	+	A	1	0	0	0	1	-
gi|29165615|ref|NC_002745.2|	555382	+	A	0	0	0	0	2	-
gi|29165615|ref|NC_002745.2|	555394	+	C	1	0	0	0	0	-
gi|29165615|ref|NC_002745.2|	555400	+	C	3	0	0	0	0	-
gi|29165615|ref|NC_002745.2|	555401	+	A	0	1	0	1	1	-
gi|29165615|ref|NC_002745.2|	555437	+	U	0	1	0	0	0	-
gi|29165615|ref|NC_002745.2|	555450	+	U	40	8	11	6	12	-
gi|29165615|ref|NC_002745.2|	555473	+	U	2	2	2	1	0	-
gi|29165615|ref|NC_002745.2|	555474	+	G	4	7	6	5	11	-
gi|29165615|ref|NC_002745.2|	555475	+	U	2502	2241	1275	977	1934	SArRNA07
gi|29165615|ref|NC_002745.2|	555476	+	C	1	15	3	1	9	SArRNA07
gi|29165615|ref|NC_002745.2|	555477	+	U	7	6	2	4	4	SArRNA07
gi|29165615|ref|NC_002745.2|	555478	+	G	1	0	1	0	1	SArRNA07
gi|29165615|ref|NC_002745.2|	555479	+	G	3	0	0	0	2	SArRNA07
gi|29165615|ref|NC_002745.2|	555480	+	U	4	5	3	0	0	SArRNA07
gi|29165615|ref|NC_002745.2|	555481	+	G	5	10	4	2	2	SArRNA07
gi|29165615|ref|NC_002745.2|	555482	+	A	0	0	0	1	0	SArRNA07
gi|29165615|ref|NC_002745.2|	555483	+	C	34	29	12	6	21	SArRNA07
gi|29165615|ref|NC_002745.2|	555484	+	U	8	22	3	2	25	SArRNA07
gi|29165615|ref|NC_002745.2|	555485	+	A	30	44	31	10	27	SArRNA07
gi|29165615|ref|NC_002745.2|	555486	+	U	1	1	0	2	1	SArRNA07
gi|29165615|ref|NC_002745.2|	555487	+	A	1	0	0	1	0	SArRNA07
gi|29165615|ref|NC_002745.2|	555488	+	G	0	0	0	1	0	SArRNA07
gi|29165615|ref|NC_002745.2|	555489	+	C	19	24	16	8	21	SArRNA07
gi|29165615|ref|NC_002745.2|	555490	+	A	4	2	1	2	4	SArRNA07
gi|29165615|ref|NC_002745.2|	555491	+	A	9	4	2	1	4	SArRNA07
gi|29165615|ref|NC_002745.2|	555492	+	G	0	2	0	0	1	SArRNA07
gi|29165615|ref|NC_002745.2|	555493	+	G	1	6	1	1	2	SArRNA07
gi|29165615|ref|NC_002745.2|	555494	+	A	0	0	1	1	1	SArRNA07
gi|29165615|ref|NC_002745.2|	555495	+	G	0	1	1	0	1	SArRNA07
gi|29165615|ref|NC_002745.2|	555497	+	U	0	1	0	0	1	SArRNA07
gi|29165615|ref|NC_002745.2|	555498	+	C	4	16	4	0	13	SArRNA07
gi|29165615|ref|NC_002745.2|	555499	+	A	2	6	0	1	6	SArRNA07
gi|29165615|ref|NC_002745.2|	555500	+	C	68	107	63	56	92	SArRNA07
gi|29165615|ref|NC_002745.2|	555501	+	A	7	25	5	7	11	SArRNA07
gi|29165615|ref|NC_002745.2|	555502	+	C	28	65	26	32	33	SArRNA07
gi|29165615|ref|NC_002745.2|	555503	+	C	8	31	9	15	7	SArRNA07
gi|29165615|ref|NC_002745.2|	555504	+	U	6	3	0	0	4	SArRNA07
gi|29165615|ref|NC_002745.2|	555505	+	G	0	1	0	4	0	SArRNA07
gi|29165615|ref|NC_002745.2|	555506	+	U	0	0	0	0	1	SArRNA07
gi|29165615|ref|NC_002745.2|	555507	+	U	0	2	1	1	1	SArRNA07
gi|29165615|ref|NC_002745.2|	555508	+	C	7	6	4	1	3	SArRNA07
gi|29165615|ref|NC_002745.2|	555509	+	C	1	23	1	0	9	SArRNA07
gi|29165615|ref|NC_002745.2|	555510	+	C	11	31	9	5	24	SArRNA07
gi|29165615|ref|NC_002745.2|	555511	+	A	3	7	1	0	6	SArRNA07
gi|29165615|ref|NC_002745.2|	555512	+	U	0	5	0	0	0	SArRNA07
gi|29165615|ref|NC_002745.2|	555513	+	G	2	2	0	0	0	SArRNA07
gi|29165615|ref|NC_002745.2|	555514	+	C	23	46	4	5	37	SArRNA07
gi|29165615|ref|NC_002745.2|	555515	+	C	70	92	45	26	54	SArRNA07
gi|29165615|ref|NC_002745.2|	555516	+	G	19	17	10	4	16	SArRNA07
gi|29165615|ref|NC_002745.2|	555517	+	A	2	2	1	1	1	SArRNA07
gi|29165615|ref|NC_002745.2|	555519	+	C	24	41	18	9	19	SArRNA07
gi|29165615|ref|NC_002745.2|	555520	+	A	2	9	0	2	6	SArRNA07

gi|29165615|ref|NC_002745.2|	555521	+	C	3	9	1	0	5	SArRNA07
gi|29165615|ref|NC_002745.2|	555522	+	A	1	7	0	1	3	SArRNA07
gi|29165615|ref|NC_002745.2|	555523	+	G	2	11	0	1	3	SArRNA07
gi|29165615|ref|NC_002745.2|	555524	+	A	0	4	1	1	1	SArRNA07
gi|29165615|ref|NC_002745.2|	555525	+	A	1	1	0	1	0	SArRNA07
gi|29165615|ref|NC_002745.2|	555526	+	G	0	0	0	0	2	SArRNA07
gi|29165615|ref|NC_002745.2|	555527	+	U	1	0	0	0	2	SArRNA07
gi|29165615|ref|NC_002745.2|	555528	+	U	2	12	3	0	2	SArRNA07
gi|29165615|ref|NC_002745.2|	555529	+	A	3	0	1	0	2	SArRNA07
gi|29165615|ref|NC_002745.2|	555530	+	A	1	0	0	1	1	SArRNA07
gi|29165615|ref|NC_002745.2|	555532	+	C	2	7	4	5	5	SArRNA07
gi|29165615|ref|NC_002745.2|	555533	+	U	1	2	1	2	0	SArRNA07
gi|29165615|ref|NC_002745.2|	555534	+	C	2	0	0	1	0	SArRNA07
gi|29165615|ref|NC_002745.2|	555535	+	C	2	2	1	0	3	SArRNA07
gi|29165615|ref|NC_002745.2|	555536	+	U	0	2	0	0	0	SArRNA07
gi|29165615|ref|NC_002745.2|	555537	+	U	1	1	0	0	0	SArRNA07
gi|29165615|ref|NC_002745.2|	555538	+	A	0	2	0	0	0	SArRNA07
gi|29165615|ref|NC_002745.2|	555540	+	C	1	3	2	0	1	SArRNA07
gi|29165615|ref|NC_002745.2|	555541	+	G	0	0	0	0	3	SArRNA07
gi|29165615|ref|NC_002745.2|	555542	+	U	1	0	0	0	1	SArRNA07
gi|29165615|ref|NC_002745.2|	555543	+	C	0	7	0	0	4	SArRNA07
gi|29165615|ref|NC_002745.2|	555544	+	G	0	7	0	3	2	SArRNA07
gi|29165615|ref|NC_002745.2|	555545	+	A	0	3	0	0	1	SArRNA07
gi|29165615|ref|NC_002745.2|	555547	+	G	0	0	0	0	1	SArRNA07
gi|29165615|ref|NC_002745.2|	555548	+	G	0	2	1	0	0	SArRNA07
gi|29165615|ref|NC_002745.2|	555549	+	U	0	2	0	0	0	SArRNA07
gi|29165615|ref|NC_002745.2|	555551	+	G	0	0	0	0	1	SArRNA07
gi|29165615|ref|NC_002745.2|	555559	+	U	1	0	0	0	0	SArRNA07
gi|29165615|ref|NC_002745.2|	555560	+	A	0	0	0	0	1	SArRNA07
gi|29165615|ref|NC_002745.2|	555561	+	C	2	2	2	0	4	SArRNA07
gi|29165615|ref|NC_002745.2|	555562	+	G	1	6	1	2	1	SArRNA07
gi|29165615|ref|NC_002745.2|	555563	+	U	0	0	0	0	1	SArRNA07
gi|29165615|ref|NC_002745.2|	555564	+	U	1	5	0	0	2	SArRNA07
gi|29165615|ref|NC_002745.2|	555565	+	C	6	2	3	3	3	SArRNA07
gi|29165615|ref|NC_002745.2|	555566	+	C	0	5	1	0	0	SArRNA07
gi|29165615|ref|NC_002745.2|	555567	+	G	0	1	0	0	2	SArRNA07
gi|29165615|ref|NC_002745.2|	555568	+	C	2	4	2	0	2	SArRNA07
gi|29165615|ref|NC_002745.2|	555569	+	U	0	1	0	1	0	SArRNA07
gi|29165615|ref|NC_002745.2|	555570	+	A	0	0	1	0	0	SArRNA07
gi|29165615|ref|NC_002745.2|	555590	+	A	1	30	8	8	12	-
gi|29165615|ref|NC_002745.2|	555591	+	A	2	69	35	21	48	-
gi|29165615|ref|NC_002745.2|	555592	+	A	3	79	17	19	58	-
gi|29165615|ref|NC_002745.2|	555593	+	A	1	58	11	14	39	-
gi|29165615|ref|NC_002745.2|	555594	+	A	1	10	7	3	13	-
gi|29165615|ref|NC_002745.2|	555595	+	A	0	2	1	0	0	-
gi|29165615|ref|NC_002745.2|	555995	+	A	0	0	1	1	0	-
gi|29165615|ref|NC_002745.2|	562867	+	U	0	1	0	1	0	SA0483
gi|29165615|ref|NC_002745.2|	563273	+	A	2	0	0	0	0	SA0483
gi|29165615|ref|NC_002745.2|	563305	+	U	0	0	0	1	0	SA0483
gi|29165615|ref|NC_002745.2|	563308	+	G	1	0	0	0	0	SA0483
gi|29165615|ref|NC_002745.2|	563320	+	U	0	1	0	0	0	SA0483
gi|29165615|ref|NC_002745.2|	563323	+	G	1	0	0	0	0	SA0483
gi|29165615|ref|NC_002745.2|	564328	+	G	1	0	0	0	0	SA0483
gi|29165615|ref|NC_002745.2|	568629	+	A	0	3	3	0	1	-
gi|29165615|ref|NC_002745.2|	568630	+	U	0	0	0	0	1	-
gi|29165615|ref|NC_002745.2|	572406	+	A	0	1	0	1	0	SA0488
gi|29165615|ref|NC_002745.2|	574124	+	A	0	1	1	0	0	SA0491
gi|29165615|ref|NC_002745.2|	575215	+	U	3	1	0	0	0	SA0493
gi|29165615|ref|NC_002745.2|	575216	+	U	2	0	0	0	1	SA0493
gi|29165615|ref|NC_002745.2|	575217	+	A	1	1	0	0	0	SA0493
gi|29165615|ref|NC_002745.2|	576827	+	U	0	0	0	2	0	SA0496
gi|29165615|ref|NC_002745.2|	579519	+	G	0	0	1	0	1	-
gi|29165615|ref|NC_002745.2|	585777	+	A	0	1	0	0	0	SA0501
gi|29165615|ref|NC_002745.2|	589764	+	C	0	1	0	0	0	SA0505
gi|29165615|ref|NC_002745.2|	590846	+	U	4	0	0	0	1	SA0506
gi|29165615|ref|NC_002745.2|	590847	+	C	1	0	0	0	0	SA0506
gi|29165615|ref|NC_002745.2|	590848	+	A	0	1	0	0	0	SA0506
gi|29165615|ref|NC_002745.2|	590850	+	G	2	0	0	0	0	SA0506
gi|29165615|ref|NC_002745.2|	590851	+	U	1	0	0	0	0	SA0506
gi|29165615|ref|NC_002745.2|	590956	+	A	0	0	1	0	3	SA0506
gi|29165615|ref|NC_002745.2|	590957	+	A	1	2	0	0	1	SA0506
gi|29165615|ref|NC_002745.2|	594185	+	A	0	1	0	0	0	SA0508
gi|29165615|ref|NC_002745.2|	606715	+	G	0	0	0	0	1	SA0519
gi|29165615|ref|NC_002745.2|	606833	+	C	0	0	0	1	0	SA0519
gi|29165615|ref|NC_002745.2|	606964	+	C	0	0	0	1	0	SA0519
gi|29165615|ref|NC_002745.2|	606970	+	A	0	0	0	0	1	SA0519
gi|29165615|ref|NC_002745.2|	607166	+	C	0	0	0	1	0	SA0519
gi|29165615|ref|NC_002745.2|	607190	+	U	0	0	1	0	0	SA0519
gi|29165615|ref|NC_002745.2|	607193	+	A	0	0	0	1	0	SA0519
gi|29165615|ref|NC_002745.2|	607203	+	U	0	0	0	0	1	SA0519
gi|29165615|ref|NC_002745.2|	607223	+	U	1	0	0	0	0	SA0519

gi|29165615|ref|NC_002745.2|	607225 +	U	0	0	0	0	1	SA0519
gi|29165615|ref|NC_002745.2|	607226 +	U	0	0	1	0	0	SA0519
gi|29165615|ref|NC_002745.2|	607229 +	A	0	0	0	0	1	SA0519
gi|29165615|ref|NC_002745.2|	607244 +	U	0	1	0	0	0	SA0519
gi|29165615|ref|NC_002745.2|	607253 +	C	0	2	0	0	0	SA0519
gi|29165615|ref|NC_002745.2|	607258 +	A	0	0	0	0	1	SA0519
gi|29165615|ref|NC_002745.2|	607261 +	A	0	0	1	1	2	SA0519
gi|29165615|ref|NC_002745.2|	607262 +	A	0	1	0	0	1	SA0519
gi|29165615|ref|NC_002745.2|	607271 +	A	0	1	0	0	1	SA0519
gi|29165615|ref|NC_002745.2|	607273 +	G	0	0	0	0	1	SA0519
gi|29165615|ref|NC_002745.2|	607274 +	A	0	0	0	1	3	SA0519
gi|29165615|ref|NC_002745.2|	607276 +	G	0	1	0	1	0	SA0519
gi|29165615|ref|NC_002745.2|	607277 +	A	0	2	0	3	2	SA0519
gi|29165615|ref|NC_002745.2|	607280 +	A	0	0	0	1	0	SA0519
gi|29165615|ref|NC_002745.2|	607289 +	A	0	0	0	1	0	SA0519
gi|29165615|ref|NC_002745.2|	607365 +	A	0	1	0	0	0	SA0519
gi|29165615|ref|NC_002745.2|	607377 +	C	0	0	1	0	0	SA0519
gi|29165615|ref|NC_002745.2|	607379 +	C	0	1	0	1	0	SA0519
gi|29165615|ref|NC_002745.2|	607380 +	A	0	0	0	1	0	SA0519
gi|29165615|ref|NC_002745.2|	607381 +	G	0	0	0	0	1	SA0519
gi|29165615|ref|NC_002745.2|	607382 +	A	0	2	0	0	0	SA0519
gi|29165615|ref|NC_002745.2|	607384 +	U	0	0	1	0	1	SA0519
gi|29165615|ref|NC_002745.2|	607385 +	C	0	0	0	0	1	SA0519
gi|29165615|ref|NC_002745.2|	607388 +	A	1	3	0	1	2	SA0519
gi|29165615|ref|NC_002745.2|	607389 +	C	0	7	0	0	0	SA0519
gi|29165615|ref|NC_002745.2|	607390 +	A	0	0	1	0	2	SA0519
gi|29165615|ref|NC_002745.2|	607392 +	C	1	2	0	1	3	SA0519
gi|29165615|ref|NC_002745.2|	607393 +	G	0	1	0	0	1	SA0519
gi|29165615|ref|NC_002745.2|	607395 +	U	0	1	0	0	0	SA0519
gi|29165615|ref|NC_002745.2|	607396 +	U	0	0	0	1	0	SA0519
gi|29165615|ref|NC_002745.2|	607397 +	C	0	3	0	1	2	SA0519
gi|29165615|ref|NC_002745.2|	607398 +	A	0	2	0	0	0	SA0519
gi|29165615|ref|NC_002745.2|	607399 +	G	0	2	0	0	0	SA0519
gi|29165615|ref|NC_002745.2|	607400 +	A	0	1	0	0	1	SA0519
gi|29165615|ref|NC_002745.2|	607401 +	C	0	0	0	0	1	SA0519
gi|29165615|ref|NC_002745.2|	607402 +	U	0	1	2	0	1	SA0519
gi|29165615|ref|NC_002745.2|	607403 +	C	0	0	0	0	1	SA0519
gi|29165615|ref|NC_002745.2|	607406 +	A	0	2	0	1	2	SA0519
gi|29165615|ref|NC_002745.2|	607407 +	U	0	5	0	0	0	SA0519
gi|29165615|ref|NC_002745.2|	607408 +	A	0	1	0	0	1	SA0519
gi|29165615|ref|NC_002745.2|	607410 +	C	0	1	1	1	2	SA0519
gi|29165615|ref|NC_002745.2|	607411 +	G	0	1	0	0	1	SA0519
gi|29165615|ref|NC_002745.2|	607413 +	C	0	0	1	0	0	SA0519
gi|29165615|ref|NC_002745.2|	607415 +	C	0	1	0	1	0	SA0519
gi|29165615|ref|NC_002745.2|	607418 +	A	1	2	0	0	1	SA0519
gi|29165615|ref|NC_002745.2|	607420 +	U	0	0	1	0	1	SA0519
gi|29165615|ref|NC_002745.2|	607421 +	C	0	0	0	0	1	SA0519
gi|29165615|ref|NC_002745.2|	607424 +	A	0	2	0	0	0	SA0519
gi|29165615|ref|NC_002745.2|	607425 +	U	0	1	0	0	0	SA0519
gi|29165615|ref|NC_002745.2|	607426 +	A	0	0	1	0	0	SA0519
gi|29165615|ref|NC_002745.2|	607428 +	U	0	1	0	1	3	SA0519
gi|29165615|ref|NC_002745.2|	607429 +	G	1	0	0	0	1	SA0519
gi|29165615|ref|NC_002745.2|	607432 +	U	0	0	0	1	0	SA0519
gi|29165615|ref|NC_002745.2|	607433 +	C	0	3	0	2	1	SA0519
gi|29165615|ref|NC_002745.2|	607434 +	A	0	1	0	0	0	SA0519
gi|29165615|ref|NC_002745.2|	607435 +	G	0	1	0	0	0	SA0519
gi|29165615|ref|NC_002745.2|	607436 +	A	0	3	0	0	1	SA0519
gi|29165615|ref|NC_002745.2|	607437 +	C	0	1	0	0	2	SA0519
gi|29165615|ref|NC_002745.2|	607438 +	U	0	1	2	0	2	SA0519
gi|29165615|ref|NC_002745.2|	607439 +	C	0	1	1	0	3	SA0519
gi|29165615|ref|NC_002745.2|	607440 +	A	0	0	0	1	0	SA0519
gi|29165615|ref|NC_002745.2|	607442 +	A	0	2	0	0	1	SA0519
gi|29165615|ref|NC_002745.2|	607443 +	U	0	7	0	0	0	SA0519
gi|29165615|ref|NC_002745.2|	607444 +	A	0	1	1	0	2	SA0519
gi|29165615|ref|NC_002745.2|	607446 +	C	0	3	1	1	3	SA0519
gi|29165615|ref|NC_002745.2|	607447 +	G	1	0	0	0	1	SA0519
gi|29165615|ref|NC_002745.2|	607450 +	U	0	0	0	1	0	SA0519
gi|29165615|ref|NC_002745.2|	607451 +	C	0	0	0	0	1	SA0519
gi|29165615|ref|NC_002745.2|	607465 +	G	0	1	0	0	0	SA0519
gi|29165615|ref|NC_002745.2|	607466 +	A	0	3	0	0	2	SA0519
gi|29165615|ref|NC_002745.2|	607467 +	C	0	2	0	0	2	SA0519
gi|29165615|ref|NC_002745.2|	607468 +	U	0	0	2	0	1	SA0519
gi|29165615|ref|NC_002745.2|	607469 +	C	0	1	1	0	3	SA0519
gi|29165615|ref|NC_002745.2|	607470 +	A	0	0	0	1	0	SA0519
gi|29165615|ref|NC_002745.2|	607472 +	A	0	2	0	1	2	SA0519
gi|29165615|ref|NC_002745.2|	607473 +	C	0	6	0	0	0	SA0519
gi|29165615|ref|NC_002745.2|	607474 +	A	0	1	0	0	0	SA0519
gi|29165615|ref|NC_002745.2|	607476 +	C	0	2	1	0	2	SA0519
gi|29165615|ref|NC_002745.2|	607477 +	G	0	1	0	0	2	SA0519
gi|29165615|ref|NC_002745.2|	607479 +	C	0	0	1	0	0	SA0519
gi|29165615|ref|NC_002745.2|	607480 +	U	0	0	0	1	0	SA0519

gi|29165615|ref|NC_002745.2|	607481	+	C	0	2	0	2	2	SA0519
gi|29165615|ref|NC_002745.2|	607482	+	A	0	1	0	0	1	SA0519
gi|29165615|ref|NC_002745.2|	607489	+	G	0	1	0	0	0	SA0519
gi|29165615|ref|NC_002745.2|	607491	+	U	0	0	1	0	1	SA0519
gi|29165615|ref|NC_002745.2|	607492	+	A	0	1	0	0	0	SA0519
gi|29165615|ref|NC_002745.2|	607494	+	U	1	1	0	0	0	SA0519
gi|29165615|ref|NC_002745.2|	607495	+	G	0	1	0	0	0	SA0519
gi|29165615|ref|NC_002745.2|	607499	+	C	0	2	0	2	0	SA0519
gi|29165615|ref|NC_002745.2|	607501	+	G	0	0	0	0	1	SA0519
gi|29165615|ref|NC_002745.2|	607502	+	A	0	2	0	0	0	SA0519
gi|29165615|ref|NC_002745.2|	607503	+	U	0	1	0	0	1	SA0519
gi|29165615|ref|NC_002745.2|	607505	+	C	0	1	1	0	2	SA0519
gi|29165615|ref|NC_002745.2|	607506	+	G	0	0	0	1	0	SA0519
gi|29165615|ref|NC_002745.2|	607507	+	G	0	1	0	0	0	SA0519
gi|29165615|ref|NC_002745.2|	607508	+	A	1	5	0	3	2	SA0519
gi|29165615|ref|NC_002745.2|	607509	+	C	0	6	1	0	1	SA0519
gi|29165615|ref|NC_002745.2|	607510	+	A	0	1	1	0	0	SA0519
gi|29165615|ref|NC_002745.2|	607512	+	C	1	3	1	0	0	SA0519
gi|29165615|ref|NC_002745.2|	607513	+	G	0	1	0	0	0	SA0519
gi|29165615|ref|NC_002745.2|	607515	+	C	0	0	1	0	0	SA0519
gi|29165615|ref|NC_002745.2|	607517	+	C	0	3	0	3	1	SA0519
gi|29165615|ref|NC_002745.2|	607518	+	A	0	0	0	1	0	SA0519
gi|29165615|ref|NC_002745.2|	607519	+	G	0	0	0	0	1	SA0519
gi|29165615|ref|NC_002745.2|	607523	+	C	0	1	0	0	0	SA0519
gi|29165615|ref|NC_002745.2|	607526	+	A	0	0	0	1	1	SA0519
gi|29165615|ref|NC_002745.2|	607527	+	C	0	6	0	0	0	SA0519
gi|29165615|ref|NC_002745.2|	607528	+	A	0	0	0	0	1	SA0519
gi|29165615|ref|NC_002745.2|	607530	+	C	0	0	0	0	1	SA0519
gi|29165615|ref|NC_002745.2|	607535	+	C	0	0	0	1	0	SA0519
gi|29165615|ref|NC_002745.2|	607538	+	A	0	2	0	0	1	SA0519
gi|29165615|ref|NC_002745.2|	607540	+	U	0	0	1	0	1	SA0519
gi|29165615|ref|NC_002745.2|	607541	+	C	0	0	0	0	1	SA0519
gi|29165615|ref|NC_002745.2|	607544	+	A	0	2	0	0	1	SA0519
gi|29165615|ref|NC_002745.2|	607545	+	U	0	7	0	0	0	SA0519
gi|29165615|ref|NC_002745.2|	607546	+	A	0	1	1	0	2	SA0519
gi|29165615|ref|NC_002745.2|	607548	+	C	0	3	1	1	3	SA0519
gi|29165615|ref|NC_002745.2|	607549	+	G	1	0	0	0	1	SA0519
gi|29165615|ref|NC_002745.2|	607552	+	U	0	0	0	1	0	SA0519
gi|29165615|ref|NC_002745.2|	607553	+	C	0	0	0	0	1	SA0519
gi|29165615|ref|NC_002745.2|	607565	+	G	0	2	0	0	0	SA0519
gi|29165615|ref|NC_002745.2|	607567	+	G	0	2	0	0	1	SA0519
gi|29165615|ref|NC_002745.2|	607568	+	A	0	2	0	0	1	SA0519
gi|29165615|ref|NC_002745.2|	607569	+	C	0	2	0	0	2	SA0519
gi|29165615|ref|NC_002745.2|	607570	+	U	0	0	2	0	1	SA0519
gi|29165615|ref|NC_002745.2|	607571	+	C	0	1	1	0	3	SA0519
gi|29165615|ref|NC_002745.2|	607572	+	A	0	0	0	1	0	SA0519
gi|29165615|ref|NC_002745.2|	607574	+	A	1	3	0	2	3	SA0519
gi|29165615|ref|NC_002745.2|	607575	+	C	0	5	0	0	0	SA0519
gi|29165615|ref|NC_002745.2|	607576	+	A	0	0	1	0	0	SA0519
gi|29165615|ref|NC_002745.2|	607578	+	C	1	2	0	0	0	SA0519
gi|29165615|ref|NC_002745.2|	607579	+	G	0	1	0	0	0	SA0519
gi|29165615|ref|NC_002745.2|	607581	+	C	0	0	1	0	0	SA0519
gi|29165615|ref|NC_002745.2|	607582	+	U	0	1	0	0	0	SA0519
gi|29165615|ref|NC_002745.2|	607583	+	C	0	3	0	3	0	SA0519
gi|29165615|ref|NC_002745.2|	607584	+	A	0	0	0	1	0	SA0519
gi|29165615|ref|NC_002745.2|	607586	+	A	1	2	0	0	1	SA0519
gi|29165615|ref|NC_002745.2|	607587	+	U	0	2	0	0	1	SA0519
gi|29165615|ref|NC_002745.2|	607588	+	U	0	0	1	0	1	SA0519
gi|29165615|ref|NC_002745.2|	607589	+	C	0	0	1	0	3	SA0519
gi|29165615|ref|NC_002745.2|	607590	+	A	0	0	0	1	0	SA0519
gi|29165615|ref|NC_002745.2|	607591	+	G	0	1	0	0	0	SA0519
gi|29165615|ref|NC_002745.2|	607592	+	A	0	3	0	0	1	SA0519
gi|29165615|ref|NC_002745.2|	607593	+	C	0	1	1	0	1	SA0519
gi|29165615|ref|NC_002745.2|	607594	+	A	0	1	1	0	1	SA0519
gi|29165615|ref|NC_002745.2|	607596	+	U	0	1	0	0	2	SA0519
gi|29165615|ref|NC_002745.2|	607597	+	G	1	1	0	0	0	SA0519
gi|29165615|ref|NC_002745.2|	607598	+	A	1	0	0	0	0	SA0519
gi|29165615|ref|NC_002745.2|	607601	+	C	0	4	0	4	0	SA0519
gi|29165615|ref|NC_002745.2|	607602	+	A	0	1	0	0	0	SA0519
gi|29165615|ref|NC_002745.2|	607603	+	G	0	2	0	0	1	SA0519
gi|29165615|ref|NC_002745.2|	607604	+	A	0	2	0	0	1	SA0519
gi|29165615|ref|NC_002745.2|	607605	+	C	0	2	0	0	2	SA0519
gi|29165615|ref|NC_002745.2|	607606	+	U	0	0	2	0	1	SA0519
gi|29165615|ref|NC_002745.2|	607607	+	C	0	1	1	0	3	SA0519
gi|29165615|ref|NC_002745.2|	607608	+	A	0	0	0	1	0	SA0519
gi|29165615|ref|NC_002745.2|	607609	+	G	0	1	0	0	0	SA0519
gi|29165615|ref|NC_002745.2|	607610	+	A	1	5	0	3	2	SA0519
gi|29165615|ref|NC_002745.2|	607611	+	C	0	6	1	0	1	SA0519
gi|29165615|ref|NC_002745.2|	607612	+	A	0	1	1	0	0	SA0519
gi|29165615|ref|NC_002745.2|	607614	+	C	1	3	1	0	0	SA0519
gi|29165615|ref|NC_002745.2|	607615	+	G	0	1	0	0	0	SA0519

gi|29165615|ref|NC_002745.2|	607617	+	C	0	0	1	0	0	SA0519
gi|29165615|ref|NC_002745.2|	607619	+	C	0	3	0	3	1	SA0519
gi|29165615|ref|NC_002745.2|	607620	+	A	0	1	0	1	0	SA0519
gi|29165615|ref|NC_002745.2|	607621	+	G	0	1	0	0	1	SA0519
gi|29165615|ref|NC_002745.2|	607625	+	C	0	2	0	0	0	SA0519
gi|29165615|ref|NC_002745.2|	607628	+	A	1	1	0	1	1	SA0519
gi|29165615|ref|NC_002745.2|	607629	+	C	0	6	0	0	0	SA0519
gi|29165615|ref|NC_002745.2|	607630	+	A	0	0	0	0	1	SA0519
gi|29165615|ref|NC_002745.2|	607632	+	C	1	1	0	0	1	SA0519
gi|29165615|ref|NC_002745.2|	607633	+	G	0	1	0	0	0	SA0519
gi|29165615|ref|NC_002745.2|	607637	+	C	0	1	0	0	1	SA0519
gi|29165615|ref|NC_002745.2|	607638	+	A	0	1	0	0	0	SA0519
gi|29165615|ref|NC_002745.2|	607646	+	A	0	3	0	1	1	SA0519
gi|29165615|ref|NC_002745.2|	607647	+	U	0	5	0	0	0	SA0519
gi|29165615|ref|NC_002745.2|	607648	+	A	0	1	0	0	1	SA0519
gi|29165615|ref|NC_002745.2|	607650	+	C	0	2	1	1	2	SA0519
gi|29165615|ref|NC_002745.2|	607654	+	U	0	0	0	1	0	SA0519
gi|29165615|ref|NC_002745.2|	607655	+	C	0	1	0	0	2	SA0519
gi|29165615|ref|NC_002745.2|	607656	+	A	0	0	0	1	1	SA0519
gi|29165615|ref|NC_002745.2|	607658	+	A	0	1	0	0	0	SA0519
gi|29165615|ref|NC_002745.2|	607660	+	U	0	0	0	0	1	SA0519
gi|29165615|ref|NC_002745.2|	607665	+	U	0	1	0	0	0	SA0519
gi|29165615|ref|NC_002745.2|	607666	+	A	0	0	0	0	1	SA0519
gi|29165615|ref|NC_002745.2|	607673	+	C	0	2	0	0	0	SA0519
gi|29165615|ref|NC_002745.2|	607676	+	A	0	1	0	0	0	SA0519
gi|29165615|ref|NC_002745.2|	607677	+	C	0	0	0	0	1	SA0519
gi|29165615|ref|NC_002745.2|	607686	+	C	0	1	1	0	0	SA0519
gi|29165615|ref|NC_002745.2|	607689	+	C	0	0	1	0	0	SA0519
gi|29165615|ref|NC_002745.2|	607691	+	C	0	1	0	1	0	SA0519
gi|29165615|ref|NC_002745.2|	607692	+	A	0	0	0	1	0	SA0519
gi|29165615|ref|NC_002745.2|	607693	+	G	0	0	0	0	1	SA0519
gi|29165615|ref|NC_002745.2|	607694	+	A	0	2	0	0	0	SA0519
gi|29165615|ref|NC_002745.2|	607696	+	U	0	0	1	0	1	SA0519
gi|29165615|ref|NC_002745.2|	607697	+	C	0	0	0	0	1	SA0519
gi|29165615|ref|NC_002745.2|	607700	+	A	1	3	0	1	2	SA0519
gi|29165615|ref|NC_002745.2|	607701	+	C	0	7	0	0	0	SA0519
gi|29165615|ref|NC_002745.2|	607702	+	A	0	0	1	0	2	SA0519
gi|29165615|ref|NC_002745.2|	607704	+	C	1	2	0	1	3	SA0519
gi|29165615|ref|NC_002745.2|	607705	+	G	0	1	0	0	1	SA0519
gi|29165615|ref|NC_002745.2|	607707	+	U	0	1	0	0	0	SA0519
gi|29165615|ref|NC_002745.2|	607708	+	U	0	0	0	1	0	SA0519
gi|29165615|ref|NC_002745.2|	607709	+	C	0	3	0	1	2	SA0519
gi|29165615|ref|NC_002745.2|	607710	+	A	0	2	0	0	0	SA0519
gi|29165615|ref|NC_002745.2|	607711	+	G	0	2	0	0	0	SA0519
gi|29165615|ref|NC_002745.2|	607712	+	A	0	1	0	0	1	SA0519
gi|29165615|ref|NC_002745.2|	607713	+	C	0	0	0	0	1	SA0519
gi|29165615|ref|NC_002745.2|	607714	+	U	0	0	0	0	1	SA0519
gi|29165615|ref|NC_002745.2|	607715	+	C	0	1	1	0	2	SA0519
gi|29165615|ref|NC_002745.2|	607716	+	A	0	0	0	1	0	SA0519
gi|29165615|ref|NC_002745.2|	607718	+	A	1	3	0	1	2	SA0519
gi|29165615|ref|NC_002745.2|	607719	+	U	0	6	0	0	0	SA0519
gi|29165615|ref|NC_002745.2|	607720	+	A	0	2	1	0	2	SA0519
gi|29165615|ref|NC_002745.2|	607722	+	C	1	3	1	1	2	SA0519
gi|29165615|ref|NC_002745.2|	607723	+	G	1	1	0	0	0	SA0519
gi|29165615|ref|NC_002745.2|	607725	+	C	0	0	1	0	0	SA0519
gi|29165615|ref|NC_002745.2|	607727	+	C	0	5	0	4	1	SA0519
gi|29165615|ref|NC_002745.2|	607728	+	A	0	0	0	1	0	SA0519
gi|29165615|ref|NC_002745.2|	607729	+	G	0	1	0	0	1	SA0519
gi|29165615|ref|NC_002745.2|	607730	+	A	0	2	0	0	0	SA0519
gi|29165615|ref|NC_002745.2|	607731	+	U	0	2	0	0	2	SA0519
gi|29165615|ref|NC_002745.2|	607732	+	U	0	0	2	0	1	SA0519
gi|29165615|ref|NC_002745.2|	607733	+	C	0	1	1	0	3	SA0519
gi|29165615|ref|NC_002745.2|	607734	+	A	0	0	0	1	0	SA0519
gi|29165615|ref|NC_002745.2|	607735	+	G	0	1	0	0	0	SA0519
gi|29165615|ref|NC_002745.2|	607736	+	A	1	5	0	3	2	SA0519
gi|29165615|ref|NC_002745.2|	607737	+	C	0	6	1	0	1	SA0519
gi|29165615|ref|NC_002745.2|	607738	+	A	0	1	1	0	0	SA0519
gi|29165615|ref|NC_002745.2|	607740	+	C	1	3	1	0	0	SA0519
gi|29165615|ref|NC_002745.2|	607741	+	G	0	1	0	0	0	SA0519
gi|29165615|ref|NC_002745.2|	607743	+	C	0	0	1	0	0	SA0519
gi|29165615|ref|NC_002745.2|	607745	+	C	0	3	0	3	1	SA0519
gi|29165615|ref|NC_002745.2|	607746	+	A	0	1	0	1	0	SA0519
gi|29165615|ref|NC_002745.2|	607747	+	G	0	1	0	0	1	SA0519
gi|29165615|ref|NC_002745.2|	607748	+	A	0	3	0	0	0	SA0519
gi|29165615|ref|NC_002745.2|	607749	+	U	0	1	0	0	1	SA0519
gi|29165615|ref|NC_002745.2|	607750	+	U	0	1	2	0	1	SA0519
gi|29165615|ref|NC_002745.2|	607751	+	C	0	1	0	0	1	SA0519
gi|29165615|ref|NC_002745.2|	607753	+	G	0	1	0	0	0	SA0519
gi|29165615|ref|NC_002745.2|	607754	+	A	1	2	0	2	1	SA0519
gi|29165615|ref|NC_002745.2|	607755	+	C	0	5	1	0	1	SA0519
gi|29165615|ref|NC_002745.2|	607756	+	A	0	1	0	0	1	SA0519

gi|29165615|ref|NC_002745.2|	607758	+	C	1	2	1	0	0	SA0519
gi|29165615|ref|NC_002745.2|	607759	+	G	0	1	0	0	1	SA0519
gi|29165615|ref|NC_002745.2|	607761	+	U	0	1	0	0	0	SA0519
gi|29165615|ref|NC_002745.2|	607762	+	U	0	0	0	1	0	SA0519
gi|29165615|ref|NC_002745.2|	607763	+	C	0	1	0	1	2	SA0519
gi|29165615|ref|NC_002745.2|	607764	+	A	0	1	0	1	1	SA0519
gi|29165615|ref|NC_002745.2|	607765	+	G	0	1	0	0	1	SA0519
gi|29165615|ref|NC_002745.2|	607766	+	A	0	1	0	0	0	SA0519
gi|29165615|ref|NC_002745.2|	607768	+	U	0	1	0	0	1	SA0519
gi|29165615|ref|NC_002745.2|	607769	+	C	0	1	0	0	0	SA0519
gi|29165615|ref|NC_002745.2|	607771	+	G	0	1	0	0	0	SA0519
gi|29165615|ref|NC_002745.2|	607772	+	A	1	3	0	2	0	SA0519
gi|29165615|ref|NC_002745.2|	607773	+	U	0	1	1	0	1	SA0519
gi|29165615|ref|NC_002745.2|	607774	+	A	0	1	1	0	2	SA0519
gi|29165615|ref|NC_002745.2|	607776	+	C	1	2	0	0	0	SA0519
gi|29165615|ref|NC_002745.2|	607777	+	G	0	1	0	0	0	SA0519
gi|29165615|ref|NC_002745.2|	607781	+	C	0	5	0	4	1	SA0519
gi|29165615|ref|NC_002745.2|	607782	+	A	0	0	0	1	0	SA0519
gi|29165615|ref|NC_002745.2|	607783	+	G	0	1	0	0	1	SA0519
gi|29165615|ref|NC_002745.2|	607784	+	A	0	2	0	0	0	SA0519
gi|29165615|ref|NC_002745.2|	607785	+	U	0	2	0	0	2	SA0519
gi|29165615|ref|NC_002745.2|	607786	+	U	0	0	2	0	1	SA0519
gi|29165615|ref|NC_002745.2|	607787	+	C	0	2	1	0	3	SA0519
gi|29165615|ref|NC_002745.2|	607788	+	A	0	0	0	1	0	SA0519
gi|29165615|ref|NC_002745.2|	607790	+	A	1	3	0	2	2	SA0519
gi|29165615|ref|NC_002745.2|	607791	+	C	0	6	0	0	0	SA0519
gi|29165615|ref|NC_002745.2|	607792	+	A	0	1	1	0	1	SA0519
gi|29165615|ref|NC_002745.2|	607794	+	C	1	3	1	1	3	SA0519
gi|29165615|ref|NC_002745.2|	607795	+	G	0	1	0	0	0	SA0519
gi|29165615|ref|NC_002745.2|	607798	+	U	0	0	0	1	0	SA0519
gi|29165615|ref|NC_002745.2|	607799	+	C	0	3	0	3	2	SA0519
gi|29165615|ref|NC_002745.2|	607800	+	A	0	0	0	1	0	SA0519
gi|29165615|ref|NC_002745.2|	607802	+	A	0	2	0	0	1	SA0519
gi|29165615|ref|NC_002745.2|	607803	+	U	0	2	0	0	1	SA0519
gi|29165615|ref|NC_002745.2|	607804	+	U	0	0	1	0	2	SA0519
gi|29165615|ref|NC_002745.2|	607805	+	C	0	0	1	0	3	SA0519
gi|29165615|ref|NC_002745.2|	607806	+	A	0	0	0	1	0	SA0519
gi|29165615|ref|NC_002745.2|	607808	+	A	0	3	0	0	0	SA0519
gi|29165615|ref|NC_002745.2|	607809	+	U	0	2	0	0	0	SA0519
gi|29165615|ref|NC_002745.2|	607810	+	A	0	1	1	0	1	SA0519
gi|29165615|ref|NC_002745.2|	607812	+	C	0	3	1	0	2	SA0519
gi|29165615|ref|NC_002745.2|	607813	+	G	1	0	0	0	1	SA0519
gi|29165615|ref|NC_002745.2|	607814	+	A	0	1	0	0	0	SA0519
gi|29165615|ref|NC_002745.2|	607815	+	C	0	0	1	0	0	SA0519
gi|29165615|ref|NC_002745.2|	607816	+	U	0	0	0	1	0	SA0519
gi|29165615|ref|NC_002745.2|	607817	+	C	0	5	0	3	1	SA0519
gi|29165615|ref|NC_002745.2|	607818	+	A	0	1	0	1	0	SA0519
gi|29165615|ref|NC_002745.2|	607819	+	G	0	1	0	0	1	SA0519
gi|29165615|ref|NC_002745.2|	607820	+	A	0	4	0	0	2	SA0519
gi|29165615|ref|NC_002745.2|	607821	+	C	0	1	0	0	2	SA0519
gi|29165615|ref|NC_002745.2|	607822	+	U	0	1	2	0	2	SA0519
gi|29165615|ref|NC_002745.2|	607823	+	C	0	2	1	0	3	SA0519
gi|29165615|ref|NC_002745.2|	607824	+	A	0	0	0	1	0	SA0519
gi|29165615|ref|NC_002745.2|	607826	+	A	1	3	0	1	2	SA0519
gi|29165615|ref|NC_002745.2|	607827	+	C	0	7	0	0	0	SA0519
gi|29165615|ref|NC_002745.2|	607828	+	A	0	0	1	0	2	SA0519
gi|29165615|ref|NC_002745.2|	607830	+	C	1	2	0	1	3	SA0519
gi|29165615|ref|NC_002745.2|	607831	+	G	0	1	0	0	1	SA0519
gi|29165615|ref|NC_002745.2|	607833	+	U	0	1	0	0	0	SA0519
gi|29165615|ref|NC_002745.2|	607834	+	U	0	0	0	1	0	SA0519
gi|29165615|ref|NC_002745.2|	607835	+	C	0	0	0	0	1	SA0519
gi|29165615|ref|NC_002745.2|	607847	+	G	0	0	0	1	0	SA0519
gi|29165615|ref|NC_002745.2|	607848	+	C	0	1	0	0	0	SA0519
gi|29165615|ref|NC_002745.2|	607849	+	G	0	1	0	0	1	SA0519
gi|29165615|ref|NC_002745.2|	607850	+	A	0	1	0	0	0	SA0519
gi|29165615|ref|NC_002745.2|	607851	+	C	0	0	0	0	1	SA0519
gi|29165615|ref|NC_002745.2|	607853	+	C	0	2	0	0	0	SA0519
gi|29165615|ref|NC_002745.2|	607856	+	A	1	1	0	1	1	SA0519
gi|29165615|ref|NC_002745.2|	607857	+	C	0	6	0	0	0	SA0519
gi|29165615|ref|NC_002745.2|	607858	+	A	0	1	0	0	1	SA0519
gi|29165615|ref|NC_002745.2|	607860	+	C	0	2	1	0	0	SA0519
gi|29165615|ref|NC_002745.2|	607861	+	G	0	0	0	0	1	SA0519
gi|29165615|ref|NC_002745.2|	607865	+	C	0	0	0	0	1	SA0519
gi|29165615|ref|NC_002745.2|	607866	+	A	0	1	0	0	0	SA0519
gi|29165615|ref|NC_002745.2|	607867	+	G	0	0	0	0	1	SA0519
gi|29165615|ref|NC_002745.2|	607868	+	A	0	1	0	0	0	SA0519
gi|29165615|ref|NC_002745.2|	607870	+	U	0	1	0	0	0	SA0519
gi|29165615|ref|NC_002745.2|	607950	+	A	0	0	1	0	0	SA0519
gi|29165615|ref|NC_002745.2|	609402	+	A	0	0	0	0	1	SA0520
gi|29165615|ref|NC_002745.2|	610498	+	G	0	0	0	0	1	SA0520
gi|29165615|ref|NC_002745.2|	610733	+	A	0	1	0	1	0	SA0520

gi|29165615|ref|NC_002745.2|	610747	+	U	0	0	0	1	0	SA0520
gi|29165615|ref|NC_002745.2|	610831	+	A	0	0	0	0	1	SA0520
gi|29165615|ref|NC_002745.2|	610874	+	C	1	0	0	0	0	SA0520
gi|29165615|ref|NC_002745.2|	611086	+	A	0	0	0	0	1	SA0520
gi|29165615|ref|NC_002745.2|	611161	+	G	0	0	0	0	1	SA0520
gi|29165615|ref|NC_002745.2|	611279	+	C	0	0	0	1	0	SA0520
gi|29165615|ref|NC_002745.2|	611396	+	A	0	1	0	1	0	SA0520
gi|29165615|ref|NC_002745.2|	611410	+	U	0	0	0	1	0	SA0520
gi|29165615|ref|NC_002745.2|	611416	+	A	0	0	0	0	1	SA0520
gi|29165615|ref|NC_002745.2|	611427	+	C	0	1	0	0	0	SA0520
gi|29165615|ref|NC_002745.2|	611438	+	C	0	0	0	0	1	SA0520
gi|29165615|ref|NC_002745.2|	611444	+	A	0	1	0	0	0	SA0520
gi|29165615|ref|NC_002745.2|	611574	+	A	0	0	1	0	0	SA0520
gi|29165615|ref|NC_002745.2|	611612	+	C	0	0	0	1	0	SA0520
gi|29165615|ref|NC_002745.2|	611636	+	U	0	0	1	0	0	SA0520
gi|29165615|ref|NC_002745.2|	611639	+	A	0	0	0	1	0	SA0520
gi|29165615|ref|NC_002745.2|	611649	+	U	0	0	0	0	1	SA0520
gi|29165615|ref|NC_002745.2|	611669	+	U	1	0	0	0	0	SA0520
gi|29165615|ref|NC_002745.2|	611671	+	U	0	0	0	0	1	SA0520
gi|29165615|ref|NC_002745.2|	611675	+	A	0	0	0	0	1	SA0520
gi|29165615|ref|NC_002745.2|	611690	+	U	0	1	0	0	0	SA0520
gi|29165615|ref|NC_002745.2|	611699	+	C	0	2	0	0	0	SA0520
gi|29165615|ref|NC_002745.2|	611704	+	A	0	0	0	0	1	SA0520
gi|29165615|ref|NC_002745.2|	611707	+	A	0	0	1	1	2	SA0520
gi|29165615|ref|NC_002745.2|	611708	+	A	0	1	0	0	1	SA0520
gi|29165615|ref|NC_002745.2|	611717	+	A	0	1	0	0	1	SA0520
gi|29165615|ref|NC_002745.2|	611719	+	G	0	0	0	0	1	SA0520
gi|29165615|ref|NC_002745.2|	611720	+	A	0	0	0	1	3	SA0520
gi|29165615|ref|NC_002745.2|	611722	+	G	0	1	0	1	0	SA0520
gi|29165615|ref|NC_002745.2|	611723	+	A	0	2	0	3	2	SA0520
gi|29165615|ref|NC_002745.2|	611726	+	A	0	0	0	1	0	SA0520
gi|29165615|ref|NC_002745.2|	611735	+	A	0	0	0	1	0	SA0520
gi|29165615|ref|NC_002745.2|	611816	+	A	0	2	0	0	0	SA0520
gi|29165615|ref|NC_002745.2|	611818	+	A	0	1	0	0	0	SA0520
gi|29165615|ref|NC_002745.2|	611820	+	C	0	2	1	0	0	SA0520
gi|29165615|ref|NC_002745.2|	611823	+	C	0	0	1	0	0	SA0520
gi|29165615|ref|NC_002745.2|	611825	+	C	0	1	0	1	0	SA0520
gi|29165615|ref|NC_002745.2|	611826	+	A	0	0	0	1	0	SA0520
gi|29165615|ref|NC_002745.2|	611827	+	G	0	0	0	0	1	SA0520
gi|29165615|ref|NC_002745.2|	611828	+	A	0	2	0	0	0	SA0520
gi|29165615|ref|NC_002745.2|	611830	+	U	0	0	0	0	1	SA0520
gi|29165615|ref|NC_002745.2|	611831	+	C	0	1	1	0	2	SA0520
gi|29165615|ref|NC_002745.2|	611832	+	G	0	0	0	1	0	SA0520
gi|29165615|ref|NC_002745.2|	611833	+	G	0	1	0	0	0	SA0520
gi|29165615|ref|NC_002745.2|	611834	+	A	1	3	0	2	1	SA0520
gi|29165615|ref|NC_002745.2|	611835	+	C	0	2	1	0	1	SA0520
gi|29165615|ref|NC_002745.2|	611836	+	A	0	0	1	0	1	SA0520
gi|29165615|ref|NC_002745.2|	611838	+	C	1	2	0	0	0	SA0520
gi|29165615|ref|NC_002745.2|	611839	+	G	0	1	0	0	1	SA0520
gi|29165615|ref|NC_002745.2|	611840	+	A	0	1	0	0	0	SA0520
gi|29165615|ref|NC_002745.2|	611841	+	U	0	1	0	0	0	SA0520
gi|29165615|ref|NC_002745.2|	611842	+	U	0	0	0	1	0	SA0520
gi|29165615|ref|NC_002745.2|	611843	+	C	0	5	0	3	1	SA0520
gi|29165615|ref|NC_002745.2|	611844	+	A	0	1	0	1	0	SA0520
gi|29165615|ref|NC_002745.2|	611845	+	G	0	1	0	0	1	SA0520
gi|29165615|ref|NC_002745.2|	611846	+	A	0	4	0	0	2	SA0520
gi|29165615|ref|NC_002745.2|	611847	+	C	0	1	0	0	2	SA0520
gi|29165615|ref|NC_002745.2|	611848	+	U	0	1	2	0	2	SA0520
gi|29165615|ref|NC_002745.2|	611849	+	C	0	2	1	0	3	SA0520
gi|29165615|ref|NC_002745.2|	611850	+	A	0	0	0	1	0	SA0520
gi|29165615|ref|NC_002745.2|	611852	+	A	0	1	0	2	0	SA0520
gi|29165615|ref|NC_002745.2|	611857	+	G	0	0	0	0	1	SA0520
gi|29165615|ref|NC_002745.2|	611858	+	A	0	1	0	0	0	SA0520
gi|29165615|ref|NC_002745.2|	611859	+	U	0	1	0	0	0	SA0520
gi|29165615|ref|NC_002745.2|	611860	+	U	0	0	0	1	0	SA0520
gi|29165615|ref|NC_002745.2|	611861	+	C	0	2	0	2	2	SA0520
gi|29165615|ref|NC_002745.2|	611862	+	A	0	1	0	0	1	SA0520
gi|29165615|ref|NC_002745.2|	611863	+	G	0	1	0	0	0	SA0520
gi|29165615|ref|NC_002745.2|	611864	+	A	0	1	0	0	1	SA0520
gi|29165615|ref|NC_002745.2|	611865	+	C	0	1	0	0	0	SA0520
gi|29165615|ref|NC_002745.2|	611866	+	U	0	1	0	0	0	SA0520
gi|29165615|ref|NC_002745.2|	611875	+	G	0	0	0	0	1	SA0520
gi|29165615|ref|NC_002745.2|	611877	+	U	0	0	1	0	0	SA0520
gi|29165615|ref|NC_002745.2|	611879	+	C	0	2	0	1	1	SA0520
gi|29165615|ref|NC_002745.2|	611880	+	A	0	1	0	0	1	SA0520
gi|29165615|ref|NC_002745.2|	611881	+	G	0	0	0	0	1	SA0520
gi|29165615|ref|NC_002745.2|	611882	+	A	0	1	0	0	0	SA0520
gi|29165615|ref|NC_002745.2|	611883	+	U	0	1	0	0	0	SA0520
gi|29165615|ref|NC_002745.2|	611884	+	U	0	1	0	0	0	SA0520
gi|29165615|ref|NC_002745.2|	611893	+	G	0	0	0	0	1	SA0520
gi|29165615|ref|NC_002745.2|	611895	+	U	0	0	1	0	0	SA0520

gi|29165615|ref|NC_002745.2|	611897 +	C	0	2	0	1	1	SA0520
gi|29165615|ref|NC_002745.2|	611898 +	A	0	1	0	0	1	SA0520
gi|29165615|ref|NC_002745.2|	611899 +	G	0	0	0	0	1	SA0520
gi|29165615|ref|NC_002745.2|	611900 +	A	0	1	0	0	0	SA0520
gi|29165615|ref|NC_002745.2|	611901 +	U	0	1	0	0	0	SA0520
gi|29165615|ref|NC_002745.2|	611902 +	U	0	0	0	0	1	SA0520
gi|29165615|ref|NC_002745.2|	611903 +	C	0	1	0	0	0	SA0520
gi|29165615|ref|NC_002745.2|	611905 +	G	0	1	0	0	0	SA0520
gi|29165615|ref|NC_002745.2|	611906 +	A	1	1	0	0	1	SA0520
gi|29165615|ref|NC_002745.2|	611907 +	U	0	1	1	0	1	SA0520
gi|29165615|ref|NC_002745.2|	611908 +	A	0	1	0	0	2	SA0520
gi|29165615|ref|NC_002745.2|	611910 +	U	1	1	0	0	0	SA0520
gi|29165615|ref|NC_002745.2|	611911 +	G	0	1	0	0	0	SA0520
gi|29165615|ref|NC_002745.2|	611915 +	C	0	5	0	3	0	SA0520
gi|29165615|ref|NC_002745.2|	611916 +	A	0	0	0	1	0	SA0520
gi|29165615|ref|NC_002745.2|	611917 +	G	0	0	0	0	1	SA0520
gi|29165615|ref|NC_002745.2|	611918 +	A	1	3	0	0	1	SA0520
gi|29165615|ref|NC_002745.2|	611919 +	U	0	2	0	0	2	SA0520
gi|29165615|ref|NC_002745.2|	611920 +	U	0	0	2	0	1	SA0520
gi|29165615|ref|NC_002745.2|	611921 +	C	0	1	1	0	3	SA0520
gi|29165615|ref|NC_002745.2|	611922 +	A	0	0	0	1	0	SA0520
gi|29165615|ref|NC_002745.2|	611924 +	A	0	2	0	1	2	SA0520
gi|29165615|ref|NC_002745.2|	611925 +	C	0	6	0	0	0	SA0520
gi|29165615|ref|NC_002745.2|	611926 +	A	0	0	1	0	0	SA0520
gi|29165615|ref|NC_002745.2|	611928 +	C	0	1	0	1	3	SA0520
gi|29165615|ref|NC_002745.2|	611929 +	G	0	1	0	0	1	SA0520
gi|29165615|ref|NC_002745.2|	611932 +	U	0	0	0	1	0	SA0520
gi|29165615|ref|NC_002745.2|	611933 +	C	0	1	0	0	1	SA0520
gi|29165615|ref|NC_002745.2|	611936 +	A	0	1	0	0	2	SA0520
gi|29165615|ref|NC_002745.2|	611938 +	U	0	0	1	0	1	SA0520
gi|29165615|ref|NC_002745.2|	611939 +	C	0	0	0	0	1	SA0520
gi|29165615|ref|NC_002745.2|	611942 +	A	0	2	0	0	0	SA0520
gi|29165615|ref|NC_002745.2|	611943 +	U	0	1	0	0	0	SA0520
gi|29165615|ref|NC_002745.2|	611944 +	A	0	1	0	0	0	SA0520
gi|29165615|ref|NC_002745.2|	611946 +	U	0	2	1	0	2	SA0520
gi|29165615|ref|NC_002745.2|	611947 +	G	0	0	0	0	1	SA0520
gi|29165615|ref|NC_002745.2|	611949 +	C	0	1	0	0	0	SA0520
gi|29165615|ref|NC_002745.2|	611950 +	U	0	0	0	1	0	SA0520
gi|29165615|ref|NC_002745.2|	611951 +	C	0	2	0	1	2	SA0520
gi|29165615|ref|NC_002745.2|	611952 +	A	0	1	0	0	1	SA0520
gi|29165615|ref|NC_002745.2|	611953 +	G	0	2	0	0	1	SA0520
gi|29165615|ref|NC_002745.2|	611954 +	A	0	2	0	0	1	SA0520
gi|29165615|ref|NC_002745.2|	611955 +	C	0	0	0	0	1	SA0520
gi|29165615|ref|NC_002745.2|	611956 +	U	0	1	0	0	1	SA0520
gi|29165615|ref|NC_002745.2|	611957 +	C	0	1	0	0	0	SA0520
gi|29165615|ref|NC_002745.2|	611960 +	A	1	1	0	1	1	SA0520
gi|29165615|ref|NC_002745.2|	611961 +	U	0	6	0	0	0	SA0520
gi|29165615|ref|NC_002745.2|	611962 +	A	0	1	0	0	1	SA0520
gi|29165615|ref|NC_002745.2|	611964 +	C	1	2	1	0	0	SA0520
gi|29165615|ref|NC_002745.2|	611965 +	G	0	1	0	0	1	SA0520
gi|29165615|ref|NC_002745.2|	611967 +	U	0	1	0	0	0	SA0520
gi|29165615|ref|NC_002745.2|	611968 +	U	0	0	0	1	0	SA0520
gi|29165615|ref|NC_002745.2|	611969 +	C	0	1	0	1	2	SA0520
gi|29165615|ref|NC_002745.2|	611970 +	A	0	1	0	1	0	SA0520
gi|29165615|ref|NC_002745.2|	611971 +	G	0	0	0	0	1	SA0520
gi|29165615|ref|NC_002745.2|	611972 +	A	0	2	0	0	1	SA0520
gi|29165615|ref|NC_002745.2|	611974 +	U	0	1	0	0	1	SA0520
gi|29165615|ref|NC_002745.2|	611975 +	C	0	1	1	0	2	SA0520
gi|29165615|ref|NC_002745.2|	611976 +	A	0	0	0	1	0	SA0520
gi|29165615|ref|NC_002745.2|	611978 +	A	1	1	0	0	1	SA0520
gi|29165615|ref|NC_002745.2|	611979 +	U	0	1	0	0	0	SA0520
gi|29165615|ref|NC_002745.2|	611980 +	A	0	0	1	0	1	SA0520
gi|29165615|ref|NC_002745.2|	611982 +	C	1	2	0	0	0	SA0520
gi|29165615|ref|NC_002745.2|	611983 +	G	0	1	0	0	1	SA0520
gi|29165615|ref|NC_002745.2|	611984 +	A	0	1	0	0	0	SA0520
gi|29165615|ref|NC_002745.2|	611985 +	U	0	1	0	0	0	SA0520
gi|29165615|ref|NC_002745.2|	611986 +	U	0	0	0	1	0	SA0520
gi|29165615|ref|NC_002745.2|	611987 +	C	0	3	0	1	2	SA0520
gi|29165615|ref|NC_002745.2|	611988 +	A	0	1	0	1	0	SA0520
gi|29165615|ref|NC_002745.2|	611989 +	G	0	1	0	0	1	SA0520
gi|29165615|ref|NC_002745.2|	611990 +	A	0	2	0	0	1	SA0520
gi|29165615|ref|NC_002745.2|	611991 +	C	0	0	0	0	1	SA0520
gi|29165615|ref|NC_002745.2|	611992 +	U	0	0	0	0	1	SA0520
gi|29165615|ref|NC_002745.2|	611993 +	C	0	1	1	0	2	SA0520
gi|29165615|ref|NC_002745.2|	611994 +	A	0	0	0	1	0	SA0520
gi|29165615|ref|NC_002745.2|	611995 +	G	0	1	0	0	0	SA0520
gi|29165615|ref|NC_002745.2|	611996 +	A	1	1	0	3	3	SA0520
gi|29165615|ref|NC_002745.2|	611997 +	C	0	6	1	0	1	SA0520
gi|29165615|ref|NC_002745.2|	611998 +	A	0	1	1	0	2	SA0520
gi|29165615|ref|NC_002745.2|	612000 +	C	1	2	0	0	0	SA0520
gi|29165615|ref|NC_002745.2|	612001 +	G	0	1	0	0	0	SA0520

gi|29165615|ref|NC_002745.2|	612005	+	C	0	5	0	3	0 SA0520
gi|29165615|ref|NC_002745.2|	612006	+	A	0	0	0	1	0 SA0520
gi|29165615|ref|NC_002745.2|	612007	+	G	0	0	0	0	1 SA0520
gi|29165615|ref|NC_002745.2|	612008	+	A	1	3	0	0	1 SA0520
gi|29165615|ref|NC_002745.2|	612009	+	U	0	2	0	0	2 SA0520
gi|29165615|ref|NC_002745.2|	612010	+	U	0	0	2	0	1 SA0520
gi|29165615|ref|NC_002745.2|	612011	+	C	0	1	1	0	3 SA0520
gi|29165615|ref|NC_002745.2|	612012	+	A	0	0	0	1	0 SA0520
gi|29165615|ref|NC_002745.2|	612014	+	A	0	3	0	1	1 SA0520
gi|29165615|ref|NC_002745.2|	612015	+	C	0	7	0	0	2 SA0520
gi|29165615|ref|NC_002745.2|	612016	+	A	0	1	1	0	2 SA0520
gi|29165615|ref|NC_002745.2|	612018	+	C	0	3	1	1	3 SA0520
gi|29165615|ref|NC_002745.2|	612019	+	G	1	0	0	0	1 SA0520
gi|29165615|ref|NC_002745.2|	612022	+	U	0	0	0	1	0 SA0520
gi|29165615|ref|NC_002745.2|	612023	+	C	0	3	0	1	2 SA0520
gi|29165615|ref|NC_002745.2|	612024	+	A	0	2	0	0	0 SA0520
gi|29165615|ref|NC_002745.2|	612025	+	G	0	1	0	0	0 SA0520
gi|29165615|ref|NC_002745.2|	612026	+	A	0	1	0	0	1 SA0520
gi|29165615|ref|NC_002745.2|	612027	+	C	0	0	0	0	1 SA0520
gi|29165615|ref|NC_002745.2|	612032	+	A	0	2	0	0	0 SA0520
gi|29165615|ref|NC_002745.2|	612034	+	A	0	1	0	0	0 SA0520
gi|29165615|ref|NC_002745.2|	612036	+	C	0	2	1	0	0 SA0520
gi|29165615|ref|NC_002745.2|	612039	+	C	0	0	1	0	0 SA0520
gi|29165615|ref|NC_002745.2|	612041	+	C	0	1	0	1	0 SA0520
gi|29165615|ref|NC_002745.2|	612042	+	A	0	0	0	1	0 SA0520
gi|29165615|ref|NC_002745.2|	612043	+	G	0	0	0	0	1 SA0520
gi|29165615|ref|NC_002745.2|	612044	+	A	0	2	0	0	0 SA0520
gi|29165615|ref|NC_002745.2|	612046	+	U	0	0	1	0	1 SA0520
gi|29165615|ref|NC_002745.2|	612047	+	C	0	0	0	0	1 SA0520
gi|29165615|ref|NC_002745.2|	612050	+	A	1	3	0	1	2 SA0520
gi|29165615|ref|NC_002745.2|	612051	+	C	0	7	0	0	0 SA0520
gi|29165615|ref|NC_002745.2|	612052	+	A	0	0	1	0	2 SA0520
gi|29165615|ref|NC_002745.2|	612054	+	C	1	2	0	1	3 SA0520
gi|29165615|ref|NC_002745.2|	612055	+	G	0	1	0	0	1 SA0520
gi|29165615|ref|NC_002745.2|	612057	+	U	0	1	0	0	0 SA0520
gi|29165615|ref|NC_002745.2|	612058	+	U	0	0	0	1	0 SA0520
gi|29165615|ref|NC_002745.2|	612059	+	C	0	3	0	2	1 SA0520
gi|29165615|ref|NC_002745.2|	612060	+	A	0	1	0	0	0 SA0520
gi|29165615|ref|NC_002745.2|	612061	+	G	0	1	0	0	0 SA0520
gi|29165615|ref|NC_002745.2|	612062	+	A	0	3	0	0	1 SA0520
gi|29165615|ref|NC_002745.2|	612063	+	C	0	1	0	0	2 SA0520
gi|29165615|ref|NC_002745.2|	612064	+	U	0	0	1	0	2 SA0520
gi|29165615|ref|NC_002745.2|	612065	+	C	0	0	1	0	3 SA0520
gi|29165615|ref|NC_002745.2|	612066	+	A	0	0	0	1	0 SA0520
gi|29165615|ref|NC_002745.2|	612068	+	A	0	3	0	0	0 SA0520
gi|29165615|ref|NC_002745.2|	612069	+	U	0	2	0	0	0 SA0520
gi|29165615|ref|NC_002745.2|	612070	+	A	0	1	1	0	1 SA0520
gi|29165615|ref|NC_002745.2|	612072	+	C	0	3	1	0	2 SA0520
gi|29165615|ref|NC_002745.2|	612073	+	G	1	0	0	0	1 SA0520
gi|29165615|ref|NC_002745.2|	612074	+	A	0	1	0	0	0 SA0520
gi|29165615|ref|NC_002745.2|	612075	+	C	0	0	1	0	0 SA0520
gi|29165615|ref|NC_002745.2|	612076	+	U	0	0	0	1	0 SA0520
gi|29165615|ref|NC_002745.2|	612077	+	C	0	5	0	3	1 SA0520
gi|29165615|ref|NC_002745.2|	612078	+	A	0	1	0	1	0 SA0520
gi|29165615|ref|NC_002745.2|	612079	+	G	0	1	0	0	1 SA0520
gi|29165615|ref|NC_002745.2|	612080	+	A	0	4	0	0	2 SA0520
gi|29165615|ref|NC_002745.2|	612081	+	C	0	1	0	0	2 SA0520
gi|29165615|ref|NC_002745.2|	612082	+	U	0	1	2	0	2 SA0520
gi|29165615|ref|NC_002745.2|	612083	+	C	0	2	1	0	3 SA0520
gi|29165615|ref|NC_002745.2|	612084	+	A	0	0	0	1	0 SA0520
gi|29165615|ref|NC_002745.2|	612086	+	A	1	3	0	1	2 SA0520
gi|29165615|ref|NC_002745.2|	612087	+	C	0	7	0	0	0 SA0520
gi|29165615|ref|NC_002745.2|	612088	+	A	0	0	1	0	2 SA0520
gi|29165615|ref|NC_002745.2|	612090	+	C	1	2	0	1	3 SA0520
gi|29165615|ref|NC_002745.2|	612091	+	G	0	1	0	0	1 SA0520
gi|29165615|ref|NC_002745.2|	612093	+	U	0	1	0	0	0 SA0520
gi|29165615|ref|NC_002745.2|	612094	+	U	0	0	0	1	0 SA0520
gi|29165615|ref|NC_002745.2|	612095	+	C	0	3	0	2	1 SA0520
gi|29165615|ref|NC_002745.2|	612096	+	A	0	1	0	0	0 SA0520
gi|29165615|ref|NC_002745.2|	612097	+	G	0	1	0	0	0 SA0520
gi|29165615|ref|NC_002745.2|	612098	+	A	0	3	0	0	1 SA0520
gi|29165615|ref|NC_002745.2|	612099	+	C	0	1	0	0	2 SA0520
gi|29165615|ref|NC_002745.2|	612100	+	U	0	1	2	0	2 SA0520
gi|29165615|ref|NC_002745.2|	612101	+	C	0	1	1	0	3 SA0520
gi|29165615|ref|NC_002745.2|	612102	+	A	0	0	0	1	0 SA0520
gi|29165615|ref|NC_002745.2|	612104	+	A	0	2	0	0	1 SA0520
gi|29165615|ref|NC_002745.2|	612105	+	U	0	7	0	0	0 SA0520
gi|29165615|ref|NC_002745.2|	612106	+	A	0	1	1	0	0 SA0520
gi|29165615|ref|NC_002745.2|	612108	+	C	0	3	1	1	3 SA0520
gi|29165615|ref|NC_002745.2|	612109	+	G	0	0	0	0	1 SA0520
gi|29165615|ref|NC_002745.2|	612111	+	C	0	1	0	0	0 SA0520

gi|29165615|ref|NC_002745.2|	612112	+	U	0	0	0	1	0	SA0520
gi|29165615|ref|NC_002745.2|	612113	+	C	0	2	0	1	2	SA0520
gi|29165615|ref|NC_002745.2|	612114	+	A	0	1	0	0	1	SA0520
gi|29165615|ref|NC_002745.2|	612115	+	G	0	2	0	0	1	SA0520
gi|29165615|ref|NC_002745.2|	612116	+	A	0	2	0	0	1	SA0520
gi|29165615|ref|NC_002745.2|	612117	+	C	0	0	0	0	1	SA0520
gi|29165615|ref|NC_002745.2|	612118	+	U	0	1	0	0	1	SA0520
gi|29165615|ref|NC_002745.2|	612119	+	C	0	1	0	0	0	SA0520
gi|29165615|ref|NC_002745.2|	612121	+	G	0	1	0	0	0	SA0520
gi|29165615|ref|NC_002745.2|	612122	+	A	1	3	0	2	0	SA0520
gi|29165615|ref|NC_002745.2|	612123	+	U	0	1	1	0	1	SA0520
gi|29165615|ref|NC_002745.2|	612124	+	A	0	2	0	0	2	SA0520
gi|29165615|ref|NC_002745.2|	612126	+	C	1	2	1	0	0	SA0520
gi|29165615|ref|NC_002745.2|	612127	+	G	0	1	0	0	1	SA0520
gi|29165615|ref|NC_002745.2|	612128	+	A	0	1	0	0	0	SA0520
gi|29165615|ref|NC_002745.2|	612129	+	U	0	0	1	0	0	SA0520
gi|29165615|ref|NC_002745.2|	612131	+	C	0	3	0	3	1	SA0520
gi|29165615|ref|NC_002745.2|	612132	+	A	0	1	0	1	0	SA0520
gi|29165615|ref|NC_002745.2|	612133	+	G	0	1	0	0	1	SA0520
gi|29165615|ref|NC_002745.2|	612134	+	A	0	3	0	0	0	SA0520
gi|29165615|ref|NC_002745.2|	612135	+	U	0	1	0	0	1	SA0520
gi|29165615|ref|NC_002745.2|	612136	+	U	0	0	0	0	1	SA0520
gi|29165615|ref|NC_002745.2|	612137	+	C	0	1	1	0	2	SA0520
gi|29165615|ref|NC_002745.2|	612138	+	A	0	0	0	1	0	SA0520
gi|29165615|ref|NC_002745.2|	612139	+	G	0	1	0	0	0	SA0520
gi|29165615|ref|NC_002745.2|	612140	+	A	1	4	0	3	1	SA0520
gi|29165615|ref|NC_002745.2|	612141	+	C	0	0	1	0	1	SA0520
gi|29165615|ref|NC_002745.2|	612144	+	C	1	1	0	0	0	SA0520
gi|29165615|ref|NC_002745.2|	612145	+	G	0	1	0	0	0	SA0520
gi|29165615|ref|NC_002745.2|	612146	+	A	0	1	0	0	0	SA0520
gi|29165615|ref|NC_002745.2|	612147	+	U	0	0	1	0	0	SA0520
gi|29165615|ref|NC_002745.2|	612149	+	C	0	2	0	1	1	SA0520
gi|29165615|ref|NC_002745.2|	612150	+	A	0	0	0	1	0	SA0520
gi|29165615|ref|NC_002745.2|	612151	+	G	0	1	0	0	1	SA0520
gi|29165615|ref|NC_002745.2|	612152	+	A	0	1	0	0	1	SA0520
gi|29165615|ref|NC_002745.2|	612155	+	C	0	1	0	0	0	SA0520
gi|29165615|ref|NC_002745.2|	612157	+	G	0	1	0	0	0	SA0520
gi|29165615|ref|NC_002745.2|	612158	+	A	1	3	0	2	1	SA0520
gi|29165615|ref|NC_002745.2|	612159	+	C	0	1	1	0	1	SA0520
gi|29165615|ref|NC_002745.2|	612160	+	A	0	1	0	0	2	SA0520
gi|29165615|ref|NC_002745.2|	612162	+	U	1	1	0	0	0	SA0520
gi|29165615|ref|NC_002745.2|	612163	+	G	0	1	0	0	0	SA0520
gi|29165615|ref|NC_002745.2|	612167	+	C	0	5	0	4	1	SA0520
gi|29165615|ref|NC_002745.2|	612168	+	A	0	0	0	1	0	SA0520
gi|29165615|ref|NC_002745.2|	612169	+	G	0	1	0	0	1	SA0520
gi|29165615|ref|NC_002745.2|	612170	+	A	0	2	0	0	0	SA0520
gi|29165615|ref|NC_002745.2|	612171	+	U	0	2	0	0	2	SA0520
gi|29165615|ref|NC_002745.2|	612172	+	U	0	0	2	0	1	SA0520
gi|29165615|ref|NC_002745.2|	612173	+	C	0	2	1	0	3	SA0520
gi|29165615|ref|NC_002745.2|	612174	+	A	0	0	0	1	0	SA0520
gi|29165615|ref|NC_002745.2|	612176	+	A	1	3	0	2	2	SA0520
gi|29165615|ref|NC_002745.2|	612177	+	C	0	6	0	0	0	SA0520
gi|29165615|ref|NC_002745.2|	612178	+	A	0	1	0	0	1	SA0520
gi|29165615|ref|NC_002745.2|	612180	+	C	0	2	1	1	2	SA0520
gi|29165615|ref|NC_002745.2|	612181	+	G	0	0	0	0	1	SA0520
gi|29165615|ref|NC_002745.2|	612183	+	C	0	0	1	0	0	SA0520
gi|29165615|ref|NC_002745.2|	612184	+	U	0	0	0	1	0	SA0520
gi|29165615|ref|NC_002745.2|	612185	+	C	0	1	0	1	2	SA0520
gi|29165615|ref|NC_002745.2|	612186	+	A	0	1	0	1	0	SA0520
gi|29165615|ref|NC_002745.2|	612187	+	G	0	0	0	0	1	SA0520
gi|29165615|ref|NC_002745.2|	612188	+	A	0	2	0	0	1	SA0520
gi|29165615|ref|NC_002745.2|	612190	+	U	0	1	0	0	1	SA0520
gi|29165615|ref|NC_002745.2|	612204	+	A	0	2	1	0	2	SA0520
gi|29165615|ref|NC_002745.2|	612205	+	G	0	0	0	0	1	SA0520
gi|29165615|ref|NC_002745.2|	612207	+	C	0	1	0	0	0	SA0520
gi|29165615|ref|NC_002745.2|	612208	+	U	0	0	0	1	0	SA0520
gi|29165615|ref|NC_002745.2|	612209	+	C	0	2	0	1	2	SA0520
gi|29165615|ref|NC_002745.2|	612210	+	A	0	1	0	0	1	SA0520
gi|29165615|ref|NC_002745.2|	612211	+	G	0	2	0	0	1	SA0520
gi|29165615|ref|NC_002745.2|	612212	+	A	0	2	0	0	1	SA0520
gi|29165615|ref|NC_002745.2|	612213	+	C	0	0	0	0	1	SA0520
gi|29165615|ref|NC_002745.2|	612214	+	U	0	1	0	0	1	SA0520
gi|29165615|ref|NC_002745.2|	612215	+	C	0	1	0	0	0	SA0520
gi|29165615|ref|NC_002745.2|	612217	+	G	0	1	0	0	0	SA0520
gi|29165615|ref|NC_002745.2|	612218	+	A	1	3	0	2	0	SA0520
gi|29165615|ref|NC_002745.2|	612219	+	U	0	1	1	0	1	SA0520
gi|29165615|ref|NC_002745.2|	612220	+	A	0	1	1	0	2	SA0520
gi|29165615|ref|NC_002745.2|	612222	+	C	1	2	0	0	0	SA0520
gi|29165615|ref|NC_002745.2|	612223	+	G	0	1	0	0	0	SA0520
gi|29165615|ref|NC_002745.2|	612227	+	C	0	5	0	4	1	SA0520
gi|29165615|ref|NC_002745.2|	612228	+	A	0	0	0	1	0	SA0520

gi|29165615|ref|NC_002745.2|	612229	+	G	0	0	0	0	1	SA0520
gi|29165615|ref|NC_002745.2|	612230	+	A	0	2	0	0	0	SA0520
gi|29165615|ref|NC_002745.2|	612231	+	U	0	1	0	0	1	SA0520
gi|29165615|ref|NC_002745.2|	612233	+	C	0	2	1	0	2	SA0520
gi|29165615|ref|NC_002745.2|	612234	+	A	0	0	0	1	0	SA0520
gi|29165615|ref|NC_002745.2|	612236	+	A	1	1	0	1	2	SA0520
gi|29165615|ref|NC_002745.2|	612237	+	C	0	6	0	0	0	SA0520
gi|29165615|ref|NC_002745.2|	612238	+	A	0	0	1	0	0	SA0520
gi|29165615|ref|NC_002745.2|	612240	+	C	1	2	0	0	1	SA0520
gi|29165615|ref|NC_002745.2|	612241	+	G	0	1	0	0	0	SA0520
gi|29165615|ref|NC_002745.2|	612245	+	C	0	3	0	3	0	SA0520
gi|29165615|ref|NC_002745.2|	612246	+	A	0	0	0	1	0	SA0520
gi|29165615|ref|NC_002745.2|	612248	+	A	0	1	0	0	0	SA0520
gi|29165615|ref|NC_002745.2|	612249	+	U	0	2	0	0	1	SA0520
gi|29165615|ref|NC_002745.2|	612250	+	U	0	0	1	0	1	SA0520
gi|29165615|ref|NC_002745.2|	612251	+	C	0	0	1	0	3	SA0520
gi|29165615|ref|NC_002745.2|	612252	+	G	0	0	0	1	0	SA0520
gi|29165615|ref|NC_002745.2|	612254	+	A	0	4	0	2	1	SA0520
gi|29165615|ref|NC_002745.2|	612255	+	C	0	1	0	0	0	SA0520
gi|29165615|ref|NC_002745.2|	612256	+	A	0	0	1	0	0	SA0520
gi|29165615|ref|NC_002745.2|	612258	+	C	0	1	0	0	0	SA0520
gi|29165615|ref|NC_002745.2|	612260	+	A	0	1	0	0	0	SA0520
gi|29165615|ref|NC_002745.2|	612261	+	C	0	0	1	0	0	SA0520
gi|29165615|ref|NC_002745.2|	612263	+	C	0	3	0	1	0	SA0520
gi|29165615|ref|NC_002745.2|	612264	+	A	0	1	0	0	0	SA0520
gi|29165615|ref|NC_002745.2|	612265	+	G	0	1	0	0	1	SA0520
gi|29165615|ref|NC_002745.2|	612266	+	A	0	2	0	0	1	SA0520
gi|29165615|ref|NC_002745.2|	612267	+	C	0	0	0	0	1	SA0520
gi|29165615|ref|NC_002745.2|	612269	+	C	0	1	0	0	0	SA0520
gi|29165615|ref|NC_002745.2|	612271	+	G	0	1	0	0	0	SA0520
gi|29165615|ref|NC_002745.2|	612272	+	A	1	2	0	1	2	SA0520
gi|29165615|ref|NC_002745.2|	612273	+	C	0	5	1	0	1	SA0520
gi|29165615|ref|NC_002745.2|	612274	+	A	0	1	0	0	1	SA0520
gi|29165615|ref|NC_002745.2|	612276	+	U	1	1	0	0	0	SA0520
gi|29165615|ref|NC_002745.2|	612277	+	G	0	1	0	0	1	SA0520
gi|29165615|ref|NC_002745.2|	612280	+	U	0	0	0	1	0	SA0520
gi|29165615|ref|NC_002745.2|	612281	+	C	0	3	0	3	2	SA0520
gi|29165615|ref|NC_002745.2|	612282	+	A	0	0	0	1	0	SA0520
gi|29165615|ref|NC_002745.2|	612284	+	A	0	2	0	0	1	SA0520
gi|29165615|ref|NC_002745.2|	612285	+	U	0	2	0	0	1	SA0520
gi|29165615|ref|NC_002745.2|	612286	+	U	0	1	2	0	2	SA0520
gi|29165615|ref|NC_002745.2|	612287	+	C	0	1	1	0	3	SA0520
gi|29165615|ref|NC_002745.2|	612288	+	A	0	0	0	1	0	SA0520
gi|29165615|ref|NC_002745.2|	612290	+	A	0	2	0	0	1	SA0520
gi|29165615|ref|NC_002745.2|	612291	+	U	0	7	0	0	0	SA0520
gi|29165615|ref|NC_002745.2|	612292	+	A	0	1	1	0	2	SA0520
gi|29165615|ref|NC_002745.2|	612294	+	C	0	3	1	1	3	SA0520
gi|29165615|ref|NC_002745.2|	612295	+	G	1	0	0	0	1	SA0520
gi|29165615|ref|NC_002745.2|	612298	+	U	0	0	0	1	0	SA0520
gi|29165615|ref|NC_002745.2|	612299	+	C	0	3	0	1	2	SA0520
gi|29165615|ref|NC_002745.2|	612300	+	A	0	2	0	0	0	SA0520
gi|29165615|ref|NC_002745.2|	612301	+	G	0	2	0	0	0	SA0520
gi|29165615|ref|NC_002745.2|	612302	+	A	0	1	0	0	1	SA0520
gi|29165615|ref|NC_002745.2|	612303	+	C	0	0	0	0	1	SA0520
gi|29165615|ref|NC_002745.2|	612304	+	U	0	0	0	0	1	SA0520
gi|29165615|ref|NC_002745.2|	612305	+	C	0	1	1	0	2	SA0520
gi|29165615|ref|NC_002745.2|	612306	+	A	0	0	0	1	0	SA0520
gi|29165615|ref|NC_002745.2|	612308	+	A	1	3	0	1	2	SA0520
gi|29165615|ref|NC_002745.2|	612309	+	U	0	6	0	0	0	SA0520
gi|29165615|ref|NC_002745.2|	612310	+	A	0	1	1	0	0	SA0520
gi|29165615|ref|NC_002745.2|	612312	+	C	1	3	1	0	0	SA0520
gi|29165615|ref|NC_002745.2|	612313	+	G	0	1	0	0	0	SA0520
gi|29165615|ref|NC_002745.2|	612315	+	C	0	0	1	0	0	SA0520
gi|29165615|ref|NC_002745.2|	612316	+	U	0	0	0	1	0	SA0520
gi|29165615|ref|NC_002745.2|	612317	+	C	0	3	0	4	2	SA0520
gi|29165615|ref|NC_002745.2|	612318	+	A	0	0	0	1	0	SA0520
gi|29165615|ref|NC_002745.2|	612319	+	G	0	1	0	0	1	SA0520
gi|29165615|ref|NC_002745.2|	612320	+	A	0	3	0	0	1	SA0520
gi|29165615|ref|NC_002745.2|	612321	+	U	0	2	0	0	1	SA0520
gi|29165615|ref|NC_002745.2|	612322	+	U	0	1	2	0	2	SA0520
gi|29165615|ref|NC_002745.2|	612323	+	C	0	2	1	0	3	SA0520
gi|29165615|ref|NC_002745.2|	612324	+	A	0	0	0	1	0	SA0520
gi|29165615|ref|NC_002745.2|	612326	+	A	0	1	0	2	0	SA0520
gi|29165615|ref|NC_002745.2|	612328	+	A	0	0	0	0	2	SA0520
gi|29165615|ref|NC_002745.2|	612330	+	C	0	0	0	0	2	SA0520
gi|29165615|ref|NC_002745.2|	612331	+	G	0	0	0	0	1	SA0520
gi|29165615|ref|NC_002745.2|	612335	+	C	0	4	0	1	0	SA0520
gi|29165615|ref|NC_002745.2|	612336	+	A	0	1	0	0	1	SA0520
gi|29165615|ref|NC_002745.2|	612351	+	C	0	0	1	0	0	SA0520
gi|29165615|ref|NC_002745.2|	612353	+	C	0	1	0	1	0	SA0520
gi|29165615|ref|NC_002745.2|	612354	+	A	0	0	0	1	0	SA0520

gi|29165615|ref|NC_002745.2|	612355	+	G	0	0	0	0	1	SA0520
gi|29165615|ref|NC_002745.2|	612356	+	A	0	2	0	0	0	SA0520
gi|29165615|ref|NC_002745.2|	612358	+	U	0	0	1	0	1	SA0520
gi|29165615|ref|NC_002745.2|	612359	+	C	0	0	0	0	1	SA0520
gi|29165615|ref|NC_002745.2|	612362	+	A	1	3	0	1	2	SA0520
gi|29165615|ref|NC_002745.2|	612363	+	C	0	7	0	0	0	SA0520
gi|29165615|ref|NC_002745.2|	612364	+	A	0	0	1	0	2	SA0520
gi|29165615|ref|NC_002745.2|	612366	+	C	1	2	0	1	3	SA0520
gi|29165615|ref|NC_002745.2|	612367	+	G	0	1	0	0	1	SA0520
gi|29165615|ref|NC_002745.2|	612369	+	U	0	1	0	0	0	SA0520
gi|29165615|ref|NC_002745.2|	612370	+	U	0	0	0	1	0	SA0520
gi|29165615|ref|NC_002745.2|	612371	+	C	0	3	0	1	2	SA0520
gi|29165615|ref|NC_002745.2|	612372	+	A	0	2	0	0	0	SA0520
gi|29165615|ref|NC_002745.2|	612373	+	G	0	2	0	0	0	SA0520
gi|29165615|ref|NC_002745.2|	612374	+	A	0	1	0	0	1	SA0520
gi|29165615|ref|NC_002745.2|	612375	+	C	0	0	0	0	1	SA0520
gi|29165615|ref|NC_002745.2|	612376	+	U	0	0	0	0	1	SA0520
gi|29165615|ref|NC_002745.2|	612377	+	C	0	1	1	0	2	SA0520
gi|29165615|ref|NC_002745.2|	612378	+	A	0	0	0	1	0	SA0520
gi|29165615|ref|NC_002745.2|	612380	+	A	0	2	0	2	1	SA0520
gi|29165615|ref|NC_002745.2|	612382	+	A	0	1	0	0	0	SA0520
gi|29165615|ref|NC_002745.2|	612384	+	C	0	1	1	0	0	SA0520
gi|29165615|ref|NC_002745.2|	612387	+	C	0	0	1	0	0	SA0520
gi|29165615|ref|NC_002745.2|	612389	+	C	0	3	0	1	0	SA0520
gi|29165615|ref|NC_002745.2|	612390	+	A	0	0	0	1	0	SA0520
gi|29165615|ref|NC_002745.2|	612391	+	G	0	0	0	0	1	SA0520
gi|29165615|ref|NC_002745.2|	612392	+	A	1	3	0	0	1	SA0520
gi|29165615|ref|NC_002745.2|	612394	+	U	0	0	1	0	1	SA0520
gi|29165615|ref|NC_002745.2|	612474	+	A	0	0	1	0	0	SA0520
gi|29165615|ref|NC_002745.2|	612508	+	A	0	1	0	0	0	SA0520
gi|29165615|ref|NC_002745.2|	612526	+	A	0	1	0	0	0	SA0520
gi|29165615|ref|NC_002745.2|	615151	+	G	0	0	0	0	1	SA0521
gi|29165615|ref|NC_002745.2|	615383	+	A	0	1	0	1	0	SA0521
gi|29165615|ref|NC_002745.2|	615397	+	U	0	0	0	1	0	SA0521
gi|29165615|ref|NC_002745.2|	615403	+	A	0	0	0	0	1	SA0521
gi|29165615|ref|NC_002745.2|	615414	+	C	0	1	0	0	0	SA0521
gi|29165615|ref|NC_002745.2|	615425	+	C	0	0	0	0	1	SA0521
gi|29165615|ref|NC_002745.2|	615431	+	A	0	1	0	0	0	SA0521
gi|29165615|ref|NC_002745.2|	615561	+	A	0	0	1	0	0	SA0521
gi|29165615|ref|NC_002745.2|	615599	+	C	0	0	0	1	0	SA0521
gi|29165615|ref|NC_002745.2|	615623	+	U	0	0	1	0	0	SA0521
gi|29165615|ref|NC_002745.2|	615626	+	A	0	0	0	1	0	SA0521
gi|29165615|ref|NC_002745.2|	615636	+	U	0	0	0	0	1	SA0521
gi|29165615|ref|NC_002745.2|	615656	+	U	1	0	0	0	0	SA0521
gi|29165615|ref|NC_002745.2|	615658	+	U	0	0	0	0	1	SA0521
gi|29165615|ref|NC_002745.2|	615659	+	U	0	0	1	0	0	SA0521
gi|29165615|ref|NC_002745.2|	615662	+	A	0	0	0	0	1	SA0521
gi|29165615|ref|NC_002745.2|	615677	+	U	0	1	0	0	0	SA0521
gi|29165615|ref|NC_002745.2|	615686	+	C	0	2	0	0	0	SA0521
gi|29165615|ref|NC_002745.2|	615691	+	A	0	0	0	0	1	SA0521
gi|29165615|ref|NC_002745.2|	615694	+	A	0	0	1	1	2	SA0521
gi|29165615|ref|NC_002745.2|	615695	+	A	0	1	0	0	1	SA0521
gi|29165615|ref|NC_002745.2|	615704	+	A	0	1	0	0	1	SA0521
gi|29165615|ref|NC_002745.2|	615706	+	G	0	0	0	0	1	SA0521
gi|29165615|ref|NC_002745.2|	615707	+	A	0	0	0	1	3	SA0521
gi|29165615|ref|NC_002745.2|	615709	+	G	0	1	0	1	0	SA0521
gi|29165615|ref|NC_002745.2|	615710	+	A	0	2	0	3	2	SA0521
gi|29165615|ref|NC_002745.2|	615713	+	A	0	0	0	1	0	SA0521
gi|29165615|ref|NC_002745.2|	615722	+	A	0	0	0	1	0	SA0521
gi|29165615|ref|NC_002745.2|	615797	+	C	0	1	0	0	1	SA0521
gi|29165615|ref|NC_002745.2|	615798	+	A	0	1	0	0	1	SA0521
gi|29165615|ref|NC_002745.2|	615799	+	U	0	0	0	0	1	SA0521
gi|29165615|ref|NC_002745.2|	615800	+	C	0	1	1	0	2	SA0521
gi|29165615|ref|NC_002745.2|	615801	+	A	0	0	0	1	0	SA0521
gi|29165615|ref|NC_002745.2|	615802	+	G	0	1	0	0	0	SA0521
gi|29165615|ref|NC_002745.2|	615803	+	A	0	2	0	1	1	SA0521
gi|29165615|ref|NC_002745.2|	615804	+	C	0	1	1	0	1	SA0521
gi|29165615|ref|NC_002745.2|	615808	+	G	0	0	0	0	1	SA0521
gi|29165615|ref|NC_002745.2|	615809	+	A	0	1	0	0	0	SA0521
gi|29165615|ref|NC_002745.2|	615810	+	U	0	0	1	0	0	SA0521
gi|29165615|ref|NC_002745.2|	615811	+	U	0	0	0	1	0	SA0521
gi|29165615|ref|NC_002745.2|	615812	+	C	0	3	0	1	1	SA0521
gi|29165615|ref|NC_002745.2|	615813	+	A	0	0	0	1	0	SA0521
gi|29165615|ref|NC_002745.2|	615814	+	G	0	1	0	0	0	SA0521
gi|29165615|ref|NC_002745.2|	615815	+	A	0	1	0	0	1	SA0521
gi|29165615|ref|NC_002745.2|	615817	+	U	0	0	0	0	1	SA0521
gi|29165615|ref|NC_002745.2|	615818	+	C	0	1	1	0	2	SA0521
gi|29165615|ref|NC_002745.2|	615819	+	A	0	0	0	1	0	SA0521
gi|29165615|ref|NC_002745.2|	615820	+	G	0	1	0	0	0	SA0521
gi|29165615|ref|NC_002745.2|	615821	+	A	1	4	0	0	1	SA0521
gi|29165615|ref|NC_002745.2|	615822	+	C	0	2	1	0	1	SA0521

gi|29165615|ref|NC_002745.2|	615823 +	A	0	1	1	0	1	SA0521
gi|29165615|ref|NC_002745.2|	615825 +	U	1	2	0	0	0	SA0521
gi|29165615|ref|NC_002745.2|	615826 +	G	1	1	0	0	1	SA0521
gi|29165615|ref|NC_002745.2|	615827 +	A	1	1	0	0	0	SA0521
gi|29165615|ref|NC_002745.2|	615830 +	C	0	4	0	4	0	SA0521
gi|29165615|ref|NC_002745.2|	615831 +	A	0	1	0	0	0	SA0521
gi|29165615|ref|NC_002745.2|	615832 +	G	0	2	0	0	1	SA0521
gi|29165615|ref|NC_002745.2|	615833 +	A	0	2	0	0	1	SA0521
gi|29165615|ref|NC_002745.2|	615834 +	C	0	2	0	0	2	SA0521
gi|29165615|ref|NC_002745.2|	615835 +	U	0	0	2	0	1	SA0521
gi|29165615|ref|NC_002745.2|	615836 +	C	0	1	1	0	3	SA0521
gi|29165615|ref|NC_002745.2|	615837 +	A	0	0	0	1	0	SA0521
gi|29165615|ref|NC_002745.2|	615839 +	A	1	3	0	2	3	SA0521
gi|29165615|ref|NC_002745.2|	615840 +	C	0	5	0	0	0	SA0521
gi|29165615|ref|NC_002745.2|	615841 +	A	0	1	0	0	0	SA0521
gi|29165615|ref|NC_002745.2|	615843 +	C	0	1	1	0	0	SA0521
gi|29165615|ref|NC_002745.2|	615846 +	C	0	0	1	0	0	SA0521
gi|29165615|ref|NC_002745.2|	615848 +	C	0	3	0	1	0	SA0521
gi|29165615|ref|NC_002745.2|	615849 +	A	0	0	0	1	0	SA0521
gi|29165615|ref|NC_002745.2|	615850 +	G	0	0	0	0	1	SA0521
gi|29165615|ref|NC_002745.2|	615851 +	A	1	3	0	0	1	SA0521
gi|29165615|ref|NC_002745.2|	615853 +	U	0	0	1	0	1	SA0521
gi|29165615|ref|NC_002745.2|	615854 +	C	0	0	0	0	1	SA0521
gi|29165615|ref|NC_002745.2|	615856 +	G	0	1	0	0	0	SA0521
gi|29165615|ref|NC_002745.2|	615857 +	A	0	3	0	1	1	SA0521
gi|29165615|ref|NC_002745.2|	615858 +	C	0	1	1	0	1	SA0521
gi|29165615|ref|NC_002745.2|	615862 +	G	0	0	0	0	1	SA0521
gi|29165615|ref|NC_002745.2|	615863 +	A	0	1	0	0	0	SA0521
gi|29165615|ref|NC_002745.2|	615864 +	U	0	1	0	0	0	SA0521
gi|29165615|ref|NC_002745.2|	615865 +	U	0	0	0	1	0	SA0521
gi|29165615|ref|NC_002745.2|	615866 +	C	0	2	0	1	2	SA0521
gi|29165615|ref|NC_002745.2|	615867 +	A	0	1	0	0	1	SA0521
gi|29165615|ref|NC_002745.2|	615868 +	G	0	2	0	0	1	SA0521
gi|29165615|ref|NC_002745.2|	615869 +	A	0	2	0	0	1	SA0521
gi|29165615|ref|NC_002745.2|	615870 +	C	0	0	0	0	1	SA0521
gi|29165615|ref|NC_002745.2|	615871 +	U	0	1	0	0	1	SA0521
gi|29165615|ref|NC_002745.2|	615872 +	C	0	1	0	0	0	SA0521
gi|29165615|ref|NC_002745.2|	615874 +	G	0	1	0	0	0	SA0521
gi|29165615|ref|NC_002745.2|	615875 +	A	1	3	0	2	0	SA0521
gi|29165615|ref|NC_002745.2|	615876 +	U	0	1	1	0	1	SA0521
gi|29165615|ref|NC_002745.2|	615877 +	A	0	1	1	0	2	SA0521
gi|29165615|ref|NC_002745.2|	615879 +	C	1	2	0	0	0	SA0521
gi|29165615|ref|NC_002745.2|	615880 +	G	0	1	0	0	0	SA0521
gi|29165615|ref|NC_002745.2|	615884 +	C	0	5	0	3	0	SA0521
gi|29165615|ref|NC_002745.2|	615885 +	A	0	0	0	1	0	SA0521
gi|29165615|ref|NC_002745.2|	615886 +	G	0	0	0	0	1	SA0521
gi|29165615|ref|NC_002745.2|	615887 +	A	1	3	0	0	1	SA0521
gi|29165615|ref|NC_002745.2|	615888 +	U	0	2	0	0	2	SA0521
gi|29165615|ref|NC_002745.2|	615889 +	U	0	0	2	0	1	SA0521
gi|29165615|ref|NC_002745.2|	615890 +	C	0	1	1	0	3	SA0521
gi|29165615|ref|NC_002745.2|	615891 +	A	0	0	0	1	0	SA0521
gi|29165615|ref|NC_002745.2|	615893 +	A	0	3	0	1	1	SA0521
gi|29165615|ref|NC_002745.2|	615894 +	C	0	7	0	0	2	SA0521
gi|29165615|ref|NC_002745.2|	615895 +	A	0	1	1	0	2	SA0521
gi|29165615|ref|NC_002745.2|	615897 +	C	0	3	1	1	3	SA0521
gi|29165615|ref|NC_002745.2|	615898 +	G	1	0	0	0	1	SA0521
gi|29165615|ref|NC_002745.2|	615901 +	U	0	0	0	1	0	SA0521
gi|29165615|ref|NC_002745.2|	615902 +	C	0	3	0	2	1	SA0521
gi|29165615|ref|NC_002745.2|	615903 +	A	0	1	0	0	0	SA0521
gi|29165615|ref|NC_002745.2|	615904 +	G	0	1	0	0	0	SA0521
gi|29165615|ref|NC_002745.2|	615905 +	A	0	3	0	0	1	SA0521
gi|29165615|ref|NC_002745.2|	615906 +	C	0	1	0	0	2	SA0521
gi|29165615|ref|NC_002745.2|	615907 +	U	0	0	1	0	2	SA0521
gi|29165615|ref|NC_002745.2|	615908 +	C	0	0	1	0	3	SA0521
gi|29165615|ref|NC_002745.2|	615909 +	A	0	0	0	1	0	SA0521
gi|29165615|ref|NC_002745.2|	615911 +	A	0	3	0	0	0	SA0521
gi|29165615|ref|NC_002745.2|	615912 +	U	0	2	0	0	0	SA0521
gi|29165615|ref|NC_002745.2|	615913 +	A	0	0	1	0	1	SA0521
gi|29165615|ref|NC_002745.2|	615915 +	C	1	2	0	1	3	SA0521
gi|29165615|ref|NC_002745.2|	615916 +	G	1	1	0	0	0	SA0521
gi|29165615|ref|NC_002745.2|	615917 +	A	1	0	0	0	0	SA0521
gi|29165615|ref|NC_002745.2|	615919 +	U	0	0	0	1	0	SA0521
gi|29165615|ref|NC_002745.2|	615920 +	C	0	5	0	4	1	SA0521
gi|29165615|ref|NC_002745.2|	615921 +	A	0	0	0	1	0	SA0521
gi|29165615|ref|NC_002745.2|	615922 +	G	0	1	0	0	0	SA0521
gi|29165615|ref|NC_002745.2|	615923 +	A	0	3	0	0	2	SA0521
gi|29165615|ref|NC_002745.2|	615924 +	C	0	2	0	0	2	SA0521
gi|29165615|ref|NC_002745.2|	615925 +	U	0	0	2	0	1	SA0521
gi|29165615|ref|NC_002745.2|	615926 +	C	0	1	1	0	3	SA0521
gi|29165615|ref|NC_002745.2|	615927 +	A	0	0	0	1	0	SA0521
gi|29165615|ref|NC_002745.2|	615929 +	A	0	3	0	1	1	SA0521

gi|29165615|ref|NC_002745.2|	615930	+	C	0	7	0	0	2	SA0521
gi|29165615|ref|NC_002745.2|	615931	+	A	0	1	1	0	2	SA0521
gi|29165615|ref|NC_002745.2|	615933	+	C	0	3	1	1	3	SA0521
gi|29165615|ref|NC_002745.2|	615934	+	G	1	0	0	0	1	SA0521
gi|29165615|ref|NC_002745.2|	615937	+	U	0	0	0	1	0	SA0521
gi|29165615|ref|NC_002745.2|	615938	+	C	0	3	0	1	2	SA0521
gi|29165615|ref|NC_002745.2|	615939	+	A	0	2	0	0	0	SA0521
gi|29165615|ref|NC_002745.2|	615940	+	G	0	1	0	0	0	SA0521
gi|29165615|ref|NC_002745.2|	615941	+	A	0	1	0	0	1	SA0521
gi|29165615|ref|NC_002745.2|	615942	+	C	0	0	0	0	1	SA0521
gi|29165615|ref|NC_002745.2|	615947	+	A	0	2	0	0	0	SA0521
gi|29165615|ref|NC_002745.2|	615949	+	A	0	1	0	0	0	SA0521
gi|29165615|ref|NC_002745.2|	615951	+	C	0	2	1	0	0	SA0521
gi|29165615|ref|NC_002745.2|	615954	+	C	0	0	1	0	0	SA0521
gi|29165615|ref|NC_002745.2|	615956	+	C	0	1	0	1	0	SA0521
gi|29165615|ref|NC_002745.2|	615957	+	A	0	0	0	1	0	SA0521
gi|29165615|ref|NC_002745.2|	615958	+	G	0	0	0	0	1	SA0521
gi|29165615|ref|NC_002745.2|	615959	+	A	0	2	0	0	0	SA0521
gi|29165615|ref|NC_002745.2|	615961	+	U	0	0	1	0	1	SA0521
gi|29165615|ref|NC_002745.2|	615962	+	C	0	0	0	0	1	SA0521
gi|29165615|ref|NC_002745.2|	615965	+	A	1	3	0	1	2	SA0521
gi|29165615|ref|NC_002745.2|	615966	+	C	0	7	0	0	0	SA0521
gi|29165615|ref|NC_002745.2|	615967	+	A	0	0	1	0	2	SA0521
gi|29165615|ref|NC_002745.2|	615969	+	C	1	2	0	1	3	SA0521
gi|29165615|ref|NC_002745.2|	615970	+	G	0	1	0	0	1	SA0521
gi|29165615|ref|NC_002745.2|	615972	+	U	0	1	0	0	0	SA0521
gi|29165615|ref|NC_002745.2|	615973	+	U	0	0	0	1	0	SA0521
gi|29165615|ref|NC_002745.2|	615974	+	C	0	3	0	1	2	SA0521
gi|29165615|ref|NC_002745.2|	615975	+	A	0	2	0	0	0	SA0521
gi|29165615|ref|NC_002745.2|	615976	+	G	0	2	0	0	0	SA0521
gi|29165615|ref|NC_002745.2|	615977	+	A	0	1	0	0	1	SA0521
gi|29165615|ref|NC_002745.2|	615978	+	C	0	0	0	0	1	SA0521
gi|29165615|ref|NC_002745.2|	615979	+	U	0	0	0	0	1	SA0521
gi|29165615|ref|NC_002745.2|	615980	+	C	0	1	1	0	2	SA0521
gi|29165615|ref|NC_002745.2|	615981	+	A	0	0	0	1	0	SA0521
gi|29165615|ref|NC_002745.2|	615983	+	A	1	3	0	1	2	SA0521
gi|29165615|ref|NC_002745.2|	615984	+	U	0	6	0	0	0	SA0521
gi|29165615|ref|NC_002745.2|	615985	+	A	0	1	1	0	0	SA0521
gi|29165615|ref|NC_002745.2|	615987	+	C	1	3	1	0	0	SA0521
gi|29165615|ref|NC_002745.2|	615988	+	G	0	1	0	0	0	SA0521
gi|29165615|ref|NC_002745.2|	615990	+	C	0	0	1	0	0	SA0521
gi|29165615|ref|NC_002745.2|	615991	+	U	0	0	0	1	0	SA0521
gi|29165615|ref|NC_002745.2|	615992	+	C	0	3	0	4	2	SA0521
gi|29165615|ref|NC_002745.2|	615993	+	A	0	0	0	1	0	SA0521
gi|29165615|ref|NC_002745.2|	615994	+	G	0	1	0	0	1	SA0521
gi|29165615|ref|NC_002745.2|	615995	+	A	0	3	0	0	1	SA0521
gi|29165615|ref|NC_002745.2|	615996	+	U	0	2	0	0	1	SA0521
gi|29165615|ref|NC_002745.2|	615997	+	U	0	1	2	0	2	SA0521
gi|29165615|ref|NC_002745.2|	615998	+	C	0	2	1	0	3	SA0521
gi|29165615|ref|NC_002745.2|	615999	+	A	0	0	0	1	0	SA0521
gi|29165615|ref|NC_002745.2|	616001	+	A	1	3	0	1	2	SA0521
gi|29165615|ref|NC_002745.2|	616002	+	C	0	7	0	0	0	SA0521
gi|29165615|ref|NC_002745.2|	616003	+	A	0	0	1	0	2	SA0521
gi|29165615|ref|NC_002745.2|	616005	+	C	1	2	0	1	3	SA0521
gi|29165615|ref|NC_002745.2|	616006	+	G	0	1	0	0	1	SA0521
gi|29165615|ref|NC_002745.2|	616008	+	U	0	1	0	0	0	SA0521
gi|29165615|ref|NC_002745.2|	616009	+	U	0	0	0	1	0	SA0521
gi|29165615|ref|NC_002745.2|	616010	+	C	0	3	0	1	2	SA0521
gi|29165615|ref|NC_002745.2|	616011	+	A	0	2	0	0	0	SA0521
gi|29165615|ref|NC_002745.2|	616012	+	G	0	2	0	0	0	SA0521
gi|29165615|ref|NC_002745.2|	616013	+	A	0	1	0	0	1	SA0521
gi|29165615|ref|NC_002745.2|	616014	+	C	0	0	0	0	1	SA0521
gi|29165615|ref|NC_002745.2|	616015	+	U	0	0	0	0	1	SA0521
gi|29165615|ref|NC_002745.2|	616016	+	C	0	1	1	0	2	SA0521
gi|29165615|ref|NC_002745.2|	616017	+	A	0	0	0	1	0	SA0521
gi|29165615|ref|NC_002745.2|	616019	+	A	0	2	0	2	1	SA0521
gi|29165615|ref|NC_002745.2|	616021	+	A	0	0	1	0	0	SA0521
gi|29165615|ref|NC_002745.2|	616023	+	C	1	2	0	0	0	SA0521
gi|29165615|ref|NC_002745.2|	616024	+	G	0	1	0	0	0	SA0521
gi|29165615|ref|NC_002745.2|	616026	+	C	0	0	1	0	0	SA0521
gi|29165615|ref|NC_002745.2|	616027	+	U	0	1	0	0	0	SA0521
gi|29165615|ref|NC_002745.2|	616028	+	C	0	3	0	3	0	SA0521
gi|29165615|ref|NC_002745.2|	616029	+	A	0	0	0	1	0	SA0521
gi|29165615|ref|NC_002745.2|	616031	+	A	1	2	0	0	1	SA0521
gi|29165615|ref|NC_002745.2|	616032	+	U	0	2	0	0	1	SA0521
gi|29165615|ref|NC_002745.2|	616033	+	U	0	0	2	0	1	SA0521
gi|29165615|ref|NC_002745.2|	616034	+	C	0	1	1	0	3	SA0521
gi|29165615|ref|NC_002745.2|	616035	+	A	0	0	0	1	0	SA0521
gi|29165615|ref|NC_002745.2|	616037	+	A	0	3	0	0	2	SA0521
gi|29165615|ref|NC_002745.2|	616038	+	C	0	7	0	0	0	SA0521
gi|29165615|ref|NC_002745.2|	616039	+	A	0	0	1	0	0	SA0521

gi|29165615|ref|NC_002745.2|	616041	+	U	0	1	0	1	3	SA0521
gi|29165615|ref|NC_002745.2|	616042	+	G	1	0	0	0	1	SA0521
gi|29165615|ref|NC_002745.2|	616045	+	U	0	0	0	1	0	SA0521
gi|29165615|ref|NC_002745.2|	616046	+	C	0	3	0	2	1	SA0521
gi|29165615|ref|NC_002745.2|	616047	+	A	0	1	0	0	0	SA0521
gi|29165615|ref|NC_002745.2|	616048	+	G	0	1	0	0	0	SA0521
gi|29165615|ref|NC_002745.2|	616049	+	A	0	3	0	0	1	SA0521
gi|29165615|ref|NC_002745.2|	616050	+	C	0	1	0	0	2	SA0521
gi|29165615|ref|NC_002745.2|	616051	+	U	0	0	1	0	2	SA0521
gi|29165615|ref|NC_002745.2|	616052	+	C	0	0	1	0	3	SA0521
gi|29165615|ref|NC_002745.2|	616053	+	A	0	0	0	1	0	SA0521
gi|29165615|ref|NC_002745.2|	616055	+	A	0	2	0	1	1	SA0521
gi|29165615|ref|NC_002745.2|	616056	+	U	0	1	0	0	0	SA0521
gi|29165615|ref|NC_002745.2|	616057	+	A	0	0	1	0	1	SA0521
gi|29165615|ref|NC_002745.2|	616059	+	C	0	1	0	0	2	SA0521
gi|29165615|ref|NC_002745.2|	616060	+	G	1	0	0	0	0	SA0521
gi|29165615|ref|NC_002745.2|	616062	+	C	0	0	1	0	0	SA0521
gi|29165615|ref|NC_002745.2|	616064	+	C	0	5	0	2	0	SA0521
gi|29165615|ref|NC_002745.2|	616067	+	A	1	3	0	0	2	SA0521
gi|29165615|ref|NC_002745.2|	616068	+	C	0	1	0	0	2	SA0521
gi|29165615|ref|NC_002745.2|	616069	+	U	0	0	1	0	1	SA0521
gi|29165615|ref|NC_002745.2|	616070	+	C	0	0	1	0	3	SA0521
gi|29165615|ref|NC_002745.2|	616071	+	A	0	0	0	1	0	SA0521
gi|29165615|ref|NC_002745.2|	616072	+	G	0	1	0	0	0	SA0521
gi|29165615|ref|NC_002745.2|	616073	+	A	0	3	0	0	1	SA0521
gi|29165615|ref|NC_002745.2|	616074	+	C	0	1	1	0	1	SA0521
gi|29165615|ref|NC_002745.2|	616075	+	A	0	0	1	0	0	SA0521
gi|29165615|ref|NC_002745.2|	616077	+	U	0	1	0	0	0	SA0521
gi|29165615|ref|NC_002745.2|	616078	+	G	1	0	0	0	1	SA0521
gi|29165615|ref|NC_002745.2|	616079	+	A	0	1	0	0	0	SA0521
gi|29165615|ref|NC_002745.2|	616080	+	C	0	0	1	0	0	SA0521
gi|29165615|ref|NC_002745.2|	616081	+	U	0	0	0	1	0	SA0521
gi|29165615|ref|NC_002745.2|	616082	+	C	0	3	0	1	2	SA0521
gi|29165615|ref|NC_002745.2|	616083	+	A	0	1	0	1	0	SA0521
gi|29165615|ref|NC_002745.2|	616084	+	G	0	1	0	0	1	SA0521
gi|29165615|ref|NC_002745.2|	616085	+	A	0	2	0	0	1	SA0521
gi|29165615|ref|NC_002745.2|	616086	+	C	0	0	0	0	1	SA0521
gi|29165615|ref|NC_002745.2|	616087	+	U	0	1	2	0	1	SA0521
gi|29165615|ref|NC_002745.2|	616088	+	C	0	1	0	0	1	SA0521
gi|29165615|ref|NC_002745.2|	616090	+	G	0	1	0	0	0	SA0521
gi|29165615|ref|NC_002745.2|	616091	+	A	1	2	0	2	1	SA0521
gi|29165615|ref|NC_002745.2|	616092	+	C	0	5	1	0	1	SA0521
gi|29165615|ref|NC_002745.2|	616093	+	A	0	2	0	0	2	SA0521
gi|29165615|ref|NC_002745.2|	616095	+	C	1	2	1	1	2	SA0521
gi|29165615|ref|NC_002745.2|	616096	+	G	0	1	0	0	1	SA0521
gi|29165615|ref|NC_002745.2|	616100	+	C	0	2	0	2	1	SA0521
gi|29165615|ref|NC_002745.2|	616101	+	A	0	1	0	0	0	SA0521
gi|29165615|ref|NC_002745.2|	616109	+	A	0	2	0	1	1	SA0521
gi|29165615|ref|NC_002745.2|	616116	+	C	0	0	1	0	0	SA0521
gi|29165615|ref|NC_002745.2|	616118	+	C	0	2	0	0	0	SA0521
gi|29165615|ref|NC_002745.2|	616119	+	A	0	0	0	1	0	SA0521
gi|29165615|ref|NC_002745.2|	616121	+	A	0	1	0	0	1	SA0521
gi|29165615|ref|NC_002745.2|	616126	+	G	0	1	0	0	0	SA0521
gi|29165615|ref|NC_002745.2|	616127	+	A	0	3	0	1	1	SA0521
gi|29165615|ref|NC_002745.2|	616128	+	C	0	1	1	0	1	SA0521
gi|29165615|ref|NC_002745.2|	616129	+	A	0	1	0	0	2	SA0521
gi|29165615|ref|NC_002745.2|	616131	+	U	0	0	0	0	2	SA0521
gi|29165615|ref|NC_002745.2|	616132	+	G	0	1	0	0	1	SA0521
gi|29165615|ref|NC_002745.2|	616134	+	U	0	1	0	0	0	SA0521
gi|29165615|ref|NC_002745.2|	616135	+	U	0	0	0	1	0	SA0521
gi|29165615|ref|NC_002745.2|	616136	+	C	0	3	0	1	2	SA0521
gi|29165615|ref|NC_002745.2|	616137	+	A	0	2	0	0	0	SA0521
gi|29165615|ref|NC_002745.2|	616138	+	G	0	2	0	0	0	SA0521
gi|29165615|ref|NC_002745.2|	616139	+	A	0	1	0	0	1	SA0521
gi|29165615|ref|NC_002745.2|	616140	+	C	0	0	0	0	1	SA0521
gi|29165615|ref|NC_002745.2|	616141	+	U	0	0	0	0	1	SA0521
gi|29165615|ref|NC_002745.2|	616142	+	C	0	1	1	0	2	SA0521
gi|29165615|ref|NC_002745.2|	616143	+	A	0	0	0	1	0	SA0521
gi|29165615|ref|NC_002745.2|	616145	+	A	1	3	0	1	2	SA0521
gi|29165615|ref|NC_002745.2|	616146	+	U	0	6	0	0	0	SA0521
gi|29165615|ref|NC_002745.2|	616147	+	A	0	2	1	0	2	SA0521
gi|29165615|ref|NC_002745.2|	616149	+	C	1	3	1	1	2	SA0521
gi|29165615|ref|NC_002745.2|	616150	+	G	1	1	0	0	0	SA0521
gi|29165615|ref|NC_002745.2|	616152	+	C	0	0	1	0	0	SA0521
gi|29165615|ref|NC_002745.2|	616154	+	C	0	5	0	3	0	SA0521
gi|29165615|ref|NC_002745.2|	616155	+	A	0	0	0	1	0	SA0521
gi|29165615|ref|NC_002745.2|	616156	+	G	0	0	0	0	1	SA0521
gi|29165615|ref|NC_002745.2|	616157	+	A	1	3	0	0	1	SA0521
gi|29165615|ref|NC_002745.2|	616158	+	U	0	2	0	0	2	SA0521
gi|29165615|ref|NC_002745.2|	616159	+	U	0	0	2	0	1	SA0521
gi|29165615|ref|NC_002745.2|	616160	+	C	0	1	1	0	3	SA0521

gi|29165615|ref|NC_002745.2|	616161	+	A	0	0	0	1	0	SA0521
gi|29165615|ref|NC_002745.2|	616163	+	A	0	3	0	1	1	SA0521
gi|29165615|ref|NC_002745.2|	616164	+	C	0	7	0	0	2	SA0521
gi|29165615|ref|NC_002745.2|	616165	+	A	0	1	1	0	2	SA0521
gi|29165615|ref|NC_002745.2|	616167	+	C	0	3	1	1	3	SA0521
gi|29165615|ref|NC_002745.2|	616168	+	G	1	0	0	0	1	SA0521
gi|29165615|ref|NC_002745.2|	616171	+	U	0	0	0	1	0	SA0521
gi|29165615|ref|NC_002745.2|	616172	+	C	0	3	0	2	1	SA0521
gi|29165615|ref|NC_002745.2|	616173	+	A	0	1	0	0	0	SA0521
gi|29165615|ref|NC_002745.2|	616174	+	G	0	1	0	0	0	SA0521
gi|29165615|ref|NC_002745.2|	616175	+	A	0	3	0	0	1	SA0521
gi|29165615|ref|NC_002745.2|	616176	+	C	0	1	0	0	2	SA0521
gi|29165615|ref|NC_002745.2|	616177	+	U	0	0	1	0	2	SA0521
gi|29165615|ref|NC_002745.2|	616178	+	C	0	0	1	0	3	SA0521
gi|29165615|ref|NC_002745.2|	616179	+	A	0	0	0	1	0	SA0521
gi|29165615|ref|NC_002745.2|	616181	+	A	0	2	0	1	1	SA0521
gi|29165615|ref|NC_002745.2|	616182	+	U	0	1	0	0	0	SA0521
gi|29165615|ref|NC_002745.2|	616183	+	A	0	0	1	0	0	SA0521
gi|29165615|ref|NC_002745.2|	616185	+	C	0	1	0	0	0	SA0521
gi|29165615|ref|NC_002745.2|	616187	+	A	0	1	0	0	0	SA0521
gi|29165615|ref|NC_002745.2|	616188	+	C	0	0	1	0	0	SA0521
gi|29165615|ref|NC_002745.2|	616189	+	U	0	0	0	1	0	SA0521
gi|29165615|ref|NC_002745.2|	616190	+	C	0	3	0	1	1	SA0521
gi|29165615|ref|NC_002745.2|	616191	+	A	0	0	0	1	0	SA0521
gi|29165615|ref|NC_002745.2|	616192	+	G	0	1	0	0	0	SA0521
gi|29165615|ref|NC_002745.2|	616193	+	A	0	1	0	0	1	SA0521
gi|29165615|ref|NC_002745.2|	616195	+	U	0	0	1	0	1	SA0521
gi|29165615|ref|NC_002745.2|	616196	+	C	0	0	0	0	1	SA0521
gi|29165615|ref|NC_002745.2|	616198	+	G	0	1	0	0	0	SA0521
gi|29165615|ref|NC_002745.2|	616199	+	A	0	3	0	1	1	SA0521
gi|29165615|ref|NC_002745.2|	616200	+	C	0	1	1	0	1	SA0521
gi|29165615|ref|NC_002745.2|	616204	+	G	0	0	0	0	1	SA0521
gi|29165615|ref|NC_002745.2|	616206	+	U	0	1	0	0	0	SA0521
gi|29165615|ref|NC_002745.2|	616207	+	U	0	0	0	1	0	SA0521
gi|29165615|ref|NC_002745.2|	616208	+	C	0	0	0	0	1	SA0521
gi|29165615|ref|NC_002745.2|	616209	+	A	0	1	0	0	1	SA0521
gi|29165615|ref|NC_002745.2|	616293	+	A	0	0	1	0	0	SA0521
gi|29165615|ref|NC_002745.2|	616327	+	A	0	1	0	0	0	SA0521
gi|29165615|ref|NC_002745.2|	616345	+	A	0	1	0	0	0	SA0521
gi|29165615|ref|NC_002745.2|	634572	+	A	0	0	1	1	1	SA0541
gi|29165615|ref|NC_002745.2|	640713	+	U	0	0	0	1	0	-
gi|29165615|ref|NC_002745.2|	640714	+	G	0	0	2	0	1	-
gi|29165615|ref|NC_002745.2|	640715	+	A	0	1	1	0	0	-
gi|29165615|ref|NC_002745.2|	642582	+	U	0	0	0	1	0	SA0548
gi|29165615|ref|NC_002745.2|	651583	+	C	0	0	0	1	0	-
gi|29165615|ref|NC_002745.2|	651596	+	U	0	1	0	0	0	-
gi|29165615|ref|NC_002745.2|	651611	+	G	0	0	0	0	1	-
gi|29165615|ref|NC_002745.2|	651617	+	U	0	0	1	0	0	-
gi|29165615|ref|NC_002745.2|	651634	+	A	0	0	0	0	1	-
gi|29165615|ref|NC_002745.2|	651641	+	A	0	0	0	0	1	-
gi|29165615|ref|NC_002745.2|	651644	+	C	0	0	0	1	0	-
gi|29165615|ref|NC_002745.2|	651684	+	A	0	0	0	0	1	-
gi|29165615|ref|NC_002745.2|	651686	+	C	0	0	1	0	0	-
gi|29165615|ref|NC_002745.2|	651687	+	A	0	0	0	1	0	-
gi|29165615|ref|NC_002745.2|	651692	+	G	0	0	0	1	0	-
gi|29165615|ref|NC_002745.2|	651700	+	A	0	0	0	5	0	-
gi|29165615|ref|NC_002745.2|	651701	+	A	0	0	0	1	0	-
gi|29165615|ref|NC_002745.2|	651702	+	A	0	0	0	0	1	-
gi|29165615|ref|NC_002745.2|	651705	+	A	0	1	0	3	2	-
gi|29165615|ref|NC_002745.2|	651706	+	A	0	0	0	1	0	-
gi|29165615|ref|NC_002745.2|	651712	+	A	1	0	0	0	0	-
gi|29165615|ref|NC_002745.2|	651754	+	G	0	0	1	0	0	-
gi|29165615|ref|NC_002745.2|	651758	+	A	0	0	0	0	1	-
gi|29165615|ref|NC_002745.2|	651767	+	C	1	2	0	0	0	-
gi|29165615|ref|NC_002745.2|	651768	+	U	0	1	0	0	0	-
gi|29165615|ref|NC_002745.2|	651770	+	A	0	3	5	10	5	-
gi|29165615|ref|NC_002745.2|	651771	+	C	0	0	0	1	0	-
gi|29165615|ref|NC_002745.2|	651773	+	A	0	0	0	0	4	-
gi|29165615|ref|NC_002745.2|	651784	+	G	3	0	0	0	0	-
gi|29165615|ref|NC_002745.2|	651800	+	G	0	2	0	0	0	-
gi|29165615|ref|NC_002745.2|	651815	+	A	0	0	0	0	1	-
gi|29165615|ref|NC_002745.2|	651816	+	A	0	2	0	1	0	-
gi|29165615|ref|NC_002745.2|	651824	+	U	0	0	0	0	1	-
gi|29165615|ref|NC_002745.2|	659124	+	G	0	0	0	1	0	-
gi|29165615|ref|NC_002745.2|	659125	+	C	0	0	1	0	0	-
gi|29165615|ref|NC_002745.2|	662134	+	A	0	0	0	0	1	SA0568
gi|29165615|ref|NC_002745.2|	662797	+	A	0	2	0	0	0	SA0568
gi|29165615|ref|NC_002745.2|	674669	+	A	0	0	1	0	0	SA0582
gi|29165615|ref|NC_002745.2|	678805	+	C	1	1	0	0	0	-
gi|29165615|ref|NC_002745.2|	678806	+	C	0	0	1	0	0	-
gi|29165615|ref|NC_002745.2|	678810	+	U	0	0	0	0	1	-

gi|29165615|ref|NC_002745.2|	678819	+	G	0	1	0	0	0	-
gi|29165615|ref|NC_002745.2|	678820	+	U	0	0	1	0	1	-
gi|29165615|ref|NC_002745.2|	678821	+	A	0	0	0	0	1	-
gi|29165615|ref|NC_002745.2|	678827	+	U	0	1	0	0	0	-
gi|29165615|ref|NC_002745.2|	678845	+	A	0	0	0	0	1	-
gi|29165615|ref|NC_002745.2|	678877	+	A	0	0	0	1	0	-
gi|29165615|ref|NC_002745.2|	678893	+	A	0	1	0	0	0	-
gi|29165615|ref|NC_002745.2|	679104	+	U	0	0	0	0	1	-
gi|29165615|ref|NC_002745.2|	679451	+	U	0	1	0	0	0	-
gi|29165615|ref|NC_002745.2|	679618	+	A	0	0	0	0	1	-
gi|29165615|ref|NC_002745.2|	679619	+	U	0	1	0	0	0	-
gi|29165615|ref|NC_002745.2|	679620	+	A	0	5	2	6	2	-
gi|29165615|ref|NC_002745.2|	679627	+	C	0	0	0	0	1	-
gi|29165615|ref|NC_002745.2|	679632	+	C	2	1	0	0	0	-
gi|29165615|ref|NC_002745.2|	679641	+	U	1	1	1	1	1	-
gi|29165615|ref|NC_002745.2|	679645	+	G	0	0	0	0	1	-
gi|29165615|ref|NC_002745.2|	687960	+	A	0	1	0	0	0	-
gi|29165615|ref|NC_002745.2|	694447	+	U	0	0	0	0	3	-
gi|29165615|ref|NC_002745.2|	697834	+	A	0	1	1	0	0	SA0602
gi|29165615|ref|NC_002745.2|	705846	+	U	0	0	0	1	0	-
gi|29165615|ref|NC_002745.2|	705984	+	A	2	2	2	0	2	-
gi|29165615|ref|NC_002745.2|	709128	+	G	0	1	0	0	0	SA0615
gi|29165615|ref|NC_002745.2|	709129	+	A	0	0	1	0	0	SA0615
gi|29165615|ref|NC_002745.2|	710396	+	A	0	1	0	0	0	SA0616
gi|29165615|ref|NC_002745.2|	710697	+	C	0	1	0	0	0	SA0616
gi|29165615|ref|NC_002745.2|	723141	+	A	1	0	1	0	0	SA0626
gi|29165615|ref|NC_002745.2|	727592	+	A	0	0	0	1	0	SA0632
gi|29165615|ref|NC_002745.2|	727717	+	A	0	0	1	0	0	SA0632
gi|29165615|ref|NC_002745.2|	727718	+	U	0	1	0	0	2	SA0632
gi|29165615|ref|NC_002745.2|	727719	+	A	2	3	2	0	0	SA0632
gi|29165615|ref|NC_002745.2|	727720	+	A	1	2	0	3	0	SA0632
gi|29165615|ref|NC_002745.2|	727722	+	G	0	1	0	0	0	SA0632
gi|29165615|ref|NC_002745.2|	734204	+	U	0	1	0	0	0	SA0640
gi|29165615|ref|NC_002745.2|	747687	+	U	0	0	0	0	1	SA0654
gi|29165615|ref|NC_002745.2|	763716	+	A	0	2	0	0	0	-
gi|29165615|ref|NC_002745.2|	767951	+	A	0	0	0	0	1	SA0674
gi|29165615|ref|NC_002745.2|	768981	+	C	0	0	0	0	1	SA0675
gi|29165615|ref|NC_002745.2|	769509	+	C	0	0	1	0	0	SA0675
gi|29165615|ref|NC_002745.2|	772712	+	G	0	0	1	0	0	SA0677
gi|29165615|ref|NC_002745.2|	772713	+	A	0	0	1	0	0	SA0677
gi|29165615|ref|NC_002745.2|	773185	+	A	0	1	0	0	0	SA0677
gi|29165615|ref|NC_002745.2|	776151	+	A	0	0	0	1	0	-
gi|29165615|ref|NC_002745.2|	776171	+	U	1	0	0	2	0	-
gi|29165615|ref|NC_002745.2|	776178	+	U	0	0	0	0	2	-
gi|29165615|ref|NC_002745.2|	776183	+	C	0	1	0	1	0	-
gi|29165615|ref|NC_002745.2|	776188	+	C	0	0	0	1	2	-
gi|29165615|ref|NC_002745.2|	776189	+	C	0	0	0	0	1	-
gi|29165615|ref|NC_002745.2|	776190	+	A	0	1	0	0	0	-
gi|29165615|ref|NC_002745.2|	776195	+	G	0	0	0	0	1	-
gi|29165615|ref|NC_002745.2|	776196	+	C	0	1	2	0	0	-
gi|29165615|ref|NC_002745.2|	776197	+	A	0	0	2	0	0	-
gi|29165615|ref|NC_002745.2|	776198	+	U	3	2	3	1	10	-
gi|29165615|ref|NC_002745.2|	776203	+	U	0	0	0	1	0	-
gi|29165615|ref|NC_002745.2|	776204	+	G	0	0	0	0	1	-
gi|29165615|ref|NC_002745.2|	776205	+	U	0	0	0	1	0	-
gi|29165615|ref|NC_002745.2|	776206	+	A	0	0	1	1	0	-
gi|29165615|ref|NC_002745.2|	776207	+	G	0	1	0	2	0	-
gi|29165615|ref|NC_002745.2|	776208	+	A	0	1	2	2	0	-
gi|29165615|ref|NC_002745.2|	776209	+	A	2	0	1	1	0	-
gi|29165615|ref|NC_002745.2|	776210	+	U	0	0	1	3	1	-
gi|29165615|ref|NC_002745.2|	776211	+	U	0	0	0	0	1	-
gi|29165615|ref|NC_002745.2|	776214	+	U	0	0	1	0	0	-
gi|29165615|ref|NC_002745.2|	776215	+	U	0	3	1	4	1	-
gi|29165615|ref|NC_002745.2|	776216	+	U	0	1	1	1	1	-
gi|29165615|ref|NC_002745.2|	776219	+	G	0	1	0	0	1	-
gi|29165615|ref|NC_002745.2|	776220	+	A	0	1	2	0	1	-
gi|29165615|ref|NC_002745.2|	776224	+	U	1	0	0	0	0	-
gi|29165615|ref|NC_002745.2|	776225	+	C	2	0	0	0	0	-
gi|29165615|ref|NC_002745.2|	776226	+	U	0	2	0	0	0	-
gi|29165615|ref|NC_002745.2|	776230	+	U	0	1	0	1	1	-
gi|29165615|ref|NC_002745.2|	776232	+	U	1	1	0	0	0	-
gi|29165615|ref|NC_002745.2|	776236	+	G	0	0	1	0	0	-
gi|29165615|ref|NC_002745.2|	776245	+	C	0	1	1	0	0	-
gi|29165615|ref|NC_002745.2|	776247	+	A	0	0	1	0	0	-
gi|29165615|ref|NC_002745.2|	776250	+	A	0	0	1	0	0	-
gi|29165615|ref|NC_002745.2|	776254	+	A	0	0	0	0	2	-
gi|29165615|ref|NC_002745.2|	776265	+	U	0	0	0	0	1	-
gi|29165615|ref|NC_002745.2|	776268	+	C	0	0	1	0	0	-
gi|29165615|ref|NC_002745.2|	776270	+	A	0	0	0	0	4	-
gi|29165615|ref|NC_002745.2|	776275	+	A	0	0	1	0	0	-
gi|29165615|ref|NC_002745.2|	776280	+	C	1	3	4	2	1	-

gi|29165615|ref|NC_002745.2|	776282	+	U	0	1	0	0	0 -
gi|29165615|ref|NC_002745.2|	776287	+	U	0	0	0	0	1 -
gi|29165615|ref|NC_002745.2|	776288	+	C	0	1	0	2	1 -
gi|29165615|ref|NC_002745.2|	776292	+	U	3	2	0	0	1 -
gi|29165615|ref|NC_002745.2|	776293	+	A	0	0	1	0	1 -
gi|29165615|ref|NC_002745.2|	776295	+	U	0	0	1	0	1 -
gi|29165615|ref|NC_002745.2|	776297	+	C	0	0	1	1	1 -
gi|29165615|ref|NC_002745.2|	776298	+	C	1	0	0	0	0 -
gi|29165615|ref|NC_002745.2|	776299	+	A	0	1	1	0	0 -
gi|29165615|ref|NC_002745.2|	776300	+	A	1	1	2	0	0 -
gi|29165615|ref|NC_002745.2|	776302	+	A	0	0	0	0	1 -
gi|29165615|ref|NC_002745.2|	776303	+	U	2	0	0	0	0 -
gi|29165615|ref|NC_002745.2|	776307	+	U	6	2	0	0	3 -
gi|29165615|ref|NC_002745.2|	776310	+	G	0	0	0	0	1 -
gi|29165615|ref|NC_002745.2|	776312	+	A	0	0	1	0	1 -
gi|29165615|ref|NC_002745.2|	776313	+	G	0	1	0	0	0 -
gi|29165615|ref|NC_002745.2|	776316	+	C	0	1	1	0	0 -
gi|29165615|ref|NC_002745.2|	776317	+	A	0	1	0	1	0 -
gi|29165615|ref|NC_002745.2|	776322	+	A	0	0	1	0	0 -
gi|29165615|ref|NC_002745.2|	776323	+	C	2	3	0	0	1 -
gi|29165615|ref|NC_002745.2|	777144	+	U	0	0	0	3	0 -
gi|29165615|ref|NC_002745.2|	777150	+	A	0	0	1	0	0 -
gi|29165615|ref|NC_002745.2|	777162	+	A	0	0	0	2	1 -
gi|29165615|ref|NC_002745.2|	777163	+	A	0	0	0	1	0 -
gi|29165615|ref|NC_002745.2|	777166	+	U	0	0	0	1	0 -
gi|29165615|ref|NC_002745.2|	777167	+	C	0	1	0	0	0 -
gi|29165615|ref|NC_002745.2|	777168	+	U	0	0	0	1	0 -
gi|29165615|ref|NC_002745.2|	777175	+	A	0	0	0	0	1 -
gi|29165615|ref|NC_002745.2|	777180	+	A	0	1	0	0	0 -
gi|29165615|ref|NC_002745.2|	777218	+	A	0	0	0	2	1 -
gi|29165615|ref|NC_002745.2|	777219	+	A	0	0	0	1	0 -
gi|29165615|ref|NC_002745.2|	777222	+	U	0	1	0	0	0 -
gi|29165615|ref|NC_002745.2|	777225	+	A	1	0	0	0	0 -
gi|29165615|ref|NC_002745.2|	777306	+	C	0	0	0	1	0 -
gi|29165615|ref|NC_002745.2|	777319	+	U	0	1	0	0	0 -
gi|29165615|ref|NC_002745.2|	777334	+	G	0	0	0	0	1 -
gi|29165615|ref|NC_002745.2|	777340	+	U	0	0	1	0	0 -
gi|29165615|ref|NC_002745.2|	777357	+	A	0	0	0	0	1 -
gi|29165615|ref|NC_002745.2|	777364	+	A	0	0	0	0	1 -
gi|29165615|ref|NC_002745.2|	777367	+	C	0	0	0	1	0 -
gi|29165615|ref|NC_002745.2|	777383	+	U	0	0	0	0	1 -
gi|29165615|ref|NC_002745.2|	777386	+	U	0	1	0	0	0 -
gi|29165615|ref|NC_002745.2|	777388	+	A	0	2	0	0	0 -
gi|29165615|ref|NC_002745.2|	777422	+	C	0	0	0	3	0 -
gi|29165615|ref|NC_002745.2|	777428	+	A	0	0	1	0	0 -
gi|29165615|ref|NC_002745.2|	777440	+	A	0	0	0	2	1 -
gi|29165615|ref|NC_002745.2|	777441	+	A	0	0	0	1	0 -
gi|29165615|ref|NC_002745.2|	777444	+	U	0	1	0	0	0 -
gi|29165615|ref|NC_002745.2|	777447	+	A	1	0	0	0	0 -
gi|29165615|ref|NC_002745.2|	777449	+	A	0	1	0	0	1 -
gi|29165615|ref|NC_002745.2|	777460	+	G	3	0	0	0	0 -
gi|29165615|ref|NC_002745.2|	777476	+	G	0	2	0	0	0 -
gi|29165615|ref|NC_002745.2|	777491	+	A	0	0	0	0	1 -
gi|29165615|ref|NC_002745.2|	777492	+	A	0	2	0	1	0 -
gi|29165615|ref|NC_002745.2|	777494	+	A	0	0	1	0	0 -
gi|29165615|ref|NC_002745.2|	777525	+	U	0	1	0	0	0 -
gi|29165615|ref|NC_002745.2|	785578	+	A	0	0	0	2	1 -
gi|29165615|ref|NC_002745.2|	787936	+	A	0	1	0	0	0 SA0688
gi|29165615|ref|NC_002745.2|	789356	+	U	1	0	0	0	0 SA0690
gi|29165615|ref|NC_002745.2|	789357	+	U	0	0	1	0	0 SA0690
gi|29165615|ref|NC_002745.2|	790402	+	A	0	0	0	0	1 SA0691
gi|29165615|ref|NC_002745.2|	790719	+	A	0	0	0	0	1 SA0691
gi|29165615|ref|NC_002745.2|	791056	+	A	0	0	0	2	1 -
gi|29165615|ref|NC_002745.2|	791062	+	U	0	0	0	1	0 -
gi|29165615|ref|NC_002745.2|	791069	+	A	0	0	0	0	1 -
gi|29165615|ref|NC_002745.2|	791074	+	A	0	1	0	0	0 -
gi|29165615|ref|NC_002745.2|	807426	+	U	1	0	0	0	0 SA0708
gi|29165615|ref|NC_002745.2|	807428	+	C	0	0	1	0	0 SA0708
gi|29165615|ref|NC_002745.2|	817936	+	G	0	0	1	0	0 -
gi|29165615|ref|NC_002745.2|	817960	+	U	0	0	1	0	0 -
gi|29165615|ref|NC_002745.2|	818052	+	A	0	0	0	1	0 -
gi|29165615|ref|NC_002745.2|	818087	+	C	0	0	0	1	0 -
gi|29165615|ref|NC_002745.2|	818092	+	C	0	0	0	1	2 -
gi|29165615|ref|NC_002745.2|	818093	+	C	0	0	0	0	1 -
gi|29165615|ref|NC_002745.2|	818099	+	G	0	0	0	0	1 -
gi|29165615|ref|NC_002745.2|	818100	+	C	0	1	2	0	0 -
gi|29165615|ref|NC_002745.2|	818101	+	A	0	0	1	0	0 -
gi|29165615|ref|NC_002745.2|	818102	+	U	3	2	3	1	10 -
gi|29165615|ref|NC_002745.2|	818107	+	U	0	0	0	1	0 -
gi|29165615|ref|NC_002745.2|	818108	+	G	0	0	0	0	1 -
gi|29165615|ref|NC_002745.2|	818109	+	U	0	0	0	1	0 -

gi|29165615|ref|NC_002745.2|	818110 +	A	0	0	1	1	0	-
gi|29165615|ref|NC_002745.2|	818111 +	G	0	1	0	2	0	-
gi|29165615|ref|NC_002745.2|	818112 +	A	0	1	2	2	0	-
gi|29165615|ref|NC_002745.2|	818113 +	A	2	0	1	1	0	-
gi|29165615|ref|NC_002745.2|	818114 +	U	0	0	1	3	1	-
gi|29165615|ref|NC_002745.2|	818115 +	U	0	0	0	0	1	-
gi|29165615|ref|NC_002745.2|	818118 +	U	0	0	1	0	0	-
gi|29165615|ref|NC_002745.2|	818119 +	U	0	3	1	4	1	-
gi|29165615|ref|NC_002745.2|	818120 +	U	0	1	1	1	1	-
gi|29165615|ref|NC_002745.2|	818123 +	G	0	1	0	0	1	-
gi|29165615|ref|NC_002745.2|	818124 +	A	0	1	1	2	0	-
gi|29165615|ref|NC_002745.2|	818134 +	U	1	1	0	0	0	-
gi|29165615|ref|NC_002745.2|	818136 +	U	0	0	1	0	0	-
gi|29165615|ref|NC_002745.2|	818144 +	C	0	1	0	1	2	-
gi|29165615|ref|NC_002745.2|	818147 +	C	0	0	0	1	2	-
gi|29165615|ref|NC_002745.2|	818148 +	C	0	0	0	0	1	-
gi|29165615|ref|NC_002745.2|	818149 +	A	0	1	0	0	0	-
gi|29165615|ref|NC_002745.2|	818154 +	G	0	0	0	0	1	-
gi|29165615|ref|NC_002745.2|	818155 +	C	0	1	2	0	0	-
gi|29165615|ref|NC_002745.2|	818156 +	A	0	0	2	0	0	-
gi|29165615|ref|NC_002745.2|	818157 +	U	3	2	3	1	10	-
gi|29165615|ref|NC_002745.2|	818162 +	U	0	0	0	1	0	-
gi|29165615|ref|NC_002745.2|	818163 +	G	0	0	0	0	1	-
gi|29165615|ref|NC_002745.2|	818164 +	U	0	0	0	1	0	-
gi|29165615|ref|NC_002745.2|	818169 +	U	0	0	1	2	0	-
gi|29165615|ref|NC_002745.2|	818174 +	U	0	3	1	3	1	-
gi|29165615|ref|NC_002745.2|	818175 +	U	0	0	0	0	1	-
gi|29165615|ref|NC_002745.2|	818178 +	G	0	1	0	0	0	-
gi|29165615|ref|NC_002745.2|	818179 +	A	0	0	0	1	0	-
gi|29165615|ref|NC_002745.2|	823371 +	G	0	0	1	0	0	-
gi|29165615|ref|NC_002745.2|	823447 +	A	0	2	0	0	0	-
gi|29165615|ref|NC_002745.2|	823536 +	C	0	0	1	0	3	-
gi|29165615|ref|NC_002745.2|	823544 +	U	0	1	1	2	0	-
gi|29165615|ref|NC_002745.2|	823545 +	A	0	0	0	1	0	-
gi|29165615|ref|NC_002745.2|	823553 +	U	0	1	1	0	0	-
gi|29165615|ref|NC_002745.2|	823554 +	U	0	3	0	0	2	-
gi|29165615|ref|NC_002745.2|	823559 +	U	0	2	1	1	1	-
gi|29165615|ref|NC_002745.2|	823560 +	A	0	0	0	2	0	-
gi|29165615|ref|NC_002745.2|	823566 +	C	0	0	0	1	0	-
gi|29165615|ref|NC_002745.2|	823567 +	U	0	0	0	1	0	-
gi|29165615|ref|NC_002745.2|	823585 +	C	0	0	0	1	0	-
gi|29165615|ref|NC_002745.2|	823592 +	G	0	0	0	0	1	-
gi|29165615|ref|NC_002745.2|	823593 +	C	0	1	2	0	0	-
gi|29165615|ref|NC_002745.2|	823594 +	A	0	0	2	0	0	-
gi|29165615|ref|NC_002745.2|	823595 +	U	3	2	3	1	10	-
gi|29165615|ref|NC_002745.2|	823600 +	U	0	0	0	1	0	-
gi|29165615|ref|NC_002745.2|	823601 +	G	0	0	0	0	1	-
gi|29165615|ref|NC_002745.2|	823602 +	U	0	0	0	1	0	-
gi|29165615|ref|NC_002745.2|	823603 +	A	0	0	1	1	0	-
gi|29165615|ref|NC_002745.2|	823604 +	G	0	1	0	2	0	-
gi|29165615|ref|NC_002745.2|	823606 +	A	0	0	1	0	0	-
gi|29165615|ref|NC_002745.2|	823607 +	U	0	0	1	3	1	-
gi|29165615|ref|NC_002745.2|	823608 +	U	0	0	0	0	1	-
gi|29165615|ref|NC_002745.2|	823612 +	U	0	3	1	3	1	-
gi|29165615|ref|NC_002745.2|	823613 +	U	0	0	1	0	1	-
gi|29165615|ref|NC_002745.2|	823624 +	C	0	0	0	1	0	-
gi|29165615|ref|NC_002745.2|	823625 +	U	0	0	0	1	0	-
gi|29165615|ref|NC_002745.2|	823638 +	A	0	0	0	1	0	-
gi|29165615|ref|NC_002745.2|	823643 +	C	0	0	1	1	2	-
gi|29165615|ref|NC_002745.2|	823644 +	C	0	1	0	2	1	-
gi|29165615|ref|NC_002745.2|	823645 +	A	0	5	1	0	1	-
gi|29165615|ref|NC_002745.2|	823646 +	A	0	2	0	2	1	-
gi|29165615|ref|NC_002745.2|	823647 +	C	2	0	0	1	0	-
gi|29165615|ref|NC_002745.2|	823648 +	U	0	1	0	1	0	-
gi|29165615|ref|NC_002745.2|	823649 +	U	0	1	0	0	0	-
gi|29165615|ref|NC_002745.2|	823650 +	G	0	0	0	2	0	-
gi|29165615|ref|NC_002745.2|	823651 +	C	0	0	0	0	1	-
gi|29165615|ref|NC_002745.2|	823652 +	A	0	0	0	0	2	-
gi|29165615|ref|NC_002745.2|	823653 +	U	0	2	1	0	0	-
gi|29165615|ref|NC_002745.2|	823654 +	U	0	0	0	1	0	-
gi|29165615|ref|NC_002745.2|	823661 +	A	0	2	0	0	0	-
gi|29165615|ref|NC_002745.2|	823670 +	A	0	0	0	0	1	-
gi|29165615|ref|NC_002745.2|	823671 +	A	0	0	0	0	1	-
gi|29165615|ref|NC_002745.2|	823683 +	C	0	0	0	1	0	-
gi|29165615|ref|NC_002745.2|	823684 +	U	0	0	0	1	0	-
gi|29165615|ref|NC_002745.2|	823697 +	A	0	0	0	1	0	-
gi|29165615|ref|NC_002745.2|	823702 +	C	0	0	1	1	2	-
gi|29165615|ref|NC_002745.2|	823703 +	C	0	1	0	2	1	-
gi|29165615|ref|NC_002745.2|	823704 +	A	0	5	1	0	1	-
gi|29165615|ref|NC_002745.2|	823705 +	A	0	2	0	2	1	-
gi|29165615|ref|NC_002745.2|	823706 +	C	2	0	0	1	0	-

gi|29165615|ref|NC_002745.2|	823707 +	U	0	1	0	1	0	-
gi|29165615|ref|NC_002745.2|	823708 +	U	0	1	0	0	0	-
gi|29165615|ref|NC_002745.2|	823709 +	G	0	0	0	2	0	-
gi|29165615|ref|NC_002745.2|	823710 +	C	0	0	0	0	1	-
gi|29165615|ref|NC_002745.2|	823711 +	A	0	0	0	0	2	-
gi|29165615|ref|NC_002745.2|	823712 +	U	0	2	1	0	0	-
gi|29165615|ref|NC_002745.2|	823713 +	U	0	0	0	1	0	-
gi|29165615|ref|NC_002745.2|	823720 +	A	0	2	0	0	0	-
gi|29165615|ref|NC_002745.2|	823729 +	A	0	0	0	0	1	-
gi|29165615|ref|NC_002745.2|	823730 +	A	0	0	0	0	1	-
gi|29165615|ref|NC_002745.2|	823742 +	C	0	0	0	1	0	-
gi|29165615|ref|NC_002745.2|	823743 +	U	0	0	0	1	0	-
gi|29165615|ref|NC_002745.2|	823761 +	C	0	0	0	1	0	-
gi|29165615|ref|NC_002745.2|	823768 +	G	0	0	0	0	1	-
gi|29165615|ref|NC_002745.2|	823769 +	C	0	1	2	0	0	-
gi|29165615|ref|NC_002745.2|	823770 +	A	0	0	2	0	0	-
gi|29165615|ref|NC_002745.2|	823771 +	U	3	2	3	1	10	-
gi|29165615|ref|NC_002745.2|	823776 +	U	0	0	0	1	0	-
gi|29165615|ref|NC_002745.2|	823777 +	G	0	0	0	0	1	-
gi|29165615|ref|NC_002745.2|	823778 +	U	0	0	0	1	0	-
gi|29165615|ref|NC_002745.2|	823779 +	A	0	0	1	1	0	-
gi|29165615|ref|NC_002745.2|	823780 +	G	0	1	0	2	0	-
gi|29165615|ref|NC_002745.2|	823781 +	A	0	1	2	2	0	-
gi|29165615|ref|NC_002745.2|	823782 +	A	2	0	1	1	0	-
gi|29165615|ref|NC_002745.2|	823783 +	U	0	0	1	3	1	-
gi|29165615|ref|NC_002745.2|	823784 +	U	0	0	0	0	1	-
gi|29165615|ref|NC_002745.2|	823787 +	U	0	0	1	0	0	-
gi|29165615|ref|NC_002745.2|	823788 +	U	0	3	1	4	1	-
gi|29165615|ref|NC_002745.2|	823789 +	U	0	1	1	1	1	-
gi|29165615|ref|NC_002745.2|	823792 +	G	0	0	0	0	1	-
gi|29165615|ref|NC_002745.2|	823795 +	A	0	0	0	1	0	-
gi|29165615|ref|NC_002745.2|	823800 +	C	0	0	0	1	0	-
gi|29165615|ref|NC_002745.2|	823801 +	U	0	0	0	1	0	-
gi|29165615|ref|NC_002745.2|	823814 +	A	0	0	0	1	0	-
gi|29165615|ref|NC_002745.2|	823819 +	C	0	0	1	1	2	-
gi|29165615|ref|NC_002745.2|	823820 +	C	0	1	0	2	1	-
gi|29165615|ref|NC_002745.2|	823821 +	A	0	5	1	0	1	-
gi|29165615|ref|NC_002745.2|	823822 +	A	0	2	0	2	1	-
gi|29165615|ref|NC_002745.2|	823823 +	C	2	0	0	1	0	-
gi|29165615|ref|NC_002745.2|	823824 +	U	0	1	0	1	0	-
gi|29165615|ref|NC_002745.2|	823825 +	U	0	1	0	0	0	-
gi|29165615|ref|NC_002745.2|	823826 +	G	0	0	0	2	0	-
gi|29165615|ref|NC_002745.2|	823827 +	C	0	0	0	0	1	-
gi|29165615|ref|NC_002745.2|	823828 +	A	0	0	0	0	2	-
gi|29165615|ref|NC_002745.2|	823829 +	U	0	2	1	0	0	-
gi|29165615|ref|NC_002745.2|	823830 +	U	0	0	0	1	0	-
gi|29165615|ref|NC_002745.2|	823837 +	A	0	2	0	0	0	-
gi|29165615|ref|NC_002745.2|	823846 +	A	0	0	0	0	1	-
gi|29165615|ref|NC_002745.2|	823847 +	A	0	0	0	0	1	-
gi|29165615|ref|NC_002745.2|	823851 +	C	0	0	0	0	1	-
gi|29165615|ref|NC_002745.2|	823852 +	A	0	1	1	0	0	-
gi|29165615|ref|NC_002745.2|	823856 +	U	1	0	0	0	0	-
gi|29165615|ref|NC_002745.2|	823857 +	C	2	0	0	0	0	-
gi|29165615|ref|NC_002745.2|	823858 +	U	0	2	0	0	0	-
gi|29165615|ref|NC_002745.2|	823862 +	U	0	1	0	1	1	-
gi|29165615|ref|NC_002745.2|	823864 +	U	1	1	0	0	0	-
gi|29165615|ref|NC_002745.2|	823868 +	G	0	0	1	0	0	-
gi|29165615|ref|NC_002745.2|	823877 +	C	0	0	1	0	0	-
gi|29165615|ref|NC_002745.2|	823879 +	A	0	0	1	0	0	-
gi|29165615|ref|NC_002745.2|	823882 +	G	0	0	1	0	0	-
gi|29165615|ref|NC_002745.2|	823886 +	A	0	0	0	0	2	-
gi|29165615|ref|NC_002745.2|	823900 +	C	0	0	1	0	0	-
gi|29165615|ref|NC_002745.2|	823902 +	A	0	0	0	0	4	-
gi|29165615|ref|NC_002745.2|	823914 +	U	0	1	0	0	0	-
gi|29165615|ref|NC_002745.2|	823954 +	A	0	0	1	0	0	-
gi|29165615|ref|NC_002745.2|	826299 +	U	0	0	1	1	0	SA0722
gi|29165615|ref|NC_002745.2|	830256 +	A	0	0	0	1	0	SA0725
gi|29165615|ref|NC_002745.2|	830864 +	A	0	0	0	1	0	-
gi|29165615|ref|NC_002745.2|	830884 +	U	1	0	0	0	0	-
gi|29165615|ref|NC_002745.2|	830911 +	C	0	0	1	0	3	-
gi|29165615|ref|NC_002745.2|	830917 +	U	0	0	1	0	0	-
gi|29165615|ref|NC_002745.2|	830919 +	U	0	1	0	1	3	-
gi|29165615|ref|NC_002745.2|	830928 +	U	0	1	1	0	0	-
gi|29165615|ref|NC_002745.2|	830929 +	U	0	4	0	0	2	-
gi|29165615|ref|NC_002745.2|	830933 +	G	0	0	1	0	0	-
gi|29165615|ref|NC_002745.2|	830934 +	U	0	3	0	1	0	-
gi|29165615|ref|NC_002745.2|	830944 +	U	1	1	0	0	0	-
gi|29165615|ref|NC_002745.2|	830952 +	C	0	1	0	0	0	-
gi|29165615|ref|NC_002745.2|	830954 +	C	0	0	1	2	1	-
gi|29165615|ref|NC_002745.2|	830958 +	C	0	0	0	0	1	-
gi|29165615|ref|NC_002745.2|	830959 +	A	0	0	0	1	0	-

gi|29165615|ref|NC_002745.2|	830967	+	C	0	0	1	0	3	-
gi|29165615|ref|NC_002745.2|	830973	+	U	0	0	1	0	0	-
gi|29165615|ref|NC_002745.2|	830975	+	U	0	1	0	1	3	-
gi|29165615|ref|NC_002745.2|	830984	+	U	0	1	1	0	0	-
gi|29165615|ref|NC_002745.2|	830985	+	U	0	4	0	0	2	-
gi|29165615|ref|NC_002745.2|	830989	+	G	0	0	1	0	0	-
gi|29165615|ref|NC_002745.2|	830990	+	U	0	3	0	1	0	-
gi|29165615|ref|NC_002745.2|	831000	+	U	1	1	0	0	0	-
gi|29165615|ref|NC_002745.2|	831008	+	C	0	1	0	0	0	-
gi|29165615|ref|NC_002745.2|	831010	+	C	0	0	1	2	1	-
gi|29165615|ref|NC_002745.2|	831014	+	C	0	0	0	0	1	-
gi|29165615|ref|NC_002745.2|	831015	+	A	0	0	0	1	0	-
gi|29165615|ref|NC_002745.2|	831023	+	C	0	0	1	0	3	-
gi|29165615|ref|NC_002745.2|	831029	+	U	0	0	1	0	0	-
gi|29165615|ref|NC_002745.2|	831031	+	U	0	1	0	1	3	-
gi|29165615|ref|NC_002745.2|	831040	+	U	0	1	1	0	0	-
gi|29165615|ref|NC_002745.2|	831041	+	U	0	4	0	0	2	-
gi|29165615|ref|NC_002745.2|	831045	+	G	0	0	1	0	0	-
gi|29165615|ref|NC_002745.2|	831046	+	U	0	3	0	1	0	-
gi|29165615|ref|NC_002745.2|	831056	+	U	1	1	0	0	0	-
gi|29165615|ref|NC_002745.2|	831064	+	C	0	1	0	0	0	-
gi|29165615|ref|NC_002745.2|	831066	+	C	0	0	1	2	1	-
gi|29165615|ref|NC_002745.2|	831070	+	C	0	0	0	0	1	-
gi|29165615|ref|NC_002745.2|	831071	+	A	0	0	0	1	0	-
gi|29165615|ref|NC_002745.2|	831079	+	C	0	0	1	0	3	-
gi|29165615|ref|NC_002745.2|	831085	+	U	0	0	1	0	0	-
gi|29165615|ref|NC_002745.2|	831087	+	U	0	1	0	1	3	-
gi|29165615|ref|NC_002745.2|	831096	+	U	0	1	1	0	0	-
gi|29165615|ref|NC_002745.2|	831097	+	U	0	4	0	0	2	-
gi|29165615|ref|NC_002745.2|	831101	+	G	0	0	1	0	0	-
gi|29165615|ref|NC_002745.2|	831102	+	U	0	3	0	1	0	-
gi|29165615|ref|NC_002745.2|	831112	+	U	1	1	0	0	0	-
gi|29165615|ref|NC_002745.2|	831120	+	C	0	1	0	0	0	-
gi|29165615|ref|NC_002745.2|	831122	+	C	0	0	1	2	1	-
gi|29165615|ref|NC_002745.2|	831126	+	C	0	0	0	0	1	-
gi|29165615|ref|NC_002745.2|	831127	+	A	0	0	0	1	0	-
gi|29165615|ref|NC_002745.2|	831135	+	C	0	0	1	0	3	-
gi|29165615|ref|NC_002745.2|	831143	+	U	0	1	0	2	1	-
gi|29165615|ref|NC_002745.2|	831152	+	U	0	1	2	1	0	-
gi|29165615|ref|NC_002745.2|	831153	+	U	0	3	0	1	2	-
gi|29165615|ref|NC_002745.2|	831157	+	G	0	0	1	0	0	-
gi|29165615|ref|NC_002745.2|	831158	+	C	0	2	0	0	0	-
gi|29165615|ref|NC_002745.2|	831164	+	U	0	0	0	0	1	-
gi|29165615|ref|NC_002745.2|	831170	+	U	0	0	1	0	0	-
gi|29165615|ref|NC_002745.2|	831178	+	C	0	1	0	1	0	-
gi|29165615|ref|NC_002745.2|	831183	+	C	0	0	1	1	2	-
gi|29165615|ref|NC_002745.2|	831184	+	C	0	0	0	2	1	-
gi|29165615|ref|NC_002745.2|	831185	+	A	0	5	0	0	1	-
gi|29165615|ref|NC_002745.2|	831186	+	A	0	1	0	1	1	-
gi|29165615|ref|NC_002745.2|	831187	+	C	1	0	0	1	0	-
gi|29165615|ref|NC_002745.2|	831188	+	U	0	1	0	0	0	-
gi|29165615|ref|NC_002745.2|	831189	+	U	0	1	0	0	0	-
gi|29165615|ref|NC_002745.2|	831190	+	G	0	0	0	2	0	-
gi|29165615|ref|NC_002745.2|	831191	+	C	0	0	0	0	1	-
gi|29165615|ref|NC_002745.2|	831193	+	U	0	1	0	1	0	-
gi|29165615|ref|NC_002745.2|	831201	+	A	0	2	0	0	0	-
gi|29165615|ref|NC_002745.2|	831204	+	A	0	2	0	0	0	-
gi|29165615|ref|NC_002745.2|	831210	+	A	0	0	0	0	1	-
gi|29165615|ref|NC_002745.2|	831211	+	A	0	0	0	0	1	-
gi|29165615|ref|NC_002745.2|	831226	+	U	0	1	0	1	1	-
gi|29165615|ref|NC_002745.2|	831246	+	A	0	0	1	0	0	-
gi|29165615|ref|NC_002745.2|	831250	+	G	0	0	0	0	2	-
gi|29165615|ref|NC_002745.2|	831264	+	C	0	0	1	0	0	-
gi|29165615|ref|NC_002745.2|	831266	+	A	0	0	0	0	3	-
gi|29165615|ref|NC_002745.2|	832700	+	G	0	1	0	0	0	SA0727
gi|29165615|ref|NC_002745.2|	832701	+	C	0	0	2	0	0	SA0727
gi|29165615|ref|NC_002745.2|	832702	+	A	0	0	1	0	0	SA0727
gi|29165615|ref|NC_002745.2|	835299	+	G	0	0	0	0	1	SA0729
gi|29165615|ref|NC_002745.2|	846715	+	A	0	0	1	0	0	-
gi|29165615|ref|NC_002745.2|	850399	+	C	0	0	0	0	1	SA0742
gi|29165615|ref|NC_002745.2|	850401	+	G	0	0	0	0	1	SA0742
gi|29165615|ref|NC_002745.2|	850417	+	C	0	0	0	0	1	SA0742
gi|29165615|ref|NC_002745.2|	850419	+	G	0	0	0	0	1	SA0742
gi|29165615|ref|NC_002745.2|	850435	+	C	0	0	0	0	1	SA0742
gi|29165615|ref|NC_002745.2|	850437	+	G	0	0	0	0	1	SA0742
gi|29165615|ref|NC_002745.2|	850482	+	A	0	0	0	0	2	SA0742
gi|29165615|ref|NC_002745.2|	850484	+	C	0	0	0	0	2	SA0742
gi|29165615|ref|NC_002745.2|	850485	+	G	0	0	0	0	1	SA0742
gi|29165615|ref|NC_002745.2|	850489	+	C	0	4	0	1	0	SA0742
gi|29165615|ref|NC_002745.2|	850490	+	A	0	1	0	0	1	SA0742
gi|29165615|ref|NC_002745.2|	850500	+	A	0	1	0	0	0	SA0742

gi|29165615|ref|NC_002745.2|	850502 +	U	0	1	1	0	0	SA0742
gi|29165615|ref|NC_002745.2|	850510 +	A	0	1	0	0	0	SA0742
gi|29165615|ref|NC_002745.2|	850517 +	U	0	1	0	0	0	SA0742
gi|29165615|ref|NC_002745.2|	850518 +	A	0	0	0	0	2	SA0742
gi|29165615|ref|NC_002745.2|	850520 +	C	0	0	0	0	2	SA0742
gi|29165615|ref|NC_002745.2|	850521 +	G	0	0	0	0	1	SA0742
gi|29165615|ref|NC_002745.2|	850525 +	C	0	2	0	0	0	SA0742
gi|29165615|ref|NC_002745.2|	850526 +	C	0	1	0	0	0	SA0742
gi|29165615|ref|NC_002745.2|	850527 +	G	0	2	0	0	0	SA0742
gi|29165615|ref|NC_002745.2|	850528 +	A	0	1	0	0	1	SA0742
gi|29165615|ref|NC_002745.2|	850529 +	C	0	0	0	0	1	SA0742
gi|29165615|ref|NC_002745.2|	850530 +	U	0	0	0	0	1	SA0742
gi|29165615|ref|NC_002745.2|	850531 +	C	0	1	1	0	2	SA0742
gi|29165615|ref|NC_002745.2|	850532 +	A	0	0	0	1	0	SA0742
gi|29165615|ref|NC_002745.2|	850534 +	A	1	3	0	1	2	SA0742
gi|29165615|ref|NC_002745.2|	850535 +	U	0	6	0	0	0	SA0742
gi|29165615|ref|NC_002745.2|	850536 +	A	0	1	1	0	0	SA0742
gi|29165615|ref|NC_002745.2|	850538 +	C	1	3	1	0	0	SA0742
gi|29165615|ref|NC_002745.2|	850541 +	C	0	0	1	0	0	SA0742
gi|29165615|ref|NC_002745.2|	850543 +	C	0	1	0	1	0	SA0742
gi|29165615|ref|NC_002745.2|	850544 +	A	0	0	0	1	0	SA0742
gi|29165615|ref|NC_002745.2|	850545 +	G	0	0	0	0	1	SA0742
gi|29165615|ref|NC_002745.2|	850546 +	A	0	2	0	0	0	SA0742
gi|29165615|ref|NC_002745.2|	850548 +	U	0	0	0	0	1	SA0742
gi|29165615|ref|NC_002745.2|	850549 +	C	0	1	1	0	2	SA0742
gi|29165615|ref|NC_002745.2|	850550 +	A	0	0	0	1	0	SA0742
gi|29165615|ref|NC_002745.2|	850552 +	A	1	1	0	0	1	SA0742
gi|29165615|ref|NC_002745.2|	850553 +	C	0	1	0	0	0	SA0742
gi|29165615|ref|NC_002745.2|	850554 +	A	0	0	0	0	1	SA0742
gi|29165615|ref|NC_002745.2|	850563 +	G	0	1	0	0	0	SA0742
gi|29165615|ref|NC_002745.2|	850564 +	A	0	1	0	0	1	SA0742
gi|29165615|ref|NC_002745.2|	850566 +	U	0	0	0	0	1	SA0742
gi|29165615|ref|NC_002745.2|	850567 +	C	0	1	1	0	2	SA0742
gi|29165615|ref|NC_002745.2|	850568 +	A	0	0	0	1	0	SA0742
gi|29165615|ref|NC_002745.2|	850570 +	A	0	1	0	1	0	SA0742
gi|29165615|ref|NC_002745.2|	850572 +	A	0	0	0	0	1	SA0742
gi|29165615|ref|NC_002745.2|	850579 +	C	0	2	0	0	0	SA0742
gi|29165615|ref|NC_002745.2|	850582 +	A	0	1	0	0	0	SA0742
gi|29165615|ref|NC_002745.2|	850583 +	C	0	0	0	0	1	SA0742
gi|29165615|ref|NC_002745.2|	850588 +	A	0	1	0	0	1	SA0742
gi|29165615|ref|NC_002745.2|	850589 +	C	0	5	0	0	0	SA0742
gi|29165615|ref|NC_002745.2|	850590 +	A	0	1	0	0	0	SA0742
gi|29165615|ref|NC_002745.2|	850592 +	U	0	1	1	0	0	SA0742
gi|29165615|ref|NC_002745.2|	850600 +	A	0	1	0	0	0	SA0742
gi|29165615|ref|NC_002745.2|	850602 +	U	0	0	0	0	1	SA0742
gi|29165615|ref|NC_002745.2|	850607 +	U	0	1	0	0	0	SA0742
gi|29165615|ref|NC_002745.2|	850608 +	A	0	0	0	0	1	SA0742
gi|29165615|ref|NC_002745.2|	850615 +	C	0	2	0	0	0	SA0742
gi|29165615|ref|NC_002745.2|	850618 +	A	0	1	0	0	0	SA0742
gi|29165615|ref|NC_002745.2|	850619 +	C	0	0	0	0	1	SA0742
gi|29165615|ref|NC_002745.2|	850624 +	A	0	1	0	0	1	SA0742
gi|29165615|ref|NC_002745.2|	850625 +	C	0	5	0	0	0	SA0742
gi|29165615|ref|NC_002745.2|	850626 +	A	0	1	0	0	0	SA0742
gi|29165615|ref|NC_002745.2|	850628 +	U	0	1	1	0	0	SA0742
gi|29165615|ref|NC_002745.2|	850636 +	A	0	1	0	0	0	SA0742
gi|29165615|ref|NC_002745.2|	850638 +	U	0	0	0	0	1	SA0742
gi|29165615|ref|NC_002745.2|	850643 +	U	0	1	0	0	0	SA0742
gi|29165615|ref|NC_002745.2|	850644 +	A	0	0	0	0	1	SA0742
gi|29165615|ref|NC_002745.2|	850651 +	C	0	2	0	0	0	SA0742
gi|29165615|ref|NC_002745.2|	850654 +	A	0	1	0	0	0	SA0742
gi|29165615|ref|NC_002745.2|	850655 +	C	0	0	0	0	1	SA0742
gi|29165615|ref|NC_002745.2|	850660 +	A	0	1	0	0	1	SA0742
gi|29165615|ref|NC_002745.2|	850661 +	C	0	5	0	0	0	SA0742
gi|29165615|ref|NC_002745.2|	850662 +	A	0	1	0	0	0	SA0742
gi|29165615|ref|NC_002745.2|	850664 +	U	0	1	1	0	0	SA0742
gi|29165615|ref|NC_002745.2|	850669 +	C	0	0	0	0	1	SA0742
gi|29165615|ref|NC_002745.2|	850670 +	A	0	1	0	0	0	SA0742
gi|29165615|ref|NC_002745.2|	850678 +	A	0	2	0	1	0	SA0742
gi|29165615|ref|NC_002745.2|	850696 +	A	0	1	0	0	1	SA0742
gi|29165615|ref|NC_002745.2|	850701 +	G	0	0	0	0	1	SA0742
gi|29165615|ref|NC_002745.2|	850704 +	U	0	0	0	1	0	SA0742
gi|29165615|ref|NC_002745.2|	850705 +	C	0	0	0	0	1	SA0742
gi|29165615|ref|NC_002745.2|	850708 +	A	0	1	0	0	1	SA0742
gi|29165615|ref|NC_002745.2|	850710 +	U	0	1	0	0	1	SA0742
gi|29165615|ref|NC_002745.2|	850715 +	U	0	1	0	0	0	SA0742
gi|29165615|ref|NC_002745.2|	850716 +	A	0	0	0	0	2	SA0742
gi|29165615|ref|NC_002745.2|	850718 +	C	0	0	0	0	2	SA0742
gi|29165615|ref|NC_002745.2|	850719 +	G	0	0	0	0	1	SA0742
gi|29165615|ref|NC_002745.2|	850723 +	C	0	2	0	0	0	SA0742
gi|29165615|ref|NC_002745.2|	850724 +	C	0	1	0	0	0	SA0742
gi|29165615|ref|NC_002745.2|	850725 +	G	0	2	0	0	0	SA0742

gi|29165615|ref|NC_002745.2|	850726	+	A	0	1	0	0	1 SA0742
gi|29165615|ref|NC_002745.2|	850727	+	C	0	0	0	0	1 SA0742
gi|29165615|ref|NC_002745.2|	850728	+	U	0	0	0	0	1 SA0742
gi|29165615|ref|NC_002745.2|	850729	+	C	0	1	1	0	2 SA0742
gi|29165615|ref|NC_002745.2|	850730	+	A	0	0	0	1	0 SA0742
gi|29165615|ref|NC_002745.2|	850732	+	A	1	3	0	1	2 SA0742
gi|29165615|ref|NC_002745.2|	850733	+	U	0	6	0	0	0 SA0742
gi|29165615|ref|NC_002745.2|	850734	+	A	0	1	1	0	0 SA0742
gi|29165615|ref|NC_002745.2|	850736	+	C	1	3	1	0	0 SA0742
gi|29165615|ref|NC_002745.2|	850737	+	G	0	1	0	0	0 SA0742
gi|29165615|ref|NC_002745.2|	850739	+	C	0	0	1	0	0 SA0742
gi|29165615|ref|NC_002745.2|	850741	+	C	0	3	0	3	1 SA0742
gi|29165615|ref|NC_002745.2|	850742	+	A	0	1	0	1	0 SA0742
gi|29165615|ref|NC_002745.2|	850743	+	G	0	1	0	0	1 SA0742
gi|29165615|ref|NC_002745.2|	850744	+	A	0	3	0	0	0 SA0742
gi|29165615|ref|NC_002745.2|	850745	+	U	0	1	0	0	1 SA0742
gi|29165615|ref|NC_002745.2|	850746	+	U	0	0	0	0	1 SA0742
gi|29165615|ref|NC_002745.2|	850747	+	C	0	1	1	0	2 SA0742
gi|29165615|ref|NC_002745.2|	850748	+	A	0	0	0	1	0 SA0742
gi|29165615|ref|NC_002745.2|	850749	+	G	0	1	0	0	0 SA0742
gi|29165615|ref|NC_002745.2|	850750	+	A	1	1	0	3	3 SA0742
gi|29165615|ref|NC_002745.2|	850751	+	C	0	6	1	0	1 SA0742
gi|29165615|ref|NC_002745.2|	850752	+	A	0	2	0	0	2 SA0742
gi|29165615|ref|NC_002745.2|	850754	+	C	1	2	1	0	0 SA0742
gi|29165615|ref|NC_002745.2|	850755	+	G	0	1	0	0	0 SA0742
gi|29165615|ref|NC_002745.2|	850759	+	C	0	1	0	1	0 SA0742
gi|29165615|ref|NC_002745.2|	850760	+	A	0	0	0	1	0 SA0742
gi|29165615|ref|NC_002745.2|	850761	+	G	0	0	0	0	1 SA0742
gi|29165615|ref|NC_002745.2|	850762	+	A	0	2	0	0	0 SA0742
gi|29165615|ref|NC_002745.2|	850764	+	U	0	0	0	0	1 SA0742
gi|29165615|ref|NC_002745.2|	850765	+	C	0	1	1	0	2 SA0742
gi|29165615|ref|NC_002745.2|	850766	+	A	0	0	0	1	0 SA0742
gi|29165615|ref|NC_002745.2|	850768	+	A	0	1	0	1	0 SA0742
gi|29165615|ref|NC_002745.2|	850770	+	A	0	0	0	0	1 SA0742
gi|29165615|ref|NC_002745.2|	850777	+	C	0	2	0	0	0 SA0742
gi|29165615|ref|NC_002745.2|	850780	+	A	0	1	0	0	0 SA0742
gi|29165615|ref|NC_002745.2|	850781	+	C	0	0	0	0	1 SA0742
gi|29165615|ref|NC_002745.2|	850786	+	A	0	1	0	0	1 SA0742
gi|29165615|ref|NC_002745.2|	850787	+	C	0	5	0	0	0 SA0742
gi|29165615|ref|NC_002745.2|	850788	+	A	0	1	0	0	0 SA0742
gi|29165615|ref|NC_002745.2|	850790	+	U	0	1	1	0	0 SA0742
gi|29165615|ref|NC_002745.2|	850795	+	C	0	0	0	0	1 SA0742
gi|29165615|ref|NC_002745.2|	850796	+	A	0	1	0	0	0 SA0742
gi|29165615|ref|NC_002745.2|	850797	+	G	0	0	0	0	1 SA0742
gi|29165615|ref|NC_002745.2|	850798	+	A	0	1	0	0	0 SA0742
gi|29165615|ref|NC_002745.2|	850800	+	U	0	0	0	0	1 SA0742
gi|29165615|ref|NC_002745.2|	850801	+	C	0	1	0	0	0 SA0742
gi|29165615|ref|NC_002745.2|	850804	+	A	1	3	0	2	1 SA0742
gi|29165615|ref|NC_002745.2|	850805	+	U	0	1	0	0	0 SA0742
gi|29165615|ref|NC_002745.2|	850808	+	C	1	1	0	0	0 SA0742
gi|29165615|ref|NC_002745.2|	850809	+	G	0	1	0	0	0 SA0742
gi|29165615|ref|NC_002745.2|	850810	+	A	0	1	0	0	0 SA0742
gi|29165615|ref|NC_002745.2|	850811	+	U	0	0	1	0	0 SA0742
gi|29165615|ref|NC_002745.2|	850813	+	C	0	3	0	1	0 SA0742
gi|29165615|ref|NC_002745.2|	850814	+	A	0	0	0	1	0 SA0742
gi|29165615|ref|NC_002745.2|	850815	+	G	0	0	0	0	1 SA0742
gi|29165615|ref|NC_002745.2|	850816	+	A	1	3	0	0	1 SA0742
gi|29165615|ref|NC_002745.2|	850818	+	U	0	0	1	0	1 SA0742
gi|29165615|ref|NC_002745.2|	850819	+	C	0	0	0	0	1 SA0742
gi|29165615|ref|NC_002745.2|	850821	+	G	0	1	0	0	0 SA0742
gi|29165615|ref|NC_002745.2|	850822	+	A	0	3	0	1	1 SA0742
gi|29165615|ref|NC_002745.2|	850823	+	C	0	1	1	0	1 SA0742
gi|29165615|ref|NC_002745.2|	850827	+	G	0	0	0	0	1 SA0742
gi|29165615|ref|NC_002745.2|	850828	+	A	0	1	0	0	0 SA0742
gi|29165615|ref|NC_002745.2|	850829	+	U	0	1	0	0	0 SA0742
gi|29165615|ref|NC_002745.2|	850830	+	U	0	0	0	1	0 SA0742
gi|29165615|ref|NC_002745.2|	850831	+	C	0	2	0	1	2 SA0742
gi|29165615|ref|NC_002745.2|	850832	+	A	0	1	0	0	1 SA0742
gi|29165615|ref|NC_002745.2|	850833	+	G	0	1	0	0	0 SA0742
gi|29165615|ref|NC_002745.2|	850836	+	U	0	0	0	0	1 SA0742
gi|29165615|ref|NC_002745.2|	850837	+	C	0	1	0	0	0 SA0742
gi|29165615|ref|NC_002745.2|	850840	+	A	0	2	0	1	0 SA0742
gi|29165615|ref|NC_002745.2|	850844	+	C	1	1	0	0	0 SA0742
gi|29165615|ref|NC_002745.2|	850845	+	G	0	1	0	0	0 SA0742
gi|29165615|ref|NC_002745.2|	850849	+	C	0	1	0	1	0 SA0742
gi|29165615|ref|NC_002745.2|	850852	+	A	1	1	0	0	0 SA0742
gi|29165615|ref|NC_002745.2|	850855	+	C	0	0	1	0	2 SA0742
gi|29165615|ref|NC_002745.2|	850856	+	C	0	0	0	1	0 SA0742
gi|29165615|ref|NC_002745.2|	850857	+	G	0	1	0	0	0 SA0742
gi|29165615|ref|NC_002745.2|	850858	+	A	0	3	0	0	1 SA0742
gi|29165615|ref|NC_002745.2|	850859	+	C	0	1	1	0	1 SA0742

gi|29165615|ref|NC_002745.2|	850860 +	A	0	0	1	0	0	SA0742
gi|29165615|ref|NC_002745.2|	850862 +	U	0	1	0	0	0	SA0742
gi|29165615|ref|NC_002745.2|	850863 +	G	1	0	0	0	1	SA0742
gi|29165615|ref|NC_002745.2|	850864 +	A	0	1	0	0	0	SA0742
gi|29165615|ref|NC_002745.2|	850865 +	C	0	0	1	0	0	SA0742
gi|29165615|ref|NC_002745.2|	850866 +	U	0	0	0	1	0	SA0742
gi|29165615|ref|NC_002745.2|	850867 +	C	0	3	0	1	2	SA0742
gi|29165615|ref|NC_002745.2|	850868 +	A	0	1	0	1	0	SA0742
gi|29165615|ref|NC_002745.2|	850869 +	G	0	1	0	0	1	SA0742
gi|29165615|ref|NC_002745.2|	850870 +	A	0	1	0	0	0	SA0742
gi|29165615|ref|NC_002745.2|	850871 +	C	0	0	0	0	1	SA0742
gi|29165615|ref|NC_002745.2|	850873 +	C	0	1	0	0	0	SA0742
gi|29165615|ref|NC_002745.2|	850876 +	A	1	1	0	1	1	SA0742
gi|29165615|ref|NC_002745.2|	850877 +	C	0	5	0	0	0	SA0742
gi|29165615|ref|NC_002745.2|	850878 +	A	0	1	0	0	1	SA0742
gi|29165615|ref|NC_002745.2|	850880 +	C	0	1	1	0	0	SA0742
gi|29165615|ref|NC_002745.2|	850881 +	G	0	0	0	0	1	SA0742
gi|29165615|ref|NC_002745.2|	850885 +	C	0	0	0	0	1	SA0742
gi|29165615|ref|NC_002745.2|	850886 +	A	0	1	0	0	0	SA0742
gi|29165615|ref|NC_002745.2|	850894 +	A	0	2	0	1	0	SA0742
gi|29165615|ref|NC_002745.2|	850898 +	C	1	1	0	0	0	SA0742
gi|29165615|ref|NC_002745.2|	850899 +	G	0	1	0	0	0	SA0742
gi|29165615|ref|NC_002745.2|	850903 +	C	0	1	0	0	0	SA0742
gi|29165615|ref|NC_002745.2|	850949 +	G	0	0	1	0	1	SA0742
gi|29165615|ref|NC_002745.2|	850950 +	A	0	2	0	0	1	SA0742
gi|29165615|ref|NC_002745.2|	850952 +	U	0	1	1	0	0	SA0742
gi|29165615|ref|NC_002745.2|	850953 +	G	0	1	0	0	1	SA0742
gi|29165615|ref|NC_002745.2|	850955 +	U	0	1	0	0	0	SA0742
gi|29165615|ref|NC_002745.2|	850956 +	U	0	0	0	1	0	SA0742
gi|29165615|ref|NC_002745.2|	850957 +	C	0	1	0	1	2	SA0742
gi|29165615|ref|NC_002745.2|	850958 +	A	0	1	0	1	0	SA0742
gi|29165615|ref|NC_002745.2|	850959 +	G	0	0	0	0	1	SA0742
gi|29165615|ref|NC_002745.2|	850960 +	A	0	1	0	0	0	SA0742
gi|29165615|ref|NC_002745.2|	850961 +	U	0	1	0	0	0	SA0742
gi|29165615|ref|NC_002745.2|	850962 +	U	0	1	1	0	1	SA0742
gi|29165615|ref|NC_002745.2|	850968 +	A	0	0	0	0	1	SA0742
gi|29165615|ref|NC_002745.2|	850970 +	C	0	0	0	1	0	SA0742
gi|29165615|ref|NC_002745.2|	850971 +	G	0	1	0	0	1	SA0742
gi|29165615|ref|NC_002745.2|	850975 +	C	0	4	0	2	0	SA0742
gi|29165615|ref|NC_002745.2|	850976 +	A	0	0	0	0	1	SA0742
gi|29165615|ref|NC_002745.2|	850978 +	A	0	0	0	0	1	SA0742
gi|29165615|ref|NC_002745.2|	850979 +	A	0	2	0	0	1	SA0742
gi|29165615|ref|NC_002745.2|	850980 +	U	0	0	0	0	1	SA0742
gi|29165615|ref|NC_002745.2|	850981 +	C	0	0	1	0	2	SA0742
gi|29165615|ref|NC_002745.2|	850982 +	A	0	0	0	1	0	SA0742
gi|29165615|ref|NC_002745.2|	850984 +	A	0	4	0	0	0	SA0742
gi|29165615|ref|NC_002745.2|	850985 +	U	0	1	0	0	0	SA0742
gi|29165615|ref|NC_002745.2|	850990 +	A	0	1	0	0	0	SA0742
gi|29165615|ref|NC_002745.2|	850991 +	C	0	0	1	0	0	SA0742
gi|29165615|ref|NC_002745.2|	850993 +	C	0	3	0	1	0	SA0742
gi|29165615|ref|NC_002745.2|	850994 +	A	0	1	0	0	0	SA0742
gi|29165615|ref|NC_002745.2|	850995 +	G	0	1	0	0	1	SA0742
gi|29165615|ref|NC_002745.2|	850996 +	A	0	2	0	0	1	SA0742
gi|29165615|ref|NC_002745.2|	850997 +	C	0	0	0	0	1	SA0742
gi|29165615|ref|NC_002745.2|	850999 +	C	0	1	0	0	0	SA0742
gi|29165615|ref|NC_002745.2|	851001 +	G	0	1	0	0	0	SA0742
gi|29165615|ref|NC_002745.2|	851002 +	A	0	1	0	2	0	SA0742
gi|29165615|ref|NC_002745.2|	851003 +	C	0	0	1	0	1	SA0742
gi|29165615|ref|NC_002745.2|	851004 +	A	0	1	0	0	1	SA0742
gi|29165615|ref|NC_002745.2|	851007 +	G	0	1	0	0	0	SA0742
gi|29165615|ref|NC_002745.2|	851011 +	C	0	1	0	1	0	SA0742
gi|29165615|ref|NC_002745.2|	851014 +	A	1	2	0	0	1	SA0742
gi|29165615|ref|NC_002745.2|	851016 +	U	0	0	0	0	1	SA0742
gi|29165615|ref|NC_002745.2|	851017 +	C	0	0	1	0	2	SA0742
gi|29165615|ref|NC_002745.2|	851018 +	A	0	0	0	1	0	SA0742
gi|29165615|ref|NC_002745.2|	851020 +	A	0	2	0	0	1	SA0742
gi|29165615|ref|NC_002745.2|	851021 +	U	0	2	0	0	0	SA0742
gi|29165615|ref|NC_002745.2|	851022 +	A	0	0	1	0	0	SA0742
gi|29165615|ref|NC_002745.2|	851024 +	U	0	1	0	0	0	SA0742
gi|29165615|ref|NC_002745.2|	851025 +	G	1	0	0	0	1	SA0742
gi|29165615|ref|NC_002745.2|	851026 +	A	0	1	0	0	0	SA0742
gi|29165615|ref|NC_002745.2|	851027 +	C	0	0	1	0	0	SA0742
gi|29165615|ref|NC_002745.2|	851028 +	U	0	0	0	1	0	SA0742
gi|29165615|ref|NC_002745.2|	851029 +	C	0	3	0	2	1	SA0742
gi|29165615|ref|NC_002745.2|	851030 +	A	0	1	0	1	0	SA0742
gi|29165615|ref|NC_002745.2|	851031 +	G	0	1	0	0	1	SA0742
gi|29165615|ref|NC_002745.2|	851032 +	A	0	2	0	0	1	SA0742
gi|29165615|ref|NC_002745.2|	851033 +	C	0	1	0	0	1	SA0742
gi|29165615|ref|NC_002745.2|	851034 +	U	0	1	2	0	1	SA0742
gi|29165615|ref|NC_002745.2|	851035 +	C	0	1	0	0	1	SA0742
gi|29165615|ref|NC_002745.2|	851038 +	A	0	1	0	2	0	SA0742

gi|29165615|ref|NC_002745.2|	851040	+	A	0	0	0	0	1	SA0742
gi|29165615|ref|NC_002745.2|	851042	+	C	0	0	0	1	0	SA0742
gi|29165615|ref|NC_002745.2|	851047	+	C	0	2	0	2	0	SA0742
gi|29165615|ref|NC_002745.2|	851048	+	A	0	0	0	0	1	SA0742
gi|29165615|ref|NC_002745.2|	851051	+	A	0	1	0	0	0	SA0742
gi|29165615|ref|NC_002745.2|	851063	+	C	0	0	1	0	0	SA0742
gi|29165615|ref|NC_002745.2|	851065	+	C	0	1	0	1	0	SA0742
gi|29165615|ref|NC_002745.2|	851066	+	C	0	0	0	1	0	SA0742
gi|29165615|ref|NC_002745.2|	851067	+	G	0	1	0	0	1	SA0742
gi|29165615|ref|NC_002745.2|	851068	+	A	0	2	0	0	0	SA0742
gi|29165615|ref|NC_002745.2|	851069	+	U	0	1	0	0	0	SA0742
gi|29165615|ref|NC_002745.2|	851070	+	U	0	1	2	0	1	SA0742
gi|29165615|ref|NC_002745.2|	851071	+	C	0	1	0	0	1	SA0742
gi|29165615|ref|NC_002745.2|	851074	+	A	0	1	0	2	0	SA0742
gi|29165615|ref|NC_002745.2|	851076	+	A	0	0	0	0	1	SA0742
gi|29165615|ref|NC_002745.2|	851078	+	C	0	0	0	1	0	SA0742
gi|29165615|ref|NC_002745.2|	851083	+	C	0	2	0	2	0	SA0742
gi|29165615|ref|NC_002745.2|	851084	+	A	0	0	0	0	1	SA0742
gi|29165615|ref|NC_002745.2|	851087	+	A	0	1	0	0	0	SA0742
gi|29165615|ref|NC_002745.2|	851094	+	A	0	1	0	0	0	SA0742
gi|29165615|ref|NC_002745.2|	851096	+	U	0	1	1	0	0	SA0742
gi|29165615|ref|NC_002745.2|	851101	+	C	0	0	0	0	1	SA0742
gi|29165615|ref|NC_002745.2|	851102	+	C	0	0	0	0	1	SA0742
gi|29165615|ref|NC_002745.2|	851103	+	G	0	0	0	0	1	SA0742
gi|29165615|ref|NC_002745.2|	851104	+	A	0	1	0	0	0	SA0742
gi|29165615|ref|NC_002745.2|	856732	+	U	0	0	1	0	0	-
gi|29165615|ref|NC_002745.2|	856778	+	G	0	0	0	0	1	-
gi|29165615|ref|NC_002745.2|	856779	+	G	0	1	0	0	0	-
gi|29165615|ref|NC_002745.2|	856780	+	A	0	0	1	0	0	-
gi|29165615|ref|NC_002745.2|	856782	+	G	1	2	0	0	0	-
gi|29165615|ref|NC_002745.2|	856783	+	U	0	3	1	0	0	-
gi|29165615|ref|NC_002745.2|	856784	+	A	0	0	1	0	0	-
gi|29165615|ref|NC_002745.2|	856785	+	U	1	0	0	0	0	-
gi|29165615|ref|NC_002745.2|	856787	+	C	0	11	3	2	3	-
gi|29165615|ref|NC_002745.2|	856788	+	A	0	3	1	0	1	-
gi|29165615|ref|NC_002745.2|	856789	+	A	0	6	1	2	1	-
gi|29165615|ref|NC_002745.2|	856790	+	U	0	9	4	4	5	-
gi|29165615|ref|NC_002745.2|	856791	+	A	0	16	9	3	10	SA0747
gi|29165615|ref|NC_002745.2|	856792	+	U	0	3	1	0	1	SA0747
gi|29165615|ref|NC_002745.2|	856793	+	G	0	4	2	1	1	SA0747
gi|29165615|ref|NC_002745.2|	856794	+	A	1	35	9	4	15	SA0747
gi|29165615|ref|NC_002745.2|	856795	+	A	0	22	12	3	8	SA0747
gi|29165615|ref|NC_002745.2|	856796	+	U	0	1	2	1	0	SA0747
gi|29165615|ref|NC_002745.2|	856797	+	A	0	4	1	0	0	SA0747
gi|29165615|ref|NC_002745.2|	856798	+	A	0	4	3	0	1	SA0747
gi|29165615|ref|NC_002745.2|	856799	+	C	0	1	1	0	0	SA0747
gi|29165615|ref|NC_002745.2|	856800	+	G	0	1	1	0	0	SA0747
gi|29165615|ref|NC_002745.2|	856801	+	G	0	19	2	3	17	SA0747
gi|29165615|ref|NC_002745.2|	856802	+	U	4	1	1	1	2	SA0747
gi|29165615|ref|NC_002745.2|	856803	+	A	0	18	9	5	13	SA0747
gi|29165615|ref|NC_002745.2|	856804	+	C	3	35	21	14	19	SA0747
gi|29165615|ref|NC_002745.2|	856805	+	A	1	21	11	4	11	SA0747
gi|29165615|ref|NC_002745.2|	856806	+	G	0	7	4	2	7	SA0747
gi|29165615|ref|NC_002745.2|	856807	+	U	4	7	5	6	3	SA0747
gi|29165615|ref|NC_002745.2|	856808	+	U	0	9	3	4	8	SA0747
gi|29165615|ref|NC_002745.2|	856809	+	A	0	44	12	11	16	SA0747
gi|29165615|ref|NC_002745.2|	856810	+	A	2	29	15	5	6	SA0747
gi|29165615|ref|NC_002745.2|	856811	+	A	2	48	2	4	14	SA0747
gi|29165615|ref|NC_002745.2|	856812	+	U	0	1	0	1	0	SA0747
gi|29165615|ref|NC_002745.2|	856813	+	G	0	1	0	0	4	SA0747
gi|29165615|ref|NC_002745.2|	856814	+	G	0	3	1	2	10	SA0747
gi|29165615|ref|NC_002745.2|	856815	+	U	0	5	0	2	3	SA0747
gi|29165615|ref|NC_002745.2|	856816	+	U	0	2	1	1	2	SA0747
gi|29165615|ref|NC_002745.2|	856817	+	U	1	9	0	1	1	SA0747
gi|29165615|ref|NC_002745.2|	856818	+	A	1	33	4	13	13	SA0747
gi|29165615|ref|NC_002745.2|	856819	+	A	0	6	3	3	3	SA0747
gi|29165615|ref|NC_002745.2|	856820	+	U	0	6	3	0	2	SA0747
gi|29165615|ref|NC_002745.2|	856821	+	G	0	2	1	3	4	SA0747
gi|29165615|ref|NC_002745.2|	856822	+	C	1	125	15	42	61	SA0747
gi|29165615|ref|NC_002745.2|	856823	+	A	2	56	5	18	45	SA0747
gi|29165615|ref|NC_002745.2|	856824	+	G	1	31	8	11	8	SA0747
gi|29165615|ref|NC_002745.2|	856825	+	A	1	36	8	21	12	SA0747
gi|29165615|ref|NC_002745.2|	856826	+	A	0	4	3	6	3	SA0747
gi|29165615|ref|NC_002745.2|	856827	+	A	0	5	3	2	0	SA0747
gi|29165615|ref|NC_002745.2|	856828	+	A	0	4	1	1	2	SA0747
gi|29165615|ref|NC_002745.2|	856829	+	A	0	0	0	0	1	SA0747
gi|29165615|ref|NC_002745.2|	856830	+	G	0	1	0	0	0	SA0747
gi|29165615|ref|NC_002745.2|	856861	+	G	0	4	1	2	1	SA0747
gi|29165615|ref|NC_002745.2|	856864	+	A	0	1	1	1	0	SA0747
gi|29165615|ref|NC_002745.2|	856865	+	C	0	1	0	0	1	SA0747
gi|29165615|ref|NC_002745.2|	856893	+	G	1	0	0	0	0	SA0747

gi|29165615|ref|NC_002745.2|	856894 +	A	0	2	0	2	1	SA0747
gi|29165615|ref|NC_002745.2|	856895 +	A	0	0	1	0	0	SA0747
gi|29165615|ref|NC_002745.2|	856896 +	G	0	1	0	0	1	SA0747
gi|29165615|ref|NC_002745.2|	856897 +	A	1	0	0	1	0	SA0747
gi|29165615|ref|NC_002745.2|	856900 +	G	0	5	0	3	4	SA0747
gi|29165615|ref|NC_002745.2|	856901 +	A	0	2	0	0	0	SA0747
gi|29165615|ref|NC_002745.2|	856903 +	A	0	0	1	1	0	SA0747
gi|29165615|ref|NC_002745.2|	857273 +	A	1	0	0	0	0	-
gi|29165615|ref|NC_002745.2|	873945 +	A	0	0	0	1	0	-
gi|29165615|ref|NC_002745.2|	873965 +	U	1	0	0	2	0	-
gi|29165615|ref|NC_002745.2|	873972 +	U	0	0	0	0	2	-
gi|29165615|ref|NC_002745.2|	873977 +	C	0	0	0	1	0	-
gi|29165615|ref|NC_002745.2|	873982 +	C	0	0	1	1	2	-
gi|29165615|ref|NC_002745.2|	873983 +	C	0	1	0	2	1	-
gi|29165615|ref|NC_002745.2|	873984 +	A	0	5	1	0	1	-
gi|29165615|ref|NC_002745.2|	873985 +	A	0	2	0	1	1	-
gi|29165615|ref|NC_002745.2|	873986 +	C	1	0	0	1	0	-
gi|29165615|ref|NC_002745.2|	873987 +	U	0	1	0	1	0	-
gi|29165615|ref|NC_002745.2|	873988 +	U	0	1	0	0	0	-
gi|29165615|ref|NC_002745.2|	873990 +	C	0	0	0	0	1	-
gi|29165615|ref|NC_002745.2|	873991 +	A	0	0	0	0	2	-
gi|29165615|ref|NC_002745.2|	873992 +	U	0	2	1	0	0	-
gi|29165615|ref|NC_002745.2|	873993 +	U	0	0	0	1	0	-
gi|29165615|ref|NC_002745.2|	874000 +	A	0	2	0	0	0	-
gi|29165615|ref|NC_002745.2|	874005 +	U	0	0	0	1	0	-
gi|29165615|ref|NC_002745.2|	874009 +	G	0	0	0	0	1	-
gi|29165615|ref|NC_002745.2|	874010 +	A	0	0	0	0	1	-
gi|29165615|ref|NC_002745.2|	874017 +	U	0	0	0	1	0	-
gi|29165615|ref|NC_002745.2|	874021 +	U	0	0	0	0	1	-
gi|29165615|ref|NC_002745.2|	874027 +	U	0	0	1	0	0	-
gi|29165615|ref|NC_002745.2|	874040 +	C	0	0	0	1	2	-
gi|29165615|ref|NC_002745.2|	874041 +	C	0	0	0	0	1	-
gi|29165615|ref|NC_002745.2|	874042 +	A	0	1	0	0	0	-
gi|29165615|ref|NC_002745.2|	874047 +	G	0	0	0	0	1	-
gi|29165615|ref|NC_002745.2|	874048 +	C	0	1	2	0	0	-
gi|29165615|ref|NC_002745.2|	874049 +	A	0	0	2	0	0	-
gi|29165615|ref|NC_002745.2|	874050 +	U	3	2	3	1	10	-
gi|29165615|ref|NC_002745.2|	874055 +	U	0	0	0	1	0	-
gi|29165615|ref|NC_002745.2|	874056 +	G	0	0	0	0	1	-
gi|29165615|ref|NC_002745.2|	874057 +	U	0	0	0	1	0	-
gi|29165615|ref|NC_002745.2|	874058 +	A	0	0	1	1	0	-
gi|29165615|ref|NC_002745.2|	874059 +	G	0	1	0	2	0	-
gi|29165615|ref|NC_002745.2|	874060 +	A	0	1	2	2	0	-
gi|29165615|ref|NC_002745.2|	874061 +	A	2	0	1	1	0	-
gi|29165615|ref|NC_002745.2|	874062 +	U	0	0	1	3	1	-
gi|29165615|ref|NC_002745.2|	874063 +	U	0	0	0	0	1	-
gi|29165615|ref|NC_002745.2|	874066 +	U	0	0	1	0	0	-
gi|29165615|ref|NC_002745.2|	874067 +	U	0	3	1	4	1	-
gi|29165615|ref|NC_002745.2|	874068 +	U	0	1	1	1	1	-
gi|29165615|ref|NC_002745.2|	874071 +	G	0	1	0	0	1	-
gi|29165615|ref|NC_002745.2|	874072 +	A	0	1	2	0	1	-
gi|29165615|ref|NC_002745.2|	874076 +	U	1	0	0	0	0	-
gi|29165615|ref|NC_002745.2|	874077 +	C	2	0	0	0	0	-
gi|29165615|ref|NC_002745.2|	874078 +	U	0	2	0	0	0	-
gi|29165615|ref|NC_002745.2|	874082 +	U	0	1	0	1	1	-
gi|29165615|ref|NC_002745.2|	874084 +	U	1	1	0	0	0	-
gi|29165615|ref|NC_002745.2|	874088 +	G	0	0	1	0	0	-
gi|29165615|ref|NC_002745.2|	874097 +	C	0	1	1	0	0	-
gi|29165615|ref|NC_002745.2|	874099 +	A	0	0	1	0	0	-
gi|29165615|ref|NC_002745.2|	874102 +	A	0	0	1	0	0	-
gi|29165615|ref|NC_002745.2|	874106 +	A	0	0	0	0	2	-
gi|29165615|ref|NC_002745.2|	874117 +	U	0	0	0	0	1	-
gi|29165615|ref|NC_002745.2|	874120 +	C	0	0	1	0	0	-
gi|29165615|ref|NC_002745.2|	874122 +	A	0	0	0	0	4	-
gi|29165615|ref|NC_002745.2|	874127 +	A	0	0	1	0	0	-
gi|29165615|ref|NC_002745.2|	874132 +	C	1	3	4	2	1	-
gi|29165615|ref|NC_002745.2|	874134 +	U	0	1	0	0	0	-
gi|29165615|ref|NC_002745.2|	874139 +	U	0	0	0	0	1	-
gi|29165615|ref|NC_002745.2|	874140 +	C	0	1	0	2	1	-
gi|29165615|ref|NC_002745.2|	874144 +	U	3	2	0	0	1	-
gi|29165615|ref|NC_002745.2|	874145 +	A	0	0	1	0	1	-
gi|29165615|ref|NC_002745.2|	874147 +	U	0	0	1	0	2	-
gi|29165615|ref|NC_002745.2|	874149 +	C	0	0	1	1	3	-
gi|29165615|ref|NC_002745.2|	874150 +	C	1	0	0	0	0	-
gi|29165615|ref|NC_002745.2|	874151 +	A	0	1	1	0	0	-
gi|29165615|ref|NC_002745.2|	874152 +	A	1	1	2	0	0	-
gi|29165615|ref|NC_002745.2|	874154 +	A	0	0	0	0	1	-
gi|29165615|ref|NC_002745.2|	874155 +	U	2	0	0	0	0	-
gi|29165615|ref|NC_002745.2|	874159 +	U	6	2	0	0	3	-
gi|29165615|ref|NC_002745.2|	874162 +	G	0	0	0	0	1	-
gi|29165615|ref|NC_002745.2|	874164 +	A	0	0	1	0	1	-

gi|29165615|ref|NC_002745.2|	874165	+	G	0	1	0	0	0	-
gi|29165615|ref|NC_002745.2|	874168	+	C	0	1	1	0	0	-
gi|29165615|ref|NC_002745.2|	874174	+	A	0	1	0	2	0	-
gi|29165615|ref|NC_002745.2|	874230	+	A	0	2	0	0	0	-
gi|29165615|ref|NC_002745.2|	874270	+	A	0	0	0	1	0	-
gi|29165615|ref|NC_002745.2|	874303	+	C	0	1	0	0	0	-
gi|29165615|ref|NC_002745.2|	874305	+	C	0	0	1	2	0	-
gi|29165615|ref|NC_002745.2|	874309	+	C	0	0	0	0	1	-
gi|29165615|ref|NC_002745.2|	874310	+	A	0	0	0	1	0	-
gi|29165615|ref|NC_002745.2|	874318	+	C	0	0	1	0	3	-
gi|29165615|ref|NC_002745.2|	874326	+	U	0	1	0	2	1	-
gi|29165615|ref|NC_002745.2|	874335	+	U	0	1	2	1	0	-
gi|29165615|ref|NC_002745.2|	874336	+	U	0	3	0	1	2	-
gi|29165615|ref|NC_002745.2|	874341	+	C	0	2	0	0	0	-
gi|29165615|ref|NC_002745.2|	874351	+	U	1	1	0	0	0	-
gi|29165615|ref|NC_002745.2|	874359	+	C	0	1	0	0	0	-
gi|29165615|ref|NC_002745.2|	874361	+	C	0	0	1	2	1	-
gi|29165615|ref|NC_002745.2|	874365	+	C	0	0	0	0	1	-
gi|29165615|ref|NC_002745.2|	874366	+	A	0	0	0	1	0	-
gi|29165615|ref|NC_002745.2|	874380	+	U	0	0	1	0	0	-
gi|29165615|ref|NC_002745.2|	874382	+	U	0	0	0	0	2	-
gi|29165615|ref|NC_002745.2|	874397	+	U	0	3	0	1	0	-
gi|29165615|ref|NC_002745.2|	875373	+	G	0	1	0	0	0	-
gi|29165615|ref|NC_002745.2|	875406	+	C	2	0	0	0	0	-
gi|29165615|ref|NC_002745.2|	878534	+	A	0	0	0	0	1	SA0772
gi|29165615|ref|NC_002745.2|	878540	+	A	0	0	0	1	0	SA0772
gi|29165615|ref|NC_002745.2|	878544	+	U	0	0	0	1	0	SA0772
gi|29165615|ref|NC_002745.2|	881136	+	A	0	0	1	0	0	SA0775
gi|29165615|ref|NC_002745.2|	882133	+	C	0	0	0	0	1	SA0775
gi|29165615|ref|NC_002745.2|	882137	+	U	0	0	0	1	1	SA0775
gi|29165615|ref|NC_002745.2|	884391	+	C	0	0	1	0	0	SA0778
gi|29165615|ref|NC_002745.2|	885485	+	U	0	0	0	0	1	SA0778
gi|29165615|ref|NC_002745.2|	885489	+	C	0	0	0	1	1	SA0778
gi|29165615|ref|NC_002745.2|	887509	+	U	0	1	0	0	0	SA0780
gi|29165615|ref|NC_002745.2|	888878	+	U	0	1	0	0	0	-
gi|29165615|ref|NC_002745.2|	888921	+	U	0	1	0	0	0	-
gi|29165615|ref|NC_002745.2|	888923	+	A	1	0	0	0	0	-
gi|29165615|ref|NC_002745.2|	888936	+	U	0	1	0	0	0	-
gi|29165615|ref|NC_002745.2|	888979	+	U	0	1	0	0	0	-
gi|29165615|ref|NC_002745.2|	889007	+	U	0	1	0	0	0	-
gi|29165615|ref|NC_002745.2|	889092	+	U	0	1	0	0	0	-
gi|29165615|ref|NC_002745.2|	889094	+	A	1	0	0	0	0	-
gi|29165615|ref|NC_002745.2|	892669	+	A	0	0	1	0	1	SA0785
gi|29165615|ref|NC_002745.2|	893882	+	A	0	0	0	1	0	-
gi|29165615|ref|NC_002745.2|	893887	+	A	15	15	14	7	8	-
gi|29165615|ref|NC_002745.2|	893892	+	A	1	1	0	0	1	-
gi|29165615|ref|NC_002745.2|	893895	+	C	0	2	1	0	1	-
gi|29165615|ref|NC_002745.2|	893935	+	A	0	0	0	2	1	SA0787
gi|29165615|ref|NC_002745.2|	893940	+	A	0	0	0	1	1	SA0787
gi|29165615|ref|NC_002745.2|	893946	+	A	0	0	0	0	1	SA0787
gi|29165615|ref|NC_002745.2|	893948	+	A	0	0	1	0	0	SA0787
gi|29165615|ref|NC_002745.2|	893949	+	A	0	0	0	1	0	SA0787
gi|29165615|ref|NC_002745.2|	893952	+	U	0	0	1	0	0	SA0787
gi|29165615|ref|NC_002745.2|	893954	+	A	0	0	0	0	2	SA0787
gi|29165615|ref|NC_002745.2|	893955	+	A	0	2	0	0	0	SA0787
gi|29165615|ref|NC_002745.2|	893957	+	U	0	0	1	0	0	SA0787
gi|29165615|ref|NC_002745.2|	893958	+	A	0	0	1	0	0	SA0787
gi|29165615|ref|NC_002745.2|	893960	+	A	0	0	2	0	0	SA0787
gi|29165615|ref|NC_002745.2|	893961	+	U	0	1	2	0	2	SA0787
gi|29165615|ref|NC_002745.2|	893962	+	A	0	1	0	0	2	SA0787
gi|29165615|ref|NC_002745.2|	893963	+	A	0	0	0	0	1	SA0787
gi|29165615|ref|NC_002745.2|	893964	+	A	0	1	0	0	0	SA0787
gi|29165615|ref|NC_002745.2|	893965	+	C	0	3	0	0	0	SA0787
gi|29165615|ref|NC_002745.2|	893967	+	A	0	0	0	0	2	SA0787
gi|29165615|ref|NC_002745.2|	893969	+	G	0	0	0	0	1	SA0787
gi|29165615|ref|NC_002745.2|	893970	+	A	2	6	1	1	6	SA0787
gi|29165615|ref|NC_002745.2|	893971	+	A	0	0	1	0	0	SA0787
gi|29165615|ref|NC_002745.2|	893976	+	A	0	1	3	0	2	SA0787
gi|29165615|ref|NC_002745.2|	893979	+	U	0	0	2	0	0	SA0787
gi|29165615|ref|NC_002745.2|	893982	+	U	0	1	0	0	1	SA0787
gi|29165615|ref|NC_002745.2|	893983	+	U	0	0	1	0	0	SA0787
gi|29165615|ref|NC_002745.2|	893987	+	A	0	2	1	0	2	SA0787
gi|29165615|ref|NC_002745.2|	893988	+	A	0	0	4	1	0	SA0787
gi|29165615|ref|NC_002745.2|	893991	+	G	0	0	0	0	1	SA0787
gi|29165615|ref|NC_002745.2|	893992	+	A	0	0	0	1	0	SA0787
gi|29165615|ref|NC_002745.2|	893993	+	A	0	1	0	0	0	SA0787
gi|29165615|ref|NC_002745.2|	893995	+	A	0	0	1	0	1	SA0787
gi|29165615|ref|NC_002745.2|	893996	+	A	1	1	2	1	3	SA0787
gi|29165615|ref|NC_002745.2|	894001	+	A	0	0	1	0	0	SA0787
gi|29165615|ref|NC_002745.2|	894003	+	C	1	0	0	0	1	SA0787
gi|29165615|ref|NC_002745.2|	894011	+	A	0	0	2	0	0	SA0787

gi|29165615|ref|NC_002745.2|	894017	+	G	0	1	0	0	0	SA0787
gi|29165615|ref|NC_002745.2|	894022	+	A	0	0	1	0	0	SA0787
gi|29165615|ref|NC_002745.2|	894024	+	C	0	0	0	0	1	SA0787
gi|29165615|ref|NC_002745.2|	894026	+	U	0	1	0	0	0	SA0787
gi|29165615|ref|NC_002745.2|	894035	+	U	0	0	1	0	0	SA0787
gi|29165615|ref|NC_002745.2|	894056	+	G	0	1	0	0	0	SA0787
gi|29165615|ref|NC_002745.2|	894073	+	A	0	0	0	1	0	SA0787
gi|29165615|ref|NC_002745.2|	894074	+	A	0	1	0	0	0	SA0787
gi|29165615|ref|NC_002745.2|	894118	+	A	0	1	0	0	0	SA0787
gi|29165615|ref|NC_002745.2|	894119	+	A	0	1	0	0	0	SA0787
gi|29165615|ref|NC_002745.2|	894150	+	C	0	1	0	0	0	SA0787
gi|29165615|ref|NC_002745.2|	894160	+	U	0	1	0	0	0	SA0787
gi|29165615|ref|NC_002745.2|	894255	+	U	0	0	0	0	1	SA0787
gi|29165615|ref|NC_002745.2|	894315	+	C	1	0	0	0	0	SA0787
gi|29165615|ref|NC_002745.2|	894426	+	U	0	1	0	0	0	SA0787
gi|29165615|ref|NC_002745.2|	894427	+	A	0	0	1	0	0	SA0787
gi|29165615|ref|NC_002745.2|	894462	+	A	0	1	0	0	0	SA0787
gi|29165615|ref|NC_002745.2|	894481	+	U	0	2	0	0	0	SA0787
gi|29165615|ref|NC_002745.2|	894489	+	A	0	1	0	0	0	SA0787
gi|29165615|ref|NC_002745.2|	894492	+	A	0	0	1	0	0	SA0787
gi|29165615|ref|NC_002745.2|	894584	+	C	0	0	0	0	1	SA0787
gi|29165615|ref|NC_002745.2|	894660	+	U	0	0	2	0	0	SA0787
gi|29165615|ref|NC_002745.2|	894661	+	A	0	0	0	0	1	SA0787
gi|29165615|ref|NC_002745.2|	894683	+	C	1	0	0	0	0	SA0787
gi|29165615|ref|NC_002745.2|	894746	+	A	0	0	1	0	0	SA0787
gi|29165615|ref|NC_002745.2|	894773	+	A	0	0	1	0	0	SA0787
gi|29165615|ref|NC_002745.2|	894774	+	G	0	0	1	0	0	SA0787
gi|29165615|ref|NC_002745.2|	894908	+	A	0	0	0	0	1	SA0787
gi|29165615|ref|NC_002745.2|	894925	+	U	0	0	0	0	1	SA0787
gi|29165615|ref|NC_002745.2|	894930	+	G	0	0	0	0	2	SA0787
gi|29165615|ref|NC_002745.2|	894960	+	U	0	0	0	0	1	SA0787
gi|29165615|ref|NC_002745.2|	894963	+	G	0	0	0	0	1	SA0787
gi|29165615|ref|NC_002745.2|	894987	+	U	0	0	1	0	0	SA0787
gi|29165615|ref|NC_002745.2|	895005	+	A	0	0	1	0	0	SA0787
gi|29165615|ref|NC_002745.2|	895038	+	C	0	0	0	1	0	SA0787
gi|29165615|ref|NC_002745.2|	895088	+	A	0	1	0	0	0	SA0787
gi|29165615|ref|NC_002745.2|	895099	+	A	0	1	0	1	0	SA0787
gi|29165615|ref|NC_002745.2|	895103	+	A	0	0	1	0	0	SA0787
gi|29165615|ref|NC_002745.2|	895134	+	A	0	0	1	0	0	SA0787
gi|29165615|ref|NC_002745.2|	895148	+	C	0	0	0	1	0	SA0787
gi|29165615|ref|NC_002745.2|	895155	+	U	0	0	0	1	0	SA0787
gi|29165615|ref|NC_002745.2|	895215	+	U	0	0	1	0	0	SA0787
gi|29165615|ref|NC_002745.2|	895223	+	U	0	0	0	1	0	SA0787
gi|29165615|ref|NC_002745.2|	895224	+	A	0	0	0	0	1	SA0787
gi|29165615|ref|NC_002745.2|	895239	+	A	0	0	0	0	1	-
gi|29165615|ref|NC_002745.2|	895245	+	U	0	0	0	0	1	-
gi|29165615|ref|NC_002745.2|	895247	+	U	0	0	0	1	0	-
gi|29165615|ref|NC_002745.2|	895250	+	U	0	0	0	0	1	-
gi|29165615|ref|NC_002745.2|	895253	+	A	0	0	0	1	2	-
gi|29165615|ref|NC_002745.2|	895254	+	A	0	1	0	1	0	-
gi|29165615|ref|NC_002745.2|	895255	+	A	0	0	0	1	0	-
gi|29165615|ref|NC_002745.2|	895256	+	A	1	0	0	0	0	-
gi|29165615|ref|NC_002745.2|	895260	+	U	0	1	0	0	0	-
gi|29165615|ref|NC_002745.2|	895267	+	A	0	0	0	0	1	-
gi|29165615|ref|NC_002745.2|	895269	+	U	0	1	2	3	0	-
gi|29165615|ref|NC_002745.2|	895270	+	U	0	0	1	0	2	-
gi|29165615|ref|NC_002745.2|	895281	+	C	2	0	0	0	0	-
gi|29165615|ref|NC_002745.2|	896180	+	C	0	0	0	0	1	SA0790
gi|29165615|ref|NC_002745.2|	897662	+	A	0	1	0	0	0	SA0791
gi|29165615|ref|NC_002745.2|	898880	+	U	0	0	0	1	0	SA0793
gi|29165615|ref|NC_002745.2|	900278	+	A	0	1	2	0	1	SA0794
gi|29165615|ref|NC_002745.2|	900279	+	A	0	1	1	1	3	SA0794
gi|29165615|ref|NC_002745.2|	907784	+	A	0	0	0	1	0	SA0803
gi|29165615|ref|NC_002745.2|	907788	+	A	0	0	1	0	0	SA0803
gi|29165615|ref|NC_002745.2|	922728	+	G	0	0	1	0	0	SA0818
gi|29165615|ref|NC_002745.2|	922729	+	C	0	0	0	1	0	SA0818
gi|29165615|ref|NC_002745.2|	922733	+	C	0	0	1	0	0	SA0818
gi|29165615|ref|NC_002745.2|	922734	+	A	0	0	0	0	1	SA0818
gi|29165615|ref|NC_002745.2|	922736	+	G	0	0	0	0	1	SA0818
gi|29165615|ref|NC_002745.2|	924583	+	G	0	0	0	0	1	-
gi|29165615|ref|NC_002745.2|	932887	+	A	0	0	0	0	1	SA0827
gi|29165615|ref|NC_002745.2|	933363	+	A	0	1	1	0	1	SA0827
gi|29165615|ref|NC_002745.2|	935101	+	A	0	0	0	0	1	SA0827
gi|29165615|ref|NC_002745.2|	943745	+	A	0	1	0	0	0	-
gi|29165615|ref|NC_002745.2|	944089	+	G	0	0	0	0	1	-
gi|29165615|ref|NC_002745.2|	944090	+	C	0	1	0	0	0	-
gi|29165615|ref|NC_002745.2|	944091	+	A	0	0	2	0	0	-
gi|29165615|ref|NC_002745.2|	944092	+	U	3	2	3	1	9	-
gi|29165615|ref|NC_002745.2|	944097	+	U	0	0	0	1	0	-
gi|29165615|ref|NC_002745.2|	944098	+	G	0	0	0	0	1	-
gi|29165615|ref|NC_002745.2|	944099	+	U	0	0	0	1	0	-

gi|29165615|ref|NC_002745.2|	944100	+	A	0	0	1	1	0	-
gi|29165615|ref|NC_002745.2|	944101	+	G	0	1	0	2	0	-
gi|29165615|ref|NC_002745.2|	944103	+	A	0	0	1	0	0	-
gi|29165615|ref|NC_002745.2|	944104	+	U	0	0	1	3	1	-
gi|29165615|ref|NC_002745.2|	944105	+	U	0	0	0	0	1	-
gi|29165615|ref|NC_002745.2|	944108	+	U	0	0	1	0	0	-
gi|29165615|ref|NC_002745.2|	944109	+	U	0	2	1	4	1	-
gi|29165615|ref|NC_002745.2|	944110	+	U	0	1	0	1	1	-
gi|29165615|ref|NC_002745.2|	944113	+	G	0	1	0	0	1	-
gi|29165615|ref|NC_002745.2|	944114	+	A	0	0	1	1	0	-
gi|29165615|ref|NC_002745.2|	944120	+	U	0	0	0	0	1	-
gi|29165615|ref|NC_002745.2|	945057	+	A	0	0	0	0	1	SA0834
gi|29165615|ref|NC_002745.2|	945062	+	U	1	0	0	0	0	SA0834
gi|29165615|ref|NC_002745.2|	945228	+	A	0	1	0	0	0	SA0834
gi|29165615|ref|NC_002745.2|	947254	+	U	0	0	0	1	0	SA0835
gi|29165615|ref|NC_002745.2|	947257	+	G	1	0	0	0	0	SA0835
gi|29165615|ref|NC_002745.2|	947269	+	U	0	1	0	0	0	SA0835
gi|29165615|ref|NC_002745.2|	947276	+	A	1	0	0	0	0	SA0835
gi|29165615|ref|NC_002745.2|	948478	+	G	1	0	0	0	0	SA0835
gi|29165615|ref|NC_002745.2|	951855	+	G	1	0	0	0	0	SA0838
gi|29165615|ref|NC_002745.2|	953470	+	U	0	0	0	0	1	SA0840
gi|29165615|ref|NC_002745.2|	953471	+	G	0	0	0	0	1	SA0840
gi|29165615|ref|NC_002745.2|	957032	+	U	1	0	0	0	0	SA0843
gi|29165615|ref|NC_002745.2|	972053	+	A	0	0	0	1	0	SA0857
gi|29165615|ref|NC_002745.2|	980291	+	C	3	0	0	0	0	SA0866
gi|29165615|ref|NC_002745.2|	981414	+	A	0	0	0	0	1	SA0867
gi|29165615|ref|NC_002745.2|	987015	+	U	0	0	1	0	0	SA0871
gi|29165615|ref|NC_002745.2|	993453	+	G	0	1	0	0	0	SA0876
gi|29165615|ref|NC_002745.2|	993455	+	U	0	0	1	0	1	SA0876
gi|29165615|ref|NC_002745.2|	994468	+	G	0	1	0	0	0	SA0877
gi|29165615|ref|NC_002745.2|	1000532	+	A	0	0	0	0	1	SA0880
gi|29165615|ref|NC_002745.2|	1003687	+	A	0	0	1	0	1	-
gi|29165615|ref|NC_002745.2|	1010752	+	A	0	1	0	1	0	-
gi|29165615|ref|NC_002745.2|	1010770	+	A	1	2	2	3	4	-
gi|29165615|ref|NC_002745.2|	1010771	+	U	0	2	0	0	1	-
gi|29165615|ref|NC_002745.2|	1012174	+	U	0	0	1	0	0	SA0891
gi|29165615|ref|NC_002745.2|	1016940	+	A	0	1	2	0	0	SA0896
gi|29165615|ref|NC_002745.2|	1033397	+	A	0	1	0	0	0	SA0909
gi|29165615|ref|NC_002745.2|	1033756	+	U	0	1	0	0	0	SA0909
gi|29165615|ref|NC_002745.2|	1034618	+	C	0	0	0	1	0	-
gi|29165615|ref|NC_002745.2|	1039951	+	A	0	0	1	0	0	-
gi|29165615|ref|NC_002745.2|	1040035	+	G	0	0	0	0	1	-
gi|29165615|ref|NC_002745.2|	1040036	+	C	0	1	2	0	0	-
gi|29165615|ref|NC_002745.2|	1040037	+	A	0	0	2	0	0	-
gi|29165615|ref|NC_002745.2|	1040038	+	U	3	2	3	1	10	-
gi|29165615|ref|NC_002745.2|	1040043	+	U	0	0	0	1	0	-
gi|29165615|ref|NC_002745.2|	1040044	+	G	0	0	0	0	1	-
gi|29165615|ref|NC_002745.2|	1040045	+	U	0	0	0	1	0	-
gi|29165615|ref|NC_002745.2|	1040046	+	A	0	0	1	1	0	-
gi|29165615|ref|NC_002745.2|	1040047	+	G	0	1	0	2	0	-
gi|29165615|ref|NC_002745.2|	1040048	+	A	0	1	2	2	0	-
gi|29165615|ref|NC_002745.2|	1040049	+	A	2	0	1	1	0	-
gi|29165615|ref|NC_002745.2|	1040050	+	U	0	0	1	3	1	-
gi|29165615|ref|NC_002745.2|	1040051	+	U	0	0	0	0	1	-
gi|29165615|ref|NC_002745.2|	1040054	+	U	0	0	1	0	0	-
gi|29165615|ref|NC_002745.2|	1040055	+	U	0	3	1	4	1	-
gi|29165615|ref|NC_002745.2|	1040056	+	U	0	1	1	1	1	-
gi|29165615|ref|NC_002745.2|	1040059	+	G	0	0	0	0	1	-
gi|29165615|ref|NC_002745.2|	1040060	+	A	0	0	1	0	0	-
gi|29165615|ref|NC_002745.2|	1040062	+	A	0	0	0	1	0	-
gi|29165615|ref|NC_002745.2|	1040066	+	U	0	0	0	0	1	-
gi|29165615|ref|NC_002745.2|	1040072	+	U	1	0	0	0	0	-
gi|29165615|ref|NC_002745.2|	1040078	+	C	0	0	1	0	0	-
gi|29165615|ref|NC_002745.2|	1040086	+	C	0	0	0	0	1	-
gi|29165615|ref|NC_002745.2|	1040087	+	A	0	0	0	1	0	-
gi|29165615|ref|NC_002745.2|	1040094	+	A	0	1	0	1	0	-
gi|29165615|ref|NC_002745.2|	1040095	+	C	0	0	1	0	3	-
gi|29165615|ref|NC_002745.2|	1040103	+	U	0	1	1	2	0	-
gi|29165615|ref|NC_002745.2|	1040112	+	U	0	1	2	0	0	-
gi|29165615|ref|NC_002745.2|	1040113	+	U	0	4	0	0	2	-
gi|29165615|ref|NC_002745.2|	1040118	+	U	0	1	0	1	0	-
gi|29165615|ref|NC_002745.2|	1040122	+	C	1	0	0	0	0	-
gi|29165615|ref|NC_002745.2|	1040128	+	U	0	1	0	1	1	-
gi|29165615|ref|NC_002745.2|	1040130	+	U	1	1	0	0	0	-
gi|29165615|ref|NC_002745.2|	1040143	+	C	0	1	1	0	0	-
gi|29165615|ref|NC_002745.2|	1040145	+	A	0	0	1	0	0	-
gi|29165615|ref|NC_002745.2|	1040148	+	A	0	0	1	0	0	-
gi|29165615|ref|NC_002745.2|	1040392	+	A	0	0	0	1	0	-
gi|29165615|ref|NC_002745.2|	1040442	+	C	0	1	1	0	0	-
gi|29165615|ref|NC_002745.2|	1040444	+	A	0	0	1	0	0	-
gi|29165615|ref|NC_002745.2|	1040447	+	A	0	0	1	0	0	-

gi|29165615|ref|NC_002745.2|	1040451	+	A	0	0	0	0	2	-
gi|29165615|ref|NC_002745.2|	1040462	+	U	0	0	0	0	1	-
gi|29165615|ref|NC_002745.2|	1040465	+	C	0	0	1	0	0	-
gi|29165615|ref|NC_002745.2|	1040467	+	A	0	0	0	0	4	-
gi|29165615|ref|NC_002745.2|	1040472	+	A	0	0	1	0	0	-
gi|29165615|ref|NC_002745.2|	1040477	+	C	1	3	4	2	1	-
gi|29165615|ref|NC_002745.2|	1040479	+	U	0	1	0	0	0	-
gi|29165615|ref|NC_002745.2|	1040485	+	C	0	1	0	1	1	-
gi|29165615|ref|NC_002745.2|	1040489	+	U	3	2	0	0	1	-
gi|29165615|ref|NC_002745.2|	1040490	+	A	0	0	0	0	1	-
gi|29165615|ref|NC_002745.2|	1040492	+	U	0	0	1	0	1	-
gi|29165615|ref|NC_002745.2|	1040494	+	C	0	0	1	1	1	-
gi|29165615|ref|NC_002745.2|	1040495	+	C	1	0	0	0	0	-
gi|29165615|ref|NC_002745.2|	1040496	+	A	0	1	1	0	0	-
gi|29165615|ref|NC_002745.2|	1040497	+	U	1	1	2	0	0	-
gi|29165615|ref|NC_002745.2|	1040499	+	A	0	0	0	0	1	-
gi|29165615|ref|NC_002745.2|	1040500	+	U	2	0	0	0	0	-
gi|29165615|ref|NC_002745.2|	1040504	+	U	6	2	0	0	3	-
gi|29165615|ref|NC_002745.2|	1040507	+	G	0	0	0	0	1	-
gi|29165615|ref|NC_002745.2|	1040509	+	A	0	0	1	0	1	-
gi|29165615|ref|NC_002745.2|	1040510	+	G	0	1	0	0	0	-
gi|29165615|ref|NC_002745.2|	1040513	+	C	0	1	1	0	0	-
gi|29165615|ref|NC_002745.2|	1040514	+	A	0	1	0	1	0	-
gi|29165615|ref|NC_002745.2|	1040519	+	A	0	0	1	0	0	-
gi|29165615|ref|NC_002745.2|	1041845	+	U	0	0	1	0	0	SA0916
gi|29165615|ref|NC_002745.2|	1044549	+	A	0	0	0	0	1	SA0920
gi|29165615|ref|NC_002745.2|	1046506	+	A	0	1	0	0	0	SA0921
gi|29165615|ref|NC_002745.2|	1048810	+	U	0	0	1	0	0	SA0923
gi|29165615|ref|NC_002745.2|	1057324	+	A	0	1	0	0	0	SA0931
gi|29165615|ref|NC_002745.2|	1057325	+	U	0	0	0	1	0	SA0931
gi|29165615|ref|NC_002745.2|	1066503	+	A	0	0	0	1	0	-
gi|29165615|ref|NC_002745.2|	1066553	+	C	0	1	1	0	0	-
gi|29165615|ref|NC_002745.2|	1066555	+	A	0	0	1	0	0	-
gi|29165615|ref|NC_002745.2|	1066558	+	A	0	0	1	0	0	-
gi|29165615|ref|NC_002745.2|	1066562	+	A	0	0	0	0	2	-
gi|29165615|ref|NC_002745.2|	1066573	+	U	0	0	0	0	1	-
gi|29165615|ref|NC_002745.2|	1066576	+	C	0	0	1	0	0	-
gi|29165615|ref|NC_002745.2|	1066578	+	A	0	0	0	0	4	-
gi|29165615|ref|NC_002745.2|	1066583	+	A	0	0	1	0	0	-
gi|29165615|ref|NC_002745.2|	1066588	+	C	1	3	4	2	1	-
gi|29165615|ref|NC_002745.2|	1066590	+	U	0	1	0	0	0	-
gi|29165615|ref|NC_002745.2|	1066595	+	U	0	0	0	0	1	-
gi|29165615|ref|NC_002745.2|	1066596	+	C	0	1	0	2	1	-
gi|29165615|ref|NC_002745.2|	1066600	+	U	3	2	0	0	1	-
gi|29165615|ref|NC_002745.2|	1066601	+	A	0	0	1	0	1	-
gi|29165615|ref|NC_002745.2|	1066603	+	U	0	0	1	0	1	-
gi|29165615|ref|NC_002745.2|	1066605	+	C	0	0	1	1	1	-
gi|29165615|ref|NC_002745.2|	1066606	+	C	1	0	0	0	0	-
gi|29165615|ref|NC_002745.2|	1066607	+	A	0	1	1	0	0	-
gi|29165615|ref|NC_002745.2|	1066608	+	A	1	1	2	0	0	-
gi|29165615|ref|NC_002745.2|	1066610	+	A	0	0	0	0	1	-
gi|29165615|ref|NC_002745.2|	1066611	+	U	2	0	0	0	0	-
gi|29165615|ref|NC_002745.2|	1066615	+	U	6	2	0	0	3	-
gi|29165615|ref|NC_002745.2|	1066618	+	G	0	0	0	0	1	-
gi|29165615|ref|NC_002745.2|	1066620	+	A	0	0	1	0	1	-
gi|29165615|ref|NC_002745.2|	1066621	+	G	0	1	0	0	0	-
gi|29165615|ref|NC_002745.2|	1066624	+	C	0	1	1	0	0	-
gi|29165615|ref|NC_002745.2|	1066625	+	A	0	1	0	1	0	-
gi|29165615|ref|NC_002745.2|	1066630	+	A	0	0	1	0	0	-
gi|29165615|ref|NC_002745.2|	1066631	+	C	2	3	0	0	1	-
gi|29165615|ref|NC_002745.2|	1073752	+	A	0	0	1	0	0	SA0945
gi|29165615|ref|NC_002745.2|	1073923	+	A	0	1	0	0	0	SA0945
gi|29165615|ref|NC_002745.2|	1077274	+	U	1	0	0	0	0	SA0950
gi|29165615|ref|NC_002745.2|	1081451	+	A	0	1	0	0	0	SA0954
gi|29165615|ref|NC_002745.2|	1085625	+	G	0	0	0	0	1	SA0958
gi|29165615|ref|NC_002745.2|	1086037	+	A	0	0	0	0	1	-
gi|29165615|ref|NC_002745.2|	1086138	+	U	4	0	0	0	1	SA0959
gi|29165615|ref|NC_002745.2|	1086139	+	C	1	0	0	0	0	SA0959
gi|29165615|ref|NC_002745.2|	1086140	+	A	0	1	0	0	0	SA0959
gi|29165615|ref|NC_002745.2|	1086142	+	G	2	0	0	0	0	SA0959
gi|29165615|ref|NC_002745.2|	1086143	+	U	1	0	0	0	0	SA0959
gi|29165615|ref|NC_002745.2|	1086146	+	A	2	0	0	0	0	SA0959
gi|29165615|ref|NC_002745.2|	1086322	+	G	0	1	0	0	0	SA0959
gi|29165615|ref|NC_002745.2|	1091925	+	U	0	1	0	0	0	SA0963
gi|29165615|ref|NC_002745.2|	1092145	+	A	0	2	0	0	1	SA0963
gi|29165615|ref|NC_002745.2|	1092151	+	A	0	0	0	0	2	SA0963
gi|29165615|ref|NC_002745.2|	1093472	+	A	0	1	0	0	0	SA0963
gi|29165615|ref|NC_002745.2|	1094631	+	A	0	0	0	0	1	SA0963
gi|29165615|ref|NC_002745.2|	1102206	+	A	0	0	0	0	1	SA0973
gi|29165615|ref|NC_002745.2|	1113062	+	A	0	2	0	1	1	-
gi|29165615|ref|NC_002745.2|	1115527	+	A	1	1	0	0	0	SA0986

gi|29165615|ref|NC_002745.2|	1115528	+	A	0	0	0	0	1	SA0986
gi|29165615|ref|NC_002745.2|	1115532	+	G	0	0	1	0	0	SA0986
gi|29165615|ref|NC_002745.2|	1115536	+	C	0	0	0	0	1	SA0986
gi|29165615|ref|NC_002745.2|	1115538	+	U	0	0	1	0	0	SA0986
gi|29165615|ref|NC_002745.2|	1136963	+	A	0	0	0	0	1	-
gi|29165615|ref|NC_002745.2|	1137086	+	A	0	0	0	0	1	-
gi|29165615|ref|NC_002745.2|	1137088	+	C	0	0	1	0	0	-
gi|29165615|ref|NC_002745.2|	1137089	+	A	0	0	0	1	0	-
gi|29165615|ref|NC_002745.2|	1137094	+	G	0	0	0	1	0	-
gi|29165615|ref|NC_002745.2|	1137107	+	A	0	0	0	2	1	-
gi|29165615|ref|NC_002745.2|	1137108	+	A	0	0	0	1	0	-
gi|29165615|ref|NC_002745.2|	1137111	+	U	0	1	0	0	0	-
gi|29165615|ref|NC_002745.2|	1137165	+	A	0	0	0	2	1	-
gi|29165615|ref|NC_002745.2|	1137171	+	A	0	0	0	1	0	-
gi|29165615|ref|NC_002745.2|	1137178	+	A	0	0	0	0	1	-
gi|29165615|ref|NC_002745.2|	1137183	+	A	0	1	0	0	0	-
gi|29165615|ref|NC_002745.2|	1137221	+	A	0	0	0	2	1	-
gi|29165615|ref|NC_002745.2|	1137228	+	A	1	0	0	0	0	-
gi|29165615|ref|NC_002745.2|	1137238	+	C	0	1	0	0	0	-
gi|29165615|ref|NC_002745.2|	1137272	+	A	0	0	0	0	1	-
gi|29165615|ref|NC_002745.2|	1137296	+	A	0	1	0	0	0	-
gi|29165615|ref|NC_002745.2|	1137308	+	C	0	0	2	0	0	-
gi|29165615|ref|NC_002745.2|	1137328	+	A	0	0	0	0	1	-
gi|29165615|ref|NC_002745.2|	1137343	+	A	0	0	0	0	2	-
gi|29165615|ref|NC_002745.2|	1137479	+	A	0	1	0	0	3	-
gi|29165615|ref|NC_002745.2|	1137504	+	U	0	0	0	1	0	-
gi|29165615|ref|NC_002745.2|	1139060	+	A	0	0	0	1	0	-
gi|29165615|ref|NC_002745.2|	1139065	+	A	15	15	14	7	8	-
gi|29165615|ref|NC_002745.2|	1139070	+	A	1	1	0	0	1	-
gi|29165615|ref|NC_002745.2|	1139073	+	C	0	2	1	0	1	-
gi|29165615|ref|NC_002745.2|	1139113	+	A	0	0	0	2	1	SA1006
gi|29165615|ref|NC_002745.2|	1139118	+	A	0	0	0	1	1	SA1006
gi|29165615|ref|NC_002745.2|	1139124	+	A	0	0	0	0	1	SA1006
gi|29165615|ref|NC_002745.2|	1139126	+	A	0	0	1	0	0	SA1006
gi|29165615|ref|NC_002745.2|	1139127	+	A	0	0	0	1	0	SA1006
gi|29165615|ref|NC_002745.2|	1139130	+	U	0	0	1	0	0	SA1006
gi|29165615|ref|NC_002745.2|	1139132	+	A	0	0	0	0	2	SA1006
gi|29165615|ref|NC_002745.2|	1139133	+	A	0	2	0	0	0	SA1006
gi|29165615|ref|NC_002745.2|	1139135	+	U	0	0	1	0	0	SA1006
gi|29165615|ref|NC_002745.2|	1139136	+	A	0	0	1	0	0	SA1006
gi|29165615|ref|NC_002745.2|	1139138	+	A	0	0	2	0	0	SA1006
gi|29165615|ref|NC_002745.2|	1139139	+	U	0	1	2	0	2	SA1006
gi|29165615|ref|NC_002745.2|	1139140	+	A	0	1	0	0	2	SA1006
gi|29165615|ref|NC_002745.2|	1139141	+	A	0	0	0	0	1	SA1006
gi|29165615|ref|NC_002745.2|	1139142	+	A	0	1	0	0	0	SA1006
gi|29165615|ref|NC_002745.2|	1139143	+	C	0	3	0	0	0	SA1006
gi|29165615|ref|NC_002745.2|	1139145	+	A	0	0	0	0	2	SA1006
gi|29165615|ref|NC_002745.2|	1139147	+	G	0	0	0	0	1	SA1006
gi|29165615|ref|NC_002745.2|	1139148	+	A	2	6	1	1	6	SA1006
gi|29165615|ref|NC_002745.2|	1139149	+	A	0	0	1	0	0	SA1006
gi|29165615|ref|NC_002745.2|	1139154	+	A	0	1	3	0	2	SA1006
gi|29165615|ref|NC_002745.2|	1139157	+	U	0	0	2	0	0	SA1006
gi|29165615|ref|NC_002745.2|	1139160	+	U	0	1	0	0	1	SA1006
gi|29165615|ref|NC_002745.2|	1139161	+	U	0	0	1	0	0	SA1006
gi|29165615|ref|NC_002745.2|	1139165	+	A	0	2	1	0	2	SA1006
gi|29165615|ref|NC_002745.2|	1139166	+	A	0	0	4	1	0	SA1006
gi|29165615|ref|NC_002745.2|	1139169	+	G	0	0	0	0	1	SA1006
gi|29165615|ref|NC_002745.2|	1139170	+	A	0	0	0	1	0	SA1006
gi|29165615|ref|NC_002745.2|	1139171	+	A	0	1	0	0	0	SA1006
gi|29165615|ref|NC_002745.2|	1139173	+	A	0	0	1	0	1	SA1006
gi|29165615|ref|NC_002745.2|	1139174	+	A	1	1	2	1	3	SA1006
gi|29165615|ref|NC_002745.2|	1139179	+	A	0	0	1	0	0	SA1006
gi|29165615|ref|NC_002745.2|	1139181	+	C	1	0	0	0	1	SA1006
gi|29165615|ref|NC_002745.2|	1139189	+	A	0	0	2	0	0	SA1006
gi|29165615|ref|NC_002745.2|	1139195	+	G	0	1	0	0	0	SA1006
gi|29165615|ref|NC_002745.2|	1139200	+	A	0	0	1	0	0	SA1006
gi|29165615|ref|NC_002745.2|	1139202	+	C	0	0	0	0	1	SA1006
gi|29165615|ref|NC_002745.2|	1139204	+	U	0	1	0	0	0	SA1006
gi|29165615|ref|NC_002745.2|	1139213	+	U	0	0	1	0	0	SA1006
gi|29165615|ref|NC_002745.2|	1139234	+	G	0	1	0	0	0	SA1006
gi|29165615|ref|NC_002745.2|	1139251	+	A	0	0	0	1	0	SA1006
gi|29165615|ref|NC_002745.2|	1139252	+	A	0	1	0	0	0	SA1006
gi|29165615|ref|NC_002745.2|	1139296	+	A	0	1	0	0	0	SA1006
gi|29165615|ref|NC_002745.2|	1139297	+	A	0	1	0	0	0	SA1006
gi|29165615|ref|NC_002745.2|	1139328	+	C	0	1	0	0	0	SA1006
gi|29165615|ref|NC_002745.2|	1139338	+	U	0	1	0	0	0	SA1006
gi|29165615|ref|NC_002745.2|	1139433	+	U	0	0	0	0	1	SA1006
gi|29165615|ref|NC_002745.2|	1139493	+	C	1	0	0	0	0	SA1006
gi|29165615|ref|NC_002745.2|	1139604	+	U	0	1	0	0	0	SA1006
gi|29165615|ref|NC_002745.2|	1139605	+	A	0	0	1	0	0	SA1006
gi|29165615|ref|NC_002745.2|	1139640	+	A	0	1	0	0	0	SA1006

gi|29165615|ref|NC_002745.2|	1139659	+	U	0	2	0	0	0	SA1006
gi|29165615|ref|NC_002745.2|	1139667	+	A	0	1	0	0	0	SA1006
gi|29165615|ref|NC_002745.2|	1139670	+	A	0	0	1	0	0	SA1006
gi|29165615|ref|NC_002745.2|	1139762	+	C	0	0	0	0	1	SA1006
gi|29165615|ref|NC_002745.2|	1139838	+	U	0	0	2	0	0	SA1006
gi|29165615|ref|NC_002745.2|	1139839	+	A	0	0	0	0	1	SA1006
gi|29165615|ref|NC_002745.2|	1139861	+	C	1	0	0	0	0	SA1006
gi|29165615|ref|NC_002745.2|	1139924	+	A	0	0	1	0	0	SA1006
gi|29165615|ref|NC_002745.2|	1139951	+	A	0	0	1	0	0	SA1006
gi|29165615|ref|NC_002745.2|	1139952	+	G	0	0	1	0	0	SA1006
gi|29165615|ref|NC_002745.2|	1140086	+	A	0	0	0	0	1	SA1006
gi|29165615|ref|NC_002745.2|	1140103	+	U	0	0	0	0	1	SA1006
gi|29165615|ref|NC_002745.2|	1140108	+	G	0	0	0	0	2	SA1006
gi|29165615|ref|NC_002745.2|	1140138	+	U	0	0	0	0	1	SA1006
gi|29165615|ref|NC_002745.2|	1140141	+	G	0	0	0	0	1	SA1006
gi|29165615|ref|NC_002745.2|	1140165	+	U	0	0	1	0	0	SA1006
gi|29165615|ref|NC_002745.2|	1140183	+	A	0	0	1	0	0	SA1006
gi|29165615|ref|NC_002745.2|	1140216	+	C	0	0	0	1	0	SA1006
gi|29165615|ref|NC_002745.2|	1140266	+	A	0	1	0	0	0	SA1006
gi|29165615|ref|NC_002745.2|	1140277	+	A	0	1	0	1	0	SA1006
gi|29165615|ref|NC_002745.2|	1140281	+	A	0	0	1	0	0	SA1006
gi|29165615|ref|NC_002745.2|	1140312	+	A	0	0	1	0	0	SA1006
gi|29165615|ref|NC_002745.2|	1140326	+	C	0	0	0	1	0	SA1006
gi|29165615|ref|NC_002745.2|	1140333	+	U	0	0	0	1	0	SA1006
gi|29165615|ref|NC_002745.2|	1140393	+	U	0	0	1	0	0	SA1006
gi|29165615|ref|NC_002745.2|	1140401	+	U	0	0	0	1	0	SA1006
gi|29165615|ref|NC_002745.2|	1140402	+	A	0	0	0	0	1	SA1006
gi|29165615|ref|NC_002745.2|	1140417	+	A	0	0	0	0	1	-
gi|29165615|ref|NC_002745.2|	1140423	+	U	0	0	0	0	1	-
gi|29165615|ref|NC_002745.2|	1140425	+	U	0	0	0	1	0	-
gi|29165615|ref|NC_002745.2|	1140428	+	U	0	0	0	0	1	-
gi|29165615|ref|NC_002745.2|	1140431	+	A	0	0	0	1	2	-
gi|29165615|ref|NC_002745.2|	1140432	+	A	0	1	0	1	0	-
gi|29165615|ref|NC_002745.2|	1140433	+	A	0	0	0	1	0	-
gi|29165615|ref|NC_002745.2|	1140434	+	A	1	0	0	0	0	-
gi|29165615|ref|NC_002745.2|	1140438	+	U	0	1	0	0	0	-
gi|29165615|ref|NC_002745.2|	1140445	+	A	0	0	0	0	1	-
gi|29165615|ref|NC_002745.2|	1140447	+	U	0	1	2	3	0	-
gi|29165615|ref|NC_002745.2|	1140448	+	U	0	0	1	0	2	-
gi|29165615|ref|NC_002745.2|	1140456	+	C	0	0	0	0	1	-
gi|29165615|ref|NC_002745.2|	1142689	+	G	0	0	1	0	0	-
gi|29165615|ref|NC_002745.2|	1142693	+	A	0	0	0	0	1	-
gi|29165615|ref|NC_002745.2|	1142702	+	C	1	2	0	0	0	-
gi|29165615|ref|NC_002745.2|	1142708	+	A	0	0	0	0	1	-
gi|29165615|ref|NC_002745.2|	1142710	+	A	0	0	1	0	0	-
gi|29165615|ref|NC_002745.2|	1142735	+	A	0	0	0	0	1	-
gi|29165615|ref|NC_002745.2|	1142737	+	C	0	0	1	0	0	-
gi|29165615|ref|NC_002745.2|	1142738	+	A	0	0	0	1	0	-
gi|29165615|ref|NC_002745.2|	1142743	+	G	0	0	0	1	0	-
gi|29165615|ref|NC_002745.2|	1142751	+	A	0	0	0	5	0	-
gi|29165615|ref|NC_002745.2|	1142752	+	A	0	0	0	1	0	-
gi|29165615|ref|NC_002745.2|	1142753	+	A	0	0	0	0	1	-
gi|29165615|ref|NC_002745.2|	1142756	+	A	0	1	0	3	2	-
gi|29165615|ref|NC_002745.2|	1142757	+	A	0	0	0	1	0	-
gi|29165615|ref|NC_002745.2|	1142760	+	U	0	0	0	1	0	-
gi|29165615|ref|NC_002745.2|	1142761	+	C	0	1	0	0	0	-
gi|29165615|ref|NC_002745.2|	1142762	+	U	0	0	0	1	0	-
gi|29165615|ref|NC_002745.2|	1142769	+	A	0	0	0	0	1	-
gi|29165615|ref|NC_002745.2|	1142774	+	A	0	1	0	0	0	-
gi|29165615|ref|NC_002745.2|	1142790	+	A	0	0	0	0	1	-
gi|29165615|ref|NC_002745.2|	1142792	+	C	0	0	1	0	0	-
gi|29165615|ref|NC_002745.2|	1142806	+	A	0	0	0	5	0	-
gi|29165615|ref|NC_002745.2|	1142807	+	A	0	0	0	1	0	-
gi|29165615|ref|NC_002745.2|	1142808	+	A	0	0	0	0	1	-
gi|29165615|ref|NC_002745.2|	1142811	+	A	0	1	0	3	2	-
gi|29165615|ref|NC_002745.2|	1142812	+	A	0	0	0	1	0	-
gi|29165615|ref|NC_002745.2|	1146683	+	U	0	0	1	0	0	SA1012
gi|29165615|ref|NC_002745.2|	1147245	+	A	0	0	0	0	1	SA1013
gi|29165615|ref|NC_002745.2|	1147451	+	A	0	0	0	1	0	SA1013
gi|29165615|ref|NC_002745.2|	1149270	+	U	0	0	0	1	0	SA1014
gi|29165615|ref|NC_002745.2|	1149734	+	U	0	0	2	1	0	-
gi|29165615|ref|NC_002745.2|	1153108	+	U	0	1	0	0	0	SA1018
gi|29165615|ref|NC_002745.2|	1156758	+	C	0	0	0	0	1	SA1022
gi|29165615|ref|NC_002745.2|	1165682	+	U	0	0	1	0	0	SA1028
gi|29165615|ref|NC_002745.2|	1165683	+	U	0	1	0	0	2	SA1028
gi|29165615|ref|NC_002745.2|	1165684	+	C	2	3	2	0	0	SA1028
gi|29165615|ref|NC_002745.2|	1165685	+	A	1	2	0	3	0	SA1028
gi|29165615|ref|NC_002745.2|	1165687	+	G	0	1	0	0	0	SA1028
gi|29165615|ref|NC_002745.2|	1165688	+	A	0	0	1	0	0	SA1028
gi|29165615|ref|NC_002745.2|	1170486	+	A	0	0	0	1	0	SA1035
gi|29165615|ref|NC_002745.2|	1170487	+	A	1	0	0	0	0	SA1035

gi|29165615|ref|NC_002745.2|	1170593	+	A	0	0	1	1	0	SA1035
gi|29165615|ref|NC_002745.2|	1175426	+	G	0	0	0	0	1	-
gi|29165615|ref|NC_002745.2|	1175445	+	C	0	0	0	0	1	-
gi|29165615|ref|NC_002745.2|	1175528	+	C	0	0	0	0	1	-
gi|29165615|ref|NC_002745.2|	1175705	+	G	1	0	0	0	0	-
gi|29165615|ref|NC_002745.2|	1175757	+	U	0	1	0	0	0	-
gi|29165615|ref|NC_002745.2|	1177160	+	G	0	2	0	0	0	SA1040
gi|29165615|ref|NC_002745.2|	1181537	+	U	0	0	0	0	1	SA1044
gi|29165615|ref|NC_002745.2|	1181870	+	A	0	0	1	1	3	SA1044
gi|29165615|ref|NC_002745.2|	1181871	+	U	0	1	0	1	0	SA1044
gi|29165615|ref|NC_002745.2|	1181937	+	A	1	0	0	0	0	SA1044
gi|29165615|ref|NC_002745.2|	1198510	+	A	0	0	0	0	1	SA1059
gi|29165615|ref|NC_002745.2|	1199790	+	A	0	1	1	0	0	SA1060
gi|29165615|ref|NC_002745.2|	1201839	+	A	0	0	0	0	1	SA1062
gi|29165615|ref|NC_002745.2|	1208703	+	G	0	0	1	0	0	SA1069
gi|29165615|ref|NC_002745.2|	1208704	+	U	0	2	1	0	3	SA1069
gi|29165615|ref|NC_002745.2|	1208705	+	A	0	0	1	0	0	SA1069
gi|29165615|ref|NC_002745.2|	1208707	+	U	0	3	2	0	0	SA1069
gi|29165615|ref|NC_002745.2|	1213020	+	A	1	0	1	0	0	SA1072
gi|29165615|ref|NC_002745.2|	1215642	+	A	0	0	1	0	0	-
gi|29165615|ref|NC_002745.2|	1215681	+	A	0	0	1	1	0	-
gi|29165615|ref|NC_002745.2|	1215688	+	U	0	0	1	0	0	-
gi|29165615|ref|NC_002745.2|	1215706	+	A	0	0	1	0	0	-
gi|29165615|ref|NC_002745.2|	1215745	+	U	0	0	1	1	0	-
gi|29165615|ref|NC_002745.2|	1215752	+	U	0	0	1	0	0	-
gi|29165615|ref|NC_002745.2|	1217909	+	A	0	0	0	0	1	SA1077
gi|29165615|ref|NC_002745.2|	1223245	+	C	0	0	1	0	0	SA1080
gi|29165615|ref|NC_002745.2|	1223704	+	A	0	0	0	1	0	-
gi|29165615|ref|NC_002745.2|	1224995	+	A	0	1	0	0	0	SA1082
gi|29165615|ref|NC_002745.2|	1233115	+	A	0	1	0	0	0	SA1089
gi|29165615|ref|NC_002745.2|	1233116	+	G	1	0	0	0	1	SA1089
gi|29165615|ref|NC_002745.2|	1238887	+	A	1	0	1	0	0	SA1093
gi|29165615|ref|NC_002745.2|	1240062	+	A	0	0	2	0	0	SA1094
gi|29165615|ref|NC_002745.2|	1240063	+	U	0	0	0	0	1	SA1094
gi|29165615|ref|NC_002745.2|	1254223	+	C	0	1	0	0	0	SA1107
gi|29165615|ref|NC_002745.2|	1254696	+	A	0	0	1	2	2	SA1107
gi|29165615|ref|NC_002745.2|	1256014	+	G	0	1	0	0	0	SA1107
gi|29165615|ref|NC_002745.2|	1256015	+	A	0	0	0	0	2	SA1107
gi|29165615|ref|NC_002745.2|	1261413	+	C	1	0	0	0	0	SA1112
gi|29165615|ref|NC_002745.2|	1261416	+	G	2	0	0	0	0	SA1112
gi|29165615|ref|NC_002745.2|	1261420	+	A	2	0	0	0	0	SA1112
gi|29165615|ref|NC_002745.2|	1267325	+	A	0	2	0	1	1	SA1117
gi|29165615|ref|NC_002745.2|	1278407	+	U	0	0	0	0	1	SA1125
gi|29165615|ref|NC_002745.2|	1280682	+	C	0	0	1	0	0	SA1128
gi|29165615|ref|NC_002745.2|	1280683	+	U	0	0	0	1	0	SA1128
gi|29165615|ref|NC_002745.2|	1281110	+	A	0	0	1	0	0	SA1128
gi|29165615|ref|NC_002745.2|	1282098	+	U	0	0	0	0	1	SA1129
gi|29165615|ref|NC_002745.2|	1282516	+	A	0	0	0	0	1	SA1129
gi|29165615|ref|NC_002745.2|	1288406	+	A	0	1	0	2	0	SA1134
gi|29165615|ref|NC_002745.2|	1288681	+	A	0	0	0	0	1	SA1134
gi|29165615|ref|NC_002745.2|	1298965	+	C	0	0	0	1	0	SA1141
gi|29165615|ref|NC_002745.2|	1301084	+	A	0	0	0	0	1	-
gi|29165615|ref|NC_002745.2|	1302423	+	A	0	0	1	0	0	SA1144
gi|29165615|ref|NC_002745.2|	1302425	+	U	1	0	0	0	0	SA1144
gi|29165615|ref|NC_002745.2|	1313815	+	U	0	1	0	0	0	SAS039
gi|29165615|ref|NC_002745.2|	1316814	+	G	1	0	1	0	0	SA1155
gi|29165615|ref|NC_002745.2|	1316816	+	A	0	0	0	1	1	SA1155
gi|29165615|ref|NC_002745.2|	1319163	+	U	0	0	3	0	5	SA1157
gi|29165615|ref|NC_002745.2|	1319164	+	G	0	0	1	6	2	SA1157
gi|29165615|ref|NC_002745.2|	1319165	+	C	0	1	0	2	0	SA1157
gi|29165615|ref|NC_002745.2|	1319166	+	G	0	0	0	0	2	SA1157
gi|29165615|ref|NC_002745.2|	1319167	+	U	1	3	2	9	17	SA1157
gi|29165615|ref|NC_002745.2|	1320056	+	U	0	1	0	0	1	SA1158
gi|29165615|ref|NC_002745.2|	1323224	+	A	0	1	0	0	0	-
gi|29165615|ref|NC_002745.2|	1332057	+	A	2	0	0	0	0	-
gi|29165615|ref|NC_002745.2|	1333706	+	C	1	0	0	0	0	-
gi|29165615|ref|NC_002745.2|	1339293	+	G	0	0	1	0	0	SA1177
gi|29165615|ref|NC_002745.2|	1339294	+	A	0	0	0	1	0	SA1177
gi|29165615|ref|NC_002745.2|	1339297	+	A	0	0	1	0	0	SA1177
gi|29165615|ref|NC_002745.2|	1339298	+	A	0	1	0	0	0	SA1177
gi|29165615|ref|NC_002745.2|	1346662	+	A	0	0	0	1	0	-
gi|29165615|ref|NC_002745.2|	1346983	+	G	0	0	1	0	0	SA1183
gi|29165615|ref|NC_002745.2|	1346996	+	U	0	0	1	0	0	SA1183
gi|29165615|ref|NC_002745.2|	1348616	+	C	0	0	0	1	0	-
gi|29165615|ref|NC_002745.2|	1348629	+	U	0	1	0	0	0	-
gi|29165615|ref|NC_002745.2|	1348644	+	G	0	0	0	0	1	-
gi|29165615|ref|NC_002745.2|	1348650	+	U	0	0	1	0	0	-
gi|29165615|ref|NC_002745.2|	1348667	+	A	0	0	0	0	1	-
gi|29165615|ref|NC_002745.2|	1348674	+	A	0	0	0	0	1	-
gi|29165615|ref|NC_002745.2|	1348677	+	C	0	0	0	1	0	-
gi|29165615|ref|NC_002745.2|	1348717	+	A	0	0	0	0	1	-

gi|29165615|ref|NC_002745.2|	1348719	+	C	0	0	1	0	0	-
gi|29165615|ref|NC_002745.2|	1348720	+	A	0	0	0	1	0	-
gi|29165615|ref|NC_002745.2|	1348725	+	G	0	0	0	1	0	-
gi|29165615|ref|NC_002745.2|	1348733	+	A	0	0	0	5	0	-
gi|29165615|ref|NC_002745.2|	1348734	+	A	0	0	0	1	0	-
gi|29165615|ref|NC_002745.2|	1348735	+	A	0	0	0	0	1	-
gi|29165615|ref|NC_002745.2|	1348738	+	A	0	1	0	1	1	-
gi|29165615|ref|NC_002745.2|	1348739	+	A	0	0	0	1	0	-
gi|29165615|ref|NC_002745.2|	1348745	+	A	1	0	0	0	0	-
gi|29165615|ref|NC_002745.2|	1348787	+	G	0	0	1	0	0	-
gi|29165615|ref|NC_002745.2|	1348791	+	A	0	0	0	0	1	-
gi|29165615|ref|NC_002745.2|	1348800	+	C	1	2	0	0	0	-
gi|29165615|ref|NC_002745.2|	1348806	+	A	0	0	0	0	1	-
gi|29165615|ref|NC_002745.2|	1348808	+	A	0	0	1	0	0	-
gi|29165615|ref|NC_002745.2|	1348837	+	A	0	0	0	3	0	-
gi|29165615|ref|NC_002745.2|	1348843	+	A	0	0	1	0	0	-
gi|29165615|ref|NC_002745.2|	1348855	+	A	0	0	0	2	1	-
gi|29165615|ref|NC_002745.2|	1348856	+	A	0	0	0	1	0	-
gi|29165615|ref|NC_002745.2|	1348859	+	U	0	0	0	1	0	-
gi|29165615|ref|NC_002745.2|	1348860	+	C	0	1	0	0	0	-
gi|29165615|ref|NC_002745.2|	1348861	+	U	0	0	0	1	0	-
gi|29165615|ref|NC_002745.2|	1348868	+	A	0	0	0	0	1	-
gi|29165615|ref|NC_002745.2|	1348873	+	A	0	1	0	0	0	-
gi|29165615|ref|NC_002745.2|	1348885	+	C	0	0	2	0	0	-
gi|29165615|ref|NC_002745.2|	1348905	+	G	0	0	0	0	1	-
gi|29165615|ref|NC_002745.2|	1348915	+	U	0	1	0	0	0	-
gi|29165615|ref|NC_002745.2|	1348917	+	A	0	3	5	10	5	-
gi|29165615|ref|NC_002745.2|	1348918	+	C	0	0	0	1	0	-
gi|29165615|ref|NC_002745.2|	1348920	+	A	0	0	0	0	4	-
gi|29165615|ref|NC_002745.2|	1348931	+	G	3	0	0	0	0	-
gi|29165615|ref|NC_002745.2|	1348947	+	G	0	2	0	0	0	-
gi|29165615|ref|NC_002745.2|	1348962	+	A	0	0	0	0	1	-
gi|29165615|ref|NC_002745.2|	1348963	+	A	0	2	0	1	0	-
gi|29165615|ref|NC_002745.2|	1348971	+	U	0	0	0	0	1	-
gi|29165615|ref|NC_002745.2|	1351957	+	U	0	1	0	0	0	SA1184
gi|29165615|ref|NC_002745.2|	1353200	+	G	0	0	0	0	1	-
gi|29165615|ref|NC_002745.2|	1353203	+	G	0	1	0	2	0	-
gi|29165615|ref|NC_002745.2|	1353205	+	A	0	0	1	0	0	-
gi|29165615|ref|NC_002745.2|	1353206	+	U	0	0	1	3	1	-
gi|29165615|ref|NC_002745.2|	1353207	+	U	0	0	0	0	1	-
gi|29165615|ref|NC_002745.2|	1353211	+	U	0	2	1	3	1	-
gi|29165615|ref|NC_002745.2|	1353212	+	A	0	0	0	0	1	-
gi|29165615|ref|NC_002745.2|	1353218	+	A	0	0	0	1	0	-
gi|29165615|ref|NC_002745.2|	1355748	+	A	0	0	1	0	0	SA1188
gi|29165615|ref|NC_002745.2|	1356092	+	C	0	0	1	0	0	SA1188
gi|29165615|ref|NC_002745.2|	1356093	+	A	0	1	0	0	0	SA1188
gi|29165615|ref|NC_002745.2|	1356094	+	A	0	1	0	0	0	SA1188
gi|29165615|ref|NC_002745.2|	1356408	+	G	2	1	0	0	0	SA1189
gi|29165615|ref|NC_002745.2|	1356716	+	U	1	0	0	0	0	SA1189
gi|29165615|ref|NC_002745.2|	1356948	+	U	0	2	0	0	1	SA1189
gi|29165615|ref|NC_002745.2|	1359600	+	U	0	0	1	0	0	SA1190
gi|29165615|ref|NC_002745.2|	1375176	+	A	0	0	0	0	2	SA1201
gi|29165615|ref|NC_002745.2|	1395507	+	G	0	0	1	0	0	-
gi|29165615|ref|NC_002745.2|	1395508	+	U	0	1	0	1	0	-
gi|29165615|ref|NC_002745.2|	1395512	+	C	1	0	0	0	0	-
gi|29165615|ref|NC_002745.2|	1395838	+	C	0	1	0	0	0	SA1222
gi|29165615|ref|NC_002745.2|	1397175	+	A	0	2	0	0	0	SA1224
gi|29165615|ref|NC_002745.2|	1397176	+	A	0	3	6	0	2	SA1224
gi|29165615|ref|NC_002745.2|	1397177	+	U	0	7	7	2	3	SA1224
gi|29165615|ref|NC_002745.2|	1397180	+	G	0	1	0	0	0	SA1224
gi|29165615|ref|NC_002745.2|	1397184	+	G	0	0	0	0	2	SA1224
gi|29165615|ref|NC_002745.2|	1398690	+	U	0	0	0	1	0	-
gi|29165615|ref|NC_002745.2|	1398691	+	G	0	0	0	0	1	-
gi|29165615|ref|NC_002745.2|	1398692	+	U	0	0	0	1	0	-
gi|29165615|ref|NC_002745.2|	1398693	+	A	0	0	1	1	0	-
gi|29165615|ref|NC_002745.2|	1398694	+	G	0	1	0	2	0	-
gi|29165615|ref|NC_002745.2|	1398695	+	A	0	1	2	2	0	-
gi|29165615|ref|NC_002745.2|	1398696	+	A	2	0	0	1	0	-
gi|29165615|ref|NC_002745.2|	1398697	+	U	0	0	1	2	0	-
gi|29165615|ref|NC_002745.2|	1398727	+	C	0	0	1	2	0	-
gi|29165615|ref|NC_002745.2|	1398790	+	C	0	0	0	0	1	-
gi|29165615|ref|NC_002745.2|	1398791	+	A	0	0	0	0	1	-
gi|29165615|ref|NC_002745.2|	1398799	+	U	0	1	0	0	0	-
gi|29165615|ref|NC_002745.2|	1398807	+	A	0	2	0	0	0	-
gi|29165615|ref|NC_002745.2|	1398810	+	A	0	2	0	0	0	-
gi|29165615|ref|NC_002745.2|	1398875	+	A	0	0	1	0	0	-
gi|29165615|ref|NC_002745.2|	1398880	+	C	1	2	2	2	1	-
gi|29165615|ref|NC_002745.2|	1398882	+	U	0	1	0	0	0	-
gi|29165615|ref|NC_002745.2|	1398892	+	U	0	1	0	0	0	-
gi|29165615|ref|NC_002745.2|	1400752	+	A	0	0	0	1	0	SA1225
gi|29165615|ref|NC_002745.2|	1436274	+	A	0	1	0	0	0	-

gi|29165615|ref|NC_002745.2|	1437032	+	A	0	0	0	1	0	-
gi|29165615|ref|NC_002745.2|	1437052	+	U	1	0	0	2	0	-
gi|29165615|ref|NC_002745.2|	1437059	+	U	0	0	0	0	2	-
gi|29165615|ref|NC_002745.2|	1437064	+	C	0	0	0	1	0	-
gi|29165615|ref|NC_002745.2|	1437069	+	C	0	0	1	1	2	-
gi|29165615|ref|NC_002745.2|	1437070	+	C	0	1	0	2	1	-
gi|29165615|ref|NC_002745.2|	1437071	+	A	0	5	1	0	1	-
gi|29165615|ref|NC_002745.2|	1437072	+	A	0	2	0	2	1	-
gi|29165615|ref|NC_002745.2|	1437073	+	C	2	0	0	1	0	-
gi|29165615|ref|NC_002745.2|	1437074	+	U	0	1	0	0	0	-
gi|29165615|ref|NC_002745.2|	1437075	+	U	0	1	0	0	0	-
gi|29165615|ref|NC_002745.2|	1437076	+	G	0	0	0	2	0	-
gi|29165615|ref|NC_002745.2|	1437077	+	C	0	0	0	0	1	-
gi|29165615|ref|NC_002745.2|	1437078	+	A	0	0	0	0	2	-
gi|29165615|ref|NC_002745.2|	1437079	+	U	0	2	1	0	0	-
gi|29165615|ref|NC_002745.2|	1437080	+	U	0	0	0	1	0	-
gi|29165615|ref|NC_002745.2|	1437087	+	A	0	2	0	0	0	-
gi|29165615|ref|NC_002745.2|	1437092	+	U	0	0	0	1	0	-
gi|29165615|ref|NC_002745.2|	1437097	+	A	0	0	0	0	1	-
gi|29165615|ref|NC_002745.2|	1437108	+	U	0	0	0	0	1	-
gi|29165615|ref|NC_002745.2|	1437120	+	U	0	0	1	0	0	-
gi|29165615|ref|NC_002745.2|	1437128	+	C	0	0	0	0	1	-
gi|29165615|ref|NC_002745.2|	1437129	+	A	0	0	0	1	0	-
gi|29165615|ref|NC_002745.2|	1437136	+	A	0	1	0	1	0	-
gi|29165615|ref|NC_002745.2|	1437137	+	C	0	0	1	0	3	-
gi|29165615|ref|NC_002745.2|	1437145	+	U	0	1	1	2	0	-
gi|29165615|ref|NC_002745.2|	1437146	+	A	0	0	0	1	0	-
gi|29165615|ref|NC_002745.2|	1437154	+	U	0	1	2	1	0	-
gi|29165615|ref|NC_002745.2|	1437155	+	U	0	4	0	1	2	-
gi|29165615|ref|NC_002745.2|	1437159	+	G	0	0	1	0	0	-
gi|29165615|ref|NC_002745.2|	1437160	+	U	0	1	0	1	0	-
gi|29165615|ref|NC_002745.2|	1437164	+	C	1	0	0	0	0	-
gi|29165615|ref|NC_002745.2|	1437165	+	U	2	0	0	0	0	-
gi|29165615|ref|NC_002745.2|	1437166	+	U	0	2	0	0	0	-
gi|29165615|ref|NC_002745.2|	1437170	+	U	0	1	0	1	1	-
gi|29165615|ref|NC_002745.2|	1437172	+	U	1	1	0	0	0	-
gi|29165615|ref|NC_002745.2|	1437176	+	G	0	0	1	0	0	-
gi|29165615|ref|NC_002745.2|	1437185	+	C	0	1	1	0	0	-
gi|29165615|ref|NC_002745.2|	1437187	+	A	0	0	1	0	0	-
gi|29165615|ref|NC_002745.2|	1437190	+	A	0	0	1	0	0	-
gi|29165615|ref|NC_002745.2|	1437194	+	A	0	0	0	0	2	-
gi|29165615|ref|NC_002745.2|	1437205	+	U	0	0	0	0	1	-
gi|29165615|ref|NC_002745.2|	1437208	+	C	0	0	1	0	0	-
gi|29165615|ref|NC_002745.2|	1437210	+	A	0	0	0	0	4	-
gi|29165615|ref|NC_002745.2|	1437215	+	A	0	0	1	0	0	-
gi|29165615|ref|NC_002745.2|	1437220	+	C	1	3	4	2	1	-
gi|29165615|ref|NC_002745.2|	1437222	+	U	0	1	0	0	0	-
gi|29165615|ref|NC_002745.2|	1437227	+	U	0	0	0	0	1	-
gi|29165615|ref|NC_002745.2|	1437228	+	C	0	1	0	2	1	-
gi|29165615|ref|NC_002745.2|	1437232	+	U	3	2	0	0	1	-
gi|29165615|ref|NC_002745.2|	1437233	+	A	0	0	1	0	1	-
gi|29165615|ref|NC_002745.2|	1437235	+	U	0	0	1	0	2	-
gi|29165615|ref|NC_002745.2|	1437237	+	C	0	0	1	1	3	-
gi|29165615|ref|NC_002745.2|	1437238	+	C	1	0	0	0	0	-
gi|29165615|ref|NC_002745.2|	1437239	+	A	0	1	1	0	0	-
gi|29165615|ref|NC_002745.2|	1437240	+	A	1	1	2	0	0	-
gi|29165615|ref|NC_002745.2|	1437242	+	A	0	0	0	0	1	-
gi|29165615|ref|NC_002745.2|	1437243	+	U	2	0	0	0	0	-
gi|29165615|ref|NC_002745.2|	1437247	+	U	6	2	0	0	3	-
gi|29165615|ref|NC_002745.2|	1437250	+	G	0	0	0	0	1	-
gi|29165615|ref|NC_002745.2|	1437252	+	A	0	0	1	0	1	-
gi|29165615|ref|NC_002745.2|	1437253	+	G	0	1	0	0	0	-
gi|29165615|ref|NC_002745.2|	1437256	+	C	0	1	1	0	0	-
gi|29165615|ref|NC_002745.2|	1437262	+	A	0	1	0	2	0	-
gi|29165615|ref|NC_002745.2|	1439942	+	U	0	0	0	1	0	-
gi|29165615|ref|NC_002745.2|	1440173	+	U	0	0	0	1	0	-
gi|29165615|ref|NC_002745.2|	1440404	+	U	0	0	0	1	0	-
gi|29165615|ref|NC_002745.2|	1497173	+	C	0	0	1	0	0	-
gi|29165615|ref|NC_002745.2|	1497174	+	U	0	0	0	1	0	-
gi|29165615|ref|NC_002745.2|	1513534	+	A	0	0	0	0	1	-
gi|29165615|ref|NC_002745.2|	1525792	+	U	1	0	0	0	0	-
gi|29165615|ref|NC_002745.2|	1526143	+	U	0	1	0	0	0	-
gi|29165615|ref|NC_002745.2|	1529914	+	U	1	0	0	0	0	-
gi|29165615|ref|NC_002745.2|	1530265	+	U	0	1	0	0	0	-
gi|29165615|ref|NC_002745.2|	1548951	+	A	0	0	0	0	3	-
gi|29165615|ref|NC_002745.2|	1550356	+	C	0	0	1	0	0	-
gi|29165615|ref|NC_002745.2|	1570767	+	A	0	0	1	1	0	SA1361
gi|29165615|ref|NC_002745.2|	1616410	+	A	0	0	0	1	0	-
gi|29165615|ref|NC_002745.2|	1616411	+	U	0	1	0	0	0	-
gi|29165615|ref|NC_002745.2|	1618430	+	G	0	0	1	0	0	-
gi|29165615|ref|NC_002745.2|	1618551	+	A	0	0	0	1	0	-

gi|29165615|ref|NC_002745.2|	1618571 +	U	1	0	0	2	0	SAS048
gi|29165615|ref|NC_002745.2|	1618578 +	U	0	0	0	0	2	SAS048
gi|29165615|ref|NC_002745.2|	1618583 +	C	0	0	0	1	0	SAS048
gi|29165615|ref|NC_002745.2|	1618588 +	C	0	0	1	1	2	SAS048
gi|29165615|ref|NC_002745.2|	1618589 +	C	0	1	0	2	1	SAS048
gi|29165615|ref|NC_002745.2|	1618590 +	A	0	5	1	0	1	SAS048
gi|29165615|ref|NC_002745.2|	1618591 +	A	0	2	0	2	1	SAS048
gi|29165615|ref|NC_002745.2|	1618592 +	C	2	0	0	1	0	SAS048
gi|29165615|ref|NC_002745.2|	1618593 +	U	0	1	0	0	0	SAS048
gi|29165615|ref|NC_002745.2|	1618594 +	U	0	1	0	0	0	SAS048
gi|29165615|ref|NC_002745.2|	1618595 +	G	0	0	0	2	0	SAS048
gi|29165615|ref|NC_002745.2|	1618596 +	C	0	0	0	0	1	SAS048
gi|29165615|ref|NC_002745.2|	1618597 +	A	0	0	0	0	2	SAS048
gi|29165615|ref|NC_002745.2|	1618598 +	U	0	2	1	0	0	SAS048
gi|29165615|ref|NC_002745.2|	1618599 +	U	0	0	0	1	0	SAS048
gi|29165615|ref|NC_002745.2|	1618606 +	A	0	2	0	0	0	SAS048
gi|29165615|ref|NC_002745.2|	1618609 +	A	0	2	0	0	0	SAS048
gi|29165615|ref|NC_002745.2|	1618611 +	U	0	0	0	1	0	SAS048
gi|29165615|ref|NC_002745.2|	1618615 +	G	0	0	0	0	1	SAS048
gi|29165615|ref|NC_002745.2|	1618616 +	A	0	0	0	0	1	SAS048
gi|29165615|ref|NC_002745.2|	1618628 +	C	0	0	0	1	0	SAS048
gi|29165615|ref|NC_002745.2|	1618629 +	U	0	0	0	1	0	SAS048
gi|29165615|ref|NC_002745.2|	1618642 +	A	0	0	0	1	0	SAS048
gi|29165615|ref|NC_002745.2|	1618647 +	C	0	0	0	1	2	SAS048
gi|29165615|ref|NC_002745.2|	1618648 +	C	0	0	0	0	1	SAS048
gi|29165615|ref|NC_002745.2|	1618649 +	A	0	1	0	0	0	SAS048
gi|29165615|ref|NC_002745.2|	1618654 +	G	0	0	0	0	1	SAS048
gi|29165615|ref|NC_002745.2|	1618655 +	C	0	1	2	0	0	SAS048
gi|29165615|ref|NC_002745.2|	1618656 +	A	0	0	2	0	0	SAS048
gi|29165615|ref|NC_002745.2|	1618657 +	U	3	2	3	1	10	SAS048
gi|29165615|ref|NC_002745.2|	1618662 +	U	0	0	0	1	0	SAS048
gi|29165615|ref|NC_002745.2|	1618663 +	G	0	0	0	0	1	SAS048
gi|29165615|ref|NC_002745.2|	1618664 +	U	0	0	0	1	0	SAS048
gi|29165615|ref|NC_002745.2|	1618665 +	A	0	0	1	1	0	SAS048
gi|29165615|ref|NC_002745.2|	1618666 +	G	0	1	0	2	0	SAS048
gi|29165615|ref|NC_002745.2|	1618667 +	A	0	1	2	2	0	SAS048
gi|29165615|ref|NC_002745.2|	1618668 +	A	2	0	1	1	0	SAS048
gi|29165615|ref|NC_002745.2|	1618669 +	U	0	0	1	3	1	SAS048
gi|29165615|ref|NC_002745.2|	1618670 +	U	0	0	0	0	1	SAS048
gi|29165615|ref|NC_002745.2|	1618673 +	U	0	0	1	0	0	SAS048
gi|29165615|ref|NC_002745.2|	1618674 +	U	0	3	1	4	1	SAS048
gi|29165615|ref|NC_002745.2|	1618675 +	U	0	1	1	1	1	SAS048
gi|29165615|ref|NC_002745.2|	1618678 +	G	0	1	0	0	1	SAS048
gi|29165615|ref|NC_002745.2|	1618679 +	A	0	1	2	0	1	SAS048
gi|29165615|ref|NC_002745.2|	1618733 +	A	0	0	0	0	1	-
gi|29165615|ref|NC_002745.2|	1633082 +	C	0	0	0	1	0	-
gi|29165615|ref|NC_002745.2|	1633095 +	U	0	1	0	0	0	-
gi|29165615|ref|NC_002745.2|	1633110 +	G	0	0	0	0	1	-
gi|29165615|ref|NC_002745.2|	1633116 +	U	0	0	1	0	0	-
gi|29165615|ref|NC_002745.2|	1633133 +	A	0	0	0	0	1	-
gi|29165615|ref|NC_002745.2|	1633140 +	A	0	0	0	0	1	-
gi|29165615|ref|NC_002745.2|	1633143 +	C	0	0	0	1	0	-
gi|29165615|ref|NC_002745.2|	1633183 +	A	0	0	0	0	1	-
gi|29165615|ref|NC_002745.2|	1633185 +	C	0	0	1	0	0	-
gi|29165615|ref|NC_002745.2|	1633186 +	A	0	0	0	1	0	-
gi|29165615|ref|NC_002745.2|	1633191 +	G	0	0	0	1	0	-
gi|29165615|ref|NC_002745.2|	1633199 +	A	0	0	0	5	0	-
gi|29165615|ref|NC_002745.2|	1633200 +	A	0	0	0	1	0	-
gi|29165615|ref|NC_002745.2|	1633201 +	A	0	0	0	0	1	-
gi|29165615|ref|NC_002745.2|	1633204 +	A	0	1	0	3	2	-
gi|29165615|ref|NC_002745.2|	1633205 +	A	0	0	0	1	0	-
gi|29165615|ref|NC_002745.2|	1633210 +	U	0	0	0	1	0	-
gi|29165615|ref|NC_002745.2|	1633211 +	A	1	0	0	0	0	-
gi|29165615|ref|NC_002745.2|	1633236 +	C	0	0	2	0	0	-
gi|29165615|ref|NC_002745.2|	1633256 +	G	0	0	0	0	1	-
gi|29165615|ref|NC_002745.2|	1633351 +	U	0	1	0	0	0	-
gi|29165615|ref|NC_002745.2|	1633368 +	U	0	1	0	0	0	-
gi|29165615|ref|NC_002745.2|	1633369 +	A	0	1	0	0	0	-
gi|29165615|ref|NC_002745.2|	1633375 +	A	0	1	0	0	0	-
gi|29165615|ref|NC_002745.2|	1648919 +	C	0	0	1	0	0	-
gi|29165615|ref|NC_002745.2|	1652908 +	C	0	0	0	1	0	-
gi|29165615|ref|NC_002745.2|	1652916 +	C	0	1	0	0	0	-
gi|29165615|ref|NC_002745.2|	1652917 +	A	0	0	2	0	0	-
gi|29165615|ref|NC_002745.2|	1652918 +	U	3	2	3	1	9	-
gi|29165615|ref|NC_002745.2|	1652923 +	U	0	0	0	1	0	-
gi|29165615|ref|NC_002745.2|	1652924 +	G	0	0	0	0	1	-
gi|29165615|ref|NC_002745.2|	1652925 +	U	0	0	0	1	0	-
gi|29165615|ref|NC_002745.2|	1652926 +	A	0	0	1	1	0	-
gi|29165615|ref|NC_002745.2|	1652927 +	G	0	1	0	2	0	-
gi|29165615|ref|NC_002745.2|	1652929 +	A	0	0	1	0	0	-
gi|29165615|ref|NC_002745.2|	1652930 +	U	0	0	1	3	1	-

gi|29165615|ref|NC_002745.2|	1652931	+	U	0	0	0	0	1	-
gi|29165615|ref|NC_002745.2|	1652935	+	U	0	2	1	3	1	-
gi|29165615|ref|NC_002745.2|	1652936	+	U	0	0	1	0	1	-
gi|29165615|ref|NC_002745.2|	1652939	+	G	0	1	0	0	1	-
gi|29165615|ref|NC_002745.2|	1652940	+	A	0	1	2	0	1	-
gi|29165615|ref|NC_002745.2|	1652959	+	C	0	0	1	0	0	-
gi|29165615|ref|NC_002745.2|	1652976	+	C	0	0	1	0	3	-
gi|29165615|ref|NC_002745.2|	1652984	+	A	0	1	0	1	0	-
gi|29165615|ref|NC_002745.2|	1652993	+	U	0	1	2	0	0	-
gi|29165615|ref|NC_002745.2|	1653027	+	C	0	0	0	2	0	-
gi|29165615|ref|NC_002745.2|	1653055	+	U	0	0	0	0	1	-
gi|29165615|ref|NC_002745.2|	1653059	+	A	0	0	1	1	0	-
gi|29165615|ref|NC_002745.2|	1653069	+	U	1	1	0	0	0	-
gi|29165615|ref|NC_002745.2|	1653071	+	U	0	0	1	0	0	-
gi|29165615|ref|NC_002745.2|	1653083	+	C	0	0	0	2	0	-
gi|29165615|ref|NC_002745.2|	1653084	+	A	0	3	0	0	1	-
gi|29165615|ref|NC_002745.2|	1653097	+	U	0	1	0	0	0	-
gi|29165615|ref|NC_002745.2|	1653111	+	U	0	1	0	0	0	-
gi|29165615|ref|NC_002745.2|	1653141	+	C	0	0	0	1	0	-
gi|29165615|ref|NC_002745.2|	1653149	+	C	0	1	0	0	0	-
gi|29165615|ref|NC_002745.2|	1653150	+	A	0	0	2	0	0	-
gi|29165615|ref|NC_002745.2|	1653151	+	U	3	2	3	1	9	-
gi|29165615|ref|NC_002745.2|	1653156	+	U	0	0	0	1	0	-
gi|29165615|ref|NC_002745.2|	1653157	+	G	0	0	0	0	1	-
gi|29165615|ref|NC_002745.2|	1653158	+	U	0	0	0	1	0	-
gi|29165615|ref|NC_002745.2|	1653163	+	U	0	0	1	2	0	-
gi|29165615|ref|NC_002745.2|	1653168	+	U	0	3	1	3	1	-
gi|29165615|ref|NC_002745.2|	1653169	+	U	0	0	0	0	1	-
gi|29165615|ref|NC_002745.2|	1653172	+	G	0	1	0	0	1	-
gi|29165615|ref|NC_002745.2|	1653173	+	A	0	0	1	2	0	-
gi|29165615|ref|NC_002745.2|	1682381	+	U	0	0	0	0	1	-
gi|29165615|ref|NC_002745.2|	1692032	+	A	0	1	0	0	0	-
gi|29165615|ref|NC_002745.2|	1698500	+	A	0	0	0	1	0	-
gi|29165615|ref|NC_002745.2|	1729536	+	G	0	2	0	0	0	-
gi|29165615|ref|NC_002745.2|	1729551	+	A	0	0	0	0	1	-
gi|29165615|ref|NC_002745.2|	1729552	+	A	0	2	0	1	0	-
gi|29165615|ref|NC_002745.2|	1732818	+	U	0	1	0	0	0	SA1519
gi|29165615|ref|NC_002745.2|	1733160	+	G	0	0	0	0	1	SA1519
gi|29165615|ref|NC_002745.2|	1745273	+	A	0	0	1	0	0	-
gi|29165615|ref|NC_002745.2|	1745285	+	A	0	0	0	2	1	-
gi|29165615|ref|NC_002745.2|	1745321	+	A	0	0	0	0	1	-
gi|29165615|ref|NC_002745.2|	1745323	+	C	0	0	1	0	0	-
gi|29165615|ref|NC_002745.2|	1745324	+	A	0	0	0	1	0	-
gi|29165615|ref|NC_002745.2|	1745329	+	G	0	0	0	1	0	-
gi|29165615|ref|NC_002745.2|	1749603	+	U	0	1	0	0	0	-
gi|29165615|ref|NC_002745.2|	1749614	+	A	0	0	0	0	1	-
gi|29165615|ref|NC_002745.2|	1761690	+	A	0	0	0	1	0	-
gi|29165615|ref|NC_002745.2|	1761695	+	A	15	15	14	7	8	-
gi|29165615|ref|NC_002745.2|	1761700	+	A	1	1	0	0	1	-
gi|29165615|ref|NC_002745.2|	1761703	+	C	0	2	1	0	1	-
gi|29165615|ref|NC_002745.2|	1761743	+	A	0	0	0	2	1	SA1541
gi|29165615|ref|NC_002745.2|	1761748	+	A	0	0	0	1	1	SA1541
gi|29165615|ref|NC_002745.2|	1761754	+	A	0	0	0	0	1	SA1541
gi|29165615|ref|NC_002745.2|	1761756	+	A	0	0	1	0	0	SA1541
gi|29165615|ref|NC_002745.2|	1761757	+	A	0	0	0	1	0	SA1541
gi|29165615|ref|NC_002745.2|	1761760	+	U	0	0	1	0	0	SA1541
gi|29165615|ref|NC_002745.2|	1761762	+	A	0	0	0	0	2	SA1541
gi|29165615|ref|NC_002745.2|	1761763	+	A	0	2	0	0	0	SA1541
gi|29165615|ref|NC_002745.2|	1761765	+	U	0	0	1	0	0	SA1541
gi|29165615|ref|NC_002745.2|	1761766	+	A	0	0	1	0	0	SA1541
gi|29165615|ref|NC_002745.2|	1761768	+	A	0	0	2	0	0	SA1541
gi|29165615|ref|NC_002745.2|	1761769	+	U	0	1	2	0	2	SA1541
gi|29165615|ref|NC_002745.2|	1761770	+	A	0	1	0	0	2	SA1541
gi|29165615|ref|NC_002745.2|	1761771	+	A	0	0	0	0	1	SA1541
gi|29165615|ref|NC_002745.2|	1761772	+	A	0	1	0	0	0	SA1541
gi|29165615|ref|NC_002745.2|	1761773	+	C	0	3	0	0	0	SA1541
gi|29165615|ref|NC_002745.2|	1761775	+	A	0	0	0	0	2	SA1541
gi|29165615|ref|NC_002745.2|	1761777	+	G	0	0	0	0	1	SA1541
gi|29165615|ref|NC_002745.2|	1761778	+	A	2	6	1	1	6	SA1541
gi|29165615|ref|NC_002745.2|	1761779	+	A	0	0	1	0	0	SA1541
gi|29165615|ref|NC_002745.2|	1761784	+	A	0	1	3	0	2	SA1541
gi|29165615|ref|NC_002745.2|	1761787	+	U	0	0	2	0	0	SA1541
gi|29165615|ref|NC_002745.2|	1761790	+	U	0	1	0	0	1	SA1541
gi|29165615|ref|NC_002745.2|	1761791	+	U	0	0	1	0	0	SA1541
gi|29165615|ref|NC_002745.2|	1761795	+	A	0	2	1	0	2	SA1541
gi|29165615|ref|NC_002745.2|	1761796	+	A	0	0	4	1	0	SA1541
gi|29165615|ref|NC_002745.2|	1761799	+	G	0	0	0	0	1	SA1541
gi|29165615|ref|NC_002745.2|	1761800	+	A	0	0	0	1	0	SA1541
gi|29165615|ref|NC_002745.2|	1761801	+	A	0	1	0	0	0	SA1541
gi|29165615|ref|NC_002745.2|	1761803	+	A	0	0	1	0	1	SA1541
gi|29165615|ref|NC_002745.2|	1761804	+	A	1	1	2	1	3	SA1541

gi|29165615|ref|NC_002745.2|	1761809	+	A	0	0	1	0	0	SA1541
gi|29165615|ref|NC_002745.2|	1761811	+	C	1	0	0	0	1	SA1541
gi|29165615|ref|NC_002745.2|	1761819	+	A	0	0	2	0	0	SA1541
gi|29165615|ref|NC_002745.2|	1761825	+	G	0	1	0	0	0	SA1541
gi|29165615|ref|NC_002745.2|	1761830	+	A	0	0	1	0	0	SA1541
gi|29165615|ref|NC_002745.2|	1761832	+	C	0	0	0	0	1	SA1541
gi|29165615|ref|NC_002745.2|	1761834	+	U	0	1	0	0	0	SA1541
gi|29165615|ref|NC_002745.2|	1761843	+	U	0	0	1	0	0	SA1541
gi|29165615|ref|NC_002745.2|	1761864	+	G	0	1	0	0	0	SA1541
gi|29165615|ref|NC_002745.2|	1761881	+	A	0	0	0	1	0	SA1541
gi|29165615|ref|NC_002745.2|	1761882	+	A	0	1	0	0	0	SA1541
gi|29165615|ref|NC_002745.2|	1761926	+	A	0	1	0	0	0	SA1541
gi|29165615|ref|NC_002745.2|	1761927	+	A	0	1	0	0	0	SA1541
gi|29165615|ref|NC_002745.2|	1761958	+	C	0	1	0	0	0	SA1541
gi|29165615|ref|NC_002745.2|	1761968	+	U	0	1	0	0	0	SA1541
gi|29165615|ref|NC_002745.2|	1762063	+	U	0	0	0	0	1	SA1541
gi|29165615|ref|NC_002745.2|	1762123	+	C	1	0	0	0	0	SA1541
gi|29165615|ref|NC_002745.2|	1762234	+	U	0	1	0	0	0	SA1541
gi|29165615|ref|NC_002745.2|	1762235	+	A	0	0	1	0	0	SA1541
gi|29165615|ref|NC_002745.2|	1762270	+	A	0	1	0	0	0	SA1541
gi|29165615|ref|NC_002745.2|	1762289	+	U	0	2	0	0	0	SA1541
gi|29165615|ref|NC_002745.2|	1762297	+	A	0	1	0	0	0	SA1541
gi|29165615|ref|NC_002745.2|	1762300	+	A	0	0	1	0	0	SA1541
gi|29165615|ref|NC_002745.2|	1762392	+	C	0	0	0	0	1	SA1541
gi|29165615|ref|NC_002745.2|	1762468	+	U	0	0	2	0	0	SA1541
gi|29165615|ref|NC_002745.2|	1762469	+	A	0	0	0	0	1	SA1541
gi|29165615|ref|NC_002745.2|	1762491	+	C	1	0	0	0	0	SA1541
gi|29165615|ref|NC_002745.2|	1762554	+	A	0	0	1	0	0	SA1541
gi|29165615|ref|NC_002745.2|	1762581	+	A	0	0	1	0	0	SA1541
gi|29165615|ref|NC_002745.2|	1762582	+	G	0	0	1	0	0	SA1541
gi|29165615|ref|NC_002745.2|	1762716	+	A	0	0	0	0	1	SA1541
gi|29165615|ref|NC_002745.2|	1762733	+	U	0	0	0	0	1	SA1541
gi|29165615|ref|NC_002745.2|	1762738	+	G	0	0	0	0	2	SA1541
gi|29165615|ref|NC_002745.2|	1762768	+	U	0	0	0	0	1	SA1541
gi|29165615|ref|NC_002745.2|	1762771	+	G	0	0	0	0	1	SA1541
gi|29165615|ref|NC_002745.2|	1762795	+	U	0	0	1	0	0	SA1541
gi|29165615|ref|NC_002745.2|	1762813	+	A	0	0	1	0	0	SA1541
gi|29165615|ref|NC_002745.2|	1762846	+	C	0	0	0	1	0	SA1541
gi|29165615|ref|NC_002745.2|	1762896	+	A	0	1	0	0	0	SA1541
gi|29165615|ref|NC_002745.2|	1762907	+	A	0	1	0	1	0	SA1541
gi|29165615|ref|NC_002745.2|	1762911	+	A	0	0	1	0	0	SA1541
gi|29165615|ref|NC_002745.2|	1762942	+	A	0	0	1	0	0	SA1541
gi|29165615|ref|NC_002745.2|	1762956	+	C	0	0	0	1	0	SA1541
gi|29165615|ref|NC_002745.2|	1762963	+	U	0	0	0	1	0	SA1541
gi|29165615|ref|NC_002745.2|	1763023	+	U	0	0	1	0	0	SA1541
gi|29165615|ref|NC_002745.2|	1763031	+	U	0	0	0	1	0	SA1541
gi|29165615|ref|NC_002745.2|	1763032	+	A	0	0	0	0	1	SA1541
gi|29165615|ref|NC_002745.2|	1763047	+	A	0	0	0	0	1	-
gi|29165615|ref|NC_002745.2|	1763053	+	U	0	0	0	0	1	-
gi|29165615|ref|NC_002745.2|	1763055	+	U	0	0	0	1	0	-
gi|29165615|ref|NC_002745.2|	1763058	+	U	0	0	0	0	1	-
gi|29165615|ref|NC_002745.2|	1763061	+	A	0	0	0	1	2	-
gi|29165615|ref|NC_002745.2|	1763062	+	A	0	1	0	1	0	-
gi|29165615|ref|NC_002745.2|	1763063	+	A	0	0	0	1	0	-
gi|29165615|ref|NC_002745.2|	1763064	+	A	1	0	0	0	0	-
gi|29165615|ref|NC_002745.2|	1763068	+	U	0	1	0	0	0	-
gi|29165615|ref|NC_002745.2|	1763075	+	A	0	0	0	0	1	-
gi|29165615|ref|NC_002745.2|	1763077	+	U	0	1	2	3	0	-
gi|29165615|ref|NC_002745.2|	1763078	+	U	0	0	1	0	2	-
gi|29165615|ref|NC_002745.2|	1763086	+	C	0	0	0	0	1	-
gi|29165615|ref|NC_002745.2|	1764024	+	A	0	1	0	0	0	-
gi|29165615|ref|NC_002745.2|	1775033	+	A	0	0	0	0	1	SA1551
gi|29165615|ref|NC_002745.2|	1783281	+	C	0	1	0	0	0	SA1556
gi|29165615|ref|NC_002745.2|	1788220	+	U	0	0	1	0	0	-
gi|29165615|ref|NC_002745.2|	1788379	+	U	0	0	1	0	0	-
gi|29165615|ref|NC_002745.2|	1788538	+	U	0	0	1	0	0	-
gi|29165615|ref|NC_002745.2|	1804084	+	A	0	0	0	0	1	-
gi|29165615|ref|NC_002745.2|	1804086	+	C	0	0	1	0	0	-
gi|29165615|ref|NC_002745.2|	1804087	+	A	0	0	0	1	0	-
gi|29165615|ref|NC_002745.2|	1804092	+	G	0	0	0	1	0	-
gi|29165615|ref|NC_002745.2|	1804100	+	A	0	0	0	5	0	-
gi|29165615|ref|NC_002745.2|	1804101	+	A	0	0	0	1	0	-
gi|29165615|ref|NC_002745.2|	1804102	+	A	0	0	0	0	1	-
gi|29165615|ref|NC_002745.2|	1804105	+	A	0	1	0	3	2	-
gi|29165615|ref|NC_002745.2|	1804106	+	A	0	0	0	1	0	-
gi|29165615|ref|NC_002745.2|	1804109	+	U	0	0	0	1	0	-
gi|29165615|ref|NC_002745.2|	1804110	+	C	0	1	0	0	0	-
gi|29165615|ref|NC_002745.2|	1804111	+	U	0	0	0	1	0	-
gi|29165615|ref|NC_002745.2|	1804118	+	A	0	0	0	0	1	-
gi|29165615|ref|NC_002745.2|	1804123	+	A	0	1	0	0	0	-
gi|29165615|ref|NC_002745.2|	1804155	+	A	0	0	0	0	1	-

gi|29165615|ref|NC_002745.2|	1804164	+	C	1	2	0	0	0	-
gi|29165615|ref|NC_002745.2|	1804165	+	U	0	1	0	0	0	-
gi|29165615|ref|NC_002745.2|	1804198	+	A	0	0	0	0	1	-
gi|29165615|ref|NC_002745.2|	1831961	+	A	1	2	0	1	2	SA1592
gi|29165615|ref|NC_002745.2|	1837971	+	U	0	0	1	0	0	SA1600
gi|29165615|ref|NC_002745.2|	1839609	+	C	0	0	0	1	0	-
gi|29165615|ref|NC_002745.2|	1841020	+	C	0	1	0	0	0	-
gi|29165615|ref|NC_002745.2|	1851921	+	C	1	0	0	0	0	SA1617
gi|29165615|ref|NC_002745.2|	1851923	+	C	0	1	0	0	0	SA1617
gi|29165615|ref|NC_002745.2|	1853893	+	A	0	0	0	0	1	SA1619
gi|29165615|ref|NC_002745.2|	1856436	+	C	0	0	0	0	1	-
gi|29165615|ref|NC_002745.2|	1856471	+	G	0	0	0	1	0	-
gi|29165615|ref|NC_002745.2|	1856476	+	A	0	0	0	1	0	-
gi|29165615|ref|NC_002745.2|	1856477	+	A	0	1	0	0	0	-
gi|29165615|ref|NC_002745.2|	1856485	+	G	0	1	0	0	1	-
gi|29165615|ref|NC_002745.2|	1856852	+	U	0	1	0	0	0	-
gi|29165615|ref|NC_002745.2|	1857014	+	A	0	0	0	1	0	-
gi|29165615|ref|NC_002745.2|	1857110	+	A	0	0	1	0	0	-
gi|29165615|ref|NC_002745.2|	1857154	+	U	2	0	0	0	0	-
gi|29165615|ref|NC_002745.2|	1857526	+	A	0	0	0	0	1	-
gi|29165615|ref|NC_002745.2|	1858547	+	U	1	0	0	0	0	-
gi|29165615|ref|NC_002745.2|	1859205	+	C	0	1	0	0	0	-
gi|29165615|ref|NC_002745.2|	1860676	+	U	0	0	1	0	0	-
gi|29165615|ref|NC_002745.2|	1860742	+	U	2	0	0	1	0	-
gi|29165615|ref|NC_002745.2|	1860760	+	A	0	0	1	0	0	-
gi|29165615|ref|NC_002745.2|	1860765	+	A	1	0	3	4	1	-
gi|29165615|ref|NC_002745.2|	1860773	+	G	0	0	1	0	0	-
gi|29165615|ref|NC_002745.2|	1866113	+	G	0	1	0	0	0	SA1633
gi|29165615|ref|NC_002745.2|	1866182	+	A	0	0	0	0	1	SA1633
gi|29165615|ref|NC_002745.2|	1876847	+	C	1	0	0	0	0	-
gi|29165615|ref|NC_002745.2|	1876849	+	C	1	1	0	0	0	-
gi|29165615|ref|NC_002745.2|	1879348	+	G	0	0	0	1	0	-
gi|29165615|ref|NC_002745.2|	1880111	+	G	0	0	0	1	0	-
gi|29165615|ref|NC_002745.2|	1893223	+	A	0	0	0	0	1	SA1659
gi|29165615|ref|NC_002745.2|	1904897	+	C	3	0	0	0	0	SA1668
gi|29165615|ref|NC_002745.2|	1904900	+	G	0	2	0	0	0	SA1668
gi|29165615|ref|NC_002745.2|	1907795	+	C	0	0	0	0	1	-
gi|29165615|ref|NC_002745.2|	1907800	+	A	0	1	0	0	0	-
gi|29165615|ref|NC_002745.2|	1907838	+	A	0	0	0	2	1	-
gi|29165615|ref|NC_002745.2|	1907842	+	U	0	1	0	0	0	-
gi|29165615|ref|NC_002745.2|	1914610	+	A	0	1	0	0	0	-
gi|29165615|ref|NC_002745.2|	1914782	+	A	0	0	0	1	0	-
gi|29165615|ref|NC_002745.2|	1914787	+	A	15	15	14	7	8	-
gi|29165615|ref|NC_002745.2|	1914792	+	A	1	1	0	0	1	-
gi|29165615|ref|NC_002745.2|	1914795	+	C	0	2	1	0	1	-
gi|29165615|ref|NC_002745.2|	1914835	+	A	0	0	0	2	1	SA1677
gi|29165615|ref|NC_002745.2|	1914840	+	A	0	0	0	1	1	SA1677
gi|29165615|ref|NC_002745.2|	1914846	+	A	0	0	0	0	1	SA1677
gi|29165615|ref|NC_002745.2|	1914848	+	A	0	0	1	0	0	SA1677
gi|29165615|ref|NC_002745.2|	1914849	+	A	0	0	0	1	0	SA1677
gi|29165615|ref|NC_002745.2|	1914852	+	U	0	0	1	0	0	SA1677
gi|29165615|ref|NC_002745.2|	1914854	+	A	0	0	0	0	2	SA1677
gi|29165615|ref|NC_002745.2|	1914855	+	A	0	2	0	0	0	SA1677
gi|29165615|ref|NC_002745.2|	1914857	+	U	0	0	1	0	0	SA1677
gi|29165615|ref|NC_002745.2|	1914858	+	A	0	0	1	0	0	SA1677
gi|29165615|ref|NC_002745.2|	1914860	+	A	0	0	2	0	0	SA1677
gi|29165615|ref|NC_002745.2|	1914861	+	U	0	1	2	0	2	SA1677
gi|29165615|ref|NC_002745.2|	1914862	+	A	0	1	0	0	2	SA1677
gi|29165615|ref|NC_002745.2|	1914863	+	A	0	0	0	0	1	SA1677
gi|29165615|ref|NC_002745.2|	1914864	+	A	0	1	0	0	0	SA1677
gi|29165615|ref|NC_002745.2|	1914865	+	C	0	3	0	0	0	SA1677
gi|29165615|ref|NC_002745.2|	1914867	+	A	0	0	0	0	2	SA1677
gi|29165615|ref|NC_002745.2|	1914869	+	G	0	0	0	0	1	SA1677
gi|29165615|ref|NC_002745.2|	1914870	+	A	2	6	1	1	6	SA1677
gi|29165615|ref|NC_002745.2|	1914871	+	A	0	0	1	0	0	SA1677
gi|29165615|ref|NC_002745.2|	1914876	+	A	0	1	3	0	2	SA1677
gi|29165615|ref|NC_002745.2|	1914879	+	U	0	0	2	0	0	SA1677
gi|29165615|ref|NC_002745.2|	1914882	+	U	0	1	0	0	1	SA1677
gi|29165615|ref|NC_002745.2|	1914883	+	U	0	0	1	0	0	SA1677
gi|29165615|ref|NC_002745.2|	1914887	+	A	0	2	1	0	2	SA1677
gi|29165615|ref|NC_002745.2|	1914888	+	A	0	0	4	1	0	SA1677
gi|29165615|ref|NC_002745.2|	1914891	+	G	0	0	0	0	1	SA1677
gi|29165615|ref|NC_002745.2|	1914892	+	A	0	0	0	1	0	SA1677
gi|29165615|ref|NC_002745.2|	1914893	+	A	0	1	0	0	0	SA1677
gi|29165615|ref|NC_002745.2|	1914895	+	A	0	0	1	0	1	SA1677
gi|29165615|ref|NC_002745.2|	1914896	+	A	1	1	2	1	3	SA1677
gi|29165615|ref|NC_002745.2|	1914901	+	A	0	0	1	0	0	SA1677
gi|29165615|ref|NC_002745.2|	1914903	+	C	1	0	0	0	1	SA1677
gi|29165615|ref|NC_002745.2|	1914911	+	A	0	0	2	0	0	SA1677
gi|29165615|ref|NC_002745.2|	1914917	+	G	0	1	0	0	0	SA1677
gi|29165615|ref|NC_002745.2|	1914922	+	A	0	0	1	0	0	SA1677

gi|29165615|ref|NC_002745.2|	1914924 +	C	0	0	0	0	1	SA1677
gi|29165615|ref|NC_002745.2|	1914926 +	U	0	1	0	0	0	SA1677
gi|29165615|ref|NC_002745.2|	1914935 +	U	0	0	1	0	0	SA1677
gi|29165615|ref|NC_002745.2|	1914956 +	G	0	1	0	0	0	SA1677
gi|29165615|ref|NC_002745.2|	1914973 +	A	0	0	0	1	0	SA1677
gi|29165615|ref|NC_002745.2|	1914974 +	A	0	1	0	0	0	SA1677
gi|29165615|ref|NC_002745.2|	1915018 +	A	0	1	0	0	0	SA1677
gi|29165615|ref|NC_002745.2|	1915019 +	A	0	1	0	0	0	SA1677
gi|29165615|ref|NC_002745.2|	1915050 +	C	0	1	0	0	0	SA1677
gi|29165615|ref|NC_002745.2|	1915060 +	U	0	1	0	0	0	SA1677
gi|29165615|ref|NC_002745.2|	1915155 +	U	0	0	0	0	1	SA1677
gi|29165615|ref|NC_002745.2|	1915215 +	C	1	0	0	0	0	SA1677
gi|29165615|ref|NC_002745.2|	1915326 +	U	0	1	0	0	0	SA1677
gi|29165615|ref|NC_002745.2|	1915327 +	A	0	0	1	0	0	SA1677
gi|29165615|ref|NC_002745.2|	1915362 +	A	0	1	0	0	0	SA1677
gi|29165615|ref|NC_002745.2|	1915381 +	U	0	2	0	0	0	SA1677
gi|29165615|ref|NC_002745.2|	1915389 +	A	0	1	0	0	0	SA1677
gi|29165615|ref|NC_002745.2|	1915392 +	A	0	0	1	0	0	SA1677
gi|29165615|ref|NC_002745.2|	1915484 +	C	0	0	0	0	1	SA1677
gi|29165615|ref|NC_002745.2|	1915560 +	U	0	0	2	0	0	SA1677
gi|29165615|ref|NC_002745.2|	1915561 +	A	0	0	0	0	1	SA1677
gi|29165615|ref|NC_002745.2|	1915583 +	C	1	0	0	0	0	SA1677
gi|29165615|ref|NC_002745.2|	1915646 +	A	0	0	1	0	0	SA1677
gi|29165615|ref|NC_002745.2|	1915673 +	A	0	0	1	0	0	SA1677
gi|29165615|ref|NC_002745.2|	1915674 +	G	0	0	1	0	0	SA1677
gi|29165615|ref|NC_002745.2|	1915808 +	A	0	0	0	0	1	SA1677
gi|29165615|ref|NC_002745.2|	1915825 +	U	0	0	0	0	1	SA1677
gi|29165615|ref|NC_002745.2|	1915830 +	G	0	0	0	0	2	SA1677
gi|29165615|ref|NC_002745.2|	1915860 +	U	0	0	0	0	1	SA1677
gi|29165615|ref|NC_002745.2|	1915863 +	G	0	0	0	0	1	SA1677
gi|29165615|ref|NC_002745.2|	1915887 +	U	0	0	1	0	0	SA1677
gi|29165615|ref|NC_002745.2|	1915905 +	A	0	0	1	0	0	SA1677
gi|29165615|ref|NC_002745.2|	1915938 +	C	0	0	0	1	0	SA1677
gi|29165615|ref|NC_002745.2|	1915988 +	A	0	1	0	0	0	SA1677
gi|29165615|ref|NC_002745.2|	1915999 +	A	0	1	0	1	0	SA1677
gi|29165615|ref|NC_002745.2|	1916003 +	A	0	0	1	0	0	SA1677
gi|29165615|ref|NC_002745.2|	1916034 +	A	0	0	1	0	0	SA1677
gi|29165615|ref|NC_002745.2|	1916048 +	C	0	0	0	1	0	SA1677
gi|29165615|ref|NC_002745.2|	1916055 +	U	0	0	0	1	0	SA1677
gi|29165615|ref|NC_002745.2|	1916115 +	U	0	0	1	0	0	SA1677
gi|29165615|ref|NC_002745.2|	1916123 +	U	0	0	0	1	0	SA1677
gi|29165615|ref|NC_002745.2|	1916124 +	A	0	0	0	0	1	SA1677
gi|29165615|ref|NC_002745.2|	1916139 +	A	0	0	0	0	1	-
gi|29165615|ref|NC_002745.2|	1916145 +	U	0	0	0	0	1	-
gi|29165615|ref|NC_002745.2|	1916147 +	U	0	0	0	1	0	-
gi|29165615|ref|NC_002745.2|	1916150 +	U	0	0	0	0	1	-
gi|29165615|ref|NC_002745.2|	1916153 +	A	0	0	0	1	2	-
gi|29165615|ref|NC_002745.2|	1916154 +	A	0	1	0	1	0	-
gi|29165615|ref|NC_002745.2|	1916155 +	A	0	0	0	1	0	-
gi|29165615|ref|NC_002745.2|	1916156 +	A	1	0	0	0	0	-
gi|29165615|ref|NC_002745.2|	1916160 +	U	0	1	0	0	0	-
gi|29165615|ref|NC_002745.2|	1916167 +	A	0	0	0	0	1	-
gi|29165615|ref|NC_002745.2|	1916169 +	U	0	1	2	3	0	-
gi|29165615|ref|NC_002745.2|	1916170 +	U	0	0	1	0	2	-
gi|29165615|ref|NC_002745.2|	1916178 +	C	0	0	0	0	1	-
gi|29165615|ref|NC_002745.2|	1918749 +	U	2	0	0	0	0	-
gi|29165615|ref|NC_002745.2|	1918787 +	C	0	1	0	0	0	-
gi|29165615|ref|NC_002745.2|	1919024 +	U	0	0	0	1	0	-
gi|29165615|ref|NC_002745.2|	1919165 +	C	0	0	0	0	2	-
gi|29165615|ref|NC_002745.2|	1919167 +	U	1	0	0	0	0	-
gi|29165615|ref|NC_002745.2|	1919187 +	C	0	0	1	0	0	-
gi|29165615|ref|NC_002745.2|	1919274 +	A	0	0	0	0	1	-
gi|29165615|ref|NC_002745.2|	1919306 +	C	0	1	0	0	0	-
gi|29165615|ref|NC_002745.2|	1919317 +	G	0	1	0	0	0	-
gi|29165615|ref|NC_002745.2|	1919776 +	C	1	0	0	0	0	-
gi|29165615|ref|NC_002745.2|	1919779 +	G	0	1	0	0	0	-
gi|29165615|ref|NC_002745.2|	1919780 +	U	0	1	1	0	0	-
gi|29165615|ref|NC_002745.2|	1919782 +	G	0	1	0	0	0	-
gi|29165615|ref|NC_002745.2|	1919815 +	U	0	0	1	0	0	-
gi|29165615|ref|NC_002745.2|	1919824 +	U	0	1	0	3	0	-
gi|29165615|ref|NC_002745.2|	1919836 +	A	0	0	1	0	0	-
gi|29165615|ref|NC_002745.2|	1919837 +	C	0	1	0	0	0	-
gi|29165615|ref|NC_002745.2|	1919838 +	A	2	1	0	0	0	-
gi|29165615|ref|NC_002745.2|	1919839 +	G	1	0	0	0	0	-
gi|29165615|ref|NC_002745.2|	1919840 +	U	1	1	1	0	0	-
gi|29165615|ref|NC_002745.2|	1919841 +	G	0	0	0	0	1	-
gi|29165615|ref|NC_002745.2|	1919871 +	G	0	1	0	0	0	-
gi|29165615|ref|NC_002745.2|	1919872 +	G	0	1	0	0	0	-
gi|29165615|ref|NC_002745.2|	1919873 +	A	0	1	0	0	0	-
gi|29165615|ref|NC_002745.2|	1919874 +	A	0	0	0	1	0	-
gi|29165615|ref|NC_002745.2|	1919875 +	C	0	1	1	0	1	-

gi|29165615|ref|NC_002745.2|	1919965	+	A	1	0	1	0	0	-
gi|29165615|ref|NC_002745.2|	1919966	+	C	1	2	3	2	0	-
gi|29165615|ref|NC_002745.2|	1919967	+	C	1	1	0	2	0	-
gi|29165615|ref|NC_002745.2|	1919968	+	A	0	0	1	0	0	-
gi|29165615|ref|NC_002745.2|	1919971	+	C	0	0	0	2	0	-
gi|29165615|ref|NC_002745.2|	1919991	+	G	1	0	0	0	0	-
gi|29165615|ref|NC_002745.2|	1920001	+	C	0	0	1	0	0	-
gi|29165615|ref|NC_002745.2|	1920003	+	G	0	0	1	0	0	-
gi|29165615|ref|NC_002745.2|	1920004	+	C	0	1	0	0	0	-
gi|29165615|ref|NC_002745.2|	1920007	+	C	3	0	1	1	0	-
gi|29165615|ref|NC_002745.2|	1920008	+	U	6	8	4	3	1	-
gi|29165615|ref|NC_002745.2|	1920056	+	U	0	0	1	0	0	-
gi|29165615|ref|NC_002745.2|	1920058	+	G	0	0	0	0	1	-
gi|29165615|ref|NC_002745.2|	1920108	+	C	0	1	0	0	0	-
gi|29165615|ref|NC_002745.2|	1920109	+	C	1	1	0	0	0	-
gi|29165615|ref|NC_002745.2|	1920111	+	U	0	0	0	1	0	-
gi|29165615|ref|NC_002745.2|	1920112	+	U	1	2	0	0	0	-
gi|29165615|ref|NC_002745.2|	1920113	+	C	0	0	0	1	0	-
gi|29165615|ref|NC_002745.2|	1920139	+	C	0	0	0	2	0	-
gi|29165615|ref|NC_002745.2|	1920164	+	G	0	1	0	0	0	-
gi|29165615|ref|NC_002745.2|	1920165	+	C	0	1	0	0	0	-
gi|29165615|ref|NC_002745.2|	1920278	+	U	1	0	0	0	0	-
gi|29165615|ref|NC_002745.2|	1920378	+	U	0	3	0	0	0	-
gi|29165615|ref|NC_002745.2|	1920386	+	C	1	0	0	0	0	-
gi|29165615|ref|NC_002745.2|	1920389	+	C	3	2	1	1	0	-
gi|29165615|ref|NC_002745.2|	1920435	+	U	1	0	0	0	0	-
gi|29165615|ref|NC_002745.2|	1920437	+	U	0	1	0	0	0	-
gi|29165615|ref|NC_002745.2|	1920474	+	G	17	17	8	17	4	-
gi|29165615|ref|NC_002745.2|	1920508	+	A	0	0	0	2	0	-
gi|29165615|ref|NC_002745.2|	1920512	+	U	0	0	1	0	0	-
gi|29165615|ref|NC_002745.2|	1920560	+	G	0	0	0	0	1	-
gi|29165615|ref|NC_002745.2|	1920565	+	U	0	0	1	0	0	-
gi|29165615|ref|NC_002745.2|	1920623	+	U	3	0	0	0	0	-
gi|29165615|ref|NC_002745.2|	1920651	+	A	0	1	1	0	0	-
gi|29165615|ref|NC_002745.2|	1920656	+	U	0	1	0	0	0	-
gi|29165615|ref|NC_002745.2|	1920673	+	C	0	0	0	1	0	-
gi|29165615|ref|NC_002745.2|	1920674	+	C	0	1	0	0	0	-
gi|29165615|ref|NC_002745.2|	1920762	+	U	0	1	0	0	0	-
gi|29165615|ref|NC_002745.2|	1920779	+	A	1	1	0	0	0	-
gi|29165615|ref|NC_002745.2|	1920781	+	C	0	1	0	0	0	-
gi|29165615|ref|NC_002745.2|	1920782	+	C	2	1	0	0	2	-
gi|29165615|ref|NC_002745.2|	1920783	+	U	2	1	0	4	1	-
gi|29165615|ref|NC_002745.2|	1920784	+	U	2	2	1	0	0	-
gi|29165615|ref|NC_002745.2|	1920785	+	U	0	0	0	1	1	-
gi|29165615|ref|NC_002745.2|	1920787	+	C	0	0	0	1	0	-
gi|29165615|ref|NC_002745.2|	1920788	+	C	0	0	1	1	0	-
gi|29165615|ref|NC_002745.2|	1920802	+	U	1	0	0	0	0	-
gi|29165615|ref|NC_002745.2|	1920831	+	U	0	1	0	0	0	-
gi|29165615|ref|NC_002745.2|	1920865	+	U	0	1	0	0	0	-
gi|29165615|ref|NC_002745.2|	1920867	+	U	1	0	0	0	0	-
gi|29165615|ref|NC_002745.2|	1920869	+	A	0	1	1	0	0	-
gi|29165615|ref|NC_002745.2|	1920870	+	C	0	1	0	0	0	-
gi|29165615|ref|NC_002745.2|	1920929	+	C	0	2	0	0	0	-
gi|29165615|ref|NC_002745.2|	1920930	+	C	0	2	0	0	0	-
gi|29165615|ref|NC_002745.2|	1920931	+	C	0	1	0	0	0	-
gi|29165615|ref|NC_002745.2|	1920932	+	C	0	0	0	0	1	-
gi|29165615|ref|NC_002745.2|	1920946	+	G	6	4	4	6	1	-
gi|29165615|ref|NC_002745.2|	1920947	+	C	0	1	0	0	1	-
gi|29165615|ref|NC_002745.2|	1920948	+	U	0	0	0	0	2	-
gi|29165615|ref|NC_002745.2|	1920949	+	C	0	0	3	0	1	-
gi|29165615|ref|NC_002745.2|	1920950	+	U	0	2	0	0	0	-
gi|29165615|ref|NC_002745.2|	1920951	+	A	0	0	1	0	0	-
gi|29165615|ref|NC_002745.2|	1920952	+	C	3	0	1	0	0	-
gi|29165615|ref|NC_002745.2|	1920974	+	G	0	0	2	0	0	-
gi|29165615|ref|NC_002745.2|	1920975	+	C	2	0	0	0	0	-
gi|29165615|ref|NC_002745.2|	1920976	+	U	0	1	0	0	0	-
gi|29165615|ref|NC_002745.2|	1920978	+	G	1	0	0	0	0	-
gi|29165615|ref|NC_002745.2|	1921011	+	U	0	0	0	0	1	-
gi|29165615|ref|NC_002745.2|	1921196	+	U	1	0	0	0	1	-
gi|29165615|ref|NC_002745.2|	1921197	+	U	0	0	0	1	0	-
gi|29165615|ref|NC_002745.2|	1921199	+	A	0	0	0	1	0	-
gi|29165615|ref|NC_002745.2|	1921209	+	C	0	0	0	1	0	-
gi|29165615|ref|NC_002745.2|	1921225	+	A	0	1	0	0	0	-
gi|29165615|ref|NC_002745.2|	1921233	+	G	1	1	0	0	0	-
gi|29165615|ref|NC_002745.2|	1921255	+	C	0	1	0	0	0	-
gi|29165615|ref|NC_002745.2|	1921263	+	A	12	22	23	23	1	-
gi|29165615|ref|NC_002745.2|	1921266	+	A	0	1	0	0	1	-
gi|29165615|ref|NC_002745.2|	1921269	+	G	0	0	0	1	0	-
gi|29165615|ref|NC_002745.2|	1921275	+	U	0	0	0	1	0	-
gi|29165615|ref|NC_002745.2|	1921277	+	A	1	0	0	0	0	-
gi|29165615|ref|NC_002745.2|	1921346	+	A	0	0	0	1	0	-

gi|29165615|ref|NC_002745.2|	1921663	+	U	0	0	0	1	0	-
gi|29165615|ref|NC_002745.2|	1921842	+	U	0	3	0	0	0	-
gi|29165615|ref|NC_002745.2|	1922757	+	A	1	3	1	0	1	-
gi|29165615|ref|NC_002745.2|	1922758	+	A	2	2	1	2	3	-
gi|29165615|ref|NC_002745.2|	1922876	+	G	0	0	0	1	0	-
gi|29165615|ref|NC_002745.2|	1922905	+	G	0	0	0	1	0	-
gi|29165615|ref|NC_002745.2|	1922945	+	G	1	0	2	0	0	-
gi|29165615|ref|NC_002745.2|	1922946	+	U	1	1	1	1	4	-
gi|29165615|ref|NC_002745.2|	1922947	+	C	0	2	1	2	0	-
gi|29165615|ref|NC_002745.2|	1922948	+	C	2	3	1	2	0	-
gi|29165615|ref|NC_002745.2|	1922949	+	C	0	0	0	0	1	-
gi|29165615|ref|NC_002745.2|	1923035	+	A	0	3	0	0	0	-
gi|29165615|ref|NC_002745.2|	1923037	+	C	1	0	0	0	0	-
gi|29165615|ref|NC_002745.2|	1923039	+	C	2	0	0	0	0	-
gi|29165615|ref|NC_002745.2|	1923067	+	C	3	7	1	3	0	-
gi|29165615|ref|NC_002745.2|	1923068	+	G	7	19	5	12	5	-
gi|29165615|ref|NC_002745.2|	1923069	+	U	26	24	15	23	6	-
gi|29165615|ref|NC_002745.2|	1923070	+	G	0	3	1	1	0	-
gi|29165615|ref|NC_002745.2|	1923071	+	G	3	12	0	5	2	-
gi|29165615|ref|NC_002745.2|	1923072	+	A	3	13	6	9	4	-
gi|29165615|ref|NC_002745.2|	1923073	+	C	19	23	6	19	4	-
gi|29165615|ref|NC_002745.2|	1923074	+	U	8	6	1	2	4	-
gi|29165615|ref|NC_002745.2|	1923075	+	A	2	2	1	0	0	-
gi|29165615|ref|NC_002745.2|	1923076	+	C	2	4	1	4	0	-
gi|29165615|ref|NC_002745.2|	1923077	+	C	0	5	0	1	0	-
gi|29165615|ref|NC_002745.2|	1923078	+	A	0	0	0	4	0	-
gi|29165615|ref|NC_002745.2|	1923079	+	G	0	1	1	2	0	-
gi|29165615|ref|NC_002745.2|	1923099	+	U	0	1	0	0	0	-
gi|29165615|ref|NC_002745.2|	1923102	+	C	0	0	0	2	0	-
gi|29165615|ref|NC_002745.2|	1923110	+	U	0	1	0	0	0	-
gi|29165615|ref|NC_002745.2|	1923151	+	C	0	2	0	0	0	-
gi|29165615|ref|NC_002745.2|	1923181	+	U	0	0	0	1	0	-
gi|29165615|ref|NC_002745.2|	1923294	+	A	0	1	0	0	0	-
gi|29165615|ref|NC_002745.2|	1923299	+	C	0	0	0	2	0	-
gi|29165615|ref|NC_002745.2|	1923305	+	A	0	0	1	0	0	-
gi|29165615|ref|NC_002745.2|	1923306	+	C	0	1	0	0	0	-
gi|29165615|ref|NC_002745.2|	1923311	+	A	0	0	1	1	1	-
gi|29165615|ref|NC_002745.2|	1923372	+	G	0	1	0	0	0	-
gi|29165615|ref|NC_002745.2|	1923389	+	U	0	0	0	1	0	-
gi|29165615|ref|NC_002745.2|	1923390	+	A	0	1	0	0	0	-
gi|29165615|ref|NC_002745.2|	1923422	+	U	0	0	3	0	0	-
gi|29165615|ref|NC_002745.2|	1923423	+	G	0	0	0	0	1	-
gi|29165615|ref|NC_002745.2|	1923424	+	U	1	0	0	0	1	-
gi|29165615|ref|NC_002745.2|	1923425	+	U	2	2	0	3	1	-
gi|29165615|ref|NC_002745.2|	1923427	+	U	0	1	1	0	1	-
gi|29165615|ref|NC_002745.2|	1923454	+	G	0	0	0	1	0	-
gi|29165615|ref|NC_002745.2|	1923521	+	G	0	1	0	0	0	-
gi|29165615|ref|NC_002745.2|	1923647	+	A	0	0	1	0	0	-
gi|29165615|ref|NC_002745.2|	1945724	+	U	0	0	0	0	1	SA1699
gi|29165615|ref|NC_002745.2|	1950618	+	C	0	0	1	1	2	-
gi|29165615|ref|NC_002745.2|	1950619	+	C	0	0	0	2	1	-
gi|29165615|ref|NC_002745.2|	1950620	+	A	0	5	0	0	1	-
gi|29165615|ref|NC_002745.2|	1950621	+	A	0	1	0	1	1	-
gi|29165615|ref|NC_002745.2|	1950622	+	C	1	0	0	0	0	-
gi|29165615|ref|NC_002745.2|	1950625	+	G	0	0	0	2	0	-
gi|29165615|ref|NC_002745.2|	1950628	+	U	0	0	0	1	0	-
gi|29165615|ref|NC_002745.2|	1950645	+	A	0	0	0	0	1	-
gi|29165615|ref|NC_002745.2|	1950646	+	G	0	0	0	0	1	-
gi|29165615|ref|NC_002745.2|	1950651	+	A	0	0	0	1	0	-
gi|29165615|ref|NC_002745.2|	1950661	+	U	1	1	0	0	0	-
gi|29165615|ref|NC_002745.2|	1950684	+	C	0	0	1	0	3	-
gi|29165615|ref|NC_002745.2|	1950692	+	U	0	1	1	2	0	-
gi|29165615|ref|NC_002745.2|	1950693	+	A	0	0	0	1	0	-
gi|29165615|ref|NC_002745.2|	1950701	+	U	0	1	2	1	0	-
gi|29165615|ref|NC_002745.2|	1950702	+	U	0	4	0	1	2	-
gi|29165615|ref|NC_002745.2|	1950707	+	U	0	2	1	1	1	-
gi|29165615|ref|NC_002745.2|	1950708	+	C	0	0	1	2	0	-
gi|29165615|ref|NC_002745.2|	1950714	+	C	0	0	0	1	0	-
gi|29165615|ref|NC_002745.2|	1950715	+	U	0	0	0	1	0	-
gi|29165615|ref|NC_002745.2|	1950726	+	C	0	0	1	0	0	-
gi|29165615|ref|NC_002745.2|	1950734	+	C	0	0	0	0	1	-
gi|29165615|ref|NC_002745.2|	1950735	+	A	0	0	0	1	0	-
gi|29165615|ref|NC_002745.2|	1950742	+	A	0	1	0	1	0	-
gi|29165615|ref|NC_002745.2|	1950743	+	C	0	0	1	0	3	-
gi|29165615|ref|NC_002745.2|	1950751	+	U	0	1	1	2	0	-
gi|29165615|ref|NC_002745.2|	1950752	+	A	0	0	0	1	0	-
gi|29165615|ref|NC_002745.2|	1950760	+	U	0	1	2	1	0	-
gi|29165615|ref|NC_002745.2|	1950761	+	U	0	4	0	1	2	-
gi|29165615|ref|NC_002745.2|	1950765	+	G	0	0	1	0	0	-
gi|29165615|ref|NC_002745.2|	1950766	+	U	0	3	0	1	0	-
gi|29165615|ref|NC_002745.2|	1950770	+	C	1	0	0	0	0	-

gi|29165615|ref|NC_002745.2|	1950772 +	U	0	2	0	0	0 -
gi|29165615|ref|NC_002745.2|	1950776 +	U	0	1	0	1	1 -
gi|29165615|ref|NC_002745.2|	1950778 +	U	1	1	0	0	0 -
gi|29165615|ref|NC_002745.2|	1950791 +	C	0	1	1	0	0 -
gi|29165615|ref|NC_002745.2|	1950793 +	A	0	0	1	0	0 -
gi|29165615|ref|NC_002745.2|	1950796 +	A	0	0	1	0	0 -
gi|29165615|ref|NC_002745.2|	1950800 +	A	0	0	0	0	2 -
gi|29165615|ref|NC_002745.2|	1950816 +	A	0	0	0	0	2 -
gi|29165615|ref|NC_002745.2|	1950828 +	U	0	1	0	0	0 -
gi|29165615|ref|NC_002745.2|	1952039 +	A	0	0	0	1	0 -
gi|29165615|ref|NC_002745.2|	1952059 +	U	1	0	0	2	0 -
gi|29165615|ref|NC_002745.2|	1952066 +	U	0	0	0	0	2 -
gi|29165615|ref|NC_002745.2|	1952071 +	C	0	0	0	1	0 -
gi|29165615|ref|NC_002745.2|	1952076 +	C	0	0	0	1	0 -
gi|29165615|ref|NC_002745.2|	1952083 +	G	0	0	0	0	1 -
gi|29165615|ref|NC_002745.2|	1952084 +	C	0	1	2	0	0 -
gi|29165615|ref|NC_002745.2|	1952085 +	A	0	0	2	0	0 -
gi|29165615|ref|NC_002745.2|	1952086 +	U	3	2	3	1	10 -
gi|29165615|ref|NC_002745.2|	1952091 +	U	0	0	0	1	0 -
gi|29165615|ref|NC_002745.2|	1952092 +	G	0	0	0	0	1 -
gi|29165615|ref|NC_002745.2|	1952093 +	U	0	0	0	1	0 -
gi|29165615|ref|NC_002745.2|	1952094 +	A	0	0	1	1	0 -
gi|29165615|ref|NC_002745.2|	1952095 +	G	0	1	0	2	0 -
gi|29165615|ref|NC_002745.2|	1952096 +	A	0	1	2	2	0 -
gi|29165615|ref|NC_002745.2|	1952097 +	A	2	0	1	1	0 -
gi|29165615|ref|NC_002745.2|	1952098 +	U	0	0	1	3	1 -
gi|29165615|ref|NC_002745.2|	1952099 +	U	0	0	0	0	1 -
gi|29165615|ref|NC_002745.2|	1952102 +	U	0	0	1	0	0 -
gi|29165615|ref|NC_002745.2|	1952103 +	U	0	2	1	4	1 -
gi|29165615|ref|NC_002745.2|	1952104 +	U	0	1	1	1	1 -
gi|29165615|ref|NC_002745.2|	1952115 +	C	0	0	0	1	0 -
gi|29165615|ref|NC_002745.2|	1952116 +	U	0	0	0	1	0 -
gi|29165615|ref|NC_002745.2|	1952129 +	A	0	0	0	1	0 -
gi|29165615|ref|NC_002745.2|	1952134 +	C	0	0	1	1	2 -
gi|29165615|ref|NC_002745.2|	1952135 +	C	0	1	0	2	1 -
gi|29165615|ref|NC_002745.2|	1952136 +	A	0	5	1	0	1 -
gi|29165615|ref|NC_002745.2|	1952137 +	A	0	2	0	2	1 -
gi|29165615|ref|NC_002745.2|	1952138 +	C	2	0	0	1	0 -
gi|29165615|ref|NC_002745.2|	1952139 +	U	0	1	0	0	0 -
gi|29165615|ref|NC_002745.2|	1952140 +	U	0	1	0	0	0 -
gi|29165615|ref|NC_002745.2|	1952141 +	G	0	0	0	2	0 -
gi|29165615|ref|NC_002745.2|	1952142 +	C	0	0	0	0	1 -
gi|29165615|ref|NC_002745.2|	1952143 +	A	0	0	0	0	2 -
gi|29165615|ref|NC_002745.2|	1952144 +	U	0	2	1	0	0 -
gi|29165615|ref|NC_002745.2|	1952145 +	U	0	0	0	1	0 -
gi|29165615|ref|NC_002745.2|	1952152 +	A	0	2	0	0	0 -
gi|29165615|ref|NC_002745.2|	1952155 +	A	0	2	0	0	0 -
gi|29165615|ref|NC_002745.2|	1952157 +	U	0	0	0	1	0 -
gi|29165615|ref|NC_002745.2|	1952161 +	G	0	0	0	0	1 -
gi|29165615|ref|NC_002745.2|	1952162 +	A	0	0	0	0	1 -
gi|29165615|ref|NC_002745.2|	1952172 +	C	2	0	0	0	0 -
gi|29165615|ref|NC_002745.2|	1952173 +	U	0	2	0	0	0 -
gi|29165615|ref|NC_002745.2|	1952177 +	U	0	1	0	1	1 -
gi|29165615|ref|NC_002745.2|	1952179 +	U	1	1	0	0	0 -
gi|29165615|ref|NC_002745.2|	1952183 +	G	0	0	1	0	0 -
gi|29165615|ref|NC_002745.2|	1952192 +	C	0	1	1	0	0 -
gi|29165615|ref|NC_002745.2|	1952194 +	A	0	0	1	0	0 -
gi|29165615|ref|NC_002745.2|	1952197 +	A	0	0	1	0	0 -
gi|29165615|ref|NC_002745.2|	1952201 +	A	0	0	0	0	2 -
gi|29165615|ref|NC_002745.2|	1952212 +	U	0	0	0	0	1 -
gi|29165615|ref|NC_002745.2|	1952215 +	C	0	0	1	0	0 -
gi|29165615|ref|NC_002745.2|	1952217 +	A	0	0	0	0	4 -
gi|29165615|ref|NC_002745.2|	1952222 +	A	0	0	1	0	0 -
gi|29165615|ref|NC_002745.2|	1952227 +	C	1	3	4	2	1 -
gi|29165615|ref|NC_002745.2|	1952229 +	U	0	1	0	0	0 -
gi|29165615|ref|NC_002745.2|	1952234 +	U	0	0	0	0	1 -
gi|29165615|ref|NC_002745.2|	1952235 +	C	0	1	0	2	1 -
gi|29165615|ref|NC_002745.2|	1952239 +	U	3	2	0	0	1 -
gi|29165615|ref|NC_002745.2|	1952240 +	A	0	0	1	0	1 -
gi|29165615|ref|NC_002745.2|	1952242 +	U	0	0	1	0	2 -
gi|29165615|ref|NC_002745.2|	1952244 +	C	0	0	1	1	3 -
gi|29165615|ref|NC_002745.2|	1952245 +	C	1	0	0	0	0 -
gi|29165615|ref|NC_002745.2|	1952246 +	A	0	1	1	0	0 -
gi|29165615|ref|NC_002745.2|	1952247 +	A	1	1	2	0	0 -
gi|29165615|ref|NC_002745.2|	1952249 +	A	0	0	0	0	1 -
gi|29165615|ref|NC_002745.2|	1952250 +	U	2	0	0	0	0 -
gi|29165615|ref|NC_002745.2|	1952254 +	U	6	2	0	0	3 -
gi|29165615|ref|NC_002745.2|	1952257 +	G	0	0	0	0	1 -
gi|29165615|ref|NC_002745.2|	1952259 +	A	0	0	1	0	1 -
gi|29165615|ref|NC_002745.2|	1952260 +	G	0	1	0	0	0 -
gi|29165615|ref|NC_002745.2|	1952269 +	A	0	0	0	1	0 -

gi|29165615|ref|NC_002745.2|	1952270	+	C	0	1	0	0	0	-
gi|29165615|ref|NC_002745.2|	1955300	+	A	2	0	0	0	0	-
gi|29165615|ref|NC_002745.2|	1961416	+	U	0	0	0	0	2	-
gi|29165615|ref|NC_002745.2|	1961421	+	C	0	0	0	1	0	-
gi|29165615|ref|NC_002745.2|	1961426	+	C	0	0	1	1	2	-
gi|29165615|ref|NC_002745.2|	1961427	+	C	0	0	0	2	1	-
gi|29165615|ref|NC_002745.2|	1961428	+	A	0	2	0	0	0	-
gi|29165615|ref|NC_002745.2|	1961433	+	G	0	0	0	0	1	-
gi|29165615|ref|NC_002745.2|	1961434	+	C	0	1	2	0	0	-
gi|29165615|ref|NC_002745.2|	1961435	+	A	0	0	1	0	0	-
gi|29165615|ref|NC_002745.2|	1961436	+	U	3	2	3	1	10	-
gi|29165615|ref|NC_002745.2|	1961441	+	U	0	0	0	1	0	-
gi|29165615|ref|NC_002745.2|	1961442	+	G	0	0	0	0	1	-
gi|29165615|ref|NC_002745.2|	1961443	+	U	0	0	0	1	0	-
gi|29165615|ref|NC_002745.2|	1961448	+	U	0	0	1	2	0	-
gi|29165615|ref|NC_002745.2|	1961471	+	U	1	0	1	0	0	-
gi|29165615|ref|NC_002745.2|	1961477	+	C	0	0	1	0	0	-
gi|29165615|ref|NC_002745.2|	1961485	+	C	0	0	0	0	1	-
gi|29165615|ref|NC_002745.2|	1961486	+	A	0	0	0	1	0	-
gi|29165615|ref|NC_002745.2|	1961517	+	U	0	2	0	0	0	-
gi|29165615|ref|NC_002745.2|	1961527	+	U	1	1	0	0	0	-
gi|29165615|ref|NC_002745.2|	1961535	+	C	0	1	0	0	0	-
gi|29165615|ref|NC_002745.2|	1961537	+	C	0	0	1	2	1	-
gi|29165615|ref|NC_002745.2|	1961541	+	C	0	0	0	0	1	-
gi|29165615|ref|NC_002745.2|	1961542	+	A	0	0	0	1	0	-
gi|29165615|ref|NC_002745.2|	1961572	+	G	0	0	1	0	0	-
gi|29165615|ref|NC_002745.2|	1961596	+	C	0	1	1	0	0	-
gi|29165615|ref|NC_002745.2|	1961598	+	A	0	0	1	0	0	-
gi|29165615|ref|NC_002745.2|	1961601	+	A	0	0	1	0	0	-
gi|29165615|ref|NC_002745.2|	1961605	+	A	0	0	0	0	2	-
gi|29165615|ref|NC_002745.2|	1961619	+	C	0	0	1	0	0	-
gi|29165615|ref|NC_002745.2|	1961621	+	A	0	0	0	0	3	-
gi|29165615|ref|NC_002745.2|	1961626	+	A	0	0	1	0	0	-
gi|29165615|ref|NC_002745.2|	1961631	+	C	1	3	4	2	1	-
gi|29165615|ref|NC_002745.2|	1961633	+	U	0	1	0	0	0	-
gi|29165615|ref|NC_002745.2|	1961639	+	C	0	1	0	1	1	-
gi|29165615|ref|NC_002745.2|	1961643	+	U	3	0	0	0	0	-
gi|29165615|ref|NC_002745.2|	1961644	+	A	0	0	0	0	1	-
gi|29165615|ref|NC_002745.2|	1965626	+	A	0	0	1	0	1	-
gi|29165615|ref|NC_002745.2|	1983764	+	A	0	0	1	0	0	-
gi|29165615|ref|NC_002745.2|	1989694	+	C	0	0	0	0	1	SA1736
gi|29165615|ref|NC_002745.2|	1989698	+	U	0	0	0	0	2	SA1736
gi|29165615|ref|NC_002745.2|	1991920	+	A	0	0	0	0	1	-
gi|29165615|ref|NC_002745.2|	1991922	+	A	0	0	1	0	0	-
gi|29165615|ref|NC_002745.2|	1991930	+	U	0	0	0	1	0	-
gi|29165615|ref|NC_002745.2|	1991936	+	C	0	1	0	1	1	-
gi|29165615|ref|NC_002745.2|	1991938	+	U	0	1	0	0	0	-
gi|29165615|ref|NC_002745.2|	1991947	+	A	0	0	0	2	0	-
gi|29165615|ref|NC_002745.2|	1991953	+	A	0	0	0	0	1	-
gi|29165615|ref|NC_002745.2|	1992037	+	U	0	0	0	1	0	-
gi|29165615|ref|NC_002745.2|	1992040	+	U	0	0	1	0	0	-
gi|29165615|ref|NC_002745.2|	1992273	+	C	0	0	0	0	1	-
gi|29165615|ref|NC_002745.2|	1992391	+	G	1	0	0	0	0	-
gi|29165615|ref|NC_002745.2|	1992415	+	A	0	0	1	1	1	-
gi|29165615|ref|NC_002745.2|	1992420	+	A	0	1	0	3	2	-
gi|29165615|ref|NC_002745.2|	1992421	+	A	0	1	1	0	0	-
gi|29165615|ref|NC_002745.2|	1992422	+	A	0	1	0	0	2	-
gi|29165615|ref|NC_002745.2|	1996455	+	A	0	0	1	0	0	SA1743
gi|29165615|ref|NC_002745.2|	2004059	+	A	0	0	0	0	1	-
gi|29165615|ref|NC_002745.2|	2004386	+	G	0	0	0	0	1	-
gi|29165615|ref|NC_002745.2|	2005433	+	A	0	0	1	0	0	-
gi|29165615|ref|NC_002745.2|	2007524	+	A	0	0	0	1	0	-
gi|29165615|ref|NC_002745.2|	2007935	+	C	0	0	1	0	0	SA1755
gi|29165615|ref|NC_002745.2|	2046415	+	A	0	1	0	0	1	-
gi|29165615|ref|NC_002745.2|	2054568	+	U	0	1	0	0	0	-
gi|29165615|ref|NC_002745.2|	2054569	+	U	0	1	0	0	0	-
gi|29165615|ref|NC_002745.2|	2054571	+	C	0	0	0	0	1	-
gi|29165615|ref|NC_002745.2|	2054573	+	U	0	1	0	0	0	-
gi|29165615|ref|NC_002745.2|	2054581	+	U	0	2	0	0	0	-
gi|29165615|ref|NC_002745.2|	2054584	+	A	0	2	0	0	0	-
gi|29165615|ref|NC_002745.2|	2054586	+	U	0	0	0	1	0	-
gi|29165615|ref|NC_002745.2|	2054591	+	A	0	0	0	0	1	-
gi|29165615|ref|NC_002745.2|	2054617	+	C	0	0	0	1	0	-
gi|29165615|ref|NC_002745.2|	2054630	+	C	0	1	2	0	0	-
gi|29165615|ref|NC_002745.2|	2054631	+	A	0	0	1	0	0	-
gi|29165615|ref|NC_002745.2|	2054632	+	U	3	2	3	1	10	-
gi|29165615|ref|NC_002745.2|	2054637	+	U	0	0	0	1	0	-
gi|29165615|ref|NC_002745.2|	2054638	+	G	0	0	0	0	1	-
gi|29165615|ref|NC_002745.2|	2054639	+	U	0	0	0	1	0	-
gi|29165615|ref|NC_002745.2|	2054640	+	A	0	0	1	1	0	-
gi|29165615|ref|NC_002745.2|	2054641	+	G	0	1	0	2	0	-

gi|29165615|ref|NC_002745.2|	2054642	+	A	0	1	2	2	0	-
gi|29165615|ref|NC_002745.2|	2054643	+	A	2	0	1	1	0	-
gi|29165615|ref|NC_002745.2|	2054644	+	U	0	0	1	3	1	-
gi|29165615|ref|NC_002745.2|	2054645	+	U	0	0	0	0	1	-
gi|29165615|ref|NC_002745.2|	2054648	+	U	0	0	1	0	0	-
gi|29165615|ref|NC_002745.2|	2054649	+	U	0	3	1	4	1	-
gi|29165615|ref|NC_002745.2|	2054650	+	U	0	1	1	1	1	-
gi|29165615|ref|NC_002745.2|	2054653	+	G	0	1	0	0	1	-
gi|29165615|ref|NC_002745.2|	2054654	+	A	0	1	1	0	0	-
gi|29165615|ref|NC_002745.2|	2054661	+	C	0	0	0	1	0	-
gi|29165615|ref|NC_002745.2|	2054662	+	U	0	0	0	1	0	-
gi|29165615|ref|NC_002745.2|	2054675	+	C	0	0	0	1	0	-
gi|29165615|ref|NC_002745.2|	2054680	+	C	0	0	1	0	2	-
gi|29165615|ref|NC_002745.2|	2054681	+	C	0	1	0	2	1	-
gi|29165615|ref|NC_002745.2|	2054682	+	A	0	5	1	0	1	-
gi|29165615|ref|NC_002745.2|	2054683	+	A	0	1	0	2	1	-
gi|29165615|ref|NC_002745.2|	2054684	+	C	1	0	0	1	0	-
gi|29165615|ref|NC_002745.2|	2054685	+	U	0	1	0	1	0	-
gi|29165615|ref|NC_002745.2|	2054686	+	U	0	1	0	0	0	-
gi|29165615|ref|NC_002745.2|	2054688	+	C	0	0	0	0	1	-
gi|29165615|ref|NC_002745.2|	2054689	+	A	0	0	0	0	2	-
gi|29165615|ref|NC_002745.2|	2054690	+	U	0	2	1	0	3	-
gi|29165615|ref|NC_002745.2|	2054691	+	U	0	0	0	1	0	-
gi|29165615|ref|NC_002745.2|	2054695	+	U	0	1	0	0	0	-
gi|29165615|ref|NC_002745.2|	2054698	+	U	0	2	0	0	0	-
gi|29165615|ref|NC_002745.2|	2054701	+	A	0	2	0	0	0	-
gi|29165615|ref|NC_002745.2|	2054703	+	U	0	0	0	1	0	-
gi|29165615|ref|NC_002745.2|	2054707	+	G	0	0	0	0	1	-
gi|29165615|ref|NC_002745.2|	2054708	+	A	0	0	0	0	1	-
gi|29165615|ref|NC_002745.2|	2054712	+	C	0	0	0	0	1	-
gi|29165615|ref|NC_002745.2|	2054713	+	A	0	1	1	0	0	-
gi|29165615|ref|NC_002745.2|	2054717	+	U	1	0	0	0	0	-
gi|29165615|ref|NC_002745.2|	2054718	+	C	2	0	0	0	0	-
gi|29165615|ref|NC_002745.2|	2054719	+	U	0	2	0	0	0	-
gi|29165615|ref|NC_002745.2|	2054723	+	U	0	1	0	1	1	-
gi|29165615|ref|NC_002745.2|	2054725	+	U	1	1	0	0	0	-
gi|29165615|ref|NC_002745.2|	2054729	+	G	0	0	1	0	0	-
gi|29165615|ref|NC_002745.2|	2059670	+	G	0	1	0	0	0	SA1818
gi|29165615|ref|NC_002745.2|	2075965	+	G	0	0	1	0	0	-
gi|29165615|ref|NC_002745.2|	2076004	+	A	0	0	1	0	0	-
gi|29165615|ref|NC_002745.2|	2076148	+	A	0	0	1	0	0	-
gi|29165615|ref|NC_002745.2|	2077432	+	A	0	2	0	0	0	-
gi|29165615|ref|NC_002745.2|	2077472	+	A	0	0	0	1	0	-
gi|29165615|ref|NC_002745.2|	2077507	+	C	0	0	0	1	0	-
gi|29165615|ref|NC_002745.2|	2077512	+	C	0	0	1	1	2	-
gi|29165615|ref|NC_002745.2|	2077513	+	C	0	1	0	2	1	-
gi|29165615|ref|NC_002745.2|	2077514	+	A	0	5	1	0	1	-
gi|29165615|ref|NC_002745.2|	2077515	+	A	0	2	0	2	1	-
gi|29165615|ref|NC_002745.2|	2077516	+	C	2	0	0	1	0	-
gi|29165615|ref|NC_002745.2|	2077517	+	U	0	1	0	1	0	-
gi|29165615|ref|NC_002745.2|	2077522	+	U	0	0	0	0	3	-
gi|29165615|ref|NC_002745.2|	2077527	+	U	0	1	0	0	0	-
gi|29165615|ref|NC_002745.2|	2077541	+	U	0	1	0	0	0	-
gi|29165615|ref|NC_002745.2|	2077569	+	C	0	1	0	1	1	-
gi|29165615|ref|NC_002745.2|	2077573	+	U	3	1	0	0	0	-
gi|29165615|ref|NC_002745.2|	2077574	+	A	0	0	0	0	1	-
gi|29165615|ref|NC_002745.2|	2090175	+	A	12	11	13	9	20	-
gi|29165615|ref|NC_002745.2|	2094338	+	G	0	0	0	0	1	-
gi|29165615|ref|NC_002745.2|	2094379	+	A	0	1	0	0	3	-
gi|29165615|ref|NC_002745.2|	2100060	+	U	0	0	0	1	0	SA1859
gi|29165615|ref|NC_002745.2|	2102682	+	A	0	0	0	0	1	SA1862
gi|29165615|ref|NC_002745.2|	2108534	+	U	2	0	0	0	0	-
gi|29165615|ref|NC_002745.2|	2108790	+	U	2	0	0	0	0	-
gi|29165615|ref|NC_002745.2|	2108811	+	G	0	1	0	0	0	-
gi|29165615|ref|NC_002745.2|	2109046	+	U	2	0	0	0	0	-
gi|29165615|ref|NC_002745.2|	2109084	+	C	0	1	0	0	0	-
gi|29165615|ref|NC_002745.2|	2109321	+	U	0	0	0	1	0	-
gi|29165615|ref|NC_002745.2|	2109462	+	C	0	0	0	0	2	-
gi|29165615|ref|NC_002745.2|	2109464	+	U	1	0	0	0	0	-
gi|29165615|ref|NC_002745.2|	2109484	+	C	0	0	1	0	0	-
gi|29165615|ref|NC_002745.2|	2109571	+	A	0	0	0	0	1	-
gi|29165615|ref|NC_002745.2|	2109603	+	C	0	1	0	0	0	-
gi|29165615|ref|NC_002745.2|	2109614	+	G	0	1	0	0	0	-
gi|29165615|ref|NC_002745.2|	2110073	+	C	1	0	0	0	0	-
gi|29165615|ref|NC_002745.2|	2110076	+	G	0	1	0	0	0	-
gi|29165615|ref|NC_002745.2|	2110077	+	U	0	1	1	0	0	-
gi|29165615|ref|NC_002745.2|	2110079	+	G	0	1	0	0	0	-
gi|29165615|ref|NC_002745.2|	2110112	+	U	0	0	1	0	0	-
gi|29165615|ref|NC_002745.2|	2110121	+	U	0	1	0	3	0	-
gi|29165615|ref|NC_002745.2|	2110133	+	A	0	0	1	0	0	-
gi|29165615|ref|NC_002745.2|	2110134	+	C	0	1	0	0	0	-

gi|29165615|ref|NC_002745.2|	2110135 +	A	2	1	0	0	0	-
gi|29165615|ref|NC_002745.2|	2110136 +	G	1	0	0	0	0	-
gi|29165615|ref|NC_002745.2|	2110137 +	U	1	1	1	0	0	-
gi|29165615|ref|NC_002745.2|	2110138 +	G	0	0	0	0	1	-
gi|29165615|ref|NC_002745.2|	2110168 +	G	0	1	0	0	0	-
gi|29165615|ref|NC_002745.2|	2110169 +	G	0	1	0	0	0	-
gi|29165615|ref|NC_002745.2|	2110170 +	A	0	1	0	0	0	-
gi|29165615|ref|NC_002745.2|	2110171 +	A	0	0	0	1	0	-
gi|29165615|ref|NC_002745.2|	2110172 +	C	0	1	1	0	1	-
gi|29165615|ref|NC_002745.2|	2110262 +	A	1	0	1	0	0	-
gi|29165615|ref|NC_002745.2|	2110263 +	C	1	2	3	2	0	-
gi|29165615|ref|NC_002745.2|	2110264 +	C	1	1	0	2	0	-
gi|29165615|ref|NC_002745.2|	2110265 +	A	0	0	1	0	0	-
gi|29165615|ref|NC_002745.2|	2110268 +	C	0	0	0	2	0	-
gi|29165615|ref|NC_002745.2|	2110288 +	G	1	0	0	0	0	-
gi|29165615|ref|NC_002745.2|	2110298 +	C	0	0	1	0	0	-
gi|29165615|ref|NC_002745.2|	2110300 +	G	0	0	1	0	0	-
gi|29165615|ref|NC_002745.2|	2110301 +	C	0	1	0	0	0	-
gi|29165615|ref|NC_002745.2|	2110304 +	C	3	0	1	1	0	-
gi|29165615|ref|NC_002745.2|	2110305 +	U	6	8	4	3	1	-
gi|29165615|ref|NC_002745.2|	2110353 +	U	0	0	1	0	0	-
gi|29165615|ref|NC_002745.2|	2110355 +	G	0	0	0	0	1	-
gi|29165615|ref|NC_002745.2|	2110405 +	C	0	1	0	0	0	-
gi|29165615|ref|NC_002745.2|	2110406 +	C	1	1	0	0	0	-
gi|29165615|ref|NC_002745.2|	2110408 +	U	0	0	0	1	0	-
gi|29165615|ref|NC_002745.2|	2110409 +	U	1	2	0	0	0	-
gi|29165615|ref|NC_002745.2|	2110410 +	C	0	0	0	1	0	-
gi|29165615|ref|NC_002745.2|	2110436 +	C	0	0	0	2	0	-
gi|29165615|ref|NC_002745.2|	2110461 +	G	0	1	0	0	0	-
gi|29165615|ref|NC_002745.2|	2110462 +	C	0	1	0	0	0	-
gi|29165615|ref|NC_002745.2|	2110575 +	U	1	0	0	0	0	-
gi|29165615|ref|NC_002745.2|	2110675 +	U	0	3	0	0	0	-
gi|29165615|ref|NC_002745.2|	2110683 +	C	1	0	0	0	0	-
gi|29165615|ref|NC_002745.2|	2110686 +	C	3	2	1	1	0	-
gi|29165615|ref|NC_002745.2|	2110732 +	U	1	0	0	0	0	-
gi|29165615|ref|NC_002745.2|	2110734 +	U	0	1	0	0	0	-
gi|29165615|ref|NC_002745.2|	2110771 +	G	17	17	8	17	4	-
gi|29165615|ref|NC_002745.2|	2110805 +	A	0	0	0	2	0	-
gi|29165615|ref|NC_002745.2|	2110809 +	U	0	0	1	0	0	-
gi|29165615|ref|NC_002745.2|	2110857 +	G	0	0	0	0	1	-
gi|29165615|ref|NC_002745.2|	2110862 +	U	0	0	1	0	0	-
gi|29165615|ref|NC_002745.2|	2110920 +	U	3	0	0	0	0	-
gi|29165615|ref|NC_002745.2|	2110948 +	A	0	1	1	0	0	-
gi|29165615|ref|NC_002745.2|	2110953 +	U	0	1	0	0	0	-
gi|29165615|ref|NC_002745.2|	2110970 +	C	0	0	0	1	0	-
gi|29165615|ref|NC_002745.2|	2110971 +	C	0	1	0	0	0	-
gi|29165615|ref|NC_002745.2|	2111059 +	U	0	1	0	0	0	-
gi|29165615|ref|NC_002745.2|	2111076 +	A	1	1	0	0	0	-
gi|29165615|ref|NC_002745.2|	2111078 +	C	0	1	0	0	0	-
gi|29165615|ref|NC_002745.2|	2111079 +	C	2	1	0	0	2	-
gi|29165615|ref|NC_002745.2|	2111080 +	U	2	1	0	4	1	-
gi|29165615|ref|NC_002745.2|	2111081 +	U	2	2	1	0	0	-
gi|29165615|ref|NC_002745.2|	2111082 +	U	0	0	0	1	1	-
gi|29165615|ref|NC_002745.2|	2111084 +	C	0	0	0	1	0	-
gi|29165615|ref|NC_002745.2|	2111085 +	C	0	0	1	1	0	-
gi|29165615|ref|NC_002745.2|	2111099 +	U	1	0	0	0	0	-
gi|29165615|ref|NC_002745.2|	2111128 +	U	0	1	0	0	0	-
gi|29165615|ref|NC_002745.2|	2111162 +	U	0	1	0	0	0	-
gi|29165615|ref|NC_002745.2|	2111164 +	U	1	0	0	0	0	-
gi|29165615|ref|NC_002745.2|	2111166 +	A	0	1	1	0	0	-
gi|29165615|ref|NC_002745.2|	2111167 +	C	0	1	0	0	0	-
gi|29165615|ref|NC_002745.2|	2111226 +	C	0	2	0	0	0	-
gi|29165615|ref|NC_002745.2|	2111227 +	C	0	2	0	0	0	-
gi|29165615|ref|NC_002745.2|	2111228 +	C	0	1	0	0	0	-
gi|29165615|ref|NC_002745.2|	2111229 +	C	0	0	0	0	1	-
gi|29165615|ref|NC_002745.2|	2111243 +	G	6	4	4	6	1	-
gi|29165615|ref|NC_002745.2|	2111244 +	C	0	1	0	0	1	-
gi|29165615|ref|NC_002745.2|	2111245 +	U	0	0	0	0	2	-
gi|29165615|ref|NC_002745.2|	2111246 +	C	0	0	3	0	1	-
gi|29165615|ref|NC_002745.2|	2111247 +	U	0	2	0	0	0	-
gi|29165615|ref|NC_002745.2|	2111248 +	A	0	0	1	0	0	-
gi|29165615|ref|NC_002745.2|	2111249 +	C	3	0	1	0	0	-
gi|29165615|ref|NC_002745.2|	2111271 +	G	0	0	2	0	0	-
gi|29165615|ref|NC_002745.2|	2111272 +	C	2	0	0	0	0	-
gi|29165615|ref|NC_002745.2|	2111273 +	U	0	1	0	0	0	-
gi|29165615|ref|NC_002745.2|	2111275 +	G	1	0	0	0	0	-
gi|29165615|ref|NC_002745.2|	2111308 +	U	0	0	0	0	1	-
gi|29165615|ref|NC_002745.2|	2111493 +	U	1	0	0	0	1	-
gi|29165615|ref|NC_002745.2|	2111494 +	U	0	0	0	1	0	-
gi|29165615|ref|NC_002745.2|	2111496 +	A	0	0	0	1	0	-
gi|29165615|ref|NC_002745.2|	2111506 +	C	0	0	0	1	0	-

gi|29165615|ref|NC_002745.2|	2111522	+	A	0	1	0	0	0	-
gi|29165615|ref|NC_002745.2|	2111530	+	G	1	1	0	0	0	-
gi|29165615|ref|NC_002745.2|	2111552	+	C	0	1	0	0	0	-
gi|29165615|ref|NC_002745.2|	2111560	+	A	12	22	23	23	1	-
gi|29165615|ref|NC_002745.2|	2111563	+	A	0	1	0	0	1	-
gi|29165615|ref|NC_002745.2|	2111566	+	G	0	0	0	1	0	-
gi|29165615|ref|NC_002745.2|	2111572	+	U	0	0	0	1	0	-
gi|29165615|ref|NC_002745.2|	2111574	+	A	1	0	0	0	0	-
gi|29165615|ref|NC_002745.2|	2111643	+	A	0	0	0	1	0	-
gi|29165615|ref|NC_002745.2|	2111960	+	U	0	0	0	1	0	-
gi|29165615|ref|NC_002745.2|	2112139	+	U	0	3	0	0	0	-
gi|29165615|ref|NC_002745.2|	2113050	+	A	1	3	1	0	1	-
gi|29165615|ref|NC_002745.2|	2113051	+	A	2	2	1	2	3	-
gi|29165615|ref|NC_002745.2|	2113169	+	G	0	0	0	1	0	-
gi|29165615|ref|NC_002745.2|	2113198	+	G	0	0	0	1	0	-
gi|29165615|ref|NC_002745.2|	2113238	+	G	1	0	2	0	0	-
gi|29165615|ref|NC_002745.2|	2113239	+	U	1	1	1	1	4	-
gi|29165615|ref|NC_002745.2|	2113240	+	C	0	2	1	2	0	-
gi|29165615|ref|NC_002745.2|	2113241	+	C	2	3	1	2	0	-
gi|29165615|ref|NC_002745.2|	2113242	+	C	0	0	0	0	1	-
gi|29165615|ref|NC_002745.2|	2113328	+	A	0	3	0	0	0	-
gi|29165615|ref|NC_002745.2|	2113330	+	C	1	0	0	0	0	-
gi|29165615|ref|NC_002745.2|	2113332	+	C	2	0	0	0	0	-
gi|29165615|ref|NC_002745.2|	2113360	+	C	3	7	1	3	0	-
gi|29165615|ref|NC_002745.2|	2113361	+	G	7	19	5	12	5	-
gi|29165615|ref|NC_002745.2|	2113362	+	U	26	24	15	23	6	-
gi|29165615|ref|NC_002745.2|	2113363	+	G	0	3	1	1	0	-
gi|29165615|ref|NC_002745.2|	2113364	+	G	3	12	0	5	2	-
gi|29165615|ref|NC_002745.2|	2113365	+	A	3	13	6	9	4	-
gi|29165615|ref|NC_002745.2|	2113366	+	C	19	23	6	19	4	-
gi|29165615|ref|NC_002745.2|	2113367	+	U	8	6	1	2	4	-
gi|29165615|ref|NC_002745.2|	2113368	+	A	2	2	1	0	0	-
gi|29165615|ref|NC_002745.2|	2113369	+	C	2	4	1	4	0	-
gi|29165615|ref|NC_002745.2|	2113370	+	C	0	5	0	1	0	-
gi|29165615|ref|NC_002745.2|	2113371	+	A	0	0	0	4	0	-
gi|29165615|ref|NC_002745.2|	2113372	+	G	0	1	1	2	0	-
gi|29165615|ref|NC_002745.2|	2113392	+	U	0	1	0	0	0	-
gi|29165615|ref|NC_002745.2|	2113395	+	C	0	0	0	2	0	-
gi|29165615|ref|NC_002745.2|	2113403	+	U	0	1	0	0	0	-
gi|29165615|ref|NC_002745.2|	2113444	+	C	0	2	0	0	0	-
gi|29165615|ref|NC_002745.2|	2113474	+	U	0	0	0	1	0	-
gi|29165615|ref|NC_002745.2|	2113587	+	A	0	1	0	0	0	-
gi|29165615|ref|NC_002745.2|	2113592	+	C	0	0	0	2	0	-
gi|29165615|ref|NC_002745.2|	2113598	+	A	0	0	1	0	0	-
gi|29165615|ref|NC_002745.2|	2113599	+	C	0	1	0	0	0	-
gi|29165615|ref|NC_002745.2|	2113604	+	A	0	0	1	1	1	-
gi|29165615|ref|NC_002745.2|	2113665	+	G	0	1	0	0	0	-
gi|29165615|ref|NC_002745.2|	2113682	+	U	0	0	0	1	0	-
gi|29165615|ref|NC_002745.2|	2113683	+	A	0	1	0	0	0	-
gi|29165615|ref|NC_002745.2|	2113715	+	U	0	0	3	0	0	-
gi|29165615|ref|NC_002745.2|	2113716	+	G	0	0	0	0	1	-
gi|29165615|ref|NC_002745.2|	2113717	+	U	1	0	0	0	1	-
gi|29165615|ref|NC_002745.2|	2113718	+	U	2	2	0	3	1	-
gi|29165615|ref|NC_002745.2|	2113720	+	U	0	1	1	0	1	-
gi|29165615|ref|NC_002745.2|	2113747	+	G	0	0	0	1	0	-
gi|29165615|ref|NC_002745.2|	2113814	+	G	0	1	0	0	0	-
gi|29165615|ref|NC_002745.2|	2113940	+	A	0	0	1	0	0	-
gi|29165615|ref|NC_002745.2|	2120241	+	A	0	0	1	0	0	-
gi|29165615|ref|NC_002745.2|	2134919	+	C	1	1	0	0	0	-
gi|29165615|ref|NC_002745.2|	2134920	+	C	0	0	1	0	0	-
gi|29165615|ref|NC_002745.2|	2134924	+	U	0	0	0	0	1	-
gi|29165615|ref|NC_002745.2|	2134933	+	G	0	1	0	0	0	-
gi|29165615|ref|NC_002745.2|	2134934	+	U	0	0	1	0	1	-
gi|29165615|ref|NC_002745.2|	2134935	+	A	0	0	0	0	1	-
gi|29165615|ref|NC_002745.2|	2134941	+	U	0	1	0	0	0	-
gi|29165615|ref|NC_002745.2|	2134959	+	A	0	0	0	0	1	-
gi|29165615|ref|NC_002745.2|	2134991	+	A	0	0	0	1	0	-
gi|29165615|ref|NC_002745.2|	2135007	+	A	0	1	0	0	0	-
gi|29165615|ref|NC_002745.2|	2135218	+	U	0	0	0	0	1	-
gi|29165615|ref|NC_002745.2|	2135565	+	U	0	1	0	0	0	-
gi|29165615|ref|NC_002745.2|	2135732	+	A	0	0	0	0	1	-
gi|29165615|ref|NC_002745.2|	2135733	+	U	0	1	0	0	0	-
gi|29165615|ref|NC_002745.2|	2135734	+	A	0	5	2	6	2	-
gi|29165615|ref|NC_002745.2|	2135741	+	C	0	0	0	0	1	-
gi|29165615|ref|NC_002745.2|	2135746	+	C	2	1	0	0	0	-
gi|29165615|ref|NC_002745.2|	2135755	+	U	1	1	1	1	1	-
gi|29165615|ref|NC_002745.2|	2135759	+	G	0	0	0	0	1	-
gi|29165615|ref|NC_002745.2|	2143902	+	C	1	0	1	0	0	SA1891
gi|29165615|ref|NC_002745.2|	2143904	+	A	0	0	0	1	1	SA1891
gi|29165615|ref|NC_002745.2|	2145315	+	U	3	0	0	0	0	-
gi|29165615|ref|NC_002745.2|	2145332	+	A	0	0	0	0	1	-

gi|29165615|ref|NC_002745.2|	2145357 +	U	3	0	0	0	0	-
gi|29165615|ref|NC_002745.2|	2145374 +	A	0	0	0	0	1	-
gi|29165615|ref|NC_002745.2|	2145400 +	A	0	0	0	1	0	-
gi|29165615|ref|NC_002745.2|	2145402 +	C	0	0	1	0	0	-
gi|29165615|ref|NC_002745.2|	2145410 +	G	0	0	0	0	1	-
gi|29165615|ref|NC_002745.2|	2145413 +	U	0	0	0	1	0	-
gi|29165615|ref|NC_002745.2|	2145417 +	U	0	0	0	0	1	-
gi|29165615|ref|NC_002745.2|	2145424 +	C	0	1	0	0	0	-
gi|29165615|ref|NC_002745.2|	2145427 +	U	0	0	0	1	0	-
gi|29165615|ref|NC_002745.2|	2145429 +	A	1	0	0	0	0	-
gi|29165615|ref|NC_002745.2|	2145431 +	G	1	0	0	0	0	-
gi|29165615|ref|NC_002745.2|	2145437 +	U	0	0	0	0	1	-
gi|29165615|ref|NC_002745.2|	2145439 +	G	0	0	0	0	1	-
gi|29165615|ref|NC_002745.2|	2145446 +	U	0	1	0	0	2	-
gi|29165615|ref|NC_002745.2|	2145452 +	G	0	0	1	0	1	-
gi|29165615|ref|NC_002745.2|	2145455 +	A	0	0	0	1	0	-
gi|29165615|ref|NC_002745.2|	2145456 +	U	1	0	0	0	0	-
gi|29165615|ref|NC_002745.2|	2145464 +	U	0	0	1	0	0	-
gi|29165615|ref|NC_002745.2|	2145467 +	G	1	0	0	0	0	-
gi|29165615|ref|NC_002745.2|	2145471 +	U	1	0	1	0	0	-
gi|29165615|ref|NC_002745.2|	2145474 +	A	0	0	0	0	1	-
gi|29165615|ref|NC_002745.2|	2145500 +	A	0	0	0	1	0	-
gi|29165615|ref|NC_002745.2|	2145502 +	C	0	0	1	0	0	-
gi|29165615|ref|NC_002745.2|	2145510 +	G	0	0	0	0	1	-
gi|29165615|ref|NC_002745.2|	2145513 +	U	0	0	0	1	0	-
gi|29165615|ref|NC_002745.2|	2145517 +	U	0	0	0	0	1	-
gi|29165615|ref|NC_002745.2|	2145524 +	C	0	1	0	0	0	-
gi|29165615|ref|NC_002745.2|	2145527 +	U	0	0	0	1	0	-
gi|29165615|ref|NC_002745.2|	2145529 +	A	1	0	0	0	0	-
gi|29165615|ref|NC_002745.2|	2145531 +	G	1	0	0	0	0	-
gi|29165615|ref|NC_002745.2|	2145537 +	U	0	0	0	0	1	-
gi|29165615|ref|NC_002745.2|	2145539 +	G	0	0	0	0	1	-
gi|29165615|ref|NC_002745.2|	2145546 +	U	0	1	0	0	2	-
gi|29165615|ref|NC_002745.2|	2145552 +	G	0	0	1	0	1	-
gi|29165615|ref|NC_002745.2|	2145555 +	A	0	0	0	1	0	-
gi|29165615|ref|NC_002745.2|	2145556 +	U	1	0	0	0	0	-
gi|29165615|ref|NC_002745.2|	2145564 +	U	0	0	1	0	0	-
gi|29165615|ref|NC_002745.2|	2145567 +	G	1	0	0	0	0	-
gi|29165615|ref|NC_002745.2|	2145571 +	U	1	0	1	0	0	-
gi|29165615|ref|NC_002745.2|	2145574 +	A	0	0	0	0	1	-
gi|29165615|ref|NC_002745.2|	2145600 +	A	0	0	0	1	0	-
gi|29165615|ref|NC_002745.2|	2145602 +	C	0	0	1	0	0	-
gi|29165615|ref|NC_002745.2|	2145610 +	G	0	0	0	0	1	-
gi|29165615|ref|NC_002745.2|	2145613 +	U	0	0	0	1	0	-
gi|29165615|ref|NC_002745.2|	2145617 +	U	0	0	0	0	1	-
gi|29165615|ref|NC_002745.2|	2145624 +	C	0	1	0	0	0	-
gi|29165615|ref|NC_002745.2|	2145627 +	U	0	0	0	1	0	-
gi|29165615|ref|NC_002745.2|	2145629 +	A	1	0	0	0	0	-
gi|29165615|ref|NC_002745.2|	2145631 +	G	1	0	0	0	0	-
gi|29165615|ref|NC_002745.2|	2145637 +	U	0	0	0	0	1	-
gi|29165615|ref|NC_002745.2|	2145639 +	G	0	0	0	0	1	-
gi|29165615|ref|NC_002745.2|	2145646 +	U	0	1	0	0	2	-
gi|29165615|ref|NC_002745.2|	2145652 +	G	0	0	1	0	1	-
gi|29165615|ref|NC_002745.2|	2145655 +	A	0	0	0	1	0	-
gi|29165615|ref|NC_002745.2|	2145656 +	U	1	0	0	0	0	-
gi|29165615|ref|NC_002745.2|	2154996 +	U	0	1	0	0	0	-
gi|29165615|ref|NC_002745.2|	2160546 +	A	0	0	0	1	0	-
gi|29165615|ref|NC_002745.2|	2183589 +	C	0	0	1	0	0	-
gi|29165615|ref|NC_002745.2|	2183591 +	A	0	0	1	0	0	-
gi|29165615|ref|NC_002745.2|	2183594 +	A	0	0	1	0	0	-
gi|29165615|ref|NC_002745.2|	2183598 +	A	0	0	0	0	2	-
gi|29165615|ref|NC_002745.2|	2190063 +	A	0	0	0	0	1	SA1940
gi|29165615|ref|NC_002745.2|	2190067 +	A	0	1	0	0	0	SA1940
gi|29165615|ref|NC_002745.2|	2190068 +	A	0	1	0	0	0	SA1940
gi|29165615|ref|NC_002745.2|	2190077 +	A	0	1	0	1	0	SA1940
gi|29165615|ref|NC_002745.2|	2190081 +	U	0	0	1	0	0	SA1940
gi|29165615|ref|NC_002745.2|	2190082 +	U	0	0	0	1	0	SA1940
gi|29165615|ref|NC_002745.2|	2190209 +	U	0	0	1	0	0	SA1940
gi|29165615|ref|NC_002745.2|	2190427 +	A	0	0	0	0	1	SA1940
gi|29165615|ref|NC_002745.2|	2206994 +	A	0	0	0	1	0	SAS069
gi|29165615|ref|NC_002745.2|	2207070 +	A	0	1	0	0	0	SAS069
gi|29165615|ref|NC_002745.2|	2207074 +	A	0	1	0	0	0	SAS069
gi|29165615|ref|NC_002745.2|	2207098 +	U	0	1	0	0	0	SAS069
gi|29165615|ref|NC_002745.2|	2207145 +	U	0	0	1	1	0	SAS069
gi|29165615|ref|NC_002745.2|	2207146 +	U	0	1	0	0	0	SAS069
gi|29165615|ref|NC_002745.2|	2207147 +	A	0	0	0	0	1	SAS069
gi|29165615|ref|NC_002745.2|	2207150 +	A	0	0	1	0	0	SAS069
gi|29165615|ref|NC_002745.2|	2207155 +	U	0	0	1	0	0	SAS069
gi|29165615|ref|NC_002745.2|	2207156 +	U	0	0	0	1	0	SAS069
gi|29165615|ref|NC_002745.2|	2207164 +	G	0	1	0	0	0	SAS069
gi|29165615|ref|NC_002745.2|	2207211 +	A	0	0	0	0	1	SAS070

gi|29165615|ref|NC_002745.2|	2207243	+	U	0	0	0	1	0	SAS070
gi|29165615|ref|NC_002745.2|	2207671	+	U	0	0	0	0	2	-
gi|29165615|ref|NC_002745.2|	2207747	+	A	0	0	1	0	0	-
gi|29165615|ref|NC_002745.2|	2208105	+	A	0	1	0	0	0	-
gi|29165615|ref|NC_002745.2|	2216269	+	A	0	3	3	0	1	SA1961
gi|29165615|ref|NC_002745.2|	2216270	+	U	0	0	0	0	1	SA1961
gi|29165615|ref|NC_002745.2|	2230745	+	U	2	0	0	0	0	-
gi|29165615|ref|NC_002745.2|	2230783	+	C	0	1	0	0	0	-
gi|29165615|ref|NC_002745.2|	2231020	+	U	0	0	0	1	0	-
gi|29165615|ref|NC_002745.2|	2231161	+	C	0	0	0	0	2	-
gi|29165615|ref|NC_002745.2|	2231163	+	U	1	0	0	0	0	-
gi|29165615|ref|NC_002745.2|	2231183	+	C	0	0	1	0	0	-
gi|29165615|ref|NC_002745.2|	2231270	+	A	0	0	0	0	1	-
gi|29165615|ref|NC_002745.2|	2231302	+	C	0	1	0	0	0	-
gi|29165615|ref|NC_002745.2|	2231313	+	G	0	1	0	0	0	-
gi|29165615|ref|NC_002745.2|	2231772	+	C	1	0	0	0	0	-
gi|29165615|ref|NC_002745.2|	2231775	+	G	0	1	0	0	0	-
gi|29165615|ref|NC_002745.2|	2231776	+	U	0	1	1	0	0	-
gi|29165615|ref|NC_002745.2|	2231778	+	G	0	1	0	0	0	-
gi|29165615|ref|NC_002745.2|	2231811	+	U	0	0	1	0	0	-
gi|29165615|ref|NC_002745.2|	2231820	+	U	0	1	0	3	0	-
gi|29165615|ref|NC_002745.2|	2231832	+	A	0	0	1	0	0	-
gi|29165615|ref|NC_002745.2|	2231833	+	C	0	1	0	0	0	-
gi|29165615|ref|NC_002745.2|	2231834	+	A	2	1	0	0	0	-
gi|29165615|ref|NC_002745.2|	2231835	+	G	1	0	0	0	0	-
gi|29165615|ref|NC_002745.2|	2231836	+	U	1	1	1	0	0	-
gi|29165615|ref|NC_002745.2|	2231837	+	G	0	0	0	0	1	-
gi|29165615|ref|NC_002745.2|	2231867	+	G	0	1	0	0	0	-
gi|29165615|ref|NC_002745.2|	2231868	+	G	0	1	0	0	0	-
gi|29165615|ref|NC_002745.2|	2231869	+	A	0	1	0	0	0	-
gi|29165615|ref|NC_002745.2|	2231870	+	A	0	0	0	1	0	-
gi|29165615|ref|NC_002745.2|	2231871	+	C	0	1	1	0	1	-
gi|29165615|ref|NC_002745.2|	2231961	+	A	1	0	1	0	0	-
gi|29165615|ref|NC_002745.2|	2231962	+	C	1	2	3	2	0	-
gi|29165615|ref|NC_002745.2|	2231963	+	C	1	1	0	2	0	-
gi|29165615|ref|NC_002745.2|	2231964	+	A	0	0	1	0	0	-
gi|29165615|ref|NC_002745.2|	2231967	+	C	0	0	0	2	0	-
gi|29165615|ref|NC_002745.2|	2231987	+	G	1	0	0	0	0	-
gi|29165615|ref|NC_002745.2|	2231997	+	C	0	0	1	0	0	-
gi|29165615|ref|NC_002745.2|	2231999	+	G	0	0	1	0	0	-
gi|29165615|ref|NC_002745.2|	2232000	+	C	0	1	0	0	0	-
gi|29165615|ref|NC_002745.2|	2232003	+	C	3	0	1	1	0	-
gi|29165615|ref|NC_002745.2|	2232004	+	U	6	8	4	3	1	-
gi|29165615|ref|NC_002745.2|	2232052	+	U	0	0	1	0	0	-
gi|29165615|ref|NC_002745.2|	2232054	+	G	0	0	0	0	1	-
gi|29165615|ref|NC_002745.2|	2232104	+	C	0	1	0	0	0	-
gi|29165615|ref|NC_002745.2|	2232105	+	C	1	1	0	0	0	-
gi|29165615|ref|NC_002745.2|	2232107	+	U	0	0	0	1	0	-
gi|29165615|ref|NC_002745.2|	2232108	+	U	1	2	0	0	0	-
gi|29165615|ref|NC_002745.2|	2232109	+	C	0	0	0	1	0	-
gi|29165615|ref|NC_002745.2|	2232135	+	C	0	0	0	2	0	-
gi|29165615|ref|NC_002745.2|	2232160	+	G	0	1	0	0	0	-
gi|29165615|ref|NC_002745.2|	2232161	+	C	0	1	0	0	0	-
gi|29165615|ref|NC_002745.2|	2232274	+	U	1	0	0	0	0	-
gi|29165615|ref|NC_002745.2|	2232374	+	U	0	3	0	0	0	-
gi|29165615|ref|NC_002745.2|	2232382	+	C	1	0	0	0	0	-
gi|29165615|ref|NC_002745.2|	2232385	+	C	3	2	1	1	0	-
gi|29165615|ref|NC_002745.2|	2232431	+	U	1	0	0	0	0	-
gi|29165615|ref|NC_002745.2|	2232433	+	U	0	1	0	0	0	-
gi|29165615|ref|NC_002745.2|	2232470	+	G	17	17	8	17	4	-
gi|29165615|ref|NC_002745.2|	2232504	+	A	0	0	0	2	0	-
gi|29165615|ref|NC_002745.2|	2232508	+	U	0	0	1	0	0	-
gi|29165615|ref|NC_002745.2|	2232556	+	G	0	0	0	0	1	-
gi|29165615|ref|NC_002745.2|	2232561	+	U	0	0	1	0	0	-
gi|29165615|ref|NC_002745.2|	2232619	+	U	3	0	0	0	0	-
gi|29165615|ref|NC_002745.2|	2232647	+	A	0	1	1	0	0	-
gi|29165615|ref|NC_002745.2|	2232652	+	U	0	1	0	0	0	-
gi|29165615|ref|NC_002745.2|	2232669	+	C	0	0	0	1	0	-
gi|29165615|ref|NC_002745.2|	2232670	+	C	0	1	0	0	0	-
gi|29165615|ref|NC_002745.2|	2232758	+	U	0	1	0	0	0	-
gi|29165615|ref|NC_002745.2|	2232775	+	A	1	1	0	0	0	-
gi|29165615|ref|NC_002745.2|	2232777	+	C	0	1	0	0	0	-
gi|29165615|ref|NC_002745.2|	2232778	+	C	2	1	0	0	2	-
gi|29165615|ref|NC_002745.2|	2232779	+	U	2	1	0	4	1	-
gi|29165615|ref|NC_002745.2|	2232780	+	U	2	2	1	0	0	-
gi|29165615|ref|NC_002745.2|	2232781	+	U	0	0	0	1	1	-
gi|29165615|ref|NC_002745.2|	2232783	+	C	0	0	0	1	0	-
gi|29165615|ref|NC_002745.2|	2232784	+	C	0	0	1	1	0	-
gi|29165615|ref|NC_002745.2|	2232798	+	U	1	0	0	0	0	-
gi|29165615|ref|NC_002745.2|	2232827	+	U	0	1	0	0	0	-
gi|29165615|ref|NC_002745.2|	2232861	+	U	0	1	0	0	0	-

gi|29165615|ref|NC_002745.2|	2232863	+	U	1	0	0	0	0	-
gi|29165615|ref|NC_002745.2|	2232865	+	A	0	1	1	0	0	-
gi|29165615|ref|NC_002745.2|	2232866	+	C	0	1	0	0	0	-
gi|29165615|ref|NC_002745.2|	2232925	+	C	0	2	0	0	0	-
gi|29165615|ref|NC_002745.2|	2232926	+	C	0	2	0	0	0	-
gi|29165615|ref|NC_002745.2|	2232927	+	C	0	1	0	0	0	-
gi|29165615|ref|NC_002745.2|	2232928	+	C	0	0	0	0	1	-
gi|29165615|ref|NC_002745.2|	2232942	+	G	6	4	4	6	1	-
gi|29165615|ref|NC_002745.2|	2232943	+	C	0	1	0	0	1	-
gi|29165615|ref|NC_002745.2|	2232944	+	U	0	0	0	0	2	-
gi|29165615|ref|NC_002745.2|	2232945	+	C	0	0	3	0	1	-
gi|29165615|ref|NC_002745.2|	2232946	+	U	0	2	0	0	0	-
gi|29165615|ref|NC_002745.2|	2232947	+	A	0	0	1	0	0	-
gi|29165615|ref|NC_002745.2|	2232948	+	C	3	0	1	0	0	-
gi|29165615|ref|NC_002745.2|	2232970	+	G	0	0	2	0	0	-
gi|29165615|ref|NC_002745.2|	2232971	+	C	2	0	0	0	0	-
gi|29165615|ref|NC_002745.2|	2232972	+	U	0	1	0	0	0	-
gi|29165615|ref|NC_002745.2|	2232974	+	G	1	0	0	0	0	-
gi|29165615|ref|NC_002745.2|	2233007	+	U	0	0	0	0	1	-
gi|29165615|ref|NC_002745.2|	2233192	+	U	1	0	0	0	1	-
gi|29165615|ref|NC_002745.2|	2233193	+	U	0	0	0	1	0	-
gi|29165615|ref|NC_002745.2|	2233195	+	A	0	0	0	1	0	-
gi|29165615|ref|NC_002745.2|	2233205	+	C	0	0	0	1	0	-
gi|29165615|ref|NC_002745.2|	2233221	+	A	0	1	0	0	0	-
gi|29165615|ref|NC_002745.2|	2233229	+	G	1	1	0	0	0	-
gi|29165615|ref|NC_002745.2|	2233251	+	C	0	1	0	0	0	-
gi|29165615|ref|NC_002745.2|	2233259	+	A	12	22	23	23	1	-
gi|29165615|ref|NC_002745.2|	2233262	+	A	0	1	0	0	1	-
gi|29165615|ref|NC_002745.2|	2233265	+	G	0	0	0	1	0	-
gi|29165615|ref|NC_002745.2|	2233271	+	U	0	0	0	1	0	-
gi|29165615|ref|NC_002745.2|	2233273	+	A	1	0	0	0	0	-
gi|29165615|ref|NC_002745.2|	2233342	+	A	0	0	0	1	0	-
gi|29165615|ref|NC_002745.2|	2233659	+	U	0	0	0	1	0	-
gi|29165615|ref|NC_002745.2|	2233838	+	U	0	3	0	0	0	-
gi|29165615|ref|NC_002745.2|	2234749	+	A	1	3	1	0	1	-
gi|29165615|ref|NC_002745.2|	2234750	+	A	2	2	1	2	3	-
gi|29165615|ref|NC_002745.2|	2234868	+	G	0	0	0	1	0	-
gi|29165615|ref|NC_002745.2|	2234897	+	G	0	0	0	1	0	-
gi|29165615|ref|NC_002745.2|	2234937	+	G	1	0	2	0	0	-
gi|29165615|ref|NC_002745.2|	2234938	+	U	1	1	1	1	4	-
gi|29165615|ref|NC_002745.2|	2234939	+	C	0	2	1	2	0	-
gi|29165615|ref|NC_002745.2|	2234940	+	C	2	3	1	2	0	-
gi|29165615|ref|NC_002745.2|	2234941	+	C	0	0	0	0	1	-
gi|29165615|ref|NC_002745.2|	2235027	+	A	0	3	0	0	0	-
gi|29165615|ref|NC_002745.2|	2235029	+	C	1	0	0	0	0	-
gi|29165615|ref|NC_002745.2|	2235031	+	C	2	0	0	0	0	-
gi|29165615|ref|NC_002745.2|	2235059	+	C	3	7	1	3	0	-
gi|29165615|ref|NC_002745.2|	2235060	+	G	7	19	5	12	5	-
gi|29165615|ref|NC_002745.2|	2235061	+	U	26	24	15	23	6	-
gi|29165615|ref|NC_002745.2|	2235062	+	G	0	3	1	1	0	-
gi|29165615|ref|NC_002745.2|	2235063	+	G	3	12	0	5	2	-
gi|29165615|ref|NC_002745.2|	2235064	+	A	3	13	6	9	4	-
gi|29165615|ref|NC_002745.2|	2235065	+	C	19	23	6	19	4	-
gi|29165615|ref|NC_002745.2|	2235066	+	U	8	6	1	2	4	-
gi|29165615|ref|NC_002745.2|	2235067	+	A	2	2	1	0	0	-
gi|29165615|ref|NC_002745.2|	2235068	+	C	2	4	1	4	0	-
gi|29165615|ref|NC_002745.2|	2235069	+	C	0	5	0	1	0	-
gi|29165615|ref|NC_002745.2|	2235070	+	A	0	0	0	4	0	-
gi|29165615|ref|NC_002745.2|	2235071	+	G	0	1	1	2	0	-
gi|29165615|ref|NC_002745.2|	2235091	+	U	0	1	0	0	0	-
gi|29165615|ref|NC_002745.2|	2235094	+	C	0	0	0	2	0	-
gi|29165615|ref|NC_002745.2|	2235102	+	U	0	1	0	0	0	-
gi|29165615|ref|NC_002745.2|	2235143	+	C	0	2	0	0	0	-
gi|29165615|ref|NC_002745.2|	2235173	+	U	0	0	0	1	0	-
gi|29165615|ref|NC_002745.2|	2235286	+	A	0	1	0	0	0	-
gi|29165615|ref|NC_002745.2|	2235291	+	C	0	0	0	2	0	-
gi|29165615|ref|NC_002745.2|	2235297	+	A	0	0	1	0	0	-
gi|29165615|ref|NC_002745.2|	2235298	+	C	0	1	0	0	0	-
gi|29165615|ref|NC_002745.2|	2235303	+	A	0	0	1	1	1	-
gi|29165615|ref|NC_002745.2|	2235364	+	G	0	1	0	0	0	-
gi|29165615|ref|NC_002745.2|	2235381	+	U	0	0	0	1	0	-
gi|29165615|ref|NC_002745.2|	2235382	+	A	0	1	0	0	0	-
gi|29165615|ref|NC_002745.2|	2235414	+	U	0	0	3	0	0	-
gi|29165615|ref|NC_002745.2|	2235415	+	G	0	0	0	0	1	-
gi|29165615|ref|NC_002745.2|	2235416	+	U	1	0	0	0	1	-
gi|29165615|ref|NC_002745.2|	2235417	+	U	2	2	0	3	1	-
gi|29165615|ref|NC_002745.2|	2235419	+	U	0	1	1	0	1	-
gi|29165615|ref|NC_002745.2|	2235446	+	G	0	0	0	1	0	-
gi|29165615|ref|NC_002745.2|	2235513	+	G	0	1	0	0	0	-
gi|29165615|ref|NC_002745.2|	2235639	+	A	0	0	1	0	0	-
gi|29165615|ref|NC_002745.2|	2242748	+	A	0	1	0	0	2	-

gi|29165615|ref|NC_002745.2|	2244106	+	A	0	0	1	0	0	SAS074
gi|29165615|ref|NC_002745.2|	2248466	+	U	0	1	0	0	0	-
gi|29165615|ref|NC_002745.2|	2275335	+	U	0	0	0	0	1	SA2003
gi|29165615|ref|NC_002745.2|	2275363	+	A	0	0	1	0	1	SA2003
gi|29165615|ref|NC_002745.2|	2275845	+	A	0	0	1	2	2	SA2003
gi|29165615|ref|NC_002745.2|	2281864	+	A	0	0	0	1	0	SA2009
gi|29165615|ref|NC_002745.2|	2281865	+	U	0	0	0	1	1	SA2009
gi|29165615|ref|NC_002745.2|	2281867	+	U	0	1	0	0	0	SA2009
gi|29165615|ref|NC_002745.2|	2281871	+	A	0	0	1	0	0	SA2009
gi|29165615|ref|NC_002745.2|	2281874	+	G	0	0	0	1	0	SA2009
gi|29165615|ref|NC_002745.2|	2281875	+	C	0	0	0	2	0	SA2009
gi|29165615|ref|NC_002745.2|	2287683	+	A	0	0	0	1	0	-
gi|29165615|ref|NC_002745.2|	2288192	+	A	0	0	0	0	1	-
gi|29165615|ref|NC_002745.2|	2289694	+	A	0	0	0	0	3	-
gi|29165615|ref|NC_002745.2|	2291769	+	G	0	0	0	0	1	-
gi|29165615|ref|NC_002745.2|	2315549	+	A	0	0	0	1	0	-
gi|29165615|ref|NC_002745.2|	2315569	+	U	1	0	0	2	0	SAS080
gi|29165615|ref|NC_002745.2|	2315576	+	U	0	0	0	0	2	SAS080
gi|29165615|ref|NC_002745.2|	2315581	+	C	0	0	0	1	0	SAS080
gi|29165615|ref|NC_002745.2|	2315586	+	C	0	0	1	1	2	SAS080
gi|29165615|ref|NC_002745.2|	2315587	+	C	0	1	0	2	1	SAS080
gi|29165615|ref|NC_002745.2|	2315588	+	A	0	5	1	0	1	SAS080
gi|29165615|ref|NC_002745.2|	2315589	+	A	0	2	0	2	1	SAS080
gi|29165615|ref|NC_002745.2|	2315590	+	C	2	0	0	1	0	SAS080
gi|29165615|ref|NC_002745.2|	2315591	+	U	0	1	0	0	0	SAS080
gi|29165615|ref|NC_002745.2|	2315592	+	U	0	1	0	0	0	SAS080
gi|29165615|ref|NC_002745.2|	2315593	+	G	0	0	0	2	0	SAS080
gi|29165615|ref|NC_002745.2|	2315594	+	C	0	0	0	0	1	SAS080
gi|29165615|ref|NC_002745.2|	2315595	+	A	0	0	0	0	2	SAS080
gi|29165615|ref|NC_002745.2|	2315596	+	U	0	2	1	0	0	SAS080
gi|29165615|ref|NC_002745.2|	2315597	+	U	0	0	0	1	0	SAS080
gi|29165615|ref|NC_002745.2|	2315604	+	A	0	2	0	0	0	SAS080
gi|29165615|ref|NC_002745.2|	2315607	+	A	0	2	0	0	0	SAS080
gi|29165615|ref|NC_002745.2|	2315609	+	U	0	0	0	1	0	SAS080
gi|29165615|ref|NC_002745.2|	2315613	+	G	0	0	0	0	1	SAS080
gi|29165615|ref|NC_002745.2|	2315614	+	A	0	0	0	0	1	SAS080
gi|29165615|ref|NC_002745.2|	2315619	+	A	0	0	1	0	0	SAS080
gi|29165615|ref|NC_002745.2|	2315625	+	U	0	0	0	0	1	SAS080
gi|29165615|ref|NC_002745.2|	2315631	+	U	1	0	1	0	0	SAS080
gi|29165615|ref|NC_002745.2|	2315637	+	C	0	0	1	0	0	SAS080
gi|29165615|ref|NC_002745.2|	2315645	+	C	0	0	0	0	1	SAS080
gi|29165615|ref|NC_002745.2|	2315646	+	A	0	0	0	1	0	SAS080
gi|29165615|ref|NC_002745.2|	2315653	+	A	0	1	0	1	0	SAS080
gi|29165615|ref|NC_002745.2|	2315654	+	C	0	0	1	0	3	SAS080
gi|29165615|ref|NC_002745.2|	2315662	+	U	0	1	1	2	0	SAS080
gi|29165615|ref|NC_002745.2|	2315663	+	A	0	0	0	1	0	SAS080
gi|29165615|ref|NC_002745.2|	2315671	+	U	0	1	2	1	0	-
gi|29165615|ref|NC_002745.2|	2315672	+	U	0	4	0	1	2	-
gi|29165615|ref|NC_002745.2|	2315676	+	G	0	0	1	0	0	-
gi|29165615|ref|NC_002745.2|	2315677	+	U	0	1	0	1	0	-
gi|29165615|ref|NC_002745.2|	2315681	+	C	1	0	0	0	0	-
gi|29165615|ref|NC_002745.2|	2315682	+	U	2	0	0	0	0	-
gi|29165615|ref|NC_002745.2|	2315683	+	U	0	2	0	0	0	-
gi|29165615|ref|NC_002745.2|	2315687	+	U	0	1	0	1	1	-
gi|29165615|ref|NC_002745.2|	2315689	+	U	1	1	0	0	0	-
gi|29165615|ref|NC_002745.2|	2315693	+	G	0	0	1	0	0	-
gi|29165615|ref|NC_002745.2|	2315702	+	C	0	1	1	0	0	-
gi|29165615|ref|NC_002745.2|	2315704	+	A	0	0	1	0	0	-
gi|29165615|ref|NC_002745.2|	2315707	+	A	0	0	1	0	0	-
gi|29165615|ref|NC_002745.2|	2315711	+	A	0	0	0	0	2	-
gi|29165615|ref|NC_002745.2|	2315722	+	U	0	0	0	0	1	-
gi|29165615|ref|NC_002745.2|	2315725	+	C	0	0	1	0	0	-
gi|29165615|ref|NC_002745.2|	2315727	+	A	0	0	0	0	4	-
gi|29165615|ref|NC_002745.2|	2315732	+	A	0	0	1	0	0	-
gi|29165615|ref|NC_002745.2|	2315737	+	C	1	3	4	2	1	-
gi|29165615|ref|NC_002745.2|	2315739	+	U	0	1	0	0	0	-
gi|29165615|ref|NC_002745.2|	2315744	+	U	0	0	0	0	1	-
gi|29165615|ref|NC_002745.2|	2315745	+	A	0	1	0	2	1	-
gi|29165615|ref|NC_002745.2|	2315749	+	U	3	2	0	0	1	-
gi|29165615|ref|NC_002745.2|	2315750	+	A	0	0	1	0	1	-
gi|29165615|ref|NC_002745.2|	2315752	+	U	0	0	1	0	2	-
gi|29165615|ref|NC_002745.2|	2315754	+	C	0	0	1	1	3	-
gi|29165615|ref|NC_002745.2|	2315755	+	C	1	0	0	0	0	-
gi|29165615|ref|NC_002745.2|	2315756	+	A	0	1	1	0	0	-
gi|29165615|ref|NC_002745.2|	2315767	+	G	0	0	0	0	1	-
gi|29165615|ref|NC_002745.2|	2315769	+	A	0	0	1	0	1	-
gi|29165615|ref|NC_002745.2|	2315770	+	G	0	1	0	0	0	-
gi|29165615|ref|NC_002745.2|	2315779	+	A	0	0	0	1	0	-
gi|29165615|ref|NC_002745.2|	2317071	+	A	0	0	0	1	0	SAS081
gi|29165615|ref|NC_002745.2|	2317584	+	G	2	0	0	0	0	SA2055
gi|29165615|ref|NC_002745.2|	2324388	+	A	0	0	1	1	1	SA2060

gi|29165615|ref|NC_002745.2|	2324389	+	A	0	5	2	3	2	SA2060
gi|29165615|ref|NC_002745.2|	2324390	+	A	0	6	1	0	3	SA2060
gi|29165615|ref|NC_002745.2|	2324392	+	A	0	14	0	0	3	SA2060
gi|29165615|ref|NC_002745.2|	2326390	+	C	0	0	1	1	2	-
gi|29165615|ref|NC_002745.2|	2326391	+	C	0	0	0	2	1	-
gi|29165615|ref|NC_002745.2|	2326392	+	A	0	2	0	0	0	-
gi|29165615|ref|NC_002745.2|	2326397	+	G	0	0	0	0	1	-
gi|29165615|ref|NC_002745.2|	2326398	+	C	0	1	2	0	0	-
gi|29165615|ref|NC_002745.2|	2326399	+	A	0	0	1	0	0	-
gi|29165615|ref|NC_002745.2|	2326400	+	U	3	2	3	1	10	-
gi|29165615|ref|NC_002745.2|	2326405	+	U	0	0	0	1	0	-
gi|29165615|ref|NC_002745.2|	2326406	+	G	0	0	0	0	1	-
gi|29165615|ref|NC_002745.2|	2326407	+	U	0	0	0	1	0	-
gi|29165615|ref|NC_002745.2|	2326408	+	A	0	0	1	0	0	-
gi|29165615|ref|NC_002745.2|	2326410	+	A	0	0	0	1	0	-
gi|29165615|ref|NC_002745.2|	2326411	+	A	0	0	1	0	0	-
gi|29165615|ref|NC_002745.2|	2326412	+	U	0	0	2	3	1	-
gi|29165615|ref|NC_002745.2|	2326416	+	U	0	0	1	1	0	-
gi|29165615|ref|NC_002745.2|	2326417	+	U	0	4	1	4	2	-
gi|29165615|ref|NC_002745.2|	2326418	+	U	0	1	0	0	1	-
gi|29165615|ref|NC_002745.2|	2326421	+	G	0	1	0	0	1	-
gi|29165615|ref|NC_002745.2|	2326422	+	A	0	0	1	2	0	-
gi|29165615|ref|NC_002745.2|	2326432	+	U	1	1	0	0	0	-
gi|29165615|ref|NC_002745.2|	2326440	+	C	0	1	0	0	0	-
gi|29165615|ref|NC_002745.2|	2326442	+	C	0	0	1	2	1	-
gi|29165615|ref|NC_002745.2|	2326446	+	C	0	0	0	0	1	-
gi|29165615|ref|NC_002745.2|	2326447	+	A	0	0	0	1	0	-
gi|29165615|ref|NC_002745.2|	2326455	+	C	0	0	1	0	3	-
gi|29165615|ref|NC_002745.2|	2326461	+	U	0	0	0	1	0	-
gi|29165615|ref|NC_002745.2|	2326463	+	U	0	1	0	1	0	-
gi|29165615|ref|NC_002745.2|	2326472	+	U	0	1	1	0	0	-
gi|29165615|ref|NC_002745.2|	2326473	+	U	0	3	0	0	2	-
gi|29165615|ref|NC_002745.2|	2326477	+	G	0	0	1	0	0	-
gi|29165615|ref|NC_002745.2|	2326478	+	U	0	3	0	1	0	-
gi|29165615|ref|NC_002745.2|	2326488	+	U	1	1	0	0	0	-
gi|29165615|ref|NC_002745.2|	2342164	+	A	1	0	0	0	0	-
gi|29165615|ref|NC_002745.2|	2352165	+	G	0	0	1	0	0	-
gi|29165615|ref|NC_002745.2|	2352741	+	U	3	0	0	0	0	SA2093
gi|29165615|ref|NC_002745.2|	2352912	+	A	0	0	2	1	0	SA2093
gi|29165615|ref|NC_002745.2|	2352970	+	U	0	3	0	0	3	SA2093
gi|29165615|ref|NC_002745.2|	2353085	+	A	0	0	2	1	1	SA2093
gi|29165615|ref|NC_002745.2|	2353086	+	A	0	0	3	0	1	SA2093
gi|29165615|ref|NC_002745.2|	2353087	+	C	0	1	0	0	0	SA2093
gi|29165615|ref|NC_002745.2|	2353090	+	A	0	0	1	0	0	SA2093
gi|29165615|ref|NC_002745.2|	2353098	+	C	1	0	0	0	0	SA2093
gi|29165615|ref|NC_002745.2|	2353119	+	U	0	1	0	0	0	SA2093
gi|29165615|ref|NC_002745.2|	2353120	+	A	0	0	2	0	0	SA2093
gi|29165615|ref|NC_002745.2|	2353164	+	C	0	0	1	2	0	SA2093
gi|29165615|ref|NC_002745.2|	2353199	+	U	0	1	0	0	0	SA2093
gi|29165615|ref|NC_002745.2|	2353201	+	C	0	1	0	0	0	SA2093
gi|29165615|ref|NC_002745.2|	2353202	+	A	0	0	1	0	0	SA2093
gi|29165615|ref|NC_002745.2|	2353207	+	G	1	0	0	0	0	SA2093
gi|29165615|ref|NC_002745.2|	2353208	+	A	0	1	0	0	0	SA2093
gi|29165615|ref|NC_002745.2|	2373376	+	U	0	0	0	1	0	-
gi|29165615|ref|NC_002745.2|	2376394	+	U	0	1	0	0	0	SA2113
gi|29165615|ref|NC_002745.2|	2401172	+	G	0	1	0	0	0	SA2135
gi|29165615|ref|NC_002745.2|	2404743	+	C	0	0	1	0	0	-
gi|29165615|ref|NC_002745.2|	2417780	+	U	0	0	3	0	0	SA2152
gi|29165615|ref|NC_002745.2|	2417781	+	C	0	1	0	0	0	SA2152
gi|29165615|ref|NC_002745.2|	2440457	+	G	0	0	0	0	1	SA2171
gi|29165615|ref|NC_002745.2|	2440487	+	C	0	0	0	0	1	SA2171
gi|29165615|ref|NC_002745.2|	2440490	+	G	0	0	0	1	0	SA2171
gi|29165615|ref|NC_002745.2|	2440491	+	A	0	0	0	1	0	SA2171
gi|29165615|ref|NC_002745.2|	2440614	+	A	0	0	1	1	1	-
gi|29165615|ref|NC_002745.2|	2440619	+	A	0	1	0	3	2	-
gi|29165615|ref|NC_002745.2|	2440620	+	A	0	1	1	0	0	-
gi|29165615|ref|NC_002745.2|	2440621	+	A	0	1	0	0	2	-
gi|29165615|ref|NC_002745.2|	2440681	+	C	0	0	1	0	0	-
gi|29165615|ref|NC_002745.2|	2440742	+	C	0	0	1	0	0	-
gi|29165615|ref|NC_002745.2|	2440803	+	C	0	0	1	0	0	-
gi|29165615|ref|NC_002745.2|	2443818	+	A	0	0	0	1	0	SA2175
gi|29165615|ref|NC_002745.2|	2475526	+	U	0	0	0	0	3	-
gi|29165615|ref|NC_002745.2|	2476226	+	A	0	0	0	1	0	SA2206
gi|29165615|ref|NC_002745.2|	2476228	+	A	0	1	0	1	2	SA2206
gi|29165615|ref|NC_002745.2|	2476230	+	C	0	1	0	1	3	SA2206
gi|29165615|ref|NC_002745.2|	2476231	+	A	0	2	0	1	2	SA2206
gi|29165615|ref|NC_002745.2|	2476232	+	A	1	2	1	2	1	SA2206
gi|29165615|ref|NC_002745.2|	2476233	+	C	0	0	1	0	2	SA2206
gi|29165615|ref|NC_002745.2|	2479405	+	G	0	0	0	0	1	-
gi|29165615|ref|NC_002745.2|	2480756	+	A	0	3	0	0	0	SA2209
gi|29165615|ref|NC_002745.2|	2487030	+	A	0	0	0	0	2	-

gi|29165615|ref|NC_002745.2|	2487032	+	A	0	2	1	4	3	-
gi|29165615|ref|NC_002745.2|	2487033	+	U	0	0	0	1	0	-
gi|29165615|ref|NC_002745.2|	2487034	+	A	0	1	2	0	1	-
gi|29165615|ref|NC_002745.2|	2487035	+	A	0	0	0	1	0	-
gi|29165615|ref|NC_002745.2|	2487039	+	A	0	0	1	0	0	-
gi|29165615|ref|NC_002745.2|	2487043	+	A	0	0	0	0	1	-
gi|29165615|ref|NC_002745.2|	2487044	+	A	0	0	0	0	2	-
gi|29165615|ref|NC_002745.2|	2490884	+	G	0	0	1	0	0	-
gi|29165615|ref|NC_002745.2|	2490908	+	U	0	0	1	0	0	-
gi|29165615|ref|NC_002745.2|	2490960	+	A	0	2	0	0	0	-
gi|29165615|ref|NC_002745.2|	2490985	+	U	1	1	0	0	0	-
gi|29165615|ref|NC_002745.2|	2490993	+	C	0	1	0	0	0	-
gi|29165615|ref|NC_002745.2|	2490995	+	C	0	0	1	2	1	-
gi|29165615|ref|NC_002745.2|	2490999	+	C	0	0	0	0	1	-
gi|29165615|ref|NC_002745.2|	2491000	+	A	0	0	0	1	0	-
gi|29165615|ref|NC_002745.2|	2491008	+	C	0	0	1	0	3	-
gi|29165615|ref|NC_002745.2|	2491016	+	U	0	0	0	0	1	-
gi|29165615|ref|NC_002745.2|	2491031	+	C	0	2	0	0	0	-
gi|29165615|ref|NC_002745.2|	2491041	+	U	1	1	0	0	0	-
gi|29165615|ref|NC_002745.2|	2491043	+	U	0	0	1	0	0	-
gi|29165615|ref|NC_002745.2|	2491049	+	C	0	0	0	1	0	-
gi|29165615|ref|NC_002745.2|	2491051	+	C	0	1	0	1	2	-
gi|29165615|ref|NC_002745.2|	2491054	+	C	0	0	1	0	2	-
gi|29165615|ref|NC_002745.2|	2491055	+	C	0	0	0	2	1	-
gi|29165615|ref|NC_002745.2|	2491056	+	A	0	5	0	0	1	-
gi|29165615|ref|NC_002745.2|	2491057	+	A	0	1	0	1	1	-
gi|29165615|ref|NC_002745.2|	2491058	+	C	0	0	0	1	0	-
gi|29165615|ref|NC_002745.2|	2491306	+	A	0	0	0	0	2	-
gi|29165615|ref|NC_002745.2|	2491308	+	A	0	2	1	4	3	-
gi|29165615|ref|NC_002745.2|	2491309	+	U	0	0	0	1	0	-
gi|29165615|ref|NC_002745.2|	2491310	+	A	0	1	2	0	1	-
gi|29165615|ref|NC_002745.2|	2491311	+	A	0	0	0	1	0	-
gi|29165615|ref|NC_002745.2|	2491315	+	A	0	0	1	0	0	-
gi|29165615|ref|NC_002745.2|	2491319	+	A	0	0	0	0	1	-
gi|29165615|ref|NC_002745.2|	2491320	+	A	0	0	0	0	2	-
gi|29165615|ref|NC_002745.2|	2498321	+	U	0	1	0	0	0	SA2226
gi|29165615|ref|NC_002745.2|	2504078	+	A	0	1	0	0	0	SA2230
gi|29165615|ref|NC_002745.2|	2526656	+	C	0	0	0	1	0	-
gi|29165615|ref|NC_002745.2|	2526669	+	U	0	1	0	0	0	-
gi|29165615|ref|NC_002745.2|	2526684	+	G	0	0	0	0	1	-
gi|29165615|ref|NC_002745.2|	2526690	+	U	0	0	1	0	0	-
gi|29165615|ref|NC_002745.2|	2526707	+	A	0	0	0	0	1	-
gi|29165615|ref|NC_002745.2|	2526714	+	A	0	0	0	0	1	-
gi|29165615|ref|NC_002745.2|	2526717	+	C	0	0	0	1	0	-
gi|29165615|ref|NC_002745.2|	2526733	+	U	0	0	0	0	1	-
gi|29165615|ref|NC_002745.2|	2526736	+	U	0	1	0	0	0	-
gi|29165615|ref|NC_002745.2|	2526738	+	A	0	2	0	0	0	-
gi|29165615|ref|NC_002745.2|	2526745	+	G	0	0	0	0	1	-
gi|29165615|ref|NC_002745.2|	2526754	+	G	3	0	0	0	0	-
gi|29165615|ref|NC_002745.2|	2526770	+	G	0	2	0	0	0	-
gi|29165615|ref|NC_002745.2|	2526785	+	A	0	0	0	0	1	-
gi|29165615|ref|NC_002745.2|	2526786	+	A	0	2	0	1	0	-
gi|29165615|ref|NC_002745.2|	2538645	+	A	1	0	0	0	0	-
gi|29165615|ref|NC_002745.2|	2541511	+	U	0	0	1	0	0	-
gi|29165615|ref|NC_002745.2|	2542637	+	U	0	0	1	0	0	-
gi|29165615|ref|NC_002745.2|	2546016	+	U	0	0	1	1	0	-
gi|29165615|ref|NC_002745.2|	2549274	+	U	0	0	1	1	0	-
gi|29165615|ref|NC_002745.2|	2550291	+	U	0	0	1	1	0	-
gi|29165615|ref|NC_002745.2|	2560655	+	C	0	0	1	0	0	-
gi|29165615|ref|NC_002745.2|	2561039	+	C	0	0	1	0	0	-
gi|29165615|ref|NC_002745.2|	2561423	+	C	0	0	1	0	0	-
gi|29165615|ref|NC_002745.2|	2561807	+	C	0	0	1	0	0	-
gi|29165615|ref|NC_002745.2|	2562191	+	C	0	0	1	0	0	-
gi|29165615|ref|NC_002745.2|	2567300	+	C	1	1	0	0	0	-
gi|29165615|ref|NC_002745.2|	2567301	+	C	0	0	1	0	0	-
gi|29165615|ref|NC_002745.2|	2567305	+	U	0	0	0	0	1	-
gi|29165615|ref|NC_002745.2|	2567314	+	G	0	1	0	0	0	-
gi|29165615|ref|NC_002745.2|	2567315	+	U	0	0	1	0	1	-
gi|29165615|ref|NC_002745.2|	2567316	+	A	0	0	0	0	1	-
gi|29165615|ref|NC_002745.2|	2567322	+	U	0	1	0	0	0	-
gi|29165615|ref|NC_002745.2|	2567340	+	A	0	0	0	0	1	-
gi|29165615|ref|NC_002745.2|	2567372	+	A	0	0	0	1	0	-
gi|29165615|ref|NC_002745.2|	2567388	+	A	0	1	0	0	0	-
gi|29165615|ref|NC_002745.2|	2567599	+	U	0	0	0	0	1	-
gi|29165615|ref|NC_002745.2|	2567946	+	U	0	1	0	0	0	-
gi|29165615|ref|NC_002745.2|	2568113	+	A	0	0	0	0	1	-
gi|29165615|ref|NC_002745.2|	2568114	+	U	0	1	0	0	0	-
gi|29165615|ref|NC_002745.2|	2568115	+	A	0	5	2	6	2	-
gi|29165615|ref|NC_002745.2|	2568122	+	C	0	0	0	0	1	-
gi|29165615|ref|NC_002745.2|	2568127	+	C	2	1	0	0	0	-
gi|29165615|ref|NC_002745.2|	2568136	+	U	1	1	1	1	1	-

gi|29165615|ref|NC_002745.2|	2568140 +	G	0	0	0	0	1	-
gi|29165615|ref|NC_002745.2|	2568603 +	G	0	0	0	1	0	-
gi|29165615|ref|NC_002745.2|	2568687 +	G	0	0	0	1	0	-
gi|29165615|ref|NC_002745.2|	2569376 +	G	0	0	1	0	0	-
gi|29165615|ref|NC_002745.2|	2572169 +	G	0	0	0	1	0	-
gi|29165615|ref|NC_002745.2|	2572211 +	G	0	0	0	1	0	-
gi|29165615|ref|NC_002745.2|	2572253 +	G	0	0	0	1	0	-
gi|29165615|ref|NC_002745.2|	2572295 +	G	0	0	0	1	0	-
gi|29165615|ref|NC_002745.2|	2573026 +	G	0	0	1	0	0	-
gi|29165615|ref|NC_002745.2|	2587120 +	C	0	2	0	0	0	SA2302
gi|29165615|ref|NC_002745.2|	2587121 +	G	0	3	6	0	2	SA2302
gi|29165615|ref|NC_002745.2|	2587122 +	U	0	7	7	2	3	SA2302
gi|29165615|ref|NC_002745.2|	2587125 +	G	0	1	0	0	0	SA2302
gi|29165615|ref|NC_002745.2|	2587129 +	G	0	0	0	0	2	SA2302
gi|29165615|ref|NC_002745.2|	2589335 +	U	0	2	1	1	5	SA2304
gi|29165615|ref|NC_002745.2|	2589336 +	A	1	2	0	4	2	SA2304
gi|29165615|ref|NC_002745.2|	2600119 +	A	0	0	0	0	1	SA2315
gi|29165615|ref|NC_002745.2|	2621356 +	A	2	0	0	0	0	SA2336
gi|29165615|ref|NC_002745.2|	2621403 +	U	0	1	0	0	0	SA2336
gi|29165615|ref|NC_002745.2|	2621406 +	G	1	0	0	0	0	SA2336
gi|29165615|ref|NC_002745.2|	2621410 +	A	1	0	0	0	0	SA2336
gi|29165615|ref|NC_002745.2|	2634837 +	C	0	0	0	0	1	-
gi|29165615|ref|NC_002745.2|	2634947 +	A	0	0	0	1	0	-
gi|29165615|ref|NC_002745.2|	2634957 +	U	0	0	0	2	1	-
gi|29165615|ref|NC_002745.2|	2634964 +	C	0	0	0	0	1	-
gi|29165615|ref|NC_002745.2|	2635074 +	A	0	0	0	1	0	-
gi|29165615|ref|NC_002745.2|	2635084 +	U	0	0	0	2	1	-
gi|29165615|ref|NC_002745.2|	2635091 +	U	0	0	0	0	1	-
gi|29165615|ref|NC_002745.2|	2635136 +	C	0	0	0	1	0	-
gi|29165615|ref|NC_002745.2|	2635149 +	U	0	1	0	0	0	-
gi|29165615|ref|NC_002745.2|	2635164 +	G	0	0	0	0	1	-
gi|29165615|ref|NC_002745.2|	2635170 +	A	0	0	1	0	0	-
gi|29165615|ref|NC_002745.2|	2635187 +	A	0	0	0	0	1	-
gi|29165615|ref|NC_002745.2|	2635194 +	A	0	0	0	0	1	-
gi|29165615|ref|NC_002745.2|	2635197 +	C	0	0	0	1	0	-
gi|29165615|ref|NC_002745.2|	2635213 +	U	0	0	0	0	1	-
gi|29165615|ref|NC_002745.2|	2635216 +	U	0	1	0	0	0	-
gi|29165615|ref|NC_002745.2|	2635218 +	A	0	1	0	0	0	-
gi|29165615|ref|NC_002745.2|	2635225 +	G	0	0	0	0	1	-
gi|29165615|ref|NC_002745.2|	2635234 +	G	3	0	0	0	0	-
gi|29165615|ref|NC_002745.2|	2635250 +	G	0	2	0	0	0	-
gi|29165615|ref|NC_002745.2|	2635265 +	A	0	0	0	0	1	-
gi|29165615|ref|NC_002745.2|	2635266 +	A	0	2	0	1	0	-
gi|29165615|ref|NC_002745.2|	2635268 +	A	0	0	1	0	0	-
gi|29165615|ref|NC_002745.2|	2636359 +	G	0	0	0	0	1	-
gi|29165615|ref|NC_002745.2|	2636362 +	U	0	0	0	1	0	-
gi|29165615|ref|NC_002745.2|	2652710 +	C	0	0	0	1	0	-
gi|29165615|ref|NC_002745.2|	2652723 +	U	0	1	0	0	0	-
gi|29165615|ref|NC_002745.2|	2652738 +	G	0	0	0	0	1	-
gi|29165615|ref|NC_002745.2|	2652744 +	U	0	0	1	0	0	-
gi|29165615|ref|NC_002745.2|	2652761 +	A	0	0	0	0	1	-
gi|29165615|ref|NC_002745.2|	2652768 +	A	0	0	0	0	1	-
gi|29165615|ref|NC_002745.2|	2652771 +	C	0	0	0	1	0	-
gi|29165615|ref|NC_002745.2|	2652787 +	U	0	0	0	0	1	-
gi|29165615|ref|NC_002745.2|	2652790 +	U	0	1	0	0	0	-
gi|29165615|ref|NC_002745.2|	2652792 +	A	0	2	0	0	0	-
gi|29165615|ref|NC_002745.2|	2652799 +	G	0	0	0	0	1	-
gi|29165615|ref|NC_002745.2|	2652808 +	G	3	0	0	0	0	-
gi|29165615|ref|NC_002745.2|	2652824 +	G	0	2	0	0	0	-
gi|29165615|ref|NC_002745.2|	2652839 +	A	0	0	0	0	1	-
gi|29165615|ref|NC_002745.2|	2652840 +	A	0	2	0	1	0	-
gi|29165615|ref|NC_002745.2|	2715891 +	U	3	1	0	0	0	SA2420
gi|29165615|ref|NC_002745.2|	2715892 +	U	2	0	0	0	1	SA2420
gi|29165615|ref|NC_002745.2|	2715893 +	A	1	1	0	0	0	SA2420
gi|29165615|ref|NC_002745.2|	2727931 +	A	0	0	1	0	0	-
gi|29165615|ref|NC_002745.2|	2744201 +	U	0	1	1	0	0	-
gi|29165615|ref|NC_002745.2|	2744202 +	U	0	0	1	1	1	-
gi|29165615|ref|NC_002745.2|	2774054 +	A	0	0	0	1	0	-
gi|29165615|ref|NC_002745.2|	2774074 +	U	1	0	0	2	0	SAS090
gi|29165615|ref|NC_002745.2|	2774081 +	U	0	0	0	0	2	SAS090
gi|29165615|ref|NC_002745.2|	2774086 +	C	0	0	0	1	0	SAS090
gi|29165615|ref|NC_002745.2|	2774091 +	C	0	0	0	1	2	SAS090
gi|29165615|ref|NC_002745.2|	2774092 +	C	0	0	0	0	1	SAS090
gi|29165615|ref|NC_002745.2|	2774098 +	G	0	0	0	0	1	SAS090
gi|29165615|ref|NC_002745.2|	2774099 +	C	0	1	2	0	0	SAS090
gi|29165615|ref|NC_002745.2|	2774100 +	A	0	0	1	0	0	SAS090
gi|29165615|ref|NC_002745.2|	2774101 +	U	3	2	3	1	10	SAS090
gi|29165615|ref|NC_002745.2|	2774106 +	U	0	0	0	1	0	SAS090
gi|29165615|ref|NC_002745.2|	2774107 +	G	0	0	0	0	1	SAS090
gi|29165615|ref|NC_002745.2|	2774108 +	U	0	0	0	1	0	SAS090
gi|29165615|ref|NC_002745.2|	2774113 +	U	0	0	1	2	0	SAS090

gi|29165615|ref|NC_002745.2|	2774118	+	U	0	3	1	3	1	SAS090
gi|29165615|ref|NC_002745.2|	2774119	+	U	0	0	0	0	1	SAS090
gi|29165615|ref|NC_002745.2|	2803490	+	A	0	1	0	0	0	SA2492
gi|29165615|ref|NC_002745.2|	2803550	+	G	0	0	1	0	0	SA2492
gi|29165615|ref|NC_002745.2|	2803551	+	A	0	0	0	1	0	SA2492
gi|29165615|ref|NC_002745.2|	2803554	+	A	0	0	1	0	0	SA2492
gi|29165615|ref|NC_002745.2|	2803555	+	A	0	1	0	0	0	SA2492
gi|29165615|ref|NC_002745.2|	2803618	+	A	0	1	0	0	1	SA2492
gi|29165615|ref|NC_002745.2|	2803791	+	C	0	1	0	0	0	SA2492
gi|29165615|ref|NC_002745.2|	2806386	+	G	0	1	0	0	0	-
gi|29165615|ref|NC_002745.2|	2806439	+	U	0	0	1	0	0	-
gi|29165615|ref|NC_002745.2|	2806483	+	G	0	0	0	0	1	-
gi|29165615|ref|NC_002745.2|	2806484	+	G	0	1	0	0	0	-
gi|29165615|ref|NC_002745.2|	2806485	+	A	0	0	1	0	0	-
gi|29165615|ref|NC_002745.2|	2806487	+	G	1	2	0	0	0	-
gi|29165615|ref|NC_002745.2|	2806488	+	U	0	3	1	0	0	-
gi|29165615|ref|NC_002745.2|	2806489	+	U	0	0	1	0	0	-
gi|29165615|ref|NC_002745.2|	2806490	+	U	1	0	0	0	0	-
gi|29165615|ref|NC_002745.2|	2806492	+	C	0	11	3	2	3	-
gi|29165615|ref|NC_002745.2|	2806493	+	U	0	3	1	0	1	-
gi|29165615|ref|NC_002745.2|	2806494	+	A	0	6	1	2	1	-
gi|29165615|ref|NC_002745.2|	2806495	+	U	0	9	4	4	5	-
gi|29165615|ref|NC_002745.2|	2806496	+	A	0	16	9	3	10	SA2494
gi|29165615|ref|NC_002745.2|	2806497	+	U	0	3	1	0	1	SA2494
gi|29165615|ref|NC_002745.2|	2806498	+	G	0	4	2	1	1	SA2494
gi|29165615|ref|NC_002745.2|	2806499	+	A	1	35	9	4	15	SA2494
gi|29165615|ref|NC_002745.2|	2806500	+	A	0	22	12	3	8	SA2494
gi|29165615|ref|NC_002745.2|	2806501	+	U	0	1	2	1	0	SA2494
gi|29165615|ref|NC_002745.2|	2806502	+	A	0	4	1	0	0	SA2494
gi|29165615|ref|NC_002745.2|	2806503	+	A	0	4	3	0	1	SA2494
gi|29165615|ref|NC_002745.2|	2806504	+	C	0	1	1	0	0	SA2494
gi|29165615|ref|NC_002745.2|	2806505	+	G	0	1	1	0	0	SA2494
gi|29165615|ref|NC_002745.2|	2806506	+	G	0	19	2	3	17	SA2494
gi|29165615|ref|NC_002745.2|	2806507	+	U	4	1	1	1	2	SA2494
gi|29165615|ref|NC_002745.2|	2806508	+	A	0	18	9	5	13	SA2494
gi|29165615|ref|NC_002745.2|	2806509	+	C	3	35	21	14	19	SA2494
gi|29165615|ref|NC_002745.2|	2806510	+	A	1	21	11	4	11	SA2494
gi|29165615|ref|NC_002745.2|	2806511	+	G	0	7	4	2	7	SA2494
gi|29165615|ref|NC_002745.2|	2806512	+	U	4	7	5	6	3	SA2494
gi|29165615|ref|NC_002745.2|	2806513	+	A	0	9	3	4	8	SA2494
gi|29165615|ref|NC_002745.2|	2806514	+	A	0	46	13	11	18	SA2494
gi|29165615|ref|NC_002745.2|	2806515	+	A	2	30	17	7	8	SA2494
gi|29165615|ref|NC_002745.2|	2806516	+	A	4	48	2	4	14	SA2494
gi|29165615|ref|NC_002745.2|	2806517	+	U	0	1	0	1	0	SA2494
gi|29165615|ref|NC_002745.2|	2806518	+	G	0	1	0	0	4	SA2494
gi|29165615|ref|NC_002745.2|	2806519	+	G	0	3	1	2	10	SA2494
gi|29165615|ref|NC_002745.2|	2806520	+	U	0	5	0	2	3	SA2494
gi|29165615|ref|NC_002745.2|	2806521	+	U	0	2	1	1	2	SA2494
gi|29165615|ref|NC_002745.2|	2806522	+	U	1	9	0	1	1	SA2494
gi|29165615|ref|NC_002745.2|	2806523	+	A	1	33	4	13	13	SA2494
gi|29165615|ref|NC_002745.2|	2806524	+	A	0	6	3	3	3	SA2494
gi|29165615|ref|NC_002745.2|	2806525	+	C	0	6	3	0	2	SA2494
gi|29165615|ref|NC_002745.2|	2806526	+	G	0	2	1	3	4	SA2494
gi|29165615|ref|NC_002745.2|	2806527	+	C	1	125	15	42	61	SA2494
gi|29165615|ref|NC_002745.2|	2806528	+	A	2	56	5	18	45	SA2494
gi|29165615|ref|NC_002745.2|	2806529	+	G	1	31	8	11	8	SA2494
gi|29165615|ref|NC_002745.2|	2806530	+	A	1	36	8	21	12	SA2494
gi|29165615|ref|NC_002745.2|	2806531	+	A	0	4	3	6	3	SA2494
gi|29165615|ref|NC_002745.2|	2806532	+	A	0	5	3	2	0	SA2494
gi|29165615|ref|NC_002745.2|	2806533	+	A	0	4	1	1	2	SA2494
gi|29165615|ref|NC_002745.2|	2806534	+	A	0	0	0	0	1	SA2494
gi|29165615|ref|NC_002745.2|	2806535	+	G	0	1	0	0	0	SA2494
gi|29165615|ref|NC_002745.2|	2806566	+	G	0	4	1	2	1	SA2494
gi|29165615|ref|NC_002745.2|	2808646	+	A	0	0	0	1	0	-
gi|29165615|ref|NC_002745.2|	2808696	+	C	0	1	1	0	0	SAS092
gi|29165615|ref|NC_002745.2|	2808698	+	A	0	0	1	0	0	SAS092
gi|29165615|ref|NC_002745.2|	2808701	+	A	0	0	1	0	0	SAS092
gi|29165615|ref|NC_002745.2|	2808705	+	A	0	0	0	0	2	SAS092
gi|29165615|ref|NC_002745.2|	2808716	+	U	0	0	0	0	1	SAS092
gi|29165615|ref|NC_002745.2|	2808719	+	C	0	0	1	0	0	SAS092
gi|29165615|ref|NC_002745.2|	2808721	+	A	0	0	0	0	4	SAS092
gi|29165615|ref|NC_002745.2|	2808726	+	A	0	0	1	0	0	SAS092
gi|29165615|ref|NC_002745.2|	2808731	+	C	1	3	4	2	1	SAS092
gi|29165615|ref|NC_002745.2|	2808733	+	U	0	1	0	0	0	SAS092
gi|29165615|ref|NC_002745.2|	2808738	+	U	0	0	0	0	1	SAS092
gi|29165615|ref|NC_002745.2|	2808739	+	C	0	1	0	2	1	SAS092
gi|29165615|ref|NC_002745.2|	2808743	+	U	3	2	0	0	1	SAS092
gi|29165615|ref|NC_002745.2|	2808744	+	A	0	0	1	0	1	SAS092
gi|29165615|ref|NC_002745.2|	2808746	+	U	0	0	1	0	2	SAS092
gi|29165615|ref|NC_002745.2|	2808748	+	C	0	0	1	1	3	SAS092
gi|29165615|ref|NC_002745.2|	2808749	+	C	1	0	0	0	0	SAS092

gi|29165615|ref|NC_002745.2|	2808750	+	A	0	1	1	0	0	SAS092
gi|29165615|ref|NC_002745.2|	2808751	+	A	1	1	2	0	0	SAS092
gi|29165615|ref|NC_002745.2|	2808753	+	A	0	0	0	0	1	SAS092
gi|29165615|ref|NC_002745.2|	2808754	+	U	2	0	0	0	0	SAS092
gi|29165615|ref|NC_002745.2|	2808758	+	U	6	2	0	0	3	SAS092
gi|29165615|ref|NC_002745.2|	2808761	+	G	0	0	0	0	1	SAS092
gi|29165615|ref|NC_002745.2|	2808763	+	A	0	0	1	0	1	SAS092
gi|29165615|ref|NC_002745.2|	2808764	+	G	0	1	0	0	0	SAS092
gi|29165615|ref|NC_002745.2|	2808773	+	A	0	1	0	2	0	SAS092
gi|29165615|ref|NC_002745.2|	10516	-	A	0	0	0	1	0	SA0007
gi|29165615|ref|NC_002745.2|	24916	-	C	0	0	1	0	0	-
gi|29165615|ref|NC_002745.2|	37879	-	U	0	0	1	0	0	SA0028
gi|29165615|ref|NC_002745.2|	51114	-	U	0	0	0	1	0	SA0042
gi|29165615|ref|NC_002745.2|	51121	-	A	0	0	0	0	1	SA0042
gi|29165615|ref|NC_002745.2|	52171	-	G	0	0	0	0	1	SA0043
gi|29165615|ref|NC_002745.2|	62279	-	A	0	0	0	0	1	SA0055
gi|29165615|ref|NC_002745.2|	72093	-	U	0	0	0	1	0	SA0065
gi|29165615|ref|NC_002745.2|	73920	-	U	0	1	0	0	0	SA0066
gi|29165615|ref|NC_002745.2|	89536	-	A	0	0	0	2	1	-
gi|29165615|ref|NC_002745.2|	89555	-	C	0	0	0	0	1	-
gi|29165615|ref|NC_002745.2|	89573	-	C	0	1	0	0	0	-
gi|29165615|ref|NC_002745.2|	89587	-	A	1	0	0	0	0	-
gi|29165615|ref|NC_002745.2|	89604	-	C	0	0	0	1	2	-
gi|29165615|ref|NC_002745.2|	89608	-	U	1	0	0	0	0	-
gi|29165615|ref|NC_002745.2|	89619	-	A	2	0	0	0	0	SA0079
gi|29165615|ref|NC_002745.2|	89635	-	G	0	1	0	0	0	SA0079
gi|29165615|ref|NC_002745.2|	89656	-	A	1	3	0	0	0	SA0079
gi|29165615|ref|NC_002745.2|	89657	-	A	2	1	0	0	0	SA0079
gi|29165615|ref|NC_002745.2|	89660	-	U	0	1	0	0	1	SA0079
gi|29165615|ref|NC_002745.2|	89661	-	U	1	0	1	0	0	SA0079
gi|29165615|ref|NC_002745.2|	89663	-	C	0	0	1	0	0	SA0079
gi|29165615|ref|NC_002745.2|	89666	-	A	0	0	0	0	1	SA0079
gi|29165615|ref|NC_002745.2|	89669	-	U	0	0	0	0	1	SA0079
gi|29165615|ref|NC_002745.2|	89673	-	U	1	0	0	0	0	SA0079
gi|29165615|ref|NC_002745.2|	89674	-	U	0	0	1	0	0	SA0079
gi|29165615|ref|NC_002745.2|	89679	-	A	0	2	0	0	0	SA0079
gi|29165615|ref|NC_002745.2|	89680	-	A	0	2	0	2	0	SA0079
gi|29165615|ref|NC_002745.2|	89681	-	C	3	0	1	0	1	SA0079
gi|29165615|ref|NC_002745.2|	89682	-	U	1	0	0	0	1	SA0079
gi|29165615|ref|NC_002745.2|	89683	-	U	0	0	0	1	0	SA0079
gi|29165615|ref|NC_002745.2|	89684	-	A	1	2	0	0	0	SA0079
gi|29165615|ref|NC_002745.2|	89685	-	A	0	0	1	0	1	SA0079
gi|29165615|ref|NC_002745.2|	89686	-	C	0	0	1	2	0	SA0079
gi|29165615|ref|NC_002745.2|	89687	-	A	2	2	1	5	4	SA0079
gi|29165615|ref|NC_002745.2|	89693	-	A	0	1	1	0	0	SA0079
gi|29165615|ref|NC_002745.2|	89694	-	A	0	0	1	0	0	SA0079
gi|29165615|ref|NC_002745.2|	89695	-	G	0	0	0	1	0	SA0079
gi|29165615|ref|NC_002745.2|	89697	-	A	0	0	2	0	0	SA0079
gi|29165615|ref|NC_002745.2|	89698	-	G	0	0	2	0	0	SA0079
gi|29165615|ref|NC_002745.2|	89699	-	A	0	1	0	0	1	SA0079
gi|29165615|ref|NC_002745.2|	89700	-	A	0	0	0	0	1	SA0079
gi|29165615|ref|NC_002745.2|	89702	-	A	0	1	1	0	3	SA0079
gi|29165615|ref|NC_002745.2|	89703	-	A	1	0	4	1	4	SA0079
gi|29165615|ref|NC_002745.2|	89704	-	G	0	0	2	0	1	SA0079
gi|29165615|ref|NC_002745.2|	89705	-	U	0	0	0	1	0	SA0079
gi|29165615|ref|NC_002745.2|	89707	-	U	0	2	1	0	0	SA0079
gi|29165615|ref|NC_002745.2|	89708	-	A	0	1	1	0	2	SA0079
gi|29165615|ref|NC_002745.2|	89709	-	A	0	2	0	1	4	SA0079
gi|29165615|ref|NC_002745.2|	89710	-	A	0	1	0	0	0	SA0079
gi|29165615|ref|NC_002745.2|	89711	-	C	0	0	1	0	0	SA0079
gi|29165615|ref|NC_002745.2|	89719	-	U	1	0	0	0	0	SA0079
gi|29165615|ref|NC_002745.2|	89720	-	A	0	0	0	0	1	SA0079
gi|29165615|ref|NC_002745.2|	89728	-	C	0	0	1	0	0	SA0079
gi|29165615|ref|NC_002745.2|	89729	-	G	0	0	0	1	0	SA0079
gi|29165615|ref|NC_002745.2|	89734	-	A	0	0	0	0	1	SA0079
gi|29165615|ref|NC_002745.2|	89736	-	U	0	0	0	0	1	SA0079
gi|29165615|ref|NC_002745.2|	89740	-	C	1	0	0	0	0	SA0079
gi|29165615|ref|NC_002745.2|	89746	-	U	0	0	1	1	0	SA0079
gi|29165615|ref|NC_002745.2|	89750	-	A	0	0	1	1	0	SA0079
gi|29165615|ref|NC_002745.2|	89751	-	A	0	0	0	1	0	SA0079
gi|29165615|ref|NC_002745.2|	89752	-	G	0	0	0	0	1	SA0079
gi|29165615|ref|NC_002745.2|	89780	-	A	0	0	1	0	0	SA0079
gi|29165615|ref|NC_002745.2|	89781	-	A	0	0	0	0	1	SA0079
gi|29165615|ref|NC_002745.2|	89787	-	C	0	1	0	0	0	SA0079
gi|29165615|ref|NC_002745.2|	89800	-	C	0	0	1	0	0	SA0079
gi|29165615|ref|NC_002745.2|	89818	-	A	0	0	1	0	0	SA0079
gi|29165615|ref|NC_002745.2|	89822	-	C	1	0	0	0	0	SA0079
gi|29165615|ref|NC_002745.2|	89834	-	C	0	1	0	0	0	SA0079
gi|29165615|ref|NC_002745.2|	89844	-	A	0	0	0	1	0	SA0079
gi|29165615|ref|NC_002745.2|	89847	-	C	0	0	0	1	0	SA0079
gi|29165615|ref|NC_002745.2|	89858	-	A	0	0	1	0	0	SA0079

gi|29165615|ref|NC_002745.2|	89859	-	G	0	1	0	0	0 SA0079
gi|29165615|ref|NC_002745.2|	89866	-	A	0	2	0	0	0 SA0079
gi|29165615|ref|NC_002745.2|	89867	-	A	0	0	1	0	0 SA0079
gi|29165615|ref|NC_002745.2|	89871	-	A	0	1	0	0	0 SA0079
gi|29165615|ref|NC_002745.2|	89878	-	A	0	0	0	0	1 SA0079
gi|29165615|ref|NC_002745.2|	89881	-	C	0	0	0	0	2 SA0079
gi|29165615|ref|NC_002745.2|	89904	-	A	0	2	0	1	0 SA0079
gi|29165615|ref|NC_002745.2|	89905	-	A	0	0	0	0	1 SA0079
gi|29165615|ref|NC_002745.2|	89920	-	G	0	2	0	0	0 SA0079
gi|29165615|ref|NC_002745.2|	89947	-	A	0	0	0	0	3 SA0079
gi|29165615|ref|NC_002745.2|	89962	-	G	0	0	0	0	1 -
gi|29165615|ref|NC_002745.2|	89982	-	C	0	0	2	0	0 -
gi|29165615|ref|NC_002745.2|	90044	-	U	0	0	1	0	0 -
gi|29165615|ref|NC_002745.2|	90050	-	G	0	0	0	0	1 -
gi|29165615|ref|NC_002745.2|	90065	-	U	0	1	0	0	0 -
gi|29165615|ref|NC_002745.2|	90078	-	C	0	0	0	1	0 -
gi|29165615|ref|NC_002745.2|	90110	-	A	0	1	0	0	0 -
gi|29165615|ref|NC_002745.2|	90115	-	C	0	1	0	0	0 -
gi|29165615|ref|NC_002745.2|	90127	-	A	0	0	0	0	1 -
gi|29165615|ref|NC_002745.2|	90128	-	G	0	0	1	0	0 -
gi|29165615|ref|NC_002745.2|	90146	-	A	0	0	1	0	0 -
gi|29165615|ref|NC_002745.2|	90151	-	U	0	0	0	0	1 -
gi|29165615|ref|NC_002745.2|	91368	-	A	0	0	1	0	0 SA0081
gi|29165615|ref|NC_002745.2|	91371	-	A	0	1	0	0	0 SA0081
gi|29165615|ref|NC_002745.2|	91372	-	A	0	0	0	0	1 SA0081
gi|29165615|ref|NC_002745.2|	91373	-	U	0	0	0	0	1 SA0081
gi|29165615|ref|NC_002745.2|	95842	-	A	0	0	0	2	1 SA0085
gi|29165615|ref|NC_002745.2|	95861	-	C	0	0	0	0	1 SA0085
gi|29165615|ref|NC_002745.2|	95879	-	C	0	1	0	0	0 SA0085
gi|29165615|ref|NC_002745.2|	95893	-	A	1	0	0	0	0 SA0085
gi|29165615|ref|NC_002745.2|	95910	-	C	0	0	0	1	2 SA0085
gi|29165615|ref|NC_002745.2|	95914	-	U	1	0	0	0	0 SA0085
gi|29165615|ref|NC_002745.2|	95925	-	C	2	0	0	0	0 SA0085
gi|29165615|ref|NC_002745.2|	95941	-	G	0	1	0	0	0 SA0085
gi|29165615|ref|NC_002745.2|	95963	-	A	1	3	0	0	0 SA0085
gi|29165615|ref|NC_002745.2|	95964	-	A	2	1	0	0	0 SA0085
gi|29165615|ref|NC_002745.2|	95967	-	U	0	1	0	0	1 SA0085
gi|29165615|ref|NC_002745.2|	95968	-	U	1	0	1	0	0 SA0085
gi|29165615|ref|NC_002745.2|	95970	-	C	0	0	1	0	0 SA0085
gi|29165615|ref|NC_002745.2|	95973	-	A	0	0	0	0	1 SA0085
gi|29165615|ref|NC_002745.2|	95976	-	U	0	0	0	0	1 SA0085
gi|29165615|ref|NC_002745.2|	95980	-	U	1	0	0	0	0 SA0085
gi|29165615|ref|NC_002745.2|	95981	-	U	0	0	1	0	0 SA0085
gi|29165615|ref|NC_002745.2|	95986	-	A	0	2	0	0	0 SA0085
gi|29165615|ref|NC_002745.2|	95987	-	A	0	2	0	2	0 SA0085
gi|29165615|ref|NC_002745.2|	95988	-	C	3	0	1	0	1 SA0085
gi|29165615|ref|NC_002745.2|	95989	-	U	1	0	0	0	1 SA0085
gi|29165615|ref|NC_002745.2|	95990	-	U	0	0	0	1	0 SA0085
gi|29165615|ref|NC_002745.2|	95991	-	A	1	2	0	0	0 SA0085
gi|29165615|ref|NC_002745.2|	95992	-	A	0	0	1	0	1 SA0085
gi|29165615|ref|NC_002745.2|	95993	-	C	0	0	1	2	0 SA0085
gi|29165615|ref|NC_002745.2|	95994	-	A	2	2	1	5	4 SA0085
gi|29165615|ref|NC_002745.2|	96000	-	A	0	1	1	0	0 SA0085
gi|29165615|ref|NC_002745.2|	96001	-	A	0	0	1	0	0 SA0085
gi|29165615|ref|NC_002745.2|	96002	-	G	0	0	0	1	0 SA0085
gi|29165615|ref|NC_002745.2|	96004	-	A	0	0	2	0	0 SA0085
gi|29165615|ref|NC_002745.2|	96005	-	G	0	0	2	0	0 SA0085
gi|29165615|ref|NC_002745.2|	96006	-	A	0	1	0	0	1 SA0085
gi|29165615|ref|NC_002745.2|	96007	-	A	0	0	0	0	1 SA0085
gi|29165615|ref|NC_002745.2|	96009	-	A	0	1	1	0	3 SA0085
gi|29165615|ref|NC_002745.2|	96010	-	A	1	0	4	1	4 SA0085
gi|29165615|ref|NC_002745.2|	96011	-	G	0	0	2	0	1 SA0085
gi|29165615|ref|NC_002745.2|	96012	-	U	0	0	0	1	0 SA0085
gi|29165615|ref|NC_002745.2|	96014	-	U	0	2	1	0	0 SA0085
gi|29165615|ref|NC_002745.2|	96015	-	A	0	1	1	0	2 SA0085
gi|29165615|ref|NC_002745.2|	96016	-	A	0	2	0	1	4 SA0085
gi|29165615|ref|NC_002745.2|	96017	-	A	0	1	0	0	0 SA0085
gi|29165615|ref|NC_002745.2|	96018	-	C	0	0	1	0	0 SA0085
gi|29165615|ref|NC_002745.2|	96026	-	U	1	0	0	0	0 SA0085
gi|29165615|ref|NC_002745.2|	96027	-	A	0	0	0	0	1 SA0085
gi|29165615|ref|NC_002745.2|	96035	-	C	0	0	1	0	0 SA0085
gi|29165615|ref|NC_002745.2|	96036	-	G	0	0	0	1	0 SA0085
gi|29165615|ref|NC_002745.2|	96041	-	A	0	0	0	0	1 SA0085
gi|29165615|ref|NC_002745.2|	96043	-	U	0	0	0	0	1 SA0085
gi|29165615|ref|NC_002745.2|	96047	-	C	1	0	0	0	0 SA0085
gi|29165615|ref|NC_002745.2|	96053	-	U	0	0	1	1	0 SA0085
gi|29165615|ref|NC_002745.2|	96057	-	A	0	0	1	1	0 SA0085
gi|29165615|ref|NC_002745.2|	96058	-	A	0	0	0	1	0 SA0085
gi|29165615|ref|NC_002745.2|	96059	-	G	0	0	0	0	1 SA0085
gi|29165615|ref|NC_002745.2|	96087	-	A	0	0	1	0	0 SA0085
gi|29165615|ref|NC_002745.2|	96088	-	A	0	0	0	0	1 SA0085

gi|29165615|ref|NC_002745.2|	96094	-	C	0	1	0	0	0	SA0085
gi|29165615|ref|NC_002745.2|	96107	-	C	0	0	1	0	0	SA0085
gi|29165615|ref|NC_002745.2|	96125	-	A	0	0	1	0	0	SA0085
gi|29165615|ref|NC_002745.2|	96129	-	C	1	0	0	0	0	SA0085
gi|29165615|ref|NC_002745.2|	96141	-	C	0	1	0	0	0	SA0085
gi|29165615|ref|NC_002745.2|	96151	-	A	0	0	0	1	0	SA0085
gi|29165615|ref|NC_002745.2|	96154	-	C	0	0	0	1	0	SA0085
gi|29165615|ref|NC_002745.2|	96165	-	A	0	0	1	0	0	SA0085
gi|29165615|ref|NC_002745.2|	96166	-	G	0	1	0	0	0	SA0085
gi|29165615|ref|NC_002745.2|	96173	-	A	0	2	0	0	0	SA0085
gi|29165615|ref|NC_002745.2|	96174	-	A	0	0	1	0	0	SA0085
gi|29165615|ref|NC_002745.2|	96178	-	A	0	1	0	0	0	SA0085
gi|29165615|ref|NC_002745.2|	96185	-	A	0	0	0	0	1	SA0085
gi|29165615|ref|NC_002745.2|	96188	-	G	0	0	0	0	2	SA0085
gi|29165615|ref|NC_002745.2|	96239	-	A	0	1	0	0	0	SA0085
gi|29165615|ref|NC_002745.2|	96244	-	C	0	1	0	0	0	SA0085
gi|29165615|ref|NC_002745.2|	96256	-	A	0	0	0	0	1	SA0085
gi|29165615|ref|NC_002745.2|	96257	-	G	0	0	1	0	0	SA0085
gi|29165615|ref|NC_002745.2|	96275	-	A	0	0	1	0	0	SA0085
gi|29165615|ref|NC_002745.2|	96280	-	U	0	0	0	0	1	SA0085
gi|29165615|ref|NC_002745.2|	104316	-	A	0	0	0	2	0	-
gi|29165615|ref|NC_002745.2|	105138	-	A	0	0	0	2	0	-
gi|29165615|ref|NC_002745.2|	105973	-	A	0	0	0	2	0	-
gi|29165615|ref|NC_002745.2|	107642	-	A	0	0	0	2	0	-
gi|29165615|ref|NC_002745.2|	122761	-	A	0	1	0	0	0	SA0107
gi|29165615|ref|NC_002745.2|	122766	-	A	0	0	1	0	0	SA0107
gi|29165615|ref|NC_002745.2|	122960	-	C	0	0	0	1	0	SA0107
gi|29165615|ref|NC_002745.2|	122961	-	A	0	2	0	0	1	SA0107
gi|29165615|ref|NC_002745.2|	122963	-	A	0	1	1	0	0	SA0107
gi|29165615|ref|NC_002745.2|	122964	-	C	0	2	0	0	1	SA0107
gi|29165615|ref|NC_002745.2|	122965	-	A	0	0	0	0	3	SA0107
gi|29165615|ref|NC_002745.2|	122966	-	A	0	3	1	0	1	SA0107
gi|29165615|ref|NC_002745.2|	122967	-	C	1	4	1	2	3	SA0107
gi|29165615|ref|NC_002745.2|	122968	-	G	1	8	4	2	9	SA0107
gi|29165615|ref|NC_002745.2|	122969	-	G	1	1	0	0	1	SA0107
gi|29165615|ref|NC_002745.2|	122970	-	C	1	2	0	1	2	SA0107
gi|29165615|ref|NC_002745.2|	122971	-	A	0	3	2	1	2	SA0107
gi|29165615|ref|NC_002745.2|	122972	-	G	0	0	0	2	0	SA0107
gi|29165615|ref|NC_002745.2|	122973	-	A	0	4	0	0	0	SA0107
gi|29165615|ref|NC_002745.2|	122974	-	A	0	0	2	1	2	SA0107
gi|29165615|ref|NC_002745.2|	122975	-	G	1	0	0	0	0	SA0107
gi|29165615|ref|NC_002745.2|	122976	-	A	0	0	0	1	1	SA0107
gi|29165615|ref|NC_002745.2|	122977	-	A	0	2	0	1	1	SA0107
gi|29165615|ref|NC_002745.2|	122978	-	A	0	5	0	0	0	SA0107
gi|29165615|ref|NC_002745.2|	122979	-	U	0	0	0	6	7	SA0107
gi|29165615|ref|NC_002745.2|	122980	-	G	0	7	2	3	5	SA0107
gi|29165615|ref|NC_002745.2|	122981	-	G	0	1	0	0	0	SA0107
gi|29165615|ref|NC_002745.2|	122982	-	U	0	2	0	0	3	SA0107
gi|29165615|ref|NC_002745.2|	122983	-	C	1	6	4	2	2	SA0107
gi|29165615|ref|NC_002745.2|	122984	-	C	0	0	1	1	3	SA0107
gi|29165615|ref|NC_002745.2|	122985	-	G	0	2	0	0	1	SA0107
gi|29165615|ref|NC_002745.2|	122987	-	A	0	1	1	0	1	SA0107
gi|29165615|ref|NC_002745.2|	122988	-	C	0	3	1	0	1	SA0107
gi|29165615|ref|NC_002745.2|	122989	-	A	0	1	0	0	3	SA0107
gi|29165615|ref|NC_002745.2|	122990	-	A	0	3	1	0	1	SA0107
gi|29165615|ref|NC_002745.2|	122991	-	C	2	4	2	3	3	SA0107
gi|29165615|ref|NC_002745.2|	122992	-	G	1	1	1	0	2	SA0107
gi|29165615|ref|NC_002745.2|	122993	-	G	1	1	0	0	3	SA0107
gi|29165615|ref|NC_002745.2|	122994	-	C	1	3	0	1	2	SA0107
gi|29165615|ref|NC_002745.2|	122995	-	A	0	4	2	2	2	SA0107
gi|29165615|ref|NC_002745.2|	122996	-	G	0	0	0	2	0	SA0107
gi|29165615|ref|NC_002745.2|	122997	-	A	0	4	0	0	1	SA0107
gi|29165615|ref|NC_002745.2|	122998	-	A	0	0	3	1	2	SA0107
gi|29165615|ref|NC_002745.2|	122999	-	G	1	0	0	0	0	SA0107
gi|29165615|ref|NC_002745.2|	123000	-	A	0	0	0	1	2	SA0107
gi|29165615|ref|NC_002745.2|	123001	-	A	0	2	0	1	1	SA0107
gi|29165615|ref|NC_002745.2|	123002	-	A	0	5	0	0	0	SA0107
gi|29165615|ref|NC_002745.2|	123003	-	U	1	0	0	6	8	SA0107
gi|29165615|ref|NC_002745.2|	123004	-	G	0	7	2	3	5	SA0107
gi|29165615|ref|NC_002745.2|	123006	-	U	0	2	0	0	3	SA0107
gi|29165615|ref|NC_002745.2|	123007	-	C	1	6	5	2	3	SA0107
gi|29165615|ref|NC_002745.2|	123008	-	C	0	0	3	0	3	SA0107
gi|29165615|ref|NC_002745.2|	123009	-	G	0	2	0	0	1	SA0107
gi|29165615|ref|NC_002745.2|	123011	-	A	0	1	1	0	1	SA0107
gi|29165615|ref|NC_002745.2|	123012	-	C	0	3	1	0	1	SA0107
gi|29165615|ref|NC_002745.2|	123013	-	A	0	1	0	0	3	SA0107
gi|29165615|ref|NC_002745.2|	123014	-	A	0	3	1	0	1	SA0107
gi|29165615|ref|NC_002745.2|	123015	-	C	2	4	2	3	3	SA0107
gi|29165615|ref|NC_002745.2|	123016	-	A	1	7	4	2	8	SA0107
gi|29165615|ref|NC_002745.2|	123017	-	A	4	5	1	2	8	SA0107
gi|29165615|ref|NC_002745.2|	123018	-	C	1	7	1	5	10	SA0107

gi|29165615|ref|NC_002745.2|	123019 -	A	0	4	2	4	6	SA0107
gi|29165615|ref|NC_002745.2|	123020 -	G	0	0	0	2	1	SA0107
gi|29165615|ref|NC_002745.2|	123021 -	A	0	5	0	0	2	SA0107
gi|29165615|ref|NC_002745.2|	123022 -	A	0	1	3	3	2	SA0107
gi|29165615|ref|NC_002745.2|	123023 -	G	1	0	0	1	4	SA0107
gi|29165615|ref|NC_002745.2|	123024 -	A	0	0	1	1	3	SA0107
gi|29165615|ref|NC_002745.2|	123025 -	A	0	2	0	0	1	SA0107
gi|29165615|ref|NC_002745.2|	123026 -	A	0	2	0	0	0	SA0107
gi|29165615|ref|NC_002745.2|	123027 -	U	0	0	0	0	3	SA0107
gi|29165615|ref|NC_002745.2|	123028 -	G	0	5	0	2	4	SA0107
gi|29165615|ref|NC_002745.2|	123029 -	G	0	1	0	0	0	SA0107
gi|29165615|ref|NC_002745.2|	123030 -	U	0	0	0	0	3	SA0107
gi|29165615|ref|NC_002745.2|	123031 -	C	1	5	2	0	4	SA0107
gi|29165615|ref|NC_002745.2|	123032 -	C	0	0	3	0	3	SA0107
gi|29165615|ref|NC_002745.2|	123033 -	G	0	1	0	0	0	SA0107
gi|29165615|ref|NC_002745.2|	123034 -	A	0	0	0	0	1	SA0107
gi|29165615|ref|NC_002745.2|	123035 -	A	0	1	0	0	1	SA0107
gi|29165615|ref|NC_002745.2|	123036 -	C	0	3	1	0	0	SA0107
gi|29165615|ref|NC_002745.2|	123037 -	A	0	1	0	0	1	SA0107
gi|29165615|ref|NC_002745.2|	123039 -	C	1	0	1	1	0	SA0107
gi|29165615|ref|NC_002745.2|	123040 -	G	1	0	1	0	1	SA0107
gi|29165615|ref|NC_002745.2|	123041 -	G	1	1	0	0	3	SA0107
gi|29165615|ref|NC_002745.2|	123042 -	C	1	3	0	1	2	SA0107
gi|29165615|ref|NC_002745.2|	123043 -	A	0	4	2	2	2	SA0107
gi|29165615|ref|NC_002745.2|	123044 -	G	0	0	0	2	0	SA0107
gi|29165615|ref|NC_002745.2|	123045 -	A	0	4	0	0	1	SA0107
gi|29165615|ref|NC_002745.2|	123046 -	A	0	0	3	1	2	SA0107
gi|29165615|ref|NC_002745.2|	123047 -	G	1	0	0	0	0	SA0107
gi|29165615|ref|NC_002745.2|	123048 -	A	0	0	0	1	2	SA0107
gi|29165615|ref|NC_002745.2|	123049 -	A	0	2	0	1	1	SA0107
gi|29165615|ref|NC_002745.2|	123050 -	A	0	5	0	0	0	SA0107
gi|29165615|ref|NC_002745.2|	123051 -	C	1	0	0	6	7	SA0107
gi|29165615|ref|NC_002745.2|	123052 -	G	0	7	2	3	5	SA0107
gi|29165615|ref|NC_002745.2|	123054 -	U	0	2	0	0	3	SA0107
gi|29165615|ref|NC_002745.2|	123055 -	C	1	5	4	2	1	SA0107
gi|29165615|ref|NC_002745.2|	123056 -	C	0	0	1	0	3	SA0107
gi|29165615|ref|NC_002745.2|	123057 -	A	0	2	0	0	1	SA0107
gi|29165615|ref|NC_002745.2|	123059 -	A	0	1	1	0	0	SA0107
gi|29165615|ref|NC_002745.2|	123060 -	C	0	2	0	0	1	SA0107
gi|29165615|ref|NC_002745.2|	123061 -	A	0	0	0	0	3	SA0107
gi|29165615|ref|NC_002745.2|	123062 -	A	0	3	1	0	1	SA0107
gi|29165615|ref|NC_002745.2|	123063 -	C	1	4	1	2	3	SA0107
gi|29165615|ref|NC_002745.2|	123064 -	A	0	8	4	2	8	SA0107
gi|29165615|ref|NC_002745.2|	123065 -	A	3	4	1	2	7	SA0107
gi|29165615|ref|NC_002745.2|	123066 -	C	0	6	1	4	9	SA0107
gi|29165615|ref|NC_002745.2|	123067 -	A	0	5	2	6	4	SA0107
gi|29165615|ref|NC_002745.2|	123068 -	G	0	0	0	0	1	SA0107
gi|29165615|ref|NC_002745.2|	123069 -	A	0	1	0	0	2	SA0107
gi|29165615|ref|NC_002745.2|	123070 -	A	0	1	3	2	2	SA0107
gi|29165615|ref|NC_002745.2|	123071 -	G	0	0	0	1	4	SA0107
gi|29165615|ref|NC_002745.2|	123072 -	A	0	0	1	0	2	SA0107
gi|29165615|ref|NC_002745.2|	123073 -	A	0	0	0	1	0	SA0107
gi|29165615|ref|NC_002745.2|	123074 -	A	0	3	0	0	0	SA0107
gi|29165615|ref|NC_002745.2|	123075 -	U	0	0	0	1	0	SA0107
gi|29165615|ref|NC_002745.2|	123076 -	G	0	1	1	1	1	SA0107
gi|29165615|ref|NC_002745.2|	123077 -	G	0	1	0	0	0	SA0107
gi|29165615|ref|NC_002745.2|	123078 -	U	0	1	0	0	2	SA0107
gi|29165615|ref|NC_002745.2|	123079 -	C	1	3	1	2	3	SA0107
gi|29165615|ref|NC_002745.2|	123080 -	C	0	0	2	0	0	SA0107
gi|29165615|ref|NC_002745.2|	123081 -	G	0	1	0	0	0	SA0107
gi|29165615|ref|NC_002745.2|	123082 -	A	0	0	0	0	1	SA0107
gi|29165615|ref|NC_002745.2|	123083 -	A	0	1	0	0	1	SA0107
gi|29165615|ref|NC_002745.2|	123084 -	C	0	3	1	0	0	SA0107
gi|29165615|ref|NC_002745.2|	123085 -	A	0	1	0	0	1	SA0107
gi|29165615|ref|NC_002745.2|	123087 -	C	1	0	1	1	0	SA0107
gi|29165615|ref|NC_002745.2|	123088 -	G	1	0	1	0	1	SA0107
gi|29165615|ref|NC_002745.2|	123089 -	G	1	1	0	0	3	SA0107
gi|29165615|ref|NC_002745.2|	123090 -	C	1	3	0	1	2	SA0107
gi|29165615|ref|NC_002745.2|	123091 -	A	0	4	2	2	2	SA0107
gi|29165615|ref|NC_002745.2|	123092 -	G	0	0	0	2	0	SA0107
gi|29165615|ref|NC_002745.2|	123093 -	A	0	4	0	0	1	SA0107
gi|29165615|ref|NC_002745.2|	123094 -	A	0	0	3	1	2	SA0107
gi|29165615|ref|NC_002745.2|	123095 -	G	1	0	0	0	0	SA0107
gi|29165615|ref|NC_002745.2|	123096 -	A	0	0	0	1	2	SA0107
gi|29165615|ref|NC_002745.2|	123097 -	A	0	2	0	1	1	SA0107
gi|29165615|ref|NC_002745.2|	123098 -	A	0	5	0	0	0	SA0107
gi|29165615|ref|NC_002745.2|	123099 -	U	1	0	0	6	8	SA0107
gi|29165615|ref|NC_002745.2|	123100 -	G	0	7	2	3	5	SA0107
gi|29165615|ref|NC_002745.2|	123102 -	U	0	2	0	0	3	SA0107
gi|29165615|ref|NC_002745.2|	123103 -	C	1	6	5	2	3	SA0107
gi|29165615|ref|NC_002745.2|	123104 -	C	0	0	3	0	3	SA0107

gi|29165615|ref|NC_002745.2|	123105	-	A	0	2	0	0	1	SA0107
gi|29165615|ref|NC_002745.2|	123107	-	A	0	1	1	0	0	SA0107
gi|29165615|ref|NC_002745.2|	123108	-	A	0	2	0	0	1	SA0107
gi|29165615|ref|NC_002745.2|	123109	-	A	0	0	0	0	3	SA0107
gi|29165615|ref|NC_002745.2|	123110	-	A	0	3	1	0	1	SA0107
gi|29165615|ref|NC_002745.2|	123111	-	C	1	4	1	2	3	SA0107
gi|29165615|ref|NC_002745.2|	123112	-	A	0	7	4	2	7	SA0107
gi|29165615|ref|NC_002745.2|	123113	-	A	3	4	1	3	7	SA0107
gi|29165615|ref|NC_002745.2|	123114	-	C	0	7	1	4	9	SA0107
gi|29165615|ref|NC_002745.2|	123115	-	A	0	3	1	6	5	SA0107
gi|29165615|ref|NC_002745.2|	123116	-	G	0	0	0	0	1	SA0107
gi|29165615|ref|NC_002745.2|	123117	-	A	0	1	0	0	2	SA0107
gi|29165615|ref|NC_002745.2|	123118	-	A	0	1	2	2	1	SA0107
gi|29165615|ref|NC_002745.2|	123119	-	G	0	0	0	1	4	SA0107
gi|29165615|ref|NC_002745.2|	123120	-	A	0	1	1	0	2	SA0107
gi|29165615|ref|NC_002745.2|	123125	-	G	0	1	0	0	0	SA0107
gi|29165615|ref|NC_002745.2|	123126	-	U	0	0	0	0	2	SA0107
gi|29165615|ref|NC_002745.2|	123127	-	C	1	2	1	0	3	SA0107
gi|29165615|ref|NC_002745.2|	123128	-	C	0	0	2	0	0	SA0107
gi|29165615|ref|NC_002745.2|	123129	-	G	0	1	0	0	0	SA0107
gi|29165615|ref|NC_002745.2|	123130	-	A	0	0	0	0	1	SA0107
gi|29165615|ref|NC_002745.2|	123131	-	A	0	1	0	0	1	SA0107
gi|29165615|ref|NC_002745.2|	123132	-	C	0	3	1	0	0	SA0107
gi|29165615|ref|NC_002745.2|	123133	-	A	0	1	0	0	1	SA0107
gi|29165615|ref|NC_002745.2|	123135	-	C	1	0	1	1	0	SA0107
gi|29165615|ref|NC_002745.2|	123136	-	G	1	0	1	0	1	SA0107
gi|29165615|ref|NC_002745.2|	123137	-	G	1	1	0	0	3	SA0107
gi|29165615|ref|NC_002745.2|	123138	-	C	1	3	0	1	2	SA0107
gi|29165615|ref|NC_002745.2|	123139	-	A	0	4	2	2	2	SA0107
gi|29165615|ref|NC_002745.2|	123140	-	G	0	0	0	2	0	SA0107
gi|29165615|ref|NC_002745.2|	123141	-	A	0	4	0	0	1	SA0107
gi|29165615|ref|NC_002745.2|	123142	-	A	0	0	3	1	2	SA0107
gi|29165615|ref|NC_002745.2|	123143	-	G	1	0	0	0	0	SA0107
gi|29165615|ref|NC_002745.2|	123144	-	A	0	0	0	1	2	SA0107
gi|29165615|ref|NC_002745.2|	123145	-	A	0	2	0	1	1	SA0107
gi|29165615|ref|NC_002745.2|	123146	-	A	0	5	0	0	0	SA0107
gi|29165615|ref|NC_002745.2|	123147	-	C	1	0	0	6	7	SA0107
gi|29165615|ref|NC_002745.2|	123148	-	G	0	7	2	3	5	SA0107
gi|29165615|ref|NC_002745.2|	123150	-	U	0	2	0	0	3	SA0107
gi|29165615|ref|NC_002745.2|	123151	-	C	1	5	4	2	1	SA0107
gi|29165615|ref|NC_002745.2|	123152	-	C	0	0	1	0	3	SA0107
gi|29165615|ref|NC_002745.2|	123153	-	A	0	2	0	0	1	SA0107
gi|29165615|ref|NC_002745.2|	123155	-	A	0	1	1	0	0	SA0107
gi|29165615|ref|NC_002745.2|	123156	-	C	0	2	0	0	1	SA0107
gi|29165615|ref|NC_002745.2|	123157	-	A	0	0	0	0	3	SA0107
gi|29165615|ref|NC_002745.2|	123158	-	A	0	3	1	0	1	SA0107
gi|29165615|ref|NC_002745.2|	123159	-	C	1	4	1	2	3	SA0107
gi|29165615|ref|NC_002745.2|	123160	-	G	0	1	2	0	2	SA0107
gi|29165615|ref|NC_002745.2|	123161	-	G	1	2	0	0	1	SA0107
gi|29165615|ref|NC_002745.2|	123162	-	C	1	4	1	1	2	SA0107
gi|29165615|ref|NC_002745.2|	123163	-	A	0	3	3	1	2	SA0107
gi|29165615|ref|NC_002745.2|	123164	-	G	0	0	0	2	1	SA0107
gi|29165615|ref|NC_002745.2|	123165	-	A	0	4	0	0	0	SA0107
gi|29165615|ref|NC_002745.2|	123166	-	A	0	0	2	1	2	SA0107
gi|29165615|ref|NC_002745.2|	123167	-	G	1	0	0	0	0	SA0107
gi|29165615|ref|NC_002745.2|	123168	-	A	0	0	0	1	1	SA0107
gi|29165615|ref|NC_002745.2|	123169	-	A	0	2	0	1	1	SA0107
gi|29165615|ref|NC_002745.2|	123170	-	A	0	5	0	0	0	SA0107
gi|29165615|ref|NC_002745.2|	123171	-	U	0	0	0	6	7	SA0107
gi|29165615|ref|NC_002745.2|	123172	-	G	0	7	2	3	5	SA0107
gi|29165615|ref|NC_002745.2|	123173	-	G	0	1	0	0	0	SA0107
gi|29165615|ref|NC_002745.2|	123174	-	U	0	2	0	0	3	SA0107
gi|29165615|ref|NC_002745.2|	123175	-	C	1	6	4	2	2	SA0107
gi|29165615|ref|NC_002745.2|	123176	-	C	0	0	1	1	3	SA0107
gi|29165615|ref|NC_002745.2|	123177	-	A	0	2	0	0	1	SA0107
gi|29165615|ref|NC_002745.2|	123179	-	A	0	1	1	0	0	SA0107
gi|29165615|ref|NC_002745.2|	123180	-	A	0	2	0	0	1	SA0107
gi|29165615|ref|NC_002745.2|	123181	-	A	0	0	0	0	3	SA0107
gi|29165615|ref|NC_002745.2|	123182	-	A	0	3	1	0	1	SA0107
gi|29165615|ref|NC_002745.2|	123183	-	C	1	4	1	2	3	SA0107
gi|29165615|ref|NC_002745.2|	123184	-	A	0	7	4	2	7	SA0107
gi|29165615|ref|NC_002745.2|	123185	-	A	3	4	1	3	7	SA0107
gi|29165615|ref|NC_002745.2|	123186	-	C	0	7	1	4	9	SA0107
gi|29165615|ref|NC_002745.2|	123187	-	A	0	3	1	6	5	SA0107
gi|29165615|ref|NC_002745.2|	123188	-	G	0	0	0	0	1	SA0107
gi|29165615|ref|NC_002745.2|	123189	-	A	0	1	0	0	2	SA0107
gi|29165615|ref|NC_002745.2|	123190	-	A	0	1	2	2	1	SA0107
gi|29165615|ref|NC_002745.2|	123191	-	G	0	0	0	1	4	SA0107
gi|29165615|ref|NC_002745.2|	123192	-	G	0	1	1	0	1	SA0107
gi|29165615|ref|NC_002745.2|	123278	-	A	0	2	0	0	0	SA0107
gi|29165615|ref|NC_002745.2|	123279	-	C	0	0	0	0	1	SA0107

gi|29165615|ref|NC_002745.2|	123280	-	U	2	0	0	0	0	SA0107
gi|29165615|ref|NC_002745.2|	123281	-	U	0	0	2	1	2	SA0107
gi|29165615|ref|NC_002745.2|	123282	-	C	3	0	0	0	0	SA0107
gi|29165615|ref|NC_002745.2|	123285	-	C	0	0	2	0	0	SA0107
gi|29165615|ref|NC_002745.2|	123287	-	A	1	0	0	0	0	SA0107
gi|29165615|ref|NC_002745.2|	123288	-	U	0	0	1	0	0	SA0107
gi|29165615|ref|NC_002745.2|	123291	-	A	0	1	0	0	0	SA0107
gi|29165615|ref|NC_002745.2|	123293	-	C	0	2	0	0	0	SA0107
gi|29165615|ref|NC_002745.2|	123304	-	U	0	1	0	0	0	SA0107
gi|29165615|ref|NC_002745.2|	123306	-	C	0	0	0	0	1	SA0107
gi|29165615|ref|NC_002745.2|	123307	-	A	0	2	0	0	0	SA0107
gi|29165615|ref|NC_002745.2|	123308	-	A	0	1	1	0	0	SA0107
gi|29165615|ref|NC_002745.2|	123309	-	U	0	0	0	1	1	SA0107
gi|29165615|ref|NC_002745.2|	123310	-	C	1	1	1	3	2	SA0107
gi|29165615|ref|NC_002745.2|	123311	-	C	0	3	1	1	1	SA0107
gi|29165615|ref|NC_002745.2|	123312	-	A	0	1	0	0	0	SA0107
gi|29165615|ref|NC_002745.2|	123314	-	U	0	1	0	0	0	SA0107
gi|29165615|ref|NC_002745.2|	123316	-	A	1	1	2	0	2	SA0107
gi|29165615|ref|NC_002745.2|	123339	-	A	0	0	1	0	0	SA0107
gi|29165615|ref|NC_002745.2|	123341	-	C	1	3	2	3	1	SA0107
gi|29165615|ref|NC_002745.2|	123342	-	A	0	5	6	8	17	SA0107
gi|29165615|ref|NC_002745.2|	123343	-	A	0	2	1	2	4	SA0107
gi|29165615|ref|NC_002745.2|	123344	-	C	1	1	1	2	4	SA0107
gi|29165615|ref|NC_002745.2|	123345	-	A	0	13	12	8	23	SA0107
gi|29165615|ref|NC_002745.2|	123346	-	A	0	12	4	6	13	SA0107
gi|29165615|ref|NC_002745.2|	123347	-	G	0	0	1	1	1	SA0107
gi|29165615|ref|NC_002745.2|	123348	-	A	0	3	1	2	4	SA0107
gi|29165615|ref|NC_002745.2|	123349	-	A	0	0	0	1	1	SA0107
gi|29165615|ref|NC_002745.2|	123350	-	A	0	0	0	1	0	SA0107
gi|29165615|ref|NC_002745.2|	123351	-	C	1	1	1	2	5	SA0107
gi|29165615|ref|NC_002745.2|	123352	-	A	0	0	2	1	8	SA0107
gi|29165615|ref|NC_002745.2|	123353	-	A	0	0	1	0	0	SA0107
gi|29165615|ref|NC_002745.2|	123354	-	C	0	0	3	1	5	SA0107
gi|29165615|ref|NC_002745.2|	123355	-	U	0	1	1	1	4	SA0107
gi|29165615|ref|NC_002745.2|	123356	-	U	0	0	0	0	1	SA0107
gi|29165615|ref|NC_002745.2|	123357	-	A	0	1	1	1	3	SA0107
gi|29165615|ref|NC_002745.2|	123358	-	A	1	9	6	1	5	SA0107
gi|29165615|ref|NC_002745.2|	123359	-	A	0	2	0	0	1	SA0107
gi|29165615|ref|NC_002745.2|	123360	-	C	0	4	1	0	0	SA0107
gi|29165615|ref|NC_002745.2|	123363	-	U	0	3	0	0	3	SA0107
gi|29165615|ref|NC_002745.2|	123364	-	A	1	1	1	0	4	SA0107
gi|29165615|ref|NC_002745.2|	123365	-	G	0	1	0	2	0	SA0107
gi|29165615|ref|NC_002745.2|	123366	-	G	0	1	0	1	0	SA0107
gi|29165615|ref|NC_002745.2|	123367	-	C	0	1	1	1	1	SA0107
gi|29165615|ref|NC_002745.2|	123368	-	G	0	2	0	0	0	SA0107
gi|29165615|ref|NC_002745.2|	123369	-	A	0	0	1	0	0	SA0107
gi|29165615|ref|NC_002745.2|	123370	-	A	0	0	0	2	1	SA0107
gi|29165615|ref|NC_002745.2|	123371	-	A	0	5	7	4	11	SA0107
gi|29165615|ref|NC_002745.2|	123373	-	C	1	2	0	0	0	SA0107
gi|29165615|ref|NC_002745.2|	123374	-	C	0	0	0	0	1	SA0107
gi|29165615|ref|NC_002745.2|	123375	-	A	0	0	1	0	0	SA0107
gi|29165615|ref|NC_002745.2|	123376	-	C	0	1	1	0	1	SA0107
gi|29165615|ref|NC_002745.2|	123377	-	G	0	0	1	0	0	SA0107
gi|29165615|ref|NC_002745.2|	123378	-	A	0	2	0	0	3	SA0107
gi|29165615|ref|NC_002745.2|	123379	-	A	0	0	0	0	1	SA0107
gi|29165615|ref|NC_002745.2|	123380	-	C	0	3	1	1	10	SA0107
gi|29165615|ref|NC_002745.2|	123382	-	C	0	1	3	0	1	SA0107
gi|29165615|ref|NC_002745.2|	123383	-	U	0	1	1	0	0	SA0107
gi|29165615|ref|NC_002745.2|	123384	-	A	0	3	5	1	4	SA0107
gi|29165615|ref|NC_002745.2|	123385	-	A	0	9	8	3	9	SA0107
gi|29165615|ref|NC_002745.2|	123386	-	G	0	1	0	0	4	SA0107
gi|29165615|ref|NC_002745.2|	123387	-	U	0	5	1	0	6	SA0107
gi|29165615|ref|NC_002745.2|	123388	-	A	0	2	3	3	2	SA0107
gi|29165615|ref|NC_002745.2|	123389	-	A	0	4	0	1	1	SA0107
gi|29165615|ref|NC_002745.2|	123390	-	A	0	0	0	5	3	SA0107
gi|29165615|ref|NC_002745.2|	123391	-	U	1	8	1	4	2	SA0107
gi|29165615|ref|NC_002745.2|	123392	-	U	0	0	0	1	1	SA0107
gi|29165615|ref|NC_002745.2|	123393	-	G	0	0	0	0	2	SA0107
gi|29165615|ref|NC_002745.2|	123395	-	A	0	1	0	0	1	SA0107
gi|29165615|ref|NC_002745.2|	123396	-	A	0	1	0	0	1	SA0107
gi|29165615|ref|NC_002745.2|	123400	-	C	0	0	0	1	1	SA0107
gi|29165615|ref|NC_002745.2|	123403	-	A	0	0	0	1	4	SA0107
gi|29165615|ref|NC_002745.2|	123404	-	G	0	1	0	0	1	SA0107
gi|29165615|ref|NC_002745.2|	123435	-	U	0	0	0	0	1	SA0107
gi|29165615|ref|NC_002745.2|	123436	-	A	0	0	0	0	2	SA0107
gi|29165615|ref|NC_002745.2|	123437	-	G	0	2	2	1	0	SA0107
gi|29165615|ref|NC_002745.2|	123438	-	A	0	1	0	0	0	SA0107
gi|29165615|ref|NC_002745.2|	123439	-	A	0	3	0	0	1	SA0107
gi|29165615|ref|NC_002745.2|	123440	-	A	0	1	0	0	2	SA0107
gi|29165615|ref|NC_002745.2|	123441	-	A	0	2	0	0	1	SA0107
gi|29165615|ref|NC_002745.2|	123442	-	U	1	3	0	1	1	SA0107

gi|29165615|ref|NC_002745.2|	123467	-	C	1	1	0	1	0	SA0107
gi|29165615|ref|NC_002745.2|	123480	-	C	0	0	0	0	1	SA0107
gi|29165615|ref|NC_002745.2|	123481	-	A	0	1	0	0	0	SA0107
gi|29165615|ref|NC_002745.2|	123482	-	A	0	1	0	0	0	SA0107
gi|29165615|ref|NC_002745.2|	123483	-	U	0	0	0	1	1	SA0107
gi|29165615|ref|NC_002745.2|	123484	-	C	1	1	1	3	2	SA0107
gi|29165615|ref|NC_002745.2|	123485	-	C	0	2	1	0	0	SA0107
gi|29165615|ref|NC_002745.2|	123486	-	G	0	1	0	0	0	SA0107
gi|29165615|ref|NC_002745.2|	123488	-	A	0	1	0	0	1	SA0107
gi|29165615|ref|NC_002745.2|	123490	-	A	1	1	2	0	2	SA0107
gi|29165615|ref|NC_002745.2|	123491	-	A	0	0	1	0	4	SA0107
gi|29165615|ref|NC_002745.2|	123492	-	G	0	0	2	0	1	SA0107
gi|29165615|ref|NC_002745.2|	123493	-	U	0	2	4	4	7	SA0107
gi|29165615|ref|NC_002745.2|	123494	-	U	0	0	1	0	1	SA0107
gi|29165615|ref|NC_002745.2|	123495	-	C	0	0	2	1	0	SA0107
gi|29165615|ref|NC_002745.2|	123496	-	U	0	2	1	0	2	SA0107
gi|29165615|ref|NC_002745.2|	123497	-	A	0	2	2	2	3	SA0107
gi|29165615|ref|NC_002745.2|	123498	-	A	0	1	0	3	1	SA0107
gi|29165615|ref|NC_002745.2|	123499	-	A	0	3	2	2	4	SA0107
gi|29165615|ref|NC_002745.2|	123501	-	U	0	0	0	0	1	SA0107
gi|29165615|ref|NC_002745.2|	123502	-	A	1	0	0	1	0	SA0107
gi|29165615|ref|NC_002745.2|	123503	-	U	0	0	0	0	1	SA0107
gi|29165615|ref|NC_002745.2|	123504	-	C	0	0	1	0	0	SA0107
gi|29165615|ref|NC_002745.2|	123505	-	U	0	2	0	2	1	SA0107
gi|29165615|ref|NC_002745.2|	123507	-	U	0	1	1	0	3	SA0107
gi|29165615|ref|NC_002745.2|	123508	-	C	4	3	1	2	7	SA0107
gi|29165615|ref|NC_002745.2|	123513	-	A	0	0	1	0	0	SA0107
gi|29165615|ref|NC_002745.2|	123515	-	C	1	3	2	3	1	SA0107
gi|29165615|ref|NC_002745.2|	123516	-	A	0	5	6	8	17	SA0107
gi|29165615|ref|NC_002745.2|	123517	-	A	0	2	1	2	4	SA0107
gi|29165615|ref|NC_002745.2|	123518	-	C	1	1	1	2	4	SA0107
gi|29165615|ref|NC_002745.2|	123519	-	A	0	13	12	8	23	SA0107
gi|29165615|ref|NC_002745.2|	123520	-	A	0	12	4	6	13	SA0107
gi|29165615|ref|NC_002745.2|	123521	-	G	0	0	1	1	1	SA0107
gi|29165615|ref|NC_002745.2|	123522	-	A	0	3	1	2	4	SA0107
gi|29165615|ref|NC_002745.2|	123523	-	A	0	0	0	1	1	SA0107
gi|29165615|ref|NC_002745.2|	123524	-	A	0	0	0	1	0	SA0107
gi|29165615|ref|NC_002745.2|	123525	-	C	1	1	1	2	5	SA0107
gi|29165615|ref|NC_002745.2|	123526	-	A	0	0	2	1	8	SA0107
gi|29165615|ref|NC_002745.2|	123527	-	A	0	0	1	0	0	SA0107
gi|29165615|ref|NC_002745.2|	123528	-	C	0	0	3	1	5	SA0107
gi|29165615|ref|NC_002745.2|	123529	-	U	0	1	1	1	4	SA0107
gi|29165615|ref|NC_002745.2|	123530	-	U	0	0	0	0	1	SA0107
gi|29165615|ref|NC_002745.2|	123531	-	U	0	1	1	1	3	SA0107
gi|29165615|ref|NC_002745.2|	123532	-	A	1	9	6	1	5	SA0107
gi|29165615|ref|NC_002745.2|	123533	-	A	0	2	0	0	1	SA0107
gi|29165615|ref|NC_002745.2|	123534	-	C	0	4	1	0	0	SA0107
gi|29165615|ref|NC_002745.2|	123537	-	U	0	3	0	0	3	SA0107
gi|29165615|ref|NC_002745.2|	123538	-	A	1	1	1	0	4	SA0107
gi|29165615|ref|NC_002745.2|	123539	-	G	0	1	0	2	0	SA0107
gi|29165615|ref|NC_002745.2|	123540	-	U	0	1	0	1	0	SA0107
gi|29165615|ref|NC_002745.2|	123541	-	C	0	1	1	1	1	SA0107
gi|29165615|ref|NC_002745.2|	123542	-	G	0	2	0	0	0	SA0107
gi|29165615|ref|NC_002745.2|	123543	-	A	0	0	1	0	0	SA0107
gi|29165615|ref|NC_002745.2|	123544	-	A	0	0	0	2	1	SA0107
gi|29165615|ref|NC_002745.2|	123545	-	A	0	5	7	4	11	SA0107
gi|29165615|ref|NC_002745.2|	123547	-	C	1	2	0	0	0	SA0107
gi|29165615|ref|NC_002745.2|	123548	-	C	0	0	0	0	1	SA0107
gi|29165615|ref|NC_002745.2|	123549	-	A	0	0	1	0	0	SA0107
gi|29165615|ref|NC_002745.2|	123550	-	C	0	1	1	0	1	SA0107
gi|29165615|ref|NC_002745.2|	123551	-	G	0	0	1	0	0	SA0107
gi|29165615|ref|NC_002745.2|	123552	-	A	0	2	0	0	3	SA0107
gi|29165615|ref|NC_002745.2|	123553	-	A	0	0	0	0	1	SA0107
gi|29165615|ref|NC_002745.2|	123554	-	C	0	3	1	1	10	SA0107
gi|29165615|ref|NC_002745.2|	123556	-	C	0	1	3	0	1	SA0107
gi|29165615|ref|NC_002745.2|	123557	-	U	0	1	1	0	0	SA0107
gi|29165615|ref|NC_002745.2|	123558	-	A	0	3	5	1	4	SA0107
gi|29165615|ref|NC_002745.2|	123559	-	A	0	9	8	3	9	SA0107
gi|29165615|ref|NC_002745.2|	123560	-	G	0	1	0	0	4	SA0107
gi|29165615|ref|NC_002745.2|	123561	-	C	0	5	1	0	6	SA0107
gi|29165615|ref|NC_002745.2|	123562	-	A	0	2	3	3	2	SA0107
gi|29165615|ref|NC_002745.2|	123563	-	A	0	4	0	1	1	SA0107
gi|29165615|ref|NC_002745.2|	123564	-	A	0	0	0	5	3	SA0107
gi|29165615|ref|NC_002745.2|	123565	-	U	1	8	1	4	2	SA0107
gi|29165615|ref|NC_002745.2|	123566	-	U	0	0	0	1	1	SA0107
gi|29165615|ref|NC_002745.2|	123567	-	A	0	0	0	0	2	SA0107
gi|29165615|ref|NC_002745.2|	123569	-	A	0	1	0	0	1	SA0107
gi|29165615|ref|NC_002745.2|	123570	-	A	0	1	0	0	1	SA0107
gi|29165615|ref|NC_002745.2|	123574	-	C	0	0	0	1	1	SA0107
gi|29165615|ref|NC_002745.2|	123577	-	A	0	0	0	1	4	SA0107
gi|29165615|ref|NC_002745.2|	123578	-	G	0	1	0	0	1	SA0107

gi|29165615|ref|NC_002745.2|	123613	-	A	0	1	0	0	0 SA0107
gi|29165615|ref|NC_002745.2|	123614	-	A	0	1	0	0	0 SA0107
gi|29165615|ref|NC_002745.2|	123616	-	U	1	0	0	0	0 SA0107
gi|29165615|ref|NC_002745.2|	123630	-	C	3	0	0	0	0 SA0107
gi|29165615|ref|NC_002745.2|	123633	-	C	0	0	2	0	0 SA0107
gi|29165615|ref|NC_002745.2|	123635	-	A	1	0	0	0	0 SA0107
gi|29165615|ref|NC_002745.2|	123636	-	U	0	0	1	0	0 SA0107
gi|29165615|ref|NC_002745.2|	123639	-	A	0	1	0	0	0 SA0107
gi|29165615|ref|NC_002745.2|	123641	-	C	0	2	0	0	0 SA0107
gi|29165615|ref|NC_002745.2|	123652	-	U	0	1	0	0	0 SA0107
gi|29165615|ref|NC_002745.2|	123655	-	A	0	1	0	0	0 SA0107
gi|29165615|ref|NC_002745.2|	123656	-	A	0	0	1	0	0 SA0107
gi|29165615|ref|NC_002745.2|	123659	-	C	0	1	0	1	1 SA0107
gi|29165615|ref|NC_002745.2|	123662	-	A	0	0	0	0	1 SA0107
gi|29165615|ref|NC_002745.2|	123665	-	A	0	0	1	0	3 SA0107
gi|29165615|ref|NC_002745.2|	123666	-	G	0	0	2	0	1 SA0107
gi|29165615|ref|NC_002745.2|	123667	-	U	0	2	4	4	7 SA0107
gi|29165615|ref|NC_002745.2|	123668	-	U	0	0	1	0	1 SA0107
gi|29165615|ref|NC_002745.2|	123669	-	C	0	0	2	1	0 SA0107
gi|29165615|ref|NC_002745.2|	123670	-	U	0	2	1	0	1 SA0107
gi|29165615|ref|NC_002745.2|	123671	-	A	0	2	2	2	3 SA0107
gi|29165615|ref|NC_002745.2|	123672	-	A	0	1	0	3	1 SA0107
gi|29165615|ref|NC_002745.2|	123673	-	A	0	3	2	2	4 SA0107
gi|29165615|ref|NC_002745.2|	123675	-	U	0	0	0	0	1 SA0107
gi|29165615|ref|NC_002745.2|	123676	-	A	1	0	0	1	0 SA0107
gi|29165615|ref|NC_002745.2|	123677	-	U	0	0	0	0	1 SA0107
gi|29165615|ref|NC_002745.2|	123678	-	C	0	0	1	0	0 SA0107
gi|29165615|ref|NC_002745.2|	123679	-	U	0	2	0	2	1 SA0107
gi|29165615|ref|NC_002745.2|	123681	-	C	0	1	1	0	3 SA0107
gi|29165615|ref|NC_002745.2|	123682	-	C	4	3	1	2	7 SA0107
gi|29165615|ref|NC_002745.2|	123792	-	U	0	0	0	0	1 SA0107
gi|29165615|ref|NC_002745.2|	123793	-	A	0	0	0	0	2 SA0107
gi|29165615|ref|NC_002745.2|	123794	-	G	0	2	2	1	0 SA0107
gi|29165615|ref|NC_002745.2|	123795	-	A	0	1	0	0	0 SA0107
gi|29165615|ref|NC_002745.2|	123796	-	A	0	2	0	0	1 SA0107
gi|29165615|ref|NC_002745.2|	123797	-	A	0	1	0	0	2 SA0107
gi|29165615|ref|NC_002745.2|	123798	-	U	0	2	0	0	1 SA0107
gi|29165615|ref|NC_002745.2|	123799	-	U	1	3	0	1	1 SA0107
gi|29165615|ref|NC_002745.2|	123809	-	A	0	2	0	0	0 SA0107
gi|29165615|ref|NC_002745.2|	123810	-	U	0	0	0	0	1 SA0107
gi|29165615|ref|NC_002745.2|	123811	-	U	2	0	0	0	0 SA0107
gi|29165615|ref|NC_002745.2|	123812	-	U	0	0	2	1	2 SA0107
gi|29165615|ref|NC_002745.2|	123824	-	C	1	1	0	1	0 SA0107
gi|29165615|ref|NC_002745.2|	123848	-	A	0	0	0	0	1 SA0107
gi|29165615|ref|NC_002745.2|	123853	-	U	0	0	1	0	2 SA0107
gi|29165615|ref|NC_002745.2|	123872	-	C	0	0	1	0	2 SA0107
gi|29165615|ref|NC_002745.2|	123873	-	A	1	2	1	2	1 SA0107
gi|29165615|ref|NC_002745.2|	123874	-	A	0	2	0	1	2 SA0107
gi|29165615|ref|NC_002745.2|	123875	-	C	0	1	0	1	3 SA0107
gi|29165615|ref|NC_002745.2|	123877	-	C	0	1	0	1	2 SA0107
gi|29165615|ref|NC_002745.2|	123879	-	A	0	0	0	1	0 SA0107
gi|29165615|ref|NC_002745.2|	123972	-	U	1	3	2	9	17 SA0107
gi|29165615|ref|NC_002745.2|	123973	-	G	0	0	0	0	2 SA0107
gi|29165615|ref|NC_002745.2|	123974	-	C	0	1	0	2	0 SA0107
gi|29165615|ref|NC_002745.2|	123975	-	U	0	0	1	6	2 SA0107
gi|29165615|ref|NC_002745.2|	123976	-	U	0	0	3	0	5 SA0107
gi|29165615|ref|NC_002745.2|	124006	-	U	0	0	0	0	1 -
gi|29165615|ref|NC_002745.2|	124007	-	A	0	0	2	0	0 -
gi|29165615|ref|NC_002745.2|	125201	-	A	5	4	2	3	1 -
gi|29165615|ref|NC_002745.2|	125202	-	U	0	0	2	0	0 -
gi|29165615|ref|NC_002745.2|	128205	-	A	0	0	0	0	2 SA0111
gi|29165615|ref|NC_002745.2|	128206	-	U	0	0	1	0	0 SA0111
gi|29165615|ref|NC_002745.2|	149820	-	G	0	1	0	0	0 -
gi|29165615|ref|NC_002745.2|	149851	-	U	0	1	0	0	0 -
gi|29165615|ref|NC_002745.2|	149853	-	C	1	3	4	2	1 -
gi|29165615|ref|NC_002745.2|	149858	-	A	0	0	1	0	0 -
gi|29165615|ref|NC_002745.2|	149863	-	A	0	0	0	0	3 -
gi|29165615|ref|NC_002745.2|	149865	-	C	0	0	1	0	0 -
gi|29165615|ref|NC_002745.2|	157017	-	A	0	1	0	0	1 SA0137
gi|29165615|ref|NC_002745.2|	157018	-	A	0	2	0	0	0 SA0137
gi|29165615|ref|NC_002745.2|	170757	-	C	0	0	0	1	0 -
gi|29165615|ref|NC_002745.2|	188313	-	C	0	1	0	0	0 -
gi|29165615|ref|NC_002745.2|	188314	-	A	0	1	0	2	0 -
gi|29165615|ref|NC_002745.2|	188320	-	C	0	1	1	0	0 -
gi|29165615|ref|NC_002745.2|	188323	-	G	0	1	0	0	0 -
gi|29165615|ref|NC_002745.2|	188324	-	A	0	0	1	0	1 -
gi|29165615|ref|NC_002745.2|	188326	-	G	0	0	0	0	1 -
gi|29165615|ref|NC_002745.2|	188329	-	U	6	2	0	0	3 -
gi|29165615|ref|NC_002745.2|	188333	-	U	2	0	0	0	0 -
gi|29165615|ref|NC_002745.2|	188334	-	A	0	0	0	0	1 -
gi|29165615|ref|NC_002745.2|	188336	-	A	1	1	2	0	0 -

gi|29165615|ref|NC_002745.2|	188337	-	A	0	1	1	0	0	-
gi|29165615|ref|NC_002745.2|	188338	-	C	1	0	0	0	0	-
gi|29165615|ref|NC_002745.2|	188339	-	C	0	0	1	1	3	-
gi|29165615|ref|NC_002745.2|	188341	-	U	0	0	1	0	2	-
gi|29165615|ref|NC_002745.2|	188343	-	A	0	0	1	0	1	-
gi|29165615|ref|NC_002745.2|	188344	-	U	3	2	0	0	1	-
gi|29165615|ref|NC_002745.2|	188348	-	C	0	1	0	2	1	-
gi|29165615|ref|NC_002745.2|	188349	-	U	0	0	0	0	1	-
gi|29165615|ref|NC_002745.2|	188354	-	U	0	1	0	0	0	-
gi|29165615|ref|NC_002745.2|	188356	-	C	1	3	4	2	1	-
gi|29165615|ref|NC_002745.2|	188361	-	A	0	0	1	0	0	-
gi|29165615|ref|NC_002745.2|	188366	-	A	0	0	0	0	4	-
gi|29165615|ref|NC_002745.2|	188368	-	C	0	0	1	0	0	-
gi|29165615|ref|NC_002745.2|	188371	-	U	0	0	0	0	1	-
gi|29165615|ref|NC_002745.2|	188382	-	A	0	0	0	0	2	-
gi|29165615|ref|NC_002745.2|	188386	-	A	0	0	1	0	0	-
gi|29165615|ref|NC_002745.2|	188389	-	A	0	0	1	0	0	SAS005
gi|29165615|ref|NC_002745.2|	188391	-	C	0	1	1	0	0	SAS005
gi|29165615|ref|NC_002745.2|	188400	-	G	0	0	1	0	0	SAS005
gi|29165615|ref|NC_002745.2|	188404	-	U	1	1	0	0	0	SAS005
gi|29165615|ref|NC_002745.2|	188406	-	U	0	1	0	1	1	SAS005
gi|29165615|ref|NC_002745.2|	188410	-	U	0	2	0	0	0	SAS005
gi|29165615|ref|NC_002745.2|	188411	-	C	2	0	0	0	0	SAS005
gi|29165615|ref|NC_002745.2|	188412	-	U	1	0	0	0	0	SAS005
gi|29165615|ref|NC_002745.2|	188416	-	A	0	1	2	0	1	SAS005
gi|29165615|ref|NC_002745.2|	188417	-	G	0	1	0	0	1	SAS005
gi|29165615|ref|NC_002745.2|	188420	-	U	0	1	1	1	1	SAS005
gi|29165615|ref|NC_002745.2|	188421	-	U	0	3	1	4	1	SAS005
gi|29165615|ref|NC_002745.2|	188422	-	U	0	0	1	0	0	SAS005
gi|29165615|ref|NC_002745.2|	188425	-	U	0	0	0	0	1	SAS005
gi|29165615|ref|NC_002745.2|	188426	-	U	0	0	1	3	1	SAS005
gi|29165615|ref|NC_002745.2|	188427	-	A	2	0	1	1	0	SAS005
gi|29165615|ref|NC_002745.2|	188428	-	A	0	1	2	2	0	SAS005
gi|29165615|ref|NC_002745.2|	188429	-	G	0	1	0	2	0	SAS005
gi|29165615|ref|NC_002745.2|	188430	-	A	0	0	1	1	0	SAS005
gi|29165615|ref|NC_002745.2|	188431	-	U	0	0	0	1	0	SAS005
gi|29165615|ref|NC_002745.2|	188432	-	G	0	0	0	0	1	SAS005
gi|29165615|ref|NC_002745.2|	188433	-	U	0	0	0	1	0	SAS005
gi|29165615|ref|NC_002745.2|	188438	-	U	3	2	3	1	10	SAS005
gi|29165615|ref|NC_002745.2|	188439	-	A	0	0	2	0	0	SAS005
gi|29165615|ref|NC_002745.2|	188440	-	C	0	1	2	0	0	SAS005
gi|29165615|ref|NC_002745.2|	188441	-	G	0	0	0	0	1	SAS005
gi|29165615|ref|NC_002745.2|	188446	-	A	0	1	0	0	0	SAS005
gi|29165615|ref|NC_002745.2|	188447	-	C	0	0	0	0	1	SAS005
gi|29165615|ref|NC_002745.2|	188448	-	C	0	0	0	1	2	SAS005
gi|29165615|ref|NC_002745.2|	188453	-	A	0	0	0	1	0	SAS005
gi|29165615|ref|NC_002745.2|	188466	-	U	0	0	0	1	0	SAS005
gi|29165615|ref|NC_002745.2|	188467	-	C	0	0	0	1	0	SAS005
gi|29165615|ref|NC_002745.2|	188479	-	A	0	0	0	0	1	SAS005
gi|29165615|ref|NC_002745.2|	188480	-	G	0	0	0	0	1	SAS005
gi|29165615|ref|NC_002745.2|	188484	-	U	0	0	0	1	0	SAS005
gi|29165615|ref|NC_002745.2|	188486	-	A	0	2	0	0	0	SAS005
gi|29165615|ref|NC_002745.2|	188489	-	A	0	2	0	0	0	SAS005
gi|29165615|ref|NC_002745.2|	188496	-	U	0	0	0	1	0	SAS005
gi|29165615|ref|NC_002745.2|	188497	-	U	0	2	1	0	0	SAS005
gi|29165615|ref|NC_002745.2|	188498	-	A	0	0	0	0	2	SAS005
gi|29165615|ref|NC_002745.2|	188499	-	C	0	0	0	0	1	SAS005
gi|29165615|ref|NC_002745.2|	188500	-	G	0	0	0	2	0	SAS005
gi|29165615|ref|NC_002745.2|	188501	-	U	0	1	0	0	0	SAS005
gi|29165615|ref|NC_002745.2|	188502	-	U	0	1	0	0	0	SAS005
gi|29165615|ref|NC_002745.2|	188503	-	C	2	0	0	1	0	SAS005
gi|29165615|ref|NC_002745.2|	188504	-	A	0	2	0	2	1	SAS005
gi|29165615|ref|NC_002745.2|	188505	-	A	0	5	1	0	1	SAS005
gi|29165615|ref|NC_002745.2|	188506	-	C	0	1	0	2	1	SAS005
gi|29165615|ref|NC_002745.2|	188507	-	C	0	0	1	1	2	SAS005
gi|29165615|ref|NC_002745.2|	188512	-	C	0	0	0	1	0	SAS005
gi|29165615|ref|NC_002745.2|	188517	-	U	0	0	0	0	2	SAS005
gi|29165615|ref|NC_002745.2|	188524	-	U	1	0	0	2	0	SAS005
gi|29165615|ref|NC_002745.2|	188544	-	A	0	0	0	1	0	-
gi|29165615|ref|NC_002745.2|	209553	-	G	0	0	0	0	1	SA0179
gi|29165615|ref|NC_002745.2|	209555	-	A	0	0	0	0	1	SA0179
gi|29165615|ref|NC_002745.2|	209556	-	C	0	0	1	0	0	SA0179
gi|29165615|ref|NC_002745.2|	209560	-	U	0	0	0	1	0	SA0179
gi|29165615|ref|NC_002745.2|	209561	-	G	0	0	1	0	0	SA0179
gi|29165615|ref|NC_002745.2|	211953	-	A	0	0	1	1	1	SA0180
gi|29165615|ref|NC_002745.2|	215222	-	A	0	0	1	0	0	SA0183
gi|29165615|ref|NC_002745.2|	215224	-	U	0	0	0	0	1	SA0183
gi|29165615|ref|NC_002745.2|	215454	-	U	0	1	0	0	0	SA0183
gi|29165615|ref|NC_002745.2|	215456	-	U	0	1	0	0	0	SA0183
gi|29165615|ref|NC_002745.2|	215541	-	A	0	1	2	0	2	SA0183
gi|29165615|ref|NC_002745.2|	215542	-	A	0	1	0	0	0	SA0183

gi|29165615|ref|NC_002745.2|	215550	-	G	0	0	0	0	1	SA0183
gi|29165615|ref|NC_002745.2|	215557	-	A	0	1	0	0	0	SA0183
gi|29165615|ref|NC_002745.2|	215639	-	C	0	0	1	0	0	SA0183
gi|29165615|ref|NC_002745.2|	215644	-	A	0	1	0	0	0	SA0183
gi|29165615|ref|NC_002745.2|	215885	-	A	0	2	0	0	0	SA0183
gi|29165615|ref|NC_002745.2|	215886	-	A	0	0	1	0	1	SA0183
gi|29165615|ref|NC_002745.2|	215911	-	U	0	0	0	1	0	SA0183
gi|29165615|ref|NC_002745.2|	215976	-	C	0	0	0	0	1	SA0183
gi|29165615|ref|NC_002745.2|	215980	-	C	0	0	0	0	1	SA0183
gi|29165615|ref|NC_002745.2|	216194	-	A	0	0	0	0	1	SA0183
gi|29165615|ref|NC_002745.2|	216477	-	G	0	0	2	0	0	SA0183
gi|29165615|ref|NC_002745.2|	216478	-	G	0	0	0	0	1	SA0183
gi|29165615|ref|NC_002745.2|	216480	-	U	1	1	1	0	0	SA0183
gi|29165615|ref|NC_002745.2|	216481	-	U	0	0	1	0	0	SA0183
gi|29165615|ref|NC_002745.2|	216482	-	U	0	1	2	0	1	SA0183
gi|29165615|ref|NC_002745.2|	216483	-	U	0	0	1	0	0	SA0183
gi|29165615|ref|NC_002745.2|	216803	-	U	1	0	0	0	0	SA0183
gi|29165615|ref|NC_002745.2|	216804	-	C	5	0	0	0	0	SA0183
gi|29165615|ref|NC_002745.2|	216805	-	C	0	0	1	0	0	SA0183
gi|29165615|ref|NC_002745.2|	216806	-	A	0	0	1	0	0	SA0183
gi|29165615|ref|NC_002745.2|	216807	-	U	7	0	0	0	0	SA0183
gi|29165615|ref|NC_002745.2|	216808	-	U	0	1	0	0	0	SA0183
gi|29165615|ref|NC_002745.2|	216809	-	G	0	0	1	0	0	SA0183
gi|29165615|ref|NC_002745.2|	216810	-	U	0	1	1	0	0	SA0183
gi|29165615|ref|NC_002745.2|	216812	-	A	0	1	3	0	4	SA0183
gi|29165615|ref|NC_002745.2|	216813	-	U	2	2	2	1	2	SA0183
gi|29165615|ref|NC_002745.2|	216814	-	C	0	1	1	0	0	SA0183
gi|29165615|ref|NC_002745.2|	216821	-	U	1	2	1	0	1	SA0183
gi|29165615|ref|NC_002745.2|	216823	-	G	0	1	0	0	0	SA0183
gi|29165615|ref|NC_002745.2|	216838	-	C	0	2	0	0	0	SA0183
gi|29165615|ref|NC_002745.2|	216839	-	U	0	3	0	6	2	SA0183
gi|29165615|ref|NC_002745.2|	216840	-	G	0	0	3	1	4	SA0183
gi|29165615|ref|NC_002745.2|	216841	-	G	0	0	0	1	0	SA0183
gi|29165615|ref|NC_002745.2|	217031	-	U	0	0	2	0	1	-
gi|29165615|ref|NC_002745.2|	233673	-	U	0	0	1	0	0	SA0198
gi|29165615|ref|NC_002745.2|	233674	-	U	1	0	0	0	0	SA0198
gi|29165615|ref|NC_002745.2|	276837	-	A	0	0	1	0	0	-
gi|29165615|ref|NC_002745.2|	279347	-	G	0	0	0	1	0	-
gi|29165615|ref|NC_002745.2|	283167	-	U	0	0	0	1	0	SA0233
gi|29165615|ref|NC_002745.2|	283232	-	C	0	0	0	0	1	SA0233
gi|29165615|ref|NC_002745.2|	283236	-	C	0	0	0	0	1	SA0233
gi|29165615|ref|NC_002745.2|	311555	-	U	0	0	0	0	1	-
gi|29165615|ref|NC_002745.2|	311556	-	U	0	3	1	3	1	-
gi|29165615|ref|NC_002745.2|	311560	-	U	0	0	0	0	1	-
gi|29165615|ref|NC_002745.2|	311561	-	U	0	0	1	3	1	-
gi|29165615|ref|NC_002745.2|	311562	-	A	0	0	1	0	0	-
gi|29165615|ref|NC_002745.2|	311564	-	G	0	1	0	2	0	-
gi|29165615|ref|NC_002745.2|	311565	-	A	0	0	1	1	0	-
gi|29165615|ref|NC_002745.2|	311566	-	U	0	0	0	1	0	-
gi|29165615|ref|NC_002745.2|	311567	-	G	0	0	0	0	1	-
gi|29165615|ref|NC_002745.2|	311568	-	U	0	0	0	1	0	-
gi|29165615|ref|NC_002745.2|	311573	-	U	3	2	3	1	9	-
gi|29165615|ref|NC_002745.2|	311574	-	A	0	0	2	0	0	-
gi|29165615|ref|NC_002745.2|	311575	-	C	0	1	0	0	0	-
gi|29165615|ref|NC_002745.2|	311583	-	C	0	0	0	1	0	-
gi|29165615|ref|NC_002745.2|	311594	-	U	0	0	1	0	0	-
gi|29165615|ref|NC_002745.2|	311596	-	U	1	1	0	0	0	-
gi|29165615|ref|NC_002745.2|	311606	-	A	0	0	1	1	0	-
gi|29165615|ref|NC_002745.2|	311607	-	G	0	1	0	0	1	-
gi|29165615|ref|NC_002745.2|	311610	-	U	0	1	1	0	1	-
gi|29165615|ref|NC_002745.2|	311611	-	U	0	2	1	4	1	-
gi|29165615|ref|NC_002745.2|	311615	-	U	0	0	0	0	1	-
gi|29165615|ref|NC_002745.2|	311616	-	U	0	0	1	3	1	-
gi|29165615|ref|NC_002745.2|	311617	-	A	0	0	1	0	0	-
gi|29165615|ref|NC_002745.2|	311618	-	A	0	0	0	1	0	-
gi|29165615|ref|NC_002745.2|	311619	-	G	0	1	0	2	0	-
gi|29165615|ref|NC_002745.2|	311620	-	A	0	0	1	1	0	-
gi|29165615|ref|NC_002745.2|	311621	-	U	0	0	0	1	0	-
gi|29165615|ref|NC_002745.2|	311622	-	G	0	0	0	0	1	-
gi|29165615|ref|NC_002745.2|	311623	-	U	0	0	0	1	0	-
gi|29165615|ref|NC_002745.2|	311628	-	U	3	2	3	1	10	-
gi|29165615|ref|NC_002745.2|	311629	-	A	0	0	2	0	0	-
gi|29165615|ref|NC_002745.2|	311630	-	C	0	1	2	0	0	-
gi|29165615|ref|NC_002745.2|	311631	-	G	0	0	0	0	1	-
gi|29165615|ref|NC_002745.2|	311636	-	A	0	1	0	0	0	-
gi|29165615|ref|NC_002745.2|	311637	-	C	0	0	0	0	1	-
gi|29165615|ref|NC_002745.2|	311638	-	C	0	0	0	1	2	-
gi|29165615|ref|NC_002745.2|	311641	-	C	0	1	0	1	2	-
gi|29165615|ref|NC_002745.2|	311649	-	U	0	0	1	0	0	-
gi|29165615|ref|NC_002745.2|	311651	-	U	1	1	0	0	0	-
gi|29165615|ref|NC_002745.2|	311661	-	A	0	1	1	2	0	-

gi|29165615|ref|NC_002745.2|	311662 -	G	0	1	0	0	1	-
gi|29165615|ref|NC_002745.2|	311665 -	U	0	1	1	1	1	-
gi|29165615|ref|NC_002745.2|	311666 -	U	0	3	1	4	1	-
gi|29165615|ref|NC_002745.2|	311667 -	U	0	0	1	0	0	-
gi|29165615|ref|NC_002745.2|	311670 -	U	0	0	0	0	1	-
gi|29165615|ref|NC_002745.2|	311671 -	U	0	0	1	3	1	-
gi|29165615|ref|NC_002745.2|	311672 -	A	2	0	1	1	0	-
gi|29165615|ref|NC_002745.2|	311673 -	A	0	1	2	2	0	-
gi|29165615|ref|NC_002745.2|	311674 -	G	0	1	0	2	0	-
gi|29165615|ref|NC_002745.2|	311675 -	A	0	0	1	1	0	-
gi|29165615|ref|NC_002745.2|	311676 -	U	0	0	0	1	0	-
gi|29165615|ref|NC_002745.2|	311677 -	G	0	0	0	0	1	-
gi|29165615|ref|NC_002745.2|	311678 -	U	0	0	0	1	0	-
gi|29165615|ref|NC_002745.2|	311683 -	U	3	2	3	1	9	-
gi|29165615|ref|NC_002745.2|	311684 -	A	0	0	2	0	0	-
gi|29165615|ref|NC_002745.2|	311685 -	C	0	1	0	0	0	-
gi|29165615|ref|NC_002745.2|	311693 -	C	0	0	0	1	0	-
gi|29165615|ref|NC_002745.2|	311728 -	A	0	0	0	1	0	-
gi|29165615|ref|NC_002745.2|	321472 -	A	0	1	0	0	0	SA0266
gi|29165615|ref|NC_002745.2|	326247 -	A	0	0	1	0	0	SA0270
gi|29165615|ref|NC_002745.2|	360089 -	U	4	0	0	0	0	SA0304
gi|29165615|ref|NC_002745.2|	387961 -	U	0	2	0	0	0	-
gi|29165615|ref|NC_002745.2|	387966 -	U	0	4	0	0	2	-
gi|29165615|ref|NC_002745.2|	387967 -	U	0	1	2	0	0	-
gi|29165615|ref|NC_002745.2|	387976 -	U	0	1	1	2	0	-
gi|29165615|ref|NC_002745.2|	387984 -	C	0	0	1	0	3	-
gi|29165615|ref|NC_002745.2|	387985 -	A	0	1	0	1	0	-
gi|29165615|ref|NC_002745.2|	387992 -	A	0	0	0	1	0	-
gi|29165615|ref|NC_002745.2|	387993 -	C	0	0	0	0	1	-
gi|29165615|ref|NC_002745.2|	388001 -	C	0	0	1	0	0	-
gi|29165615|ref|NC_002745.2|	388007 -	U	1	0	0	0	0	-
gi|29165615|ref|NC_002745.2|	388013 -	U	0	0	0	0	1	-
gi|29165615|ref|NC_002745.2|	388017 -	U	0	0	0	1	0	-
gi|29165615|ref|NC_002745.2|	388048 -	C	0	0	0	1	0	-
gi|29165615|ref|NC_002745.2|	388049 -	A	0	1	0	1	1	-
gi|29165615|ref|NC_002745.2|	388050 -	A	0	5	0	0	2	-
gi|29165615|ref|NC_002745.2|	388051 -	C	0	1	0	2	1	-
gi|29165615|ref|NC_002745.2|	388052 -	C	0	0	1	1	2	-
gi|29165615|ref|NC_002745.2|	388055 -	C	0	1	0	1	2	-
gi|29165615|ref|NC_002745.2|	388057 -	C	0	0	0	1	0	-
gi|29165615|ref|NC_002745.2|	388063 -	U	0	0	1	0	0	-
gi|29165615|ref|NC_002745.2|	388065 -	U	1	1	0	0	0	-
gi|29165615|ref|NC_002745.2|	426654 -	G	0	0	0	0	1	-
gi|29165615|ref|NC_002745.2|	426658 -	U	1	1	1	1	1	-
gi|29165615|ref|NC_002745.2|	426667 -	C	2	1	0	0	0	-
gi|29165615|ref|NC_002745.2|	426672 -	C	0	0	0	0	1	-
gi|29165615|ref|NC_002745.2|	426679 -	A	0	5	2	6	2	-
gi|29165615|ref|NC_002745.2|	426680 -	U	0	1	0	0	0	-
gi|29165615|ref|NC_002745.2|	426681 -	A	0	0	0	0	1	-
gi|29165615|ref|NC_002745.2|	426848 -	U	0	1	0	0	0	-
gi|29165615|ref|NC_002745.2|	427195 -	U	0	0	0	0	1	-
gi|29165615|ref|NC_002745.2|	427406 -	A	0	1	0	0	0	-
gi|29165615|ref|NC_002745.2|	427422 -	A	0	0	0	1	0	-
gi|29165615|ref|NC_002745.2|	427454 -	A	0	0	0	0	1	-
gi|29165615|ref|NC_002745.2|	427472 -	U	0	1	0	0	0	-
gi|29165615|ref|NC_002745.2|	427478 -	A	0	0	0	0	1	-
gi|29165615|ref|NC_002745.2|	427479 -	U	0	0	1	0	1	-
gi|29165615|ref|NC_002745.2|	427480 -	G	0	1	0	0	0	-
gi|29165615|ref|NC_002745.2|	427489 -	U	0	0	0	0	1	-
gi|29165615|ref|NC_002745.2|	427493 -	C	0	0	1	0	0	-
gi|29165615|ref|NC_002745.2|	427494 -	C	1	1	0	0	0	-
gi|29165615|ref|NC_002745.2|	450935 -	G	0	0	1	0	0	-
gi|29165615|ref|NC_002745.2|	450943 -	A	1	0	3	4	1	-
gi|29165615|ref|NC_002745.2|	450948 -	A	0	0	1	0	0	-
gi|29165615|ref|NC_002745.2|	450966 -	U	2	0	0	1	0	-
gi|29165615|ref|NC_002745.2|	451032 -	U	0	0	1	0	0	-
gi|29165615|ref|NC_002745.2|	452503 -	C	0	1	0	0	0	-
gi|29165615|ref|NC_002745.2|	453149 -	U	1	0	0	0	0	-
gi|29165615|ref|NC_002745.2|	459243 -	A	0	0	0	2	0	-
gi|29165615|ref|NC_002745.2|	506397 -	A	0	0	1	0	0	-
gi|29165615|ref|NC_002745.2|	506523 -	G	0	1	0	0	0	-
gi|29165615|ref|NC_002745.2|	506590 -	G	0	0	0	1	0	-
gi|29165615|ref|NC_002745.2|	506617 -	U	0	1	1	0	1	-
gi|29165615|ref|NC_002745.2|	506619 -	U	2	2	0	3	1	-
gi|29165615|ref|NC_002745.2|	506620 -	U	1	0	0	0	1	-
gi|29165615|ref|NC_002745.2|	506621 -	G	0	0	0	0	1	-
gi|29165615|ref|NC_002745.2|	506622 -	U	0	0	3	0	0	-
gi|29165615|ref|NC_002745.2|	506654 -	A	0	1	0	0	0	-
gi|29165615|ref|NC_002745.2|	506655 -	U	0	0	0	1	0	-
gi|29165615|ref|NC_002745.2|	506672 -	G	0	1	0	0	0	-
gi|29165615|ref|NC_002745.2|	506733 -	A	0	0	1	1	1	-

gi|29165615|ref|NC_002745.2|	506738 -	C	0	1	0	0	0 -
gi|29165615|ref|NC_002745.2|	506739 -	A	0	0	1	0	0 -
gi|29165615|ref|NC_002745.2|	506745 -	C	0	0	0	2	0 -
gi|29165615|ref|NC_002745.2|	506750 -	A	0	1	0	0	0 -
gi|29165615|ref|NC_002745.2|	506863 -	U	0	0	0	1	0 -
gi|29165615|ref|NC_002745.2|	506893 -	C	0	2	0	0	0 -
gi|29165615|ref|NC_002745.2|	506934 -	U	0	1	0	0	0 -
gi|29165615|ref|NC_002745.2|	506942 -	C	0	0	0	2	0 -
gi|29165615|ref|NC_002745.2|	506945 -	U	0	1	0	0	0 -
gi|29165615|ref|NC_002745.2|	506965 -	G	0	1	1	2	0 -
gi|29165615|ref|NC_002745.2|	506966 -	A	0	0	0	4	0 -
gi|29165615|ref|NC_002745.2|	506967 -	C	0	5	0	1	0 -
gi|29165615|ref|NC_002745.2|	506968 -	C	2	4	1	4	0 -
gi|29165615|ref|NC_002745.2|	506969 -	A	2	2	1	0	0 -
gi|29165615|ref|NC_002745.2|	506970 -	U	8	6	1	2	4 -
gi|29165615|ref|NC_002745.2|	506971 -	C	19	23	6	19	4 -
gi|29165615|ref|NC_002745.2|	506972 -	A	3	13	6	9	4 -
gi|29165615|ref|NC_002745.2|	506973 -	G	3	12	0	5	2 -
gi|29165615|ref|NC_002745.2|	506974 -	G	0	3	1	1	0 -
gi|29165615|ref|NC_002745.2|	506975 -	U	26	24	15	23	6 -
gi|29165615|ref|NC_002745.2|	506976 -	G	7	19	5	12	5 -
gi|29165615|ref|NC_002745.2|	506977 -	C	3	7	1	3	0 -
gi|29165615|ref|NC_002745.2|	507005 -	C	2	0	0	0	0 -
gi|29165615|ref|NC_002745.2|	507007 -	C	1	0	0	0	0 -
gi|29165615|ref|NC_002745.2|	507009 -	A	0	3	0	0	0 -
gi|29165615|ref|NC_002745.2|	507095 -	C	0	0	0	0	1 -
gi|29165615|ref|NC_002745.2|	507096 -	C	2	3	1	2	0 -
gi|29165615|ref|NC_002745.2|	507097 -	C	0	2	1	2	0 -
gi|29165615|ref|NC_002745.2|	507098 -	U	1	1	1	1	4 -
gi|29165615|ref|NC_002745.2|	507099 -	G	1	0	2	0	0 -
gi|29165615|ref|NC_002745.2|	507139 -	G	0	0	0	1	0 -
gi|29165615|ref|NC_002745.2|	507168 -	G	0	0	0	1	0 -
gi|29165615|ref|NC_002745.2|	507286 -	A	2	2	1	2	3 -
gi|29165615|ref|NC_002745.2|	507287 -	A	1	3	1	0	1 -
gi|29165615|ref|NC_002745.2|	508202 -	U	0	3	0	0	0 -
gi|29165615|ref|NC_002745.2|	508381 -	U	0	0	0	1	0 -
gi|29165615|ref|NC_002745.2|	508698 -	A	0	0	0	1	0 -
gi|29165615|ref|NC_002745.2|	508767 -	A	1	0	0	0	0 -
gi|29165615|ref|NC_002745.2|	508769 -	U	0	0	0	1	0 -
gi|29165615|ref|NC_002745.2|	508775 -	G	0	0	0	1	0 -
gi|29165615|ref|NC_002745.2|	508778 -	A	0	1	0	0	1 -
gi|29165615|ref|NC_002745.2|	508781 -	A	12	22	23	23	1 -
gi|29165615|ref|NC_002745.2|	508789 -	C	0	1	0	0	0 -
gi|29165615|ref|NC_002745.2|	508811 -	G	1	1	0	0	0 -
gi|29165615|ref|NC_002745.2|	508819 -	A	0	1	0	0	0 -
gi|29165615|ref|NC_002745.2|	508835 -	C	0	0	0	1	0 -
gi|29165615|ref|NC_002745.2|	508845 -	A	0	0	0	1	0 -
gi|29165615|ref|NC_002745.2|	508847 -	U	0	0	0	1	0 -
gi|29165615|ref|NC_002745.2|	508848 -	U	1	0	0	0	1 -
gi|29165615|ref|NC_002745.2|	509033 -	U	0	0	0	0	1 -
gi|29165615|ref|NC_002745.2|	509066 -	G	1	0	0	0	0 -
gi|29165615|ref|NC_002745.2|	509068 -	U	0	1	0	0	0 -
gi|29165615|ref|NC_002745.2|	509069 -	C	2	0	0	0	0 -
gi|29165615|ref|NC_002745.2|	509070 -	G	0	0	2	0	0 -
gi|29165615|ref|NC_002745.2|	509092 -	C	3	0	1	0	0 -
gi|29165615|ref|NC_002745.2|	509093 -	A	0	0	1	0	0 -
gi|29165615|ref|NC_002745.2|	509094 -	U	0	2	0	0	0 -
gi|29165615|ref|NC_002745.2|	509095 -	C	0	0	3	0	1 -
gi|29165615|ref|NC_002745.2|	509096 -	U	0	0	0	0	2 -
gi|29165615|ref|NC_002745.2|	509097 -	C	0	1	0	0	1 -
gi|29165615|ref|NC_002745.2|	509098 -	G	6	4	4	6	1 -
gi|29165615|ref|NC_002745.2|	509112 -	C	0	0	0	0	1 -
gi|29165615|ref|NC_002745.2|	509113 -	C	0	1	0	0	0 -
gi|29165615|ref|NC_002745.2|	509114 -	C	0	2	0	0	0 -
gi|29165615|ref|NC_002745.2|	509115 -	C	0	2	0	0	0 -
gi|29165615|ref|NC_002745.2|	509174 -	C	0	1	0	0	0 -
gi|29165615|ref|NC_002745.2|	509175 -	A	0	1	1	0	0 -
gi|29165615|ref|NC_002745.2|	509177 -	U	1	0	0	0	0 -
gi|29165615|ref|NC_002745.2|	509179 -	U	0	1	0	0	0 -
gi|29165615|ref|NC_002745.2|	509213 -	U	0	1	0	0	0 -
gi|29165615|ref|NC_002745.2|	509242 -	U	1	0	0	0	0 -
gi|29165615|ref|NC_002745.2|	509256 -	C	0	0	1	1	0 -
gi|29165615|ref|NC_002745.2|	509257 -	C	0	0	0	1	0 -
gi|29165615|ref|NC_002745.2|	509259 -	U	0	0	0	1	1 -
gi|29165615|ref|NC_002745.2|	509260 -	U	2	2	1	0	0 -
gi|29165615|ref|NC_002745.2|	509261 -	U	2	1	0	4	1 -
gi|29165615|ref|NC_002745.2|	509262 -	C	2	1	0	0	2 -
gi|29165615|ref|NC_002745.2|	509263 -	C	0	1	0	0	0 -
gi|29165615|ref|NC_002745.2|	509265 -	A	1	1	0	0	0 -
gi|29165615|ref|NC_002745.2|	509282 -	U	0	1	0	0	0 -
gi|29165615|ref|NC_002745.2|	509370 -	C	0	1	0	0	0 -

gi|29165615|ref|NC_002745.2|	509371 -	C	0	0	0	1	0	-
gi|29165615|ref|NC_002745.2|	509388 -	U	0	1	0	0	0	-
gi|29165615|ref|NC_002745.2|	509393 -	A	0	1	1	0	0	-
gi|29165615|ref|NC_002745.2|	509421 -	U	3	0	0	0	0	-
gi|29165615|ref|NC_002745.2|	509479 -	U	0	0	1	0	0	-
gi|29165615|ref|NC_002745.2|	509484 -	G	0	0	0	0	1	-
gi|29165615|ref|NC_002745.2|	509532 -	U	0	0	1	0	0	-
gi|29165615|ref|NC_002745.2|	509536 -	A	0	0	0	2	0	-
gi|29165615|ref|NC_002745.2|	509570 -	G	17	17	8	17	4	-
gi|29165615|ref|NC_002745.2|	509607 -	U	0	1	0	0	0	-
gi|29165615|ref|NC_002745.2|	509609 -	U	1	0	0	0	0	-
gi|29165615|ref|NC_002745.2|	509655 -	C	3	2	1	1	0	-
gi|29165615|ref|NC_002745.2|	509658 -	C	1	0	0	0	0	-
gi|29165615|ref|NC_002745.2|	509666 -	U	0	3	0	0	0	-
gi|29165615|ref|NC_002745.2|	509766 -	U	1	0	0	0	0	-
gi|29165615|ref|NC_002745.2|	509879 -	C	0	1	0	0	0	-
gi|29165615|ref|NC_002745.2|	509880 -	G	0	1	0	0	0	-
gi|29165615|ref|NC_002745.2|	509905 -	C	0	0	0	2	0	-
gi|29165615|ref|NC_002745.2|	509931 -	C	0	0	0	1	0	-
gi|29165615|ref|NC_002745.2|	509932 -	U	1	2	0	0	0	-
gi|29165615|ref|NC_002745.2|	509933 -	U	0	0	0	1	0	-
gi|29165615|ref|NC_002745.2|	509935 -	C	1	1	0	0	0	-
gi|29165615|ref|NC_002745.2|	509936 -	C	0	1	0	0	0	-
gi|29165615|ref|NC_002745.2|	509986 -	G	0	0	0	0	1	-
gi|29165615|ref|NC_002745.2|	509988 -	U	0	0	1	0	0	-
gi|29165615|ref|NC_002745.2|	510036 -	U	6	8	4	3	1	-
gi|29165615|ref|NC_002745.2|	510037 -	C	3	0	1	1	0	-
gi|29165615|ref|NC_002745.2|	510040 -	C	0	1	0	0	0	-
gi|29165615|ref|NC_002745.2|	510041 -	G	0	0	1	0	0	-
gi|29165615|ref|NC_002745.2|	510043 -	C	0	0	1	0	0	-
gi|29165615|ref|NC_002745.2|	510053 -	G	1	0	0	0	0	-
gi|29165615|ref|NC_002745.2|	510073 -	C	0	0	0	2	0	-
gi|29165615|ref|NC_002745.2|	510076 -	A	0	0	1	0	0	-
gi|29165615|ref|NC_002745.2|	510077 -	C	1	1	0	2	0	-
gi|29165615|ref|NC_002745.2|	510078 -	C	1	2	3	2	0	-
gi|29165615|ref|NC_002745.2|	510079 -	A	1	0	1	0	0	-
gi|29165615|ref|NC_002745.2|	510169 -	C	0	1	1	0	1	-
gi|29165615|ref|NC_002745.2|	510170 -	A	0	0	0	1	0	-
gi|29165615|ref|NC_002745.2|	510171 -	A	0	1	0	0	0	-
gi|29165615|ref|NC_002745.2|	510172 -	G	0	1	0	0	0	-
gi|29165615|ref|NC_002745.2|	510173 -	G	0	1	0	0	0	-
gi|29165615|ref|NC_002745.2|	510203 -	G	0	0	0	0	1	-
gi|29165615|ref|NC_002745.2|	510204 -	U	1	1	1	0	0	-
gi|29165615|ref|NC_002745.2|	510205 -	G	1	0	0	0	0	-
gi|29165615|ref|NC_002745.2|	510206 -	A	2	1	0	0	0	-
gi|29165615|ref|NC_002745.2|	510207 -	C	0	1	0	0	0	-
gi|29165615|ref|NC_002745.2|	510208 -	A	0	0	1	0	0	-
gi|29165615|ref|NC_002745.2|	510220 -	U	0	1	0	3	0	-
gi|29165615|ref|NC_002745.2|	510229 -	U	0	0	1	0	0	-
gi|29165615|ref|NC_002745.2|	510262 -	G	0	1	0	0	0	-
gi|29165615|ref|NC_002745.2|	510264 -	U	0	1	1	0	0	-
gi|29165615|ref|NC_002745.2|	510265 -	G	0	1	0	0	0	-
gi|29165615|ref|NC_002745.2|	510268 -	C	1	0	0	0	0	-
gi|29165615|ref|NC_002745.2|	510727 -	G	0	1	0	0	0	-
gi|29165615|ref|NC_002745.2|	510738 -	C	0	1	0	0	0	-
gi|29165615|ref|NC_002745.2|	510770 -	A	0	0	0	0	1	-
gi|29165615|ref|NC_002745.2|	510857 -	C	0	0	1	0	0	-
gi|29165615|ref|NC_002745.2|	510877 -	U	1	0	0	0	0	-
gi|29165615|ref|NC_002745.2|	510879 -	C	0	0	0	0	2	-
gi|29165615|ref|NC_002745.2|	511020 -	U	0	0	0	1	0	-
gi|29165615|ref|NC_002745.2|	511257 -	C	0	1	0	0	0	-
gi|29165615|ref|NC_002745.2|	511295 -	U	2	0	0	0	0	-
gi|29165615|ref|NC_002745.2|	511553 -	G	0	1	0	0	0	-
gi|29165615|ref|NC_002745.2|	511574 -	U	2	0	0	0	0	-
gi|29165615|ref|NC_002745.2|	511830 -	G	0	1	0	0	0	-
gi|29165615|ref|NC_002745.2|	511851 -	U	2	0	0	0	0	-
gi|29165615|ref|NC_002745.2|	549689 -	C	0	1	0	0	0	-
gi|29165615|ref|NC_002745.2|	549727 -	U	2	0	0	0	0	-
gi|29165615|ref|NC_002745.2|	550825 -	A	0	0	1	0	0	-
gi|29165615|ref|NC_002745.2|	550951 -	G	0	1	0	0	0	-
gi|29165615|ref|NC_002745.2|	551018 -	G	0	0	0	1	0	-
gi|29165615|ref|NC_002745.2|	551045 -	U	0	1	1	0	1	-
gi|29165615|ref|NC_002745.2|	551047 -	U	2	2	0	3	1	-
gi|29165615|ref|NC_002745.2|	551048 -	U	1	0	0	0	1	-
gi|29165615|ref|NC_002745.2|	551049 -	G	0	0	0	0	1	-
gi|29165615|ref|NC_002745.2|	551050 -	U	0	0	3	0	0	-
gi|29165615|ref|NC_002745.2|	551082 -	A	0	1	0	0	0	-
gi|29165615|ref|NC_002745.2|	551083 -	U	0	0	0	1	0	-
gi|29165615|ref|NC_002745.2|	551100 -	G	0	1	0	0	0	-
gi|29165615|ref|NC_002745.2|	551161 -	A	0	0	1	1	1	-
gi|29165615|ref|NC_002745.2|	551166 -	C	0	1	0	0	0	-

gi|29165615|ref|NC_002745.2|	551167	-	A	0	0	1	0	0 -
gi|29165615|ref|NC_002745.2|	551173	-	C	0	0	0	2	0 -
gi|29165615|ref|NC_002745.2|	551178	-	A	0	1	0	0	0 -
gi|29165615|ref|NC_002745.2|	551291	-	U	0	0	0	1	0 -
gi|29165615|ref|NC_002745.2|	551321	-	C	0	2	0	0	0 -
gi|29165615|ref|NC_002745.2|	551362	-	U	0	1	0	0	0 -
gi|29165615|ref|NC_002745.2|	551370	-	C	0	0	0	2	0 -
gi|29165615|ref|NC_002745.2|	551373	-	U	0	1	0	0	0 -
gi|29165615|ref|NC_002745.2|	551393	-	G	0	1	1	2	0 -
gi|29165615|ref|NC_002745.2|	551394	-	A	0	0	0	4	0 -
gi|29165615|ref|NC_002745.2|	551395	-	C	0	5	0	1	0 -
gi|29165615|ref|NC_002745.2|	551396	-	C	2	4	1	4	0 -
gi|29165615|ref|NC_002745.2|	551397	-	A	2	2	1	0	0 -
gi|29165615|ref|NC_002745.2|	551398	-	U	8	6	1	2	4 -
gi|29165615|ref|NC_002745.2|	551399	-	C	19	23	6	19	4 -
gi|29165615|ref|NC_002745.2|	551400	-	A	3	13	6	9	4 -
gi|29165615|ref|NC_002745.2|	551401	-	G	3	12	0	5	2 -
gi|29165615|ref|NC_002745.2|	551402	-	G	0	3	1	1	0 -
gi|29165615|ref|NC_002745.2|	551403	-	U	26	24	15	23	6 -
gi|29165615|ref|NC_002745.2|	551404	-	G	7	19	5	12	5 -
gi|29165615|ref|NC_002745.2|	551405	-	C	3	7	1	3	0 -
gi|29165615|ref|NC_002745.2|	551433	-	C	2	0	0	0	0 -
gi|29165615|ref|NC_002745.2|	551435	-	C	1	0	0	0	0 -
gi|29165615|ref|NC_002745.2|	551437	-	A	0	3	0	0	0 -
gi|29165615|ref|NC_002745.2|	551523	-	C	0	0	0	0	1 -
gi|29165615|ref|NC_002745.2|	551524	-	C	2	3	1	2	0 -
gi|29165615|ref|NC_002745.2|	551525	-	C	0	2	1	2	0 -
gi|29165615|ref|NC_002745.2|	551526	-	U	1	1	1	1	4 -
gi|29165615|ref|NC_002745.2|	551527	-	G	1	0	2	0	0 -
gi|29165615|ref|NC_002745.2|	551567	-	G	0	0	0	1	0 -
gi|29165615|ref|NC_002745.2|	551596	-	G	0	0	0	1	0 -
gi|29165615|ref|NC_002745.2|	551714	-	A	2	2	1	2	3 -
gi|29165615|ref|NC_002745.2|	551715	-	A	1	3	1	0	1 -
gi|29165615|ref|NC_002745.2|	552493	-	U	0	3	0	0	0 -
gi|29165615|ref|NC_002745.2|	552672	-	U	0	0	0	1	0 -
gi|29165615|ref|NC_002745.2|	552989	-	A	0	0	0	1	0 -
gi|29165615|ref|NC_002745.2|	553058	-	A	1	0	0	0	0 -
gi|29165615|ref|NC_002745.2|	553060	-	U	0	0	0	1	0 -
gi|29165615|ref|NC_002745.2|	553066	-	G	0	0	0	1	0 -
gi|29165615|ref|NC_002745.2|	553069	-	A	0	1	0	0	1 -
gi|29165615|ref|NC_002745.2|	553072	-	A	12	22	23	23	1 -
gi|29165615|ref|NC_002745.2|	553080	-	C	0	1	0	0	0 -
gi|29165615|ref|NC_002745.2|	553102	-	G	1	1	0	0	0 -
gi|29165615|ref|NC_002745.2|	553110	-	A	0	1	0	0	0 -
gi|29165615|ref|NC_002745.2|	553126	-	C	0	0	0	1	0 -
gi|29165615|ref|NC_002745.2|	553136	-	A	0	0	0	1	0 -
gi|29165615|ref|NC_002745.2|	553138	-	U	0	0	0	1	0 -
gi|29165615|ref|NC_002745.2|	553139	-	U	1	0	0	0	1 -
gi|29165615|ref|NC_002745.2|	553324	-	U	0	0	0	0	1 -
gi|29165615|ref|NC_002745.2|	553357	-	G	1	0	0	0	0 -
gi|29165615|ref|NC_002745.2|	553359	-	U	0	1	0	0	0 -
gi|29165615|ref|NC_002745.2|	553360	-	C	2	0	0	0	0 -
gi|29165615|ref|NC_002745.2|	553361	-	G	0	0	2	0	0 -
gi|29165615|ref|NC_002745.2|	553383	-	C	3	0	1	0	0 -
gi|29165615|ref|NC_002745.2|	553384	-	A	0	0	1	0	0 -
gi|29165615|ref|NC_002745.2|	553385	-	U	0	2	0	0	0 -
gi|29165615|ref|NC_002745.2|	553386	-	C	0	0	3	0	1 -
gi|29165615|ref|NC_002745.2|	553387	-	U	0	0	0	0	2 -
gi|29165615|ref|NC_002745.2|	553388	-	C	0	1	0	0	1 -
gi|29165615|ref|NC_002745.2|	553389	-	G	6	4	4	6	1 -
gi|29165615|ref|NC_002745.2|	553403	-	C	0	0	0	0	1 -
gi|29165615|ref|NC_002745.2|	553404	-	C	0	1	0	0	0 -
gi|29165615|ref|NC_002745.2|	553405	-	C	0	2	0	0	0 -
gi|29165615|ref|NC_002745.2|	553406	-	C	0	2	0	0	0 -
gi|29165615|ref|NC_002745.2|	553465	-	C	0	1	0	0	0 -
gi|29165615|ref|NC_002745.2|	553466	-	A	0	1	1	0	0 -
gi|29165615|ref|NC_002745.2|	553468	-	U	1	0	0	0	0 -
gi|29165615|ref|NC_002745.2|	553470	-	U	0	1	0	0	0 -
gi|29165615|ref|NC_002745.2|	553504	-	U	0	1	0	0	0 -
gi|29165615|ref|NC_002745.2|	553533	-	U	1	0	0	0	0 -
gi|29165615|ref|NC_002745.2|	553547	-	C	0	0	1	1	0 -
gi|29165615|ref|NC_002745.2|	553548	-	C	0	0	0	1	0 -
gi|29165615|ref|NC_002745.2|	553550	-	U	0	0	0	1	1 -
gi|29165615|ref|NC_002745.2|	553551	-	U	2	2	1	0	0 -
gi|29165615|ref|NC_002745.2|	553552	-	U	2	1	0	4	1 -
gi|29165615|ref|NC_002745.2|	553553	-	C	2	1	0	0	2 -
gi|29165615|ref|NC_002745.2|	553554	-	C	0	1	0	0	0 -
gi|29165615|ref|NC_002745.2|	553556	-	A	1	1	0	0	0 -
gi|29165615|ref|NC_002745.2|	553573	-	U	0	1	0	0	0 -
gi|29165615|ref|NC_002745.2|	553661	-	C	0	1	0	0	0 -
gi|29165615|ref|NC_002745.2|	553662	-	C	0	0	0	1	0 -

gi|29165615|ref|NC_002745.2|	553679 -	U	0	1	0	0	0	-
gi|29165615|ref|NC_002745.2|	553684 -	A	0	1	1	0	0	-
gi|29165615|ref|NC_002745.2|	553712 -	U	3	0	0	0	0	-
gi|29165615|ref|NC_002745.2|	553770 -	U	0	0	1	0	0	-
gi|29165615|ref|NC_002745.2|	553775 -	G	0	0	0	0	1	-
gi|29165615|ref|NC_002745.2|	553823 -	U	0	0	1	0	0	-
gi|29165615|ref|NC_002745.2|	553827 -	A	0	0	0	2	0	-
gi|29165615|ref|NC_002745.2|	553861 -	G	17	17	8	17	4	-
gi|29165615|ref|NC_002745.2|	553898 -	U	0	1	0	0	0	-
gi|29165615|ref|NC_002745.2|	553900 -	U	1	0	0	0	0	-
gi|29165615|ref|NC_002745.2|	553946 -	C	3	2	1	1	0	-
gi|29165615|ref|NC_002745.2|	553949 -	C	1	0	0	0	0	-
gi|29165615|ref|NC_002745.2|	553957 -	U	0	3	0	0	0	-
gi|29165615|ref|NC_002745.2|	554057 -	U	1	0	0	0	0	-
gi|29165615|ref|NC_002745.2|	554170 -	C	0	1	0	0	0	-
gi|29165615|ref|NC_002745.2|	554171 -	G	0	1	0	0	0	-
gi|29165615|ref|NC_002745.2|	554196 -	C	0	0	0	2	0	-
gi|29165615|ref|NC_002745.2|	554222 -	C	0	0	0	1	0	-
gi|29165615|ref|NC_002745.2|	554223 -	U	1	2	0	0	0	-
gi|29165615|ref|NC_002745.2|	554224 -	U	0	0	0	1	0	-
gi|29165615|ref|NC_002745.2|	554226 -	C	1	1	0	0	0	-
gi|29165615|ref|NC_002745.2|	554227 -	C	0	1	0	0	0	-
gi|29165615|ref|NC_002745.2|	554277 -	G	0	0	0	0	1	-
gi|29165615|ref|NC_002745.2|	554279 -	U	0	0	1	0	0	-
gi|29165615|ref|NC_002745.2|	554327 -	U	6	8	4	3	1	-
gi|29165615|ref|NC_002745.2|	554328 -	C	3	0	1	1	0	-
gi|29165615|ref|NC_002745.2|	554331 -	C	0	1	0	0	0	-
gi|29165615|ref|NC_002745.2|	554332 -	G	0	0	1	0	0	-
gi|29165615|ref|NC_002745.2|	554334 -	C	0	0	1	0	0	-
gi|29165615|ref|NC_002745.2|	554344 -	G	1	0	0	0	0	-
gi|29165615|ref|NC_002745.2|	554364 -	C	0	0	0	2	0	-
gi|29165615|ref|NC_002745.2|	554367 -	A	0	0	1	0	0	-
gi|29165615|ref|NC_002745.2|	554368 -	C	1	1	0	2	0	-
gi|29165615|ref|NC_002745.2|	554369 -	C	1	2	3	2	0	-
gi|29165615|ref|NC_002745.2|	554370 -	A	1	0	1	0	0	-
gi|29165615|ref|NC_002745.2|	554460 -	C	0	1	1	0	1	-
gi|29165615|ref|NC_002745.2|	554461 -	A	0	0	0	1	0	-
gi|29165615|ref|NC_002745.2|	554462 -	A	0	1	0	0	0	-
gi|29165615|ref|NC_002745.2|	554463 -	G	0	1	0	0	0	-
gi|29165615|ref|NC_002745.2|	554464 -	G	0	1	0	0	0	-
gi|29165615|ref|NC_002745.2|	554494 -	G	0	0	0	0	1	-
gi|29165615|ref|NC_002745.2|	554495 -	U	1	1	1	0	0	-
gi|29165615|ref|NC_002745.2|	554496 -	G	1	0	0	0	0	-
gi|29165615|ref|NC_002745.2|	554497 -	A	2	1	0	0	0	-
gi|29165615|ref|NC_002745.2|	554498 -	C	0	1	0	0	0	-
gi|29165615|ref|NC_002745.2|	554499 -	A	0	0	1	0	0	-
gi|29165615|ref|NC_002745.2|	554511 -	U	0	1	0	3	0	-
gi|29165615|ref|NC_002745.2|	554520 -	U	0	0	1	0	0	-
gi|29165615|ref|NC_002745.2|	554553 -	G	0	1	0	0	0	-
gi|29165615|ref|NC_002745.2|	554555 -	U	0	1	1	0	0	-
gi|29165615|ref|NC_002745.2|	554556 -	G	0	1	0	0	0	-
gi|29165615|ref|NC_002745.2|	554559 -	C	1	0	0	0	0	-
gi|29165615|ref|NC_002745.2|	555018 -	G	0	1	0	0	0	-
gi|29165615|ref|NC_002745.2|	555029 -	C	0	1	0	0	0	-
gi|29165615|ref|NC_002745.2|	555061 -	A	0	0	0	0	1	-
gi|29165615|ref|NC_002745.2|	555148 -	C	0	0	1	0	0	-
gi|29165615|ref|NC_002745.2|	555168 -	U	1	0	0	0	0	-
gi|29165615|ref|NC_002745.2|	555170 -	C	0	0	0	0	2	-
gi|29165615|ref|NC_002745.2|	555311 -	U	0	0	0	1	0	-
gi|29165615|ref|NC_002745.2|	555548 -	C	0	1	0	0	0	-
gi|29165615|ref|NC_002745.2|	555586 -	U	2	0	0	0	0	-
gi|29165615|ref|NC_002745.2|	555842 -	G	0	1	0	0	0	-
gi|29165615|ref|NC_002745.2|	592188 -	C	0	0	0	1	0	-
gi|29165615|ref|NC_002745.2|	595468 -	A	0	1	0	0	0	-
gi|29165615|ref|NC_002745.2|	601907 -	A	0	0	1	0	0	SA0514
gi|29165615|ref|NC_002745.2|	651595 -	A	0	1	0	2	0	-
gi|29165615|ref|NC_002745.2|	651601 -	C	0	1	1	0	0	-
gi|29165615|ref|NC_002745.2|	651604 -	G	0	1	0	0	0	-
gi|29165615|ref|NC_002745.2|	651605 -	A	0	0	1	0	1	-
gi|29165615|ref|NC_002745.2|	651607 -	G	0	0	0	0	1	-
gi|29165615|ref|NC_002745.2|	651610 -	U	6	2	0	0	3	-
gi|29165615|ref|NC_002745.2|	651614 -	U	2	0	0	0	0	-
gi|29165615|ref|NC_002745.2|	651615 -	A	0	0	0	0	1	-
gi|29165615|ref|NC_002745.2|	651617 -	A	1	1	2	0	0	-
gi|29165615|ref|NC_002745.2|	651618 -	A	0	1	1	0	0	-
gi|29165615|ref|NC_002745.2|	651619 -	C	1	0	0	0	0	-
gi|29165615|ref|NC_002745.2|	651620 -	C	0	0	1	1	3	-
gi|29165615|ref|NC_002745.2|	651622 -	U	0	0	1	0	2	-
gi|29165615|ref|NC_002745.2|	651624 -	A	0	0	1	0	1	-
gi|29165615|ref|NC_002745.2|	651625 -	U	3	2	0	0	1	-
gi|29165615|ref|NC_002745.2|	651629 -	C	0	1	0	2	1	-

gi|29165615|ref|NC_002745.2|	651630	-	U	0	0	0	0	1	-
gi|29165615|ref|NC_002745.2|	651635	-	U	0	1	0	0	0	-
gi|29165615|ref|NC_002745.2|	651637	-	C	1	3	4	2	1	-
gi|29165615|ref|NC_002745.2|	651642	-	A	0	0	1	0	0	-
gi|29165615|ref|NC_002745.2|	651647	-	A	0	0	0	0	4	-
gi|29165615|ref|NC_002745.2|	651649	-	C	0	0	1	0	0	-
gi|29165615|ref|NC_002745.2|	651652	-	U	0	0	0	0	1	-
gi|29165615|ref|NC_002745.2|	651663	-	A	0	0	0	0	2	-
gi|29165615|ref|NC_002745.2|	651667	-	A	0	0	1	0	0	-
gi|29165615|ref|NC_002745.2|	651670	-	A	0	0	1	0	0	-
gi|29165615|ref|NC_002745.2|	651672	-	C	0	0	1	0	0	-
gi|29165615|ref|NC_002745.2|	651681	-	G	0	0	1	0	0	-
gi|29165615|ref|NC_002745.2|	651685	-	U	1	1	0	0	0	-
gi|29165615|ref|NC_002745.2|	651687	-	U	0	1	0	1	1	-
gi|29165615|ref|NC_002745.2|	651691	-	U	0	2	0	0	0	-
gi|29165615|ref|NC_002745.2|	651692	-	C	2	0	0	0	0	-
gi|29165615|ref|NC_002745.2|	651693	-	U	1	0	0	0	0	-
gi|29165615|ref|NC_002745.2|	651697	-	A	0	1	2	0	1	-
gi|29165615|ref|NC_002745.2|	651698	-	G	0	1	0	0	1	-
gi|29165615|ref|NC_002745.2|	651701	-	U	0	1	1	1	1	-
gi|29165615|ref|NC_002745.2|	651702	-	U	0	3	1	4	1	-
gi|29165615|ref|NC_002745.2|	651703	-	U	0	0	1	0	0	-
gi|29165615|ref|NC_002745.2|	651706	-	U	0	0	0	0	1	-
gi|29165615|ref|NC_002745.2|	651707	-	U	0	0	1	3	1	-
gi|29165615|ref|NC_002745.2|	651708	-	A	2	0	1	1	0	-
gi|29165615|ref|NC_002745.2|	651709	-	A	0	1	2	2	0	-
gi|29165615|ref|NC_002745.2|	651710	-	G	0	1	0	2	0	-
gi|29165615|ref|NC_002745.2|	651711	-	A	0	0	1	1	0	-
gi|29165615|ref|NC_002745.2|	651712	-	U	0	0	0	1	0	-
gi|29165615|ref|NC_002745.2|	651713	-	G	0	0	0	0	1	-
gi|29165615|ref|NC_002745.2|	651714	-	U	0	0	0	1	0	-
gi|29165615|ref|NC_002745.2|	651719	-	U	3	2	3	1	10	-
gi|29165615|ref|NC_002745.2|	651720	-	A	0	0	2	0	0	-
gi|29165615|ref|NC_002745.2|	651721	-	C	0	1	2	0	0	-
gi|29165615|ref|NC_002745.2|	651722	-	G	0	0	0	0	1	-
gi|29165615|ref|NC_002745.2|	651727	-	A	0	1	0	0	0	-
gi|29165615|ref|NC_002745.2|	651728	-	C	0	0	0	0	1	-
gi|29165615|ref|NC_002745.2|	651729	-	C	0	0	0	1	2	-
gi|29165615|ref|NC_002745.2|	651734	-	A	0	0	0	1	0	-
gi|29165615|ref|NC_002745.2|	651747	-	U	0	0	0	1	0	-
gi|29165615|ref|NC_002745.2|	651748	-	C	0	0	0	1	0	-
gi|29165615|ref|NC_002745.2|	651754	-	C	0	0	1	2	0	-
gi|29165615|ref|NC_002745.2|	651755	-	U	0	2	1	1	1	-
gi|29165615|ref|NC_002745.2|	651760	-	U	0	4	0	1	2	-
gi|29165615|ref|NC_002745.2|	651761	-	U	0	1	2	1	0	-
gi|29165615|ref|NC_002745.2|	651769	-	A	0	0	0	1	0	-
gi|29165615|ref|NC_002745.2|	651770	-	U	0	1	1	2	0	-
gi|29165615|ref|NC_002745.2|	651778	-	C	0	0	1	0	3	-
gi|29165615|ref|NC_002745.2|	651779	-	A	0	1	0	1	0	-
gi|29165615|ref|NC_002745.2|	651786	-	A	0	0	0	1	0	-
gi|29165615|ref|NC_002745.2|	651787	-	C	0	0	0	0	1	-
gi|29165615|ref|NC_002745.2|	651795	-	U	0	0	1	0	0	-
gi|29165615|ref|NC_002745.2|	651805	-	U	1	0	0	2	0	-
gi|29165615|ref|NC_002745.2|	651825	-	A	0	0	0	1	0	-
gi|29165615|ref|NC_002745.2|	662158	-	C	0	0	1	0	0	-
gi|29165615|ref|NC_002745.2|	666185	-	A	0	1	0	0	0	-
gi|29165615|ref|NC_002745.2|	668408	-	A	0	0	2	1	0	SA0574
gi|29165615|ref|NC_002745.2|	669117	-	A	0	1	0	0	0	SA0576
gi|29165615|ref|NC_002745.2|	678244	-	C	2	0	0	0	0	-
gi|29165615|ref|NC_002745.2|	678247	-	C	0	0	0	0	1	-
gi|29165615|ref|NC_002745.2|	678255	-	U	0	0	1	0	2	-
gi|29165615|ref|NC_002745.2|	678256	-	U	0	1	2	3	0	-
gi|29165615|ref|NC_002745.2|	678258	-	A	0	0	0	0	1	-
gi|29165615|ref|NC_002745.2|	678265	-	U	0	1	0	0	0	-
gi|29165615|ref|NC_002745.2|	678269	-	A	1	0	0	0	0	-
gi|29165615|ref|NC_002745.2|	678270	-	A	0	0	0	1	0	-
gi|29165615|ref|NC_002745.2|	678271	-	A	0	1	0	1	0	-
gi|29165615|ref|NC_002745.2|	678272	-	A	0	0	0	1	2	-
gi|29165615|ref|NC_002745.2|	678275	-	U	0	0	0	0	1	-
gi|29165615|ref|NC_002745.2|	678278	-	U	0	0	0	1	0	-
gi|29165615|ref|NC_002745.2|	678280	-	U	0	0	0	0	1	-
gi|29165615|ref|NC_002745.2|	678286	-	A	0	0	0	0	1	-
gi|29165615|ref|NC_002745.2|	678301	-	A	0	0	0	0	1	SA0586
gi|29165615|ref|NC_002745.2|	678302	-	U	0	0	0	1	0	SA0586
gi|29165615|ref|NC_002745.2|	678310	-	U	0	0	1	0	0	SA0586
gi|29165615|ref|NC_002745.2|	678370	-	U	0	0	0	1	0	SA0586
gi|29165615|ref|NC_002745.2|	678377	-	C	0	0	0	1	0	SA0586
gi|29165615|ref|NC_002745.2|	678391	-	A	0	0	1	0	0	SA0586
gi|29165615|ref|NC_002745.2|	678422	-	A	0	0	1	0	0	SA0586
gi|29165615|ref|NC_002745.2|	678426	-	A	0	1	0	1	0	SA0586
gi|29165615|ref|NC_002745.2|	678437	-	A	0	1	0	0	0	SA0586

gi|29165615|ref|NC_002745.2|	678487	-	C	0	0	0	1	0	SA0586
gi|29165615|ref|NC_002745.2|	678520	-	A	0	0	1	0	0	SA0586
gi|29165615|ref|NC_002745.2|	678538	-	U	0	0	1	0	0	SA0586
gi|29165615|ref|NC_002745.2|	678562	-	G	0	0	0	0	1	SA0586
gi|29165615|ref|NC_002745.2|	678565	-	U	0	0	0	0	1	SA0586
gi|29165615|ref|NC_002745.2|	678595	-	G	0	0	0	0	2	SA0586
gi|29165615|ref|NC_002745.2|	678600	-	U	0	0	0	0	1	SA0586
gi|29165615|ref|NC_002745.2|	678617	-	A	0	0	0	0	1	SA0586
gi|29165615|ref|NC_002745.2|	678751	-	G	0	0	1	0	0	SA0586
gi|29165615|ref|NC_002745.2|	678752	-	A	0	0	1	0	0	SA0586
gi|29165615|ref|NC_002745.2|	678779	-	A	0	0	1	0	0	SA0586
gi|29165615|ref|NC_002745.2|	678842	-	C	1	0	0	0	0	SA0586
gi|29165615|ref|NC_002745.2|	678864	-	A	0	0	0	0	1	SA0586
gi|29165615|ref|NC_002745.2|	678865	-	U	0	0	2	0	0	SA0586
gi|29165615|ref|NC_002745.2|	678941	-	C	0	0	0	0	1	SA0586
gi|29165615|ref|NC_002745.2|	679033	-	A	0	0	1	0	0	SA0586
gi|29165615|ref|NC_002745.2|	679036	-	A	0	1	0	0	0	SA0586
gi|29165615|ref|NC_002745.2|	679044	-	U	0	2	0	0	0	SA0586
gi|29165615|ref|NC_002745.2|	679063	-	A	0	1	0	0	0	SA0586
gi|29165615|ref|NC_002745.2|	679098	-	A	0	0	1	0	0	SA0586
gi|29165615|ref|NC_002745.2|	679099	-	U	0	1	0	0	0	SA0586
gi|29165615|ref|NC_002745.2|	679210	-	C	1	0	0	0	0	SA0586
gi|29165615|ref|NC_002745.2|	679270	-	U	0	0	0	0	1	SA0586
gi|29165615|ref|NC_002745.2|	679365	-	U	0	1	0	0	0	SA0586
gi|29165615|ref|NC_002745.2|	679375	-	C	0	1	0	0	0	SA0586
gi|29165615|ref|NC_002745.2|	679406	-	A	0	1	0	0	0	SA0586
gi|29165615|ref|NC_002745.2|	679407	-	A	0	1	0	0	0	SA0586
gi|29165615|ref|NC_002745.2|	679451	-	A	0	1	0	0	0	SA0586
gi|29165615|ref|NC_002745.2|	679452	-	A	0	0	0	1	0	SA0586
gi|29165615|ref|NC_002745.2|	679469	-	G	0	1	0	0	0	SA0586
gi|29165615|ref|NC_002745.2|	679490	-	U	0	0	1	0	0	SA0586
gi|29165615|ref|NC_002745.2|	679499	-	U	0	1	0	0	0	SA0586
gi|29165615|ref|NC_002745.2|	679501	-	C	0	0	0	0	1	SA0586
gi|29165615|ref|NC_002745.2|	679503	-	A	0	0	1	0	0	SA0586
gi|29165615|ref|NC_002745.2|	679508	-	G	0	1	0	0	0	SA0586
gi|29165615|ref|NC_002745.2|	679514	-	A	0	0	2	0	0	SA0586
gi|29165615|ref|NC_002745.2|	679522	-	C	1	0	0	0	1	SA0586
gi|29165615|ref|NC_002745.2|	679524	-	A	0	0	1	0	0	SA0586
gi|29165615|ref|NC_002745.2|	679529	-	A	1	1	2	1	3	SA0586
gi|29165615|ref|NC_002745.2|	679530	-	A	0	0	1	0	1	SA0586
gi|29165615|ref|NC_002745.2|	679532	-	A	0	1	0	0	0	SA0586
gi|29165615|ref|NC_002745.2|	679533	-	A	0	0	0	1	0	SA0586
gi|29165615|ref|NC_002745.2|	679534	-	G	0	0	0	0	1	SA0586
gi|29165615|ref|NC_002745.2|	679537	-	A	0	0	4	1	0	SA0586
gi|29165615|ref|NC_002745.2|	679538	-	A	0	2	1	0	2	SA0586
gi|29165615|ref|NC_002745.2|	679542	-	U	0	0	1	0	0	SA0586
gi|29165615|ref|NC_002745.2|	679543	-	U	0	1	0	0	1	SA0586
gi|29165615|ref|NC_002745.2|	679546	-	U	0	0	2	0	0	SA0586
gi|29165615|ref|NC_002745.2|	679549	-	A	0	1	3	0	2	SA0586
gi|29165615|ref|NC_002745.2|	679554	-	A	0	0	1	0	0	SA0586
gi|29165615|ref|NC_002745.2|	679555	-	A	2	6	1	1	6	SA0586
gi|29165615|ref|NC_002745.2|	679556	-	G	0	0	0	0	1	SA0586
gi|29165615|ref|NC_002745.2|	679558	-	A	0	0	0	0	2	SA0586
gi|29165615|ref|NC_002745.2|	679560	-	C	0	3	0	0	0	SA0586
gi|29165615|ref|NC_002745.2|	679561	-	A	0	1	0	0	0	SA0586
gi|29165615|ref|NC_002745.2|	679562	-	A	0	0	0	0	1	SA0586
gi|29165615|ref|NC_002745.2|	679563	-	A	0	1	0	0	2	SA0586
gi|29165615|ref|NC_002745.2|	679564	-	U	0	1	2	0	2	SA0586
gi|29165615|ref|NC_002745.2|	679565	-	A	0	0	2	0	0	SA0586
gi|29165615|ref|NC_002745.2|	679567	-	A	0	0	1	0	0	SA0586
gi|29165615|ref|NC_002745.2|	679568	-	U	0	0	1	0	0	SA0586
gi|29165615|ref|NC_002745.2|	679570	-	A	0	2	0	0	0	SA0586
gi|29165615|ref|NC_002745.2|	679571	-	A	0	0	0	0	2	SA0586
gi|29165615|ref|NC_002745.2|	679573	-	U	0	0	1	0	0	SA0586
gi|29165615|ref|NC_002745.2|	679576	-	A	0	0	0	1	0	SA0586
gi|29165615|ref|NC_002745.2|	679577	-	A	0	0	1	0	0	SA0586
gi|29165615|ref|NC_002745.2|	679579	-	A	0	0	0	0	1	SA0586
gi|29165615|ref|NC_002745.2|	679585	-	A	0	0	0	1	1	SA0586
gi|29165615|ref|NC_002745.2|	679590	-	A	0	0	0	2	1	SA0586
gi|29165615|ref|NC_002745.2|	679630	-	C	0	2	1	0	1	-
gi|29165615|ref|NC_002745.2|	679633	-	A	1	1	0	0	1	-
gi|29165615|ref|NC_002745.2|	679638	-	A	15	15	14	7	8	-
gi|29165615|ref|NC_002745.2|	679643	-	A	0	0	0	1	0	-
gi|29165615|ref|NC_002745.2|	680377	-	U	1	0	0	0	0	-
gi|29165615|ref|NC_002745.2|	680401	-	C	0	1	0	0	0	-
gi|29165615|ref|NC_002745.2|	680406	-	U	0	1	0	0	0	-
gi|29165615|ref|NC_002745.2|	680412	-	U	0	0	0	0	1	-
gi|29165615|ref|NC_002745.2|	680510	-	U	1	0	0	0	0	-
gi|29165615|ref|NC_002745.2|	680534	-	C	0	1	0	0	0	-
gi|29165615|ref|NC_002745.2|	680539	-	U	0	1	0	0	0	-
gi|29165615|ref|NC_002745.2|	680545	-	U	0	0	0	0	1	-

gi|29165615|ref|NC_002745.2|	681107 -	A	1	0	0	0	0	SA0587
gi|29165615|ref|NC_002745.2|	681131 -	C	1	1	0	0	0	SA0587
gi|29165615|ref|NC_002745.2|	681133 -	C	1	0	0	0	0	SA0587
gi|29165615|ref|NC_002745.2|	681170 -	A	0	1	0	0	0	SA0587
gi|29165615|ref|NC_002745.2|	715997 -	C	1	0	0	0	0	SA0620
gi|29165615|ref|NC_002745.2|	716005 -	A	0	0	1	0	0	SA0620
gi|29165615|ref|NC_002745.2|	716008 -	U	0	1	0	0	0	SA0620
gi|29165615|ref|NC_002745.2|	716009 -	A	0	0	3	0	1	SA0620
gi|29165615|ref|NC_002745.2|	716010 -	A	0	0	2	1	1	SA0620
gi|29165615|ref|NC_002745.2|	716213 -	A	0	0	1	0	0	SA0620
gi|29165615|ref|NC_002745.2|	716399 -	A	0	0	1	0	0	SA0620
gi|29165615|ref|NC_002745.2|	729499 -	A	0	0	0	1	0	SA0635
gi|29165615|ref|NC_002745.2|	756503 -	U	0	1	0	0	0	SA0661
gi|29165615|ref|NC_002745.2|	776158 -	A	0	0	1	0	0	-
gi|29165615|ref|NC_002745.2|	776160 -	A	0	2	0	1	0	-
gi|29165615|ref|NC_002745.2|	776161 -	A	0	0	0	0	1	-
gi|29165615|ref|NC_002745.2|	776176 -	G	0	2	0	0	0	-
gi|29165615|ref|NC_002745.2|	776192 -	G	3	0	0	0	0	-
gi|29165615|ref|NC_002745.2|	776205 -	A	1	0	0	0	0	-
gi|29165615|ref|NC_002745.2|	776206 -	U	0	0	0	1	0	-
gi|29165615|ref|NC_002745.2|	776211 -	A	0	0	0	1	0	-
gi|29165615|ref|NC_002745.2|	776212 -	A	0	1	0	3	2	-
gi|29165615|ref|NC_002745.2|	776215 -	A	0	0	0	0	1	-
gi|29165615|ref|NC_002745.2|	776216 -	A	0	0	0	1	0	-
gi|29165615|ref|NC_002745.2|	776217 -	A	0	0	0	5	0	-
gi|29165615|ref|NC_002745.2|	776225 -	G	0	0	0	1	0	-
gi|29165615|ref|NC_002745.2|	776230 -	A	0	0	0	1	0	-
gi|29165615|ref|NC_002745.2|	776231 -	C	0	0	1	0	0	-
gi|29165615|ref|NC_002745.2|	776233 -	A	0	0	0	0	1	-
gi|29165615|ref|NC_002745.2|	776273 -	C	0	0	0	1	0	-
gi|29165615|ref|NC_002745.2|	776276 -	A	0	0	0	0	1	-
gi|29165615|ref|NC_002745.2|	776283 -	A	0	0	0	0	1	-
gi|29165615|ref|NC_002745.2|	776300 -	U	0	0	1	0	0	-
gi|29165615|ref|NC_002745.2|	776306 -	G	0	0	0	0	1	-
gi|29165615|ref|NC_002745.2|	776321 -	U	0	1	0	0	0	-
gi|29165615|ref|NC_002745.2|	776334 -	C	0	0	0	1	0	-
gi|29165615|ref|NC_002745.2|	777147 -	U	0	0	0	0	1	-
gi|29165615|ref|NC_002745.2|	777153 -	A	0	0	0	1	0	-
gi|29165615|ref|NC_002745.2|	777158 -	A	0	0	0	0	1	-
gi|29165615|ref|NC_002745.2|	777159 -	G	0	0	0	0	1	-
gi|29165615|ref|NC_002745.2|	777163 -	U	0	0	0	1	0	-
gi|29165615|ref|NC_002745.2|	777165 -	A	0	2	0	0	0	-
gi|29165615|ref|NC_002745.2|	777168 -	A	0	2	0	0	0	-
gi|29165615|ref|NC_002745.2|	777175 -	U	0	0	0	1	0	-
gi|29165615|ref|NC_002745.2|	777176 -	U	0	2	1	0	0	-
gi|29165615|ref|NC_002745.2|	777177 -	A	0	0	0	0	2	-
gi|29165615|ref|NC_002745.2|	777178 -	C	0	0	0	0	1	-
gi|29165615|ref|NC_002745.2|	777179 -	G	0	0	0	2	0	-
gi|29165615|ref|NC_002745.2|	777180 -	U	0	1	0	0	0	-
gi|29165615|ref|NC_002745.2|	777181 -	U	0	1	0	0	0	-
gi|29165615|ref|NC_002745.2|	777182 -	C	2	0	0	1	0	-
gi|29165615|ref|NC_002745.2|	777183 -	A	0	2	0	2	1	-
gi|29165615|ref|NC_002745.2|	777184 -	A	0	5	1	0	1	-
gi|29165615|ref|NC_002745.2|	777185 -	C	0	1	0	2	1	-
gi|29165615|ref|NC_002745.2|	777186 -	C	0	0	1	1	2	-
gi|29165615|ref|NC_002745.2|	777189 -	C	0	1	0	1	2	-
gi|29165615|ref|NC_002745.2|	777191 -	C	0	0	0	1	0	-
gi|29165615|ref|NC_002745.2|	777197 -	U	0	0	1	0	0	-
gi|29165615|ref|NC_002745.2|	777199 -	U	1	1	0	0	0	-
gi|29165615|ref|NC_002745.2|	777209 -	A	0	0	0	1	0	-
gi|29165615|ref|NC_002745.2|	777213 -	U	0	1	0	0	0	-
gi|29165615|ref|NC_002745.2|	777237 -	U	0	1	0	1	0	-
gi|29165615|ref|NC_002745.2|	777238 -	C	2	0	0	1	0	-
gi|29165615|ref|NC_002745.2|	777239 -	A	0	2	0	2	1	-
gi|29165615|ref|NC_002745.2|	777240 -	A	0	5	1	0	1	-
gi|29165615|ref|NC_002745.2|	777241 -	C	0	1	0	2	1	-
gi|29165615|ref|NC_002745.2|	777242 -	C	0	0	1	1	2	-
gi|29165615|ref|NC_002745.2|	777247 -	C	0	0	0	1	0	-
gi|29165615|ref|NC_002745.2|	777282 -	A	0	0	0	1	0	-
gi|29165615|ref|NC_002745.2|	777324 -	C	0	1	1	0	0	-
gi|29165615|ref|NC_002745.2|	777327 -	G	0	1	0	0	0	-
gi|29165615|ref|NC_002745.2|	777328 -	A	0	0	1	0	1	-
gi|29165615|ref|NC_002745.2|	777330 -	G	0	0	0	0	1	-
gi|29165615|ref|NC_002745.2|	777333 -	U	6	2	0	0	3	-
gi|29165615|ref|NC_002745.2|	777337 -	U	2	0	0	0	0	-
gi|29165615|ref|NC_002745.2|	777338 -	A	0	0	0	0	1	-
gi|29165615|ref|NC_002745.2|	777340 -	A	1	1	2	0	0	-
gi|29165615|ref|NC_002745.2|	777341 -	A	0	1	1	0	0	-
gi|29165615|ref|NC_002745.2|	777342 -	C	1	0	0	0	0	-
gi|29165615|ref|NC_002745.2|	777343 -	C	0	0	1	1	3	-
gi|29165615|ref|NC_002745.2|	777345 -	U	0	0	1	0	2	-

gi|29165615|ref|NC_002745.2|	777347 -	A	0	0	1	0	1	-
gi|29165615|ref|NC_002745.2|	777348 -	U	3	2	0	0	1	-
gi|29165615|ref|NC_002745.2|	777352 -	C	0	1	0	2	1	-
gi|29165615|ref|NC_002745.2|	777353 -	U	0	0	0	0	1	-
gi|29165615|ref|NC_002745.2|	777358 -	U	0	1	0	0	0	-
gi|29165615|ref|NC_002745.2|	777360 -	C	1	3	4	2	1	-
gi|29165615|ref|NC_002745.2|	777365 -	A	0	0	1	0	0	-
gi|29165615|ref|NC_002745.2|	777370 -	A	0	0	0	0	4	-
gi|29165615|ref|NC_002745.2|	777372 -	C	0	0	1	0	0	-
gi|29165615|ref|NC_002745.2|	777375 -	U	0	0	0	0	1	-
gi|29165615|ref|NC_002745.2|	777386 -	A	0	0	0	0	2	-
gi|29165615|ref|NC_002745.2|	777390 -	A	0	0	1	0	0	-
gi|29165615|ref|NC_002745.2|	777393 -	A	0	0	1	0	0	-
gi|29165615|ref|NC_002745.2|	777395 -	C	0	1	1	0	0	-
gi|29165615|ref|NC_002745.2|	777431 -	A	0	0	1	1	0	-
gi|29165615|ref|NC_002745.2|	777436 -	A	0	0	0	0	1	-
gi|29165615|ref|NC_002745.2|	777437 -	G	0	0	0	0	1	-
gi|29165615|ref|NC_002745.2|	777441 -	U	0	0	0	1	0	-
gi|29165615|ref|NC_002745.2|	777443 -	A	0	2	0	0	0	-
gi|29165615|ref|NC_002745.2|	777446 -	A	0	2	0	0	0	-
gi|29165615|ref|NC_002745.2|	777453 -	U	0	0	0	1	0	-
gi|29165615|ref|NC_002745.2|	777454 -	U	0	2	1	0	0	-
gi|29165615|ref|NC_002745.2|	777455 -	A	0	0	0	0	2	-
gi|29165615|ref|NC_002745.2|	777456 -	C	0	0	0	0	1	-
gi|29165615|ref|NC_002745.2|	777457 -	G	0	0	0	2	0	-
gi|29165615|ref|NC_002745.2|	777458 -	U	0	1	0	0	0	-
gi|29165615|ref|NC_002745.2|	777459 -	U	0	1	0	0	0	-
gi|29165615|ref|NC_002745.2|	777460 -	C	2	0	0	1	0	-
gi|29165615|ref|NC_002745.2|	777461 -	A	0	2	0	2	1	-
gi|29165615|ref|NC_002745.2|	777462 -	A	0	5	1	0	1	-
gi|29165615|ref|NC_002745.2|	777463 -	C	0	1	0	2	1	-
gi|29165615|ref|NC_002745.2|	777464 -	C	0	0	1	1	2	-
gi|29165615|ref|NC_002745.2|	777469 -	C	0	0	0	1	0	-
gi|29165615|ref|NC_002745.2|	777474 -	U	0	0	0	0	2	-
gi|29165615|ref|NC_002745.2|	777481 -	U	1	0	0	2	0	-
gi|29165615|ref|NC_002745.2|	777501 -	A	0	0	0	1	0	-
gi|29165615|ref|NC_002745.2|	777597 -	G	0	0	1	0	0	-
gi|29165615|ref|NC_002745.2|	778608 -	A	0	0	0	0	1	SA0681
gi|29165615|ref|NC_002745.2|	779709 -	C	0	0	0	1	0	SA0682
gi|29165615|ref|NC_002745.2|	780368 -	C	0	2	1	0	0	SA0682
gi|29165615|ref|NC_002745.2|	791075 -	U	0	0	0	1	0	-
gi|29165615|ref|NC_002745.2|	791076 -	C	2	0	0	0	0	-
gi|29165615|ref|NC_002745.2|	791077 -	A	0	2	0	1	1	-
gi|29165615|ref|NC_002745.2|	791078 -	A	0	5	0	0	1	-
gi|29165615|ref|NC_002745.2|	791079 -	C	0	0	0	2	1	-
gi|29165615|ref|NC_002745.2|	791080 -	C	0	0	1	0	2	-
gi|29165615|ref|NC_002745.2|	791083 -	C	0	1	0	1	2	-
gi|29165615|ref|NC_002745.2|	791091 -	U	0	0	1	0	0	-
gi|29165615|ref|NC_002745.2|	791093 -	U	1	1	0	0	0	-
gi|29165615|ref|NC_002745.2|	791103 -	A	0	0	0	1	0	-
gi|29165615|ref|NC_002745.2|	791107 -	U	0	1	0	0	0	-
gi|29165615|ref|NC_002745.2|	791108 -	U	0	1	0	1	1	-
gi|29165615|ref|NC_002745.2|	791109 -	U	0	0	0	1	0	-
gi|29165615|ref|NC_002745.2|	791113 -	U	0	0	1	0	1	-
gi|29165615|ref|NC_002745.2|	791115 -	A	0	0	0	1	0	-
gi|29165615|ref|NC_002745.2|	791119 -	G	0	0	0	0	1	-
gi|29165615|ref|NC_002745.2|	791120 -	U	0	0	0	1	0	-
gi|29165615|ref|NC_002745.2|	791483 -	A	0	2	1	0	0	SA0692
gi|29165615|ref|NC_002745.2|	799672 -	A	0	0	2	0	0	SA0701
gi|29165615|ref|NC_002745.2|	818008 -	U	0	1	0	0	0	-
gi|29165615|ref|NC_002745.2|	818025 -	U	0	1	0	0	0	-
gi|29165615|ref|NC_002745.2|	818112 -	U	0	1	0	0	0	-
gi|29165615|ref|NC_002745.2|	818115 -	A	0	0	0	1	0	-
gi|29165615|ref|NC_002745.2|	818116 -	A	0	0	0	2	1	-
gi|29165615|ref|NC_002745.2|	818129 -	G	0	0	0	1	0	-
gi|29165615|ref|NC_002745.2|	818134 -	A	0	0	0	1	0	-
gi|29165615|ref|NC_002745.2|	818135 -	C	0	0	1	0	0	-
gi|29165615|ref|NC_002745.2|	818137 -	A	0	0	0	0	1	-
gi|29165615|ref|NC_002745.2|	818153 -	A	0	1	0	0	0	-
gi|29165615|ref|NC_002745.2|	818158 -	A	0	0	0	0	1	-
gi|29165615|ref|NC_002745.2|	818164 -	A	0	0	1	0	0	-
gi|29165615|ref|NC_002745.2|	818165 -	U	0	0	0	1	0	-
gi|29165615|ref|NC_002745.2|	818166 -	C	0	1	0	0	0	-
gi|29165615|ref|NC_002745.2|	818167 -	U	0	0	0	1	0	-
gi|29165615|ref|NC_002745.2|	818170 -	A	0	0	0	1	0	-
gi|29165615|ref|NC_002745.2|	818171 -	A	0	1	0	3	2	-
gi|29165615|ref|NC_002745.2|	818174 -	A	0	0	0	0	1	-
gi|29165615|ref|NC_002745.2|	818175 -	A	0	0	0	1	0	-
gi|29165615|ref|NC_002745.2|	818176 -	A	0	0	0	5	0	-
gi|29165615|ref|NC_002745.2|	818184 -	G	0	0	0	1	0	-
gi|29165615|ref|NC_002745.2|	818189 -	A	0	0	0	1	0	-

gi|29165615|ref|NC_002745.2|	818208 -	U	0	1	0	0	0 -
gi|29165615|ref|NC_002745.2|	823436 -	A	0	1	0	0	0 -
gi|29165615|ref|NC_002745.2|	823442 -	A	0	1	0	0	0 -
gi|29165615|ref|NC_002745.2|	823443 -	U	0	1	0	0	0 -
gi|29165615|ref|NC_002745.2|	823460 -	U	0	1	0	0	0 -
gi|29165615|ref|NC_002745.2|	823541 -	A	0	0	0	0	3 -
gi|29165615|ref|NC_002745.2|	823556 -	A	0	0	0	0	1 -
gi|29165615|ref|NC_002745.2|	823560 -	U	0	0	1	0	0 -
gi|29165615|ref|NC_002745.2|	823602 -	A	1	0	0	0	0 -
gi|29165615|ref|NC_002745.2|	823608 -	A	0	0	0	1	0 -
gi|29165615|ref|NC_002745.2|	823609 -	A	0	1	0	1	1 -
gi|29165615|ref|NC_002745.2|	823612 -	A	0	0	0	0	1 -
gi|29165615|ref|NC_002745.2|	823613 -	A	0	0	0	1	0 -
gi|29165615|ref|NC_002745.2|	823614 -	A	0	0	0	5	0 -
gi|29165615|ref|NC_002745.2|	823622 -	G	0	0	0	1	0 -
gi|29165615|ref|NC_002745.2|	823627 -	A	0	0	0	1	0 -
gi|29165615|ref|NC_002745.2|	823628 -	C	0	0	1	0	0 -
gi|29165615|ref|NC_002745.2|	823630 -	A	0	0	0	0	1 -
gi|29165615|ref|NC_002745.2|	823663 -	U	0	1	0	0	0 -
gi|29165615|ref|NC_002745.2|	823666 -	A	0	0	0	1	0 -
gi|29165615|ref|NC_002745.2|	823667 -	A	0	0	0	2	1 -
gi|29165615|ref|NC_002745.2|	823679 -	A	0	0	1	0	0 -
gi|29165615|ref|NC_002745.2|	823685 -	C	0	0	0	3	0 -
gi|29165615|ref|NC_002745.2|	823722 -	U	0	1	0	0	0 -
gi|29165615|ref|NC_002745.2|	823725 -	A	0	0	0	1	0 -
gi|29165615|ref|NC_002745.2|	823726 -	A	0	0	0	2	1 -
gi|29165615|ref|NC_002745.2|	823738 -	A	0	0	1	0	0 -
gi|29165615|ref|NC_002745.2|	823744 -	C	0	0	0	3	0 -
gi|29165615|ref|NC_002745.2|	823778 -	A	1	0	0	0	0 -
gi|29165615|ref|NC_002745.2|	823784 -	A	0	0	0	1	0 -
gi|29165615|ref|NC_002745.2|	823785 -	A	0	1	0	1	1 -
gi|29165615|ref|NC_002745.2|	823788 -	A	0	0	0	0	1 -
gi|29165615|ref|NC_002745.2|	823789 -	A	0	0	0	1	0 -
gi|29165615|ref|NC_002745.2|	823790 -	A	0	0	0	5	0 -
gi|29165615|ref|NC_002745.2|	823798 -	G	0	0	0	1	0 -
gi|29165615|ref|NC_002745.2|	823803 -	A	0	0	0	1	0 -
gi|29165615|ref|NC_002745.2|	823804 -	C	0	0	1	0	0 -
gi|29165615|ref|NC_002745.2|	823806 -	A	0	0	0	0	1 -
gi|29165615|ref|NC_002745.2|	823839 -	U	0	1	0	0	0 -
gi|29165615|ref|NC_002745.2|	823842 -	A	0	0	0	1	0 -
gi|29165615|ref|NC_002745.2|	823843 -	A	0	0	0	2	1 -
gi|29165615|ref|NC_002745.2|	823855 -	A	0	0	1	0	0 -
gi|29165615|ref|NC_002745.2|	823861 -	C	0	0	0	3	0 -
gi|29165615|ref|NC_002745.2|	823905 -	C	0	0	0	1	0 -
gi|29165615|ref|NC_002745.2|	823908 -	A	0	0	0	0	1 -
gi|29165615|ref|NC_002745.2|	830874 -	A	0	0	0	0	1 -
gi|29165615|ref|NC_002745.2|	830889 -	G	0	2	0	0	0 -
gi|29165615|ref|NC_002745.2|	830931 -	G	0	0	0	0	1 -
gi|29165615|ref|NC_002745.2|	830934 -	A	0	0	0	1	0 -
gi|29165615|ref|NC_002745.2|	830935 -	G	0	0	1	0	0 -
gi|29165615|ref|NC_002745.2|	830951 -	C	0	0	2	0	0 -
gi|29165615|ref|NC_002745.2|	830963 -	A	0	1	0	0	0 -
gi|29165615|ref|NC_002745.2|	830987 -	G	0	0	0	0	1 -
gi|29165615|ref|NC_002745.2|	830990 -	A	0	0	0	1	0 -
gi|29165615|ref|NC_002745.2|	830991 -	G	0	0	1	0	0 -
gi|29165615|ref|NC_002745.2|	831007 -	C	0	0	2	0	0 -
gi|29165615|ref|NC_002745.2|	831019 -	A	0	1	0	0	0 -
gi|29165615|ref|NC_002745.2|	831043 -	G	0	0	0	0	1 -
gi|29165615|ref|NC_002745.2|	831046 -	A	0	0	0	1	0 -
gi|29165615|ref|NC_002745.2|	831047 -	G	0	0	1	0	0 -
gi|29165615|ref|NC_002745.2|	831063 -	C	0	0	2	0	0 -
gi|29165615|ref|NC_002745.2|	831075 -	A	0	1	0	0	0 -
gi|29165615|ref|NC_002745.2|	831099 -	G	0	0	0	0	1 -
gi|29165615|ref|NC_002745.2|	831102 -	A	0	0	0	1	0 -
gi|29165615|ref|NC_002745.2|	831103 -	G	0	0	1	0	0 -
gi|29165615|ref|NC_002745.2|	831119 -	C	0	0	2	0	0 -
gi|29165615|ref|NC_002745.2|	831131 -	A	0	1	0	0	0 -
gi|29165615|ref|NC_002745.2|	831155 -	G	0	0	0	0	1 -
gi|29165615|ref|NC_002745.2|	831175 -	C	0	0	2	0	0 -
gi|29165615|ref|NC_002745.2|	831200 -	A	1	0	0	0	0 -
gi|29165615|ref|NC_002745.2|	831201 -	U	0	0	0	1	0 -
gi|29165615|ref|NC_002745.2|	831206 -	A	0	0	0	1	0 -
gi|29165615|ref|NC_002745.2|	831207 -	A	0	1	0	3	2 -
gi|29165615|ref|NC_002745.2|	831219 -	A	0	0	1	0	0 -
gi|29165615|ref|NC_002745.2|	831225 -	U	0	0	0	3	0 -
gi|29165615|ref|NC_002745.2|	831269 -	C	0	0	0	1	0 -
gi|29165615|ref|NC_002745.2|	831279 -	A	0	0	0	0	1 -
gi|29165615|ref|NC_002745.2|	834752 -	C	0	1	0	0	0 -
gi|29165615|ref|NC_002745.2|	873952 -	A	0	0	1	0	0 -
gi|29165615|ref|NC_002745.2|	873954 -	A	0	2	0	1	0 -
gi|29165615|ref|NC_002745.2|	873955 -	A	0	0	0	0	1 -

gi|29165615|ref|NC_002745.2|	873970	-	G	0	2	0	0	0	-
gi|29165615|ref|NC_002745.2|	873986	-	G	3	0	0	0	0	-
gi|29165615|ref|NC_002745.2|	873997	-	A	0	1	0	0	1	-
gi|29165615|ref|NC_002745.2|	873999	-	A	1	0	0	0	0	-
gi|29165615|ref|NC_002745.2|	874002	-	U	0	1	0	0	0	-
gi|29165615|ref|NC_002745.2|	874005	-	A	0	0	0	1	0	-
gi|29165615|ref|NC_002745.2|	874006	-	A	0	0	0	2	1	-
gi|29165615|ref|NC_002745.2|	874018	-	A	0	0	1	0	0	-
gi|29165615|ref|NC_002745.2|	874024	-	A	0	0	0	3	0	-
gi|29165615|ref|NC_002745.2|	874063	-	A	0	0	0	1	0	-
gi|29165615|ref|NC_002745.2|	874064	-	A	0	1	0	3	2	-
gi|29165615|ref|NC_002745.2|	874067	-	A	0	0	0	0	1	-
gi|29165615|ref|NC_002745.2|	874068	-	A	0	0	0	1	0	-
gi|29165615|ref|NC_002745.2|	874069	-	A	0	0	0	5	0	-
gi|29165615|ref|NC_002745.2|	874077	-	G	0	0	0	1	0	-
gi|29165615|ref|NC_002745.2|	874082	-	A	0	0	0	1	0	-
gi|29165615|ref|NC_002745.2|	874083	-	C	0	0	1	0	0	-
gi|29165615|ref|NC_002745.2|	874085	-	A	0	0	0	0	1	-
gi|29165615|ref|NC_002745.2|	874125	-	C	0	0	0	1	0	-
gi|29165615|ref|NC_002745.2|	874128	-	A	0	0	0	0	1	-
gi|29165615|ref|NC_002745.2|	874135	-	A	0	0	0	0	1	-
gi|29165615|ref|NC_002745.2|	874152	-	U	0	0	1	0	0	-
gi|29165615|ref|NC_002745.2|	874158	-	G	0	0	0	0	1	-
gi|29165615|ref|NC_002745.2|	874173	-	U	0	1	0	0	0	-
gi|29165615|ref|NC_002745.2|	874186	-	C	0	0	0	1	0	-
gi|29165615|ref|NC_002745.2|	874225	-	A	0	1	0	0	0	-
gi|29165615|ref|NC_002745.2|	874243	-	U	0	1	0	0	0	-
gi|29165615|ref|NC_002745.2|	874329	-	C	1	2	0	0	0	-
gi|29165615|ref|NC_002745.2|	874338	-	G	0	0	0	0	1	-
gi|29165615|ref|NC_002745.2|	874358	-	C	0	0	2	0	0	-
gi|29165615|ref|NC_002745.2|	874370	-	A	0	1	0	0	0	-
gi|29165615|ref|NC_002745.2|	874394	-	G	0	0	0	0	1	-
gi|29165615|ref|NC_002745.2|	874397	-	A	0	0	0	1	0	-
gi|29165615|ref|NC_002745.2|	875036	-	U	1	0	0	0	0	-
gi|29165615|ref|NC_002745.2|	881160	-	U	0	0	0	0	1	-
gi|29165615|ref|NC_002745.2|	884414	-	A	0	1	0	1	3	-
gi|29165615|ref|NC_002745.2|	893880	-	G	0	0	0	0	1	-
gi|29165615|ref|NC_002745.2|	893884	-	U	1	1	1	1	1	-
gi|29165615|ref|NC_002745.2|	893893	-	C	2	1	0	0	0	-
gi|29165615|ref|NC_002745.2|	893898	-	C	0	0	0	0	1	-
gi|29165615|ref|NC_002745.2|	893905	-	A	0	5	2	6	2	-
gi|29165615|ref|NC_002745.2|	893906	-	U	0	1	0	0	0	-
gi|29165615|ref|NC_002745.2|	893907	-	A	0	0	0	0	1	-
gi|29165615|ref|NC_002745.2|	894074	-	U	0	1	0	0	0	-
gi|29165615|ref|NC_002745.2|	894421	-	U	0	0	0	0	1	-
gi|29165615|ref|NC_002745.2|	894632	-	A	0	1	0	0	0	-
gi|29165615|ref|NC_002745.2|	894648	-	A	0	0	0	1	0	-
gi|29165615|ref|NC_002745.2|	894680	-	A	0	0	0	0	1	-
gi|29165615|ref|NC_002745.2|	894698	-	U	0	1	0	0	0	-
gi|29165615|ref|NC_002745.2|	894704	-	A	0	0	0	0	1	-
gi|29165615|ref|NC_002745.2|	894705	-	U	0	0	1	0	1	-
gi|29165615|ref|NC_002745.2|	894706	-	G	0	1	0	0	0	-
gi|29165615|ref|NC_002745.2|	894715	-	U	0	0	0	0	1	-
gi|29165615|ref|NC_002745.2|	894719	-	C	0	0	1	0	0	-
gi|29165615|ref|NC_002745.2|	894720	-	C	1	1	0	0	0	-
gi|29165615|ref|NC_002745.2|	915203	-	A	0	1	0	0	0	SA0811
gi|29165615|ref|NC_002745.2|	916672	-	G	0	0	0	0	1	SA0813
gi|29165615|ref|NC_002745.2|	924580	-	C	0	2	0	0	0	-
gi|29165615|ref|NC_002745.2|	924594	-	A	0	0	0	1	0	-
gi|29165615|ref|NC_002745.2|	924595	-	U	0	0	0	1	0	-
gi|29165615|ref|NC_002745.2|	924626	-	U	1	1	0	0	0	-
gi|29165615|ref|NC_002745.2|	944109	-	A	0	0	0	0	1	SAS024
gi|29165615|ref|NC_002745.2|	944110	-	A	0	0	0	1	0	SAS024
gi|29165615|ref|NC_002745.2|	944111	-	A	0	0	0	5	0	SAS024
gi|29165615|ref|NC_002745.2|	944119	-	G	0	0	0	1	0	SAS024
gi|29165615|ref|NC_002745.2|	944124	-	A	0	0	0	1	0	SAS024
gi|29165615|ref|NC_002745.2|	944125	-	C	0	0	1	0	0	SAS024
gi|29165615|ref|NC_002745.2|	944127	-	A	0	0	0	0	1	SAS024
gi|29165615|ref|NC_002745.2|	957670	-	U	0	0	1	0	1	SA0844
gi|29165615|ref|NC_002745.2|	968130	-	A	2	2	2	0	2	-
gi|29165615|ref|NC_002745.2|	980060	-	G	1	0	0	0	1	-
gi|29165615|ref|NC_002745.2|	980061	-	A	0	1	0	0	0	-
gi|29165615|ref|NC_002745.2|	991119	-	G	0	0	0	1	0	SA0875
gi|29165615|ref|NC_002745.2|	1002774	-	G	1	0	0	0	0	SAtRNA17
gi|29165615|ref|NC_002745.2|	1002783	-	A	1	0	0	0	0	SAtRNA17
gi|29165615|ref|NC_002745.2|	1002796	-	U	14	14	18	11	14	SAtRNA17
gi|29165615|ref|NC_002745.2|	1004357	-	A	0	1	0	0	0	SA0884
gi|29165615|ref|NC_002745.2|	1004358	-	U	0	0	0	0	1	SA0884
gi|29165615|ref|NC_002745.2|	1006415	-	A	0	0	1	0	0	-
gi|29165615|ref|NC_002745.2|	1006481	-	G	0	0	0	0	1	-
gi|29165615|ref|NC_002745.2|	1006784	-	A	0	0	1	0	0	-

gi|29165615|ref|NC_002745.2|	1006850 -	A	0	0	0	0	1	-
gi|29165615|ref|NC_002745.2|	1007979 -	A	0	0	0	1	0	-
gi|29165615|ref|NC_002745.2|	1027917 -	A	0	1	0	0	0	SA0905
gi|29165615|ref|NC_002745.2|	1028424 -	A	0	1	0	0	0	SA0905
gi|29165615|ref|NC_002745.2|	1029945 -	U	1	0	0	0	0	SA0905
gi|29165615|ref|NC_002745.2|	1034572 -	A	0	0	0	0	1	-
gi|29165615|ref|NC_002745.2|	1039984 -	A	0	1	0	0	3	-
gi|29165615|ref|NC_002745.2|	1040039 -	A	0	0	0	0	1	-
gi|29165615|ref|NC_002745.2|	1040055 -	A	0	0	0	0	1	-
gi|29165615|ref|NC_002745.2|	1040056 -	A	0	0	0	1	0	-
gi|29165615|ref|NC_002745.2|	1040057 -	A	0	0	0	5	0	-
gi|29165615|ref|NC_002745.2|	1040065 -	G	0	0	0	1	0	-
gi|29165615|ref|NC_002745.2|	1040070 -	A	0	0	0	1	0	-
gi|29165615|ref|NC_002745.2|	1040071 -	C	0	0	1	0	0	-
gi|29165615|ref|NC_002745.2|	1040073 -	A	0	0	0	0	1	-
gi|29165615|ref|NC_002745.2|	1040098 -	A	0	0	1	0	0	-
gi|29165615|ref|NC_002745.2|	1040100 -	A	0	0	0	0	1	-
gi|29165615|ref|NC_002745.2|	1040106 -	C	1	2	0	0	0	-
gi|29165615|ref|NC_002745.2|	1040115 -	A	0	0	0	0	1	-
gi|29165615|ref|NC_002745.2|	1040119 -	G	0	0	1	0	0	-
gi|29165615|ref|NC_002745.2|	1040171 -	A	0	0	0	1	0	-
gi|29165615|ref|NC_002745.2|	1040174 -	A	0	0	0	0	1	-
gi|29165615|ref|NC_002745.2|	1040399 -	A	0	0	1	0	0	-
gi|29165615|ref|NC_002745.2|	1040401 -	A	0	2	0	1	0	-
gi|29165615|ref|NC_002745.2|	1040402 -	A	0	0	0	0	1	-
gi|29165615|ref|NC_002745.2|	1040417 -	G	0	2	0	0	0	-
gi|29165615|ref|NC_002745.2|	1040433 -	G	3	0	0	0	0	-
gi|29165615|ref|NC_002745.2|	1040442 -	G	0	0	0	0	1	-
gi|29165615|ref|NC_002745.2|	1040449 -	A	0	2	0	0	0	-
gi|29165615|ref|NC_002745.2|	1040451 -	U	0	1	0	0	0	-
gi|29165615|ref|NC_002745.2|	1040454 -	U	0	0	0	0	1	-
gi|29165615|ref|NC_002745.2|	1040470 -	C	0	0	0	1	0	-
gi|29165615|ref|NC_002745.2|	1040473 -	A	0	0	0	0	1	-
gi|29165615|ref|NC_002745.2|	1040480 -	A	0	0	0	0	1	-
gi|29165615|ref|NC_002745.2|	1040497 -	A	0	0	1	0	0	-
gi|29165615|ref|NC_002745.2|	1040503 -	G	0	0	0	0	1	-
gi|29165615|ref|NC_002745.2|	1040518 -	U	0	1	0	0	0	-
gi|29165615|ref|NC_002745.2|	1040531 -	C	0	0	0	1	0	-
gi|29165615|ref|NC_002745.2|	1040959 -	U	0	1	0	0	1	SA0915
gi|29165615|ref|NC_002745.2|	1054108 -	A	0	1	1	0	0	SA0928
gi|29165615|ref|NC_002745.2|	1056859 -	A	1	2	0	4	2	SAS030
gi|29165615|ref|NC_002745.2|	1056860 -	U	0	2	1	1	5	SAS030
gi|29165615|ref|NC_002745.2|	1066510 -	A	0	0	1	0	0	-
gi|29165615|ref|NC_002745.2|	1066512 -	A	0	2	0	1	0	-
gi|29165615|ref|NC_002745.2|	1066513 -	A	0	0	0	0	1	-
gi|29165615|ref|NC_002745.2|	1066528 -	G	0	2	0	0	0	-
gi|29165615|ref|NC_002745.2|	1066544 -	G	3	0	0	0	0	-
gi|29165615|ref|NC_002745.2|	1066553 -	G	0	0	0	0	1	-
gi|29165615|ref|NC_002745.2|	1066560 -	A	0	2	0	0	0	-
gi|29165615|ref|NC_002745.2|	1066562 -	U	0	1	0	0	0	-
gi|29165615|ref|NC_002745.2|	1066565 -	U	0	0	0	0	1	-
gi|29165615|ref|NC_002745.2|	1066581 -	C	0	0	0	1	0	-
gi|29165615|ref|NC_002745.2|	1066584 -	A	0	0	0	0	1	-
gi|29165615|ref|NC_002745.2|	1066591 -	A	0	0	0	0	1	-
gi|29165615|ref|NC_002745.2|	1066608 -	U	0	0	1	0	0	-
gi|29165615|ref|NC_002745.2|	1066614 -	G	0	0	0	0	1	-
gi|29165615|ref|NC_002745.2|	1066629 -	U	0	1	0	0	0	-
gi|29165615|ref|NC_002745.2|	1066642 -	C	0	0	0	1	0	-
gi|29165615|ref|NC_002745.2|	1084209 -	A	0	1	0	2	0	SA0957
gi|29165615|ref|NC_002745.2|	1104773 -	A	0	1	0	0	0	SA0976
gi|29165615|ref|NC_002745.2|	1137099 -	A	0	1	2	1	1	-
gi|29165615|ref|NC_002745.2|	1137100 -	G	0	1	0	0	1	-
gi|29165615|ref|NC_002745.2|	1137103 -	U	0	1	1	1	1	-
gi|29165615|ref|NC_002745.2|	1137104 -	U	0	3	1	4	1	-
gi|29165615|ref|NC_002745.2|	1137105 -	U	0	0	1	0	0	-
gi|29165615|ref|NC_002745.2|	1137108 -	U	0	0	0	0	1	-
gi|29165615|ref|NC_002745.2|	1137109 -	U	0	0	1	3	1	-
gi|29165615|ref|NC_002745.2|	1137110 -	A	2	0	1	1	0	-
gi|29165615|ref|NC_002745.2|	1137111 -	A	0	1	2	2	0	-
gi|29165615|ref|NC_002745.2|	1137112 -	G	0	1	0	2	0	-
gi|29165615|ref|NC_002745.2|	1137113 -	A	0	0	1	1	0	-
gi|29165615|ref|NC_002745.2|	1137114 -	U	0	0	0	1	0	-
gi|29165615|ref|NC_002745.2|	1137115 -	G	0	0	0	0	1	-
gi|29165615|ref|NC_002745.2|	1137116 -	U	0	0	0	1	0	-
gi|29165615|ref|NC_002745.2|	1137121 -	U	3	2	3	1	10	-
gi|29165615|ref|NC_002745.2|	1137122 -	A	0	0	1	0	0	-
gi|29165615|ref|NC_002745.2|	1137123 -	C	0	1	2	0	0	-
gi|29165615|ref|NC_002745.2|	1137124 -	G	0	0	0	0	1	-
gi|29165615|ref|NC_002745.2|	1137130 -	C	0	0	0	0	1	-
gi|29165615|ref|NC_002745.2|	1137131 -	C	0	0	0	1	2	-
gi|29165615|ref|NC_002745.2|	1137136 -	C	0	0	0	1	0	-

gi|29165615|ref|NC_002745.2|	1137141 -	U	0	0	0	0	2 -
gi|29165615|ref|NC_002745.2|	1137144 -	U	0	0	1	0	0 -
gi|29165615|ref|NC_002745.2|	1137174 -	U	0	1	1	0	0 -
gi|29165615|ref|NC_002745.2|	1137179 -	U	0	0	0	0	3 -
gi|29165615|ref|NC_002745.2|	1137184 -	U	0	0	0	1	0 -
gi|29165615|ref|NC_002745.2|	1137186 -	A	0	1	0	2	1 -
gi|29165615|ref|NC_002745.2|	1137187 -	A	0	4	1	0	1 -
gi|29165615|ref|NC_002745.2|	1137188 -	C	0	1	0	2	1 -
gi|29165615|ref|NC_002745.2|	1137189 -	C	0	0	0	0	2 -
gi|29165615|ref|NC_002745.2|	1137200 -	U	0	0	1	0	0 -
gi|29165615|ref|NC_002745.2|	1137202 -	U	1	1	0	0	0 -
gi|29165615|ref|NC_002745.2|	1137212 -	A	0	0	0	1	0 -
gi|29165615|ref|NC_002745.2|	1137217 -	G	0	0	0	0	1 -
gi|29165615|ref|NC_002745.2|	1137230 -	U	0	1	1	0	0 -
gi|29165615|ref|NC_002745.2|	1137235 -	U	0	0	0	0	3 -
gi|29165615|ref|NC_002745.2|	1137240 -	U	0	0	0	1	0 -
gi|29165615|ref|NC_002745.2|	1137241 -	C	1	0	0	1	0 -
gi|29165615|ref|NC_002745.2|	1137242 -	A	0	1	0	2	1 -
gi|29165615|ref|NC_002745.2|	1137243 -	A	0	5	1	0	1 -
gi|29165615|ref|NC_002745.2|	1137244 -	C	0	1	0	2	1 -
gi|29165615|ref|NC_002745.2|	1137245 -	C	0	0	1	0	2 -
gi|29165615|ref|NC_002745.2|	1137250 -	C	0	0	0	1	0 -
gi|29165615|ref|NC_002745.2|	1137268 -	C	0	0	1	0	0 -
gi|29165615|ref|NC_002745.2|	1137274 -	U	0	3	0	1	2 -
gi|29165615|ref|NC_002745.2|	1137275 -	U	0	1	2	1	0 -
gi|29165615|ref|NC_002745.2|	1137282 -	U	0	0	1	0	0 -
gi|29165615|ref|NC_002745.2|	1137284 -	U	0	1	0	1	0 -
gi|29165615|ref|NC_002745.2|	1137290 -	U	0	1	0	0	0 -
gi|29165615|ref|NC_002745.2|	1137291 -	A	0	0	0	0	2 -
gi|29165615|ref|NC_002745.2|	1137292 -	C	0	4	1	5	2 -
gi|29165615|ref|NC_002745.2|	1137293 -	A	0	1	0	1	0 -
gi|29165615|ref|NC_002745.2|	1137300 -	A	0	0	0	1	0 -
gi|29165615|ref|NC_002745.2|	1137301 -	C	0	0	0	0	1 -
gi|29165615|ref|NC_002745.2|	1137305 -	C	0	0	0	0	1 -
gi|29165615|ref|NC_002745.2|	1137307 -	C	0	1	0	0	0 -
gi|29165615|ref|NC_002745.2|	1137315 -	U	1	1	0	0	0 -
gi|29165615|ref|NC_002745.2|	1137325 -	C	0	2	0	0	0 -
gi|29165615|ref|NC_002745.2|	1137330 -	U	0	3	0	1	2 -
gi|29165615|ref|NC_002745.2|	1137331 -	U	0	1	2	1	0 -
gi|29165615|ref|NC_002745.2|	1137338 -	U	0	0	1	0	0 -
gi|29165615|ref|NC_002745.2|	1137340 -	U	0	1	0	1	0 -
gi|29165615|ref|NC_002745.2|	1137346 -	U	0	1	0	0	0 -
gi|29165615|ref|NC_002745.2|	1137347 -	A	0	0	0	0	2 -
gi|29165615|ref|NC_002745.2|	1137348 -	C	0	4	1	5	2 -
gi|29165615|ref|NC_002745.2|	1137349 -	A	0	1	0	1	0 -
gi|29165615|ref|NC_002745.2|	1137356 -	A	0	0	0	1	0 -
gi|29165615|ref|NC_002745.2|	1137357 -	C	0	0	0	0	1 -
gi|29165615|ref|NC_002745.2|	1137361 -	A	0	0	0	0	1 -
gi|29165615|ref|NC_002745.2|	1137397 -	A	0	0	0	1	0 -
gi|29165615|ref|NC_002745.2|	1137437 -	A	0	2	0	0	0 -
gi|29165615|ref|NC_002745.2|	1137488 -	U	0	0	1	0	0 -
gi|29165615|ref|NC_002745.2|	1137512 -	G	0	0	1	0	0 -
gi|29165615|ref|NC_002745.2|	1139058 -	G	0	0	0	0	1 -
gi|29165615|ref|NC_002745.2|	1139062 -	U	1	1	1	1	1 -
gi|29165615|ref|NC_002745.2|	1139071 -	C	2	1	0	0	0 -
gi|29165615|ref|NC_002745.2|	1139076 -	C	0	0	0	0	1 -
gi|29165615|ref|NC_002745.2|	1139083 -	A	0	5	2	6	2 -
gi|29165615|ref|NC_002745.2|	1139084 -	U	0	1	0	0	0 -
gi|29165615|ref|NC_002745.2|	1139085 -	A	0	0	0	0	1 -
gi|29165615|ref|NC_002745.2|	1139252 -	U	0	1	0	0	0 -
gi|29165615|ref|NC_002745.2|	1139599 -	U	0	0	0	0	1 -
gi|29165615|ref|NC_002745.2|	1139810 -	A	0	1	0	0	0 -
gi|29165615|ref|NC_002745.2|	1139826 -	A	0	0	0	1	0 -
gi|29165615|ref|NC_002745.2|	1139858 -	A	0	0	0	0	1 -
gi|29165615|ref|NC_002745.2|	1139876 -	U	0	1	0	0	0 -
gi|29165615|ref|NC_002745.2|	1139882 -	A	0	0	0	0	1 -
gi|29165615|ref|NC_002745.2|	1139883 -	U	0	0	1	0	1 -
gi|29165615|ref|NC_002745.2|	1139884 -	G	0	1	0	0	0 -
gi|29165615|ref|NC_002745.2|	1139893 -	U	0	0	0	0	1 -
gi|29165615|ref|NC_002745.2|	1139897 -	C	0	0	1	0	0 -
gi|29165615|ref|NC_002745.2|	1139898 -	C	1	1	0	0	0 -
gi|29165615|ref|NC_002745.2|	1142640 -	U	0	1	0	0	0 -
gi|29165615|ref|NC_002745.2|	1142690 -	U	0	2	1	1	1 -
gi|29165615|ref|NC_002745.2|	1142691 -	G	0	0	1	0	0 -
gi|29165615|ref|NC_002745.2|	1142695 -	U	0	4	0	1	2 -
gi|29165615|ref|NC_002745.2|	1142696 -	U	0	1	2	1	0 -
gi|29165615|ref|NC_002745.2|	1142704 -	A	0	0	0	1	0 -
gi|29165615|ref|NC_002745.2|	1142705 -	U	0	1	1	2	0 -
gi|29165615|ref|NC_002745.2|	1142713 -	C	0	0	1	0	3 -
gi|29165615|ref|NC_002745.2|	1142714 -	A	0	1	0	1	0 -
gi|29165615|ref|NC_002745.2|	1142721 -	A	0	0	0	1	0 -

gi|29165615|ref|NC_002745.2|	1142722	-	C	0	0	0	0	1	-
gi|29165615|ref|NC_002745.2|	1142730	-	C	0	0	1	0	0	-
gi|29165615|ref|NC_002745.2|	1142736	-	U	1	0	0	0	0	-
gi|29165615|ref|NC_002745.2|	1142742	-	U	0	0	0	0	1	-
gi|29165615|ref|NC_002745.2|	1142746	-	A	0	0	0	1	0	-
gi|29165615|ref|NC_002745.2|	1142748	-	A	0	0	1	0	0	SAS036
gi|29165615|ref|NC_002745.2|	1142749	-	G	0	0	0	0	1	SAS036
gi|29165615|ref|NC_002745.2|	1142752	-	U	0	2	1	1	1	SAS036
gi|29165615|ref|NC_002745.2|	1142753	-	U	0	3	1	5	2	SAS036
gi|29165615|ref|NC_002745.2|	1142754	-	U	0	0	1	1	0	SAS036
gi|29165615|ref|NC_002745.2|	1142757	-	U	0	0	0	0	1	SAS036
gi|29165615|ref|NC_002745.2|	1142758	-	U	0	0	2	3	1	SAS036
gi|29165615|ref|NC_002745.2|	1142759	-	A	0	0	1	0	0	SAS036
gi|29165615|ref|NC_002745.2|	1142760	-	A	0	0	0	1	0	SAS036
gi|29165615|ref|NC_002745.2|	1142761	-	G	0	1	0	2	0	SAS036
gi|29165615|ref|NC_002745.2|	1142762	-	A	0	0	1	1	0	SAS036
gi|29165615|ref|NC_002745.2|	1142763	-	U	0	0	0	1	0	SAS036
gi|29165615|ref|NC_002745.2|	1142764	-	G	0	0	0	0	1	SAS036
gi|29165615|ref|NC_002745.2|	1142765	-	U	0	0	0	1	0	SAS036
gi|29165615|ref|NC_002745.2|	1142770	-	U	3	2	3	1	10	SAS036
gi|29165615|ref|NC_002745.2|	1142771	-	A	0	0	1	0	0	SAS036
gi|29165615|ref|NC_002745.2|	1142772	-	C	0	1	2	0	0	SAS036
gi|29165615|ref|NC_002745.2|	1142773	-	G	0	0	0	0	1	SAS036
gi|29165615|ref|NC_002745.2|	1142778	-	A	0	2	0	0	0	SAS036
gi|29165615|ref|NC_002745.2|	1142779	-	C	0	0	0	2	1	SAS036
gi|29165615|ref|NC_002745.2|	1142780	-	C	0	0	1	1	2	SAS036
gi|29165615|ref|NC_002745.2|	1142783	-	C	0	1	0	1	2	SAS036
gi|29165615|ref|NC_002745.2|	1142785	-	C	0	0	0	1	0	SAS036
gi|29165615|ref|NC_002745.2|	1142791	-	U	0	0	1	0	0	SAS036
gi|29165615|ref|NC_002745.2|	1142793	-	U	1	1	0	0	0	SAS036
gi|29165615|ref|NC_002745.2|	1142803	-	A	0	0	0	1	0	SAS036
gi|29165615|ref|NC_002745.2|	1142807	-	U	0	1	0	0	0	SAS036
gi|29165615|ref|NC_002745.2|	1142808	-	U	0	1	0	1	1	SAS036
gi|29165615|ref|NC_002745.2|	1142809	-	U	0	0	0	1	0	SAS036
gi|29165615|ref|NC_002745.2|	1142813	-	U	0	0	1	0	1	SAS036
gi|29165615|ref|NC_002745.2|	1142815	-	A	0	0	0	1	0	SAS036
gi|29165615|ref|NC_002745.2|	1142819	-	G	0	0	0	0	1	SAS036
gi|29165615|ref|NC_002745.2|	1142820	-	U	0	0	0	1	0	SAS036
gi|29165615|ref|NC_002745.2|	1142825	-	U	3	2	3	1	10	SAS036
gi|29165615|ref|NC_002745.2|	1142826	-	A	0	0	1	0	0	SAS036
gi|29165615|ref|NC_002745.2|	1142827	-	C	0	1	2	0	0	SAS036
gi|29165615|ref|NC_002745.2|	1142828	-	G	0	0	0	0	1	SAS036
gi|29165615|ref|NC_002745.2|	1142833	-	A	0	2	0	0	0	SAS036
gi|29165615|ref|NC_002745.2|	1142834	-	C	0	0	0	2	1	SAS036
gi|29165615|ref|NC_002745.2|	1142835	-	C	0	0	1	1	2	SAS036
gi|29165615|ref|NC_002745.2|	1142840	-	A	0	0	0	1	0	SAS036
gi|29165615|ref|NC_002745.2|	1151839	-	A	0	0	1	0	0	-
gi|29165615|ref|NC_002745.2|	1165435	-	A	0	1	0	0	0	-
gi|29165615|ref|NC_002745.2|	1175296	-	C	0	1	0	0	0	SA1038
gi|29165615|ref|NC_002745.2|	1175472	-	U	0	0	1	0	0	-
gi|29165615|ref|NC_002745.2|	1175732	-	A	0	0	1	1	1	-
gi|29165615|ref|NC_002745.2|	1207434	-	A	0	1	0	0	0	-
gi|29165615|ref|NC_002745.2|	1207698	-	U	0	0	1	0	1	-
gi|29165615|ref|NC_002745.2|	1228337	-	G	0	0	0	0	1	SA1085
gi|29165615|ref|NC_002745.2|	1253350	-	C	0	1	0	0	0	-
gi|29165615|ref|NC_002745.2|	1281134	-	U	0	0	0	0	1	-
gi|29165615|ref|NC_002745.2|	1290736	-	A	0	1	0	0	0	-
gi|29165615|ref|NC_002745.2|	1312288	-	C	0	0	0	1	0	-
gi|29165615|ref|NC_002745.2|	1320635	-	A	0	0	2	0	0	-
gi|29165615|ref|NC_002745.2|	1346387	-	G	0	0	0	1	1	SA1182
gi|29165615|ref|NC_002745.2|	1348637	-	G	0	1	0	0	0	-
gi|29165615|ref|NC_002745.2|	1348651	-	A	0	1	1	0	0	-
gi|29165615|ref|NC_002745.2|	1348652	-	C	1	0	0	0	0	-
gi|29165615|ref|NC_002745.2|	1348653	-	C	0	0	1	1	3	-
gi|29165615|ref|NC_002745.2|	1348655	-	U	0	0	1	0	2	-
gi|29165615|ref|NC_002745.2|	1348657	-	A	0	0	1	0	1	-
gi|29165615|ref|NC_002745.2|	1348658	-	U	3	2	0	0	1	-
gi|29165615|ref|NC_002745.2|	1348662	-	C	0	1	0	2	1	-
gi|29165615|ref|NC_002745.2|	1348663	-	U	0	0	0	0	1	-
gi|29165615|ref|NC_002745.2|	1348668	-	U	0	1	0	0	0	-
gi|29165615|ref|NC_002745.2|	1348670	-	C	1	3	4	2	1	-
gi|29165615|ref|NC_002745.2|	1348675	-	A	0	0	1	0	0	-
gi|29165615|ref|NC_002745.2|	1348680	-	A	0	0	0	0	4	-
gi|29165615|ref|NC_002745.2|	1348682	-	C	0	0	1	0	0	-
gi|29165615|ref|NC_002745.2|	1348685	-	U	0	0	0	0	1	-
gi|29165615|ref|NC_002745.2|	1348696	-	A	0	0	0	0	2	-
gi|29165615|ref|NC_002745.2|	1348700	-	A	0	0	1	0	0	-
gi|29165615|ref|NC_002745.2|	1348703	-	A	0	0	1	0	0	-
gi|29165615|ref|NC_002745.2|	1348705	-	C	0	1	1	0	0	-
gi|29165615|ref|NC_002745.2|	1348714	-	G	0	0	1	0	0	-
gi|29165615|ref|NC_002745.2|	1348718	-	U	1	1	0	0	0	-

gi|29165615|ref|NC_002745.2|	1348720 -	U	0	1	0	1	1 -	
gi|29165615|ref|NC_002745.2|	1348724 -	U	0	2	0	0	0 -	
gi|29165615|ref|NC_002745.2|	1348725 -	C	2	0	0	0	0 -	
gi|29165615|ref|NC_002745.2|	1348726 -	U	1	0	0	0	0 -	
gi|29165615|ref|NC_002745.2|	1348730 -	A	0	1	2	0	1 -	
gi|29165615|ref|NC_002745.2|	1348731 -	G	0	1	0	0	1 -	
gi|29165615|ref|NC_002745.2|	1348734 -	U	0	1	1	1	1 -	
gi|29165615|ref|NC_002745.2|	1348735 -	U	0	3	1	4	1 -	
gi|29165615|ref|NC_002745.2|	1348736 -	U	0	0	1	0	0 -	
gi|29165615|ref|NC_002745.2|	1348739 -	U	0	0	0	0	1 -	
gi|29165615|ref|NC_002745.2|	1348740 -	U	0	0	1	3	1 -	
gi|29165615|ref|NC_002745.2|	1348741 -	A	2	0	1	1	0 -	
gi|29165615|ref|NC_002745.2|	1348742 -	A	0	1	2	2	0 -	
gi|29165615|ref|NC_002745.2|	1348743 -	G	0	1	0	2	0 -	
gi|29165615|ref|NC_002745.2|	1348744 -	A	0	0	1	1	0 -	
gi|29165615|ref|NC_002745.2|	1348745 -	U	0	0	0	1	0 -	
gi|29165615|ref|NC_002745.2|	1348746 -	G	0	0	0	0	1 -	
gi|29165615|ref|NC_002745.2|	1348747 -	U	0	0	0	1	0 -	
gi|29165615|ref|NC_002745.2|	1348752 -	U	3	2	3	1	10 -	
gi|29165615|ref|NC_002745.2|	1348753 -	A	0	0	2	0	0 -	
gi|29165615|ref|NC_002745.2|	1348754 -	C	0	1	2	0	0 -	
gi|29165615|ref|NC_002745.2|	1348755 -	G	0	0	0	0	1 -	
gi|29165615|ref|NC_002745.2|	1348762 -	C	0	0	0	1	0 -	
gi|29165615|ref|NC_002745.2|	1348780 -	U	0	0	0	1	0 -	
gi|29165615|ref|NC_002745.2|	1348781 -	C	0	0	0	1	0 -	
gi|29165615|ref|NC_002745.2|	1348787 -	C	0	0	0	2	0 -	
gi|29165615|ref|NC_002745.2|	1348788 -	U	0	2	1	1	1 -	
gi|29165615|ref|NC_002745.2|	1348793 -	U	0	4	0	1	2 -	
gi|29165615|ref|NC_002745.2|	1348794 -	U	0	1	2	1	0 -	
gi|29165615|ref|NC_002745.2|	1348802 -	A	0	0	0	1	0 -	
gi|29165615|ref|NC_002745.2|	1348803 -	U	0	1	1	2	0 -	
gi|29165615|ref|NC_002745.2|	1348811 -	C	0	0	1	0	3 -	
gi|29165615|ref|NC_002745.2|	1348812 -	A	0	1	0	1	0 -	
gi|29165615|ref|NC_002745.2|	1348819 -	A	0	0	0	1	0 -	
gi|29165615|ref|NC_002745.2|	1348820 -	C	0	0	0	0	1 -	
gi|29165615|ref|NC_002745.2|	1348828 -	C	0	0	1	0	0 -	
gi|29165615|ref|NC_002745.2|	1348834 -	U	1	0	0	0	0 -	
gi|29165615|ref|NC_002745.2|	1348840 -	U	0	0	0	0	1 -	
gi|29165615|ref|NC_002745.2|	1348844 -	U	0	0	0	1	0 -	
gi|29165615|ref|NC_002745.2|	1348851 -	A	0	0	0	0	1 -	
gi|29165615|ref|NC_002745.2|	1348852 -	G	0	0	0	0	1 -	
gi|29165615|ref|NC_002745.2|	1348856 -	U	0	0	0	1	0 -	
gi|29165615|ref|NC_002745.2|	1348858 -	A	0	2	0	0	0 -	
gi|29165615|ref|NC_002745.2|	1348861 -	A	0	2	0	0	0 -	
gi|29165615|ref|NC_002745.2|	1348868 -	U	0	0	0	1	0 -	
gi|29165615|ref|NC_002745.2|	1348869 -	U	0	2	1	0	0 -	
gi|29165615|ref|NC_002745.2|	1348870 -	A	0	0	0	0	2 -	
gi|29165615|ref|NC_002745.2|	1348871 -	C	0	0	0	0	1 -	
gi|29165615|ref|NC_002745.2|	1348872 -	G	0	0	0	2	0 -	
gi|29165615|ref|NC_002745.2|	1348873 -	U	0	1	0	0	0 -	
gi|29165615|ref|NC_002745.2|	1348874 -	U	0	1	0	0	0 -	
gi|29165615|ref|NC_002745.2|	1348875 -	C	2	0	0	1	0 -	
gi|29165615|ref|NC_002745.2|	1348876 -	A	0	2	0	2	1 -	
gi|29165615|ref|NC_002745.2|	1348877 -	A	0	5	1	0	1 -	
gi|29165615|ref|NC_002745.2|	1348878 -	C	0	1	0	2	1 -	
gi|29165615|ref|NC_002745.2|	1348879 -	C	0	0	1	1	2 -	
gi|29165615|ref|NC_002745.2|	1348882 -	C	0	1	0	1	2 -	
gi|29165615|ref|NC_002745.2|	1348884 -	C	0	0	0	1	0 -	
gi|29165615|ref|NC_002745.2|	1348890 -	U	0	0	1	0	0 -	
gi|29165615|ref|NC_002745.2|	1348892 -	U	1	1	0	0	0 -	
gi|29165615|ref|NC_002745.2|	1348902 -	C	0	2	0	0	0 -	
gi|29165615|ref|NC_002745.2|	1348908 -	U	0	0	1	0	0 -	
gi|29165615|ref|NC_002745.2|	1348917 -	U	0	0	0	0	1 -	
gi|29165615|ref|NC_002745.2|	1348942 -	U	0	0	1	0	0 -	
gi|29165615|ref|NC_002745.2|	1348952 -	U	1	0	0	2	0 -	
gi|29165615|ref|NC_002745.2|	1348972 -	A	0	0	0	1	0 -	
gi|29165615|ref|NC_002745.2|	1353226 -	A	0	0	0	1	0 -	
gi|29165615|ref|NC_002745.2|	1353227 -	C	0	0	1	0	0 -	
gi|29165615|ref|NC_002745.2|	1353229 -	A	0	0	0	0	1 -	
gi|29165615|ref|NC_002745.2|	1353257 -	C	0	0	0	1	0 -	
gi|29165615|ref|NC_002745.2|	1353258 -	A	0	3	5	10	5 -	
gi|29165615|ref|NC_002745.2|	1353260 -	U	0	1	0	0	0 -	
gi|29165615|ref|NC_002745.2|	1353261 -	C	1	2	0	0	0 -	
gi|29165615|ref|NC_002745.2|	1353273 -	A	0	0	0	1	0 -	
gi|29165615|ref|NC_002745.2|	1353378 -	A	0	0	0	0	1 -	
gi|29165615|ref|NC_002745.2|	1384326 -	A	0	0	0	1	0 -	
gi|29165615|ref|NC_002745.2|	1388350 -	A	0	2	1	0	1 -	
gi|29165615|ref|NC_002745.2|	1395324 -	A	0	1	0	0	2 -	
gi|29165615|ref|NC_002745.2|	1395325 -	A	0	1	1	0	0 -	
gi|29165615|ref|NC_002745.2|	1395326 -	A	0	1	0	3	2 -	
gi|29165615|ref|NC_002745.2|	1395331 -	A	0	0	1	1	1 -	

gi|29165615|ref|NC_002745.2|	1395355	-	G	1	0	0	0	0	-
gi|29165615|ref|NC_002745.2|	1395588	-	C	0	0	0	0	1	-
gi|29165615|ref|NC_002745.2|	1398717	-	A	0	0	0	1	0	-
gi|29165615|ref|NC_002745.2|	1398813	-	A	0	0	0	2	1	-
gi|29165615|ref|NC_002745.2|	1398825	-	A	0	0	1	0	0	-
gi|29165615|ref|NC_002745.2|	1398831	-	U	0	0	0	3	0	-
gi|29165615|ref|NC_002745.2|	1398900	-	U	0	0	1	0	0	-
gi|29165615|ref|NC_002745.2|	1398906	-	G	0	0	0	0	1	-
gi|29165615|ref|NC_002745.2|	1399018	-	A	0	1	0	0	2	-
gi|29165615|ref|NC_002745.2|	1399019	-	A	0	1	1	0	0	-
gi|29165615|ref|NC_002745.2|	1399020	-	A	0	1	0	3	2	-
gi|29165615|ref|NC_002745.2|	1399025	-	A	0	0	1	1	1	-
gi|29165615|ref|NC_002745.2|	1399049	-	G	1	0	0	0	0	-
gi|29165615|ref|NC_002745.2|	1408920	-	A	0	0	1	1	0	SA1234
gi|29165615|ref|NC_002745.2|	1408922	-	U	0	2	0	0	0	SA1234
gi|29165615|ref|NC_002745.2|	1408923	-	G	0	5	0	3	4	SA1234
gi|29165615|ref|NC_002745.2|	1408926	-	A	1	0	0	1	0	SA1234
gi|29165615|ref|NC_002745.2|	1408927	-	G	0	1	0	0	1	SA1234
gi|29165615|ref|NC_002745.2|	1408928	-	A	0	0	1	0	0	SA1234
gi|29165615|ref|NC_002745.2|	1408929	-	A	0	2	0	2	1	SA1234
gi|29165615|ref|NC_002745.2|	1408930	-	C	1	0	0	0	0	SA1234
gi|29165615|ref|NC_002745.2|	1408958	-	C	0	1	0	0	1	SA1234
gi|29165615|ref|NC_002745.2|	1408959	-	A	0	1	1	1	0	SA1234
gi|29165615|ref|NC_002745.2|	1409012	-	A	3	23	1	1	9	SA1234
gi|29165615|ref|NC_002745.2|	1409013	-	A	0	7	6	3	3	SA1234
gi|29165615|ref|NC_002745.2|	1409014	-	A	0	14	3	0	6	SA1234
gi|29165615|ref|NC_002745.2|	1409015	-	U	0	9	3	4	8	SA1234
gi|29165615|ref|NC_002745.2|	1409016	-	U	4	7	5	6	3	SA1234
gi|29165615|ref|NC_002745.2|	1409017	-	G	0	7	4	2	7	SA1234
gi|29165615|ref|NC_002745.2|	1409018	-	A	1	21	11	4	11	SA1234
gi|29165615|ref|NC_002745.2|	1409019	-	C	3	35	21	14	19	SA1234
gi|29165615|ref|NC_002745.2|	1409020	-	A	0	18	9	5	13	SA1234
gi|29165615|ref|NC_002745.2|	1409021	-	U	4	1	1	1	2	SA1234
gi|29165615|ref|NC_002745.2|	1409022	-	G	0	19	2	3	17	SA1234
gi|29165615|ref|NC_002745.2|	1409023	-	G	0	1	1	0	0	SA1234
gi|29165615|ref|NC_002745.2|	1409024	-	A	0	1	1	0	0	SA1234
gi|29165615|ref|NC_002745.2|	1409025	-	A	0	4	3	0	1	SA1234
gi|29165615|ref|NC_002745.2|	1415025	-	U	0	0	0	0	1	SA1240
gi|29165615|ref|NC_002745.2|	1421093	-	U	0	0	0	0	1	SA1245
gi|29165615|ref|NC_002745.2|	1422143	-	A	0	1	0	0	0	-
gi|29165615|ref|NC_002745.2|	1422150	-	U	0	1	0	0	0	-
gi|29165615|ref|NC_002745.2|	1422158	-	C	1	0	0	0	0	-
gi|29165615|ref|NC_002745.2|	1430449	-	G	2	0	0	0	0	SA1257
gi|29165615|ref|NC_002745.2|	1437039	-	A	0	0	1	0	0	-
gi|29165615|ref|NC_002745.2|	1437041	-	A	0	2	0	1	0	-
gi|29165615|ref|NC_002745.2|	1437042	-	A	0	0	0	0	1	-
gi|29165615|ref|NC_002745.2|	1437057	-	G	0	2	0	0	0	-
gi|29165615|ref|NC_002745.2|	1437073	-	G	3	0	0	0	0	-
gi|29165615|ref|NC_002745.2|	1437084	-	A	0	1	0	0	1	-
gi|29165615|ref|NC_002745.2|	1437086	-	A	1	0	0	0	0	-
gi|29165615|ref|NC_002745.2|	1437089	-	U	0	1	0	0	0	-
gi|29165615|ref|NC_002745.2|	1437092	-	A	0	0	0	1	0	-
gi|29165615|ref|NC_002745.2|	1437093	-	A	0	0	0	2	1	-
gi|29165615|ref|NC_002745.2|	1437105	-	A	0	0	1	0	0	-
gi|29165615|ref|NC_002745.2|	1437111	-	A	0	0	0	3	0	-
gi|29165615|ref|NC_002745.2|	1437142	-	A	0	0	0	0	2	-
gi|29165615|ref|NC_002745.2|	1437148	-	C	1	2	0	0	0	-
gi|29165615|ref|NC_002745.2|	1437157	-	A	0	0	0	0	1	-
gi|29165615|ref|NC_002745.2|	1437161	-	G	0	0	1	0	0	-
gi|29165615|ref|NC_002745.2|	1437213	-	C	0	0	0	1	0	-
gi|29165615|ref|NC_002745.2|	1437216	-	A	0	0	0	0	1	-
gi|29165615|ref|NC_002745.2|	1437223	-	A	0	0	0	0	1	-
gi|29165615|ref|NC_002745.2|	1437240	-	U	0	0	1	0	0	-
gi|29165615|ref|NC_002745.2|	1437246	-	G	0	0	0	0	1	-
gi|29165615|ref|NC_002745.2|	1437261	-	U	0	1	0	0	0	-
gi|29165615|ref|NC_002745.2|	1437274	-	C	0	0	0	1	0	-
gi|29165615|ref|NC_002745.2|	1440079	-	A	0	0	0	1	0	SA1267
gi|29165615|ref|NC_002745.2|	1440102	-	A	0	1	0	0	0	SA1267
gi|29165615|ref|NC_002745.2|	1440103	-	A	0	1	0	0	0	SA1267
gi|29165615|ref|NC_002745.2|	1440310	-	A	0	0	0	1	0	SA1267
gi|29165615|ref|NC_002745.2|	1440333	-	A	0	1	0	0	0	SA1267
gi|29165615|ref|NC_002745.2|	1440334	-	A	0	1	0	0	0	SA1267
gi|29165615|ref|NC_002745.2|	1440541	-	A	0	0	0	1	0	SA1267
gi|29165615|ref|NC_002745.2|	1440564	-	A	0	1	0	0	0	SA1267
gi|29165615|ref|NC_002745.2|	1440565	-	A	0	1	0	0	0	SA1267
gi|29165615|ref|NC_002745.2|	1446151	-	A	0	1	0	0	0	SA1267
gi|29165615|ref|NC_002745.2|	1447599	-	C	0	1	0	1	1	SA1267
gi|29165615|ref|NC_002745.2|	1451440	-	A	0	1	0	0	0	SA1267
gi|29165615|ref|NC_002745.2|	1454761	-	A	0	1	0	0	0	SA1267
gi|29165615|ref|NC_002745.2|	1454763	-	A	0	0	1	0	0	SA1267
gi|29165615|ref|NC_002745.2|	1457785	-	A	0	1	0	0	0	SA1267

gi|29165615|ref|NC_002745.2|	1457787	-	A	0	0	1	0	0	SA1267
gi|29165615|ref|NC_002745.2|	1464163	-	A	1	0	0	0	0	SA1268
gi|29165615|ref|NC_002745.2|	1470808	-	A	0	0	0	0	1	SA1269
gi|29165615|ref|NC_002745.2|	1470813	-	A	0	1	0	0	0	SA1269
gi|29165615|ref|NC_002745.2|	1478398	-	A	0	1	0	0	0	SA1274
gi|29165615|ref|NC_002745.2|	1479318	-	C	0	1	0	0	0	SA1274
gi|29165615|ref|NC_002745.2|	1482069	-	A	0	0	0	1	0	SA1276
gi|29165615|ref|NC_002745.2|	1482070	-	G	0	0	0	1	0	SA1276
gi|29165615|ref|NC_002745.2|	1482073	-	C	0	0	0	0	1	SA1276
gi|29165615|ref|NC_002745.2|	1489583	-	U	0	1	0	0	0	SA1284
gi|29165615|ref|NC_002745.2|	1501720	-	A	1	0	0	0	0	SA1295
gi|29165615|ref|NC_002745.2|	1509659	-	C	0	1	0	0	0	SA1303
gi|29165615|ref|NC_002745.2|	1510883	-	A	0	2	0	2	2	SA1305
gi|29165615|ref|NC_002745.2|	1510884	-	A	0	1	0	0	3	SA1305
gi|29165615|ref|NC_002745.2|	1510885	-	A	0	2	0	0	0	SA1305
gi|29165615|ref|NC_002745.2|	1518995	-	A	0	0	0	0	2	SA1312
gi|29165615|ref|NC_002745.2|	1519016	-	A	0	0	0	0	2	SA1312
gi|29165615|ref|NC_002745.2|	1519485	-	A	0	0	1	0	0	SA1312
gi|29165615|ref|NC_002745.2|	1519665	-	A	0	0	1	0	0	SA1312
gi|29165615|ref|NC_002745.2|	1523850	-	G	1	0	1	0	1	-
gi|29165615|ref|NC_002745.2|	1523856	-	G	0	1	0	0	0	-
gi|29165615|ref|NC_002745.2|	1523863	-	C	0	1	0	0	0	-
gi|29165615|ref|NC_002745.2|	1523865	-	A	0	2	0	0	0	-
gi|29165615|ref|NC_002745.2|	1523866	-	U	1	0	2	0	0	-
gi|29165615|ref|NC_002745.2|	1523868	-	U	0	0	0	1	0	-
gi|29165615|ref|NC_002745.2|	1523872	-	A	0	1	0	0	0	-
gi|29165615|ref|NC_002745.2|	1523874	-	C	0	2	1	0	0	-
gi|29165615|ref|NC_002745.2|	1523877	-	C	0	0	0	1	0	-
gi|29165615|ref|NC_002745.2|	1523878	-	C	0	0	0	1	0	-
gi|29165615|ref|NC_002745.2|	1523886	-	G	0	1	0	0	1	-
gi|29165615|ref|NC_002745.2|	1523888	-	A	0	1	0	0	1	-
gi|29165615|ref|NC_002745.2|	1523890	-	U	0	1	1	2	0	-
gi|29165615|ref|NC_002745.2|	1523892	-	A	1	0	0	0	0	-
gi|29165615|ref|NC_002745.2|	1523897	-	C	0	0	0	0	1	-
gi|29165615|ref|NC_002745.2|	1523898	-	G	1	0	1	0	0	-
gi|29165615|ref|NC_002745.2|	1523901	-	C	0	1	0	0	0	-
gi|29165615|ref|NC_002745.2|	1523903	-	A	1	0	0	0	0	-
gi|29165615|ref|NC_002745.2|	1523905	-	C	0	1	0	0	0	-
gi|29165615|ref|NC_002745.2|	1524654	-	G	0	0	1	0	0	SA1317
gi|29165615|ref|NC_002745.2|	1524667	-	U	0	0	0	0	1	SA1317
gi|29165615|ref|NC_002745.2|	1524672	-	U	0	1	0	0	0	SA1317
gi|29165615|ref|NC_002745.2|	1524674	-	A	0	0	0	0	1	SA1317
gi|29165615|ref|NC_002745.2|	1524683	-	G	0	0	0	2	0	SA1317
gi|29165615|ref|NC_002745.2|	1524691	-	A	0	0	0	0	1	SA1317
gi|29165615|ref|NC_002745.2|	1524692	-	G	0	0	0	0	1	SA1317
gi|29165615|ref|NC_002745.2|	1524694	-	A	0	0	0	0	1	SA1317
gi|29165615|ref|NC_002745.2|	1524697	-	G	0	1	0	0	0	SA1317
gi|29165615|ref|NC_002745.2|	1524707	-	U	0	0	1	0	0	SA1317
gi|29165615|ref|NC_002745.2|	1524708	-	C	0	1	0	2	1	SA1317
gi|29165615|ref|NC_002745.2|	1524712	-	A	0	0	0	0	1	SA1317
gi|29165615|ref|NC_002745.2|	1524718	-	A	0	4	5	4	12	SA1317
gi|29165615|ref|NC_002745.2|	1524719	-	G	0	1	0	0	0	SA1317
gi|29165615|ref|NC_002745.2|	1524722	-	A	0	4	0	0	2	SA1317
gi|29165615|ref|NC_002745.2|	1524723	-	U	0	0	0	0	1	SA1317
gi|29165615|ref|NC_002745.2|	1524724	-	U	0	0	1	1	0	SA1317
gi|29165615|ref|NC_002745.2|	1524725	-	A	0	4	0	1	0	SA1317
gi|29165615|ref|NC_002745.2|	1524727	-	C	0	3	0	1	0	SA1317
gi|29165615|ref|NC_002745.2|	1524728	-	U	0	0	0	0	1	SA1317
gi|29165615|ref|NC_002745.2|	1524751	-	A	0	1	0	0	0	SA1317
gi|29165615|ref|NC_002745.2|	1524779	-	C	0	0	0	1	0	SA1317
gi|29165615|ref|NC_002745.2|	1524793	-	U	1	0	0	0	0	SA1317
gi|29165615|ref|NC_002745.2|	1524828	-	U	0	1	0	0	0	SA1317
gi|29165615|ref|NC_002745.2|	1524829	-	U	0	0	0	0	1	SA1317
gi|29165615|ref|NC_002745.2|	1524857	-	U	0	0	0	1	0	SA1317
gi|29165615|ref|NC_002745.2|	1524878	-	A	0	1	0	0	0	SA1317
gi|29165615|ref|NC_002745.2|	1524880	-	A	0	1	0	0	0	SA1317
gi|29165615|ref|NC_002745.2|	1524908	-	A	0	0	2	0	0	SA1317
gi|29165615|ref|NC_002745.2|	1524934	-	U	1	0	0	0	0	SA1317
gi|29165615|ref|NC_002745.2|	1524951	-	A	0	1	0	0	0	SA1317
gi|29165615|ref|NC_002745.2|	1524955	-	C	1	0	0	0	0	SA1317
gi|29165615|ref|NC_002745.2|	1525164	-	C	0	1	0	0	0	-
gi|29165615|ref|NC_002745.2|	1525818	-	A	0	0	1	0	1	SA1318
gi|29165615|ref|NC_002745.2|	1525819	-	G	0	0	0	1	0	SA1318
gi|29165615|ref|NC_002745.2|	1525828	-	A	0	1	0	0	0	SA1318
gi|29165615|ref|NC_002745.2|	1525874	-	A	0	1	0	0	0	SA1318
gi|29165615|ref|NC_002745.2|	1526074	-	G	0	1	0	0	0	SA1318
gi|29165615|ref|NC_002745.2|	1526158	-	C	0	0	1	0	0	SA1318
gi|29165615|ref|NC_002745.2|	1526190	-	A	0	1	0	0	0	SA1318
gi|29165615|ref|NC_002745.2|	1526214	-	C	0	0	2	0	0	SA1318
gi|29165615|ref|NC_002745.2|	1526219	-	U	0	1	0	0	0	SA1318
gi|29165615|ref|NC_002745.2|	1526220	-	U	0	0	0	0	1	SA1318

gi|29165615|ref|NC_002745.2|	1526325	-	U	1	0	0	0	0	SA1318
gi|29165615|ref|NC_002745.2|	1526342	-	A	0	1	0	0	0	SA1318
gi|29165615|ref|NC_002745.2|	1526346	-	C	1	0	0	0	0	SA1318
gi|29165615|ref|NC_002745.2|	1527008	-	G	0	0	1	0	0	SA1319
gi|29165615|ref|NC_002745.2|	1527021	-	U	0	0	0	0	1	SA1319
gi|29165615|ref|NC_002745.2|	1527026	-	U	0	1	0	0	0	SA1319
gi|29165615|ref|NC_002745.2|	1527028	-	A	0	0	0	0	1	SA1319
gi|29165615|ref|NC_002745.2|	1527037	-	G	0	0	0	2	0	SA1319
gi|29165615|ref|NC_002745.2|	1527045	-	A	0	0	0	0	1	SA1319
gi|29165615|ref|NC_002745.2|	1527046	-	G	0	0	0	0	1	SA1319
gi|29165615|ref|NC_002745.2|	1527048	-	A	0	0	0	0	1	SA1319
gi|29165615|ref|NC_002745.2|	1527051	-	G	0	1	0	0	0	SA1319
gi|29165615|ref|NC_002745.2|	1527061	-	U	0	0	1	0	0	SA1319
gi|29165615|ref|NC_002745.2|	1527062	-	C	0	1	0	2	1	SA1319
gi|29165615|ref|NC_002745.2|	1527066	-	A	0	0	0	0	1	SA1319
gi|29165615|ref|NC_002745.2|	1527072	-	A	0	4	5	4	12	SA1319
gi|29165615|ref|NC_002745.2|	1527073	-	G	0	1	0	0	0	SA1319
gi|29165615|ref|NC_002745.2|	1527076	-	A	0	4	0	0	2	SA1319
gi|29165615|ref|NC_002745.2|	1527077	-	A	0	0	0	0	1	SA1319
gi|29165615|ref|NC_002745.2|	1527078	-	U	0	0	1	1	0	SA1319
gi|29165615|ref|NC_002745.2|	1527079	-	A	0	4	0	1	0	SA1319
gi|29165615|ref|NC_002745.2|	1527081	-	C	0	3	0	1	0	SA1319
gi|29165615|ref|NC_002745.2|	1527082	-	U	0	0	0	0	1	SA1319
gi|29165615|ref|NC_002745.2|	1527105	-	A	0	1	0	0	0	SA1319
gi|29165615|ref|NC_002745.2|	1527133	-	C	0	0	0	1	0	SA1319
gi|29165615|ref|NC_002745.2|	1527147	-	U	1	0	0	0	0	SA1319
gi|29165615|ref|NC_002745.2|	1527182	-	U	0	1	0	0	0	SA1319
gi|29165615|ref|NC_002745.2|	1527183	-	U	0	0	0	0	1	SA1319
gi|29165615|ref|NC_002745.2|	1527211	-	U	0	0	0	1	0	SA1319
gi|29165615|ref|NC_002745.2|	1527232	-	A	0	1	0	0	0	SA1319
gi|29165615|ref|NC_002745.2|	1527234	-	A	0	1	0	0	0	SA1319
gi|29165615|ref|NC_002745.2|	1527262	-	A	0	0	2	0	0	SA1319
gi|29165615|ref|NC_002745.2|	1527288	-	U	1	0	0	0	0	SA1319
gi|29165615|ref|NC_002745.2|	1527305	-	A	0	1	0	0	0	SA1319
gi|29165615|ref|NC_002745.2|	1527309	-	C	1	0	0	0	0	SA1319
gi|29165615|ref|NC_002745.2|	1529285	-	C	0	1	0	0	0	SA1320
gi|29165615|ref|NC_002745.2|	1529940	-	A	0	0	1	0	1	SA1321
gi|29165615|ref|NC_002745.2|	1529941	-	G	0	0	0	1	0	SA1321
gi|29165615|ref|NC_002745.2|	1529950	-	A	0	1	0	0	0	SA1321
gi|29165615|ref|NC_002745.2|	1529996	-	A	0	1	0	0	0	SA1321
gi|29165615|ref|NC_002745.2|	1530196	-	G	0	1	0	0	0	SA1321
gi|29165615|ref|NC_002745.2|	1530280	-	C	0	0	1	0	0	SA1321
gi|29165615|ref|NC_002745.2|	1530312	-	A	0	1	0	0	0	SA1321
gi|29165615|ref|NC_002745.2|	1530336	-	C	0	0	2	0	0	SA1321
gi|29165615|ref|NC_002745.2|	1530341	-	U	0	1	0	0	0	SA1321
gi|29165615|ref|NC_002745.2|	1530342	-	U	0	0	0	0	1	SA1321
gi|29165615|ref|NC_002745.2|	1530447	-	U	1	0	0	0	0	SA1321
gi|29165615|ref|NC_002745.2|	1530464	-	A	0	1	0	0	0	SA1321
gi|29165615|ref|NC_002745.2|	1530468	-	C	1	0	0	0	0	SA1321
gi|29165615|ref|NC_002745.2|	1531069	-	G	0	0	1	0	0	SA1322
gi|29165615|ref|NC_002745.2|	1534872	-	A	0	0	1	0	0	SA1326
gi|29165615|ref|NC_002745.2|	1539626	-	A	0	0	0	0	1	SA1333
gi|29165615|ref|NC_002745.2|	1550512	-	A	0	1	0	0	0	-
gi|29165615|ref|NC_002745.2|	1566663	-	A	0	0	0	0	2	SA1357
gi|29165615|ref|NC_002745.2|	1566669	-	A	0	2	0	0	1	SA1357
gi|29165615|ref|NC_002745.2|	1568764	-	G	2	1	0	0	0	SA1359
gi|29165615|ref|NC_002745.2|	1568766	-	A	0	1	0	0	0	SA1359
gi|29165615|ref|NC_002745.2|	1569161	-	G	1	0	0	0	0	SA1360
gi|29165615|ref|NC_002745.2|	1575717	-	A	1	0	0	0	0	SA1367
gi|29165615|ref|NC_002745.2|	1576165	-	A	0	1	0	1	1	SA1367
gi|29165615|ref|NC_002745.2|	1576166	-	A	0	0	0	2	7	SA1367
gi|29165615|ref|NC_002745.2|	1576167	-	G	1	0	0	0	0	SA1367
gi|29165615|ref|NC_002745.2|	1584137	-	U	1	0	0	0	0	SA1379
gi|29165615|ref|NC_002745.2|	1585714	-	C	1	0	0	0	0	-
gi|29165615|ref|NC_002745.2|	1588611	-	A	0	3	0	1	3	SA1382
gi|29165615|ref|NC_002745.2|	1590542	-	C	1	0	0	0	1	SA1385
gi|29165615|ref|NC_002745.2|	1590547	-	A	0	1	0	0	1	SA1385
gi|29165615|ref|NC_002745.2|	1590548	-	A	0	2	0	0	0	SA1385
gi|29165615|ref|NC_002745.2|	1590855	-	C	0	0	1	0	0	SA1385
gi|29165615|ref|NC_002745.2|	1592241	-	U	1	0	0	0	0	SA1387
gi|29165615|ref|NC_002745.2|	1595985	-	U	0	0	1	0	1	SA1390
gi|29165615|ref|NC_002745.2|	1595987	-	U	0	1	0	0	0	SA1390
gi|29165615|ref|NC_002745.2|	1598717	-	G	2	1	0	0	0	SA1392
gi|29165615|ref|NC_002745.2|	1598719	-	A	0	1	0	0	0	SA1392
gi|29165615|ref|NC_002745.2|	1601803	-	U	0	0	1	0	2	-
gi|29165615|ref|NC_002745.2|	1601804	-	U	0	1	2	3	0	-
gi|29165615|ref|NC_002745.2|	1601806	-	A	0	0	0	0	1	-
gi|29165615|ref|NC_002745.2|	1611105	-	A	0	1	0	1	0	SA1406
gi|29165615|ref|NC_002745.2|	1611106	-	G	0	0	0	0	1	SA1406
gi|29165615|ref|NC_002745.2|	1618463	-	A	0	1	0	0	3	-
gi|29165615|ref|NC_002745.2|	1618558	-	A	0	0	1	0	0	-

gi|29165615|ref|NC_002745.2|	1618560	-	A	0	2	0	1	0	-
gi|29165615|ref|NC_002745.2|	1618561	-	A	0	0	0	0	1	-
gi|29165615|ref|NC_002745.2|	1618576	-	G	0	2	0	0	0	-
gi|29165615|ref|NC_002745.2|	1618603	-	A	0	1	0	0	1	-
gi|29165615|ref|NC_002745.2|	1618605	-	A	1	0	0	0	0	-
gi|29165615|ref|NC_002745.2|	1618608	-	U	0	1	0	0	0	-
gi|29165615|ref|NC_002745.2|	1618611	-	A	0	0	0	1	0	-
gi|29165615|ref|NC_002745.2|	1618612	-	A	0	0	0	2	1	-
gi|29165615|ref|NC_002745.2|	1618624	-	A	0	0	1	0	0	-
gi|29165615|ref|NC_002745.2|	1618630	-	C	0	0	0	3	0	-
gi|29165615|ref|NC_002745.2|	1618664	-	A	1	0	0	0	0	-
gi|29165615|ref|NC_002745.2|	1618670	-	A	0	0	0	1	0	-
gi|29165615|ref|NC_002745.2|	1618671	-	A	0	1	0	3	2	-
gi|29165615|ref|NC_002745.2|	1618674	-	A	0	0	0	0	1	-
gi|29165615|ref|NC_002745.2|	1618675	-	A	0	0	0	1	0	-
gi|29165615|ref|NC_002745.2|	1618676	-	A	0	0	0	5	0	-
gi|29165615|ref|NC_002745.2|	1618684	-	G	0	0	0	1	0	-
gi|29165615|ref|NC_002745.2|	1618689	-	A	0	0	0	1	0	-
gi|29165615|ref|NC_002745.2|	1618690	-	C	0	0	1	0	0	-
gi|29165615|ref|NC_002745.2|	1618692	-	A	0	0	0	0	1	-
gi|29165615|ref|NC_002745.2|	1620435	-	G	0	1	0	0	2	SA1413
gi|29165615|ref|NC_002745.2|	1620497	-	A	1	2	0	0	1	SA1413
gi|29165615|ref|NC_002745.2|	1620498	-	A	0	0	1	0	3	SA1413
gi|29165615|ref|NC_002745.2|	1633094	-	A	0	1	0	2	0	-
gi|29165615|ref|NC_002745.2|	1633100	-	C	0	1	1	0	0	-
gi|29165615|ref|NC_002745.2|	1633103	-	G	0	1	0	0	0	-
gi|29165615|ref|NC_002745.2|	1633104	-	A	0	0	1	0	1	-
gi|29165615|ref|NC_002745.2|	1633106	-	G	0	0	0	0	1	-
gi|29165615|ref|NC_002745.2|	1633109	-	U	6	2	0	0	3	-
gi|29165615|ref|NC_002745.2|	1633113	-	U	2	0	0	0	0	-
gi|29165615|ref|NC_002745.2|	1633114	-	A	0	0	0	0	1	-
gi|29165615|ref|NC_002745.2|	1633116	-	A	1	1	2	0	0	-
gi|29165615|ref|NC_002745.2|	1633117	-	A	0	1	1	0	0	-
gi|29165615|ref|NC_002745.2|	1633118	-	C	1	0	0	0	0	-
gi|29165615|ref|NC_002745.2|	1633119	-	C	0	0	1	1	3	-
gi|29165615|ref|NC_002745.2|	1633121	-	U	0	0	1	0	2	-
gi|29165615|ref|NC_002745.2|	1633123	-	A	0	0	1	0	1	-
gi|29165615|ref|NC_002745.2|	1633124	-	U	3	2	0	0	1	-
gi|29165615|ref|NC_002745.2|	1633128	-	C	0	1	0	2	1	-
gi|29165615|ref|NC_002745.2|	1633129	-	U	0	0	0	0	1	-
gi|29165615|ref|NC_002745.2|	1633134	-	U	0	1	0	0	0	-
gi|29165615|ref|NC_002745.2|	1633136	-	C	1	3	4	2	1	-
gi|29165615|ref|NC_002745.2|	1633141	-	A	0	0	1	0	0	-
gi|29165615|ref|NC_002745.2|	1633146	-	A	0	0	0	0	6	-
gi|29165615|ref|NC_002745.2|	1633148	-	C	0	0	1	0	0	-
gi|29165615|ref|NC_002745.2|	1633151	-	U	0	0	0	0	1	-
gi|29165615|ref|NC_002745.2|	1633162	-	A	0	0	0	0	2	-
gi|29165615|ref|NC_002745.2|	1633166	-	A	0	0	1	0	0	-
gi|29165615|ref|NC_002745.2|	1633169	-	A	0	0	1	0	0	-
gi|29165615|ref|NC_002745.2|	1633171	-	C	0	1	1	0	0	-
gi|29165615|ref|NC_002745.2|	1633180	-	G	0	0	1	0	0	-
gi|29165615|ref|NC_002745.2|	1633184	-	U	1	1	0	0	0	-
gi|29165615|ref|NC_002745.2|	1633186	-	U	0	1	0	1	1	-
gi|29165615|ref|NC_002745.2|	1633190	-	U	0	2	0	0	0	-
gi|29165615|ref|NC_002745.2|	1633191	-	C	2	0	0	0	0	-
gi|29165615|ref|NC_002745.2|	1633192	-	U	1	0	0	0	0	-
gi|29165615|ref|NC_002745.2|	1633196	-	A	0	1	2	0	1	-
gi|29165615|ref|NC_002745.2|	1633197	-	G	0	1	0	0	1	-
gi|29165615|ref|NC_002745.2|	1633200	-	U	0	1	1	1	1	-
gi|29165615|ref|NC_002745.2|	1633201	-	U	0	3	1	4	1	-
gi|29165615|ref|NC_002745.2|	1633202	-	U	0	0	1	0	0	-
gi|29165615|ref|NC_002745.2|	1633205	-	U	0	0	0	0	1	-
gi|29165615|ref|NC_002745.2|	1633206	-	U	0	0	1	3	1	-
gi|29165615|ref|NC_002745.2|	1633207	-	A	2	0	1	1	0	-
gi|29165615|ref|NC_002745.2|	1633208	-	A	0	1	2	2	0	-
gi|29165615|ref|NC_002745.2|	1633209	-	G	0	1	0	2	0	-
gi|29165615|ref|NC_002745.2|	1633210	-	A	0	0	1	1	0	-
gi|29165615|ref|NC_002745.2|	1633211	-	U	0	0	0	1	0	-
gi|29165615|ref|NC_002745.2|	1633212	-	G	0	0	0	0	1	-
gi|29165615|ref|NC_002745.2|	1633213	-	U	0	0	0	1	0	-
gi|29165615|ref|NC_002745.2|	1633218	-	U	3	2	3	1	10	-
gi|29165615|ref|NC_002745.2|	1633219	-	A	0	0	2	0	0	-
gi|29165615|ref|NC_002745.2|	1633220	-	C	0	1	2	0	0	-
gi|29165615|ref|NC_002745.2|	1633221	-	G	0	0	0	0	1	-
gi|29165615|ref|NC_002745.2|	1633226	-	A	0	1	0	0	0	-
gi|29165615|ref|NC_002745.2|	1633227	-	C	0	0	0	0	1	-
gi|29165615|ref|NC_002745.2|	1633228	-	C	0	0	0	1	2	-
gi|29165615|ref|NC_002745.2|	1633233	-	C	0	0	0	1	0	-
gi|29165615|ref|NC_002745.2|	1633241	-	U	0	0	1	0	0	-
gi|29165615|ref|NC_002745.2|	1633247	-	U	0	0	0	0	1	-
gi|29165615|ref|NC_002745.2|	1633253	-	C	0	2	0	0	0	-

gi|29165615|ref|NC_002745.2|	1633259	-	U	0	0	1	0	0	-
gi|29165615|ref|NC_002745.2|	1633266	-	A	0	0	1	0	0	-
gi|29165615|ref|NC_002745.2|	1633267	-	A	0	0	0	1	0	-
gi|29165615|ref|NC_002745.2|	1633268	-	U	0	1	0	2	1	-
gi|29165615|ref|NC_002745.2|	1633276	-	C	0	0	1	0	3	-
gi|29165615|ref|NC_002745.2|	1633284	-	A	0	0	0	1	0	-
gi|29165615|ref|NC_002745.2|	1633285	-	C	0	0	0	0	1	-
gi|29165615|ref|NC_002745.2|	1633289	-	C	0	0	1	2	0	-
gi|29165615|ref|NC_002745.2|	1633324	-	A	0	0	0	1	0	-
gi|29165615|ref|NC_002745.2|	1633364	-	A	0	2	0	0	0	-
gi|29165615|ref|NC_002745.2|	1633440	-	G	0	0	1	0	0	-
gi|29165615|ref|NC_002745.2|	1637984	-	G	0	1	0	0	0	SA1434
gi|29165615|ref|NC_002745.2|	1639114	-	A	1	0	1	0	0	SA1435
gi|29165615|ref|NC_002745.2|	1641406	-	A	0	0	1	0	0	SA1438
gi|29165615|ref|NC_002745.2|	1644528	-	A	0	2	1	0	1	SA1442
gi|29165615|ref|NC_002745.2|	1652072	-	U	0	0	1	0	0	SA1448
gi|29165615|ref|NC_002745.2|	1652177	-	U	0	0	1	0	0	SA1448
gi|29165615|ref|NC_002745.2|	1652845	-	U	0	1	0	0	0	-
gi|29165615|ref|NC_002745.2|	1652882	-	A	0	0	1	0	0	-
gi|29165615|ref|NC_002745.2|	1652884	-	A	0	2	0	1	0	-
gi|29165615|ref|NC_002745.2|	1652885	-	A	0	0	0	0	1	-
gi|29165615|ref|NC_002745.2|	1652931	-	A	0	0	0	1	0	-
gi|29165615|ref|NC_002745.2|	1652932	-	A	0	1	0	1	1	-
gi|29165615|ref|NC_002745.2|	1652935	-	A	0	0	0	0	1	-
gi|29165615|ref|NC_002745.2|	1652936	-	A	0	0	0	1	0	-
gi|29165615|ref|NC_002745.2|	1652937	-	U	0	0	0	3	0	-
gi|29165615|ref|NC_002745.2|	1652945	-	G	0	0	0	1	0	-
gi|29165615|ref|NC_002745.2|	1652950	-	A	0	0	0	1	0	-
gi|29165615|ref|NC_002745.2|	1652951	-	C	0	0	1	0	0	-
gi|29165615|ref|NC_002745.2|	1652953	-	A	0	0	0	0	1	-
gi|29165615|ref|NC_002745.2|	1652996	-	A	0	0	0	0	1	-
gi|29165615|ref|NC_002745.2|	1653088	-	A	0	1	0	0	0	-
gi|29165615|ref|NC_002745.2|	1653093	-	A	0	0	0	0	1	-
gi|29165615|ref|NC_002745.2|	1653100	-	A	0	0	0	1	0	-
gi|29165615|ref|NC_002745.2|	1653164	-	A	0	0	0	1	0	-
gi|29165615|ref|NC_002745.2|	1653165	-	A	0	1	0	1	1	-
gi|29165615|ref|NC_002745.2|	1653168	-	A	0	0	0	0	1	-
gi|29165615|ref|NC_002745.2|	1653169	-	A	0	0	0	1	0	-
gi|29165615|ref|NC_002745.2|	1653178	-	G	0	0	0	1	0	-
gi|29165615|ref|NC_002745.2|	1653202	-	A	0	1	0	0	0	-
gi|29165615|ref|NC_002745.2|	1653225	-	A	0	0	0	0	1	-
gi|29165615|ref|NC_002745.2|	1654786	-	C	0	1	0	0	0	SA1450
gi|29165615|ref|NC_002745.2|	1654797	-	A	0	1	0	0	0	SA1450
gi|29165615|ref|NC_002745.2|	1657480	-	U	0	0	0	1	0	SA1452
gi|29165615|ref|NC_002745.2|	1657484	-	A	0	0	0	1	0	SA1452
gi|29165615|ref|NC_002745.2|	1657490	-	A	0	0	0	0	1	SA1452
gi|29165615|ref|NC_002745.2|	1660302	-	A	0	0	2	0	0	SA1455
gi|29165615|ref|NC_002745.2|	1661112	-	A	2	0	0	0	0	SA1456
gi|29165615|ref|NC_002745.2|	1663368	-	A	0	1	0	0	0	SA1457
gi|29165615|ref|NC_002745.2|	1663404	-	U	0	1	0	1	0	SA1457
gi|29165615|ref|NC_002745.2|	1663405	-	A	0	0	1	1	3	SA1457
gi|29165615|ref|NC_002745.2|	1667481	-	A	0	0	0	0	1	SA1460
gi|29165615|ref|NC_002745.2|	1667569	-	A	0	1	0	0	0	SA1460
gi|29165615|ref|NC_002745.2|	1668841	-	C	0	0	0	0	1	SA1462
gi|29165615|ref|NC_002745.2|	1668842	-	U	0	0	0	0	1	SA1462
gi|29165615|ref|NC_002745.2|	1678719	-	A	0	1	0	1	1	SA1470
gi|29165615|ref|NC_002745.2|	1679807	-	A	0	0	1	0	0	-
gi|29165615|ref|NC_002745.2|	1679808	-	A	0	0	0	1	0	-
gi|29165615|ref|NC_002745.2|	1682405	-	C	0	0	1	0	0	SA1475
gi|29165615|ref|NC_002745.2|	1693106	-	A	0	0	1	0	0	SA1487
gi|29165615|ref|NC_002745.2|	1693110	-	A	0	0	0	1	0	SA1487
gi|29165615|ref|NC_002745.2|	1693564	-	A	0	1	0	0	1	SA1487
gi|29165615|ref|NC_002745.2|	1695603	-	A	0	1	0	0	0	SA1488
gi|29165615|ref|NC_002745.2|	1696293	-	C	0	1	1	0	0	SA1488
gi|29165615|ref|NC_002745.2|	1706685	-	A	0	0	0	0	1	SA1498
gi|29165615|ref|NC_002745.2|	1707886	-	A	0	0	2	0	0	SA1499
gi|29165615|ref|NC_002745.2|	1708395	-	U	0	3	2	0	0	SA1499
gi|29165615|ref|NC_002745.2|	1708397	-	A	0	0	1	0	0	SA1499
gi|29165615|ref|NC_002745.2|	1708398	-	U	0	2	1	0	3	SA1499
gi|29165615|ref|NC_002745.2|	1708399	-	G	0	0	1	0	0	SA1499
gi|29165615|ref|NC_002745.2|	1708760	-	C	0	0	1	0	0	SA1499
gi|29165615|ref|NC_002745.2|	1716343	-	C	0	1	0	0	0	-
gi|29165615|ref|NC_002745.2|	1717442	-	G	0	2	0	0	0	SA1508
gi|29165615|ref|NC_002745.2|	1717447	-	C	0	0	1	0	0	SA1508
gi|29165615|ref|NC_002745.2|	1717449	-	U	0	0	0	1	0	SA1508
gi|29165615|ref|NC_002745.2|	1720383	-	A	0	0	1	0	0	SA1510
gi|29165615|ref|NC_002745.2|	1720384	-	C	0	0	2	0	0	SA1510
gi|29165615|ref|NC_002745.2|	1720385	-	G	0	1	0	0	0	SA1510
gi|29165615|ref|NC_002745.2|	1729085	-	U	0	1	0	0	0	SA1516
gi|29165615|ref|NC_002745.2|	1729096	-	U	0	0	1	0	1	SA1516
gi|29165615|ref|NC_002745.2|	1729605	-	U	0	2	0	0	0	-

gi|29165615|ref|NC_002745.2|	1736386	-	A	0	2	0	0	1	SA1521
gi|29165615|ref|NC_002745.2|	1736408	-	U	0	2	0	0	1	SA1521
gi|29165615|ref|NC_002745.2|	1736409	-	A	1	2	2	3	4	SA1521
gi|29165615|ref|NC_002745.2|	1741057	-	A	0	1	0	0	0	SA1525
gi|29165615|ref|NC_002745.2|	1745304	-	U	0	1	0	0	0	-
gi|29165615|ref|NC_002745.2|	1745305	-	C	1	0	0	1	0	-
gi|29165615|ref|NC_002745.2|	1745306	-	A	0	1	0	1	1	-
gi|29165615|ref|NC_002745.2|	1745307	-	A	0	5	0	0	1	-
gi|29165615|ref|NC_002745.2|	1745308	-	C	0	0	0	2	1	-
gi|29165615|ref|NC_002745.2|	1745309	-	C	0	0	1	0	2	-
gi|29165615|ref|NC_002745.2|	1745314	-	C	0	0	0	1	0	-
gi|29165615|ref|NC_002745.2|	1745322	-	U	0	0	1	0	0	-
gi|29165615|ref|NC_002745.2|	1745334	-	A	0	1	1	0	0	-
gi|29165615|ref|NC_002745.2|	1745335	-	G	0	1	0	0	1	-
gi|29165615|ref|NC_002745.2|	1745338	-	U	0	0	1	0	1	-
gi|29165615|ref|NC_002745.2|	1745339	-	U	0	3	1	3	1	-
gi|29165615|ref|NC_002745.2|	1745343	-	U	0	0	0	0	1	-
gi|29165615|ref|NC_002745.2|	1745344	-	U	0	0	1	3	1	-
gi|29165615|ref|NC_002745.2|	1745345	-	A	0	0	1	0	0	-
gi|29165615|ref|NC_002745.2|	1745347	-	G	0	1	0	2	0	-
gi|29165615|ref|NC_002745.2|	1745348	-	A	0	0	1	1	0	-
gi|29165615|ref|NC_002745.2|	1745349	-	U	0	0	0	1	0	-
gi|29165615|ref|NC_002745.2|	1745350	-	G	0	0	0	0	1	-
gi|29165615|ref|NC_002745.2|	1745351	-	U	0	0	0	1	0	-
gi|29165615|ref|NC_002745.2|	1745356	-	U	3	2	3	1	10	-
gi|29165615|ref|NC_002745.2|	1745357	-	A	0	0	1	0	0	-
gi|29165615|ref|NC_002745.2|	1745358	-	C	0	1	2	0	0	-
gi|29165615|ref|NC_002745.2|	1761688	-	G	0	0	0	0	1	-
gi|29165615|ref|NC_002745.2|	1761692	-	U	1	1	1	1	1	-
gi|29165615|ref|NC_002745.2|	1761701	-	C	2	1	0	0	0	-
gi|29165615|ref|NC_002745.2|	1761706	-	C	0	0	0	0	1	-
gi|29165615|ref|NC_002745.2|	1761713	-	A	0	5	2	6	2	-
gi|29165615|ref|NC_002745.2|	1761714	-	U	0	1	0	0	0	-
gi|29165615|ref|NC_002745.2|	1761715	-	A	0	0	0	0	1	-
gi|29165615|ref|NC_002745.2|	1761882	-	U	0	1	0	0	0	-
gi|29165615|ref|NC_002745.2|	1762229	-	U	0	0	0	0	1	-
gi|29165615|ref|NC_002745.2|	1762440	-	A	0	1	0	0	0	-
gi|29165615|ref|NC_002745.2|	1762456	-	A	0	0	0	1	0	-
gi|29165615|ref|NC_002745.2|	1762488	-	A	0	0	0	0	1	-
gi|29165615|ref|NC_002745.2|	1762506	-	U	0	1	0	0	0	-
gi|29165615|ref|NC_002745.2|	1762512	-	A	0	0	0	0	1	-
gi|29165615|ref|NC_002745.2|	1762513	-	U	0	0	1	0	1	-
gi|29165615|ref|NC_002745.2|	1762514	-	G	0	1	0	0	0	-
gi|29165615|ref|NC_002745.2|	1762523	-	U	0	0	0	0	1	-
gi|29165615|ref|NC_002745.2|	1762527	-	C	0	0	1	0	0	-
gi|29165615|ref|NC_002745.2|	1762528	-	C	1	1	0	0	0	-
gi|29165615|ref|NC_002745.2|	1770208	-	A	0	1	3	0	4	SA1547
gi|29165615|ref|NC_002745.2|	1770209	-	U	1	2	2	1	2	SA1547
gi|29165615|ref|NC_002745.2|	1770210	-	U	0	1	1	0	0	SA1547
gi|29165615|ref|NC_002745.2|	1773736	-	A	0	1	0	0	0	SA1550
gi|29165615|ref|NC_002745.2|	1775151	-	A	0	0	1	1	1	-
gi|29165615|ref|NC_002745.2|	1780451	-	G	0	1	0	0	0	SA1554
gi|29165615|ref|NC_002745.2|	1781465	-	U	0	0	0	1	0	SA1554
gi|29165615|ref|NC_002745.2|	1786397	-	A	0	2	0	2	2	SA1558
gi|29165615|ref|NC_002745.2|	1786398	-	A	0	1	0	0	3	SA1558
gi|29165615|ref|NC_002745.2|	1786399	-	A	0	2	0	0	0	SA1558
gi|29165615|ref|NC_002745.2|	1788228	-	A	0	0	0	1	0	SA1559
gi|29165615|ref|NC_002745.2|	1788313	-	A	1	0	0	0	0	SA1559
gi|29165615|ref|NC_002745.2|	1788387	-	A	0	0	0	1	0	SA1559
gi|29165615|ref|NC_002745.2|	1788472	-	A	1	0	0	0	0	SA1559
gi|29165615|ref|NC_002745.2|	1788546	-	A	0	0	0	1	0	SA1559
gi|29165615|ref|NC_002745.2|	1789550	-	A	0	0	1	0	0	SA1561
gi|29165615|ref|NC_002745.2|	1794972	-	U	0	0	1	0	0	SA1563
gi|29165615|ref|NC_002745.2|	1794974	-	C	0	0	0	0	1	SA1563
gi|29165615|ref|NC_002745.2|	1794978	-	C	0	0	1	0	0	SA1563
gi|29165615|ref|NC_002745.2|	1794982	-	A	0	0	0	0	1	SA1563
gi|29165615|ref|NC_002745.2|	1794983	-	A	1	1	0	0	0	SA1563
gi|29165615|ref|NC_002745.2|	1804097	-	A	0	1	2	1	1	-
gi|29165615|ref|NC_002745.2|	1804098	-	G	0	1	0	0	1	-
gi|29165615|ref|NC_002745.2|	1804101	-	U	0	1	1	1	1	-
gi|29165615|ref|NC_002745.2|	1804102	-	U	0	3	1	4	1	-
gi|29165615|ref|NC_002745.2|	1804103	-	U	0	0	1	0	0	-
gi|29165615|ref|NC_002745.2|	1804106	-	U	0	0	0	0	1	-
gi|29165615|ref|NC_002745.2|	1804107	-	U	0	0	1	3	1	-
gi|29165615|ref|NC_002745.2|	1804108	-	A	2	0	1	1	0	-
gi|29165615|ref|NC_002745.2|	1804109	-	A	0	1	2	2	0	-
gi|29165615|ref|NC_002745.2|	1804110	-	G	0	1	0	2	0	-
gi|29165615|ref|NC_002745.2|	1804111	-	A	0	0	1	1	0	-
gi|29165615|ref|NC_002745.2|	1804112	-	U	0	0	0	1	0	-
gi|29165615|ref|NC_002745.2|	1804113	-	G	0	0	0	0	1	-
gi|29165615|ref|NC_002745.2|	1804114	-	U	0	0	0	1	0	-

gi|29165615|ref|NC_002745.2|	1804119	-	U	3	2	3	1	10	-
gi|29165615|ref|NC_002745.2|	1804120	-	A	0	0	2	0	0	-
gi|29165615|ref|NC_002745.2|	1804121	-	C	0	1	2	0	0	-
gi|29165615|ref|NC_002745.2|	1804122	-	G	0	0	0	0	1	-
gi|29165615|ref|NC_002745.2|	1804127	-	A	0	2	0	0	0	-
gi|29165615|ref|NC_002745.2|	1804128	-	C	0	0	0	2	1	-
gi|29165615|ref|NC_002745.2|	1804129	-	C	0	0	1	1	2	-
gi|29165615|ref|NC_002745.2|	1804132	-	C	0	1	0	1	2	-
gi|29165615|ref|NC_002745.2|	1804134	-	C	0	0	0	1	0	-
gi|29165615|ref|NC_002745.2|	1804140	-	U	0	0	1	0	0	-
gi|29165615|ref|NC_002745.2|	1804142	-	U	1	1	0	0	0	-
gi|29165615|ref|NC_002745.2|	1804152	-	C	0	2	0	0	0	-
gi|29165615|ref|NC_002745.2|	1804209	-	A	0	0	0	1	0	-
gi|29165615|ref|NC_002745.2|	1804211	-	A	0	0	1	0	0	-
gi|29165615|ref|NC_002745.2|	1804212	-	G	0	0	0	0	1	-
gi|29165615|ref|NC_002745.2|	1804215	-	U	0	1	1	1	1	-
gi|29165615|ref|NC_002745.2|	1804216	-	U	0	3	1	4	1	-
gi|29165615|ref|NC_002745.2|	1804217	-	U	0	0	1	0	0	-
gi|29165615|ref|NC_002745.2|	1804220	-	A	0	0	0	0	1	-
gi|29165615|ref|NC_002745.2|	1804221	-	U	0	0	1	3	1	-
gi|29165615|ref|NC_002745.2|	1804337	-	U	0	0	1	0	0	-
gi|29165615|ref|NC_002745.2|	1816865	-	A	0	1	1	0	0	SA1579
gi|29165615|ref|NC_002745.2|	1816868	-	A	0	0	0	1	0	SA1579
gi|29165615|ref|NC_002745.2|	1828498	-	G	1	0	1	0	1	-
gi|29165615|ref|NC_002745.2|	1828504	-	G	0	1	0	0	0	-
gi|29165615|ref|NC_002745.2|	1828511	-	C	0	1	0	0	0	-
gi|29165615|ref|NC_002745.2|	1828513	-	U	0	2	0	0	0	-
gi|29165615|ref|NC_002745.2|	1828514	-	U	1	0	2	0	0	-
gi|29165615|ref|NC_002745.2|	1828516	-	U	0	0	0	1	0	-
gi|29165615|ref|NC_002745.2|	1828520	-	A	0	1	0	0	0	-
gi|29165615|ref|NC_002745.2|	1828522	-	C	0	2	1	0	0	-
gi|29165615|ref|NC_002745.2|	1828525	-	C	0	0	0	1	0	-
gi|29165615|ref|NC_002745.2|	1828526	-	C	0	0	0	1	0	-
gi|29165615|ref|NC_002745.2|	1828534	-	G	0	1	0	0	1	-
gi|29165615|ref|NC_002745.2|	1828536	-	A	0	1	0	0	1	-
gi|29165615|ref|NC_002745.2|	1828538	-	U	0	1	1	2	0	-
gi|29165615|ref|NC_002745.2|	1828540	-	A	1	0	0	0	0	-
gi|29165615|ref|NC_002745.2|	1828545	-	C	0	0	0	0	1	-
gi|29165615|ref|NC_002745.2|	1828546	-	G	1	0	1	0	0	-
gi|29165615|ref|NC_002745.2|	1828549	-	C	0	1	0	0	0	-
gi|29165615|ref|NC_002745.2|	1828551	-	A	1	0	0	0	0	-
gi|29165615|ref|NC_002745.2|	1828553	-	C	0	1	0	0	0	-
gi|29165615|ref|NC_002745.2|	1829575	-	A	1	0	0	0	0	SA1590
gi|29165615|ref|NC_002745.2|	1832767	-	C	1	0	0	0	0	SA1593
gi|29165615|ref|NC_002745.2|	1835520	-	A	1	1	0	0	0	SA1597
gi|29165615|ref|NC_002745.2|	1837685	-	U	0	1	0	0	0	-
gi|29165615|ref|NC_002745.2|	1839343	-	U	0	1	0	0	0	-
gi|29165615|ref|NC_002745.2|	1839364	-	A	0	1	0	0	2	-
gi|29165615|ref|NC_002745.2|	1839365	-	A	0	1	1	0	0	-
gi|29165615|ref|NC_002745.2|	1839366	-	A	0	1	0	3	2	-
gi|29165615|ref|NC_002745.2|	1839371	-	A	0	0	1	1	1	-
gi|29165615|ref|NC_002745.2|	1839395	-	G	1	0	0	0	0	-
gi|29165615|ref|NC_002745.2|	1839513	-	C	0	0	0	0	1	-
gi|29165615|ref|NC_002745.2|	1839621	-	U	0	0	1	0	0	-
gi|29165615|ref|NC_002745.2|	1839624	-	U	0	0	0	1	0	-
gi|29165615|ref|NC_002745.2|	1839708	-	A	0	0	0	0	1	-
gi|29165615|ref|NC_002745.2|	1839714	-	A	0	0	0	2	0	-
gi|29165615|ref|NC_002745.2|	1839723	-	U	0	1	0	0	0	-
gi|29165615|ref|NC_002745.2|	1839725	-	C	0	1	0	1	1	-
gi|29165615|ref|NC_002745.2|	1839731	-	U	0	0	0	1	0	-
gi|29165615|ref|NC_002745.2|	1839739	-	A	0	0	1	0	0	-
gi|29165615|ref|NC_002745.2|	1839741	-	A	0	0	0	0	1	-
gi|29165615|ref|NC_002745.2|	1844628	-	C	2	0	0	0	0	-
gi|29165615|ref|NC_002745.2|	1856833	-	A	0	1	0	0	0	-
gi|29165615|ref|NC_002745.2|	1856848	-	A	0	0	0	0	3	-
gi|29165615|ref|NC_002745.2|	1857010	-	U	0	0	0	1	0	SA1624
gi|29165615|ref|NC_002745.2|	1857042	-	A	0	0	0	0	1	SA1624
gi|29165615|ref|NC_002745.2|	1857155	-	U	0	1	0	0	0	SA1624
gi|29165615|ref|NC_002745.2|	1857179	-	A	0	1	0	0	0	SA1624
gi|29165615|ref|NC_002745.2|	1857183	-	A	0	1	0	0	0	SA1624
gi|29165615|ref|NC_002745.2|	1857259	-	A	0	0	0	1	0	SA1624
gi|29165615|ref|NC_002745.2|	1857381	-	U	1	0	0	0	0	SA1624
gi|29165615|ref|NC_002745.2|	1857451	-	G	0	1	0	0	1	SA1624
gi|29165615|ref|NC_002745.2|	1857480	-	C	0	0	0	1	0	-
gi|29165615|ref|NC_002745.2|	1859116	-	C	1	0	0	0	0	SA1625
gi|29165615|ref|NC_002745.2|	1859117	-	C	1	0	0	0	0	SA1625
gi|29165615|ref|NC_002745.2|	1859119	-	U	4	0	0	0	0	SA1625
gi|29165615|ref|NC_002745.2|	1859132	-	C	0	0	1	0	0	SA1625
gi|29165615|ref|NC_002745.2|	1859137	-	A	1	0	0	0	2	SA1625
gi|29165615|ref|NC_002745.2|	1859138	-	A	0	0	2	0	1	SA1625
gi|29165615|ref|NC_002745.2|	1859139	-	A	0	0	0	0	1	SA1625

gi|29165615|ref|NC_002745.2|	1859141	-	A	1	1	0	0	1	SA1625
gi|29165615|ref|NC_002745.2|	1859142	-	G	0	1	0	0	0	SA1625
gi|29165615|ref|NC_002745.2|	1859143	-	C	0	0	0	0	2	SA1625
gi|29165615|ref|NC_002745.2|	1859144	-	A	0	0	0	0	1	SA1625
gi|29165615|ref|NC_002745.2|	1859145	-	A	0	2	0	0	1	SA1625
gi|29165615|ref|NC_002745.2|	1859146	-	A	0	1	0	0	0	SA1625
gi|29165615|ref|NC_002745.2|	1859147	-	C	0	0	0	0	1	SA1625
gi|29165615|ref|NC_002745.2|	1859148	-	A	0	1	2	4	1	SA1625
gi|29165615|ref|NC_002745.2|	1859149	-	C	0	1	1	0	1	SA1625
gi|29165615|ref|NC_002745.2|	1859150	-	A	0	0	1	0	0	SA1625
gi|29165615|ref|NC_002745.2|	1859151	-	U	0	2	0	0	2	SA1625
gi|29165615|ref|NC_002745.2|	1859152	-	A	1	0	0	0	1	SA1625_SA1626
gi|29165615|ref|NC_002745.2|	1859153	-	A	0	1	0	0	0	SA1625_SA1626
gi|29165615|ref|NC_002745.2|	1859155	-	G	1	0	0	0	0	SA1625_SA1626
gi|29165615|ref|NC_002745.2|	1859156	-	A	0	0	2	0	2	SA1625_SA1626
gi|29165615|ref|NC_002745.2|	1859157	-	G	0	1	0	0	0	SA1625_SA1626
gi|29165615|ref|NC_002745.2|	1859168	-	U	5	0	0	0	0	SA1626
gi|29165615|ref|NC_002745.2|	1859170	-	G	5	0	1	1	0	SA1626
gi|29165615|ref|NC_002745.2|	1859171	-	G	4	0	0	0	0	SA1626
gi|29165615|ref|NC_002745.2|	1859172	-	G	1	0	0	0	0	SA1626
gi|29165615|ref|NC_002745.2|	1859173	-	U	1	0	0	0	0	SA1626
gi|29165615|ref|NC_002745.2|	1859174	-	U	3	0	0	0	0	SA1626
gi|29165615|ref|NC_002745.2|	1859175	-	C	2	0	3	0	0	SA1626
gi|29165615|ref|NC_002745.2|	1859176	-	A	2	0	1	0	0	SA1626
gi|29165615|ref|NC_002745.2|	1859177	-	A	1	0	0	0	0	SA1626
gi|29165615|ref|NC_002745.2|	1859178	-	G	0	0	0	0	1	SA1626
gi|29165615|ref|NC_002745.2|	1859181	-	A	0	2	1	3	1	SA1626
gi|29165615|ref|NC_002745.2|	1859182	-	G	1	0	0	0	0	SA1626
gi|29165615|ref|NC_002745.2|	1859183	-	U	1	0	0	0	0	SA1626
gi|29165615|ref|NC_002745.2|	1859188	-	A	0	0	0	1	0	SA1626
gi|29165615|ref|NC_002745.2|	1859189	-	C	2	0	0	0	0	SA1626
gi|29165615|ref|NC_002745.2|	1859190	-	G	2	0	0	0	0	SA1626
gi|29165615|ref|NC_002745.2|	1859192	-	A	2	0	0	0	0	SA1626
gi|29165615|ref|NC_002745.2|	1859193	-	A	0	2	0	0	5	SA1626
gi|29165615|ref|NC_002745.2|	1859194	-	C	0	1	2	1	0	SA1626
gi|29165615|ref|NC_002745.2|	1859195	-	U	2	3	1	0	1	SA1626
gi|29165615|ref|NC_002745.2|	1859197	-	A	0	3	1	0	0	SA1626
gi|29165615|ref|NC_002745.2|	1859198	-	A	1	16	8	8	7	SA1626
gi|29165615|ref|NC_002745.2|	1859199	-	G	0	1	1	0	0	SA1626
gi|29165615|ref|NC_002745.2|	1859202	-	C	1	1	3	2	1	SA1626
gi|29165615|ref|NC_002745.2|	1859203	-	A	0	2	1	1	1	SA1626
gi|29165615|ref|NC_002745.2|	1859204	-	A	0	0	2	1	0	SA1626
gi|29165615|ref|NC_002745.2|	1859205	-	G	0	1	0	0	0	SA1626
gi|29165615|ref|NC_002745.2|	1859206	-	U	1	0	0	0	0	SA1626
gi|29165615|ref|NC_002745.2|	1859207	-	U	0	0	0	0	1	SA1626
gi|29165615|ref|NC_002745.2|	1859210	-	A	0	0	2	1	0	SA1626
gi|29165615|ref|NC_002745.2|	1859215	-	C	1	0	0	0	0	SA1626
gi|29165615|ref|NC_002745.2|	1859217	-	A	0	1	0	0	2	SA1626
gi|29165615|ref|NC_002745.2|	1859219	-	A	0	0	0	1	1	SA1626
gi|29165615|ref|NC_002745.2|	1859220	-	G	0	0	0	0	1	SA1626
gi|29165615|ref|NC_002745.2|	1859225	-	A	0	1	0	0	0	SA1626
gi|29165615|ref|NC_002745.2|	1859226	-	G	0	0	1	0	0	SA1626
gi|29165615|ref|NC_002745.2|	1859227	-	C	1	1	0	0	0	SA1626
gi|29165615|ref|NC_002745.2|	1859231	-	A	0	1	0	0	0	SA1626
gi|29165615|ref|NC_002745.2|	1859234	-	A	0	0	0	0	1	SA1626
gi|29165615|ref|NC_002745.2|	1859243	-	A	0	0	0	0	1	SA1626
gi|29165615|ref|NC_002745.2|	1859246	-	A	1	0	0	0	0	SA1626
gi|29165615|ref|NC_002745.2|	1859252	-	A	0	0	0	1	0	SA1626
gi|29165615|ref|NC_002745.2|	1859258	-	U	0	0	0	1	0	SA1626
gi|29165615|ref|NC_002745.2|	1859285	-	C	0	0	1	0	0	SA1626
gi|29165615|ref|NC_002745.2|	1859291	-	U	0	0	0	0	1	SA1626
gi|29165615|ref|NC_002745.2|	1859321	-	A	0	0	1	0	0	SA1626
gi|29165615|ref|NC_002745.2|	1859324	-	A	0	1	0	0	0	SA1626
gi|29165615|ref|NC_002745.2|	1859327	-	C	0	0	0	1	1	SA1626
gi|29165615|ref|NC_002745.2|	1859332	-	G	0	0	1	0	0	SA1626
gi|29165615|ref|NC_002745.2|	1859335	-	A	0	0	1	0	0	SA1626
gi|29165615|ref|NC_002745.2|	1859340	-	U	1	1	0	0	0	SA1626
gi|29165615|ref|NC_002745.2|	1859351	-	A	0	1	0	0	0	SA1626
gi|29165615|ref|NC_002745.2|	1859381	-	A	0	0	0	1	1	SA1626
gi|29165615|ref|NC_002745.2|	1859426	-	A	0	0	1	0	0	SA1626
gi|29165615|ref|NC_002745.2|	1859434	-	A	0	1	0	0	0	SA1626
gi|29165615|ref|NC_002745.2|	1859475	-	G	0	0	0	0	1	SA1626
gi|29165615|ref|NC_002745.2|	1859487	-	C	0	0	0	0	1	SA1626
gi|29165615|ref|NC_002745.2|	1859679	-	C	2	0	0	0	0	SA1626
gi|29165615|ref|NC_002745.2|	1859719	-	C	3	0	0	0	0	SA1626
gi|29165615|ref|NC_002745.2|	1859759	-	A	0	1	0	0	0	SA1626
gi|29165615|ref|NC_002745.2|	1859769	-	U	0	0	0	1	0	SA1626
gi|29165615|ref|NC_002745.2|	1859770	-	A	0	1	0	0	0	SA1626
gi|29165615|ref|NC_002745.2|	1859778	-	G	0	0	1	0	0	SA1626
gi|29165615|ref|NC_002745.2|	1859780	-	C	0	1	0	0	0	SA1626
gi|29165615|ref|NC_002745.2|	1859782	-	A	1	0	0	0	0	SA1626

gi|29165615|ref|NC_002745.2|	1859828	-	C	0	2	0	0	0	SA1626
gi|29165615|ref|NC_002745.2|	1859906	-	U	1	0	0	0	0	SA1626
gi|29165615|ref|NC_002745.2|	1859930	-	A	0	0	0	0	1	SA1626
gi|29165615|ref|NC_002745.2|	1859952	-	C	0	0	0	1	0	SA1626
gi|29165615|ref|NC_002745.2|	1859989	-	U	0	0	0	0	1	SA1626
gi|29165615|ref|NC_002745.2|	1860022	-	C	0	0	0	0	1	SA1626
gi|29165615|ref|NC_002745.2|	1860034	-	U	1	0	0	0	0	SA1626
gi|29165615|ref|NC_002745.2|	1860048	-	A	0	0	0	0	1	SA1626
gi|29165615|ref|NC_002745.2|	1860192	-	C	0	0	0	0	1	SA1626
gi|29165615|ref|NC_002745.2|	1860203	-	U	1	0	0	0	0	SA1626
gi|29165615|ref|NC_002745.2|	1860221	-	U	0	1	0	0	0	SA1626
gi|29165615|ref|NC_002745.2|	1860257	-	A	0	0	0	0	1	SA1626
gi|29165615|ref|NC_002745.2|	1860258	-	A	0	1	0	0	0	SA1626
gi|29165615|ref|NC_002745.2|	1860259	-	C	0	1	0	0	0	SA1626
gi|29165615|ref|NC_002745.2|	1860279	-	A	0	1	0	0	0	SA1626
gi|29165615|ref|NC_002745.2|	1860307	-	U	0	0	1	0	0	SA1626
gi|29165615|ref|NC_002745.2|	1860309	-	U	1	0	0	0	0	SA1626
gi|29165615|ref|NC_002745.2|	1860311	-	A	0	1	0	2	1	SA1626
gi|29165615|ref|NC_002745.2|	1860317	-	A	0	0	1	1	0	SA1626
gi|29165615|ref|NC_002745.2|	1860329	-	G	0	0	0	1	0	SA1626
gi|29165615|ref|NC_002745.2|	1860337	-	U	0	0	0	1	0	SA1626
gi|29165615|ref|NC_002745.2|	1860350	-	A	0	0	0	0	1	SA1626
gi|29165615|ref|NC_002745.2|	1860434	-	A	0	0	0	0	1	SA1626
gi|29165615|ref|NC_002745.2|	1860547	-	A	0	0	0	1	1	SA1626
gi|29165615|ref|NC_002745.2|	1860548	-	A	0	1	2	0	0	SA1626
gi|29165615|ref|NC_002745.2|	1860550	-	A	0	0	0	1	0	SA1626
gi|29165615|ref|NC_002745.2|	1860553	-	A	0	1	0	0	0	SA1626
gi|29165615|ref|NC_002745.2|	1860557	-	C	0	0	0	1	0	SA1626
gi|29165615|ref|NC_002745.2|	1860583	-	U	0	0	1	0	0	SA1626
gi|29165615|ref|NC_002745.2|	1860585	-	A	0	0	1	0	0	SA1626
gi|29165615|ref|NC_002745.2|	1860593	-	U	0	0	1	0	0	SA1626
gi|29165615|ref|NC_002745.2|	1860602	-	A	0	1	0	0	0	SA1626
gi|29165615|ref|NC_002745.2|	1860604	-	U	0	0	0	0	1	SA1626
gi|29165615|ref|NC_002745.2|	1860617	-	C	0	0	0	0	1	SA1626
gi|29165615|ref|NC_002745.2|	1860624	-	A	0	0	1	0	0	SA1626
gi|29165615|ref|NC_002745.2|	1860625	-	C	0	0	0	0	1	SA1626
gi|29165615|ref|NC_002745.2|	1860626	-	A	2	0	1	0	0	SA1626
gi|29165615|ref|NC_002745.2|	1860627	-	A	0	0	3	0	2	SA1626
gi|29165615|ref|NC_002745.2|	1860633	-	A	0	0	1	0	0	SA1626
gi|29165615|ref|NC_002745.2|	1860634	-	A	0	0	1	1	1	SA1626
gi|29165615|ref|NC_002745.2|	1860638	-	A	0	2	1	0	2	SA1626
gi|29165615|ref|NC_002745.2|	1860639	-	G	0	0	0	0	1	SA1626
gi|29165615|ref|NC_002745.2|	1860642	-	A	0	1	0	0	0	SA1626
gi|29165615|ref|NC_002745.2|	1860643	-	G	0	1	0	0	0	SA1626
gi|29165615|ref|NC_002745.2|	1860644	-	C	0	1	0	0	2	SA1626
gi|29165615|ref|NC_002745.2|	1860648	-	A	0	5	0	1	1	SA1626
gi|29165615|ref|NC_002745.2|	1860649	-	G	0	1	0	0	0	SA1626
gi|29165615|ref|NC_002745.2|	1860651	-	U	0	1	1	0	0	SA1626
gi|29165615|ref|NC_002745.2|	1860656	-	U	0	1	1	0	1	SA1626
gi|29165615|ref|NC_002745.2|	1860657	-	U	0	1	1	0	0	SA1626
gi|29165615|ref|NC_002745.2|	1860658	-	A	0	4	1	1	5	SA1626
gi|29165615|ref|NC_002745.2|	1860659	-	A	0	1	0	1	1	SA1626
gi|29165615|ref|NC_002745.2|	1860660	-	A	0	10	3	2	15	SA1626
gi|29165615|ref|NC_002745.2|	1860661	-	A	1	16	11	6	17	SA1626
gi|29165615|ref|NC_002745.2|	1860662	-	A	1	2	1	2	5	SA1626
gi|29165615|ref|NC_002745.2|	1860663	-	A	0	4	5	1	5	SA1626
gi|29165615|ref|NC_002745.2|	1860664	-	U	0	1	0	1	0	SA1626
gi|29165615|ref|NC_002745.2|	1860665	-	A	1	6	3	0	3	SA1626
gi|29165615|ref|NC_002745.2|	1860667	-	A	0	1	0	0	0	SA1626
gi|29165615|ref|NC_002745.2|	1860668	-	U	0	1	1	0	0	SA1626
gi|29165615|ref|NC_002745.2|	1860677	-	A	1	0	0	0	0	SA1626
gi|29165615|ref|NC_002745.2|	1860682	-	G	0	1	0	0	0	SA1626
gi|29165615|ref|NC_002745.2|	1860691	-	A	0	4	2	0	1	SA1626
gi|29165615|ref|NC_002745.2|	1860692	-	A	0	22	12	9	15	SA1626
gi|29165615|ref|NC_002745.2|	1860693	-	A	2	12	3	10	14	SA1626
gi|29165615|ref|NC_002745.2|	1860694	-	A	1	20	19	23	35	SA1626
gi|29165615|ref|NC_002745.2|	1860695	-	A	0	23	6	7	19	SA1626
gi|29165615|ref|NC_002745.2|	1860697	-	U	0	0	1	0	0	SA1626
gi|29165615|ref|NC_002745.2|	1860699	-	A	1	0	2	0	2	SA1626
gi|29165615|ref|NC_002745.2|	1860700	-	U	0	3	2	1	1	SA1626
gi|29165615|ref|NC_002745.2|	1860701	-	U	0	0	0	1	0	SA1626
gi|29165615|ref|NC_002745.2|	1860702	-	A	0	1	0	0	1	SA1626
gi|29165615|ref|NC_002745.2|	1860703	-	U	0	1	1	1	3	SA1626
gi|29165615|ref|NC_002745.2|	1860706	-	G	0	1	0	0	0	SA1626
gi|29165615|ref|NC_002745.2|	1860708	-	A	0	0	1	0	0	SA1626
gi|29165615|ref|NC_002745.2|	1860709	-	C	0	0	0	1	0	-
gi|29165615|ref|NC_002745.2|	1860710	-	A	0	0	0	0	2	-
gi|29165615|ref|NC_002745.2|	1860714	-	A	0	0	0	0	1	-
gi|29165615|ref|NC_002745.2|	1860715	-	G	1	1	0	0	0	-
gi|29165615|ref|NC_002745.2|	1860718	-	G	0	0	2	0	0	-
gi|29165615|ref|NC_002745.2|	1860726	-	C	0	0	1	0	0	-

gi|29165615|ref|NC_002745.2|	1860727	-	G	0	1	0	0	0	-
gi|29165615|ref|NC_002745.2|	1860730	-	U	0	1	4	0	2	-
gi|29165615|ref|NC_002745.2|	1860737	-	A	0	0	0	1	0	-
gi|29165615|ref|NC_002745.2|	1860738	-	A	0	0	1	0	2	-
gi|29165615|ref|NC_002745.2|	1860739	-	U	0	1	0	1	1	-
gi|29165615|ref|NC_002745.2|	1860740	-	A	0	1	2	1	0	-
gi|29165615|ref|NC_002745.2|	1860741	-	G	0	0	0	1	0	-
gi|29165615|ref|NC_002745.2|	1860742	-	A	0	0	0	1	0	-
gi|29165615|ref|NC_002745.2|	1860743	-	G	0	1	0	0	0	-
gi|29165615|ref|NC_002745.2|	1860747	-	C	0	0	1	0	0	-
gi|29165615|ref|NC_002745.2|	1860748	-	A	0	0	1	0	0	-
gi|29165615|ref|NC_002745.2|	1860749	-	C	0	0	0	0	1	-
gi|29165615|ref|NC_002745.2|	1860750	-	A	1	0	0	0	1	-
gi|29165615|ref|NC_002745.2|	1860751	-	U	0	1	3	0	2	-
gi|29165615|ref|NC_002745.2|	1860752	-	U	0	0	0	2	1	-
gi|29165615|ref|NC_002745.2|	1860753	-	A	15	66	45	42	56	-
gi|29165615|ref|NC_002745.2|	1860754	-	U	0	0	3	0	1	-
gi|29165615|ref|NC_002745.2|	1860756	-	U	0	2	3	0	2	-
gi|29165615|ref|NC_002745.2|	1869845	-	A	0	3	0	0	0	SA1637
gi|29165615|ref|NC_002745.2|	1871184	-	G	0	0	0	0	1	-
gi|29165615|ref|NC_002745.2|	1876142	-	C	0	0	0	2	0	-
gi|29165615|ref|NC_002745.2|	1876143	-	G	0	0	0	1	0	-
gi|29165615|ref|NC_002745.2|	1876146	-	A	0	0	1	0	0	-
gi|29165615|ref|NC_002745.2|	1876150	-	U	0	1	0	0	0	-
gi|29165615|ref|NC_002745.2|	1876152	-	U	0	0	0	1	1	-
gi|29165615|ref|NC_002745.2|	1876153	-	G	0	0	0	1	0	-
gi|29165615|ref|NC_002745.2|	1876474	-	U	0	0	0	3	0	SA1642
gi|29165615|ref|NC_002745.2|	1876475	-	A	0	3	3	11	7	SA1642
gi|29165615|ref|NC_002745.2|	1876476	-	G	0	4	7	8	1	SA1642
gi|29165615|ref|NC_002745.2|	1878291	-	U	0	0	1	0	0	SA1644
gi|29165615|ref|NC_002745.2|	1878294	-	U	0	0	0	2	0	SA1644
gi|29165615|ref|NC_002745.2|	1878295	-	U	0	0	0	1	1	SA1644
gi|29165615|ref|NC_002745.2|	1878296	-	A	0	2	4	0	2	SA1644
gi|29165615|ref|NC_002745.2|	1878366	-	A	0	0	0	1	0	SA1644
gi|29165615|ref|NC_002745.2|	1878973	-	C	1	0	0	0	0	-
gi|29165615|ref|NC_002745.2|	1879362	-	A	0	0	0	0	1	SA1646
gi|29165615|ref|NC_002745.2|	1880125	-	A	0	0	0	0	1	SA1647
gi|29165615|ref|NC_002745.2|	1882046	-	C	0	0	1	0	0	SAtRNA20
gi|29165615|ref|NC_002745.2|	1882061	-	G	0	2	0	0	0	SAtRNA20
gi|29165615|ref|NC_002745.2|	1882115	-	G	1	0	0	0	0	SAtRNA21
gi|29165615|ref|NC_002745.2|	1882124	-	A	1	0	0	0	0	SAtRNA21
gi|29165615|ref|NC_002745.2|	1882137	-	U	14	14	18	11	14	SAtRNA21
gi|29165615|ref|NC_002745.2|	1882228	-	G	4	6	6	1	3	SAtRNA22
gi|29165615|ref|NC_002745.2|	1882273	-	U	1	0	0	0	0	SAtRNA23
gi|29165615|ref|NC_002745.2|	1882274	-	U	0	0	0	2	0	SAtRNA23
gi|29165615|ref|NC_002745.2|	1882278	-	G	2	0	0	0	0	SAtRNA23
gi|29165615|ref|NC_002745.2|	1882279	-	C	2	0	0	0	0	SAtRNA23
gi|29165615|ref|NC_002745.2|	1882281	-	U	0	0	0	1	0	SAtRNA23
gi|29165615|ref|NC_002745.2|	1882320	-	G	9	7	7	4	9	-
gi|29165615|ref|NC_002745.2|	1882405	-	G	0	1	0	0	0	SAtRNA24
gi|29165615|ref|NC_002745.2|	1882411	-	C	0	0	0	0	1	-
gi|29165615|ref|NC_002745.2|	1882459	-	G	0	0	0	0	1	SAtRNA25
gi|29165615|ref|NC_002745.2|	1882460	-	C	0	0	0	0	1	SAtRNA25
gi|29165615|ref|NC_002745.2|	1882461	-	A	0	0	0	1	0	SAtRNA25
gi|29165615|ref|NC_002745.2|	1882462	-	C	1	1	0	0	0	SAtRNA25
gi|29165615|ref|NC_002745.2|	1882476	-	A	0	0	0	1	0	SAtRNA25
gi|29165615|ref|NC_002745.2|	1882484	-	A	0	0	0	0	1	SAtRNA25
gi|29165615|ref|NC_002745.2|	1882502	-	U	1	0	0	0	0	-
gi|29165615|ref|NC_002745.2|	1882518	-	U	3	3	9	4	5	-
gi|29165615|ref|NC_002745.2|	1882591	-	G	0	0	0	0	1	SAtRNA26
gi|29165615|ref|NC_002745.2|	1882592	-	C	22	11	11	6	11	SAtRNA26
gi|29165615|ref|NC_002745.2|	1894215	-	U	1	0	0	0	0	SA1660
gi|29165615|ref|NC_002745.2|	1894217	-	A	0	0	1	0	0	SA1660
gi|29165615|ref|NC_002745.2|	1896540	-	A	0	0	0	0	1	SA1661
gi|29165615|ref|NC_002745.2|	1896706	-	A	1	0	0	0	0	SA1661
gi|29165615|ref|NC_002745.2|	1896707	-	A	0	0	0	1	0	SA1661
gi|29165615|ref|NC_002745.2|	1898008	-	G	0	1	0	0	0	SA1662
gi|29165615|ref|NC_002745.2|	1898206	-	A	0	0	0	0	1	SA1662
gi|29165615|ref|NC_002745.2|	1899538	-	A	0	2	0	0	0	-
gi|29165615|ref|NC_002745.2|	1899543	-	A	0	1	0	0	0	-
gi|29165615|ref|NC_002745.2|	1899545	-	A	0	1	0	0	0	-
gi|29165615|ref|NC_002745.2|	1899694	-	A	0	2	0	0	0	-
gi|29165615|ref|NC_002745.2|	1899699	-	A	0	1	0	0	0	-
gi|29165615|ref|NC_002745.2|	1899701	-	A	0	1	0	0	0	-
gi|29165615|ref|NC_002745.2|	1907739	-	U	0	0	1	0	1	-
gi|29165615|ref|NC_002745.2|	1907741	-	A	0	0	0	0	1	-
gi|29165615|ref|NC_002745.2|	1907742	-	U	3	2	0	0	1	-
gi|29165615|ref|NC_002745.2|	1907746	-	U	0	1	0	2	1	-
gi|29165615|ref|NC_002745.2|	1907747	-	U	0	0	0	0	1	-
gi|29165615|ref|NC_002745.2|	1907752	-	U	0	1	0	0	0	-
gi|29165615|ref|NC_002745.2|	1907787	-	A	0	0	0	1	0	-

gi|29165615|ref|NC_002745.2|	1907788	-	U	0	0	0	1	3	-
gi|29165615|ref|NC_002745.2|	1907819	-	U	1	1	0	0	0	-
gi|29165615|ref|NC_002745.2|	1907860	-	A	0	0	0	0	1	-
gi|29165615|ref|NC_002745.2|	1907861	-	C	0	0	0	0	1	-
gi|29165615|ref|NC_002745.2|	1907862	-	C	0	0	0	0	2	-
gi|29165615|ref|NC_002745.2|	1907907	-	U	0	0	0	1	0	-
gi|29165615|ref|NC_002745.2|	1910881	-	U	0	1	0	0	0	SA1674
gi|29165615|ref|NC_002745.2|	1910889	-	A	0	1	1	1	0	SA1674
gi|29165615|ref|NC_002745.2|	1912681	-	A	0	0	1	0	0	SA1675
gi|29165615|ref|NC_002745.2|	1914780	-	G	0	0	0	0	1	-
gi|29165615|ref|NC_002745.2|	1914784	-	U	1	1	1	1	1	-
gi|29165615|ref|NC_002745.2|	1914793	-	C	2	1	0	0	0	-
gi|29165615|ref|NC_002745.2|	1914798	-	C	0	0	0	0	1	-
gi|29165615|ref|NC_002745.2|	1914805	-	A	0	5	2	6	2	-
gi|29165615|ref|NC_002745.2|	1914806	-	U	0	1	0	0	0	-
gi|29165615|ref|NC_002745.2|	1914807	-	A	0	0	0	0	1	-
gi|29165615|ref|NC_002745.2|	1914974	-	U	0	1	0	0	0	-
gi|29165615|ref|NC_002745.2|	1915321	-	U	0	0	0	0	1	-
gi|29165615|ref|NC_002745.2|	1915532	-	A	0	1	0	0	0	-
gi|29165615|ref|NC_002745.2|	1915548	-	A	0	0	0	1	0	-
gi|29165615|ref|NC_002745.2|	1915580	-	A	0	0	0	0	1	-
gi|29165615|ref|NC_002745.2|	1915598	-	U	0	1	0	0	0	-
gi|29165615|ref|NC_002745.2|	1915604	-	A	0	0	0	0	1	-
gi|29165615|ref|NC_002745.2|	1915605	-	U	0	0	1	0	1	-
gi|29165615|ref|NC_002745.2|	1915606	-	G	0	1	0	0	0	-
gi|29165615|ref|NC_002745.2|	1915615	-	U	0	0	0	0	1	-
gi|29165615|ref|NC_002745.2|	1915619	-	C	0	0	1	0	0	-
gi|29165615|ref|NC_002745.2|	1915620	-	C	1	1	0	0	0	-
gi|29165615|ref|NC_002745.2|	1916373	-	G	3	0	0	2	0	SAtRNA27
gi|29165615|ref|NC_002745.2|	1916431	-	A	0	0	0	0	1	SAtRNA28
gi|29165615|ref|NC_002745.2|	1916437	-	A	0	2	0	0	0	SAtRNA28
gi|29165615|ref|NC_002745.2|	1916438	-	U	0	1	0	0	0	SAtRNA28
gi|29165615|ref|NC_002745.2|	1916474	-	C	0	1	0	0	0	SAtRNA28
gi|29165615|ref|NC_002745.2|	1916486	-	G	0	3	0	1	1	SAtRNA28
gi|29165615|ref|NC_002745.2|	1916519	-	A	0	0	0	0	1	-
gi|29165615|ref|NC_002745.2|	1916525	-	A	0	2	0	0	0	-
gi|29165615|ref|NC_002745.2|	1916526	-	U	0	1	0	0	0	-
gi|29165615|ref|NC_002745.2|	1916574	-	G	0	3	0	0	1	-
gi|29165615|ref|NC_002745.2|	1916609	-	A	0	0	0	0	1	SAtRNA29
gi|29165615|ref|NC_002745.2|	1916615	-	A	0	2	0	0	0	SAtRNA29
gi|29165615|ref|NC_002745.2|	1916616	-	U	0	1	0	0	0	SAtRNA29
gi|29165615|ref|NC_002745.2|	1916652	-	C	0	1	0	0	0	SAtRNA29
gi|29165615|ref|NC_002745.2|	1916664	-	G	0	3	0	1	1	SAtRNA29
gi|29165615|ref|NC_002745.2|	1916778	-	C	0	0	0	0	1	SAtRNA31
gi|29165615|ref|NC_002745.2|	1916780	-	C	0	1	0	0	0	SAtRNA31
gi|29165615|ref|NC_002745.2|	1916782	-	G	0	1	0	0	1	SAtRNA31
gi|29165615|ref|NC_002745.2|	1916783	-	C	0	1	2	1	0	SAtRNA31
gi|29165615|ref|NC_002745.2|	1916785	-	U	0	1	0	0	0	SAtRNA31
gi|29165615|ref|NC_002745.2|	1916786	-	C	1	3	0	1	1	SAtRNA31
gi|29165615|ref|NC_002745.2|	1916792	-	C	1	0	0	0	0	SAtRNA31
gi|29165615|ref|NC_002745.2|	1916798	-	A	1	1	0	0	0	SAtRNA31
gi|29165615|ref|NC_002745.2|	1916799	-	C	0	0	1	1	1	SAtRNA31
gi|29165615|ref|NC_002745.2|	1916811	-	C	0	0	0	1	0	SAtRNA31
gi|29165615|ref|NC_002745.2|	1916822	-	U	3	10	6	5	7	SAtRNA31
gi|29165615|ref|NC_002745.2|	1916860	-	U	1	0	0	0	0	SAtRNA32
gi|29165615|ref|NC_002745.2|	1916861	-	U	0	0	0	2	0	SAtRNA32
gi|29165615|ref|NC_002745.2|	1916865	-	G	2	0	0	0	0	SAtRNA32
gi|29165615|ref|NC_002745.2|	1916866	-	C	2	0	0	0	0	SAtRNA32
gi|29165615|ref|NC_002745.2|	1916868	-	U	0	0	0	1	0	SAtRNA32
gi|29165615|ref|NC_002745.2|	1916907	-	G	9	7	7	4	9	-
gi|29165615|ref|NC_002745.2|	1917029	-	C	1	0	0	0	0	SAtRNA34
gi|29165615|ref|NC_002745.2|	1917037	-	C	1	0	0	0	0	SAtRNA34
gi|29165615|ref|NC_002745.2|	1917043	-	U	1	0	0	0	0	SAtRNA34
gi|29165615|ref|NC_002745.2|	1917078	-	G	0	2	0	1	0	SAtRNA34
gi|29165615|ref|NC_002745.2|	1917127	-	U	1	0	0	0	0	SAtRNA35
gi|29165615|ref|NC_002745.2|	1917128	-	C	0	0	1	1	0	SAtRNA35
gi|29165615|ref|NC_002745.2|	1917239	-	G	0	1	0	0	0	SAtRNA36
gi|29165615|ref|NC_002745.2|	1917245	-	U	0	0	0	0	1	-
gi|29165615|ref|NC_002745.2|	1917295	-	G	0	0	0	0	1	SAtRNA37
gi|29165615|ref|NC_002745.2|	1917296	-	C	0	0	0	0	1	SAtRNA37
gi|29165615|ref|NC_002745.2|	1917297	-	A	0	0	0	1	0	SAtRNA37
gi|29165615|ref|NC_002745.2|	1917298	-	C	1	1	0	0	0	SAtRNA37
gi|29165615|ref|NC_002745.2|	1917312	-	A	0	0	0	1	0	SAtRNA37
gi|29165615|ref|NC_002745.2|	1917320	-	A	0	0	0	0	1	SAtRNA37
gi|29165615|ref|NC_002745.2|	1917338	-	U	1	0	0	0	0	-
gi|29165615|ref|NC_002745.2|	1917354	-	U	3	3	9	4	5	-
gi|29165615|ref|NC_002745.2|	1917427	-	G	0	0	0	0	1	SAtRNA38
gi|29165615|ref|NC_002745.2|	1917428	-	C	22	11	11	6	11	SAtRNA38
gi|29165615|ref|NC_002745.2|	1917478	-	C	0	0	0	0	2	SAtRNA39
gi|29165615|ref|NC_002745.2|	1917504	-	G	0	1	0	0	0	SAtRNA39
gi|29165615|ref|NC_002745.2|	1917519	-	C	1	0	0	0	0	SAtRNA39

gi|29165615|ref|NC_002745.2|	1917535	-	G	1	3	0	0	0	SAtRNA39
gi|29165615|ref|NC_002745.2|	1917541	-	A	0	0	0	0	1	-
gi|29165615|ref|NC_002745.2|	1917543	-	A	0	0	1	0	1	-
gi|29165615|ref|NC_002745.2|	1917544	-	U	1	0	0	0	1	-
gi|29165615|ref|NC_002745.2|	1917611	-	G	0	0	0	0	1	SAtRNA40
gi|29165615|ref|NC_002745.2|	1917612	-	C	0	0	0	0	1	SAtRNA40
gi|29165615|ref|NC_002745.2|	1917613	-	A	0	0	0	1	0	SAtRNA40
gi|29165615|ref|NC_002745.2|	1917614	-	C	1	1	0	0	0	SAtRNA40
gi|29165615|ref|NC_002745.2|	1917628	-	A	0	0	0	1	0	SAtRNA40
gi|29165615|ref|NC_002745.2|	1917636	-	A	0	0	0	0	1	SAtRNA40
gi|29165615|ref|NC_002745.2|	1917691	-	G	0	0	0	0	1	SAtRNA41
gi|29165615|ref|NC_002745.2|	1917692	-	C	0	0	0	0	2	SAtRNA41
gi|29165615|ref|NC_002745.2|	1917708	-	A	0	0	0	0	1	SAtRNA41
gi|29165615|ref|NC_002745.2|	1917718	-	G	0	1	0	0	0	SAtRNA41
gi|29165615|ref|NC_002745.2|	1917733	-	C	1	0	0	0	0	SAtRNA41
gi|29165615|ref|NC_002745.2|	1917749	-	G	1	3	0	0	0	SAtRNA41
gi|29165615|ref|NC_002745.2|	1917755	-	A	0	0	0	0	1	-
gi|29165615|ref|NC_002745.2|	1917757	-	A	0	0	1	0	1	-
gi|29165615|ref|NC_002745.2|	1917758	-	U	1	0	0	0	1	-
gi|29165615|ref|NC_002745.2|	1917927	-	U	1	0	0	0	0	SAtRNA43
gi|29165615|ref|NC_002745.2|	1917972	-	C	2	0	0	0	0	SAtRNA44
gi|29165615|ref|NC_002745.2|	1917976	-	C	1	0	0	0	0	SAtRNA44
gi|29165615|ref|NC_002745.2|	1917990	-	G	3	0	0	0	0	SAtRNA44
gi|29165615|ref|NC_002745.2|	1917995	-	A	1	0	0	0	0	SAtRNA44
gi|29165615|ref|NC_002745.2|	1917998	-	U	0	0	1	0	0	SAtRNA44
gi|29165615|ref|NC_002745.2|	1917999	-	U	0	0	0	2	1	SAtRNA44
gi|29165615|ref|NC_002745.2|	1918000	-	U	2	0	0	0	0	SAtRNA44
gi|29165615|ref|NC_002745.2|	1918007	-	C	0	0	0	0	2	SAtRNA44
gi|29165615|ref|NC_002745.2|	1918013	-	G	0	0	0	0	1	SAtRNA44
gi|29165615|ref|NC_002745.2|	1918021	-	C	1	0	0	0	0	SAtRNA44
gi|29165615|ref|NC_002745.2|	1918028	-	G	0	0	1	0	0	SAtRNA44
gi|29165615|ref|NC_002745.2|	1918029	-	G	0	1	0	0	0	SAtRNA44
gi|29165615|ref|NC_002745.2|	1918030	-	G	1	1	0	1	1	SAtRNA44
gi|29165615|ref|NC_002745.2|	1918031	-	G	1287	1680	1600	664	1174	SAtRNA44
gi|29165615|ref|NC_002745.2|	1918032	-	U	9	5	4	2	4	-
gi|29165615|ref|NC_002745.2|	1918081	-	A	0	0	2	0	0	SAtRNA45
gi|29165615|ref|NC_002745.2|	1918122	-	C	2	2	1	1	2	SAtRNA45
gi|29165615|ref|NC_002745.2|	1918157	-	U	1	0	0	0	0	SAtRNA46
gi|29165615|ref|NC_002745.2|	1918158	-	U	0	0	1	0	0	SAtRNA46
gi|29165615|ref|NC_002745.2|	1918163	-	A	1	0	0	0	0	SAtRNA46
gi|29165615|ref|NC_002745.2|	1918164	-	A	0	1	0	0	1	SAtRNA46
gi|29165615|ref|NC_002745.2|	1918165	-	C	0	0	0	0	1	SAtRNA46
gi|29165615|ref|NC_002745.2|	1918166	-	U	0	1	0	0	0	SAtRNA46
gi|29165615|ref|NC_002745.2|	1918180	-	C	0	1	0	0	0	SAtRNA46
gi|29165615|ref|NC_002745.2|	1918193	-	C	0	0	0	1	0	SAtRNA46
gi|29165615|ref|NC_002745.2|	1918205	-	G	18	9	7	3	13	SAtRNA46
gi|29165615|ref|NC_002745.2|	1918208	-	U	0	2	0	0	0	-
gi|29165615|ref|NC_002745.2|	1918257	-	C	0	0	1	0	0	SAtRNA47
gi|29165615|ref|NC_002745.2|	1918384	-	A	0	2	0	0	0	SAtRNA48
gi|29165615|ref|NC_002745.2|	1918392	-	A	0	2	0	0	0	SAtRNA48
gi|29165615|ref|NC_002745.2|	1918394	-	G	0	0	0	0	1	SAtRNA48
gi|29165615|ref|NC_002745.2|	1918397	-	G	11	6	10	6	3	SAtRNA48
gi|29165615|ref|NC_002745.2|	1918482	-	G	3	0	0	2	0	SAtRNA49
gi|29165615|ref|NC_002745.2|	1918508	-	U	1	0	0	0	0	SAtRNA50
gi|29165615|ref|NC_002745.2|	1918522	-	C	1	0	0	0	0	SAtRNA50
gi|29165615|ref|NC_002745.2|	1918523	-	U	0	1	0	0	0	SAtRNA50
gi|29165615|ref|NC_002745.2|	1918524	-	A	1	0	0	0	0	SAtRNA50
gi|29165615|ref|NC_002745.2|	1918610	-	U	1	0	0	0	0	SAtRNA51
gi|29165615|ref|NC_002745.2|	1918611	-	C	0	0	1	1	0	SAtRNA51
gi|29165615|ref|NC_002745.2|	1918684	-	C	1	0	0	0	0	SAtRNA52
gi|29165615|ref|NC_002745.2|	1918699	-	C	0	0	0	0	2	SAtRNA52
gi|29165615|ref|NC_002745.2|	1918703	-	C	1	0	0	0	0	SAtRNA52
gi|29165615|ref|NC_002745.2|	1918705	-	G	0	0	0	0	1	SAtRNA52
gi|29165615|ref|NC_002745.2|	1918719	-	C	0	0	0	0	1	SAtRNA52
gi|29165615|ref|NC_002745.2|	1918721	-	A	0	0	1	0	0	SAtRNA52
gi|29165615|ref|NC_002745.2|	1918724	-	C	0	1	0	0	0	SAtRNA52
gi|29165615|ref|NC_002745.2|	1918734	-	G	14	12	14	5	8	SAtRNA52
gi|29165615|ref|NC_002745.2|	1918766	-	U	0	0	0	1	0	SArRNA08
gi|29165615|ref|NC_002745.2|	1918767	-	C	2	2	1	0	1	SArRNA08
gi|29165615|ref|NC_002745.2|	1918768	-	G	0	1	0	0	2	SArRNA08
gi|29165615|ref|NC_002745.2|	1918769	-	C	0	5	1	0	0	SArRNA08
gi|29165615|ref|NC_002745.2|	1918770	-	C	6	2	3	3	3	SArRNA08
gi|29165615|ref|NC_002745.2|	1918771	-	U	1	5	0	0	2	SArRNA08
gi|29165615|ref|NC_002745.2|	1918772	-	U	0	0	0	0	1	SArRNA08
gi|29165615|ref|NC_002745.2|	1918773	-	G	1	6	1	2	1	SArRNA08
gi|29165615|ref|NC_002745.2|	1918774	-	C	2	2	2	0	4	SArRNA08
gi|29165615|ref|NC_002745.2|	1918775	-	A	0	0	0	0	1	SArRNA08
gi|29165615|ref|NC_002745.2|	1918776	-	U	1	0	0	0	0	SArRNA08
gi|29165615|ref|NC_002745.2|	1918784	-	G	0	0	0	0	1	SArRNA08
gi|29165615|ref|NC_002745.2|	1918786	-	U	0	2	0	0	0	SArRNA08
gi|29165615|ref|NC_002745.2|	1918787	-	G	0	2	1	0	0	SArRNA08

gi|29165615|ref|NC_002745.2|	1918788	-	G	0	0	0	0	1	SArRNA08
gi|29165615|ref|NC_002745.2|	1918790	-	A	0	3	0	0	1	SArRNA08
gi|29165615|ref|NC_002745.2|	1918791	-	G	0	7	0	3	2	SArRNA08
gi|29165615|ref|NC_002745.2|	1918792	-	C	0	7	0	0	4	SArRNA08
gi|29165615|ref|NC_002745.2|	1918793	-	U	1	0	0	0	1	SArRNA08
gi|29165615|ref|NC_002745.2|	1918794	-	G	0	0	0	0	3	SArRNA08
gi|29165615|ref|NC_002745.2|	1918795	-	C	1	3	2	0	1	SArRNA08
gi|29165615|ref|NC_002745.2|	1918797	-	A	0	2	0	0	0	SArRNA08
gi|29165615|ref|NC_002745.2|	1918798	-	U	1	1	0	0	0	SArRNA08
gi|29165615|ref|NC_002745.2|	1918799	-	U	0	2	0	0	0	SArRNA08
gi|29165615|ref|NC_002745.2|	1918800	-	C	2	2	1	0	3	SArRNA08
gi|29165615|ref|NC_002745.2|	1918801	-	C	2	0	0	1	0	SArRNA08
gi|29165615|ref|NC_002745.2|	1918802	-	U	1	2	1	2	0	SArRNA08
gi|29165615|ref|NC_002745.2|	1918803	-	C	2	7	4	5	5	SArRNA08
gi|29165615|ref|NC_002745.2|	1918805	-	A	1	0	0	1	1	SArRNA08
gi|29165615|ref|NC_002745.2|	1918806	-	A	3	0	1	0	2	SArRNA08
gi|29165615|ref|NC_002745.2|	1918807	-	U	2	12	3	0	2	SArRNA08
gi|29165615|ref|NC_002745.2|	1918808	-	U	1	0	0	0	2	SArRNA08
gi|29165615|ref|NC_002745.2|	1918809	-	G	0	0	0	0	2	SArRNA08
gi|29165615|ref|NC_002745.2|	1918810	-	A	1	1	0	1	0	SArRNA08
gi|29165615|ref|NC_002745.2|	1918811	-	A	0	4	1	1	1	SArRNA08
gi|29165615|ref|NC_002745.2|	1918812	-	G	2	11	0	1	3	SArRNA08
gi|29165615|ref|NC_002745.2|	1918813	-	A	1	7	0	1	3	SArRNA08
gi|29165615|ref|NC_002745.2|	1918814	-	C	3	9	1	0	5	SArRNA08
gi|29165615|ref|NC_002745.2|	1918815	-	A	2	9	0	2	6	SArRNA08
gi|29165615|ref|NC_002745.2|	1918816	-	C	24	41	18	9	19	SArRNA08
gi|29165615|ref|NC_002745.2|	1918818	-	A	2	2	1	1	1	SArRNA08
gi|29165615|ref|NC_002745.2|	1918819	-	G	19	17	10	4	16	SArRNA08
gi|29165615|ref|NC_002745.2|	1918820	-	C	70	92	45	26	54	SArRNA08
gi|29165615|ref|NC_002745.2|	1918821	-	C	23	46	4	5	37	SArRNA08
gi|29165615|ref|NC_002745.2|	1918822	-	G	2	2	0	0	0	SArRNA08
gi|29165615|ref|NC_002745.2|	1918823	-	U	0	5	0	0	0	SArRNA08
gi|29165615|ref|NC_002745.2|	1918824	-	A	3	7	1	0	6	SArRNA08
gi|29165615|ref|NC_002745.2|	1918825	-	C	11	31	9	5	24	SArRNA08
gi|29165615|ref|NC_002745.2|	1918826	-	C	1	23	1	0	9	SArRNA08
gi|29165615|ref|NC_002745.2|	1918827	-	C	7	6	4	1	3	SArRNA08
gi|29165615|ref|NC_002745.2|	1918828	-	U	0	2	1	1	1	SArRNA08
gi|29165615|ref|NC_002745.2|	1918829	-	U	0	0	0	0	1	SArRNA08
gi|29165615|ref|NC_002745.2|	1918830	-	G	0	1	0	4	0	SArRNA08
gi|29165615|ref|NC_002745.2|	1918831	-	U	6	3	0	0	4	SArRNA08
gi|29165615|ref|NC_002745.2|	1918832	-	C	8	31	9	15	7	SArRNA08
gi|29165615|ref|NC_002745.2|	1918833	-	C	28	65	26	32	33	SArRNA08
gi|29165615|ref|NC_002745.2|	1918834	-	A	7	25	5	7	11	SArRNA08
gi|29165615|ref|NC_002745.2|	1918835	-	C	68	107	63	56	92	SArRNA08
gi|29165615|ref|NC_002745.2|	1918836	-	A	2	6	0	1	6	SArRNA08
gi|29165615|ref|NC_002745.2|	1918837	-	C	4	16	4	0	13	SArRNA08
gi|29165615|ref|NC_002745.2|	1918838	-	U	0	1	0	0	1	SArRNA08
gi|29165615|ref|NC_002745.2|	1918840	-	G	0	1	1	0	1	SArRNA08
gi|29165615|ref|NC_002745.2|	1918841	-	A	0	0	1	1	1	SArRNA08
gi|29165615|ref|NC_002745.2|	1918842	-	G	1	6	1	1	2	SArRNA08
gi|29165615|ref|NC_002745.2|	1918843	-	G	0	2	0	0	1	SArRNA08
gi|29165615|ref|NC_002745.2|	1918844	-	A	9	4	2	1	4	SArRNA08
gi|29165615|ref|NC_002745.2|	1918845	-	A	4	2	1	2	4	SArRNA08
gi|29165615|ref|NC_002745.2|	1918846	-	C	19	24	16	8	21	SArRNA08
gi|29165615|ref|NC_002745.2|	1918847	-	G	0	0	0	1	0	SArRNA08
gi|29165615|ref|NC_002745.2|	1918848	-	A	1	0	0	1	0	SArRNA08
gi|29165615|ref|NC_002745.2|	1918849	-	U	1	1	0	2	1	SArRNA08
gi|29165615|ref|NC_002745.2|	1918850	-	A	30	44	31	10	27	SArRNA08
gi|29165615|ref|NC_002745.2|	1918851	-	U	8	22	3	2	25	SArRNA08
gi|29165615|ref|NC_002745.2|	1918852	-	C	34	29	12	6	21	SArRNA08
gi|29165615|ref|NC_002745.2|	1918853	-	A	0	0	0	1	0	SArRNA08
gi|29165615|ref|NC_002745.2|	1918854	-	G	5	10	4	2	2	SArRNA08
gi|29165615|ref|NC_002745.2|	1918855	-	U	4	5	3	0	0	SArRNA08
gi|29165615|ref|NC_002745.2|	1918856	-	G	3	0	0	0	2	SArRNA08
gi|29165615|ref|NC_002745.2|	1918857	-	G	1	0	1	0	1	SArRNA08
gi|29165615|ref|NC_002745.2|	1918858	-	U	7	6	2	4	4	SArRNA08
gi|29165615|ref|NC_002745.2|	1918859	-	C	1	15	3	1	9	SArRNA08
gi|29165615|ref|NC_002745.2|	1918860	-	U	2502	2241	1275	977	1934	SArRNA08
gi|29165615|ref|NC_002745.2|	1918861	-	G	4	7	6	5	11	-
gi|29165615|ref|NC_002745.2|	1918862	-	U	2	2	2	1	0	-
gi|29165615|ref|NC_002745.2|	1918885	-	U	40	8	11	6	12	-
gi|29165615|ref|NC_002745.2|	1918898	-	U	0	1	0	0	0	-
gi|29165615|ref|NC_002745.2|	1918934	-	A	0	1	0	1	1	-
gi|29165615|ref|NC_002745.2|	1918935	-	C	3	0	0	0	0	-
gi|29165615|ref|NC_002745.2|	1918941	-	C	1	0	0	0	0	-
gi|29165615|ref|NC_002745.2|	1918953	-	A	0	0	0	0	2	-
gi|29165615|ref|NC_002745.2|	1918957	-	A	1	0	0	0	1	-
gi|29165615|ref|NC_002745.2|	1918958	-	U	1	0	0	0	0	-
gi|29165615|ref|NC_002745.2|	1918960	-	A	0	0	1	0	0	-
gi|29165615|ref|NC_002745.2|	1918961	-	G	0	0	0	1	0	-
gi|29165615|ref|NC_002745.2|	1918968	-	A	0	0	1	0	0	-

gi|29165615|ref|NC_002745.2|	1918970	-	G	0	0	1	0	0	-
gi|29165615|ref|NC_002745.2|	1918971	-	U	1	0	0	1	0	-
gi|29165615|ref|NC_002745.2|	1918972	-	G	2	0	0	0	0	-
gi|29165615|ref|NC_002745.2|	1918973	-	U	0	0	0	0	1	-
gi|29165615|ref|NC_002745.2|	1918975	-	C	2	0	0	0	0	-
gi|29165615|ref|NC_002745.2|	1918976	-	A	0	1	1	0	0	-
gi|29165615|ref|NC_002745.2|	1918978	-	U	1	0	0	0	1	-
gi|29165615|ref|NC_002745.2|	1918983	-	C	0	1	1	2	0	-
gi|29165615|ref|NC_002745.2|	1918984	-	G	0	0	0	0	1	-
gi|29165615|ref|NC_002745.2|	1918986	-	A	1	0	0	0	0	-
gi|29165615|ref|NC_002745.2|	1918987	-	G	1	3	1	2	1	-
gi|29165615|ref|NC_002745.2|	1918988	-	G	0	0	0	0	1	-
gi|29165615|ref|NC_002745.2|	1918992	-	A	2	1	0	0	0	-
gi|29165615|ref|NC_002745.2|	1918993	-	G	3	1	0	1	4	-
gi|29165615|ref|NC_002745.2|	1918994	-	C	0	3	0	0	1	-
gi|29165615|ref|NC_002745.2|	1918996	-	U	0	0	0	0	1	-
gi|29165615|ref|NC_002745.2|	1918999	-	A	0	2	0	0	0	-
gi|29165615|ref|NC_002745.2|	1919000	-	U	2	1	1	0	0	-
gi|29165615|ref|NC_002745.2|	1919001	-	A	0	7	0	0	6	-
gi|29165615|ref|NC_002745.2|	1919003	-	U	0	0	1	0	0	-
gi|29165615|ref|NC_002745.2|	1919004	-	U	0	4	0	0	0	-
gi|29165615|ref|NC_002745.2|	1919008	-	G	0	0	0	1	2	-
gi|29165615|ref|NC_002745.2|	1919010	-	A	4	0	1	1	0	-
gi|29165615|ref|NC_002745.2|	1919011	-	G	3	1	0	1	1	-
gi|29165615|ref|NC_002745.2|	1919012	-	U	2	0	1	0	0	-
gi|29165615|ref|NC_002745.2|	1919013	-	A	2	1	0	1	0	-
gi|29165615|ref|NC_002745.2|	1919014	-	G	2	0	1	4	1	-
gi|29165615|ref|NC_002745.2|	1919015	-	A	0	2	0	0	0	-
gi|29165615|ref|NC_002745.2|	1919016	-	A	0	0	2	1	2	-
gi|29165615|ref|NC_002745.2|	1919017	-	A	0	3	1	0	1	-
gi|29165615|ref|NC_002745.2|	1919018	-	C	1	3	0	0	1	-
gi|29165615|ref|NC_002745.2|	1919019	-	U	4	2	0	0	1	-
gi|29165615|ref|NC_002745.2|	1919020	-	C	3	6	3	2	2	-
gi|29165615|ref|NC_002745.2|	1919021	-	C	27	28	12	10	13	-
gi|29165615|ref|NC_002745.2|	1919022	-	C	22	19	8	8	13	SArRNA09
gi|29165615|ref|NC_002745.2|	1919023	-	U	6	7	4	3	5	SArRNA09
gi|29165615|ref|NC_002745.2|	1919024	-	A	3	11	0	1	4	SArRNA09
gi|29165615|ref|NC_002745.2|	1919025	-	G	2	1	5	3	0	SArRNA09
gi|29165615|ref|NC_002745.2|	1919026	-	A	0	0	0	0	1	SArRNA09
gi|29165615|ref|NC_002745.2|	1919027	-	A	0	0	0	1	0	SArRNA09
gi|29165615|ref|NC_002745.2|	1919029	-	A	1	0	0	0	2	SArRNA09
gi|29165615|ref|NC_002745.2|	1919030	-	U	0	0	2	0	0	SArRNA09
gi|29165615|ref|NC_002745.2|	1919031	-	U	0	0	0	1	0	SArRNA09
gi|29165615|ref|NC_002745.2|	1919033	-	G	4	0	0	0	0	SArRNA09
gi|29165615|ref|NC_002745.2|	1919034	-	C	14	5	0	4	1	SArRNA09
gi|29165615|ref|NC_002745.2|	1919035	-	U	1	0	0	0	0	SArRNA09
gi|29165615|ref|NC_002745.2|	1919036	-	U	0	2	0	0	0	SArRNA09
gi|29165615|ref|NC_002745.2|	1919037	-	C	1	0	0	0	1	SArRNA09
gi|29165615|ref|NC_002745.2|	1919038	-	A	0	0	2	1	0	SArRNA09
gi|29165615|ref|NC_002745.2|	1919040	-	C	1	3	1	0	1	SArRNA09
gi|29165615|ref|NC_002745.2|	1919041	-	C	0	2	0	0	1	SArRNA09
gi|29165615|ref|NC_002745.2|	1919042	-	C	1	2	0	1	1	SArRNA09
gi|29165615|ref|NC_002745.2|	1919043	-	U	1	0	1	0	1	SArRNA09
gi|29165615|ref|NC_002745.2|	1919044	-	U	0	1	0	0	0	SArRNA09
gi|29165615|ref|NC_002745.2|	1919045	-	U	0	3	0	0	0	SArRNA09
gi|29165615|ref|NC_002745.2|	1919046	-	A	0	0	0	1	0	SArRNA09
gi|29165615|ref|NC_002745.2|	1919054	-	A	1	5	1	0	1	SArRNA09
gi|29165615|ref|NC_002745.2|	1919055	-	C	2	6	1	2	3	SArRNA09
gi|29165615|ref|NC_002745.2|	1919056	-	U	0	2	0	0	0	SArRNA09
gi|29165615|ref|NC_002745.2|	1919057	-	C	0	2	1	0	2	SArRNA09
gi|29165615|ref|NC_002745.2|	1919058	-	C	1	9	1	1	3	SArRNA09
gi|29165615|ref|NC_002745.2|	1919059	-	C	6	7	4	6	11	SArRNA09
gi|29165615|ref|NC_002745.2|	1919060	-	C	32	29	17	8	26	SArRNA09
gi|29165615|ref|NC_002745.2|	1919061	-	C	23	26	14	13	25	SArRNA09
gi|29165615|ref|NC_002745.2|	1919062	-	C	16	21	14	11	19	SArRNA09
gi|29165615|ref|NC_002745.2|	1919063	-	G	1	0	0	0	0	SArRNA09
gi|29165615|ref|NC_002745.2|	1919064	-	A	0	1	0	0	1	SArRNA09
gi|29165615|ref|NC_002745.2|	1919065	-	A	0	0	1	0	0	SArRNA09
gi|29165615|ref|NC_002745.2|	1919066	-	G	0	0	0	1	0	SArRNA09
gi|29165615|ref|NC_002745.2|	1919068	-	A	1	1	0	0	1	SArRNA09
gi|29165615|ref|NC_002745.2|	1919069	-	C	0	1	0	2	1	SArRNA09
gi|29165615|ref|NC_002745.2|	1919071	-	A	0	1	1	0	2	SArRNA09
gi|29165615|ref|NC_002745.2|	1919072	-	A	2	3	0	0	0	SArRNA09
gi|29165615|ref|NC_002745.2|	1919073	-	U	0	11	1	3	5	SArRNA09
gi|29165615|ref|NC_002745.2|	1919074	-	C	3	19	0	1	12	SArRNA09
gi|29165615|ref|NC_002745.2|	1919075	-	U	0	7	1	1	1	SArRNA09
gi|29165615|ref|NC_002745.2|	1919076	-	A	2	3	2	0	1	SArRNA09
gi|29165615|ref|NC_002745.2|	1919077	-	C	1	5	1	1	4	SArRNA09
gi|29165615|ref|NC_002745.2|	1919078	-	G	1	1	0	0	0	SArRNA09
gi|29165615|ref|NC_002745.2|	1919079	-	A	0	2	0	0	0	SArRNA09
gi|29165615|ref|NC_002745.2|	1919080	-	A	0	0	0	0	1	SArRNA09

gi|29165615|ref|NC_002745.2|	1919081	-	A	1	3	0	0	1 SArRNA09
gi|29165615|ref|NC_002745.2|	1919082	-	G	0	1	0	0	0 SArRNA09
gi|29165615|ref|NC_002745.2|	1919083	-	U	0	1	0	0	0 SArRNA09
gi|29165615|ref|NC_002745.2|	1919084	-	C	0	1	0	0	0 SArRNA09
gi|29165615|ref|NC_002745.2|	1919086	-	U	1	0	0	0	6 SArRNA09
gi|29165615|ref|NC_002745.2|	1919087	-	G	1	7	0	0	1 SArRNA09
gi|29165615|ref|NC_002745.2|	1919088	-	A	0	9	1	0	3 SArRNA09
gi|29165615|ref|NC_002745.2|	1919089	-	A	4	23	0	1	10 SArRNA09
gi|29165615|ref|NC_002745.2|	1919090	-	U	1	17	1	1	3 SArRNA09
gi|29165615|ref|NC_002745.2|	1919091	-	A	1	2	1	0	0 SArRNA09
gi|29165615|ref|NC_002745.2|	1919092	-	G	0	0	0	0	2 SArRNA09
gi|29165615|ref|NC_002745.2|	1919094	-	G	3	11	3	4	7 SArRNA09
gi|29165615|ref|NC_002745.2|	1919095	-	C	17	20	1	5	4 SArRNA09
gi|29165615|ref|NC_002745.2|	1919096	-	A	2	0	0	0	0 SArRNA09
gi|29165615|ref|NC_002745.2|	1919097	-	G	3	7	0	1	0 SArRNA09
gi|29165615|ref|NC_002745.2|	1919098	-	G	1	1	0	1	3 SArRNA09
gi|29165615|ref|NC_002745.2|	1919099	-	U	0	1	0	1	1 SArRNA09
gi|29165615|ref|NC_002745.2|	1919101	-	U	0	1	0	0	0 SArRNA09
gi|29165615|ref|NC_002745.2|	1919102	-	G	0	1	0	0	0 SArRNA09
gi|29165615|ref|NC_002745.2|	1919104	-	A	3	8	2	0	0 SArRNA09
gi|29165615|ref|NC_002745.2|	1919105	-	U	2	17	0	1	8 SArRNA09
gi|29165615|ref|NC_002745.2|	1919106	-	C	2	9	0	0	3 SArRNA09
gi|29165615|ref|NC_002745.2|	1919108	-	A	0	1	1	0	0 SArRNA09
gi|29165615|ref|NC_002745.2|	1919109	-	U	6	4	0	0	4 SArRNA09
gi|29165615|ref|NC_002745.2|	1919110	-	G	0	1	0	0	3 SArRNA09
gi|29165615|ref|NC_002745.2|	1919112	-	G	0	0	0	1	0 SArRNA09
gi|29165615|ref|NC_002745.2|	1919113	-	U	3	3	3	0	1 SArRNA09
gi|29165615|ref|NC_002745.2|	1919114	-	C	2	4	1	0	1 SArRNA09
gi|29165615|ref|NC_002745.2|	1919115	-	G	1	2	1	0	1 SArRNA09
gi|29165615|ref|NC_002745.2|	1919116	-	A	4	1	0	0	2 SArRNA09
gi|29165615|ref|NC_002745.2|	1919117	-	U	1	1	0	0	3 SArRNA09
gi|29165615|ref|NC_002745.2|	1919118	-	A	1	5	0	1	3 SArRNA09
gi|29165615|ref|NC_002745.2|	1919119	-	C	0	4	0	3	1 SArRNA09
gi|29165615|ref|NC_002745.2|	1919121	-	G	1	8	0	1	3 SArRNA09
gi|29165615|ref|NC_002745.2|	1919122	-	C	2	7	4	1	1 SArRNA09
gi|29165615|ref|NC_002745.2|	1919123	-	A	1	25	1	0	12 SArRNA09
gi|29165615|ref|NC_002745.2|	1919124	-	A	3	18	0	0	8 SArRNA09
gi|29165615|ref|NC_002745.2|	1919125	-	C	5	10	0	3	2 SArRNA09
gi|29165615|ref|NC_002745.2|	1919126	-	C	1	6	0	0	2 SArRNA09
gi|29165615|ref|NC_002745.2|	1919128	-	U	0	0	0	0	1 SArRNA09
gi|29165615|ref|NC_002745.2|	1919129	-	G	0	2	0	1	1 SArRNA09
gi|29165615|ref|NC_002745.2|	1919130	-	C	4	2	1	1	0 SArRNA09
gi|29165615|ref|NC_002745.2|	1919131	-	U	1	4	0	0	7 SArRNA09
gi|29165615|ref|NC_002745.2|	1919132	-	G	1	3	1	0	8 SArRNA09
gi|29165615|ref|NC_002745.2|	1919133	-	U	4	5	1	0	4 SArRNA09
gi|29165615|ref|NC_002745.2|	1919134	-	U	6	0	3	1	9 SArRNA09
gi|29165615|ref|NC_002745.2|	1919135	-	G	4	1	0	3	11 SArRNA09
gi|29165615|ref|NC_002745.2|	1919136	-	A	26	5	4	9	33 SArRNA09
gi|29165615|ref|NC_002745.2|	1919137	-	C	312	108	47	55	199 SArRNA09
gi|29165615|ref|NC_002745.2|	1919138	-	C	12	12	4	4	11 SArRNA09
gi|29165615|ref|NC_002745.2|	1919139	-	A	8	7	5	3	10 SArRNA09
gi|29165615|ref|NC_002745.2|	1919140	-	U	5	7	3	3	12 SArRNA09
gi|29165615|ref|NC_002745.2|	1919141	-	G	7	9	2	5	5 SArRNA09
gi|29165615|ref|NC_002745.2|	1919142	-	U	8	9	3	1	12 SArRNA09
gi|29165615|ref|NC_002745.2|	1919143	-	G	2	3	3	6	6 SArRNA09
gi|29165615|ref|NC_002745.2|	1919144	-	G	1	5	3	2	2 SArRNA09
gi|29165615|ref|NC_002745.2|	1919145	-	U	3	3	0	1	7 SArRNA09
gi|29165615|ref|NC_002745.2|	1919146	-	C	8	1	2	4	6 SArRNA09
gi|29165615|ref|NC_002745.2|	1919147	-	U	4	1	5	4	6 SArRNA09
gi|29165615|ref|NC_002745.2|	1919148	-	C	8	5	5	1	7 SArRNA09
gi|29165615|ref|NC_002745.2|	1919149	-	C	11	6	4	2	8 SArRNA09
gi|29165615|ref|NC_002745.2|	1919150	-	A	13	10	1	4	10 SArRNA09
gi|29165615|ref|NC_002745.2|	1919151	-	U	9	19	8	7	25 SArRNA09
gi|29165615|ref|NC_002745.2|	1919152	-	A	4	21	3	7	12 SArRNA09
gi|29165615|ref|NC_002745.2|	1919153	-	C	3	9	4	3	6 SArRNA09
gi|29165615|ref|NC_002745.2|	1919154	-	A	23	2	3	1	7 SArRNA09
gi|29165615|ref|NC_002745.2|	1919155	-	G	14	1	0	0	3 SArRNA09
gi|29165615|ref|NC_002745.2|	1919156	-	G	4	0	0	2	2 SArRNA09
gi|29165615|ref|NC_002745.2|	1919157	-	U	12	11	1	0	4 SArRNA09
gi|29165615|ref|NC_002745.2|	1919158	-	A	14	9	2	3	5 SArRNA09
gi|29165615|ref|NC_002745.2|	1919159	-	G	6	0	0	4	8 SArRNA09
gi|29165615|ref|NC_002745.2|	1919160	-	G	1	2	0	2	1 SArRNA09
gi|29165615|ref|NC_002745.2|	1919161	-	G	2	5	1	0	4 SArRNA09
gi|29165615|ref|NC_002745.2|	1919162	-	C	9	10	3	6	15 SArRNA09
gi|29165615|ref|NC_002745.2|	1919163	-	C	3	9	0	0	3 SArRNA09
gi|29165615|ref|NC_002745.2|	1919164	-	A	3	1	0	1	0 SArRNA09
gi|29165615|ref|NC_002745.2|	1919165	-	G	0	0	0	1	2 SArRNA09
gi|29165615|ref|NC_002745.2|	1919166	-	G	4	2	0	0	1 SArRNA09
gi|29165615|ref|NC_002745.2|	1919167	-	A	2	4	0	2	1 SArRNA09
gi|29165615|ref|NC_002745.2|	1919168	-	G	0	0	0	0	2 SArRNA09
gi|29165615|ref|NC_002745.2|	1919169	-	A	2	1	1	1	1 SArRNA09

gi|29165615|ref|NC_002745.2|	1919170	-	G	2	9	2	2	8	SArRNA09
gi|29165615|ref|NC_002745.2|	1919171	-	C	7	11	2	2	3	SArRNA09
gi|29165615|ref|NC_002745.2|	1919172	-	A	8	9	1	0	1	SArRNA09
gi|29165615|ref|NC_002745.2|	1919173	-	U	1	14	3	1	3	SArRNA09
gi|29165615|ref|NC_002745.2|	1919174	-	G	2	3	1	0	4	SArRNA09
gi|29165615|ref|NC_002745.2|	1919175	-	A	2	4	1	1	3	SArRNA09
gi|29165615|ref|NC_002745.2|	1919176	-	U	7	8	3	4	0	SArRNA09
gi|29165615|ref|NC_002745.2|	1919177	-	U	4	4	4	1	3	SArRNA09
gi|29165615|ref|NC_002745.2|	1919178	-	C	4	4	0	1	6	SArRNA09
gi|29165615|ref|NC_002745.2|	1919179	-	C	6	1	4	0	4	SArRNA09
gi|29165615|ref|NC_002745.2|	1919180	-	U	3	3	0	1	2	SArRNA09
gi|29165615|ref|NC_002745.2|	1919181	-	G	0	2	0	0	1	SArRNA09
gi|29165615|ref|NC_002745.2|	1919182	-	U	2	1	1	0	4	SArRNA09
gi|29165615|ref|NC_002745.2|	1919183	-	C	1	1	2	2	3	SArRNA09
gi|29165615|ref|NC_002745.2|	1919184	-	G	5	4	1	0	1	SArRNA09
gi|29165615|ref|NC_002745.2|	1919185	-	A	6	3	1	5	7	SArRNA09
gi|29165615|ref|NC_002745.2|	1919186	-	G	5	1	2	4	6	SArRNA09
gi|29165615|ref|NC_002745.2|	1919187	-	G	1	2	1	0	1	SArRNA09
gi|29165615|ref|NC_002745.2|	1919188	-	A	3	2	1	3	10	SArRNA09
gi|29165615|ref|NC_002745.2|	1919189	-	G	2	3	2	2	0	SArRNA09
gi|29165615|ref|NC_002745.2|	1919190	-	A	0	2	1	0	1	SArRNA09
gi|29165615|ref|NC_002745.2|	1919191	-	G	12	7	2	4	9	SArRNA09
gi|29165615|ref|NC_002745.2|	1919192	-	U	7	8	3	3	7	SArRNA09
gi|29165615|ref|NC_002745.2|	1919193	-	U	10	5	1	3	12	SArRNA09
gi|29165615|ref|NC_002745.2|	1919194	-	U	10	9	3	3	6	SArRNA09
gi|29165615|ref|NC_002745.2|	1919195	-	A	13	64	3	6	24	SArRNA09
gi|29165615|ref|NC_002745.2|	1919196	-	A	10	6	2	2	6	SArRNA09
gi|29165615|ref|NC_002745.2|	1919197	-	A	0	2	0	3	1	SArRNA09
gi|29165615|ref|NC_002745.2|	1919198	-	G	1	1	1	0	1	SArRNA09
gi|29165615|ref|NC_002745.2|	1919199	-	G	0	2	0	0	1	SArRNA09
gi|29165615|ref|NC_002745.2|	1919200	-	A	1	1	0	0	2	SArRNA09
gi|29165615|ref|NC_002745.2|	1919201	-	U	1	11	2	6	6	SArRNA09
gi|29165615|ref|NC_002745.2|	1919202	-	G	6	5	2	1	3	SArRNA09
gi|29165615|ref|NC_002745.2|	1919203	-	C	6	4	0	1	2	SArRNA09
gi|29165615|ref|NC_002745.2|	1919204	-	G	0	1	0	0	0	SArRNA09
gi|29165615|ref|NC_002745.2|	1919205	-	G	1	2	0	0	1	SArRNA09
gi|29165615|ref|NC_002745.2|	1919206	-	G	0	1	0	0	2	SArRNA09
gi|29165615|ref|NC_002745.2|	1919207	-	U	6	2	2	0	1	SArRNA09
gi|29165615|ref|NC_002745.2|	1919208	-	G	0	2	1	0	0	SArRNA09
gi|29165615|ref|NC_002745.2|	1919209	-	C	3	3	2	1	3	SArRNA09
gi|29165615|ref|NC_002745.2|	1919210	-	U	8	1	0	0	4	SArRNA09
gi|29165615|ref|NC_002745.2|	1919211	-	G	2	15	3	4	5	SArRNA09
gi|29165615|ref|NC_002745.2|	1919212	-	C	11	10	2	8	11	SArRNA09
gi|29165615|ref|NC_002745.2|	1919213	-	C	2	8	1	0	5	SArRNA09
gi|29165615|ref|NC_002745.2|	1919214	-	U	1	0	2	1	2	SArRNA09
gi|29165615|ref|NC_002745.2|	1919215	-	A	4	17	2	2	7	SArRNA09
gi|29165615|ref|NC_002745.2|	1919216	-	U	8	25	1	0	5	SArRNA09
gi|29165615|ref|NC_002745.2|	1919217	-	C	6	17	0	1	4	SArRNA09
gi|29165615|ref|NC_002745.2|	1919218	-	C	1	11	0	1	6	SArRNA09
gi|29165615|ref|NC_002745.2|	1919219	-	C	3	9	3	2	6	SArRNA09
gi|29165615|ref|NC_002745.2|	1919220	-	U	0	0	0	0	1	SArRNA09
gi|29165615|ref|NC_002745.2|	1919221	-	G	2	1	0	0	0	SArRNA09
gi|29165615|ref|NC_002745.2|	1919222	-	G	1	2	0	0	1	SArRNA09
gi|29165615|ref|NC_002745.2|	1919223	-	C	4	3	0	0	7	SArRNA09
gi|29165615|ref|NC_002745.2|	1919224	-	U	0	1	0	0	3	SArRNA09
gi|29165615|ref|NC_002745.2|	1919225	-	U	2	0	0	0	0	SArRNA09
gi|29165615|ref|NC_002745.2|	1919226	-	G	0	3	0	0	2	SArRNA09
gi|29165615|ref|NC_002745.2|	1919227	-	A	2	7	2	3	1	SArRNA09
gi|29165615|ref|NC_002745.2|	1919228	-	C	4	4	0	4	0	SArRNA09
gi|29165615|ref|NC_002745.2|	1919229	-	A	4	2	1	2	3	SArRNA09
gi|29165615|ref|NC_002745.2|	1919231	-	A	1	2	1	0	2	SArRNA09
gi|29165615|ref|NC_002745.2|	1919232	-	G	1	3	1	2	0	SArRNA09
gi|29165615|ref|NC_002745.2|	1919233	-	U	3	6	2	2	10	SArRNA09
gi|29165615|ref|NC_002745.2|	1919234	-	G	6	1	2	3	1	SArRNA09
gi|29165615|ref|NC_002745.2|	1919235	-	C	1	9	4	1	4	SArRNA09
gi|29165615|ref|NC_002745.2|	1919236	-	U	1	2	2	1	1	SArRNA09
gi|29165615|ref|NC_002745.2|	1919237	-	G	6	8	3	2	3	SArRNA09
gi|29165615|ref|NC_002745.2|	1919238	-	C	6	17	1	4	16	SArRNA09
gi|29165615|ref|NC_002745.2|	1919239	-	A	2	1	0	0	0	SArRNA09
gi|29165615|ref|NC_002745.2|	1919240	-	A	1	3	0	0	0	SArRNA09
gi|29165615|ref|NC_002745.2|	1919242	-	A	1	8	2	0	6	SArRNA09
gi|29165615|ref|NC_002745.2|	1919243	-	C	15	29	8	12	22	SArRNA09
gi|29165615|ref|NC_002745.2|	1919244	-	U	0	2	0	0	6	SArRNA09
gi|29165615|ref|NC_002745.2|	1919245	-	U	2	0	0	0	2	SArRNA09
gi|29165615|ref|NC_002745.2|	1919246	-	G	0	1	0	1	1	SArRNA09
gi|29165615|ref|NC_002745.2|	1919247	-	G	1	1	0	0	0	SArRNA09
gi|29165615|ref|NC_002745.2|	1919248	-	G	1	0	0	1	2	SArRNA09
gi|29165615|ref|NC_002745.2|	1919249	-	U	2	16	3	1	12	SArRNA09
gi|29165615|ref|NC_002745.2|	1919250	-	C	7	13	0	3	10	SArRNA09
gi|29165615|ref|NC_002745.2|	1919251	-	G	1	1	2	1	3	SArRNA09
gi|29165615|ref|NC_002745.2|	1919252	-	A	7	3	2	2	11	SArRNA09

gi|29165615|ref|NC_002745.2|	1919253	-	G	8	12	4	1	7	SArRNA09
gi|29165615|ref|NC_002745.2|	1919254	-	C	17	18	6	3	11	SArRNA09
gi|29165615|ref|NC_002745.2|	1919255	-	G	2	5	2	2	3	SArRNA09
gi|29165615|ref|NC_002745.2|	1919256	-	C	4	4	1	3	2	SArRNA09
gi|29165615|ref|NC_002745.2|	1919257	-	A	2	4	1	1	1	SArRNA09
gi|29165615|ref|NC_002745.2|	1919258	-	U	29	38	14	9	21	SArRNA09
gi|29165615|ref|NC_002745.2|	1919259	-	G	3	10	0	1	4	SArRNA09
gi|29165615|ref|NC_002745.2|	1919260	-	G	7	4	2	3	1	SArRNA09
gi|29165615|ref|NC_002745.2|	1919261	-	C	16	7	3	2	10	SArRNA09
gi|29165615|ref|NC_002745.2|	1919262	-	G	7	8	3	0	3	SArRNA09
gi|29165615|ref|NC_002745.2|	1919263	-	A	4	15	0	2	10	SArRNA09
gi|29165615|ref|NC_002745.2|	1919264	-	A	6	6	2	2	9	SArRNA09
gi|29165615|ref|NC_002745.2|	1919265	-	A	9	6	2	3	4	SArRNA09
gi|29165615|ref|NC_002745.2|	1919266	-	U	3	6	0	3	7	SArRNA09
gi|29165615|ref|NC_002745.2|	1919267	-	U	9	2	5	4	11	SArRNA09
gi|29165615|ref|NC_002745.2|	1919268	-	A	5	3	2	1	9	SArRNA09
gi|29165615|ref|NC_002745.2|	1919269	-	C	1	8	4	0	9	SArRNA09
gi|29165615|ref|NC_002745.2|	1919270	-	C	5	10	2	1	7	SArRNA09
gi|29165615|ref|NC_002745.2|	1919271	-	C	18	13	2	4	13	SArRNA09
gi|29165615|ref|NC_002745.2|	1919272	-	G	14	28	3	11	28	SArRNA09
gi|29165615|ref|NC_002745.2|	1919273	-	C	106	105	44	53	73	SArRNA09
gi|29165615|ref|NC_002745.2|	1919274	-	U	0	3	0	0	2	SArRNA09
gi|29165615|ref|NC_002745.2|	1919275	-	U	1	0	0	0	0	SArRNA09
gi|29165615|ref|NC_002745.2|	1919276	-	G	24	0	7	1	3	SArRNA09
gi|29165615|ref|NC_002745.2|	1919277	-	U	12	2	1	0	0	SArRNA09
gi|29165615|ref|NC_002745.2|	1919278	-	C	7	0	0	1	3	SArRNA09
gi|29165615|ref|NC_002745.2|	1919279	-	G	1	2	0	0	3	SArRNA09
gi|29165615|ref|NC_002745.2|	1919280	-	G	1	2	0	1	2	SArRNA09
gi|29165615|ref|NC_002745.2|	1919281	-	G	2	3	3	0	7	SArRNA09
gi|29165615|ref|NC_002745.2|	1919282	-	U	2	0	1	1	5	SArRNA09
gi|29165615|ref|NC_002745.2|	1919283	-	U	13	3	2	3	3	SArRNA09
gi|29165615|ref|NC_002745.2|	1919284	-	G	10	10	5	3	9	SArRNA09
gi|29165615|ref|NC_002745.2|	1919285	-	G	12	3	2	6	13	SArRNA09
gi|29165615|ref|NC_002745.2|	1919286	-	G	4	3	1	2	0	SArRNA09
gi|29165615|ref|NC_002745.2|	1919287	-	A	6	6	0	6	10	SArRNA09
gi|29165615|ref|NC_002745.2|	1919288	-	A	6	7	1	1	6	SArRNA09
gi|29165615|ref|NC_002745.2|	1919289	-	C	24	15	10	8	17	SArRNA09
gi|29165615|ref|NC_002745.2|	1919290	-	C	28	26	10	9	23	SArRNA09
gi|29165615|ref|NC_002745.2|	1919291	-	C	21	28	5	9	17	SArRNA09
gi|29165615|ref|NC_002745.2|	1919292	-	U	26	17	10	5	9	SArRNA09
gi|29165615|ref|NC_002745.2|	1919293	-	G	21	21	8	11	17	SArRNA09
gi|29165615|ref|NC_002745.2|	1919294	-	G	21	19	14	6	9	SArRNA09
gi|29165615|ref|NC_002745.2|	1919295	-	C	13	22	6	5	19	SArRNA09
gi|29165615|ref|NC_002745.2|	1919296	-	U	31	50	6	14	34	SArRNA09
gi|29165615|ref|NC_002745.2|	1919297	-	G	3	8	1	2	6	SArRNA09
gi|29165615|ref|NC_002745.2|	1919298	-	A	6	1	1	3	3	SArRNA09
gi|29165615|ref|NC_002745.2|	1919299	-	U	6	4	5	4	12	SArRNA09
gi|29165615|ref|NC_002745.2|	1919300	-	G	10	13	3	3	10	SArRNA09
gi|29165615|ref|NC_002745.2|	1919301	-	U	8	3	4	4	17	SArRNA09
gi|29165615|ref|NC_002745.2|	1919302	-	C	3	8	2	4	5	SArRNA09
gi|29165615|ref|NC_002745.2|	1919303	-	G	3	4	1	1	1	SArRNA09
gi|29165615|ref|NC_002745.2|	1919304	-	G	32	10	5	11	15	SArRNA09
gi|29165615|ref|NC_002745.2|	1919305	-	G	4	5	4	3	3	SArRNA09
gi|29165615|ref|NC_002745.2|	1919306	-	G	0	1	0	0	1	SArRNA09
gi|29165615|ref|NC_002745.2|	1919307	-	U	26	16	9	10	13	SArRNA09
gi|29165615|ref|NC_002745.2|	1919308	-	C	59	19	14	10	20	SArRNA09
gi|29165615|ref|NC_002745.2|	1919309	-	C	49	12	8	7	26	SArRNA09
gi|29165615|ref|NC_002745.2|	1919310	-	U	27	9	6	8	14	SArRNA09
gi|29165615|ref|NC_002745.2|	1919311	-	A	8	16	7	5	17	SArRNA09
gi|29165615|ref|NC_002745.2|	1919312	-	C	23	25	12	8	15	SArRNA09
gi|29165615|ref|NC_002745.2|	1919313	-	G	5	5	2	2	9	SArRNA09
gi|29165615|ref|NC_002745.2|	1919314	-	C	10	16	6	3	9	SArRNA09
gi|29165615|ref|NC_002745.2|	1919315	-	U	8	2	3	3	9	SArRNA09
gi|29165615|ref|NC_002745.2|	1919316	-	A	21	29	4	7	14	SArRNA09
gi|29165615|ref|NC_002745.2|	1919317	-	C	39	40	4	15	40	SArRNA09
gi|29165615|ref|NC_002745.2|	1919318	-	U	2	3	1	1	7	SArRNA09
gi|29165615|ref|NC_002745.2|	1919319	-	C	0	4	3	1	1	SArRNA09
gi|29165615|ref|NC_002745.2|	1919320	-	G	0	0	0	1	1	SArRNA09
gi|29165615|ref|NC_002745.2|	1919321	-	G	1	2	0	0	1	SArRNA09
gi|29165615|ref|NC_002745.2|	1919322	-	C	5	9	5	4	2	SArRNA09
gi|29165615|ref|NC_002745.2|	1919323	-	U	0	1	0	0	3	SArRNA09
gi|29165615|ref|NC_002745.2|	1919324	-	G	2	0	0	0	0	SArRNA09
gi|29165615|ref|NC_002745.2|	1919325	-	U	0	0	0	0	1	SArRNA09
gi|29165615|ref|NC_002745.2|	1919326	-	A	1	0	0	1	0	SArRNA09
gi|29165615|ref|NC_002745.2|	1919327	-	G	6	30	5	4	12	SArRNA09
gi|29165615|ref|NC_002745.2|	1919328	-	C	5	18	1	6	18	SArRNA09
gi|29165615|ref|NC_002745.2|	1919329	-	U	3	2	1	0	4	SArRNA09
gi|29165615|ref|NC_002745.2|	1919330	-	C	2	7	2	3	4	SArRNA09
gi|29165615|ref|NC_002745.2|	1919331	-	C	3	4	2	1	3	SArRNA09
gi|29165615|ref|NC_002745.2|	1919332	-	A	6	5	0	1	3	SArRNA09
gi|29165615|ref|NC_002745.2|	1919333	-	C	12	22	2	7	28	SArRNA09

gi|29165615|ref|NC_002745.2|	1919336	-	U	56	109	5	2	58	SArRNA09
gi|29165615|ref|NC_002745.2|	1919337	-	U	2	16	1	2	5	SArRNA09
gi|29165615|ref|NC_002745.2|	1919338	-	U	0	0	0	0	1	SArRNA09
gi|29165615|ref|NC_002745.2|	1919339	-	G	1	0	0	0	1	SArRNA09
gi|29165615|ref|NC_002745.2|	1919340	-	G	1	0	0	0	0	SArRNA09
gi|29165615|ref|NC_002745.2|	1919344	-	G	0	0	1	0	0	SArRNA09
gi|29165615|ref|NC_002745.2|	1919345	-	G	0	1	0	0	0	SArRNA09
gi|29165615|ref|NC_002745.2|	1919346	-	C	3	2	0	0	1	SArRNA09
gi|29165615|ref|NC_002745.2|	1919347	-	A	0	0	0	1	0	SArRNA09
gi|29165615|ref|NC_002745.2|	1919348	-	G	1	0	0	0	0	SArRNA09
gi|29165615|ref|NC_002745.2|	1919349	-	C	0	1	1	2	2	SArRNA09
gi|29165615|ref|NC_002745.2|	1919350	-	U	0	0	0	1	0	SArRNA09
gi|29165615|ref|NC_002745.2|	1919351	-	A	8	12	0	1	6	SArRNA09
gi|29165615|ref|NC_002745.2|	1919352	-	C	11	17	8	7	9	SArRNA09
gi|29165615|ref|NC_002745.2|	1919353	-	A	5	6	0	4	1	SArRNA09
gi|29165615|ref|NC_002745.2|	1919354	-	C	8	17	4	1	14	SArRNA09
gi|29165615|ref|NC_002745.2|	1919355	-	U	0	1	0	0	0	SArRNA09
gi|29165615|ref|NC_002745.2|	1919358	-	A	0	2	0	0	2	SArRNA09
gi|29165615|ref|NC_002745.2|	1919360	-	A	0	0	0	1	1	SArRNA09
gi|29165615|ref|NC_002745.2|	1919362	-	C	0	0	0	1	0	SArRNA09
gi|29165615|ref|NC_002745.2|	1919363	-	C	0	1	1	0	0	SArRNA09
gi|29165615|ref|NC_002745.2|	1919364	-	C	3	1	1	0	0	SArRNA09
gi|29165615|ref|NC_002745.2|	1919365	-	C	12	4	4	2	4	SArRNA09
gi|29165615|ref|NC_002745.2|	1919366	-	C	0	3	2	1	2	SArRNA09
gi|29165615|ref|NC_002745.2|	1919367	-	U	0	1	1	0	0	SArRNA09
gi|29165615|ref|NC_002745.2|	1919368	-	C	5	1	0	0	0	SArRNA09
gi|29165615|ref|NC_002745.2|	1919369	-	U	0	1	0	0	0	SArRNA09
gi|29165615|ref|NC_002745.2|	1919376	-	A	0	1	0	0	0	SArRNA09
gi|29165615|ref|NC_002745.2|	1919377	-	C	0	1	0	2	2	SArRNA09
gi|29165615|ref|NC_002745.2|	1919378	-	A	1	0	0	0	0	SArRNA09
gi|29165615|ref|NC_002745.2|	1919381	-	A	0	0	0	1	0	SArRNA09
gi|29165615|ref|NC_002745.2|	1919385	-	G	2	1	0	0	0	SArRNA09
gi|29165615|ref|NC_002745.2|	1919386	-	C	0	1	1	0	0	SArRNA09
gi|29165615|ref|NC_002745.2|	1919387	-	C	1	0	0	0	0	SArRNA09
gi|29165615|ref|NC_002745.2|	1919388	-	C	0	1	1	0	0	SArRNA09
gi|29165615|ref|NC_002745.2|	1919389	-	C	1	2	0	0	0	SArRNA09
gi|29165615|ref|NC_002745.2|	1919390	-	A	0	0	0	0	1	SArRNA09
gi|29165615|ref|NC_002745.2|	1919392	-	C	0	1	0	0	0	SArRNA09
gi|29165615|ref|NC_002745.2|	1919398	-	U	1	0	0	1	0	SArRNA09
gi|29165615|ref|NC_002745.2|	1919400	-	G	0	0	0	1	0	SArRNA09
gi|29165615|ref|NC_002745.2|	1919402	-	C	0	1	0	0	0	SArRNA09
gi|29165615|ref|NC_002745.2|	1919403	-	A	0	1	0	0	0	SArRNA09
gi|29165615|ref|NC_002745.2|	1919404	-	A	2	1	0	0	0	SArRNA09
gi|29165615|ref|NC_002745.2|	1919405	-	C	0	1	0	0	0	SArRNA09
gi|29165615|ref|NC_002745.2|	1919407	-	C	0	0	0	0	1	SArRNA09
gi|29165615|ref|NC_002745.2|	1919409	-	C	1	1	0	0	0	SArRNA09
gi|29165615|ref|NC_002745.2|	1919411	-	A	0	0	0	0	1	SArRNA09
gi|29165615|ref|NC_002745.2|	1919417	-	A	0	0	0	0	1	SArRNA09
gi|29165615|ref|NC_002745.2|	1919418	-	A	0	0	0	0	1	SArRNA09
gi|29165615|ref|NC_002745.2|	1919422	-	A	0	0	0	0	2	SArRNA09
gi|29165615|ref|NC_002745.2|	1919434	-	C	0	0	1	0	0	SArRNA09
gi|29165615|ref|NC_002745.2|	1919435	-	C	0	1	0	2	1	SArRNA09
gi|29165615|ref|NC_002745.2|	1919438	-	G	1	1	0	0	0	SArRNA09
gi|29165615|ref|NC_002745.2|	1919441	-	A	1	0	0	0	0	SArRNA09
gi|29165615|ref|NC_002745.2|	1919444	-	C	1	2	0	0	0	SArRNA09
gi|29165615|ref|NC_002745.2|	1919448	-	C	1	0	0	0	0	SArRNA09
gi|29165615|ref|NC_002745.2|	1919449	-	A	1	0	0	0	0	SArRNA09
gi|29165615|ref|NC_002745.2|	1919452	-	A	0	2	0	0	1	SArRNA09
gi|29165615|ref|NC_002745.2|	1919453	-	A	0	0	0	0	1	SArRNA09
gi|29165615|ref|NC_002745.2|	1919460	-	A	0	0	1	0	2	SArRNA09
gi|29165615|ref|NC_002745.2|	1919462	-	G	0	0	0	0	2	SArRNA09
gi|29165615|ref|NC_002745.2|	1919463	-	A	0	0	1	0	1	SArRNA09
gi|29165615|ref|NC_002745.2|	1919465	-	C	3	2	1	0	5	SArRNA09
gi|29165615|ref|NC_002745.2|	1919466	-	U	0	0	0	0	2	SArRNA09
gi|29165615|ref|NC_002745.2|	1919467	-	G	1	0	0	0	1	SArRNA09
gi|29165615|ref|NC_002745.2|	1919468	-	A	1	0	0	0	0	SArRNA09
gi|29165615|ref|NC_002745.2|	1919469	-	A	3	4	3	0	1	SArRNA09
gi|29165615|ref|NC_002745.2|	1919470	-	C	7	9	0	6	5	SArRNA09
gi|29165615|ref|NC_002745.2|	1919471	-	A	1	0	0	0	0	SArRNA09
gi|29165615|ref|NC_002745.2|	1919472	-	U	0	1	0	0	1	SArRNA09
gi|29165615|ref|NC_002745.2|	1919474	-	C	1	0	0	0	0	SArRNA09
gi|29165615|ref|NC_002745.2|	1919476	-	G	0	0	0	0	2	SArRNA09
gi|29165615|ref|NC_002745.2|	1919477	-	A	0	2	0	0	0	SArRNA09
gi|29165615|ref|NC_002745.2|	1919478	-	G	0	1	0	1	0	SArRNA09
gi|29165615|ref|NC_002745.2|	1919479	-	C	0	3	0	1	0	SArRNA09
gi|29165615|ref|NC_002745.2|	1919480	-	G	0	1	0	0	0	SArRNA09
gi|29165615|ref|NC_002745.2|	1919481	-	U	6	0	2	2	4	SArRNA09
gi|29165615|ref|NC_002745.2|	1919487	-	C	1	1	0	0	0	SArRNA09
gi|29165615|ref|NC_002745.2|	1919490	-	G	0	1	1	0	0	SArRNA09
gi|29165615|ref|NC_002745.2|	1919491	-	G	0	0	0	0	1	SArRNA09
gi|29165615|ref|NC_002745.2|	1919493	-	A	0	1	0	0	0	SArRNA09

gi|29165615|ref|NC_002745.2|	1919494	-	A	0	1	1	0	4	SArRNA09
gi|29165615|ref|NC_002745.2|	1919495	-	U	6	7	4	4	12	SArRNA09
gi|29165615|ref|NC_002745.2|	1919496	-	A	4	1	4	3	5	SArRNA09
gi|29165615|ref|NC_002745.2|	1919497	-	C	1	2	0	0	1	SArRNA09
gi|29165615|ref|NC_002745.2|	1919498	-	G	2	3	1	2	6	SArRNA09
gi|29165615|ref|NC_002745.2|	1919499	-	G	1	5	2	0	1	SArRNA09
gi|29165615|ref|NC_002745.2|	1919500	-	A	5	0	1	0	1	SArRNA09
gi|29165615|ref|NC_002745.2|	1919501	-	A	4	6	0	3	4	SArRNA09
gi|29165615|ref|NC_002745.2|	1919502	-	A	1	1	3	0	1	SArRNA09
gi|29165615|ref|NC_002745.2|	1919503	-	U	0	4	0	0	3	SArRNA09
gi|29165615|ref|NC_002745.2|	1919504	-	G	4	3	0	1	3	SArRNA09
gi|29165615|ref|NC_002745.2|	1919506	-	G	3	2	1	2	7	SArRNA09
gi|29165615|ref|NC_002745.2|	1919507	-	A	1	3	0	0	2	SArRNA09
gi|29165615|ref|NC_002745.2|	1919508	-	G	1	2	5	2	5	SArRNA09
gi|29165615|ref|NC_002745.2|	1919509	-	A	7	4	3	2	6	SArRNA09
gi|29165615|ref|NC_002745.2|	1919510	-	U	3	4	2	2	7	SArRNA09
gi|29165615|ref|NC_002745.2|	1919511	-	A	2	5	5	1	3	SArRNA09
gi|29165615|ref|NC_002745.2|	1919512	-	C	5	5	2	0	4	SArRNA09
gi|29165615|ref|NC_002745.2|	1919513	-	U	1	3	4	3	3	SArRNA09
gi|29165615|ref|NC_002745.2|	1919514	-	U	0	1	1	0	4	SArRNA09
gi|29165615|ref|NC_002745.2|	1919515	-	A	0	4	4	1	4	SArRNA09
gi|29165615|ref|NC_002745.2|	1919516	-	C	2	4	1	4	3	SArRNA09
gi|29165615|ref|NC_002745.2|	1919517	-	U	0	2	1	0	1	SArRNA09
gi|29165615|ref|NC_002745.2|	1919518	-	A	9	70	8	4	20	SArRNA09
gi|29165615|ref|NC_002745.2|	1919519	-	A	1	4	1	0	1	SArRNA09
gi|29165615|ref|NC_002745.2|	1919520	-	A	0	2	0	0	1	SArRNA09
gi|29165615|ref|NC_002745.2|	1919521	-	G	0	2	1	0	1	SArRNA09
gi|29165615|ref|NC_002745.2|	1919522	-	G	6	4	4	2	6	SArRNA09
gi|29165615|ref|NC_002745.2|	1919523	-	U	0	3	1	1	3	SArRNA09
gi|29165615|ref|NC_002745.2|	1919524	-	U	3	2	2	4	7	SArRNA09
gi|29165615|ref|NC_002745.2|	1919525	-	G	3	13	2	0	8	SArRNA09
gi|29165615|ref|NC_002745.2|	1919526	-	G	4	6	0	2	5	SArRNA09
gi|29165615|ref|NC_002745.2|	1919527	-	U	3	2	2	1	6	SArRNA09
gi|29165615|ref|NC_002745.2|	1919528	-	A	3	7	4	3	5	SArRNA09
gi|29165615|ref|NC_002745.2|	1919529	-	A	3	3	0	1	3	SArRNA09
gi|29165615|ref|NC_002745.2|	1919531	-	A	1	1	0	0	1	SArRNA09
gi|29165615|ref|NC_002745.2|	1919532	-	C	13	2	5	3	6	SArRNA09
gi|29165615|ref|NC_002745.2|	1919533	-	U	0	0	1	0	0	SArRNA09
gi|29165615|ref|NC_002745.2|	1919534	-	C	3	1	0	2	2	SArRNA09
gi|29165615|ref|NC_002745.2|	1919535	-	C	1	6	2	0	1	SArRNA09
gi|29165615|ref|NC_002745.2|	1919536	-	C	6	2	2	1	1	SArRNA09
gi|29165615|ref|NC_002745.2|	1919537	-	U	1	2	0	0	9	SArRNA09
gi|29165615|ref|NC_002745.2|	1919538	-	U	0	3	0	1	0	SArRNA09
gi|29165615|ref|NC_002745.2|	1919539	-	G	0	1	0	0	1	SArRNA09
gi|29165615|ref|NC_002745.2|	1919540	-	G	0	1	0	0	0	SArRNA09
gi|29165615|ref|NC_002745.2|	1919541	-	A	2	0	0	0	0	SArRNA09
gi|29165615|ref|NC_002745.2|	1919542	-	A	0	0	0	0	1	SArRNA09
gi|29165615|ref|NC_002745.2|	1919543	-	A	0	0	0	0	1	SArRNA09
gi|29165615|ref|NC_002745.2|	1919544	-	C	0	2	0	2	0	SArRNA09
gi|29165615|ref|NC_002745.2|	1919545	-	U	3	1	1	0	0	SArRNA09
gi|29165615|ref|NC_002745.2|	1919546	-	C	3	1	0	0	2	SArRNA09
gi|29165615|ref|NC_002745.2|	1919548	-	C	1	0	0	0	0	SArRNA09
gi|29165615|ref|NC_002745.2|	1919549	-	G	5	1	2	0	0	SArRNA09
gi|29165615|ref|NC_002745.2|	1919550	-	G	0	0	0	1	0	SArRNA09
gi|29165615|ref|NC_002745.2|	1919551	-	A	1	1	1	0	1	SArRNA09
gi|29165615|ref|NC_002745.2|	1919552	-	G	5	1	0	0	0	SArRNA09
gi|29165615|ref|NC_002745.2|	1919553	-	G	0	1	2	0	0	SArRNA09
gi|29165615|ref|NC_002745.2|	1919554	-	C	1	5	0	1	4	SArRNA09
gi|29165615|ref|NC_002745.2|	1919556	-	A	0	0	0	0	1	SArRNA09
gi|29165615|ref|NC_002745.2|	1919557	-	U	2	1	2	2	1	SArRNA09
gi|29165615|ref|NC_002745.2|	1919558	-	G	0	1	0	0	1	SArRNA09
gi|29165615|ref|NC_002745.2|	1919559	-	G	0	1	0	0	0	SArRNA09
gi|29165615|ref|NC_002745.2|	1919560	-	A	4	7	1	1	7	SArRNA09
gi|29165615|ref|NC_002745.2|	1919561	-	A	17	29	0	5	14	SArRNA09
gi|29165615|ref|NC_002745.2|	1919562	-	A	0	0	1	0	0	SArRNA09
gi|29165615|ref|NC_002745.2|	1919563	-	A	0	1	0	1	2	SArRNA09
gi|29165615|ref|NC_002745.2|	1919565	-	C	4	0	1	1	3	SArRNA09
gi|29165615|ref|NC_002745.2|	1919566	-	C	0	4	2	0	0	SArRNA09
gi|29165615|ref|NC_002745.2|	1919567	-	U	0	1	0	1	0	SArRNA09
gi|29165615|ref|NC_002745.2|	1919568	-	C	0	3	0	0	0	SArRNA09
gi|29165615|ref|NC_002745.2|	1919569	-	C	1	1	0	0	0	SArRNA09
gi|29165615|ref|NC_002745.2|	1919571	-	C	0	0	1	0	2	SArRNA09
gi|29165615|ref|NC_002745.2|	1919572	-	U	1	0	0	0	0	SArRNA09
gi|29165615|ref|NC_002745.2|	1919575	-	C	0	0	0	0	1	SArRNA09
gi|29165615|ref|NC_002745.2|	1919578	-	G	1	0	0	0	0	SArRNA09
gi|29165615|ref|NC_002745.2|	1919579	-	G	0	2	0	0	1	SArRNA09
gi|29165615|ref|NC_002745.2|	1919580	-	U	5	1	2	1	2	SArRNA09
gi|29165615|ref|NC_002745.2|	1919581	-	C	6	7	1	0	0	SArRNA09
gi|29165615|ref|NC_002745.2|	1919582	-	A	0	2	0	1	0	SArRNA09
gi|29165615|ref|NC_002745.2|	1919583	-	G	0	2	3	0	1	SArRNA09
gi|29165615|ref|NC_002745.2|	1919584	-	U	1	3	1	0	4	SArRNA09

gi|29165615|ref|NC_002745.2|	1919585	-	U	4	5	0	1	3	SArRNA09
gi|29165615|ref|NC_002745.2|	1919586	-	U	3	1	0	3	3	SArRNA09
gi|29165615|ref|NC_002745.2|	1919587	-	G	0	0	1	0	0	SArRNA09
gi|29165615|ref|NC_002745.2|	1919588	-	A	0	3	1	1	2	SArRNA09
gi|29165615|ref|NC_002745.2|	1919589	-	C	9	3	2	2	1	SArRNA09
gi|29165615|ref|NC_002745.2|	1919590	-	G	2	1	0	0	3	SArRNA09
gi|29165615|ref|NC_002745.2|	1919591	-	G	3	3	0	0	1	SArRNA09
gi|29165615|ref|NC_002745.2|	1919592	-	G	4	1	0	0	3	SArRNA09
gi|29165615|ref|NC_002745.2|	1919593	-	C	1	5	2	2	1	SArRNA09
gi|29165615|ref|NC_002745.2|	1919594	-	G	0	2	0	1	0	SArRNA09
gi|29165615|ref|NC_002745.2|	1919595	-	G	0	0	0	2	3	SArRNA09
gi|29165615|ref|NC_002745.2|	1919596	-	A	0	1	1	1	1	SArRNA09
gi|29165615|ref|NC_002745.2|	1919597	-	C	5	0	1	0	2	SArRNA09
gi|29165615|ref|NC_002745.2|	1919598	-	U	4	1	0	1	6	SArRNA09
gi|29165615|ref|NC_002745.2|	1919599	-	G	1	0	0	1	1	SArRNA09
gi|29165615|ref|NC_002745.2|	1919600	-	U	0	0	0	4	2	SArRNA09
gi|29165615|ref|NC_002745.2|	1919601	-	G	3	1	0	3	5	SArRNA09
gi|29165615|ref|NC_002745.2|	1919602	-	A	5	9	2	5	1	SArRNA09
gi|29165615|ref|NC_002745.2|	1919603	-	C	16	22	5	10	20	SArRNA09
gi|29165615|ref|NC_002745.2|	1919604	-	A	4	5	2	2	3	SArRNA09
gi|29165615|ref|NC_002745.2|	1919605	-	G	1	3	3	0	0	SArRNA09
gi|29165615|ref|NC_002745.2|	1919606	-	A	3	5	4	1	2	SArRNA09
gi|29165615|ref|NC_002745.2|	1919607	-	G	5	3	0	0	2	SArRNA09
gi|29165615|ref|NC_002745.2|	1919608	-	G	0	0	1	1	0	SArRNA09
gi|29165615|ref|NC_002745.2|	1919609	-	G	0	0	0	0	1	SArRNA09
gi|29165615|ref|NC_002745.2|	1919610	-	U	0	0	0	0	1	SArRNA09
gi|29165615|ref|NC_002745.2|	1919611	-	G	0	0	0	0	1	SArRNA09
gi|29165615|ref|NC_002745.2|	1919614	-	G	0	2	1	1	3	SArRNA09
gi|29165615|ref|NC_002745.2|	1919615	-	C	1	2	4	1	2	SArRNA09
gi|29165615|ref|NC_002745.2|	1919616	-	U	2	2	2	0	3	SArRNA09
gi|29165615|ref|NC_002745.2|	1919617	-	A	3	1	0	1	3	SArRNA09
gi|29165615|ref|NC_002745.2|	1919618	-	U	2	6	0	0	4	SArRNA09
gi|29165615|ref|NC_002745.2|	1919619	-	U	2	1	2	1	5	SArRNA09
gi|29165615|ref|NC_002745.2|	1919620	-	C	6	2	0	3	1	SArRNA09
gi|29165615|ref|NC_002745.2|	1919621	-	A	1	4	2	0	3	SArRNA09
gi|29165615|ref|NC_002745.2|	1919622	-	C	5	3	0	0	1	SArRNA09
gi|29165615|ref|NC_002745.2|	1919623	-	C	0	1	0	0	0	SArRNA09
gi|29165615|ref|NC_002745.2|	1919624	-	A	1	0	0	0	0	SArRNA09
gi|29165615|ref|NC_002745.2|	1919625	-	C	1	1	0	0	0	SArRNA09
gi|29165615|ref|NC_002745.2|	1919626	-	G	0	2	0	0	1	SArRNA09
gi|29165615|ref|NC_002745.2|	1919627	-	C	2	2	0	1	1	SArRNA09
gi|29165615|ref|NC_002745.2|	1919628	-	C	3	1	1	1	1	SArRNA09
gi|29165615|ref|NC_002745.2|	1919629	-	C	7	11	4	1	7	SArRNA09
gi|29165615|ref|NC_002745.2|	1919630	-	A	0	1	0	1	2	SArRNA09
gi|29165615|ref|NC_002745.2|	1919631	-	A	0	0	1	1	2	SArRNA09
gi|29165615|ref|NC_002745.2|	1919632	-	U	0	2	0	1	5	SArRNA09
gi|29165615|ref|NC_002745.2|	1919633	-	C	4	5	2	0	8	SArRNA09
gi|29165615|ref|NC_002745.2|	1919634	-	U	3	0	1	1	4	SArRNA09
gi|29165615|ref|NC_002745.2|	1919635	-	U	3	0	0	0	3	SArRNA09
gi|29165615|ref|NC_002745.2|	1919636	-	U	5	1	3	1	0	SArRNA09
gi|29165615|ref|NC_002745.2|	1919637	-	C	5	1	0	0	2	SArRNA09
gi|29165615|ref|NC_002745.2|	1919639	-	G	1	0	0	0	0	SArRNA09
gi|29165615|ref|NC_002745.2|	1919640	-	U	0	0	0	2	3	SArRNA09
gi|29165615|ref|NC_002745.2|	1919641	-	U	6	3	0	1	3	SArRNA09
gi|29165615|ref|NC_002745.2|	1919642	-	G	2	1	0	0	0	SArRNA09
gi|29165615|ref|NC_002745.2|	1919643	-	U	0	1	1	0	5	SArRNA09
gi|29165615|ref|NC_002745.2|	1919644	-	G	2	0	0	0	0	SArRNA09
gi|29165615|ref|NC_002745.2|	1919645	-	U	1	0	0	0	0	SArRNA09
gi|29165615|ref|NC_002745.2|	1919646	-	C	1	1	0	1	2	SArRNA09
gi|29165615|ref|NC_002745.2|	1919648	-	A	2	5	0	2	2	SArRNA09
gi|29165615|ref|NC_002745.2|	1919649	-	U	3	2	0	3	3	SArRNA09
gi|29165615|ref|NC_002745.2|	1919650	-	C	5	18	9	5	11	SArRNA09
gi|29165615|ref|NC_002745.2|	1919651	-	C	1	9	2	2	3	SArRNA09
gi|29165615|ref|NC_002745.2|	1919652	-	C	10	12	6	2	3	SArRNA09
gi|29165615|ref|NC_002745.2|	1919653	-	A	15	11	3	3	9	SArRNA09
gi|29165615|ref|NC_002745.2|	1919654	-	U	53	28	14	13	40	SArRNA09
gi|29165615|ref|NC_002745.2|	1919655	-	C	14	14	4	9	15	SArRNA09
gi|29165615|ref|NC_002745.2|	1919656	-	A	0	2	1	0	3	SArRNA09
gi|29165615|ref|NC_002745.2|	1919657	-	U	7	6	4	1	5	SArRNA09
gi|29165615|ref|NC_002745.2|	1919658	-	A	3	6	2	0	3	SArRNA09
gi|29165615|ref|NC_002745.2|	1919659	-	G	6	2	0	3	5	SArRNA09
gi|29165615|ref|NC_002745.2|	1919660	-	G	0	2	1	0	1	SArRNA09
gi|29165615|ref|NC_002745.2|	1919661	-	G	1	2	0	0	1	SArRNA09
gi|29165615|ref|NC_002745.2|	1919662	-	U	3	8	0	1	4	SArRNA09
gi|29165615|ref|NC_002745.2|	1919663	-	G	5	6	0	1	4	SArRNA09
gi|29165615|ref|NC_002745.2|	1919664	-	G	1	3	1	0	4	SArRNA09
gi|29165615|ref|NC_002745.2|	1919665	-	U	17	9	2	1	12	SArRNA09
gi|29165615|ref|NC_002745.2|	1919666	-	C	4	7	2	2	7	SArRNA09
gi|29165615|ref|NC_002745.2|	1919667	-	G	0	1	0	1	1	SArRNA09
gi|29165615|ref|NC_002745.2|	1919668	-	C	1	1	1	1	5	SArRNA09
gi|29165615|ref|NC_002745.2|	1919669	-	G	2	0	2	2	1	SArRNA09

gi|29165615|ref|NC_002745.2|	1919670	-	G	2	1	1	1	6	SArRNA09
gi|29165615|ref|NC_002745.2|	1919671	-	A	0	2	2	2	2	SArRNA09
gi|29165615|ref|NC_002745.2|	1919672	-	G	1	1	1	0	2	SArRNA09
gi|29165615|ref|NC_002745.2|	1919673	-	G	1	2	0	0	1	SArRNA09
gi|29165615|ref|NC_002745.2|	1919674	-	U	0	2	0	2	1	SArRNA09
gi|29165615|ref|NC_002745.2|	1919675	-	G	1	0	0	0	2	SArRNA09
gi|29165615|ref|NC_002745.2|	1919676	-	C	0	1	1	0	1	SArRNA09
gi|29165615|ref|NC_002745.2|	1919677	-	A	1	5	0	0	2	SArRNA09
gi|29165615|ref|NC_002745.2|	1919678	-	U	1	1	0	0	2	SArRNA09
gi|29165615|ref|NC_002745.2|	1919679	-	U	0	5	2	1	5	SArRNA09
gi|29165615|ref|NC_002745.2|	1919680	-	C	6	6	1	1	2	SArRNA09
gi|29165615|ref|NC_002745.2|	1919681	-	G	2	0	1	1	2	SArRNA09
gi|29165615|ref|NC_002745.2|	1919682	-	A	3	4	0	0	2	SArRNA09
gi|29165615|ref|NC_002745.2|	1919683	-	U	5	1	3	5	5	SArRNA09
gi|29165615|ref|NC_002745.2|	1919684	-	C	2	7	3	1	6	SArRNA09
gi|29165615|ref|NC_002745.2|	1919685	-	G	0	1	0	0	2	SArRNA09
gi|29165615|ref|NC_002745.2|	1919686	-	C	1	3	0	1	5	SArRNA09
gi|29165615|ref|NC_002745.2|	1919687	-	G	0	0	0	0	2	SArRNA09
gi|29165615|ref|NC_002745.2|	1919688	-	A	0	0	1	1	1	SArRNA09
gi|29165615|ref|NC_002745.2|	1919689	-	G	1	0	0	0	0	SArRNA09
gi|29165615|ref|NC_002745.2|	1919690	-	U	1	0	2	1	3	SArRNA09
gi|29165615|ref|NC_002745.2|	1919691	-	G	0	0	0	1	1	SArRNA09
gi|29165615|ref|NC_002745.2|	1919692	-	C	4	0	0	1	2	SArRNA09
gi|29165615|ref|NC_002745.2|	1919694	-	A	1	0	0	2	2	SArRNA09
gi|29165615|ref|NC_002745.2|	1919695	-	A	1	1	0	1	4	SArRNA09
gi|29165615|ref|NC_002745.2|	1919696	-	G	0	0	0	1	0	SArRNA09
gi|29165615|ref|NC_002745.2|	1919697	-	U	5	1	1	0	2	SArRNA09
gi|29165615|ref|NC_002745.2|	1919698	-	U	11	3	0	1	7	SArRNA09
gi|29165615|ref|NC_002745.2|	1919699	-	U	1	4	3	1	5	SArRNA09
gi|29165615|ref|NC_002745.2|	1919700	-	C	6	4	1	1	6	SArRNA09
gi|29165615|ref|NC_002745.2|	1919701	-	C	13	6	3	8	11	SArRNA09
gi|29165615|ref|NC_002745.2|	1919703	-	A	5	6	2	1	4	SArRNA09
gi|29165615|ref|NC_002745.2|	1919704	-	G	10	4	5	6	8	SArRNA09
gi|29165615|ref|NC_002745.2|	1919705	-	G	4	2	0	3	5	SArRNA09
gi|29165615|ref|NC_002745.2|	1919706	-	A	7	8	4	2	5	SArRNA09
gi|29165615|ref|NC_002745.2|	1919707	-	U	17	8	3	5	8	SArRNA09
gi|29165615|ref|NC_002745.2|	1919708	-	G	6	1	2	5	5	SArRNA09
gi|29165615|ref|NC_002745.2|	1919709	-	G	3	3	2	1	1	SArRNA09
gi|29165615|ref|NC_002745.2|	1919710	-	A	2	3	1	2	4	SArRNA09
gi|29165615|ref|NC_002745.2|	1919711	-	U	15	24	5	14	11	SArRNA09
gi|29165615|ref|NC_002745.2|	1919712	-	A	162	13	47	10	13	SArRNA09
gi|29165615|ref|NC_002745.2|	1919713	-	G	141	9	56	5	15	SArRNA09
gi|29165615|ref|NC_002745.2|	1919714	-	G	4	4	3	3	6	SArRNA09
gi|29165615|ref|NC_002745.2|	1919715	-	A	14	28	4	11	23	SArRNA09
gi|29165615|ref|NC_002745.2|	1919716	-	C	35	76	12	23	51	SArRNA09
gi|29165615|ref|NC_002745.2|	1919717	-	A	3	2	0	0	3	SArRNA09
gi|29165615|ref|NC_002745.2|	1919718	-	U	5	13	3	2	3	SArRNA09
gi|29165615|ref|NC_002745.2|	1919719	-	G	6	4	0	1	10	SArRNA09
gi|29165615|ref|NC_002745.2|	1919720	-	U	6	2	0	4	4	SArRNA09
gi|29165615|ref|NC_002745.2|	1919721	-	U	3	8	3	5	7	SArRNA09
gi|29165615|ref|NC_002745.2|	1919722	-	C	11	8	3	1	4	SArRNA09
gi|29165615|ref|NC_002745.2|	1919723	-	G	1	2	0	0	0	SArRNA09
gi|29165615|ref|NC_002745.2|	1919724	-	A	0	2	0	0	0	SArRNA09
gi|29165615|ref|NC_002745.2|	1919725	-	C	1	1	0	0	3	SArRNA09
gi|29165615|ref|NC_002745.2|	1919726	-	A	0	2	0	0	1	SArRNA09
gi|29165615|ref|NC_002745.2|	1919727	-	C	0	1	0	2	0	SArRNA09
gi|29165615|ref|NC_002745.2|	1919729	-	G	1	1	0	0	2	SArRNA09
gi|29165615|ref|NC_002745.2|	1919730	-	C	11	22	6	5	10	SArRNA09
gi|29165615|ref|NC_002745.2|	1919731	-	U	2	5	2	1	2	SArRNA09
gi|29165615|ref|NC_002745.2|	1919732	-	U	6	6	2	0	1	SArRNA09
gi|29165615|ref|NC_002745.2|	1919733	-	A	2	15	1	0	7	SArRNA09
gi|29165615|ref|NC_002745.2|	1919734	-	A	0	0	1	1	0	SArRNA09
gi|29165615|ref|NC_002745.2|	1919735	-	A	1	4	1	0	3	SArRNA09
gi|29165615|ref|NC_002745.2|	1919736	-	G	0	0	0	1	1	SArRNA09
gi|29165615|ref|NC_002745.2|	1919738	-	U	0	0	0	0	3	SArRNA09
gi|29165615|ref|NC_002745.2|	1919739	-	A	1	2	2	2	4	SArRNA09
gi|29165615|ref|NC_002745.2|	1919740	-	U	10	3	1	1	6	SArRNA09
gi|29165615|ref|NC_002745.2|	1919741	-	A	5	2	1	0	8	SArRNA09
gi|29165615|ref|NC_002745.2|	1919742	-	G	0	1	0	0	1	SArRNA09
gi|29165615|ref|NC_002745.2|	1919743	-	U	0	5	0	1	4	SArRNA09
gi|29165615|ref|NC_002745.2|	1919744	-	C	16	13	10	6	6	SArRNA09
gi|29165615|ref|NC_002745.2|	1919745	-	C	1	2	0	0	1	SArRNA09
gi|29165615|ref|NC_002745.2|	1919747	-	A	3	1	0	0	0	SArRNA09
gi|29165615|ref|NC_002745.2|	1919748	-	U	2	0	0	2	1	SArRNA09
gi|29165615|ref|NC_002745.2|	1919749	-	G	1	1	1	0	0	SArRNA09
gi|29165615|ref|NC_002745.2|	1919750	-	U	2	5	0	2	5	SArRNA09
gi|29165615|ref|NC_002745.2|	1919751	-	C	2	3	4	5	2	SArRNA09
gi|29165615|ref|NC_002745.2|	1919752	-	A	0	4	0	0	3	SArRNA09
gi|29165615|ref|NC_002745.2|	1919753	-	U	6	4	0	0	4	SArRNA09
gi|29165615|ref|NC_002745.2|	1919754	-	U	2	1	0	0	0	SArRNA09
gi|29165615|ref|NC_002745.2|	1919755	-	U	6	2	1	2	3	SArRNA09

gi|29165615|ref|NC_002745.2|	1919756	-	C	8	6	3	1	7	SArRNA09
gi|29165615|ref|NC_002745.2|	1919757	-	G	7	4	0	0	4	SArRNA09
gi|29165615|ref|NC_002745.2|	1919758	-	A	3	0	2	0	4	SArRNA09
gi|29165615|ref|NC_002745.2|	1919759	-	G	3	2	1	2	2	SArRNA09
gi|29165615|ref|NC_002745.2|	1919760	-	G	1	1	0	0	1	SArRNA09
gi|29165615|ref|NC_002745.2|	1919761	-	U	6	3	1	1	5	SArRNA09
gi|29165615|ref|NC_002745.2|	1919762	-	G	2	4	2	1	4	SArRNA09
gi|29165615|ref|NC_002745.2|	1919763	-	C	17	22	4	24	10	SArRNA09
gi|29165615|ref|NC_002745.2|	1919764	-	C	6	1	3	1	6	SArRNA09
gi|29165615|ref|NC_002745.2|	1919765	-	C	19	5	2	3	4	SArRNA09
gi|29165615|ref|NC_002745.2|	1919766	-	C	7	9	2	3	17	SArRNA09
gi|29165615|ref|NC_002745.2|	1919767	-	A	5	3	3	1	6	SArRNA09
gi|29165615|ref|NC_002745.2|	1919768	-	G	10	7	2	0	4	SArRNA09
gi|29165615|ref|NC_002745.2|	1919769	-	A	3	3	1	0	5	SArRNA09
gi|29165615|ref|NC_002745.2|	1919770	-	A	5	3	2	2	3	SArRNA09
gi|29165615|ref|NC_002745.2|	1919771	-	A	1	0	1	2	3	SArRNA09
gi|29165615|ref|NC_002745.2|	1919772	-	G	0	4	1	0	1	SArRNA09
gi|29165615|ref|NC_002745.2|	1919773	-	G	1	5	0	1	2	SArRNA09
gi|29165615|ref|NC_002745.2|	1919774	-	C	7	2	1	0	4	SArRNA09
gi|29165615|ref|NC_002745.2|	1919775	-	A	5	1	2	0	2	SArRNA09
gi|29165615|ref|NC_002745.2|	1919776	-	G	2	4	4	1	4	SArRNA09
gi|29165615|ref|NC_002745.2|	1919777	-	G	5	4	0	1	12	SArRNA09
gi|29165615|ref|NC_002745.2|	1919778	-	A	5	5	2	0	1	SArRNA09
gi|29165615|ref|NC_002745.2|	1919779	-	C	20	23	3	1	11	SArRNA09
gi|29165615|ref|NC_002745.2|	1919780	-	A	6	3	2	1	2	SArRNA09
gi|29165615|ref|NC_002745.2|	1919781	-	G	1	3	0	2	1	SArRNA09
gi|29165615|ref|NC_002745.2|	1919782	-	C	9	2	2	5	2	SArRNA09
gi|29165615|ref|NC_002745.2|	1919783	-	G	0	2	0	0	1	SArRNA09
gi|29165615|ref|NC_002745.2|	1919784	-	C	9	1	3	5	7	SArRNA09
gi|29165615|ref|NC_002745.2|	1919785	-	C	5	7	4	2	4	SArRNA09
gi|29165615|ref|NC_002745.2|	1919786	-	C	10	10	5	2	10	SArRNA09
gi|29165615|ref|NC_002745.2|	1919787	-	A	6	3	1	0	3	SArRNA09
gi|29165615|ref|NC_002745.2|	1919788	-	U	7	5	0	1	6	SArRNA09
gi|29165615|ref|NC_002745.2|	1919789	-	U	6	2	2	0	6	SArRNA09
gi|29165615|ref|NC_002745.2|	1919790	-	G	1	1	1	1	0	SArRNA09
gi|29165615|ref|NC_002745.2|	1919791	-	G	0	1	0	0	1	SArRNA09
gi|29165615|ref|NC_002745.2|	1919792	-	A	7	4	1	4	7	SArRNA09
gi|29165615|ref|NC_002745.2|	1919793	-	C	13	13	1	3	0	SArRNA09
gi|29165615|ref|NC_002745.2|	1919794	-	G	2	4	2	0	7	SArRNA09
gi|29165615|ref|NC_002745.2|	1919795	-	U	4	5	3	3	5	SArRNA09
gi|29165615|ref|NC_002745.2|	1919796	-	A	6	9	6	4	7	SArRNA09
gi|29165615|ref|NC_002745.2|	1919797	-	G	10	12	3	4	5	SArRNA09
gi|29165615|ref|NC_002745.2|	1919798	-	A	10	14	0	2	9	SArRNA09
gi|29165615|ref|NC_002745.2|	1919799	-	A	4	3	2	2	4	SArRNA09
gi|29165615|ref|NC_002745.2|	1919800	-	G	1	0	2	5	3	SArRNA09
gi|29165615|ref|NC_002745.2|	1919801	-	U	6	3	4	2	2	SArRNA09
gi|29165615|ref|NC_002745.2|	1919802	-	G	2	1	0	1	1	SArRNA09
gi|29165615|ref|NC_002745.2|	1919803	-	U	6	6	0	1	2	SArRNA09
gi|29165615|ref|NC_002745.2|	1919804	-	C	3	3	0	3	1	SArRNA09
gi|29165615|ref|NC_002745.2|	1919805	-	C	1	1	3	1	3	SArRNA09
gi|29165615|ref|NC_002745.2|	1919806	-	A	1	2	2	1	0	SArRNA09
gi|29165615|ref|NC_002745.2|	1919807	-	U	5	9	2	0	5	SArRNA09
gi|29165615|ref|NC_002745.2|	1919808	-	G	0	2	1	0	0	SArRNA09
gi|29165615|ref|NC_002745.2|	1919809	-	A	0	2	0	0	1	SArRNA09
gi|29165615|ref|NC_002745.2|	1919810	-	U	8	7	2	7	11	SArRNA09
gi|29165615|ref|NC_002745.2|	1919811	-	A	20	10	6	2	10	SArRNA09
gi|29165615|ref|NC_002745.2|	1919812	-	C	14	37	3	3	25	SArRNA09
gi|29165615|ref|NC_002745.2|	1919813	-	U	2	2	0	0	4	SArRNA09
gi|29165615|ref|NC_002745.2|	1919814	-	A	15	147	24	3	35	SArRNA09
gi|29165615|ref|NC_002745.2|	1919815	-	A	5	6	3	6	7	SArRNA09
gi|29165615|ref|NC_002745.2|	1919816	-	A	0	0	1	0	2	SArRNA09
gi|29165615|ref|NC_002745.2|	1919817	-	G	0	3	1	0	0	SArRNA09
gi|29165615|ref|NC_002745.2|	1919818	-	U	4	1	0	1	2	SArRNA09
gi|29165615|ref|NC_002745.2|	1919819	-	G	1	2	0	0	1	SArRNA09
gi|29165615|ref|NC_002745.2|	1919820	-	G	1	6	1	0	2	SArRNA09
gi|29165615|ref|NC_002745.2|	1919821	-	C	19	22	8	5	12	SArRNA09
gi|29165615|ref|NC_002745.2|	1919822	-	U	5	13	1	3	8	SArRNA09
gi|29165615|ref|NC_002745.2|	1919823	-	C	11	9	3	1	9	SArRNA09
gi|29165615|ref|NC_002745.2|	1919824	-	A	5	7	4	1	6	SArRNA09
gi|29165615|ref|NC_002745.2|	1919825	-	G	6	3	4	4	7	SArRNA09
gi|29165615|ref|NC_002745.2|	1919826	-	A	1	5	1	0	4	SArRNA09
gi|29165615|ref|NC_002745.2|	1919827	-	G	4	0	3	1	0	SArRNA09
gi|29165615|ref|NC_002745.2|	1919828	-	A	2	3	0	2	1	SArRNA09
gi|29165615|ref|NC_002745.2|	1919829	-	G	8	12	3	0	9	SArRNA09
gi|29165615|ref|NC_002745.2|	1919830	-	C	52	42	16	10	27	SArRNA09
gi|29165615|ref|NC_002745.2|	1919831	-	A	1	6	5	1	6	SArRNA09
gi|29165615|ref|NC_002745.2|	1919832	-	A	42	48	24	10	26	SArRNA09
gi|29165615|ref|NC_002745.2|	1919833	-	C	49	48	13	17	34	SArRNA09
gi|29165615|ref|NC_002745.2|	1919834	-	U	1	4	2	1	6	SArRNA09
gi|29165615|ref|NC_002745.2|	1919835	-	C	6	13	2	2	2	SArRNA09
gi|29165615|ref|NC_002745.2|	1919836	-	U	0	2	0	1	2	SArRNA09

gi|29165615|ref|NC_002745.2|	1919837	-	G	0	0	0	0	1	SArRNA09
gi|29165615|ref|NC_002745.2|	1919838	-	U	2	3	0	1	1	SArRNA09
gi|29165615|ref|NC_002745.2|	1919839	-	C	12	11	3	3	5	SArRNA09
gi|29165615|ref|NC_002745.2|	1919840	-	A	1	5	3	1	2	SArRNA09
gi|29165615|ref|NC_002745.2|	1919841	-	C	8	8	6	7	6	SArRNA09
gi|29165615|ref|NC_002745.2|	1919842	-	G	0	2	0	0	1	SArRNA09
gi|29165615|ref|NC_002745.2|	1919843	-	G	0	2	0	0	0	SArRNA09
gi|29165615|ref|NC_002745.2|	1919844	-	G	6	26	0	0	13	SArRNA09
gi|29165615|ref|NC_002745.2|	1919845	-	U	3	10	4	1	6	SArRNA09
gi|29165615|ref|NC_002745.2|	1919846	-	U	3	10	4	7	6	SArRNA09
gi|29165615|ref|NC_002745.2|	1919847	-	U	10	34	17	11	18	SArRNA09
gi|29165615|ref|NC_002745.2|	1919848	-	A	36	228	142	235	273	SArRNA09
gi|29165615|ref|NC_002745.2|	1919849	-	G	14	17	4	7	18	SArRNA09
gi|29165615|ref|NC_002745.2|	1919850	-	C	10	33	12	15	24	SArRNA09
gi|29165615|ref|NC_002745.2|	1919851	-	A	4	12	12	7	19	SArRNA09
gi|29165615|ref|NC_002745.2|	1919852	-	A	0	17	10	9	11	SArRNA09
gi|29165615|ref|NC_002745.2|	1919853	-	U	3	6	6	3	7	SArRNA09
gi|29165615|ref|NC_002745.2|	1919854	-	G	7	9	2	0	2	SArRNA09
gi|29165615|ref|NC_002745.2|	1919855	-	C	6	4	1	2	4	SArRNA09
gi|29165615|ref|NC_002745.2|	1919856	-	G	10	0	0	0	5	SArRNA09
gi|29165615|ref|NC_002745.2|	1919857	-	G	1	0	0	0	1	SArRNA09
gi|29165615|ref|NC_002745.2|	1919858	-	A	15	6	4	1	6	SArRNA09
gi|29165615|ref|NC_002745.2|	1919859	-	A	20	25	10	5	18	SArRNA09
gi|29165615|ref|NC_002745.2|	1919860	-	A	3	5	3	3	8	SArRNA09
gi|29165615|ref|NC_002745.2|	1919861	-	G	10	2	7	1	15	SArRNA09
gi|29165615|ref|NC_002745.2|	1919862	-	C	4	5	3	1	6	SArRNA09
gi|29165615|ref|NC_002745.2|	1919863	-	A	7	14	6	4	11	SArRNA09
gi|29165615|ref|NC_002745.2|	1919864	-	C	41	46	19	9	37	SArRNA09
gi|29165615|ref|NC_002745.2|	1919865	-	G	14	17	3	1	19	SArRNA09
gi|29165615|ref|NC_002745.2|	1919866	-	C	11	15	1	1	8	SArRNA09
gi|29165615|ref|NC_002745.2|	1919867	-	C	20	35	18	5	28	SArRNA09
gi|29165615|ref|NC_002745.2|	1919868	-	C	65	32	10	9	35	SArRNA09
gi|29165615|ref|NC_002745.2|	1919869	-	A	0	8	0	0	6	SArRNA09
gi|29165615|ref|NC_002745.2|	1919870	-	G	4	6	0	1	2	SArRNA09
gi|29165615|ref|NC_002745.2|	1919871	-	C	1	4	2	1	1	SArRNA09
gi|29165615|ref|NC_002745.2|	1919872	-	C	3	6	0	0	2	SArRNA09
gi|29165615|ref|NC_002745.2|	1919873	-	U	1	3	0	0	0	SArRNA09
gi|29165615|ref|NC_002745.2|	1919874	-	U	0	2	1	0	0	SArRNA09
gi|29165615|ref|NC_002745.2|	1919875	-	G	0	0	0	0	1	SArRNA09
gi|29165615|ref|NC_002745.2|	1919876	-	A	3	1	0	2	0	SArRNA09
gi|29165615|ref|NC_002745.2|	1919877	-	A	2	4	0	1	3	SArRNA09
gi|29165615|ref|NC_002745.2|	1919878	-	U	3	0	2	1	3	SArRNA09
gi|29165615|ref|NC_002745.2|	1919879	-	G	1	1	4	0	3	SArRNA09
gi|29165615|ref|NC_002745.2|	1919880	-	G	1	1	0	0	2	SArRNA09
gi|29165615|ref|NC_002745.2|	1919881	-	G	5	10	0	0	4	SArRNA09
gi|29165615|ref|NC_002745.2|	1919882	-	C	21	18	3	6	13	SArRNA09
gi|29165615|ref|NC_002745.2|	1919883	-	U	10	9	2	1	9	SArRNA09
gi|29165615|ref|NC_002745.2|	1919884	-	G	12	4	1	0	6	SArRNA09
gi|29165615|ref|NC_002745.2|	1919885	-	U	8	4	3	1	11	SArRNA09
gi|29165615|ref|NC_002745.2|	1919886	-	U	9	12	3	4	12	SArRNA09
gi|29165615|ref|NC_002745.2|	1919887	-	C	75	135	30	39	92	SArRNA09
gi|29165615|ref|NC_002745.2|	1919888	-	C	5	17	1	3	10	SArRNA09
gi|29165615|ref|NC_002745.2|	1919889	-	U	1	1	0	1	2	SArRNA09
gi|29165615|ref|NC_002745.2|	1919890	-	U	0	4	3	0	2	SArRNA09
gi|29165615|ref|NC_002745.2|	1919891	-	A	11	67	7	3	37	SArRNA09
gi|29165615|ref|NC_002745.2|	1919892	-	A	2	4	3	4	7	SArRNA09
gi|29165615|ref|NC_002745.2|	1919893	-	A	1	8	1	4	4	SArRNA09
gi|29165615|ref|NC_002745.2|	1919894	-	G	0	0	1	1	1	SArRNA09
gi|29165615|ref|NC_002745.2|	1919895	-	C	2	0	0	0	1	SArRNA09
gi|29165615|ref|NC_002745.2|	1919897	-	A	0	1	0	0	0	SArRNA09
gi|29165615|ref|NC_002745.2|	1919898	-	U	0	1	0	0	1	SArRNA09
gi|29165615|ref|NC_002745.2|	1919899	-	G	0	0	0	0	1	SArRNA09
gi|29165615|ref|NC_002745.2|	1919901	-	A	0	0	0	1	0	SArRNA09
gi|29165615|ref|NC_002745.2|	1919903	-	U	0	2	0	0	0	SArRNA09
gi|29165615|ref|NC_002745.2|	1919904	-	C	3	0	0	0	1	SArRNA09
gi|29165615|ref|NC_002745.2|	1919909	-	C	0	0	1	0	0	SArRNA09
gi|29165615|ref|NC_002745.2|	1919910	-	A	1	2	0	0	0	SArRNA09
gi|29165615|ref|NC_002745.2|	1919911	-	A	0	0	1	0	2	SArRNA09
gi|29165615|ref|NC_002745.2|	1919913	-	A	1	1	2	2	1	SArRNA09
gi|29165615|ref|NC_002745.2|	1919914	-	U	0	0	0	0	2	SArRNA09
gi|29165615|ref|NC_002745.2|	1919916	-	A	0	0	0	0	3	SArRNA09
gi|29165615|ref|NC_002745.2|	1919917	-	A	0	0	0	1	0	SArRNA09
gi|29165615|ref|NC_002745.2|	1919918	-	U	0	0	0	0	1	SArRNA09
gi|29165615|ref|NC_002745.2|	1919919	-	G	1	0	0	0	0	SArRNA09
gi|29165615|ref|NC_002745.2|	1919922	-	G	0	1	0	0	0	SArRNA09
gi|29165615|ref|NC_002745.2|	1919923	-	G	0	1	0	0	0	SArRNA09
gi|29165615|ref|NC_002745.2|	1919925	-	G	0	2	0	0	0	SArRNA09
gi|29165615|ref|NC_002745.2|	1919927	-	C	1	1	0	0	2	SArRNA09
gi|29165615|ref|NC_002745.2|	1919929	-	A	0	1	0	0	0	SArRNA09
gi|29165615|ref|NC_002745.2|	1919930	-	A	0	1	0	0	0	SArRNA09
gi|29165615|ref|NC_002745.2|	1919933	-	A	0	1	1	0	0	SArRNA09

gi|29165615|ref|NC_002745.2|	1919934	-	C	5	2	0	1	1 SArRNA09
gi|29165615|ref|NC_002745.2|	1919935	-	C	0	1	0	0	1 SArRNA09
gi|29165615|ref|NC_002745.2|	1919937	-	C	4	3	0	0	1 SArRNA09
gi|29165615|ref|NC_002745.2|	1919947	-	C	0	0	0	0	2 SArRNA09
gi|29165615|ref|NC_002745.2|	1919952	-	A	0	0	1	0	0 SArRNA09
gi|29165615|ref|NC_002745.2|	1919954	-	G	0	1	0	0	0 SArRNA09
gi|29165615|ref|NC_002745.2|	1919956	-	G	0	0	0	1	0 SArRNA09
gi|29165615|ref|NC_002745.2|	1919958	-	C	0	0	0	0	2 SArRNA09
gi|29165615|ref|NC_002745.2|	1919961	-	C	0	1	0	0	0 SArRNA09
gi|29165615|ref|NC_002745.2|	1919962	-	G	0	1	0	0	0 SArRNA09
gi|29165615|ref|NC_002745.2|	1919963	-	A	0	0	1	0	0 SArRNA09
gi|29165615|ref|NC_002745.2|	1919964	-	U	0	0	0	0	1 SArRNA09
gi|29165615|ref|NC_002745.2|	1919965	-	U	0	0	0	1	1 SArRNA09
gi|29165615|ref|NC_002745.2|	1919967	-	G	0	0	0	0	2 SArRNA09
gi|29165615|ref|NC_002745.2|	1919968	-	U	0	1	1	0	0 SArRNA09
gi|29165615|ref|NC_002745.2|	1919969	-	G	0	1	0	0	0 SArRNA09
gi|29165615|ref|NC_002745.2|	1919970	-	A	0	0	1	0	0 SArRNA09
gi|29165615|ref|NC_002745.2|	1919971	-	G	1	1	0	0	1 SArRNA09
gi|29165615|ref|NC_002745.2|	1919972	-	G	0	1	0	0	0 SArRNA09
gi|29165615|ref|NC_002745.2|	1919973	-	A	1	2	0	0	0 SArRNA09
gi|29165615|ref|NC_002745.2|	1919974	-	G	0	0	0	1	0 SArRNA09
gi|29165615|ref|NC_002745.2|	1919976	-	A	0	1	0	0	0 SArRNA09
gi|29165615|ref|NC_002745.2|	1919977	-	U	0	1	0	0	0 SArRNA09
gi|29165615|ref|NC_002745.2|	1919979	-	G	0	1	0	0	0 SArRNA09
gi|29165615|ref|NC_002745.2|	1919981	-	A	0	0	0	1	2 SArRNA09
gi|29165615|ref|NC_002745.2|	1919983	-	G	2	8	5	1	5 SArRNA09
gi|29165615|ref|NC_002745.2|	1919990	-	G	0	0	0	1	0 SArRNA09
gi|29165615|ref|NC_002745.2|	1919991	-	C	0	1	0	0	0 SArRNA09
gi|29165615|ref|NC_002745.2|	1919992	-	C	1	1	0	0	0 SArRNA09
gi|29165615|ref|NC_002745.2|	1919993	-	C	2	1	0	0	1 SArRNA09
gi|29165615|ref|NC_002745.2|	1919995	-	U	0	0	1	0	0 SArRNA09
gi|29165615|ref|NC_002745.2|	1919996	-	C	1	1	0	0	0 SArRNA09
gi|29165615|ref|NC_002745.2|	1919997	-	C	0	1	0	0	0 SArRNA09
gi|29165615|ref|NC_002745.2|	1919998	-	G	1	0	0	0	0 SArRNA09
gi|29165615|ref|NC_002745.2|	1920002	-	U	0	1	0	0	0 SArRNA09
gi|29165615|ref|NC_002745.2|	1920003	-	C	0	0	1	0	1 SArRNA09
gi|29165615|ref|NC_002745.2|	1920005	-	G	1	0	0	0	0 SArRNA09
gi|29165615|ref|NC_002745.2|	1920006	-	G	0	1	1	0	1 SArRNA09
gi|29165615|ref|NC_002745.2|	1920008	-	A	2	1	0	0	0 SArRNA09
gi|29165615|ref|NC_002745.2|	1920009	-	U	2	0	2	0	1 SArRNA09
gi|29165615|ref|NC_002745.2|	1920010	-	A	1	1	1	0	0 SArRNA09
gi|29165615|ref|NC_002745.2|	1920011	-	U	0	1	0	0	0 SArRNA09
gi|29165615|ref|NC_002745.2|	1920012	-	G	1	0	0	0	0 SArRNA09
gi|29165615|ref|NC_002745.2|	1920014	-	A	1	0	1	1	0 SArRNA09
gi|29165615|ref|NC_002745.2|	1920015	-	G	0	2	0	1	1 SArRNA09
gi|29165615|ref|NC_002745.2|	1920016	-	U	0	1	2	0	0 SArRNA09
gi|29165615|ref|NC_002745.2|	1920017	-	G	0	0	0	0	1 SArRNA09
gi|29165615|ref|NC_002745.2|	1920018	-	G	2	3	1	0	1 SArRNA09
gi|29165615|ref|NC_002745.2|	1920019	-	A	0	0	2	0	1 SArRNA09
gi|29165615|ref|NC_002745.2|	1920020	-	A	1	2	1	0	1 SArRNA09
gi|29165615|ref|NC_002745.2|	1920021	-	U	2	1	0	0	1 SArRNA09
gi|29165615|ref|NC_002745.2|	1920022	-	G	0	0	1	0	2 SArRNA09
gi|29165615|ref|NC_002745.2|	1920023	-	C	2	1	2	0	3 SArRNA09
gi|29165615|ref|NC_002745.2|	1920024	-	C	2	1	1	0	4 SArRNA09
gi|29165615|ref|NC_002745.2|	1920025	-	A	1	0	0	0	0 SArRNA09
gi|29165615|ref|NC_002745.2|	1920026	-	A	3	3	0	0	3 SArRNA09
gi|29165615|ref|NC_002745.2|	1920027	-	A	1	1	0	0	2 SArRNA09
gi|29165615|ref|NC_002745.2|	1920028	-	U	3	0	3	0	3 SArRNA09
gi|29165615|ref|NC_002745.2|	1920029	-	C	3	3	0	2	5 SArRNA09
gi|29165615|ref|NC_002745.2|	1920030	-	G	3	1	2	1	0 SArRNA09
gi|29165615|ref|NC_002745.2|	1920031	-	U	0	0	2	0	1 SArRNA09
gi|29165615|ref|NC_002745.2|	1920032	-	C	2	0	0	0	3 SArRNA09
gi|29165615|ref|NC_002745.2|	1920033	-	U	5	1	1	2	2 SArRNA09
gi|29165615|ref|NC_002745.2|	1920034	-	C	30	0	1	0	2 SArRNA09
gi|29165615|ref|NC_002745.2|	1920035	-	U	167	6	10	2	7 SArRNA09
gi|29165615|ref|NC_002745.2|	1920036	-	G	1	0	0	0	1 SArRNA09
gi|29165615|ref|NC_002745.2|	1920037	-	G	0	0	0	0	1 SArRNA09
gi|29165615|ref|NC_002745.2|	1920040	-	A	4	0	2	1	1 SArRNA09
gi|29165615|ref|NC_002745.2|	1920041	-	C	11	2	1	4	1 SArRNA09
gi|29165615|ref|NC_002745.2|	1920042	-	A	5	2	1	0	3 SArRNA09
gi|29165615|ref|NC_002745.2|	1920043	-	A	2	1	3	0	3 SArRNA09
gi|29165615|ref|NC_002745.2|	1920044	-	A	0	1	0	2	1 SArRNA09
gi|29165615|ref|NC_002745.2|	1920045	-	A	4	0	0	0	3 SArRNA09
gi|29165615|ref|NC_002745.2|	1920046	-	A	1	0	1	0	1 SArRNA09
gi|29165615|ref|NC_002745.2|	1920047	-	C	2	0	1	1	4 SArRNA09
gi|29165615|ref|NC_002745.2|	1920048	-	U	4	4	0	0	2 SArRNA09
gi|29165615|ref|NC_002745.2|	1920049	-	A	1	2	1	0	0 SArRNA09
gi|29165615|ref|NC_002745.2|	1920050	-	U	0	1	2	2	3 SArRNA09
gi|29165615|ref|NC_002745.2|	1920051	-	U	0	0	0	0	3 SArRNA09
gi|29165615|ref|NC_002745.2|	1920052	-	U	2	0	0	1	1 SArRNA09
gi|29165615|ref|NC_002745.2|	1920054	-	U	0	0	1	1	2 SArRNA09

gi|29165615|ref|NC_002745.2|	1920055 -	C	1	0	2	0	0	SArRNA09
gi|29165615|ref|NC_002745.2|	1920056 -	A	0	0	1	0	1	SArRNA09
gi|29165615|ref|NC_002745.2|	1920057 -	G	0	2	3	2	0	SArRNA09
gi|29165615|ref|NC_002745.2|	1920058 -	C	2	0	1	0	1	SArRNA09
gi|29165615|ref|NC_002745.2|	1920059 -	G	0	1	0	0	2	SArRNA09
gi|29165615|ref|NC_002745.2|	1920060 -	A	2	1	0	1	3	SArRNA09
gi|29165615|ref|NC_002745.2|	1920061 -	A	6	5	5	1	2	SArRNA09
gi|29165615|ref|NC_002745.2|	1920062 -	C	3	1	3	2	9	SArRNA09
gi|29165615|ref|NC_002745.2|	1920063 -	C	0	1	1	1	5	SArRNA09
gi|29165615|ref|NC_002745.2|	1920064 -	C	3	3	2	3	6	SArRNA09
gi|29165615|ref|NC_002745.2|	1920065 -	G	0	0	1	2	0	SArRNA09
gi|29165615|ref|NC_002745.2|	1920066 -	G	0	1	0	0	2	SArRNA09
gi|29165615|ref|NC_002745.2|	1920067 -	A	3	4	0	0	0	SArRNA09
gi|29165615|ref|NC_002745.2|	1920068 -	U	1	3	0	0	2	SArRNA09
gi|29165615|ref|NC_002745.2|	1920069 -	A	4	7	3	1	3	SArRNA09
gi|29165615|ref|NC_002745.2|	1920070 -	A	3	2	0	1	1	SArRNA09
gi|29165615|ref|NC_002745.2|	1920071 -	G	2	2	1	8	1	SArRNA09
gi|29165615|ref|NC_002745.2|	1920072 -	U	2	1	2	2	0	SArRNA09
gi|29165615|ref|NC_002745.2|	1920073 -	G	1	0	2	0	2	SArRNA09
gi|29165615|ref|NC_002745.2|	1920074 -	A	5	1	0	2	2	SArRNA09
gi|29165615|ref|NC_002745.2|	1920075 -	C	1	1	2	1	3	SArRNA09
gi|29165615|ref|NC_002745.2|	1920076 -	G	2	0	1	0	2	SArRNA09
gi|29165615|ref|NC_002745.2|	1920077 -	C	1	0	0	0	1	SArRNA09
gi|29165615|ref|NC_002745.2|	1920078 -	C	3	2	0	2	1	SArRNA09
gi|29165615|ref|NC_002745.2|	1920079 -	G	0	0	0	1	1	SArRNA09
gi|29165615|ref|NC_002745.2|	1920080 -	A	1	0	0	0	1	SArRNA09
gi|29165615|ref|NC_002745.2|	1920081 -	G	1	3	0	0	1	SArRNA09
gi|29165615|ref|NC_002745.2|	1920082 -	A	0	2	2	0	1	SArRNA09
gi|29165615|ref|NC_002745.2|	1920083 -	A	1	1	0	3	0	SArRNA09
gi|29165615|ref|NC_002745.2|	1920084 -	G	1	0	0	0	2	SArRNA09
gi|29165615|ref|NC_002745.2|	1920085 -	A	0	0	1	1	0	SArRNA09
gi|29165615|ref|NC_002745.2|	1920086 -	C	2	2	1	2	0	SArRNA09
gi|29165615|ref|NC_002745.2|	1920087 -	C	6	1	0	1	3	SArRNA09
gi|29165615|ref|NC_002745.2|	1920089 -	G	8	3	0	2	6	SArRNA09
gi|29165615|ref|NC_002745.2|	1920090 -	C	30	21	11	17	19	SArRNA09
gi|29165615|ref|NC_002745.2|	1920091 -	A	1	1	2	0	2	SArRNA09
gi|29165615|ref|NC_002745.2|	1920093 -	U	1	0	0	0	0	SArRNA09
gi|29165615|ref|NC_002745.2|	1920095 -	G	0	0	0	0	1	SArRNA09
gi|29165615|ref|NC_002745.2|	1920096 -	G	0	1	0	0	0	SArRNA09
gi|29165615|ref|NC_002745.2|	1920100 -	U	1	0	1	0	1	SArRNA09
gi|29165615|ref|NC_002745.2|	1920101 -	U	2	0	1	0	0	SArRNA09
gi|29165615|ref|NC_002745.2|	1920102 -	C	2	0	0	0	2	SArRNA09
gi|29165615|ref|NC_002745.2|	1920103 -	U	0	1	0	0	3	SArRNA09
gi|29165615|ref|NC_002745.2|	1920110 -	G	0	0	0	0	1	SArRNA09
gi|29165615|ref|NC_002745.2|	1920112 -	A	1	0	1	1	3	SArRNA09
gi|29165615|ref|NC_002745.2|	1920113 -	G	1	1	0	0	3	SArRNA09
gi|29165615|ref|NC_002745.2|	1920114 -	A	1	0	2	1	1	SArRNA09
gi|29165615|ref|NC_002745.2|	1920116 -	G	2	0	0	2	1	SArRNA09
gi|29165615|ref|NC_002745.2|	1920117 -	G	1	1	1	0	0	SArRNA09
gi|29165615|ref|NC_002745.2|	1920118 -	C	2	1	0	3	3	SArRNA09
gi|29165615|ref|NC_002745.2|	1920119 -	U	1	2	2	1	2	SArRNA09
gi|29165615|ref|NC_002745.2|	1920120 -	U	2	2	0	4	2	SArRNA09
gi|29165615|ref|NC_002745.2|	1920121 -	C	6	3	2	1	0	SArRNA09
gi|29165615|ref|NC_002745.2|	1920122 -	A	0	1	0	1	1	SArRNA09
gi|29165615|ref|NC_002745.2|	1920123 -	A	1	1	0	0	0	SArRNA09
gi|29165615|ref|NC_002745.2|	1920124 -	U	1	0	0	0	2	SArRNA09
gi|29165615|ref|NC_002745.2|	1920125 -	G	0	1	1	0	1	SArRNA09
gi|29165615|ref|NC_002745.2|	1920126 -	C	1	1	0	0	0	SArRNA09
gi|29165615|ref|NC_002745.2|	1920127 -	C	1	0	0	0	0	SArRNA09
gi|29165615|ref|NC_002745.2|	1920128 -	C	0	2	0	1	1	SArRNA09
gi|29165615|ref|NC_002745.2|	1920129 -	C	4	8	1	1	6	SArRNA09
gi|29165615|ref|NC_002745.2|	1920131 -	G	0	0	1	0	1	SArRNA09
gi|29165615|ref|NC_002745.2|	1920133 -	A	0	1	0	0	1	SArRNA09
gi|29165615|ref|NC_002745.2|	1920134 -	A	4	1	0	0	4	SArRNA09
gi|29165615|ref|NC_002745.2|	1920135 -	A	0	0	0	0	1	SArRNA09
gi|29165615|ref|NC_002745.2|	1920136 -	A	2	1	0	0	1	SArRNA09
gi|29165615|ref|NC_002745.2|	1920138 -	G	0	0	0	0	1	SArRNA09
gi|29165615|ref|NC_002745.2|	1920140 -	C	2	1	0	2	1	SArRNA09
gi|29165615|ref|NC_002745.2|	1920141 -	U	0	1	2	4	4	SArRNA09
gi|29165615|ref|NC_002745.2|	1920142 -	C	1	1	1	2	0	SArRNA09
gi|29165615|ref|NC_002745.2|	1920144 -	A	1	1	0	0	0	SArRNA09
gi|29165615|ref|NC_002745.2|	1920145 -	G	0	1	0	0	0	SArRNA09
gi|29165615|ref|NC_002745.2|	1920146 -	G	0	0	0	0	1	SArRNA09
gi|29165615|ref|NC_002745.2|	1920147 -	A	7	2	0	2	2	SArRNA09
gi|29165615|ref|NC_002745.2|	1920148 -	A	0	1	3	0	0	SArRNA09
gi|29165615|ref|NC_002745.2|	1920149 -	U	0	0	0	1	4	SArRNA09
gi|29165615|ref|NC_002745.2|	1920150 -	U	0	0	0	1	0	SArRNA09
gi|29165615|ref|NC_002745.2|	1920151 -	G	1	1	0	0	1	SArRNA09
gi|29165615|ref|NC_002745.2|	1920152 -	C	3	1	0	1	2	SArRNA09
gi|29165615|ref|NC_002745.2|	1920153 -	U	1	0	2	0	2	SArRNA09
gi|29165615|ref|NC_002745.2|	1920154 -	C	1	0	0	2	1	SArRNA09

gi|29165615|ref|NC_002745.2|	1920156	-	C	1	2	1	1	1	SArRNA09
gi|29165615|ref|NC_002745.2|	1920158	-	A	3	2	0	0	0	SArRNA09
gi|29165615|ref|NC_002745.2|	1920159	-	G	0	4	0	2	0	SArRNA09
gi|29165615|ref|NC_002745.2|	1920160	-	C	3	1	1	1	0	SArRNA09
gi|29165615|ref|NC_002745.2|	1920161	-	G	1	0	0	1	0	SArRNA09
gi|29165615|ref|NC_002745.2|	1920163	-	G	1	2	0	1	0	SArRNA09
gi|29165615|ref|NC_002745.2|	1920164	-	C	4	3	0	1	4	SArRNA09
gi|29165615|ref|NC_002745.2|	1920165	-	G	0	0	0	0	1	SArRNA09
gi|29165615|ref|NC_002745.2|	1920166	-	A	0	2	0	2	0	SArRNA09
gi|29165615|ref|NC_002745.2|	1920167	-	G	1	2	0	1	1	SArRNA09
gi|29165615|ref|NC_002745.2|	1920168	-	U	5	2	4	6	3	SArRNA09
gi|29165615|ref|NC_002745.2|	1920169	-	G	0	1	0	0	1	SArRNA09
gi|29165615|ref|NC_002745.2|	1920170	-	G	0	0	0	1	1	SArRNA09
gi|29165615|ref|NC_002745.2|	1920171	-	A	3	4	1	4	2	SArRNA09
gi|29165615|ref|NC_002745.2|	1920172	-	A	2	15	3	13	8	SArRNA09
gi|29165615|ref|NC_002745.2|	1920173	-	U	3	9	1	2	5	SArRNA09
gi|29165615|ref|NC_002745.2|	1920174	-	C	9	11	1	3	9	SArRNA09
gi|29165615|ref|NC_002745.2|	1920175	-	U	1	3	0	0	0	SArRNA09
gi|29165615|ref|NC_002745.2|	1920176	-	U	1	1	0	2	5	SArRNA09
gi|29165615|ref|NC_002745.2|	1920177	-	A	15	54	8	5	19	SArRNA09
gi|29165615|ref|NC_002745.2|	1920178	-	A	8	3	4	3	5	SArRNA09
gi|29165615|ref|NC_002745.2|	1920179	-	G	4	3	0	0	3	SArRNA09
gi|29165615|ref|NC_002745.2|	1920180	-	A	1	2	0	1	3	SArRNA09
gi|29165615|ref|NC_002745.2|	1920181	-	G	1	1	0	0	1	SArRNA09
gi|29165615|ref|NC_002745.2|	1920182	-	U	0	0	0	1	3	SArRNA09
gi|29165615|ref|NC_002745.2|	1920183	-	A	0	1	0	0	0	SArRNA09
gi|29165615|ref|NC_002745.2|	1920184	-	G	1	1	0	0	0	SArRNA09
gi|29165615|ref|NC_002745.2|	1920185	-	A	0	3	1	0	3	SArRNA09
gi|29165615|ref|NC_002745.2|	1920186	-	A	1	5	2	0	1	SArRNA09
gi|29165615|ref|NC_002745.2|	1920187	-	C	18	18	7	11	13	SArRNA09
gi|29165615|ref|NC_002745.2|	1920188	-	U	0	0	0	1	1	SArRNA09
gi|29165615|ref|NC_002745.2|	1920189	-	G	1	0	0	0	0	SArRNA09
gi|29165615|ref|NC_002745.2|	1920191	-	U	3	7	0	0	2	SArRNA09
gi|29165615|ref|NC_002745.2|	1920192	-	G	1	2	0	2	1	SArRNA09
gi|29165615|ref|NC_002745.2|	1920193	-	G	0	1	0	1	0	SArRNA09
gi|29165615|ref|NC_002745.2|	1920194	-	A	2	12	1	1	3	SArRNA09
gi|29165615|ref|NC_002745.2|	1920195	-	C	15	23	5	8	10	SArRNA09
gi|29165615|ref|NC_002745.2|	1920196	-	A	0	13	1	0	3	SArRNA09
gi|29165615|ref|NC_002745.2|	1920197	-	C	4	8	0	0	6	SArRNA09
gi|29165615|ref|NC_002745.2|	1920199	-	G	1	8	0	2	4	SArRNA09
gi|29165615|ref|NC_002745.2|	1920200	-	C	6	21	2	2	6	SArRNA09
gi|29165615|ref|NC_002745.2|	1920201	-	C	1	1	1	0	4	SArRNA09
gi|29165615|ref|NC_002745.2|	1920202	-	A	1	16	1	0	6	SArRNA09
gi|29165615|ref|NC_002745.2|	1920203	-	A	1	7	1	0	5	SArRNA09
gi|29165615|ref|NC_002745.2|	1920204	-	A	0	1	0	1	4	SArRNA09
gi|29165615|ref|NC_002745.2|	1920205	-	C	5	6	2	1	4	SArRNA09
gi|29165615|ref|NC_002745.2|	1920206	-	G	1	1	0	1	2	SArRNA09
gi|29165615|ref|NC_002745.2|	1920207	-	C	0	1	0	0	0	SArRNA09
gi|29165615|ref|NC_002745.2|	1920208	-	C	1	0	0	0	1	SArRNA09
gi|29165615|ref|NC_002745.2|	1920210	-	U	1	7	1	0	2	SArRNA09
gi|29165615|ref|NC_002745.2|	1920211	-	G	0	4	0	0	0	SArRNA09
gi|29165615|ref|NC_002745.2|	1920212	-	C	2	1	0	0	1	SArRNA09
gi|29165615|ref|NC_002745.2|	1920213	-	C	0	6	0	0	0	SArRNA09
gi|29165615|ref|NC_002745.2|	1920214	-	C	0	0	1	0	1	SArRNA09
gi|29165615|ref|NC_002745.2|	1920219	-	A	3	1	1	1	2	SArRNA09
gi|29165615|ref|NC_002745.2|	1920220	-	U	0	3	1	1	1	SArRNA09
gi|29165615|ref|NC_002745.2|	1920221	-	A	13	72	1	2	15	SArRNA09
gi|29165615|ref|NC_002745.2|	1920222	-	A	3	0	3	1	4	SArRNA09
gi|29165615|ref|NC_002745.2|	1920223	-	A	4	3	0	3	3	SArRNA09
gi|29165615|ref|NC_002745.2|	1920224	-	A	0	3	2	2	7	SArRNA09
gi|29165615|ref|NC_002745.2|	1920225	-	G	1	1	4	3	0	SArRNA09
gi|29165615|ref|NC_002745.2|	1920226	-	A	0	1	3	1	3	SArRNA09
gi|29165615|ref|NC_002745.2|	1920227	-	U	4	5	1	3	6	SArRNA09
gi|29165615|ref|NC_002745.2|	1920228	-	A	4	8	1	2	5	SArRNA09
gi|29165615|ref|NC_002745.2|	1920229	-	G	0	3	1	4	1	SArRNA09
gi|29165615|ref|NC_002745.2|	1920230	-	A	2	4	1	0	5	SArRNA09
gi|29165615|ref|NC_002745.2|	1920231	-	U	1	9	2	0	3	SArRNA09
gi|29165615|ref|NC_002745.2|	1920232	-	C	1	4	0	1	5	SArRNA09
gi|29165615|ref|NC_002745.2|	1920233	-	U	2	1	0	1	2	SArRNA09
gi|29165615|ref|NC_002745.2|	1920234	-	C	3	1	3	1	2	SArRNA09
gi|29165615|ref|NC_002745.2|	1920235	-	C	2	0	1	0	0	SArRNA09
gi|29165615|ref|NC_002745.2|	1920237	-	A	1	1	0	0	1	SArRNA09
gi|29165615|ref|NC_002745.2|	1920238	-	A	1	1	0	0	0	SArRNA09
gi|29165615|ref|NC_002745.2|	1920239	-	A	1	0	0	0	1	SArRNA09
gi|29165615|ref|NC_002745.2|	1920240	-	A	1	0	1	0	0	SArRNA09
gi|29165615|ref|NC_002745.2|	1920241	-	G	1	0	1	0	1	SArRNA09
gi|29165615|ref|NC_002745.2|	1920242	-	A	0	0	0	0	1	SArRNA09
gi|29165615|ref|NC_002745.2|	1920243	-	G	0	0	0	1	0	SArRNA09
gi|29165615|ref|NC_002745.2|	1920244	-	C	2	3	1	0	0	SArRNA09
gi|29165615|ref|NC_002745.2|	1920246	-	G	0	0	0	1	0	SArRNA09
gi|29165615|ref|NC_002745.2|	1920247	-	U	0	0	1	0	1	SArRNA09

gi|29165615|ref|NC_002745.2|	1920248	-	C	2	1	0	2	1	SArRNA09
gi|29165615|ref|NC_002745.2|	1920249	-	A	2	1	0	1	0	SArRNA09
gi|29165615|ref|NC_002745.2|	1920250	-	C	3	5	2	0	2	SArRNA09
gi|29165615|ref|NC_002745.2|	1920251	-	A	2	0	0	0	1	SArRNA09
gi|29165615|ref|NC_002745.2|	1920252	-	C	2	2	2	1	0	SArRNA09
gi|29165615|ref|NC_002745.2|	1920253	-	U	0	1	0	0	1	SArRNA09
gi|29165615|ref|NC_002745.2|	1920254	-	U	0	0	1	1	0	SArRNA09
gi|29165615|ref|NC_002745.2|	1920255	-	U	3	0	0	0	0	SArRNA09
gi|29165615|ref|NC_002745.2|	1920256	-	A	0	1	0	0	1	SArRNA09
gi|29165615|ref|NC_002745.2|	1920259	-	U	0	0	1	0	0	SArRNA09
gi|29165615|ref|NC_002745.2|	1920260	-	G	1	1	0	0	1	SArRNA09
gi|29165615|ref|NC_002745.2|	1920261	-	C	2	0	0	0	0	SArRNA09
gi|29165615|ref|NC_002745.2|	1920262	-	U	2	0	1	0	1	SArRNA09
gi|29165615|ref|NC_002745.2|	1920263	-	G	0	0	0	0	1	SArRNA09
gi|29165615|ref|NC_002745.2|	1920265	-	G	0	1	1	0	0	SArRNA09
gi|29165615|ref|NC_002745.2|	1920266	-	C	0	1	0	0	1	SArRNA09
gi|29165615|ref|NC_002745.2|	1920267	-	U	0	1	0	0	1	SArRNA09
gi|29165615|ref|NC_002745.2|	1920268	-	U	1	1	1	0	0	SArRNA09
gi|29165615|ref|NC_002745.2|	1920269	-	C	0	1	0	0	0	SArRNA09
gi|29165615|ref|NC_002745.2|	1920270	-	U	0	0	0	0	1	SArRNA09
gi|29165615|ref|NC_002745.2|	1920271	-	G	1	0	0	0	1	SArRNA09
gi|29165615|ref|NC_002745.2|	1920272	-	U	0	0	0	0	3	SArRNA09
gi|29165615|ref|NC_002745.2|	1920274	-	U	1	0	0	0	0	SArRNA09
gi|29165615|ref|NC_002745.2|	1920275	-	U	0	0	1	1	1	SArRNA09
gi|29165615|ref|NC_002745.2|	1920276	-	A	3	1	0	0	2	SArRNA09
gi|29165615|ref|NC_002745.2|	1920277	-	C	1	0	1	2	2	SArRNA09
gi|29165615|ref|NC_002745.2|	1920278	-	A	1	0	0	0	3	SArRNA09
gi|29165615|ref|NC_002745.2|	1920279	-	G	0	1	0	0	0	SArRNA09
gi|29165615|ref|NC_002745.2|	1920280	-	A	2	0	0	0	0	SArRNA09
gi|29165615|ref|NC_002745.2|	1920281	-	A	1	0	0	0	0	SArRNA09
gi|29165615|ref|NC_002745.2|	1920286	-	G	1	1	1	0	0	SArRNA09
gi|29165615|ref|NC_002745.2|	1920287	-	G	1	1	1	1	0	SArRNA09
gi|29165615|ref|NC_002745.2|	1920288	-	U	1	8	2	2	6	SArRNA09
gi|29165615|ref|NC_002745.2|	1920289	-	A	1	0	0	0	0	SArRNA09
gi|29165615|ref|NC_002745.2|	1920290	-	G	1	3	1	0	0	SArRNA09
gi|29165615|ref|NC_002745.2|	1920291	-	U	0	0	0	0	1	SArRNA09
gi|29165615|ref|NC_002745.2|	1920292	-	G	0	0	0	0	1	SArRNA09
gi|29165615|ref|NC_002745.2|	1920293	-	U	2	0	3	0	0	SArRNA09
gi|29165615|ref|NC_002745.2|	1920294	-	C	1	0	1	0	0	SArRNA09
gi|29165615|ref|NC_002745.2|	1920296	-	A	4	0	0	1	2	SArRNA09
gi|29165615|ref|NC_002745.2|	1920297	-	G	0	0	1	0	1	SArRNA09
gi|29165615|ref|NC_002745.2|	1920298	-	U	1	2	0	1	2	SArRNA09
gi|29165615|ref|NC_002745.2|	1920299	-	C	1	1	1	0	5	SArRNA09
gi|29165615|ref|NC_002745.2|	1920300	-	G	1	1	0	0	0	SArRNA09
gi|29165615|ref|NC_002745.2|	1920301	-	G	0	2	0	1	0	SArRNA09
gi|29165615|ref|NC_002745.2|	1920302	-	A	3	2	0	0	2	SArRNA09
gi|29165615|ref|NC_002745.2|	1920303	-	A	0	6	0	0	1	SArRNA09
gi|29165615|ref|NC_002745.2|	1920304	-	U	3	2	0	0	6	SArRNA09
gi|29165615|ref|NC_002745.2|	1920305	-	U	1	0	0	0	1	SArRNA09
gi|29165615|ref|NC_002745.2|	1920306	-	G	1	0	0	0	5	SArRNA09
gi|29165615|ref|NC_002745.2|	1920307	-	C	0	0	0	0	1	SArRNA09
gi|29165615|ref|NC_002745.2|	1920308	-	U	0	1	1	0	1	SArRNA09
gi|29165615|ref|NC_002745.2|	1920309	-	C	0	2	0	0	1	SArRNA09
gi|29165615|ref|NC_002745.2|	1920310	-	A	0	1	1	0	0	SArRNA09
gi|29165615|ref|NC_002745.2|	1920312	-	G	2	0	0	1	0	SArRNA09
gi|29165615|ref|NC_002745.2|	1920313	-	G	0	0	0	1	0	SArRNA09
gi|29165615|ref|NC_002745.2|	1920314	-	C	1	4	0	0	0	SArRNA09
gi|29165615|ref|NC_002745.2|	1920315	-	C	1	2	0	0	2	SArRNA09
gi|29165615|ref|NC_002745.2|	1920316	-	U	1	1	1	0	1	SArRNA09
gi|29165615|ref|NC_002745.2|	1920317	-	A	4	3	1	1	1	SArRNA09
gi|29165615|ref|NC_002745.2|	1920318	-	A	0	0	0	0	1	SArRNA09
gi|29165615|ref|NC_002745.2|	1920319	-	A	1	1	0	0	1	SArRNA09
gi|29165615|ref|NC_002745.2|	1920322	-	G	2	0	0	0	1	SArRNA09
gi|29165615|ref|NC_002745.2|	1920323	-	A	2	1	0	1	1	SArRNA09
gi|29165615|ref|NC_002745.2|	1920324	-	U	2	3	0	1	1	SArRNA09
gi|29165615|ref|NC_002745.2|	1920326	-	A	0	1	1	0	2	SArRNA09
gi|29165615|ref|NC_002745.2|	1920327	-	U	0	1	0	0	0	SArRNA09
gi|29165615|ref|NC_002745.2|	1920329	-	A	0	1	0	0	3	SArRNA09
gi|29165615|ref|NC_002745.2|	1920330	-	G	0	0	0	0	1	SArRNA09
gi|29165615|ref|NC_002745.2|	1920331	-	U	0	1	0	1	0	SArRNA09
gi|29165615|ref|NC_002745.2|	1920332	-	C	4	0	0	1	1	SArRNA09
gi|29165615|ref|NC_002745.2|	1920333	-	G	1	1	0	0	1	SArRNA09
gi|29165615|ref|NC_002745.2|	1920335	-	A	1	1	0	0	1	SArRNA09
gi|29165615|ref|NC_002745.2|	1920336	-	A	2	4	0	0	3	SArRNA09
gi|29165615|ref|NC_002745.2|	1920337	-	U	2	0	0	0	2	SArRNA09
gi|29165615|ref|NC_002745.2|	1920338	-	G	0	0	0	3	1	SArRNA09
gi|29165615|ref|NC_002745.2|	1920339	-	A	3	0	0	0	2	SArRNA09
gi|29165615|ref|NC_002745.2|	1920340	-	C	4	3	0	1	0	SArRNA09
gi|29165615|ref|NC_002745.2|	1920342	-	A	0	0	0	0	1	SArRNA09
gi|29165615|ref|NC_002745.2|	1920343	-	A	0	2	0	0	1	SArRNA09
gi|29165615|ref|NC_002745.2|	1920344	-	U	2	6	1	0	2	SArRNA09

gi|29165615|ref|NC_002745.2|	1920345 -	C	1	6	1	2	12	SArRNA09
gi|29165615|ref|NC_002745.2|	1920346 -	U	1	0	0	0	0	SArRNA09
gi|29165615|ref|NC_002745.2|	1920347 -	G	1	2	0	1	0	SArRNA09
gi|29165615|ref|NC_002745.2|	1920348 -	C	1	0	0	0	2	SArRNA09
gi|29165615|ref|NC_002745.2|	1920349 -	A	0	2	0	0	1	SArRNA09
gi|29165615|ref|NC_002745.2|	1920350 -	C	0	7	2	0	3	SArRNA09
gi|29165615|ref|NC_002745.2|	1920351 -	G	0	1	0	0	1	SArRNA09
gi|29165615|ref|NC_002745.2|	1920352 -	U	0	0	0	0	2	SArRNA09
gi|29165615|ref|NC_002745.2|	1920353 -	U	3	2	0	0	1	SArRNA09
gi|29165615|ref|NC_002745.2|	1920354 -	A	1	3	3	0	5	SArRNA09
gi|29165615|ref|NC_002745.2|	1920355 -	G	1	3	0	0	1	SArRNA09
gi|29165615|ref|NC_002745.2|	1920356 -	G	2	1	0	0	1	SArRNA09
gi|29165615|ref|NC_002745.2|	1920357 -	U	1	1	0	0	1	SArRNA09
gi|29165615|ref|NC_002745.2|	1920358 -	U	0	0	2	3	3	SArRNA09
gi|29165615|ref|NC_002745.2|	1920359 -	A	0	2	3	0	2	SArRNA09
gi|29165615|ref|NC_002745.2|	1920360 -	G	1	3	0	0	3	SArRNA09
gi|29165615|ref|NC_002745.2|	1920361 -	C	4	1	3	0	4	SArRNA09
gi|29165615|ref|NC_002745.2|	1920362 -	G	0	1	0	0	0	SArRNA09
gi|29165615|ref|NC_002745.2|	1920363 -	U	0	1	0	0	0	SArRNA09
gi|29165615|ref|NC_002745.2|	1920368 -	A	0	1	0	0	0	SArRNA09
gi|29165615|ref|NC_002745.2|	1920369 -	G	1	4	0	0	1	SArRNA09
gi|29165615|ref|NC_002745.2|	1920370 -	C	3	0	1	2	3	SArRNA09
gi|29165615|ref|NC_002745.2|	1920372 -	G	0	1	0	0	0	SArRNA09
gi|29165615|ref|NC_002745.2|	1920373 -	A	2	0	2	1	1	SArRNA09
gi|29165615|ref|NC_002745.2|	1920374 -	U	5	5	0	3	3	SArRNA09
gi|29165615|ref|NC_002745.2|	1920375 -	A	2	5	0	1	5	SArRNA09
gi|29165615|ref|NC_002745.2|	1920376 -	G	0	1	0	1	1	SArRNA09
gi|29165615|ref|NC_002745.2|	1920377 -	G	0	0	0	1	0	SArRNA09
gi|29165615|ref|NC_002745.2|	1920378 -	A	1	3	2	0	1	SArRNA09
gi|29165615|ref|NC_002745.2|	1920379 -	U	7	2	5	0	1	SArRNA09
gi|29165615|ref|NC_002745.2|	1920380 -	G	2	1	0	0	3	SArRNA09
gi|29165615|ref|NC_002745.2|	1920381 -	A	0	1	1	0	1	SArRNA09
gi|29165615|ref|NC_002745.2|	1920382 -	C	3	0	0	1	0	SArRNA09
gi|29165615|ref|NC_002745.2|	1920383 -	G	1	4	4	1	1	SArRNA09
gi|29165615|ref|NC_002745.2|	1920384 -	C	1	12	0	0	4	SArRNA09
gi|29165615|ref|NC_002745.2|	1920386 -	G	0	0	1	0	1	SArRNA09
gi|29165615|ref|NC_002745.2|	1920388 -	G	0	0	0	0	1	SArRNA09
gi|29165615|ref|NC_002745.2|	1920389 -	G	1	3	3	0	2	SArRNA09
gi|29165615|ref|NC_002745.2|	1920390 -	G	0	2	0	1	0	SArRNA09
gi|29165615|ref|NC_002745.2|	1920391 -	G	0	1	1	0	2	SArRNA09
gi|29165615|ref|NC_002745.2|	1920392 -	U	7	12	0	2	1	SArRNA09
gi|29165615|ref|NC_002745.2|	1920393 -	A	3	2	1	2	2	SArRNA09
gi|29165615|ref|NC_002745.2|	1920394 -	G	6	10	3	2	6	SArRNA09
gi|29165615|ref|NC_002745.2|	1920395 -	C	8	5	0	3	2	SArRNA09
gi|29165615|ref|NC_002745.2|	1920396 -	U	4	3	1	2	5	SArRNA09
gi|29165615|ref|NC_002745.2|	1920397 -	A	9	75	22	3	28	SArRNA09
gi|29165615|ref|NC_002745.2|	1920398 -	A	2	3	1	1	4	SArRNA09
gi|29165615|ref|NC_002745.2|	1920399 -	U	5	7	3	5	7	SArRNA09
gi|29165615|ref|NC_002745.2|	1920400 -	U	6	8	3	0	1	SArRNA09
gi|29165615|ref|NC_002745.2|	1920401 -	U	8	4	1	0	3	SArRNA09
gi|29165615|ref|NC_002745.2|	1920402 -	U	0	4	4	2	2	SArRNA09
gi|29165615|ref|NC_002745.2|	1920403 -	G	1	2	2	3	4	SArRNA09
gi|29165615|ref|NC_002745.2|	1920404 -	C	4	3	2	1	5	SArRNA09
gi|29165615|ref|NC_002745.2|	1920405 -	U	6	1	2	2	1	SArRNA09
gi|29165615|ref|NC_002745.2|	1920406 -	A	3	11	1	2	13	SArRNA09
gi|29165615|ref|NC_002745.2|	1920407 -	A	4	1	1	2	5	SArRNA09
gi|29165615|ref|NC_002745.2|	1920408 -	U	6	8	1	2	6	SArRNA09
gi|29165615|ref|NC_002745.2|	1920409 -	A	3	1	1	1	0	SArRNA09
gi|29165615|ref|NC_002745.2|	1920410 -	U	4	5	2	3	6	SArRNA09
gi|29165615|ref|NC_002745.2|	1920411 -	C	6	4	1	2	6	SArRNA09
gi|29165615|ref|NC_002745.2|	1920412 -	C	1	3	1	2	2	SArRNA09
gi|29165615|ref|NC_002745.2|	1920413 -	A	1	5	0	2	3	SArRNA09
gi|29165615|ref|NC_002745.2|	1920414 -	C	15	13	3	2	13	SArRNA09
gi|29165615|ref|NC_002745.2|	1920415 -	C	0	4	1	2	6	SArRNA09
gi|29165615|ref|NC_002745.2|	1920416 -	A	0	1	0	0	0	SArRNA09
gi|29165615|ref|NC_002745.2|	1920417 -	U	1	2	0	4	2	SArRNA09
gi|29165615|ref|NC_002745.2|	1920418 -	G	0	1	0	0	1	SArRNA09
gi|29165615|ref|NC_002745.2|	1920419 -	U	7	3	0	1	1	SArRNA09
gi|29165615|ref|NC_002745.2|	1920420 -	C	2	0	0	1	0	SArRNA09
gi|29165615|ref|NC_002745.2|	1920421 -	C	2	1	0	2	1	SArRNA09
gi|29165615|ref|NC_002745.2|	1920422 -	U	4	2	0	0	0	SArRNA09
gi|29165615|ref|NC_002745.2|	1920423 -	U	3	0	3	0	2	SArRNA09
gi|29165615|ref|NC_002745.2|	1920424 -	A	1	1	2	0	4	SArRNA09
gi|29165615|ref|NC_002745.2|	1920425 -	U	0	4	0	3	4	SArRNA09
gi|29165615|ref|NC_002745.2|	1920426 -	A	2	5	0	2	2	SArRNA09
gi|29165615|ref|NC_002745.2|	1920427 -	G	3	0	2	0	2	SArRNA09
gi|29165615|ref|NC_002745.2|	1920428 -	U	0	1	1	1	2	SArRNA09
gi|29165615|ref|NC_002745.2|	1920429 -	U	1	0	0	1	3	SArRNA09
gi|29165615|ref|NC_002745.2|	1920430 -	G	0	1	0	1	0	SArRNA09
gi|29165615|ref|NC_002745.2|	1920431 -	G	1	0	1	0	1	SArRNA09
gi|29165615|ref|NC_002745.2|	1920432 -	A	1	3	0	0	2	SArRNA09

gi|29165615|ref|NC_002745.2|	1920433	-	C	7	7	4	1	1	SArRNA09
gi|29165615|ref|NC_002745.2|	1920434	-	A	0	1	0	0	2	SArRNA09
gi|29165615|ref|NC_002745.2|	1920435	-	A	1	0	0	1	5	SArRNA09
gi|29165615|ref|NC_002745.2|	1920436	-	U	1	1	1	0	0	SArRNA09
gi|29165615|ref|NC_002745.2|	1920437	-	A	1	1	1	5	5	SArRNA09
gi|29165615|ref|NC_002745.2|	1920438	-	G	0	1	1	2	3	SArRNA09
gi|29165615|ref|NC_002745.2|	1920439	-	G	2	3	1	1	2	SArRNA09
gi|29165615|ref|NC_002745.2|	1920440	-	U	5	6	4	1	1	SArRNA09
gi|29165615|ref|NC_002745.2|	1920441	-	A	4	6	1	1	6	SArRNA09
gi|29165615|ref|NC_002745.2|	1920442	-	G	17	21	12	8	17	SArRNA09
gi|29165615|ref|NC_002745.2|	1920443	-	C	5	6	4	3	12	SArRNA09
gi|29165615|ref|NC_002745.2|	1920444	-	G	0	1	0	0	0	SArRNA09
gi|29165615|ref|NC_002745.2|	1920445	-	G	0	2	1	0	1	SArRNA09
gi|29165615|ref|NC_002745.2|	1920446	-	A	1	2	3	0	3	SArRNA09
gi|29165615|ref|NC_002745.2|	1920447	-	U	15	13	3	11	8	SArRNA09
gi|29165615|ref|NC_002745.2|	1920448	-	G	5	6	2	1	2	SArRNA09
gi|29165615|ref|NC_002745.2|	1920449	-	C	4	1	2	1	7	SArRNA09
gi|29165615|ref|NC_002745.2|	1920450	-	G	2	1	1	0	2	SArRNA09
gi|29165615|ref|NC_002745.2|	1920451	-	G	7	16	1	3	7	SArRNA09
gi|29165615|ref|NC_002745.2|	1920452	-	A	6	14	2	3	6	SArRNA09
gi|29165615|ref|NC_002745.2|	1920453	-	C	29	46	11	9	29	SArRNA09
gi|29165615|ref|NC_002745.2|	1920454	-	A	1	5	0	0	8	SArRNA09
gi|29165615|ref|NC_002745.2|	1920455	-	G	7	5	3	1	6	SArRNA09
gi|29165615|ref|NC_002745.2|	1920456	-	C	11	7	2	5	10	SArRNA09
gi|29165615|ref|NC_002745.2|	1920457	-	C	0	3	0	1	2	SArRNA09
gi|29165615|ref|NC_002745.2|	1920458	-	G	3	1	0	1	2	SArRNA09
gi|29165615|ref|NC_002745.2|	1920459	-	G	0	1	0	1	1	SArRNA09
gi|29165615|ref|NC_002745.2|	1920460	-	A	3	1	2	0	0	SArRNA09
gi|29165615|ref|NC_002745.2|	1920461	-	G	6	14	7	1	9	SArRNA09
gi|29165615|ref|NC_002745.2|	1920462	-	U	5	6	4	0	3	SArRNA09
gi|29165615|ref|NC_002745.2|	1920463	-	C	4	2	2	4	1	SArRNA09
gi|29165615|ref|NC_002745.2|	1920464	-	G	0	0	0	0	1	SArRNA09
gi|29165615|ref|NC_002745.2|	1920465	-	A	10	4	1	3	3	SArRNA09
gi|29165615|ref|NC_002745.2|	1920466	-	A	6	5	1	1	5	SArRNA09
gi|29165615|ref|NC_002745.2|	1920467	-	U	2	11	3	1	11	SArRNA09
gi|29165615|ref|NC_002745.2|	1920468	-	C	5	14	0	5	8	SArRNA09
gi|29165615|ref|NC_002745.2|	1920469	-	C	1	2	0	2	0	SArRNA09
gi|29165615|ref|NC_002745.2|	1920470	-	U	0	3	0	0	1	SArRNA09
gi|29165615|ref|NC_002745.2|	1920471	-	G	0	3	0	1	0	SArRNA09
gi|29165615|ref|NC_002745.2|	1920472	-	G	4	2	1	0	1	SArRNA09
gi|29165615|ref|NC_002745.2|	1920473	-	G	2	4	1	1	0	SArRNA09
gi|29165615|ref|NC_002745.2|	1920474	-	C	1	8	0	2	2	SArRNA09
gi|29165615|ref|NC_002745.2|	1920475	-	U	4	0	1	0	4	SArRNA09
gi|29165615|ref|NC_002745.2|	1920476	-	G	15	5	4	6	6	SArRNA09
gi|29165615|ref|NC_002745.2|	1920477	-	A	12	10	4	6	13	SArRNA09
gi|29165615|ref|NC_002745.2|	1920478	-	U	6	12	8	8	13	SArRNA09
gi|29165615|ref|NC_002745.2|	1920479	-	U	11	12	8	1	16	SArRNA09
gi|29165615|ref|NC_002745.2|	1920480	-	G	12	7	4	5	13	SArRNA09
gi|29165615|ref|NC_002745.2|	1920481	-	G	10	8	1	5	7	SArRNA09
gi|29165615|ref|NC_002745.2|	1920482	-	G	6	3	3	3	3	SArRNA09
gi|29165615|ref|NC_002745.2|	1920483	-	U	15	13	7	4	17	SArRNA09
gi|29165615|ref|NC_002745.2|	1920484	-	C	2	6	2	1	4	SArRNA09
gi|29165615|ref|NC_002745.2|	1920485	-	U	8	11	3	5	5	SArRNA09
gi|29165615|ref|NC_002745.2|	1920486	-	C	10	12	3	5	12	SArRNA09
gi|29165615|ref|NC_002745.2|	1920487	-	G	7	12	7	7	16	SArRNA09
gi|29165615|ref|NC_002745.2|	1920488	-	C	17	24	2	9	20	SArRNA09
gi|29165615|ref|NC_002745.2|	1920489	-	C	4	5	0	1	5	SArRNA09
gi|29165615|ref|NC_002745.2|	1920490	-	U	2	2	0	1	5	SArRNA09
gi|29165615|ref|NC_002745.2|	1920491	-	G	2	3	0	0	7	SArRNA09
gi|29165615|ref|NC_002745.2|	1920492	-	C	4	2	2	0	4	SArRNA09
gi|29165615|ref|NC_002745.2|	1920493	-	U	3	8	2	0	2	SArRNA09
gi|29165615|ref|NC_002745.2|	1920494	-	C	0	0	1	0	2	SArRNA09
gi|29165615|ref|NC_002745.2|	1920495	-	G	2	1	1	2	2	SArRNA09
gi|29165615|ref|NC_002745.2|	1920496	-	G	1	3	0	3	3	SArRNA09
gi|29165615|ref|NC_002745.2|	1920497	-	A	11	9	5	5	13	SArRNA09
gi|29165615|ref|NC_002745.2|	1920498	-	A	9	20	2	7	10	SArRNA09
gi|29165615|ref|NC_002745.2|	1920499	-	G	15	13	3	4	13	SArRNA09
gi|29165615|ref|NC_002745.2|	1920500	-	G	6	6	5	5	11	SArRNA09
gi|29165615|ref|NC_002745.2|	1920501	-	A	11	1	2	4	13	SArRNA09
gi|29165615|ref|NC_002745.2|	1920502	-	G	3	1	5	3	9	SArRNA09
gi|29165615|ref|NC_002745.2|	1920503	-	A	4	5	3	2	10	SArRNA09
gi|29165615|ref|NC_002745.2|	1920504	-	C	3	1	1	3	7	SArRNA09
gi|29165615|ref|NC_002745.2|	1920505	-	C	2	2	0	0	2	SArRNA09
gi|29165615|ref|NC_002745.2|	1920506	-	U	2	2	0	0	1	SArRNA09
gi|29165615|ref|NC_002745.2|	1920507	-	U	5	3	0	1	7	SArRNA09
gi|29165615|ref|NC_002745.2|	1920508	-	U	10	11	6	9	19	SArRNA09
gi|29165615|ref|NC_002745.2|	1920509	-	G	22	16	15	11	35	SArRNA09
gi|29165615|ref|NC_002745.2|	1920510	-	G	36	22	13	7	23	SArRNA09
gi|29165615|ref|NC_002745.2|	1920511	-	A	37	36	14	11	39	SArRNA09
gi|29165615|ref|NC_002745.2|	1920512	-	A	16	19	9	7	19	SArRNA09
gi|29165615|ref|NC_002745.2|	1920513	-	U	34	38	15	23	27	SArRNA09

gi|29165615|ref|NC_002745.2|	1920514	-	C	12	17	4	9	21	SArRNA09
gi|29165615|ref|NC_002745.2|	1920515	-	A	7	13	6	7	18	SArRNA09
gi|29165615|ref|NC_002745.2|	1920516	-	G	0	2	0	2	0	SArRNA09
gi|29165615|ref|NC_002745.2|	1920517	-	U	25	16	11	7	23	SArRNA09
gi|29165615|ref|NC_002745.2|	1920518	-	U	38	31	16	22	35	SArRNA09
gi|29165615|ref|NC_002745.2|	1920519	-	A	28	31	15	10	23	SArRNA09
gi|29165615|ref|NC_002745.2|	1920520	-	G	26	20	10	18	25	SArRNA09
gi|29165615|ref|NC_002745.2|	1920521	-	C	60	40	14	15	27	SArRNA09
gi|29165615|ref|NC_002745.2|	1920522	-	C	1	4	0	0	1	SArRNA09
gi|29165615|ref|NC_002745.2|	1920523	-	A	1	2	1	1	1	SArRNA09
gi|29165615|ref|NC_002745.2|	1920524	-	C	2	3	2	0	0	SArRNA09
gi|29165615|ref|NC_002745.2|	1920525	-	C	0	1	0	0	1	SArRNA09
gi|29165615|ref|NC_002745.2|	1920526	-	U	0	1	0	1	0	SArRNA09
gi|29165615|ref|NC_002745.2|	1920527	-	G	1	3	1	2	1	SArRNA09
gi|29165615|ref|NC_002745.2|	1920528	-	C	3	1	2	0	0	SArRNA09
gi|29165615|ref|NC_002745.2|	1920529	-	C	3	5	0	1	6	SArRNA09
gi|29165615|ref|NC_002745.2|	1920530	-	C	26	10	5	4	9	SArRNA09
gi|29165615|ref|NC_002745.2|	1920531	-	U	0	1	0	0	1	SArRNA09
gi|29165615|ref|NC_002745.2|	1920532	-	A	0	2	1	0	1	SArRNA09
gi|29165615|ref|NC_002745.2|	1920533	-	A	0	3	0	0	3	SArRNA09
gi|29165615|ref|NC_002745.2|	1920534	-	G	0	0	0	0	1	SArRNA09
gi|29165615|ref|NC_002745.2|	1920535	-	A	0	0	0	1	1	SArRNA09
gi|29165615|ref|NC_002745.2|	1920536	-	G	0	0	0	0	2	SArRNA09
gi|29165615|ref|NC_002745.2|	1920537	-	U	0	0	1	2	1	SArRNA09
gi|29165615|ref|NC_002745.2|	1920538	-	G	6	2	1	1	0	SArRNA09
gi|29165615|ref|NC_002745.2|	1920539	-	G	0	0	1	0	1	SArRNA09
gi|29165615|ref|NC_002745.2|	1920541	-	C	0	1	0	0	0	SArRNA09
gi|29165615|ref|NC_002745.2|	1920543	-	G	0	2	0	2	2	SArRNA09
gi|29165615|ref|NC_002745.2|	1920544	-	A	0	1	0	0	1	SArRNA09
gi|29165615|ref|NC_002745.2|	1920545	-	A	1	3	0	0	2	SArRNA09
gi|29165615|ref|NC_002745.2|	1920546	-	A	2	1	1	1	3	SArRNA09
gi|29165615|ref|NC_002745.2|	1920547	-	G	4	6	3	1	2	SArRNA09
gi|29165615|ref|NC_002745.2|	1920548	-	C	8	9	0	6	4	SArRNA09
gi|29165615|ref|NC_002745.2|	1920549	-	G	9	5	1	0	2	SArRNA09
gi|29165615|ref|NC_002745.2|	1920550	-	A	35	35	8	11	16	SArRNA09
gi|29165615|ref|NC_002745.2|	1920551	-	U	30	30	15	13	17	SArRNA09
gi|29165615|ref|NC_002745.2|	1920552	-	G	35	26	10	12	15	SArRNA09
gi|29165615|ref|NC_002745.2|	1920553	-	A	7	11	4	3	11	SArRNA09
gi|29165615|ref|NC_002745.2|	1920554	-	G	11	8	6	5	5	SArRNA09
gi|29165615|ref|NC_002745.2|	1920555	-	U	10	5	7	2	10	SArRNA09
gi|29165615|ref|NC_002745.2|	1920556	-	G	9	7	2	4	8	SArRNA09
gi|29165615|ref|NC_002745.2|	1920557	-	U	10	11	2	7	9	SArRNA09
gi|29165615|ref|NC_002745.2|	1920558	-	G	5	5	4	1	8	SArRNA09
gi|29165615|ref|NC_002745.2|	1920559	-	G	9	5	5	2	5	SArRNA09
gi|29165615|ref|NC_002745.2|	1920560	-	C	43	37	8	14	20	SArRNA09
gi|29165615|ref|NC_002745.2|	1920561	-	C	10	6	5	5	1	SArRNA09
gi|29165615|ref|NC_002745.2|	1920562	-	G	2	4	0	0	0	SArRNA09
gi|29165615|ref|NC_002745.2|	1920563	-	U	10	12	3	4	9	SArRNA09
gi|29165615|ref|NC_002745.2|	1920564	-	A	24	20	8	6	10	SArRNA09
gi|29165615|ref|NC_002745.2|	1920565	-	A	25	18	12	17	15	SArRNA09
gi|29165615|ref|NC_002745.2|	1920566	-	G	8	9	7	3	17	SArRNA09
gi|29165615|ref|NC_002745.2|	1920567	-	A	11	6	5	4	8	SArRNA09
gi|29165615|ref|NC_002745.2|	1920568	-	G	17	13	10	5	18	SArRNA09
gi|29165615|ref|NC_002745.2|	1920569	-	U	16	14	5	8	15	SArRNA09
gi|29165615|ref|NC_002745.2|	1920570	-	G	16	13	7	9	19	SArRNA09
gi|29165615|ref|NC_002745.2|	1920571	-	A	10	21	7	6	25	SArRNA09
gi|29165615|ref|NC_002745.2|	1920572	-	A	20	9	11	9	22	SArRNA09
gi|29165615|ref|NC_002745.2|	1920573	-	G	29	26	10	9	11	SArRNA09
gi|29165615|ref|NC_002745.2|	1920574	-	A	8	10	2	7	8	SArRNA09
gi|29165615|ref|NC_002745.2|	1920575	-	U	21	21	8	12	19	SArRNA09
gi|29165615|ref|NC_002745.2|	1920576	-	U	6	6	5	2	11	SArRNA09
gi|29165615|ref|NC_002745.2|	1920577	-	C	4	2	1	2	8	SArRNA09
gi|29165615|ref|NC_002745.2|	1920578	-	G	0	1	1	1	4	SArRNA09
gi|29165615|ref|NC_002745.2|	1920579	-	C	5	4	4	4	10	SArRNA09
gi|29165615|ref|NC_002745.2|	1920580	-	G	1	3	0	2	3	SArRNA09
gi|29165615|ref|NC_002745.2|	1920581	-	A	6	4	1	2	4	SArRNA09
gi|29165615|ref|NC_002745.2|	1920582	-	G	9	8	2	0	3	SArRNA09
gi|29165615|ref|NC_002745.2|	1920583	-	G	7	1	1	3	1	SArRNA09
gi|29165615|ref|NC_002745.2|	1920584	-	U	4	1	3	2	1	SArRNA09
gi|29165615|ref|NC_002745.2|	1920585	-	G	4	1	2	2	2	SArRNA09
gi|29165615|ref|NC_002745.2|	1920586	-	U	2	6	3	4	6	SArRNA09
gi|29165615|ref|NC_002745.2|	1920587	-	A	9	8	6	9	11	SArRNA09
gi|29165615|ref|NC_002745.2|	1920588	-	C	10	6	4	3	7	SArRNA09
gi|29165615|ref|NC_002745.2|	1920589	-	A	6	15	6	4	7	SArRNA09
gi|29165615|ref|NC_002745.2|	1920590	-	G	20	20	5	5	19	SArRNA09
gi|29165615|ref|NC_002745.2|	1920591	-	G	4	3	5	4	6	SArRNA09
gi|29165615|ref|NC_002745.2|	1920592	-	A	9	7	5	3	8	SArRNA09
gi|29165615|ref|NC_002745.2|	1920593	-	A	9	22	2	2	7	SArRNA09
gi|29165615|ref|NC_002745.2|	1920594	-	U	4	20	5	6	10	SArRNA09
gi|29165615|ref|NC_002745.2|	1920595	-	G	24	13	6	7	24	SArRNA09
gi|29165615|ref|NC_002745.2|	1920596	-	C	8	7	6	4	12	SArRNA09

gi|29165615|ref|NC_002745.2|	1920597 -	U	12	11	6	9	12	SArRNA09
gi|29165615|ref|NC_002745.2|	1920598 -	A	2	12	4	3	11	SArRNA09
gi|29165615|ref|NC_002745.2|	1920599 -	G	4	14	1	1	4	SArRNA09
gi|29165615|ref|NC_002745.2|	1920600 -	U	3	4	0	4	3	SArRNA09
gi|29165615|ref|NC_002745.2|	1920601 -	A	4	8	3	2	4	SArRNA09
gi|29165615|ref|NC_002745.2|	1920602 -	C	12	15	3	13	6	SArRNA09
gi|29165615|ref|NC_002745.2|	1920603 -	G	0	1	0	0	1	SArRNA09
gi|29165615|ref|NC_002745.2|	1920604 -	A	2	0	1	0	3	SArRNA09
gi|29165615|ref|NC_002745.2|	1920605 -	A	0	0	1	0	2	SArRNA09
gi|29165615|ref|NC_002745.2|	1920606 -	G	8	26	4	6	10	SArRNA09
gi|29165615|ref|NC_002745.2|	1920607 -	U	7	3	1	2	7	SArRNA09
gi|29165615|ref|NC_002745.2|	1920608 -	U	3	7	6	5	4	SArRNA09
gi|29165615|ref|NC_002745.2|	1920609 -	G	4	2	3	2	2	SArRNA09
gi|29165615|ref|NC_002745.2|	1920610 -	C	9	2	1	1	2	SArRNA09
gi|29165615|ref|NC_002745.2|	1920611 -	G	0	2	0	2	0	SArRNA09
gi|29165615|ref|NC_002745.2|	1920612 -	G	1	1	0	0	0	SArRNA09
gi|29165615|ref|NC_002745.2|	1920613 -	G	1	1	0	0	0	SArRNA09
gi|29165615|ref|NC_002745.2|	1920614 -	A	6	4	3	2	4	SArRNA09
gi|29165615|ref|NC_002745.2|	1920615 -	A	9	9	2	2	9	SArRNA09
gi|29165615|ref|NC_002745.2|	1920616 -	U	2	6	0	0	7	SArRNA09
gi|29165615|ref|NC_002745.2|	1920617 -	C	2	2	2	1	3	SArRNA09
gi|29165615|ref|NC_002745.2|	1920618 -	U	1	0	1	0	1	SArRNA09
gi|29165615|ref|NC_002745.2|	1920619 -	U	0	1	0	0	1	SArRNA09
gi|29165615|ref|NC_002745.2|	1920620 -	G	6	5	5	3	8	SArRNA09
gi|29165615|ref|NC_002745.2|	1920621 -	C	11	9	2	2	8	SArRNA09
gi|29165615|ref|NC_002745.2|	1920622 -	G	3	2	0	0	2	SArRNA09
gi|29165615|ref|NC_002745.2|	1920623 -	A	9	2	0	1	1	SArRNA09
gi|29165615|ref|NC_002745.2|	1920624 -	G	2	4	2	2	4	SArRNA09
gi|29165615|ref|NC_002745.2|	1920625 -	A	8	3	8	3	4	SArRNA09
gi|29165615|ref|NC_002745.2|	1920626 -	G	3	7	6	2	3	SArRNA09
gi|29165615|ref|NC_002745.2|	1920627 -	G	1	1	0	1	0	SArRNA09
gi|29165615|ref|NC_002745.2|	1920628 -	A	2	2	1	0	6	SArRNA09
gi|29165615|ref|NC_002745.2|	1920629 -	U	6	7	0	4	3	SArRNA09
gi|29165615|ref|NC_002745.2|	1920630 -	G	12	12	4	10	9	SArRNA09
gi|29165615|ref|NC_002745.2|	1920631 -	G	1	1	1	3	1	SArRNA09
gi|29165615|ref|NC_002745.2|	1920632 -	U	4	6	1	2	2	SArRNA09
gi|29165615|ref|NC_002745.2|	1920633 -	A	6	11	6	3	19	SArRNA09
gi|29165615|ref|NC_002745.2|	1920634 -	A	9	7	2	2	7	SArRNA09
gi|29165615|ref|NC_002745.2|	1920635 -	C	13	7	4	4	4	SArRNA09
gi|29165615|ref|NC_002745.2|	1920636 -	A	5	2	3	2	3	SArRNA09
gi|29165615|ref|NC_002745.2|	1920637 -	G	5	5	7	4	15	SArRNA09
gi|29165615|ref|NC_002745.2|	1920638 -	G	7	3	2	1	2	SArRNA09
gi|29165615|ref|NC_002745.2|	1920639 -	U	8	2	2	0	2	SArRNA09
gi|29165615|ref|NC_002745.2|	1920640 -	U	4	4	1	3	8	SArRNA09
gi|29165615|ref|NC_002745.2|	1920641 -	U	2	3	1	5	6	SArRNA09
gi|29165615|ref|NC_002745.2|	1920642 -	C	2	4	2	3	2	SArRNA09
gi|29165615|ref|NC_002745.2|	1920643 -	C	8	6	4	6	7	SArRNA09
gi|29165615|ref|NC_002745.2|	1920644 -	U	0	1	1	2	1	SArRNA09
gi|29165615|ref|NC_002745.2|	1920645 -	G	2	1	0	0	0	SArRNA09
gi|29165615|ref|NC_002745.2|	1920646 -	U	5	2	0	2	1	SArRNA09
gi|29165615|ref|NC_002745.2|	1920647 -	U	2	3	2	2	2	SArRNA09
gi|29165615|ref|NC_002745.2|	1920648 -	A	1	2	0	0	1	SArRNA09
gi|29165615|ref|NC_002745.2|	1920649 -	G	3	2	1	0	3	SArRNA09
gi|29165615|ref|NC_002745.2|	1920650 -	G	2	1	1	1	0	SArRNA09
gi|29165615|ref|NC_002745.2|	1920651 -	U	4	2	3	5	3	SArRNA09
gi|29165615|ref|NC_002745.2|	1920652 -	G	6	2	1	1	4	SArRNA09
gi|29165615|ref|NC_002745.2|	1920653 -	U	8	8	2	3	14	SArRNA09
gi|29165615|ref|NC_002745.2|	1920654 -	C	3	7	1	3	3	SArRNA09
gi|29165615|ref|NC_002745.2|	1920655 -	G	1	0	0	1	3	SArRNA09
gi|29165615|ref|NC_002745.2|	1920656 -	A	50	62	23	20	59	SArRNA09
gi|29165615|ref|NC_002745.2|	1920657 -	A	65	83	39	25	103	SArRNA09
gi|29165615|ref|NC_002745.2|	1920658 -	G	98	83	32	40	98	SArRNA09
gi|29165615|ref|NC_002745.2|	1920659 -	C	108	115	57	58	139	SArRNA09
gi|29165615|ref|NC_002745.2|	1920660 -	C	72	67	34	25	60	SArRNA09
gi|29165615|ref|NC_002745.2|	1920661 -	A	51	46	28	20	53	SArRNA09
gi|29165615|ref|NC_002745.2|	1920662 -	U	50	61	20	20	44	SArRNA09
gi|29165615|ref|NC_002745.2|	1920663 -	U	72	54	42	27	87	SArRNA09
gi|29165615|ref|NC_002745.2|	1920664 -	A	165	81	41	33	108	SArRNA09
gi|29165615|ref|NC_002745.2|	1920665 -	U	451	92	51	40	143	SArRNA09
gi|29165615|ref|NC_002745.2|	1920666 -	A	128	116	74	50	179	SArRNA09
gi|29165615|ref|NC_002745.2|	1920667 -	C	157	127	50	37	137	SArRNA09
gi|29165615|ref|NC_002745.2|	1920668 -	A	69	59	47	26	106	SArRNA09
gi|29165615|ref|NC_002745.2|	1920669 -	A	49	65	40	20	72	SArRNA09
gi|29165615|ref|NC_002745.2|	1920670 -	A	38	44	30	19	55	SArRNA09
gi|29165615|ref|NC_002745.2|	1920671 -	U	51	60	37	19	78	SArRNA09
gi|29165615|ref|NC_002745.2|	1920672 -	C	71	65	39	32	107	SArRNA09
gi|29165615|ref|NC_002745.2|	1920673 -	G	5	13	6	2	17	SArRNA09
gi|29165615|ref|NC_002745.2|	1920674 -	G	11	4	4	3	6	SArRNA09
gi|29165615|ref|NC_002745.2|	1920675 -	G	10	15	4	4	9	SArRNA09
gi|29165615|ref|NC_002745.2|	1920676 -	G	92	142	34	28	133	SArRNA09
gi|29165615|ref|NC_002745.2|	1920677 -	C	227	264	51	55	276	SArRNA09

gi|29165615|ref|NC_002745.2|	1920678	-	C	74	75	19	24	94	SArRNA09
gi|29165615|ref|NC_002745.2|	1920679	-	A	50	43	21	13	40	SArRNA09
gi|29165615|ref|NC_002745.2|	1920680	-	U	39	69	45	57	100	SArRNA09
gi|29165615|ref|NC_002745.2|	1920681	-	G	81	138	62	54	122	SArRNA09
gi|29165615|ref|NC_002745.2|	1920682	-	U	120	152	77	66	164	SArRNA09
gi|29165615|ref|NC_002745.2|	1920683	-	A	109	188	71	53	193	SArRNA09
gi|29165615|ref|NC_002745.2|	1920684	-	A	108	87	66	48	89	SArRNA09
gi|29165615|ref|NC_002745.2|	1920685	-	A	59	57	29	25	81	SArRNA09
gi|29165615|ref|NC_002745.2|	1920686	-	A	30	45	26	16	52	SArRNA09
gi|29165615|ref|NC_002745.2|	1920687	-	G	34	51	23	27	49	SArRNA09
gi|29165615|ref|NC_002745.2|	1920688	-	C	49	32	22	17	66	SArRNA09
gi|29165615|ref|NC_002745.2|	1920689	-	C	22	29	20	11	44	SArRNA09
gi|29165615|ref|NC_002745.2|	1920690	-	G	13	9	8	8	14	SArRNA09
gi|29165615|ref|NC_002745.2|	1920691	-	C	44	42	28	23	24	SArRNA09
gi|29165615|ref|NC_002745.2|	1920692	-	G	13	11	1	0	3	SArRNA09
gi|29165615|ref|NC_002745.2|	1920693	-	U	31	50	17	11	42	SArRNA09
gi|29165615|ref|NC_002745.2|	1920694	-	C	47	58	26	20	82	SArRNA09
gi|29165615|ref|NC_002745.2|	1920695	-	A	22	25	22	13	42	SArRNA09
gi|29165615|ref|NC_002745.2|	1920696	-	C	125	92	45	43	112	SArRNA09
gi|29165615|ref|NC_002745.2|	1920697	-	A	71	47	15	30	41	SArRNA09
gi|29165615|ref|NC_002745.2|	1920698	-	G	27	34	24	22	46	SArRNA09
gi|29165615|ref|NC_002745.2|	1920699	-	U	68	65	38	22	72	SArRNA09
gi|29165615|ref|NC_002745.2|	1920700	-	G	67	93	42	36	113	SArRNA09
gi|29165615|ref|NC_002745.2|	1920701	-	A	104	102	61	36	146	SArRNA09
gi|29165615|ref|NC_002745.2|	1920702	-	G	152	167	88	92	208	SArRNA09
gi|29165615|ref|NC_002745.2|	1920703	-	C	714	707	287	514	634	SArRNA09
gi|29165615|ref|NC_002745.2|	1920704	-	U	98	58	35	23	99	SArRNA09
gi|29165615|ref|NC_002745.2|	1920705	-	G	45	43	23	22	59	SArRNA09
gi|29165615|ref|NC_002745.2|	1920706	-	A	43	49	22	25	52	SArRNA09
gi|29165615|ref|NC_002745.2|	1920707	-	U	183	142	99	218	363	SArRNA09
gi|29165615|ref|NC_002745.2|	1920708	-	C	183	242	98	89	164	SArRNA09
gi|29165615|ref|NC_002745.2|	1920709	-	A	48	43	29	28	87	SArRNA09
gi|29165615|ref|NC_002745.2|	1920710	-	C	87	84	32	26	85	SArRNA09
gi|29165615|ref|NC_002745.2|	1920711	-	U	40	38	23	24	66	SArRNA09
gi|29165615|ref|NC_002745.2|	1920712	-	C	71	59	30	18	101	SArRNA09
gi|29165615|ref|NC_002745.2|	1920713	-	G	11	15	4	3	13	SArRNA09
gi|29165615|ref|NC_002745.2|	1920714	-	A	19	54	12	10	38	SArRNA09
gi|29165615|ref|NC_002745.2|	1920715	-	U	65	140	87	69	205	SArRNA09
gi|29165615|ref|NC_002745.2|	1920716	-	A	114	368	68	46	279	SArRNA09
gi|29165615|ref|NC_002745.2|	1920717	-	A	38	71	30	22	65	SArRNA09
gi|29165615|ref|NC_002745.2|	1920718	-	U	90	91	54	36	120	SArRNA09
gi|29165615|ref|NC_002745.2|	1920719	-	G	57	74	31	28	120	SArRNA09
gi|29165615|ref|NC_002745.2|	1920720	-	C	164	110	73	88	190	SArRNA09
gi|29165615|ref|NC_002745.2|	1920721	-	G	40	29	13	21	40	SArRNA09
gi|29165615|ref|NC_002745.2|	1920722	-	U	33	25	11	16	63	SArRNA09
gi|29165615|ref|NC_002745.2|	1920723	-	G	30	19	14	19	60	SArRNA09
gi|29165615|ref|NC_002745.2|	1920724	-	A	89	69	48	69	132	SArRNA09
gi|29165615|ref|NC_002745.2|	1920725	-	G	958	595	533	1258	2364	SArRNA09
gi|29165615|ref|NC_002745.2|	1920726	-	A	108	82	64	88	131	SArRNA09
gi|29165615|ref|NC_002745.2|	1920727	-	A	119	89	59	47	171	SArRNA09
gi|29165615|ref|NC_002745.2|	1920728	-	A	132	84	45	53	155	SArRNA09
gi|29165615|ref|NC_002745.2|	1920729	-	U	128	100	60	49	194	SArRNA09
gi|29165615|ref|NC_002745.2|	1920730	-	U	109	76	58	42	179	SArRNA09
gi|29165615|ref|NC_002745.2|	1920731	-	U	123	111	62	62	227	SArRNA09
gi|29165615|ref|NC_002745.2|	1920732	-	A	223	204	132	106	364	SArRNA09
gi|29165615|ref|NC_002745.2|	1920733	-	C	484	386	226	169	424	SArRNA09
gi|29165615|ref|NC_002745.2|	1920734	-	U	89	54	26	31	123	SArRNA09
gi|29165615|ref|NC_002745.2|	1920735	-	A	106	121	66	55	205	SArRNA09
gi|29165615|ref|NC_002745.2|	1920736	-	C	288	240	164	158	315	SArRNA09
gi|29165615|ref|NC_002745.2|	1920737	-	C	131	123	68	94	186	SArRNA09
gi|29165615|ref|NC_002745.2|	1920738	-	G	57	35	27	13	65	SArRNA09
gi|29165615|ref|NC_002745.2|	1920739	-	A	144	176	47	57	134	SArRNA09
gi|29165615|ref|NC_002745.2|	1920740	-	C	400	533	245	246	467	SArRNA09
gi|29165615|ref|NC_002745.2|	1920741	-	G	55	48	18	15	45	SArRNA09
gi|29165615|ref|NC_002745.2|	1920742	-	A	58	64	19	22	68	SArRNA09
gi|29165615|ref|NC_002745.2|	1920743	-	A	13	34	18	6	27	SArRNA09
gi|29165615|ref|NC_002745.2|	1920744	-	G	312	403	146	169	330	SArRNA09
gi|29165615|ref|NC_002745.2|	1920745	-	A	66	55	26	38	118	SArRNA09
gi|29165615|ref|NC_002745.2|	1920746	-	U	67	75	49	40	144	SArRNA09
gi|29165615|ref|NC_002745.2|	1920747	-	U	80	39	34	25	119	SArRNA09
gi|29165615|ref|NC_002745.2|	1920748	-	C	91	83	47	52	184	SArRNA09
gi|29165615|ref|NC_002745.2|	1920749	-	G	41	26	12	27	46	SArRNA09
gi|29165615|ref|NC_002745.2|	1920750	-	G	6	9	5	3	6	SArRNA09
gi|29165615|ref|NC_002745.2|	1920751	-	U	34	35	25	39	50	SArRNA09
gi|29165615|ref|NC_002745.2|	1920752	-	U	51	34	32	28	91	SArRNA09
gi|29165615|ref|NC_002745.2|	1920753	-	G	45	7	14	8	30	SArRNA09
gi|29165615|ref|NC_002745.2|	1920754	-	U	51	24	23	24	33	SArRNA09
gi|29165615|ref|NC_002745.2|	1920755	-	A	34	19	11	11	32	SArRNA09
gi|29165615|ref|NC_002745.2|	1920756	-	G	6	19	8	9	20	SArRNA09
gi|29165615|ref|NC_002745.2|	1920757	-	G	5	7	8	8	20	SArRNA09
gi|29165615|ref|NC_002745.2|	1920758	-	A	323	56	22	36	74	SArRNA09

gi|29165615|ref|NC_002745.2|	1920759	-	U	1890	212	137	143	279	SArRNA09
gi|29165615|ref|NC_002745.2|	1920760	-	C	1250	580	338	329	760	SArRNA09
gi|29165615|ref|NC_002745.2|	1920761	-	A	250	159	42	34	140	SArRNA09
gi|29165615|ref|NC_002745.2|	1920762	-	A	259	111	62	77	176	SArRNA09
gi|29165615|ref|NC_002745.2|	1920763	-	C	1002	196	135	151	410	SArRNA09
gi|29165615|ref|NC_002745.2|	1920764	-	A	93	50	55	45	161	SArRNA09
gi|29165615|ref|NC_002745.2|	1920765	-	G	128	91	65	62	160	SArRNA09
gi|29165615|ref|NC_002745.2|	1920766	-	A	207	145	108	111	287	SArRNA09
gi|29165615|ref|NC_002745.2|	1920767	-	C	478	302	227	232	493	SArRNA09
gi|29165615|ref|NC_002745.2|	1920768	-	C	291	175	105	101	338	SArRNA09
gi|29165615|ref|NC_002745.2|	1920769	-	C	83	86	40	48	127	SArRNA09
gi|29165615|ref|NC_002745.2|	1920770	-	G	28	17	5	17	31	SArRNA09
gi|29165615|ref|NC_002745.2|	1920771	-	U	40	55	18	15	60	SArRNA09
gi|29165615|ref|NC_002745.2|	1920772	-	U	123	87	49	46	98	SArRNA09
gi|29165615|ref|NC_002745.2|	1920773	-	G	514	62	46	36	78	SArRNA09
gi|29165615|ref|NC_002745.2|	1920774	-	C	9042	427	333	314	477	SArRNA09
gi|29165615|ref|NC_002745.2|	1920775	-	G	144	12	8	5	19	SArRNA09
gi|29165615|ref|NC_002745.2|	1920776	-	G	63	17	14	22	36	SArRNA09
gi|29165615|ref|NC_002745.2|	1920777	-	U	1319	440	275	257	685	SArRNA09
gi|29165615|ref|NC_002745.2|	1920778	-	G	245	153	120	106	285	SArRNA09
gi|29165615|ref|NC_002745.2|	1920779	-	U	717	191	136	111	314	SArRNA09
gi|29165615|ref|NC_002745.2|	1920780	-	A	538	210	182	157	427	SArRNA09
gi|29165615|ref|NC_002745.2|	1920781	-	G	395	246	145	169	495	SArRNA09
gi|29165615|ref|NC_002745.2|	1920782	-	G	177	115	76	93	186	SArRNA09
gi|29165615|ref|NC_002745.2|	1920783	-	A	320	188	124	117	334	SArRNA09
gi|29165615|ref|NC_002745.2|	1920784	-	A	265	242	134	141	310	SArRNA09
gi|29165615|ref|NC_002745.2|	1920785	-	A	245	213	109	125	263	SArRNA09
gi|29165615|ref|NC_002745.2|	1920786	-	A	400	341	169	198	418	SArRNA09
gi|29165615|ref|NC_002745.2|	1920787	-	G	454	345	188	206	505	SArRNA09
gi|29165615|ref|NC_002745.2|	1920788	-	G	800	708	397	544	894	SArRNA09
gi|29165615|ref|NC_002745.2|	1920789	-	U	751	572	298	316	655	SArRNA09
gi|29165615|ref|NC_002745.2|	1920790	-	G	1404	969	490	728	1118	SArRNA09
gi|29165615|ref|NC_002745.2|	1920791	-	A	490	350	197	251	525	SArRNA09
gi|29165615|ref|NC_002745.2|	1920792	-	A	552	445	228	247	466	SArRNA09
gi|29165615|ref|NC_002745.2|	1920793	-	U	671	528	253	331	640	SArRNA09
gi|29165615|ref|NC_002745.2|	1920794	-	U	388	283	211	187	495	SArRNA09
gi|29165615|ref|NC_002745.2|	1920795	-	G	452	318	196	200	533	SArRNA09
gi|29165615|ref|NC_002745.2|	1920796	-	U	565	283	201	177	472	SArRNA09
gi|29165615|ref|NC_002745.2|	1920797	-	A	441	287	188	192	435	SArRNA09
gi|29165615|ref|NC_002745.2|	1920798	-	U	817	637	329	313	600	SArRNA09
gi|29165615|ref|NC_002745.2|	1920799	-	A	416	426	186	206	426	SArRNA09
gi|29165615|ref|NC_002745.2|	1920800	-	U	446	805	517	339	851	SArRNA09
gi|29165615|ref|NC_002745.2|	1920801	-	A	455	1097	359	183	825	SArRNA09
gi|29165615|ref|NC_002745.2|	1920802	-	A	351	227	130	125	338	SArRNA09
gi|29165615|ref|NC_002745.2|	1920803	-	A	413	257	132	115	304	SArRNA09
gi|29165615|ref|NC_002745.2|	1920804	-	A	254	167	90	111	256	SArRNA09
gi|29165615|ref|NC_002745.2|	1920805	-	C	235	174	87	84	271	SArRNA09
gi|29165615|ref|NC_002745.2|	1920806	-	C	354	282	165	135	263	SArRNA09
gi|29165615|ref|NC_002745.2|	1920807	-	C	531	407	230	198	374	SArRNA09
gi|29165615|ref|NC_002745.2|	1920808	-	U	284	209	117	109	284	SArRNA09
gi|29165615|ref|NC_002745.2|	1920809	-	G	301	266	136	154	397	SArRNA09
gi|29165615|ref|NC_002745.2|	1920810	-	G	316	222	99	123	330	SArRNA09
gi|29165615|ref|NC_002745.2|	1920811	-	A	316	214	114	147	300	SArRNA09
gi|29165615|ref|NC_002745.2|	1920812	-	A	451	230	97	108	264	SArRNA09
gi|29165615|ref|NC_002745.2|	1920813	-	U	1706	176	135	143	249	SArRNA09
gi|29165615|ref|NC_002745.2|	1920814	-	C	805	166	161	130	350	SArRNA09
gi|29165615|ref|NC_002745.2|	1920815	-	G	108	56	11	31	74	SArRNA09
gi|29165615|ref|NC_002745.2|	1920816	-	A	126	104	37	53	62	SArRNA09
gi|29165615|ref|NC_002745.2|	1920817	-	C	417	292	162	134	193	SArRNA09
gi|29165615|ref|NC_002745.2|	1920818	-	C	189	123	52	46	97	SArRNA09
gi|29165615|ref|NC_002745.2|	1920819	-	A	238	163	81	69	107	SArRNA09
gi|29165615|ref|NC_002745.2|	1920820	-	C	915	512	368	299	530	SArRNA09
gi|29165615|ref|NC_002745.2|	1920821	-	C	353	220	136	125	196	SArRNA09
gi|29165615|ref|NC_002745.2|	1920822	-	A	15	20	5	8	15	SArRNA09
gi|29165615|ref|NC_002745.2|	1920823	-	G	19	6	5	9	13	SArRNA09
gi|29165615|ref|NC_002745.2|	1920824	-	A	189	80	50	34	62	SArRNA09
gi|29165615|ref|NC_002745.2|	1920825	-	C	374	180	91	89	113	SArRNA09
gi|29165615|ref|NC_002745.2|	1920826	-	C	132	153	48	46	66	SArRNA09
gi|29165615|ref|NC_002745.2|	1920827	-	C	145	145	51	48	65	SArRNA09
gi|29165615|ref|NC_002745.2|	1920828	-	G	4	4	3	2	1	SArRNA09
gi|29165615|ref|NC_002745.2|	1920829	-	A	22	33	9	5	21	SArRNA09
gi|29165615|ref|NC_002745.2|	1920830	-	C	164	171	49	80	110	SArRNA09
gi|29165615|ref|NC_002745.2|	1920831	-	A	23	20	3	7	15	SArRNA09
gi|29165615|ref|NC_002745.2|	1920832	-	A	10	13	6	8	12	SArRNA09
gi|29165615|ref|NC_002745.2|	1920833	-	A	11	4	0	2	7	SArRNA09
gi|29165615|ref|NC_002745.2|	1920834	-	G	11	27	7	8	3	SArRNA09
gi|29165615|ref|NC_002745.2|	1920835	-	G	17	26	4	7	7	SArRNA09
gi|29165615|ref|NC_002745.2|	1920836	-	G	14	9	3	10	9	SArRNA09
gi|29165615|ref|NC_002745.2|	1920837	-	A	51	46	14	17	29	SArRNA09
gi|29165615|ref|NC_002745.2|	1920838	-	A	49	72	19	22	37	SArRNA09
gi|29165615|ref|NC_002745.2|	1920839	-	A	54	36	14	24	27	SArRNA09

gi|29165615|ref|NC_002745.2|	1920840 -	G	44	43	24	30	41	SArRNA09
gi|29165615|ref|NC_002745.2|	1920841 -	C	62	34	15	19	38	SArRNA09
gi|29165615|ref|NC_002745.2|	1920842 -	U	69	56	32	55	44	SArRNA09
gi|29165615|ref|NC_002745.2|	1920843 -	U	50	43	19	29	43	SArRNA09
gi|29165615|ref|NC_002745.2|	1920844 -	G	12	13	6	10	10	SArRNA09
gi|29165615|ref|NC_002745.2|	1920845 -	U	28	28	15	25	35	SArRNA09
gi|29165615|ref|NC_002745.2|	1920846 -	G	29	43	17	17	13	SArRNA09
gi|29165615|ref|NC_002745.2|	1920847 -	C	171	102	67	60	90	SArRNA09
gi|29165615|ref|NC_002745.2|	1920848 -	C	57	39	9	5	36	SArRNA09
gi|29165615|ref|NC_002745.2|	1920849 -	U	9	13	4	2	12	SArRNA09
gi|29165615|ref|NC_002745.2|	1920850 -	G	18	16	1	11	16	SArRNA09
gi|29165615|ref|NC_002745.2|	1920851 -	G	26	25	12	13	24	SArRNA09
gi|29165615|ref|NC_002745.2|	1920852 -	A	38	36	16	28	43	SArRNA09
gi|29165615|ref|NC_002745.2|	1920853 -	A	49	56	18	26	54	SArRNA09
gi|29165615|ref|NC_002745.2|	1920854 -	U	76	85	26	43	84	SArRNA09
gi|29165615|ref|NC_002745.2|	1920855 -	A	190	128	85	78	198	SArRNA09
gi|29165615|ref|NC_002745.2|	1920856 -	G	143	87	53	61	112	SArRNA09
gi|29165615|ref|NC_002745.2|	1920857 -	U	253	139	73	96	190	SArRNA09
gi|29165615|ref|NC_002745.2|	1920858 -	G	144	111	57	53	139	SArRNA09
gi|29165615|ref|NC_002745.2|	1920859 -	G	148	91	46	69	139	SArRNA09
gi|29165615|ref|NC_002745.2|	1920860 -	G	23	15	6	14	13	SArRNA09
gi|29165615|ref|NC_002745.2|	1920861 -	U	48	50	13	16	17	SArRNA09
gi|29165615|ref|NC_002745.2|	1920862 -	A	26	44	9	16	19	SArRNA09
gi|29165615|ref|NC_002745.2|	1920863 -	C	70	73	44	31	41	SArRNA09
gi|29165615|ref|NC_002745.2|	1920864 -	A	104	63	21	36	56	SArRNA09
gi|29165615|ref|NC_002745.2|	1920865 -	A	173	70	32	65	104	SArRNA09
gi|29165615|ref|NC_002745.2|	1920866 -	G	14	14	7	1	12	SArRNA09
gi|29165615|ref|NC_002745.2|	1920867 -	A	367	270	111	162	180	SArRNA09
gi|29165615|ref|NC_002745.2|	1920868 -	C	1680	1188	549	620	777	SArRNA09
gi|29165615|ref|NC_002745.2|	1920869 -	U	287	205	80	83	128	SArRNA09
gi|29165615|ref|NC_002745.2|	1920870 -	G	60	34	22	22	47	SArRNA09
gi|29165615|ref|NC_002745.2|	1920871 -	A	54	36	13	18	53	SArRNA09
gi|29165615|ref|NC_002745.2|	1920872 -	G	170	112	69	63	74	SArRNA09
gi|29165615|ref|NC_002745.2|	1920873 -	G	104	64	38	46	50	SArRNA09
gi|29165615|ref|NC_002745.2|	1920874 -	G	13	9	5	13	12	SArRNA09
gi|29165615|ref|NC_002745.2|	1920875 -	U	12	11	5	10	18	SArRNA09
gi|29165615|ref|NC_002745.2|	1920876 -	U	33	26	10	16	38	SArRNA09
gi|29165615|ref|NC_002745.2|	1920877 -	C	125	113	39	59	55	SArRNA09
gi|29165615|ref|NC_002745.2|	1920878 -	A	29	28	11	16	24	SArRNA09
gi|29165615|ref|NC_002745.2|	1920879 -	A	20	19	11	12	19	SArRNA09
gi|29165615|ref|NC_002745.2|	1920880 -	U	59	53	17	22	37	SArRNA09
gi|29165615|ref|NC_002745.2|	1920881 -	U	30	24	21	19	45	SArRNA09
gi|29165615|ref|NC_002745.2|	1920882 -	U	60	38	16	34	49	SArRNA09
gi|29165615|ref|NC_002745.2|	1920883 -	A	115	103	43	48	94	SArRNA09
gi|29165615|ref|NC_002745.2|	1920884 -	A	72	55	20	31	55	SArRNA09
gi|29165615|ref|NC_002745.2|	1920885 -	U	81	62	27	30	69	SArRNA09
gi|29165615|ref|NC_002745.2|	1920886 -	U	40	30	25	38	53	SArRNA09
gi|29165615|ref|NC_002745.2|	1920887 -	A	52	24	13	20	35	SArRNA09
gi|29165615|ref|NC_002745.2|	1920888 -	A	83	78	34	37	67	SArRNA09
gi|29165615|ref|NC_002745.2|	1920889 -	C	409	436	175	254	197	SArRNA09
gi|29165615|ref|NC_002745.2|	1920890 -	C	52	38	11	19	26	SArRNA09
gi|29165615|ref|NC_002745.2|	1920891 -	G	20	11	2	4	10	SArRNA09
gi|29165615|ref|NC_002745.2|	1920892 -	U	47	47	21	22	39	SArRNA09
gi|29165615|ref|NC_002745.2|	1920893 -	A	236	237	84	150	159	SArRNA09
gi|29165615|ref|NC_002745.2|	1920894 -	A	569	466	239	312	316	SArRNA09
gi|29165615|ref|NC_002745.2|	1920895 -	G	178	149	57	78	119	SArRNA09
gi|29165615|ref|NC_002745.2|	1920896 -	C	211	145	53	72	109	SArRNA09
gi|29165615|ref|NC_002745.2|	1920897 -	C	100	47	18	34	41	SArRNA09
gi|29165615|ref|NC_002745.2|	1920898 -	U	64	59	26	42	55	SArRNA09
gi|29165615|ref|NC_002745.2|	1920899 -	C	219	234	101	139	100	SArRNA09
gi|29165615|ref|NC_002745.2|	1920900 -	A	69	59	23	43	50	SArRNA09
gi|29165615|ref|NC_002745.2|	1920901 -	A	36	43	13	20	35	SArRNA09
gi|29165615|ref|NC_002745.2|	1920902 -	A	30	36	8	18	22	SArRNA09
gi|29165615|ref|NC_002745.2|	1920903 -	C	113	113	69	57	51	SArRNA09
gi|29165615|ref|NC_002745.2|	1920904 -	A	5	11	7	8	16	SArRNA09
gi|29165615|ref|NC_002745.2|	1920905 -	G	9	14	6	6	6	SArRNA09
gi|29165615|ref|NC_002745.2|	1920906 -	A	54	37	15	19	29	SArRNA09
gi|29165615|ref|NC_002745.2|	1920907 -	C	131	96	60	51	59	SArRNA09
gi|29165615|ref|NC_002745.2|	1920908 -	U	15	21	9	12	13	SArRNA09
gi|29165615|ref|NC_002745.2|	1920909 -	U	17	19	6	10	10	SArRNA09
gi|29165615|ref|NC_002745.2|	1920910 -	A	23	31	13	13	18	SArRNA09
gi|29165615|ref|NC_002745.2|	1920911 -	A	62	57	19	19	36	SArRNA09
gi|29165615|ref|NC_002745.2|	1920912 -	G	21	16	0	11	17	SArRNA09
gi|29165615|ref|NC_002745.2|	1920913 -	C	45	17	10	7	32	SArRNA09
gi|29165615|ref|NC_002745.2|	1920914 -	C	23	8	1	8	12	SArRNA09
gi|29165615|ref|NC_002745.2|	1920915 -	A	14	6	1	6	8	SArRNA09
gi|29165615|ref|NC_002745.2|	1920916 -	U	33	42	13	18	25	SArRNA09
gi|29165615|ref|NC_002745.2|	1920917 -	U	10	12	4	6	4	SArRNA09
gi|29165615|ref|NC_002745.2|	1920918 -	G	4	3	1	2	4	SArRNA09
gi|29165615|ref|NC_002745.2|	1920919 -	G	4	2	0	2	4	SArRNA09
gi|29165615|ref|NC_002745.2|	1920920 -	G	88	113	52	47	56	SArRNA09

gi|29165615|ref|NC_002745.2|	1920921	-	C	558	587	222	330	303	SArRNA09
gi|29165615|ref|NC_002745.2|	1920922	-	U	10	8	3	10	14	SArRNA09
gi|29165615|ref|NC_002745.2|	1920923	-	C	39	29	11	20	19	SArRNA09
gi|29165615|ref|NC_002745.2|	1920924	-	U	35	29	16	10	37	SArRNA09
gi|29165615|ref|NC_002745.2|	1920925	-	C	52	47	25	18	37	SArRNA09
gi|29165615|ref|NC_002745.2|	1920926	-	C	93	50	18	30	30	SArRNA09
gi|29165615|ref|NC_002745.2|	1920927	-	C	216	140	65	85	93	SArRNA09
gi|29165615|ref|NC_002745.2|	1920928	-	C	636	397	286	203	277	SArRNA09
gi|29165615|ref|NC_002745.2|	1920929	-	G	4	2	0	1	3	SArRNA09
gi|29165615|ref|NC_002745.2|	1920930	-	G	1	3	0	0	1	SArRNA09
gi|29165615|ref|NC_002745.2|	1920931	-	G	2	2	0	0	1	SArRNA09
gi|29165615|ref|NC_002745.2|	1920932	-	G	3	0	0	0	1	SArRNA09
gi|29165615|ref|NC_002745.2|	1920933	-	A	5	3	1	0	3	SArRNA09
gi|29165615|ref|NC_002745.2|	1920934	-	G	2	1	0	2	2	SArRNA09
gi|29165615|ref|NC_002745.2|	1920935	-	C	6	2	3	3	4	SArRNA09
gi|29165615|ref|NC_002745.2|	1920936	-	A	7	1	0	4	2	SArRNA09
gi|29165615|ref|NC_002745.2|	1920937	-	G	1	2	0	0	0	SArRNA09
gi|29165615|ref|NC_002745.2|	1920938	-	G	1	0	2	3	0	SArRNA09
gi|29165615|ref|NC_002745.2|	1920939	-	U	4	2	1	3	3	SArRNA09
gi|29165615|ref|NC_002745.2|	1920940	-	U	1	4	0	1	2	SArRNA09
gi|29165615|ref|NC_002745.2|	1920941	-	U	8	9	3	2	8	SArRNA09
gi|29165615|ref|NC_002745.2|	1920942	-	G	1	0	0	0	0	SArRNA09
gi|29165615|ref|NC_002745.2|	1920943	-	U	5	5	1	1	2	SArRNA09
gi|29165615|ref|NC_002745.2|	1920944	-	C	35	38	14	10	16	SArRNA09
gi|29165615|ref|NC_002745.2|	1920945	-	A	31	11	2	13	14	SArRNA09
gi|29165615|ref|NC_002745.2|	1920946	-	C	76	36	13	13	20	SArRNA09
gi|29165615|ref|NC_002745.2|	1920947	-	G	6	4	4	4	11	SArRNA09
gi|29165615|ref|NC_002745.2|	1920948	-	A	10	11	3	11	5	SArRNA09
gi|29165615|ref|NC_002745.2|	1920949	-	G	23	27	9	9	8	SArRNA09
gi|29165615|ref|NC_002745.2|	1920950	-	A	3	24	2	7	11	SArRNA09
gi|29165615|ref|NC_002745.2|	1920951	-	U	18	16	1	6	15	SArRNA09
gi|29165615|ref|NC_002745.2|	1920952	-	G	14	8	5	5	6	SArRNA09
gi|29165615|ref|NC_002745.2|	1920953	-	G	5	2	0	1	3	SArRNA09
gi|29165615|ref|NC_002745.2|	1920954	-	A	21	12	3	10	10	SArRNA09
gi|29165615|ref|NC_002745.2|	1920955	-	G	11	11	4	8	4	SArRNA09
gi|29165615|ref|NC_002745.2|	1920956	-	G	1	3	1	3	6	SArRNA09
gi|29165615|ref|NC_002745.2|	1920957	-	U	17	6	5	6	9	SArRNA09
gi|29165615|ref|NC_002745.2|	1920958	-	U	19	5	3	5	8	SArRNA09
gi|29165615|ref|NC_002745.2|	1920959	-	A	9	8	6	2	4	SArRNA09
gi|29165615|ref|NC_002745.2|	1920960	-	U	32	67	24	21	19	SArRNA09
gi|29165615|ref|NC_002745.2|	1920961	-	U	10	10	1	10	8	SArRNA09
gi|29165615|ref|NC_002745.2|	1920962	-	A	3	8	2	2	7	SArRNA09
gi|29165615|ref|NC_002745.2|	1920963	-	G	12	20	6	2	6	SArRNA09
gi|29165615|ref|NC_002745.2|	1920964	-	U	10	18	7	12	15	SArRNA09
gi|29165615|ref|NC_002745.2|	1920965	-	A	19	8	5	4	8	SArRNA09
gi|29165615|ref|NC_002745.2|	1920966	-	G	87	90	43	38	48	SArRNA09
gi|29165615|ref|NC_002745.2|	1920967	-	U	6	5	3	2	7	SArRNA09
gi|29165615|ref|NC_002745.2|	1920968	-	G	5	1	2	5	8	SArRNA09
gi|29165615|ref|NC_002745.2|	1920969	-	A	9	15	3	4	6	SArRNA09
gi|29165615|ref|NC_002745.2|	1920970	-	A	16	17	5	5	9	SArRNA09
gi|29165615|ref|NC_002745.2|	1920971	-	C	65	72	36	25	22	SArRNA09
gi|29165615|ref|NC_002745.2|	1920972	-	U	5	3	1	0	1	SArRNA09
gi|29165615|ref|NC_002745.2|	1920973	-	C	5	7	3	2	2	SArRNA09
gi|29165615|ref|NC_002745.2|	1920974	-	C	17	19	11	10	7	SArRNA09
gi|29165615|ref|NC_002745.2|	1920975	-	G	11	10	2	3	5	SArRNA09
gi|29165615|ref|NC_002745.2|	1920976	-	A	6	8	1	1	4	SArRNA09
gi|29165615|ref|NC_002745.2|	1920977	-	U	7	18	1	4	3	SArRNA09
gi|29165615|ref|NC_002745.2|	1920978	-	C	73	29	10	10	17	SArRNA09
gi|29165615|ref|NC_002745.2|	1920979	-	G	6	8	0	3	4	SArRNA09
gi|29165615|ref|NC_002745.2|	1920980	-	G	1	1	1	1	4	SArRNA09
gi|29165615|ref|NC_002745.2|	1920981	-	G	2	1	5	1	2	SArRNA09
gi|29165615|ref|NC_002745.2|	1920982	-	A	7	12	1	0	7	SArRNA09
gi|29165615|ref|NC_002745.2|	1920983	-	U	9	14	3	5	11	SArRNA09
gi|29165615|ref|NC_002745.2|	1920984	-	U	7	9	5	4	4	SArRNA09
gi|29165615|ref|NC_002745.2|	1920985	-	U	13	7	5	6	10	SArRNA09
gi|29165615|ref|NC_002745.2|	1920986	-	C	28	19	4	11	14	SArRNA09
gi|29165615|ref|NC_002745.2|	1920987	-	G	7	6	4	4	3	SArRNA09
gi|29165615|ref|NC_002745.2|	1920988	-	A	7	7	6	3	2	SArRNA09
gi|29165615|ref|NC_002745.2|	1920989	-	U	9	9	4	6	9	SArRNA09
gi|29165615|ref|NC_002745.2|	1920990	-	A	23	204	51	17	63	SArRNA09
gi|29165615|ref|NC_002745.2|	1920991	-	A	6	7	1	3	7	SArRNA09
gi|29165615|ref|NC_002745.2|	1920992	-	A	6	7	1	2	3	SArRNA09
gi|29165615|ref|NC_002745.2|	1920993	-	G	1499	1071	669	908	645	SArRNA09
gi|29165615|ref|NC_002745.2|	1920994	-	C	8	9	3	4	6	SArRNA09
gi|29165615|ref|NC_002745.2|	1920995	-	C	4	5	0	0	2	SArRNA09
gi|29165615|ref|NC_002745.2|	1920996	-	U	13	9	2	4	6	SArRNA09
gi|29165615|ref|NC_002745.2|	1920997	-	C	35	39	14	22	20	SArRNA09
gi|29165615|ref|NC_002745.2|	1920998	-	U	3	5	1	5	1	SArRNA09
gi|29165615|ref|NC_002745.2|	1920999	-	C	6	9	6	3	9	SArRNA09
gi|29165615|ref|NC_002745.2|	1921000	-	U	2	4	1	0	2	SArRNA09
gi|29165615|ref|NC_002745.2|	1921001	-	U	7	0	0	2	2	SArRNA09

gi|29165615|ref|NC_002745.2|	1921002	-	G	7	1	1	1	1	SArRNA09
gi|29165615|ref|NC_002745.2|	1921003	-	G	3	1	0	0	1	SArRNA09
gi|29165615|ref|NC_002745.2|	1921004	-	U	4	6	1	1	3	SArRNA09
gi|29165615|ref|NC_002745.2|	1921005	-	C	7	14	2	1	8	SArRNA09
gi|29165615|ref|NC_002745.2|	1921006	-	G	2	0	1	1	1	SArRNA09
gi|29165615|ref|NC_002745.2|	1921007	-	A	5	0	1	2	3	SArRNA09
gi|29165615|ref|NC_002745.2|	1921008	-	U	12	19	13	13	18	SArRNA09
gi|29165615|ref|NC_002745.2|	1921009	-	A	23	20	4	7	14	SArRNA09
gi|29165615|ref|NC_002745.2|	1921010	-	G	11	13	5	3	9	SArRNA09
gi|29165615|ref|NC_002745.2|	1921011	-	A	10	3	4	2	3	SArRNA09
gi|29165615|ref|NC_002745.2|	1921012	-	G	8	3	0	1	7	SArRNA09
gi|29165615|ref|NC_002745.2|	1921013	-	G	6	6	5	7	0	SArRNA09
gi|29165615|ref|NC_002745.2|	1921014	-	U	10	9	1	4	3	SArRNA09
gi|29165615|ref|NC_002745.2|	1921015	-	C	15	10	6	3	7	SArRNA09
gi|29165615|ref|NC_002745.2|	1921016	-	C	11	6	6	3	3	SArRNA09
gi|29165615|ref|NC_002745.2|	1921017	-	A	4	6	2	3	9	SArRNA09
gi|29165615|ref|NC_002745.2|	1921018	-	A	18	22	3	5	3	SArRNA09
gi|29165615|ref|NC_002745.2|	1921019	-	G	11	11	3	10	6	SArRNA09
gi|29165615|ref|NC_002745.2|	1921020	-	C	7	15	2	3	4	SArRNA09
gi|29165615|ref|NC_002745.2|	1921021	-	U	3	3	0	0	1	SArRNA09
gi|29165615|ref|NC_002745.2|	1921022	-	A	8	54	6	5	10	SArRNA09
gi|29165615|ref|NC_002745.2|	1921023	-	A	31	40	15	8	14	SArRNA09
gi|29165615|ref|NC_002745.2|	1921024	-	C	127	100	35	49	54	SArRNA09
gi|29165615|ref|NC_002745.2|	1921025	-	C	18	26	4	1	10	SArRNA09
gi|29165615|ref|NC_002745.2|	1921026	-	U	7	4	0	1	5	SArRNA09
gi|29165615|ref|NC_002745.2|	1921027	-	U	7	10	5	10	4	SArRNA09
gi|29165615|ref|NC_002745.2|	1921028	-	A	25	125	24	11	35	SArRNA09
gi|29165615|ref|NC_002745.2|	1921029	-	A	16	17	5	1	8	SArRNA09
gi|29165615|ref|NC_002745.2|	1921030	-	A	7	4	2	3	9	SArRNA09
gi|29165615|ref|NC_002745.2|	1921031	-	G	3	6	2	2	1	SArRNA09
gi|29165615|ref|NC_002745.2|	1921032	-	A	6	1	1	0	5	SArRNA09
gi|29165615|ref|NC_002745.2|	1921033	-	G	4	1	1	4	4	SArRNA09
gi|29165615|ref|NC_002745.2|	1921034	-	G	1	12	3	3	4	SArRNA09
gi|29165615|ref|NC_002745.2|	1921035	-	C	13	10	7	2	5	SArRNA09
gi|29165615|ref|NC_002745.2|	1921036	-	G	0	0	0	1	1	SArRNA09
gi|29165615|ref|NC_002745.2|	1921037	-	A	1	1	0	0	4	SArRNA09
gi|29165615|ref|NC_002745.2|	1921038	-	U	4	6	3	2	3	SArRNA09
gi|29165615|ref|NC_002745.2|	1921039	-	G	10	7	4	3	8	SArRNA09
gi|29165615|ref|NC_002745.2|	1921040	-	G	1	0	0	1	1	SArRNA09
gi|29165615|ref|NC_002745.2|	1921042	-	A	6	3	1	1	5	SArRNA09
gi|29165615|ref|NC_002745.2|	1921043	-	G	1	5	1	0	3	SArRNA09
gi|29165615|ref|NC_002745.2|	1921044	-	U	32	43	7	10	12	SArRNA09
gi|29165615|ref|NC_002745.2|	1921045	-	C	41	30	8	12	27	SArRNA09
gi|29165615|ref|NC_002745.2|	1921046	-	A	31	34	14	23	17	SArRNA09
gi|29165615|ref|NC_002745.2|	1921047	-	A	108	108	34	42	62	SArRNA09
gi|29165615|ref|NC_002745.2|	1921048	-	G	46	49	16	20	30	SArRNA09
gi|29165615|ref|NC_002745.2|	1921049	-	U	23	16	9	8	13	SArRNA09
gi|29165615|ref|NC_002745.2|	1921050	-	A	16	16	8	7	16	SArRNA09
gi|29165615|ref|NC_002745.2|	1921051	-	G	11	15	6	4	7	SArRNA09
gi|29165615|ref|NC_002745.2|	1921052	-	G	54	48	14	27	33	SArRNA09
gi|29165615|ref|NC_002745.2|	1921053	-	C	147	96	45	43	57	SArRNA09
gi|29165615|ref|NC_002745.2|	1921054	-	G	5	8	2	0	4	SArRNA09
gi|29165615|ref|NC_002745.2|	1921055	-	A	12	16	9	13	18	SArRNA09
gi|29165615|ref|NC_002745.2|	1921056	-	G	9	20	18	22	17	SArRNA09
gi|29165615|ref|NC_002745.2|	1921057	-	U	36	68	27	57	43	SArRNA09
gi|29165615|ref|NC_002745.2|	1921058	-	G	7	14	2	9	14	SArRNA09
gi|29165615|ref|NC_002745.2|	1921059	-	A	14	15	8	6	12	SArRNA09
gi|29165615|ref|NC_002745.2|	1921060	-	A	19	31	14	16	29	SArRNA09
gi|29165615|ref|NC_002745.2|	1921061	-	A	7	6	5	5	7	SArRNA09
gi|29165615|ref|NC_002745.2|	1921062	-	A	10	8	4	3	10	SArRNA09
gi|29165615|ref|NC_002745.2|	1921063	-	G	10	8	2	9	12	SArRNA09
gi|29165615|ref|NC_002745.2|	1921064	-	U	30	28	5	20	24	SArRNA09
gi|29165615|ref|NC_002745.2|	1921065	-	U	16	7	3	3	15	SArRNA09
gi|29165615|ref|NC_002745.2|	1921066	-	G	13	18	7	13	17	SArRNA09
gi|29165615|ref|NC_002745.2|	1921067	-	C	45	34	24	29	37	SArRNA09
gi|29165615|ref|NC_002745.2|	1921068	-	A	12	14	0	4	8	SArRNA09
gi|29165615|ref|NC_002745.2|	1921069	-	U	28	25	11	14	25	SArRNA09
gi|29165615|ref|NC_002745.2|	1921070	-	U	7	9	4	6	5	SArRNA09
gi|29165615|ref|NC_002745.2|	1921071	-	C	7	7	5	0	8	SArRNA09
gi|29165615|ref|NC_002745.2|	1921072	-	A	2	4	2	4	5	SArRNA09
gi|29165615|ref|NC_002745.2|	1921073	-	G	2	4	2	2	6	SArRNA09
gi|29165615|ref|NC_002745.2|	1921074	-	C	27	21	8	5	8	SArRNA09
gi|29165615|ref|NC_002745.2|	1921075	-	C	5	8	0	0	3	SArRNA09
gi|29165615|ref|NC_002745.2|	1921076	-	A	2	4	0	1	2	SArRNA09
gi|29165615|ref|NC_002745.2|	1921077	-	A	14	14	9	15	9	SArRNA09
gi|29165615|ref|NC_002745.2|	1921078	-	G	23	23	12	12	19	SArRNA09
gi|29165615|ref|NC_002745.2|	1921079	-	C	31	53	22	9	31	SArRNA09
gi|29165615|ref|NC_002745.2|	1921080	-	C	7	14	6	3	5	SArRNA09
gi|29165615|ref|NC_002745.2|	1921081	-	A	2	2	1	0	0	SArRNA09
gi|29165615|ref|NC_002745.2|	1921082	-	G	7	7	3	5	5	SArRNA09
gi|29165615|ref|NC_002745.2|	1921083	-	G	4	3	1	4	2	SArRNA09

gi|29165615|ref|NC_002745.2|	1921084	-	A	8	10	0	1	5	SArRNA09
gi|29165615|ref|NC_002745.2|	1921085	-	G	5	5	2	0	5	SArRNA09
gi|29165615|ref|NC_002745.2|	1921086	-	G	0	0	0	0	1	SArRNA09
gi|29165615|ref|NC_002745.2|	1921087	-	U	3	3	0	1	5	SArRNA09
gi|29165615|ref|NC_002745.2|	1921088	-	A	3	2	1	0	0	SArRNA09
gi|29165615|ref|NC_002745.2|	1921089	-	A	6	4	2	0	4	SArRNA09
gi|29165615|ref|NC_002745.2|	1921090	-	G	2	4	0	1	2	SArRNA09
gi|29165615|ref|NC_002745.2|	1921091	-	U	4	2	1	2	4	SArRNA09
gi|29165615|ref|NC_002745.2|	1921092	-	C	6	8	0	1	5	SArRNA09
gi|29165615|ref|NC_002745.2|	1921093	-	A	38	35	9	18	19	SArRNA09
gi|29165615|ref|NC_002745.2|	1921094	-	C	99	96	36	43	59	SArRNA09
gi|29165615|ref|NC_002745.2|	1921095	-	A	4	3	1	1	4	SArRNA09
gi|29165615|ref|NC_002745.2|	1921096	-	A	55	53	17	19	38	SArRNA09
gi|29165615|ref|NC_002745.2|	1921097	-	U	18	13	7	2	18	SArRNA09
gi|29165615|ref|NC_002745.2|	1921098	-	G	14	6	5	3	11	SArRNA09
gi|29165615|ref|NC_002745.2|	1921099	-	G	8	9	4	4	14	SArRNA09
gi|29165615|ref|NC_002745.2|	1921100	-	A	18	5	0	5	8	SArRNA09
gi|29165615|ref|NC_002745.2|	1921101	-	C	21	21	7	7	14	SArRNA09
gi|29165615|ref|NC_002745.2|	1921102	-	U	0	2	0	1	2	SArRNA09
gi|29165615|ref|NC_002745.2|	1921103	-	U	0	0	0	0	2	SArRNA09
gi|29165615|ref|NC_002745.2|	1921104	-	G	1	1	0	0	0	SArRNA09
gi|29165615|ref|NC_002745.2|	1921105	-	A	3	3	0	0	4	SArRNA09
gi|29165615|ref|NC_002745.2|	1921106	-	A	0	2	1	0	2	SArRNA09
gi|29165615|ref|NC_002745.2|	1921107	-	G	0	3	0	1	0	SArRNA09
gi|29165615|ref|NC_002745.2|	1921108	-	U	2	1	1	0	1	SArRNA09
gi|29165615|ref|NC_002745.2|	1921109	-	U	3	1	5	0	6	SArRNA09
gi|29165615|ref|NC_002745.2|	1921110	-	G	4	0	5	6	5	SArRNA09
gi|29165615|ref|NC_002745.2|	1921111	-	G	1	6	1	1	2	SArRNA09
gi|29165615|ref|NC_002745.2|	1921112	-	A	17	25	3	4	16	SArRNA09
gi|29165615|ref|NC_002745.2|	1921113	-	C	26	37	8	11	18	SArRNA09
gi|29165615|ref|NC_002745.2|	1921114	-	U	9	6	2	2	5	SArRNA09
gi|29165615|ref|NC_002745.2|	1921115	-	G	2	0	0	2	4	SArRNA09
gi|29165615|ref|NC_002745.2|	1921116	-	G	1	1	1	3	1	SArRNA09
gi|29165615|ref|NC_002745.2|	1921117	-	U	13	6	3	6	3	SArRNA09
gi|29165615|ref|NC_002745.2|	1921118	-	U	3	0	1	2	3	SArRNA09
gi|29165615|ref|NC_002745.2|	1921119	-	C	9	4	0	1	1	SArRNA09
gi|29165615|ref|NC_002745.2|	1921120	-	C	4	14	3	3	0	SArRNA09
gi|29165615|ref|NC_002745.2|	1921121	-	C	26	31	9	14	13	SArRNA09
gi|29165615|ref|NC_002745.2|	1921122	-	A	5	1	0	1	0	SArRNA09
gi|29165615|ref|NC_002745.2|	1921123	-	U	1	3	1	0	1	SArRNA09
gi|29165615|ref|NC_002745.2|	1921124	-	C	2	1	0	1	0	SArRNA09
gi|29165615|ref|NC_002745.2|	1921126	-	A	1	0	0	2	1	SArRNA09
gi|29165615|ref|NC_002745.2|	1921127	-	G	2	3	0	0	1	SArRNA09
gi|29165615|ref|NC_002745.2|	1921128	-	U	2	1	0	1	0	SArRNA09
gi|29165615|ref|NC_002745.2|	1921129	-	G	1	1	0	0	0	SArRNA09
gi|29165615|ref|NC_002745.2|	1921130	-	G	0	0	0	0	1	SArRNA09
gi|29165615|ref|NC_002745.2|	1921132	-	C	4	6	0	0	3	SArRNA09
gi|29165615|ref|NC_002745.2|	1921133	-	C	0	1	0	0	1	SArRNA09
gi|29165615|ref|NC_002745.2|	1921134	-	A	2	1	0	0	0	SArRNA09
gi|29165615|ref|NC_002745.2|	1921135	-	A	6	1	0	0	4	SArRNA09
gi|29165615|ref|NC_002745.2|	1921136	-	A	2	1	0	0	2	SArRNA09
gi|29165615|ref|NC_002745.2|	1921137	-	G	182	102	70	74	75	SArRNA09
gi|29165615|ref|NC_002745.2|	1921138	-	C	21	12	6	2	3	SArRNA09
gi|29165615|ref|NC_002745.2|	1921139	-	C	11	6	4	1	7	SArRNA09
gi|29165615|ref|NC_002745.2|	1921140	-	C	47	44	14	21	26	SArRNA09
gi|29165615|ref|NC_002745.2|	1921141	-	A	1	2	0	2	1	SArRNA09
gi|29165615|ref|NC_002745.2|	1921142	-	G	12	16	2	7	5	SArRNA09
gi|29165615|ref|NC_002745.2|	1921143	-	A	3	3	0	0	0	SArRNA09
gi|29165615|ref|NC_002745.2|	1921144	-	U	1	1	1	0	1	SArRNA09
gi|29165615|ref|NC_002745.2|	1921145	-	G	6	3	1	2	2	SArRNA09
gi|29165615|ref|NC_002745.2|	1921146	-	C	1	3	1	0	1	SArRNA09
gi|29165615|ref|NC_002745.2|	1921148	-	G	0	0	0	0	1	SArRNA09
gi|29165615|ref|NC_002745.2|	1921149	-	G	0	1	0	0	0	SArRNA09
gi|29165615|ref|NC_002745.2|	1921150	-	U	0	1	0	1	2	SArRNA09
gi|29165615|ref|NC_002745.2|	1921151	-	U	4	1	0	0	5	SArRNA09
gi|29165615|ref|NC_002745.2|	1921152	-	U	2	2	1	3	3	SArRNA09
gi|29165615|ref|NC_002745.2|	1921153	-	A	13	11	3	6	9	SArRNA09
gi|29165615|ref|NC_002745.2|	1921154	-	U	13	4	7	3	7	SArRNA09
gi|29165615|ref|NC_002745.2|	1921155	-	G	9	5	3	1	5	SArRNA09
gi|29165615|ref|NC_002745.2|	1921156	-	A	9	10	7	7	9	SArRNA09
gi|29165615|ref|NC_002745.2|	1921157	-	U	9	10	11	5	9	SArRNA09
gi|29165615|ref|NC_002745.2|	1921158	-	U	13	6	3	1	8	SArRNA09
gi|29165615|ref|NC_002745.2|	1921159	-	U	8	5	7	2	15	SArRNA09
gi|29165615|ref|NC_002745.2|	1921160	-	G	8	1	3	6	9	SArRNA09
gi|29165615|ref|NC_002745.2|	1921161	-	C	9	5	1	2	5	SArRNA09
gi|29165615|ref|NC_002745.2|	1921162	-	G	4	2	0	0	2	SArRNA09
gi|29165615|ref|NC_002745.2|	1921163	-	G	3	4	1	2	2	SArRNA09
gi|29165615|ref|NC_002745.2|	1921164	-	G	5	2	0	5	1	SArRNA09
gi|29165615|ref|NC_002745.2|	1921165	-	A	9	16	3	9	8	SArRNA09
gi|29165615|ref|NC_002745.2|	1921166	-	U	14	16	11	17	20	SArRNA09
gi|29165615|ref|NC_002745.2|	1921167	-	A	8	22	2	4	7	SArRNA09

gi|29165615|ref|NC_002745.2|	1921168	-	A	4	9	3	3	4	SArRNA09
gi|29165615|ref|NC_002745.2|	1921169	-	G	2	2	0	4	1	SArRNA09
gi|29165615|ref|NC_002745.2|	1921170	-	U	9	11	7	3	4	SArRNA09
gi|29165615|ref|NC_002745.2|	1921171	-	C	48	35	13	15	17	SArRNA09
gi|29165615|ref|NC_002745.2|	1921172	-	U	31	16	4	9	8	SArRNA09
gi|29165615|ref|NC_002745.2|	1921173	-	G	4	0	1	2	0	SArRNA09
gi|29165615|ref|NC_002745.2|	1921174	-	A	1	2	1	1	1	SArRNA09
gi|29165615|ref|NC_002745.2|	1921175	-	G	3	10	4	4	1	SArRNA09
gi|29165615|ref|NC_002745.2|	1921176	-	C	40	21	21	18	13	SArRNA09
gi|29165615|ref|NC_002745.2|	1921177	-	G	0	2	0	0	1	SArRNA09
gi|29165615|ref|NC_002745.2|	1921178	-	A	21	14	5	15	12	SArRNA09
gi|29165615|ref|NC_002745.2|	1921179	-	A	10	9	7	14	14	SArRNA09
gi|29165615|ref|NC_002745.2|	1921180	-	A	27	12	5	7	17	SArRNA09
gi|29165615|ref|NC_002745.2|	1921181	-	G	28	10	4	6	12	SArRNA09
gi|29165615|ref|NC_002745.2|	1921182	-	C	51	26	7	11	28	SArRNA09
gi|29165615|ref|NC_002745.2|	1921183	-	G	7	7	2	7	9	SArRNA09
gi|29165615|ref|NC_002745.2|	1921184	-	A	23	20	8	13	15	SArRNA09
gi|29165615|ref|NC_002745.2|	1921185	-	U	38	42	22	23	29	SArRNA09
gi|29165615|ref|NC_002745.2|	1921186	-	G	33	20	6	9	18	SArRNA09
gi|29165615|ref|NC_002745.2|	1921187	-	C	169	21	11	18	21	SArRNA09
gi|29165615|ref|NC_002745.2|	1921188	-	C	1122	81	54	59	67	SArRNA09
gi|29165615|ref|NC_002745.2|	1921189	-	G	34	8	2	2	1	SArRNA09
gi|29165615|ref|NC_002745.2|	1921190	-	A	14	10	3	0	12	SArRNA09
gi|29165615|ref|NC_002745.2|	1921191	-	G	1	6	2	2	6	SArRNA09
gi|29165615|ref|NC_002745.2|	1921192	-	G	3	1	0	1	2	SArRNA09
gi|29165615|ref|NC_002745.2|	1921193	-	U	6	3	4	1	5	SArRNA09
gi|29165615|ref|NC_002745.2|	1921194	-	G	5	3	7	4	7	SArRNA09
gi|29165615|ref|NC_002745.2|	1921195	-	U	8	5	2	10	6	SArRNA09
gi|29165615|ref|NC_002745.2|	1921196	-	A	23	48	6	4	32	SArRNA09
gi|29165615|ref|NC_002745.2|	1921197	-	A	39	24	9	6	26	SArRNA09
gi|29165615|ref|NC_002745.2|	1921198	-	A	35	17	8	7	18	SArRNA09
gi|29165615|ref|NC_002745.2|	1921199	-	U	16	10	7	10	24	SArRNA09
gi|29165615|ref|NC_002745.2|	1921200	-	G	17	18	7	15	24	SArRNA09
gi|29165615|ref|NC_002745.2|	1921201	-	A	22	18	9	3	14	SArRNA09
gi|29165615|ref|NC_002745.2|	1921202	-	C	29	32	10	10	19	SArRNA09
gi|29165615|ref|NC_002745.2|	1921203	-	G	19	21	5	6	9	SArRNA09
gi|29165615|ref|NC_002745.2|	1921204	-	A	23	11	7	8	22	SArRNA09
gi|29165615|ref|NC_002745.2|	1921205	-	A	11	7	5	12	13	SArRNA09
gi|29165615|ref|NC_002745.2|	1921206	-	U	30	26	9	8	17	SArRNA09
gi|29165615|ref|NC_002745.2|	1921207	-	U	18	17	9	10	23	SArRNA09
gi|29165615|ref|NC_002745.2|	1921208	-	G	20	13	11	10	21	SArRNA09
gi|29165615|ref|NC_002745.2|	1921209	-	G	7	4	4	3	0	SArRNA09
gi|29165615|ref|NC_002745.2|	1921210	-	A	16	4	7	4	9	SArRNA09
gi|29165615|ref|NC_002745.2|	1921211	-	A	24	31	8	9	21	SArRNA09
gi|29165615|ref|NC_002745.2|	1921212	-	C	35	15	13	13	27	SArRNA09
gi|29165615|ref|NC_002745.2|	1921213	-	G	5	3	3	5	4	SArRNA09
gi|29165615|ref|NC_002745.2|	1921214	-	U	20	10	3	9	9	SArRNA09
gi|29165615|ref|NC_002745.2|	1921215	-	A	26	18	3	4	15	SArRNA09
gi|29165615|ref|NC_002745.2|	1921216	-	G	52	48	20	34	32	SArRNA09
gi|29165615|ref|NC_002745.2|	1921217	-	U	29	17	7	9	12	SArRNA09
gi|29165615|ref|NC_002745.2|	1921218	-	U	12	16	9	6	18	SArRNA09
gi|29165615|ref|NC_002745.2|	1921219	-	U	15	17	9	8	19	SArRNA09
gi|29165615|ref|NC_002745.2|	1921220	-	A	36	19	9	11	22	SArRNA09
gi|29165615|ref|NC_002745.2|	1921221	-	G	26	27	14	25	30	SArRNA09
gi|29165615|ref|NC_002745.2|	1921222	-	C	19	13	5	10	15	SArRNA09
gi|29165615|ref|NC_002745.2|	1921223	-	A	18	6	7	2	7	SArRNA09
gi|29165615|ref|NC_002745.2|	1921224	-	U	3	5	1	5	15	SArRNA09
gi|29165615|ref|NC_002745.2|	1921225	-	U	1	2	3	2	10	SArRNA09
gi|29165615|ref|NC_002745.2|	1921226	-	G	17	2	1	6	5	SArRNA09
gi|29165615|ref|NC_002745.2|	1921227	-	A	5	4	2	4	5	SArRNA09
gi|29165615|ref|NC_002745.2|	1921228	-	G	8	9	2	4	4	SArRNA09
gi|29165615|ref|NC_002745.2|	1921229	-	C	3	6	4	3	3	SArRNA09
gi|29165615|ref|NC_002745.2|	1921230	-	G	4	1	2	1	3	SArRNA09
gi|29165615|ref|NC_002745.2|	1921231	-	G	4	12	2	4	1	SArRNA09
gi|29165615|ref|NC_002745.2|	1921232	-	C	26	15	5	6	9	SArRNA09
gi|29165615|ref|NC_002745.2|	1921233	-	C	9	15	4	5	10	SArRNA09
gi|29165615|ref|NC_002745.2|	1921234	-	A	3	5	0	2	4	SArRNA09
gi|29165615|ref|NC_002745.2|	1921235	-	A	3	4	2	2	3	SArRNA09
gi|29165615|ref|NC_002745.2|	1921236	-	G	2	4	0	3	2	SArRNA09
gi|29165615|ref|NC_002745.2|	1921237	-	U	8	9	1	1	5	SArRNA09
gi|29165615|ref|NC_002745.2|	1921238	-	A	39	304	57	12	81	SArRNA09
gi|29165615|ref|NC_002745.2|	1921239	-	A	22	12	8	13	6	SArRNA09
gi|29165615|ref|NC_002745.2|	1921240	-	G	8	8	2	1	6	SArRNA09
gi|29165615|ref|NC_002745.2|	1921241	-	A	2	8	1	4	2	SArRNA09
gi|29165615|ref|NC_002745.2|	1921242	-	U	8	6	2	8	4	SArRNA09
gi|29165615|ref|NC_002745.2|	1921243	-	G	16	17	6	7	8	SArRNA09
gi|29165615|ref|NC_002745.2|	1921244	-	U	4	8	1	1	7	SArRNA09
gi|29165615|ref|NC_002745.2|	1921245	-	U	16	72	36	73	53	SArRNA09
gi|29165615|ref|NC_002745.2|	1921246	-	U	44	145	94	150	124	SArRNA09
gi|29165615|ref|NC_002745.2|	1921247	-	U	19	117	92	100	108	SArRNA09
gi|29165615|ref|NC_002745.2|	1921248	-	C	20	16	4	7	9	SArRNA09

gi|29165615|ref|NC_002745.2|	1921249	-	C	10	13	6	8	10	SArRNA09
gi|29165615|ref|NC_002745.2|	1921250	-	G	1	1	0	1	0	SArRNA09
gi|29165615|ref|NC_002745.2|	1921251	-	U	4	5	2	2	1	SArRNA09
gi|29165615|ref|NC_002745.2|	1921252	-	G	2	0	2	0	0	SArRNA09
gi|29165615|ref|NC_002745.2|	1921253	-	C	7	3	2	0	0	SArRNA09
gi|29165615|ref|NC_002745.2|	1921255	-	G	1	0	0	0	0	SArRNA09
gi|29165615|ref|NC_002745.2|	1921256	-	U	2	0	0	1	0	SArRNA09
gi|29165615|ref|NC_002745.2|	1921257	-	A	6	3	2	1	2	SArRNA09
gi|29165615|ref|NC_002745.2|	1921258	-	G	1	0	0	1	0	SArRNA09
gi|29165615|ref|NC_002745.2|	1921259	-	U	8	2	4	4	7	SArRNA09
gi|29165615|ref|NC_002745.2|	1921260	-	G	10	2	1	2	7	SArRNA09
gi|29165615|ref|NC_002745.2|	1921261	-	G	5	10	5	0	4	SArRNA09
gi|29165615|ref|NC_002745.2|	1921262	-	G	1	0	1	1	0	SArRNA09
gi|29165615|ref|NC_002745.2|	1921263	-	U	0	3	1	0	1	SArRNA09
gi|29165615|ref|NC_002745.2|	1921264	-	A	2	3	2	2	6	SArRNA09
gi|29165615|ref|NC_002745.2|	1921265	-	A	2	5	1	3	6	SArRNA09
gi|29165615|ref|NC_002745.2|	1921266	-	U	0	2	3	1	5	SArRNA09
gi|29165615|ref|NC_002745.2|	1921267	-	U	4	2	0	3	2	SArRNA09
gi|29165615|ref|NC_002745.2|	1921268	-	G	7	2	5	2	7	SArRNA09
gi|29165615|ref|NC_002745.2|	1921269	-	C	35	18	9	16	24	SArRNA09
gi|29165615|ref|NC_002745.2|	1921270	-	C	4	3	5	5	4	SArRNA09
gi|29165615|ref|NC_002745.2|	1921271	-	C	6	5	1	3	12	SArRNA09
gi|29165615|ref|NC_002745.2|	1921272	-	G	3	3	0	0	4	SArRNA09
gi|29165615|ref|NC_002745.2|	1921273	-	A	2	3	1	1	2	SArRNA09
gi|29165615|ref|NC_002745.2|	1921274	-	G	9	3	1	2	6	SArRNA09
gi|29165615|ref|NC_002745.2|	1921275	-	A	7	11	3	6	3	SArRNA09
gi|29165615|ref|NC_002745.2|	1921276	-	C	10	6	5	3	7	SArRNA09
gi|29165615|ref|NC_002745.2|	1921277	-	U	30	16	13	7	26	SArRNA09
gi|29165615|ref|NC_002745.2|	1921278	-	G	4	5	2	2	5	SArRNA09
gi|29165615|ref|NC_002745.2|	1921279	-	A	11	3	2	4	6	SArRNA09
gi|29165615|ref|NC_002745.2|	1921280	-	U	19	18	16	15	19	SArRNA09
gi|29165615|ref|NC_002745.2|	1921281	-	G	16	10	7	4	12	SArRNA09
gi|29165615|ref|NC_002745.2|	1921282	-	A	8	11	4	6	10	SArRNA09
gi|29165615|ref|NC_002745.2|	1921283	-	A	8	13	7	13	25	SArRNA09
gi|29165615|ref|NC_002745.2|	1921284	-	C	28	28	6	17	29	SArRNA09
gi|29165615|ref|NC_002745.2|	1921285	-	A	7	7	4	2	15	SArRNA09
gi|29165615|ref|NC_002745.2|	1921286	-	U	16	35	17	24	44	SArRNA09
gi|29165615|ref|NC_002745.2|	1921287	-	U	4	1	5	7	6	SArRNA09
gi|29165615|ref|NC_002745.2|	1921288	-	C	7	8	6	7	7	SArRNA09
gi|29165615|ref|NC_002745.2|	1921289	-	G	7	3	1	0	6	SArRNA09
gi|29165615|ref|NC_002745.2|	1921290	-	U	5	5	2	3	6	SArRNA09
gi|29165615|ref|NC_002745.2|	1921291	-	G	4	6	2	0	6	SArRNA09
gi|29165615|ref|NC_002745.2|	1921292	-	U	17	8	4	7	11	SArRNA09
gi|29165615|ref|NC_002745.2|	1921293	-	G	4	4	2	4	7	SArRNA09
gi|29165615|ref|NC_002745.2|	1921294	-	C	6	10	5	1	11	SArRNA09
gi|29165615|ref|NC_002745.2|	1921295	-	C	8	4	3	1	13	SArRNA09
gi|29165615|ref|NC_002745.2|	1921296	-	A	9	6	3	1	10	SArRNA09
gi|29165615|ref|NC_002745.2|	1921297	-	A	12	2	3	2	6	SArRNA09
gi|29165615|ref|NC_002745.2|	1921298	-	A	4	3	0	4	11	SArRNA09
gi|29165615|ref|NC_002745.2|	1921299	-	G	8	6	6	3	7	SArRNA09
gi|29165615|ref|NC_002745.2|	1921300	-	U	10	12	2	7	17	SArRNA09
gi|29165615|ref|NC_002745.2|	1921301	-	C	27	34	8	15	25	SArRNA09
gi|29165615|ref|NC_002745.2|	1921302	-	C	39	23	12	15	28	SArRNA09
gi|29165615|ref|NC_002745.2|	1921303	-	A	12	13	1	4	12	SArRNA09
gi|29165615|ref|NC_002745.2|	1921304	-	A	20	23	8	10	21	SArRNA09
gi|29165615|ref|NC_002745.2|	1921305	-	G	17	13	7	5	9	SArRNA09
gi|29165615|ref|NC_002745.2|	1921306	-	A	8	22	2	1	16	SArRNA09
gi|29165615|ref|NC_002745.2|	1921307	-	U	12	37	21	18	43	SArRNA09
gi|29165615|ref|NC_002745.2|	1921308	-	A	7	53	4	9	31	SArRNA09
gi|29165615|ref|NC_002745.2|	1921309	-	A	10	3	6	5	21	SArRNA09
gi|29165615|ref|NC_002745.2|	1921310	-	A	3	4	3	3	17	SArRNA09
gi|29165615|ref|NC_002745.2|	1921311	-	G	4	5	4	5	7	SArRNA09
gi|29165615|ref|NC_002745.2|	1921312	-	U	8	11	3	3	13	SArRNA09
gi|29165615|ref|NC_002745.2|	1921313	-	G	9	2	1	8	9	SArRNA09
gi|29165615|ref|NC_002745.2|	1921314	-	A	10	9	4	5	12	SArRNA09
gi|29165615|ref|NC_002745.2|	1921315	-	G	4	7	4	8	10	SArRNA09
gi|29165615|ref|NC_002745.2|	1921316	-	G	1	4	2	1	4	SArRNA09
gi|29165615|ref|NC_002745.2|	1921317	-	G	1	0	0	0	4	SArRNA09
gi|29165615|ref|NC_002745.2|	1921318	-	G	1	2	2	3	8	SArRNA09
gi|29165615|ref|NC_002745.2|	1921319	-	A	16	2	2	3	8	SArRNA09
gi|29165615|ref|NC_002745.2|	1921320	-	A	10	7	7	4	3	SArRNA09
gi|29165615|ref|NC_002745.2|	1921321	-	G	9	7	6	8	15	SArRNA09
gi|29165615|ref|NC_002745.2|	1921322	-	G	9	6	5	2	13	SArRNA09
gi|29165615|ref|NC_002745.2|	1921323	-	C	15	1	7	4	13	SArRNA09
gi|29165615|ref|NC_002745.2|	1921324	-	C	14	10	4	7	7	SArRNA09
gi|29165615|ref|NC_002745.2|	1921325	-	C	28	13	7	9	15	SArRNA09
gi|29165615|ref|NC_002745.2|	1921326	-	C	53	28	14	27	30	SArRNA09
gi|29165615|ref|NC_002745.2|	1921327	-	A	9	12	3	0	6	SArRNA09
gi|29165615|ref|NC_002745.2|	1921328	-	C	41	37	12	11	25	SArRNA09
gi|29165615|ref|NC_002745.2|	1921329	-	G	3	1	1	1	2	SArRNA09
gi|29165615|ref|NC_002745.2|	1921330	-	A	3	3	1	0	3	SArRNA09

gi|29165615|ref|NC_002745.2|	1921331	-	A	5	0	0	0	2 SArRNA09
gi|29165615|ref|NC_002745.2|	1921332	-	A	5	2	0	0	6 SArRNA09
gi|29165615|ref|NC_002745.2|	1921333	-	A	12	10	5	3	16 SArRNA09
gi|29165615|ref|NC_002745.2|	1921334	-	G	7	2	1	1	5 SArRNA09
gi|29165615|ref|NC_002745.2|	1921335	-	U	13	7	9	0	14 SArRNA09
gi|29165615|ref|NC_002745.2|	1921336	-	G	9	9	5	10	10 SArRNA09
gi|29165615|ref|NC_002745.2|	1921337	-	G	1	2	2	1	2 SArRNA09
gi|29165615|ref|NC_002745.2|	1921338	-	A	6	8	2	3	11 SArRNA09
gi|29165615|ref|NC_002745.2|	1921339	-	A	11	4	4	5	15 SArRNA09
gi|29165615|ref|NC_002745.2|	1921340	-	A	8	10	2	6	10 SArRNA09
gi|29165615|ref|NC_002745.2|	1921341	-	G	4	2	4	2	7 SArRNA09
gi|29165615|ref|NC_002745.2|	1921342	-	G	2	2	1	0	5 SArRNA09
gi|29165615|ref|NC_002745.2|	1921343	-	G	1	2	0	0	4 SArRNA09
gi|29165615|ref|NC_002745.2|	1921344	-	A	11	6	2	2	6 SArRNA09
gi|29165615|ref|NC_002745.2|	1921345	-	G	15	9	7	10	12 SArRNA09
gi|29165615|ref|NC_002745.2|	1921346	-	U	24	12	9	8	20 SArRNA09
gi|29165615|ref|NC_002745.2|	1921347	-	G	35	23	11	8	27 SArRNA09
gi|29165615|ref|NC_002745.2|	1921348	-	C	113	46	16	20	74 SArRNA09
gi|29165615|ref|NC_002745.2|	1921349	-	C	30	14	7	2	19 SArRNA09
gi|29165615|ref|NC_002745.2|	1921350	-	A	15	17	1	4	24 SArRNA09
gi|29165615|ref|NC_002745.2|	1921351	-	U	11	14	3	9	6 SArRNA09
gi|29165615|ref|NC_002745.2|	1921352	-	G	7	2	3	2	5 SArRNA09
gi|29165615|ref|NC_002745.2|	1921353	-	A	6	10	5	3	11 SArRNA09
gi|29165615|ref|NC_002745.2|	1921354	-	C	33	26	11	7	17 SArRNA09
gi|29165615|ref|NC_002745.2|	1921355	-	C	43	27	6	13	20 SArRNA09
gi|29165615|ref|NC_002745.2|	1921356	-	A	4	3	2	3	7 SArRNA09
gi|29165615|ref|NC_002745.2|	1921357	-	A	6	4	0	7	6 SArRNA09
gi|29165615|ref|NC_002745.2|	1921358	-	G	9	7	3	4	5 SArRNA09
gi|29165615|ref|NC_002745.2|	1921359	-	U	9	8	2	5	11 SArRNA09
gi|29165615|ref|NC_002745.2|	1921360	-	G	6	4	0	1	10 SArRNA09
gi|29165615|ref|NC_002745.2|	1921361	-	A	8	6	5	3	8 SArRNA09
gi|29165615|ref|NC_002745.2|	1921362	-	U	17	17	6	13	23 SArRNA09
gi|29165615|ref|NC_002745.2|	1921363	-	A	16	14	5	6	14 SArRNA09
gi|29165615|ref|NC_002745.2|	1921364	-	G	11	7	2	5	9 SArRNA09
gi|29165615|ref|NC_002745.2|	1921365	-	C	18	17	6	3	11 SArRNA09
gi|29165615|ref|NC_002745.2|	1921366	-	C	8	14	3	1	4 SArRNA09
gi|29165615|ref|NC_002745.2|	1921367	-	A	5	6	1	1	2 SArRNA09
gi|29165615|ref|NC_002745.2|	1921368	-	G	7	2	4	4	2 SArRNA09
gi|29165615|ref|NC_002745.2|	1921369	-	U	9	1	0	3	6 SArRNA09
gi|29165615|ref|NC_002745.2|	1921370	-	G	7	1	0	0	1 SArRNA09
gi|29165615|ref|NC_002745.2|	1921371	-	A	7	6	3	1	6 SArRNA09
gi|29165615|ref|NC_002745.2|	1921372	-	U	20	11	4	6	11 SArRNA09
gi|29165615|ref|NC_002745.2|	1921373	-	C	46	37	7	11	16 SArRNA09
gi|29165615|ref|NC_002745.2|	1921374	-	U	9	8	4	4	18 SArRNA09
gi|29165615|ref|NC_002745.2|	1921375	-	C	7	8	2	6	7 SArRNA09
gi|29165615|ref|NC_002745.2|	1921376	-	U	18	19	2	13	11 SArRNA09
gi|29165615|ref|NC_002745.2|	1921377	-	C	31	27	10	10	32 SArRNA09
gi|29165615|ref|NC_002745.2|	1921378	-	A	4	3	2	1	5 SArRNA09
gi|29165615|ref|NC_002745.2|	1921379	-	U	10	27	6	8	16 SArRNA09
gi|29165615|ref|NC_002745.2|	1921380	-	A	15	153	14	8	47 SArRNA09
gi|29165615|ref|NC_002745.2|	1921381	-	A	3	4	2	4	4 SArRNA09
gi|29165615|ref|NC_002745.2|	1921382	-	A	6	2	2	1	5 SArRNA09
gi|29165615|ref|NC_002745.2|	1921383	-	U	7	12	2	1	17 SArRNA09
gi|29165615|ref|NC_002745.2|	1921384	-	C	10	3	1	7	9 SArRNA09
gi|29165615|ref|NC_002745.2|	1921385	-	G	1	1	2	1	3 SArRNA09
gi|29165615|ref|NC_002745.2|	1921386	-	G	2	3	0	1	3 SArRNA09
gi|29165615|ref|NC_002745.2|	1921387	-	A	1	4	3	3	6 SArRNA09
gi|29165615|ref|NC_002745.2|	1921388	-	A	3	2	3	1	4 SArRNA09
gi|29165615|ref|NC_002745.2|	1921389	-	U	5	8	3	4	6 SArRNA09
gi|29165615|ref|NC_002745.2|	1921390	-	C	5	2	4	3	7 SArRNA09
gi|29165615|ref|NC_002745.2|	1921391	-	C	1	1	1	4	2 SArRNA09
gi|29165615|ref|NC_002745.2|	1921392	-	U	5	9	2	1	8 SArRNA09
gi|29165615|ref|NC_002745.2|	1921393	-	C	15	24	11	24	21 SArRNA09
gi|29165615|ref|NC_002745.2|	1921394	-	U	1	3	4	5	7 SArRNA09
gi|29165615|ref|NC_002745.2|	1921395	-	A	8	9	4	3	7 SArRNA09
gi|29165615|ref|NC_002745.2|	1921396	-	C	42	28	13	22	17 SArRNA09
gi|29165615|ref|NC_002745.2|	1921397	-	C	18	28	9	11	21 SArRNA09
gi|29165615|ref|NC_002745.2|	1921398	-	A	3	2	0	1	5 SArRNA09
gi|29165615|ref|NC_002745.2|	1921399	-	G	6	0	1	2	8 SArRNA09
gi|29165615|ref|NC_002745.2|	1921400	-	G	0	1	0	1	0 SArRNA09
gi|29165615|ref|NC_002745.2|	1921401	-	A	0	1	0	0	1 SArRNA09
gi|29165615|ref|NC_002745.2|	1921402	-	G	0	1	1	0	3 SArRNA09
gi|29165615|ref|NC_002745.2|	1921403	-	G	0	2	0	0	1 SArRNA09
gi|29165615|ref|NC_002745.2|	1921404	-	G	0	0	0	0	2 SArRNA09
gi|29165615|ref|NC_002745.2|	1921405	-	U	4	5	1	6	5 SArRNA09
gi|29165615|ref|NC_002745.2|	1921406	-	C	5	20	3	3	4 SArRNA09
gi|29165615|ref|NC_002745.2|	1921407	-	U	6	4	1	3	7 SArRNA09
gi|29165615|ref|NC_002745.2|	1921408	-	A	3	6	0	1	6 SArRNA09
gi|29165615|ref|NC_002745.2|	1921409	-	A	6	3	0	1	4 SArRNA09
gi|29165615|ref|NC_002745.2|	1921410	-	G	3	5	1	2	6 SArRNA09
gi|29165615|ref|NC_002745.2|	1921411	-	G	0	1	1	0	0 SArRNA09

gi|29165615|ref|NC_002745.2|	1921412	-	C	6	1	0	1	1 SArRNA09
gi|29165615|ref|NC_002745.2|	1921413	-	U	5	2	0	1	0 SArRNA09
gi|29165615|ref|NC_002745.2|	1921414	-	G	0	4	1	0	2 SArRNA09
gi|29165615|ref|NC_002745.2|	1921415	-	C	3	0	3	1	3 SArRNA09
gi|29165615|ref|NC_002745.2|	1921416	-	C	6	1	2	0	1 SArRNA09
gi|29165615|ref|NC_002745.2|	1921417	-	U	0	2	2	0	2 SArRNA09
gi|29165615|ref|NC_002745.2|	1921418	-	U	3	1	0	1	1 SArRNA09
gi|29165615|ref|NC_002745.2|	1921419	-	A	2	7	0	1	6 SArRNA09
gi|29165615|ref|NC_002745.2|	1921420	-	A	0	4	0	1	3 SArRNA09
gi|29165615|ref|NC_002745.2|	1921421	-	A	3	1	2	0	0 SArRNA09
gi|29165615|ref|NC_002745.2|	1921422	-	G	2	2	0	3	2 SArRNA09
gi|29165615|ref|NC_002745.2|	1921423	-	U	6	1	0	3	3 SArRNA09
gi|29165615|ref|NC_002745.2|	1921424	-	G	2	3	2	5	4 SArRNA09
gi|29165615|ref|NC_002745.2|	1921425	-	C	22	13	6	12	16 SArRNA09
gi|29165615|ref|NC_002745.2|	1921426	-	A	14	10	2	4	6 SArRNA09
gi|29165615|ref|NC_002745.2|	1921427	-	C	21	25	9	12	15 SArRNA09
gi|29165615|ref|NC_002745.2|	1921428	-	G	4	2	0	1	0 SArRNA09
gi|29165615|ref|NC_002745.2|	1921429	-	A	6	3	0	3	7 SArRNA09
gi|29165615|ref|NC_002745.2|	1921430	-	G	12	6	5	9	6 SArRNA09
gi|29165615|ref|NC_002745.2|	1921431	-	G	4	4	1	0	6 SArRNA09
gi|29165615|ref|NC_002745.2|	1921432	-	C	5	8	3	3	8 SArRNA09
gi|29165615|ref|NC_002745.2|	1921433	-	A	10	5	1	6	4 SArRNA09
gi|29165615|ref|NC_002745.2|	1921434	-	G	8	6	0	2	12 SArRNA09
gi|29165615|ref|NC_002745.2|	1921435	-	C	7	3	0	2	6 SArRNA09
gi|29165615|ref|NC_002745.2|	1921436	-	A	5	5	3	6	4 SArRNA09
gi|29165615|ref|NC_002745.2|	1921437	-	U	40	12	2	12	13 SArRNA09
gi|29165615|ref|NC_002745.2|	1921438	-	G	11	10	3	3	0 SArRNA09
gi|29165615|ref|NC_002745.2|	1921439	-	A	1	1	0	0	3 SArRNA09
gi|29165615|ref|NC_002745.2|	1921440	-	G	2	1	0	0	2 SArRNA09
gi|29165615|ref|NC_002745.2|	1921441	-	U	11	6	2	2	4 SArRNA09
gi|29165615|ref|NC_002745.2|	1921442	-	C	24	9	2	7	7 SArRNA09
gi|29165615|ref|NC_002745.2|	1921443	-	C	8	7	1	1	6 SArRNA09
gi|29165615|ref|NC_002745.2|	1921444	-	U	4	3	0	1	3 SArRNA09
gi|29165615|ref|NC_002745.2|	1921445	-	A	5	11	6	9	4 SArRNA09
gi|29165615|ref|NC_002745.2|	1921446	-	G	4	5	7	3	7 SArRNA09
gi|29165615|ref|NC_002745.2|	1921447	-	G	0	1	0	0	0 SArRNA09
gi|29165615|ref|NC_002745.2|	1921448	-	U	1	2	0	3	3 SArRNA09
gi|29165615|ref|NC_002745.2|	1921449	-	G	2	2	0	5	0 SArRNA09
gi|29165615|ref|NC_002745.2|	1921450	-	A	3	1	0	1	3 SArRNA09
gi|29165615|ref|NC_002745.2|	1921451	-	G	2	1	0	1	1 SArRNA09
gi|29165615|ref|NC_002745.2|	1921452	-	U	2	6	0	1	4 SArRNA09
gi|29165615|ref|NC_002745.2|	1921453	-	U	4	2	1	0	1 SArRNA09
gi|29165615|ref|NC_002745.2|	1921454	-	C	1	2	2	2	0 SArRNA09
gi|29165615|ref|NC_002745.2|	1921455	-	U	13	0	1	7	4 SArRNA09
gi|29165615|ref|NC_002745.2|	1921456	-	C	5	9	3	3	5 SArRNA09
gi|29165615|ref|NC_002745.2|	1921457	-	U	2	5	1	4	2 SArRNA09
gi|29165615|ref|NC_002745.2|	1921458	-	C	6	8	2	1	3 SArRNA09
gi|29165615|ref|NC_002745.2|	1921459	-	U	7	2	1	0	0 SArRNA09
gi|29165615|ref|NC_002745.2|	1921460	-	G	2	0	0	3	0 SArRNA09
gi|29165615|ref|NC_002745.2|	1921461	-	U	2	1	0	1	1 SArRNA09
gi|29165615|ref|NC_002745.2|	1921462	-	U	2	2	0	0	1 SArRNA09
gi|29165615|ref|NC_002745.2|	1921463	-	G	1	1	0	1	0 SArRNA09
gi|29165615|ref|NC_002745.2|	1921464	-	U	4	4	1	1	2 SArRNA09
gi|29165615|ref|NC_002745.2|	1921465	-	A	9	0	1	2	2 SArRNA09
gi|29165615|ref|NC_002745.2|	1921466	-	A	9	5	1	4	6 SArRNA09
gi|29165615|ref|NC_002745.2|	1921467	-	A	12	9	0	1	2 SArRNA09
gi|29165615|ref|NC_002745.2|	1921468	-	A	2	2	1	0	0 SArRNA09
gi|29165615|ref|NC_002745.2|	1921469	-	G	2	0	1	0	6 SArRNA09
gi|29165615|ref|NC_002745.2|	1921470	-	C	4	3	1	1	1 SArRNA09
gi|29165615|ref|NC_002745.2|	1921471	-	U	4	4	1	2	1 SArRNA09
gi|29165615|ref|NC_002745.2|	1921472	-	G	4	1	1	1	1 SArRNA09
gi|29165615|ref|NC_002745.2|	1921473	-	A	5	3	0	4	2 SArRNA09
gi|29165615|ref|NC_002745.2|	1921474	-	U	3	4	1	2	1 SArRNA09
gi|29165615|ref|NC_002745.2|	1921475	-	G	13	13	1	7	7 SArRNA09
gi|29165615|ref|NC_002745.2|	1921476	-	U	3	8	0	0	9 SArRNA09
gi|29165615|ref|NC_002745.2|	1921477	-	C	9	7	3	1	4 SArRNA09
gi|29165615|ref|NC_002745.2|	1921478	-	C	5	7	4	7	6 SArRNA09
gi|29165615|ref|NC_002745.2|	1921479	-	U	4	10	1	4	7 SArRNA09
gi|29165615|ref|NC_002745.2|	1921480	-	A	1	14	2	5	8 SArRNA09
gi|29165615|ref|NC_002745.2|	1921481	-	A	6	4	0	2	4 SArRNA09
gi|29165615|ref|NC_002745.2|	1921482	-	U	11	3	6	5	7 SArRNA09
gi|29165615|ref|NC_002745.2|	1921483	-	A	10	10	0	2	6 SArRNA09
gi|29165615|ref|NC_002745.2|	1921484	-	A	7	4	1	4	9 SArRNA09
gi|29165615|ref|NC_002745.2|	1921485	-	U	7	2	0	0	5 SArRNA09
gi|29165615|ref|NC_002745.2|	1921486	-	G	2	2	2	2	2 SArRNA09
gi|29165615|ref|NC_002745.2|	1921487	-	G	0	2	0	1	3 SArRNA09
gi|29165615|ref|NC_002745.2|	1921488	-	A	3	0	1	1	3 SArRNA09
gi|29165615|ref|NC_002745.2|	1921489	-	A	3	7	3	3	1 SArRNA09
gi|29165615|ref|NC_002745.2|	1921490	-	G	7	7	1	6	4 SArRNA09
gi|29165615|ref|NC_002745.2|	1921491	-	A	3	9	4	0	1 SArRNA09
gi|29165615|ref|NC_002745.2|	1921492	-	A	3	0	1	1	3 SArRNA09

gi|29165615|ref|NC_002745.2|	1921493	-	A	3	1	1	2	6	SArRNA09
gi|29165615|ref|NC_002745.2|	1921494	-	C	7	18	0	4	7	SArRNA09
gi|29165615|ref|NC_002745.2|	1921495	-	U	0	1	0	0	1	SArRNA09
gi|29165615|ref|NC_002745.2|	1921496	-	A	25	31	10	22	25	SArRNA09
gi|29165615|ref|NC_002745.2|	1921497	-	A	9	14	6	4	3	SArRNA09
gi|29165615|ref|NC_002745.2|	1921498	-	G	15	4	2	6	13	SArRNA09
gi|29165615|ref|NC_002745.2|	1921499	-	U	4	4	2	2	2	SArRNA09
gi|29165615|ref|NC_002745.2|	1921500	-	A	2	3	0	0	3	SArRNA09
gi|29165615|ref|NC_002745.2|	1921501	-	G	2	3	1	3	2	SArRNA09
gi|29165615|ref|NC_002745.2|	1921502	-	A	3	5	2	1	3	SArRNA09
gi|29165615|ref|NC_002745.2|	1921503	-	A	8	9	0	0	0	SArRNA09
gi|29165615|ref|NC_002745.2|	1921504	-	A	1	5	2	2	2	SArRNA09
gi|29165615|ref|NC_002745.2|	1921505	-	G	7	1	1	0	5	SArRNA09
gi|29165615|ref|NC_002745.2|	1921506	-	G	0	0	1	1	0	SArRNA09
gi|29165615|ref|NC_002745.2|	1921507	-	U	3	4	2	1	1	SArRNA09
gi|29165615|ref|NC_002745.2|	1921508	-	C	7	9	7	5	5	SArRNA09
gi|29165615|ref|NC_002745.2|	1921509	-	U	2	3	1	0	0	SArRNA09
gi|29165615|ref|NC_002745.2|	1921510	-	A	3	5	1	1	1	SArRNA09
gi|29165615|ref|NC_002745.2|	1921511	-	C	9	4	3	4	2	SArRNA09
gi|29165615|ref|NC_002745.2|	1921512	-	U	6	1	1	3	0	SArRNA09
gi|29165615|ref|NC_002745.2|	1921513	-	A	1	9	0	0	8	SArRNA09
gi|29165615|ref|NC_002745.2|	1921514	-	A	0	1	0	0	2	SArRNA09
gi|29165615|ref|NC_002745.2|	1921515	-	G	0	1	1	0	0	SArRNA09
gi|29165615|ref|NC_002745.2|	1921516	-	C	1	3	1	1	0	SArRNA09
gi|29165615|ref|NC_002745.2|	1921517	-	A	0	0	0	0	1	SArRNA09
gi|29165615|ref|NC_002745.2|	1921518	-	G	0	1	0	2	1	SArRNA09
gi|29165615|ref|NC_002745.2|	1921519	-	A	0	1	0	0	2	SArRNA09
gi|29165615|ref|NC_002745.2|	1921520	-	U	2	6	1	2	0	SArRNA09
gi|29165615|ref|NC_002745.2|	1921521	-	U	7	3	0	1	3	SArRNA09
gi|29165615|ref|NC_002745.2|	1921522	-	A	2	3	0	1	0	SArRNA09
gi|29165615|ref|NC_002745.2|	1921523	-	C	6	1	1	1	2	SArRNA09
gi|29165615|ref|NC_002745.2|	1921524	-	A	5	0	1	1	0	SArRNA09
gi|29165615|ref|NC_002745.2|	1921525	-	G	0	1	0	1	2	SArRNA09
gi|29165615|ref|NC_002745.2|	1921526	-	C	7	4	1	3	4	SArRNA09
gi|29165615|ref|NC_002745.2|	1921527	-	A	1	1	1	0	2	SArRNA09
gi|29165615|ref|NC_002745.2|	1921528	-	G	0	2	1	0	0	SArRNA09
gi|29165615|ref|NC_002745.2|	1921529	-	G	3	2	3	2	0	SArRNA09
gi|29165615|ref|NC_002745.2|	1921530	-	A	2	2	0	0	0	SArRNA09
gi|29165615|ref|NC_002745.2|	1921531	-	A	2	7	0	0	0	SArRNA09
gi|29165615|ref|NC_002745.2|	1921532	-	A	1	10	0	2	3	SArRNA09
gi|29165615|ref|NC_002745.2|	1921533	-	C	8	16	4	6	8	SArRNA09
gi|29165615|ref|NC_002745.2|	1921534	-	A	1	0	0	1	1	SArRNA09
gi|29165615|ref|NC_002745.2|	1921535	-	U	10	14	5	4	7	SArRNA09
gi|29165615|ref|NC_002745.2|	1921536	-	U	1	2	0	1	0	SArRNA09
gi|29165615|ref|NC_002745.2|	1921537	-	G	1	5	0	0	2	SArRNA09
gi|29165615|ref|NC_002745.2|	1921538	-	A	1	1	0	2	2	SArRNA09
gi|29165615|ref|NC_002745.2|	1921539	-	G	1	5	1	4	0	SArRNA09
gi|29165615|ref|NC_002745.2|	1921540	-	G	1	9	2	2	6	SArRNA09
gi|29165615|ref|NC_002745.2|	1921541	-	C	3	12	1	1	2	SArRNA09
gi|29165615|ref|NC_002745.2|	1921542	-	A	2	0	1	2	0	SArRNA09
gi|29165615|ref|NC_002745.2|	1921543	-	U	6	2	1	0	6	SArRNA09
gi|29165615|ref|NC_002745.2|	1921544	-	A	0	2	0	0	3	SArRNA09
gi|29165615|ref|NC_002745.2|	1921545	-	U	2	2	0	0	1	SArRNA09
gi|29165615|ref|NC_002745.2|	1921546	-	C	2	3	1	3	4	SArRNA09
gi|29165615|ref|NC_002745.2|	1921547	-	U	0	2	0	0	2	SArRNA09
gi|29165615|ref|NC_002745.2|	1921548	-	C	7	5	3	4	5	SArRNA09
gi|29165615|ref|NC_002745.2|	1921549	-	A	8	5	3	5	4	SArRNA09
gi|29165615|ref|NC_002745.2|	1921550	-	C	22	22	7	5	10	SArRNA09
gi|29165615|ref|NC_002745.2|	1921553	-	G	2	1	1	0	0	SArRNA09
gi|29165615|ref|NC_002745.2|	1921554	-	A	0	5	0	1	0	SArRNA09
gi|29165615|ref|NC_002745.2|	1921555	-	U	2	5	0	3	0	SArRNA09
gi|29165615|ref|NC_002745.2|	1921556	-	G	1	1	0	1	0	SArRNA09
gi|29165615|ref|NC_002745.2|	1921557	-	U	1	0	1	0	0	SArRNA09
gi|29165615|ref|NC_002745.2|	1921558	-	U	0	2	2	0	1	SArRNA09
gi|29165615|ref|NC_002745.2|	1921559	-	G	1	0	0	0	3	SArRNA09
gi|29165615|ref|NC_002745.2|	1921560	-	G	1	1	0	2	2	SArRNA09
gi|29165615|ref|NC_002745.2|	1921562	-	G	0	0	1	0	1	SArRNA09
gi|29165615|ref|NC_002745.2|	1921563	-	U	6	4	2	2	0	SArRNA09
gi|29165615|ref|NC_002745.2|	1921564	-	U	5	0	2	1	2	SArRNA09
gi|29165615|ref|NC_002745.2|	1921565	-	G	3	2	1	0	0	SArRNA09
gi|29165615|ref|NC_002745.2|	1921566	-	U	2	1	3	0	1	SArRNA09
gi|29165615|ref|NC_002745.2|	1921567	-	U	0	0	0	1	3	SArRNA09
gi|29165615|ref|NC_002745.2|	1921568	-	C	3	6	2	1	3	SArRNA09
gi|29165615|ref|NC_002745.2|	1921569	-	G	1	1	1	1	1	SArRNA09
gi|29165615|ref|NC_002745.2|	1921570	-	U	0	1	1	0	1	SArRNA09
gi|29165615|ref|NC_002745.2|	1921571	-	U	5	3	0	2	5	SArRNA09
gi|29165615|ref|NC_002745.2|	1921572	-	C	10	6	3	2	5	SArRNA09
gi|29165615|ref|NC_002745.2|	1921573	-	G	1	2	0	0	0	SArRNA09
gi|29165615|ref|NC_002745.2|	1921574	-	A	3	2	1	1	0	SArRNA09
gi|29165615|ref|NC_002745.2|	1921575	-	A	7	4	1	1	3	SArRNA09
gi|29165615|ref|NC_002745.2|	1921576	-	C	6	1	3	1	4	SArRNA09

gi|29165615|ref|NC_002745.2|	1921577	-	A	5	3	1	1	5	SArRNA09
gi|29165615|ref|NC_002745.2|	1921578	-	A	7	4	4	4	6	SArRNA09
gi|29165615|ref|NC_002745.2|	1921579	-	C	16	2	2	7	4	SArRNA09
gi|29165615|ref|NC_002745.2|	1921580	-	C	1	2	1	2	4	SArRNA09
gi|29165615|ref|NC_002745.2|	1921581	-	A	3	2	3	2	2	SArRNA09
gi|29165615|ref|NC_002745.2|	1921582	-	A	2	7	3	1	3	SArRNA09
gi|29165615|ref|NC_002745.2|	1921583	-	A	2	2	1	3	1	SArRNA09
gi|29165615|ref|NC_002745.2|	1921584	-	C	15	11	3	7	7	SArRNA09
gi|29165615|ref|NC_002745.2|	1921585	-	C	3	4	0	3	5	SArRNA09
gi|29165615|ref|NC_002745.2|	1921586	-	C	10	8	1	2	2	SArRNA09
gi|29165615|ref|NC_002745.2|	1921587	-	G	0	1	0	0	0	SArRNA09
gi|29165615|ref|NC_002745.2|	1921588	-	A	1	0	0	1	0	SArRNA09
gi|29165615|ref|NC_002745.2|	1921589	-	G	0	0	1	0	0	SArRNA09
gi|29165615|ref|NC_002745.2|	1921590	-	A	5	1	2	0	0	SArRNA09
gi|29165615|ref|NC_002745.2|	1921591	-	A	0	1	1	0	1	SArRNA09
gi|29165615|ref|NC_002745.2|	1921592	-	G	1	0	0	0	0	SArRNA09
gi|29165615|ref|NC_002745.2|	1921593	-	G	2	0	0	0	0	SArRNA09
gi|29165615|ref|NC_002745.2|	1921594	-	G	0	0	0	1	2	SArRNA09
gi|29165615|ref|NC_002745.2|	1921595	-	C	7	3	1	1	3	SArRNA09
gi|29165615|ref|NC_002745.2|	1921596	-	A	1	2	0	0	3	SArRNA09
gi|29165615|ref|NC_002745.2|	1921597	-	A	3	1	0	3	4	SArRNA09
gi|29165615|ref|NC_002745.2|	1921598	-	A	1	1	1	0	0	SArRNA09
gi|29165615|ref|NC_002745.2|	1921599	-	G	5	5	0	1	3	SArRNA09
gi|29165615|ref|NC_002745.2|	1921600	-	C	5	6	1	2	3	SArRNA09
gi|29165615|ref|NC_002745.2|	1921601	-	G	1	2	1	2	0	SArRNA09
gi|29165615|ref|NC_002745.2|	1921602	-	A	1	5	0	1	5	SArRNA09
gi|29165615|ref|NC_002745.2|	1921603	-	G	6	5	1	1	8	SArRNA09
gi|29165615|ref|NC_002745.2|	1921604	-	C	16	15	4	8	19	SArRNA09
gi|29165615|ref|NC_002745.2|	1921605	-	G	1	1	0	0	1	SArRNA09
gi|29165615|ref|NC_002745.2|	1921606	-	G	2	0	1	0	1	SArRNA09
gi|29165615|ref|NC_002745.2|	1921607	-	C	7	4	4	2	2	SArRNA09
gi|29165615|ref|NC_002745.2|	1921608	-	G	1	0	1	0	0	SArRNA09
gi|29165615|ref|NC_002745.2|	1921609	-	A	7	3	3	1	5	SArRNA09
gi|29165615|ref|NC_002745.2|	1921610	-	U	9	5	2	2	7	SArRNA09
gi|29165615|ref|NC_002745.2|	1921611	-	G	3	6	2	2	4	SArRNA09
gi|29165615|ref|NC_002745.2|	1921612	-	A	6	2	1	1	4	SArRNA09
gi|29165615|ref|NC_002745.2|	1921613	-	U	5	3	4	5	5	SArRNA09
gi|29165615|ref|NC_002745.2|	1921614	-	U	7	5	3	5	7	SArRNA09
gi|29165615|ref|NC_002745.2|	1921615	-	C	5	2	2	4	7	SArRNA09
gi|29165615|ref|NC_002745.2|	1921616	-	C	16	5	5	2	3	SArRNA09
gi|29165615|ref|NC_002745.2|	1921617	-	C	27	20	9	13	8	SArRNA09
gi|29165615|ref|NC_002745.2|	1921618	-	U	1	8	0	1	1	SArRNA09
gi|29165615|ref|NC_002745.2|	1921620	-	A	1	3	1	1	0	SArRNA09
gi|29165615|ref|NC_002745.2|	1921621	-	G	0	3	1	0	0	SArRNA09
gi|29165615|ref|NC_002745.2|	1921622	-	C	2	6	1	0	4	SArRNA09
gi|29165615|ref|NC_002745.2|	1921623	-	U	2	0	2	0	4	SArRNA09
gi|29165615|ref|NC_002745.2|	1921624	-	U	2	6	4	1	4	SArRNA09
gi|29165615|ref|NC_002745.2|	1921625	-	A	9	124	10	3	21	SArRNA09
gi|29165615|ref|NC_002745.2|	1921626	-	A	1	1	1	1	2	SArRNA09
gi|29165615|ref|NC_002745.2|	1921627	-	A	2	3	0	0	0	SArRNA09
gi|29165615|ref|NC_002745.2|	1921628	-	A	0	2	0	0	0	SArRNA09
gi|29165615|ref|NC_002745.2|	1921629	-	G	0	4	1	1	0	SArRNA09
gi|29165615|ref|NC_002745.2|	1921630	-	A	1	1	0	3	0	SArRNA09
gi|29165615|ref|NC_002745.2|	1921631	-	A	0	2	0	2	1	SArRNA09
gi|29165615|ref|NC_002745.2|	1921632	-	A	1	1	0	0	1	SArRNA09
gi|29165615|ref|NC_002745.2|	1921633	-	G	0	2	0	3	5	SArRNA09
gi|29165615|ref|NC_002745.2|	1921634	-	A	1	0	0	1	1	SArRNA09
gi|29165615|ref|NC_002745.2|	1921635	-	G	2	2	2	3	2	SArRNA09
gi|29165615|ref|NC_002745.2|	1921636	-	A	38	45	11	18	15	SArRNA09
gi|29165615|ref|NC_002745.2|	1921637	-	A	4	10	0	0	4	SArRNA09
gi|29165615|ref|NC_002745.2|	1921638	-	G	0	1	0	0	2	SArRNA09
gi|29165615|ref|NC_002745.2|	1921639	-	G	1	0	1	0	0	SArRNA09
gi|29165615|ref|NC_002745.2|	1921640	-	A	0	5	2	1	1	SArRNA09
gi|29165615|ref|NC_002745.2|	1921641	-	G	5	6	4	6	4	SArRNA09
gi|29165615|ref|NC_002745.2|	1921642	-	G	0	4	1	1	3	SArRNA09
gi|29165615|ref|NC_002745.2|	1921643	-	C	11	8	8	3	12	SArRNA09
gi|29165615|ref|NC_002745.2|	1921644	-	C	10	14	3	6	8	SArRNA09
gi|29165615|ref|NC_002745.2|	1921645	-	C	12	8	4	2	5	SArRNA09
gi|29165615|ref|NC_002745.2|	1921646	-	A	0	2	1	0	1	SArRNA09
gi|29165615|ref|NC_002745.2|	1921647	-	U	2	5	1	0	6	SArRNA09
gi|29165615|ref|NC_002745.2|	1921648	-	G	1	3	0	0	1	SArRNA09
gi|29165615|ref|NC_002745.2|	1921649	-	A	0	1	0	2	2	SArRNA09
gi|29165615|ref|NC_002745.2|	1921650	-	U	0	1	0	1	2	SArRNA09
gi|29165615|ref|NC_002745.2|	1921651	-	U	0	0	0	0	1	SArRNA09
gi|29165615|ref|NC_002745.2|	1921652	-	C	2	9	1	2	0	SArRNA09
gi|29165615|ref|NC_002745.2|	1921653	-	U	3	3	5	3	2	SArRNA09
gi|29165615|ref|NC_002745.2|	1921654	-	A	4	13	2	0	7	SArRNA09
gi|29165615|ref|NC_002745.2|	1921655	-	C	11	12	4	9	11	SArRNA09
gi|29165615|ref|NC_002745.2|	1921656	-	A	0	2	3	0	0	SArRNA09
gi|29165615|ref|NC_002745.2|	1921657	-	A	1	0	0	1	0	SArRNA09
gi|29165615|ref|NC_002745.2|	1921658	-	A	3	1	0	0	1	SArRNA09

gi|29165615|ref|NC_002745.2|	1921659	-	G	1	1	0	0	0	SArRNA09
gi|29165615|ref|NC_002745.2|	1921660	-	U	0	1	1	1	1	SArRNA09
gi|29165615|ref|NC_002745.2|	1921661	-	C	7	4	0	1	2	SArRNA09
gi|29165615|ref|NC_002745.2|	1921662	-	A	1	0	1	0	1	SArRNA09
gi|29165615|ref|NC_002745.2|	1921663	-	A	7	8	3	1	8	SArRNA09
gi|29165615|ref|NC_002745.2|	1921664	-	G	14	6	2	6	6	SArRNA09
gi|29165615|ref|NC_002745.2|	1921665	-	A	7	9	1	1	5	SArRNA09
gi|29165615|ref|NC_002745.2|	1921666	-	G	6	11	3	1	5	SArRNA09
gi|29165615|ref|NC_002745.2|	1921667	-	G	67	174	20	22	67	SArRNA09
gi|29165615|ref|NC_002745.2|	1921668	-	C	7	18	5	8	2	SArRNA09
gi|29165615|ref|NC_002745.2|	1921669	-	C	8	10	2	3	7	SArRNA09
gi|29165615|ref|NC_002745.2|	1921670	-	C	27	23	6	9	7	SArRNA09
gi|29165615|ref|NC_002745.2|	1921671	-	A	14	20	4	5	9	SArRNA09
gi|29165615|ref|NC_002745.2|	1921672	-	C	44	27	21	16	18	SArRNA09
gi|29165615|ref|NC_002745.2|	1921673	-	A	14	22	2	2	7	SArRNA09
gi|29165615|ref|NC_002745.2|	1921674	-	C	24	17	4	12	13	SArRNA09
gi|29165615|ref|NC_002745.2|	1921675	-	G	0	2	0	1	0	SArRNA09
gi|29165615|ref|NC_002745.2|	1921676	-	G	2	2	0	0	0	SArRNA09
gi|29165615|ref|NC_002745.2|	1921677	-	A	131	231	47	58	77	SArRNA09
gi|29165615|ref|NC_002745.2|	1921678	-	A	25	60	18	8	6	SArRNA09
gi|29165615|ref|NC_002745.2|	1921679	-	G	8	7	0	6	2	SArRNA09
gi|29165615|ref|NC_002745.2|	1921680	-	A	13	8	5	3	5	SArRNA09
gi|29165615|ref|NC_002745.2|	1921681	-	C	18	9	6	2	7	SArRNA09
gi|29165615|ref|NC_002745.2|	1921682	-	U	11	7	3	5	7	SArRNA09
gi|29165615|ref|NC_002745.2|	1921683	-	A	3	6	2	0	5	SArRNA09
gi|29165615|ref|NC_002745.2|	1921684	-	U	5	3	1	0	7	SArRNA09
gi|29165615|ref|NC_002745.2|	1921685	-	A	14	9	1	5	7	SArRNA09
gi|29165615|ref|NC_002745.2|	1921686	-	C	28	25	9	10	15	SArRNA09
gi|29165615|ref|NC_002745.2|	1921687	-	G	6	1	1	3	4	SArRNA09
gi|29165615|ref|NC_002745.2|	1921688	-	A	3	9	1	1	2	SArRNA09
gi|29165615|ref|NC_002745.2|	1921689	-	U	22	18	3	5	17	SArRNA09
gi|29165615|ref|NC_002745.2|	1921690	-	A	20	33	6	11	22	SArRNA09
gi|29165615|ref|NC_002745.2|	1921691	-	C	31	48	4	10	36	SArRNA09
gi|29165615|ref|NC_002745.2|	1921692	-	A	38	31	4	7	30	SArRNA09
gi|29165615|ref|NC_002745.2|	1921693	-	U	8	16	3	1	17	SArRNA09
gi|29165615|ref|NC_002745.2|	1921694	-	A	7	17	2	7	12	SArRNA09
gi|29165615|ref|NC_002745.2|	1921695	-	A	16	6	4	5	8	SArRNA09
gi|29165615|ref|NC_002745.2|	1921696	-	G	10	6	2	9	8	SArRNA09
gi|29165615|ref|NC_002745.2|	1921697	-	U	5	3	2	3	5	SArRNA09
gi|29165615|ref|NC_002745.2|	1921698	-	G	6	0	6	6	4	SArRNA09
gi|29165615|ref|NC_002745.2|	1921699	-	U	13	3	6	4	10	SArRNA09
gi|29165615|ref|NC_002745.2|	1921700	-	A	7	7	3	0	4	SArRNA09
gi|29165615|ref|NC_002745.2|	1921701	-	U	28	30	8	11	22	SArRNA09
gi|29165615|ref|NC_002745.2|	1921702	-	A	2	7	1	0	4	SArRNA09
gi|29165615|ref|NC_002745.2|	1921703	-	G	9	19	5	6	8	SArRNA09
gi|29165615|ref|NC_002745.2|	1921704	-	C	33	44	19	20	22	SArRNA09
gi|29165615|ref|NC_002745.2|	1921705	-	U	0	7	0	1	2	SArRNA09
gi|29165615|ref|NC_002745.2|	1921706	-	A	0	8	1	1	6	SArRNA09
gi|29165615|ref|NC_002745.2|	1921707	-	U	25	23	13	11	16	SArRNA09
gi|29165615|ref|NC_002745.2|	1921708	-	U	9	7	3	2	5	SArRNA09
gi|29165615|ref|NC_002745.2|	1921709	-	G	2	4	0	1	5	SArRNA09
gi|29165615|ref|NC_002745.2|	1921710	-	U	4	7	1	4	8	SArRNA09
gi|29165615|ref|NC_002745.2|	1921711	-	A	27	24	7	10	9	SArRNA09
gi|29165615|ref|NC_002745.2|	1921712	-	C	132	85	45	52	59	SArRNA09
gi|29165615|ref|NC_002745.2|	1921713	-	U	5	13	2	2	2	SArRNA09
gi|29165615|ref|NC_002745.2|	1921714	-	G	2	1	2	0	7	SArRNA09
gi|29165615|ref|NC_002745.2|	1921715	-	U	7	6	4	2	3	SArRNA09
gi|29165615|ref|NC_002745.2|	1921716	-	A	16	14	6	3	5	SArRNA09
gi|29165615|ref|NC_002745.2|	1921717	-	U	42	29	13	18	31	SArRNA09
gi|29165615|ref|NC_002745.2|	1921718	-	U	17	5	4	2	6	SArRNA09
gi|29165615|ref|NC_002745.2|	1921719	-	G	8	6	7	21	17	SArRNA09
gi|29165615|ref|NC_002745.2|	1921720	-	A	12	9	2	9	8	SArRNA09
gi|29165615|ref|NC_002745.2|	1921721	-	G	24	18	11	13	11	SArRNA09
gi|29165615|ref|NC_002745.2|	1921722	-	U	10	19	7	4	16	SArRNA09
gi|29165615|ref|NC_002745.2|	1921723	-	A	4	11	5	4	11	SArRNA09
gi|29165615|ref|NC_002745.2|	1921724	-	C	2	1	3	2	9	SArRNA09
gi|29165615|ref|NC_002745.2|	1921725	-	G	3	1	0	2	1	SArRNA09
gi|29165615|ref|NC_002745.2|	1921726	-	A	23	47	20	25	24	SArRNA09
gi|29165615|ref|NC_002745.2|	1921727	-	C	14	21	10	10	14	SArRNA09
gi|29165615|ref|NC_002745.2|	1921728	-	C	30	32	9	13	32	SArRNA09
gi|29165615|ref|NC_002745.2|	1921729	-	C	21	22	3	1	31	SArRNA09
gi|29165615|ref|NC_002745.2|	1921730	-	A	3	7	1	0	5	SArRNA09
gi|29165615|ref|NC_002745.2|	1921731	-	A	31	58	10	9	28	SArRNA09
gi|29165615|ref|NC_002745.2|	1921732	-	A	0	6	0	0	3	SArRNA09
gi|29165615|ref|NC_002745.2|	1921733	-	G	3	1	0	2	2	SArRNA09
gi|29165615|ref|NC_002745.2|	1921734	-	G	0	0	5	0	2	SArRNA09
gi|29165615|ref|NC_002745.2|	1921735	-	G	0	2	1	0	4	SArRNA09
gi|29165615|ref|NC_002745.2|	1921736	-	G	3	4	1	1	4	SArRNA09
gi|29165615|ref|NC_002745.2|	1921737	-	U	6	9	0	2	3	SArRNA09
gi|29165615|ref|NC_002745.2|	1921738	-	A	5	15	0	2	4	SArRNA09
gi|29165615|ref|NC_002745.2|	1921739	-	A	5	7	1	4	4	SArRNA09

gi|29165615|ref|NC_002745.2|	1921740 -	G	10	23	5	4	14	SArRNA09
gi|29165615|ref|NC_002745.2|	1921741 -	C	54	64	16	32	28	SArRNA09
gi|29165615|ref|NC_002745.2|	1921742 -	C	5	10	4	6	11	SArRNA09
gi|29165615|ref|NC_002745.2|	1921743 -	U	3	3	2	5	3	SArRNA09
gi|29165615|ref|NC_002745.2|	1921744 -	U	4	3	3	4	3	SArRNA09
gi|29165615|ref|NC_002745.2|	1921745 -	U	5	4	3	2	6	SArRNA09
gi|29165615|ref|NC_002745.2|	1921746 -	A	23	32	19	12	11	SArRNA09
gi|29165615|ref|NC_002745.2|	1921747 -	G	53	58	20	16	25	SArRNA09
gi|29165615|ref|NC_002745.2|	1921748 -	A	21	15	1	7	10	SArRNA09
gi|29165615|ref|NC_002745.2|	1921749 -	G	21	35	12	20	27	SArRNA09
gi|29165615|ref|NC_002745.2|	1921750 -	A	15	19	2	4	19	SArRNA09
gi|29165615|ref|NC_002745.2|	1921751 -	C	30	28	14	23	28	SArRNA09
gi|29165615|ref|NC_002745.2|	1921752 -	C	17	36	3	7	19	SArRNA09
gi|29165615|ref|NC_002745.2|	1921753 -	U	3	5	1	4	3	SArRNA09
gi|29165615|ref|NC_002745.2|	1921754 -	A	6	7	1	5	16	SArRNA09
gi|29165615|ref|NC_002745.2|	1921755 -	G	27	43	19	18	30	SArRNA09
gi|29165615|ref|NC_002745.2|	1921756 -	U	13	28	14	7	12	SArRNA09
gi|29165615|ref|NC_002745.2|	1921757 -	U	5	3	5	4	10	SArRNA09
gi|29165615|ref|NC_002745.2|	1921758 -	U	5	4	1	0	9	SArRNA09
gi|29165615|ref|NC_002745.2|	1921759 -	C	22	7	10	6	18	SArRNA09
gi|29165615|ref|NC_002745.2|	1921760 -	G	1	3	3	0	3	SArRNA09
gi|29165615|ref|NC_002745.2|	1921761 -	A	13	11	4	6	8	SArRNA09
gi|29165615|ref|NC_002745.2|	1921762 -	A	12	15	11	13	18	SArRNA09
gi|29165615|ref|NC_002745.2|	1921763 -	U	26	42	20	42	43	SArRNA09
gi|29165615|ref|NC_002745.2|	1921764 -	G	33	25	10	18	30	SArRNA09
gi|29165615|ref|NC_002745.2|	1921765 -	A	29	8	14	14	32	SArRNA09
gi|29165615|ref|NC_002745.2|	1921766 -	A	24	24	4	6	24	SArRNA09
gi|29165615|ref|NC_002745.2|	1921767 -	U	36	27	10	11	25	SArRNA09
gi|29165615|ref|NC_002745.2|	1921768 -	G	9	8	1	5	9	SArRNA09
gi|29165615|ref|NC_002745.2|	1921769 -	U	19	8	3	7	14	SArRNA09
gi|29165615|ref|NC_002745.2|	1921770 -	C	26	13	5	4	5	SArRNA09
gi|29165615|ref|NC_002745.2|	1921771 -	G	13	12	13	12	14	SArRNA09
gi|29165615|ref|NC_002745.2|	1921772 -	A	54	35	34	21	76	SArRNA09
gi|29165615|ref|NC_002745.2|	1921773 -	G	70	35	27	24	76	SArRNA09
gi|29165615|ref|NC_002745.2|	1921774 -	G	15	13	8	10	14	SArRNA09
gi|29165615|ref|NC_002745.2|	1921775 -	G	11	11	4	2	8	SArRNA09
gi|29165615|ref|NC_002745.2|	1921776 -	G	4	12	5	4	10	SArRNA09
gi|29165615|ref|NC_002745.2|	1921777 -	U	21	14	4	15	19	SArRNA09
gi|29165615|ref|NC_002745.2|	1921778 -	U	53	20	20	21	44	SArRNA09
gi|29165615|ref|NC_002745.2|	1921779 -	U	42	34	23	22	55	SArRNA09
gi|29165615|ref|NC_002745.2|	1921780 -	C	65	29	16	11	32	SArRNA09
gi|29165615|ref|NC_002745.2|	1921781 -	G	44	24	14	21	23	SArRNA09
gi|29165615|ref|NC_002745.2|	1921782 -	U	57	37	21	30	59	SArRNA09
gi|29165615|ref|NC_002745.2|	1921783 -	A	56	37	8	15	44	SArRNA09
gi|29165615|ref|NC_002745.2|	1921784 -	U	113	122	65	65	114	SArRNA09
gi|29165615|ref|NC_002745.2|	1921785 -	A	31	32	16	16	39	SArRNA09
gi|29165615|ref|NC_002745.2|	1921786 -	G	34	19	4	8	22	SArRNA09
gi|29165615|ref|NC_002745.2|	1921787 -	C	34	36	18	22	23	SArRNA09
gi|29165615|ref|NC_002745.2|	1921788 -	A	26	8	7	13	17	SArRNA09
gi|29165615|ref|NC_002745.2|	1921789 -	G	29	15	7	13	22	SArRNA09
gi|29165615|ref|NC_002745.2|	1921790 -	C	50	42	22	36	46	SArRNA09
gi|29165615|ref|NC_002745.2|	1921791 -	A	1007	878	350	1165	819	SArRNA09
gi|29165615|ref|NC_002745.2|	1921792 -	A	34	48	14	28	45	SArRNA09
gi|29165615|ref|NC_002745.2|	1921793 -	U	41	41	12	23	47	SArRNA09
gi|29165615|ref|NC_002745.2|	1921794 -	C	24	34	13	24	42	SArRNA09
gi|29165615|ref|NC_002745.2|	1921795 -	A	20	12	8	14	22	SArRNA09
gi|29165615|ref|NC_002745.2|	1921796 -	U	23	51	23	21	33	SArRNA09
gi|29165615|ref|NC_002745.2|	1921797 -	U	27	32	19	8	37	SArRNA09
gi|29165615|ref|NC_002745.2|	1921798 -	G	35	19	9	13	20	SArRNA09
gi|29165615|ref|NC_002745.2|	1921799 -	C	29	27	18	16	22	SArRNA09
gi|29165615|ref|NC_002745.2|	1921800 -	A	15	6	3	5	12	SArRNA09
gi|29165615|ref|NC_002745.2|	1921801 -	G	18	22	11	9	22	SArRNA09
gi|29165615|ref|NC_002745.2|	1921802 -	G	4	6	4	4	9	SArRNA09
gi|29165615|ref|NC_002745.2|	1921803 -	A	13	17	4	2	14	SArRNA09
gi|29165615|ref|NC_002745.2|	1921804 -	A	18	14	4	12	11	SArRNA09
gi|29165615|ref|NC_002745.2|	1921805 -	G	16	9	11	11	23	SArRNA09
gi|29165615|ref|NC_002745.2|	1921806 -	U	9	6	12	13	31	SArRNA09
gi|29165615|ref|NC_002745.2|	1921807 -	A	10	10	3	3	16	SArRNA09
gi|29165615|ref|NC_002745.2|	1921808 -	G	94	77	31	42	70	SArRNA09
gi|29165615|ref|NC_002745.2|	1921809 -	C	51	83	17	12	80	SArRNA09
gi|29165615|ref|NC_002745.2|	1921810 -	C	8	6	5	2	12	SArRNA09
gi|29165615|ref|NC_002745.2|	1921811 -	G	11	21	11	6	11	SArRNA09
gi|29165615|ref|NC_002745.2|	1921812 -	A	130	32	11	5	15	SArRNA09
gi|29165615|ref|NC_002745.2|	1921813 -	A	90	18	12	7	23	SArRNA09
gi|29165615|ref|NC_002745.2|	1921814 -	G	57	34	20	12	33	SArRNA09
gi|29165615|ref|NC_002745.2|	1921815 -	A	14	17	3	3	4	SArRNA09
gi|29165615|ref|NC_002745.2|	1921816 -	U	41	27	11	11	31	SArRNA09
gi|29165615|ref|NC_002745.2|	1921817 -	C	46	35	14	21	35	SArRNA09
gi|29165615|ref|NC_002745.2|	1921818 -	A	30	41	13	11	20	SArRNA09
gi|29165615|ref|NC_002745.2|	1921819 -	C	57	52	31	20	45	SArRNA09
gi|29165615|ref|NC_002745.2|	1921820 -	G	7	10	4	3	13	SArRNA09

gi|29165615|ref|NC_002745.2|	1921821	-	G	1	8	3	1	4	SArRNA09
gi|29165615|ref|NC_002745.2|	1921822	-	U	25	26	8	9	24	SArRNA09
gi|29165615|ref|NC_002745.2|	1921823	-	U	19	19	6	0	12	SArRNA09
gi|29165615|ref|NC_002745.2|	1921824	-	C	39	84	13	10	50	SArRNA09
gi|29165615|ref|NC_002745.2|	1921825	-	C	37	16	7	2	12	SArRNA09
gi|29165615|ref|NC_002745.2|	1921826	-	G	0	0	2	0	0	SArRNA09
gi|29165615|ref|NC_002745.2|	1921827	-	U	4	1	3	1	8	SArRNA09
gi|29165615|ref|NC_002745.2|	1921828	-	A	108	14	30	12	27	SArRNA09
gi|29165615|ref|NC_002745.2|	1921829	-	G	78	30	17	12	26	SArRNA09
gi|29165615|ref|NC_002745.2|	1921830	-	G	29	18	6	9	13	SArRNA09
gi|29165615|ref|NC_002745.2|	1921831	-	U	47	16	9	7	22	SArRNA09
gi|29165615|ref|NC_002745.2|	1921832	-	G	23	23	13	12	27	SArRNA09
gi|29165615|ref|NC_002745.2|	1921833	-	G	44	47	28	20	39	SArRNA09
gi|29165615|ref|NC_002745.2|	1921834	-	C	199	85	55	40	80	SArRNA09
gi|29165615|ref|NC_002745.2|	1921835	-	A	55	41	28	20	48	SArRNA09
gi|29165615|ref|NC_002745.2|	1921836	-	C	305	178	109	69	115	SArRNA09
gi|29165615|ref|NC_002745.2|	1921837	-	G	137	34	26	16	29	SArRNA09
gi|29165615|ref|NC_002745.2|	1921838	-	C	1179	89	111	44	96	SArRNA09
gi|29165615|ref|NC_002745.2|	1921839	-	G	839	183	146	59	118	SArRNA09
gi|29165615|ref|NC_002745.2|	1921840	-	G	412	34	33	21	36	SArRNA09
gi|29165615|ref|NC_002745.2|	1921841	-	G	119	23	14	13	16	SArRNA09
gi|29165615|ref|NC_002745.2|	1921842	-	A	230	64	26	13	38	SArRNA09
gi|29165615|ref|NC_002745.2|	1921843	-	A	422	149	104	47	98	SArRNA09
gi|29165615|ref|NC_002745.2|	1921844	-	U	3981	458	739	280	397	SArRNA09
gi|29165615|ref|NC_002745.2|	1921845	-	U	748	265	365	187	307	SArRNA09
gi|29165615|ref|NC_002745.2|	1921846	-	A	835	841	506	578	829	SArRNA09
gi|29165615|ref|NC_002745.2|	1921847	-	U	2637	1112	902	775	1163	SArRNA09
gi|29165615|ref|NC_002745.2|	1921848	-	U	3050	1134	903	873	1058	SArRNA09
gi|29165615|ref|NC_002745.2|	1921849	-	G	3105	1040	814	698	995	SArRNA09
gi|29165615|ref|NC_002745.2|	1921850	-	A	18974	1553	1313	1189	1437	SArRNA09
gi|29165615|ref|NC_002745.2|	1921851	-	A	8496	3514	2335	2876	3068	SArRNA09
gi|29165615|ref|NC_002745.2|	1921852	-	U	2404	4642	3181	3137	4527	SArRNA09
gi|29165615|ref|NC_002745.2|	1921853	-	U	4688426	3458875	2860835	2114435	3322169	SArRNA09
gi|29165615|ref|NC_002745.2|	1921854	-	A	13289	9658	6342	5268	7428	SArRNA09
gi|29165615|ref|NC_002745.2|	1921855	-	G	236	167	138	146	174	SArRNA09
gi|29165615|ref|NC_002745.2|	1921856	-	A	77	25	17	21	19	-
gi|29165615|ref|NC_002745.2|	1921857	-	U	56	21	23	51	27	-
gi|29165615|ref|NC_002745.2|	1921858	-	A	19	10	4	26	14	-
gi|29165615|ref|NC_002745.2|	1921859	-	C	22	4	3	15	6	-
gi|29165615|ref|NC_002745.2|	1921860	-	A	5	3	3	32	0	-
gi|29165615|ref|NC_002745.2|	1921861	-	A	12	2	3	55	7	-
gi|29165615|ref|NC_002745.2|	1921862	-	A	7	5	3	33	4	-
gi|29165615|ref|NC_002745.2|	1921863	-	G	4	0	2	27	4	-
gi|29165615|ref|NC_002745.2|	1921864	-	G	0	0	0	10	1	-
gi|29165615|ref|NC_002745.2|	1921865	-	U	4	0	2	0	1	-
gi|29165615|ref|NC_002745.2|	1921866	-	G	2	2	5	33	4	-
gi|29165615|ref|NC_002745.2|	1921867	-	G	5	1	2	23	4	-
gi|29165615|ref|NC_002745.2|	1921868	-	U	1	0	0	5	0	-
gi|29165615|ref|NC_002745.2|	1921869	-	A	4	5	0	14	14	-
gi|29165615|ref|NC_002745.2|	1921870	-	A	2	5	1	34	0	-
gi|29165615|ref|NC_002745.2|	1921871	-	A	3	2	0	17	2	-
gi|29165615|ref|NC_002745.2|	1921872	-	A	1	1	1	17	2	-
gi|29165615|ref|NC_002745.2|	1921873	-	A	0	2	0	13	3	-
gi|29165615|ref|NC_002745.2|	1921874	-	U	2	0	0	3	0	-
gi|29165615|ref|NC_002745.2|	1921875	-	A	4	0	1	13	3	-
gi|29165615|ref|NC_002745.2|	1921876	-	U	0	0	0	1	0	-
gi|29165615|ref|NC_002745.2|	1921877	-	U	1	2	0	0	1	-
gi|29165615|ref|NC_002745.2|	1921878	-	C	5	2	0	6	0	-
gi|29165615|ref|NC_002745.2|	1921879	-	A	2	4	2	66	1	-
gi|29165615|ref|NC_002745.2|	1921880	-	G	0	0	0	1	0	-
gi|29165615|ref|NC_002745.2|	1921882	-	U	1	0	0	0	0	-
gi|29165615|ref|NC_002745.2|	1921883	-	U	1	0	1	0	0	-
gi|29165615|ref|NC_002745.2|	1921884	-	G	1	0	0	1	0	-
gi|29165615|ref|NC_002745.2|	1921885	-	C	3	0	0	2	1	-
gi|29165615|ref|NC_002745.2|	1921886	-	A	5	0	1	11	2	-
gi|29165615|ref|NC_002745.2|	1921887	-	A	1	2	1	6	3	-
gi|29165615|ref|NC_002745.2|	1921888	-	U	1	1	0	4	0	-
gi|29165615|ref|NC_002745.2|	1921889	-	U	1	0	0	3	1	-
gi|29165615|ref|NC_002745.2|	1921890	-	G	0	1	0	4	0	-
gi|29165615|ref|NC_002745.2|	1921891	-	U	0	0	0	4	0	-
gi|29165615|ref|NC_002745.2|	1921892	-	A	0	0	0	7	0	-
gi|29165615|ref|NC_002745.2|	1921893	-	C	3	0	0	29	1	-
gi|29165615|ref|NC_002745.2|	1921894	-	U	1	1	0	7	0	-
gi|29165615|ref|NC_002745.2|	1921895	-	U	0	0	1	12	1	-
gi|29165615|ref|NC_002745.2|	1921896	-	C	2	0	0	7	1	-
gi|29165615|ref|NC_002745.2|	1921897	-	A	2	2	0	36	1	-
gi|29165615|ref|NC_002745.2|	1921898	-	A	3	0	0	86	1	-
gi|29165615|ref|NC_002745.2|	1921899	-	U	0	0	0	11	0	-
gi|29165615|ref|NC_002745.2|	1921900	-	G	0	0	0	4	0	-
gi|29165615|ref|NC_002745.2|	1921901	-	C	0	0	0	2	1	-
gi|29165615|ref|NC_002745.2|	1921902	-	A	0	0	0	2	1	-

gi|29165615|ref|NC_002745.2|	1921903 -	A	3	1	2	28	0	-
gi|29165615|ref|NC_002745.2|	1921904 -	G	3	0	0	3	0	-
gi|29165615|ref|NC_002745.2|	1921905 -	A	0	0	0	6	0	-
gi|29165615|ref|NC_002745.2|	1921906 -	A	0	0	0	5	0	-
gi|29165615|ref|NC_002745.2|	1921907 -	A	0	0	1	20	0	-
gi|29165615|ref|NC_002745.2|	1921908 -	A	1	0	0	44	0	-
gi|29165615|ref|NC_002745.2|	1921909 -	U	0	1	1	6	0	-
gi|29165615|ref|NC_002745.2|	1921910 -	A	1	2	1	37	2	-
gi|29165615|ref|NC_002745.2|	1921911 -	U	1	0	0	1	1	-
gi|29165615|ref|NC_002745.2|	1921912 -	U	0	0	0	0	1	-
gi|29165615|ref|NC_002745.2|	1921915 -	U	0	0	0	1	0	-
gi|29165615|ref|NC_002745.2|	1921916 -	C	2	1	1	7	0	-
gi|29165615|ref|NC_002745.2|	1921918 -	A	0	3	3	5	1	-
gi|29165615|ref|NC_002745.2|	1921919 -	A	1	0	0	5	0	-
gi|29165615|ref|NC_002745.2|	1921920 -	A	0	0	1	2	0	-
gi|29165615|ref|NC_002745.2|	1921921 -	U	0	1	0	3	0	-
gi|29165615|ref|NC_002745.2|	1921922 -	U	0	2	2	1	0	-
gi|29165615|ref|NC_002745.2|	1921923 -	U	0	1	2	8	0	-
gi|29165615|ref|NC_002745.2|	1921924 -	G	2	1	3	39	3	-
gi|29165615|ref|NC_002745.2|	1921925 -	C	14	62	29	374	44	-
gi|29165615|ref|NC_002745.2|	1921926 -	G	0	0	0	2	0	-
gi|29165615|ref|NC_002745.2|	1921927 -	C	2	0	1	0	1	-
gi|29165615|ref|NC_002745.2|	1921928 -	A	1	0	3	10	0	-
gi|29165615|ref|NC_002745.2|	1921929 -	A	2	0	1	19	2	-
gi|29165615|ref|NC_002745.2|	1921930 -	U	1	0	1	0	1	-
gi|29165615|ref|NC_002745.2|	1921931 -	A	0	3	1	20	2	-
gi|29165615|ref|NC_002745.2|	1921932 -	A	0	0	0	0	1	-
gi|29165615|ref|NC_002745.2|	1921933 -	U	1	0	1	0	0	-
gi|29165615|ref|NC_002745.2|	1921934 -	U	0	0	2	0	0	-
gi|29165615|ref|NC_002745.2|	1921935 -	A	0	1	0	1	0	-
gi|29165615|ref|NC_002745.2|	1921936 -	G	1	0	0	0	0	-
gi|29165615|ref|NC_002745.2|	1921937 -	A	0	0	1	0	0	-
gi|29165615|ref|NC_002745.2|	1921938 -	A	2	0	1	1	0	-
gi|29165615|ref|NC_002745.2|	1921939 -	C	3	0	0	0	0	-
gi|29165615|ref|NC_002745.2|	1921940 -	A	1	0	0	0	0	-
gi|29165615|ref|NC_002745.2|	1921942 -	U	0	0	1	0	0	-
gi|29165615|ref|NC_002745.2|	1921943 -	C	2	0	0	1	0	-
gi|29165615|ref|NC_002745.2|	1921944 -	A	7	1	2	1	4	-
gi|29165615|ref|NC_002745.2|	1921945 -	C	2	2	1	1	3	-
gi|29165615|ref|NC_002745.2|	1921946 -	A	4	0	0	0	0	-
gi|29165615|ref|NC_002745.2|	1921947 -	A	6	0	0	1	1	-
gi|29165615|ref|NC_002745.2|	1921949 -	A	0	0	0	1	0	-
gi|29165615|ref|NC_002745.2|	1921950 -	A	0	0	0	3	0	-
gi|29165615|ref|NC_002745.2|	1921951 -	A	0	1	2	2	0	-
gi|29165615|ref|NC_002745.2|	1921952 -	G	1	0	0	5	0	-
gi|29165615|ref|NC_002745.2|	1921953 -	C	0	0	0	2	1	-
gi|29165615|ref|NC_002745.2|	1921954 -	U	1	0	0	0	0	-
gi|29165615|ref|NC_002745.2|	1921955 -	U	3	1	0	0	0	-
gi|29165615|ref|NC_002745.2|	1921959 -	A	1	0	0	1	0	-
gi|29165615|ref|NC_002745.2|	1921961 -	C	0	0	0	1	0	-
gi|29165615|ref|NC_002745.2|	1921963 -	C	1	1	0	2	1	-
gi|29165615|ref|NC_002745.2|	1921964 -	U	0	1	0	9	2	-
gi|29165615|ref|NC_002745.2|	1921965 -	A	5	14	2	56	12	-
gi|29165615|ref|NC_002745.2|	1921966 -	A	8	4	3	112	9	-
gi|29165615|ref|NC_002745.2|	1921967 -	U	2	4	1	19	1	-
gi|29165615|ref|NC_002745.2|	1921968 -	A	3	11	4	89	10	-
gi|29165615|ref|NC_002745.2|	1921969 -	A	9	23	6	185	16	-
gi|29165615|ref|NC_002745.2|	1921970 -	A	3	17	3	135	9	-
gi|29165615|ref|NC_002745.2|	1921971 -	G	8	22	10	187	24	-
gi|29165615|ref|NC_002745.2|	1921972 -	A	4	26	10	263	28	-
gi|29165615|ref|NC_002745.2|	1921973 -	A	8	48	14	417	49	-
gi|29165615|ref|NC_002745.2|	1921974 -	U	9	23	16	165	27	-
gi|29165615|ref|NC_002745.2|	1921975 -	A	3	2	0	11	0	-
gi|29165615|ref|NC_002745.2|	1921976 -	C	1	1	0	2	0	-
gi|29165615|ref|NC_002745.2|	1921977 -	U	1	0	0	1	0	-
gi|29165615|ref|NC_002745.2|	1921978 -	U	0	3	0	4	0	-
gi|29165615|ref|NC_002745.2|	1921979 -	A	1	2	3	14	3	-
gi|29165615|ref|NC_002745.2|	1921980 -	A	1	4	3	31	2	-
gi|29165615|ref|NC_002745.2|	1921981 -	G	4	0	1	8	1	-
gi|29165615|ref|NC_002745.2|	1921982 -	U	1	0	0	0	0	-
gi|29165615|ref|NC_002745.2|	1921983 -	U	1	0	0	0	0	-
gi|29165615|ref|NC_002745.2|	1921984 -	C	0	0	0	1	0	-
gi|29165615|ref|NC_002745.2|	1921985 -	G	0	1	0	2	0	-
gi|29165615|ref|NC_002745.2|	1921986 -	A	0	0	1	3	0	-
gi|29165615|ref|NC_002745.2|	1921987 -	A	1	1	1	11	0	-
gi|29165615|ref|NC_002745.2|	1921988 -	U	2	1	1	3	0	-
gi|29165615|ref|NC_002745.2|	1921989 -	A	0	4	2	3	4	-
gi|29165615|ref|NC_002745.2|	1921990 -	A	0	9	1	30	5	-
gi|29165615|ref|NC_002745.2|	1921991 -	A	1	4	0	36	3	-
gi|29165615|ref|NC_002745.2|	1921992 -	U	2	0	1	5	0	-
gi|29165615|ref|NC_002745.2|	1921993 -	U	0	0	1	0	0	-

gi|29165615|ref|NC_002745.2|	1921995	-	U	0	0	0	1	0	-
gi|29165615|ref|NC_002745.2|	1921996	-	G	0	0	0	2	1	-
gi|29165615|ref|NC_002745.2|	1921997	-	A	0	1	1	11	2	-
gi|29165615|ref|NC_002745.2|	1921998	-	G	0	0	0	2	0	-
gi|29165615|ref|NC_002745.2|	1922000	-	A	0	1	0	2	0	-
gi|29165615|ref|NC_002745.2|	1922001	-	A	1	0	1	2	1	-
gi|29165615|ref|NC_002745.2|	1922003	-	A	0	0	0	0	1	-
gi|29165615|ref|NC_002745.2|	1922004	-	A	0	1	0	8	0	-
gi|29165615|ref|NC_002745.2|	1922006	-	U	0	0	0	1	0	-
gi|29165615|ref|NC_002745.2|	1922007	-	G	0	0	0	0	1	-
gi|29165615|ref|NC_002745.2|	1922008	-	A	0	0	0	2	0	-
gi|29165615|ref|NC_002745.2|	1922009	-	G	1	2	0	4	1	-
gi|29165615|ref|NC_002745.2|	1922010	-	C	3	1	1	1	3	-
gi|29165615|ref|NC_002745.2|	1922011	-	C	0	0	0	0	2	-
gi|29165615|ref|NC_002745.2|	1922012	-	A	0	0	1	0	0	-
gi|29165615|ref|NC_002745.2|	1922013	-	A	1	1	0	4	1	-
gi|29165615|ref|NC_002745.2|	1922014	-	A	0	5	2	13	1	-
gi|29165615|ref|NC_002745.2|	1922015	-	A	1	1	1	8	1	-
gi|29165615|ref|NC_002745.2|	1922016	-	C	2	0	0	5	1	-
gi|29165615|ref|NC_002745.2|	1922017	-	G	1	0	0	1	0	-
gi|29165615|ref|NC_002745.2|	1922018	-	A	0	0	0	1	1	-
gi|29165615|ref|NC_002745.2|	1922019	-	A	1	0	0	3	2	-
gi|29165615|ref|NC_002745.2|	1922020	-	C	4	1	2	9	3	-
gi|29165615|ref|NC_002745.2|	1922021	-	C	1	2	0	1	0	-
gi|29165615|ref|NC_002745.2|	1922022	-	A	0	1	0	1	0	-
gi|29165615|ref|NC_002745.2|	1922023	-	U	0	0	1	0	0	-
gi|29165615|ref|NC_002745.2|	1922025	-	U	0	1	1	0	0	-
gi|29165615|ref|NC_002745.2|	1922027	-	A	0	2	2	7	1	-
gi|29165615|ref|NC_002745.2|	1922028	-	G	0	4	3	5	3	-
gi|29165615|ref|NC_002745.2|	1922029	-	A	0	0	1	1	0	-
gi|29165615|ref|NC_002745.2|	1922030	-	U	0	0	0	2	0	-
gi|29165615|ref|NC_002745.2|	1922031	-	A	0	0	0	3	0	-
gi|29165615|ref|NC_002745.2|	1922033	-	A	0	1	0	4	0	-
gi|29165615|ref|NC_002745.2|	1922034	-	A	1	3	0	2	0	-
gi|29165615|ref|NC_002745.2|	1922035	-	A	0	0	0	2	0	-
gi|29165615|ref|NC_002745.2|	1922036	-	A	0	0	1	7	0	-
gi|29165615|ref|NC_002745.2|	1922038	-	G	0	0	0	1	0	-
gi|29165615|ref|NC_002745.2|	1922039	-	A	0	0	0	1	0	-
gi|29165615|ref|NC_002745.2|	1922040	-	A	0	0	1	2	0	-
gi|29165615|ref|NC_002745.2|	1922041	-	U	2	0	0	1	0	-
gi|29165615|ref|NC_002745.2|	1922042	-	G	0	0	0	1	0	-
gi|29165615|ref|NC_002745.2|	1922044	-	A	0	1	1	1	0	-
gi|29165615|ref|NC_002745.2|	1922045	-	U	0	0	0	0	1	-
gi|29165615|ref|NC_002745.2|	1922046	-	A	1	1	1	4	3	-
gi|29165615|ref|NC_002745.2|	1922047	-	G	1	1	2	2	1	-
gi|29165615|ref|NC_002745.2|	1922048	-	A	0	2	0	3	2	-
gi|29165615|ref|NC_002745.2|	1922049	-	U	1	0	1	2	1	-
gi|29165615|ref|NC_002745.2|	1922050	-	C	2	2	2	9	6	-
gi|29165615|ref|NC_002745.2|	1922051	-	A	1136	220	223	625	436	-
gi|29165615|ref|NC_002745.2|	1922052	-	A	7	2	5	4	2	-
gi|29165615|ref|NC_002745.2|	1922053	-	A	2	1	0	1	0	-
gi|29165615|ref|NC_002745.2|	1922054	-	A	0	2	1	0	0	-
gi|29165615|ref|NC_002745.2|	1922055	-	G	0	1	1	0	0	-
gi|29165615|ref|NC_002745.2|	1922059	-	C	1	0	2	2	0	-
gi|29165615|ref|NC_002745.2|	1922060	-	A	0	0	1	0	0	-
gi|29165615|ref|NC_002745.2|	1922061	-	U	0	1	0	0	0	-
gi|29165615|ref|NC_002745.2|	1922062	-	G	1	0	0	0	0	-
gi|29165615|ref|NC_002745.2|	1922064	-	U	0	0	1	0	0	-
gi|29165615|ref|NC_002745.2|	1922065	-	U	0	0	0	0	1	-
gi|29165615|ref|NC_002745.2|	1922067	-	U	0	0	0	2	2	-
gi|29165615|ref|NC_002745.2|	1922068	-	U	0	2	1	5	1	-
gi|29165615|ref|NC_002745.2|	1922070	-	C	0	1	0	0	0	SAtRNA53
gi|29165615|ref|NC_002745.2|	1922071	-	C	0	0	0	1	0	SAtRNA53
gi|29165615|ref|NC_002745.2|	1922072	-	A	0	1	0	0	0	SAtRNA53
gi|29165615|ref|NC_002745.2|	1922073	-	C	1	0	0	0	2	SAtRNA53
gi|29165615|ref|NC_002745.2|	1922075	-	U	1	0	0	0	0	SAtRNA53
gi|29165615|ref|NC_002745.2|	1922085	-	C	2	0	0	0	0	SAtRNA53
gi|29165615|ref|NC_002745.2|	1922089	-	C	1	0	0	0	0	SAtRNA53
gi|29165615|ref|NC_002745.2|	1922103	-	G	3	0	0	0	0	SAtRNA53
gi|29165615|ref|NC_002745.2|	1922108	-	A	1	0	0	0	0	SAtRNA53
gi|29165615|ref|NC_002745.2|	1922111	-	U	0	0	1	0	0	SAtRNA53
gi|29165615|ref|NC_002745.2|	1922112	-	U	0	0	0	2	1	SAtRNA53
gi|29165615|ref|NC_002745.2|	1922113	-	U	2	0	0	0	0	SAtRNA53
gi|29165615|ref|NC_002745.2|	1922120	-	C	0	0	0	0	2	SAtRNA53
gi|29165615|ref|NC_002745.2|	1922126	-	G	0	0	0	0	1	SAtRNA53
gi|29165615|ref|NC_002745.2|	1922134	-	C	1	0	0	0	0	SAtRNA53
gi|29165615|ref|NC_002745.2|	1922141	-	G	0	0	1	0	0	SAtRNA53
gi|29165615|ref|NC_002745.2|	1922142	-	G	0	1	0	0	0	SAtRNA53
gi|29165615|ref|NC_002745.2|	1922143	-	G	1	1	0	1	1	SAtRNA53
gi|29165615|ref|NC_002745.2|	1922144	-	G	1287	1680	1600	664	1174	SAtRNA53
gi|29165615|ref|NC_002745.2|	1922145	-	U	9	5	4	2	4	-

gi|29165615|ref|NC_002745.2|	1922154	-	A	1	0	0	0	0	-
gi|29165615|ref|NC_002745.2|	1922204	-	A	1	0	0	0	0	SAtRNA54
gi|29165615|ref|NC_002745.2|	1922220	-	U	0	0	0	0	1	SAtRNA54
gi|29165615|ref|NC_002745.2|	1922232	-	U	1	0	0	0	0	SAtRNA54
gi|29165615|ref|NC_002745.2|	1922239	-	G	9	2	1	1	2	SAtRNA54
gi|29165615|ref|NC_002745.2|	1922243	-	U	0	0	1	0	0	-
gi|29165615|ref|NC_002745.2|	1922264	-	U	8	1	3	3	1	-
gi|29165615|ref|NC_002745.2|	1922265	-	U	0	0	0	0	1	-
gi|29165615|ref|NC_002745.2|	1922298	-	G	0	0	1	0	0	-
gi|29165615|ref|NC_002745.2|	1922299	-	A	0	1	0	0	0	-
gi|29165615|ref|NC_002745.2|	1922300	-	A	0	1	0	0	0	-
gi|29165615|ref|NC_002745.2|	1922301	-	G	0	0	2	0	0	-
gi|29165615|ref|NC_002745.2|	1922303	-	A	0	1	2	0	1	-
gi|29165615|ref|NC_002745.2|	1922304	-	G	0	1	1	0	3	-
gi|29165615|ref|NC_002745.2|	1922307	-	U	0	0	1	0	0	-
gi|29165615|ref|NC_002745.2|	1922308	-	U	0	0	1	0	0	-
gi|29165615|ref|NC_002745.2|	1922312	-	C	2	0	0	0	0	-
gi|29165615|ref|NC_002745.2|	1922320	-	A	0	0	0	0	1	-
gi|29165615|ref|NC_002745.2|	1922322	-	A	0	1	0	0	1	-
gi|29165615|ref|NC_002745.2|	1922324	-	A	0	0	3	2	2	-
gi|29165615|ref|NC_002745.2|	1922325	-	G	1	0	3	3	5	-
gi|29165615|ref|NC_002745.2|	1922326	-	G	7	42	52	13	17	-
gi|29165615|ref|NC_002745.2|	1922334	-	C	0	1	0	0	0	SArRNA10
gi|29165615|ref|NC_002745.2|	1922335	-	C	0	0	0	0	2	SArRNA10
gi|29165615|ref|NC_002745.2|	1922336	-	U	0	0	0	0	1	SArRNA10
gi|29165615|ref|NC_002745.2|	1922337	-	C	8	2	1	2	0	SArRNA10
gi|29165615|ref|NC_002745.2|	1922338	-	C	2	0	0	0	0	SArRNA10
gi|29165615|ref|NC_002745.2|	1922353	-	G	0	1	0	0	0	SArRNA10
gi|29165615|ref|NC_002745.2|	1922372	-	C	1	0	0	0	0	SArRNA10
gi|29165615|ref|NC_002745.2|	1922399	-	G	0	1	0	0	0	SArRNA10
gi|29165615|ref|NC_002745.2|	1922401	-	U	1	0	0	0	0	SArRNA10
gi|29165615|ref|NC_002745.2|	1922402	-	G	0	0	1	0	0	SArRNA10
gi|29165615|ref|NC_002745.2|	1922403	-	G	1	0	0	0	0	SArRNA10
gi|29165615|ref|NC_002745.2|	1922405	-	A	0	2	0	0	0	SArRNA10
gi|29165615|ref|NC_002745.2|	1922406	-	G	6	3	2	2	6	SArRNA10
gi|29165615|ref|NC_002745.2|	1922407	-	C	8	11	0	3	4	SArRNA10
gi|29165615|ref|NC_002745.2|	1922408	-	U	0	0	2	1	0	SArRNA10
gi|29165615|ref|NC_002745.2|	1922409	-	G	19	16	8	4	7	SArRNA10
gi|29165615|ref|NC_002745.2|	1922410	-	C	62	37	21	17	22	SArRNA10
gi|29165615|ref|NC_002745.2|	1922411	-	C	4	5	3	4	4	SArRNA10
gi|29165615|ref|NC_002745.2|	1922412	-	G	8	6	3	2	7	SArRNA10
gi|29165615|ref|NC_002745.2|	1922413	-	A	4	7	0	0	9	SArRNA10
gi|29165615|ref|NC_002745.2|	1922414	-	U	16	26	3	3	23	SArRNA10
gi|29165615|ref|NC_002745.2|	1922415	-	C	203	158	56	42	112	SArRNA10
gi|29165615|ref|NC_002745.2|	1922416	-	G	1	0	3	1	1	SArRNA10
gi|29165615|ref|NC_002745.2|	1922417	-	A	3	1	2	1	1	SArRNA10
gi|29165615|ref|NC_002745.2|	1922418	-	G	3	0	1	1	4	SArRNA10
gi|29165615|ref|NC_002745.2|	1922419	-	G	5	5	2	0	0	SArRNA10
gi|29165615|ref|NC_002745.2|	1922420	-	A	1	11	0	1	4	SArRNA10
gi|29165615|ref|NC_002745.2|	1922421	-	U	6	8	0	3	5	SArRNA10
gi|29165615|ref|NC_002745.2|	1922422	-	U	0	1	0	0	4	SArRNA10
gi|29165615|ref|NC_002745.2|	1922423	-	U	6	3	3	0	8	SArRNA10
gi|29165615|ref|NC_002745.2|	1922424	-	U	10	2	4	4	4	SArRNA10
gi|29165615|ref|NC_002745.2|	1922425	-	C	3	7	2	1	6	SArRNA10
gi|29165615|ref|NC_002745.2|	1922426	-	C	17	31	6	11	22	SArRNA10
gi|29165615|ref|NC_002745.2|	1922427	-	A	5	7	0	2	8	SArRNA10
gi|29165615|ref|NC_002745.2|	1922428	-	A	13	30	3	5	11	SArRNA10
gi|29165615|ref|NC_002745.2|	1922429	-	U	1	4	0	1	3	SArRNA10
gi|29165615|ref|NC_002745.2|	1922430	-	G	3	4	2	0	2	SArRNA10
gi|29165615|ref|NC_002745.2|	1922431	-	A	1	0	0	0	4	SArRNA10
gi|29165615|ref|NC_002745.2|	1922432	-	G	2	0	0	1	0	SArRNA10
gi|29165615|ref|NC_002745.2|	1922433	-	G	0	0	0	0	1	SArRNA10
gi|29165615|ref|NC_002745.2|	1922434	-	U	1	4	0	0	0	SArRNA10
gi|29165615|ref|NC_002745.2|	1922436	-	G	5	6	1	1	2	SArRNA10
gi|29165615|ref|NC_002745.2|	1922437	-	C	10	15	4	5	4	SArRNA10
gi|29165615|ref|NC_002745.2|	1922438	-	C	7	14	1	5	5	SArRNA10
gi|29165615|ref|NC_002745.2|	1922439	-	G	0	1	1	0	0	SArRNA10
gi|29165615|ref|NC_002745.2|	1922440	-	A	0	1	0	0	0	SArRNA10
gi|29165615|ref|NC_002745.2|	1922441	-	A	3	6	2	2	3	SArRNA10
gi|29165615|ref|NC_002745.2|	1922442	-	G	21	19	10	6	9	SArRNA10
gi|29165615|ref|NC_002745.2|	1922443	-	C	18	20	4	7	16	SArRNA10
gi|29165615|ref|NC_002745.2|	1922444	-	C	3	6	3	2	11	SArRNA10
gi|29165615|ref|NC_002745.2|	1922445	-	C	41	30	16	8	19	SArRNA10
gi|29165615|ref|NC_002745.2|	1922446	-	A	11	18	2	4	6	SArRNA10
gi|29165615|ref|NC_002745.2|	1922447	-	C	37	43	21	14	45	SArRNA10
gi|29165615|ref|NC_002745.2|	1922448	-	A	1	3	0	0	1	SArRNA10
gi|29165615|ref|NC_002745.2|	1922449	-	A	1	0	1	1	1	SArRNA10
gi|29165615|ref|NC_002745.2|	1922450	-	U	0	0	0	0	3	SArRNA10
gi|29165615|ref|NC_002745.2|	1922451	-	G	0	0	0	1	0	SArRNA10
gi|29165615|ref|NC_002745.2|	1922452	-	U	0	0	0	0	1	SArRNA10
gi|29165615|ref|NC_002745.2|	1922454	-	U	0	0	0	1	0	SArRNA10

gi|29165615|ref|NC_002745.2|	1922457 -	G	1	0	0	0	0	SArRNA10
gi|29165615|ref|NC_002745.2|	1922458 -	A	2	0	0	0	0	SArRNA10
gi|29165615|ref|NC_002745.2|	1922461 -	A	0	1	3	0	1	SArRNA10
gi|29165615|ref|NC_002745.2|	1922462 -	C	4	13	4	9	15	SArRNA10
gi|29165615|ref|NC_002745.2|	1922463 -	C	0	3	1	0	2	SArRNA10
gi|29165615|ref|NC_002745.2|	1922464 -	A	2	3	1	0	0	SArRNA10
gi|29165615|ref|NC_002745.2|	1922465 -	C	5	1	0	2	0	SArRNA10
gi|29165615|ref|NC_002745.2|	1922467 -	C	4	2	2	1	1	SArRNA10
gi|29165615|ref|NC_002745.2|	1922470 -	C	0	1	0	0	0	SArRNA10
gi|29165615|ref|NC_002745.2|	1922473 -	G	0	3	0	0	0	SArRNA10
gi|29165615|ref|NC_002745.2|	1922474 -	C	0	1	0	0	1	SArRNA10
gi|29165615|ref|NC_002745.2|	1922475 -	C	0	3	0	0	0	SArRNA10
gi|29165615|ref|NC_002745.2|	1922476 -	A	0	1	0	0	0	SArRNA10
gi|29165615|ref|NC_002745.2|	1922477 -	C	1	3	1	0	4	SArRNA10
gi|29165615|ref|NC_002745.2|	1922479 -	C	0	3	0	0	0	SArRNA10
gi|29165615|ref|NC_002745.2|	1922484 -	U	0	0	0	0	3	SArRNA10
gi|29165615|ref|NC_002745.2|	1922485 -	C	2	3	0	0	0	SArRNA10
gi|29165615|ref|NC_002745.2|	1922486 -	U	1	0	0	0	0	SArRNA10
gi|29165615|ref|NC_002745.2|	1922491 -	C	0	0	0	0	1	SArRNA10
gi|29165615|ref|NC_002745.2|	1922492 -	C	0	0	1	0	0	SArRNA10
gi|29165615|ref|NC_002745.2|	1922493 -	U	2	1	0	0	2	SArRNA10
gi|29165615|ref|NC_002745.2|	1922495 -	G	2	0	0	0	1	SArRNA10
gi|29165615|ref|NC_002745.2|	1922496 -	C	1	0	0	2	0	SArRNA10
gi|29165615|ref|NC_002745.2|	1922497 -	A	0	3	0	0	0	SArRNA10
gi|29165615|ref|NC_002745.2|	1922498 -	U	1	5	0	1	1	SArRNA10
gi|29165615|ref|NC_002745.2|	1922499 -	A	1	5	0	3	0	SArRNA10
gi|29165615|ref|NC_002745.2|	1922500 -	A	0	1	0	0	0	SArRNA10
gi|29165615|ref|NC_002745.2|	1922502 -	U	0	0	0	0	1	SArRNA10
gi|29165615|ref|NC_002745.2|	1922503 -	G	1	3	1	0	0	SArRNA10
gi|29165615|ref|NC_002745.2|	1922504 -	G	0	2	0	0	1	SArRNA10
gi|29165615|ref|NC_002745.2|	1922505 -	C	0	0	0	2	1	SArRNA10
gi|29165615|ref|NC_002745.2|	1922506 -	A	0	0	0	1	0	SArRNA10
gi|29165615|ref|NC_002745.2|	1922507 -	U	0	0	0	0	1	SArRNA10
gi|29165615|ref|NC_002745.2|	1922508 -	C	3	2	0	0	2	SArRNA10
gi|29165615|ref|NC_002745.2|	1922509 -	G	0	2	0	0	0	SArRNA10
gi|29165615|ref|NC_002745.2|	1922510 -	U	2	1	0	0	2	SArRNA10
gi|29165615|ref|NC_002745.2|	1922511 -	A	2	3	0	0	1	SArRNA10
gi|29165615|ref|NC_002745.2|	1922512 -	C	4	2	0	2	2	SArRNA10
gi|29165615|ref|NC_002745.2|	1922514 -	A	0	3	0	1	1	SArRNA10
gi|29165615|ref|NC_002745.2|	1922515 -	C	1	1	0	0	3	SArRNA10
gi|29165615|ref|NC_002745.2|	1922516 -	U	1	3	0	2	2	SArRNA10
gi|29165615|ref|NC_002745.2|	1922517 -	A	2	1	0	0	2	SArRNA10
gi|29165615|ref|NC_002745.2|	1922518 -	G	0	0	0	0	1	SArRNA10
gi|29165615|ref|NC_002745.2|	1922519 -	A	0	0	0	0	1	SArRNA10
gi|29165615|ref|NC_002745.2|	1922520 -	U	0	4	0	1	3	SArRNA10
gi|29165615|ref|NC_002745.2|	1922521 -	G	0	1	0	0	1	SArRNA10
gi|29165615|ref|NC_002745.2|	1922524 -	A	1	11	1	0	5	SArRNA10
gi|29165615|ref|NC_002745.2|	1922525 -	A	0	1	0	0	1	SArRNA10
gi|29165615|ref|NC_002745.2|	1922526 -	U	0	1	0	0	0	SArRNA10
gi|29165615|ref|NC_002745.2|	1922527 -	G	0	1	1	0	2	SArRNA10
gi|29165615|ref|NC_002745.2|	1922528 -	A	0	1	0	1	0	SArRNA10
gi|29165615|ref|NC_002745.2|	1922529 -	U	0	1	0	0	3	SArRNA10
gi|29165615|ref|NC_002745.2|	1922530 -	C	4	6	3	4	4	SArRNA10
gi|29165615|ref|NC_002745.2|	1922531 -	G	3	5	0	1	0	SArRNA10
gi|29165615|ref|NC_002745.2|	1922532 -	C	0	1	0	0	2	SArRNA10
gi|29165615|ref|NC_002745.2|	1922533 -	U	1	1	1	0	2	SArRNA10
gi|29165615|ref|NC_002745.2|	1922534 -	A	0	5	0	1	8	SArRNA10
gi|29165615|ref|NC_002745.2|	1922535 -	A	0	3	0	2	7	SArRNA10
gi|29165615|ref|NC_002745.2|	1922536 -	G	0	3	1	1	1	SArRNA10
gi|29165615|ref|NC_002745.2|	1922537 -	G	0	0	0	0	1	SArRNA10
gi|29165615|ref|NC_002745.2|	1922538 -	U	0	1	0	0	2	SArRNA10
gi|29165615|ref|NC_002745.2|	1922539 -	C	2	2	0	0	2	SArRNA10
gi|29165615|ref|NC_002745.2|	1922540 -	G	3	1	0	1	1	SArRNA10
gi|29165615|ref|NC_002745.2|	1922541 -	A	3	3	0	2	2	SArRNA10
gi|29165615|ref|NC_002745.2|	1922542 -	A	0	0	0	1	3	SArRNA10
gi|29165615|ref|NC_002745.2|	1922543 -	G	4	8	1	3	4	SArRNA10
gi|29165615|ref|NC_002745.2|	1922544 -	U	0	0	2	0	1	SArRNA10
gi|29165615|ref|NC_002745.2|	1922545 -	A	2	2	0	1	0	SArRNA10
gi|29165615|ref|NC_002745.2|	1922546 -	C	1	0	0	1	0	SArRNA10
gi|29165615|ref|NC_002745.2|	1922547 -	A	0	2	2	1	1	SArRNA10
gi|29165615|ref|NC_002745.2|	1922548 -	U	0	2	0	0	0	SArRNA10
gi|29165615|ref|NC_002745.2|	1922549 -	C	3	2	0	0	0	SArRNA10
gi|29165615|ref|NC_002745.2|	1922551 -	G	2	7	0	4	2	SArRNA10
gi|29165615|ref|NC_002745.2|	1922552 -	C	32	30	9	13	26	SArRNA10
gi|29165615|ref|NC_002745.2|	1922553 -	U	1	3	0	0	1	SArRNA10
gi|29165615|ref|NC_002745.2|	1922554 -	C	2	2	0	0	3	SArRNA10
gi|29165615|ref|NC_002745.2|	1922555 -	A	0	1	0	0	1	SArRNA10
gi|29165615|ref|NC_002745.2|	1922556 -	A	0	2	0	0	1	SArRNA10
gi|29165615|ref|NC_002745.2|	1922557 -	C	0	7	1	1	2	SArRNA10
gi|29165615|ref|NC_002745.2|	1922559 -	U	0	1	0	0	0	SArRNA10
gi|29165615|ref|NC_002745.2|	1922561 -	U	1	0	0	1	0	SArRNA10

gi|29165615|ref|NC_002745.2|	1922563	-	A	0	0	0	0	2	SArRNA10
gi|29165615|ref|NC_002745.2|	1922566	-	U	0	2	0	0	0	SArRNA10
gi|29165615|ref|NC_002745.2|	1922568	-	A	0	1	0	0	0	SArRNA10
gi|29165615|ref|NC_002745.2|	1922569	-	G	0	0	1	0	0	SArRNA10
gi|29165615|ref|NC_002745.2|	1922570	-	G	0	0	0	0	1	SArRNA10
gi|29165615|ref|NC_002745.2|	1922571	-	C	3	5	4	2	6	SArRNA10
gi|29165615|ref|NC_002745.2|	1922572	-	U	0	3	2	2	1	SArRNA10
gi|29165615|ref|NC_002745.2|	1922573	-	U	2	3	2	0	1	SArRNA10
gi|29165615|ref|NC_002745.2|	1922574	-	G	0	1	0	2	1	SArRNA10
gi|29165615|ref|NC_002745.2|	1922575	-	A	2	1	3	0	3	SArRNA10
gi|29165615|ref|NC_002745.2|	1922576	-	C	1	8	0	0	1	SArRNA10
gi|29165615|ref|NC_002745.2|	1922577	-	U	2	1	3	0	1	SArRNA10
gi|29165615|ref|NC_002745.2|	1922578	-	C	3	6	3	1	5	SArRNA10
gi|29165615|ref|NC_002745.2|	1922579	-	U	3	1	0	0	1	SArRNA10
gi|29165615|ref|NC_002745.2|	1922580	-	U	0	0	1	0	1	SArRNA10
gi|29165615|ref|NC_002745.2|	1922581	-	G	4	6	1	0	5	SArRNA10
gi|29165615|ref|NC_002745.2|	1922582	-	U	2	4	0	1	0	SArRNA10
gi|29165615|ref|NC_002745.2|	1922583	-	U	0	1	1	1	2	SArRNA10
gi|29165615|ref|NC_002745.2|	1922584	-	G	2	0	0	0	0	SArRNA10
gi|29165615|ref|NC_002745.2|	1922585	-	A	1	3	1	0	2	SArRNA10
gi|29165615|ref|NC_002745.2|	1922586	-	A	0	1	0	0	3	SArRNA10
gi|29165615|ref|NC_002745.2|	1922587	-	A	2	3	0	0	1	SArRNA10
gi|29165615|ref|NC_002745.2|	1922588	-	U	1	3	1	0	2	SArRNA10
gi|29165615|ref|NC_002745.2|	1922589	-	A	0	3	1	1	1	SArRNA10
gi|29165615|ref|NC_002745.2|	1922590	-	C	2	3	1	1	1	SArRNA10
gi|29165615|ref|NC_002745.2|	1922591	-	C	14	11	7	5	8	SArRNA10
gi|29165615|ref|NC_002745.2|	1922592	-	C	14	29	12	13	14	SArRNA10
gi|29165615|ref|NC_002745.2|	1922593	-	U	14	20	3	13	13	SArRNA10
gi|29165615|ref|NC_002745.2|	1922594	-	A	9	16	1	3	11	SArRNA10
gi|29165615|ref|NC_002745.2|	1922595	-	A	0	2	0	0	3	SArRNA10
gi|29165615|ref|NC_002745.2|	1922596	-	A	2	2	0	1	1	SArRNA10
gi|29165615|ref|NC_002745.2|	1922597	-	C	0	0	0	0	1	SArRNA10
gi|29165615|ref|NC_002745.2|	1922598	-	G	2	0	0	1	0	SArRNA10
gi|29165615|ref|NC_002745.2|	1922599	-	A	1	0	2	1	1	SArRNA10
gi|29165615|ref|NC_002745.2|	1922600	-	A	2	1	1	1	4	SArRNA10
gi|29165615|ref|NC_002745.2|	1922601	-	C	11	9	6	5	3	SArRNA10
gi|29165615|ref|NC_002745.2|	1922602	-	U	0	1	0	0	1	SArRNA10
gi|29165615|ref|NC_002745.2|	1922603	-	G	1	0	0	1	2	SArRNA10
gi|29165615|ref|NC_002745.2|	1922604	-	G	0	1	0	0	0	SArRNA10
gi|29165615|ref|NC_002745.2|	1922605	-	A	1	1	0	0	1	SArRNA10
gi|29165615|ref|NC_002745.2|	1922607	-	C	0	3	0	0	0	SArRNA10
gi|29165615|ref|NC_002745.2|	1922608	-	G	0	0	0	0	4	SArRNA10
gi|29165615|ref|NC_002745.2|	1922609	-	C	3	4	2	2	6	SArRNA10
gi|29165615|ref|NC_002745.2|	1922610	-	C	2	1	0	0	2	SArRNA10
gi|29165615|ref|NC_002745.2|	1922611	-	A	3	5	2	3	2	SArRNA10
gi|29165615|ref|NC_002745.2|	1922612	-	A	6	7	3	3	6	SArRNA10
gi|29165615|ref|NC_002745.2|	1922613	-	A	0	0	0	0	2	SArRNA10
gi|29165615|ref|NC_002745.2|	1922614	-	G	0	0	0	0	2	SArRNA10
gi|29165615|ref|NC_002745.2|	1922615	-	C	2	2	3	3	3	SArRNA10
gi|29165615|ref|NC_002745.2|	1922616	-	G	0	2	0	0	0	SArRNA10
gi|29165615|ref|NC_002745.2|	1922617	-	A	1	8	1	0	3	SArRNA10
gi|29165615|ref|NC_002745.2|	1922618	-	C	10	5	3	4	1	SArRNA10
gi|29165615|ref|NC_002745.2|	1922619	-	G	1	1	0	1	0	SArRNA10
gi|29165615|ref|NC_002745.2|	1922620	-	G	0	2	0	0	0	SArRNA10
gi|29165615|ref|NC_002745.2|	1922621	-	G	0	0	0	2	0	SArRNA10
gi|29165615|ref|NC_002745.2|	1922622	-	A	4	20	0	2	10	SArRNA10
gi|29165615|ref|NC_002745.2|	1922623	-	A	3	17	0	1	6	SArRNA10
gi|29165615|ref|NC_002745.2|	1922624	-	A	1	1	0	2	3	SArRNA10
gi|29165615|ref|NC_002745.2|	1922625	-	C	3	11	2	1	3	SArRNA10
gi|29165615|ref|NC_002745.2|	1922626	-	A	2	9	3	6	6	SArRNA10
gi|29165615|ref|NC_002745.2|	1922627	-	U	4	3	2	2	6	SArRNA10
gi|29165615|ref|NC_002745.2|	1922628	-	A	25	213	56	12	114	SArRNA10
gi|29165615|ref|NC_002745.2|	1922629	-	A	18	17	8	5	10	SArRNA10
gi|29165615|ref|NC_002745.2|	1922630	-	C	51	41	21	15	29	SArRNA10
gi|29165615|ref|NC_002745.2|	1922631	-	A	1	6	0	0	1	SArRNA10
gi|29165615|ref|NC_002745.2|	1922632	-	G	15	11	6	7	2	SArRNA10
gi|29165615|ref|NC_002745.2|	1922633	-	G	6	6	0	1	3	SArRNA10
gi|29165615|ref|NC_002745.2|	1922634	-	U	0	5	1	1	1	SArRNA10
gi|29165615|ref|NC_002745.2|	1922635	-	A	0	7	0	0	1	SArRNA10
gi|29165615|ref|NC_002745.2|	1922636	-	A	1	7	4	4	5	SArRNA10
gi|29165615|ref|NC_002745.2|	1922637	-	C	14	14	4	8	16	SArRNA10
gi|29165615|ref|NC_002745.2|	1922638	-	A	0	2	0	0	0	SArRNA10
gi|29165615|ref|NC_002745.2|	1922639	-	U	1	5	3	5	3	SArRNA10
gi|29165615|ref|NC_002745.2|	1922640	-	C	5	3	4	0	2	SArRNA10
gi|29165615|ref|NC_002745.2|	1922641	-	G	1	0	0	0	0	SArRNA10
gi|29165615|ref|NC_002745.2|	1922642	-	U	0	0	1	0	0	SArRNA10
gi|29165615|ref|NC_002745.2|	1922643	-	G	4	2	3	2	2	SArRNA10
gi|29165615|ref|NC_002745.2|	1922644	-	C	0	5	1	3	0	SArRNA10
gi|29165615|ref|NC_002745.2|	1922645	-	A	4	12	0	0	6	SArRNA10
gi|29165615|ref|NC_002745.2|	1922646	-	C	46	29	17	14	30	SArRNA10
gi|29165615|ref|NC_002745.2|	1922647	-	A	6	7	2	0	8	SArRNA10

gi|29165615|ref|NC_002745.2|	1922648	-	C	6	7	0	2	3	SArRNA10
gi|29165615|ref|NC_002745.2|	1922649	-	A	6	2	0	3	4	SArRNA10
gi|29165615|ref|NC_002745.2|	1922650	-	U	8	9	3	5	6	SArRNA10
gi|29165615|ref|NC_002745.2|	1922651	-	C	8	9	1	2	2	SArRNA10
gi|29165615|ref|NC_002745.2|	1922652	-	G	2	1	0	1	1	SArRNA10
gi|29165615|ref|NC_002745.2|	1922653	-	G	0	1	0	1	2	SArRNA10
gi|29165615|ref|NC_002745.2|	1922654	-	G	1	1	1	1	0	SArRNA10
gi|29165615|ref|NC_002745.2|	1922655	-	U	1	4	0	0	0	SArRNA10
gi|29165615|ref|NC_002745.2|	1922656	-	U	6	1	0	0	5	SArRNA10
gi|29165615|ref|NC_002745.2|	1922657	-	U	5	7	3	7	13	SArRNA10
gi|29165615|ref|NC_002745.2|	1922658	-	A	9	5	1	8	13	SArRNA10
gi|29165615|ref|NC_002745.2|	1922659	-	G	16	10	6	10	11	SArRNA10
gi|29165615|ref|NC_002745.2|	1922660	-	U	11	7	6	6	11	SArRNA10
gi|29165615|ref|NC_002745.2|	1922661	-	A	7	4	2	3	4	SArRNA10
gi|29165615|ref|NC_002745.2|	1922662	-	U	7	7	3	1	4	SArRNA10
gi|29165615|ref|NC_002745.2|	1922663	-	U	5	3	2	0	2	SArRNA10
gi|29165615|ref|NC_002745.2|	1922664	-	C	2	4	4	1	7	SArRNA10
gi|29165615|ref|NC_002745.2|	1922665	-	C	4	4	0	1	5	SArRNA10
gi|29165615|ref|NC_002745.2|	1922666	-	C	7	13	3	5	3	SArRNA10
gi|29165615|ref|NC_002745.2|	1922667	-	C	22	17	5	11	13	SArRNA10
gi|29165615|ref|NC_002745.2|	1922668	-	G	0	1	0	1	1	SArRNA10
gi|29165615|ref|NC_002745.2|	1922669	-	U	4	4	0	0	1	SArRNA10
gi|29165615|ref|NC_002745.2|	1922670	-	A	4	4	0	1	4	SArRNA10
gi|29165615|ref|NC_002745.2|	1922671	-	C	6	15	0	6	4	SArRNA10
gi|29165615|ref|NC_002745.2|	1922672	-	U	5	2	0	1	5	SArRNA10
gi|29165615|ref|NC_002745.2|	1922673	-	A	7	11	3	8	3	SArRNA10
gi|29165615|ref|NC_002745.2|	1922674	-	C	11	20	4	8	10	SArRNA10
gi|29165615|ref|NC_002745.2|	1922675	-	U	9	8	6	6	10	SArRNA10
gi|29165615|ref|NC_002745.2|	1922676	-	A	7	14	2	6	10	SArRNA10
gi|29165615|ref|NC_002745.2|	1922677	-	A	7	20	2	3	10	SArRNA10
gi|29165615|ref|NC_002745.2|	1922678	-	A	0	0	0	2	4	SArRNA10
gi|29165615|ref|NC_002745.2|	1922679	-	C	4	1	0	1	4	SArRNA10
gi|29165615|ref|NC_002745.2|	1922680	-	U	0	3	1	3	1	SArRNA10
gi|29165615|ref|NC_002745.2|	1922681	-	G	2	2	5	2	2	SArRNA10
gi|29165615|ref|NC_002745.2|	1922682	-	C	3	3	2	2	7	SArRNA10
gi|29165615|ref|NC_002745.2|	1922683	-	A	10	4	2	2	8	SArRNA10
gi|29165615|ref|NC_002745.2|	1922684	-	G	3	2	0	1	8	SArRNA10
gi|29165615|ref|NC_002745.2|	1922685	-	U	3	7	1	1	3	SArRNA10
gi|29165615|ref|NC_002745.2|	1922686	-	A	1	4	2	3	1	SArRNA10
gi|29165615|ref|NC_002745.2|	1922687	-	G	2	3	0	3	2	SArRNA10
gi|29165615|ref|NC_002745.2|	1922689	-	G	0	1	0	0	0	SArRNA10
gi|29165615|ref|NC_002745.2|	1922690	-	G	0	0	0	2	0	SArRNA10
gi|29165615|ref|NC_002745.2|	1922691	-	U	3	3	1	0	0	SArRNA10
gi|29165615|ref|NC_002745.2|	1922692	-	G	8	5	3	3	6	SArRNA10
gi|29165615|ref|NC_002745.2|	1922693	-	G	2	1	0	1	1	SArRNA10
gi|29165615|ref|NC_002745.2|	1922694	-	A	10	16	3	7	7	SArRNA10
gi|29165615|ref|NC_002745.2|	1922695	-	A	16	14	13	15	20	SArRNA10
gi|29165615|ref|NC_002745.2|	1922696	-	G	1	6	3	2	5	SArRNA10
gi|29165615|ref|NC_002745.2|	1922697	-	G	4	8	1	3	2	SArRNA10
gi|29165615|ref|NC_002745.2|	1922698	-	A	4	3	0	5	2	SArRNA10
gi|29165615|ref|NC_002745.2|	1922699	-	G	11	3	3	6	2	SArRNA10
gi|29165615|ref|NC_002745.2|	1922700	-	G	5	10	1	0	4	SArRNA10
gi|29165615|ref|NC_002745.2|	1922701	-	C	11	13	4	5	3	SArRNA10
gi|29165615|ref|NC_002745.2|	1922702	-	C	7	3	5	4	4	SArRNA10
gi|29165615|ref|NC_002745.2|	1922703	-	A	2	8	1	0	6	SArRNA10
gi|29165615|ref|NC_002745.2|	1922704	-	A	15	50	2	3	17	SArRNA10
gi|29165615|ref|NC_002745.2|	1922705	-	A	5	6	0	3	6	SArRNA10
gi|29165615|ref|NC_002745.2|	1922706	-	C	8	19	6	3	15	SArRNA10
gi|29165615|ref|NC_002745.2|	1922707	-	A	1	4	2	1	1	SArRNA10
gi|29165615|ref|NC_002745.2|	1922708	-	G	6	3	1	1	2	SArRNA10
gi|29165615|ref|NC_002745.2|	1922709	-	U	1	3	3	2	4	SArRNA10
gi|29165615|ref|NC_002745.2|	1922710	-	G	1	4	0	1	1	SArRNA10
gi|29165615|ref|NC_002745.2|	1922711	-	G	7	3	0	0	1	SArRNA10
gi|29165615|ref|NC_002745.2|	1922712	-	C	3	5	0	0	4	SArRNA10
gi|29165615|ref|NC_002745.2|	1922713	-	C	1	0	0	0	0	SArRNA10
gi|29165615|ref|NC_002745.2|	1922714	-	G	2	0	0	0	0	SArRNA10
gi|29165615|ref|NC_002745.2|	1922715	-	U	6	1	1	1	1	SArRNA10
gi|29165615|ref|NC_002745.2|	1922716	-	C	3	7	1	2	3	SArRNA10
gi|29165615|ref|NC_002745.2|	1922717	-	A	1	3	0	0	1	SArRNA10
gi|29165615|ref|NC_002745.2|	1922718	-	G	0	3	0	1	1	SArRNA10
gi|29165615|ref|NC_002745.2|	1922719	-	U	0	0	3	2	1	SArRNA10
gi|29165615|ref|NC_002745.2|	1922720	-	U	1	3	0	0	5	SArRNA10
gi|29165615|ref|NC_002745.2|	1922721	-	G	9	14	1	1	7	SArRNA10
gi|29165615|ref|NC_002745.2|	1922722	-	A	4	4	1	0	2	SArRNA10
gi|29165615|ref|NC_002745.2|	1922723	-	A	3	4	0	3	1	SArRNA10
gi|29165615|ref|NC_002745.2|	1922724	-	U	2	4	0	0	5	SArRNA10
gi|29165615|ref|NC_002745.2|	1922725	-	C	4	8	2	1	1	SArRNA10
gi|29165615|ref|NC_002745.2|	1922726	-	U	1	5	2	0	2	SArRNA10
gi|29165615|ref|NC_002745.2|	1922727	-	C	2	7	0	5	5	SArRNA10
gi|29165615|ref|NC_002745.2|	1922728	-	A	3	1	2	4	2	SArRNA10
gi|29165615|ref|NC_002745.2|	1922729	-	C	12	14	8	4	10	SArRNA10

gi|29165615|ref|NC_002745.2|	1922730	-	G	1	3	1	0	3 SArRNA10
gi|29165615|ref|NC_002745.2|	1922731	-	G	1	2	0	1	1 SArRNA10
gi|29165615|ref|NC_002745.2|	1922732	-	G	0	3	2	1	2 SArRNA10
gi|29165615|ref|NC_002745.2|	1922733	-	U	2	12	1	1	4 SArRNA10
gi|29165615|ref|NC_002745.2|	1922734	-	U	2	1	1	1	1 SArRNA10
gi|29165615|ref|NC_002745.2|	1922735	-	G	5	5	1	2	4 SArRNA10
gi|29165615|ref|NC_002745.2|	1922736	-	A	8	5	4	4	4 SArRNA10
gi|29165615|ref|NC_002745.2|	1922737	-	A	11	3	0	3	3 SArRNA10
gi|29165615|ref|NC_002745.2|	1922738	-	U	4	13	0	3	9 SArRNA10
gi|29165615|ref|NC_002745.2|	1922739	-	U	8	5	3	0	5 SArRNA10
gi|29165615|ref|NC_002745.2|	1922740	-	A	7	3	1	5	11 SArRNA10
gi|29165615|ref|NC_002745.2|	1922741	-	C	11	5	2	8	9 SArRNA10
gi|29165615|ref|NC_002745.2|	1922742	-	U	21	28	4	8	7 SArRNA10
gi|29165615|ref|NC_002745.2|	1922743	-	A	10	15	4	6	7 SArRNA10
gi|29165615|ref|NC_002745.2|	1922744	-	C	13	7	9	4	11 SArRNA10
gi|29165615|ref|NC_002745.2|	1922745	-	C	4	9	0	1	3 SArRNA10
gi|29165615|ref|NC_002745.2|	1922746	-	G	5	6	3	1	2 SArRNA10
gi|29165615|ref|NC_002745.2|	1922747	-	U	5	4	2	0	2 SArRNA10
gi|29165615|ref|NC_002745.2|	1922748	-	U	6	8	0	5	1 SArRNA10
gi|29165615|ref|NC_002745.2|	1922749	-	G	1	2	0	0	3 SArRNA10
gi|29165615|ref|NC_002745.2|	1922750	-	A	0	6	0	1	2 SArRNA10
gi|29165615|ref|NC_002745.2|	1922751	-	U	0	2	0	1	3 SArRNA10
gi|29165615|ref|NC_002745.2|	1922752	-	U	0	0	0	1	0 SArRNA10
gi|29165615|ref|NC_002745.2|	1922753	-	C	0	0	0	0	2 SArRNA10
gi|29165615|ref|NC_002745.2|	1922755	-	A	1	0	0	0	0 SArRNA10
gi|29165615|ref|NC_002745.2|	1922756	-	A	2	8	0	1	1 SArRNA10
gi|29165615|ref|NC_002745.2|	1922757	-	U	5	7	0	0	2 SArRNA10
gi|29165615|ref|NC_002745.2|	1922758	-	U	4	8	2	4	6 SArRNA10
gi|29165615|ref|NC_002745.2|	1922759	-	C	13	2	1	2	3 SArRNA10
gi|29165615|ref|NC_002745.2|	1922760	-	C	24	14	2	4	4 SArRNA10
gi|29165615|ref|NC_002745.2|	1922761	-	C	34	3	8	4	7 SArRNA10
gi|29165615|ref|NC_002745.2|	1922762	-	A	6	5	2	3	8 SArRNA10
gi|29165615|ref|NC_002745.2|	1922763	-	A	14	11	0	3	2 SArRNA10
gi|29165615|ref|NC_002745.2|	1922764	-	C	23	2	2	3	2 SArRNA10
gi|29165615|ref|NC_002745.2|	1922765	-	G	5	4	0	1	1 SArRNA10
gi|29165615|ref|NC_002745.2|	1922766	-	C	12	4	2	1	1 SArRNA10
gi|29165615|ref|NC_002745.2|	1922767	-	G	2	0	0	0	1 SArRNA10
gi|29165615|ref|NC_002745.2|	1922768	-	A	8	2	1	1	2 SArRNA10
gi|29165615|ref|NC_002745.2|	1922769	-	G	13	2	1	1	1 SArRNA10
gi|29165615|ref|NC_002745.2|	1922770	-	C	34	4	2	5	4 SArRNA10
gi|29165615|ref|NC_002745.2|	1922771	-	A	16	0	1	0	2 SArRNA10
gi|29165615|ref|NC_002745.2|	1922772	-	A	4	0	0	0	0 SArRNA10
gi|29165615|ref|NC_002745.2|	1922773	-	C	0	2	0	0	0 SArRNA10
gi|29165615|ref|NC_002745.2|	1922774	-	G	2	6	0	0	1 SArRNA10
gi|29165615|ref|NC_002745.2|	1922775	-	C	3	3	0	0	0 SArRNA10
gi|29165615|ref|NC_002745.2|	1922776	-	C	1	4	1	1	1 SArRNA10
gi|29165615|ref|NC_002745.2|	1922777	-	C	2	4	0	0	2 SArRNA10
gi|29165615|ref|NC_002745.2|	1922778	-	U	2	0	0	0	0 SArRNA10
gi|29165615|ref|NC_002745.2|	1922779	-	G	0	0	0	0	1 SArRNA10
gi|29165615|ref|NC_002745.2|	1922780	-	A	1	1	0	0	3 SArRNA10
gi|29165615|ref|NC_002745.2|	1922781	-	A	0	0	1	0	0 SArRNA10
gi|29165615|ref|NC_002745.2|	1922782	-	U	0	0	1	0	0 SArRNA10
gi|29165615|ref|NC_002745.2|	1922785	-	G	0	0	1	5	1 SArRNA10
gi|29165615|ref|NC_002745.2|	1922790	-	U	1	2	0	0	0 SArRNA10
gi|29165615|ref|NC_002745.2|	1922792	-	G	1	0	0	0	2 SArRNA10
gi|29165615|ref|NC_002745.2|	1922793	-	A	1	0	0	0	0 SArRNA10
gi|29165615|ref|NC_002745.2|	1922795	-	U	2	0	0	0	0 SArRNA10
gi|29165615|ref|NC_002745.2|	1922802	-	C	1	1	0	0	0 SArRNA10
gi|29165615|ref|NC_002745.2|	1922804	-	C	0	1	0	1	1 SArRNA10
gi|29165615|ref|NC_002745.2|	1922807	-	C	1	0	0	0	0 SArRNA10
gi|29165615|ref|NC_002745.2|	1922808	-	U	0	0	0	0	2 SArRNA10
gi|29165615|ref|NC_002745.2|	1922809	-	G	0	0	0	0	1 SArRNA10
gi|29165615|ref|NC_002745.2|	1922810	-	C	2	0	0	0	0 SArRNA10
gi|29165615|ref|NC_002745.2|	1922811	-	U	1	0	0	0	0 SArRNA10
gi|29165615|ref|NC_002745.2|	1922813	-	U	3	0	0	0	0 SArRNA10
gi|29165615|ref|NC_002745.2|	1922814	-	U	0	0	0	0	3 SArRNA10
gi|29165615|ref|NC_002745.2|	1922816	-	G	3	1	2	0	0 SArRNA10
gi|29165615|ref|NC_002745.2|	1922817	-	U	1	1	0	0	0 SArRNA10
gi|29165615|ref|NC_002745.2|	1922818	-	A	2	0	0	0	2 SArRNA10
gi|29165615|ref|NC_002745.2|	1922819	-	C	0	0	0	0	1 SArRNA10
gi|29165615|ref|NC_002745.2|	1922820	-	G	2	0	0	0	0 SArRNA10
gi|29165615|ref|NC_002745.2|	1922821	-	U	1	0	2	1	0 SArRNA10
gi|29165615|ref|NC_002745.2|	1922822	-	G	2	1	0	1	1 SArRNA10
gi|29165615|ref|NC_002745.2|	1922824	-	U	4	1	0	0	1 SArRNA10
gi|29165615|ref|NC_002745.2|	1922825	-	G	0	1	0	0	2 SArRNA10
gi|29165615|ref|NC_002745.2|	1922826	-	G	0	1	0	0	2 SArRNA10
gi|29165615|ref|NC_002745.2|	1922827	-	A	4	2	0	0	1 SArRNA10
gi|29165615|ref|NC_002745.2|	1922828	-	C	21	35	8	18	16 SArRNA10
gi|29165615|ref|NC_002745.2|	1922829	-	A	0	1	1	0	0 SArRNA10
gi|29165615|ref|NC_002745.2|	1922830	-	G	1	1	1	0	1 SArRNA10
gi|29165615|ref|NC_002745.2|	1922831	-	U	0	0	0	0	1 SArRNA10

gi|29165615|ref|NC_002745.2|	1922832	-	G	0	1	0	0	3	SArRNA10
gi|29165615|ref|NC_002745.2|	1922833	-	A	0	1	0	0	3	SArRNA10
gi|29165615|ref|NC_002745.2|	1922834	-	A	0	1	0	0	0	SArRNA10
gi|29165615|ref|NC_002745.2|	1922835	-	A	3	1	0	0	1	SArRNA10
gi|29165615|ref|NC_002745.2|	1922836	-	C	9	0	1	0	0	SArRNA10
gi|29165615|ref|NC_002745.2|	1922837	-	A	0	0	1	0	1	SArRNA10
gi|29165615|ref|NC_002745.2|	1922838	-	G	0	1	0	0	0	SArRNA10
gi|29165615|ref|NC_002745.2|	1922839	-	G	0	0	0	0	2	SArRNA10
gi|29165615|ref|NC_002745.2|	1922841	-	G	0	1	0	0	0	SArRNA10
gi|29165615|ref|NC_002745.2|	1922843	-	C	3	1	0	0	1	SArRNA10
gi|29165615|ref|NC_002745.2|	1922845	-	U	0	1	0	0	0	SArRNA10
gi|29165615|ref|NC_002745.2|	1922846	-	C	0	0	1	0	3	SArRNA10
gi|29165615|ref|NC_002745.2|	1922847	-	C	0	0	0	1	2	SArRNA10
gi|29165615|ref|NC_002745.2|	1922848	-	C	4	6	1	1	2	SArRNA10
gi|29165615|ref|NC_002745.2|	1922849	-	C	14	4	1	5	3	SArRNA10
gi|29165615|ref|NC_002745.2|	1922850	-	U	2	1	1	0	0	SArRNA10
gi|29165615|ref|NC_002745.2|	1922851	-	U	1	1	0	0	0	SArRNA10
gi|29165615|ref|NC_002745.2|	1922852	-	C	33	7	4	6	4	SArRNA10
gi|29165615|ref|NC_002745.2|	1922853	-	C	2	0	0	1	0	SArRNA10
gi|29165615|ref|NC_002745.2|	1922854	-	G	0	0	0	1	0	SArRNA10
gi|29165615|ref|NC_002745.2|	1922856	-	G	0	0	1	0	0	SArRNA10
gi|29165615|ref|NC_002745.2|	1922857	-	A	1	0	0	1	1	SArRNA10
gi|29165615|ref|NC_002745.2|	1922858	-	U	3	0	1	1	0	SArRNA10
gi|29165615|ref|NC_002745.2|	1922859	-	A	0	2	2	1	1	SArRNA10
gi|29165615|ref|NC_002745.2|	1922860	-	G	2	1	0	0	3	SArRNA10
gi|29165615|ref|NC_002745.2|	1922861	-	A	0	0	1	1	1	SArRNA10
gi|29165615|ref|NC_002745.2|	1922862	-	G	2	1	1	1	1	SArRNA10
gi|29165615|ref|NC_002745.2|	1922863	-	A	2	1	0	0	0	SArRNA10
gi|29165615|ref|NC_002745.2|	1922864	-	U	1	2	0	0	2	SArRNA10
gi|29165615|ref|NC_002745.2|	1922865	-	C	5	1	0	1	1	SArRNA10
gi|29165615|ref|NC_002745.2|	1922866	-	U	0	0	0	0	4	SArRNA10
gi|29165615|ref|NC_002745.2|	1922867	-	C	1	0	0	3	2	SArRNA10
gi|29165615|ref|NC_002745.2|	1922868	-	A	5	2	2	0	0	SArRNA10
gi|29165615|ref|NC_002745.2|	1922869	-	A	0	1	0	1	1	SArRNA10
gi|29165615|ref|NC_002745.2|	1922870	-	C	3	2	1	0	2	SArRNA10
gi|29165615|ref|NC_002745.2|	1922873	-	U	0	3	0	0	1	SArRNA10
gi|29165615|ref|NC_002745.2|	1922874	-	U	0	3	1	0	1	SArRNA10
gi|29165615|ref|NC_002745.2|	1922875	-	U	2	0	2	1	2	SArRNA10
gi|29165615|ref|NC_002745.2|	1922876	-	C	1	4	0	1	1	SArRNA10
gi|29165615|ref|NC_002745.2|	1922877	-	C	1	2	0	0	0	SArRNA10
gi|29165615|ref|NC_002745.2|	1922878	-	U	0	0	0	2	0	SArRNA10
gi|29165615|ref|NC_002745.2|	1922879	-	A	0	8	2	2	0	SArRNA10
gi|29165615|ref|NC_002745.2|	1922880	-	C	7	5	5	6	8	SArRNA10
gi|29165615|ref|NC_002745.2|	1922881	-	A	0	0	0	0	1	SArRNA10
gi|29165615|ref|NC_002745.2|	1922883	-	U	1	1	1	0	0	SArRNA10
gi|29165615|ref|NC_002745.2|	1922884	-	U	4	4	1	4	0	SArRNA10
gi|29165615|ref|NC_002745.2|	1922885	-	C	2	6	1	1	1	SArRNA10
gi|29165615|ref|NC_002745.2|	1922886	-	U	0	2	2	0	0	SArRNA10
gi|29165615|ref|NC_002745.2|	1922887	-	A	0	0	0	0	1	SArRNA10
gi|29165615|ref|NC_002745.2|	1922888	-	A	1	0	0	1	1	SArRNA10
gi|29165615|ref|NC_002745.2|	1922889	-	A	5	2	1	3	2	SArRNA10
gi|29165615|ref|NC_002745.2|	1922890	-	C	2	2	3	11	3	SArRNA10
gi|29165615|ref|NC_002745.2|	1922891	-	C	1	0	1	0	0	SArRNA10
gi|29165615|ref|NC_002745.2|	1922892	-	A	0	0	1	0	0	SArRNA10
gi|29165615|ref|NC_002745.2|	1922893	-	U	2	3	2	3	1	SArRNA10
gi|29165615|ref|NC_002745.2|	1922894	-	U	1	4	0	1	2	SArRNA10
gi|29165615|ref|NC_002745.2|	1922895	-	C	7	2	1	5	6	SArRNA10
gi|29165615|ref|NC_002745.2|	1922896	-	C	7	3	0	1	1	SArRNA10
gi|29165615|ref|NC_002745.2|	1922897	-	A	0	2	1	0	1	SArRNA10
gi|29165615|ref|NC_002745.2|	1922898	-	A	1	3	2	1	2	SArRNA10
gi|29165615|ref|NC_002745.2|	1922899	-	G	2	0	0	0	1	SArRNA10
gi|29165615|ref|NC_002745.2|	1922900	-	A	1	2	1	2	1	SArRNA10
gi|29165615|ref|NC_002745.2|	1922901	-	A	3	0	1	0	2	SArRNA10
gi|29165615|ref|NC_002745.2|	1922902	-	G	8	2	1	3	4	SArRNA10
gi|29165615|ref|NC_002745.2|	1922903	-	C	2	0	2	0	1	SArRNA10
gi|29165615|ref|NC_002745.2|	1922904	-	G	4	1	0	0	1	SArRNA10
gi|29165615|ref|NC_002745.2|	1922905	-	C	9	7	1	2	4	SArRNA10
gi|29165615|ref|NC_002745.2|	1922906	-	A	0	2	0	0	0	SArRNA10
gi|29165615|ref|NC_002745.2|	1922907	-	A	15	5	1	4	5	SArRNA10
gi|29165615|ref|NC_002745.2|	1922908	-	C	1	2	0	1	0	SArRNA10
gi|29165615|ref|NC_002745.2|	1922909	-	G	2	3	0	1	0	SArRNA10
gi|29165615|ref|NC_002745.2|	1922910	-	A	1	0	0	2	0	SArRNA10
gi|29165615|ref|NC_002745.2|	1922911	-	A	1	1	1	1	2	SArRNA10
gi|29165615|ref|NC_002745.2|	1922912	-	G	1	2	0	0	1	SArRNA10
gi|29165615|ref|NC_002745.2|	1922913	-	C	1	7	0	1	1	SArRNA10
gi|29165615|ref|NC_002745.2|	1922914	-	U	1	0	1	1	3	SArRNA10
gi|29165615|ref|NC_002745.2|	1922915	-	U	0	2	0	0	2	SArRNA10
gi|29165615|ref|NC_002745.2|	1922916	-	A	6	14	3	2	7	SArRNA10
gi|29165615|ref|NC_002745.2|	1922917	-	A	3	6	0	0	2	SArRNA10
gi|29165615|ref|NC_002745.2|	1922918	-	U	1	2	0	0	0	SArRNA10
gi|29165615|ref|NC_002745.2|	1922919	-	U	0	1	1	0	1	SArRNA10

gi|29165615|ref|NC_002745.2|	1922920	-	U	0	2	0	0	1	SArRNA10
gi|29165615|ref|NC_002745.2|	1922921	-	G	0	0	0	1	0	SArRNA10
gi|29165615|ref|NC_002745.2|	1922922	-	G	1	1	0	1	0	SArRNA10
gi|29165615|ref|NC_002745.2|	1922923	-	U	0	1	0	0	0	SArRNA10
gi|29165615|ref|NC_002745.2|	1922924	-	G	1	0	0	1	0	SArRNA10
gi|29165615|ref|NC_002745.2|	1922926	-	A	4	2	3	3	1	SArRNA10
gi|29165615|ref|NC_002745.2|	1922927	-	C	11	4	4	4	3	SArRNA10
gi|29165615|ref|NC_002745.2|	1922928	-	G	4	1	1	3	3	SArRNA10
gi|29165615|ref|NC_002745.2|	1922929	-	A	2	1	0	2	4	SArRNA10
gi|29165615|ref|NC_002745.2|	1922930	-	G	2	1	1	1	1	SArRNA10
gi|29165615|ref|NC_002745.2|	1922931	-	G	0	2	0	0	2	SArRNA10
gi|29165615|ref|NC_002745.2|	1922932	-	U	10	0	1	5	2	SArRNA10
gi|29165615|ref|NC_002745.2|	1922933	-	G	0	1	1	0	3	SArRNA10
gi|29165615|ref|NC_002745.2|	1922934	-	G	3	1	0	0	1	SArRNA10
gi|29165615|ref|NC_002745.2|	1922935	-	C	9	3	2	4	4	SArRNA10
gi|29165615|ref|NC_002745.2|	1922936	-	G	5	0	1	0	0	SArRNA10
gi|29165615|ref|NC_002745.2|	1922937	-	A	2	7	0	1	3	SArRNA10
gi|29165615|ref|NC_002745.2|	1922938	-	A	4	9	3	3	8	SArRNA10
gi|29165615|ref|NC_002745.2|	1922939	-	C	19	9	6	5	9	SArRNA10
gi|29165615|ref|NC_002745.2|	1922940	-	A	8	6	1	3	2	SArRNA10
gi|29165615|ref|NC_002745.2|	1922941	-	C	6	8	2	4	6	SArRNA10
gi|29165615|ref|NC_002745.2|	1922942	-	G	1	1	5	0	8	SArRNA10
gi|29165615|ref|NC_002745.2|	1922943	-	C	8	6	1	1	5	SArRNA10
gi|29165615|ref|NC_002745.2|	1922944	-	C	8	5	2	2	4	SArRNA10
gi|29165615|ref|NC_002745.2|	1922945	-	C	21	10	6	2	21	SArRNA10
gi|29165615|ref|NC_002745.2|	1922946	-	A	0	2	0	0	1	SArRNA10
gi|29165615|ref|NC_002745.2|	1922947	-	G	2	2	0	0	1	SArRNA10
gi|29165615|ref|NC_002745.2|	1922948	-	G	0	0	2	0	0	SArRNA10
gi|29165615|ref|NC_002745.2|	1922950	-	G	0	0	0	0	2	SArRNA10
gi|29165615|ref|NC_002745.2|	1922951	-	C	5	7	0	0	0	SArRNA10
gi|29165615|ref|NC_002745.2|	1922952	-	A	0	0	0	0	1	SArRNA10
gi|29165615|ref|NC_002745.2|	1922953	-	G	5	6	2	2	2	SArRNA10
gi|29165615|ref|NC_002745.2|	1922954	-	U	3	3	0	0	2	SArRNA10
gi|29165615|ref|NC_002745.2|	1922955	-	U	2	6	1	0	2	SArRNA10
gi|29165615|ref|NC_002745.2|	1922956	-	A	3	20	3	1	11	SArRNA10
gi|29165615|ref|NC_002745.2|	1922957	-	A	4	4	4	3	2	SArRNA10
gi|29165615|ref|NC_002745.2|	1922958	-	G	1	1	0	1	2	SArRNA10
gi|29165615|ref|NC_002745.2|	1922959	-	G	1	1	1	2	0	SArRNA10
gi|29165615|ref|NC_002745.2|	1922960	-	A	0	0	2	1	3	SArRNA10
gi|29165615|ref|NC_002745.2|	1922961	-	A	0	5	0	0	1	SArRNA10
gi|29165615|ref|NC_002745.2|	1922962	-	A	0	1	0	0	3	SArRNA10
gi|29165615|ref|NC_002745.2|	1922963	-	C	0	3	0	0	1	SArRNA10
gi|29165615|ref|NC_002745.2|	1922964	-	U	1	6	0	1	0	SArRNA10
gi|29165615|ref|NC_002745.2|	1922965	-	C	1	0	1	1	0	SArRNA10
gi|29165615|ref|NC_002745.2|	1922966	-	A	1	4	0	1	6	SArRNA10
gi|29165615|ref|NC_002745.2|	1922967	-	A	0	9	1	0	5	SArRNA10
gi|29165615|ref|NC_002745.2|	1922968	-	A	1	0	1	0	0	SArRNA10
gi|29165615|ref|NC_002745.2|	1922969	-	G	1	2	0	2	4	SArRNA10
gi|29165615|ref|NC_002745.2|	1922970	-	U	2	0	0	1	1	SArRNA10
gi|29165615|ref|NC_002745.2|	1922971	-	U	0	1	0	0	0	SArRNA10
gi|29165615|ref|NC_002745.2|	1922972	-	G	0	2	0	1	1	SArRNA10
gi|29165615|ref|NC_002745.2|	1922973	-	G	0	0	0	0	1	SArRNA10
gi|29165615|ref|NC_002745.2|	1922974	-	A	1	0	0	0	1	SArRNA10
gi|29165615|ref|NC_002745.2|	1922975	-	A	3	4	1	2	3	SArRNA10
gi|29165615|ref|NC_002745.2|	1922976	-	C	4	5	0	0	1	SArRNA10
gi|29165615|ref|NC_002745.2|	1922977	-	G	0	2	0	0	2	SArRNA10
gi|29165615|ref|NC_002745.2|	1922978	-	C	0	2	0	3	1	SArRNA10
gi|29165615|ref|NC_002745.2|	1922979	-	C	1	1	1	0	0	SArRNA10
gi|29165615|ref|NC_002745.2|	1922980	-	A	1	1	1	0	2	SArRNA10
gi|29165615|ref|NC_002745.2|	1922981	-	G	0	9	0	0	2	SArRNA10
gi|29165615|ref|NC_002745.2|	1922982	-	C	7	2	1	1	4	SArRNA10
gi|29165615|ref|NC_002745.2|	1922983	-	A	0	4	0	0	0	SArRNA10
gi|29165615|ref|NC_002745.2|	1922984	-	U	2	6	3	3	7	SArRNA10
gi|29165615|ref|NC_002745.2|	1922985	-	G	1	0	0	1	1	SArRNA10
gi|29165615|ref|NC_002745.2|	1922986	-	A	1	0	0	0	0	SArRNA10
gi|29165615|ref|NC_002745.2|	1922988	-	G	3	0	0	1	1	SArRNA10
gi|29165615|ref|NC_002745.2|	1922989	-	G	1	0	0	0	1	SArRNA10
gi|29165615|ref|NC_002745.2|	1922990	-	G	0	0	0	0	1	SArRNA10
gi|29165615|ref|NC_002745.2|	1922991	-	U	3	10	1	1	2	SArRNA10
gi|29165615|ref|NC_002745.2|	1922992	-	C	10	35	7	11	26	SArRNA10
gi|29165615|ref|NC_002745.2|	1922993	-	C	4	1	0	0	0	SArRNA10
gi|29165615|ref|NC_002745.2|	1922994	-	G	1	2	0	0	2	SArRNA10
gi|29165615|ref|NC_002745.2|	1922995	-	C	5	4	6	2	10	SArRNA10
gi|29165615|ref|NC_002745.2|	1922996	-	C	4	1	1	0	2	SArRNA10
gi|29165615|ref|NC_002745.2|	1922997	-	U	2	0	2	0	0	SArRNA10
gi|29165615|ref|NC_002745.2|	1922998	-	C	4	4	0	1	2	SArRNA10
gi|29165615|ref|NC_002745.2|	1922999	-	A	3	2	0	1	0	SArRNA10
gi|29165615|ref|NC_002745.2|	1923000	-	C	5	6	0	0	2	SArRNA10
gi|29165615|ref|NC_002745.2|	1923001	-	G	0	0	0	1	0	SArRNA10
gi|29165615|ref|NC_002745.2|	1923002	-	A	0	5	0	2	5	SArRNA10
gi|29165615|ref|NC_002745.2|	1923003	-	A	3	1	1	3	4	SArRNA10

gi|29165615|ref|NC_002745.2|	1923004 -	U	2	5	1	1	3	SArRNA10
gi|29165615|ref|NC_002745.2|	1923005 -	U	3	3	0	0	2	SArRNA10
gi|29165615|ref|NC_002745.2|	1923006 -	A	5	0	0	0	4	SArRNA10
gi|29165615|ref|NC_002745.2|	1923007 -	C	3	4	1	2	1	SArRNA10
gi|29165615|ref|NC_002745.2|	1923008 -	G	0	0	0	0	4	SArRNA10
gi|29165615|ref|NC_002745.2|	1923009 -	C	5	4	1	0	3	SArRNA10
gi|29165615|ref|NC_002745.2|	1923010 -	A	3	2	0	0	0	SArRNA10
gi|29165615|ref|NC_002745.2|	1923011 -	A	3	6	0	1	5	SArRNA10
gi|29165615|ref|NC_002745.2|	1923012 -	U	3	5	0	2	1	SArRNA10
gi|29165615|ref|NC_002745.2|	1923013 -	C	2	4	0	3	7	SArRNA10
gi|29165615|ref|NC_002745.2|	1923014 -	G	2	0	0	1	1	SArRNA10
gi|29165615|ref|NC_002745.2|	1923015 -	A	3	5	2	1	6	SArRNA10
gi|29165615|ref|NC_002745.2|	1923016 -	C	7	16	1	6	10	SArRNA10
gi|29165615|ref|NC_002745.2|	1923018 -	U	0	5	2	1	5	SArRNA10
gi|29165615|ref|NC_002745.2|	1923019 -	C	10	4	6	4	10	SArRNA10
gi|29165615|ref|NC_002745.2|	1923020 -	G	1	0	0	0	0	SArRNA10
gi|29165615|ref|NC_002745.2|	1923021 -	U	6	0	1	2	5	SArRNA10
gi|29165615|ref|NC_002745.2|	1923022 -	G	5	4	2	4	11	SArRNA10
gi|29165615|ref|NC_002745.2|	1923023 -	A	16	10	7	3	16	SArRNA10
gi|29165615|ref|NC_002745.2|	1923024 -	U	12	6	3	5	11	SArRNA10
gi|29165615|ref|NC_002745.2|	1923025 -	U	22	12	3	3	10	SArRNA10
gi|29165615|ref|NC_002745.2|	1923026 -	C	11	4	4	3	10	SArRNA10
gi|29165615|ref|NC_002745.2|	1923027 -	C	15	3	3	3	9	SArRNA10
gi|29165615|ref|NC_002745.2|	1923028 -	C	15	5	6	2	12	SArRNA10
gi|29165615|ref|NC_002745.2|	1923029 -	C	11	8	5	7	22	SArRNA10
gi|29165615|ref|NC_002745.2|	1923030 -	G	4	10	2	4	7	SArRNA10
gi|29165615|ref|NC_002745.2|	1923031 -	C	18	35	5	10	27	SArRNA10
gi|29165615|ref|NC_002745.2|	1923032 -	C	2	5	0	1	3	SArRNA10
gi|29165615|ref|NC_002745.2|	1923033 -	U	3	1	2	1	3	SArRNA10
gi|29165615|ref|NC_002745.2|	1923034 -	U	0	3	0	1	1	SArRNA10
gi|29165615|ref|NC_002745.2|	1923035 -	U	0	0	0	0	3	SArRNA10
gi|29165615|ref|NC_002745.2|	1923036 -	G	0	1	0	0	1	SArRNA10
gi|29165615|ref|NC_002745.2|	1923040 -	G	0	1	0	0	0	SArRNA10
gi|29165615|ref|NC_002745.2|	1923041 -	A	3	0	1	0	2	SArRNA10
gi|29165615|ref|NC_002745.2|	1923042 -	U	7	2	0	0	7	SArRNA10
gi|29165615|ref|NC_002745.2|	1923043 -	U	2	3	3	1	12	SArRNA10
gi|29165615|ref|NC_002745.2|	1923044 -	G	5	0	3	4	11	SArRNA10
gi|29165615|ref|NC_002745.2|	1923045 -	U	8	5	5	3	8	SArRNA10
gi|29165615|ref|NC_002745.2|	1923046 -	G	8	5	5	2	11	SArRNA10
gi|29165615|ref|NC_002745.2|	1923047 -	A	13	10	4	1	9	SArRNA10
gi|29165615|ref|NC_002745.2|	1923048 -	A	6	6	1	0	4	SArRNA10
gi|29165615|ref|NC_002745.2|	1923049 -	U	8	3	0	6	9	SArRNA10
gi|29165615|ref|NC_002745.2|	1923050 -	C	9	9	4	7	8	SArRNA10
gi|29165615|ref|NC_002745.2|	1923051 -	G	1	0	1	1	2	SArRNA10
gi|29165615|ref|NC_002745.2|	1923052 -	U	4	4	2	1	4	SArRNA10
gi|29165615|ref|NC_002745.2|	1923053 -	G	2	0	0	0	1	SArRNA10
gi|29165615|ref|NC_002745.2|	1923054 -	A	5	2	1	0	2	SArRNA10
gi|29165615|ref|NC_002745.2|	1923055 -	G	2	7	0	2	3	SArRNA10
gi|29165615|ref|NC_002745.2|	1923056 -	U	11	12	3	4	9	SArRNA10
gi|29165615|ref|NC_002745.2|	1923057 -	A	8	1	4	9	6	SArRNA10
gi|29165615|ref|NC_002745.2|	1923058 -	G	9	22	5	12	20	SArRNA10
gi|29165615|ref|NC_002745.2|	1923059 -	C	46	62	14	20	40	SArRNA10
gi|29165615|ref|NC_002745.2|	1923060 -	A	9	50	15	4	33	SArRNA10
gi|29165615|ref|NC_002745.2|	1923061 -	A	38	30	13	13	15	SArRNA10
gi|29165615|ref|NC_002745.2|	1923062 -	A	5	5	5	3	5	SArRNA10
gi|29165615|ref|NC_002745.2|	1923063 -	U	10	27	13	18	36	SArRNA10
gi|29165615|ref|NC_002745.2|	1923064 -	G	11	7	9	14	16	SArRNA10
gi|29165615|ref|NC_002745.2|	1923065 -	C	18	15	6	4	5	SArRNA10
gi|29165615|ref|NC_002745.2|	1923066 -	C	0	3	0	1	5	SArRNA10
gi|29165615|ref|NC_002745.2|	1923067 -	G	4	1	0	2	2	SArRNA10
gi|29165615|ref|NC_002745.2|	1923068 -	C	6	6	3	4	10	SArRNA10
gi|29165615|ref|NC_002745.2|	1923069 -	A	6	7	2	3	9	SArRNA10
gi|29165615|ref|NC_002745.2|	1923070 -	C	18	13	3	10	7	SArRNA10
gi|29165615|ref|NC_002745.2|	1923071 -	C	6	5	2	1	0	SArRNA10
gi|29165615|ref|NC_002745.2|	1923072 -	U	7	0	1	2	2	SArRNA10
gi|29165615|ref|NC_002745.2|	1923073 -	G	0	1	0	2	2	SArRNA10
gi|29165615|ref|NC_002745.2|	1923074 -	A	6	5	1	2	2	SArRNA10
gi|29165615|ref|NC_002745.2|	1923075 -	U	3	0	3	1	2	SArRNA10
gi|29165615|ref|NC_002745.2|	1923076 -	G	0	3	0	0	2	SArRNA10
gi|29165615|ref|NC_002745.2|	1923077 -	G	2	1	0	0	2	SArRNA10
gi|29165615|ref|NC_002745.2|	1923078 -	U	7	7	0	0	5	SArRNA10
gi|29165615|ref|NC_002745.2|	1923079 -	C	11	17	2	4	13	SArRNA10
gi|29165615|ref|NC_002745.2|	1923080 -	C	5	9	3	0	7	SArRNA10
gi|29165615|ref|NC_002745.2|	1923081 -	C	6	22	0	0	8	SArRNA10
gi|29165615|ref|NC_002745.2|	1923082 -	A	0	2	0	1	3	SArRNA10
gi|29165615|ref|NC_002745.2|	1923083 -	U	7	3	0	1	7	SArRNA10
gi|29165615|ref|NC_002745.2|	1923084 -	A	3	0	1	0	0	SArRNA10
gi|29165615|ref|NC_002745.2|	1923086 -	A	0	0	1	0	1	SArRNA10
gi|29165615|ref|NC_002745.2|	1923088 -	U	0	0	0	0	1	SArRNA10
gi|29165615|ref|NC_002745.2|	1923090 -	G	1	0	0	0	0	SArRNA10
gi|29165615|ref|NC_002745.2|	1923092 -	A	1	2	0	0	0	SArRNA10

gi|29165615|ref|NC_002745.2|	1923093 -	C	0	2	0	0	1	SArRNA10
gi|29165615|ref|NC_002745.2|	1923094 -	A	0	0	1	0	0	SArRNA10
gi|29165615|ref|NC_002745.2|	1923099 -	A	0	0	0	1	0	SArRNA10
gi|29165615|ref|NC_002745.2|	1923100 -	G	0	0	0	0	1	SArRNA10
gi|29165615|ref|NC_002745.2|	1923108 -	A	1	0	0	0	0	SArRNA10
gi|29165615|ref|NC_002745.2|	1923110 -	A	1	0	0	1	0	SArRNA10
gi|29165615|ref|NC_002745.2|	1923111 -	G	2	1	1	1	4	SArRNA10
gi|29165615|ref|NC_002745.2|	1923112 -	C	0	4	0	0	0	SArRNA10
gi|29165615|ref|NC_002745.2|	1923114 -	U	0	1	0	1	0	SArRNA10
gi|29165615|ref|NC_002745.2|	1923116 -	U	3	2	1	2	4	SArRNA10
gi|29165615|ref|NC_002745.2|	1923117 -	A	1	2	0	1	0	SArRNA10
gi|29165615|ref|NC_002745.2|	1923118 -	G	4	2	0	0	2	SArRNA10
gi|29165615|ref|NC_002745.2|	1923119 -	U	3	0	0	0	0	SArRNA10
gi|29165615|ref|NC_002745.2|	1923120 -	C	0	2	1	1	1	SArRNA10
gi|29165615|ref|NC_002745.2|	1923122 -	C	0	0	2	1	0	SArRNA10
gi|29165615|ref|NC_002745.2|	1923123 -	A	0	0	1	0	1	SArRNA10
gi|29165615|ref|NC_002745.2|	1923124 -	G	0	1	1	1	0	SArRNA10
gi|29165615|ref|NC_002745.2|	1923125 -	U	3	5	0	0	1	SArRNA10
gi|29165615|ref|NC_002745.2|	1923126 -	C	9	3	2	2	6	SArRNA10
gi|29165615|ref|NC_002745.2|	1923127 -	A	5	0	2	0	3	SArRNA10
gi|29165615|ref|NC_002745.2|	1923128 -	A	2	1	1	0	1	SArRNA10
gi|29165615|ref|NC_002745.2|	1923129 -	U	0	2	1	2	1	SArRNA10
gi|29165615|ref|NC_002745.2|	1923130 -	G	0	0	1	1	1	SArRNA10
gi|29165615|ref|NC_002745.2|	1923131 -	U	2	1	0	1	2	SArRNA10
gi|29165615|ref|NC_002745.2|	1923132 -	C	0	3	0	1	2	SArRNA10
gi|29165615|ref|NC_002745.2|	1923133 -	U	0	3	1	0	1	SArRNA10
gi|29165615|ref|NC_002745.2|	1923134 -	G	0	0	0	1	0	SArRNA10
gi|29165615|ref|NC_002745.2|	1923136 -	U	0	0	0	1	3	SArRNA10
gi|29165615|ref|NC_002745.2|	1923137 -	C	1	1	0	0	0	SArRNA10
gi|29165615|ref|NC_002745.2|	1923138 -	U	1	0	0	0	1	SArRNA10
gi|29165615|ref|NC_002745.2|	1923139 -	U	0	0	3	0	1	SArRNA10
gi|29165615|ref|NC_002745.2|	1923140 -	U	1	0	0	0	0	SArRNA10
gi|29165615|ref|NC_002745.2|	1923141 -	C	6	1	1	0	1	SArRNA10
gi|29165615|ref|NC_002745.2|	1923142 -	A	5	2	0	1	2	SArRNA10
gi|29165615|ref|NC_002745.2|	1923143 -	G	2	2	0	1	1	SArRNA10
gi|29165615|ref|NC_002745.2|	1923144 -	C	3	0	1	1	2	SArRNA10
gi|29165615|ref|NC_002745.2|	1923145 -	G	0	0	0	1	1	SArRNA10
gi|29165615|ref|NC_002745.2|	1923147 -	A	1	0	1	0	0	SArRNA10
gi|29165615|ref|NC_002745.2|	1923148 -	A	0	1	0	1	2	SArRNA10
gi|29165615|ref|NC_002745.2|	1923149 -	G	3	0	0	1	2	SArRNA10
gi|29165615|ref|NC_002745.2|	1923150 -	C	0	1	0	1	0	SArRNA10
gi|29165615|ref|NC_002745.2|	1923151 -	G	3	0	0	2	1	SArRNA10
gi|29165615|ref|NC_002745.2|	1923152 -	G	0	0	0	0	1	SArRNA10
gi|29165615|ref|NC_002745.2|	1923153 -	U	0	0	1	1	1	SArRNA10
gi|29165615|ref|NC_002745.2|	1923154 -	G	0	5	2	1	4	SArRNA10
gi|29165615|ref|NC_002745.2|	1923155 -	A	0	0	0	0	1	SArRNA10
gi|29165615|ref|NC_002745.2|	1923156 -	C	1	7	0	0	2	SArRNA10
gi|29165615|ref|NC_002745.2|	1923157 -	C	3	1	0	0	1	SArRNA10
gi|29165615|ref|NC_002745.2|	1923158 -	A	2	2	0	0	1	SArRNA10
gi|29165615|ref|NC_002745.2|	1923159 -	C	0	3	0	1	1	SArRNA10
gi|29165615|ref|NC_002745.2|	1923160 -	A	2	3	1	0	0	SArRNA10
gi|29165615|ref|NC_002745.2|	1923161 -	A	0	2	0	0	1	SArRNA10
gi|29165615|ref|NC_002745.2|	1923162 -	G	0	1	0	0	1	SArRNA10
gi|29165615|ref|NC_002745.2|	1923163 -	G	0	0	0	1	1	SArRNA10
gi|29165615|ref|NC_002745.2|	1923164 -	A	2	2	1	0	1	SArRNA10
gi|29165615|ref|NC_002745.2|	1923165 -	G	2	2	0	1	1	SArRNA10
gi|29165615|ref|NC_002745.2|	1923166 -	G	0	0	0	1	2	SArRNA10
gi|29165615|ref|NC_002745.2|	1923167 -	U	3	1	0	2	1	SArRNA10
gi|29165615|ref|NC_002745.2|	1923168 -	A	1	1	1	1	3	SArRNA10
gi|29165615|ref|NC_002745.2|	1923169 -	U	1	1	1	1	2	SArRNA10
gi|29165615|ref|NC_002745.2|	1923170 -	A	2	0	0	0	1	SArRNA10
gi|29165615|ref|NC_002745.2|	1923171 -	G	2	2	0	0	0	SArRNA10
gi|29165615|ref|NC_002745.2|	1923172 -	A	1	0	0	0	0	SArRNA10
gi|29165615|ref|NC_002745.2|	1923173 -	G	0	3	0	0	2	SArRNA10
gi|29165615|ref|NC_002745.2|	1923174 -	A	1	2	0	0	2	SArRNA10
gi|29165615|ref|NC_002745.2|	1923175 -	C	0	0	1	0	1	SArRNA10
gi|29165615|ref|NC_002745.2|	1923176 -	G	0	0	0	0	2	SArRNA10
gi|29165615|ref|NC_002745.2|	1923177 -	C	3	2	0	0	0	SArRNA10
gi|29165615|ref|NC_002745.2|	1923179 -	U	5	2	1	2	0	SArRNA10
gi|29165615|ref|NC_002745.2|	1923180 -	A	0	6	0	1	1	SArRNA10
gi|29165615|ref|NC_002745.2|	1923181 -	A	0	0	0	0	1	SArRNA10
gi|29165615|ref|NC_002745.2|	1923182 -	A	3	0	0	0	1	SArRNA10
gi|29165615|ref|NC_002745.2|	1923184 -	U	5	1	0	0	0	SArRNA10
gi|29165615|ref|NC_002745.2|	1923185 -	G	1	3	0	2	1	SArRNA10
gi|29165615|ref|NC_002745.2|	1923186 -	G	0	4	0	1	7	SArRNA10
gi|29165615|ref|NC_002745.2|	1923187 -	C	2	0	0	1	1	SArRNA10
gi|29165615|ref|NC_002745.2|	1923188 -	G	0	1	0	0	1	SArRNA10
gi|29165615|ref|NC_002745.2|	1923189 -	A	3	4	2	1	3	SArRNA10
gi|29165615|ref|NC_002745.2|	1923190 -	U	2	3	1	1	3	SArRNA10
gi|29165615|ref|NC_002745.2|	1923191 -	G	3	4	1	2	4	SArRNA10
gi|29165615|ref|NC_002745.2|	1923192 -	U	2	4	0	1	2	SArRNA10

gi|29165615|ref|NC_002745.2|	1923193 -	G	3	2	0	1	2	SArRNA10
gi|29165615|ref|NC_002745.2|	1923194 -	U	0	1	0	1	2	SArRNA10
gi|29165615|ref|NC_002745.2|	1923195 -	A	1	5	2	4	3	SArRNA10
gi|29165615|ref|NC_002745.2|	1923196 -	C	4	2	3	3	6	SArRNA10
gi|29165615|ref|NC_002745.2|	1923197 -	C	1	3	0	4	1	SArRNA10
gi|29165615|ref|NC_002745.2|	1923200 -	A	0	1	0	0	1	SArRNA10
gi|29165615|ref|NC_002745.2|	1923201 -	A	2	0	0	0	0	SArRNA10
gi|29165615|ref|NC_002745.2|	1923202 -	G	0	1	0	1	0	SArRNA10
gi|29165615|ref|NC_002745.2|	1923204 -	U	2	0	0	0	2	SArRNA10
gi|29165615|ref|NC_002745.2|	1923205 -	G	1	1	0	0	0	SArRNA10
gi|29165615|ref|NC_002745.2|	1923206 -	A	1	0	1	0	0	SArRNA10
gi|29165615|ref|NC_002745.2|	1923207 -	A	0	2	0	1	0	SArRNA10
gi|29165615|ref|NC_002745.2|	1923208 -	A	0	0	0	0	1	SArRNA10
gi|29165615|ref|NC_002745.2|	1923209 -	G	0	0	0	1	1	SArRNA10
gi|29165615|ref|NC_002745.2|	1923210 -	G	3	0	0	0	1	SArRNA10
gi|29165615|ref|NC_002745.2|	1923211 -	A	1	1	1	0	1	SArRNA10
gi|29165615|ref|NC_002745.2|	1923212 -	G	11	1	2	1	2	SArRNA10
gi|29165615|ref|NC_002745.2|	1923213 -	A	1	4	1	2	1	SArRNA10
gi|29165615|ref|NC_002745.2|	1923214 -	A	4	2	2	1	1	SArRNA10
gi|29165615|ref|NC_002745.2|	1923215 -	G	1	3	0	1	1	SArRNA10
gi|29165615|ref|NC_002745.2|	1923216 -	A	11	12	2	3	5	SArRNA10
gi|29165615|ref|NC_002745.2|	1923217 -	C	5	4	5	2	2	SArRNA10
gi|29165615|ref|NC_002745.2|	1923218 -	G	1	0	1	0	1	SArRNA10
gi|29165615|ref|NC_002745.2|	1923219 -	U	3	2	0	0	0	SArRNA10
gi|29165615|ref|NC_002745.2|	1923220 -	G	3	0	0	0	0	SArRNA10
gi|29165615|ref|NC_002745.2|	1923221 -	A	6	2	1	2	1	SArRNA10
gi|29165615|ref|NC_002745.2|	1923222 -	G	10	2	0	0	1	SArRNA10
gi|29165615|ref|NC_002745.2|	1923223 -	U	2	3	1	1	3	SArRNA10
gi|29165615|ref|NC_002745.2|	1923224 -	U	10	6	1	2	3	SArRNA10
gi|29165615|ref|NC_002745.2|	1923225 -	C	41	6	3	1	8	SArRNA10
gi|29165615|ref|NC_002745.2|	1923226 -	A	24	8	0	3	7	SArRNA10
gi|29165615|ref|NC_002745.2|	1923227 -	A	4	1	2	1	3	SArRNA10
gi|29165615|ref|NC_002745.2|	1923228 -	A	2	1	0	2	5	SArRNA10
gi|29165615|ref|NC_002745.2|	1923229 -	A	0	0	2	0	3	SArRNA10
gi|29165615|ref|NC_002745.2|	1923230 -	G	0	0	0	0	1	SArRNA10
gi|29165615|ref|NC_002745.2|	1923231 -	G	0	0	0	0	3	SArRNA10
gi|29165615|ref|NC_002745.2|	1923232 -	U	7	5	4	2	3	SArRNA10
gi|29165615|ref|NC_002745.2|	1923233 -	C	12	11	6	3	13	SArRNA10
gi|29165615|ref|NC_002745.2|	1923234 -	A	2	3	1	0	3	SArRNA10
gi|29165615|ref|NC_002745.2|	1923235 -	A	0	3	1	0	2	SArRNA10
gi|29165615|ref|NC_002745.2|	1923236 -	A	5	0	3	0	1	SArRNA10
gi|29165615|ref|NC_002745.2|	1923237 -	G	0	4	1	3	6	SArRNA10
gi|29165615|ref|NC_002745.2|	1923238 -	G	0	1	1	0	2	SArRNA10
gi|29165615|ref|NC_002745.2|	1923239 -	U	6	7	3	1	4	SArRNA10
gi|29165615|ref|NC_002745.2|	1923240 -	U	0	5	3	5	8	SArRNA10
gi|29165615|ref|NC_002745.2|	1923241 -	A	22	31	13	11	29	SArRNA10
gi|29165615|ref|NC_002745.2|	1923242 -	C	133	94	41	36	76	SArRNA10
gi|29165615|ref|NC_002745.2|	1923243 -	U	3	4	8	5	10	SArRNA10
gi|29165615|ref|NC_002745.2|	1923244 -	G	17	11	2	10	15	SArRNA10
gi|29165615|ref|NC_002745.2|	1923245 -	G	11	12	2	6	6	SArRNA10
gi|29165615|ref|NC_002745.2|	1923246 -	G	6	6	1	2	6	SArRNA10
gi|29165615|ref|NC_002745.2|	1923247 -	A	2	4	0	1	3	SArRNA10
gi|29165615|ref|NC_002745.2|	1923248 -	G	7	3	2	5	12	SArRNA10
gi|29165615|ref|NC_002745.2|	1923249 -	G	15	9	12	3	18	SArRNA10
gi|29165615|ref|NC_002745.2|	1923250 -	U	12	13	5	7	23	SArRNA10
gi|29165615|ref|NC_002745.2|	1923251 -	G	22	21	14	8	23	SArRNA10
gi|29165615|ref|NC_002745.2|	1923252 -	C	40	22	21	20	31	SArRNA10
gi|29165615|ref|NC_002745.2|	1923253 -	C	23	25	13	10	28	SArRNA10
gi|29165615|ref|NC_002745.2|	1923254 -	A	15	16	21	8	24	SArRNA10
gi|29165615|ref|NC_002745.2|	1923255 -	A	38	38	16	19	42	SArRNA10
gi|29165615|ref|NC_002745.2|	1923256 -	C	85	77	33	38	62	SArRNA10
gi|29165615|ref|NC_002745.2|	1923257 -	U	31	16	15	8	39	SArRNA10
gi|29165615|ref|NC_002745.2|	1923258 -	C	23	19	14	13	15	SArRNA10
gi|29165615|ref|NC_002745.2|	1923259 -	G	8	9	3	3	13	SArRNA10
gi|29165615|ref|NC_002745.2|	1923260 -	G	10	23	5	8	17	SArRNA10
gi|29165615|ref|NC_002745.2|	1923261 -	C	31	17	9	13	19	SArRNA10
gi|29165615|ref|NC_002745.2|	1923262 -	A	19	22	12	9	29	SArRNA10
gi|29165615|ref|NC_002745.2|	1923263 -	C	43	14	16	26	19	SArRNA10
gi|29165615|ref|NC_002745.2|	1923264 -	C	8	5	3	8	17	SArRNA10
gi|29165615|ref|NC_002745.2|	1923265 -	C	24	18	5	5	14	SArRNA10
gi|29165615|ref|NC_002745.2|	1923266 -	G	38	21	15	13	29	SArRNA10
gi|29165615|ref|NC_002745.2|	1923267 -	A	54	31	19	20	43	SArRNA10
gi|29165615|ref|NC_002745.2|	1923268 -	A	43	44	23	20	52	SArRNA10
gi|29165615|ref|NC_002745.2|	1923269 -	A	52	48	35	16	59	SArRNA10
gi|29165615|ref|NC_002745.2|	1923270 -	G	41	38	15	33	52	SArRNA10
gi|29165615|ref|NC_002745.2|	1923271 -	U	55	34	30	21	46	SArRNA10
gi|29165615|ref|NC_002745.2|	1923272 -	G	34	40	21	18	45	SArRNA10
gi|29165615|ref|NC_002745.2|	1923273 -	U	44	45	22	16	58	SArRNA10
gi|29165615|ref|NC_002745.2|	1923274 -	A	55	44	38	33	82	SArRNA10
gi|29165615|ref|NC_002745.2|	1923275 -	G	25	39	28	26	47	SArRNA10
gi|29165615|ref|NC_002745.2|	1923276 -	U	90	48	26	26	80	SArRNA10

gi|29165615|ref|NC_002745.2|	1923277	-	C	170	187	80	66	137	SArRNA10
gi|29165615|ref|NC_002745.2|	1923278	-	U	28	25	13	19	33	SArRNA10
gi|29165615|ref|NC_002745.2|	1923279	-	G	3	3	6	2	3	SArRNA10
gi|29165615|ref|NC_002745.2|	1923280	-	A	3	3	1	5	10	SArRNA10
gi|29165615|ref|NC_002745.2|	1923281	-	A	10	13	6	8	12	SArRNA10
gi|29165615|ref|NC_002745.2|	1923282	-	U	27	53	19	21	40	SArRNA10
gi|29165615|ref|NC_002745.2|	1923283	-	U	36	29	15	20	60	SArRNA10
gi|29165615|ref|NC_002745.2|	1923284	-	U	70	88	36	41	99	SArRNA10
gi|29165615|ref|NC_002745.2|	1923285	-	U	88	67	58	59	132	SArRNA10
gi|29165615|ref|NC_002745.2|	1923286	-	U	125	105	93	48	143	SArRNA10
gi|29165615|ref|NC_002745.2|	1923287	-	U	139	97	67	65	162	SArRNA10
gi|29165615|ref|NC_002745.2|	1923288	-	G	39	44	21	28	63	SArRNA10
gi|29165615|ref|NC_002745.2|	1923289	-	G	22	15	10	15	18	SArRNA10
gi|29165615|ref|NC_002745.2|	1923290	-	C	29	24	15	14	20	SArRNA10
gi|29165615|ref|NC_002745.2|	1923291	-	G	5	5	2	4	5	SArRNA10
gi|29165615|ref|NC_002745.2|	1923292	-	G	5	7	1	6	6	SArRNA10
gi|29165615|ref|NC_002745.2|	1923293	-	A	26	8	7	5	17	SArRNA10
gi|29165615|ref|NC_002745.2|	1923294	-	U	61	29	18	12	40	SArRNA10
gi|29165615|ref|NC_002745.2|	1923295	-	G	38	14	11	16	32	SArRNA10
gi|29165615|ref|NC_002745.2|	1923296	-	C	113	13	17	16	37	SArRNA10
gi|29165615|ref|NC_002745.2|	1923297	-	G	36	16	8	8	19	SArRNA10
gi|29165615|ref|NC_002745.2|	1923298	-	C	76	32	32	30	40	SArRNA10
gi|29165615|ref|NC_002745.2|	1923299	-	G	19	18	8	19	28	SArRNA10
gi|29165615|ref|NC_002745.2|	1923300	-	C	132	53	37	34	71	SArRNA10
gi|29165615|ref|NC_002745.2|	1923301	-	G	29	9	6	13	16	SArRNA10
gi|29165615|ref|NC_002745.2|	1923302	-	A	53	36	15	17	52	SArRNA10
gi|29165615|ref|NC_002745.2|	1923303	-	A	130	27	26	25	61	SArRNA10
gi|29165615|ref|NC_002745.2|	1923304	-	A	123	48	24	33	45	SArRNA10
gi|29165615|ref|NC_002745.2|	1923305	-	U	118	45	35	36	95	SArRNA10
gi|29165615|ref|NC_002745.2|	1923306	-	G	50	50	27	28	93	SArRNA10
gi|29165615|ref|NC_002745.2|	1923307	-	C	95	43	39	39	120	SArRNA10
gi|29165615|ref|NC_002745.2|	1923308	-	G	11	15	4	4	10	SArRNA10
gi|29165615|ref|NC_002745.2|	1923309	-	G	24	19	16	16	30	SArRNA10
gi|29165615|ref|NC_002745.2|	1923310	-	G	24	11	9	6	17	SArRNA10
gi|29165615|ref|NC_002745.2|	1923311	-	U	86	47	12	15	80	SArRNA10
gi|29165615|ref|NC_002745.2|	1923312	-	U	130	58	43	34	112	SArRNA10
gi|29165615|ref|NC_002745.2|	1923313	-	A	200	89	61	65	152	SArRNA10
gi|29165615|ref|NC_002745.2|	1923314	-	U	277	152	136	125	202	SArRNA10
gi|29165615|ref|NC_002745.2|	1923315	-	U	148	142	83	95	245	SArRNA10
gi|29165615|ref|NC_002745.2|	1923316	-	A	191	149	87	89	209	SArRNA10
gi|29165615|ref|NC_002745.2|	1923317	-	A	130	70	76	57	163	SArRNA10
gi|29165615|ref|NC_002745.2|	1923318	-	G	127	85	69	57	177	SArRNA10
gi|29165615|ref|NC_002745.2|	1923319	-	G	257	170	70	78	234	SArRNA10
gi|29165615|ref|NC_002745.2|	1923320	-	C	615	390	182	243	499	SArRNA10
gi|29165615|ref|NC_002745.2|	1923321	-	C	229	190	66	77	182	SArRNA10
gi|29165615|ref|NC_002745.2|	1923322	-	U	80	68	40	47	96	SArRNA10
gi|29165615|ref|NC_002745.2|	1923323	-	A	71	38	37	37	99	SArRNA10
gi|29165615|ref|NC_002745.2|	1923324	-	U	106	63	41	54	109	SArRNA10
gi|29165615|ref|NC_002745.2|	1923325	-	U	89	67	30	43	111	SArRNA10
gi|29165615|ref|NC_002745.2|	1923326	-	G	92	50	42	32	99	SArRNA10
gi|29165615|ref|NC_002745.2|	1923327	-	C	82	37	35	37	80	SArRNA10
gi|29165615|ref|NC_002745.2|	1923328	-	G	15	7	5	6	10	SArRNA10
gi|29165615|ref|NC_002745.2|	1923329	-	A	50	27	20	15	45	SArRNA10
gi|29165615|ref|NC_002745.2|	1923330	-	A	51	57	29	23	86	SArRNA10
gi|29165615|ref|NC_002745.2|	1923331	-	C	62	59	34	27	85	SArRNA10
gi|29165615|ref|NC_002745.2|	1923332	-	G	27	24	10	12	34	SArRNA10
gi|29165615|ref|NC_002745.2|	1923333	-	G	35	34	10	20	35	SArRNA10
gi|29165615|ref|NC_002745.2|	1923334	-	U	212	296	73	95	211	SArRNA10
gi|29165615|ref|NC_002745.2|	1923335	-	G	113	107	48	53	89	SArRNA10
gi|29165615|ref|NC_002745.2|	1923336	-	G	29	23	20	17	44	SArRNA10
gi|29165615|ref|NC_002745.2|	1923337	-	A	44	23	22	27	69	SArRNA10
gi|29165615|ref|NC_002745.2|	1923338	-	U	83	99	54	55	136	SArRNA10
gi|29165615|ref|NC_002745.2|	1923339	-	G	152	98	51	57	149	SArRNA10
gi|29165615|ref|NC_002745.2|	1923340	-	C	156	87	49	72	151	SArRNA10
gi|29165615|ref|NC_002745.2|	1923341	-	A	129	89	51	55	137	SArRNA10
gi|29165615|ref|NC_002745.2|	1923342	-	U	204	222	115	101	261	SArRNA10
gi|29165615|ref|NC_002745.2|	1923343	-	A	112	109	52	48	161	SArRNA10
gi|29165615|ref|NC_002745.2|	1923344	-	A	31	37	17	18	26	SArRNA10
gi|29165615|ref|NC_002745.2|	1923345	-	U	124	92	61	69	143	SArRNA10
gi|29165615|ref|NC_002745.2|	1923346	-	G	67	56	32	33	94	SArRNA10
gi|29165615|ref|NC_002745.2|	1923347	-	G	61	45	23	27	67	SArRNA10
gi|29165615|ref|NC_002745.2|	1923348	-	C	45	31	17	26	43	SArRNA10
gi|29165615|ref|NC_002745.2|	1923349	-	G	60	70	26	22	83	SArRNA10
gi|29165615|ref|NC_002745.2|	1923350	-	C	122	79	42	40	99	SArRNA10
gi|29165615|ref|NC_002745.2|	1923351	-	C	104	43	20	26	73	SArRNA10
gi|29165615|ref|NC_002745.2|	1923352	-	G	19	13	7	7	10	SArRNA10
gi|29165615|ref|NC_002745.2|	1923353	-	A	79	49	34	36	80	SArRNA10
gi|29165615|ref|NC_002745.2|	1923354	-	C	119	59	50	33	125	SArRNA10
gi|29165615|ref|NC_002745.2|	1923355	-	G	62	32	26	30	55	SArRNA10
gi|29165615|ref|NC_002745.2|	1923356	-	A	75	52	31	34	95	SArRNA10
gi|29165615|ref|NC_002745.2|	1923357	-	C	136	95	49	52	140	SArRNA10

gi|29165615|ref|NC_002745.2|	1923358	-	C	87	66	36	46	98	SArRNA10
gi|29165615|ref|NC_002745.2|	1923359	-	G	46	31	29	17	59	SArRNA10
gi|29165615|ref|NC_002745.2|	1923360	-	U	35	11	16	11	38	SArRNA10
gi|29165615|ref|NC_002745.2|	1923361	-	G	6	2	7	2	9	SArRNA10
gi|29165615|ref|NC_002745.2|	1923362	-	C	19	10	9	12	28	SArRNA10
gi|29165615|ref|NC_002745.2|	1923363	-	A	9	9	3	7	32	SArRNA10
gi|29165615|ref|NC_002745.2|	1923364	-	U	11	7	9	12	37	SArRNA10
gi|29165615|ref|NC_002745.2|	1923365	-	C	6	5	3	8	21	SArRNA10
gi|29165615|ref|NC_002745.2|	1923366	-	A	4	5	3	2	7	SArRNA10
gi|29165615|ref|NC_002745.2|	1923367	-	A	21	13	6	6	30	SArRNA10
gi|29165615|ref|NC_002745.2|	1923368	-	U	23	15	14	15	70	SArRNA10
gi|29165615|ref|NC_002745.2|	1923369	-	C	39	18	17	16	91	SArRNA10
gi|29165615|ref|NC_002745.2|	1923370	-	G	6	3	4	1	16	SArRNA10
gi|29165615|ref|NC_002745.2|	1923371	-	G	11	15	8	8	28	SArRNA10
gi|29165615|ref|NC_002745.2|	1923372	-	C	89	62	45	55	128	SArRNA10
gi|29165615|ref|NC_002745.2|	1923373	-	A	29	10	16	14	51	SArRNA10
gi|29165615|ref|NC_002745.2|	1923374	-	C	81	51	32	38	96	SArRNA10
gi|29165615|ref|NC_002745.2|	1923375	-	C	23	15	10	11	42	SArRNA10
gi|29165615|ref|NC_002745.2|	1923376	-	G	1	0	3	1	5	SArRNA10
gi|29165615|ref|NC_002745.2|	1923377	-	A	8	7	0	6	14	SArRNA10
gi|29165615|ref|NC_002745.2|	1923378	-	A	17	8	4	13	23	SArRNA10
gi|29165615|ref|NC_002745.2|	1923379	-	A	24	12	14	14	31	SArRNA10
gi|29165615|ref|NC_002745.2|	1923380	-	G	11	5	10	4	18	SArRNA10
gi|29165615|ref|NC_002745.2|	1923381	-	A	31	37	26	16	68	SArRNA10
gi|29165615|ref|NC_002745.2|	1923382	-	C	114	63	42	58	121	SArRNA10
gi|29165615|ref|NC_002745.2|	1923383	-	U	108	45	30	40	91	SArRNA10
gi|29165615|ref|NC_002745.2|	1923384	-	A	83	96	42	46	137	SArRNA10
gi|29165615|ref|NC_002745.2|	1923385	-	A	30	20	17	20	38	SArRNA10
gi|29165615|ref|NC_002745.2|	1923386	-	U	73	52	35	36	87	SArRNA10
gi|29165615|ref|NC_002745.2|	1923387	-	C	386	342	203	208	362	SArRNA10
gi|29165615|ref|NC_002745.2|	1923388	-	C	45	46	27	19	72	SArRNA10
gi|29165615|ref|NC_002745.2|	1923389	-	A	16	19	8	5	35	SArRNA10
gi|29165615|ref|NC_002745.2|	1923390	-	U	28	42	17	15	56	SArRNA10
gi|29165615|ref|NC_002745.2|	1923391	-	G	10	18	7	4	19	SArRNA10
gi|29165615|ref|NC_002745.2|	1923392	-	G	24	44	18	19	40	SArRNA10
gi|29165615|ref|NC_002745.2|	1923393	-	C	137	85	44	56	102	SArRNA10
gi|29165615|ref|NC_002745.2|	1923394	-	A	43	34	17	24	49	SArRNA10
gi|29165615|ref|NC_002745.2|	1923395	-	G	74	62	28	50	72	SArRNA10
gi|29165615|ref|NC_002745.2|	1923396	-	U	73	43	14	25	58	SArRNA10
gi|29165615|ref|NC_002745.2|	1923397	-	U	85	62	37	49	121	SArRNA10
gi|29165615|ref|NC_002745.2|	1923398	-	C	117	97	36	49	98	SArRNA10
gi|29165615|ref|NC_002745.2|	1923399	-	U	75	28	25	38	56	SArRNA10
gi|29165615|ref|NC_002745.2|	1923400	-	A	85	69	46	46	120	SArRNA10
gi|29165615|ref|NC_002745.2|	1923401	-	C	224	161	102	98	176	SArRNA10
gi|29165615|ref|NC_002745.2|	1923402	-	A	53	37	25	22	59	SArRNA10
gi|29165615|ref|NC_002745.2|	1923403	-	C	94	53	39	39	70	SArRNA10
gi|29165615|ref|NC_002745.2|	1923404	-	G	11	7	5	2	13	SArRNA10
gi|29165615|ref|NC_002745.2|	1923405	-	U	18	15	6	6	21	SArRNA10
gi|29165615|ref|NC_002745.2|	1923406	-	G	31	38	7	8	36	SArRNA10
gi|29165615|ref|NC_002745.2|	1923407	-	U	88	81	28	38	117	SArRNA10
gi|29165615|ref|NC_002745.2|	1923408	-	C	167	163	24	36	213	SArRNA10
gi|29165615|ref|NC_002745.2|	1923409	-	A	50	51	28	21	70	SArRNA10
gi|29165615|ref|NC_002745.2|	1923410	-	A	41	61	21	24	63	SArRNA10
gi|29165615|ref|NC_002745.2|	1923411	-	U	80	44	43	41	84	SArRNA10
gi|29165615|ref|NC_002745.2|	1923412	-	G	38	39	25	24	53	SArRNA10
gi|29165615|ref|NC_002745.2|	1923413	-	A	42	26	24	26	59	SArRNA10
gi|29165615|ref|NC_002745.2|	1923414	-	A	30	22	14	26	51	SArRNA10
gi|29165615|ref|NC_002745.2|	1923415	-	U	80	57	31	34	115	SArRNA10
gi|29165615|ref|NC_002745.2|	1923416	-	G	57	39	27	49	80	SArRNA10
gi|29165615|ref|NC_002745.2|	1923417	-	U	75	44	28	40	82	SArRNA10
gi|29165615|ref|NC_002745.2|	1923418	-	G	12	19	13	15	24	SArRNA10
gi|29165615|ref|NC_002745.2|	1923419	-	U	91	64	50	46	79	SArRNA10
gi|29165615|ref|NC_002745.2|	1923420	-	A	100	120	34	61	95	SArRNA10
gi|29165615|ref|NC_002745.2|	1923421	-	U	169	173	71	88	154	SArRNA10
gi|29165615|ref|NC_002745.2|	1923422	-	A	103	133	41	56	135	SArRNA10
gi|29165615|ref|NC_002745.2|	1923423	-	C	260	250	65	131	213	SArRNA10
gi|29165615|ref|NC_002745.2|	1923424	-	A	45	44	17	18	50	SArRNA10
gi|29165615|ref|NC_002745.2|	1923425	-	A	55	39	24	31	72	SArRNA10
gi|29165615|ref|NC_002745.2|	1923426	-	G	57	70	21	32	67	SArRNA10
gi|29165615|ref|NC_002745.2|	1923427	-	A	30	30	14	17	40	SArRNA10
gi|29165615|ref|NC_002745.2|	1923428	-	A	32	28	21	15	52	SArRNA10
gi|29165615|ref|NC_002745.2|	1923429	-	G	111	83	47	52	84	SArRNA10
gi|29165615|ref|NC_002745.2|	1923430	-	G	15	18	11	5	13	SArRNA10
gi|29165615|ref|NC_002745.2|	1923431	-	G	18	33	13	10	21	SArRNA10
gi|29165615|ref|NC_002745.2|	1923432	-	A	469	260	191	250	272	SArRNA10
gi|29165615|ref|NC_002745.2|	1923433	-	U	140	101	42	55	121	SArRNA10
gi|29165615|ref|NC_002745.2|	1923434	-	U	46	33	19	44	76	SArRNA10
gi|29165615|ref|NC_002745.2|	1923435	-	A	15	11	6	6	15	SArRNA10
gi|29165615|ref|NC_002745.2|	1923436	-	U	25	36	22	18	48	SArRNA10
gi|29165615|ref|NC_002745.2|	1923437	-	U	25	29	12	16	55	SArRNA10
gi|29165615|ref|NC_002745.2|	1923438	-	G	16	16	7	17	19	SArRNA10

gi|29165615|ref|NC_002745.2|	1923439	-	U	46	22	19	15	40	SArRNA10
gi|29165615|ref|NC_002745.2|	1923440	-	C	55	43	36	22	46	SArRNA10
gi|29165615|ref|NC_002745.2|	1923441	-	U	91	67	25	35	61	SArRNA10
gi|29165615|ref|NC_002745.2|	1923442	-	C	402	305	150	152	272	SArRNA10
gi|29165615|ref|NC_002745.2|	1923443	-	A	112	955	115	29	409	SArRNA10
gi|29165615|ref|NC_002745.2|	1923444	-	A	32	44	12	28	46	SArRNA10
gi|29165615|ref|NC_002745.2|	1923445	-	A	23	18	8	24	35	SArRNA10
gi|29165615|ref|NC_002745.2|	1923446	-	A	29	19	13	13	28	SArRNA10
gi|29165615|ref|NC_002745.2|	1923447	-	U	87	78	79	56	132	SArRNA10
gi|29165615|ref|NC_002745.2|	1923448	-	G	49	47	39	43	88	SArRNA10
gi|29165615|ref|NC_002745.2|	1923449	-	C	55	50	20	29	76	SArRNA10
gi|29165615|ref|NC_002745.2|	1923450	-	U	89	58	45	51	99	SArRNA10
gi|29165615|ref|NC_002745.2|	1923451	-	A	123	74	52	53	158	SArRNA10
gi|29165615|ref|NC_002745.2|	1923452	-	G	100	64	50	53	105	SArRNA10
gi|29165615|ref|NC_002745.2|	1923453	-	G	56	40	23	26	39	SArRNA10
gi|29165615|ref|NC_002745.2|	1923454	-	C	189	108	69	80	107	SArRNA10
gi|29165615|ref|NC_002745.2|	1923455	-	U	15	12	13	11	9	SArRNA10
gi|29165615|ref|NC_002745.2|	1923456	-	U	16	7	6	10	29	SArRNA10
gi|29165615|ref|NC_002745.2|	1923457	-	C	77	27	24	13	37	SArRNA10
gi|29165615|ref|NC_002745.2|	1923458	-	U	117	60	25	32	76	SArRNA10
gi|29165615|ref|NC_002745.2|	1923459	-	G	237	86	56	56	159	SArRNA10
gi|29165615|ref|NC_002745.2|	1923460	-	G	74	58	56	127	81	SArRNA10
gi|29165615|ref|NC_002745.2|	1923461	-	A	153	111	61	88	114	SArRNA10
gi|29165615|ref|NC_002745.2|	1923462	-	A	116	76	33	61	101	SArRNA10
gi|29165615|ref|NC_002745.2|	1923463	-	G	143	79	41	52	67	SArRNA10
gi|29165615|ref|NC_002745.2|	1923464	-	U	88	58	31	48	78	SArRNA10
gi|29165615|ref|NC_002745.2|	1923465	-	A	104	71	44	50	93	SArRNA10
gi|29165615|ref|NC_002745.2|	1923466	-	G	100	78	51	66	84	SArRNA10
gi|29165615|ref|NC_002745.2|	1923467	-	U	94	67	48	45	115	SArRNA10
gi|29165615|ref|NC_002745.2|	1923468	-	G	131	119	58	72	112	SArRNA10
gi|29165615|ref|NC_002745.2|	1923469	-	A	78	65	40	51	84	SArRNA10
gi|29165615|ref|NC_002745.2|	1923470	-	G	63	53	41	39	48	SArRNA10
gi|29165615|ref|NC_002745.2|	1923471	-	U	59	50	17	22	54	SArRNA10
gi|29165615|ref|NC_002745.2|	1923472	-	G	22	21	15	14	38	SArRNA10
gi|29165615|ref|NC_002745.2|	1923473	-	C	34	29	18	24	35	SArRNA10
gi|29165615|ref|NC_002745.2|	1923474	-	G	12	18	8	6	13	SArRNA10
gi|29165615|ref|NC_002745.2|	1923475	-	C	90	58	30	27	78	SArRNA10
gi|29165615|ref|NC_002745.2|	1923476	-	C	50	29	19	38	52	SArRNA10
gi|29165615|ref|NC_002745.2|	1923477	-	G	27	7	8	11	19	SArRNA10
gi|29165615|ref|NC_002745.2|	1923478	-	C	87	53	27	43	83	SArRNA10
gi|29165615|ref|NC_002745.2|	1923479	-	A	39	25	12	27	46	SArRNA10
gi|29165615|ref|NC_002745.2|	1923480	-	A	77	64	31	30	80	SArRNA10
gi|29165615|ref|NC_002745.2|	1923481	-	C	226	107	51	72	121	SArRNA10
gi|29165615|ref|NC_002745.2|	1923482	-	G	33	27	17	22	38	SArRNA10
gi|29165615|ref|NC_002745.2|	1923483	-	A	45	39	13	20	40	SArRNA10
gi|29165615|ref|NC_002745.2|	1923484	-	G	47	36	18	26	37	SArRNA10
gi|29165615|ref|NC_002745.2|	1923485	-	G	90	63	29	38	76	SArRNA10
gi|29165615|ref|NC_002745.2|	1923486	-	C	106	68	28	25	92	SArRNA10
gi|29165615|ref|NC_002745.2|	1923487	-	A	69	23	25	26	50	SArRNA10
gi|29165615|ref|NC_002745.2|	1923488	-	G	46	45	18	23	45	SArRNA10
gi|29165615|ref|NC_002745.2|	1923489	-	U	60	31	18	15	37	SArRNA10
gi|29165615|ref|NC_002745.2|	1923490	-	C	46	21	14	17	31	SArRNA10
gi|29165615|ref|NC_002745.2|	1923491	-	C	35	9	6	17	16	SArRNA10
gi|29165615|ref|NC_002745.2|	1923492	-	G	1	0	0	4	2	SArRNA10
gi|29165615|ref|NC_002745.2|	1923493	-	A	22	9	12	6	16	SArRNA10
gi|29165615|ref|NC_002745.2|	1923494	-	A	28	16	10	18	36	SArRNA10
gi|29165615|ref|NC_002745.2|	1923495	-	A	67	22	17	18	45	SArRNA10
gi|29165615|ref|NC_002745.2|	1923496	-	G	43	31	14	25	58	SArRNA10
gi|29165615|ref|NC_002745.2|	1923497	-	C	66	30	20	26	52	SArRNA10
gi|29165615|ref|NC_002745.2|	1923498	-	G	27	20	4	14	41	SArRNA10
gi|29165615|ref|NC_002745.2|	1923499	-	G	114	66	26	43	102	SArRNA10
gi|29165615|ref|NC_002745.2|	1923500	-	G	147	76	40	71	157	SArRNA10
gi|29165615|ref|NC_002745.2|	1923501	-	U	351	182	56	126	157	SArRNA10
gi|29165615|ref|NC_002745.2|	1923502	-	A	186	241	52	53	210	SArRNA10
gi|29165615|ref|NC_002745.2|	1923503	-	A	139	101	40	66	81	SArRNA10
gi|29165615|ref|NC_002745.2|	1923504	-	C	218	121	48	68	114	SArRNA10
gi|29165615|ref|NC_002745.2|	1923505	-	G	134	45	35	45	90	SArRNA10
gi|29165615|ref|NC_002745.2|	1923506	-	C	182	67	35	53	111	SArRNA10
gi|29165615|ref|NC_002745.2|	1923507	-	C	80	29	14	39	59	SArRNA10
gi|29165615|ref|NC_002745.2|	1923508	-	U	34	16	13	17	25	SArRNA10
gi|29165615|ref|NC_002745.2|	1923509	-	U	189	144	74	109	137	SArRNA10
gi|29165615|ref|NC_002745.2|	1923510	-	C	1125	685	259	374	472	SArRNA10
gi|29165615|ref|NC_002745.2|	1923511	-	U	75	57	18	44	56	SArRNA10
gi|29165615|ref|NC_002745.2|	1923512	-	A	125	419	50	54	177	SArRNA10
gi|29165615|ref|NC_002745.2|	1923513	-	A	37	26	18	12	25	SArRNA10
gi|29165615|ref|NC_002745.2|	1923514	-	G	37	16	12	17	33	SArRNA10
gi|29165615|ref|NC_002745.2|	1923515	-	G	59	27	19	16	32	SArRNA10
gi|29165615|ref|NC_002745.2|	1923516	-	G	43	29	19	27	44	SArRNA10
gi|29165615|ref|NC_002745.2|	1923517	-	A	77	61	32	35	60	SArRNA10
gi|29165615|ref|NC_002745.2|	1923518	-	U	106	106	40	59	85	SArRNA10
gi|29165615|ref|NC_002745.2|	1923519	-	G	169	103	51	99	185	SArRNA10

gi|29165615|ref|NC_002745.2|	1923520 -	A	194	120	53	91	176	SArRNA10
gi|29165615|ref|NC_002745.2|	1923521 -	C	193	122	46	76	129	SArRNA10
gi|29165615|ref|NC_002745.2|	1923522 -	G	110	63	30	40	71	SArRNA10
gi|29165615|ref|NC_002745.2|	1923523 -	A	187	114	59	66	108	SArRNA10
gi|29165615|ref|NC_002745.2|	1923524 -	C	241	82	50	60	97	SArRNA10
gi|29165615|ref|NC_002745.2|	1923525 -	G	72	52	19	26	45	SArRNA10
gi|29165615|ref|NC_002745.2|	1923526 -	G	62	34	22	29	45	SArRNA10
gi|29165615|ref|NC_002745.2|	1923527 -	A	414	178	120	145	174	SArRNA10
gi|29165615|ref|NC_002745.2|	1923528 -	G	143	89	38	81	95	SArRNA10
gi|29165615|ref|NC_002745.2|	1923529 -	G	68	20	9	28	43	SArRNA10
gi|29165615|ref|NC_002745.2|	1923530 -	G	49	46	13	21	35	SArRNA10
gi|29165615|ref|NC_002745.2|	1923531 -	C	281	173	90	141	129	SArRNA10
gi|29165615|ref|NC_002745.2|	1923532 -	A	50	41	20	31	56	SArRNA10
gi|29165615|ref|NC_002745.2|	1923533 -	U	140	88	39	44	100	SArRNA10
gi|29165615|ref|NC_002745.2|	1923534 -	C	163	71	46	50	116	SArRNA10
gi|29165615|ref|NC_002745.2|	1923535 -	C	76	34	9	10	32	SArRNA10
gi|29165615|ref|NC_002745.2|	1923536 -	U	134	72	21	26	53	SArRNA10
gi|29165615|ref|NC_002745.2|	1923537 -	C	95	56	20	32	37	SArRNA10
gi|29165615|ref|NC_002745.2|	1923538 -	A	17	12	4	11	13	SArRNA10
gi|29165615|ref|NC_002745.2|	1923539 -	G	27	24	9	3	12	SArRNA10
gi|29165615|ref|NC_002745.2|	1923540 -	A	26	14	10	16	17	SArRNA10
gi|29165615|ref|NC_002745.2|	1923541 -	C	129	50	16	21	38	SArRNA10
gi|29165615|ref|NC_002745.2|	1923542 -	C	24	20	11	4	20	SArRNA10
gi|29165615|ref|NC_002745.2|	1923543 -	U	22	15	7	12	20	SArRNA10
gi|29165615|ref|NC_002745.2|	1923544 -	G	24	6	6	16	27	SArRNA10
gi|29165615|ref|NC_002745.2|	1923545 -	G	16	20	15	13	24	SArRNA10
gi|29165615|ref|NC_002745.2|	1923546 -	C	67	44	19	14	33	SArRNA10
gi|29165615|ref|NC_002745.2|	1923547 -	A	26	28	18	16	29	SArRNA10
gi|29165615|ref|NC_002745.2|	1923548 -	C	199	144	42	75	110	SArRNA10
gi|29165615|ref|NC_002745.2|	1923549 -	A	68	24	10	13	27	SArRNA10
gi|29165615|ref|NC_002745.2|	1923550 -	G	55	43	19	32	40	SArRNA10
gi|29165615|ref|NC_002745.2|	1923551 -	A	76	39	26	39	38	SArRNA10
gi|29165615|ref|NC_002745.2|	1923552 -	G	26	17	12	13	20	SArRNA10
gi|29165615|ref|NC_002745.2|	1923553 -	U	50	39	18	31	21	SArRNA10
gi|29165615|ref|NC_002745.2|	1923554 -	C	63	52	23	26	35	SArRNA10
gi|29165615|ref|NC_002745.2|	1923555 -	A	29	18	12	12	9	SArRNA10
gi|29165615|ref|NC_002745.2|	1923556 -	A	65	65	15	25	33	SArRNA10
gi|29165615|ref|NC_002745.2|	1923557 -	G	23	18	6	8	24	SArRNA10
gi|29165615|ref|NC_002745.2|	1923558 -	G	7	9	8	11	29	SArRNA10
gi|29165615|ref|NC_002745.2|	1923559 -	U	54	19	9	15	39	SArRNA10
gi|29165615|ref|NC_002745.2|	1923560 -	C	57	35	19	18	24	SArRNA10
gi|29165615|ref|NC_002745.2|	1923561 -	A	18	35	7	10	19	SArRNA10
gi|29165615|ref|NC_002745.2|	1923562 -	C	145	96	31	49	57	SArRNA10
gi|29165615|ref|NC_002745.2|	1923563 -	A	16	26	10	9	15	SArRNA10
gi|29165615|ref|NC_002745.2|	1923564 -	C	71	61	15	19	23	SArRNA10
gi|29165615|ref|NC_002745.2|	1923565 -	C	29	22	7	10	16	SArRNA10
gi|29165615|ref|NC_002745.2|	1923566 -	G	6	2	1	1	6	SArRNA10
gi|29165615|ref|NC_002745.2|	1923567 -	G	24	21	10	12	6	SArRNA10
gi|29165615|ref|NC_002745.2|	1923568 -	C	95	57	34	34	59	SArRNA10
gi|29165615|ref|NC_002745.2|	1923569 -	U	71	31	10	9	34	SArRNA10
gi|29165615|ref|NC_002745.2|	1923570 -	A	49	22	13	16	21	SArRNA10
gi|29165615|ref|NC_002745.2|	1923571 -	G	22	6	2	11	19	SArRNA10
gi|29165615|ref|NC_002745.2|	1923572 -	U	34	11	8	13	17	SArRNA10
gi|29165615|ref|NC_002745.2|	1923573 -	G	19	7	2	4	22	SArRNA10
gi|29165615|ref|NC_002745.2|	1923574 -	G	16	12	2	5	11	SArRNA10
gi|29165615|ref|NC_002745.2|	1923575 -	G	19	14	8	7	16	SArRNA10
gi|29165615|ref|NC_002745.2|	1923576 -	A	29	15	16	10	29	SArRNA10
gi|29165615|ref|NC_002745.2|	1923577 -	G	53	28	13	16	32	SArRNA10
gi|29165615|ref|NC_002745.2|	1923578 -	A	91	25	15	26	68	SArRNA10
gi|29165615|ref|NC_002745.2|	1923579 -	G	56	36	22	15	44	SArRNA10
gi|29165615|ref|NC_002745.2|	1923580 -	U	71	51	33	45	58	SArRNA10
gi|29165615|ref|NC_002745.2|	1923581 -	C	93	53	28	39	72	SArRNA10
gi|29165615|ref|NC_002745.2|	1923582 -	C	47	65	19	51	46	SArRNA10
gi|29165615|ref|NC_002745.2|	1923583 -	A	10	9	5	5	11	SArRNA10
gi|29165615|ref|NC_002745.2|	1923584 -	G	26	34	5	10	22	SArRNA10
gi|29165615|ref|NC_002745.2|	1923585 -	C	35	60	18	14	31	SArRNA10
gi|29165615|ref|NC_002745.2|	1923586 -	C	10	8	3	2	4	SArRNA10
gi|29165615|ref|NC_002745.2|	1923587 -	G	12	8	1	0	4	SArRNA10
gi|29165615|ref|NC_002745.2|	1923588 -	A	13	13	1	3	5	SArRNA10
gi|29165615|ref|NC_002745.2|	1923589 -	U	49	50	28	52	39	SArRNA10
gi|29165615|ref|NC_002745.2|	1923590 -	A	33	32	8	8	19	SArRNA10
gi|29165615|ref|NC_002745.2|	1923591 -	C	76	28	13	16	11	SArRNA10
gi|29165615|ref|NC_002745.2|	1923592 -	G	16	5	2	5	8	SArRNA10
gi|29165615|ref|NC_002745.2|	1923593 -	U	35	17	10	8	30	SArRNA10
gi|29165615|ref|NC_002745.2|	1923594 -	A	52	31	16	16	34	SArRNA10
gi|29165615|ref|NC_002745.2|	1923595 -	G	162	159	80	79	93	SArRNA10
gi|29165615|ref|NC_002745.2|	1923596 -	C	125	111	39	43	98	SArRNA10
gi|29165615|ref|NC_002745.2|	1923597 -	A	57	23	12	26	44	SArRNA10
gi|29165615|ref|NC_002745.2|	1923598 -	A	46	24	17	14	39	SArRNA10
gi|29165615|ref|NC_002745.2|	1923599 -	C	78	35	24	16	36	SArRNA10
gi|29165615|ref|NC_002745.2|	1923600 -	G	27	6	5	10	23	SArRNA10

gi|29165615|ref|NC_002745.2|	1923601 -	G	45	10	4	6	19	SArRNA10
gi|29165615|ref|NC_002745.2|	1923602 -	A	95	46	31	26	33	SArRNA10
gi|29165615|ref|NC_002745.2|	1923603 -	A	95	69	30	46	51	SArRNA10
gi|29165615|ref|NC_002745.2|	1923604 -	C	148	80	49	51	91	SArRNA10
gi|29165615|ref|NC_002745.2|	1923605 -	C	19	18	7	9	10	SArRNA10
gi|29165615|ref|NC_002745.2|	1923606 -	A	8	5	5	8	12	SArRNA10
gi|29165615|ref|NC_002745.2|	1923607 -	U	60	50	31	39	34	SArRNA10
gi|29165615|ref|NC_002745.2|	1923608 -	U	27	28	10	12	18	SArRNA10
gi|29165615|ref|NC_002745.2|	1923609 -	C	33	17	15	5	20	SArRNA10
gi|29165615|ref|NC_002745.2|	1923610 -	G	3	2	0	0	3	SArRNA10
gi|29165615|ref|NC_002745.2|	1923611 -	G	18	21	5	6	12	SArRNA10
gi|29165615|ref|NC_002745.2|	1923612 -	C	94	98	31	57	42	SArRNA10
gi|29165615|ref|NC_002745.2|	1923613 -	A	47	14	6	5	7	SArRNA10
gi|29165615|ref|NC_002745.2|	1923614 -	A	18	11	6	4	11	SArRNA10
gi|29165615|ref|NC_002745.2|	1923615 -	U	16	6	6	4	13	SArRNA10
gi|29165615|ref|NC_002745.2|	1923616 -	G	11	6	2	1	10	SArRNA10
gi|29165615|ref|NC_002745.2|	1923617 -	G	0	0	1	4	3	SArRNA10
gi|29165615|ref|NC_002745.2|	1923618 -	A	16	15	5	4	8	SArRNA10
gi|29165615|ref|NC_002745.2|	1923619 -	A	18	12	8	8	20	SArRNA10
gi|29165615|ref|NC_002745.2|	1923620 -	U	18	10	6	6	19	SArRNA10
gi|29165615|ref|NC_002745.2|	1923621 -	G	20	6	4	4	14	SArRNA10
gi|29165615|ref|NC_002745.2|	1923622 -	G	0	3	0	2	0	SArRNA10
gi|29165615|ref|NC_002745.2|	1923623 -	U	3	3	0	5	0	SArRNA10
gi|29165615|ref|NC_002745.2|	1923624 -	U	2	5	0	2	1	SArRNA10
gi|29165615|ref|NC_002745.2|	1923625 -	G	11	3	6	4	3	SArRNA10
gi|29165615|ref|NC_002745.2|	1923626 -	A	18	13	4	4	8	SArRNA10
gi|29165615|ref|NC_002745.2|	1923627 -	U	39	14	11	8	19	SArRNA10
gi|29165615|ref|NC_002745.2|	1923628 -	C	108	116	34	56	51	SArRNA10
gi|29165615|ref|NC_002745.2|	1923629 -	G	1	1	3	0	3	SArRNA10
gi|29165615|ref|NC_002745.2|	1923630 -	A	8	3	7	6	8	SArRNA10
gi|29165615|ref|NC_002745.2|	1923631 -	U	24	8	7	17	20	SArRNA10
gi|29165615|ref|NC_002745.2|	1923632 -	U	33	18	14	10	15	SArRNA10
gi|29165615|ref|NC_002745.2|	1923633 -	A	17	27	8	15	18	SArRNA10
gi|29165615|ref|NC_002745.2|	1923634 -	C	34	19	7	12	9	SArRNA10
gi|29165615|ref|NC_002745.2|	1923635 -	G	7	3	0	0	0	SArRNA10
gi|29165615|ref|NC_002745.2|	1923636 -	U	6	11	3	3	6	SArRNA10
gi|29165615|ref|NC_002745.2|	1923637 -	C	11	18	9	5	9	SArRNA10
gi|29165615|ref|NC_002745.2|	1923638 -	G	3	3	0	0	2	SArRNA10
gi|29165615|ref|NC_002745.2|	1923639 -	C	34	11	3	3	13	SArRNA10
gi|29165615|ref|NC_002745.2|	1923640 -	G	10	8	2	9	9	SArRNA10
gi|29165615|ref|NC_002745.2|	1923641 -	C	42	27	12	7	32	SArRNA10
gi|29165615|ref|NC_002745.2|	1923642 -	C	20	8	0	2	12	SArRNA10
gi|29165615|ref|NC_002745.2|	1923643 -	U	7	5	1	1	4	SArRNA10
gi|29165615|ref|NC_002745.2|	1923644 -	A	5	11	4	3	6	SArRNA10
gi|29165615|ref|NC_002745.2|	1923645 -	G	8	15	2	5	5	SArRNA10
gi|29165615|ref|NC_002745.2|	1923646 -	G	2	1	0	6	2	SArRNA10
gi|29165615|ref|NC_002745.2|	1923647 -	U	10	6	3	14	11	SArRNA10
gi|29165615|ref|NC_002745.2|	1923648 -	A	13	23	8	9	16	SArRNA10
gi|29165615|ref|NC_002745.2|	1923649 -	G	20	13	4	7	18	SArRNA10
gi|29165615|ref|NC_002745.2|	1923650 -	A	20	13	4	2	11	SArRNA10
gi|29165615|ref|NC_002745.2|	1923651 -	U	23	17	10	8	15	SArRNA10
gi|29165615|ref|NC_002745.2|	1923652 -	A	16	6	10	2	18	SArRNA10
gi|29165615|ref|NC_002745.2|	1923653 -	U	17	9	3	8	15	SArRNA10
gi|29165615|ref|NC_002745.2|	1923654 -	U	15	8	5	4	13	SArRNA10
gi|29165615|ref|NC_002745.2|	1923655 -	C	14	3	2	4	22	SArRNA10
gi|29165615|ref|NC_002745.2|	1923656 -	A	12	13	9	2	15	SArRNA10
gi|29165615|ref|NC_002745.2|	1923657 -	C	40	43	23	15	25	SArRNA10
gi|29165615|ref|NC_002745.2|	1923658 -	U	3	4	2	2	5	SArRNA10
gi|29165615|ref|NC_002745.2|	1923659 -	G	1	1	1	1	3	SArRNA10
gi|29165615|ref|NC_002745.2|	1923660 -	U	10	5	2	5	10	SArRNA10
gi|29165615|ref|NC_002745.2|	1923661 -	C	16	22	5	4	10	SArRNA10
gi|29165615|ref|NC_002745.2|	1923662 -	G	3	5	2	6	2	SArRNA10
gi|29165615|ref|NC_002745.2|	1923663 -	U	8	6	2	0	14	SArRNA10
gi|29165615|ref|NC_002745.2|	1923664 -	U	22	15	17	10	26	SArRNA10
gi|29165615|ref|NC_002745.2|	1923665 -	C	103	101	20	39	60	SArRNA10
gi|29165615|ref|NC_002745.2|	1923666 -	U	40	11	9	6	15	SArRNA10
gi|29165615|ref|NC_002745.2|	1923667 -	G	2	2	3	3	5	SArRNA10
gi|29165615|ref|NC_002745.2|	1923668 -	G	1	13	2	1	2	SArRNA10
gi|29165615|ref|NC_002745.2|	1923669 -	C	10	11	5	2	7	SArRNA10
gi|29165615|ref|NC_002745.2|	1923670 -	A	2	0	0	0	4	SArRNA10
gi|29165615|ref|NC_002745.2|	1923671 -	G	14	17	19	15	19	SArRNA10
gi|29165615|ref|NC_002745.2|	1923672 -	A	29	15	15	14	27	SArRNA10
gi|29165615|ref|NC_002745.2|	1923673 -	A	227	105	66	139	324	SArRNA10
gi|29165615|ref|NC_002745.2|	1923674 -	A	24	16	5	13	25	SArRNA10
gi|29165615|ref|NC_002745.2|	1923675 -	G	21	18	15	12	23	SArRNA10
gi|29165615|ref|NC_002745.2|	1923676 -	U	6	10	4	9	36	SArRNA10
gi|29165615|ref|NC_002745.2|	1923677 -	G	76	75	26	49	76	SArRNA10
gi|29165615|ref|NC_002745.2|	1923678 -	A	20	17	12	13	35	SArRNA10
gi|29165615|ref|NC_002745.2|	1923679 -	A	26	16	12	10	31	SArRNA10
gi|29165615|ref|NC_002745.2|	1923680 -	A	18	21	8	8	22	SArRNA10
gi|29165615|ref|NC_002745.2|	1923681 -	A	15	12	11	13	13	SArRNA10

gi|29165615|ref|NC_002745.2|	1923682	-	C	12	14	3	7	16	SArRNA10
gi|29165615|ref|NC_002745.2|	1923683	-	U	2	6	0	2	5	SArRNA10
gi|29165615|ref|NC_002745.2|	1923684	-	U	4	3	2	3	16	SArRNA10
gi|29165615|ref|NC_002745.2|	1923685	-	G	5	5	3	2	15	SArRNA10
gi|29165615|ref|NC_002745.2|	1923686	-	G	20	10	2	2	26	SArRNA10
gi|29165615|ref|NC_002745.2|	1923687	-	U	26	18	10	16	25	SArRNA10
gi|29165615|ref|NC_002745.2|	1923688	-	A	83	47	17	22	58	SArRNA10
gi|29165615|ref|NC_002745.2|	1923689	-	C	40	20	17	19	54	SArRNA10
gi|29165615|ref|NC_002745.2|	1923690	-	G	12	9	7	2	19	SArRNA10
gi|29165615|ref|NC_002745.2|	1923691	-	C	12	6	2	5	15	SArRNA10
gi|29165615|ref|NC_002745.2|	1923692	-	C	6	4	2	4	7	SArRNA10
gi|29165615|ref|NC_002745.2|	1923693	-	A	9	13	8	13	14	SArRNA10
gi|29165615|ref|NC_002745.2|	1923694	-	A	7	6	1	2	5	SArRNA10
gi|29165615|ref|NC_002745.2|	1923695	-	G	1	0	0	1	1	SArRNA10
gi|29165615|ref|NC_002745.2|	1923696	-	U	4	3	0	0	4	SArRNA10
gi|29165615|ref|NC_002745.2|	1923697	-	U	2	1	0	0	0	SArRNA10
gi|29165615|ref|NC_002745.2|	1923698	-	U	3	0	1	2	4	SArRNA10
gi|29165615|ref|NC_002745.2|	1923699	-	U	0	1	0	0	0	SArRNA10
gi|29165615|ref|NC_002745.2|	1923700	-	A	1	0	0	1	0	SArRNA10
gi|29165615|ref|NC_002745.2|	1923701	-	U	0	6	8	2	8	SArRNA10
gi|29165615|ref|NC_002745.2|	1923702	-	A	13	26	6	7	35	SArRNA10
gi|29165615|ref|NC_002745.2|	1923703	-	A	12	3	1	4	18	SArRNA10
gi|29165615|ref|NC_002745.2|	1923704	-	U	8	16	6	4	23	SArRNA10
gi|29165615|ref|NC_002745.2|	1923705	-	A	10	5	3	2	23	SArRNA10
gi|29165615|ref|NC_002745.2|	1923706	-	G	8	6	2	2	14	SArRNA10
gi|29165615|ref|NC_002745.2|	1923707	-	G	13	12	4	5	25	SArRNA10
gi|29165615|ref|NC_002745.2|	1923708	-	C	54	19	10	17	61	SArRNA10
gi|29165615|ref|NC_002745.2|	1923709	-	C	29	8	10	9	14	SArRNA10
gi|29165615|ref|NC_002745.2|	1923710	-	A	11	3	5	5	20	SArRNA10
gi|29165615|ref|NC_002745.2|	1923711	-	U	49	32	18	15	52	SArRNA10
gi|29165615|ref|NC_002745.2|	1923712	-	A	21	12	3	5	31	SArRNA10
gi|29165615|ref|NC_002745.2|	1923713	-	A	4	6	2	3	4	SArRNA10
gi|29165615|ref|NC_002745.2|	1923714	-	U	7	3	1	2	8	SArRNA10
gi|29165615|ref|NC_002745.2|	1923715	-	C	5	10	5	3	10	SArRNA10
gi|29165615|ref|NC_002745.2|	1923716	-	G	2	1	0	0	0	SArRNA10
gi|29165615|ref|NC_002745.2|	1923717	-	A	2	2	2	1	2	SArRNA10
gi|29165615|ref|NC_002745.2|	1923718	-	G	4	1	0	1	2	SArRNA10
gi|29165615|ref|NC_002745.2|	1923719	-	G	0	3	2	2	5	SArRNA10
gi|29165615|ref|NC_002745.2|	1923720	-	C	4	4	1	2	4	SArRNA10
gi|29165615|ref|NC_002745.2|	1923721	-	C	2	4	0	0	0	SArRNA10
gi|29165615|ref|NC_002745.2|	1923722	-	A	4	0	3	0	6	SArRNA10
gi|29165615|ref|NC_002745.2|	1923723	-	A	11	6	6	2	4	SArRNA10
gi|29165615|ref|NC_002745.2|	1923724	-	A	7	3	3	2	8	SArRNA10
gi|29165615|ref|NC_002745.2|	1923725	-	G	9	4	5	5	13	SArRNA10
gi|29165615|ref|NC_002745.2|	1923726	-	G	19	5	3	5	14	SArRNA10
gi|29165615|ref|NC_002745.2|	1923727	-	G	26	14	8	6	17	SArRNA10
gi|29165615|ref|NC_002745.2|	1923728	-	C	25	9	12	5	19	SArRNA10
gi|29165615|ref|NC_002745.2|	1923729	-	U	12	2	5	6	7	SArRNA10
gi|29165615|ref|NC_002745.2|	1923730	-	U	10	1	2	2	6	SArRNA10
gi|29165615|ref|NC_002745.2|	1923731	-	C	7	3	2	1	6	SArRNA10
gi|29165615|ref|NC_002745.2|	1923732	-	A	4	4	1	2	4	SArRNA10
gi|29165615|ref|NC_002745.2|	1923733	-	A	6	8	1	2	7	SArRNA10
gi|29165615|ref|NC_002745.2|	1923734	-	U	5	2	0	1	2	SArRNA10
gi|29165615|ref|NC_002745.2|	1923735	-	A	8	1	2	2	3	SArRNA10
gi|29165615|ref|NC_002745.2|	1923736	-	G	6	1	4	1	4	SArRNA10
gi|29165615|ref|NC_002745.2|	1923737	-	G	3	2	0	0	3	SArRNA10
gi|29165615|ref|NC_002745.2|	1923738	-	G	3	5	1	0	6	SArRNA10
gi|29165615|ref|NC_002745.2|	1923739	-	U	10	9	2	7	7	SArRNA10
gi|29165615|ref|NC_002745.2|	1923740	-	C	17	22	8	14	25	SArRNA10
gi|29165615|ref|NC_002745.2|	1923741	-	A	1	3	0	4	3	SArRNA10
gi|29165615|ref|NC_002745.2|	1923742	-	G	18	10	1	7	5	SArRNA10
gi|29165615|ref|NC_002745.2|	1923743	-	A	4	6	1	2	7	SArRNA10
gi|29165615|ref|NC_002745.2|	1923744	-	A	10	9	5	3	12	SArRNA10
gi|29165615|ref|NC_002745.2|	1923745	-	U	14	4	2	8	15	SArRNA10
gi|29165615|ref|NC_002745.2|	1923746	-	A	11	3	4	2	9	SArRNA10
gi|29165615|ref|NC_002745.2|	1923747	-	U	8	12	1	3	10	SArRNA10
gi|29165615|ref|NC_002745.2|	1923748	-	C	49	29	18	17	18	SArRNA10
gi|29165615|ref|NC_002745.2|	1923749	-	C	13	6	4	2	5	SArRNA10
gi|29165615|ref|NC_002745.2|	1923750	-	A	9	5	2	1	8	SArRNA10
gi|29165615|ref|NC_002745.2|	1923751	-	U	13	7	4	4	6	SArRNA10
gi|29165615|ref|NC_002745.2|	1923752	-	C	13	16	5	3	13	SArRNA10
gi|29165615|ref|NC_002745.2|	1923753	-	C	5	5	2	1	6	SArRNA10
gi|29165615|ref|NC_002745.2|	1923754	-	A	0	4	0	2	3	SArRNA10
gi|29165615|ref|NC_002745.2|	1923755	-	A	1	2	0	0	4	SArRNA10
gi|29165615|ref|NC_002745.2|	1923756	-	U	5	2	1	0	1	SArRNA10
gi|29165615|ref|NC_002745.2|	1923757	-	A	2	2	0	0	6	SArRNA10
gi|29165615|ref|NC_002745.2|	1923758	-	G	0	0	1	0	1	SArRNA10
gi|29165615|ref|NC_002745.2|	1923759	-	G	0	0	0	0	1	SArRNA10
gi|29165615|ref|NC_002745.2|	1923760	-	U	7	4	2	2	2	SArRNA10
gi|29165615|ref|NC_002745.2|	1923761	-	G	4	1	2	3	4	SArRNA10
gi|29165615|ref|NC_002745.2|	1923762	-	C	9	1	3	2	5	SArRNA10

gi|29165615|ref|NC_002745.2|	1923763 -	A	12	6	1	4	6	SArRNA10
gi|29165615|ref|NC_002745.2|	1923764 -	C	55	28	10	27	29	SArRNA10
gi|29165615|ref|NC_002745.2|	1923765 -	A	12	50	6	2	24	SArRNA10
gi|29165615|ref|NC_002745.2|	1923766 -	A	5	8	2	5	4	SArRNA10
gi|29165615|ref|NC_002745.2|	1923767 -	U	2	1	1	3	1	SArRNA10
gi|29165615|ref|NC_002745.2|	1923768 -	G	1	2	0	0	2	SArRNA10
gi|29165615|ref|NC_002745.2|	1923769 -	A	0	1	1	2	0	SArRNA10
gi|29165615|ref|NC_002745.2|	1923770 -	G	5	1	2	1	3	SArRNA10
gi|29165615|ref|NC_002745.2|	1923771 -	U	22	8	9	8	5	SArRNA10
gi|29165615|ref|NC_002745.2|	1923772 -	G	1	2	0	0	1	SArRNA10
gi|29165615|ref|NC_002745.2|	1923773 -	G	2	0	1	0	1	SArRNA10
gi|29165615|ref|NC_002745.2|	1923774 -	G	8	13	7	9	5	SArRNA10
gi|29165615|ref|NC_002745.2|	1923775 -	C	89	46	41	42	59	SArRNA10
gi|29165615|ref|NC_002745.2|	1923776 -	A	6	1	0	1	4	SArRNA10
gi|29165615|ref|NC_002745.2|	1923777 -	G	23	4	6	5	8	SArRNA10
gi|29165615|ref|NC_002745.2|	1923778 -	G	15	6	3	5	10	SArRNA10
gi|29165615|ref|NC_002745.2|	1923779 -	C	26	4	9	14	13	SArRNA10
gi|29165615|ref|NC_002745.2|	1923780 -	G	10	0	4	1	5	SArRNA10
gi|29165615|ref|NC_002745.2|	1923781 -	G	20	5	4	1	11	SArRNA10
gi|29165615|ref|NC_002745.2|	1923782 -	C	47	10	12	10	28	SArRNA10
gi|29165615|ref|NC_002745.2|	1923783 -	G	3	2	1	5	1	SArRNA10
gi|29165615|ref|NC_002745.2|	1923784 -	A	17	20	2	5	4	SArRNA10
gi|29165615|ref|NC_002745.2|	1923785 -	U	27	16	11	5	13	SArRNA10
gi|29165615|ref|NC_002745.2|	1923786 -	U	13	7	4	2	4	SArRNA10
gi|29165615|ref|NC_002745.2|	1923787 -	G	14	3	1	4	3	SArRNA10
gi|29165615|ref|NC_002745.2|	1923788 -	U	39	8	2	4	7	SArRNA10
gi|29165615|ref|NC_002745.2|	1923789 -	A	15	7	6	10	10	SArRNA10
gi|29165615|ref|NC_002745.2|	1923790 -	G	13	3	3	2	11	SArRNA10
gi|29165615|ref|NC_002745.2|	1923791 -	U	18	6	6	3	6	SArRNA10
gi|29165615|ref|NC_002745.2|	1923792 -	C	8	8	1	4	2	SArRNA10
gi|29165615|ref|NC_002745.2|	1923793 -	U	0	2	2	1	4	SArRNA10
gi|29165615|ref|NC_002745.2|	1923794 -	C	5	3	2	2	4	SArRNA10
gi|29165615|ref|NC_002745.2|	1923795 -	U	0	1	0	0	0	SArRNA10
gi|29165615|ref|NC_002745.2|	1923796 -	U	4	2	1	3	3	SArRNA10
gi|29165615|ref|NC_002745.2|	1923797 -	C	2	0	2	2	8	SArRNA10
gi|29165615|ref|NC_002745.2|	1923798 -	G	0	2	0	1	1	SArRNA10
gi|29165615|ref|NC_002745.2|	1923799 -	U	7	2	0	1	1	SArRNA10
gi|29165615|ref|NC_002745.2|	1923800 -	U	9	1	1	3	7	SArRNA10
gi|29165615|ref|NC_002745.2|	1923801 -	C	15	18	3	8	9	SArRNA10
gi|29165615|ref|NC_002745.2|	1923802 -	G	0	1	0	0	1	SArRNA10
gi|29165615|ref|NC_002745.2|	1923803 -	A	5	1	3	3	2	SArRNA10
gi|29165615|ref|NC_002745.2|	1923804 -	A	6	6	2	3	9	SArRNA10
gi|29165615|ref|NC_002745.2|	1923805 -	G	2	4	1	0	6	SArRNA10
gi|29165615|ref|NC_002745.2|	1923806 -	A	1	2	0	4	8	SArRNA10
gi|29165615|ref|NC_002745.2|	1923807 -	G	3	1	1	0	2	SArRNA10
gi|29165615|ref|NC_002745.2|	1923808 -	C	54	16	5	7	18	SArRNA10
gi|29165615|ref|NC_002745.2|	1923809 -	A	13	4	3	4	8	SArRNA10
gi|29165615|ref|NC_002745.2|	1923810 -	G	9	14	6	8	8	SArRNA10
gi|29165615|ref|NC_002745.2|	1923811 -	G	10	10	8	4	11	SArRNA10
gi|29165615|ref|NC_002745.2|	1923812 -	C	21	16	7	5	28	SArRNA10
gi|29165615|ref|NC_002745.2|	1923813 -	A	13	29	7	6	17	SArRNA10
gi|29165615|ref|NC_002745.2|	1923814 -	A	15	5	5	2	8	SArRNA10
gi|29165615|ref|NC_002745.2|	1923815 -	G	9	4	3	3	4	SArRNA10
gi|29165615|ref|NC_002745.2|	1923816 -	C	33	19	8	6	15	SArRNA10
gi|29165615|ref|NC_002745.2|	1923817 -	G	6	1	3	2	2	SArRNA10
gi|29165615|ref|NC_002745.2|	1923818 -	A	7	3	3	3	4	SArRNA10
gi|29165615|ref|NC_002745.2|	1923819 -	G	23	21	8	9	17	SArRNA10
gi|29165615|ref|NC_002745.2|	1923820 -	C	64	37	15	26	42	SArRNA10
gi|29165615|ref|NC_002745.2|	1923821 -	U	28	12	4	7	19	SArRNA10
gi|29165615|ref|NC_002745.2|	1923822 -	G	5	11	4	2	2	SArRNA10
gi|29165615|ref|NC_002745.2|	1923823 -	A	4	8	3	1	7	SArRNA10
gi|29165615|ref|NC_002745.2|	1923824 -	A	9	18	4	7	12	SArRNA10
gi|29165615|ref|NC_002745.2|	1923825 -	C	57	45	17	24	33	SArRNA10
gi|29165615|ref|NC_002745.2|	1923826 -	G	6	7	1	7	5	SArRNA10
gi|29165615|ref|NC_002745.2|	1923827 -	U	5	2	3	3	3	SArRNA10
gi|29165615|ref|NC_002745.2|	1923828 -	A	59	44	19	17	47	SArRNA10
gi|29165615|ref|NC_002745.2|	1923829 -	C	229	156	58	98	147	SArRNA10
gi|29165615|ref|NC_002745.2|	1923830 -	A	66	56	12	12	37	SArRNA10
gi|29165615|ref|NC_002745.2|	1923831 -	U	18	34	4	7	20	SArRNA10
gi|29165615|ref|NC_002745.2|	1923832 -	A	15	31	5	2	17	SArRNA10
gi|29165615|ref|NC_002745.2|	1923833 -	A	11	15	6	7	6	SArRNA10
gi|29165615|ref|NC_002745.2|	1923834 -	U	7	14	1	1	7	SArRNA10
gi|29165615|ref|NC_002745.2|	1923835 -	C	6	9	3	3	16	SArRNA10
gi|29165615|ref|NC_002745.2|	1923836 -	C	1	0	0	2	0	SArRNA10
gi|29165615|ref|NC_002745.2|	1923837 -	G	1	0	1	0	1	SArRNA10
gi|29165615|ref|NC_002745.2|	1923838 -	U	1	0	0	0	1	SArRNA10
gi|29165615|ref|NC_002745.2|	1923839 -	G	1	0	0	0	1	SArRNA10
gi|29165615|ref|NC_002745.2|	1923840 -	C	3	1	0	0	2	SArRNA10
gi|29165615|ref|NC_002745.2|	1923842 -	G	0	2	0	0	1	SArRNA10
gi|29165615|ref|NC_002745.2|	1923843 -	C	3	0	2	1	0	SArRNA10
gi|29165615|ref|NC_002745.2|	1923845 -	G	20	0	1	1	2	SArRNA10

gi|29165615|ref|NC_002745.2|	1923846	-	U	18	2	2	0	2	SArRNA10
gi|29165615|ref|NC_002745.2|	1923847	-	C	13	1	0	1	2	SArRNA10
gi|29165615|ref|NC_002745.2|	1923848	-	G	42	5	5	0	3	SArRNA10
gi|29165615|ref|NC_002745.2|	1923849	-	C	101	4	6	0	9	SArRNA10
gi|29165615|ref|NC_002745.2|	1923850	-	A	7	0	5	2	1	SArRNA10
gi|29165615|ref|NC_002745.2|	1923851	-	A	11	2	2	1	0	SArRNA10
gi|29165615|ref|NC_002745.2|	1923852	-	G	14	0	1	5	2	SArRNA10
gi|29165615|ref|NC_002745.2|	1923853	-	U	11	3	0	4	2	SArRNA10
gi|29165615|ref|NC_002745.2|	1923854	-	A	13	0	5	3	2	SArRNA10
gi|29165615|ref|NC_002745.2|	1923855	-	G	5	5	2	2	4	SArRNA10
gi|29165615|ref|NC_002745.2|	1923856	-	G	5	2	0	1	2	SArRNA10
gi|29165615|ref|NC_002745.2|	1923857	-	A	8	3	4	0	4	SArRNA10
gi|29165615|ref|NC_002745.2|	1923858	-	C	29	2	5	2	6	SArRNA10
gi|29165615|ref|NC_002745.2|	1923859	-	U	9	0	2	2	4	SArRNA10
gi|29165615|ref|NC_002745.2|	1923860	-	C	26	1	3	1	2	SArRNA10
gi|29165615|ref|NC_002745.2|	1923861	-	G	6	0	0	0	1	SArRNA10
gi|29165615|ref|NC_002745.2|	1923862	-	G	13	2	0	3	1	SArRNA10
gi|29165615|ref|NC_002745.2|	1923863	-	U	10	5	4	2	8	SArRNA10
gi|29165615|ref|NC_002745.2|	1923864	-	C	37	17	10	8	20	SArRNA10
gi|29165615|ref|NC_002745.2|	1923865	-	C	8	0	2	0	2	SArRNA10
gi|29165615|ref|NC_002745.2|	1923866	-	U	4	0	1	0	4	SArRNA10
gi|29165615|ref|NC_002745.2|	1923867	-	A	5	1	0	2	3	SArRNA10
gi|29165615|ref|NC_002745.2|	1923868	-	G	14	2	2	1	6	SArRNA10
gi|29165615|ref|NC_002745.2|	1923869	-	U	12	2	0	1	4	SArRNA10
gi|29165615|ref|NC_002745.2|	1923870	-	U	8	0	0	3	5	SArRNA10
gi|29165615|ref|NC_002745.2|	1923871	-	U	37	6	3	1	15	SArRNA10
gi|29165615|ref|NC_002745.2|	1923872	-	G	5	4	2	3	3	SArRNA10
gi|29165615|ref|NC_002745.2|	1923873	-	A	15	3	3	3	8	SArRNA10
gi|29165615|ref|NC_002745.2|	1923874	-	G	10	5	5	5	12	SArRNA10
gi|29165615|ref|NC_002745.2|	1923875	-	A	13	0	4	6	6	SArRNA10
gi|29165615|ref|NC_002745.2|	1923876	-	G	20	2	6	12	12	SArRNA10
gi|29165615|ref|NC_002745.2|	1923877	-	G	13	6	3	4	11	SArRNA10
gi|29165615|ref|NC_002745.2|	1923878	-	U	55	6	6	12	16	SArRNA10
gi|29165615|ref|NC_002745.2|	1923879	-	A	51	5	12	10	8	SArRNA10
gi|29165615|ref|NC_002745.2|	1923880	-	U	61	14	19	23	25	SArRNA10
gi|29165615|ref|NC_002745.2|	1923881	-	U	164	14	39	27	34	SArRNA10
gi|29165615|ref|NC_002745.2|	1923882	-	U	610	52	163	67	119	SArRNA10
gi|29165615|ref|NC_002745.2|	1923883	-	U	440756	11429	58051	10561	12323	SArRNA10
gi|29165615|ref|NC_002745.2|	1923884	-	U	128816	3410	40795	3380	3824	-
gi|29165615|ref|NC_002745.2|	1923885	-	A	19984	566	8000	630	919	-
gi|29165615|ref|NC_002745.2|	1923886	-	A	895	406	3481	459	1038	-
gi|29165615|ref|NC_002745.2|	1923887	-	U	121	370	1228	630	972	-
gi|29165615|ref|NC_002745.2|	1923888	-	A	105	1422	601	2312	3778	-
gi|29165615|ref|NC_002745.2|	1923889	-	C	160	1860	812	3271	5259	-
gi|29165615|ref|NC_002745.2|	1923890	-	U	20	290	120	650	821	-
gi|29165615|ref|NC_002745.2|	1923891	-	A	459	6162	2598	11041	16052	-
gi|29165615|ref|NC_002745.2|	1923892	-	C	95	1334	554	2375	3232	-
gi|29165615|ref|NC_002745.2|	1923893	-	A	51	654	267	992	1393	-
gi|29165615|ref|NC_002745.2|	1923894	-	A	140	1794	671	2660	3656	-
gi|29165615|ref|NC_002745.2|	1923895	-	A	27	311	122	535	723	-
gi|29165615|ref|NC_002745.2|	1923896	-	C	5	77	23	138	156	-
gi|29165615|ref|NC_002745.2|	1923897	-	U	1	12	2	16	26	-
gi|29165615|ref|NC_002745.2|	1923898	-	A	15	194	77	253	472	-
gi|29165615|ref|NC_002745.2|	1923899	-	A	27	483	191	605	1085	-
gi|29165615|ref|NC_002745.2|	1923900	-	U	17	159	68	211	348	-
gi|29165615|ref|NC_002745.2|	1923901	-	C	62	864	353	1152	2058	-
gi|29165615|ref|NC_002745.2|	1923902	-	G	72	1167	427	1302	2372	-
gi|29165615|ref|NC_002745.2|	1923903	-	A	39	588	266	680	1544	-
gi|29165615|ref|NC_002745.2|	1923904	-	G	14	104	37	134	273	-
gi|29165615|ref|NC_002745.2|	1923905	-	U	6	62	29	68	359	-
gi|29165615|ref|NC_002745.2|	1923906	-	A	150	1778	944	2409	5056	-
gi|29165615|ref|NC_002745.2|	1923907	-	U	29	455	241	567	1206	-
gi|29165615|ref|NC_002745.2|	1923908	-	U	71	663	306	1025	1803	-
gi|29165615|ref|NC_002745.2|	1923909	-	U	177	2450	1176	2819	5923	-
gi|29165615|ref|NC_002745.2|	1923910	-	A	652	9769	4105	11799	22778	-
gi|29165615|ref|NC_002745.2|	1923911	-	U	581	9005	4237	9137	18497	-
gi|29165615|ref|NC_002745.2|	1923912	-	G	126	1881	925	1908	4827	-
gi|29165615|ref|NC_002745.2|	1923913	-	A	41	743	319	699	1579	-
gi|29165615|ref|NC_002745.2|	1923914	-	U	4	145	62	120	262	-
gi|29165615|ref|NC_002745.2|	1923915	-	U	13	235	108	248	456	-
gi|29165615|ref|NC_002745.2|	1923916	-	U	12	246	145	246	506	-
gi|29165615|ref|NC_002745.2|	1923917	-	A	16	189	116	223	392	-
gi|29165615|ref|NC_002745.2|	1923918	-	U	17	205	116	180	298	-
gi|29165615|ref|NC_002745.2|	1923919	-	U	13	141	50	154	228	-
gi|29165615|ref|NC_002745.2|	1923920	-	A	10	100	54	141	231	-
gi|29165615|ref|NC_002745.2|	1923921	-	C	5	131	52	124	235	-
gi|29165615|ref|NC_002745.2|	1923922	-	A	23	505	145	427	892	-
gi|29165615|ref|NC_002745.2|	1923923	-	A	50	1002	283	991	1965	-
gi|29165615|ref|NC_002745.2|	1923924	-	A	3	135	42	149	204	-
gi|29165615|ref|NC_002745.2|	1923925	-	C	2	15	7	27	48	-
gi|29165615|ref|NC_002745.2|	1923926	-	A	1	61	30	55	105	-

gi|29165615|ref|NC_002745.2|	1923927	-	U	2	5	1	3	9	-
gi|29165615|ref|NC_002745.2|	1923928	-	U	0	2	2	3	6	-
gi|29165615|ref|NC_002745.2|	1923929	-	G	4	32	14	36	48	-
gi|29165615|ref|NC_002745.2|	1923930	-	A	3	54	15	32	45	-
gi|29165615|ref|NC_002745.2|	1923931	-	A	0	43	16	34	61	-
gi|29165615|ref|NC_002745.2|	1923932	-	U	1	16	6	24	22	-
gi|29165615|ref|NC_002745.2|	1923933	-	A	1	18	6	15	38	-
gi|29165615|ref|NC_002745.2|	1923934	-	U	4	10	1	5	10	-
gi|29165615|ref|NC_002745.2|	1923935	-	C	0	20	10	27	39	-
gi|29165615|ref|NC_002745.2|	1923936	-	A	0	41	14	50	59	-
gi|29165615|ref|NC_002745.2|	1923937	-	A	0	31	17	22	43	-
gi|29165615|ref|NC_002745.2|	1923938	-	U	0	3	1	2	3	-
gi|29165615|ref|NC_002745.2|	1923939	-	G	0	2	1	1	2	-
gi|29165615|ref|NC_002745.2|	1923940	-	C	1	8	5	7	6	-
gi|29165615|ref|NC_002745.2|	1923941	-	A	2	28	6	5	25	-
gi|29165615|ref|NC_002745.2|	1923942	-	A	3	83	11	26	98	-
gi|29165615|ref|NC_002745.2|	1923943	-	A	0	48	10	48	91	-
gi|29165615|ref|NC_002745.2|	1923944	-	A	3	92	22	117	219	-
gi|29165615|ref|NC_002745.2|	1923945	-	A	18	283	63	293	593	-
gi|29165615|ref|NC_002745.2|	1923946	-	C	31	557	173	494	1037	-
gi|29165615|ref|NC_002745.2|	1923947	-	C	16	202	87	229	387	-
gi|29165615|ref|NC_002745.2|	1923948	-	U	6	93	31	91	120	-
gi|29165615|ref|NC_002745.2|	1923949	-	U	0	12	3	15	24	-
gi|29165615|ref|NC_002745.2|	1923950	-	A	8	113	45	113	168	-
gi|29165615|ref|NC_002745.2|	1923951	-	A	10	137	39	150	278	-
gi|29165615|ref|NC_002745.2|	1923952	-	U	0	6	4	5	6	-
gi|29165615|ref|NC_002745.2|	1923953	-	U	2	34	14	39	46	-
gi|29165615|ref|NC_002745.2|	1923954	-	G	0	14	6	17	21	-
gi|29165615|ref|NC_002745.2|	1923955	-	C	0	2	9	3	5	-
gi|29165615|ref|NC_002745.2|	1923956	-	A	4	52	19	37	75	-
gi|29165615|ref|NC_002745.2|	1923957	-	A	6	121	38	126	200	-
gi|29165615|ref|NC_002745.2|	1923958	-	C	0	21	6	19	24	-
gi|29165615|ref|NC_002745.2|	1923959	-	U	3	52	11	48	77	-
gi|29165615|ref|NC_002745.2|	1923960	-	G	0	25	10	25	25	-
gi|29165615|ref|NC_002745.2|	1923961	-	U	0	0	0	4	2	-
gi|29165615|ref|NC_002745.2|	1923962	-	A	9	168	76	131	252	-
gi|29165615|ref|NC_002745.2|	1923963	-	U	9	121	57	95	175	-
gi|29165615|ref|NC_002745.2|	1923964	-	A	30	694	296	468	885	-
gi|29165615|ref|NC_002745.2|	1923965	-	A	12	117	58	86	160	-
gi|29165615|ref|NC_002745.2|	1923966	-	C	4	51	34	46	58	-
gi|29165615|ref|NC_002745.2|	1923967	-	A	8	107	76	100	166	-
gi|29165615|ref|NC_002745.2|	1923968	-	G	15	351	143	245	509	-
gi|29165615|ref|NC_002745.2|	1923969	-	U	1	16	8	15	25	-
gi|29165615|ref|NC_002745.2|	1923970	-	A	9	120	68	95	204	-
gi|29165615|ref|NC_002745.2|	1923971	-	A	18	331	187	337	562	-
gi|29165615|ref|NC_002745.2|	1923972	-	G	25	349	192	268	521	-
gi|29165615|ref|NC_002745.2|	1923973	-	U	27	357	170	239	520	-
gi|29165615|ref|NC_002745.2|	1923974	-	C	70	1473	732	776	2100	-
gi|29165615|ref|NC_002745.2|	1923975	-	A	6395	28137	20043	16082	34402	-
gi|29165615|ref|NC_002745.2|	1923976	-	A	10	63	29	41	79	-
gi|29165615|ref|NC_002745.2|	1923977	-	A	0	12	8	6	14	-
gi|29165615|ref|NC_002745.2|	1923978	-	A	0	13	9	10	10	-
gi|29165615|ref|NC_002745.2|	1923979	-	G	0	3	0	0	1	-
gi|29165615|ref|NC_002745.2|	1923980	-	U	0	0	0	0	2	-
gi|29165615|ref|NC_002745.2|	1923981	-	U	1	1	0	0	0	-
gi|29165615|ref|NC_002745.2|	1923982	-	A	0	6	0	5	2	-
gi|29165615|ref|NC_002745.2|	1923983	-	C	0	2	4	2	2	-
gi|29165615|ref|NC_002745.2|	1923984	-	A	0	3	3	2	2	-
gi|29165615|ref|NC_002745.2|	1923985	-	A	0	4	3	3	6	-
gi|29165615|ref|NC_002745.2|	1923986	-	G	0	0	0	0	1	-
gi|29165615|ref|NC_002745.2|	1923987	-	C	0	0	1	0	0	-
gi|29165615|ref|NC_002745.2|	1923988	-	A	0	5	2	0	8	-
gi|29165615|ref|NC_002745.2|	1923989	-	A	0	5	3	1	1	-
gi|29165615|ref|NC_002745.2|	1923991	-	C	0	0	1	0	0	-
gi|29165615|ref|NC_002745.2|	1923992	-	A	0	0	0	0	1	-
gi|29165615|ref|NC_002745.2|	1924010	-	A	0	0	0	2	1	-
gi|29165615|ref|NC_002745.2|	1926209	-	A	0	1	0	0	0	SA1680
gi|29165615|ref|NC_002745.2|	1926294	-	U	0	0	0	2	0	SA1680
gi|29165615|ref|NC_002745.2|	1929831	-	A	0	1	0	0	1	SA1683
gi|29165615|ref|NC_002745.2|	1944179	-	A	0	0	1	0	0	SA1696
gi|29165615|ref|NC_002745.2|	1944180	-	U	0	0	1	0	0	SA1696
gi|29165615|ref|NC_002745.2|	1950630	-	C	0	0	0	1	0	-
gi|29165615|ref|NC_002745.2|	1950633	-	A	0	1	0	0	1	-
gi|29165615|ref|NC_002745.2|	1950641	-	A	0	0	0	1	0	-
gi|29165615|ref|NC_002745.2|	1950642	-	A	0	1	0	3	2	-
gi|29165615|ref|NC_002745.2|	1950680	-	A	0	1	0	0	0	-
gi|29165615|ref|NC_002745.2|	1950704	-	A	0	0	0	0	1	-
gi|29165615|ref|NC_002745.2|	1950708	-	G	0	0	1	0	0	-
gi|29165615|ref|NC_002745.2|	1950746	-	A	0	0	1	0	0	-
gi|29165615|ref|NC_002745.2|	1950748	-	A	0	2	0	1	1	-
gi|29165615|ref|NC_002745.2|	1950753	-	U	0	1	0	0	0	-

gi|29165615|ref|NC_002745.2|	1950754 -	C	1	2	0	0	0	-
gi|29165615|ref|NC_002745.2|	1950763 -	A	0	0	0	0	1	-
gi|29165615|ref|NC_002745.2|	1950767 -	G	0	0	1	0	0	-
gi|29165615|ref|NC_002745.2|	1950819 -	C	0	0	0	1	0	-
gi|29165615|ref|NC_002745.2|	1952048 -	A	0	2	0	1	0	-
gi|29165615|ref|NC_002745.2|	1952049 -	A	0	0	0	0	1	-
gi|29165615|ref|NC_002745.2|	1952064 -	G	0	2	0	0	0	-
gi|29165615|ref|NC_002745.2|	1952080 -	G	3	0	0	0	0	-
gi|29165615|ref|NC_002745.2|	1952093 -	A	1	0	0	0	0	-
gi|29165615|ref|NC_002745.2|	1952099 -	A	0	0	0	1	0	-
gi|29165615|ref|NC_002745.2|	1952100 -	A	0	1	0	1	1	-
gi|29165615|ref|NC_002745.2|	1952103 -	A	0	0	0	0	1	-
gi|29165615|ref|NC_002745.2|	1952104 -	A	0	0	0	1	0	-
gi|29165615|ref|NC_002745.2|	1952105 -	A	0	0	0	5	0	-
gi|29165615|ref|NC_002745.2|	1952113 -	G	0	0	0	1	0	-
gi|29165615|ref|NC_002745.2|	1952118 -	A	0	0	0	1	0	-
gi|29165615|ref|NC_002745.2|	1952119 -	C	0	0	1	0	0	-
gi|29165615|ref|NC_002745.2|	1952121 -	A	0	0	0	0	1	-
gi|29165615|ref|NC_002745.2|	1952154 -	U	0	1	0	0	0	-
gi|29165615|ref|NC_002745.2|	1952157 -	A	0	0	0	1	0	-
gi|29165615|ref|NC_002745.2|	1952158 -	A	0	0	0	2	1	-
gi|29165615|ref|NC_002745.2|	1952170 -	A	0	0	1	0	0	-
gi|29165615|ref|NC_002745.2|	1952176 -	A	0	0	0	3	0	-
gi|29165615|ref|NC_002745.2|	1952220 -	C	0	0	0	1	0	-
gi|29165615|ref|NC_002745.2|	1952223 -	A	0	0	0	0	1	-
gi|29165615|ref|NC_002745.2|	1952230 -	A	0	0	0	0	1	-
gi|29165615|ref|NC_002745.2|	1952247 -	U	0	0	1	0	0	-
gi|29165615|ref|NC_002745.2|	1952253 -	G	0	0	0	0	1	-
gi|29165615|ref|NC_002745.2|	1952268 -	U	0	1	0	0	0	-
gi|29165615|ref|NC_002745.2|	1952281 -	C	0	0	0	1	0	-
gi|29165615|ref|NC_002745.2|	1958801 -	U	0	6	2	8	7	-
gi|29165615|ref|NC_002745.2|	1961400 -	A	0	0	1	0	0	-
gi|29165615|ref|NC_002745.2|	1961402 -	A	0	2	0	1	0	-
gi|29165615|ref|NC_002745.2|	1961403 -	U	0	0	0	0	1	-
gi|29165615|ref|NC_002745.2|	1961438 -	C	0	0	0	1	0	-
gi|29165615|ref|NC_002745.2|	1961441 -	A	0	1	0	0	1	-
gi|29165615|ref|NC_002745.2|	1961446 -	U	0	1	0	0	0	-
gi|29165615|ref|NC_002745.2|	1961449 -	A	0	0	0	1	0	-
gi|29165615|ref|NC_002745.2|	1961450 -	A	0	1	0	3	2	-
gi|29165615|ref|NC_002745.2|	1961463 -	G	0	0	0	1	0	-
gi|29165615|ref|NC_002745.2|	1961491 -	C	0	1	0	0	0	-
gi|29165615|ref|NC_002745.2|	1961497 -	A	0	0	1	0	0	-
gi|29165615|ref|NC_002745.2|	1961499 -	A	0	2	0	1	3	-
gi|29165615|ref|NC_002745.2|	1961501 -	C	0	0	0	1	0	-
gi|29165615|ref|NC_002745.2|	1961502 -	A	0	3	5	10	5	-
gi|29165615|ref|NC_002745.2|	1961504 -	U	0	1	0	0	0	-
gi|29165615|ref|NC_002745.2|	1961505 -	C	1	2	0	0	0	-
gi|29165615|ref|NC_002745.2|	1961515 -	U	0	1	0	0	0	-
gi|29165615|ref|NC_002745.2|	1961526 -	U	0	0	0	1	0	-
gi|29165615|ref|NC_002745.2|	1961534 -	C	0	0	2	0	0	-
gi|29165615|ref|NC_002745.2|	1961546 -	A	0	1	0	0	0	-
gi|29165615|ref|NC_002745.2|	1961571 -	U	0	1	0	0	0	-
gi|29165615|ref|NC_002745.2|	1961582 -	U	0	0	0	1	0	-
gi|29165615|ref|NC_002745.2|	1961590 -	C	0	0	2	0	0	-
gi|29165615|ref|NC_002745.2|	1961624 -	C	0	0	0	1	0	-
gi|29165615|ref|NC_002745.2|	1961627 -	A	0	0	0	0	1	-
gi|29165615|ref|NC_002745.2|	1961634 -	A	0	0	0	0	1	-
gi|29165615|ref|NC_002745.2|	1961651 -	U	0	0	1	0	0	-
gi|29165615|ref|NC_002745.2|	1961657 -	G	0	0	0	0	1	-
gi|29165615|ref|NC_002745.2|	1963823 -	A	0	1	0	0	0	SA1716
gi|29165615|ref|NC_002745.2|	1970095 -	A	1	0	0	0	0	SA1720
gi|29165615|ref|NC_002745.2|	1971660 -	A	0	1	1	0	1	SA1721
gi|29165615|ref|NC_002745.2|	1978547 -	U	0	1	0	0	0	SA1727
gi|29165615|ref|NC_002745.2|	1984952 -	U	0	0	0	0	1	SA1732
gi|29165615|ref|NC_002745.2|	1992052 -	C	0	0	0	1	0	SAS055
gi|29165615|ref|NC_002745.2|	2004193 -	G	0	0	0	0	1	SA1751
gi|29165615|ref|NC_002745.2|	2004195 -	U	0	0	0	0	1	SA1751
gi|29165615|ref|NC_002745.2|	2004214 -	A	0	0	0	0	1	SA1751
gi|29165615|ref|NC_002745.2|	2004216 -	A	0	0	1	0	0	SA1751
gi|29165615|ref|NC_002745.2|	2004231 -	C	0	0	0	1	0	SA1751
gi|29165615|ref|NC_002745.2|	2004258 -	C	0	0	1	0	0	SA1751
gi|29165615|ref|NC_002745.2|	2004520 -	G	0	0	0	0	1	SA1751
gi|29165615|ref|NC_002745.2|	2004522 -	U	0	0	0	0	1	SA1751
gi|29165615|ref|NC_002745.2|	2004541 -	A	0	0	0	0	1	SA1751
gi|29165615|ref|NC_002745.2|	2004543 -	A	0	0	1	0	0	SA1751
gi|29165615|ref|NC_002745.2|	2004558 -	C	0	0	0	1	0	SA1751
gi|29165615|ref|NC_002745.2|	2004585 -	C	0	0	1	0	0	SA1751
gi|29165615|ref|NC_002745.2|	2007959 -	U	0	0	0	0	1	-
gi|29165615|ref|NC_002745.2|	2011184 -	G	2	0	0	0	0	-
gi|29165615|ref|NC_002745.2|	2013385 -	A	1	0	0	0	0	SAS061
gi|29165615|ref|NC_002745.2|	2015264 -	U	1	0	0	0	0	SA1764

gi|29165615|ref|NC_002745.2|	2036049	-	C	0	0	1	0	0	SA1791
gi|29165615|ref|NC_002745.2|	2036051	-	A	0	0	0	1	0	SA1791
gi|29165615|ref|NC_002745.2|	2040496	-	U	0	1	0	0	0	SA1795
gi|29165615|ref|NC_002745.2|	2042954	-	A	0	0	0	1	0	SA1801
gi|29165615|ref|NC_002745.2|	2044306	-	A	0	0	0	1	0	SA1804
gi|29165615|ref|NC_002745.2|	2054599	-	A	0	0	1	0	0	-
gi|29165615|ref|NC_002745.2|	2054605	-	A	0	0	0	3	0	-
gi|29165615|ref|NC_002745.2|	2054642	-	U	0	1	0	0	0	-
gi|29165615|ref|NC_002745.2|	2054659	-	G	0	0	0	1	0	-
gi|29165615|ref|NC_002745.2|	2054664	-	A	0	0	0	1	0	-
gi|29165615|ref|NC_002745.2|	2054665	-	C	0	0	1	0	0	-
gi|29165615|ref|NC_002745.2|	2054667	-	A	0	0	0	0	1	-
gi|29165615|ref|NC_002745.2|	2054687	-	C	0	1	0	0	0	-
gi|29165615|ref|NC_002745.2|	2054697	-	A	1	0	0	0	0	-
gi|29165615|ref|NC_002745.2|	2054704	-	A	0	0	0	2	1	-
gi|29165615|ref|NC_002745.2|	2054716	-	A	0	0	1	0	0	-
gi|29165615|ref|NC_002745.2|	2054722	-	C	0	0	0	3	0	-
gi|29165615|ref|NC_002745.2|	2058184	-	U	0	0	1	0	0	SA1817
gi|29165615|ref|NC_002745.2|	2058187	-	A	0	0	0	2	0	SA1817
gi|29165615|ref|NC_002745.2|	2058188	-	U	0	0	0	1	1	SA1817
gi|29165615|ref|NC_002745.2|	2058189	-	A	0	2	4	0	2	SA1817
gi|29165615|ref|NC_002745.2|	2058217	-	U	0	0	0	3	0	SA1817
gi|29165615|ref|NC_002745.2|	2058218	-	A	0	3	3	11	7	SA1817
gi|29165615|ref|NC_002745.2|	2058219	-	G	0	4	7	8	1	SA1817
gi|29165615|ref|NC_002745.2|	2058259	-	A	0	0	0	1	0	SA1817
gi|29165615|ref|NC_002745.2|	2062355	-	A	1	0	0	0	0	SA1820
gi|29165615|ref|NC_002745.2|	2073555	-	C	0	1	0	0	0	SA1836
gi|29165615|ref|NC_002745.2|	2073557	-	C	1	0	0	0	0	SA1836
gi|29165615|ref|NC_002745.2|	2075990	-	A	0	0	1	0	0	SA1839
gi|29165615|ref|NC_002745.2|	2076001	-	A	0	0	1	0	0	SA1839
gi|29165615|ref|NC_002745.2|	2076020	-	C	1	0	0	0	0	SA1839
gi|29165615|ref|NC_002745.2|	2076040	-	A	0	0	1	0	0	SA1839
gi|29165615|ref|NC_002745.2|	2076059	-	C	1	0	0	0	0	SA1839
gi|29165615|ref|NC_002745.2|	2076118	-	A	0	0	1	0	0	SA1839
gi|29165615|ref|NC_002745.2|	2076173	-	A	0	0	1	0	0	SA1839
gi|29165615|ref|NC_002745.2|	2076203	-	C	1	0	0	0	0	SA1839
gi|29165615|ref|NC_002745.2|	2077529	-	A	1	0	0	0	0	-
gi|29165615|ref|NC_002745.2|	2077532	-	U	0	1	0	0	0	-
gi|29165615|ref|NC_002745.2|	2077535	-	A	0	0	0	1	0	-
gi|29165615|ref|NC_002745.2|	2077536	-	A	0	0	0	2	1	-
gi|29165615|ref|NC_002745.2|	2079338	-	U	0	0	0	0	1	-
gi|29165615|ref|NC_002745.2|	2094349	-	A	0	0	0	1	0	-
gi|29165615|ref|NC_002745.2|	2094350	-	U	0	0	0	1	1	-
gi|29165615|ref|NC_002745.2|	2094358	-	C	0	0	1	0	3	-
gi|29165615|ref|NC_002745.2|	2094366	-	A	0	0	0	1	0	-
gi|29165615|ref|NC_002745.2|	2094367	-	C	0	0	0	0	1	-
gi|29165615|ref|NC_002745.2|	2094371	-	C	0	0	1	2	0	-
gi|29165615|ref|NC_002745.2|	2094412	-	G	0	0	1	0	0	-
gi|29165615|ref|NC_002745.2|	2108447	-	A	0	0	0	0	1	-
gi|29165615|ref|NC_002745.2|	2108453	-	A	0	0	0	0	2	-
gi|29165615|ref|NC_002745.2|	2108454	-	A	0	0	0	0	2	-
gi|29165615|ref|NC_002745.2|	2108460	-	A	0	0	1	0	0	-
gi|29165615|ref|NC_002745.2|	2108525	-	G	0	2	1	0	0	-
gi|29165615|ref|NC_002745.2|	2108526	-	A	1	10	6	3	13	-
gi|29165615|ref|NC_002745.2|	2108527	-	A	1	58	11	14	39	-
gi|29165615|ref|NC_002745.2|	2108528	-	A	3	79	17	19	57	-
gi|29165615|ref|NC_002745.2|	2108529	-	A	2	68	35	21	48	-
gi|29165615|ref|NC_002745.2|	2108530	-	A	1	30	8	8	12	-
gi|29165615|ref|NC_002745.2|	2108550	-	A	0	0	1	0	0	-
gi|29165615|ref|NC_002745.2|	2108551	-	U	0	1	0	1	0	-
gi|29165615|ref|NC_002745.2|	2108552	-	C	2	4	2	0	2	-
gi|29165615|ref|NC_002745.2|	2108553	-	G	0	1	0	0	2	-
gi|29165615|ref|NC_002745.2|	2108554	-	C	0	5	1	0	0	-
gi|29165615|ref|NC_002745.2|	2108555	-	C	6	2	3	3	3	-
gi|29165615|ref|NC_002745.2|	2108556	-	U	1	5	0	0	2	-
gi|29165615|ref|NC_002745.2|	2108557	-	U	0	0	0	0	1	-
gi|29165615|ref|NC_002745.2|	2108558	-	G	1	6	1	2	1	-
gi|29165615|ref|NC_002745.2|	2108559	-	C	2	2	2	0	4	-
gi|29165615|ref|NC_002745.2|	2108560	-	A	0	0	0	0	1	-
gi|29165615|ref|NC_002745.2|	2108561	-	U	1	0	0	0	0	-
gi|29165615|ref|NC_002745.2|	2108725	-	A	0	0	0	0	1	-
gi|29165615|ref|NC_002745.2|	2108731	-	A	0	0	0	0	2	-
gi|29165615|ref|NC_002745.2|	2108732	-	A	0	0	0	0	2	-
gi|29165615|ref|NC_002745.2|	2108738	-	A	0	0	1	0	0	-
gi|29165615|ref|NC_002745.2|	2108806	-	A	0	0	1	0	0	-
gi|29165615|ref|NC_002745.2|	2108807	-	U	0	3	0	1	0	-
gi|29165615|ref|NC_002745.2|	2108808	-	C	2	4	2	0	2	-
gi|29165615|ref|NC_002745.2|	2108809	-	G	0	1	0	0	2	-
gi|29165615|ref|NC_002745.2|	2108810	-	C	0	5	1	0	0	-
gi|29165615|ref|NC_002745.2|	2108811	-	C	6	2	3	3	3	-
gi|29165615|ref|NC_002745.2|	2108812	-	U	1	5	0	0	2	-

gi|29165615|ref|NC_002745.2|	2108813 -	U	0	0	0	0	1	-
gi|29165615|ref|NC_002745.2|	2108814 -	G	1	6	1	2	1	-
gi|29165615|ref|NC_002745.2|	2108815 -	C	2	2	2	0	4	-
gi|29165615|ref|NC_002745.2|	2108816 -	A	0	0	0	0	1	-
gi|29165615|ref|NC_002745.2|	2108817 -	U	1	0	0	0	0	-
gi|29165615|ref|NC_002745.2|	2108981 -	A	0	0	0	0	1	-
gi|29165615|ref|NC_002745.2|	2108987 -	A	0	0	0	0	2	-
gi|29165615|ref|NC_002745.2|	2108988 -	A	0	0	0	0	2	-
gi|29165615|ref|NC_002745.2|	2108994 -	A	0	0	1	0	0	-
gi|29165615|ref|NC_002745.2|	2109062 -	A	0	0	1	0	0	SArRNA11
gi|29165615|ref|NC_002745.2|	2109063 -	U	0	3	0	1	0	SArRNA11
gi|29165615|ref|NC_002745.2|	2109064 -	C	2	4	2	0	2	SArRNA11
gi|29165615|ref|NC_002745.2|	2109065 -	G	0	1	0	0	2	SArRNA11
gi|29165615|ref|NC_002745.2|	2109066 -	C	0	5	1	0	0	SArRNA11
gi|29165615|ref|NC_002745.2|	2109067 -	C	6	2	3	3	3	SArRNA11
gi|29165615|ref|NC_002745.2|	2109068 -	U	1	5	0	0	2	SArRNA11
gi|29165615|ref|NC_002745.2|	2109069 -	U	0	0	0	0	1	SArRNA11
gi|29165615|ref|NC_002745.2|	2109070 -	G	1	6	1	2	1	SArRNA11
gi|29165615|ref|NC_002745.2|	2109071 -	C	2	2	2	0	4	SArRNA11
gi|29165615|ref|NC_002745.2|	2109072 -	A	0	0	0	0	1	SArRNA11
gi|29165615|ref|NC_002745.2|	2109073 -	U	1	0	0	0	0	SArRNA11
gi|29165615|ref|NC_002745.2|	2109081 -	G	0	0	0	0	1	SArRNA11
gi|29165615|ref|NC_002745.2|	2109083 -	U	0	2	0	0	0	SArRNA11
gi|29165615|ref|NC_002745.2|	2109084 -	G	0	2	1	0	0	SArRNA11
gi|29165615|ref|NC_002745.2|	2109085 -	G	0	0	0	0	1	SArRNA11
gi|29165615|ref|NC_002745.2|	2109087 -	A	0	3	0	0	1	SArRNA11
gi|29165615|ref|NC_002745.2|	2109088 -	G	0	7	0	3	2	SArRNA11
gi|29165615|ref|NC_002745.2|	2109089 -	C	0	7	0	0	4	SArRNA11
gi|29165615|ref|NC_002745.2|	2109090 -	U	1	0	0	0	1	SArRNA11
gi|29165615|ref|NC_002745.2|	2109091 -	G	0	0	0	0	3	SArRNA11
gi|29165615|ref|NC_002745.2|	2109092 -	C	1	3	2	0	1	SArRNA11
gi|29165615|ref|NC_002745.2|	2109094 -	A	0	2	0	0	0	SArRNA11
gi|29165615|ref|NC_002745.2|	2109095 -	U	1	1	0	0	0	SArRNA11
gi|29165615|ref|NC_002745.2|	2109096 -	U	0	2	0	0	0	SArRNA11
gi|29165615|ref|NC_002745.2|	2109097 -	C	2	2	1	0	3	SArRNA11
gi|29165615|ref|NC_002745.2|	2109098 -	C	2	0	0	1	0	SArRNA11
gi|29165615|ref|NC_002745.2|	2109099 -	U	1	2	1	2	0	SArRNA11
gi|29165615|ref|NC_002745.2|	2109100 -	C	2	7	4	5	5	SArRNA11
gi|29165615|ref|NC_002745.2|	2109102 -	A	1	0	0	1	1	SArRNA11
gi|29165615|ref|NC_002745.2|	2109103 -	A	3	0	1	0	2	SArRNA11
gi|29165615|ref|NC_002745.2|	2109104 -	U	2	12	3	0	2	SArRNA11
gi|29165615|ref|NC_002745.2|	2109105 -	U	1	0	0	0	2	SArRNA11
gi|29165615|ref|NC_002745.2|	2109106 -	G	0	0	0	0	2	SArRNA11
gi|29165615|ref|NC_002745.2|	2109107 -	A	1	1	0	1	0	SArRNA11
gi|29165615|ref|NC_002745.2|	2109108 -	A	0	4	1	1	1	SArRNA11
gi|29165615|ref|NC_002745.2|	2109109 -	G	2	11	0	1	3	SArRNA11
gi|29165615|ref|NC_002745.2|	2109110 -	A	1	7	0	1	3	SArRNA11
gi|29165615|ref|NC_002745.2|	2109111 -	C	3	9	1	0	5	SArRNA11
gi|29165615|ref|NC_002745.2|	2109112 -	A	2	9	0	2	6	SArRNA11
gi|29165615|ref|NC_002745.2|	2109113 -	C	24	41	18	9	19	SArRNA11
gi|29165615|ref|NC_002745.2|	2109115 -	A	2	2	1	1	1	SArRNA11
gi|29165615|ref|NC_002745.2|	2109116 -	G	19	17	10	4	16	SArRNA11
gi|29165615|ref|NC_002745.2|	2109117 -	C	70	92	45	26	54	SArRNA11
gi|29165615|ref|NC_002745.2|	2109118 -	C	23	46	4	5	37	SArRNA11
gi|29165615|ref|NC_002745.2|	2109119 -	G	2	2	0	0	0	SArRNA11
gi|29165615|ref|NC_002745.2|	2109120 -	U	0	5	0	0	0	SArRNA11
gi|29165615|ref|NC_002745.2|	2109121 -	A	3	7	1	0	6	SArRNA11
gi|29165615|ref|NC_002745.2|	2109122 -	C	11	31	9	5	24	SArRNA11
gi|29165615|ref|NC_002745.2|	2109123 -	C	1	23	1	0	9	SArRNA11
gi|29165615|ref|NC_002745.2|	2109124 -	C	7	6	4	1	3	SArRNA11
gi|29165615|ref|NC_002745.2|	2109125 -	U	0	2	1	1	1	SArRNA11
gi|29165615|ref|NC_002745.2|	2109126 -	U	0	0	0	0	1	SArRNA11
gi|29165615|ref|NC_002745.2|	2109127 -	G	0	1	0	4	0	SArRNA11
gi|29165615|ref|NC_002745.2|	2109128 -	U	6	3	0	0	4	SArRNA11
gi|29165615|ref|NC_002745.2|	2109129 -	C	8	31	9	15	7	SArRNA11
gi|29165615|ref|NC_002745.2|	2109130 -	C	28	65	26	32	33	SArRNA11
gi|29165615|ref|NC_002745.2|	2109131 -	A	7	25	5	7	11	SArRNA11
gi|29165615|ref|NC_002745.2|	2109132 -	C	68	107	63	56	92	SArRNA11
gi|29165615|ref|NC_002745.2|	2109133 -	A	2	6	0	1	6	SArRNA11
gi|29165615|ref|NC_002745.2|	2109134 -	C	4	16	4	0	13	SArRNA11
gi|29165615|ref|NC_002745.2|	2109135 -	U	0	1	0	0	1	SArRNA11
gi|29165615|ref|NC_002745.2|	2109137 -	G	0	1	1	0	1	SArRNA11
gi|29165615|ref|NC_002745.2|	2109138 -	A	0	0	1	1	1	SArRNA11
gi|29165615|ref|NC_002745.2|	2109139 -	G	1	6	1	1	2	SArRNA11
gi|29165615|ref|NC_002745.2|	2109140 -	G	0	2	0	0	1	SArRNA11
gi|29165615|ref|NC_002745.2|	2109141 -	A	9	4	2	1	4	SArRNA11
gi|29165615|ref|NC_002745.2|	2109142 -	A	4	2	1	2	4	SArRNA11
gi|29165615|ref|NC_002745.2|	2109143 -	C	19	24	16	8	21	SArRNA11
gi|29165615|ref|NC_002745.2|	2109144 -	G	0	0	0	1	0	SArRNA11
gi|29165615|ref|NC_002745.2|	2109145 -	A	1	0	0	1	0	SArRNA11
gi|29165615|ref|NC_002745.2|	2109146 -	U	1	1	0	2	1	SArRNA11

gi|29165615|ref|NC_002745.2|	2109147	-	A	30	44	31	10	27	SArRNA11
gi|29165615|ref|NC_002745.2|	2109148	-	U	8	22	3	2	25	SArRNA11
gi|29165615|ref|NC_002745.2|	2109149	-	C	34	29	12	6	21	SArRNA11
gi|29165615|ref|NC_002745.2|	2109150	-	A	0	0	0	1	0	SArRNA11
gi|29165615|ref|NC_002745.2|	2109151	-	G	5	10	4	2	2	SArRNA11
gi|29165615|ref|NC_002745.2|	2109152	-	U	4	5	3	0	0	SArRNA11
gi|29165615|ref|NC_002745.2|	2109153	-	G	3	0	0	0	2	SArRNA11
gi|29165615|ref|NC_002745.2|	2109154	-	G	1	0	1	0	1	SArRNA11
gi|29165615|ref|NC_002745.2|	2109155	-	U	7	6	2	4	4	SArRNA11
gi|29165615|ref|NC_002745.2|	2109156	-	C	1	15	3	1	9	SArRNA11
gi|29165615|ref|NC_002745.2|	2109157	-	U	2502	2241	1275	977	1934	SArRNA11
gi|29165615|ref|NC_002745.2|	2109158	-	G	4	7	6	5	11	-
gi|29165615|ref|NC_002745.2|	2109159	-	U	2	2	2	1	0	-
gi|29165615|ref|NC_002745.2|	2109182	-	U	40	8	11	6	12	-
gi|29165615|ref|NC_002745.2|	2109195	-	U	0	1	0	0	0	-
gi|29165615|ref|NC_002745.2|	2109231	-	A	0	1	0	1	1	-
gi|29165615|ref|NC_002745.2|	2109232	-	C	3	0	0	0	0	-
gi|29165615|ref|NC_002745.2|	2109238	-	C	1	0	0	0	0	-
gi|29165615|ref|NC_002745.2|	2109250	-	A	0	0	0	0	2	-
gi|29165615|ref|NC_002745.2|	2109254	-	A	1	0	0	0	1	-
gi|29165615|ref|NC_002745.2|	2109255	-	U	1	0	0	0	0	-
gi|29165615|ref|NC_002745.2|	2109257	-	A	0	0	1	0	0	-
gi|29165615|ref|NC_002745.2|	2109258	-	G	0	0	0	1	0	-
gi|29165615|ref|NC_002745.2|	2109265	-	A	0	0	1	0	0	-
gi|29165615|ref|NC_002745.2|	2109267	-	G	0	0	1	0	0	-
gi|29165615|ref|NC_002745.2|	2109268	-	U	1	0	0	1	0	-
gi|29165615|ref|NC_002745.2|	2109269	-	G	2	0	0	0	0	-
gi|29165615|ref|NC_002745.2|	2109270	-	U	0	0	0	0	1	-
gi|29165615|ref|NC_002745.2|	2109272	-	C	2	0	0	0	0	-
gi|29165615|ref|NC_002745.2|	2109273	-	A	0	1	1	0	0	-
gi|29165615|ref|NC_002745.2|	2109275	-	U	1	0	0	0	1	-
gi|29165615|ref|NC_002745.2|	2109280	-	C	0	1	1	2	0	-
gi|29165615|ref|NC_002745.2|	2109281	-	G	0	0	0	0	1	-
gi|29165615|ref|NC_002745.2|	2109283	-	A	1	0	0	0	0	-
gi|29165615|ref|NC_002745.2|	2109284	-	G	1	3	1	2	1	-
gi|29165615|ref|NC_002745.2|	2109285	-	G	0	0	0	0	1	-
gi|29165615|ref|NC_002745.2|	2109289	-	A	2	1	0	0	0	-
gi|29165615|ref|NC_002745.2|	2109290	-	G	3	1	0	1	4	-
gi|29165615|ref|NC_002745.2|	2109291	-	C	0	3	0	0	1	-
gi|29165615|ref|NC_002745.2|	2109293	-	U	0	0	0	0	1	-
gi|29165615|ref|NC_002745.2|	2109296	-	A	0	2	0	0	0	-
gi|29165615|ref|NC_002745.2|	2109297	-	U	2	1	1	0	0	-
gi|29165615|ref|NC_002745.2|	2109298	-	A	0	7	0	0	6	-
gi|29165615|ref|NC_002745.2|	2109300	-	U	0	0	1	0	0	-
gi|29165615|ref|NC_002745.2|	2109301	-	U	0	4	0	0	0	-
gi|29165615|ref|NC_002745.2|	2109305	-	G	0	0	0	1	2	-
gi|29165615|ref|NC_002745.2|	2109307	-	A	4	0	1	1	0	-
gi|29165615|ref|NC_002745.2|	2109308	-	G	3	1	0	1	1	-
gi|29165615|ref|NC_002745.2|	2109309	-	U	2	0	1	0	0	-
gi|29165615|ref|NC_002745.2|	2109310	-	A	2	1	0	1	0	-
gi|29165615|ref|NC_002745.2|	2109311	-	G	2	0	1	4	1	-
gi|29165615|ref|NC_002745.2|	2109312	-	A	0	2	0	0	0	-
gi|29165615|ref|NC_002745.2|	2109313	-	A	0	0	2	1	2	-
gi|29165615|ref|NC_002745.2|	2109314	-	A	0	3	1	0	1	-
gi|29165615|ref|NC_002745.2|	2109315	-	C	1	3	0	0	1	-
gi|29165615|ref|NC_002745.2|	2109316	-	U	4	2	0	0	1	-
gi|29165615|ref|NC_002745.2|	2109317	-	C	3	6	3	2	2	-
gi|29165615|ref|NC_002745.2|	2109318	-	C	27	28	12	10	13	-
gi|29165615|ref|NC_002745.2|	2109319	-	C	22	19	8	8	13	SArRNA12
gi|29165615|ref|NC_002745.2|	2109320	-	U	6	7	4	3	5	SArRNA12
gi|29165615|ref|NC_002745.2|	2109321	-	A	3	11	0	1	4	SArRNA12
gi|29165615|ref|NC_002745.2|	2109322	-	G	2	1	5	3	0	SArRNA12
gi|29165615|ref|NC_002745.2|	2109323	-	A	0	0	0	0	1	SArRNA12
gi|29165615|ref|NC_002745.2|	2109324	-	A	0	0	0	1	0	SArRNA12
gi|29165615|ref|NC_002745.2|	2109326	-	A	1	0	0	0	2	SArRNA12
gi|29165615|ref|NC_002745.2|	2109327	-	U	0	0	2	0	0	SArRNA12
gi|29165615|ref|NC_002745.2|	2109328	-	U	0	0	0	1	0	SArRNA12
gi|29165615|ref|NC_002745.2|	2109330	-	G	4	0	0	0	0	SArRNA12
gi|29165615|ref|NC_002745.2|	2109331	-	C	14	5	0	4	1	SArRNA12
gi|29165615|ref|NC_002745.2|	2109332	-	U	1	0	0	0	0	SArRNA12
gi|29165615|ref|NC_002745.2|	2109333	-	U	0	2	0	0	0	SArRNA12
gi|29165615|ref|NC_002745.2|	2109334	-	C	1	0	0	0	1	SArRNA12
gi|29165615|ref|NC_002745.2|	2109335	-	A	0	0	2	1	0	SArRNA12
gi|29165615|ref|NC_002745.2|	2109337	-	C	1	3	1	0	1	SArRNA12
gi|29165615|ref|NC_002745.2|	2109338	-	C	0	2	0	0	1	SArRNA12
gi|29165615|ref|NC_002745.2|	2109339	-	C	1	2	0	1	1	SArRNA12
gi|29165615|ref|NC_002745.2|	2109340	-	U	1	0	1	0	1	SArRNA12
gi|29165615|ref|NC_002745.2|	2109341	-	U	0	1	0	0	0	SArRNA12
gi|29165615|ref|NC_002745.2|	2109342	-	U	0	3	0	0	0	SArRNA12
gi|29165615|ref|NC_002745.2|	2109343	-	A	0	0	0	1	0	SArRNA12
gi|29165615|ref|NC_002745.2|	2109351	-	A	1	5	1	0	1	SArRNA12

gi|29165615|ref|NC_002745.2|	2109352	-	C	2	6	1	2	3	SArRNA12
gi|29165615|ref|NC_002745.2|	2109353	-	U	0	2	0	0	0	SArRNA12
gi|29165615|ref|NC_002745.2|	2109354	-	C	0	2	1	0	2	SArRNA12
gi|29165615|ref|NC_002745.2|	2109355	-	C	1	9	1	1	3	SArRNA12
gi|29165615|ref|NC_002745.2|	2109356	-	C	6	7	4	6	11	SArRNA12
gi|29165615|ref|NC_002745.2|	2109357	-	C	32	29	17	8	26	SArRNA12
gi|29165615|ref|NC_002745.2|	2109358	-	C	23	26	14	13	25	SArRNA12
gi|29165615|ref|NC_002745.2|	2109359	-	C	16	21	14	11	19	SArRNA12
gi|29165615|ref|NC_002745.2|	2109360	-	G	1	0	0	0	0	SArRNA12
gi|29165615|ref|NC_002745.2|	2109361	-	A	0	1	0	0	1	SArRNA12
gi|29165615|ref|NC_002745.2|	2109362	-	A	0	0	1	0	0	SArRNA12
gi|29165615|ref|NC_002745.2|	2109363	-	G	0	0	0	1	0	SArRNA12
gi|29165615|ref|NC_002745.2|	2109365	-	A	1	1	0	0	1	SArRNA12
gi|29165615|ref|NC_002745.2|	2109366	-	C	0	1	0	2	1	SArRNA12
gi|29165615|ref|NC_002745.2|	2109368	-	A	0	1	1	0	2	SArRNA12
gi|29165615|ref|NC_002745.2|	2109369	-	A	2	3	0	0	0	SArRNA12
gi|29165615|ref|NC_002745.2|	2109370	-	U	0	11	1	3	5	SArRNA12
gi|29165615|ref|NC_002745.2|	2109371	-	C	3	19	0	1	12	SArRNA12
gi|29165615|ref|NC_002745.2|	2109372	-	U	0	7	1	1	1	SArRNA12
gi|29165615|ref|NC_002745.2|	2109373	-	A	2	3	2	0	1	SArRNA12
gi|29165615|ref|NC_002745.2|	2109374	-	C	1	5	1	1	4	SArRNA12
gi|29165615|ref|NC_002745.2|	2109375	-	G	1	1	0	0	0	SArRNA12
gi|29165615|ref|NC_002745.2|	2109376	-	A	0	2	0	0	0	SArRNA12
gi|29165615|ref|NC_002745.2|	2109377	-	A	0	0	0	0	1	SArRNA12
gi|29165615|ref|NC_002745.2|	2109378	-	A	1	3	0	0	1	SArRNA12
gi|29165615|ref|NC_002745.2|	2109379	-	G	0	1	0	0	0	SArRNA12
gi|29165615|ref|NC_002745.2|	2109380	-	U	0	1	0	0	0	SArRNA12
gi|29165615|ref|NC_002745.2|	2109381	-	C	0	1	0	0	0	SArRNA12
gi|29165615|ref|NC_002745.2|	2109383	-	U	1	0	0	0	6	SArRNA12
gi|29165615|ref|NC_002745.2|	2109384	-	G	1	7	0	0	1	SArRNA12
gi|29165615|ref|NC_002745.2|	2109385	-	A	0	9	1	0	3	SArRNA12
gi|29165615|ref|NC_002745.2|	2109386	-	A	4	23	0	1	10	SArRNA12
gi|29165615|ref|NC_002745.2|	2109387	-	U	1	17	1	1	3	SArRNA12
gi|29165615|ref|NC_002745.2|	2109388	-	A	1	2	1	0	0	SArRNA12
gi|29165615|ref|NC_002745.2|	2109389	-	G	0	0	0	0	2	SArRNA12
gi|29165615|ref|NC_002745.2|	2109391	-	G	3	11	3	4	7	SArRNA12
gi|29165615|ref|NC_002745.2|	2109392	-	C	17	20	1	5	4	SArRNA12
gi|29165615|ref|NC_002745.2|	2109393	-	A	2	0	0	0	0	SArRNA12
gi|29165615|ref|NC_002745.2|	2109394	-	G	3	7	0	1	0	SArRNA12
gi|29165615|ref|NC_002745.2|	2109395	-	G	1	1	0	1	3	SArRNA12
gi|29165615|ref|NC_002745.2|	2109396	-	U	0	1	0	1	1	SArRNA12
gi|29165615|ref|NC_002745.2|	2109398	-	U	0	1	0	0	0	SArRNA12
gi|29165615|ref|NC_002745.2|	2109399	-	G	0	1	0	0	0	SArRNA12
gi|29165615|ref|NC_002745.2|	2109401	-	A	3	8	2	0	0	SArRNA12
gi|29165615|ref|NC_002745.2|	2109402	-	U	2	17	0	1	8	SArRNA12
gi|29165615|ref|NC_002745.2|	2109403	-	C	2	9	0	0	3	SArRNA12
gi|29165615|ref|NC_002745.2|	2109405	-	A	0	1	1	0	0	SArRNA12
gi|29165615|ref|NC_002745.2|	2109406	-	U	6	4	0	0	4	SArRNA12
gi|29165615|ref|NC_002745.2|	2109407	-	G	0	1	0	0	3	SArRNA12
gi|29165615|ref|NC_002745.2|	2109409	-	G	0	0	0	1	0	SArRNA12
gi|29165615|ref|NC_002745.2|	2109410	-	U	3	3	3	0	1	SArRNA12
gi|29165615|ref|NC_002745.2|	2109411	-	C	2	4	1	0	1	SArRNA12
gi|29165615|ref|NC_002745.2|	2109412	-	G	1	2	1	0	1	SArRNA12
gi|29165615|ref|NC_002745.2|	2109413	-	A	4	1	0	0	2	SArRNA12
gi|29165615|ref|NC_002745.2|	2109414	-	U	1	1	0	0	3	SArRNA12
gi|29165615|ref|NC_002745.2|	2109415	-	A	1	5	0	1	3	SArRNA12
gi|29165615|ref|NC_002745.2|	2109416	-	C	0	4	0	3	1	SArRNA12
gi|29165615|ref|NC_002745.2|	2109418	-	G	1	8	0	1	3	SArRNA12
gi|29165615|ref|NC_002745.2|	2109419	-	C	2	7	4	1	1	SArRNA12
gi|29165615|ref|NC_002745.2|	2109420	-	A	1	25	1	0	12	SArRNA12
gi|29165615|ref|NC_002745.2|	2109421	-	A	3	18	0	0	8	SArRNA12
gi|29165615|ref|NC_002745.2|	2109422	-	C	5	10	0	3	2	SArRNA12
gi|29165615|ref|NC_002745.2|	2109423	-	C	1	6	0	0	2	SArRNA12
gi|29165615|ref|NC_002745.2|	2109425	-	U	0	0	0	0	1	SArRNA12
gi|29165615|ref|NC_002745.2|	2109426	-	G	0	2	0	1	1	SArRNA12
gi|29165615|ref|NC_002745.2|	2109427	-	C	4	2	1	1	0	SArRNA12
gi|29165615|ref|NC_002745.2|	2109428	-	U	1	4	0	0	7	SArRNA12
gi|29165615|ref|NC_002745.2|	2109429	-	G	1	3	1	0	8	SArRNA12
gi|29165615|ref|NC_002745.2|	2109430	-	U	4	5	1	0	4	SArRNA12
gi|29165615|ref|NC_002745.2|	2109431	-	U	6	0	3	1	9	SArRNA12
gi|29165615|ref|NC_002745.2|	2109432	-	G	4	1	0	3	11	SArRNA12
gi|29165615|ref|NC_002745.2|	2109433	-	A	26	5	4	9	33	SArRNA12
gi|29165615|ref|NC_002745.2|	2109434	-	C	312	108	47	55	199	SArRNA12
gi|29165615|ref|NC_002745.2|	2109435	-	C	12	12	4	4	11	SArRNA12
gi|29165615|ref|NC_002745.2|	2109436	-	A	8	7	5	3	10	SArRNA12
gi|29165615|ref|NC_002745.2|	2109437	-	U	5	7	3	3	12	SArRNA12
gi|29165615|ref|NC_002745.2|	2109438	-	G	7	9	2	5	5	SArRNA12
gi|29165615|ref|NC_002745.2|	2109439	-	U	8	9	3	1	12	SArRNA12
gi|29165615|ref|NC_002745.2|	2109440	-	G	2	3	3	6	6	SArRNA12
gi|29165615|ref|NC_002745.2|	2109441	-	G	1	5	3	2	2	SArRNA12
gi|29165615|ref|NC_002745.2|	2109442	-	U	3	3	0	1	7	SArRNA12

gi|29165615|ref|NC_002745.2|	2109443 -	C	8	1	2	4	6 SArRNA12
gi|29165615|ref|NC_002745.2|	2109444 -	U	4	1	5	4	6 SArRNA12
gi|29165615|ref|NC_002745.2|	2109445 -	C	8	5	5	1	7 SArRNA12
gi|29165615|ref|NC_002745.2|	2109446 -	C	11	6	4	2	8 SArRNA12
gi|29165615|ref|NC_002745.2|	2109447 -	A	13	10	1	4	10 SArRNA12
gi|29165615|ref|NC_002745.2|	2109448 -	U	9	19	8	7	25 SArRNA12
gi|29165615|ref|NC_002745.2|	2109449 -	A	4	21	3	7	12 SArRNA12
gi|29165615|ref|NC_002745.2|	2109450 -	C	3	9	4	3	6 SArRNA12
gi|29165615|ref|NC_002745.2|	2109451 -	A	23	2	3	1	7 SArRNA12
gi|29165615|ref|NC_002745.2|	2109452 -	G	14	1	0	0	3 SArRNA12
gi|29165615|ref|NC_002745.2|	2109453 -	G	4	0	0	2	2 SArRNA12
gi|29165615|ref|NC_002745.2|	2109454 -	U	12	11	1	0	4 SArRNA12
gi|29165615|ref|NC_002745.2|	2109455 -	A	14	9	2	3	5 SArRNA12
gi|29165615|ref|NC_002745.2|	2109456 -	G	6	0	0	4	8 SArRNA12
gi|29165615|ref|NC_002745.2|	2109457 -	G	1	2	0	2	1 SArRNA12
gi|29165615|ref|NC_002745.2|	2109458 -	G	2	5	1	0	4 SArRNA12
gi|29165615|ref|NC_002745.2|	2109459 -	C	9	10	3	6	15 SArRNA12
gi|29165615|ref|NC_002745.2|	2109460 -	C	3	9	0	0	3 SArRNA12
gi|29165615|ref|NC_002745.2|	2109461 -	A	3	1	0	1	0 SArRNA12
gi|29165615|ref|NC_002745.2|	2109462 -	G	0	0	0	1	2 SArRNA12
gi|29165615|ref|NC_002745.2|	2109463 -	G	4	2	0	0	1 SArRNA12
gi|29165615|ref|NC_002745.2|	2109464 -	A	2	4	0	2	1 SArRNA12
gi|29165615|ref|NC_002745.2|	2109465 -	G	0	0	0	0	2 SArRNA12
gi|29165615|ref|NC_002745.2|	2109466 -	A	2	1	1	1	1 SArRNA12
gi|29165615|ref|NC_002745.2|	2109467 -	G	2	9	2	2	8 SArRNA12
gi|29165615|ref|NC_002745.2|	2109468 -	C	7	11	2	2	3 SArRNA12
gi|29165615|ref|NC_002745.2|	2109469 -	A	8	9	1	0	1 SArRNA12
gi|29165615|ref|NC_002745.2|	2109470 -	U	1	14	3	1	3 SArRNA12
gi|29165615|ref|NC_002745.2|	2109471 -	G	2	3	1	0	4 SArRNA12
gi|29165615|ref|NC_002745.2|	2109472 -	A	2	4	1	1	3 SArRNA12
gi|29165615|ref|NC_002745.2|	2109473 -	U	7	8	3	4	0 SArRNA12
gi|29165615|ref|NC_002745.2|	2109474 -	U	4	4	4	1	3 SArRNA12
gi|29165615|ref|NC_002745.2|	2109475 -	C	4	4	0	1	6 SArRNA12
gi|29165615|ref|NC_002745.2|	2109476 -	C	6	1	4	0	4 SArRNA12
gi|29165615|ref|NC_002745.2|	2109477 -	U	3	3	0	1	2 SArRNA12
gi|29165615|ref|NC_002745.2|	2109478 -	G	0	2	0	0	1 SArRNA12
gi|29165615|ref|NC_002745.2|	2109479 -	U	2	1	1	0	4 SArRNA12
gi|29165615|ref|NC_002745.2|	2109480 -	C	1	1	2	2	3 SArRNA12
gi|29165615|ref|NC_002745.2|	2109481 -	G	5	4	1	0	1 SArRNA12
gi|29165615|ref|NC_002745.2|	2109482 -	A	6	3	1	5	7 SArRNA12
gi|29165615|ref|NC_002745.2|	2109483 -	G	5	1	2	4	6 SArRNA12
gi|29165615|ref|NC_002745.2|	2109484 -	G	1	2	1	0	1 SArRNA12
gi|29165615|ref|NC_002745.2|	2109485 -	A	3	2	1	3	10 SArRNA12
gi|29165615|ref|NC_002745.2|	2109486 -	G	2	3	2	2	0 SArRNA12
gi|29165615|ref|NC_002745.2|	2109487 -	A	0	2	1	0	1 SArRNA12
gi|29165615|ref|NC_002745.2|	2109488 -	G	12	7	2	4	9 SArRNA12
gi|29165615|ref|NC_002745.2|	2109489 -	U	7	8	3	3	7 SArRNA12
gi|29165615|ref|NC_002745.2|	2109490 -	U	10	5	1	3	12 SArRNA12
gi|29165615|ref|NC_002745.2|	2109491 -	U	10	9	3	3	6 SArRNA12
gi|29165615|ref|NC_002745.2|	2109492 -	A	13	64	3	6	24 SArRNA12
gi|29165615|ref|NC_002745.2|	2109493 -	A	10	6	2	2	6 SArRNA12
gi|29165615|ref|NC_002745.2|	2109494 -	A	0	2	0	3	1 SArRNA12
gi|29165615|ref|NC_002745.2|	2109495 -	G	1	1	1	0	1 SArRNA12
gi|29165615|ref|NC_002745.2|	2109496 -	G	0	2	0	0	1 SArRNA12
gi|29165615|ref|NC_002745.2|	2109497 -	A	1	1	0	0	2 SArRNA12
gi|29165615|ref|NC_002745.2|	2109498 -	U	1	11	2	6	6 SArRNA12
gi|29165615|ref|NC_002745.2|	2109499 -	G	6	5	2	1	3 SArRNA12
gi|29165615|ref|NC_002745.2|	2109500 -	C	6	4	0	1	2 SArRNA12
gi|29165615|ref|NC_002745.2|	2109501 -	G	0	1	0	0	0 SArRNA12
gi|29165615|ref|NC_002745.2|	2109502 -	G	1	2	0	0	1 SArRNA12
gi|29165615|ref|NC_002745.2|	2109503 -	G	0	1	0	0	2 SArRNA12
gi|29165615|ref|NC_002745.2|	2109504 -	U	6	2	2	0	1 SArRNA12
gi|29165615|ref|NC_002745.2|	2109505 -	G	0	2	1	0	0 SArRNA12
gi|29165615|ref|NC_002745.2|	2109506 -	C	3	3	2	1	3 SArRNA12
gi|29165615|ref|NC_002745.2|	2109507 -	U	8	1	0	0	4 SArRNA12
gi|29165615|ref|NC_002745.2|	2109508 -	G	2	15	3	4	5 SArRNA12
gi|29165615|ref|NC_002745.2|	2109509 -	C	11	10	2	8	11 SArRNA12
gi|29165615|ref|NC_002745.2|	2109510 -	C	2	8	1	0	5 SArRNA12
gi|29165615|ref|NC_002745.2|	2109511 -	U	1	0	2	1	2 SArRNA12
gi|29165615|ref|NC_002745.2|	2109512 -	A	4	17	2	2	7 SArRNA12
gi|29165615|ref|NC_002745.2|	2109513 -	U	8	25	1	0	5 SArRNA12
gi|29165615|ref|NC_002745.2|	2109514 -	C	6	17	0	1	4 SArRNA12
gi|29165615|ref|NC_002745.2|	2109515 -	C	1	11	0	1	6 SArRNA12
gi|29165615|ref|NC_002745.2|	2109516 -	C	3	9	3	2	6 SArRNA12
gi|29165615|ref|NC_002745.2|	2109517 -	U	0	0	0	0	1 SArRNA12
gi|29165615|ref|NC_002745.2|	2109518 -	G	2	1	0	0	0 SArRNA12
gi|29165615|ref|NC_002745.2|	2109519 -	G	1	2	0	0	1 SArRNA12
gi|29165615|ref|NC_002745.2|	2109520 -	C	4	3	0	0	7 SArRNA12
gi|29165615|ref|NC_002745.2|	2109521 -	U	0	1	0	0	3 SArRNA12
gi|29165615|ref|NC_002745.2|	2109522 -	U	2	0	0	0	0 SArRNA12
gi|29165615|ref|NC_002745.2|	2109523 -	G	0	3	0	0	2 SArRNA12

gi|29165615|ref|NC_002745.2|	2109524	-	A	2	7	2	3	1	SArRNA12
gi|29165615|ref|NC_002745.2|	2109525	-	C	4	4	0	4	0	SArRNA12
gi|29165615|ref|NC_002745.2|	2109526	-	A	4	2	1	2	3	SArRNA12
gi|29165615|ref|NC_002745.2|	2109528	-	A	1	2	1	0	2	SArRNA12
gi|29165615|ref|NC_002745.2|	2109529	-	G	1	3	1	2	0	SArRNA12
gi|29165615|ref|NC_002745.2|	2109530	-	U	3	6	2	2	10	SArRNA12
gi|29165615|ref|NC_002745.2|	2109531	-	G	6	1	2	3	1	SArRNA12
gi|29165615|ref|NC_002745.2|	2109532	-	C	1	9	4	1	4	SArRNA12
gi|29165615|ref|NC_002745.2|	2109533	-	U	1	2	2	1	1	SArRNA12
gi|29165615|ref|NC_002745.2|	2109534	-	G	6	8	3	2	3	SArRNA12
gi|29165615|ref|NC_002745.2|	2109535	-	C	6	17	1	4	16	SArRNA12
gi|29165615|ref|NC_002745.2|	2109536	-	A	2	1	0	0	0	SArRNA12
gi|29165615|ref|NC_002745.2|	2109537	-	A	1	3	0	0	0	SArRNA12
gi|29165615|ref|NC_002745.2|	2109539	-	A	1	8	2	0	6	SArRNA12
gi|29165615|ref|NC_002745.2|	2109540	-	C	15	29	8	12	22	SArRNA12
gi|29165615|ref|NC_002745.2|	2109541	-	U	0	2	0	0	6	SArRNA12
gi|29165615|ref|NC_002745.2|	2109542	-	U	2	0	0	0	2	SArRNA12
gi|29165615|ref|NC_002745.2|	2109543	-	G	0	1	0	1	1	SArRNA12
gi|29165615|ref|NC_002745.2|	2109544	-	G	1	1	0	0	0	SArRNA12
gi|29165615|ref|NC_002745.2|	2109545	-	G	1	0	0	1	2	SArRNA12
gi|29165615|ref|NC_002745.2|	2109546	-	U	2	16	3	1	12	SArRNA12
gi|29165615|ref|NC_002745.2|	2109547	-	C	7	13	0	3	10	SArRNA12
gi|29165615|ref|NC_002745.2|	2109548	-	G	1	1	2	1	3	SArRNA12
gi|29165615|ref|NC_002745.2|	2109549	-	A	7	3	2	2	11	SArRNA12
gi|29165615|ref|NC_002745.2|	2109550	-	G	8	12	4	1	7	SArRNA12
gi|29165615|ref|NC_002745.2|	2109551	-	C	17	18	6	3	11	SArRNA12
gi|29165615|ref|NC_002745.2|	2109552	-	G	2	5	2	2	3	SArRNA12
gi|29165615|ref|NC_002745.2|	2109553	-	C	4	4	1	3	2	SArRNA12
gi|29165615|ref|NC_002745.2|	2109554	-	A	2	4	1	1	1	SArRNA12
gi|29165615|ref|NC_002745.2|	2109555	-	U	29	38	14	9	21	SArRNA12
gi|29165615|ref|NC_002745.2|	2109556	-	G	3	10	0	1	4	SArRNA12
gi|29165615|ref|NC_002745.2|	2109557	-	G	7	4	2	3	1	SArRNA12
gi|29165615|ref|NC_002745.2|	2109558	-	C	16	7	3	2	10	SArRNA12
gi|29165615|ref|NC_002745.2|	2109559	-	G	7	8	3	0	3	SArRNA12
gi|29165615|ref|NC_002745.2|	2109560	-	A	4	15	0	2	10	SArRNA12
gi|29165615|ref|NC_002745.2|	2109561	-	A	6	6	2	2	9	SArRNA12
gi|29165615|ref|NC_002745.2|	2109562	-	A	9	6	2	3	4	SArRNA12
gi|29165615|ref|NC_002745.2|	2109563	-	U	3	6	0	3	7	SArRNA12
gi|29165615|ref|NC_002745.2|	2109564	-	U	9	2	5	4	11	SArRNA12
gi|29165615|ref|NC_002745.2|	2109565	-	A	5	3	2	1	9	SArRNA12
gi|29165615|ref|NC_002745.2|	2109566	-	C	1	8	4	0	9	SArRNA12
gi|29165615|ref|NC_002745.2|	2109567	-	C	5	10	2	1	7	SArRNA12
gi|29165615|ref|NC_002745.2|	2109568	-	C	18	13	2	4	13	SArRNA12
gi|29165615|ref|NC_002745.2|	2109569	-	G	14	28	3	11	28	SArRNA12
gi|29165615|ref|NC_002745.2|	2109570	-	C	106	105	44	53	73	SArRNA12
gi|29165615|ref|NC_002745.2|	2109571	-	U	0	3	0	0	2	SArRNA12
gi|29165615|ref|NC_002745.2|	2109572	-	U	1	0	0	0	0	SArRNA12
gi|29165615|ref|NC_002745.2|	2109573	-	G	24	0	7	1	3	SArRNA12
gi|29165615|ref|NC_002745.2|	2109574	-	U	12	2	1	0	0	SArRNA12
gi|29165615|ref|NC_002745.2|	2109575	-	C	7	0	0	1	3	SArRNA12
gi|29165615|ref|NC_002745.2|	2109576	-	G	1	2	0	0	3	SArRNA12
gi|29165615|ref|NC_002745.2|	2109577	-	G	1	2	0	1	2	SArRNA12
gi|29165615|ref|NC_002745.2|	2109578	-	G	2	3	3	0	7	SArRNA12
gi|29165615|ref|NC_002745.2|	2109579	-	U	2	0	1	1	5	SArRNA12
gi|29165615|ref|NC_002745.2|	2109580	-	U	13	3	2	3	3	SArRNA12
gi|29165615|ref|NC_002745.2|	2109581	-	G	10	10	5	3	9	SArRNA12
gi|29165615|ref|NC_002745.2|	2109582	-	G	12	3	2	6	13	SArRNA12
gi|29165615|ref|NC_002745.2|	2109583	-	G	4	3	1	2	0	SArRNA12
gi|29165615|ref|NC_002745.2|	2109584	-	A	6	6	0	6	10	SArRNA12
gi|29165615|ref|NC_002745.2|	2109585	-	A	6	7	1	1	6	SArRNA12
gi|29165615|ref|NC_002745.2|	2109586	-	C	24	15	10	8	17	SArRNA12
gi|29165615|ref|NC_002745.2|	2109587	-	C	28	26	10	9	23	SArRNA12
gi|29165615|ref|NC_002745.2|	2109588	-	C	21	28	5	9	17	SArRNA12
gi|29165615|ref|NC_002745.2|	2109589	-	U	26	17	10	5	9	SArRNA12
gi|29165615|ref|NC_002745.2|	2109590	-	G	21	21	8	11	17	SArRNA12
gi|29165615|ref|NC_002745.2|	2109591	-	G	21	19	14	6	9	SArRNA12
gi|29165615|ref|NC_002745.2|	2109592	-	C	13	22	6	5	19	SArRNA12
gi|29165615|ref|NC_002745.2|	2109593	-	U	31	50	6	14	34	SArRNA12
gi|29165615|ref|NC_002745.2|	2109594	-	G	3	8	1	2	6	SArRNA12
gi|29165615|ref|NC_002745.2|	2109595	-	A	6	1	1	3	3	SArRNA12
gi|29165615|ref|NC_002745.2|	2109596	-	U	6	4	5	4	12	SArRNA12
gi|29165615|ref|NC_002745.2|	2109597	-	G	10	13	3	3	10	SArRNA12
gi|29165615|ref|NC_002745.2|	2109598	-	U	8	3	4	4	17	SArRNA12
gi|29165615|ref|NC_002745.2|	2109599	-	C	3	8	2	4	5	SArRNA12
gi|29165615|ref|NC_002745.2|	2109600	-	G	3	4	1	1	1	SArRNA12
gi|29165615|ref|NC_002745.2|	2109601	-	G	32	10	5	11	15	SArRNA12
gi|29165615|ref|NC_002745.2|	2109602	-	G	4	5	4	3	3	SArRNA12
gi|29165615|ref|NC_002745.2|	2109603	-	G	0	1	0	0	1	SArRNA12
gi|29165615|ref|NC_002745.2|	2109604	-	U	26	16	9	10	13	SArRNA12
gi|29165615|ref|NC_002745.2|	2109605	-	C	59	19	14	10	20	SArRNA12
gi|29165615|ref|NC_002745.2|	2109606	-	C	49	12	8	7	26	SArRNA12

gi|29165615|ref|NC_002745.2|	2109607	-	U	27	9	6	8	14 SArRNA12
gi|29165615|ref|NC_002745.2|	2109608	-	A	8	16	7	5	17 SArRNA12
gi|29165615|ref|NC_002745.2|	2109609	-	C	23	25	12	8	15 SArRNA12
gi|29165615|ref|NC_002745.2|	2109610	-	G	5	5	2	2	9 SArRNA12
gi|29165615|ref|NC_002745.2|	2109611	-	C	10	16	6	3	9 SArRNA12
gi|29165615|ref|NC_002745.2|	2109612	-	U	8	2	3	3	9 SArRNA12
gi|29165615|ref|NC_002745.2|	2109613	-	A	21	29	4	7	14 SArRNA12
gi|29165615|ref|NC_002745.2|	2109614	-	C	39	40	4	15	40 SArRNA12
gi|29165615|ref|NC_002745.2|	2109615	-	U	2	3	1	1	7 SArRNA12
gi|29165615|ref|NC_002745.2|	2109616	-	C	0	4	3	1	1 SArRNA12
gi|29165615|ref|NC_002745.2|	2109617	-	G	0	0	0	1	1 SArRNA12
gi|29165615|ref|NC_002745.2|	2109618	-	G	1	2	0	0	1 SArRNA12
gi|29165615|ref|NC_002745.2|	2109619	-	C	5	9	5	4	2 SArRNA12
gi|29165615|ref|NC_002745.2|	2109620	-	U	0	1	0	0	3 SArRNA12
gi|29165615|ref|NC_002745.2|	2109621	-	G	2	0	0	0	0 SArRNA12
gi|29165615|ref|NC_002745.2|	2109622	-	U	0	0	0	0	1 SArRNA12
gi|29165615|ref|NC_002745.2|	2109623	-	A	1	0	0	1	0 SArRNA12
gi|29165615|ref|NC_002745.2|	2109624	-	G	6	30	5	4	12 SArRNA12
gi|29165615|ref|NC_002745.2|	2109625	-	C	5	18	1	6	18 SArRNA12
gi|29165615|ref|NC_002745.2|	2109626	-	U	3	2	1	0	4 SArRNA12
gi|29165615|ref|NC_002745.2|	2109627	-	C	2	7	2	3	4 SArRNA12
gi|29165615|ref|NC_002745.2|	2109628	-	C	3	4	2	1	3 SArRNA12
gi|29165615|ref|NC_002745.2|	2109629	-	A	6	5	0	1	3 SArRNA12
gi|29165615|ref|NC_002745.2|	2109630	-	C	12	22	2	7	28 SArRNA12
gi|29165615|ref|NC_002745.2|	2109633	-	U	56	109	5	2	58 SArRNA12
gi|29165615|ref|NC_002745.2|	2109634	-	U	2	16	1	2	5 SArRNA12
gi|29165615|ref|NC_002745.2|	2109635	-	U	0	0	0	0	1 SArRNA12
gi|29165615|ref|NC_002745.2|	2109636	-	G	1	0	0	0	1 SArRNA12
gi|29165615|ref|NC_002745.2|	2109637	-	G	1	0	0	0	0 SArRNA12
gi|29165615|ref|NC_002745.2|	2109641	-	G	0	0	1	0	0 SArRNA12
gi|29165615|ref|NC_002745.2|	2109642	-	G	0	1	0	0	0 SArRNA12
gi|29165615|ref|NC_002745.2|	2109643	-	C	3	2	0	0	1 SArRNA12
gi|29165615|ref|NC_002745.2|	2109644	-	A	0	0	0	1	0 SArRNA12
gi|29165615|ref|NC_002745.2|	2109645	-	G	1	0	0	0	0 SArRNA12
gi|29165615|ref|NC_002745.2|	2109646	-	C	0	1	1	2	2 SArRNA12
gi|29165615|ref|NC_002745.2|	2109647	-	U	0	0	0	1	0 SArRNA12
gi|29165615|ref|NC_002745.2|	2109648	-	A	8	12	0	1	6 SArRNA12
gi|29165615|ref|NC_002745.2|	2109649	-	C	11	17	8	7	9 SArRNA12
gi|29165615|ref|NC_002745.2|	2109650	-	A	5	6	0	4	1 SArRNA12
gi|29165615|ref|NC_002745.2|	2109651	-	C	8	17	4	1	14 SArRNA12
gi|29165615|ref|NC_002745.2|	2109652	-	U	0	1	0	0	0 SArRNA12
gi|29165615|ref|NC_002745.2|	2109655	-	A	0	2	0	0	2 SArRNA12
gi|29165615|ref|NC_002745.2|	2109657	-	A	0	0	0	1	1 SArRNA12
gi|29165615|ref|NC_002745.2|	2109659	-	C	0	0	0	1	0 SArRNA12
gi|29165615|ref|NC_002745.2|	2109660	-	C	0	1	1	0	0 SArRNA12
gi|29165615|ref|NC_002745.2|	2109661	-	C	3	1	1	0	0 SArRNA12
gi|29165615|ref|NC_002745.2|	2109662	-	C	12	4	4	2	4 SArRNA12
gi|29165615|ref|NC_002745.2|	2109663	-	C	0	3	2	1	2 SArRNA12
gi|29165615|ref|NC_002745.2|	2109664	-	U	0	1	1	0	0 SArRNA12
gi|29165615|ref|NC_002745.2|	2109665	-	C	5	1	0	0	0 SArRNA12
gi|29165615|ref|NC_002745.2|	2109666	-	U	0	1	0	0	0 SArRNA12
gi|29165615|ref|NC_002745.2|	2109673	-	A	0	1	0	0	0 SArRNA12
gi|29165615|ref|NC_002745.2|	2109674	-	C	0	1	0	2	2 SArRNA12
gi|29165615|ref|NC_002745.2|	2109675	-	A	1	0	0	0	0 SArRNA12
gi|29165615|ref|NC_002745.2|	2109678	-	A	0	0	0	1	0 SArRNA12
gi|29165615|ref|NC_002745.2|	2109682	-	G	2	1	0	0	0 SArRNA12
gi|29165615|ref|NC_002745.2|	2109683	-	C	0	1	1	0	0 SArRNA12
gi|29165615|ref|NC_002745.2|	2109684	-	C	1	0	0	0	0 SArRNA12
gi|29165615|ref|NC_002745.2|	2109685	-	C	0	1	1	0	0 SArRNA12
gi|29165615|ref|NC_002745.2|	2109686	-	C	1	2	0	0	0 SArRNA12
gi|29165615|ref|NC_002745.2|	2109687	-	A	0	0	0	0	1 SArRNA12
gi|29165615|ref|NC_002745.2|	2109689	-	C	0	1	0	0	0 SArRNA12
gi|29165615|ref|NC_002745.2|	2109695	-	U	1	0	0	1	0 SArRNA12
gi|29165615|ref|NC_002745.2|	2109697	-	G	0	0	0	1	0 SArRNA12
gi|29165615|ref|NC_002745.2|	2109699	-	C	0	1	0	0	0 SArRNA12
gi|29165615|ref|NC_002745.2|	2109700	-	A	0	1	0	0	0 SArRNA12
gi|29165615|ref|NC_002745.2|	2109701	-	A	2	1	0	0	0 SArRNA12
gi|29165615|ref|NC_002745.2|	2109702	-	C	0	1	0	0	0 SArRNA12
gi|29165615|ref|NC_002745.2|	2109704	-	C	0	0	0	0	1 SArRNA12
gi|29165615|ref|NC_002745.2|	2109706	-	C	1	1	0	0	0 SArRNA12
gi|29165615|ref|NC_002745.2|	2109708	-	A	0	0	0	0	1 SArRNA12
gi|29165615|ref|NC_002745.2|	2109714	-	A	0	0	0	0	1 SArRNA12
gi|29165615|ref|NC_002745.2|	2109715	-	A	0	0	0	0	1 SArRNA12
gi|29165615|ref|NC_002745.2|	2109719	-	A	0	0	0	0	2 SArRNA12
gi|29165615|ref|NC_002745.2|	2109731	-	C	0	0	1	0	0 SArRNA12
gi|29165615|ref|NC_002745.2|	2109732	-	C	0	1	0	2	1 SArRNA12
gi|29165615|ref|NC_002745.2|	2109735	-	G	1	1	0	0	0 SArRNA12
gi|29165615|ref|NC_002745.2|	2109738	-	A	1	0	0	0	0 SArRNA12
gi|29165615|ref|NC_002745.2|	2109741	-	C	1	2	0	0	0 SArRNA12
gi|29165615|ref|NC_002745.2|	2109745	-	C	1	0	0	0	0 SArRNA12
gi|29165615|ref|NC_002745.2|	2109746	-	A	1	0	0	0	0 SArRNA12

gi|29165615|ref|NC_002745.2|	2109749	-	A	0	2	0	0	1	SArRNA12
gi|29165615|ref|NC_002745.2|	2109750	-	A	0	0	0	0	1	SArRNA12
gi|29165615|ref|NC_002745.2|	2109757	-	A	0	0	1	0	2	SArRNA12
gi|29165615|ref|NC_002745.2|	2109759	-	G	0	0	0	0	2	SArRNA12
gi|29165615|ref|NC_002745.2|	2109760	-	A	0	0	1	0	1	SArRNA12
gi|29165615|ref|NC_002745.2|	2109762	-	C	3	2	1	0	5	SArRNA12
gi|29165615|ref|NC_002745.2|	2109763	-	U	0	0	0	0	2	SArRNA12
gi|29165615|ref|NC_002745.2|	2109764	-	G	1	0	0	0	1	SArRNA12
gi|29165615|ref|NC_002745.2|	2109765	-	A	1	0	0	0	0	SArRNA12
gi|29165615|ref|NC_002745.2|	2109766	-	A	3	4	3	0	1	SArRNA12
gi|29165615|ref|NC_002745.2|	2109767	-	C	7	9	0	6	5	SArRNA12
gi|29165615|ref|NC_002745.2|	2109768	-	A	1	0	0	0	0	SArRNA12
gi|29165615|ref|NC_002745.2|	2109769	-	U	0	1	0	0	1	SArRNA12
gi|29165615|ref|NC_002745.2|	2109771	-	C	1	0	0	0	0	SArRNA12
gi|29165615|ref|NC_002745.2|	2109773	-	G	0	0	0	0	2	SArRNA12
gi|29165615|ref|NC_002745.2|	2109774	-	A	0	2	0	0	0	SArRNA12
gi|29165615|ref|NC_002745.2|	2109775	-	G	0	1	0	1	0	SArRNA12
gi|29165615|ref|NC_002745.2|	2109776	-	C	0	3	0	1	0	SArRNA12
gi|29165615|ref|NC_002745.2|	2109777	-	G	0	1	0	0	0	SArRNA12
gi|29165615|ref|NC_002745.2|	2109778	-	U	6	0	2	2	4	SArRNA12
gi|29165615|ref|NC_002745.2|	2109784	-	C	1	1	0	0	0	SArRNA12
gi|29165615|ref|NC_002745.2|	2109787	-	G	0	1	1	0	0	SArRNA12
gi|29165615|ref|NC_002745.2|	2109788	-	G	0	0	0	0	1	SArRNA12
gi|29165615|ref|NC_002745.2|	2109790	-	A	0	1	0	0	0	SArRNA12
gi|29165615|ref|NC_002745.2|	2109791	-	A	0	1	1	0	4	SArRNA12
gi|29165615|ref|NC_002745.2|	2109792	-	U	6	7	4	4	12	SArRNA12
gi|29165615|ref|NC_002745.2|	2109793	-	A	4	1	4	3	5	SArRNA12
gi|29165615|ref|NC_002745.2|	2109794	-	C	1	2	0	0	1	SArRNA12
gi|29165615|ref|NC_002745.2|	2109795	-	G	2	3	1	2	6	SArRNA12
gi|29165615|ref|NC_002745.2|	2109796	-	G	1	5	2	0	1	SArRNA12
gi|29165615|ref|NC_002745.2|	2109797	-	A	5	0	1	0	1	SArRNA12
gi|29165615|ref|NC_002745.2|	2109798	-	A	4	6	0	3	4	SArRNA12
gi|29165615|ref|NC_002745.2|	2109799	-	A	1	1	3	0	1	SArRNA12
gi|29165615|ref|NC_002745.2|	2109800	-	U	0	4	0	0	3	SArRNA12
gi|29165615|ref|NC_002745.2|	2109801	-	G	4	3	0	1	3	SArRNA12
gi|29165615|ref|NC_002745.2|	2109803	-	G	3	2	1	2	7	SArRNA12
gi|29165615|ref|NC_002745.2|	2109804	-	A	1	3	0	0	2	SArRNA12
gi|29165615|ref|NC_002745.2|	2109805	-	G	1	2	5	2	5	SArRNA12
gi|29165615|ref|NC_002745.2|	2109806	-	A	7	4	3	2	6	SArRNA12
gi|29165615|ref|NC_002745.2|	2109807	-	U	3	4	2	2	7	SArRNA12
gi|29165615|ref|NC_002745.2|	2109808	-	A	2	5	5	1	3	SArRNA12
gi|29165615|ref|NC_002745.2|	2109809	-	C	5	5	2	0	4	SArRNA12
gi|29165615|ref|NC_002745.2|	2109810	-	U	1	3	4	3	3	SArRNA12
gi|29165615|ref|NC_002745.2|	2109811	-	U	0	1	1	0	4	SArRNA12
gi|29165615|ref|NC_002745.2|	2109812	-	A	0	4	4	1	4	SArRNA12
gi|29165615|ref|NC_002745.2|	2109813	-	C	2	4	1	4	3	SArRNA12
gi|29165615|ref|NC_002745.2|	2109814	-	U	0	2	1	0	1	SArRNA12
gi|29165615|ref|NC_002745.2|	2109815	-	A	9	70	8	4	20	SArRNA12
gi|29165615|ref|NC_002745.2|	2109816	-	A	1	4	1	0	1	SArRNA12
gi|29165615|ref|NC_002745.2|	2109817	-	A	0	2	0	0	1	SArRNA12
gi|29165615|ref|NC_002745.2|	2109818	-	G	0	2	1	0	1	SArRNA12
gi|29165615|ref|NC_002745.2|	2109819	-	G	6	4	4	2	6	SArRNA12
gi|29165615|ref|NC_002745.2|	2109820	-	U	0	3	1	1	3	SArRNA12
gi|29165615|ref|NC_002745.2|	2109821	-	U	3	2	2	4	7	SArRNA12
gi|29165615|ref|NC_002745.2|	2109822	-	G	3	13	2	0	8	SArRNA12
gi|29165615|ref|NC_002745.2|	2109823	-	G	4	6	0	2	5	SArRNA12
gi|29165615|ref|NC_002745.2|	2109824	-	U	3	2	2	1	6	SArRNA12
gi|29165615|ref|NC_002745.2|	2109825	-	A	3	7	4	3	5	SArRNA12
gi|29165615|ref|NC_002745.2|	2109826	-	A	3	3	0	1	3	SArRNA12
gi|29165615|ref|NC_002745.2|	2109828	-	A	1	1	0	0	1	SArRNA12
gi|29165615|ref|NC_002745.2|	2109829	-	C	13	2	5	3	6	SArRNA12
gi|29165615|ref|NC_002745.2|	2109830	-	U	0	0	1	0	0	SArRNA12
gi|29165615|ref|NC_002745.2|	2109831	-	C	3	1	0	2	2	SArRNA12
gi|29165615|ref|NC_002745.2|	2109832	-	C	1	6	2	0	1	SArRNA12
gi|29165615|ref|NC_002745.2|	2109833	-	C	6	2	2	1	1	SArRNA12
gi|29165615|ref|NC_002745.2|	2109834	-	U	1	2	0	0	9	SArRNA12
gi|29165615|ref|NC_002745.2|	2109835	-	U	0	3	0	1	0	SArRNA12
gi|29165615|ref|NC_002745.2|	2109836	-	G	0	1	0	0	1	SArRNA12
gi|29165615|ref|NC_002745.2|	2109837	-	G	0	1	0	0	0	SArRNA12
gi|29165615|ref|NC_002745.2|	2109838	-	A	2	0	0	0	0	SArRNA12
gi|29165615|ref|NC_002745.2|	2109839	-	A	0	0	0	0	1	SArRNA12
gi|29165615|ref|NC_002745.2|	2109840	-	A	0	0	0	0	1	SArRNA12
gi|29165615|ref|NC_002745.2|	2109841	-	C	0	2	0	2	0	SArRNA12
gi|29165615|ref|NC_002745.2|	2109842	-	U	3	1	1	0	0	SArRNA12
gi|29165615|ref|NC_002745.2|	2109843	-	C	3	1	0	0	2	SArRNA12
gi|29165615|ref|NC_002745.2|	2109845	-	C	1	0	0	0	0	SArRNA12
gi|29165615|ref|NC_002745.2|	2109846	-	G	5	1	2	0	0	SArRNA12
gi|29165615|ref|NC_002745.2|	2109847	-	G	0	0	0	1	0	SArRNA12
gi|29165615|ref|NC_002745.2|	2109848	-	A	1	1	1	0	1	SArRNA12
gi|29165615|ref|NC_002745.2|	2109849	-	G	5	1	0	0	0	SArRNA12
gi|29165615|ref|NC_002745.2|	2109850	-	G	0	1	2	0	0	SArRNA12

gi|29165615|ref|NC_002745.2|	2109851	-	C	1	5	0	1	4	SArRNA12
gi|29165615|ref|NC_002745.2|	2109853	-	A	0	0	0	0	1	SArRNA12
gi|29165615|ref|NC_002745.2|	2109854	-	U	2	1	2	2	1	SArRNA12
gi|29165615|ref|NC_002745.2|	2109855	-	G	0	1	0	0	1	SArRNA12
gi|29165615|ref|NC_002745.2|	2109856	-	G	0	1	0	0	0	SArRNA12
gi|29165615|ref|NC_002745.2|	2109857	-	A	4	7	1	1	7	SArRNA12
gi|29165615|ref|NC_002745.2|	2109858	-	A	17	29	0	5	14	SArRNA12
gi|29165615|ref|NC_002745.2|	2109859	-	A	0	0	1	0	0	SArRNA12
gi|29165615|ref|NC_002745.2|	2109860	-	A	0	1	0	1	2	SArRNA12
gi|29165615|ref|NC_002745.2|	2109862	-	C	4	0	1	1	3	SArRNA12
gi|29165615|ref|NC_002745.2|	2109863	-	C	0	4	2	0	0	SArRNA12
gi|29165615|ref|NC_002745.2|	2109864	-	U	0	1	0	1	0	SArRNA12
gi|29165615|ref|NC_002745.2|	2109865	-	C	0	3	0	0	0	SArRNA12
gi|29165615|ref|NC_002745.2|	2109866	-	C	1	1	0	0	0	SArRNA12
gi|29165615|ref|NC_002745.2|	2109868	-	C	0	0	1	0	2	SArRNA12
gi|29165615|ref|NC_002745.2|	2109869	-	U	1	0	0	0	0	SArRNA12
gi|29165615|ref|NC_002745.2|	2109872	-	C	0	0	0	0	1	SArRNA12
gi|29165615|ref|NC_002745.2|	2109875	-	G	1	0	0	0	0	SArRNA12
gi|29165615|ref|NC_002745.2|	2109876	-	G	0	2	0	0	1	SArRNA12
gi|29165615|ref|NC_002745.2|	2109877	-	U	5	1	2	1	2	SArRNA12
gi|29165615|ref|NC_002745.2|	2109878	-	C	6	7	1	0	0	SArRNA12
gi|29165615|ref|NC_002745.2|	2109879	-	A	0	2	0	1	0	SArRNA12
gi|29165615|ref|NC_002745.2|	2109880	-	G	0	2	3	0	1	SArRNA12
gi|29165615|ref|NC_002745.2|	2109881	-	U	1	3	1	0	4	SArRNA12
gi|29165615|ref|NC_002745.2|	2109882	-	U	4	5	0	1	3	SArRNA12
gi|29165615|ref|NC_002745.2|	2109883	-	U	3	1	0	3	3	SArRNA12
gi|29165615|ref|NC_002745.2|	2109884	-	G	0	0	1	0	0	SArRNA12
gi|29165615|ref|NC_002745.2|	2109885	-	A	0	3	1	1	2	SArRNA12
gi|29165615|ref|NC_002745.2|	2109886	-	C	9	3	2	2	1	SArRNA12
gi|29165615|ref|NC_002745.2|	2109887	-	G	2	1	0	0	3	SArRNA12
gi|29165615|ref|NC_002745.2|	2109888	-	G	3	3	0	0	1	SArRNA12
gi|29165615|ref|NC_002745.2|	2109889	-	G	4	1	0	0	3	SArRNA12
gi|29165615|ref|NC_002745.2|	2109890	-	C	1	5	2	2	1	SArRNA12
gi|29165615|ref|NC_002745.2|	2109891	-	G	0	2	0	1	0	SArRNA12
gi|29165615|ref|NC_002745.2|	2109892	-	G	0	0	0	2	3	SArRNA12
gi|29165615|ref|NC_002745.2|	2109893	-	A	0	1	1	1	1	SArRNA12
gi|29165615|ref|NC_002745.2|	2109894	-	C	5	0	1	0	2	SArRNA12
gi|29165615|ref|NC_002745.2|	2109895	-	U	4	1	0	1	6	SArRNA12
gi|29165615|ref|NC_002745.2|	2109896	-	G	1	0	0	1	1	SArRNA12
gi|29165615|ref|NC_002745.2|	2109897	-	U	0	0	0	4	2	SArRNA12
gi|29165615|ref|NC_002745.2|	2109898	-	G	3	1	0	3	5	SArRNA12
gi|29165615|ref|NC_002745.2|	2109899	-	A	5	9	2	5	1	SArRNA12
gi|29165615|ref|NC_002745.2|	2109900	-	C	16	22	5	10	20	SArRNA12
gi|29165615|ref|NC_002745.2|	2109901	-	A	4	5	2	2	3	SArRNA12
gi|29165615|ref|NC_002745.2|	2109902	-	G	1	3	3	0	0	SArRNA12
gi|29165615|ref|NC_002745.2|	2109903	-	A	3	5	4	1	2	SArRNA12
gi|29165615|ref|NC_002745.2|	2109904	-	G	5	3	0	0	2	SArRNA12
gi|29165615|ref|NC_002745.2|	2109905	-	G	0	0	1	1	0	SArRNA12
gi|29165615|ref|NC_002745.2|	2109906	-	G	0	0	0	0	1	SArRNA12
gi|29165615|ref|NC_002745.2|	2109907	-	U	0	0	0	0	1	SArRNA12
gi|29165615|ref|NC_002745.2|	2109908	-	G	0	0	0	0	1	SArRNA12
gi|29165615|ref|NC_002745.2|	2109911	-	G	0	2	1	1	3	SArRNA12
gi|29165615|ref|NC_002745.2|	2109912	-	C	1	2	4	1	2	SArRNA12
gi|29165615|ref|NC_002745.2|	2109913	-	U	2	2	2	0	3	SArRNA12
gi|29165615|ref|NC_002745.2|	2109914	-	A	3	1	0	1	3	SArRNA12
gi|29165615|ref|NC_002745.2|	2109915	-	U	2	6	0	0	4	SArRNA12
gi|29165615|ref|NC_002745.2|	2109916	-	U	2	1	2	1	5	SArRNA12
gi|29165615|ref|NC_002745.2|	2109917	-	C	6	2	0	3	1	SArRNA12
gi|29165615|ref|NC_002745.2|	2109918	-	A	1	4	2	0	3	SArRNA12
gi|29165615|ref|NC_002745.2|	2109919	-	C	5	3	0	0	1	SArRNA12
gi|29165615|ref|NC_002745.2|	2109920	-	C	0	1	0	0	0	SArRNA12
gi|29165615|ref|NC_002745.2|	2109921	-	A	1	0	0	0	0	SArRNA12
gi|29165615|ref|NC_002745.2|	2109922	-	C	1	1	0	0	0	SArRNA12
gi|29165615|ref|NC_002745.2|	2109923	-	G	0	2	0	0	1	SArRNA12
gi|29165615|ref|NC_002745.2|	2109924	-	C	2	2	0	1	1	SArRNA12
gi|29165615|ref|NC_002745.2|	2109925	-	C	3	1	1	1	1	SArRNA12
gi|29165615|ref|NC_002745.2|	2109926	-	C	7	11	4	1	7	SArRNA12
gi|29165615|ref|NC_002745.2|	2109927	-	A	0	1	0	1	2	SArRNA12
gi|29165615|ref|NC_002745.2|	2109928	-	A	0	0	1	1	2	SArRNA12
gi|29165615|ref|NC_002745.2|	2109929	-	U	0	2	0	1	5	SArRNA12
gi|29165615|ref|NC_002745.2|	2109930	-	C	4	5	2	0	8	SArRNA12
gi|29165615|ref|NC_002745.2|	2109931	-	U	3	0	1	1	4	SArRNA12
gi|29165615|ref|NC_002745.2|	2109932	-	U	3	0	0	0	3	SArRNA12
gi|29165615|ref|NC_002745.2|	2109933	-	U	5	1	3	1	0	SArRNA12
gi|29165615|ref|NC_002745.2|	2109934	-	C	5	1	0	0	2	SArRNA12
gi|29165615|ref|NC_002745.2|	2109936	-	G	1	0	0	0	0	SArRNA12
gi|29165615|ref|NC_002745.2|	2109937	-	U	0	0	0	2	3	SArRNA12
gi|29165615|ref|NC_002745.2|	2109938	-	U	6	3	0	1	3	SArRNA12
gi|29165615|ref|NC_002745.2|	2109939	-	G	2	1	0	0	0	SArRNA12
gi|29165615|ref|NC_002745.2|	2109940	-	U	0	1	1	0	5	SArRNA12
gi|29165615|ref|NC_002745.2|	2109941	-	G	2	0	0	0	0	SArRNA12

gi|29165615|ref|NC_002745.2|	2109942	-	U	1	0	0	0	0	SArRNA12
gi|29165615|ref|NC_002745.2|	2109943	-	C	1	1	0	1	2	SArRNA12
gi|29165615|ref|NC_002745.2|	2109945	-	A	2	5	0	2	2	SArRNA12
gi|29165615|ref|NC_002745.2|	2109946	-	U	3	2	0	3	3	SArRNA12
gi|29165615|ref|NC_002745.2|	2109947	-	C	5	18	9	5	11	SArRNA12
gi|29165615|ref|NC_002745.2|	2109948	-	C	1	9	2	2	3	SArRNA12
gi|29165615|ref|NC_002745.2|	2109949	-	C	10	12	6	2	3	SArRNA12
gi|29165615|ref|NC_002745.2|	2109950	-	A	15	11	3	3	9	SArRNA12
gi|29165615|ref|NC_002745.2|	2109951	-	U	53	28	14	13	40	SArRNA12
gi|29165615|ref|NC_002745.2|	2109952	-	C	14	14	4	9	15	SArRNA12
gi|29165615|ref|NC_002745.2|	2109953	-	A	0	2	1	0	3	SArRNA12
gi|29165615|ref|NC_002745.2|	2109954	-	U	7	6	4	1	5	SArRNA12
gi|29165615|ref|NC_002745.2|	2109955	-	A	3	6	2	0	3	SArRNA12
gi|29165615|ref|NC_002745.2|	2109956	-	G	6	2	0	3	5	SArRNA12
gi|29165615|ref|NC_002745.2|	2109957	-	G	0	2	1	0	1	SArRNA12
gi|29165615|ref|NC_002745.2|	2109958	-	G	1	2	0	0	1	SArRNA12
gi|29165615|ref|NC_002745.2|	2109959	-	U	3	8	0	1	4	SArRNA12
gi|29165615|ref|NC_002745.2|	2109960	-	G	5	6	0	1	4	SArRNA12
gi|29165615|ref|NC_002745.2|	2109961	-	G	1	3	1	0	4	SArRNA12
gi|29165615|ref|NC_002745.2|	2109962	-	U	17	9	2	1	12	SArRNA12
gi|29165615|ref|NC_002745.2|	2109963	-	C	4	7	2	2	7	SArRNA12
gi|29165615|ref|NC_002745.2|	2109964	-	G	0	1	0	1	1	SArRNA12
gi|29165615|ref|NC_002745.2|	2109965	-	C	1	1	1	1	5	SArRNA12
gi|29165615|ref|NC_002745.2|	2109966	-	G	2	0	2	2	1	SArRNA12
gi|29165615|ref|NC_002745.2|	2109967	-	G	2	1	1	1	6	SArRNA12
gi|29165615|ref|NC_002745.2|	2109968	-	A	0	2	2	2	2	SArRNA12
gi|29165615|ref|NC_002745.2|	2109969	-	G	1	1	1	0	2	SArRNA12
gi|29165615|ref|NC_002745.2|	2109970	-	G	1	2	0	0	1	SArRNA12
gi|29165615|ref|NC_002745.2|	2109971	-	U	0	2	0	2	1	SArRNA12
gi|29165615|ref|NC_002745.2|	2109972	-	G	1	0	0	0	2	SArRNA12
gi|29165615|ref|NC_002745.2|	2109973	-	C	0	1	1	0	1	SArRNA12
gi|29165615|ref|NC_002745.2|	2109974	-	A	1	5	0	0	2	SArRNA12
gi|29165615|ref|NC_002745.2|	2109975	-	U	1	1	0	0	2	SArRNA12
gi|29165615|ref|NC_002745.2|	2109976	-	U	0	5	2	1	5	SArRNA12
gi|29165615|ref|NC_002745.2|	2109977	-	C	6	6	1	1	2	SArRNA12
gi|29165615|ref|NC_002745.2|	2109978	-	G	2	0	1	1	2	SArRNA12
gi|29165615|ref|NC_002745.2|	2109979	-	A	3	4	0	0	2	SArRNA12
gi|29165615|ref|NC_002745.2|	2109980	-	U	5	1	3	5	5	SArRNA12
gi|29165615|ref|NC_002745.2|	2109981	-	C	2	7	3	1	6	SArRNA12
gi|29165615|ref|NC_002745.2|	2109982	-	G	0	1	0	0	2	SArRNA12
gi|29165615|ref|NC_002745.2|	2109983	-	C	1	3	0	1	5	SArRNA12
gi|29165615|ref|NC_002745.2|	2109984	-	G	0	0	0	0	2	SArRNA12
gi|29165615|ref|NC_002745.2|	2109985	-	A	0	0	1	1	1	SArRNA12
gi|29165615|ref|NC_002745.2|	2109986	-	G	1	0	0	0	0	SArRNA12
gi|29165615|ref|NC_002745.2|	2109987	-	U	1	0	2	1	3	SArRNA12
gi|29165615|ref|NC_002745.2|	2109988	-	G	0	0	0	1	1	SArRNA12
gi|29165615|ref|NC_002745.2|	2109989	-	C	4	0	0	1	2	SArRNA12
gi|29165615|ref|NC_002745.2|	2109991	-	A	1	0	0	2	2	SArRNA12
gi|29165615|ref|NC_002745.2|	2109992	-	A	1	1	0	1	4	SArRNA12
gi|29165615|ref|NC_002745.2|	2109993	-	G	0	0	0	1	0	SArRNA12
gi|29165615|ref|NC_002745.2|	2109994	-	U	5	1	1	0	2	SArRNA12
gi|29165615|ref|NC_002745.2|	2109995	-	U	11	3	0	1	7	SArRNA12
gi|29165615|ref|NC_002745.2|	2109996	-	U	1	4	3	1	5	SArRNA12
gi|29165615|ref|NC_002745.2|	2109997	-	C	6	4	1	1	6	SArRNA12
gi|29165615|ref|NC_002745.2|	2109998	-	C	13	6	3	8	11	SArRNA12
gi|29165615|ref|NC_002745.2|	2110000	-	A	5	6	2	1	4	SArRNA12
gi|29165615|ref|NC_002745.2|	2110001	-	G	10	4	5	6	8	SArRNA12
gi|29165615|ref|NC_002745.2|	2110002	-	G	4	2	0	3	5	SArRNA12
gi|29165615|ref|NC_002745.2|	2110003	-	A	7	8	4	2	5	SArRNA12
gi|29165615|ref|NC_002745.2|	2110004	-	U	17	8	3	5	8	SArRNA12
gi|29165615|ref|NC_002745.2|	2110005	-	G	6	1	2	5	5	SArRNA12
gi|29165615|ref|NC_002745.2|	2110006	-	G	3	3	2	1	1	SArRNA12
gi|29165615|ref|NC_002745.2|	2110007	-	A	2	3	1	2	4	SArRNA12
gi|29165615|ref|NC_002745.2|	2110008	-	U	15	24	5	14	11	SArRNA12
gi|29165615|ref|NC_002745.2|	2110009	-	A	162	13	47	10	13	SArRNA12
gi|29165615|ref|NC_002745.2|	2110010	-	G	141	9	56	5	15	SArRNA12
gi|29165615|ref|NC_002745.2|	2110011	-	G	4	4	3	3	6	SArRNA12
gi|29165615|ref|NC_002745.2|	2110012	-	A	14	28	4	11	23	SArRNA12
gi|29165615|ref|NC_002745.2|	2110013	-	C	35	76	12	23	51	SArRNA12
gi|29165615|ref|NC_002745.2|	2110014	-	A	3	2	0	0	3	SArRNA12
gi|29165615|ref|NC_002745.2|	2110015	-	U	5	13	3	2	3	SArRNA12
gi|29165615|ref|NC_002745.2|	2110016	-	G	6	4	0	1	10	SArRNA12
gi|29165615|ref|NC_002745.2|	2110017	-	U	6	2	0	4	4	SArRNA12
gi|29165615|ref|NC_002745.2|	2110018	-	U	3	8	3	5	7	SArRNA12
gi|29165615|ref|NC_002745.2|	2110019	-	C	11	8	3	1	4	SArRNA12
gi|29165615|ref|NC_002745.2|	2110020	-	G	1	2	0	0	0	SArRNA12
gi|29165615|ref|NC_002745.2|	2110021	-	A	0	2	0	0	0	SArRNA12
gi|29165615|ref|NC_002745.2|	2110022	-	C	1	1	0	0	3	SArRNA12
gi|29165615|ref|NC_002745.2|	2110023	-	A	0	2	0	0	1	SArRNA12
gi|29165615|ref|NC_002745.2|	2110024	-	C	0	1	0	2	0	SArRNA12
gi|29165615|ref|NC_002745.2|	2110026	-	G	1	1	0	0	2	SArRNA12

gi|29165615|ref|NC_002745.2|	2110027 -	C	11	22	6	5	10	SArRNA12
gi|29165615|ref|NC_002745.2|	2110028 -	U	2	5	2	1	2	SArRNA12
gi|29165615|ref|NC_002745.2|	2110029 -	U	6	6	2	0	1	SArRNA12
gi|29165615|ref|NC_002745.2|	2110030 -	A	2	15	1	0	7	SArRNA12
gi|29165615|ref|NC_002745.2|	2110031 -	A	0	0	1	1	0	SArRNA12
gi|29165615|ref|NC_002745.2|	2110032 -	A	1	4	1	0	3	SArRNA12
gi|29165615|ref|NC_002745.2|	2110033 -	G	0	0	0	1	1	SArRNA12
gi|29165615|ref|NC_002745.2|	2110035 -	U	0	0	0	0	3	SArRNA12
gi|29165615|ref|NC_002745.2|	2110036 -	A	1	2	2	2	4	SArRNA12
gi|29165615|ref|NC_002745.2|	2110037 -	U	10	3	1	1	6	SArRNA12
gi|29165615|ref|NC_002745.2|	2110038 -	A	5	2	1	0	8	SArRNA12
gi|29165615|ref|NC_002745.2|	2110039 -	G	0	1	0	0	1	SArRNA12
gi|29165615|ref|NC_002745.2|	2110040 -	U	0	5	0	1	4	SArRNA12
gi|29165615|ref|NC_002745.2|	2110041 -	C	16	13	10	6	6	SArRNA12
gi|29165615|ref|NC_002745.2|	2110042 -	C	1	2	0	0	1	SArRNA12
gi|29165615|ref|NC_002745.2|	2110044 -	A	3	1	0	0	0	SArRNA12
gi|29165615|ref|NC_002745.2|	2110045 -	U	2	0	0	2	1	SArRNA12
gi|29165615|ref|NC_002745.2|	2110046 -	G	1	1	1	0	0	SArRNA12
gi|29165615|ref|NC_002745.2|	2110047 -	U	2	5	0	2	5	SArRNA12
gi|29165615|ref|NC_002745.2|	2110048 -	C	2	3	4	5	2	SArRNA12
gi|29165615|ref|NC_002745.2|	2110049 -	A	0	4	0	0	3	SArRNA12
gi|29165615|ref|NC_002745.2|	2110050 -	U	6	4	0	0	4	SArRNA12
gi|29165615|ref|NC_002745.2|	2110051 -	U	2	1	0	0	0	SArRNA12
gi|29165615|ref|NC_002745.2|	2110052 -	U	6	2	1	2	3	SArRNA12
gi|29165615|ref|NC_002745.2|	2110053 -	C	8	6	3	1	7	SArRNA12
gi|29165615|ref|NC_002745.2|	2110054 -	G	7	4	0	0	4	SArRNA12
gi|29165615|ref|NC_002745.2|	2110055 -	A	3	0	2	0	4	SArRNA12
gi|29165615|ref|NC_002745.2|	2110056 -	G	3	2	1	2	2	SArRNA12
gi|29165615|ref|NC_002745.2|	2110057 -	G	1	1	0	0	1	SArRNA12
gi|29165615|ref|NC_002745.2|	2110058 -	U	6	3	1	1	5	SArRNA12
gi|29165615|ref|NC_002745.2|	2110059 -	G	2	4	2	1	4	SArRNA12
gi|29165615|ref|NC_002745.2|	2110060 -	C	17	22	4	24	10	SArRNA12
gi|29165615|ref|NC_002745.2|	2110061 -	C	6	1	3	1	6	SArRNA12
gi|29165615|ref|NC_002745.2|	2110062 -	C	19	5	2	3	4	SArRNA12
gi|29165615|ref|NC_002745.2|	2110063 -	C	7	9	2	3	17	SArRNA12
gi|29165615|ref|NC_002745.2|	2110064 -	A	5	3	3	1	6	SArRNA12
gi|29165615|ref|NC_002745.2|	2110065 -	G	10	7	2	0	4	SArRNA12
gi|29165615|ref|NC_002745.2|	2110066 -	A	3	3	1	0	5	SArRNA12
gi|29165615|ref|NC_002745.2|	2110067 -	A	5	3	2	2	3	SArRNA12
gi|29165615|ref|NC_002745.2|	2110068 -	A	1	0	1	2	3	SArRNA12
gi|29165615|ref|NC_002745.2|	2110069 -	G	0	4	1	0	1	SArRNA12
gi|29165615|ref|NC_002745.2|	2110070 -	G	1	5	0	1	2	SArRNA12
gi|29165615|ref|NC_002745.2|	2110071 -	C	7	2	1	0	4	SArRNA12
gi|29165615|ref|NC_002745.2|	2110072 -	A	5	1	2	0	2	SArRNA12
gi|29165615|ref|NC_002745.2|	2110073 -	G	2	4	4	1	4	SArRNA12
gi|29165615|ref|NC_002745.2|	2110074 -	G	5	4	0	1	12	SArRNA12
gi|29165615|ref|NC_002745.2|	2110075 -	A	5	5	2	0	1	SArRNA12
gi|29165615|ref|NC_002745.2|	2110076 -	C	20	23	3	1	11	SArRNA12
gi|29165615|ref|NC_002745.2|	2110077 -	A	6	3	2	1	2	SArRNA12
gi|29165615|ref|NC_002745.2|	2110078 -	G	1	3	0	2	1	SArRNA12
gi|29165615|ref|NC_002745.2|	2110079 -	C	9	2	2	5	2	SArRNA12
gi|29165615|ref|NC_002745.2|	2110080 -	G	0	2	0	0	1	SArRNA12
gi|29165615|ref|NC_002745.2|	2110081 -	C	9	1	3	5	7	SArRNA12
gi|29165615|ref|NC_002745.2|	2110082 -	C	5	7	4	2	4	SArRNA12
gi|29165615|ref|NC_002745.2|	2110083 -	C	10	10	5	2	10	SArRNA12
gi|29165615|ref|NC_002745.2|	2110084 -	A	6	3	1	0	3	SArRNA12
gi|29165615|ref|NC_002745.2|	2110085 -	U	7	5	0	1	6	SArRNA12
gi|29165615|ref|NC_002745.2|	2110086 -	U	6	2	2	0	6	SArRNA12
gi|29165615|ref|NC_002745.2|	2110087 -	G	1	1	1	1	0	SArRNA12
gi|29165615|ref|NC_002745.2|	2110088 -	G	0	1	0	0	1	SArRNA12
gi|29165615|ref|NC_002745.2|	2110089 -	A	7	4	1	4	7	SArRNA12
gi|29165615|ref|NC_002745.2|	2110090 -	C	13	13	1	3	0	SArRNA12
gi|29165615|ref|NC_002745.2|	2110091 -	G	2	4	2	0	7	SArRNA12
gi|29165615|ref|NC_002745.2|	2110092 -	U	4	5	3	3	5	SArRNA12
gi|29165615|ref|NC_002745.2|	2110093 -	A	6	9	6	4	7	SArRNA12
gi|29165615|ref|NC_002745.2|	2110094 -	G	10	12	3	4	5	SArRNA12
gi|29165615|ref|NC_002745.2|	2110095 -	A	10	14	0	2	9	SArRNA12
gi|29165615|ref|NC_002745.2|	2110096 -	A	4	3	2	2	4	SArRNA12
gi|29165615|ref|NC_002745.2|	2110097 -	G	1	0	2	5	3	SArRNA12
gi|29165615|ref|NC_002745.2|	2110098 -	U	6	3	4	2	2	SArRNA12
gi|29165615|ref|NC_002745.2|	2110099 -	G	2	1	0	1	1	SArRNA12
gi|29165615|ref|NC_002745.2|	2110100 -	U	6	6	0	1	2	SArRNA12
gi|29165615|ref|NC_002745.2|	2110101 -	C	3	3	0	3	1	SArRNA12
gi|29165615|ref|NC_002745.2|	2110102 -	C	1	1	3	1	3	SArRNA12
gi|29165615|ref|NC_002745.2|	2110103 -	A	1	2	2	1	0	SArRNA12
gi|29165615|ref|NC_002745.2|	2110104 -	U	5	9	2	0	5	SArRNA12
gi|29165615|ref|NC_002745.2|	2110105 -	G	0	2	1	0	0	SArRNA12
gi|29165615|ref|NC_002745.2|	2110106 -	A	0	2	0	0	1	SArRNA12
gi|29165615|ref|NC_002745.2|	2110107 -	U	8	7	2	7	11	SArRNA12
gi|29165615|ref|NC_002745.2|	2110108 -	A	20	10	6	2	10	SArRNA12
gi|29165615|ref|NC_002745.2|	2110109 -	C	14	37	3	3	25	SArRNA12

gi|29165615|ref|NC_002745.2|	2110110	-	U	2	2	0	0	4 SArRNA12
gi|29165615|ref|NC_002745.2|	2110111	-	A	15	147	24	3	35 SArRNA12
gi|29165615|ref|NC_002745.2|	2110112	-	A	5	6	3	6	7 SArRNA12
gi|29165615|ref|NC_002745.2|	2110113	-	A	0	0	1	0	2 SArRNA12
gi|29165615|ref|NC_002745.2|	2110114	-	G	0	3	1	0	0 SArRNA12
gi|29165615|ref|NC_002745.2|	2110115	-	U	4	1	0	1	2 SArRNA12
gi|29165615|ref|NC_002745.2|	2110116	-	G	1	2	0	0	1 SArRNA12
gi|29165615|ref|NC_002745.2|	2110117	-	G	1	6	1	0	2 SArRNA12
gi|29165615|ref|NC_002745.2|	2110118	-	C	19	22	8	5	12 SArRNA12
gi|29165615|ref|NC_002745.2|	2110119	-	U	5	13	1	3	8 SArRNA12
gi|29165615|ref|NC_002745.2|	2110120	-	C	11	9	3	1	9 SArRNA12
gi|29165615|ref|NC_002745.2|	2110121	-	A	5	7	4	1	6 SArRNA12
gi|29165615|ref|NC_002745.2|	2110122	-	G	6	3	4	4	7 SArRNA12
gi|29165615|ref|NC_002745.2|	2110123	-	A	1	5	1	0	4 SArRNA12
gi|29165615|ref|NC_002745.2|	2110124	-	G	4	0	3	1	0 SArRNA12
gi|29165615|ref|NC_002745.2|	2110125	-	A	2	3	0	2	1 SArRNA12
gi|29165615|ref|NC_002745.2|	2110126	-	G	8	12	3	0	9 SArRNA12
gi|29165615|ref|NC_002745.2|	2110127	-	C	52	42	16	10	27 SArRNA12
gi|29165615|ref|NC_002745.2|	2110128	-	A	1	6	5	1	6 SArRNA12
gi|29165615|ref|NC_002745.2|	2110129	-	A	42	48	24	10	26 SArRNA12
gi|29165615|ref|NC_002745.2|	2110130	-	C	49	48	13	17	34 SArRNA12
gi|29165615|ref|NC_002745.2|	2110131	-	U	1	4	2	1	6 SArRNA12
gi|29165615|ref|NC_002745.2|	2110132	-	C	6	13	2	2	2 SArRNA12
gi|29165615|ref|NC_002745.2|	2110133	-	U	0	2	0	1	2 SArRNA12
gi|29165615|ref|NC_002745.2|	2110134	-	G	0	0	0	0	1 SArRNA12
gi|29165615|ref|NC_002745.2|	2110135	-	U	2	3	0	1	1 SArRNA12
gi|29165615|ref|NC_002745.2|	2110136	-	C	12	11	3	3	5 SArRNA12
gi|29165615|ref|NC_002745.2|	2110137	-	A	1	5	3	1	2 SArRNA12
gi|29165615|ref|NC_002745.2|	2110138	-	C	8	8	6	7	6 SArRNA12
gi|29165615|ref|NC_002745.2|	2110139	-	G	0	2	0	0	1 SArRNA12
gi|29165615|ref|NC_002745.2|	2110140	-	G	0	2	0	0	0 SArRNA12
gi|29165615|ref|NC_002745.2|	2110141	-	G	6	26	0	0	13 SArRNA12
gi|29165615|ref|NC_002745.2|	2110142	-	U	3	10	4	1	6 SArRNA12
gi|29165615|ref|NC_002745.2|	2110143	-	U	3	10	4	7	6 SArRNA12
gi|29165615|ref|NC_002745.2|	2110144	-	U	10	34	17	11	18 SArRNA12
gi|29165615|ref|NC_002745.2|	2110145	-	A	36	228	142	235	273 SArRNA12
gi|29165615|ref|NC_002745.2|	2110146	-	G	14	17	4	7	18 SArRNA12
gi|29165615|ref|NC_002745.2|	2110147	-	C	10	33	12	15	24 SArRNA12
gi|29165615|ref|NC_002745.2|	2110148	-	A	4	12	12	7	19 SArRNA12
gi|29165615|ref|NC_002745.2|	2110149	-	A	0	17	10	9	11 SArRNA12
gi|29165615|ref|NC_002745.2|	2110150	-	U	3	6	6	3	7 SArRNA12
gi|29165615|ref|NC_002745.2|	2110151	-	G	7	9	2	0	2 SArRNA12
gi|29165615|ref|NC_002745.2|	2110152	-	C	6	4	1	2	4 SArRNA12
gi|29165615|ref|NC_002745.2|	2110153	-	G	10	0	0	0	5 SArRNA12
gi|29165615|ref|NC_002745.2|	2110154	-	G	1	0	0	0	1 SArRNA12
gi|29165615|ref|NC_002745.2|	2110155	-	A	15	6	4	1	6 SArRNA12
gi|29165615|ref|NC_002745.2|	2110156	-	A	20	25	10	5	18 SArRNA12
gi|29165615|ref|NC_002745.2|	2110157	-	A	3	5	3	3	8 SArRNA12
gi|29165615|ref|NC_002745.2|	2110158	-	G	10	2	7	1	15 SArRNA12
gi|29165615|ref|NC_002745.2|	2110159	-	C	4	5	3	1	6 SArRNA12
gi|29165615|ref|NC_002745.2|	2110160	-	A	7	14	6	4	11 SArRNA12
gi|29165615|ref|NC_002745.2|	2110161	-	C	41	46	19	9	37 SArRNA12
gi|29165615|ref|NC_002745.2|	2110162	-	G	14	17	3	1	19 SArRNA12
gi|29165615|ref|NC_002745.2|	2110163	-	C	11	15	1	1	8 SArRNA12
gi|29165615|ref|NC_002745.2|	2110164	-	C	20	35	18	5	28 SArRNA12
gi|29165615|ref|NC_002745.2|	2110165	-	C	65	32	10	9	35 SArRNA12
gi|29165615|ref|NC_002745.2|	2110166	-	A	0	8	0	0	6 SArRNA12
gi|29165615|ref|NC_002745.2|	2110167	-	G	4	6	0	1	2 SArRNA12
gi|29165615|ref|NC_002745.2|	2110168	-	C	1	4	2	1	1 SArRNA12
gi|29165615|ref|NC_002745.2|	2110169	-	C	3	6	0	0	2 SArRNA12
gi|29165615|ref|NC_002745.2|	2110170	-	U	1	3	0	0	0 SArRNA12
gi|29165615|ref|NC_002745.2|	2110171	-	U	0	2	1	0	0 SArRNA12
gi|29165615|ref|NC_002745.2|	2110172	-	G	0	0	0	0	1 SArRNA12
gi|29165615|ref|NC_002745.2|	2110173	-	A	3	1	0	2	0 SArRNA12
gi|29165615|ref|NC_002745.2|	2110174	-	A	2	4	0	1	3 SArRNA12
gi|29165615|ref|NC_002745.2|	2110175	-	U	3	0	2	1	3 SArRNA12
gi|29165615|ref|NC_002745.2|	2110176	-	G	1	1	4	0	3 SArRNA12
gi|29165615|ref|NC_002745.2|	2110177	-	G	1	1	0	0	2 SArRNA12
gi|29165615|ref|NC_002745.2|	2110178	-	G	5	10	0	0	4 SArRNA12
gi|29165615|ref|NC_002745.2|	2110179	-	C	21	18	3	6	13 SArRNA12
gi|29165615|ref|NC_002745.2|	2110180	-	U	10	9	2	1	9 SArRNA12
gi|29165615|ref|NC_002745.2|	2110181	-	G	12	4	1	0	6 SArRNA12
gi|29165615|ref|NC_002745.2|	2110182	-	U	8	4	3	1	11 SArRNA12
gi|29165615|ref|NC_002745.2|	2110183	-	U	9	12	3	4	12 SArRNA12
gi|29165615|ref|NC_002745.2|	2110184	-	C	75	135	30	39	92 SArRNA12
gi|29165615|ref|NC_002745.2|	2110185	-	C	5	17	1	3	10 SArRNA12
gi|29165615|ref|NC_002745.2|	2110186	-	U	1	1	0	1	2 SArRNA12
gi|29165615|ref|NC_002745.2|	2110187	-	U	0	4	3	0	2 SArRNA12
gi|29165615|ref|NC_002745.2|	2110188	-	A	11	67	7	3	37 SArRNA12
gi|29165615|ref|NC_002745.2|	2110189	-	A	2	4	3	4	7 SArRNA12
gi|29165615|ref|NC_002745.2|	2110190	-	A	1	8	1	4	4 SArRNA12

gi|29165615|ref|NC_002745.2|	2110191	-	G	0	0	1	1	1	SArRNA12
gi|29165615|ref|NC_002745.2|	2110192	-	C	2	0	0	0	1	SArRNA12
gi|29165615|ref|NC_002745.2|	2110194	-	A	0	1	0	0	0	SArRNA12
gi|29165615|ref|NC_002745.2|	2110195	-	U	0	1	0	0	1	SArRNA12
gi|29165615|ref|NC_002745.2|	2110196	-	G	0	0	0	0	1	SArRNA12
gi|29165615|ref|NC_002745.2|	2110198	-	A	0	0	0	1	0	SArRNA12
gi|29165615|ref|NC_002745.2|	2110200	-	U	0	2	0	0	0	SArRNA12
gi|29165615|ref|NC_002745.2|	2110201	-	C	3	0	0	0	1	SArRNA12
gi|29165615|ref|NC_002745.2|	2110206	-	C	0	0	1	0	0	SArRNA12
gi|29165615|ref|NC_002745.2|	2110207	-	A	1	2	0	0	0	SArRNA12
gi|29165615|ref|NC_002745.2|	2110208	-	A	0	0	1	0	2	SArRNA12
gi|29165615|ref|NC_002745.2|	2110210	-	A	1	1	2	2	1	SArRNA12
gi|29165615|ref|NC_002745.2|	2110211	-	U	0	0	0	0	2	SArRNA12
gi|29165615|ref|NC_002745.2|	2110213	-	A	0	0	0	0	3	SArRNA12
gi|29165615|ref|NC_002745.2|	2110214	-	A	0	0	0	1	0	SArRNA12
gi|29165615|ref|NC_002745.2|	2110215	-	U	0	0	0	0	1	SArRNA12
gi|29165615|ref|NC_002745.2|	2110216	-	G	1	0	0	0	0	SArRNA12
gi|29165615|ref|NC_002745.2|	2110219	-	G	0	1	0	0	0	SArRNA12
gi|29165615|ref|NC_002745.2|	2110220	-	G	0	1	0	0	0	SArRNA12
gi|29165615|ref|NC_002745.2|	2110222	-	G	0	2	0	0	0	SArRNA12
gi|29165615|ref|NC_002745.2|	2110224	-	C	1	1	0	0	2	SArRNA12
gi|29165615|ref|NC_002745.2|	2110226	-	A	0	1	0	0	0	SArRNA12
gi|29165615|ref|NC_002745.2|	2110227	-	A	0	1	0	0	0	SArRNA12
gi|29165615|ref|NC_002745.2|	2110230	-	A	0	1	1	0	0	SArRNA12
gi|29165615|ref|NC_002745.2|	2110231	-	C	5	2	0	1	1	SArRNA12
gi|29165615|ref|NC_002745.2|	2110232	-	C	0	1	0	0	1	SArRNA12
gi|29165615|ref|NC_002745.2|	2110234	-	C	4	3	0	0	1	SArRNA12
gi|29165615|ref|NC_002745.2|	2110244	-	C	0	0	0	0	2	SArRNA12
gi|29165615|ref|NC_002745.2|	2110249	-	A	0	0	1	0	0	SArRNA12
gi|29165615|ref|NC_002745.2|	2110251	-	G	0	1	0	0	0	SArRNA12
gi|29165615|ref|NC_002745.2|	2110253	-	G	0	0	0	1	0	SArRNA12
gi|29165615|ref|NC_002745.2|	2110255	-	C	0	0	0	0	2	SArRNA12
gi|29165615|ref|NC_002745.2|	2110258	-	C	0	1	0	0	0	SArRNA12
gi|29165615|ref|NC_002745.2|	2110259	-	G	0	1	0	0	0	SArRNA12
gi|29165615|ref|NC_002745.2|	2110260	-	A	0	0	1	0	0	SArRNA12
gi|29165615|ref|NC_002745.2|	2110261	-	U	0	0	0	0	1	SArRNA12
gi|29165615|ref|NC_002745.2|	2110262	-	U	0	0	0	1	1	SArRNA12
gi|29165615|ref|NC_002745.2|	2110264	-	G	0	0	0	0	2	SArRNA12
gi|29165615|ref|NC_002745.2|	2110265	-	U	0	1	1	0	0	SArRNA12
gi|29165615|ref|NC_002745.2|	2110266	-	G	0	1	0	0	0	SArRNA12
gi|29165615|ref|NC_002745.2|	2110267	-	A	0	0	1	0	0	SArRNA12
gi|29165615|ref|NC_002745.2|	2110268	-	G	1	1	0	0	1	SArRNA12
gi|29165615|ref|NC_002745.2|	2110269	-	G	0	1	0	0	0	SArRNA12
gi|29165615|ref|NC_002745.2|	2110270	-	A	1	2	0	0	0	SArRNA12
gi|29165615|ref|NC_002745.2|	2110271	-	G	0	0	0	1	0	SArRNA12
gi|29165615|ref|NC_002745.2|	2110273	-	A	0	1	0	0	0	SArRNA12
gi|29165615|ref|NC_002745.2|	2110274	-	U	0	1	0	0	0	SArRNA12
gi|29165615|ref|NC_002745.2|	2110276	-	G	0	1	0	0	0	SArRNA12
gi|29165615|ref|NC_002745.2|	2110278	-	A	0	0	0	1	2	SArRNA12
gi|29165615|ref|NC_002745.2|	2110280	-	G	2	8	5	1	5	SArRNA12
gi|29165615|ref|NC_002745.2|	2110287	-	G	0	0	0	1	0	SArRNA12
gi|29165615|ref|NC_002745.2|	2110288	-	C	0	1	0	0	0	SArRNA12
gi|29165615|ref|NC_002745.2|	2110289	-	C	1	1	0	0	0	SArRNA12
gi|29165615|ref|NC_002745.2|	2110290	-	C	2	1	0	0	1	SArRNA12
gi|29165615|ref|NC_002745.2|	2110292	-	U	0	0	1	0	0	SArRNA12
gi|29165615|ref|NC_002745.2|	2110293	-	C	1	1	0	0	0	SArRNA12
gi|29165615|ref|NC_002745.2|	2110294	-	C	0	1	0	0	0	SArRNA12
gi|29165615|ref|NC_002745.2|	2110295	-	G	1	0	0	0	0	SArRNA12
gi|29165615|ref|NC_002745.2|	2110299	-	U	0	1	0	0	0	SArRNA12
gi|29165615|ref|NC_002745.2|	2110300	-	C	0	0	1	0	1	SArRNA12
gi|29165615|ref|NC_002745.2|	2110302	-	G	1	0	0	0	0	SArRNA12
gi|29165615|ref|NC_002745.2|	2110303	-	G	0	1	1	0	1	SArRNA12
gi|29165615|ref|NC_002745.2|	2110305	-	A	2	1	0	0	0	SArRNA12
gi|29165615|ref|NC_002745.2|	2110306	-	U	2	0	2	0	1	SArRNA12
gi|29165615|ref|NC_002745.2|	2110307	-	A	1	1	1	0	0	SArRNA12
gi|29165615|ref|NC_002745.2|	2110308	-	U	0	1	0	0	0	SArRNA12
gi|29165615|ref|NC_002745.2|	2110309	-	G	1	0	0	0	0	SArRNA12
gi|29165615|ref|NC_002745.2|	2110311	-	A	1	0	1	1	0	SArRNA12
gi|29165615|ref|NC_002745.2|	2110312	-	G	0	2	0	1	1	SArRNA12
gi|29165615|ref|NC_002745.2|	2110313	-	U	0	1	2	0	0	SArRNA12
gi|29165615|ref|NC_002745.2|	2110314	-	G	0	0	0	0	1	SArRNA12
gi|29165615|ref|NC_002745.2|	2110315	-	G	2	3	1	0	1	SArRNA12
gi|29165615|ref|NC_002745.2|	2110316	-	A	0	0	2	0	1	SArRNA12
gi|29165615|ref|NC_002745.2|	2110317	-	A	1	2	1	0	1	SArRNA12
gi|29165615|ref|NC_002745.2|	2110318	-	U	2	1	0	0	1	SArRNA12
gi|29165615|ref|NC_002745.2|	2110319	-	G	0	0	1	0	2	SArRNA12
gi|29165615|ref|NC_002745.2|	2110320	-	C	2	1	2	0	3	SArRNA12
gi|29165615|ref|NC_002745.2|	2110321	-	C	2	1	1	0	4	SArRNA12
gi|29165615|ref|NC_002745.2|	2110322	-	A	1	0	0	0	0	SArRNA12
gi|29165615|ref|NC_002745.2|	2110323	-	A	3	3	0	0	3	SArRNA12
gi|29165615|ref|NC_002745.2|	2110324	-	A	1	1	0	0	2	SArRNA12

gi|29165615|ref|NC_002745.2|	2110325	-	U	3	0	3	0	3	SArRNA12
gi|29165615|ref|NC_002745.2|	2110326	-	C	3	3	0	2	5	SArRNA12
gi|29165615|ref|NC_002745.2|	2110327	-	G	3	1	2	1	0	SArRNA12
gi|29165615|ref|NC_002745.2|	2110328	-	U	0	0	2	0	1	SArRNA12
gi|29165615|ref|NC_002745.2|	2110329	-	C	2	0	0	0	3	SArRNA12
gi|29165615|ref|NC_002745.2|	2110330	-	U	5	1	1	2	2	SArRNA12
gi|29165615|ref|NC_002745.2|	2110331	-	C	30	0	1	0	2	SArRNA12
gi|29165615|ref|NC_002745.2|	2110332	-	U	167	6	10	2	7	SArRNA12
gi|29165615|ref|NC_002745.2|	2110333	-	G	1	0	0	0	1	SArRNA12
gi|29165615|ref|NC_002745.2|	2110334	-	G	0	0	0	0	1	SArRNA12
gi|29165615|ref|NC_002745.2|	2110337	-	A	4	0	2	1	1	SArRNA12
gi|29165615|ref|NC_002745.2|	2110338	-	C	11	2	1	4	1	SArRNA12
gi|29165615|ref|NC_002745.2|	2110339	-	A	5	2	1	0	3	SArRNA12
gi|29165615|ref|NC_002745.2|	2110340	-	A	2	1	3	0	3	SArRNA12
gi|29165615|ref|NC_002745.2|	2110341	-	A	0	1	0	2	1	SArRNA12
gi|29165615|ref|NC_002745.2|	2110342	-	A	4	0	0	0	3	SArRNA12
gi|29165615|ref|NC_002745.2|	2110343	-	A	1	0	1	0	1	SArRNA12
gi|29165615|ref|NC_002745.2|	2110344	-	C	2	0	1	1	4	SArRNA12
gi|29165615|ref|NC_002745.2|	2110345	-	U	4	4	0	0	2	SArRNA12
gi|29165615|ref|NC_002745.2|	2110346	-	A	1	2	1	0	0	SArRNA12
gi|29165615|ref|NC_002745.2|	2110347	-	U	0	1	2	2	3	SArRNA12
gi|29165615|ref|NC_002745.2|	2110348	-	U	0	0	0	0	3	SArRNA12
gi|29165615|ref|NC_002745.2|	2110349	-	U	2	0	0	1	1	SArRNA12
gi|29165615|ref|NC_002745.2|	2110351	-	U	0	0	1	1	2	SArRNA12
gi|29165615|ref|NC_002745.2|	2110352	-	C	1	0	2	0	0	SArRNA12
gi|29165615|ref|NC_002745.2|	2110353	-	A	0	0	1	0	1	SArRNA12
gi|29165615|ref|NC_002745.2|	2110354	-	G	0	2	3	2	0	SArRNA12
gi|29165615|ref|NC_002745.2|	2110355	-	C	2	0	1	0	1	SArRNA12
gi|29165615|ref|NC_002745.2|	2110356	-	G	0	1	0	0	2	SArRNA12
gi|29165615|ref|NC_002745.2|	2110357	-	A	2	1	0	1	3	SArRNA12
gi|29165615|ref|NC_002745.2|	2110358	-	A	6	5	5	1	2	SArRNA12
gi|29165615|ref|NC_002745.2|	2110359	-	C	3	1	3	2	9	SArRNA12
gi|29165615|ref|NC_002745.2|	2110360	-	C	0	1	1	1	5	SArRNA12
gi|29165615|ref|NC_002745.2|	2110361	-	C	3	3	2	3	6	SArRNA12
gi|29165615|ref|NC_002745.2|	2110362	-	G	0	0	1	2	0	SArRNA12
gi|29165615|ref|NC_002745.2|	2110363	-	G	0	1	0	0	2	SArRNA12
gi|29165615|ref|NC_002745.2|	2110364	-	A	3	4	0	0	0	SArRNA12
gi|29165615|ref|NC_002745.2|	2110365	-	U	1	3	0	0	2	SArRNA12
gi|29165615|ref|NC_002745.2|	2110366	-	A	4	7	3	1	3	SArRNA12
gi|29165615|ref|NC_002745.2|	2110367	-	A	3	2	0	1	1	SArRNA12
gi|29165615|ref|NC_002745.2|	2110368	-	G	2	2	1	8	1	SArRNA12
gi|29165615|ref|NC_002745.2|	2110369	-	U	2	1	2	2	0	SArRNA12
gi|29165615|ref|NC_002745.2|	2110370	-	G	1	0	2	0	2	SArRNA12
gi|29165615|ref|NC_002745.2|	2110371	-	A	5	1	0	2	2	SArRNA12
gi|29165615|ref|NC_002745.2|	2110372	-	C	1	1	2	1	3	SArRNA12
gi|29165615|ref|NC_002745.2|	2110373	-	G	2	0	1	0	2	SArRNA12
gi|29165615|ref|NC_002745.2|	2110374	-	C	1	0	0	0	1	SArRNA12
gi|29165615|ref|NC_002745.2|	2110375	-	C	3	2	0	2	1	SArRNA12
gi|29165615|ref|NC_002745.2|	2110376	-	G	0	0	0	1	1	SArRNA12
gi|29165615|ref|NC_002745.2|	2110377	-	A	1	0	0	0	1	SArRNA12
gi|29165615|ref|NC_002745.2|	2110378	-	G	1	3	0	0	1	SArRNA12
gi|29165615|ref|NC_002745.2|	2110379	-	A	0	2	2	0	1	SArRNA12
gi|29165615|ref|NC_002745.2|	2110380	-	A	1	1	0	3	0	SArRNA12
gi|29165615|ref|NC_002745.2|	2110381	-	G	1	0	0	0	2	SArRNA12
gi|29165615|ref|NC_002745.2|	2110382	-	A	0	0	1	1	0	SArRNA12
gi|29165615|ref|NC_002745.2|	2110383	-	C	2	2	1	2	0	SArRNA12
gi|29165615|ref|NC_002745.2|	2110384	-	C	6	1	0	1	3	SArRNA12
gi|29165615|ref|NC_002745.2|	2110386	-	G	8	3	0	2	6	SArRNA12
gi|29165615|ref|NC_002745.2|	2110387	-	C	30	21	11	17	19	SArRNA12
gi|29165615|ref|NC_002745.2|	2110388	-	A	1	1	2	0	2	SArRNA12
gi|29165615|ref|NC_002745.2|	2110390	-	U	1	0	0	0	0	SArRNA12
gi|29165615|ref|NC_002745.2|	2110392	-	G	0	0	0	0	1	SArRNA12
gi|29165615|ref|NC_002745.2|	2110393	-	G	0	1	0	0	0	SArRNA12
gi|29165615|ref|NC_002745.2|	2110397	-	U	1	0	1	0	1	SArRNA12
gi|29165615|ref|NC_002745.2|	2110398	-	U	2	0	1	0	0	SArRNA12
gi|29165615|ref|NC_002745.2|	2110399	-	C	2	0	0	0	2	SArRNA12
gi|29165615|ref|NC_002745.2|	2110400	-	U	0	1	0	0	3	SArRNA12
gi|29165615|ref|NC_002745.2|	2110407	-	G	0	0	0	0	1	SArRNA12
gi|29165615|ref|NC_002745.2|	2110409	-	A	1	0	1	1	3	SArRNA12
gi|29165615|ref|NC_002745.2|	2110410	-	G	1	1	0	0	3	SArRNA12
gi|29165615|ref|NC_002745.2|	2110411	-	A	1	0	2	1	1	SArRNA12
gi|29165615|ref|NC_002745.2|	2110413	-	G	2	0	0	2	1	SArRNA12
gi|29165615|ref|NC_002745.2|	2110414	-	G	1	1	1	0	0	SArRNA12
gi|29165615|ref|NC_002745.2|	2110415	-	C	2	1	0	3	3	SArRNA12
gi|29165615|ref|NC_002745.2|	2110416	-	U	1	2	2	1	2	SArRNA12
gi|29165615|ref|NC_002745.2|	2110417	-	U	2	2	0	4	2	SArRNA12
gi|29165615|ref|NC_002745.2|	2110418	-	C	6	3	2	1	0	SArRNA12
gi|29165615|ref|NC_002745.2|	2110419	-	A	0	1	0	1	1	SArRNA12
gi|29165615|ref|NC_002745.2|	2110420	-	A	1	1	0	0	0	SArRNA12
gi|29165615|ref|NC_002745.2|	2110421	-	U	1	0	0	0	2	SArRNA12
gi|29165615|ref|NC_002745.2|	2110422	-	G	0	1	1	0	1	SArRNA12

gi|29165615|ref|NC_002745.2|	2110423 -	C	1	1	0	0	0	SArRNA12
gi|29165615|ref|NC_002745.2|	2110424 -	C	1	0	0	0	0	SArRNA12
gi|29165615|ref|NC_002745.2|	2110425 -	C	0	2	0	1	1	SArRNA12
gi|29165615|ref|NC_002745.2|	2110426 -	C	4	8	1	1	6	SArRNA12
gi|29165615|ref|NC_002745.2|	2110428 -	G	0	0	1	0	1	SArRNA12
gi|29165615|ref|NC_002745.2|	2110430 -	A	0	1	0	0	1	SArRNA12
gi|29165615|ref|NC_002745.2|	2110431 -	A	4	1	0	0	4	SArRNA12
gi|29165615|ref|NC_002745.2|	2110432 -	A	0	0	0	0	1	SArRNA12
gi|29165615|ref|NC_002745.2|	2110433 -	A	2	1	0	0	1	SArRNA12
gi|29165615|ref|NC_002745.2|	2110435 -	G	0	0	0	0	1	SArRNA12
gi|29165615|ref|NC_002745.2|	2110437 -	C	2	1	0	2	1	SArRNA12
gi|29165615|ref|NC_002745.2|	2110438 -	U	0	1	2	4	4	SArRNA12
gi|29165615|ref|NC_002745.2|	2110439 -	C	1	1	1	2	0	SArRNA12
gi|29165615|ref|NC_002745.2|	2110441 -	A	1	1	0	0	0	SArRNA12
gi|29165615|ref|NC_002745.2|	2110442 -	G	0	1	0	0	0	SArRNA12
gi|29165615|ref|NC_002745.2|	2110443 -	G	0	0	0	0	1	SArRNA12
gi|29165615|ref|NC_002745.2|	2110444 -	A	7	2	0	2	2	SArRNA12
gi|29165615|ref|NC_002745.2|	2110445 -	A	0	1	3	0	0	SArRNA12
gi|29165615|ref|NC_002745.2|	2110446 -	U	0	0	0	1	4	SArRNA12
gi|29165615|ref|NC_002745.2|	2110447 -	U	0	0	0	1	0	SArRNA12
gi|29165615|ref|NC_002745.2|	2110448 -	G	1	1	0	0	1	SArRNA12
gi|29165615|ref|NC_002745.2|	2110449 -	C	3	1	0	1	2	SArRNA12
gi|29165615|ref|NC_002745.2|	2110450 -	U	1	0	2	0	2	SArRNA12
gi|29165615|ref|NC_002745.2|	2110451 -	C	1	0	0	2	1	SArRNA12
gi|29165615|ref|NC_002745.2|	2110453 -	C	1	2	1	1	1	SArRNA12
gi|29165615|ref|NC_002745.2|	2110455 -	A	3	2	0	0	0	SArRNA12
gi|29165615|ref|NC_002745.2|	2110456 -	G	0	4	0	2	0	SArRNA12
gi|29165615|ref|NC_002745.2|	2110457 -	C	3	1	1	1	0	SArRNA12
gi|29165615|ref|NC_002745.2|	2110458 -	G	1	0	0	1	0	SArRNA12
gi|29165615|ref|NC_002745.2|	2110460 -	G	1	2	0	1	0	SArRNA12
gi|29165615|ref|NC_002745.2|	2110461 -	C	4	3	0	1	4	SArRNA12
gi|29165615|ref|NC_002745.2|	2110462 -	G	0	0	0	0	1	SArRNA12
gi|29165615|ref|NC_002745.2|	2110463 -	A	0	2	0	2	0	SArRNA12
gi|29165615|ref|NC_002745.2|	2110464 -	G	1	2	0	1	1	SArRNA12
gi|29165615|ref|NC_002745.2|	2110465 -	U	5	2	4	6	3	SArRNA12
gi|29165615|ref|NC_002745.2|	2110466 -	G	0	1	0	0	1	SArRNA12
gi|29165615|ref|NC_002745.2|	2110467 -	G	0	0	0	1	1	SArRNA12
gi|29165615|ref|NC_002745.2|	2110468 -	A	3	4	1	4	2	SArRNA12
gi|29165615|ref|NC_002745.2|	2110469 -	A	2	15	3	13	8	SArRNA12
gi|29165615|ref|NC_002745.2|	2110470 -	U	3	9	1	2	5	SArRNA12
gi|29165615|ref|NC_002745.2|	2110471 -	C	9	11	1	3	9	SArRNA12
gi|29165615|ref|NC_002745.2|	2110472 -	U	1	3	0	0	0	SArRNA12
gi|29165615|ref|NC_002745.2|	2110473 -	U	1	1	0	2	5	SArRNA12
gi|29165615|ref|NC_002745.2|	2110474 -	A	15	54	8	5	19	SArRNA12
gi|29165615|ref|NC_002745.2|	2110475 -	A	8	3	4	3	5	SArRNA12
gi|29165615|ref|NC_002745.2|	2110476 -	G	4	3	0	0	3	SArRNA12
gi|29165615|ref|NC_002745.2|	2110477 -	A	1	2	0	1	3	SArRNA12
gi|29165615|ref|NC_002745.2|	2110478 -	G	1	1	0	0	1	SArRNA12
gi|29165615|ref|NC_002745.2|	2110479 -	U	0	0	0	1	3	SArRNA12
gi|29165615|ref|NC_002745.2|	2110480 -	A	0	1	0	0	0	SArRNA12
gi|29165615|ref|NC_002745.2|	2110481 -	G	1	1	0	0	0	SArRNA12
gi|29165615|ref|NC_002745.2|	2110482 -	A	0	3	1	0	3	SArRNA12
gi|29165615|ref|NC_002745.2|	2110483 -	A	1	5	2	0	1	SArRNA12
gi|29165615|ref|NC_002745.2|	2110484 -	C	18	18	7	11	13	SArRNA12
gi|29165615|ref|NC_002745.2|	2110485 -	U	0	0	0	1	1	SArRNA12
gi|29165615|ref|NC_002745.2|	2110486 -	G	1	0	0	0	0	SArRNA12
gi|29165615|ref|NC_002745.2|	2110488 -	U	3	7	0	0	2	SArRNA12
gi|29165615|ref|NC_002745.2|	2110489 -	G	1	2	0	2	1	SArRNA12
gi|29165615|ref|NC_002745.2|	2110490 -	G	0	1	0	1	0	SArRNA12
gi|29165615|ref|NC_002745.2|	2110491 -	A	2	12	1	1	3	SArRNA12
gi|29165615|ref|NC_002745.2|	2110492 -	C	15	23	5	8	10	SArRNA12
gi|29165615|ref|NC_002745.2|	2110493 -	A	0	13	1	0	3	SArRNA12
gi|29165615|ref|NC_002745.2|	2110494 -	C	4	8	0	0	6	SArRNA12
gi|29165615|ref|NC_002745.2|	2110496 -	G	1	8	0	2	4	SArRNA12
gi|29165615|ref|NC_002745.2|	2110497 -	C	6	21	2	2	6	SArRNA12
gi|29165615|ref|NC_002745.2|	2110498 -	C	1	1	1	0	4	SArRNA12
gi|29165615|ref|NC_002745.2|	2110499 -	A	1	16	1	0	6	SArRNA12
gi|29165615|ref|NC_002745.2|	2110500 -	A	1	7	1	0	5	SArRNA12
gi|29165615|ref|NC_002745.2|	2110501 -	A	0	1	0	1	4	SArRNA12
gi|29165615|ref|NC_002745.2|	2110502 -	C	5	6	2	1	4	SArRNA12
gi|29165615|ref|NC_002745.2|	2110503 -	G	1	1	0	1	2	SArRNA12
gi|29165615|ref|NC_002745.2|	2110504 -	C	0	1	0	0	0	SArRNA12
gi|29165615|ref|NC_002745.2|	2110505 -	C	1	0	0	0	1	SArRNA12
gi|29165615|ref|NC_002745.2|	2110507 -	U	1	7	1	0	2	SArRNA12
gi|29165615|ref|NC_002745.2|	2110508 -	G	0	4	0	0	0	SArRNA12
gi|29165615|ref|NC_002745.2|	2110509 -	C	2	1	0	0	1	SArRNA12
gi|29165615|ref|NC_002745.2|	2110510 -	C	0	6	0	0	0	SArRNA12
gi|29165615|ref|NC_002745.2|	2110511 -	C	0	0	1	0	1	SArRNA12
gi|29165615|ref|NC_002745.2|	2110516 -	A	3	1	1	1	2	SArRNA12
gi|29165615|ref|NC_002745.2|	2110517 -	U	0	3	1	1	1	SArRNA12
gi|29165615|ref|NC_002745.2|	2110518 -	A	13	72	1	2	15	SArRNA12

gi|29165615|ref|NC_002745.2|	2110519 -	A	3	0	3	1	4	SArRNA12
gi|29165615|ref|NC_002745.2|	2110520 -	A	4	3	0	3	3	SArRNA12
gi|29165615|ref|NC_002745.2|	2110521 -	A	0	3	2	2	7	SArRNA12
gi|29165615|ref|NC_002745.2|	2110522 -	G	1	1	4	3	0	SArRNA12
gi|29165615|ref|NC_002745.2|	2110523 -	A	0	1	3	1	3	SArRNA12
gi|29165615|ref|NC_002745.2|	2110524 -	U	4	5	1	3	6	SArRNA12
gi|29165615|ref|NC_002745.2|	2110525 -	A	4	8	1	2	5	SArRNA12
gi|29165615|ref|NC_002745.2|	2110526 -	G	0	3	1	4	1	SArRNA12
gi|29165615|ref|NC_002745.2|	2110527 -	A	2	4	1	0	5	SArRNA12
gi|29165615|ref|NC_002745.2|	2110528 -	U	1	9	2	0	3	SArRNA12
gi|29165615|ref|NC_002745.2|	2110529 -	C	1	4	0	1	5	SArRNA12
gi|29165615|ref|NC_002745.2|	2110530 -	U	2	1	0	1	2	SArRNA12
gi|29165615|ref|NC_002745.2|	2110531 -	C	3	1	3	1	2	SArRNA12
gi|29165615|ref|NC_002745.2|	2110532 -	C	2	0	1	0	0	SArRNA12
gi|29165615|ref|NC_002745.2|	2110534 -	A	1	1	0	0	1	SArRNA12
gi|29165615|ref|NC_002745.2|	2110535 -	A	1	1	0	0	0	SArRNA12
gi|29165615|ref|NC_002745.2|	2110536 -	A	1	0	0	0	1	SArRNA12
gi|29165615|ref|NC_002745.2|	2110537 -	A	1	0	1	0	0	SArRNA12
gi|29165615|ref|NC_002745.2|	2110538 -	G	1	0	1	0	1	SArRNA12
gi|29165615|ref|NC_002745.2|	2110539 -	A	0	0	0	0	1	SArRNA12
gi|29165615|ref|NC_002745.2|	2110540 -	G	0	0	0	1	0	SArRNA12
gi|29165615|ref|NC_002745.2|	2110541 -	C	2	3	1	0	0	SArRNA12
gi|29165615|ref|NC_002745.2|	2110543 -	G	0	0	0	1	0	SArRNA12
gi|29165615|ref|NC_002745.2|	2110544 -	U	0	0	1	0	1	SArRNA12
gi|29165615|ref|NC_002745.2|	2110545 -	C	2	1	0	2	1	SArRNA12
gi|29165615|ref|NC_002745.2|	2110546 -	A	2	1	0	1	0	SArRNA12
gi|29165615|ref|NC_002745.2|	2110547 -	C	3	5	2	0	2	SArRNA12
gi|29165615|ref|NC_002745.2|	2110548 -	A	2	0	0	0	1	SArRNA12
gi|29165615|ref|NC_002745.2|	2110549 -	C	2	2	2	1	0	SArRNA12
gi|29165615|ref|NC_002745.2|	2110550 -	U	0	1	0	0	1	SArRNA12
gi|29165615|ref|NC_002745.2|	2110551 -	U	0	0	1	1	0	SArRNA12
gi|29165615|ref|NC_002745.2|	2110552 -	U	3	0	0	0	0	SArRNA12
gi|29165615|ref|NC_002745.2|	2110553 -	A	0	1	0	0	1	SArRNA12
gi|29165615|ref|NC_002745.2|	2110556 -	U	0	0	1	0	0	SArRNA12
gi|29165615|ref|NC_002745.2|	2110557 -	G	1	1	0	0	1	SArRNA12
gi|29165615|ref|NC_002745.2|	2110558 -	C	2	0	0	0	0	SArRNA12
gi|29165615|ref|NC_002745.2|	2110559 -	U	2	0	1	0	1	SArRNA12
gi|29165615|ref|NC_002745.2|	2110560 -	G	0	0	0	0	1	SArRNA12
gi|29165615|ref|NC_002745.2|	2110562 -	G	0	1	1	0	0	SArRNA12
gi|29165615|ref|NC_002745.2|	2110563 -	C	0	1	0	0	1	SArRNA12
gi|29165615|ref|NC_002745.2|	2110564 -	U	0	1	0	0	1	SArRNA12
gi|29165615|ref|NC_002745.2|	2110565 -	U	1	1	1	0	0	SArRNA12
gi|29165615|ref|NC_002745.2|	2110566 -	C	0	1	0	0	0	SArRNA12
gi|29165615|ref|NC_002745.2|	2110567 -	U	0	0	0	0	1	SArRNA12
gi|29165615|ref|NC_002745.2|	2110568 -	G	1	0	0	0	1	SArRNA12
gi|29165615|ref|NC_002745.2|	2110569 -	U	0	0	0	0	3	SArRNA12
gi|29165615|ref|NC_002745.2|	2110571 -	U	1	0	0	0	0	SArRNA12
gi|29165615|ref|NC_002745.2|	2110572 -	U	0	0	1	1	1	SArRNA12
gi|29165615|ref|NC_002745.2|	2110573 -	A	3	1	0	0	2	SArRNA12
gi|29165615|ref|NC_002745.2|	2110574 -	C	1	0	1	2	2	SArRNA12
gi|29165615|ref|NC_002745.2|	2110575 -	A	1	0	0	0	3	SArRNA12
gi|29165615|ref|NC_002745.2|	2110576 -	G	0	1	0	0	0	SArRNA12
gi|29165615|ref|NC_002745.2|	2110577 -	A	2	0	0	0	0	SArRNA12
gi|29165615|ref|NC_002745.2|	2110578 -	A	1	0	0	0	0	SArRNA12
gi|29165615|ref|NC_002745.2|	2110583 -	G	1	1	1	0	0	SArRNA12
gi|29165615|ref|NC_002745.2|	2110584 -	G	1	1	1	1	0	SArRNA12
gi|29165615|ref|NC_002745.2|	2110585 -	U	1	8	2	2	6	SArRNA12
gi|29165615|ref|NC_002745.2|	2110586 -	A	1	0	0	0	0	SArRNA12
gi|29165615|ref|NC_002745.2|	2110587 -	G	1	3	1	0	0	SArRNA12
gi|29165615|ref|NC_002745.2|	2110588 -	U	0	0	0	0	1	SArRNA12
gi|29165615|ref|NC_002745.2|	2110589 -	G	0	0	0	0	1	SArRNA12
gi|29165615|ref|NC_002745.2|	2110590 -	U	2	0	3	0	0	SArRNA12
gi|29165615|ref|NC_002745.2|	2110591 -	C	1	0	1	0	0	SArRNA12
gi|29165615|ref|NC_002745.2|	2110593 -	A	4	0	0	1	2	SArRNA12
gi|29165615|ref|NC_002745.2|	2110594 -	G	0	0	1	0	1	SArRNA12
gi|29165615|ref|NC_002745.2|	2110595 -	U	1	2	0	1	2	SArRNA12
gi|29165615|ref|NC_002745.2|	2110596 -	C	1	1	1	0	5	SArRNA12
gi|29165615|ref|NC_002745.2|	2110597 -	G	1	1	0	0	0	SArRNA12
gi|29165615|ref|NC_002745.2|	2110598 -	G	0	2	0	1	0	SArRNA12
gi|29165615|ref|NC_002745.2|	2110599 -	A	3	2	0	0	2	SArRNA12
gi|29165615|ref|NC_002745.2|	2110600 -	A	0	6	0	0	1	SArRNA12
gi|29165615|ref|NC_002745.2|	2110601 -	U	3	2	0	0	6	SArRNA12
gi|29165615|ref|NC_002745.2|	2110602 -	U	1	0	0	0	1	SArRNA12
gi|29165615|ref|NC_002745.2|	2110603 -	G	1	0	0	0	5	SArRNA12
gi|29165615|ref|NC_002745.2|	2110604 -	C	0	0	0	0	1	SArRNA12
gi|29165615|ref|NC_002745.2|	2110605 -	U	0	1	1	0	1	SArRNA12
gi|29165615|ref|NC_002745.2|	2110606 -	C	0	2	0	0	1	SArRNA12
gi|29165615|ref|NC_002745.2|	2110607 -	A	0	1	1	0	0	SArRNA12
gi|29165615|ref|NC_002745.2|	2110609 -	G	2	0	0	1	0	SArRNA12
gi|29165615|ref|NC_002745.2|	2110610 -	G	0	0	0	1	0	SArRNA12
gi|29165615|ref|NC_002745.2|	2110611 -	C	1	4	0	0	0	SArRNA12

gi|29165615|ref|NC_002745.2|	2110612 -	C	1	2	0	0	2	SArRNA12
gi|29165615|ref|NC_002745.2|	2110613 -	U	1	1	1	0	1	SArRNA12
gi|29165615|ref|NC_002745.2|	2110614 -	A	4	3	1	1	1	SArRNA12
gi|29165615|ref|NC_002745.2|	2110615 -	A	0	0	0	0	1	SArRNA12
gi|29165615|ref|NC_002745.2|	2110616 -	A	1	1	0	0	1	SArRNA12
gi|29165615|ref|NC_002745.2|	2110619 -	G	2	0	0	0	1	SArRNA12
gi|29165615|ref|NC_002745.2|	2110620 -	A	2	1	0	1	1	SArRNA12
gi|29165615|ref|NC_002745.2|	2110621 -	U	2	3	0	1	1	SArRNA12
gi|29165615|ref|NC_002745.2|	2110623 -	A	0	1	1	0	2	SArRNA12
gi|29165615|ref|NC_002745.2|	2110624 -	U	0	1	0	0	0	SArRNA12
gi|29165615|ref|NC_002745.2|	2110626 -	A	0	1	0	0	3	SArRNA12
gi|29165615|ref|NC_002745.2|	2110627 -	G	0	0	0	0	1	SArRNA12
gi|29165615|ref|NC_002745.2|	2110628 -	U	0	1	0	1	0	SArRNA12
gi|29165615|ref|NC_002745.2|	2110629 -	C	4	0	0	1	1	SArRNA12
gi|29165615|ref|NC_002745.2|	2110630 -	G	1	1	0	0	1	SArRNA12
gi|29165615|ref|NC_002745.2|	2110632 -	A	1	1	0	0	1	SArRNA12
gi|29165615|ref|NC_002745.2|	2110633 -	A	2	4	0	0	3	SArRNA12
gi|29165615|ref|NC_002745.2|	2110634 -	U	2	0	0	0	2	SArRNA12
gi|29165615|ref|NC_002745.2|	2110635 -	G	0	0	0	3	1	SArRNA12
gi|29165615|ref|NC_002745.2|	2110636 -	A	3	0	0	0	2	SArRNA12
gi|29165615|ref|NC_002745.2|	2110637 -	C	4	3	0	1	0	SArRNA12
gi|29165615|ref|NC_002745.2|	2110639 -	A	0	0	0	0	1	SArRNA12
gi|29165615|ref|NC_002745.2|	2110640 -	A	0	2	0	0	1	SArRNA12
gi|29165615|ref|NC_002745.2|	2110641 -	U	2	6	1	0	2	SArRNA12
gi|29165615|ref|NC_002745.2|	2110642 -	C	1	6	1	2	12	SArRNA12
gi|29165615|ref|NC_002745.2|	2110643 -	U	1	0	0	0	0	SArRNA12
gi|29165615|ref|NC_002745.2|	2110644 -	G	1	2	0	1	0	SArRNA12
gi|29165615|ref|NC_002745.2|	2110645 -	C	1	0	0	0	2	SArRNA12
gi|29165615|ref|NC_002745.2|	2110646 -	A	0	2	0	0	1	SArRNA12
gi|29165615|ref|NC_002745.2|	2110647 -	C	0	7	2	0	3	SArRNA12
gi|29165615|ref|NC_002745.2|	2110648 -	G	0	1	0	0	1	SArRNA12
gi|29165615|ref|NC_002745.2|	2110649 -	U	0	0	0	0	2	SArRNA12
gi|29165615|ref|NC_002745.2|	2110650 -	U	3	2	0	0	1	SArRNA12
gi|29165615|ref|NC_002745.2|	2110651 -	A	1	3	3	0	5	SArRNA12
gi|29165615|ref|NC_002745.2|	2110652 -	G	1	3	0	0	1	SArRNA12
gi|29165615|ref|NC_002745.2|	2110653 -	G	2	1	0	0	1	SArRNA12
gi|29165615|ref|NC_002745.2|	2110654 -	U	1	1	0	0	1	SArRNA12
gi|29165615|ref|NC_002745.2|	2110655 -	U	0	0	2	3	3	SArRNA12
gi|29165615|ref|NC_002745.2|	2110656 -	A	0	2	3	0	2	SArRNA12
gi|29165615|ref|NC_002745.2|	2110657 -	G	1	3	0	0	3	SArRNA12
gi|29165615|ref|NC_002745.2|	2110658 -	C	4	1	3	0	4	SArRNA12
gi|29165615|ref|NC_002745.2|	2110659 -	G	0	1	0	0	0	SArRNA12
gi|29165615|ref|NC_002745.2|	2110660 -	U	0	1	0	0	0	SArRNA12
gi|29165615|ref|NC_002745.2|	2110665 -	A	0	1	0	0	0	SArRNA12
gi|29165615|ref|NC_002745.2|	2110666 -	G	1	4	0	0	1	SArRNA12
gi|29165615|ref|NC_002745.2|	2110667 -	C	3	0	1	2	3	SArRNA12
gi|29165615|ref|NC_002745.2|	2110669 -	G	0	1	0	0	0	SArRNA12
gi|29165615|ref|NC_002745.2|	2110670 -	A	2	0	2	1	1	SArRNA12
gi|29165615|ref|NC_002745.2|	2110671 -	U	5	5	0	3	3	SArRNA12
gi|29165615|ref|NC_002745.2|	2110672 -	A	2	5	0	1	5	SArRNA12
gi|29165615|ref|NC_002745.2|	2110673 -	G	0	1	0	1	1	SArRNA12
gi|29165615|ref|NC_002745.2|	2110674 -	G	0	0	0	1	0	SArRNA12
gi|29165615|ref|NC_002745.2|	2110675 -	A	1	3	2	0	1	SArRNA12
gi|29165615|ref|NC_002745.2|	2110676 -	U	7	2	5	0	1	SArRNA12
gi|29165615|ref|NC_002745.2|	2110677 -	G	2	1	0	0	3	SArRNA12
gi|29165615|ref|NC_002745.2|	2110678 -	A	0	1	1	0	1	SArRNA12
gi|29165615|ref|NC_002745.2|	2110679 -	C	3	0	0	1	0	SArRNA12
gi|29165615|ref|NC_002745.2|	2110680 -	G	1	4	4	1	1	SArRNA12
gi|29165615|ref|NC_002745.2|	2110681 -	C	1	12	0	0	4	SArRNA12
gi|29165615|ref|NC_002745.2|	2110683 -	G	0	0	1	0	1	SArRNA12
gi|29165615|ref|NC_002745.2|	2110685 -	G	0	0	0	0	1	SArRNA12
gi|29165615|ref|NC_002745.2|	2110686 -	G	1	3	3	0	2	SArRNA12
gi|29165615|ref|NC_002745.2|	2110687 -	G	0	2	0	1	0	SArRNA12
gi|29165615|ref|NC_002745.2|	2110688 -	G	0	1	1	0	2	SArRNA12
gi|29165615|ref|NC_002745.2|	2110689 -	U	7	12	0	2	1	SArRNA12
gi|29165615|ref|NC_002745.2|	2110690 -	A	3	2	1	2	2	SArRNA12
gi|29165615|ref|NC_002745.2|	2110691 -	G	6	10	3	2	6	SArRNA12
gi|29165615|ref|NC_002745.2|	2110692 -	C	8	5	0	3	2	SArRNA12
gi|29165615|ref|NC_002745.2|	2110693 -	U	4	3	1	2	5	SArRNA12
gi|29165615|ref|NC_002745.2|	2110694 -	A	9	75	22	3	28	SArRNA12
gi|29165615|ref|NC_002745.2|	2110695 -	A	2	3	1	1	4	SArRNA12
gi|29165615|ref|NC_002745.2|	2110696 -	U	5	7	3	5	7	SArRNA12
gi|29165615|ref|NC_002745.2|	2110697 -	U	6	8	3	0	1	SArRNA12
gi|29165615|ref|NC_002745.2|	2110698 -	U	8	4	1	0	3	SArRNA12
gi|29165615|ref|NC_002745.2|	2110699 -	U	0	4	4	2	2	SArRNA12
gi|29165615|ref|NC_002745.2|	2110700 -	G	1	2	2	3	4	SArRNA12
gi|29165615|ref|NC_002745.2|	2110701 -	C	4	3	2	1	5	SArRNA12
gi|29165615|ref|NC_002745.2|	2110702 -	U	6	1	2	2	1	SArRNA12
gi|29165615|ref|NC_002745.2|	2110703 -	A	3	11	1	2	13	SArRNA12
gi|29165615|ref|NC_002745.2|	2110704 -	A	4	1	1	2	5	SArRNA12
gi|29165615|ref|NC_002745.2|	2110705 -	U	6	8	1	2	6	SArRNA12

gi|29165615|ref|NC_002745.2|	2110706	-	A	3	1	1	1	0	SArRNA12
gi|29165615|ref|NC_002745.2|	2110707	-	U	4	5	2	3	6	SArRNA12
gi|29165615|ref|NC_002745.2|	2110708	-	C	6	4	1	2	6	SArRNA12
gi|29165615|ref|NC_002745.2|	2110709	-	C	1	3	1	2	2	SArRNA12
gi|29165615|ref|NC_002745.2|	2110710	-	A	1	5	0	2	3	SArRNA12
gi|29165615|ref|NC_002745.2|	2110711	-	C	15	13	3	2	13	SArRNA12
gi|29165615|ref|NC_002745.2|	2110712	-	C	0	4	1	2	6	SArRNA12
gi|29165615|ref|NC_002745.2|	2110713	-	A	0	1	0	0	0	SArRNA12
gi|29165615|ref|NC_002745.2|	2110714	-	U	1	2	0	4	2	SArRNA12
gi|29165615|ref|NC_002745.2|	2110715	-	G	0	1	0	0	1	SArRNA12
gi|29165615|ref|NC_002745.2|	2110716	-	U	7	3	0	1	1	SArRNA12
gi|29165615|ref|NC_002745.2|	2110717	-	C	2	0	0	1	0	SArRNA12
gi|29165615|ref|NC_002745.2|	2110718	-	C	2	1	0	2	1	SArRNA12
gi|29165615|ref|NC_002745.2|	2110719	-	U	4	2	0	0	0	SArRNA12
gi|29165615|ref|NC_002745.2|	2110720	-	U	3	0	3	0	2	SArRNA12
gi|29165615|ref|NC_002745.2|	2110721	-	A	1	1	2	0	4	SArRNA12
gi|29165615|ref|NC_002745.2|	2110722	-	U	0	4	0	3	4	SArRNA12
gi|29165615|ref|NC_002745.2|	2110723	-	A	2	5	0	2	2	SArRNA12
gi|29165615|ref|NC_002745.2|	2110724	-	G	3	0	2	0	2	SArRNA12
gi|29165615|ref|NC_002745.2|	2110725	-	U	0	1	1	1	2	SArRNA12
gi|29165615|ref|NC_002745.2|	2110726	-	U	1	0	0	1	3	SArRNA12
gi|29165615|ref|NC_002745.2|	2110727	-	G	0	1	0	1	0	SArRNA12
gi|29165615|ref|NC_002745.2|	2110728	-	G	1	0	1	0	1	SArRNA12
gi|29165615|ref|NC_002745.2|	2110729	-	A	1	3	0	0	2	SArRNA12
gi|29165615|ref|NC_002745.2|	2110730	-	C	7	7	4	1	1	SArRNA12
gi|29165615|ref|NC_002745.2|	2110731	-	A	0	1	0	0	2	SArRNA12
gi|29165615|ref|NC_002745.2|	2110732	-	A	1	0	0	1	5	SArRNA12
gi|29165615|ref|NC_002745.2|	2110733	-	U	1	1	1	0	0	SArRNA12
gi|29165615|ref|NC_002745.2|	2110734	-	A	1	1	1	5	5	SArRNA12
gi|29165615|ref|NC_002745.2|	2110735	-	G	0	1	1	2	3	SArRNA12
gi|29165615|ref|NC_002745.2|	2110736	-	G	2	3	1	1	2	SArRNA12
gi|29165615|ref|NC_002745.2|	2110737	-	U	5	6	4	1	1	SArRNA12
gi|29165615|ref|NC_002745.2|	2110738	-	A	4	6	1	1	6	SArRNA12
gi|29165615|ref|NC_002745.2|	2110739	-	G	17	21	12	8	17	SArRNA12
gi|29165615|ref|NC_002745.2|	2110740	-	C	5	6	4	3	12	SArRNA12
gi|29165615|ref|NC_002745.2|	2110741	-	G	0	1	0	0	0	SArRNA12
gi|29165615|ref|NC_002745.2|	2110742	-	G	0	2	1	0	1	SArRNA12
gi|29165615|ref|NC_002745.2|	2110743	-	A	1	2	3	0	3	SArRNA12
gi|29165615|ref|NC_002745.2|	2110744	-	U	15	13	3	11	8	SArRNA12
gi|29165615|ref|NC_002745.2|	2110745	-	G	5	6	2	1	2	SArRNA12
gi|29165615|ref|NC_002745.2|	2110746	-	C	4	1	2	1	7	SArRNA12
gi|29165615|ref|NC_002745.2|	2110747	-	G	2	1	1	0	2	SArRNA12
gi|29165615|ref|NC_002745.2|	2110748	-	G	7	16	1	3	7	SArRNA12
gi|29165615|ref|NC_002745.2|	2110749	-	A	6	14	2	3	6	SArRNA12
gi|29165615|ref|NC_002745.2|	2110750	-	C	29	46	11	9	29	SArRNA12
gi|29165615|ref|NC_002745.2|	2110751	-	A	1	5	0	0	8	SArRNA12
gi|29165615|ref|NC_002745.2|	2110752	-	G	7	5	3	1	6	SArRNA12
gi|29165615|ref|NC_002745.2|	2110753	-	C	11	7	2	5	10	SArRNA12
gi|29165615|ref|NC_002745.2|	2110754	-	C	0	3	0	1	2	SArRNA12
gi|29165615|ref|NC_002745.2|	2110755	-	G	3	1	0	1	2	SArRNA12
gi|29165615|ref|NC_002745.2|	2110756	-	G	0	1	0	1	1	SArRNA12
gi|29165615|ref|NC_002745.2|	2110757	-	A	3	1	2	0	0	SArRNA12
gi|29165615|ref|NC_002745.2|	2110758	-	G	6	14	7	1	9	SArRNA12
gi|29165615|ref|NC_002745.2|	2110759	-	U	5	6	4	0	3	SArRNA12
gi|29165615|ref|NC_002745.2|	2110760	-	C	4	2	2	4	1	SArRNA12
gi|29165615|ref|NC_002745.2|	2110761	-	G	0	0	0	0	1	SArRNA12
gi|29165615|ref|NC_002745.2|	2110762	-	A	10	4	1	3	3	SArRNA12
gi|29165615|ref|NC_002745.2|	2110763	-	A	6	5	1	1	5	SArRNA12
gi|29165615|ref|NC_002745.2|	2110764	-	U	2	11	3	1	11	SArRNA12
gi|29165615|ref|NC_002745.2|	2110765	-	C	5	14	0	5	8	SArRNA12
gi|29165615|ref|NC_002745.2|	2110766	-	C	1	2	0	2	0	SArRNA12
gi|29165615|ref|NC_002745.2|	2110767	-	U	0	3	0	0	1	SArRNA12
gi|29165615|ref|NC_002745.2|	2110768	-	G	0	3	0	1	0	SArRNA12
gi|29165615|ref|NC_002745.2|	2110769	-	G	4	2	1	0	1	SArRNA12
gi|29165615|ref|NC_002745.2|	2110770	-	G	2	4	1	1	0	SArRNA12
gi|29165615|ref|NC_002745.2|	2110771	-	C	1	8	0	2	2	SArRNA12
gi|29165615|ref|NC_002745.2|	2110772	-	U	4	0	1	0	4	SArRNA12
gi|29165615|ref|NC_002745.2|	2110773	-	G	15	5	4	6	6	SArRNA12
gi|29165615|ref|NC_002745.2|	2110774	-	A	12	10	4	6	13	SArRNA12
gi|29165615|ref|NC_002745.2|	2110775	-	U	6	12	8	8	13	SArRNA12
gi|29165615|ref|NC_002745.2|	2110776	-	U	11	12	8	1	16	SArRNA12
gi|29165615|ref|NC_002745.2|	2110777	-	G	12	7	4	5	13	SArRNA12
gi|29165615|ref|NC_002745.2|	2110778	-	G	10	8	1	5	7	SArRNA12
gi|29165615|ref|NC_002745.2|	2110779	-	G	6	3	3	3	3	SArRNA12
gi|29165615|ref|NC_002745.2|	2110780	-	U	15	13	7	4	17	SArRNA12
gi|29165615|ref|NC_002745.2|	2110781	-	C	2	6	2	1	4	SArRNA12
gi|29165615|ref|NC_002745.2|	2110782	-	U	8	11	3	5	5	SArRNA12
gi|29165615|ref|NC_002745.2|	2110783	-	C	10	12	3	5	12	SArRNA12
gi|29165615|ref|NC_002745.2|	2110784	-	G	7	12	7	7	16	SArRNA12
gi|29165615|ref|NC_002745.2|	2110785	-	C	17	24	2	9	20	SArRNA12
gi|29165615|ref|NC_002745.2|	2110786	-	C	4	5	0	1	5	SArRNA12

gi|29165615|ref|NC_002745.2|	2110787	-	U	2	2	0	1	5	SArRNA12
gi|29165615|ref|NC_002745.2|	2110788	-	G	2	3	0	0	7	SArRNA12
gi|29165615|ref|NC_002745.2|	2110789	-	C	4	2	2	0	4	SArRNA12
gi|29165615|ref|NC_002745.2|	2110790	-	U	3	8	2	0	2	SArRNA12
gi|29165615|ref|NC_002745.2|	2110791	-	C	0	0	1	0	2	SArRNA12
gi|29165615|ref|NC_002745.2|	2110792	-	G	2	1	1	2	2	SArRNA12
gi|29165615|ref|NC_002745.2|	2110793	-	G	1	3	0	3	3	SArRNA12
gi|29165615|ref|NC_002745.2|	2110794	-	A	11	9	5	5	13	SArRNA12
gi|29165615|ref|NC_002745.2|	2110795	-	A	9	20	2	7	10	SArRNA12
gi|29165615|ref|NC_002745.2|	2110796	-	G	15	13	3	4	13	SArRNA12
gi|29165615|ref|NC_002745.2|	2110797	-	G	6	6	5	5	11	SArRNA12
gi|29165615|ref|NC_002745.2|	2110798	-	A	11	1	2	4	13	SArRNA12
gi|29165615|ref|NC_002745.2|	2110799	-	G	3	1	5	3	9	SArRNA12
gi|29165615|ref|NC_002745.2|	2110800	-	A	4	5	3	2	10	SArRNA12
gi|29165615|ref|NC_002745.2|	2110801	-	C	3	1	1	3	7	SArRNA12
gi|29165615|ref|NC_002745.2|	2110802	-	C	2	2	0	0	2	SArRNA12
gi|29165615|ref|NC_002745.2|	2110803	-	U	2	2	0	0	1	SArRNA12
gi|29165615|ref|NC_002745.2|	2110804	-	U	5	3	0	1	7	SArRNA12
gi|29165615|ref|NC_002745.2|	2110805	-	U	10	11	6	9	19	SArRNA12
gi|29165615|ref|NC_002745.2|	2110806	-	G	22	16	15	11	35	SArRNA12
gi|29165615|ref|NC_002745.2|	2110807	-	G	36	22	13	7	23	SArRNA12
gi|29165615|ref|NC_002745.2|	2110808	-	A	37	36	14	11	39	SArRNA12
gi|29165615|ref|NC_002745.2|	2110809	-	A	16	19	9	7	19	SArRNA12
gi|29165615|ref|NC_002745.2|	2110810	-	U	34	38	15	23	27	SArRNA12
gi|29165615|ref|NC_002745.2|	2110811	-	C	12	17	4	9	21	SArRNA12
gi|29165615|ref|NC_002745.2|	2110812	-	A	7	13	6	7	18	SArRNA12
gi|29165615|ref|NC_002745.2|	2110813	-	G	0	2	0	2	0	SArRNA12
gi|29165615|ref|NC_002745.2|	2110814	-	U	25	16	11	7	23	SArRNA12
gi|29165615|ref|NC_002745.2|	2110815	-	U	38	31	16	22	35	SArRNA12
gi|29165615|ref|NC_002745.2|	2110816	-	A	28	31	15	10	23	SArRNA12
gi|29165615|ref|NC_002745.2|	2110817	-	G	26	20	10	18	25	SArRNA12
gi|29165615|ref|NC_002745.2|	2110818	-	C	60	40	14	15	27	SArRNA12
gi|29165615|ref|NC_002745.2|	2110819	-	C	1	4	0	0	1	SArRNA12
gi|29165615|ref|NC_002745.2|	2110820	-	A	1	2	1	1	1	SArRNA12
gi|29165615|ref|NC_002745.2|	2110821	-	C	2	3	2	0	0	SArRNA12
gi|29165615|ref|NC_002745.2|	2110822	-	C	0	1	0	0	1	SArRNA12
gi|29165615|ref|NC_002745.2|	2110823	-	U	0	1	0	1	0	SArRNA12
gi|29165615|ref|NC_002745.2|	2110824	-	G	1	3	1	2	1	SArRNA12
gi|29165615|ref|NC_002745.2|	2110825	-	C	3	1	2	0	0	SArRNA12
gi|29165615|ref|NC_002745.2|	2110826	-	C	3	5	0	1	6	SArRNA12
gi|29165615|ref|NC_002745.2|	2110827	-	C	26	10	5	4	9	SArRNA12
gi|29165615|ref|NC_002745.2|	2110828	-	U	0	1	0	0	1	SArRNA12
gi|29165615|ref|NC_002745.2|	2110829	-	A	0	2	1	0	1	SArRNA12
gi|29165615|ref|NC_002745.2|	2110830	-	A	0	3	0	0	3	SArRNA12
gi|29165615|ref|NC_002745.2|	2110831	-	G	0	0	0	0	1	SArRNA12
gi|29165615|ref|NC_002745.2|	2110832	-	A	0	0	0	1	1	SArRNA12
gi|29165615|ref|NC_002745.2|	2110833	-	G	0	0	0	0	2	SArRNA12
gi|29165615|ref|NC_002745.2|	2110834	-	U	0	0	1	2	1	SArRNA12
gi|29165615|ref|NC_002745.2|	2110835	-	G	6	2	1	1	0	SArRNA12
gi|29165615|ref|NC_002745.2|	2110836	-	G	0	0	1	0	1	SArRNA12
gi|29165615|ref|NC_002745.2|	2110838	-	C	0	1	0	0	0	SArRNA12
gi|29165615|ref|NC_002745.2|	2110840	-	G	0	2	0	2	2	SArRNA12
gi|29165615|ref|NC_002745.2|	2110841	-	A	0	1	0	0	1	SArRNA12
gi|29165615|ref|NC_002745.2|	2110842	-	A	1	3	0	0	2	SArRNA12
gi|29165615|ref|NC_002745.2|	2110843	-	A	2	1	1	1	3	SArRNA12
gi|29165615|ref|NC_002745.2|	2110844	-	G	4	6	3	1	2	SArRNA12
gi|29165615|ref|NC_002745.2|	2110845	-	C	8	9	0	6	4	SArRNA12
gi|29165615|ref|NC_002745.2|	2110846	-	G	9	5	1	0	2	SArRNA12
gi|29165615|ref|NC_002745.2|	2110847	-	A	35	35	8	11	16	SArRNA12
gi|29165615|ref|NC_002745.2|	2110848	-	U	30	30	15	13	17	SArRNA12
gi|29165615|ref|NC_002745.2|	2110849	-	G	35	26	10	12	15	SArRNA12
gi|29165615|ref|NC_002745.2|	2110850	-	A	7	11	4	3	11	SArRNA12
gi|29165615|ref|NC_002745.2|	2110851	-	G	11	8	6	5	5	SArRNA12
gi|29165615|ref|NC_002745.2|	2110852	-	U	10	5	7	2	10	SArRNA12
gi|29165615|ref|NC_002745.2|	2110853	-	G	9	7	2	4	8	SArRNA12
gi|29165615|ref|NC_002745.2|	2110854	-	U	10	11	2	7	9	SArRNA12
gi|29165615|ref|NC_002745.2|	2110855	-	G	5	5	4	1	8	SArRNA12
gi|29165615|ref|NC_002745.2|	2110856	-	G	9	5	5	2	5	SArRNA12
gi|29165615|ref|NC_002745.2|	2110857	-	C	43	37	8	14	20	SArRNA12
gi|29165615|ref|NC_002745.2|	2110858	-	C	10	6	5	5	1	SArRNA12
gi|29165615|ref|NC_002745.2|	2110859	-	G	2	4	0	0	0	SArRNA12
gi|29165615|ref|NC_002745.2|	2110860	-	U	10	12	3	4	9	SArRNA12
gi|29165615|ref|NC_002745.2|	2110861	-	A	24	20	8	6	10	SArRNA12
gi|29165615|ref|NC_002745.2|	2110862	-	A	25	18	12	17	15	SArRNA12
gi|29165615|ref|NC_002745.2|	2110863	-	G	8	9	7	3	17	SArRNA12
gi|29165615|ref|NC_002745.2|	2110864	-	A	11	6	5	4	8	SArRNA12
gi|29165615|ref|NC_002745.2|	2110865	-	G	17	13	10	5	18	SArRNA12
gi|29165615|ref|NC_002745.2|	2110866	-	U	16	14	5	8	15	SArRNA12
gi|29165615|ref|NC_002745.2|	2110867	-	G	16	13	7	9	19	SArRNA12
gi|29165615|ref|NC_002745.2|	2110868	-	A	10	21	7	6	25	SArRNA12
gi|29165615|ref|NC_002745.2|	2110869	-	A	20	9	11	9	22	SArRNA12

gi|29165615|ref|NC_002745.2|	2110870	-	G	29	26	10	9	11 SArRNA12
gi|29165615|ref|NC_002745.2|	2110871	-	A	8	10	2	7	8 SArRNA12
gi|29165615|ref|NC_002745.2|	2110872	-	U	21	21	8	12	19 SArRNA12
gi|29165615|ref|NC_002745.2|	2110873	-	U	6	6	5	2	11 SArRNA12
gi|29165615|ref|NC_002745.2|	2110874	-	C	4	2	1	2	8 SArRNA12
gi|29165615|ref|NC_002745.2|	2110875	-	G	0	1	1	1	4 SArRNA12
gi|29165615|ref|NC_002745.2|	2110876	-	C	5	4	4	4	10 SArRNA12
gi|29165615|ref|NC_002745.2|	2110877	-	G	1	3	0	2	3 SArRNA12
gi|29165615|ref|NC_002745.2|	2110878	-	A	6	4	1	2	4 SArRNA12
gi|29165615|ref|NC_002745.2|	2110879	-	G	9	8	2	0	3 SArRNA12
gi|29165615|ref|NC_002745.2|	2110880	-	G	7	1	1	3	1 SArRNA12
gi|29165615|ref|NC_002745.2|	2110881	-	U	4	1	3	2	1 SArRNA12
gi|29165615|ref|NC_002745.2|	2110882	-	G	4	1	2	2	2 SArRNA12
gi|29165615|ref|NC_002745.2|	2110883	-	U	2	6	3	4	6 SArRNA12
gi|29165615|ref|NC_002745.2|	2110884	-	A	9	8	6	9	11 SArRNA12
gi|29165615|ref|NC_002745.2|	2110885	-	C	10	6	4	3	7 SArRNA12
gi|29165615|ref|NC_002745.2|	2110886	-	A	6	15	6	4	7 SArRNA12
gi|29165615|ref|NC_002745.2|	2110887	-	G	20	20	5	5	19 SArRNA12
gi|29165615|ref|NC_002745.2|	2110888	-	G	4	3	5	4	6 SArRNA12
gi|29165615|ref|NC_002745.2|	2110889	-	A	9	7	5	3	8 SArRNA12
gi|29165615|ref|NC_002745.2|	2110890	-	A	9	22	2	2	7 SArRNA12
gi|29165615|ref|NC_002745.2|	2110891	-	U	4	20	5	6	10 SArRNA12
gi|29165615|ref|NC_002745.2|	2110892	-	G	24	13	6	7	24 SArRNA12
gi|29165615|ref|NC_002745.2|	2110893	-	C	8	7	6	4	12 SArRNA12
gi|29165615|ref|NC_002745.2|	2110894	-	U	12	11	6	9	12 SArRNA12
gi|29165615|ref|NC_002745.2|	2110895	-	A	2	12	4	3	11 SArRNA12
gi|29165615|ref|NC_002745.2|	2110896	-	G	4	14	1	1	4 SArRNA12
gi|29165615|ref|NC_002745.2|	2110897	-	U	3	4	0	4	3 SArRNA12
gi|29165615|ref|NC_002745.2|	2110898	-	A	4	8	3	2	4 SArRNA12
gi|29165615|ref|NC_002745.2|	2110899	-	C	12	15	3	13	6 SArRNA12
gi|29165615|ref|NC_002745.2|	2110900	-	G	0	1	0	0	1 SArRNA12
gi|29165615|ref|NC_002745.2|	2110901	-	A	2	0	1	0	3 SArRNA12
gi|29165615|ref|NC_002745.2|	2110902	-	A	0	0	1	0	2 SArRNA12
gi|29165615|ref|NC_002745.2|	2110903	-	G	8	26	4	6	10 SArRNA12
gi|29165615|ref|NC_002745.2|	2110904	-	U	7	3	1	2	7 SArRNA12
gi|29165615|ref|NC_002745.2|	2110905	-	U	3	7	6	5	4 SArRNA12
gi|29165615|ref|NC_002745.2|	2110906	-	G	4	2	3	2	2 SArRNA12
gi|29165615|ref|NC_002745.2|	2110907	-	C	9	2	1	1	2 SArRNA12
gi|29165615|ref|NC_002745.2|	2110908	-	G	0	2	0	2	0 SArRNA12
gi|29165615|ref|NC_002745.2|	2110909	-	G	1	1	0	0	0 SArRNA12
gi|29165615|ref|NC_002745.2|	2110910	-	G	1	1	0	0	0 SArRNA12
gi|29165615|ref|NC_002745.2|	2110911	-	A	6	4	3	2	4 SArRNA12
gi|29165615|ref|NC_002745.2|	2110912	-	A	9	9	2	2	9 SArRNA12
gi|29165615|ref|NC_002745.2|	2110913	-	U	2	6	0	0	7 SArRNA12
gi|29165615|ref|NC_002745.2|	2110914	-	C	2	2	2	1	3 SArRNA12
gi|29165615|ref|NC_002745.2|	2110915	-	U	1	0	1	0	1 SArRNA12
gi|29165615|ref|NC_002745.2|	2110916	-	U	0	1	0	0	1 SArRNA12
gi|29165615|ref|NC_002745.2|	2110917	-	G	6	5	5	3	8 SArRNA12
gi|29165615|ref|NC_002745.2|	2110918	-	C	11	9	2	2	8 SArRNA12
gi|29165615|ref|NC_002745.2|	2110919	-	G	3	2	0	0	2 SArRNA12
gi|29165615|ref|NC_002745.2|	2110920	-	A	9	2	0	1	1 SArRNA12
gi|29165615|ref|NC_002745.2|	2110921	-	G	2	4	2	2	4 SArRNA12
gi|29165615|ref|NC_002745.2|	2110922	-	A	8	3	8	3	4 SArRNA12
gi|29165615|ref|NC_002745.2|	2110923	-	G	3	7	6	2	3 SArRNA12
gi|29165615|ref|NC_002745.2|	2110924	-	G	1	1	0	1	0 SArRNA12
gi|29165615|ref|NC_002745.2|	2110925	-	A	2	2	1	0	6 SArRNA12
gi|29165615|ref|NC_002745.2|	2110926	-	U	6	7	0	4	3 SArRNA12
gi|29165615|ref|NC_002745.2|	2110927	-	G	12	12	4	10	9 SArRNA12
gi|29165615|ref|NC_002745.2|	2110928	-	G	1	1	1	3	1 SArRNA12
gi|29165615|ref|NC_002745.2|	2110929	-	U	4	6	1	2	2 SArRNA12
gi|29165615|ref|NC_002745.2|	2110930	-	A	6	11	6	3	19 SArRNA12
gi|29165615|ref|NC_002745.2|	2110931	-	A	9	7	2	2	7 SArRNA12
gi|29165615|ref|NC_002745.2|	2110932	-	C	13	7	4	4	4 SArRNA12
gi|29165615|ref|NC_002745.2|	2110933	-	A	5	2	3	2	3 SArRNA12
gi|29165615|ref|NC_002745.2|	2110934	-	G	5	5	7	4	15 SArRNA12
gi|29165615|ref|NC_002745.2|	2110935	-	G	7	3	2	1	2 SArRNA12
gi|29165615|ref|NC_002745.2|	2110936	-	U	8	2	2	0	2 SArRNA12
gi|29165615|ref|NC_002745.2|	2110937	-	U	4	4	1	3	8 SArRNA12
gi|29165615|ref|NC_002745.2|	2110938	-	U	2	3	1	5	6 SArRNA12
gi|29165615|ref|NC_002745.2|	2110939	-	C	2	4	2	3	2 SArRNA12
gi|29165615|ref|NC_002745.2|	2110940	-	C	8	6	4	6	7 SArRNA12
gi|29165615|ref|NC_002745.2|	2110941	-	U	0	1	1	2	1 SArRNA12
gi|29165615|ref|NC_002745.2|	2110942	-	G	2	1	0	0	0 SArRNA12
gi|29165615|ref|NC_002745.2|	2110943	-	U	5	2	0	2	1 SArRNA12
gi|29165615|ref|NC_002745.2|	2110944	-	U	2	3	2	2	2 SArRNA12
gi|29165615|ref|NC_002745.2|	2110945	-	A	1	2	0	0	1 SArRNA12
gi|29165615|ref|NC_002745.2|	2110946	-	G	3	2	1	0	3 SArRNA12
gi|29165615|ref|NC_002745.2|	2110947	-	G	2	1	1	1	0 SArRNA12
gi|29165615|ref|NC_002745.2|	2110948	-	U	4	2	3	5	3 SArRNA12
gi|29165615|ref|NC_002745.2|	2110949	-	G	6	2	1	1	4 SArRNA12
gi|29165615|ref|NC_002745.2|	2110950	-	U	8	8	2	3	14 SArRNA12

gi|29165615|ref|NC_002745.2|	2110951	-	C	3	7	1	3	3	SArRNA12
gi|29165615|ref|NC_002745.2|	2110952	-	G	1	0	0	1	3	SArRNA12
gi|29165615|ref|NC_002745.2|	2110953	-	A	50	62	23	20	59	SArRNA12
gi|29165615|ref|NC_002745.2|	2110954	-	A	65	83	39	25	103	SArRNA12
gi|29165615|ref|NC_002745.2|	2110955	-	G	98	83	32	40	98	SArRNA12
gi|29165615|ref|NC_002745.2|	2110956	-	C	108	115	57	58	139	SArRNA12
gi|29165615|ref|NC_002745.2|	2110957	-	C	72	67	34	25	60	SArRNA12
gi|29165615|ref|NC_002745.2|	2110958	-	A	51	46	28	20	53	SArRNA12
gi|29165615|ref|NC_002745.2|	2110959	-	U	50	61	20	20	44	SArRNA12
gi|29165615|ref|NC_002745.2|	2110960	-	U	72	54	42	27	87	SArRNA12
gi|29165615|ref|NC_002745.2|	2110961	-	A	165	81	41	33	108	SArRNA12
gi|29165615|ref|NC_002745.2|	2110962	-	U	451	92	51	40	143	SArRNA12
gi|29165615|ref|NC_002745.2|	2110963	-	A	128	116	74	50	179	SArRNA12
gi|29165615|ref|NC_002745.2|	2110964	-	C	157	127	50	37	137	SArRNA12
gi|29165615|ref|NC_002745.2|	2110965	-	A	69	59	47	26	106	SArRNA12
gi|29165615|ref|NC_002745.2|	2110966	-	A	49	65	40	20	72	SArRNA12
gi|29165615|ref|NC_002745.2|	2110967	-	A	38	44	30	19	55	SArRNA12
gi|29165615|ref|NC_002745.2|	2110968	-	U	51	60	37	19	78	SArRNA12
gi|29165615|ref|NC_002745.2|	2110969	-	C	71	65	39	32	107	SArRNA12
gi|29165615|ref|NC_002745.2|	2110970	-	G	5	13	6	2	17	SArRNA12
gi|29165615|ref|NC_002745.2|	2110971	-	G	11	4	4	3	6	SArRNA12
gi|29165615|ref|NC_002745.2|	2110972	-	G	10	15	4	4	9	SArRNA12
gi|29165615|ref|NC_002745.2|	2110973	-	G	92	142	34	28	133	SArRNA12
gi|29165615|ref|NC_002745.2|	2110974	-	C	227	264	51	55	276	SArRNA12
gi|29165615|ref|NC_002745.2|	2110975	-	C	74	75	19	24	94	SArRNA12
gi|29165615|ref|NC_002745.2|	2110976	-	A	50	43	21	13	40	SArRNA12
gi|29165615|ref|NC_002745.2|	2110977	-	U	39	69	45	57	100	SArRNA12
gi|29165615|ref|NC_002745.2|	2110978	-	G	81	138	62	54	122	SArRNA12
gi|29165615|ref|NC_002745.2|	2110979	-	U	120	152	77	66	164	SArRNA12
gi|29165615|ref|NC_002745.2|	2110980	-	A	109	188	71	53	193	SArRNA12
gi|29165615|ref|NC_002745.2|	2110981	-	A	108	87	66	48	89	SArRNA12
gi|29165615|ref|NC_002745.2|	2110982	-	A	59	57	29	25	81	SArRNA12
gi|29165615|ref|NC_002745.2|	2110983	-	A	30	45	26	16	52	SArRNA12
gi|29165615|ref|NC_002745.2|	2110984	-	G	34	51	23	27	49	SArRNA12
gi|29165615|ref|NC_002745.2|	2110985	-	C	49	32	22	17	66	SArRNA12
gi|29165615|ref|NC_002745.2|	2110986	-	C	22	29	20	11	44	SArRNA12
gi|29165615|ref|NC_002745.2|	2110987	-	G	13	9	8	8	14	SArRNA12
gi|29165615|ref|NC_002745.2|	2110988	-	C	44	42	28	23	24	SArRNA12
gi|29165615|ref|NC_002745.2|	2110989	-	G	13	11	1	0	3	SArRNA12
[truncated: 801,006 more chars]
