# Supplementary material for: Transcriptome-Wide Analyses of 5′-Ends in RNase J Mutants of a Gram-Positive Pathogen Reveal a Role in RNA Maturation, Regulation and Degradation
Source: PLoS Genet. 2014 Feb 27;10(2):e1004207. doi: 10.1371/journal.pgen.1004207 (PMC3937233; doi:10.1371/journal.pgen.1004207)
Supplement: Table S9 — List of oligos used in addition to the oligos shown in Table 8. (PDF) [file pgen.1004207.s011.pdf]

Table S9, Additional oligos:

Name	Sequence
**pRLYT-J1AGA-1**	
RNaseJ1-L-Xho-F1	CTGATCTCGAGTATCTTAACAACCTTTTTACTG
J1-AGA-NAR-FUS-R1	CTTCGGCGCCTGCTGTTATAAATAGGCCAACAAT
J1-AGA-NAR-FUS-F1	ATAACAGCAGGCGCCGAAGACCATATAGGCGGTG
J1-INT-Xho-R3	TGACCCTCGAGTAGCGTGATATGGGTAGACG
**pRLYT-J2AGA-3**	
J2-AGA-Nar-F1	CCTTACAGCAGGCGCCGAGCACGCGATTGGTG
J2-AGA-Nar-R1	TCGGCGCCTGCTGTAAGGAATATACCCTTCAA
RNaseJ2-Xho-F1	ACGTGCTCGAGGTCGTCGTGAAATTGGACA
J2-INT-Xho-R5	CCTCACCTCGAGATTTCAGACCATTCTATGCGT
**pRLYE-85RL-Kan-1**	
SA1885-L-Xho-F1	CATCTGCTCGAGGATGAACCATTATTGAAACCAC
SA1885-L-Bam-R1	CCTCTGGATCCTAGCATTAGATAAGATGTAAGCG
SA1885-R-Bam-F1	CACTTGGATCCGTATGCGGTTATATCATTCTGTG
SA1885-R-Nar-R1	CACTTAGGCGCCGATATTCGAGCCTTTTTACAC
Kan-Bam-F1	TCAAGGATCCCAGCGAACCATTTGAG
Kan-Bgl-R1	TGAGGAGATCTTTTTAGACATCTAAATCTAGG
**pRLYC9-J1ERYS-1, pRLYT9-J1ERYS-1, and pRLYE9-J1CATS-1**	
J1-INT-Nar-F4	TTCACGGCGCCCTACCAAACTTTTTATCCCCTC
RNaseJ1-L-Xba-R1	CAGAATCTAGACCTCCAAGTGCATATACACC
RNaseJ1-R-Eco-F1	CAGATGAATTCTATCATTGAAACATTACAACC
J1-INT-Xho-R3	TGACCCTCGAGTAGCGTGATATGGGTAGACG
***CAT194*-cassette**	
CAT-Bam-F1	GTATCAGGATCCATGTATTCTCAAGATAAGAAAG
CAT-Bgl-R1	GAGCATAGATCTTCTTCAACTAACGGGGC
***ermC*-cassette**	
ERY-Bam-F1	ACTATGGATCCTTTAAGAACTTTCTTTTTTTAC
ERY-Bgl-R1	TATCAAGATCTCACAAAAAATAGGCACACG
**pJ1 and pJ1^{AGA}**	
J1-Sal-F3	GAACGGTCGACATTGACAAAAATCACCCAT
J1-Bam-R3	GAAGCGGGATCCGTTCAGTCAACTACTGCC

**pJ2**	
J2-Sal-F4	GAGTTCGTCGACATCACACAGAGCATTAGAAG
J2-Bam-R4	GAGTAAGGATCCAAACAGACGATGAAAGAGG
**RnpB end-determination**	
rnpB-RT-R1	CCGTTAAACAAGTGCTCC
rnpB-INT-Eco-F2	GGTAACGGAATTCAACGTATAAACGAG
rnpB-INT-Hind-R1	GAGGAGAAGCTTGTTTACCGCGTTCCACCTT
SA1279-INTHind-R1	GAGGAGAAGCTTTTTCTAAGTTTGA
**Northern blot probes**	
RnpB-prb-R1	TACAATCATCTCAGACTGTGTGAGCATGGACTTTCCTCT
SA1075-prb-R2	TTCAGTTACTTTATCAGCGTCTACACCTAAACGGTCAAC
S5rRNA-prb-R1	TTAACTTCTGTGTTCGGCATGGGAACAGGTGTGACCTCC
