# Supplementary material for: Transcriptome-Wide Analyses of 5′-Ends in RNase J Mutants of a Gram-Positive Pathogen Reveal a Role in RNA Maturation, Regulation and Degradation
Source: PLoS Genet. 2014 Feb 27;10(2):e1004207. doi: 10.1371/journal.pgen.1004207 (PMC3937233; doi:10.1371/journal.pgen.1004207)
Supplement: Table S1 — A full EMOTE data-set of WT and RNase J mutant reads, mapping to unique locations on the S. aureus N315 chromosome. (PDF) [file pgen.1004207.s003.pdf]

N315 chromosome assembly	position	strand	5'nt	WT	dJ1	dJ2	dJ1dJ2	J1AGA	ORF
gi|29165615|ref|NC_002745.2|	313	+	G	0	0	0	1	2	-
gi|29165615|ref|NC_002745.2|	314	+	A	0	0	0	0	1	-
gi|29165615|ref|NC_002745.2|	491	+	A	0	0	1	0	0	-
gi|29165615|ref|NC_002745.2|	507	+	G	0	0	0	1	0	-
gi|29165615|ref|NC_002745.2|	524	+	A	0	1	0	0	0	SA0001
gi|29165615|ref|NC_002745.2|	529	+	G	0	0	0	0	1	SA0001
gi|29165615|ref|NC_002745.2|	530	+	A	0	0	0	2	0	SA0001
gi|29165615|ref|NC_002745.2|	569	+	U	1	0	0	0	0	SA0001
gi|29165615|ref|NC_002745.2|	718	+	G	0	0	0	0	1	SA0001
gi|29165615|ref|NC_002745.2|	749	+	U	0	1	0	0	0	SA0001
gi|29165615|ref|NC_002745.2|	770	+	A	0	0	0	0	1	SA0001
gi|29165615|ref|NC_002745.2|	791	+	C	0	1	0	0	0	SA0001
gi|29165615|ref|NC_002745.2|	847	+	C	0	0	1	0	0	SA0001
gi|29165615|ref|NC_002745.2|	893	+	U	0	0	0	1	0	SA0001
gi|29165615|ref|NC_002745.2|	907	+	A	0	1	0	0	0	SA0001
gi|29165615|ref|NC_002745.2|	925	+	G	0	0	1	0	0	SA0001
gi|29165615|ref|NC_002745.2|	953	+	C	1	0	0	0	0	SA0001
gi|29165615|ref|NC_002745.2|	977	+	U	0	1	0	0	0	SA0001
gi|29165615|ref|NC_002745.2|	1088	+	C	1	0	0	0	0	SA0001
gi|29165615|ref|NC_002745.2|	1175	+	A	0	0	0	0	1	SA0001
gi|29165615|ref|NC_002745.2|	1234	+	G	0	1	0	0	0	SA0001
gi|29165615|ref|NC_002745.2|	1240	+	C	1	0	0	0	0	SA0001
gi|29165615|ref|NC_002745.2|	1258	+	A	0	0	1	0	0	SA0001
gi|29165615|ref|NC_002745.2|	1299	+	A	0	1	0	0	0	SA0001
gi|29165615|ref|NC_002745.2|	1319	+	C	1	0	0	0	0	SA0001
gi|29165615|ref|NC_002745.2|	1320	+	A	1	0	0	0	0	SA0001
gi|29165615|ref|NC_002745.2|	1406	+	A	0	0	0	0	1	SA0001
gi|29165615|ref|NC_002745.2|	1415	+	A	0	1	0	0	0	SA0001
gi|29165615|ref|NC_002745.2|	1438	+	U	0	0	0	0	1	SA0001
gi|29165615|ref|NC_002745.2|	1445	+	C	0	0	0	0	1	SA0001
gi|29165615|ref|NC_002745.2|	1570	+	C	0	0	0	0	1	SA0001
gi|29165615|ref|NC_002745.2|	1583	+	C	0	0	0	1	0	SA0001
gi|29165615|ref|NC_002745.2|	1615	+	A	0	0	1	0	0	SA0001
gi|29165615|ref|NC_002745.2|	1678	+	A	0	1	0	0	0	SA0001
gi|29165615|ref|NC_002745.2|	1903	+	C	0	1	0	0	0	-
gi|29165615|ref|NC_002745.2|	1957	+	A	0	1	0	0	0	-
gi|29165615|ref|NC_002745.2|	2130	+	U	0	0	0	0	1	-
gi|29165615|ref|NC_002745.2|	2176	+	A	0	0	0	1	0	SA0002
gi|29165615|ref|NC_002745.2|	2195	+	C	0	0	0	0	1	SA0002
gi|29165615|ref|NC_002745.2|	2218	+	U	1	0	0	0	0	SA0002
gi|29165615|ref|NC_002745.2|	2339	+	G	1	0	0	0	0	SA0002
gi|29165615|ref|NC_002745.2|	2426	+	C	1	0	0	0	0	SA0002
gi|29165615|ref|NC_002745.2|	2427	+	C	0	0	0	0	2	SA0002
gi|29165615|ref|NC_002745.2|	2461	+	A	0	0	1	0	0	SA0002
gi|29165615|ref|NC_002745.2|	2883	+	G	1	0	0	0	0	SA0002
gi|29165615|ref|NC_002745.2|	2924	+	C	1	0	0	0	0	SA0002
gi|29165615|ref|NC_002745.2|	2939	+	A	0	0	0	1	0	SA0002
gi|29165615|ref|NC_002745.2|	2958	+	A	0	1	0	0	0	SA0002
gi|29165615|ref|NC_002745.2|	2967	+	U	2	0	0	0	0	SA0002
gi|29165615|ref|NC_002745.2|	2970	+	A	1	0	0	0	0	SA0002
gi|29165615|ref|NC_002745.2|	2975	+	G	1	0	0	0	0	SA0002
gi|29165615|ref|NC_002745.2|	3000	+	C	0	0	2	0	0	SA0002
gi|29165615|ref|NC_002745.2|	3088	+	A	0	0	0	0	1	SA0002
gi|29165615|ref|NC_002745.2|	3649	+	A	0	1	0	0	0	-
gi|29165615|ref|NC_002745.2|	3650	+	A	2	2	1	0	1	-
gi|29165615|ref|NC_002745.2|	3652	+	G	0	1	0	0	0	-
gi|29165615|ref|NC_002745.2|	3656	+	C	0	0	1	0	0	-
gi|29165615|ref|NC_002745.2|	3663	+	A	0	0	0	0	1	-
gi|29165615|ref|NC_002745.2|	3677	+	U	0	1	0	0	0	SA0003
gi|29165615|ref|NC_002745.2|	3749	+	A	0	0	0	0	1	SA0003
gi|29165615|ref|NC_002745.2|	3760	+	C	0	0	0	1	0	SA0003
gi|29165615|ref|NC_002745.2|	3778	+	C	2	0	0	0	0	SA0003
gi|29165615|ref|NC_002745.2|	3791	+	U	0	0	0	1	0	SA0003
gi|29165615|ref|NC_002745.2|	3796	+	A	0	0	0	0	1	SA0003
gi|29165615|ref|NC_002745.2|	4081	+	A	0	0	0	0	1	SA0004
gi|29165615|ref|NC_002745.2|	4095	+	C	0	0	1	1	0	SA0004
gi|29165615|ref|NC_002745.2|	4128	+	G	0	2	0	0	1	SA0004
gi|29165615|ref|NC_002745.2|	4138	+	A	0	0	1	0	0	SA0004
gi|29165615|ref|NC_002745.2|	4139	+	U	0	0	1	0	0	SA0004
gi|29165615|ref|NC_002745.2|	4140	+	A	0	0	1	0	0	SA0004
gi|29165615|ref|NC_002745.2|	4142	+	A	1	0	0	0	0	SA0004
gi|29165615|ref|NC_002745.2|	4144	+	A	1	0	0	0	0	SA0004
gi|29165615|ref|NC_002745.2|	4275	+	A	0	0	0	1	1	SA0004
gi|29165615|ref|NC_002745.2|	4387	+	A	2	0	0	0	0	SA0004
gi|29165615|ref|NC_002745.2|	4392	+	A	0	0	0	0	1	SA0004
gi|29165615|ref|NC_002745.2|	4393	+	A	0	2	0	0	0	SA0004
gi|29165615|ref|NC_002745.2|	4399	+	A	0	0	1	0	0	SA0004
gi|29165615|ref|NC_002745.2|	4400	+	C	0	1	0	0	0	SA0004
gi|29165615|ref|NC_002745.2|	4431	+	G	0	1	0	0	0	SA0004
gi|29165615|ref|NC_002745.2|	4438	+	C	0	0	0	0	1	SA0004

gi|29165615|ref|NC_002745.2|	4447	+	A	0	0	0	0	1	SA0004
gi|29165615|ref|NC_002745.2|	4625	+	G	0	0	0	0	1	SA0004
gi|29165615|ref|NC_002745.2|	4677	+	A	0	0	0	1	0	SA0004
gi|29165615|ref|NC_002745.2|	4685	+	A	0	0	0	0	1	SA0004
gi|29165615|ref|NC_002745.2|	4753	+	C	0	0	0	0	1	SA0004
gi|29165615|ref|NC_002745.2|	4840	+	A	1	0	0	0	0	SA0004
gi|29165615|ref|NC_002745.2|	4859	+	C	0	0	1	0	0	SA0004
gi|29165615|ref|NC_002745.2|	4890	+	C	0	0	1	0	0	SA0004
gi|29165615|ref|NC_002745.2|	4900	+	U	0	0	1	0	0	SA0004
gi|29165615|ref|NC_002745.2|	4970	+	A	0	0	0	0	1	SA0004
gi|29165615|ref|NC_002745.2|	4975	+	U	1	0	0	1	1	SA0004
gi|29165615|ref|NC_002745.2|	4976	+	G	0	0	0	1	0	SA0004
gi|29165615|ref|NC_002745.2|	4977	+	A	0	1	0	0	0	SA0004
gi|29165615|ref|NC_002745.2|	4979	+	U	1	0	0	0	0	SA0004
gi|29165615|ref|NC_002745.2|	4987	+	A	0	2	0	1	1	SA0004
gi|29165615|ref|NC_002745.2|	4992	+	U	0	2	0	1	1	SA0004
gi|29165615|ref|NC_002745.2|	4994	+	U	0	0	0	0	1	SA0004
gi|29165615|ref|NC_002745.2|	4997	+	U	0	0	1	1	0	SA0004
gi|29165615|ref|NC_002745.2|	4998	+	A	0	2	0	0	0	SA0004
gi|29165615|ref|NC_002745.2|	5000	+	U	0	0	1	0	0	SA0004
gi|29165615|ref|NC_002745.2|	5001	+	A	0	0	1	0	0	SA0004
gi|29165615|ref|NC_002745.2|	5002	+	A	0	1	0	1	0	SA0004
gi|29165615|ref|NC_002745.2|	5003	+	U	0	0	0	1	0	SA0004
gi|29165615|ref|NC_002745.2|	5004	+	C	0	0	1	0	0	SA0004
gi|29165615|ref|NC_002745.2|	5006	+	A	3	0	0	1	0	SA0004
gi|29165615|ref|NC_002745.2|	5008	+	G	1	0	0	0	0	SA0004
gi|29165615|ref|NC_002745.2|	5009	+	U	0	0	1	0	0	SA0004
gi|29165615|ref|NC_002745.2|	5010	+	G	0	1	0	0	0	SA0004
gi|29165615|ref|NC_002745.2|	5011	+	A	0	1	4	2	0	SA0004
gi|29165615|ref|NC_002745.2|	5012	+	A	0	0	2	0	0	SA0004
gi|29165615|ref|NC_002745.2|	5013	+	A	0	1	1	0	2	SA0004
gi|29165615|ref|NC_002745.2|	5017	+	U	0	0	2	0	0	SA0004
gi|29165615|ref|NC_002745.2|	5018	+	A	0	3	3	2	1	SA0004
gi|29165615|ref|NC_002745.2|	5020	+	A	1	0	0	0	0	SA0004
gi|29165615|ref|NC_002745.2|	5023	+	A	0	0	1	0	0	SA0004
gi|29165615|ref|NC_002745.2|	5025	+	C	0	1	0	0	0	-
gi|29165615|ref|NC_002745.2|	5027	+	G	0	1	0	1	0	-
gi|29165615|ref|NC_002745.2|	5028	+	A	0	2	2	1	1	-
gi|29165615|ref|NC_002745.2|	5032	+	C	0	1	0	0	0	-
gi|29165615|ref|NC_002745.2|	5034	+	A	1	1	0	0	0	SA0005
gi|29165615|ref|NC_002745.2|	5035	+	U	2	0	0	0	0	SA0005
gi|29165615|ref|NC_002745.2|	5038	+	U	1	0	0	0	0	SA0005
gi|29165615|ref|NC_002745.2|	5059	+	A	0	0	1	0	0	SA0005
gi|29165615|ref|NC_002745.2|	5068	+	A	0	0	0	0	4	SA0005
gi|29165615|ref|NC_002745.2|	5070	+	A	0	0	0	1	0	SA0005
gi|29165615|ref|NC_002745.2|	5074	+	A	1	0	1	0	0	SA0005
gi|29165615|ref|NC_002745.2|	5101	+	A	0	1	0	0	0	SA0005
gi|29165615|ref|NC_002745.2|	5153	+	A	1	0	0	0	0	SA0005
gi|29165615|ref|NC_002745.2|	5173	+	U	1	0	0	0	0	SA0005
gi|29165615|ref|NC_002745.2|	5184	+	A	0	1	0	0	0	SA0005
gi|29165615|ref|NC_002745.2|	5198	+	U	0	1	0	1	0	SA0005
gi|29165615|ref|NC_002745.2|	5209	+	C	1	0	0	0	0	SA0005
gi|29165615|ref|NC_002745.2|	5225	+	A	0	0	1	0	0	SA0005
gi|29165615|ref|NC_002745.2|	5251	+	A	2	1	0	0	0	SA0005
gi|29165615|ref|NC_002745.2|	5254	+	A	0	1	0	0	0	SA0005
gi|29165615|ref|NC_002745.2|	5255	+	U	0	0	1	0	0	SA0005
gi|29165615|ref|NC_002745.2|	5257	+	A	0	0	0	0	1	SA0005
gi|29165615|ref|NC_002745.2|	5263	+	U	1	0	0	0	0	SA0005
gi|29165615|ref|NC_002745.2|	5288	+	U	1	0	0	0	0	SA0005
gi|29165615|ref|NC_002745.2|	5333	+	A	0	0	0	0	1	SA0005
gi|29165615|ref|NC_002745.2|	5339	+	U	0	1	0	0	0	SA0005
gi|29165615|ref|NC_002745.2|	5352	+	C	1	0	0	0	0	SA0005
gi|29165615|ref|NC_002745.2|	5367	+	U	0	1	0	0	0	SA0005
gi|29165615|ref|NC_002745.2|	5475	+	A	3	0	0	0	0	SA0005
gi|29165615|ref|NC_002745.2|	5586	+	A	0	1	0	0	0	SA0005
gi|29165615|ref|NC_002745.2|	5597	+	U	0	1	0	0	0	SA0005
gi|29165615|ref|NC_002745.2|	5603	+	C	0	0	1	0	0	SA0005
gi|29165615|ref|NC_002745.2|	5611	+	A	0	0	1	0	0	SA0005
gi|29165615|ref|NC_002745.2|	5616	+	U	0	0	0	1	0	SA0005
gi|29165615|ref|NC_002745.2|	5617	+	U	1	0	0	0	0	SA0005
gi|29165615|ref|NC_002745.2|	5661	+	C	0	0	1	0	0	SA0005
gi|29165615|ref|NC_002745.2|	5670	+	U	0	0	1	0	0	SA0005
gi|29165615|ref|NC_002745.2|	5675	+	A	0	0	0	1	0	SA0005
gi|29165615|ref|NC_002745.2|	5689	+	A	0	0	1	0	0	SA0005
gi|29165615|ref|NC_002745.2|	5695	+	A	1	0	0	0	0	SA0005
gi|29165615|ref|NC_002745.2|	5700	+	A	0	0	0	0	1	SA0005
gi|29165615|ref|NC_002745.2|	5703	+	G	1	0	0	0	0	SA0005
gi|29165615|ref|NC_002745.2|	5714	+	U	1	0	0	0	0	SA0005
gi|29165615|ref|NC_002745.2|	5743	+	U	1	0	0	0	0	SA0005
gi|29165615|ref|NC_002745.2|	5754	+	A	0	0	1	0	0	SA0005
gi|29165615|ref|NC_002745.2|	5758	+	A	1	0	1	0	0	SA0005

gi|29165615|ref|NC_002745.2|	5759 +	A	1	0	0	0	0	SA0005
gi|29165615|ref|NC_002745.2|	5760 +	A	0	0	2	0	0	SA0005
gi|29165615|ref|NC_002745.2|	5762 +	U	2	1	0	0	1	SA0005
gi|29165615|ref|NC_002745.2|	5764 +	A	0	0	0	0	1	SA0005
gi|29165615|ref|NC_002745.2|	5770 +	C	1	0	0	0	0	SA0005
gi|29165615|ref|NC_002745.2|	5775 +	C	0	0	0	1	0	SA0005
gi|29165615|ref|NC_002745.2|	5780 +	U	1	0	0	0	0	SA0005
gi|29165615|ref|NC_002745.2|	5784 +	C	0	0	1	0	0	SA0005
gi|29165615|ref|NC_002745.2|	5785 +	C	0	0	1	0	0	SA0005
gi|29165615|ref|NC_002745.2|	5794 +	U	0	0	0	2	0	SA0005
gi|29165615|ref|NC_002745.2|	5796 +	C	1	0	0	0	0	SA0005
gi|29165615|ref|NC_002745.2|	5808 +	G	0	0	1	0	1	SA0005
gi|29165615|ref|NC_002745.2|	5821 +	U	0	1	1	0	0	SA0005
gi|29165615|ref|NC_002745.2|	5824 +	A	0	1	0	0	1	SA0005
gi|29165615|ref|NC_002745.2|	5826 +	A	1	2	2	0	2	SA0005
gi|29165615|ref|NC_002745.2|	5827 +	U	0	1	0	0	0	SA0005
gi|29165615|ref|NC_002745.2|	5839 +	A	0	0	0	0	1	SA0005
gi|29165615|ref|NC_002745.2|	5842 +	A	2	0	0	0	0	SA0005
gi|29165615|ref|NC_002745.2|	5843 +	C	1	1	1	0	0	SA0005
gi|29165615|ref|NC_002745.2|	5845 +	C	1	0	0	0	0	SA0005
gi|29165615|ref|NC_002745.2|	5848 +	G	0	0	1	0	0	SA0005
gi|29165615|ref|NC_002745.2|	5857 +	C	0	0	0	1	0	SA0005
gi|29165615|ref|NC_002745.2|	5861 +	U	0	0	0	1	0	SA0005
gi|29165615|ref|NC_002745.2|	5862 +	C	2	0	0	0	0	SA0005
gi|29165615|ref|NC_002745.2|	5863 +	U	4	0	0	0	0	SA0005
gi|29165615|ref|NC_002745.2|	5869 +	C	0	0	0	1	0	SA0005
gi|29165615|ref|NC_002745.2|	5874 +	G	0	1	0	0	0	SA0005
gi|29165615|ref|NC_002745.2|	5875 +	C	2	6	1	2	2	SA0005
gi|29165615|ref|NC_002745.2|	5876 +	A	1	0	1	0	0	SA0005
gi|29165615|ref|NC_002745.2|	5878 +	A	0	1	0	0	0	SA0005
gi|29165615|ref|NC_002745.2|	5879 +	U	1	2	2	0	0	SA0005
gi|29165615|ref|NC_002745.2|	5880 +	A	0	0	0	0	1	SA0005
gi|29165615|ref|NC_002745.2|	5881 +	A	1	0	0	0	0	SA0005
gi|29165615|ref|NC_002745.2|	5882 +	C	0	0	1	0	0	SA0005
gi|29165615|ref|NC_002745.2|	5883 +	A	2	0	1	0	0	SA0005
gi|29165615|ref|NC_002745.2|	5887 +	A	0	0	0	0	1	SA0005
gi|29165615|ref|NC_002745.2|	5888 +	U	0	0	1	0	0	SA0005
gi|29165615|ref|NC_002745.2|	5890 +	C	1	0	1	0	0	SA0005
gi|29165615|ref|NC_002745.2|	5892 +	U	4	0	0	0	2	SA0005
gi|29165615|ref|NC_002745.2|	5893 +	A	1	0	1	0	0	SA0005
gi|29165615|ref|NC_002745.2|	5895 +	G	0	0	2	1	0	SA0005
gi|29165615|ref|NC_002745.2|	5896 +	A	0	1	0	0	0	SA0005
gi|29165615|ref|NC_002745.2|	5897 +	A	0	0	0	0	1	SA0005
gi|29165615|ref|NC_002745.2|	5900 +	U	5	1	0	0	0	SA0005
gi|29165615|ref|NC_002745.2|	5905 +	C	1	0	0	0	0	SA0005
gi|29165615|ref|NC_002745.2|	5913 +	G	0	0	1	0	0	SA0005
gi|29165615|ref|NC_002745.2|	5914 +	A	0	1	0	0	1	SA0005
gi|29165615|ref|NC_002745.2|	5915 +	C	1	0	0	0	0	SA0005
gi|29165615|ref|NC_002745.2|	5917 +	G	1	4	0	1	6	SA0005
gi|29165615|ref|NC_002745.2|	5920 +	U	1	0	1	0	0	SA0005
gi|29165615|ref|NC_002745.2|	5923 +	A	0	0	0	1	0	SA0005
gi|29165615|ref|NC_002745.2|	5925 +	C	1	1	0	0	0	SA0005
gi|29165615|ref|NC_002745.2|	5926 +	G	0	0	0	0	1	SA0005
gi|29165615|ref|NC_002745.2|	5927 +	U	1	0	0	0	3	SA0005
gi|29165615|ref|NC_002745.2|	5929 +	C	0	0	1	0	0	SA0005
gi|29165615|ref|NC_002745.2|	5932 +	U	1	1	1	1	0	SA0005
gi|29165615|ref|NC_002745.2|	5934 +	A	1	0	0	0	0	SA0005
gi|29165615|ref|NC_002745.2|	5939 +	U	2	0	0	0	0	SA0005
gi|29165615|ref|NC_002745.2|	5947 +	A	1	1	0	0	1	SA0005
gi|29165615|ref|NC_002745.2|	5956 +	G	1	0	0	0	0	SA0005
gi|29165615|ref|NC_002745.2|	5983 +	A	0	1	0	0	0	SA0005
gi|29165615|ref|NC_002745.2|	6002 +	U	1	0	0	0	0	SA0005
gi|29165615|ref|NC_002745.2|	6044 +	A	0	1	0	0	0	SA0005
gi|29165615|ref|NC_002745.2|	6081 +	A	0	0	1	0	1	SA0005
gi|29165615|ref|NC_002745.2|	6091 +	A	0	0	0	1	0	SA0005
gi|29165615|ref|NC_002745.2|	6176 +	A	5	0	0	0	0	SA0005
gi|29165615|ref|NC_002745.2|	6195 +	A	0	0	0	0	1	SA0005
gi|29165615|ref|NC_002745.2|	6196 +	U	1	0	0	0	0	SA0005
gi|29165615|ref|NC_002745.2|	6277 +	C	1	0	0	0	0	SA0005
gi|29165615|ref|NC_002745.2|	6336 +	G	1	0	0	0	0	SA0005
gi|29165615|ref|NC_002745.2|	6500 +	C	2	0	0	0	0	SA0005
gi|29165615|ref|NC_002745.2|	6544 +	U	1	0	0	0	0	SA0005
gi|29165615|ref|NC_002745.2|	6555 +	G	0	1	0	0	0	SA0005
gi|29165615|ref|NC_002745.2|	6578 +	U	1	0	0	0	0	SA0005
gi|29165615|ref|NC_002745.2|	6609 +	C	1	0	0	0	0	SA0005
gi|29165615|ref|NC_002745.2|	6658 +	C	1	0	0	0	0	SA0005
gi|29165615|ref|NC_002745.2|	6731 +	A	0	0	0	0	1	SA0005
gi|29165615|ref|NC_002745.2|	6772 +	A	1	0	0	0	0	SA0005
gi|29165615|ref|NC_002745.2|	6798 +	G	0	0	1	0	0	SA0005
gi|29165615|ref|NC_002745.2|	6804 +	U	1	0	0	0	0	SA0005
gi|29165615|ref|NC_002745.2|	6861 +	G	0	0	0	0	1	SA0005

gi|29165615|ref|NC_002745.2|	6868	+	U	1	0	0	0	0	SA0005
gi|29165615|ref|NC_002745.2|	6885	+	U	1	0	0	0	0	SA0005
gi|29165615|ref|NC_002745.2|	6910	+	U	0	1	0	0	0	SA0005
gi|29165615|ref|NC_002745.2|	6927	+	U	0	0	0	0	1	SA0005
gi|29165615|ref|NC_002745.2|	6934	+	A	0	0	0	1	1	SA0005
gi|29165615|ref|NC_002745.2|	6949	+	A	0	0	0	0	1	SA0005
gi|29165615|ref|NC_002745.2|	6952	+	C	0	1	0	0	0	SA0005
gi|29165615|ref|NC_002745.2|	6954	+	A	0	0	0	0	1	SA0005
gi|29165615|ref|NC_002745.2|	6961	+	A	0	1	0	0	0	SA0005
gi|29165615|ref|NC_002745.2|	6964	+	U	0	0	0	0	1	SA0005
gi|29165615|ref|NC_002745.2|	7040	+	A	1	0	2	0	1	SA0006
gi|29165615|ref|NC_002745.2|	7041	+	A	0	0	1	0	0	SA0006
gi|29165615|ref|NC_002745.2|	7054	+	A	5	0	0	0	1	SA0006
gi|29165615|ref|NC_002745.2|	7061	+	U	1	0	0	0	0	SA0006
gi|29165615|ref|NC_002745.2|	7066	+	C	1	0	0	0	0	SA0006
gi|29165615|ref|NC_002745.2|	7081	+	C	0	0	1	0	0	SA0006
gi|29165615|ref|NC_002745.2|	7082	+	G	1	0	0	0	0	SA0006
gi|29165615|ref|NC_002745.2|	7085	+	G	1	0	0	0	0	SA0006
gi|29165615|ref|NC_002745.2|	7090	+	U	1	0	0	0	0	SA0006
gi|29165615|ref|NC_002745.2|	7093	+	U	1	0	0	0	0	SA0006
gi|29165615|ref|NC_002745.2|	7162	+	U	0	0	1	0	0	SA0006
gi|29165615|ref|NC_002745.2|	7165	+	A	0	1	0	0	0	SA0006
gi|29165615|ref|NC_002745.2|	7182	+	C	0	0	0	0	1	SA0006
gi|29165615|ref|NC_002745.2|	7202	+	A	0	1	0	0	0	SA0006
gi|29165615|ref|NC_002745.2|	7216	+	U	0	1	0	0	0	SA0006
gi|29165615|ref|NC_002745.2|	7272	+	A	0	1	0	0	1	SA0006
gi|29165615|ref|NC_002745.2|	7395	+	A	0	0	1	0	0	SA0006
gi|29165615|ref|NC_002745.2|	7405	+	A	0	0	0	1	0	SA0006
gi|29165615|ref|NC_002745.2|	7421	+	U	0	0	0	0	1	SA0006
gi|29165615|ref|NC_002745.2|	7462	+	A	1	0	0	0	0	SA0006
gi|29165615|ref|NC_002745.2|	7486	+	C	0	0	0	1	0	SA0006
gi|29165615|ref|NC_002745.2|	7528	+	U	1	0	0	0	0	SA0006
gi|29165615|ref|NC_002745.2|	7534	+	U	2	1	0	0	0	SA0006
gi|29165615|ref|NC_002745.2|	7550	+	U	1	0	0	0	0	SA0006
gi|29165615|ref|NC_002745.2|	7630	+	U	2	0	0	0	0	SA0006
gi|29165615|ref|NC_002745.2|	7660	+	C	1	0	0	0	0	SA0006
gi|29165615|ref|NC_002745.2|	7666	+	C	1	0	0	0	0	SA0006
gi|29165615|ref|NC_002745.2|	7678	+	U	0	0	0	0	1	SA0006
gi|29165615|ref|NC_002745.2|	7750	+	U	2	0	0	0	0	SA0006
gi|29165615|ref|NC_002745.2|	7756	+	A	1	0	0	0	0	SA0006
gi|29165615|ref|NC_002745.2|	7813	+	A	0	1	0	0	0	SA0006
gi|29165615|ref|NC_002745.2|	7816	+	C	1	0	0	0	0	SA0006
gi|29165615|ref|NC_002745.2|	7825	+	U	1	0	0	0	0	SA0006
gi|29165615|ref|NC_002745.2|	7838	+	A	0	0	0	0	1	SA0006
gi|29165615|ref|NC_002745.2|	7843	+	U	0	1	0	0	0	SA0006
gi|29165615|ref|NC_002745.2|	7846	+	U	1	0	0	0	0	SA0006
gi|29165615|ref|NC_002745.2|	7864	+	A	0	0	0	2	0	SA0006
gi|29165615|ref|NC_002745.2|	7982	+	U	1	0	0	0	0	SA0006
gi|29165615|ref|NC_002745.2|	7991	+	A	1	0	0	0	0	SA0006
gi|29165615|ref|NC_002745.2|	8006	+	U	0	0	0	0	1	SA0006
gi|29165615|ref|NC_002745.2|	8017	+	U	0	0	0	1	0	SA0006
gi|29165615|ref|NC_002745.2|	8056	+	C	0	0	0	0	1	SA0006
gi|29165615|ref|NC_002745.2|	8112	+	A	0	0	0	1	0	SA0006
gi|29165615|ref|NC_002745.2|	8113	+	A	1	0	0	0	0	SA0006
gi|29165615|ref|NC_002745.2|	8119	+	G	0	0	1	0	0	SA0006
gi|29165615|ref|NC_002745.2|	8123	+	A	1	0	0	0	0	SA0006
gi|29165615|ref|NC_002745.2|	8125	+	C	1	0	0	0	0	SA0006
gi|29165615|ref|NC_002745.2|	8127	+	A	0	1	0	0	0	SA0006
gi|29165615|ref|NC_002745.2|	8159	+	U	1	0	0	0	0	SA0006
gi|29165615|ref|NC_002745.2|	8244	+	C	0	0	0	0	1	SA0006
gi|29165615|ref|NC_002745.2|	8265	+	C	0	0	1	0	0	SA0006
gi|29165615|ref|NC_002745.2|	8278	+	U	1	0	0	0	0	SA0006
gi|29165615|ref|NC_002745.2|	8289	+	C	1	0	0	0	0	SA0006
gi|29165615|ref|NC_002745.2|	8312	+	A	0	1	0	0	0	SA0006
gi|29165615|ref|NC_002745.2|	8324	+	A	0	0	0	0	1	SA0006
gi|29165615|ref|NC_002745.2|	8365	+	A	0	0	0	0	3	SA0006
gi|29165615|ref|NC_002745.2|	8370	+	G	0	0	0	1	0	SA0006
gi|29165615|ref|NC_002745.2|	8383	+	C	0	0	0	1	0	SA0006
gi|29165615|ref|NC_002745.2|	8384	+	U	1	0	0	0	0	SA0006
gi|29165615|ref|NC_002745.2|	8405	+	A	0	1	0	0	0	SA0006
gi|29165615|ref|NC_002745.2|	8411	+	U	1	0	0	0	0	SA0006
gi|29165615|ref|NC_002745.2|	8428	+	A	2	0	0	0	0	SA0006
gi|29165615|ref|NC_002745.2|	8441	+	U	2	0	0	0	0	SA0006
gi|29165615|ref|NC_002745.2|	8523	+	A	0	0	0	0	1	SA0006
gi|29165615|ref|NC_002745.2|	8545	+	A	0	0	0	1	0	SA0006
gi|29165615|ref|NC_002745.2|	8552	+	C	1	0	0	0	0	SA0006
gi|29165615|ref|NC_002745.2|	8590	+	A	0	0	1	0	0	SA0006
gi|29165615|ref|NC_002745.2|	8673	+	C	1	0	0	0	0	SA0006
gi|29165615|ref|NC_002745.2|	8695	+	A	0	1	0	0	0	SA0006
gi|29165615|ref|NC_002745.2|	8714	+	U	0	0	1	0	1	SA0006
gi|29165615|ref|NC_002745.2|	8719	+	G	2	0	0	0	1	SA0006

gi|29165615|ref|NC_002745.2|	8722 +	A	1	0	0	0	0	SA0006
gi|29165615|ref|NC_002745.2|	8728 +	U	2	0	0	0	0	SA0006
gi|29165615|ref|NC_002745.2|	8745 +	C	0	1	0	0	0	SA0006
gi|29165615|ref|NC_002745.2|	8752 +	A	1	0	0	0	0	SA0006
gi|29165615|ref|NC_002745.2|	8761 +	C	1	0	0	0	0	SA0006
gi|29165615|ref|NC_002745.2|	8767 +	U	1	0	0	0	0	SA0006
gi|29165615|ref|NC_002745.2|	8777 +	U	0	0	0	0	2	SA0006
gi|29165615|ref|NC_002745.2|	8782 +	U	0	0	0	0	1	SA0006
gi|29165615|ref|NC_002745.2|	8788 +	A	0	1	0	0	0	SA0006
gi|29165615|ref|NC_002745.2|	8811 +	A	1	0	0	0	0	SA0006
gi|29165615|ref|NC_002745.2|	8911 +	A	0	1	0	0	0	SA0006
gi|29165615|ref|NC_002745.2|	9022 +	U	1	0	0	0	0	SA0006
gi|29165615|ref|NC_002745.2|	9061 +	C	0	0	0	1	0	SA0006
gi|29165615|ref|NC_002745.2|	9064 +	C	1	0	0	0	0	SA0006
gi|29165615|ref|NC_002745.2|	9070 +	G	1	0	0	0	0	SA0006
gi|29165615|ref|NC_002745.2|	9136 +	G	1	0	0	0	0	SA0006
gi|29165615|ref|NC_002745.2|	9200 +	U	0	1	0	0	0	SA0006
gi|29165615|ref|NC_002745.2|	9313 +	C	1	0	0	0	0	SA0006
gi|29165615|ref|NC_002745.2|	9315 +	A	2	0	0	0	0	SA0006
gi|29165615|ref|NC_002745.2|	9353 +	U	1	0	0	0	0	SA0006
gi|29165615|ref|NC_002745.2|	9434 +	A	0	0	0	1	0	SA0006
gi|29165615|ref|NC_002745.2|	9438 +	G	0	0	0	0	1	SA0006
gi|29165615|ref|NC_002745.2|	9441 +	G	0	1	0	0	0	SA0006
gi|29165615|ref|NC_002745.2|	9499 +	G	0	0	0	0	1	SA0006
gi|29165615|ref|NC_002745.2|	9537 +	A	0	1	0	0	0	SA0006
gi|29165615|ref|NC_002745.2|	9552 +	A	0	0	0	0	1	SA0006
gi|29165615|ref|NC_002745.2|	9612 +	C	1	0	0	0	0	SA0006
gi|29165615|ref|NC_002745.2|	9621 +	A	0	0	1	0	0	SA0006
gi|29165615|ref|NC_002745.2|	10858 +	A	0	0	0	0	1	-
gi|29165615|ref|NC_002745.2|	10861 +	A	0	0	0	2	1	-
gi|29165615|ref|NC_002745.2|	10862 +	A	3	17	5	33	23	-
gi|29165615|ref|NC_002745.2|	10863 +	G	1	6	6	9	11	-
gi|29165615|ref|NC_002745.2|	10864 +	G	0	0	0	1	0	-
gi|29165615|ref|NC_002745.2|	10873 +	C	0	0	0	0	1	-
gi|29165615|ref|NC_002745.2|	10874 +	U	0	2	0	0	1	-
gi|29165615|ref|NC_002745.2|	10875 +	U	0	0	0	0	2	-
gi|29165615|ref|NC_002745.2|	10876 +	A	0	1	0	3	0	-
gi|29165615|ref|NC_002745.2|	10877 +	A	0	0	0	1	0	-
gi|29165615|ref|NC_002745.2|	10879 +	A	0	0	0	1	0	SA0008
gi|29165615|ref|NC_002745.2|	10880 +	U	0	0	1	1	2	SA0008
gi|29165615|ref|NC_002745.2|	10881 +	A	0	1	1	0	0	SA0008
gi|29165615|ref|NC_002745.2|	10882 +	A	0	1	0	0	0	SA0008
gi|29165615|ref|NC_002745.2|	10884 +	C	0	0	1	0	0	SA0008
gi|29165615|ref|NC_002745.2|	10897 +	U	0	0	0	1	0	SA0008
gi|29165615|ref|NC_002745.2|	10909 +	U	0	1	0	2	6	SA0008
gi|29165615|ref|NC_002745.2|	10910 +	A	0	0	0	3	0	SA0008
gi|29165615|ref|NC_002745.2|	10919 +	G	0	1	0	0	0	SA0008
gi|29165615|ref|NC_002745.2|	10920 +	U	0	0	0	1	0	SA0008
gi|29165615|ref|NC_002745.2|	10929 +	A	0	0	0	0	2	SA0008
gi|29165615|ref|NC_002745.2|	10931 +	C	0	0	0	1	0	SA0008
gi|29165615|ref|NC_002745.2|	10932 +	A	0	0	0	1	0	SA0008
gi|29165615|ref|NC_002745.2|	10933 +	A	0	1	0	0	0	SA0008
gi|29165615|ref|NC_002745.2|	10937 +	A	0	0	0	0	1	SA0008
gi|29165615|ref|NC_002745.2|	10939 +	G	0	0	0	0	1	SA0008
gi|29165615|ref|NC_002745.2|	10956 +	A	0	3	0	3	4	SA0008
gi|29165615|ref|NC_002745.2|	10959 +	A	0	0	0	1	1	SA0008
gi|29165615|ref|NC_002745.2|	10962 +	A	0	0	1	1	1	SA0008
gi|29165615|ref|NC_002745.2|	10963 +	C	0	0	0	5	1	SA0008
gi|29165615|ref|NC_002745.2|	10965 +	A	0	1	0	1	0	SA0008
gi|29165615|ref|NC_002745.2|	10966 +	U	0	1	0	0	0	SA0008
gi|29165615|ref|NC_002745.2|	10967 +	C	0	0	0	1	0	SA0008
gi|29165615|ref|NC_002745.2|	10969 +	A	0	1	0	0	0	SA0008
gi|29165615|ref|NC_002745.2|	10970 +	A	0	0	0	0	1	SA0008
gi|29165615|ref|NC_002745.2|	10976 +	A	0	1	0	2	1	SA0008
gi|29165615|ref|NC_002745.2|	10978 +	A	0	2	0	0	0	SA0008
gi|29165615|ref|NC_002745.2|	10992 +	G	0	0	1	0	0	SA0008
gi|29165615|ref|NC_002745.2|	11009 +	A	0	0	0	0	1	SA0008
gi|29165615|ref|NC_002745.2|	11012 +	G	0	0	1	0	0	SA0008
gi|29165615|ref|NC_002745.2|	11035 +	A	0	0	0	1	0	SA0008
gi|29165615|ref|NC_002745.2|	11039 +	A	0	1	0	1	1	SA0008
gi|29165615|ref|NC_002745.2|	11043 +	U	0	0	0	3	0	SA0008
gi|29165615|ref|NC_002745.2|	11044 +	G	0	0	0	0	1	SA0008
gi|29165615|ref|NC_002745.2|	11045 +	A	0	0	0	1	2	SA0008
gi|29165615|ref|NC_002745.2|	11078 +	G	0	0	0	0	1	SA0008
gi|29165615|ref|NC_002745.2|	11109 +	G	0	0	0	0	1	SA0008
gi|29165615|ref|NC_002745.2|	11110 +	C	0	0	0	1	0	SA0008
gi|29165615|ref|NC_002745.2|	11120 +	A	0	0	0	0	1	SA0008
gi|29165615|ref|NC_002745.2|	11125 +	C	0	0	0	1	0	SA0008
gi|29165615|ref|NC_002745.2|	11126 +	A	0	1	0	0	0	SA0008
gi|29165615|ref|NC_002745.2|	11131 +	A	0	0	0	0	2	SA0008
gi|29165615|ref|NC_002745.2|	11144 +	C	0	0	0	1	0	SA0008

gi|29165615|ref|NC_002745.2|	11191	+	G	0	0	0	1	0	SA0008
gi|29165615|ref|NC_002745.2|	11197	+	A	0	1	0	0	0	SA0008
gi|29165615|ref|NC_002745.2|	11206	+	C	0	0	0	3	0	SA0008
gi|29165615|ref|NC_002745.2|	11212	+	A	0	0	0	0	2	SA0008
gi|29165615|ref|NC_002745.2|	11215	+	A	0	1	0	0	2	SA0008
gi|29165615|ref|NC_002745.2|	11218	+	U	0	2	0	0	0	SA0008
gi|29165615|ref|NC_002745.2|	11223	+	A	0	0	0	1	0	SA0008
gi|29165615|ref|NC_002745.2|	11255	+	U	0	0	0	1	2	SA0008
gi|29165615|ref|NC_002745.2|	11256	+	G	0	2	0	1	1	SA0008
gi|29165615|ref|NC_002745.2|	11257	+	A	0	1	0	0	1	SA0008
gi|29165615|ref|NC_002745.2|	11258	+	G	0	1	0	2	0	SA0008
gi|29165615|ref|NC_002745.2|	11259	+	A	0	2	0	3	7	SA0008
gi|29165615|ref|NC_002745.2|	11261	+	A	0	0	0	0	1	SA0008
gi|29165615|ref|NC_002745.2|	11262	+	A	1	0	0	0	0	SA0008
gi|29165615|ref|NC_002745.2|	11268	+	A	0	1	0	1	0	SA0008
gi|29165615|ref|NC_002745.2|	11272	+	U	0	2	0	4	0	SA0008
gi|29165615|ref|NC_002745.2|	11273	+	U	0	0	0	2	0	SA0008
gi|29165615|ref|NC_002745.2|	11275	+	A	1	2	0	1	2	SA0008
gi|29165615|ref|NC_002745.2|	11276	+	U	0	2	0	4	7	SA0008
gi|29165615|ref|NC_002745.2|	11277	+	A	0	8	2	7	5	SA0008
gi|29165615|ref|NC_002745.2|	11278	+	A	0	0	0	5	0	SA0008
gi|29165615|ref|NC_002745.2|	11279	+	A	0	0	0	1	1	SA0008
gi|29165615|ref|NC_002745.2|	11281	+	G	0	2	0	4	0	SA0008
gi|29165615|ref|NC_002745.2|	11282	+	A	1	0	0	2	1	SA0008
gi|29165615|ref|NC_002745.2|	11284	+	C	0	0	0	1	1	SA0008
gi|29165615|ref|NC_002745.2|	11286	+	U	0	3	0	5	1	SA0008
gi|29165615|ref|NC_002745.2|	11287	+	A	0	0	0	0	1	SA0008
gi|29165615|ref|NC_002745.2|	11289	+	U	0	0	2	0	0	SA0008
gi|29165615|ref|NC_002745.2|	11291	+	U	0	0	0	0	2	SA0008
gi|29165615|ref|NC_002745.2|	11295	+	G	0	0	0	2	0	SA0008
gi|29165615|ref|NC_002745.2|	11296	+	A	0	0	0	0	1	SA0008
gi|29165615|ref|NC_002745.2|	11301	+	C	0	1	0	0	0	SA0008
gi|29165615|ref|NC_002745.2|	11304	+	A	0	2	0	1	1	SA0008
gi|29165615|ref|NC_002745.2|	11305	+	C	0	0	0	1	0	SA0008
gi|29165615|ref|NC_002745.2|	11306	+	A	0	0	1	4	0	SA0008
gi|29165615|ref|NC_002745.2|	11307	+	A	0	0	0	0	1	SA0008
gi|29165615|ref|NC_002745.2|	11320	+	G	0	0	0	0	1	SA0008
gi|29165615|ref|NC_002745.2|	11327	+	C	0	0	0	1	1	SA0008
gi|29165615|ref|NC_002745.2|	11330	+	G	1	0	0	0	0	SA0008
gi|29165615|ref|NC_002745.2|	11340	+	G	0	2	0	0	0	SA0008
gi|29165615|ref|NC_002745.2|	11344	+	U	0	0	0	1	0	SA0008
gi|29165615|ref|NC_002745.2|	11345	+	U	0	0	0	1	0	SA0008
gi|29165615|ref|NC_002745.2|	11354	+	U	1	0	0	0	0	SA0008
gi|29165615|ref|NC_002745.2|	11362	+	G	0	0	0	1	0	SA0008
gi|29165615|ref|NC_002745.2|	11391	+	C	0	0	1	0	0	SA0008
gi|29165615|ref|NC_002745.2|	11396	+	G	0	0	0	1	0	SA0008
gi|29165615|ref|NC_002745.2|	11414	+	A	0	0	0	0	1	SA0008
gi|29165615|ref|NC_002745.2|	11427	+	A	0	0	0	0	2	SA0008
gi|29165615|ref|NC_002745.2|	11429	+	A	0	1	0	0	0	SA0008
gi|29165615|ref|NC_002745.2|	11436	+	U	0	1	0	0	1	SA0008
gi|29165615|ref|NC_002745.2|	11438	+	G	0	0	2	0	0	SA0008
gi|29165615|ref|NC_002745.2|	11456	+	A	0	0	0	0	1	SA0008
gi|29165615|ref|NC_002745.2|	11486	+	G	0	1	0	0	1	SA0008
gi|29165615|ref|NC_002745.2|	11487	+	U	0	0	0	0	1	SA0008
gi|29165615|ref|NC_002745.2|	11491	+	C	0	1	0	0	0	SA0008
gi|29165615|ref|NC_002745.2|	11531	+	C	0	0	0	1	0	SA0008
gi|29165615|ref|NC_002745.2|	11579	+	A	0	1	0	0	0	SA0008
gi|29165615|ref|NC_002745.2|	11603	+	C	0	0	0	1	0	SA0008
gi|29165615|ref|NC_002745.2|	11665	+	A	0	0	0	1	0	SA0008
gi|29165615|ref|NC_002745.2|	11690	+	C	0	0	1	0	0	SA0008
gi|29165615|ref|NC_002745.2|	11692	+	U	0	0	1	0	0	SA0008
gi|29165615|ref|NC_002745.2|	11778	+	A	0	0	0	1	2	SA0008
gi|29165615|ref|NC_002745.2|	11797	+	C	0	1	0	1	0	SA0008
gi|29165615|ref|NC_002745.2|	11811	+	A	0	0	0	1	0	SA0008
gi|29165615|ref|NC_002745.2|	11852	+	C	0	0	0	1	0	SA0008
gi|29165615|ref|NC_002745.2|	11854	+	A	0	0	0	0	1	SA0008
gi|29165615|ref|NC_002745.2|	11934	+	A	0	0	0	1	0	SA0008
gi|29165615|ref|NC_002745.2|	12067	+	G	0	0	0	0	1	SA0008
gi|29165615|ref|NC_002745.2|	12098	+	C	0	1	0	0	0	SA0008
gi|29165615|ref|NC_002745.2|	12107	+	C	0	1	0	0	0	SA0008
gi|29165615|ref|NC_002745.2|	12143	+	A	0	0	0	1	0	SA0008
gi|29165615|ref|NC_002745.2|	12146	+	A	0	0	0	0	1	SA0008
gi|29165615|ref|NC_002745.2|	12150	+	C	0	0	0	1	0	SA0008
gi|29165615|ref|NC_002745.2|	12152	+	U	0	0	0	1	3	SA0008
gi|29165615|ref|NC_002745.2|	12182	+	G	0	0	0	0	2	SA0008
gi|29165615|ref|NC_002745.2|	12183	+	U	0	0	0	1	0	SA0008
gi|29165615|ref|NC_002745.2|	12185	+	A	0	0	0	3	0	SA0008
gi|29165615|ref|NC_002745.2|	12188	+	A	0	0	0	0	1	SA0008
gi|29165615|ref|NC_002745.2|	12189	+	A	0	0	0	0	1	SA0008
gi|29165615|ref|NC_002745.2|	12190	+	A	0	0	0	1	0	SA0008
gi|29165615|ref|NC_002745.2|	12196	+	G	0	0	0	2	0	SA0008

gi|29165615|ref|NC_002745.2|	12197	+	A	0	0	0	4	0	SA0008
gi|29165615|ref|NC_002745.2|	12198	+	A	0	1	0	0	0	SA0008
gi|29165615|ref|NC_002745.2|	12199	+	A	0	1	0	2	6	SA0008
gi|29165615|ref|NC_002745.2|	12270	+	A	0	0	0	3	0	SA0008
gi|29165615|ref|NC_002745.2|	12292	+	A	0	1	0	0	0	SA0008
gi|29165615|ref|NC_002745.2|	12293	+	A	0	1	0	0	0	SA0008
gi|29165615|ref|NC_002745.2|	12368	+	C	0	0	0	0	1	SA0008
gi|29165615|ref|NC_002745.2|	12480	+	A	3	5	2	3	7	-
gi|29165615|ref|NC_002745.2|	12481	+	A	0	1	0	0	0	-
gi|29165615|ref|NC_002745.2|	12484	+	U	0	0	0	0	1	-
gi|29165615|ref|NC_002745.2|	12497	+	U	0	0	1	0	0	-
gi|29165615|ref|NC_002745.2|	12508	+	A	0	0	0	1	0	-
gi|29165615|ref|NC_002745.2|	12593	+	A	0	2	1	0	0	-
gi|29165615|ref|NC_002745.2|	12594	+	A	0	0	0	0	1	-
gi|29165615|ref|NC_002745.2|	12595	+	A	0	0	1	4	2	-
gi|29165615|ref|NC_002745.2|	12605	+	G	0	0	1	0	0	-
gi|29165615|ref|NC_002745.2|	12608	+	A	0	0	1	0	0	-
gi|29165615|ref|NC_002745.2|	12615	+	U	0	0	1	0	0	-
gi|29165615|ref|NC_002745.2|	12619	+	U	1	0	0	0	0	-
gi|29165615|ref|NC_002745.2|	12621	+	U	0	3	0	0	0	-
gi|29165615|ref|NC_002745.2|	12622	+	A	0	0	0	0	1	-
gi|29165615|ref|NC_002745.2|	12623	+	G	0	0	0	1	1	-
gi|29165615|ref|NC_002745.2|	12624	+	U	134	94	70	24	48	-
gi|29165615|ref|NC_002745.2|	12625	+	G	4	2	4	2	5	-
gi|29165615|ref|NC_002745.2|	12626	+	A	1	0	0	0	0	-
gi|29165615|ref|NC_002745.2|	12662	+	A	0	1	0	0	0	-
gi|29165615|ref|NC_002745.2|	12664	+	U	0	0	1	0	0	-
gi|29165615|ref|NC_002745.2|	12667	+	U	0	1	1	0	1	-
gi|29165615|ref|NC_002745.2|	12670	+	A	0	0	1	0	0	-
gi|29165615|ref|NC_002745.2|	12694	+	C	2	0	0	0	0	-
gi|29165615|ref|NC_002745.2|	12707	+	G	0	0	0	1	0	-
gi|29165615|ref|NC_002745.2|	12738	+	A	0	2	0	0	0	-
gi|29165615|ref|NC_002745.2|	12759	+	U	0	0	2	0	0	-
gi|29165615|ref|NC_002745.2|	12760	+	A	0	0	1	0	0	-
gi|29165615|ref|NC_002745.2|	12761	+	A	0	2	1	0	0	-
gi|29165615|ref|NC_002745.2|	12762	+	A	0	1	0	0	3	-
gi|29165615|ref|NC_002745.2|	12764	+	G	0	2	0	1	0	-
gi|29165615|ref|NC_002745.2|	12765	+	A	5	69	55	5	36	-
gi|29165615|ref|NC_002745.2|	12766	+	U	0	3	0	0	4	-
gi|29165615|ref|NC_002745.2|	12767	+	A	1	3	2	0	1	-
gi|29165615|ref|NC_002745.2|	12768	+	A	1	12	13	2	12	-
gi|29165615|ref|NC_002745.2|	12769	+	U	1	1	0	0	1	-
gi|29165615|ref|NC_002745.2|	12770	+	U	0	1	0	0	1	-
gi|29165615|ref|NC_002745.2|	12771	+	A	0	2	4	0	1	-
gi|29165615|ref|NC_002745.2|	12772	+	U	0	3	1	0	1	-
gi|29165615|ref|NC_002745.2|	12776	+	G	0	0	0	1	1	-
gi|29165615|ref|NC_002745.2|	12778	+	A	0	1	1	1	3	-
gi|29165615|ref|NC_002745.2|	12780	+	A	0	0	0	1	0	-
gi|29165615|ref|NC_002745.2|	12781	+	G	0	1	0	0	0	-
gi|29165615|ref|NC_002745.2|	12782	+	G	0	0	0	0	1	-
gi|29165615|ref|NC_002745.2|	12786	+	A	0	3	1	0	0	-
gi|29165615|ref|NC_002745.2|	12789	+	G	0	0	1	0	0	-
gi|29165615|ref|NC_002745.2|	12792	+	A	0	0	1	1	0	-
gi|29165615|ref|NC_002745.2|	12793	+	A	0	0	1	0	0	SA0009
gi|29165615|ref|NC_002745.2|	12800	+	A	0	1	0	0	0	SA0009
gi|29165615|ref|NC_002745.2|	12812	+	U	0	0	0	0	1	SA0009
gi|29165615|ref|NC_002745.2|	12815	+	G	0	1	0	0	0	SA0009
gi|29165615|ref|NC_002745.2|	12816	+	A	0	0	1	0	1	SA0009
gi|29165615|ref|NC_002745.2|	12827	+	A	0	0	0	0	1	SA0009
gi|29165615|ref|NC_002745.2|	12842	+	A	0	0	1	0	0	SA0009
gi|29165615|ref|NC_002745.2|	12883	+	A	0	1	0	0	0	SA0009
gi|29165615|ref|NC_002745.2|	12938	+	A	0	0	0	0	1	SA0009
gi|29165615|ref|NC_002745.2|	12946	+	C	2	0	0	0	0	SA0009
gi|29165615|ref|NC_002745.2|	12952	+	A	0	0	0	0	1	SA0009
gi|29165615|ref|NC_002745.2|	12957	+	A	0	0	0	0	1	SA0009
gi|29165615|ref|NC_002745.2|	12968	+	U	1	0	0	0	0	SA0009
gi|29165615|ref|NC_002745.2|	12985	+	A	0	0	0	2	1	SA0009
gi|29165615|ref|NC_002745.2|	12986	+	A	0	1	0	0	1	SA0009
gi|29165615|ref|NC_002745.2|	12988	+	A	0	1	0	0	2	SA0009
gi|29165615|ref|NC_002745.2|	12989	+	A	0	1	3	0	3	SA0009
gi|29165615|ref|NC_002745.2|	12991	+	G	0	0	1	0	0	SA0009
gi|29165615|ref|NC_002745.2|	12992	+	A	0	1	3	0	1	SA0009
gi|29165615|ref|NC_002745.2|	13037	+	A	0	1	0	0	0	SA0009
gi|29165615|ref|NC_002745.2|	13052	+	A	0	0	0	0	1	SA0009
gi|29165615|ref|NC_002745.2|	13073	+	A	0	0	1	0	2	SA0009
gi|29165615|ref|NC_002745.2|	13077	+	U	0	0	0	0	1	SA0009
gi|29165615|ref|NC_002745.2|	13079	+	A	0	1	0	0	0	SA0009
gi|29165615|ref|NC_002745.2|	13080	+	A	0	0	0	0	1	SA0009
gi|29165615|ref|NC_002745.2|	13081	+	A	0	1	0	0	0	SA0009
gi|29165615|ref|NC_002745.2|	13084	+	A	0	1	0	0	0	SA0009
gi|29165615|ref|NC_002745.2|	13114	+	A	0	1	0	0	0	SA0009

gi|29165615|ref|NC_002745.2|	13184	+	G	1	0	0	0	0	SA0009
gi|29165615|ref|NC_002745.2|	13210	+	C	0	0	0	0	1	SA0009
gi|29165615|ref|NC_002745.2|	13226	+	A	0	1	0	0	0	SA0009
gi|29165615|ref|NC_002745.2|	13229	+	A	0	2	2	0	0	SA0009
gi|29165615|ref|NC_002745.2|	13234	+	A	0	1	1	0	0	SA0009
gi|29165615|ref|NC_002745.2|	13241	+	C	0	0	1	0	1	SA0009
gi|29165615|ref|NC_002745.2|	13244	+	A	0	3	2	0	0	SA0009
gi|29165615|ref|NC_002745.2|	13247	+	U	1	0	0	0	0	SA0009
gi|29165615|ref|NC_002745.2|	13251	+	U	0	1	1	0	0	SA0009
gi|29165615|ref|NC_002745.2|	13275	+	G	1	0	0	0	0	SA0009
gi|29165615|ref|NC_002745.2|	13283	+	U	2	0	0	0	0	SA0009
gi|29165615|ref|NC_002745.2|	13288	+	U	0	0	0	0	2	SA0009
gi|29165615|ref|NC_002745.2|	13292	+	C	0	3	0	0	0	SA0009
gi|29165615|ref|NC_002745.2|	13310	+	U	0	1	0	0	0	SA0009
gi|29165615|ref|NC_002745.2|	13334	+	U	0	0	1	0	0	SA0009
gi|29165615|ref|NC_002745.2|	13336	+	A	0	3	0	0	0	SA0009
gi|29165615|ref|NC_002745.2|	13342	+	A	0	0	1	0	0	SA0009
gi|29165615|ref|NC_002745.2|	13345	+	C	0	0	0	0	3	SA0009
gi|29165615|ref|NC_002745.2|	13369	+	G	0	2	0	0	0	SA0009
gi|29165615|ref|NC_002745.2|	13376	+	U	0	0	1	0	0	SA0009
gi|29165615|ref|NC_002745.2|	13413	+	U	0	0	0	1	0	SA0009
gi|29165615|ref|NC_002745.2|	13457	+	A	0	1	0	0	0	SA0009
gi|29165615|ref|NC_002745.2|	13460	+	A	0	0	1	0	0	SA0009
gi|29165615|ref|NC_002745.2|	13475	+	C	0	1	0	0	1	SA0009
gi|29165615|ref|NC_002745.2|	13481	+	C	1	0	0	0	0	SA0009
gi|29165615|ref|NC_002745.2|	13551	+	A	0	1	0	0	0	SA0009
gi|29165615|ref|NC_002745.2|	13742	+	A	0	1	0	0	0	SA0009
gi|29165615|ref|NC_002745.2|	13766	+	G	0	0	0	0	1	SA0009
gi|29165615|ref|NC_002745.2|	13818	+	A	1	0	0	0	0	SA0009
gi|29165615|ref|NC_002745.2|	13851	+	C	0	0	0	1	0	SA0009
gi|29165615|ref|NC_002745.2|	13862	+	C	0	0	0	0	1	SA0009
gi|29165615|ref|NC_002745.2|	13869	+	C	1	0	0	0	0	SA0009
gi|29165615|ref|NC_002745.2|	13870	+	C	0	0	0	0	1	SA0009
gi|29165615|ref|NC_002745.2|	13871	+	A	0	0	0	0	1	SA0009
gi|29165615|ref|NC_002745.2|	13959	+	U	0	0	1	0	0	SA0009
gi|29165615|ref|NC_002745.2|	13988	+	A	0	2	0	0	0	SA0009
gi|29165615|ref|NC_002745.2|	14350	+	A	0	0	0	0	1	-
gi|29165615|ref|NC_002745.2|	14676	+	U	0	0	1	0	0	-
gi|29165615|ref|NC_002745.2|	14679	+	A	3	4	1	1	2	-
gi|29165615|ref|NC_002745.2|	14695	+	U	2	0	1	0	0	-
gi|29165615|ref|NC_002745.2|	14701	+	A	1	1	1	0	0	-
gi|29165615|ref|NC_002745.2|	14851	+	A	0	1	0	1	0	SA0010
gi|29165615|ref|NC_002745.2|	14852	+	A	0	0	0	1	0	SA0010
gi|29165615|ref|NC_002745.2|	14867	+	U	0	1	0	0	0	SA0010
gi|29165615|ref|NC_002745.2|	14875	+	U	0	0	0	1	0	SA0010
gi|29165615|ref|NC_002745.2|	14964	+	A	2	0	0	0	0	SA0010
gi|29165615|ref|NC_002745.2|	14974	+	U	1	0	0	0	0	SA0010
gi|29165615|ref|NC_002745.2|	14980	+	U	0	0	1	0	0	SA0010
gi|29165615|ref|NC_002745.2|	14985	+	A	0	0	0	1	0	SA0010
gi|29165615|ref|NC_002745.2|	15011	+	G	0	0	1	0	0	SA0010
gi|29165615|ref|NC_002745.2|	15045	+	U	0	0	0	1	0	SA0010
gi|29165615|ref|NC_002745.2|	15061	+	A	0	0	0	0	1	SA0010
gi|29165615|ref|NC_002745.2|	15082	+	C	0	2	0	0	0	SA0010
gi|29165615|ref|NC_002745.2|	15103	+	C	0	0	0	0	1	SA0010
gi|29165615|ref|NC_002745.2|	15107	+	C	2	0	0	0	0	SA0010
gi|29165615|ref|NC_002745.2|	15128	+	U	0	1	0	0	0	SA0010
gi|29165615|ref|NC_002745.2|	15156	+	G	0	1	0	0	0	SA0010
gi|29165615|ref|NC_002745.2|	15250	+	U	1	0	0	0	0	SA0010
gi|29165615|ref|NC_002745.2|	15338	+	A	0	0	1	0	0	SA0010
gi|29165615|ref|NC_002745.2|	15500	+	A	0	0	1	0	0	SAS001
gi|29165615|ref|NC_002745.2|	15516	+	U	2	0	0	0	0	SAS001
gi|29165615|ref|NC_002745.2|	15573	+	C	0	0	0	0	1	SAS001
gi|29165615|ref|NC_002745.2|	15883	+	C	0	1	0	0	0	-
gi|29165615|ref|NC_002745.2|	15886	+	U	0	0	1	0	0	-
gi|29165615|ref|NC_002745.2|	15888	+	A	1	0	1	0	0	-
gi|29165615|ref|NC_002745.2|	16275	+	A	0	0	0	0	1	SA0011
gi|29165615|ref|NC_002745.2|	16333	+	C	0	1	0	0	0	SA0011
gi|29165615|ref|NC_002745.2|	16488	+	U	0	0	0	1	0	SA0011
gi|29165615|ref|NC_002745.2|	16489	+	A	0	0	0	0	1	SA0011
gi|29165615|ref|NC_002745.2|	16621	+	A	0	0	0	0	1	SA0011
gi|29165615|ref|NC_002745.2|	16645	+	G	0	4	0	0	0	SA0011
gi|29165615|ref|NC_002745.2|	16652	+	A	1	0	0	0	0	SA0011
gi|29165615|ref|NC_002745.2|	16721	+	G	0	0	0	0	1	SA0011
gi|29165615|ref|NC_002745.2|	16780	+	C	0	1	0	0	0	SA0011
gi|29165615|ref|NC_002745.2|	17065	+	C	0	1	0	0	0	SA0011
gi|29165615|ref|NC_002745.2|	17361	+	C	1	5	4	2	2	SA0012
gi|29165615|ref|NC_002745.2|	17362	+	U	0	0	0	1	0	SA0012
gi|29165615|ref|NC_002745.2|	17363	+	A	6	4	8	2	5	SA0012
gi|29165615|ref|NC_002745.2|	17370	+	A	0	1	0	0	0	SA0012
gi|29165615|ref|NC_002745.2|	17372	+	A	0	2	0	0	0	SA0012
gi|29165615|ref|NC_002745.2|	17398	+	A	0	1	0	0	0	SA0012

gi|29165615|ref|NC_002745.2|	17399 +	A	0	2	1	0	0	SA0012
gi|29165615|ref|NC_002745.2|	17401 +	A	0	0	0	1	1	SA0012
gi|29165615|ref|NC_002745.2|	17528 +	A	0	2	0	0	0	SA0012
gi|29165615|ref|NC_002745.2|	17531 +	A	0	1	1	1	3	SA0012
gi|29165615|ref|NC_002745.2|	17532 +	A	0	0	0	0	1	SA0012
gi|29165615|ref|NC_002745.2|	17534 +	U	0	0	3	3	4	SA0012
gi|29165615|ref|NC_002745.2|	17535 +	C	0	0	1	0	1	SA0012
gi|29165615|ref|NC_002745.2|	17536 +	A	0	1	0	0	0	SA0012
gi|29165615|ref|NC_002745.2|	17539 +	A	0	0	2	1	1	SA0012
gi|29165615|ref|NC_002745.2|	17540 +	C	0	0	0	0	1	SA0012
gi|29165615|ref|NC_002745.2|	17542 +	A	0	0	0	0	1	SA0012
gi|29165615|ref|NC_002745.2|	17573 +	A	0	0	0	1	0	SA0012
gi|29165615|ref|NC_002745.2|	17575 +	U	1	0	0	0	0	SA0012
gi|29165615|ref|NC_002745.2|	17586 +	U	0	1	2	3	3	SA0012
gi|29165615|ref|NC_002745.2|	17601 +	U	0	1	0	0	0	SA0012
gi|29165615|ref|NC_002745.2|	17608 +	A	0	1	0	0	0	SA0012
gi|29165615|ref|NC_002745.2|	17634 +	C	0	0	0	1	0	SA0012
gi|29165615|ref|NC_002745.2|	17641 +	A	0	0	1	1	0	SA0012
gi|29165615|ref|NC_002745.2|	17653 +	A	0	3	0	0	0	SA0012
gi|29165615|ref|NC_002745.2|	17660 +	A	0	1	0	0	0	SA0012
gi|29165615|ref|NC_002745.2|	17714 +	U	0	0	0	0	1	SA0012
gi|29165615|ref|NC_002745.2|	17715 +	U	1	0	1	0	1	SA0012
gi|29165615|ref|NC_002745.2|	17791 +	C	0	1	1	0	0	SA0012
gi|29165615|ref|NC_002745.2|	17795 +	C	0	0	1	0	0	SA0012
gi|29165615|ref|NC_002745.2|	17797 +	A	0	0	0	0	1	SA0012
gi|29165615|ref|NC_002745.2|	17798 +	A	0	1	0	0	1	SA0012
gi|29165615|ref|NC_002745.2|	17812 +	G	0	1	0	0	0	SA0012
gi|29165615|ref|NC_002745.2|	17813 +	A	0	1	0	0	0	SA0012
gi|29165615|ref|NC_002745.2|	17816 +	A	0	0	1	0	0	SA0012
gi|29165615|ref|NC_002745.2|	17830 +	C	0	1	0	0	0	SA0012
gi|29165615|ref|NC_002745.2|	17852 +	U	0	0	1	0	0	SA0012
gi|29165615|ref|NC_002745.2|	17932 +	C	0	2	0	0	0	SA0012
gi|29165615|ref|NC_002745.2|	17966 +	A	0	1	0	0	0	SA0012
gi|29165615|ref|NC_002745.2|	17967 +	A	0	1	0	0	1	SA0012
gi|29165615|ref|NC_002745.2|	17968 +	A	0	0	1	0	0	SA0012
gi|29165615|ref|NC_002745.2|	18028 +	G	0	0	0	2	0	SA0012
gi|29165615|ref|NC_002745.2|	18029 +	A	0	0	0	2	0	SA0012
gi|29165615|ref|NC_002745.2|	18035 +	A	0	1	0	0	0	SA0012
gi|29165615|ref|NC_002745.2|	18044 +	A	0	0	1	0	0	SA0012
gi|29165615|ref|NC_002745.2|	18045 +	U	0	0	0	1	0	SA0012
gi|29165615|ref|NC_002745.2|	18052 +	U	0	1	0	0	0	SA0012
gi|29165615|ref|NC_002745.2|	18054 +	U	1	0	0	0	0	SA0012
gi|29165615|ref|NC_002745.2|	18055 +	A	0	2	1	1	1	SA0012
gi|29165615|ref|NC_002745.2|	18056 +	U	0	1	0	1	0	SA0012
gi|29165615|ref|NC_002745.2|	18074 +	A	0	0	0	1	0	SA0012
gi|29165615|ref|NC_002745.2|	18076 +	U	0	1	0	0	0	SA0012
gi|29165615|ref|NC_002745.2|	18113 +	U	0	0	1	0	0	SA0012
gi|29165615|ref|NC_002745.2|	18125 +	A	1	0	0	0	0	SA0012
gi|29165615|ref|NC_002745.2|	18127 +	G	0	1	1	5	5	SA0012
gi|29165615|ref|NC_002745.2|	18150 +	U	0	0	0	0	1	SA0012
gi|29165615|ref|NC_002745.2|	18157 +	C	2	0	0	0	1	SA0012
gi|29165615|ref|NC_002745.2|	18163 +	U	1	0	0	0	0	SA0012
gi|29165615|ref|NC_002745.2|	18167 +	G	1	0	0	0	0	SA0012
gi|29165615|ref|NC_002745.2|	18170 +	A	0	2	0	0	0	SA0012
gi|29165615|ref|NC_002745.2|	18210 +	U	1	0	0	0	0	SA0012
gi|29165615|ref|NC_002745.2|	18294 +	A	0	0	1	0	0	SA0012
gi|29165615|ref|NC_002745.2|	18300 +	A	0	1	0	0	0	SA0012
gi|29165615|ref|NC_002745.2|	18302 +	U	0	0	0	0	4	SA0012
gi|29165615|ref|NC_002745.2|	18308 +	A	0	0	0	0	1	SA0012
gi|29165615|ref|NC_002745.2|	18312 +	G	0	0	1	0	0	SA0012
gi|29165615|ref|NC_002745.2|	18391 +	A	0	2	0	0	0	SA0013
gi|29165615|ref|NC_002745.2|	18411 +	G	0	1	0	0	0	SA0013
gi|29165615|ref|NC_002745.2|	18426 +	U	0	1	4	2	2	SA0013
gi|29165615|ref|NC_002745.2|	18439 +	A	0	0	0	0	1	SA0013
gi|29165615|ref|NC_002745.2|	18478 +	A	1	0	0	0	0	SA0013
gi|29165615|ref|NC_002745.2|	18492 +	A	0	0	0	1	0	SA0013
gi|29165615|ref|NC_002745.2|	18508 +	A	0	0	0	0	1	SA0013
gi|29165615|ref|NC_002745.2|	18545 +	C	0	1	0	0	0	SA0013
gi|29165615|ref|NC_002745.2|	18554 +	A	0	0	0	0	1	SA0013
gi|29165615|ref|NC_002745.2|	18581 +	U	0	1	0	0	0	SA0013
gi|29165615|ref|NC_002745.2|	18594 +	U	0	1	0	0	0	SA0013
gi|29165615|ref|NC_002745.2|	18645 +	U	0	0	0	1	0	SA0013
gi|29165615|ref|NC_002745.2|	18675 +	A	0	2	0	0	0	SA0013
gi|29165615|ref|NC_002745.2|	18703 +	C	0	0	1	1	1	SA0013
gi|29165615|ref|NC_002745.2|	18704 +	A	0	0	0	0	1	SA0013
gi|29165615|ref|NC_002745.2|	18709 +	G	0	0	0	1	0	SA0013
gi|29165615|ref|NC_002745.2|	18716 +	U	1	0	0	0	0	SA0013
gi|29165615|ref|NC_002745.2|	18718 +	A	0	0	1	0	3	SA0013
gi|29165615|ref|NC_002745.2|	18728 +	A	0	0	1	0	0	SA0013
gi|29165615|ref|NC_002745.2|	18734 +	A	0	1	1	1	1	SA0013
gi|29165615|ref|NC_002745.2|	18805 +	G	0	0	0	1	0	SA0013

gi|29165615|ref|NC_002745.2|	18813 +	A	0	1	0	0	0	SA0013
gi|29165615|ref|NC_002745.2|	18814 +	C	0	1	0	0	0	SA0013
gi|29165615|ref|NC_002745.2|	18815 +	A	0	0	0	0	1	SA0013
gi|29165615|ref|NC_002745.2|	18818 +	C	0	0	0	0	1	SA0013
gi|29165615|ref|NC_002745.2|	18825 +	A	0	0	0	1	0	SA0013
gi|29165615|ref|NC_002745.2|	18833 +	A	0	0	1	0	1	SA0013
gi|29165615|ref|NC_002745.2|	18835 +	A	0	1	0	0	0	SA0013
gi|29165615|ref|NC_002745.2|	18841 +	A	0	1	1	0	0	SA0013
gi|29165615|ref|NC_002745.2|	18854 +	C	1	0	0	0	0	SA0013
gi|29165615|ref|NC_002745.2|	18855 +	G	0	0	0	0	1	SA0013
gi|29165615|ref|NC_002745.2|	18856 +	A	0	0	0	1	1	SA0013
gi|29165615|ref|NC_002745.2|	18857 +	C	0	0	0	1	0	SA0013
gi|29165615|ref|NC_002745.2|	18912 +	U	1	0	0	0	0	SA0013
gi|29165615|ref|NC_002745.2|	19020 +	A	0	0	0	0	1	SA0013
gi|29165615|ref|NC_002745.2|	19021 +	A	0	0	0	1	1	SA0013
gi|29165615|ref|NC_002745.2|	19023 +	A	0	2	2	3	6	SA0013
gi|29165615|ref|NC_002745.2|	19024 +	A	0	2	0	0	0	SA0013
gi|29165615|ref|NC_002745.2|	19025 +	U	0	1	0	0	1	SA0013
gi|29165615|ref|NC_002745.2|	19031 +	C	1	0	0	0	0	SA0013
gi|29165615|ref|NC_002745.2|	19049 +	A	0	0	1	0	0	SA0013
gi|29165615|ref|NC_002745.2|	19079 +	A	0	0	1	0	0	SA0013
gi|29165615|ref|NC_002745.2|	19081 +	A	0	1	0	0	0	SA0013
gi|29165615|ref|NC_002745.2|	19083 +	U	2	0	0	1	0	SA0013
gi|29165615|ref|NC_002745.2|	19089 +	U	1	0	0	0	0	SA0013
gi|29165615|ref|NC_002745.2|	19090 +	U	1	0	0	0	0	SA0013
gi|29165615|ref|NC_002745.2|	19111 +	A	0	2	0	0	0	SA0013
gi|29165615|ref|NC_002745.2|	19114 +	A	0	0	0	0	1	SA0013
gi|29165615|ref|NC_002745.2|	19136 +	A	0	1	0	0	0	SA0013
gi|29165615|ref|NC_002745.2|	19211 +	C	0	1	0	0	0	SA0013
gi|29165615|ref|NC_002745.2|	19218 +	A	0	1	0	0	0	SA0013
gi|29165615|ref|NC_002745.2|	19219 +	A	0	0	1	2	1	SA0013
gi|29165615|ref|NC_002745.2|	19220 +	G	0	0	0	1	0	SA0013
gi|29165615|ref|NC_002745.2|	19221 +	U	0	0	0	0	1	SA0013
gi|29165615|ref|NC_002745.2|	19223 +	U	0	0	0	1	0	SA0013
gi|29165615|ref|NC_002745.2|	19225 +	A	0	0	1	0	0	SA0013
gi|29165615|ref|NC_002745.2|	19233 +	U	1	0	0	0	0	SA0013
gi|29165615|ref|NC_002745.2|	19247 +	G	0	0	2	0	0	SA0013
gi|29165615|ref|NC_002745.2|	19263 +	G	0	4	0	0	0	SA0013
gi|29165615|ref|NC_002745.2|	19272 +	A	0	0	0	1	1	SA0013
gi|29165615|ref|NC_002745.2|	19273 +	C	1	0	0	0	0	SA0013
gi|29165615|ref|NC_002745.2|	19277 +	C	0	1	0	0	0	SA0013
gi|29165615|ref|NC_002745.2|	19286 +	G	1	3	0	0	0	SA0013
gi|29165615|ref|NC_002745.2|	19295 +	U	1	0	0	0	0	SA0013
gi|29165615|ref|NC_002745.2|	19296 +	G	0	1	0	0	0	SA0013
gi|29165615|ref|NC_002745.2|	19326 +	A	0	1	0	0	0	SA0013
gi|29165615|ref|NC_002745.2|	19391 +	U	0	0	0	0	1	SA0013
gi|29165615|ref|NC_002745.2|	19392 +	C	0	0	0	1	0	SA0013
gi|29165615|ref|NC_002745.2|	19410 +	A	0	1	0	0	0	SA0013
gi|29165615|ref|NC_002745.2|	19466 +	C	0	1	0	0	0	SA0013
gi|29165615|ref|NC_002745.2|	19467 +	A	0	0	0	0	1	SA0013
gi|29165615|ref|NC_002745.2|	19469 +	U	0	2	0	0	0	SA0013
gi|29165615|ref|NC_002745.2|	19470 +	A	0	0	1	0	0	SA0013
gi|29165615|ref|NC_002745.2|	19472 +	G	0	0	0	0	1	SA0013
gi|29165615|ref|NC_002745.2|	19473 +	A	0	3	0	0	0	SA0013
gi|29165615|ref|NC_002745.2|	19489 +	A	0	0	0	0	1	SA0013
gi|29165615|ref|NC_002745.2|	19493 +	A	0	0	2	0	0	SA0013
gi|29165615|ref|NC_002745.2|	19501 +	U	0	0	0	0	1	SA0013
gi|29165615|ref|NC_002745.2|	19505 +	A	0	1	0	0	0	SA0013
gi|29165615|ref|NC_002745.2|	19512 +	A	0	1	0	0	1	SA0013
gi|29165615|ref|NC_002745.2|	19517 +	G	0	0	0	1	0	SA0013
gi|29165615|ref|NC_002745.2|	19518 +	A	0	3	1	6	0	SA0013
gi|29165615|ref|NC_002745.2|	19519 +	U	0	0	2	0	1	SA0013
gi|29165615|ref|NC_002745.2|	19520 +	U	1	0	0	0	0	SA0013
gi|29165615|ref|NC_002745.2|	19606 +	G	0	0	0	1	0	SA0013
gi|29165615|ref|NC_002745.2|	19613 +	A	3	0	0	0	1	SA0013
gi|29165615|ref|NC_002745.2|	19616 +	U	0	1	0	0	0	SA0013
gi|29165615|ref|NC_002745.2|	19617 +	C	0	0	0	0	1	SA0013
gi|29165615|ref|NC_002745.2|	19620 +	A	0	1	0	1	0	SA0013
gi|29165615|ref|NC_002745.2|	19624 +	A	0	0	0	0	1	SA0013
gi|29165615|ref|NC_002745.2|	19627 +	G	0	1	0	0	0	SA0013
gi|29165615|ref|NC_002745.2|	19630 +	A	0	0	0	0	1	SA0013
gi|29165615|ref|NC_002745.2|	19631 +	A	0	1	0	0	0	SA0013
gi|29165615|ref|NC_002745.2|	19643 +	U	0	0	0	0	1	SA0013
gi|29165615|ref|NC_002745.2|	19651 +	C	0	0	1	0	0	SA0013
gi|29165615|ref|NC_002745.2|	19657 +	A	0	1	0	0	0	SA0013
gi|29165615|ref|NC_002745.2|	19664 +	G	2	0	0	0	0	SA0013
gi|29165615|ref|NC_002745.2|	19676 +	U	0	0	1	0	0	SA0013
gi|29165615|ref|NC_002745.2|	19681 +	A	0	1	0	0	0	SA0013
gi|29165615|ref|NC_002745.2|	19685 +	C	1	0	0	0	0	SA0013
gi|29165615|ref|NC_002745.2|	19689 +	G	0	0	1	0	0	SA0013
gi|29165615|ref|NC_002745.2|	19690 +	U	0	1	0	0	0	SA0013

gi|29165615|ref|NC_002745.2|	19691	+	U	1	0	0	0	0	SA0013
gi|29165615|ref|NC_002745.2|	19693	+	A	0	0	1	0	2	SA0013
gi|29165615|ref|NC_002745.2|	19694	+	U	0	0	0	1	0	SA0013
gi|29165615|ref|NC_002745.2|	19695	+	A	0	0	0	2	0	SA0013
gi|29165615|ref|NC_002745.2|	19696	+	U	0	1	0	0	0	SA0013
gi|29165615|ref|NC_002745.2|	19698	+	U	0	0	1	0	0	SA0013
gi|29165615|ref|NC_002745.2|	19720	+	A	0	2	0	0	0	SA0013
gi|29165615|ref|NC_002745.2|	19748	+	A	0	0	1	0	0	SA0013
gi|29165615|ref|NC_002745.2|	19749	+	A	0	0	0	0	1	SA0013
gi|29165615|ref|NC_002745.2|	19751	+	A	1	0	0	0	0	SA0013
gi|29165615|ref|NC_002745.2|	19758	+	A	0	0	1	0	0	SA0013
gi|29165615|ref|NC_002745.2|	19760	+	C	0	1	0	0	0	SA0013
gi|29165615|ref|NC_002745.2|	19772	+	U	1	0	0	0	0	SA0013
gi|29165615|ref|NC_002745.2|	19795	+	A	0	1	0	0	0	SA0013
gi|29165615|ref|NC_002745.2|	19803	+	A	0	0	0	0	1	SA0013
gi|29165615|ref|NC_002745.2|	19854	+	A	0	1	0	0	0	SA0013
gi|29165615|ref|NC_002745.2|	19923	+	U	1	0	0	0	0	SA0013
gi|29165615|ref|NC_002745.2|	19997	+	C	0	0	0	0	1	SA0013
gi|29165615|ref|NC_002745.2|	20016	+	A	0	0	0	0	1	SA0013
gi|29165615|ref|NC_002745.2|	20017	+	A	0	1	0	0	1	SA0013
gi|29165615|ref|NC_002745.2|	20022	+	U	0	0	1	0	0	SA0013
gi|29165615|ref|NC_002745.2|	20023	+	C	0	1	0	0	0	SA0013
gi|29165615|ref|NC_002745.2|	20032	+	A	0	0	0	0	2	SA0013
gi|29165615|ref|NC_002745.2|	20033	+	C	0	0	0	0	1	SA0013
gi|29165615|ref|NC_002745.2|	20039	+	C	2	0	0	0	0	SA0013
gi|29165615|ref|NC_002745.2|	20062	+	A	0	0	0	1	0	SA0013
gi|29165615|ref|NC_002745.2|	20072	+	G	1	12	3	9	8	SA0013
gi|29165615|ref|NC_002745.2|	20073	+	U	0	0	0	0	1	SA0013
gi|29165615|ref|NC_002745.2|	20074	+	A	0	0	1	0	0	SA0013
gi|29165615|ref|NC_002745.2|	20085	+	A	0	0	0	0	1	SA0013
gi|29165615|ref|NC_002745.2|	20088	+	U	0	1	0	0	0	SA0013
gi|29165615|ref|NC_002745.2|	20089	+	G	1	0	0	0	0	SA0013
gi|29165615|ref|NC_002745.2|	20090	+	U	2	0	0	0	0	SA0013
gi|29165615|ref|NC_002745.2|	20092	+	G	0	0	1	0	1	SA0013
gi|29165615|ref|NC_002745.2|	20094	+	U	0	0	1	0	0	SA0013
gi|29165615|ref|NC_002745.2|	20096	+	C	0	0	0	0	1	SA0013
gi|29165615|ref|NC_002745.2|	20105	+	A	0	0	0	1	0	SA0013
gi|29165615|ref|NC_002745.2|	20109	+	U	0	1	0	0	1	SA0013
gi|29165615|ref|NC_002745.2|	20110	+	A	0	0	1	0	0	SA0013
gi|29165615|ref|NC_002745.2|	20114	+	U	2	0	0	0	0	SA0013
gi|29165615|ref|NC_002745.2|	20116	+	A	0	1	0	0	0	SA0013
gi|29165615|ref|NC_002745.2|	20120	+	G	1	0	0	0	0	SA0013
gi|29165615|ref|NC_002745.2|	20121	+	U	1	0	0	0	0	SA0013
gi|29165615|ref|NC_002745.2|	20131	+	C	0	0	0	1	0	SA0013
gi|29165615|ref|NC_002745.2|	20134	+	U	0	0	0	1	0	SA0013
gi|29165615|ref|NC_002745.2|	20135	+	C	1	0	0	0	0	SA0013
gi|29165615|ref|NC_002745.2|	20138	+	U	1	0	0	0	0	SA0013
gi|29165615|ref|NC_002745.2|	20139	+	C	0	0	1	0	0	SA0013
gi|29165615|ref|NC_002745.2|	20141	+	G	0	0	0	0	1	SA0013
gi|29165615|ref|NC_002745.2|	20142	+	U	0	0	1	0	0	SA0013
gi|29165615|ref|NC_002745.2|	20143	+	U	1	0	0	0	0	SA0013
gi|29165615|ref|NC_002745.2|	20153	+	U	0	0	0	1	0	SA0013
gi|29165615|ref|NC_002745.2|	20154	+	U	0	0	0	1	0	SA0013
gi|29165615|ref|NC_002745.2|	20155	+	C	0	0	0	0	1	SA0013
gi|29165615|ref|NC_002745.2|	20156	+	A	0	0	1	0	0	SA0013
gi|29165615|ref|NC_002745.2|	20158	+	U	0	0	1	0	0	SA0013
gi|29165615|ref|NC_002745.2|	20162	+	C	1	1	0	0	0	SA0013
gi|29165615|ref|NC_002745.2|	20167	+	G	0	1	0	0	0	SA0013
gi|29165615|ref|NC_002745.2|	20173	+	C	1	0	0	0	0	SA0013
gi|29165615|ref|NC_002745.2|	20174	+	U	0	0	1	0	0	SA0013
gi|29165615|ref|NC_002745.2|	20177	+	G	6	0	0	0	0	SA0013
gi|29165615|ref|NC_002745.2|	20178	+	U	2	1	0	0	0	SA0013
gi|29165615|ref|NC_002745.2|	20179	+	G	0	0	2	0	0	SA0013
gi|29165615|ref|NC_002745.2|	20180	+	G	1	0	0	0	0	SA0013
gi|29165615|ref|NC_002745.2|	20181	+	C	0	0	0	0	1	SA0013
gi|29165615|ref|NC_002745.2|	20187	+	A	0	1	0	0	0	SA0013
gi|29165615|ref|NC_002745.2|	20188	+	C	0	0	0	0	1	SA0013
gi|29165615|ref|NC_002745.2|	20189	+	A	2	0	0	0	4	SA0013
gi|29165615|ref|NC_002745.2|	20191	+	U	1	0	0	0	1	SA0013
gi|29165615|ref|NC_002745.2|	20192	+	U	1	0	0	0	0	SA0013
gi|29165615|ref|NC_002745.2|	20195	+	C	0	0	0	0	1	SA0013
gi|29165615|ref|NC_002745.2|	20196	+	C	0	0	0	0	1	SA0013
gi|29165615|ref|NC_002745.2|	20197	+	A	0	1	0	0	0	SA0013
gi|29165615|ref|NC_002745.2|	20207	+	C	0	0	0	0	1	SA0013
gi|29165615|ref|NC_002745.2|	20212	+	C	0	0	0	0	1	SA0013
gi|29165615|ref|NC_002745.2|	20225	+	C	1	0	0	1	0	SA0013
gi|29165615|ref|NC_002745.2|	20240	+	U	3	0	0	1	0	SA0013
gi|29165615|ref|NC_002745.2|	20249	+	U	0	0	1	0	0	SA0013
gi|29165615|ref|NC_002745.2|	20250	+	A	0	0	2	0	0	SA0013
gi|29165615|ref|NC_002745.2|	20251	+	C	0	0	0	0	1	SA0013
gi|29165615|ref|NC_002745.2|	20254	+	C	0	0	0	0	1	SA0013

gi|29165615|ref|NC_002745.2|	20255	+	A	0	1	0	0	0	SA0013
gi|29165615|ref|NC_002745.2|	20258	+	C	0	0	0	0	2	SA0013
gi|29165615|ref|NC_002745.2|	20260	+	A	0	1	0	0	0	SA0013
gi|29165615|ref|NC_002745.2|	20264	+	C	0	0	0	0	1	SA0013
gi|29165615|ref|NC_002745.2|	20267	+	A	0	0	0	1	3	SA0013
gi|29165615|ref|NC_002745.2|	20268	+	A	0	0	0	0	2	SA0013
gi|29165615|ref|NC_002745.2|	20269	+	C	1	3	1	2	0	SA0013
gi|29165615|ref|NC_002745.2|	20270	+	A	0	0	0	0	1	SA0013
gi|29165615|ref|NC_002745.2|	20277	+	U	0	1	0	0	0	SA0013
gi|29165615|ref|NC_002745.2|	20280	+	G	0	2	0	0	2	SA0013
gi|29165615|ref|NC_002745.2|	20281	+	A	0	1	0	0	0	SA0013
gi|29165615|ref|NC_002745.2|	20291	+	C	0	0	0	0	1	SA0013
gi|29165615|ref|NC_002745.2|	20296	+	A	1	0	0	0	0	SA0014
gi|29165615|ref|NC_002745.2|	20310	+	C	1	0	0	0	0	SA0014
gi|29165615|ref|NC_002745.2|	20318	+	U	0	1	0	0	0	SA0014
gi|29165615|ref|NC_002745.2|	20319	+	A	0	0	0	0	1	SA0014
gi|29165615|ref|NC_002745.2|	20320	+	A	1	0	1	0	0	SA0014
gi|29165615|ref|NC_002745.2|	20331	+	A	0	0	0	1	0	SA0014
gi|29165615|ref|NC_002745.2|	20335	+	A	0	0	1	1	1	SA0014
gi|29165615|ref|NC_002745.2|	20341	+	A	0	0	0	1	0	SA0014
gi|29165615|ref|NC_002745.2|	20365	+	A	1	0	0	0	0	SA0014
gi|29165615|ref|NC_002745.2|	20368	+	C	1	1	0	0	0	SA0014
gi|29165615|ref|NC_002745.2|	20369	+	A	0	1	0	0	0	SA0014
gi|29165615|ref|NC_002745.2|	20371	+	A	1	0	0	0	0	SA0014
gi|29165615|ref|NC_002745.2|	20379	+	U	0	0	1	0	0	SA0014
gi|29165615|ref|NC_002745.2|	20383	+	U	0	0	0	0	1	SA0014
gi|29165615|ref|NC_002745.2|	20389	+	A	0	0	0	1	1	SA0014
gi|29165615|ref|NC_002745.2|	20391	+	A	0	2	0	1	0	SA0014
gi|29165615|ref|NC_002745.2|	20398	+	C	0	0	0	1	0	SA0014
gi|29165615|ref|NC_002745.2|	20418	+	A	2	0	0	0	0	SA0014
gi|29165615|ref|NC_002745.2|	20426	+	A	0	0	0	1	0	SA0014
gi|29165615|ref|NC_002745.2|	20428	+	A	0	0	0	0	1	SA0014
gi|29165615|ref|NC_002745.2|	20429	+	A	0	1	0	0	1	SA0014
gi|29165615|ref|NC_002745.2|	20439	+	C	0	1	0	0	0	SA0014
gi|29165615|ref|NC_002745.2|	20441	+	G	0	0	0	0	1	SA0014
gi|29165615|ref|NC_002745.2|	20442	+	A	1	0	1	0	2	SA0014
gi|29165615|ref|NC_002745.2|	20445	+	A	2	0	0	0	0	SA0014
gi|29165615|ref|NC_002745.2|	20450	+	U	0	1	0	0	0	SA0014
gi|29165615|ref|NC_002745.2|	20457	+	C	0	0	0	0	1	SA0014
gi|29165615|ref|NC_002745.2|	20465	+	C	0	0	0	1	0	SA0014
gi|29165615|ref|NC_002745.2|	20473	+	A	0	0	0	1	0	SA0014
gi|29165615|ref|NC_002745.2|	20510	+	A	0	0	1	0	0	SA0014
gi|29165615|ref|NC_002745.2|	20533	+	C	1	0	0	0	0	SA0014
gi|29165615|ref|NC_002745.2|	20540	+	U	0	0	0	1	0	SA0014
gi|29165615|ref|NC_002745.2|	20570	+	A	0	0	1	0	0	SA0014
gi|29165615|ref|NC_002745.2|	20623	+	A	0	0	0	0	2	SA0014
gi|29165615|ref|NC_002745.2|	20626	+	A	0	1	0	0	0	SA0014
gi|29165615|ref|NC_002745.2|	20637	+	G	0	2	0	0	0	SA0014
gi|29165615|ref|NC_002745.2|	20638	+	A	0	0	1	0	0	SA0014
gi|29165615|ref|NC_002745.2|	20651	+	A	0	2	0	0	0	SA0014
gi|29165615|ref|NC_002745.2|	20654	+	U	0	0	1	0	0	SA0014
gi|29165615|ref|NC_002745.2|	20660	+	C	1	0	0	0	0	SA0014
gi|29165615|ref|NC_002745.2|	20673	+	A	0	0	0	0	2	SA0014
gi|29165615|ref|NC_002745.2|	20689	+	U	0	1	0	0	0	SA0014
gi|29165615|ref|NC_002745.2|	20692	+	A	0	1	0	0	0	SA0014
gi|29165615|ref|NC_002745.2|	20694	+	A	0	0	1	0	0	SA0014
gi|29165615|ref|NC_002745.2|	20698	+	A	0	0	0	0	1	SA0014
gi|29165615|ref|NC_002745.2|	20710	+	C	0	0	1	0	0	SA0014
gi|29165615|ref|NC_002745.2|	20719	+	U	0	0	0	1	0	SA0014
gi|29165615|ref|NC_002745.2|	20728	+	U	0	0	0	0	1	SA0014
gi|29165615|ref|NC_002745.2|	20735	+	A	0	0	1	0	0	SA0014
gi|29165615|ref|NC_002745.2|	20737	+	A	0	0	0	0	1	SA0014
gi|29165615|ref|NC_002745.2|	20738	+	A	0	1	0	0	0	SA0014
gi|29165615|ref|NC_002745.2|	20744	+	G	0	2	0	1	0	-
gi|29165615|ref|NC_002745.2|	20745	+	A	0	0	0	0	1	-
gi|29165615|ref|NC_002745.2|	20748	+	G	0	1	0	0	0	-
gi|29165615|ref|NC_002745.2|	20749	+	A	0	1	0	0	0	-
gi|29165615|ref|NC_002745.2|	20751	+	A	0	0	0	1	0	-
gi|29165615|ref|NC_002745.2|	20755	+	G	0	0	0	0	1	-
gi|29165615|ref|NC_002745.2|	20778	+	A	0	0	0	1	0	SA0015
gi|29165615|ref|NC_002745.2|	20794	+	C	0	1	0	0	0	SA0015
gi|29165615|ref|NC_002745.2|	20800	+	C	0	0	0	0	1	SA0015
gi|29165615|ref|NC_002745.2|	20801	+	C	0	0	0	0	1	SA0015
gi|29165615|ref|NC_002745.2|	20803	+	C	0	0	0	0	1	SA0015
gi|29165615|ref|NC_002745.2|	20810	+	A	0	1	0	1	0	SA0015
gi|29165615|ref|NC_002745.2|	20813	+	A	0	1	0	0	0	SA0015
gi|29165615|ref|NC_002745.2|	20897	+	U	1	0	0	0	0	SA0015
gi|29165615|ref|NC_002745.2|	20910	+	C	0	0	0	1	0	SA0015
gi|29165615|ref|NC_002745.2|	20947	+	G	0	0	0	0	1	SA0015
gi|29165615|ref|NC_002745.2|	21061	+	A	0	1	0	0	0	SA0015
gi|29165615|ref|NC_002745.2|	21103	+	C	0	0	0	1	0	SA0015

gi|29165615|ref|NC_002745.2|	21113	+	A	0	4	0	0	1	SA0015
gi|29165615|ref|NC_002745.2|	21115	+	C	1	0	0	0	0	SA0015
gi|29165615|ref|NC_002745.2|	21127	+	C	0	0	0	1	0	SA0015
gi|29165615|ref|NC_002745.2|	21141	+	U	0	0	1	0	0	SA0015
gi|29165615|ref|NC_002745.2|	21182	+	C	0	1	0	0	0	SA0015
gi|29165615|ref|NC_002745.2|	21183	+	G	0	0	0	1	0	SA0015
gi|29165615|ref|NC_002745.2|	21185	+	U	0	0	0	0	1	SA0015
gi|29165615|ref|NC_002745.2|	21193	+	G	0	0	0	1	0	SA0015
gi|29165615|ref|NC_002745.2|	21263	+	U	3	0	0	0	0	SA0015
gi|29165615|ref|NC_002745.2|	21277	+	U	0	0	0	3	0	SA0015
gi|29165615|ref|NC_002745.2|	21306	+	U	0	0	0	0	1	SA0015
gi|29165615|ref|NC_002745.2|	21327	+	A	2	0	0	0	0	SA0015
gi|29165615|ref|NC_002745.2|	21370	+	A	0	1	0	0	0	SA0015
gi|29165615|ref|NC_002745.2|	21387	+	U	0	1	0	0	0	SA0015
gi|29165615|ref|NC_002745.2|	21391	+	C	0	0	0	1	0	SA0015
gi|29165615|ref|NC_002745.2|	21398	+	C	1	0	0	0	0	SA0015
gi|29165615|ref|NC_002745.2|	21400	+	C	1	0	0	0	0	SA0015
gi|29165615|ref|NC_002745.2|	21408	+	U	0	1	0	0	0	SA0015
gi|29165615|ref|NC_002745.2|	21409	+	G	0	1	0	0	0	SA0015
gi|29165615|ref|NC_002745.2|	21429	+	A	1	0	0	0	0	SA0015
gi|29165615|ref|NC_002745.2|	21440	+	C	0	0	0	1	0	SA0015
gi|29165615|ref|NC_002745.2|	21471	+	U	0	0	0	1	0	SA0015
gi|29165615|ref|NC_002745.2|	21475	+	G	0	1	0	0	1	SA0015
gi|29165615|ref|NC_002745.2|	21482	+	U	0	0	0	0	1	SA0015
gi|29165615|ref|NC_002745.2|	21497	+	U	0	0	0	1	0	SA0015
gi|29165615|ref|NC_002745.2|	21506	+	A	0	0	0	1	0	SA0015
gi|29165615|ref|NC_002745.2|	21517	+	A	0	0	0	0	1	SA0015
gi|29165615|ref|NC_002745.2|	21532	+	A	0	1	0	0	0	SA0015
gi|29165615|ref|NC_002745.2|	21558	+	C	0	0	0	1	0	SA0015
gi|29165615|ref|NC_002745.2|	21648	+	U	0	0	0	1	0	SA0015
gi|29165615|ref|NC_002745.2|	21650	+	C	1	0	0	0	0	SA0015
gi|29165615|ref|NC_002745.2|	21739	+	C	0	0	0	1	0	SA0015
gi|29165615|ref|NC_002745.2|	21753	+	U	3	0	0	0	0	SA0015
gi|29165615|ref|NC_002745.2|	21759	+	U	1	0	0	0	0	SA0015
gi|29165615|ref|NC_002745.2|	21761	+	C	0	0	2	0	0	SA0015
gi|29165615|ref|NC_002745.2|	21895	+	C	1	0	0	0	0	SA0015
gi|29165615|ref|NC_002745.2|	21960	+	C	0	1	0	0	0	SA0015
gi|29165615|ref|NC_002745.2|	21988	+	G	0	1	0	0	0	SA0015
gi|29165615|ref|NC_002745.2|	22055	+	U	1	0	0	0	0	SA0015
gi|29165615|ref|NC_002745.2|	22197	+	A	0	1	0	0	1	-
gi|29165615|ref|NC_002745.2|	22344	+	C	0	0	1	0	0	-
gi|29165615|ref|NC_002745.2|	22415	+	A	0	1	1	1	0	-
gi|29165615|ref|NC_002745.2|	22416	+	U	0	0	0	0	1	-
gi|29165615|ref|NC_002745.2|	22417	+	G	57	9	23	8	20	-
gi|29165615|ref|NC_002745.2|	22418	+	G	1	2	2	1	2	-
gi|29165615|ref|NC_002745.2|	22421	+	A	0	0	0	0	2	-
gi|29165615|ref|NC_002745.2|	22422	+	A	1	0	0	0	0	-
gi|29165615|ref|NC_002745.2|	22423	+	A	0	0	1	0	0	-
gi|29165615|ref|NC_002745.2|	22429	+	A	1	1	1	0	0	-
gi|29165615|ref|NC_002745.2|	22431	+	A	0	1	0	0	0	-
gi|29165615|ref|NC_002745.2|	22432	+	A	0	0	0	0	1	-
gi|29165615|ref|NC_002745.2|	22434	+	U	2	0	0	0	0	-
gi|29165615|ref|NC_002745.2|	22447	+	C	0	0	1	0	0	-
gi|29165615|ref|NC_002745.2|	22466	+	G	1	0	0	0	0	SA0016
gi|29165615|ref|NC_002745.2|	22475	+	C	1	0	0	0	0	SA0016
gi|29165615|ref|NC_002745.2|	22488	+	A	0	1	0	0	0	SA0016
gi|29165615|ref|NC_002745.2|	22494	+	A	0	0	0	0	2	SA0016
gi|29165615|ref|NC_002745.2|	22502	+	A	0	0	1	0	0	SA0016
gi|29165615|ref|NC_002745.2|	22523	+	C	1	0	0	1	0	SA0016
gi|29165615|ref|NC_002745.2|	22527	+	C	1	0	0	0	0	SA0016
gi|29165615|ref|NC_002745.2|	22530	+	A	1	0	0	0	0	SA0016
gi|29165615|ref|NC_002745.2|	22531	+	U	0	0	0	0	1	SA0016
gi|29165615|ref|NC_002745.2|	22548	+	C	3	0	0	0	0	SA0016
gi|29165615|ref|NC_002745.2|	22549	+	A	1	0	0	0	0	SA0016
gi|29165615|ref|NC_002745.2|	22554	+	G	1	0	1	0	1	SA0016
gi|29165615|ref|NC_002745.2|	22558	+	U	1	0	0	0	0	SA0016
gi|29165615|ref|NC_002745.2|	22559	+	A	1	0	0	0	0	SA0016
gi|29165615|ref|NC_002745.2|	22573	+	C	0	0	0	0	1	SA0016
gi|29165615|ref|NC_002745.2|	22591	+	A	1	0	0	0	0	SA0016
gi|29165615|ref|NC_002745.2|	22594	+	A	4	0	0	0	0	SA0016
gi|29165615|ref|NC_002745.2|	22600	+	A	0	0	1	0	0	SA0016
gi|29165615|ref|NC_002745.2|	22639	+	A	0	2	0	0	0	SA0016
gi|29165615|ref|NC_002745.2|	22646	+	G	1	0	0	0	0	SA0016
gi|29165615|ref|NC_002745.2|	22654	+	U	1	0	0	0	0	SA0016
gi|29165615|ref|NC_002745.2|	22661	+	G	1	0	0	0	0	SA0016
gi|29165615|ref|NC_002745.2|	22698	+	A	0	1	0	0	0	SA0016
gi|29165615|ref|NC_002745.2|	22710	+	A	0	0	1	0	0	SA0016
gi|29165615|ref|NC_002745.2|	22720	+	U	2	0	0	0	0	SA0016
gi|29165615|ref|NC_002745.2|	22779	+	C	0	1	0	0	0	SA0016
gi|29165615|ref|NC_002745.2|	22803	+	U	3	0	0	0	0	SA0016
gi|29165615|ref|NC_002745.2|	22822	+	U	1	0	0	0	0	SA0016

gi|29165615|ref|NC_002745.2|	22853	+	U	0	0	0	0	1	SA0016
gi|29165615|ref|NC_002745.2|	22881	+	U	2	0	0	0	0	SA0016
gi|29165615|ref|NC_002745.2|	22908	+	A	0	0	1	0	0	SA0016
gi|29165615|ref|NC_002745.2|	22909	+	A	0	0	0	0	1	SA0016
gi|29165615|ref|NC_002745.2|	22921	+	A	0	0	1	1	0	SA0016
gi|29165615|ref|NC_002745.2|	22941	+	A	0	0	1	0	0	SA0016
gi|29165615|ref|NC_002745.2|	22955	+	U	0	0	0	1	0	SA0016
gi|29165615|ref|NC_002745.2|	22973	+	A	0	0	0	0	1	SA0016
gi|29165615|ref|NC_002745.2|	23039	+	G	0	1	0	0	0	SA0016
gi|29165615|ref|NC_002745.2|	23063	+	A	0	1	0	0	0	SA0016
gi|29165615|ref|NC_002745.2|	23104	+	C	1	0	0	0	0	SA0016
gi|29165615|ref|NC_002745.2|	23109	+	G	0	1	0	0	0	SA0016
gi|29165615|ref|NC_002745.2|	23143	+	U	0	1	0	0	0	SA0016
gi|29165615|ref|NC_002745.2|	23145	+	C	0	1	0	0	0	SA0016
gi|29165615|ref|NC_002745.2|	23146	+	A	0	0	0	0	1	SA0016
gi|29165615|ref|NC_002745.2|	23149	+	U	0	0	0	0	1	SA0016
gi|29165615|ref|NC_002745.2|	23157	+	U	0	0	1	0	0	SA0016
gi|29165615|ref|NC_002745.2|	23174	+	A	2	0	0	0	0	SA0016
gi|29165615|ref|NC_002745.2|	23178	+	C	0	0	0	2	0	SA0016
gi|29165615|ref|NC_002745.2|	23190	+	C	0	0	1	0	0	SA0016
gi|29165615|ref|NC_002745.2|	23193	+	U	0	0	0	0	2	SA0016
gi|29165615|ref|NC_002745.2|	23199	+	C	1	0	0	0	0	SA0016
gi|29165615|ref|NC_002745.2|	23212	+	U	1	0	0	0	0	SA0016
gi|29165615|ref|NC_002745.2|	23247	+	C	1	0	0	0	0	SA0016
gi|29165615|ref|NC_002745.2|	23253	+	C	1	0	0	0	0	SA0016
gi|29165615|ref|NC_002745.2|	23286	+	A	0	0	0	1	0	SA0016
gi|29165615|ref|NC_002745.2|	23353	+	U	3	0	0	0	0	SA0016
gi|29165615|ref|NC_002745.2|	23359	+	U	3	0	0	0	0	SA0016
gi|29165615|ref|NC_002745.2|	23365	+	A	1	0	0	0	0	SA0016
gi|29165615|ref|NC_002745.2|	23372	+	U	1	0	0	0	0	SA0016
gi|29165615|ref|NC_002745.2|	23382	+	U	0	0	0	1	0	SA0016
gi|29165615|ref|NC_002745.2|	23383	+	U	4	0	0	0	0	SA0016
gi|29165615|ref|NC_002745.2|	23385	+	U	2	0	0	0	0	SA0016
gi|29165615|ref|NC_002745.2|	23396	+	U	1	0	0	0	0	SA0016
gi|29165615|ref|NC_002745.2|	23397	+	C	1	0	0	0	0	SA0016
gi|29165615|ref|NC_002745.2|	23446	+	U	0	0	1	0	0	SA0016
gi|29165615|ref|NC_002745.2|	23461	+	C	0	0	0	0	1	SA0016
gi|29165615|ref|NC_002745.2|	23529	+	A	0	1	0	0	0	SA0016
gi|29165615|ref|NC_002745.2|	24147	+	U	0	0	0	1	0	-
gi|29165615|ref|NC_002745.2|	24148	+	G	0	3	1	0	1	-
gi|29165615|ref|NC_002745.2|	24439	+	C	0	0	1	0	0	-
gi|29165615|ref|NC_002745.2|	24789	+	A	0	1	1	0	0	-
gi|29165615|ref|NC_002745.2|	24821	+	C	0	0	0	1	0	-
gi|29165615|ref|NC_002745.2|	24827	+	A	0	0	1	0	0	-
gi|29165615|ref|NC_002745.2|	24828	+	A	0	0	1	2	1	-
gi|29165615|ref|NC_002745.2|	24829	+	C	0	0	1	0	0	-
gi|29165615|ref|NC_002745.2|	24838	+	U	0	0	1	0	0	-
gi|29165615|ref|NC_002745.2|	24867	+	A	0	0	0	1	0	-
gi|29165615|ref|NC_002745.2|	24872	+	A	0	0	0	1	0	-
gi|29165615|ref|NC_002745.2|	24875	+	G	0	0	1	0	0	-
gi|29165615|ref|NC_002745.2|	24876	+	A	0	1	0	0	0	-
gi|29165615|ref|NC_002745.2|	24878	+	A	0	0	1	0	0	-
gi|29165615|ref|NC_002745.2|	24879	+	A	0	0	2	1	0	-
gi|29165615|ref|NC_002745.2|	24880	+	A	0	0	1	0	2	-
gi|29165615|ref|NC_002745.2|	24881	+	G	0	1	0	0	0	-
gi|29165615|ref|NC_002745.2|	24882	+	A	0	1	0	0	1	-
gi|29165615|ref|NC_002745.2|	24883	+	A	0	0	0	1	0	-
gi|29165615|ref|NC_002745.2|	24886	+	A	0	0	0	1	0	-
gi|29165615|ref|NC_002745.2|	24887	+	A	0	0	0	1	2	-
gi|29165615|ref|NC_002745.2|	24888	+	A	0	0	0	0	1	-
gi|29165615|ref|NC_002745.2|	24889	+	A	0	1	0	0	0	-
gi|29165615|ref|NC_002745.2|	24892	+	G	0	0	1	0	2	-
gi|29165615|ref|NC_002745.2|	24895	+	A	0	3	1	0	2	-
gi|29165615|ref|NC_002745.2|	24896	+	A	0	0	0	0	1	-
gi|29165615|ref|NC_002745.2|	24897	+	A	0	0	0	0	1	-
gi|29165615|ref|NC_002745.2|	24898	+	A	0	1	1	0	0	-
gi|29165615|ref|NC_002745.2|	24900	+	A	0	6	3	4	1	-
gi|29165615|ref|NC_002745.2|	24901	+	A	0	3	1	0	6	-
gi|29165615|ref|NC_002745.2|	24902	+	U	0	0	0	1	0	-
gi|29165615|ref|NC_002745.2|	24904	+	U	0	1	0	1	1	-
gi|29165615|ref|NC_002745.2|	24905	+	U	0	1	0	0	1	-
gi|29165615|ref|NC_002745.2|	24906	+	A	4	47	22	14	41	-
gi|29165615|ref|NC_002745.2|	24907	+	A	0	5	0	2	3	-
gi|29165615|ref|NC_002745.2|	24908	+	U	0	1	0	0	0	-
gi|29165615|ref|NC_002745.2|	24909	+	G	0	3	3	1	5	-
gi|29165615|ref|NC_002745.2|	24910	+	A	1	0	2	0	4	-
gi|29165615|ref|NC_002745.2|	24911	+	U	0	2	1	0	0	-
gi|29165615|ref|NC_002745.2|	24913	+	U	0	1	2	0	4	-
gi|29165615|ref|NC_002745.2|	24914	+	A	0	11	5	5	14	-
gi|29165615|ref|NC_002745.2|	24915	+	A	0	6	2	2	11	-
gi|29165615|ref|NC_002745.2|	24916	+	G	0	19	13	5	10	-

gi|29165615|ref|NC_002745.2|	24917 +	A	0	17	12	11	19	-
gi|29165615|ref|NC_002745.2|	24918 +	A	0	11	10	2	9	-
gi|29165615|ref|NC_002745.2|	24919 +	A	0	4	2	1	1	-
gi|29165615|ref|NC_002745.2|	24920 +	A	0	1	3	0	0	-
gi|29165615|ref|NC_002745.2|	24921 +	G	0	1	0	0	2	-
gi|29165615|ref|NC_002745.2|	24922 +	A	0	1	4	0	4	-
gi|29165615|ref|NC_002745.2|	24924 +	G	0	0	0	0	1	-
gi|29165615|ref|NC_002745.2|	24925 +	U	0	1	0	0	3	-
gi|29165615|ref|NC_002745.2|	24930 +	G	0	0	0	1	0	SA0017
gi|29165615|ref|NC_002745.2|	24931 +	C	0	3	1	0	0	SA0017
gi|29165615|ref|NC_002745.2|	24932 +	A	0	6	1	3	3	SA0017
gi|29165615|ref|NC_002745.2|	24933 +	A	0	1	0	2	1	SA0017
gi|29165615|ref|NC_002745.2|	24934 +	A	0	0	2	0	0	SA0017
gi|29165615|ref|NC_002745.2|	24935 +	U	0	0	0	0	1	SA0017
gi|29165615|ref|NC_002745.2|	24937 +	G	0	1	0	0	0	SA0017
gi|29165615|ref|NC_002745.2|	24938 +	C	0	0	1	0	1	SA0017
gi|29165615|ref|NC_002745.2|	24939 +	U	0	2	0	0	0	SA0017
gi|29165615|ref|NC_002745.2|	24941 +	G	0	0	1	0	0	SA0017
gi|29165615|ref|NC_002745.2|	24942 +	A	0	1	0	1	1	SA0017
gi|29165615|ref|NC_002745.2|	24943 +	A	0	1	1	1	3	SA0017
gi|29165615|ref|NC_002745.2|	24944 +	A	0	1	1	0	0	SA0017
gi|29165615|ref|NC_002745.2|	24945 +	A	0	0	1	0	0	SA0017
gi|29165615|ref|NC_002745.2|	24948 +	U	0	0	1	0	0	SA0017
gi|29165615|ref|NC_002745.2|	24951 +	U	0	1	0	0	1	SA0017
gi|29165615|ref|NC_002745.2|	24954 +	A	0	1	0	0	0	SA0017
gi|29165615|ref|NC_002745.2|	24958 +	G	0	1	1	0	0	SA0017
gi|29165615|ref|NC_002745.2|	24959 +	A	1	0	0	0	0	SA0017
gi|29165615|ref|NC_002745.2|	24962 +	A	0	0	0	0	1	SA0017
gi|29165615|ref|NC_002745.2|	24965 +	A	0	0	0	0	2	SA0017
gi|29165615|ref|NC_002745.2|	24966 +	A	0	0	0	1	0	SA0017
gi|29165615|ref|NC_002745.2|	24972 +	G	0	0	1	0	0	SA0017
gi|29165615|ref|NC_002745.2|	24973 +	A	1	7	1	1	1	SA0017
gi|29165615|ref|NC_002745.2|	24975 +	U	0	0	0	0	1	SA0017
gi|29165615|ref|NC_002745.2|	24980 +	A	0	0	0	1	0	SA0017
gi|29165615|ref|NC_002745.2|	24988 +	G	0	0	1	0	0	SA0017
gi|29165615|ref|NC_002745.2|	24989 +	A	0	3	0	0	4	SA0017
gi|29165615|ref|NC_002745.2|	24998 +	U	0	0	0	0	1	SA0017
gi|29165615|ref|NC_002745.2|	25000 +	A	0	1	0	0	0	SA0017
gi|29165615|ref|NC_002745.2|	25001 +	A	0	3	1	1	3	SA0017
gi|29165615|ref|NC_002745.2|	25002 +	A	0	1	0	0	2	SA0017
gi|29165615|ref|NC_002745.2|	25003 +	A	1	1	0	0	2	SA0017
gi|29165615|ref|NC_002745.2|	25004 +	A	0	0	0	0	1	SA0017
gi|29165615|ref|NC_002745.2|	25007 +	A	0	1	0	0	2	SA0017
gi|29165615|ref|NC_002745.2|	25010 +	G	0	0	1	0	1	SA0017
gi|29165615|ref|NC_002745.2|	25016 +	A	1	0	0	0	0	SA0017
gi|29165615|ref|NC_002745.2|	25025 +	G	1	0	0	0	0	SA0017
gi|29165615|ref|NC_002745.2|	25032 +	C	0	0	0	0	1	SA0017
gi|29165615|ref|NC_002745.2|	25033 +	G	0	1	1	0	1	SA0017
gi|29165615|ref|NC_002745.2|	25039 +	A	0	0	0	0	1	SA0017
gi|29165615|ref|NC_002745.2|	25049 +	U	0	1	0	0	0	SA0017
gi|29165615|ref|NC_002745.2|	25050 +	C	0	0	1	0	0	SA0017
gi|29165615|ref|NC_002745.2|	25060 +	U	0	0	0	0	1	SA0017
gi|29165615|ref|NC_002745.2|	25064 +	A	0	1	0	0	0	SA0017
gi|29165615|ref|NC_002745.2|	25086 +	A	0	0	0	1	0	SA0017
gi|29165615|ref|NC_002745.2|	25091 +	A	0	0	0	1	0	SA0017
gi|29165615|ref|NC_002745.2|	25093 +	A	0	0	1	0	0	SA0017
gi|29165615|ref|NC_002745.2|	25103 +	C	0	0	0	0	1	SA0017
gi|29165615|ref|NC_002745.2|	25107 +	U	0	0	0	0	1	SA0017
gi|29165615|ref|NC_002745.2|	25111 +	G	0	0	1	0	0	SA0017
gi|29165615|ref|NC_002745.2|	25112 +	A	0	0	0	0	1	SA0017
gi|29165615|ref|NC_002745.2|	25128 +	U	0	1	0	0	0	SA0017
gi|29165615|ref|NC_002745.2|	25146 +	A	0	0	1	0	0	SA0017
gi|29165615|ref|NC_002745.2|	25150 +	G	0	1	0	0	0	SA0017
gi|29165615|ref|NC_002745.2|	25156 +	C	0	0	0	0	1	SA0017
gi|29165615|ref|NC_002745.2|	25164 +	A	0	0	0	0	1	SA0017
gi|29165615|ref|NC_002745.2|	25165 +	A	0	0	0	0	1	SA0017
gi|29165615|ref|NC_002745.2|	25178 +	A	0	1	0	0	0	SA0017
gi|29165615|ref|NC_002745.2|	25189 +	A	0	1	0	0	0	SA0017
gi|29165615|ref|NC_002745.2|	25376 +	C	0	0	0	0	2	SA0017
gi|29165615|ref|NC_002745.2|	25378 +	A	0	0	0	1	1	SA0017
gi|29165615|ref|NC_002745.2|	25380 +	U	0	0	1	0	0	SA0017
gi|29165615|ref|NC_002745.2|	25381 +	A	0	1	0	0	1	SA0017
gi|29165615|ref|NC_002745.2|	25382 +	A	0	0	0	0	2	SA0017
gi|29165615|ref|NC_002745.2|	25383 +	A	0	1	0	0	0	SA0017
gi|29165615|ref|NC_002745.2|	25394 +	A	0	2	0	0	0	SA0017
gi|29165615|ref|NC_002745.2|	25397 +	A	0	1	1	0	2	SA0017
gi|29165615|ref|NC_002745.2|	25398 +	U	0	0	0	0	1	SA0017
gi|29165615|ref|NC_002745.2|	25402 +	G	0	0	0	0	1	SA0017
gi|29165615|ref|NC_002745.2|	25403 +	A	0	1	0	0	0	SA0017
gi|29165615|ref|NC_002745.2|	25405 +	U	0	1	0	0	0	SA0017
gi|29165615|ref|NC_002745.2|	25407 +	A	0	1	0	0	0	SA0017

gi|29165615|ref|NC_002745.2|	25408	+	A	0	1	0	0	0	SA0017
gi|29165615|ref|NC_002745.2|	25418	+	A	0	2	0	0	0	SA0017
gi|29165615|ref|NC_002745.2|	25432	+	C	0	1	0	0	0	SA0017
gi|29165615|ref|NC_002745.2|	25461	+	A	0	1	0	1	0	SA0017
gi|29165615|ref|NC_002745.2|	25462	+	A	0	1	0	0	0	SA0017
gi|29165615|ref|NC_002745.2|	25465	+	A	0	1	0	0	0	SA0017
gi|29165615|ref|NC_002745.2|	25466	+	C	2	0	1	0	0	SA0017
gi|29165615|ref|NC_002745.2|	25468	+	C	0	0	0	0	1	SA0017
gi|29165615|ref|NC_002745.2|	25472	+	A	0	0	0	0	1	SA0017
gi|29165615|ref|NC_002745.2|	25474	+	C	0	0	0	0	1	SA0017
gi|29165615|ref|NC_002745.2|	25476	+	U	0	0	0	1	0	SA0017
gi|29165615|ref|NC_002745.2|	25477	+	U	0	0	1	0	0	SA0017
gi|29165615|ref|NC_002745.2|	25478	+	U	0	0	0	0	1	SA0017
gi|29165615|ref|NC_002745.2|	25479	+	A	0	2	1	1	3	SA0017
gi|29165615|ref|NC_002745.2|	25482	+	A	0	1	1	1	3	SA0017
gi|29165615|ref|NC_002745.2|	25485	+	A	0	1	1	0	0	SA0017
gi|29165615|ref|NC_002745.2|	25486	+	A	0	2	0	0	0	SA0017
gi|29165615|ref|NC_002745.2|	25487	+	C	0	2	0	0	0	SA0017
gi|29165615|ref|NC_002745.2|	25489	+	G	0	1	0	0	3	SA0017
gi|29165615|ref|NC_002745.2|	25490	+	U	0	3	0	0	1	SA0017
gi|29165615|ref|NC_002745.2|	25496	+	G	0	0	1	0	0	SA0017
gi|29165615|ref|NC_002745.2|	25507	+	U	0	0	2	0	0	SA0017
gi|29165615|ref|NC_002745.2|	25509	+	U	0	1	0	0	0	SA0017
gi|29165615|ref|NC_002745.2|	25515	+	U	1	0	0	0	0	SA0017
gi|29165615|ref|NC_002745.2|	25565	+	A	0	0	0	0	1	SA0017
gi|29165615|ref|NC_002745.2|	25621	+	C	0	0	0	1	0	SA0017
gi|29165615|ref|NC_002745.2|	25644	+	A	0	0	1	0	0	-
gi|29165615|ref|NC_002745.2|	25665	+	A	0	2	0	0	0	SA0018
gi|29165615|ref|NC_002745.2|	25682	+	C	0	1	0	0	0	SA0018
gi|29165615|ref|NC_002745.2|	25790	+	A	0	1	0	0	0	SA0018
gi|29165615|ref|NC_002745.2|	25794	+	U	0	0	0	1	0	SA0018
gi|29165615|ref|NC_002745.2|	25859	+	A	0	0	0	0	1	SA0018
gi|29165615|ref|NC_002745.2|	25878	+	A	0	0	0	0	1	SA0018
gi|29165615|ref|NC_002745.2|	25880	+	A	0	1	1	0	0	SA0018
gi|29165615|ref|NC_002745.2|	25887	+	A	0	0	0	1	0	SA0018
gi|29165615|ref|NC_002745.2|	25894	+	U	0	1	0	0	0	SA0018
gi|29165615|ref|NC_002745.2|	26177	+	U	0	1	3	0	0	SA0018
gi|29165615|ref|NC_002745.2|	26178	+	A	0	0	0	1	0	SA0018
gi|29165615|ref|NC_002745.2|	26180	+	A	0	2	0	0	0	SA0018
gi|29165615|ref|NC_002745.2|	26190	+	U	0	3	0	0	0	SA0018
gi|29165615|ref|NC_002745.2|	26192	+	A	0	0	1	0	0	SA0018
gi|29165615|ref|NC_002745.2|	26230	+	A	0	0	0	1	0	SA0018
gi|29165615|ref|NC_002745.2|	26235	+	C	0	0	2	1	0	SA0018
gi|29165615|ref|NC_002745.2|	26249	+	U	0	0	1	0	0	SA0018
gi|29165615|ref|NC_002745.2|	26321	+	A	0	0	0	0	1	SA0018
gi|29165615|ref|NC_002745.2|	26324	+	C	0	0	1	0	0	SA0018
gi|29165615|ref|NC_002745.2|	26356	+	A	0	3	0	0	1	SA0018
gi|29165615|ref|NC_002745.2|	26401	+	C	0	1	0	0	0	SA0018
gi|29165615|ref|NC_002745.2|	26431	+	A	0	0	0	1	0	SA0018
gi|29165615|ref|NC_002745.2|	26432	+	A	0	1	0	1	0	SA0018
gi|29165615|ref|NC_002745.2|	26436	+	U	0	0	0	1	0	SA0018
gi|29165615|ref|NC_002745.2|	26440	+	C	0	0	0	0	1	SA0018
gi|29165615|ref|NC_002745.2|	26444	+	A	1	0	0	0	0	SA0018
gi|29165615|ref|NC_002745.2|	26467	+	G	0	0	0	0	1	SA0018
gi|29165615|ref|NC_002745.2|	26519	+	U	0	2	0	0	0	SA0018
gi|29165615|ref|NC_002745.2|	26524	+	C	0	0	0	0	1	SA0018
gi|29165615|ref|NC_002745.2|	26597	+	A	0	0	1	0	0	SA0018
gi|29165615|ref|NC_002745.2|	26630	+	A	0	1	0	0	0	SA0018
gi|29165615|ref|NC_002745.2|	26634	+	U	0	0	0	1	1	SA0018
gi|29165615|ref|NC_002745.2|	26639	+	U	0	0	0	0	1	SA0018
gi|29165615|ref|NC_002745.2|	26644	+	G	0	0	1	1	1	SA0018
gi|29165615|ref|NC_002745.2|	26645	+	A	0	0	0	0	1	SA0018
gi|29165615|ref|NC_002745.2|	26649	+	A	0	1	0	0	0	SA0018
gi|29165615|ref|NC_002745.2|	26650	+	A	0	0	1	0	1	SA0018
gi|29165615|ref|NC_002745.2|	26651	+	A	2	4	0	1	0	SA0018
gi|29165615|ref|NC_002745.2|	26653	+	G	0	0	0	0	1	SA0018
gi|29165615|ref|NC_002745.2|	26654	+	A	0	0	1	0	0	SA0018
gi|29165615|ref|NC_002745.2|	26655	+	A	0	0	0	0	1	SA0018
gi|29165615|ref|NC_002745.2|	26656	+	G	0	1	0	0	0	SA0018
gi|29165615|ref|NC_002745.2|	26657	+	A	0	1	0	0	1	SA0018
gi|29165615|ref|NC_002745.2|	26659	+	G	0	1	0	0	0	SA0018
gi|29165615|ref|NC_002745.2|	26664	+	U	0	0	0	0	1	SA0018
gi|29165615|ref|NC_002745.2|	26667	+	A	0	1	0	0	0	SA0018
gi|29165615|ref|NC_002745.2|	26668	+	A	0	0	1	0	0	SA0018
gi|29165615|ref|NC_002745.2|	26669	+	U	0	0	0	0	2	SA0018
gi|29165615|ref|NC_002745.2|	26679	+	U	0	1	0	0	0	SA0018
gi|29165615|ref|NC_002745.2|	26701	+	G	0	0	0	1	0	SA0018
gi|29165615|ref|NC_002745.2|	26708	+	G	0	0	1	0	0	SA0018
gi|29165615|ref|NC_002745.2|	26709	+	A	0	2	2	0	0	SA0018
gi|29165615|ref|NC_002745.2|	26711	+	U	0	0	2	0	0	SA0018
gi|29165615|ref|NC_002745.2|	26723	+	A	1	0	0	0	0	SA0018

gi|29165615|ref|NC_002745.2|	26730	+	U	0	1	0	0	0	SA0018
gi|29165615|ref|NC_002745.2|	26749	+	G	0	1	0	0	0	SA0018
gi|29165615|ref|NC_002745.2|	26826	+	U	0	1	0	0	0	SA0018
gi|29165615|ref|NC_002745.2|	26830	+	A	0	0	0	0	1	SA0018
gi|29165615|ref|NC_002745.2|	26831	+	A	0	1	0	0	0	SA0018
gi|29165615|ref|NC_002745.2|	26837	+	A	0	0	2	0	0	SA0018
gi|29165615|ref|NC_002745.2|	26838	+	C	0	1	0	0	0	SA0018
gi|29165615|ref|NC_002745.2|	26870	+	A	0	0	0	0	1	SA0018
gi|29165615|ref|NC_002745.2|	27118	+	A	0	0	0	0	2	SA0018
gi|29165615|ref|NC_002745.2|	27133	+	G	1	0	0	0	0	SA0018
gi|29165615|ref|NC_002745.2|	27160	+	U	0	1	0	0	0	SA0018
gi|29165615|ref|NC_002745.2|	27173	+	A	0	0	0	0	1	SA0018
gi|29165615|ref|NC_002745.2|	27192	+	C	1	0	0	0	0	SA0018
gi|29165615|ref|NC_002745.2|	27203	+	A	0	0	0	0	1	SA0018
gi|29165615|ref|NC_002745.2|	27210	+	U	0	1	0	0	0	SA0018
gi|29165615|ref|NC_002745.2|	27215	+	A	0	2	0	0	0	SA0018
gi|29165615|ref|NC_002745.2|	27219	+	A	0	1	0	0	0	SA0018
gi|29165615|ref|NC_002745.2|	27257	+	C	0	1	0	0	0	SA0018
gi|29165615|ref|NC_002745.2|	27297	+	U	0	1	0	0	0	SA0018
gi|29165615|ref|NC_002745.2|	27304	+	A	1	0	0	0	0	SA0018
gi|29165615|ref|NC_002745.2|	27305	+	A	0	3	0	0	3	SA0018
gi|29165615|ref|NC_002745.2|	27321	+	A	0	0	1	0	0	SA0018
gi|29165615|ref|NC_002745.2|	27331	+	A	0	0	0	0	1	SA0018
gi|29165615|ref|NC_002745.2|	27369	+	A	0	0	1	0	0	SA0018
gi|29165615|ref|NC_002745.2|	27370	+	G	0	1	0	0	0	SA0018
gi|29165615|ref|NC_002745.2|	27377	+	A	0	2	0	0	0	SA0018
gi|29165615|ref|NC_002745.2|	27386	+	U	0	0	1	0	0	SA0018
gi|29165615|ref|NC_002745.2|	27431	+	C	0	0	0	0	1	SA0018
gi|29165615|ref|NC_002745.2|	27472	+	U	0	0	0	0	1	SA0018_SA0019
gi|29165615|ref|NC_002745.2|	27490	+	A	0	1	0	0	0	SA0019
gi|29165615|ref|NC_002745.2|	27498	+	U	0	0	0	0	1	SA0019
gi|29165615|ref|NC_002745.2|	27517	+	G	0	1	0	0	0	SA0019
gi|29165615|ref|NC_002745.2|	27537	+	A	0	0	1	0	0	SA0019
gi|29165615|ref|NC_002745.2|	27560	+	G	0	0	0	0	2	SA0019
gi|29165615|ref|NC_002745.2|	27589	+	U	0	0	0	1	0	SA0019
gi|29165615|ref|NC_002745.2|	27621	+	C	1	0	0	0	0	SA0019
gi|29165615|ref|NC_002745.2|	27627	+	C	0	0	0	1	1	SA0019
gi|29165615|ref|NC_002745.2|	27645	+	C	0	2	0	0	0	SA0019
gi|29165615|ref|NC_002745.2|	27717	+	U	0	1	0	0	0	SA0019
gi|29165615|ref|NC_002745.2|	27767	+	C	0	1	0	0	0	SA0019
gi|29165615|ref|NC_002745.2|	27804	+	U	0	3	0	0	0	SA0019
gi|29165615|ref|NC_002745.2|	27814	+	U	0	0	0	0	1	SA0019
gi|29165615|ref|NC_002745.2|	27820	+	A	0	0	0	0	1	SA0019
gi|29165615|ref|NC_002745.2|	27823	+	A	0	1	0	0	0	SA0019
gi|29165615|ref|NC_002745.2|	27865	+	C	0	1	0	0	1	SA0019
gi|29165615|ref|NC_002745.2|	27897	+	A	0	1	0	0	0	SA0019
gi|29165615|ref|NC_002745.2|	27979	+	C	0	1	0	0	0	SA0019
gi|29165615|ref|NC_002745.2|	27991	+	A	0	0	0	0	1	SA0019
gi|29165615|ref|NC_002745.2|	28010	+	A	0	0	1	0	0	SA0019
gi|29165615|ref|NC_002745.2|	28013	+	G	0	0	0	0	1	SA0019
gi|29165615|ref|NC_002745.2|	28029	+	C	0	2	0	0	0	SA0019
gi|29165615|ref|NC_002745.2|	28068	+	A	0	0	0	0	1	SA0019
gi|29165615|ref|NC_002745.2|	28082	+	A	0	0	0	0	1	SA0019
gi|29165615|ref|NC_002745.2|	28422	+	A	0	0	0	1	0	SA0019
gi|29165615|ref|NC_002745.2|	28427	+	C	0	0	0	1	0	SA0019
gi|29165615|ref|NC_002745.2|	28493	+	A	0	0	1	0	0	SA0019
gi|29165615|ref|NC_002745.2|	28508	+	A	0	0	1	0	0	SA0019
gi|29165615|ref|NC_002745.2|	28519	+	A	0	0	1	0	0	SA0019
gi|29165615|ref|NC_002745.2|	28543	+	U	0	0	0	0	1	SA0019
gi|29165615|ref|NC_002745.2|	28569	+	U	1	0	0	0	0	SA0019
gi|29165615|ref|NC_002745.2|	28588	+	A	0	0	0	0	1	SA0019
gi|29165615|ref|NC_002745.2|	28595	+	C	0	1	0	0	0	SA0019
gi|29165615|ref|NC_002745.2|	28598	+	A	0	1	0	0	0	SA0019
gi|29165615|ref|NC_002745.2|	28613	+	C	0	1	0	0	0	SA0019
gi|29165615|ref|NC_002745.2|	28617	+	C	0	0	0	1	0	SA0019
gi|29165615|ref|NC_002745.2|	28619	+	A	0	1	0	0	0	SA0019
gi|29165615|ref|NC_002745.2|	28624	+	A	1	0	0	0	0	SA0019
gi|29165615|ref|NC_002745.2|	28625	+	G	1	0	0	0	0	SA0019
gi|29165615|ref|NC_002745.2|	28632	+	A	0	1	0	0	2	SA0019
gi|29165615|ref|NC_002745.2|	28644	+	A	0	2	1	0	0	SA0019
gi|29165615|ref|NC_002745.2|	28646	+	U	0	2	0	0	1	SA0019
gi|29165615|ref|NC_002745.2|	28655	+	G	0	0	0	1	0	SA0019
gi|29165615|ref|NC_002745.2|	28662	+	A	0	1	0	0	1	SA0019
gi|29165615|ref|NC_002745.2|	28663	+	C	0	0	1	0	0	SA0019
gi|29165615|ref|NC_002745.2|	28812	+	A	0	1	0	0	0	SA0020
gi|29165615|ref|NC_002745.2|	28815	+	U	0	0	0	0	1	SA0020
gi|29165615|ref|NC_002745.2|	28837	+	C	1	0	0	0	0	SA0020
gi|29165615|ref|NC_002745.2|	28852	+	C	2	0	0	0	0	SA0020
gi|29165615|ref|NC_002745.2|	28874	+	U	0	0	0	0	1	SA0020
gi|29165615|ref|NC_002745.2|	28875	+	A	0	1	0	0	2	SA0020
gi|29165615|ref|NC_002745.2|	28890	+	A	0	2	0	0	0	SA0020

gi|29165615|ref|NC_002745.2|	28907 +	G	0	0	0	1	0	SA0020
gi|29165615|ref|NC_002745.2|	28921 +	C	1	0	0	0	0	SA0020
gi|29165615|ref|NC_002745.2|	28947 +	A	0	0	0	0	1	SA0020
gi|29165615|ref|NC_002745.2|	28978 +	A	0	3	0	0	2	SA0020
gi|29165615|ref|NC_002745.2|	28982 +	G	0	0	0	1	0	SA0020
gi|29165615|ref|NC_002745.2|	29015 +	C	0	1	0	0	0	SA0020
gi|29165615|ref|NC_002745.2|	29080 +	A	0	0	0	0	1	SA0020
gi|29165615|ref|NC_002745.2|	29124 +	A	0	0	0	0	2	SA0020
gi|29165615|ref|NC_002745.2|	29148 +	A	0	0	0	0	1	SA0020
gi|29165615|ref|NC_002745.2|	29215 +	A	1	1	0	0	0	SA0020
gi|29165615|ref|NC_002745.2|	29217 +	A	0	1	0	0	0	SA0020
gi|29165615|ref|NC_002745.2|	29253 +	A	1	16	3	0	14	SA0020
gi|29165615|ref|NC_002745.2|	29258 +	A	0	1	0	0	0	SA0020
gi|29165615|ref|NC_002745.2|	29263 +	G	0	0	0	0	1	SA0020
gi|29165615|ref|NC_002745.2|	29264 +	A	0	0	1	0	0	SA0020
gi|29165615|ref|NC_002745.2|	29276 +	A	0	0	0	1	0	SA0020
gi|29165615|ref|NC_002745.2|	29284 +	A	0	0	1	0	0	SA0020
gi|29165615|ref|NC_002745.2|	29286 +	A	0	2	0	0	0	SA0020
gi|29165615|ref|NC_002745.2|	29287 +	U	0	1	0	0	0	SA0020
gi|29165615|ref|NC_002745.2|	29296 +	G	0	0	0	0	1	SA0020
gi|29165615|ref|NC_002745.2|	29303 +	U	0	1	0	0	0	SA0020
gi|29165615|ref|NC_002745.2|	29310 +	A	0	1	0	0	0	SA0020
gi|29165615|ref|NC_002745.2|	29315 +	A	0	0	1	0	0	SA0020
gi|29165615|ref|NC_002745.2|	29336 +	A	0	0	0	0	1	SA0020
gi|29165615|ref|NC_002745.2|	29457 +	C	0	0	1	0	0	SA0020
gi|29165615|ref|NC_002745.2|	29796 +	A	0	0	1	0	0	-
gi|29165615|ref|NC_002745.2|	29797 +	G	0	5	2	2	9	-
gi|29165615|ref|NC_002745.2|	29801 +	U	1	0	0	0	0	-
gi|29165615|ref|NC_002745.2|	29808 +	C	0	2	0	0	0	-
gi|29165615|ref|NC_002745.2|	29809 +	A	0	1	0	0	0	-
gi|29165615|ref|NC_002745.2|	29812 +	U	0	0	0	0	1	-
gi|29165615|ref|NC_002745.2|	29825 +	U	0	0	0	1	0	-
gi|29165615|ref|NC_002745.2|	29828 +	A	0	0	0	0	1	-
gi|29165615|ref|NC_002745.2|	29830 +	U	0	1	0	0	0	-
gi|29165615|ref|NC_002745.2|	29836 +	A	0	0	1	2	1	-
gi|29165615|ref|NC_002745.2|	29838 +	A	0	0	1	0	0	-
gi|29165615|ref|NC_002745.2|	29842 +	G	0	0	1	0	0	-
gi|29165615|ref|NC_002745.2|	29849 +	A	0	0	0	1	0	-
gi|29165615|ref|NC_002745.2|	29860 +	C	0	0	0	1	1	-
gi|29165615|ref|NC_002745.2|	29861 +	A	0	2	0	0	2	-
gi|29165615|ref|NC_002745.2|	29863 +	A	0	0	0	1	1	-
gi|29165615|ref|NC_002745.2|	29864 +	A	0	1	0	0	0	-
gi|29165615|ref|NC_002745.2|	29865 +	U	0	0	1	0	2	-
gi|29165615|ref|NC_002745.2|	29876 +	U	0	0	0	0	1	-
gi|29165615|ref|NC_002745.2|	29886 +	C	0	0	0	1	0	-
gi|29165615|ref|NC_002745.2|	29891 +	A	0	0	1	0	0	-
gi|29165615|ref|NC_002745.2|	29896 +	G	0	0	0	0	1	-
gi|29165615|ref|NC_002745.2|	29898 +	U	0	0	0	1	0	-
gi|29165615|ref|NC_002745.2|	29907 +	U	0	0	1	0	0	-
gi|29165615|ref|NC_002745.2|	29921 +	U	0	0	1	0	0	-
gi|29165615|ref|NC_002745.2|	29967 +	U	0	0	0	0	1	-
gi|29165615|ref|NC_002745.2|	29974 +	A	1	1	0	2	0	-
gi|29165615|ref|NC_002745.2|	29978 +	G	0	0	1	0	0	-
gi|29165615|ref|NC_002745.2|	30006 +	U	0	0	0	0	1	SA0021
gi|29165615|ref|NC_002745.2|	30031 +	C	0	0	1	0	0	SA0021
gi|29165615|ref|NC_002745.2|	30037 +	U	2	0	0	0	0	SA0021
gi|29165615|ref|NC_002745.2|	30043 +	A	0	1	0	0	0	SA0021
gi|29165615|ref|NC_002745.2|	30044 +	A	0	1	0	0	1	SA0021
gi|29165615|ref|NC_002745.2|	30045 +	A	0	0	1	1	1	SA0021
gi|29165615|ref|NC_002745.2|	30046 +	A	0	1	0	0	0	SA0021
gi|29165615|ref|NC_002745.2|	30056 +	U	0	1	0	0	0	SA0021
gi|29165615|ref|NC_002745.2|	30059 +	U	0	0	0	1	0	SA0021
gi|29165615|ref|NC_002745.2|	30060 +	C	0	0	1	0	0	SA0021
gi|29165615|ref|NC_002745.2|	30093 +	A	0	1	1	0	0	SA0021
gi|29165615|ref|NC_002745.2|	30131 +	A	0	0	0	1	0	SA0021
gi|29165615|ref|NC_002745.2|	30133 +	A	0	1	1	0	0	SA0021
gi|29165615|ref|NC_002745.2|	30136 +	U	0	0	0	0	1	SA0021
gi|29165615|ref|NC_002745.2|	30143 +	U	0	1	0	0	0	SA0021
gi|29165615|ref|NC_002745.2|	30144 +	A	0	0	1	0	0	SA0021
gi|29165615|ref|NC_002745.2|	30150 +	G	0	0	0	0	1	SA0021
gi|29165615|ref|NC_002745.2|	30166 +	U	0	1	0	0	0	SA0021
gi|29165615|ref|NC_002745.2|	30196 +	C	1	0	0	0	0	SA0021
gi|29165615|ref|NC_002745.2|	30214 +	C	1	0	0	0	0	SA0021
gi|29165615|ref|NC_002745.2|	30236 +	U	0	0	0	1	0	SA0021
gi|29165615|ref|NC_002745.2|	30267 +	A	0	0	0	0	1	SA0021
gi|29165615|ref|NC_002745.2|	30286 +	U	1	0	0	0	0	SA0021
gi|29165615|ref|NC_002745.2|	30318 +	G	0	0	1	0	0	SA0021
gi|29165615|ref|NC_002745.2|	30319 +	C	0	1	1	2	1	SA0021
gi|29165615|ref|NC_002745.2|	30343 +	A	1	0	0	0	0	SA0021
gi|29165615|ref|NC_002745.2|	30369 +	C	0	0	1	0	1	SA0021
gi|29165615|ref|NC_002745.2|	30370 +	A	0	0	0	0	1	SA0021

gi|29165615|ref|NC_002745.2|	30371	+	A	0	0	1	0	0	SA0021
gi|29165615|ref|NC_002745.2|	30373	+	U	0	0	1	0	0	SA0021
gi|29165615|ref|NC_002745.2|	30376	+	A	0	3	0	2	0	SA0021
gi|29165615|ref|NC_002745.2|	30377	+	U	1	11	4	7	8	SA0021
gi|29165615|ref|NC_002745.2|	30378	+	A	0	0	1	1	2	SA0021
gi|29165615|ref|NC_002745.2|	30379	+	U	0	0	1	0	0	SA0021
gi|29165615|ref|NC_002745.2|	30382	+	U	1	2	0	1	1	SA0021
gi|29165615|ref|NC_002745.2|	30383	+	C	0	1	0	0	0	SA0021
gi|29165615|ref|NC_002745.2|	30384	+	C	0	0	0	0	1	SA0021
gi|29165615|ref|NC_002745.2|	30385	+	A	0	0	1	0	1	SA0021
gi|29165615|ref|NC_002745.2|	30388	+	A	0	1	1	0	3	SA0021
gi|29165615|ref|NC_002745.2|	30389	+	U	0	0	0	0	1	SA0021
gi|29165615|ref|NC_002745.2|	30390	+	A	0	0	1	1	1	SA0021
gi|29165615|ref|NC_002745.2|	30391	+	A	0	12	2	7	7	SA0021
gi|29165615|ref|NC_002745.2|	30392	+	C	0	0	1	0	0	SA0021
gi|29165615|ref|NC_002745.2|	30394	+	A	2	7	2	5	4	SA0021
gi|29165615|ref|NC_002745.2|	30395	+	U	1	6	1	1	2	SA0021
gi|29165615|ref|NC_002745.2|	30396	+	A	2	3	2	4	2	SA0021
gi|29165615|ref|NC_002745.2|	30397	+	A	0	10	3	7	8	SA0021
gi|29165615|ref|NC_002745.2|	30398	+	G	0	4	2	4	3	SA0021
gi|29165615|ref|NC_002745.2|	30399	+	A	0	16	7	7	5	SA0021
gi|29165615|ref|NC_002745.2|	30400	+	A	0	1	0	0	0	SA0021
gi|29165615|ref|NC_002745.2|	30401	+	G	0	0	0	0	1	SA0021
gi|29165615|ref|NC_002745.2|	30402	+	U	1	0	3	2	0	SA0021
gi|29165615|ref|NC_002745.2|	30403	+	U	1	0	0	0	0	SA0021
gi|29165615|ref|NC_002745.2|	30404	+	U	1	1	0	1	0	SA0021
gi|29165615|ref|NC_002745.2|	30405	+	A	0	0	1	0	0	SA0021
gi|29165615|ref|NC_002745.2|	30406	+	C	0	1	0	0	0	SA0021
gi|29165615|ref|NC_002745.2|	30407	+	G	0	1	0	0	2	SA0021
gi|29165615|ref|NC_002745.2|	30408	+	A	1	4	3	7	2	SA0021
gi|29165615|ref|NC_002745.2|	30409	+	U	0	0	0	1	0	SA0021
gi|29165615|ref|NC_002745.2|	30410	+	U	1	0	0	0	0	SA0021
gi|29165615|ref|NC_002745.2|	30411	+	U	0	0	0	1	0	SA0021
gi|29165615|ref|NC_002745.2|	30412	+	U	0	1	0	0	0	SA0021
gi|29165615|ref|NC_002745.2|	30413	+	A	0	0	0	1	0	SA0021
gi|29165615|ref|NC_002745.2|	30414	+	A	0	4	0	0	2	SA0021
gi|29165615|ref|NC_002745.2|	30417	+	G	3	2	1	0	2	SA0021
gi|29165615|ref|NC_002745.2|	30419	+	U	0	0	0	1	0	SA0021
gi|29165615|ref|NC_002745.2|	30420	+	A	0	0	1	0	0	SA0021
gi|29165615|ref|NC_002745.2|	30421	+	C	0	0	1	0	0	SA0021
gi|29165615|ref|NC_002745.2|	30424	+	G	1	0	0	0	0	SA0021
gi|29165615|ref|NC_002745.2|	30425	+	U	2	0	0	0	0	SA0021
gi|29165615|ref|NC_002745.2|	30430	+	U	1	0	0	0	0	SA0021
gi|29165615|ref|NC_002745.2|	30436	+	A	1	0	0	0	0	SA0021
gi|29165615|ref|NC_002745.2|	30440	+	U	0	0	0	0	2	SA0021
gi|29165615|ref|NC_002745.2|	30444	+	A	0	0	1	0	0	SA0021
gi|29165615|ref|NC_002745.2|	30447	+	G	0	1	0	0	0	SA0021
gi|29165615|ref|NC_002745.2|	30449	+	U	1	0	0	1	0	SA0021
gi|29165615|ref|NC_002745.2|	30450	+	A	0	0	2	0	0	SA0021
gi|29165615|ref|NC_002745.2|	30453	+	A	0	0	0	1	2	SA0021
gi|29165615|ref|NC_002745.2|	30454	+	U	2	0	0	0	0	SA0021
gi|29165615|ref|NC_002745.2|	30456	+	C	0	0	1	0	0	SA0021
gi|29165615|ref|NC_002745.2|	30462	+	A	0	1	0	0	0	SA0021
gi|29165615|ref|NC_002745.2|	30464	+	C	0	1	0	0	0	SA0021
gi|29165615|ref|NC_002745.2|	30471	+	U	0	0	0	0	1	SA0021
gi|29165615|ref|NC_002745.2|	30472	+	U	0	0	0	0	1	SA0021
gi|29165615|ref|NC_002745.2|	30473	+	U	1	0	0	0	0	SA0021
gi|29165615|ref|NC_002745.2|	30479	+	U	1	0	0	0	0	SA0021
gi|29165615|ref|NC_002745.2|	30481	+	A	0	1	1	2	2	SA0021
gi|29165615|ref|NC_002745.2|	30485	+	U	0	0	1	0	0	SA0021
gi|29165615|ref|NC_002745.2|	30486	+	A	0	1	1	0	0	SA0021
gi|29165615|ref|NC_002745.2|	30487	+	A	1	2	1	2	0	SA0021
gi|29165615|ref|NC_002745.2|	30491	+	U	0	0	0	0	1	SA0021
gi|29165615|ref|NC_002745.2|	30492	+	G	0	0	0	1	0	SA0021
gi|29165615|ref|NC_002745.2|	30493	+	A	1	0	1	0	0	SA0021
gi|29165615|ref|NC_002745.2|	30496	+	U	0	1	0	1	0	SA0021
gi|29165615|ref|NC_002745.2|	30499	+	A	1	0	0	1	1	SA0021
gi|29165615|ref|NC_002745.2|	30500	+	U	0	1	0	0	0	SA0021
gi|29165615|ref|NC_002745.2|	30501	+	A	0	0	1	0	0	SA0021
gi|29165615|ref|NC_002745.2|	30504	+	U	0	0	1	0	0	SA0021
gi|29165615|ref|NC_002745.2|	30507	+	A	0	0	1	0	0	SA0021
gi|29165615|ref|NC_002745.2|	30509	+	A	0	1	0	1	0	SA0021
gi|29165615|ref|NC_002745.2|	30510	+	A	1	0	1	0	0	SA0021
gi|29165615|ref|NC_002745.2|	30516	+	C	1	0	0	0	0	SA0021
gi|29165615|ref|NC_002745.2|	30523	+	C	2	0	0	0	0	SA0021
gi|29165615|ref|NC_002745.2|	30531	+	A	0	0	0	0	1	SA0021
gi|29165615|ref|NC_002745.2|	30533	+	G	0	0	1	1	0	SA0021
gi|29165615|ref|NC_002745.2|	30534	+	A	0	1	0	3	0	SA0021
gi|29165615|ref|NC_002745.2|	30535	+	A	0	0	0	1	0	SA0021
gi|29165615|ref|NC_002745.2|	30536	+	A	0	1	0	0	0	SA0021
gi|29165615|ref|NC_002745.2|	30538	+	A	1	0	0	0	0	SA0021

gi|29165615|ref|NC_002745.2|	30542 +	U	0	1	0	3	0	SA0021
gi|29165615|ref|NC_002745.2|	30543 +	A	0	4	1	0	0	SA0021
gi|29165615|ref|NC_002745.2|	30544 +	U	0	0	0	0	1	SA0021
gi|29165615|ref|NC_002745.2|	30548 +	A	0	0	0	0	1	SA0021
gi|29165615|ref|NC_002745.2|	30550 +	G	1	0	0	0	0	SA0021
gi|29165615|ref|NC_002745.2|	30551 +	C	0	0	0	1	0	SA0021
gi|29165615|ref|NC_002745.2|	30560 +	U	3	0	0	0	0	SA0021
gi|29165615|ref|NC_002745.2|	30561 +	C	0	2	0	0	0	SA0021
gi|29165615|ref|NC_002745.2|	30567 +	U	0	0	0	2	0	SA0021
gi|29165615|ref|NC_002745.2|	30574 +	A	0	0	1	0	2	SA0021
gi|29165615|ref|NC_002745.2|	30575 +	G	1	0	0	0	0	SA0021
gi|29165615|ref|NC_002745.2|	30576 +	G	0	1	1	0	1	SA0021
gi|29165615|ref|NC_002745.2|	30580 +	C	0	3	0	0	0	SA0021
gi|29165615|ref|NC_002745.2|	30615 +	A	0	0	0	0	1	SA0021
gi|29165615|ref|NC_002745.2|	30636 +	C	0	1	0	0	0	SA0021
gi|29165615|ref|NC_002745.2|	30646 +	A	0	0	0	2	0	SA0021
gi|29165615|ref|NC_002745.2|	30649 +	A	0	1	1	0	0	SA0021
gi|29165615|ref|NC_002745.2|	30652 +	A	0	1	0	0	1	SA0021
gi|29165615|ref|NC_002745.2|	30653 +	U	0	0	0	1	0	SA0021
gi|29165615|ref|NC_002745.2|	30654 +	A	0	0	0	1	0	SA0021
gi|29165615|ref|NC_002745.2|	30655 +	A	0	7	0	0	6	SA0021
gi|29165615|ref|NC_002745.2|	30656 +	C	0	2	1	1	3	SA0021
gi|29165615|ref|NC_002745.2|	30657 +	A	0	0	0	2	0	SA0021
gi|29165615|ref|NC_002745.2|	30658 +	U	0	0	1	0	0	SA0021
gi|29165615|ref|NC_002745.2|	30660 +	A	0	0	0	0	1	SA0021
gi|29165615|ref|NC_002745.2|	30661 +	A	0	0	0	0	1	SA0021
gi|29165615|ref|NC_002745.2|	30663 +	G	0	1	0	0	0	SA0021
gi|29165615|ref|NC_002745.2|	30669 +	G	0	3	1	1	0	SA0021
gi|29165615|ref|NC_002745.2|	30670 +	C	0	1	1	0	0	SA0021
gi|29165615|ref|NC_002745.2|	30671 +	G	0	0	1	0	0	SA0021
gi|29165615|ref|NC_002745.2|	30695 +	G	0	1	0	0	0	SA0021
gi|29165615|ref|NC_002745.2|	30700 +	A	0	0	0	0	1	SA0021
gi|29165615|ref|NC_002745.2|	30712 +	A	0	1	1	0	0	SA0021
gi|29165615|ref|NC_002745.2|	30724 +	A	0	0	0	1	0	SA0021
gi|29165615|ref|NC_002745.2|	30728 +	A	0	1	0	0	0	SA0021
gi|29165615|ref|NC_002745.2|	30765 +	A	0	0	0	0	1	SA0021
gi|29165615|ref|NC_002745.2|	31138 +	G	0	0	0	1	0	SA0022
gi|29165615|ref|NC_002745.2|	31233 +	A	1	0	0	0	0	SA0022
gi|29165615|ref|NC_002745.2|	31439 +	A	0	1	0	0	0	SA0022
gi|29165615|ref|NC_002745.2|	31450 +	C	0	0	0	1	0	SA0022
gi|29165615|ref|NC_002745.2|	31462 +	C	0	0	0	0	1	SA0022
gi|29165615|ref|NC_002745.2|	31467 +	A	0	0	0	1	0	SA0022
gi|29165615|ref|NC_002745.2|	31539 +	A	0	0	1	1	0	SA0022
gi|29165615|ref|NC_002745.2|	31542 +	A	0	0	0	1	0	SA0022
gi|29165615|ref|NC_002745.2|	31543 +	C	0	1	0	0	0	SA0022
gi|29165615|ref|NC_002745.2|	31544 +	A	0	0	0	0	1	SA0022
gi|29165615|ref|NC_002745.2|	31753 +	U	0	0	0	1	0	SA0022
gi|29165615|ref|NC_002745.2|	31789 +	A	0	0	0	0	1	SA0022
gi|29165615|ref|NC_002745.2|	32130 +	A	0	1	0	0	0	SA0022
gi|29165615|ref|NC_002745.2|	32380 +	A	0	0	1	0	0	SA0022
gi|29165615|ref|NC_002745.2|	32483 +	A	0	1	0	0	0	SA0022
gi|29165615|ref|NC_002745.2|	32518 +	U	0	1	0	0	0	SA0022
gi|29165615|ref|NC_002745.2|	32536 +	A	0	1	0	0	0	SA0022
gi|29165615|ref|NC_002745.2|	32767 +	A	0	0	1	0	0	SA0022
gi|29165615|ref|NC_002745.2|	32929 +	C	0	1	0	0	0	SA0022
gi|29165615|ref|NC_002745.2|	32935 +	U	0	0	0	0	1	SA0022
gi|29165615|ref|NC_002745.2|	32976 +	A	0	1	0	0	0	SA0022
gi|29165615|ref|NC_002745.2|	32984 +	G	0	1	0	0	0	SA0022
gi|29165615|ref|NC_002745.2|	33015 +	A	0	1	0	1	0	SA0022
gi|29165615|ref|NC_002745.2|	33016 +	C	0	0	0	1	0	SA0022
gi|29165615|ref|NC_002745.2|	33258 +	A	0	0	0	0	1	SA0022
gi|29165615|ref|NC_002745.2|	33561 +	A	0	0	1	0	0	-
gi|29165615|ref|NC_002745.2|	33622 +	G	0	5	0	2	2	-
gi|29165615|ref|NC_002745.2|	33667 +	A	0	0	0	1	0	-
gi|29165615|ref|NC_002745.2|	33670 +	A	0	0	0	0	1	-
gi|29165615|ref|NC_002745.2|	33687 +	A	0	0	1	0	0	-
gi|29165615|ref|NC_002745.2|	33697 +	A	0	1	0	0	0	SA0023
gi|29165615|ref|NC_002745.2|	33703 +	C	1	0	0	0	0	SA0023
gi|29165615|ref|NC_002745.2|	33740 +	A	0	0	1	0	0	SA0023
gi|29165615|ref|NC_002745.2|	33757 +	A	0	1	0	0	0	SA0023
gi|29165615|ref|NC_002745.2|	33770 +	U	0	1	0	0	0	SA0023
gi|29165615|ref|NC_002745.2|	33815 +	A	0	1	0	0	0	SA0023
gi|29165615|ref|NC_002745.2|	33839 +	A	0	0	0	0	1	SA0023
gi|29165615|ref|NC_002745.2|	33841 +	A	0	0	0	0	1	SA0023
gi|29165615|ref|NC_002745.2|	33856 +	A	0	0	1	0	0	SA0023
gi|29165615|ref|NC_002745.2|	33899 +	C	0	0	1	0	0	SA0023
gi|29165615|ref|NC_002745.2|	33902 +	U	0	1	0	0	0	SA0023
gi|29165615|ref|NC_002745.2|	33921 +	A	0	1	0	0	0	SA0023
gi|29165615|ref|NC_002745.2|	33923 +	A	0	1	0	0	0	SA0023
gi|29165615|ref|NC_002745.2|	33976 +	C	0	0	0	0	1	SA0023
gi|29165615|ref|NC_002745.2|	33981 +	C	1	0	0	0	0	SA0023

gi|29165615|ref|NC_002745.2|	34002	+	U	0	1	0	0	0	SA0023
gi|29165615|ref|NC_002745.2|	34090	+	A	0	0	0	0	1	SA0023
gi|29165615|ref|NC_002745.2|	34091	+	C	0	1	0	0	1	SA0023
gi|29165615|ref|NC_002745.2|	34139	+	A	0	0	0	0	1	SA0023
gi|29165615|ref|NC_002745.2|	87141	+	C	0	0	1	0	0	-
gi|29165615|ref|NC_002745.2|	87146	+	A	0	0	1	0	0	-
gi|29165615|ref|NC_002745.2|	87149	+	U	0	0	1	0	0	-
gi|29165615|ref|NC_002745.2|	87177	+	G	0	0	0	0	1	-
gi|29165615|ref|NC_002745.2|	87223	+	A	0	1	0	0	0	-
gi|29165615|ref|NC_002745.2|	87225	+	A	2	4	2	0	1	-
gi|29165615|ref|NC_002745.2|	87476	+	A	0	0	0	0	1	-
gi|29165615|ref|NC_002745.2|	87485	+	A	0	0	0	1	0	-
gi|29165615|ref|NC_002745.2|	87553	+	C	0	1	0	0	0	-
gi|29165615|ref|NC_002745.2|	87593	+	A	0	0	1	0	0	-
gi|29165615|ref|NC_002745.2|	87601	+	A	0	0	0	0	1	-
gi|29165615|ref|NC_002745.2|	87607	+	A	0	1	0	0	0	-
gi|29165615|ref|NC_002745.2|	87614	+	A	0	1	0	1	3	-
gi|29165615|ref|NC_002745.2|	87616	+	A	0	0	1	0	0	-
gi|29165615|ref|NC_002745.2|	87629	+	C	0	0	0	0	1	-
gi|29165615|ref|NC_002745.2|	87640	+	A	0	0	1	0	0	-
gi|29165615|ref|NC_002745.2|	87751	+	A	0	1	0	0	0	SA0078
gi|29165615|ref|NC_002745.2|	87853	+	C	0	1	0	0	0	SA0078
gi|29165615|ref|NC_002745.2|	87964	+	C	0	0	1	0	0	SA0078
gi|29165615|ref|NC_002745.2|	88033	+	U	0	1	0	0	0	SA0078
gi|29165615|ref|NC_002745.2|	88148	+	A	0	0	0	0	1	SA0078
gi|29165615|ref|NC_002745.2|	88221	+	A	0	1	0	0	0	SA0078
gi|29165615|ref|NC_002745.2|	88512	+	U	0	0	0	1	0	SA0078
gi|29165615|ref|NC_002745.2|	88514	+	U	0	0	0	0	2	SA0078
gi|29165615|ref|NC_002745.2|	88517	+	G	0	0	0	1	0	SA0078
gi|29165615|ref|NC_002745.2|	88518	+	A	0	0	1	0	0	SA0078
gi|29165615|ref|NC_002745.2|	88541	+	U	0	0	0	1	0	SA0078
gi|29165615|ref|NC_002745.2|	88542	+	A	0	1	0	0	0	SA0078
gi|29165615|ref|NC_002745.2|	88543	+	U	0	0	0	1	0	SA0078
gi|29165615|ref|NC_002745.2|	88556	+	A	0	0	0	2	0	SA0078
gi|29165615|ref|NC_002745.2|	88557	+	A	0	0	0	1	0	SA0078
gi|29165615|ref|NC_002745.2|	88559	+	A	0	0	0	0	2	SA0078
gi|29165615|ref|NC_002745.2|	88562	+	A	0	1	0	0	1	SA0078
gi|29165615|ref|NC_002745.2|	88565	+	A	0	1	0	0	0	SA0078
gi|29165615|ref|NC_002745.2|	88566	+	C	0	1	0	0	0	SA0078
gi|29165615|ref|NC_002745.2|	88568	+	U	0	0	1	0	2	SA0078
gi|29165615|ref|NC_002745.2|	88569	+	U	0	0	1	0	0	SA0078
gi|29165615|ref|NC_002745.2|	88570	+	A	0	2	2	1	0	SA0078
gi|29165615|ref|NC_002745.2|	88571	+	G	0	0	0	0	1	SA0078
gi|29165615|ref|NC_002745.2|	88572	+	A	0	1	1	0	0	SA0078
gi|29165615|ref|NC_002745.2|	88573	+	G	0	0	0	0	1	SA0078
gi|29165615|ref|NC_002745.2|	88574	+	A	0	2	1	4	0	SA0078
gi|29165615|ref|NC_002745.2|	88577	+	U	0	0	0	1	0	SA0078
gi|29165615|ref|NC_002745.2|	88578	+	U	0	0	1	0	0	SA0078
gi|29165615|ref|NC_002745.2|	88580	+	G	0	0	1	0	0	SA0078
gi|29165615|ref|NC_002745.2|	88581	+	A	0	1	1	0	0	SA0078
gi|29165615|ref|NC_002745.2|	88585	+	G	1	0	0	0	0	SA0078
gi|29165615|ref|NC_002745.2|	88587	+	C	0	0	0	0	2	SA0078
gi|29165615|ref|NC_002745.2|	88627	+	C	0	0	0	1	0	SA0078
gi|29165615|ref|NC_002745.2|	88636	+	A	0	1	0	0	1	SA0078
gi|29165615|ref|NC_002745.2|	88641	+	A	0	1	0	0	0	SA0078
gi|29165615|ref|NC_002745.2|	88648	+	U	0	0	0	1	0	SA0078
gi|29165615|ref|NC_002745.2|	88650	+	C	0	0	1	0	0	SA0078
gi|29165615|ref|NC_002745.2|	88652	+	A	0	1	0	1	1	SA0078
gi|29165615|ref|NC_002745.2|	88653	+	U	2	0	0	1	1	SA0078
gi|29165615|ref|NC_002745.2|	88662	+	U	0	1	0	0	0	SA0078
gi|29165615|ref|NC_002745.2|	88665	+	G	0	0	0	1	0	SA0078
gi|29165615|ref|NC_002745.2|	88676	+	A	0	1	0	0	0	SA0078
gi|29165615|ref|NC_002745.2|	88691	+	C	0	0	0	1	0	SA0078
gi|29165615|ref|NC_002745.2|	88693	+	U	2	0	0	0	0	SA0078
gi|29165615|ref|NC_002745.2|	88695	+	C	0	0	0	0	2	SA0078
gi|29165615|ref|NC_002745.2|	88696	+	A	0	0	1	0	0	SA0078
gi|29165615|ref|NC_002745.2|	88699	+	U	0	0	0	0	1	SA0078
gi|29165615|ref|NC_002745.2|	88709	+	C	0	0	0	0	1	SA0078
gi|29165615|ref|NC_002745.2|	88712	+	A	0	0	1	0	1	SA0078
gi|29165615|ref|NC_002745.2|	88716	+	C	0	0	0	0	2	SA0078
gi|29165615|ref|NC_002745.2|	88718	+	A	0	1	1	1	2	SA0078
gi|29165615|ref|NC_002745.2|	88724	+	C	0	2	0	1	0	SA0078
gi|29165615|ref|NC_002745.2|	88731	+	A	0	0	0	0	2	SA0078
gi|29165615|ref|NC_002745.2|	88739	+	A	0	1	0	0	0	SA0078
gi|29165615|ref|NC_002745.2|	88743	+	A	0	0	0	0	1	SA0078
gi|29165615|ref|NC_002745.2|	88750	+	A	1	0	0	0	0	SA0078
gi|29165615|ref|NC_002745.2|	88761	+	U	1	0	0	0	0	SA0078
gi|29165615|ref|NC_002745.2|	88762	+	A	0	0	1	0	0	SA0078
gi|29165615|ref|NC_002745.2|	88770	+	A	0	0	1	0	0	SA0078
gi|29165615|ref|NC_002745.2|	88775	+	A	0	0	0	1	0	SA0078
gi|29165615|ref|NC_002745.2|	88783	+	U	1	0	0	0	0	SA0078

gi|29165615|ref|NC_002745.2|	88789 +	U	0	1	0	0	0	SA0078
gi|29165615|ref|NC_002745.2|	88792 +	U	0	1	0	0	0	SA0078
gi|29165615|ref|NC_002745.2|	88802 +	U	0	0	0	0	1	SA0078
gi|29165615|ref|NC_002745.2|	88805 +	G	1	1	0	0	0	SA0078
gi|29165615|ref|NC_002745.2|	88816 +	U	0	0	1	0	0	SA0078
gi|29165615|ref|NC_002745.2|	88869 +	U	0	0	0	1	0	SA0078
gi|29165615|ref|NC_002745.2|	88907 +	A	0	1	0	0	0	SA0078
gi|29165615|ref|NC_002745.2|	88951 +	A	0	0	0	0	1	SA0078
gi|29165615|ref|NC_002745.2|	89041 +	C	0	1	0	0	0	SA0078
gi|29165615|ref|NC_002745.2|	89273 +	C	0	0	0	1	0	SA0078
gi|29165615|ref|NC_002745.2|	89346 +	U	0	1	0	0	0	SA0078
gi|29165615|ref|NC_002745.2|	89372 +	C	0	0	0	0	1	SA0078
gi|29165615|ref|NC_002745.2|	89382 +	A	0	0	1	0	0	SA0078
gi|29165615|ref|NC_002745.2|	89396 +	A	0	0	0	2	0	SA0078
gi|29165615|ref|NC_002745.2|	89400 +	A	0	0	0	0	1	SA0078
gi|29165615|ref|NC_002745.2|	89408 +	A	0	1	0	0	0	SA0078
gi|29165615|ref|NC_002745.2|	89418 +	G	0	0	0	0	1	SA0078
gi|29165615|ref|NC_002745.2|	89419 +	A	0	1	0	1	0	SA0078
gi|29165615|ref|NC_002745.2|	89420 +	A	0	1	0	0	0	SA0078
gi|29165615|ref|NC_002745.2|	89421 +	A	0	0	0	1	0	SA0078
gi|29165615|ref|NC_002745.2|	89427 +	C	0	0	0	1	0	-
gi|29165615|ref|NC_002745.2|	89432 +	A	0	0	0	0	1	-
gi|29165615|ref|NC_002745.2|	89433 +	U	0	0	1	0	0	-
gi|29165615|ref|NC_002745.2|	89439 +	A	0	1	0	0	0	-
gi|29165615|ref|NC_002745.2|	89468 +	C	0	1	0	1	0	-
gi|29165615|ref|NC_002745.2|	89626 +	U	1	0	0	0	0	-
gi|29165615|ref|NC_002745.2|	90376 +	A	0	1	0	0	0	-
gi|29165615|ref|NC_002745.2|	91497 +	A	0	0	2	0	1	-
gi|29165615|ref|NC_002745.2|	92838 +	A	0	1	0	0	0	SA0083
gi|29165615|ref|NC_002745.2|	93165 +	A	0	0	1	0	0	SA0083
gi|29165615|ref|NC_002745.2|	93884 +	C	0	0	0	0	1	SA0083
gi|29165615|ref|NC_002745.2|	94345 +	A	3	0	0	0	0	SA0084
gi|29165615|ref|NC_002745.2|	94348 +	A	0	1	0	0	0	SA0084
gi|29165615|ref|NC_002745.2|	94413 +	A	0	0	0	0	1	SA0084
gi|29165615|ref|NC_002745.2|	94480 +	U	0	1	0	0	0	SA0084
gi|29165615|ref|NC_002745.2|	94486 +	A	0	1	0	0	0	SA0084
gi|29165615|ref|NC_002745.2|	94523 +	U	0	0	0	0	1	SA0084
gi|29165615|ref|NC_002745.2|	95113 +	A	0	1	0	0	0	SA0084
gi|29165615|ref|NC_002745.2|	95230 +	A	0	0	0	0	1	-
gi|29165615|ref|NC_002745.2|	97033 +	G	0	0	1	0	0	-
gi|29165615|ref|NC_002745.2|	97175 +	U	1	0	0	0	0	SA0086
gi|29165615|ref|NC_002745.2|	97267 +	C	0	0	0	1	0	SA0086
gi|29165615|ref|NC_002745.2|	97277 +	A	0	3	0	2	3	SA0086
gi|29165615|ref|NC_002745.2|	97280 +	U	0	0	0	0	2	SA0086
gi|29165615|ref|NC_002745.2|	97283 +	A	0	0	0	0	2	SA0086
gi|29165615|ref|NC_002745.2|	97289 +	C	0	0	1	2	1	SA0086
gi|29165615|ref|NC_002745.2|	97307 +	A	0	0	0	1	0	SA0086
gi|29165615|ref|NC_002745.2|	97311 +	A	0	1	0	1	1	SA0086
gi|29165615|ref|NC_002745.2|	97322 +	A	0	0	0	0	1	SA0086
gi|29165615|ref|NC_002745.2|	97340 +	A	0	0	1	0	0	-
gi|29165615|ref|NC_002745.2|	97374 +	C	0	2	0	0	0	-
gi|29165615|ref|NC_002745.2|	97419 +	U	2	0	0	0	0	SA0087
gi|29165615|ref|NC_002745.2|	97480 +	U	1	0	0	0	0	SA0087
gi|29165615|ref|NC_002745.2|	97484 +	G	1	1	1	1	0	SA0087
gi|29165615|ref|NC_002745.2|	97504 +	U	0	0	1	0	0	SA0087
gi|29165615|ref|NC_002745.2|	97656 +	A	0	0	0	0	2	SA0087
gi|29165615|ref|NC_002745.2|	97759 +	A	0	1	0	0	0	-
gi|29165615|ref|NC_002745.2|	97764 +	U	0	1	0	0	0	-
gi|29165615|ref|NC_002745.2|	97765 +	A	0	1	0	0	0	-
gi|29165615|ref|NC_002745.2|	97768 +	U	0	0	0	0	1	-
gi|29165615|ref|NC_002745.2|	97785 +	A	0	0	1	1	1	-
gi|29165615|ref|NC_002745.2|	97801 +	U	0	1	0	0	0	-
gi|29165615|ref|NC_002745.2|	97803 +	A	0	2	0	0	0	-
gi|29165615|ref|NC_002745.2|	97804 +	A	0	0	1	0	0	-
gi|29165615|ref|NC_002745.2|	97805 +	U	0	0	0	1	0	-
gi|29165615|ref|NC_002745.2|	97808 +	G	0	0	0	0	1	-
gi|29165615|ref|NC_002745.2|	97832 +	U	0	0	0	1	0	-
gi|29165615|ref|NC_002745.2|	97844 +	C	0	1	1	0	0	-
gi|29165615|ref|NC_002745.2|	97845 +	A	0	0	0	1	0	-
gi|29165615|ref|NC_002745.2|	97854 +	A	0	0	0	0	1	-
gi|29165615|ref|NC_002745.2|	97856 +	A	0	1	0	0	0	-
gi|29165615|ref|NC_002745.2|	97874 +	A	0	0	0	1	0	SA0088
gi|29165615|ref|NC_002745.2|	98029 +	U	0	0	1	0	0	SA0088
gi|29165615|ref|NC_002745.2|	98210 +	A	0	0	0	1	0	SA0088
gi|29165615|ref|NC_002745.2|	98227 +	A	0	0	0	0	1	SA0088
gi|29165615|ref|NC_002745.2|	98228 +	A	0	0	0	1	0	SA0088
gi|29165615|ref|NC_002745.2|	98237 +	G	0	1	0	0	0	SA0088
gi|29165615|ref|NC_002745.2|	98238 +	U	0	0	0	0	1	SA0088
gi|29165615|ref|NC_002745.2|	98248 +	G	0	1	0	0	0	SA0088
gi|29165615|ref|NC_002745.2|	98249 +	A	0	0	0	0	1	SA0088
gi|29165615|ref|NC_002745.2|	98300 +	A	0	1	0	0	6	SA0088

gi|29165615|ref|NC_002745.2|	98301	+	A	0	1	0	0	0	SA0088
gi|29165615|ref|NC_002745.2|	98303	+	A	0	0	1	1	1	SA0088
gi|29165615|ref|NC_002745.2|	98304	+	U	0	1	0	1	1	SA0088
gi|29165615|ref|NC_002745.2|	98306	+	U	0	1	1	0	0	SA0088
gi|29165615|ref|NC_002745.2|	98307	+	A	0	0	0	0	1	SA0088
gi|29165615|ref|NC_002745.2|	98349	+	U	0	0	0	0	1	SA0088
gi|29165615|ref|NC_002745.2|	98350	+	A	0	0	1	1	2	SA0088
gi|29165615|ref|NC_002745.2|	98353	+	G	0	0	0	1	1	SA0088
gi|29165615|ref|NC_002745.2|	98355	+	A	0	0	1	0	0	SA0088
gi|29165615|ref|NC_002745.2|	98714	+	A	1	6	3	3	3	-
gi|29165615|ref|NC_002745.2|	98715	+	A	0	0	0	1	0	-
gi|29165615|ref|NC_002745.2|	98716	+	G	0	0	0	1	0	-
gi|29165615|ref|NC_002745.2|	98726	+	A	0	0	2	0	0	-
gi|29165615|ref|NC_002745.2|	98734	+	A	0	0	1	0	0	-
gi|29165615|ref|NC_002745.2|	98743	+	G	0	0	0	0	2	-
gi|29165615|ref|NC_002745.2|	98748	+	C	0	0	0	0	1	-
gi|29165615|ref|NC_002745.2|	98756	+	A	0	1	0	0	0	SA0089
gi|29165615|ref|NC_002745.2|	98757	+	A	0	1	0	0	0	SA0089
gi|29165615|ref|NC_002745.2|	99278	+	A	0	1	0	0	0	SA0089
gi|29165615|ref|NC_002745.2|	99280	+	A	0	0	1	0	0	SA0089
gi|29165615|ref|NC_002745.2|	99355	+	A	0	1	0	0	0	SA0089
gi|29165615|ref|NC_002745.2|	99359	+	A	0	0	0	0	1	SA0089
gi|29165615|ref|NC_002745.2|	99371	+	A	0	0	0	0	1	SA0089
gi|29165615|ref|NC_002745.2|	99416	+	A	0	1	0	1	0	SA0089
gi|29165615|ref|NC_002745.2|	99936	+	A	0	1	0	0	0	SA0089
gi|29165615|ref|NC_002745.2|	100158	+	A	0	0	1	0	0	SA0089
gi|29165615|ref|NC_002745.2|	100302	+	A	0	0	1	0	0	SA0089
gi|29165615|ref|NC_002745.2|	100366	+	A	0	2	0	0	0	SA0089
gi|29165615|ref|NC_002745.2|	100408	+	A	0	1	0	0	0	SA0089
gi|29165615|ref|NC_002745.2|	100437	+	C	0	0	0	1	0	SA0089
gi|29165615|ref|NC_002745.2|	100617	+	G	0	0	1	0	0	SA0089
gi|29165615|ref|NC_002745.2|	100627	+	A	0	1	0	0	0	SA0089
gi|29165615|ref|NC_002745.2|	100636	+	A	0	1	0	0	0	SA0089
gi|29165615|ref|NC_002745.2|	100717	+	A	0	1	0	0	0	SA0089
gi|29165615|ref|NC_002745.2|	100837	+	C	0	0	0	1	0	SA0089
gi|29165615|ref|NC_002745.2|	100890	+	U	1	0	0	0	0	SA0089
gi|29165615|ref|NC_002745.2|	100906	+	A	0	0	1	0	0	SA0089
gi|29165615|ref|NC_002745.2|	100908	+	A	0	0	0	0	1	SA0089
gi|29165615|ref|NC_002745.2|	101038	+	U	0	1	0	0	0	SA0089
gi|29165615|ref|NC_002745.2|	101350	+	A	0	1	0	0	0	SA0089
gi|29165615|ref|NC_002745.2|	101359	+	A	0	1	0	0	0	SA0089
gi|29165615|ref|NC_002745.2|	101378	+	A	0	0	0	0	1	SA0089
gi|29165615|ref|NC_002745.2|	101382	+	U	0	0	1	0	0	SA0089
gi|29165615|ref|NC_002745.2|	101388	+	A	1	0	0	0	0	SA0089
gi|29165615|ref|NC_002745.2|	101389	+	A	10	9	21	9	7	SA0089
gi|29165615|ref|NC_002745.2|	101390	+	U	1	0	0	0	0	SA0089
gi|29165615|ref|NC_002745.2|	101391	+	G	0	1	0	0	0	SA0089
gi|29165615|ref|NC_002745.2|	101397	+	G	0	0	0	1	0	SA0089
gi|29165615|ref|NC_002745.2|	101413	+	A	0	0	0	0	2	SA0089
gi|29165615|ref|NC_002745.2|	101416	+	G	0	0	0	1	0	SA0089
gi|29165615|ref|NC_002745.2|	101417	+	U	0	0	0	0	2	SA0089
gi|29165615|ref|NC_002745.2|	101421	+	U	0	0	1	0	0	SA0089
gi|29165615|ref|NC_002745.2|	101449	+	A	1	0	0	0	0	SA0089
gi|29165615|ref|NC_002745.2|	101466	+	A	0	0	0	0	1	SA0089
gi|29165615|ref|NC_002745.2|	101485	+	G	1	0	0	0	0	SA0089
gi|29165615|ref|NC_002745.2|	101655	+	U	0	1	0	0	0	SA0089
gi|29165615|ref|NC_002745.2|	101969	+	U	0	0	1	0	0	SA0090
gi|29165615|ref|NC_002745.2|	101975	+	C	0	0	0	1	0	SA0090
gi|29165615|ref|NC_002745.2|	102037	+	A	1	0	0	0	0	SA0090
gi|29165615|ref|NC_002745.2|	102047	+	A	0	0	1	0	0	SA0090
gi|29165615|ref|NC_002745.2|	102048	+	U	0	0	0	0	1	SA0090
gi|29165615|ref|NC_002745.2|	102058	+	U	1	0	0	0	0	SA0090
gi|29165615|ref|NC_002745.2|	102059	+	A	1	0	0	0	0	SA0090
gi|29165615|ref|NC_002745.2|	102062	+	U	1	0	0	0	0	SA0090
gi|29165615|ref|NC_002745.2|	102066	+	U	0	0	1	0	0	SA0090
gi|29165615|ref|NC_002745.2|	102076	+	U	0	0	1	0	0	SA0090
gi|29165615|ref|NC_002745.2|	102078	+	G	0	1	0	0	0	SA0090
gi|29165615|ref|NC_002745.2|	102080	+	U	0	1	1	0	0	SA0090
gi|29165615|ref|NC_002745.2|	102116	+	C	0	0	0	1	0	SA0090
gi|29165615|ref|NC_002745.2|	102169	+	U	0	0	0	1	0	SA0090
gi|29165615|ref|NC_002745.2|	102184	+	U	1	0	0	0	0	SA0090
gi|29165615|ref|NC_002745.2|	102217	+	A	0	0	0	1	0	SA0090
gi|29165615|ref|NC_002745.2|	102300	+	U	0	1	1	1	2	SA0090
gi|29165615|ref|NC_002745.2|	102301	+	C	0	0	1	0	1	SA0090
gi|29165615|ref|NC_002745.2|	102306	+	G	0	0	1	2	0	SA0090
gi|29165615|ref|NC_002745.2|	102312	+	A	1	0	1	0	0	SA0090
gi|29165615|ref|NC_002745.2|	102313	+	C	1	0	0	0	0	SA0090
gi|29165615|ref|NC_002745.2|	102325	+	U	0	1	0	0	0	SA0090
gi|29165615|ref|NC_002745.2|	102358	+	A	0	1	0	0	0	SA0090
gi|29165615|ref|NC_002745.2|	102690	+	A	1	0	0	0	0	SA0091
gi|29165615|ref|NC_002745.2|	103818	+	A	0	1	0	0	0	-

gi|29165615|ref|NC_002745.2|	103849	+	U	0	0	0	0	1	-
gi|29165615|ref|NC_002745.2|	104664	+	A	0	0	1	0	0	-
gi|29165615|ref|NC_002745.2|	105480	+	A	0	0	0	0	1	-
gi|29165615|ref|NC_002745.2|	105523	+	U	1	0	0	0	0	SA0094
gi|29165615|ref|NC_002745.2|	105702	+	A	0	0	1	0	0	SA0094
gi|29165615|ref|NC_002745.2|	106371	+	G	0	0	1	0	0	SA0095
gi|29165615|ref|NC_002745.2|	107191	+	U	0	0	0	1	0	SA0096
gi|29165615|ref|NC_002745.2|	107990	+	G	1	0	0	0	0	-
gi|29165615|ref|NC_002745.2|	108008	+	A	0	0	1	0	0	-
gi|29165615|ref|NC_002745.2|	108019	+	A	3	10	1	6	8	-
gi|29165615|ref|NC_002745.2|	108389	+	A	0	0	0	0	1	SA0097
gi|29165615|ref|NC_002745.2|	108515	+	U	3	0	0	0	0	SA0097
gi|29165615|ref|NC_002745.2|	108672	+	U	0	2	0	0	0	SA0097
gi|29165615|ref|NC_002745.2|	108842	+	C	1	0	0	0	0	SA0097
gi|29165615|ref|NC_002745.2|	109640	+	C	0	0	0	0	1	SA0097
gi|29165615|ref|NC_002745.2|	109809	+	A	0	0	0	1	0	SA0097
gi|29165615|ref|NC_002745.2|	109923	+	U	0	0	1	0	0	SA0097
gi|29165615|ref|NC_002745.2|	109948	+	G	0	0	0	1	0	SA0097
gi|29165615|ref|NC_002745.2|	109952	+	C	0	0	0	0	1	SA0097
gi|29165615|ref|NC_002745.2|	109971	+	C	2	0	0	0	0	SA0097
gi|29165615|ref|NC_002745.2|	110360	+	A	0	0	2	0	0	-
gi|29165615|ref|NC_002745.2|	111356	+	A	0	1	0	0	0	SA0098
gi|29165615|ref|NC_002745.2|	111451	+	A	0	0	1	0	0	SA0098
gi|29165615|ref|NC_002745.2|	111571	+	G	1	0	0	0	0	SA0098
gi|29165615|ref|NC_002745.2|	111611	+	A	0	1	0	0	0	SA0098
gi|29165615|ref|NC_002745.2|	111696	+	C	1	0	0	0	0	SA0099
gi|29165615|ref|NC_002745.2|	111722	+	C	0	1	0	0	0	SA0099
gi|29165615|ref|NC_002745.2|	111752	+	A	0	0	0	0	1	SA0099
gi|29165615|ref|NC_002745.2|	111776	+	G	0	0	0	0	1	SA0099
gi|29165615|ref|NC_002745.2|	111798	+	U	1	0	0	0	0	SA0099
gi|29165615|ref|NC_002745.2|	111882	+	C	0	0	0	0	1	SA0099
gi|29165615|ref|NC_002745.2|	111942	+	U	0	1	0	0	0	SA0099
gi|29165615|ref|NC_002745.2|	112050	+	G	1	0	0	0	0	SA0099
gi|29165615|ref|NC_002745.2|	112051	+	C	1	0	0	0	0	SA0099
gi|29165615|ref|NC_002745.2|	112405	+	A	0	1	0	0	0	SA0099
gi|29165615|ref|NC_002745.2|	112466	+	G	0	0	1	0	0	SA0099
gi|29165615|ref|NC_002745.2|	112540	+	C	1	0	0	0	0	SA0099
gi|29165615|ref|NC_002745.2|	112936	+	C	0	0	0	0	1	SA0099
gi|29165615|ref|NC_002745.2|	114287	+	A	0	1	1	0	0	-
gi|29165615|ref|NC_002745.2|	114306	+	G	2	0	0	0	0	-
gi|29165615|ref|NC_002745.2|	114980	+	A	1	0	0	0	0	-
gi|29165615|ref|NC_002745.2|	115015	+	A	0	1	2	0	0	-
gi|29165615|ref|NC_002745.2|	115035	+	U	0	0	0	1	0	-
gi|29165615|ref|NC_002745.2|	115532	+	G	0	0	0	0	1	-
gi|29165615|ref|NC_002745.2|	118353	+	U	0	0	0	0	1	-
gi|29165615|ref|NC_002745.2|	118356	+	A	0	1	1	0	0	-
gi|29165615|ref|NC_002745.2|	118866	+	A	0	0	0	2	0	SA0104
gi|29165615|ref|NC_002745.2|	119214	+	A	0	0	0	1	0	SA0104
gi|29165615|ref|NC_002745.2|	119820	+	A	0	0	1	0	0	-
gi|29165615|ref|NC_002745.2|	119996	+	U	0	1	0	0	0	SA0105
gi|29165615|ref|NC_002745.2|	120009	+	C	0	0	0	0	1	SA0105
gi|29165615|ref|NC_002745.2|	120029	+	C	0	0	1	0	0	SA0105
gi|29165615|ref|NC_002745.2|	120102	+	A	0	0	1	0	0	SA0105
gi|29165615|ref|NC_002745.2|	120127	+	C	0	0	1	0	0	SA0105
gi|29165615|ref|NC_002745.2|	120131	+	G	1	0	0	0	0	SA0105
gi|29165615|ref|NC_002745.2|	120159	+	A	0	0	0	0	1	SA0105
gi|29165615|ref|NC_002745.2|	120256	+	A	0	0	0	1	0	SA0105
gi|29165615|ref|NC_002745.2|	120299	+	A	0	0	0	0	1	SA0105
gi|29165615|ref|NC_002745.2|	120323	+	A	1	0	0	0	0	SA0105
gi|29165615|ref|NC_002745.2|	120343	+	A	0	0	0	0	1	SA0105
gi|29165615|ref|NC_002745.2|	120377	+	U	0	1	0	0	0	SA0105
gi|29165615|ref|NC_002745.2|	120379	+	U	0	1	0	1	0	SA0105
gi|29165615|ref|NC_002745.2|	120409	+	A	0	1	1	1	0	SA0105
gi|29165615|ref|NC_002745.2|	120417	+	U	0	0	0	0	1	SA0105
gi|29165615|ref|NC_002745.2|	120443	+	C	0	0	0	1	0	SA0105
gi|29165615|ref|NC_002745.2|	120444	+	A	0	0	1	0	0	SA0105
gi|29165615|ref|NC_002745.2|	120466	+	A	1	0	0	0	0	SA0105
gi|29165615|ref|NC_002745.2|	120474	+	A	0	0	0	0	1	-
gi|29165615|ref|NC_002745.2|	120564	+	A	0	1	4	0	3	-
gi|29165615|ref|NC_002745.2|	120596	+	U	0	0	0	1	0	-
gi|29165615|ref|NC_002745.2|	120598	+	U	0	0	1	0	0	-
gi|29165615|ref|NC_002745.2|	120599	+	A	0	1	0	0	0	-
gi|29165615|ref|NC_002745.2|	120602	+	A	0	0	2	0	0	-
gi|29165615|ref|NC_002745.2|	120642	+	A	0	0	1	0	0	-
gi|29165615|ref|NC_002745.2|	120644	+	A	6	12	7	7	16	-
gi|29165615|ref|NC_002745.2|	120646	+	G	0	0	1	0	0	-
gi|29165615|ref|NC_002745.2|	120657	+	A	0	1	0	0	1	-
gi|29165615|ref|NC_002745.2|	120664	+	A	0	0	1	0	0	-
gi|29165615|ref|NC_002745.2|	120665	+	A	1	0	0	0	0	-
gi|29165615|ref|NC_002745.2|	120670	+	A	0	0	0	1	0	-
gi|29165615|ref|NC_002745.2|	120676	+	G	0	0	1	0	0	-

gi|29165615|ref|NC_002745.2|	120680	+	U	0	0	2	2	2	-
gi|29165615|ref|NC_002745.2|	120682	+	G	0	0	0	1	0	-
gi|29165615|ref|NC_002745.2|	120687	+	A	0	2	1	0	1	-
gi|29165615|ref|NC_002745.2|	120688	+	U	0	1	0	0	1	-
gi|29165615|ref|NC_002745.2|	120689	+	C	0	2	0	0	0	-
gi|29165615|ref|NC_002745.2|	120690	+	A	0	0	0	0	4	-
gi|29165615|ref|NC_002745.2|	120692	+	A	0	5	13	19	9	-
gi|29165615|ref|NC_002745.2|	120693	+	A	0	7	4	4	3	-
gi|29165615|ref|NC_002745.2|	120694	+	U	0	1	1	0	0	-
gi|29165615|ref|NC_002745.2|	120696	+	A	0	0	1	0	2	-
gi|29165615|ref|NC_002745.2|	120698	+	A	0	0	1	0	0	-
gi|29165615|ref|NC_002745.2|	120699	+	A	0	1	0	2	2	-
gi|29165615|ref|NC_002745.2|	120703	+	U	0	0	0	0	1	-
gi|29165615|ref|NC_002745.2|	120704	+	U	0	1	0	1	0	-
gi|29165615|ref|NC_002745.2|	120705	+	U	0	1	0	0	0	-
gi|29165615|ref|NC_002745.2|	120706	+	U	0	4	4	3	3	-
gi|29165615|ref|NC_002745.2|	120707	+	A	0	3	2	3	6	-
gi|29165615|ref|NC_002745.2|	120708	+	U	0	1	0	2	0	-
gi|29165615|ref|NC_002745.2|	120709	+	A	0	3	6	3	1	-
gi|29165615|ref|NC_002745.2|	120710	+	U	0	1	0	1	2	-
gi|29165615|ref|NC_002745.2|	120711	+	A	0	1	2	4	2	-
gi|29165615|ref|NC_002745.2|	120712	+	A	0	0	0	4	0	-
gi|29165615|ref|NC_002745.2|	120713	+	A	0	1	1	4	1	-
gi|29165615|ref|NC_002745.2|	120714	+	G	0	0	1	0	0	-
gi|29165615|ref|NC_002745.2|	120715	+	A	0	0	2	5	2	-
gi|29165615|ref|NC_002745.2|	120716	+	G	0	1	0	1	1	-
gi|29165615|ref|NC_002745.2|	120720	+	G	0	0	0	0	1	-
gi|29165615|ref|NC_002745.2|	120723	+	U	0	0	0	0	1	-
gi|29165615|ref|NC_002745.2|	120724	+	C	0	6	2	1	2	-
gi|29165615|ref|NC_002745.2|	120725	+	A	0	0	2	1	0	-
gi|29165615|ref|NC_002745.2|	120727	+	U	0	3	0	2	3	-
gi|29165615|ref|NC_002745.2|	120728	+	A	0	2	1	0	0	SA0106
gi|29165615|ref|NC_002745.2|	120730	+	G	0	4	1	1	0	SA0106
gi|29165615|ref|NC_002745.2|	120731	+	A	0	0	0	3	1	SA0106
gi|29165615|ref|NC_002745.2|	120732	+	C	0	0	0	3	0	SA0106
gi|29165615|ref|NC_002745.2|	120734	+	C	0	2	1	1	0	SA0106
gi|29165615|ref|NC_002745.2|	120735	+	U	2	2	0	1	0	SA0106
gi|29165615|ref|NC_002745.2|	120736	+	A	0	0	0	1	1	SA0106
gi|29165615|ref|NC_002745.2|	120737	+	C	2	0	0	0	0	SA0106
gi|29165615|ref|NC_002745.2|	120738	+	U	1	0	0	0	0	SA0106
gi|29165615|ref|NC_002745.2|	120739	+	U	0	3	4	2	2	SA0106
gi|29165615|ref|NC_002745.2|	120741	+	C	1	0	0	0	0	SA0106
gi|29165615|ref|NC_002745.2|	120743	+	G	0	0	1	0	0	SA0106
gi|29165615|ref|NC_002745.2|	120748	+	U	0	0	1	0	0	SA0106
gi|29165615|ref|NC_002745.2|	120750	+	C	1	0	0	0	0	SA0106
gi|29165615|ref|NC_002745.2|	120753	+	U	0	1	0	0	0	SA0106
gi|29165615|ref|NC_002745.2|	120754	+	C	0	0	0	1	0	SA0106
gi|29165615|ref|NC_002745.2|	120756	+	A	0	0	1	2	0	SA0106
gi|29165615|ref|NC_002745.2|	120758	+	A	0	0	0	0	1	SA0106
gi|29165615|ref|NC_002745.2|	120759	+	A	0	1	0	0	0	SA0106
gi|29165615|ref|NC_002745.2|	120762	+	U	0	1	1	1	1	SA0106
gi|29165615|ref|NC_002745.2|	120765	+	G	0	0	0	1	1	SA0106
gi|29165615|ref|NC_002745.2|	120766	+	A	0	1	0	0	0	SA0106
gi|29165615|ref|NC_002745.2|	120771	+	C	0	1	0	0	0	SA0106
gi|29165615|ref|NC_002745.2|	120774	+	C	0	0	1	0	0	SA0106
gi|29165615|ref|NC_002745.2|	120775	+	C	1	0	0	0	0	SA0106
gi|29165615|ref|NC_002745.2|	120776	+	U	0	0	0	1	1	SA0106
gi|29165615|ref|NC_002745.2|	120777	+	U	3	2	2	0	2	SA0106
gi|29165615|ref|NC_002745.2|	120778	+	A	0	1	1	0	0	SA0106
gi|29165615|ref|NC_002745.2|	120779	+	G	0	0	1	0	0	SA0106
gi|29165615|ref|NC_002745.2|	120780	+	U	3	0	0	1	0	SA0106
gi|29165615|ref|NC_002745.2|	120782	+	G	0	0	1	0	0	SA0106
gi|29165615|ref|NC_002745.2|	120783	+	C	0	0	0	0	1	SA0106
gi|29165615|ref|NC_002745.2|	120786	+	C	0	0	2	3	1	SA0106
gi|29165615|ref|NC_002745.2|	120787	+	A	0	0	0	0	2	SA0106
gi|29165615|ref|NC_002745.2|	120791	+	C	0	0	0	1	0	SA0106
gi|29165615|ref|NC_002745.2|	120792	+	C	0	0	0	0	1	SA0106
gi|29165615|ref|NC_002745.2|	120795	+	U	0	0	1	1	0	SA0106
gi|29165615|ref|NC_002745.2|	120796	+	U	0	1	1	1	1	SA0106
gi|29165615|ref|NC_002745.2|	120801	+	U	1	0	2	0	0	SA0106
gi|29165615|ref|NC_002745.2|	120802	+	A	1	1	3	1	0	SA0106
gi|29165615|ref|NC_002745.2|	120803	+	U	0	10	1	3	4	SA0106
gi|29165615|ref|NC_002745.2|	120804	+	U	0	1	3	1	1	SA0106
gi|29165615|ref|NC_002745.2|	120805	+	U	0	6	8	5	7	SA0106
gi|29165615|ref|NC_002745.2|	120806	+	U	0	0	0	0	5	SA0106
gi|29165615|ref|NC_002745.2|	120807	+	U	0	0	6	0	1	SA0106
gi|29165615|ref|NC_002745.2|	120808	+	A	0	11	10	6	4	SA0106
gi|29165615|ref|NC_002745.2|	120809	+	U	0	1	0	2	2	SA0106
gi|29165615|ref|NC_002745.2|	120828	+	A	0	21	7	8	11	SA0106
gi|29165615|ref|NC_002745.2|	120829	+	A	0	10	12	6	8	SA0106
gi|29165615|ref|NC_002745.2|	120830	+	A	0	2	2	0	0	SA0106

gi|29165615|ref|NC_002745.2|	120831 +	U	0	0	0	1	2	SA0106
gi|29165615|ref|NC_002745.2|	120832 +	G	0	1	0	2	1	SA0106
gi|29165615|ref|NC_002745.2|	120833 +	A	0	3	1	0	1	SA0106
gi|29165615|ref|NC_002745.2|	120834 +	A	0	7	0	2	1	SA0106
gi|29165615|ref|NC_002745.2|	120835 +	A	1	3	1	0	1	SA0106
gi|29165615|ref|NC_002745.2|	120837 +	G	0	6	2	1	2	SA0106
gi|29165615|ref|NC_002745.2|	120838 +	C	0	4	3	6	6	SA0106
gi|29165615|ref|NC_002745.2|	120839 +	A	0	4	1	0	1	SA0106
gi|29165615|ref|NC_002745.2|	120840 +	U	0	1	2	1	2	SA0106
gi|29165615|ref|NC_002745.2|	120841 +	U	0	2	0	1	0	SA0106
gi|29165615|ref|NC_002745.2|	120842 +	U	0	0	3	0	3	SA0106
gi|29165615|ref|NC_002745.2|	120846 +	C	0	2	1	2	1	SA0106
gi|29165615|ref|NC_002745.2|	120847 +	A	2	0	0	0	0	SA0106
gi|29165615|ref|NC_002745.2|	120848 +	G	0	0	0	1	0	SA0106
gi|29165615|ref|NC_002745.2|	120849 +	C	0	4	4	2	1	SA0106
gi|29165615|ref|NC_002745.2|	120850 +	A	0	1	0	0	0	SA0106
gi|29165615|ref|NC_002745.2|	120851 +	U	0	1	0	0	1	SA0106
gi|29165615|ref|NC_002745.2|	120854 +	A	0	1	2	0	1	SA0106
gi|29165615|ref|NC_002745.2|	120855 +	C	1	0	0	0	0	SA0106
gi|29165615|ref|NC_002745.2|	120856 +	A	0	2	1	0	1	SA0106
gi|29165615|ref|NC_002745.2|	120857 +	A	0	0	0	1	0	SA0106
gi|29165615|ref|NC_002745.2|	120858 +	C	0	0	0	1	0	SA0106
gi|29165615|ref|NC_002745.2|	120859 +	U	0	1	0	0	0	SA0106
gi|29165615|ref|NC_002745.2|	120869 +	A	0	0	3	0	0	SA0106
gi|29165615|ref|NC_002745.2|	120870 +	C	3	0	0	0	4	SA0106
gi|29165615|ref|NC_002745.2|	120871 +	A	0	0	1	0	0	SA0106
gi|29165615|ref|NC_002745.2|	120872 +	U	0	0	0	1	0	SA0106
gi|29165615|ref|NC_002745.2|	120875 +	A	0	15	8	14	12	SA0106
gi|29165615|ref|NC_002745.2|	120877 +	U	0	0	3	0	0	SA0106
gi|29165615|ref|NC_002745.2|	120879 +	U	2	0	3	0	0	SA0106
gi|29165615|ref|NC_002745.2|	120881 +	G	1	7	8	6	3	SA0106
gi|29165615|ref|NC_002745.2|	120882 +	C	2	5	6	2	4	SA0106
gi|29165615|ref|NC_002745.2|	120883 +	U	0	2	0	0	3	SA0106
gi|29165615|ref|NC_002745.2|	120884 +	U	0	0	2	0	0	SA0106
gi|29165615|ref|NC_002745.2|	120886 +	A	0	2	1	0	1	SA0106
gi|29165615|ref|NC_002745.2|	120887 +	U	0	2	2	0	0	SA0106
gi|29165615|ref|NC_002745.2|	120889 +	U	0	0	1	0	1	SA0106
gi|29165615|ref|NC_002745.2|	120891 +	U	2	21	16	25	24	SA0106
gi|29165615|ref|NC_002745.2|	120892 +	A	0	6	12	3	4	SA0106
gi|29165615|ref|NC_002745.2|	120893 +	U	0	0	0	4	3	SA0106
gi|29165615|ref|NC_002745.2|	120894 +	U	1	0	1	0	0	SA0106
gi|29165615|ref|NC_002745.2|	120895 +	U	3	7	5	2	8	SA0106
gi|29165615|ref|NC_002745.2|	120896 +	G	3	64	66	59	61	SA0106
gi|29165615|ref|NC_002745.2|	120897 +	A	1	47	39	40	23	SA0106
gi|29165615|ref|NC_002745.2|	120898 +	A	0	1	1	3	1	SA0106
gi|29165615|ref|NC_002745.2|	120899 +	U	1	0	0	0	0	SA0106
gi|29165615|ref|NC_002745.2|	120900 +	U	2	0	1	0	0	SA0106
gi|29165615|ref|NC_002745.2|	120902 +	C	1	0	1	0	2	SA0106
gi|29165615|ref|NC_002745.2|	120903 +	C	1	0	1	2	0	SA0106
gi|29165615|ref|NC_002745.2|	120908 +	C	0	0	1	3	1	SA0106
gi|29165615|ref|NC_002745.2|	120910 +	U	0	1	1	1	1	SA0106
gi|29165615|ref|NC_002745.2|	120913 +	A	0	0	1	0	0	SA0106
gi|29165615|ref|NC_002745.2|	120915 +	C	0	0	1	0	0	SA0106
gi|29165615|ref|NC_002745.2|	120918 +	C	0	0	0	1	0	SA0106
gi|29165615|ref|NC_002745.2|	120919 +	A	0	1	0	0	0	SA0106
gi|29165615|ref|NC_002745.2|	120921 +	G	0	0	1	0	0	SA0106
gi|29165615|ref|NC_002745.2|	120924 +	C	0	0	2	3	2	SA0106
gi|29165615|ref|NC_002745.2|	120926 +	A	0	2	1	2	2	SA0106
gi|29165615|ref|NC_002745.2|	120927 +	U	0	1	0	0	0	SA0106
gi|29165615|ref|NC_002745.2|	120929 +	A	0	1	1	0	0	SA0106
gi|29165615|ref|NC_002745.2|	120933 +	A	0	4	1	2	2	SA0106
gi|29165615|ref|NC_002745.2|	120936 +	G	0	1	0	0	0	SA0106
gi|29165615|ref|NC_002745.2|	120937 +	C	0	1	2	1	1	SA0106
gi|29165615|ref|NC_002745.2|	120939 +	U	3	3	0	2	2	SA0106
gi|29165615|ref|NC_002745.2|	120942 +	U	1	0	0	0	0	SA0106
gi|29165615|ref|NC_002745.2|	120951 +	U	1	0	0	0	2	SA0106
gi|29165615|ref|NC_002745.2|	120955 +	C	0	0	0	0	1	SA0106
gi|29165615|ref|NC_002745.2|	120957 +	C	0	0	0	0	1	SA0106
gi|29165615|ref|NC_002745.2|	120960 +	U	1	0	0	1	0	SA0106
gi|29165615|ref|NC_002745.2|	120963 +	G	0	1	0	3	1	SA0106
gi|29165615|ref|NC_002745.2|	120964 +	A	0	0	1	0	0	SA0106
gi|29165615|ref|NC_002745.2|	120967 +	U	1	0	0	0	0	SA0106
gi|29165615|ref|NC_002745.2|	120969 +	U	2	0	0	0	0	SA0106
gi|29165615|ref|NC_002745.2|	120972 +	U	1	0	0	0	0	SA0106
gi|29165615|ref|NC_002745.2|	120973 +	U	0	1	1	0	0	SA0106
gi|29165615|ref|NC_002745.2|	120974 +	U	0	0	0	0	1	SA0106
gi|29165615|ref|NC_002745.2|	120978 +	U	1	0	0	0	0	SA0106
gi|29165615|ref|NC_002745.2|	120980 +	G	0	0	0	0	1	SA0106
gi|29165615|ref|NC_002745.2|	120981 +	C	0	0	1	0	0	SA0106
gi|29165615|ref|NC_002745.2|	120988 +	G	0	3	2	1	1	SA0106
gi|29165615|ref|NC_002745.2|	120989 +	U	1	0	0	0	1	SA0106

gi|29165615|ref|NC_002745.2|	120991	+	A	0	1	0	0	0	SA0106
gi|29165615|ref|NC_002745.2|	120995	+	A	0	2	0	0	0	SA0106
gi|29165615|ref|NC_002745.2|	120999	+	U	0	0	0	1	0	SA0106
gi|29165615|ref|NC_002745.2|	121001	+	U	0	1	0	0	1	SA0106
gi|29165615|ref|NC_002745.2|	121002	+	C	0	3	6	3	2	SA0106
gi|29165615|ref|NC_002745.2|	121004	+	A	0	0	3	0	2	SA0106
gi|29165615|ref|NC_002745.2|	121006	+	U	0	1	0	0	0	SA0106
gi|29165615|ref|NC_002745.2|	121007	+	A	0	6	4	4	1	SA0106
gi|29165615|ref|NC_002745.2|	121008	+	A	0	2	1	1	0	SA0106
gi|29165615|ref|NC_002745.2|	121009	+	A	0	1	3	2	2	SA0106
gi|29165615|ref|NC_002745.2|	121010	+	A	0	1	0	2	0	SA0106
gi|29165615|ref|NC_002745.2|	121011	+	C	1	1	0	0	0	SA0106
gi|29165615|ref|NC_002745.2|	121012	+	A	1	0	0	0	0	SA0106
gi|29165615|ref|NC_002745.2|	121014	+	G	0	1	1	1	0	SA0106
gi|29165615|ref|NC_002745.2|	121015	+	A	0	12	3	10	8	SA0106
gi|29165615|ref|NC_002745.2|	121016	+	C	0	3	1	0	3	SA0106
gi|29165615|ref|NC_002745.2|	121017	+	A	0	0	0	1	1	SA0106
gi|29165615|ref|NC_002745.2|	121018	+	A	3	0	1	0	1	SA0106
gi|29165615|ref|NC_002745.2|	121019	+	U	0	1	0	0	1	SA0106
gi|29165615|ref|NC_002745.2|	121020	+	U	0	1	0	0	0	SA0106
gi|29165615|ref|NC_002745.2|	121022	+	U	0	3	2	1	3	SA0106
gi|29165615|ref|NC_002745.2|	121023	+	C	0	0	1	1	0	SA0106
gi|29165615|ref|NC_002745.2|	121025	+	A	0	0	1	0	2	SA0106
gi|29165615|ref|NC_002745.2|	121026	+	U	0	2	0	0	1	SA0106
gi|29165615|ref|NC_002745.2|	121027	+	U	0	0	1	0	0	SA0106
gi|29165615|ref|NC_002745.2|	121032	+	A	0	0	1	0	1	SA0106
gi|29165615|ref|NC_002745.2|	121035	+	A	0	0	1	0	0	SA0106
gi|29165615|ref|NC_002745.2|	121039	+	U	0	1	0	1	1	SA0106
gi|29165615|ref|NC_002745.2|	121040	+	A	0	2	0	0	2	SA0106
gi|29165615|ref|NC_002745.2|	121043	+	G	0	0	0	2	0	SA0106
gi|29165615|ref|NC_002745.2|	121044	+	C	0	3	1	4	2	SA0106
gi|29165615|ref|NC_002745.2|	121045	+	A	0	2	1	1	0	SA0106
gi|29165615|ref|NC_002745.2|	121047	+	G	0	1	0	0	0	SA0106
gi|29165615|ref|NC_002745.2|	121048	+	U	0	0	0	0	1	SA0106
gi|29165615|ref|NC_002745.2|	121049	+	A	0	0	0	0	1	SA0106
gi|29165615|ref|NC_002745.2|	121052	+	U	0	0	0	1	0	SA0106
gi|29165615|ref|NC_002745.2|	121053	+	C	0	0	0	2	0	SA0106
gi|29165615|ref|NC_002745.2|	121054	+	A	0	0	1	0	0	SA0106
gi|29165615|ref|NC_002745.2|	121055	+	G	0	1	0	0	0	SA0106
gi|29165615|ref|NC_002745.2|	121056	+	A	0	1	1	1	0	SA0106
gi|29165615|ref|NC_002745.2|	121058	+	G	0	0	1	0	0	SA0106
gi|29165615|ref|NC_002745.2|	121059	+	A	0	0	3	2	0	SA0106
gi|29165615|ref|NC_002745.2|	121060	+	C	1	0	2	0	0	SA0106
gi|29165615|ref|NC_002745.2|	121062	+	A	0	1	0	0	0	SA0106
gi|29165615|ref|NC_002745.2|	121070	+	C	0	1	0	0	0	SA0106
gi|29165615|ref|NC_002745.2|	121071	+	A	1	0	0	0	0	SA0106
gi|29165615|ref|NC_002745.2|	121075	+	A	0	1	0	0	0	SA0106
gi|29165615|ref|NC_002745.2|	121076	+	U	0	1	0	0	0	SA0106
gi|29165615|ref|NC_002745.2|	121081	+	A	0	0	0	0	1	SA0106
gi|29165615|ref|NC_002745.2|	121083	+	U	1	0	0	0	0	SA0106
gi|29165615|ref|NC_002745.2|	121086	+	G	0	2	0	0	0	SA0106
gi|29165615|ref|NC_002745.2|	121087	+	A	0	0	1	0	0	SA0106
gi|29165615|ref|NC_002745.2|	121089	+	U	0	0	1	0	1	SA0106
gi|29165615|ref|NC_002745.2|	121153	+	A	0	0	1	0	0	SA0106
gi|29165615|ref|NC_002745.2|	121160	+	A	0	1	0	0	0	SA0106
gi|29165615|ref|NC_002745.2|	121164	+	A	0	0	2	1	1	SA0106
gi|29165615|ref|NC_002745.2|	121165	+	A	0	1	0	0	2	SA0106
gi|29165615|ref|NC_002745.2|	121170	+	G	0	3	2	0	0	SA0106
gi|29165615|ref|NC_002745.2|	121171	+	A	0	2	0	1	0	SA0106
gi|29165615|ref|NC_002745.2|	121173	+	U	0	0	1	0	0	SA0106
gi|29165615|ref|NC_002745.2|	121185	+	A	0	0	1	0	0	SA0106
gi|29165615|ref|NC_002745.2|	121187	+	G	0	1	0	0	0	SA0106
gi|29165615|ref|NC_002745.2|	121191	+	C	0	1	0	0	0	SA0106
gi|29165615|ref|NC_002745.2|	121197	+	U	0	1	0	0	0	SA0106
gi|29165615|ref|NC_002745.2|	121208	+	G	1	1	0	0	0	SA0106
gi|29165615|ref|NC_002745.2|	121213	+	U	1	0	0	0	0	SA0106
gi|29165615|ref|NC_002745.2|	121243	+	U	0	0	1	0	0	SA0106
gi|29165615|ref|NC_002745.2|	121328	+	C	0	1	0	0	0	SA0106
gi|29165615|ref|NC_002745.2|	121337	+	A	0	1	0	0	0	SA0106
gi|29165615|ref|NC_002745.2|	121352	+	U	0	0	0	0	1	SA0106
gi|29165615|ref|NC_002745.2|	121356	+	U	0	1	1	0	0	SA0106
gi|29165615|ref|NC_002745.2|	121371	+	U	0	0	2	0	0	SA0106
gi|29165615|ref|NC_002745.2|	121375	+	U	0	0	1	0	0	SA0106
gi|29165615|ref|NC_002745.2|	121421	+	A	0	1	0	0	0	SA0106
gi|29165615|ref|NC_002745.2|	121429	+	A	0	0	0	0	1	SA0106
gi|29165615|ref|NC_002745.2|	121446	+	U	1	0	0	0	0	SA0106
gi|29165615|ref|NC_002745.2|	121450	+	A	0	0	0	0	1	SA0106
gi|29165615|ref|NC_002745.2|	121454	+	A	0	0	0	1	0	SA0106
gi|29165615|ref|NC_002745.2|	121457	+	A	0	0	1	0	0	SA0106
gi|29165615|ref|NC_002745.2|	121482	+	A	0	0	1	0	0	SA0106
gi|29165615|ref|NC_002745.2|	121504	+	A	0	1	0	0	0	SA0106

gi|29165615|ref|NC_002745.2|	121538	+	A	0	0	1	1	0	SA0106
gi|29165615|ref|NC_002745.2|	121558	+	A	0	0	0	0	1	SA0106
gi|29165615|ref|NC_002745.2|	121578	+	A	0	0	0	1	0	SA0106
gi|29165615|ref|NC_002745.2|	121588	+	A	0	1	0	0	0	SA0106
gi|29165615|ref|NC_002745.2|	121593	+	C	0	0	0	1	0	SA0106
gi|29165615|ref|NC_002745.2|	121595	+	C	0	1	0	0	0	SA0106
gi|29165615|ref|NC_002745.2|	121597	+	U	0	0	0	1	0	SA0106
gi|29165615|ref|NC_002745.2|	121599	+	A	0	0	1	0	0	SA0106
gi|29165615|ref|NC_002745.2|	121601	+	G	0	1	0	0	0	SA0106
gi|29165615|ref|NC_002745.2|	121603	+	A	0	1	0	0	0	SA0106
gi|29165615|ref|NC_002745.2|	121641	+	C	0	0	1	1	0	SA0106
gi|29165615|ref|NC_002745.2|	121680	+	U	0	0	0	1	0	SA0106
gi|29165615|ref|NC_002745.2|	121720	+	A	0	1	0	0	1	SA0106
gi|29165615|ref|NC_002745.2|	121724	+	C	0	1	0	0	0	SA0106
gi|29165615|ref|NC_002745.2|	121726	+	A	1	0	0	0	0	SA0106
gi|29165615|ref|NC_002745.2|	121737	+	C	2	0	0	0	0	SA0106
gi|29165615|ref|NC_002745.2|	121762	+	A	0	0	1	0	1	SA0106
gi|29165615|ref|NC_002745.2|	121765	+	U	0	0	0	1	0	SA0106
gi|29165615|ref|NC_002745.2|	121835	+	A	0	1	0	0	1	SA0106
gi|29165615|ref|NC_002745.2|	121841	+	A	0	0	0	1	0	SA0106
gi|29165615|ref|NC_002745.2|	121847	+	A	0	0	0	1	0	SA0106
gi|29165615|ref|NC_002745.2|	121849	+	A	0	1	0	0	0	SA0106
gi|29165615|ref|NC_002745.2|	121871	+	U	1	0	0	0	0	SA0106
gi|29165615|ref|NC_002745.2|	121889	+	G	0	0	0	0	1	SA0106
gi|29165615|ref|NC_002745.2|	121890	+	A	0	0	1	0	0	SA0106
gi|29165615|ref|NC_002745.2|	121902	+	C	0	0	1	0	0	SA0106
gi|29165615|ref|NC_002745.2|	121915	+	U	0	1	0	0	0	SA0106
gi|29165615|ref|NC_002745.2|	121920	+	C	0	0	0	0	1	SA0106
gi|29165615|ref|NC_002745.2|	121935	+	C	0	0	0	0	1	SA0106
gi|29165615|ref|NC_002745.2|	121936	+	U	0	0	0	2	0	SA0106
gi|29165615|ref|NC_002745.2|	121937	+	A	0	2	0	1	1	SA0106
gi|29165615|ref|NC_002745.2|	121942	+	U	0	0	1	0	0	SA0106
gi|29165615|ref|NC_002745.2|	121943	+	A	0	0	1	0	1	SA0106
gi|29165615|ref|NC_002745.2|	121944	+	U	2	0	1	0	0	SA0106
gi|29165615|ref|NC_002745.2|	121948	+	A	1	0	1	0	0	SA0106
gi|29165615|ref|NC_002745.2|	121962	+	C	0	0	1	0	0	SA0106
gi|29165615|ref|NC_002745.2|	121987	+	U	0	1	0	0	0	SA0106
gi|29165615|ref|NC_002745.2|	122016	+	C	1	1	0	0	0	SA0106
gi|29165615|ref|NC_002745.2|	122073	+	C	0	0	0	0	1	SA0106
gi|29165615|ref|NC_002745.2|	122077	+	A	0	0	0	0	1	SA0106
gi|29165615|ref|NC_002745.2|	122082	+	C	0	0	0	0	1	SA0106
gi|29165615|ref|NC_002745.2|	122088	+	U	0	0	1	0	0	SA0106
gi|29165615|ref|NC_002745.2|	122093	+	G	0	1	0	0	0	SA0106
gi|29165615|ref|NC_002745.2|	122096	+	A	0	1	0	0	0	SA0106
gi|29165615|ref|NC_002745.2|	122099	+	G	0	0	0	0	1	SA0106
gi|29165615|ref|NC_002745.2|	122100	+	U	1	2	0	0	1	SA0106
gi|29165615|ref|NC_002745.2|	122111	+	A	0	1	0	0	0	SA0106
gi|29165615|ref|NC_002745.2|	122114	+	U	0	0	0	1	1	SA0106
gi|29165615|ref|NC_002745.2|	122148	+	C	0	1	0	0	0	SA0106
gi|29165615|ref|NC_002745.2|	122199	+	C	1	0	0	0	0	SA0106
gi|29165615|ref|NC_002745.2|	122243	+	A	0	1	0	0	0	SA0106
gi|29165615|ref|NC_002745.2|	122249	+	A	0	0	0	0	1	SA0106
gi|29165615|ref|NC_002745.2|	122256	+	A	0	0	0	0	1	SA0106
gi|29165615|ref|NC_002745.2|	122261	+	A	0	2	0	0	0	SA0106
gi|29165615|ref|NC_002745.2|	122264	+	A	0	2	0	1	0	SA0106
gi|29165615|ref|NC_002745.2|	122268	+	U	0	0	1	0	0	SA0106
gi|29165615|ref|NC_002745.2|	122326	+	A	0	1	0	0	0	-
gi|29165615|ref|NC_002745.2|	122624	+	A	3	0	0	0	0	-
gi|29165615|ref|NC_002745.2|	123293	+	G	0	1	0	1	2	-
gi|29165615|ref|NC_002745.2|	123295	+	U	0	1	0	0	3	-
gi|29165615|ref|NC_002745.2|	123304	+	A	0	0	1	0	0	-
gi|29165615|ref|NC_002745.2|	123344	+	G	0	0	1	1	0	-
gi|29165615|ref|NC_002745.2|	123467	+	G	0	2	0	1	0	-
gi|29165615|ref|NC_002745.2|	123518	+	G	0	0	0	0	1	-
gi|29165615|ref|NC_002745.2|	123588	+	A	0	0	0	0	1	-
gi|29165615|ref|NC_002745.2|	123825	+	A	0	0	2	0	0	-
gi|29165615|ref|NC_002745.2|	126932	+	A	0	0	1	0	0	-
gi|29165615|ref|NC_002745.2|	127207	+	G	0	1	0	0	0	-
gi|29165615|ref|NC_002745.2|	129364	+	A	1	0	0	0	0	SA0112
gi|29165615|ref|NC_002745.2|	129700	+	C	0	0	2	0	0	SA0112
gi|29165615|ref|NC_002745.2|	129748	+	A	0	0	1	1	0	SA0112
gi|29165615|ref|NC_002745.2|	130013	+	A	0	0	1	0	0	SA0113
gi|29165615|ref|NC_002745.2|	130018	+	U	0	0	2	0	0	SA0113
gi|29165615|ref|NC_002745.2|	130080	+	C	0	0	0	1	0	SA0113
gi|29165615|ref|NC_002745.2|	130549	+	C	0	0	0	1	0	SA0113
gi|29165615|ref|NC_002745.2|	130760	+	G	0	1	0	0	0	-
gi|29165615|ref|NC_002745.2|	130856	+	A	0	1	0	0	0	SA0114
gi|29165615|ref|NC_002745.2|	130899	+	C	0	0	0	1	0	SA0114
gi|29165615|ref|NC_002745.2|	130905	+	A	0	1	0	0	0	SA0114
gi|29165615|ref|NC_002745.2|	130938	+	A	0	0	0	0	1	SA0114
gi|29165615|ref|NC_002745.2|	132865	+	A	0	0	0	0	1	SA0115

gi|29165615|ref|NC_002745.2|	134841 +	C	0	0	0	0	1	SA0116
gi|29165615|ref|NC_002745.2|	134847 +	G	0	0	1	0	0	SA0116
gi|29165615|ref|NC_002745.2|	135150 +	G	1	0	0	0	0	SA0116
gi|29165615|ref|NC_002745.2|	135209 +	A	1	0	0	0	0	SA0116
gi|29165615|ref|NC_002745.2|	135831 +	U	0	0	0	0	1	SA0117
gi|29165615|ref|NC_002745.2|	135865 +	C	0	0	0	0	1	SA0117
gi|29165615|ref|NC_002745.2|	136384 +	A	0	0	0	0	1	SA0117
gi|29165615|ref|NC_002745.2|	137445 +	C	0	0	1	0	0	SA0118
gi|29165615|ref|NC_002745.2|	137491 +	G	0	3	2	0	2	SA0118
gi|29165615|ref|NC_002745.2|	137561 +	A	0	0	0	0	1	SA0118
gi|29165615|ref|NC_002745.2|	137569 +	U	0	0	0	1	0	SA0118
gi|29165615|ref|NC_002745.2|	137587 +	A	0	0	0	0	1	SA0118
gi|29165615|ref|NC_002745.2|	137599 +	G	0	0	0	2	0	SA0118
gi|29165615|ref|NC_002745.2|	137639 +	C	0	0	0	1	0	SA0118
gi|29165615|ref|NC_002745.2|	138531 +	A	0	0	1	0	0	SA0119
gi|29165615|ref|NC_002745.2|	138639 +	A	0	0	0	1	0	SA0119
gi|29165615|ref|NC_002745.2|	138640 +	A	0	0	0	0	1	SA0119
gi|29165615|ref|NC_002745.2|	138644 +	C	0	0	1	0	0	SA0119
gi|29165615|ref|NC_002745.2|	138648 +	A	0	1	0	0	0	SA0119
gi|29165615|ref|NC_002745.2|	138759 +	A	0	0	0	1	0	SA0119
gi|29165615|ref|NC_002745.2|	139260 +	A	0	0	0	1	1	SA0120
gi|29165615|ref|NC_002745.2|	139323 +	U	0	1	0	0	0	SA0120
gi|29165615|ref|NC_002745.2|	139372 +	C	0	0	1	0	0	SA0120
gi|29165615|ref|NC_002745.2|	139378 +	G	0	0	0	1	0	SA0120
gi|29165615|ref|NC_002745.2|	139380 +	U	0	2	0	0	0	SA0120
gi|29165615|ref|NC_002745.2|	139780 +	A	0	0	0	0	1	SA0120
gi|29165615|ref|NC_002745.2|	139800 +	A	0	1	0	0	0	SA0120
gi|29165615|ref|NC_002745.2|	139878 +	A	1	0	0	0	0	SA0120
gi|29165615|ref|NC_002745.2|	139885 +	C	0	1	1	0	0	SA0120
gi|29165615|ref|NC_002745.2|	139895 +	A	0	0	0	0	1	SA0120
gi|29165615|ref|NC_002745.2|	139904 +	A	0	0	1	0	0	SA0120
gi|29165615|ref|NC_002745.2|	139931 +	C	0	0	1	0	0	SA0120
gi|29165615|ref|NC_002745.2|	139965 +	A	0	0	1	0	0	SA0120
gi|29165615|ref|NC_002745.2|	139969 +	G	3	3	4	6	2	SA0120
gi|29165615|ref|NC_002745.2|	139975 +	U	0	0	0	1	0	SA0120
gi|29165615|ref|NC_002745.2|	140163 +	A	0	1	0	0	0	-
gi|29165615|ref|NC_002745.2|	140773 +	C	1	0	0	0	0	SA0121
gi|29165615|ref|NC_002745.2|	140989 +	U	0	0	0	0	1	-
gi|29165615|ref|NC_002745.2|	140991 +	A	0	3	0	1	0	-
gi|29165615|ref|NC_002745.2|	140992 +	U	0	2	0	0	1	-
gi|29165615|ref|NC_002745.2|	140993 +	U	2	0	0	3	4	-
gi|29165615|ref|NC_002745.2|	140994 +	A	0	5	5	6	8	-
gi|29165615|ref|NC_002745.2|	140995 +	A	0	3	0	1	1	-
gi|29165615|ref|NC_002745.2|	140998 +	G	0	3	1	5	0	-
gi|29165615|ref|NC_002745.2|	141000 +	A	0	1	0	0	0	-
gi|29165615|ref|NC_002745.2|	141003 +	A	0	1	0	0	1	-
gi|29165615|ref|NC_002745.2|	141010 +	A	0	0	2	0	0	-
gi|29165615|ref|NC_002745.2|	141015 +	U	1	0	0	0	0	-
gi|29165615|ref|NC_002745.2|	141022 +	U	0	1	0	0	0	-
gi|29165615|ref|NC_002745.2|	141033 +	A	0	0	1	0	0	SA0122
gi|29165615|ref|NC_002745.2|	141035 +	A	0	0	0	0	2	SA0122
gi|29165615|ref|NC_002745.2|	141039 +	U	0	2	0	0	0	SA0122
gi|29165615|ref|NC_002745.2|	141042 +	C	0	0	0	2	0	SA0122
gi|29165615|ref|NC_002745.2|	141043 +	A	0	1	0	0	0	SA0122
gi|29165615|ref|NC_002745.2|	141049 +	A	0	1	0	0	0	SA0122
gi|29165615|ref|NC_002745.2|	141072 +	G	0	0	0	2	0	SA0122
gi|29165615|ref|NC_002745.2|	141073 +	U	0	0	0	0	1	SA0122
gi|29165615|ref|NC_002745.2|	141446 +	C	0	1	0	0	0	SA0122
gi|29165615|ref|NC_002745.2|	141601 +	A	0	0	0	1	0	SA0122
gi|29165615|ref|NC_002745.2|	141630 +	C	0	1	0	0	0	SA0122
gi|29165615|ref|NC_002745.2|	141631 +	A	0	1	0	0	0	SA0122
gi|29165615|ref|NC_002745.2|	143212 +	A	0	0	0	0	1	SA0124
gi|29165615|ref|NC_002745.2|	145683 +	A	0	1	0	0	0	SA0126
gi|29165615|ref|NC_002745.2|	146140 +	U	0	0	0	1	0	SA0126
gi|29165615|ref|NC_002745.2|	146171 +	C	0	0	0	0	1	SA0126
gi|29165615|ref|NC_002745.2|	146236 +	A	0	0	0	0	1	SA0126
gi|29165615|ref|NC_002745.2|	146287 +	A	0	0	0	1	0	SA0126
gi|29165615|ref|NC_002745.2|	146519 +	G	1	0	0	0	0	SA0127
gi|29165615|ref|NC_002745.2|	147227 +	A	0	0	0	1	0	SA0127
gi|29165615|ref|NC_002745.2|	147240 +	A	0	1	0	0	0	SA0127
gi|29165615|ref|NC_002745.2|	147416 +	A	0	0	0	2	0	SA0127
gi|29165615|ref|NC_002745.2|	147464 +	U	0	1	0	0	0	SA0127
gi|29165615|ref|NC_002745.2|	147487 +	G	0	1	0	0	1	SA0127
gi|29165615|ref|NC_002745.2|	147488 +	A	0	0	1	0	0	SA0127
gi|29165615|ref|NC_002745.2|	147515 +	A	0	0	0	0	1	SA0127
gi|29165615|ref|NC_002745.2|	147523 +	A	0	0	0	0	1	SA0127
gi|29165615|ref|NC_002745.2|	147627 +	A	0	1	0	1	1	SA0127
gi|29165615|ref|NC_002745.2|	147633 +	C	0	0	0	1	0	SA0127
gi|29165615|ref|NC_002745.2|	147787 +	U	0	1	0	0	0	-
gi|29165615|ref|NC_002745.2|	148025 +	G	0	0	1	0	0	-
gi|29165615|ref|NC_002745.2|	148027 +	A	1	5	0	3	2	-

gi|29165615|ref|NC_002745.2|	148029	+	A	0	0	0	0	1	-
gi|29165615|ref|NC_002745.2|	148037	+	A	0	0	0	1	0	-
gi|29165615|ref|NC_002745.2|	148049	+	A	0	0	1	1	0	-
gi|29165615|ref|NC_002745.2|	148067	+	A	0	0	0	1	0	SA0128
gi|29165615|ref|NC_002745.2|	148070	+	A	1	0	0	0	4	SA0128
gi|29165615|ref|NC_002745.2|	148099	+	A	0	0	0	1	0	SA0128
gi|29165615|ref|NC_002745.2|	148131	+	C	0	0	0	1	0	SA0128
gi|29165615|ref|NC_002745.2|	148148	+	C	0	0	0	1	0	SA0128
gi|29165615|ref|NC_002745.2|	148150	+	A	0	2	0	0	0	SA0128
gi|29165615|ref|NC_002745.2|	148370	+	G	0	2	0	0	0	SA0128
gi|29165615|ref|NC_002745.2|	148582	+	A	0	1	0	0	1	SA0128
gi|29165615|ref|NC_002745.2|	148589	+	G	0	0	0	1	0	SA0128
gi|29165615|ref|NC_002745.2|	148941	+	U	0	0	1	0	0	-
gi|29165615|ref|NC_002745.2|	148988	+	G	0	1	4	2	2	-
gi|29165615|ref|NC_002745.2|	149000	+	U	0	1	0	0	0	-
gi|29165615|ref|NC_002745.2|	149002	+	A	0	0	0	1	0	-
gi|29165615|ref|NC_002745.2|	149006	+	A	0	0	1	0	0	-
gi|29165615|ref|NC_002745.2|	149016	+	A	0	2	0	0	0	-
gi|29165615|ref|NC_002745.2|	149049	+	A	0	0	0	0	1	SA0129
gi|29165615|ref|NC_002745.2|	149060	+	C	0	0	1	0	0	SA0129
gi|29165615|ref|NC_002745.2|	149118	+	A	0	0	0	1	0	SA0129
gi|29165615|ref|NC_002745.2|	149184	+	G	0	0	1	0	0	SA0129
gi|29165615|ref|NC_002745.2|	149288	+	A	0	0	1	0	0	SA0129
gi|29165615|ref|NC_002745.2|	149422	+	C	0	0	0	1	0	SA0129
gi|29165615|ref|NC_002745.2|	149491	+	A	0	0	0	0	1	SA0129
gi|29165615|ref|NC_002745.2|	149532	+	C	0	0	0	2	0	SA0129
gi|29165615|ref|NC_002745.2|	149583	+	A	0	0	1	0	0	SA0129
gi|29165615|ref|NC_002745.2|	149599	+	A	0	0	1	0	0	SA0129
gi|29165615|ref|NC_002745.2|	149604	+	C	0	0	2	0	2	SA0129
gi|29165615|ref|NC_002745.2|	149616	+	A	0	1	0	0	0	SA0129
gi|29165615|ref|NC_002745.2|	149617	+	G	0	0	0	0	1	SA0129
gi|29165615|ref|NC_002745.2|	149620	+	A	0	0	1	0	0	SA0129
gi|29165615|ref|NC_002745.2|	149621	+	C	0	1	0	0	0	SA0129
gi|29165615|ref|NC_002745.2|	149622	+	A	0	0	0	1	0	SA0129
gi|29165615|ref|NC_002745.2|	149623	+	A	0	0	0	1	0	SA0129
gi|29165615|ref|NC_002745.2|	149625	+	A	0	0	0	0	1	SA0129
gi|29165615|ref|NC_002745.2|	149626	+	A	0	0	1	0	0	SA0129
gi|29165615|ref|NC_002745.2|	149627	+	A	0	0	1	0	1	SA0129
gi|29165615|ref|NC_002745.2|	149629	+	U	2	0	1	0	0	SA0129
gi|29165615|ref|NC_002745.2|	149638	+	C	0	0	1	0	0	SA0129
gi|29165615|ref|NC_002745.2|	149659	+	C	0	1	0	0	0	SA0129
gi|29165615|ref|NC_002745.2|	149661	+	A	0	0	0	1	0	SA0129
gi|29165615|ref|NC_002745.2|	149663	+	U	0	2	1	0	3	SA0129
gi|29165615|ref|NC_002745.2|	149664	+	A	0	0	2	0	2	SA0129
gi|29165615|ref|NC_002745.2|	149665	+	U	0	1	0	1	2	SA0129
gi|29165615|ref|NC_002745.2|	149666	+	U	0	0	1	0	0	SA0129
gi|29165615|ref|NC_002745.2|	149667	+	C	0	0	0	0	1	SA0129
gi|29165615|ref|NC_002745.2|	149674	+	C	0	0	1	0	0	SA0129
gi|29165615|ref|NC_002745.2|	149683	+	C	0	0	1	0	0	SA0129
gi|29165615|ref|NC_002745.2|	150784	+	U	0	0	1	0	0	-
gi|29165615|ref|NC_002745.2|	150877	+	G	0	4	0	0	2	-
gi|29165615|ref|NC_002745.2|	150891	+	A	0	1	0	0	0	-
gi|29165615|ref|NC_002745.2|	150892	+	C	0	1	0	0	0	-
gi|29165615|ref|NC_002745.2|	150897	+	C	0	0	0	0	2	-
gi|29165615|ref|NC_002745.2|	150904	+	C	0	1	0	0	0	-
gi|29165615|ref|NC_002745.2|	151330	+	A	0	1	0	0	0	SA0131
gi|29165615|ref|NC_002745.2|	151404	+	A	0	0	0	0	1	SA0131
gi|29165615|ref|NC_002745.2|	151527	+	G	0	0	0	0	1	SA0131
gi|29165615|ref|NC_002745.2|	151775	+	A	0	0	0	0	1	SA0132
gi|29165615|ref|NC_002745.2|	151898	+	A	0	0	0	0	1	SA0132
gi|29165615|ref|NC_002745.2|	151914	+	G	0	0	0	1	0	SA0132
gi|29165615|ref|NC_002745.2|	151918	+	C	0	0	0	0	1	SA0132
gi|29165615|ref|NC_002745.2|	151961	+	A	0	0	1	0	0	SA0132
gi|29165615|ref|NC_002745.2|	152312	+	G	0	0	1	0	0	SA0132
gi|29165615|ref|NC_002745.2|	152690	+	A	0	0	0	0	1	SA0132
gi|29165615|ref|NC_002745.2|	152706	+	G	1	0	0	0	2	SA0132
gi|29165615|ref|NC_002745.2|	152768	+	A	0	1	0	0	0	SA0132
gi|29165615|ref|NC_002745.2|	152810	+	G	0	0	0	1	0	SA0132
gi|29165615|ref|NC_002745.2|	153021	+	U	0	1	0	0	0	-
gi|29165615|ref|NC_002745.2|	153023	+	U	1	0	0	0	0	-
gi|29165615|ref|NC_002745.2|	153026	+	A	0	1	0	0	0	-
gi|29165615|ref|NC_002745.2|	153032	+	A	0	0	0	0	1	-
gi|29165615|ref|NC_002745.2|	153036	+	U	0	0	1	2	1	-
gi|29165615|ref|NC_002745.2|	153037	+	A	0	0	0	2	0	-
gi|29165615|ref|NC_002745.2|	153038	+	A	0	3	0	0	0	-
gi|29165615|ref|NC_002745.2|	153039	+	A	0	3	3	0	0	-
gi|29165615|ref|NC_002745.2|	153040	+	U	1	3	1	0	5	-
gi|29165615|ref|NC_002745.2|	153041	+	A	0	8	6	3	6	-
gi|29165615|ref|NC_002745.2|	153042	+	A	0	1	0	0	1	-
gi|29165615|ref|NC_002745.2|	153043	+	A	0	0	2	0	0	-
gi|29165615|ref|NC_002745.2|	153044	+	G	0	0	0	0	1	-

gi|29165615|ref|NC_002745.2|	153050	+	A	0	1	0	0	0	-
gi|29165615|ref|NC_002745.2|	153056	+	C	0	1	0	0	0	-
gi|29165615|ref|NC_002745.2|	153062	+	G	0	0	0	0	1	-
gi|29165615|ref|NC_002745.2|	153063	+	A	0	1	0	0	0	-
gi|29165615|ref|NC_002745.2|	153064	+	G	0	1	0	0	0	-
gi|29165615|ref|NC_002745.2|	153067	+	G	0	0	0	0	1	-
gi|29165615|ref|NC_002745.2|	153070	+	A	0	1	0	0	0	-
gi|29165615|ref|NC_002745.2|	153073	+	A	0	0	2	1	0	SA0133
gi|29165615|ref|NC_002745.2|	153075	+	G	0	1	0	0	0	SA0133
gi|29165615|ref|NC_002745.2|	153076	+	A	0	0	0	0	1	SA0133
gi|29165615|ref|NC_002745.2|	153082	+	G	0	0	0	1	0	SA0133
gi|29165615|ref|NC_002745.2|	153085	+	A	0	0	0	0	1	SA0133
gi|29165615|ref|NC_002745.2|	153088	+	U	0	0	0	1	0	SA0133
gi|29165615|ref|NC_002745.2|	153718	+	G	0	0	0	0	1	SA0133
gi|29165615|ref|NC_002745.2|	153725	+	C	0	0	1	1	0	SA0133
gi|29165615|ref|NC_002745.2|	153734	+	A	0	0	0	0	1	SA0133
gi|29165615|ref|NC_002745.2|	153738	+	U	0	0	1	1	0	-
gi|29165615|ref|NC_002745.2|	153752	+	G	0	0	0	0	1	-
gi|29165615|ref|NC_002745.2|	153768	+	A	0	1	0	0	0	SA0134
gi|29165615|ref|NC_002745.2|	153873	+	U	0	0	1	0	0	SA0134
gi|29165615|ref|NC_002745.2|	154103	+	A	0	0	0	0	1	SA0134
gi|29165615|ref|NC_002745.2|	154111	+	G	0	0	0	0	1	SA0134
gi|29165615|ref|NC_002745.2|	154218	+	U	1	0	0	0	0	SA0134
gi|29165615|ref|NC_002745.2|	154233	+	A	1	4	0	0	2	SA0134
gi|29165615|ref|NC_002745.2|	154264	+	A	0	0	0	0	1	SA0134
gi|29165615|ref|NC_002745.2|	154266	+	C	0	1	0	0	0	SA0134
gi|29165615|ref|NC_002745.2|	154326	+	A	0	1	0	0	1	SA0134
gi|29165615|ref|NC_002745.2|	154348	+	G	1	0	0	0	0	SA0134
gi|29165615|ref|NC_002745.2|	154433	+	U	0	0	0	0	1	SA0134
gi|29165615|ref|NC_002745.2|	154439	+	A	1	0	0	0	0	SA0134
gi|29165615|ref|NC_002745.2|	154441	+	C	0	0	0	1	1	SA0134
gi|29165615|ref|NC_002745.2|	154473	+	C	0	0	1	0	0	SA0134
gi|29165615|ref|NC_002745.2|	154483	+	A	0	0	1	0	0	SA0134
gi|29165615|ref|NC_002745.2|	154493	+	U	2	0	0	0	0	SA0134
gi|29165615|ref|NC_002745.2|	154531	+	A	0	0	0	1	0	SA0134
gi|29165615|ref|NC_002745.2|	154567	+	A	0	0	1	0	0	SA0134
gi|29165615|ref|NC_002745.2|	154610	+	U	2	0	0	0	0	SA0134
gi|29165615|ref|NC_002745.2|	154676	+	U	0	1	0	0	0	SA0134
gi|29165615|ref|NC_002745.2|	154723	+	U	0	1	0	0	0	SA0134
gi|29165615|ref|NC_002745.2|	154867	+	G	0	0	0	0	1	SA0134
gi|29165615|ref|NC_002745.2|	158944	+	G	0	0	0	0	1	SA0139
gi|29165615|ref|NC_002745.2|	159413	+	G	0	1	0	0	0	SA0139
gi|29165615|ref|NC_002745.2|	159509	+	G	0	1	0	0	0	SA0139
gi|29165615|ref|NC_002745.2|	159774	+	A	0	0	1	0	0	SA0139
gi|29165615|ref|NC_002745.2|	159794	+	A	0	0	0	0	1	SA0139
gi|29165615|ref|NC_002745.2|	160294	+	C	0	0	1	0	0	SA0139
gi|29165615|ref|NC_002745.2|	160397	+	A	0	11	11	11	12	-
gi|29165615|ref|NC_002745.2|	160398	+	A	0	0	2	0	0	-
gi|29165615|ref|NC_002745.2|	160654	+	C	0	0	1	0	0	SA0140
gi|29165615|ref|NC_002745.2|	160669	+	U	0	0	1	0	0	SA0140
gi|29165615|ref|NC_002745.2|	160741	+	A	0	3	0	0	0	SA0140
gi|29165615|ref|NC_002745.2|	160768	+	C	0	0	0	0	1	SA0140
gi|29165615|ref|NC_002745.2|	160786	+	A	0	0	1	0	0	SA0140
gi|29165615|ref|NC_002745.2|	160843	+	G	0	0	0	1	0	SA0140
gi|29165615|ref|NC_002745.2|	161009	+	G	0	0	1	0	0	SA0140
gi|29165615|ref|NC_002745.2|	161099	+	A	0	0	0	0	3	SA0140
gi|29165615|ref|NC_002745.2|	161122	+	C	0	0	0	0	1	SA0140
gi|29165615|ref|NC_002745.2|	161151	+	U	0	0	0	2	0	SA0140
gi|29165615|ref|NC_002745.2|	161193	+	U	0	0	1	0	0	SA0140
gi|29165615|ref|NC_002745.2|	161279	+	C	0	0	0	0	1	SA0140
gi|29165615|ref|NC_002745.2|	161344	+	A	0	1	0	0	0	SA0140
gi|29165615|ref|NC_002745.2|	161482	+	U	0	0	1	0	0	SA0140
gi|29165615|ref|NC_002745.2|	161484	+	U	2	0	0	0	0	SA0140
gi|29165615|ref|NC_002745.2|	161528	+	A	0	0	1	0	0	SA0140
gi|29165615|ref|NC_002745.2|	161534	+	A	0	0	1	0	0	SA0140
gi|29165615|ref|NC_002745.2|	161542	+	C	0	0	1	1	0	SA0140
gi|29165615|ref|NC_002745.2|	161545	+	A	0	0	0	0	2	SA0140
gi|29165615|ref|NC_002745.2|	161550	+	A	0	1	0	0	0	SA0140
gi|29165615|ref|NC_002745.2|	161555	+	A	0	1	0	0	0	SA0140
gi|29165615|ref|NC_002745.2|	161595	+	U	0	0	1	0	0	SA0140
gi|29165615|ref|NC_002745.2|	161606	+	C	0	0	0	1	0	SA0140
gi|29165615|ref|NC_002745.2|	161607	+	A	0	0	1	0	0	SA0140
gi|29165615|ref|NC_002745.2|	161616	+	U	2	0	0	0	0	SA0140
gi|29165615|ref|NC_002745.2|	161724	+	A	0	0	0	0	1	SA0140
gi|29165615|ref|NC_002745.2|	161825	+	G	1	0	0	0	0	SA0140
gi|29165615|ref|NC_002745.2|	161826	+	C	0	0	0	0	1	SA0140
gi|29165615|ref|NC_002745.2|	161832	+	U	1	0	0	0	0	SA0140
gi|29165615|ref|NC_002745.2|	162099	+	A	0	2	0	1	0	-
gi|29165615|ref|NC_002745.2|	162106	+	U	0	0	0	1	0	-
gi|29165615|ref|NC_002745.2|	162110	+	A	0	1	0	0	0	-
gi|29165615|ref|NC_002745.2|	162112	+	G	0	0	0	0	1	-

gi|29165615|ref|NC_002745.2|	162127	+	G	0	0	1	0	0	SA0141
gi|29165615|ref|NC_002745.2|	162128	+	A	0	1	1	0	0	SA0141
gi|29165615|ref|NC_002745.2|	162136	+	A	0	1	0	0	0	SA0141
gi|29165615|ref|NC_002745.2|	162140	+	A	0	0	0	0	1	SA0141
gi|29165615|ref|NC_002745.2|	162156	+	U	0	0	1	1	1	SA0141
gi|29165615|ref|NC_002745.2|	162157	+	A	0	2	0	0	0	SA0141
gi|29165615|ref|NC_002745.2|	162163	+	A	0	1	0	0	1	SA0141
gi|29165615|ref|NC_002745.2|	162168	+	A	0	0	0	0	1	SA0141
gi|29165615|ref|NC_002745.2|	162184	+	A	0	0	0	1	0	SA0141
gi|29165615|ref|NC_002745.2|	162191	+	A	0	1	0	0	0	SA0141
gi|29165615|ref|NC_002745.2|	162194	+	U	0	1	0	1	0	SA0141
gi|29165615|ref|NC_002745.2|	162203	+	G	0	0	0	0	1	SA0141
gi|29165615|ref|NC_002745.2|	162216	+	G	0	0	1	0	2	SA0141
gi|29165615|ref|NC_002745.2|	162217	+	A	0	2	0	0	1	SA0141
gi|29165615|ref|NC_002745.2|	162226	+	G	0	0	0	1	0	SA0141
gi|29165615|ref|NC_002745.2|	162374	+	A	0	0	0	0	2	SA0141
gi|29165615|ref|NC_002745.2|	162376	+	A	0	1	0	1	0	SA0141
gi|29165615|ref|NC_002745.2|	162379	+	A	0	0	0	0	1	SA0141
gi|29165615|ref|NC_002745.2|	162380	+	A	0	0	0	1	0	SA0141
gi|29165615|ref|NC_002745.2|	162382	+	A	0	0	1	0	0	SA0141
gi|29165615|ref|NC_002745.2|	162388	+	U	0	0	1	0	0	SA0141
gi|29165615|ref|NC_002745.2|	162397	+	A	0	0	0	1	1	SA0141
gi|29165615|ref|NC_002745.2|	162398	+	A	0	0	1	3	0	SA0141
gi|29165615|ref|NC_002745.2|	162399	+	A	0	0	1	0	1	SA0141
gi|29165615|ref|NC_002745.2|	162403	+	G	0	0	0	1	0	SA0141
gi|29165615|ref|NC_002745.2|	162404	+	A	0	7	1	2	0	SA0141
gi|29165615|ref|NC_002745.2|	162418	+	G	0	0	0	0	1	SA0141
gi|29165615|ref|NC_002745.2|	162419	+	A	0	1	1	0	0	SA0141
gi|29165615|ref|NC_002745.2|	162421	+	A	0	1	0	1	0	SA0141
gi|29165615|ref|NC_002745.2|	162422	+	A	0	0	0	0	1	SA0141
gi|29165615|ref|NC_002745.2|	162423	+	A	0	2	0	1	1	SA0141
gi|29165615|ref|NC_002745.2|	162424	+	A	0	1	1	0	0	SA0141
gi|29165615|ref|NC_002745.2|	162427	+	G	0	0	0	0	1	SA0141
gi|29165615|ref|NC_002745.2|	162428	+	U	0	0	0	0	2	SA0141
gi|29165615|ref|NC_002745.2|	162429	+	A	0	1	0	1	0	SA0141
gi|29165615|ref|NC_002745.2|	162433	+	G	0	0	1	0	0	SA0141
gi|29165615|ref|NC_002745.2|	162434	+	U	0	0	0	2	0	SA0141
gi|29165615|ref|NC_002745.2|	162436	+	G	0	0	1	0	0	SA0141
gi|29165615|ref|NC_002745.2|	162438	+	G	0	1	0	0	1	SA0141
gi|29165615|ref|NC_002745.2|	162439	+	A	0	0	0	0	1	SA0141
gi|29165615|ref|NC_002745.2|	162442	+	A	0	0	0	1	1	SA0141
gi|29165615|ref|NC_002745.2|	162443	+	G	0	1	0	0	0	SA0141
gi|29165615|ref|NC_002745.2|	162444	+	U	0	0	0	0	2	SA0141
gi|29165615|ref|NC_002745.2|	162449	+	U	0	0	1	0	0	SA0141
gi|29165615|ref|NC_002745.2|	162467	+	A	0	1	0	0	0	SA0141
gi|29165615|ref|NC_002745.2|	162472	+	A	0	2	1	0	0	SA0141
gi|29165615|ref|NC_002745.2|	162482	+	U	0	1	0	0	1	SA0141
gi|29165615|ref|NC_002745.2|	162485	+	A	0	1	0	0	0	SA0141
gi|29165615|ref|NC_002745.2|	162499	+	A	0	0	0	1	0	SA0141
gi|29165615|ref|NC_002745.2|	162509	+	A	0	1	1	0	1	SA0141
gi|29165615|ref|NC_002745.2|	162650	+	A	0	1	0	0	0	SA0141
gi|29165615|ref|NC_002745.2|	162653	+	C	1	0	0	0	0	SA0141
gi|29165615|ref|NC_002745.2|	162657	+	G	0	1	0	0	0	SA0141
gi|29165615|ref|NC_002745.2|	162658	+	C	0	0	1	1	2	SA0141
gi|29165615|ref|NC_002745.2|	162659	+	A	0	0	1	0	0	SA0141
gi|29165615|ref|NC_002745.2|	162678	+	A	0	1	0	0	1	SA0141
gi|29165615|ref|NC_002745.2|	162695	+	A	0	1	0	0	0	SA0141
gi|29165615|ref|NC_002745.2|	162718	+	G	0	0	0	0	1	SA0141
gi|29165615|ref|NC_002745.2|	162734	+	U	0	1	0	0	0	SA0141
gi|29165615|ref|NC_002745.2|	162742	+	A	0	0	0	0	1	SA0141
gi|29165615|ref|NC_002745.2|	162746	+	U	0	0	1	0	0	SA0141
gi|29165615|ref|NC_002745.2|	162747	+	A	0	0	0	0	1	SA0141
gi|29165615|ref|NC_002745.2|	162748	+	A	0	1	0	1	0	SA0141
gi|29165615|ref|NC_002745.2|	162754	+	G	0	0	0	0	1	SA0141
gi|29165615|ref|NC_002745.2|	162755	+	A	0	3	0	0	1	SA0141
gi|29165615|ref|NC_002745.2|	162756	+	A	0	1	0	0	0	SA0141
gi|29165615|ref|NC_002745.2|	162757	+	A	2	1	1	1	1	SA0141
gi|29165615|ref|NC_002745.2|	162758	+	G	0	3	3	1	2	SA0141
gi|29165615|ref|NC_002745.2|	162759	+	A	0	4	1	0	5	SA0141
gi|29165615|ref|NC_002745.2|	162760	+	G	0	9	0	1	5	SA0141
gi|29165615|ref|NC_002745.2|	162761	+	A	0	5	5	0	0	SA0141
gi|29165615|ref|NC_002745.2|	162762	+	A	0	0	1	0	0	SA0141
gi|29165615|ref|NC_002745.2|	162776	+	A	0	1	0	1	2	SA0141
gi|29165615|ref|NC_002745.2|	162782	+	A	0	1	0	0	0	SA0141
gi|29165615|ref|NC_002745.2|	162783	+	A	0	2	0	0	0	SA0141
gi|29165615|ref|NC_002745.2|	162796	+	C	0	1	0	0	0	SA0141
gi|29165615|ref|NC_002745.2|	162799	+	A	0	0	0	0	1	SA0141
gi|29165615|ref|NC_002745.2|	162806	+	A	0	1	0	1	1	SA0141
gi|29165615|ref|NC_002745.2|	162808	+	G	0	1	0	0	0	SA0141
gi|29165615|ref|NC_002745.2|	162820	+	C	0	1	1	0	0	SA0141
gi|29165615|ref|NC_002745.2|	162826	+	G	0	0	0	0	1	SA0141

gi|29165615|ref|NC_002745.2|	162851	+	G	0	1	0	0	0	SA0141
gi|29165615|ref|NC_002745.2|	162878	+	A	0	0	0	0	1	SA0141
gi|29165615|ref|NC_002745.2|	162884	+	A	0	0	0	0	2	SA0141
gi|29165615|ref|NC_002745.2|	163150	+	A	0	1	0	0	0	SA0142
gi|29165615|ref|NC_002745.2|	163175	+	A	0	0	1	0	0	SA0142
gi|29165615|ref|NC_002745.2|	163190	+	U	0	0	1	0	0	SA0142
gi|29165615|ref|NC_002745.2|	163209	+	A	0	1	0	0	0	SA0142
gi|29165615|ref|NC_002745.2|	163267	+	U	0	0	0	0	1	SA0142
gi|29165615|ref|NC_002745.2|	163273	+	A	0	0	0	1	0	SA0142
gi|29165615|ref|NC_002745.2|	163317	+	A	0	0	0	1	0	SA0142
gi|29165615|ref|NC_002745.2|	163324	+	A	0	1	0	0	0	SA0142
gi|29165615|ref|NC_002745.2|	163332	+	A	0	1	0	0	0	SA0142
gi|29165615|ref|NC_002745.2|	163362	+	A	0	2	1	0	0	SA0142
gi|29165615|ref|NC_002745.2|	163390	+	U	0	0	1	0	0	SA0142
gi|29165615|ref|NC_002745.2|	163419	+	G	0	1	0	0	0	SA0142
gi|29165615|ref|NC_002745.2|	163436	+	A	0	0	0	1	0	SA0142
gi|29165615|ref|NC_002745.2|	163438	+	U	1	0	0	0	0	SA0142
gi|29165615|ref|NC_002745.2|	163441	+	A	0	1	0	1	0	SA0142
gi|29165615|ref|NC_002745.2|	163445	+	U	0	0	1	0	0	SA0142
gi|29165615|ref|NC_002745.2|	163491	+	A	0	0	0	0	1	SA0142
gi|29165615|ref|NC_002745.2|	163495	+	U	1	0	0	0	0	SA0142
gi|29165615|ref|NC_002745.2|	163681	+	A	0	0	1	1	0	SA0142
gi|29165615|ref|NC_002745.2|	163739	+	A	0	2	0	0	0	SA0142
gi|29165615|ref|NC_002745.2|	164336	+	G	1	0	0	0	0	-
gi|29165615|ref|NC_002745.2|	165447	+	A	0	0	0	1	0	SA0143
gi|29165615|ref|NC_002745.2|	165464	+	A	0	1	0	0	0	SA0143
gi|29165615|ref|NC_002745.2|	165721	+	A	0	0	0	1	0	SA0143
gi|29165615|ref|NC_002745.2|	165769	+	C	0	0	0	1	0	SA0143
gi|29165615|ref|NC_002745.2|	165816	+	A	0	1	0	0	0	SA0143
gi|29165615|ref|NC_002745.2|	165845	+	U	0	1	0	0	0	SA0143
gi|29165615|ref|NC_002745.2|	165877	+	A	0	0	0	1	0	SA0143
gi|29165615|ref|NC_002745.2|	165921	+	G	0	0	1	0	0	SA0143
gi|29165615|ref|NC_002745.2|	165931	+	U	0	0	1	0	0	SA0143
gi|29165615|ref|NC_002745.2|	165956	+	C	0	0	0	0	1	SA0143
gi|29165615|ref|NC_002745.2|	165959	+	U	0	2	3	0	1	SA0143
gi|29165615|ref|NC_002745.2|	165961	+	A	0	4	7	4	6	SA0143
gi|29165615|ref|NC_002745.2|	165962	+	U	0	2	0	0	0	SA0143
gi|29165615|ref|NC_002745.2|	165965	+	A	0	2	0	1	0	SA0143
gi|29165615|ref|NC_002745.2|	165970	+	C	0	1	0	0	1	SA0143
gi|29165615|ref|NC_002745.2|	166000	+	A	0	0	1	0	0	SA0143
gi|29165615|ref|NC_002745.2|	166222	+	C	0	0	0	1	0	SA0143
gi|29165615|ref|NC_002745.2|	166228	+	A	0	1	0	1	0	SA0143
gi|29165615|ref|NC_002745.2|	166267	+	C	0	1	0	0	0	SA0143
gi|29165615|ref|NC_002745.2|	166391	+	A	0	0	0	1	0	SA0143
gi|29165615|ref|NC_002745.2|	166767	+	U	0	0	0	0	1	SA0143
gi|29165615|ref|NC_002745.2|	166781	+	A	0	1	0	0	0	SA0143
gi|29165615|ref|NC_002745.2|	166859	+	A	0	1	0	0	0	SA0143
gi|29165615|ref|NC_002745.2|	166901	+	A	0	0	1	0	0	SA0143
gi|29165615|ref|NC_002745.2|	167040	+	C	0	0	1	0	0	SA0143
gi|29165615|ref|NC_002745.2|	167342	+	A	1	0	0	0	0	-
gi|29165615|ref|NC_002745.2|	167362	+	A	0	1	0	0	0	-
gi|29165615|ref|NC_002745.2|	167616	+	A	0	0	0	0	1	SA0144
gi|29165615|ref|NC_002745.2|	167667	+	U	1	0	0	0	0	SA0144
gi|29165615|ref|NC_002745.2|	167761	+	U	0	0	0	1	0	SA0144
gi|29165615|ref|NC_002745.2|	167776	+	A	0	0	0	1	0	SA0144
gi|29165615|ref|NC_002745.2|	167849	+	G	0	0	1	0	0	SA0144
gi|29165615|ref|NC_002745.2|	168080	+	A	0	0	0	1	0	-
gi|29165615|ref|NC_002745.2|	168144	+	A	0	0	0	1	0	SA0145
gi|29165615|ref|NC_002745.2|	168147	+	U	0	0	0	1	0	SA0145
gi|29165615|ref|NC_002745.2|	168173	+	U	1	0	0	0	0	SA0145
gi|29165615|ref|NC_002745.2|	168325	+	G	2	0	0	0	0	SA0145
gi|29165615|ref|NC_002745.2|	168362	+	U	0	0	0	0	1	SA0145
gi|29165615|ref|NC_002745.2|	168367	+	A	0	0	0	0	1	SA0145
gi|29165615|ref|NC_002745.2|	168466	+	G	1	0	0	0	0	SA0145
gi|29165615|ref|NC_002745.2|	168494	+	A	1	0	0	0	0	SA0145
gi|29165615|ref|NC_002745.2|	168773	+	U	1	0	0	0	0	SA0145
gi|29165615|ref|NC_002745.2|	168854	+	C	0	0	0	0	1	SA0146
gi|29165615|ref|NC_002745.2|	168872	+	A	0	1	0	0	0	SA0146
gi|29165615|ref|NC_002745.2|	169157	+	C	0	0	1	0	0	SA0146
gi|29165615|ref|NC_002745.2|	169210	+	A	0	1	0	0	0	SA0146
gi|29165615|ref|NC_002745.2|	169741	+	C	0	0	1	0	0	SA0147
gi|29165615|ref|NC_002745.2|	169782	+	A	0	0	5	0	0	SA0147
gi|29165615|ref|NC_002745.2|	169803	+	A	0	2	0	0	0	SA0147
gi|29165615|ref|NC_002745.2|	169849	+	U	0	0	1	0	0	SA0147
gi|29165615|ref|NC_002745.2|	170008	+	C	1	0	0	0	0	SA0147
gi|29165615|ref|NC_002745.2|	170037	+	C	0	0	0	0	1	SA0147
gi|29165615|ref|NC_002745.2|	170097	+	C	0	0	0	0	1	SA0147
gi|29165615|ref|NC_002745.2|	170246	+	U	1	0	0	0	0	SA0147
gi|29165615|ref|NC_002745.2|	170281	+	C	0	1	0	0	0	SA0147
gi|29165615|ref|NC_002745.2|	170409	+	A	0	1	0	0	0	SA0147
gi|29165615|ref|NC_002745.2|	170415	+	A	0	0	1	0	0	SA0147

gi|29165615|ref|NC_002745.2|	170608 +	G	0	0	0	1	0	SA0147
gi|29165615|ref|NC_002745.2|	170853 +	C	0	1	0	0	0	SA0147
gi|29165615|ref|NC_002745.2|	170855 +	A	0	0	0	0	1	SA0147
gi|29165615|ref|NC_002745.2|	170885 +	U	1	0	1	0	0	SA0147
gi|29165615|ref|NC_002745.2|	170896 +	U	1	0	0	0	0	SA0147
gi|29165615|ref|NC_002745.2|	170920 +	C	1	0	0	0	0	SA0147
gi|29165615|ref|NC_002745.2|	170929 +	C	1	0	0	0	0	SA0147
gi|29165615|ref|NC_002745.2|	171010 +	U	0	0	1	0	0	SA0147
gi|29165615|ref|NC_002745.2|	171012 +	A	0	1	0	0	0	SA0147
gi|29165615|ref|NC_002745.2|	171235 +	C	1	0	0	0	0	SA0147
gi|29165615|ref|NC_002745.2|	171375 +	A	0	0	1	0	0	SA0147
gi|29165615|ref|NC_002745.2|	171537 +	A	0	0	1	0	0	SA0148
gi|29165615|ref|NC_002745.2|	171544 +	U	1	0	0	0	0	SA0148
gi|29165615|ref|NC_002745.2|	171554 +	G	1	0	0	0	0	SA0148
gi|29165615|ref|NC_002745.2|	171558 +	U	0	1	0	0	0	SA0148
gi|29165615|ref|NC_002745.2|	171753 +	U	1	0	0	0	0	SA0148
gi|29165615|ref|NC_002745.2|	171941 +	U	1	0	0	0	0	SA0148
gi|29165615|ref|NC_002745.2|	171973 +	U	1	0	0	0	0	SA0148
gi|29165615|ref|NC_002745.2|	171974 +	U	2	0	0	0	0	SA0148
gi|29165615|ref|NC_002745.2|	172014 +	A	0	0	0	1	0	SA0148
gi|29165615|ref|NC_002745.2|	172016 +	U	1	0	0	0	0	SA0148
gi|29165615|ref|NC_002745.2|	172022 +	A	1	0	0	0	0	SA0148
gi|29165615|ref|NC_002745.2|	172046 +	U	2	0	0	0	0	SA0148
gi|29165615|ref|NC_002745.2|	172057 +	C	1	0	0	0	0	SA0148
gi|29165615|ref|NC_002745.2|	172074 +	G	1	0	0	0	0	SA0148
gi|29165615|ref|NC_002745.2|	172090 +	U	0	0	0	1	0	SA0148
gi|29165615|ref|NC_002745.2|	172107 +	A	0	0	0	0	1	SA0148
gi|29165615|ref|NC_002745.2|	172415 +	U	2	0	0	0	0	-
gi|29165615|ref|NC_002745.2|	172478 +	U	0	1	0	0	0	SA0149
gi|29165615|ref|NC_002745.2|	172538 +	U	2	0	0	0	0	SA0149
gi|29165615|ref|NC_002745.2|	172636 +	U	0	0	0	0	1	SA0149
gi|29165615|ref|NC_002745.2|	172666 +	A	0	0	0	0	1	SA0149
gi|29165615|ref|NC_002745.2|	172674 +	A	1	0	0	0	0	SA0149
gi|29165615|ref|NC_002745.2|	172785 +	C	1	0	0	0	0	SA0149
gi|29165615|ref|NC_002745.2|	172865 +	U	0	1	0	0	0	SA0149
gi|29165615|ref|NC_002745.2|	172896 +	U	0	0	0	1	0	SA0149
gi|29165615|ref|NC_002745.2|	172928 +	U	1	0	0	0	0	SA0149
gi|29165615|ref|NC_002745.2|	172946 +	U	1	0	0	0	0	SA0149
gi|29165615|ref|NC_002745.2|	172960 +	A	1	0	0	0	0	SA0149
gi|29165615|ref|NC_002745.2|	173202 +	U	1	0	0	0	0	SA0149
gi|29165615|ref|NC_002745.2|	173227 +	G	1	0	0	0	0	SA0149
gi|29165615|ref|NC_002745.2|	173252 +	U	1	0	0	0	0	SA0149
gi|29165615|ref|NC_002745.2|	173321 +	C	0	0	1	0	0	SA0149
gi|29165615|ref|NC_002745.2|	173355 +	U	1	0	0	0	0	SA0149
gi|29165615|ref|NC_002745.2|	173363 +	A	0	0	0	2	0	SA0149
gi|29165615|ref|NC_002745.2|	173444 +	U	0	1	0	0	0	SA0149
gi|29165615|ref|NC_002745.2|	173518 +	U	1	0	0	0	0	SA0149
gi|29165615|ref|NC_002745.2|	173527 +	U	0	0	1	0	0	SA0149
gi|29165615|ref|NC_002745.2|	173559 +	A	1	0	0	0	0	SA0150
gi|29165615|ref|NC_002745.2|	173563 +	G	0	0	0	1	0	SA0150
gi|29165615|ref|NC_002745.2|	173578 +	A	0	0	1	0	0	SA0150
gi|29165615|ref|NC_002745.2|	173663 +	C	1	0	0	0	0	SA0150
gi|29165615|ref|NC_002745.2|	173695 +	U	0	0	1	0	0	SA0150
gi|29165615|ref|NC_002745.2|	173847 +	A	0	1	0	0	0	SA0150
gi|29165615|ref|NC_002745.2|	174152 +	A	0	0	0	1	0	SA0150
gi|29165615|ref|NC_002745.2|	174170 +	A	0	0	1	0	0	SA0150
gi|29165615|ref|NC_002745.2|	174194 +	A	0	0	0	1	0	SA0150
gi|29165615|ref|NC_002745.2|	174236 +	U	1	0	0	0	0	SA0150
gi|29165615|ref|NC_002745.2|	174296 +	C	0	0	0	0	1	SA0150
gi|29165615|ref|NC_002745.2|	174414 +	C	1	0	0	0	0	SA0150
gi|29165615|ref|NC_002745.2|	174426 +	U	0	0	1	0	0	SA0150
gi|29165615|ref|NC_002745.2|	174483 +	U	1	0	0	0	0	SA0150
gi|29165615|ref|NC_002745.2|	174559 +	G	0	0	0	1	0	SA0150
gi|29165615|ref|NC_002745.2|	174585 +	C	1	0	0	0	0	SA0150
gi|29165615|ref|NC_002745.2|	174590 +	C	1	0	0	0	0	SA0150
gi|29165615|ref|NC_002745.2|	174607 +	A	0	0	0	1	0	SA0150
gi|29165615|ref|NC_002745.2|	174617 +	G	1	0	0	0	0	SA0150
gi|29165615|ref|NC_002745.2|	174626 +	A	0	0	0	1	0	SA0150
gi|29165615|ref|NC_002745.2|	174680 +	G	0	0	0	1	0	SA0151
gi|29165615|ref|NC_002745.2|	174792 +	U	0	0	1	0	0	SA0151
gi|29165615|ref|NC_002745.2|	174986 +	G	1	0	0	0	0	SA0151
gi|29165615|ref|NC_002745.2|	175096 +	U	0	1	0	0	0	SA0151
gi|29165615|ref|NC_002745.2|	175140 +	G	0	0	1	0	0	SA0151
gi|29165615|ref|NC_002745.2|	175360 +	A	1	0	0	0	0	SA0152
gi|29165615|ref|NC_002745.2|	175753 +	A	1	0	0	0	0	SA0152
gi|29165615|ref|NC_002745.2|	175835 +	A	2	0	0	0	0	SA0152
gi|29165615|ref|NC_002745.2|	175851 +	A	1	0	0	0	0	SA0152
gi|29165615|ref|NC_002745.2|	175891 +	A	0	0	0	0	1	SA0152
gi|29165615|ref|NC_002745.2|	176189 +	C	1	0	0	0	0	SA0152
gi|29165615|ref|NC_002745.2|	176332 +	A	1	0	0	0	0	SA0152
gi|29165615|ref|NC_002745.2|	176376 +	A	1	0	0	0	0	SA0152

gi|29165615|ref|NC_002745.2|	176578	+	C	0	0	0	0	1	SA0153
gi|29165615|ref|NC_002745.2|	176588	+	A	0	0	1	0	0	SA0153
gi|29165615|ref|NC_002745.2|	176592	+	C	2	0	0	0	0	SA0153
gi|29165615|ref|NC_002745.2|	176614	+	U	1	0	0	0	0	SA0153
gi|29165615|ref|NC_002745.2|	176615	+	G	0	0	1	0	0	SA0153
gi|29165615|ref|NC_002745.2|	176908	+	U	1	0	0	0	0	SA0153
gi|29165615|ref|NC_002745.2|	176989	+	U	0	0	0	0	2	SA0153
gi|29165615|ref|NC_002745.2|	177160	+	U	0	1	0	0	0	SA0153
gi|29165615|ref|NC_002745.2|	177310	+	U	0	0	0	0	1	SA0153
gi|29165615|ref|NC_002745.2|	177334	+	U	0	0	1	0	0	SA0153
gi|29165615|ref|NC_002745.2|	177477	+	A	1	0	0	0	0	SA0153
gi|29165615|ref|NC_002745.2|	177508	+	U	0	0	0	0	1	SA0153
gi|29165615|ref|NC_002745.2|	177534	+	U	1	0	0	0	0	SA0153
gi|29165615|ref|NC_002745.2|	177546	+	A	1	0	0	0	0	SA0153
gi|29165615|ref|NC_002745.2|	177944	+	A	0	1	1	0	0	SA0154
gi|29165615|ref|NC_002745.2|	178018	+	U	0	0	1	0	0	SA0154
gi|29165615|ref|NC_002745.2|	179442	+	C	0	0	0	0	1	SA0155
gi|29165615|ref|NC_002745.2|	179825	+	U	1	0	0	0	0	SA0155
gi|29165615|ref|NC_002745.2|	179843	+	C	2	0	0	0	0	SA0155
gi|29165615|ref|NC_002745.2|	179881	+	C	1	0	0	0	0	SA0155
gi|29165615|ref|NC_002745.2|	180167	+	U	2	0	0	0	0	SA0156
gi|29165615|ref|NC_002745.2|	180226	+	A	0	0	0	1	0	SA0156
gi|29165615|ref|NC_002745.2|	180328	+	U	0	0	0	1	0	SA0156
gi|29165615|ref|NC_002745.2|	180340	+	G	0	2	0	0	0	SA0156
gi|29165615|ref|NC_002745.2|	180359	+	C	1	0	0	0	0	SA0156
gi|29165615|ref|NC_002745.2|	180611	+	U	1	0	0	0	0	SA0157
gi|29165615|ref|NC_002745.2|	180733	+	C	0	0	1	0	0	SA0157
gi|29165615|ref|NC_002745.2|	181293	+	U	0	0	1	0	0	SA0157
gi|29165615|ref|NC_002745.2|	181373	+	U	1	0	0	0	0	SA0157
gi|29165615|ref|NC_002745.2|	181469	+	A	1	0	0	0	0	-
gi|29165615|ref|NC_002745.2|	181552	+	A	0	0	0	1	0	SA0158
gi|29165615|ref|NC_002745.2|	181702	+	A	0	0	1	0	0	SA0158
gi|29165615|ref|NC_002745.2|	181838	+	G	0	0	0	1	0	SA0158
gi|29165615|ref|NC_002745.2|	181934	+	C	2	0	0	0	0	SA0158
gi|29165615|ref|NC_002745.2|	181982	+	C	0	0	1	0	0	SA0158
gi|29165615|ref|NC_002745.2|	182264	+	U	1	0	0	0	0	SA0158
gi|29165615|ref|NC_002745.2|	182658	+	A	0	0	0	0	1	SA0158
gi|29165615|ref|NC_002745.2|	182733	+	U	0	1	0	0	0	SA0158
gi|29165615|ref|NC_002745.2|	182741	+	U	1	0	0	0	0	SA0158
gi|29165615|ref|NC_002745.2|	182775	+	U	0	0	0	0	1	-
gi|29165615|ref|NC_002745.2|	182787	+	G	0	1	0	1	0	-
gi|29165615|ref|NC_002745.2|	182788	+	A	0	2	0	0	0	-
gi|29165615|ref|NC_002745.2|	182986	+	A	0	2	0	0	0	SA0159
gi|29165615|ref|NC_002745.2|	183010	+	A	1	0	0	0	0	SA0159
gi|29165615|ref|NC_002745.2|	183019	+	A	0	0	0	1	0	SA0159
gi|29165615|ref|NC_002745.2|	183022	+	A	0	0	0	1	0	SA0159
gi|29165615|ref|NC_002745.2|	183025	+	A	0	0	0	0	1	SA0159
gi|29165615|ref|NC_002745.2|	183039	+	G	0	1	0	0	0	SA0159
gi|29165615|ref|NC_002745.2|	183041	+	C	0	0	0	1	0	SA0159
gi|29165615|ref|NC_002745.2|	183152	+	A	1	0	0	0	0	SA0159
gi|29165615|ref|NC_002745.2|	183198	+	A	0	1	0	0	0	SA0159
gi|29165615|ref|NC_002745.2|	183209	+	A	1	0	0	0	0	SA0159
gi|29165615|ref|NC_002745.2|	183210	+	U	0	0	0	1	0	SA0159
gi|29165615|ref|NC_002745.2|	183240	+	G	0	0	0	0	1	SA0159
gi|29165615|ref|NC_002745.2|	183385	+	A	0	0	1	0	0	SA0159
gi|29165615|ref|NC_002745.2|	183400	+	U	0	1	0	0	0	SA0159
gi|29165615|ref|NC_002745.2|	183402	+	A	0	0	0	1	0	SA0159
gi|29165615|ref|NC_002745.2|	183435	+	C	0	0	0	1	2	SA0159
gi|29165615|ref|NC_002745.2|	183447	+	C	1	0	0	0	0	SA0159
gi|29165615|ref|NC_002745.2|	183458	+	U	0	0	0	1	1	SA0159
gi|29165615|ref|NC_002745.2|	183460	+	U	1	0	0	0	0	SA0159
gi|29165615|ref|NC_002745.2|	183493	+	U	0	1	0	0	0	SA0159
gi|29165615|ref|NC_002745.2|	183517	+	A	1	0	0	0	0	SA0159
gi|29165615|ref|NC_002745.2|	183534	+	U	0	0	2	0	0	SA0159
gi|29165615|ref|NC_002745.2|	183609	+	A	0	0	1	0	0	SA0159
gi|29165615|ref|NC_002745.2|	183659	+	C	0	1	0	0	0	SA0159
gi|29165615|ref|NC_002745.2|	183781	+	U	0	1	0	0	0	SA0159
gi|29165615|ref|NC_002745.2|	184833	+	G	0	0	0	1	0	-
gi|29165615|ref|NC_002745.2|	185090	+	G	0	0	0	0	1	-
gi|29165615|ref|NC_002745.2|	185106	+	G	1	0	0	0	0	-
gi|29165615|ref|NC_002745.2|	185107	+	A	0	0	0	0	1	-
gi|29165615|ref|NC_002745.2|	185108	+	A	0	1	0	1	0	-
gi|29165615|ref|NC_002745.2|	185111	+	A	1	26	8	16	16	-
gi|29165615|ref|NC_002745.2|	185129	+	A	0	1	0	0	0	-
gi|29165615|ref|NC_002745.2|	185132	+	A	0	2	0	0	0	-
gi|29165615|ref|NC_002745.2|	185137	+	U	0	0	0	0	1	-
gi|29165615|ref|NC_002745.2|	185148	+	G	0	0	1	0	0	-
gi|29165615|ref|NC_002745.2|	185152	+	U	0	0	0	1	0	-
gi|29165615|ref|NC_002745.2|	185154	+	U	0	1	0	2	1	-
gi|29165615|ref|NC_002745.2|	185155	+	A	0	0	0	1	1	-
gi|29165615|ref|NC_002745.2|	185158	+	A	0	0	0	0	1	-

gi|29165615|ref|NC_002745.2|	185162 +	C	0	1	0	2	0	-
gi|29165615|ref|NC_002745.2|	185165 +	A	0	1	0	0	0	-
gi|29165615|ref|NC_002745.2|	185168 +	A	0	0	0	0	1	-
gi|29165615|ref|NC_002745.2|	185171 +	A	0	0	0	0	1	-
gi|29165615|ref|NC_002745.2|	185172 +	A	0	0	0	1	1	-
gi|29165615|ref|NC_002745.2|	185174 +	G	0	0	0	0	1	-
gi|29165615|ref|NC_002745.2|	185176 +	A	0	0	1	0	2	-
gi|29165615|ref|NC_002745.2|	185178 +	A	0	1	0	0	2	-
gi|29165615|ref|NC_002745.2|	185180 +	A	0	0	0	0	1	-
gi|29165615|ref|NC_002745.2|	185182 +	A	0	0	0	1	0	-
gi|29165615|ref|NC_002745.2|	185186 +	U	0	1	0	0	0	SA0162
gi|29165615|ref|NC_002745.2|	185188 +	G	0	0	1	1	4	SA0162
gi|29165615|ref|NC_002745.2|	185189 +	C	0	0	0	1	3	SA0162
gi|29165615|ref|NC_002745.2|	185192 +	U	0	2	1	0	1	SA0162
gi|29165615|ref|NC_002745.2|	185194 +	A	0	1	0	0	1	SA0162
gi|29165615|ref|NC_002745.2|	185198 +	U	0	0	0	2	0	SA0162
gi|29165615|ref|NC_002745.2|	185202 +	A	0	0	0	0	2	SA0162
gi|29165615|ref|NC_002745.2|	185204 +	A	0	3	0	0	0	SA0162
gi|29165615|ref|NC_002745.2|	185206 +	U	0	1	0	0	0	SA0162
gi|29165615|ref|NC_002745.2|	185208 +	U	0	1	0	0	0	SA0162
gi|29165615|ref|NC_002745.2|	185210 +	U	0	0	0	0	1	SA0162
gi|29165615|ref|NC_002745.2|	185212 +	G	0	5	0	0	1	SA0162
gi|29165615|ref|NC_002745.2|	185213 +	C	0	0	1	0	0	SA0162
gi|29165615|ref|NC_002745.2|	185214 +	A	0	0	0	0	1	SA0162
gi|29165615|ref|NC_002745.2|	185216 +	A	0	2	1	1	2	SA0162
gi|29165615|ref|NC_002745.2|	185217 +	G	0	1	0	0	0	SA0162
gi|29165615|ref|NC_002745.2|	185218 +	A	0	2	0	2	1	SA0162
gi|29165615|ref|NC_002745.2|	185222 +	A	0	0	0	1	0	SA0162
gi|29165615|ref|NC_002745.2|	185225 +	G	0	1	0	0	2	SA0162
gi|29165615|ref|NC_002745.2|	185226 +	U	0	1	0	2	1	SA0162
gi|29165615|ref|NC_002745.2|	185231 +	U	0	0	0	0	1	SA0162
gi|29165615|ref|NC_002745.2|	185232 +	U	0	0	0	0	2	SA0162
gi|29165615|ref|NC_002745.2|	185233 +	A	0	4	0	1	0	SA0162
gi|29165615|ref|NC_002745.2|	185234 +	U	0	1	0	3	3	SA0162
gi|29165615|ref|NC_002745.2|	185235 +	C	0	2	0	0	1	SA0162
gi|29165615|ref|NC_002745.2|	185236 +	A	0	0	0	0	2	SA0162
gi|29165615|ref|NC_002745.2|	185240 +	G	0	3	0	0	0	SA0162
gi|29165615|ref|NC_002745.2|	185243 +	A	0	2	0	0	0	SA0162
gi|29165615|ref|NC_002745.2|	185248 +	G	0	1	0	0	0	SA0162
gi|29165615|ref|NC_002745.2|	185249 +	U	0	0	1	1	0	SA0162
gi|29165615|ref|NC_002745.2|	185250 +	U	0	0	0	1	0	SA0162
gi|29165615|ref|NC_002745.2|	185258 +	G	0	0	0	1	0	SA0162
gi|29165615|ref|NC_002745.2|	185259 +	C	0	0	0	1	0	SA0162
gi|29165615|ref|NC_002745.2|	185267 +	A	0	0	0	1	0	SA0162
gi|29165615|ref|NC_002745.2|	185269 +	A	0	0	0	1	1	SA0162
gi|29165615|ref|NC_002745.2|	185270 +	C	1	14	3	9	18	SA0162
gi|29165615|ref|NC_002745.2|	185271 +	A	0	1	0	0	4	SA0162
gi|29165615|ref|NC_002745.2|	185272 +	A	0	0	0	1	0	SA0162
gi|29165615|ref|NC_002745.2|	185292 +	A	0	0	0	0	1	SA0162
gi|29165615|ref|NC_002745.2|	185293 +	A	0	0	0	0	1	SA0162
gi|29165615|ref|NC_002745.2|	185298 +	A	0	0	0	0	2	SA0162
gi|29165615|ref|NC_002745.2|	185299 +	G	0	0	0	0	1	SA0162
gi|29165615|ref|NC_002745.2|	185308 +	U	0	0	0	0	2	SA0162
gi|29165615|ref|NC_002745.2|	185313 +	U	0	0	1	0	0	SA0162
gi|29165615|ref|NC_002745.2|	185316 +	U	0	1	0	0	0	SA0162
gi|29165615|ref|NC_002745.2|	185317 +	A	0	1	0	0	0	SA0162
gi|29165615|ref|NC_002745.2|	185318 +	C	0	0	0	0	2	SA0162
gi|29165615|ref|NC_002745.2|	185320 +	A	0	0	1	2	2	SA0162
gi|29165615|ref|NC_002745.2|	185321 +	G	0	0	0	0	2	SA0162
gi|29165615|ref|NC_002745.2|	185322 +	A	0	0	0	1	0	SA0162
gi|29165615|ref|NC_002745.2|	185323 +	G	0	1	0	1	0	SA0162
gi|29165615|ref|NC_002745.2|	185325 +	A	0	0	0	1	0	SA0162
gi|29165615|ref|NC_002745.2|	185326 +	A	0	0	1	0	1	SA0162
gi|29165615|ref|NC_002745.2|	185327 +	A	0	2	0	5	3	SA0162
gi|29165615|ref|NC_002745.2|	185328 +	A	0	2	1	0	1	SA0162
gi|29165615|ref|NC_002745.2|	185339 +	U	0	1	0	1	4	SA0162
gi|29165615|ref|NC_002745.2|	185342 +	A	0	1	0	0	0	SA0162
gi|29165615|ref|NC_002745.2|	185387 +	U	0	0	0	1	0	SA0162
gi|29165615|ref|NC_002745.2|	185395 +	A	0	2	0	1	3	SA0162
gi|29165615|ref|NC_002745.2|	185398 +	U	0	0	0	0	1	SA0162
gi|29165615|ref|NC_002745.2|	185402 +	A	0	0	0	1	0	SA0162
gi|29165615|ref|NC_002745.2|	185432 +	A	0	0	2	1	0	SA0162
gi|29165615|ref|NC_002745.2|	185441 +	U	1	0	0	0	0	SA0162
gi|29165615|ref|NC_002745.2|	185452 +	G	0	0	0	0	1	SA0162
gi|29165615|ref|NC_002745.2|	185455 +	A	0	0	0	0	1	SA0162
gi|29165615|ref|NC_002745.2|	185456 +	A	0	0	0	0	1	SA0162
gi|29165615|ref|NC_002745.2|	185457 +	A	0	0	0	1	0	SA0162
gi|29165615|ref|NC_002745.2|	185458 +	A	0	3	1	0	4	SA0162
gi|29165615|ref|NC_002745.2|	185461 +	G	0	1	0	0	0	SA0162
gi|29165615|ref|NC_002745.2|	185462 +	C	0	1	0	0	1	SA0162
gi|29165615|ref|NC_002745.2|	185464 +	A	0	0	0	0	1	SA0162

gi|29165615|ref|NC_002745.2|	185466	+	G	0	0	1	0	0	SA0162
gi|29165615|ref|NC_002745.2|	185476	+	U	1	0	0	0	0	SA0162
gi|29165615|ref|NC_002745.2|	185477	+	U	0	1	0	0	0	SA0162
gi|29165615|ref|NC_002745.2|	185489	+	A	0	0	0	0	2	SA0162
gi|29165615|ref|NC_002745.2|	185496	+	U	0	0	0	1	0	SA0162
gi|29165615|ref|NC_002745.2|	185520	+	U	0	0	0	0	1	SA0162
gi|29165615|ref|NC_002745.2|	185521	+	C	0	1	0	0	0	SA0162
gi|29165615|ref|NC_002745.2|	185585	+	A	0	1	0	0	0	SA0162
gi|29165615|ref|NC_002745.2|	185588	+	A	0	1	2	0	0	SA0162
gi|29165615|ref|NC_002745.2|	185608	+	A	0	0	0	0	1	SA0162
gi|29165615|ref|NC_002745.2|	185614	+	G	0	1	0	0	0	SA0162
gi|29165615|ref|NC_002745.2|	185660	+	A	0	0	1	0	0	SA0162
gi|29165615|ref|NC_002745.2|	185671	+	C	0	0	0	1	0	SA0162
gi|29165615|ref|NC_002745.2|	185769	+	U	0	1	0	0	0	SA0162
gi|29165615|ref|NC_002745.2|	185791	+	C	0	0	0	0	1	SA0162
gi|29165615|ref|NC_002745.2|	185803	+	G	0	0	0	0	1	SA0162
gi|29165615|ref|NC_002745.2|	185883	+	G	0	0	0	0	1	SA0162
gi|29165615|ref|NC_002745.2|	186022	+	U	0	0	0	0	1	SA0162
gi|29165615|ref|NC_002745.2|	186024	+	A	0	0	0	0	1	SA0162
gi|29165615|ref|NC_002745.2|	186029	+	U	0	0	0	0	1	SA0162
gi|29165615|ref|NC_002745.2|	186033	+	A	0	0	0	1	0	SA0162
gi|29165615|ref|NC_002745.2|	186052	+	U	0	0	0	0	1	SA0162
gi|29165615|ref|NC_002745.2|	186071	+	U	0	1	0	0	0	SA0162
gi|29165615|ref|NC_002745.2|	186079	+	C	0	0	0	0	1	SA0162
gi|29165615|ref|NC_002745.2|	186081	+	U	0	1	0	0	0	SA0162
gi|29165615|ref|NC_002745.2|	186082	+	G	0	0	0	0	1	SA0162
gi|29165615|ref|NC_002745.2|	186086	+	A	0	0	0	0	1	SA0162
gi|29165615|ref|NC_002745.2|	186087	+	A	0	1	0	0	0	SA0162
gi|29165615|ref|NC_002745.2|	186091	+	U	0	1	0	0	0	SA0162
gi|29165615|ref|NC_002745.2|	186092	+	A	0	0	0	1	0	SA0162
gi|29165615|ref|NC_002745.2|	186093	+	U	0	1	0	1	1	SA0162
gi|29165615|ref|NC_002745.2|	186094	+	G	0	0	0	1	2	SA0162
gi|29165615|ref|NC_002745.2|	186095	+	A	0	3	1	0	6	SA0162
gi|29165615|ref|NC_002745.2|	186096	+	U	0	1	0	2	1	SA0162
gi|29165615|ref|NC_002745.2|	186119	+	A	0	1	0	0	1	SA0162
gi|29165615|ref|NC_002745.2|	186132	+	U	0	1	0	0	0	SA0162
gi|29165615|ref|NC_002745.2|	186136	+	A	1	0	1	0	3	SA0162
gi|29165615|ref|NC_002745.2|	186137	+	A	0	2	0	4	1	SA0162
gi|29165615|ref|NC_002745.2|	186178	+	C	0	1	0	0	0	SA0162
gi|29165615|ref|NC_002745.2|	186191	+	A	0	0	0	0	1	SA0162
gi|29165615|ref|NC_002745.2|	186192	+	U	0	0	0	0	1	SA0162
gi|29165615|ref|NC_002745.2|	186194	+	A	0	0	0	0	1	SA0162
gi|29165615|ref|NC_002745.2|	186204	+	A	0	1	0	2	1	SA0162
gi|29165615|ref|NC_002745.2|	186208	+	C	0	0	0	1	1	SA0162
gi|29165615|ref|NC_002745.2|	186209	+	A	0	0	0	1	0	SA0162
gi|29165615|ref|NC_002745.2|	186210	+	A	0	0	0	0	1	SA0162
gi|29165615|ref|NC_002745.2|	186213	+	A	0	0	0	0	1	SA0162
gi|29165615|ref|NC_002745.2|	186217	+	A	0	0	0	0	1	SA0162
gi|29165615|ref|NC_002745.2|	186218	+	U	0	0	0	1	0	SA0162
gi|29165615|ref|NC_002745.2|	186226	+	G	0	1	0	1	0	SA0162
gi|29165615|ref|NC_002745.2|	186227	+	C	1	0	0	3	0	SA0162
gi|29165615|ref|NC_002745.2|	186228	+	A	0	0	1	0	0	SA0162
gi|29165615|ref|NC_002745.2|	186229	+	A	0	0	0	2	0	SA0162
gi|29165615|ref|NC_002745.2|	186232	+	G	0	0	0	0	4	SA0162
gi|29165615|ref|NC_002745.2|	186233	+	A	0	0	0	0	2	SA0162
gi|29165615|ref|NC_002745.2|	186236	+	C	0	0	0	0	1	SA0162
gi|29165615|ref|NC_002745.2|	186239	+	A	0	0	0	1	0	SA0162
gi|29165615|ref|NC_002745.2|	186241	+	G	0	1	0	0	0	SA0162
gi|29165615|ref|NC_002745.2|	186246	+	A	0	0	0	0	1	SA0162
gi|29165615|ref|NC_002745.2|	186247	+	A	0	0	0	0	1	SA0162
gi|29165615|ref|NC_002745.2|	186254	+	C	0	1	1	0	0	SA0162
gi|29165615|ref|NC_002745.2|	186258	+	U	0	0	0	0	2	SA0162
gi|29165615|ref|NC_002745.2|	186260	+	G	0	1	0	0	1	SA0162
gi|29165615|ref|NC_002745.2|	186261	+	U	0	0	0	1	0	SA0162
gi|29165615|ref|NC_002745.2|	186268	+	U	0	0	0	0	1	SA0162
gi|29165615|ref|NC_002745.2|	186269	+	U	0	1	0	0	0	SA0162
gi|29165615|ref|NC_002745.2|	186277	+	A	0	1	0	1	0	SA0162
gi|29165615|ref|NC_002745.2|	186280	+	G	0	0	0	1	0	SA0162
gi|29165615|ref|NC_002745.2|	186281	+	G	0	0	1	0	1	SA0162
gi|29165615|ref|NC_002745.2|	186282	+	A	0	0	0	1	0	SA0162
gi|29165615|ref|NC_002745.2|	186284	+	U	0	0	0	1	1	SA0162
gi|29165615|ref|NC_002745.2|	186285	+	A	0	1	0	0	0	SA0162
gi|29165615|ref|NC_002745.2|	186288	+	U	0	0	0	1	0	SA0162
gi|29165615|ref|NC_002745.2|	186295	+	U	0	1	0	0	0	SA0162
gi|29165615|ref|NC_002745.2|	186310	+	A	0	2	0	1	0	SA0162
gi|29165615|ref|NC_002745.2|	186313	+	U	0	1	0	0	0	SA0162
gi|29165615|ref|NC_002745.2|	186316	+	A	0	0	0	0	1	SA0162
gi|29165615|ref|NC_002745.2|	186327	+	A	0	1	0	0	0	SA0162
gi|29165615|ref|NC_002745.2|	186332	+	A	0	1	0	0	0	SA0162
gi|29165615|ref|NC_002745.2|	186358	+	A	0	0	0	0	1	SA0162
gi|29165615|ref|NC_002745.2|	186464	+	A	0	1	0	0	0	SA0162

gi|29165615|ref|NC_002745.2|	186567	+	A	0	0	0	0	1	SA0162
gi|29165615|ref|NC_002745.2|	186570	+	A	0	1	0	0	0	SA0162
gi|29165615|ref|NC_002745.2|	186579	+	C	0	1	0	0	0	SA0162
gi|29165615|ref|NC_002745.2|	186582	+	U	0	0	0	0	1	SA0162
gi|29165615|ref|NC_002745.2|	186602	+	C	0	1	0	0	0	SA0162
gi|29165615|ref|NC_002745.2|	186605	+	U	0	1	1	0	1	SA0162
gi|29165615|ref|NC_002745.2|	186612	+	C	0	0	0	0	1	SA0162
gi|29165615|ref|NC_002745.2|	186614	+	A	0	0	0	1	0	SA0162
gi|29165615|ref|NC_002745.2|	186615	+	U	0	0	0	1	1	SA0162
gi|29165615|ref|NC_002745.2|	186617	+	A	0	0	0	1	0	SA0162
gi|29165615|ref|NC_002745.2|	186618	+	A	0	0	1	3	5	SA0162
gi|29165615|ref|NC_002745.2|	186619	+	C	0	2	0	1	2	SA0162
gi|29165615|ref|NC_002745.2|	186621	+	A	0	0	0	0	1	SA0162
gi|29165615|ref|NC_002745.2|	186625	+	A	0	0	0	4	3	SA0162
gi|29165615|ref|NC_002745.2|	186626	+	A	0	1	0	0	1	SA0162
gi|29165615|ref|NC_002745.2|	186627	+	A	0	0	0	2	1	SA0162
gi|29165615|ref|NC_002745.2|	186628	+	A	0	0	0	0	2	SA0162
gi|29165615|ref|NC_002745.2|	186629	+	A	0	1	0	1	1	SA0162
gi|29165615|ref|NC_002745.2|	186630	+	U	0	0	0	0	1	SA0162
gi|29165615|ref|NC_002745.2|	186631	+	A	0	0	0	1	1	SA0162
gi|29165615|ref|NC_002745.2|	186632	+	U	0	0	1	0	2	SA0162
gi|29165615|ref|NC_002745.2|	186633	+	U	0	0	0	1	1	SA0162
gi|29165615|ref|NC_002745.2|	186634	+	U	0	0	0	0	2	SA0162
gi|29165615|ref|NC_002745.2|	186635	+	A	0	0	0	3	1	SA0162
gi|29165615|ref|NC_002745.2|	186636	+	U	0	0	0	1	0	SA0162
gi|29165615|ref|NC_002745.2|	186637	+	A	0	0	0	0	2	SA0162
gi|29165615|ref|NC_002745.2|	186638	+	U	0	0	0	0	1	SA0162
gi|29165615|ref|NC_002745.2|	186640	+	G	0	0	0	1	3	SA0162
gi|29165615|ref|NC_002745.2|	186641	+	A	0	0	0	2	3	SA0162
gi|29165615|ref|NC_002745.2|	186642	+	U	0	0	1	0	0	SA0162
gi|29165615|ref|NC_002745.2|	186643	+	A	0	1	0	0	0	SA0162
gi|29165615|ref|NC_002745.2|	186644	+	C	0	1	0	2	5	SA0162
gi|29165615|ref|NC_002745.2|	186649	+	A	0	0	0	1	0	SA0162
gi|29165615|ref|NC_002745.2|	186653	+	C	0	0	0	1	0	SA0162
gi|29165615|ref|NC_002745.2|	186657	+	A	0	0	0	6	1	SA0162
gi|29165615|ref|NC_002745.2|	186658	+	A	0	0	0	10	0	SA0162
gi|29165615|ref|NC_002745.2|	186668	+	A	0	0	0	0	4	SA0162
gi|29165615|ref|NC_002745.2|	186671	+	A	0	5	1	8	4	SA0162
gi|29165615|ref|NC_002745.2|	186672	+	G	0	0	0	0	1	SA0162
gi|29165615|ref|NC_002745.2|	186673	+	A	0	1	0	2	3	-
gi|29165615|ref|NC_002745.2|	186674	+	A	0	0	0	2	3	-
gi|29165615|ref|NC_002745.2|	186675	+	U	0	0	0	1	0	-
gi|29165615|ref|NC_002745.2|	186676	+	A	0	0	0	4	4	-
gi|29165615|ref|NC_002745.2|	186677	+	A	0	0	0	3	2	-
gi|29165615|ref|NC_002745.2|	186678	+	A	0	0	0	2	2	-
gi|29165615|ref|NC_002745.2|	186679	+	U	0	0	0	1	0	-
gi|29165615|ref|NC_002745.2|	186681	+	U	0	0	0	1	1	-
gi|29165615|ref|NC_002745.2|	186684	+	U	1	2	0	7	2	-
gi|29165615|ref|NC_002745.2|	186689	+	G	0	0	0	2	0	-
gi|29165615|ref|NC_002745.2|	186697	+	U	0	1	0	0	0	-
gi|29165615|ref|NC_002745.2|	186802	+	A	0	0	0	0	1	-
gi|29165615|ref|NC_002745.2|	186805	+	A	0	1	1	0	2	-
gi|29165615|ref|NC_002745.2|	187103	+	A	0	2	0	0	2	-
gi|29165615|ref|NC_002745.2|	187104	+	U	0	1	1	0	3	-
gi|29165615|ref|NC_002745.2|	187105	+	A	0	8	5	5	3	-
gi|29165615|ref|NC_002745.2|	187106	+	A	0	1	0	0	1	-
gi|29165615|ref|NC_002745.2|	187110	+	A	0	0	1	0	0	-
gi|29165615|ref|NC_002745.2|	187117	+	G	0	1	0	0	0	-
gi|29165615|ref|NC_002745.2|	187206	+	U	0	1	0	0	0	-
gi|29165615|ref|NC_002745.2|	187385	+	A	0	0	0	0	1	SA0163
gi|29165615|ref|NC_002745.2|	187401	+	A	0	0	0	0	1	SA0163
gi|29165615|ref|NC_002745.2|	187402	+	A	0	0	0	1	0	SA0163
gi|29165615|ref|NC_002745.2|	187607	+	G	0	1	0	0	0	SA0163
gi|29165615|ref|NC_002745.2|	187912	+	A	0	1	0	0	0	SA0163
gi|29165615|ref|NC_002745.2|	187926	+	U	0	1	0	2	0	SA0163
gi|29165615|ref|NC_002745.2|	187967	+	C	0	0	0	1	0	SA0163
gi|29165615|ref|NC_002745.2|	188136	+	A	0	1	0	0	0	SA0163
gi|29165615|ref|NC_002745.2|	188170	+	A	0	0	0	0	1	SA0163
gi|29165615|ref|NC_002745.2|	188207	+	C	0	1	0	0	0	SA0163
gi|29165615|ref|NC_002745.2|	188285	+	C	0	1	0	0	0	-
gi|29165615|ref|NC_002745.2|	188649	+	A	0	0	0	0	2	-
gi|29165615|ref|NC_002745.2|	188652	+	A	0	0	0	0	1	-
gi|29165615|ref|NC_002745.2|	188678	+	A	0	0	1	0	0	-
gi|29165615|ref|NC_002745.2|	188682	+	A	0	0	0	1	0	-
gi|29165615|ref|NC_002745.2|	188928	+	C	0	1	0	0	0	-
gi|29165615|ref|NC_002745.2|	188929	+	A	5	4	6	2	8	-
gi|29165615|ref|NC_002745.2|	189001	+	C	0	0	0	0	1	SA0165
gi|29165615|ref|NC_002745.2|	189024	+	G	0	0	1	0	0	SA0165
gi|29165615|ref|NC_002745.2|	189048	+	A	0	1	0	0	0	SA0165
gi|29165615|ref|NC_002745.2|	189211	+	U	0	0	0	0	1	SA0165
gi|29165615|ref|NC_002745.2|	189223	+	A	0	0	0	0	1	SA0165

gi|29165615|ref|NC_002745.2|	189304 +	A	0	1	0	0	0	SA0165
gi|29165615|ref|NC_002745.2|	189313 +	A	0	1	0	0	0	SA0165
gi|29165615|ref|NC_002745.2|	189343 +	A	0	0	1	0	0	SA0165
gi|29165615|ref|NC_002745.2|	189490 +	A	0	0	0	0	1	-
gi|29165615|ref|NC_002745.2|	189495 +	U	0	0	0	1	0	-
gi|29165615|ref|NC_002745.2|	189798 +	G	2	0	0	0	0	-
gi|29165615|ref|NC_002745.2|	191579 +	A	0	1	0	0	0	SA0168
gi|29165615|ref|NC_002745.2|	191718 +	G	0	0	1	0	0	SA0168
gi|29165615|ref|NC_002745.2|	191840 +	C	0	0	0	2	0	SA0168
gi|29165615|ref|NC_002745.2|	192008 +	A	0	0	0	0	1	SA0168
gi|29165615|ref|NC_002745.2|	192082 +	U	2	0	0	0	0	SA0168
gi|29165615|ref|NC_002745.2|	192220 +	A	0	0	0	2	0	SA0168
gi|29165615|ref|NC_002745.2|	192226 +	U	0	0	0	1	0	SA0168
gi|29165615|ref|NC_002745.2|	192229 +	U	0	0	1	0	0	SA0168
gi|29165615|ref|NC_002745.2|	192240 +	A	0	0	0	0	1	SA0168
gi|29165615|ref|NC_002745.2|	192433 +	G	0	0	0	0	1	SA0169
gi|29165615|ref|NC_002745.2|	192856 +	U	0	0	1	0	0	SA0169
gi|29165615|ref|NC_002745.2|	193148 +	A	0	1	0	0	0	SA0169
gi|29165615|ref|NC_002745.2|	193184 +	G	0	0	0	1	0	SA0169
gi|29165615|ref|NC_002745.2|	193239 +	C	0	0	0	1	0	SA0169
gi|29165615|ref|NC_002745.2|	193279 +	A	1	0	0	0	0	SA0169
gi|29165615|ref|NC_002745.2|	193519 +	G	0	4	1	4	2	-
gi|29165615|ref|NC_002745.2|	193522 +	A	0	0	0	1	0	-
gi|29165615|ref|NC_002745.2|	193528 +	U	1	2	0	1	0	-
gi|29165615|ref|NC_002745.2|	193529 +	A	0	2	0	0	0	-
gi|29165615|ref|NC_002745.2|	193562 +	A	0	0	0	1	0	SA0170
gi|29165615|ref|NC_002745.2|	193581 +	A	0	0	1	0	0	SA0170
gi|29165615|ref|NC_002745.2|	193582 +	A	1	2	0	0	0	SA0170
gi|29165615|ref|NC_002745.2|	193585 +	A	0	5	3	1	2	SA0170
gi|29165615|ref|NC_002745.2|	193595 +	A	0	0	1	0	1	SA0170
gi|29165615|ref|NC_002745.2|	193598 +	C	0	1	1	0	0	SA0170
gi|29165615|ref|NC_002745.2|	193599 +	U	0	0	0	1	0	SA0170
gi|29165615|ref|NC_002745.2|	193602 +	U	0	0	0	1	0	SA0170
gi|29165615|ref|NC_002745.2|	193604 +	A	0	0	0	0	1	SA0170
gi|29165615|ref|NC_002745.2|	193662 +	A	0	1	0	2	0	SA0170
gi|29165615|ref|NC_002745.2|	193668 +	A	0	0	0	1	0	SA0170
gi|29165615|ref|NC_002745.2|	193684 +	U	0	0	1	0	0	SA0170
gi|29165615|ref|NC_002745.2|	193695 +	U	0	1	0	0	0	SA0170
gi|29165615|ref|NC_002745.2|	193699 +	G	1	0	0	0	0	SA0170
gi|29165615|ref|NC_002745.2|	193735 +	C	0	1	0	0	0	SA0170
gi|29165615|ref|NC_002745.2|	193736 +	A	0	1	0	0	0	SA0170
gi|29165615|ref|NC_002745.2|	193742 +	U	0	0	0	0	1	SA0170
gi|29165615|ref|NC_002745.2|	193772 +	A	0	0	0	1	0	SA0170
gi|29165615|ref|NC_002745.2|	193777 +	A	0	1	0	0	0	SA0170
gi|29165615|ref|NC_002745.2|	193789 +	A	0	1	0	0	0	SA0170
gi|29165615|ref|NC_002745.2|	193793 +	A	0	1	0	0	0	SA0170
gi|29165615|ref|NC_002745.2|	193797 +	A	0	0	0	1	0	SA0170
gi|29165615|ref|NC_002745.2|	193799 +	G	0	0	0	1	0	SA0170
gi|29165615|ref|NC_002745.2|	193800 +	U	0	0	1	1	0	SA0170
gi|29165615|ref|NC_002745.2|	193802 +	A	0	0	0	1	0	SA0170
gi|29165615|ref|NC_002745.2|	193806 +	A	0	1	0	1	0	SA0170
gi|29165615|ref|NC_002745.2|	193808 +	A	0	0	2	2	0	SA0170
gi|29165615|ref|NC_002745.2|	193812 +	A	0	0	0	1	0	SA0170
gi|29165615|ref|NC_002745.2|	193837 +	A	0	1	0	0	0	SA0170
gi|29165615|ref|NC_002745.2|	193839 +	A	0	1	0	0	0	SA0170
gi|29165615|ref|NC_002745.2|	193840 +	A	0	0	0	1	2	SA0170
gi|29165615|ref|NC_002745.2|	193906 +	A	0	0	0	1	0	SA0170
gi|29165615|ref|NC_002745.2|	193915 +	G	0	0	0	1	0	-
gi|29165615|ref|NC_002745.2|	193919 +	C	0	1	0	0	0	-
gi|29165615|ref|NC_002745.2|	193925 +	U	0	0	0	0	1	-
gi|29165615|ref|NC_002745.2|	193926 +	A	0	1	0	0	0	-
gi|29165615|ref|NC_002745.2|	193971 +	A	1	0	0	0	0	-
gi|29165615|ref|NC_002745.2|	193986 +	U	2	0	0	0	0	-
gi|29165615|ref|NC_002745.2|	194058 +	C	0	0	0	0	2	SA0171
gi|29165615|ref|NC_002745.2|	195453 +	A	0	0	0	0	1	-
gi|29165615|ref|NC_002745.2|	195456 +	A	0	1	0	0	0	-
gi|29165615|ref|NC_002745.2|	195678 +	G	0	1	0	0	0	SA0172
gi|29165615|ref|NC_002745.2|	195682 +	C	0	0	1	0	1	SA0172
gi|29165615|ref|NC_002745.2|	195692 +	A	0	0	0	0	1	SA0172
gi|29165615|ref|NC_002745.2|	195718 +	U	0	1	0	0	0	SA0172
gi|29165615|ref|NC_002745.2|	195719 +	A	0	0	1	0	0	SA0172
gi|29165615|ref|NC_002745.2|	195872 +	G	0	1	0	0	0	SA0172
gi|29165615|ref|NC_002745.2|	196034 +	U	0	1	0	0	0	SA0172
gi|29165615|ref|NC_002745.2|	196115 +	C	0	1	0	0	1	SA0172
gi|29165615|ref|NC_002745.2|	196117 +	A	0	0	1	0	0	SA0172
gi|29165615|ref|NC_002745.2|	196118 +	A	0	2	0	0	0	SA0172
gi|29165615|ref|NC_002745.2|	196124 +	A	0	0	1	0	2	SA0172
gi|29165615|ref|NC_002745.2|	196149 +	U	0	0	0	1	0	SA0172
gi|29165615|ref|NC_002745.2|	196172 +	U	0	0	0	1	0	SA0172
gi|29165615|ref|NC_002745.2|	196174 +	A	0	1	0	0	0	SA0172
gi|29165615|ref|NC_002745.2|	196343 +	C	0	1	0	0	0	SA0172

gi|29165615|ref|NC_002745.2|	196355	+	A	0	0	0	1	0	SA0172
gi|29165615|ref|NC_002745.2|	196361	+	A	0	0	0	0	1	SA0172
gi|29165615|ref|NC_002745.2|	196369	+	A	0	0	1	0	0	SA0172
gi|29165615|ref|NC_002745.2|	196384	+	U	0	0	0	0	1	SA0172
gi|29165615|ref|NC_002745.2|	196390	+	A	0	0	0	0	1	SA0172
gi|29165615|ref|NC_002745.2|	196416	+	A	0	0	0	0	1	SA0172
gi|29165615|ref|NC_002745.2|	196536	+	A	2	0	0	0	0	SA0172
gi|29165615|ref|NC_002745.2|	196538	+	A	0	1	0	0	0	SA0172
gi|29165615|ref|NC_002745.2|	196546	+	U	0	0	3	0	0	SA0172
gi|29165615|ref|NC_002745.2|	196547	+	A	0	0	1	1	0	SA0172
gi|29165615|ref|NC_002745.2|	196548	+	A	0	0	0	0	1	SA0172
gi|29165615|ref|NC_002745.2|	196561	+	A	0	1	0	0	0	SA0172
gi|29165615|ref|NC_002745.2|	196572	+	C	0	0	0	1	0	SA0172
gi|29165615|ref|NC_002745.2|	196575	+	U	0	0	0	0	1	SA0172
gi|29165615|ref|NC_002745.2|	196600	+	A	0	0	1	0	0	SA0172
gi|29165615|ref|NC_002745.2|	196603	+	A	0	1	0	0	0	SA0172
gi|29165615|ref|NC_002745.2|	196623	+	U	0	0	1	0	0	SA0172
gi|29165615|ref|NC_002745.2|	196625	+	G	0	0	1	0	0	SA0172
gi|29165615|ref|NC_002745.2|	196626	+	U	0	0	1	0	0	SA0172
gi|29165615|ref|NC_002745.2|	196638	+	U	3	0	1	0	0	SA0172
gi|29165615|ref|NC_002745.2|	196642	+	A	0	0	1	0	0	SA0172
gi|29165615|ref|NC_002745.2|	196646	+	U	1	1	0	0	1	SA0172
gi|29165615|ref|NC_002745.2|	196649	+	U	0	2	0	0	0	SA0172
gi|29165615|ref|NC_002745.2|	196651	+	A	0	0	0	0	1	SA0172
gi|29165615|ref|NC_002745.2|	196652	+	C	0	1	0	0	0	SA0172
gi|29165615|ref|NC_002745.2|	196654	+	A	0	0	0	1	0	SA0172
gi|29165615|ref|NC_002745.2|	196655	+	A	0	0	0	1	0	SA0172
gi|29165615|ref|NC_002745.2|	196660	+	A	0	0	1	0	0	SA0172
gi|29165615|ref|NC_002745.2|	196664	+	U	1	0	0	0	1	SA0172
gi|29165615|ref|NC_002745.2|	196677	+	U	2	0	0	0	0	SA0172
gi|29165615|ref|NC_002745.2|	196695	+	G	0	0	1	0	0	SA0172
gi|29165615|ref|NC_002745.2|	196702	+	U	0	1	0	0	0	SA0172
gi|29165615|ref|NC_002745.2|	196788	+	A	0	1	0	0	0	-
gi|29165615|ref|NC_002745.2|	196845	+	A	0	0	0	0	1	-
gi|29165615|ref|NC_002745.2|	196857	+	A	0	0	0	1	0	-
gi|29165615|ref|NC_002745.2|	196896	+	G	5	1	2	0	1	-
gi|29165615|ref|NC_002745.2|	196947	+	C	1	0	0	0	0	-
gi|29165615|ref|NC_002745.2|	197014	+	A	0	1	0	0	0	-
gi|29165615|ref|NC_002745.2|	197067	+	U	0	0	0	0	1	-
gi|29165615|ref|NC_002745.2|	197084	+	U	0	0	1	0	0	-
gi|29165615|ref|NC_002745.2|	197104	+	A	0	1	0	0	0	-
gi|29165615|ref|NC_002745.2|	197152	+	U	1	0	0	0	0	-
gi|29165615|ref|NC_002745.2|	197174	+	A	0	0	0	1	0	-
gi|29165615|ref|NC_002745.2|	197179	+	A	0	1	0	0	0	-
gi|29165615|ref|NC_002745.2|	197187	+	A	0	0	0	0	1	-
gi|29165615|ref|NC_002745.2|	197251	+	A	0	0	1	0	0	SA0173
gi|29165615|ref|NC_002745.2|	197468	+	A	0	0	1	0	0	SA0173
gi|29165615|ref|NC_002745.2|	197535	+	U	0	0	0	1	0	SA0173
gi|29165615|ref|NC_002745.2|	197536	+	A	2	0	0	0	0	SA0173
gi|29165615|ref|NC_002745.2|	197537	+	A	0	0	0	0	2	SA0173
gi|29165615|ref|NC_002745.2|	197811	+	U	2	0	0	0	0	SA0173
gi|29165615|ref|NC_002745.2|	197837	+	U	1	0	0	0	0	SA0173
gi|29165615|ref|NC_002745.2|	197843	+	A	1	0	0	0	0	SA0173
gi|29165615|ref|NC_002745.2|	197858	+	U	0	0	1	0	0	SA0173
gi|29165615|ref|NC_002745.2|	198001	+	G	1	0	0	0	0	SA0173
gi|29165615|ref|NC_002745.2|	198211	+	C	0	0	0	0	1	SA0173
gi|29165615|ref|NC_002745.2|	198216	+	U	1	0	0	0	0	SA0173
gi|29165615|ref|NC_002745.2|	198246	+	U	1	0	0	0	0	SA0173
gi|29165615|ref|NC_002745.2|	198256	+	G	0	0	1	0	0	SA0173
gi|29165615|ref|NC_002745.2|	198263	+	U	2	0	0	0	0	SA0173
gi|29165615|ref|NC_002745.2|	198272	+	A	2	0	1	0	0	SA0173
gi|29165615|ref|NC_002745.2|	198316	+	G	0	1	0	0	0	SA0173
gi|29165615|ref|NC_002745.2|	198325	+	G	0	0	0	1	0	SA0173
gi|29165615|ref|NC_002745.2|	198339	+	A	0	0	1	0	0	SA0173
gi|29165615|ref|NC_002745.2|	198363	+	G	2	0	0	0	0	SA0173
gi|29165615|ref|NC_002745.2|	198384	+	A	0	0	0	0	1	SA0173
gi|29165615|ref|NC_002745.2|	198488	+	A	0	1	0	0	0	SA0173
gi|29165615|ref|NC_002745.2|	198534	+	G	0	0	0	0	1	SA0173
gi|29165615|ref|NC_002745.2|	198796	+	A	0	0	0	1	0	SA0173
gi|29165615|ref|NC_002745.2|	198820	+	A	0	1	2	0	1	SA0173
gi|29165615|ref|NC_002745.2|	198841	+	U	0	0	0	0	1	SA0173
gi|29165615|ref|NC_002745.2|	198847	+	G	0	0	1	0	0	SA0173
gi|29165615|ref|NC_002745.2|	198848	+	A	1	0	0	0	0	SA0173
gi|29165615|ref|NC_002745.2|	198862	+	A	0	1	0	0	0	SA0173
gi|29165615|ref|NC_002745.2|	198864	+	U	0	0	0	1	0	SA0173
gi|29165615|ref|NC_002745.2|	198872	+	C	0	1	0	0	0	SA0173
gi|29165615|ref|NC_002745.2|	199031	+	U	1	0	0	0	0	SA0173
gi|29165615|ref|NC_002745.2|	199104	+	C	1	0	0	0	0	SA0173
gi|29165615|ref|NC_002745.2|	199120	+	A	0	2	0	0	0	SA0173
gi|29165615|ref|NC_002745.2|	199169	+	G	0	0	0	0	1	SA0173
gi|29165615|ref|NC_002745.2|	199196	+	A	0	1	0	0	0	SA0173

gi|29165615|ref|NC_002745.2|	199261	+	C	1	0	0	0	0	SA0173
gi|29165615|ref|NC_002745.2|	199287	+	A	1	0	0	0	0	SA0173
gi|29165615|ref|NC_002745.2|	199330	+	A	0	2	1	2	3	SA0173
gi|29165615|ref|NC_002745.2|	199331	+	A	0	0	1	0	0	SA0173
gi|29165615|ref|NC_002745.2|	199340	+	C	1	0	0	0	0	SA0173
gi|29165615|ref|NC_002745.2|	199365	+	G	1	0	0	0	0	SA0173
gi|29165615|ref|NC_002745.2|	199367	+	U	2	0	0	0	0	SA0173
gi|29165615|ref|NC_002745.2|	199393	+	C	1	0	0	0	0	SA0173
gi|29165615|ref|NC_002745.2|	199409	+	G	1	0	0	0	0	SA0173
gi|29165615|ref|NC_002745.2|	199428	+	A	0	0	1	0	0	SA0173
gi|29165615|ref|NC_002745.2|	199441	+	A	0	0	0	1	0	SA0173
gi|29165615|ref|NC_002745.2|	199513	+	A	0	0	0	1	0	SA0173
gi|29165615|ref|NC_002745.2|	199517	+	U	0	0	1	0	0	SA0173
gi|29165615|ref|NC_002745.2|	199518	+	A	0	1	0	0	0	SA0173
gi|29165615|ref|NC_002745.2|	199523	+	A	1	0	0	0	0	SA0173
gi|29165615|ref|NC_002745.2|	199532	+	A	0	0	1	0	0	SA0173
gi|29165615|ref|NC_002745.2|	199542	+	U	0	0	0	0	1	SA0173
gi|29165615|ref|NC_002745.2|	199604	+	U	0	0	1	0	0	SA0173
gi|29165615|ref|NC_002745.2|	199624	+	A	0	0	1	0	0	SA0173
gi|29165615|ref|NC_002745.2|	199649	+	C	1	0	0	0	0	SA0173
gi|29165615|ref|NC_002745.2|	199784	+	C	1	0	0	0	0	SA0173
gi|29165615|ref|NC_002745.2|	199942	+	C	0	0	0	0	1	SA0173
gi|29165615|ref|NC_002745.2|	199955	+	A	0	0	1	0	0	SA0173
gi|29165615|ref|NC_002745.2|	199963	+	C	1	0	0	0	0	SA0173
gi|29165615|ref|NC_002745.2|	200075	+	U	0	0	0	1	0	SA0173
gi|29165615|ref|NC_002745.2|	200182	+	U	0	1	0	0	0	SA0173
gi|29165615|ref|NC_002745.2|	200195	+	C	0	0	1	0	0	SA0173
gi|29165615|ref|NC_002745.2|	200284	+	C	2	0	0	0	0	SA0173
gi|29165615|ref|NC_002745.2|	200364	+	A	0	0	1	0	0	SA0173
gi|29165615|ref|NC_002745.2|	200444	+	A	0	1	0	0	0	SA0173
gi|29165615|ref|NC_002745.2|	200500	+	A	0	0	0	0	1	SA0173
gi|29165615|ref|NC_002745.2|	200521	+	C	0	1	0	0	0	SA0173
gi|29165615|ref|NC_002745.2|	200525	+	U	1	0	0	0	0	SA0173
gi|29165615|ref|NC_002745.2|	200533	+	U	1	0	0	0	0	SA0173
gi|29165615|ref|NC_002745.2|	200624	+	U	5	0	0	0	0	SA0173
gi|29165615|ref|NC_002745.2|	200711	+	C	0	0	0	0	1	SA0173
gi|29165615|ref|NC_002745.2|	200783	+	A	1	0	0	0	0	SA0173
gi|29165615|ref|NC_002745.2|	200867	+	U	0	0	0	1	0	SA0173
gi|29165615|ref|NC_002745.2|	200872	+	U	1	0	0	0	0	SA0173
gi|29165615|ref|NC_002745.2|	200873	+	U	1	0	0	0	0	SA0173
gi|29165615|ref|NC_002745.2|	200879	+	U	1	0	0	0	0	SA0173
gi|29165615|ref|NC_002745.2|	200881	+	A	0	0	0	0	1	SA0173
gi|29165615|ref|NC_002745.2|	200882	+	G	4	0	0	0	0	SA0173
gi|29165615|ref|NC_002745.2|	200886	+	G	1	0	0	0	0	SA0173
gi|29165615|ref|NC_002745.2|	200896	+	U	0	0	1	0	0	SA0173
gi|29165615|ref|NC_002745.2|	200903	+	G	1	0	0	0	0	SA0173
gi|29165615|ref|NC_002745.2|	200920	+	G	0	0	0	1	0	SA0173
gi|29165615|ref|NC_002745.2|	200924	+	A	1	0	0	0	0	SA0173
gi|29165615|ref|NC_002745.2|	200929	+	G	1	0	0	0	0	SA0173
gi|29165615|ref|NC_002745.2|	200947	+	A	0	0	1	0	0	SA0173
gi|29165615|ref|NC_002745.2|	200959	+	C	0	0	0	0	1	SA0173
gi|29165615|ref|NC_002745.2|	201005	+	U	1	0	0	0	0	SA0173
gi|29165615|ref|NC_002745.2|	201009	+	U	2	0	0	0	0	SA0173
gi|29165615|ref|NC_002745.2|	201313	+	G	0	0	1	0	0	SA0173
gi|29165615|ref|NC_002745.2|	201328	+	U	0	1	0	0	0	SA0173
gi|29165615|ref|NC_002745.2|	201330	+	A	0	0	0	1	0	SA0173
gi|29165615|ref|NC_002745.2|	201382	+	G	0	2	1	0	0	SA0173
gi|29165615|ref|NC_002745.2|	201521	+	U	1	0	0	0	0	SA0173
gi|29165615|ref|NC_002745.2|	201529	+	G	1	0	0	0	0	SA0173
gi|29165615|ref|NC_002745.2|	201549	+	U	0	0	0	1	0	SA0173
gi|29165615|ref|NC_002745.2|	201550	+	C	0	2	0	0	0	SA0173
gi|29165615|ref|NC_002745.2|	201551	+	A	1	0	0	0	0	SA0173
gi|29165615|ref|NC_002745.2|	201552	+	U	0	0	0	0	1	SA0173
gi|29165615|ref|NC_002745.2|	201608	+	U	2	0	0	0	0	SA0173
gi|29165615|ref|NC_002745.2|	201610	+	G	1	0	0	0	0	SA0173
gi|29165615|ref|NC_002745.2|	201611	+	A	4	0	0	0	0	SA0173
gi|29165615|ref|NC_002745.2|	201612	+	U	2	0	0	0	0	SA0173
gi|29165615|ref|NC_002745.2|	201619	+	G	1	0	0	0	0	SA0173
gi|29165615|ref|NC_002745.2|	201632	+	U	0	0	1	0	0	SA0173
gi|29165615|ref|NC_002745.2|	201693	+	G	1	0	0	0	0	SA0173
gi|29165615|ref|NC_002745.2|	201773	+	G	0	0	0	1	0	SA0173
gi|29165615|ref|NC_002745.2|	201776	+	C	0	1	0	0	0	SA0173
gi|29165615|ref|NC_002745.2|	201821	+	U	1	0	0	0	0	SA0173
gi|29165615|ref|NC_002745.2|	201825	+	A	0	1	0	0	0	SA0173
gi|29165615|ref|NC_002745.2|	201890	+	A	0	0	0	0	1	SA0173
gi|29165615|ref|NC_002745.2|	201893	+	A	0	0	1	0	0	SA0173
gi|29165615|ref|NC_002745.2|	201901	+	G	0	0	0	1	0	SA0173
gi|29165615|ref|NC_002745.2|	201910	+	C	0	0	0	0	1	SA0173
gi|29165615|ref|NC_002745.2|	201981	+	C	1	0	0	0	0	SA0173
gi|29165615|ref|NC_002745.2|	202010	+	A	2	0	0	0	0	SA0173
gi|29165615|ref|NC_002745.2|	202022	+	U	2	0	0	0	0	SA0173

gi|29165615|ref|NC_002745.2|	202072	+	G	0	1	0	0	0	SA0173
gi|29165615|ref|NC_002745.2|	202146	+	U	1	0	0	0	0	SA0173
gi|29165615|ref|NC_002745.2|	202160	+	C	0	1	0	0	0	SA0173
gi|29165615|ref|NC_002745.2|	202224	+	G	1	0	0	0	0	SA0173
gi|29165615|ref|NC_002745.2|	202282	+	U	0	0	0	0	1	SA0173
gi|29165615|ref|NC_002745.2|	202355	+	A	1	0	0	0	0	SA0173
gi|29165615|ref|NC_002745.2|	202426	+	G	0	0	1	0	0	SA0173
gi|29165615|ref|NC_002745.2|	202444	+	A	0	0	0	0	1	SA0173
gi|29165615|ref|NC_002745.2|	202450	+	U	1	1	0	0	0	SA0173
gi|29165615|ref|NC_002745.2|	202460	+	U	1	0	0	0	0	SA0173
gi|29165615|ref|NC_002745.2|	202469	+	A	1	0	0	0	0	SA0173
gi|29165615|ref|NC_002745.2|	202475	+	U	2	1	0	0	0	SA0173
gi|29165615|ref|NC_002745.2|	202476	+	G	0	0	0	0	2	SA0173
gi|29165615|ref|NC_002745.2|	202478	+	C	3	0	0	0	2	SA0173
gi|29165615|ref|NC_002745.2|	202491	+	A	0	0	0	0	1	SA0173
gi|29165615|ref|NC_002745.2|	202503	+	U	1	0	1	0	0	SA0173
gi|29165615|ref|NC_002745.2|	202507	+	C	0	1	0	0	0	SA0173
gi|29165615|ref|NC_002745.2|	202517	+	U	1	0	0	0	0	SA0173
gi|29165615|ref|NC_002745.2|	202526	+	C	1	0	0	0	0	SA0173
gi|29165615|ref|NC_002745.2|	202529	+	G	0	0	0	1	0	SA0173
gi|29165615|ref|NC_002745.2|	202532	+	A	0	0	1	0	0	SA0173
gi|29165615|ref|NC_002745.2|	202584	+	G	0	0	0	0	1	SA0173
gi|29165615|ref|NC_002745.2|	202592	+	A	0	0	0	0	1	SA0173
gi|29165615|ref|NC_002745.2|	202605	+	A	0	0	0	1	0	SA0173
gi|29165615|ref|NC_002745.2|	202613	+	U	1	0	0	0	0	SA0173
gi|29165615|ref|NC_002745.2|	202619	+	A	0	1	0	0	0	SA0173
gi|29165615|ref|NC_002745.2|	202627	+	G	0	1	1	0	1	SA0173
gi|29165615|ref|NC_002745.2|	202643	+	C	1	0	0	0	0	SA0173
gi|29165615|ref|NC_002745.2|	202646	+	C	1	0	0	0	1	SA0173
gi|29165615|ref|NC_002745.2|	202649	+	A	0	0	0	0	1	SA0173
gi|29165615|ref|NC_002745.2|	202661	+	A	0	0	1	1	0	SA0173
gi|29165615|ref|NC_002745.2|	202665	+	U	0	0	1	0	0	SA0173
gi|29165615|ref|NC_002745.2|	202668	+	A	0	1	0	2	0	SA0173
gi|29165615|ref|NC_002745.2|	202674	+	A	0	0	0	0	1	SA0173
gi|29165615|ref|NC_002745.2|	202675	+	A	0	0	0	0	1	SA0173
gi|29165615|ref|NC_002745.2|	202687	+	G	0	0	1	0	0	SA0173
gi|29165615|ref|NC_002745.2|	202690	+	A	0	0	1	1	0	SA0173
gi|29165615|ref|NC_002745.2|	202692	+	A	0	0	0	0	1	SA0173
gi|29165615|ref|NC_002745.2|	202693	+	G	1	0	0	0	0	SA0173
gi|29165615|ref|NC_002745.2|	202694	+	U	0	0	0	1	0	SA0173
gi|29165615|ref|NC_002745.2|	202695	+	U	0	0	1	0	0	SA0173
gi|29165615|ref|NC_002745.2|	202708	+	A	0	1	0	0	0	SA0173
gi|29165615|ref|NC_002745.2|	202713	+	A	0	0	1	0	0	SA0173
gi|29165615|ref|NC_002745.2|	202715	+	U	1	0	0	0	0	SA0173
gi|29165615|ref|NC_002745.2|	202718	+	A	0	0	0	1	0	SA0173
gi|29165615|ref|NC_002745.2|	202720	+	G	0	0	0	0	2	SA0173
gi|29165615|ref|NC_002745.2|	202721	+	A	0	0	0	0	1	SA0173
gi|29165615|ref|NC_002745.2|	202724	+	U	0	0	0	0	1	SA0173
gi|29165615|ref|NC_002745.2|	202726	+	G	0	0	0	1	0	SA0173
gi|29165615|ref|NC_002745.2|	202727	+	A	0	0	0	1	0	SA0173
gi|29165615|ref|NC_002745.2|	202729	+	A	0	0	1	1	0	SA0173
gi|29165615|ref|NC_002745.2|	202731	+	U	1	0	0	0	0	SA0173
gi|29165615|ref|NC_002745.2|	202732	+	G	0	0	0	1	1	SA0173
gi|29165615|ref|NC_002745.2|	202734	+	A	0	0	1	1	1	SA0173
gi|29165615|ref|NC_002745.2|	202735	+	A	0	1	0	0	0	SA0173
gi|29165615|ref|NC_002745.2|	202736	+	U	1	0	0	0	0	SA0173
gi|29165615|ref|NC_002745.2|	202738	+	U	1	0	0	0	0	SA0173
gi|29165615|ref|NC_002745.2|	202741	+	G	0	0	1	0	0	SA0173
gi|29165615|ref|NC_002745.2|	202742	+	C	3	0	0	0	0	SA0173
gi|29165615|ref|NC_002745.2|	202744	+	A	0	1	0	0	0	SA0173
gi|29165615|ref|NC_002745.2|	202745	+	U	1	0	0	0	0	SA0173
gi|29165615|ref|NC_002745.2|	202748	+	G	1	0	0	0	0	SA0173
gi|29165615|ref|NC_002745.2|	202750	+	G	0	0	0	0	1	SA0173
gi|29165615|ref|NC_002745.2|	202760	+	A	0	0	0	1	0	SA0173
gi|29165615|ref|NC_002745.2|	202769	+	U	1	0	0	0	0	SA0173
gi|29165615|ref|NC_002745.2|	202782	+	C	1	0	0	0	0	SA0173
gi|29165615|ref|NC_002745.2|	202784	+	U	0	0	1	0	0	SA0173
gi|29165615|ref|NC_002745.2|	202805	+	A	1	0	0	0	0	SA0173
gi|29165615|ref|NC_002745.2|	202856	+	U	1	0	0	0	0	SA0173
gi|29165615|ref|NC_002745.2|	202861	+	G	0	0	1	0	0	SA0173
gi|29165615|ref|NC_002745.2|	202892	+	C	1	0	0	0	0	SA0173
gi|29165615|ref|NC_002745.2|	202941	+	U	1	0	0	0	0	SA0173
gi|29165615|ref|NC_002745.2|	202959	+	U	0	0	0	0	1	SA0173
gi|29165615|ref|NC_002745.2|	202982	+	A	0	1	0	0	0	SA0173
gi|29165615|ref|NC_002745.2|	203051	+	A	0	0	0	0	1	SA0173
gi|29165615|ref|NC_002745.2|	203223	+	A	0	1	0	0	0	SA0173
gi|29165615|ref|NC_002745.2|	203232	+	U	1	0	0	0	0	SA0173
gi|29165615|ref|NC_002745.2|	203240	+	C	0	0	0	0	1	SA0173
gi|29165615|ref|NC_002745.2|	203242	+	G	1	0	0	0	0	SA0173
gi|29165615|ref|NC_002745.2|	203303	+	A	0	0	0	1	0	SA0173
gi|29165615|ref|NC_002745.2|	203307	+	U	2	0	0	0	0	SA0173

gi|29165615|ref|NC_002745.2|	203328 +	A	0	0	0	0	1	SA0173
gi|29165615|ref|NC_002745.2|	203331 +	U	0	0	0	1	0	SA0173
gi|29165615|ref|NC_002745.2|	203332 +	A	1	0	0	0	0	SA0173
gi|29165615|ref|NC_002745.2|	203379 +	A	1	0	0	0	0	SA0173
gi|29165615|ref|NC_002745.2|	203398 +	C	0	1	0	0	0	SA0173
gi|29165615|ref|NC_002745.2|	203403 +	C	1	1	0	0	0	SA0173
gi|29165615|ref|NC_002745.2|	203409 +	U	1	0	0	0	0	SA0173
gi|29165615|ref|NC_002745.2|	203411 +	G	1	0	0	0	0	SA0173
gi|29165615|ref|NC_002745.2|	203412 +	U	4	0	0	0	0	SA0173
gi|29165615|ref|NC_002745.2|	203414 +	U	3	0	0	0	0	SA0173
gi|29165615|ref|NC_002745.2|	203427 +	U	1	0	0	1	0	SA0173
gi|29165615|ref|NC_002745.2|	203436 +	A	0	0	0	0	1	SA0173
gi|29165615|ref|NC_002745.2|	203466 +	A	0	0	0	1	0	SA0173
gi|29165615|ref|NC_002745.2|	203534 +	U	0	0	1	0	0	SA0173
gi|29165615|ref|NC_002745.2|	203810 +	G	0	0	0	0	2	SA0173
gi|29165615|ref|NC_002745.2|	203816 +	U	1	0	0	0	0	SA0173
gi|29165615|ref|NC_002745.2|	203825 +	A	1	0	0	0	0	SA0173
gi|29165615|ref|NC_002745.2|	203829 +	A	0	0	0	0	2	SA0173
gi|29165615|ref|NC_002745.2|	203835 +	A	1	0	0	0	0	SA0173
gi|29165615|ref|NC_002745.2|	203840 +	A	0	1	0	0	0	SA0173
gi|29165615|ref|NC_002745.2|	203853 +	U	0	0	0	0	2	SA0173
gi|29165615|ref|NC_002745.2|	204018 +	U	1	0	1	0	0	SA0173
gi|29165615|ref|NC_002745.2|	204084 +	C	1	0	0	0	0	SA0173
gi|29165615|ref|NC_002745.2|	204185 +	U	1	0	0	0	0	SA0173
gi|29165615|ref|NC_002745.2|	204290 +	U	0	0	0	1	0	SA0173
gi|29165615|ref|NC_002745.2|	204342 +	A	0	0	0	0	1	SA0173
gi|29165615|ref|NC_002745.2|	204656 +	C	0	1	0	0	0	SA0174
gi|29165615|ref|NC_002745.2|	204665 +	A	1	0	0	1	0	SA0174
gi|29165615|ref|NC_002745.2|	204667 +	C	1	0	0	0	0	SA0174
gi|29165615|ref|NC_002745.2|	204808 +	C	0	1	0	0	0	SA0174
gi|29165615|ref|NC_002745.2|	204861 +	C	0	0	0	1	0	SA0174
gi|29165615|ref|NC_002745.2|	204920 +	A	0	0	0	2	0	SA0174
gi|29165615|ref|NC_002745.2|	204930 +	A	0	1	0	0	0	SA0174
gi|29165615|ref|NC_002745.2|	204931 +	U	0	0	1	0	0	SA0174
gi|29165615|ref|NC_002745.2|	204934 +	A	0	0	0	0	1	SA0174
gi|29165615|ref|NC_002745.2|	204937 +	U	0	1	0	0	0	SA0174
gi|29165615|ref|NC_002745.2|	204940 +	G	0	1	0	0	0	SA0174
gi|29165615|ref|NC_002745.2|	204946 +	U	0	3	0	2	1	SA0174
gi|29165615|ref|NC_002745.2|	204947 +	U	0	1	0	0	0	SA0174
gi|29165615|ref|NC_002745.2|	204948 +	U	0	2	1	2	5	SA0174
gi|29165615|ref|NC_002745.2|	204949 +	A	0	0	0	4	1	SA0174
gi|29165615|ref|NC_002745.2|	204958 +	G	0	0	0	2	0	SA0174
gi|29165615|ref|NC_002745.2|	204959 +	U	0	0	0	1	0	SA0174
gi|29165615|ref|NC_002745.2|	204977 +	C	0	0	0	1	0	SA0174
gi|29165615|ref|NC_002745.2|	206932 +	C	1	0	0	0	0	-
gi|29165615|ref|NC_002745.2|	208252 +	C	0	0	0	0	1	-
gi|29165615|ref|NC_002745.2|	209299 +	A	0	0	1	0	0	-
gi|29165615|ref|NC_002745.2|	209894 +	G	0	0	0	1	0	-
gi|29165615|ref|NC_002745.2|	210857 +	A	0	1	0	0	0	-
gi|29165615|ref|NC_002745.2|	211016 +	C	0	0	1	0	0	-
gi|29165615|ref|NC_002745.2|	214909 +	U	0	1	0	0	0	-
gi|29165615|ref|NC_002745.2|	217070 +	U	0	0	1	0	0	-
gi|29165615|ref|NC_002745.2|	217389 +	G	0	0	2	3	3	-
gi|29165615|ref|NC_002745.2|	217593 +	C	0	0	0	1	0	SA0184
gi|29165615|ref|NC_002745.2|	217603 +	A	0	1	0	1	0	SA0184
gi|29165615|ref|NC_002745.2|	217604 +	A	0	1	0	0	2	SA0184
gi|29165615|ref|NC_002745.2|	217605 +	A	0	2	0	0	0	SA0184
gi|29165615|ref|NC_002745.2|	217608 +	U	0	0	0	1	4	SA0184
gi|29165615|ref|NC_002745.2|	217609 +	G	0	0	0	1	0	SA0184
gi|29165615|ref|NC_002745.2|	217610 +	A	0	1	1	1	1	SA0184
gi|29165615|ref|NC_002745.2|	217611 +	A	0	1	0	0	0	SA0184
gi|29165615|ref|NC_002745.2|	217612 +	C	0	0	1	0	0	SA0184
gi|29165615|ref|NC_002745.2|	217613 +	A	0	3	2	2	4	SA0184
gi|29165615|ref|NC_002745.2|	217616 +	U	0	0	0	2	1	SA0184
gi|29165615|ref|NC_002745.2|	217617 +	G	0	0	1	1	0	SA0184
gi|29165615|ref|NC_002745.2|	217618 +	A	0	3	2	1	8	SA0184
gi|29165615|ref|NC_002745.2|	217619 +	C	0	0	0	0	2	SA0184
gi|29165615|ref|NC_002745.2|	217631 +	A	0	0	0	0	1	SA0184
gi|29165615|ref|NC_002745.2|	217648 +	U	0	0	0	0	1	SA0184
gi|29165615|ref|NC_002745.2|	217663 +	U	0	0	0	0	1	SA0184
gi|29165615|ref|NC_002745.2|	217687 +	G	0	1	0	0	0	SA0184
gi|29165615|ref|NC_002745.2|	217690 +	C	0	0	1	0	0	SA0184
gi|29165615|ref|NC_002745.2|	218473 +	C	0	0	1	0	0	SA0184
gi|29165615|ref|NC_002745.2|	218476 +	A	0	0	0	0	1	SA0184
gi|29165615|ref|NC_002745.2|	218484 +	G	0	0	0	1	0	SA0184
gi|29165615|ref|NC_002745.2|	218640 +	A	0	0	0	0	1	SA0185
gi|29165615|ref|NC_002745.2|	218648 +	A	0	1	0	0	0	SA0185
gi|29165615|ref|NC_002745.2|	218654 +	A	0	1	0	0	0	SA0185
gi|29165615|ref|NC_002745.2|	218693 +	U	0	0	0	0	1	SA0185
gi|29165615|ref|NC_002745.2|	218707 +	C	0	0	0	1	0	SA0185
gi|29165615|ref|NC_002745.2|	218730 +	G	0	1	0	0	0	SA0185

gi|29165615|ref|NC_002745.2|	218874 +	G	0	0	0	1	0	SA0185
gi|29165615|ref|NC_002745.2|	219172 +	U	0	0	0	1	0	SA0185
gi|29165615|ref|NC_002745.2|	219189 +	A	0	0	0	1	0	SA0185
gi|29165615|ref|NC_002745.2|	219205 +	C	0	0	0	1	0	SA0185
gi|29165615|ref|NC_002745.2|	219245 +	U	0	1	0	0	0	SA0185
gi|29165615|ref|NC_002745.2|	219381 +	G	0	0	0	1	0	SA0185
gi|29165615|ref|NC_002745.2|	219401 +	A	0	1	0	0	3	-
gi|29165615|ref|NC_002745.2|	219404 +	U	0	0	0	3	2	-
gi|29165615|ref|NC_002745.2|	219405 +	A	0	0	1	0	2	-
gi|29165615|ref|NC_002745.2|	219412 +	C	0	1	0	0	1	SA0186
gi|29165615|ref|NC_002745.2|	219414 +	A	0	0	0	0	2	SA0186
gi|29165615|ref|NC_002745.2|	219415 +	A	0	0	1	1	0	SA0186
gi|29165615|ref|NC_002745.2|	219417 +	A	0	0	0	1	1	SA0186
gi|29165615|ref|NC_002745.2|	219418 +	A	0	2	0	0	1	SA0186
gi|29165615|ref|NC_002745.2|	219428 +	G	0	0	0	0	1	SA0186
gi|29165615|ref|NC_002745.2|	219434 +	C	0	0	0	0	1	SA0186
gi|29165615|ref|NC_002745.2|	219436 +	A	1	0	0	0	0	SA0186
gi|29165615|ref|NC_002745.2|	219447 +	C	0	0	1	0	0	SA0186
gi|29165615|ref|NC_002745.2|	219465 +	A	0	0	0	1	0	SA0186
gi|29165615|ref|NC_002745.2|	219472 +	U	1	0	0	0	0	SA0186
gi|29165615|ref|NC_002745.2|	219523 +	U	0	0	0	0	1	SA0186
gi|29165615|ref|NC_002745.2|	219528 +	U	0	1	0	0	0	SA0186
gi|29165615|ref|NC_002745.2|	219540 +	A	0	1	0	0	0	SA0186
gi|29165615|ref|NC_002745.2|	219786 +	A	0	0	0	1	0	SA0186
gi|29165615|ref|NC_002745.2|	219800 +	A	0	1	0	0	0	SA0186
gi|29165615|ref|NC_002745.2|	219804 +	C	0	1	0	0	0	SA0186
gi|29165615|ref|NC_002745.2|	219834 +	U	0	1	0	0	0	SA0186
gi|29165615|ref|NC_002745.2|	219835 +	U	0	0	0	1	0	SA0186
gi|29165615|ref|NC_002745.2|	219843 +	C	0	0	1	0	0	SA0186
gi|29165615|ref|NC_002745.2|	219929 +	A	0	0	0	0	1	SA0186
gi|29165615|ref|NC_002745.2|	219942 +	U	0	0	0	0	1	SA0186
gi|29165615|ref|NC_002745.2|	220012 +	A	0	0	0	0	1	SA0186
gi|29165615|ref|NC_002745.2|	220054 +	A	0	1	0	0	0	SA0186
gi|29165615|ref|NC_002745.2|	220081 +	A	0	0	0	0	2	SA0186
gi|29165615|ref|NC_002745.2|	220105 +	A	0	0	0	0	1	SA0186
gi|29165615|ref|NC_002745.2|	220118 +	C	0	1	0	0	0	SA0186
gi|29165615|ref|NC_002745.2|	220153 +	G	0	0	0	0	1	SA0186
gi|29165615|ref|NC_002745.2|	220159 +	A	0	0	0	0	1	SA0186
gi|29165615|ref|NC_002745.2|	220170 +	A	0	0	0	1	0	SA0186
gi|29165615|ref|NC_002745.2|	220246 +	A	0	0	0	1	0	SA0186
gi|29165615|ref|NC_002745.2|	220262 +	A	0	0	1	0	0	SA0186
gi|29165615|ref|NC_002745.2|	220313 +	A	0	0	1	0	1	SA0186
gi|29165615|ref|NC_002745.2|	220581 +	U	0	0	0	1	0	SA0186
gi|29165615|ref|NC_002745.2|	220688 +	A	0	0	0	1	0	SA0186
gi|29165615|ref|NC_002745.2|	220736 +	A	0	0	0	0	1	SA0186
gi|29165615|ref|NC_002745.2|	220767 +	C	0	1	0	0	0	SA0186
gi|29165615|ref|NC_002745.2|	220788 +	C	0	0	0	2	0	SA0186
gi|29165615|ref|NC_002745.2|	220841 +	A	0	0	0	0	1	SA0186
gi|29165615|ref|NC_002745.2|	220885 +	A	0	0	1	0	0	SA0187
gi|29165615|ref|NC_002745.2|	220960 +	A	0	0	0	0	2	SA0187
gi|29165615|ref|NC_002745.2|	220973 +	C	0	1	0	0	0	SA0187
gi|29165615|ref|NC_002745.2|	221164 +	A	0	0	1	0	0	SA0187
gi|29165615|ref|NC_002745.2|	221224 +	G	0	1	0	0	0	SA0187
gi|29165615|ref|NC_002745.2|	221261 +	U	0	0	1	0	0	SA0187
gi|29165615|ref|NC_002745.2|	221359 +	C	0	0	0	0	1	SA0187
gi|29165615|ref|NC_002745.2|	221415 +	G	0	0	0	1	0	SA0187
gi|29165615|ref|NC_002745.2|	221475 +	U	0	0	1	1	3	SA0187
gi|29165615|ref|NC_002745.2|	221476 +	A	0	0	1	1	0	SA0187
gi|29165615|ref|NC_002745.2|	221509 +	A	0	1	0	0	0	SA0187
gi|29165615|ref|NC_002745.2|	221610 +	C	0	0	0	1	0	SA0187
gi|29165615|ref|NC_002745.2|	221617 +	G	0	0	0	0	1	SA0187
gi|29165615|ref|NC_002745.2|	221638 +	G	0	0	0	0	1	SA0187
gi|29165615|ref|NC_002745.2|	221692 +	C	0	0	0	1	0	SA0187
gi|29165615|ref|NC_002745.2|	222368 +	U	0	0	0	0	1	-
gi|29165615|ref|NC_002745.2|	222399 +	A	4	7	6	8	10	-
gi|29165615|ref|NC_002745.2|	222400 +	A	0	0	0	0	1	-
gi|29165615|ref|NC_002745.2|	222455 +	A	0	0	1	0	0	SA0189
gi|29165615|ref|NC_002745.2|	222483 +	A	0	1	0	0	0	SA0189
gi|29165615|ref|NC_002745.2|	222552 +	U	0	1	0	0	0	SA0189
gi|29165615|ref|NC_002745.2|	222612 +	G	0	0	0	0	1	SA0189
gi|29165615|ref|NC_002745.2|	222659 +	U	0	0	2	0	0	SA0189
gi|29165615|ref|NC_002745.2|	222778 +	A	0	2	0	0	0	SA0189
gi|29165615|ref|NC_002745.2|	223038 +	A	0	0	1	0	0	SA0189
gi|29165615|ref|NC_002745.2|	223050 +	A	0	0	0	0	1	SA0189
gi|29165615|ref|NC_002745.2|	223081 +	U	0	1	0	0	0	SA0189
gi|29165615|ref|NC_002745.2|	223126 +	A	0	0	0	0	1	SA0189
gi|29165615|ref|NC_002745.2|	223165 +	U	0	0	0	0	1	SA0189
gi|29165615|ref|NC_002745.2|	223200 +	G	0	1	0	0	0	SA0189
gi|29165615|ref|NC_002745.2|	223209 +	U	1	0	0	0	0	SA0189
gi|29165615|ref|NC_002745.2|	223211 +	A	0	1	0	0	0	SA0189
gi|29165615|ref|NC_002745.2|	223215 +	A	0	1	0	0	0	SA0189

gi|29165615|ref|NC_002745.2|	223229	+	C	0	1	0	0	0	SA0189
gi|29165615|ref|NC_002745.2|	223351	+	G	0	2	2	0	0	SA0189
gi|29165615|ref|NC_002745.2|	223352	+	A	0	1	0	0	0	SA0189
gi|29165615|ref|NC_002745.2|	223357	+	A	0	1	0	0	1	SA0189
gi|29165615|ref|NC_002745.2|	223360	+	A	0	1	0	0	1	SA0189
gi|29165615|ref|NC_002745.2|	223361	+	A	1	0	0	0	0	SA0189
gi|29165615|ref|NC_002745.2|	223362	+	A	0	0	0	0	1	SA0189
gi|29165615|ref|NC_002745.2|	223367	+	C	0	1	0	0	0	SA0189
gi|29165615|ref|NC_002745.2|	223369	+	A	0	0	1	0	0	SA0189
gi|29165615|ref|NC_002745.2|	223371	+	G	0	0	0	0	1	SA0189
gi|29165615|ref|NC_002745.2|	223511	+	C	0	0	0	1	0	SA0189
gi|29165615|ref|NC_002745.2|	223585	+	C	0	1	0	0	0	SA0189
gi|29165615|ref|NC_002745.2|	223591	+	A	0	0	1	0	0	SA0189
gi|29165615|ref|NC_002745.2|	223670	+	A	0	1	0	0	0	SA0189
gi|29165615|ref|NC_002745.2|	223691	+	A	0	0	0	0	1	SA0189
gi|29165615|ref|NC_002745.2|	223696	+	U	0	0	0	1	0	SA0189
gi|29165615|ref|NC_002745.2|	223701	+	A	0	0	0	0	1	SA0189
gi|29165615|ref|NC_002745.2|	223750	+	U	0	0	0	0	1	SA0189
gi|29165615|ref|NC_002745.2|	223753	+	A	0	0	0	0	1	SA0189
gi|29165615|ref|NC_002745.2|	223765	+	A	0	0	0	0	1	SA0189
gi|29165615|ref|NC_002745.2|	223767	+	A	0	0	1	1	0	SA0189
gi|29165615|ref|NC_002745.2|	223793	+	A	0	0	0	0	1	SA0189
gi|29165615|ref|NC_002745.2|	223877	+	A	0	0	1	0	0	SA0189
gi|29165615|ref|NC_002745.2|	224129	+	C	0	1	0	0	0	SA0189
gi|29165615|ref|NC_002745.2|	224135	+	C	0	1	0	0	0	SA0189
gi|29165615|ref|NC_002745.2|	224163	+	A	0	0	0	1	0	SA0189
gi|29165615|ref|NC_002745.2|	224168	+	A	0	0	1	0	0	SA0189
gi|29165615|ref|NC_002745.2|	224171	+	U	0	1	0	0	0	SA0189
gi|29165615|ref|NC_002745.2|	224195	+	A	0	0	0	0	1	SA0189
gi|29165615|ref|NC_002745.2|	224196	+	A	0	0	0	0	1	SA0189
gi|29165615|ref|NC_002745.2|	224206	+	U	0	1	0	0	0	SA0189
gi|29165615|ref|NC_002745.2|	224244	+	U	0	0	1	0	0	SA0189
gi|29165615|ref|NC_002745.2|	224295	+	A	0	1	0	0	0	SA0189
gi|29165615|ref|NC_002745.2|	224299	+	C	0	0	0	0	1	SA0189
gi|29165615|ref|NC_002745.2|	224313	+	U	0	0	0	2	0	SA0189
gi|29165615|ref|NC_002745.2|	224342	+	C	0	0	1	0	0	SA0189
gi|29165615|ref|NC_002745.2|	224352	+	A	0	0	0	1	0	SA0189
gi|29165615|ref|NC_002745.2|	224361	+	C	0	0	1	0	0	SA0189
gi|29165615|ref|NC_002745.2|	224363	+	A	0	1	1	0	0	SA0189
gi|29165615|ref|NC_002745.2|	224375	+	A	0	1	0	1	0	SA0189
gi|29165615|ref|NC_002745.2|	224377	+	A	0	0	0	0	2	SA0189
gi|29165615|ref|NC_002745.2|	224390	+	A	0	1	0	0	0	SA0189
gi|29165615|ref|NC_002745.2|	224425	+	G	0	0	1	0	0	SA0189
gi|29165615|ref|NC_002745.2|	224429	+	C	0	1	0	0	0	SA0189
gi|29165615|ref|NC_002745.2|	224430	+	A	0	0	0	2	0	SA0189
gi|29165615|ref|NC_002745.2|	224432	+	U	0	0	0	0	1	SA0189
gi|29165615|ref|NC_002745.2|	224442	+	C	0	1	0	0	0	SA0189
gi|29165615|ref|NC_002745.2|	224444	+	G	0	1	0	0	0	SA0189
gi|29165615|ref|NC_002745.2|	224453	+	A	0	0	0	0	2	SA0189
gi|29165615|ref|NC_002745.2|	224455	+	U	0	1	0	0	0	SA0189
gi|29165615|ref|NC_002745.2|	224458	+	A	0	0	0	0	1	SA0189
gi|29165615|ref|NC_002745.2|	224459	+	A	0	1	1	0	0	SA0189
gi|29165615|ref|NC_002745.2|	224460	+	A	0	1	0	0	0	SA0189
gi|29165615|ref|NC_002745.2|	224461	+	A	0	0	1	0	0	SA0189
gi|29165615|ref|NC_002745.2|	224462	+	A	0	0	0	0	1	SA0189
gi|29165615|ref|NC_002745.2|	224463	+	A	0	0	0	0	1	SA0189
gi|29165615|ref|NC_002745.2|	224464	+	G	0	2	0	0	1	SA0189
gi|29165615|ref|NC_002745.2|	224465	+	A	0	3	1	0	1	SA0189
gi|29165615|ref|NC_002745.2|	224466	+	A	0	0	1	0	0	SA0189
gi|29165615|ref|NC_002745.2|	224469	+	U	0	0	1	1	1	SA0189
gi|29165615|ref|NC_002745.2|	224470	+	A	0	0	1	0	0	SA0189
gi|29165615|ref|NC_002745.2|	224471	+	U	0	0	2	0	0	SA0189
gi|29165615|ref|NC_002745.2|	224477	+	C	0	0	0	0	1	SA0189
gi|29165615|ref|NC_002745.2|	224479	+	U	0	1	0	0	0	SA0189
gi|29165615|ref|NC_002745.2|	224486	+	A	1	1	0	0	0	SA0189
gi|29165615|ref|NC_002745.2|	224492	+	A	0	0	1	0	0	SA0189
gi|29165615|ref|NC_002745.2|	224494	+	A	0	0	0	0	1	SA0189
gi|29165615|ref|NC_002745.2|	224592	+	A	0	0	1	0	0	SA0189
gi|29165615|ref|NC_002745.2|	224626	+	A	0	1	0	0	0	SA0189
gi|29165615|ref|NC_002745.2|	224669	+	A	0	0	0	1	0	SA0189
gi|29165615|ref|NC_002745.2|	224671	+	A	0	1	0	0	0	SA0189
gi|29165615|ref|NC_002745.2|	224699	+	U	0	0	0	0	1	SA0189
gi|29165615|ref|NC_002745.2|	224708	+	C	0	0	0	0	1	SA0189
gi|29165615|ref|NC_002745.2|	224724	+	U	0	0	1	0	0	SA0189
gi|29165615|ref|NC_002745.2|	224727	+	A	0	0	1	0	0	SA0189
gi|29165615|ref|NC_002745.2|	224744	+	A	0	1	0	0	2	SA0189
gi|29165615|ref|NC_002745.2|	224750	+	A	0	0	1	1	0	SA0189
gi|29165615|ref|NC_002745.2|	224756	+	A	0	1	0	1	0	SA0189
gi|29165615|ref|NC_002745.2|	224757	+	A	0	0	0	1	0	SA0189
gi|29165615|ref|NC_002745.2|	224758	+	A	0	3	0	1	3	SA0189
gi|29165615|ref|NC_002745.2|	224761	+	G	0	2	0	2	0	SA0189

gi|29165615|ref|NC_002745.2|	224762	+	A	0	2	1	1	1	SA0189
gi|29165615|ref|NC_002745.2|	224763	+	A	0	1	0	0	0	SA0189
gi|29165615|ref|NC_002745.2|	224769	+	G	0	1	0	0	0	SA0189
gi|29165615|ref|NC_002745.2|	224776	+	G	0	0	0	0	1	SA0189
gi|29165615|ref|NC_002745.2|	224782	+	A	0	0	0	1	0	SA0189
gi|29165615|ref|NC_002745.2|	224785	+	A	0	0	0	1	0	SA0189
gi|29165615|ref|NC_002745.2|	224790	+	G	0	0	1	0	0	SA0189
gi|29165615|ref|NC_002745.2|	224795	+	A	0	0	0	1	0	SA0189
gi|29165615|ref|NC_002745.2|	224797	+	A	0	1	0	0	0	SA0189
gi|29165615|ref|NC_002745.2|	224798	+	U	0	0	0	1	0	SA0189
gi|29165615|ref|NC_002745.2|	224810	+	U	0	0	0	1	0	SA0189
gi|29165615|ref|NC_002745.2|	224814	+	A	0	1	0	0	0	SA0189
gi|29165615|ref|NC_002745.2|	224827	+	G	0	2	0	0	0	SA0189
gi|29165615|ref|NC_002745.2|	224828	+	A	0	2	0	6	0	SA0189
gi|29165615|ref|NC_002745.2|	224829	+	A	0	0	0	1	0	SA0189
gi|29165615|ref|NC_002745.2|	224834	+	A	0	0	0	0	1	SA0189
gi|29165615|ref|NC_002745.2|	224835	+	A	0	1	0	0	0	SA0189
gi|29165615|ref|NC_002745.2|	224837	+	C	0	0	0	1	0	SA0189
gi|29165615|ref|NC_002745.2|	224858	+	A	0	1	0	0	0	SA0189
gi|29165615|ref|NC_002745.2|	224885	+	C	0	0	0	0	1	SA0189
gi|29165615|ref|NC_002745.2|	224907	+	A	0	0	0	1	0	SA0189
gi|29165615|ref|NC_002745.2|	224919	+	A	0	0	0	1	0	SA0189
gi|29165615|ref|NC_002745.2|	224931	+	C	0	0	0	0	1	SA0189
gi|29165615|ref|NC_002745.2|	224969	+	A	0	1	0	0	0	SA0189
gi|29165615|ref|NC_002745.2|	224981	+	A	0	0	0	1	0	SA0189
gi|29165615|ref|NC_002745.2|	224982	+	A	0	2	0	1	0	SA0189
gi|29165615|ref|NC_002745.2|	224986	+	G	0	0	0	0	1	SA0189
gi|29165615|ref|NC_002745.2|	224988	+	U	0	0	0	1	0	SA0189
gi|29165615|ref|NC_002745.2|	224996	+	A	0	1	0	0	1	SA0189
gi|29165615|ref|NC_002745.2|	225001	+	A	0	1	0	0	0	SA0189
gi|29165615|ref|NC_002745.2|	225014	+	C	0	0	0	1	0	SA0189
gi|29165615|ref|NC_002745.2|	225049	+	G	0	0	1	0	0	SA0189
gi|29165615|ref|NC_002745.2|	226022	+	U	0	0	1	0	0	-
gi|29165615|ref|NC_002745.2|	226031	+	A	0	0	0	1	0	-
gi|29165615|ref|NC_002745.2|	226038	+	A	0	0	0	2	1	-
gi|29165615|ref|NC_002745.2|	226042	+	U	0	1	0	0	0	-
gi|29165615|ref|NC_002745.2|	226043	+	A	0	0	0	0	1	-
gi|29165615|ref|NC_002745.2|	226044	+	A	0	1	0	0	0	-
gi|29165615|ref|NC_002745.2|	226045	+	A	0	0	0	1	0	-
gi|29165615|ref|NC_002745.2|	226452	+	G	0	1	0	0	0	SA0191
gi|29165615|ref|NC_002745.2|	227213	+	A	0	1	0	0	0	SA0192
gi|29165615|ref|NC_002745.2|	227275	+	C	0	0	0	0	1	SA0192
gi|29165615|ref|NC_002745.2|	227276	+	A	0	1	0	1	0	SA0192
gi|29165615|ref|NC_002745.2|	227285	+	C	0	0	0	0	1	SA0192
gi|29165615|ref|NC_002745.2|	227302	+	A	0	0	0	0	1	SA0192
gi|29165615|ref|NC_002745.2|	227308	+	G	0	1	0	0	0	SA0192
gi|29165615|ref|NC_002745.2|	227536	+	A	0	0	1	0	0	SA0192
gi|29165615|ref|NC_002745.2|	227550	+	A	0	2	0	0	0	SA0192
gi|29165615|ref|NC_002745.2|	227557	+	A	0	1	1	0	0	SA0192
gi|29165615|ref|NC_002745.2|	227558	+	A	0	1	0	0	0	SA0192
gi|29165615|ref|NC_002745.2|	227561	+	U	0	0	0	1	2	SA0192
gi|29165615|ref|NC_002745.2|	227562	+	U	0	0	0	0	1	SA0192
gi|29165615|ref|NC_002745.2|	227617	+	A	0	0	0	0	1	SA0192
gi|29165615|ref|NC_002745.2|	227640	+	A	1	0	0	0	0	SA0192
gi|29165615|ref|NC_002745.2|	227645	+	U	0	0	1	0	0	SA0192
gi|29165615|ref|NC_002745.2|	227741	+	A	0	0	1	0	0	SA0192
gi|29165615|ref|NC_002745.2|	227936	+	A	0	0	0	1	0	SA0193
gi|29165615|ref|NC_002745.2|	227941	+	U	1	0	0	0	0	SA0193
gi|29165615|ref|NC_002745.2|	228078	+	A	0	1	0	0	0	SA0193
gi|29165615|ref|NC_002745.2|	228102	+	A	0	0	1	0	0	SA0193
gi|29165615|ref|NC_002745.2|	228124	+	G	0	0	0	0	1	SA0193
gi|29165615|ref|NC_002745.2|	228255	+	A	0	1	0	0	0	SA0193
gi|29165615|ref|NC_002745.2|	228260	+	A	0	0	0	0	1	SA0193
gi|29165615|ref|NC_002745.2|	228308	+	A	0	0	0	0	1	SA0193
gi|29165615|ref|NC_002745.2|	228666	+	A	0	0	2	1	0	SA0193
gi|29165615|ref|NC_002745.2|	228667	+	G	0	1	0	0	0	SA0193
gi|29165615|ref|NC_002745.2|	228671	+	U	0	0	0	1	0	SA0193
gi|29165615|ref|NC_002745.2|	228681	+	G	0	1	0	0	0	SA0193
gi|29165615|ref|NC_002745.2|	228828	+	A	0	1	0	0	0	SA0193
gi|29165615|ref|NC_002745.2|	228830	+	A	0	0	1	0	0	SA0193
gi|29165615|ref|NC_002745.2|	228836	+	C	0	0	1	1	0	SA0193
gi|29165615|ref|NC_002745.2|	228934	+	U	0	0	0	0	1	SA0193
gi|29165615|ref|NC_002745.2|	228953	+	C	0	0	0	1	0	SA0193
gi|29165615|ref|NC_002745.2|	228957	+	A	0	0	0	0	1	SA0193
gi|29165615|ref|NC_002745.2|	229221	+	A	0	0	0	1	0	-
gi|29165615|ref|NC_002745.2|	229223	+	A	0	0	0	1	0	-
gi|29165615|ref|NC_002745.2|	229239	+	G	2	0	1	0	1	-
gi|29165615|ref|NC_002745.2|	229240	+	A	1	0	0	0	0	-
gi|29165615|ref|NC_002745.2|	229254	+	G	1	0	0	0	0	-
gi|29165615|ref|NC_002745.2|	229270	+	A	0	0	0	0	4	SA0194
gi|29165615|ref|NC_002745.2|	229271	+	C	0	0	0	1	0	SA0194

gi|29165615|ref|NC_002745.2|	229272	+	A	0	3	0	1	4	SA0194
gi|29165615|ref|NC_002745.2|	229273	+	U	0	2	0	0	1	SA0194
gi|29165615|ref|NC_002745.2|	229274	+	A	0	1	0	1	0	SA0194
gi|29165615|ref|NC_002745.2|	229278	+	A	0	3	0	0	0	SA0194
gi|29165615|ref|NC_002745.2|	229286	+	U	0	0	0	0	1	SA0194
gi|29165615|ref|NC_002745.2|	229310	+	A	0	0	1	0	0	SA0194
gi|29165615|ref|NC_002745.2|	229317	+	C	0	2	0	0	0	SA0194
gi|29165615|ref|NC_002745.2|	229319	+	U	0	3	1	0	0	SA0194
gi|29165615|ref|NC_002745.2|	229320	+	U	0	0	0	2	0	SA0194
gi|29165615|ref|NC_002745.2|	229322	+	A	0	0	0	0	1	SA0194
gi|29165615|ref|NC_002745.2|	229323	+	U	0	0	0	1	0	SA0194
gi|29165615|ref|NC_002745.2|	229329	+	C	0	2	1	2	1	SA0194
gi|29165615|ref|NC_002745.2|	229332	+	U	0	0	1	0	0	SA0194
gi|29165615|ref|NC_002745.2|	229333	+	A	0	3	1	0	0	SA0194
gi|29165615|ref|NC_002745.2|	229335	+	A	0	0	1	1	0	SA0194
gi|29165615|ref|NC_002745.2|	229336	+	U	0	1	0	0	0	SA0194
gi|29165615|ref|NC_002745.2|	229337	+	A	0	1	0	0	2	SA0194
gi|29165615|ref|NC_002745.2|	229339	+	U	0	0	0	1	0	SA0194
gi|29165615|ref|NC_002745.2|	229341	+	G	0	0	0	4	0	SA0194
gi|29165615|ref|NC_002745.2|	229342	+	A	0	1	0	0	0	SA0194
gi|29165615|ref|NC_002745.2|	229344	+	G	1	0	1	0	0	SA0194
gi|29165615|ref|NC_002745.2|	229345	+	U	0	0	1	0	0	SA0194
gi|29165615|ref|NC_002745.2|	229346	+	A	0	4	2	2	5	SA0194
gi|29165615|ref|NC_002745.2|	229349	+	G	0	0	0	1	0	SA0194
gi|29165615|ref|NC_002745.2|	229350	+	U	0	0	0	2	0	SA0194
gi|29165615|ref|NC_002745.2|	229351	+	U	0	0	2	0	0	SA0194
gi|29165615|ref|NC_002745.2|	229352	+	U	4	0	0	0	0	SA0194
gi|29165615|ref|NC_002745.2|	229354	+	G	0	0	0	0	1	SA0194
gi|29165615|ref|NC_002745.2|	229362	+	U	1	0	0	0	0	SA0194
gi|29165615|ref|NC_002745.2|	229367	+	G	0	3	0	0	0	SA0194
gi|29165615|ref|NC_002745.2|	229368	+	U	2	0	1	0	0	SA0194
gi|29165615|ref|NC_002745.2|	229371	+	C	3	0	0	0	0	SA0194
gi|29165615|ref|NC_002745.2|	229375	+	U	0	0	1	0	0	SA0194
gi|29165615|ref|NC_002745.2|	229377	+	U	0	1	0	0	0	SA0194
gi|29165615|ref|NC_002745.2|	229379	+	G	0	0	0	0	1	SA0194
gi|29165615|ref|NC_002745.2|	229380	+	C	2	0	0	0	0	SA0194
gi|29165615|ref|NC_002745.2|	229383	+	U	0	0	0	0	1	SA0194
gi|29165615|ref|NC_002745.2|	229387	+	U	0	0	1	0	0	SA0194
gi|29165615|ref|NC_002745.2|	231065	+	A	0	1	0	0	0	SA0195
gi|29165615|ref|NC_002745.2|	231132	+	A	0	0	0	0	1	SA0195
gi|29165615|ref|NC_002745.2|	231784	+	C	0	1	0	0	0	SA0197
gi|29165615|ref|NC_002745.2|	231786	+	A	0	1	0	0	0	SA0197
gi|29165615|ref|NC_002745.2|	235384	+	U	1	0	0	0	0	SA0199
gi|29165615|ref|NC_002745.2|	236043	+	G	0	0	0	0	1	SA0199
gi|29165615|ref|NC_002745.2|	236453	+	A	0	0	0	2	0	SA0200
gi|29165615|ref|NC_002745.2|	236744	+	C	0	0	3	0	0	SA0200
gi|29165615|ref|NC_002745.2|	237731	+	U	0	0	0	0	1	SA0201
gi|29165615|ref|NC_002745.2|	237841	+	A	1	0	0	0	0	SA0201
gi|29165615|ref|NC_002745.2|	238285	+	A	0	1	0	0	0	SA0201
gi|29165615|ref|NC_002745.2|	238287	+	G	0	1	0	1	1	SA0201
gi|29165615|ref|NC_002745.2|	238375	+	A	0	1	0	0	0	SA0201
gi|29165615|ref|NC_002745.2|	238525	+	A	0	0	0	0	1	SA0201
gi|29165615|ref|NC_002745.2|	239052	+	A	0	0	0	0	1	SA0201
gi|29165615|ref|NC_002745.2|	239239	+	A	0	1	0	0	0	SA0201
gi|29165615|ref|NC_002745.2|	239772	+	A	1	0	0	0	0	SA0202
gi|29165615|ref|NC_002745.2|	239776	+	G	0	0	0	0	1	SA0202
gi|29165615|ref|NC_002745.2|	239784	+	A	0	0	0	0	1	SA0202
gi|29165615|ref|NC_002745.2|	239875	+	A	0	0	0	1	0	SA0202
gi|29165615|ref|NC_002745.2|	239910	+	A	0	0	0	1	0	SA0202
gi|29165615|ref|NC_002745.2|	239953	+	U	1	0	0	0	0	SA0202
gi|29165615|ref|NC_002745.2|	239962	+	A	0	0	0	0	1	SA0202
gi|29165615|ref|NC_002745.2|	239970	+	A	0	0	1	0	0	SA0202
gi|29165615|ref|NC_002745.2|	239977	+	A	0	1	0	0	0	SA0202
gi|29165615|ref|NC_002745.2|	239991	+	U	1	0	0	0	0	SA0202
gi|29165615|ref|NC_002745.2|	240233	+	U	0	0	0	1	0	SA0202
gi|29165615|ref|NC_002745.2|	240924	+	A	0	0	2	0	0	SA0202
gi|29165615|ref|NC_002745.2|	241022	+	G	0	0	0	1	0	SA0202
gi|29165615|ref|NC_002745.2|	241048	+	A	0	1	0	0	0	SA0202
gi|29165615|ref|NC_002745.2|	241104	+	U	1	0	0	0	0	SA0202
gi|29165615|ref|NC_002745.2|	241106	+	C	0	0	0	1	0	SA0202
gi|29165615|ref|NC_002745.2|	241193	+	A	0	0	0	1	0	SA0202
gi|29165615|ref|NC_002745.2|	241218	+	C	0	0	0	1	0	SA0202
gi|29165615|ref|NC_002745.2|	241231	+	A	0	3	0	0	0	SA0202
gi|29165615|ref|NC_002745.2|	241549	+	C	3	0	0	0	0	-
gi|29165615|ref|NC_002745.2|	241915	+	A	0	1	0	0	0	-
gi|29165615|ref|NC_002745.2|	241927	+	G	0	1	0	0	0	-
gi|29165615|ref|NC_002745.2|	242657	+	A	0	0	0	0	1	-
gi|29165615|ref|NC_002745.2|	243058	+	U	0	0	1	0	0	-
gi|29165615|ref|NC_002745.2|	243155	+	U	0	0	0	0	2	-
gi|29165615|ref|NC_002745.2|	243461	+	A	0	0	0	1	0	-
gi|29165615|ref|NC_002745.2|	243547	+	A	0	0	0	0	1	SA0205

gi|29165615|ref|NC_002745.2|	243566	+	A	0	1	0	1	2	SA0205
gi|29165615|ref|NC_002745.2|	243567	+	C	0	0	0	1	1	SA0205
gi|29165615|ref|NC_002745.2|	243568	+	A	0	0	1	0	0	SA0205
gi|29165615|ref|NC_002745.2|	243571	+	C	0	1	2	0	0	SA0205
gi|29165615|ref|NC_002745.2|	243572	+	A	0	2	0	0	0	SA0205
gi|29165615|ref|NC_002745.2|	243573	+	A	0	0	0	1	1	SA0205
gi|29165615|ref|NC_002745.2|	243575	+	A	0	0	0	1	0	SA0205
gi|29165615|ref|NC_002745.2|	243579	+	G	0	0	0	1	0	SA0205
gi|29165615|ref|NC_002745.2|	243580	+	U	0	0	0	1	1	SA0205
gi|29165615|ref|NC_002745.2|	243581	+	U	0	0	0	0	1	SA0205
gi|29165615|ref|NC_002745.2|	243583	+	A	0	0	0	1	0	SA0205
gi|29165615|ref|NC_002745.2|	243585	+	C	0	0	1	1	0	SA0205
gi|29165615|ref|NC_002745.2|	243590	+	A	0	1	1	1	0	SA0205
gi|29165615|ref|NC_002745.2|	243593	+	C	0	0	1	0	0	SA0205
gi|29165615|ref|NC_002745.2|	243594	+	C	0	0	0	1	0	SA0205
gi|29165615|ref|NC_002745.2|	243595	+	G	0	3	0	0	1	SA0205
gi|29165615|ref|NC_002745.2|	243596	+	A	1	2	3	8	4	SA0205
gi|29165615|ref|NC_002745.2|	243598	+	A	0	1	0	2	4	SA0205
gi|29165615|ref|NC_002745.2|	243599	+	A	0	2	1	4	2	SA0205
gi|29165615|ref|NC_002745.2|	243600	+	A	0	1	0	3	3	SA0205
gi|29165615|ref|NC_002745.2|	243603	+	G	0	0	0	1	0	SA0205
gi|29165615|ref|NC_002745.2|	243604	+	A	0	1	0	2	0	SA0205
gi|29165615|ref|NC_002745.2|	243605	+	C	0	1	1	0	0	SA0205
gi|29165615|ref|NC_002745.2|	243606	+	A	0	2	0	1	0	SA0205
gi|29165615|ref|NC_002745.2|	243608	+	A	0	0	1	0	0	SA0205
gi|29165615|ref|NC_002745.2|	243611	+	A	0	0	0	1	0	SA0205
gi|29165615|ref|NC_002745.2|	243616	+	A	0	1	0	0	0	SA0205
gi|29165615|ref|NC_002745.2|	243617	+	A	0	1	0	0	0	SA0205
gi|29165615|ref|NC_002745.2|	243618	+	A	0	1	0	0	0	SA0205
gi|29165615|ref|NC_002745.2|	243627	+	A	0	0	0	1	0	SA0205
gi|29165615|ref|NC_002745.2|	243628	+	C	0	0	0	0	1	SA0205
gi|29165615|ref|NC_002745.2|	243632	+	C	0	0	0	1	0	SA0205
gi|29165615|ref|NC_002745.2|	243633	+	A	0	0	0	1	2	SA0205
gi|29165615|ref|NC_002745.2|	243634	+	A	0	2	0	1	1	SA0205
gi|29165615|ref|NC_002745.2|	243651	+	U	0	0	0	1	0	SA0205
gi|29165615|ref|NC_002745.2|	243657	+	G	0	1	0	0	0	SA0205
gi|29165615|ref|NC_002745.2|	243681	+	C	0	0	0	0	1	SA0205
gi|29165615|ref|NC_002745.2|	243682	+	A	1	0	0	0	1	SA0205
gi|29165615|ref|NC_002745.2|	243694	+	U	0	1	0	0	0	SA0205
gi|29165615|ref|NC_002745.2|	243715	+	U	0	0	0	1	0	SA0205
gi|29165615|ref|NC_002745.2|	243722	+	U	0	0	0	1	0	SA0205
gi|29165615|ref|NC_002745.2|	243727	+	U	0	0	1	0	0	SA0205
gi|29165615|ref|NC_002745.2|	243883	+	G	0	0	0	1	0	SA0205
gi|29165615|ref|NC_002745.2|	243985	+	G	0	0	1	0	0	SA0205
gi|29165615|ref|NC_002745.2|	244376	+	U	0	0	0	0	1	-
gi|29165615|ref|NC_002745.2|	244377	+	A	0	30	19	18	30	-
gi|29165615|ref|NC_002745.2|	244388	+	C	0	0	0	0	1	-
gi|29165615|ref|NC_002745.2|	244399	+	A	0	0	0	0	1	-
gi|29165615|ref|NC_002745.2|	244425	+	U	0	0	0	2	1	-
gi|29165615|ref|NC_002745.2|	244428	+	A	0	1	0	0	0	-
gi|29165615|ref|NC_002745.2|	244430	+	A	0	0	0	0	1	-
gi|29165615|ref|NC_002745.2|	244433	+	G	0	0	0	0	2	-
gi|29165615|ref|NC_002745.2|	244434	+	U	0	4	0	1	4	-
gi|29165615|ref|NC_002745.2|	244440	+	A	0	2	0	0	0	-
gi|29165615|ref|NC_002745.2|	244445	+	U	0	0	1	0	0	-
gi|29165615|ref|NC_002745.2|	244453	+	C	0	0	0	0	1	SA0206
gi|29165615|ref|NC_002745.2|	244460	+	A	0	0	0	0	1	SA0206
gi|29165615|ref|NC_002745.2|	244464	+	U	0	0	0	0	1	SA0206
gi|29165615|ref|NC_002745.2|	244470	+	C	0	1	0	0	2	SA0206
gi|29165615|ref|NC_002745.2|	244479	+	A	0	1	0	0	0	SA0206
gi|29165615|ref|NC_002745.2|	244482	+	A	0	0	0	0	1	SA0206
gi|29165615|ref|NC_002745.2|	244483	+	C	0	1	0	2	0	SA0206
gi|29165615|ref|NC_002745.2|	244492	+	A	0	0	0	0	1	SA0206
gi|29165615|ref|NC_002745.2|	244498	+	A	0	0	0	1	0	SA0206
gi|29165615|ref|NC_002745.2|	244516	+	U	0	1	0	0	0	SA0206
gi|29165615|ref|NC_002745.2|	244537	+	A	0	0	0	0	1	SA0206
gi|29165615|ref|NC_002745.2|	244540	+	A	0	1	0	0	0	SA0206
gi|29165615|ref|NC_002745.2|	244575	+	A	0	1	0	0	0	SA0206
gi|29165615|ref|NC_002745.2|	244582	+	C	0	1	0	0	0	SA0206
gi|29165615|ref|NC_002745.2|	244585	+	C	0	0	0	0	1	SA0206
gi|29165615|ref|NC_002745.2|	244586	+	A	0	0	1	0	0	SA0206
gi|29165615|ref|NC_002745.2|	244589	+	A	0	0	0	0	1	SA0206
gi|29165615|ref|NC_002745.2|	244633	+	A	0	1	1	1	2	SA0206
gi|29165615|ref|NC_002745.2|	244634	+	U	0	0	1	1	1	SA0206
gi|29165615|ref|NC_002745.2|	244635	+	A	0	0	1	0	0	SA0206
gi|29165615|ref|NC_002745.2|	244641	+	G	0	1	0	0	0	SA0206
gi|29165615|ref|NC_002745.2|	244649	+	C	0	1	1	0	0	SA0206
gi|29165615|ref|NC_002745.2|	244666	+	C	0	1	0	0	0	SA0206
gi|29165615|ref|NC_002745.2|	244671	+	A	0	0	1	2	1	SA0206
gi|29165615|ref|NC_002745.2|	244686	+	A	0	1	0	0	0	SA0206
gi|29165615|ref|NC_002745.2|	244687	+	U	0	1	0	0	0	SA0206

gi|29165615|ref|NC_002745.2|	244705 +	C	0	1	0	1	3	SA0206
gi|29165615|ref|NC_002745.2|	244706 +	A	0	1	0	0	0	SA0206
gi|29165615|ref|NC_002745.2|	244708 +	U	0	1	0	0	0	SA0206
gi|29165615|ref|NC_002745.2|	244714 +	C	0	0	0	0	1	SA0206
gi|29165615|ref|NC_002745.2|	244716 +	C	0	0	0	0	2	SA0206
gi|29165615|ref|NC_002745.2|	244718 +	U	0	0	0	1	0	SA0206
gi|29165615|ref|NC_002745.2|	244719 +	A	0	0	1	0	0	SA0206
gi|29165615|ref|NC_002745.2|	244727 +	A	0	0	0	0	1	SA0206
gi|29165615|ref|NC_002745.2|	244731 +	G	0	0	0	2	1	SA0206
gi|29165615|ref|NC_002745.2|	244732 +	A	0	0	1	0	2	SA0206
gi|29165615|ref|NC_002745.2|	244735 +	A	0	2	0	0	2	SA0206
gi|29165615|ref|NC_002745.2|	244737 +	A	0	0	1	0	0	SA0206
gi|29165615|ref|NC_002745.2|	244740 +	G	0	0	0	0	2	SA0206
gi|29165615|ref|NC_002745.2|	244741 +	C	1	0	0	0	2	SA0206
gi|29165615|ref|NC_002745.2|	244742 +	A	0	1	0	0	0	SA0206
gi|29165615|ref|NC_002745.2|	244749 +	C	0	1	0	0	0	SA0206
gi|29165615|ref|NC_002745.2|	244758 +	C	0	0	0	0	1	SA0206
gi|29165615|ref|NC_002745.2|	244765 +	U	0	1	0	1	0	SA0206
gi|29165615|ref|NC_002745.2|	244767 +	A	0	0	0	1	0	SA0206
gi|29165615|ref|NC_002745.2|	244768 +	A	0	3	0	0	0	SA0206
gi|29165615|ref|NC_002745.2|	244769 +	U	0	0	0	1	1	SA0206
gi|29165615|ref|NC_002745.2|	244770 +	A	0	1	0	0	0	SA0206
gi|29165615|ref|NC_002745.2|	244773 +	A	0	0	0	0	1	SA0206
gi|29165615|ref|NC_002745.2|	244777 +	A	0	4	2	1	1	SA0206
gi|29165615|ref|NC_002745.2|	244778 +	G	0	0	0	0	1	SA0206
gi|29165615|ref|NC_002745.2|	244779 +	A	0	0	1	0	0	SA0206
gi|29165615|ref|NC_002745.2|	244784 +	A	0	0	1	1	1	SA0206
gi|29165615|ref|NC_002745.2|	244786 +	A	0	1	0	0	0	SA0206
gi|29165615|ref|NC_002745.2|	244788 +	A	0	0	0	0	2	SA0206
gi|29165615|ref|NC_002745.2|	244789 +	A	0	1	0	2	1	SA0206
gi|29165615|ref|NC_002745.2|	244792 +	U	0	1	0	1	0	SA0206
gi|29165615|ref|NC_002745.2|	244793 +	C	0	0	0	1	0	SA0206
gi|29165615|ref|NC_002745.2|	244801 +	C	0	1	0	0	3	SA0206
gi|29165615|ref|NC_002745.2|	244808 +	A	0	3	2	0	1	SA0206
gi|29165615|ref|NC_002745.2|	244810 +	U	0	0	0	0	6	SA0206
gi|29165615|ref|NC_002745.2|	244812 +	U	0	1	0	0	0	SA0206
gi|29165615|ref|NC_002745.2|	244814 +	A	0	3	0	0	3	SA0206
gi|29165615|ref|NC_002745.2|	244821 +	A	0	0	0	0	1	SA0206
gi|29165615|ref|NC_002745.2|	244822 +	C	0	2	0	0	0	SA0206
gi|29165615|ref|NC_002745.2|	244824 +	G	0	1	0	0	0	SA0206
gi|29165615|ref|NC_002745.2|	244843 +	C	0	0	0	1	0	SA0206
gi|29165615|ref|NC_002745.2|	244847 +	A	0	0	0	0	1	SA0206
gi|29165615|ref|NC_002745.2|	244853 +	A	0	0	0	0	1	SA0206
gi|29165615|ref|NC_002745.2|	244861 +	G	0	1	0	0	0	SA0206
gi|29165615|ref|NC_002745.2|	244958 +	A	0	0	0	0	1	SA0206
gi|29165615|ref|NC_002745.2|	244967 +	G	0	1	0	0	0	SA0206
gi|29165615|ref|NC_002745.2|	244983 +	A	0	0	0	1	0	SA0206
gi|29165615|ref|NC_002745.2|	244988 +	A	0	0	0	0	1	SA0206
gi|29165615|ref|NC_002745.2|	244989 +	C	0	0	1	0	0	SA0206
gi|29165615|ref|NC_002745.2|	244991 +	U	0	1	0	2	0	SA0206
gi|29165615|ref|NC_002745.2|	244992 +	A	0	0	0	0	1	SA0206
gi|29165615|ref|NC_002745.2|	245001 +	A	0	1	0	0	0	SA0206
gi|29165615|ref|NC_002745.2|	245009 +	G	0	1	0	0	0	SA0206
gi|29165615|ref|NC_002745.2|	245010 +	A	0	1	0	0	0	SA0206
gi|29165615|ref|NC_002745.2|	245023 +	C	0	0	1	1	1	SA0206
gi|29165615|ref|NC_002745.2|	245025 +	C	0	2	0	1	0	SA0206
gi|29165615|ref|NC_002745.2|	245026 +	A	0	0	0	1	0	SA0206
gi|29165615|ref|NC_002745.2|	245029 +	A	1	0	0	0	0	SA0206
gi|29165615|ref|NC_002745.2|	245038 +	A	0	1	0	0	0	SA0206
gi|29165615|ref|NC_002745.2|	245041 +	C	0	1	0	0	0	SA0206
gi|29165615|ref|NC_002745.2|	245050 +	U	0	0	0	0	2	SA0206
gi|29165615|ref|NC_002745.2|	245087 +	U	0	0	0	0	1	SA0206
gi|29165615|ref|NC_002745.2|	245096 +	C	0	0	0	0	1	SA0206
gi|29165615|ref|NC_002745.2|	245110 +	A	0	0	1	0	0	SA0206
gi|29165615|ref|NC_002745.2|	245112 +	A	0	0	0	0	1	SA0206
gi|29165615|ref|NC_002745.2|	245113 +	U	0	0	3	0	2	SA0206
gi|29165615|ref|NC_002745.2|	245115 +	U	0	0	0	0	1	SA0206
gi|29165615|ref|NC_002745.2|	245122 +	C	0	0	0	0	2	SA0206
gi|29165615|ref|NC_002745.2|	245123 +	C	0	1	0	0	0	SA0206
gi|29165615|ref|NC_002745.2|	245128 +	A	0	1	0	0	0	SA0206
gi|29165615|ref|NC_002745.2|	245160 +	C	0	0	0	0	1	SA0206
gi|29165615|ref|NC_002745.2|	245162 +	A	0	1	0	0	0	SA0206
gi|29165615|ref|NC_002745.2|	245166 +	A	0	0	0	0	1	SA0206
gi|29165615|ref|NC_002745.2|	245169 +	A	0	1	0	0	0	SA0206
gi|29165615|ref|NC_002745.2|	245170 +	A	0	1	0	0	1	SA0206
gi|29165615|ref|NC_002745.2|	245175 +	U	0	0	0	0	1	SA0206
gi|29165615|ref|NC_002745.2|	245176 +	U	0	0	1	0	0	SA0206
gi|29165615|ref|NC_002745.2|	245184 +	A	0	0	1	0	0	SA0206
gi|29165615|ref|NC_002745.2|	245197 +	A	0	1	0	0	0	SA0206
gi|29165615|ref|NC_002745.2|	245202 +	U	0	1	0	0	0	SA0206
gi|29165615|ref|NC_002745.2|	245205 +	A	0	0	0	0	1	SA0206

gi|29165615|ref|NC_002745.2|	245211	+	G	0	0	0	1	0	SA0206
gi|29165615|ref|NC_002745.2|	245212	+	G	0	0	0	0	1	SA0206
gi|29165615|ref|NC_002745.2|	245213	+	A	0	0	1	0	0	SA0206
gi|29165615|ref|NC_002745.2|	245217	+	C	0	0	1	0	2	SA0206
gi|29165615|ref|NC_002745.2|	245225	+	U	0	0	3	1	1	SA0206
gi|29165615|ref|NC_002745.2|	245226	+	A	0	2	1	0	0	SA0206
gi|29165615|ref|NC_002745.2|	245227	+	A	0	0	0	0	2	SA0206
gi|29165615|ref|NC_002745.2|	245230	+	U	0	0	0	0	1	SA0206
gi|29165615|ref|NC_002745.2|	245231	+	A	0	0	0	0	1	SA0206
gi|29165615|ref|NC_002745.2|	245232	+	C	0	0	0	3	2	SA0206
gi|29165615|ref|NC_002745.2|	245233	+	A	0	2	1	0	4	SA0206
gi|29165615|ref|NC_002745.2|	245234	+	U	0	1	2	3	5	SA0206
gi|29165615|ref|NC_002745.2|	245235	+	A	0	0	2	5	9	SA0206
gi|29165615|ref|NC_002745.2|	245236	+	A	0	2	1	1	2	SA0206
gi|29165615|ref|NC_002745.2|	245237	+	U	0	0	0	0	2	SA0206
gi|29165615|ref|NC_002745.2|	245238	+	A	0	2	1	1	5	SA0206
gi|29165615|ref|NC_002745.2|	245239	+	A	0	0	0	0	8	SA0206
gi|29165615|ref|NC_002745.2|	245240	+	A	0	3	0	1	0	SA0206
gi|29165615|ref|NC_002745.2|	245241	+	A	0	3	0	1	5	SA0206
gi|29165615|ref|NC_002745.2|	245242	+	A	1	20	7	13	37	SA0206
gi|29165615|ref|NC_002745.2|	245243	+	A	0	1	0	2	4	SA0206
gi|29165615|ref|NC_002745.2|	245244	+	U	0	1	2	0	0	SA0206
gi|29165615|ref|NC_002745.2|	245245	+	U	0	0	0	1	0	SA0206
gi|29165615|ref|NC_002745.2|	245246	+	U	0	0	0	2	3	SA0206
gi|29165615|ref|NC_002745.2|	245247	+	G	0	2	0	0	6	SA0206
gi|29165615|ref|NC_002745.2|	245248	+	A	1	6	7	10	38	SA0206
gi|29165615|ref|NC_002745.2|	245249	+	A	0	13	1	9	35	SA0206
gi|29165615|ref|NC_002745.2|	245250	+	A	0	2	0	4	7	SA0206
gi|29165615|ref|NC_002745.2|	245251	+	A	0	0	0	0	4	SA0206
gi|29165615|ref|NC_002745.2|	245252	+	G	0	1	0	2	3	SA0206
gi|29165615|ref|NC_002745.2|	245253	+	U	0	2	0	2	2	SA0206
gi|29165615|ref|NC_002745.2|	245254	+	U	0	0	0	1	0	SA0206
gi|29165615|ref|NC_002745.2|	245255	+	A	0	1	0	0	3	SA0206
gi|29165615|ref|NC_002745.2|	245256	+	A	0	1	0	0	3	SA0206
gi|29165615|ref|NC_002745.2|	245257	+	A	0	0	0	1	0	SA0206
gi|29165615|ref|NC_002745.2|	245258	+	A	0	0	0	0	1	SA0206
gi|29165615|ref|NC_002745.2|	245261	+	U	0	0	1	0	0	SA0206
gi|29165615|ref|NC_002745.2|	245281	+	A	0	1	0	0	0	SA0206
gi|29165615|ref|NC_002745.2|	245300	+	A	0	0	1	0	0	SA0206
gi|29165615|ref|NC_002745.2|	245309	+	C	0	0	1	0	0	SA0206
gi|29165615|ref|NC_002745.2|	245359	+	A	0	1	0	0	0	SA0206
gi|29165615|ref|NC_002745.2|	245407	+	A	0	0	0	0	1	SA0206
gi|29165615|ref|NC_002745.2|	245457	+	C	0	0	0	0	1	SA0206
gi|29165615|ref|NC_002745.2|	245460	+	G	0	1	0	0	0	SA0206
gi|29165615|ref|NC_002745.2|	245473	+	A	0	0	0	0	1	SA0206
gi|29165615|ref|NC_002745.2|	245478	+	A	0	0	0	1	0	SA0206
gi|29165615|ref|NC_002745.2|	245494	+	A	0	0	0	1	0	SA0206
gi|29165615|ref|NC_002745.2|	245501	+	U	0	0	0	0	1	SA0206
gi|29165615|ref|NC_002745.2|	245503	+	A	0	0	0	1	0	SA0206
gi|29165615|ref|NC_002745.2|	245521	+	A	0	0	0	0	1	SA0206
gi|29165615|ref|NC_002745.2|	245527	+	C	0	1	0	0	0	SA0206
gi|29165615|ref|NC_002745.2|	245560	+	U	0	1	0	0	0	SA0207
gi|29165615|ref|NC_002745.2|	245562	+	U	0	0	0	1	0	SA0207
gi|29165615|ref|NC_002745.2|	245563	+	C	0	1	0	0	1	SA0207
gi|29165615|ref|NC_002745.2|	245564	+	U	0	0	0	0	2	SA0207
gi|29165615|ref|NC_002745.2|	245565	+	A	0	3	0	7	9	SA0207
gi|29165615|ref|NC_002745.2|	245566	+	A	0	0	1	2	6	SA0207
gi|29165615|ref|NC_002745.2|	245567	+	A	0	2	0	0	4	SA0207
gi|29165615|ref|NC_002745.2|	245568	+	A	0	3	1	0	9	SA0207
gi|29165615|ref|NC_002745.2|	245571	+	U	0	0	0	0	1	SA0207
gi|29165615|ref|NC_002745.2|	245572	+	U	0	0	0	1	3	SA0207
gi|29165615|ref|NC_002745.2|	245573	+	A	0	2	0	2	4	SA0207
gi|29165615|ref|NC_002745.2|	245574	+	A	0	0	0	1	0	SA0207
gi|29165615|ref|NC_002745.2|	245582	+	C	0	0	0	0	1	SA0207
gi|29165615|ref|NC_002745.2|	245584	+	C	0	0	0	2	0	SA0207
gi|29165615|ref|NC_002745.2|	245586	+	U	0	0	1	0	1	SA0207
gi|29165615|ref|NC_002745.2|	245595	+	G	0	1	0	1	1	SA0207
gi|29165615|ref|NC_002745.2|	245596	+	U	0	0	1	0	2	SA0207
gi|29165615|ref|NC_002745.2|	245601	+	U	0	2	0	0	4	SA0207
gi|29165615|ref|NC_002745.2|	245607	+	A	0	0	0	0	5	SA0207
gi|29165615|ref|NC_002745.2|	245608	+	U	0	1	0	0	0	SA0207
gi|29165615|ref|NC_002745.2|	245609	+	C	0	0	0	0	1	SA0207
gi|29165615|ref|NC_002745.2|	245610	+	G	0	0	0	0	2	SA0207
gi|29165615|ref|NC_002745.2|	245611	+	U	0	2	1	2	7	SA0207
gi|29165615|ref|NC_002745.2|	245612	+	A	0	2	0	0	2	SA0207
gi|29165615|ref|NC_002745.2|	245613	+	A	0	1	0	0	3	SA0207
gi|29165615|ref|NC_002745.2|	245617	+	C	0	0	0	0	1	SA0207
gi|29165615|ref|NC_002745.2|	245618	+	A	0	0	0	0	1	SA0207
gi|29165615|ref|NC_002745.2|	245635	+	C	0	0	0	0	1	SA0207
gi|29165615|ref|NC_002745.2|	245643	+	G	0	0	0	1	0	SA0207
gi|29165615|ref|NC_002745.2|	245650	+	A	0	0	0	0	1	SA0207

gi|29165615|ref|NC_002745.2|	245652 +	A	0	1	0	0	0	SA0207
gi|29165615|ref|NC_002745.2|	245661 +	A	0	2	0	0	0	SA0207
gi|29165615|ref|NC_002745.2|	245742 +	A	0	1	0	0	0	SA0207
gi|29165615|ref|NC_002745.2|	245757 +	A	0	0	0	0	1	SA0207
gi|29165615|ref|NC_002745.2|	245760 +	A	0	0	0	0	1	SA0207
gi|29165615|ref|NC_002745.2|	245815 +	A	0	1	0	0	1	SA0207
gi|29165615|ref|NC_002745.2|	245819 +	U	0	0	0	0	1	SA0207
gi|29165615|ref|NC_002745.2|	245851 +	C	0	0	1	0	1	SA0207
gi|29165615|ref|NC_002745.2|	245875 +	A	0	0	0	1	1	SA0207
gi|29165615|ref|NC_002745.2|	245878 +	A	0	0	0	0	1	SA0207
gi|29165615|ref|NC_002745.2|	245879 +	U	0	0	0	0	1	SA0207
gi|29165615|ref|NC_002745.2|	245882 +	U	0	0	1	0	0	SA0207
gi|29165615|ref|NC_002745.2|	245933 +	U	0	1	0	0	0	SA0207
gi|29165615|ref|NC_002745.2|	246013 +	A	0	1	0	0	0	SA0207
gi|29165615|ref|NC_002745.2|	246103 +	A	0	0	0	0	1	SA0207
gi|29165615|ref|NC_002745.2|	246112 +	A	0	0	0	0	1	SA0207
gi|29165615|ref|NC_002745.2|	246190 +	U	0	0	0	0	1	SA0207
gi|29165615|ref|NC_002745.2|	246202 +	A	0	0	0	0	2	SA0207
gi|29165615|ref|NC_002745.2|	246229 +	U	0	1	0	0	0	SA0207
gi|29165615|ref|NC_002745.2|	246238 +	A	0	4	0	0	0	SA0207
gi|29165615|ref|NC_002745.2|	246243 +	A	0	1	0	0	0	SA0207
gi|29165615|ref|NC_002745.2|	246245 +	A	0	0	0	0	1	SA0207
gi|29165615|ref|NC_002745.2|	246255 +	A	1	0	0	0	0	SA0207
gi|29165615|ref|NC_002745.2|	246258 +	A	0	1	0	0	0	SA0207
gi|29165615|ref|NC_002745.2|	246259 +	A	0	0	0	0	1	SA0207
gi|29165615|ref|NC_002745.2|	246282 +	U	0	0	0	1	0	SA0207
gi|29165615|ref|NC_002745.2|	246283 +	A	0	1	0	0	0	SA0207
gi|29165615|ref|NC_002745.2|	246312 +	A	0	0	0	0	1	SA0207
gi|29165615|ref|NC_002745.2|	246322 +	U	0	1	0	0	0	SA0207
gi|29165615|ref|NC_002745.2|	246323 +	C	0	1	0	0	0	SA0207
gi|29165615|ref|NC_002745.2|	246324 +	A	0	0	1	0	0	SA0207
gi|29165615|ref|NC_002745.2|	246325 +	U	0	0	0	0	1	SA0207
gi|29165615|ref|NC_002745.2|	246326 +	G	0	0	0	2	0	SA0207
gi|29165615|ref|NC_002745.2|	246327 +	A	0	1	0	0	0	SA0207
gi|29165615|ref|NC_002745.2|	246334 +	U	0	0	0	0	1	SA0207
gi|29165615|ref|NC_002745.2|	246337 +	U	0	0	0	0	1	SA0207
gi|29165615|ref|NC_002745.2|	246338 +	U	0	0	0	0	1	SA0207
gi|29165615|ref|NC_002745.2|	246341 +	A	0	0	0	1	0	SA0207
gi|29165615|ref|NC_002745.2|	246358 +	A	0	1	0	0	1	SA0207
gi|29165615|ref|NC_002745.2|	246361 +	U	0	0	0	2	0	SA0207
gi|29165615|ref|NC_002745.2|	246364 +	U	0	0	0	0	1	SA0207
gi|29165615|ref|NC_002745.2|	246365 +	C	0	1	0	0	0	SA0207
gi|29165615|ref|NC_002745.2|	246371 +	A	0	0	0	0	1	SA0207
gi|29165615|ref|NC_002745.2|	246378 +	A	0	0	0	1	0	SA0207
gi|29165615|ref|NC_002745.2|	246389 +	A	0	1	0	0	2	SA0207
gi|29165615|ref|NC_002745.2|	246391 +	A	0	0	0	1	0	SA0207
gi|29165615|ref|NC_002745.2|	246393 +	C	0	0	0	1	3	SA0207
gi|29165615|ref|NC_002745.2|	246395 +	A	0	0	0	0	1	SA0207
gi|29165615|ref|NC_002745.2|	246396 +	G	0	1	0	0	0	SA0207
gi|29165615|ref|NC_002745.2|	246400 +	C	0	0	0	0	2	SA0207
gi|29165615|ref|NC_002745.2|	246401 +	G	0	0	0	1	0	SA0207
gi|29165615|ref|NC_002745.2|	246402 +	U	0	0	0	1	0	SA0207
gi|29165615|ref|NC_002745.2|	246404 +	U	0	0	0	0	1	SA0207
gi|29165615|ref|NC_002745.2|	246406 +	G	0	2	0	0	0	SA0207
gi|29165615|ref|NC_002745.2|	246407 +	U	0	1	2	0	0	SA0207
gi|29165615|ref|NC_002745.2|	246408 +	A	0	1	0	0	0	SA0207
gi|29165615|ref|NC_002745.2|	246411 +	G	0	1	0	0	0	SA0207
gi|29165615|ref|NC_002745.2|	246413 +	U	0	1	0	0	0	SA0207
gi|29165615|ref|NC_002745.2|	246417 +	G	0	0	1	1	0	SA0207
gi|29165615|ref|NC_002745.2|	246419 +	A	0	1	0	0	0	SA0207
gi|29165615|ref|NC_002745.2|	246426 +	A	0	1	0	0	2	SA0207
gi|29165615|ref|NC_002745.2|	246428 +	A	0	0	0	1	0	SA0207
gi|29165615|ref|NC_002745.2|	246431 +	A	0	1	0	0	0	SA0207
gi|29165615|ref|NC_002745.2|	246435 +	A	0	1	0	0	0	SA0207
gi|29165615|ref|NC_002745.2|	246445 +	G	0	0	1	0	0	SA0207
gi|29165615|ref|NC_002745.2|	246451 +	C	0	1	0	0	0	SA0207
gi|29165615|ref|NC_002745.2|	246452 +	U	0	0	0	0	1	SA0207
gi|29165615|ref|NC_002745.2|	246461 +	A	0	0	0	0	1	SA0207
gi|29165615|ref|NC_002745.2|	246465 +	C	0	0	1	0	0	SA0207
gi|29165615|ref|NC_002745.2|	246469 +	G	0	0	0	0	1	SA0207
gi|29165615|ref|NC_002745.2|	246472 +	U	0	1	0	0	0	SA0207
gi|29165615|ref|NC_002745.2|	246488 +	A	0	1	0	1	0	SA0207
gi|29165615|ref|NC_002745.2|	246489 +	U	0	1	0	0	0	SA0207
gi|29165615|ref|NC_002745.2|	246491 +	U	0	1	0	0	0	SA0207
gi|29165615|ref|NC_002745.2|	246493 +	A	0	1	0	0	0	SA0207
gi|29165615|ref|NC_002745.2|	246497 +	U	0	0	0	0	1	SA0207
gi|29165615|ref|NC_002745.2|	246498 +	A	0	0	0	0	1	SA0207
gi|29165615|ref|NC_002745.2|	246499 +	G	0	0	0	0	1	SA0207
gi|29165615|ref|NC_002745.2|	246501 +	A	0	0	0	0	1	SA0207
gi|29165615|ref|NC_002745.2|	246502 +	A	0	1	0	0	0	SA0207
gi|29165615|ref|NC_002745.2|	246504 +	C	0	0	0	0	1	SA0207

gi|29165615|ref|NC_002745.2|	246505	+	A	0	0	0	0	1	SA0207
gi|29165615|ref|NC_002745.2|	246506	+	U	0	0	0	1	2	SA0207
gi|29165615|ref|NC_002745.2|	246508	+	A	0	0	0	0	2	SA0207
gi|29165615|ref|NC_002745.2|	246509	+	G	0	0	0	0	1	SA0207
gi|29165615|ref|NC_002745.2|	246517	+	C	0	0	0	1	0	SA0207
gi|29165615|ref|NC_002745.2|	246564	+	U	0	0	0	1	0	SA0207
gi|29165615|ref|NC_002745.2|	246576	+	A	0	0	0	0	1	SA0207
gi|29165615|ref|NC_002745.2|	246583	+	A	0	0	0	2	1	SA0207
gi|29165615|ref|NC_002745.2|	246597	+	G	0	0	0	1	0	SA0207
gi|29165615|ref|NC_002745.2|	246621	+	A	0	0	0	1	0	SA0207
gi|29165615|ref|NC_002745.2|	246622	+	A	0	1	0	0	0	SA0207
gi|29165615|ref|NC_002745.2|	246627	+	A	0	0	0	1	0	SA0207
gi|29165615|ref|NC_002745.2|	246647	+	A	0	0	0	0	1	SA0207
gi|29165615|ref|NC_002745.2|	246650	+	U	1	0	0	0	0	SA0207
gi|29165615|ref|NC_002745.2|	246661	+	C	0	0	0	0	1	SA0207
gi|29165615|ref|NC_002745.2|	246704	+	G	0	1	0	0	0	SA0207
gi|29165615|ref|NC_002745.2|	246713	+	A	0	1	0	0	0	SA0207
gi|29165615|ref|NC_002745.2|	246728	+	A	0	1	0	0	0	SA0207
gi|29165615|ref|NC_002745.2|	246775	+	U	0	1	0	0	0	SA0207
gi|29165615|ref|NC_002745.2|	246784	+	A	0	1	0	0	0	SA0207
gi|29165615|ref|NC_002745.2|	246792	+	A	0	1	0	0	0	SA0207
gi|29165615|ref|NC_002745.2|	246805	+	C	1	1	0	0	0	SA0207
gi|29165615|ref|NC_002745.2|	246806	+	A	0	0	1	0	0	SA0207
gi|29165615|ref|NC_002745.2|	246810	+	A	0	1	0	0	0	SA0207
gi|29165615|ref|NC_002745.2|	246811	+	A	0	4	1	0	3	SA0207
gi|29165615|ref|NC_002745.2|	246816	+	A	0	0	0	5	0	SA0207
gi|29165615|ref|NC_002745.2|	246822	+	G	0	2	0	0	0	SA0207
gi|29165615|ref|NC_002745.2|	246863	+	U	0	0	0	0	1	SA0208
gi|29165615|ref|NC_002745.2|	246934	+	C	0	1	0	0	0	SA0208
gi|29165615|ref|NC_002745.2|	246939	+	U	0	0	0	1	0	SA0208
gi|29165615|ref|NC_002745.2|	246946	+	U	1	56	23	38	119	SA0208
gi|29165615|ref|NC_002745.2|	246947	+	U	0	9	2	4	17	SA0208
gi|29165615|ref|NC_002745.2|	246948	+	U	0	1	0	0	1	SA0208
gi|29165615|ref|NC_002745.2|	246949	+	U	0	1	0	0	0	SA0208
gi|29165615|ref|NC_002745.2|	246950	+	A	0	1	0	0	0	SA0208
gi|29165615|ref|NC_002745.2|	246952	+	U	0	0	0	0	1	SA0208
gi|29165615|ref|NC_002745.2|	246953	+	U	0	0	1	0	0	SA0208
gi|29165615|ref|NC_002745.2|	246954	+	C	0	0	0	0	1	SA0208
gi|29165615|ref|NC_002745.2|	246956	+	G	0	0	0	1	0	SA0208
gi|29165615|ref|NC_002745.2|	246957	+	U	3	106	28	37	190	SA0208
gi|29165615|ref|NC_002745.2|	246958	+	A	0	34	0	11	62	SA0208
gi|29165615|ref|NC_002745.2|	246959	+	U	0	0	0	0	1	SA0208
gi|29165615|ref|NC_002745.2|	246960	+	U	0	1	0	1	2	SA0208
gi|29165615|ref|NC_002745.2|	246961	+	U	0	1	0	0	0	SA0208
gi|29165615|ref|NC_002745.2|	246962	+	U	0	1	0	0	0	SA0208
gi|29165615|ref|NC_002745.2|	246964	+	U	0	0	0	0	1	SA0208
gi|29165615|ref|NC_002745.2|	246965	+	A	0	0	0	0	1	SA0208
gi|29165615|ref|NC_002745.2|	246966	+	G	0	0	0	1	0	SA0208
gi|29165615|ref|NC_002745.2|	246967	+	C	0	1	1	5	2	SA0208
gi|29165615|ref|NC_002745.2|	246968	+	U	0	0	0	0	1	SA0208
gi|29165615|ref|NC_002745.2|	246969	+	U	0	0	0	2	0	SA0208
gi|29165615|ref|NC_002745.2|	246970	+	U	0	1	0	1	1	SA0208
gi|29165615|ref|NC_002745.2|	246972	+	U	0	2	1	1	4	SA0208
gi|29165615|ref|NC_002745.2|	246973	+	A	1	5	0	3	6	SA0208
gi|29165615|ref|NC_002745.2|	246974	+	A	1	6	2	11	10	SA0208
gi|29165615|ref|NC_002745.2|	246975	+	A	0	0	0	0	6	SA0208
gi|29165615|ref|NC_002745.2|	246976	+	U	0	3	0	1	3	SA0208
gi|29165615|ref|NC_002745.2|	246977	+	A	0	1	0	1	3	SA0208
gi|29165615|ref|NC_002745.2|	246978	+	U	0	0	0	0	1	SA0208
gi|29165615|ref|NC_002745.2|	246981	+	G	0	4	4	3	18	SA0208
gi|29165615|ref|NC_002745.2|	246982	+	U	0	0	1	0	1	SA0208
gi|29165615|ref|NC_002745.2|	246990	+	G	0	0	0	0	1	SA0208
gi|29165615|ref|NC_002745.2|	247025	+	G	0	0	0	0	1	SA0208
gi|29165615|ref|NC_002745.2|	247050	+	G	0	0	0	1	0	SA0208
gi|29165615|ref|NC_002745.2|	247052	+	A	0	0	0	1	0	SA0208
gi|29165615|ref|NC_002745.2|	247055	+	A	0	0	0	1	0	SA0208
gi|29165615|ref|NC_002745.2|	247076	+	U	0	0	0	1	0	SA0208
gi|29165615|ref|NC_002745.2|	247077	+	U	0	0	0	1	0	SA0208
gi|29165615|ref|NC_002745.2|	247081	+	A	0	0	0	1	0	SA0208
gi|29165615|ref|NC_002745.2|	247088	+	C	0	0	0	0	1	SA0208
gi|29165615|ref|NC_002745.2|	247091	+	U	0	0	0	0	1	SA0208
gi|29165615|ref|NC_002745.2|	247108	+	A	0	0	0	1	0	SA0208
gi|29165615|ref|NC_002745.2|	247121	+	A	0	0	0	1	0	SA0208
gi|29165615|ref|NC_002745.2|	247145	+	G	0	0	0	0	1	SA0208
gi|29165615|ref|NC_002745.2|	247149	+	A	0	0	0	0	1	SA0208
gi|29165615|ref|NC_002745.2|	247153	+	A	0	1	0	0	0	SA0208
gi|29165615|ref|NC_002745.2|	247160	+	C	0	0	0	0	1	SA0208
gi|29165615|ref|NC_002745.2|	247229	+	U	0	0	0	1	0	SA0208
gi|29165615|ref|NC_002745.2|	247233	+	U	0	0	0	0	1	SA0208
gi|29165615|ref|NC_002745.2|	247248	+	U	0	0	0	0	1	SA0208
gi|29165615|ref|NC_002745.2|	247264	+	U	0	0	0	0	1	SA0208

gi|29165615|ref|NC_002745.2|	247267 +	G	0	0	0	0	1	SA0208
gi|29165615|ref|NC_002745.2|	247274 +	G	0	2	0	0	0	SA0208
gi|29165615|ref|NC_002745.2|	247296 +	U	0	0	0	0	1	SA0208
gi|29165615|ref|NC_002745.2|	247304 +	G	0	0	0	0	1	SA0208
gi|29165615|ref|NC_002745.2|	247312 +	G	0	0	0	1	0	SA0208
gi|29165615|ref|NC_002745.2|	247313 +	A	0	1	0	0	0	SA0208
gi|29165615|ref|NC_002745.2|	247315 +	A	0	0	0	1	0	SA0208
gi|29165615|ref|NC_002745.2|	247316 +	C	0	0	0	0	1	SA0208
gi|29165615|ref|NC_002745.2|	247318 +	C	0	0	1	0	0	SA0208
gi|29165615|ref|NC_002745.2|	247320 +	C	0	0	0	0	6	SA0208
gi|29165615|ref|NC_002745.2|	247321 +	A	0	1	0	0	0	SA0208
gi|29165615|ref|NC_002745.2|	247322 +	U	0	2	0	1	3	SA0208
gi|29165615|ref|NC_002745.2|	247324 +	A	0	3	1	3	2	SA0208
gi|29165615|ref|NC_002745.2|	247325 +	G	1	1	0	1	9	SA0208
gi|29165615|ref|NC_002745.2|	247326 +	A	0	3	2	9	10	SA0208
gi|29165615|ref|NC_002745.2|	247327 +	A	0	0	0	0	1	SA0208
gi|29165615|ref|NC_002745.2|	247330 +	G	0	0	0	7	4	SA0208
gi|29165615|ref|NC_002745.2|	247331 +	G	0	1	0	1	3	SA0208
gi|29165615|ref|NC_002745.2|	247332 +	U	0	2	0	1	8	SA0208
gi|29165615|ref|NC_002745.2|	247333 +	U	0	1	0	0	0	SA0208
gi|29165615|ref|NC_002745.2|	247335 +	G	0	5	0	0	2	SA0208
gi|29165615|ref|NC_002745.2|	247338 +	U	0	0	0	0	1	SA0208
gi|29165615|ref|NC_002745.2|	247341 +	A	0	1	0	0	1	SA0208
gi|29165615|ref|NC_002745.2|	247342 +	U	0	1	0	0	0	SA0208
gi|29165615|ref|NC_002745.2|	247344 +	A	0	1	0	0	1	SA0208
gi|29165615|ref|NC_002745.2|	247349 +	A	0	2	0	1	1	SA0208
gi|29165615|ref|NC_002745.2|	247350 +	A	0	3	0	0	1	SA0208
gi|29165615|ref|NC_002745.2|	247351 +	A	0	3	0	2	4	SA0208
gi|29165615|ref|NC_002745.2|	247354 +	G	0	0	0	0	1	SA0208
gi|29165615|ref|NC_002745.2|	247355 +	U	0	3	0	7	19	SA0208
gi|29165615|ref|NC_002745.2|	247356 +	U	0	0	0	0	2	SA0208
gi|29165615|ref|NC_002745.2|	247357 +	A	0	0	0	0	2	SA0208
gi|29165615|ref|NC_002745.2|	247358 +	U	0	1	0	0	3	SA0208
gi|29165615|ref|NC_002745.2|	247359 +	U	0	0	0	1	0	SA0208
gi|29165615|ref|NC_002745.2|	247361 +	A	0	2	0	0	0	SA0208
gi|29165615|ref|NC_002745.2|	247363 +	A	0	0	0	0	2	SA0208
gi|29165615|ref|NC_002745.2|	247364 +	A	0	4	0	0	3	SA0208
gi|29165615|ref|NC_002745.2|	247365 +	U	0	0	0	0	2	SA0208
gi|29165615|ref|NC_002745.2|	247375 +	G	0	0	0	0	1	SA0208
gi|29165615|ref|NC_002745.2|	247381 +	A	0	0	0	0	2	SA0208
gi|29165615|ref|NC_002745.2|	247390 +	C	0	0	0	2	0	SA0208
gi|29165615|ref|NC_002745.2|	247394 +	G	0	0	0	0	1	SA0208
gi|29165615|ref|NC_002745.2|	247395 +	U	0	0	0	0	1	SA0208
gi|29165615|ref|NC_002745.2|	247398 +	U	0	0	0	0	1	SA0208
gi|29165615|ref|NC_002745.2|	247399 +	U	0	1	0	0	0	SA0208
gi|29165615|ref|NC_002745.2|	247402 +	U	0	1	0	0	0	SA0208
gi|29165615|ref|NC_002745.2|	247405 +	G	0	0	0	0	1	SA0208
gi|29165615|ref|NC_002745.2|	247407 +	C	0	0	0	0	1	SA0208
gi|29165615|ref|NC_002745.2|	247437 +	U	0	1	0	0	0	SA0208
gi|29165615|ref|NC_002745.2|	247439 +	C	0	0	0	0	1	SA0208
gi|29165615|ref|NC_002745.2|	247446 +	C	0	1	0	1	2	SA0208
gi|29165615|ref|NC_002745.2|	247463 +	G	0	1	0	0	0	SA0208
gi|29165615|ref|NC_002745.2|	247467 +	U	0	0	0	0	1	SA0208
gi|29165615|ref|NC_002745.2|	247473 +	U	0	0	0	0	1	SA0208
gi|29165615|ref|NC_002745.2|	247475 +	A	0	0	0	0	1	SA0208
gi|29165615|ref|NC_002745.2|	247479 +	A	0	1	0	0	0	SA0208
gi|29165615|ref|NC_002745.2|	247480 +	C	0	0	0	0	1	SA0208
gi|29165615|ref|NC_002745.2|	247481 +	C	0	0	0	0	1	SA0208
gi|29165615|ref|NC_002745.2|	247495 +	U	0	0	0	0	1	SA0208
gi|29165615|ref|NC_002745.2|	247499 +	A	0	1	0	0	2	SA0208
gi|29165615|ref|NC_002745.2|	247502 +	U	0	1	0	0	0	SA0208
gi|29165615|ref|NC_002745.2|	247504 +	U	0	0	0	0	1	SA0208
gi|29165615|ref|NC_002745.2|	247544 +	U	0	0	0	0	1	SA0208
gi|29165615|ref|NC_002745.2|	247545 +	U	0	0	0	0	2	SA0208
gi|29165615|ref|NC_002745.2|	247558 +	A	0	0	0	1	0	SA0208
gi|29165615|ref|NC_002745.2|	247559 +	A	0	0	1	0	1	SA0208
gi|29165615|ref|NC_002745.2|	247560 +	U	0	1	0	1	0	SA0208
gi|29165615|ref|NC_002745.2|	247562 +	U	0	0	0	0	1	SA0208
gi|29165615|ref|NC_002745.2|	247571 +	U	0	0	0	0	1	SA0208
gi|29165615|ref|NC_002745.2|	247578 +	A	0	0	0	1	3	SA0208
gi|29165615|ref|NC_002745.2|	247584 +	A	0	0	0	0	1	SA0208
gi|29165615|ref|NC_002745.2|	247591 +	U	0	0	0	0	1	SA0208
gi|29165615|ref|NC_002745.2|	247596 +	U	0	0	0	0	1	SA0208
gi|29165615|ref|NC_002745.2|	247604 +	G	0	0	0	0	1	SA0208
gi|29165615|ref|NC_002745.2|	247605 +	A	0	0	0	0	1	SA0208
gi|29165615|ref|NC_002745.2|	247606 +	U	0	0	1	0	0	SA0208
gi|29165615|ref|NC_002745.2|	247609 +	U	0	0	1	0	0	SA0208
gi|29165615|ref|NC_002745.2|	247613 +	C	0	0	0	0	1	SA0208
gi|29165615|ref|NC_002745.2|	247622 +	U	0	0	1	0	0	SA0208
gi|29165615|ref|NC_002745.2|	247638 +	C	0	0	2	0	0	SA0208
gi|29165615|ref|NC_002745.2|	247646 +	A	0	0	0	0	1	SA0208

gi|29165615|ref|NC_002745.2|	247649 +	G	0	0	0	0	1	SA0208
gi|29165615|ref|NC_002745.2|	247658 +	U	0	0	0	0	1	SA0208
gi|29165615|ref|NC_002745.2|	247661 +	G	0	1	0	0	0	SA0208
gi|29165615|ref|NC_002745.2|	247663 +	A	0	0	0	0	2	SA0208
gi|29165615|ref|NC_002745.2|	247676 +	A	0	1	1	0	0	SA0208
gi|29165615|ref|NC_002745.2|	247679 +	G	0	1	0	0	0	SA0208
gi|29165615|ref|NC_002745.2|	247697 +	G	0	0	0	0	1	SA0208
gi|29165615|ref|NC_002745.2|	247702 +	C	0	2	0	0	0	SA0208
gi|29165615|ref|NC_002745.2|	247767 +	C	0	0	0	1	0	SA0208
gi|29165615|ref|NC_002745.2|	247770 +	A	0	0	0	1	0	SA0208
gi|29165615|ref|NC_002745.2|	247812 +	C	0	0	0	0	2	SA0208
gi|29165615|ref|NC_002745.2|	247852 +	U	0	0	0	0	1	SA0208
gi|29165615|ref|NC_002745.2|	247856 +	C	0	0	0	0	1	SA0208
gi|29165615|ref|NC_002745.2|	247857 +	A	0	0	0	0	1	SA0208
gi|29165615|ref|NC_002745.2|	247863 +	C	0	0	1	0	0	SA0208
gi|29165615|ref|NC_002745.2|	247872 +	U	0	0	0	0	1	SA0208
gi|29165615|ref|NC_002745.2|	247874 +	A	0	0	0	0	1	SA0208
gi|29165615|ref|NC_002745.2|	247875 +	A	0	0	0	1	4	SA0208
gi|29165615|ref|NC_002745.2|	247878 +	A	0	1	0	1	0	SA0208
gi|29165615|ref|NC_002745.2|	247881 +	U	0	2	0	0	4	SA0208
gi|29165615|ref|NC_002745.2|	247882 +	U	0	0	0	0	2	SA0208
gi|29165615|ref|NC_002745.2|	247883 +	A	0	3	1	1	4	SA0208
gi|29165615|ref|NC_002745.2|	247884 +	A	0	2	1	0	2	SA0208
gi|29165615|ref|NC_002745.2|	247885 +	U	0	1	0	0	1	SA0208
gi|29165615|ref|NC_002745.2|	247887 +	U	0	0	0	0	1	SA0208
gi|29165615|ref|NC_002745.2|	247888 +	U	0	0	0	0	1	SA0208
gi|29165615|ref|NC_002745.2|	247890 +	U	0	0	1	0	1	SA0208
gi|29165615|ref|NC_002745.2|	247891 +	U	0	0	0	1	0	SA0208
gi|29165615|ref|NC_002745.2|	247894 +	U	0	0	0	0	2	SA0208
gi|29165615|ref|NC_002745.2|	247896 +	U	0	1	0	0	1	SA0208
gi|29165615|ref|NC_002745.2|	247898 +	U	0	0	0	0	1	SA0208
gi|29165615|ref|NC_002745.2|	247899 +	U	0	0	0	0	1	SA0208
gi|29165615|ref|NC_002745.2|	247900 +	U	0	0	0	0	2	SA0208
gi|29165615|ref|NC_002745.2|	247901 +	A	0	1	1	0	6	SA0208
gi|29165615|ref|NC_002745.2|	247902 +	A	0	0	0	0	1	SA0208
gi|29165615|ref|NC_002745.2|	247904 +	A	0	0	0	0	1	SA0208
gi|29165615|ref|NC_002745.2|	247905 +	A	0	0	1	0	0	SA0208
gi|29165615|ref|NC_002745.2|	247908 +	G	0	0	0	1	0	SA0208
gi|29165615|ref|NC_002745.2|	247914 +	C	0	1	0	0	0	SA0208
gi|29165615|ref|NC_002745.2|	247933 +	U	0	1	0	0	0	SA0208
gi|29165615|ref|NC_002745.2|	247941 +	G	0	0	0	0	1	SA0208
gi|29165615|ref|NC_002745.2|	247951 +	C	0	0	0	1	1	SA0208
gi|29165615|ref|NC_002745.2|	247966 +	G	0	0	0	0	1	SA0208
gi|29165615|ref|NC_002745.2|	247976 +	A	0	0	0	0	2	SA0208
gi|29165615|ref|NC_002745.2|	247977 +	C	0	0	0	0	2	SA0208
gi|29165615|ref|NC_002745.2|	247979 +	U	0	0	0	0	1	SA0208
gi|29165615|ref|NC_002745.2|	247982 +	G	0	0	0	0	2	SA0208
gi|29165615|ref|NC_002745.2|	247983 +	A	0	0	0	0	3	SA0208
gi|29165615|ref|NC_002745.2|	247989 +	A	0	0	1	0	1	SA0208
gi|29165615|ref|NC_002745.2|	247990 +	C	0	0	0	0	1	SA0208
gi|29165615|ref|NC_002745.2|	247992 +	A	0	0	0	0	4	SA0208
gi|29165615|ref|NC_002745.2|	247995 +	U	0	0	0	2	0	SA0208
gi|29165615|ref|NC_002745.2|	247998 +	A	0	1	0	0	0	SA0208
gi|29165615|ref|NC_002745.2|	248006 +	G	0	0	0	0	1	SA0208
gi|29165615|ref|NC_002745.2|	248008 +	A	0	0	0	0	2	SA0208
gi|29165615|ref|NC_002745.2|	248009 +	G	0	0	0	0	1	SA0208
gi|29165615|ref|NC_002745.2|	248011 +	U	0	0	0	0	1	SA0208
gi|29165615|ref|NC_002745.2|	248022 +	U	0	0	0	0	1	SA0208
gi|29165615|ref|NC_002745.2|	248028 +	G	0	2	0	1	5	SA0208
gi|29165615|ref|NC_002745.2|	248029 +	A	0	0	0	0	1	SA0208
gi|29165615|ref|NC_002745.2|	248030 +	U	0	2	0	0	0	SA0208
gi|29165615|ref|NC_002745.2|	248120 +	C	0	0	0	1	0	SA0209
gi|29165615|ref|NC_002745.2|	248133 +	A	0	1	0	0	0	SA0209
gi|29165615|ref|NC_002745.2|	248138 +	U	0	0	0	2	0	SA0209
gi|29165615|ref|NC_002745.2|	248139 +	A	0	0	0	1	0	SA0209
gi|29165615|ref|NC_002745.2|	248147 +	U	0	0	0	1	0	SA0209
gi|29165615|ref|NC_002745.2|	248148 +	U	0	1	0	0	1	SA0209
gi|29165615|ref|NC_002745.2|	248206 +	C	0	0	0	1	0	SA0209
gi|29165615|ref|NC_002745.2|	248224 +	A	0	0	0	1	0	SA0209
gi|29165615|ref|NC_002745.2|	248267 +	A	0	0	0	0	2	SA0209
gi|29165615|ref|NC_002745.2|	248271 +	U	0	0	0	0	1	SA0209
gi|29165615|ref|NC_002745.2|	248275 +	C	0	2	0	0	0	SA0209
gi|29165615|ref|NC_002745.2|	248290 +	A	0	0	0	1	0	SA0209
gi|29165615|ref|NC_002745.2|	248297 +	U	0	1	0	0	1	SA0209
gi|29165615|ref|NC_002745.2|	248317 +	A	0	1	0	0	0	SA0209
gi|29165615|ref|NC_002745.2|	248319 +	A	0	0	0	0	1	SA0209
gi|29165615|ref|NC_002745.2|	248328 +	C	0	0	0	0	1	SA0209
gi|29165615|ref|NC_002745.2|	248346 +	A	0	2	0	0	0	SA0209
gi|29165615|ref|NC_002745.2|	248397 +	A	0	0	0	0	1	SA0209
gi|29165615|ref|NC_002745.2|	248399 +	A	0	1	0	0	0	SA0209
gi|29165615|ref|NC_002745.2|	248420 +	A	0	0	0	1	0	SA0209

gi|29165615|ref|NC_002745.2|	248481	+	A	0	1	0	0	0	SA0209
gi|29165615|ref|NC_002745.2|	248502	+	A	0	0	0	0	1	SA0209
gi|29165615|ref|NC_002745.2|	248510	+	A	0	0	0	1	0	SA0209
gi|29165615|ref|NC_002745.2|	248542	+	A	0	0	0	0	1	SA0209
gi|29165615|ref|NC_002745.2|	248601	+	C	0	0	0	0	1	SA0209
gi|29165615|ref|NC_002745.2|	248609	+	A	0	0	0	0	1	SA0209
gi|29165615|ref|NC_002745.2|	248617	+	A	0	0	0	0	1	SA0209
gi|29165615|ref|NC_002745.2|	248619	+	U	0	1	0	0	0	SA0209
gi|29165615|ref|NC_002745.2|	248620	+	C	0	0	0	2	0	SA0209
gi|29165615|ref|NC_002745.2|	248623	+	C	0	0	0	0	1	SA0209
gi|29165615|ref|NC_002745.2|	248628	+	A	0	1	0	0	0	SA0209
gi|29165615|ref|NC_002745.2|	248637	+	G	0	0	0	1	0	SA0209
gi|29165615|ref|NC_002745.2|	248683	+	A	0	1	0	0	0	SA0209
gi|29165615|ref|NC_002745.2|	248695	+	C	0	1	0	0	0	SA0209
gi|29165615|ref|NC_002745.2|	248734	+	A	1	0	0	0	3	SA0209
gi|29165615|ref|NC_002745.2|	248749	+	A	0	0	0	0	1	SA0209
gi|29165615|ref|NC_002745.2|	248751	+	A	0	0	0	0	1	SA0209
gi|29165615|ref|NC_002745.2|	248756	+	A	0	1	0	0	0	SA0209
gi|29165615|ref|NC_002745.2|	248774	+	A	0	1	0	1	3	SA0209
gi|29165615|ref|NC_002745.2|	248777	+	C	0	0	0	0	1	SA0209
gi|29165615|ref|NC_002745.2|	248778	+	A	0	1	0	0	0	SA0209
gi|29165615|ref|NC_002745.2|	248779	+	C	0	0	0	1	0	SA0209
gi|29165615|ref|NC_002745.2|	248802	+	U	0	0	0	0	1	SA0209
gi|29165615|ref|NC_002745.2|	248804	+	U	0	0	0	0	1	SA0209
gi|29165615|ref|NC_002745.2|	248805	+	A	0	0	0	1	0	SA0209
gi|29165615|ref|NC_002745.2|	248806	+	U	0	0	0	1	0	SA0209
gi|29165615|ref|NC_002745.2|	248807	+	U	0	0	0	1	0	SA0209
gi|29165615|ref|NC_002745.2|	248809	+	A	0	0	0	0	1	SA0209
gi|29165615|ref|NC_002745.2|	248812	+	A	0	0	0	1	0	SA0209
gi|29165615|ref|NC_002745.2|	248813	+	U	0	0	0	0	1	SA0209
gi|29165615|ref|NC_002745.2|	248821	+	C	0	0	0	2	0	SA0209
gi|29165615|ref|NC_002745.2|	248822	+	A	0	0	0	0	1	SA0209
gi|29165615|ref|NC_002745.2|	248823	+	A	0	0	0	1	0	SA0209
gi|29165615|ref|NC_002745.2|	248826	+	A	0	0	1	0	0	SA0209
gi|29165615|ref|NC_002745.2|	248827	+	A	0	0	0	0	4	SA0209
gi|29165615|ref|NC_002745.2|	248828	+	U	0	1	0	0	0	SA0209
gi|29165615|ref|NC_002745.2|	248831	+	C	0	0	0	1	2	SA0209
gi|29165615|ref|NC_002745.2|	248851	+	C	0	0	0	0	1	SA0209
gi|29165615|ref|NC_002745.2|	248863	+	C	0	0	0	0	1	SA0209
gi|29165615|ref|NC_002745.2|	248867	+	A	0	0	0	0	1	SA0209
gi|29165615|ref|NC_002745.2|	248872	+	U	0	0	0	0	1	SA0209
gi|29165615|ref|NC_002745.2|	248875	+	C	0	0	0	0	1	SA0209
gi|29165615|ref|NC_002745.2|	248891	+	C	0	0	0	0	1	SA0209
gi|29165615|ref|NC_002745.2|	248892	+	U	0	1	0	0	0	SA0209
gi|29165615|ref|NC_002745.2|	248896	+	A	0	0	0	0	4	SA0209
gi|29165615|ref|NC_002745.2|	248900	+	C	0	1	0	0	0	SA0209
gi|29165615|ref|NC_002745.2|	248902	+	A	0	2	0	0	0	SA0209
gi|29165615|ref|NC_002745.2|	248905	+	U	0	0	0	0	1	SA0209
gi|29165615|ref|NC_002745.2|	248907	+	G	0	0	0	0	1	SA0209
gi|29165615|ref|NC_002745.2|	248910	+	U	0	0	0	0	1	SA0209
gi|29165615|ref|NC_002745.2|	248914	+	G	0	0	0	0	1	SA0209
gi|29165615|ref|NC_002745.2|	248915	+	U	0	1	0	0	0	SA0209
gi|29165615|ref|NC_002745.2|	248916	+	U	0	1	0	1	0	SA0209
gi|29165615|ref|NC_002745.2|	248919	+	A	0	0	0	3	1	SA0209
gi|29165615|ref|NC_002745.2|	248921	+	A	0	0	0	0	1	SA0209
gi|29165615|ref|NC_002745.2|	248922	+	A	0	0	2	0	0	SA0209
gi|29165615|ref|NC_002745.2|	248923	+	C	0	0	0	0	1	SA0209
gi|29165615|ref|NC_002745.2|	248926	+	G	0	0	0	1	0	SA0209
gi|29165615|ref|NC_002745.2|	248928	+	G	0	0	0	3	0	SA0209
gi|29165615|ref|NC_002745.2|	248929	+	C	0	1	0	3	0	SA0209
gi|29165615|ref|NC_002745.2|	248930	+	G	0	0	0	1	0	SA0209
gi|29165615|ref|NC_002745.2|	248931	+	A	0	1	0	3	1	SA0209
gi|29165615|ref|NC_002745.2|	248932	+	C	0	0	0	1	0	SA0209
gi|29165615|ref|NC_002745.2|	248941	+	A	0	2	0	0	0	SA0209
gi|29165615|ref|NC_002745.2|	248942	+	G	0	1	0	1	0	SA0209
gi|29165615|ref|NC_002745.2|	248943	+	U	0	0	0	5	8	-
gi|29165615|ref|NC_002745.2|	248944	+	U	0	0	0	1	0	-
gi|29165615|ref|NC_002745.2|	248945	+	U	0	0	0	1	1	-
gi|29165615|ref|NC_002745.2|	248946	+	G	0	2	0	6	13	-
gi|29165615|ref|NC_002745.2|	248947	+	A	0	2	1	9	23	-
gi|29165615|ref|NC_002745.2|	248948	+	A	0	0	1	1	3	-
gi|29165615|ref|NC_002745.2|	248949	+	A	0	2	0	0	1	-
gi|29165615|ref|NC_002745.2|	248950	+	U	0	0	0	1	0	-
gi|29165615|ref|NC_002745.2|	248966	+	A	0	2	0	0	0	-
gi|29165615|ref|NC_002745.2|	248970	+	A	0	0	0	0	1	-
gi|29165615|ref|NC_002745.2|	248972	+	A	0	1	0	1	0	-
gi|29165615|ref|NC_002745.2|	248975	+	G	0	1	0	0	1	-
gi|29165615|ref|NC_002745.2|	248981	+	C	0	1	0	1	0	-
gi|29165615|ref|NC_002745.2|	248982	+	U	0	0	1	0	0	-
gi|29165615|ref|NC_002745.2|	248984	+	A	0	0	0	0	1	-
gi|29165615|ref|NC_002745.2|	248988	+	C	0	0	0	1	0	-

gi|29165615|ref|NC_002745.2|	248989 +	G	0	0	0	0	1	-
gi|29165615|ref|NC_002745.2|	248990 +	A	0	0	0	1	0	-
gi|29165615|ref|NC_002745.2|	248993 +	A	0	1	0	6	2	-
gi|29165615|ref|NC_002745.2|	248994 +	A	0	1	0	1	1	-
gi|29165615|ref|NC_002745.2|	248995 +	G	0	0	0	0	1	-
gi|29165615|ref|NC_002745.2|	248996 +	A	0	0	0	0	1	-
gi|29165615|ref|NC_002745.2|	248997 +	U	0	0	0	1	0	-
gi|29165615|ref|NC_002745.2|	248998 +	U	0	0	0	0	1	-
gi|29165615|ref|NC_002745.2|	248999 +	A	0	0	0	1	4	-
gi|29165615|ref|NC_002745.2|	249000 +	A	0	0	0	0	4	-
gi|29165615|ref|NC_002745.2|	249001 +	A	0	0	0	1	0	-
gi|29165615|ref|NC_002745.2|	249002 +	A	0	0	0	1	0	-
gi|29165615|ref|NC_002745.2|	249007 +	G	0	0	1	1	3	-
gi|29165615|ref|NC_002745.2|	249009 +	C	0	0	0	1	0	-
gi|29165615|ref|NC_002745.2|	249011 +	U	0	0	0	1	0	-
gi|29165615|ref|NC_002745.2|	249016 +	A	0	0	0	0	1	SA0210
gi|29165615|ref|NC_002745.2|	249019 +	A	0	0	0	0	2	SA0210
gi|29165615|ref|NC_002745.2|	249020 +	C	0	0	0	0	1	SA0210
gi|29165615|ref|NC_002745.2|	249021 +	G	0	0	0	0	1	SA0210
gi|29165615|ref|NC_002745.2|	249022 +	A	0	0	0	0	1	SA0210
gi|29165615|ref|NC_002745.2|	249024 +	U	0	1	0	0	0	SA0210
gi|29165615|ref|NC_002745.2|	249029 +	U	0	0	0	1	0	SA0210
gi|29165615|ref|NC_002745.2|	249032 +	G	0	2	0	0	0	SA0210
gi|29165615|ref|NC_002745.2|	249033 +	A	0	2	0	0	0	SA0210
gi|29165615|ref|NC_002745.2|	249034 +	A	0	4	0	0	1	SA0210
gi|29165615|ref|NC_002745.2|	249036 +	C	0	0	1	0	0	SA0210
gi|29165615|ref|NC_002745.2|	249052 +	A	0	1	0	0	1	SA0210
gi|29165615|ref|NC_002745.2|	249060 +	U	0	1	0	0	1	SA0210
gi|29165615|ref|NC_002745.2|	249064 +	A	0	1	0	0	2	SA0210
gi|29165615|ref|NC_002745.2|	249087 +	A	0	1	0	0	0	SA0210
gi|29165615|ref|NC_002745.2|	249093 +	A	0	0	0	0	1	SA0210
gi|29165615|ref|NC_002745.2|	249094 +	A	0	1	0	0	0	SA0210
gi|29165615|ref|NC_002745.2|	249095 +	A	0	1	0	0	0	SA0210
gi|29165615|ref|NC_002745.2|	249126 +	C	0	0	1	0	0	SA0210
gi|29165615|ref|NC_002745.2|	249136 +	G	1	1	0	2	3	SA0210
gi|29165615|ref|NC_002745.2|	249148 +	G	0	0	0	0	1	SA0210
gi|29165615|ref|NC_002745.2|	249168 +	C	0	0	0	1	0	SA0210
gi|29165615|ref|NC_002745.2|	249257 +	G	0	0	0	1	0	SA0210
gi|29165615|ref|NC_002745.2|	249260 +	A	0	0	0	0	1	SA0210
gi|29165615|ref|NC_002745.2|	249265 +	A	0	0	0	1	0	SA0210
gi|29165615|ref|NC_002745.2|	249268 +	G	0	0	0	0	1	SA0210
gi|29165615|ref|NC_002745.2|	249272 +	C	0	0	0	0	1	SA0210
gi|29165615|ref|NC_002745.2|	249298 +	A	0	0	0	0	1	SA0210
gi|29165615|ref|NC_002745.2|	249326 +	C	0	0	0	0	2	SA0210
gi|29165615|ref|NC_002745.2|	249331 +	G	0	1	0	0	0	SA0210
gi|29165615|ref|NC_002745.2|	249339 +	A	0	0	0	0	1	SA0210
gi|29165615|ref|NC_002745.2|	249340 +	A	0	0	0	0	1	SA0210
gi|29165615|ref|NC_002745.2|	249343 +	A	0	1	0	1	2	SA0210
gi|29165615|ref|NC_002745.2|	249345 +	G	0	0	0	0	1	SA0210
gi|29165615|ref|NC_002745.2|	249352 +	A	0	0	0	1	0	SA0210
gi|29165615|ref|NC_002745.2|	249356 +	C	0	0	0	0	1	SA0210
gi|29165615|ref|NC_002745.2|	249358 +	A	0	0	0	0	1	SA0210
gi|29165615|ref|NC_002745.2|	249363 +	A	0	0	0	0	1	SA0210
gi|29165615|ref|NC_002745.2|	249365 +	C	0	0	0	0	1	SA0210
gi|29165615|ref|NC_002745.2|	249373 +	A	0	0	0	0	1	SA0210
gi|29165615|ref|NC_002745.2|	249382 +	A	0	3	0	0	0	SA0210
gi|29165615|ref|NC_002745.2|	249386 +	G	0	0	0	1	0	SA0210
gi|29165615|ref|NC_002745.2|	249391 +	C	0	0	0	2	0	SA0210
gi|29165615|ref|NC_002745.2|	249395 +	A	0	1	0	0	0	SA0210
gi|29165615|ref|NC_002745.2|	249400 +	U	0	0	0	0	1	SA0210
gi|29165615|ref|NC_002745.2|	249406 +	C	0	1	0	0	0	SA0210
gi|29165615|ref|NC_002745.2|	249423 +	A	0	0	0	1	0	SA0210
gi|29165615|ref|NC_002745.2|	249427 +	C	0	1	0	0	0	SA0210
gi|29165615|ref|NC_002745.2|	249429 +	A	0	0	0	1	0	SA0210
gi|29165615|ref|NC_002745.2|	249440 +	G	0	0	0	0	1	SA0210
gi|29165615|ref|NC_002745.2|	249441 +	U	0	0	0	0	1	SA0210
gi|29165615|ref|NC_002745.2|	249453 +	A	0	0	0	0	1	SA0210
gi|29165615|ref|NC_002745.2|	249485 +	G	1	0	0	0	0	SA0210
gi|29165615|ref|NC_002745.2|	249499 +	C	0	1	0	0	0	SA0210
gi|29165615|ref|NC_002745.2|	249502 +	A	0	1	0	0	0	SA0210
gi|29165615|ref|NC_002745.2|	249504 +	A	0	0	0	1	0	SA0210
gi|29165615|ref|NC_002745.2|	249511 +	G	0	0	0	1	0	SA0210
gi|29165615|ref|NC_002745.2|	249545 +	C	0	0	0	0	1	SA0210
gi|29165615|ref|NC_002745.2|	249548 +	U	0	0	0	0	1	SA0210
gi|29165615|ref|NC_002745.2|	249549 +	A	0	0	0	0	1	SA0210
gi|29165615|ref|NC_002745.2|	249553 +	G	0	0	0	0	1	SA0210
gi|29165615|ref|NC_002745.2|	249558 +	U	0	0	0	0	1	SA0210
gi|29165615|ref|NC_002745.2|	249568 +	G	0	0	0	0	1	SA0210
gi|29165615|ref|NC_002745.2|	249569 +	C	0	0	0	0	1	SA0210
gi|29165615|ref|NC_002745.2|	249571 +	U	0	0	0	1	0	SA0210
gi|29165615|ref|NC_002745.2|	249581 +	C	0	0	0	0	2	SA0210

gi|29165615|ref|NC_002745.2|	249582 +	G	0	0	0	0	1	SA0210
gi|29165615|ref|NC_002745.2|	249591 +	G	0	1	0	0	0	SA0210
gi|29165615|ref|NC_002745.2|	249600 +	U	0	0	0	0	1	SA0210
gi|29165615|ref|NC_002745.2|	249601 +	U	0	0	0	1	1	SA0210
gi|29165615|ref|NC_002745.2|	249606 +	A	0	0	0	1	0	SA0210
gi|29165615|ref|NC_002745.2|	249609 +	A	0	1	0	0	1	SA0210
gi|29165615|ref|NC_002745.2|	249610 +	G	0	1	0	1	0	SA0210
gi|29165615|ref|NC_002745.2|	249613 +	U	0	0	1	1	0	SA0210
gi|29165615|ref|NC_002745.2|	249618 +	G	0	0	0	0	1	SA0210
gi|29165615|ref|NC_002745.2|	249625 +	U	2	0	0	0	0	SA0210
gi|29165615|ref|NC_002745.2|	249637 +	A	0	0	0	1	1	SA0210
gi|29165615|ref|NC_002745.2|	249641 +	U	0	0	0	0	1	SA0210
gi|29165615|ref|NC_002745.2|	249643 +	A	0	0	0	1	0	SA0210
gi|29165615|ref|NC_002745.2|	249644 +	A	0	2	0	0	3	SA0210
gi|29165615|ref|NC_002745.2|	249646 +	A	0	0	0	2	0	SA0210
gi|29165615|ref|NC_002745.2|	249647 +	A	0	0	0	0	1	SA0210
gi|29165615|ref|NC_002745.2|	249649 +	C	0	0	0	1	0	SA0210
gi|29165615|ref|NC_002745.2|	249652 +	C	0	0	0	1	0	SA0210
gi|29165615|ref|NC_002745.2|	249654 +	U	0	0	0	1	0	SA0210
gi|29165615|ref|NC_002745.2|	249659 +	C	0	1	0	0	1	SA0210
gi|29165615|ref|NC_002745.2|	249661 +	G	0	0	0	0	1	SA0210
gi|29165615|ref|NC_002745.2|	249663 +	A	0	0	0	1	0	SA0210
gi|29165615|ref|NC_002745.2|	249677 +	C	0	0	0	0	1	SA0210
gi|29165615|ref|NC_002745.2|	249684 +	U	0	0	0	0	1	SA0210
gi|29165615|ref|NC_002745.2|	249685 +	C	0	2	0	0	0	SA0210
gi|29165615|ref|NC_002745.2|	249690 +	U	0	1	0	0	0	SA0210
gi|29165615|ref|NC_002745.2|	249692 +	A	0	1	0	2	1	SA0210
gi|29165615|ref|NC_002745.2|	249694 +	U	0	0	0	0	1	SA0210
gi|29165615|ref|NC_002745.2|	249695 +	U	0	0	0	0	2	SA0210
gi|29165615|ref|NC_002745.2|	249697 +	A	0	0	1	0	0	SA0210
gi|29165615|ref|NC_002745.2|	249698 +	C	0	0	0	2	2	SA0210
gi|29165615|ref|NC_002745.2|	249699 +	A	0	0	0	1	0	SA0210
gi|29165615|ref|NC_002745.2|	249701 +	U	0	0	1	0	0	SA0210
gi|29165615|ref|NC_002745.2|	249702 +	U	0	0	0	0	2	SA0210
gi|29165615|ref|NC_002745.2|	249704 +	A	0	0	0	1	0	SA0210
gi|29165615|ref|NC_002745.2|	249709 +	U	0	0	0	1	0	SA0210
gi|29165615|ref|NC_002745.2|	249712 +	G	0	0	0	0	1	SA0210
gi|29165615|ref|NC_002745.2|	249719 +	G	0	2	0	0	0	SA0210
gi|29165615|ref|NC_002745.2|	249730 +	A	0	2	0	0	0	SA0210
gi|29165615|ref|NC_002745.2|	249734 +	A	0	0	0	0	2	SA0210
gi|29165615|ref|NC_002745.2|	249738 +	C	0	1	0	0	0	SA0210
gi|29165615|ref|NC_002745.2|	249748 +	A	0	0	0	0	1	SA0210
gi|29165615|ref|NC_002745.2|	249750 +	C	0	0	0	0	1	SA0210
gi|29165615|ref|NC_002745.2|	249753 +	U	0	0	0	0	2	SA0210
gi|29165615|ref|NC_002745.2|	249759 +	A	0	0	0	0	1	SA0210
gi|29165615|ref|NC_002745.2|	249765 +	U	0	1	0	0	0	SA0210
gi|29165615|ref|NC_002745.2|	249778 +	U	0	0	0	0	2	SA0210
gi|29165615|ref|NC_002745.2|	249781 +	U	0	0	0	1	0	SA0210
gi|29165615|ref|NC_002745.2|	249783 +	A	0	1	0	0	0	SA0210
gi|29165615|ref|NC_002745.2|	249784 +	G	0	0	0	0	2	SA0210
gi|29165615|ref|NC_002745.2|	249785 +	A	0	0	1	0	0	SA0210
gi|29165615|ref|NC_002745.2|	249789 +	G	0	0	1	0	0	SA0210
gi|29165615|ref|NC_002745.2|	249794 +	A	1	0	0	0	1	SA0210
gi|29165615|ref|NC_002745.2|	249795 +	G	0	0	0	0	1	SA0210
gi|29165615|ref|NC_002745.2|	249797 +	C	0	0	0	0	1	SA0210
gi|29165615|ref|NC_002745.2|	249800 +	A	0	0	1	0	1	SA0210
gi|29165615|ref|NC_002745.2|	249803 +	G	0	1	0	0	0	SA0210
gi|29165615|ref|NC_002745.2|	249805 +	U	0	0	0	0	1	SA0210
gi|29165615|ref|NC_002745.2|	249808 +	U	0	0	0	0	2	SA0210
gi|29165615|ref|NC_002745.2|	249809 +	U	0	1	0	0	1	SA0210
gi|29165615|ref|NC_002745.2|	249810 +	A	0	0	0	1	0	SA0210
gi|29165615|ref|NC_002745.2|	249812 +	C	0	0	0	0	4	SA0210
gi|29165615|ref|NC_002745.2|	249813 +	A	1	1	0	0	0	SA0210
gi|29165615|ref|NC_002745.2|	249815 +	G	0	1	0	0	0	SA0210
gi|29165615|ref|NC_002745.2|	249816 +	A	0	2	0	0	3	SA0210
gi|29165615|ref|NC_002745.2|	249820 +	A	0	0	0	0	1	SA0210
gi|29165615|ref|NC_002745.2|	249821 +	A	0	0	0	0	1	SA0210
gi|29165615|ref|NC_002745.2|	249825 +	A	0	0	0	0	1	SA0210
gi|29165615|ref|NC_002745.2|	249830 +	C	0	0	0	1	0	SA0210
gi|29165615|ref|NC_002745.2|	249834 +	U	0	0	0	2	0	SA0210
gi|29165615|ref|NC_002745.2|	249840 +	A	0	0	0	0	1	SA0210
gi|29165615|ref|NC_002745.2|	249863 +	A	0	0	0	0	1	SA0210
gi|29165615|ref|NC_002745.2|	249869 +	U	0	0	0	0	1	SA0210
gi|29165615|ref|NC_002745.2|	249870 +	G	0	11	0	1	4	SA0210
gi|29165615|ref|NC_002745.2|	249871 +	G	0	0	0	1	1	SA0210
gi|29165615|ref|NC_002745.2|	249885 +	C	0	0	0	0	1	SA0210
gi|29165615|ref|NC_002745.2|	249887 +	A	0	0	0	0	1	SA0210
gi|29165615|ref|NC_002745.2|	249918 +	C	0	0	0	0	1	SA0210
gi|29165615|ref|NC_002745.2|	249929 +	G	0	0	0	0	1	SA0210
gi|29165615|ref|NC_002745.2|	249931 +	A	0	0	0	1	0	SA0210
gi|29165615|ref|NC_002745.2|	249936 +	A	0	0	0	0	1	SA0210

gi|29165615|ref|NC_002745.2|	249950 +	A	0	0	0	0	1	SA0210
gi|29165615|ref|NC_002745.2|	249952 +	G	0	0	0	0	2	SA0210
gi|29165615|ref|NC_002745.2|	249981 +	U	0	0	0	0	1	SA0210
gi|29165615|ref|NC_002745.2|	249986 +	A	0	2	0	0	0	SA0210
gi|29165615|ref|NC_002745.2|	250000 +	G	0	0	0	0	1	SA0210
gi|29165615|ref|NC_002745.2|	250015 +	C	0	1	0	0	1	SA0210
gi|29165615|ref|NC_002745.2|	250036 +	A	1	3	0	0	0	SA0210
gi|29165615|ref|NC_002745.2|	250044 +	A	0	0	0	0	1	SA0210
gi|29165615|ref|NC_002745.2|	250051 +	U	0	0	0	0	1	SA0210
gi|29165615|ref|NC_002745.2|	250065 +	A	0	1	0	0	0	SA0210
gi|29165615|ref|NC_002745.2|	250067 +	C	0	2	0	0	0	SA0210
gi|29165615|ref|NC_002745.2|	250071 +	C	0	0	0	1	0	SA0210
gi|29165615|ref|NC_002745.2|	250084 +	U	0	0	0	1	0	SA0210
gi|29165615|ref|NC_002745.2|	250086 +	U	0	0	0	0	1	SA0210
gi|29165615|ref|NC_002745.2|	250094 +	A	0	2	0	0	0	-
gi|29165615|ref|NC_002745.2|	250107 +	G	0	1	0	1	0	-
gi|29165615|ref|NC_002745.2|	250108 +	A	1	6	1	0	5	-
gi|29165615|ref|NC_002745.2|	250110 +	G	0	1	0	0	0	-
gi|29165615|ref|NC_002745.2|	250113 +	C	0	0	0	1	0	-
gi|29165615|ref|NC_002745.2|	250124 +	A	0	1	0	0	0	SA0211
gi|29165615|ref|NC_002745.2|	250125 +	A	0	1	0	0	3	SA0211
gi|29165615|ref|NC_002745.2|	250129 +	A	0	0	0	1	0	SA0211
gi|29165615|ref|NC_002745.2|	250146 +	U	0	0	0	0	1	SA0211
gi|29165615|ref|NC_002745.2|	250151 +	G	0	0	0	0	2	SA0211
gi|29165615|ref|NC_002745.2|	250152 +	U	0	1	0	0	3	SA0211
gi|29165615|ref|NC_002745.2|	250157 +	U	0	0	0	0	1	SA0211
gi|29165615|ref|NC_002745.2|	250177 +	C	0	0	0	0	1	SA0211
gi|29165615|ref|NC_002745.2|	250196 +	A	0	1	0	0	0	SA0211
gi|29165615|ref|NC_002745.2|	250207 +	U	0	1	0	0	0	SA0211
gi|29165615|ref|NC_002745.2|	250220 +	U	0	0	0	1	0	SA0211
gi|29165615|ref|NC_002745.2|	250227 +	U	0	0	0	0	1	SA0211
gi|29165615|ref|NC_002745.2|	250230 +	U	0	0	0	1	0	SA0211
gi|29165615|ref|NC_002745.2|	250232 +	A	0	0	1	0	1	SA0211
gi|29165615|ref|NC_002745.2|	250233 +	U	0	0	0	1	1	SA0211
gi|29165615|ref|NC_002745.2|	250242 +	G	0	0	0	0	1	SA0211
gi|29165615|ref|NC_002745.2|	250246 +	A	0	0	0	0	1	SA0211
gi|29165615|ref|NC_002745.2|	250248 +	U	0	0	0	1	0	SA0211
gi|29165615|ref|NC_002745.2|	250249 +	G	0	0	0	0	1	SA0211
gi|29165615|ref|NC_002745.2|	250271 +	U	0	2	0	1	0	SA0211
gi|29165615|ref|NC_002745.2|	250281 +	A	0	0	0	0	1	SA0211
gi|29165615|ref|NC_002745.2|	250291 +	C	0	0	0	0	1	SA0211
gi|29165615|ref|NC_002745.2|	250297 +	G	0	0	0	0	1	SA0211
gi|29165615|ref|NC_002745.2|	250300 +	C	0	0	0	0	1	SA0211
gi|29165615|ref|NC_002745.2|	250313 +	U	0	0	0	0	1	SA0211
gi|29165615|ref|NC_002745.2|	250318 +	G	0	0	0	1	1	SA0211
gi|29165615|ref|NC_002745.2|	250327 +	A	0	1	0	0	0	SA0211
gi|29165615|ref|NC_002745.2|	250344 +	A	0	0	0	0	1	SA0211
gi|29165615|ref|NC_002745.2|	250348 +	C	0	0	0	0	1	SA0211
gi|29165615|ref|NC_002745.2|	250361 +	C	0	0	0	1	0	SA0211
gi|29165615|ref|NC_002745.2|	250459 +	A	0	0	0	0	1	SA0211
gi|29165615|ref|NC_002745.2|	250460 +	A	0	1	0	0	1	SA0211
gi|29165615|ref|NC_002745.2|	250467 +	A	0	1	0	0	0	SA0211
gi|29165615|ref|NC_002745.2|	250472 +	U	0	0	0	1	0	SA0211
gi|29165615|ref|NC_002745.2|	250473 +	A	0	1	0	0	0	SA0211
gi|29165615|ref|NC_002745.2|	250491 +	U	0	0	0	1	0	SA0211
gi|29165615|ref|NC_002745.2|	250507 +	G	0	0	0	0	1	SA0211
gi|29165615|ref|NC_002745.2|	250530 +	U	0	1	0	0	0	SA0211
gi|29165615|ref|NC_002745.2|	250555 +	U	0	0	0	0	1	SA0211
gi|29165615|ref|NC_002745.2|	250558 +	G	0	0	0	1	0	SA0211
gi|29165615|ref|NC_002745.2|	250570 +	C	0	0	1	0	3	SA0211
gi|29165615|ref|NC_002745.2|	250573 +	G	0	0	0	0	1	SA0211
gi|29165615|ref|NC_002745.2|	250574 +	C	0	0	0	0	1	SA0211
gi|29165615|ref|NC_002745.2|	250576 +	A	0	0	0	0	2	SA0211
gi|29165615|ref|NC_002745.2|	250579 +	C	0	0	0	1	0	SA0211
gi|29165615|ref|NC_002745.2|	250585 +	C	0	0	0	1	0	SA0211
gi|29165615|ref|NC_002745.2|	250588 +	A	0	1	0	0	0	SA0211
gi|29165615|ref|NC_002745.2|	250609 +	U	0	0	0	1	1	SA0211
gi|29165615|ref|NC_002745.2|	250613 +	C	0	0	0	0	1	SA0211
gi|29165615|ref|NC_002745.2|	250615 +	A	0	1	0	0	0	SA0211
gi|29165615|ref|NC_002745.2|	250628 +	A	0	1	0	0	0	SA0211
gi|29165615|ref|NC_002745.2|	250647 +	C	0	0	0	0	1	SA0211
gi|29165615|ref|NC_002745.2|	250661 +	A	0	0	0	0	1	SA0211
gi|29165615|ref|NC_002745.2|	250665 +	G	0	0	0	0	1	SA0211
gi|29165615|ref|NC_002745.2|	250680 +	G	0	0	0	0	1	SA0211
gi|29165615|ref|NC_002745.2|	250690 +	G	0	0	0	1	0	SA0211
gi|29165615|ref|NC_002745.2|	250691 +	G	0	0	0	1	0	SA0211
gi|29165615|ref|NC_002745.2|	250695 +	A	0	0	0	0	1	SA0211
gi|29165615|ref|NC_002745.2|	250707 +	G	1	0	0	0	0	SA0211
gi|29165615|ref|NC_002745.2|	250709 +	A	0	0	0	0	1	SA0211
gi|29165615|ref|NC_002745.2|	250725 +	A	0	1	0	0	0	SA0211
gi|29165615|ref|NC_002745.2|	250726 +	A	1	0	0	0	0	SA0211

gi|29165615|ref|NC_002745.2|	250729	+	U	0	0	0	1	0	SA0211
gi|29165615|ref|NC_002745.2|	250731	+	U	0	0	0	1	1	SA0211
gi|29165615|ref|NC_002745.2|	250733	+	A	0	0	0	1	1	SA0211
gi|29165615|ref|NC_002745.2|	250734	+	U	0	0	0	1	0	SA0211
gi|29165615|ref|NC_002745.2|	250735	+	C	0	1	0	0	0	SA0211
gi|29165615|ref|NC_002745.2|	250737	+	A	1	0	0	0	2	SA0211
gi|29165615|ref|NC_002745.2|	250738	+	U	0	0	0	1	0	SA0211
gi|29165615|ref|NC_002745.2|	250743	+	C	0	0	0	1	0	SA0211
gi|29165615|ref|NC_002745.2|	250746	+	A	0	0	0	1	0	SA0211
gi|29165615|ref|NC_002745.2|	250747	+	C	0	1	0	0	0	SA0211
gi|29165615|ref|NC_002745.2|	250748	+	A	0	0	0	0	1	SA0211
gi|29165615|ref|NC_002745.2|	250750	+	C	0	0	0	0	1	SA0211
gi|29165615|ref|NC_002745.2|	250751	+	C	0	1	1	0	1	SA0211
gi|29165615|ref|NC_002745.2|	250756	+	C	0	0	0	1	1	SA0211
gi|29165615|ref|NC_002745.2|	250759	+	A	0	1	0	1	2	SA0211
gi|29165615|ref|NC_002745.2|	250760	+	U	0	0	0	0	1	SA0211
gi|29165615|ref|NC_002745.2|	250763	+	A	0	0	0	0	2	SA0211
gi|29165615|ref|NC_002745.2|	250764	+	U	0	0	0	0	1	SA0211
gi|29165615|ref|NC_002745.2|	250773	+	A	0	1	0	0	0	SA0211
gi|29165615|ref|NC_002745.2|	250775	+	C	0	1	0	0	0	SA0211
gi|29165615|ref|NC_002745.2|	250781	+	A	0	1	0	0	0	SA0211
gi|29165615|ref|NC_002745.2|	250783	+	C	0	0	0	0	1	SA0211
gi|29165615|ref|NC_002745.2|	250784	+	A	0	0	0	1	1	SA0211
gi|29165615|ref|NC_002745.2|	250787	+	C	0	0	0	0	1	SA0211
gi|29165615|ref|NC_002745.2|	250788	+	U	0	1	0	1	0	SA0211
gi|29165615|ref|NC_002745.2|	250789	+	A	0	0	0	0	1	SA0211
gi|29165615|ref|NC_002745.2|	250790	+	A	0	0	0	3	0	SA0211
gi|29165615|ref|NC_002745.2|	250791	+	A	0	1	0	0	0	SA0211
gi|29165615|ref|NC_002745.2|	250793	+	U	0	1	0	0	0	SA0211
gi|29165615|ref|NC_002745.2|	250802	+	A	0	0	0	1	0	SA0211
gi|29165615|ref|NC_002745.2|	250809	+	U	0	0	0	0	1	SA0211
gi|29165615|ref|NC_002745.2|	250810	+	G	0	0	0	1	0	SA0211
gi|29165615|ref|NC_002745.2|	250813	+	A	0	0	0	1	0	SA0211
gi|29165615|ref|NC_002745.2|	250815	+	U	0	0	0	1	0	SA0211
gi|29165615|ref|NC_002745.2|	250818	+	U	0	0	0	1	0	SA0211
gi|29165615|ref|NC_002745.2|	250819	+	U	0	0	0	2	0	SA0211
gi|29165615|ref|NC_002745.2|	250820	+	A	0	1	0	3	0	SA0211
gi|29165615|ref|NC_002745.2|	250821	+	U	0	0	0	1	1	SA0211
gi|29165615|ref|NC_002745.2|	250825	+	A	0	0	0	2	0	SA0211
gi|29165615|ref|NC_002745.2|	250826	+	C	0	1	0	0	0	SA0211
gi|29165615|ref|NC_002745.2|	250828	+	U	0	0	0	0	1	SA0211
gi|29165615|ref|NC_002745.2|	250832	+	C	0	0	0	1	0	SA0211
gi|29165615|ref|NC_002745.2|	250834	+	A	0	0	0	1	0	SA0211
gi|29165615|ref|NC_002745.2|	250835	+	A	0	1	0	0	2	SA0211
gi|29165615|ref|NC_002745.2|	250837	+	C	0	0	0	1	0	SA0211
gi|29165615|ref|NC_002745.2|	250839	+	A	0	0	0	0	1	SA0211
gi|29165615|ref|NC_002745.2|	250841	+	C	0	0	1	0	0	SA0211
gi|29165615|ref|NC_002745.2|	250844	+	G	0	0	0	1	0	SA0211
gi|29165615|ref|NC_002745.2|	250845	+	C	0	0	0	1	0	SA0211
gi|29165615|ref|NC_002745.2|	250846	+	A	0	0	0	0	1	SA0211
gi|29165615|ref|NC_002745.2|	250848	+	G	0	1	0	0	1	SA0211
gi|29165615|ref|NC_002745.2|	250851	+	G	0	1	0	0	0	SA0211
gi|29165615|ref|NC_002745.2|	250855	+	G	0	0	0	0	1	SA0211
gi|29165615|ref|NC_002745.2|	250856	+	A	0	0	1	0	2	SA0211
gi|29165615|ref|NC_002745.2|	250857	+	A	0	1	0	0	1	SA0211
gi|29165615|ref|NC_002745.2|	250859	+	G	0	0	0	0	1	SA0211
gi|29165615|ref|NC_002745.2|	250860	+	U	0	0	0	3	0	SA0211
gi|29165615|ref|NC_002745.2|	250861	+	U	0	0	0	0	1	SA0211
gi|29165615|ref|NC_002745.2|	250872	+	A	0	0	0	1	0	SA0211
gi|29165615|ref|NC_002745.2|	250873	+	A	0	0	0	1	1	SA0211
gi|29165615|ref|NC_002745.2|	250874	+	A	0	0	0	0	1	SA0211
gi|29165615|ref|NC_002745.2|	250875	+	U	0	0	0	0	1	SA0211
gi|29165615|ref|NC_002745.2|	250876	+	A	0	1	0	0	0	SA0211
gi|29165615|ref|NC_002745.2|	250877	+	U	0	1	0	1	1	SA0211
gi|29165615|ref|NC_002745.2|	250879	+	A	0	0	0	1	1	SA0211
gi|29165615|ref|NC_002745.2|	250882	+	G	0	0	0	0	1	SA0211
gi|29165615|ref|NC_002745.2|	250883	+	A	0	0	0	3	6	SA0211
gi|29165615|ref|NC_002745.2|	250886	+	A	1	0	0	4	1	SA0211
gi|29165615|ref|NC_002745.2|	250887	+	U	0	0	0	0	2	SA0211
gi|29165615|ref|NC_002745.2|	250888	+	A	0	0	0	1	1	SA0211
gi|29165615|ref|NC_002745.2|	250889	+	A	0	0	1	0	1	SA0211
gi|29165615|ref|NC_002745.2|	250893	+	A	0	0	0	0	1	SA0211
gi|29165615|ref|NC_002745.2|	250900	+	A	0	0	0	1	0	SA0211
gi|29165615|ref|NC_002745.2|	250907	+	C	0	1	0	1	3	SA0211
gi|29165615|ref|NC_002745.2|	250908	+	A	0	0	0	1	0	SA0211
gi|29165615|ref|NC_002745.2|	250910	+	G	0	0	0	0	1	SA0211
gi|29165615|ref|NC_002745.2|	250926	+	C	0	0	0	1	0	SA0211
gi|29165615|ref|NC_002745.2|	250930	+	U	0	0	0	0	2	SA0211
gi|29165615|ref|NC_002745.2|	250934	+	U	0	0	0	1	0	SA0211
gi|29165615|ref|NC_002745.2|	250935	+	U	0	0	0	0	1	SA0211
gi|29165615|ref|NC_002745.2|	250938	+	A	0	0	0	0	1	SA0211

gi|29165615|ref|NC_002745.2|	250939 +	U	0	0	0	1	1	SA0211
gi|29165615|ref|NC_002745.2|	250942 +	G	0	1	0	1	2	SA0211
gi|29165615|ref|NC_002745.2|	250943 +	A	0	0	1	1	2	SA0211
gi|29165615|ref|NC_002745.2|	250944 +	A	0	0	0	0	2	SA0211
gi|29165615|ref|NC_002745.2|	250945 +	A	0	0	0	0	1	SA0211
gi|29165615|ref|NC_002745.2|	250946 +	U	1	3	0	2	4	SA0211
gi|29165615|ref|NC_002745.2|	250949 +	A	0	0	0	3	0	SA0211
gi|29165615|ref|NC_002745.2|	250950 +	U	0	0	0	1	0	SA0211
gi|29165615|ref|NC_002745.2|	250951 +	G	0	0	0	0	1	SA0211
gi|29165615|ref|NC_002745.2|	250954 +	C	0	1	0	3	1	SA0211
gi|29165615|ref|NC_002745.2|	250955 +	C	0	2	1	0	2	SA0211
gi|29165615|ref|NC_002745.2|	250956 +	C	0	0	0	0	1	SA0211
gi|29165615|ref|NC_002745.2|	250957 +	C	0	2	1	0	1	SA0211
gi|29165615|ref|NC_002745.2|	250964 +	G	0	1	0	0	0	SA0211
gi|29165615|ref|NC_002745.2|	250965 +	A	0	0	0	2	0	SA0211
gi|29165615|ref|NC_002745.2|	250966 +	A	0	1	0	0	0	SA0211
gi|29165615|ref|NC_002745.2|	250967 +	C	0	0	0	0	2	SA0211
gi|29165615|ref|NC_002745.2|	250968 +	U	0	0	0	0	2	SA0211
gi|29165615|ref|NC_002745.2|	250969 +	A	0	0	0	0	1	SA0211
gi|29165615|ref|NC_002745.2|	250970 +	U	0	0	1	1	1	SA0211
gi|29165615|ref|NC_002745.2|	250971 +	U	0	0	0	0	1	SA0211
gi|29165615|ref|NC_002745.2|	250972 +	U	0	0	0	0	1	SA0211
gi|29165615|ref|NC_002745.2|	250974 +	U	0	3	0	0	0	SA0211
gi|29165615|ref|NC_002745.2|	250975 +	G	0	4	0	0	0	SA0211
gi|29165615|ref|NC_002745.2|	250976 +	A	0	4	1	16	8	SA0211
gi|29165615|ref|NC_002745.2|	250977 +	A	0	0	0	0	1	SA0211
gi|29165615|ref|NC_002745.2|	250978 +	A	0	0	1	3	0	SA0211
gi|29165615|ref|NC_002745.2|	250979 +	G	0	0	0	0	4	SA0211
gi|29165615|ref|NC_002745.2|	250980 +	C	0	0	0	1	3	SA0211
gi|29165615|ref|NC_002745.2|	250981 +	A	0	0	0	2	0	SA0211
gi|29165615|ref|NC_002745.2|	250982 +	A	0	2	0	6	3	SA0211
gi|29165615|ref|NC_002745.2|	250983 +	A	0	0	2	0	0	SA0211
gi|29165615|ref|NC_002745.2|	250984 +	G	0	1	0	0	0	SA0211
gi|29165615|ref|NC_002745.2|	250985 +	C	0	0	0	2	0	SA0211
gi|29165615|ref|NC_002745.2|	250986 +	U	0	2	0	0	1	SA0211
gi|29165615|ref|NC_002745.2|	250987 +	A	0	0	0	0	1	SA0211
gi|29165615|ref|NC_002745.2|	250988 +	A	0	2	0	0	2	SA0211
gi|29165615|ref|NC_002745.2|	250989 +	U	0	1	0	1	3	SA0211
gi|29165615|ref|NC_002745.2|	250990 +	G	0	0	0	0	1	SA0211
gi|29165615|ref|NC_002745.2|	250991 +	U	0	0	1	0	1	SA0211
gi|29165615|ref|NC_002745.2|	250993 +	G	0	0	0	0	1	SA0211
gi|29165615|ref|NC_002745.2|	250995 +	G	0	1	0	0	1	SA0211
gi|29165615|ref|NC_002745.2|	250998 +	U	0	2	0	0	3	SA0211
gi|29165615|ref|NC_002745.2|	250999 +	A	0	1	0	0	1	SA0211
gi|29165615|ref|NC_002745.2|	251000 +	A	0	0	0	1	1	SA0211
gi|29165615|ref|NC_002745.2|	251001 +	C	0	0	0	1	1	SA0211
gi|29165615|ref|NC_002745.2|	251002 +	G	0	1	1	1	0	SA0211
gi|29165615|ref|NC_002745.2|	251003 +	A	0	0	0	2	3	SA0211
gi|29165615|ref|NC_002745.2|	251004 +	A	0	0	0	0	1	SA0211
gi|29165615|ref|NC_002745.2|	251005 +	G	0	0	1	1	1	SA0211
gi|29165615|ref|NC_002745.2|	251006 +	A	0	0	1	0	6	SA0211
gi|29165615|ref|NC_002745.2|	251007 +	C	0	0	0	0	2	SA0211
gi|29165615|ref|NC_002745.2|	251008 +	A	0	2	0	1	5	SA0211
gi|29165615|ref|NC_002745.2|	251009 +	U	0	0	0	0	3	SA0211
gi|29165615|ref|NC_002745.2|	251011 +	G	0	0	0	0	1	SA0211
gi|29165615|ref|NC_002745.2|	251012 +	C	0	0	0	2	4	SA0211
gi|29165615|ref|NC_002745.2|	251013 +	U	0	0	0	0	1	SA0211
gi|29165615|ref|NC_002745.2|	251016 +	U	0	0	1	0	1	SA0211
gi|29165615|ref|NC_002745.2|	251017 +	G	0	0	0	1	0	SA0211
gi|29165615|ref|NC_002745.2|	251018 +	A	0	0	0	0	1	SA0211
gi|29165615|ref|NC_002745.2|	251021 +	G	0	1	0	0	0	SA0211
gi|29165615|ref|NC_002745.2|	251022 +	A	0	0	0	0	2	SA0211
gi|29165615|ref|NC_002745.2|	251023 +	C	0	0	2	0	0	SA0211
gi|29165615|ref|NC_002745.2|	251024 +	A	0	1	0	0	0	SA0211
gi|29165615|ref|NC_002745.2|	251026 +	G	0	0	0	0	1	SA0211
gi|29165615|ref|NC_002745.2|	251027 +	C	0	0	0	0	1	SA0211
gi|29165615|ref|NC_002745.2|	251028 +	G	0	0	0	0	1	SA0211
gi|29165615|ref|NC_002745.2|	251029 +	C	0	0	0	0	1	SA0211
gi|29165615|ref|NC_002745.2|	251031 +	U	0	1	1	0	1	SA0211
gi|29165615|ref|NC_002745.2|	251032 +	A	0	1	0	1	0	SA0211
gi|29165615|ref|NC_002745.2|	251033 +	A	0	0	0	0	2	SA0211
gi|29165615|ref|NC_002745.2|	251034 +	C	0	0	0	0	1	SA0211
gi|29165615|ref|NC_002745.2|	251035 +	U	0	1	0	0	3	SA0211
gi|29165615|ref|NC_002745.2|	251036 +	U	0	2	0	0	2	SA0211
gi|29165615|ref|NC_002745.2|	251037 +	U	0	1	0	0	0	SA0211
gi|29165615|ref|NC_002745.2|	251038 +	G	0	0	0	1	1	SA0211
gi|29165615|ref|NC_002745.2|	251040 +	C	1	0	1	0	0	SA0211
gi|29165615|ref|NC_002745.2|	251041 +	A	0	2	0	1	1	SA0211
gi|29165615|ref|NC_002745.2|	251042 +	A	0	0	0	0	1	SA0211
gi|29165615|ref|NC_002745.2|	251056 +	A	0	0	0	0	1	SA0211
gi|29165615|ref|NC_002745.2|	251058 +	A	0	1	0	0	0	SA0211

gi|29165615|ref|NC_002745.2|	251060	+	A	0	0	0	1	0	SA0211
gi|29165615|ref|NC_002745.2|	251078	+	C	0	0	1	0	0	SA0211
gi|29165615|ref|NC_002745.2|	251084	+	A	1	3	0	0	0	SA0211
gi|29165615|ref|NC_002745.2|	251150	+	A	0	0	0	1	0	SA0211
gi|29165615|ref|NC_002745.2|	251151	+	A	0	0	0	0	1	SA0211
gi|29165615|ref|NC_002745.2|	251157	+	A	0	0	0	1	0	SA0211
gi|29165615|ref|NC_002745.2|	251168	+	A	0	0	0	1	0	-
gi|29165615|ref|NC_002745.2|	251169	+	U	0	0	0	2	0	-
gi|29165615|ref|NC_002745.2|	251170	+	A	0	0	0	2	1	-
gi|29165615|ref|NC_002745.2|	251172	+	A	0	0	0	0	1	-
gi|29165615|ref|NC_002745.2|	251178	+	A	0	0	0	0	1	-
gi|29165615|ref|NC_002745.2|	251179	+	A	0	0	0	1	0	-
gi|29165615|ref|NC_002745.2|	251180	+	A	0	4	0	0	1	-
gi|29165615|ref|NC_002745.2|	251184	+	U	0	0	0	1	0	-
gi|29165615|ref|NC_002745.2|	251185	+	C	0	0	0	0	2	-
gi|29165615|ref|NC_002745.2|	251186	+	A	1	0	0	0	0	-
gi|29165615|ref|NC_002745.2|	251187	+	A	0	0	0	1	0	-
gi|29165615|ref|NC_002745.2|	251191	+	A	0	0	0	1	0	-
gi|29165615|ref|NC_002745.2|	251193	+	A	0	0	0	1	2	-
gi|29165615|ref|NC_002745.2|	251194	+	A	0	0	0	0	1	-
gi|29165615|ref|NC_002745.2|	251195	+	A	0	0	0	0	1	-
gi|29165615|ref|NC_002745.2|	251197	+	A	0	0	0	1	0	-
gi|29165615|ref|NC_002745.2|	251199	+	G	0	1	0	1	1	-
gi|29165615|ref|NC_002745.2|	251200	+	G	0	0	0	0	2	-
gi|29165615|ref|NC_002745.2|	251208	+	U	0	0	0	1	0	-
gi|29165615|ref|NC_002745.2|	251210	+	C	0	0	0	1	0	-
gi|29165615|ref|NC_002745.2|	251211	+	A	0	0	0	0	1	-
gi|29165615|ref|NC_002745.2|	251217	+	A	0	0	0	1	0	SA0212
gi|29165615|ref|NC_002745.2|	251218	+	A	0	1	0	0	0	SA0212
gi|29165615|ref|NC_002745.2|	251219	+	U	0	0	0	0	2	SA0212
gi|29165615|ref|NC_002745.2|	251220	+	A	0	0	0	1	0	SA0212
gi|29165615|ref|NC_002745.2|	251222	+	G	0	0	0	0	1	SA0212
gi|29165615|ref|NC_002745.2|	251225	+	U	0	0	0	1	0	SA0212
gi|29165615|ref|NC_002745.2|	251228	+	U	0	0	0	0	1	SA0212
gi|29165615|ref|NC_002745.2|	251233	+	G	0	0	0	0	1	SA0212
gi|29165615|ref|NC_002745.2|	251239	+	U	0	0	0	0	1	SA0212
gi|29165615|ref|NC_002745.2|	251242	+	U	0	0	0	0	1	SA0212
gi|29165615|ref|NC_002745.2|	251243	+	A	0	0	0	0	1	SA0212
gi|29165615|ref|NC_002745.2|	251244	+	C	0	0	0	1	0	SA0212
gi|29165615|ref|NC_002745.2|	251245	+	G	0	1	0	4	0	SA0212
gi|29165615|ref|NC_002745.2|	251246	+	A	0	0	0	1	0	SA0212
gi|29165615|ref|NC_002745.2|	251249	+	A	0	0	0	0	1	SA0212
gi|29165615|ref|NC_002745.2|	251250	+	A	0	0	1	1	0	SA0212
gi|29165615|ref|NC_002745.2|	251251	+	A	0	0	0	1	1	SA0212
gi|29165615|ref|NC_002745.2|	251252	+	A	2	10	1	5	20	SA0212
gi|29165615|ref|NC_002745.2|	251255	+	U	0	0	0	0	1	SA0212
gi|29165615|ref|NC_002745.2|	251257	+	G	0	0	0	1	2	SA0212
gi|29165615|ref|NC_002745.2|	251258	+	A	0	0	0	5	4	SA0212
gi|29165615|ref|NC_002745.2|	251260	+	G	0	0	0	2	0	SA0212
gi|29165615|ref|NC_002745.2|	251261	+	A	0	0	0	0	1	SA0212
gi|29165615|ref|NC_002745.2|	251262	+	U	0	2	0	1	0	SA0212
gi|29165615|ref|NC_002745.2|	251263	+	A	0	1	0	0	1	SA0212
gi|29165615|ref|NC_002745.2|	251264	+	U	0	0	0	0	1	SA0212
gi|29165615|ref|NC_002745.2|	251266	+	U	0	1	0	0	1	SA0212
gi|29165615|ref|NC_002745.2|	251269	+	G	1	0	0	1	0	SA0212
gi|29165615|ref|NC_002745.2|	251270	+	A	0	0	1	3	1	SA0212
gi|29165615|ref|NC_002745.2|	251271	+	U	0	1	0	0	0	SA0212
gi|29165615|ref|NC_002745.2|	251272	+	U	0	1	0	0	0	SA0212
gi|29165615|ref|NC_002745.2|	251273	+	A	0	0	0	0	1	SA0212
gi|29165615|ref|NC_002745.2|	251277	+	C	0	3	0	0	0	SA0212
gi|29165615|ref|NC_002745.2|	251285	+	C	0	1	0	0	0	SA0212
gi|29165615|ref|NC_002745.2|	251286	+	U	0	1	0	0	0	SA0212
gi|29165615|ref|NC_002745.2|	251293	+	G	0	0	0	0	1	SA0212
gi|29165615|ref|NC_002745.2|	251295	+	U	0	1	0	0	0	SA0212
gi|29165615|ref|NC_002745.2|	251297	+	U	0	0	0	1	0	SA0212
gi|29165615|ref|NC_002745.2|	251299	+	A	0	0	1	0	0	SA0212
gi|29165615|ref|NC_002745.2|	251303	+	A	0	0	0	0	1	SA0212
gi|29165615|ref|NC_002745.2|	251308	+	G	0	1	0	0	0	SA0212
gi|29165615|ref|NC_002745.2|	251312	+	C	0	0	0	1	0	SA0212
gi|29165615|ref|NC_002745.2|	251319	+	U	0	1	0	1	0	SA0212
gi|29165615|ref|NC_002745.2|	251320	+	A	0	0	0	0	1	SA0212
gi|29165615|ref|NC_002745.2|	251324	+	C	0	1	0	0	0	SA0212
gi|29165615|ref|NC_002745.2|	251325	+	A	0	1	0	0	0	SA0212
gi|29165615|ref|NC_002745.2|	251332	+	A	0	0	0	1	0	SA0212
gi|29165615|ref|NC_002745.2|	251333	+	A	0	0	0	1	0	SA0212
gi|29165615|ref|NC_002745.2|	251359	+	G	0	0	0	0	1	SA0212
gi|29165615|ref|NC_002745.2|	251363	+	A	1	3	0	0	4	SA0212
gi|29165615|ref|NC_002745.2|	251371	+	A	0	0	0	1	0	SA0212
gi|29165615|ref|NC_002745.2|	251374	+	C	0	0	0	1	0	SA0212
gi|29165615|ref|NC_002745.2|	251377	+	C	0	0	0	0	2	SA0212
gi|29165615|ref|NC_002745.2|	251388	+	G	0	0	0	0	2	SA0212

gi|29165615|ref|NC_002745.2|	251395	+	A	0	3	0	0	1	SA0212
gi|29165615|ref|NC_002745.2|	251399	+	C	0	1	0	0	0	SA0212
gi|29165615|ref|NC_002745.2|	251402	+	A	0	0	0	0	1	SA0212
gi|29165615|ref|NC_002745.2|	251409	+	C	0	0	0	0	1	SA0212
gi|29165615|ref|NC_002745.2|	251414	+	A	0	0	0	1	0	SA0212
gi|29165615|ref|NC_002745.2|	251416	+	A	0	1	0	2	0	SA0212
gi|29165615|ref|NC_002745.2|	251417	+	U	0	0	0	1	0	SA0212
gi|29165615|ref|NC_002745.2|	251421	+	U	0	0	0	0	1	SA0212
gi|29165615|ref|NC_002745.2|	251431	+	U	0	0	0	1	0	SA0212
gi|29165615|ref|NC_002745.2|	251443	+	C	1	0	0	3	0	SA0212
gi|29165615|ref|NC_002745.2|	251449	+	U	0	0	0	0	1	SA0212
gi|29165615|ref|NC_002745.2|	251450	+	C	0	0	0	2	0	SA0212
gi|29165615|ref|NC_002745.2|	251452	+	C	0	0	0	2	0	SA0212
gi|29165615|ref|NC_002745.2|	251453	+	C	0	1	0	0	0	SA0212
gi|29165615|ref|NC_002745.2|	251457	+	U	0	0	0	0	2	SA0212
gi|29165615|ref|NC_002745.2|	251460	+	G	0	0	0	0	2	SA0212
gi|29165615|ref|NC_002745.2|	251463	+	A	0	0	0	1	0	SA0212
gi|29165615|ref|NC_002745.2|	251464	+	G	0	0	0	2	0	SA0212
gi|29165615|ref|NC_002745.2|	251472	+	A	0	0	0	0	1	SA0212
gi|29165615|ref|NC_002745.2|	251479	+	G	0	0	0	1	0	SA0212
gi|29165615|ref|NC_002745.2|	251483	+	A	0	1	0	0	0	SA0212
gi|29165615|ref|NC_002745.2|	251489	+	U	0	1	0	0	0	SA0212
gi|29165615|ref|NC_002745.2|	251494	+	A	0	0	0	1	0	SA0212
gi|29165615|ref|NC_002745.2|	251495	+	A	0	0	0	1	0	SA0212
gi|29165615|ref|NC_002745.2|	251496	+	A	0	0	0	2	0	SA0212
gi|29165615|ref|NC_002745.2|	251497	+	A	0	0	0	0	1	SA0212
gi|29165615|ref|NC_002745.2|	251498	+	C	0	0	0	1	0	SA0212
gi|29165615|ref|NC_002745.2|	251502	+	C	0	0	1	0	0	SA0212
gi|29165615|ref|NC_002745.2|	251505	+	U	0	0	0	0	1	SA0212
gi|29165615|ref|NC_002745.2|	251510	+	C	0	0	0	1	0	SA0212
gi|29165615|ref|NC_002745.2|	251513	+	A	0	0	0	0	1	SA0212
gi|29165615|ref|NC_002745.2|	251515	+	C	0	0	0	1	0	SA0212
gi|29165615|ref|NC_002745.2|	251517	+	A	0	0	0	0	1	SA0212
gi|29165615|ref|NC_002745.2|	251519	+	U	0	0	1	0	0	SA0212
gi|29165615|ref|NC_002745.2|	251522	+	A	0	1	0	0	0	SA0212
gi|29165615|ref|NC_002745.2|	251524	+	G	0	0	0	0	1	SA0212
gi|29165615|ref|NC_002745.2|	251545	+	U	0	0	0	0	1	SA0212
gi|29165615|ref|NC_002745.2|	251548	+	G	0	0	0	1	0	SA0212
gi|29165615|ref|NC_002745.2|	251549	+	G	0	0	0	1	0	SA0212
gi|29165615|ref|NC_002745.2|	251551	+	A	0	0	0	0	1	SA0212
gi|29165615|ref|NC_002745.2|	251560	+	U	0	0	0	0	1	SA0212
gi|29165615|ref|NC_002745.2|	251566	+	G	0	0	0	1	0	SA0212
gi|29165615|ref|NC_002745.2|	251570	+	C	0	0	0	0	1	SA0212
gi|29165615|ref|NC_002745.2|	251583	+	U	0	0	0	1	0	SA0212
gi|29165615|ref|NC_002745.2|	251585	+	A	1	0	0	0	0	SA0212
gi|29165615|ref|NC_002745.2|	251597	+	C	0	0	0	1	0	SA0212
gi|29165615|ref|NC_002745.2|	251605	+	C	0	1	0	0	0	SA0212
gi|29165615|ref|NC_002745.2|	251631	+	U	0	0	0	1	0	SA0212
gi|29165615|ref|NC_002745.2|	251645	+	A	0	0	0	1	1	SA0212
gi|29165615|ref|NC_002745.2|	251650	+	U	0	0	0	0	1	SA0212
gi|29165615|ref|NC_002745.2|	251657	+	C	0	0	0	1	0	SA0212
gi|29165615|ref|NC_002745.2|	251673	+	A	0	0	0	0	1	SA0212
gi|29165615|ref|NC_002745.2|	251675	+	C	0	0	0	0	1	SA0212
gi|29165615|ref|NC_002745.2|	251689	+	G	1	0	0	0	0	SA0212
gi|29165615|ref|NC_002745.2|	251692	+	C	0	0	0	0	1	SA0212
gi|29165615|ref|NC_002745.2|	251694	+	A	0	0	0	0	1	SA0212
gi|29165615|ref|NC_002745.2|	251704	+	A	0	1	0	0	2	SA0212
gi|29165615|ref|NC_002745.2|	251705	+	U	0	1	0	0	0	SA0212
gi|29165615|ref|NC_002745.2|	251709	+	C	0	0	0	1	0	SA0212
gi|29165615|ref|NC_002745.2|	251717	+	U	0	1	0	0	0	SA0212
gi|29165615|ref|NC_002745.2|	251723	+	C	0	0	0	0	1	SA0212
gi|29165615|ref|NC_002745.2|	251726	+	G	0	0	0	1	0	SA0212
gi|29165615|ref|NC_002745.2|	251731	+	U	0	0	0	0	1	SA0212
gi|29165615|ref|NC_002745.2|	251732	+	U	1	0	0	0	0	SA0212
gi|29165615|ref|NC_002745.2|	251735	+	U	0	0	0	1	0	SA0212
gi|29165615|ref|NC_002745.2|	251755	+	U	0	0	0	1	0	SA0212
gi|29165615|ref|NC_002745.2|	251757	+	A	0	0	0	0	1	SA0212
gi|29165615|ref|NC_002745.2|	251767	+	G	0	0	1	0	0	SA0212
gi|29165615|ref|NC_002745.2|	251771	+	C	0	0	0	1	0	SA0212
gi|29165615|ref|NC_002745.2|	251773	+	A	0	0	0	1	1	SA0212
gi|29165615|ref|NC_002745.2|	251774	+	C	0	0	2	1	0	SA0212
gi|29165615|ref|NC_002745.2|	251775	+	A	0	0	0	1	1	SA0212
gi|29165615|ref|NC_002745.2|	251777	+	A	0	0	0	0	2	SA0212
gi|29165615|ref|NC_002745.2|	251778	+	U	0	0	1	0	0	SA0212
gi|29165615|ref|NC_002745.2|	251779	+	G	0	0	0	3	0	SA0212
gi|29165615|ref|NC_002745.2|	251782	+	U	0	0	0	1	1	SA0212
gi|29165615|ref|NC_002745.2|	251784	+	U	0	0	0	0	1	SA0212
gi|29165615|ref|NC_002745.2|	251786	+	U	0	0	0	1	0	SA0212
gi|29165615|ref|NC_002745.2|	251787	+	C	0	0	1	0	0	SA0212
gi|29165615|ref|NC_002745.2|	251789	+	G	0	1	0	0	1	SA0212
gi|29165615|ref|NC_002745.2|	251792	+	C	0	0	0	1	0	SA0212

gi|29165615|ref|NC_002745.2|	251793 +	U	0	0	0	0	1	SA0212
gi|29165615|ref|NC_002745.2|	251795 +	A	0	1	0	0	0	SA0212
gi|29165615|ref|NC_002745.2|	251798 +	U	0	0	0	0	1	SA0212
gi|29165615|ref|NC_002745.2|	251808 +	U	0	0	0	1	0	SA0212
gi|29165615|ref|NC_002745.2|	251813 +	U	0	0	0	0	1	SA0212
gi|29165615|ref|NC_002745.2|	251818 +	U	0	3	0	0	1	SA0212
gi|29165615|ref|NC_002745.2|	251820 +	G	0	0	0	0	1	SA0212
gi|29165615|ref|NC_002745.2|	251830 +	G	0	0	0	0	1	SA0212
gi|29165615|ref|NC_002745.2|	251832 +	U	0	0	0	0	1	SA0212
gi|29165615|ref|NC_002745.2|	251837 +	U	0	0	0	1	0	SA0212
gi|29165615|ref|NC_002745.2|	251850 +	C	0	0	0	1	0	SA0212
gi|29165615|ref|NC_002745.2|	251851 +	A	0	0	0	1	0	SA0212
gi|29165615|ref|NC_002745.2|	251856 +	A	0	0	0	1	0	SA0212
gi|29165615|ref|NC_002745.2|	251872 +	A	0	1	0	0	0	SA0212
gi|29165615|ref|NC_002745.2|	251875 +	C	0	1	0	0	0	SA0212
gi|29165615|ref|NC_002745.2|	251876 +	A	0	0	0	0	1	SA0212
gi|29165615|ref|NC_002745.2|	251884 +	C	0	0	0	1	0	SA0212
gi|29165615|ref|NC_002745.2|	251885 +	A	0	0	0	0	1	SA0212
gi|29165615|ref|NC_002745.2|	251891 +	A	0	0	0	0	1	SA0212
gi|29165615|ref|NC_002745.2|	251893 +	G	0	0	0	0	1	SA0212
gi|29165615|ref|NC_002745.2|	251896 +	A	0	1	0	0	0	SA0212
gi|29165615|ref|NC_002745.2|	251902 +	A	0	0	0	2	1	SA0212
gi|29165615|ref|NC_002745.2|	251905 +	A	0	0	0	0	1	SA0212
gi|29165615|ref|NC_002745.2|	251907 +	U	0	0	0	0	1	SA0212
gi|29165615|ref|NC_002745.2|	251908 +	C	0	4	0	0	0	SA0212
gi|29165615|ref|NC_002745.2|	251909 +	A	0	2	0	0	0	SA0212
gi|29165615|ref|NC_002745.2|	251911 +	G	0	0	0	1	1	SA0212
gi|29165615|ref|NC_002745.2|	251912 +	A	0	0	0	0	2	SA0212
gi|29165615|ref|NC_002745.2|	251913 +	A	0	0	0	1	0	SA0212
gi|29165615|ref|NC_002745.2|	251914 +	A	0	1	0	0	0	SA0212
gi|29165615|ref|NC_002745.2|	251915 +	A	0	1	0	0	1	SA0212
gi|29165615|ref|NC_002745.2|	251916 +	U	0	0	0	0	2	SA0212
gi|29165615|ref|NC_002745.2|	251919 +	A	0	0	0	2	1	SA0212
gi|29165615|ref|NC_002745.2|	251920 +	A	0	0	0	0	1	SA0212
gi|29165615|ref|NC_002745.2|	251921 +	A	0	3	0	2	2	SA0212
gi|29165615|ref|NC_002745.2|	251922 +	U	0	3	1	1	2	SA0212
gi|29165615|ref|NC_002745.2|	251923 +	A	0	3	0	0	2	SA0212
gi|29165615|ref|NC_002745.2|	251924 +	U	0	1	3	1	0	SA0212
gi|29165615|ref|NC_002745.2|	251927 +	A	0	0	0	1	0	SA0212
gi|29165615|ref|NC_002745.2|	251931 +	U	1	0	0	0	0	SA0212
gi|29165615|ref|NC_002745.2|	251932 +	C	0	1	0	0	1	SA0212
gi|29165615|ref|NC_002745.2|	251934 +	A	0	0	0	1	0	SA0212
gi|29165615|ref|NC_002745.2|	251935 +	A	0	0	0	1	0	SA0212
gi|29165615|ref|NC_002745.2|	251936 +	C	0	1	0	0	0	SA0212
gi|29165615|ref|NC_002745.2|	251940 +	U	0	0	0	1	2	SA0212
gi|29165615|ref|NC_002745.2|	251944 +	C	0	1	0	0	0	SA0212
gi|29165615|ref|NC_002745.2|	251945 +	A	0	1	0	0	0	SA0212
gi|29165615|ref|NC_002745.2|	251949 +	A	0	0	0	0	2	SA0212
gi|29165615|ref|NC_002745.2|	251950 +	U	1	1	1	5	4	SA0212
gi|29165615|ref|NC_002745.2|	251951 +	A	0	1	0	0	0	SA0212
gi|29165615|ref|NC_002745.2|	251955 +	U	0	1	0	0	2	SA0212
gi|29165615|ref|NC_002745.2|	251956 +	A	0	1	0	1	1	SA0212
gi|29165615|ref|NC_002745.2|	251957 +	A	0	0	1	0	0	SA0212
gi|29165615|ref|NC_002745.2|	251959 +	G	0	0	0	1	0	SA0212
gi|29165615|ref|NC_002745.2|	251968 +	A	0	0	0	1	0	SA0212
gi|29165615|ref|NC_002745.2|	251978 +	C	0	1	0	0	0	SA0212
gi|29165615|ref|NC_002745.2|	251980 +	U	0	0	0	0	1	SA0212
gi|29165615|ref|NC_002745.2|	251989 +	G	0	0	0	0	1	SA0212
gi|29165615|ref|NC_002745.2|	251997 +	U	0	0	0	0	1	SA0212
gi|29165615|ref|NC_002745.2|	252017 +	G	0	0	0	0	1	SA0212
gi|29165615|ref|NC_002745.2|	252021 +	A	0	1	0	0	0	SA0212
gi|29165615|ref|NC_002745.2|	252024 +	U	0	0	0	1	2	SA0212
gi|29165615|ref|NC_002745.2|	252025 +	A	0	0	0	0	1	SA0212
gi|29165615|ref|NC_002745.2|	252029 +	U	0	1	0	0	0	SA0212
gi|29165615|ref|NC_002745.2|	252034 +	C	0	1	0	1	1	SA0212
gi|29165615|ref|NC_002745.2|	252036 +	A	0	5	0	0	2	SA0212
gi|29165615|ref|NC_002745.2|	252043 +	A	0	1	0	0	0	SA0212
gi|29165615|ref|NC_002745.2|	252047 +	A	0	3	0	1	1	SA0212
gi|29165615|ref|NC_002745.2|	252052 +	U	0	1	0	0	1	SA0212
gi|29165615|ref|NC_002745.2|	252056 +	A	0	0	0	0	1	SA0212
gi|29165615|ref|NC_002745.2|	252058 +	U	0	0	0	1	0	SA0212
gi|29165615|ref|NC_002745.2|	252060 +	U	0	0	0	0	1	SA0212
gi|29165615|ref|NC_002745.2|	252062 +	U	0	1	0	0	0	SA0212
gi|29165615|ref|NC_002745.2|	252063 +	A	0	0	0	1	0	SA0212
gi|29165615|ref|NC_002745.2|	252067 +	A	0	1	0	0	0	SA0212
gi|29165615|ref|NC_002745.2|	252074 +	A	0	0	0	3	0	SA0212
gi|29165615|ref|NC_002745.2|	252080 +	A	0	0	0	1	0	SA0212
gi|29165615|ref|NC_002745.2|	252090 +	U	0	1	0	0	0	SA0212
gi|29165615|ref|NC_002745.2|	252102 +	A	0	1	0	0	0	SA0212
gi|29165615|ref|NC_002745.2|	252153 +	C	0	1	0	0	0	SA0212
gi|29165615|ref|NC_002745.2|	252184 +	G	0	0	0	0	1	-

gi|29165615|ref|NC_002745.2|	252185	+	A	0	0	0	1	0	-
gi|29165615|ref|NC_002745.2|	252203	+	A	0	0	0	0	1	-
gi|29165615|ref|NC_002745.2|	252216	+	U	0	1	0	0	1	-
gi|29165615|ref|NC_002745.2|	252286	+	C	0	0	0	0	2	-
gi|29165615|ref|NC_002745.2|	252292	+	A	0	0	0	0	1	-
gi|29165615|ref|NC_002745.2|	252898	+	U	0	0	0	0	1	-
gi|29165615|ref|NC_002745.2|	252936	+	C	0	0	0	0	1	-
gi|29165615|ref|NC_002745.2|	253099	+	G	0	0	1	0	0	-
gi|29165615|ref|NC_002745.2|	253179	+	A	0	1	0	0	0	-
gi|29165615|ref|NC_002745.2|	253191	+	U	0	0	0	0	1	-
gi|29165615|ref|NC_002745.2|	253243	+	A	0	0	0	1	1	-
gi|29165615|ref|NC_002745.2|	253435	+	A	0	0	0	0	2	SA0214
gi|29165615|ref|NC_002745.2|	253494	+	C	0	1	0	0	0	SA0214
gi|29165615|ref|NC_002745.2|	253508	+	A	0	1	0	0	0	SA0214
gi|29165615|ref|NC_002745.2|	253561	+	G	0	0	0	0	1	SA0214
gi|29165615|ref|NC_002745.2|	253562	+	U	0	0	0	1	1	SA0214
gi|29165615|ref|NC_002745.2|	253563	+	U	0	0	0	0	1	SA0214
gi|29165615|ref|NC_002745.2|	253586	+	A	0	0	0	1	0	SA0214
gi|29165615|ref|NC_002745.2|	253589	+	G	0	0	0	0	3	SA0214
gi|29165615|ref|NC_002745.2|	253590	+	U	2	63	33	52	128	SA0214
gi|29165615|ref|NC_002745.2|	253591	+	U	0	19	5	12	36	SA0214
gi|29165615|ref|NC_002745.2|	253592	+	U	0	7	2	10	11	SA0214
gi|29165615|ref|NC_002745.2|	253593	+	U	0	0	1	3	4	SA0214
gi|29165615|ref|NC_002745.2|	253594	+	A	0	4	0	1	3	SA0214
gi|29165615|ref|NC_002745.2|	253595	+	A	0	1	0	0	1	SA0214
gi|29165615|ref|NC_002745.2|	253596	+	U	0	0	0	0	2	SA0214
gi|29165615|ref|NC_002745.2|	253597	+	U	0	0	1	0	0	SA0214
gi|29165615|ref|NC_002745.2|	253598	+	A	0	0	1	0	0	SA0214
gi|29165615|ref|NC_002745.2|	253601	+	G	0	1	0	0	0	SA0214
gi|29165615|ref|NC_002745.2|	253602	+	G	0	1	0	1	1	SA0214
gi|29165615|ref|NC_002745.2|	253603	+	A	0	0	0	2	0	SA0214
gi|29165615|ref|NC_002745.2|	253604	+	U	0	0	0	0	1	SA0214
gi|29165615|ref|NC_002745.2|	253605	+	U	0	0	0	1	0	SA0214
gi|29165615|ref|NC_002745.2|	253607	+	G	0	0	0	0	1	SA0214
gi|29165615|ref|NC_002745.2|	253608	+	U	0	0	1	0	0	SA0214
gi|29165615|ref|NC_002745.2|	253617	+	A	0	0	0	1	0	SA0214
gi|29165615|ref|NC_002745.2|	253623	+	G	0	0	0	0	1	SA0214
gi|29165615|ref|NC_002745.2|	253624	+	U	0	1	0	0	0	SA0214
gi|29165615|ref|NC_002745.2|	253636	+	A	0	0	0	0	1	SA0214
gi|29165615|ref|NC_002745.2|	253647	+	U	0	0	0	0	2	SA0214
gi|29165615|ref|NC_002745.2|	253649	+	C	0	0	1	0	0	SA0214
gi|29165615|ref|NC_002745.2|	253715	+	U	0	1	0	0	0	SA0214
gi|29165615|ref|NC_002745.2|	253758	+	A	0	0	0	2	0	SA0214
gi|29165615|ref|NC_002745.2|	253761	+	C	0	0	0	0	1	SA0214
gi|29165615|ref|NC_002745.2|	253818	+	U	0	0	1	0	0	SA0214
gi|29165615|ref|NC_002745.2|	253838	+	A	0	0	0	0	1	SA0214
gi|29165615|ref|NC_002745.2|	253897	+	U	0	0	0	1	0	SA0214
gi|29165615|ref|NC_002745.2|	253899	+	U	0	0	0	0	1	SA0214
gi|29165615|ref|NC_002745.2|	253900	+	C	0	0	0	0	1	SA0214
gi|29165615|ref|NC_002745.2|	254020	+	U	0	0	0	0	1	SA0214
gi|29165615|ref|NC_002745.2|	254064	+	U	0	0	1	1	0	SA0214
gi|29165615|ref|NC_002745.2|	254111	+	U	0	0	0	1	0	SA0214
gi|29165615|ref|NC_002745.2|	254127	+	U	0	0	1	0	0	SA0214
gi|29165615|ref|NC_002745.2|	254134	+	U	0	0	0	0	1	SA0214
gi|29165615|ref|NC_002745.2|	254164	+	C	0	0	0	0	2	SA0214
gi|29165615|ref|NC_002745.2|	254171	+	G	0	0	0	0	1	SA0214
gi|29165615|ref|NC_002745.2|	254172	+	A	0	0	0	1	0	SA0214
gi|29165615|ref|NC_002745.2|	254203	+	G	0	1	0	0	0	SA0214
gi|29165615|ref|NC_002745.2|	254299	+	U	0	0	0	0	1	SA0214
gi|29165615|ref|NC_002745.2|	254330	+	U	0	1	0	0	0	SA0214
gi|29165615|ref|NC_002745.2|	254403	+	A	0	2	0	0	0	SA0214
gi|29165615|ref|NC_002745.2|	254413	+	U	0	0	0	1	0	SA0214
gi|29165615|ref|NC_002745.2|	254421	+	A	0	2	0	0	0	SA0214
gi|29165615|ref|NC_002745.2|	254631	+	G	0	0	0	0	1	SA0214
gi|29165615|ref|NC_002745.2|	254646	+	A	0	0	0	1	0	SA0214
gi|29165615|ref|NC_002745.2|	255819	+	G	0	0	1	0	0	-
gi|29165615|ref|NC_002745.2|	255902	+	G	0	0	0	0	1	-
gi|29165615|ref|NC_002745.2|	256728	+	U	0	2	0	0	0	-
gi|29165615|ref|NC_002745.2|	256909	+	A	0	0	1	0	0	-
gi|29165615|ref|NC_002745.2|	256934	+	A	0	1	0	0	0	-
gi|29165615|ref|NC_002745.2|	257000	+	U	0	0	0	0	1	-
gi|29165615|ref|NC_002745.2|	257516	+	G	0	0	0	0	1	-
gi|29165615|ref|NC_002745.2|	257609	+	U	0	1	0	0	0	-
gi|29165615|ref|NC_002745.2|	258651	+	A	0	0	0	1	0	-
gi|29165615|ref|NC_002745.2|	258781	+	A	0	0	0	0	1	-
gi|29165615|ref|NC_002745.2|	258785	+	C	0	1	0	1	0	-
gi|29165615|ref|NC_002745.2|	258809	+	G	0	2	1	2	3	-
gi|29165615|ref|NC_002745.2|	258810	+	A	0	0	0	1	0	-
gi|29165615|ref|NC_002745.2|	258825	+	C	0	0	0	0	1	-
gi|29165615|ref|NC_002745.2|	258844	+	U	0	0	0	1	0	-
gi|29165615|ref|NC_002745.2|	258847	+	A	0	0	1	0	0	-

gi|29165615|ref|NC_002745.2|	258852	+	U	0	1	0	0	0	-
gi|29165615|ref|NC_002745.2|	258860	+	U	0	0	0	1	2	-
gi|29165615|ref|NC_002745.2|	258866	+	A	0	0	0	1	0	SA0218
gi|29165615|ref|NC_002745.2|	258876	+	C	0	0	0	0	1	SA0218
gi|29165615|ref|NC_002745.2|	258882	+	A	0	0	0	0	1	SA0218
gi|29165615|ref|NC_002745.2|	258883	+	A	0	0	0	1	0	SA0218
gi|29165615|ref|NC_002745.2|	258884	+	A	0	0	1	0	0	SA0218
gi|29165615|ref|NC_002745.2|	258887	+	C	0	2	0	0	0	SA0218
gi|29165615|ref|NC_002745.2|	258907	+	A	0	1	0	0	0	SA0218
gi|29165615|ref|NC_002745.2|	258915	+	A	0	0	0	0	1	SA0218
gi|29165615|ref|NC_002745.2|	258927	+	A	0	0	0	1	0	SA0218
gi|29165615|ref|NC_002745.2|	258976	+	U	1	0	0	0	0	SA0218
gi|29165615|ref|NC_002745.2|	259026	+	U	1	0	0	0	0	SA0218
gi|29165615|ref|NC_002745.2|	259669	+	A	0	0	0	0	1	SA0218
gi|29165615|ref|NC_002745.2|	259713	+	C	1	0	0	0	0	SA0218
gi|29165615|ref|NC_002745.2|	259732	+	A	0	2	0	0	0	SA0218
gi|29165615|ref|NC_002745.2|	260175	+	U	0	0	0	0	1	SA0218
gi|29165615|ref|NC_002745.2|	260214	+	G	1	0	0	0	0	SA0218
gi|29165615|ref|NC_002745.2|	260755	+	A	0	0	0	1	0	SA0218
gi|29165615|ref|NC_002745.2|	261264	+	A	0	1	0	0	0	SA0219
gi|29165615|ref|NC_002745.2|	261540	+	A	0	0	0	0	2	SA0219
gi|29165615|ref|NC_002745.2|	261542	+	A	0	0	0	0	2	SA0219
gi|29165615|ref|NC_002745.2|	261561	+	A	0	0	0	1	0	SA0219
gi|29165615|ref|NC_002745.2|	261572	+	A	0	1	0	0	0	SA0219
gi|29165615|ref|NC_002745.2|	261594	+	C	0	0	0	1	0	SA0219
gi|29165615|ref|NC_002745.2|	262188	+	A	1	2	5	3	1	-
gi|29165615|ref|NC_002745.2|	262197	+	G	0	1	0	0	0	-
gi|29165615|ref|NC_002745.2|	262344	+	C	0	0	0	1	0	SA0220
gi|29165615|ref|NC_002745.2|	262451	+	A	0	0	0	0	1	SA0220
gi|29165615|ref|NC_002745.2|	262453	+	U	1	0	0	0	1	SA0220
gi|29165615|ref|NC_002745.2|	262454	+	A	0	0	0	1	0	SA0220
gi|29165615|ref|NC_002745.2|	262455	+	A	0	0	0	0	1	SA0220
gi|29165615|ref|NC_002745.2|	262558	+	C	0	0	1	1	0	SA0220
gi|29165615|ref|NC_002745.2|	262600	+	U	0	1	0	0	0	SA0220
gi|29165615|ref|NC_002745.2|	262753	+	U	0	0	0	1	0	SA0220
gi|29165615|ref|NC_002745.2|	262765	+	A	0	0	1	0	0	SA0220
gi|29165615|ref|NC_002745.2|	262766	+	C	0	0	1	0	0	SA0220
gi|29165615|ref|NC_002745.2|	262769	+	U	0	2	0	0	0	SA0220
gi|29165615|ref|NC_002745.2|	262770	+	A	0	2	0	0	1	SA0220
gi|29165615|ref|NC_002745.2|	262776	+	A	0	1	0	0	0	SA0220
gi|29165615|ref|NC_002745.2|	262778	+	A	0	0	0	1	0	SA0220
gi|29165615|ref|NC_002745.2|	262783	+	C	0	1	1	0	2	SA0220
gi|29165615|ref|NC_002745.2|	262784	+	C	0	0	0	1	0	SA0220
gi|29165615|ref|NC_002745.2|	262785	+	U	0	0	0	0	1	SA0220
gi|29165615|ref|NC_002745.2|	262797	+	A	0	1	0	0	0	SA0220
gi|29165615|ref|NC_002745.2|	262822	+	A	0	0	0	1	0	SA0220
gi|29165615|ref|NC_002745.2|	262832	+	A	0	1	1	0	0	SA0220
gi|29165615|ref|NC_002745.2|	262842	+	C	0	0	0	2	0	SA0220
gi|29165615|ref|NC_002745.2|	262843	+	A	0	0	0	2	0	SA0220
gi|29165615|ref|NC_002745.2|	262850	+	G	0	0	1	0	0	SA0220
gi|29165615|ref|NC_002745.2|	262851	+	A	0	1	0	0	0	SA0220
gi|29165615|ref|NC_002745.2|	262880	+	A	0	1	0	0	0	SA0220
gi|29165615|ref|NC_002745.2|	262885	+	U	0	1	0	0	0	SA0220
gi|29165615|ref|NC_002745.2|	262887	+	A	0	1	0	0	0	SA0220
gi|29165615|ref|NC_002745.2|	262893	+	G	0	1	0	0	0	SA0220
gi|29165615|ref|NC_002745.2|	262913	+	U	0	0	1	0	0	SA0220
gi|29165615|ref|NC_002745.2|	262916	+	A	0	1	0	0	0	SA0220
gi|29165615|ref|NC_002745.2|	262955	+	U	0	1	0	1	0	SA0220
gi|29165615|ref|NC_002745.2|	262966	+	A	0	0	1	0	0	SA0220
gi|29165615|ref|NC_002745.2|	263043	+	A	0	0	0	0	1	SA0220
gi|29165615|ref|NC_002745.2|	263050	+	C	0	0	0	0	1	SA0220
gi|29165615|ref|NC_002745.2|	263087	+	G	0	0	0	1	0	SA0220
gi|29165615|ref|NC_002745.2|	263095	+	U	0	0	0	0	1	SA0220
gi|29165615|ref|NC_002745.2|	263164	+	U	1	0	0	0	0	SA0220
gi|29165615|ref|NC_002745.2|	263167	+	U	0	0	1	0	0	SA0220
gi|29165615|ref|NC_002745.2|	263217	+	A	0	0	1	0	0	SA0220
gi|29165615|ref|NC_002745.2|	263221	+	C	0	1	0	0	0	SA0220
gi|29165615|ref|NC_002745.2|	263225	+	C	0	0	1	0	0	SA0220
gi|29165615|ref|NC_002745.2|	263229	+	A	0	0	0	0	2	SA0220
gi|29165615|ref|NC_002745.2|	263230	+	C	0	0	0	1	0	SA0220
gi|29165615|ref|NC_002745.2|	263232	+	A	0	1	0	0	0	SA0220
gi|29165615|ref|NC_002745.2|	263243	+	U	0	0	0	1	0	SA0220
gi|29165615|ref|NC_002745.2|	263248	+	G	0	1	0	0	0	SA0220
gi|29165615|ref|NC_002745.2|	263257	+	G	0	0	0	1	0	SA0220
gi|29165615|ref|NC_002745.2|	263263	+	A	0	0	0	1	0	SA0220
gi|29165615|ref|NC_002745.2|	263265	+	G	0	0	1	0	0	SA0220
gi|29165615|ref|NC_002745.2|	263283	+	U	0	0	1	0	0	SA0220
gi|29165615|ref|NC_002745.2|	263292	+	U	0	0	0	1	0	SA0220
gi|29165615|ref|NC_002745.2|	263293	+	C	0	0	0	1	1	SA0220
gi|29165615|ref|NC_002745.2|	263397	+	U	0	0	0	0	1	SA0220
gi|29165615|ref|NC_002745.2|	263415	+	A	0	1	0	0	0	SA0220

gi|29165615|ref|NC_002745.2|	263600	+	U	0	0	0	1	0	SA0220
gi|29165615|ref|NC_002745.2|	263793	+	C	0	0	1	0	0	SA0220
gi|29165615|ref|NC_002745.2|	263814	+	G	0	0	0	0	1	SA0220
gi|29165615|ref|NC_002745.2|	264540	+	A	0	1	0	0	0	-
gi|29165615|ref|NC_002745.2|	264646	+	G	0	1	0	1	0	-
gi|29165615|ref|NC_002745.2|	264663	+	G	0	0	1	0	0	-
gi|29165615|ref|NC_002745.2|	264685	+	C	0	0	0	0	2	SA0222
gi|29165615|ref|NC_002745.2|	264737	+	A	1	0	1	0	0	SA0222
gi|29165615|ref|NC_002745.2|	264770	+	A	1	0	0	0	0	SA0222
gi|29165615|ref|NC_002745.2|	264796	+	A	0	0	3	0	0	SA0222
gi|29165615|ref|NC_002745.2|	264847	+	G	0	0	1	0	0	SA0222
gi|29165615|ref|NC_002745.2|	265088	+	A	0	0	2	0	0	SA0222
gi|29165615|ref|NC_002745.2|	265164	+	A	0	1	1	0	0	SA0222
gi|29165615|ref|NC_002745.2|	265165	+	A	0	0	1	0	0	SA0222
gi|29165615|ref|NC_002745.2|	265172	+	A	0	0	1	0	0	SA0222
gi|29165615|ref|NC_002745.2|	265252	+	G	0	0	0	0	2	SA0222
gi|29165615|ref|NC_002745.2|	265256	+	U	0	0	0	0	1	SA0222
gi|29165615|ref|NC_002745.2|	265271	+	A	0	0	0	0	1	SA0222
gi|29165615|ref|NC_002745.2|	265276	+	G	0	2	1	0	1	SA0222
gi|29165615|ref|NC_002745.2|	265334	+	C	1	0	0	0	0	SA0222
gi|29165615|ref|NC_002745.2|	265534	+	A	0	1	0	0	0	SA0222
gi|29165615|ref|NC_002745.2|	265584	+	A	0	0	0	0	1	SA0222
gi|29165615|ref|NC_002745.2|	265747	+	G	0	0	0	0	1	SA0222
gi|29165615|ref|NC_002745.2|	265950	+	A	0	2	0	0	0	SA0222
gi|29165615|ref|NC_002745.2|	266674	+	C	0	1	0	0	0	-
gi|29165615|ref|NC_002745.2|	271526	+	U	0	0	1	0	0	-
gi|29165615|ref|NC_002745.2|	275654	+	U	0	3	1	0	0	-
gi|29165615|ref|NC_002745.2|	275657	+	A	3	5	7	4	6	-
gi|29165615|ref|NC_002745.2|	275669	+	G	0	0	1	0	0	-
gi|29165615|ref|NC_002745.2|	275678	+	U	0	0	0	0	1	-
gi|29165615|ref|NC_002745.2|	275708	+	A	1	0	0	0	0	-
gi|29165615|ref|NC_002745.2|	275735	+	A	0	0	1	0	0	-
gi|29165615|ref|NC_002745.2|	275820	+	G	0	0	1	0	0	SA0228
gi|29165615|ref|NC_002745.2|	275821	+	A	0	0	0	0	1	SA0228
gi|29165615|ref|NC_002745.2|	275827	+	U	0	1	0	0	0	SA0228
gi|29165615|ref|NC_002745.2|	275828	+	C	0	0	0	0	1	SA0228
gi|29165615|ref|NC_002745.2|	275830	+	A	0	0	0	0	1	SA0228
gi|29165615|ref|NC_002745.2|	275835	+	C	0	1	2	1	1	SA0228
gi|29165615|ref|NC_002745.2|	275836	+	A	0	2	0	1	0	SA0228
gi|29165615|ref|NC_002745.2|	275837	+	A	0	1	0	0	0	SA0228
gi|29165615|ref|NC_002745.2|	275863	+	C	0	0	0	1	0	SA0228
gi|29165615|ref|NC_002745.2|	275887	+	A	0	0	0	0	1	SA0228
gi|29165615|ref|NC_002745.2|	275890	+	C	0	0	0	1	0	SA0228
gi|29165615|ref|NC_002745.2|	275912	+	A	0	0	1	1	1	SA0228
gi|29165615|ref|NC_002745.2|	275916	+	A	0	0	0	0	1	SA0228
gi|29165615|ref|NC_002745.2|	275920	+	C	0	0	1	0	0	SA0228
gi|29165615|ref|NC_002745.2|	275926	+	G	0	0	0	0	1	SA0228
gi|29165615|ref|NC_002745.2|	275927	+	U	0	0	0	1	0	SA0228
gi|29165615|ref|NC_002745.2|	275970	+	G	0	1	0	0	0	SA0228
gi|29165615|ref|NC_002745.2|	275990	+	U	0	1	0	0	0	SA0228
gi|29165615|ref|NC_002745.2|	275997	+	C	0	1	1	0	0	SA0228
gi|29165615|ref|NC_002745.2|	276004	+	A	0	0	1	2	1	SA0228
gi|29165615|ref|NC_002745.2|	276006	+	A	0	1	0	0	1	SA0228
gi|29165615|ref|NC_002745.2|	276010	+	A	0	1	4	4	5	SA0228
gi|29165615|ref|NC_002745.2|	276011	+	A	0	2	0	0	0	SA0228
gi|29165615|ref|NC_002745.2|	276012	+	G	0	0	0	0	1	SA0228
gi|29165615|ref|NC_002745.2|	276013	+	C	0	3	2	3	1	SA0228
gi|29165615|ref|NC_002745.2|	276015	+	G	0	2	0	2	0	SA0228
gi|29165615|ref|NC_002745.2|	276019	+	A	0	2	0	1	0	SA0228
gi|29165615|ref|NC_002745.2|	276020	+	A	0	0	1	0	0	SA0228
gi|29165615|ref|NC_002745.2|	276024	+	U	0	0	0	0	1	SA0228
gi|29165615|ref|NC_002745.2|	276027	+	A	0	1	0	0	0	SA0228
gi|29165615|ref|NC_002745.2|	276029	+	A	0	0	1	0	0	SA0228
gi|29165615|ref|NC_002745.2|	276120	+	A	0	0	0	1	0	SA0228
gi|29165615|ref|NC_002745.2|	276127	+	C	0	0	0	0	1	SA0228
gi|29165615|ref|NC_002745.2|	276162	+	A	0	0	0	0	1	SA0228
gi|29165615|ref|NC_002745.2|	276213	+	C	0	0	0	2	1	SA0228
gi|29165615|ref|NC_002745.2|	276214	+	C	1	0	0	0	0	SA0228
gi|29165615|ref|NC_002745.2|	276253	+	U	1	0	0	0	0	SA0228
gi|29165615|ref|NC_002745.2|	276258	+	A	0	1	0	0	0	SA0228
gi|29165615|ref|NC_002745.2|	276259	+	A	0	1	0	0	0	SA0228
gi|29165615|ref|NC_002745.2|	276276	+	G	0	1	0	0	0	SA0228
gi|29165615|ref|NC_002745.2|	276280	+	U	0	0	0	0	1	SA0228
gi|29165615|ref|NC_002745.2|	276294	+	A	2	0	0	0	0	SA0228
gi|29165615|ref|NC_002745.2|	276316	+	U	0	0	0	0	1	SA0228
gi|29165615|ref|NC_002745.2|	276416	+	A	1	0	0	0	0	SA0228
gi|29165615|ref|NC_002745.2|	276425	+	A	0	0	1	0	0	SA0228
gi|29165615|ref|NC_002745.2|	276432	+	C	0	0	0	0	1	SA0228
gi|29165615|ref|NC_002745.2|	276448	+	U	0	0	1	0	0	SA0228
gi|29165615|ref|NC_002745.2|	276451	+	U	0	1	0	1	0	SA0228
gi|29165615|ref|NC_002745.2|	276452	+	C	0	0	0	0	1	SA0228

gi|29165615|ref|NC_002745.2|	276453	+	A	0	1	0	1	0	SA0228
gi|29165615|ref|NC_002745.2|	276456	+	A	0	1	0	2	0	SA0228
gi|29165615|ref|NC_002745.2|	276457	+	A	0	0	0	1	0	SA0228
gi|29165615|ref|NC_002745.2|	276462	+	A	0	2	0	3	1	SA0228
gi|29165615|ref|NC_002745.2|	276463	+	A	0	0	1	0	0	SA0228
gi|29165615|ref|NC_002745.2|	276464	+	A	0	1	0	1	0	SA0228
gi|29165615|ref|NC_002745.2|	276468	+	A	0	0	1	3	0	SA0228
gi|29165615|ref|NC_002745.2|	276474	+	A	0	6	0	0	2	SA0228
gi|29165615|ref|NC_002745.2|	276475	+	U	0	1	0	0	0	SA0228
gi|29165615|ref|NC_002745.2|	276478	+	U	0	0	0	1	0	SA0228
gi|29165615|ref|NC_002745.2|	276480	+	A	0	1	0	1	0	SA0228
gi|29165615|ref|NC_002745.2|	276484	+	G	0	1	0	0	1	SA0228
gi|29165615|ref|NC_002745.2|	276485	+	A	0	2	2	3	5	SA0228
gi|29165615|ref|NC_002745.2|	276486	+	U	0	2	0	0	0	SA0228
gi|29165615|ref|NC_002745.2|	276487	+	U	0	0	0	0	1	SA0228
gi|29165615|ref|NC_002745.2|	276490	+	U	1	0	0	0	0	SA0228
gi|29165615|ref|NC_002745.2|	276535	+	C	0	0	1	0	0	SA0228
gi|29165615|ref|NC_002745.2|	276553	+	U	1	0	0	0	0	SA0228
gi|29165615|ref|NC_002745.2|	276554	+	C	0	1	0	1	0	SA0228
gi|29165615|ref|NC_002745.2|	276555	+	G	0	0	0	0	1	SA0228
gi|29165615|ref|NC_002745.2|	276583	+	A	0	0	0	1	1	SA0228
gi|29165615|ref|NC_002745.2|	276584	+	U	0	0	1	0	0	SA0228
gi|29165615|ref|NC_002745.2|	276589	+	U	0	1	0	1	0	SA0228
gi|29165615|ref|NC_002745.2|	276590	+	C	0	2	0	0	0	SA0228
gi|29165615|ref|NC_002745.2|	276598	+	G	1	0	0	0	0	SA0228
gi|29165615|ref|NC_002745.2|	276605	+	G	1	0	0	0	0	SA0228
gi|29165615|ref|NC_002745.2|	276689	+	C	0	0	0	1	0	SA0228
gi|29165615|ref|NC_002745.2|	276691	+	A	0	1	0	0	0	SA0228
gi|29165615|ref|NC_002745.2|	276719	+	A	0	0	0	0	1	SA0228
gi|29165615|ref|NC_002745.2|	276720	+	U	0	0	0	0	2	SA0228
gi|29165615|ref|NC_002745.2|	276722	+	C	0	0	1	0	5	SA0228
gi|29165615|ref|NC_002745.2|	278246	+	A	0	0	1	0	0	-
gi|29165615|ref|NC_002745.2|	278478	+	G	0	1	1	0	0	-
gi|29165615|ref|NC_002745.2|	278878	+	U	0	0	0	1	0	-
gi|29165615|ref|NC_002745.2|	278887	+	A	0	0	0	0	1	-
gi|29165615|ref|NC_002745.2|	278895	+	A	0	0	1	0	0	-
gi|29165615|ref|NC_002745.2|	279720	+	C	0	0	1	0	0	-
gi|29165615|ref|NC_002745.2|	280271	+	U	0	1	0	0	0	-
gi|29165615|ref|NC_002745.2|	280556	+	A	0	1	0	0	0	-
gi|29165615|ref|NC_002745.2|	280954	+	U	0	1	0	0	0	-
gi|29165615|ref|NC_002745.2|	281240	+	A	0	0	0	1	0	-
gi|29165615|ref|NC_002745.2|	281786	+	C	0	1	0	0	0	SA0232
gi|29165615|ref|NC_002745.2|	284466	+	A	0	2	0	2	0	-
gi|29165615|ref|NC_002745.2|	284469	+	A	0	0	0	0	1	-
gi|29165615|ref|NC_002745.2|	284475	+	U	0	0	0	0	1	-
gi|29165615|ref|NC_002745.2|	284535	+	A	0	0	0	1	0	SA0234
gi|29165615|ref|NC_002745.2|	284550	+	U	1	0	0	0	0	SA0234
gi|29165615|ref|NC_002745.2|	284556	+	U	0	0	0	1	0	SA0234
gi|29165615|ref|NC_002745.2|	284690	+	A	0	0	0	2	0	SA0234
gi|29165615|ref|NC_002745.2|	284700	+	A	1	0	0	0	0	SA0234
gi|29165615|ref|NC_002745.2|	284729	+	G	1	0	0	0	0	SA0234
gi|29165615|ref|NC_002745.2|	284796	+	C	1	0	0	0	0	SA0234
gi|29165615|ref|NC_002745.2|	284828	+	A	0	0	0	0	1	SA0234
gi|29165615|ref|NC_002745.2|	284849	+	A	0	0	1	0	0	SA0234
gi|29165615|ref|NC_002745.2|	284892	+	C	0	1	0	0	0	SA0234
gi|29165615|ref|NC_002745.2|	284931	+	U	1	0	0	0	0	SA0234
gi|29165615|ref|NC_002745.2|	284941	+	U	0	0	0	1	0	SA0234
gi|29165615|ref|NC_002745.2|	285377	+	G	0	0	1	0	0	SA0234
gi|29165615|ref|NC_002745.2|	286775	+	A	0	0	0	1	0	SA0235
gi|29165615|ref|NC_002745.2|	287842	+	A	0	0	1	0	0	SA0235
gi|29165615|ref|NC_002745.2|	288300	+	U	0	0	0	0	1	SA0236
gi|29165615|ref|NC_002745.2|	289437	+	U	0	1	0	0	0	SA0238
gi|29165615|ref|NC_002745.2|	290424	+	A	0	0	0	1	0	SA0239
gi|29165615|ref|NC_002745.2|	290435	+	A	0	1	0	0	0	SA0239
gi|29165615|ref|NC_002745.2|	290652	+	U	1	0	0	0	0	SA0239
gi|29165615|ref|NC_002745.2|	291079	+	G	0	0	0	1	0	SA0239
gi|29165615|ref|NC_002745.2|	291181	+	U	0	0	0	1	0	SAS008
gi|29165615|ref|NC_002745.2|	291193	+	U	0	0	0	0	1	SAS008
gi|29165615|ref|NC_002745.2|	291194	+	A	0	2	4	4	2	SAS008
gi|29165615|ref|NC_002745.2|	291195	+	A	0	0	0	0	1	SAS008
gi|29165615|ref|NC_002745.2|	291393	+	A	0	1	0	0	0	SA0240
gi|29165615|ref|NC_002745.2|	292142	+	G	0	0	0	1	0	SA0240
gi|29165615|ref|NC_002745.2|	292168	+	A	0	0	0	1	0	SA0240
gi|29165615|ref|NC_002745.2|	292324	+	G	0	2	0	0	0	SA0240
gi|29165615|ref|NC_002745.2|	292432	+	A	0	0	1	0	0	-
gi|29165615|ref|NC_002745.2|	292659	+	A	0	3	2	1	3	-
gi|29165615|ref|NC_002745.2|	292669	+	A	0	0	1	0	0	-
gi|29165615|ref|NC_002745.2|	292674	+	G	0	0	1	1	0	-
gi|29165615|ref|NC_002745.2|	292678	+	A	0	1	0	0	0	-
gi|29165615|ref|NC_002745.2|	292686	+	A	0	0	0	0	1	-
gi|29165615|ref|NC_002745.2|	292693	+	U	0	0	0	1	0	-

gi|29165615|ref|NC_002745.2|	292721	+	U	0	1	0	0	0	-
gi|29165615|ref|NC_002745.2|	292781	+	U	0	0	1	0	0	-
gi|29165615|ref|NC_002745.2|	292787	+	A	0	0	0	0	1	-
gi|29165615|ref|NC_002745.2|	292788	+	U	0	0	0	0	1	-
gi|29165615|ref|NC_002745.2|	292790	+	A	0	0	1	0	0	-
gi|29165615|ref|NC_002745.2|	292792	+	C	0	0	0	0	1	-
gi|29165615|ref|NC_002745.2|	292837	+	G	0	2	0	0	0	-
gi|29165615|ref|NC_002745.2|	292841	+	A	0	2	0	0	0	-
gi|29165615|ref|NC_002745.2|	292880	+	A	0	0	0	0	1	-
gi|29165615|ref|NC_002745.2|	292883	+	A	0	0	1	0	0	-
gi|29165615|ref|NC_002745.2|	292966	+	A	0	0	1	0	0	SA0241
gi|29165615|ref|NC_002745.2|	293108	+	U	0	0	0	1	0	SA0241
gi|29165615|ref|NC_002745.2|	293112	+	A	0	0	1	0	1	SA0241
gi|29165615|ref|NC_002745.2|	293114	+	A	0	1	0	0	1	SA0241
gi|29165615|ref|NC_002745.2|	293115	+	A	0	1	0	0	0	SA0241
gi|29165615|ref|NC_002745.2|	293116	+	A	0	0	0	0	2	SA0241
gi|29165615|ref|NC_002745.2|	293117	+	A	0	2	0	1	3	SA0241
gi|29165615|ref|NC_002745.2|	293118	+	A	0	0	1	0	0	SA0241
gi|29165615|ref|NC_002745.2|	293119	+	U	1	0	0	1	0	SA0241
gi|29165615|ref|NC_002745.2|	293123	+	A	0	2	0	0	1	SA0241
gi|29165615|ref|NC_002745.2|	293124	+	C	0	1	0	0	0	SA0241
gi|29165615|ref|NC_002745.2|	293125	+	A	0	0	1	0	0	SA0241
gi|29165615|ref|NC_002745.2|	293126	+	U	0	0	1	0	0	SA0241
gi|29165615|ref|NC_002745.2|	293128	+	A	0	6	0	1	4	SA0241
gi|29165615|ref|NC_002745.2|	293129	+	C	1	13	6	2	10	SA0241
gi|29165615|ref|NC_002745.2|	293130	+	A	0	1	1	0	2	SA0241
gi|29165615|ref|NC_002745.2|	293131	+	G	0	0	0	1	0	SA0241
gi|29165615|ref|NC_002745.2|	293133	+	U	0	0	0	0	1	SA0241
gi|29165615|ref|NC_002745.2|	293135	+	A	0	0	0	1	0	SA0241
gi|29165615|ref|NC_002745.2|	293136	+	A	0	1	0	0	0	SA0241
gi|29165615|ref|NC_002745.2|	293167	+	C	0	1	0	0	0	SA0241
gi|29165615|ref|NC_002745.2|	293181	+	U	0	0	0	0	1	SA0241
gi|29165615|ref|NC_002745.2|	293219	+	U	0	0	0	1	0	SA0241
gi|29165615|ref|NC_002745.2|	293288	+	A	0	0	1	0	0	SA0241
gi|29165615|ref|NC_002745.2|	293293	+	A	0	0	0	0	2	SA0241
gi|29165615|ref|NC_002745.2|	293434	+	A	0	2	0	1	0	SA0241
gi|29165615|ref|NC_002745.2|	293435	+	A	0	2	0	0	0	SA0241
gi|29165615|ref|NC_002745.2|	293436	+	U	0	1	0	0	0	SA0241
gi|29165615|ref|NC_002745.2|	293440	+	A	0	1	0	0	0	SA0241
gi|29165615|ref|NC_002745.2|	293442	+	A	0	1	1	0	2	SA0241
gi|29165615|ref|NC_002745.2|	293452	+	G	0	0	0	2	0	SA0241
gi|29165615|ref|NC_002745.2|	293453	+	A	0	1	1	0	2	SA0241
gi|29165615|ref|NC_002745.2|	293457	+	U	0	0	1	0	0	SA0241
gi|29165615|ref|NC_002745.2|	293464	+	G	0	0	0	0	1	SA0241
gi|29165615|ref|NC_002745.2|	293477	+	A	0	0	0	0	1	SA0241
gi|29165615|ref|NC_002745.2|	293481	+	A	0	0	0	2	2	SA0241
gi|29165615|ref|NC_002745.2|	293482	+	A	0	0	0	1	0	SA0241
gi|29165615|ref|NC_002745.2|	293519	+	A	0	1	0	0	0	SA0241
gi|29165615|ref|NC_002745.2|	293589	+	A	0	1	0	0	0	SA0241
gi|29165615|ref|NC_002745.2|	293645	+	C	0	0	0	1	0	SA0242
gi|29165615|ref|NC_002745.2|	293689	+	U	0	0	1	0	0	SA0242
gi|29165615|ref|NC_002745.2|	293690	+	G	0	0	2	0	0	SA0242
gi|29165615|ref|NC_002745.2|	293736	+	U	1	0	0	0	0	SA0242
gi|29165615|ref|NC_002745.2|	293775	+	A	1	0	0	0	0	SA0242
gi|29165615|ref|NC_002745.2|	293781	+	A	0	0	0	0	1	SA0242
gi|29165615|ref|NC_002745.2|	293821	+	A	0	0	0	0	1	SA0242
gi|29165615|ref|NC_002745.2|	293851	+	A	0	0	1	0	0	SA0242
gi|29165615|ref|NC_002745.2|	294056	+	G	0	1	0	0	0	SA0242
gi|29165615|ref|NC_002745.2|	294077	+	C	0	1	0	0	0	SA0242
gi|29165615|ref|NC_002745.2|	294105	+	C	1	0	0	0	0	SA0242
gi|29165615|ref|NC_002745.2|	294227	+	A	1	0	0	0	0	SA0242
gi|29165615|ref|NC_002745.2|	294275	+	A	0	2	0	0	1	SA0242
gi|29165615|ref|NC_002745.2|	294350	+	A	0	1	0	0	0	SA0242
gi|29165615|ref|NC_002745.2|	294351	+	U	0	0	0	1	0	SA0242
gi|29165615|ref|NC_002745.2|	294401	+	G	0	1	0	0	0	SA0242
gi|29165615|ref|NC_002745.2|	294432	+	U	0	1	0	0	0	SA0242
gi|29165615|ref|NC_002745.2|	294459	+	G	1	0	0	0	0	SA0242
gi|29165615|ref|NC_002745.2|	294460	+	U	0	1	0	0	0	SA0242
gi|29165615|ref|NC_002745.2|	294529	+	U	0	0	0	0	1	SA0242
gi|29165615|ref|NC_002745.2|	294545	+	U	0	3	0	0	0	SA0242
gi|29165615|ref|NC_002745.2|	294555	+	A	0	1	0	0	0	SA0242
gi|29165615|ref|NC_002745.2|	294557	+	G	0	1	0	0	0	SA0242
gi|29165615|ref|NC_002745.2|	294629	+	A	0	1	1	0	0	SA0242
gi|29165615|ref|NC_002745.2|	294683	+	A	0	1	0	0	0	SA0243
gi|29165615|ref|NC_002745.2|	294702	+	U	0	0	0	1	0	SA0243
gi|29165615|ref|NC_002745.2|	294709	+	C	0	1	0	0	0	SA0243
gi|29165615|ref|NC_002745.2|	294713	+	U	0	0	1	0	0	SA0243
gi|29165615|ref|NC_002745.2|	294734	+	U	1	0	0	0	0	SA0243
gi|29165615|ref|NC_002745.2|	294758	+	G	0	0	1	0	0	SA0243
gi|29165615|ref|NC_002745.2|	294771	+	C	1	0	0	0	0	SA0243
gi|29165615|ref|NC_002745.2|	294815	+	G	0	1	1	0	2	SA0243

gi|29165615|ref|NC_002745.2|	294821 +	A	0	0	0	0	1	SA0243
gi|29165615|ref|NC_002745.2|	294824 +	G	0	2	0	0	0	SA0243
gi|29165615|ref|NC_002745.2|	294825 +	A	0	0	0	0	1	SA0243
gi|29165615|ref|NC_002745.2|	294826 +	A	0	0	0	2	0	SA0243
gi|29165615|ref|NC_002745.2|	294833 +	A	0	0	0	0	4	SA0243
gi|29165615|ref|NC_002745.2|	294862 +	C	0	0	0	0	1	SA0243
gi|29165615|ref|NC_002745.2|	294870 +	C	0	1	0	0	0	SA0243
gi|29165615|ref|NC_002745.2|	294873 +	U	0	1	0	0	0	SA0243
gi|29165615|ref|NC_002745.2|	294892 +	U	1	0	0	0	0	SA0243
gi|29165615|ref|NC_002745.2|	294893 +	U	0	1	0	0	0	SA0243
gi|29165615|ref|NC_002745.2|	294900 +	U	1	0	0	0	0	SA0243
gi|29165615|ref|NC_002745.2|	294908 +	U	0	1	0	0	0	SA0243
gi|29165615|ref|NC_002745.2|	294909 +	A	0	0	0	0	1	SA0243
gi|29165615|ref|NC_002745.2|	294914 +	U	0	0	0	1	0	SA0243
gi|29165615|ref|NC_002745.2|	294918 +	U	0	1	1	0	0	SA0243
gi|29165615|ref|NC_002745.2|	294922 +	U	1	0	0	0	0	SA0243
gi|29165615|ref|NC_002745.2|	294923 +	C	1	1	0	0	0	SA0243
gi|29165615|ref|NC_002745.2|	294928 +	C	0	0	0	1	0	SA0243
gi|29165615|ref|NC_002745.2|	294929 +	C	0	1	0	0	1	SA0243
gi|29165615|ref|NC_002745.2|	294932 +	U	0	0	1	0	1	SA0243
gi|29165615|ref|NC_002745.2|	294934 +	A	0	1	0	0	0	SA0243
gi|29165615|ref|NC_002745.2|	294947 +	C	0	1	0	0	0	SA0243
gi|29165615|ref|NC_002745.2|	294967 +	U	0	1	0	0	0	SA0243
gi|29165615|ref|NC_002745.2|	294970 +	U	0	1	0	0	0	SA0243
gi|29165615|ref|NC_002745.2|	295086 +	G	0	1	0	0	0	SA0243
gi|29165615|ref|NC_002745.2|	295104 +	U	1	0	0	0	0	SA0243
gi|29165615|ref|NC_002745.2|	295124 +	A	0	0	0	0	1	SA0243
gi|29165615|ref|NC_002745.2|	295138 +	U	1	0	0	0	0	SA0243
gi|29165615|ref|NC_002745.2|	295240 +	A	0	0	1	0	0	SA0243
gi|29165615|ref|NC_002745.2|	295294 +	A	0	0	0	0	1	SA0243
gi|29165615|ref|NC_002745.2|	295315 +	A	0	1	0	0	0	SA0243
gi|29165615|ref|NC_002745.2|	295343 +	A	0	0	0	0	1	SA0243
gi|29165615|ref|NC_002745.2|	295382 +	C	0	1	0	0	0	SA0243
gi|29165615|ref|NC_002745.2|	295410 +	A	0	0	0	0	1	SA0243
gi|29165615|ref|NC_002745.2|	295418 +	A	0	2	0	0	0	SA0243
gi|29165615|ref|NC_002745.2|	295438 +	U	0	0	1	0	0	SA0243
gi|29165615|ref|NC_002745.2|	295453 +	A	0	0	0	0	1	SA0243
gi|29165615|ref|NC_002745.2|	295481 +	U	0	1	0	0	0	SA0243
gi|29165615|ref|NC_002745.2|	295546 +	U	0	0	1	0	0	SA0243
gi|29165615|ref|NC_002745.2|	295560 +	A	0	0	0	0	1	SA0243
gi|29165615|ref|NC_002745.2|	295703 +	U	0	0	0	1	0	SA0243
gi|29165615|ref|NC_002745.2|	295782 +	A	0	1	0	0	0	SA0243
gi|29165615|ref|NC_002745.2|	296222 +	A	0	1	0	0	0	SA0243
gi|29165615|ref|NC_002745.2|	296228 +	C	0	0	0	1	0	SA0243
gi|29165615|ref|NC_002745.2|	296230 +	A	0	2	0	0	1	SA0243
gi|29165615|ref|NC_002745.2|	296261 +	A	0	0	0	1	0	SA0243
gi|29165615|ref|NC_002745.2|	296809 +	C	0	0	1	1	0	SA0244
gi|29165615|ref|NC_002745.2|	296825 +	A	0	1	0	0	0	SA0244
gi|29165615|ref|NC_002745.2|	296882 +	A	0	0	1	0	0	SA0244
gi|29165615|ref|NC_002745.2|	296893 +	A	0	0	0	1	0	SA0244
gi|29165615|ref|NC_002745.2|	297029 +	U	1	0	0	0	0	SA0244
gi|29165615|ref|NC_002745.2|	297039 +	U	0	0	0	0	1	SA0244
gi|29165615|ref|NC_002745.2|	297558 +	U	0	0	0	0	1	SA0244
gi|29165615|ref|NC_002745.2|	297566 +	C	0	0	0	0	1	SA0244
gi|29165615|ref|NC_002745.2|	297567 +	A	0	1	0	0	0	SA0244
gi|29165615|ref|NC_002745.2|	297810 +	A	0	2	0	0	0	SA0244
gi|29165615|ref|NC_002745.2|	298087 +	U	0	1	0	0	0	-
gi|29165615|ref|NC_002745.2|	298158 +	A	0	0	1	0	0	-
gi|29165615|ref|NC_002745.2|	298184 +	A	0	1	0	0	0	-
gi|29165615|ref|NC_002745.2|	298189 +	A	0	0	0	0	1	-
gi|29165615|ref|NC_002745.2|	298193 +	A	0	2	0	2	0	-
gi|29165615|ref|NC_002745.2|	298194 +	A	0	0	0	1	1	-
gi|29165615|ref|NC_002745.2|	298200 +	A	0	0	0	0	1	-
gi|29165615|ref|NC_002745.2|	298204 +	A	0	0	1	0	0	-
gi|29165615|ref|NC_002745.2|	298207 +	A	0	1	0	0	0	-
gi|29165615|ref|NC_002745.2|	298208 +	A	0	0	0	1	0	-
gi|29165615|ref|NC_002745.2|	298210 +	U	0	0	0	0	1	-
gi|29165615|ref|NC_002745.2|	298215 +	A	0	1	0	0	0	-
gi|29165615|ref|NC_002745.2|	298231 +	G	0	0	2	0	0	-
gi|29165615|ref|NC_002745.2|	298242 +	U	1	0	0	0	0	SA0245
gi|29165615|ref|NC_002745.2|	298243 +	G	0	2	0	0	0	SA0245
gi|29165615|ref|NC_002745.2|	298263 +	C	0	1	0	0	0	SA0245
gi|29165615|ref|NC_002745.2|	298265 +	G	0	0	0	1	0	SA0245
gi|29165615|ref|NC_002745.2|	298354 +	A	0	0	0	0	1	SA0245
gi|29165615|ref|NC_002745.2|	298366 +	A	0	0	0	1	0	SA0245
gi|29165615|ref|NC_002745.2|	298385 +	A	0	0	0	1	0	SA0245
gi|29165615|ref|NC_002745.2|	298450 +	U	1	0	0	0	0	SA0245
gi|29165615|ref|NC_002745.2|	298526 +	G	0	1	1	0	1	SA0245
gi|29165615|ref|NC_002745.2|	298527 +	A	0	0	0	1	0	SA0245
gi|29165615|ref|NC_002745.2|	298608 +	A	0	0	0	0	1	SA0245
gi|29165615|ref|NC_002745.2|	298611 +	A	0	2	0	2	1	SA0245

gi|29165615|ref|NC_002745.2|	298612	+	A	0	0	0	0	1	SA0245
gi|29165615|ref|NC_002745.2|	298613	+	A	0	0	0	2	0	SA0245
gi|29165615|ref|NC_002745.2|	298614	+	A	0	3	1	0	0	SA0245
gi|29165615|ref|NC_002745.2|	298615	+	U	0	1	1	0	0	SA0245
gi|29165615|ref|NC_002745.2|	298616	+	A	0	1	0	0	0	SA0245
gi|29165615|ref|NC_002745.2|	298619	+	C	0	2	0	0	3	SA0245
gi|29165615|ref|NC_002745.2|	298620	+	A	0	1	0	0	2	SA0245
gi|29165615|ref|NC_002745.2|	298673	+	A	0	0	1	0	0	SA0245
gi|29165615|ref|NC_002745.2|	298679	+	A	0	0	0	1	0	SA0245
gi|29165615|ref|NC_002745.2|	298692	+	A	0	0	0	0	1	SA0245
gi|29165615|ref|NC_002745.2|	298764	+	A	0	0	0	0	1	SA0245
gi|29165615|ref|NC_002745.2|	298776	+	A	0	0	0	1	0	SA0245
gi|29165615|ref|NC_002745.2|	298793	+	A	0	0	0	1	0	SA0245
gi|29165615|ref|NC_002745.2|	298808	+	A	0	0	1	0	0	SA0245
gi|29165615|ref|NC_002745.2|	298815	+	U	0	0	1	0	0	SA0245
gi|29165615|ref|NC_002745.2|	298819	+	U	1	0	0	0	0	SA0245
gi|29165615|ref|NC_002745.2|	298824	+	C	0	0	1	0	1	SA0245
gi|29165615|ref|NC_002745.2|	298835	+	A	0	0	1	0	0	SA0245
gi|29165615|ref|NC_002745.2|	298842	+	A	0	0	2	0	0	SA0245
gi|29165615|ref|NC_002745.2|	298845	+	C	0	1	0	0	1	SA0245
gi|29165615|ref|NC_002745.2|	298847	+	A	0	1	0	0	0	SA0245
gi|29165615|ref|NC_002745.2|	298863	+	U	1	0	0	0	0	SA0245
gi|29165615|ref|NC_002745.2|	298904	+	U	0	0	1	0	0	SA0245
gi|29165615|ref|NC_002745.2|	298922	+	A	0	0	1	0	1	SA0245
gi|29165615|ref|NC_002745.2|	298926	+	C	0	0	0	0	1	SA0245
gi|29165615|ref|NC_002745.2|	299126	+	A	0	2	0	0	0	SA0246
gi|29165615|ref|NC_002745.2|	299177	+	U	0	0	0	0	1	SA0246
gi|29165615|ref|NC_002745.2|	299225	+	A	0	0	0	1	0	SA0246
gi|29165615|ref|NC_002745.2|	299255	+	U	0	0	1	0	0	SA0246
gi|29165615|ref|NC_002745.2|	299259	+	A	0	0	0	1	0	SA0246
gi|29165615|ref|NC_002745.2|	299262	+	U	0	1	0	0	0	SA0246
gi|29165615|ref|NC_002745.2|	299274	+	A	0	0	0	0	1	SA0246
gi|29165615|ref|NC_002745.2|	299320	+	A	1	0	0	0	0	SA0246
gi|29165615|ref|NC_002745.2|	299392	+	U	0	1	0	0	0	SA0246
gi|29165615|ref|NC_002745.2|	299424	+	A	0	0	0	0	1	SA0246
gi|29165615|ref|NC_002745.2|	299442	+	A	0	0	0	0	1	SA0246
gi|29165615|ref|NC_002745.2|	299502	+	A	0	1	1	0	0	SA0246
gi|29165615|ref|NC_002745.2|	299504	+	A	0	1	0	0	0	SA0246
gi|29165615|ref|NC_002745.2|	299545	+	A	2	0	0	1	0	SA0246
gi|29165615|ref|NC_002745.2|	299601	+	C	0	1	0	0	0	SA0246
gi|29165615|ref|NC_002745.2|	299614	+	C	0	0	0	0	1	SA0246
gi|29165615|ref|NC_002745.2|	299673	+	A	0	0	0	0	1	SA0246
gi|29165615|ref|NC_002745.2|	299681	+	U	3	0	0	0	0	SA0246
gi|29165615|ref|NC_002745.2|	299709	+	C	0	2	0	0	0	SA0246
gi|29165615|ref|NC_002745.2|	299712	+	U	1	0	0	0	0	SA0246
gi|29165615|ref|NC_002745.2|	299746	+	C	0	0	1	0	0	SA0246
gi|29165615|ref|NC_002745.2|	299750	+	U	0	0	1	0	0	SA0246
gi|29165615|ref|NC_002745.2|	299755	+	U	0	0	0	0	1	SA0246
gi|29165615|ref|NC_002745.2|	299831	+	C	0	1	0	0	0	SA0246
gi|29165615|ref|NC_002745.2|	299843	+	A	1	0	0	1	1	SA0246
gi|29165615|ref|NC_002745.2|	299852	+	A	0	1	0	0	0	SA0246
gi|29165615|ref|NC_002745.2|	299857	+	A	0	0	1	0	0	SA0246
gi|29165615|ref|NC_002745.2|	299859	+	U	0	0	0	1	0	SA0246
gi|29165615|ref|NC_002745.2|	299866	+	U	2	0	0	0	0	SA0246
gi|29165615|ref|NC_002745.2|	299868	+	U	0	1	0	0	0	SA0246
gi|29165615|ref|NC_002745.2|	299889	+	A	0	1	0	0	0	SA0246
gi|29165615|ref|NC_002745.2|	299915	+	A	1	0	0	0	0	SA0246
gi|29165615|ref|NC_002745.2|	299920	+	U	1	0	0	0	0	SA0246
gi|29165615|ref|NC_002745.2|	299952	+	A	0	1	0	0	1	SA0246
gi|29165615|ref|NC_002745.2|	299956	+	U	0	0	0	0	1	SA0246
gi|29165615|ref|NC_002745.2|	299957	+	A	0	1	2	0	1	SA0246
gi|29165615|ref|NC_002745.2|	299961	+	A	0	1	0	1	0	SA0246
gi|29165615|ref|NC_002745.2|	299962	+	A	0	0	0	0	1	SA0246
gi|29165615|ref|NC_002745.2|	299973	+	U	0	0	0	0	1	SA0246
gi|29165615|ref|NC_002745.2|	299976	+	A	0	2	0	0	0	-
gi|29165615|ref|NC_002745.2|	300025	+	A	0	1	0	0	0	SA0247
gi|29165615|ref|NC_002745.2|	300085	+	A	0	0	0	1	0	SA0247
gi|29165615|ref|NC_002745.2|	300127	+	C	0	1	0	0	1	SA0247
gi|29165615|ref|NC_002745.2|	300150	+	A	0	2	0	0	0	SA0247
gi|29165615|ref|NC_002745.2|	300151	+	U	0	0	0	1	0	SA0247
gi|29165615|ref|NC_002745.2|	300208	+	A	0	0	1	1	0	SA0247
gi|29165615|ref|NC_002745.2|	300214	+	C	0	0	0	0	1	SA0247
gi|29165615|ref|NC_002745.2|	300229	+	U	0	1	0	0	0	SA0247
gi|29165615|ref|NC_002745.2|	300238	+	A	0	0	0	0	1	SA0247
gi|29165615|ref|NC_002745.2|	300257	+	U	0	0	1	0	0	SA0247
gi|29165615|ref|NC_002745.2|	300265	+	A	1	0	0	0	0	SA0247
gi|29165615|ref|NC_002745.2|	300284	+	U	0	0	1	0	0	SA0247
gi|29165615|ref|NC_002745.2|	300301	+	A	0	1	0	0	0	SA0247
gi|29165615|ref|NC_002745.2|	300303	+	C	0	1	0	0	0	SA0247
gi|29165615|ref|NC_002745.2|	300331	+	A	0	0	0	0	1	SA0247
gi|29165615|ref|NC_002745.2|	300338	+	A	1	1	0	0	0	SA0247

gi|29165615|ref|NC_002745.2|	300342	+	A	0	0	1	0	0	SA0247
gi|29165615|ref|NC_002745.2|	300354	+	A	0	0	0	0	1	SA0247
gi|29165615|ref|NC_002745.2|	300358	+	U	0	0	0	0	1	SA0247
gi|29165615|ref|NC_002745.2|	300415	+	A	0	0	0	1	0	SA0247
gi|29165615|ref|NC_002745.2|	300427	+	C	0	0	1	0	0	SA0247
gi|29165615|ref|NC_002745.2|	300554	+	A	0	1	0	0	0	SA0247
gi|29165615|ref|NC_002745.2|	300699	+	U	0	1	0	0	0	SA0247
gi|29165615|ref|NC_002745.2|	300707	+	A	1	0	0	0	0	SA0247
gi|29165615|ref|NC_002745.2|	300712	+	U	0	0	0	1	1	SA0247
gi|29165615|ref|NC_002745.2|	300715	+	A	0	0	0	1	0	SA0247
gi|29165615|ref|NC_002745.2|	300720	+	A	0	0	0	0	2	SA0247
gi|29165615|ref|NC_002745.2|	300721	+	A	0	0	0	1	0	SA0247
gi|29165615|ref|NC_002745.2|	300739	+	U	0	1	0	0	0	SA0247
gi|29165615|ref|NC_002745.2|	300751	+	A	0	1	0	0	0	SA0247
gi|29165615|ref|NC_002745.2|	300775	+	U	0	1	0	0	0	SA0247
gi|29165615|ref|NC_002745.2|	300815	+	C	0	0	0	1	0	SA0247
gi|29165615|ref|NC_002745.2|	300837	+	C	0	0	0	0	1	SA0247
gi|29165615|ref|NC_002745.2|	300846	+	A	0	0	0	0	1	SA0247
gi|29165615|ref|NC_002745.2|	300848	+	U	1	0	0	0	0	SA0247
gi|29165615|ref|NC_002745.2|	300861	+	U	0	0	0	1	0	SA0247
gi|29165615|ref|NC_002745.2|	300862	+	U	1	0	0	0	0	SA0247
gi|29165615|ref|NC_002745.2|	300868	+	G	1	0	0	0	0	SA0247
gi|29165615|ref|NC_002745.2|	300869	+	U	1	0	0	0	0	SA0247
gi|29165615|ref|NC_002745.2|	300880	+	U	1	0	0	0	0	SA0247
gi|29165615|ref|NC_002745.2|	300928	+	G	1	0	0	0	0	SA0247
gi|29165615|ref|NC_002745.2|	300930	+	C	0	0	0	0	1	SA0247
gi|29165615|ref|NC_002745.2|	300934	+	C	0	1	0	0	0	SA0247
gi|29165615|ref|NC_002745.2|	300937	+	G	0	0	1	0	0	SA0247
gi|29165615|ref|NC_002745.2|	300938	+	U	1	0	0	0	0	SA0247
gi|29165615|ref|NC_002745.2|	300940	+	A	0	2	0	0	0	SA0247
gi|29165615|ref|NC_002745.2|	300942	+	A	0	1	0	0	1	SA0247
gi|29165615|ref|NC_002745.2|	300952	+	C	0	0	0	0	1	SA0247
gi|29165615|ref|NC_002745.2|	300959	+	U	0	0	0	0	1	SA0247
gi|29165615|ref|NC_002745.2|	300960	+	G	0	1	0	0	0	SA0247
gi|29165615|ref|NC_002745.2|	300964	+	C	0	0	0	0	1	SA0247
gi|29165615|ref|NC_002745.2|	300968	+	A	0	0	1	0	0	SA0247
gi|29165615|ref|NC_002745.2|	300970	+	A	0	0	0	1	0	SA0247
gi|29165615|ref|NC_002745.2|	300972	+	C	1	0	0	0	0	SA0247
gi|29165615|ref|NC_002745.2|	300975	+	C	0	1	0	0	0	SA0247
gi|29165615|ref|NC_002745.2|	300980	+	C	0	0	0	0	1	SA0247
gi|29165615|ref|NC_002745.2|	300982	+	A	0	0	1	0	0	SA0247
gi|29165615|ref|NC_002745.2|	300987	+	A	0	1	0	1	0	SA0247
gi|29165615|ref|NC_002745.2|	300989	+	A	0	0	0	1	1	SA0247
gi|29165615|ref|NC_002745.2|	300991	+	C	0	6	5	4	2	SA0247
gi|29165615|ref|NC_002745.2|	300992	+	U	0	2	0	1	0	SA0247
gi|29165615|ref|NC_002745.2|	301002	+	U	0	0	0	1	0	SA0247
gi|29165615|ref|NC_002745.2|	301017	+	C	0	0	0	1	1	SA0247
gi|29165615|ref|NC_002745.2|	301018	+	C	0	0	2	0	0	SA0247
gi|29165615|ref|NC_002745.2|	301019	+	A	0	2	0	0	1	SA0247
gi|29165615|ref|NC_002745.2|	301023	+	U	0	1	1	2	1	SA0247
gi|29165615|ref|NC_002745.2|	301024	+	A	1	0	0	1	0	SA0247
gi|29165615|ref|NC_002745.2|	301025	+	C	0	0	3	0	0	SA0247
gi|29165615|ref|NC_002745.2|	301027	+	C	0	1	0	0	0	SA0247
gi|29165615|ref|NC_002745.2|	301028	+	U	0	0	0	1	0	SA0247
gi|29165615|ref|NC_002745.2|	301029	+	G	0	0	1	0	0	SA0247
gi|29165615|ref|NC_002745.2|	301031	+	A	0	1	0	0	1	SA0247
gi|29165615|ref|NC_002745.2|	301033	+	C	1	3	5	3	1	SA0247
gi|29165615|ref|NC_002745.2|	301034	+	A	2	0	0	0	0	SA0247
gi|29165615|ref|NC_002745.2|	301038	+	G	0	0	1	0	0	SA0247
gi|29165615|ref|NC_002745.2|	301039	+	G	0	2	3	1	1	SA0247
gi|29165615|ref|NC_002745.2|	301040	+	U	0	0	0	1	0	SA0247
gi|29165615|ref|NC_002745.2|	301041	+	A	0	0	0	1	0	SA0247
gi|29165615|ref|NC_002745.2|	301042	+	U	1	0	0	0	0	SA0247
gi|29165615|ref|NC_002745.2|	301043	+	U	0	2	0	0	1	SA0247
gi|29165615|ref|NC_002745.2|	301044	+	A	1	3	1	0	0	SA0247
gi|29165615|ref|NC_002745.2|	301045	+	A	0	0	0	0	1	SA0247
gi|29165615|ref|NC_002745.2|	301047	+	G	0	0	2	0	0	SA0247
gi|29165615|ref|NC_002745.2|	301048	+	A	0	0	0	1	2	SA0247
gi|29165615|ref|NC_002745.2|	301049	+	G	0	0	0	0	1	SA0247
gi|29165615|ref|NC_002745.2|	301050	+	A	0	0	0	3	3	SA0247
gi|29165615|ref|NC_002745.2|	301051	+	A	0	3	1	1	0	SA0247
gi|29165615|ref|NC_002745.2|	301052	+	A	0	0	1	1	0	SA0247
gi|29165615|ref|NC_002745.2|	301053	+	A	0	1	0	0	2	SA0247
gi|29165615|ref|NC_002745.2|	301054	+	A	1	1	0	0	1	SA0247
gi|29165615|ref|NC_002745.2|	301058	+	A	1	0	0	0	0	SA0247
gi|29165615|ref|NC_002745.2|	301181	+	A	0	1	0	0	0	SA0247
gi|29165615|ref|NC_002745.2|	301247	+	A	0	0	0	0	1	SA0247
gi|29165615|ref|NC_002745.2|	301292	+	G	0	0	0	0	1	SA0247
gi|29165615|ref|NC_002745.2|	301309	+	A	0	0	1	0	1	SA0247
gi|29165615|ref|NC_002745.2|	301330	+	A	0	0	1	0	0	SA0247
gi|29165615|ref|NC_002745.2|	301337	+	A	0	0	1	0	0	SA0247

gi|29165615|ref|NC_002745.2|	301388 +	C	0	0	0	0	1	SA0247
gi|29165615|ref|NC_002745.2|	301391 +	A	0	3	2	2	1	SA0247
gi|29165615|ref|NC_002745.2|	301399 +	U	0	0	1	0	0	SA0247
gi|29165615|ref|NC_002745.2|	301474 +	A	0	1	0	0	0	SA0247
gi|29165615|ref|NC_002745.2|	301528 +	U	1	0	0	0	0	SA0247
gi|29165615|ref|NC_002745.2|	301539 +	A	0	2	0	0	0	SA0247
gi|29165615|ref|NC_002745.2|	301556 +	C	0	1	0	0	0	SA0247
gi|29165615|ref|NC_002745.2|	301588 +	A	0	1	0	0	1	SA0247
gi|29165615|ref|NC_002745.2|	301598 +	A	0	0	0	1	1	SA0247
gi|29165615|ref|NC_002745.2|	301599 +	A	0	0	1	0	0	SA0247
gi|29165615|ref|NC_002745.2|	301600 +	A	0	0	0	1	1	SA0247
gi|29165615|ref|NC_002745.2|	301602 +	G	0	1	0	0	0	SA0247
gi|29165615|ref|NC_002745.2|	301603 +	U	0	2	2	0	5	SA0247
gi|29165615|ref|NC_002745.2|	301604 +	C	0	0	0	0	1	SA0247
gi|29165615|ref|NC_002745.2|	301633 +	A	0	1	0	0	0	SA0247
gi|29165615|ref|NC_002745.2|	301634 +	U	0	1	0	0	0	SA0247
gi|29165615|ref|NC_002745.2|	301635 +	C	1	0	0	0	2	SA0247
gi|29165615|ref|NC_002745.2|	301637 +	A	0	1	0	0	1	SA0247
gi|29165615|ref|NC_002745.2|	301638 +	G	0	1	0	0	0	SA0247
gi|29165615|ref|NC_002745.2|	301639 +	A	0	1	0	2	0	SA0247
gi|29165615|ref|NC_002745.2|	301642 +	G	0	2	0	0	1	SA0247
gi|29165615|ref|NC_002745.2|	301643 +	C	0	7	4	0	3	SA0247
gi|29165615|ref|NC_002745.2|	301644 +	C	0	2	0	0	1	SA0247
gi|29165615|ref|NC_002745.2|	301645 +	G	0	1	1	0	0	SA0247
gi|29165615|ref|NC_002745.2|	301646 +	A	0	1	5	0	2	SA0247
gi|29165615|ref|NC_002745.2|	301648 +	C	1	9	5	2	6	SA0247
gi|29165615|ref|NC_002745.2|	301649 +	A	0	4	2	2	2	SA0247
gi|29165615|ref|NC_002745.2|	301650 +	A	0	0	1	0	0	SA0247
gi|29165615|ref|NC_002745.2|	301652 +	U	0	4	3	0	6	SA0247
gi|29165615|ref|NC_002745.2|	301653 +	G	0	5	1	0	2	SA0247
gi|29165615|ref|NC_002745.2|	301654 +	A	0	4	1	3	3	SA0247
gi|29165615|ref|NC_002745.2|	301673 +	A	0	0	1	0	0	SA0247
gi|29165615|ref|NC_002745.2|	301691 +	U	0	1	0	0	0	-
gi|29165615|ref|NC_002745.2|	301735 +	A	0	0	0	1	0	SA0248
gi|29165615|ref|NC_002745.2|	301736 +	A	0	1	0	0	0	SA0248
gi|29165615|ref|NC_002745.2|	301766 +	A	0	0	0	1	0	SA0248
gi|29165615|ref|NC_002745.2|	301774 +	A	0	0	0	0	1	SA0248
gi|29165615|ref|NC_002745.2|	301865 +	A	0	0	0	1	1	SA0248
gi|29165615|ref|NC_002745.2|	301885 +	U	0	0	0	1	0	SA0248
gi|29165615|ref|NC_002745.2|	301910 +	C	0	1	0	0	0	SA0248
gi|29165615|ref|NC_002745.2|	301924 +	U	1	0	0	0	0	SA0248
gi|29165615|ref|NC_002745.2|	301945 +	A	0	1	0	0	0	SA0248
gi|29165615|ref|NC_002745.2|	301960 +	A	0	2	0	0	2	SA0248
gi|29165615|ref|NC_002745.2|	301980 +	U	0	0	0	0	1	SA0248
gi|29165615|ref|NC_002745.2|	302007 +	U	0	0	0	0	1	SA0248
gi|29165615|ref|NC_002745.2|	302021 +	U	0	0	1	0	0	SA0248
gi|29165615|ref|NC_002745.2|	302022 +	U	0	1	0	0	0	SA0248
gi|29165615|ref|NC_002745.2|	302034 +	C	0	1	0	0	0	SA0248
gi|29165615|ref|NC_002745.2|	302059 +	A	0	0	0	0	1	SA0248
gi|29165615|ref|NC_002745.2|	302060 +	G	0	0	0	1	0	SA0248
gi|29165615|ref|NC_002745.2|	302080 +	G	0	1	0	0	0	SA0248
gi|29165615|ref|NC_002745.2|	302087 +	G	0	1	0	1	1	SA0248
gi|29165615|ref|NC_002745.2|	302088 +	U	0	1	0	0	0	SA0248
gi|29165615|ref|NC_002745.2|	302147 +	U	0	1	0	0	0	SA0248
gi|29165615|ref|NC_002745.2|	302149 +	A	0	1	0	0	2	SA0248
gi|29165615|ref|NC_002745.2|	302151 +	A	0	1	1	0	1	SA0248
gi|29165615|ref|NC_002745.2|	302154 +	C	0	1	0	0	0	SA0248
gi|29165615|ref|NC_002745.2|	302156 +	A	0	0	1	0	1	SA0248
gi|29165615|ref|NC_002745.2|	302162 +	A	0	0	1	1	0	SA0248
gi|29165615|ref|NC_002745.2|	302163 +	U	0	0	0	1	0	SA0248
gi|29165615|ref|NC_002745.2|	302168 +	A	0	0	0	0	1	SA0248
gi|29165615|ref|NC_002745.2|	302172 +	U	0	0	0	0	1	SA0248
gi|29165615|ref|NC_002745.2|	302174 +	A	0	0	0	1	1	SA0248
gi|29165615|ref|NC_002745.2|	302180 +	A	0	1	0	0	0	SA0248
gi|29165615|ref|NC_002745.2|	302184 +	A	0	3	0	0	0	SA0248
gi|29165615|ref|NC_002745.2|	302187 +	U	0	0	0	0	1	SA0248
gi|29165615|ref|NC_002745.2|	302188 +	C	0	0	1	0	0	SA0248
gi|29165615|ref|NC_002745.2|	302190 +	A	0	0	1	0	0	SA0248
gi|29165615|ref|NC_002745.2|	302192 +	A	0	0	0	0	1	SA0248
gi|29165615|ref|NC_002745.2|	302204 +	C	0	0	0	0	1	SA0248
gi|29165615|ref|NC_002745.2|	302208 +	A	0	1	0	0	0	SA0248
gi|29165615|ref|NC_002745.2|	302211 +	A	0	0	2	0	0	SA0248
gi|29165615|ref|NC_002745.2|	302213 +	A	0	0	0	0	1	SA0248
gi|29165615|ref|NC_002745.2|	302214 +	A	0	0	1	0	0	SA0248
gi|29165615|ref|NC_002745.2|	302217 +	A	0	0	3	2	3	SA0248
gi|29165615|ref|NC_002745.2|	302223 +	A	0	0	1	0	0	SA0248
gi|29165615|ref|NC_002745.2|	302225 +	U	0	0	0	0	1	SA0248
gi|29165615|ref|NC_002745.2|	302232 +	A	0	0	0	1	0	SA0248
gi|29165615|ref|NC_002745.2|	302233 +	A	0	0	0	1	0	SA0248
gi|29165615|ref|NC_002745.2|	302234 +	G	0	0	1	1	0	SA0248
gi|29165615|ref|NC_002745.2|	302235 +	A	0	1	2	1	0	SA0248

gi|29165615|ref|NC_002745.2|	302236	+	A	0	1	0	0	0	SA0248
gi|29165615|ref|NC_002745.2|	302240	+	A	0	0	0	1	0	SA0248
gi|29165615|ref|NC_002745.2|	302257	+	A	0	1	0	0	0	SA0248
gi|29165615|ref|NC_002745.2|	302259	+	U	0	0	0	0	1	SA0248
gi|29165615|ref|NC_002745.2|	302267	+	A	0	1	0	0	0	SA0248
gi|29165615|ref|NC_002745.2|	302289	+	A	0	1	0	0	0	SA0248
gi|29165615|ref|NC_002745.2|	302291	+	C	0	1	0	0	0	SA0248
gi|29165615|ref|NC_002745.2|	302294	+	A	0	0	0	1	0	SA0248
gi|29165615|ref|NC_002745.2|	302295	+	U	0	0	0	0	1	SA0248
gi|29165615|ref|NC_002745.2|	302300	+	G	0	1	0	0	0	SA0248
gi|29165615|ref|NC_002745.2|	302328	+	C	0	0	0	0	1	SA0248
gi|29165615|ref|NC_002745.2|	302351	+	A	1	0	0	0	0	SA0248
gi|29165615|ref|NC_002745.2|	302414	+	A	0	1	0	0	0	SA0248
gi|29165615|ref|NC_002745.2|	302505	+	U	0	0	0	1	0	SA0248
gi|29165615|ref|NC_002745.2|	302537	+	C	0	0	1	0	0	SA0248
gi|29165615|ref|NC_002745.2|	302632	+	G	0	0	0	1	0	SA0248
gi|29165615|ref|NC_002745.2|	302681	+	C	0	1	0	0	0	SA0248
gi|29165615|ref|NC_002745.2|	302692	+	C	0	0	0	0	1	SA0248
gi|29165615|ref|NC_002745.2|	302701	+	U	0	0	0	0	1	SA0248
gi|29165615|ref|NC_002745.2|	302729	+	C	0	0	0	0	1	SA0248
gi|29165615|ref|NC_002745.2|	302731	+	A	0	1	1	0	1	SA0248
gi|29165615|ref|NC_002745.2|	302748	+	C	0	1	0	0	0	SA0248
gi|29165615|ref|NC_002745.2|	302789	+	A	0	1	0	0	0	SA0248
gi|29165615|ref|NC_002745.2|	302822	+	G	0	0	0	0	2	SA0248
gi|29165615|ref|NC_002745.2|	302836	+	G	0	0	1	0	0	SA0248
gi|29165615|ref|NC_002745.2|	302991	+	C	0	1	0	0	0	SA0248
gi|29165615|ref|NC_002745.2|	303014	+	U	0	1	0	0	0	SA0248
gi|29165615|ref|NC_002745.2|	303030	+	U	0	1	0	0	0	SA0248
gi|29165615|ref|NC_002745.2|	303049	+	A	1	0	0	0	0	SA0248
gi|29165615|ref|NC_002745.2|	303062	+	A	0	0	0	0	1	SA0248
gi|29165615|ref|NC_002745.2|	303076	+	A	0	1	0	0	0	SA0248
gi|29165615|ref|NC_002745.2|	303088	+	U	1	0	0	0	0	SA0248
gi|29165615|ref|NC_002745.2|	303100	+	U	0	0	1	0	0	SA0248
gi|29165615|ref|NC_002745.2|	303167	+	A	0	1	0	0	0	SA0248
gi|29165615|ref|NC_002745.2|	303186	+	C	0	0	1	0	0	SA0248
gi|29165615|ref|NC_002745.2|	303206	+	A	0	0	0	0	1	SA0248
gi|29165615|ref|NC_002745.2|	303212	+	A	0	1	0	0	0	SA0248
gi|29165615|ref|NC_002745.2|	303214	+	G	0	1	0	0	0	SA0248
gi|29165615|ref|NC_002745.2|	303215	+	A	0	0	0	0	1	SA0248
gi|29165615|ref|NC_002745.2|	303228	+	A	0	2	0	0	0	SA0248
gi|29165615|ref|NC_002745.2|	303236	+	A	0	1	0	0	0	SA0248
gi|29165615|ref|NC_002745.2|	303238	+	C	0	0	1	0	0	SA0248
gi|29165615|ref|NC_002745.2|	303242	+	U	0	0	1	0	0	SA0248
gi|29165615|ref|NC_002745.2|	303251	+	C	0	0	0	0	1	SA0248
gi|29165615|ref|NC_002745.2|	303372	+	U	0	0	0	1	0	SA0248
gi|29165615|ref|NC_002745.2|	303402	+	A	0	0	0	1	0	SA0248
gi|29165615|ref|NC_002745.2|	303408	+	A	0	1	0	0	0	SA0248
gi|29165615|ref|NC_002745.2|	303522	+	C	0	0	0	1	0	-
gi|29165615|ref|NC_002745.2|	303567	+	G	0	2	0	1	0	-
gi|29165615|ref|NC_002745.2|	303611	+	A	0	0	0	0	1	SA0249
gi|29165615|ref|NC_002745.2|	303618	+	A	0	0	0	0	1	SA0249
gi|29165615|ref|NC_002745.2|	303623	+	A	0	0	1	0	0	SA0249
gi|29165615|ref|NC_002745.2|	303624	+	U	0	0	0	0	1	SA0249
gi|29165615|ref|NC_002745.2|	303635	+	C	0	0	1	0	0	SA0249
gi|29165615|ref|NC_002745.2|	303638	+	C	0	0	0	0	2	SA0249
gi|29165615|ref|NC_002745.2|	303651	+	A	0	0	0	0	1	SA0249
gi|29165615|ref|NC_002745.2|	303660	+	U	0	0	0	0	1	SA0249
gi|29165615|ref|NC_002745.2|	303694	+	G	0	1	0	0	0	SA0249
gi|29165615|ref|NC_002745.2|	303717	+	A	0	1	0	0	0	SA0249
gi|29165615|ref|NC_002745.2|	303769	+	A	0	0	0	0	1	SA0249
gi|29165615|ref|NC_002745.2|	303770	+	A	0	2	0	0	0	SA0249
gi|29165615|ref|NC_002745.2|	303794	+	A	0	0	0	0	1	SA0249
gi|29165615|ref|NC_002745.2|	303962	+	G	0	1	0	0	0	SA0249
gi|29165615|ref|NC_002745.2|	303973	+	G	0	0	1	0	0	SA0249
gi|29165615|ref|NC_002745.2|	304021	+	U	0	0	0	1	0	SA0249
gi|29165615|ref|NC_002745.2|	304473	+	A	0	2	0	1	0	-
gi|29165615|ref|NC_002745.2|	304474	+	C	0	2	2	5	0	-
gi|29165615|ref|NC_002745.2|	304475	+	A	0	0	0	0	1	-
gi|29165615|ref|NC_002745.2|	304477	+	A	16	122	61	69	113	-
gi|29165615|ref|NC_002745.2|	304478	+	A	0	3	0	2	2	-
gi|29165615|ref|NC_002745.2|	304479	+	U	4	0	0	0	0	-
gi|29165615|ref|NC_002745.2|	304486	+	U	0	0	1	0	0	-
gi|29165615|ref|NC_002745.2|	304492	+	A	0	0	0	1	0	-
gi|29165615|ref|NC_002745.2|	304511	+	C	0	0	0	0	3	SA0250
gi|29165615|ref|NC_002745.2|	304521	+	U	0	0	0	0	1	SA0250
gi|29165615|ref|NC_002745.2|	304581	+	A	0	0	1	0	0	SA0250
gi|29165615|ref|NC_002745.2|	304584	+	U	0	0	0	1	0	SA0250
gi|29165615|ref|NC_002745.2|	304588	+	A	0	0	0	1	0	SA0250
gi|29165615|ref|NC_002745.2|	304589	+	A	0	0	0	0	1	SA0250
gi|29165615|ref|NC_002745.2|	304632	+	U	0	0	0	0	1	SA0250
gi|29165615|ref|NC_002745.2|	304640	+	A	0	0	1	0	0	SA0250

gi|29165615|ref|NC_002745.2|	304643 +	U	0	0	2	0	0	SA0250
gi|29165615|ref|NC_002745.2|	304660 +	A	0	0	0	1	0	SA0250
gi|29165615|ref|NC_002745.2|	304665 +	C	0	1	0	0	0	SA0250
gi|29165615|ref|NC_002745.2|	304671 +	U	0	1	0	0	0	SA0250
gi|29165615|ref|NC_002745.2|	304749 +	C	0	0	0	0	1	SA0250
gi|29165615|ref|NC_002745.2|	304757 +	C	0	0	0	0	1	SA0250
gi|29165615|ref|NC_002745.2|	304780 +	A	0	0	0	2	0	SA0250
gi|29165615|ref|NC_002745.2|	304795 +	C	0	0	0	0	1	SA0250
gi|29165615|ref|NC_002745.2|	304809 +	U	2	0	0	0	0	SA0250
gi|29165615|ref|NC_002745.2|	304860 +	A	0	0	0	0	1	SA0250
gi|29165615|ref|NC_002745.2|	304892 +	A	0	1	0	0	0	SA0250
gi|29165615|ref|NC_002745.2|	304896 +	G	0	0	0	0	1	SA0250
gi|29165615|ref|NC_002745.2|	304900 +	A	0	1	0	0	0	SA0250
gi|29165615|ref|NC_002745.2|	304901 +	A	0	1	0	0	0	SA0250
gi|29165615|ref|NC_002745.2|	304924 +	A	0	0	0	1	0	SA0250
gi|29165615|ref|NC_002745.2|	304936 +	C	0	0	0	1	0	SA0250
gi|29165615|ref|NC_002745.2|	304953 +	U	0	1	0	0	0	SA0250
gi|29165615|ref|NC_002745.2|	304954 +	U	1	0	0	0	0	SA0250
gi|29165615|ref|NC_002745.2|	304970 +	A	2	0	0	0	0	SA0250
gi|29165615|ref|NC_002745.2|	305021 +	C	0	0	0	1	1	SA0250
gi|29165615|ref|NC_002745.2|	305028 +	A	0	0	0	0	1	SA0250
gi|29165615|ref|NC_002745.2|	305054 +	U	1	0	0	0	0	SA0250
gi|29165615|ref|NC_002745.2|	305061 +	C	0	0	1	0	0	SA0250
gi|29165615|ref|NC_002745.2|	305062 +	A	0	0	1	0	0	SA0250
gi|29165615|ref|NC_002745.2|	305063 +	C	1	0	0	1	0	SA0250
gi|29165615|ref|NC_002745.2|	305064 +	U	1	0	0	0	0	SA0250
gi|29165615|ref|NC_002745.2|	305075 +	A	0	0	1	0	0	SA0250
gi|29165615|ref|NC_002745.2|	305078 +	G	0	0	0	1	0	SA0250
gi|29165615|ref|NC_002745.2|	305109 +	C	0	1	0	0	0	SA0250
gi|29165615|ref|NC_002745.2|	305112 +	U	0	0	0	0	1	SA0250
gi|29165615|ref|NC_002745.2|	305132 +	C	0	0	0	1	0	SA0250
gi|29165615|ref|NC_002745.2|	305147 +	A	0	0	1	0	0	SA0250
gi|29165615|ref|NC_002745.2|	305163 +	A	0	0	0	1	0	SA0250
gi|29165615|ref|NC_002745.2|	305282 +	A	0	0	0	0	1	SA0250
gi|29165615|ref|NC_002745.2|	305473 +	U	0	0	0	0	1	SA0250
gi|29165615|ref|NC_002745.2|	305478 +	U	1	0	0	0	0	SA0250
gi|29165615|ref|NC_002745.2|	305564 +	U	0	0	1	0	0	SA0250
gi|29165615|ref|NC_002745.2|	305688 +	U	0	0	0	0	1	SA0250
gi|29165615|ref|NC_002745.2|	305724 +	A	0	1	0	0	0	SA0250
gi|29165615|ref|NC_002745.2|	305792 +	C	0	1	0	0	0	SA0250
gi|29165615|ref|NC_002745.2|	305798 +	A	1	0	0	0	1	SA0250
gi|29165615|ref|NC_002745.2|	305805 +	U	0	0	1	0	0	SA0250
gi|29165615|ref|NC_002745.2|	305806 +	A	0	0	0	1	0	SA0250
gi|29165615|ref|NC_002745.2|	305807 +	G	1	0	2	0	0	SA0250
gi|29165615|ref|NC_002745.2|	305808 +	A	0	0	1	2	1	SA0250
gi|29165615|ref|NC_002745.2|	305809 +	U	0	1	0	0	0	SA0250
gi|29165615|ref|NC_002745.2|	305812 +	A	0	1	0	1	0	SA0250
gi|29165615|ref|NC_002745.2|	305813 +	G	0	0	1	0	0	SA0250
gi|29165615|ref|NC_002745.2|	305814 +	A	0	0	0	2	0	SA0250
gi|29165615|ref|NC_002745.2|	305815 +	G	0	0	1	0	0	SA0250
gi|29165615|ref|NC_002745.2|	305818 +	A	0	0	0	0	1	SA0250
gi|29165615|ref|NC_002745.2|	305834 +	U	0	1	0	0	0	SA0250
gi|29165615|ref|NC_002745.2|	305862 +	C	1	0	0	0	0	SA0250
gi|29165615|ref|NC_002745.2|	305874 +	A	0	1	0	0	0	SA0250
gi|29165615|ref|NC_002745.2|	305879 +	A	0	0	1	0	0	SA0250
gi|29165615|ref|NC_002745.2|	305892 +	A	2	0	0	2	0	SA0250
gi|29165615|ref|NC_002745.2|	305894 +	G	0	1	0	0	0	SA0250
gi|29165615|ref|NC_002745.2|	305896 +	C	0	0	0	1	0	SA0250
gi|29165615|ref|NC_002745.2|	305919 +	C	0	0	0	1	0	SA0250
gi|29165615|ref|NC_002745.2|	305925 +	U	0	0	1	0	0	SA0250
gi|29165615|ref|NC_002745.2|	305933 +	C	0	0	2	0	0	SA0250
gi|29165615|ref|NC_002745.2|	305940 +	U	1	0	0	0	0	SA0250
gi|29165615|ref|NC_002745.2|	305969 +	A	0	0	0	1	0	SA0250
gi|29165615|ref|NC_002745.2|	305970 +	C	0	0	1	0	0	SA0250
gi|29165615|ref|NC_002745.2|	305973 +	A	0	4	0	0	1	SA0250
gi|29165615|ref|NC_002745.2|	305977 +	A	0	0	1	0	2	SA0250
gi|29165615|ref|NC_002745.2|	305991 +	A	0	0	0	1	0	SA0250
gi|29165615|ref|NC_002745.2|	305993 +	A	0	1	0	0	0	SA0250
gi|29165615|ref|NC_002745.2|	306048 +	A	0	0	0	2	0	SA0250
gi|29165615|ref|NC_002745.2|	306060 +	G	1	0	0	0	0	SA0250
gi|29165615|ref|NC_002745.2|	306105 +	A	0	0	0	2	0	SA0250
gi|29165615|ref|NC_002745.2|	306148 +	A	0	0	0	0	1	SA0250
gi|29165615|ref|NC_002745.2|	306195 +	C	0	1	0	0	0	SA0250
gi|29165615|ref|NC_002745.2|	306236 +	G	0	0	0	0	1	SA0250
gi|29165615|ref|NC_002745.2|	306242 +	C	0	1	0	0	0	SA0250
gi|29165615|ref|NC_002745.2|	306244 +	A	0	0	1	0	0	SA0250
gi|29165615|ref|NC_002745.2|	306251 +	G	1	0	0	0	0	SA0250
gi|29165615|ref|NC_002745.2|	306256 +	A	0	0	0	1	0	SA0250
gi|29165615|ref|NC_002745.2|	306266 +	C	0	0	0	1	0	SA0251
gi|29165615|ref|NC_002745.2|	306315 +	A	0	0	1	0	0	SA0251
gi|29165615|ref|NC_002745.2|	306316 +	U	0	0	0	0	1	SA0251

gi|29165615|ref|NC_002745.2|	306323	+	A	0	0	0	1	0	SA0251
gi|29165615|ref|NC_002745.2|	306390	+	C	1	0	0	0	1	SA0251
gi|29165615|ref|NC_002745.2|	306396	+	A	0	0	1	0	1	SA0251
gi|29165615|ref|NC_002745.2|	306409	+	U	0	0	0	1	0	SA0251
gi|29165615|ref|NC_002745.2|	306425	+	U	1	0	0	0	0	SA0251
gi|29165615|ref|NC_002745.2|	306434	+	A	0	0	0	1	0	SA0251
gi|29165615|ref|NC_002745.2|	306479	+	C	1	0	0	0	0	SA0251
gi|29165615|ref|NC_002745.2|	306505	+	C	1	0	0	0	0	SA0251
gi|29165615|ref|NC_002745.2|	306506	+	A	1	0	0	0	0	SA0251
gi|29165615|ref|NC_002745.2|	306546	+	A	1	0	0	0	0	SA0251
gi|29165615|ref|NC_002745.2|	306555	+	U	1	0	0	0	0	SA0251
gi|29165615|ref|NC_002745.2|	306557	+	U	2	0	0	0	0	SA0251
gi|29165615|ref|NC_002745.2|	306562	+	C	0	0	1	0	0	SA0251
gi|29165615|ref|NC_002745.2|	306583	+	G	1	0	0	0	0	SA0251
gi|29165615|ref|NC_002745.2|	306590	+	C	1	0	0	1	0	SA0251
gi|29165615|ref|NC_002745.2|	306656	+	U	1	0	0	0	0	SA0251
gi|29165615|ref|NC_002745.2|	306669	+	U	0	1	0	0	0	SA0251
gi|29165615|ref|NC_002745.2|	306683	+	C	1	0	0	0	0	SA0251
gi|29165615|ref|NC_002745.2|	306706	+	C	0	0	0	1	0	SA0251
gi|29165615|ref|NC_002745.2|	306707	+	A	0	0	0	0	1	SA0251
gi|29165615|ref|NC_002745.2|	306738	+	U	0	0	0	0	1	SA0251
gi|29165615|ref|NC_002745.2|	306765	+	A	1	1	0	0	1	SA0251
gi|29165615|ref|NC_002745.2|	306770	+	C	0	1	0	0	0	SA0251
gi|29165615|ref|NC_002745.2|	306782	+	A	0	0	0	1	0	SA0251
gi|29165615|ref|NC_002745.2|	306792	+	A	0	0	0	0	1	SA0251
gi|29165615|ref|NC_002745.2|	306793	+	A	0	0	0	1	0	SA0251
gi|29165615|ref|NC_002745.2|	306801	+	A	0	3	0	0	0	SA0251
gi|29165615|ref|NC_002745.2|	306844	+	C	0	1	0	0	0	SA0251
gi|29165615|ref|NC_002745.2|	306869	+	A	0	0	0	1	0	SA0251
gi|29165615|ref|NC_002745.2|	306881	+	U	0	0	0	1	0	SA0251
gi|29165615|ref|NC_002745.2|	306882	+	U	0	0	0	2	2	SA0251
gi|29165615|ref|NC_002745.2|	306883	+	A	0	0	0	4	1	SA0251
gi|29165615|ref|NC_002745.2|	306908	+	A	0	0	0	1	0	SA0251
gi|29165615|ref|NC_002745.2|	307092	+	U	0	0	0	1	1	-
gi|29165615|ref|NC_002745.2|	307093	+	A	0	6	0	6	1	-
gi|29165615|ref|NC_002745.2|	307094	+	A	0	0	0	1	0	-
gi|29165615|ref|NC_002745.2|	307095	+	A	0	0	0	1	0	-
gi|29165615|ref|NC_002745.2|	307099	+	A	0	0	0	1	0	-
gi|29165615|ref|NC_002745.2|	307103	+	U	0	0	0	1	0	-
gi|29165615|ref|NC_002745.2|	307106	+	G	0	0	0	1	0	-
gi|29165615|ref|NC_002745.2|	307109	+	G	0	0	0	1	0	-
gi|29165615|ref|NC_002745.2|	307128	+	A	0	0	0	1	0	SA0252
gi|29165615|ref|NC_002745.2|	307137	+	C	0	0	0	1	0	SA0252
gi|29165615|ref|NC_002745.2|	307145	+	C	0	1	0	0	0	SA0252
gi|29165615|ref|NC_002745.2|	307190	+	U	0	0	0	1	0	SA0252
gi|29165615|ref|NC_002745.2|	307204	+	A	0	1	0	0	0	SA0252
gi|29165615|ref|NC_002745.2|	307208	+	G	0	0	0	1	0	SA0252
gi|29165615|ref|NC_002745.2|	307209	+	A	0	0	0	0	1	SA0252
gi|29165615|ref|NC_002745.2|	307210	+	A	0	0	0	1	0	SA0252
gi|29165615|ref|NC_002745.2|	307211	+	U	0	3	0	0	1	SA0252
gi|29165615|ref|NC_002745.2|	307212	+	C	0	0	0	1	0	SA0252
gi|29165615|ref|NC_002745.2|	307236	+	C	0	0	0	1	0	SA0252
gi|29165615|ref|NC_002745.2|	307242	+	U	0	0	0	1	0	SA0252
gi|29165615|ref|NC_002745.2|	307248	+	G	0	0	0	1	0	SA0252
gi|29165615|ref|NC_002745.2|	307254	+	U	0	10	5	11	4	SA0252
gi|29165615|ref|NC_002745.2|	307255	+	A	0	6	4	28	15	SA0252
gi|29165615|ref|NC_002745.2|	307256	+	U	0	0	0	1	0	SA0252
gi|29165615|ref|NC_002745.2|	307257	+	U	0	0	0	2	0	SA0252
gi|29165615|ref|NC_002745.2|	307258	+	A	0	0	1	1	0	SA0252
gi|29165615|ref|NC_002745.2|	307259	+	U	0	0	0	1	0	SA0252
gi|29165615|ref|NC_002745.2|	307263	+	U	0	0	0	1	0	SA0252
gi|29165615|ref|NC_002745.2|	307264	+	U	0	0	1	0	0	SA0252
gi|29165615|ref|NC_002745.2|	307265	+	G	0	0	0	0	2	SA0252
gi|29165615|ref|NC_002745.2|	307266	+	U	0	0	2	0	2	SA0252
gi|29165615|ref|NC_002745.2|	307267	+	A	0	3	1	3	0	SA0252
gi|29165615|ref|NC_002745.2|	307268	+	U	0	1	0	0	0	SA0252
gi|29165615|ref|NC_002745.2|	307269	+	U	0	0	0	1	0	SA0252
gi|29165615|ref|NC_002745.2|	307270	+	A	0	0	1	0	2	SA0252
gi|29165615|ref|NC_002745.2|	307271	+	U	0	0	0	1	0	SA0252
gi|29165615|ref|NC_002745.2|	307272	+	U	0	1	0	0	0	SA0252
gi|29165615|ref|NC_002745.2|	307281	+	G	0	0	1	0	0	SA0252
gi|29165615|ref|NC_002745.2|	307282	+	U	1	0	0	0	0	SA0252
gi|29165615|ref|NC_002745.2|	307285	+	U	0	0	0	0	1	SA0252
gi|29165615|ref|NC_002745.2|	307308	+	A	0	0	0	1	0	SA0252
gi|29165615|ref|NC_002745.2|	307313	+	G	0	0	0	1	0	SA0252
gi|29165615|ref|NC_002745.2|	307314	+	G	0	0	0	2	1	SA0252
gi|29165615|ref|NC_002745.2|	307333	+	C	0	0	0	0	1	SA0252
gi|29165615|ref|NC_002745.2|	307342	+	A	0	0	0	1	0	SA0252
gi|29165615|ref|NC_002745.2|	307404	+	U	0	0	0	0	1	SA0252
gi|29165615|ref|NC_002745.2|	307409	+	A	0	0	0	0	1	SA0252
gi|29165615|ref|NC_002745.2|	307410	+	U	0	1	0	0	0	SA0252

gi|29165615|ref|NC_002745.2|	307436	+	A	0	1	0	0	0	SA0252
gi|29165615|ref|NC_002745.2|	307453	+	U	0	0	0	1	0	SA0252
gi|29165615|ref|NC_002745.2|	307511	+	G	0	1	0	0	0	SA0252
gi|29165615|ref|NC_002745.2|	307535	+	G	0	0	0	0	1	SA0252
gi|29165615|ref|NC_002745.2|	307556	+	C	0	0	0	0	1	SA0253
gi|29165615|ref|NC_002745.2|	307573	+	C	1	0	0	0	0	SA0253
gi|29165615|ref|NC_002745.2|	307582	+	U	0	1	0	0	0	SA0253
gi|29165615|ref|NC_002745.2|	307656	+	U	0	1	0	0	0	SA0253
gi|29165615|ref|NC_002745.2|	307682	+	A	0	0	1	0	0	SA0253
gi|29165615|ref|NC_002745.2|	307708	+	C	0	0	0	0	1	SA0253
gi|29165615|ref|NC_002745.2|	307720	+	A	0	1	0	0	0	SA0253
gi|29165615|ref|NC_002745.2|	307729	+	A	0	0	0	1	0	SA0253
gi|29165615|ref|NC_002745.2|	307734	+	A	0	1	0	0	0	SA0253
gi|29165615|ref|NC_002745.2|	307736	+	A	0	0	0	1	0	SA0253
gi|29165615|ref|NC_002745.2|	307765	+	A	0	0	0	0	1	SA0253
gi|29165615|ref|NC_002745.2|	307770	+	G	0	0	0	0	1	SA0253
gi|29165615|ref|NC_002745.2|	307781	+	U	0	0	0	0	1	SA0253
gi|29165615|ref|NC_002745.2|	307794	+	U	0	0	0	1	0	SA0253
gi|29165615|ref|NC_002745.2|	307798	+	A	0	1	0	0	0	SA0253
gi|29165615|ref|NC_002745.2|	307802	+	A	1	0	0	0	0	SA0253
gi|29165615|ref|NC_002745.2|	307836	+	A	0	0	0	0	1	SA0253
gi|29165615|ref|NC_002745.2|	307894	+	C	0	1	0	0	1	SA0253
gi|29165615|ref|NC_002745.2|	307930	+	C	0	1	0	0	0	SA0253
gi|29165615|ref|NC_002745.2|	308002	+	U	0	0	0	1	0	SA0253
gi|29165615|ref|NC_002745.2|	308040	+	U	0	0	0	1	0	SA0253
gi|29165615|ref|NC_002745.2|	308050	+	G	0	1	0	1	0	SA0253
gi|29165615|ref|NC_002745.2|	308057	+	A	0	1	0	0	0	SA0253
gi|29165615|ref|NC_002745.2|	308063	+	G	0	3	0	0	0	SA0253
gi|29165615|ref|NC_002745.2|	308069	+	U	0	0	1	0	0	SA0253
gi|29165615|ref|NC_002745.2|	308078	+	U	1	0	0	0	0	SA0253
gi|29165615|ref|NC_002745.2|	308079	+	A	0	0	1	0	0	SA0253
gi|29165615|ref|NC_002745.2|	308097	+	G	1	0	0	0	0	SA0253
gi|29165615|ref|NC_002745.2|	308170	+	C	2	0	0	0	0	SA0253
gi|29165615|ref|NC_002745.2|	308172	+	A	0	1	0	0	0	SA0253
gi|29165615|ref|NC_002745.2|	308815	+	A	0	1	0	0	0	-
gi|29165615|ref|NC_002745.2|	309159	+	A	0	1	0	0	0	-
gi|29165615|ref|NC_002745.2|	309162	+	U	0	0	1	0	0	-
gi|29165615|ref|NC_002745.2|	309163	+	A	0	17	4	29	13	-
gi|29165615|ref|NC_002745.2|	309164	+	A	0	1	0	0	0	-
gi|29165615|ref|NC_002745.2|	309189	+	C	0	0	1	0	0	-
gi|29165615|ref|NC_002745.2|	309247	+	U	0	0	0	1	0	SA0255
gi|29165615|ref|NC_002745.2|	309273	+	A	0	0	0	1	0	SA0255
gi|29165615|ref|NC_002745.2|	309291	+	G	0	1	0	0	0	SA0255
gi|29165615|ref|NC_002745.2|	309334	+	C	0	1	0	0	0	SA0255
gi|29165615|ref|NC_002745.2|	309350	+	G	0	1	0	0	0	SA0255
gi|29165615|ref|NC_002745.2|	309398	+	A	0	0	0	0	1	SA0255
gi|29165615|ref|NC_002745.2|	309415	+	G	0	0	0	1	0	SA0255
gi|29165615|ref|NC_002745.2|	309416	+	A	0	0	0	0	2	SA0255
gi|29165615|ref|NC_002745.2|	309417	+	A	0	0	0	1	0	SA0255
gi|29165615|ref|NC_002745.2|	309421	+	U	0	1	0	0	0	SA0255
gi|29165615|ref|NC_002745.2|	309434	+	U	0	1	0	1	1	SA0255
gi|29165615|ref|NC_002745.2|	309438	+	C	0	1	0	1	2	SA0255
gi|29165615|ref|NC_002745.2|	309440	+	A	0	0	0	0	1	SA0255
gi|29165615|ref|NC_002745.2|	309447	+	C	0	0	0	0	1	SA0255
gi|29165615|ref|NC_002745.2|	309449	+	A	0	1	0	1	0	SA0255
gi|29165615|ref|NC_002745.2|	309450	+	U	0	0	0	2	0	SA0255
gi|29165615|ref|NC_002745.2|	309452	+	A	0	0	1	1	0	SA0255
gi|29165615|ref|NC_002745.2|	309453	+	A	0	0	1	2	1	SA0255
gi|29165615|ref|NC_002745.2|	309454	+	U	0	0	0	0	1	SA0255
gi|29165615|ref|NC_002745.2|	309456	+	A	0	0	0	1	0	SA0255
gi|29165615|ref|NC_002745.2|	309457	+	A	0	0	0	1	0	SA0255
gi|29165615|ref|NC_002745.2|	309459	+	A	1	7	3	20	12	SA0255
gi|29165615|ref|NC_002745.2|	309460	+	A	0	2	0	2	2	SA0255
gi|29165615|ref|NC_002745.2|	309463	+	C	0	0	0	2	0	SA0255
gi|29165615|ref|NC_002745.2|	309464	+	U	0	1	0	0	0	SA0255
gi|29165615|ref|NC_002745.2|	309466	+	A	0	0	0	0	1	SA0255
gi|29165615|ref|NC_002745.2|	309469	+	U	0	0	0	1	0	SA0255
gi|29165615|ref|NC_002745.2|	309470	+	C	0	0	0	0	1	SA0255
gi|29165615|ref|NC_002745.2|	309481	+	A	0	1	0	0	0	SA0255
gi|29165615|ref|NC_002745.2|	309484	+	A	0	0	0	1	0	SA0255
gi|29165615|ref|NC_002745.2|	309487	+	C	0	0	0	0	1	SA0255
gi|29165615|ref|NC_002745.2|	309490	+	A	0	2	0	2	1	SA0255
gi|29165615|ref|NC_002745.2|	309493	+	A	0	0	1	1	0	SA0255
gi|29165615|ref|NC_002745.2|	309497	+	U	0	1	0	0	0	SA0255
gi|29165615|ref|NC_002745.2|	309498	+	A	0	1	0	1	0	SA0255
gi|29165615|ref|NC_002745.2|	309499	+	A	0	0	0	3	3	SA0255
gi|29165615|ref|NC_002745.2|	309503	+	C	0	0	0	0	1	SA0255
gi|29165615|ref|NC_002745.2|	309507	+	G	0	1	0	0	0	SA0255
gi|29165615|ref|NC_002745.2|	309509	+	U	0	0	0	1	1	SA0255
gi|29165615|ref|NC_002745.2|	309510	+	A	0	0	0	1	0	SA0255
gi|29165615|ref|NC_002745.2|	309511	+	C	0	1	0	2	0	SA0255

gi|29165615|ref|NC_002745.2|	309520 +	C	0	1	0	0	0	SA0255
gi|29165615|ref|NC_002745.2|	309525 +	C	0	0	0	1	2	SA0255
gi|29165615|ref|NC_002745.2|	309533 +	A	0	1	0	0	0	SA0255
gi|29165615|ref|NC_002745.2|	309540 +	A	0	1	1	1	0	SA0255
gi|29165615|ref|NC_002745.2|	309543 +	C	0	0	0	1	0	SA0255
gi|29165615|ref|NC_002745.2|	309546 +	A	0	0	0	0	1	SA0255
gi|29165615|ref|NC_002745.2|	309548 +	U	0	0	0	1	0	SA0255
gi|29165615|ref|NC_002745.2|	309549 +	U	0	1	0	0	0	SA0255
gi|29165615|ref|NC_002745.2|	309551 +	A	0	1	0	0	0	SA0255
gi|29165615|ref|NC_002745.2|	309555 +	A	0	0	1	0	0	SA0255
gi|29165615|ref|NC_002745.2|	309558 +	G	0	1	0	0	0	SA0255
gi|29165615|ref|NC_002745.2|	309564 +	A	0	2	0	0	0	SA0255
gi|29165615|ref|NC_002745.2|	309590 +	A	0	0	0	0	1	SA0255
gi|29165615|ref|NC_002745.2|	309591 +	G	0	0	0	0	1	SA0255
gi|29165615|ref|NC_002745.2|	309599 +	A	0	2	0	4	0	SA0255
gi|29165615|ref|NC_002745.2|	309605 +	A	0	0	0	0	1	SA0255
gi|29165615|ref|NC_002745.2|	309608 +	U	0	0	0	1	0	SA0255
gi|29165615|ref|NC_002745.2|	309612 +	A	0	1	0	0	0	SA0255
gi|29165615|ref|NC_002745.2|	309614 +	A	0	0	0	2	2	SA0255
gi|29165615|ref|NC_002745.2|	309615 +	G	0	0	0	1	0	SA0255
gi|29165615|ref|NC_002745.2|	309617 +	G	0	0	0	1	0	SA0255
gi|29165615|ref|NC_002745.2|	309618 +	A	0	0	0	1	0	SA0255
gi|29165615|ref|NC_002745.2|	309620 +	A	0	0	0	1	0	SA0255
gi|29165615|ref|NC_002745.2|	309621 +	A	0	3	0	0	1	SA0255
gi|29165615|ref|NC_002745.2|	309624 +	G	0	1	1	2	0	SA0255
gi|29165615|ref|NC_002745.2|	309625 +	U	0	1	0	0	1	SA0255
gi|29165615|ref|NC_002745.2|	309626 +	A	0	0	0	1	0	SA0255
gi|29165615|ref|NC_002745.2|	309649 +	C	0	0	1	0	0	SA0255
gi|29165615|ref|NC_002745.2|	309655 +	A	0	2	0	0	0	SA0255
gi|29165615|ref|NC_002745.2|	309658 +	A	0	2	0	0	0	SA0255
gi|29165615|ref|NC_002745.2|	309661 +	C	0	0	0	1	1	SA0255
gi|29165615|ref|NC_002745.2|	309666 +	G	0	1	0	0	0	SA0255
gi|29165615|ref|NC_002745.2|	309678 +	U	0	1	0	0	0	SA0255
gi|29165615|ref|NC_002745.2|	309686 +	U	0	0	0	1	0	SA0255
gi|29165615|ref|NC_002745.2|	309729 +	C	0	1	0	0	0	SA0255
gi|29165615|ref|NC_002745.2|	309760 +	A	0	1	0	0	0	SA0255
gi|29165615|ref|NC_002745.2|	309767 +	C	0	0	0	0	1	SA0255
gi|29165615|ref|NC_002745.2|	309768 +	G	0	0	0	0	1	SA0255
gi|29165615|ref|NC_002745.2|	309769 +	U	0	0	0	1	0	SA0255
gi|29165615|ref|NC_002745.2|	309771 +	A	0	1	0	0	0	SA0255
gi|29165615|ref|NC_002745.2|	309775 +	C	0	0	0	1	0	SA0255
gi|29165615|ref|NC_002745.2|	309787 +	A	0	0	0	1	0	SA0255
gi|29165615|ref|NC_002745.2|	309819 +	A	0	2	0	1	0	SA0255
gi|29165615|ref|NC_002745.2|	309820 +	A	0	0	0	0	3	SA0255
gi|29165615|ref|NC_002745.2|	309823 +	A	0	0	0	0	1	SA0255
gi|29165615|ref|NC_002745.2|	309825 +	C	3	0	0	0	0	SA0255
gi|29165615|ref|NC_002745.2|	309827 +	U	0	1	0	0	0	SA0255
gi|29165615|ref|NC_002745.2|	309829 +	U	0	0	0	1	0	SA0255
gi|29165615|ref|NC_002745.2|	309861 +	C	0	1	0	0	0	SA0255
gi|29165615|ref|NC_002745.2|	309873 +	C	1	0	0	0	0	SA0255
gi|29165615|ref|NC_002745.2|	309883 +	G	0	0	0	2	1	SA0255
gi|29165615|ref|NC_002745.2|	309887 +	C	1	1	0	2	0	SA0255
gi|29165615|ref|NC_002745.2|	309913 +	G	0	0	0	0	1	SA0255
gi|29165615|ref|NC_002745.2|	309922 +	C	0	1	0	0	0	SA0255
gi|29165615|ref|NC_002745.2|	309932 +	A	0	0	0	2	0	SA0255
gi|29165615|ref|NC_002745.2|	309933 +	A	0	0	1	0	0	SA0255
gi|29165615|ref|NC_002745.2|	309935 +	A	0	1	0	1	0	SA0255
gi|29165615|ref|NC_002745.2|	309936 +	G	0	0	0	0	1	SA0255
gi|29165615|ref|NC_002745.2|	309938 +	A	0	0	0	1	0	SA0255
gi|29165615|ref|NC_002745.2|	309939 +	G	0	0	0	0	1	SA0255
gi|29165615|ref|NC_002745.2|	309940 +	A	0	0	0	1	1	SA0255
gi|29165615|ref|NC_002745.2|	309941 +	A	0	2	0	0	0	SA0255
gi|29165615|ref|NC_002745.2|	309942 +	C	0	0	0	1	0	SA0255
gi|29165615|ref|NC_002745.2|	309945 +	A	0	1	1	4	3	SA0255
gi|29165615|ref|NC_002745.2|	309948 +	A	0	0	0	0	1	SA0255
gi|29165615|ref|NC_002745.2|	309951 +	A	1	3	0	4	3	SA0255
gi|29165615|ref|NC_002745.2|	309954 +	G	0	0	0	1	0	SA0255
gi|29165615|ref|NC_002745.2|	309955 +	A	0	0	0	1	0	SA0255
gi|29165615|ref|NC_002745.2|	309957 +	A	0	1	0	0	1	SA0255
gi|29165615|ref|NC_002745.2|	309958 +	U	0	0	0	1	0	SA0255
gi|29165615|ref|NC_002745.2|	309959 +	C	0	0	0	1	0	SA0255
gi|29165615|ref|NC_002745.2|	309963 +	A	0	0	0	1	0	SA0255
gi|29165615|ref|NC_002745.2|	309964 +	C	0	0	0	1	0	SA0255
gi|29165615|ref|NC_002745.2|	309965 +	G	0	1	0	0	0	SA0255
gi|29165615|ref|NC_002745.2|	309966 +	A	0	0	0	2	3	SA0255
gi|29165615|ref|NC_002745.2|	309969 +	G	0	0	0	0	1	SA0255
gi|29165615|ref|NC_002745.2|	309970 +	A	0	7	2	15	6	SA0255
gi|29165615|ref|NC_002745.2|	309973 +	A	0	1	0	1	1	SA0255
gi|29165615|ref|NC_002745.2|	309976 +	U	0	1	0	4	0	SA0255
gi|29165615|ref|NC_002745.2|	309977 +	U	0	1	0	1	0	SA0255
gi|29165615|ref|NC_002745.2|	309983 +	U	0	1	0	0	0	SA0255

gi|29165615|ref|NC_002745.2|	309984	+	A	0	1	0	2	1	SA0255
gi|29165615|ref|NC_002745.2|	309985	+	A	0	0	2	0	0	SA0255
gi|29165615|ref|NC_002745.2|	309986	+	A	0	2	2	3	1	SA0255
gi|29165615|ref|NC_002745.2|	309987	+	A	0	0	0	1	2	SA0255
gi|29165615|ref|NC_002745.2|	309988	+	U	0	0	2	1	1	SA0255
gi|29165615|ref|NC_002745.2|	309990	+	U	0	3	0	1	0	SA0255
gi|29165615|ref|NC_002745.2|	309991	+	U	0	1	0	0	1	SA0255
gi|29165615|ref|NC_002745.2|	309992	+	U	0	0	1	1	0	SA0255
gi|29165615|ref|NC_002745.2|	309993	+	A	0	1	0	1	2	SA0255
gi|29165615|ref|NC_002745.2|	309994	+	A	0	4	0	10	7	SA0255
gi|29165615|ref|NC_002745.2|	309995	+	A	0	1	0	2	1	SA0255
gi|29165615|ref|NC_002745.2|	309996	+	A	0	0	0	0	1	SA0255
gi|29165615|ref|NC_002745.2|	309997	+	A	0	1	0	1	1	SA0255
gi|29165615|ref|NC_002745.2|	309999	+	U	0	0	0	2	0	SA0255
gi|29165615|ref|NC_002745.2|	310001	+	G	0	0	0	2	2	SA0255
gi|29165615|ref|NC_002745.2|	310002	+	G	0	2	0	4	2	-
gi|29165615|ref|NC_002745.2|	310003	+	A	0	2	0	3	1	-
gi|29165615|ref|NC_002745.2|	310006	+	G	0	0	0	2	0	-
gi|29165615|ref|NC_002745.2|	310007	+	U	0	0	1	1	1	-
gi|29165615|ref|NC_002745.2|	310014	+	A	0	0	0	0	1	-
gi|29165615|ref|NC_002745.2|	310015	+	U	0	0	0	1	0	-
gi|29165615|ref|NC_002745.2|	310016	+	A	0	1	0	1	0	-
gi|29165615|ref|NC_002745.2|	310017	+	A	0	0	0	1	1	SA0256
gi|29165615|ref|NC_002745.2|	310020	+	A	0	0	0	1	0	SA0256
gi|29165615|ref|NC_002745.2|	310023	+	A	0	0	0	1	1	SA0256
gi|29165615|ref|NC_002745.2|	310025	+	A	0	1	0	0	1	SA0256
gi|29165615|ref|NC_002745.2|	310032	+	C	0	0	0	0	1	SA0256
gi|29165615|ref|NC_002745.2|	310033	+	A	0	0	0	2	1	SA0256
gi|29165615|ref|NC_002745.2|	310034	+	A	0	2	0	1	0	SA0256
gi|29165615|ref|NC_002745.2|	310035	+	A	0	0	0	1	2	SA0256
gi|29165615|ref|NC_002745.2|	310036	+	A	0	0	0	0	1	SA0256
gi|29165615|ref|NC_002745.2|	310037	+	U	0	0	0	1	0	SA0256
gi|29165615|ref|NC_002745.2|	310040	+	U	0	0	0	1	2	SA0256
gi|29165615|ref|NC_002745.2|	310041	+	A	0	0	0	1	0	SA0256
gi|29165615|ref|NC_002745.2|	310045	+	G	0	1	0	3	0	SA0256
gi|29165615|ref|NC_002745.2|	310070	+	A	0	1	0	0	0	SA0256
gi|29165615|ref|NC_002745.2|	310120	+	U	0	1	0	0	0	SA0256
gi|29165615|ref|NC_002745.2|	310155	+	A	0	0	0	2	0	SA0256
gi|29165615|ref|NC_002745.2|	310185	+	U	1	0	0	0	0	SA0256
gi|29165615|ref|NC_002745.2|	310198	+	A	0	0	1	0	1	SA0256
gi|29165615|ref|NC_002745.2|	310201	+	G	0	0	1	0	0	SA0256
gi|29165615|ref|NC_002745.2|	310202	+	U	0	0	0	1	0	SA0256
gi|29165615|ref|NC_002745.2|	310204	+	U	0	0	0	1	0	SA0256
gi|29165615|ref|NC_002745.2|	310209	+	U	0	0	0	0	1	SA0256
gi|29165615|ref|NC_002745.2|	310212	+	U	0	1	0	0	0	SA0256
gi|29165615|ref|NC_002745.2|	310224	+	A	0	1	0	0	0	SA0256
gi|29165615|ref|NC_002745.2|	310227	+	G	0	0	1	0	0	SA0256
gi|29165615|ref|NC_002745.2|	310232	+	U	0	0	1	0	0	SA0256
gi|29165615|ref|NC_002745.2|	310234	+	U	0	0	1	0	0	SA0256
gi|29165615|ref|NC_002745.2|	310241	+	G	1	0	0	0	0	SA0256
gi|29165615|ref|NC_002745.2|	310244	+	U	0	0	0	1	0	SA0256
gi|29165615|ref|NC_002745.2|	310246	+	A	0	0	0	0	1	SA0256
gi|29165615|ref|NC_002745.2|	310248	+	G	0	0	0	1	0	SA0256
gi|29165615|ref|NC_002745.2|	310250	+	A	0	0	1	0	0	SA0256
gi|29165615|ref|NC_002745.2|	310251	+	A	0	0	0	2	1	SA0256
gi|29165615|ref|NC_002745.2|	310252	+	U	1	0	0	0	0	SA0256
gi|29165615|ref|NC_002745.2|	310255	+	G	0	1	2	0	0	SA0256
gi|29165615|ref|NC_002745.2|	310258	+	U	0	0	1	0	0	SA0256
gi|29165615|ref|NC_002745.2|	310262	+	A	0	0	0	1	1	SA0256
gi|29165615|ref|NC_002745.2|	310263	+	U	0	0	1	0	0	SA0256
gi|29165615|ref|NC_002745.2|	310266	+	U	0	2	0	0	1	SA0256
gi|29165615|ref|NC_002745.2|	310268	+	A	1	0	0	0	0	SA0256
gi|29165615|ref|NC_002745.2|	310270	+	G	0	1	0	0	0	SA0256
gi|29165615|ref|NC_002745.2|	310273	+	C	0	1	0	0	0	SA0256
gi|29165615|ref|NC_002745.2|	310275	+	U	1	0	0	0	0	SA0256
gi|29165615|ref|NC_002745.2|	310277	+	G	1	0	0	0	1	SA0256
gi|29165615|ref|NC_002745.2|	310291	+	G	0	0	0	0	1	SA0256
gi|29165615|ref|NC_002745.2|	310296	+	U	0	1	0	0	0	SA0256
gi|29165615|ref|NC_002745.2|	310300	+	C	0	0	0	1	0	SA0256
gi|29165615|ref|NC_002745.2|	310311	+	G	0	1	0	0	0	SA0256
gi|29165615|ref|NC_002745.2|	310321	+	C	0	1	0	0	0	SA0256
gi|29165615|ref|NC_002745.2|	310330	+	A	0	0	1	1	1	SA0256
gi|29165615|ref|NC_002745.2|	310333	+	G	0	0	0	0	2	SA0256
gi|29165615|ref|NC_002745.2|	310335	+	C	1	0	0	0	0	SA0256
gi|29165615|ref|NC_002745.2|	310354	+	U	0	0	0	1	0	SA0256
gi|29165615|ref|NC_002745.2|	310356	+	U	0	0	0	0	1	SA0256
gi|29165615|ref|NC_002745.2|	310360	+	A	0	3	0	0	1	SA0256
gi|29165615|ref|NC_002745.2|	310363	+	A	0	0	0	2	0	SA0256
gi|29165615|ref|NC_002745.2|	310367	+	A	0	0	1	0	1	SA0256
gi|29165615|ref|NC_002745.2|	310368	+	A	0	0	1	0	1	SA0256
gi|29165615|ref|NC_002745.2|	310378	+	G	0	1	0	3	1	SA0256

gi|29165615|ref|NC_002745.2|	310379 +	U	0	0	0	2	2	SA0256
gi|29165615|ref|NC_002745.2|	310380 +	A	0	0	0	3	1	SA0256
gi|29165615|ref|NC_002745.2|	310383 +	G	0	0	0	3	0	SA0256
gi|29165615|ref|NC_002745.2|	310384 +	A	0	0	0	3	2	SA0256
gi|29165615|ref|NC_002745.2|	310385 +	A	0	2	0	1	0	SA0256
gi|29165615|ref|NC_002745.2|	310386 +	C	0	0	0	1	0	SA0256
gi|29165615|ref|NC_002745.2|	310396 +	C	0	1	0	0	0	SA0256
gi|29165615|ref|NC_002745.2|	310402 +	C	0	0	0	0	1	SA0256
gi|29165615|ref|NC_002745.2|	310403 +	A	0	0	0	0	1	SA0256
gi|29165615|ref|NC_002745.2|	310405 +	A	0	0	0	0	1	SA0256
gi|29165615|ref|NC_002745.2|	310406 +	U	0	0	0	1	3	SA0256
gi|29165615|ref|NC_002745.2|	310407 +	U	0	0	1	0	0	SA0256
gi|29165615|ref|NC_002745.2|	310409 +	U	0	0	0	1	0	SA0256
gi|29165615|ref|NC_002745.2|	310411 +	A	0	1	0	0	0	SA0256
gi|29165615|ref|NC_002745.2|	310413 +	A	0	0	0	1	0	SA0256
gi|29165615|ref|NC_002745.2|	310418 +	A	0	0	0	0	1	SA0256
gi|29165615|ref|NC_002745.2|	310426 +	U	0	1	0	0	0	SA0256
gi|29165615|ref|NC_002745.2|	310429 +	C	0	2	0	0	0	SA0256
gi|29165615|ref|NC_002745.2|	310431 +	A	0	2	0	3	0	SA0256
gi|29165615|ref|NC_002745.2|	310432 +	A	0	1	1	2	1	SA0256
gi|29165615|ref|NC_002745.2|	310433 +	A	0	1	0	2	3	SA0256
gi|29165615|ref|NC_002745.2|	310434 +	C	0	1	0	2	2	SA0256
gi|29165615|ref|NC_002745.2|	310435 +	A	0	0	0	0	1	SA0256
gi|29165615|ref|NC_002745.2|	310436 +	U	0	0	0	1	0	SA0256
gi|29165615|ref|NC_002745.2|	310438 +	A	0	0	0	2	0	SA0256
gi|29165615|ref|NC_002745.2|	310444 +	G	0	0	0	0	1	SA0256
gi|29165615|ref|NC_002745.2|	310445 +	A	0	0	0	3	0	SA0256
gi|29165615|ref|NC_002745.2|	310447 +	U	0	0	0	0	1	SA0256
gi|29165615|ref|NC_002745.2|	310449 +	A	0	0	0	0	1	SA0256
gi|29165615|ref|NC_002745.2|	310451 +	A	0	5	1	2	0	SA0256
gi|29165615|ref|NC_002745.2|	310452 +	A	0	0	0	1	2	SA0256
gi|29165615|ref|NC_002745.2|	310453 +	A	0	1	1	0	1	SA0256
gi|29165615|ref|NC_002745.2|	310454 +	U	0	0	0	0	1	SA0256
gi|29165615|ref|NC_002745.2|	310455 +	A	0	0	0	0	1	SA0256
gi|29165615|ref|NC_002745.2|	310456 +	G	0	0	0	1	0	SA0256
gi|29165615|ref|NC_002745.2|	310457 +	A	0	0	0	1	0	SA0256
gi|29165615|ref|NC_002745.2|	310458 +	G	0	0	0	0	1	SA0256
gi|29165615|ref|NC_002745.2|	310459 +	A	0	2	0	2	1	SA0256
gi|29165615|ref|NC_002745.2|	310460 +	A	0	0	0	2	1	SA0256
gi|29165615|ref|NC_002745.2|	310462 +	U	0	0	0	1	1	SA0256
gi|29165615|ref|NC_002745.2|	310465 +	U	0	0	1	2	3	SA0256
gi|29165615|ref|NC_002745.2|	310466 +	C	0	0	0	0	1	SA0256
gi|29165615|ref|NC_002745.2|	310467 +	G	0	1	1	0	0	SA0256
gi|29165615|ref|NC_002745.2|	310468 +	A	0	3	0	1	4	SA0256
gi|29165615|ref|NC_002745.2|	310471 +	A	0	0	0	0	1	SA0256
gi|29165615|ref|NC_002745.2|	310505 +	A	0	0	0	1	0	SA0256
gi|29165615|ref|NC_002745.2|	310521 +	A	1	0	0	0	0	SA0256
gi|29165615|ref|NC_002745.2|	310523 +	A	0	0	0	0	1	SA0256
gi|29165615|ref|NC_002745.2|	310538 +	U	0	0	0	1	0	SA0256
gi|29165615|ref|NC_002745.2|	310552 +	A	0	0	0	0	1	SA0256
gi|29165615|ref|NC_002745.2|	310566 +	U	0	0	0	1	0	SA0256
gi|29165615|ref|NC_002745.2|	310591 +	A	0	0	0	0	1	SA0256
gi|29165615|ref|NC_002745.2|	310621 +	A	0	1	0	0	0	SA0256
gi|29165615|ref|NC_002745.2|	310623 +	C	0	1	0	0	0	SA0256
gi|29165615|ref|NC_002745.2|	310657 +	A	0	1	0	1	0	SA0256
gi|29165615|ref|NC_002745.2|	310667 +	C	0	0	1	0	0	SA0256
gi|29165615|ref|NC_002745.2|	310700 +	U	0	1	0	0	0	SA0256
gi|29165615|ref|NC_002745.2|	310725 +	A	0	0	0	0	1	SA0256
gi|29165615|ref|NC_002745.2|	310746 +	U	0	1	0	2	0	SA0256
gi|29165615|ref|NC_002745.2|	310747 +	A	0	0	0	1	0	SA0256
gi|29165615|ref|NC_002745.2|	310779 +	G	0	1	0	0	0	SA0256
gi|29165615|ref|NC_002745.2|	310780 +	A	0	1	0	0	0	SA0256
gi|29165615|ref|NC_002745.2|	310785 +	C	0	0	0	0	1	SA0256
gi|29165615|ref|NC_002745.2|	310787 +	A	0	2	0	1	1	SA0256
gi|29165615|ref|NC_002745.2|	310815 +	C	0	0	0	1	0	SA0256
gi|29165615|ref|NC_002745.2|	310820 +	U	0	1	0	0	0	SA0256
gi|29165615|ref|NC_002745.2|	310821 +	G	0	0	0	1	0	SA0256
gi|29165615|ref|NC_002745.2|	310824 +	C	0	1	0	0	1	SA0256
gi|29165615|ref|NC_002745.2|	310825 +	A	0	2	0	0	0	SA0256
gi|29165615|ref|NC_002745.2|	310826 +	A	0	0	0	0	1	SA0256
gi|29165615|ref|NC_002745.2|	310839 +	U	0	0	0	0	2	SA0256
gi|29165615|ref|NC_002745.2|	310847 +	C	0	0	0	1	0	SA0256
gi|29165615|ref|NC_002745.2|	310848 +	U	0	0	2	0	0	SA0256
gi|29165615|ref|NC_002745.2|	310857 +	A	0	1	0	0	0	SA0256
gi|29165615|ref|NC_002745.2|	310861 +	U	2	0	0	0	0	SA0256
gi|29165615|ref|NC_002745.2|	310864 +	U	0	0	0	0	2	SA0256
gi|29165615|ref|NC_002745.2|	310921 +	A	0	0	0	1	0	SA0256
gi|29165615|ref|NC_002745.2|	310922 +	G	1	0	0	1	1	SA0256
gi|29165615|ref|NC_002745.2|	310954 +	A	0	0	0	1	0	SA0256
gi|29165615|ref|NC_002745.2|	310987 +	C	0	1	0	0	0	SA0256
gi|29165615|ref|NC_002745.2|	310988 +	U	0	1	0	0	0	SA0256

gi|29165615|ref|NC_002745.2|	311011	+	A	0	1	0	0	0	SA0256
gi|29165615|ref|NC_002745.2|	311017	+	G	0	0	0	2	0	SA0256
gi|29165615|ref|NC_002745.2|	311024	+	U	0	0	1	0	0	SA0256
gi|29165615|ref|NC_002745.2|	311040	+	C	0	0	0	0	1	SA0256
gi|29165615|ref|NC_002745.2|	311041	+	C	0	0	1	0	0	SA0256
gi|29165615|ref|NC_002745.2|	311052	+	A	0	0	0	1	0	SA0256
gi|29165615|ref|NC_002745.2|	311060	+	U	0	0	1	0	0	SA0256
gi|29165615|ref|NC_002745.2|	311093	+	A	0	0	1	0	0	SA0256
gi|29165615|ref|NC_002745.2|	311098	+	C	0	0	1	0	1	SA0256
gi|29165615|ref|NC_002745.2|	311120	+	U	0	0	0	0	1	SA0256
gi|29165615|ref|NC_002745.2|	311128	+	U	1	0	0	0	0	SA0256
gi|29165615|ref|NC_002745.2|	311133	+	C	0	0	0	0	1	SA0256
gi|29165615|ref|NC_002745.2|	311138	+	U	0	0	0	0	1	SA0256
gi|29165615|ref|NC_002745.2|	311146	+	A	0	2	0	1	1	SA0256
gi|29165615|ref|NC_002745.2|	311163	+	G	0	0	0	0	1	SA0256
gi|29165615|ref|NC_002745.2|	311176	+	U	0	0	0	1	0	SA0256
gi|29165615|ref|NC_002745.2|	311178	+	G	0	0	0	0	1	SA0256
gi|29165615|ref|NC_002745.2|	311189	+	U	0	0	0	0	1	SA0256
gi|29165615|ref|NC_002745.2|	311234	+	A	0	0	0	0	1	SA0256
gi|29165615|ref|NC_002745.2|	311242	+	A	0	0	0	1	0	SA0256
gi|29165615|ref|NC_002745.2|	311245	+	C	0	0	0	1	0	SA0256
gi|29165615|ref|NC_002745.2|	311263	+	U	0	0	0	0	1	SA0256
gi|29165615|ref|NC_002745.2|	311332	+	A	0	1	0	0	0	SA0256
gi|29165615|ref|NC_002745.2|	311375	+	U	0	0	0	1	0	SA0256
gi|29165615|ref|NC_002745.2|	311447	+	A	0	1	0	1	0	SA0256
gi|29165615|ref|NC_002745.2|	311465	+	A	0	0	0	0	1	-
gi|29165615|ref|NC_002745.2|	311466	+	A	0	0	0	0	1	-
gi|29165615|ref|NC_002745.2|	311468	+	U	0	0	0	3	2	-
gi|29165615|ref|NC_002745.2|	311760	+	A	0	0	0	1	0	-
gi|29165615|ref|NC_002745.2|	311823	+	C	0	0	1	0	0	-
gi|29165615|ref|NC_002745.2|	311989	+	A	0	0	0	1	0	-
gi|29165615|ref|NC_002745.2|	312004	+	U	0	0	0	1	0	-
gi|29165615|ref|NC_002745.2|	312054	+	G	0	2	0	0	0	-
gi|29165615|ref|NC_002745.2|	312141	+	A	0	1	0	0	0	-
gi|29165615|ref|NC_002745.2|	313501	+	U	1	0	0	0	0	-
gi|29165615|ref|NC_002745.2|	316469	+	U	0	1	0	0	0	-
gi|29165615|ref|NC_002745.2|	316745	+	A	0	0	1	0	2	-
gi|29165615|ref|NC_002745.2|	316747	+	G	2	1	0	0	2	-
gi|29165615|ref|NC_002745.2|	316748	+	U	0	0	0	1	0	-
gi|29165615|ref|NC_002745.2|	316773	+	C	0	0	1	0	0	-
gi|29165615|ref|NC_002745.2|	316782	+	A	0	0	0	0	1	-
gi|29165615|ref|NC_002745.2|	316838	+	G	0	0	1	0	0	SA0262
gi|29165615|ref|NC_002745.2|	316846	+	G	0	0	0	1	0	SA0262
gi|29165615|ref|NC_002745.2|	316847	+	A	0	0	0	0	1	SA0262
gi|29165615|ref|NC_002745.2|	316848	+	A	0	0	1	0	1	SA0262
gi|29165615|ref|NC_002745.2|	316852	+	A	0	0	0	0	1	SA0262
gi|29165615|ref|NC_002745.2|	316853	+	A	0	2	0	0	0	SA0262
gi|29165615|ref|NC_002745.2|	316860	+	A	0	1	0	0	0	SA0262
gi|29165615|ref|NC_002745.2|	316861	+	G	0	0	1	0	0	SA0262
gi|29165615|ref|NC_002745.2|	316862	+	A	0	0	1	0	0	SA0262
gi|29165615|ref|NC_002745.2|	316863	+	A	0	0	0	0	1	SA0262
gi|29165615|ref|NC_002745.2|	316865	+	A	0	1	0	0	1	SA0262
gi|29165615|ref|NC_002745.2|	316867	+	G	0	1	0	0	0	SA0262
gi|29165615|ref|NC_002745.2|	316868	+	A	0	1	0	0	0	SA0262
gi|29165615|ref|NC_002745.2|	316869	+	G	0	0	0	0	1	SA0262
gi|29165615|ref|NC_002745.2|	316872	+	C	1	0	0	0	0	SA0262
gi|29165615|ref|NC_002745.2|	316873	+	G	0	1	1	1	1	SA0262
gi|29165615|ref|NC_002745.2|	316874	+	A	0	9	2	4	9	SA0262
gi|29165615|ref|NC_002745.2|	316875	+	A	0	1	0	0	0	SA0262
gi|29165615|ref|NC_002745.2|	316880	+	G	0	0	1	0	0	SA0262
gi|29165615|ref|NC_002745.2|	316899	+	A	0	1	0	0	0	SA0262
gi|29165615|ref|NC_002745.2|	316910	+	A	0	0	1	0	0	SA0262
gi|29165615|ref|NC_002745.2|	316953	+	C	0	1	0	0	0	SA0262
gi|29165615|ref|NC_002745.2|	316981	+	A	0	0	0	0	1	SA0262
gi|29165615|ref|NC_002745.2|	316985	+	A	0	1	0	0	0	SA0262
gi|29165615|ref|NC_002745.2|	316992	+	A	0	0	0	0	1	SA0262
gi|29165615|ref|NC_002745.2|	317006	+	A	0	1	0	0	0	SA0262
gi|29165615|ref|NC_002745.2|	317009	+	A	1	0	0	0	0	SA0262
gi|29165615|ref|NC_002745.2|	317045	+	U	1	0	0	0	0	SA0262
gi|29165615|ref|NC_002745.2|	317103	+	A	0	1	0	0	0	SA0262
gi|29165615|ref|NC_002745.2|	317137	+	A	0	1	0	0	0	SA0262
gi|29165615|ref|NC_002745.2|	317146	+	A	0	0	0	2	2	SA0262
gi|29165615|ref|NC_002745.2|	317157	+	G	0	1	0	0	1	SA0262
gi|29165615|ref|NC_002745.2|	318875	+	G	0	1	0	0	0	-
gi|29165615|ref|NC_002745.2|	318876	+	U	1	0	0	0	0	-
gi|29165615|ref|NC_002745.2|	318877	+	G	0	3	3	2	2	-
gi|29165615|ref|NC_002745.2|	318878	+	A	0	1	1	0	1	-
gi|29165615|ref|NC_002745.2|	318879	+	A	0	0	0	1	0	-
gi|29165615|ref|NC_002745.2|	318915	+	G	0	0	1	0	0	-
gi|29165615|ref|NC_002745.2|	318918	+	C	0	0	0	0	1	-
gi|29165615|ref|NC_002745.2|	318932	+	C	1	0	0	0	0	-

gi|29165615|ref|NC_002745.2|	318935 +	U	0	0	0	0	1	SA0264
gi|29165615|ref|NC_002745.2|	318936 +	G	0	0	0	0	1	SA0264
gi|29165615|ref|NC_002745.2|	318944 +	G	0	0	1	0	0	SA0264
gi|29165615|ref|NC_002745.2|	318964 +	A	0	0	1	0	0	SA0264
gi|29165615|ref|NC_002745.2|	318967 +	A	0	1	0	0	0	SA0264
gi|29165615|ref|NC_002745.2|	318969 +	U	0	0	0	0	1	SA0264
gi|29165615|ref|NC_002745.2|	318978 +	U	0	0	2	0	0	SA0264
gi|29165615|ref|NC_002745.2|	318988 +	A	0	0	1	0	0	SA0264
gi|29165615|ref|NC_002745.2|	319084 +	U	0	0	1	0	0	SA0264
gi|29165615|ref|NC_002745.2|	319728 +	U	1	0	0	0	0	SA0264
gi|29165615|ref|NC_002745.2|	319865 +	A	0	0	0	0	1	SA0264
gi|29165615|ref|NC_002745.2|	319912 +	A	0	0	0	1	0	SA0264
gi|29165615|ref|NC_002745.2|	319934 +	G	0	0	0	1	0	-
gi|29165615|ref|NC_002745.2|	319935 +	A	0	0	0	1	0	-
gi|29165615|ref|NC_002745.2|	319999 +	C	0	0	0	1	0	-
gi|29165615|ref|NC_002745.2|	320022 +	A	0	0	0	1	0	-
gi|29165615|ref|NC_002745.2|	320203 +	A	2	0	0	1	0	-
gi|29165615|ref|NC_002745.2|	320469 +	A	0	0	0	1	0	SA0265
gi|29165615|ref|NC_002745.2|	320490 +	C	0	0	0	0	1	SA0265
gi|29165615|ref|NC_002745.2|	320809 +	G	1	0	0	0	0	SA0265
gi|29165615|ref|NC_002745.2|	320842 +	A	0	0	0	1	0	SA0265
gi|29165615|ref|NC_002745.2|	321266 +	A	0	0	0	0	1	-
gi|29165615|ref|NC_002745.2|	321299 +	A	0	0	0	0	1	-
gi|29165615|ref|NC_002745.2|	322029 +	G	0	0	1	1	0	-
gi|29165615|ref|NC_002745.2|	322692 +	U	0	0	1	0	0	-
gi|29165615|ref|NC_002745.2|	323080 +	U	0	0	0	0	1	-
gi|29165615|ref|NC_002745.2|	326999 +	A	3	0	0	0	0	-
gi|29165615|ref|NC_002745.2|	327026 +	C	1	0	0	0	0	-
gi|29165615|ref|NC_002745.2|	327044 +	A	0	1	1	1	1	-
gi|29165615|ref|NC_002745.2|	327045 +	A	0	0	0	0	1	-
gi|29165615|ref|NC_002745.2|	327049 +	A	0	0	1	0	0	-
gi|29165615|ref|NC_002745.2|	327050 +	A	0	0	0	0	1	-
gi|29165615|ref|NC_002745.2|	327051 +	A	0	0	0	0	2	-
gi|29165615|ref|NC_002745.2|	327052 +	A	0	0	0	0	3	-
gi|29165615|ref|NC_002745.2|	327054 +	A	0	0	0	1	0	-
gi|29165615|ref|NC_002745.2|	327056 +	U	0	1	0	0	0	-
gi|29165615|ref|NC_002745.2|	327057 +	C	0	0	0	2	0	-
gi|29165615|ref|NC_002745.2|	327058 +	A	0	0	1	0	0	-
gi|29165615|ref|NC_002745.2|	327061 +	A	0	0	0	0	1	-
gi|29165615|ref|NC_002745.2|	327063 +	G	0	1	1	0	0	-
gi|29165615|ref|NC_002745.2|	327067 +	C	0	0	0	0	1	-
gi|29165615|ref|NC_002745.2|	327076 +	G	0	0	1	1	0	SA0271
gi|29165615|ref|NC_002745.2|	327078 +	A	0	0	0	0	1	SA0271
gi|29165615|ref|NC_002745.2|	327082 +	A	0	0	0	2	3	SA0271
gi|29165615|ref|NC_002745.2|	327085 +	A	0	1	0	0	5	SA0271
gi|29165615|ref|NC_002745.2|	327086 +	A	0	0	0	2	0	SA0271
gi|29165615|ref|NC_002745.2|	327087 +	G	0	0	1	2	0	SA0271
gi|29165615|ref|NC_002745.2|	327088 +	A	1	0	1	0	1	SA0271
gi|29165615|ref|NC_002745.2|	327090 +	G	0	1	0	0	0	SA0271
gi|29165615|ref|NC_002745.2|	327093 +	U	0	1	0	1	0	SA0271
gi|29165615|ref|NC_002745.2|	327094 +	C	0	0	0	0	1	SA0271
gi|29165615|ref|NC_002745.2|	327095 +	C	0	0	0	1	0	SA0271
gi|29165615|ref|NC_002745.2|	327103 +	A	1	0	0	0	0	SA0271
gi|29165615|ref|NC_002745.2|	327106 +	A	1	1	0	0	0	SA0271
gi|29165615|ref|NC_002745.2|	327113 +	A	0	0	0	1	0	SA0271
gi|29165615|ref|NC_002745.2|	327114 +	A	1	0	0	0	0	SA0271
gi|29165615|ref|NC_002745.2|	327118 +	C	0	0	0	1	1	SA0271
gi|29165615|ref|NC_002745.2|	327119 +	A	0	0	0	2	0	SA0271
gi|29165615|ref|NC_002745.2|	327137 +	C	0	0	1	0	0	SA0271
gi|29165615|ref|NC_002745.2|	327139 +	G	0	0	0	1	0	SA0271
gi|29165615|ref|NC_002745.2|	327157 +	U	0	0	0	0	1	SA0271
gi|29165615|ref|NC_002745.2|	327163 +	G	0	0	0	0	1	SA0271
gi|29165615|ref|NC_002745.2|	327164 +	A	0	0	1	1	0	SA0271
gi|29165615|ref|NC_002745.2|	327168 +	A	0	0	0	1	0	SA0271
gi|29165615|ref|NC_002745.2|	327176 +	C	0	1	0	0	1	SA0271
gi|29165615|ref|NC_002745.2|	327179 +	A	1	0	0	0	0	SA0271
gi|29165615|ref|NC_002745.2|	327187 +	A	0	0	2	0	1	SA0271
gi|29165615|ref|NC_002745.2|	327194 +	C	0	0	1	0	0	SA0271
gi|29165615|ref|NC_002745.2|	327261 +	A	0	0	1	0	0	SA0271
gi|29165615|ref|NC_002745.2|	327263 +	A	0	0	1	0	0	SA0271
gi|29165615|ref|NC_002745.2|	327269 +	C	0	0	1	0	0	SA0271
gi|29165615|ref|NC_002745.2|	327271 +	C	0	0	0	0	1	SA0271
gi|29165615|ref|NC_002745.2|	327284 +	A	0	4	0	3	2	SA0271
gi|29165615|ref|NC_002745.2|	327285 +	A	0	5	0	1	0	SA0271
gi|29165615|ref|NC_002745.2|	327286 +	A	0	1	1	0	0	SA0271
gi|29165615|ref|NC_002745.2|	327288 +	U	0	0	0	1	0	SA0271
gi|29165615|ref|NC_002745.2|	327289 +	A	0	0	0	1	0	SA0271
gi|29165615|ref|NC_002745.2|	327290 +	A	0	2	0	2	2	SA0271
gi|29165615|ref|NC_002745.2|	327291 +	A	0	5	0	0	0	SA0271
gi|29165615|ref|NC_002745.2|	327294 +	A	0	0	0	0	1	SA0271
gi|29165615|ref|NC_002745.2|	327295 +	C	0	0	0	1	0	SA0271

gi|29165615|ref|NC_002745.2|	327300	+	G	0	0	0	1	0	SA0271
gi|29165615|ref|NC_002745.2|	327301	+	A	0	1	0	1	0	SA0271
gi|29165615|ref|NC_002745.2|	327302	+	A	0	0	1	0	0	SA0271
gi|29165615|ref|NC_002745.2|	327311	+	C	0	1	0	0	0	SA0271
gi|29165615|ref|NC_002745.2|	327643	+	A	0	0	0	1	0	SA0272
gi|29165615|ref|NC_002745.2|	327681	+	C	1	0	0	0	0	SA0272
gi|29165615|ref|NC_002745.2|	327828	+	U	0	0	0	1	0	SA0272
gi|29165615|ref|NC_002745.2|	328162	+	A	0	0	0	0	1	SA0272
gi|29165615|ref|NC_002745.2|	328277	+	C	0	0	0	0	2	SA0272
gi|29165615|ref|NC_002745.2|	328551	+	U	0	0	0	0	1	SA0272
gi|29165615|ref|NC_002745.2|	328782	+	A	0	0	1	0	0	SA0272
gi|29165615|ref|NC_002745.2|	328805	+	G	1	0	0	0	0	SA0272
gi|29165615|ref|NC_002745.2|	328877	+	C	0	0	1	0	0	SA0272
gi|29165615|ref|NC_002745.2|	328928	+	A	0	1	0	0	0	SA0272
gi|29165615|ref|NC_002745.2|	329092	+	A	0	0	0	0	1	SA0272
gi|29165615|ref|NC_002745.2|	329131	+	U	1	0	0	0	0	SA0272
gi|29165615|ref|NC_002745.2|	329208	+	U	1	0	0	0	0	SA0272
gi|29165615|ref|NC_002745.2|	329670	+	A	0	1	0	0	0	SA0272
gi|29165615|ref|NC_002745.2|	329680	+	G	0	0	0	0	1	SA0272
gi|29165615|ref|NC_002745.2|	329684	+	A	0	0	1	0	0	SA0272
gi|29165615|ref|NC_002745.2|	329851	+	U	1	0	0	0	0	SA0272
gi|29165615|ref|NC_002745.2|	329968	+	U	0	0	1	0	0	SA0272
gi|29165615|ref|NC_002745.2|	329971	+	U	0	0	0	1	0	SA0272
gi|29165615|ref|NC_002745.2|	329973	+	U	1	0	0	0	0	SA0272
gi|29165615|ref|NC_002745.2|	330476	+	U	0	0	0	0	1	SA0272
gi|29165615|ref|NC_002745.2|	330481	+	U	0	2	2	0	0	SA0273
gi|29165615|ref|NC_002745.2|	330483	+	G	1	0	0	0	1	SA0273
gi|29165615|ref|NC_002745.2|	330659	+	A	0	0	0	0	1	SA0273
gi|29165615|ref|NC_002745.2|	330666	+	A	0	0	1	0	0	SA0273
gi|29165615|ref|NC_002745.2|	330682	+	A	0	1	0	0	0	SA0273
gi|29165615|ref|NC_002745.2|	330687	+	U	0	1	0	0	0	SA0273
gi|29165615|ref|NC_002745.2|	330945	+	A	0	1	0	0	0	SA0274
gi|29165615|ref|NC_002745.2|	331085	+	C	0	0	0	0	1	SA0274
gi|29165615|ref|NC_002745.2|	332358	+	A	0	0	0	1	0	SA0275
gi|29165615|ref|NC_002745.2|	332597	+	G	0	0	0	0	1	SA0276
gi|29165615|ref|NC_002745.2|	333765	+	U	1	0	0	0	0	SA0276
gi|29165615|ref|NC_002745.2|	334495	+	A	0	1	1	1	1	SA0276
gi|29165615|ref|NC_002745.2|	335180	+	G	0	0	0	1	0	SA0276
gi|29165615|ref|NC_002745.2|	335400	+	U	1	0	0	0	0	SA0276
gi|29165615|ref|NC_002745.2|	335409	+	A	0	0	0	2	0	SA0276
gi|29165615|ref|NC_002745.2|	335434	+	A	0	0	1	1	0	SA0276
gi|29165615|ref|NC_002745.2|	335459	+	G	0	0	0	1	0	SA0276
gi|29165615|ref|NC_002745.2|	335475	+	U	0	0	0	0	1	SA0276
gi|29165615|ref|NC_002745.2|	335612	+	G	0	0	1	0	0	SA0276
gi|29165615|ref|NC_002745.2|	336640	+	A	1	0	0	0	0	SA0276
gi|29165615|ref|NC_002745.2|	336780	+	C	0	0	0	1	0	SA0276
gi|29165615|ref|NC_002745.2|	337260	+	G	1	0	0	0	0	SA0277
gi|29165615|ref|NC_002745.2|	337287	+	U	1	0	0	0	0	SA0277
gi|29165615|ref|NC_002745.2|	337390	+	G	0	0	0	1	0	-
gi|29165615|ref|NC_002745.2|	338011	+	A	0	0	0	2	0	SA0279
gi|29165615|ref|NC_002745.2|	338362	+	A	0	1	0	0	0	SA0279
gi|29165615|ref|NC_002745.2|	338519	+	A	0	1	0	0	0	SA0280
gi|29165615|ref|NC_002745.2|	338582	+	C	0	1	0	0	0	SA0280
gi|29165615|ref|NC_002745.2|	338695	+	G	0	0	0	1	0	SA0280
gi|29165615|ref|NC_002745.2|	338697	+	G	0	1	0	2	0	SA0280
gi|29165615|ref|NC_002745.2|	338724	+	U	1	0	0	0	0	SA0281
gi|29165615|ref|NC_002745.2|	338766	+	U	0	0	0	0	1	SA0281
gi|29165615|ref|NC_002745.2|	339050	+	A	0	0	0	0	1	SA0281
gi|29165615|ref|NC_002745.2|	339116	+	G	0	0	0	1	0	SA0281
gi|29165615|ref|NC_002745.2|	339175	+	U	2	0	0	0	0	SA0281
gi|29165615|ref|NC_002745.2|	339219	+	A	0	0	0	1	0	SA0281
gi|29165615|ref|NC_002745.2|	339379	+	A	0	0	1	0	0	SA0281
gi|29165615|ref|NC_002745.2|	339495	+	A	0	0	0	1	0	SA0281
gi|29165615|ref|NC_002745.2|	339723	+	U	1	0	0	0	0	SA0281
gi|29165615|ref|NC_002745.2|	339904	+	U	0	0	0	0	2	SA0281
gi|29165615|ref|NC_002745.2|	339905	+	G	0	3	1	3	0	SA0281
gi|29165615|ref|NC_002745.2|	339906	+	A	0	1	0	2	2	SA0281
gi|29165615|ref|NC_002745.2|	339907	+	A	0	0	0	0	1	SA0281
gi|29165615|ref|NC_002745.2|	339934	+	A	0	0	0	0	1	SA0281
gi|29165615|ref|NC_002745.2|	340004	+	G	0	0	3	0	0	SA0281
gi|29165615|ref|NC_002745.2|	340018	+	U	0	0	1	0	0	SA0281
gi|29165615|ref|NC_002745.2|	340039	+	U	0	0	0	1	0	SA0281
gi|29165615|ref|NC_002745.2|	340048	+	A	0	1	0	0	0	SA0281
gi|29165615|ref|NC_002745.2|	340072	+	C	0	0	0	1	0	SA0281
gi|29165615|ref|NC_002745.2|	340080	+	A	0	0	0	1	0	SA0281
gi|29165615|ref|NC_002745.2|	340120	+	A	0	2	0	0	0	SA0281
gi|29165615|ref|NC_002745.2|	340133	+	G	0	0	0	1	0	SA0281
gi|29165615|ref|NC_002745.2|	340134	+	A	0	0	0	1	1	SA0281
gi|29165615|ref|NC_002745.2|	340155	+	A	0	0	0	1	0	SA0281
gi|29165615|ref|NC_002745.2|	340160	+	U	0	1	0	0	0	SA0281
gi|29165615|ref|NC_002745.2|	340251	+	C	1	0	0	0	0	SA0281

gi|29165615|ref|NC_002745.2|	340356	+	A	0	1	0	0	0	SA0281
gi|29165615|ref|NC_002745.2|	340395	+	A	1	0	0	0	0	SA0281
gi|29165615|ref|NC_002745.2|	340432	+	A	0	0	0	0	1	SA0281
gi|29165615|ref|NC_002745.2|	340446	+	A	0	0	0	0	1	SA0281
gi|29165615|ref|NC_002745.2|	340454	+	A	0	1	0	0	0	SA0281
gi|29165615|ref|NC_002745.2|	340499	+	A	0	0	1	0	0	SA0281
gi|29165615|ref|NC_002745.2|	340506	+	A	0	2	2	0	0	SA0281
gi|29165615|ref|NC_002745.2|	340512	+	A	0	2	5	4	6	SA0281
gi|29165615|ref|NC_002745.2|	340513	+	A	0	0	0	1	3	SA0281
gi|29165615|ref|NC_002745.2|	340518	+	A	0	1	1	1	1	SA0281
gi|29165615|ref|NC_002745.2|	340520	+	G	0	0	0	0	1	SA0281
gi|29165615|ref|NC_002745.2|	340523	+	A	0	1	1	1	1	SA0281
gi|29165615|ref|NC_002745.2|	340524	+	A	0	3	1	3	0	SA0281
gi|29165615|ref|NC_002745.2|	340525	+	A	0	0	0	1	0	SA0281
gi|29165615|ref|NC_002745.2|	340526	+	A	0	0	0	1	0	SA0281
gi|29165615|ref|NC_002745.2|	340527	+	G	0	0	0	1	1	SA0281
gi|29165615|ref|NC_002745.2|	340528	+	A	0	0	0	0	1	SA0281
gi|29165615|ref|NC_002745.2|	340529	+	A	0	1	0	0	0	SA0281
gi|29165615|ref|NC_002745.2|	340537	+	A	0	2	1	2	0	SA0281
gi|29165615|ref|NC_002745.2|	340540	+	A	1	1	1	3	2	SA0281
gi|29165615|ref|NC_002745.2|	340541	+	A	0	0	1	2	2	SA0281
gi|29165615|ref|NC_002745.2|	340544	+	U	3	1	0	0	1	SA0281
gi|29165615|ref|NC_002745.2|	340545	+	U	0	1	0	0	0	SA0281
gi|29165615|ref|NC_002745.2|	340546	+	A	0	2	0	0	0	SA0281
gi|29165615|ref|NC_002745.2|	340547	+	A	0	2	3	3	1	SA0281
gi|29165615|ref|NC_002745.2|	340548	+	A	1	0	0	0	0	SA0281
gi|29165615|ref|NC_002745.2|	340549	+	U	0	0	0	1	0	SA0281
gi|29165615|ref|NC_002745.2|	340550	+	A	0	1	0	0	0	SA0281
gi|29165615|ref|NC_002745.2|	340553	+	U	1	1	0	0	2	SA0281
gi|29165615|ref|NC_002745.2|	340554	+	A	0	0	0	1	0	SA0281
gi|29165615|ref|NC_002745.2|	340556	+	A	0	0	0	1	0	-
gi|29165615|ref|NC_002745.2|	340570	+	C	0	0	0	1	0	SA0282
gi|29165615|ref|NC_002745.2|	340576	+	A	0	1	0	1	0	SA0282
gi|29165615|ref|NC_002745.2|	341003	+	U	0	1	0	0	0	SA0282
gi|29165615|ref|NC_002745.2|	341681	+	C	0	0	1	0	0	SA0283
gi|29165615|ref|NC_002745.2|	341841	+	G	0	0	2	0	0	SA0283
gi|29165615|ref|NC_002745.2|	341907	+	C	1	0	0	0	0	SA0283
gi|29165615|ref|NC_002745.2|	341979	+	A	0	1	0	0	1	-
gi|29165615|ref|NC_002745.2|	341981	+	G	0	1	0	0	0	-
gi|29165615|ref|NC_002745.2|	342029	+	G	0	0	0	1	0	-
gi|29165615|ref|NC_002745.2|	342031	+	G	0	0	0	1	0	-
gi|29165615|ref|NC_002745.2|	342142	+	A	0	0	0	1	0	SA0284
gi|29165615|ref|NC_002745.2|	342451	+	U	0	0	0	1	0	SA0284
gi|29165615|ref|NC_002745.2|	342496	+	A	0	1	0	0	0	SA0284
gi|29165615|ref|NC_002745.2|	342508	+	U	0	1	0	0	0	SA0284
gi|29165615|ref|NC_002745.2|	342529	+	U	0	0	0	0	1	SA0284
gi|29165615|ref|NC_002745.2|	342534	+	U	0	0	0	0	1	SA0284
gi|29165615|ref|NC_002745.2|	342710	+	A	0	0	1	0	0	-
gi|29165615|ref|NC_002745.2|	342788	+	A	0	0	0	2	0	-
gi|29165615|ref|NC_002745.2|	342790	+	A	0	3	5	0	3	-
gi|29165615|ref|NC_002745.2|	342817	+	U	0	0	1	0	0	-
gi|29165615|ref|NC_002745.2|	342868	+	A	0	1	0	0	0	SA0285
gi|29165615|ref|NC_002745.2|	342949	+	A	0	0	0	0	1	SA0285
gi|29165615|ref|NC_002745.2|	342950	+	A	0	1	0	0	0	SA0285
gi|29165615|ref|NC_002745.2|	343671	+	G	1	0	0	2	0	SA0286
gi|29165615|ref|NC_002745.2|	343705	+	G	0	0	1	0	0	SA0287
gi|29165615|ref|NC_002745.2|	343706	+	A	0	0	0	1	0	SA0287
gi|29165615|ref|NC_002745.2|	344348	+	G	0	0	0	0	1	SA0288
gi|29165615|ref|NC_002745.2|	344469	+	U	0	0	1	0	0	SA0288
gi|29165615|ref|NC_002745.2|	344607	+	U	0	1	0	0	0	SA0288
gi|29165615|ref|NC_002745.2|	344609	+	U	0	0	1	0	0	SA0288
gi|29165615|ref|NC_002745.2|	344615	+	A	0	0	1	0	0	SA0288
gi|29165615|ref|NC_002745.2|	344636	+	A	2	0	0	0	0	SA0288
gi|29165615|ref|NC_002745.2|	344646	+	C	0	0	1	0	0	SA0288
gi|29165615|ref|NC_002745.2|	346256	+	C	0	0	0	1	0	-
gi|29165615|ref|NC_002745.2|	346295	+	A	0	0	0	0	1	-
gi|29165615|ref|NC_002745.2|	346461	+	A	1	0	0	0	0	SA0291
gi|29165615|ref|NC_002745.2|	346466	+	C	2	0	0	0	0	SA0291
gi|29165615|ref|NC_002745.2|	346538	+	U	0	0	0	1	0	SA0291
gi|29165615|ref|NC_002745.2|	346565	+	A	0	2	0	0	0	SA0291
gi|29165615|ref|NC_002745.2|	346792	+	A	1	0	0	0	0	-
gi|29165615|ref|NC_002745.2|	346794	+	A	0	0	0	1	0	-
gi|29165615|ref|NC_002745.2|	346797	+	A	0	0	0	0	1	-
gi|29165615|ref|NC_002745.2|	346845	+	A	0	0	0	0	1	-
gi|29165615|ref|NC_002745.2|	346851	+	G	0	0	1	1	0	-
gi|29165615|ref|NC_002745.2|	346855	+	U	0	1	0	0	0	-
gi|29165615|ref|NC_002745.2|	346859	+	U	0	0	1	0	0	-
gi|29165615|ref|NC_002745.2|	346868	+	A	0	1	0	1	1	SA0292
gi|29165615|ref|NC_002745.2|	346874	+	A	0	0	0	1	0	SA0292
gi|29165615|ref|NC_002745.2|	346877	+	A	0	0	0	2	1	SA0292
gi|29165615|ref|NC_002745.2|	346889	+	A	0	1	0	0	3	SA0292

gi|29165615|ref|NC_002745.2|	346899 +	U	0	0	0	1	0	SA0292
gi|29165615|ref|NC_002745.2|	346903 +	U	0	0	0	0	1	SA0292
gi|29165615|ref|NC_002745.2|	347043 +	U	0	0	1	0	0	SA0292
gi|29165615|ref|NC_002745.2|	347096 +	A	0	0	0	0	1	SA0292
gi|29165615|ref|NC_002745.2|	347137 +	U	0	0	0	0	1	SA0292
gi|29165615|ref|NC_002745.2|	350396 +	U	0	0	0	0	1	-
gi|29165615|ref|NC_002745.2|	350397 +	G	4	4	8	4	5	-
gi|29165615|ref|NC_002745.2|	350405 +	A	0	0	1	0	0	-
gi|29165615|ref|NC_002745.2|	350412 +	A	0	0	0	1	0	-
gi|29165615|ref|NC_002745.2|	350434 +	A	0	0	1	0	0	SA0295
gi|29165615|ref|NC_002745.2|	350442 +	A	0	1	0	0	1	SA0295
gi|29165615|ref|NC_002745.2|	350462 +	C	0	0	0	0	1	SA0295
gi|29165615|ref|NC_002745.2|	350500 +	A	0	1	0	0	0	SA0295
gi|29165615|ref|NC_002745.2|	350503 +	A	0	0	0	1	0	SA0295
gi|29165615|ref|NC_002745.2|	350653 +	C	1	0	0	0	0	SA0295
gi|29165615|ref|NC_002745.2|	350654 +	A	0	0	0	0	1	SA0295
gi|29165615|ref|NC_002745.2|	350690 +	A	0	0	0	0	1	SA0295
gi|29165615|ref|NC_002745.2|	350789 +	A	0	6	3	5	3	SA0295
gi|29165615|ref|NC_002745.2|	350790 +	U	0	0	0	0	1	SA0295
gi|29165615|ref|NC_002745.2|	350868 +	C	0	0	0	1	0	SA0295
gi|29165615|ref|NC_002745.2|	350897 +	A	0	1	0	0	0	SA0295
gi|29165615|ref|NC_002745.2|	350901 +	C	0	0	0	1	0	SA0295
gi|29165615|ref|NC_002745.2|	350958 +	A	0	1	0	0	0	SA0295
gi|29165615|ref|NC_002745.2|	350973 +	C	0	0	0	0	1	SA0295
gi|29165615|ref|NC_002745.2|	351041 +	A	3	0	0	0	0	SA0295
gi|29165615|ref|NC_002745.2|	351120 +	A	0	0	0	0	1	SA0295
gi|29165615|ref|NC_002745.2|	351155 +	A	0	1	0	0	0	SA0295
gi|29165615|ref|NC_002745.2|	351278 +	C	2	0	0	0	0	SA0295
gi|29165615|ref|NC_002745.2|	351445 +	A	0	1	0	0	0	-
gi|29165615|ref|NC_002745.2|	351454 +	G	0	0	1	1	1	-
gi|29165615|ref|NC_002745.2|	351472 +	A	1	1	1	2	1	-
gi|29165615|ref|NC_002745.2|	351473 +	U	0	0	1	0	1	-
gi|29165615|ref|NC_002745.2|	351474 +	A	0	12	7	14	12	-
gi|29165615|ref|NC_002745.2|	351475 +	A	0	1	0	0	0	-
gi|29165615|ref|NC_002745.2|	351481 +	U	0	0	0	0	1	-
gi|29165615|ref|NC_002745.2|	351486 +	A	0	1	0	0	0	-
gi|29165615|ref|NC_002745.2|	351487 +	A	0	1	0	0	2	-
gi|29165615|ref|NC_002745.2|	351498 +	C	0	0	0	0	1	-
gi|29165615|ref|NC_002745.2|	351510 +	U	0	1	0	0	0	-
gi|29165615|ref|NC_002745.2|	351524 +	A	0	1	0	0	1	-
gi|29165615|ref|NC_002745.2|	351529 +	A	0	0	0	0	1	-
gi|29165615|ref|NC_002745.2|	351530 +	A	0	1	0	1	1	-
gi|29165615|ref|NC_002745.2|	351533 +	A	0	0	0	0	2	-
gi|29165615|ref|NC_002745.2|	351537 +	U	0	0	3	0	1	-
gi|29165615|ref|NC_002745.2|	351539 +	U	0	0	0	0	1	-
gi|29165615|ref|NC_002745.2|	351540 +	A	0	1	1	0	0	-
gi|29165615|ref|NC_002745.2|	351544 +	C	0	1	0	0	0	-
gi|29165615|ref|NC_002745.2|	351546 +	U	0	0	0	1	0	-
gi|29165615|ref|NC_002745.2|	351548 +	G	0	0	2	0	0	-
gi|29165615|ref|NC_002745.2|	351549 +	U	0	0	0	0	1	-
gi|29165615|ref|NC_002745.2|	351551 +	G	0	1	0	0	1	-
gi|29165615|ref|NC_002745.2|	351559 +	A	0	1	0	0	0	-
gi|29165615|ref|NC_002745.2|	351561 +	A	0	0	0	1	1	-
gi|29165615|ref|NC_002745.2|	351569 +	G	0	0	0	0	1	SA0296
gi|29165615|ref|NC_002745.2|	351632 +	A	0	0	1	0	0	SA0296
gi|29165615|ref|NC_002745.2|	351767 +	A	0	1	0	0	0	SA0296
gi|29165615|ref|NC_002745.2|	351783 +	A	0	0	0	0	1	SA0296
gi|29165615|ref|NC_002745.2|	351799 +	A	0	0	0	1	2	SA0296
gi|29165615|ref|NC_002745.2|	351802 +	A	0	0	0	0	1	SA0296
gi|29165615|ref|NC_002745.2|	351805 +	A	0	0	1	0	0	SA0296
gi|29165615|ref|NC_002745.2|	351806 +	G	0	0	0	2	0	SA0296
gi|29165615|ref|NC_002745.2|	351807 +	A	0	0	0	0	1	SA0296
gi|29165615|ref|NC_002745.2|	351821 +	G	0	1	0	0	0	SA0296
gi|29165615|ref|NC_002745.2|	351822 +	A	0	0	0	0	1	SA0296
gi|29165615|ref|NC_002745.2|	351825 +	C	0	1	1	0	1	SA0296
gi|29165615|ref|NC_002745.2|	351828 +	A	0	1	0	0	4	SA0296
gi|29165615|ref|NC_002745.2|	351829 +	C	0	0	0	2	0	SA0296
gi|29165615|ref|NC_002745.2|	351836 +	A	0	0	1	0	0	SA0296
gi|29165615|ref|NC_002745.2|	351847 +	A	0	0	0	0	1	SA0296
gi|29165615|ref|NC_002745.2|	351855 +	C	0	0	1	0	0	SA0296
gi|29165615|ref|NC_002745.2|	351940 +	A	0	0	1	1	0	SA0296
gi|29165615|ref|NC_002745.2|	352006 +	U	0	0	0	0	1	SA0296
gi|29165615|ref|NC_002745.2|	352009 +	C	0	0	0	0	1	SA0296
gi|29165615|ref|NC_002745.2|	352057 +	A	0	1	0	0	0	SA0296
gi|29165615|ref|NC_002745.2|	352059 +	U	0	2	0	0	0	SA0296
gi|29165615|ref|NC_002745.2|	352141 +	C	0	0	0	0	1	SA0296
gi|29165615|ref|NC_002745.2|	352231 +	C	0	0	0	1	0	SA0296
gi|29165615|ref|NC_002745.2|	352348 +	U	1	0	0	0	0	SA0296
gi|29165615|ref|NC_002745.2|	352398 +	G	0	1	0	0	0	SA0296
gi|29165615|ref|NC_002745.2|	352438 +	C	1	0	0	0	0	SA0296
gi|29165615|ref|NC_002745.2|	352457 +	A	1	0	0	0	0	SA0296

gi|29165615|ref|NC_002745.2|	352473 +	U	2	0	0	0	0	SA0296
gi|29165615|ref|NC_002745.2|	352477 +	U	2	0	0	0	0	SA0296
gi|29165615|ref|NC_002745.2|	352480 +	C	0	0	1	0	0	SA0296
gi|29165615|ref|NC_002745.2|	352483 +	A	0	0	0	1	0	SA0296
gi|29165615|ref|NC_002745.2|	352513 +	C	1	0	0	0	0	SA0296
gi|29165615|ref|NC_002745.2|	352523 +	A	0	0	0	0	1	SA0296
gi|29165615|ref|NC_002745.2|	352544 +	U	0	0	0	0	2	SA0296
gi|29165615|ref|NC_002745.2|	352558 +	G	0	0	1	0	0	SA0296
gi|29165615|ref|NC_002745.2|	352559 +	A	0	0	0	0	1	SA0296
gi|29165615|ref|NC_002745.2|	352561 +	G	0	1	0	0	0	SA0296
gi|29165615|ref|NC_002745.2|	352562 +	U	0	0	1	0	1	SA0296
gi|29165615|ref|NC_002745.2|	352563 +	U	1	0	0	0	0	SA0296
gi|29165615|ref|NC_002745.2|	352564 +	U	0	1	0	0	0	SA0296
gi|29165615|ref|NC_002745.2|	352578 +	A	0	0	0	0	1	SA0296
gi|29165615|ref|NC_002745.2|	352579 +	C	0	0	0	0	1	SA0296
gi|29165615|ref|NC_002745.2|	352580 +	A	0	2	0	0	0	SA0296
gi|29165615|ref|NC_002745.2|	352584 +	A	0	1	0	0	0	SA0296
gi|29165615|ref|NC_002745.2|	352586 +	A	0	0	0	3	2	SA0296
gi|29165615|ref|NC_002745.2|	352587 +	A	0	1	1	0	1	SA0296
gi|29165615|ref|NC_002745.2|	352588 +	A	0	0	1	0	4	SA0296
gi|29165615|ref|NC_002745.2|	352592 +	A	0	0	0	1	0	SA0296
gi|29165615|ref|NC_002745.2|	352598 +	G	1	0	1	0	0	SA0296
gi|29165615|ref|NC_002745.2|	352602 +	A	0	0	0	0	1	SA0296
gi|29165615|ref|NC_002745.2|	352605 +	G	0	0	0	0	1	SA0296
gi|29165615|ref|NC_002745.2|	352610 +	U	2	0	0	0	0	SA0296
gi|29165615|ref|NC_002745.2|	352824 +	A	0	0	0	1	0	SA0297
gi|29165615|ref|NC_002745.2|	352957 +	A	0	0	0	0	1	SA0297
gi|29165615|ref|NC_002745.2|	352979 +	U	0	0	0	0	1	SA0297
gi|29165615|ref|NC_002745.2|	352990 +	A	0	1	0	0	0	SA0297
gi|29165615|ref|NC_002745.2|	352998 +	A	0	0	0	0	1	SA0297
gi|29165615|ref|NC_002745.2|	353041 +	C	0	0	0	1	0	SA0297
gi|29165615|ref|NC_002745.2|	353046 +	A	0	1	0	0	0	SA0297
gi|29165615|ref|NC_002745.2|	353051 +	A	1	0	0	0	0	SA0297
gi|29165615|ref|NC_002745.2|	353103 +	U	0	0	0	0	1	SA0297
gi|29165615|ref|NC_002745.2|	353104 +	C	0	0	0	1	0	SA0297
gi|29165615|ref|NC_002745.2|	353195 +	A	0	0	1	0	0	SA0297
gi|29165615|ref|NC_002745.2|	353208 +	A	0	0	0	0	1	SA0297
gi|29165615|ref|NC_002745.2|	353210 +	U	0	0	0	1	0	SA0297
gi|29165615|ref|NC_002745.2|	353219 +	A	0	0	1	1	0	SA0297
gi|29165615|ref|NC_002745.2|	353238 +	G	0	0	0	1	0	SA0297
gi|29165615|ref|NC_002745.2|	353269 +	A	0	1	0	0	0	SA0297
gi|29165615|ref|NC_002745.2|	353451 +	U	1	0	1	1	0	-
gi|29165615|ref|NC_002745.2|	353452 +	A	1	0	1	0	2	-
gi|29165615|ref|NC_002745.2|	353475 +	G	0	0	0	1	0	-
gi|29165615|ref|NC_002745.2|	353486 +	A	1	0	0	0	0	-
gi|29165615|ref|NC_002745.2|	353489 +	A	0	0	0	0	1	-
gi|29165615|ref|NC_002745.2|	353496 +	U	0	0	1	0	0	-
gi|29165615|ref|NC_002745.2|	353499 +	U	0	0	1	0	0	-
gi|29165615|ref|NC_002745.2|	353517 +	G	0	0	0	1	0	SA0298
gi|29165615|ref|NC_002745.2|	353550 +	G	3	13	2	1	2	SA0298
gi|29165615|ref|NC_002745.2|	353551 +	U	12	27	49	25	41	SA0298
gi|29165615|ref|NC_002745.2|	353552 +	C	0	8	13	2	4	SA0298
gi|29165615|ref|NC_002745.2|	353553 +	U	0	0	1	1	0	SA0298
gi|29165615|ref|NC_002745.2|	353557 +	U	1	1	1	0	1	SA0298
gi|29165615|ref|NC_002745.2|	353558 +	G	0	2	1	0	0	SA0298
gi|29165615|ref|NC_002745.2|	353559 +	A	2	7	9	5	7	SA0298
gi|29165615|ref|NC_002745.2|	353560 +	U	1	0	0	0	1	SA0298
gi|29165615|ref|NC_002745.2|	353564 +	U	0	0	0	0	1	SA0298
gi|29165615|ref|NC_002745.2|	353565 +	A	0	1	2	1	0	SA0298
gi|29165615|ref|NC_002745.2|	353566 +	C	0	0	0	0	1	SA0298
gi|29165615|ref|NC_002745.2|	353567 +	A	0	2	0	0	0	SA0298
gi|29165615|ref|NC_002745.2|	353571 +	U	0	0	0	1	0	SA0298
gi|29165615|ref|NC_002745.2|	353574 +	A	3	3	2	4	0	SA0298
gi|29165615|ref|NC_002745.2|	353575 +	C	0	13	11	9	5	SA0298
gi|29165615|ref|NC_002745.2|	353576 +	A	0	11	9	5	12	SA0298
gi|29165615|ref|NC_002745.2|	353577 +	U	0	1	2	0	1	SA0298
gi|29165615|ref|NC_002745.2|	353578 +	A	0	1	1	0	0	SA0298
gi|29165615|ref|NC_002745.2|	353580 +	A	0	5	0	0	2	SA0298
gi|29165615|ref|NC_002745.2|	353581 +	A	0	0	0	0	1	SA0298
gi|29165615|ref|NC_002745.2|	353584 +	C	0	0	0	0	1	SA0298
gi|29165615|ref|NC_002745.2|	353585 +	A	0	0	0	1	0	SA0298
gi|29165615|ref|NC_002745.2|	353588 +	U	1	0	0	0	1	SA0298
gi|29165615|ref|NC_002745.2|	353589 +	A	0	0	2	0	0	SA0298
gi|29165615|ref|NC_002745.2|	353598 +	C	2	0	0	0	0	SA0298
gi|29165615|ref|NC_002745.2|	353612 +	A	1	0	0	0	0	SA0298
gi|29165615|ref|NC_002745.2|	353613 +	U	5	0	0	0	0	SA0298
gi|29165615|ref|NC_002745.2|	353622 +	G	0	0	2	0	0	SA0298
gi|29165615|ref|NC_002745.2|	353623 +	C	0	0	1	0	0	SA0298
gi|29165615|ref|NC_002745.2|	353626 +	C	0	0	0	2	0	SA0298
gi|29165615|ref|NC_002745.2|	353628 +	A	0	1	0	0	0	SA0298
gi|29165615|ref|NC_002745.2|	353632 +	C	0	0	0	0	1	SA0298

gi|29165615|ref|NC_002745.2|	353636 +	A	0	0	0	0	1	SA0298
gi|29165615|ref|NC_002745.2|	353638 +	U	0	0	1	0	0	SA0298
gi|29165615|ref|NC_002745.2|	353640 +	U	0	2	0	2	1	SA0298
gi|29165615|ref|NC_002745.2|	353641 +	U	0	0	1	0	0	SA0298
gi|29165615|ref|NC_002745.2|	353643 +	G	0	0	1	0	0	SA0298
gi|29165615|ref|NC_002745.2|	353646 +	G	0	1	0	0	0	SA0298
gi|29165615|ref|NC_002745.2|	353648 +	A	0	0	0	1	0	SA0298
gi|29165615|ref|NC_002745.2|	353650 +	C	0	1	0	1	0	SA0298
gi|29165615|ref|NC_002745.2|	353651 +	A	0	0	0	1	1	SA0298
gi|29165615|ref|NC_002745.2|	353655 +	A	0	0	0	1	0	SA0298
gi|29165615|ref|NC_002745.2|	353658 +	A	0	1	0	0	0	SA0298
gi|29165615|ref|NC_002745.2|	353660 +	A	0	0	0	0	1	SA0298
gi|29165615|ref|NC_002745.2|	353661 +	U	0	0	2	1	0	SA0298
gi|29165615|ref|NC_002745.2|	353665 +	U	0	0	0	1	0	SA0298
gi|29165615|ref|NC_002745.2|	353672 +	C	0	0	0	0	1	SA0298
gi|29165615|ref|NC_002745.2|	353678 +	A	0	0	0	0	1	SA0298
gi|29165615|ref|NC_002745.2|	353683 +	G	0	1	1	0	1	SA0298
gi|29165615|ref|NC_002745.2|	353684 +	U	0	1	2	0	2	SA0298
gi|29165615|ref|NC_002745.2|	353685 +	A	0	2	2	4	3	SA0298
gi|29165615|ref|NC_002745.2|	353687 +	U	0	0	0	1	0	SA0298
gi|29165615|ref|NC_002745.2|	353688 +	A	0	2	0	2	2	SA0298
gi|29165615|ref|NC_002745.2|	353689 +	A	0	0	0	1	1	SA0298
gi|29165615|ref|NC_002745.2|	353690 +	A	0	0	0	0	1	SA0298
gi|29165615|ref|NC_002745.2|	353691 +	U	1	0	0	1	0	SA0298
gi|29165615|ref|NC_002745.2|	353693 +	U	0	0	0	0	2	SA0298
gi|29165615|ref|NC_002745.2|	353694 +	U	0	0	1	0	1	SA0298
gi|29165615|ref|NC_002745.2|	353695 +	U	0	0	0	0	1	SA0298
gi|29165615|ref|NC_002745.2|	353696 +	A	0	0	0	0	1	SA0298
gi|29165615|ref|NC_002745.2|	353697 +	G	0	1	0	0	1	SA0298
gi|29165615|ref|NC_002745.2|	353698 +	A	0	0	2	1	1	SA0298
gi|29165615|ref|NC_002745.2|	353699 +	A	0	0	1	0	1	SA0298
gi|29165615|ref|NC_002745.2|	353701 +	A	0	1	0	1	3	SA0298
gi|29165615|ref|NC_002745.2|	353703 +	C	0	0	0	1	6	SA0298
gi|29165615|ref|NC_002745.2|	353704 +	U	0	0	1	0	1	SA0298
gi|29165615|ref|NC_002745.2|	353708 +	A	0	0	0	1	0	SA0298
gi|29165615|ref|NC_002745.2|	353711 +	C	0	0	1	0	0	SA0298
gi|29165615|ref|NC_002745.2|	353714 +	U	1	1	0	0	0	SA0298
gi|29165615|ref|NC_002745.2|	353732 +	U	1	0	0	0	0	SA0298
gi|29165615|ref|NC_002745.2|	353738 +	U	1	0	0	0	0	SA0298
gi|29165615|ref|NC_002745.2|	353741 +	C	0	0	0	0	1	SA0298
gi|29165615|ref|NC_002745.2|	353746 +	G	0	0	0	1	0	SA0298
gi|29165615|ref|NC_002745.2|	353756 +	A	0	0	1	0	0	SA0298
gi|29165615|ref|NC_002745.2|	353761 +	C	1	0	0	0	0	SA0298
gi|29165615|ref|NC_002745.2|	353764 +	U	1	0	0	0	0	SA0298
gi|29165615|ref|NC_002745.2|	353788 +	U	0	1	0	0	0	SA0298
gi|29165615|ref|NC_002745.2|	353790 +	G	0	0	0	0	1	SA0298
gi|29165615|ref|NC_002745.2|	353803 +	C	0	0	0	0	1	SA0298
gi|29165615|ref|NC_002745.2|	353825 +	A	0	0	0	1	0	SA0298
gi|29165615|ref|NC_002745.2|	353847 +	A	1	0	0	0	0	SA0298
gi|29165615|ref|NC_002745.2|	353850 +	A	0	1	0	0	0	SA0298
gi|29165615|ref|NC_002745.2|	353856 +	U	0	0	0	1	0	SA0298
gi|29165615|ref|NC_002745.2|	353865 +	A	0	0	0	1	0	SA0298
gi|29165615|ref|NC_002745.2|	353867 +	A	0	1	0	0	0	SA0298
gi|29165615|ref|NC_002745.2|	353879 +	A	0	1	0	0	0	SA0298
gi|29165615|ref|NC_002745.2|	354009 +	A	0	1	0	0	0	SA0298
gi|29165615|ref|NC_002745.2|	354064 +	U	0	0	1	0	0	SA0298
gi|29165615|ref|NC_002745.2|	354288 +	A	0	0	0	0	1	SA0298
gi|29165615|ref|NC_002745.2|	354289 +	C	0	1	1	0	0	SA0298
gi|29165615|ref|NC_002745.2|	354290 +	A	0	1	0	0	0	SA0298
gi|29165615|ref|NC_002745.2|	354304 +	G	1	0	0	0	0	SA0298
gi|29165615|ref|NC_002745.2|	354448 +	U	0	2	0	0	0	SA0298
gi|29165615|ref|NC_002745.2|	354769 +	A	0	0	0	0	1	-
gi|29165615|ref|NC_002745.2|	355665 +	A	0	0	0	1	0	-
gi|29165615|ref|NC_002745.2|	356026 +	C	0	1	0	0	0	SA0301
gi|29165615|ref|NC_002745.2|	356214 +	A	0	0	0	0	1	SA0301
gi|29165615|ref|NC_002745.2|	356257 +	A	0	0	0	0	1	SA0301
gi|29165615|ref|NC_002745.2|	356412 +	A	0	0	1	0	0	SA0301
gi|29165615|ref|NC_002745.2|	356723 +	U	0	1	0	0	0	SA0301
gi|29165615|ref|NC_002745.2|	357888 +	C	0	0	1	0	0	SA0302
gi|29165615|ref|NC_002745.2|	359700 +	C	1	0	0	0	0	-
gi|29165615|ref|NC_002745.2|	359781 +	U	0	1	0	0	0	-
gi|29165615|ref|NC_002745.2|	360765 +	A	0	1	0	3	0	-
gi|29165615|ref|NC_002745.2|	360805 +	A	0	0	1	0	0	-
gi|29165615|ref|NC_002745.2|	360810 +	U	0	0	0	0	1	-
gi|29165615|ref|NC_002745.2|	360812 +	A	0	0	1	0	0	-
gi|29165615|ref|NC_002745.2|	360818 +	U	0	2	0	0	0	-
gi|29165615|ref|NC_002745.2|	360825 +	A	0	0	0	0	2	-
gi|29165615|ref|NC_002745.2|	360831 +	A	0	0	0	0	1	-
gi|29165615|ref|NC_002745.2|	360864 +	C	0	0	0	1	0	SA0305
gi|29165615|ref|NC_002745.2|	360906 +	A	0	0	0	0	1	SA0305
gi|29165615|ref|NC_002745.2|	361056 +	U	0	0	1	0	0	SA0305

gi|29165615|ref|NC_002745.2|	361137 +	U	0	0	0	1	0	SA0305
gi|29165615|ref|NC_002745.2|	361155 +	A	0	0	1	0	0	SA0305
gi|29165615|ref|NC_002745.2|	361173 +	A	0	1	0	0	0	SA0305
gi|29165615|ref|NC_002745.2|	361213 +	A	0	0	0	0	3	SA0305
gi|29165615|ref|NC_002745.2|	361214 +	U	0	2	0	0	0	SA0305
gi|29165615|ref|NC_002745.2|	361229 +	A	0	1	0	0	0	SA0305
gi|29165615|ref|NC_002745.2|	361258 +	U	0	1	0	0	0	SA0305
gi|29165615|ref|NC_002745.2|	361273 +	G	0	0	0	0	1	SA0305
gi|29165615|ref|NC_002745.2|	361274 +	A	0	0	0	0	1	SA0305
gi|29165615|ref|NC_002745.2|	361275 +	A	0	0	0	0	1	SA0305
gi|29165615|ref|NC_002745.2|	361277 +	A	0	0	0	2	0	SA0305
gi|29165615|ref|NC_002745.2|	361278 +	A	0	1	0	0	1	SA0305
gi|29165615|ref|NC_002745.2|	361280 +	C	0	0	0	1	0	SA0305
gi|29165615|ref|NC_002745.2|	361281 +	A	0	0	0	1	0	SA0305
gi|29165615|ref|NC_002745.2|	361289 +	A	0	0	0	0	1	SA0305
gi|29165615|ref|NC_002745.2|	361290 +	U	0	0	0	1	1	SA0305
gi|29165615|ref|NC_002745.2|	361293 +	A	0	0	0	1	1	SA0305
gi|29165615|ref|NC_002745.2|	361294 +	U	0	0	0	0	1	SA0305
gi|29165615|ref|NC_002745.2|	361307 +	C	0	1	0	0	0	SA0305
gi|29165615|ref|NC_002745.2|	361308 +	A	0	0	0	0	2	SA0305
gi|29165615|ref|NC_002745.2|	361309 +	U	0	0	0	0	2	SA0305
gi|29165615|ref|NC_002745.2|	361314 +	C	0	3	3	7	13	SA0305
gi|29165615|ref|NC_002745.2|	361315 +	A	0	0	0	2	1	SA0305
gi|29165615|ref|NC_002745.2|	361320 +	A	0	0	0	2	0	SA0305
gi|29165615|ref|NC_002745.2|	361326 +	G	0	1	0	0	0	SA0305
gi|29165615|ref|NC_002745.2|	361340 +	C	0	0	1	0	0	SA0305
gi|29165615|ref|NC_002745.2|	361346 +	A	0	0	0	1	0	SA0305
gi|29165615|ref|NC_002745.2|	361352 +	A	0	2	0	1	0	SA0305
gi|29165615|ref|NC_002745.2|	361353 +	A	0	4	2	0	2	SA0305
gi|29165615|ref|NC_002745.2|	361354 +	U	0	1	0	1	0	SA0305
gi|29165615|ref|NC_002745.2|	361355 +	A	0	0	0	0	2	SA0305
gi|29165615|ref|NC_002745.2|	361359 +	C	0	0	0	1	0	SA0305
gi|29165615|ref|NC_002745.2|	361362 +	U	2	0	0	0	0	SA0305
gi|29165615|ref|NC_002745.2|	361363 +	U	0	0	1	0	0	SA0305
gi|29165615|ref|NC_002745.2|	361365 +	A	0	0	0	0	1	SA0305
gi|29165615|ref|NC_002745.2|	361367 +	C	0	0	2	0	0	SA0305
gi|29165615|ref|NC_002745.2|	361406 +	G	0	0	1	0	0	SA0305
gi|29165615|ref|NC_002745.2|	361408 +	C	0	0	0	1	0	SA0305
gi|29165615|ref|NC_002745.2|	361485 +	U	0	0	0	1	0	SA0305
gi|29165615|ref|NC_002745.2|	361486 +	A	0	0	0	1	0	SA0305
gi|29165615|ref|NC_002745.2|	361507 +	U	0	0	1	0	0	SA0305
gi|29165615|ref|NC_002745.2|	361541 +	U	0	0	0	0	1	SA0305
gi|29165615|ref|NC_002745.2|	361653 +	C	0	0	0	1	0	SA0305
gi|29165615|ref|NC_002745.2|	361678 +	A	0	0	0	0	1	SA0305
gi|29165615|ref|NC_002745.2|	361686 +	C	0	0	0	0	1	SA0305
gi|29165615|ref|NC_002745.2|	361706 +	C	0	0	0	1	0	SA0305
gi|29165615|ref|NC_002745.2|	361737 +	A	0	1	0	0	0	-
gi|29165615|ref|NC_002745.2|	361747 +	G	0	2	0	0	0	-
gi|29165615|ref|NC_002745.2|	361807 +	C	1	0	0	0	0	-
gi|29165615|ref|NC_002745.2|	361826 +	C	0	0	0	0	1	-
gi|29165615|ref|NC_002745.2|	361988 +	U	0	1	0	0	0	-
gi|29165615|ref|NC_002745.2|	362165 +	A	0	1	0	0	0	-
gi|29165615|ref|NC_002745.2|	362196 +	A	1	0	0	0	0	-
gi|29165615|ref|NC_002745.2|	362248 +	A	0	1	0	0	0	-
gi|29165615|ref|NC_002745.2|	362280 +	A	0	1	0	0	0	-
gi|29165615|ref|NC_002745.2|	362436 +	C	0	1	0	0	0	-
gi|29165615|ref|NC_002745.2|	362627 +	A	0	1	0	0	0	-
gi|29165615|ref|NC_002745.2|	362711 +	U	0	0	1	0	0	-
gi|29165615|ref|NC_002745.2|	362865 +	A	0	1	0	0	0	-
gi|29165615|ref|NC_002745.2|	362890 +	G	1	22	19	4	13	-
gi|29165615|ref|NC_002745.2|	362891 +	A	1	4	0	1	0	-
gi|29165615|ref|NC_002745.2|	362899 +	U	0	0	0	0	1	-
gi|29165615|ref|NC_002745.2|	362901 +	A	0	0	2	0	0	-
gi|29165615|ref|NC_002745.2|	362902 +	A	0	0	1	0	0	-
gi|29165615|ref|NC_002745.2|	362903 +	A	0	0	1	0	0	-
gi|29165615|ref|NC_002745.2|	362905 +	A	0	0	0	0	1	-
gi|29165615|ref|NC_002745.2|	362908 +	G	0	0	1	0	0	-
gi|29165615|ref|NC_002745.2|	362909 +	A	0	0	1	0	1	-
gi|29165615|ref|NC_002745.2|	362910 +	C	0	0	0	0	1	-
gi|29165615|ref|NC_002745.2|	362915 +	G	0	0	0	0	1	-
gi|29165615|ref|NC_002745.2|	362919 +	U	0	0	0	1	0	-
gi|29165615|ref|NC_002745.2|	362924 +	A	0	1	0	0	0	-
gi|29165615|ref|NC_002745.2|	362926 +	U	0	0	0	1	0	-
gi|29165615|ref|NC_002745.2|	362931 +	U	0	0	0	2	1	SA0307
gi|29165615|ref|NC_002745.2|	362935 +	C	0	0	1	0	0	SA0307
gi|29165615|ref|NC_002745.2|	362941 +	G	0	0	1	0	0	SA0307
gi|29165615|ref|NC_002745.2|	362950 +	U	0	0	0	0	1	SA0307
gi|29165615|ref|NC_002745.2|	362968 +	C	0	1	0	0	0	SA0307
gi|29165615|ref|NC_002745.2|	363009 +	G	0	0	1	0	0	SA0307
gi|29165615|ref|NC_002745.2|	363016 +	C	0	0	1	0	1	SA0307
gi|29165615|ref|NC_002745.2|	363027 +	G	0	1	0	0	0	SA0307

gi|29165615|ref|NC_002745.2|	363055	+	C	0	0	0	0	1	SA0307
gi|29165615|ref|NC_002745.2|	363075	+	A	0	1	0	0	0	SA0307
gi|29165615|ref|NC_002745.2|	363136	+	A	0	0	0	1	0	SA0307
gi|29165615|ref|NC_002745.2|	363151	+	C	0	1	0	0	0	SA0307
gi|29165615|ref|NC_002745.2|	363158	+	A	0	0	0	0	1	SA0307
gi|29165615|ref|NC_002745.2|	363207	+	G	0	1	0	0	0	SA0307
gi|29165615|ref|NC_002745.2|	363231	+	A	0	0	0	0	1	SA0307
gi|29165615|ref|NC_002745.2|	363289	+	U	2	0	0	0	0	SA0307
gi|29165615|ref|NC_002745.2|	363291	+	A	0	0	0	0	1	SA0307
gi|29165615|ref|NC_002745.2|	363310	+	U	0	1	0	0	0	SA0307
gi|29165615|ref|NC_002745.2|	363319	+	C	0	1	1	0	1	SA0307
gi|29165615|ref|NC_002745.2|	363328	+	C	0	2	0	0	0	SA0307
gi|29165615|ref|NC_002745.2|	363353	+	U	0	1	0	0	1	SA0307
gi|29165615|ref|NC_002745.2|	363384	+	A	0	1	0	0	0	SA0307
gi|29165615|ref|NC_002745.2|	363387	+	C	0	0	0	0	1	SA0307
gi|29165615|ref|NC_002745.2|	363405	+	C	0	0	0	0	1	SA0307
gi|29165615|ref|NC_002745.2|	363419	+	A	0	1	2	0	0	SA0307
gi|29165615|ref|NC_002745.2|	363426	+	A	0	0	0	0	1	SA0307
gi|29165615|ref|NC_002745.2|	363486	+	G	0	0	0	2	0	SA0307
gi|29165615|ref|NC_002745.2|	363489	+	A	0	1	0	0	0	SA0307
gi|29165615|ref|NC_002745.2|	363492	+	U	0	1	0	0	0	SA0307
gi|29165615|ref|NC_002745.2|	363593	+	U	0	1	0	0	0	SA0307
gi|29165615|ref|NC_002745.2|	365226	+	A	0	0	0	0	2	-
gi|29165615|ref|NC_002745.2|	365372	+	A	0	0	0	0	1	-
gi|29165615|ref|NC_002745.2|	365374	+	A	0	1	0	0	0	-
gi|29165615|ref|NC_002745.2|	365375	+	A	0	1	1	0	0	-
gi|29165615|ref|NC_002745.2|	365377	+	A	0	1	0	0	0	-
gi|29165615|ref|NC_002745.2|	365382	+	A	0	1	0	0	0	-
gi|29165615|ref|NC_002745.2|	365385	+	U	0	0	0	1	0	-
gi|29165615|ref|NC_002745.2|	365400	+	G	0	3	0	0	0	-
gi|29165615|ref|NC_002745.2|	365401	+	G	1	7	2	1	6	-
gi|29165615|ref|NC_002745.2|	365402	+	U	3	3	6	0	7	-
gi|29165615|ref|NC_002745.2|	365403	+	G	56	165	175	61	189	-
gi|29165615|ref|NC_002745.2|	365404	+	C	0	0	4	0	3	-
gi|29165615|ref|NC_002745.2|	365405	+	A	0	0	1	0	2	-
gi|29165615|ref|NC_002745.2|	365407	+	U	0	0	0	0	1	-
gi|29165615|ref|NC_002745.2|	365409	+	G	0	1	0	0	0	-
gi|29165615|ref|NC_002745.2|	365411	+	U	0	0	0	0	1	-
gi|29165615|ref|NC_002745.2|	365414	+	U	0	2	2	0	0	-
gi|29165615|ref|NC_002745.2|	365415	+	C	1	0	1	2	0	-
gi|29165615|ref|NC_002745.2|	365418	+	U	0	0	2	0	0	-
gi|29165615|ref|NC_002745.2|	365421	+	C	0	0	1	0	0	-
gi|29165615|ref|NC_002745.2|	365432	+	C	0	0	1	0	0	-
gi|29165615|ref|NC_002745.2|	365435	+	A	0	1	0	1	0	-
gi|29165615|ref|NC_002745.2|	365436	+	A	0	0	0	2	0	-
gi|29165615|ref|NC_002745.2|	365437	+	A	0	1	0	0	0	-
gi|29165615|ref|NC_002745.2|	365438	+	A	0	0	1	1	0	-
gi|29165615|ref|NC_002745.2|	365439	+	A	0	2	0	0	0	-
gi|29165615|ref|NC_002745.2|	365440	+	U	0	0	0	1	0	-
gi|29165615|ref|NC_002745.2|	365452	+	G	0	0	0	1	0	-
gi|29165615|ref|NC_002745.2|	365453	+	A	0	0	0	0	1	-
gi|29165615|ref|NC_002745.2|	365457	+	U	0	0	1	2	0	-
gi|29165615|ref|NC_002745.2|	365460	+	G	0	0	1	0	1	SA0309
gi|29165615|ref|NC_002745.2|	365462	+	U	0	0	1	0	0	SA0309
gi|29165615|ref|NC_002745.2|	365463	+	A	0	1	1	0	1	SA0309
gi|29165615|ref|NC_002745.2|	365464	+	A	0	0	0	0	1	SA0309
gi|29165615|ref|NC_002745.2|	365465	+	G	0	0	0	1	0	SA0309
gi|29165615|ref|NC_002745.2|	365466	+	A	1	2	1	2	2	SA0309
gi|29165615|ref|NC_002745.2|	365468	+	G	1	0	1	0	0	SA0309
gi|29165615|ref|NC_002745.2|	365469	+	A	0	0	0	0	1	SA0309
gi|29165615|ref|NC_002745.2|	365470	+	C	0	0	1	0	0	SA0309
gi|29165615|ref|NC_002745.2|	365474	+	A	0	3	0	2	0	SA0309
gi|29165615|ref|NC_002745.2|	365476	+	G	0	0	1	0	0	SA0309
gi|29165615|ref|NC_002745.2|	365477	+	A	0	0	0	2	5	SA0309
gi|29165615|ref|NC_002745.2|	365478	+	A	1	1	0	0	0	SA0309
gi|29165615|ref|NC_002745.2|	365480	+	G	0	0	0	0	1	SA0309
gi|29165615|ref|NC_002745.2|	365481	+	A	0	1	1	0	1	SA0309
gi|29165615|ref|NC_002745.2|	365483	+	A	0	1	0	0	1	SA0309
gi|29165615|ref|NC_002745.2|	365486	+	A	0	1	0	0	0	SA0309
gi|29165615|ref|NC_002745.2|	365487	+	U	0	0	1	0	2	SA0309
gi|29165615|ref|NC_002745.2|	365488	+	A	0	1	1	0	1	SA0309
gi|29165615|ref|NC_002745.2|	365489	+	G	0	1	1	0	0	SA0309
gi|29165615|ref|NC_002745.2|	365490	+	U	0	1	1	1	4	SA0309
gi|29165615|ref|NC_002745.2|	365491	+	A	0	5	1	0	2	SA0309
gi|29165615|ref|NC_002745.2|	365492	+	U	0	0	1	0	0	SA0309
gi|29165615|ref|NC_002745.2|	365493	+	U	0	1	1	0	0	SA0309
gi|29165615|ref|NC_002745.2|	365494	+	A	0	0	2	1	1	SA0309
gi|29165615|ref|NC_002745.2|	365495	+	G	0	2	2	2	2	SA0309
gi|29165615|ref|NC_002745.2|	365496	+	A	0	14	12	8	14	SA0309
gi|29165615|ref|NC_002745.2|	365497	+	A	0	0	0	1	2	SA0309
gi|29165615|ref|NC_002745.2|	365498	+	A	0	0	4	0	2	SA0309

gi|29165615|ref|NC_002745.2|	365499 +	G	1	2	0	1	2	SA0309
gi|29165615|ref|NC_002745.2|	365500 +	U	2	27	25	18	46	SA0309
gi|29165615|ref|NC_002745.2|	365501 +	A	2	12	9	3	18	SA0309
gi|29165615|ref|NC_002745.2|	365502 +	U	0	1	1	2	5	SA0309
gi|29165615|ref|NC_002745.2|	365503 +	U	0	2	3	2	0	SA0309
gi|29165615|ref|NC_002745.2|	365504 +	C	0	2	0	1	0	SA0309
gi|29165615|ref|NC_002745.2|	365505 +	A	0	0	0	1	2	SA0309
gi|29165615|ref|NC_002745.2|	365506 +	A	0	2	0	0	2	SA0309
gi|29165615|ref|NC_002745.2|	365507 +	U	0	4	2	1	1	SA0309
gi|29165615|ref|NC_002745.2|	365508 +	A	0	0	0	0	1	SA0309
gi|29165615|ref|NC_002745.2|	365509 +	G	0	1	0	0	0	SA0309
gi|29165615|ref|NC_002745.2|	365511 +	C	0	0	1	0	0	SA0309
gi|29165615|ref|NC_002745.2|	365513 +	U	0	1	0	0	1	SA0309
gi|29165615|ref|NC_002745.2|	365518 +	U	0	0	0	0	1	SA0309
gi|29165615|ref|NC_002745.2|	365519 +	C	0	0	0	1	0	SA0309
gi|29165615|ref|NC_002745.2|	365520 +	A	0	0	0	1	0	SA0309
gi|29165615|ref|NC_002745.2|	365521 +	G	0	0	0	0	2	SA0309
gi|29165615|ref|NC_002745.2|	365529 +	G	0	1	0	0	0	SA0309
gi|29165615|ref|NC_002745.2|	365534 +	C	0	0	0	0	1	SA0309
gi|29165615|ref|NC_002745.2|	365535 +	A	0	0	0	0	1	SA0309
gi|29165615|ref|NC_002745.2|	365540 +	U	1	0	0	0	0	SA0309
gi|29165615|ref|NC_002745.2|	365557 +	G	0	1	0	0	0	SA0309
gi|29165615|ref|NC_002745.2|	365569 +	U	0	1	0	0	0	SA0309
gi|29165615|ref|NC_002745.2|	365573 +	A	0	0	0	1	0	SA0309
gi|29165615|ref|NC_002745.2|	365578 +	A	0	0	1	0	0	SA0309
gi|29165615|ref|NC_002745.2|	365589 +	C	0	0	1	0	0	SA0309
gi|29165615|ref|NC_002745.2|	365590 +	A	0	1	0	1	0	SA0309
gi|29165615|ref|NC_002745.2|	365597 +	C	3	7	0	0	0	SA0309
gi|29165615|ref|NC_002745.2|	365599 +	G	0	1	0	0	0	SA0309
gi|29165615|ref|NC_002745.2|	365611 +	A	0	3	0	0	0	SA0309
gi|29165615|ref|NC_002745.2|	365612 +	C	0	2	1	1	0	SA0309
gi|29165615|ref|NC_002745.2|	365614 +	C	1	0	2	0	0	SA0309
gi|29165615|ref|NC_002745.2|	365615 +	U	0	0	0	0	1	SA0309
gi|29165615|ref|NC_002745.2|	365616 +	A	0	9	2	2	15	SA0309
gi|29165615|ref|NC_002745.2|	365617 +	A	0	0	3	0	1	SA0309
gi|29165615|ref|NC_002745.2|	365618 +	A	0	1	1	0	0	SA0309
gi|29165615|ref|NC_002745.2|	365626 +	G	0	1	0	0	0	SA0309
gi|29165615|ref|NC_002745.2|	365627 +	G	0	1	1	0	3	SA0309
gi|29165615|ref|NC_002745.2|	365628 +	A	1	12	4	3	27	SA0309
gi|29165615|ref|NC_002745.2|	365629 +	G	0	5	3	1	3	SA0309
gi|29165615|ref|NC_002745.2|	365630 +	A	1	4	3	0	3	SA0309
gi|29165615|ref|NC_002745.2|	365631 +	A	0	1	0	1	0	SA0309
gi|29165615|ref|NC_002745.2|	365632 +	C	0	0	2	2	4	SA0309
gi|29165615|ref|NC_002745.2|	365635 +	G	0	2	0	0	0	SA0309
gi|29165615|ref|NC_002745.2|	365638 +	A	0	0	0	0	2	SA0309
gi|29165615|ref|NC_002745.2|	365650 +	U	0	0	1	0	0	SA0309
gi|29165615|ref|NC_002745.2|	365669 +	G	0	0	0	1	0	SA0309
gi|29165615|ref|NC_002745.2|	365688 +	G	2	0	0	0	0	SA0309
gi|29165615|ref|NC_002745.2|	365698 +	A	0	1	0	0	0	SA0309
gi|29165615|ref|NC_002745.2|	365699 +	A	0	1	0	0	0	SA0309
gi|29165615|ref|NC_002745.2|	365720 +	C	0	0	0	1	0	SA0309
gi|29165615|ref|NC_002745.2|	365730 +	A	0	0	0	1	0	SA0309
gi|29165615|ref|NC_002745.2|	365755 +	C	1	0	0	0	0	SA0309
gi|29165615|ref|NC_002745.2|	365757 +	A	0	0	0	0	1	SA0309
gi|29165615|ref|NC_002745.2|	365768 +	A	0	0	0	0	1	SA0309
gi|29165615|ref|NC_002745.2|	365773 +	A	0	0	0	0	1	SA0309
gi|29165615|ref|NC_002745.2|	365775 +	A	0	0	0	1	0	SA0309
gi|29165615|ref|NC_002745.2|	365778 +	A	0	0	0	1	0	SA0309
gi|29165615|ref|NC_002745.2|	365789 +	A	0	0	0	1	0	SA0309
gi|29165615|ref|NC_002745.2|	365795 +	A	0	0	0	1	0	SA0309
gi|29165615|ref|NC_002745.2|	365800 +	C	0	0	1	0	0	SA0309
gi|29165615|ref|NC_002745.2|	365805 +	A	0	1	0	0	0	SA0309
gi|29165615|ref|NC_002745.2|	365819 +	A	0	0	1	0	0	SA0309
gi|29165615|ref|NC_002745.2|	365846 +	A	0	0	0	1	0	SA0309
gi|29165615|ref|NC_002745.2|	365847 +	A	0	1	0	0	0	SA0309
gi|29165615|ref|NC_002745.2|	365849 +	A	0	1	0	0	0	SA0309
gi|29165615|ref|NC_002745.2|	365851 +	A	0	0	1	0	0	SA0309
gi|29165615|ref|NC_002745.2|	365870 +	A	0	2	1	0	0	SA0309
gi|29165615|ref|NC_002745.2|	365876 +	A	0	1	0	0	0	SA0309
gi|29165615|ref|NC_002745.2|	365909 +	A	0	1	0	0	0	SA0309
gi|29165615|ref|NC_002745.2|	365921 +	C	0	1	0	0	0	SA0309
gi|29165615|ref|NC_002745.2|	365925 +	A	0	1	0	0	0	SA0309
gi|29165615|ref|NC_002745.2|	365960 +	C	0	0	0	1	0	SA0309
gi|29165615|ref|NC_002745.2|	366008 +	C	0	1	0	0	0	SA0309
gi|29165615|ref|NC_002745.2|	366016 +	A	0	1	0	0	0	SA0309
gi|29165615|ref|NC_002745.2|	366019 +	U	0	0	0	0	1	SA0309
gi|29165615|ref|NC_002745.2|	366022 +	A	0	1	1	2	1	SA0309
gi|29165615|ref|NC_002745.2|	366025 +	A	0	0	1	0	0	SA0309
gi|29165615|ref|NC_002745.2|	366028 +	C	0	0	0	1	2	SA0309
gi|29165615|ref|NC_002745.2|	366029 +	C	0	0	2	0	0	SA0309
gi|29165615|ref|NC_002745.2|	366030 +	C	0	0	1	0	1	SA0309

gi|29165615|ref|NC_002745.2|	366034 +	U	0	2	0	0	3	SA0309
gi|29165615|ref|NC_002745.2|	366035 +	C	0	1	0	0	0	SA0309
gi|29165615|ref|NC_002745.2|	366037 +	A	0	2	0	0	1	SA0309
gi|29165615|ref|NC_002745.2|	366038 +	A	0	1	0	1	0	SA0309
gi|29165615|ref|NC_002745.2|	366040 +	G	0	1	0	0	0	SA0309
gi|29165615|ref|NC_002745.2|	366041 +	A	0	1	0	4	2	SA0309
gi|29165615|ref|NC_002745.2|	366043 +	A	0	1	0	0	2	SA0309
gi|29165615|ref|NC_002745.2|	366046 +	A	0	0	0	0	1	SA0309
gi|29165615|ref|NC_002745.2|	366055 +	A	0	0	1	0	0	SA0309
gi|29165615|ref|NC_002745.2|	366064 +	A	0	0	1	0	0	SA0309
gi|29165615|ref|NC_002745.2|	366077 +	C	1	0	0	0	0	SA0309
gi|29165615|ref|NC_002745.2|	366085 +	G	0	0	0	1	0	SA0309
gi|29165615|ref|NC_002745.2|	366086 +	A	0	0	0	0	1	SA0309
gi|29165615|ref|NC_002745.2|	366090 +	A	0	1	0	0	0	SA0309
gi|29165615|ref|NC_002745.2|	366091 +	C	0	0	1	0	0	SA0309
gi|29165615|ref|NC_002745.2|	366105 +	A	0	0	0	1	0	SA0309
gi|29165615|ref|NC_002745.2|	366106 +	G	0	0	1	0	0	SA0309
gi|29165615|ref|NC_002745.2|	366110 +	C	0	1	0	0	0	SA0309
gi|29165615|ref|NC_002745.2|	366117 +	A	0	0	0	0	1	SA0309
gi|29165615|ref|NC_002745.2|	366121 +	G	0	0	0	0	1	SA0309
gi|29165615|ref|NC_002745.2|	366128 +	A	0	0	0	1	0	SA0309
gi|29165615|ref|NC_002745.2|	366134 +	U	0	1	0	0	1	SA0309
gi|29165615|ref|NC_002745.2|	366135 +	A	0	3	0	0	0	SA0309
gi|29165615|ref|NC_002745.2|	366142 +	A	0	0	0	1	0	SA0309
gi|29165615|ref|NC_002745.2|	366178 +	C	0	0	0	0	1	SA0309
gi|29165615|ref|NC_002745.2|	366200 +	A	0	1	0	0	0	SA0309
gi|29165615|ref|NC_002745.2|	366202 +	C	0	1	1	0	0	SA0309
gi|29165615|ref|NC_002745.2|	366204 +	U	0	1	0	0	0	SA0309
gi|29165615|ref|NC_002745.2|	366210 +	U	1	0	0	0	0	SA0309
gi|29165615|ref|NC_002745.2|	366213 +	U	0	0	1	0	0	SA0309
gi|29165615|ref|NC_002745.2|	366222 +	C	0	1	0	0	0	SA0309
gi|29165615|ref|NC_002745.2|	366227 +	A	0	0	0	1	0	SA0309
gi|29165615|ref|NC_002745.2|	366230 +	A	0	0	0	0	1	SA0309
gi|29165615|ref|NC_002745.2|	366238 +	G	0	1	0	0	0	SA0309
gi|29165615|ref|NC_002745.2|	366241 +	A	0	0	1	0	0	SA0309
gi|29165615|ref|NC_002745.2|	366242 +	A	0	1	0	0	0	SA0309
gi|29165615|ref|NC_002745.2|	366244 +	A	0	1	0	0	0	SA0309
gi|29165615|ref|NC_002745.2|	366245 +	A	0	1	0	0	0	SA0309
gi|29165615|ref|NC_002745.2|	366247 +	A	0	0	0	1	0	SA0309
gi|29165615|ref|NC_002745.2|	366248 +	C	0	1	0	0	0	SA0309
gi|29165615|ref|NC_002745.2|	366250 +	G	1	0	0	0	0	SA0309
gi|29165615|ref|NC_002745.2|	366251 +	A	0	1	1	1	1	SA0309
gi|29165615|ref|NC_002745.2|	366253 +	A	0	1	1	0	0	SA0309
gi|29165615|ref|NC_002745.2|	366255 +	U	0	0	1	0	0	SA0309
gi|29165615|ref|NC_002745.2|	366256 +	A	0	1	0	0	0	SA0309
gi|29165615|ref|NC_002745.2|	366257 +	A	0	1	0	1	0	SA0309
gi|29165615|ref|NC_002745.2|	366258 +	A	0	1	2	3	2	SA0309
gi|29165615|ref|NC_002745.2|	366259 +	C	0	1	0	0	0	SA0309
gi|29165615|ref|NC_002745.2|	366261 +	A	0	1	2	0	0	SA0309
gi|29165615|ref|NC_002745.2|	366262 +	C	0	1	0	1	0	SA0309
gi|29165615|ref|NC_002745.2|	366263 +	U	0	1	0	0	1	SA0309
gi|29165615|ref|NC_002745.2|	366264 +	A	0	1	2	3	0	SA0309
gi|29165615|ref|NC_002745.2|	366265 +	A	0	1	0	1	0	SA0309
gi|29165615|ref|NC_002745.2|	366267 +	C	0	0	2	0	5	SA0309
gi|29165615|ref|NC_002745.2|	366268 +	A	0	2	0	1	0	SA0309
gi|29165615|ref|NC_002745.2|	366269 +	A	0	1	0	0	0	SA0309
gi|29165615|ref|NC_002745.2|	366271 +	G	0	1	0	0	0	SA0309
gi|29165615|ref|NC_002745.2|	366275 +	C	1	0	0	1	0	SA0309
gi|29165615|ref|NC_002745.2|	366279 +	U	0	0	1	0	0	SA0309
gi|29165615|ref|NC_002745.2|	366280 +	C	0	1	1	0	0	SA0309
gi|29165615|ref|NC_002745.2|	366282 +	A	0	0	1	0	0	SA0309
gi|29165615|ref|NC_002745.2|	366291 +	A	0	1	0	0	0	SA0309
gi|29165615|ref|NC_002745.2|	366297 +	U	1	1	0	1	0	SA0309
gi|29165615|ref|NC_002745.2|	366302 +	C	0	0	0	1	0	SA0309
gi|29165615|ref|NC_002745.2|	366304 +	A	0	0	0	1	0	SA0309
gi|29165615|ref|NC_002745.2|	366307 +	A	0	2	2	1	1	SA0309
gi|29165615|ref|NC_002745.2|	366309 +	U	0	0	1	0	0	SA0309
gi|29165615|ref|NC_002745.2|	366310 +	G	0	1	0	0	0	SA0309
gi|29165615|ref|NC_002745.2|	366319 +	G	0	0	0	0	1	SA0309
gi|29165615|ref|NC_002745.2|	366320 +	A	0	0	1	0	0	SA0309
gi|29165615|ref|NC_002745.2|	366329 +	A	0	1	0	0	0	SA0309
gi|29165615|ref|NC_002745.2|	366334 +	C	0	0	1	0	0	SA0309
gi|29165615|ref|NC_002745.2|	366338 +	C	0	1	1	0	1	SA0309
gi|29165615|ref|NC_002745.2|	366342 +	U	1	0	0	1	0	SA0309
gi|29165615|ref|NC_002745.2|	366343 +	A	0	1	0	0	0	SA0309
gi|29165615|ref|NC_002745.2|	366348 +	G	0	0	0	0	1	SA0309
gi|29165615|ref|NC_002745.2|	366349 +	A	0	0	1	0	0	SA0309
gi|29165615|ref|NC_002745.2|	366352 +	C	0	0	0	1	1	SA0309
gi|29165615|ref|NC_002745.2|	366358 +	C	0	0	0	0	1	SA0309
gi|29165615|ref|NC_002745.2|	366361 +	C	0	1	0	0	0	SA0309
gi|29165615|ref|NC_002745.2|	366362 +	C	1	0	0	1	2	SA0309

gi|29165615|ref|NC_002745.2|	366363 +	A	0	1	0	0	0	SA0309
gi|29165615|ref|NC_002745.2|	366366 +	U	0	0	1	0	0	SA0309
gi|29165615|ref|NC_002745.2|	366368 +	A	0	0	0	0	1	SA0309
gi|29165615|ref|NC_002745.2|	366370 +	A	0	0	0	2	2	SA0309
gi|29165615|ref|NC_002745.2|	366371 +	A	0	1	0	2	0	SA0309
gi|29165615|ref|NC_002745.2|	366372 +	A	3	5	1	0	11	SA0309
gi|29165615|ref|NC_002745.2|	366373 +	U	0	0	3	0	1	SA0309
gi|29165615|ref|NC_002745.2|	366376 +	C	0	0	1	0	1	SA0309
gi|29165615|ref|NC_002745.2|	366380 +	U	0	0	0	1	0	SA0309
gi|29165615|ref|NC_002745.2|	366382 +	G	0	0	1	0	0	SA0309
gi|29165615|ref|NC_002745.2|	366386 +	U	1	0	0	0	0	SA0309
gi|29165615|ref|NC_002745.2|	366387 +	U	0	0	0	0	1	SA0309
gi|29165615|ref|NC_002745.2|	366388 +	G	0	0	0	1	0	SA0309
gi|29165615|ref|NC_002745.2|	366395 +	G	1	0	0	0	0	SA0309
gi|29165615|ref|NC_002745.2|	366396 +	A	0	0	1	0	0	SA0309
gi|29165615|ref|NC_002745.2|	366397 +	U	0	0	1	0	1	SA0309
gi|29165615|ref|NC_002745.2|	366399 +	U	0	0	1	0	0	SA0309
gi|29165615|ref|NC_002745.2|	366400 +	U	0	0	1	0	0	SA0309
gi|29165615|ref|NC_002745.2|	366401 +	U	0	1	2	0	0	SA0309
gi|29165615|ref|NC_002745.2|	366402 +	A	0	2	0	0	1	SA0309
gi|29165615|ref|NC_002745.2|	366405 +	A	0	0	1	0	0	SA0309
gi|29165615|ref|NC_002745.2|	366407 +	U	1	0	0	0	0	SA0309
gi|29165615|ref|NC_002745.2|	366409 +	G	0	0	2	0	0	SA0309
gi|29165615|ref|NC_002745.2|	366416 +	A	0	1	0	0	0	SA0309
gi|29165615|ref|NC_002745.2|	366419 +	A	0	0	0	0	1	SA0309
gi|29165615|ref|NC_002745.2|	366422 +	C	0	0	1	1	0	SA0309
gi|29165615|ref|NC_002745.2|	366430 +	U	0	0	0	0	1	SA0309
gi|29165615|ref|NC_002745.2|	366431 +	U	0	1	0	0	0	SA0309
gi|29165615|ref|NC_002745.2|	366437 +	C	0	1	0	0	0	SA0309
gi|29165615|ref|NC_002745.2|	366440 +	A	0	0	1	0	0	SA0309
gi|29165615|ref|NC_002745.2|	366442 +	U	0	0	1	0	0	SA0309
gi|29165615|ref|NC_002745.2|	366443 +	A	2	0	1	0	2	SA0309
gi|29165615|ref|NC_002745.2|	366459 +	A	0	0	1	0	0	SA0309
gi|29165615|ref|NC_002745.2|	366461 +	U	0	0	0	1	0	SA0309
gi|29165615|ref|NC_002745.2|	366463 +	A	0	1	0	2	0	SA0309
gi|29165615|ref|NC_002745.2|	366464 +	A	0	3	1	3	1	SA0309
gi|29165615|ref|NC_002745.2|	366467 +	U	0	1	1	2	0	SA0309
gi|29165615|ref|NC_002745.2|	366468 +	U	0	3	0	0	0	SA0309
gi|29165615|ref|NC_002745.2|	366469 +	A	0	2	0	0	0	SA0309
gi|29165615|ref|NC_002745.2|	366471 +	C	0	0	0	1	0	SA0309
gi|29165615|ref|NC_002745.2|	366475 +	G	0	1	0	0	0	SA0309
gi|29165615|ref|NC_002745.2|	366477 +	A	0	0	0	0	1	SA0309
gi|29165615|ref|NC_002745.2|	366479 +	U	0	0	1	0	0	SA0309
gi|29165615|ref|NC_002745.2|	366483 +	A	0	0	0	0	1	SA0309
gi|29165615|ref|NC_002745.2|	366496 +	A	0	0	0	1	0	SA0309
gi|29165615|ref|NC_002745.2|	366504 +	U	0	0	0	0	1	SA0309
gi|29165615|ref|NC_002745.2|	366505 +	C	0	0	1	0	0	SA0309
gi|29165615|ref|NC_002745.2|	366509 +	C	0	0	0	1	0	SA0309
gi|29165615|ref|NC_002745.2|	366513 +	U	0	0	0	0	1	SA0309
gi|29165615|ref|NC_002745.2|	366533 +	A	0	0	1	1	0	SA0309
gi|29165615|ref|NC_002745.2|	366539 +	A	0	0	1	0	0	SA0309
gi|29165615|ref|NC_002745.2|	366541 +	C	0	0	0	0	1	SA0309
gi|29165615|ref|NC_002745.2|	366545 +	C	0	0	0	0	1	SA0309
gi|29165615|ref|NC_002745.2|	366554 +	U	0	0	0	0	1	SA0309
gi|29165615|ref|NC_002745.2|	366557 +	A	0	0	0	0	1	SA0309
gi|29165615|ref|NC_002745.2|	366559 +	U	0	1	0	0	1	SA0309
gi|29165615|ref|NC_002745.2|	366560 +	A	2	0	0	0	0	SA0309
gi|29165615|ref|NC_002745.2|	366562 +	U	0	0	0	0	1	SA0309
gi|29165615|ref|NC_002745.2|	366563 +	A	0	0	0	1	1	SA0309
gi|29165615|ref|NC_002745.2|	366564 +	C	0	0	0	0	1	SA0309
gi|29165615|ref|NC_002745.2|	366565 +	A	1	0	0	0	1	SA0309
gi|29165615|ref|NC_002745.2|	366566 +	U	0	1	0	0	0	SA0309
gi|29165615|ref|NC_002745.2|	366568 +	A	0	0	0	2	1	SA0309
gi|29165615|ref|NC_002745.2|	366569 +	A	0	1	0	2	0	SA0309
gi|29165615|ref|NC_002745.2|	366570 +	A	1	0	0	0	0	SA0309
gi|29165615|ref|NC_002745.2|	366572 +	G	0	0	1	0	0	SA0309
gi|29165615|ref|NC_002745.2|	366573 +	U	0	1	0	0	0	SA0309
gi|29165615|ref|NC_002745.2|	366598 +	C	0	1	0	0	0	SA0309
gi|29165615|ref|NC_002745.2|	366604 +	G	0	0	1	0	0	SA0309
gi|29165615|ref|NC_002745.2|	366605 +	C	0	0	0	1	1	SA0309
gi|29165615|ref|NC_002745.2|	366609 +	A	0	1	0	0	0	SA0309
gi|29165615|ref|NC_002745.2|	366612 +	C	0	0	0	0	1	SA0309
gi|29165615|ref|NC_002745.2|	366613 +	G	0	0	0	0	1	SA0309
gi|29165615|ref|NC_002745.2|	366616 +	C	0	2	0	0	0	SA0309
gi|29165615|ref|NC_002745.2|	366617 +	A	0	1	0	0	0	SA0309
gi|29165615|ref|NC_002745.2|	366620 +	A	0	0	0	0	1	SA0309
gi|29165615|ref|NC_002745.2|	366625 +	U	0	1	0	0	1	SA0309
gi|29165615|ref|NC_002745.2|	366632 +	A	0	0	1	0	1	SA0309
gi|29165615|ref|NC_002745.2|	366634 +	A	0	1	0	2	0	SA0309
gi|29165615|ref|NC_002745.2|	366635 +	C	0	0	1	2	0	SA0309
gi|29165615|ref|NC_002745.2|	366638 +	A	0	0	1	0	0	SA0309

gi|29165615|ref|NC_002745.2|	366639 +	U	0	0	0	1	0	SA0309
gi|29165615|ref|NC_002745.2|	366640 +	A	0	1	0	0	0	SA0309
gi|29165615|ref|NC_002745.2|	366641 +	A	0	0	0	0	1	SA0309
gi|29165615|ref|NC_002745.2|	366644 +	G	0	1	0	0	0	SA0309
gi|29165615|ref|NC_002745.2|	366647 +	U	0	0	4	0	1	SA0309
gi|29165615|ref|NC_002745.2|	366648 +	C	1	1	0	0	1	SA0309
gi|29165615|ref|NC_002745.2|	366656 +	A	0	0	0	1	0	SA0309
gi|29165615|ref|NC_002745.2|	366658 +	U	0	1	0	0	0	SA0309
gi|29165615|ref|NC_002745.2|	366665 +	C	0	0	1	0	1	SA0309
gi|29165615|ref|NC_002745.2|	366670 +	A	0	0	1	0	0	SA0309
gi|29165615|ref|NC_002745.2|	366675 +	G	0	0	1	0	0	SA0309
gi|29165615|ref|NC_002745.2|	366676 +	G	0	0	0	0	1	SA0309
gi|29165615|ref|NC_002745.2|	366677 +	U	1	0	0	0	0	SA0309
gi|29165615|ref|NC_002745.2|	366680 +	A	0	0	1	0	0	SA0309
gi|29165615|ref|NC_002745.2|	366681 +	U	0	0	1	0	0	SA0309
gi|29165615|ref|NC_002745.2|	366682 +	C	0	1	1	0	0	SA0309
gi|29165615|ref|NC_002745.2|	366683 +	U	0	0	0	0	1	SA0309
gi|29165615|ref|NC_002745.2|	366693 +	U	0	0	0	0	2	SA0309
gi|29165615|ref|NC_002745.2|	366710 +	C	0	0	1	0	1	SA0309
gi|29165615|ref|NC_002745.2|	366712 +	A	0	4	0	0	0	SA0309
gi|29165615|ref|NC_002745.2|	366720 +	A	0	0	1	0	0	SA0309
gi|29165615|ref|NC_002745.2|	366740 +	A	0	1	0	0	0	SA0309
gi|29165615|ref|NC_002745.2|	366745 +	A	0	0	0	0	1	SA0309
gi|29165615|ref|NC_002745.2|	366748 +	A	0	0	0	1	0	SA0309
gi|29165615|ref|NC_002745.2|	366768 +	U	1	0	0	0	0	SA0309
gi|29165615|ref|NC_002745.2|	366786 +	A	0	1	0	0	0	SA0309
gi|29165615|ref|NC_002745.2|	366787 +	A	0	1	0	0	0	SA0309
gi|29165615|ref|NC_002745.2|	366831 +	A	0	0	1	0	0	SA0309
gi|29165615|ref|NC_002745.2|	366851 +	C	0	1	0	0	0	SA0309
gi|29165615|ref|NC_002745.2|	366865 +	C	0	0	1	0	0	SA0309
gi|29165615|ref|NC_002745.2|	366869 +	C	0	0	1	0	0	SA0309
gi|29165615|ref|NC_002745.2|	366888 +	U	0	0	1	2	0	SA0309
gi|29165615|ref|NC_002745.2|	366893 +	A	1	0	0	0	0	SA0309
gi|29165615|ref|NC_002745.2|	366894 +	A	1	0	0	0	0	SA0309
gi|29165615|ref|NC_002745.2|	366907 +	A	0	0	0	0	1	SA0309
gi|29165615|ref|NC_002745.2|	366921 +	A	0	1	0	0	0	SA0309
gi|29165615|ref|NC_002745.2|	366923 +	A	1	0	1	0	0	SA0309
gi|29165615|ref|NC_002745.2|	366925 +	C	0	2	0	0	1	SA0309
gi|29165615|ref|NC_002745.2|	366926 +	G	1	1	0	0	0	SA0309
gi|29165615|ref|NC_002745.2|	366928 +	U	0	1	0	0	0	SA0309
gi|29165615|ref|NC_002745.2|	366930 +	U	0	1	0	0	0	SA0309
gi|29165615|ref|NC_002745.2|	366931 +	A	0	0	0	1	0	SA0309
gi|29165615|ref|NC_002745.2|	366935 +	G	0	0	0	0	2	SA0309
gi|29165615|ref|NC_002745.2|	366939 +	C	0	0	1	1	0	SA0309
gi|29165615|ref|NC_002745.2|	366943 +	U	0	0	1	0	0	SA0309
gi|29165615|ref|NC_002745.2|	366950 +	A	0	1	1	0	0	SA0309
gi|29165615|ref|NC_002745.2|	366961 +	G	0	0	1	0	0	SA0309
gi|29165615|ref|NC_002745.2|	366962 +	G	0	0	0	0	1	SA0309
gi|29165615|ref|NC_002745.2|	366966 +	A	0	0	0	0	1	SA0309
gi|29165615|ref|NC_002745.2|	366967 +	A	0	1	0	0	0	SA0309
gi|29165615|ref|NC_002745.2|	366970 +	C	0	0	1	1	0	SA0309
gi|29165615|ref|NC_002745.2|	366971 +	A	0	1	0	0	0	SA0309
gi|29165615|ref|NC_002745.2|	366981 +	U	0	2	0	0	0	SA0309
gi|29165615|ref|NC_002745.2|	366983 +	A	0	0	0	0	1	SA0309
gi|29165615|ref|NC_002745.2|	366984 +	A	0	0	0	0	1	SA0309
gi|29165615|ref|NC_002745.2|	366987 +	A	0	1	1	0	3	SA0309
gi|29165615|ref|NC_002745.2|	366988 +	U	0	0	0	0	2	SA0309
gi|29165615|ref|NC_002745.2|	366992 +	C	0	0	0	1	0	SA0309
gi|29165615|ref|NC_002745.2|	366993 +	A	0	0	0	0	1	SA0309
gi|29165615|ref|NC_002745.2|	366994 +	A	0	0	0	3	0	SA0309
gi|29165615|ref|NC_002745.2|	366998 +	A	0	0	0	1	0	SA0309
gi|29165615|ref|NC_002745.2|	366999 +	G	0	1	0	0	0	SA0309
gi|29165615|ref|NC_002745.2|	367000 +	A	0	1	0	0	0	SA0309
gi|29165615|ref|NC_002745.2|	367004 +	A	0	0	1	2	0	SA0309
gi|29165615|ref|NC_002745.2|	367005 +	C	0	0	0	0	1	SA0309
gi|29165615|ref|NC_002745.2|	367007 +	U	0	0	0	0	1	SA0309
gi|29165615|ref|NC_002745.2|	367010 +	A	0	5	0	0	0	SA0309
gi|29165615|ref|NC_002745.2|	367011 +	C	0	1	0	0	1	SA0309
gi|29165615|ref|NC_002745.2|	367014 +	U	0	0	0	1	0	SA0309
gi|29165615|ref|NC_002745.2|	367016 +	U	0	0	1	0	1	SA0309
gi|29165615|ref|NC_002745.2|	367017 +	A	0	0	1	1	1	SA0309
gi|29165615|ref|NC_002745.2|	367019 +	A	0	0	1	0	1	SA0309
gi|29165615|ref|NC_002745.2|	367024 +	G	0	0	0	0	1	SA0309
gi|29165615|ref|NC_002745.2|	367025 +	U	0	0	0	2	0	SA0309
gi|29165615|ref|NC_002745.2|	367027 +	A	0	0	0	0	1	SA0309
gi|29165615|ref|NC_002745.2|	367029 +	U	3	0	0	0	0	SA0309
gi|29165615|ref|NC_002745.2|	367035 +	C	0	0	0	1	0	SA0309
gi|29165615|ref|NC_002745.2|	367039 +	A	0	0	0	1	0	SA0309
gi|29165615|ref|NC_002745.2|	367045 +	A	0	1	0	0	0	SA0309
gi|29165615|ref|NC_002745.2|	367048 +	U	0	2	0	0	0	SA0309
gi|29165615|ref|NC_002745.2|	367049 +	C	0	0	0	1	0	SA0309

gi|29165615|ref|NC_002745.2|	367052	+	A	0	0	0	3	2	SA0309
gi|29165615|ref|NC_002745.2|	367056	+	U	0	0	1	0	0	SA0309
gi|29165615|ref|NC_002745.2|	367057	+	A	0	0	0	1	0	SA0309
gi|29165615|ref|NC_002745.2|	367065	+	C	0	0	1	0	0	SA0309
gi|29165615|ref|NC_002745.2|	367095	+	A	0	4	2	2	4	SA0309
gi|29165615|ref|NC_002745.2|	367116	+	G	0	2	0	1	0	SA0309
gi|29165615|ref|NC_002745.2|	367117	+	A	0	0	1	0	0	SA0309
gi|29165615|ref|NC_002745.2|	367118	+	A	0	2	0	0	0	SA0309
gi|29165615|ref|NC_002745.2|	367120	+	C	0	0	1	0	0	SA0309
gi|29165615|ref|NC_002745.2|	367123	+	A	0	1	1	0	1	SA0309
gi|29165615|ref|NC_002745.2|	367132	+	U	0	1	0	0	0	SA0309
gi|29165615|ref|NC_002745.2|	367134	+	U	2	0	0	0	0	SA0309
gi|29165615|ref|NC_002745.2|	367144	+	A	0	0	0	0	1	SA0309
gi|29165615|ref|NC_002745.2|	367145	+	C	1	0	0	0	0	SA0309
gi|29165615|ref|NC_002745.2|	367163	+	C	0	0	1	0	0	SA0309
gi|29165615|ref|NC_002745.2|	367174	+	G	0	0	0	0	1	SA0309
gi|29165615|ref|NC_002745.2|	367184	+	A	0	1	1	0	0	SA0309
gi|29165615|ref|NC_002745.2|	367189	+	G	0	0	0	0	1	SA0309
gi|29165615|ref|NC_002745.2|	367226	+	G	1	0	0	0	0	SA0309
gi|29165615|ref|NC_002745.2|	367228	+	A	0	1	0	0	0	SA0309
gi|29165615|ref|NC_002745.2|	367265	+	A	0	2	0	0	0	SA0309
gi|29165615|ref|NC_002745.2|	367266	+	U	0	0	1	0	0	SA0309
gi|29165615|ref|NC_002745.2|	367307	+	A	0	1	0	0	0	SA0309
gi|29165615|ref|NC_002745.2|	367330	+	A	0	0	0	0	1	SA0309
gi|29165615|ref|NC_002745.2|	367375	+	A	0	3	0	0	0	SA0309
gi|29165615|ref|NC_002745.2|	367379	+	U	0	1	0	0	0	SA0309
gi|29165615|ref|NC_002745.2|	367455	+	C	0	1	0	0	0	SA0309
gi|29165615|ref|NC_002745.2|	367466	+	G	0	1	0	0	0	SA0309
gi|29165615|ref|NC_002745.2|	367475	+	A	0	0	0	0	1	SA0309
gi|29165615|ref|NC_002745.2|	367518	+	A	0	1	0	0	0	SA0309
gi|29165615|ref|NC_002745.2|	367522	+	A	0	0	0	1	0	SA0309
gi|29165615|ref|NC_002745.2|	367542	+	U	0	0	0	0	1	-
gi|29165615|ref|NC_002745.2|	367559	+	A	0	0	1	0	0	-
gi|29165615|ref|NC_002745.2|	367561	+	G	0	0	0	1	0	-
gi|29165615|ref|NC_002745.2|	367635	+	A	0	0	0	0	1	-
gi|29165615|ref|NC_002745.2|	367958	+	C	1	0	0	0	0	-
gi|29165615|ref|NC_002745.2|	367966	+	A	0	0	1	0	0	-
gi|29165615|ref|NC_002745.2|	368014	+	A	0	1	0	0	0	-
gi|29165615|ref|NC_002745.2|	369212	+	G	0	0	0	0	1	-
gi|29165615|ref|NC_002745.2|	369344	+	A	0	0	0	1	0	-
gi|29165615|ref|NC_002745.2|	369461	+	G	0	1	0	0	0	-
gi|29165615|ref|NC_002745.2|	369738	+	U	0	0	1	0	0	-
gi|29165615|ref|NC_002745.2|	369836	+	U	0	0	1	0	0	-
gi|29165615|ref|NC_002745.2|	369965	+	G	0	1	1	0	0	-
gi|29165615|ref|NC_002745.2|	369986	+	A	0	0	0	0	1	-
gi|29165615|ref|NC_002745.2|	369998	+	U	0	0	1	0	0	-
gi|29165615|ref|NC_002745.2|	370034	+	A	0	0	0	0	1	-
gi|29165615|ref|NC_002745.2|	370039	+	A	0	0	1	0	0	-
gi|29165615|ref|NC_002745.2|	370042	+	A	0	0	0	0	1	-
gi|29165615|ref|NC_002745.2|	370055	+	A	0	0	1	0	0	-
gi|29165615|ref|NC_002745.2|	370057	+	A	0	1	2	0	1	-
gi|29165615|ref|NC_002745.2|	370058	+	U	0	0	0	1	0	-
gi|29165615|ref|NC_002745.2|	370060	+	U	0	0	1	0	0	-
gi|29165615|ref|NC_002745.2|	370069	+	A	0	0	0	1	0	-
gi|29165615|ref|NC_002745.2|	370074	+	A	0	0	0	0	1	-
gi|29165615|ref|NC_002745.2|	370075	+	A	0	0	0	0	1	-
gi|29165615|ref|NC_002745.2|	370076	+	A	0	0	0	0	1	-
gi|29165615|ref|NC_002745.2|	370080	+	U	0	1	0	1	1	-
gi|29165615|ref|NC_002745.2|	370092	+	U	0	0	0	0	1	-
gi|29165615|ref|NC_002745.2|	370093	+	G	3	12	10	17	20	-
gi|29165615|ref|NC_002745.2|	370094	+	G	1	1	1	0	0	-
gi|29165615|ref|NC_002745.2|	370095	+	G	0	0	0	1	0	-
gi|29165615|ref|NC_002745.2|	370096	+	U	0	0	1	0	0	-
gi|29165615|ref|NC_002745.2|	370097	+	A	0	0	0	1	0	-
gi|29165615|ref|NC_002745.2|	370098	+	U	0	1	0	0	1	-
gi|29165615|ref|NC_002745.2|	370101	+	G	0	4	0	0	0	-
gi|29165615|ref|NC_002745.2|	370102	+	A	1	0	0	0	0	-
gi|29165615|ref|NC_002745.2|	370138	+	U	0	1	0	0	0	-
gi|29165615|ref|NC_002745.2|	370190	+	C	0	0	0	1	0	SA0312
gi|29165615|ref|NC_002745.2|	370265	+	A	0	1	0	0	0	SA0312
gi|29165615|ref|NC_002745.2|	370680	+	A	1	0	0	0	0	SA0312
gi|29165615|ref|NC_002745.2|	370931	+	A	0	1	0	0	0	SA0312
gi|29165615|ref|NC_002745.2|	370938	+	G	0	1	0	0	0	SA0312
gi|29165615|ref|NC_002745.2|	371056	+	A	1	0	0	0	0	SA0312
gi|29165615|ref|NC_002745.2|	371091	+	G	0	0	0	1	0	SA0312
gi|29165615|ref|NC_002745.2|	371163	+	G	0	0	0	1	0	SA0312
gi|29165615|ref|NC_002745.2|	371186	+	A	0	3	0	0	1	-
gi|29165615|ref|NC_002745.2|	371193	+	A	0	0	1	0	0	-
gi|29165615|ref|NC_002745.2|	371195	+	A	0	1	0	0	0	-
gi|29165615|ref|NC_002745.2|	371203	+	A	0	1	0	0	1	-
gi|29165615|ref|NC_002745.2|	371210	+	G	0	0	1	0	0	-

gi|29165615|ref|NC_002745.2|	371213	+	G	0	1	0	0	0	SA0313
gi|29165615|ref|NC_002745.2|	371217	+	A	0	0	1	0	0	SA0313
gi|29165615|ref|NC_002745.2|	371219	+	G	0	0	0	1	0	SA0313
gi|29165615|ref|NC_002745.2|	371224	+	C	0	0	0	0	1	SA0313
gi|29165615|ref|NC_002745.2|	371230	+	A	0	0	2	0	0	SA0313
gi|29165615|ref|NC_002745.2|	371233	+	U	0	0	0	0	1	SA0313
gi|29165615|ref|NC_002745.2|	371235	+	U	0	0	0	0	1	SA0313
gi|29165615|ref|NC_002745.2|	371241	+	G	0	0	1	0	0	SA0313
gi|29165615|ref|NC_002745.2|	371245	+	A	3	0	0	0	0	SA0313
gi|29165615|ref|NC_002745.2|	371254	+	A	0	0	0	2	0	SA0313
gi|29165615|ref|NC_002745.2|	371259	+	U	0	0	1	0	0	SA0313
gi|29165615|ref|NC_002745.2|	371275	+	C	0	1	0	0	0	SA0313
gi|29165615|ref|NC_002745.2|	371342	+	A	0	0	1	0	0	SA0313
gi|29165615|ref|NC_002745.2|	371359	+	U	1	0	0	0	0	SA0313
gi|29165615|ref|NC_002745.2|	371565	+	A	0	0	0	1	0	SA0314
gi|29165615|ref|NC_002745.2|	371578	+	A	0	0	0	1	0	SA0314
gi|29165615|ref|NC_002745.2|	371659	+	A	0	1	0	0	0	SA0314
gi|29165615|ref|NC_002745.2|	371827	+	C	0	0	0	1	0	SA0314
gi|29165615|ref|NC_002745.2|	371887	+	A	0	0	0	1	0	SA0314
gi|29165615|ref|NC_002745.2|	371910	+	A	0	0	1	0	0	SA0314
gi|29165615|ref|NC_002745.2|	371937	+	C	0	0	0	0	1	SA0314
gi|29165615|ref|NC_002745.2|	372256	+	C	0	0	1	1	0	SA0314
gi|29165615|ref|NC_002745.2|	372361	+	U	0	0	0	0	1	SA0315
gi|29165615|ref|NC_002745.2|	373056	+	A	0	1	0	0	0	SA0315
gi|29165615|ref|NC_002745.2|	373102	+	G	3	0	0	0	0	SA0315
gi|29165615|ref|NC_002745.2|	373201	+	A	0	2	1	0	0	SA0315
gi|29165615|ref|NC_002745.2|	373213	+	A	0	0	0	0	1	SA0315
gi|29165615|ref|NC_002745.2|	373232	+	A	0	1	0	0	0	SA0315
gi|29165615|ref|NC_002745.2|	373351	+	A	0	0	0	1	0	SA0316
gi|29165615|ref|NC_002745.2|	373443	+	U	0	0	0	0	1	SA0316
gi|29165615|ref|NC_002745.2|	373467	+	U	1	0	0	0	0	SA0316
gi|29165615|ref|NC_002745.2|	373684	+	A	1	0	0	0	0	SA0316
gi|29165615|ref|NC_002745.2|	373721	+	U	0	0	0	0	1	SA0316
gi|29165615|ref|NC_002745.2|	373756	+	G	0	0	0	0	1	SA0316
gi|29165615|ref|NC_002745.2|	373794	+	U	1	0	0	0	0	SA0316
gi|29165615|ref|NC_002745.2|	373831	+	A	2	0	0	0	0	SA0316
gi|29165615|ref|NC_002745.2|	373870	+	A	1	0	0	0	0	SA0316
gi|29165615|ref|NC_002745.2|	373952	+	U	0	0	1	0	0	SA0316
gi|29165615|ref|NC_002745.2|	373962	+	U	1	0	0	0	0	SA0316
gi|29165615|ref|NC_002745.2|	373992	+	A	0	0	1	0	0	SA0316
gi|29165615|ref|NC_002745.2|	374042	+	G	0	0	0	2	3	SA0316
gi|29165615|ref|NC_002745.2|	374096	+	A	0	0	0	1	0	SA0316
gi|29165615|ref|NC_002745.2|	374178	+	U	2	0	0	0	0	SA0316
gi|29165615|ref|NC_002745.2|	374286	+	U	1	0	0	0	0	-
gi|29165615|ref|NC_002745.2|	374314	+	U	0	2	0	0	0	-
gi|29165615|ref|NC_002745.2|	374318	+	U	0	0	0	0	1	-
gi|29165615|ref|NC_002745.2|	374331	+	A	0	0	0	1	0	-
gi|29165615|ref|NC_002745.2|	374334	+	A	0	0	0	0	1	-
gi|29165615|ref|NC_002745.2|	374343	+	A	0	0	0	1	0	-
gi|29165615|ref|NC_002745.2|	374498	+	A	0	0	0	0	1	-
gi|29165615|ref|NC_002745.2|	374500	+	G	0	0	0	1	0	-
gi|29165615|ref|NC_002745.2|	374564	+	A	0	10	2	3	2	-
gi|29165615|ref|NC_002745.2|	374565	+	A	4	1	2	0	1	-
gi|29165615|ref|NC_002745.2|	374566	+	U	0	0	1	0	0	-
gi|29165615|ref|NC_002745.2|	374567	+	C	3	5	5	2	3	-
gi|29165615|ref|NC_002745.2|	374568	+	A	2	3	2	3	11	-
gi|29165615|ref|NC_002745.2|	374569	+	A	0	0	0	0	1	-
gi|29165615|ref|NC_002745.2|	374570	+	U	0	0	0	0	1	-
gi|29165615|ref|NC_002745.2|	374571	+	A	0	0	1	0	0	-
gi|29165615|ref|NC_002745.2|	374572	+	G	0	0	0	0	1	-
gi|29165615|ref|NC_002745.2|	374575	+	A	0	2	0	0	0	-
gi|29165615|ref|NC_002745.2|	374583	+	G	0	0	1	0	1	-
gi|29165615|ref|NC_002745.2|	374584	+	A	0	0	0	0	1	-
gi|29165615|ref|NC_002745.2|	374586	+	A	0	0	0	0	1	-
gi|29165615|ref|NC_002745.2|	374596	+	G	0	0	1	0	0	SA0317
gi|29165615|ref|NC_002745.2|	374598	+	A	0	0	0	1	0	SA0317
gi|29165615|ref|NC_002745.2|	374599	+	U	0	0	0	0	1	SA0317
gi|29165615|ref|NC_002745.2|	374604	+	A	0	0	0	0	1	SA0317
gi|29165615|ref|NC_002745.2|	374606	+	G	0	0	0	1	0	SA0317
gi|29165615|ref|NC_002745.2|	374608	+	A	0	1	1	0	1	SA0317
gi|29165615|ref|NC_002745.2|	374610	+	U	0	0	1	0	0	SA0317
gi|29165615|ref|NC_002745.2|	374612	+	G	0	0	2	0	0	SA0317
gi|29165615|ref|NC_002745.2|	374617	+	C	0	1	0	0	0	SA0317
gi|29165615|ref|NC_002745.2|	374659	+	A	1	0	0	0	0	SA0317
gi|29165615|ref|NC_002745.2|	374721	+	U	0	1	0	0	0	SA0317
gi|29165615|ref|NC_002745.2|	374722	+	U	0	0	0	0	1	SA0317
gi|29165615|ref|NC_002745.2|	374824	+	A	0	1	0	0	0	SA0317
gi|29165615|ref|NC_002745.2|	374853	+	U	0	0	1	0	0	SA0317
gi|29165615|ref|NC_002745.2|	374864	+	A	0	0	0	0	1	SA0317
gi|29165615|ref|NC_002745.2|	374870	+	G	0	0	1	0	0	SA0317
gi|29165615|ref|NC_002745.2|	374916	+	U	0	0	1	0	0	SA0317

gi|29165615|ref|NC_002745.2|	374918	+	U	1	0	0	0	0	SA0317
gi|29165615|ref|NC_002745.2|	374933	+	C	0	0	0	1	0	SA0317
gi|29165615|ref|NC_002745.2|	375010	+	C	0	0	0	1	0	SA0317
gi|29165615|ref|NC_002745.2|	375042	+	A	1	0	0	0	0	SA0317
gi|29165615|ref|NC_002745.2|	375050	+	U	1	0	0	0	0	SA0317
gi|29165615|ref|NC_002745.2|	375058	+	A	1	0	0	0	0	SA0317
gi|29165615|ref|NC_002745.2|	375116	+	A	0	0	0	0	2	SA0317
gi|29165615|ref|NC_002745.2|	375122	+	A	0	1	0	0	0	SA0317
gi|29165615|ref|NC_002745.2|	375147	+	C	0	0	0	1	0	SA0317
gi|29165615|ref|NC_002745.2|	375234	+	G	0	2	0	0	0	SA0317
gi|29165615|ref|NC_002745.2|	375285	+	U	0	0	0	1	0	SA0317
gi|29165615|ref|NC_002745.2|	375290	+	A	1	0	0	0	0	SA0317
gi|29165615|ref|NC_002745.2|	375471	+	A	0	0	1	0	0	SA0317
gi|29165615|ref|NC_002745.2|	375513	+	U	0	0	0	1	0	SA0317
gi|29165615|ref|NC_002745.2|	375514	+	A	0	0	1	0	0	SA0317
gi|29165615|ref|NC_002745.2|	375516	+	G	0	0	2	1	0	SA0317
gi|29165615|ref|NC_002745.2|	375517	+	U	0	1	0	0	0	SA0317
gi|29165615|ref|NC_002745.2|	375528	+	C	0	0	0	1	0	SA0317
gi|29165615|ref|NC_002745.2|	378239	+	G	0	0	0	0	2	-
gi|29165615|ref|NC_002745.2|	378423	+	A	0	0	0	0	1	-
gi|29165615|ref|NC_002745.2|	379398	+	U	0	0	1	0	0	-
gi|29165615|ref|NC_002745.2|	379555	+	G	0	0	1	0	0	-
gi|29165615|ref|NC_002745.2|	379947	+	G	0	0	0	1	0	-
gi|29165615|ref|NC_002745.2|	379948	+	A	0	0	0	0	1	-
gi|29165615|ref|NC_002745.2|	380063	+	C	0	0	1	0	2	SA0322
gi|29165615|ref|NC_002745.2|	380145	+	A	0	2	0	0	0	SA0322
gi|29165615|ref|NC_002745.2|	380182	+	A	1	0	0	0	0	SA0322
gi|29165615|ref|NC_002745.2|	380191	+	C	0	0	0	0	1	SA0322
gi|29165615|ref|NC_002745.2|	380234	+	G	0	1	0	0	0	SA0322
gi|29165615|ref|NC_002745.2|	380292	+	G	0	0	0	0	1	SA0322
gi|29165615|ref|NC_002745.2|	380360	+	C	1	0	0	0	0	SA0322
gi|29165615|ref|NC_002745.2|	380511	+	A	0	1	0	0	0	SA0323
gi|29165615|ref|NC_002745.2|	380512	+	A	0	0	1	0	0	SA0323
gi|29165615|ref|NC_002745.2|	380519	+	U	0	1	0	0	0	SA0323
gi|29165615|ref|NC_002745.2|	380520	+	A	0	0	0	0	1	SA0323
gi|29165615|ref|NC_002745.2|	380551	+	G	0	0	1	0	0	SA0323
gi|29165615|ref|NC_002745.2|	380586	+	C	0	0	1	0	0	SA0323
gi|29165615|ref|NC_002745.2|	380611	+	A	0	0	0	0	1	SA0323
gi|29165615|ref|NC_002745.2|	380614	+	A	0	0	0	0	1	SA0323
gi|29165615|ref|NC_002745.2|	380621	+	U	0	0	0	0	1	SA0323
gi|29165615|ref|NC_002745.2|	380631	+	G	0	1	0	0	0	SA0323
gi|29165615|ref|NC_002745.2|	380632	+	A	0	0	0	0	1	SA0323
gi|29165615|ref|NC_002745.2|	380639	+	G	0	0	1	0	0	SA0323
gi|29165615|ref|NC_002745.2|	380640	+	A	0	0	0	1	0	SA0323
gi|29165615|ref|NC_002745.2|	380650	+	C	0	0	1	0	0	SA0323
gi|29165615|ref|NC_002745.2|	380678	+	C	0	0	2	0	0	SA0323
gi|29165615|ref|NC_002745.2|	380682	+	U	0	0	1	0	2	SA0323
gi|29165615|ref|NC_002745.2|	380688	+	C	0	1	5	1	1	SA0323
gi|29165615|ref|NC_002745.2|	380689	+	U	0	1	0	0	0	SA0323
gi|29165615|ref|NC_002745.2|	380690	+	A	0	1	0	0	0	SA0323
gi|29165615|ref|NC_002745.2|	380701	+	G	0	0	2	0	2	SA0323
gi|29165615|ref|NC_002745.2|	380712	+	U	0	1	0	0	0	SA0323
gi|29165615|ref|NC_002745.2|	380717	+	G	0	0	0	0	1	SA0323
gi|29165615|ref|NC_002745.2|	380859	+	A	0	0	0	0	1	SA0323
gi|29165615|ref|NC_002745.2|	380903	+	A	0	1	0	0	0	SA0323
gi|29165615|ref|NC_002745.2|	381013	+	U	0	0	1	0	1	SA0323
gi|29165615|ref|NC_002745.2|	381041	+	A	0	1	0	0	0	SA0323
gi|29165615|ref|NC_002745.2|	381072	+	A	0	0	0	0	1	SA0323
gi|29165615|ref|NC_002745.2|	381297	+	C	0	1	0	0	0	SA0323
gi|29165615|ref|NC_002745.2|	381400	+	A	0	1	0	0	0	SA0323
gi|29165615|ref|NC_002745.2|	381406	+	A	0	1	0	0	0	SA0323
gi|29165615|ref|NC_002745.2|	381464	+	U	0	2	0	0	2	SA0323
gi|29165615|ref|NC_002745.2|	381472	+	C	0	1	0	0	0	SA0323
gi|29165615|ref|NC_002745.2|	381577	+	G	0	0	0	0	3	SA0323
gi|29165615|ref|NC_002745.2|	381578	+	A	0	1	3	0	1	SA0323
gi|29165615|ref|NC_002745.2|	381605	+	A	0	1	0	1	0	SA0323
gi|29165615|ref|NC_002745.2|	381609	+	G	0	0	1	0	2	SA0323
gi|29165615|ref|NC_002745.2|	381611	+	A	0	0	1	2	0	SA0323
gi|29165615|ref|NC_002745.2|	381713	+	A	0	1	0	0	0	SA0323
gi|29165615|ref|NC_002745.2|	381982	+	U	1	0	0	0	0	SA0324
gi|29165615|ref|NC_002745.2|	382033	+	A	0	1	0	0	2	SA0324
gi|29165615|ref|NC_002745.2|	382034	+	A	0	2	1	0	0	SA0324
gi|29165615|ref|NC_002745.2|	382036	+	A	0	0	1	0	0	SA0324
gi|29165615|ref|NC_002745.2|	382037	+	U	0	0	0	1	0	SA0324
gi|29165615|ref|NC_002745.2|	382043	+	A	0	0	0	2	0	SA0324
gi|29165615|ref|NC_002745.2|	382045	+	A	0	1	0	0	0	SA0324
gi|29165615|ref|NC_002745.2|	382078	+	A	0	0	1	0	0	SA0324
gi|29165615|ref|NC_002745.2|	382082	+	A	0	0	0	1	0	SA0324
gi|29165615|ref|NC_002745.2|	382108	+	C	0	2	0	0	0	SA0324
gi|29165615|ref|NC_002745.2|	382113	+	A	0	1	0	0	0	SA0324
gi|29165615|ref|NC_002745.2|	382149	+	G	1	0	0	0	1	SA0324

gi|29165615|ref|NC_002745.2|	382153	+	A	0	1	0	0	0	SA0324
gi|29165615|ref|NC_002745.2|	382159	+	A	0	1	0	0	0	SA0324
gi|29165615|ref|NC_002745.2|	382172	+	A	0	0	0	0	1	SA0324
gi|29165615|ref|NC_002745.2|	382206	+	A	0	0	0	0	1	SA0324
gi|29165615|ref|NC_002745.2|	382223	+	A	0	0	0	1	0	SA0324
gi|29165615|ref|NC_002745.2|	384137	+	A	0	1	2	1	6	-
gi|29165615|ref|NC_002745.2|	384294	+	A	0	1	0	0	0	SA0326
gi|29165615|ref|NC_002745.2|	384455	+	A	0	2	2	0	3	SA0326
gi|29165615|ref|NC_002745.2|	384607	+	A	0	0	0	1	0	SA0326
gi|29165615|ref|NC_002745.2|	384610	+	U	0	1	0	0	0	SA0326
gi|29165615|ref|NC_002745.2|	384613	+	A	0	0	0	1	0	SA0326
gi|29165615|ref|NC_002745.2|	384615	+	G	0	1	0	1	1	SA0326
gi|29165615|ref|NC_002745.2|	384636	+	G	0	0	1	0	0	SA0326
gi|29165615|ref|NC_002745.2|	384639	+	U	0	0	1	0	0	SA0326
gi|29165615|ref|NC_002745.2|	384642	+	G	0	2	0	0	0	SA0326
gi|29165615|ref|NC_002745.2|	384904	+	C	0	0	0	0	1	SA0326
gi|29165615|ref|NC_002745.2|	385189	+	G	0	1	0	0	0	SA0327
gi|29165615|ref|NC_002745.2|	385229	+	A	0	0	0	1	0	SA0327
gi|29165615|ref|NC_002745.2|	385553	+	A	0	0	0	1	0	SA0327
gi|29165615|ref|NC_002745.2|	385555	+	A	0	1	0	0	0	SA0327
gi|29165615|ref|NC_002745.2|	385566	+	C	0	0	0	0	1	SA0327
gi|29165615|ref|NC_002745.2|	385633	+	A	0	0	0	1	0	SA0327
gi|29165615|ref|NC_002745.2|	385639	+	U	0	0	0	1	0	SA0327
gi|29165615|ref|NC_002745.2|	385926	+	U	0	1	0	0	0	SA0327
gi|29165615|ref|NC_002745.2|	386090	+	G	0	0	1	0	0	SA0327
gi|29165615|ref|NC_002745.2|	386094	+	A	0	0	1	0	0	SA0327
gi|29165615|ref|NC_002745.2|	386297	+	A	0	0	0	1	0	SA0328
gi|29165615|ref|NC_002745.2|	386299	+	U	0	0	0	1	0	SA0328
gi|29165615|ref|NC_002745.2|	386366	+	U	1	0	0	0	0	SA0328
gi|29165615|ref|NC_002745.2|	387293	+	G	0	1	0	0	0	-
gi|29165615|ref|NC_002745.2|	387335	+	U	0	0	0	0	1	-
gi|29165615|ref|NC_002745.2|	387791	+	C	0	1	0	0	0	-
gi|29165615|ref|NC_002745.2|	387869	+	G	0	0	0	0	1	-
gi|29165615|ref|NC_002745.2|	387918	+	A	0	1	0	0	0	-
gi|29165615|ref|NC_002745.2|	388110	+	A	0	15	5	6	11	-
gi|29165615|ref|NC_002745.2|	388111	+	A	0	0	3	1	0	-
gi|29165615|ref|NC_002745.2|	388123	+	A	0	0	1	0	0	-
gi|29165615|ref|NC_002745.2|	388131	+	A	0	0	1	0	0	-
gi|29165615|ref|NC_002745.2|	388132	+	A	0	0	0	0	1	-
gi|29165615|ref|NC_002745.2|	388134	+	G	0	1	0	0	0	-
gi|29165615|ref|NC_002745.2|	388135	+	A	0	1	0	0	1	-
gi|29165615|ref|NC_002745.2|	388176	+	C	0	1	0	0	0	SA0330
gi|29165615|ref|NC_002745.2|	388187	+	A	0	0	0	1	0	SA0330
gi|29165615|ref|NC_002745.2|	388259	+	A	1	0	0	0	0	SA0330
gi|29165615|ref|NC_002745.2|	388273	+	U	1	0	0	0	0	SA0330
gi|29165615|ref|NC_002745.2|	388297	+	A	0	0	1	0	0	SA0330
gi|29165615|ref|NC_002745.2|	388315	+	A	0	0	3	0	0	SA0330
gi|29165615|ref|NC_002745.2|	388316	+	A	0	0	1	0	0	SA0330
gi|29165615|ref|NC_002745.2|	388319	+	A	0	0	0	0	1	SA0330
gi|29165615|ref|NC_002745.2|	388320	+	G	0	2	0	0	0	SA0330
gi|29165615|ref|NC_002745.2|	388322	+	A	0	0	0	1	1	SA0330
gi|29165615|ref|NC_002745.2|	388329	+	C	0	1	0	0	0	SA0330
gi|29165615|ref|NC_002745.2|	388336	+	C	0	0	1	0	0	SA0330
gi|29165615|ref|NC_002745.2|	388342	+	G	0	0	0	0	1	SA0330
gi|29165615|ref|NC_002745.2|	388348	+	G	0	0	1	0	0	SA0330
gi|29165615|ref|NC_002745.2|	388367	+	G	0	1	0	0	0	SA0330
gi|29165615|ref|NC_002745.2|	388369	+	A	0	0	0	1	0	SA0330
gi|29165615|ref|NC_002745.2|	388376	+	A	0	0	1	0	0	SA0330
gi|29165615|ref|NC_002745.2|	388411	+	A	0	0	0	0	1	SA0330
gi|29165615|ref|NC_002745.2|	388421	+	C	0	0	0	0	2	SA0330
gi|29165615|ref|NC_002745.2|	388442	+	C	0	0	0	1	0	SA0330
gi|29165615|ref|NC_002745.2|	388450	+	A	0	0	2	0	0	SA0330
gi|29165615|ref|NC_002745.2|	388452	+	A	0	0	1	0	4	SA0330
gi|29165615|ref|NC_002745.2|	388492	+	A	0	1	0	0	0	SA0330
gi|29165615|ref|NC_002745.2|	388589	+	G	0	0	0	1	0	SA0330
gi|29165615|ref|NC_002745.2|	388602	+	A	0	0	1	0	0	SA0330
gi|29165615|ref|NC_002745.2|	388610	+	U	0	0	0	0	1	SA0330
gi|29165615|ref|NC_002745.2|	389404	+	A	0	0	1	0	0	SA0331
gi|29165615|ref|NC_002745.2|	389789	+	C	0	0	1	0	0	SA0331
gi|29165615|ref|NC_002745.2|	389814	+	A	0	1	0	0	0	SA0331
gi|29165615|ref|NC_002745.2|	389828	+	A	0	0	1	0	1	-
gi|29165615|ref|NC_002745.2|	389834	+	U	0	0	0	2	0	-
gi|29165615|ref|NC_002745.2|	390360	+	A	0	1	0	0	0	SA0332
gi|29165615|ref|NC_002745.2|	390766	+	A	0	0	1	0	0	SA0332
gi|29165615|ref|NC_002745.2|	390957	+	A	0	0	1	1	0	SA0332
gi|29165615|ref|NC_002745.2|	391225	+	U	1	0	0	0	0	SA0333
gi|29165615|ref|NC_002745.2|	391259	+	G	0	1	0	0	0	SA0333
gi|29165615|ref|NC_002745.2|	391895	+	G	0	0	0	1	0	SA0333
gi|29165615|ref|NC_002745.2|	391939	+	A	1	0	0	0	0	SA0333
gi|29165615|ref|NC_002745.2|	391945	+	A	0	1	0	0	0	SA0333
gi|29165615|ref|NC_002745.2|	392024	+	G	0	0	0	1	0	SA0333

gi|29165615|ref|NC_002745.2|	392044	+	G	0	2	3	2	1	SA0333
gi|29165615|ref|NC_002745.2|	392066	+	A	0	0	1	0	0	SA0333
gi|29165615|ref|NC_002745.2|	392077	+	U	0	1	0	0	0	SA0333
gi|29165615|ref|NC_002745.2|	392078	+	A	3	5	0	0	4	SA0333
gi|29165615|ref|NC_002745.2|	392080	+	U	2	0	0	0	0	SA0333
gi|29165615|ref|NC_002745.2|	392293	+	A	0	0	1	0	2	SA0333
gi|29165615|ref|NC_002745.2|	392295	+	A	0	0	2	3	5	SA0333
gi|29165615|ref|NC_002745.2|	392297	+	A	2	1	6	6	1	SA0333
gi|29165615|ref|NC_002745.2|	392304	+	C	0	1	0	0	0	SA0333
gi|29165615|ref|NC_002745.2|	392312	+	A	0	0	1	0	0	SA0333
gi|29165615|ref|NC_002745.2|	392335	+	G	1	6	1	2	1	SA0333
gi|29165615|ref|NC_002745.2|	392336	+	U	0	1	0	0	0	SA0333
gi|29165615|ref|NC_002745.2|	392344	+	G	0	0	0	1	0	SA0333
gi|29165615|ref|NC_002745.2|	392396	+	A	0	1	0	0	0	SA0333
gi|29165615|ref|NC_002745.2|	392809	+	C	2	0	0	0	0	-
gi|29165615|ref|NC_002745.2|	394268	+	U	0	1	0	0	0	-
gi|29165615|ref|NC_002745.2|	394485	+	G	0	0	1	0	0	-
gi|29165615|ref|NC_002745.2|	394632	+	A	8	16	10	11	7	-
gi|29165615|ref|NC_002745.2|	394633	+	A	0	0	1	0	0	-
gi|29165615|ref|NC_002745.2|	394634	+	G	0	0	0	0	1	-
gi|29165615|ref|NC_002745.2|	394653	+	U	0	1	0	0	0	SA0337
gi|29165615|ref|NC_002745.2|	394707	+	C	0	0	0	1	0	SA0337
gi|29165615|ref|NC_002745.2|	394755	+	C	0	0	1	0	0	SA0337
gi|29165615|ref|NC_002745.2|	394783	+	A	0	1	0	0	0	SA0337
gi|29165615|ref|NC_002745.2|	394827	+	U	0	0	0	0	1	SA0337
gi|29165615|ref|NC_002745.2|	394833	+	U	2	0	0	0	0	SA0337
gi|29165615|ref|NC_002745.2|	394834	+	G	0	0	0	0	2	SA0337
gi|29165615|ref|NC_002745.2|	394835	+	A	0	0	0	0	1	SA0337
gi|29165615|ref|NC_002745.2|	394838	+	A	0	1	0	0	0	SA0337
gi|29165615|ref|NC_002745.2|	394854	+	A	0	1	0	0	0	SA0338
gi|29165615|ref|NC_002745.2|	394857	+	C	0	0	0	0	1	SA0338
gi|29165615|ref|NC_002745.2|	394859	+	A	0	0	0	2	0	SA0338
gi|29165615|ref|NC_002745.2|	394860	+	A	0	0	1	0	0	SA0338
gi|29165615|ref|NC_002745.2|	394861	+	G	0	2	0	0	0	SA0338
gi|29165615|ref|NC_002745.2|	394862	+	A	0	0	1	0	0	SA0338
gi|29165615|ref|NC_002745.2|	394863	+	U	0	2	0	0	0	SA0338
gi|29165615|ref|NC_002745.2|	394873	+	A	0	0	0	0	1	SA0338
gi|29165615|ref|NC_002745.2|	394876	+	U	1	0	0	0	0	SA0338
gi|29165615|ref|NC_002745.2|	394880	+	A	1	0	0	0	0	SA0338
gi|29165615|ref|NC_002745.2|	394889	+	A	0	1	0	0	0	SA0338
gi|29165615|ref|NC_002745.2|	394894	+	U	0	0	0	0	1	SA0338
gi|29165615|ref|NC_002745.2|	394899	+	G	0	0	1	0	0	SA0338
gi|29165615|ref|NC_002745.2|	394906	+	U	0	1	0	0	0	SA0338
gi|29165615|ref|NC_002745.2|	394913	+	A	0	2	0	0	0	SA0338
gi|29165615|ref|NC_002745.2|	394915	+	U	0	1	0	0	0	SA0338
gi|29165615|ref|NC_002745.2|	394918	+	U	0	1	0	0	0	SA0338
gi|29165615|ref|NC_002745.2|	394919	+	A	0	1	0	0	0	SA0338
gi|29165615|ref|NC_002745.2|	394921	+	G	0	0	1	1	1	SA0338
gi|29165615|ref|NC_002745.2|	394923	+	A	0	1	0	0	0	SA0338
gi|29165615|ref|NC_002745.2|	394924	+	A	0	0	1	0	0	SA0338
gi|29165615|ref|NC_002745.2|	394927	+	U	1	0	1	0	0	SA0338
gi|29165615|ref|NC_002745.2|	394930	+	A	0	3	1	3	0	SA0338
gi|29165615|ref|NC_002745.2|	394947	+	A	0	0	0	0	1	SA0338
gi|29165615|ref|NC_002745.2|	394953	+	A	0	1	2	2	1	SA0338
gi|29165615|ref|NC_002745.2|	394960	+	C	0	0	0	1	0	SA0338
gi|29165615|ref|NC_002745.2|	394983	+	A	0	2	0	0	0	SA0338
gi|29165615|ref|NC_002745.2|	394993	+	U	0	0	1	0	0	SA0338
gi|29165615|ref|NC_002745.2|	395028	+	G	0	0	0	0	1	SA0338
gi|29165615|ref|NC_002745.2|	395083	+	U	1	0	0	0	0	SA0338
gi|29165615|ref|NC_002745.2|	395084	+	A	0	1	0	0	0	SA0338
gi|29165615|ref|NC_002745.2|	395097	+	A	0	1	0	0	0	SA0338
gi|29165615|ref|NC_002745.2|	395115	+	C	0	0	0	0	1	SA0338
gi|29165615|ref|NC_002745.2|	395135	+	A	0	0	0	1	0	SA0338
gi|29165615|ref|NC_002745.2|	395163	+	A	0	0	1	0	0	SA0338
gi|29165615|ref|NC_002745.2|	395179	+	U	0	1	1	0	0	SA0338
gi|29165615|ref|NC_002745.2|	395186	+	A	0	1	1	0	0	SA0338
gi|29165615|ref|NC_002745.2|	395193	+	U	0	0	1	0	0	SA0338
gi|29165615|ref|NC_002745.2|	395197	+	U	0	0	0	1	0	SA0338
gi|29165615|ref|NC_002745.2|	395204	+	G	0	0	1	0	0	SA0338
gi|29165615|ref|NC_002745.2|	395205	+	C	0	0	0	1	0	SA0338
gi|29165615|ref|NC_002745.2|	395215	+	C	0	0	1	0	0	SA0338
gi|29165615|ref|NC_002745.2|	395237	+	A	0	0	0	1	0	SA0338
gi|29165615|ref|NC_002745.2|	395398	+	A	0	4	0	0	0	SA0338
gi|29165615|ref|NC_002745.2|	395421	+	A	0	0	0	0	1	SA0338
gi|29165615|ref|NC_002745.2|	395460	+	A	0	1	0	0	0	SA0338
gi|29165615|ref|NC_002745.2|	395479	+	A	0	0	0	1	0	SA0338
gi|29165615|ref|NC_002745.2|	395493	+	C	1	0	0	0	0	SA0338
gi|29165615|ref|NC_002745.2|	395506	+	C	0	1	0	0	0	SA0338
gi|29165615|ref|NC_002745.2|	395537	+	A	0	0	0	1	0	SA0338
gi|29165615|ref|NC_002745.2|	395544	+	A	0	0	0	0	1	SA0338
gi|29165615|ref|NC_002745.2|	395566	+	A	0	0	0	0	1	-

gi|29165615|ref|NC_002745.2|	395583	+	G	0	1	0	0	0	-
gi|29165615|ref|NC_002745.2|	395624	+	A	0	1	0	0	0	SA0339
gi|29165615|ref|NC_002745.2|	395645	+	A	0	0	1	0	0	SA0339
gi|29165615|ref|NC_002745.2|	395692	+	U	0	0	2	0	0	SA0339
gi|29165615|ref|NC_002745.2|	395974	+	A	0	0	0	0	2	SA0339
gi|29165615|ref|NC_002745.2|	395975	+	A	0	0	0	0	1	SA0339
gi|29165615|ref|NC_002745.2|	395976	+	A	0	1	1	0	0	SA0339
gi|29165615|ref|NC_002745.2|	395977	+	C	0	0	0	1	0	SA0339
gi|29165615|ref|NC_002745.2|	395979	+	U	0	0	1	0	0	SA0339
gi|29165615|ref|NC_002745.2|	395983	+	C	0	1	0	0	0	SA0339
gi|29165615|ref|NC_002745.2|	396069	+	A	0	0	0	0	1	SA0339
gi|29165615|ref|NC_002745.2|	396123	+	C	0	1	0	0	0	SA0339
gi|29165615|ref|NC_002745.2|	396173	+	A	0	1	0	0	0	SA0339
gi|29165615|ref|NC_002745.2|	396236	+	A	0	0	1	0	0	SA0339
gi|29165615|ref|NC_002745.2|	396365	+	A	1	0	0	0	0	SA0339
gi|29165615|ref|NC_002745.2|	396397	+	C	1	0	0	0	0	SA0339
gi|29165615|ref|NC_002745.2|	396405	+	A	0	1	0	0	0	SA0339
gi|29165615|ref|NC_002745.2|	396419	+	U	0	0	0	1	0	SA0339
gi|29165615|ref|NC_002745.2|	396437	+	A	0	0	1	0	0	SA0340
gi|29165615|ref|NC_002745.2|	396589	+	U	0	0	1	0	0	SA0340
gi|29165615|ref|NC_002745.2|	396592	+	A	0	1	0	0	0	SA0340
gi|29165615|ref|NC_002745.2|	396620	+	U	0	0	0	0	1	SA0340
gi|29165615|ref|NC_002745.2|	396665	+	U	0	0	0	0	1	SA0340
gi|29165615|ref|NC_002745.2|	396691	+	C	0	1	0	0	0	SA0340
gi|29165615|ref|NC_002745.2|	396696	+	C	0	1	0	0	0	SA0340
gi|29165615|ref|NC_002745.2|	396784	+	C	2	0	0	0	0	SA0340
gi|29165615|ref|NC_002745.2|	396793	+	A	0	0	2	0	8	SA0340
gi|29165615|ref|NC_002745.2|	396794	+	U	0	1	0	0	0	SA0340
gi|29165615|ref|NC_002745.2|	396828	+	G	0	0	2	0	0	SA0340
gi|29165615|ref|NC_002745.2|	396835	+	A	0	1	0	0	0	SA0340
gi|29165615|ref|NC_002745.2|	396881	+	U	0	0	0	0	1	SA0340
gi|29165615|ref|NC_002745.2|	396885	+	U	0	2	1	0	0	SA0340
gi|29165615|ref|NC_002745.2|	396888	+	U	0	1	0	0	0	SA0340
gi|29165615|ref|NC_002745.2|	396890	+	G	0	2	4	6	2	SA0340
gi|29165615|ref|NC_002745.2|	396891	+	U	0	36	35	27	36	SA0340
gi|29165615|ref|NC_002745.2|	396892	+	C	0	1	1	0	1	SA0340
gi|29165615|ref|NC_002745.2|	396895	+	U	0	0	0	0	2	SA0340
gi|29165615|ref|NC_002745.2|	396899	+	U	0	1	0	0	0	SA0340
gi|29165615|ref|NC_002745.2|	396903	+	U	2	1	6	2	4	SA0340
gi|29165615|ref|NC_002745.2|	396904	+	A	0	7	2	0	2	SA0340
gi|29165615|ref|NC_002745.2|	396905	+	U	0	2	3	2	2	SA0340
gi|29165615|ref|NC_002745.2|	396909	+	G	0	3	0	0	2	SA0340
gi|29165615|ref|NC_002745.2|	396933	+	U	1	0	0	0	0	SA0340
gi|29165615|ref|NC_002745.2|	396947	+	A	0	0	0	0	2	SA0340
gi|29165615|ref|NC_002745.2|	396956	+	G	0	0	1	0	0	SA0340
gi|29165615|ref|NC_002745.2|	396965	+	U	0	0	1	0	0	SA0340
gi|29165615|ref|NC_002745.2|	397104	+	A	0	0	1	0	0	-
gi|29165615|ref|NC_002745.2|	397151	+	A	0	0	2	0	0	-
gi|29165615|ref|NC_002745.2|	397154	+	G	0	0	0	0	1	-
gi|29165615|ref|NC_002745.2|	397179	+	G	0	0	0	1	0	-
gi|29165615|ref|NC_002745.2|	397228	+	A	0	0	1	0	0	-
gi|29165615|ref|NC_002745.2|	397265	+	A	0	1	0	0	0	-
gi|29165615|ref|NC_002745.2|	398132	+	A	1	0	0	0	0	-
gi|29165615|ref|NC_002745.2|	398283	+	G	0	0	0	0	1	-
gi|29165615|ref|NC_002745.2|	398596	+	A	1	0	0	0	0	-
gi|29165615|ref|NC_002745.2|	398597	+	G	0	0	1	0	0	-
gi|29165615|ref|NC_002745.2|	398620	+	A	0	0	0	0	1	-
gi|29165615|ref|NC_002745.2|	398760	+	G	1	0	0	0	0	-
gi|29165615|ref|NC_002745.2|	398762	+	G	2	1	3	1	0	-
gi|29165615|ref|NC_002745.2|	398763	+	A	0	0	0	1	0	-
gi|29165615|ref|NC_002745.2|	398766	+	U	1	0	0	0	0	-
gi|29165615|ref|NC_002745.2|	398774	+	G	0	0	1	0	0	-
gi|29165615|ref|NC_002745.2|	398816	+	A	0	0	1	0	0	-
gi|29165615|ref|NC_002745.2|	398888	+	G	2	3	4	1	2	-
gi|29165615|ref|NC_002745.2|	398889	+	G	1	7	3	0	2	-
gi|29165615|ref|NC_002745.2|	398892	+	G	0	1	0	0	0	-
gi|29165615|ref|NC_002745.2|	398899	+	C	0	0	1	1	0	-
gi|29165615|ref|NC_002745.2|	398900	+	A	0	0	0	2	0	-
gi|29165615|ref|NC_002745.2|	398901	+	U	0	1	0	0	0	-
gi|29165615|ref|NC_002745.2|	398908	+	G	0	0	1	0	0	-
gi|29165615|ref|NC_002745.2|	398914	+	G	0	0	1	0	0	-
gi|29165615|ref|NC_002745.2|	398916	+	A	0	1	0	0	0	-
gi|29165615|ref|NC_002745.2|	398922	+	A	0	0	1	0	0	-
gi|29165615|ref|NC_002745.2|	398924	+	A	0	0	0	0	1	-
gi|29165615|ref|NC_002745.2|	398981	+	U	0	0	0	2	0	-
gi|29165615|ref|NC_002745.2|	398982	+	U	0	0	1	0	0	-
gi|29165615|ref|NC_002745.2|	398983	+	U	0	1	0	0	0	-
gi|29165615|ref|NC_002745.2|	398984	+	G	0	18	15	20	14	-
gi|29165615|ref|NC_002745.2|	398985	+	U	0	1	1	1	0	-
gi|29165615|ref|NC_002745.2|	398993	+	A	0	0	0	0	1	-
gi|29165615|ref|NC_002745.2|	398994	+	U	0	0	0	0	1	-

gi|29165615|ref|NC_002745.2|	398996 +	A	0	1	0	0	0	-
gi|29165615|ref|NC_002745.2|	399000 +	G	0	1	0	0	0	-
gi|29165615|ref|NC_002745.2|	399001 +	A	0	0	0	3	0	-
gi|29165615|ref|NC_002745.2|	399005 +	A	0	1	0	0	0	-
gi|29165615|ref|NC_002745.2|	399006 +	A	0	0	0	1	0	-
gi|29165615|ref|NC_002745.2|	399007 +	A	0	2	1	4	0	-
gi|29165615|ref|NC_002745.2|	399008 +	A	0	0	0	1	0	-
gi|29165615|ref|NC_002745.2|	399012 +	A	0	1	1	0	1	-
gi|29165615|ref|NC_002745.2|	399013 +	A	0	0	1	0	0	-
gi|29165615|ref|NC_002745.2|	399014 +	C	0	0	0	0	1	-
gi|29165615|ref|NC_002745.2|	399015 +	A	0	0	0	0	1	-
gi|29165615|ref|NC_002745.2|	399035 +	U	0	0	0	2	0	-
gi|29165615|ref|NC_002745.2|	399036 +	A	0	0	0	3	0	-
gi|29165615|ref|NC_002745.2|	399053 +	C	0	0	0	1	0	-
gi|29165615|ref|NC_002745.2|	399061 +	U	0	0	1	0	0	-
gi|29165615|ref|NC_002745.2|	399068 +	G	0	1	0	0	0	-
gi|29165615|ref|NC_002745.2|	399069 +	A	0	1	1	0	0	-
gi|29165615|ref|NC_002745.2|	399070 +	C	0	0	0	1	0	-
gi|29165615|ref|NC_002745.2|	399077 +	G	0	1	0	0	0	-
gi|29165615|ref|NC_002745.2|	399083 +	A	1	1	0	0	0	-
gi|29165615|ref|NC_002745.2|	399085 +	A	0	1	0	0	0	-
gi|29165615|ref|NC_002745.2|	399089 +	U	0	0	2	1	1	-
gi|29165615|ref|NC_002745.2|	399091 +	U	0	0	0	0	1	-
gi|29165615|ref|NC_002745.2|	399101 +	G	0	0	1	0	0	-
gi|29165615|ref|NC_002745.2|	399102 +	G	1	0	0	1	0	-
gi|29165615|ref|NC_002745.2|	399103 +	A	0	0	0	1	0	-
gi|29165615|ref|NC_002745.2|	399115 +	C	0	0	0	0	1	-
gi|29165615|ref|NC_002745.2|	399120 +	C	0	0	0	0	1	SA0342
gi|29165615|ref|NC_002745.2|	399121 +	G	0	1	0	0	1	SA0342
gi|29165615|ref|NC_002745.2|	399141 +	C	0	0	0	0	3	SA0342
gi|29165615|ref|NC_002745.2|	399143 +	U	1	0	0	0	0	SA0342
gi|29165615|ref|NC_002745.2|	399177 +	U	0	0	1	0	0	SA0342
gi|29165615|ref|NC_002745.2|	399178 +	U	1	0	0	1	0	SA0342
gi|29165615|ref|NC_002745.2|	399189 +	C	0	0	1	0	0	SA0342
gi|29165615|ref|NC_002745.2|	399215 +	A	0	1	0	0	0	SA0342
gi|29165615|ref|NC_002745.2|	399221 +	C	0	0	0	0	1	SA0342
gi|29165615|ref|NC_002745.2|	399226 +	U	0	0	0	0	1	SA0342
gi|29165615|ref|NC_002745.2|	399325 +	U	1	0	0	0	0	SA0342
gi|29165615|ref|NC_002745.2|	399341 +	C	0	0	1	0	0	SA0342
gi|29165615|ref|NC_002745.2|	399375 +	U	0	0	1	0	0	SA0342
gi|29165615|ref|NC_002745.2|	399376 +	A	3	0	0	0	0	SA0342
gi|29165615|ref|NC_002745.2|	399382 +	U	1	0	0	0	0	SA0342
gi|29165615|ref|NC_002745.2|	399408 +	C	1	0	0	0	0	SA0342
gi|29165615|ref|NC_002745.2|	399451 +	U	0	0	1	0	0	SA0342
gi|29165615|ref|NC_002745.2|	399485 +	C	0	0	0	1	0	SA0342
gi|29165615|ref|NC_002745.2|	399567 +	U	0	0	0	1	0	SA0342
gi|29165615|ref|NC_002745.2|	399589 +	U	1	0	0	0	0	SA0342
gi|29165615|ref|NC_002745.2|	399594 +	C	1	0	0	0	0	SA0342
gi|29165615|ref|NC_002745.2|	399595 +	U	1	0	0	0	0	SA0342
gi|29165615|ref|NC_002745.2|	399604 +	U	0	0	0	0	1	SA0342
gi|29165615|ref|NC_002745.2|	399613 +	G	0	1	0	0	0	SA0342
gi|29165615|ref|NC_002745.2|	399627 +	C	0	0	0	1	0	SA0342
gi|29165615|ref|NC_002745.2|	399630 +	G	0	0	0	1	0	SA0342
gi|29165615|ref|NC_002745.2|	399645 +	C	1	0	1	0	0	SA0342
gi|29165615|ref|NC_002745.2|	399646 +	A	2	0	0	1	0	SA0342
gi|29165615|ref|NC_002745.2|	399647 +	U	0	0	1	0	0	SA0342
gi|29165615|ref|NC_002745.2|	399649 +	U	1	0	0	0	0	SA0342
gi|29165615|ref|NC_002745.2|	399659 +	U	0	1	0	0	0	SA0342
gi|29165615|ref|NC_002745.2|	399662 +	C	0	0	0	0	1	SA0342
gi|29165615|ref|NC_002745.2|	399667 +	U	0	1	0	0	0	SA0342
gi|29165615|ref|NC_002745.2|	399674 +	G	0	0	0	0	1	SA0342
gi|29165615|ref|NC_002745.2|	399681 +	C	0	0	1	0	0	SA0342
gi|29165615|ref|NC_002745.2|	399690 +	A	0	1	0	0	0	SA0342
gi|29165615|ref|NC_002745.2|	399700 +	U	0	1	0	0	0	SA0342
gi|29165615|ref|NC_002745.2|	399702 +	A	0	1	1	0	0	SA0342
gi|29165615|ref|NC_002745.2|	399705 +	G	1	0	0	0	0	SA0342
gi|29165615|ref|NC_002745.2|	399707 +	G	0	0	1	0	0	SA0342
gi|29165615|ref|NC_002745.2|	399708 +	A	0	1	0	0	0	SA0342
gi|29165615|ref|NC_002745.2|	399709 +	A	0	1	0	0	0	SA0342
gi|29165615|ref|NC_002745.2|	399710 +	A	0	0	0	0	1	SA0342
gi|29165615|ref|NC_002745.2|	399711 +	U	0	0	1	0	0	SA0342
gi|29165615|ref|NC_002745.2|	399714 +	U	2	0	1	0	0	SA0342
gi|29165615|ref|NC_002745.2|	399715 +	U	1	0	0	0	0	SA0342
gi|29165615|ref|NC_002745.2|	399717 +	C	1	0	0	0	0	SA0342
gi|29165615|ref|NC_002745.2|	399725 +	A	0	0	0	0	1	SA0342
gi|29165615|ref|NC_002745.2|	399726 +	U	0	0	1	0	0	SA0342
gi|29165615|ref|NC_002745.2|	399727 +	U	1	0	0	0	0	SA0342
gi|29165615|ref|NC_002745.2|	399729 +	C	0	0	0	1	0	SA0342
gi|29165615|ref|NC_002745.2|	399731 +	C	0	1	0	0	0	SA0342
gi|29165615|ref|NC_002745.2|	399742 +	U	2	0	0	0	0	SA0342
gi|29165615|ref|NC_002745.2|	399743 +	G	0	0	0	0	1	SA0342

gi|29165615|ref|NC_002745.2|	399756 +	U	1	0	0	0	0	SA0342
gi|29165615|ref|NC_002745.2|	399762 +	A	0	0	2	0	0	SA0342
gi|29165615|ref|NC_002745.2|	399763 +	G	0	1	0	0	0	SA0342
gi|29165615|ref|NC_002745.2|	399764 +	G	0	0	0	0	1	SA0342
gi|29165615|ref|NC_002745.2|	399765 +	A	0	1	0	0	1	SA0342
gi|29165615|ref|NC_002745.2|	399780 +	A	0	0	0	0	1	SA0342
gi|29165615|ref|NC_002745.2|	399788 +	U	0	1	0	0	0	SA0342
gi|29165615|ref|NC_002745.2|	399794 +	G	0	1	0	0	0	SA0342
gi|29165615|ref|NC_002745.2|	399796 +	A	1	0	0	0	0	SA0342
gi|29165615|ref|NC_002745.2|	399798 +	A	0	0	0	1	0	SA0342
gi|29165615|ref|NC_002745.2|	399801 +	U	0	0	0	0	1	SA0342
gi|29165615|ref|NC_002745.2|	399821 +	U	0	0	0	0	1	SA0342
gi|29165615|ref|NC_002745.2|	399853 +	U	0	0	0	0	1	SA0342
gi|29165615|ref|NC_002745.2|	399865 +	C	0	0	0	0	1	SA0342
gi|29165615|ref|NC_002745.2|	399915 +	A	0	0	0	1	0	SA0342
gi|29165615|ref|NC_002745.2|	399918 +	U	0	0	1	0	0	SA0342
gi|29165615|ref|NC_002745.2|	399920 +	A	0	0	2	0	0	SA0342
gi|29165615|ref|NC_002745.2|	399921 +	A	0	3	1	0	2	SA0342
gi|29165615|ref|NC_002745.2|	399922 +	U	0	1	0	0	0	SA0342
gi|29165615|ref|NC_002745.2|	399927 +	A	0	0	1	0	0	SA0342
gi|29165615|ref|NC_002745.2|	399930 +	C	1	0	0	0	0	SA0342
gi|29165615|ref|NC_002745.2|	399931 +	A	0	0	1	0	0	SA0342
gi|29165615|ref|NC_002745.2|	399956 +	A	0	0	2	0	0	SA0342
gi|29165615|ref|NC_002745.2|	399972 +	C	0	0	2	0	0	SA0342
gi|29165615|ref|NC_002745.2|	399976 +	U	0	0	1	0	0	SA0342
gi|29165615|ref|NC_002745.2|	399978 +	U	0	0	1	0	0	SA0342
gi|29165615|ref|NC_002745.2|	399980 +	A	0	1	0	0	0	SA0342
gi|29165615|ref|NC_002745.2|	399985 +	U	0	1	0	0	0	SA0342
gi|29165615|ref|NC_002745.2|	400060 +	A	0	0	0	0	1	SA0342
gi|29165615|ref|NC_002745.2|	400089 +	U	1	0	0	0	0	SA0342
gi|29165615|ref|NC_002745.2|	400110 +	A	0	0	1	0	0	SA0342
gi|29165615|ref|NC_002745.2|	400111 +	A	0	0	0	0	1	SA0342
gi|29165615|ref|NC_002745.2|	400113 +	U	0	0	0	0	1	SA0342
gi|29165615|ref|NC_002745.2|	400152 +	C	2	0	0	0	0	SA0342
gi|29165615|ref|NC_002745.2|	400652 +	A	0	1	0	0	0	-
gi|29165615|ref|NC_002745.2|	403509 +	U	0	0	1	0	0	-
gi|29165615|ref|NC_002745.2|	403773 +	A	0	3	1	1	0	-
gi|29165615|ref|NC_002745.2|	408065 +	A	0	0	1	0	0	-
gi|29165615|ref|NC_002745.2|	408105 +	A	0	8	6	8	8	-
gi|29165615|ref|NC_002745.2|	408178 +	A	0	0	0	0	2	SA0348
gi|29165615|ref|NC_002745.2|	408180 +	G	0	0	1	0	0	SA0348
gi|29165615|ref|NC_002745.2|	408184 +	A	0	1	0	0	0	SA0348
gi|29165615|ref|NC_002745.2|	408211 +	A	0	1	0	0	0	SA0348
gi|29165615|ref|NC_002745.2|	408213 +	A	0	0	1	0	0	SA0348
gi|29165615|ref|NC_002745.2|	408238 +	G	0	0	1	0	0	SA0348
gi|29165615|ref|NC_002745.2|	408288 +	C	0	0	1	0	0	SA0348
gi|29165615|ref|NC_002745.2|	408295 +	G	0	0	1	0	0	SA0348
gi|29165615|ref|NC_002745.2|	408296 +	A	0	1	1	0	0	SA0348
gi|29165615|ref|NC_002745.2|	408309 +	A	0	0	0	0	1	SA0348
gi|29165615|ref|NC_002745.2|	408330 +	C	0	0	0	0	1	SA0348
gi|29165615|ref|NC_002745.2|	408346 +	U	0	2	0	0	0	SA0348
gi|29165615|ref|NC_002745.2|	408349 +	U	0	0	1	0	0	SA0348
gi|29165615|ref|NC_002745.2|	408469 +	A	0	0	0	0	1	SA0348
gi|29165615|ref|NC_002745.2|	408490 +	A	0	0	1	0	1	SA0348
gi|29165615|ref|NC_002745.2|	408525 +	A	0	1	0	0	1	SA0348
gi|29165615|ref|NC_002745.2|	408532 +	C	0	0	0	1	0	SA0348
gi|29165615|ref|NC_002745.2|	408539 +	A	0	0	1	0	1	SA0348
gi|29165615|ref|NC_002745.2|	408576 +	A	0	0	0	0	1	SA0348
gi|29165615|ref|NC_002745.2|	408606 +	C	0	0	0	0	1	SA0348
gi|29165615|ref|NC_002745.2|	408615 +	A	0	1	0	0	0	SA0348
gi|29165615|ref|NC_002745.2|	408628 +	U	0	0	0	0	2	SA0348
gi|29165615|ref|NC_002745.2|	408641 +	A	0	0	1	0	0	SA0348
gi|29165615|ref|NC_002745.2|	408724 +	A	0	0	0	0	1	SA0348
gi|29165615|ref|NC_002745.2|	408754 +	A	0	0	1	0	0	SA0348
gi|29165615|ref|NC_002745.2|	408758 +	U	0	1	0	0	0	SA0348
gi|29165615|ref|NC_002745.2|	408805 +	A	0	0	2	0	0	SA0348
gi|29165615|ref|NC_002745.2|	408991 +	U	0	1	0	0	0	-
gi|29165615|ref|NC_002745.2|	408992 +	A	0	1	0	1	1	-
gi|29165615|ref|NC_002745.2|	408993 +	U	0	0	0	1	0	-
gi|29165615|ref|NC_002745.2|	408995 +	A	0	0	0	1	0	-
gi|29165615|ref|NC_002745.2|	409000 +	U	3	0	0	0	1	-
gi|29165615|ref|NC_002745.2|	409001 +	C	3	0	0	0	0	-
gi|29165615|ref|NC_002745.2|	409004 +	U	0	0	0	1	0	-
gi|29165615|ref|NC_002745.2|	409005 +	G	4	0	1	0	0	-
gi|29165615|ref|NC_002745.2|	409025 +	A	0	1	1	0	0	-
gi|29165615|ref|NC_002745.2|	409045 +	A	0	1	1	0	0	-
gi|29165615|ref|NC_002745.2|	409048 +	A	0	0	0	0	1	-
gi|29165615|ref|NC_002745.2|	409051 +	A	0	0	1	0	0	-
gi|29165615|ref|NC_002745.2|	409063 +	U	0	0	1	0	0	-
gi|29165615|ref|NC_002745.2|	409076 +	U	0	0	0	0	1	-
gi|29165615|ref|NC_002745.2|	409077 +	A	0	0	1	0	0	-

gi|29165615|ref|NC_002745.2|	409083	+	A	0	1	1	0	0	-
gi|29165615|ref|NC_002745.2|	409084	+	U	0	0	1	0	0	-
gi|29165615|ref|NC_002745.2|	409093	+	A	0	0	0	0	1	-
gi|29165615|ref|NC_002745.2|	409094	+	A	0	0	1	0	0	-
gi|29165615|ref|NC_002745.2|	409097	+	A	0	1	0	0	0	-
gi|29165615|ref|NC_002745.2|	409104	+	A	0	1	1	0	0	-
gi|29165615|ref|NC_002745.2|	409110	+	U	0	0	1	0	0	-
gi|29165615|ref|NC_002745.2|	409117	+	G	0	1	0	0	0	-
gi|29165615|ref|NC_002745.2|	409181	+	A	0	1	0	0	0	SA0349
gi|29165615|ref|NC_002745.2|	409195	+	A	0	0	0	1	0	SA0349
gi|29165615|ref|NC_002745.2|	409196	+	A	0	0	1	0	0	SA0349
gi|29165615|ref|NC_002745.2|	409204	+	C	0	1	0	0	0	SA0349
gi|29165615|ref|NC_002745.2|	409263	+	C	0	0	0	1	0	SA0349
gi|29165615|ref|NC_002745.2|	409357	+	A	1	0	0	0	0	SA0349
gi|29165615|ref|NC_002745.2|	409363	+	U	0	2	5	0	4	SA0349
gi|29165615|ref|NC_002745.2|	409364	+	G	0	4	0	1	5	SA0349
gi|29165615|ref|NC_002745.2|	409365	+	A	0	4	3	3	2	SA0349
gi|29165615|ref|NC_002745.2|	409368	+	U	0	0	2	0	1	SA0349
gi|29165615|ref|NC_002745.2|	409372	+	U	0	0	0	0	1	SA0349
gi|29165615|ref|NC_002745.2|	409379	+	G	0	0	2	0	0	SA0349
gi|29165615|ref|NC_002745.2|	409380	+	U	0	1	0	0	0	SA0349
gi|29165615|ref|NC_002745.2|	409381	+	U	0	0	1	0	0	SA0349
gi|29165615|ref|NC_002745.2|	409383	+	A	0	0	0	1	0	SA0349
gi|29165615|ref|NC_002745.2|	409384	+	U	0	0	0	1	0	SA0349
gi|29165615|ref|NC_002745.2|	409387	+	U	0	0	2	0	1	SA0349
gi|29165615|ref|NC_002745.2|	409391	+	U	0	0	0	0	1	SA0349
gi|29165615|ref|NC_002745.2|	409392	+	A	0	0	1	0	0	SA0349
gi|29165615|ref|NC_002745.2|	409394	+	G	1	1	1	0	0	SA0349
gi|29165615|ref|NC_002745.2|	409397	+	G	0	2	0	0	0	SA0349
gi|29165615|ref|NC_002745.2|	409398	+	A	0	1	1	3	1	SA0349
gi|29165615|ref|NC_002745.2|	409400	+	U	0	1	0	0	0	SA0349
gi|29165615|ref|NC_002745.2|	409401	+	U	0	0	0	0	1	SA0349
gi|29165615|ref|NC_002745.2|	409402	+	U	1	0	0	1	0	SA0349
gi|29165615|ref|NC_002745.2|	409403	+	A	0	1	0	0	0	SA0349
gi|29165615|ref|NC_002745.2|	409406	+	U	0	0	0	1	0	SA0349
gi|29165615|ref|NC_002745.2|	409407	+	A	0	0	0	0	1	SA0349
gi|29165615|ref|NC_002745.2|	409408	+	A	0	0	1	0	0	SA0349
gi|29165615|ref|NC_002745.2|	409410	+	U	0	0	0	0	1	SA0349
gi|29165615|ref|NC_002745.2|	409413	+	G	0	0	0	0	1	SA0349
gi|29165615|ref|NC_002745.2|	409414	+	G	0	1	2	0	2	SA0349
gi|29165615|ref|NC_002745.2|	409415	+	C	0	0	0	0	1	SA0349
gi|29165615|ref|NC_002745.2|	409417	+	U	0	1	0	0	0	SA0349
gi|29165615|ref|NC_002745.2|	409418	+	U	0	1	0	0	0	SA0349
gi|29165615|ref|NC_002745.2|	409419	+	A	0	3	1	2	0	SA0349
gi|29165615|ref|NC_002745.2|	409420	+	G	0	1	0	0	1	SA0349
gi|29165615|ref|NC_002745.2|	409421	+	U	0	0	0	0	3	SA0349
gi|29165615|ref|NC_002745.2|	409424	+	U	2	0	0	0	0	SA0349
gi|29165615|ref|NC_002745.2|	409425	+	G	0	0	0	1	0	SA0349
gi|29165615|ref|NC_002745.2|	409426	+	A	0	2	0	0	0	SA0349
gi|29165615|ref|NC_002745.2|	409427	+	A	0	1	0	0	0	SA0349
gi|29165615|ref|NC_002745.2|	409434	+	A	0	0	0	1	0	SA0349
gi|29165615|ref|NC_002745.2|	409439	+	A	0	0	1	0	0	SA0349
gi|29165615|ref|NC_002745.2|	409532	+	A	0	1	0	0	0	SA0349
gi|29165615|ref|NC_002745.2|	409566	+	G	0	0	1	0	1	SA0349
gi|29165615|ref|NC_002745.2|	409761	+	A	0	2	0	0	0	SA0349
gi|29165615|ref|NC_002745.2|	409762	+	A	0	0	1	0	0	SA0349
gi|29165615|ref|NC_002745.2|	409998	+	G	0	0	0	2	0	SA0349
gi|29165615|ref|NC_002745.2|	410003	+	C	0	0	0	0	1	SA0349
gi|29165615|ref|NC_002745.2|	410005	+	G	0	0	0	0	1	SA0349
gi|29165615|ref|NC_002745.2|	410016	+	U	2	1	0	0	0	-
gi|29165615|ref|NC_002745.2|	410019	+	A	0	0	0	1	0	-
gi|29165615|ref|NC_002745.2|	410030	+	A	0	0	1	0	0	-
gi|29165615|ref|NC_002745.2|	410035	+	A	2	0	0	0	0	-
gi|29165615|ref|NC_002745.2|	410052	+	A	0	0	0	1	0	SA0350
gi|29165615|ref|NC_002745.2|	410060	+	A	0	0	1	0	0	SA0350
gi|29165615|ref|NC_002745.2|	410064	+	A	0	1	0	0	0	SA0350
gi|29165615|ref|NC_002745.2|	410067	+	A	0	0	1	0	0	SA0350
gi|29165615|ref|NC_002745.2|	410068	+	U	0	1	0	1	0	SA0350
gi|29165615|ref|NC_002745.2|	410070	+	U	0	0	1	0	0	SA0350
gi|29165615|ref|NC_002745.2|	410075	+	G	1	2	2	0	2	SA0350
gi|29165615|ref|NC_002745.2|	410076	+	A	0	1	1	1	0	SA0350
gi|29165615|ref|NC_002745.2|	410078	+	A	0	0	0	0	1	SA0350
gi|29165615|ref|NC_002745.2|	410079	+	U	0	0	1	1	1	SA0350
gi|29165615|ref|NC_002745.2|	410080	+	G	23	28	14	18	35	SA0350
gi|29165615|ref|NC_002745.2|	410081	+	A	1	8	8	1	6	SA0350
gi|29165615|ref|NC_002745.2|	410082	+	A	5	6	8	4	4	SA0350
gi|29165615|ref|NC_002745.2|	410083	+	A	3	9	13	6	9	SA0350
gi|29165615|ref|NC_002745.2|	410084	+	A	0	1	1	0	0	SA0350
gi|29165615|ref|NC_002745.2|	410085	+	A	1	0	1	2	0	SA0350
gi|29165615|ref|NC_002745.2|	410086	+	A	0	0	2	0	0	SA0350
gi|29165615|ref|NC_002745.2|	410088	+	A	0	1	0	0	0	SA0350

gi|29165615|ref|NC_002745.2|	410090 +	C	0	0	1	0	1	SA0350
gi|29165615|ref|NC_002745.2|	410117 +	A	0	1	0	0	0	SA0350
gi|29165615|ref|NC_002745.2|	410121 +	U	0	0	0	0	1	SA0350
gi|29165615|ref|NC_002745.2|	410123 +	A	0	0	0	1	0	SA0350
gi|29165615|ref|NC_002745.2|	410125 +	A	0	0	1	0	0	SA0350
gi|29165615|ref|NC_002745.2|	410147 +	A	0	0	0	0	1	SA0350
gi|29165615|ref|NC_002745.2|	410157 +	A	0	1	0	0	0	SA0350
gi|29165615|ref|NC_002745.2|	410192 +	U	0	0	0	0	1	SA0350
gi|29165615|ref|NC_002745.2|	410196 +	A	0	0	0	1	0	SA0350
gi|29165615|ref|NC_002745.2|	410199 +	A	0	0	1	0	0	SA0350
gi|29165615|ref|NC_002745.2|	410202 +	A	0	2	0	0	0	SA0350
gi|29165615|ref|NC_002745.2|	410203 +	A	0	0	1	0	0	SA0350
gi|29165615|ref|NC_002745.2|	410206 +	A	0	4	3	0	1	SA0350
gi|29165615|ref|NC_002745.2|	410207 +	A	0	1	1	0	0	SA0350
gi|29165615|ref|NC_002745.2|	410209 +	A	0	1	0	0	0	SA0350
gi|29165615|ref|NC_002745.2|	410210 +	A	0	0	0	1	0	SA0350
gi|29165615|ref|NC_002745.2|	410224 +	U	0	0	0	0	1	SA0350
gi|29165615|ref|NC_002745.2|	410228 +	G	0	0	0	1	0	SA0350
gi|29165615|ref|NC_002745.2|	410235 +	C	0	0	0	1	0	SA0350
gi|29165615|ref|NC_002745.2|	410242 +	A	1	1	0	0	0	SA0350
gi|29165615|ref|NC_002745.2|	410246 +	G	0	1	0	0	0	-
gi|29165615|ref|NC_002745.2|	410247 +	A	0	1	1	0	0	-
gi|29165615|ref|NC_002745.2|	410249 +	A	0	0	2	0	0	-
gi|29165615|ref|NC_002745.2|	410252 +	G	0	0	0	0	1	-
gi|29165615|ref|NC_002745.2|	410266 +	A	0	1	0	0	0	SA0351
gi|29165615|ref|NC_002745.2|	410284 +	U	1	0	0	0	0	SA0351
gi|29165615|ref|NC_002745.2|	410308 +	U	0	2	0	0	0	SA0351
gi|29165615|ref|NC_002745.2|	410318 +	C	0	0	1	0	2	SA0351
gi|29165615|ref|NC_002745.2|	410320 +	A	0	0	0	0	1	SA0351
gi|29165615|ref|NC_002745.2|	410324 +	C	0	1	0	0	0	SA0351
gi|29165615|ref|NC_002745.2|	410330 +	C	0	1	0	0	0	SA0351
gi|29165615|ref|NC_002745.2|	410348 +	A	0	0	0	0	2	SA0351
gi|29165615|ref|NC_002745.2|	410349 +	C	0	0	2	0	0	SA0351
gi|29165615|ref|NC_002745.2|	410357 +	U	1	0	0	0	0	SA0351
gi|29165615|ref|NC_002745.2|	410360 +	C	0	1	0	0	0	SA0351
gi|29165615|ref|NC_002745.2|	410366 +	U	0	0	0	1	0	SA0351
gi|29165615|ref|NC_002745.2|	410372 +	C	0	0	1	0	0	SA0351
gi|29165615|ref|NC_002745.2|	410385 +	U	3	0	0	0	0	SA0351
gi|29165615|ref|NC_002745.2|	410415 +	A	0	1	0	0	0	SA0351
gi|29165615|ref|NC_002745.2|	410449 +	C	0	0	0	0	1	SA0351
gi|29165615|ref|NC_002745.2|	410456 +	C	0	0	1	0	0	SA0351
gi|29165615|ref|NC_002745.2|	410476 +	G	1	0	0	0	0	SA0351
gi|29165615|ref|NC_002745.2|	410478 +	C	1	0	0	0	0	SA0351
gi|29165615|ref|NC_002745.2|	410506 +	G	0	0	0	3	0	SA0351
gi|29165615|ref|NC_002745.2|	410528 +	U	1	0	0	0	0	SA0351
gi|29165615|ref|NC_002745.2|	410539 +	A	0	0	0	1	0	SA0351
gi|29165615|ref|NC_002745.2|	410566 +	G	0	0	1	0	0	SA0351
gi|29165615|ref|NC_002745.2|	410571 +	U	0	0	0	0	1	SA0351
gi|29165615|ref|NC_002745.2|	410649 +	A	0	0	0	0	1	SA0351
gi|29165615|ref|NC_002745.2|	410664 +	C	1	0	0	0	0	SA0351
gi|29165615|ref|NC_002745.2|	410709 +	A	0	1	0	0	0	SA0351
gi|29165615|ref|NC_002745.2|	410718 +	A	0	0	1	0	0	SA0351
gi|29165615|ref|NC_002745.2|	410755 +	A	0	2	0	0	0	SA0351
gi|29165615|ref|NC_002745.2|	410769 +	U	0	0	0	1	0	SA0351
gi|29165615|ref|NC_002745.2|	410773 +	G	0	0	1	0	0	SA0351
gi|29165615|ref|NC_002745.2|	410785 +	C	0	0	0	0	1	SA0351
gi|29165615|ref|NC_002745.2|	410796 +	U	0	1	0	0	0	SA0351
gi|29165615|ref|NC_002745.2|	410799 +	U	1	0	0	0	0	SA0351
gi|29165615|ref|NC_002745.2|	410801 +	A	0	0	0	1	0	SA0351
gi|29165615|ref|NC_002745.2|	410802 +	C	0	0	0	1	0	SA0351
gi|29165615|ref|NC_002745.2|	410805 +	U	1	0	0	0	0	SA0351
gi|29165615|ref|NC_002745.2|	410807 +	A	0	0	1	0	0	SA0351
gi|29165615|ref|NC_002745.2|	410809 +	G	0	0	0	1	0	SA0351
gi|29165615|ref|NC_002745.2|	410811 +	A	1	0	0	0	0	SA0351
gi|29165615|ref|NC_002745.2|	410813 +	A	0	0	0	2	0	SA0351
gi|29165615|ref|NC_002745.2|	410818 +	C	0	0	0	0	1	SA0351
gi|29165615|ref|NC_002745.2|	410820 +	A	0	0	2	1	1	SA0351
gi|29165615|ref|NC_002745.2|	410821 +	A	0	0	1	0	0	SA0351
gi|29165615|ref|NC_002745.2|	410824 +	U	0	0	0	1	0	SA0351
gi|29165615|ref|NC_002745.2|	410829 +	G	1	0	0	0	0	SA0351
gi|29165615|ref|NC_002745.2|	410835 +	A	0	0	0	0	1	SA0351
gi|29165615|ref|NC_002745.2|	410840 +	A	0	0	1	0	0	SA0351
gi|29165615|ref|NC_002745.2|	410848 +	A	0	0	0	1	2	SA0351
gi|29165615|ref|NC_002745.2|	410849 +	C	1	0	0	0	0	SA0351
gi|29165615|ref|NC_002745.2|	410854 +	A	0	0	1	0	0	SA0351
gi|29165615|ref|NC_002745.2|	410855 +	A	0	0	0	1	0	SA0351
gi|29165615|ref|NC_002745.2|	410857 +	A	0	1	0	0	0	SA0351
gi|29165615|ref|NC_002745.2|	410863 +	C	0	0	1	1	2	SA0351
gi|29165615|ref|NC_002745.2|	410873 +	C	1	1	0	0	0	SA0351
gi|29165615|ref|NC_002745.2|	410874 +	U	1	0	0	0	0	SA0351
gi|29165615|ref|NC_002745.2|	410881 +	G	0	0	1	0	0	SA0351

gi|29165615|ref|NC_002745.2|	410883 +	U	2	0	0	0	0	SA0351
gi|29165615|ref|NC_002745.2|	410884 +	G	1	0	0	0	0	SA0351
gi|29165615|ref|NC_002745.2|	410885 +	A	0	1	0	0	0	SA0351
gi|29165615|ref|NC_002745.2|	410900 +	A	0	0	1	0	0	SA0351
gi|29165615|ref|NC_002745.2|	410904 +	U	0	1	0	0	0	SA0351
gi|29165615|ref|NC_002745.2|	410907 +	U	1	0	0	0	0	SA0351
gi|29165615|ref|NC_002745.2|	410914 +	A	0	0	0	0	1	SA0351
gi|29165615|ref|NC_002745.2|	410915 +	A	0	0	0	1	0	SA0351
gi|29165615|ref|NC_002745.2|	410916 +	A	0	0	1	0	0	SA0351
gi|29165615|ref|NC_002745.2|	410918 +	U	0	1	1	0	0	SA0351
gi|29165615|ref|NC_002745.2|	410919 +	A	0	1	0	0	1	SA0351
gi|29165615|ref|NC_002745.2|	410925 +	G	1	0	0	0	0	SA0351
gi|29165615|ref|NC_002745.2|	410933 +	A	0	0	0	2	1	SA0351
gi|29165615|ref|NC_002745.2|	410935 +	U	0	0	0	0	2	SA0351
gi|29165615|ref|NC_002745.2|	410939 +	C	1	0	0	0	0	SA0351
gi|29165615|ref|NC_002745.2|	410950 +	G	0	2	0	0	1	SA0351
gi|29165615|ref|NC_002745.2|	410957 +	A	2	0	1	0	0	SA0351
gi|29165615|ref|NC_002745.2|	410968 +	A	0	0	1	0	0	SA0351
gi|29165615|ref|NC_002745.2|	410969 +	U	0	0	1	0	0	SA0351
gi|29165615|ref|NC_002745.2|	410976 +	A	0	0	0	0	1	SA0351
gi|29165615|ref|NC_002745.2|	410978 +	A	0	0	0	3	1	SA0351
gi|29165615|ref|NC_002745.2|	410979 +	A	1	0	0	0	0	SA0351
gi|29165615|ref|NC_002745.2|	410980 +	A	1	2	0	0	1	SA0351
gi|29165615|ref|NC_002745.2|	410984 +	A	0	0	1	0	0	SA0351
gi|29165615|ref|NC_002745.2|	410986 +	G	0	0	1	0	0	SA0351
gi|29165615|ref|NC_002745.2|	410987 +	A	0	0	1	0	1	SA0351
gi|29165615|ref|NC_002745.2|	410990 +	A	0	2	0	1	3	SA0351
gi|29165615|ref|NC_002745.2|	410991 +	A	0	1	0	0	0	SA0351
gi|29165615|ref|NC_002745.2|	410996 +	C	0	1	1	0	1	SA0351
gi|29165615|ref|NC_002745.2|	411005 +	A	0	1	5	3	1	SA0351
gi|29165615|ref|NC_002745.2|	411008 +	A	1	0	0	0	0	SA0351
gi|29165615|ref|NC_002745.2|	411010 +	G	0	0	0	0	1	SA0351
gi|29165615|ref|NC_002745.2|	411011 +	A	1	3	3	3	2	SA0351
gi|29165615|ref|NC_002745.2|	411014 +	A	0	0	2	3	2	SA0351
gi|29165615|ref|NC_002745.2|	411015 +	U	0	0	1	2	0	SA0351
gi|29165615|ref|NC_002745.2|	411016 +	A	0	1	0	1	0	SA0351
gi|29165615|ref|NC_002745.2|	411022 +	A	1	0	0	0	0	SA0351
gi|29165615|ref|NC_002745.2|	411034 +	G	0	1	0	0	0	SA0351
gi|29165615|ref|NC_002745.2|	411044 +	U	1	0	0	0	0	SA0351
gi|29165615|ref|NC_002745.2|	411047 +	A	0	0	0	1	0	SA0351
gi|29165615|ref|NC_002745.2|	411051 +	A	1	0	0	0	0	SA0351
gi|29165615|ref|NC_002745.2|	411053 +	C	1	0	0	0	0	SA0351
gi|29165615|ref|NC_002745.2|	411055 +	G	0	0	0	1	0	SA0351
gi|29165615|ref|NC_002745.2|	411062 +	A	0	0	1	1	1	SA0351
gi|29165615|ref|NC_002745.2|	411063 +	U	0	1	0	0	1	SA0351
gi|29165615|ref|NC_002745.2|	411064 +	C	0	1	0	0	0	SA0351
gi|29165615|ref|NC_002745.2|	411066 +	A	1	2	1	1	1	SA0351
gi|29165615|ref|NC_002745.2|	411068 +	U	0	0	0	1	0	SA0351
gi|29165615|ref|NC_002745.2|	411069 +	A	0	0	1	0	0	SA0351
gi|29165615|ref|NC_002745.2|	411072 +	U	1	0	0	1	0	SA0351
gi|29165615|ref|NC_002745.2|	411073 +	A	0	1	3	2	3	SA0351
gi|29165615|ref|NC_002745.2|	411074 +	G	0	1	0	0	0	SA0351
gi|29165615|ref|NC_002745.2|	411075 +	A	0	1	2	2	0	SA0351
gi|29165615|ref|NC_002745.2|	411076 +	A	1	1	2	1	1	SA0351
gi|29165615|ref|NC_002745.2|	411077 +	C	0	3	0	2	1	SA0351
gi|29165615|ref|NC_002745.2|	411079 +	A	0	0	1	0	0	SA0351
gi|29165615|ref|NC_002745.2|	411080 +	C	0	0	0	2	0	SA0351
gi|29165615|ref|NC_002745.2|	411083 +	A	0	0	0	1	0	SA0351
gi|29165615|ref|NC_002745.2|	411084 +	U	0	1	0	0	0	SA0351
gi|29165615|ref|NC_002745.2|	411085 +	G	0	1	0	2	0	SA0351
gi|29165615|ref|NC_002745.2|	411086 +	A	2	11	6	11	6	SA0351
gi|29165615|ref|NC_002745.2|	411087 +	A	0	4	5	0	0	SA0351
gi|29165615|ref|NC_002745.2|	411089 +	U	4	2	0	0	0	SA0351
gi|29165615|ref|NC_002745.2|	411090 +	A	0	0	3	0	0	SA0351
gi|29165615|ref|NC_002745.2|	411092 +	U	0	0	2	0	0	SA0351
gi|29165615|ref|NC_002745.2|	411093 +	A	0	6	6	5	1	SA0351
gi|29165615|ref|NC_002745.2|	411094 +	G	0	0	1	0	0	SA0351
gi|29165615|ref|NC_002745.2|	411095 +	G	0	4	0	2	3	SA0351
gi|29165615|ref|NC_002745.2|	411096 +	A	0	7	4	8	6	SA0351
gi|29165615|ref|NC_002745.2|	411097 +	U	0	0	2	1	0	SA0351
gi|29165615|ref|NC_002745.2|	411098 +	U	1	0	0	0	0	SA0351
gi|29165615|ref|NC_002745.2|	411099 +	A	1	4	0	0	2	SA0351
gi|29165615|ref|NC_002745.2|	411100 +	U	0	6	2	1	2	SA0351
gi|29165615|ref|NC_002745.2|	411101 +	C	1	3	4	1	1	SA0351
gi|29165615|ref|NC_002745.2|	411102 +	A	0	2	4	3	1	SA0351
gi|29165615|ref|NC_002745.2|	411103 +	A	0	1	4	3	3	SA0351
gi|29165615|ref|NC_002745.2|	411104 +	C	2	0	0	0	0	SA0351
gi|29165615|ref|NC_002745.2|	411105 +	A	0	1	0	2	0	SA0351
gi|29165615|ref|NC_002745.2|	411106 +	U	4	8	1	6	6	SA0351
gi|29165615|ref|NC_002745.2|	411107 +	A	2	0	1	0	0	SA0351
gi|29165615|ref|NC_002745.2|	411108 +	U	0	0	0	1	0	SA0351

gi|29165615|ref|NC_002745.2|	411110 +	U	1	0	0	0	1	SA0351
gi|29165615|ref|NC_002745.2|	411111 +	U	0	0	0	0	1	SA0351
gi|29165615|ref|NC_002745.2|	411112 +	A	0	0	0	1	1	SA0351
gi|29165615|ref|NC_002745.2|	411113 +	C	4	0	1	0	1	SA0351
gi|29165615|ref|NC_002745.2|	411114 +	U	0	0	1	1	0	SA0351
gi|29165615|ref|NC_002745.2|	411116 +	C	3	0	0	0	0	SA0351
gi|29165615|ref|NC_002745.2|	411119 +	G	0	2	0	0	0	SA0351
gi|29165615|ref|NC_002745.2|	411120 +	U	1	0	0	0	0	SA0351
gi|29165615|ref|NC_002745.2|	411121 +	G	0	3	1	0	0	SA0351
gi|29165615|ref|NC_002745.2|	411123 +	G	5	0	0	2	0	SA0351
gi|29165615|ref|NC_002745.2|	411124 +	C	0	0	0	1	0	SA0351
gi|29165615|ref|NC_002745.2|	411125 +	A	1	0	0	2	0	SA0351
gi|29165615|ref|NC_002745.2|	411126 +	A	0	1	0	0	0	SA0351
gi|29165615|ref|NC_002745.2|	411129 +	A	0	0	0	0	1	SA0351
gi|29165615|ref|NC_002745.2|	411131 +	U	0	0	1	0	0	SA0351
gi|29165615|ref|NC_002745.2|	411132 +	A	2	0	1	0	0	SA0351
gi|29165615|ref|NC_002745.2|	411133 +	C	0	0	0	1	0	SA0351
gi|29165615|ref|NC_002745.2|	411137 +	C	1	0	0	0	0	SA0351
gi|29165615|ref|NC_002745.2|	411138 +	U	1	1	0	0	0	SA0351
gi|29165615|ref|NC_002745.2|	411142 +	A	0	0	0	1	0	SA0351
gi|29165615|ref|NC_002745.2|	411143 +	C	0	0	2	1	0	SA0351
gi|29165615|ref|NC_002745.2|	411144 +	A	0	2	0	0	0	SA0351
gi|29165615|ref|NC_002745.2|	411146 +	U	0	0	0	0	1	SA0351
gi|29165615|ref|NC_002745.2|	411147 +	U	0	3	3	2	5	SA0351
gi|29165615|ref|NC_002745.2|	411148 +	A	0	0	0	1	0	SA0351
gi|29165615|ref|NC_002745.2|	411149 +	A	0	1	1	0	0	SA0351
gi|29165615|ref|NC_002745.2|	411156 +	U	1	0	0	0	1	SA0351
gi|29165615|ref|NC_002745.2|	411158 +	U	1	0	0	0	0	SA0351
gi|29165615|ref|NC_002745.2|	411159 +	G	0	1	0	0	0	SA0351
gi|29165615|ref|NC_002745.2|	411164 +	C	1	0	0	0	0	SA0351
gi|29165615|ref|NC_002745.2|	411167 +	C	1	0	0	0	0	SA0351
gi|29165615|ref|NC_002745.2|	411168 +	U	2	0	0	0	0	SA0351
gi|29165615|ref|NC_002745.2|	411171 +	A	2	0	1	0	0	SA0351
gi|29165615|ref|NC_002745.2|	411172 +	U	1	0	0	0	1	SA0351
gi|29165615|ref|NC_002745.2|	411176 +	C	1	0	0	0	0	SA0351
gi|29165615|ref|NC_002745.2|	411180 +	U	2	0	0	0	0	SA0351
gi|29165615|ref|NC_002745.2|	411184 +	A	0	1	0	0	0	SA0351
gi|29165615|ref|NC_002745.2|	411194 +	A	0	0	1	0	0	SA0351
gi|29165615|ref|NC_002745.2|	411197 +	U	2	0	0	0	0	SA0351
gi|29165615|ref|NC_002745.2|	411199 +	G	0	0	0	0	1	SA0351
gi|29165615|ref|NC_002745.2|	411200 +	A	0	0	0	1	1	SA0351
gi|29165615|ref|NC_002745.2|	411202 +	C	0	0	1	0	0	SA0351
gi|29165615|ref|NC_002745.2|	411206 +	G	0	4	1	1	1	SA0351
gi|29165615|ref|NC_002745.2|	411208 +	U	0	1	0	0	0	SA0351
gi|29165615|ref|NC_002745.2|	411209 +	U	2	0	0	0	0	SA0351
gi|29165615|ref|NC_002745.2|	411210 +	U	0	0	0	1	0	SA0351
gi|29165615|ref|NC_002745.2|	411212 +	U	1	0	0	1	0	SA0351
gi|29165615|ref|NC_002745.2|	411213 +	C	1	0	0	0	0	SA0351
gi|29165615|ref|NC_002745.2|	411219 +	C	1	0	1	0	0	SA0351
gi|29165615|ref|NC_002745.2|	411221 +	A	0	0	1	1	0	SA0351
gi|29165615|ref|NC_002745.2|	411223 +	G	0	0	1	0	0	SA0351
gi|29165615|ref|NC_002745.2|	411224 +	U	0	1	1	0	1	SA0351
gi|29165615|ref|NC_002745.2|	411226 +	A	0	0	1	0	0	SA0351
gi|29165615|ref|NC_002745.2|	411227 +	C	1	0	0	0	0	SA0351
gi|29165615|ref|NC_002745.2|	411228 +	A	0	0	1	0	0	SA0351
gi|29165615|ref|NC_002745.2|	411242 +	A	0	0	0	1	0	SA0351
gi|29165615|ref|NC_002745.2|	411244 +	G	0	0	1	0	0	SA0351
gi|29165615|ref|NC_002745.2|	411245 +	U	0	1	0	1	0	SA0351
gi|29165615|ref|NC_002745.2|	411247 +	C	0	3	1	0	0	SA0351
gi|29165615|ref|NC_002745.2|	411249 +	A	1	0	0	0	0	SA0351
gi|29165615|ref|NC_002745.2|	411254 +	G	0	0	0	2	0	SA0351
gi|29165615|ref|NC_002745.2|	411272 +	A	0	1	0	0	0	SA0351
gi|29165615|ref|NC_002745.2|	411296 +	A	0	0	0	1	0	SA0351
gi|29165615|ref|NC_002745.2|	411316 +	C	0	0	0	0	1	SA0351
gi|29165615|ref|NC_002745.2|	411317 +	A	0	0	1	0	0	SA0351
gi|29165615|ref|NC_002745.2|	411338 +	U	0	0	0	1	0	SA0351
gi|29165615|ref|NC_002745.2|	411439 +	A	0	0	1	0	0	-
gi|29165615|ref|NC_002745.2|	411789 +	U	1	0	0	0	0	-
gi|29165615|ref|NC_002745.2|	411792 +	A	6	3	2	7	1	-
gi|29165615|ref|NC_002745.2|	411793 +	U	1	0	0	0	0	-
gi|29165615|ref|NC_002745.2|	411795 +	G	10	2	3	5	7	-
gi|29165615|ref|NC_002745.2|	411805 +	A	0	5	16	3	3	-
gi|29165615|ref|NC_002745.2|	411806 +	A	0	0	1	0	0	-
gi|29165615|ref|NC_002745.2|	411812 +	U	1	0	0	0	0	-
gi|29165615|ref|NC_002745.2|	411813 +	U	3	0	0	0	0	-
gi|29165615|ref|NC_002745.2|	411824 +	C	0	2	0	0	0	-
gi|29165615|ref|NC_002745.2|	411829 +	U	0	0	0	0	1	-
gi|29165615|ref|NC_002745.2|	411839 +	U	1	0	0	0	0	-
gi|29165615|ref|NC_002745.2|	411850 +	G	4	0	0	1	1	-
gi|29165615|ref|NC_002745.2|	411852 +	C	2	0	0	0	0	-
gi|29165615|ref|NC_002745.2|	411854 +	A	3	0	0	0	0	-

gi|29165615|ref|NC_002745.2|	411855	+	C	1	0	0	0	0	-
gi|29165615|ref|NC_002745.2|	411857	+	A	0	0	0	1	0	-
gi|29165615|ref|NC_002745.2|	411872	+	A	0	3	3	0	2	-
gi|29165615|ref|NC_002745.2|	411873	+	A	0	0	1	1	0	-
gi|29165615|ref|NC_002745.2|	411874	+	A	0	3	2	1	1	-
gi|29165615|ref|NC_002745.2|	411875	+	A	0	2	0	2	0	SA0352
gi|29165615|ref|NC_002745.2|	411878	+	A	0	0	1	0	0	SA0352
gi|29165615|ref|NC_002745.2|	411880	+	A	0	1	1	0	0	SA0352
gi|29165615|ref|NC_002745.2|	411882	+	C	0	0	1	0	1	SA0352
gi|29165615|ref|NC_002745.2|	411883	+	A	0	1	1	0	0	SA0352
gi|29165615|ref|NC_002745.2|	411887	+	G	0	0	0	1	0	SA0352
gi|29165615|ref|NC_002745.2|	411888	+	A	1	1	2	0	0	SA0352
gi|29165615|ref|NC_002745.2|	411893	+	A	1	0	0	0	0	SA0352
gi|29165615|ref|NC_002745.2|	411905	+	C	1	0	0	0	0	SA0352
gi|29165615|ref|NC_002745.2|	411906	+	G	0	0	1	0	0	SA0352
gi|29165615|ref|NC_002745.2|	411911	+	A	0	1	0	0	0	SA0352
gi|29165615|ref|NC_002745.2|	411912	+	A	0	0	0	0	1	SA0352
gi|29165615|ref|NC_002745.2|	411914	+	A	1	0	0	0	0	SA0352
gi|29165615|ref|NC_002745.2|	411916	+	U	3	0	0	0	0	SA0352
gi|29165615|ref|NC_002745.2|	411923	+	G	1	0	0	0	0	SA0352
gi|29165615|ref|NC_002745.2|	411927	+	C	0	0	0	1	0	SA0352
gi|29165615|ref|NC_002745.2|	411932	+	A	0	0	1	0	0	SA0352
gi|29165615|ref|NC_002745.2|	411933	+	A	0	0	1	0	0	SA0352
gi|29165615|ref|NC_002745.2|	411947	+	C	0	1	0	0	0	SA0352
gi|29165615|ref|NC_002745.2|	411973	+	A	0	1	0	0	0	SA0352
gi|29165615|ref|NC_002745.2|	411986	+	U	0	1	0	0	0	SA0352
gi|29165615|ref|NC_002745.2|	411987	+	U	1	0	0	1	0	SA0352
gi|29165615|ref|NC_002745.2|	411992	+	G	0	0	0	1	0	SA0352
gi|29165615|ref|NC_002745.2|	411993	+	C	0	0	0	0	1	SA0352
gi|29165615|ref|NC_002745.2|	411994	+	A	0	0	1	0	0	SA0352
gi|29165615|ref|NC_002745.2|	411995	+	A	1	0	1	0	0	SA0352
gi|29165615|ref|NC_002745.2|	411996	+	A	0	0	0	1	0	SA0352
gi|29165615|ref|NC_002745.2|	411999	+	A	0	0	1	0	0	SA0352
gi|29165615|ref|NC_002745.2|	412010	+	C	1	0	0	0	1	SA0352
gi|29165615|ref|NC_002745.2|	412017	+	U	1	0	0	0	0	SA0352
gi|29165615|ref|NC_002745.2|	412034	+	G	1	0	0	0	0	SA0352
gi|29165615|ref|NC_002745.2|	412051	+	C	0	0	0	1	0	SA0352
gi|29165615|ref|NC_002745.2|	412054	+	C	1	0	0	1	2	SA0352
gi|29165615|ref|NC_002745.2|	412057	+	C	0	0	0	0	1	SA0352
gi|29165615|ref|NC_002745.2|	412069	+	U	2	0	0	0	0	SA0352
gi|29165615|ref|NC_002745.2|	412070	+	A	0	0	1	0	0	SA0352
gi|29165615|ref|NC_002745.2|	412072	+	A	0	1	0	0	0	SA0352
gi|29165615|ref|NC_002745.2|	412074	+	C	1	0	0	0	0	SA0352
gi|29165615|ref|NC_002745.2|	412076	+	G	0	0	0	0	2	SA0352
gi|29165615|ref|NC_002745.2|	412077	+	A	0	1	0	6	1	SA0352
gi|29165615|ref|NC_002745.2|	412079	+	A	0	0	0	0	1	SA0352
gi|29165615|ref|NC_002745.2|	412080	+	A	0	0	0	0	1	SA0352
gi|29165615|ref|NC_002745.2|	412081	+	C	0	1	0	0	0	SA0352
gi|29165615|ref|NC_002745.2|	412092	+	C	1	0	0	0	0	SA0352
gi|29165615|ref|NC_002745.2|	412098	+	A	0	0	0	1	0	SA0352
gi|29165615|ref|NC_002745.2|	412110	+	U	4	0	0	0	0	SA0352
gi|29165615|ref|NC_002745.2|	412118	+	A	0	0	1	1	0	SA0352
gi|29165615|ref|NC_002745.2|	412120	+	C	0	0	0	0	1	SA0352
gi|29165615|ref|NC_002745.2|	412131	+	U	2	0	1	0	0	SA0352
gi|29165615|ref|NC_002745.2|	412133	+	A	0	0	0	1	0	SA0352
gi|29165615|ref|NC_002745.2|	412134	+	U	1	0	0	0	0	SA0352
gi|29165615|ref|NC_002745.2|	412141	+	C	1	0	0	0	0	SA0352
gi|29165615|ref|NC_002745.2|	412149	+	U	1	0	0	0	0	SA0352
gi|29165615|ref|NC_002745.2|	412151	+	C	2	0	0	3	0	SA0352
gi|29165615|ref|NC_002745.2|	412159	+	C	0	0	0	0	1	SA0352
gi|29165615|ref|NC_002745.2|	412169	+	U	0	2	0	0	0	SA0352
gi|29165615|ref|NC_002745.2|	412209	+	A	0	0	1	1	0	SA0353
gi|29165615|ref|NC_002745.2|	412224	+	A	0	0	0	0	1	SA0353
gi|29165615|ref|NC_002745.2|	412234	+	C	1	0	0	0	0	SA0353
gi|29165615|ref|NC_002745.2|	412247	+	C	1	0	0	0	0	SA0353
gi|29165615|ref|NC_002745.2|	412248	+	C	0	0	1	0	0	SA0353
gi|29165615|ref|NC_002745.2|	412253	+	C	1	0	0	0	0	SA0353
gi|29165615|ref|NC_002745.2|	412274	+	C	0	0	0	1	0	SA0353
gi|29165615|ref|NC_002745.2|	412278	+	C	0	0	1	0	0	SA0353
gi|29165615|ref|NC_002745.2|	412279	+	A	0	0	0	0	1	SA0353
gi|29165615|ref|NC_002745.2|	412281	+	U	1	1	0	0	0	SA0353
gi|29165615|ref|NC_002745.2|	412283	+	U	3	0	0	0	0	SA0353
gi|29165615|ref|NC_002745.2|	412284	+	U	1	0	0	0	0	SA0353
gi|29165615|ref|NC_002745.2|	412306	+	A	2	0	2	0	0	SA0353
gi|29165615|ref|NC_002745.2|	412329	+	A	1	0	0	0	0	SA0353
gi|29165615|ref|NC_002745.2|	412374	+	A	0	0	0	0	1	SA0353
gi|29165615|ref|NC_002745.2|	412375	+	A	0	1	0	0	0	SA0353
gi|29165615|ref|NC_002745.2|	412385	+	U	1	0	0	0	0	SA0353
gi|29165615|ref|NC_002745.2|	412386	+	A	0	1	0	0	0	SA0353
gi|29165615|ref|NC_002745.2|	412387	+	U	0	0	1	0	0	SA0353
gi|29165615|ref|NC_002745.2|	412390	+	A	0	3	0	0	0	SA0353

gi|29165615|ref|NC_002745.2|	412394 +	G	0	0	0	1	0	SA0353
gi|29165615|ref|NC_002745.2|	412395 +	U	1	0	0	0	0	SA0353
gi|29165615|ref|NC_002745.2|	412406 +	G	0	0	0	0	1	SA0353
gi|29165615|ref|NC_002745.2|	412423 +	C	0	0	1	0	0	SA0353
gi|29165615|ref|NC_002745.2|	412431 +	U	0	1	0	1	0	SA0353
gi|29165615|ref|NC_002745.2|	412436 +	A	1	0	0	0	0	SA0353
gi|29165615|ref|NC_002745.2|	412438 +	G	0	0	1	0	0	SA0353
gi|29165615|ref|NC_002745.2|	412439 +	A	2	0	3	4	0	SA0353
gi|29165615|ref|NC_002745.2|	412440 +	A	1	0	2	1	1	SA0353
gi|29165615|ref|NC_002745.2|	412441 +	A	0	0	2	1	0	SA0353
gi|29165615|ref|NC_002745.2|	412472 +	A	0	1	0	0	0	SA0353
gi|29165615|ref|NC_002745.2|	412509 +	A	1	0	0	0	0	SA0353
gi|29165615|ref|NC_002745.2|	412516 +	C	1	0	0	0	0	SA0353
gi|29165615|ref|NC_002745.2|	412523 +	A	0	0	1	0	0	SA0353
gi|29165615|ref|NC_002745.2|	412533 +	A	0	0	1	0	0	SA0353
gi|29165615|ref|NC_002745.2|	412537 +	C	0	0	0	1	0	SA0353
gi|29165615|ref|NC_002745.2|	412538 +	A	1	0	0	0	0	SA0353
gi|29165615|ref|NC_002745.2|	412544 +	A	0	0	0	0	1	SA0353
gi|29165615|ref|NC_002745.2|	412553 +	A	0	0	0	0	1	SA0353
gi|29165615|ref|NC_002745.2|	412554 +	A	1	0	0	0	0	SA0353
gi|29165615|ref|NC_002745.2|	412555 +	G	0	0	0	0	1	SA0353
gi|29165615|ref|NC_002745.2|	412556 +	A	0	0	0	0	1	SA0353
gi|29165615|ref|NC_002745.2|	412558 +	U	0	0	0	1	0	SA0353
gi|29165615|ref|NC_002745.2|	412565 +	A	2	0	0	0	0	SA0353
gi|29165615|ref|NC_002745.2|	412572 +	C	0	0	0	0	1	SA0353
gi|29165615|ref|NC_002745.2|	412578 +	U	1	0	0	0	0	SA0353
gi|29165615|ref|NC_002745.2|	412582 +	C	0	1	0	2	0	SA0353
gi|29165615|ref|NC_002745.2|	412583 +	A	0	1	0	0	0	SA0353
gi|29165615|ref|NC_002745.2|	412585 +	U	0	0	0	0	1	SA0353
gi|29165615|ref|NC_002745.2|	412586 +	C	3	0	0	0	0	SA0353
gi|29165615|ref|NC_002745.2|	412588 +	G	0	1	0	0	0	SA0353
gi|29165615|ref|NC_002745.2|	412589 +	G	2	0	0	0	0	SA0353
gi|29165615|ref|NC_002745.2|	412590 +	A	0	0	0	1	0	SA0353
gi|29165615|ref|NC_002745.2|	412595 +	A	0	0	2	2	0	SA0353
gi|29165615|ref|NC_002745.2|	412596 +	C	0	0	2	0	0	SA0353
gi|29165615|ref|NC_002745.2|	412597 +	A	0	1	0	0	1	SA0353
gi|29165615|ref|NC_002745.2|	412598 +	A	0	0	0	0	1	SA0353
gi|29165615|ref|NC_002745.2|	412600 +	U	0	0	0	1	0	SA0353
gi|29165615|ref|NC_002745.2|	412601 +	C	0	0	3	2	1	SA0353
gi|29165615|ref|NC_002745.2|	412602 +	G	0	2	0	0	1	SA0353
gi|29165615|ref|NC_002745.2|	412603 +	U	0	0	1	0	0	SA0353
gi|29165615|ref|NC_002745.2|	412604 +	A	1	2	2	3	1	SA0353
gi|29165615|ref|NC_002745.2|	412605 +	C	1	0	0	0	0	SA0353
gi|29165615|ref|NC_002745.2|	412609 +	A	0	1	0	0	0	SA0353
gi|29165615|ref|NC_002745.2|	412620 +	C	1	0	0	0	0	SA0353
gi|29165615|ref|NC_002745.2|	412624 +	A	0	0	1	0	0	SA0353
gi|29165615|ref|NC_002745.2|	412627 +	C	1	0	0	0	0	SA0353
gi|29165615|ref|NC_002745.2|	412629 +	A	0	0	0	0	1	SA0353
gi|29165615|ref|NC_002745.2|	412631 +	C	0	0	0	0	1	SA0353
gi|29165615|ref|NC_002745.2|	412638 +	U	0	1	0	0	0	SA0353
gi|29165615|ref|NC_002745.2|	412639 +	C	0	0	1	0	0	SA0353
gi|29165615|ref|NC_002745.2|	412649 +	A	0	1	0	0	0	SA0353
gi|29165615|ref|NC_002745.2|	412661 +	C	0	0	1	0	0	SA0353
gi|29165615|ref|NC_002745.2|	412663 +	A	0	0	0	1	0	SA0353
gi|29165615|ref|NC_002745.2|	412688 +	C	1	1	0	1	1	SA0353
gi|29165615|ref|NC_002745.2|	412689 +	A	0	1	2	1	1	SA0353
gi|29165615|ref|NC_002745.2|	412691 +	U	0	0	1	0	0	SA0353
gi|29165615|ref|NC_002745.2|	412692 +	C	3	20	23	23	8	SA0353
gi|29165615|ref|NC_002745.2|	412693 +	U	0	4	4	3	1	SA0353
gi|29165615|ref|NC_002745.2|	412694 +	A	0	20	22	10	14	SA0353
gi|29165615|ref|NC_002745.2|	412695 +	A	0	9	12	2	10	SA0353
gi|29165615|ref|NC_002745.2|	412696 +	U	0	0	0	0	1	-
gi|29165615|ref|NC_002745.2|	412697 +	A	0	3	4	3	6	-
gi|29165615|ref|NC_002745.2|	412698 +	A	1	61	43	27	32	-
gi|29165615|ref|NC_002745.2|	412699 +	A	4	79	46	23	64	-
gi|29165615|ref|NC_002745.2|	412700 +	A	1	55	34	18	34	-
gi|29165615|ref|NC_002745.2|	412701 +	A	3	28	19	18	26	-
gi|29165615|ref|NC_002745.2|	412702 +	U	0	2	2	6	2	-
gi|29165615|ref|NC_002745.2|	412703 +	U	0	0	0	0	1	-
gi|29165615|ref|NC_002745.2|	412704 +	A	2	79	31	31	39	-
gi|29165615|ref|NC_002745.2|	412705 +	A	0	18	15	11	14	-
gi|29165615|ref|NC_002745.2|	412706 +	C	0	4	1	2	3	-
gi|29165615|ref|NC_002745.2|	412707 +	G	0	7	5	1	3	-
gi|29165615|ref|NC_002745.2|	412708 +	A	1	48	26	30	27	-
gi|29165615|ref|NC_002745.2|	412709 +	A	0	43	17	22	41	-
gi|29165615|ref|NC_002745.2|	412710 +	A	4	50	27	24	42	-
gi|29165615|ref|NC_002745.2|	412711 +	U	0	4	1	2	4	-
gi|29165615|ref|NC_002745.2|	412712 +	U	0	4	0	0	3	-
gi|29165615|ref|NC_002745.2|	412713 +	A	8	108	38	48	72	-
gi|29165615|ref|NC_002745.2|	412714 +	A	2	63	22	35	56	-
gi|29165615|ref|NC_002745.2|	412715 +	A	0	13	5	3	11	-

gi|29165615|ref|NC_002745.2|	412716	+	G	0	4	1	1	4	-
gi|29165615|ref|NC_002745.2|	412717	+	C	0	8	4	4	4	-
gi|29165615|ref|NC_002745.2|	412718	+	G	0	1	2	2	6	-
gi|29165615|ref|NC_002745.2|	412719	+	G	0	1	2	0	7	-
gi|29165615|ref|NC_002745.2|	412723	+	A	1	5	1	0	2	-
gi|29165615|ref|NC_002745.2|	412724	+	A	0	1	1	0	1	-
gi|29165615|ref|NC_002745.2|	412725	+	U	2	5	2	1	3	-
gi|29165615|ref|NC_002745.2|	412726	+	U	1	0	0	1	2	-
gi|29165615|ref|NC_002745.2|	412727	+	A	0	8	2	0	2	-
gi|29165615|ref|NC_002745.2|	412728	+	U	1	0	1	0	0	-
gi|29165615|ref|NC_002745.2|	412729	+	C	0	1	1	0	3	-
gi|29165615|ref|NC_002745.2|	412730	+	A	0	2	0	0	2	-
gi|29165615|ref|NC_002745.2|	412731	+	A	0	1	0	1	3	-
gi|29165615|ref|NC_002745.2|	412733	+	G	0	0	0	0	1	-
gi|29165615|ref|NC_002745.2|	412734	+	G	0	4	0	1	3	-
gi|29165615|ref|NC_002745.2|	412735	+	A	0	8	1	2	9	-
gi|29165615|ref|NC_002745.2|	412738	+	C	0	0	1	1	1	-
gi|29165615|ref|NC_002745.2|	412739	+	A	0	1	0	0	0	-
gi|29165615|ref|NC_002745.2|	412742	+	C	0	1	0	0	0	-
gi|29165615|ref|NC_002745.2|	412743	+	A	0	5	0	3	2	-
gi|29165615|ref|NC_002745.2|	412745	+	U	0	0	1	1	3	-
gi|29165615|ref|NC_002745.2|	412746	+	C	0	5	0	2	2	-
gi|29165615|ref|NC_002745.2|	412747	+	A	0	1	0	0	0	SA0354
gi|29165615|ref|NC_002745.2|	412749	+	G	0	1	0	0	0	SA0354
gi|29165615|ref|NC_002745.2|	412750	+	G	0	0	0	0	1	SA0354
gi|29165615|ref|NC_002745.2|	412760	+	C	0	0	0	0	1	SA0354
gi|29165615|ref|NC_002745.2|	412764	+	A	0	1	0	0	0	SA0354
gi|29165615|ref|NC_002745.2|	412767	+	A	0	0	0	1	0	SA0354
gi|29165615|ref|NC_002745.2|	412784	+	A	0	0	0	1	0	SA0354
gi|29165615|ref|NC_002745.2|	412785	+	A	0	1	0	0	0	SA0354
gi|29165615|ref|NC_002745.2|	412822	+	A	0	0	1	0	0	SA0354
gi|29165615|ref|NC_002745.2|	412825	+	G	0	2	0	0	0	SA0354
gi|29165615|ref|NC_002745.2|	412832	+	A	0	1	0	0	0	SA0354
gi|29165615|ref|NC_002745.2|	412842	+	A	0	0	1	0	0	SA0354
gi|29165615|ref|NC_002745.2|	412848	+	A	0	0	0	1	0	SA0354
gi|29165615|ref|NC_002745.2|	412852	+	C	0	0	0	0	1	SA0354
gi|29165615|ref|NC_002745.2|	412854	+	U	0	1	0	1	0	SA0354
gi|29165615|ref|NC_002745.2|	412857	+	U	0	0	0	0	1	SA0354
gi|29165615|ref|NC_002745.2|	412858	+	A	0	0	0	0	2	SA0354
gi|29165615|ref|NC_002745.2|	412866	+	A	1	0	0	0	0	SA0354
gi|29165615|ref|NC_002745.2|	412870	+	G	0	0	0	0	1	SA0354
gi|29165615|ref|NC_002745.2|	412881	+	A	0	0	0	1	0	SA0354
gi|29165615|ref|NC_002745.2|	412980	+	A	0	0	1	0	0	SA0354
gi|29165615|ref|NC_002745.2|	413193	+	U	0	1	0	0	0	-
gi|29165615|ref|NC_002745.2|	413237	+	C	0	0	0	0	1	-
gi|29165615|ref|NC_002745.2|	415128	+	C	0	1	0	1	0	-
gi|29165615|ref|NC_002745.2|	415224	+	C	0	0	0	0	1	SA0357
gi|29165615|ref|NC_002745.2|	415469	+	G	0	0	0	1	0	SA0357
gi|29165615|ref|NC_002745.2|	415472	+	C	0	0	1	0	0	SA0357
gi|29165615|ref|NC_002745.2|	415878	+	A	0	0	0	0	1	-
gi|29165615|ref|NC_002745.2|	416635	+	A	3	0	0	1	1	-
gi|29165615|ref|NC_002745.2|	417374	+	A	0	0	0	0	1	-
gi|29165615|ref|NC_002745.2|	417405	+	U	0	0	1	0	0	-
gi|29165615|ref|NC_002745.2|	417491	+	A	0	1	0	0	0	-
gi|29165615|ref|NC_002745.2|	417890	+	U	1	0	0	0	0	-
gi|29165615|ref|NC_002745.2|	417893	+	U	0	0	1	0	0	-
gi|29165615|ref|NC_002745.2|	417903	+	G	0	0	0	1	0	-
gi|29165615|ref|NC_002745.2|	417904	+	U	0	0	0	1	0	-
gi|29165615|ref|NC_002745.2|	417905	+	A	0	0	2	1	0	-
gi|29165615|ref|NC_002745.2|	417906	+	A	0	0	2	0	0	-
gi|29165615|ref|NC_002745.2|	417908	+	U	1	0	0	0	1	-
gi|29165615|ref|NC_002745.2|	417909	+	A	1	6	8	0	1	-
gi|29165615|ref|NC_002745.2|	417911	+	G	0	0	0	1	0	-
gi|29165615|ref|NC_002745.2|	417913	+	G	0	0	0	0	1	-
gi|29165615|ref|NC_002745.2|	417915	+	U	0	0	0	1	0	-
gi|29165615|ref|NC_002745.2|	417921	+	U	0	0	0	1	0	-
gi|29165615|ref|NC_002745.2|	417923	+	A	14	1	1	2	5	-
gi|29165615|ref|NC_002745.2|	417924	+	G	1	0	0	0	0	-
gi|29165615|ref|NC_002745.2|	417925	+	A	12	9	0	12	14	-
gi|29165615|ref|NC_002745.2|	417926	+	A	223	38	25	63	65	-
gi|29165615|ref|NC_002745.2|	417927	+	U	0	0	0	0	1	-
gi|29165615|ref|NC_002745.2|	417928	+	A	18	9	5	19	8	-
gi|29165615|ref|NC_002745.2|	417929	+	A	0	2	1	1	0	-
gi|29165615|ref|NC_002745.2|	417930	+	C	1	0	0	1	1	-
gi|29165615|ref|NC_002745.2|	417931	+	G	0	0	0	0	2	-
gi|29165615|ref|NC_002745.2|	417932	+	C	0	0	0	2	2	-
gi|29165615|ref|NC_002745.2|	417933	+	A	2	0	2	2	3	-
gi|29165615|ref|NC_002745.2|	417934	+	A	0	0	0	1	1	-
gi|29165615|ref|NC_002745.2|	417935	+	A	1	2	0	0	2	-
gi|29165615|ref|NC_002745.2|	417936	+	A	0	0	0	0	2	-
gi|29165615|ref|NC_002745.2|	417937	+	U	0	1	0	0	0	-

gi|29165615|ref|NC_002745.2|	417938 +	A	1	2	2	2	1	-
gi|29165615|ref|NC_002745.2|	417939 +	A	0	0	0	2	1	-
gi|29165615|ref|NC_002745.2|	417940 +	A	0	1	1	2	3	-
gi|29165615|ref|NC_002745.2|	417942 +	A	1	1	1	3	1	-
gi|29165615|ref|NC_002745.2|	417943 +	G	1	1	0	2	1	-
gi|29165615|ref|NC_002745.2|	417944 +	A	1	1	1	2	2	-
gi|29165615|ref|NC_002745.2|	417945 +	A	3	1	0	1	0	-
gi|29165615|ref|NC_002745.2|	417946 +	A	0	2	0	2	3	-
gi|29165615|ref|NC_002745.2|	417948 +	A	0	0	0	0	1	-
gi|29165615|ref|NC_002745.2|	417949 +	G	0	0	0	1	0	-
gi|29165615|ref|NC_002745.2|	417950 +	G	2	0	0	0	0	-
gi|29165615|ref|NC_002745.2|	417951 +	A	0	0	1	0	1	-
gi|29165615|ref|NC_002745.2|	417952 +	G	1	1	0	0	2	-
gi|29165615|ref|NC_002745.2|	417953 +	A	2	0	1	0	2	-
gi|29165615|ref|NC_002745.2|	417954 +	C	1	0	0	0	0	-
gi|29165615|ref|NC_002745.2|	417956 +	G	1	3	0	0	0	-
gi|29165615|ref|NC_002745.2|	417957 +	A	1	1	0	4	0	-
gi|29165615|ref|NC_002745.2|	417958 +	A	0	0	0	2	0	-
gi|29165615|ref|NC_002745.2|	417959 +	A	0	1	0	0	0	-
gi|29165615|ref|NC_002745.2|	417960 +	A	0	0	0	1	0	-
gi|29165615|ref|NC_002745.2|	417961 +	U	2	0	0	0	0	-
gi|29165615|ref|NC_002745.2|	417963 +	A	0	2	1	0	0	SA0360
gi|29165615|ref|NC_002745.2|	417964 +	U	2	0	3	2	5	SA0360
gi|29165615|ref|NC_002745.2|	417965 +	G	3	0	1	0	0	SA0360
gi|29165615|ref|NC_002745.2|	417966 +	U	0	1	1	2	0	SA0360
gi|29165615|ref|NC_002745.2|	417970 +	G	1	3	2	1	6	SA0360
gi|29165615|ref|NC_002745.2|	417971 +	A	0	0	0	1	2	SA0360
gi|29165615|ref|NC_002745.2|	417973 +	U	2	0	0	0	0	SA0360
gi|29165615|ref|NC_002745.2|	417974 +	U	3	0	1	0	0	SA0360
gi|29165615|ref|NC_002745.2|	417975 +	A	1	0	0	0	0	SA0360
gi|29165615|ref|NC_002745.2|	417979 +	G	1	1	0	1	2	SA0360
gi|29165615|ref|NC_002745.2|	417980 +	A	4	1	0	1	7	SA0360
gi|29165615|ref|NC_002745.2|	417984 +	U	3	0	1	0	2	SA0360
gi|29165615|ref|NC_002745.2|	417985 +	U	3	0	0	0	0	SA0360
gi|29165615|ref|NC_002745.2|	417988 +	U	1	0	0	0	1	SA0360
gi|29165615|ref|NC_002745.2|	417989 +	U	0	0	0	1	0	SA0360
gi|29165615|ref|NC_002745.2|	417995 +	U	2	0	0	0	0	SA0360
gi|29165615|ref|NC_002745.2|	417997 +	G	1	0	0	0	0	SA0360
gi|29165615|ref|NC_002745.2|	418000 +	U	1	0	0	0	0	SA0360
gi|29165615|ref|NC_002745.2|	418012 +	C	2	0	0	0	0	SA0360
gi|29165615|ref|NC_002745.2|	418043 +	A	1	0	0	0	0	SA0360
gi|29165615|ref|NC_002745.2|	418054 +	U	1	0	0	0	0	SA0360
gi|29165615|ref|NC_002745.2|	418055 +	A	0	0	1	0	0	SA0360
gi|29165615|ref|NC_002745.2|	418060 +	A	0	0	1	0	0	SA0360
gi|29165615|ref|NC_002745.2|	418066 +	U	1	0	0	0	0	SA0360
gi|29165615|ref|NC_002745.2|	418076 +	U	2	0	0	0	0	SA0360
gi|29165615|ref|NC_002745.2|	418079 +	U	0	0	0	0	1	SA0360
gi|29165615|ref|NC_002745.2|	418081 +	G	0	0	0	0	1	SA0360
gi|29165615|ref|NC_002745.2|	418103 +	A	1	0	0	0	0	SA0360
gi|29165615|ref|NC_002745.2|	418105 +	U	1	0	0	0	0	SA0360
gi|29165615|ref|NC_002745.2|	418163 +	A	1	0	0	0	0	SA0360
gi|29165615|ref|NC_002745.2|	418173 +	A	0	0	1	0	0	SA0360
gi|29165615|ref|NC_002745.2|	418332 +	A	0	0	0	1	0	-
gi|29165615|ref|NC_002745.2|	418366 +	A	0	0	0	0	1	-
gi|29165615|ref|NC_002745.2|	418440 +	A	0	0	0	1	0	-
gi|29165615|ref|NC_002745.2|	418565 +	A	0	1	0	0	0	-
gi|29165615|ref|NC_002745.2|	418613 +	G	1	1	1	1	1	-
gi|29165615|ref|NC_002745.2|	418614 +	A	0	0	0	0	1	-
gi|29165615|ref|NC_002745.2|	418618 +	A	0	2	0	0	0	SA0361
gi|29165615|ref|NC_002745.2|	418619 +	C	0	1	0	0	0	SA0361
gi|29165615|ref|NC_002745.2|	418631 +	A	0	0	0	0	1	SA0361
gi|29165615|ref|NC_002745.2|	418671 +	C	0	1	0	0	0	SA0361
gi|29165615|ref|NC_002745.2|	418789 +	A	0	1	0	0	0	SA0361
gi|29165615|ref|NC_002745.2|	418926 +	A	0	0	0	1	0	SA0361
gi|29165615|ref|NC_002745.2|	418927 +	A	0	0	0	1	0	SA0361
gi|29165615|ref|NC_002745.2|	419009 +	A	0	0	1	0	0	SA0361
gi|29165615|ref|NC_002745.2|	419015 +	C	0	0	0	1	0	SA0361
gi|29165615|ref|NC_002745.2|	419020 +	A	1	0	0	0	0	SA0361
gi|29165615|ref|NC_002745.2|	419032 +	U	1	0	0	0	0	SA0361
gi|29165615|ref|NC_002745.2|	419034 +	A	0	0	1	0	0	SA0361
gi|29165615|ref|NC_002745.2|	419039 +	G	0	0	0	2	0	SA0361
gi|29165615|ref|NC_002745.2|	419040 +	U	0	1	1	1	3	SA0361
gi|29165615|ref|NC_002745.2|	419041 +	A	0	1	0	0	0	SA0361
gi|29165615|ref|NC_002745.2|	419043 +	A	0	0	0	1	0	SA0361
gi|29165615|ref|NC_002745.2|	419050 +	C	0	0	0	1	2	SA0361
gi|29165615|ref|NC_002745.2|	419052 +	U	0	1	0	1	3	SA0361
gi|29165615|ref|NC_002745.2|	419053 +	A	0	1	1	2	0	SA0361
gi|29165615|ref|NC_002745.2|	419054 +	G	0	1	0	0	0	SA0361
gi|29165615|ref|NC_002745.2|	419055 +	A	0	0	0	1	1	SA0361
gi|29165615|ref|NC_002745.2|	419060 +	U	0	1	0	0	0	SA0361
gi|29165615|ref|NC_002745.2|	419061 +	U	0	0	0	0	1	SA0361

gi|29165615|ref|NC_002745.2|	419062 +	U	0	0	0	0	2	SA0361
gi|29165615|ref|NC_002745.2|	419065 +	G	0	0	0	1	0	SA0361
gi|29165615|ref|NC_002745.2|	419067 +	A	0	0	0	2	0	SA0361
gi|29165615|ref|NC_002745.2|	419073 +	U	0	0	0	0	3	SA0361
gi|29165615|ref|NC_002745.2|	419083 +	A	0	0	0	1	0	SA0361
gi|29165615|ref|NC_002745.2|	419085 +	A	0	0	0	0	1	SA0361
gi|29165615|ref|NC_002745.2|	419088 +	C	0	0	1	0	0	SA0361
gi|29165615|ref|NC_002745.2|	419099 +	G	0	0	1	0	0	SA0361
gi|29165615|ref|NC_002745.2|	419100 +	A	0	1	2	6	8	SA0361
gi|29165615|ref|NC_002745.2|	419101 +	U	1	1	1	0	0	SA0361
gi|29165615|ref|NC_002745.2|	419102 +	A	0	0	0	1	0	SA0361
gi|29165615|ref|NC_002745.2|	419103 +	U	0	0	0	1	0	SA0361
gi|29165615|ref|NC_002745.2|	419106 +	U	0	0	0	1	0	SA0361
gi|29165615|ref|NC_002745.2|	419127 +	U	0	0	1	0	1	SA0361
gi|29165615|ref|NC_002745.2|	419129 +	A	0	0	1	0	0	SA0361
gi|29165615|ref|NC_002745.2|	419132 +	C	0	0	0	0	1	SA0361
gi|29165615|ref|NC_002745.2|	419134 +	U	0	1	0	0	0	SA0361
gi|29165615|ref|NC_002745.2|	419141 +	A	0	0	1	0	0	SA0361
gi|29165615|ref|NC_002745.2|	419153 +	A	0	0	0	1	0	SA0361
gi|29165615|ref|NC_002745.2|	419157 +	A	0	1	0	0	0	SA0361
gi|29165615|ref|NC_002745.2|	419164 +	A	0	1	0	0	0	SA0361
gi|29165615|ref|NC_002745.2|	419442 +	C	1	0	0	0	0	-
gi|29165615|ref|NC_002745.2|	419653 +	A	0	0	1	0	1	-
gi|29165615|ref|NC_002745.2|	419729 +	C	0	0	0	0	1	-
gi|29165615|ref|NC_002745.2|	420448 +	U	0	0	1	0	0	-
gi|29165615|ref|NC_002745.2|	422492 +	U	0	0	0	1	0	-
gi|29165615|ref|NC_002745.2|	422846 +	A	0	0	1	0	0	-
gi|29165615|ref|NC_002745.2|	422867 +	C	0	1	0	0	0	-
gi|29165615|ref|NC_002745.2|	422880 +	A	0	0	1	0	0	-
gi|29165615|ref|NC_002745.2|	422896 +	U	0	1	0	0	0	-
gi|29165615|ref|NC_002745.2|	422914 +	U	0	1	0	0	0	-
gi|29165615|ref|NC_002745.2|	422916 +	A	0	1	0	0	0	-
gi|29165615|ref|NC_002745.2|	423077 +	U	0	1	0	0	0	-
gi|29165615|ref|NC_002745.2|	423118 +	U	0	1	0	0	0	-
gi|29165615|ref|NC_002745.2|	423162 +	A	0	0	1	0	0	-
gi|29165615|ref|NC_002745.2|	423268 +	A	0	0	0	0	1	-
gi|29165615|ref|NC_002745.2|	423279 +	A	0	0	0	0	1	-
gi|29165615|ref|NC_002745.2|	423281 +	A	0	3	1	0	0	-
gi|29165615|ref|NC_002745.2|	423296 +	U	0	0	0	0	1	-
gi|29165615|ref|NC_002745.2|	423298 +	G	0	0	0	0	1	-
gi|29165615|ref|NC_002745.2|	423312 +	U	0	3	0	0	0	-
gi|29165615|ref|NC_002745.2|	423313 +	A	0	0	0	0	1	-
gi|29165615|ref|NC_002745.2|	423317 +	G	0	1	0	0	0	-
gi|29165615|ref|NC_002745.2|	423327 +	A	0	0	0	1	0	-
gi|29165615|ref|NC_002745.2|	423362 +	G	0	0	0	1	0	-
gi|29165615|ref|NC_002745.2|	423381 +	A	0	1	0	0	0	-
gi|29165615|ref|NC_002745.2|	423442 +	A	0	0	2	0	0	-
gi|29165615|ref|NC_002745.2|	423452 +	G	0	0	0	0	1	-
gi|29165615|ref|NC_002745.2|	423491 +	U	0	0	0	0	1	-
gi|29165615|ref|NC_002745.2|	423541 +	A	0	0	0	0	1	-
gi|29165615|ref|NC_002745.2|	423548 +	G	0	0	1	0	0	-
gi|29165615|ref|NC_002745.2|	423562 +	C	0	1	0	0	0	-
gi|29165615|ref|NC_002745.2|	423563 +	A	0	0	0	0	1	-
gi|29165615|ref|NC_002745.2|	423566 +	A	8	5	3	2	9	-
gi|29165615|ref|NC_002745.2|	423567 +	U	0	1	0	0	2	-
gi|29165615|ref|NC_002745.2|	423576 +	U	0	2	0	0	0	-
gi|29165615|ref|NC_002745.2|	423577 +	U	0	1	0	0	0	-
gi|29165615|ref|NC_002745.2|	423582 +	A	0	1	0	0	0	-
gi|29165615|ref|NC_002745.2|	423586 +	G	0	0	0	0	1	-
gi|29165615|ref|NC_002745.2|	423587 +	U	0	0	0	0	1	-
gi|29165615|ref|NC_002745.2|	423588 +	A	0	0	2	0	0	-
gi|29165615|ref|NC_002745.2|	423589 +	A	0	0	1	0	1	-
gi|29165615|ref|NC_002745.2|	423590 +	A	0	1	0	0	0	-
gi|29165615|ref|NC_002745.2|	423593 +	A	0	0	0	0	1	-
gi|29165615|ref|NC_002745.2|	423599 +	G	1	1	2	0	2	-
gi|29165615|ref|NC_002745.2|	423600 +	A	0	1	0	0	1	-
gi|29165615|ref|NC_002745.2|	423602 +	U	0	0	0	0	1	-
gi|29165615|ref|NC_002745.2|	423606 +	U	0	0	0	1	0	-
gi|29165615|ref|NC_002745.2|	423665 +	A	0	0	1	0	0	SA0367
gi|29165615|ref|NC_002745.2|	423677 +	U	0	1	0	0	0	SA0367
gi|29165615|ref|NC_002745.2|	423678 +	A	0	0	1	0	1	SA0367
gi|29165615|ref|NC_002745.2|	423685 +	G	0	1	0	0	0	SA0367
gi|29165615|ref|NC_002745.2|	423695 +	A	0	2	0	0	0	SA0367
gi|29165615|ref|NC_002745.2|	423756 +	U	0	1	0	0	0	SA0367
gi|29165615|ref|NC_002745.2|	423787 +	A	0	0	0	1	0	SA0367
gi|29165615|ref|NC_002745.2|	423797 +	A	0	1	0	0	0	SA0367
gi|29165615|ref|NC_002745.2|	423809 +	A	0	1	0	0	0	SA0367
gi|29165615|ref|NC_002745.2|	423814 +	U	0	1	0	0	0	SA0367
gi|29165615|ref|NC_002745.2|	423818 +	U	0	0	1	0	0	SA0367
gi|29165615|ref|NC_002745.2|	423841 +	G	0	1	0	0	0	SA0367
gi|29165615|ref|NC_002745.2|	423842 +	U	0	1	0	0	0	SA0367

gi|29165615|ref|NC_002745.2|	423871	+	U	0	0	1	0	0	SA0367
gi|29165615|ref|NC_002745.2|	423908	+	C	0	0	0	0	1	SA0367
gi|29165615|ref|NC_002745.2|	423918	+	A	0	0	0	0	1	SA0367
gi|29165615|ref|NC_002745.2|	423945	+	C	0	1	0	0	0	SA0367
gi|29165615|ref|NC_002745.2|	424198	+	U	0	0	0	0	1	SA0367
gi|29165615|ref|NC_002745.2|	424216	+	U	2	0	0	0	0	SA0367
gi|29165615|ref|NC_002745.2|	424228	+	A	0	0	0	0	1	SA0367
gi|29165615|ref|NC_002745.2|	424265	+	G	0	1	0	0	0	SA0367
gi|29165615|ref|NC_002745.2|	424271	+	A	0	0	1	0	0	SA0367
gi|29165615|ref|NC_002745.2|	424274	+	C	0	1	0	0	0	SA0367
gi|29165615|ref|NC_002745.2|	424434	+	C	0	2	0	0	0	-
gi|29165615|ref|NC_002745.2|	425935	+	C	0	0	0	2	0	-
gi|29165615|ref|NC_002745.2|	426182	+	A	0	1	0	0	0	-
gi|29165615|ref|NC_002745.2|	426350	+	C	0	0	0	0	1	-
gi|29165615|ref|NC_002745.2|	429660	+	A	0	0	0	1	0	-
gi|29165615|ref|NC_002745.2|	430265	+	U	0	0	1	0	0	-
gi|29165615|ref|NC_002745.2|	430799	+	U	0	0	1	0	1	-
gi|29165615|ref|NC_002745.2|	430800	+	A	0	0	0	1	1	-
gi|29165615|ref|NC_002745.2|	430801	+	A	0	1	4	3	1	-
gi|29165615|ref|NC_002745.2|	430802	+	A	10	26	28	26	12	-
gi|29165615|ref|NC_002745.2|	430803	+	U	0	2	1	1	0	-
gi|29165615|ref|NC_002745.2|	430804	+	A	1	4	3	2	0	-
gi|29165615|ref|NC_002745.2|	430809	+	A	0	0	0	2	0	-
gi|29165615|ref|NC_002745.2|	430811	+	A	0	2	0	0	0	-
gi|29165615|ref|NC_002745.2|	430813	+	A	0	1	0	0	1	-
gi|29165615|ref|NC_002745.2|	430815	+	A	0	0	1	0	0	-
gi|29165615|ref|NC_002745.2|	430816	+	C	0	1	0	2	0	-
gi|29165615|ref|NC_002745.2|	430817	+	U	0	0	0	0	3	-
gi|29165615|ref|NC_002745.2|	430818	+	C	1	5	5	2	2	-
gi|29165615|ref|NC_002745.2|	430819	+	A	1	0	1	0	0	-
gi|29165615|ref|NC_002745.2|	430820	+	U	0	0	1	0	1	-
gi|29165615|ref|NC_002745.2|	430821	+	A	0	0	0	1	1	-
gi|29165615|ref|NC_002745.2|	430822	+	U	0	0	2	0	2	-
gi|29165615|ref|NC_002745.2|	430823	+	A	0	1	2	1	0	-
gi|29165615|ref|NC_002745.2|	430827	+	U	0	0	0	2	0	-
gi|29165615|ref|NC_002745.2|	430828	+	A	0	1	0	3	1	-
gi|29165615|ref|NC_002745.2|	430829	+	A	0	1	1	1	1	-
gi|29165615|ref|NC_002745.2|	430830	+	A	0	1	1	1	0	-
gi|29165615|ref|NC_002745.2|	430831	+	G	1	0	2	2	1	-
gi|29165615|ref|NC_002745.2|	430832	+	A	0	1	1	0	0	-
gi|29165615|ref|NC_002745.2|	430838	+	G	0	0	0	1	0	-
gi|29165615|ref|NC_002745.2|	430839	+	C	0	0	1	0	0	-
gi|29165615|ref|NC_002745.2|	430840	+	U	0	1	0	1	0	-
gi|29165615|ref|NC_002745.2|	430841	+	U	0	0	1	0	1	-
gi|29165615|ref|NC_002745.2|	430843	+	A	0	1	0	2	2	-
gi|29165615|ref|NC_002745.2|	430844	+	G	0	4	1	2	0	-
gi|29165615|ref|NC_002745.2|	430845	+	A	0	1	1	2	0	-
gi|29165615|ref|NC_002745.2|	430847	+	G	0	1	0	0	0	-
gi|29165615|ref|NC_002745.2|	430848	+	U	0	0	0	1	0	-
gi|29165615|ref|NC_002745.2|	430852	+	U	0	0	0	1	0	-
gi|29165615|ref|NC_002745.2|	430872	+	C	1	0	0	0	0	-
gi|29165615|ref|NC_002745.2|	430894	+	A	0	0	1	0	1	-
gi|29165615|ref|NC_002745.2|	430895	+	U	0	1	0	0	0	-
gi|29165615|ref|NC_002745.2|	430921	+	U	0	1	0	0	1	-
gi|29165615|ref|NC_002745.2|	430950	+	U	0	0	0	2	0	-
gi|29165615|ref|NC_002745.2|	430955	+	A	0	0	1	0	0	-
gi|29165615|ref|NC_002745.2|	430963	+	C	1	0	0	0	0	-
gi|29165615|ref|NC_002745.2|	430969	+	U	0	0	0	2	0	-
gi|29165615|ref|NC_002745.2|	430991	+	C	1	0	3	0	0	-
gi|29165615|ref|NC_002745.2|	430992	+	A	0	1	2	0	0	-
gi|29165615|ref|NC_002745.2|	431010	+	U	0	0	0	1	1	-
gi|29165615|ref|NC_002745.2|	431018	+	C	0	1	0	0	0	-
gi|29165615|ref|NC_002745.2|	431028	+	C	1	0	0	0	0	-
gi|29165615|ref|NC_002745.2|	431030	+	C	0	2	0	1	1	-
gi|29165615|ref|NC_002745.2|	431044	+	A	0	0	1	0	0	-
gi|29165615|ref|NC_002745.2|	431045	+	A	1	0	0	0	0	-
gi|29165615|ref|NC_002745.2|	431047	+	U	1	0	0	0	0	-
gi|29165615|ref|NC_002745.2|	431048	+	A	0	0	0	1	0	-
gi|29165615|ref|NC_002745.2|	431049	+	U	0	0	0	2	0	-
gi|29165615|ref|NC_002745.2|	431050	+	U	0	0	1	0	0	-
gi|29165615|ref|NC_002745.2|	431051	+	A	0	0	0	3	0	-
gi|29165615|ref|NC_002745.2|	431052	+	U	0	1	1	0	0	-
gi|29165615|ref|NC_002745.2|	431054	+	U	0	1	0	0	0	-
gi|29165615|ref|NC_002745.2|	431073	+	U	0	3	2	2	4	-
gi|29165615|ref|NC_002745.2|	431075	+	U	2	1	0	0	0	-
gi|29165615|ref|NC_002745.2|	431076	+	C	0	4	0	0	2	-
gi|29165615|ref|NC_002745.2|	431077	+	U	2	0	0	0	0	-
gi|29165615|ref|NC_002745.2|	431078	+	A	0	0	0	1	0	-
gi|29165615|ref|NC_002745.2|	431079	+	G	0	2	0	0	1	-
gi|29165615|ref|NC_002745.2|	431080	+	C	2	2	2	2	1	-
gi|29165615|ref|NC_002745.2|	431081	+	A	1	0	0	0	0	-

gi|29165615|ref|NC_002745.2|	431082 +	C	0	2	3	1	1	-
gi|29165615|ref|NC_002745.2|	431083 +	C	0	0	2	0	1	-
gi|29165615|ref|NC_002745.2|	431084 +	A	0	2	0	0	0	-
gi|29165615|ref|NC_002745.2|	431085 +	A	0	1	2	0	0	-
gi|29165615|ref|NC_002745.2|	431088 +	U	0	0	0	0	1	-
gi|29165615|ref|NC_002745.2|	431089 +	A	0	0	3	0	0	-
gi|29165615|ref|NC_002745.2|	431090 +	A	1	11	10	8	10	-
gi|29165615|ref|NC_002745.2|	431091 +	G	0	1	0	0	0	-
gi|29165615|ref|NC_002745.2|	431092 +	A	0	1	2	1	1	-
gi|29165615|ref|NC_002745.2|	431094 +	C	0	1	0	1	3	-
gi|29165615|ref|NC_002745.2|	431095 +	A	0	1	0	1	4	-
gi|29165615|ref|NC_002745.2|	431096 +	A	1	2	2	0	0	-
gi|29165615|ref|NC_002745.2|	431097 +	A	0	4	0	0	1	-
gi|29165615|ref|NC_002745.2|	431098 +	A	0	1	1	0	0	-
gi|29165615|ref|NC_002745.2|	431101 +	U	0	0	1	1	1	-
gi|29165615|ref|NC_002745.2|	431104 +	C	0	4	6	5	1	-
gi|29165615|ref|NC_002745.2|	431105 +	A	0	1	2	0	0	-
gi|29165615|ref|NC_002745.2|	431106 +	C	0	1	1	0	1	-
gi|29165615|ref|NC_002745.2|	431107 +	A	0	0	1	0	0	-
gi|29165615|ref|NC_002745.2|	431108 +	C	1	2	0	1	0	-
gi|29165615|ref|NC_002745.2|	431109 +	A	0	0	2	0	0	-
gi|29165615|ref|NC_002745.2|	431110 +	A	0	3	4	2	2	-
gi|29165615|ref|NC_002745.2|	431111 +	A	0	12	1	0	9	-
gi|29165615|ref|NC_002745.2|	431112 +	U	0	2	0	0	2	-
gi|29165615|ref|NC_002745.2|	431113 +	C	0	1	0	0	0	-
gi|29165615|ref|NC_002745.2|	431114 +	U	0	0	0	0	1	-
gi|29165615|ref|NC_002745.2|	431115 +	G	0	2	0	0	2	-
gi|29165615|ref|NC_002745.2|	431116 +	A	0	7	5	8	8	-
gi|29165615|ref|NC_002745.2|	431118 +	G	0	5	4	0	1	-
gi|29165615|ref|NC_002745.2|	431119 +	A	0	0	1	0	1	-
gi|29165615|ref|NC_002745.2|	431121 +	G	0	1	0	0	2	-
gi|29165615|ref|NC_002745.2|	431123 +	U	0	1	0	0	0	-
gi|29165615|ref|NC_002745.2|	431125 +	U	0	0	0	0	1	-
gi|29165615|ref|NC_002745.2|	431126 +	A	0	1	1	1	1	-
gi|29165615|ref|NC_002745.2|	431127 +	A	0	4	0	0	0	-
gi|29165615|ref|NC_002745.2|	431133 +	G	0	1	0	0	0	SA0373
gi|29165615|ref|NC_002745.2|	431140 +	U	0	0	1	0	0	SA0373
gi|29165615|ref|NC_002745.2|	431142 +	G	0	1	0	0	0	SA0373
gi|29165615|ref|NC_002745.2|	431143 +	G	0	0	0	1	0	SA0373
gi|29165615|ref|NC_002745.2|	431147 +	A	0	4	0	0	0	SA0373
gi|29165615|ref|NC_002745.2|	431148 +	A	0	2	0	0	1	SA0373
gi|29165615|ref|NC_002745.2|	431149 +	A	0	0	1	0	0	SA0373
gi|29165615|ref|NC_002745.2|	431150 +	A	0	2	0	0	1	SA0373
gi|29165615|ref|NC_002745.2|	431153 +	A	0	1	0	0	0	SA0373
gi|29165615|ref|NC_002745.2|	431154 +	A	0	1	0	0	0	SA0373
gi|29165615|ref|NC_002745.2|	431217 +	A	0	0	1	0	0	SA0373
gi|29165615|ref|NC_002745.2|	431219 +	U	2	0	0	0	0	SA0373
gi|29165615|ref|NC_002745.2|	431221 +	A	1	0	0	0	0	SA0373
gi|29165615|ref|NC_002745.2|	431229 +	U	0	1	0	0	0	SA0373
gi|29165615|ref|NC_002745.2|	431230 +	U	1	0	0	0	0	SA0373
gi|29165615|ref|NC_002745.2|	431232 +	A	0	2	1	0	0	SA0373
gi|29165615|ref|NC_002745.2|	431234 +	G	0	0	0	0	1	SA0373
gi|29165615|ref|NC_002745.2|	431235 +	A	0	3	0	1	1	SA0373
gi|29165615|ref|NC_002745.2|	431239 +	A	0	0	1	1	0	SA0373
gi|29165615|ref|NC_002745.2|	431246 +	U	0	1	0	0	1	SA0373
gi|29165615|ref|NC_002745.2|	431269 +	A	0	1	0	0	0	SA0373
gi|29165615|ref|NC_002745.2|	431279 +	G	0	2	0	0	0	SA0373
gi|29165615|ref|NC_002745.2|	431286 +	A	0	0	0	0	1	SA0373
gi|29165615|ref|NC_002745.2|	431293 +	U	0	0	0	0	1	SA0373
gi|29165615|ref|NC_002745.2|	431294 +	A	0	0	1	0	0	SA0373
gi|29165615|ref|NC_002745.2|	431300 +	U	0	0	0	1	0	SA0373
gi|29165615|ref|NC_002745.2|	431302 +	A	0	0	0	1	0	SA0373
gi|29165615|ref|NC_002745.2|	431305 +	C	4	0	0	0	0	SA0373
gi|29165615|ref|NC_002745.2|	431308 +	C	1	0	0	0	0	SA0373
gi|29165615|ref|NC_002745.2|	431309 +	C	0	1	0	0	0	SA0373
gi|29165615|ref|NC_002745.2|	431314 +	U	0	1	0	0	0	SA0373
gi|29165615|ref|NC_002745.2|	431318 +	A	0	0	1	0	0	SA0373
gi|29165615|ref|NC_002745.2|	431323 +	C	1	0	0	0	0	SA0373
gi|29165615|ref|NC_002745.2|	431326 +	U	2	0	0	0	0	SA0373
gi|29165615|ref|NC_002745.2|	431327 +	C	2	0	0	0	0	SA0373
gi|29165615|ref|NC_002745.2|	431328 +	A	0	0	0	0	1	SA0373
gi|29165615|ref|NC_002745.2|	431332 +	C	0	0	0	0	1	SA0373
gi|29165615|ref|NC_002745.2|	431338 +	U	0	1	0	0	0	SA0373
gi|29165615|ref|NC_002745.2|	431342 +	U	0	1	0	0	0	SA0373
gi|29165615|ref|NC_002745.2|	431398 +	G	0	1	0	0	0	SA0373
gi|29165615|ref|NC_002745.2|	431403 +	U	0	1	0	0	0	SA0373
gi|29165615|ref|NC_002745.2|	431440 +	C	0	0	1	0	0	SA0373
gi|29165615|ref|NC_002745.2|	431445 +	A	0	0	0	1	1	SA0373
gi|29165615|ref|NC_002745.2|	431458 +	C	0	0	0	0	1	SA0373
gi|29165615|ref|NC_002745.2|	431472 +	U	0	0	0	0	1	SA0373
gi|29165615|ref|NC_002745.2|	431473 +	C	1	0	0	0	1	SA0373

gi|29165615|ref|NC_002745.2|	431476	+	A	1	0	0	0	1	SA0373
gi|29165615|ref|NC_002745.2|	431515	+	A	0	1	0	0	0	SA0373
gi|29165615|ref|NC_002745.2|	431525	+	U	0	1	0	0	0	SA0373
gi|29165615|ref|NC_002745.2|	431553	+	C	0	0	0	1	0	SA0373
gi|29165615|ref|NC_002745.2|	431564	+	U	0	1	1	0	0	SA0373
gi|29165615|ref|NC_002745.2|	431574	+	G	0	0	2	0	0	SA0373
gi|29165615|ref|NC_002745.2|	431584	+	U	1	0	0	0	0	SA0373
gi|29165615|ref|NC_002745.2|	431596	+	A	0	1	0	0	0	SA0373
gi|29165615|ref|NC_002745.2|	431632	+	C	0	0	0	1	0	SA0373
gi|29165615|ref|NC_002745.2|	431635	+	G	0	0	1	0	0	SA0373
gi|29165615|ref|NC_002745.2|	431636	+	U	0	0	0	1	0	SA0373
gi|29165615|ref|NC_002745.2|	431638	+	U	1	0	0	0	0	SA0373
gi|29165615|ref|NC_002745.2|	431650	+	C	2	0	0	0	0	SA0373
gi|29165615|ref|NC_002745.2|	431678	+	A	0	0	0	1	0	SA0373
gi|29165615|ref|NC_002745.2|	431700	+	G	0	0	0	0	1	SA0373
gi|29165615|ref|NC_002745.2|	431711	+	A	0	1	0	0	1	SA0374
gi|29165615|ref|NC_002745.2|	431714	+	A	0	4	1	0	2	SA0374
gi|29165615|ref|NC_002745.2|	431717	+	U	0	0	0	0	1	SA0374
gi|29165615|ref|NC_002745.2|	431719	+	A	0	1	0	0	0	SA0374
gi|29165615|ref|NC_002745.2|	431728	+	U	0	0	0	1	0	SA0374
gi|29165615|ref|NC_002745.2|	431730	+	U	0	0	1	0	2	SA0374
gi|29165615|ref|NC_002745.2|	431731	+	U	0	0	0	1	1	SA0374
gi|29165615|ref|NC_002745.2|	431732	+	C	0	0	2	0	1	SA0374
gi|29165615|ref|NC_002745.2|	431734	+	A	0	1	0	1	0	SA0374
gi|29165615|ref|NC_002745.2|	431738	+	C	0	0	0	1	0	SA0374
gi|29165615|ref|NC_002745.2|	431739	+	U	0	0	1	0	0	SA0374
gi|29165615|ref|NC_002745.2|	431742	+	U	2	0	0	0	0	SA0374
gi|29165615|ref|NC_002745.2|	431744	+	G	0	0	1	0	0	SA0374
gi|29165615|ref|NC_002745.2|	431746	+	U	0	1	0	0	0	SA0374
gi|29165615|ref|NC_002745.2|	431761	+	C	0	0	0	0	1	SA0374
gi|29165615|ref|NC_002745.2|	431765	+	U	0	0	0	0	1	SA0374
gi|29165615|ref|NC_002745.2|	431768	+	G	0	1	2	0	0	SA0374
gi|29165615|ref|NC_002745.2|	431793	+	U	0	0	0	1	0	SA0374
gi|29165615|ref|NC_002745.2|	431795	+	A	0	0	1	0	1	SA0374
gi|29165615|ref|NC_002745.2|	431812	+	A	0	0	1	0	0	SA0374
gi|29165615|ref|NC_002745.2|	431813	+	A	0	1	0	0	2	SA0374
gi|29165615|ref|NC_002745.2|	431814	+	U	1	0	0	0	0	SA0374
gi|29165615|ref|NC_002745.2|	431817	+	C	1	0	1	0	0	SA0374
gi|29165615|ref|NC_002745.2|	431821	+	C	1	0	0	0	0	SA0374
gi|29165615|ref|NC_002745.2|	431835	+	A	0	1	4	2	1	SA0374
gi|29165615|ref|NC_002745.2|	431837	+	A	0	0	1	0	0	SA0374
gi|29165615|ref|NC_002745.2|	431838	+	U	0	0	1	0	1	SA0374
gi|29165615|ref|NC_002745.2|	431840	+	U	0	2	0	0	0	SA0374
gi|29165615|ref|NC_002745.2|	431843	+	A	1	0	0	0	0	SA0374
gi|29165615|ref|NC_002745.2|	431862	+	U	0	0	0	0	1	SA0374
gi|29165615|ref|NC_002745.2|	431863	+	U	0	0	0	0	1	SA0374
gi|29165615|ref|NC_002745.2|	431874	+	A	0	0	0	0	2	SA0374
gi|29165615|ref|NC_002745.2|	431876	+	A	0	1	0	0	0	SA0374
gi|29165615|ref|NC_002745.2|	431878	+	A	0	0	1	1	0	SA0374
gi|29165615|ref|NC_002745.2|	431879	+	G	1	0	0	0	0	SA0374
gi|29165615|ref|NC_002745.2|	431894	+	U	0	1	0	0	0	SA0374
gi|29165615|ref|NC_002745.2|	431897	+	C	0	0	0	0	1	SA0374
gi|29165615|ref|NC_002745.2|	431899	+	A	1	0	0	0	0	SA0374
gi|29165615|ref|NC_002745.2|	431900	+	A	1	1	0	0	0	SA0374
gi|29165615|ref|NC_002745.2|	431901	+	U	0	1	0	0	0	SA0374
gi|29165615|ref|NC_002745.2|	431906	+	C	1	0	0	0	0	SA0374
gi|29165615|ref|NC_002745.2|	431916	+	C	0	0	1	0	0	SA0374
gi|29165615|ref|NC_002745.2|	431917	+	A	2	0	0	0	1	SA0374
gi|29165615|ref|NC_002745.2|	431941	+	U	1	0	0	0	0	SA0374
gi|29165615|ref|NC_002745.2|	431945	+	A	0	1	0	0	0	SA0374
gi|29165615|ref|NC_002745.2|	431984	+	C	0	0	0	1	0	SA0374
gi|29165615|ref|NC_002745.2|	432045	+	U	0	0	0	0	1	SA0374
gi|29165615|ref|NC_002745.2|	432050	+	C	1	0	0	0	0	SA0374
gi|29165615|ref|NC_002745.2|	432051	+	C	1	0	0	0	0	SA0374
gi|29165615|ref|NC_002745.2|	432053	+	C	1	0	0	0	0	SA0374
gi|29165615|ref|NC_002745.2|	432062	+	A	0	0	0	0	1	SA0374
gi|29165615|ref|NC_002745.2|	432063	+	C	1	0	0	0	0	SA0374
gi|29165615|ref|NC_002745.2|	432064	+	G	0	1	0	0	0	SA0374
gi|29165615|ref|NC_002745.2|	432068	+	A	0	1	0	0	0	SA0374
gi|29165615|ref|NC_002745.2|	432070	+	U	2	0	0	0	0	SA0374
gi|29165615|ref|NC_002745.2|	432075	+	U	0	0	1	0	0	SA0374
gi|29165615|ref|NC_002745.2|	432082	+	C	1	0	0	0	0	SA0374
gi|29165615|ref|NC_002745.2|	432102	+	C	0	0	0	1	0	SA0374
gi|29165615|ref|NC_002745.2|	432109	+	A	0	0	1	0	0	SA0374
gi|29165615|ref|NC_002745.2|	432111	+	U	1	0	0	0	0	SA0374
gi|29165615|ref|NC_002745.2|	432117	+	A	1	0	0	0	0	SA0374
gi|29165615|ref|NC_002745.2|	432123	+	C	1	0	0	2	0	SA0374
gi|29165615|ref|NC_002745.2|	432124	+	U	0	0	0	0	1	SA0374
gi|29165615|ref|NC_002745.2|	432138	+	C	0	0	1	0	0	SA0374
gi|29165615|ref|NC_002745.2|	432141	+	A	0	0	0	0	3	SA0374
gi|29165615|ref|NC_002745.2|	432142	+	G	0	0	1	0	0	SA0374

gi|29165615|ref|NC_002745.2|	432147	+	A	0	0	0	0	1	SA0374
gi|29165615|ref|NC_002745.2|	432177	+	U	1	0	0	0	0	SA0374
gi|29165615|ref|NC_002745.2|	432182	+	A	0	0	0	1	0	SA0374
gi|29165615|ref|NC_002745.2|	432188	+	A	0	0	0	2	1	SA0374
gi|29165615|ref|NC_002745.2|	432190	+	C	0	1	0	0	0	SA0374
gi|29165615|ref|NC_002745.2|	432191	+	A	0	3	0	0	0	SA0374
gi|29165615|ref|NC_002745.2|	432199	+	U	0	0	0	0	1	SA0374
gi|29165615|ref|NC_002745.2|	432222	+	G	1	1	3	1	3	SA0374
gi|29165615|ref|NC_002745.2|	432223	+	A	0	0	0	0	1	SA0374
gi|29165615|ref|NC_002745.2|	432224	+	U	0	0	2	0	2	SA0374
gi|29165615|ref|NC_002745.2|	432225	+	U	0	1	0	0	0	SA0374
gi|29165615|ref|NC_002745.2|	432226	+	U	0	0	0	1	0	SA0374
gi|29165615|ref|NC_002745.2|	432227	+	A	0	1	2	1	0	SA0374
gi|29165615|ref|NC_002745.2|	432228	+	U	0	0	1	0	0	SA0374
gi|29165615|ref|NC_002745.2|	432229	+	U	0	0	0	1	0	SA0374
gi|29165615|ref|NC_002745.2|	432230	+	A	0	0	2	1	1	SA0374
gi|29165615|ref|NC_002745.2|	432231	+	A	0	0	0	0	1	SA0374
gi|29165615|ref|NC_002745.2|	432233	+	A	0	0	0	1	0	SA0374
gi|29165615|ref|NC_002745.2|	432234	+	G	0	0	1	0	0	SA0374
gi|29165615|ref|NC_002745.2|	432235	+	U	0	1	1	1	2	SA0374
gi|29165615|ref|NC_002745.2|	432236	+	A	0	0	0	0	1	SA0374
gi|29165615|ref|NC_002745.2|	432237	+	U	1	0	0	0	0	SA0374
gi|29165615|ref|NC_002745.2|	432240	+	C	0	2	2	0	0	SA0374
gi|29165615|ref|NC_002745.2|	432243	+	U	0	1	0	0	0	SA0374
gi|29165615|ref|NC_002745.2|	432245	+	U	0	0	2	0	0	SA0374
gi|29165615|ref|NC_002745.2|	432246	+	U	0	3	0	3	0	SA0374
gi|29165615|ref|NC_002745.2|	432247	+	A	0	1	0	0	1	SA0374
gi|29165615|ref|NC_002745.2|	432249	+	U	0	0	0	0	1	SA0374
gi|29165615|ref|NC_002745.2|	432252	+	G	1	0	0	0	0	SA0374
gi|29165615|ref|NC_002745.2|	432253	+	A	0	0	1	0	1	SA0374
gi|29165615|ref|NC_002745.2|	432257	+	G	0	0	0	0	1	SA0374
gi|29165615|ref|NC_002745.2|	432258	+	U	0	1	1	0	0	SA0374
gi|29165615|ref|NC_002745.2|	432260	+	U	0	0	0	0	1	SA0374
gi|29165615|ref|NC_002745.2|	432262	+	A	0	0	0	0	1	SA0374
gi|29165615|ref|NC_002745.2|	432263	+	G	0	1	0	0	0	SA0374
gi|29165615|ref|NC_002745.2|	432265	+	A	0	1	0	0	1	SA0374
gi|29165615|ref|NC_002745.2|	432267	+	C	1	0	1	0	0	SA0374
gi|29165615|ref|NC_002745.2|	432269	+	A	0	0	3	1	1	SA0374
gi|29165615|ref|NC_002745.2|	432270	+	U	0	1	1	1	0	SA0374
gi|29165615|ref|NC_002745.2|	432282	+	U	1	0	0	0	0	SA0374
gi|29165615|ref|NC_002745.2|	432283	+	A	0	0	1	0	0	SA0374
gi|29165615|ref|NC_002745.2|	432284	+	C	0	0	1	0	0	SA0374
gi|29165615|ref|NC_002745.2|	432285	+	U	1	0	0	0	0	SA0374
gi|29165615|ref|NC_002745.2|	432290	+	A	0	0	1	0	0	SA0374
gi|29165615|ref|NC_002745.2|	432302	+	A	1	0	0	0	0	SA0374
gi|29165615|ref|NC_002745.2|	432305	+	C	0	0	0	0	1	SA0374
gi|29165615|ref|NC_002745.2|	432311	+	A	0	0	1	0	0	SA0374
gi|29165615|ref|NC_002745.2|	432316	+	U	0	1	0	0	0	SA0374
gi|29165615|ref|NC_002745.2|	432318	+	C	0	1	0	0	0	SA0374
gi|29165615|ref|NC_002745.2|	432320	+	G	0	0	0	0	1	SA0374
gi|29165615|ref|NC_002745.2|	432322	+	U	0	0	0	0	2	SA0374
gi|29165615|ref|NC_002745.2|	432325	+	G	0	0	0	0	1	SA0374
gi|29165615|ref|NC_002745.2|	432335	+	C	0	1	0	0	0	SA0374
gi|29165615|ref|NC_002745.2|	432336	+	C	1	0	0	0	1	SA0374
gi|29165615|ref|NC_002745.2|	432337	+	A	1	0	0	0	0	SA0374
gi|29165615|ref|NC_002745.2|	432339	+	U	0	0	0	0	1	SA0374
gi|29165615|ref|NC_002745.2|	432342	+	C	1	0	0	0	0	SA0374
gi|29165615|ref|NC_002745.2|	432345	+	U	0	0	0	0	1	SA0374
gi|29165615|ref|NC_002745.2|	432346	+	U	1	0	0	0	0	SA0374
gi|29165615|ref|NC_002745.2|	432348	+	G	0	0	1	0	1	SA0374
gi|29165615|ref|NC_002745.2|	432349	+	A	0	0	0	1	0	SA0374
gi|29165615|ref|NC_002745.2|	432354	+	C	0	0	1	0	0	SA0374
gi|29165615|ref|NC_002745.2|	432358	+	A	1	0	0	0	0	SA0374
gi|29165615|ref|NC_002745.2|	432366	+	U	1	0	0	0	0	SA0374
gi|29165615|ref|NC_002745.2|	432369	+	A	0	1	0	0	1	SA0374
gi|29165615|ref|NC_002745.2|	432372	+	U	0	0	0	0	1	SA0374
gi|29165615|ref|NC_002745.2|	432375	+	C	1	0	0	1	1	SA0374
gi|29165615|ref|NC_002745.2|	432380	+	A	0	0	0	0	1	SA0374
gi|29165615|ref|NC_002745.2|	432384	+	U	0	0	0	0	1	SA0374
gi|29165615|ref|NC_002745.2|	432385	+	A	0	0	0	0	2	SA0374
gi|29165615|ref|NC_002745.2|	432387	+	U	0	0	1	0	0	SA0374
gi|29165615|ref|NC_002745.2|	432389	+	U	0	0	0	0	1	SA0374
gi|29165615|ref|NC_002745.2|	432394	+	U	0	1	0	0	0	SA0374
gi|29165615|ref|NC_002745.2|	432396	+	U	1	0	1	0	1	SA0374
gi|29165615|ref|NC_002745.2|	432398	+	U	0	1	0	0	1	SA0374
gi|29165615|ref|NC_002745.2|	432399	+	U	0	0	0	1	0	SA0374
gi|29165615|ref|NC_002745.2|	432400	+	A	0	1	0	0	0	SA0374
gi|29165615|ref|NC_002745.2|	432405	+	U	0	1	0	0	0	SA0374
gi|29165615|ref|NC_002745.2|	432408	+	U	0	1	0	0	0	SA0374
gi|29165615|ref|NC_002745.2|	432409	+	U	0	1	1	0	0	SA0374
gi|29165615|ref|NC_002745.2|	432410	+	A	0	0	0	0	1	SA0374

gi|29165615|ref|NC_002745.2|	432412	+	U	0	1	1	0	0	SA0374
gi|29165615|ref|NC_002745.2|	432415	+	A	0	0	1	0	0	SA0374
gi|29165615|ref|NC_002745.2|	432422	+	U	0	1	0	0	0	SA0374
gi|29165615|ref|NC_002745.2|	432423	+	C	0	0	1	0	0	SA0374
gi|29165615|ref|NC_002745.2|	432426	+	C	1	0	0	0	0	SA0374
gi|29165615|ref|NC_002745.2|	432446	+	A	0	1	0	0	0	SA0374
gi|29165615|ref|NC_002745.2|	432448	+	U	0	0	1	0	0	SA0374
gi|29165615|ref|NC_002745.2|	432450	+	A	0	1	0	0	0	SA0374
gi|29165615|ref|NC_002745.2|	432464	+	A	0	0	1	0	0	SA0374
gi|29165615|ref|NC_002745.2|	432468	+	U	1	0	0	0	0	SA0374
gi|29165615|ref|NC_002745.2|	432475	+	A	0	0	0	1	0	SA0374
gi|29165615|ref|NC_002745.2|	432476	+	A	1	0	0	0	0	SA0374
gi|29165615|ref|NC_002745.2|	432488	+	A	0	0	0	0	1	SA0374
gi|29165615|ref|NC_002745.2|	432496	+	U	0	1	0	0	0	SA0374
gi|29165615|ref|NC_002745.2|	432501	+	C	2	0	0	0	1	SA0374
gi|29165615|ref|NC_002745.2|	432526	+	U	0	0	1	0	0	SA0374
gi|29165615|ref|NC_002745.2|	432529	+	U	0	1	0	0	0	SA0374
gi|29165615|ref|NC_002745.2|	432531	+	U	0	0	1	0	0	SA0374
gi|29165615|ref|NC_002745.2|	432535	+	C	0	1	0	0	0	SA0374
gi|29165615|ref|NC_002745.2|	432540	+	C	0	0	1	0	0	SA0374
gi|29165615|ref|NC_002745.2|	432602	+	A	0	0	1	0	2	SA0374
gi|29165615|ref|NC_002745.2|	432603	+	A	0	1	3	0	5	SA0374
gi|29165615|ref|NC_002745.2|	432604	+	U	0	0	2	1	0	SA0374
gi|29165615|ref|NC_002745.2|	432605	+	A	0	1	4	2	2	SA0374
gi|29165615|ref|NC_002745.2|	432606	+	A	0	0	1	0	0	SA0374
gi|29165615|ref|NC_002745.2|	432608	+	G	0	0	0	0	1	SA0374
gi|29165615|ref|NC_002745.2|	432609	+	U	0	1	0	0	0	SA0374
gi|29165615|ref|NC_002745.2|	432612	+	U	0	0	0	0	1	SA0374
gi|29165615|ref|NC_002745.2|	432617	+	G	0	0	1	0	0	SA0374
gi|29165615|ref|NC_002745.2|	432623	+	G	0	2	0	0	0	SA0374
gi|29165615|ref|NC_002745.2|	432624	+	U	0	0	0	1	0	SA0374
gi|29165615|ref|NC_002745.2|	432629	+	U	0	0	0	0	1	SA0374
gi|29165615|ref|NC_002745.2|	432630	+	U	0	0	1	0	0	SA0374
gi|29165615|ref|NC_002745.2|	432631	+	A	2	0	0	0	0	SA0374
gi|29165615|ref|NC_002745.2|	432633	+	U	1	0	0	0	0	SA0374
gi|29165615|ref|NC_002745.2|	432638	+	A	0	1	0	0	0	SA0374
gi|29165615|ref|NC_002745.2|	432639	+	U	0	0	0	0	1	SA0374
gi|29165615|ref|NC_002745.2|	432642	+	G	0	1	0	0	0	SA0374
gi|29165615|ref|NC_002745.2|	432643	+	U	0	1	1	0	0	SA0374
gi|29165615|ref|NC_002745.2|	432651	+	U	2	0	0	0	0	SA0374
gi|29165615|ref|NC_002745.2|	432654	+	C	1	0	0	0	0	SA0374
gi|29165615|ref|NC_002745.2|	432655	+	U	0	1	0	0	1	SA0374
gi|29165615|ref|NC_002745.2|	432689	+	C	0	1	1	0	0	SA0374
gi|29165615|ref|NC_002745.2|	432692	+	G	0	0	1	0	0	SA0374
gi|29165615|ref|NC_002745.2|	432693	+	U	0	0	1	0	0	SA0374
gi|29165615|ref|NC_002745.2|	432695	+	U	0	0	0	1	0	SA0374
gi|29165615|ref|NC_002745.2|	432705	+	C	0	0	2	0	0	SA0374
gi|29165615|ref|NC_002745.2|	432715	+	U	0	1	0	0	0	SA0374
gi|29165615|ref|NC_002745.2|	432757	+	A	0	1	0	0	1	SA0374
gi|29165615|ref|NC_002745.2|	432758	+	C	0	0	1	0	0	SA0374
gi|29165615|ref|NC_002745.2|	432762	+	U	0	0	0	1	0	SA0374
gi|29165615|ref|NC_002745.2|	432772	+	A	0	0	0	0	2	SA0374
gi|29165615|ref|NC_002745.2|	432773	+	A	0	0	1	0	0	SA0374
gi|29165615|ref|NC_002745.2|	432777	+	A	0	0	0	1	0	SA0374
gi|29165615|ref|NC_002745.2|	432783	+	A	0	1	0	1	0	SA0374
gi|29165615|ref|NC_002745.2|	432788	+	U	0	0	0	2	0	SA0374
gi|29165615|ref|NC_002745.2|	432789	+	U	0	2	0	0	0	SA0374
gi|29165615|ref|NC_002745.2|	432790	+	A	0	0	1	0	0	SA0374
gi|29165615|ref|NC_002745.2|	432791	+	A	0	0	1	0	0	SA0374
gi|29165615|ref|NC_002745.2|	432792	+	U	0	1	0	0	1	SA0374
gi|29165615|ref|NC_002745.2|	432793	+	U	2	1	0	0	0	SA0374
gi|29165615|ref|NC_002745.2|	432795	+	U	0	0	1	0	0	SA0374
gi|29165615|ref|NC_002745.2|	432798	+	C	2	0	0	0	0	SA0374
gi|29165615|ref|NC_002745.2|	432812	+	U	0	1	0	0	0	SA0374
gi|29165615|ref|NC_002745.2|	432816	+	G	0	1	0	0	0	SA0374
gi|29165615|ref|NC_002745.2|	432822	+	G	0	0	0	1	0	SA0374
gi|29165615|ref|NC_002745.2|	432831	+	C	1	0	0	0	0	SA0374
gi|29165615|ref|NC_002745.2|	432839	+	C	0	0	0	1	1	SA0374
gi|29165615|ref|NC_002745.2|	432840	+	A	0	1	0	0	0	SA0374
gi|29165615|ref|NC_002745.2|	432841	+	A	0	0	1	0	1	SA0374
gi|29165615|ref|NC_002745.2|	432842	+	G	0	0	2	0	0	SA0374
gi|29165615|ref|NC_002745.2|	432843	+	C	0	0	1	1	0	SA0374
gi|29165615|ref|NC_002745.2|	432844	+	A	0	0	0	0	1	SA0374
gi|29165615|ref|NC_002745.2|	432859	+	U	0	1	0	1	0	SA0374
gi|29165615|ref|NC_002745.2|	432860	+	G	0	0	0	0	1	SA0374
gi|29165615|ref|NC_002745.2|	432923	+	A	0	0	1	0	0	SA0374
gi|29165615|ref|NC_002745.2|	432928	+	C	0	0	1	0	0	SA0374
gi|29165615|ref|NC_002745.2|	432929	+	U	1	0	0	0	1	SA0374
gi|29165615|ref|NC_002745.2|	432930	+	U	0	0	1	0	3	SA0374
gi|29165615|ref|NC_002745.2|	432932	+	U	0	0	0	0	1	SA0374
gi|29165615|ref|NC_002745.2|	432939	+	G	0	0	0	0	1	SA0374

gi|29165615|ref|NC_002745.2|	432940 +	A	0	0	0	1	0	SA0374
gi|29165615|ref|NC_002745.2|	432942 +	U	1	0	0	0	0	SA0374
gi|29165615|ref|NC_002745.2|	432945 +	A	0	1	1	0	0	SA0374
gi|29165615|ref|NC_002745.2|	432946 +	G	1	0	1	0	0	SA0374
gi|29165615|ref|NC_002745.2|	432950 +	A	0	1	1	0	1	SA0374
gi|29165615|ref|NC_002745.2|	432951 +	A	0	0	2	0	0	SA0374
gi|29165615|ref|NC_002745.2|	432953 +	C	0	0	0	1	0	SA0374
gi|29165615|ref|NC_002745.2|	432954 +	A	0	1	0	1	0	SA0374
gi|29165615|ref|NC_002745.2|	432955 +	A	1	1	1	1	1	SA0374
gi|29165615|ref|NC_002745.2|	432957 +	C	0	1	0	0	0	SA0374
gi|29165615|ref|NC_002745.2|	432959 +	G	0	2	0	1	0	SA0374
gi|29165615|ref|NC_002745.2|	432960 +	A	0	1	1	1	1	SA0374
gi|29165615|ref|NC_002745.2|	432961 +	A	0	0	1	0	0	SA0374
gi|29165615|ref|NC_002745.2|	432962 +	G	0	3	1	0	1	SA0374
gi|29165615|ref|NC_002745.2|	432963 +	A	0	0	0	1	0	SA0374
gi|29165615|ref|NC_002745.2|	432965 +	A	0	2	3	0	0	SA0374
gi|29165615|ref|NC_002745.2|	432966 +	A	0	0	2	1	0	SA0374
gi|29165615|ref|NC_002745.2|	432970 +	G	0	0	3	0	0	SA0374
gi|29165615|ref|NC_002745.2|	432971 +	A	0	1	0	0	0	SA0374
gi|29165615|ref|NC_002745.2|	432972 +	A	0	1	0	0	0	SA0374
gi|29165615|ref|NC_002745.2|	432974 +	U	0	0	0	1	0	SA0374
gi|29165615|ref|NC_002745.2|	432975 +	A	0	3	2	0	0	SA0374
gi|29165615|ref|NC_002745.2|	432978 +	A	0	2	4	4	1	-
gi|29165615|ref|NC_002745.2|	432979 +	U	0	0	0	0	1	-
gi|29165615|ref|NC_002745.2|	432980 +	A	0	0	1	3	0	-
gi|29165615|ref|NC_002745.2|	432982 +	C	0	0	0	3	2	-
gi|29165615|ref|NC_002745.2|	432983 +	U	0	0	0	1	0	-
gi|29165615|ref|NC_002745.2|	432984 +	A	9	54	32	32	41	-
gi|29165615|ref|NC_002745.2|	432985 +	A	0	3	3	3	2	-
gi|29165615|ref|NC_002745.2|	432986 +	U	0	0	0	0	1	-
gi|29165615|ref|NC_002745.2|	432988 +	A	1	1	6	2	3	-
gi|29165615|ref|NC_002745.2|	432989 +	A	0	1	1	0	0	-
gi|29165615|ref|NC_002745.2|	432992 +	U	0	1	0	0	1	-
gi|29165615|ref|NC_002745.2|	432993 +	G	0	0	0	1	1	-
gi|29165615|ref|NC_002745.2|	432994 +	A	0	0	4	2	1	-
gi|29165615|ref|NC_002745.2|	432995 +	A	0	8	1	0	2	-
gi|29165615|ref|NC_002745.2|	432996 +	A	0	2	0	1	0	-
gi|29165615|ref|NC_002745.2|	432999 +	U	0	1	0	0	0	-
gi|29165615|ref|NC_002745.2|	433000 +	G	0	1	0	0	0	-
gi|29165615|ref|NC_002745.2|	433001 +	G	1	1	0	2	2	-
gi|29165615|ref|NC_002745.2|	433002 +	A	0	2	0	0	1	-
gi|29165615|ref|NC_002745.2|	433003 +	G	1	0	0	0	0	-
gi|29165615|ref|NC_002745.2|	433005 +	C	0	0	0	0	1	-
gi|29165615|ref|NC_002745.2|	433007 +	G	0	0	0	0	1	-
gi|29165615|ref|NC_002745.2|	433008 +	U	0	0	1	1	0	-
gi|29165615|ref|NC_002745.2|	433009 +	U	3	0	0	0	0	-
gi|29165615|ref|NC_002745.2|	433010 +	U	0	1	0	0	0	-
gi|29165615|ref|NC_002745.2|	433012 +	U	2	0	0	0	0	-
gi|29165615|ref|NC_002745.2|	433021 +	A	0	0	1	0	0	SA0375
gi|29165615|ref|NC_002745.2|	433025 +	U	0	0	0	0	1	SA0375
gi|29165615|ref|NC_002745.2|	433028 +	A	0	2	0	0	2	SA0375
gi|29165615|ref|NC_002745.2|	433041 +	U	0	0	0	1	0	SA0375
gi|29165615|ref|NC_002745.2|	433045 +	U	0	0	1	0	0	SA0375
gi|29165615|ref|NC_002745.2|	433047 +	A	0	0	0	0	1	SA0375
gi|29165615|ref|NC_002745.2|	433052 +	U	0	1	0	0	0	SA0375
gi|29165615|ref|NC_002745.2|	433071 +	C	0	1	0	0	0	SA0375
gi|29165615|ref|NC_002745.2|	433141 +	A	0	1	0	0	0	SA0375
gi|29165615|ref|NC_002745.2|	433142 +	U	1	0	0	0	0	SA0375
gi|29165615|ref|NC_002745.2|	433150 +	U	2	0	0	0	0	SA0375
gi|29165615|ref|NC_002745.2|	433151 +	U	1	0	0	0	0	SA0375
gi|29165615|ref|NC_002745.2|	433152 +	A	0	0	1	0	0	SA0375
gi|29165615|ref|NC_002745.2|	433153 +	U	1	0	0	0	1	SA0375
gi|29165615|ref|NC_002745.2|	433156 +	C	3	0	0	0	1	SA0375
gi|29165615|ref|NC_002745.2|	433177 +	C	0	0	0	1	0	SA0375
gi|29165615|ref|NC_002745.2|	433183 +	C	1	0	0	0	0	SA0375
gi|29165615|ref|NC_002745.2|	433188 +	A	0	0	0	0	1	SA0375
gi|29165615|ref|NC_002745.2|	433198 +	C	0	0	1	0	0	SA0375
gi|29165615|ref|NC_002745.2|	433203 +	G	0	0	0	0	1	SA0375
gi|29165615|ref|NC_002745.2|	433206 +	C	2	0	0	0	0	SA0375
gi|29165615|ref|NC_002745.2|	433207 +	G	0	0	1	0	0	SA0375
gi|29165615|ref|NC_002745.2|	433208 +	U	1	0	0	0	0	SA0375
gi|29165615|ref|NC_002745.2|	433209 +	C	0	1	0	0	0	SA0375
gi|29165615|ref|NC_002745.2|	433240 +	U	0	0	0	0	1	SA0375
gi|29165615|ref|NC_002745.2|	433246 +	U	1	0	0	0	0	SA0375
gi|29165615|ref|NC_002745.2|	433261 +	A	1	0	0	0	0	SA0375
gi|29165615|ref|NC_002745.2|	433263 +	G	0	1	0	0	0	SA0375
gi|29165615|ref|NC_002745.2|	433298 +	C	0	1	0	0	0	SA0375
gi|29165615|ref|NC_002745.2|	433310 +	A	0	1	0	0	1	SA0375
gi|29165615|ref|NC_002745.2|	433312 +	U	1	1	0	0	0	SA0375
gi|29165615|ref|NC_002745.2|	433324 +	C	1	0	0	0	0	SA0375
gi|29165615|ref|NC_002745.2|	433325 +	A	0	0	0	0	1	SA0375

gi|29165615|ref|NC_002745.2|	433327	+	A	0	1	0	0	0	SA0375
gi|29165615|ref|NC_002745.2|	433330	+	A	1	0	0	0	0	SA0375
gi|29165615|ref|NC_002745.2|	433336	+	U	0	1	0	0	0	SA0375
gi|29165615|ref|NC_002745.2|	433337	+	U	1	0	0	0	0	SA0375
gi|29165615|ref|NC_002745.2|	433338	+	U	1	0	0	0	0	SA0375
gi|29165615|ref|NC_002745.2|	433341	+	G	1	0	0	0	0	SA0375
gi|29165615|ref|NC_002745.2|	433343	+	A	0	1	0	1	0	SA0375
gi|29165615|ref|NC_002745.2|	433347	+	G	0	1	0	1	0	SA0375
gi|29165615|ref|NC_002745.2|	433348	+	A	0	1	0	3	0	SA0375
gi|29165615|ref|NC_002745.2|	433349	+	A	0	1	0	0	0	SA0375
gi|29165615|ref|NC_002745.2|	433350	+	G	0	0	0	1	0	SA0375
gi|29165615|ref|NC_002745.2|	433351	+	C	1	0	0	0	0	SA0375
gi|29165615|ref|NC_002745.2|	433353	+	U	0	1	0	0	0	SA0375
gi|29165615|ref|NC_002745.2|	433354	+	U	0	1	0	0	0	SA0375
gi|29165615|ref|NC_002745.2|	433355	+	A	1	0	0	0	0	SA0375
gi|29165615|ref|NC_002745.2|	433357	+	U	0	1	0	0	0	SA0375
gi|29165615|ref|NC_002745.2|	433367	+	C	0	1	0	0	0	SA0375
gi|29165615|ref|NC_002745.2|	433370	+	U	1	0	0	0	0	SA0375
gi|29165615|ref|NC_002745.2|	433388	+	U	0	1	0	0	0	SA0375
gi|29165615|ref|NC_002745.2|	433410	+	A	0	0	0	0	1	SA0375
gi|29165615|ref|NC_002745.2|	433412	+	C	0	1	0	0	0	SA0375
gi|29165615|ref|NC_002745.2|	433413	+	U	0	1	0	0	0	SA0375
gi|29165615|ref|NC_002745.2|	433448	+	U	0	1	0	0	0	SA0375
gi|29165615|ref|NC_002745.2|	433467	+	A	0	0	1	0	0	SA0375
gi|29165615|ref|NC_002745.2|	433470	+	A	0	0	1	0	1	SA0375
gi|29165615|ref|NC_002745.2|	433484	+	G	0	0	1	0	0	SA0375
gi|29165615|ref|NC_002745.2|	433495	+	A	0	1	0	0	0	SA0375
gi|29165615|ref|NC_002745.2|	433514	+	G	1	0	0	0	0	SA0375
gi|29165615|ref|NC_002745.2|	433521	+	A	1	0	0	0	2	SA0375
gi|29165615|ref|NC_002745.2|	433534	+	C	0	0	0	1	0	SA0375
gi|29165615|ref|NC_002745.2|	433535	+	A	0	0	1	0	0	SA0375
gi|29165615|ref|NC_002745.2|	433536	+	G	0	0	0	0	3	SA0375
gi|29165615|ref|NC_002745.2|	433540	+	A	0	0	0	1	0	SA0375
gi|29165615|ref|NC_002745.2|	433542	+	A	0	5	1	0	3	SA0375
gi|29165615|ref|NC_002745.2|	433550	+	A	0	0	0	3	0	SA0375
gi|29165615|ref|NC_002745.2|	433551	+	A	0	1	0	0	0	SA0375
gi|29165615|ref|NC_002745.2|	433555	+	A	0	0	0	0	1	SA0375
gi|29165615|ref|NC_002745.2|	433557	+	A	0	0	1	0	0	SA0375
gi|29165615|ref|NC_002745.2|	433558	+	A	0	2	0	1	2	SA0375
gi|29165615|ref|NC_002745.2|	433559	+	G	0	0	0	1	0	SA0375
gi|29165615|ref|NC_002745.2|	433560	+	A	0	2	1	0	0	SA0375
gi|29165615|ref|NC_002745.2|	433565	+	A	0	1	0	1	0	SA0375
gi|29165615|ref|NC_002745.2|	433566	+	A	0	1	0	0	0	SA0375
gi|29165615|ref|NC_002745.2|	433568	+	G	0	0	1	0	0	SA0375
gi|29165615|ref|NC_002745.2|	433569	+	U	0	0	1	0	1	SA0375
gi|29165615|ref|NC_002745.2|	433570	+	U	0	0	0	0	2	SA0375
gi|29165615|ref|NC_002745.2|	433576	+	U	0	0	2	0	0	SA0375
gi|29165615|ref|NC_002745.2|	433593	+	C	0	0	0	0	1	SA0375
gi|29165615|ref|NC_002745.2|	433594	+	U	1	0	0	0	0	SA0375
gi|29165615|ref|NC_002745.2|	433604	+	U	0	0	0	1	0	SA0375
gi|29165615|ref|NC_002745.2|	433609	+	C	0	1	0	1	0	SA0375
gi|29165615|ref|NC_002745.2|	433614	+	A	0	0	0	1	1	SA0375
gi|29165615|ref|NC_002745.2|	433618	+	A	0	2	0	0	0	SA0375
gi|29165615|ref|NC_002745.2|	433619	+	U	0	1	0	0	0	SA0375
gi|29165615|ref|NC_002745.2|	433624	+	A	0	2	1	1	0	SA0375
gi|29165615|ref|NC_002745.2|	433625	+	A	0	0	0	1	0	SA0375
gi|29165615|ref|NC_002745.2|	433626	+	A	0	0	0	1	0	SA0375
gi|29165615|ref|NC_002745.2|	433630	+	U	1	1	2	2	2	SA0375
gi|29165615|ref|NC_002745.2|	433631	+	U	1	0	0	1	2	SA0375
gi|29165615|ref|NC_002745.2|	433632	+	A	0	0	0	0	1	SA0375
gi|29165615|ref|NC_002745.2|	433633	+	U	0	1	0	0	0	SA0375
gi|29165615|ref|NC_002745.2|	433634	+	C	0	0	0	1	0	SA0375
gi|29165615|ref|NC_002745.2|	433636	+	A	0	3	0	1	0	SA0375
gi|29165615|ref|NC_002745.2|	433637	+	A	0	0	0	0	2	SA0375
gi|29165615|ref|NC_002745.2|	433640	+	C	1	0	0	0	0	SA0375
gi|29165615|ref|NC_002745.2|	433641	+	C	1	0	0	0	0	SA0375
gi|29165615|ref|NC_002745.2|	433647	+	G	0	0	0	1	0	SA0375
gi|29165615|ref|NC_002745.2|	433651	+	C	2	0	0	0	1	SA0375
gi|29165615|ref|NC_002745.2|	433652	+	A	0	1	0	0	0	SA0375
gi|29165615|ref|NC_002745.2|	433653	+	A	0	1	0	0	0	SA0375
gi|29165615|ref|NC_002745.2|	433654	+	A	1	0	0	0	1	SA0375
gi|29165615|ref|NC_002745.2|	433656	+	G	0	0	0	1	0	SA0375
gi|29165615|ref|NC_002745.2|	433659	+	G	0	1	0	0	0	SA0375
gi|29165615|ref|NC_002745.2|	433662	+	C	2	0	0	0	0	SA0375
gi|29165615|ref|NC_002745.2|	433663	+	A	0	1	0	0	0	SA0375
gi|29165615|ref|NC_002745.2|	433667	+	U	1	0	0	0	0	SA0375
gi|29165615|ref|NC_002745.2|	433673	+	A	0	0	1	0	0	SA0375
gi|29165615|ref|NC_002745.2|	433678	+	U	1	0	0	0	0	SA0375
gi|29165615|ref|NC_002745.2|	433681	+	C	1	0	0	0	0	SA0375
gi|29165615|ref|NC_002745.2|	433682	+	C	0	2	0	0	0	SA0375
gi|29165615|ref|NC_002745.2|	433684	+	C	2	0	0	0	0	SA0375

gi|29165615|ref|NC_002745.2|	433696 +	U	1	0	0	0	0	SA0375
gi|29165615|ref|NC_002745.2|	433701 +	A	0	1	0	0	2	SA0375
gi|29165615|ref|NC_002745.2|	433703 +	A	2	0	0	0	1	SA0375
gi|29165615|ref|NC_002745.2|	433705 +	A	0	1	2	4	2	SA0375
gi|29165615|ref|NC_002745.2|	433707 +	A	0	0	0	0	1	SA0375
gi|29165615|ref|NC_002745.2|	433708 +	C	1	2	1	0	0	SA0375
gi|29165615|ref|NC_002745.2|	433711 +	A	2	0	0	0	0	SA0375
gi|29165615|ref|NC_002745.2|	433715 +	U	1	0	0	0	0	SA0375
gi|29165615|ref|NC_002745.2|	433723 +	A	0	0	1	0	0	SA0375
gi|29165615|ref|NC_002745.2|	433724 +	A	0	0	1	0	0	SA0375
gi|29165615|ref|NC_002745.2|	433727 +	A	0	4	0	0	0	SA0375
gi|29165615|ref|NC_002745.2|	433728 +	U	0	2	1	0	3	SA0375
gi|29165615|ref|NC_002745.2|	433733 +	C	1	1	0	0	0	SA0375
gi|29165615|ref|NC_002745.2|	433744 +	U	2	0	0	0	0	SA0375
gi|29165615|ref|NC_002745.2|	433762 +	A	1	0	0	0	0	SA0375
gi|29165615|ref|NC_002745.2|	433775 +	U	0	1	0	0	0	SA0375
gi|29165615|ref|NC_002745.2|	433779 +	U	0	1	0	0	0	SA0375
gi|29165615|ref|NC_002745.2|	433793 +	C	0	0	0	1	0	SA0375
gi|29165615|ref|NC_002745.2|	433797 +	C	0	0	1	0	0	SA0375
gi|29165615|ref|NC_002745.2|	433808 +	U	2	0	0	0	0	SA0375
gi|29165615|ref|NC_002745.2|	433809 +	A	0	0	1	0	0	SA0375
gi|29165615|ref|NC_002745.2|	433811 +	U	0	0	0	1	0	SA0375
gi|29165615|ref|NC_002745.2|	433823 +	C	0	1	0	0	0	SA0375
gi|29165615|ref|NC_002745.2|	433830 +	A	0	1	0	0	0	SA0375
gi|29165615|ref|NC_002745.2|	433834 +	C	0	1	0	0	0	SA0375
gi|29165615|ref|NC_002745.2|	433848 +	A	0	0	0	1	0	SA0375
gi|29165615|ref|NC_002745.2|	433850 +	C	0	0	1	0	0	SA0375
gi|29165615|ref|NC_002745.2|	433852 +	U	1	0	0	0	0	SA0375
gi|29165615|ref|NC_002745.2|	433853 +	A	0	0	0	1	0	SA0375
gi|29165615|ref|NC_002745.2|	433855 +	C	0	0	0	1	0	SA0375
gi|29165615|ref|NC_002745.2|	433857 +	A	0	1	0	0	0	SA0375
gi|29165615|ref|NC_002745.2|	433862 +	A	0	0	1	0	0	SA0375
gi|29165615|ref|NC_002745.2|	433879 +	U	0	1	0	0	0	SA0375
gi|29165615|ref|NC_002745.2|	433882 +	U	2	1	0	3	0	SA0375
gi|29165615|ref|NC_002745.2|	433884 +	G	0	1	0	0	1	SA0375
gi|29165615|ref|NC_002745.2|	433885 +	A	1	0	0	0	0	SA0375
gi|29165615|ref|NC_002745.2|	433888 +	C	1	0	0	0	0	SA0375
gi|29165615|ref|NC_002745.2|	433899 +	A	1	0	0	0	0	SA0375
gi|29165615|ref|NC_002745.2|	433913 +	U	2	0	1	0	0	SA0375
gi|29165615|ref|NC_002745.2|	433914 +	A	1	0	0	0	0	SA0375
gi|29165615|ref|NC_002745.2|	433917 +	G	0	0	1	0	0	SA0375
gi|29165615|ref|NC_002745.2|	433920 +	C	0	0	0	1	0	SA0375
gi|29165615|ref|NC_002745.2|	433924 +	G	1	0	0	0	0	SA0375
gi|29165615|ref|NC_002745.2|	433926 +	U	0	0	0	0	1	SA0375
gi|29165615|ref|NC_002745.2|	433929 +	A	0	1	0	0	0	SA0375
gi|29165615|ref|NC_002745.2|	433930 +	U	1	0	0	0	0	SA0375
gi|29165615|ref|NC_002745.2|	433935 +	A	0	0	1	0	0	SA0375
gi|29165615|ref|NC_002745.2|	433936 +	C	1	0	0	0	1	SA0375
gi|29165615|ref|NC_002745.2|	433948 +	U	1	0	0	0	0	SA0375
gi|29165615|ref|NC_002745.2|	433958 +	U	0	0	0	1	0	SA0375
gi|29165615|ref|NC_002745.2|	433959 +	G	0	1	0	0	0	SA0375
gi|29165615|ref|NC_002745.2|	433969 +	A	0	0	0	0	1	SA0375
gi|29165615|ref|NC_002745.2|	433971 +	A	1	2	0	0	0	SA0375
gi|29165615|ref|NC_002745.2|	433972 +	U	0	0	1	0	0	SA0375
gi|29165615|ref|NC_002745.2|	433977 +	G	0	0	0	1	0	SA0375
gi|29165615|ref|NC_002745.2|	433978 +	C	0	0	2	1	2	SA0375
gi|29165615|ref|NC_002745.2|	433983 +	U	0	0	0	0	1	SA0375
gi|29165615|ref|NC_002745.2|	433986 +	G	1	0	0	0	0	SA0375
gi|29165615|ref|NC_002745.2|	433987 +	A	1	0	0	0	0	SA0375
gi|29165615|ref|NC_002745.2|	434000 +	A	0	0	0	0	1	SA0375
gi|29165615|ref|NC_002745.2|	434001 +	G	0	0	1	0	0	SA0375
gi|29165615|ref|NC_002745.2|	434006 +	C	0	0	0	0	2	SA0375
gi|29165615|ref|NC_002745.2|	434009 +	A	0	0	0	0	2	SA0375
gi|29165615|ref|NC_002745.2|	434010 +	C	0	0	0	1	1	SA0375
gi|29165615|ref|NC_002745.2|	434011 +	A	0	1	0	0	0	SA0375
gi|29165615|ref|NC_002745.2|	434019 +	G	0	0	1	0	0	SA0375
gi|29165615|ref|NC_002745.2|	434023 +	U	1	0	0	2	0	SA0375
gi|29165615|ref|NC_002745.2|	434029 +	C	1	0	0	0	0	SA0375
gi|29165615|ref|NC_002745.2|	434039 +	U	1	0	0	0	0	SA0375
gi|29165615|ref|NC_002745.2|	434045 +	A	0	0	0	0	1	SA0375
gi|29165615|ref|NC_002745.2|	434056 +	A	0	0	0	0	1	SA0375
gi|29165615|ref|NC_002745.2|	434068 +	C	1	0	0	0	0	SA0375
gi|29165615|ref|NC_002745.2|	434074 +	C	0	1	0	0	0	SA0375
gi|29165615|ref|NC_002745.2|	434075 +	U	0	0	0	0	1	SA0375
gi|29165615|ref|NC_002745.2|	434077 +	C	1	0	0	0	0	SA0375
gi|29165615|ref|NC_002745.2|	434079 +	G	0	2	0	0	0	SA0375
gi|29165615|ref|NC_002745.2|	434080 +	G	0	0	0	0	1	SA0375
gi|29165615|ref|NC_002745.2|	434083 +	G	0	0	1	0	0	SA0375
gi|29165615|ref|NC_002745.2|	434095 +	U	1	0	0	0	0	SA0375
gi|29165615|ref|NC_002745.2|	434107 +	U	1	0	0	0	0	SA0375
gi|29165615|ref|NC_002745.2|	434110 +	U	2	0	0	0	0	SA0375

gi|29165615|ref|NC_002745.2|	434112	+	G	0	1	0	0	0	SA0375
gi|29165615|ref|NC_002745.2|	434114	+	A	1	0	0	0	0	SA0375
gi|29165615|ref|NC_002745.2|	434115	+	G	0	0	0	1	0	SA0375
gi|29165615|ref|NC_002745.2|	434117	+	U	1	0	0	0	0	SA0375
gi|29165615|ref|NC_002745.2|	434118	+	A	1	0	0	0	0	SA0375
gi|29165615|ref|NC_002745.2|	434127	+	A	0	1	0	0	0	SA0375
gi|29165615|ref|NC_002745.2|	434128	+	G	1	0	0	0	0	SA0375
gi|29165615|ref|NC_002745.2|	434131	+	C	0	1	0	0	0	SA0375
gi|29165615|ref|NC_002745.2|	434135	+	U	1	0	0	0	0	SA0375
gi|29165615|ref|NC_002745.2|	434137	+	C	1	0	0	0	0	SA0375
gi|29165615|ref|NC_002745.2|	434139	+	A	0	1	0	0	0	SA0375
gi|29165615|ref|NC_002745.2|	434140	+	C	1	0	0	0	0	SA0375
gi|29165615|ref|NC_002745.2|	434143	+	A	0	0	1	0	0	SA0375
gi|29165615|ref|NC_002745.2|	434146	+	U	0	0	0	0	1	SA0375
gi|29165615|ref|NC_002745.2|	434151	+	C	0	0	0	1	1	SA0375
gi|29165615|ref|NC_002745.2|	434152	+	A	0	1	0	0	0	SA0375
gi|29165615|ref|NC_002745.2|	434156	+	U	0	0	0	1	0	SA0375
gi|29165615|ref|NC_002745.2|	434160	+	C	2	0	2	0	0	SA0375
gi|29165615|ref|NC_002745.2|	434161	+	A	0	0	0	0	1	SA0375
gi|29165615|ref|NC_002745.2|	434162	+	A	0	1	0	0	0	SA0375
gi|29165615|ref|NC_002745.2|	434164	+	A	1	0	0	0	0	SA0375
gi|29165615|ref|NC_002745.2|	434165	+	U	0	1	0	0	0	SA0375
gi|29165615|ref|NC_002745.2|	434167	+	A	0	0	0	0	1	SA0375
gi|29165615|ref|NC_002745.2|	434169	+	G	0	0	1	0	0	SA0375
gi|29165615|ref|NC_002745.2|	434172	+	U	1	0	0	0	0	SA0375
gi|29165615|ref|NC_002745.2|	434173	+	A	1	0	0	0	0	SA0375
gi|29165615|ref|NC_002745.2|	434174	+	C	0	0	0	0	1	SA0375
gi|29165615|ref|NC_002745.2|	434176	+	G	1	0	0	0	0	SA0375
gi|29165615|ref|NC_002745.2|	434178	+	G	1	0	0	0	0	SA0375
gi|29165615|ref|NC_002745.2|	434179	+	G	3	0	1	0	1	SA0375
gi|29165615|ref|NC_002745.2|	434181	+	A	1	0	0	0	0	SA0375
gi|29165615|ref|NC_002745.2|	434182	+	U	2	0	0	0	0	SA0375
gi|29165615|ref|NC_002745.2|	434184	+	G	0	1	0	0	0	SA0375
gi|29165615|ref|NC_002745.2|	434185	+	G	1	0	0	0	0	SA0375
gi|29165615|ref|NC_002745.2|	434187	+	U	3	0	0	0	0	SA0375
gi|29165615|ref|NC_002745.2|	434188	+	C	4	0	0	0	0	SA0375
gi|29165615|ref|NC_002745.2|	434189	+	U	1	0	0	0	0	SA0375
gi|29165615|ref|NC_002745.2|	434191	+	U	1	0	0	0	0	SA0375
gi|29165615|ref|NC_002745.2|	434192	+	A	0	0	0	0	1	SA0375
gi|29165615|ref|NC_002745.2|	434195	+	U	1	0	0	0	0	SA0375
gi|29165615|ref|NC_002745.2|	434197	+	C	0	0	0	2	0	SA0375
gi|29165615|ref|NC_002745.2|	434198	+	G	0	2	0	1	1	SA0375
gi|29165615|ref|NC_002745.2|	434199	+	A	0	1	0	0	0	SA0375
gi|29165615|ref|NC_002745.2|	434203	+	A	0	1	0	0	0	SA0375
gi|29165615|ref|NC_002745.2|	434204	+	A	1	0	0	0	0	SA0375
gi|29165615|ref|NC_002745.2|	434206	+	A	0	1	0	0	0	SA0375
gi|29165615|ref|NC_002745.2|	434211	+	U	1	0	0	0	0	SA0375
gi|29165615|ref|NC_002745.2|	434217	+	G	2	0	0	0	0	SA0375
gi|29165615|ref|NC_002745.2|	434220	+	C	1	0	0	0	0	SA0375
gi|29165615|ref|NC_002745.2|	434221	+	G	2	0	0	0	0	SA0375
gi|29165615|ref|NC_002745.2|	434222	+	U	4	1	0	1	0	SA0375
gi|29165615|ref|NC_002745.2|	434223	+	U	0	0	0	1	0	SA0375
gi|29165615|ref|NC_002745.2|	434224	+	A	1	0	1	0	0	SA0375
gi|29165615|ref|NC_002745.2|	434225	+	C	0	0	1	0	0	SA0375
gi|29165615|ref|NC_002745.2|	434226	+	U	0	0	0	0	4	SA0375
gi|29165615|ref|NC_002745.2|	434227	+	U	0	0	2	0	0	SA0375
gi|29165615|ref|NC_002745.2|	434228	+	C	0	0	0	0	1	SA0375
gi|29165615|ref|NC_002745.2|	434229	+	C	1	0	0	0	0	SA0375
gi|29165615|ref|NC_002745.2|	434232	+	G	0	1	0	0	0	SA0375
gi|29165615|ref|NC_002745.2|	434236	+	A	1	0	0	0	0	SA0375
gi|29165615|ref|NC_002745.2|	434237	+	C	0	0	0	1	0	SA0375
gi|29165615|ref|NC_002745.2|	434238	+	A	0	0	0	0	1	SA0375
gi|29165615|ref|NC_002745.2|	434240	+	A	2	0	0	0	0	SA0375
gi|29165615|ref|NC_002745.2|	434241	+	G	1	0	0	0	0	SA0375
gi|29165615|ref|NC_002745.2|	434242	+	C	0	0	0	1	1	SA0375
gi|29165615|ref|NC_002745.2|	434245	+	C	4	0	0	0	1	SA0375
gi|29165615|ref|NC_002745.2|	434246	+	U	3	0	0	0	0	SA0375
gi|29165615|ref|NC_002745.2|	434248	+	A	1	0	0	1	0	SA0375
gi|29165615|ref|NC_002745.2|	434249	+	G	3	0	0	1	0	SA0375
gi|29165615|ref|NC_002745.2|	434250	+	A	0	1	0	0	0	SA0375
gi|29165615|ref|NC_002745.2|	434251	+	A	2	0	0	0	0	SA0375
gi|29165615|ref|NC_002745.2|	434252	+	A	0	0	1	0	2	SA0375
gi|29165615|ref|NC_002745.2|	434253	+	U	0	2	0	1	0	SA0375
gi|29165615|ref|NC_002745.2|	434254	+	U	1	0	1	0	0	SA0375
gi|29165615|ref|NC_002745.2|	434255	+	U	0	1	0	1	1	SA0375
gi|29165615|ref|NC_002745.2|	434257	+	U	8	0	1	0	0	SA0375
gi|29165615|ref|NC_002745.2|	434258	+	U	0	0	0	1	0	SA0375
gi|29165615|ref|NC_002745.2|	434259	+	C	0	0	2	1	0	SA0375
gi|29165615|ref|NC_002745.2|	434260	+	C	1	0	2	0	1	SA0375
gi|29165615|ref|NC_002745.2|	434261	+	U	2	0	0	0	0	SA0375
gi|29165615|ref|NC_002745.2|	434262	+	G	1	0	0	0	0	SA0375

gi|29165615|ref|NC_002745.2|	434263 +	A	0	0	0	1	0	SA0375
gi|29165615|ref|NC_002745.2|	434264 +	A	2	0	0	0	0	SA0375
gi|29165615|ref|NC_002745.2|	434267 +	U	1	0	0	0	0	SA0375
gi|29165615|ref|NC_002745.2|	434268 +	A	0	1	0	0	0	SA0375
gi|29165615|ref|NC_002745.2|	434269 +	U	1	0	0	0	2	SA0375
gi|29165615|ref|NC_002745.2|	434270 +	C	0	0	0	2	0	SA0375
gi|29165615|ref|NC_002745.2|	434271 +	G	0	0	0	0	1	SA0375
gi|29165615|ref|NC_002745.2|	434281 +	C	1	0	0	0	1	SA0375
gi|29165615|ref|NC_002745.2|	434282 +	G	0	1	0	0	0	SA0375
gi|29165615|ref|NC_002745.2|	434283 +	G	0	0	1	0	0	SA0375
gi|29165615|ref|NC_002745.2|	434284 +	C	1	0	0	1	0	SA0375
gi|29165615|ref|NC_002745.2|	434285 +	U	0	0	1	0	0	SA0375
gi|29165615|ref|NC_002745.2|	434286 +	U	0	1	3	0	2	SA0375
gi|29165615|ref|NC_002745.2|	434288 +	U	4	1	0	0	1	SA0375
gi|29165615|ref|NC_002745.2|	434290 +	A	0	0	0	1	0	SA0375
gi|29165615|ref|NC_002745.2|	434291 +	A	0	1	0	0	0	SA0375
gi|29165615|ref|NC_002745.2|	434293 +	G	1	0	0	0	0	SA0375
gi|29165615|ref|NC_002745.2|	434296 +	C	1	0	1	0	0	SA0375
gi|29165615|ref|NC_002745.2|	434297 +	G	1	0	0	0	0	SA0375
gi|29165615|ref|NC_002745.2|	434299 +	U	2	0	1	0	0	SA0375
gi|29165615|ref|NC_002745.2|	434301 +	C	0	0	0	0	2	SA0375
gi|29165615|ref|NC_002745.2|	434302 +	A	0	0	0	0	2	SA0375
gi|29165615|ref|NC_002745.2|	434303 +	A	0	0	1	1	0	SA0375
gi|29165615|ref|NC_002745.2|	434308 +	C	0	0	1	0	0	SA0375
gi|29165615|ref|NC_002745.2|	434310 +	A	0	0	2	0	0	SA0375
gi|29165615|ref|NC_002745.2|	434311 +	U	0	0	2	0	1	SA0375
gi|29165615|ref|NC_002745.2|	434315 +	C	0	0	1	0	1	SA0375
gi|29165615|ref|NC_002745.2|	434316 +	C	1	1	1	0	0	SA0375
gi|29165615|ref|NC_002745.2|	434317 +	A	0	0	0	1	0	SA0375
gi|29165615|ref|NC_002745.2|	434318 +	A	3	0	0	0	0	SA0375
gi|29165615|ref|NC_002745.2|	434319 +	U	0	0	0	0	1	SA0375
gi|29165615|ref|NC_002745.2|	434320 +	U	1	1	0	0	0	SA0375
gi|29165615|ref|NC_002745.2|	434321 +	A	1	0	0	1	1	SA0375
gi|29165615|ref|NC_002745.2|	434322 +	A	1	0	0	1	0	SA0375
gi|29165615|ref|NC_002745.2|	434323 +	U	2	1	0	0	0	SA0375
gi|29165615|ref|NC_002745.2|	434326 +	G	2	0	1	0	0	SA0375
gi|29165615|ref|NC_002745.2|	434327 +	C	2	0	1	0	1	SA0375
gi|29165615|ref|NC_002745.2|	434328 +	G	1	0	0	0	0	SA0375
gi|29165615|ref|NC_002745.2|	434329 +	G	3	0	0	1	0	SA0375
gi|29165615|ref|NC_002745.2|	434331 +	G	0	0	1	0	0	SA0375
gi|29165615|ref|NC_002745.2|	434332 +	U	1	0	0	0	0	SA0375
gi|29165615|ref|NC_002745.2|	434339 +	U	1	0	1	0	0	SA0375
gi|29165615|ref|NC_002745.2|	434342 +	U	2	0	0	0	0	SA0375
gi|29165615|ref|NC_002745.2|	434348 +	U	2	0	0	0	0	SA0375
gi|29165615|ref|NC_002745.2|	434349 +	U	0	0	0	1	0	SA0375
gi|29165615|ref|NC_002745.2|	434351 +	U	0	1	0	1	0	SA0375
gi|29165615|ref|NC_002745.2|	434355 +	G	1	0	0	0	0	SA0375
gi|29165615|ref|NC_002745.2|	434360 +	A	0	1	0	0	0	SA0375
gi|29165615|ref|NC_002745.2|	434368 +	U	2	0	1	0	0	SA0375
gi|29165615|ref|NC_002745.2|	434370 +	A	0	1	0	0	0	SA0375
gi|29165615|ref|NC_002745.2|	434372 +	A	0	2	0	1	1	SA0375
gi|29165615|ref|NC_002745.2|	434373 +	G	0	0	1	0	0	SA0375
gi|29165615|ref|NC_002745.2|	434377 +	U	0	0	0	1	0	SA0375
gi|29165615|ref|NC_002745.2|	434380 +	G	0	0	1	0	0	SA0375
gi|29165615|ref|NC_002745.2|	434381 +	C	2	0	1	0	1	SA0375
gi|29165615|ref|NC_002745.2|	434382 +	G	0	0	0	0	1	SA0375
gi|29165615|ref|NC_002745.2|	434387 +	A	0	0	1	0	1	SA0375
gi|29165615|ref|NC_002745.2|	434389 +	C	0	0	0	0	1	SA0375
gi|29165615|ref|NC_002745.2|	434391 +	C	0	0	0	3	1	SA0375
gi|29165615|ref|NC_002745.2|	434393 +	A	2	0	1	0	1	SA0375
gi|29165615|ref|NC_002745.2|	434394 +	U	0	0	0	1	1	SA0375
gi|29165615|ref|NC_002745.2|	434396 +	U	0	0	0	1	0	SA0375
gi|29165615|ref|NC_002745.2|	434397 +	A	0	0	2	0	0	SA0375
gi|29165615|ref|NC_002745.2|	434399 +	A	0	0	1	0	0	SA0375
gi|29165615|ref|NC_002745.2|	434401 +	G	4	0	0	0	0	SA0375
gi|29165615|ref|NC_002745.2|	434402 +	U	1	0	0	0	0	SA0375
gi|29165615|ref|NC_002745.2|	434404 +	U	1	1	0	0	0	SA0375
gi|29165615|ref|NC_002745.2|	434410 +	C	4	0	0	0	1	SA0375
gi|29165615|ref|NC_002745.2|	434411 +	U	2	0	0	0	0	SA0375
gi|29165615|ref|NC_002745.2|	434413 +	C	1	0	0	0	0	SA0375
gi|29165615|ref|NC_002745.2|	434417 +	U	0	0	0	0	1	SA0375
gi|29165615|ref|NC_002745.2|	434430 +	C	0	0	0	0	1	SA0375
gi|29165615|ref|NC_002745.2|	434434 +	C	0	1	0	0	0	SA0375
gi|29165615|ref|NC_002745.2|	434437 +	A	1	0	0	0	0	SA0375
gi|29165615|ref|NC_002745.2|	434440 +	A	0	1	0	0	0	SA0375
gi|29165615|ref|NC_002745.2|	434445 +	C	0	1	1	0	1	SA0375
gi|29165615|ref|NC_002745.2|	434448 +	A	0	1	0	1	0	SA0375
gi|29165615|ref|NC_002745.2|	434454 +	A	0	0	1	0	0	SA0375
gi|29165615|ref|NC_002745.2|	434457 +	G	0	0	0	1	0	SA0375
gi|29165615|ref|NC_002745.2|	434474 +	A	0	1	0	0	0	SA0375
gi|29165615|ref|NC_002745.2|	434483 +	A	1	0	0	0	0	-

gi|29165615|ref|NC_002745.2|	434492	+	G	0	1	0	1	0	-
gi|29165615|ref|NC_002745.2|	434501	+	A	0	0	0	0	1	-
gi|29165615|ref|NC_002745.2|	434503	+	A	0	2	0	0	1	-
gi|29165615|ref|NC_002745.2|	434511	+	A	1	0	0	0	0	SA0376
gi|29165615|ref|NC_002745.2|	434516	+	A	0	0	1	0	0	SA0376
gi|29165615|ref|NC_002745.2|	434535	+	C	0	0	0	0	1	SA0376
gi|29165615|ref|NC_002745.2|	434537	+	U	2	0	0	0	0	SA0376
gi|29165615|ref|NC_002745.2|	434558	+	A	0	0	1	0	0	SA0376
gi|29165615|ref|NC_002745.2|	434592	+	A	0	0	0	0	1	SA0376
gi|29165615|ref|NC_002745.2|	434600	+	U	2	0	0	0	0	SA0376
gi|29165615|ref|NC_002745.2|	434601	+	U	0	1	0	0	0	SA0376
gi|29165615|ref|NC_002745.2|	434608	+	A	0	0	0	0	1	SA0376
gi|29165615|ref|NC_002745.2|	434609	+	A	0	1	0	1	1	SA0376
gi|29165615|ref|NC_002745.2|	434659	+	A	0	1	0	0	0	SA0376
gi|29165615|ref|NC_002745.2|	434670	+	U	1	0	0	0	0	SA0376
gi|29165615|ref|NC_002745.2|	434678	+	U	0	0	0	0	1	SA0376
gi|29165615|ref|NC_002745.2|	434719	+	A	0	0	0	1	0	SA0376
gi|29165615|ref|NC_002745.2|	434726	+	A	0	0	0	0	1	SA0376
gi|29165615|ref|NC_002745.2|	434727	+	A	0	0	0	1	0	SA0376
gi|29165615|ref|NC_002745.2|	434749	+	U	0	0	2	0	0	SA0376
gi|29165615|ref|NC_002745.2|	434762	+	U	2	0	0	0	0	SA0376
gi|29165615|ref|NC_002745.2|	434765	+	C	0	0	0	0	1	SA0376
gi|29165615|ref|NC_002745.2|	434770	+	U	1	0	0	0	0	SA0376
gi|29165615|ref|NC_002745.2|	434776	+	U	1	0	0	0	0	SA0376
gi|29165615|ref|NC_002745.2|	434786	+	A	0	2	0	0	0	SA0376
gi|29165615|ref|NC_002745.2|	434799	+	A	0	1	0	0	0	SA0376
gi|29165615|ref|NC_002745.2|	434802	+	G	0	1	0	0	0	SA0376
gi|29165615|ref|NC_002745.2|	434803	+	U	3	0	0	0	0	SA0376
gi|29165615|ref|NC_002745.2|	434806	+	A	1	0	0	0	0	SA0376
gi|29165615|ref|NC_002745.2|	434807	+	A	1	0	0	0	0	SA0376
gi|29165615|ref|NC_002745.2|	434808	+	C	0	1	0	0	0	SA0376
gi|29165615|ref|NC_002745.2|	434821	+	G	0	0	0	1	0	SA0376
gi|29165615|ref|NC_002745.2|	434825	+	A	0	0	0	0	1	SA0376
gi|29165615|ref|NC_002745.2|	434841	+	A	0	1	0	0	0	SA0376
gi|29165615|ref|NC_002745.2|	434843	+	U	0	0	0	0	1	SA0376
gi|29165615|ref|NC_002745.2|	434845	+	A	0	0	0	0	1	SA0376
gi|29165615|ref|NC_002745.2|	434850	+	A	0	0	0	1	0	SA0376
gi|29165615|ref|NC_002745.2|	434863	+	U	1	0	0	0	1	SA0376
gi|29165615|ref|NC_002745.2|	434890	+	C	0	0	0	1	0	SA0376
gi|29165615|ref|NC_002745.2|	434893	+	U	0	0	1	0	0	SA0376
gi|29165615|ref|NC_002745.2|	434925	+	A	0	0	1	0	0	SA0376
gi|29165615|ref|NC_002745.2|	434941	+	A	1	0	0	0	0	SA0376
gi|29165615|ref|NC_002745.2|	434960	+	A	1	0	1	0	1	SA0376
gi|29165615|ref|NC_002745.2|	434965	+	C	0	0	0	0	1	SA0376
gi|29165615|ref|NC_002745.2|	434984	+	A	1	0	0	0	0	SA0376
gi|29165615|ref|NC_002745.2|	434985	+	G	0	0	0	0	1	SA0376
gi|29165615|ref|NC_002745.2|	434986	+	A	0	0	1	0	0	SA0376
gi|29165615|ref|NC_002745.2|	434994	+	C	3	0	0	0	0	SA0376
gi|29165615|ref|NC_002745.2|	435001	+	U	1	0	0	0	0	SA0376
gi|29165615|ref|NC_002745.2|	435004	+	A	0	0	0	0	1	SA0376
gi|29165615|ref|NC_002745.2|	435010	+	U	1	0	0	0	0	SA0376
gi|29165615|ref|NC_002745.2|	435014	+	A	0	1	0	0	0	SA0376
gi|29165615|ref|NC_002745.2|	435018	+	C	0	0	0	0	1	SA0376
gi|29165615|ref|NC_002745.2|	435019	+	A	0	0	1	0	0	SA0376
gi|29165615|ref|NC_002745.2|	435022	+	C	1	0	0	0	0	SA0376
gi|29165615|ref|NC_002745.2|	435031	+	G	0	0	0	1	0	SA0376
gi|29165615|ref|NC_002745.2|	435033	+	C	0	0	1	1	0	SA0376
gi|29165615|ref|NC_002745.2|	435038	+	A	0	0	0	0	1	SA0376
gi|29165615|ref|NC_002745.2|	435047	+	U	1	1	0	0	0	SA0376
gi|29165615|ref|NC_002745.2|	435052	+	A	0	0	0	0	1	SA0376
gi|29165615|ref|NC_002745.2|	435056	+	A	1	0	0	0	0	SA0376
gi|29165615|ref|NC_002745.2|	435060	+	A	0	1	0	0	0	SA0376
gi|29165615|ref|NC_002745.2|	435064	+	A	0	0	0	1	0	SA0376
gi|29165615|ref|NC_002745.2|	435067	+	U	1	0	0	0	0	SA0376
gi|29165615|ref|NC_002745.2|	435069	+	G	0	1	0	0	0	SA0376
gi|29165615|ref|NC_002745.2|	435071	+	A	0	0	0	1	0	SA0376
gi|29165615|ref|NC_002745.2|	435073	+	G	0	0	0	0	1	SA0376
gi|29165615|ref|NC_002745.2|	435075	+	C	0	1	0	0	0	SA0376
gi|29165615|ref|NC_002745.2|	435076	+	G	0	1	0	0	0	SA0376
gi|29165615|ref|NC_002745.2|	435085	+	A	0	1	0	0	0	SA0376
gi|29165615|ref|NC_002745.2|	435087	+	U	0	0	0	0	1	SA0376
gi|29165615|ref|NC_002745.2|	435095	+	A	0	0	0	1	0	SA0376
gi|29165615|ref|NC_002745.2|	435098	+	A	2	1	0	0	1	SA0376
gi|29165615|ref|NC_002745.2|	435101	+	G	1	0	0	0	0	SA0376
gi|29165615|ref|NC_002745.2|	435103	+	C	0	0	0	1	0	SA0376
gi|29165615|ref|NC_002745.2|	435110	+	A	1	0	0	0	0	SA0376
gi|29165615|ref|NC_002745.2|	435112	+	A	0	0	0	0	1	SA0376
gi|29165615|ref|NC_002745.2|	435116	+	U	1	0	0	0	0	SA0376
gi|29165615|ref|NC_002745.2|	435119	+	C	1	0	1	0	0	SA0376
gi|29165615|ref|NC_002745.2|	435121	+	A	0	0	1	0	0	SA0376
gi|29165615|ref|NC_002745.2|	435124	+	U	0	1	0	0	0	SA0376

gi|29165615|ref|NC_002745.2|	435126 +	G	0	1	0	0	0	SA0376
gi|29165615|ref|NC_002745.2|	435127 +	A	1	0	1	0	1	SA0376
gi|29165615|ref|NC_002745.2|	435128 +	A	0	0	0	0	1	SA0376
gi|29165615|ref|NC_002745.2|	435129 +	A	0	1	1	0	0	SA0376
gi|29165615|ref|NC_002745.2|	435130 +	U	0	2	0	0	1	SA0376
gi|29165615|ref|NC_002745.2|	435131 +	U	0	1	0	2	0	SA0376
gi|29165615|ref|NC_002745.2|	435132 +	G	1	1	0	0	1	SA0376
gi|29165615|ref|NC_002745.2|	435133 +	A	0	1	0	0	1	SA0376
gi|29165615|ref|NC_002745.2|	435135 +	A	0	0	1	1	1	SA0376
gi|29165615|ref|NC_002745.2|	435136 +	A	1	0	0	0	0	SA0376
gi|29165615|ref|NC_002745.2|	435138 +	A	0	0	1	0	1	SA0376
gi|29165615|ref|NC_002745.2|	435139 +	U	9	0	0	0	0	SA0376
gi|29165615|ref|NC_002745.2|	435140 +	U	2	0	1	1	0	SA0376
gi|29165615|ref|NC_002745.2|	435141 +	C	0	1	0	0	1	SA0376
gi|29165615|ref|NC_002745.2|	435143 +	U	8	0	1	0	1	SA0376
gi|29165615|ref|NC_002745.2|	435144 +	C	1	0	2	1	1	SA0376
gi|29165615|ref|NC_002745.2|	435145 +	A	0	0	1	0	0	SA0376
gi|29165615|ref|NC_002745.2|	435147 +	C	0	1	0	0	0	SA0376
gi|29165615|ref|NC_002745.2|	435149 +	C	0	0	1	0	0	SA0376
gi|29165615|ref|NC_002745.2|	435151 +	U	0	1	0	0	0	SA0376
gi|29165615|ref|NC_002745.2|	435155 +	C	3	0	0	0	0	SA0376
gi|29165615|ref|NC_002745.2|	435156 +	G	0	1	0	0	0	SA0376
gi|29165615|ref|NC_002745.2|	435157 +	A	1	0	0	0	0	SA0376
gi|29165615|ref|NC_002745.2|	435160 +	G	0	0	0	0	1	SA0376
gi|29165615|ref|NC_002745.2|	435162 +	C	1	0	0	0	0	SA0376
gi|29165615|ref|NC_002745.2|	435163 +	G	1	0	0	0	0	SA0376
gi|29165615|ref|NC_002745.2|	435166 +	U	3	0	0	0	0	SA0376
gi|29165615|ref|NC_002745.2|	435168 +	U	0	0	1	0	0	SA0376
gi|29165615|ref|NC_002745.2|	435169 +	U	5	0	0	0	1	SA0376
gi|29165615|ref|NC_002745.2|	435170 +	A	2	0	1	0	0	SA0376
gi|29165615|ref|NC_002745.2|	435173 +	U	2	0	0	0	0	SA0376
gi|29165615|ref|NC_002745.2|	435175 +	C	0	0	0	1	0	SA0376
gi|29165615|ref|NC_002745.2|	435176 +	G	0	0	0	0	1	SA0376
gi|29165615|ref|NC_002745.2|	435177 +	A	1	0	0	0	0	SA0376
gi|29165615|ref|NC_002745.2|	435178 +	U	4	0	0	0	0	SA0376
gi|29165615|ref|NC_002745.2|	435182 +	U	3	0	0	0	0	SA0376
gi|29165615|ref|NC_002745.2|	435185 +	U	1	0	0	0	0	SA0376
gi|29165615|ref|NC_002745.2|	435186 +	G	0	0	0	1	0	SA0376
gi|29165615|ref|NC_002745.2|	435188 +	C	0	0	0	0	1	SA0376
gi|29165615|ref|NC_002745.2|	435190 +	U	3	0	1	1	0	SA0376
gi|29165615|ref|NC_002745.2|	435193 +	A	0	1	0	0	0	SA0376
gi|29165615|ref|NC_002745.2|	435194 +	U	0	0	0	0	1	SA0376
gi|29165615|ref|NC_002745.2|	435196 +	C	5	1	2	2	0	SA0376
gi|29165615|ref|NC_002745.2|	435197 +	A	1	0	0	0	1	SA0376
gi|29165615|ref|NC_002745.2|	435198 +	U	0	1	0	0	0	SA0376
gi|29165615|ref|NC_002745.2|	435199 +	C	7	0	0	1	0	SA0376
gi|29165615|ref|NC_002745.2|	435200 +	U	1	1	0	0	1	SA0376
gi|29165615|ref|NC_002745.2|	435201 +	G	0	0	0	0	1	SA0376
gi|29165615|ref|NC_002745.2|	435202 +	U	11	0	0	0	1	SA0376
gi|29165615|ref|NC_002745.2|	435203 +	U	3	1	0	0	0	SA0376
gi|29165615|ref|NC_002745.2|	435204 +	G	3	0	0	0	0	SA0376
gi|29165615|ref|NC_002745.2|	435205 +	U	5	0	1	0	0	SA0376
gi|29165615|ref|NC_002745.2|	435206 +	U	2	0	0	0	0	SA0376
gi|29165615|ref|NC_002745.2|	435209 +	U	0	0	0	0	1	SA0376
gi|29165615|ref|NC_002745.2|	435210 +	G	1	0	0	0	0	SA0376
gi|29165615|ref|NC_002745.2|	435212 +	A	0	0	1	0	0	SA0376
gi|29165615|ref|NC_002745.2|	435214 +	U	0	0	0	1	1	SA0376
gi|29165615|ref|NC_002745.2|	435219 +	C	0	0	0	1	0	SA0376
gi|29165615|ref|NC_002745.2|	435220 +	A	0	1	0	0	0	SA0376
gi|29165615|ref|NC_002745.2|	435221 +	U	0	0	0	1	0	SA0376
gi|29165615|ref|NC_002745.2|	435222 +	A	0	2	0	1	0	SA0376
gi|29165615|ref|NC_002745.2|	435226 +	C	0	0	1	0	0	SA0376
gi|29165615|ref|NC_002745.2|	435227 +	A	1	0	0	0	0	SA0376
gi|29165615|ref|NC_002745.2|	435230 +	U	0	1	0	0	0	SA0376
gi|29165615|ref|NC_002745.2|	435231 +	G	1	0	0	0	0	SA0376
gi|29165615|ref|NC_002745.2|	435234 +	G	1	0	1	0	0	SA0376
gi|29165615|ref|NC_002745.2|	435235 +	A	0	0	0	0	1	SA0376
gi|29165615|ref|NC_002745.2|	435237 +	C	1	1	3	1	1	SA0376
gi|29165615|ref|NC_002745.2|	435238 +	A	0	1	0	0	0	SA0376
gi|29165615|ref|NC_002745.2|	435243 +	A	0	1	0	0	0	SA0376
gi|29165615|ref|NC_002745.2|	435244 +	C	0	0	0	0	1	SA0376
gi|29165615|ref|NC_002745.2|	435246 +	U	0	0	1	0	0	SA0376
gi|29165615|ref|NC_002745.2|	435251 +	C	0	1	0	0	0	SA0376
gi|29165615|ref|NC_002745.2|	435254 +	U	2	0	0	0	0	SA0376
gi|29165615|ref|NC_002745.2|	435255 +	G	1	0	0	0	0	SA0376
gi|29165615|ref|NC_002745.2|	435270 +	C	1	1	0	0	0	SA0376
gi|29165615|ref|NC_002745.2|	435274 +	G	1	0	1	0	0	SA0376
gi|29165615|ref|NC_002745.2|	435275 +	U	0	0	1	0	0	SA0376
gi|29165615|ref|NC_002745.2|	435278 +	A	1	0	0	1	0	SA0376
gi|29165615|ref|NC_002745.2|	435281 +	U	1	0	0	0	0	SA0376
gi|29165615|ref|NC_002745.2|	435282 +	G	0	0	0	0	2	SA0376

gi|29165615|ref|NC_002745.2|	435287	+	C	0	0	0	1	0	SA0376
gi|29165615|ref|NC_002745.2|	435291	+	A	0	0	0	0	1	SA0376
gi|29165615|ref|NC_002745.2|	435303	+	C	0	1	0	0	0	SA0376
gi|29165615|ref|NC_002745.2|	435305	+	A	0	1	0	1	0	SA0376
gi|29165615|ref|NC_002745.2|	435314	+	A	0	1	0	0	0	SA0376
gi|29165615|ref|NC_002745.2|	435321	+	A	0	2	0	0	0	SA0376
gi|29165615|ref|NC_002745.2|	435365	+	U	0	0	0	0	1	SA0376
gi|29165615|ref|NC_002745.2|	435368	+	A	0	1	0	0	0	SA0376
gi|29165615|ref|NC_002745.2|	435387	+	C	0	2	0	0	0	SA0376
gi|29165615|ref|NC_002745.2|	435398	+	A	1	0	0	0	0	SA0376
gi|29165615|ref|NC_002745.2|	435405	+	A	0	3	1	0	0	SA0376
gi|29165615|ref|NC_002745.2|	435419	+	A	0	0	0	0	1	SA0376
gi|29165615|ref|NC_002745.2|	435436	+	A	0	0	0	0	2	SA0376
gi|29165615|ref|NC_002745.2|	435448	+	C	0	0	1	0	0	SA0376
gi|29165615|ref|NC_002745.2|	435468	+	U	0	0	0	1	0	SA0376
gi|29165615|ref|NC_002745.2|	435477	+	C	0	0	1	0	0	SA0376
gi|29165615|ref|NC_002745.2|	435484	+	C	0	0	1	0	0	SA0376
gi|29165615|ref|NC_002745.2|	435493	+	C	0	1	0	0	0	SA0376
gi|29165615|ref|NC_002745.2|	435495	+	G	0	0	0	0	1	SA0376
gi|29165615|ref|NC_002745.2|	435497	+	C	1	0	0	0	0	SA0376
gi|29165615|ref|NC_002745.2|	435507	+	U	1	0	0	0	0	SA0376
gi|29165615|ref|NC_002745.2|	435508	+	C	0	1	1	0	0	SA0376
gi|29165615|ref|NC_002745.2|	435514	+	C	1	0	0	0	0	SA0376
gi|29165615|ref|NC_002745.2|	435517	+	A	0	1	0	0	0	SA0376
gi|29165615|ref|NC_002745.2|	435529	+	C	1	0	0	0	0	SA0376
gi|29165615|ref|NC_002745.2|	435531	+	A	0	0	0	0	1	SA0376
gi|29165615|ref|NC_002745.2|	435538	+	C	0	1	0	0	0	SA0376
gi|29165615|ref|NC_002745.2|	435540	+	C	0	1	0	0	0	SA0376
gi|29165615|ref|NC_002745.2|	435557	+	A	0	0	0	0	1	SA0376
gi|29165615|ref|NC_002745.2|	435560	+	A	0	0	0	1	0	SA0376
gi|29165615|ref|NC_002745.2|	435565	+	A	0	0	0	1	0	SA0376
gi|29165615|ref|NC_002745.2|	435569	+	C	1	0	2	0	1	SA0376
gi|29165615|ref|NC_002745.2|	435570	+	A	1	0	0	0	0	SA0376
gi|29165615|ref|NC_002745.2|	435584	+	A	0	0	0	0	1	SA0376
gi|29165615|ref|NC_002745.2|	435585	+	A	0	0	1	0	1	SA0376
gi|29165615|ref|NC_002745.2|	435594	+	A	0	1	2	0	0	SA0376
gi|29165615|ref|NC_002745.2|	435598	+	A	0	0	0	0	3	SA0376
gi|29165615|ref|NC_002745.2|	435601	+	C	1	0	0	0	1	SA0376
gi|29165615|ref|NC_002745.2|	435602	+	A	0	0	1	2	1	SA0376
gi|29165615|ref|NC_002745.2|	435604	+	U	0	1	0	0	0	SA0376
gi|29165615|ref|NC_002745.2|	435607	+	U	0	3	0	0	0	SA0376
gi|29165615|ref|NC_002745.2|	435608	+	U	0	1	0	0	0	SA0376
gi|29165615|ref|NC_002745.2|	435610	+	A	0	2	0	0	0	SA0376
gi|29165615|ref|NC_002745.2|	435613	+	A	0	0	1	0	0	SA0376
gi|29165615|ref|NC_002745.2|	435626	+	A	0	1	0	0	0	SA0376
gi|29165615|ref|NC_002745.2|	435627	+	U	0	0	0	0	2	SA0376
gi|29165615|ref|NC_002745.2|	435628	+	U	1	0	1	0	0	SA0376
gi|29165615|ref|NC_002745.2|	435629	+	A	1	0	0	0	0	SA0376
gi|29165615|ref|NC_002745.2|	435640	+	U	0	1	0	0	0	SA0376
gi|29165615|ref|NC_002745.2|	435652	+	A	1	0	1	0	0	SA0376
gi|29165615|ref|NC_002745.2|	435656	+	U	1	0	0	0	0	SA0376
gi|29165615|ref|NC_002745.2|	435661	+	U	1	0	0	0	0	SA0376
gi|29165615|ref|NC_002745.2|	435664	+	G	1	0	0	0	0	SA0376
gi|29165615|ref|NC_002745.2|	435679	+	C	0	1	0	0	0	SA0376
gi|29165615|ref|NC_002745.2|	435682	+	G	1	0	0	0	0	SA0376
gi|29165615|ref|NC_002745.2|	435683	+	A	3	0	0	0	0	SA0376
gi|29165615|ref|NC_002745.2|	435684	+	C	0	1	0	0	0	SA0376
gi|29165615|ref|NC_002745.2|	435685	+	C	0	0	0	1	0	SA0376
gi|29165615|ref|NC_002745.2|	435693	+	G	0	0	0	0	1	SA0376
gi|29165615|ref|NC_002745.2|	435702	+	G	0	0	1	1	0	SA0376
gi|29165615|ref|NC_002745.2|	435703	+	U	0	1	0	0	0	SA0376
gi|29165615|ref|NC_002745.2|	435705	+	C	0	1	1	0	1	SA0376
gi|29165615|ref|NC_002745.2|	435709	+	G	0	1	0	0	0	SA0376
gi|29165615|ref|NC_002745.2|	435711	+	G	0	1	0	0	0	SA0376
gi|29165615|ref|NC_002745.2|	435716	+	U	0	0	0	0	1	SA0376
gi|29165615|ref|NC_002745.2|	435717	+	A	0	1	0	0	0	SA0376
gi|29165615|ref|NC_002745.2|	435718	+	C	0	1	0	1	0	SA0376
gi|29165615|ref|NC_002745.2|	435721	+	A	0	0	0	1	1	SA0376
gi|29165615|ref|NC_002745.2|	435724	+	A	0	0	0	0	1	SA0376
gi|29165615|ref|NC_002745.2|	435729	+	C	0	0	1	1	0	SA0376
gi|29165615|ref|NC_002745.2|	435735	+	A	0	3	0	0	0	SA0376
gi|29165615|ref|NC_002745.2|	435738	+	G	0	0	0	0	1	SA0376
gi|29165615|ref|NC_002745.2|	435746	+	A	0	1	0	0	0	SA0376
gi|29165615|ref|NC_002745.2|	435749	+	A	0	0	1	0	0	SA0376
gi|29165615|ref|NC_002745.2|	435760	+	U	1	0	0	0	0	SA0376
gi|29165615|ref|NC_002745.2|	435787	+	U	0	0	0	0	1	SA0376
gi|29165615|ref|NC_002745.2|	435865	+	A	1	0	0	0	0	SA0376
gi|29165615|ref|NC_002745.2|	435870	+	C	0	0	0	0	1	SA0376
gi|29165615|ref|NC_002745.2|	435876	+	G	0	0	0	0	1	SA0376
gi|29165615|ref|NC_002745.2|	435905	+	C	0	2	1	0	0	SA0376
gi|29165615|ref|NC_002745.2|	435946	+	U	0	1	0	0	0	SA0376

gi|29165615|ref|NC_002745.2|	436026	+	A	0	0	1	0	0	SA0376
gi|29165615|ref|NC_002745.2|	436029	+	A	0	0	1	0	0	SA0376
gi|29165615|ref|NC_002745.2|	437402	+	U	0	0	1	1	0	-
gi|29165615|ref|NC_002745.2|	438085	+	A	0	1	0	0	0	-
gi|29165615|ref|NC_002745.2|	438149	+	A	0	1	0	0	0	-
gi|29165615|ref|NC_002745.2|	438177	+	A	0	0	0	0	1	-
gi|29165615|ref|NC_002745.2|	438212	+	U	0	1	0	0	0	-
gi|29165615|ref|NC_002745.2|	438268	+	C	0	0	0	0	2	-
gi|29165615|ref|NC_002745.2|	438304	+	G	0	0	1	0	0	-
gi|29165615|ref|NC_002745.2|	438548	+	A	0	0	0	1	0	-
gi|29165615|ref|NC_002745.2|	438723	+	U	0	1	0	0	0	-
gi|29165615|ref|NC_002745.2|	438774	+	G	0	0	0	0	1	-
gi|29165615|ref|NC_002745.2|	438785	+	U	0	0	0	0	1	-
gi|29165615|ref|NC_002745.2|	439012	+	U	0	1	0	0	0	-
gi|29165615|ref|NC_002745.2|	439813	+	A	0	0	1	0	0	-
gi|29165615|ref|NC_002745.2|	440274	+	A	1	0	0	0	0	-
gi|29165615|ref|NC_002745.2|	441084	+	A	0	1	0	0	0	-
gi|29165615|ref|NC_002745.2|	442444	+	A	0	0	0	1	0	SA0383
gi|29165615|ref|NC_002745.2|	442542	+	G	0	1	0	0	0	SA0383
gi|29165615|ref|NC_002745.2|	442594	+	A	0	0	0	0	1	SA0383
gi|29165615|ref|NC_002745.2|	443057	+	A	0	0	0	0	1	SA0384
gi|29165615|ref|NC_002745.2|	443710	+	A	0	1	0	0	0	SA0384
gi|29165615|ref|NC_002745.2|	443992	+	A	0	6	1	3	0	SA0384
gi|29165615|ref|NC_002745.2|	444611	+	C	0	0	0	0	1	SA0385
gi|29165615|ref|NC_002745.2|	445624	+	A	0	1	0	0	2	-
gi|29165615|ref|NC_002745.2|	446317	+	G	0	2	1	0	3	SA0386
gi|29165615|ref|NC_002745.2|	446318	+	A	0	0	0	0	2	SA0386
gi|29165615|ref|NC_002745.2|	447066	+	A	0	1	0	0	0	SA0387
gi|29165615|ref|NC_002745.2|	447079	+	A	0	0	0	0	1	SA0387
gi|29165615|ref|NC_002745.2|	447245	+	G	1	0	0	0	0	SA0387
gi|29165615|ref|NC_002745.2|	447389	+	A	0	0	1	0	0	SA0387
gi|29165615|ref|NC_002745.2|	447974	+	A	0	0	1	0	0	SA0388
gi|29165615|ref|NC_002745.2|	448202	+	A	0	1	0	0	0	SA0388
gi|29165615|ref|NC_002745.2|	448212	+	G	0	0	0	0	1	SA0388
gi|29165615|ref|NC_002745.2|	448295	+	A	0	1	0	0	0	SA0388
gi|29165615|ref|NC_002745.2|	448301	+	A	0	0	0	0	1	SA0388
gi|29165615|ref|NC_002745.2|	448347	+	A	0	0	0	1	1	SA0388
gi|29165615|ref|NC_002745.2|	448717	+	U	0	0	1	0	1	-
gi|29165615|ref|NC_002745.2|	448728	+	C	0	0	1	0	0	-
gi|29165615|ref|NC_002745.2|	449531	+	A	0	1	0	0	1	SA0389
gi|29165615|ref|NC_002745.2|	450093	+	G	0	0	0	1	0	SA0390
gi|29165615|ref|NC_002745.2|	450221	+	A	0	0	0	1	0	SA0390
gi|29165615|ref|NC_002745.2|	450246	+	G	0	0	0	0	1	SA0390
gi|29165615|ref|NC_002745.2|	450276	+	G	0	0	1	0	0	SA0390
gi|29165615|ref|NC_002745.2|	450290	+	G	0	0	0	0	2	SA0390
gi|29165615|ref|NC_002745.2|	450316	+	U	0	0	0	0	1	SA0390
gi|29165615|ref|NC_002745.2|	450331	+	U	0	1	0	0	0	SA0390
gi|29165615|ref|NC_002745.2|	450390	+	U	0	0	1	0	0	SA0390
gi|29165615|ref|NC_002745.2|	450518	+	G	0	1	0	0	0	SA0390
gi|29165615|ref|NC_002745.2|	450520	+	A	0	0	0	0	1	SA0390
gi|29165615|ref|NC_002745.2|	450521	+	A	0	0	0	1	0	SA0390
gi|29165615|ref|NC_002745.2|	450527	+	A	0	0	0	1	0	SA0390
gi|29165615|ref|NC_002745.2|	450529	+	A	0	0	1	0	0	SA0390
gi|29165615|ref|NC_002745.2|	450542	+	G	0	0	1	0	0	SA0390
gi|29165615|ref|NC_002745.2|	450575	+	U	0	1	0	0	0	SA0390
gi|29165615|ref|NC_002745.2|	450587	+	A	0	1	0	0	0	SA0390
gi|29165615|ref|NC_002745.2|	450593	+	A	0	0	0	1	0	SA0390
gi|29165615|ref|NC_002745.2|	450597	+	C	1	0	0	0	0	SA0390
gi|29165615|ref|NC_002745.2|	450599	+	A	0	0	1	0	0	SA0390
gi|29165615|ref|NC_002745.2|	450657	+	G	0	0	0	1	0	SA0390
gi|29165615|ref|NC_002745.2|	450658	+	A	0	0	1	0	0	SA0390
gi|29165615|ref|NC_002745.2|	450674	+	A	0	0	1	0	0	SA0390
gi|29165615|ref|NC_002745.2|	450675	+	A	0	0	1	0	0	SA0390
gi|29165615|ref|NC_002745.2|	450733	+	G	0	2	0	0	0	SA0390
gi|29165615|ref|NC_002745.2|	450734	+	U	0	0	1	0	0	SA0390
gi|29165615|ref|NC_002745.2|	450735	+	A	0	0	1	0	0	SA0390
gi|29165615|ref|NC_002745.2|	451364	+	A	0	0	0	0	1	SA0391
gi|29165615|ref|NC_002745.2|	451455	+	C	0	0	2	0	1	SA0391
gi|29165615|ref|NC_002745.2|	451460	+	A	0	0	1	0	0	SA0391
gi|29165615|ref|NC_002745.2|	451462	+	C	0	0	0	0	1	SA0391
gi|29165615|ref|NC_002745.2|	451471	+	C	0	0	0	0	1	SA0391
gi|29165615|ref|NC_002745.2|	451774	+	A	0	1	0	0	0	SA0391
gi|29165615|ref|NC_002745.2|	451828	+	U	0	2	0	0	0	SA0391
gi|29165615|ref|NC_002745.2|	452319	+	C	0	0	0	1	0	SA0391
gi|29165615|ref|NC_002745.2|	452325	+	C	1	1	0	1	0	SA0391
gi|29165615|ref|NC_002745.2|	452341	+	A	0	0	1	0	0	SA0391
gi|29165615|ref|NC_002745.2|	452344	+	G	0	0	0	1	0	SA0391
gi|29165615|ref|NC_002745.2|	452397	+	C	0	1	0	0	0	SA0391
gi|29165615|ref|NC_002745.2|	452400	+	A	1	0	0	0	0	SA0391
gi|29165615|ref|NC_002745.2|	452404	+	A	0	0	0	0	1	SA0391
gi|29165615|ref|NC_002745.2|	452405	+	U	0	0	0	1	1	SA0391

gi|29165615|ref|NC_002745.2|	452623	+	G	0	0	1	0	0	SA0392
gi|29165615|ref|NC_002745.2|	452898	+	A	0	1	0	0	0	SA0392
gi|29165615|ref|NC_002745.2|	453043	+	A	0	1	0	0	0	SA0392
gi|29165615|ref|NC_002745.2|	453152	+	G	1	0	0	0	0	SA0392
gi|29165615|ref|NC_002745.2|	453174	+	C	1	0	0	0	0	SA0392
gi|29165615|ref|NC_002745.2|	453177	+	A	2	0	0	0	0	SA0392
gi|29165615|ref|NC_002745.2|	453192	+	A	1	0	0	0	0	SA0392
gi|29165615|ref|NC_002745.2|	453228	+	A	1	0	1	0	1	SA0392
gi|29165615|ref|NC_002745.2|	453245	+	U	0	1	0	0	0	SA0392
gi|29165615|ref|NC_002745.2|	453247	+	U	0	0	0	0	1	SA0392
gi|29165615|ref|NC_002745.2|	453360	+	A	0	1	0	0	0	SA0392
gi|29165615|ref|NC_002745.2|	453412	+	A	0	0	1	0	0	SA0392
gi|29165615|ref|NC_002745.2|	453460	+	C	0	0	0	1	0	SA0392
gi|29165615|ref|NC_002745.2|	453637	+	G	0	1	0	0	0	SA0392
gi|29165615|ref|NC_002745.2|	453638	+	G	0	0	0	0	1	SA0392
gi|29165615|ref|NC_002745.2|	454082	+	G	5	1	4	0	1	-
gi|29165615|ref|NC_002745.2|	454097	+	C	1	0	0	0	0	-
gi|29165615|ref|NC_002745.2|	454116	+	U	0	0	1	0	0	-
gi|29165615|ref|NC_002745.2|	454119	+	A	0	0	1	0	0	-
gi|29165615|ref|NC_002745.2|	454139	+	A	0	0	1	0	0	-
gi|29165615|ref|NC_002745.2|	454203	+	A	0	0	1	0	0	SA0393
gi|29165615|ref|NC_002745.2|	454411	+	A	1	1	0	0	0	SA0393
gi|29165615|ref|NC_002745.2|	454776	+	G	0	0	0	1	0	SA0393
gi|29165615|ref|NC_002745.2|	454789	+	A	0	1	0	0	0	SA0393
gi|29165615|ref|NC_002745.2|	454797	+	A	0	1	0	0	0	SA0393
gi|29165615|ref|NC_002745.2|	454829	+	A	0	1	0	0	0	-
gi|29165615|ref|NC_002745.2|	454858	+	A	0	0	1	0	0	SA0394
gi|29165615|ref|NC_002745.2|	455109	+	C	0	0	1	0	0	SA0394
gi|29165615|ref|NC_002745.2|	455470	+	U	0	0	1	0	0	SA0394
gi|29165615|ref|NC_002745.2|	455632	+	U	1	0	0	0	0	SA0394
gi|29165615|ref|NC_002745.2|	455857	+	A	0	0	0	0	2	SA0394
gi|29165615|ref|NC_002745.2|	455872	+	U	1	0	0	0	0	SA0394
gi|29165615|ref|NC_002745.2|	455957	+	A	0	2	0	0	0	SA0394
gi|29165615|ref|NC_002745.2|	455971	+	C	1	0	0	0	0	SA0394
gi|29165615|ref|NC_002745.2|	456019	+	A	0	0	0	0	1	SA0394
gi|29165615|ref|NC_002745.2|	458570	+	U	0	0	1	0	0	SA0397
gi|29165615|ref|NC_002745.2|	458585	+	A	0	0	0	0	1	SA0397
gi|29165615|ref|NC_002745.2|	464531	+	G	0	0	0	0	1	-
gi|29165615|ref|NC_002745.2|	464614	+	U	1	0	0	0	0	-
gi|29165615|ref|NC_002745.2|	464615	+	G	2	1	1	0	1	-
gi|29165615|ref|NC_002745.2|	464647	+	U	1	0	0	0	0	-
gi|29165615|ref|NC_002745.2|	464653	+	A	0	1	0	0	0	-
gi|29165615|ref|NC_002745.2|	464660	+	A	0	0	1	0	0	-
gi|29165615|ref|NC_002745.2|	464675	+	A	0	0	0	1	0	-
gi|29165615|ref|NC_002745.2|	464677	+	A	0	1	0	1	0	-
gi|29165615|ref|NC_002745.2|	464708	+	U	0	1	0	0	0	SA0405
gi|29165615|ref|NC_002745.2|	465622	+	A	0	0	1	0	0	SA0406
gi|29165615|ref|NC_002745.2|	465914	+	A	0	0	1	0	0	SA0406
gi|29165615|ref|NC_002745.2|	465938	+	A	0	2	0	0	0	SA0406
gi|29165615|ref|NC_002745.2|	465960	+	A	0	1	0	0	0	SA0406
gi|29165615|ref|NC_002745.2|	466099	+	A	0	0	0	1	0	SA0406
gi|29165615|ref|NC_002745.2|	466686	+	C	0	1	0	0	0	SA0406
gi|29165615|ref|NC_002745.2|	466700	+	C	1	0	0	0	0	SA0406
gi|29165615|ref|NC_002745.2|	466868	+	A	0	0	0	1	0	SA0407
gi|29165615|ref|NC_002745.2|	466869	+	G	0	0	0	1	0	SA0407
gi|29165615|ref|NC_002745.2|	466880	+	U	0	0	0	1	0	SA0407
gi|29165615|ref|NC_002745.2|	466884	+	G	1	0	0	0	0	SA0407
gi|29165615|ref|NC_002745.2|	466948	+	G	1	0	0	0	0	SA0407
gi|29165615|ref|NC_002745.2|	466976	+	U	2	0	0	0	0	SA0407
gi|29165615|ref|NC_002745.2|	466979	+	A	1	0	0	0	0	SA0407
gi|29165615|ref|NC_002745.2|	466986	+	U	1	0	0	0	0	SA0407
gi|29165615|ref|NC_002745.2|	467002	+	A	0	1	0	0	0	SA0407
gi|29165615|ref|NC_002745.2|	467047	+	A	0	0	0	0	1	SA0407
gi|29165615|ref|NC_002745.2|	467120	+	A	1	0	0	0	0	SA0407
gi|29165615|ref|NC_002745.2|	467252	+	A	0	0	0	0	1	SA0408
gi|29165615|ref|NC_002745.2|	467254	+	A	0	0	0	1	0	SA0408
gi|29165615|ref|NC_002745.2|	467333	+	U	0	1	0	0	0	SA0408
gi|29165615|ref|NC_002745.2|	467839	+	A	0	0	0	1	0	-
gi|29165615|ref|NC_002745.2|	468111	+	A	0	0	0	0	1	-
gi|29165615|ref|NC_002745.2|	468368	+	G	0	0	1	0	0	SA0410
gi|29165615|ref|NC_002745.2|	468486	+	A	1	0	0	0	0	SA0410
gi|29165615|ref|NC_002745.2|	468538	+	A	0	0	1	0	0	SA0410
gi|29165615|ref|NC_002745.2|	468591	+	U	0	1	0	0	0	SA0410
gi|29165615|ref|NC_002745.2|	468681	+	U	0	0	0	0	1	SA0410
gi|29165615|ref|NC_002745.2|	468685	+	U	0	0	1	0	0	SA0410
gi|29165615|ref|NC_002745.2|	468686	+	A	0	0	0	0	1	SA0410
gi|29165615|ref|NC_002745.2|	468687	+	A	0	1	0	0	0	SA0410
gi|29165615|ref|NC_002745.2|	468699	+	U	0	0	1	0	0	SA0410
gi|29165615|ref|NC_002745.2|	468704	+	G	0	0	0	1	0	SA0410
gi|29165615|ref|NC_002745.2|	468706	+	A	0	0	0	0	1	SA0410
gi|29165615|ref|NC_002745.2|	468721	+	G	0	0	1	0	0	SA0410

gi|29165615|ref|NC_002745.2|	468729	+	A	0	0	0	1	0	SA0410
gi|29165615|ref|NC_002745.2|	468730	+	A	0	0	1	0	0	SA0410
gi|29165615|ref|NC_002745.2|	468769	+	G	0	1	1	2	0	SA0410
gi|29165615|ref|NC_002745.2|	468770	+	A	0	0	0	0	3	SA0410
gi|29165615|ref|NC_002745.2|	468771	+	C	0	1	0	0	0	SA0410
gi|29165615|ref|NC_002745.2|	468801	+	U	0	1	0	0	0	SA0410
gi|29165615|ref|NC_002745.2|	468802	+	C	0	0	1	0	0	SA0410
gi|29165615|ref|NC_002745.2|	468822	+	A	0	0	0	0	1	SA0410
gi|29165615|ref|NC_002745.2|	468829	+	A	0	0	0	1	0	SA0410
gi|29165615|ref|NC_002745.2|	468831	+	A	0	0	0	0	1	SA0410
gi|29165615|ref|NC_002745.2|	468834	+	C	1	0	0	0	0	SA0410
gi|29165615|ref|NC_002745.2|	468835	+	G	0	0	0	1	0	SA0410
gi|29165615|ref|NC_002745.2|	469133	+	A	0	0	0	0	1	SA0410
gi|29165615|ref|NC_002745.2|	469240	+	A	0	1	0	0	0	SA0410
gi|29165615|ref|NC_002745.2|	470642	+	U	3	2	1	0	3	-
gi|29165615|ref|NC_002745.2|	470643	+	U	0	0	0	1	0	-
gi|29165615|ref|NC_002745.2|	470644	+	A	2	17	7	8	24	-
gi|29165615|ref|NC_002745.2|	470645	+	U	0	1	0	1	0	-
gi|29165615|ref|NC_002745.2|	470646	+	A	0	2	3	2	3	-
gi|29165615|ref|NC_002745.2|	470647	+	A	0	0	0	1	1	-
gi|29165615|ref|NC_002745.2|	470648	+	G	0	0	0	0	1	-
gi|29165615|ref|NC_002745.2|	470652	+	A	0	5	0	0	2	-
gi|29165615|ref|NC_002745.2|	470653	+	A	0	5	4	5	6	-
gi|29165615|ref|NC_002745.2|	470654	+	A	1	5	6	8	1	-
gi|29165615|ref|NC_002745.2|	470655	+	A	0	1	5	5	13	-
gi|29165615|ref|NC_002745.2|	470656	+	C	3	12	0	5	9	-
gi|29165615|ref|NC_002745.2|	470657	+	G	0	3	2	1	0	-
gi|29165615|ref|NC_002745.2|	470658	+	A	9	58	54	39	42	-
gi|29165615|ref|NC_002745.2|	470659	+	A	2	26	18	14	21	-
gi|29165615|ref|NC_002745.2|	470660	+	U	0	1	0	0	2	-
gi|29165615|ref|NC_002745.2|	470661	+	A	0	1	2	3	0	-
gi|29165615|ref|NC_002745.2|	470662	+	A	0	0	1	0	0	-
gi|29165615|ref|NC_002745.2|	470663	+	C	3	0	0	2	0	-
gi|29165615|ref|NC_002745.2|	470664	+	A	1	0	0	0	0	-
gi|29165615|ref|NC_002745.2|	470665	+	C	1	0	0	0	0	-
gi|29165615|ref|NC_002745.2|	470680	+	A	0	1	0	0	0	-
gi|29165615|ref|NC_002745.2|	470684	+	A	0	1	0	0	0	-
gi|29165615|ref|NC_002745.2|	470736	+	A	0	1	0	0	1	-
gi|29165615|ref|NC_002745.2|	470810	+	U	1	0	0	0	0	-
gi|29165615|ref|NC_002745.2|	470811	+	A	0	1	0	0	0	-
gi|29165615|ref|NC_002745.2|	470840	+	G	0	0	1	0	0	-
gi|29165615|ref|NC_002745.2|	470841	+	C	2	4	3	1	1	-
gi|29165615|ref|NC_002745.2|	470844	+	U	0	0	1	0	0	-
gi|29165615|ref|NC_002745.2|	470846	+	A	0	0	1	0	0	-
gi|29165615|ref|NC_002745.2|	470847	+	U	0	4	1	1	2	-
gi|29165615|ref|NC_002745.2|	470849	+	U	0	0	1	0	1	-
gi|29165615|ref|NC_002745.2|	470882	+	U	0	0	1	0	0	-
gi|29165615|ref|NC_002745.2|	470883	+	A	0	1	0	0	0	-
gi|29165615|ref|NC_002745.2|	470884	+	C	0	0	0	2	0	-
gi|29165615|ref|NC_002745.2|	470885	+	A	0	1	0	0	1	-
gi|29165615|ref|NC_002745.2|	470886	+	U	1	0	0	0	0	-
gi|29165615|ref|NC_002745.2|	470887	+	A	0	1	0	0	0	-
gi|29165615|ref|NC_002745.2|	470890	+	C	0	1	0	0	0	-
gi|29165615|ref|NC_002745.2|	470892	+	A	0	0	0	0	1	-
gi|29165615|ref|NC_002745.2|	470894	+	U	0	0	1	1	0	-
gi|29165615|ref|NC_002745.2|	470898	+	C	0	2	2	0	0	-
gi|29165615|ref|NC_002745.2|	470900	+	A	0	0	1	0	0	-
gi|29165615|ref|NC_002745.2|	470901	+	G	0	0	0	1	0	-
gi|29165615|ref|NC_002745.2|	470902	+	A	0	1	0	0	1	-
gi|29165615|ref|NC_002745.2|	470903	+	G	0	1	0	0	0	-
gi|29165615|ref|NC_002745.2|	470904	+	A	0	2	1	2	0	-
gi|29165615|ref|NC_002745.2|	470907	+	A	1	0	2	0	0	-
gi|29165615|ref|NC_002745.2|	470908	+	G	2	24	8	4	16	-
gi|29165615|ref|NC_002745.2|	470909	+	A	1	11	5	3	1	-
gi|29165615|ref|NC_002745.2|	470910	+	A	0	2	4	1	0	-
gi|29165615|ref|NC_002745.2|	470912	+	U	0	0	1	0	1	-
gi|29165615|ref|NC_002745.2|	470913	+	A	0	0	1	0	2	-
gi|29165615|ref|NC_002745.2|	470914	+	A	0	2	1	0	1	-
gi|29165615|ref|NC_002745.2|	470915	+	G	0	1	0	0	0	-
gi|29165615|ref|NC_002745.2|	470916	+	G	0	3	1	1	4	-
gi|29165615|ref|NC_002745.2|	470917	+	A	0	0	1	0	0	-
gi|29165615|ref|NC_002745.2|	470918	+	G	0	0	0	1	0	-
gi|29165615|ref|NC_002745.2|	470925	+	C	0	2	0	0	0	-
gi|29165615|ref|NC_002745.2|	470932	+	A	0	1	0	0	0	SA0411
gi|29165615|ref|NC_002745.2|	470945	+	C	0	1	0	0	0	SA0411
gi|29165615|ref|NC_002745.2|	470957	+	U	0	0	0	0	1	SA0411
gi|29165615|ref|NC_002745.2|	470983	+	G	0	0	1	0	0	SA0411
gi|29165615|ref|NC_002745.2|	471008	+	C	0	0	1	0	0	SA0411
gi|29165615|ref|NC_002745.2|	471040	+	U	0	1	0	0	0	SA0411
gi|29165615|ref|NC_002745.2|	471045	+	A	0	1	0	0	0	SA0411
gi|29165615|ref|NC_002745.2|	471046	+	C	0	0	0	0	2	SA0411

gi|29165615|ref|NC_002745.2|	471047	+	A	0	3	2	1	1	SA0411
gi|29165615|ref|NC_002745.2|	471056	+	A	0	0	0	0	1	SA0411
gi|29165615|ref|NC_002745.2|	471063	+	U	0	34	21	2	35	SA0411
gi|29165615|ref|NC_002745.2|	471064	+	A	1	13	4	4	24	SA0411
gi|29165615|ref|NC_002745.2|	471066	+	U	0	0	0	1	0	SA0411
gi|29165615|ref|NC_002745.2|	471067	+	A	0	0	0	0	1	SA0411
gi|29165615|ref|NC_002745.2|	471072	+	C	0	2	0	0	0	SA0411
gi|29165615|ref|NC_002745.2|	471075	+	U	0	0	1	0	1	SA0411
gi|29165615|ref|NC_002745.2|	471081	+	G	0	0	0	1	1	SA0411
gi|29165615|ref|NC_002745.2|	471087	+	U	0	1	0	0	2	SA0411
gi|29165615|ref|NC_002745.2|	471088	+	C	0	1	0	1	3	SA0411
gi|29165615|ref|NC_002745.2|	471089	+	G	0	0	0	1	1	SA0411
gi|29165615|ref|NC_002745.2|	471090	+	G	0	8	3	7	5	SA0411
gi|29165615|ref|NC_002745.2|	471101	+	C	0	0	0	0	2	SA0411
gi|29165615|ref|NC_002745.2|	471102	+	A	0	1	0	0	0	SA0411
gi|29165615|ref|NC_002745.2|	471103	+	A	0	0	0	0	2	SA0411
gi|29165615|ref|NC_002745.2|	471104	+	C	2	0	0	0	0	SA0411
gi|29165615|ref|NC_002745.2|	471108	+	U	0	9	3	1	7	SA0411
gi|29165615|ref|NC_002745.2|	471109	+	C	0	2	0	0	0	SA0411
gi|29165615|ref|NC_002745.2|	471122	+	A	0	2	0	0	1	SA0411
gi|29165615|ref|NC_002745.2|	471125	+	A	0	0	0	0	1	SA0411
gi|29165615|ref|NC_002745.2|	471129	+	A	0	1	0	0	1	SA0411
gi|29165615|ref|NC_002745.2|	471130	+	U	0	1	0	0	0	SA0411
gi|29165615|ref|NC_002745.2|	471133	+	U	0	0	0	0	1	SA0411
gi|29165615|ref|NC_002745.2|	471137	+	U	1	0	0	0	0	SA0411
gi|29165615|ref|NC_002745.2|	471142	+	G	0	2	0	1	1	SA0411
gi|29165615|ref|NC_002745.2|	471143	+	U	0	0	0	0	3	SA0411
gi|29165615|ref|NC_002745.2|	471167	+	C	0	0	0	1	0	SA0411
gi|29165615|ref|NC_002745.2|	471186	+	A	0	1	0	0	0	SA0411
gi|29165615|ref|NC_002745.2|	471310	+	C	0	0	0	0	2	SA0411
gi|29165615|ref|NC_002745.2|	471324	+	U	1	0	0	0	0	SA0411
gi|29165615|ref|NC_002745.2|	471341	+	C	0	0	1	0	0	SA0411
gi|29165615|ref|NC_002745.2|	471372	+	C	0	0	0	0	1	SA0411
gi|29165615|ref|NC_002745.2|	471374	+	C	0	0	0	0	1	SA0411
gi|29165615|ref|NC_002745.2|	471390	+	U	0	1	0	0	0	SA0411
gi|29165615|ref|NC_002745.2|	471393	+	C	1	0	0	0	0	SA0411
gi|29165615|ref|NC_002745.2|	471407	+	U	0	0	1	0	0	SA0411
gi|29165615|ref|NC_002745.2|	471455	+	G	0	0	1	0	0	SA0411
gi|29165615|ref|NC_002745.2|	471479	+	C	0	0	1	0	0	SA0411
gi|29165615|ref|NC_002745.2|	471495	+	C	0	3	0	0	0	SA0411
gi|29165615|ref|NC_002745.2|	471530	+	U	0	0	0	0	1	SA0411
gi|29165615|ref|NC_002745.2|	471564	+	U	0	1	0	0	0	SA0411
gi|29165615|ref|NC_002745.2|	471578	+	A	0	0	1	0	0	SA0411
gi|29165615|ref|NC_002745.2|	471583	+	A	0	0	1	0	0	SA0411
gi|29165615|ref|NC_002745.2|	471590	+	A	0	0	1	0	0	SA0411
gi|29165615|ref|NC_002745.2|	471621	+	A	0	3	0	0	0	SA0411
gi|29165615|ref|NC_002745.2|	471648	+	U	0	1	0	0	0	SA0411
gi|29165615|ref|NC_002745.2|	471672	+	U	0	0	0	0	1	SA0411
gi|29165615|ref|NC_002745.2|	471705	+	U	1	0	0	0	1	SA0411
gi|29165615|ref|NC_002745.2|	471726	+	U	0	0	0	0	1	SA0411
gi|29165615|ref|NC_002745.2|	471727	+	A	0	0	0	1	0	SA0411
gi|29165615|ref|NC_002745.2|	471742	+	U	0	1	0	0	0	SA0411
gi|29165615|ref|NC_002745.2|	471772	+	A	0	0	0	0	1	SA0411
gi|29165615|ref|NC_002745.2|	471813	+	U	0	0	0	0	1	SA0411
gi|29165615|ref|NC_002745.2|	471926	+	A	0	0	0	0	1	SA0411
gi|29165615|ref|NC_002745.2|	471933	+	A	0	0	0	0	1	SA0411
gi|29165615|ref|NC_002745.2|	471961	+	C	0	0	0	0	1	SA0411
gi|29165615|ref|NC_002745.2|	471991	+	U	0	0	0	1	0	SA0411
gi|29165615|ref|NC_002745.2|	471996	+	U	0	0	0	1	0	SA0411
gi|29165615|ref|NC_002745.2|	472161	+	U	0	0	0	0	2	SA0411
gi|29165615|ref|NC_002745.2|	472171	+	C	0	1	0	0	0	SA0411
gi|29165615|ref|NC_002745.2|	472177	+	C	0	0	0	0	2	SA0411
gi|29165615|ref|NC_002745.2|	472179	+	A	0	1	0	0	0	SA0411
gi|29165615|ref|NC_002745.2|	472180	+	U	0	0	0	0	1	SA0411
gi|29165615|ref|NC_002745.2|	472185	+	U	0	0	0	0	2	SA0411
gi|29165615|ref|NC_002745.2|	472188	+	A	0	2	0	0	0	SA0411
gi|29165615|ref|NC_002745.2|	472195	+	A	0	1	0	0	0	SA0411
gi|29165615|ref|NC_002745.2|	472198	+	A	0	0	0	0	1	SA0411
gi|29165615|ref|NC_002745.2|	472200	+	A	0	1	0	0	0	SA0411
gi|29165615|ref|NC_002745.2|	472204	+	U	0	0	0	0	1	SA0411
gi|29165615|ref|NC_002745.2|	472205	+	A	0	0	0	0	3	SA0411
gi|29165615|ref|NC_002745.2|	472224	+	C	1	0	0	0	0	SA0411
gi|29165615|ref|NC_002745.2|	472231	+	U	0	0	0	0	1	SA0411
gi|29165615|ref|NC_002745.2|	472258	+	C	0	1	0	0	0	SA0411
gi|29165615|ref|NC_002745.2|	472260	+	U	1	0	0	1	0	SA0411
gi|29165615|ref|NC_002745.2|	472271	+	G	0	0	0	1	0	SA0411
gi|29165615|ref|NC_002745.2|	472272	+	G	0	5	0	0	1	SA0411
gi|29165615|ref|NC_002745.2|	472273	+	C	0	0	0	1	2	SA0411
gi|29165615|ref|NC_002745.2|	472286	+	A	1	0	0	0	0	SA0411
gi|29165615|ref|NC_002745.2|	472287	+	U	0	0	1	0	0	SA0411
gi|29165615|ref|NC_002745.2|	472293	+	U	1	0	0	0	0	SA0411

gi|29165615|ref|NC_002745.2|	472312 +	U	0	0	0	0	1	SA0411
gi|29165615|ref|NC_002745.2|	472345 +	U	0	0	0	0	1	SA0411
gi|29165615|ref|NC_002745.2|	472347 +	A	0	1	0	0	0	SA0411
gi|29165615|ref|NC_002745.2|	472356 +	A	0	0	0	0	1	SA0411
gi|29165615|ref|NC_002745.2|	472423 +	U	0	0	0	0	1	-
gi|29165615|ref|NC_002745.2|	472446 +	A	0	1	0	0	0	SA0412
gi|29165615|ref|NC_002745.2|	472449 +	C	0	2	0	0	0	SA0412
gi|29165615|ref|NC_002745.2|	472458 +	A	0	0	0	0	1	SA0412
gi|29165615|ref|NC_002745.2|	472473 +	U	0	0	0	0	1	SA0412
gi|29165615|ref|NC_002745.2|	472499 +	A	0	0	1	0	0	SA0412
gi|29165615|ref|NC_002745.2|	472728 +	A	0	0	0	0	1	SA0412
gi|29165615|ref|NC_002745.2|	472731 +	A	0	0	0	0	1	SA0412
gi|29165615|ref|NC_002745.2|	472733 +	A	0	0	0	0	1	SA0412
gi|29165615|ref|NC_002745.2|	472741 +	A	0	0	0	0	1	SA0412
gi|29165615|ref|NC_002745.2|	472763 +	U	0	0	0	1	0	SA0412
gi|29165615|ref|NC_002745.2|	472780 +	U	0	0	0	1	0	SA0412
gi|29165615|ref|NC_002745.2|	472782 +	U	0	0	1	0	0	SA0412
gi|29165615|ref|NC_002745.2|	472794 +	C	0	0	0	0	1	SA0412
gi|29165615|ref|NC_002745.2|	472802 +	C	0	1	0	0	0	SA0412
gi|29165615|ref|NC_002745.2|	472815 +	C	0	0	0	0	2	SA0412
gi|29165615|ref|NC_002745.2|	472847 +	U	0	0	0	1	0	SA0412
gi|29165615|ref|NC_002745.2|	472885 +	A	0	1	0	0	0	SA0412
gi|29165615|ref|NC_002745.2|	472893 +	A	1	0	0	0	0	SA0412
gi|29165615|ref|NC_002745.2|	472903 +	C	0	0	0	0	1	SA0412
gi|29165615|ref|NC_002745.2|	472914 +	A	0	0	1	0	1	SA0412
gi|29165615|ref|NC_002745.2|	472919 +	C	0	0	1	0	0	SA0412
gi|29165615|ref|NC_002745.2|	472921 +	A	0	0	0	0	1	SA0412
gi|29165615|ref|NC_002745.2|	472928 +	A	0	0	0	0	2	SA0412
gi|29165615|ref|NC_002745.2|	472939 +	A	0	2	0	0	0	SA0412
gi|29165615|ref|NC_002745.2|	472940 +	C	0	0	0	0	1	SA0412
gi|29165615|ref|NC_002745.2|	472944 +	A	0	0	0	1	0	SA0412
gi|29165615|ref|NC_002745.2|	472945 +	U	1	0	0	0	0	SA0412
gi|29165615|ref|NC_002745.2|	472950 +	A	0	0	0	1	0	SA0412
gi|29165615|ref|NC_002745.2|	472954 +	A	0	0	0	0	1	SA0412
gi|29165615|ref|NC_002745.2|	472973 +	A	0	1	0	0	0	SA0412
gi|29165615|ref|NC_002745.2|	472985 +	G	0	0	0	0	1	SA0412
gi|29165615|ref|NC_002745.2|	472999 +	C	0	1	0	0	1	SA0412
gi|29165615|ref|NC_002745.2|	473016 +	U	0	0	0	1	0	SA0412
gi|29165615|ref|NC_002745.2|	473027 +	G	0	1	0	0	0	SA0412
gi|29165615|ref|NC_002745.2|	473036 +	U	0	0	0	1	0	SA0412
gi|29165615|ref|NC_002745.2|	473039 +	A	1	0	0	0	0	SA0412
gi|29165615|ref|NC_002745.2|	473040 +	C	0	0	0	0	1	SA0412
gi|29165615|ref|NC_002745.2|	473042 +	A	0	0	0	1	1	SA0412
gi|29165615|ref|NC_002745.2|	473044 +	A	0	0	0	0	1	SA0412
gi|29165615|ref|NC_002745.2|	473045 +	G	0	0	0	1	0	SA0412
gi|29165615|ref|NC_002745.2|	473048 +	C	0	0	0	0	1	SA0412
gi|29165615|ref|NC_002745.2|	473059 +	G	0	0	1	0	0	SA0412
gi|29165615|ref|NC_002745.2|	473060 +	A	0	0	1	0	1	SA0412
gi|29165615|ref|NC_002745.2|	473061 +	U	0	1	0	0	0	SA0412
gi|29165615|ref|NC_002745.2|	473070 +	U	0	1	0	0	0	SA0412
gi|29165615|ref|NC_002745.2|	473073 +	C	0	0	0	0	2	SA0412
gi|29165615|ref|NC_002745.2|	473075 +	A	0	0	1	0	0	SA0412
gi|29165615|ref|NC_002745.2|	473079 +	A	0	0	0	1	0	SA0412
gi|29165615|ref|NC_002745.2|	473090 +	A	0	1	0	0	1	SA0412
gi|29165615|ref|NC_002745.2|	473091 +	C	0	1	0	0	0	SA0412
gi|29165615|ref|NC_002745.2|	473092 +	G	0	0	0	0	1	SA0412
gi|29165615|ref|NC_002745.2|	473093 +	A	0	0	0	0	2	SA0412
gi|29165615|ref|NC_002745.2|	473094 +	U	0	0	0	1	0	SA0412
gi|29165615|ref|NC_002745.2|	473096 +	G	0	0	0	3	4	SA0412
gi|29165615|ref|NC_002745.2|	473099 +	U	0	2	0	2	2	SA0412
gi|29165615|ref|NC_002745.2|	473100 +	C	1	0	0	0	2	SA0412
gi|29165615|ref|NC_002745.2|	473101 +	A	0	1	0	0	0	SA0412
gi|29165615|ref|NC_002745.2|	473102 +	G	0	3	1	5	3	SA0412
gi|29165615|ref|NC_002745.2|	473103 +	U	2	3	1	2	8	SA0412
gi|29165615|ref|NC_002745.2|	473104 +	A	0	2	2	8	4	SA0412
gi|29165615|ref|NC_002745.2|	473109 +	C	0	1	0	0	0	SA0412
gi|29165615|ref|NC_002745.2|	473114 +	U	0	0	1	0	1	SA0412
gi|29165615|ref|NC_002745.2|	473116 +	U	3	0	0	0	1	SA0412
gi|29165615|ref|NC_002745.2|	473117 +	U	0	0	0	1	0	SA0412
gi|29165615|ref|NC_002745.2|	473118 +	C	0	1	0	1	3	SA0412
gi|29165615|ref|NC_002745.2|	473119 +	A	0	0	0	1	3	SA0412
gi|29165615|ref|NC_002745.2|	473120 +	A	0	4	0	0	13	SA0412
gi|29165615|ref|NC_002745.2|	473121 +	U	0	3	0	0	1	SA0412
gi|29165615|ref|NC_002745.2|	473124 +	A	0	0	0	1	1	SA0412
gi|29165615|ref|NC_002745.2|	473128 +	G	1	0	0	0	0	SA0412
gi|29165615|ref|NC_002745.2|	473129 +	G	0	0	0	1	3	SA0412
gi|29165615|ref|NC_002745.2|	473130 +	A	0	1	0	0	1	SA0412
gi|29165615|ref|NC_002745.2|	473133 +	A	2	2	1	1	3	SA0412
gi|29165615|ref|NC_002745.2|	473134 +	C	0	3	0	2	2	SA0412
gi|29165615|ref|NC_002745.2|	473136 +	A	1	0	0	0	0	SA0412
gi|29165615|ref|NC_002745.2|	473138 +	C	0	0	0	1	2	SA0412

gi|29165615|ref|NC_002745.2|	473139	+	A	0	0	0	1	1	SA0412
gi|29165615|ref|NC_002745.2|	473142	+	C	0	1	1	0	0	SA0412
gi|29165615|ref|NC_002745.2|	473148	+	U	1	0	0	0	2	SA0412
gi|29165615|ref|NC_002745.2|	473149	+	U	0	1	0	0	0	SA0412
gi|29165615|ref|NC_002745.2|	473150	+	G	0	0	1	1	0	SA0412
gi|29165615|ref|NC_002745.2|	473151	+	A	0	1	0	0	1	SA0412
gi|29165615|ref|NC_002745.2|	473155	+	A	0	0	0	0	1	SA0412
gi|29165615|ref|NC_002745.2|	473157	+	A	0	1	0	0	0	SA0412
gi|29165615|ref|NC_002745.2|	473159	+	C	0	0	1	0	1	SA0412
gi|29165615|ref|NC_002745.2|	473160	+	U	13	0	0	0	1	SA0412
gi|29165615|ref|NC_002745.2|	473161	+	U	3	0	0	0	0	SA0412
gi|29165615|ref|NC_002745.2|	473163	+	U	3	0	1	1	0	SA0412
gi|29165615|ref|NC_002745.2|	473166	+	C	3	0	0	0	0	SA0412
gi|29165615|ref|NC_002745.2|	473167	+	G	0	1	2	0	0	SA0412
gi|29165615|ref|NC_002745.2|	473168	+	U	0	2	0	0	0	SA0412
gi|29165615|ref|NC_002745.2|	473169	+	U	2	1	0	0	0	SA0412
gi|29165615|ref|NC_002745.2|	473170	+	A	0	1	0	0	0	SA0412
gi|29165615|ref|NC_002745.2|	473172	+	C	0	1	0	1	2	SA0412
gi|29165615|ref|NC_002745.2|	473176	+	U	5	0	0	0	0	SA0412
gi|29165615|ref|NC_002745.2|	473178	+	G	0	0	0	0	1	SA0412
gi|29165615|ref|NC_002745.2|	473195	+	U	0	0	0	0	1	SA0412
gi|29165615|ref|NC_002745.2|	473200	+	U	0	0	1	1	0	SA0412
gi|29165615|ref|NC_002745.2|	473202	+	C	1	0	0	0	0	SA0412
gi|29165615|ref|NC_002745.2|	473203	+	A	0	0	0	0	2	SA0412
gi|29165615|ref|NC_002745.2|	473206	+	A	0	1	0	0	0	SA0412
gi|29165615|ref|NC_002745.2|	473207	+	C	0	1	0	0	1	SA0412
gi|29165615|ref|NC_002745.2|	473208	+	A	0	1	0	0	0	SA0412
gi|29165615|ref|NC_002745.2|	473218	+	U	0	2	0	0	1	SA0412
gi|29165615|ref|NC_002745.2|	473222	+	C	0	0	1	0	0	SA0412
gi|29165615|ref|NC_002745.2|	473224	+	A	0	0	0	0	1	SA0412
gi|29165615|ref|NC_002745.2|	473225	+	C	0	1	0	0	0	SA0412
gi|29165615|ref|NC_002745.2|	473226	+	A	2	0	0	0	3	SA0412
gi|29165615|ref|NC_002745.2|	473227	+	U	0	0	0	0	1	SA0412
gi|29165615|ref|NC_002745.2|	473231	+	U	0	1	0	0	1	SA0412
gi|29165615|ref|NC_002745.2|	473234	+	A	1	0	0	0	0	SA0412
gi|29165615|ref|NC_002745.2|	473238	+	A	1	0	0	1	0	SA0412
gi|29165615|ref|NC_002745.2|	473241	+	A	1	0	1	0	0	SA0412
gi|29165615|ref|NC_002745.2|	473243	+	U	0	0	0	2	0	SA0412
gi|29165615|ref|NC_002745.2|	473244	+	U	3	0	1	0	0	SA0412
gi|29165615|ref|NC_002745.2|	473249	+	A	0	1	0	0	1	SA0412
gi|29165615|ref|NC_002745.2|	473256	+	U	0	0	0	0	1	SA0412
gi|29165615|ref|NC_002745.2|	473265	+	A	0	1	0	0	0	SA0412
gi|29165615|ref|NC_002745.2|	473278	+	U	1	0	0	0	0	SA0412
gi|29165615|ref|NC_002745.2|	473281	+	U	1	0	0	0	1	SA0412
gi|29165615|ref|NC_002745.2|	473295	+	C	0	1	0	0	0	SA0412
gi|29165615|ref|NC_002745.2|	473320	+	A	0	0	1	0	0	SA0412
gi|29165615|ref|NC_002745.2|	473332	+	G	0	0	1	0	0	SA0412
gi|29165615|ref|NC_002745.2|	473335	+	U	0	2	0	0	0	SA0412
gi|29165615|ref|NC_002745.2|	473403	+	U	0	0	1	0	0	SA0412
gi|29165615|ref|NC_002745.2|	473461	+	A	0	0	0	0	1	SA0412
gi|29165615|ref|NC_002745.2|	473490	+	C	0	0	1	0	0	SA0412
gi|29165615|ref|NC_002745.2|	473507	+	A	0	0	0	0	1	SA0412
gi|29165615|ref|NC_002745.2|	473508	+	A	0	0	0	1	0	SA0412
gi|29165615|ref|NC_002745.2|	473513	+	A	0	0	0	2	0	SA0412
gi|29165615|ref|NC_002745.2|	473514	+	A	1	0	0	2	2	SA0412
gi|29165615|ref|NC_002745.2|	473518	+	U	0	1	0	0	0	SA0412
gi|29165615|ref|NC_002745.2|	473519	+	A	0	0	1	0	0	SA0412
gi|29165615|ref|NC_002745.2|	473527	+	U	0	0	0	1	0	SA0412
gi|29165615|ref|NC_002745.2|	473538	+	U	1	0	0	0	0	SA0412
gi|29165615|ref|NC_002745.2|	473561	+	C	0	0	0	1	0	SA0412
gi|29165615|ref|NC_002745.2|	473577	+	A	0	0	0	0	1	SA0412
gi|29165615|ref|NC_002745.2|	473580	+	A	0	0	1	0	0	SA0412
gi|29165615|ref|NC_002745.2|	473583	+	A	0	0	0	0	1	SA0412
gi|29165615|ref|NC_002745.2|	473590	+	U	0	0	0	0	1	SA0412
gi|29165615|ref|NC_002745.2|	473598	+	C	0	0	0	1	0	SA0412
gi|29165615|ref|NC_002745.2|	473612	+	U	0	0	0	1	0	SA0412
gi|29165615|ref|NC_002745.2|	473614	+	A	0	1	0	0	0	SA0412
gi|29165615|ref|NC_002745.2|	473615	+	A	0	0	0	1	0	SA0412
gi|29165615|ref|NC_002745.2|	473619	+	A	0	0	0	1	2	SA0412
gi|29165615|ref|NC_002745.2|	473620	+	C	0	0	0	1	0	SA0412
gi|29165615|ref|NC_002745.2|	473622	+	C	0	2	0	0	0	SA0412
gi|29165615|ref|NC_002745.2|	473631	+	U	0	0	0	0	1	SA0412
gi|29165615|ref|NC_002745.2|	473632	+	U	0	1	0	0	0	SA0412
gi|29165615|ref|NC_002745.2|	473636	+	G	0	0	0	0	2	SA0412
gi|29165615|ref|NC_002745.2|	473637	+	A	0	0	0	2	2	SA0412
gi|29165615|ref|NC_002745.2|	473638	+	A	0	0	0	1	1	SA0412
gi|29165615|ref|NC_002745.2|	473643	+	A	0	0	1	1	0	SA0412
gi|29165615|ref|NC_002745.2|	473644	+	U	1	0	0	0	0	SA0412
gi|29165615|ref|NC_002745.2|	473657	+	C	0	1	0	0	0	SA0412
gi|29165615|ref|NC_002745.2|	473661	+	U	0	1	0	0	2	SA0412
gi|29165615|ref|NC_002745.2|	473662	+	G	0	4	0	0	2	SA0412

gi|29165615|ref|NC_002745.2|	473681 +	C	1	0	0	0	0	SA0412
gi|29165615|ref|NC_002745.2|	473683 +	A	0	1	0	0	0	SA0412
gi|29165615|ref|NC_002745.2|	473730 +	U	2	0	0	0	0	SA0412
gi|29165615|ref|NC_002745.2|	473761 +	U	0	0	0	0	1	SA0412
gi|29165615|ref|NC_002745.2|	473832 +	C	1	0	0	0	0	SA0412
gi|29165615|ref|NC_002745.2|	473844 +	C	1	0	0	0	0	SA0412
gi|29165615|ref|NC_002745.2|	473845 +	G	1	0	0	0	0	SA0412
gi|29165615|ref|NC_002745.2|	473847 +	C	1	0	0	0	0	SA0412
gi|29165615|ref|NC_002745.2|	473891 +	G	0	0	0	0	1	SA0412
gi|29165615|ref|NC_002745.2|	473899 +	A	0	0	0	1	0	SA0412
gi|29165615|ref|NC_002745.2|	473902 +	A	0	0	0	1	0	SA0412
gi|29165615|ref|NC_002745.2|	473906 +	C	0	0	0	2	0	SA0412
gi|29165615|ref|NC_002745.2|	473908 +	A	1	0	0	0	0	SA0412
gi|29165615|ref|NC_002745.2|	473909 +	C	0	0	0	0	1	SA0412
gi|29165615|ref|NC_002745.2|	473947 +	G	0	0	0	1	0	SA0412
gi|29165615|ref|NC_002745.2|	473948 +	A	0	2	0	0	0	SA0412
gi|29165615|ref|NC_002745.2|	473953 +	U	0	1	0	0	0	SA0412
gi|29165615|ref|NC_002745.2|	473957 +	G	0	0	0	0	1	SA0412
gi|29165615|ref|NC_002745.2|	473971 +	G	0	1	0	0	0	SA0412
gi|29165615|ref|NC_002745.2|	473989 +	U	1	0	0	0	0	SA0412
gi|29165615|ref|NC_002745.2|	474037 +	A	0	0	0	0	1	SA0412
gi|29165615|ref|NC_002745.2|	474038 +	A	0	0	0	0	1	SA0412
gi|29165615|ref|NC_002745.2|	474048 +	C	2	0	0	0	0	SA0412
gi|29165615|ref|NC_002745.2|	474063 +	U	1	2	0	0	0	SA0412
gi|29165615|ref|NC_002745.2|	474066 +	A	0	0	1	1	1	SA0412
gi|29165615|ref|NC_002745.2|	474067 +	A	0	2	0	0	2	SA0412
gi|29165615|ref|NC_002745.2|	474071 +	A	0	0	0	1	0	SA0412
gi|29165615|ref|NC_002745.2|	474073 +	A	0	1	0	0	3	SA0412
gi|29165615|ref|NC_002745.2|	474076 +	G	0	1	0	0	0	SA0412
gi|29165615|ref|NC_002745.2|	474078 +	U	1	0	0	0	0	SA0412
gi|29165615|ref|NC_002745.2|	474081 +	A	0	0	0	1	0	SA0412
gi|29165615|ref|NC_002745.2|	474083 +	A	0	0	0	0	1	SA0412
gi|29165615|ref|NC_002745.2|	474091 +	A	0	0	0	0	1	SA0412
gi|29165615|ref|NC_002745.2|	474151 +	U	0	0	0	1	0	SA0412
gi|29165615|ref|NC_002745.2|	474192 +	U	0	0	0	1	0	SA0412
gi|29165615|ref|NC_002745.2|	474267 +	A	2	0	0	0	0	SA0412
gi|29165615|ref|NC_002745.2|	474334 +	G	1	2	1	1	1	SA0412
gi|29165615|ref|NC_002745.2|	474335 +	A	0	0	0	1	0	SA0412
gi|29165615|ref|NC_002745.2|	474336 +	U	0	0	0	0	1	SA0412
gi|29165615|ref|NC_002745.2|	474349 +	U	0	1	0	0	0	SA0412
gi|29165615|ref|NC_002745.2|	474350 +	C	1	0	0	0	0	SA0412
gi|29165615|ref|NC_002745.2|	474352 +	A	0	0	0	1	0	SA0412
gi|29165615|ref|NC_002745.2|	474353 +	A	0	0	0	1	0	SA0412
gi|29165615|ref|NC_002745.2|	474378 +	C	0	0	0	1	0	SA0412
gi|29165615|ref|NC_002745.2|	474382 +	U	2	0	1	0	0	SA0412
gi|29165615|ref|NC_002745.2|	474385 +	G	1	0	0	0	0	SA0412
gi|29165615|ref|NC_002745.2|	474393 +	C	0	0	0	0	1	SA0412
gi|29165615|ref|NC_002745.2|	474416 +	G	0	0	0	0	1	SA0412
gi|29165615|ref|NC_002745.2|	474440 +	A	1	0	0	0	0	SA0412
gi|29165615|ref|NC_002745.2|	474446 +	G	1	0	0	0	0	SA0412
gi|29165615|ref|NC_002745.2|	474469 +	A	0	0	1	0	0	SA0412
gi|29165615|ref|NC_002745.2|	474471 +	U	0	2	0	0	0	SA0412
gi|29165615|ref|NC_002745.2|	474475 +	A	0	1	0	0	0	SA0412
gi|29165615|ref|NC_002745.2|	474491 +	G	0	0	0	1	0	SA0412
gi|29165615|ref|NC_002745.2|	474501 +	C	0	1	0	0	0	SA0412
gi|29165615|ref|NC_002745.2|	474502 +	A	0	1	0	0	0	SA0412
gi|29165615|ref|NC_002745.2|	474503 +	U	0	0	0	0	1	SA0412
gi|29165615|ref|NC_002745.2|	474504 +	U	1	0	0	0	0	SA0412
gi|29165615|ref|NC_002745.2|	474514 +	G	1	0	1	0	0	SA0412
gi|29165615|ref|NC_002745.2|	474525 +	U	0	0	0	0	1	SA0412
gi|29165615|ref|NC_002745.2|	474528 +	C	0	0	0	0	1	SA0412
gi|29165615|ref|NC_002745.2|	474532 +	A	1	0	0	0	0	SA0412
gi|29165615|ref|NC_002745.2|	474533 +	C	0	0	0	0	1	SA0412
gi|29165615|ref|NC_002745.2|	474549 +	G	1	0	0	0	0	SA0412
gi|29165615|ref|NC_002745.2|	474550 +	U	1	0	0	0	0	SA0412
gi|29165615|ref|NC_002745.2|	474570 +	U	1	0	0	0	0	SA0412
gi|29165615|ref|NC_002745.2|	474571 +	U	0	0	1	0	0	SA0412
gi|29165615|ref|NC_002745.2|	474574 +	U	1	0	0	0	0	SA0412
gi|29165615|ref|NC_002745.2|	474575 +	C	0	0	1	0	0	SA0412
gi|29165615|ref|NC_002745.2|	474577 +	U	1	0	0	0	0	SA0412
gi|29165615|ref|NC_002745.2|	474583 +	U	0	0	0	0	1	SA0412
gi|29165615|ref|NC_002745.2|	474585 +	A	0	0	1	0	0	SA0412
gi|29165615|ref|NC_002745.2|	474587 +	C	0	1	0	0	0	SA0412
gi|29165615|ref|NC_002745.2|	474589 +	A	0	0	0	0	1	SA0412
gi|29165615|ref|NC_002745.2|	474590 +	G	0	0	0	0	1	SA0412
gi|29165615|ref|NC_002745.2|	474593 +	G	0	1	0	3	1	SA0412
gi|29165615|ref|NC_002745.2|	474594 +	A	1	0	0	1	0	SA0412
gi|29165615|ref|NC_002745.2|	474595 +	A	0	0	0	0	1	SA0412
gi|29165615|ref|NC_002745.2|	474596 +	G	0	1	0	0	0	SA0412
gi|29165615|ref|NC_002745.2|	474597 +	A	0	0	0	0	2	SA0412
gi|29165615|ref|NC_002745.2|	474600 +	C	0	0	0	1	0	SA0412

gi|29165615|ref|NC_002745.2|	474601 +	A	0	1	0	0	0	SA0412
gi|29165615|ref|NC_002745.2|	474612 +	C	0	0	0	1	0	SA0412
gi|29165615|ref|NC_002745.2|	474636 +	C	0	1	0	0	0	SA0412
gi|29165615|ref|NC_002745.2|	474686 +	U	0	1	0	0	0	SA0412
gi|29165615|ref|NC_002745.2|	474693 +	A	0	0	0	0	1	SA0412
gi|29165615|ref|NC_002745.2|	474696 +	G	1	0	0	0	0	SA0412
gi|29165615|ref|NC_002745.2|	474699 +	U	1	0	0	0	0	SA0412
gi|29165615|ref|NC_002745.2|	474711 +	G	1	0	0	0	0	SA0412
gi|29165615|ref|NC_002745.2|	474717 +	C	0	0	0	1	0	SA0412
gi|29165615|ref|NC_002745.2|	474723 +	U	1	0	0	0	0	SA0412
gi|29165615|ref|NC_002745.2|	474727 +	C	0	0	0	0	1	SA0412
gi|29165615|ref|NC_002745.2|	474729 +	A	0	0	0	0	1	SA0412
gi|29165615|ref|NC_002745.2|	474741 +	G	0	0	1	0	0	SA0412
gi|29165615|ref|NC_002745.2|	474748 +	U	0	1	0	0	0	SA0412
gi|29165615|ref|NC_002745.2|	474750 +	A	0	0	0	1	0	SA0412
gi|29165615|ref|NC_002745.2|	474761 +	U	0	0	0	0	1	SA0412
gi|29165615|ref|NC_002745.2|	474762 +	U	1	0	0	0	0	SA0412
gi|29165615|ref|NC_002745.2|	474765 +	U	0	0	1	0	0	SA0412
gi|29165615|ref|NC_002745.2|	474770 +	A	0	0	0	1	1	SA0412
gi|29165615|ref|NC_002745.2|	474779 +	U	0	0	1	0	0	SA0412
gi|29165615|ref|NC_002745.2|	474790 +	G	0	0	0	1	0	SA0412
gi|29165615|ref|NC_002745.2|	474791 +	C	0	0	0	0	1	SA0412
gi|29165615|ref|NC_002745.2|	474795 +	U	0	0	0	0	1	SA0412
gi|29165615|ref|NC_002745.2|	474797 +	G	0	0	0	0	1	SA0412
gi|29165615|ref|NC_002745.2|	474798 +	C	0	1	0	0	0	SA0412
gi|29165615|ref|NC_002745.2|	474799 +	A	0	0	0	0	1	SA0412
gi|29165615|ref|NC_002745.2|	474800 +	C	0	0	0	0	1	SA0412
gi|29165615|ref|NC_002745.2|	474802 +	A	0	0	0	0	1	SA0412
gi|29165615|ref|NC_002745.2|	474810 +	A	1	1	0	0	0	SA0412
gi|29165615|ref|NC_002745.2|	474811 +	U	0	1	0	1	0	SA0412
gi|29165615|ref|NC_002745.2|	474813 +	U	0	3	0	0	0	SA0412
gi|29165615|ref|NC_002745.2|	474814 +	A	0	1	1	1	0	SA0412
gi|29165615|ref|NC_002745.2|	474815 +	C	0	1	0	5	1	SA0412
gi|29165615|ref|NC_002745.2|	474816 +	A	1	2	1	0	2	SA0412
gi|29165615|ref|NC_002745.2|	474817 +	A	0	2	0	1	0	SA0412
gi|29165615|ref|NC_002745.2|	474818 +	U	2	1	1	1	0	SA0412
gi|29165615|ref|NC_002745.2|	474819 +	A	0	1	0	1	0	SA0412
gi|29165615|ref|NC_002745.2|	474821 +	U	0	1	0	0	0	SA0412
gi|29165615|ref|NC_002745.2|	474822 +	A	3	0	0	1	0	SA0412
gi|29165615|ref|NC_002745.2|	474824 +	G	3	0	0	0	1	SA0412
gi|29165615|ref|NC_002745.2|	474826 +	G	3	3	1	0	2	SA0412
gi|29165615|ref|NC_002745.2|	474827 +	U	2	1	0	0	0	SA0412
gi|29165615|ref|NC_002745.2|	474828 +	C	1	0	0	0	0	SA0412
gi|29165615|ref|NC_002745.2|	474829 +	G	0	0	0	0	1	SA0412
gi|29165615|ref|NC_002745.2|	474830 +	A	0	0	1	0	0	SA0412
gi|29165615|ref|NC_002745.2|	474836 +	G	0	0	0	0	1	SA0412
gi|29165615|ref|NC_002745.2|	474837 +	C	1	0	0	0	0	SA0412
gi|29165615|ref|NC_002745.2|	474842 +	C	0	0	0	1	2	SA0412
gi|29165615|ref|NC_002745.2|	474844 +	U	0	1	0	0	0	SA0412
gi|29165615|ref|NC_002745.2|	474846 +	U	0	0	2	0	0	SA0412
gi|29165615|ref|NC_002745.2|	474847 +	U	0	1	0	0	1	SA0412
gi|29165615|ref|NC_002745.2|	474848 +	U	1	1	0	0	0	SA0412
gi|29165615|ref|NC_002745.2|	474850 +	C	0	0	0	0	1	SA0412
gi|29165615|ref|NC_002745.2|	474851 +	G	0	0	0	1	1	SA0412
gi|29165615|ref|NC_002745.2|	474852 +	G	0	0	2	2	0	SA0412
gi|29165615|ref|NC_002745.2|	474853 +	A	0	0	0	0	1	SA0412
gi|29165615|ref|NC_002745.2|	474856 +	U	0	0	0	3	0	SA0412
gi|29165615|ref|NC_002745.2|	474857 +	G	0	1	0	0	0	SA0412
gi|29165615|ref|NC_002745.2|	474858 +	G	0	0	0	2	1	SA0412
gi|29165615|ref|NC_002745.2|	474859 +	G	0	0	0	2	0	SA0412
gi|29165615|ref|NC_002745.2|	474860 +	A	0	1	0	3	1	SA0412
gi|29165615|ref|NC_002745.2|	474861 +	A	1	0	0	0	0	SA0412
gi|29165615|ref|NC_002745.2|	474862 +	U	0	1	0	0	2	SA0412
gi|29165615|ref|NC_002745.2|	474863 +	A	0	0	0	3	1	SA0412
gi|29165615|ref|NC_002745.2|	474864 +	A	0	0	0	0	1	SA0412
gi|29165615|ref|NC_002745.2|	474865 +	A	1	2	0	0	0	SA0412
gi|29165615|ref|NC_002745.2|	474867 +	C	0	0	0	1	0	SA0412
gi|29165615|ref|NC_002745.2|	474869 +	A	0	1	0	1	2	SA0412
gi|29165615|ref|NC_002745.2|	474870 +	C	2	0	0	0	0	SA0412
gi|29165615|ref|NC_002745.2|	474875 +	A	0	0	1	0	0	SA0412
gi|29165615|ref|NC_002745.2|	474876 +	C	1	0	0	0	0	SA0412
gi|29165615|ref|NC_002745.2|	474877 +	C	0	0	0	0	1	SA0412
gi|29165615|ref|NC_002745.2|	474879 +	U	1	0	0	0	0	SA0412
gi|29165615|ref|NC_002745.2|	474881 +	A	1	0	1	0	2	SA0412
gi|29165615|ref|NC_002745.2|	474882 +	C	1	0	0	0	0	SA0412
gi|29165615|ref|NC_002745.2|	474884 +	U	1	0	1	0	1	SA0412
gi|29165615|ref|NC_002745.2|	474885 +	C	1	0	0	0	1	SA0412
gi|29165615|ref|NC_002745.2|	474886 +	A	0	0	1	0	0	SA0412
gi|29165615|ref|NC_002745.2|	474888 +	G	0	0	0	0	1	SA0412
gi|29165615|ref|NC_002745.2|	474889 +	U	1	0	1	0	1	SA0412
gi|29165615|ref|NC_002745.2|	474890 +	G	0	2	0	0	0	SA0412

gi|29165615|ref|NC_002745.2|	474891	+	U	0	0	1	0	0	SA0412
gi|29165615|ref|NC_002745.2|	474893	+	G	1	0	0	0	0	SA0412
gi|29165615|ref|NC_002745.2|	474894	+	G	1	0	0	2	0	SA0412
gi|29165615|ref|NC_002745.2|	474898	+	C	1	1	0	0	2	SA0412
gi|29165615|ref|NC_002745.2|	474900	+	U	0	0	1	0	0	SA0412
gi|29165615|ref|NC_002745.2|	474902	+	C	0	0	0	0	1	SA0412
gi|29165615|ref|NC_002745.2|	474904	+	A	0	1	0	0	0	SA0412
gi|29165615|ref|NC_002745.2|	474907	+	U	0	0	0	0	1	SA0412
gi|29165615|ref|NC_002745.2|	474909	+	A	3	0	0	0	0	SA0412
gi|29165615|ref|NC_002745.2|	474912	+	C	0	0	0	0	2	SA0412
gi|29165615|ref|NC_002745.2|	474917	+	G	0	1	0	0	0	SA0412
gi|29165615|ref|NC_002745.2|	474918	+	A	1	0	0	0	0	SA0412
gi|29165615|ref|NC_002745.2|	474919	+	U	1	0	0	1	0	SA0412
gi|29165615|ref|NC_002745.2|	474922	+	G	1	0	0	0	0	SA0412
gi|29165615|ref|NC_002745.2|	474924	+	U	0	1	0	0	1	SA0412
gi|29165615|ref|NC_002745.2|	474932	+	U	0	0	0	1	0	SA0412
gi|29165615|ref|NC_002745.2|	474933	+	U	1	0	0	0	1	SA0412
gi|29165615|ref|NC_002745.2|	474936	+	C	0	0	0	2	1	SA0412
gi|29165615|ref|NC_002745.2|	474937	+	A	0	0	0	0	1	SA0412
gi|29165615|ref|NC_002745.2|	474940	+	G	0	0	0	0	1	SA0412
gi|29165615|ref|NC_002745.2|	474941	+	C	0	0	0	1	1	SA0412
gi|29165615|ref|NC_002745.2|	474942	+	A	0	0	1	0	2	SA0412
gi|29165615|ref|NC_002745.2|	474946	+	U	0	0	1	0	0	SA0412
gi|29165615|ref|NC_002745.2|	474948	+	U	1	0	0	0	1	SA0412
gi|29165615|ref|NC_002745.2|	474950	+	A	1	3	0	0	6	SA0412
gi|29165615|ref|NC_002745.2|	474951	+	U	1	0	0	0	0	SA0412
gi|29165615|ref|NC_002745.2|	474952	+	G	1	0	0	0	0	SA0412
gi|29165615|ref|NC_002745.2|	474955	+	U	0	0	1	0	0	SA0412
gi|29165615|ref|NC_002745.2|	474957	+	C	0	1	0	0	0	SA0412
gi|29165615|ref|NC_002745.2|	474970	+	G	0	0	1	0	0	SA0412
gi|29165615|ref|NC_002745.2|	474972	+	A	0	0	0	0	1	SA0412
gi|29165615|ref|NC_002745.2|	474975	+	A	2	0	0	0	0	SA0412
gi|29165615|ref|NC_002745.2|	474983	+	A	0	0	1	0	0	SA0412
gi|29165615|ref|NC_002745.2|	474985	+	U	0	0	0	0	1	SA0412
gi|29165615|ref|NC_002745.2|	474987	+	G	3	0	0	1	0	SA0412
gi|29165615|ref|NC_002745.2|	474990	+	U	0	2	1	0	0	SA0412
gi|29165615|ref|NC_002745.2|	474992	+	C	1	0	0	0	0	SA0412
gi|29165615|ref|NC_002745.2|	474993	+	U	1	0	1	0	1	SA0412
gi|29165615|ref|NC_002745.2|	474996	+	U	2	0	0	0	0	SA0412
gi|29165615|ref|NC_002745.2|	474998	+	G	0	0	0	1	4	SA0412
gi|29165615|ref|NC_002745.2|	475003	+	U	2	0	0	0	0	SA0412
gi|29165615|ref|NC_002745.2|	475004	+	C	3	1	0	1	0	SA0412
gi|29165615|ref|NC_002745.2|	475005	+	A	3	0	0	0	0	SA0412
gi|29165615|ref|NC_002745.2|	475008	+	C	0	1	1	0	0	SA0412
gi|29165615|ref|NC_002745.2|	475010	+	C	0	0	0	1	0	SA0412
gi|29165615|ref|NC_002745.2|	475011	+	C	0	0	0	0	1	SA0412
gi|29165615|ref|NC_002745.2|	475012	+	C	0	0	0	1	0	SA0412
gi|29165615|ref|NC_002745.2|	475019	+	G	0	0	0	0	3	SA0412
gi|29165615|ref|NC_002745.2|	475028	+	A	0	0	0	0	1	SA0412
gi|29165615|ref|NC_002745.2|	475029	+	G	0	0	0	0	1	SA0412
gi|29165615|ref|NC_002745.2|	475030	+	A	0	1	2	0	0	SA0412
gi|29165615|ref|NC_002745.2|	475031	+	C	0	0	0	1	0	SA0412
gi|29165615|ref|NC_002745.2|	475034	+	U	0	0	1	0	0	SA0412
gi|29165615|ref|NC_002745.2|	475038	+	C	0	0	0	0	1	SA0412
gi|29165615|ref|NC_002745.2|	475040	+	A	0	0	0	1	0	SA0412
gi|29165615|ref|NC_002745.2|	475045	+	U	1	0	0	0	0	SA0412
gi|29165615|ref|NC_002745.2|	475047	+	A	0	0	0	1	0	SA0412
gi|29165615|ref|NC_002745.2|	475048	+	G	0	0	0	1	0	SA0412
gi|29165615|ref|NC_002745.2|	475050	+	A	0	0	0	0	1	SA0412
gi|29165615|ref|NC_002745.2|	475051	+	U	1	0	0	0	0	SA0412
gi|29165615|ref|NC_002745.2|	475055	+	G	0	0	1	0	0	SA0412
gi|29165615|ref|NC_002745.2|	475056	+	C	0	0	0	1	0	SA0412
gi|29165615|ref|NC_002745.2|	475057	+	U	0	0	1	0	0	SA0412
gi|29165615|ref|NC_002745.2|	475060	+	G	2	0	0	0	0	SA0412
gi|29165615|ref|NC_002745.2|	475062	+	A	1	0	0	0	0	SA0412
gi|29165615|ref|NC_002745.2|	475066	+	G	0	0	0	1	0	SA0412
gi|29165615|ref|NC_002745.2|	475073	+	C	0	0	1	0	0	SA0412
gi|29165615|ref|NC_002745.2|	475144	+	G	0	0	0	0	1	-
gi|29165615|ref|NC_002745.2|	475190	+	C	0	0	1	0	0	-
gi|29165615|ref|NC_002745.2|	475196	+	A	0	0	0	0	2	-
gi|29165615|ref|NC_002745.2|	475205	+	A	1	0	0	0	0	-
gi|29165615|ref|NC_002745.2|	475210	+	A	0	0	1	0	0	-
gi|29165615|ref|NC_002745.2|	475222	+	U	0	0	1	0	0	-
gi|29165615|ref|NC_002745.2|	475223	+	A	3	51	33	38	73	-
gi|29165615|ref|NC_002745.2|	475224	+	A	0	4	2	5	3	-
gi|29165615|ref|NC_002745.2|	475228	+	U	0	1	0	0	0	-
gi|29165615|ref|NC_002745.2|	475244	+	G	0	1	0	0	0	-
gi|29165615|ref|NC_002745.2|	475245	+	A	0	0	0	0	1	-
gi|29165615|ref|NC_002745.2|	475248	+	C	0	0	0	0	1	-
gi|29165615|ref|NC_002745.2|	475249	+	A	0	0	0	1	1	-
gi|29165615|ref|NC_002745.2|	475250	+	A	0	0	0	0	1	-

gi|29165615|ref|NC_002745.2|	475251	+	A	0	0	0	0	1	-
gi|29165615|ref|NC_002745.2|	475253	+	A	0	0	0	0	1	-
gi|29165615|ref|NC_002745.2|	475259	+	U	0	2	2	1	1	-
gi|29165615|ref|NC_002745.2|	475260	+	A	0	1	0	0	2	-
gi|29165615|ref|NC_002745.2|	475261	+	A	0	0	0	1	0	-
gi|29165615|ref|NC_002745.2|	475265	+	A	0	0	1	0	0	-
gi|29165615|ref|NC_002745.2|	475270	+	A	0	10	1	11	7	-
gi|29165615|ref|NC_002745.2|	475271	+	A	0	3	1	4	4	-
gi|29165615|ref|NC_002745.2|	475273	+	A	0	0	0	0	1	-
gi|29165615|ref|NC_002745.2|	475274	+	U	0	1	1	2	1	-
gi|29165615|ref|NC_002745.2|	475275	+	U	0	0	0	1	0	-
gi|29165615|ref|NC_002745.2|	475279	+	G	0	0	0	1	0	-
gi|29165615|ref|NC_002745.2|	475280	+	G	0	0	1	1	0	-
gi|29165615|ref|NC_002745.2|	475282	+	G	0	0	0	0	1	-
gi|29165615|ref|NC_002745.2|	475284	+	U	0	0	0	0	1	-
gi|29165615|ref|NC_002745.2|	475287	+	U	0	1	0	1	0	-
gi|29165615|ref|NC_002745.2|	475288	+	G	0	0	0	1	1	-
gi|29165615|ref|NC_002745.2|	475291	+	A	0	0	0	1	0	SA0413
gi|29165615|ref|NC_002745.2|	475293	+	G	0	0	0	0	3	SA0413
gi|29165615|ref|NC_002745.2|	475294	+	A	0	2	0	2	1	SA0413
gi|29165615|ref|NC_002745.2|	475296	+	A	0	2	0	0	0	SA0413
gi|29165615|ref|NC_002745.2|	475297	+	A	0	1	0	0	0	SA0413
gi|29165615|ref|NC_002745.2|	475299	+	A	0	0	0	0	1	SA0413
gi|29165615|ref|NC_002745.2|	475302	+	G	0	0	0	1	0	SA0413
gi|29165615|ref|NC_002745.2|	475303	+	A	0	0	0	1	0	SA0413
gi|29165615|ref|NC_002745.2|	475304	+	A	0	0	1	0	0	SA0413
gi|29165615|ref|NC_002745.2|	475310	+	A	0	0	0	1	1	SA0413
gi|29165615|ref|NC_002745.2|	475311	+	A	0	1	0	0	0	SA0413
gi|29165615|ref|NC_002745.2|	475315	+	G	0	0	1	0	0	SA0413
gi|29165615|ref|NC_002745.2|	475316	+	A	0	0	0	1	0	SA0413
gi|29165615|ref|NC_002745.2|	475322	+	A	0	0	0	1	0	SA0413
gi|29165615|ref|NC_002745.2|	475323	+	A	0	0	1	0	1	SA0413
gi|29165615|ref|NC_002745.2|	475325	+	U	0	0	0	0	4	SA0413
gi|29165615|ref|NC_002745.2|	475334	+	C	0	0	0	1	0	SA0413
gi|29165615|ref|NC_002745.2|	475342	+	C	0	1	0	0	0	SA0413
gi|29165615|ref|NC_002745.2|	475351	+	A	0	0	0	1	0	SA0413
gi|29165615|ref|NC_002745.2|	475353	+	A	0	0	0	1	0	SA0413
gi|29165615|ref|NC_002745.2|	475390	+	G	0	1	0	1	0	SA0413
gi|29165615|ref|NC_002745.2|	475391	+	A	1	0	0	2	1	SA0413
gi|29165615|ref|NC_002745.2|	475398	+	A	0	0	1	0	0	SA0413
gi|29165615|ref|NC_002745.2|	475400	+	G	0	0	0	1	0	SA0413
gi|29165615|ref|NC_002745.2|	475406	+	U	2	0	0	0	0	SA0413
gi|29165615|ref|NC_002745.2|	475418	+	G	0	2	0	0	0	SA0413
gi|29165615|ref|NC_002745.2|	475419	+	U	0	0	0	0	1	SA0413
gi|29165615|ref|NC_002745.2|	475423	+	C	0	0	0	1	0	SA0413
gi|29165615|ref|NC_002745.2|	475424	+	A	0	0	1	0	0	SA0413
gi|29165615|ref|NC_002745.2|	475426	+	G	0	1	0	0	0	SA0413
gi|29165615|ref|NC_002745.2|	475428	+	U	0	0	0	1	0	SA0413
gi|29165615|ref|NC_002745.2|	475442	+	C	0	0	0	1	0	SA0413
gi|29165615|ref|NC_002745.2|	475447	+	U	0	1	0	0	0	SA0413
gi|29165615|ref|NC_002745.2|	475450	+	C	0	0	1	0	0	SA0413
gi|29165615|ref|NC_002745.2|	475452	+	U	0	1	1	7	3	SA0413
gi|29165615|ref|NC_002745.2|	475455	+	G	0	0	0	2	0	SA0413
gi|29165615|ref|NC_002745.2|	475458	+	U	0	0	0	1	0	SA0413
gi|29165615|ref|NC_002745.2|	475461	+	U	0	1	0	0	0	SA0413
gi|29165615|ref|NC_002745.2|	475464	+	G	0	0	0	2	0	SA0413
gi|29165615|ref|NC_002745.2|	475465	+	A	0	1	0	1	0	SA0413
gi|29165615|ref|NC_002745.2|	475466	+	A	0	1	0	0	0	SA0413
gi|29165615|ref|NC_002745.2|	475469	+	A	0	1	0	0	0	SA0413
gi|29165615|ref|NC_002745.2|	475470	+	A	0	0	2	0	0	SA0413
gi|29165615|ref|NC_002745.2|	475472	+	G	0	0	1	0	0	SA0413
gi|29165615|ref|NC_002745.2|	475473	+	A	0	1	1	1	2	SA0413
gi|29165615|ref|NC_002745.2|	475476	+	A	0	0	0	1	1	SA0413
gi|29165615|ref|NC_002745.2|	475477	+	C	0	0	0	0	3	SA0413
gi|29165615|ref|NC_002745.2|	475480	+	A	0	8	2	4	4	SA0413
gi|29165615|ref|NC_002745.2|	475481	+	A	1	1	0	6	4	SA0413
gi|29165615|ref|NC_002745.2|	475482	+	U	0	1	0	1	0	SA0413
gi|29165615|ref|NC_002745.2|	475483	+	A	0	0	0	1	0	SA0413
gi|29165615|ref|NC_002745.2|	475485	+	U	0	0	0	1	0	SA0413
gi|29165615|ref|NC_002745.2|	475486	+	A	0	0	0	0	1	SA0413
gi|29165615|ref|NC_002745.2|	475487	+	A	0	2	0	0	3	SA0413
gi|29165615|ref|NC_002745.2|	475489	+	C	0	0	0	2	1	SA0413
gi|29165615|ref|NC_002745.2|	475491	+	A	0	0	0	3	1	SA0413
gi|29165615|ref|NC_002745.2|	475493	+	C	0	0	0	2	0	SA0413
gi|29165615|ref|NC_002745.2|	475495	+	C	0	1	1	0	0	SA0413
gi|29165615|ref|NC_002745.2|	475496	+	G	0	0	0	0	1	SA0413
gi|29165615|ref|NC_002745.2|	475498	+	U	0	0	0	1	0	SA0413
gi|29165615|ref|NC_002745.2|	475499	+	C	0	0	0	0	2	SA0413
gi|29165615|ref|NC_002745.2|	475501	+	G	0	2	0	0	0	SA0413
gi|29165615|ref|NC_002745.2|	475502	+	A	0	0	0	2	1	SA0413
gi|29165615|ref|NC_002745.2|	475507	+	G	0	0	1	0	0	SA0413

gi|29165615|ref|NC_002745.2|	475508 +	U	0	0	0	3	3	SA0413
gi|29165615|ref|NC_002745.2|	475510 +	G	1	0	0	0	0	SA0413
gi|29165615|ref|NC_002745.2|	475511 +	A	0	1	0	0	0	SA0413
gi|29165615|ref|NC_002745.2|	475514 +	C	0	1	0	0	0	SA0413
gi|29165615|ref|NC_002745.2|	475515 +	C	0	2	0	0	0	SA0413
gi|29165615|ref|NC_002745.2|	475516 +	G	0	1	0	0	0	SA0413
gi|29165615|ref|NC_002745.2|	475517 +	G	0	0	0	4	0	SA0413
gi|29165615|ref|NC_002745.2|	475518 +	U	0	2	1	2	0	SA0413
gi|29165615|ref|NC_002745.2|	475522 +	C	1	2	0	3	1	SA0413
gi|29165615|ref|NC_002745.2|	475523 +	A	0	0	2	0	1	SA0413
gi|29165615|ref|NC_002745.2|	475524 +	A	0	1	0	0	0	SA0413
gi|29165615|ref|NC_002745.2|	475525 +	G	0	0	2	0	1	SA0413
gi|29165615|ref|NC_002745.2|	475526 +	A	0	3	0	0	0	SA0413
gi|29165615|ref|NC_002745.2|	475531 +	A	1	0	1	2	0	SA0413
gi|29165615|ref|NC_002745.2|	475532 +	A	0	1	0	0	0	SA0413
gi|29165615|ref|NC_002745.2|	475539 +	C	0	0	0	1	0	SA0413
gi|29165615|ref|NC_002745.2|	475542 +	U	0	0	1	0	0	SA0413
gi|29165615|ref|NC_002745.2|	475543 +	C	0	0	0	0	1	SA0413
gi|29165615|ref|NC_002745.2|	475545 +	U	0	3	0	0	0	SA0413
gi|29165615|ref|NC_002745.2|	475546 +	A	0	1	0	9	0	SA0413
gi|29165615|ref|NC_002745.2|	475547 +	A	0	1	0	0	0	SA0413
gi|29165615|ref|NC_002745.2|	475548 +	A	0	0	2	0	2	SA0413
gi|29165615|ref|NC_002745.2|	475549 +	A	1	0	1	4	4	SA0413
gi|29165615|ref|NC_002745.2|	475550 +	U	0	1	0	1	0	SA0413
gi|29165615|ref|NC_002745.2|	475551 +	U	0	0	0	1	1	SA0413
gi|29165615|ref|NC_002745.2|	475552 +	A	0	3	0	5	1	SA0413
gi|29165615|ref|NC_002745.2|	475553 +	C	2	1	0	2	0	SA0413
gi|29165615|ref|NC_002745.2|	475554 +	A	0	0	0	0	1	SA0413
gi|29165615|ref|NC_002745.2|	475555 +	G	0	0	0	2	0	SA0413
gi|29165615|ref|NC_002745.2|	475556 +	G	0	7	1	3	0	SA0413
gi|29165615|ref|NC_002745.2|	475557 +	U	0	1	0	0	0	SA0413
gi|29165615|ref|NC_002745.2|	475558 +	A	0	1	1	0	0	SA0413
gi|29165615|ref|NC_002745.2|	475561 +	A	0	1	1	3	0	SA0413
gi|29165615|ref|NC_002745.2|	475562 +	A	0	1	0	1	0	SA0413
gi|29165615|ref|NC_002745.2|	475563 +	A	1	0	0	0	0	SA0413
gi|29165615|ref|NC_002745.2|	475570 +	A	0	1	0	0	0	SA0413
gi|29165615|ref|NC_002745.2|	475576 +	C	0	0	1	1	0	SA0413
gi|29165615|ref|NC_002745.2|	475577 +	A	0	0	0	0	1	SA0413
gi|29165615|ref|NC_002745.2|	475582 +	G	0	1	0	1	0	SA0413
gi|29165615|ref|NC_002745.2|	475583 +	A	0	0	0	1	1	SA0413
gi|29165615|ref|NC_002745.2|	475590 +	U	0	1	1	0	0	SA0413
gi|29165615|ref|NC_002745.2|	475591 +	A	0	0	1	0	0	SA0413
gi|29165615|ref|NC_002745.2|	475592 +	C	0	0	0	0	1	SA0413
gi|29165615|ref|NC_002745.2|	475602 +	U	0	0	0	0	1	SA0413
gi|29165615|ref|NC_002745.2|	475642 +	A	0	0	0	1	0	SA0413
gi|29165615|ref|NC_002745.2|	475725 +	A	0	0	0	0	1	-
gi|29165615|ref|NC_002745.2|	475821 +	A	0	1	0	0	0	-
gi|29165615|ref|NC_002745.2|	476220 +	U	0	0	1	0	0	-
gi|29165615|ref|NC_002745.2|	476223 +	A	0	1	0	0	0	-
gi|29165615|ref|NC_002745.2|	476229 +	A	0	1	0	0	0	-
gi|29165615|ref|NC_002745.2|	476234 +	A	0	3	0	2	1	-
gi|29165615|ref|NC_002745.2|	476238 +	U	0	0	0	1	0	-
gi|29165615|ref|NC_002745.2|	476240 +	A	0	0	1	0	5	-
gi|29165615|ref|NC_002745.2|	476242 +	A	0	0	1	0	0	-
gi|29165615|ref|NC_002745.2|	476243 +	A	0	1	0	2	1	-
gi|29165615|ref|NC_002745.2|	476248 +	G	0	0	0	1	0	-
gi|29165615|ref|NC_002745.2|	476249 +	A	1	0	1	0	1	-
gi|29165615|ref|NC_002745.2|	476258 +	G	0	0	1	0	0	-
gi|29165615|ref|NC_002745.2|	476266 +	A	0	0	0	0	1	-
gi|29165615|ref|NC_002745.2|	476273 +	U	1	1	0	0	0	-
gi|29165615|ref|NC_002745.2|	476288 +	A	0	0	0	2	0	-
gi|29165615|ref|NC_002745.2|	476289 +	U	1	0	0	0	0	-
gi|29165615|ref|NC_002745.2|	476291 +	G	0	0	0	0	1	-
gi|29165615|ref|NC_002745.2|	476302 +	U	0	0	0	1	0	-
gi|29165615|ref|NC_002745.2|	476311 +	A	0	0	0	0	1	-
gi|29165615|ref|NC_002745.2|	476314 +	G	0	0	0	1	0	-
gi|29165615|ref|NC_002745.2|	476315 +	A	0	0	0	0	1	-
gi|29165615|ref|NC_002745.2|	476333 +	A	0	0	0	1	0	-
gi|29165615|ref|NC_002745.2|	476342 +	G	0	0	0	0	1	SA0415
gi|29165615|ref|NC_002745.2|	476355 +	C	1	0	0	0	0	SA0415
gi|29165615|ref|NC_002745.2|	476400 +	U	0	0	0	1	1	SA0415
gi|29165615|ref|NC_002745.2|	476415 +	U	1	0	0	0	0	SA0415
gi|29165615|ref|NC_002745.2|	476430 +	C	0	0	0	0	1	SA0415
gi|29165615|ref|NC_002745.2|	476434 +	U	0	1	0	0	0	SA0415
gi|29165615|ref|NC_002745.2|	476451 +	A	0	0	0	0	1	SA0415
gi|29165615|ref|NC_002745.2|	476452 +	U	0	0	0	1	0	SA0415
gi|29165615|ref|NC_002745.2|	476454 +	U	0	1	0	0	0	SA0415
gi|29165615|ref|NC_002745.2|	476473 +	A	1	0	0	0	0	SA0415
gi|29165615|ref|NC_002745.2|	476501 +	C	1	0	0	0	0	SA0415
gi|29165615|ref|NC_002745.2|	476566 +	C	0	0	0	1	0	SA0415
gi|29165615|ref|NC_002745.2|	476614 +	U	0	0	2	0	0	SA0415

gi|29165615|ref|NC_002745.2|	476615	+	A	0	0	0	1	0	SA0415
gi|29165615|ref|NC_002745.2|	476621	+	A	0	1	0	0	0	SA0415
gi|29165615|ref|NC_002745.2|	476627	+	U	0	0	0	0	1	SA0415
gi|29165615|ref|NC_002745.2|	476684	+	A	0	0	1	0	0	SA0415
gi|29165615|ref|NC_002745.2|	476690	+	A	0	0	1	0	0	SA0415
gi|29165615|ref|NC_002745.2|	476694	+	U	0	2	3	2	4	SA0415
gi|29165615|ref|NC_002745.2|	476695	+	U	0	1	0	0	0	SA0415
gi|29165615|ref|NC_002745.2|	476715	+	A	0	0	0	1	0	SA0415
gi|29165615|ref|NC_002745.2|	476733	+	C	0	0	0	0	1	SA0415
gi|29165615|ref|NC_002745.2|	476781	+	A	1	0	0	0	0	SA0415
gi|29165615|ref|NC_002745.2|	476811	+	U	1	0	0	0	0	SA0415
gi|29165615|ref|NC_002745.2|	476862	+	U	1	0	0	0	0	SA0415
gi|29165615|ref|NC_002745.2|	476880	+	U	1	0	0	0	0	SA0415
gi|29165615|ref|NC_002745.2|	476922	+	U	0	0	0	1	0	SA0415
gi|29165615|ref|NC_002745.2|	476925	+	G	1	0	0	1	0	SA0415
gi|29165615|ref|NC_002745.2|	476926	+	C	0	2	0	0	2	SA0415
gi|29165615|ref|NC_002745.2|	476980	+	C	0	0	1	0	0	SA0415
gi|29165615|ref|NC_002745.2|	476995	+	A	0	1	0	1	1	SA0415
gi|29165615|ref|NC_002745.2|	477005	+	G	0	0	2	0	0	SA0415
gi|29165615|ref|NC_002745.2|	477017	+	U	0	1	0	0	0	-
gi|29165615|ref|NC_002745.2|	477023	+	G	0	0	0	0	1	-
gi|29165615|ref|NC_002745.2|	477027	+	U	0	1	0	0	0	-
gi|29165615|ref|NC_002745.2|	477043	+	A	0	0	0	1	0	-
gi|29165615|ref|NC_002745.2|	477065	+	A	0	0	0	2	0	SA0416
gi|29165615|ref|NC_002745.2|	477072	+	C	0	0	0	0	1	SA0416
gi|29165615|ref|NC_002745.2|	477081	+	A	0	0	0	0	1	SA0416
gi|29165615|ref|NC_002745.2|	477082	+	A	0	1	0	0	0	SA0416
gi|29165615|ref|NC_002745.2|	477114	+	A	1	0	0	0	0	SA0416
gi|29165615|ref|NC_002745.2|	477132	+	A	0	0	0	1	0	SA0416
gi|29165615|ref|NC_002745.2|	477165	+	A	0	0	1	0	0	SA0416
gi|29165615|ref|NC_002745.2|	477170	+	C	1	0	0	0	0	SA0416
gi|29165615|ref|NC_002745.2|	477189	+	C	1	0	0	0	0	SA0416
gi|29165615|ref|NC_002745.2|	477224	+	G	0	0	0	0	1	SA0416
gi|29165615|ref|NC_002745.2|	477322	+	U	1	0	0	0	0	SA0416
gi|29165615|ref|NC_002745.2|	477377	+	C	0	1	0	0	0	SA0416
gi|29165615|ref|NC_002745.2|	477379	+	U	1	0	0	0	0	SA0416
gi|29165615|ref|NC_002745.2|	477519	+	A	0	1	0	0	0	SA0416
gi|29165615|ref|NC_002745.2|	477555	+	A	0	0	0	0	1	SA0416
gi|29165615|ref|NC_002745.2|	477579	+	U	0	0	0	0	1	SA0416
gi|29165615|ref|NC_002745.2|	477587	+	A	1	1	0	0	2	SA0416
gi|29165615|ref|NC_002745.2|	477594	+	C	0	1	0	0	1	SA0416
gi|29165615|ref|NC_002745.2|	477596	+	A	0	0	0	0	1	SA0416
gi|29165615|ref|NC_002745.2|	477597	+	A	0	2	1	0	0	SA0416
gi|29165615|ref|NC_002745.2|	477598	+	U	0	0	1	0	0	SA0416
gi|29165615|ref|NC_002745.2|	477599	+	A	0	0	0	0	1	SA0416
gi|29165615|ref|NC_002745.2|	477603	+	U	1	0	0	0	0	SA0416
gi|29165615|ref|NC_002745.2|	477604	+	A	0	0	1	0	0	SA0416
gi|29165615|ref|NC_002745.2|	477639	+	G	0	2	0	0	0	SA0416
gi|29165615|ref|NC_002745.2|	477642	+	C	0	0	0	0	1	SA0416
gi|29165615|ref|NC_002745.2|	477644	+	C	0	1	0	0	0	SA0416
gi|29165615|ref|NC_002745.2|	477653	+	U	0	0	1	0	0	SA0416
gi|29165615|ref|NC_002745.2|	477657	+	A	0	0	1	1	0	SA0416
gi|29165615|ref|NC_002745.2|	477674	+	G	0	0	0	0	1	SA0416
gi|29165615|ref|NC_002745.2|	477793	+	A	0	0	0	0	1	-
gi|29165615|ref|NC_002745.2|	477950	+	G	0	0	0	0	1	-
gi|29165615|ref|NC_002745.2|	478110	+	U	0	2	0	0	0	-
gi|29165615|ref|NC_002745.2|	478112	+	A	0	1	2	0	2	-
gi|29165615|ref|NC_002745.2|	478113	+	A	0	0	2	0	0	-
gi|29165615|ref|NC_002745.2|	478122	+	A	0	1	1	0	0	-
gi|29165615|ref|NC_002745.2|	478141	+	G	0	1	0	0	0	-
gi|29165615|ref|NC_002745.2|	478171	+	C	0	0	2	0	0	SA0417
gi|29165615|ref|NC_002745.2|	478196	+	U	0	0	1	0	0	SA0417
gi|29165615|ref|NC_002745.2|	478296	+	A	0	0	1	0	0	SA0417
gi|29165615|ref|NC_002745.2|	478535	+	U	0	0	1	0	0	SA0417
gi|29165615|ref|NC_002745.2|	478579	+	C	0	1	0	0	0	SA0417
gi|29165615|ref|NC_002745.2|	478723	+	U	0	2	0	0	0	SA0417
gi|29165615|ref|NC_002745.2|	478724	+	C	0	1	0	0	0	SA0417
gi|29165615|ref|NC_002745.2|	479229	+	C	1	0	0	0	0	SA0417
gi|29165615|ref|NC_002745.2|	479254	+	G	0	0	1	0	0	SA0417
gi|29165615|ref|NC_002745.2|	479376	+	A	0	0	0	0	1	SA0417
gi|29165615|ref|NC_002745.2|	479379	+	A	0	0	1	0	2	SA0417
gi|29165615|ref|NC_002745.2|	479382	+	A	0	3	6	0	0	SA0417
gi|29165615|ref|NC_002745.2|	479384	+	A	0	1	0	0	0	SA0417
gi|29165615|ref|NC_002745.2|	479385	+	U	0	0	0	0	1	SA0417
gi|29165615|ref|NC_002745.2|	479390	+	G	0	3	0	0	3	SA0417
gi|29165615|ref|NC_002745.2|	479391	+	U	0	1	1	0	1	SA0417
gi|29165615|ref|NC_002745.2|	479392	+	A	0	1	0	0	1	SA0417
gi|29165615|ref|NC_002745.2|	479393	+	A	0	0	0	1	0	SA0417
gi|29165615|ref|NC_002745.2|	479405	+	G	0	0	1	0	0	SA0417
gi|29165615|ref|NC_002745.2|	479413	+	A	0	0	1	0	0	SA0417
gi|29165615|ref|NC_002745.2|	479415	+	U	0	0	1	0	0	SA0417

gi|29165615|ref|NC_002745.2|	479631 +	G	1	3	2	2	2	-
gi|29165615|ref|NC_002745.2|	479632 +	A	0	1	0	0	0	-
gi|29165615|ref|NC_002745.2|	479633 +	A	0	0	1	0	0	-
gi|29165615|ref|NC_002745.2|	479634 +	A	0	1	0	0	0	-
gi|29165615|ref|NC_002745.2|	479639 +	A	0	0	0	2	0	-
gi|29165615|ref|NC_002745.2|	479640 +	G	0	1	1	0	2	-
gi|29165615|ref|NC_002745.2|	479641 +	A	0	0	1	0	2	-
gi|29165615|ref|NC_002745.2|	479642 +	A	0	0	1	0	0	-
gi|29165615|ref|NC_002745.2|	479645 +	A	0	0	1	0	0	-
gi|29165615|ref|NC_002745.2|	479658 +	G	0	1	0	0	0	-
gi|29165615|ref|NC_002745.2|	479661 +	G	0	0	1	0	0	-
gi|29165615|ref|NC_002745.2|	479662 +	A	0	0	0	0	1	-
gi|29165615|ref|NC_002745.2|	479666 +	G	0	0	0	0	1	-
gi|29165615|ref|NC_002745.2|	479674 +	C	0	0	1	0	0	-
gi|29165615|ref|NC_002745.2|	479714 +	C	0	0	0	1	0	SA0418
gi|29165615|ref|NC_002745.2|	479719 +	G	0	0	0	1	0	SA0418
gi|29165615|ref|NC_002745.2|	479735 +	C	0	1	0	0	0	SA0418
gi|29165615|ref|NC_002745.2|	479760 +	U	0	0	0	1	0	SA0418
gi|29165615|ref|NC_002745.2|	479770 +	A	0	2	0	0	0	SA0418
gi|29165615|ref|NC_002745.2|	479771 +	A	0	0	0	1	1	SA0418
gi|29165615|ref|NC_002745.2|	479777 +	A	0	2	0	0	1	SA0418
gi|29165615|ref|NC_002745.2|	479783 +	A	0	1	0	0	0	SA0418
gi|29165615|ref|NC_002745.2|	479875 +	C	0	0	1	0	0	SA0418
gi|29165615|ref|NC_002745.2|	480017 +	U	0	0	1	0	0	SA0418
gi|29165615|ref|NC_002745.2|	480468 +	C	0	0	1	0	0	SA0418
gi|29165615|ref|NC_002745.2|	480502 +	A	0	0	5	0	7	SA0418
gi|29165615|ref|NC_002745.2|	480577 +	C	0	0	0	0	1	SA0418
gi|29165615|ref|NC_002745.2|	480622 +	A	0	1	0	0	0	SA0419
gi|29165615|ref|NC_002745.2|	480637 +	A	1	0	1	0	0	SA0419
gi|29165615|ref|NC_002745.2|	480665 +	G	0	0	1	0	0	SA0419
gi|29165615|ref|NC_002745.2|	480725 +	C	0	1	0	0	0	SA0419
gi|29165615|ref|NC_002745.2|	480739 +	U	0	0	0	0	1	SA0419
gi|29165615|ref|NC_002745.2|	480770 +	G	0	1	0	0	0	SA0419
gi|29165615|ref|NC_002745.2|	480771 +	U	0	1	0	0	0	SA0419
gi|29165615|ref|NC_002745.2|	480791 +	U	0	0	0	0	1	SA0419
gi|29165615|ref|NC_002745.2|	481304 +	C	0	0	0	0	1	SA0419
gi|29165615|ref|NC_002745.2|	481393 +	G	0	0	0	1	0	SA0419
gi|29165615|ref|NC_002745.2|	481462 +	C	1	0	1	0	1	SA0419
gi|29165615|ref|NC_002745.2|	481485 +	A	0	0	1	0	0	SA0419
gi|29165615|ref|NC_002745.2|	481495 +	G	0	1	0	0	0	SA0419
gi|29165615|ref|NC_002745.2|	481518 +	C	0	0	0	1	0	SA0419
gi|29165615|ref|NC_002745.2|	482002 +	A	1	1	1	0	2	-
gi|29165615|ref|NC_002745.2|	482031 +	G	0	0	0	1	0	-
gi|29165615|ref|NC_002745.2|	482096 +	G	0	1	0	0	0	SA0420
gi|29165615|ref|NC_002745.2|	482104 +	G	0	2	0	0	0	SA0420
gi|29165615|ref|NC_002745.2|	482236 +	U	3	0	0	0	0	SA0420
gi|29165615|ref|NC_002745.2|	482635 +	A	1	0	0	0	0	SA0420
gi|29165615|ref|NC_002745.2|	482753 +	C	0	0	0	1	0	SA0420
gi|29165615|ref|NC_002745.2|	482964 +	U	0	0	0	0	1	SA0420
gi|29165615|ref|NC_002745.2|	482970 +	A	0	1	0	0	0	SA0420
gi|29165615|ref|NC_002745.2|	483019 +	A	0	1	0	0	1	SA0420
gi|29165615|ref|NC_002745.2|	483025 +	A	0	0	0	0	1	SA0420
gi|29165615|ref|NC_002745.2|	483078 +	G	0	1	0	0	0	SA0421
gi|29165615|ref|NC_002745.2|	483084 +	A	0	0	0	0	1	SA0421
gi|29165615|ref|NC_002745.2|	483164 +	A	0	0	0	0	1	SA0421
gi|29165615|ref|NC_002745.2|	483204 +	A	0	0	0	0	1	SA0421
gi|29165615|ref|NC_002745.2|	483240 +	U	0	1	0	0	3	SA0421
gi|29165615|ref|NC_002745.2|	483241 +	U	0	0	0	1	0	SA0421
gi|29165615|ref|NC_002745.2|	483252 +	U	0	0	0	0	1	SA0421
gi|29165615|ref|NC_002745.2|	483255 +	U	0	0	0	0	1	SA0421
gi|29165615|ref|NC_002745.2|	483256 +	C	0	1	0	0	1	SA0421
gi|29165615|ref|NC_002745.2|	483258 +	A	0	0	0	0	1	SA0421
gi|29165615|ref|NC_002745.2|	483259 +	U	0	1	0	0	1	SA0421
gi|29165615|ref|NC_002745.2|	483265 +	A	0	1	0	0	0	SA0421
gi|29165615|ref|NC_002745.2|	483272 +	C	0	1	0	0	1	SA0421
gi|29165615|ref|NC_002745.2|	483274 +	A	0	2	0	0	0	SA0421
gi|29165615|ref|NC_002745.2|	483275 +	C	0	1	0	0	0	SA0421
gi|29165615|ref|NC_002745.2|	483276 +	C	0	0	1	0	0	SA0421
gi|29165615|ref|NC_002745.2|	483281 +	A	0	0	0	1	0	SA0421
gi|29165615|ref|NC_002745.2|	483287 +	U	0	0	0	0	1	SA0421
gi|29165615|ref|NC_002745.2|	483288 +	U	1	0	0	0	0	SA0421
gi|29165615|ref|NC_002745.2|	483290 +	U	0	1	0	0	0	SA0421
gi|29165615|ref|NC_002745.2|	483293 +	A	0	0	0	0	2	SA0421
gi|29165615|ref|NC_002745.2|	483369 +	U	1	0	0	0	0	SA0421
gi|29165615|ref|NC_002745.2|	483405 +	A	0	1	0	0	0	SA0421
gi|29165615|ref|NC_002745.2|	483416 +	G	0	0	1	0	0	SA0421
gi|29165615|ref|NC_002745.2|	483423 +	A	0	0	1	0	0	SA0421
gi|29165615|ref|NC_002745.2|	483466 +	A	1	0	0	0	0	SA0421
gi|29165615|ref|NC_002745.2|	483468 +	U	1	0	0	0	0	SA0421
gi|29165615|ref|NC_002745.2|	483489 +	U	1	0	0	0	0	SA0421
gi|29165615|ref|NC_002745.2|	483498 +	A	0	1	0	0	0	SA0421

gi|29165615|ref|NC_002745.2|	483522	+	C	0	1	0	0	0	SA0421
gi|29165615|ref|NC_002745.2|	483566	+	G	0	0	0	0	1	SA0421
gi|29165615|ref|NC_002745.2|	483575	+	G	1	0	0	0	0	SA0421
gi|29165615|ref|NC_002745.2|	483603	+	C	0	0	0	0	1	SA0421
gi|29165615|ref|NC_002745.2|	483660	+	U	1	0	0	0	0	SA0421
gi|29165615|ref|NC_002745.2|	483698	+	A	0	0	0	0	1	SA0421
gi|29165615|ref|NC_002745.2|	483738	+	U	0	0	0	0	1	-
gi|29165615|ref|NC_002745.2|	483743	+	A	0	0	1	0	1	-
gi|29165615|ref|NC_002745.2|	483745	+	G	0	0	0	0	1	-
gi|29165615|ref|NC_002745.2|	483746	+	A	0	7	4	1	7	-
gi|29165615|ref|NC_002745.2|	483748	+	U	2	1	0	0	0	-
gi|29165615|ref|NC_002745.2|	483750	+	U	0	1	1	0	1	-
gi|29165615|ref|NC_002745.2|	483751	+	G	0	1	0	0	1	-
gi|29165615|ref|NC_002745.2|	483752	+	A	0	4	1	0	9	-
gi|29165615|ref|NC_002745.2|	483753	+	A	0	1	0	2	0	-
gi|29165615|ref|NC_002745.2|	483754	+	A	0	0	0	1	0	-
gi|29165615|ref|NC_002745.2|	483755	+	G	0	0	1	0	1	-
gi|29165615|ref|NC_002745.2|	483756	+	G	1	5	2	0	9	-
gi|29165615|ref|NC_002745.2|	483757	+	A	0	1	1	0	2	-
gi|29165615|ref|NC_002745.2|	483761	+	G	0	5	0	0	1	-
gi|29165615|ref|NC_002745.2|	483762	+	A	0	0	0	2	2	-
gi|29165615|ref|NC_002745.2|	483764	+	A	0	1	3	0	3	-
gi|29165615|ref|NC_002745.2|	483765	+	G	0	2	0	2	0	-
gi|29165615|ref|NC_002745.2|	483766	+	A	0	0	4	6	2	-
gi|29165615|ref|NC_002745.2|	483767	+	A	0	1	0	0	0	SA0422
gi|29165615|ref|NC_002745.2|	483768	+	U	0	0	0	0	1	SA0422
gi|29165615|ref|NC_002745.2|	483769	+	G	1	1	0	1	8	SA0422
gi|29165615|ref|NC_002745.2|	483770	+	A	0	7	5	2	14	SA0422
gi|29165615|ref|NC_002745.2|	483771	+	A	0	3	0	2	5	SA0422
gi|29165615|ref|NC_002745.2|	483772	+	A	0	1	1	5	4	SA0422
gi|29165615|ref|NC_002745.2|	483773	+	A	0	2	1	2	5	SA0422
gi|29165615|ref|NC_002745.2|	483774	+	G	0	9	11	2	8	SA0422
gi|29165615|ref|NC_002745.2|	483775	+	A	6	70	38	24	63	SA0422
gi|29165615|ref|NC_002745.2|	483776	+	U	0	2	1	0	2	SA0422
gi|29165615|ref|NC_002745.2|	483777	+	U	0	0	0	2	1	SA0422
gi|29165615|ref|NC_002745.2|	483778	+	G	0	2	6	4	3	SA0422
gi|29165615|ref|NC_002745.2|	483779	+	A	0	5	7	1	14	SA0422
gi|29165615|ref|NC_002745.2|	483785	+	U	0	1	0	1	0	SA0422
gi|29165615|ref|NC_002745.2|	483789	+	U	0	0	0	2	0	SA0422
gi|29165615|ref|NC_002745.2|	483798	+	C	0	0	1	0	2	SA0422
gi|29165615|ref|NC_002745.2|	483799	+	A	0	0	1	0	0	SA0422
gi|29165615|ref|NC_002745.2|	483801	+	U	0	1	0	0	0	SA0422
gi|29165615|ref|NC_002745.2|	483803	+	G	0	0	0	3	0	SA0422
gi|29165615|ref|NC_002745.2|	483804	+	U	0	0	1	0	0	SA0422
gi|29165615|ref|NC_002745.2|	483805	+	A	0	0	2	1	0	SA0422
gi|29165615|ref|NC_002745.2|	483808	+	A	0	0	0	0	1	SA0422
gi|29165615|ref|NC_002745.2|	483812	+	G	0	1	0	0	0	SA0422
gi|29165615|ref|NC_002745.2|	483816	+	C	0	0	1	1	1	SA0422
gi|29165615|ref|NC_002745.2|	483831	+	A	0	0	1	0	0	SA0422
gi|29165615|ref|NC_002745.2|	483862	+	A	0	0	1	0	0	SA0422
gi|29165615|ref|NC_002745.2|	483892	+	A	0	0	0	0	1	SA0422
gi|29165615|ref|NC_002745.2|	483900	+	C	0	0	0	0	1	SA0422
gi|29165615|ref|NC_002745.2|	483905	+	A	0	0	0	0	1	SA0422
gi|29165615|ref|NC_002745.2|	483909	+	A	0	1	0	0	0	SA0422
gi|29165615|ref|NC_002745.2|	483956	+	A	0	0	0	1	1	SA0422
gi|29165615|ref|NC_002745.2|	483963	+	C	0	0	0	1	0	SA0422
gi|29165615|ref|NC_002745.2|	484112	+	G	0	0	2	1	3	SA0422
gi|29165615|ref|NC_002745.2|	484159	+	A	0	0	0	1	0	SA0422
gi|29165615|ref|NC_002745.2|	484215	+	A	0	0	0	0	1	SA0422
gi|29165615|ref|NC_002745.2|	484234	+	A	2	0	0	0	0	SA0422
gi|29165615|ref|NC_002745.2|	484279	+	U	0	0	0	1	0	SA0422
gi|29165615|ref|NC_002745.2|	484289	+	G	0	0	1	0	0	SA0422
gi|29165615|ref|NC_002745.2|	484366	+	U	2	0	0	0	0	SA0422
gi|29165615|ref|NC_002745.2|	484390	+	A	0	3	0	0	0	SA0422
gi|29165615|ref|NC_002745.2|	484408	+	U	0	0	1	1	0	SA0422
gi|29165615|ref|NC_002745.2|	484484	+	A	0	4	0	0	3	SA0422
gi|29165615|ref|NC_002745.2|	484489	+	A	0	0	0	2	0	SA0422
gi|29165615|ref|NC_002745.2|	484500	+	A	0	1	0	0	1	SA0422
gi|29165615|ref|NC_002745.2|	484503	+	C	0	1	0	0	0	SA0422
gi|29165615|ref|NC_002745.2|	484507	+	A	0	0	0	0	1	SA0422
gi|29165615|ref|NC_002745.2|	484512	+	C	0	1	0	1	0	SA0422
gi|29165615|ref|NC_002745.2|	484516	+	A	0	0	0	1	0	SA0422
gi|29165615|ref|NC_002745.2|	484517	+	A	0	2	0	0	0	SA0422
gi|29165615|ref|NC_002745.2|	484518	+	A	0	0	0	0	1	SA0422
gi|29165615|ref|NC_002745.2|	484529	+	G	0	1	0	0	0	SA0422
gi|29165615|ref|NC_002745.2|	484593	+	C	0	0	0	0	1	SA0422
gi|29165615|ref|NC_002745.2|	484783	+	G	6	7	6	4	5	-
gi|29165615|ref|NC_002745.2|	484786	+	A	0	0	1	0	0	-
gi|29165615|ref|NC_002745.2|	484815	+	U	0	0	0	1	0	-
gi|29165615|ref|NC_002745.2|	484822	+	G	0	0	0	0	1	-
gi|29165615|ref|NC_002745.2|	484842	+	C	0	0	0	1	0	-

gi|29165615|ref|NC_002745.2|	484853	+	U	0	2	0	0	0	-
gi|29165615|ref|NC_002745.2|	484854	+	A	0	0	0	1	0	-
gi|29165615|ref|NC_002745.2|	484855	+	U	0	0	1	1	1	-
gi|29165615|ref|NC_002745.2|	484856	+	A	0	0	1	0	0	-
gi|29165615|ref|NC_002745.2|	484857	+	C	0	1	0	0	0	-
gi|29165615|ref|NC_002745.2|	484858	+	A	0	0	0	0	1	-
gi|29165615|ref|NC_002745.2|	484860	+	A	0	0	1	1	0	-
gi|29165615|ref|NC_002745.2|	484861	+	A	0	0	0	1	0	-
gi|29165615|ref|NC_002745.2|	484864	+	A	0	0	2	0	0	-
gi|29165615|ref|NC_002745.2|	484866	+	A	3	3	4	0	2	-
gi|29165615|ref|NC_002745.2|	484868	+	C	0	3	3	1	3	-
gi|29165615|ref|NC_002745.2|	484869	+	G	0	0	2	0	1	-
gi|29165615|ref|NC_002745.2|	484870	+	U	0	6	6	5	6	-
gi|29165615|ref|NC_002745.2|	484871	+	A	0	4	3	3	3	-
gi|29165615|ref|NC_002745.2|	484874	+	A	0	0	0	1	0	-
gi|29165615|ref|NC_002745.2|	484875	+	U	0	0	0	1	0	-
gi|29165615|ref|NC_002745.2|	484881	+	A	1	0	1	2	0	-
gi|29165615|ref|NC_002745.2|	484882	+	G	135	137	113	161	156	-
gi|29165615|ref|NC_002745.2|	484883	+	U	0	2	4	2	1	-
gi|29165615|ref|NC_002745.2|	484884	+	A	2	0	0	1	1	-
gi|29165615|ref|NC_002745.2|	484885	+	U	0	0	0	0	1	-
gi|29165615|ref|NC_002745.2|	484887	+	C	0	0	2	1	0	-
gi|29165615|ref|NC_002745.2|	484888	+	A	0	1	0	0	0	-
gi|29165615|ref|NC_002745.2|	484890	+	A	0	0	1	0	0	-
gi|29165615|ref|NC_002745.2|	484891	+	A	0	4	0	1	0	-
gi|29165615|ref|NC_002745.2|	484892	+	A	0	0	0	0	2	-
gi|29165615|ref|NC_002745.2|	484895	+	C	0	0	0	2	1	-
gi|29165615|ref|NC_002745.2|	484897	+	G	0	6	5	6	5	-
gi|29165615|ref|NC_002745.2|	484898	+	A	0	7	3	4	5	-
gi|29165615|ref|NC_002745.2|	484899	+	U	0	0	0	1	0	-
gi|29165615|ref|NC_002745.2|	484900	+	A	0	1	0	2	0	-
gi|29165615|ref|NC_002745.2|	484902	+	A	0	1	0	0	0	-
gi|29165615|ref|NC_002745.2|	484905	+	A	0	1	1	1	1	-
gi|29165615|ref|NC_002745.2|	484908	+	G	0	0	0	0	1	-
gi|29165615|ref|NC_002745.2|	484913	+	G	0	1	0	0	0	-
gi|29165615|ref|NC_002745.2|	484932	+	G	0	1	0	0	0	-
gi|29165615|ref|NC_002745.2|	484947	+	A	0	0	0	1	0	SA0423
gi|29165615|ref|NC_002745.2|	484949	+	A	0	1	0	0	0	SA0423
gi|29165615|ref|NC_002745.2|	484950	+	A	1	1	0	0	0	SA0423
gi|29165615|ref|NC_002745.2|	484966	+	U	1	0	0	0	0	SA0423
gi|29165615|ref|NC_002745.2|	484976	+	A	0	0	0	1	0	SA0423
gi|29165615|ref|NC_002745.2|	485012	+	U	2	0	0	0	0	SA0423
gi|29165615|ref|NC_002745.2|	485032	+	U	1	0	0	0	0	SA0423
gi|29165615|ref|NC_002745.2|	485033	+	A	2	0	0	0	0	SA0423
gi|29165615|ref|NC_002745.2|	485137	+	A	0	1	0	0	0	SA0423
gi|29165615|ref|NC_002745.2|	485320	+	A	1	0	0	0	0	SA0423
gi|29165615|ref|NC_002745.2|	485323	+	C	1	0	0	0	0	SA0423
gi|29165615|ref|NC_002745.2|	485364	+	A	2	0	0	0	0	SA0423
gi|29165615|ref|NC_002745.2|	485579	+	A	0	1	0	0	0	SA0423
gi|29165615|ref|NC_002745.2|	485581	+	C	0	0	1	0	0	SA0423
gi|29165615|ref|NC_002745.2|	485597	+	C	0	0	1	0	1	SA0423
gi|29165615|ref|NC_002745.2|	485608	+	U	3	0	0	0	0	SA0423
gi|29165615|ref|NC_002745.2|	485624	+	C	1	0	0	0	0	SA0423
gi|29165615|ref|NC_002745.2|	485626	+	U	2	0	0	0	0	SA0423
gi|29165615|ref|NC_002745.2|	485635	+	G	1	0	0	0	0	SA0423
gi|29165615|ref|NC_002745.2|	485639	+	U	2	0	0	0	0	SA0423
gi|29165615|ref|NC_002745.2|	485644	+	G	0	1	0	0	0	SA0423
gi|29165615|ref|NC_002745.2|	485650	+	A	1	0	0	0	0	SA0423
gi|29165615|ref|NC_002745.2|	485651	+	U	0	1	0	0	0	SA0423
gi|29165615|ref|NC_002745.2|	485703	+	U	0	0	0	1	0	SA0423
gi|29165615|ref|NC_002745.2|	485725	+	A	1	0	0	0	0	SA0423
gi|29165615|ref|NC_002745.2|	485762	+	A	0	0	0	0	1	SA0423
gi|29165615|ref|NC_002745.2|	485873	+	A	0	1	0	0	0	SA0423
gi|29165615|ref|NC_002745.2|	485996	+	G	1	0	0	0	0	-
gi|29165615|ref|NC_002745.2|	486023	+	A	1	0	0	0	0	-
gi|29165615|ref|NC_002745.2|	486036	+	C	0	2	0	0	0	-
gi|29165615|ref|NC_002745.2|	486387	+	A	0	0	0	1	0	-
gi|29165615|ref|NC_002745.2|	486450	+	U	0	0	1	0	0	-
gi|29165615|ref|NC_002745.2|	486501	+	U	1	2	4	0	1	-
gi|29165615|ref|NC_002745.2|	486502	+	A	2	16	7	11	18	-
gi|29165615|ref|NC_002745.2|	486503	+	C	0	0	1	0	0	-
gi|29165615|ref|NC_002745.2|	486504	+	A	0	0	1	0	0	-
gi|29165615|ref|NC_002745.2|	486505	+	G	0	0	1	0	0	-
gi|29165615|ref|NC_002745.2|	486509	+	A	0	1	0	0	0	-
gi|29165615|ref|NC_002745.2|	486515	+	A	0	2	1	0	2	-
gi|29165615|ref|NC_002745.2|	486516	+	A	1	2	0	0	1	-
gi|29165615|ref|NC_002745.2|	486517	+	A	0	2	0	0	0	-
gi|29165615|ref|NC_002745.2|	486518	+	A	0	0	0	0	2	-
gi|29165615|ref|NC_002745.2|	486523	+	A	0	3	1	2	1	-
gi|29165615|ref|NC_002745.2|	486528	+	A	0	0	0	0	1	-
gi|29165615|ref|NC_002745.2|	486529	+	C	0	3	0	0	1	-

gi|29165615|ref|NC_002745.2|	486530 +	A	0	2	0	0	0	-
gi|29165615|ref|NC_002745.2|	486542 +	U	0	0	0	0	1	-
gi|29165615|ref|NC_002745.2|	486547 +	G	0	0	1	0	2	-
gi|29165615|ref|NC_002745.2|	486548 +	A	0	0	0	0	1	SA0425
gi|29165615|ref|NC_002745.2|	486550 +	G	0	1	0	0	0	SA0425
gi|29165615|ref|NC_002745.2|	486551 +	A	0	1	0	1	1	SA0425
gi|29165615|ref|NC_002745.2|	486554 +	A	0	1	3	0	1	SA0425
gi|29165615|ref|NC_002745.2|	486599 +	G	0	0	0	1	0	SA0425
gi|29165615|ref|NC_002745.2|	486621 +	A	4	0	0	0	0	SA0425
gi|29165615|ref|NC_002745.2|	486644 +	A	0	1	1	0	3	SA0425
gi|29165615|ref|NC_002745.2|	486645 +	U	0	1	0	0	0	SA0425
gi|29165615|ref|NC_002745.2|	486700 +	A	0	0	1	0	1	SA0425
gi|29165615|ref|NC_002745.2|	486713 +	A	1	0	1	0	0	SA0425
gi|29165615|ref|NC_002745.2|	486714 +	C	0	2	0	0	0	SA0425
gi|29165615|ref|NC_002745.2|	486786 +	U	0	0	1	0	0	SA0425
gi|29165615|ref|NC_002745.2|	486792 +	G	0	0	0	0	1	SA0425
gi|29165615|ref|NC_002745.2|	486844 +	A	0	3	0	0	1	SA0425
gi|29165615|ref|NC_002745.2|	486851 +	G	0	0	0	0	1	SA0425
gi|29165615|ref|NC_002745.2|	486940 +	A	0	1	0	0	0	SA0425_SA0426
gi|29165615|ref|NC_002745.2|	486942 +	A	0	0	0	0	1	SA0425_SA0426
gi|29165615|ref|NC_002745.2|	487018 +	A	0	0	1	0	0	SA0426
gi|29165615|ref|NC_002745.2|	487045 +	A	0	0	0	1	0	SA0426
gi|29165615|ref|NC_002745.2|	487049 +	U	0	1	0	0	0	SA0426
gi|29165615|ref|NC_002745.2|	487083 +	C	1	0	0	0	0	SA0426
gi|29165615|ref|NC_002745.2|	487305 +	A	0	1	0	0	0	SA0426
gi|29165615|ref|NC_002745.2|	487327 +	U	1	0	0	0	0	SA0426
gi|29165615|ref|NC_002745.2|	487571 +	A	0	0	0	0	1	-
gi|29165615|ref|NC_002745.2|	487573 +	A	0	0	1	0	0	-
gi|29165615|ref|NC_002745.2|	489538 +	A	0	1	0	0	0	-
gi|29165615|ref|NC_002745.2|	490620 +	A	0	6	2	0	1	-
gi|29165615|ref|NC_002745.2|	490630 +	A	0	1	0	0	0	-
gi|29165615|ref|NC_002745.2|	490631 +	A	0	1	0	0	0	-
gi|29165615|ref|NC_002745.2|	490646 +	A	0	0	0	0	2	-
gi|29165615|ref|NC_002745.2|	490647 +	A	0	2	0	0	0	-
gi|29165615|ref|NC_002745.2|	490648 +	A	0	1	0	2	1	-
gi|29165615|ref|NC_002745.2|	490649 +	A	0	0	0	0	2	-
gi|29165615|ref|NC_002745.2|	490676 +	A	0	0	0	0	2	SA0430
gi|29165615|ref|NC_002745.2|	490690 +	C	0	1	0	0	0	SA0430
gi|29165615|ref|NC_002745.2|	490695 +	A	0	0	0	0	1	SA0430
gi|29165615|ref|NC_002745.2|	490698 +	A	0	0	0	1	0	SA0430
gi|29165615|ref|NC_002745.2|	490705 +	U	0	1	0	0	0	SA0430
gi|29165615|ref|NC_002745.2|	490748 +	G	0	0	1	0	0	SA0430
gi|29165615|ref|NC_002745.2|	490749 +	A	0	0	1	0	1	SA0430
gi|29165615|ref|NC_002745.2|	490751 +	A	0	3	0	0	1	SA0430
gi|29165615|ref|NC_002745.2|	490752 +	A	0	0	0	0	1	SA0430
gi|29165615|ref|NC_002745.2|	490753 +	U	0	0	0	1	1	SA0430
gi|29165615|ref|NC_002745.2|	491078 +	G	0	0	0	1	0	SA0430
gi|29165615|ref|NC_002745.2|	491289 +	A	0	0	0	1	0	SA0430
gi|29165615|ref|NC_002745.2|	491361 +	G	0	1	0	0	0	SA0430
gi|29165615|ref|NC_002745.2|	491412 +	A	0	1	0	1	0	SA0430
gi|29165615|ref|NC_002745.2|	491415 +	G	0	0	0	0	1	SA0430
gi|29165615|ref|NC_002745.2|	491428 +	G	0	1	0	0	0	SA0430
gi|29165615|ref|NC_002745.2|	491833 +	U	0	1	0	0	0	SA0430
gi|29165615|ref|NC_002745.2|	491888 +	A	0	1	0	0	0	SA0430
gi|29165615|ref|NC_002745.2|	491900 +	G	0	0	0	0	1	SA0430
gi|29165615|ref|NC_002745.2|	492039 +	A	0	1	0	0	0	SA0430
gi|29165615|ref|NC_002745.2|	492147 +	U	0	0	1	0	0	SA0430
gi|29165615|ref|NC_002745.2|	492158 +	C	0	1	0	0	0	SA0430
gi|29165615|ref|NC_002745.2|	492185 +	A	0	3	1	2	0	SA0430
gi|29165615|ref|NC_002745.2|	492186 +	A	0	0	1	0	0	SA0430
gi|29165615|ref|NC_002745.2|	492213 +	C	0	0	0	1	0	SA0430
gi|29165615|ref|NC_002745.2|	492264 +	U	0	0	1	0	0	SA0430
gi|29165615|ref|NC_002745.2|	492313 +	U	0	0	1	0	0	SA0430
gi|29165615|ref|NC_002745.2|	492365 +	C	0	1	0	0	0	SA0430
gi|29165615|ref|NC_002745.2|	492411 +	A	0	0	0	2	0	SA0430
gi|29165615|ref|NC_002745.2|	492953 +	C	0	1	0	0	0	SA0430
gi|29165615|ref|NC_002745.2|	493092 +	C	0	1	0	0	0	SA0430
gi|29165615|ref|NC_002745.2|	493112 +	A	0	1	0	0	0	SA0430
gi|29165615|ref|NC_002745.2|	493114 +	A	0	0	0	0	1	SA0430
gi|29165615|ref|NC_002745.2|	493115 +	A	0	0	0	0	1	SA0430
gi|29165615|ref|NC_002745.2|	493585 +	A	0	0	0	1	0	SA0430
gi|29165615|ref|NC_002745.2|	493623 +	C	0	0	0	1	0	SA0430
gi|29165615|ref|NC_002745.2|	493631 +	G	0	1	0	0	0	SA0430
gi|29165615|ref|NC_002745.2|	493655 +	G	0	0	0	0	1	SA0430
gi|29165615|ref|NC_002745.2|	493660 +	G	2	6	2	4	6	SA0430
gi|29165615|ref|NC_002745.2|	493687 +	U	0	0	1	0	0	SA0430
gi|29165615|ref|NC_002745.2|	493688 +	G	0	0	0	1	0	SA0430
gi|29165615|ref|NC_002745.2|	493714 +	A	0	1	0	0	0	SA0430
gi|29165615|ref|NC_002745.2|	493723 +	C	0	0	0	0	1	SA0430
gi|29165615|ref|NC_002745.2|	493742 +	A	0	0	0	1	0	SA0430
gi|29165615|ref|NC_002745.2|	493762 +	G	1	0	0	0	0	SA0430

gi|29165615|ref|NC_002745.2|	493816 +	A	0	0	0	1	0	SA0430
gi|29165615|ref|NC_002745.2|	493822 +	A	0	0	0	1	1	SA0430
gi|29165615|ref|NC_002745.2|	493826 +	C	0	0	0	1	0	SA0430
gi|29165615|ref|NC_002745.2|	493827 +	U	1	0	0	0	0	SA0430
gi|29165615|ref|NC_002745.2|	493830 +	A	0	0	0	1	0	SA0430
gi|29165615|ref|NC_002745.2|	493860 +	C	0	0	0	0	2	SA0430
gi|29165615|ref|NC_002745.2|	493862 +	G	0	1	0	0	0	SA0430
gi|29165615|ref|NC_002745.2|	493915 +	G	0	1	0	0	0	SA0430
gi|29165615|ref|NC_002745.2|	494095 +	A	0	1	0	0	0	SA0430
gi|29165615|ref|NC_002745.2|	494098 +	A	0	0	0	0	1	SA0430
gi|29165615|ref|NC_002745.2|	494307 +	U	0	0	1	0	0	SA0430
gi|29165615|ref|NC_002745.2|	494365 +	U	0	1	0	0	0	SA0430
gi|29165615|ref|NC_002745.2|	494525 +	G	0	0	0	0	1	SA0430
gi|29165615|ref|NC_002745.2|	494702 +	G	0	0	0	1	0	SA0430
gi|29165615|ref|NC_002745.2|	494764 +	C	0	0	0	1	0	SA0430
gi|29165615|ref|NC_002745.2|	495079 +	C	0	2	0	0	0	SA0430
gi|29165615|ref|NC_002745.2|	495086 +	G	0	1	0	0	0	SA0430
gi|29165615|ref|NC_002745.2|	495488 +	G	0	0	0	1	0	SA0431
gi|29165615|ref|NC_002745.2|	496127 +	C	0	1	0	0	0	SA0431
gi|29165615|ref|NC_002745.2|	496177 +	A	0	1	0	0	0	SA0431
gi|29165615|ref|NC_002745.2|	496385 +	A	0	1	0	0	0	SA0431
gi|29165615|ref|NC_002745.2|	496416 +	A	0	1	0	0	0	SA0431
gi|29165615|ref|NC_002745.2|	496752 +	A	0	0	1	0	0	-
gi|29165615|ref|NC_002745.2|	496831 +	C	0	1	0	0	0	-
gi|29165615|ref|NC_002745.2|	497024 +	A	0	1	0	0	0	-
gi|29165615|ref|NC_002745.2|	497028 +	U	0	1	0	1	1	-
gi|29165615|ref|NC_002745.2|	497029 +	A	0	0	0	0	2	-
gi|29165615|ref|NC_002745.2|	497041 +	U	0	0	0	1	1	-
gi|29165615|ref|NC_002745.2|	497042 +	A	0	0	1	0	0	-
gi|29165615|ref|NC_002745.2|	497069 +	U	0	0	0	0	1	-
gi|29165615|ref|NC_002745.2|	497080 +	C	0	0	1	0	0	-
gi|29165615|ref|NC_002745.2|	497157 +	C	0	1	0	0	0	-
gi|29165615|ref|NC_002745.2|	497192 +	A	0	1	0	0	0	-
gi|29165615|ref|NC_002745.2|	497400 +	A	0	0	0	0	1	-
gi|29165615|ref|NC_002745.2|	497402 +	U	0	0	0	0	1	-
gi|29165615|ref|NC_002745.2|	497449 +	U	0	8	3	0	7	-
gi|29165615|ref|NC_002745.2|	497450 +	U	0	40	12	3	21	-
gi|29165615|ref|NC_002745.2|	497451 +	A	102	2905	1410	141	1541	-
gi|29165615|ref|NC_002745.2|	497452 +	A	2	220	75	15	184	-
gi|29165615|ref|NC_002745.2|	497453 +	G	4	108	34	17	80	-
gi|29165615|ref|NC_002745.2|	497454 +	A	2	88	31	14	93	-
gi|29165615|ref|NC_002745.2|	497455 +	C	0	7	3	0	2	-
gi|29165615|ref|NC_002745.2|	497456 +	A	0	9	4	2	15	-
gi|29165615|ref|NC_002745.2|	497457 +	U	0	1	2	0	0	-
gi|29165615|ref|NC_002745.2|	497458 +	U	0	1	0	0	2	-
gi|29165615|ref|NC_002745.2|	497459 +	A	1	1	4	0	2	-
gi|29165615|ref|NC_002745.2|	497460 +	A	0	5	1	1	7	-
gi|29165615|ref|NC_002745.2|	497461 +	G	0	7	1	1	5	-
gi|29165615|ref|NC_002745.2|	497462 +	G	0	57	13	7	33	-
gi|29165615|ref|NC_002745.2|	497463 +	G	0	20	5	1	12	-
gi|29165615|ref|NC_002745.2|	497464 +	A	1	21	4	1	12	-
gi|29165615|ref|NC_002745.2|	497465 +	G	0	4	2	1	5	-
gi|29165615|ref|NC_002745.2|	497466 +	U	1	36	10	1	17	-
gi|29165615|ref|NC_002745.2|	497467 +	G	1	51	10	11	43	-
gi|29165615|ref|NC_002745.2|	497468 +	A	2	70	19	14	52	-
gi|29165615|ref|NC_002745.2|	497469 +	A	0	12	2	2	11	-
gi|29165615|ref|NC_002745.2|	497470 +	A	1	7	0	1	2	-
gi|29165615|ref|NC_002745.2|	497471 +	U	0	2	1	0	4	-
gi|29165615|ref|NC_002745.2|	497472 +	A	0	1	1	0	0	-
gi|29165615|ref|NC_002745.2|	497473 +	U	0	1	2	0	0	-
gi|29165615|ref|NC_002745.2|	497474 +	A	0	1	0	0	0	SA0432
gi|29165615|ref|NC_002745.2|	497475 +	U	0	0	1	0	1	SA0432
gi|29165615|ref|NC_002745.2|	497476 +	G	0	1	0	0	5	SA0432
gi|29165615|ref|NC_002745.2|	497477 +	G	0	2	1	1	2	SA0432
gi|29165615|ref|NC_002745.2|	497478 +	C	0	1	2	0	0	SA0432
gi|29165615|ref|NC_002745.2|	497481 +	U	0	3	0	0	1	SA0432
gi|29165615|ref|NC_002745.2|	497482 +	A	0	4	1	1	3	SA0432
gi|29165615|ref|NC_002745.2|	497483 +	A	0	6	0	0	8	SA0432
gi|29165615|ref|NC_002745.2|	497484 +	A	0	17	5	2	12	SA0432
gi|29165615|ref|NC_002745.2|	497485 +	A	1	4	1	0	5	SA0432
gi|29165615|ref|NC_002745.2|	497486 +	A	0	2	0	1	0	SA0432
gi|29165615|ref|NC_002745.2|	497487 +	G	0	1	0	1	0	SA0432
gi|29165615|ref|NC_002745.2|	497488 +	A	0	1	0	0	0	SA0432
gi|29165615|ref|NC_002745.2|	497489 +	G	0	4	1	0	3	SA0432
gi|29165615|ref|NC_002745.2|	497490 +	A	0	4	2	2	5	SA0432
gi|29165615|ref|NC_002745.2|	497491 +	A	0	3	0	0	1	SA0432
gi|29165615|ref|NC_002745.2|	497492 +	G	0	8	3	4	4	SA0432
gi|29165615|ref|NC_002745.2|	497493 +	A	0	0	3	0	1	SA0432
gi|29165615|ref|NC_002745.2|	497495 +	G	0	0	0	0	1	SA0432
gi|29165615|ref|NC_002745.2|	497496 +	U	0	2	1	0	1	SA0432
gi|29165615|ref|NC_002745.2|	497497 +	A	0	2	0	0	1	SA0432

gi|29165615|ref|NC_002745.2|	497498 +	A	0	0	1	0	3	SA0432
gi|29165615|ref|NC_002745.2|	497499 +	A	0	2	0	0	0	SA0432
gi|29165615|ref|NC_002745.2|	497500 +	A	0	0	0	0	1	SA0432
gi|29165615|ref|NC_002745.2|	497502 +	C	0	1	0	0	0	SA0432
gi|29165615|ref|NC_002745.2|	497503 +	C	0	2	0	0	0	SA0432
gi|29165615|ref|NC_002745.2|	497504 +	A	0	0	0	0	2	SA0432
gi|29165615|ref|NC_002745.2|	497508 +	U	0	0	0	1	3	SA0432
gi|29165615|ref|NC_002745.2|	497509 +	A	0	1	0	0	1	SA0432
gi|29165615|ref|NC_002745.2|	497510 +	A	1	0	1	0	0	SA0432
gi|29165615|ref|NC_002745.2|	497514 +	C	0	17	4	1	6	SA0432
gi|29165615|ref|NC_002745.2|	497515 +	U	0	0	1	0	1	SA0432
gi|29165615|ref|NC_002745.2|	497516 +	A	0	2	1	1	1	SA0432
gi|29165615|ref|NC_002745.2|	497517 +	U	0	2	0	0	0	SA0432
gi|29165615|ref|NC_002745.2|	497518 +	U	0	1	0	0	0	SA0432
gi|29165615|ref|NC_002745.2|	497520 +	G	0	4	0	0	3	SA0432
gi|29165615|ref|NC_002745.2|	497522 +	G	0	1	0	0	0	SA0432
gi|29165615|ref|NC_002745.2|	497523 +	G	0	3	1	0	4	SA0432
gi|29165615|ref|NC_002745.2|	497524 +	A	0	11	0	1	4	SA0432
gi|29165615|ref|NC_002745.2|	497525 +	A	0	5	2	2	5	SA0432
gi|29165615|ref|NC_002745.2|	497526 +	A	0	1	0	0	1	SA0432
gi|29165615|ref|NC_002745.2|	497527 +	A	0	1	0	1	0	SA0432
gi|29165615|ref|NC_002745.2|	497529 +	A	0	0	0	0	1	SA0432
gi|29165615|ref|NC_002745.2|	497530 +	A	0	1	0	0	0	SA0432
gi|29165615|ref|NC_002745.2|	497533 +	U	0	0	0	1	0	SA0432
gi|29165615|ref|NC_002745.2|	497537 +	G	0	0	1	0	1	SA0432
gi|29165615|ref|NC_002745.2|	497538 +	A	0	3	0	0	4	SA0432
gi|29165615|ref|NC_002745.2|	497546 +	A	0	1	0	0	0	SA0432
gi|29165615|ref|NC_002745.2|	497556 +	U	0	0	1	0	0	SA0432
gi|29165615|ref|NC_002745.2|	497560 +	A	0	0	0	0	1	SA0432
gi|29165615|ref|NC_002745.2|	497562 +	G	0	0	0	0	2	SA0432
gi|29165615|ref|NC_002745.2|	497563 +	A	0	0	0	0	1	SA0432
gi|29165615|ref|NC_002745.2|	497566 +	A	0	1	0	0	1	SA0432
gi|29165615|ref|NC_002745.2|	497568 +	G	1	0	0	0	0	SA0432
gi|29165615|ref|NC_002745.2|	497569 +	U	0	0	0	0	1	SA0432
gi|29165615|ref|NC_002745.2|	497571 +	U	0	1	1	0	0	SA0432
gi|29165615|ref|NC_002745.2|	497573 +	G	0	1	0	0	0	SA0432
gi|29165615|ref|NC_002745.2|	497584 +	U	1	0	0	0	0	SA0432
gi|29165615|ref|NC_002745.2|	497586 +	A	0	1	0	1	1	SA0432
gi|29165615|ref|NC_002745.2|	497587 +	A	0	0	1	0	0	SA0432
gi|29165615|ref|NC_002745.2|	497591 +	A	0	0	0	0	1	SA0432
gi|29165615|ref|NC_002745.2|	497592 +	A	0	2	0	0	5	SA0432
gi|29165615|ref|NC_002745.2|	497595 +	U	0	7	0	0	2	SA0432
gi|29165615|ref|NC_002745.2|	497597 +	G	0	1	1	0	2	SA0432
gi|29165615|ref|NC_002745.2|	497598 +	A	0	4	4	0	9	SA0432
gi|29165615|ref|NC_002745.2|	497602 +	A	0	1	0	0	0	SA0432
gi|29165615|ref|NC_002745.2|	497604 +	A	0	0	1	0	0	SA0432
gi|29165615|ref|NC_002745.2|	497605 +	C	0	0	0	1	0	SA0432
gi|29165615|ref|NC_002745.2|	497606 +	G	0	0	0	1	0	SA0432
gi|29165615|ref|NC_002745.2|	497607 +	C	0	1	4	0	0	SA0432
gi|29165615|ref|NC_002745.2|	497610 +	U	0	2	0	0	0	SA0432
gi|29165615|ref|NC_002745.2|	497611 +	A	0	1	0	0	0	SA0432
gi|29165615|ref|NC_002745.2|	497613 +	G	0	0	0	1	0	SA0432
gi|29165615|ref|NC_002745.2|	497615 +	A	0	0	0	2	1	SA0432
gi|29165615|ref|NC_002745.2|	497619 +	A	0	1	0	0	0	SA0432
gi|29165615|ref|NC_002745.2|	497624 +	U	0	2	0	0	0	SA0432
gi|29165615|ref|NC_002745.2|	497632 +	G	0	2	0	0	0	SA0432
gi|29165615|ref|NC_002745.2|	497633 +	G	1	1	2	0	1	SA0432
gi|29165615|ref|NC_002745.2|	497639 +	U	0	0	0	0	1	SA0432
gi|29165615|ref|NC_002745.2|	497646 +	C	0	0	1	0	0	SA0432
gi|29165615|ref|NC_002745.2|	497650 +	C	0	1	0	0	0	SA0432
gi|29165615|ref|NC_002745.2|	497656 +	A	0	1	0	0	0	SA0432
gi|29165615|ref|NC_002745.2|	497661 +	A	0	0	0	0	1	SA0432
gi|29165615|ref|NC_002745.2|	497662 +	A	0	1	0	0	2	SA0432
gi|29165615|ref|NC_002745.2|	497663 +	A	0	0	0	0	1	SA0432
gi|29165615|ref|NC_002745.2|	497667 +	U	0	3	1	1	1	SA0432
gi|29165615|ref|NC_002745.2|	497668 +	C	0	0	0	0	1	SA0432
gi|29165615|ref|NC_002745.2|	497674 +	U	0	0	0	0	2	SA0432
gi|29165615|ref|NC_002745.2|	497675 +	C	0	1	0	0	0	SA0432
gi|29165615|ref|NC_002745.2|	497676 +	C	0	3	0	0	0	SA0432
gi|29165615|ref|NC_002745.2|	497678 +	G	0	1	0	0	0	SA0432
gi|29165615|ref|NC_002745.2|	497682 +	C	0	2	0	0	0	SA0432
gi|29165615|ref|NC_002745.2|	497691 +	A	0	0	1	0	0	SA0432
gi|29165615|ref|NC_002745.2|	497692 +	A	0	1	0	0	0	SA0432
gi|29165615|ref|NC_002745.2|	497697 +	A	0	0	1	0	1	SA0432
gi|29165615|ref|NC_002745.2|	497698 +	U	0	0	1	0	1	SA0432
gi|29165615|ref|NC_002745.2|	497702 +	C	0	5	1	5	0	SA0432
gi|29165615|ref|NC_002745.2|	497709 +	U	0	1	0	0	1	SA0432
gi|29165615|ref|NC_002745.2|	497710 +	U	0	1	0	0	0	SA0432
gi|29165615|ref|NC_002745.2|	497711 +	G	0	7	1	2	0	SA0432
gi|29165615|ref|NC_002745.2|	497712 +	A	0	0	0	0	1	SA0432
gi|29165615|ref|NC_002745.2|	497718 +	C	0	0	0	0	1	SA0432

gi|29165615|ref|NC_002745.2|	497724	+	C	0	1	0	0	0	SA0432
gi|29165615|ref|NC_002745.2|	497739	+	A	0	2	0	0	0	SA0432
gi|29165615|ref|NC_002745.2|	497740	+	A	0	0	0	0	1	SA0432
gi|29165615|ref|NC_002745.2|	497745	+	A	0	1	0	0	0	SA0432
gi|29165615|ref|NC_002745.2|	497747	+	G	1	0	0	0	1	SA0432
gi|29165615|ref|NC_002745.2|	497766	+	A	0	0	1	0	0	SA0432
gi|29165615|ref|NC_002745.2|	497769	+	A	0	1	0	0	0	SA0432
gi|29165615|ref|NC_002745.2|	497773	+	G	0	1	0	0	0	SA0432
gi|29165615|ref|NC_002745.2|	497775	+	A	0	1	0	1	0	SA0432
gi|29165615|ref|NC_002745.2|	497781	+	U	0	0	0	0	2	SA0432
gi|29165615|ref|NC_002745.2|	497782	+	A	1	0	0	0	0	SA0432
gi|29165615|ref|NC_002745.2|	497785	+	A	0	1	0	0	0	SA0432
gi|29165615|ref|NC_002745.2|	497786	+	C	0	0	1	0	0	SA0432
gi|29165615|ref|NC_002745.2|	497788	+	U	0	0	0	1	1	SA0432
gi|29165615|ref|NC_002745.2|	497790	+	U	0	0	0	0	1	SA0432
gi|29165615|ref|NC_002745.2|	497797	+	A	0	3	1	0	3	SA0432
gi|29165615|ref|NC_002745.2|	497798	+	U	0	1	0	0	2	SA0432
gi|29165615|ref|NC_002745.2|	497822	+	A	0	0	0	0	1	SA0432
gi|29165615|ref|NC_002745.2|	497825	+	U	0	0	0	0	1	SA0432
gi|29165615|ref|NC_002745.2|	497826	+	U	0	0	1	0	0	SA0432
gi|29165615|ref|NC_002745.2|	497833	+	G	0	1	0	0	0	SA0432
gi|29165615|ref|NC_002745.2|	497837	+	G	0	1	0	0	0	SA0432
gi|29165615|ref|NC_002745.2|	497840	+	A	0	0	0	0	1	SA0432
gi|29165615|ref|NC_002745.2|	497841	+	C	1	1	0	0	0	SA0432
gi|29165615|ref|NC_002745.2|	497854	+	A	0	0	0	1	0	SA0432
gi|29165615|ref|NC_002745.2|	497857	+	G	0	1	0	0	0	SA0432
gi|29165615|ref|NC_002745.2|	497862	+	U	0	1	0	0	2	SA0432
gi|29165615|ref|NC_002745.2|	497863	+	C	0	1	0	0	0	SA0432
gi|29165615|ref|NC_002745.2|	497864	+	A	0	0	0	0	1	SA0432
gi|29165615|ref|NC_002745.2|	497865	+	A	0	0	0	1	0	SA0432
gi|29165615|ref|NC_002745.2|	497866	+	U	0	1	0	0	0	SA0432
gi|29165615|ref|NC_002745.2|	497867	+	A	0	1	0	0	0	SA0432
gi|29165615|ref|NC_002745.2|	497871	+	U	0	1	0	0	0	SA0432
gi|29165615|ref|NC_002745.2|	497873	+	C	0	2	1	0	0	SA0432
gi|29165615|ref|NC_002745.2|	497876	+	A	0	5	1	1	5	SA0432
gi|29165615|ref|NC_002745.2|	497877	+	C	0	1	0	0	0	SA0432
gi|29165615|ref|NC_002745.2|	497878	+	A	0	1	0	0	1	SA0432
gi|29165615|ref|NC_002745.2|	497882	+	A	0	0	0	0	2	SA0432
gi|29165615|ref|NC_002745.2|	497900	+	A	0	1	0	0	0	SA0432
gi|29165615|ref|NC_002745.2|	497906	+	C	0	1	0	0	0	SA0432
gi|29165615|ref|NC_002745.2|	497913	+	A	1	0	0	0	0	SA0432
gi|29165615|ref|NC_002745.2|	497914	+	G	0	0	1	0	0	SA0432
gi|29165615|ref|NC_002745.2|	497915	+	A	0	1	0	0	0	SA0432
gi|29165615|ref|NC_002745.2|	497917	+	G	0	0	1	0	0	SA0432
gi|29165615|ref|NC_002745.2|	497918	+	U	0	1	2	0	0	SA0432
gi|29165615|ref|NC_002745.2|	497919	+	A	1	0	1	0	0	SA0432
gi|29165615|ref|NC_002745.2|	497920	+	U	0	1	1	1	0	SA0432
gi|29165615|ref|NC_002745.2|	497922	+	C	0	1	0	0	0	SA0432
gi|29165615|ref|NC_002745.2|	497924	+	C	0	2	0	0	2	SA0432
gi|29165615|ref|NC_002745.2|	497927	+	A	0	2	0	0	0	SA0432
gi|29165615|ref|NC_002745.2|	497931	+	C	0	0	1	0	0	SA0432
gi|29165615|ref|NC_002745.2|	497934	+	A	0	0	0	1	0	SA0432
gi|29165615|ref|NC_002745.2|	497936	+	A	0	0	0	0	1	SA0432
gi|29165615|ref|NC_002745.2|	497940	+	C	0	0	0	0	1	SA0432
gi|29165615|ref|NC_002745.2|	497942	+	A	0	0	1	0	0	SA0432
gi|29165615|ref|NC_002745.2|	497944	+	C	0	0	0	0	1	SA0432
gi|29165615|ref|NC_002745.2|	497945	+	A	0	0	0	0	2	SA0432
gi|29165615|ref|NC_002745.2|	497946	+	U	0	2	0	1	1	SA0432
gi|29165615|ref|NC_002745.2|	497947	+	C	0	1	0	0	0	SA0432
gi|29165615|ref|NC_002745.2|	497948	+	A	0	0	1	0	1	SA0432
gi|29165615|ref|NC_002745.2|	497949	+	U	0	0	1	0	0	SA0432
gi|29165615|ref|NC_002745.2|	497950	+	U	0	1	2	0	0	SA0432
gi|29165615|ref|NC_002745.2|	497951	+	A	0	2	1	2	2	SA0432
gi|29165615|ref|NC_002745.2|	497952	+	A	0	0	0	0	1	SA0432
gi|29165615|ref|NC_002745.2|	497953	+	U	0	1	0	0	1	SA0432
gi|29165615|ref|NC_002745.2|	497954	+	G	0	1	1	0	0	SA0432
gi|29165615|ref|NC_002745.2|	497955	+	U	0	1	1	1	1	SA0432
gi|29165615|ref|NC_002745.2|	497956	+	G	1	0	0	0	0	SA0432
gi|29165615|ref|NC_002745.2|	497957	+	A	0	3	0	2	2	SA0432
gi|29165615|ref|NC_002745.2|	497961	+	C	0	1	0	0	0	SA0432
gi|29165615|ref|NC_002745.2|	497965	+	U	0	0	0	1	0	SA0432
gi|29165615|ref|NC_002745.2|	497966	+	A	0	0	1	0	0	SA0432
gi|29165615|ref|NC_002745.2|	497968	+	G	0	0	0	0	1	SA0432
gi|29165615|ref|NC_002745.2|	497969	+	G	0	1	0	0	0	SA0432
gi|29165615|ref|NC_002745.2|	497970	+	C	1	25	7	2	18	SA0432
gi|29165615|ref|NC_002745.2|	497971	+	A	0	1	0	1	1	SA0432
gi|29165615|ref|NC_002745.2|	497972	+	U	0	0	0	0	2	SA0432
gi|29165615|ref|NC_002745.2|	497973	+	U	0	0	0	0	1	SA0432
gi|29165615|ref|NC_002745.2|	497974	+	U	0	1	1	1	0	SA0432
gi|29165615|ref|NC_002745.2|	497975	+	A	0	0	1	0	0	SA0432
gi|29165615|ref|NC_002745.2|	497976	+	U	0	3	1	1	2	SA0432

gi|29165615|ref|NC_002745.2|	497977 +	U	0	1	0	0	1	SA0432
gi|29165615|ref|NC_002745.2|	497978 +	U	0	2	0	0	3	SA0432
gi|29165615|ref|NC_002745.2|	497979 +	U	3	2	6	0	1	SA0432
gi|29165615|ref|NC_002745.2|	497980 +	C	0	0	0	0	1	SA0432
gi|29165615|ref|NC_002745.2|	497981 +	U	0	0	3	0	2	SA0432
gi|29165615|ref|NC_002745.2|	497982 +	U	1	0	1	2	0	SA0432
gi|29165615|ref|NC_002745.2|	497983 +	A	0	3	1	0	0	SA0432
gi|29165615|ref|NC_002745.2|	497984 +	C	0	3	1	0	2	SA0432
gi|29165615|ref|NC_002745.2|	497985 +	C	0	2	0	0	1	SA0432
gi|29165615|ref|NC_002745.2|	497986 +	A	0	1	0	0	6	SA0432
gi|29165615|ref|NC_002745.2|	497987 +	G	0	6	4	3	7	SA0432
gi|29165615|ref|NC_002745.2|	497988 +	C	0	1	0	1	1	SA0432
gi|29165615|ref|NC_002745.2|	497989 +	A	0	3	0	0	1	SA0432
gi|29165615|ref|NC_002745.2|	497990 +	U	0	12	12	2	18	SA0432
gi|29165615|ref|NC_002745.2|	497991 +	U	0	5	4	0	1	SA0432
gi|29165615|ref|NC_002745.2|	497992 +	A	0	5	1	0	2	SA0432
gi|29165615|ref|NC_002745.2|	497993 +	A	0	9	4	0	4	SA0432
gi|29165615|ref|NC_002745.2|	497994 +	U	0	1	0	2	0	SA0432
gi|29165615|ref|NC_002745.2|	497995 +	U	0	0	4	0	0	SA0432
gi|29165615|ref|NC_002745.2|	497996 +	G	0	1	3	0	2	SA0432
gi|29165615|ref|NC_002745.2|	497998 +	U	0	1	1	0	1	SA0432
gi|29165615|ref|NC_002745.2|	498000 +	G	0	2	0	1	2	SA0432
gi|29165615|ref|NC_002745.2|	498001 +	G	0	3	3	1	5	SA0432
gi|29165615|ref|NC_002745.2|	498002 +	A	0	5	0	0	3	SA0432
gi|29165615|ref|NC_002745.2|	498003 +	G	0	2	1	0	6	SA0432
gi|29165615|ref|NC_002745.2|	498004 +	U	0	3	0	0	2	SA0432
gi|29165615|ref|NC_002745.2|	498005 +	A	0	1	1	0	1	SA0432
gi|29165615|ref|NC_002745.2|	498006 +	G	0	1	1	0	0	SA0432
gi|29165615|ref|NC_002745.2|	498007 +	U	0	1	0	0	9	SA0432
gi|29165615|ref|NC_002745.2|	498011 +	C	0	1	0	0	0	SA0432
gi|29165615|ref|NC_002745.2|	498012 +	G	0	2	0	0	0	SA0432
gi|29165615|ref|NC_002745.2|	498013 +	U	0	2	1	0	2	SA0432
gi|29165615|ref|NC_002745.2|	498014 +	G	0	8	1	0	3	SA0432
gi|29165615|ref|NC_002745.2|	498015 +	U	1	1	0	1	0	SA0432
gi|29165615|ref|NC_002745.2|	498016 +	A	2	0	1	0	0	SA0432
gi|29165615|ref|NC_002745.2|	498018 +	U	0	1	0	0	0	SA0432
gi|29165615|ref|NC_002745.2|	498019 +	U	0	1	1	0	1	SA0432
gi|29165615|ref|NC_002745.2|	498020 +	G	0	0	1	0	0	SA0432
gi|29165615|ref|NC_002745.2|	498021 +	G	0	1	0	0	0	SA0432
gi|29165615|ref|NC_002745.2|	498024 +	G	0	0	1	0	3	SA0432
gi|29165615|ref|NC_002745.2|	498025 +	U	0	3	0	0	0	SA0432
gi|29165615|ref|NC_002745.2|	498026 +	A	0	0	0	0	1	SA0432
gi|29165615|ref|NC_002745.2|	498028 +	U	0	3	2	1	0	SA0432
gi|29165615|ref|NC_002745.2|	498029 +	C	0	0	0	0	1	SA0432
gi|29165615|ref|NC_002745.2|	498031 +	G	0	0	1	0	0	SA0432
gi|29165615|ref|NC_002745.2|	498035 +	C	0	1	1	0	0	SA0432
gi|29165615|ref|NC_002745.2|	498037 +	A	0	1	0	0	0	SA0432
gi|29165615|ref|NC_002745.2|	498040 +	C	0	0	1	0	2	SA0432
gi|29165615|ref|NC_002745.2|	498041 +	A	0	0	0	0	1	SA0432
gi|29165615|ref|NC_002745.2|	498044 +	G	0	2	0	0	0	SA0432
gi|29165615|ref|NC_002745.2|	498045 +	U	0	3	0	3	6	SA0432
gi|29165615|ref|NC_002745.2|	498046 +	C	0	0	0	1	1	SA0432
gi|29165615|ref|NC_002745.2|	498047 +	U	0	1	0	1	0	SA0432
gi|29165615|ref|NC_002745.2|	498048 +	U	0	1	1	0	0	SA0432
gi|29165615|ref|NC_002745.2|	498049 +	A	0	0	0	0	1	SA0432
gi|29165615|ref|NC_002745.2|	498052 +	U	1	1	0	0	0	SA0432
gi|29165615|ref|NC_002745.2|	498053 +	U	0	0	0	0	1	SA0432
gi|29165615|ref|NC_002745.2|	498056 +	A	0	0	0	1	1	SA0432
gi|29165615|ref|NC_002745.2|	498060 +	U	0	2	1	0	0	SA0432
gi|29165615|ref|NC_002745.2|	498061 +	A	0	0	1	0	0	SA0432
gi|29165615|ref|NC_002745.2|	498062 +	A	0	0	0	0	1	SA0432
gi|29165615|ref|NC_002745.2|	498071 +	C	0	3	0	0	3	SA0432
gi|29165615|ref|NC_002745.2|	498073 +	A	0	6	0	0	1	SA0432
gi|29165615|ref|NC_002745.2|	498074 +	U	0	0	1	0	0	SA0432
gi|29165615|ref|NC_002745.2|	498075 +	U	0	0	2	0	1	SA0432
gi|29165615|ref|NC_002745.2|	498078 +	U	0	2	0	0	1	SA0432
gi|29165615|ref|NC_002745.2|	498079 +	A	0	1	0	0	0	SA0432
gi|29165615|ref|NC_002745.2|	498083 +	C	0	1	0	0	0	SA0432
gi|29165615|ref|NC_002745.2|	498084 +	A	0	1	0	0	0	SA0432
gi|29165615|ref|NC_002745.2|	498090 +	A	0	3	0	0	2	SA0432
gi|29165615|ref|NC_002745.2|	498092 +	U	0	1	1	0	0	SA0432
gi|29165615|ref|NC_002745.2|	498096 +	C	0	2	1	0	1	SA0432
gi|29165615|ref|NC_002745.2|	498097 +	A	0	1	1	0	1	SA0432
gi|29165615|ref|NC_002745.2|	498100 +	A	0	1	0	0	0	SA0432
gi|29165615|ref|NC_002745.2|	498102 +	G	0	2	2	0	0	SA0432
gi|29165615|ref|NC_002745.2|	498104 +	A	0	1	0	2	0	SA0432
gi|29165615|ref|NC_002745.2|	498109 +	U	0	0	0	0	1	SA0432
gi|29165615|ref|NC_002745.2|	498113 +	A	0	1	2	0	1	SA0432
gi|29165615|ref|NC_002745.2|	498118 +	G	0	1	0	0	1	SA0432
gi|29165615|ref|NC_002745.2|	498122 +	U	0	1	0	0	0	SA0432
gi|29165615|ref|NC_002745.2|	498123 +	U	0	1	0	0	0	SA0432

gi|29165615|ref|NC_002745.2|	498135 +	A	0	1	0	0	0	SA0432
gi|29165615|ref|NC_002745.2|	498138 +	U	0	1	0	0	0	SA0432
gi|29165615|ref|NC_002745.2|	498140 +	A	0	1	0	0	0	SA0432
gi|29165615|ref|NC_002745.2|	498143 +	C	0	0	0	0	1	SA0432
gi|29165615|ref|NC_002745.2|	498147 +	U	0	0	1	0	0	SA0432
gi|29165615|ref|NC_002745.2|	498149 +	A	0	1	0	0	0	SA0432
gi|29165615|ref|NC_002745.2|	498155 +	C	0	0	0	0	1	SA0432
gi|29165615|ref|NC_002745.2|	498160 +	U	0	4	6	4	8	SA0432
gi|29165615|ref|NC_002745.2|	498161 +	C	0	3	1	1	4	SA0432
gi|29165615|ref|NC_002745.2|	498162 +	A	0	2	0	0	1	SA0432
gi|29165615|ref|NC_002745.2|	498163 +	A	0	11	5	5	6	SA0432
gi|29165615|ref|NC_002745.2|	498164 +	G	0	0	0	0	1	SA0432
gi|29165615|ref|NC_002745.2|	498174 +	U	0	0	0	0	1	SA0432
gi|29165615|ref|NC_002745.2|	498184 +	A	0	0	0	0	1	SA0432
gi|29165615|ref|NC_002745.2|	498192 +	U	0	0	0	0	1	SA0432
gi|29165615|ref|NC_002745.2|	498194 +	C	0	0	0	0	1	SA0432
gi|29165615|ref|NC_002745.2|	498201 +	A	0	1	0	0	0	SA0432
gi|29165615|ref|NC_002745.2|	498203 +	A	0	3	0	0	0	SA0432
gi|29165615|ref|NC_002745.2|	498208 +	A	0	1	0	0	0	SA0432
gi|29165615|ref|NC_002745.2|	498213 +	G	0	2	2	2	2	SA0432
gi|29165615|ref|NC_002745.2|	498214 +	A	0	1	0	0	3	SA0432
gi|29165615|ref|NC_002745.2|	498216 +	U	0	0	1	0	0	SA0432
gi|29165615|ref|NC_002745.2|	498217 +	A	0	2	0	0	2	SA0432
gi|29165615|ref|NC_002745.2|	498224 +	G	0	1	0	0	0	SA0432
gi|29165615|ref|NC_002745.2|	498231 +	A	0	0	0	0	1	SA0432
gi|29165615|ref|NC_002745.2|	498233 +	G	0	1	0	4	0	SA0432
gi|29165615|ref|NC_002745.2|	498236 +	U	0	1	0	0	0	SA0432
gi|29165615|ref|NC_002745.2|	498242 +	A	0	0	0	0	1	SA0432
gi|29165615|ref|NC_002745.2|	498245 +	A	0	0	0	1	1	SA0432
gi|29165615|ref|NC_002745.2|	498248 +	U	0	1	0	0	1	SA0432
gi|29165615|ref|NC_002745.2|	498257 +	G	0	1	0	0	0	SA0432
gi|29165615|ref|NC_002745.2|	498262 +	C	0	4	0	0	0	SA0432
gi|29165615|ref|NC_002745.2|	498263 +	G	0	2	0	0	6	SA0432
gi|29165615|ref|NC_002745.2|	498282 +	G	0	4	0	0	3	SA0432
gi|29165615|ref|NC_002745.2|	498283 +	A	0	1	0	0	0	SA0432
gi|29165615|ref|NC_002745.2|	498290 +	G	0	0	0	0	1	SA0432
gi|29165615|ref|NC_002745.2|	498291 +	C	0	0	0	0	1	SA0432
gi|29165615|ref|NC_002745.2|	498294 +	U	0	0	1	0	0	SA0432
gi|29165615|ref|NC_002745.2|	498296 +	A	0	2	0	1	1	SA0432
gi|29165615|ref|NC_002745.2|	498300 +	U	0	0	1	0	0	SA0432
gi|29165615|ref|NC_002745.2|	498302 +	A	2	0	1	0	1	SA0432
gi|29165615|ref|NC_002745.2|	498310 +	A	0	1	0	0	0	SA0432
gi|29165615|ref|NC_002745.2|	498315 +	C	0	1	0	0	0	SA0432
gi|29165615|ref|NC_002745.2|	498316 +	A	0	2	0	0	0	SA0432
gi|29165615|ref|NC_002745.2|	498323 +	A	0	0	0	1	0	SA0432
gi|29165615|ref|NC_002745.2|	498330 +	C	0	0	0	0	1	SA0432
gi|29165615|ref|NC_002745.2|	498334 +	U	0	1	0	0	2	SA0432
gi|29165615|ref|NC_002745.2|	498339 +	C	1	1	0	0	1	SA0432
gi|29165615|ref|NC_002745.2|	498342 +	C	0	0	0	0	1	SA0432
gi|29165615|ref|NC_002745.2|	498345 +	G	0	3	0	0	1	SA0432
gi|29165615|ref|NC_002745.2|	498346 +	U	0	1	0	0	2	SA0432
gi|29165615|ref|NC_002745.2|	498348 +	U	0	0	2	0	0	SA0432
gi|29165615|ref|NC_002745.2|	498349 +	U	1	1	0	0	1	SA0432
gi|29165615|ref|NC_002745.2|	498350 +	A	0	0	2	1	0	SA0432
gi|29165615|ref|NC_002745.2|	498351 +	C	0	1	1	1	0	SA0432
gi|29165615|ref|NC_002745.2|	498352 +	A	0	0	1	0	0	SA0432
gi|29165615|ref|NC_002745.2|	498354 +	U	0	1	0	0	0	SA0432
gi|29165615|ref|NC_002745.2|	498355 +	U	0	0	0	1	0	SA0432
gi|29165615|ref|NC_002745.2|	498357 +	U	1	0	0	0	3	SA0432
gi|29165615|ref|NC_002745.2|	498358 +	A	0	1	0	0	1	SA0432
gi|29165615|ref|NC_002745.2|	498361 +	C	0	0	0	0	1	SA0432
gi|29165615|ref|NC_002745.2|	498362 +	C	1	0	1	0	0	SA0432
gi|29165615|ref|NC_002745.2|	498364 +	A	0	3	0	0	0	SA0432
gi|29165615|ref|NC_002745.2|	498365 +	C	0	0	0	0	1	SA0432
gi|29165615|ref|NC_002745.2|	498368 +	G	0	0	0	0	1	SA0432
gi|29165615|ref|NC_002745.2|	498371 +	G	0	0	0	0	1	SA0432
gi|29165615|ref|NC_002745.2|	498378 +	U	0	1	0	0	0	SA0432
gi|29165615|ref|NC_002745.2|	498379 +	U	0	0	0	0	1	SA0432
gi|29165615|ref|NC_002745.2|	498382 +	C	0	0	1	0	1	SA0432
gi|29165615|ref|NC_002745.2|	498389 +	A	0	7	0	0	3	SA0432
gi|29165615|ref|NC_002745.2|	498390 +	U	1	0	0	0	0	SA0432
gi|29165615|ref|NC_002745.2|	498395 +	G	0	1	0	0	0	SA0432
gi|29165615|ref|NC_002745.2|	498397 +	A	0	1	0	1	1	SA0432
gi|29165615|ref|NC_002745.2|	498398 +	U	0	0	0	1	0	SA0432
gi|29165615|ref|NC_002745.2|	498401 +	U	0	0	0	1	0	SA0432
gi|29165615|ref|NC_002745.2|	498402 +	U	0	0	1	0	0	SA0432
gi|29165615|ref|NC_002745.2|	498403 +	A	1	0	0	0	0	SA0432
gi|29165615|ref|NC_002745.2|	498405 +	A	0	1	0	0	1	SA0432
gi|29165615|ref|NC_002745.2|	498411 +	C	0	0	1	0	1	SA0432
gi|29165615|ref|NC_002745.2|	498422 +	A	0	0	1	0	0	SA0432
gi|29165615|ref|NC_002745.2|	498423 +	C	0	0	0	0	1	SA0432

gi|29165615|ref|NC_002745.2|	498426	+	G	0	1	0	0	1	SA0432
gi|29165615|ref|NC_002745.2|	498428	+	C	0	0	0	0	1	SA0432
gi|29165615|ref|NC_002745.2|	498430	+	A	0	1	0	0	1	SA0432
gi|29165615|ref|NC_002745.2|	498431	+	C	0	0	0	1	5	SA0432
gi|29165615|ref|NC_002745.2|	498433	+	C	0	0	1	0	0	SA0432
gi|29165615|ref|NC_002745.2|	498436	+	U	0	1	0	0	0	SA0432
gi|29165615|ref|NC_002745.2|	498440	+	U	1	0	0	0	2	SA0432
gi|29165615|ref|NC_002745.2|	498446	+	G	0	0	0	0	2	SA0432
gi|29165615|ref|NC_002745.2|	498447	+	C	0	0	0	0	1	SA0432
gi|29165615|ref|NC_002745.2|	498448	+	A	0	1	0	0	0	SA0432
gi|29165615|ref|NC_002745.2|	498450	+	U	0	2	0	0	1	SA0432
gi|29165615|ref|NC_002745.2|	498452	+	G	0	3	0	0	2	SA0432
gi|29165615|ref|NC_002745.2|	498453	+	A	0	0	0	1	0	SA0432
gi|29165615|ref|NC_002745.2|	498458	+	C	0	1	0	0	0	SA0432
gi|29165615|ref|NC_002745.2|	498459	+	A	0	0	0	0	1	SA0432
gi|29165615|ref|NC_002745.2|	498461	+	U	0	0	0	0	1	SA0432
gi|29165615|ref|NC_002745.2|	498464	+	A	0	1	0	0	1	SA0432
gi|29165615|ref|NC_002745.2|	498466	+	G	0	1	0	0	0	SA0432
gi|29165615|ref|NC_002745.2|	498471	+	G	1	0	0	0	0	SA0432
gi|29165615|ref|NC_002745.2|	498475	+	C	0	0	0	0	1	SA0432
gi|29165615|ref|NC_002745.2|	498479	+	G	1	0	0	0	0	SA0432
gi|29165615|ref|NC_002745.2|	498487	+	G	0	0	0	0	1	SA0432
gi|29165615|ref|NC_002745.2|	498491	+	U	0	0	0	0	1	SA0432
gi|29165615|ref|NC_002745.2|	498492	+	U	1	0	0	1	1	SA0432
gi|29165615|ref|NC_002745.2|	498495	+	G	1	0	0	0	2	SA0432
gi|29165615|ref|NC_002745.2|	498500	+	A	0	0	0	0	1	SA0432
gi|29165615|ref|NC_002745.2|	498506	+	G	0	0	0	1	0	SA0432
gi|29165615|ref|NC_002745.2|	498507	+	C	0	0	1	0	0	SA0432
gi|29165615|ref|NC_002745.2|	498509	+	A	0	1	0	2	3	SA0432
gi|29165615|ref|NC_002745.2|	498513	+	C	0	0	0	1	1	SA0432
gi|29165615|ref|NC_002745.2|	498514	+	C	0	0	0	0	1	SA0432
gi|29165615|ref|NC_002745.2|	498515	+	A	0	1	0	0	3	SA0432
gi|29165615|ref|NC_002745.2|	498516	+	A	0	0	1	2	6	SA0432
gi|29165615|ref|NC_002745.2|	498521	+	U	0	0	0	0	1	SA0432
gi|29165615|ref|NC_002745.2|	498525	+	A	0	0	0	0	1	SA0432
gi|29165615|ref|NC_002745.2|	498526	+	G	0	1	0	0	0	SA0432
gi|29165615|ref|NC_002745.2|	498529	+	C	0	0	1	0	0	SA0432
gi|29165615|ref|NC_002745.2|	498530	+	U	0	0	0	0	1	SA0432
gi|29165615|ref|NC_002745.2|	498531	+	C	0	0	0	0	1	SA0432
gi|29165615|ref|NC_002745.2|	498532	+	U	0	0	1	0	0	SA0432
gi|29165615|ref|NC_002745.2|	498534	+	C	0	0	0	0	1	SA0432
gi|29165615|ref|NC_002745.2|	498537	+	C	0	0	0	1	1	SA0432
gi|29165615|ref|NC_002745.2|	498539	+	U	0	1	0	0	0	SA0432
gi|29165615|ref|NC_002745.2|	498540	+	U	0	2	0	0	0	SA0432
gi|29165615|ref|NC_002745.2|	498541	+	U	0	2	0	0	0	SA0432
gi|29165615|ref|NC_002745.2|	498543	+	G	0	1	0	0	0	SA0432
gi|29165615|ref|NC_002745.2|	498544	+	A	0	2	0	0	0	SA0432
gi|29165615|ref|NC_002745.2|	498545	+	G	0	2	0	0	0	SA0432
gi|29165615|ref|NC_002745.2|	498546	+	C	0	0	2	0	0	SA0432
gi|29165615|ref|NC_002745.2|	498548	+	U	0	3	0	0	1	SA0432
gi|29165615|ref|NC_002745.2|	498550	+	G	0	0	0	1	1	SA0432
gi|29165615|ref|NC_002745.2|	498552	+	U	0	0	0	0	1	SA0432
gi|29165615|ref|NC_002745.2|	498553	+	U	0	0	0	0	2	SA0432
gi|29165615|ref|NC_002745.2|	498554	+	G	0	0	1	0	0	SA0432
gi|29165615|ref|NC_002745.2|	498555	+	U	0	11	2	4	13	SA0432
gi|29165615|ref|NC_002745.2|	498556	+	C	0	2	1	1	2	SA0432
gi|29165615|ref|NC_002745.2|	498557	+	U	0	0	1	0	2	SA0432
gi|29165615|ref|NC_002745.2|	498558	+	A	0	0	1	1	6	SA0432
gi|29165615|ref|NC_002745.2|	498559	+	U	0	1	1	0	2	SA0432
gi|29165615|ref|NC_002745.2|	498560	+	A	0	1	1	0	0	SA0432
gi|29165615|ref|NC_002745.2|	498561	+	A	0	1	2	1	0	SA0432
gi|29165615|ref|NC_002745.2|	498562	+	A	0	0	0	0	1	SA0432
gi|29165615|ref|NC_002745.2|	498563	+	C	0	1	0	0	0	SA0432
gi|29165615|ref|NC_002745.2|	498564	+	G	0	0	0	0	2	SA0432
gi|29165615|ref|NC_002745.2|	498565	+	U	0	4	2	3	4	SA0432
gi|29165615|ref|NC_002745.2|	498566	+	C	0	1	1	3	1	SA0432
gi|29165615|ref|NC_002745.2|	498567	+	G	0	0	0	0	2	SA0432
gi|29165615|ref|NC_002745.2|	498568	+	U	0	4	1	2	3	SA0432
gi|29165615|ref|NC_002745.2|	498569	+	A	0	5	2	6	7	SA0432
gi|29165615|ref|NC_002745.2|	498570	+	A	0	2	1	2	3	SA0432
gi|29165615|ref|NC_002745.2|	498572	+	A	0	14	1	2	6	SA0432
gi|29165615|ref|NC_002745.2|	498573	+	U	0	0	1	0	3	SA0432
gi|29165615|ref|NC_002745.2|	498574	+	G	0	0	0	3	0	SA0432
gi|29165615|ref|NC_002745.2|	498575	+	G	0	3	1	2	1	SA0432
gi|29165615|ref|NC_002745.2|	498576	+	U	0	2	1	1	6	SA0432
gi|29165615|ref|NC_002745.2|	498577	+	U	0	4	2	0	2	SA0432
gi|29165615|ref|NC_002745.2|	498578	+	A	0	6	3	3	9	SA0432
gi|29165615|ref|NC_002745.2|	498579	+	A	0	4	0	0	2	SA0432
gi|29165615|ref|NC_002745.2|	498580	+	A	1	1	1	4	4	SA0432
gi|29165615|ref|NC_002745.2|	498581	+	G	0	4	4	4	4	SA0432
gi|29165615|ref|NC_002745.2|	498582	+	A	0	1	2	1	8	SA0432

gi|29165615|ref|NC_002745.2|	498583 +	A	0	1	1	0	2	SA0432
gi|29165615|ref|NC_002745.2|	498584 +	G	0	1	0	0	0	SA0432
gi|29165615|ref|NC_002745.2|	498585 +	A	0	3	1	0	0	SA0432
gi|29165615|ref|NC_002745.2|	498586 +	A	0	0	0	1	0	SA0432
gi|29165615|ref|NC_002745.2|	498587 +	G	0	0	0	0	2	SA0432
gi|29165615|ref|NC_002745.2|	498588 +	G	1	0	1	0	0	SA0432
gi|29165615|ref|NC_002745.2|	498590 +	U	0	0	1	0	0	SA0432
gi|29165615|ref|NC_002745.2|	498591 +	U	1	0	0	0	0	SA0432
gi|29165615|ref|NC_002745.2|	498592 +	G	0	0	1	1	0	SA0432
gi|29165615|ref|NC_002745.2|	498593 +	G	0	0	0	0	1	SA0432
gi|29165615|ref|NC_002745.2|	498594 +	C	0	0	0	1	0	SA0432
gi|29165615|ref|NC_002745.2|	498595 +	A	0	0	1	0	0	SA0432
gi|29165615|ref|NC_002745.2|	498596 +	U	0	3	1	0	1	SA0432
gi|29165615|ref|NC_002745.2|	498598 +	A	0	0	1	0	0	SA0432
gi|29165615|ref|NC_002745.2|	498599 +	A	0	2	1	0	1	SA0432
gi|29165615|ref|NC_002745.2|	498601 +	A	0	0	1	2	0	SA0432
gi|29165615|ref|NC_002745.2|	498602 +	U	0	2	1	1	2	SA0432
gi|29165615|ref|NC_002745.2|	498603 +	C	0	0	0	0	1	SA0432
gi|29165615|ref|NC_002745.2|	498604 +	U	0	0	2	0	0	SA0432
gi|29165615|ref|NC_002745.2|	498605 +	U	0	0	0	1	0	SA0432
gi|29165615|ref|NC_002745.2|	498606 +	G	2	0	0	0	0	SA0432
gi|29165615|ref|NC_002745.2|	498607 +	U	1	0	0	0	3	SA0432
gi|29165615|ref|NC_002745.2|	498608 +	A	0	1	0	4	2	SA0432
gi|29165615|ref|NC_002745.2|	498609 +	U	1	0	1	1	0	SA0432
gi|29165615|ref|NC_002745.2|	498610 +	U	0	1	1	1	1	SA0432
gi|29165615|ref|NC_002745.2|	498611 +	U	0	0	0	0	2	SA0432
gi|29165615|ref|NC_002745.2|	498612 +	C	0	0	1	0	1	SA0432
gi|29165615|ref|NC_002745.2|	498613 +	U	0	1	0	0	1	SA0432
gi|29165615|ref|NC_002745.2|	498615 +	C	3	1	0	0	2	SA0432
gi|29165615|ref|NC_002745.2|	498616 +	U	0	1	0	0	1	SA0432
gi|29165615|ref|NC_002745.2|	498617 +	A	0	0	2	2	2	SA0432
gi|29165615|ref|NC_002745.2|	498621 +	U	2	0	0	0	0	SA0432
gi|29165615|ref|NC_002745.2|	498624 +	G	1	0	1	0	0	SA0432
gi|29165615|ref|NC_002745.2|	498626 +	G	0	2	1	0	1	SA0432
gi|29165615|ref|NC_002745.2|	498629 +	A	0	7	3	2	1	SA0432
gi|29165615|ref|NC_002745.2|	498636 +	C	0	0	1	0	1	SA0432
gi|29165615|ref|NC_002745.2|	498638 +	G	0	0	0	0	1	SA0432
gi|29165615|ref|NC_002745.2|	498639 +	C	0	0	1	0	0	SA0432
gi|29165615|ref|NC_002745.2|	498641 +	A	0	4	1	1	0	SA0432
gi|29165615|ref|NC_002745.2|	498642 +	U	0	0	1	0	1	SA0432
gi|29165615|ref|NC_002745.2|	498643 +	G	0	0	1	0	0	SA0432
gi|29165615|ref|NC_002745.2|	498644 +	U	1	0	0	0	0	SA0432
gi|29165615|ref|NC_002745.2|	498645 +	U	1	1	0	0	0	SA0432
gi|29165615|ref|NC_002745.2|	498648 +	G	2	0	0	0	1	SA0432
gi|29165615|ref|NC_002745.2|	498650 +	G	0	1	0	0	0	SA0432
gi|29165615|ref|NC_002745.2|	498651 +	U	0	0	0	1	2	SA0432
gi|29165615|ref|NC_002745.2|	498652 +	G	1	2	1	0	3	SA0432
gi|29165615|ref|NC_002745.2|	498653 +	A	0	3	1	0	1	SA0432
gi|29165615|ref|NC_002745.2|	498654 +	A	0	1	5	0	0	SA0432
gi|29165615|ref|NC_002745.2|	498655 +	C	0	2	1	0	0	SA0432
gi|29165615|ref|NC_002745.2|	498656 +	U	0	0	0	0	2	SA0432
gi|29165615|ref|NC_002745.2|	498657 +	U	0	1	1	3	2	SA0432
gi|29165615|ref|NC_002745.2|	498658 +	A	0	2	1	0	1	SA0432
gi|29165615|ref|NC_002745.2|	498659 +	C	0	3	2	1	3	SA0432
gi|29165615|ref|NC_002745.2|	498660 +	C	1	1	1	0	1	SA0432
gi|29165615|ref|NC_002745.2|	498661 +	U	0	0	0	0	1	SA0432
gi|29165615|ref|NC_002745.2|	498662 +	U	0	0	1	0	0	SA0432
gi|29165615|ref|NC_002745.2|	498664 +	G	0	1	0	1	0	SA0432
gi|29165615|ref|NC_002745.2|	498665 +	A	0	0	3	1	4	SA0432
gi|29165615|ref|NC_002745.2|	498666 +	A	0	0	0	1	2	SA0432
gi|29165615|ref|NC_002745.2|	498667 +	A	0	6	0	0	0	SA0432
gi|29165615|ref|NC_002745.2|	498668 +	U	0	1	1	0	2	SA0432
gi|29165615|ref|NC_002745.2|	498669 +	A	0	0	2	1	0	SA0432
gi|29165615|ref|NC_002745.2|	498671 +	C	0	1	0	1	0	SA0432
gi|29165615|ref|NC_002745.2|	498672 +	C	0	0	1	0	1	SA0432
gi|29165615|ref|NC_002745.2|	498674 +	U	0	1	0	0	0	SA0432
gi|29165615|ref|NC_002745.2|	498675 +	U	1	0	0	0	0	SA0432
gi|29165615|ref|NC_002745.2|	498676 +	U	0	0	0	0	3	SA0432
gi|29165615|ref|NC_002745.2|	498677 +	A	0	0	0	0	2	SA0432
gi|29165615|ref|NC_002745.2|	498683 +	G	0	1	2	0	0	SA0432
gi|29165615|ref|NC_002745.2|	498684 +	C	0	2	0	0	0	SA0432
gi|29165615|ref|NC_002745.2|	498687 +	U	0	0	1	0	0	SA0432
gi|29165615|ref|NC_002745.2|	498690 +	C	0	0	1	0	0	SA0432
gi|29165615|ref|NC_002745.2|	498693 +	C	0	0	0	0	1	SA0432
gi|29165615|ref|NC_002745.2|	498695 +	U	0	0	1	0	0	SA0432
gi|29165615|ref|NC_002745.2|	498696 +	C	0	0	1	0	0	SA0432
gi|29165615|ref|NC_002745.2|	498698 +	U	0	2	0	0	0	SA0432
gi|29165615|ref|NC_002745.2|	498699 +	G	0	1	0	0	0	SA0432
gi|29165615|ref|NC_002745.2|	498701 +	G	0	1	0	0	0	SA0432
gi|29165615|ref|NC_002745.2|	498702 +	U	0	0	0	0	1	SA0432
gi|29165615|ref|NC_002745.2|	498706 +	G	0	1	0	0	0	SA0432

gi|29165615|ref|NC_002745.2|	498707 +	G	0	2	0	0	0	SA0432
gi|29165615|ref|NC_002745.2|	498708 +	G	0	2	0	0	0	SA0432
gi|29165615|ref|NC_002745.2|	498710 +	G	0	2	0	0	0	SA0432
gi|29165615|ref|NC_002745.2|	498711 +	C	0	1	0	0	0	SA0432
gi|29165615|ref|NC_002745.2|	498712 +	A	0	0	0	0	1	SA0432
gi|29165615|ref|NC_002745.2|	498713 +	A	0	0	2	0	0	SA0432
gi|29165615|ref|NC_002745.2|	498715 +	C	1	0	0	2	0	SA0432
gi|29165615|ref|NC_002745.2|	498716 +	G	0	0	0	0	1	SA0432
gi|29165615|ref|NC_002745.2|	498717 +	U	0	0	1	0	0	SA0432
gi|29165615|ref|NC_002745.2|	498722 +	A	0	0	0	0	1	SA0432
gi|29165615|ref|NC_002745.2|	498724 +	G	0	1	0	0	2	SA0432
gi|29165615|ref|NC_002745.2|	498725 +	A	0	2	0	0	0	SA0432
gi|29165615|ref|NC_002745.2|	498727 +	U	0	2	0	0	2	SA0432
gi|29165615|ref|NC_002745.2|	498728 +	A	0	1	0	0	0	SA0432
gi|29165615|ref|NC_002745.2|	498730 +	C	1	0	0	0	0	SA0432
gi|29165615|ref|NC_002745.2|	498731 +	G	0	1	0	0	0	SA0432
gi|29165615|ref|NC_002745.2|	498732 +	U	0	5	0	0	5	SA0432
gi|29165615|ref|NC_002745.2|	498733 +	A	0	1	0	1	0	SA0432
gi|29165615|ref|NC_002745.2|	498737 +	G	0	0	0	1	0	SA0432
gi|29165615|ref|NC_002745.2|	498738 +	G	0	1	1	0	3	SA0432
gi|29165615|ref|NC_002745.2|	498739 +	A	0	1	0	0	2	SA0432
gi|29165615|ref|NC_002745.2|	498740 +	A	0	0	0	0	1	SA0432
gi|29165615|ref|NC_002745.2|	498743 +	G	0	2	2	0	0	SA0432
gi|29165615|ref|NC_002745.2|	498750 +	U	0	1	0	0	0	SA0432
gi|29165615|ref|NC_002745.2|	498751 +	U	1	0	0	0	0	SA0432
gi|29165615|ref|NC_002745.2|	498766 +	A	0	2	0	0	0	SA0432
gi|29165615|ref|NC_002745.2|	498775 +	A	0	1	0	0	0	SA0432
gi|29165615|ref|NC_002745.2|	498776 +	A	0	5	1	0	1	SA0432
gi|29165615|ref|NC_002745.2|	498780 +	A	0	1	0	0	0	SA0432
gi|29165615|ref|NC_002745.2|	498784 +	A	0	0	1	0	0	SA0432
gi|29165615|ref|NC_002745.2|	498790 +	U	0	0	1	0	0	SA0432
gi|29165615|ref|NC_002745.2|	498793 +	G	0	1	0	0	0	SA0432
gi|29165615|ref|NC_002745.2|	498794 +	C	0	0	0	0	1	SA0432
gi|29165615|ref|NC_002745.2|	498795 +	C	0	1	0	0	0	SA0432
gi|29165615|ref|NC_002745.2|	498800 +	U	0	1	0	0	0	SA0432
gi|29165615|ref|NC_002745.2|	498812 +	A	0	0	0	1	0	SA0432
gi|29165615|ref|NC_002745.2|	498825 +	U	0	0	0	0	1	SA0432
gi|29165615|ref|NC_002745.2|	498834 +	C	0	3	0	0	0	SA0432
gi|29165615|ref|NC_002745.2|	498844 +	A	0	1	1	1	0	SA0432
gi|29165615|ref|NC_002745.2|	498851 +	G	0	0	0	1	0	SA0432
gi|29165615|ref|NC_002745.2|	498875 +	A	0	1	0	0	0	SA0432
gi|29165615|ref|NC_002745.2|	498879 +	C	0	0	1	0	0	SA0432
gi|29165615|ref|NC_002745.2|	498881 +	A	0	1	0	0	1	SA0432
gi|29165615|ref|NC_002745.2|	498884 +	G	0	1	0	0	0	SA0432
gi|29165615|ref|NC_002745.2|	498886 +	A	0	0	0	0	1	SA0432
gi|29165615|ref|NC_002745.2|	498894 +	A	0	1	0	0	0	SA0432
gi|29165615|ref|NC_002745.2|	498897 +	A	0	1	0	1	1	SA0432
gi|29165615|ref|NC_002745.2|	498900 +	A	0	1	1	2	2	SA0432
gi|29165615|ref|NC_002745.2|	498901 +	A	0	0	1	0	2	SA0432
gi|29165615|ref|NC_002745.2|	498903 +	A	0	9	0	2	12	-
gi|29165615|ref|NC_002745.2|	498904 +	A	0	9	5	2	13	-
gi|29165615|ref|NC_002745.2|	498905 +	A	0	12	6	4	9	-
gi|29165615|ref|NC_002745.2|	498906 +	A	0	3	1	0	7	-
gi|29165615|ref|NC_002745.2|	498908 +	A	0	0	0	0	1	-
gi|29165615|ref|NC_002745.2|	498912 +	A	0	0	0	0	1	-
gi|29165615|ref|NC_002745.2|	498924 +	U	0	0	0	0	2	-
gi|29165615|ref|NC_002745.2|	498940 +	U	0	2	0	0	0	-
gi|29165615|ref|NC_002745.2|	498943 +	U	0	1	0	0	0	-
gi|29165615|ref|NC_002745.2|	498946 +	G	0	0	1	0	0	-
gi|29165615|ref|NC_002745.2|	498947 +	U	0	0	1	0	0	-
gi|29165615|ref|NC_002745.2|	498950 +	U	0	1	0	0	0	-
gi|29165615|ref|NC_002745.2|	498953 +	G	0	1	0	0	0	-
gi|29165615|ref|NC_002745.2|	498954 +	G	0	0	0	1	1	-
gi|29165615|ref|NC_002745.2|	498957 +	G	0	2	0	0	4	-
gi|29165615|ref|NC_002745.2|	498958 +	U	0	0	0	1	0	-
gi|29165615|ref|NC_002745.2|	498959 +	A	0	0	1	0	0	-
gi|29165615|ref|NC_002745.2|	499080 +	A	0	0	0	1	0	SA0433
gi|29165615|ref|NC_002745.2|	499135 +	A	1	0	0	0	0	SA0433
gi|29165615|ref|NC_002745.2|	499138 +	U	0	1	0	0	0	SA0433
gi|29165615|ref|NC_002745.2|	499176 +	A	0	0	1	0	0	SA0433
gi|29165615|ref|NC_002745.2|	499244 +	U	0	0	0	0	1	SA0433
gi|29165615|ref|NC_002745.2|	499268 +	A	0	0	0	0	1	SA0433
gi|29165615|ref|NC_002745.2|	499315 +	A	0	1	0	0	0	SA0433
gi|29165615|ref|NC_002745.2|	499320 +	A	0	1	0	0	0	SA0433
gi|29165615|ref|NC_002745.2|	499332 +	A	0	0	0	1	0	SA0433
gi|29165615|ref|NC_002745.2|	499338 +	A	0	1	0	0	2	SA0433
gi|29165615|ref|NC_002745.2|	499355 +	U	0	1	0	0	0	SA0433
gi|29165615|ref|NC_002745.2|	499356 +	C	0	1	0	0	0	SA0433
gi|29165615|ref|NC_002745.2|	499358 +	U	0	0	1	0	0	SA0433
gi|29165615|ref|NC_002745.2|	499362 +	A	0	0	1	0	0	SA0433
gi|29165615|ref|NC_002745.2|	499398 +	G	0	0	1	0	0	SA0433

gi|29165615|ref|NC_002745.2|	499407	+	C	0	0	0	0	1	SA0433
gi|29165615|ref|NC_002745.2|	499427	+	A	0	0	1	0	0	SA0433
gi|29165615|ref|NC_002745.2|	499428	+	C	0	0	0	0	1	SA0433
gi|29165615|ref|NC_002745.2|	499434	+	A	0	0	0	0	2	SA0433
gi|29165615|ref|NC_002745.2|	499450	+	A	0	1	0	0	0	SA0433
gi|29165615|ref|NC_002745.2|	499462	+	U	0	0	0	1	0	SA0433
gi|29165615|ref|NC_002745.2|	499476	+	A	0	3	0	0	0	SA0433
gi|29165615|ref|NC_002745.2|	499482	+	A	0	0	0	0	1	SA0433
gi|29165615|ref|NC_002745.2|	499584	+	G	0	0	1	0	0	SA0433
gi|29165615|ref|NC_002745.2|	499600	+	U	0	1	0	0	0	SA0433
gi|29165615|ref|NC_002745.2|	499607	+	A	0	3	0	0	1	SA0433
gi|29165615|ref|NC_002745.2|	499617	+	A	0	0	0	1	2	SA0433
gi|29165615|ref|NC_002745.2|	499619	+	U	0	0	0	0	1	SA0433
gi|29165615|ref|NC_002745.2|	499620	+	U	0	0	0	0	1	SA0433
gi|29165615|ref|NC_002745.2|	499701	+	U	0	0	0	0	1	SA0433
gi|29165615|ref|NC_002745.2|	499702	+	A	0	0	0	0	1	SA0433
gi|29165615|ref|NC_002745.2|	499704	+	G	0	0	0	0	1	SA0433
gi|29165615|ref|NC_002745.2|	499707	+	A	0	0	1	0	0	SA0433
gi|29165615|ref|NC_002745.2|	499716	+	C	0	0	0	0	1	SA0433
gi|29165615|ref|NC_002745.2|	499724	+	A	0	1	0	0	0	SA0433
gi|29165615|ref|NC_002745.2|	499726	+	U	0	1	0	0	0	SA0433
gi|29165615|ref|NC_002745.2|	499731	+	A	0	4	1	0	2	SA0433
gi|29165615|ref|NC_002745.2|	499732	+	U	0	0	1	0	0	SA0433
gi|29165615|ref|NC_002745.2|	499735	+	U	0	0	1	0	2	SA0433
gi|29165615|ref|NC_002745.2|	499736	+	A	0	1	0	0	0	SA0433
gi|29165615|ref|NC_002745.2|	499738	+	U	0	1	0	0	1	SA0433
gi|29165615|ref|NC_002745.2|	499742	+	U	0	0	0	0	2	SA0433
gi|29165615|ref|NC_002745.2|	499744	+	U	0	0	0	0	1	SA0433
gi|29165615|ref|NC_002745.2|	499747	+	A	0	0	0	1	0	SA0433
gi|29165615|ref|NC_002745.2|	499756	+	A	0	2	0	0	2	SA0433
gi|29165615|ref|NC_002745.2|	499759	+	C	0	2	0	2	1	SA0433
gi|29165615|ref|NC_002745.2|	499760	+	C	0	0	0	0	1	SA0433
gi|29165615|ref|NC_002745.2|	499761	+	A	0	0	1	0	0	SA0433
gi|29165615|ref|NC_002745.2|	499762	+	A	0	1	0	0	0	SA0433
gi|29165615|ref|NC_002745.2|	499763	+	G	0	1	0	1	1	SA0433
gi|29165615|ref|NC_002745.2|	499764	+	A	1	6	1	4	2	SA0433
gi|29165615|ref|NC_002745.2|	499765	+	A	0	1	0	0	2	SA0433
gi|29165615|ref|NC_002745.2|	499766	+	U	0	1	0	0	0	SA0433
gi|29165615|ref|NC_002745.2|	499768	+	G	0	1	0	0	0	SA0433
gi|29165615|ref|NC_002745.2|	499769	+	A	0	3	0	2	3	SA0433
gi|29165615|ref|NC_002745.2|	499770	+	A	0	1	0	0	0	SA0433
gi|29165615|ref|NC_002745.2|	499771	+	U	0	2	1	0	0	SA0433
gi|29165615|ref|NC_002745.2|	499772	+	A	0	0	1	0	0	SA0433
gi|29165615|ref|NC_002745.2|	499774	+	U	0	1	1	2	0	SA0433
gi|29165615|ref|NC_002745.2|	499775	+	G	0	1	0	1	1	SA0433
gi|29165615|ref|NC_002745.2|	499776	+	U	0	12	6	3	7	SA0433
gi|29165615|ref|NC_002745.2|	499777	+	U	0	1	1	0	0	SA0433
gi|29165615|ref|NC_002745.2|	499779	+	U	0	0	1	1	0	SA0433
gi|29165615|ref|NC_002745.2|	499780	+	U	0	1	0	0	1	SA0433
gi|29165615|ref|NC_002745.2|	499782	+	A	2	2	0	2	1	SA0433
gi|29165615|ref|NC_002745.2|	499784	+	U	1	0	1	0	0	SA0433
gi|29165615|ref|NC_002745.2|	499787	+	C	0	0	0	0	1	SA0433
gi|29165615|ref|NC_002745.2|	499792	+	U	0	1	0	0	0	SA0433
gi|29165615|ref|NC_002745.2|	499793	+	C	0	0	0	0	1	SA0433
gi|29165615|ref|NC_002745.2|	499796	+	A	0	1	0	0	0	SA0433
gi|29165615|ref|NC_002745.2|	499803	+	A	0	1	0	0	0	SA0433
gi|29165615|ref|NC_002745.2|	499806	+	A	0	1	0	0	0	SA0433
gi|29165615|ref|NC_002745.2|	499810	+	U	0	0	0	0	1	SA0433
gi|29165615|ref|NC_002745.2|	499811	+	G	0	0	1	1	0	SA0433
gi|29165615|ref|NC_002745.2|	499812	+	A	0	2	0	0	0	SA0433
gi|29165615|ref|NC_002745.2|	499813	+	U	0	0	0	0	1	SA0433
gi|29165615|ref|NC_002745.2|	499814	+	G	0	0	0	0	1	SA0433
gi|29165615|ref|NC_002745.2|	499817	+	G	0	1	1	0	0	SA0433
gi|29165615|ref|NC_002745.2|	499819	+	A	0	1	0	0	0	SA0433
gi|29165615|ref|NC_002745.2|	499820	+	A	0	0	1	0	1	SA0433
gi|29165615|ref|NC_002745.2|	499821	+	A	0	0	0	1	0	SA0433
gi|29165615|ref|NC_002745.2|	499822	+	G	0	0	0	0	2	SA0433
gi|29165615|ref|NC_002745.2|	499825	+	G	0	0	0	1	0	SA0433
gi|29165615|ref|NC_002745.2|	499826	+	A	0	0	1	1	0	SA0433
gi|29165615|ref|NC_002745.2|	499827	+	C	1	0	1	0	1	SA0433
gi|29165615|ref|NC_002745.2|	499829	+	A	0	1	0	2	4	SA0433
gi|29165615|ref|NC_002745.2|	499830	+	A	2	0	0	1	0	SA0433
gi|29165615|ref|NC_002745.2|	499833	+	C	0	0	1	0	2	SA0433
gi|29165615|ref|NC_002745.2|	499835	+	A	0	0	1	0	0	SA0433
gi|29165615|ref|NC_002745.2|	499837	+	G	0	0	0	0	1	SA0433
gi|29165615|ref|NC_002745.2|	499838	+	C	0	1	0	0	0	SA0433
gi|29165615|ref|NC_002745.2|	499839	+	U	0	0	0	0	1	SA0433
gi|29165615|ref|NC_002745.2|	499840	+	U	0	0	0	1	0	SA0433
gi|29165615|ref|NC_002745.2|	499841	+	G	2	0	0	0	0	SA0433
gi|29165615|ref|NC_002745.2|	499842	+	A	0	3	1	0	0	SA0433
gi|29165615|ref|NC_002745.2|	499843	+	U	0	0	0	0	1	SA0433

gi|29165615|ref|NC_002745.2|	499844 +	U	0	0	0	0	1	SA0433
gi|29165615|ref|NC_002745.2|	499846 +	U	0	0	1	0	0	SA0433
gi|29165615|ref|NC_002745.2|	499847 +	C	0	0	0	0	1	SA0433
gi|29165615|ref|NC_002745.2|	499848 +	A	0	0	0	0	2	SA0433
gi|29165615|ref|NC_002745.2|	499849 +	U	0	1	0	0	3	SA0433
gi|29165615|ref|NC_002745.2|	499850 +	A	0	0	0	0	3	SA0433
gi|29165615|ref|NC_002745.2|	499851 +	A	0	1	0	1	0	SA0433
gi|29165615|ref|NC_002745.2|	499852 +	G	0	0	0	0	1	SA0433
gi|29165615|ref|NC_002745.2|	499853 +	U	0	1	0	1	2	SA0433
gi|29165615|ref|NC_002745.2|	499854 +	U	0	0	1	0	0	SA0433
gi|29165615|ref|NC_002745.2|	499855 +	A	0	3	1	2	7	SA0433
gi|29165615|ref|NC_002745.2|	499856 +	A	0	1	0	2	4	SA0433
gi|29165615|ref|NC_002745.2|	499859 +	G	0	2	0	3	1	SA0433
gi|29165615|ref|NC_002745.2|	499860 +	A	0	0	0	1	1	SA0433
gi|29165615|ref|NC_002745.2|	499862 +	A	0	4	1	0	1	SA0433
gi|29165615|ref|NC_002745.2|	499867 +	G	2	2	0	0	0	SA0433
gi|29165615|ref|NC_002745.2|	499868 +	A	0	0	0	1	1	SA0433
gi|29165615|ref|NC_002745.2|	499871 +	C	0	0	0	1	1	SA0433
gi|29165615|ref|NC_002745.2|	499872 +	A	0	2	0	1	0	SA0433
gi|29165615|ref|NC_002745.2|	499873 +	A	0	1	0	0	0	SA0433
gi|29165615|ref|NC_002745.2|	499877 +	C	0	0	0	0	1	SA0433
gi|29165615|ref|NC_002745.2|	499878 +	A	0	4	0	0	1	SA0433
gi|29165615|ref|NC_002745.2|	499879 +	A	0	0	0	0	1	SA0433
gi|29165615|ref|NC_002745.2|	499881 +	G	0	0	0	0	1	SA0433
gi|29165615|ref|NC_002745.2|	499884 +	G	0	0	0	0	1	SA0433
gi|29165615|ref|NC_002745.2|	499885 +	U	0	1	1	0	0	SA0433
gi|29165615|ref|NC_002745.2|	499888 +	U	0	3	0	0	0	SA0433
gi|29165615|ref|NC_002745.2|	499889 +	U	0	0	2	0	0	SA0433
gi|29165615|ref|NC_002745.2|	499890 +	A	0	1	0	0	1	SA0433
gi|29165615|ref|NC_002745.2|	499894 +	C	0	0	1	0	0	SA0433
gi|29165615|ref|NC_002745.2|	499895 +	G	2	0	0	0	0	SA0433
gi|29165615|ref|NC_002745.2|	499904 +	U	0	2	0	0	0	SA0433
gi|29165615|ref|NC_002745.2|	499906 +	G	0	1	0	0	0	SA0433
gi|29165615|ref|NC_002745.2|	499907 +	A	0	1	0	0	0	SA0433
gi|29165615|ref|NC_002745.2|	499915 +	U	0	0	1	0	0	SA0433
gi|29165615|ref|NC_002745.2|	499950 +	C	0	2	0	0	0	SA0433
gi|29165615|ref|NC_002745.2|	500077 +	A	0	5	0	0	0	SA0433
gi|29165615|ref|NC_002745.2|	500126 +	U	0	0	1	0	0	SA0433
gi|29165615|ref|NC_002745.2|	500142 +	G	0	0	0	0	1	SA0433
gi|29165615|ref|NC_002745.2|	500144 +	G	0	0	0	0	1	SA0433
gi|29165615|ref|NC_002745.2|	500206 +	A	0	0	0	0	1	SA0433
gi|29165615|ref|NC_002745.2|	500226 +	A	0	0	0	1	0	SA0433
gi|29165615|ref|NC_002745.2|	500229 +	A	0	1	0	0	0	SA0433
gi|29165615|ref|NC_002745.2|	500248 +	A	0	0	0	0	1	SA0433
gi|29165615|ref|NC_002745.2|	500359 +	A	0	0	0	0	1	SA0433
gi|29165615|ref|NC_002745.2|	500364 +	A	1	0	0	0	0	SA0433
gi|29165615|ref|NC_002745.2|	500372 +	G	0	0	0	0	1	SA0433
gi|29165615|ref|NC_002745.2|	500378 +	A	0	0	0	0	1	SA0433
gi|29165615|ref|NC_002745.2|	500385 +	A	0	2	0	1	1	SA0433
gi|29165615|ref|NC_002745.2|	500387 +	G	2	1	0	2	2	SA0433
gi|29165615|ref|NC_002745.2|	500391 +	A	0	0	0	1	0	SA0433
gi|29165615|ref|NC_002745.2|	500405 +	U	0	0	0	0	1	SA0433
gi|29165615|ref|NC_002745.2|	500409 +	U	0	0	0	0	1	SA0433
gi|29165615|ref|NC_002745.2|	500414 +	C	0	0	1	0	0	SA0433
gi|29165615|ref|NC_002745.2|	500431 +	U	1	0	0	0	0	SA0433
gi|29165615|ref|NC_002745.2|	500444 +	U	0	0	0	1	0	SA0433
gi|29165615|ref|NC_002745.2|	500451 +	A	0	1	0	0	1	SA0433
gi|29165615|ref|NC_002745.2|	500455 +	A	0	1	0	0	0	SA0433
gi|29165615|ref|NC_002745.2|	500458 +	A	0	0	1	0	0	SA0433
gi|29165615|ref|NC_002745.2|	500476 +	U	0	0	0	0	1	SA0433
gi|29165615|ref|NC_002745.2|	500481 +	C	0	0	1	0	0	SA0433
gi|29165615|ref|NC_002745.2|	500490 +	C	0	2	0	0	0	SA0433
gi|29165615|ref|NC_002745.2|	500537 +	C	0	0	0	0	1	SA0433
gi|29165615|ref|NC_002745.2|	500549 +	G	0	0	1	0	0	SA0433
gi|29165615|ref|NC_002745.2|	500552 +	G	1	1	0	0	0	SA0433
gi|29165615|ref|NC_002745.2|	500553 +	A	0	0	0	0	1	SA0433
gi|29165615|ref|NC_002745.2|	500560 +	U	0	0	0	0	1	SA0433
gi|29165615|ref|NC_002745.2|	500561 +	A	4	2	2	7	6	SA0433
gi|29165615|ref|NC_002745.2|	500562 +	C	0	1	5	2	3	SA0433
gi|29165615|ref|NC_002745.2|	500564 +	A	1	1	1	1	0	SA0433
gi|29165615|ref|NC_002745.2|	500580 +	C	0	0	0	0	1	SA0433
gi|29165615|ref|NC_002745.2|	500607 +	A	0	0	0	0	1	-
gi|29165615|ref|NC_002745.2|	500608 +	U	0	1	0	0	0	-
gi|29165615|ref|NC_002745.2|	500610 +	A	0	0	0	0	1	-
gi|29165615|ref|NC_002745.2|	500612 +	U	0	0	0	0	1	-
gi|29165615|ref|NC_002745.2|	500613 +	U	0	0	0	1	0	-
gi|29165615|ref|NC_002745.2|	500615 +	A	0	0	0	1	0	-
gi|29165615|ref|NC_002745.2|	500617 +	U	0	0	1	0	0	-
gi|29165615|ref|NC_002745.2|	500619 +	G	0	0	0	0	1	-
gi|29165615|ref|NC_002745.2|	500620 +	A	0	0	1	0	0	-
gi|29165615|ref|NC_002745.2|	500639 +	C	0	0	0	0	1	SA0434

gi|29165615|ref|NC_002745.2|	500640	+	A	0	0	0	0	1	SA0434
gi|29165615|ref|NC_002745.2|	500643	+	A	0	0	0	1	0	SA0434
gi|29165615|ref|NC_002745.2|	500644	+	A	1	0	0	0	0	SA0434
gi|29165615|ref|NC_002745.2|	500645	+	A	0	0	0	0	1	SA0434
gi|29165615|ref|NC_002745.2|	500648	+	U	0	5	0	1	1	SA0434
gi|29165615|ref|NC_002745.2|	500649	+	U	0	0	0	0	1	SA0434
gi|29165615|ref|NC_002745.2|	500654	+	A	0	1	0	0	1	SA0434
gi|29165615|ref|NC_002745.2|	500655	+	A	0	1	0	0	0	SA0434
gi|29165615|ref|NC_002745.2|	500657	+	A	0	0	0	1	0	SA0434
gi|29165615|ref|NC_002745.2|	500658	+	U	0	1	0	0	0	SA0434
gi|29165615|ref|NC_002745.2|	500666	+	G	0	0	0	0	1	SA0434
gi|29165615|ref|NC_002745.2|	500672	+	A	0	1	0	0	0	SA0434
gi|29165615|ref|NC_002745.2|	500699	+	C	0	0	0	0	1	SA0434
gi|29165615|ref|NC_002745.2|	500714	+	A	0	1	0	0	0	SA0434
gi|29165615|ref|NC_002745.2|	500721	+	C	0	0	0	0	1	SA0434
gi|29165615|ref|NC_002745.2|	500732	+	U	0	1	0	0	0	SA0434
gi|29165615|ref|NC_002745.2|	500742	+	U	0	0	0	0	2	SA0434
gi|29165615|ref|NC_002745.2|	500794	+	A	0	1	0	0	0	SA0434
gi|29165615|ref|NC_002745.2|	500886	+	A	0	0	0	0	1	SA0434
gi|29165615|ref|NC_002745.2|	500897	+	G	0	0	0	0	1	SA0434
gi|29165615|ref|NC_002745.2|	500925	+	A	0	0	1	0	0	SA0434
gi|29165615|ref|NC_002745.2|	500975	+	C	0	0	1	0	0	SA0434
gi|29165615|ref|NC_002745.2|	501003	+	C	0	0	0	1	0	SA0434
gi|29165615|ref|NC_002745.2|	501007	+	C	0	0	0	0	1	SA0434
gi|29165615|ref|NC_002745.2|	501037	+	C	0	0	0	0	1	SA0434
gi|29165615|ref|NC_002745.2|	501041	+	C	0	0	0	0	1	SA0434
gi|29165615|ref|NC_002745.2|	501113	+	G	0	0	0	0	1	SA0434
gi|29165615|ref|NC_002745.2|	501147	+	A	0	1	0	0	0	SA0434
gi|29165615|ref|NC_002745.2|	501180	+	C	0	1	0	0	0	SA0434
gi|29165615|ref|NC_002745.2|	501196	+	U	0	0	1	0	0	SA0434
gi|29165615|ref|NC_002745.2|	501211	+	A	0	0	0	0	1	SA0434
gi|29165615|ref|NC_002745.2|	501274	+	U	0	1	0	0	0	SA0434
gi|29165615|ref|NC_002745.2|	501319	+	A	0	0	0	0	1	SA0434
gi|29165615|ref|NC_002745.2|	501385	+	C	0	1	0	2	2	-
gi|29165615|ref|NC_002745.2|	501386	+	A	0	1	0	2	2	-
gi|29165615|ref|NC_002745.2|	501387	+	A	0	0	1	1	0	-
gi|29165615|ref|NC_002745.2|	501390	+	A	0	0	0	1	0	-
gi|29165615|ref|NC_002745.2|	501391	+	A	0	1	1	0	0	-
gi|29165615|ref|NC_002745.2|	501394	+	A	0	1	0	2	3	-
gi|29165615|ref|NC_002745.2|	501397	+	A	0	2	0	2	0	-
gi|29165615|ref|NC_002745.2|	501398	+	A	0	0	1	0	0	-
gi|29165615|ref|NC_002745.2|	501399	+	A	0	2	0	1	2	-
gi|29165615|ref|NC_002745.2|	501402	+	A	0	0	0	1	0	-
gi|29165615|ref|NC_002745.2|	501404	+	A	0	0	0	2	0	-
gi|29165615|ref|NC_002745.2|	501405	+	G	0	0	0	1	2	-
gi|29165615|ref|NC_002745.2|	501407	+	A	0	0	0	2	2	-
gi|29165615|ref|NC_002745.2|	501408	+	A	0	6	0	4	1	-
gi|29165615|ref|NC_002745.2|	501409	+	U	0	1	0	3	1	-
gi|29165615|ref|NC_002745.2|	501410	+	A	0	4	5	13	11	-
gi|29165615|ref|NC_002745.2|	501411	+	U	0	3	1	0	3	-
gi|29165615|ref|NC_002745.2|	501412	+	A	0	6	1	6	3	-
gi|29165615|ref|NC_002745.2|	501413	+	U	0	1	1	2	3	-
gi|29165615|ref|NC_002745.2|	501414	+	A	0	0	0	1	2	-
gi|29165615|ref|NC_002745.2|	501416	+	C	0	1	0	0	0	-
gi|29165615|ref|NC_002745.2|	501417	+	U	0	1	0	0	1	-
gi|29165615|ref|NC_002745.2|	501418	+	U	2	1	2	0	2	-
gi|29165615|ref|NC_002745.2|	501419	+	G	28	16	18	14	19	-
gi|29165615|ref|NC_002745.2|	501420	+	C	0	1	0	0	0	-
gi|29165615|ref|NC_002745.2|	501421	+	C	2	0	0	1	0	-
gi|29165615|ref|NC_002745.2|	501422	+	G	3	0	1	1	2	-
gi|29165615|ref|NC_002745.2|	501437	+	G	0	1	0	0	0	-
gi|29165615|ref|NC_002745.2|	501445	+	U	0	1	0	0	0	-
gi|29165615|ref|NC_002745.2|	501446	+	C	0	0	0	0	1	-
gi|29165615|ref|NC_002745.2|	501454	+	U	1	0	0	0	0	-
gi|29165615|ref|NC_002745.2|	501459	+	A	1	0	1	0	1	-
gi|29165615|ref|NC_002745.2|	501462	+	C	0	1	0	0	0	-
gi|29165615|ref|NC_002745.2|	501464	+	C	1	0	0	0	0	-
gi|29165615|ref|NC_002745.2|	501467	+	U	7	8	5	7	6	-
gi|29165615|ref|NC_002745.2|	501484	+	C	0	2	1	0	0	-
gi|29165615|ref|NC_002745.2|	501498	+	U	0	0	0	0	1	-
gi|29165615|ref|NC_002745.2|	501499	+	A	0	0	0	1	1	-
gi|29165615|ref|NC_002745.2|	501505	+	G	1	0	0	0	0	-
gi|29165615|ref|NC_002745.2|	501506	+	C	1	0	0	0	0	-
gi|29165615|ref|NC_002745.2|	501511	+	U	0	1	0	0	0	-
gi|29165615|ref|NC_002745.2|	501512	+	C	8	10	7	7	4	-
gi|29165615|ref|NC_002745.2|	501513	+	U	3	3	0	1	4	-
gi|29165615|ref|NC_002745.2|	501514	+	G	0	1	0	0	0	-
gi|29165615|ref|NC_002745.2|	501515	+	C	5	0	0	0	1	-
gi|29165615|ref|NC_002745.2|	501518	+	A	0	1	0	0	2	-
gi|29165615|ref|NC_002745.2|	501522	+	C	1	2	1	2	0	-
gi|29165615|ref|NC_002745.2|	501524	+	C	0	1	0	0	0	-

gi|29165615|ref|NC_002745.2|	501528 +	G	2	0	1	0	0	-
gi|29165615|ref|NC_002745.2|	501537 +	G	1	0	0	0	0	-
gi|29165615|ref|NC_002745.2|	501542 +	C	0	0	0	1	0	-
gi|29165615|ref|NC_002745.2|	501543 +	G	0	1	0	0	0	-
gi|29165615|ref|NC_002745.2|	501547 +	C	0	0	1	0	0	-
gi|29165615|ref|NC_002745.2|	501549 +	A	0	0	0	0	1	-
gi|29165615|ref|NC_002745.2|	501552 +	C	0	0	0	0	2	-
gi|29165615|ref|NC_002745.2|	501554 +	A	0	1	0	0	0	-
gi|29165615|ref|NC_002745.2|	501555 +	U	0	0	0	1	0	-
gi|29165615|ref|NC_002745.2|	501560 +	A	0	0	0	0	1	-
gi|29165615|ref|NC_002745.2|	501563 +	C	3	0	1	0	1	-
gi|29165615|ref|NC_002745.2|	501566 +	G	0	0	0	1	0	-
gi|29165615|ref|NC_002745.2|	501567 +	A	0	0	0	0	1	-
gi|29165615|ref|NC_002745.2|	501568 +	A	0	1	0	0	0	-
gi|29165615|ref|NC_002745.2|	501569 +	C	0	0	1	0	0	-
gi|29165615|ref|NC_002745.2|	501570 +	C	1	1	0	1	0	-
gi|29165615|ref|NC_002745.2|	501571 +	A	0	0	0	1	0	-
gi|29165615|ref|NC_002745.2|	501572 +	U	1	0	0	0	0	-
gi|29165615|ref|NC_002745.2|	501573 +	G	0	0	0	0	1	-
gi|29165615|ref|NC_002745.2|	501575 +	C	0	1	0	0	0	-
gi|29165615|ref|NC_002745.2|	501579 +	U	0	1	0	0	0	-
gi|29165615|ref|NC_002745.2|	501580 +	C	0	1	2	0	1	-
gi|29165615|ref|NC_002745.2|	501581 +	C	0	1	0	0	0	-
gi|29165615|ref|NC_002745.2|	501583 +	G	0	1	0	0	0	-
gi|29165615|ref|NC_002745.2|	501585 +	C	0	0	1	0	0	-
gi|29165615|ref|NC_002745.2|	501587 +	G	0	0	0	0	3	-
gi|29165615|ref|NC_002745.2|	501589 +	A	0	0	0	0	2	-
gi|29165615|ref|NC_002745.2|	501591 +	C	2	5	1	0	1	-
gi|29165615|ref|NC_002745.2|	501592 +	A	0	0	0	0	1	-
gi|29165615|ref|NC_002745.2|	501593 +	G	0	0	0	0	6	-
gi|29165615|ref|NC_002745.2|	501594 +	C	0	0	2	0	0	-
gi|29165615|ref|NC_002745.2|	501595 +	A	0	0	2	0	0	-
gi|29165615|ref|NC_002745.2|	501596 +	U	0	1	0	1	1	-
gi|29165615|ref|NC_002745.2|	501597 +	U	0	1	0	2	0	-
gi|29165615|ref|NC_002745.2|	501599 +	A	0	0	1	1	0	-
gi|29165615|ref|NC_002745.2|	501600 +	G	0	0	0	1	0	-
gi|29165615|ref|NC_002745.2|	501603 +	G	0	0	0	1	0	-
gi|29165615|ref|NC_002745.2|	501604 +	A	0	0	0	1	0	-
gi|29165615|ref|NC_002745.2|	501606 +	C	0	2	0	1	0	-
gi|29165615|ref|NC_002745.2|	501615 +	U	1	0	0	0	0	-
gi|29165615|ref|NC_002745.2|	501616 +	G	3	2	0	0	0	-
gi|29165615|ref|NC_002745.2|	501626 +	A	0	0	1	1	0	-
gi|29165615|ref|NC_002745.2|	501628 +	C	0	1	0	0	0	-
gi|29165615|ref|NC_002745.2|	501636 +	U	2	0	0	0	0	-
gi|29165615|ref|NC_002745.2|	501641 +	A	0	1	0	0	0	-
gi|29165615|ref|NC_002745.2|	501643 +	C	0	1	0	1	0	-
gi|29165615|ref|NC_002745.2|	501646 +	C	0	0	1	0	0	-
gi|29165615|ref|NC_002745.2|	501649 +	U	1	0	0	1	0	-
gi|29165615|ref|NC_002745.2|	501744 +	U	0	0	1	0	0	-
gi|29165615|ref|NC_002745.2|	501764 +	G	0	0	1	0	0	-
gi|29165615|ref|NC_002745.2|	501775 +	A	0	0	1	0	0	-
gi|29165615|ref|NC_002745.2|	501825 +	A	0	0	1	0	0	-
gi|29165615|ref|NC_002745.2|	501831 +	G	0	0	0	2	0	-
gi|29165615|ref|NC_002745.2|	501836 +	A	0	1	0	0	0	-
gi|29165615|ref|NC_002745.2|	501884 +	A	0	1	0	0	0	-
gi|29165615|ref|NC_002745.2|	501887 +	A	0	1	0	0	0	-
gi|29165615|ref|NC_002745.2|	501894 +	A	0	0	0	1	0	-
gi|29165615|ref|NC_002745.2|	501903 +	A	0	2	1	1	0	-
gi|29165615|ref|NC_002745.2|	501949 +	U	0	0	0	0	1	-
gi|29165615|ref|NC_002745.2|	501950 +	A	2	41	14	18	60	-
gi|29165615|ref|NC_002745.2|	501951 +	U	0	1	0	0	0	-
gi|29165615|ref|NC_002745.2|	501954 +	A	0	0	0	0	3	-
gi|29165615|ref|NC_002745.2|	501964 +	U	0	0	1	0	0	-
gi|29165615|ref|NC_002745.2|	501968 +	G	0	0	0	0	1	-
gi|29165615|ref|NC_002745.2|	501975 +	G	0	0	0	0	1	-
gi|29165615|ref|NC_002745.2|	501976 +	A	0	6	2	3	10	-
gi|29165615|ref|NC_002745.2|	501977 +	C	0	0	2	1	1	-
gi|29165615|ref|NC_002745.2|	501978 +	A	0	1	0	1	0	-
gi|29165615|ref|NC_002745.2|	501979 +	A	0	0	2	0	0	-
gi|29165615|ref|NC_002745.2|	501987 +	C	0	1	0	1	0	-
gi|29165615|ref|NC_002745.2|	501993 +	G	0	1	0	1	0	-
gi|29165615|ref|NC_002745.2|	502004 +	C	0	1	0	0	0	SA0435
gi|29165615|ref|NC_002745.2|	502010 +	U	0	1	0	1	0	SA0435
gi|29165615|ref|NC_002745.2|	502011 +	A	0	0	1	1	0	SA0435
gi|29165615|ref|NC_002745.2|	502014 +	U	0	0	1	0	1	SA0435
gi|29165615|ref|NC_002745.2|	502015 +	A	0	0	0	0	1	SA0435
gi|29165615|ref|NC_002745.2|	502024 +	A	0	0	1	0	1	SA0435
gi|29165615|ref|NC_002745.2|	502025 +	A	0	2	0	2	2	SA0435
gi|29165615|ref|NC_002745.2|	502026 +	C	0	0	0	1	2	SA0435
gi|29165615|ref|NC_002745.2|	502027 +	A	0	1	0	0	0	SA0435
gi|29165615|ref|NC_002745.2|	502029 +	A	0	1	0	0	0	SA0435

gi|29165615|ref|NC_002745.2|	502031 +	U	0	0	1	0	0	SA0435
gi|29165615|ref|NC_002745.2|	502035 +	A	0	4	1	1	2	SA0435
gi|29165615|ref|NC_002745.2|	502038 +	A	0	0	0	1	0	SA0435
gi|29165615|ref|NC_002745.2|	502045 +	A	0	0	0	0	1	SA0435
gi|29165615|ref|NC_002745.2|	502047 +	C	0	1	0	0	0	SA0435
gi|29165615|ref|NC_002745.2|	502052 +	A	0	1	0	0	0	SA0435
gi|29165615|ref|NC_002745.2|	502057 +	U	1	0	1	1	1	SA0435
gi|29165615|ref|NC_002745.2|	502058 +	A	0	1	0	1	1	SA0435
gi|29165615|ref|NC_002745.2|	502060 +	U	0	1	0	0	0	SA0435
gi|29165615|ref|NC_002745.2|	502061 +	G	0	0	0	1	0	SA0435
gi|29165615|ref|NC_002745.2|	502062 +	A	0	0	1	1	3	SA0435
gi|29165615|ref|NC_002745.2|	502065 +	A	0	1	0	0	0	SA0435
gi|29165615|ref|NC_002745.2|	502069 +	U	0	1	0	1	0	SA0435
gi|29165615|ref|NC_002745.2|	502073 +	G	0	2	0	0	0	SA0435
gi|29165615|ref|NC_002745.2|	502074 +	A	0	2	0	0	1	SA0435
gi|29165615|ref|NC_002745.2|	502076 +	G	0	1	0	0	0	SA0435
gi|29165615|ref|NC_002745.2|	502077 +	A	0	1	0	1	0	SA0435
gi|29165615|ref|NC_002745.2|	502080 +	A	0	0	0	1	0	SA0435
gi|29165615|ref|NC_002745.2|	502082 +	C	0	0	0	0	1	SA0435
gi|29165615|ref|NC_002745.2|	502085 +	G	0	2	0	0	1	SA0435
gi|29165615|ref|NC_002745.2|	502086 +	A	0	0	1	1	2	SA0435
gi|29165615|ref|NC_002745.2|	502087 +	A	0	1	0	0	0	SA0435
gi|29165615|ref|NC_002745.2|	502088 +	A	0	0	1	0	0	SA0435
gi|29165615|ref|NC_002745.2|	502089 +	C	0	1	0	0	0	SA0435
gi|29165615|ref|NC_002745.2|	502096 +	G	0	1	0	0	0	SA0435
gi|29165615|ref|NC_002745.2|	502097 +	C	0	0	1	0	0	SA0435
gi|29165615|ref|NC_002745.2|	502104 +	G	0	0	1	0	0	SA0435
gi|29165615|ref|NC_002745.2|	502109 +	A	0	1	0	0	1	SA0435
gi|29165615|ref|NC_002745.2|	502110 +	A	0	1	0	0	0	SA0435
gi|29165615|ref|NC_002745.2|	502118 +	C	0	0	1	1	0	SA0435
gi|29165615|ref|NC_002745.2|	502121 +	U	0	1	0	0	0	SA0435
gi|29165615|ref|NC_002745.2|	502124 +	A	0	0	0	0	1	SA0435
gi|29165615|ref|NC_002745.2|	502125 +	G	0	0	1	1	1	SA0435
gi|29165615|ref|NC_002745.2|	502126 +	A	0	1	0	0	1	SA0435
gi|29165615|ref|NC_002745.2|	502127 +	A	0	0	0	0	1	SA0435
gi|29165615|ref|NC_002745.2|	502149 +	A	0	2	3	1	0	SA0435
gi|29165615|ref|NC_002745.2|	502150 +	A	0	0	1	0	1	SA0435
gi|29165615|ref|NC_002745.2|	502152 +	U	0	1	0	0	0	SA0435
gi|29165615|ref|NC_002745.2|	502153 +	A	0	1	1	1	2	SA0435
gi|29165615|ref|NC_002745.2|	502155 +	A	0	0	0	0	1	SA0435
gi|29165615|ref|NC_002745.2|	502158 +	U	0	2	0	0	0	SA0435
gi|29165615|ref|NC_002745.2|	502159 +	A	0	1	0	0	0	SA0435
gi|29165615|ref|NC_002745.2|	502160 +	A	0	0	1	0	0	SA0435
gi|29165615|ref|NC_002745.2|	502166 +	A	0	0	1	0	0	SA0435
gi|29165615|ref|NC_002745.2|	502167 +	A	0	4	1	0	0	SA0435
gi|29165615|ref|NC_002745.2|	502168 +	A	0	1	0	1	1	SA0435
gi|29165615|ref|NC_002745.2|	502169 +	A	0	1	0	3	0	SA0435
gi|29165615|ref|NC_002745.2|	502170 +	A	0	0	0	1	0	SA0435
gi|29165615|ref|NC_002745.2|	502171 +	U	0	0	0	0	1	SA0435
gi|29165615|ref|NC_002745.2|	502173 +	A	2	22	5	8	23	SA0435
gi|29165615|ref|NC_002745.2|	502174 +	A	0	0	0	0	1	SA0435
gi|29165615|ref|NC_002745.2|	502176 +	A	0	0	1	1	0	SA0435
gi|29165615|ref|NC_002745.2|	502179 +	A	0	0	1	0	0	SA0435
gi|29165615|ref|NC_002745.2|	502186 +	C	0	1	1	0	0	SA0435
gi|29165615|ref|NC_002745.2|	502194 +	A	0	0	1	0	0	SA0435
gi|29165615|ref|NC_002745.2|	502200 +	U	0	0	0	0	1	SA0435
gi|29165615|ref|NC_002745.2|	502201 +	A	0	0	0	0	2	SA0435
gi|29165615|ref|NC_002745.2|	502204 +	A	0	0	0	0	2	SA0435
gi|29165615|ref|NC_002745.2|	502205 +	A	0	1	1	0	0	SA0435
gi|29165615|ref|NC_002745.2|	502208 +	G	0	0	2	1	1	SA0435
gi|29165615|ref|NC_002745.2|	502209 +	A	0	6	1	0	3	SA0435
gi|29165615|ref|NC_002745.2|	502210 +	A	0	2	3	3	6	SA0435
gi|29165615|ref|NC_002745.2|	502211 +	G	0	4	2	1	4	SA0435
gi|29165615|ref|NC_002745.2|	502213 +	A	0	5	2	0	2	SA0435
gi|29165615|ref|NC_002745.2|	502214 +	G	0	1	2	6	5	SA0435
gi|29165615|ref|NC_002745.2|	502215 +	A	1	34	15	17	43	SA0435
gi|29165615|ref|NC_002745.2|	502216 +	A	0	5	1	2	5	SA0435
gi|29165615|ref|NC_002745.2|	502217 +	A	0	3	2	1	1	SA0435
gi|29165615|ref|NC_002745.2|	502220 +	A	0	4	1	1	1	SA0435
gi|29165615|ref|NC_002745.2|	502221 +	A	0	6	0	1	3	SA0435
gi|29165615|ref|NC_002745.2|	502224 +	G	0	1	1	1	0	SA0435
gi|29165615|ref|NC_002745.2|	502226 +	G	0	1	0	0	0	SA0435
gi|29165615|ref|NC_002745.2|	502227 +	A	0	21	16	8	18	SA0435
gi|29165615|ref|NC_002745.2|	502228 +	U	0	1	0	0	0	SA0435
gi|29165615|ref|NC_002745.2|	502229 +	G	0	1	2	0	3	SA0435
gi|29165615|ref|NC_002745.2|	502230 +	A	1	2	3	2	3	SA0435
gi|29165615|ref|NC_002745.2|	502231 +	U	0	0	0	1	1	SA0435
gi|29165615|ref|NC_002745.2|	502232 +	A	0	3	0	1	1	SA0435
gi|29165615|ref|NC_002745.2|	502233 +	A	0	3	0	1	0	SA0435
gi|29165615|ref|NC_002745.2|	502234 +	G	0	4	0	1	0	SA0435
gi|29165615|ref|NC_002745.2|	502235 +	A	0	6	2	1	1	SA0435

gi|29165615|ref|NC_002745.2|	502236	+	C	0	0	5	4	2	SA0435
gi|29165615|ref|NC_002745.2|	502237	+	G	0	1	2	1	0	SA0435
gi|29165615|ref|NC_002745.2|	502238	+	U	0	9	4	2	0	SA0435
gi|29165615|ref|NC_002745.2|	502239	+	A	0	3	1	0	3	SA0435
gi|29165615|ref|NC_002745.2|	502241	+	U	0	1	2	2	2	SA0435
gi|29165615|ref|NC_002745.2|	502242	+	A	0	1	0	0	0	SA0435
gi|29165615|ref|NC_002745.2|	502245	+	G	0	0	0	1	0	SA0435
gi|29165615|ref|NC_002745.2|	502252	+	G	0	0	0	0	1	SA0435
gi|29165615|ref|NC_002745.2|	502255	+	U	0	0	1	0	0	SA0435
gi|29165615|ref|NC_002745.2|	502264	+	A	0	1	0	0	0	SA0435
gi|29165615|ref|NC_002745.2|	502285	+	U	0	0	1	0	1	SA0435
gi|29165615|ref|NC_002745.2|	502286	+	G	0	0	0	1	0	SA0435
gi|29165615|ref|NC_002745.2|	502288	+	A	0	0	0	1	1	SA0435
gi|29165615|ref|NC_002745.2|	502292	+	A	0	0	1	0	0	SA0435
gi|29165615|ref|NC_002745.2|	502293	+	A	0	1	0	1	0	SA0435
gi|29165615|ref|NC_002745.2|	502294	+	A	0	0	0	1	1	SA0435
gi|29165615|ref|NC_002745.2|	502300	+	U	0	0	1	0	0	SA0435
gi|29165615|ref|NC_002745.2|	502322	+	G	0	0	0	1	0	SA0435
gi|29165615|ref|NC_002745.2|	502341	+	A	0	0	0	0	1	SA0435
gi|29165615|ref|NC_002745.2|	502355	+	G	0	1	0	0	0	SA0435
gi|29165615|ref|NC_002745.2|	502372	+	U	0	0	0	0	1	SA0435
gi|29165615|ref|NC_002745.2|	502380	+	A	0	0	1	0	0	SA0435
gi|29165615|ref|NC_002745.2|	502395	+	G	0	1	0	0	0	SA0435
gi|29165615|ref|NC_002745.2|	502401	+	A	0	1	0	0	0	SA0435
gi|29165615|ref|NC_002745.2|	502565	+	G	0	1	0	0	0	-
gi|29165615|ref|NC_002745.2|	502570	+	G	0	1	2	1	6	-
gi|29165615|ref|NC_002745.2|	502571	+	U	0	0	3	0	0	-
gi|29165615|ref|NC_002745.2|	502572	+	A	4	21	21	10	19	-
gi|29165615|ref|NC_002745.2|	502573	+	A	0	1	0	1	2	-
gi|29165615|ref|NC_002745.2|	502575	+	U	0	0	0	0	1	-
gi|29165615|ref|NC_002745.2|	502577	+	U	0	0	1	0	0	-
gi|29165615|ref|NC_002745.2|	502583	+	G	0	0	0	0	1	-
gi|29165615|ref|NC_002745.2|	502586	+	G	0	0	0	1	0	-
gi|29165615|ref|NC_002745.2|	502627	+	C	0	0	1	0	0	SA0436
gi|29165615|ref|NC_002745.2|	502635	+	U	0	0	1	0	0	SA0436
gi|29165615|ref|NC_002745.2|	502639	+	G	0	0	0	0	1	SA0436
gi|29165615|ref|NC_002745.2|	502653	+	A	0	0	0	0	1	SA0436
gi|29165615|ref|NC_002745.2|	502660	+	C	0	0	1	0	0	SA0436
gi|29165615|ref|NC_002745.2|	502681	+	A	0	0	0	0	1	SA0436
gi|29165615|ref|NC_002745.2|	502685	+	C	0	0	1	0	0	SA0436
gi|29165615|ref|NC_002745.2|	502707	+	G	1	0	0	0	0	SA0436
gi|29165615|ref|NC_002745.2|	502721	+	U	0	0	0	0	1	SA0436
gi|29165615|ref|NC_002745.2|	502765	+	U	0	0	0	0	1	SA0436
gi|29165615|ref|NC_002745.2|	502786	+	C	0	1	1	0	0	SA0436
gi|29165615|ref|NC_002745.2|	502787	+	U	0	0	0	0	1	SA0436
gi|29165615|ref|NC_002745.2|	502808	+	C	0	0	0	0	1	SA0436
gi|29165615|ref|NC_002745.2|	502863	+	G	1	0	0	0	0	SA0436
gi|29165615|ref|NC_002745.2|	502901	+	A	0	0	1	0	0	SA0436
gi|29165615|ref|NC_002745.2|	502906	+	A	0	0	0	1	0	SA0436
gi|29165615|ref|NC_002745.2|	502922	+	A	0	0	0	0	1	SA0436
gi|29165615|ref|NC_002745.2|	502926	+	U	0	1	0	0	0	SA0436
gi|29165615|ref|NC_002745.2|	502929	+	A	0	1	0	0	0	SA0436
gi|29165615|ref|NC_002745.2|	502947	+	G	1	0	0	0	0	SA0436
gi|29165615|ref|NC_002745.2|	502952	+	A	0	1	0	0	0	SA0436
gi|29165615|ref|NC_002745.2|	502954	+	A	0	0	0	0	1	SA0436
gi|29165615|ref|NC_002745.2|	502958	+	U	0	0	1	1	0	SA0436
gi|29165615|ref|NC_002745.2|	502960	+	U	0	0	1	0	0	SA0436
gi|29165615|ref|NC_002745.2|	502965	+	U	0	0	0	0	1	SA0436
gi|29165615|ref|NC_002745.2|	502966	+	A	0	1	0	0	0	SA0436
gi|29165615|ref|NC_002745.2|	502967	+	U	0	0	1	0	0	SA0436
gi|29165615|ref|NC_002745.2|	502968	+	A	0	0	2	0	0	SA0436
gi|29165615|ref|NC_002745.2|	503039	+	A	0	0	1	0	0	SA0436
gi|29165615|ref|NC_002745.2|	503070	+	A	1	0	1	0	0	SA0436
gi|29165615|ref|NC_002745.2|	503072	+	A	0	0	0	0	1	SA0436
gi|29165615|ref|NC_002745.2|	503074	+	A	0	0	0	0	1	SA0436
gi|29165615|ref|NC_002745.2|	503075	+	A	0	0	1	0	0	SA0436
gi|29165615|ref|NC_002745.2|	503076	+	A	0	0	0	1	0	SA0436
gi|29165615|ref|NC_002745.2|	503099	+	G	0	1	0	0	1	SA0436
gi|29165615|ref|NC_002745.2|	503132	+	U	0	0	0	0	1	SA0436
gi|29165615|ref|NC_002745.2|	503142	+	U	0	0	0	0	1	SA0436
gi|29165615|ref|NC_002745.2|	503149	+	C	0	0	0	0	1	SA0436
gi|29165615|ref|NC_002745.2|	503150	+	G	0	0	0	1	0	SA0436
gi|29165615|ref|NC_002745.2|	503153	+	U	0	0	1	0	0	SA0436
gi|29165615|ref|NC_002745.2|	503157	+	A	0	2	1	0	0	SA0436
gi|29165615|ref|NC_002745.2|	503158	+	U	0	1	0	0	0	SA0436
gi|29165615|ref|NC_002745.2|	503162	+	U	0	0	1	0	0	SA0436
gi|29165615|ref|NC_002745.2|	503163	+	A	0	0	0	0	1	SA0436
gi|29165615|ref|NC_002745.2|	503170	+	G	0	1	0	0	0	SA0436
gi|29165615|ref|NC_002745.2|	503179	+	A	0	0	0	0	1	SA0436
gi|29165615|ref|NC_002745.2|	503183	+	A	0	1	0	0	1	SA0436
gi|29165615|ref|NC_002745.2|	503184	+	A	0	0	0	0	1	SA0436

gi|29165615|ref|NC_002745.2|	503185 +	U	0	0	1	0	0	SA0436
gi|29165615|ref|NC_002745.2|	503189 +	A	0	0	0	0	2	SA0436
gi|29165615|ref|NC_002745.2|	503210 +	U	0	0	0	0	2	SA0436
gi|29165615|ref|NC_002745.2|	503213 +	C	1	0	1	0	0	SA0436
gi|29165615|ref|NC_002745.2|	503219 +	C	0	0	1	0	0	SA0436
gi|29165615|ref|NC_002745.2|	503231 +	G	1	0	0	0	0	SA0436
gi|29165615|ref|NC_002745.2|	503232 +	U	2	0	0	1	0	SA0436
gi|29165615|ref|NC_002745.2|	503270 +	C	0	1	0	0	0	SA0436
gi|29165615|ref|NC_002745.2|	503283 +	U	0	1	0	0	0	SA0436
gi|29165615|ref|NC_002745.2|	503342 +	A	0	0	1	0	0	SA0436
gi|29165615|ref|NC_002745.2|	503377 +	C	0	2	0	0	0	SA0436
gi|29165615|ref|NC_002745.2|	503410 +	A	0	1	0	0	0	SA0436
gi|29165615|ref|NC_002745.2|	503419 +	A	0	0	0	0	1	SA0436
gi|29165615|ref|NC_002745.2|	503444 +	A	0	0	1	0	0	SA0436
gi|29165615|ref|NC_002745.2|	503453 +	A	1	0	0	0	0	SA0436
gi|29165615|ref|NC_002745.2|	503469 +	G	0	0	0	1	0	SA0436
gi|29165615|ref|NC_002745.2|	503476 +	A	0	0	1	0	0	SA0436
gi|29165615|ref|NC_002745.2|	503502 +	G	0	1	0	0	0	SA0436
gi|29165615|ref|NC_002745.2|	503513 +	U	0	0	0	1	0	SA0436
gi|29165615|ref|NC_002745.2|	503522 +	U	0	0	1	0	0	SA0436
gi|29165615|ref|NC_002745.2|	503524 +	G	1	0	0	0	1	SA0436
gi|29165615|ref|NC_002745.2|	503525 +	A	0	0	0	0	1	SA0436
gi|29165615|ref|NC_002745.2|	503531 +	A	0	0	3	0	0	SA0436
gi|29165615|ref|NC_002745.2|	503532 +	U	0	0	0	0	2	SA0436
gi|29165615|ref|NC_002745.2|	503536 +	U	3	0	0	0	0	SA0436
gi|29165615|ref|NC_002745.2|	503537 +	U	0	0	0	1	0	SA0436
gi|29165615|ref|NC_002745.2|	503538 +	A	0	2	0	0	0	SA0436
gi|29165615|ref|NC_002745.2|	503542 +	C	0	0	0	0	1	SA0436
gi|29165615|ref|NC_002745.2|	503544 +	A	0	0	0	2	0	SA0436
gi|29165615|ref|NC_002745.2|	503546 +	U	0	0	0	0	1	SA0436
gi|29165615|ref|NC_002745.2|	503563 +	G	0	0	1	0	0	SA0436
gi|29165615|ref|NC_002745.2|	503564 +	A	0	2	1	1	0	SA0436
gi|29165615|ref|NC_002745.2|	503565 +	U	0	0	0	0	1	SA0436
gi|29165615|ref|NC_002745.2|	503567 +	C	1	0	0	0	0	SA0436
gi|29165615|ref|NC_002745.2|	503568 +	A	0	0	2	0	0	SA0436
gi|29165615|ref|NC_002745.2|	503577 +	G	0	0	0	0	1	SA0436
gi|29165615|ref|NC_002745.2|	503592 +	G	1	0	0	0	0	SA0436
gi|29165615|ref|NC_002745.2|	503596 +	C	0	0	0	0	1	SA0436
gi|29165615|ref|NC_002745.2|	503617 +	U	0	0	0	0	1	SA0436
gi|29165615|ref|NC_002745.2|	503620 +	U	0	0	1	0	0	SA0436
gi|29165615|ref|NC_002745.2|	503621 +	U	0	1	0	0	0	SA0436
gi|29165615|ref|NC_002745.2|	503627 +	A	0	0	1	0	0	SA0436
gi|29165615|ref|NC_002745.2|	503628 +	A	0	2	0	0	2	SA0436
gi|29165615|ref|NC_002745.2|	503630 +	U	0	0	0	0	1	SA0436
gi|29165615|ref|NC_002745.2|	503638 +	C	0	0	0	3	1	SA0436
gi|29165615|ref|NC_002745.2|	503641 +	A	0	0	1	0	0	SA0436
gi|29165615|ref|NC_002745.2|	503651 +	A	0	0	0	2	0	SA0436
gi|29165615|ref|NC_002745.2|	503656 +	C	0	0	0	1	0	SA0436
gi|29165615|ref|NC_002745.2|	503668 +	A	0	0	1	0	0	SA0436
gi|29165615|ref|NC_002745.2|	503671 +	G	0	0	0	1	0	SA0436
gi|29165615|ref|NC_002745.2|	503681 +	C	0	1	0	0	0	SA0436
gi|29165615|ref|NC_002745.2|	503712 +	U	2	0	0	0	0	SA0436
gi|29165615|ref|NC_002745.2|	503713 +	G	0	0	1	0	0	SA0436
gi|29165615|ref|NC_002745.2|	503729 +	U	0	0	0	0	1	SA0436
gi|29165615|ref|NC_002745.2|	503760 +	A	0	1	0	0	0	SA0436
gi|29165615|ref|NC_002745.2|	503771 +	G	1	0	0	0	0	SA0436
gi|29165615|ref|NC_002745.2|	503780 +	U	1	0	0	0	0	SA0436
gi|29165615|ref|NC_002745.2|	503796 +	A	0	1	0	0	0	SA0436
gi|29165615|ref|NC_002745.2|	503800 +	U	0	1	0	0	0	SA0436
gi|29165615|ref|NC_002745.2|	503802 +	G	0	0	0	1	0	SA0436
gi|29165615|ref|NC_002745.2|	503803 +	A	0	0	0	1	0	SA0436
gi|29165615|ref|NC_002745.2|	503810 +	C	1	0	0	0	0	SA0436
gi|29165615|ref|NC_002745.2|	503817 +	A	0	0	0	0	1	SA0436
gi|29165615|ref|NC_002745.2|	503829 +	A	0	0	1	0	0	SA0436
gi|29165615|ref|NC_002745.2|	503830 +	U	0	0	0	3	0	SA0436
gi|29165615|ref|NC_002745.2|	503831 +	C	0	0	0	2	0	SA0436
gi|29165615|ref|NC_002745.2|	503833 +	A	0	0	1	0	0	SA0436
gi|29165615|ref|NC_002745.2|	503834 +	A	0	0	0	1	0	SA0436
gi|29165615|ref|NC_002745.2|	503840 +	C	0	0	0	1	0	SA0436
gi|29165615|ref|NC_002745.2|	503848 +	A	0	0	0	0	1	SA0436
gi|29165615|ref|NC_002745.2|	503849 +	U	0	0	0	0	1	SA0436
gi|29165615|ref|NC_002745.2|	503856 +	A	0	0	0	1	0	SA0436
gi|29165615|ref|NC_002745.2|	503857 +	A	0	0	1	0	1	SA0436
gi|29165615|ref|NC_002745.2|	503863 +	A	0	1	0	0	0	SA0436
gi|29165615|ref|NC_002745.2|	503868 +	G	0	0	0	0	1	SA0436
gi|29165615|ref|NC_002745.2|	503869 +	C	1	0	0	0	0	SA0436
gi|29165615|ref|NC_002745.2|	503872 +	G	0	1	0	0	0	SA0436
gi|29165615|ref|NC_002745.2|	503873 +	A	0	0	0	1	0	SA0436
gi|29165615|ref|NC_002745.2|	503879 +	C	1	0	0	0	0	SA0436
gi|29165615|ref|NC_002745.2|	503888 +	C	0	2	0	0	0	SA0436
gi|29165615|ref|NC_002745.2|	503904 +	G	0	1	0	0	0	SA0436

gi|29165615|ref|NC_002745.2|	503905 +	A	0	0	2	0	0	SA0436
gi|29165615|ref|NC_002745.2|	503906 +	A	0	0	0	0	1	SA0436
gi|29165615|ref|NC_002745.2|	503907 +	A	0	0	0	1	0	SA0436
gi|29165615|ref|NC_002745.2|	503911 +	C	2	0	0	1	0	SA0436
gi|29165615|ref|NC_002745.2|	503917 +	C	0	0	0	0	2	SA0436
gi|29165615|ref|NC_002745.2|	503927 +	U	0	0	1	0	0	SA0436
gi|29165615|ref|NC_002745.2|	503943 +	C	0	0	0	0	2	SA0436
gi|29165615|ref|NC_002745.2|	503944 +	A	0	0	0	1	0	SA0436
gi|29165615|ref|NC_002745.2|	503945 +	A	0	1	0	0	0	SA0436
gi|29165615|ref|NC_002745.2|	503946 +	U	0	0	0	2	0	SA0436
gi|29165615|ref|NC_002745.2|	503947 +	G	0	0	0	0	1	SA0436
gi|29165615|ref|NC_002745.2|	503948 +	A	0	0	0	1	1	SA0436
gi|29165615|ref|NC_002745.2|	503953 +	A	0	0	0	0	1	SA0436
gi|29165615|ref|NC_002745.2|	503954 +	A	0	0	0	0	1	SA0436
gi|29165615|ref|NC_002745.2|	503955 +	A	1	1	0	0	0	SA0436
gi|29165615|ref|NC_002745.2|	503957 +	C	1	0	0	0	0	SA0436
gi|29165615|ref|NC_002745.2|	503958 +	A	0	0	0	1	0	SA0436
gi|29165615|ref|NC_002745.2|	503959 +	C	1	0	1	0	0	SA0436
gi|29165615|ref|NC_002745.2|	503962 +	G	0	0	0	0	1	SA0436
gi|29165615|ref|NC_002745.2|	503966 +	G	0	0	1	0	0	SA0436
gi|29165615|ref|NC_002745.2|	503967 +	U	1	0	0	0	0	SA0436
gi|29165615|ref|NC_002745.2|	503968 +	U	0	0	0	0	1	SA0436
gi|29165615|ref|NC_002745.2|	503970 +	A	0	1	1	0	1	SA0436
gi|29165615|ref|NC_002745.2|	503971 +	U	0	0	0	1	0	SA0436
gi|29165615|ref|NC_002745.2|	503973 +	G	1	1	0	0	0	SA0436
gi|29165615|ref|NC_002745.2|	503974 +	C	0	1	0	0	0	SA0436
gi|29165615|ref|NC_002745.2|	503976 +	A	0	1	0	1	2	SA0436
gi|29165615|ref|NC_002745.2|	503977 +	A	0	1	1	2	0	SA0436
gi|29165615|ref|NC_002745.2|	503978 +	A	0	3	0	0	1	SA0436
gi|29165615|ref|NC_002745.2|	503980 +	U	1	0	0	0	0	SA0436
gi|29165615|ref|NC_002745.2|	503983 +	G	0	0	1	0	0	SA0436
gi|29165615|ref|NC_002745.2|	503984 +	A	2	0	2	0	0	SA0436
gi|29165615|ref|NC_002745.2|	503986 +	C	0	1	1	0	0	SA0436
gi|29165615|ref|NC_002745.2|	503990 +	U	1	0	0	0	0	SA0436
gi|29165615|ref|NC_002745.2|	503992 +	G	1	0	0	0	0	SA0436
gi|29165615|ref|NC_002745.2|	503993 +	C	0	1	0	0	6	SA0436
gi|29165615|ref|NC_002745.2|	504021 +	A	0	2	0	0	0	SA0436
gi|29165615|ref|NC_002745.2|	504058 +	G	0	0	0	1	0	SA0436
gi|29165615|ref|NC_002745.2|	504061 +	G	0	0	0	0	1	SA0436
gi|29165615|ref|NC_002745.2|	504070 +	C	0	1	0	0	0	SA0436
gi|29165615|ref|NC_002745.2|	504078 +	U	0	0	1	0	0	SA0436
gi|29165615|ref|NC_002745.2|	504092 +	U	0	0	0	0	1	SA0436
gi|29165615|ref|NC_002745.2|	504100 +	A	0	0	0	0	1	SA0436
gi|29165615|ref|NC_002745.2|	504102 +	C	0	0	0	1	0	SA0436
gi|29165615|ref|NC_002745.2|	504105 +	U	0	0	0	0	1	SA0436
gi|29165615|ref|NC_002745.2|	504112 +	A	0	1	0	0	0	SA0436
gi|29165615|ref|NC_002745.2|	504115 +	G	0	0	0	0	1	SA0436
gi|29165615|ref|NC_002745.2|	504125 +	U	1	0	0	0	0	SA0436
gi|29165615|ref|NC_002745.2|	504129 +	U	1	0	0	0	0	SA0436
gi|29165615|ref|NC_002745.2|	504151 +	A	0	1	0	0	0	SA0436
gi|29165615|ref|NC_002745.2|	504158 +	G	0	1	0	0	0	SA0436
gi|29165615|ref|NC_002745.2|	504163 +	G	0	1	0	0	0	SA0436
gi|29165615|ref|NC_002745.2|	504166 +	U	0	0	1	0	1	SA0436
gi|29165615|ref|NC_002745.2|	504167 +	A	0	1	0	0	0	SA0436
gi|29165615|ref|NC_002745.2|	504169 +	U	0	0	0	0	1	SA0436
gi|29165615|ref|NC_002745.2|	504172 +	C	0	0	0	0	2	SA0436
gi|29165615|ref|NC_002745.2|	504175 +	A	0	0	0	0	1	SA0436
gi|29165615|ref|NC_002745.2|	504176 +	A	0	2	0	0	1	SA0436
gi|29165615|ref|NC_002745.2|	504180 +	U	0	0	0	0	1	SA0436
gi|29165615|ref|NC_002745.2|	504184 +	A	0	2	0	0	0	SA0436
gi|29165615|ref|NC_002745.2|	504185 +	A	0	1	0	0	0	SA0436
gi|29165615|ref|NC_002745.2|	504187 +	G	0	1	0	0	0	SA0436
gi|29165615|ref|NC_002745.2|	504188 +	A	0	0	0	0	1	SA0436
gi|29165615|ref|NC_002745.2|	504190 +	G	1	0	0	0	0	SA0436
gi|29165615|ref|NC_002745.2|	504194 +	A	0	0	0	1	0	SA0436
gi|29165615|ref|NC_002745.2|	504198 +	U	1	0	0	0	0	SA0436
gi|29165615|ref|NC_002745.2|	504208 +	C	0	1	0	0	0	SA0436
gi|29165615|ref|NC_002745.2|	504212 +	A	1	0	0	0	0	SA0436
gi|29165615|ref|NC_002745.2|	504229 +	A	0	0	1	0	0	SA0436
gi|29165615|ref|NC_002745.2|	504238 +	A	1	0	0	0	0	SA0436
gi|29165615|ref|NC_002745.2|	504246 +	A	0	0	0	0	1	SA0436
gi|29165615|ref|NC_002745.2|	504250 +	C	0	0	0	0	1	SA0436
gi|29165615|ref|NC_002745.2|	504258 +	U	1	0	0	0	0	SA0436
gi|29165615|ref|NC_002745.2|	504262 +	G	0	0	0	0	1	SA0436
gi|29165615|ref|NC_002745.2|	504263 +	A	0	0	1	0	0	SA0436
gi|29165615|ref|NC_002745.2|	504264 +	A	0	0	0	0	1	SA0436
gi|29165615|ref|NC_002745.2|	504265 +	A	0	1	0	0	0	SA0436
gi|29165615|ref|NC_002745.2|	504266 +	C	0	1	0	1	0	SA0436
gi|29165615|ref|NC_002745.2|	504269 +	U	0	0	0	3	0	SA0436
gi|29165615|ref|NC_002745.2|	504271 +	C	2	1	0	1	0	SA0436
gi|29165615|ref|NC_002745.2|	504272 +	A	5	0	0	0	0	SA0436

gi|29165615|ref|NC_002745.2|	504280	+	G	0	0	0	0	1	SA0436
gi|29165615|ref|NC_002745.2|	504283	+	G	0	0	1	0	0	SA0436
gi|29165615|ref|NC_002745.2|	504284	+	A	0	1	0	0	0	SA0436
gi|29165615|ref|NC_002745.2|	504285	+	A	0	1	0	0	0	SA0436
gi|29165615|ref|NC_002745.2|	504291	+	A	0	1	0	0	1	SA0436
gi|29165615|ref|NC_002745.2|	504294	+	C	2	0	0	0	0	-
gi|29165615|ref|NC_002745.2|	504299	+	C	0	1	0	0	0	-
gi|29165615|ref|NC_002745.2|	504300	+	A	0	2	0	0	1	-
gi|29165615|ref|NC_002745.2|	504301	+	A	0	1	0	0	0	-
gi|29165615|ref|NC_002745.2|	504302	+	G	0	0	0	1	0	-
gi|29165615|ref|NC_002745.2|	504303	+	C	0	1	0	0	1	-
gi|29165615|ref|NC_002745.2|	504304	+	G	0	1	0	0	0	-
gi|29165615|ref|NC_002745.2|	504305	+	A	0	0	0	1	0	-
gi|29165615|ref|NC_002745.2|	504306	+	U	0	0	2	0	0	-
gi|29165615|ref|NC_002745.2|	504308	+	U	0	1	0	1	0	-
gi|29165615|ref|NC_002745.2|	504309	+	A	0	0	1	1	0	-
gi|29165615|ref|NC_002745.2|	504310	+	A	0	1	0	0	1	-
gi|29165615|ref|NC_002745.2|	504314	+	U	5	19	12	23	11	-
gi|29165615|ref|NC_002745.2|	504315	+	A	1	8	3	5	7	-
gi|29165615|ref|NC_002745.2|	504316	+	U	0	1	1	1	1	-
gi|29165615|ref|NC_002745.2|	504317	+	G	0	0	0	1	1	-
gi|29165615|ref|NC_002745.2|	504318	+	U	0	0	0	3	1	-
gi|29165615|ref|NC_002745.2|	504319	+	A	0	0	1	1	1	-
gi|29165615|ref|NC_002745.2|	504320	+	U	0	1	0	0	0	-
gi|29165615|ref|NC_002745.2|	504321	+	A	1	1	0	4	3	-
gi|29165615|ref|NC_002745.2|	504324	+	G	0	6	3	3	9	-
gi|29165615|ref|NC_002745.2|	504325	+	A	4	42	17	28	35	-
gi|29165615|ref|NC_002745.2|	504326	+	A	6	21	9	6	6	-
gi|29165615|ref|NC_002745.2|	504327	+	A	14	43	27	34	33	-
gi|29165615|ref|NC_002745.2|	504328	+	G	20	76	49	54	68	-
gi|29165615|ref|NC_002745.2|	504329	+	A	13	61	56	51	64	-
gi|29165615|ref|NC_002745.2|	504330	+	A	3	14	19	21	18	-
gi|29165615|ref|NC_002745.2|	504331	+	A	0	7	4	5	3	-
gi|29165615|ref|NC_002745.2|	504332	+	C	0	2	1	1	1	-
gi|29165615|ref|NC_002745.2|	504335	+	C	0	1	0	0	0	-
gi|29165615|ref|NC_002745.2|	504337	+	U	0	0	1	0	2	-
gi|29165615|ref|NC_002745.2|	504338	+	U	0	2	0	0	1	-
gi|29165615|ref|NC_002745.2|	504339	+	U	0	1	1	0	0	-
gi|29165615|ref|NC_002745.2|	504341	+	A	0	1	0	2	1	-
gi|29165615|ref|NC_002745.2|	504342	+	U	0	0	0	2	1	-
gi|29165615|ref|NC_002745.2|	504343	+	U	0	2	0	1	2	-
gi|29165615|ref|NC_002745.2|	504344	+	G	0	5	4	3	6	-
gi|29165615|ref|NC_002745.2|	504345	+	A	1	13	10	23	6	-
gi|29165615|ref|NC_002745.2|	504347	+	A	0	1	1	5	4	-
gi|29165615|ref|NC_002745.2|	504348	+	A	1	20	10	22	23	-
gi|29165615|ref|NC_002745.2|	504349	+	A	0	3	4	4	4	-
gi|29165615|ref|NC_002745.2|	504350	+	U	0	5	0	2	1	-
gi|29165615|ref|NC_002745.2|	504351	+	A	0	3	3	2	2	-
gi|29165615|ref|NC_002745.2|	504352	+	U	0	7	2	6	6	-
gi|29165615|ref|NC_002745.2|	504353	+	U	0	4	0	1	0	-
gi|29165615|ref|NC_002745.2|	504354	+	U	0	2	2	2	5	-
gi|29165615|ref|NC_002745.2|	504355	+	A	0	2	4	3	8	-
gi|29165615|ref|NC_002745.2|	504356	+	U	0	2	1	1	0	-
gi|29165615|ref|NC_002745.2|	504358	+	G	0	5	0	5	0	-
gi|29165615|ref|NC_002745.2|	504359	+	A	0	10	5	18	10	-
gi|29165615|ref|NC_002745.2|	504360	+	U	0	1	0	1	0	-
gi|29165615|ref|NC_002745.2|	504362	+	U	0	0	0	1	0	-
gi|29165615|ref|NC_002745.2|	504363	+	U	0	1	1	2	1	-
gi|29165615|ref|NC_002745.2|	504364	+	C	0	1	2	2	1	-
gi|29165615|ref|NC_002745.2|	504365	+	A	0	1	2	3	2	-
gi|29165615|ref|NC_002745.2|	504366	+	A	1	4	0	1	2	-
gi|29165615|ref|NC_002745.2|	504367	+	G	1	1	2	2	1	-
gi|29165615|ref|NC_002745.2|	504368	+	G	0	2	2	4	1	-
gi|29165615|ref|NC_002745.2|	504369	+	A	0	0	1	2	1	-
gi|29165615|ref|NC_002745.2|	504370	+	G	2	2	2	2	1	-
gi|29165615|ref|NC_002745.2|	504371	+	G	2	21	7	35	27	-
gi|29165615|ref|NC_002745.2|	504372	+	A	1	27	17	31	28	-
gi|29165615|ref|NC_002745.2|	504373	+	A	0	13	7	19	11	-
gi|29165615|ref|NC_002745.2|	504374	+	A	0	2	2	1	0	-
gi|29165615|ref|NC_002745.2|	504375	+	U	0	0	0	1	0	-
gi|29165615|ref|NC_002745.2|	504376	+	G	2	0	1	0	1	-
gi|29165615|ref|NC_002745.2|	504377	+	G	0	2	3	3	1	-
gi|29165615|ref|NC_002745.2|	504378	+	A	0	2	5	13	7	-
gi|29165615|ref|NC_002745.2|	504379	+	A	0	1	1	2	1	-
gi|29165615|ref|NC_002745.2|	504380	+	U	0	3	1	3	1	-
gi|29165615|ref|NC_002745.2|	504381	+	A	0	0	1	0	0	SA0437
gi|29165615|ref|NC_002745.2|	504382	+	U	0	0	1	1	0	SA0437
gi|29165615|ref|NC_002745.2|	504383	+	G	0	2	0	0	0	SA0437
gi|29165615|ref|NC_002745.2|	504384	+	C	0	1	0	0	1	SA0437
gi|29165615|ref|NC_002745.2|	504388	+	G	0	0	0	2	1	SA0437
gi|29165615|ref|NC_002745.2|	504390	+	G	0	1	0	0	0	SA0437

gi|29165615|ref|NC_002745.2|	504398 +	C	0	1	0	1	0	SA0437
gi|29165615|ref|NC_002745.2|	504401 +	G	0	1	0	0	0	SA0437
gi|29165615|ref|NC_002745.2|	504402 +	C	0	1	0	2	0	SA0437
gi|29165615|ref|NC_002745.2|	504403 +	A	0	0	1	1	0	SA0437
gi|29165615|ref|NC_002745.2|	504404 +	A	0	2	0	0	1	SA0437
gi|29165615|ref|NC_002745.2|	504405 +	C	0	1	0	0	3	SA0437
gi|29165615|ref|NC_002745.2|	504406 +	A	0	0	1	5	1	SA0437
gi|29165615|ref|NC_002745.2|	504407 +	A	0	1	1	2	0	SA0437
gi|29165615|ref|NC_002745.2|	504408 +	A	0	0	1	0	0	SA0437
gi|29165615|ref|NC_002745.2|	504409 +	U	0	0	0	0	1	SA0437
gi|29165615|ref|NC_002745.2|	504411 +	A	0	1	0	0	1	SA0437
gi|29165615|ref|NC_002745.2|	504413 +	G	0	1	0	2	0	SA0437
gi|29165615|ref|NC_002745.2|	504414 +	A	0	2	1	0	2	SA0437
gi|29165615|ref|NC_002745.2|	504416 +	A	0	0	0	0	1	SA0437
gi|29165615|ref|NC_002745.2|	504417 +	C	0	1	0	0	0	SA0437
gi|29165615|ref|NC_002745.2|	504418 +	A	0	0	1	0	0	SA0437
gi|29165615|ref|NC_002745.2|	504419 +	A	0	1	0	1	1	SA0437
gi|29165615|ref|NC_002745.2|	504424 +	A	0	0	0	0	1	SA0437
gi|29165615|ref|NC_002745.2|	504425 +	A	0	0	1	0	0	SA0437
gi|29165615|ref|NC_002745.2|	504426 +	A	0	0	0	1	0	SA0437
gi|29165615|ref|NC_002745.2|	504427 +	A	0	0	1	0	1	SA0437
gi|29165615|ref|NC_002745.2|	504428 +	A	0	0	1	0	3	SA0437
gi|29165615|ref|NC_002745.2|	504432 +	C	0	4	2	4	4	SA0437
gi|29165615|ref|NC_002745.2|	504433 +	A	0	6	1	2	6	SA0437
gi|29165615|ref|NC_002745.2|	504434 +	A	0	1	0	10	8	SA0437
gi|29165615|ref|NC_002745.2|	504435 +	A	0	1	0	2	2	SA0437
gi|29165615|ref|NC_002745.2|	504436 +	A	0	1	0	0	0	SA0437
gi|29165615|ref|NC_002745.2|	504437 +	G	0	0	0	4	5	SA0437
gi|29165615|ref|NC_002745.2|	504438 +	A	0	1	2	5	0	SA0437
gi|29165615|ref|NC_002745.2|	504441 +	A	0	12	1	0	4	SA0437
gi|29165615|ref|NC_002745.2|	504445 +	C	0	2	0	2	2	SA0437
gi|29165615|ref|NC_002745.2|	504449 +	A	0	0	0	0	2	SA0437
gi|29165615|ref|NC_002745.2|	504450 +	G	0	1	0	0	0	SA0437
gi|29165615|ref|NC_002745.2|	504451 +	A	0	0	0	3	3	SA0437
gi|29165615|ref|NC_002745.2|	504452 +	A	0	1	1	0	2	SA0437
gi|29165615|ref|NC_002745.2|	504453 +	C	0	1	0	0	0	SA0437
gi|29165615|ref|NC_002745.2|	504455 +	A	0	1	0	0	0	SA0437
gi|29165615|ref|NC_002745.2|	504456 +	G	0	0	0	1	3	SA0437
gi|29165615|ref|NC_002745.2|	504457 +	A	0	1	0	1	0	SA0437
gi|29165615|ref|NC_002745.2|	504458 +	A	1	3	3	2	0	SA0437
gi|29165615|ref|NC_002745.2|	504459 +	A	0	0	2	1	1	SA0437
gi|29165615|ref|NC_002745.2|	504460 +	A	0	2	0	1	1	SA0437
gi|29165615|ref|NC_002745.2|	504461 +	A	0	6	1	1	1	SA0437
gi|29165615|ref|NC_002745.2|	504462 +	C	1	2	0	0	2	SA0437
gi|29165615|ref|NC_002745.2|	504464 +	U	0	0	0	2	1	SA0437
gi|29165615|ref|NC_002745.2|	504465 +	A	1	4	1	5	0	SA0437
gi|29165615|ref|NC_002745.2|	504466 +	A	0	1	1	6	3	SA0437
gi|29165615|ref|NC_002745.2|	504467 +	A	0	0	0	2	1	SA0437
gi|29165615|ref|NC_002745.2|	504468 +	G	0	0	0	2	0	SA0437
gi|29165615|ref|NC_002745.2|	504470 +	A	0	0	0	1	0	SA0437
gi|29165615|ref|NC_002745.2|	504471 +	G	0	1	2	3	3	SA0437
gi|29165615|ref|NC_002745.2|	504472 +	A	0	1	0	1	2	SA0437
gi|29165615|ref|NC_002745.2|	504474 +	C	0	1	0	0	0	SA0437
gi|29165615|ref|NC_002745.2|	504475 +	G	0	1	0	1	0	SA0437
gi|29165615|ref|NC_002745.2|	504476 +	U	0	4	1	4	4	SA0437
gi|29165615|ref|NC_002745.2|	504477 +	A	0	6	1	2	4	SA0437
gi|29165615|ref|NC_002745.2|	504484 +	G	0	0	1	1	0	SA0437
gi|29165615|ref|NC_002745.2|	504486 +	A	0	0	2	0	0	SA0437
gi|29165615|ref|NC_002745.2|	504487 +	C	0	2	0	2	0	SA0437
gi|29165615|ref|NC_002745.2|	504497 +	U	0	0	0	0	2	SA0437
gi|29165615|ref|NC_002745.2|	504498 +	G	0	1	0	0	0	SA0437
gi|29165615|ref|NC_002745.2|	504502 +	U	1	0	0	0	0	SA0437
gi|29165615|ref|NC_002745.2|	504510 +	G	0	0	1	0	0	SA0437
gi|29165615|ref|NC_002745.2|	504512 +	U	0	0	1	0	0	SA0437
gi|29165615|ref|NC_002745.2|	504514 +	C	0	1	0	0	0	SA0437
gi|29165615|ref|NC_002745.2|	504524 +	U	2	0	0	0	0	SA0437
gi|29165615|ref|NC_002745.2|	504531 +	G	0	0	0	0	1	SA0437
gi|29165615|ref|NC_002745.2|	504533 +	A	2	0	0	0	0	SA0437
gi|29165615|ref|NC_002745.2|	504541 +	A	0	0	0	1	0	SA0437
gi|29165615|ref|NC_002745.2|	504543 +	G	0	2	0	0	0	SA0437
gi|29165615|ref|NC_002745.2|	504547 +	A	0	1	0	0	1	SA0437
gi|29165615|ref|NC_002745.2|	504548 +	A	0	1	0	2	0	SA0437
gi|29165615|ref|NC_002745.2|	504549 +	A	0	0	0	3	1	SA0437
gi|29165615|ref|NC_002745.2|	504550 +	U	0	0	0	2	1	SA0437
gi|29165615|ref|NC_002745.2|	504551 +	C	0	2	1	1	0	SA0437
gi|29165615|ref|NC_002745.2|	504552 +	A	0	0	0	2	1	SA0437
gi|29165615|ref|NC_002745.2|	504553 +	A	0	7	1	2	1	SA0437
gi|29165615|ref|NC_002745.2|	504554 +	A	0	0	0	2	0	SA0437
gi|29165615|ref|NC_002745.2|	504556 +	A	0	3	1	1	0	SA0437
gi|29165615|ref|NC_002745.2|	504559 +	A	0	1	0	1	1	SA0437
gi|29165615|ref|NC_002745.2|	504571 +	C	0	1	0	0	0	SA0437

gi|29165615|ref|NC_002745.2|	504572 +	A	1	1	0	1	3	SA0437
gi|29165615|ref|NC_002745.2|	504577 +	A	0	0	1	0	0	SA0437
gi|29165615|ref|NC_002745.2|	504625 +	U	0	0	1	0	0	SA0437
gi|29165615|ref|NC_002745.2|	504643 +	U	2	0	0	0	0	SA0437
gi|29165615|ref|NC_002745.2|	504644 +	U	0	1	0	0	0	SA0437
gi|29165615|ref|NC_002745.2|	504648 +	C	0	1	0	0	0	SA0437
gi|29165615|ref|NC_002745.2|	504681 +	A	0	0	0	1	0	SA0437
gi|29165615|ref|NC_002745.2|	504683 +	C	0	0	0	0	1	SA0437
gi|29165615|ref|NC_002745.2|	504695 +	G	0	1	0	0	0	SA0437
gi|29165615|ref|NC_002745.2|	504711 +	U	0	1	0	0	0	SA0438
gi|29165615|ref|NC_002745.2|	504732 +	C	0	0	0	1	0	SA0438
gi|29165615|ref|NC_002745.2|	504752 +	A	6	0	0	0	1	SA0438
gi|29165615|ref|NC_002745.2|	504772 +	A	0	0	0	0	1	SA0438
gi|29165615|ref|NC_002745.2|	504775 +	C	0	0	1	0	0	SA0438
gi|29165615|ref|NC_002745.2|	504781 +	A	0	1	0	0	0	SA0438
gi|29165615|ref|NC_002745.2|	504790 +	C	0	0	0	1	0	SA0438
gi|29165615|ref|NC_002745.2|	504804 +	G	0	2	0	0	4	SA0438
gi|29165615|ref|NC_002745.2|	504805 +	A	0	3	1	1	2	SA0438
gi|29165615|ref|NC_002745.2|	504806 +	U	0	0	0	0	3	SA0438
gi|29165615|ref|NC_002745.2|	504814 +	A	0	0	1	0	0	SA0438
gi|29165615|ref|NC_002745.2|	504815 +	A	0	0	1	0	0	SA0438
gi|29165615|ref|NC_002745.2|	504817 +	A	0	0	0	0	1	SA0438
gi|29165615|ref|NC_002745.2|	504844 +	U	1	0	0	0	0	SA0438
gi|29165615|ref|NC_002745.2|	504866 +	A	0	1	0	0	0	SA0438
gi|29165615|ref|NC_002745.2|	504869 +	A	0	1	0	0	0	SA0438
gi|29165615|ref|NC_002745.2|	504880 +	U	1	0	0	1	1	SA0438
gi|29165615|ref|NC_002745.2|	504888 +	C	1	0	0	0	0	SA0438
gi|29165615|ref|NC_002745.2|	504893 +	U	2	0	0	0	0	SA0438
gi|29165615|ref|NC_002745.2|	504894 +	A	1	0	0	0	0	SA0438
gi|29165615|ref|NC_002745.2|	504901 +	A	0	1	0	0	0	SA0438
gi|29165615|ref|NC_002745.2|	504902 +	U	1	0	0	0	0	SA0438
gi|29165615|ref|NC_002745.2|	504904 +	A	0	0	0	1	0	SA0438
gi|29165615|ref|NC_002745.2|	504911 +	U	0	0	0	1	0	SA0438
gi|29165615|ref|NC_002745.2|	504912 +	U	0	0	0	1	0	SA0438
gi|29165615|ref|NC_002745.2|	504915 +	A	0	0	0	0	1	SA0438
gi|29165615|ref|NC_002745.2|	504952 +	G	0	0	1	0	0	SA0438
gi|29165615|ref|NC_002745.2|	504960 +	G	0	0	0	0	1	SA0438
gi|29165615|ref|NC_002745.2|	504963 +	G	0	1	1	0	0	SA0438
gi|29165615|ref|NC_002745.2|	504988 +	A	0	1	0	0	0	SA0438
gi|29165615|ref|NC_002745.2|	504993 +	A	0	0	0	0	1	SA0438
gi|29165615|ref|NC_002745.2|	504995 +	G	1	0	0	0	0	SA0438
gi|29165615|ref|NC_002745.2|	505000 +	A	0	0	0	1	0	SA0438
gi|29165615|ref|NC_002745.2|	505001 +	A	0	0	1	0	0	SA0438
gi|29165615|ref|NC_002745.2|	505010 +	U	0	0	1	0	0	SA0438
gi|29165615|ref|NC_002745.2|	505012 +	U	0	1	0	0	0	SA0438
gi|29165615|ref|NC_002745.2|	505017 +	C	0	0	0	0	1	SA0438
gi|29165615|ref|NC_002745.2|	505018 +	A	1	0	0	0	0	SA0438
gi|29165615|ref|NC_002745.2|	505020 +	G	1	0	0	0	0	SA0438
gi|29165615|ref|NC_002745.2|	505024 +	U	1	0	0	0	0	SA0438
gi|29165615|ref|NC_002745.2|	505025 +	A	0	0	0	1	0	SA0438
gi|29165615|ref|NC_002745.2|	505027 +	A	1	0	0	0	0	SA0438
gi|29165615|ref|NC_002745.2|	505072 +	A	0	1	0	0	0	SA0438
gi|29165615|ref|NC_002745.2|	505077 +	C	0	0	0	0	1	SA0438
gi|29165615|ref|NC_002745.2|	505081 +	C	0	0	1	0	0	SA0438
gi|29165615|ref|NC_002745.2|	505104 +	G	0	1	0	0	0	SA0438
gi|29165615|ref|NC_002745.2|	505143 +	U	0	1	0	0	0	SA0438
gi|29165615|ref|NC_002745.2|	505154 +	A	0	1	0	0	0	SA0438
gi|29165615|ref|NC_002745.2|	505334 +	U	0	1	0	0	0	-
gi|29165615|ref|NC_002745.2|	505375 +	U	0	1	0	0	0	-
gi|29165615|ref|NC_002745.2|	505712 +	A	0	1	0	0	0	-
gi|29165615|ref|NC_002745.2|	506030 +	A	0	0	1	0	0	-
gi|29165615|ref|NC_002745.2|	506038 +	C	0	0	0	2	0	-
gi|29165615|ref|NC_002745.2|	506039 +	U	1	0	1	0	1	-
gi|29165615|ref|NC_002745.2|	506041 +	G	2	1	3	0	2	-
gi|29165615|ref|NC_002745.2|	506045 +	G	0	0	0	0	2	-
gi|29165615|ref|NC_002745.2|	508146 +	A	0	0	0	1	0	-
gi|29165615|ref|NC_002745.2|	508485 +	G	0	0	0	0	1	SArRNA02
gi|29165615|ref|NC_002745.2|	510428 +	U	0	1	0	0	0	SArRNA02
gi|29165615|ref|NC_002745.2|	512169 +	C	0	1	0	0	0	-
gi|29165615|ref|NC_002745.2|	512181 +	A	0	0	0	0	1	-
gi|29165615|ref|NC_002745.2|	512203 +	G	0	0	0	0	1	SA0439
gi|29165615|ref|NC_002745.2|	512305 +	A	0	0	0	1	0	SA0439
gi|29165615|ref|NC_002745.2|	512589 +	C	0	0	0	0	1	SA0439
gi|29165615|ref|NC_002745.2|	512607 +	U	0	1	0	0	0	SA0439
gi|29165615|ref|NC_002745.2|	512613 +	U	2	0	0	0	0	SA0439
gi|29165615|ref|NC_002745.2|	512627 +	A	0	0	1	0	0	SA0439
gi|29165615|ref|NC_002745.2|	512742 +	A	0	1	0	0	0	SA0439
gi|29165615|ref|NC_002745.2|	513010 +	U	0	0	0	0	1	SA0439
gi|29165615|ref|NC_002745.2|	513039 +	A	0	1	0	0	0	SA0439
gi|29165615|ref|NC_002745.2|	513242 +	A	0	0	1	0	0	SA0439
gi|29165615|ref|NC_002745.2|	513449 +	A	0	0	1	0	0	SA0439

gi|29165615|ref|NC_002745.2|	513468 +	A	1	0	2	2	0	SA0439
gi|29165615|ref|NC_002745.2|	513470 +	G	0	1	0	1	0	SA0439
gi|29165615|ref|NC_002745.2|	513510 +	A	0	0	0	0	1	SA0439
gi|29165615|ref|NC_002745.2|	513516 +	A	0	2	0	0	0	SA0439
gi|29165615|ref|NC_002745.2|	513517 +	A	1	0	0	0	0	SA0439
gi|29165615|ref|NC_002745.2|	513538 +	A	0	0	0	0	1	SA0439
gi|29165615|ref|NC_002745.2|	513580 +	G	0	0	0	1	0	SA0440
gi|29165615|ref|NC_002745.2|	513588 +	A	0	2	0	0	0	SA0440
gi|29165615|ref|NC_002745.2|	513591 +	G	0	0	0	0	1	SA0440
gi|29165615|ref|NC_002745.2|	513592 +	U	2	11	8	11	21	SA0440
gi|29165615|ref|NC_002745.2|	513593 +	A	0	8	9	9	14	SA0440
gi|29165615|ref|NC_002745.2|	513594 +	A	0	0	1	1	3	SA0440
gi|29165615|ref|NC_002745.2|	513595 +	U	0	0	1	0	1	SA0440
gi|29165615|ref|NC_002745.2|	513597 +	A	0	1	0	0	1	SA0440
gi|29165615|ref|NC_002745.2|	513598 +	A	0	0	1	0	0	SA0440
gi|29165615|ref|NC_002745.2|	513600 +	G	0	0	0	0	1	SA0440
gi|29165615|ref|NC_002745.2|	513601 +	A	0	1	0	3	0	SA0440
gi|29165615|ref|NC_002745.2|	513602 +	A	0	0	0	0	1	SA0440
gi|29165615|ref|NC_002745.2|	513604 +	U	0	1	0	4	6	SA0440
gi|29165615|ref|NC_002745.2|	513606 +	U	0	0	0	1	0	SA0440
gi|29165615|ref|NC_002745.2|	513607 +	A	0	0	2	0	1	SA0440
gi|29165615|ref|NC_002745.2|	513608 +	C	0	0	1	0	1	SA0440
gi|29165615|ref|NC_002745.2|	513614 +	A	0	1	0	4	2	SA0440
gi|29165615|ref|NC_002745.2|	513618 +	G	0	1	1	0	1	SA0440
gi|29165615|ref|NC_002745.2|	513619 +	U	0	0	0	3	0	SA0440
gi|29165615|ref|NC_002745.2|	513620 +	A	0	0	1	3	1	SA0440
gi|29165615|ref|NC_002745.2|	513621 +	A	0	2	0	1	2	SA0440
gi|29165615|ref|NC_002745.2|	513622 +	A	0	0	1	2	1	SA0440
gi|29165615|ref|NC_002745.2|	513623 +	A	0	2	0	0	1	SA0440
gi|29165615|ref|NC_002745.2|	513624 +	G	0	1	2	0	2	SA0440
gi|29165615|ref|NC_002745.2|	513625 +	A	1	7	10	6	3	SA0440
gi|29165615|ref|NC_002745.2|	513627 +	U	0	1	0	0	3	SA0440
gi|29165615|ref|NC_002745.2|	513628 +	A	0	0	0	1	0	SA0440
gi|29165615|ref|NC_002745.2|	513630 +	G	0	1	0	0	0	SA0440
gi|29165615|ref|NC_002745.2|	513631 +	A	0	0	1	0	0	SA0440
gi|29165615|ref|NC_002745.2|	513634 +	U	2	0	0	0	0	SA0440
gi|29165615|ref|NC_002745.2|	513637 +	U	0	0	0	1	0	SA0440
gi|29165615|ref|NC_002745.2|	513638 +	U	0	0	1	0	0	SA0440
gi|29165615|ref|NC_002745.2|	513639 +	A	0	1	1	2	2	SA0440
gi|29165615|ref|NC_002745.2|	513642 +	A	0	0	0	3	0	SA0440
gi|29165615|ref|NC_002745.2|	513647 +	A	0	0	0	1	1	SA0440
gi|29165615|ref|NC_002745.2|	513652 +	C	0	0	0	0	1	SA0440
gi|29165615|ref|NC_002745.2|	513657 +	G	0	1	0	0	0	SA0440
gi|29165615|ref|NC_002745.2|	513661 +	U	1	0	0	0	0	SA0440
gi|29165615|ref|NC_002745.2|	513671 +	U	1	0	0	0	0	SA0440
gi|29165615|ref|NC_002745.2|	513677 +	A	0	0	0	0	1	SA0440
gi|29165615|ref|NC_002745.2|	513679 +	U	0	0	1	0	0	SA0440
gi|29165615|ref|NC_002745.2|	513683 +	U	0	0	0	1	1	SA0440
gi|29165615|ref|NC_002745.2|	513684 +	A	0	0	0	2	1	SA0440
gi|29165615|ref|NC_002745.2|	513687 +	A	0	2	2	0	0	SA0440
gi|29165615|ref|NC_002745.2|	513691 +	U	0	1	3	9	8	SA0440
gi|29165615|ref|NC_002745.2|	513692 +	A	0	2	1	6	4	SA0440
gi|29165615|ref|NC_002745.2|	513693 +	U	0	0	0	1	0	SA0440
gi|29165615|ref|NC_002745.2|	513694 +	U	0	0	0	0	1	SA0440
gi|29165615|ref|NC_002745.2|	513695 +	A	1	0	0	0	2	SA0440
gi|29165615|ref|NC_002745.2|	513696 +	G	0	1	0	0	1	SA0440
gi|29165615|ref|NC_002745.2|	513697 +	A	0	3	1	4	3	SA0440
gi|29165615|ref|NC_002745.2|	513700 +	G	0	0	0	1	0	SA0440
gi|29165615|ref|NC_002745.2|	513701 +	C	0	2	0	0	0	SA0440
gi|29165615|ref|NC_002745.2|	513702 +	A	0	0	0	0	1	SA0440
gi|29165615|ref|NC_002745.2|	513705 +	G	0	0	0	0	1	SA0440
gi|29165615|ref|NC_002745.2|	513706 +	A	0	0	0	2	0	SA0440
gi|29165615|ref|NC_002745.2|	513707 +	U	0	0	1	0	0	SA0440
gi|29165615|ref|NC_002745.2|	513711 +	G	0	0	0	0	1	SA0440
gi|29165615|ref|NC_002745.2|	513712 +	A	0	0	2	5	2	SA0440
gi|29165615|ref|NC_002745.2|	513717 +	A	0	0	0	1	0	SA0440
gi|29165615|ref|NC_002745.2|	513718 +	G	0	0	0	1	0	SA0440
gi|29165615|ref|NC_002745.2|	513719 +	A	0	0	1	2	1	SA0440
gi|29165615|ref|NC_002745.2|	513724 +	A	0	1	0	0	0	SA0440
gi|29165615|ref|NC_002745.2|	513727 +	C	0	1	2	0	2	SA0440
gi|29165615|ref|NC_002745.2|	513728 +	A	1	0	0	0	0	SA0440
gi|29165615|ref|NC_002745.2|	513731 +	G	2	0	0	0	0	SA0440
gi|29165615|ref|NC_002745.2|	513732 +	U	0	1	1	0	0	SA0440
gi|29165615|ref|NC_002745.2|	513733 +	U	1	0	0	0	0	SA0440
gi|29165615|ref|NC_002745.2|	513734 +	A	0	0	0	0	1	SA0440
gi|29165615|ref|NC_002745.2|	513739 +	C	1	0	0	0	0	SA0440
gi|29165615|ref|NC_002745.2|	513741 +	G	0	0	0	0	1	SA0440
gi|29165615|ref|NC_002745.2|	513742 +	C	0	0	0	0	1	SA0440
gi|29165615|ref|NC_002745.2|	513745 +	C	2	0	0	0	1	SA0440
gi|29165615|ref|NC_002745.2|	513748 +	G	0	1	0	1	0	SA0440
gi|29165615|ref|NC_002745.2|	513749 +	A	0	2	2	0	2	SA0440

gi|29165615|ref|NC_002745.2|	513750 +	A	0	0	1	0	0	SA0440
gi|29165615|ref|NC_002745.2|	513751 +	G	0	0	1	1	0	SA0440
gi|29165615|ref|NC_002745.2|	513752 +	A	0	5	1	2	2	SA0440
gi|29165615|ref|NC_002745.2|	513754 +	A	0	1	1	0	0	SA0440
gi|29165615|ref|NC_002745.2|	513755 +	A	0	1	0	0	3	SA0440
gi|29165615|ref|NC_002745.2|	513756 +	C	0	1	0	1	0	SA0440
gi|29165615|ref|NC_002745.2|	513757 +	A	7	0	0	0	0	SA0440
gi|29165615|ref|NC_002745.2|	513758 +	U	0	0	0	1	1	SA0440
gi|29165615|ref|NC_002745.2|	513759 +	C	1	1	0	0	2	SA0440
gi|29165615|ref|NC_002745.2|	513760 +	U	1	0	0	0	0	SA0440
gi|29165615|ref|NC_002745.2|	513761 +	U	0	0	0	1	0	SA0440
gi|29165615|ref|NC_002745.2|	513762 +	G	0	1	1	0	1	SA0440
gi|29165615|ref|NC_002745.2|	513763 +	U	2	0	2	0	2	SA0440
gi|29165615|ref|NC_002745.2|	513764 +	A	3	0	0	1	1	SA0440
gi|29165615|ref|NC_002745.2|	513766 +	U	1	0	0	0	0	SA0440
gi|29165615|ref|NC_002745.2|	513767 +	A	0	0	0	1	0	SA0440
gi|29165615|ref|NC_002745.2|	513768 +	A	0	0	0	0	2	SA0440
gi|29165615|ref|NC_002745.2|	513769 +	A	0	0	1	0	0	SA0440
gi|29165615|ref|NC_002745.2|	513771 +	G	0	0	0	2	2	SA0440
gi|29165615|ref|NC_002745.2|	513772 +	U	0	0	1	0	0	SA0440
gi|29165615|ref|NC_002745.2|	513773 +	C	0	0	1	0	0	SA0440
gi|29165615|ref|NC_002745.2|	513775 +	U	0	0	0	0	1	SA0440
gi|29165615|ref|NC_002745.2|	513778 +	C	2	0	0	0	0	SA0440
gi|29165615|ref|NC_002745.2|	513780 +	G	0	1	0	0	0	SA0440
gi|29165615|ref|NC_002745.2|	513783 +	U	0	0	0	0	1	SA0440
gi|29165615|ref|NC_002745.2|	513784 +	U	0	1	1	0	0	SA0440
gi|29165615|ref|NC_002745.2|	513785 +	A	0	4	0	1	2	SA0440
gi|29165615|ref|NC_002745.2|	513786 +	A	0	0	1	0	0	SA0440
gi|29165615|ref|NC_002745.2|	513787 +	A	1	0	0	0	0	SA0440
gi|29165615|ref|NC_002745.2|	513788 +	A	0	2	2	0	1	SA0440
gi|29165615|ref|NC_002745.2|	513789 +	G	0	1	0	1	0	SA0440
gi|29165615|ref|NC_002745.2|	513790 +	A	0	1	0	5	2	SA0440
gi|29165615|ref|NC_002745.2|	513793 +	G	0	1	0	0	2	SA0440
gi|29165615|ref|NC_002745.2|	513794 +	U	0	0	0	0	2	SA0440
gi|29165615|ref|NC_002745.2|	513798 +	G	0	1	0	0	2	SA0440
gi|29165615|ref|NC_002745.2|	513802 +	U	1	0	0	0	1	SA0440
gi|29165615|ref|NC_002745.2|	513803 +	G	0	0	0	0	1	SA0440
gi|29165615|ref|NC_002745.2|	513808 +	G	1	0	0	0	0	SA0440
gi|29165615|ref|NC_002745.2|	513809 +	U	1	0	0	0	0	SA0440
gi|29165615|ref|NC_002745.2|	513810 +	G	0	0	0	0	1	SA0440
gi|29165615|ref|NC_002745.2|	513815 +	C	0	0	0	0	1	SA0440
gi|29165615|ref|NC_002745.2|	513824 +	U	0	0	0	1	0	SA0440
gi|29165615|ref|NC_002745.2|	513828 +	U	0	0	1	0	0	SA0440
gi|29165615|ref|NC_002745.2|	513835 +	C	0	2	1	0	1	SA0440
gi|29165615|ref|NC_002745.2|	513840 +	C	0	0	0	1	0	SA0440
gi|29165615|ref|NC_002745.2|	513845 +	U	0	1	0	0	0	SA0440
gi|29165615|ref|NC_002745.2|	513876 +	A	0	0	0	0	1	SA0440
gi|29165615|ref|NC_002745.2|	513922 +	C	0	1	0	0	0	SA0440
gi|29165615|ref|NC_002745.2|	513990 +	A	0	0	0	1	0	SA0440
gi|29165615|ref|NC_002745.2|	514048 +	U	1	0	0	0	0	SA0440
gi|29165615|ref|NC_002745.2|	514063 +	C	2	3	2	8	4	SA0440
gi|29165615|ref|NC_002745.2|	514064 +	A	0	0	1	0	1	SA0440
gi|29165615|ref|NC_002745.2|	514065 +	C	1	4	1	2	2	SA0440
gi|29165615|ref|NC_002745.2|	514066 +	A	16	15	14	16	11	SA0440
gi|29165615|ref|NC_002745.2|	514114 +	A	0	0	0	0	2	SA0440
gi|29165615|ref|NC_002745.2|	514131 +	A	0	1	0	0	0	SA0440
gi|29165615|ref|NC_002745.2|	514133 +	C	0	0	0	1	0	SA0440
gi|29165615|ref|NC_002745.2|	514138 +	A	0	1	0	0	1	SA0440
gi|29165615|ref|NC_002745.2|	514140 +	U	0	0	0	0	1	SA0440
gi|29165615|ref|NC_002745.2|	514142 +	U	0	0	0	1	0	SA0440
gi|29165615|ref|NC_002745.2|	514143 +	U	0	1	0	0	0	SA0440
gi|29165615|ref|NC_002745.2|	514144 +	U	1	0	0	0	3	SA0440
gi|29165615|ref|NC_002745.2|	514145 +	A	0	2	1	2	4	SA0440
gi|29165615|ref|NC_002745.2|	514146 +	G	0	2	2	0	0	SA0440
gi|29165615|ref|NC_002745.2|	514147 +	A	0	1	0	4	2	SA0440
gi|29165615|ref|NC_002745.2|	514148 +	A	1	0	0	0	1	SA0440
gi|29165615|ref|NC_002745.2|	514150 +	A	0	0	0	0	1	SA0440
gi|29165615|ref|NC_002745.2|	514151 +	G	0	1	0	2	1	SA0440
gi|29165615|ref|NC_002745.2|	514152 +	A	0	2	1	3	0	SA0440
gi|29165615|ref|NC_002745.2|	514155 +	U	0	0	0	1	0	SA0440
gi|29165615|ref|NC_002745.2|	514156 +	G	0	1	2	2	4	SA0440
gi|29165615|ref|NC_002745.2|	514157 +	A	0	0	3	2	1	SA0440
gi|29165615|ref|NC_002745.2|	514158 +	U	0	0	0	1	0	-
gi|29165615|ref|NC_002745.2|	514159 +	A	0	0	1	0	0	-
gi|29165615|ref|NC_002745.2|	514161 +	A	0	0	0	0	2	-
gi|29165615|ref|NC_002745.2|	514162 +	A	0	2	0	4	0	-
gi|29165615|ref|NC_002745.2|	514165 +	G	0	1	3	1	0	-
gi|29165615|ref|NC_002745.2|	514166 +	U	0	4	0	0	1	-
gi|29165615|ref|NC_002745.2|	514167 +	U	0	4	0	2	1	-
gi|29165615|ref|NC_002745.2|	514168 +	A	3	3	0	3	2	-
gi|29165615|ref|NC_002745.2|	514169 +	G	4	5	4	1	1	-

gi|29165615|ref|NC_002745.2|	514170	+	A	12	65	36	38	44	-
gi|29165615|ref|NC_002745.2|	514171	+	A	3	5	3	3	4	-
gi|29165615|ref|NC_002745.2|	514172	+	G	0	0	0	0	1	-
gi|29165615|ref|NC_002745.2|	514173	+	A	0	0	1	0	0	-
gi|29165615|ref|NC_002745.2|	514174	+	G	1	0	0	0	0	-
gi|29165615|ref|NC_002745.2|	514178	+	U	0	0	0	1	1	-
gi|29165615|ref|NC_002745.2|	514179	+	U	0	0	1	0	0	-
gi|29165615|ref|NC_002745.2|	514182	+	A	0	0	0	2	1	-
gi|29165615|ref|NC_002745.2|	514185	+	A	0	1	0	0	0	SA0441
gi|29165615|ref|NC_002745.2|	514189	+	A	0	0	1	1	0	SA0441
gi|29165615|ref|NC_002745.2|	514191	+	A	0	0	0	0	1	SA0441
gi|29165615|ref|NC_002745.2|	514193	+	G	0	0	0	4	1	SA0441
gi|29165615|ref|NC_002745.2|	514194	+	A	0	0	0	1	0	SA0441
gi|29165615|ref|NC_002745.2|	514197	+	A	0	0	0	1	0	SA0441
gi|29165615|ref|NC_002745.2|	514201	+	C	0	0	0	1	0	SA0441
gi|29165615|ref|NC_002745.2|	514209	+	C	0	0	1	0	0	SA0441
gi|29165615|ref|NC_002745.2|	514211	+	A	0	1	0	0	0	SA0441
gi|29165615|ref|NC_002745.2|	514213	+	A	0	0	0	1	0	SA0441
gi|29165615|ref|NC_002745.2|	514219	+	A	0	0	0	0	1	SA0441
gi|29165615|ref|NC_002745.2|	514224	+	C	0	1	0	0	0	SA0441
gi|29165615|ref|NC_002745.2|	514225	+	A	0	0	0	1	0	SA0441
gi|29165615|ref|NC_002745.2|	514227	+	G	0	1	0	0	0	SA0441
gi|29165615|ref|NC_002745.2|	514229	+	A	0	0	0	2	0	SA0441
gi|29165615|ref|NC_002745.2|	514230	+	C	0	1	0	0	0	SA0441
gi|29165615|ref|NC_002745.2|	514234	+	C	2	9	3	10	4	SA0441
gi|29165615|ref|NC_002745.2|	514235	+	A	0	0	1	1	0	SA0441
gi|29165615|ref|NC_002745.2|	514236	+	G	0	1	0	0	0	SA0441
gi|29165615|ref|NC_002745.2|	514237	+	A	0	0	0	1	0	SA0441
gi|29165615|ref|NC_002745.2|	514239	+	C	0	0	0	1	0	SA0441
gi|29165615|ref|NC_002745.2|	514242	+	C	0	0	1	0	0	SA0441
gi|29165615|ref|NC_002745.2|	514243	+	U	1	0	0	0	0	SA0441
gi|29165615|ref|NC_002745.2|	514246	+	U	0	0	0	0	1	SA0441
gi|29165615|ref|NC_002745.2|	514248	+	A	0	1	0	0	0	SA0441
gi|29165615|ref|NC_002745.2|	514249	+	A	0	1	0	1	2	SA0441
gi|29165615|ref|NC_002745.2|	514253	+	U	1	0	0	0	0	SA0441
gi|29165615|ref|NC_002745.2|	514254	+	A	0	0	0	0	1	SA0441
gi|29165615|ref|NC_002745.2|	514255	+	A	0	0	0	2	0	SA0441
gi|29165615|ref|NC_002745.2|	514260	+	A	0	0	0	1	0	SA0441
gi|29165615|ref|NC_002745.2|	514262	+	A	0	0	0	1	0	SA0441
gi|29165615|ref|NC_002745.2|	514263	+	G	0	0	0	0	1	SA0441
gi|29165615|ref|NC_002745.2|	514275	+	G	0	0	0	1	0	SA0441
gi|29165615|ref|NC_002745.2|	514281	+	A	0	0	0	1	0	SA0441
gi|29165615|ref|NC_002745.2|	514282	+	C	0	1	0	0	0	SA0441
gi|29165615|ref|NC_002745.2|	514283	+	A	0	0	1	0	0	SA0441
gi|29165615|ref|NC_002745.2|	514290	+	U	0	0	0	1	0	SA0441
gi|29165615|ref|NC_002745.2|	514295	+	A	0	0	0	0	1	SA0441
gi|29165615|ref|NC_002745.2|	514304	+	U	0	0	0	0	1	SA0441
gi|29165615|ref|NC_002745.2|	514305	+	A	0	0	0	1	0	SA0441
gi|29165615|ref|NC_002745.2|	514312	+	C	1	1	0	1	0	SA0441
gi|29165615|ref|NC_002745.2|	514322	+	U	0	0	2	0	0	SA0441
gi|29165615|ref|NC_002745.2|	514331	+	U	0	0	0	0	1	SA0441
gi|29165615|ref|NC_002745.2|	514332	+	G	0	1	0	0	0	SA0441
gi|29165615|ref|NC_002745.2|	514338	+	C	0	0	0	1	0	SA0441
gi|29165615|ref|NC_002745.2|	514348	+	A	0	1	0	0	0	SA0441
gi|29165615|ref|NC_002745.2|	514352	+	A	0	1	0	0	0	SA0441
gi|29165615|ref|NC_002745.2|	514354	+	U	2	0	0	0	0	SA0441
gi|29165615|ref|NC_002745.2|	514357	+	C	0	0	0	0	1	SA0441
gi|29165615|ref|NC_002745.2|	514359	+	G	0	0	0	1	0	SA0441
gi|29165615|ref|NC_002745.2|	514370	+	A	0	0	0	0	1	SA0441
gi|29165615|ref|NC_002745.2|	514374	+	U	0	0	0	1	0	SA0441
gi|29165615|ref|NC_002745.2|	514375	+	G	0	0	1	0	0	SA0441
gi|29165615|ref|NC_002745.2|	514388	+	A	0	1	0	0	0	SA0441
gi|29165615|ref|NC_002745.2|	514392	+	U	0	0	1	0	0	SA0441
gi|29165615|ref|NC_002745.2|	514417	+	G	0	2	0	0	0	SA0441
gi|29165615|ref|NC_002745.2|	514418	+	A	1	15	0	0	3	SA0441
gi|29165615|ref|NC_002745.2|	514447	+	C	0	1	0	0	0	SA0441
gi|29165615|ref|NC_002745.2|	514483	+	U	0	1	0	0	0	SA0441
gi|29165615|ref|NC_002745.2|	514521	+	A	0	1	0	0	0	-
gi|29165615|ref|NC_002745.2|	514522	+	A	0	1	0	0	0	-
gi|29165615|ref|NC_002745.2|	514529	+	C	0	0	0	0	1	-
gi|29165615|ref|NC_002745.2|	514532	+	C	0	0	0	0	1	-
gi|29165615|ref|NC_002745.2|	514533	+	A	0	0	0	0	1	-
gi|29165615|ref|NC_002745.2|	514539	+	A	0	0	0	1	0	-
gi|29165615|ref|NC_002745.2|	514541	+	A	0	0	0	0	1	-
gi|29165615|ref|NC_002745.2|	514548	+	A	0	0	0	0	1	-
gi|29165615|ref|NC_002745.2|	514549	+	A	0	0	0	1	0	-
gi|29165615|ref|NC_002745.2|	514554	+	U	0	0	0	0	1	-
gi|29165615|ref|NC_002745.2|	514555	+	G	0	1	3	3	1	-
gi|29165615|ref|NC_002745.2|	514556	+	U	0	0	0	0	2	-
gi|29165615|ref|NC_002745.2|	514557	+	A	0	2	0	2	0	-
gi|29165615|ref|NC_002745.2|	514560	+	A	0	1	1	0	1	-

gi|29165615|ref|NC_002745.2|	514561 +	A	0	0	0	1	0	-
gi|29165615|ref|NC_002745.2|	514568 +	A	0	1	0	1	1	-
gi|29165615|ref|NC_002745.2|	514571 +	A	0	0	4	1	1	-
gi|29165615|ref|NC_002745.2|	514572 +	A	0	4	4	4	2	-
gi|29165615|ref|NC_002745.2|	514573 +	C	0	0	1	0	0	-
gi|29165615|ref|NC_002745.2|	514574 +	A	0	1	1	1	1	-
gi|29165615|ref|NC_002745.2|	514575 +	A	0	1	0	0	0	-
gi|29165615|ref|NC_002745.2|	514576 +	U	0	0	0	0	1	-
gi|29165615|ref|NC_002745.2|	514710 +	A	0	0	0	2	0	-
gi|29165615|ref|NC_002745.2|	514718 +	U	0	1	0	0	0	-
gi|29165615|ref|NC_002745.2|	514976 +	G	0	0	0	1	0	SA0442
gi|29165615|ref|NC_002745.2|	514983 +	C	0	1	0	0	0	SA0442
gi|29165615|ref|NC_002745.2|	515086 +	A	0	0	0	1	0	SA0442
gi|29165615|ref|NC_002745.2|	515123 +	G	1	0	0	0	0	SA0442
gi|29165615|ref|NC_002745.2|	515139 +	A	0	1	0	0	0	SA0442
gi|29165615|ref|NC_002745.2|	515422 +	C	0	0	1	0	0	SA0442
gi|29165615|ref|NC_002745.2|	515465 +	A	0	0	2	0	0	SA0442
gi|29165615|ref|NC_002745.2|	515472 +	A	0	1	1	0	1	SA0442
gi|29165615|ref|NC_002745.2|	515482 +	A	0	0	0	0	1	SA0442
gi|29165615|ref|NC_002745.2|	515492 +	A	0	0	0	2	0	SA0442
gi|29165615|ref|NC_002745.2|	515506 +	A	0	0	1	0	0	SA0442
gi|29165615|ref|NC_002745.2|	515541 +	U	0	0	0	0	1	SA0442
gi|29165615|ref|NC_002745.2|	515545 +	A	1	0	0	0	0	SA0442
gi|29165615|ref|NC_002745.2|	515555 +	U	0	0	0	0	1	SA0442
gi|29165615|ref|NC_002745.2|	515566 +	A	1	0	0	0	0	SA0442
gi|29165615|ref|NC_002745.2|	515572 +	U	0	0	0	1	0	SA0442
gi|29165615|ref|NC_002745.2|	515598 +	C	0	1	0	0	0	SA0442
gi|29165615|ref|NC_002745.2|	515605 +	U	0	0	1	0	0	SA0442
gi|29165615|ref|NC_002745.2|	515620 +	U	1	0	0	0	0	SA0442
gi|29165615|ref|NC_002745.2|	515625 +	G	0	1	0	0	0	SA0442
gi|29165615|ref|NC_002745.2|	515628 +	C	1	0	0	0	0	SA0442
gi|29165615|ref|NC_002745.2|	515632 +	U	1	0	0	0	0	SA0442
gi|29165615|ref|NC_002745.2|	515633 +	C	2	0	0	0	0	SA0442
gi|29165615|ref|NC_002745.2|	515635 +	U	0	1	0	1	3	SA0442
gi|29165615|ref|NC_002745.2|	515636 +	A	0	0	0	0	1	SA0442
gi|29165615|ref|NC_002745.2|	515637 +	A	1	0	0	0	0	SA0442
gi|29165615|ref|NC_002745.2|	515640 +	A	0	0	0	1	0	SA0442
gi|29165615|ref|NC_002745.2|	515643 +	G	0	1	0	0	0	SA0442
gi|29165615|ref|NC_002745.2|	515644 +	G	1	0	0	0	0	SA0442
gi|29165615|ref|NC_002745.2|	515661 +	A	0	0	0	1	1	-
gi|29165615|ref|NC_002745.2|	515676 +	C	0	0	0	0	1	-
gi|29165615|ref|NC_002745.2|	515726 +	A	0	1	0	0	0	-
gi|29165615|ref|NC_002745.2|	515739 +	G	0	0	0	1	0	-
gi|29165615|ref|NC_002745.2|	515755 +	A	0	0	0	0	1	-
gi|29165615|ref|NC_002745.2|	515770 +	U	1	0	0	0	0	-
gi|29165615|ref|NC_002745.2|	515785 +	U	0	0	0	1	0	-
gi|29165615|ref|NC_002745.2|	515802 +	G	0	0	0	1	0	-
gi|29165615|ref|NC_002745.2|	515807 +	U	0	0	0	1	0	-
gi|29165615|ref|NC_002745.2|	515808 +	G	0	0	1	0	0	-
gi|29165615|ref|NC_002745.2|	515817 +	G	1	0	0	0	0	-
gi|29165615|ref|NC_002745.2|	515821 +	U	2	0	0	0	0	-
gi|29165615|ref|NC_002745.2|	515846 +	U	0	1	0	0	0	SA0443
gi|29165615|ref|NC_002745.2|	515849 +	C	0	1	0	0	0	SA0443
gi|29165615|ref|NC_002745.2|	515872 +	A	1	0	0	2	1	SA0443
gi|29165615|ref|NC_002745.2|	515873 +	U	1	0	0	0	0	SA0443
gi|29165615|ref|NC_002745.2|	515874 +	A	0	1	0	0	4	SA0443
gi|29165615|ref|NC_002745.2|	515875 +	A	0	0	0	1	2	SA0443
gi|29165615|ref|NC_002745.2|	515878 +	U	1	0	1	0	0	SA0443
gi|29165615|ref|NC_002745.2|	515881 +	A	0	0	0	0	2	SA0443
gi|29165615|ref|NC_002745.2|	515885 +	U	1	0	0	0	0	SA0443
gi|29165615|ref|NC_002745.2|	515886 +	A	0	0	0	0	2	SA0443
gi|29165615|ref|NC_002745.2|	515908 +	C	1	0	0	0	0	SA0443
gi|29165615|ref|NC_002745.2|	515911 +	U	1	0	0	1	0	SA0443
gi|29165615|ref|NC_002745.2|	515917 +	U	1	0	0	0	0	SA0443
gi|29165615|ref|NC_002745.2|	515918 +	A	1	0	0	0	0	SA0443
gi|29165615|ref|NC_002745.2|	515921 +	U	0	0	1	0	0	SA0443
gi|29165615|ref|NC_002745.2|	515923 +	A	0	1	0	1	0	SA0443
gi|29165615|ref|NC_002745.2|	515926 +	A	0	0	1	0	0	SA0443
gi|29165615|ref|NC_002745.2|	515932 +	U	0	0	0	1	0	SA0443
gi|29165615|ref|NC_002745.2|	515934 +	A	0	0	0	1	0	SA0443
gi|29165615|ref|NC_002745.2|	515941 +	A	0	0	0	0	1	SA0443
gi|29165615|ref|NC_002745.2|	515948 +	G	0	0	1	0	0	SA0443
gi|29165615|ref|NC_002745.2|	515985 +	A	0	0	0	1	0	SA0443
gi|29165615|ref|NC_002745.2|	515991 +	A	0	0	0	1	0	SA0443
gi|29165615|ref|NC_002745.2|	515992 +	A	1	0	0	0	0	SA0443
gi|29165615|ref|NC_002745.2|	516002 +	U	0	0	1	0	0	SA0443
gi|29165615|ref|NC_002745.2|	516035 +	A	0	0	0	0	1	SA0443
gi|29165615|ref|NC_002745.2|	516038 +	A	0	0	2	0	1	SA0443
gi|29165615|ref|NC_002745.2|	516053 +	A	2	0	0	0	0	SA0443
gi|29165615|ref|NC_002745.2|	516090 +	A	0	0	0	0	3	SA0443
gi|29165615|ref|NC_002745.2|	516098 +	A	0	0	0	0	2	SA0443

gi|29165615|ref|NC_002745.2|	516175 +	U	1	0	0	0	0	SA0443
gi|29165615|ref|NC_002745.2|	516195 +	A	0	1	0	0	0	SA0443
gi|29165615|ref|NC_002745.2|	516228 +	A	0	0	0	1	0	SA0443
gi|29165615|ref|NC_002745.2|	516234 +	A	0	0	0	0	1	SA0443
gi|29165615|ref|NC_002745.2|	516242 +	U	0	1	0	0	0	SA0443
gi|29165615|ref|NC_002745.2|	516244 +	C	2	0	0	0	0	SA0443
gi|29165615|ref|NC_002745.2|	516247 +	G	1	0	0	0	0	SA0443
gi|29165615|ref|NC_002745.2|	516250 +	G	1	0	0	0	0	SA0443
gi|29165615|ref|NC_002745.2|	516259 +	U	0	0	0	1	0	SA0443
gi|29165615|ref|NC_002745.2|	516274 +	A	0	1	0	1	0	SA0443
gi|29165615|ref|NC_002745.2|	516286 +	A	2	0	0	0	0	SA0443
gi|29165615|ref|NC_002745.2|	516306 +	C	1	0	0	0	0	SA0443
gi|29165615|ref|NC_002745.2|	516330 +	A	0	2	0	0	1	SA0443
gi|29165615|ref|NC_002745.2|	516339 +	C	0	0	0	1	0	SA0443
gi|29165615|ref|NC_002745.2|	516346 +	G	2	0	0	0	0	SA0443
gi|29165615|ref|NC_002745.2|	516374 +	A	0	1	0	0	0	SA0443
gi|29165615|ref|NC_002745.2|	516379 +	A	1	0	0	0	1	SA0443
gi|29165615|ref|NC_002745.2|	516384 +	U	0	0	0	0	1	SA0443
gi|29165615|ref|NC_002745.2|	516390 +	C	0	0	0	1	0	SA0443
gi|29165615|ref|NC_002745.2|	516441 +	A	0	1	0	0	0	SA0443
gi|29165615|ref|NC_002745.2|	516444 +	G	0	2	0	0	0	SA0443
gi|29165615|ref|NC_002745.2|	516529 +	C	1	0	0	0	0	SA0444
gi|29165615|ref|NC_002745.2|	516550 +	G	0	0	0	1	0	SA0444
gi|29165615|ref|NC_002745.2|	516584 +	A	0	0	0	1	0	SA0444
gi|29165615|ref|NC_002745.2|	516611 +	A	0	0	1	1	0	SA0444
gi|29165615|ref|NC_002745.2|	516612 +	U	0	0	1	0	0	SA0444
gi|29165615|ref|NC_002745.2|	516680 +	A	0	0	0	0	1	SA0444
gi|29165615|ref|NC_002745.2|	516773 +	A	1	0	0	0	0	SA0444
gi|29165615|ref|NC_002745.2|	516786 +	A	0	1	0	0	0	SA0444
gi|29165615|ref|NC_002745.2|	516822 +	A	0	0	0	0	1	SA0444
gi|29165615|ref|NC_002745.2|	516823 +	U	0	0	1	0	0	-
gi|29165615|ref|NC_002745.2|	516851 +	A	0	0	0	0	1	-
gi|29165615|ref|NC_002745.2|	516852 +	U	0	0	0	0	1	-
gi|29165615|ref|NC_002745.2|	516853 +	A	0	0	2	0	0	-
gi|29165615|ref|NC_002745.2|	516857 +	G	21	157	142	69	183	-
gi|29165615|ref|NC_002745.2|	516858 +	A	1	8	6	9	4	-
gi|29165615|ref|NC_002745.2|	516859 +	A	0	0	0	2	0	-
gi|29165615|ref|NC_002745.2|	516860 +	U	0	0	1	0	0	-
gi|29165615|ref|NC_002745.2|	516865 +	U	0	0	0	0	1	-
gi|29165615|ref|NC_002745.2|	516882 +	C	0	0	1	0	0	-
gi|29165615|ref|NC_002745.2|	516936 +	A	0	0	0	1	0	-
gi|29165615|ref|NC_002745.2|	516984 +	U	1	0	0	0	0	-
gi|29165615|ref|NC_002745.2|	516989 +	A	0	0	0	0	1	-
gi|29165615|ref|NC_002745.2|	517015 +	C	0	1	0	0	0	-
gi|29165615|ref|NC_002745.2|	517489 +	C	0	1	0	0	0	SA0445
gi|29165615|ref|NC_002745.2|	517532 +	C	0	1	0	0	0	SA0445
gi|29165615|ref|NC_002745.2|	517553 +	C	1	0	0	0	0	SA0445
gi|29165615|ref|NC_002745.2|	517652 +	A	0	0	1	1	1	SA0445
gi|29165615|ref|NC_002745.2|	517656 +	U	1	0	0	0	0	SA0445
gi|29165615|ref|NC_002745.2|	517657 +	A	0	1	0	0	0	SA0445
gi|29165615|ref|NC_002745.2|	517668 +	U	0	0	0	0	1	SA0445
gi|29165615|ref|NC_002745.2|	517834 +	U	0	1	0	0	0	SA0446
gi|29165615|ref|NC_002745.2|	517850 +	G	0	1	1	0	1	SA0446
gi|29165615|ref|NC_002745.2|	517964 +	C	0	0	1	0	0	SA0446
gi|29165615|ref|NC_002745.2|	518004 +	A	0	0	0	1	0	SA0446
gi|29165615|ref|NC_002745.2|	518016 +	A	0	0	0	0	2	SA0446
gi|29165615|ref|NC_002745.2|	518025 +	C	0	0	1	0	0	SA0446
gi|29165615|ref|NC_002745.2|	518028 +	G	0	0	2	0	0	SA0446
gi|29165615|ref|NC_002745.2|	518042 +	C	0	1	0	0	0	SA0446
gi|29165615|ref|NC_002745.2|	518043 +	G	0	0	0	0	1	SA0446
gi|29165615|ref|NC_002745.2|	518044 +	A	0	1	0	0	0	SA0446
gi|29165615|ref|NC_002745.2|	518087 +	C	0	0	0	0	1	SA0447
gi|29165615|ref|NC_002745.2|	518125 +	C	0	1	0	0	0	SA0447
gi|29165615|ref|NC_002745.2|	518153 +	G	1	0	0	0	0	SA0447
gi|29165615|ref|NC_002745.2|	518194 +	A	0	0	1	0	0	SA0447
gi|29165615|ref|NC_002745.2|	518276 +	A	0	1	0	0	0	SA0447
gi|29165615|ref|NC_002745.2|	518376 +	A	2	0	0	0	0	SA0447
gi|29165615|ref|NC_002745.2|	518521 +	A	0	0	0	0	1	SA0447
gi|29165615|ref|NC_002745.2|	518577 +	A	0	1	0	0	0	SA0447
gi|29165615|ref|NC_002745.2|	518890 +	A	0	0	0	0	1	SA0447
gi|29165615|ref|NC_002745.2|	519058 +	A	8	18	21	15	17	-
gi|29165615|ref|NC_002745.2|	519068 +	A	1	0	0	0	0	-
gi|29165615|ref|NC_002745.2|	519069 +	U	0	0	1	1	0	-
gi|29165615|ref|NC_002745.2|	519070 +	A	0	0	0	0	1	-
gi|29165615|ref|NC_002745.2|	519072 +	A	1	0	1	0	0	-
gi|29165615|ref|NC_002745.2|	519075 +	C	0	1	0	0	0	-
gi|29165615|ref|NC_002745.2|	519077 +	U	0	0	0	1	0	-
gi|29165615|ref|NC_002745.2|	519079 +	A	0	0	0	0	1	-
gi|29165615|ref|NC_002745.2|	519080 +	A	0	1	0	0	1	-
gi|29165615|ref|NC_002745.2|	519085 +	G	0	1	0	0	0	-
gi|29165615|ref|NC_002745.2|	519086 +	A	0	1	1	2	0	-

gi|29165615|ref|NC_002745.2|	519089 +	A	0	0	0	1	0	-
gi|29165615|ref|NC_002745.2|	519092 +	A	0	1	2	1	0	-
gi|29165615|ref|NC_002745.2|	519095 +	A	0	1	0	0	1	-
gi|29165615|ref|NC_002745.2|	519098 +	A	0	1	0	0	0	-
gi|29165615|ref|NC_002745.2|	519103 +	A	0	0	0	1	0	-
gi|29165615|ref|NC_002745.2|	519105 +	G	0	0	0	0	1	-
gi|29165615|ref|NC_002745.2|	519107 +	C	0	0	0	0	1	-
gi|29165615|ref|NC_002745.2|	519109 +	A	0	0	0	1	0	-
gi|29165615|ref|NC_002745.2|	519112 +	U	0	0	0	0	2	-
gi|29165615|ref|NC_002745.2|	519113 +	A	0	1	1	2	1	-
gi|29165615|ref|NC_002745.2|	519136 +	C	0	0	0	0	1	-
gi|29165615|ref|NC_002745.2|	519142 +	A	0	0	0	1	0	-
gi|29165615|ref|NC_002745.2|	519143 +	A	0	1	0	0	0	-
gi|29165615|ref|NC_002745.2|	519149 +	G	0	1	0	0	0	-
gi|29165615|ref|NC_002745.2|	519154 +	U	0	0	1	0	0	-
gi|29165615|ref|NC_002745.2|	519155 +	A	0	0	0	0	1	-
gi|29165615|ref|NC_002745.2|	519162 +	G	0	1	0	0	0	-
gi|29165615|ref|NC_002745.2|	519164 +	G	0	1	0	2	2	-
gi|29165615|ref|NC_002745.2|	519192 +	C	0	0	0	0	1	SA0448
gi|29165615|ref|NC_002745.2|	519201 +	C	0	0	0	1	0	SA0448
gi|29165615|ref|NC_002745.2|	519206 +	U	0	1	1	1	0	SA0448
gi|29165615|ref|NC_002745.2|	519208 +	U	0	0	0	0	1	SA0448
gi|29165615|ref|NC_002745.2|	519209 +	A	0	1	1	0	0	SA0448
gi|29165615|ref|NC_002745.2|	519216 +	C	0	1	0	0	0	SA0448
gi|29165615|ref|NC_002745.2|	519218 +	C	0	2	0	0	1	SA0448
gi|29165615|ref|NC_002745.2|	519219 +	C	0	0	3	0	0	SA0448
gi|29165615|ref|NC_002745.2|	519220 +	A	0	0	1	2	2	SA0448
gi|29165615|ref|NC_002745.2|	519221 +	A	0	1	0	1	1	SA0448
gi|29165615|ref|NC_002745.2|	519222 +	U	0	2	2	8	1	SA0448
gi|29165615|ref|NC_002745.2|	519227 +	U	0	0	0	1	0	SA0448
gi|29165615|ref|NC_002745.2|	519244 +	A	0	0	0	0	1	SA0448
gi|29165615|ref|NC_002745.2|	519254 +	C	1	0	0	0	0	SA0448
gi|29165615|ref|NC_002745.2|	519258 +	C	0	0	1	0	0	SA0448
gi|29165615|ref|NC_002745.2|	519260 +	U	0	1	0	0	1	SA0448
gi|29165615|ref|NC_002745.2|	519263 +	U	0	0	1	0	0	SA0448
gi|29165615|ref|NC_002745.2|	519264 +	C	0	0	0	0	1	SA0448
gi|29165615|ref|NC_002745.2|	519265 +	U	0	1	0	0	0	SA0448
gi|29165615|ref|NC_002745.2|	519269 +	G	0	0	1	0	0	SA0448
gi|29165615|ref|NC_002745.2|	519273 +	C	0	1	0	0	0	SA0448
gi|29165615|ref|NC_002745.2|	519288 +	C	0	1	0	0	0	SA0448
gi|29165615|ref|NC_002745.2|	519291 +	G	0	0	0	1	0	SA0448
gi|29165615|ref|NC_002745.2|	519294 +	A	1	0	0	0	0	SA0448
gi|29165615|ref|NC_002745.2|	519295 +	U	0	0	1	0	0	SA0448
gi|29165615|ref|NC_002745.2|	519301 +	A	0	1	0	0	1	SA0448
gi|29165615|ref|NC_002745.2|	519305 +	C	0	0	0	0	1	SA0448
gi|29165615|ref|NC_002745.2|	519306 +	A	0	0	0	1	0	SA0448
gi|29165615|ref|NC_002745.2|	519317 +	G	0	2	0	0	0	SA0448
gi|29165615|ref|NC_002745.2|	519335 +	A	0	1	0	0	0	SA0448
gi|29165615|ref|NC_002745.2|	519356 +	A	1	7	1	0	6	SA0448
gi|29165615|ref|NC_002745.2|	519370 +	U	0	0	0	1	0	SA0448
gi|29165615|ref|NC_002745.2|	519371 +	C	0	1	0	0	0	SA0448
gi|29165615|ref|NC_002745.2|	519383 +	A	0	1	0	0	1	SA0448
gi|29165615|ref|NC_002745.2|	519390 +	A	0	0	0	0	1	SA0448
gi|29165615|ref|NC_002745.2|	519402 +	U	0	0	1	0	0	SA0448
gi|29165615|ref|NC_002745.2|	519404 +	G	0	1	0	0	0	SA0448
gi|29165615|ref|NC_002745.2|	519409 +	G	0	0	1	0	0	SA0448
gi|29165615|ref|NC_002745.2|	519410 +	A	0	0	0	1	0	SA0448
gi|29165615|ref|NC_002745.2|	519413 +	A	0	1	0	0	1	SA0448
gi|29165615|ref|NC_002745.2|	519423 +	U	0	0	1	0	0	SA0448
gi|29165615|ref|NC_002745.2|	519444 +	U	2	0	0	0	0	SA0448
gi|29165615|ref|NC_002745.2|	519454 +	A	0	1	0	0	0	SA0448
gi|29165615|ref|NC_002745.2|	519501 +	U	1	0	0	0	0	SA0448
gi|29165615|ref|NC_002745.2|	519626 +	A	0	1	0	0	0	SA0448
gi|29165615|ref|NC_002745.2|	519766 +	A	0	0	1	0	0	SA0448
gi|29165615|ref|NC_002745.2|	519767 +	C	0	0	1	0	0	SA0448
gi|29165615|ref|NC_002745.2|	519782 +	A	0	0	0	1	0	SA0448
gi|29165615|ref|NC_002745.2|	519795 +	A	1	0	0	0	0	SA0448
gi|29165615|ref|NC_002745.2|	519808 +	A	0	1	0	0	0	SA0448
gi|29165615|ref|NC_002745.2|	519824 +	U	0	0	0	0	1	SA0448
gi|29165615|ref|NC_002745.2|	519834 +	C	1	0	0	0	0	SA0448
gi|29165615|ref|NC_002745.2|	519847 +	U	0	0	0	0	1	SA0448
gi|29165615|ref|NC_002745.2|	519869 +	U	0	0	0	0	1	SA0448
gi|29165615|ref|NC_002745.2|	519870 +	C	1	0	0	0	0	SA0448
gi|29165615|ref|NC_002745.2|	519881 +	C	0	0	1	0	0	SA0448
gi|29165615|ref|NC_002745.2|	519885 +	U	1	0	0	0	0	SA0448
gi|29165615|ref|NC_002745.2|	519888 +	U	0	0	1	0	0	SA0448
gi|29165615|ref|NC_002745.2|	519891 +	A	0	1	0	0	0	SA0448
gi|29165615|ref|NC_002745.2|	519893 +	G	2	0	0	0	0	SA0448
gi|29165615|ref|NC_002745.2|	519904 +	U	1	0	0	0	0	SA0448
gi|29165615|ref|NC_002745.2|	519912 +	U	0	0	0	1	1	SA0448
gi|29165615|ref|NC_002745.2|	519918 +	A	0	1	0	0	0	SA0448

gi|29165615|ref|NC_002745.2|	519924 +	C	0	0	0	0	1	SA0448
gi|29165615|ref|NC_002745.2|	519930 +	U	0	0	0	0	1	SA0448
gi|29165615|ref|NC_002745.2|	519933 +	G	0	0	0	1	0	SA0448
gi|29165615|ref|NC_002745.2|	519942 +	C	0	1	0	0	0	SA0448
gi|29165615|ref|NC_002745.2|	519961 +	U	0	1	0	0	0	SA0448
gi|29165615|ref|NC_002745.2|	519964 +	C	0	1	0	0	0	SA0448
gi|29165615|ref|NC_002745.2|	519968 +	U	0	0	1	0	0	SA0448
gi|29165615|ref|NC_002745.2|	519974 +	C	1	0	0	0	1	SA0448
gi|29165615|ref|NC_002745.2|	519990 +	U	0	1	0	0	0	SA0448
gi|29165615|ref|NC_002745.2|	520002 +	A	0	0	0	1	0	SA0448
gi|29165615|ref|NC_002745.2|	520015 +	C	0	0	1	0	0	SA0448
gi|29165615|ref|NC_002745.2|	520018 +	C	0	2	0	0	0	SA0448
gi|29165615|ref|NC_002745.2|	520022 +	A	0	0	1	0	0	SA0448
gi|29165615|ref|NC_002745.2|	520091 +	A	0	1	1	0	0	SA0448
gi|29165615|ref|NC_002745.2|	520108 +	A	0	0	1	0	0	SA0448
gi|29165615|ref|NC_002745.2|	520120 +	A	0	1	0	0	0	SA0448
gi|29165615|ref|NC_002745.2|	520121 +	U	0	0	1	0	0	SA0448
gi|29165615|ref|NC_002745.2|	520126 +	A	1	0	0	0	0	SA0448
gi|29165615|ref|NC_002745.2|	520144 +	U	1	1	0	1	1	SA0448
gi|29165615|ref|NC_002745.2|	520147 +	U	0	1	0	0	0	SA0448
gi|29165615|ref|NC_002745.2|	520148 +	A	0	0	0	1	0	SA0448
gi|29165615|ref|NC_002745.2|	520176 +	C	4	0	0	0	0	SA0448
gi|29165615|ref|NC_002745.2|	520186 +	U	1	0	0	0	0	SA0448
gi|29165615|ref|NC_002745.2|	520191 +	U	1	1	0	0	0	SA0448
gi|29165615|ref|NC_002745.2|	520230 +	C	0	0	1	0	0	SA0448
gi|29165615|ref|NC_002745.2|	520330 +	C	0	1	0	0	0	SA0448
gi|29165615|ref|NC_002745.2|	520411 +	C	0	0	0	0	1	SA0448
gi|29165615|ref|NC_002745.2|	520421 +	A	0	0	0	0	4	SA0448
gi|29165615|ref|NC_002745.2|	520425 +	A	0	0	0	0	1	SA0448
gi|29165615|ref|NC_002745.2|	520438 +	U	0	0	0	0	1	SA0448
gi|29165615|ref|NC_002745.2|	520467 +	U	1	0	1	0	0	SA0448
gi|29165615|ref|NC_002745.2|	520482 +	A	0	0	1	0	0	SA0448
gi|29165615|ref|NC_002745.2|	520487 +	U	0	0	0	1	0	SA0448
gi|29165615|ref|NC_002745.2|	520536 +	A	0	2	0	0	0	SA0448
gi|29165615|ref|NC_002745.2|	520553 +	G	0	0	0	1	0	SA0448
gi|29165615|ref|NC_002745.2|	520575 +	A	0	0	0	0	3	SA0448
gi|29165615|ref|NC_002745.2|	520578 +	U	1	0	0	1	0	SA0448
gi|29165615|ref|NC_002745.2|	520605 +	C	0	1	0	0	0	SA0448
gi|29165615|ref|NC_002745.2|	520618 +	U	0	1	0	0	0	SA0448
gi|29165615|ref|NC_002745.2|	520637 +	G	0	0	1	0	0	SA0448
gi|29165615|ref|NC_002745.2|	520638 +	A	0	0	0	1	0	SA0448
gi|29165615|ref|NC_002745.2|	520645 +	G	0	0	1	0	0	SA0448
gi|29165615|ref|NC_002745.2|	520646 +	A	0	1	0	1	1	SA0448
gi|29165615|ref|NC_002745.2|	520647 +	A	0	0	0	0	1	SA0448
gi|29165615|ref|NC_002745.2|	520649 +	A	0	0	1	0	0	SA0448
gi|29165615|ref|NC_002745.2|	520656 +	A	2	1	1	0	1	SA0448
gi|29165615|ref|NC_002745.2|	520657 +	U	0	0	0	1	0	SA0448
gi|29165615|ref|NC_002745.2|	520663 +	A	0	0	0	1	0	SA0448
gi|29165615|ref|NC_002745.2|	520678 +	U	0	1	0	1	1	SA0448
gi|29165615|ref|NC_002745.2|	520680 +	G	0	0	1	0	0	SA0448
gi|29165615|ref|NC_002745.2|	520689 +	A	0	0	0	0	1	SA0448
gi|29165615|ref|NC_002745.2|	520690 +	A	2	0	0	0	0	SA0448
gi|29165615|ref|NC_002745.2|	520692 +	A	0	0	1	0	0	SA0448
gi|29165615|ref|NC_002745.2|	520693 +	U	1	0	1	0	2	SA0448
gi|29165615|ref|NC_002745.2|	520704 +	C	0	1	0	0	0	SA0448
gi|29165615|ref|NC_002745.2|	520744 +	C	0	0	1	0	0	SA0448
gi|29165615|ref|NC_002745.2|	520761 +	A	1	0	0	0	0	SA0448
gi|29165615|ref|NC_002745.2|	520803 +	C	0	0	0	1	1	SA0448
gi|29165615|ref|NC_002745.2|	520895 +	G	1	0	0	0	0	SA0448
gi|29165615|ref|NC_002745.2|	520896 +	A	1	0	0	0	1	SA0448
gi|29165615|ref|NC_002745.2|	520907 +	G	0	1	0	0	0	SA0448
gi|29165615|ref|NC_002745.2|	520943 +	G	0	0	0	1	0	SA0448
gi|29165615|ref|NC_002745.2|	520960 +	A	2	0	0	0	0	SA0448
gi|29165615|ref|NC_002745.2|	520974 +	C	2	0	0	0	0	SA0448
gi|29165615|ref|NC_002745.2|	520986 +	A	0	0	1	0	0	SA0448
gi|29165615|ref|NC_002745.2|	520993 +	U	0	0	0	1	0	SA0448
gi|29165615|ref|NC_002745.2|	521021 +	G	0	1	0	0	0	SA0448
gi|29165615|ref|NC_002745.2|	521031 +	U	1	0	0	0	0	SA0448
gi|29165615|ref|NC_002745.2|	521057 +	A	0	0	0	0	1	SA0448
gi|29165615|ref|NC_002745.2|	521067 +	A	0	1	0	0	0	SA0448
gi|29165615|ref|NC_002745.2|	521068 +	A	0	1	2	0	3	SA0448
gi|29165615|ref|NC_002745.2|	521074 +	A	1	0	0	0	0	SA0448
gi|29165615|ref|NC_002745.2|	521076 +	G	0	1	0	0	0	SA0448
gi|29165615|ref|NC_002745.2|	521081 +	A	0	0	0	1	0	SA0448
gi|29165615|ref|NC_002745.2|	521082 +	U	2	0	0	1	0	SA0448
gi|29165615|ref|NC_002745.2|	521088 +	C	4	0	0	0	0	SA0448
gi|29165615|ref|NC_002745.2|	521089 +	U	1	0	0	0	0	SA0448
gi|29165615|ref|NC_002745.2|	521124 +	U	1	0	0	0	0	SA0448
gi|29165615|ref|NC_002745.2|	521127 +	C	0	1	0	0	0	SA0448
gi|29165615|ref|NC_002745.2|	521128 +	A	0	1	0	0	0	SA0448
gi|29165615|ref|NC_002745.2|	521141 +	A	0	1	0	0	0	SA0448

gi|29165615|ref|NC_002745.2|	521160 +	A	0	0	0	1	0	SA0448
gi|29165615|ref|NC_002745.2|	521176 +	A	0	1	0	0	0	-
gi|29165615|ref|NC_002745.2|	521181 +	G	0	0	0	0	1	-
gi|29165615|ref|NC_002745.2|	521182 +	A	0	1	0	1	0	-
gi|29165615|ref|NC_002745.2|	521219 +	A	0	0	0	1	0	SA0449
gi|29165615|ref|NC_002745.2|	521230 +	A	0	2	0	0	0	SA0449
gi|29165615|ref|NC_002745.2|	521339 +	A	0	2	0	0	0	SA0449
gi|29165615|ref|NC_002745.2|	521344 +	U	0	0	0	0	1	SA0449
gi|29165615|ref|NC_002745.2|	521504 +	G	0	1	0	0	0	SA0449
gi|29165615|ref|NC_002745.2|	521554 +	G	0	0	0	0	1	SA0449
gi|29165615|ref|NC_002745.2|	521566 +	U	0	0	1	0	0	SA0449
gi|29165615|ref|NC_002745.2|	521586 +	C	0	1	0	0	0	SA0449
gi|29165615|ref|NC_002745.2|	521587 +	A	0	1	0	0	0	SA0449
gi|29165615|ref|NC_002745.2|	521672 +	A	0	1	0	0	0	SA0449
gi|29165615|ref|NC_002745.2|	521699 +	A	1	0	0	0	0	SA0449
gi|29165615|ref|NC_002745.2|	521933 +	A	0	1	0	0	0	SA0449
gi|29165615|ref|NC_002745.2|	521964 +	A	0	0	0	1	0	SA0449
gi|29165615|ref|NC_002745.2|	521992 +	A	1	0	0	0	0	-
gi|29165615|ref|NC_002745.2|	522057 +	A	0	2	0	0	0	-
gi|29165615|ref|NC_002745.2|	522072 +	A	0	0	0	0	1	-
gi|29165615|ref|NC_002745.2|	522116 +	U	0	0	0	1	0	-
gi|29165615|ref|NC_002745.2|	522136 +	A	0	1	0	0	0	SA0450
gi|29165615|ref|NC_002745.2|	522140 +	C	0	0	0	1	0	SA0450
gi|29165615|ref|NC_002745.2|	522161 +	A	0	0	2	0	0	SA0450
gi|29165615|ref|NC_002745.2|	522178 +	A	0	0	0	0	1	SA0450
gi|29165615|ref|NC_002745.2|	522218 +	G	5	21	7	6	19	SA0450
gi|29165615|ref|NC_002745.2|	522219 +	A	0	0	1	0	0	SA0450
gi|29165615|ref|NC_002745.2|	522479 +	U	0	1	0	0	0	SA0450
gi|29165615|ref|NC_002745.2|	522489 +	A	0	0	0	0	1	SA0450
gi|29165615|ref|NC_002745.2|	522491 +	U	0	0	0	1	0	SA0450
gi|29165615|ref|NC_002745.2|	522503 +	G	1	0	0	0	0	SA0450
gi|29165615|ref|NC_002745.2|	522523 +	U	2	0	0	0	0	SA0450
gi|29165615|ref|NC_002745.2|	522527 +	U	0	0	0	0	1	SA0450
gi|29165615|ref|NC_002745.2|	522557 +	U	0	0	0	0	1	SA0450
gi|29165615|ref|NC_002745.2|	522560 +	A	0	0	0	0	1	SA0450
gi|29165615|ref|NC_002745.2|	522578 +	C	0	0	1	0	0	SA0450
gi|29165615|ref|NC_002745.2|	522589 +	A	0	1	0	0	0	SA0450
gi|29165615|ref|NC_002745.2|	522609 +	A	0	1	0	0	0	SA0450
gi|29165615|ref|NC_002745.2|	522620 +	A	0	0	0	0	1	SA0450
gi|29165615|ref|NC_002745.2|	522680 +	U	1	0	0	0	0	SA0451
gi|29165615|ref|NC_002745.2|	522956 +	C	1	0	0	0	0	SA0451
gi|29165615|ref|NC_002745.2|	522993 +	U	1	0	0	0	0	SA0451
gi|29165615|ref|NC_002745.2|	523032 +	A	1	0	0	0	0	SA0451
gi|29165615|ref|NC_002745.2|	523033 +	C	0	0	0	1	0	SA0451
gi|29165615|ref|NC_002745.2|	523034 +	A	0	1	0	0	0	SA0451
gi|29165615|ref|NC_002745.2|	523055 +	U	1	0	0	0	0	SA0451
gi|29165615|ref|NC_002745.2|	523056 +	U	1	0	0	0	0	SA0451
gi|29165615|ref|NC_002745.2|	523058 +	U	1	0	0	0	0	SA0451
gi|29165615|ref|NC_002745.2|	523091 +	U	1	0	0	0	0	SA0451
gi|29165615|ref|NC_002745.2|	523100 +	A	0	1	0	0	0	SA0451
gi|29165615|ref|NC_002745.2|	523146 +	G	1	0	0	0	0	SA0451
gi|29165615|ref|NC_002745.2|	523295 +	U	1	0	0	0	0	SA0451
gi|29165615|ref|NC_002745.2|	523333 +	A	1	0	0	0	0	SA0451
gi|29165615|ref|NC_002745.2|	523430 +	A	0	0	1	0	0	SA0451
gi|29165615|ref|NC_002745.2|	523530 +	A	0	0	0	0	1	SA0451
gi|29165615|ref|NC_002745.2|	523565 +	A	0	0	1	0	0	SA0451
gi|29165615|ref|NC_002745.2|	523650 +	A	0	1	0	0	0	-
gi|29165615|ref|NC_002745.2|	523652 +	U	1	1	0	0	0	-
gi|29165615|ref|NC_002745.2|	523653 +	U	1	0	1	1	2	-
gi|29165615|ref|NC_002745.2|	523654 +	G	53	118	58	119	102	-
gi|29165615|ref|NC_002745.2|	523655 +	U	0	0	2	0	0	-
gi|29165615|ref|NC_002745.2|	523658 +	C	0	0	1	0	0	-
gi|29165615|ref|NC_002745.2|	523668 +	C	0	0	0	0	1	-
gi|29165615|ref|NC_002745.2|	523700 +	A	1	2	0	0	0	SA0452
gi|29165615|ref|NC_002745.2|	523761 +	A	0	0	1	0	0	SA0452
gi|29165615|ref|NC_002745.2|	523762 +	A	0	1	0	0	0	SA0452
gi|29165615|ref|NC_002745.2|	523769 +	A	0	1	0	0	0	SA0452
gi|29165615|ref|NC_002745.2|	523796 +	A	1	0	0	0	0	SA0452
gi|29165615|ref|NC_002745.2|	523806 +	U	0	0	0	0	1	SA0452
gi|29165615|ref|NC_002745.2|	523830 +	C	0	1	0	0	0	SA0452
gi|29165615|ref|NC_002745.2|	523831 +	A	0	0	0	2	0	SA0452
gi|29165615|ref|NC_002745.2|	523836 +	C	0	1	0	0	0	SA0452
gi|29165615|ref|NC_002745.2|	523837 +	A	0	0	0	1	0	SA0452
gi|29165615|ref|NC_002745.2|	523853 +	C	0	0	0	1	0	SA0452
gi|29165615|ref|NC_002745.2|	523854 +	U	0	0	1	0	0	SA0452
gi|29165615|ref|NC_002745.2|	523856 +	A	0	0	0	1	0	SA0452
gi|29165615|ref|NC_002745.2|	523858 +	A	0	2	0	0	0	SA0452
gi|29165615|ref|NC_002745.2|	523859 +	C	0	0	0	1	0	SA0452
gi|29165615|ref|NC_002745.2|	523860 +	A	0	0	1	4	1	SA0452
gi|29165615|ref|NC_002745.2|	523861 +	U	0	1	0	0	0	SA0452
gi|29165615|ref|NC_002745.2|	523865 +	C	0	0	1	0	0	SA0452

gi|29165615|ref|NC_002745.2|	523881 +	A	0	0	0	1	1	SA0452
gi|29165615|ref|NC_002745.2|	523882 +	A	0	1	0	2	1	SA0452
gi|29165615|ref|NC_002745.2|	523888 +	C	0	1	0	0	0	SA0452
gi|29165615|ref|NC_002745.2|	523890 +	A	0	0	1	0	0	SA0452
gi|29165615|ref|NC_002745.2|	523893 +	U	0	0	0	0	1	SA0452
gi|29165615|ref|NC_002745.2|	523895 +	C	0	0	0	1	0	SA0452
gi|29165615|ref|NC_002745.2|	523902 +	A	0	1	0	0	0	SA0452
gi|29165615|ref|NC_002745.2|	523903 +	G	0	0	0	2	0	SA0452
gi|29165615|ref|NC_002745.2|	523915 +	C	0	0	0	1	0	SA0452
gi|29165615|ref|NC_002745.2|	523934 +	A	0	1	0	0	0	SA0452
gi|29165615|ref|NC_002745.2|	523948 +	G	0	1	1	0	1	-
gi|29165615|ref|NC_002745.2|	524105 +	A	0	1	2	0	0	-
gi|29165615|ref|NC_002745.2|	524150 +	U	0	0	0	1	0	-
gi|29165615|ref|NC_002745.2|	524212 +	C	0	1	0	0	0	-
gi|29165615|ref|NC_002745.2|	524218 +	A	0	3	4	1	3	-
gi|29165615|ref|NC_002745.2|	524220 +	U	0	0	0	1	0	-
gi|29165615|ref|NC_002745.2|	524223 +	G	0	1	0	0	0	-
gi|29165615|ref|NC_002745.2|	524264 +	C	0	1	0	0	0	SA0453
gi|29165615|ref|NC_002745.2|	524299 +	A	0	0	1	0	2	SA0453
gi|29165615|ref|NC_002745.2|	524305 +	A	0	0	0	0	2	SA0453
gi|29165615|ref|NC_002745.2|	524312 +	G	0	1	0	0	0	SA0453
gi|29165615|ref|NC_002745.2|	524327 +	A	0	0	0	0	1	SA0453
gi|29165615|ref|NC_002745.2|	524332 +	A	0	1	0	0	0	SA0453
gi|29165615|ref|NC_002745.2|	524333 +	U	0	0	0	0	1	SA0453
gi|29165615|ref|NC_002745.2|	524335 +	A	0	1	0	0	2	SA0453
gi|29165615|ref|NC_002745.2|	524338 +	A	0	0	0	0	1	SA0453
gi|29165615|ref|NC_002745.2|	524371 +	C	0	0	1	0	0	SA0453
gi|29165615|ref|NC_002745.2|	524395 +	G	0	0	0	0	1	SA0453
gi|29165615|ref|NC_002745.2|	524402 +	A	0	0	0	1	0	SA0453
gi|29165615|ref|NC_002745.2|	524407 +	G	0	0	0	0	1	SA0453
gi|29165615|ref|NC_002745.2|	524408 +	A	0	0	3	0	0	SA0453
gi|29165615|ref|NC_002745.2|	524409 +	A	1	0	0	0	0	SA0453
gi|29165615|ref|NC_002745.2|	524412 +	U	0	0	0	0	1	SA0453
gi|29165615|ref|NC_002745.2|	524413 +	A	0	0	0	0	1	SA0453
gi|29165615|ref|NC_002745.2|	524414 +	A	0	3	1	0	1	SA0453
gi|29165615|ref|NC_002745.2|	524432 +	A	0	1	0	0	0	SA0453
gi|29165615|ref|NC_002745.2|	524434 +	A	0	0	0	0	1	SA0453
gi|29165615|ref|NC_002745.2|	524435 +	A	0	1	0	0	0	SA0453
gi|29165615|ref|NC_002745.2|	524442 +	C	0	0	1	0	0	SA0453
gi|29165615|ref|NC_002745.2|	524444 +	C	0	2	0	0	0	SA0453
gi|29165615|ref|NC_002745.2|	524446 +	U	1	1	0	0	0	SA0453
gi|29165615|ref|NC_002745.2|	524478 +	U	0	0	0	1	1	SA0453
gi|29165615|ref|NC_002745.2|	524479 +	C	0	0	0	1	1	SA0453
gi|29165615|ref|NC_002745.2|	524484 +	A	1	0	0	1	1	SA0453
gi|29165615|ref|NC_002745.2|	524487 +	G	0	0	1	0	0	SA0453
gi|29165615|ref|NC_002745.2|	524488 +	C	0	0	0	1	0	SA0453
gi|29165615|ref|NC_002745.2|	524491 +	G	0	0	0	0	1	SA0453
gi|29165615|ref|NC_002745.2|	524492 +	G	0	1	0	1	0	SA0453
gi|29165615|ref|NC_002745.2|	524493 +	U	0	1	0	0	0	SA0453
gi|29165615|ref|NC_002745.2|	524494 +	G	0	1	0	0	0	SA0453
gi|29165615|ref|NC_002745.2|	524495 +	U	0	0	0	0	1	SA0453
gi|29165615|ref|NC_002745.2|	524497 +	A	0	0	0	0	1	SA0453
gi|29165615|ref|NC_002745.2|	524498 +	C	0	1	2	0	1	SA0453
gi|29165615|ref|NC_002745.2|	524499 +	A	0	1	0	0	0	SA0453
gi|29165615|ref|NC_002745.2|	524500 +	A	0	2	0	0	0	SA0453
gi|29165615|ref|NC_002745.2|	524504 +	C	0	1	0	0	0	SA0453
gi|29165615|ref|NC_002745.2|	524506 +	A	0	1	0	0	0	SA0453
gi|29165615|ref|NC_002745.2|	524507 +	U	0	1	0	0	0	SA0453
gi|29165615|ref|NC_002745.2|	524508 +	C	0	1	0	0	0	SA0453
gi|29165615|ref|NC_002745.2|	524510 +	A	0	1	0	1	0	SA0453
gi|29165615|ref|NC_002745.2|	524512 +	A	0	0	1	0	0	SA0453
gi|29165615|ref|NC_002745.2|	524515 +	G	0	2	0	1	0	SA0453
gi|29165615|ref|NC_002745.2|	524516 +	A	0	2	2	0	1	SA0453
gi|29165615|ref|NC_002745.2|	524531 +	C	2	0	0	1	0	SA0453
gi|29165615|ref|NC_002745.2|	524533 +	G	0	1	0	0	0	SA0453
gi|29165615|ref|NC_002745.2|	524542 +	G	0	0	1	0	0	SA0453
gi|29165615|ref|NC_002745.2|	524552 +	C	1	0	0	0	0	SA0453
gi|29165615|ref|NC_002745.2|	524561 +	C	0	1	0	0	0	SA0453
gi|29165615|ref|NC_002745.2|	524562 +	A	0	1	0	0	0	SA0453
gi|29165615|ref|NC_002745.2|	524577 +	A	0	1	2	2	0	SA0453
gi|29165615|ref|NC_002745.2|	524578 +	U	0	0	1	0	0	SA0453
gi|29165615|ref|NC_002745.2|	524580 +	G	0	0	1	1	0	SA0453
gi|29165615|ref|NC_002745.2|	524581 +	A	0	0	1	1	0	SA0453
gi|29165615|ref|NC_002745.2|	524586 +	A	0	2	0	0	1	SA0453
gi|29165615|ref|NC_002745.2|	524592 +	U	0	2	0	2	1	SA0453
gi|29165615|ref|NC_002745.2|	524593 +	G	0	3	0	2	2	SA0453
gi|29165615|ref|NC_002745.2|	524611 +	G	0	0	1	0	0	SA0453
gi|29165615|ref|NC_002745.2|	524638 +	A	0	0	0	0	2	SA0453
gi|29165615|ref|NC_002745.2|	524639 +	U	0	1	0	0	0	SA0453
gi|29165615|ref|NC_002745.2|	524653 +	C	0	0	0	0	1	SA0453
gi|29165615|ref|NC_002745.2|	524656 +	U	0	3	3	0	4	SA0453

gi|29165615|ref|NC_002745.2|	524666	+	A	1	0	0	0	0	SA0453
gi|29165615|ref|NC_002745.2|	524670	+	U	0	0	0	0	1	SA0453
gi|29165615|ref|NC_002745.2|	524694	+	A	0	0	0	1	0	SA0453
gi|29165615|ref|NC_002745.2|	524716	+	A	0	0	1	0	0	SA0453
gi|29165615|ref|NC_002745.2|	524753	+	C	0	1	0	0	0	SA0453
gi|29165615|ref|NC_002745.2|	524757	+	C	0	0	0	0	1	SA0453
gi|29165615|ref|NC_002745.2|	524789	+	U	0	1	0	0	0	SA0453
gi|29165615|ref|NC_002745.2|	524800	+	G	0	0	1	0	0	SA0453
gi|29165615|ref|NC_002745.2|	524801	+	A	0	0	0	1	0	SA0453
gi|29165615|ref|NC_002745.2|	524829	+	G	0	0	0	0	1	SA0453
gi|29165615|ref|NC_002745.2|	524830	+	U	0	1	0	0	0	SA0453
gi|29165615|ref|NC_002745.2|	524845	+	G	0	0	0	1	0	SA0453
gi|29165615|ref|NC_002745.2|	524893	+	G	0	1	0	0	0	SA0453
gi|29165615|ref|NC_002745.2|	524896	+	C	0	0	0	1	0	SA0453
gi|29165615|ref|NC_002745.2|	524909	+	C	0	0	0	1	0	SA0453
gi|29165615|ref|NC_002745.2|	524910	+	A	0	1	0	0	0	SA0453
gi|29165615|ref|NC_002745.2|	524916	+	C	0	1	0	0	0	SA0453
gi|29165615|ref|NC_002745.2|	524919	+	A	0	0	1	0	0	SA0453
gi|29165615|ref|NC_002745.2|	524920	+	C	0	0	0	0	1	SA0453
gi|29165615|ref|NC_002745.2|	524929	+	A	0	2	1	0	3	SA0453
gi|29165615|ref|NC_002745.2|	524930	+	A	0	0	0	0	1	SA0453
gi|29165615|ref|NC_002745.2|	524933	+	U	0	1	0	0	0	SA0453
gi|29165615|ref|NC_002745.2|	524934	+	A	0	0	1	0	0	SA0453
gi|29165615|ref|NC_002745.2|	524935	+	A	0	4	2	3	0	SA0453
gi|29165615|ref|NC_002745.2|	524936	+	A	0	10	1	7	9	SA0453
gi|29165615|ref|NC_002745.2|	524937	+	A	0	5	0	1	2	SA0453
gi|29165615|ref|NC_002745.2|	524938	+	A	0	1	0	0	1	SA0453
gi|29165615|ref|NC_002745.2|	524940	+	U	0	1	1	0	1	SA0453
gi|29165615|ref|NC_002745.2|	524941	+	A	0	4	3	3	4	SA0453
gi|29165615|ref|NC_002745.2|	524942	+	A	0	3	1	1	4	SA0453
gi|29165615|ref|NC_002745.2|	524943	+	U	0	1	2	1	2	SA0453
gi|29165615|ref|NC_002745.2|	524944	+	A	0	1	2	1	0	SA0453
gi|29165615|ref|NC_002745.2|	524945	+	U	0	0	0	1	1	SA0453
gi|29165615|ref|NC_002745.2|	524946	+	G	0	1	0	0	0	SA0453
gi|29165615|ref|NC_002745.2|	524947	+	U	0	1	1	0	1	SA0453
gi|29165615|ref|NC_002745.2|	524948	+	U	0	2	1	0	1	SA0453
gi|29165615|ref|NC_002745.2|	524949	+	G	1	3	1	2	1	SA0453
gi|29165615|ref|NC_002745.2|	524950	+	A	1	4	3	2	4	SA0453
gi|29165615|ref|NC_002745.2|	524951	+	A	1	4	3	1	1	SA0453
gi|29165615|ref|NC_002745.2|	524952	+	A	0	1	2	0	0	SA0453
gi|29165615|ref|NC_002745.2|	524953	+	A	0	1	0	1	4	SA0453
gi|29165615|ref|NC_002745.2|	524957	+	G	0	0	0	0	1	SA0453
gi|29165615|ref|NC_002745.2|	524958	+	U	1	0	0	0	0	SA0453
gi|29165615|ref|NC_002745.2|	524960	+	C	1	2	0	0	0	SA0453
gi|29165615|ref|NC_002745.2|	524961	+	A	1	0	0	0	1	SA0453
gi|29165615|ref|NC_002745.2|	524962	+	G	0	1	0	0	0	SA0453
gi|29165615|ref|NC_002745.2|	524963	+	A	1	0	0	0	0	SA0453
gi|29165615|ref|NC_002745.2|	524967	+	U	1	0	0	0	1	SA0453
gi|29165615|ref|NC_002745.2|	524970	+	G	0	0	0	1	0	SA0453
gi|29165615|ref|NC_002745.2|	524971	+	U	0	0	1	1	0	SA0453
gi|29165615|ref|NC_002745.2|	524973	+	A	0	2	1	0	2	SA0453
gi|29165615|ref|NC_002745.2|	524975	+	U	1	1	1	0	0	SA0453
gi|29165615|ref|NC_002745.2|	524976	+	G	0	0	0	0	1	SA0453
gi|29165615|ref|NC_002745.2|	524977	+	A	0	2	0	0	1	SA0453
gi|29165615|ref|NC_002745.2|	524981	+	G	0	5	2	2	0	SA0453
gi|29165615|ref|NC_002745.2|	524982	+	A	3	8	2	3	7	SA0453
gi|29165615|ref|NC_002745.2|	524987	+	G	0	0	0	0	1	SA0453
gi|29165615|ref|NC_002745.2|	524992	+	A	0	0	1	0	0	SA0453
gi|29165615|ref|NC_002745.2|	524994	+	U	1	0	0	0	0	SA0453
gi|29165615|ref|NC_002745.2|	524996	+	U	1	0	0	0	0	SA0453
gi|29165615|ref|NC_002745.2|	524998	+	U	0	2	2	0	0	SA0453
gi|29165615|ref|NC_002745.2|	525000	+	U	1	0	0	0	0	SA0453
gi|29165615|ref|NC_002745.2|	525008	+	C	1	0	0	1	3	SA0453
gi|29165615|ref|NC_002745.2|	525009	+	A	0	0	1	1	0	SA0453
gi|29165615|ref|NC_002745.2|	525012	+	A	0	0	0	1	0	SA0453
gi|29165615|ref|NC_002745.2|	525013	+	A	0	0	3	0	0	SA0453
gi|29165615|ref|NC_002745.2|	525015	+	A	0	0	1	0	0	SA0453
gi|29165615|ref|NC_002745.2|	525017	+	A	0	3	0	1	2	SA0453
gi|29165615|ref|NC_002745.2|	525018	+	A	0	0	2	1	3	SA0453
gi|29165615|ref|NC_002745.2|	525019	+	A	0	0	1	0	0	SA0453
gi|29165615|ref|NC_002745.2|	525021	+	C	0	0	1	0	0	SA0453
gi|29165615|ref|NC_002745.2|	525022	+	C	0	0	1	1	0	SA0453
gi|29165615|ref|NC_002745.2|	525023	+	A	0	0	1	0	0	SA0453
gi|29165615|ref|NC_002745.2|	525027	+	A	0	0	0	1	1	SA0453
gi|29165615|ref|NC_002745.2|	525028	+	A	0	0	1	0	0	SA0453
gi|29165615|ref|NC_002745.2|	525030	+	A	0	2	1	0	1	SA0453
gi|29165615|ref|NC_002745.2|	525031	+	A	0	2	0	1	2	SA0453
gi|29165615|ref|NC_002745.2|	525032	+	A	1	4	4	1	1	SA0453
gi|29165615|ref|NC_002745.2|	525033	+	U	0	2	2	0	1	SA0453
gi|29165615|ref|NC_002745.2|	525035	+	U	0	5	8	1	12	SA0453
gi|29165615|ref|NC_002745.2|	525036	+	U	0	2	1	0	2	SA0453

gi|29165615|ref|NC_002745.2|	525037	+	U	0	3	4	3	4	SA0453
gi|29165615|ref|NC_002745.2|	525038	+	A	0	7	9	3	14	SA0453
gi|29165615|ref|NC_002745.2|	525039	+	U	0	1	1	0	1	SA0453
gi|29165615|ref|NC_002745.2|	525040	+	A	0	3	2	2	0	SA0453
gi|29165615|ref|NC_002745.2|	525041	+	A	0	2	2	1	1	SA0453
gi|29165615|ref|NC_002745.2|	525042	+	U	1	2	0	0	0	SA0453
gi|29165615|ref|NC_002745.2|	525043	+	G	0	1	1	1	0	SA0453
gi|29165615|ref|NC_002745.2|	525044	+	C	4	64	34	14	43	SA0453
gi|29165615|ref|NC_002745.2|	525045	+	A	0	7	3	2	7	SA0453
gi|29165615|ref|NC_002745.2|	525046	+	G	0	1	0	0	1	SA0453
gi|29165615|ref|NC_002745.2|	525047	+	U	0	17	17	9	17	SA0453
gi|29165615|ref|NC_002745.2|	525048	+	U	0	0	0	2	0	SA0453
gi|29165615|ref|NC_002745.2|	525049	+	A	0	0	3	0	1	SA0453
gi|29165615|ref|NC_002745.2|	525050	+	A	0	1	0	4	1	SA0453
gi|29165615|ref|NC_002745.2|	525051	+	C	0	1	1	2	0	SA0453
gi|29165615|ref|NC_002745.2|	525053	+	G	0	1	2	1	1	SA0453
gi|29165615|ref|NC_002745.2|	525054	+	U	1	2	0	0	3	SA0453
gi|29165615|ref|NC_002745.2|	525056	+	G	0	0	1	0	0	SA0453
gi|29165615|ref|NC_002745.2|	525057	+	U	0	0	0	0	2	SA0453
gi|29165615|ref|NC_002745.2|	525058	+	U	0	1	0	0	0	SA0453
gi|29165615|ref|NC_002745.2|	525059	+	G	0	4	0	0	4	SA0453
gi|29165615|ref|NC_002745.2|	525060	+	U	0	2	2	1	0	SA0453
gi|29165615|ref|NC_002745.2|	525061	+	A	0	10	1	5	2	SA0453
gi|29165615|ref|NC_002745.2|	525065	+	A	0	2	3	0	3	SA0453
gi|29165615|ref|NC_002745.2|	525069	+	G	0	0	0	0	1	SA0453
gi|29165615|ref|NC_002745.2|	525070	+	U	0	0	1	0	1	SA0453
gi|29165615|ref|NC_002745.2|	525075	+	A	1	0	1	0	0	SA0453
gi|29165615|ref|NC_002745.2|	525077	+	U	0	0	0	1	0	SA0453
gi|29165615|ref|NC_002745.2|	525081	+	A	0	2	3	2	2	SA0453
gi|29165615|ref|NC_002745.2|	525087	+	A	0	1	0	0	1	SA0453
gi|29165615|ref|NC_002745.2|	525088	+	G	0	0	1	0	0	SA0453
gi|29165615|ref|NC_002745.2|	525089	+	G	0	1	0	1	2	SA0453
gi|29165615|ref|NC_002745.2|	525090	+	A	0	0	1	2	2	SA0453
gi|29165615|ref|NC_002745.2|	525092	+	A	1	0	2	2	1	SA0453
gi|29165615|ref|NC_002745.2|	525093	+	G	0	1	0	0	1	SA0453
gi|29165615|ref|NC_002745.2|	525110	+	A	0	0	0	0	1	SA0454
gi|29165615|ref|NC_002745.2|	525141	+	U	1	0	0	0	0	SA0454
gi|29165615|ref|NC_002745.2|	525168	+	A	0	0	0	0	1	SA0454
gi|29165615|ref|NC_002745.2|	525224	+	A	0	0	0	0	2	SA0454
gi|29165615|ref|NC_002745.2|	525235	+	U	1	0	0	0	0	SA0454
gi|29165615|ref|NC_002745.2|	525242	+	A	1	0	0	0	0	SA0454
gi|29165615|ref|NC_002745.2|	525248	+	A	0	0	0	2	1	SA0454
gi|29165615|ref|NC_002745.2|	525251	+	A	0	1	0	0	0	SA0454
gi|29165615|ref|NC_002745.2|	525252	+	A	0	2	0	0	0	SA0454
gi|29165615|ref|NC_002745.2|	525255	+	C	0	0	0	0	1	SA0454
gi|29165615|ref|NC_002745.2|	525261	+	A	0	1	0	2	1	SA0454
gi|29165615|ref|NC_002745.2|	525262	+	A	0	0	1	1	1	SA0454
gi|29165615|ref|NC_002745.2|	525263	+	A	1	1	0	1	0	SA0454
gi|29165615|ref|NC_002745.2|	525264	+	A	0	0	0	0	1	SA0454
gi|29165615|ref|NC_002745.2|	525267	+	A	0	2	0	0	0	SA0454
gi|29165615|ref|NC_002745.2|	525268	+	A	0	0	0	4	2	SA0454
gi|29165615|ref|NC_002745.2|	525269	+	A	0	0	0	1	0	SA0454
gi|29165615|ref|NC_002745.2|	525270	+	A	0	0	0	1	0	SA0454
gi|29165615|ref|NC_002745.2|	525272	+	U	0	0	2	1	0	SA0454
gi|29165615|ref|NC_002745.2|	525273	+	U	0	0	0	1	0	SA0454
gi|29165615|ref|NC_002745.2|	525277	+	U	0	1	0	0	0	SA0454
gi|29165615|ref|NC_002745.2|	525278	+	A	0	0	0	0	1	SA0454
gi|29165615|ref|NC_002745.2|	525279	+	C	4	0	0	0	0	SA0454
gi|29165615|ref|NC_002745.2|	525281	+	G	0	0	0	1	0	SA0454
gi|29165615|ref|NC_002745.2|	525282	+	U	1	3	2	0	3	SA0454
gi|29165615|ref|NC_002745.2|	525285	+	U	1	0	0	0	0	SA0454
gi|29165615|ref|NC_002745.2|	525287	+	A	1	0	0	0	0	SA0454
gi|29165615|ref|NC_002745.2|	525288	+	C	1	0	0	1	1	SA0454
gi|29165615|ref|NC_002745.2|	525289	+	U	0	0	2	0	0	SA0454
gi|29165615|ref|NC_002745.2|	525291	+	C	4	0	0	0	0	SA0454
gi|29165615|ref|NC_002745.2|	525294	+	C	0	0	0	0	1	SA0454
gi|29165615|ref|NC_002745.2|	525300	+	C	3	0	0	0	0	SA0454
gi|29165615|ref|NC_002745.2|	525301	+	A	1	0	1	0	0	SA0454
gi|29165615|ref|NC_002745.2|	525309	+	G	1	0	0	0	0	SA0454
gi|29165615|ref|NC_002745.2|	525312	+	U	1	0	0	0	0	SA0454
gi|29165615|ref|NC_002745.2|	525326	+	A	0	0	0	1	0	SA0454
gi|29165615|ref|NC_002745.2|	525328	+	G	0	0	0	1	0	SA0454
gi|29165615|ref|NC_002745.2|	525329	+	A	0	3	1	1	2	SA0454
gi|29165615|ref|NC_002745.2|	525331	+	G	0	1	1	0	0	SA0454
gi|29165615|ref|NC_002745.2|	525332	+	A	0	1	0	0	1	SA0454
gi|29165615|ref|NC_002745.2|	525333	+	G	0	1	0	0	0	SA0454
gi|29165615|ref|NC_002745.2|	525334	+	U	0	3	0	0	1	SA0454
gi|29165615|ref|NC_002745.2|	525335	+	A	0	1	2	1	0	SA0454
gi|29165615|ref|NC_002745.2|	525336	+	A	0	2	0	2	1	SA0454
gi|29165615|ref|NC_002745.2|	525338	+	G	1	0	0	1	0	SA0454
gi|29165615|ref|NC_002745.2|	525342	+	A	0	0	1	0	0	SA0454

gi|29165615|ref|NC_002745.2|	525347 +	A	0	1	0	0	0	SA0454
gi|29165615|ref|NC_002745.2|	525352 +	A	1	0	0	0	0	SA0454
gi|29165615|ref|NC_002745.2|	525357 +	U	1	1	2	1	0	SA0454
gi|29165615|ref|NC_002745.2|	525358 +	U	0	1	0	0	1	SA0454
gi|29165615|ref|NC_002745.2|	525359 +	A	0	0	1	0	2	SA0454
gi|29165615|ref|NC_002745.2|	525369 +	U	0	0	1	0	0	SA0454
gi|29165615|ref|NC_002745.2|	525377 +	U	0	1	0	0	0	SA0454
gi|29165615|ref|NC_002745.2|	525382 +	A	0	0	0	0	1	SA0454
gi|29165615|ref|NC_002745.2|	525384 +	A	0	0	1	0	0	SA0454
gi|29165615|ref|NC_002745.2|	525385 +	G	0	1	0	0	0	SA0454
gi|29165615|ref|NC_002745.2|	525386 +	A	0	0	0	0	1	SA0454
gi|29165615|ref|NC_002745.2|	525387 +	A	0	2	0	0	0	SA0454
gi|29165615|ref|NC_002745.2|	525390 +	A	1	0	0	0	0	SA0454
gi|29165615|ref|NC_002745.2|	525394 +	U	0	0	1	1	0	SA0454
gi|29165615|ref|NC_002745.2|	525417 +	U	1	0	0	0	0	SA0454
gi|29165615|ref|NC_002745.2|	525423 +	C	0	2	0	0	1	SA0454
gi|29165615|ref|NC_002745.2|	525429 +	U	1	0	0	0	0	SA0454
gi|29165615|ref|NC_002745.2|	525430 +	G	1	0	0	0	0	SA0454
gi|29165615|ref|NC_002745.2|	525441 +	C	0	0	0	2	0	SA0454
gi|29165615|ref|NC_002745.2|	525448 +	A	0	1	0	0	0	SA0454
gi|29165615|ref|NC_002745.2|	525451 +	A	0	1	0	0	0	SA0454
gi|29165615|ref|NC_002745.2|	525456 +	A	0	1	0	0	0	SA0454
gi|29165615|ref|NC_002745.2|	525459 +	U	0	0	0	0	1	SA0454
gi|29165615|ref|NC_002745.2|	525462 +	G	0	2	0	0	0	SA0454
gi|29165615|ref|NC_002745.2|	525463 +	U	0	1	0	0	1	SA0454
gi|29165615|ref|NC_002745.2|	525464 +	A	0	2	1	1	0	SA0454
gi|29165615|ref|NC_002745.2|	525466 +	G	0	0	1	0	0	SA0454
gi|29165615|ref|NC_002745.2|	525467 +	U	0	1	0	0	0	SA0454
gi|29165615|ref|NC_002745.2|	525488 +	G	0	3	0	0	0	SA0454
gi|29165615|ref|NC_002745.2|	525489 +	A	0	1	0	0	0	SA0454
gi|29165615|ref|NC_002745.2|	525491 +	G	0	0	1	0	0	SA0454
gi|29165615|ref|NC_002745.2|	525493 +	A	0	0	1	0	0	SA0454
gi|29165615|ref|NC_002745.2|	525495 +	A	0	0	0	1	1	SA0454
gi|29165615|ref|NC_002745.2|	525496 +	A	0	2	0	0	1	SA0454
gi|29165615|ref|NC_002745.2|	525507 +	U	0	1	0	0	0	SA0454
gi|29165615|ref|NC_002745.2|	525514 +	C	0	0	1	0	0	SA0454
gi|29165615|ref|NC_002745.2|	525525 +	A	0	1	0	0	0	SA0454
gi|29165615|ref|NC_002745.2|	525535 +	A	0	1	0	0	0	SA0454
gi|29165615|ref|NC_002745.2|	525570 +	C	1	0	0	0	0	SA0454
gi|29165615|ref|NC_002745.2|	525574 +	A	0	2	0	0	0	SA0454
gi|29165615|ref|NC_002745.2|	525580 +	G	0	1	0	0	0	SA0454
gi|29165615|ref|NC_002745.2|	525590 +	G	0	0	0	0	1	SA0454
gi|29165615|ref|NC_002745.2|	525594 +	A	0	0	0	0	1	SA0454
gi|29165615|ref|NC_002745.2|	525653 +	A	0	1	0	0	0	SA0454
gi|29165615|ref|NC_002745.2|	525695 +	U	0	0	0	0	1	SA0454
gi|29165615|ref|NC_002745.2|	525698 +	A	0	0	1	0	0	SA0454
gi|29165615|ref|NC_002745.2|	525699 +	A	0	0	1	0	0	SA0454
gi|29165615|ref|NC_002745.2|	525702 +	U	0	1	0	0	0	SA0454
gi|29165615|ref|NC_002745.2|	525727 +	G	1	0	0	0	0	SA0454
gi|29165615|ref|NC_002745.2|	525739 +	U	1	0	0	0	0	SA0454
gi|29165615|ref|NC_002745.2|	525746 +	G	0	0	2	0	0	SA0454
gi|29165615|ref|NC_002745.2|	525752 +	A	0	0	0	0	2	SA0454
gi|29165615|ref|NC_002745.2|	525806 +	G	0	0	0	0	1	SA0454
gi|29165615|ref|NC_002745.2|	525807 +	A	0	0	0	0	1	SA0454
gi|29165615|ref|NC_002745.2|	525828 +	G	0	1	0	0	0	SA0454
gi|29165615|ref|NC_002745.2|	525842 +	U	0	0	0	0	1	SA0454
gi|29165615|ref|NC_002745.2|	525843 +	A	0	4	1	1	6	SA0454
gi|29165615|ref|NC_002745.2|	525847 +	U	0	0	0	0	1	SA0454
gi|29165615|ref|NC_002745.2|	525856 +	G	0	1	0	0	0	SA0454
gi|29165615|ref|NC_002745.2|	525864 +	C	0	1	0	0	0	SA0454
gi|29165615|ref|NC_002745.2|	525876 +	A	0	0	1	1	1	SA0454
gi|29165615|ref|NC_002745.2|	525879 +	A	0	0	0	1	0	SA0454
gi|29165615|ref|NC_002745.2|	525882 +	A	0	0	0	1	0	SA0454
gi|29165615|ref|NC_002745.2|	525885 +	A	0	0	0	0	1	SA0454
gi|29165615|ref|NC_002745.2|	525888 +	A	0	0	1	1	0	SA0454
gi|29165615|ref|NC_002745.2|	525895 +	C	0	0	0	0	1	SA0454
gi|29165615|ref|NC_002745.2|	525901 +	A	1	0	0	0	0	SA0454
gi|29165615|ref|NC_002745.2|	525904 +	U	0	0	0	0	1	SA0454
gi|29165615|ref|NC_002745.2|	525913 +	U	1	0	0	0	0	SA0454
gi|29165615|ref|NC_002745.2|	525926 +	U	0	0	0	0	1	SA0454
gi|29165615|ref|NC_002745.2|	525937 +	G	0	0	0	0	2	-
gi|29165615|ref|NC_002745.2|	525944 +	U	0	1	0	0	0	-
gi|29165615|ref|NC_002745.2|	525946 +	U	1	0	0	0	0	-
gi|29165615|ref|NC_002745.2|	525958 +	U	1	0	0	0	0	SA0455
gi|29165615|ref|NC_002745.2|	525971 +	A	0	1	0	0	0	SA0455
gi|29165615|ref|NC_002745.2|	525978 +	G	0	0	1	0	0	SA0455
gi|29165615|ref|NC_002745.2|	525979 +	A	0	0	0	0	1	SA0455
gi|29165615|ref|NC_002745.2|	526004 +	A	0	1	0	0	0	SA0455
gi|29165615|ref|NC_002745.2|	526039 +	A	0	0	1	0	0	SA0455
gi|29165615|ref|NC_002745.2|	526083 +	C	1	0	0	0	0	SA0455
gi|29165615|ref|NC_002745.2|	526143 +	G	0	0	0	1	0	SA0455

gi|29165615|ref|NC_002745.2|	526146 +	U	0	0	0	0	1	SA0455
gi|29165615|ref|NC_002745.2|	526154 +	U	1	0	0	0	0	SA0455
gi|29165615|ref|NC_002745.2|	526185 +	A	0	0	1	0	0	SA0455
gi|29165615|ref|NC_002745.2|	526199 +	A	0	1	0	0	0	SA0455
gi|29165615|ref|NC_002745.2|	526203 +	A	0	1	0	0	0	SA0455
gi|29165615|ref|NC_002745.2|	526204 +	U	0	0	2	0	0	SA0455
gi|29165615|ref|NC_002745.2|	526224 +	U	0	0	1	0	0	SA0455
gi|29165615|ref|NC_002745.2|	526230 +	A	0	1	1	0	0	SA0455
gi|29165615|ref|NC_002745.2|	526232 +	U	1	0	0	0	0	SA0455
gi|29165615|ref|NC_002745.2|	526236 +	C	0	0	0	0	1	SA0455
gi|29165615|ref|NC_002745.2|	526243 +	C	3	0	0	0	0	SA0455
gi|29165615|ref|NC_002745.2|	526249 +	G	1	0	0	0	0	SA0455
gi|29165615|ref|NC_002745.2|	526293 +	G	0	0	0	0	2	SA0455
gi|29165615|ref|NC_002745.2|	526300 +	A	0	0	0	0	1	SA0455
gi|29165615|ref|NC_002745.2|	526306 +	U	0	0	0	1	1	SA0455
gi|29165615|ref|NC_002745.2|	526307 +	A	0	0	1	1	0	SA0455
gi|29165615|ref|NC_002745.2|	526319 +	G	0	0	1	0	0	SA0455
gi|29165615|ref|NC_002745.2|	526320 +	G	0	1	0	1	0	SA0455
gi|29165615|ref|NC_002745.2|	526321 +	A	0	1	0	0	0	SA0455
gi|29165615|ref|NC_002745.2|	526322 +	A	0	0	1	0	0	SA0455
gi|29165615|ref|NC_002745.2|	526328 +	A	0	0	0	0	1	SA0455
gi|29165615|ref|NC_002745.2|	526329 +	U	1	3	3	0	4	-
gi|29165615|ref|NC_002745.2|	526331 +	U	0	0	0	0	1	-
gi|29165615|ref|NC_002745.2|	526335 +	A	0	0	0	1	0	-
gi|29165615|ref|NC_002745.2|	526339 +	A	0	1	0	0	0	-
gi|29165615|ref|NC_002745.2|	526344 +	U	0	1	0	0	0	-
gi|29165615|ref|NC_002745.2|	526346 +	U	0	0	0	0	1	-
gi|29165615|ref|NC_002745.2|	526354 +	C	0	0	1	0	0	-
gi|29165615|ref|NC_002745.2|	526359 +	A	0	0	0	0	2	-
gi|29165615|ref|NC_002745.2|	526360 +	A	0	2	1	0	0	-
gi|29165615|ref|NC_002745.2|	526366 +	A	0	0	0	0	1	-
gi|29165615|ref|NC_002745.2|	526377 +	U	0	0	1	0	1	SA0456
gi|29165615|ref|NC_002745.2|	526378 +	A	0	0	0	0	1	SA0456
gi|29165615|ref|NC_002745.2|	526379 +	U	0	0	0	1	0	SA0456
gi|29165615|ref|NC_002745.2|	526380 +	A	0	0	1	0	0	SA0456
gi|29165615|ref|NC_002745.2|	526387 +	G	0	0	0	0	1	SA0456
gi|29165615|ref|NC_002745.2|	526405 +	A	0	0	0	1	0	SA0456
gi|29165615|ref|NC_002745.2|	526410 +	C	0	1	0	0	0	SA0456
gi|29165615|ref|NC_002745.2|	526412 +	G	0	0	0	0	1	SA0456
gi|29165615|ref|NC_002745.2|	526426 +	A	0	1	0	0	0	SA0456
gi|29165615|ref|NC_002745.2|	526428 +	A	0	0	0	0	1	SA0456
gi|29165615|ref|NC_002745.2|	526429 +	A	0	0	0	0	3	SA0456
gi|29165615|ref|NC_002745.2|	526430 +	A	0	5	3	1	2	SA0456
gi|29165615|ref|NC_002745.2|	526431 +	U	0	0	1	0	0	SA0456
gi|29165615|ref|NC_002745.2|	526432 +	A	0	0	1	0	1	SA0456
gi|29165615|ref|NC_002745.2|	526433 +	C	0	3	0	2	2	SA0456
gi|29165615|ref|NC_002745.2|	526434 +	A	0	0	1	1	0	SA0456
gi|29165615|ref|NC_002745.2|	526436 +	A	0	2	2	0	1	SA0456
gi|29165615|ref|NC_002745.2|	526437 +	C	0	0	0	0	1	SA0456
gi|29165615|ref|NC_002745.2|	526441 +	U	0	0	1	0	0	SA0456
gi|29165615|ref|NC_002745.2|	526444 +	A	0	0	0	1	0	SA0456
gi|29165615|ref|NC_002745.2|	526446 +	G	0	0	0	1	0	SA0456
gi|29165615|ref|NC_002745.2|	526448 +	A	0	0	0	0	1	SA0456
gi|29165615|ref|NC_002745.2|	526450 +	G	0	0	0	0	1	SA0456
gi|29165615|ref|NC_002745.2|	526451 +	A	0	2	1	0	1	SA0456
gi|29165615|ref|NC_002745.2|	526469 +	A	0	0	0	0	1	SA0456
gi|29165615|ref|NC_002745.2|	526479 +	A	0	4	0	0	0	SA0456
gi|29165615|ref|NC_002745.2|	526482 +	C	0	0	1	0	0	SA0456
gi|29165615|ref|NC_002745.2|	526488 +	U	0	0	0	0	3	SA0456
gi|29165615|ref|NC_002745.2|	526493 +	C	0	0	1	0	0	SA0456
gi|29165615|ref|NC_002745.2|	526495 +	U	0	1	0	0	0	SA0456
gi|29165615|ref|NC_002745.2|	526496 +	G	0	2	0	0	0	SA0456
gi|29165615|ref|NC_002745.2|	526497 +	A	0	1	0	2	2	SA0456
gi|29165615|ref|NC_002745.2|	526507 +	A	0	0	0	1	1	SA0456
gi|29165615|ref|NC_002745.2|	526511 +	G	0	1	1	0	0	SA0456
gi|29165615|ref|NC_002745.2|	526515 +	G	0	0	0	1	0	SA0456
gi|29165615|ref|NC_002745.2|	526521 +	C	4	1	2	0	0	SA0456
gi|29165615|ref|NC_002745.2|	526524 +	G	0	1	0	1	2	SA0456
gi|29165615|ref|NC_002745.2|	526526 +	U	0	1	0	0	4	SA0456
gi|29165615|ref|NC_002745.2|	526527 +	U	0	0	0	1	0	SA0456
gi|29165615|ref|NC_002745.2|	526529 +	U	0	0	1	0	2	SA0456
gi|29165615|ref|NC_002745.2|	526531 +	C	0	0	0	0	1	SA0456
gi|29165615|ref|NC_002745.2|	526533 +	U	0	0	0	0	1	SA0456
gi|29165615|ref|NC_002745.2|	526536 +	C	0	1	0	0	0	SA0456
gi|29165615|ref|NC_002745.2|	526544 +	A	0	0	0	0	1	SA0456
gi|29165615|ref|NC_002745.2|	526549 +	A	1	0	0	0	1	SA0456
gi|29165615|ref|NC_002745.2|	526556 +	C	1	0	0	0	0	SA0456
gi|29165615|ref|NC_002745.2|	526566 +	A	0	1	0	0	0	SA0456
gi|29165615|ref|NC_002745.2|	526583 +	C	0	0	0	0	1	SA0456
gi|29165615|ref|NC_002745.2|	526587 +	C	0	0	0	0	2	SA0456
gi|29165615|ref|NC_002745.2|	526592 +	A	0	1	0	0	0	SA0456

gi|29165615|ref|NC_002745.2|	526593	+	A	0	1	0	0	1	SA0456
gi|29165615|ref|NC_002745.2|	526596	+	C	0	0	0	0	1	SA0456
gi|29165615|ref|NC_002745.2|	526598	+	G	0	0	1	0	0	SA0456
gi|29165615|ref|NC_002745.2|	526599	+	A	0	0	0	0	2	SA0456
gi|29165615|ref|NC_002745.2|	526601	+	A	0	1	0	1	0	SA0456
gi|29165615|ref|NC_002745.2|	526603	+	G	0	1	0	0	0	SA0456
gi|29165615|ref|NC_002745.2|	526605	+	G	0	0	0	2	0	SA0456
gi|29165615|ref|NC_002745.2|	526607	+	C	0	0	1	0	0	SA0456
gi|29165615|ref|NC_002745.2|	526611	+	A	0	0	0	2	3	SA0456
gi|29165615|ref|NC_002745.2|	526613	+	A	0	1	0	0	0	SA0456
gi|29165615|ref|NC_002745.2|	526617	+	A	0	3	0	0	0	SA0456
gi|29165615|ref|NC_002745.2|	526618	+	A	0	2	1	3	0	SA0456
gi|29165615|ref|NC_002745.2|	526619	+	G	0	3	0	1	1	SA0456
gi|29165615|ref|NC_002745.2|	526641	+	A	0	0	1	0	0	SA0456
gi|29165615|ref|NC_002745.2|	526647	+	C	0	2	0	1	0	SA0456
gi|29165615|ref|NC_002745.2|	526652	+	G	0	0	0	1	0	SA0456
gi|29165615|ref|NC_002745.2|	526669	+	A	0	0	0	1	0	SA0456
gi|29165615|ref|NC_002745.2|	526933	+	U	0	1	0	0	0	-
gi|29165615|ref|NC_002745.2|	527021	+	A	0	0	0	0	1	-
gi|29165615|ref|NC_002745.2|	527027	+	A	0	0	0	1	0	-
gi|29165615|ref|NC_002745.2|	527028	+	A	0	2	1	0	0	-
gi|29165615|ref|NC_002745.2|	527033	+	G	1	8	3	6	10	-
gi|29165615|ref|NC_002745.2|	527034	+	A	1	16	25	17	23	-
gi|29165615|ref|NC_002745.2|	527035	+	C	10	104	90	47	158	-
gi|29165615|ref|NC_002745.2|	527036	+	G	60	1008	778	441	1577	-
gi|29165615|ref|NC_002745.2|	527037	+	A	0	172	129	49	122	-
gi|29165615|ref|NC_002745.2|	527038	+	A	0	36	29	13	49	-
gi|29165615|ref|NC_002745.2|	527039	+	G	0	9	2	4	7	-
gi|29165615|ref|NC_002745.2|	527040	+	U	0	1	1	1	0	-
gi|29165615|ref|NC_002745.2|	527041	+	G	0	2	8	9	16	-
gi|29165615|ref|NC_002745.2|	527042	+	U	0	0	0	1	3	-
gi|29165615|ref|NC_002745.2|	527043	+	A	1	9	8	3	13	-
gi|29165615|ref|NC_002745.2|	527044	+	A	1	69	24	27	70	-
gi|29165615|ref|NC_002745.2|	527045	+	A	3	81	36	23	70	-
gi|29165615|ref|NC_002745.2|	527046	+	A	0	15	8	12	7	-
gi|29165615|ref|NC_002745.2|	527047	+	U	2	3	4	3	6	-
gi|29165615|ref|NC_002745.2|	527048	+	U	1	5	0	5	10	-
gi|29165615|ref|NC_002745.2|	527049	+	U	0	1	7	3	4	-
gi|29165615|ref|NC_002745.2|	527050	+	A	2	15	12	7	10	-
gi|29165615|ref|NC_002745.2|	527051	+	A	0	4	7	4	5	-
gi|29165615|ref|NC_002745.2|	527052	+	U	1	2	8	1	2	-
gi|29165615|ref|NC_002745.2|	527054	+	G	0	5	1	2	2	-
gi|29165615|ref|NC_002745.2|	527055	+	G	0	2	0	0	1	-
gi|29165615|ref|NC_002745.2|	527057	+	G	1	4	0	0	1	-
gi|29165615|ref|NC_002745.2|	527058	+	U	0	3	0	1	1	-
gi|29165615|ref|NC_002745.2|	527059	+	U	0	0	1	0	1	-
gi|29165615|ref|NC_002745.2|	527060	+	A	1	4	3	0	3	-
gi|29165615|ref|NC_002745.2|	527061	+	A	0	1	1	1	1	-
gi|29165615|ref|NC_002745.2|	527064	+	U	0	1	0	0	0	-
gi|29165615|ref|NC_002745.2|	527065	+	U	0	0	2	0	0	-
gi|29165615|ref|NC_002745.2|	527066	+	C	1	0	1	0	0	-
gi|29165615|ref|NC_002745.2|	527067	+	A	0	0	0	0	1	SA0457
gi|29165615|ref|NC_002745.2|	527070	+	C	0	2	0	1	0	SA0457
gi|29165615|ref|NC_002745.2|	527072	+	A	0	3	0	1	1	SA0457
gi|29165615|ref|NC_002745.2|	527073	+	A	0	2	0	0	1	SA0457
gi|29165615|ref|NC_002745.2|	527074	+	G	0	1	2	2	1	SA0457
gi|29165615|ref|NC_002745.2|	527076	+	C	0	0	0	1	0	SA0457
gi|29165615|ref|NC_002745.2|	527077	+	A	0	1	0	0	1	SA0457
gi|29165615|ref|NC_002745.2|	527078	+	C	0	1	0	0	0	SA0457
gi|29165615|ref|NC_002745.2|	527082	+	A	0	1	0	0	0	SA0457
gi|29165615|ref|NC_002745.2|	527084	+	A	0	0	1	1	0	SA0457
gi|29165615|ref|NC_002745.2|	527085	+	A	0	0	1	0	0	SA0457
gi|29165615|ref|NC_002745.2|	527094	+	G	0	0	1	0	0	SA0457
gi|29165615|ref|NC_002745.2|	527095	+	C	0	1	1	0	0	SA0457
gi|29165615|ref|NC_002745.2|	527097	+	G	0	0	0	0	1	SA0457
gi|29165615|ref|NC_002745.2|	527098	+	G	0	0	1	0	0	SA0457
gi|29165615|ref|NC_002745.2|	527099	+	U	0	0	0	0	1	SA0457
gi|29165615|ref|NC_002745.2|	527100	+	A	0	1	0	0	1	SA0457
gi|29165615|ref|NC_002745.2|	527101	+	A	0	1	0	0	2	SA0457
gi|29165615|ref|NC_002745.2|	527103	+	G	0	1	0	0	0	SA0457
gi|29165615|ref|NC_002745.2|	527105	+	C	0	1	0	0	0	SA0457
gi|29165615|ref|NC_002745.2|	527108	+	A	0	2	1	0	1	SA0457
gi|29165615|ref|NC_002745.2|	527111	+	A	0	0	0	1	6	SA0457
gi|29165615|ref|NC_002745.2|	527114	+	G	0	0	0	0	1	SA0457
gi|29165615|ref|NC_002745.2|	527115	+	A	0	0	0	0	1	SA0457
gi|29165615|ref|NC_002745.2|	527116	+	A	0	1	1	0	0	SA0457
gi|29165615|ref|NC_002745.2|	527121	+	A	0	0	0	0	1	SA0457
gi|29165615|ref|NC_002745.2|	527122	+	A	0	3	2	0	2	SA0457
gi|29165615|ref|NC_002745.2|	527123	+	A	0	6	1	1	4	SA0457
gi|29165615|ref|NC_002745.2|	527124	+	A	0	1	0	0	4	SA0457
gi|29165615|ref|NC_002745.2|	527125	+	A	0	0	1	1	3	SA0457

gi|29165615|ref|NC_002745.2|	527127 +	U	0	0	0	0	2	SA0457
gi|29165615|ref|NC_002745.2|	527128 +	A	0	0	1	0	3	SA0457
gi|29165615|ref|NC_002745.2|	527129 +	U	1	1	0	1	2	SA0457
gi|29165615|ref|NC_002745.2|	527130 +	A	0	1	1	0	1	SA0457
gi|29165615|ref|NC_002745.2|	527131 +	A	1	2	0	0	3	SA0457
gi|29165615|ref|NC_002745.2|	527132 +	A	0	1	1	0	0	SA0457
gi|29165615|ref|NC_002745.2|	527135 +	G	0	0	1	0	0	SA0457
gi|29165615|ref|NC_002745.2|	527137 +	U	0	1	0	0	0	SA0457
gi|29165615|ref|NC_002745.2|	527138 +	A	0	0	1	0	0	SA0457
gi|29165615|ref|NC_002745.2|	527140 +	A	0	2	3	1	0	SA0457
gi|29165615|ref|NC_002745.2|	527142 +	G	0	0	0	0	1	SA0457
gi|29165615|ref|NC_002745.2|	527143 +	A	0	0	1	0	0	SA0457
gi|29165615|ref|NC_002745.2|	527145 +	G	0	0	1	0	0	SA0457
gi|29165615|ref|NC_002745.2|	527148 +	G	0	0	1	0	0	SA0457
gi|29165615|ref|NC_002745.2|	527151 +	G	0	0	0	0	2	SA0457
gi|29165615|ref|NC_002745.2|	527154 +	A	0	1	0	0	0	SA0457
gi|29165615|ref|NC_002745.2|	527156 +	A	0	1	1	0	0	SA0457
gi|29165615|ref|NC_002745.2|	527158 +	C	0	0	1	0	0	SA0457
gi|29165615|ref|NC_002745.2|	527159 +	U	0	0	1	0	1	SA0457
gi|29165615|ref|NC_002745.2|	527166 +	G	0	0	0	0	1	SA0457
gi|29165615|ref|NC_002745.2|	527167 +	A	0	2	0	0	1	SA0457
gi|29165615|ref|NC_002745.2|	527168 +	A	0	1	1	0	2	SA0457
gi|29165615|ref|NC_002745.2|	527169 +	C	0	0	0	0	1	SA0457
gi|29165615|ref|NC_002745.2|	527174 +	A	0	0	0	0	3	SA0457
gi|29165615|ref|NC_002745.2|	527176 +	U	0	0	2	0	0	SA0457
gi|29165615|ref|NC_002745.2|	527184 +	G	0	0	0	0	2	SA0457
gi|29165615|ref|NC_002745.2|	527186 +	G	0	1	0	0	0	SA0457
gi|29165615|ref|NC_002745.2|	527188 +	A	0	0	0	0	1	SA0457
gi|29165615|ref|NC_002745.2|	527193 +	U	0	0	3	0	0	SA0457
gi|29165615|ref|NC_002745.2|	527198 +	U	0	0	1	0	1	SA0457
gi|29165615|ref|NC_002745.2|	527201 +	C	1	0	0	0	0	SA0457
gi|29165615|ref|NC_002745.2|	527202 +	G	0	2	3	0	5	SA0457
gi|29165615|ref|NC_002745.2|	527203 +	A	0	0	1	0	0	SA0457
gi|29165615|ref|NC_002745.2|	527204 +	U	0	0	0	0	2	SA0457
gi|29165615|ref|NC_002745.2|	527205 +	C	0	2	1	0	0	SA0457
gi|29165615|ref|NC_002745.2|	527207 +	A	0	0	0	0	1	SA0457
gi|29165615|ref|NC_002745.2|	527208 +	G	0	1	0	0	0	SA0457
gi|29165615|ref|NC_002745.2|	527209 +	U	0	1	0	0	0	SA0457
gi|29165615|ref|NC_002745.2|	527212 +	U	0	0	1	0	0	SA0457
gi|29165615|ref|NC_002745.2|	527216 +	C	0	0	0	0	1	SA0457
gi|29165615|ref|NC_002745.2|	527219 +	C	1	0	0	0	0	SA0457
gi|29165615|ref|NC_002745.2|	527220 +	G	0	0	1	0	2	SA0457
gi|29165615|ref|NC_002745.2|	527221 +	U	0	0	0	0	1	SA0457
gi|29165615|ref|NC_002745.2|	527222 +	A	0	0	1	1	0	SA0457
gi|29165615|ref|NC_002745.2|	527224 +	G	0	2	0	0	2	SA0457
gi|29165615|ref|NC_002745.2|	527225 +	A	0	0	4	0	0	SA0457
gi|29165615|ref|NC_002745.2|	527226 +	C	0	0	1	0	0	SA0457
gi|29165615|ref|NC_002745.2|	527227 +	A	1	0	1	0	0	SA0457
gi|29165615|ref|NC_002745.2|	527249 +	A	0	0	0	2	1	SA0457
gi|29165615|ref|NC_002745.2|	527250 +	C	0	1	0	1	0	SA0457
gi|29165615|ref|NC_002745.2|	527253 +	U	0	0	2	0	0	SA0457
gi|29165615|ref|NC_002745.2|	527255 +	A	0	0	0	0	2	SA0457
gi|29165615|ref|NC_002745.2|	527265 +	U	0	1	0	1	0	SA0457
gi|29165615|ref|NC_002745.2|	527267 +	U	0	0	0	2	0	SA0457
gi|29165615|ref|NC_002745.2|	527270 +	A	0	0	1	0	0	SA0457
gi|29165615|ref|NC_002745.2|	527271 +	U	0	0	0	0	1	SA0457
gi|29165615|ref|NC_002745.2|	527272 +	A	0	1	0	0	0	SA0457
gi|29165615|ref|NC_002745.2|	527273 +	C	0	0	1	0	2	SA0457
gi|29165615|ref|NC_002745.2|	527276 +	U	0	0	0	0	3	SA0457
gi|29165615|ref|NC_002745.2|	527279 +	U	0	0	1	0	0	SA0457
gi|29165615|ref|NC_002745.2|	527280 +	C	0	7	1	1	9	SA0457
gi|29165615|ref|NC_002745.2|	527281 +	A	1	1	0	0	2	SA0457
gi|29165615|ref|NC_002745.2|	527282 +	A	0	1	0	0	0	SA0457
gi|29165615|ref|NC_002745.2|	527284 +	A	0	0	0	0	1	SA0457
gi|29165615|ref|NC_002745.2|	527285 +	G	0	2	1	1	4	SA0457
gi|29165615|ref|NC_002745.2|	527286 +	A	0	0	2	0	2	SA0457
gi|29165615|ref|NC_002745.2|	527287 +	A	0	1	1	0	0	SA0457
gi|29165615|ref|NC_002745.2|	527288 +	A	0	0	1	1	1	SA0457
gi|29165615|ref|NC_002745.2|	527289 +	C	0	1	1	0	0	SA0457
gi|29165615|ref|NC_002745.2|	527291 +	A	0	0	3	0	0	SA0457
gi|29165615|ref|NC_002745.2|	527293 +	U	0	0	1	0	0	SA0457
gi|29165615|ref|NC_002745.2|	527297 +	U	0	2	0	0	0	SA0457
gi|29165615|ref|NC_002745.2|	527299 +	C	0	0	1	0	0	SA0457
gi|29165615|ref|NC_002745.2|	527300 +	U	0	0	1	0	0	SA0457
gi|29165615|ref|NC_002745.2|	527301 +	G	0	0	1	0	1	SA0457
gi|29165615|ref|NC_002745.2|	527302 +	C	0	0	1	0	0	SA0457
gi|29165615|ref|NC_002745.2|	527304 +	C	0	0	0	0	1	SA0457
gi|29165615|ref|NC_002745.2|	527305 +	A	0	0	0	0	1	SA0457
gi|29165615|ref|NC_002745.2|	527320 +	C	0	0	0	0	1	SA0457
gi|29165615|ref|NC_002745.2|	527324 +	A	0	1	0	0	0	SA0457
gi|29165615|ref|NC_002745.2|	527326 +	C	0	0	2	0	1	SA0457

gi|29165615|ref|NC_002745.2|	527335 +	A	0	0	1	2	3	SA0457
gi|29165615|ref|NC_002745.2|	527338 +	A	0	1	0	1	0	SA0457
gi|29165615|ref|NC_002745.2|	527340 +	A	0	1	0	0	1	SA0457
gi|29165615|ref|NC_002745.2|	527343 +	G	0	2	2	0	0	SA0457
gi|29165615|ref|NC_002745.2|	527344 +	A	0	1	0	0	1	SA0457
gi|29165615|ref|NC_002745.2|	527347 +	G	0	1	0	0	0	SA0457
gi|29165615|ref|NC_002745.2|	527352 +	A	0	0	0	1	0	SA0457
gi|29165615|ref|NC_002745.2|	527355 +	A	0	1	0	0	0	SA0457
gi|29165615|ref|NC_002745.2|	527358 +	G	0	0	0	0	1	SA0457
gi|29165615|ref|NC_002745.2|	527396 +	A	0	0	0	0	1	SA0457
gi|29165615|ref|NC_002745.2|	527400 +	G	0	0	0	0	2	SA0457
gi|29165615|ref|NC_002745.2|	527408 +	G	0	0	1	0	0	SA0457
gi|29165615|ref|NC_002745.2|	527429 +	U	0	0	0	1	0	SA0457
gi|29165615|ref|NC_002745.2|	527431 +	A	0	0	0	1	0	SA0457
gi|29165615|ref|NC_002745.2|	527446 +	U	0	0	1	0	0	SA0457
gi|29165615|ref|NC_002745.2|	527452 +	C	0	0	1	0	0	SA0457
gi|29165615|ref|NC_002745.2|	527459 +	G	0	0	1	0	0	SA0457
gi|29165615|ref|NC_002745.2|	527463 +	C	0	2	0	0	0	SA0457
gi|29165615|ref|NC_002745.2|	527472 +	U	0	2	1	1	1	SA0457
gi|29165615|ref|NC_002745.2|	527478 +	U	0	0	1	0	0	SA0457
gi|29165615|ref|NC_002745.2|	527480 +	C	0	2	1	0	0	SA0457
gi|29165615|ref|NC_002745.2|	527481 +	G	0	1	0	0	0	SA0457
gi|29165615|ref|NC_002745.2|	527487 +	A	0	1	0	0	2	SA0457
gi|29165615|ref|NC_002745.2|	527501 +	G	1	0	0	0	0	SA0457
gi|29165615|ref|NC_002745.2|	527503 +	C	0	1	0	0	0	SA0457
gi|29165615|ref|NC_002745.2|	527570 +	U	0	2	0	0	0	SA0457
gi|29165615|ref|NC_002745.2|	527593 +	A	0	1	0	0	0	SA0457
gi|29165615|ref|NC_002745.2|	527618 +	A	0	2	0	0	0	SA0457
gi|29165615|ref|NC_002745.2|	527622 +	C	0	0	1	1	0	SA0457
gi|29165615|ref|NC_002745.2|	527626 +	U	0	1	0	0	0	SA0457
gi|29165615|ref|NC_002745.2|	527627 +	G	0	1	0	0	0	SA0457
gi|29165615|ref|NC_002745.2|	527632 +	A	0	0	0	0	1	SA0457
gi|29165615|ref|NC_002745.2|	527635 +	A	0	1	0	1	0	SA0457
gi|29165615|ref|NC_002745.2|	527639 +	U	0	1	0	0	0	SA0457
gi|29165615|ref|NC_002745.2|	527643 +	C	0	0	0	0	1	SA0457
gi|29165615|ref|NC_002745.2|	527654 +	U	0	0	0	0	1	SA0457
gi|29165615|ref|NC_002745.2|	527656 +	A	0	1	0	0	0	SA0457
gi|29165615|ref|NC_002745.2|	527660 +	C	0	0	0	0	1	SA0457
gi|29165615|ref|NC_002745.2|	527668 +	U	0	0	1	0	0	SA0457
gi|29165615|ref|NC_002745.2|	527684 +	A	0	0	1	1	0	SA0457
gi|29165615|ref|NC_002745.2|	527685 +	A	0	0	0	0	1	SA0457
gi|29165615|ref|NC_002745.2|	527686 +	A	0	1	0	0	1	SA0457
gi|29165615|ref|NC_002745.2|	527689 +	A	0	1	3	0	0	SA0457
gi|29165615|ref|NC_002745.2|	527690 +	U	0	0	1	0	0	SA0457
gi|29165615|ref|NC_002745.2|	527692 +	G	0	0	0	0	1	SA0457
gi|29165615|ref|NC_002745.2|	527701 +	U	0	0	0	0	1	SA0457
gi|29165615|ref|NC_002745.2|	527705 +	A	0	0	1	0	0	SA0457
gi|29165615|ref|NC_002745.2|	527712 +	C	0	1	0	0	0	SA0457
gi|29165615|ref|NC_002745.2|	527716 +	C	0	0	1	0	0	SA0457
gi|29165615|ref|NC_002745.2|	527719 +	A	0	0	1	0	0	SA0457
gi|29165615|ref|NC_002745.2|	527720 +	U	0	0	0	0	1	SA0457
gi|29165615|ref|NC_002745.2|	527721 +	G	0	1	0	0	0	SA0457
gi|29165615|ref|NC_002745.2|	527722 +	A	0	1	1	1	0	SA0457
gi|29165615|ref|NC_002745.2|	527723 +	U	0	1	0	0	0	SA0457
gi|29165615|ref|NC_002745.2|	527725 +	U	0	0	2	0	0	SA0457
gi|29165615|ref|NC_002745.2|	527726 +	U	0	0	0	1	0	SA0457
gi|29165615|ref|NC_002745.2|	527727 +	G	0	1	1	1	1	SA0457
gi|29165615|ref|NC_002745.2|	527728 +	A	1	5	1	3	1	SA0457
gi|29165615|ref|NC_002745.2|	527729 +	A	0	3	2	1	7	SA0457
gi|29165615|ref|NC_002745.2|	527730 +	G	0	3	0	3	1	SA0457
gi|29165615|ref|NC_002745.2|	527731 +	A	0	7	5	0	6	SA0457
gi|29165615|ref|NC_002745.2|	527732 +	A	0	0	1	1	1	SA0457
gi|29165615|ref|NC_002745.2|	527733 +	A	0	0	1	0	1	SA0457
gi|29165615|ref|NC_002745.2|	527734 +	U	0	0	3	0	0	SA0457
gi|29165615|ref|NC_002745.2|	527735 +	C	2	2	2	1	0	SA0457
gi|29165615|ref|NC_002745.2|	527736 +	A	0	0	0	0	1	SA0457
gi|29165615|ref|NC_002745.2|	527744 +	A	0	0	0	1	0	SA0457
gi|29165615|ref|NC_002745.2|	527749 +	A	0	4	1	0	0	SA0457
gi|29165615|ref|NC_002745.2|	527751 +	C	0	1	0	0	1	SA0457
gi|29165615|ref|NC_002745.2|	527752 +	G	0	0	3	0	0	SA0457
gi|29165615|ref|NC_002745.2|	527755 +	U	0	0	1	0	1	SA0457
gi|29165615|ref|NC_002745.2|	527758 +	U	0	0	1	0	0	SA0457
gi|29165615|ref|NC_002745.2|	527760 +	C	0	1	0	0	0	SA0457
gi|29165615|ref|NC_002745.2|	527766 +	C	1	1	0	1	1	SA0457
gi|29165615|ref|NC_002745.2|	527771 +	U	0	0	0	1	0	SA0457
gi|29165615|ref|NC_002745.2|	527779 +	C	0	1	0	0	0	SA0457
gi|29165615|ref|NC_002745.2|	527790 +	C	0	0	0	0	1	SA0457
gi|29165615|ref|NC_002745.2|	527797 +	A	0	2	0	0	0	SA0457
gi|29165615|ref|NC_002745.2|	527799 +	C	0	0	1	0	0	SA0457
gi|29165615|ref|NC_002745.2|	527807 +	C	0	0	0	0	1	SA0457
gi|29165615|ref|NC_002745.2|	527808 +	A	0	1	0	0	1	SA0457

gi|29165615|ref|NC_002745.2|	527815	+	A	0	1	0	0	0	SA0457
gi|29165615|ref|NC_002745.2|	527816	+	U	1	0	0	1	0	SA0457
gi|29165615|ref|NC_002745.2|	527820	+	G	1	0	0	0	0	SA0457
gi|29165615|ref|NC_002745.2|	527823	+	A	0	1	0	0	0	SA0457
gi|29165615|ref|NC_002745.2|	527824	+	C	0	1	0	0	0	SA0457
gi|29165615|ref|NC_002745.2|	527826	+	A	0	0	0	0	1	SA0457
gi|29165615|ref|NC_002745.2|	527845	+	C	0	1	0	0	0	SA0457
gi|29165615|ref|NC_002745.2|	527849	+	U	0	0	3	0	0	SA0457
gi|29165615|ref|NC_002745.2|	527859	+	G	0	2	4	0	0	SA0457
gi|29165615|ref|NC_002745.2|	527860	+	A	0	3	1	3	2	SA0457
gi|29165615|ref|NC_002745.2|	527861	+	C	0	0	0	1	0	SA0457
gi|29165615|ref|NC_002745.2|	527866	+	C	0	1	2	0	1	SA0457
gi|29165615|ref|NC_002745.2|	527868	+	A	0	0	0	1	0	SA0457
gi|29165615|ref|NC_002745.2|	527872	+	G	0	3	1	2	0	SA0457
gi|29165615|ref|NC_002745.2|	527873	+	U	0	0	0	2	3	SA0457
gi|29165615|ref|NC_002745.2|	527874	+	A	0	2	2	1	1	SA0457
gi|29165615|ref|NC_002745.2|	527876	+	U	0	0	1	1	0	SA0457
gi|29165615|ref|NC_002745.2|	527877	+	G	0	1	0	0	2	SA0457
gi|29165615|ref|NC_002745.2|	527878	+	A	0	1	0	0	0	SA0457
gi|29165615|ref|NC_002745.2|	527879	+	U	0	0	1	0	0	SA0457
gi|29165615|ref|NC_002745.2|	527884	+	U	0	0	1	0	0	SA0457
gi|29165615|ref|NC_002745.2|	527885	+	C	0	1	0	0	1	SA0457
gi|29165615|ref|NC_002745.2|	527899	+	U	0	0	0	0	2	SA0457
gi|29165615|ref|NC_002745.2|	527901	+	C	0	0	0	0	1	SA0457
gi|29165615|ref|NC_002745.2|	527902	+	G	0	0	0	0	1	SA0457
gi|29165615|ref|NC_002745.2|	527903	+	A	0	0	0	0	1	SA0457
gi|29165615|ref|NC_002745.2|	527904	+	A	0	0	0	0	1	SA0457
gi|29165615|ref|NC_002745.2|	527905	+	U	1	0	0	0	0	SA0457
gi|29165615|ref|NC_002745.2|	527906	+	U	0	0	0	0	1	SA0457
gi|29165615|ref|NC_002745.2|	527907	+	A	0	0	1	0	0	SA0457
gi|29165615|ref|NC_002745.2|	527908	+	A	1	0	0	0	0	SA0457
gi|29165615|ref|NC_002745.2|	527914	+	G	0	1	0	0	0	SA0457
gi|29165615|ref|NC_002745.2|	527919	+	G	0	1	0	0	0	SA0457
gi|29165615|ref|NC_002745.2|	527922	+	A	0	0	1	0	2	SA0457
gi|29165615|ref|NC_002745.2|	527926	+	G	0	0	2	0	1	SA0457
gi|29165615|ref|NC_002745.2|	527928	+	G	0	0	0	1	0	SA0457
gi|29165615|ref|NC_002745.2|	527932	+	A	0	2	0	3	1	SA0457
gi|29165615|ref|NC_002745.2|	527933	+	U	0	0	1	0	0	SA0457
gi|29165615|ref|NC_002745.2|	527934	+	G	0	1	1	0	0	SA0457
gi|29165615|ref|NC_002745.2|	527935	+	U	1	0	0	0	0	SA0457
gi|29165615|ref|NC_002745.2|	527936	+	U	0	0	1	0	0	SA0457
gi|29165615|ref|NC_002745.2|	527938	+	U	0	3	4	0	1	SA0457
gi|29165615|ref|NC_002745.2|	527939	+	U	0	0	3	1	0	SA0457
gi|29165615|ref|NC_002745.2|	527940	+	A	0	1	2	0	0	SA0457
gi|29165615|ref|NC_002745.2|	527941	+	U	0	0	1	0	0	SA0457
gi|29165615|ref|NC_002745.2|	527946	+	C	0	0	1	0	0	SA0457
gi|29165615|ref|NC_002745.2|	527948	+	G	0	1	0	0	1	SA0457
gi|29165615|ref|NC_002745.2|	527949	+	U	0	0	1	0	1	SA0457
gi|29165615|ref|NC_002745.2|	527950	+	A	0	0	2	0	0	SA0457
gi|29165615|ref|NC_002745.2|	527951	+	C	0	1	1	0	0	SA0457
gi|29165615|ref|NC_002745.2|	527956	+	A	0	1	0	1	3	SA0457
gi|29165615|ref|NC_002745.2|	527957	+	A	0	0	1	0	1	SA0457
gi|29165615|ref|NC_002745.2|	527958	+	A	0	1	0	0	0	SA0457
gi|29165615|ref|NC_002745.2|	527961	+	A	0	0	1	0	0	SA0457
gi|29165615|ref|NC_002745.2|	527962	+	A	0	4	0	1	1	SA0457
gi|29165615|ref|NC_002745.2|	527963	+	C	0	1	1	0	0	SA0457
gi|29165615|ref|NC_002745.2|	527965	+	A	1	3	1	0	2	SA0457
gi|29165615|ref|NC_002745.2|	527966	+	U	0	0	3	0	1	SA0457
gi|29165615|ref|NC_002745.2|	527967	+	A	0	0	0	0	2	SA0457
gi|29165615|ref|NC_002745.2|	527969	+	U	0	0	0	1	0	SA0457
gi|29165615|ref|NC_002745.2|	527970	+	A	0	1	0	0	2	SA0457
gi|29165615|ref|NC_002745.2|	527971	+	C	0	0	3	0	1	SA0457
gi|29165615|ref|NC_002745.2|	527972	+	G	0	2	0	0	0	SA0457
gi|29165615|ref|NC_002745.2|	527973	+	A	1	1	1	3	0	SA0457
gi|29165615|ref|NC_002745.2|	527976	+	G	0	0	0	0	2	SA0457
gi|29165615|ref|NC_002745.2|	527977	+	A	0	0	1	0	0	SA0457
gi|29165615|ref|NC_002745.2|	527978	+	A	0	1	1	0	0	SA0457
gi|29165615|ref|NC_002745.2|	527979	+	A	0	2	2	0	0	SA0457
gi|29165615|ref|NC_002745.2|	527980	+	A	0	2	0	0	1	SA0457
gi|29165615|ref|NC_002745.2|	527981	+	U	0	0	1	0	0	SA0457
gi|29165615|ref|NC_002745.2|	527982	+	G	0	0	1	1	1	SA0457
gi|29165615|ref|NC_002745.2|	527983	+	G	0	0	0	1	0	SA0457
gi|29165615|ref|NC_002745.2|	527984	+	U	0	0	0	0	1	SA0457
gi|29165615|ref|NC_002745.2|	527985	+	G	0	0	0	1	0	SA0457
gi|29165615|ref|NC_002745.2|	527986	+	C	0	9	0	2	8	SA0457
gi|29165615|ref|NC_002745.2|	527987	+	A	0	0	1	3	1	SA0457
gi|29165615|ref|NC_002745.2|	527988	+	U	0	0	0	0	1	SA0457
gi|29165615|ref|NC_002745.2|	527989	+	G	0	0	0	2	1	SA0457
gi|29165615|ref|NC_002745.2|	527990	+	U	0	0	1	2	2	SA0457
gi|29165615|ref|NC_002745.2|	527991	+	A	0	2	2	0	2	SA0457
gi|29165615|ref|NC_002745.2|	527993	+	U	0	0	0	0	1	SA0457

gi|29165615|ref|NC_002745.2|	528000	+	U	0	0	1	0	0	SA0457
gi|29165615|ref|NC_002745.2|	528001	+	C	2	0	2	0	2	SA0457
gi|29165615|ref|NC_002745.2|	528002	+	U	0	0	1	0	0	SA0457
gi|29165615|ref|NC_002745.2|	528004	+	U	1	0	0	0	3	SA0457
gi|29165615|ref|NC_002745.2|	528005	+	U	0	1	0	0	0	SA0457
gi|29165615|ref|NC_002745.2|	528006	+	G	0	0	0	2	1	SA0457
gi|29165615|ref|NC_002745.2|	528007	+	U	1	1	1	1	1	SA0457
gi|29165615|ref|NC_002745.2|	528008	+	U	0	1	0	0	0	SA0457
gi|29165615|ref|NC_002745.2|	528009	+	A	0	1	3	0	1	SA0457
gi|29165615|ref|NC_002745.2|	528010	+	A	0	1	0	2	0	SA0457
gi|29165615|ref|NC_002745.2|	528011	+	U	0	0	1	1	0	SA0457
gi|29165615|ref|NC_002745.2|	528012	+	G	0	0	2	0	1	SA0457
gi|29165615|ref|NC_002745.2|	528013	+	A	0	1	0	0	0	SA0457
gi|29165615|ref|NC_002745.2|	528015	+	G	0	0	2	0	0	SA0457
gi|29165615|ref|NC_002745.2|	528016	+	C	0	1	0	0	0	SA0457
gi|29165615|ref|NC_002745.2|	528017	+	U	0	0	0	0	2	SA0457
gi|29165615|ref|NC_002745.2|	528020	+	C	0	0	0	1	1	SA0457
gi|29165615|ref|NC_002745.2|	528021	+	G	0	0	1	0	0	SA0457
gi|29165615|ref|NC_002745.2|	528022	+	U	0	0	0	1	1	SA0457
gi|29165615|ref|NC_002745.2|	528025	+	G	0	0	0	0	1	SA0457
gi|29165615|ref|NC_002745.2|	528029	+	U	1	0	0	0	0	SA0457
gi|29165615|ref|NC_002745.2|	528030	+	A	0	4	0	0	0	SA0457
gi|29165615|ref|NC_002745.2|	528031	+	A	0	4	1	1	1	SA0457
gi|29165615|ref|NC_002745.2|	528033	+	A	0	2	0	0	0	SA0457
gi|29165615|ref|NC_002745.2|	528035	+	U	0	1	0	1	0	SA0457
gi|29165615|ref|NC_002745.2|	528036	+	A	0	0	1	0	0	SA0457
gi|29165615|ref|NC_002745.2|	528039	+	G	0	1	0	0	0	SA0457
gi|29165615|ref|NC_002745.2|	528040	+	U	0	0	2	0	0	SA0457
gi|29165615|ref|NC_002745.2|	528044	+	A	0	1	0	0	0	SA0457
gi|29165615|ref|NC_002745.2|	528048	+	U	0	0	0	0	1	SA0457
gi|29165615|ref|NC_002745.2|	528050	+	U	3	0	0	0	0	SA0457
gi|29165615|ref|NC_002745.2|	528064	+	C	0	2	0	2	0	SA0457
gi|29165615|ref|NC_002745.2|	528065	+	A	0	0	0	0	2	SA0457
gi|29165615|ref|NC_002745.2|	528076	+	U	0	1	1	0	0	SA0457
gi|29165615|ref|NC_002745.2|	528079	+	G	0	0	0	1	0	SA0457
gi|29165615|ref|NC_002745.2|	528082	+	C	0	0	0	1	0	SA0457
gi|29165615|ref|NC_002745.2|	528084	+	G	0	0	1	0	0	SA0457
gi|29165615|ref|NC_002745.2|	528085	+	A	0	1	1	0	0	SA0457
gi|29165615|ref|NC_002745.2|	528089	+	U	0	1	0	0	0	SA0457
gi|29165615|ref|NC_002745.2|	528091	+	A	0	2	0	0	0	SA0457
gi|29165615|ref|NC_002745.2|	528092	+	G	0	0	0	0	1	SA0457
gi|29165615|ref|NC_002745.2|	528093	+	G	0	0	1	0	0	SA0457
gi|29165615|ref|NC_002745.2|	528094	+	U	0	0	1	0	1	SA0457
gi|29165615|ref|NC_002745.2|	528097	+	G	0	2	2	2	2	SA0457
gi|29165615|ref|NC_002745.2|	528098	+	A	0	0	1	0	1	SA0457
gi|29165615|ref|NC_002745.2|	528100	+	A	0	2	0	0	2	SA0457
gi|29165615|ref|NC_002745.2|	528102	+	U	0	0	0	1	0	SA0457
gi|29165615|ref|NC_002745.2|	528104	+	U	0	0	0	0	1	SA0457
gi|29165615|ref|NC_002745.2|	528107	+	A	0	0	1	0	0	SA0457
gi|29165615|ref|NC_002745.2|	528108	+	G	0	0	0	0	1	SA0457
gi|29165615|ref|NC_002745.2|	528109	+	A	0	1	0	0	3	SA0457
gi|29165615|ref|NC_002745.2|	528110	+	A	0	2	0	0	0	SA0457
gi|29165615|ref|NC_002745.2|	528111	+	A	0	3	0	0	0	SA0457
gi|29165615|ref|NC_002745.2|	528114	+	A	0	2	0	1	2	SA0457
gi|29165615|ref|NC_002745.2|	528115	+	A	0	3	2	2	1	SA0457
gi|29165615|ref|NC_002745.2|	528116	+	A	0	0	0	1	1	SA0457
gi|29165615|ref|NC_002745.2|	528117	+	A	0	6	1	2	2	SA0457
gi|29165615|ref|NC_002745.2|	528120	+	G	1	0	0	0	0	SA0457
gi|29165615|ref|NC_002745.2|	528121	+	C	0	1	1	4	1	SA0457
gi|29165615|ref|NC_002745.2|	528123	+	G	0	0	1	0	0	SA0457
gi|29165615|ref|NC_002745.2|	528124	+	A	0	2	0	4	1	SA0457
gi|29165615|ref|NC_002745.2|	528127	+	U	0	2	0	0	0	SA0457
gi|29165615|ref|NC_002745.2|	528129	+	A	0	5	1	0	0	SA0457
gi|29165615|ref|NC_002745.2|	528130	+	A	0	1	0	1	0	SA0457
gi|29165615|ref|NC_002745.2|	528131	+	A	0	1	0	0	0	SA0457
gi|29165615|ref|NC_002745.2|	528132	+	G	0	4	2	1	0	SA0457
gi|29165615|ref|NC_002745.2|	528133	+	A	0	0	1	0	0	SA0457
gi|29165615|ref|NC_002745.2|	528134	+	U	0	0	1	0	0	SA0457
gi|29165615|ref|NC_002745.2|	528149	+	A	0	1	0	0	0	SA0457
gi|29165615|ref|NC_002745.2|	528151	+	A	0	0	0	1	0	SA0457
gi|29165615|ref|NC_002745.2|	528155	+	A	0	0	1	0	1	SA0457
gi|29165615|ref|NC_002745.2|	528159	+	U	0	0	0	0	1	SA0457
gi|29165615|ref|NC_002745.2|	528160	+	A	0	1	0	1	0	SA0457
gi|29165615|ref|NC_002745.2|	528161	+	U	0	1	0	1	0	SA0457
gi|29165615|ref|NC_002745.2|	528162	+	A	0	0	0	0	1	SA0457
gi|29165615|ref|NC_002745.2|	528163	+	U	0	0	0	2	0	SA0457
gi|29165615|ref|NC_002745.2|	528166	+	G	0	0	0	1	0	SA0457
gi|29165615|ref|NC_002745.2|	528168	+	G	0	0	1	0	0	SA0457
gi|29165615|ref|NC_002745.2|	528177	+	A	0	0	0	0	1	SA0457
gi|29165615|ref|NC_002745.2|	528188	+	U	0	1	0	0	0	SA0457
gi|29165615|ref|NC_002745.2|	528195	+	A	0	0	0	0	1	SA0457

gi|29165615|ref|NC_002745.2|	528196 +	U	0	0	2	0	0	SA0457
gi|29165615|ref|NC_002745.2|	528197 +	U	0	0	0	1	0	SA0457
gi|29165615|ref|NC_002745.2|	528218 +	U	0	0	2	0	0	SA0457
gi|29165615|ref|NC_002745.2|	528222 +	U	0	0	1	0	0	SA0457
gi|29165615|ref|NC_002745.2|	528229 +	G	0	0	0	0	1	SA0457
gi|29165615|ref|NC_002745.2|	528232 +	A	0	0	1	0	0	SA0457
gi|29165615|ref|NC_002745.2|	528234 +	A	0	0	0	1	0	SA0457
gi|29165615|ref|NC_002745.2|	528238 +	A	0	2	1	2	1	SA0457
gi|29165615|ref|NC_002745.2|	528239 +	A	0	0	2	0	0	SA0457
gi|29165615|ref|NC_002745.2|	528240 +	U	0	1	0	0	1	SA0457
gi|29165615|ref|NC_002745.2|	528241 +	U	0	1	0	0	2	SA0457
gi|29165615|ref|NC_002745.2|	528242 +	U	0	2	0	0	0	SA0457
gi|29165615|ref|NC_002745.2|	528243 +	A	0	6	0	2	3	SA0457
gi|29165615|ref|NC_002745.2|	528244 +	A	0	3	1	0	1	SA0457
gi|29165615|ref|NC_002745.2|	528246 +	A	0	0	1	0	1	SA0457
gi|29165615|ref|NC_002745.2|	528251 +	C	1	1	0	1	1	SA0457
gi|29165615|ref|NC_002745.2|	528254 +	C	0	1	0	0	0	SA0457
gi|29165615|ref|NC_002745.2|	528255 +	G	0	2	0	0	0	SA0457
gi|29165615|ref|NC_002745.2|	528257 +	C	0	1	0	0	0	SA0457
gi|29165615|ref|NC_002745.2|	528258 +	A	0	2	0	0	1	SA0457
gi|29165615|ref|NC_002745.2|	528259 +	A	0	1	1	0	2	SA0457
gi|29165615|ref|NC_002745.2|	528261 +	G	0	0	2	0	0	SA0457
gi|29165615|ref|NC_002745.2|	528263 +	U	0	0	0	1	0	SA0457
gi|29165615|ref|NC_002745.2|	528265 +	C	0	0	1	0	0	SA0457
gi|29165615|ref|NC_002745.2|	528266 +	A	0	1	0	0	0	SA0457
gi|29165615|ref|NC_002745.2|	528267 +	U	0	0	1	0	0	SA0457
gi|29165615|ref|NC_002745.2|	528270 +	G	0	1	0	0	0	SA0457
gi|29165615|ref|NC_002745.2|	528278 +	C	0	2	1	1	0	SA0457
gi|29165615|ref|NC_002745.2|	528279 +	A	0	3	0	0	1	SA0457
gi|29165615|ref|NC_002745.2|	528280 +	A	0	1	1	0	1	SA0457
gi|29165615|ref|NC_002745.2|	528283 +	U	0	1	0	0	0	SA0457
gi|29165615|ref|NC_002745.2|	528285 +	A	0	1	1	0	1	SA0457
gi|29165615|ref|NC_002745.2|	528287 +	U	0	1	0	0	0	SA0457
gi|29165615|ref|NC_002745.2|	528288 +	U	0	0	0	0	1	SA0457
gi|29165615|ref|NC_002745.2|	528289 +	U	1	0	1	0	0	SA0457
gi|29165615|ref|NC_002745.2|	528292 +	U	0	0	0	1	1	SA0457
gi|29165615|ref|NC_002745.2|	528293 +	A	0	0	0	0	1	SA0457
gi|29165615|ref|NC_002745.2|	528295 +	C	0	1	0	0	0	SA0457
gi|29165615|ref|NC_002745.2|	528296 +	A	0	0	2	0	0	SA0457
gi|29165615|ref|NC_002745.2|	528297 +	C	0	1	0	0	1	SA0457
gi|29165615|ref|NC_002745.2|	528298 +	C	0	0	0	0	1	SA0457
gi|29165615|ref|NC_002745.2|	528302 +	A	0	0	1	0	0	SA0457
gi|29165615|ref|NC_002745.2|	528305 +	A	0	0	1	0	0	SA0457
gi|29165615|ref|NC_002745.2|	528306 +	A	0	1	1	0	0	SA0457
gi|29165615|ref|NC_002745.2|	528312 +	G	0	0	1	0	0	SA0457
gi|29165615|ref|NC_002745.2|	528313 +	A	0	0	0	1	4	SA0457
gi|29165615|ref|NC_002745.2|	528316 +	A	0	0	0	1	0	SA0457
gi|29165615|ref|NC_002745.2|	528319 +	U	1	0	1	0	0	SA0457
gi|29165615|ref|NC_002745.2|	528321 +	U	0	0	1	0	0	SA0457
gi|29165615|ref|NC_002745.2|	528328 +	C	0	0	0	1	0	SA0457
gi|29165615|ref|NC_002745.2|	528329 +	A	1	0	0	0	1	SA0457
gi|29165615|ref|NC_002745.2|	528341 +	A	0	1	0	1	0	SA0457
gi|29165615|ref|NC_002745.2|	528342 +	A	0	1	0	0	0	SA0457
gi|29165615|ref|NC_002745.2|	528346 +	C	0	0	1	0	0	SA0457
gi|29165615|ref|NC_002745.2|	528348 +	G	0	0	1	0	0	SA0457
gi|29165615|ref|NC_002745.2|	528352 +	A	0	0	1	0	0	SA0457
gi|29165615|ref|NC_002745.2|	528353 +	C	0	0	0	1	0	SA0457
gi|29165615|ref|NC_002745.2|	528357 +	C	0	1	0	0	0	SA0457
gi|29165615|ref|NC_002745.2|	528360 +	A	0	0	1	0	0	SA0457
gi|29165615|ref|NC_002745.2|	528363 +	G	0	1	0	0	0	SA0457
gi|29165615|ref|NC_002745.2|	528365 +	C	0	0	0	1	0	SA0457
gi|29165615|ref|NC_002745.2|	528367 +	G	3	0	0	0	2	SA0457
gi|29165615|ref|NC_002745.2|	528370 +	U	0	0	0	1	0	SA0457
gi|29165615|ref|NC_002745.2|	528373 +	C	1	0	1	0	0	SA0457
gi|29165615|ref|NC_002745.2|	528380 +	A	0	0	0	0	1	SA0457
gi|29165615|ref|NC_002745.2|	528381 +	A	0	1	0	0	0	SA0457
gi|29165615|ref|NC_002745.2|	528391 +	A	0	0	0	1	0	SA0457
gi|29165615|ref|NC_002745.2|	528392 +	A	0	0	1	0	0	SA0457
gi|29165615|ref|NC_002745.2|	528394 +	C	0	0	0	0	1	SA0457
gi|29165615|ref|NC_002745.2|	528399 +	A	0	0	0	0	1	SA0457
gi|29165615|ref|NC_002745.2|	528405 +	G	0	0	0	0	1	SA0457
gi|29165615|ref|NC_002745.2|	528410 +	U	0	0	0	0	1	SA0457
gi|29165615|ref|NC_002745.2|	528418 +	A	0	0	0	0	1	SA0457
gi|29165615|ref|NC_002745.2|	528419 +	A	0	0	1	0	0	SA0457
gi|29165615|ref|NC_002745.2|	528430 +	A	0	1	0	0	0	-
gi|29165615|ref|NC_002745.2|	528431 +	U	0	1	0	0	0	-
gi|29165615|ref|NC_002745.2|	528434 +	A	0	1	0	1	0	-
gi|29165615|ref|NC_002745.2|	528440 +	G	0	0	1	0	0	-
gi|29165615|ref|NC_002745.2|	528441 +	C	0	0	1	0	0	-
gi|29165615|ref|NC_002745.2|	528447 +	U	0	0	0	0	1	-
gi|29165615|ref|NC_002745.2|	528448 +	A	0	1	0	0	3	-

gi|29165615|ref|NC_002745.2|	528450 +	A	0	1	0	0	0	-
gi|29165615|ref|NC_002745.2|	528454 +	A	0	0	0	1	0	-
gi|29165615|ref|NC_002745.2|	528455 +	U	0	0	0	0	1	-
gi|29165615|ref|NC_002745.2|	528457 +	A	0	4	0	0	1	-
gi|29165615|ref|NC_002745.2|	528465 +	U	0	1	1	2	2	-
gi|29165615|ref|NC_002745.2|	528466 +	A	0	5	3	3	4	-
gi|29165615|ref|NC_002745.2|	528468 +	U	0	0	0	1	1	-
gi|29165615|ref|NC_002745.2|	528473 +	A	1	0	0	0	0	-
gi|29165615|ref|NC_002745.2|	528476 +	U	0	1	0	0	0	-
gi|29165615|ref|NC_002745.2|	528478 +	A	0	0	1	0	2	-
gi|29165615|ref|NC_002745.2|	528480 +	C	0	0	0	1	0	-
gi|29165615|ref|NC_002745.2|	528483 +	U	0	1	0	0	1	-
gi|29165615|ref|NC_002745.2|	528487 +	A	0	1	0	0	1	-
gi|29165615|ref|NC_002745.2|	528495 +	A	0	0	1	1	0	-
gi|29165615|ref|NC_002745.2|	528500 +	C	0	8	4	6	5	-
gi|29165615|ref|NC_002745.2|	528501 +	A	0	5	3	0	2	-
gi|29165615|ref|NC_002745.2|	528507 +	A	0	0	0	0	1	-
gi|29165615|ref|NC_002745.2|	528509 +	G	0	0	0	0	1	-
gi|29165615|ref|NC_002745.2|	528510 +	G	0	0	1	1	0	-
gi|29165615|ref|NC_002745.2|	528516 +	C	0	2	1	5	4	-
gi|29165615|ref|NC_002745.2|	528517 +	A	0	1	2	1	1	-
gi|29165615|ref|NC_002745.2|	528520 +	U	0	0	0	0	1	-
gi|29165615|ref|NC_002745.2|	528521 +	U	0	1	0	2	4	-
gi|29165615|ref|NC_002745.2|	528522 +	A	0	7	5	3	6	-
gi|29165615|ref|NC_002745.2|	528523 +	U	0	6	6	7	7	-
gi|29165615|ref|NC_002745.2|	528524 +	A	0	2	4	1	1	-
gi|29165615|ref|NC_002745.2|	528525 +	U	0	0	1	0	1	-
gi|29165615|ref|NC_002745.2|	528526 +	A	0	0	0	1	0	-
gi|29165615|ref|NC_002745.2|	528527 +	U	0	0	1	0	0	-
gi|29165615|ref|NC_002745.2|	528528 +	A	0	3	2	1	2	-
gi|29165615|ref|NC_002745.2|	528529 +	A	1	7	4	6	4	-
gi|29165615|ref|NC_002745.2|	528530 +	U	0	0	0	0	4	-
gi|29165615|ref|NC_002745.2|	528531 +	A	0	0	0	0	1	-
gi|29165615|ref|NC_002745.2|	528533 +	U	0	0	1	1	1	-
gi|29165615|ref|NC_002745.2|	528536 +	U	0	0	1	0	1	-
gi|29165615|ref|NC_002745.2|	528538 +	G	0	9	5	4	7	-
gi|29165615|ref|NC_002745.2|	528541 +	U	0	0	0	0	2	-
gi|29165615|ref|NC_002745.2|	528543 +	A	0	1	0	0	0	-
gi|29165615|ref|NC_002745.2|	528545 +	U	0	4	1	1	6	-
gi|29165615|ref|NC_002745.2|	528546 +	A	0	3	0	0	0	-
gi|29165615|ref|NC_002745.2|	528547 +	A	0	1	1	0	0	-
gi|29165615|ref|NC_002745.2|	528548 +	A	0	6	0	0	0	-
gi|29165615|ref|NC_002745.2|	528549 +	U	0	0	1	0	0	-
gi|29165615|ref|NC_002745.2|	528551 +	A	0	0	1	0	0	-
gi|29165615|ref|NC_002745.2|	528559 +	A	0	1	0	0	1	-
gi|29165615|ref|NC_002745.2|	528561 +	U	0	0	1	0	0	-
gi|29165615|ref|NC_002745.2|	528564 +	A	0	1	0	0	0	-
gi|29165615|ref|NC_002745.2|	528566 +	A	0	1	1	0	0	SA0458
gi|29165615|ref|NC_002745.2|	528567 +	U	0	1	0	0	0	SA0458
gi|29165615|ref|NC_002745.2|	528568 +	G	0	2	0	0	0	SA0458
gi|29165615|ref|NC_002745.2|	528571 +	A	0	1	0	1	0	SA0458
gi|29165615|ref|NC_002745.2|	528572 +	A	0	1	0	0	0	SA0458
gi|29165615|ref|NC_002745.2|	528573 +	A	0	0	0	0	1	SA0458
gi|29165615|ref|NC_002745.2|	528576 +	A	0	0	0	0	1	SA0458
gi|29165615|ref|NC_002745.2|	528578 +	G	0	0	1	0	0	SA0458
gi|29165615|ref|NC_002745.2|	528579 +	A	0	4	1	0	2	SA0458
gi|29165615|ref|NC_002745.2|	528580 +	A	0	0	2	0	0	SA0458
gi|29165615|ref|NC_002745.2|	528581 +	U	1	0	1	0	0	SA0458
gi|29165615|ref|NC_002745.2|	528582 +	A	0	0	0	1	0	SA0458
gi|29165615|ref|NC_002745.2|	528583 +	U	0	0	0	1	0	SA0458
gi|29165615|ref|NC_002745.2|	528593 +	U	0	1	0	0	0	SA0458
gi|29165615|ref|NC_002745.2|	528595 +	A	0	0	0	0	1	SA0458
gi|29165615|ref|NC_002745.2|	528604 +	U	0	0	1	0	0	SA0458
gi|29165615|ref|NC_002745.2|	528609 +	C	0	5	0	1	2	SA0458
gi|29165615|ref|NC_002745.2|	528610 +	A	0	1	2	2	4	SA0458
gi|29165615|ref|NC_002745.2|	528613 +	G	0	1	1	0	0	SA0458
gi|29165615|ref|NC_002745.2|	528614 +	A	0	0	0	0	2	SA0458
gi|29165615|ref|NC_002745.2|	528615 +	A	0	0	1	1	0	SA0458
gi|29165615|ref|NC_002745.2|	528618 +	G	1	1	2	0	0	SA0458
gi|29165615|ref|NC_002745.2|	528619 +	A	0	0	0	0	1	SA0458
gi|29165615|ref|NC_002745.2|	528620 +	A	0	1	0	1	0	SA0458
gi|29165615|ref|NC_002745.2|	528621 +	A	1	0	1	0	0	SA0458
gi|29165615|ref|NC_002745.2|	528622 +	C	0	2	0	0	0	SA0458
gi|29165615|ref|NC_002745.2|	528623 +	G	0	1	0	1	2	SA0458
gi|29165615|ref|NC_002745.2|	528624 +	A	0	1	2	1	3	SA0458
gi|29165615|ref|NC_002745.2|	528625 +	A	0	1	0	1	1	SA0458
gi|29165615|ref|NC_002745.2|	528626 +	G	0	1	0	0	0	SA0458
gi|29165615|ref|NC_002745.2|	528627 +	C	1	0	1	0	2	SA0458
gi|29165615|ref|NC_002745.2|	528628 +	A	0	0	2	1	0	SA0458
gi|29165615|ref|NC_002745.2|	528629 +	U	0	2	0	0	0	SA0458
gi|29165615|ref|NC_002745.2|	528630 +	U	0	0	2	0	0	SA0458

gi|29165615|ref|NC_002745.2|	528631	+	A	0	0	2	0	1	SA0458
gi|29165615|ref|NC_002745.2|	528635	+	C	0	0	1	0	0	SA0458
gi|29165615|ref|NC_002745.2|	528636	+	A	1	1	0	1	2	SA0458
gi|29165615|ref|NC_002745.2|	528637	+	A	1	0	0	0	0	SA0458
gi|29165615|ref|NC_002745.2|	528638	+	G	0	1	0	1	2	SA0458
gi|29165615|ref|NC_002745.2|	528639	+	A	0	0	1	1	1	SA0458
gi|29165615|ref|NC_002745.2|	528640	+	A	1	0	1	0	0	SA0458
gi|29165615|ref|NC_002745.2|	528641	+	G	0	1	1	0	2	SA0458
gi|29165615|ref|NC_002745.2|	528642	+	U	0	0	0	0	3	SA0458
gi|29165615|ref|NC_002745.2|	528643	+	U	0	0	0	0	1	SA0458
gi|29165615|ref|NC_002745.2|	528647	+	G	0	1	0	0	0	SA0458
gi|29165615|ref|NC_002745.2|	528650	+	C	0	1	2	0	1	SA0458
gi|29165615|ref|NC_002745.2|	528651	+	A	0	1	0	0	0	SA0458
gi|29165615|ref|NC_002745.2|	528655	+	A	0	0	1	0	0	SA0458
gi|29165615|ref|NC_002745.2|	528660	+	U	1	0	0	0	0	SA0458
gi|29165615|ref|NC_002745.2|	528665	+	C	0	1	0	0	0	SA0458
gi|29165615|ref|NC_002745.2|	528667	+	A	0	0	1	0	0	SA0458
gi|29165615|ref|NC_002745.2|	528675	+	G	0	0	1	0	0	SA0458
gi|29165615|ref|NC_002745.2|	528678	+	C	0	1	0	0	0	SA0458
gi|29165615|ref|NC_002745.2|	528682	+	U	0	0	0	1	0	SA0458
gi|29165615|ref|NC_002745.2|	528684	+	A	0	1	0	0	0	SA0458
gi|29165615|ref|NC_002745.2|	528685	+	A	0	1	0	0	0	SA0458
gi|29165615|ref|NC_002745.2|	528688	+	U	0	1	0	0	0	SA0458
gi|29165615|ref|NC_002745.2|	528712	+	U	0	0	0	0	1	SA0458
gi|29165615|ref|NC_002745.2|	528722	+	G	0	1	0	0	0	SA0458
gi|29165615|ref|NC_002745.2|	528749	+	A	0	0	1	0	0	SA0458
gi|29165615|ref|NC_002745.2|	528755	+	C	0	0	1	0	0	SA0458
gi|29165615|ref|NC_002745.2|	528756	+	A	0	0	0	0	1	SA0458
gi|29165615|ref|NC_002745.2|	528758	+	C	0	1	0	0	0	SA0458
gi|29165615|ref|NC_002745.2|	528772	+	U	0	0	1	0	1	SA0458
gi|29165615|ref|NC_002745.2|	528779	+	C	0	1	0	0	0	SA0458
gi|29165615|ref|NC_002745.2|	528783	+	A	0	0	1	0	0	SA0458
gi|29165615|ref|NC_002745.2|	528793	+	A	0	1	0	0	0	SA0458
gi|29165615|ref|NC_002745.2|	528797	+	U	0	0	0	1	0	SA0458
gi|29165615|ref|NC_002745.2|	528798	+	U	0	0	0	0	1	SA0458
gi|29165615|ref|NC_002745.2|	528799	+	A	1	0	0	0	0	SA0458
gi|29165615|ref|NC_002745.2|	528801	+	U	0	0	1	0	1	SA0458
gi|29165615|ref|NC_002745.2|	528802	+	U	1	0	1	0	1	SA0458
gi|29165615|ref|NC_002745.2|	528805	+	G	0	0	1	1	0	SA0458
gi|29165615|ref|NC_002745.2|	528810	+	A	0	1	0	0	0	SA0458
gi|29165615|ref|NC_002745.2|	528824	+	G	0	0	1	0	0	SA0458
gi|29165615|ref|NC_002745.2|	528825	+	C	1	0	0	0	0	SA0458
gi|29165615|ref|NC_002745.2|	528826	+	U	0	0	1	0	0	SA0458
gi|29165615|ref|NC_002745.2|	528836	+	A	1	0	0	0	0	SA0458
gi|29165615|ref|NC_002745.2|	528844	+	U	0	1	0	0	0	SA0458
gi|29165615|ref|NC_002745.2|	528857	+	U	0	0	0	0	1	SA0458
gi|29165615|ref|NC_002745.2|	528861	+	A	0	0	0	0	1	SA0458
gi|29165615|ref|NC_002745.2|	528864	+	G	0	0	0	0	1	SA0458
gi|29165615|ref|NC_002745.2|	528870	+	C	0	0	0	2	0	SA0458
gi|29165615|ref|NC_002745.2|	528874	+	A	0	0	1	0	0	SA0458
gi|29165615|ref|NC_002745.2|	528878	+	G	0	0	1	0	0	SA0458
gi|29165615|ref|NC_002745.2|	528879	+	A	1	4	1	4	1	SA0458
gi|29165615|ref|NC_002745.2|	528880	+	U	0	0	0	1	0	SA0458
gi|29165615|ref|NC_002745.2|	528881	+	A	0	1	2	0	0	SA0458
gi|29165615|ref|NC_002745.2|	528882	+	G	0	1	1	0	0	SA0458
gi|29165615|ref|NC_002745.2|	528883	+	A	0	0	0	1	0	SA0458
gi|29165615|ref|NC_002745.2|	528884	+	A	0	1	1	0	1	SA0458
gi|29165615|ref|NC_002745.2|	528891	+	G	0	0	1	0	0	SA0458
gi|29165615|ref|NC_002745.2|	528902	+	C	0	0	0	0	1	SA0458
gi|29165615|ref|NC_002745.2|	528903	+	C	0	1	1	0	1	SA0458
gi|29165615|ref|NC_002745.2|	528904	+	A	0	0	0	0	1	SA0458
gi|29165615|ref|NC_002745.2|	528907	+	C	0	0	0	0	1	SA0458
gi|29165615|ref|NC_002745.2|	528909	+	C	0	1	0	0	0	SA0458
gi|29165615|ref|NC_002745.2|	528912	+	C	0	0	1	0	0	SA0458
gi|29165615|ref|NC_002745.2|	528916	+	A	0	0	0	0	1	SA0458
gi|29165615|ref|NC_002745.2|	528918	+	U	0	1	0	0	0	SA0458
gi|29165615|ref|NC_002745.2|	528926	+	A	0	0	0	1	0	SA0458
gi|29165615|ref|NC_002745.2|	528931	+	A	1	0	0	0	0	SA0458
gi|29165615|ref|NC_002745.2|	528932	+	A	0	1	0	0	0	SA0458
gi|29165615|ref|NC_002745.2|	528938	+	A	0	0	0	0	1	SA0458
gi|29165615|ref|NC_002745.2|	528949	+	A	0	0	0	1	0	SA0458
gi|29165615|ref|NC_002745.2|	528955	+	U	1	0	0	0	0	SA0458
gi|29165615|ref|NC_002745.2|	528968	+	G	0	0	1	0	1	SA0458
gi|29165615|ref|NC_002745.2|	528969	+	A	0	1	1	0	0	SA0458
gi|29165615|ref|NC_002745.2|	528974	+	C	0	0	0	2	0	SA0458
gi|29165615|ref|NC_002745.2|	528979	+	A	0	1	0	0	0	SA0458
gi|29165615|ref|NC_002745.2|	528981	+	C	0	1	0	0	0	SA0458
gi|29165615|ref|NC_002745.2|	528985	+	A	0	0	3	0	0	SA0458
gi|29165615|ref|NC_002745.2|	528986	+	A	0	7	1	0	5	SA0458
gi|29165615|ref|NC_002745.2|	528996	+	U	0	0	0	2	0	SA0458
gi|29165615|ref|NC_002745.2|	529002	+	A	0	1	0	1	2	SA0458

gi|29165615|ref|NC_002745.2|	529003 +	U	0	0	1	0	0	SA0458
gi|29165615|ref|NC_002745.2|	529004 +	A	0	0	0	0	1	SA0458
gi|29165615|ref|NC_002745.2|	529008 +	C	0	1	1	0	0	SA0458
gi|29165615|ref|NC_002745.2|	529010 +	A	0	1	3	1	0	SA0458
gi|29165615|ref|NC_002745.2|	529011 +	U	0	2	0	0	0	SA0458
gi|29165615|ref|NC_002745.2|	529012 +	U	0	1	0	1	0	SA0458
gi|29165615|ref|NC_002745.2|	529013 +	G	1	0	0	0	0	SA0458
gi|29165615|ref|NC_002745.2|	529014 +	A	0	1	0	0	1	SA0458
gi|29165615|ref|NC_002745.2|	529015 +	C	0	0	1	0	0	SA0458
gi|29165615|ref|NC_002745.2|	529016 +	C	0	0	4	0	0	SA0458
gi|29165615|ref|NC_002745.2|	529017 +	A	0	0	2	0	0	SA0458
gi|29165615|ref|NC_002745.2|	529018 +	C	0	0	1	0	0	SA0458
gi|29165615|ref|NC_002745.2|	529019 +	U	0	0	1	0	0	SA0458
gi|29165615|ref|NC_002745.2|	529020 +	U	0	1	2	0	0	SA0458
gi|29165615|ref|NC_002745.2|	529022 +	A	1	0	0	0	0	SA0458
gi|29165615|ref|NC_002745.2|	529024 +	G	0	0	0	1	0	SA0458
gi|29165615|ref|NC_002745.2|	529034 +	A	0	1	0	0	0	SA0458
gi|29165615|ref|NC_002745.2|	529037 +	C	0	0	0	0	2	SA0458
gi|29165615|ref|NC_002745.2|	529046 +	C	0	0	1	1	0	SA0458
gi|29165615|ref|NC_002745.2|	529047 +	A	0	1	0	0	0	SA0458
gi|29165615|ref|NC_002745.2|	529052 +	A	0	0	0	0	1	SA0458
gi|29165615|ref|NC_002745.2|	529058 +	G	0	1	0	0	0	SA0458
gi|29165615|ref|NC_002745.2|	529059 +	A	0	1	0	0	0	SA0458
gi|29165615|ref|NC_002745.2|	529073 +	C	0	0	0	0	2	SA0458
gi|29165615|ref|NC_002745.2|	529098 +	C	0	1	0	0	0	SA0458
gi|29165615|ref|NC_002745.2|	529099 +	A	0	1	0	0	0	SA0458
gi|29165615|ref|NC_002745.2|	529101 +	A	0	0	1	0	0	SA0458
gi|29165615|ref|NC_002745.2|	529104 +	A	0	0	1	0	0	SA0458
gi|29165615|ref|NC_002745.2|	529105 +	U	0	0	0	1	0	SA0458
gi|29165615|ref|NC_002745.2|	529111 +	U	0	0	0	0	2	SA0458
gi|29165615|ref|NC_002745.2|	529116 +	C	2	0	0	0	0	SA0458
gi|29165615|ref|NC_002745.2|	529117 +	A	0	0	1	0	0	SA0458
gi|29165615|ref|NC_002745.2|	529119 +	G	0	1	0	0	0	SA0458
gi|29165615|ref|NC_002745.2|	529129 +	A	0	8	0	0	3	SA0458
gi|29165615|ref|NC_002745.2|	529130 +	U	0	0	0	1	0	SA0458
gi|29165615|ref|NC_002745.2|	529131 +	U	0	0	0	0	1	SA0458
gi|29165615|ref|NC_002745.2|	529134 +	C	0	1	0	0	0	SA0458
gi|29165615|ref|NC_002745.2|	529138 +	C	0	1	0	0	0	SA0458
gi|29165615|ref|NC_002745.2|	529143 +	U	0	0	0	2	0	SA0458
gi|29165615|ref|NC_002745.2|	529144 +	A	0	0	0	3	0	SA0458
gi|29165615|ref|NC_002745.2|	529145 +	A	0	1	0	0	0	SA0458
gi|29165615|ref|NC_002745.2|	529148 +	A	0	1	1	0	0	SA0458
gi|29165615|ref|NC_002745.2|	529149 +	C	0	0	0	0	1	SA0458
gi|29165615|ref|NC_002745.2|	529158 +	C	0	1	0	0	0	SA0458
gi|29165615|ref|NC_002745.2|	529160 +	A	0	1	1	0	0	SA0458
gi|29165615|ref|NC_002745.2|	529162 +	U	0	0	0	1	0	SA0458
gi|29165615|ref|NC_002745.2|	529178 +	C	0	0	2	0	0	SA0458
gi|29165615|ref|NC_002745.2|	529180 +	U	0	0	0	1	0	SA0458
gi|29165615|ref|NC_002745.2|	529181 +	A	1	0	0	0	0	SA0458
gi|29165615|ref|NC_002745.2|	529182 +	G	0	0	1	0	0	SA0458
gi|29165615|ref|NC_002745.2|	529184 +	C	1	0	0	0	0	SA0458
gi|29165615|ref|NC_002745.2|	529185 +	C	2	0	0	0	0	SA0458
gi|29165615|ref|NC_002745.2|	529186 +	A	0	1	2	0	0	SA0458
gi|29165615|ref|NC_002745.2|	529187 +	A	0	2	0	0	2	SA0458
gi|29165615|ref|NC_002745.2|	529188 +	A	0	0	1	1	2	SA0458
gi|29165615|ref|NC_002745.2|	529189 +	U	0	0	1	1	2	SA0458
gi|29165615|ref|NC_002745.2|	529190 +	G	0	0	1	0	1	SA0458
gi|29165615|ref|NC_002745.2|	529191 +	U	1	0	0	0	0	SA0458
gi|29165615|ref|NC_002745.2|	529207 +	C	0	3	0	0	1	SA0458
gi|29165615|ref|NC_002745.2|	529233 +	C	0	0	0	0	2	SA0458
gi|29165615|ref|NC_002745.2|	529251 +	A	0	0	0	0	1	SA0458
gi|29165615|ref|NC_002745.2|	529256 +	A	0	1	0	0	0	SA0458
gi|29165615|ref|NC_002745.2|	529266 +	C	0	0	1	0	0	SA0458
gi|29165615|ref|NC_002745.2|	529295 +	U	0	0	0	0	1	SA0458
gi|29165615|ref|NC_002745.2|	529303 +	U	0	1	0	0	0	SA0458
gi|29165615|ref|NC_002745.2|	529327 +	U	1	0	0	0	0	SA0458
gi|29165615|ref|NC_002745.2|	529338 +	A	0	0	0	0	2	SA0458
gi|29165615|ref|NC_002745.2|	529374 +	A	0	2	0	0	0	SA0458
gi|29165615|ref|NC_002745.2|	529378 +	U	1	0	0	0	0	SA0458
gi|29165615|ref|NC_002745.2|	529380 +	C	0	1	0	0	0	SA0458
gi|29165615|ref|NC_002745.2|	529389 +	A	0	0	1	0	0	SA0458
gi|29165615|ref|NC_002745.2|	529405 +	A	0	0	1	0	0	SA0458
gi|29165615|ref|NC_002745.2|	529406 +	A	0	0	0	1	0	SA0458
gi|29165615|ref|NC_002745.2|	529412 +	A	0	1	0	0	0	SA0458
gi|29165615|ref|NC_002745.2|	529440 +	C	0	0	0	0	2	SA0458
gi|29165615|ref|NC_002745.2|	529443 +	A	0	1	0	0	0	SA0458
gi|29165615|ref|NC_002745.2|	529449 +	A	0	1	0	1	0	SA0458
gi|29165615|ref|NC_002745.2|	529450 +	A	0	0	1	0	0	SA0458
gi|29165615|ref|NC_002745.2|	529451 +	G	0	0	0	0	1	SA0458
gi|29165615|ref|NC_002745.2|	529452 +	A	0	1	0	1	1	SA0458
gi|29165615|ref|NC_002745.2|	529453 +	A	1	0	0	0	0	SA0458

gi|29165615|ref|NC_002745.2|	529472 +	A	0	0	1	0	0	SA0458
gi|29165615|ref|NC_002745.2|	529499 +	G	0	0	0	0	1	SA0458
gi|29165615|ref|NC_002745.2|	529502 +	A	0	0	0	0	1	SA0458
gi|29165615|ref|NC_002745.2|	529509 +	C	0	1	0	0	0	SA0458
gi|29165615|ref|NC_002745.2|	529531 +	A	0	1	0	0	1	SA0458
gi|29165615|ref|NC_002745.2|	529532 +	U	0	0	1	0	0	-
gi|29165615|ref|NC_002745.2|	529533 +	A	0	0	1	0	0	-
gi|29165615|ref|NC_002745.2|	529534 +	U	1	0	0	0	0	-
gi|29165615|ref|NC_002745.2|	529556 +	U	0	0	0	0	1	-
gi|29165615|ref|NC_002745.2|	529581 +	G	11	4	3	3	0	-
gi|29165615|ref|NC_002745.2|	529601 +	A	0	1	0	0	0	-
gi|29165615|ref|NC_002745.2|	529608 +	C	0	0	0	1	0	-
gi|29165615|ref|NC_002745.2|	529611 +	G	0	0	0	2	0	-
gi|29165615|ref|NC_002745.2|	529612 +	C	1	13	7	6	4	-
gi|29165615|ref|NC_002745.2|	529613 +	A	0	2	1	1	0	-
gi|29165615|ref|NC_002745.2|	529614 +	A	0	1	1	0	1	-
gi|29165615|ref|NC_002745.2|	529615 +	G	0	3	0	0	0	-
gi|29165615|ref|NC_002745.2|	529616 +	C	0	0	1	0	0	-
gi|29165615|ref|NC_002745.2|	529618 +	A	0	0	1	0	0	-
gi|29165615|ref|NC_002745.2|	529623 +	G	0	0	0	2	0	-
gi|29165615|ref|NC_002745.2|	529624 +	A	1	0	2	0	0	-
gi|29165615|ref|NC_002745.2|	529626 +	G	0	0	1	0	0	-
gi|29165615|ref|NC_002745.2|	529627 +	U	0	0	1	0	0	-
gi|29165615|ref|NC_002745.2|	529629 +	G	0	1	1	0	0	-
gi|29165615|ref|NC_002745.2|	529630 +	A	0	0	2	0	0	-
gi|29165615|ref|NC_002745.2|	529653 +	U	0	0	0	0	1	-
gi|29165615|ref|NC_002745.2|	529656 +	G	0	0	0	0	1	-
gi|29165615|ref|NC_002745.2|	529660 +	A	0	1	0	2	0	-
gi|29165615|ref|NC_002745.2|	529661 +	A	1	3	0	1	0	-
gi|29165615|ref|NC_002745.2|	529662 +	A	0	2	0	0	0	-
gi|29165615|ref|NC_002745.2|	529665 +	A	0	6	3	1	2	-
gi|29165615|ref|NC_002745.2|	529666 +	A	0	4	3	3	6	-
gi|29165615|ref|NC_002745.2|	529667 +	A	0	1	2	0	3	-
gi|29165615|ref|NC_002745.2|	529668 +	G	1	0	0	1	0	-
gi|29165615|ref|NC_002745.2|	529669 +	G	0	15	6	7	8	-
gi|29165615|ref|NC_002745.2|	529670 +	U	0	0	0	1	0	-
gi|29165615|ref|NC_002745.2|	529672 +	G	3	42	18	15	48	-
gi|29165615|ref|NC_002745.2|	529673 +	A	0	17	4	12	19	-
gi|29165615|ref|NC_002745.2|	529674 +	A	0	6	4	3	5	-
gi|29165615|ref|NC_002745.2|	529675 +	A	0	0	1	0	0	-
gi|29165615|ref|NC_002745.2|	529677 +	G	0	0	1	0	0	-
gi|29165615|ref|NC_002745.2|	529678 +	A	0	0	2	0	0	-
gi|29165615|ref|NC_002745.2|	529680 +	A	0	1	0	0	0	-
gi|29165615|ref|NC_002745.2|	529688 +	C	0	0	0	1	0	SA0459
gi|29165615|ref|NC_002745.2|	529696 +	U	0	0	0	1	0	SA0459
gi|29165615|ref|NC_002745.2|	529697 +	C	0	0	2	0	0	SA0459
gi|29165615|ref|NC_002745.2|	529698 +	A	0	1	0	0	0	SA0459
gi|29165615|ref|NC_002745.2|	529699 +	A	0	2	0	0	3	SA0459
gi|29165615|ref|NC_002745.2|	529701 +	C	0	2	0	0	2	SA0459
gi|29165615|ref|NC_002745.2|	529702 +	A	0	1	1	0	0	SA0459
gi|29165615|ref|NC_002745.2|	529708 +	C	0	2	2	1	0	SA0459
gi|29165615|ref|NC_002745.2|	529709 +	A	0	0	0	1	2	SA0459
gi|29165615|ref|NC_002745.2|	529710 +	A	0	0	1	0	0	SA0459
gi|29165615|ref|NC_002745.2|	529712 +	G	0	2	1	2	2	SA0459
gi|29165615|ref|NC_002745.2|	529713 +	U	1	6	5	5	4	SA0459
gi|29165615|ref|NC_002745.2|	529714 +	A	0	5	3	0	2	SA0459
gi|29165615|ref|NC_002745.2|	529715 +	A	0	2	1	0	0	SA0459
gi|29165615|ref|NC_002745.2|	529717 +	C	0	3	2	1	0	SA0459
gi|29165615|ref|NC_002745.2|	529718 +	A	0	1	1	0	0	SA0459
gi|29165615|ref|NC_002745.2|	529719 +	A	1	1	1	1	0	SA0459
gi|29165615|ref|NC_002745.2|	529721 +	C	8	9	6	1	5	SA0459
gi|29165615|ref|NC_002745.2|	529722 +	A	2	3	4	0	0	SA0459
gi|29165615|ref|NC_002745.2|	529723 +	C	0	2	6	0	4	SA0459
gi|29165615|ref|NC_002745.2|	529724 +	G	0	0	2	1	0	SA0459
gi|29165615|ref|NC_002745.2|	529725 +	U	0	1	2	0	2	SA0459
gi|29165615|ref|NC_002745.2|	529727 +	C	0	0	0	0	2	SA0459
gi|29165615|ref|NC_002745.2|	529728 +	A	0	0	0	0	1	SA0459
gi|29165615|ref|NC_002745.2|	529729 +	G	0	2	0	0	1	SA0459
gi|29165615|ref|NC_002745.2|	529730 +	A	0	6	1	3	2	SA0459
gi|29165615|ref|NC_002745.2|	529732 +	C	2	0	0	0	0	SA0459
gi|29165615|ref|NC_002745.2|	529733 +	U	0	1	0	0	0	SA0459
gi|29165615|ref|NC_002745.2|	529735 +	A	0	0	1	0	0	SA0459
gi|29165615|ref|NC_002745.2|	529736 +	A	0	2	0	0	4	SA0459
gi|29165615|ref|NC_002745.2|	529737 +	A	0	5	3	1	0	SA0459
gi|29165615|ref|NC_002745.2|	529738 +	C	0	2	2	0	5	SA0459
gi|29165615|ref|NC_002745.2|	529739 +	A	0	3	0	0	3	SA0459
gi|29165615|ref|NC_002745.2|	529740 +	A	1	2	0	1	1	SA0459
gi|29165615|ref|NC_002745.2|	529741 +	U	0	0	0	2	1	SA0459
gi|29165615|ref|NC_002745.2|	529742 +	U	0	1	1	3	2	SA0459
gi|29165615|ref|NC_002745.2|	529743 +	A	2	51	14	28	48	SA0459
gi|29165615|ref|NC_002745.2|	529744 +	A	1	16	5	10	21	SA0459

gi|29165615|ref|NC_002745.2|	529745 +	G	0	1	0	2	8	SA0459
gi|29165615|ref|NC_002745.2|	529746 +	A	0	2	2	0	1	SA0459
gi|29165615|ref|NC_002745.2|	529747 +	A	0	0	0	0	2	SA0459
gi|29165615|ref|NC_002745.2|	529748 +	A	0	0	0	0	1	SA0459
gi|29165615|ref|NC_002745.2|	529749 +	A	0	2	1	0	0	SA0459
gi|29165615|ref|NC_002745.2|	529750 +	U	0	0	1	1	0	SA0459
gi|29165615|ref|NC_002745.2|	529751 +	C	0	4	1	3	0	SA0459
gi|29165615|ref|NC_002745.2|	529753 +	G	0	0	0	1	0	SA0459
gi|29165615|ref|NC_002745.2|	529754 +	G	0	3	0	0	1	SA0459
gi|29165615|ref|NC_002745.2|	529755 +	U	1	4	2	0	1	SA0459
gi|29165615|ref|NC_002745.2|	529756 +	A	0	0	0	0	2	SA0459
gi|29165615|ref|NC_002745.2|	529760 +	U	0	0	0	0	1	SA0459
gi|29165615|ref|NC_002745.2|	529761 +	A	0	1	0	1	0	SA0459
gi|29165615|ref|NC_002745.2|	529764 +	A	0	1	0	1	0	SA0459
gi|29165615|ref|NC_002745.2|	529765 +	G	0	0	0	0	1	SA0459
gi|29165615|ref|NC_002745.2|	529766 +	C	0	0	0	2	0	SA0459
gi|29165615|ref|NC_002745.2|	529768 +	G	0	0	0	1	2	SA0459
gi|29165615|ref|NC_002745.2|	529771 +	G	0	1	0	0	0	SA0459
gi|29165615|ref|NC_002745.2|	529772 +	U	1	1	0	0	0	SA0459
gi|29165615|ref|NC_002745.2|	529774 +	U	0	2	0	0	1	SA0459
gi|29165615|ref|NC_002745.2|	529779 +	U	0	2	0	0	0	SA0459
gi|29165615|ref|NC_002745.2|	529782 +	C	0	1	0	0	0	SA0459
gi|29165615|ref|NC_002745.2|	529784 +	G	0	0	0	1	0	SA0459
gi|29165615|ref|NC_002745.2|	529785 +	U	0	0	1	0	0	SA0459
gi|29165615|ref|NC_002745.2|	529786 +	A	0	0	1	0	0	SA0459
gi|29165615|ref|NC_002745.2|	529787 +	C	1	0	0	1	0	SA0459
gi|29165615|ref|NC_002745.2|	529789 +	A	0	0	1	0	1	SA0459
gi|29165615|ref|NC_002745.2|	529790 +	A	0	1	0	1	2	SA0459
gi|29165615|ref|NC_002745.2|	529791 +	A	0	11	1	2	22	SA0459
gi|29165615|ref|NC_002745.2|	529792 +	A	0	9	0	5	3	SA0459
gi|29165615|ref|NC_002745.2|	529793 +	A	0	1	1	1	0	SA0459
gi|29165615|ref|NC_002745.2|	529795 +	G	0	1	0	0	1	SA0459
gi|29165615|ref|NC_002745.2|	529798 +	U	0	4	1	1	0	SA0459
gi|29165615|ref|NC_002745.2|	529799 +	C	1	0	0	0	2	SA0459
gi|29165615|ref|NC_002745.2|	529802 +	U	0	0	1	0	0	SA0459
gi|29165615|ref|NC_002745.2|	529804 +	A	0	1	0	0	0	SA0459
gi|29165615|ref|NC_002745.2|	529806 +	A	0	0	0	1	0	SA0459
gi|29165615|ref|NC_002745.2|	529817 +	U	0	0	0	0	1	SA0459
gi|29165615|ref|NC_002745.2|	529820 +	A	0	2	2	0	1	SA0459
gi|29165615|ref|NC_002745.2|	529821 +	A	0	1	0	0	0	SA0459
gi|29165615|ref|NC_002745.2|	529823 +	U	1	1	0	0	0	SA0459
gi|29165615|ref|NC_002745.2|	529828 +	A	0	0	0	0	3	SA0459
gi|29165615|ref|NC_002745.2|	529831 +	G	0	1	0	0	0	SA0459
gi|29165615|ref|NC_002745.2|	529834 +	A	0	0	0	0	1	SA0459
gi|29165615|ref|NC_002745.2|	529835 +	U	0	1	0	1	1	SA0459
gi|29165615|ref|NC_002745.2|	529836 +	C	0	3	0	2	0	SA0459
gi|29165615|ref|NC_002745.2|	529837 +	C	1	0	0	1	1	SA0459
gi|29165615|ref|NC_002745.2|	529839 +	U	1	2	1	0	2	SA0459
gi|29165615|ref|NC_002745.2|	529840 +	G	0	29	14	10	17	SA0459
gi|29165615|ref|NC_002745.2|	529841 +	A	3	101	68	57	73	SA0459
gi|29165615|ref|NC_002745.2|	529842 +	A	0	18	7	8	10	SA0459
gi|29165615|ref|NC_002745.2|	529843 +	G	0	2	2	0	3	SA0459
gi|29165615|ref|NC_002745.2|	529844 +	U	4	5	5	2	4	SA0459
gi|29165615|ref|NC_002745.2|	529845 +	A	0	1	1	0	1	SA0459
gi|29165615|ref|NC_002745.2|	529847 +	G	1	21	9	7	18	SA0459
gi|29165615|ref|NC_002745.2|	529848 +	U	0	5	3	0	9	SA0459
gi|29165615|ref|NC_002745.2|	529849 +	C	0	6	2	1	6	SA0459
gi|29165615|ref|NC_002745.2|	529850 +	G	0	2	0	1	1	SA0459
gi|29165615|ref|NC_002745.2|	529851 +	U	0	0	1	0	0	SA0459
gi|29165615|ref|NC_002745.2|	529852 +	A	0	2	0	0	0	SA0459
gi|29165615|ref|NC_002745.2|	529854 +	C	0	1	0	0	0	SA0459
gi|29165615|ref|NC_002745.2|	529860 +	U	0	1	0	0	0	SA0459
gi|29165615|ref|NC_002745.2|	529865 +	A	0	0	0	1	1	SA0459
gi|29165615|ref|NC_002745.2|	529866 +	A	0	1	0	0	0	SA0459
gi|29165615|ref|NC_002745.2|	529867 +	U	0	0	0	0	1	SA0459
gi|29165615|ref|NC_002745.2|	529869 +	A	0	0	1	0	0	SA0459
gi|29165615|ref|NC_002745.2|	529871 +	G	0	1	3	0	0	SA0459
gi|29165615|ref|NC_002745.2|	529872 +	C	0	1	0	0	0	SA0459
gi|29165615|ref|NC_002745.2|	529878 +	U	2	0	0	0	0	SA0459
gi|29165615|ref|NC_002745.2|	529882 +	A	0	1	0	0	0	SA0459
gi|29165615|ref|NC_002745.2|	529884 +	A	0	0	0	0	1	SA0459
gi|29165615|ref|NC_002745.2|	529886 +	C	0	0	0	0	1	SA0459
gi|29165615|ref|NC_002745.2|	529887 +	U	0	1	1	0	0	SA0459
gi|29165615|ref|NC_002745.2|	529891 +	A	0	1	1	0	1	SA0459
gi|29165615|ref|NC_002745.2|	529892 +	A	0	0	0	1	1	SA0459
gi|29165615|ref|NC_002745.2|	529894 +	G	0	1	0	0	0	SA0459
gi|29165615|ref|NC_002745.2|	529905 +	A	0	1	0	1	0	SA0459
gi|29165615|ref|NC_002745.2|	529908 +	C	0	0	0	0	1	SA0459
gi|29165615|ref|NC_002745.2|	529909 +	U	0	3	0	0	0	SA0459
gi|29165615|ref|NC_002745.2|	529912 +	C	1	4	0	0	0	SA0459
gi|29165615|ref|NC_002745.2|	529913 +	A	0	0	0	0	1	SA0459

gi|29165615|ref|NC_002745.2|	529917 +	C	0	1	0	0	0	SA0459
gi|29165615|ref|NC_002745.2|	529918 +	G	1	0	0	0	1	SA0459
gi|29165615|ref|NC_002745.2|	529921 +	C	1	1	0	0	2	SA0459
gi|29165615|ref|NC_002745.2|	529929 +	A	0	0	0	0	2	SA0459
gi|29165615|ref|NC_002745.2|	529931 +	A	0	0	1	0	1	SA0459
gi|29165615|ref|NC_002745.2|	529932 +	C	0	1	1	0	1	SA0459
gi|29165615|ref|NC_002745.2|	529933 +	C	0	2	1	1	7	SA0459
gi|29165615|ref|NC_002745.2|	529934 +	A	0	1	0	2	0	SA0459
gi|29165615|ref|NC_002745.2|	529935 +	A	0	1	1	0	0	SA0459
gi|29165615|ref|NC_002745.2|	529938 +	U	0	0	0	0	1	SA0459
gi|29165615|ref|NC_002745.2|	529940 +	C	0	0	0	1	0	SA0459
gi|29165615|ref|NC_002745.2|	529941 +	U	0	1	0	0	0	SA0459
gi|29165615|ref|NC_002745.2|	529949 +	A	0	0	1	0	0	SA0459
gi|29165615|ref|NC_002745.2|	529953 +	C	0	3	0	0	2	SA0459
gi|29165615|ref|NC_002745.2|	529954 +	U	0	1	0	0	0	SA0459
gi|29165615|ref|NC_002745.2|	529956 +	A	0	0	1	0	0	SA0459
gi|29165615|ref|NC_002745.2|	529958 +	C	0	7	0	3	3	SA0459
gi|29165615|ref|NC_002745.2|	529959 +	A	0	1	0	0	1	SA0459
gi|29165615|ref|NC_002745.2|	529960 +	A	0	3	0	1	3	SA0459
gi|29165615|ref|NC_002745.2|	529962 +	C	0	0	0	1	0	SA0459
gi|29165615|ref|NC_002745.2|	529963 +	A	0	3	0	0	0	SA0459
gi|29165615|ref|NC_002745.2|	529964 +	A	1	1	0	0	2	SA0459
gi|29165615|ref|NC_002745.2|	529965 +	U	0	1	0	0	0	SA0459
gi|29165615|ref|NC_002745.2|	529966 +	A	0	2	0	0	2	SA0459
gi|29165615|ref|NC_002745.2|	529967 +	U	0	1	1	0	1	SA0459
gi|29165615|ref|NC_002745.2|	529968 +	G	0	0	0	0	1	SA0459
gi|29165615|ref|NC_002745.2|	529971 +	U	0	0	0	0	1	SA0459
gi|29165615|ref|NC_002745.2|	529972 +	G	0	1	0	1	0	SA0459
gi|29165615|ref|NC_002745.2|	529973 +	A	0	0	0	1	0	SA0459
gi|29165615|ref|NC_002745.2|	529976 +	A	0	0	0	0	1	SA0459
gi|29165615|ref|NC_002745.2|	529978 +	C	0	3	0	0	0	SA0459
gi|29165615|ref|NC_002745.2|	529979 +	G	1	0	0	0	0	SA0459
gi|29165615|ref|NC_002745.2|	529980 +	U	0	6	0	1	0	SA0459
gi|29165615|ref|NC_002745.2|	529981 +	A	0	0	0	0	1	SA0459
gi|29165615|ref|NC_002745.2|	529982 +	C	0	9	4	3	5	SA0459
gi|29165615|ref|NC_002745.2|	529988 +	A	0	1	1	0	0	SA0459
gi|29165615|ref|NC_002745.2|	529989 +	A	0	0	0	0	1	SA0459
gi|29165615|ref|NC_002745.2|	529993 +	C	0	2	0	0	0	SA0459
gi|29165615|ref|NC_002745.2|	529995 +	A	0	1	0	0	0	SA0459
gi|29165615|ref|NC_002745.2|	529999 +	C	0	1	0	1	0	SA0459
gi|29165615|ref|NC_002745.2|	530001 +	A	0	2	0	0	0	SA0459
gi|29165615|ref|NC_002745.2|	530006 +	U	0	0	0	0	1	SA0459
gi|29165615|ref|NC_002745.2|	530010 +	U	0	0	1	0	0	SA0459
gi|29165615|ref|NC_002745.2|	530012 +	A	0	2	0	0	0	SA0459
gi|29165615|ref|NC_002745.2|	530022 +	C	0	0	0	0	1	SA0459
gi|29165615|ref|NC_002745.2|	530024 +	C	0	0	1	0	0	SA0459
gi|29165615|ref|NC_002745.2|	530025 +	U	0	1	0	0	3	SA0459
gi|29165615|ref|NC_002745.2|	530026 +	A	0	1	0	0	0	SA0459
gi|29165615|ref|NC_002745.2|	530027 +	A	0	1	0	0	0	SA0459
gi|29165615|ref|NC_002745.2|	530028 +	A	0	0	1	0	0	SA0459
gi|29165615|ref|NC_002745.2|	530029 +	G	0	0	0	0	1	SA0459
gi|29165615|ref|NC_002745.2|	530030 +	A	0	1	0	0	1	SA0459
gi|29165615|ref|NC_002745.2|	530031 +	A	0	2	0	0	0	SA0459
gi|29165615|ref|NC_002745.2|	530036 +	G	0	0	2	0	1	SA0459
gi|29165615|ref|NC_002745.2|	530046 +	A	0	0	0	1	0	SA0459
gi|29165615|ref|NC_002745.2|	530050 +	C	0	1	0	0	0	SA0459
gi|29165615|ref|NC_002745.2|	530052 +	A	0	0	0	0	2	SA0459
gi|29165615|ref|NC_002745.2|	530053 +	U	1	0	1	0	0	SA0459
gi|29165615|ref|NC_002745.2|	530054 +	U	1	1	1	0	2	SA0459
gi|29165615|ref|NC_002745.2|	530055 +	A	0	3	0	0	1	SA0459
gi|29165615|ref|NC_002745.2|	530056 +	U	0	1	0	0	0	SA0459
gi|29165615|ref|NC_002745.2|	530058 +	C	0	0	0	0	1	SA0459
gi|29165615|ref|NC_002745.2|	530059 +	A	0	1	0	0	1	SA0459
gi|29165615|ref|NC_002745.2|	530062 +	U	0	1	0	0	0	SA0459
gi|29165615|ref|NC_002745.2|	530065 +	G	0	3	0	0	1	SA0459
gi|29165615|ref|NC_002745.2|	530066 +	A	0	1	0	0	1	SA0459
gi|29165615|ref|NC_002745.2|	530068 +	G	0	1	0	0	0	SA0459
gi|29165615|ref|NC_002745.2|	530078 +	C	0	0	1	0	0	SA0459
gi|29165615|ref|NC_002745.2|	530080 +	C	0	0	0	0	1	SA0459
gi|29165615|ref|NC_002745.2|	530084 +	A	0	0	0	0	1	SA0459
gi|29165615|ref|NC_002745.2|	530085 +	C	0	0	0	0	1	SA0459
gi|29165615|ref|NC_002745.2|	530087 +	A	0	1	0	0	0	SA0459
gi|29165615|ref|NC_002745.2|	530088 +	U	0	1	0	0	0	SA0459
gi|29165615|ref|NC_002745.2|	530091 +	U	0	0	0	0	1	SA0459
gi|29165615|ref|NC_002745.2|	530095 +	G	0	6	3	3	1	SA0459
gi|29165615|ref|NC_002745.2|	530099 +	C	0	2	0	0	2	SA0459
gi|29165615|ref|NC_002745.2|	530101 +	A	0	0	0	0	1	SA0459
gi|29165615|ref|NC_002745.2|	530107 +	G	0	1	0	0	0	SA0459
gi|29165615|ref|NC_002745.2|	530112 +	C	0	0	1	0	0	SA0459
gi|29165615|ref|NC_002745.2|	530113 +	A	0	1	0	0	0	SA0459
gi|29165615|ref|NC_002745.2|	530117 +	C	1	3	0	1	0	SA0459

gi|29165615|ref|NC_002745.2|	530118 +	U	0	1	1	0	0	SA0459
gi|29165615|ref|NC_002745.2|	530120 +	A	0	0	0	0	1	SA0459
gi|29165615|ref|NC_002745.2|	530122 +	U	0	0	0	0	1	SA0459
gi|29165615|ref|NC_002745.2|	530128 +	A	0	1	0	0	0	SA0459
gi|29165615|ref|NC_002745.2|	530131 +	A	0	0	1	0	0	SA0459
gi|29165615|ref|NC_002745.2|	530132 +	A	0	1	0	2	0	SA0459
gi|29165615|ref|NC_002745.2|	530136 +	C	0	0	1	0	1	SA0459
gi|29165615|ref|NC_002745.2|	530138 +	G	0	1	0	0	0	SA0459
gi|29165615|ref|NC_002745.2|	530139 +	U	0	0	0	0	1	SA0459
gi|29165615|ref|NC_002745.2|	530140 +	U	0	0	2	0	0	SA0459
gi|29165615|ref|NC_002745.2|	530142 +	A	0	1	0	0	1	SA0459
gi|29165615|ref|NC_002745.2|	530144 +	C	7	0	2	0	0	SA0459
gi|29165615|ref|NC_002745.2|	530147 +	U	7	0	1	0	2	SA0459
gi|29165615|ref|NC_002745.2|	530165 +	C	0	0	0	0	1	SA0459
gi|29165615|ref|NC_002745.2|	530175 +	C	0	0	1	0	0	SA0459
gi|29165615|ref|NC_002745.2|	530177 +	A	0	1	0	0	0	SA0459
gi|29165615|ref|NC_002745.2|	530178 +	A	0	1	0	0	0	SA0459
gi|29165615|ref|NC_002745.2|	530179 +	A	0	2	1	0	1	SA0459
gi|29165615|ref|NC_002745.2|	530180 +	U	2	0	0	0	0	SA0459
gi|29165615|ref|NC_002745.2|	530183 +	A	0	0	0	0	2	SA0459
gi|29165615|ref|NC_002745.2|	530185 +	A	0	0	0	0	1	SA0459
gi|29165615|ref|NC_002745.2|	530186 +	A	0	1	1	0	2	SA0459
gi|29165615|ref|NC_002745.2|	530187 +	C	0	0	1	0	0	SA0459
gi|29165615|ref|NC_002745.2|	530188 +	G	0	1	0	0	0	SA0459
gi|29165615|ref|NC_002745.2|	530192 +	C	0	2	0	2	3	SA0459
gi|29165615|ref|NC_002745.2|	530193 +	A	0	2	0	0	2	SA0459
gi|29165615|ref|NC_002745.2|	530211 +	A	0	0	0	0	1	SA0459
gi|29165615|ref|NC_002745.2|	530213 +	U	0	0	0	0	1	SA0459
gi|29165615|ref|NC_002745.2|	530215 +	G	0	0	1	0	0	SA0459
gi|29165615|ref|NC_002745.2|	530220 +	U	1	0	0	0	0	SA0459
gi|29165615|ref|NC_002745.2|	530222 +	C	0	3	0	0	0	SA0459
gi|29165615|ref|NC_002745.2|	530223 +	A	0	1	0	0	0	SA0459
gi|29165615|ref|NC_002745.2|	530225 +	C	0	0	0	0	1	SA0459
gi|29165615|ref|NC_002745.2|	530226 +	U	0	0	0	0	1	SA0459
gi|29165615|ref|NC_002745.2|	530230 +	G	0	1	0	0	0	SA0459
gi|29165615|ref|NC_002745.2|	530234 +	C	0	1	0	0	0	SA0459
gi|29165615|ref|NC_002745.2|	530235 +	A	0	0	0	0	1	SA0459
gi|29165615|ref|NC_002745.2|	530236 +	A	0	1	0	0	0	SA0459
gi|29165615|ref|NC_002745.2|	530248 +	A	0	0	1	0	0	SA0459
gi|29165615|ref|NC_002745.2|	530253 +	A	1	1	0	0	0	SA0459
gi|29165615|ref|NC_002745.2|	530275 +	A	0	1	0	0	0	SA0459
gi|29165615|ref|NC_002745.2|	530281 +	G	0	1	0	0	0	SA0459
gi|29165615|ref|NC_002745.2|	530554 +	C	0	0	1	0	0	-
gi|29165615|ref|NC_002745.2|	530563 +	A	0	1	0	0	1	-
gi|29165615|ref|NC_002745.2|	530564 +	A	0	0	4	1	2	-
gi|29165615|ref|NC_002745.2|	530567 +	A	0	1	2	1	3	-
gi|29165615|ref|NC_002745.2|	530617 +	G	1	0	1	0	2	-
gi|29165615|ref|NC_002745.2|	530618 +	A	0	0	0	0	1	-
gi|29165615|ref|NC_002745.2|	530653 +	U	0	1	0	0	0	SA0460
gi|29165615|ref|NC_002745.2|	530677 +	U	0	0	1	0	0	SA0460
gi|29165615|ref|NC_002745.2|	530730 +	U	0	0	0	0	1	SA0460
gi|29165615|ref|NC_002745.2|	530734 +	A	0	0	1	2	0	SA0460
gi|29165615|ref|NC_002745.2|	530735 +	G	0	1	0	1	0	SA0460
gi|29165615|ref|NC_002745.2|	530736 +	A	0	1	0	0	1	SA0460
gi|29165615|ref|NC_002745.2|	530753 +	U	0	0	0	0	1	SA0460
gi|29165615|ref|NC_002745.2|	530755 +	A	0	0	0	0	1	SA0460
gi|29165615|ref|NC_002745.2|	530758 +	U	0	0	0	1	0	SA0460
gi|29165615|ref|NC_002745.2|	530759 +	A	0	0	0	0	1	SA0460
gi|29165615|ref|NC_002745.2|	530762 +	C	1	0	1	0	0	SA0460
gi|29165615|ref|NC_002745.2|	530764 +	A	0	0	0	1	0	SA0460
gi|29165615|ref|NC_002745.2|	530765 +	A	0	0	0	1	0	SA0460
gi|29165615|ref|NC_002745.2|	530766 +	A	0	1	0	0	0	SA0460
gi|29165615|ref|NC_002745.2|	530778 +	C	0	0	1	0	0	SA0460
gi|29165615|ref|NC_002745.2|	530779 +	A	0	0	0	0	1	SA0460
gi|29165615|ref|NC_002745.2|	530783 +	A	0	0	0	0	1	SA0460
gi|29165615|ref|NC_002745.2|	530784 +	C	0	0	2	0	0	SA0460
gi|29165615|ref|NC_002745.2|	530785 +	A	0	0	1	0	0	SA0460
gi|29165615|ref|NC_002745.2|	530786 +	A	0	1	0	0	1	SA0460
gi|29165615|ref|NC_002745.2|	530792 +	C	0	0	0	0	1	SA0460
gi|29165615|ref|NC_002745.2|	530805 +	A	0	0	0	0	1	SA0460
gi|29165615|ref|NC_002745.2|	530807 +	A	0	0	0	0	1	SA0460
gi|29165615|ref|NC_002745.2|	530808 +	A	0	0	0	0	1	SA0460
gi|29165615|ref|NC_002745.2|	530824 +	A	0	0	0	0	1	SA0460
gi|29165615|ref|NC_002745.2|	530836 +	G	0	1	0	0	0	SA0460
gi|29165615|ref|NC_002745.2|	530837 +	A	0	0	0	0	1	SA0460
gi|29165615|ref|NC_002745.2|	530841 +	A	0	1	0	0	0	SA0460
gi|29165615|ref|NC_002745.2|	530874 +	A	0	1	0	0	0	SA0460
gi|29165615|ref|NC_002745.2|	530877 +	A	0	0	0	1	0	SA0460
gi|29165615|ref|NC_002745.2|	530880 +	A	0	0	1	0	0	SA0460
gi|29165615|ref|NC_002745.2|	530881 +	C	0	0	0	1	0	SA0460
gi|29165615|ref|NC_002745.2|	530884 +	U	0	1	0	0	0	SA0460

gi|29165615|ref|NC_002745.2|	530886	+	U	0	0	2	0	0	SA0460
gi|29165615|ref|NC_002745.2|	530895	+	A	0	0	0	1	0	SA0460
gi|29165615|ref|NC_002745.2|	530908	+	C	0	1	0	0	0	SA0460
gi|29165615|ref|NC_002745.2|	530929	+	A	0	0	0	0	1	SA0460
gi|29165615|ref|NC_002745.2|	530956	+	A	0	1	0	0	0	SA0460
gi|29165615|ref|NC_002745.2|	530987	+	A	0	1	0	0	0	SA0460
gi|29165615|ref|NC_002745.2|	530993	+	A	0	2	0	0	0	SA0460
gi|29165615|ref|NC_002745.2|	530999	+	A	0	1	0	0	1	SA0460
gi|29165615|ref|NC_002745.2|	531021	+	U	0	0	0	1	0	SA0460
gi|29165615|ref|NC_002745.2|	531051	+	C	0	1	0	0	1	SA0460
gi|29165615|ref|NC_002745.2|	531064	+	G	2	0	0	0	0	SA0460
gi|29165615|ref|NC_002745.2|	531221	+	C	3	0	0	0	0	SA0461
gi|29165615|ref|NC_002745.2|	531364	+	C	0	0	0	0	1	SA0461
gi|29165615|ref|NC_002745.2|	531386	+	A	0	0	1	0	1	SA0461
gi|29165615|ref|NC_002745.2|	531405	+	A	0	2	0	0	2	SA0461
gi|29165615|ref|NC_002745.2|	531406	+	U	0	0	0	0	1	SA0461
gi|29165615|ref|NC_002745.2|	531411	+	A	0	0	0	0	1	SA0461
gi|29165615|ref|NC_002745.2|	531426	+	A	0	1	0	0	0	SA0461
gi|29165615|ref|NC_002745.2|	531431	+	U	0	0	0	1	0	SA0461
gi|29165615|ref|NC_002745.2|	531434	+	A	0	0	0	1	0	SA0461
gi|29165615|ref|NC_002745.2|	531571	+	C	0	0	0	1	0	SA0461
gi|29165615|ref|NC_002745.2|	531608	+	A	0	0	0	0	1	SA0461
gi|29165615|ref|NC_002745.2|	531628	+	A	0	1	0	0	0	SA0461
gi|29165615|ref|NC_002745.2|	531654	+	C	0	0	1	0	0	SA0461
gi|29165615|ref|NC_002745.2|	532040	+	A	0	0	0	1	0	SA0461
gi|29165615|ref|NC_002745.2|	532102	+	U	0	0	0	1	0	SA0461
gi|29165615|ref|NC_002745.2|	532133	+	A	0	0	1	0	0	SA0461
gi|29165615|ref|NC_002745.2|	532141	+	A	0	1	0	0	0	SA0461
gi|29165615|ref|NC_002745.2|	532142	+	A	0	1	0	0	0	SA0461
gi|29165615|ref|NC_002745.2|	532147	+	A	0	1	0	0	0	SA0461
gi|29165615|ref|NC_002745.2|	532150	+	A	0	1	0	0	0	SA0461
gi|29165615|ref|NC_002745.2|	532167	+	U	0	1	0	0	0	SA0461
gi|29165615|ref|NC_002745.2|	532193	+	U	0	0	0	0	1	SA0461
gi|29165615|ref|NC_002745.2|	532237	+	U	0	0	0	0	1	SA0461
gi|29165615|ref|NC_002745.2|	532475	+	C	0	0	1	0	0	SA0461
gi|29165615|ref|NC_002745.2|	532524	+	A	0	0	0	0	1	SA0461
gi|29165615|ref|NC_002745.2|	532529	+	C	4	0	0	0	0	SA0461
gi|29165615|ref|NC_002745.2|	532586	+	A	2	0	0	0	0	SA0461
gi|29165615|ref|NC_002745.2|	532604	+	U	1	0	0	0	0	SA0461
gi|29165615|ref|NC_002745.2|	532632	+	A	0	0	0	0	1	SA0461
gi|29165615|ref|NC_002745.2|	532658	+	A	0	0	0	2	0	SA0461
gi|29165615|ref|NC_002745.2|	532667	+	C	1	0	0	0	0	SA0461
gi|29165615|ref|NC_002745.2|	532678	+	A	0	0	0	1	0	SA0461
gi|29165615|ref|NC_002745.2|	532679	+	A	0	0	0	3	0	SA0461
gi|29165615|ref|NC_002745.2|	532683	+	U	0	0	0	0	1	SA0461
gi|29165615|ref|NC_002745.2|	532722	+	U	1	0	0	0	0	SA0461
gi|29165615|ref|NC_002745.2|	532780	+	A	0	0	0	0	1	SA0461
gi|29165615|ref|NC_002745.2|	532806	+	A	0	1	0	0	0	SA0461
gi|29165615|ref|NC_002745.2|	532863	+	A	0	1	0	0	0	SA0461
gi|29165615|ref|NC_002745.2|	532906	+	G	0	1	0	0	0	SA0461
gi|29165615|ref|NC_002745.2|	532926	+	A	0	0	0	0	1	SA0461
gi|29165615|ref|NC_002745.2|	532927	+	A	0	1	1	0	0	SA0461
gi|29165615|ref|NC_002745.2|	533091	+	A	0	0	0	0	1	SA0461
gi|29165615|ref|NC_002745.2|	533114	+	A	0	0	0	0	1	SA0461
gi|29165615|ref|NC_002745.2|	533115	+	C	0	0	0	0	1	SA0461
gi|29165615|ref|NC_002745.2|	533124	+	A	0	5	0	0	0	SA0461
gi|29165615|ref|NC_002745.2|	533175	+	U	0	0	0	1	0	SA0461
gi|29165615|ref|NC_002745.2|	533307	+	A	0	0	0	0	1	SA0461
gi|29165615|ref|NC_002745.2|	533313	+	A	0	0	0	1	0	SA0461
gi|29165615|ref|NC_002745.2|	533323	+	C	2	0	0	0	0	SA0461
gi|29165615|ref|NC_002745.2|	533325	+	U	0	0	1	0	0	SA0461
gi|29165615|ref|NC_002745.2|	533357	+	A	1	0	0	0	0	SA0461
gi|29165615|ref|NC_002745.2|	533366	+	U	0	0	1	0	0	SA0461
gi|29165615|ref|NC_002745.2|	533392	+	G	0	0	1	0	0	SA0461
gi|29165615|ref|NC_002745.2|	533408	+	U	0	0	0	0	1	SA0461
gi|29165615|ref|NC_002745.2|	533416	+	A	0	0	1	0	0	SA0461
gi|29165615|ref|NC_002745.2|	533433	+	A	0	1	0	0	0	SA0461
gi|29165615|ref|NC_002745.2|	533522	+	U	1	0	0	0	0	SA0461
gi|29165615|ref|NC_002745.2|	533615	+	A	0	1	0	0	0	SA0461
gi|29165615|ref|NC_002745.2|	533696	+	A	0	0	1	0	0	SA0461
gi|29165615|ref|NC_002745.2|	533722	+	A	1	0	0	0	0	SA0461
gi|29165615|ref|NC_002745.2|	533723	+	A	1	0	0	0	0	SA0461
gi|29165615|ref|NC_002745.2|	533729	+	A	0	0	0	0	1	SA0461
gi|29165615|ref|NC_002745.2|	533736	+	U	1	0	0	0	0	SA0461
gi|29165615|ref|NC_002745.2|	533821	+	G	0	1	0	0	0	SA0461
gi|29165615|ref|NC_002745.2|	533830	+	A	0	0	0	1	0	SA0461
gi|29165615|ref|NC_002745.2|	533844	+	C	2	0	0	0	0	SA0461
gi|29165615|ref|NC_002745.2|	533853	+	A	0	1	0	0	0	SA0461
gi|29165615|ref|NC_002745.2|	533858	+	A	0	0	0	1	0	SA0461
gi|29165615|ref|NC_002745.2|	533861	+	U	0	0	0	0	1	SA0461
gi|29165615|ref|NC_002745.2|	533862	+	U	0	0	0	1	0	SA0461

gi|29165615|ref|NC_002745.2|	533871 +	G	0	1	1	2	0	SA0461
gi|29165615|ref|NC_002745.2|	533875 +	A	0	0	0	0	1	SA0461
gi|29165615|ref|NC_002745.2|	533878 +	A	0	0	1	0	0	SA0461
gi|29165615|ref|NC_002745.2|	533884 +	G	0	0	0	0	1	SA0461
gi|29165615|ref|NC_002745.2|	533978 +	U	0	1	0	0	0	SA0461
gi|29165615|ref|NC_002745.2|	533979 +	U	0	0	0	0	1	SA0461
gi|29165615|ref|NC_002745.2|	534002 +	A	1	0	0	0	0	SA0461
gi|29165615|ref|NC_002745.2|	534003 +	U	2	0	0	0	0	SA0461
gi|29165615|ref|NC_002745.2|	534098 +	U	0	0	0	0	1	SA0461
gi|29165615|ref|NC_002745.2|	534125 +	U	1	0	0	0	0	SA0461
gi|29165615|ref|NC_002745.2|	534140 +	A	0	1	0	0	0	SA0461
gi|29165615|ref|NC_002745.2|	534141 +	U	0	0	0	1	0	SA0461
gi|29165615|ref|NC_002745.2|	534178 +	G	0	0	0	0	1	SA0461
gi|29165615|ref|NC_002745.2|	534195 +	C	0	0	0	1	0	SA0461
gi|29165615|ref|NC_002745.2|	534206 +	U	1	0	0	0	0	SA0461
gi|29165615|ref|NC_002745.2|	534209 +	A	0	0	0	1	0	SA0461
gi|29165615|ref|NC_002745.2|	534220 +	A	0	0	1	0	0	SA0461
gi|29165615|ref|NC_002745.2|	534233 +	G	0	0	0	1	0	SA0461
gi|29165615|ref|NC_002745.2|	534249 +	A	1	0	0	0	0	SA0461
gi|29165615|ref|NC_002745.2|	534349 +	C	0	0	0	0	1	SA0461
gi|29165615|ref|NC_002745.2|	534356 +	A	0	1	0	0	0	SA0461
gi|29165615|ref|NC_002745.2|	534458 +	U	0	1	2	0	0	SA0461
gi|29165615|ref|NC_002745.2|	534460 +	A	0	0	0	0	2	SA0461
gi|29165615|ref|NC_002745.2|	534837 +	C	1	0	0	0	0	SA0462
gi|29165615|ref|NC_002745.2|	534948 +	C	0	1	0	0	0	SA0462
gi|29165615|ref|NC_002745.2|	534989 +	U	0	0	0	0	1	SA0462
gi|29165615|ref|NC_002745.2|	535005 +	U	1	0	0	0	0	SA0462
gi|29165615|ref|NC_002745.2|	535078 +	C	0	0	0	0	1	SA0462
gi|29165615|ref|NC_002745.2|	535095 +	U	0	0	1	0	0	SA0462
gi|29165615|ref|NC_002745.2|	535106 +	U	1	0	0	0	0	SA0462
gi|29165615|ref|NC_002745.2|	535139 +	A	0	0	0	0	1	SA0462
gi|29165615|ref|NC_002745.2|	535157 +	C	0	0	0	1	0	SA0462
gi|29165615|ref|NC_002745.2|	535158 +	A	0	1	0	0	0	SA0462
gi|29165615|ref|NC_002745.2|	535178 +	C	0	1	0	0	0	SA0462
gi|29165615|ref|NC_002745.2|	535215 +	U	0	0	1	0	0	SA0462
gi|29165615|ref|NC_002745.2|	535275 +	G	0	0	0	0	1	SA0462
gi|29165615|ref|NC_002745.2|	535328 +	A	0	1	0	0	0	SA0462
gi|29165615|ref|NC_002745.2|	535331 +	G	0	0	0	0	1	SA0462
gi|29165615|ref|NC_002745.2|	535337 +	A	0	2	0	0	0	SA0462
gi|29165615|ref|NC_002745.2|	535345 +	A	1	0	0	0	0	SA0462
gi|29165615|ref|NC_002745.2|	535443 +	U	1	0	0	0	0	SA0462
gi|29165615|ref|NC_002745.2|	535454 +	C	0	0	0	1	0	SA0462
gi|29165615|ref|NC_002745.2|	535559 +	U	0	0	0	1	0	SA0462
gi|29165615|ref|NC_002745.2|	535572 +	C	0	1	0	0	0	SA0462
gi|29165615|ref|NC_002745.2|	535724 +	A	0	0	0	1	0	SA0462
gi|29165615|ref|NC_002745.2|	535744 +	A	0	1	0	3	1	SA0462
gi|29165615|ref|NC_002745.2|	535755 +	U	0	0	1	0	0	SA0462
gi|29165615|ref|NC_002745.2|	535765 +	C	0	0	1	0	0	SA0462
gi|29165615|ref|NC_002745.2|	535779 +	U	0	0	0	0	1	SA0462
gi|29165615|ref|NC_002745.2|	535786 +	G	0	1	1	2	2	SA0462
gi|29165615|ref|NC_002745.2|	535790 +	G	0	0	0	1	0	SA0462
gi|29165615|ref|NC_002745.2|	535805 +	C	0	0	0	1	0	SA0462
gi|29165615|ref|NC_002745.2|	535869 +	U	0	0	0	0	1	SA0462
gi|29165615|ref|NC_002745.2|	535940 +	A	0	1	0	0	0	SA0462
gi|29165615|ref|NC_002745.2|	535942 +	A	1	0	0	1	1	SA0462
gi|29165615|ref|NC_002745.2|	535943 +	U	0	1	0	0	0	SA0462
gi|29165615|ref|NC_002745.2|	535952 +	A	1	0	0	0	0	SA0462
gi|29165615|ref|NC_002745.2|	535959 +	A	1	0	0	0	0	SA0462
gi|29165615|ref|NC_002745.2|	535962 +	U	2	0	0	0	0	SA0462
gi|29165615|ref|NC_002745.2|	535988 +	C	0	0	0	1	0	SA0462
gi|29165615|ref|NC_002745.2|	536014 +	U	0	0	0	0	2	SA0462
gi|29165615|ref|NC_002745.2|	536043 +	U	0	0	0	0	1	SA0462
gi|29165615|ref|NC_002745.2|	536046 +	U	0	0	0	0	1	SA0462
gi|29165615|ref|NC_002745.2|	536077 +	A	0	1	0	0	0	SA0462
gi|29165615|ref|NC_002745.2|	536091 +	C	1	0	0	0	0	SA0462
gi|29165615|ref|NC_002745.2|	536113 +	U	1	0	0	0	0	SA0462
gi|29165615|ref|NC_002745.2|	536121 +	U	0	1	0	0	0	SA0462
gi|29165615|ref|NC_002745.2|	536136 +	C	0	1	0	0	0	SA0462
gi|29165615|ref|NC_002745.2|	536141 +	U	0	0	2	0	0	SA0462
gi|29165615|ref|NC_002745.2|	536149 +	U	0	1	0	0	0	SA0462
gi|29165615|ref|NC_002745.2|	536151 +	U	0	0	0	0	1	SA0462
gi|29165615|ref|NC_002745.2|	536158 +	A	0	1	1	0	1	SA0462
gi|29165615|ref|NC_002745.2|	536162 +	A	0	0	1	0	0	SA0462
gi|29165615|ref|NC_002745.2|	536178 +	A	0	0	1	0	0	SA0462
gi|29165615|ref|NC_002745.2|	536179 +	A	0	0	0	0	1	SA0462
gi|29165615|ref|NC_002745.2|	536187 +	C	0	1	1	0	0	SA0462
gi|29165615|ref|NC_002745.2|	536207 +	A	0	0	1	0	0	SA0462
gi|29165615|ref|NC_002745.2|	536222 +	A	0	0	0	0	1	SA0462
gi|29165615|ref|NC_002745.2|	536265 +	C	0	0	0	0	1	SA0463
gi|29165615|ref|NC_002745.2|	536271 +	A	0	1	0	0	0	SA0463
gi|29165615|ref|NC_002745.2|	536275 +	U	0	0	1	0	0	SA0463

gi|29165615|ref|NC_002745.2|	536301 +	G	1	0	0	0	0	SA0463
gi|29165615|ref|NC_002745.2|	536316 +	A	0	0	1	0	0	SA0463
gi|29165615|ref|NC_002745.2|	536327 +	A	0	0	1	0	0	SA0463
gi|29165615|ref|NC_002745.2|	536380 +	G	0	0	1	0	0	SA0463
gi|29165615|ref|NC_002745.2|	536481 +	A	0	0	1	0	0	SA0463
gi|29165615|ref|NC_002745.2|	536483 +	A	0	0	2	0	0	SA0463
gi|29165615|ref|NC_002745.2|	536555 +	C	0	0	1	0	0	SA0463
gi|29165615|ref|NC_002745.2|	536755 +	A	0	1	0	0	0	SA0463
gi|29165615|ref|NC_002745.2|	536871 +	A	0	0	0	0	1	SA0463
gi|29165615|ref|NC_002745.2|	536899 +	U	0	0	0	0	2	SA0463
gi|29165615|ref|NC_002745.2|	536901 +	C	0	0	0	0	1	SA0463
gi|29165615|ref|NC_002745.2|	536911 +	A	0	0	1	0	0	SA0463
gi|29165615|ref|NC_002745.2|	536924 +	C	0	1	0	0	0	SA0463
gi|29165615|ref|NC_002745.2|	536939 +	A	0	0	0	0	1	SA0463
gi|29165615|ref|NC_002745.2|	536946 +	U	0	0	0	0	1	SA0463
gi|29165615|ref|NC_002745.2|	536954 +	C	1	0	0	0	0	SA0463
gi|29165615|ref|NC_002745.2|	536977 +	G	0	0	5	3	2	SA0463
gi|29165615|ref|NC_002745.2|	536978 +	A	1	0	0	0	0	SA0463
gi|29165615|ref|NC_002745.2|	536981 +	A	0	1	0	0	0	SA0463
gi|29165615|ref|NC_002745.2|	536993 +	G	1	1	0	0	0	SA0463
gi|29165615|ref|NC_002745.2|	537027 +	U	1	0	0	0	0	SA0463
gi|29165615|ref|NC_002745.2|	537045 +	A	0	0	1	0	0	SA0463
gi|29165615|ref|NC_002745.2|	537076 +	A	0	0	0	0	1	SA0463
gi|29165615|ref|NC_002745.2|	537105 +	A	1	0	1	0	2	SA0463
gi|29165615|ref|NC_002745.2|	537115 +	A	0	0	0	0	1	SA0463
gi|29165615|ref|NC_002745.2|	537125 +	A	0	0	0	1	1	SA0463
gi|29165615|ref|NC_002745.2|	537149 +	G	0	1	0	0	0	SA0463
gi|29165615|ref|NC_002745.2|	537153 +	A	0	0	0	0	1	SA0463
gi|29165615|ref|NC_002745.2|	537192 +	U	0	0	0	1	0	SA0463
gi|29165615|ref|NC_002745.2|	537214 +	A	0	0	1	0	0	SA0463
gi|29165615|ref|NC_002745.2|	537260 +	A	0	0	0	2	1	SA0463
gi|29165615|ref|NC_002745.2|	537264 +	A	0	1	0	2	2	SA0463
gi|29165615|ref|NC_002745.2|	537265 +	A	0	0	0	1	0	SA0463
gi|29165615|ref|NC_002745.2|	537266 +	A	0	0	1	0	1	SA0463
gi|29165615|ref|NC_002745.2|	537269 +	A	0	3	0	0	0	SA0463
gi|29165615|ref|NC_002745.2|	537270 +	A	0	0	0	0	1	SA0463
gi|29165615|ref|NC_002745.2|	537277 +	U	0	0	1	0	0	SA0463
gi|29165615|ref|NC_002745.2|	537284 +	C	0	1	0	0	0	SA0463
gi|29165615|ref|NC_002745.2|	537296 +	A	0	1	1	0	0	SA0463
gi|29165615|ref|NC_002745.2|	537318 +	A	0	0	1	0	0	SA0463
gi|29165615|ref|NC_002745.2|	537355 +	U	0	0	0	1	0	SA0463
gi|29165615|ref|NC_002745.2|	537356 +	A	0	0	1	1	0	SA0463
gi|29165615|ref|NC_002745.2|	537360 +	G	0	1	0	0	0	SA0463
gi|29165615|ref|NC_002745.2|	537365 +	C	0	0	0	1	0	SA0463
gi|29165615|ref|NC_002745.2|	537366 +	G	1	0	0	0	0	SA0463
gi|29165615|ref|NC_002745.2|	537367 +	A	0	1	0	0	0	SA0463
gi|29165615|ref|NC_002745.2|	537379 +	U	0	0	0	1	0	SA0463
gi|29165615|ref|NC_002745.2|	537395 +	U	1	0	0	0	0	SA0463
gi|29165615|ref|NC_002745.2|	537411 +	A	0	0	1	0	0	SA0463
gi|29165615|ref|NC_002745.2|	537412 +	A	1	0	0	1	0	SA0463
gi|29165615|ref|NC_002745.2|	537417 +	G	0	1	0	0	0	SA0463
gi|29165615|ref|NC_002745.2|	537421 +	A	0	0	0	0	1	SA0463
gi|29165615|ref|NC_002745.2|	537423 +	U	0	0	1	0	0	SA0463
gi|29165615|ref|NC_002745.2|	537437 +	A	1	0	0	0	0	SA0464
gi|29165615|ref|NC_002745.2|	537439 +	A	0	0	4	1	1	SA0464
gi|29165615|ref|NC_002745.2|	537440 +	U	0	1	0	0	0	SA0464
gi|29165615|ref|NC_002745.2|	537442 +	A	0	0	0	1	0	SA0464
gi|29165615|ref|NC_002745.2|	537443 +	A	0	1	0	1	1	SA0464
gi|29165615|ref|NC_002745.2|	537447 +	U	0	0	0	0	1	SA0464
gi|29165615|ref|NC_002745.2|	537448 +	U	0	0	2	0	0	SA0464
gi|29165615|ref|NC_002745.2|	537449 +	A	0	2	0	5	0	SA0464
gi|29165615|ref|NC_002745.2|	537450 +	A	0	0	0	0	1	SA0464
gi|29165615|ref|NC_002745.2|	537454 +	U	2	0	0	0	0	SA0464
gi|29165615|ref|NC_002745.2|	537455 +	A	1	0	0	0	0	SA0464
gi|29165615|ref|NC_002745.2|	537462 +	U	0	0	0	0	1	SA0464
gi|29165615|ref|NC_002745.2|	537466 +	U	0	0	0	0	1	SA0464
gi|29165615|ref|NC_002745.2|	537467 +	A	0	0	0	1	0	SA0464
gi|29165615|ref|NC_002745.2|	537468 +	A	0	0	0	0	1	SA0464
gi|29165615|ref|NC_002745.2|	537472 +	G	0	0	0	0	1	SA0464
gi|29165615|ref|NC_002745.2|	537485 +	A	0	1	0	0	0	SA0464
gi|29165615|ref|NC_002745.2|	537493 +	U	0	0	0	1	2	SA0464
gi|29165615|ref|NC_002745.2|	537497 +	U	0	0	1	0	1	SA0464
gi|29165615|ref|NC_002745.2|	537501 +	C	0	1	0	0	0	SA0464
gi|29165615|ref|NC_002745.2|	537508 +	G	1	0	0	0	0	SA0464
gi|29165615|ref|NC_002745.2|	537509 +	A	0	0	0	1	0	SA0464
gi|29165615|ref|NC_002745.2|	537513 +	A	0	1	1	0	2	SA0464
gi|29165615|ref|NC_002745.2|	537514 +	C	0	0	1	0	0	SA0464
gi|29165615|ref|NC_002745.2|	537516 +	A	0	1	0	0	0	SA0464
gi|29165615|ref|NC_002745.2|	537517 +	U	0	2	1	1	0	SA0464
gi|29165615|ref|NC_002745.2|	537519 +	A	0	0	0	1	1	SA0464
gi|29165615|ref|NC_002745.2|	537523 +	G	0	0	0	0	1	SA0464

gi|29165615|ref|NC_002745.2|	537524	+	U	1	1	1	0	0	SA0464
gi|29165615|ref|NC_002745.2|	537532	+	C	1	0	0	0	0	SA0464
gi|29165615|ref|NC_002745.2|	537533	+	U	0	1	0	0	0	SA0464
gi|29165615|ref|NC_002745.2|	537543	+	U	0	1	0	0	0	SA0464
gi|29165615|ref|NC_002745.2|	537556	+	U	1	0	0	0	0	SA0464
gi|29165615|ref|NC_002745.2|	537564	+	G	0	0	1	2	0	SA0464
gi|29165615|ref|NC_002745.2|	537579	+	U	0	0	0	0	1	SA0464
gi|29165615|ref|NC_002745.2|	537592	+	U	0	0	0	0	1	SA0464
gi|29165615|ref|NC_002745.2|	537601	+	U	0	0	0	0	1	SA0464
gi|29165615|ref|NC_002745.2|	537603	+	A	0	1	0	2	3	SA0464
gi|29165615|ref|NC_002745.2|	537604	+	A	0	0	0	2	0	SA0464
gi|29165615|ref|NC_002745.2|	537608	+	A	1	0	0	0	0	SA0464
gi|29165615|ref|NC_002745.2|	537612	+	G	0	0	1	0	0	SA0464
gi|29165615|ref|NC_002745.2|	537613	+	C	0	3	2	0	0	SA0464
gi|29165615|ref|NC_002745.2|	537614	+	A	0	0	1	0	0	SA0464
gi|29165615|ref|NC_002745.2|	537615	+	U	0	0	1	0	1	SA0464
gi|29165615|ref|NC_002745.2|	537616	+	U	0	0	0	1	0	SA0464
gi|29165615|ref|NC_002745.2|	537617	+	A	0	1	0	1	0	SA0464
gi|29165615|ref|NC_002745.2|	537628	+	C	0	0	0	0	2	SA0464
gi|29165615|ref|NC_002745.2|	537636	+	G	0	0	0	0	1	SA0464
gi|29165615|ref|NC_002745.2|	537670	+	A	1	0	0	0	0	SA0464
gi|29165615|ref|NC_002745.2|	537677	+	A	0	0	0	1	0	SA0464
gi|29165615|ref|NC_002745.2|	537706	+	A	1	0	0	0	0	-
gi|29165615|ref|NC_002745.2|	537730	+	U	0	0	1	0	0	SA0465
gi|29165615|ref|NC_002745.2|	537748	+	G	0	0	0	1	0	SA0465
gi|29165615|ref|NC_002745.2|	537768	+	A	1	0	0	1	0	SA0465
gi|29165615|ref|NC_002745.2|	537770	+	C	0	0	1	0	0	SA0465
gi|29165615|ref|NC_002745.2|	537775	+	U	1	0	0	0	0	SA0465
gi|29165615|ref|NC_002745.2|	537776	+	C	1	0	0	0	0	SA0465
gi|29165615|ref|NC_002745.2|	537783	+	U	1	0	0	0	0	SA0465
gi|29165615|ref|NC_002745.2|	537786	+	A	2	0	0	0	1	SA0465
gi|29165615|ref|NC_002745.2|	537810	+	U	0	1	0	0	1	SA0465
gi|29165615|ref|NC_002745.2|	537816	+	U	0	0	0	0	2	SA0465
gi|29165615|ref|NC_002745.2|	537817	+	A	0	1	0	0	0	SA0465
gi|29165615|ref|NC_002745.2|	537822	+	C	1	0	0	0	0	SA0465
gi|29165615|ref|NC_002745.2|	537850	+	U	1	0	0	0	0	SA0465
gi|29165615|ref|NC_002745.2|	537857	+	A	0	1	0	0	0	SA0465
gi|29165615|ref|NC_002745.2|	537879	+	A	0	1	0	0	0	SA0465
gi|29165615|ref|NC_002745.2|	537942	+	A	0	2	0	0	0	SA0465
gi|29165615|ref|NC_002745.2|	537965	+	A	0	3	1	1	0	SA0465
gi|29165615|ref|NC_002745.2|	537980	+	A	0	0	1	0	0	SA0465
gi|29165615|ref|NC_002745.2|	537987	+	A	0	0	0	0	1	SA0465
gi|29165615|ref|NC_002745.2|	537988	+	A	0	1	0	0	0	SA0465
gi|29165615|ref|NC_002745.2|	537990	+	A	0	0	1	0	0	SA0465
gi|29165615|ref|NC_002745.2|	537994	+	C	0	1	0	0	0	SA0465
gi|29165615|ref|NC_002745.2|	538001	+	A	0	0	0	0	1	SA0465
gi|29165615|ref|NC_002745.2|	538002	+	A	0	0	1	0	0	SA0465
gi|29165615|ref|NC_002745.2|	538004	+	A	0	1	0	0	1	SA0465
gi|29165615|ref|NC_002745.2|	538170	+	G	0	2	1	0	2	-
gi|29165615|ref|NC_002745.2|	538171	+	G	2	0	2	4	1	-
gi|29165615|ref|NC_002745.2|	538172	+	A	1	5	1	0	2	-
gi|29165615|ref|NC_002745.2|	538173	+	A	0	1	1	0	0	-
gi|29165615|ref|NC_002745.2|	538174	+	A	0	1	0	0	0	-
gi|29165615|ref|NC_002745.2|	538175	+	A	0	1	0	0	0	-
gi|29165615|ref|NC_002745.2|	538179	+	A	0	2	0	1	0	-
gi|29165615|ref|NC_002745.2|	538182	+	C	1	0	0	0	0	-
gi|29165615|ref|NC_002745.2|	538204	+	C	0	2	0	0	0	-
gi|29165615|ref|NC_002745.2|	538206	+	U	0	1	0	0	0	-
gi|29165615|ref|NC_002745.2|	538210	+	U	0	0	1	0	0	SA0466
gi|29165615|ref|NC_002745.2|	538211	+	C	0	0	1	0	0	SA0466
gi|29165615|ref|NC_002745.2|	538223	+	G	0	0	0	1	0	SA0466
gi|29165615|ref|NC_002745.2|	538226	+	A	0	5	1	0	0	SA0466
gi|29165615|ref|NC_002745.2|	538227	+	U	0	0	0	0	1	SA0466
gi|29165615|ref|NC_002745.2|	538235	+	A	0	2	0	0	0	SA0466
gi|29165615|ref|NC_002745.2|	538247	+	C	0	1	0	0	0	SA0466
gi|29165615|ref|NC_002745.2|	538250	+	G	1	0	0	0	0	SA0466
gi|29165615|ref|NC_002745.2|	538251	+	U	0	0	0	0	1	SA0466
gi|29165615|ref|NC_002745.2|	538254	+	U	0	1	0	0	0	SA0466
gi|29165615|ref|NC_002745.2|	538260	+	G	1	0	0	0	0	SA0466
gi|29165615|ref|NC_002745.2|	538261	+	U	1	0	1	0	0	SA0466
gi|29165615|ref|NC_002745.2|	538262	+	U	0	0	0	2	0	SA0466
gi|29165615|ref|NC_002745.2|	538266	+	U	0	0	0	1	1	SA0466
gi|29165615|ref|NC_002745.2|	538272	+	C	0	1	0	0	0	SA0466
gi|29165615|ref|NC_002745.2|	538273	+	G	0	0	1	0	0	SA0466
gi|29165615|ref|NC_002745.2|	538282	+	C	0	1	0	0	0	SA0466
gi|29165615|ref|NC_002745.2|	538283	+	C	0	1	0	0	0	SA0466
gi|29165615|ref|NC_002745.2|	538292	+	A	0	1	0	0	0	SA0466
gi|29165615|ref|NC_002745.2|	538294	+	A	0	1	0	0	0	SA0466
gi|29165615|ref|NC_002745.2|	538296	+	U	0	0	0	1	1	SA0466
gi|29165615|ref|NC_002745.2|	538311	+	U	0	0	0	0	1	SA0466
gi|29165615|ref|NC_002745.2|	538317	+	A	0	1	0	1	0	SA0466

gi|29165615|ref|NC_002745.2|	538329 +	U	0	0	0	0	2	SA0466
gi|29165615|ref|NC_002745.2|	538331 +	A	0	1	0	0	0	SA0466
gi|29165615|ref|NC_002745.2|	538334 +	U	0	0	1	0	0	SA0466
gi|29165615|ref|NC_002745.2|	538340 +	A	0	0	1	0	0	SA0466
gi|29165615|ref|NC_002745.2|	538547 +	A	0	1	0	0	0	SA0466
gi|29165615|ref|NC_002745.2|	538552 +	A	0	1	1	0	1	SA0466
gi|29165615|ref|NC_002745.2|	538554 +	U	0	0	0	0	1	SA0466
gi|29165615|ref|NC_002745.2|	538556 +	C	0	0	0	1	1	SA0466
gi|29165615|ref|NC_002745.2|	538742 +	C	0	0	0	1	0	-
gi|29165615|ref|NC_002745.2|	538747 +	A	0	0	0	1	0	-
gi|29165615|ref|NC_002745.2|	538748 +	C	0	0	0	0	2	-
gi|29165615|ref|NC_002745.2|	538767 +	U	2	0	0	0	0	-
gi|29165615|ref|NC_002745.2|	538771 +	A	0	0	0	0	1	-
gi|29165615|ref|NC_002745.2|	538868 +	A	0	0	2	0	0	SA0467
gi|29165615|ref|NC_002745.2|	538877 +	A	0	0	0	0	1	SA0467
gi|29165615|ref|NC_002745.2|	538970 +	U	0	0	1	0	0	SA0467
gi|29165615|ref|NC_002745.2|	539006 +	A	1	3	0	0	1	SA0467
gi|29165615|ref|NC_002745.2|	539021 +	A	0	0	0	1	0	SA0467
gi|29165615|ref|NC_002745.2|	539023 +	A	0	0	1	0	0	SA0467
gi|29165615|ref|NC_002745.2|	539028 +	U	0	1	0	0	0	SA0467
gi|29165615|ref|NC_002745.2|	539032 +	A	0	1	0	0	0	SA0467
gi|29165615|ref|NC_002745.2|	539045 +	A	0	0	0	0	2	SA0467
gi|29165615|ref|NC_002745.2|	539070 +	C	0	0	1	0	0	SA0467
gi|29165615|ref|NC_002745.2|	539071 +	G	0	0	1	0	0	SA0467
gi|29165615|ref|NC_002745.2|	539100 +	A	0	0	1	0	0	SA0467
gi|29165615|ref|NC_002745.2|	539116 +	A	0	0	1	0	0	SA0467
gi|29165615|ref|NC_002745.2|	539127 +	U	0	1	0	0	0	SA0467
gi|29165615|ref|NC_002745.2|	539154 +	C	0	0	1	0	0	SA0467
gi|29165615|ref|NC_002745.2|	539212 +	G	0	0	0	0	1	SA0467
gi|29165615|ref|NC_002745.2|	539432 +	A	0	0	0	0	3	SA0467
gi|29165615|ref|NC_002745.2|	539438 +	A	0	1	0	0	0	SA0467
gi|29165615|ref|NC_002745.2|	539443 +	A	0	0	0	1	0	SA0467
gi|29165615|ref|NC_002745.2|	539490 +	C	0	0	0	0	1	SA0467
gi|29165615|ref|NC_002745.2|	539508 +	G	0	0	0	0	1	SA0467
gi|29165615|ref|NC_002745.2|	539527 +	U	0	0	0	0	1	SA0467
gi|29165615|ref|NC_002745.2|	539532 +	G	0	0	0	0	1	SA0467
gi|29165615|ref|NC_002745.2|	539545 +	C	1	1	0	0	0	SA0467
gi|29165615|ref|NC_002745.2|	539551 +	A	0	0	0	1	0	SA0467
gi|29165615|ref|NC_002745.2|	539559 +	C	1	0	0	0	0	SA0467
gi|29165615|ref|NC_002745.2|	539560 +	C	0	1	0	0	0	SA0467
gi|29165615|ref|NC_002745.2|	539568 +	U	0	0	0	0	1	SA0467
gi|29165615|ref|NC_002745.2|	539570 +	A	0	0	0	0	1	SA0467
gi|29165615|ref|NC_002745.2|	539599 +	C	1	0	0	0	0	SA0467
gi|29165615|ref|NC_002745.2|	539735 +	A	0	1	0	0	0	SA0467
gi|29165615|ref|NC_002745.2|	539738 +	A	1	0	0	0	0	SA0467
gi|29165615|ref|NC_002745.2|	539771 +	A	0	0	1	0	0	SA0467
gi|29165615|ref|NC_002745.2|	539784 +	A	0	0	0	0	1	SA0467
gi|29165615|ref|NC_002745.2|	539790 +	C	0	0	1	0	0	SA0467
gi|29165615|ref|NC_002745.2|	539806 +	U	1	0	0	0	0	SA0467
gi|29165615|ref|NC_002745.2|	539807 +	U	0	0	0	1	0	SA0467
gi|29165615|ref|NC_002745.2|	539810 +	C	0	0	0	0	1	SA0467
gi|29165615|ref|NC_002745.2|	539812 +	A	0	0	0	0	1	SA0467
gi|29165615|ref|NC_002745.2|	539829 +	C	1	0	0	0	0	SA0467
gi|29165615|ref|NC_002745.2|	539836 +	A	0	0	1	0	0	SA0467
gi|29165615|ref|NC_002745.2|	539840 +	A	0	1	0	0	0	SA0467
gi|29165615|ref|NC_002745.2|	539842 +	C	0	0	0	1	0	SA0467
gi|29165615|ref|NC_002745.2|	539849 +	U	1	0	0	0	0	SA0467
gi|29165615|ref|NC_002745.2|	539850 +	U	0	0	1	0	0	SA0467
gi|29165615|ref|NC_002745.2|	539855 +	U	0	0	0	0	1	SA0467
gi|29165615|ref|NC_002745.2|	539864 +	A	1	0	0	0	0	SA0467
gi|29165615|ref|NC_002745.2|	539865 +	G	0	0	1	0	0	SA0467
gi|29165615|ref|NC_002745.2|	539874 +	C	0	0	0	1	0	SA0467
gi|29165615|ref|NC_002745.2|	539875 +	G	0	0	1	0	0	SA0467
gi|29165615|ref|NC_002745.2|	539876 +	A	0	0	2	0	0	SA0467
gi|29165615|ref|NC_002745.2|	539879 +	A	1	0	0	0	0	SA0467
gi|29165615|ref|NC_002745.2|	539880 +	A	1	0	0	1	0	SA0467
gi|29165615|ref|NC_002745.2|	539889 +	A	0	0	1	0	0	SA0467
gi|29165615|ref|NC_002745.2|	539890 +	C	0	0	1	0	0	SA0467
gi|29165615|ref|NC_002745.2|	539894 +	U	2	0	0	0	0	SA0467
gi|29165615|ref|NC_002745.2|	539898 +	C	0	0	1	0	0	SA0467
gi|29165615|ref|NC_002745.2|	539899 +	U	1	0	0	0	0	SA0467
gi|29165615|ref|NC_002745.2|	539902 +	A	1	0	0	0	0	SA0467
gi|29165615|ref|NC_002745.2|	539907 +	C	1	0	0	0	0	SA0467
gi|29165615|ref|NC_002745.2|	539908 +	G	1	0	0	0	0	SA0467
gi|29165615|ref|NC_002745.2|	539922 +	A	0	0	0	0	1	SA0467
gi|29165615|ref|NC_002745.2|	539924 +	A	1	0	0	0	0	SA0467
gi|29165615|ref|NC_002745.2|	539928 +	A	0	0	1	0	0	SA0467
gi|29165615|ref|NC_002745.2|	539931 +	C	1	0	0	0	0	SA0467
gi|29165615|ref|NC_002745.2|	539935 +	U	0	0	0	0	1	SA0467
gi|29165615|ref|NC_002745.2|	539939 +	U	0	0	0	0	1	SA0467
gi|29165615|ref|NC_002745.2|	539942 +	A	0	0	0	0	1	SA0467

gi|29165615|ref|NC_002745.2|	539943 +	G	1	0	0	0	0	SA0467
gi|29165615|ref|NC_002745.2|	539944 +	A	0	0	2	0	0	SA0467
gi|29165615|ref|NC_002745.2|	539945 +	U	0	0	0	4	0	SA0467
gi|29165615|ref|NC_002745.2|	539947 +	G	2	0	0	0	0	SA0467
gi|29165615|ref|NC_002745.2|	539950 +	A	0	0	0	0	1	SA0467
gi|29165615|ref|NC_002745.2|	539956 +	C	2	0	0	0	0	SA0467
gi|29165615|ref|NC_002745.2|	539962 +	G	1	0	0	0	0	SA0467
gi|29165615|ref|NC_002745.2|	540005 +	C	0	1	0	0	0	SA0467
gi|29165615|ref|NC_002745.2|	540010 +	U	1	0	0	0	0	SA0467
gi|29165615|ref|NC_002745.2|	540026 +	A	2	0	0	1	0	SA0467
gi|29165615|ref|NC_002745.2|	540032 +	A	0	0	1	0	0	SA0467
gi|29165615|ref|NC_002745.2|	540035 +	A	1	0	0	0	0	SA0467
gi|29165615|ref|NC_002745.2|	540036 +	C	2	1	0	0	4	SA0467
gi|29165615|ref|NC_002745.2|	540037 +	A	0	1	0	1	0	SA0467
gi|29165615|ref|NC_002745.2|	540038 +	A	1	0	0	1	2	SA0467
gi|29165615|ref|NC_002745.2|	540039 +	A	5	7	3	5	3	SA0467
gi|29165615|ref|NC_002745.2|	540040 +	C	0	0	0	1	0	SA0467
gi|29165615|ref|NC_002745.2|	540041 +	A	0	0	1	0	0	SA0467
gi|29165615|ref|NC_002745.2|	540042 +	A	0	1	1	0	0	SA0467
gi|29165615|ref|NC_002745.2|	540044 +	A	1	1	1	1	1	SA0467
gi|29165615|ref|NC_002745.2|	540045 +	A	0	1	1	0	0	SA0467
gi|29165615|ref|NC_002745.2|	540046 +	A	1	0	1	0	0	SA0467
gi|29165615|ref|NC_002745.2|	540047 +	A	0	1	3	1	3	SA0467
gi|29165615|ref|NC_002745.2|	540048 +	A	0	2	0	0	1	SA0467
gi|29165615|ref|NC_002745.2|	540050 +	A	0	1	1	0	0	SA0467
gi|29165615|ref|NC_002745.2|	540051 +	U	1	0	1	0	2	SA0467
gi|29165615|ref|NC_002745.2|	540053 +	G	0	2	6	4	3	SA0467
gi|29165615|ref|NC_002745.2|	540054 +	A	1	11	7	1	6	SA0467
gi|29165615|ref|NC_002745.2|	540055 +	U	1	0	0	0	0	SA0467
gi|29165615|ref|NC_002745.2|	540056 +	U	0	0	0	1	0	SA0467
gi|29165615|ref|NC_002745.2|	540057 +	A	0	1	3	2	3	SA0467
gi|29165615|ref|NC_002745.2|	540058 +	U	0	0	0	1	0	SA0467
gi|29165615|ref|NC_002745.2|	540059 +	A	1	2	1	1	0	SA0467
gi|29165615|ref|NC_002745.2|	540060 +	A	0	6	7	4	7	SA0467
gi|29165615|ref|NC_002745.2|	540061 +	U	0	1	2	0	1	SA0467
gi|29165615|ref|NC_002745.2|	540063 +	A	0	1	2	0	5	SA0467
gi|29165615|ref|NC_002745.2|	540064 +	G	0	7	5	4	9	SA0467
gi|29165615|ref|NC_002745.2|	540065 +	U	2	2	2	1	1	SA0467
gi|29165615|ref|NC_002745.2|	540066 +	A	5	6	8	1	3	SA0467
gi|29165615|ref|NC_002745.2|	540067 +	A	3	7	13	5	8	SA0467
gi|29165615|ref|NC_002745.2|	540068 +	A	2	1	1	0	5	SA0467
gi|29165615|ref|NC_002745.2|	540069 +	A	0	1	1	2	2	SA0467
gi|29165615|ref|NC_002745.2|	540073 +	G	0	4	6	2	2	SA0467
gi|29165615|ref|NC_002745.2|	540074 +	A	0	3	2	0	1	SA0467
gi|29165615|ref|NC_002745.2|	540076 +	A	0	2	1	1	2	SA0467
gi|29165615|ref|NC_002745.2|	540077 +	U	2	0	0	0	0	SA0467
gi|29165615|ref|NC_002745.2|	540079 +	A	0	2	3	1	3	SA0467
gi|29165615|ref|NC_002745.2|	540080 +	A	0	0	2	1	3	SA0467
gi|29165615|ref|NC_002745.2|	540081 +	U	0	0	0	1	0	SA0467
gi|29165615|ref|NC_002745.2|	540084 +	C	0	1	0	0	0	-
gi|29165615|ref|NC_002745.2|	540087 +	U	0	0	0	0	1	-
gi|29165615|ref|NC_002745.2|	540089 +	U	1	0	0	0	1	SA0468
gi|29165615|ref|NC_002745.2|	540101 +	U	0	1	0	0	0	SA0468
gi|29165615|ref|NC_002745.2|	540111 +	A	0	0	1	0	0	SA0468
gi|29165615|ref|NC_002745.2|	540113 +	U	1	0	0	0	0	SA0468
gi|29165615|ref|NC_002745.2|	540119 +	C	1	0	0	0	0	SA0468
gi|29165615|ref|NC_002745.2|	540127 +	G	0	1	0	0	0	SA0468
gi|29165615|ref|NC_002745.2|	540144 +	U	0	0	0	0	1	SA0468
gi|29165615|ref|NC_002745.2|	540150 +	A	0	0	0	0	1	SA0468
gi|29165615|ref|NC_002745.2|	540153 +	G	0	0	0	0	1	SA0468
gi|29165615|ref|NC_002745.2|	540194 +	U	0	1	0	0	0	SA0468
gi|29165615|ref|NC_002745.2|	540227 +	U	1	0	0	0	0	SA0468
gi|29165615|ref|NC_002745.2|	540300 +	A	0	1	0	0	0	SA0468
gi|29165615|ref|NC_002745.2|	540425 +	U	0	0	1	0	0	SA0468
gi|29165615|ref|NC_002745.2|	540431 +	A	0	0	1	0	0	SA0468
gi|29165615|ref|NC_002745.2|	540449 +	C	1	0	0	0	0	SA0468
gi|29165615|ref|NC_002745.2|	540497 +	A	0	0	1	0	0	SA0468
gi|29165615|ref|NC_002745.2|	540511 +	U	0	0	1	0	0	SA0468
gi|29165615|ref|NC_002745.2|	540524 +	A	0	0	0	1	0	SA0468
gi|29165615|ref|NC_002745.2|	540556 +	U	0	1	0	0	0	SA0468
gi|29165615|ref|NC_002745.2|	540623 +	A	0	0	0	0	1	SA0468
gi|29165615|ref|NC_002745.2|	540636 +	U	0	1	0	0	0	-
gi|29165615|ref|NC_002745.2|	540639 +	A	0	1	0	0	0	-
gi|29165615|ref|NC_002745.2|	540654 +	A	0	0	1	0	0	-
gi|29165615|ref|NC_002745.2|	540659 +	G	1	0	0	0	0	-
gi|29165615|ref|NC_002745.2|	540689 +	A	1	0	0	0	0	-
gi|29165615|ref|NC_002745.2|	540690 +	A	1	0	0	1	1	-
gi|29165615|ref|NC_002745.2|	540691 +	U	0	0	0	1	0	-
gi|29165615|ref|NC_002745.2|	540692 +	A	2	0	1	2	0	-
gi|29165615|ref|NC_002745.2|	540695 +	A	1	0	0	0	0	-
gi|29165615|ref|NC_002745.2|	540701 +	G	0	0	1	0	0	-

gi|29165615|ref|NC_002745.2|	540702 +	U	0	0	3	1	1	-
gi|29165615|ref|NC_002745.2|	540712 +	A	0	1	0	0	0	-
gi|29165615|ref|NC_002745.2|	540713 +	U	0	0	2	2	2	-
gi|29165615|ref|NC_002745.2|	540714 +	A	0	0	0	0	2	-
gi|29165615|ref|NC_002745.2|	540718 +	A	0	0	1	1	0	-
gi|29165615|ref|NC_002745.2|	540719 +	A	0	0	1	1	5	-
gi|29165615|ref|NC_002745.2|	540723 +	U	0	0	0	1	0	-
gi|29165615|ref|NC_002745.2|	540732 +	C	0	1	0	0	0	-
gi|29165615|ref|NC_002745.2|	540745 +	A	0	0	0	0	1	-
gi|29165615|ref|NC_002745.2|	540751 +	A	0	0	1	0	0	-
gi|29165615|ref|NC_002745.2|	540752 +	A	0	0	0	1	0	-
gi|29165615|ref|NC_002745.2|	540754 +	A	0	0	0	1	0	-
gi|29165615|ref|NC_002745.2|	540755 +	A	0	1	0	0	0	-
gi|29165615|ref|NC_002745.2|	540758 +	U	0	3	0	1	0	-
gi|29165615|ref|NC_002745.2|	540760 +	G	0	0	0	1	0	-
gi|29165615|ref|NC_002745.2|	540761 +	A	1	0	0	0	0	-
gi|29165615|ref|NC_002745.2|	540766 +	G	0	0	1	0	0	-
gi|29165615|ref|NC_002745.2|	540770 +	A	0	1	1	0	0	-
gi|29165615|ref|NC_002745.2|	540772 +	A	0	0	1	0	1	-
gi|29165615|ref|NC_002745.2|	540773 +	C	0	5	4	2	2	-
gi|29165615|ref|NC_002745.2|	540774 +	U	0	0	1	0	1	-
gi|29165615|ref|NC_002745.2|	540775 +	A	1	1	1	1	1	-
gi|29165615|ref|NC_002745.2|	540776 +	A	0	1	1	0	0	-
gi|29165615|ref|NC_002745.2|	540777 +	U	1	2	1	1	0	-
gi|29165615|ref|NC_002745.2|	540778 +	A	0	1	0	0	1	-
gi|29165615|ref|NC_002745.2|	540779 +	C	5	0	0	1	0	-
gi|29165615|ref|NC_002745.2|	540780 +	G	0	1	1	1	1	-
gi|29165615|ref|NC_002745.2|	540792 +	C	0	0	1	0	0	-
gi|29165615|ref|NC_002745.2|	540796 +	G	0	0	0	1	0	-
gi|29165615|ref|NC_002745.2|	540807 +	C	0	0	0	0	1	-
gi|29165615|ref|NC_002745.2|	540810 +	U	0	0	0	0	1	-
gi|29165615|ref|NC_002745.2|	540813 +	C	0	1	0	0	0	-
gi|29165615|ref|NC_002745.2|	540824 +	A	0	0	0	1	0	-
gi|29165615|ref|NC_002745.2|	540826 +	C	0	1	1	0	0	-
gi|29165615|ref|NC_002745.2|	540840 +	C	0	1	0	0	0	-
gi|29165615|ref|NC_002745.2|	540841 +	A	0	0	0	0	1	-
gi|29165615|ref|NC_002745.2|	540844 +	U	0	0	1	0	0	-
gi|29165615|ref|NC_002745.2|	540849 +	U	0	0	1	0	0	-
gi|29165615|ref|NC_002745.2|	540850 +	U	0	0	0	1	1	-
gi|29165615|ref|NC_002745.2|	540851 +	A	3	8	6	4	6	-
gi|29165615|ref|NC_002745.2|	540853 +	U	1	0	0	0	0	-
gi|29165615|ref|NC_002745.2|	540854 +	U	0	0	2	0	0	-
gi|29165615|ref|NC_002745.2|	540859 +	U	1	0	0	0	0	-
gi|29165615|ref|NC_002745.2|	540864 +	G	0	0	0	0	1	-
gi|29165615|ref|NC_002745.2|	540865 +	A	0	0	1	0	0	-
gi|29165615|ref|NC_002745.2|	540868 +	U	0	0	2	1	1	-
gi|29165615|ref|NC_002745.2|	540869 +	A	0	0	1	0	0	-
gi|29165615|ref|NC_002745.2|	540886 +	G	0	2	0	0	0	SA0469
gi|29165615|ref|NC_002745.2|	540887 +	C	0	0	1	0	0	SA0469
gi|29165615|ref|NC_002745.2|	540894 +	C	0	0	1	0	0	SA0469
gi|29165615|ref|NC_002745.2|	540901 +	C	0	0	1	0	0	SA0469
gi|29165615|ref|NC_002745.2|	540914 +	A	0	0	1	0	0	SA0469
gi|29165615|ref|NC_002745.2|	540923 +	A	0	1	1	0	1	SA0469
gi|29165615|ref|NC_002745.2|	540933 +	U	1	0	0	0	0	SA0469
gi|29165615|ref|NC_002745.2|	540948 +	U	1	0	0	0	0	SA0469
gi|29165615|ref|NC_002745.2|	540951 +	C	1	0	0	0	0	SA0469
gi|29165615|ref|NC_002745.2|	540969 +	G	0	0	0	0	1	SA0469
gi|29165615|ref|NC_002745.2|	540989 +	A	1	0	0	0	0	SA0469
gi|29165615|ref|NC_002745.2|	540998 +	C	0	0	0	0	1	SA0469
gi|29165615|ref|NC_002745.2|	541004 +	A	0	1	0	0	0	SA0469
gi|29165615|ref|NC_002745.2|	541005 +	C	0	0	1	0	0	SA0469
gi|29165615|ref|NC_002745.2|	541026 +	A	0	0	0	1	0	SA0469
gi|29165615|ref|NC_002745.2|	541038 +	U	1	0	0	0	0	SA0469
gi|29165615|ref|NC_002745.2|	541049 +	C	1	0	0	0	0	SA0469
gi|29165615|ref|NC_002745.2|	541050 +	C	0	1	0	0	0	SA0469
gi|29165615|ref|NC_002745.2|	541066 +	U	1	0	0	0	0	SA0469
gi|29165615|ref|NC_002745.2|	541083 +	C	0	1	0	0	0	SA0469
gi|29165615|ref|NC_002745.2|	541098 +	A	0	0	0	2	0	SA0469
gi|29165615|ref|NC_002745.2|	541116 +	U	0	0	0	0	1	SA0469
gi|29165615|ref|NC_002745.2|	541118 +	U	0	0	1	0	1	SA0469
gi|29165615|ref|NC_002745.2|	541119 +	A	0	0	3	1	1	SA0469
gi|29165615|ref|NC_002745.2|	541122 +	A	2	6	3	6	2	SA0469
gi|29165615|ref|NC_002745.2|	541123 +	C	0	0	3	0	0	SA0469
gi|29165615|ref|NC_002745.2|	541124 +	A	0	1	1	0	0	SA0469
gi|29165615|ref|NC_002745.2|	541125 +	A	1	0	1	1	2	SA0469
gi|29165615|ref|NC_002745.2|	541128 +	A	0	6	11	10	4	SA0469
gi|29165615|ref|NC_002745.2|	541129 +	A	0	1	5	3	1	SA0469
gi|29165615|ref|NC_002745.2|	541130 +	A	0	1	3	0	1	SA0469
gi|29165615|ref|NC_002745.2|	541131 +	A	0	0	0	0	1	SA0469
gi|29165615|ref|NC_002745.2|	541132 +	A	0	1	0	0	2	SA0469
gi|29165615|ref|NC_002745.2|	541133 +	G	0	1	1	0	0	SA0469

gi|29165615|ref|NC_002745.2|	541134	+	A	0	7	7	3	12	SA0469
gi|29165615|ref|NC_002745.2|	541135	+	A	0	3	5	5	5	SA0469
gi|29165615|ref|NC_002745.2|	541136	+	U	0	0	0	1	0	SA0469
gi|29165615|ref|NC_002745.2|	541137	+	U	0	0	1	0	0	SA0469
gi|29165615|ref|NC_002745.2|	541138	+	A	0	4	4	8	8	SA0469
gi|29165615|ref|NC_002745.2|	541139	+	C	1	9	5	2	8	SA0469
gi|29165615|ref|NC_002745.2|	541140	+	A	0	3	5	3	4	SA0469
gi|29165615|ref|NC_002745.2|	541141	+	A	0	8	12	7	12	SA0469
gi|29165615|ref|NC_002745.2|	541142	+	A	1	10	11	14	18	SA0469
gi|29165615|ref|NC_002745.2|	541143	+	A	0	5	6	3	3	SA0469
gi|29165615|ref|NC_002745.2|	541144	+	A	0	3	1	1	1	SA0469
gi|29165615|ref|NC_002745.2|	541145	+	A	0	8	6	3	4	SA0469
gi|29165615|ref|NC_002745.2|	541146	+	U	0	0	4	2	2	SA0469
gi|29165615|ref|NC_002745.2|	541147	+	U	1	0	0	1	3	SA0469
gi|29165615|ref|NC_002745.2|	541148	+	A	0	2	2	3	4	SA0469
gi|29165615|ref|NC_002745.2|	541149	+	C	0	0	3	2	2	SA0469
gi|29165615|ref|NC_002745.2|	541151	+	G	0	0	1	1	0	SA0469
gi|29165615|ref|NC_002745.2|	541152	+	A	0	0	0	0	1	SA0469
gi|29165615|ref|NC_002745.2|	541153	+	U	0	0	1	0	0	SA0469
gi|29165615|ref|NC_002745.2|	541155	+	C	0	0	1	0	2	SA0469
gi|29165615|ref|NC_002745.2|	541158	+	C	0	1	2	0	1	SA0469
gi|29165615|ref|NC_002745.2|	541159	+	U	0	1	1	0	1	SA0469
gi|29165615|ref|NC_002745.2|	541160	+	A	0	1	1	0	0	SA0469
gi|29165615|ref|NC_002745.2|	541161	+	A	0	2	4	1	1	SA0469
gi|29165615|ref|NC_002745.2|	541162	+	A	0	3	1	1	3	SA0469
gi|29165615|ref|NC_002745.2|	541164	+	A	0	1	0	0	1	SA0469
gi|29165615|ref|NC_002745.2|	541166	+	C	0	0	0	0	2	SA0469
gi|29165615|ref|NC_002745.2|	541167	+	A	0	1	1	0	1	SA0469
gi|29165615|ref|NC_002745.2|	541169	+	A	0	1	4	0	1	SA0469
gi|29165615|ref|NC_002745.2|	541171	+	C	0	0	0	1	0	SA0469
gi|29165615|ref|NC_002745.2|	541173	+	G	0	0	1	1	0	SA0469
gi|29165615|ref|NC_002745.2|	541174	+	U	0	0	0	0	2	SA0469
gi|29165615|ref|NC_002745.2|	541176	+	U	0	1	0	0	0	SA0469
gi|29165615|ref|NC_002745.2|	541177	+	A	0	2	0	1	0	SA0469
gi|29165615|ref|NC_002745.2|	541178	+	A	0	3	1	1	4	SA0469
gi|29165615|ref|NC_002745.2|	541179	+	A	0	0	0	0	1	SA0469
gi|29165615|ref|NC_002745.2|	541180	+	A	0	0	0	2	0	SA0469
gi|29165615|ref|NC_002745.2|	541181	+	U	0	2	1	0	1	SA0469
gi|29165615|ref|NC_002745.2|	541184	+	A	0	8	6	4	9	SA0469
gi|29165615|ref|NC_002745.2|	541185	+	C	1	7	5	2	2	SA0469
gi|29165615|ref|NC_002745.2|	541186	+	G	0	1	0	1	0	SA0469
gi|29165615|ref|NC_002745.2|	541187	+	A	0	0	3	1	7	SA0469
gi|29165615|ref|NC_002745.2|	541189	+	U	0	0	0	1	0	SA0469
gi|29165615|ref|NC_002745.2|	541190	+	A	0	5	2	4	7	SA0469
gi|29165615|ref|NC_002745.2|	541191	+	A	0	6	3	1	2	SA0469
gi|29165615|ref|NC_002745.2|	541192	+	A	0	2	0	0	1	SA0469
gi|29165615|ref|NC_002745.2|	541193	+	G	0	1	2	0	1	SA0469
gi|29165615|ref|NC_002745.2|	541194	+	A	0	7	6	7	3	SA0469
gi|29165615|ref|NC_002745.2|	541195	+	A	0	1	2	0	2	SA0469
gi|29165615|ref|NC_002745.2|	541196	+	G	1	1	5	1	1	SA0469
gi|29165615|ref|NC_002745.2|	541197	+	A	2	18	23	14	27	SA0469
gi|29165615|ref|NC_002745.2|	541198	+	A	0	3	7	1	5	SA0469
gi|29165615|ref|NC_002745.2|	541199	+	G	0	1	0	1	0	SA0469
gi|29165615|ref|NC_002745.2|	541200	+	A	0	14	8	3	5	SA0469
gi|29165615|ref|NC_002745.2|	541201	+	A	1	19	15	8	11	SA0469
gi|29165615|ref|NC_002745.2|	541202	+	A	1	5	3	0	1	SA0469
gi|29165615|ref|NC_002745.2|	541203	+	A	0	1	0	1	1	SA0469
gi|29165615|ref|NC_002745.2|	541204	+	A	0	1	1	1	0	SA0469
gi|29165615|ref|NC_002745.2|	541205	+	C	0	1	0	1	0	SA0469
gi|29165615|ref|NC_002745.2|	541206	+	A	0	0	3	0	0	SA0469
gi|29165615|ref|NC_002745.2|	541207	+	A	0	5	3	5	4	SA0469
gi|29165615|ref|NC_002745.2|	541209	+	G	1	0	0	0	0	SA0469
gi|29165615|ref|NC_002745.2|	541212	+	U	0	2	1	0	0	SA0469
gi|29165615|ref|NC_002745.2|	541213	+	C	0	0	1	0	0	SA0469
gi|29165615|ref|NC_002745.2|	541215	+	U	1	0	0	0	0	SA0469
gi|29165615|ref|NC_002745.2|	541218	+	U	0	0	0	0	2	SA0469
gi|29165615|ref|NC_002745.2|	541219	+	G	0	0	1	0	0	SA0469
gi|29165615|ref|NC_002745.2|	541220	+	A	1	0	1	0	2	SA0469
gi|29165615|ref|NC_002745.2|	541222	+	U	0	1	2	0	1	SA0469
gi|29165615|ref|NC_002745.2|	541223	+	A	0	1	2	1	0	SA0469
gi|29165615|ref|NC_002745.2|	541226	+	C	0	4	1	0	0	SA0469
gi|29165615|ref|NC_002745.2|	541230	+	C	0	0	0	0	1	SA0469
gi|29165615|ref|NC_002745.2|	541232	+	A	0	8	2	1	3	SA0469
gi|29165615|ref|NC_002745.2|	541233	+	C	0	2	0	2	0	SA0469
gi|29165615|ref|NC_002745.2|	541234	+	A	0	0	0	1	0	SA0469
gi|29165615|ref|NC_002745.2|	541235	+	U	0	0	0	2	0	SA0469
gi|29165615|ref|NC_002745.2|	541236	+	U	0	1	0	0	0	SA0469
gi|29165615|ref|NC_002745.2|	541237	+	A	0	1	3	2	0	SA0469
gi|29165615|ref|NC_002745.2|	541238	+	A	0	0	2	2	0	SA0469
gi|29165615|ref|NC_002745.2|	541241	+	C	0	0	1	0	0	SA0469
gi|29165615|ref|NC_002745.2|	541242	+	C	0	0	0	0	1	SA0469

gi|29165615|ref|NC_002745.2|	541243	+	A	0	1	0	0	0	SA0469
gi|29165615|ref|NC_002745.2|	541244	+	G	0	2	0	0	0	SA0469
gi|29165615|ref|NC_002745.2|	541245	+	U	1	0	2	1	4	SA0469
gi|29165615|ref|NC_002745.2|	541248	+	U	0	1	1	2	0	SA0469
gi|29165615|ref|NC_002745.2|	541249	+	A	0	0	2	1	0	SA0469
gi|29165615|ref|NC_002745.2|	541252	+	C	0	0	1	0	0	SA0469
gi|29165615|ref|NC_002745.2|	541253	+	A	0	0	1	0	0	SA0469
gi|29165615|ref|NC_002745.2|	541254	+	U	0	0	0	0	1	SA0469
gi|29165615|ref|NC_002745.2|	541276	+	C	0	0	0	0	1	SA0469
gi|29165615|ref|NC_002745.2|	541282	+	C	1	0	0	0	0	SA0469
gi|29165615|ref|NC_002745.2|	541288	+	A	1	0	0	0	0	SA0469
gi|29165615|ref|NC_002745.2|	541291	+	A	0	0	2	0	0	SA0469
gi|29165615|ref|NC_002745.2|	541293	+	G	0	0	1	0	0	SA0469
gi|29165615|ref|NC_002745.2|	541303	+	U	0	1	1	0	0	SA0469
gi|29165615|ref|NC_002745.2|	541311	+	G	0	1	0	0	0	SA0469
gi|29165615|ref|NC_002745.2|	541313	+	A	1	0	0	0	0	SA0469
gi|29165615|ref|NC_002745.2|	541332	+	C	0	0	0	0	1	SA0469
gi|29165615|ref|NC_002745.2|	541333	+	U	0	0	0	0	1	SA0469
gi|29165615|ref|NC_002745.2|	541339	+	A	0	0	0	0	1	SA0469
gi|29165615|ref|NC_002745.2|	541343	+	A	0	1	0	1	0	SA0469
gi|29165615|ref|NC_002745.2|	541345	+	G	1	0	0	0	0	SA0469
gi|29165615|ref|NC_002745.2|	541350	+	A	0	0	1	0	1	SA0469
gi|29165615|ref|NC_002745.2|	541353	+	A	0	3	0	2	0	SA0469
gi|29165615|ref|NC_002745.2|	541355	+	A	0	1	0	0	0	SA0469
gi|29165615|ref|NC_002745.2|	541356	+	A	2	0	0	0	0	SA0469
gi|29165615|ref|NC_002745.2|	541357	+	U	0	0	1	0	0	SA0469
gi|29165615|ref|NC_002745.2|	541361	+	C	0	0	2	0	0	SA0469
gi|29165615|ref|NC_002745.2|	541363	+	U	1	0	0	0	1	SA0469
gi|29165615|ref|NC_002745.2|	541364	+	C	1	0	0	0	0	SA0469
gi|29165615|ref|NC_002745.2|	541365	+	G	0	0	0	0	1	SA0469
gi|29165615|ref|NC_002745.2|	541366	+	U	2	0	0	0	0	SA0469
gi|29165615|ref|NC_002745.2|	541368	+	U	1	0	0	0	0	SA0469
gi|29165615|ref|NC_002745.2|	541370	+	C	1	0	0	0	0	SA0469
gi|29165615|ref|NC_002745.2|	541371	+	G	1	0	0	0	0	SA0469
gi|29165615|ref|NC_002745.2|	541372	+	U	0	0	1	0	0	SA0469
gi|29165615|ref|NC_002745.2|	541374	+	U	2	0	0	0	0	SA0469
gi|29165615|ref|NC_002745.2|	541377	+	C	1	0	0	0	0	SA0469
gi|29165615|ref|NC_002745.2|	541383	+	U	1	0	0	0	0	SA0469
gi|29165615|ref|NC_002745.2|	541397	+	G	1	0	0	0	0	SA0469
gi|29165615|ref|NC_002745.2|	541398	+	A	0	1	0	0	2	SA0469
gi|29165615|ref|NC_002745.2|	541399	+	A	0	1	0	0	0	SA0469
gi|29165615|ref|NC_002745.2|	541405	+	A	2	0	0	0	0	SA0469
gi|29165615|ref|NC_002745.2|	541406	+	C	0	1	0	0	1	SA0469
gi|29165615|ref|NC_002745.2|	541407	+	A	1	0	0	0	0	SA0469
gi|29165615|ref|NC_002745.2|	541408	+	A	1	0	0	0	0	SA0469
gi|29165615|ref|NC_002745.2|	541409	+	G	0	1	0	1	0	SA0469
gi|29165615|ref|NC_002745.2|	541413	+	U	0	1	0	0	0	SA0469
gi|29165615|ref|NC_002745.2|	541414	+	A	0	3	2	2	0	SA0469
gi|29165615|ref|NC_002745.2|	541415	+	A	2	7	4	2	1	SA0469
gi|29165615|ref|NC_002745.2|	541416	+	U	2	0	0	1	2	SA0469
gi|29165615|ref|NC_002745.2|	541418	+	G	0	6	0	4	1	SA0469
gi|29165615|ref|NC_002745.2|	541419	+	A	2	35	21	50	22	SA0469
gi|29165615|ref|NC_002745.2|	541420	+	A	1	5	2	6	7	SA0469
gi|29165615|ref|NC_002745.2|	541421	+	A	0	8	10	14	2	SA0469
gi|29165615|ref|NC_002745.2|	541422	+	U	0	2	1	4	0	SA0469
gi|29165615|ref|NC_002745.2|	541424	+	G	0	2	1	1	0	SA0469
gi|29165615|ref|NC_002745.2|	541425	+	U	3	10	9	16	11	SA0469
gi|29165615|ref|NC_002745.2|	541426	+	U	1	0	1	2	1	SA0469
gi|29165615|ref|NC_002745.2|	541427	+	G	0	1	1	3	1	SA0469
gi|29165615|ref|NC_002745.2|	541428	+	A	0	1	3	0	0	SA0469
gi|29165615|ref|NC_002745.2|	541429	+	U	1	0	0	0	0	SA0469
gi|29165615|ref|NC_002745.2|	541434	+	U	1	0	0	0	1	SA0469
gi|29165615|ref|NC_002745.2|	541435	+	G	0	0	0	1	0	SA0469
gi|29165615|ref|NC_002745.2|	541436	+	A	0	1	2	2	1	SA0469
gi|29165615|ref|NC_002745.2|	541437	+	A	1	0	1	2	1	SA0469
gi|29165615|ref|NC_002745.2|	541439	+	G	0	0	1	0	1	SA0469
gi|29165615|ref|NC_002745.2|	541440	+	A	0	11	4	2	8	SA0469
gi|29165615|ref|NC_002745.2|	541441	+	U	0	0	0	0	1	SA0469
gi|29165615|ref|NC_002745.2|	541442	+	A	0	1	2	0	3	SA0469
gi|29165615|ref|NC_002745.2|	541443	+	A	1	5	1	8	6	SA0469
gi|29165615|ref|NC_002745.2|	541444	+	U	0	2	1	0	0	SA0469
gi|29165615|ref|NC_002745.2|	541445	+	A	0	0	0	1	1	SA0469
gi|29165615|ref|NC_002745.2|	541446	+	A	0	1	1	1	0	SA0469
gi|29165615|ref|NC_002745.2|	541447	+	A	0	9	3	4	1	SA0469
gi|29165615|ref|NC_002745.2|	541448	+	A	0	2	8	3	2	SA0469
gi|29165615|ref|NC_002745.2|	541449	+	A	0	43	14	14	21	SA0469
gi|29165615|ref|NC_002745.2|	541450	+	A	8	78	42	8	45	SA0469
gi|29165615|ref|NC_002745.2|	541451	+	U	0	0	2	2	2	SA0469
gi|29165615|ref|NC_002745.2|	541452	+	U	2	5	0	2	1	SA0469
gi|29165615|ref|NC_002745.2|	541453	+	C	0	14	10	12	25	SA0469
gi|29165615|ref|NC_002745.2|	541454	+	A	5	28	9	20	9	SA0469

gi|29165615|ref|NC_002745.2|	541455	+	A	0	12	11	8	6	SA0469
gi|29165615|ref|NC_002745.2|	541456	+	A	0	2	1	1	1	SA0469
gi|29165615|ref|NC_002745.2|	541457	+	G	1	3	0	2	2	SA0469
gi|29165615|ref|NC_002745.2|	541458	+	A	1	3	1	1	0	SA0469
gi|29165615|ref|NC_002745.2|	541459	+	A	2	0	0	0	0	SA0469
gi|29165615|ref|NC_002745.2|	541460	+	A	1	0	0	1	0	SA0469
gi|29165615|ref|NC_002745.2|	541461	+	U	5	0	0	0	0	SA0469
gi|29165615|ref|NC_002745.2|	541463	+	G	0	0	1	1	0	SA0469
gi|29165615|ref|NC_002745.2|	541464	+	G	0	3	1	0	0	SA0469
gi|29165615|ref|NC_002745.2|	541467	+	C	2	0	0	0	0	SA0469
gi|29165615|ref|NC_002745.2|	541471	+	G	0	1	1	0	0	SA0469
gi|29165615|ref|NC_002745.2|	541472	+	A	0	1	0	0	0	SA0469
gi|29165615|ref|NC_002745.2|	541473	+	U	1	0	0	0	0	SA0469
gi|29165615|ref|NC_002745.2|	541477	+	U	1	0	1	0	0	SA0469
gi|29165615|ref|NC_002745.2|	541478	+	A	0	0	0	0	2	SA0469
gi|29165615|ref|NC_002745.2|	541483	+	U	1	0	0	0	0	SA0469
gi|29165615|ref|NC_002745.2|	541485	+	U	1	0	0	0	0	SA0469
gi|29165615|ref|NC_002745.2|	541488	+	U	3	0	1	0	0	SA0469
gi|29165615|ref|NC_002745.2|	541501	+	U	0	0	0	1	0	SA0469
gi|29165615|ref|NC_002745.2|	541507	+	U	1	0	0	0	0	SA0469
gi|29165615|ref|NC_002745.2|	541512	+	G	1	0	0	0	1	SA0469
gi|29165615|ref|NC_002745.2|	541513	+	U	3	0	0	0	0	SA0469
gi|29165615|ref|NC_002745.2|	541516	+	A	0	0	0	0	1	SA0469
gi|29165615|ref|NC_002745.2|	541521	+	U	0	0	3	0	0	SA0469
gi|29165615|ref|NC_002745.2|	541533	+	C	0	0	0	2	0	SA0469
gi|29165615|ref|NC_002745.2|	541539	+	C	2	0	0	0	0	SA0469
gi|29165615|ref|NC_002745.2|	541552	+	C	1	0	0	0	0	SA0469
gi|29165615|ref|NC_002745.2|	541554	+	C	2	0	0	0	1	SA0469
gi|29165615|ref|NC_002745.2|	541560	+	U	1	0	0	0	1	SA0469
gi|29165615|ref|NC_002745.2|	541573	+	U	1	0	0	0	0	SA0469
gi|29165615|ref|NC_002745.2|	541578	+	C	1	0	0	0	0	SA0469
gi|29165615|ref|NC_002745.2|	541585	+	U	1	0	0	0	0	SA0469
gi|29165615|ref|NC_002745.2|	541587	+	U	1	0	0	1	0	SA0469
gi|29165615|ref|NC_002745.2|	541590	+	A	3	0	0	0	0	SA0469
gi|29165615|ref|NC_002745.2|	541595	+	U	0	1	1	0	0	SA0469
gi|29165615|ref|NC_002745.2|	541597	+	U	0	0	3	0	0	SA0469
gi|29165615|ref|NC_002745.2|	541598	+	G	0	0	1	0	0	SA0469
gi|29165615|ref|NC_002745.2|	541606	+	U	1	0	0	0	0	SA0469
gi|29165615|ref|NC_002745.2|	541608	+	G	0	0	0	0	1	SA0469
gi|29165615|ref|NC_002745.2|	541625	+	G	0	2	0	0	1	SA0469
gi|29165615|ref|NC_002745.2|	541626	+	A	0	1	1	1	0	SA0469
gi|29165615|ref|NC_002745.2|	541627	+	C	0	1	0	0	0	SA0469
gi|29165615|ref|NC_002745.2|	541629	+	U	1	1	1	0	0	SA0469
gi|29165615|ref|NC_002745.2|	541630	+	A	0	0	1	0	0	SA0469
gi|29165615|ref|NC_002745.2|	541632	+	U	0	1	0	1	1	SA0469
gi|29165615|ref|NC_002745.2|	541634	+	G	0	1	0	2	0	SA0469
gi|29165615|ref|NC_002745.2|	541635	+	A	0	4	2	2	1	SA0469
gi|29165615|ref|NC_002745.2|	541636	+	U	1	0	0	0	1	SA0469
gi|29165615|ref|NC_002745.2|	541637	+	A	0	1	1	0	1	SA0469
gi|29165615|ref|NC_002745.2|	541639	+	U	0	1	0	0	0	SA0469
gi|29165615|ref|NC_002745.2|	541641	+	C	0	2	2	2	0	SA0469
gi|29165615|ref|NC_002745.2|	541642	+	U	0	1	1	0	0	SA0469
gi|29165615|ref|NC_002745.2|	541643	+	A	0	5	2	0	1	SA0469
gi|29165615|ref|NC_002745.2|	541644	+	A	3	2	0	2	1	SA0469
gi|29165615|ref|NC_002745.2|	541645	+	G	0	0	2	2	0	SA0469
gi|29165615|ref|NC_002745.2|	541646	+	A	0	2	1	1	1	SA0469
gi|29165615|ref|NC_002745.2|	541648	+	A	0	0	0	1	0	SA0469
gi|29165615|ref|NC_002745.2|	541649	+	A	1	0	0	0	0	SA0469
gi|29165615|ref|NC_002745.2|	541650	+	A	0	0	1	0	0	SA0469
gi|29165615|ref|NC_002745.2|	541651	+	C	0	0	1	0	0	SA0469
gi|29165615|ref|NC_002745.2|	541653	+	C	3	0	0	0	0	SA0469
gi|29165615|ref|NC_002745.2|	541656	+	C	0	0	0	0	1	SA0469
gi|29165615|ref|NC_002745.2|	541659	+	G	0	0	0	0	1	SA0469
gi|29165615|ref|NC_002745.2|	541660	+	U	9	0	0	0	0	SA0469
gi|29165615|ref|NC_002745.2|	541662	+	U	1	0	0	0	0	SA0469
gi|29165615|ref|NC_002745.2|	541668	+	U	1	1	0	0	0	SA0469
gi|29165615|ref|NC_002745.2|	541669	+	U	0	0	1	0	0	SA0469
gi|29165615|ref|NC_002745.2|	541672	+	C	2	0	0	0	1	SA0469
gi|29165615|ref|NC_002745.2|	541680	+	U	0	0	1	0	0	SA0469
gi|29165615|ref|NC_002745.2|	541681	+	U	2	0	0	0	0	SA0469
gi|29165615|ref|NC_002745.2|	541683	+	A	2	0	1	0	0	SA0469
gi|29165615|ref|NC_002745.2|	541719	+	G	0	0	0	0	1	SA0469
gi|29165615|ref|NC_002745.2|	541724	+	C	0	0	0	0	1	SA0469
gi|29165615|ref|NC_002745.2|	541732	+	A	1	0	0	0	0	SA0469
gi|29165615|ref|NC_002745.2|	541746	+	U	0	1	1	0	0	SA0469
gi|29165615|ref|NC_002745.2|	541755	+	U	0	1	0	0	0	SA0469
gi|29165615|ref|NC_002745.2|	541766	+	A	0	0	0	0	2	SA0469
gi|29165615|ref|NC_002745.2|	541770	+	A	1	0	0	0	0	SA0469
gi|29165615|ref|NC_002745.2|	541774	+	U	1	0	0	0	0	SA0469
gi|29165615|ref|NC_002745.2|	541786	+	U	0	0	0	0	1	SA0469
gi|29165615|ref|NC_002745.2|	541796	+	A	0	1	0	0	0	SA0469

gi|29165615|ref|NC_002745.2|	541806 +	C	1	0	0	0	0	SA0469
gi|29165615|ref|NC_002745.2|	541820 +	C	0	0	0	0	1	SA0469
gi|29165615|ref|NC_002745.2|	541827 +	U	1	0	0	0	0	SA0469
gi|29165615|ref|NC_002745.2|	541839 +	C	1	0	0	0	0	SA0469
gi|29165615|ref|NC_002745.2|	541845 +	U	1	0	0	0	0	SA0469
gi|29165615|ref|NC_002745.2|	541857 +	G	2	0	0	0	1	SA0469
gi|29165615|ref|NC_002745.2|	541866 +	G	0	1	0	0	0	SA0469
gi|29165615|ref|NC_002745.2|	541886 +	C	0	0	0	0	1	SA0469
gi|29165615|ref|NC_002745.2|	541889 +	G	0	1	0	0	0	SA0469
gi|29165615|ref|NC_002745.2|	541922 +	C	0	0	0	0	1	SA0469
gi|29165615|ref|NC_002745.2|	541942 +	U	0	0	0	0	1	SA0469
gi|29165615|ref|NC_002745.2|	541967 +	A	0	1	0	0	0	SA0469
gi|29165615|ref|NC_002745.2|	541983 +	C	1	0	0	0	0	SA0469
gi|29165615|ref|NC_002745.2|	541989 +	U	2	0	0	0	0	SA0469
gi|29165615|ref|NC_002745.2|	541998 +	C	0	1	0	0	1	SA0469
gi|29165615|ref|NC_002745.2|	542008 +	G	1	1	0	0	0	SA0469
gi|29165615|ref|NC_002745.2|	542009 +	A	0	0	1	0	0	SA0469
gi|29165615|ref|NC_002745.2|	542010 +	A	1	0	0	0	0	SA0469
gi|29165615|ref|NC_002745.2|	542011 +	C	0	0	0	0	1	SA0469
gi|29165615|ref|NC_002745.2|	542016 +	U	0	0	0	0	1	SA0469
gi|29165615|ref|NC_002745.2|	542025 +	C	0	0	1	3	0	SA0469
gi|29165615|ref|NC_002745.2|	542026 +	A	1	0	1	0	0	SA0469
gi|29165615|ref|NC_002745.2|	542032 +	A	0	0	1	0	0	SA0469
gi|29165615|ref|NC_002745.2|	542033 +	A	0	0	0	1	0	SA0469
gi|29165615|ref|NC_002745.2|	542035 +	U	1	0	0	0	0	SA0469
gi|29165615|ref|NC_002745.2|	542060 +	A	0	0	0	0	2	SA0469
gi|29165615|ref|NC_002745.2|	542082 +	A	1	0	0	0	0	SA0469
gi|29165615|ref|NC_002745.2|	542090 +	G	0	1	0	0	0	SA0469
gi|29165615|ref|NC_002745.2|	542095 +	A	0	1	0	0	0	SA0469
gi|29165615|ref|NC_002745.2|	542097 +	U	1	0	0	0	1	SA0469
gi|29165615|ref|NC_002745.2|	542105 +	G	0	1	0	0	0	SA0469
gi|29165615|ref|NC_002745.2|	542109 +	C	1	0	0	0	0	SA0469
gi|29165615|ref|NC_002745.2|	542119 +	A	0	1	0	0	0	SA0469
gi|29165615|ref|NC_002745.2|	542121 +	C	1	0	0	0	0	SA0469
gi|29165615|ref|NC_002745.2|	542133 +	C	0	1	0	0	0	SA0469
gi|29165615|ref|NC_002745.2|	542134 +	U	0	1	0	0	0	SA0469
gi|29165615|ref|NC_002745.2|	542137 +	G	0	1	0	0	0	SA0469
gi|29165615|ref|NC_002745.2|	542142 +	A	0	2	0	0	0	SA0469
gi|29165615|ref|NC_002745.2|	542154 +	U	1	1	0	0	0	SA0469
gi|29165615|ref|NC_002745.2|	542157 +	C	1	0	0	0	0	SA0469
gi|29165615|ref|NC_002745.2|	542166 +	A	0	0	1	0	0	SA0469
gi|29165615|ref|NC_002745.2|	542176 +	U	0	0	1	0	0	SA0469
gi|29165615|ref|NC_002745.2|	542185 +	C	0	0	1	0	0	SA0469
gi|29165615|ref|NC_002745.2|	542188 +	U	1	0	0	0	0	SA0469
gi|29165615|ref|NC_002745.2|	542202 +	A	1	0	0	0	1	SA0469
gi|29165615|ref|NC_002745.2|	542209 +	A	0	0	0	0	1	SA0469
gi|29165615|ref|NC_002745.2|	542210 +	G	0	0	0	0	1	SA0469
gi|29165615|ref|NC_002745.2|	542211 +	U	2	0	1	0	0	SA0469
gi|29165615|ref|NC_002745.2|	542217 +	A	1	0	0	0	0	SA0469
gi|29165615|ref|NC_002745.2|	542222 +	G	0	0	0	0	1	SA0469
gi|29165615|ref|NC_002745.2|	542223 +	U	2	0	1	0	0	SA0469
gi|29165615|ref|NC_002745.2|	542224 +	U	1	0	0	0	1	SA0469
gi|29165615|ref|NC_002745.2|	542225 +	A	1	0	0	0	0	SA0469
gi|29165615|ref|NC_002745.2|	542226 +	C	5	0	1	1	0	SA0469
gi|29165615|ref|NC_002745.2|	542227 +	U	1	1	0	0	0	SA0469
gi|29165615|ref|NC_002745.2|	542229 +	U	2	0	0	0	0	SA0469
gi|29165615|ref|NC_002745.2|	542236 +	A	0	1	1	0	0	SA0469
gi|29165615|ref|NC_002745.2|	542237 +	C	0	1	0	0	0	SA0469
gi|29165615|ref|NC_002745.2|	542242 +	A	0	0	1	0	0	SA0469
gi|29165615|ref|NC_002745.2|	542243 +	C	0	0	1	0	1	SA0469
gi|29165615|ref|NC_002745.2|	542244 +	A	4	0	1	0	0	SA0469
gi|29165615|ref|NC_002745.2|	542246 +	G	0	1	0	0	0	SA0469
gi|29165615|ref|NC_002745.2|	542247 +	C	10	1	0	0	0	SA0469
gi|29165615|ref|NC_002745.2|	542248 +	A	0	0	0	0	1	SA0469
gi|29165615|ref|NC_002745.2|	542249 +	G	0	0	1	0	1	SA0469
gi|29165615|ref|NC_002745.2|	542252 +	G	1	0	0	0	0	SA0469
gi|29165615|ref|NC_002745.2|	542259 +	C	1	0	1	0	0	SA0469
gi|29165615|ref|NC_002745.2|	542268 +	U	0	1	0	0	0	SA0469
gi|29165615|ref|NC_002745.2|	542275 +	A	0	0	1	0	0	SA0469
gi|29165615|ref|NC_002745.2|	542280 +	A	0	0	0	0	1	SA0469
gi|29165615|ref|NC_002745.2|	542284 +	U	1	0	1	0	0	SA0469
gi|29165615|ref|NC_002745.2|	542286 +	U	2	0	0	0	0	SA0469
gi|29165615|ref|NC_002745.2|	542289 +	U	4	0	1	0	0	SA0469
gi|29165615|ref|NC_002745.2|	542294 +	A	0	0	0	0	1	SA0469
gi|29165615|ref|NC_002745.2|	542297 +	G	0	0	0	1	0	SA0469
gi|29165615|ref|NC_002745.2|	542300 +	C	0	0	1	0	0	SA0469
gi|29165615|ref|NC_002745.2|	542301 +	A	0	1	0	0	0	SA0469
gi|29165615|ref|NC_002745.2|	542318 +	A	0	0	0	0	1	SA0469
gi|29165615|ref|NC_002745.2|	542319 +	U	0	0	0	2	0	SA0469
gi|29165615|ref|NC_002745.2|	542320 +	C	1	0	0	0	0	SA0469
gi|29165615|ref|NC_002745.2|	542325 +	G	0	0	1	0	0	SA0469

gi|29165615|ref|NC_002745.2|	542326 +	U	0	1	0	0	0	SA0469
gi|29165615|ref|NC_002745.2|	542328 +	U	0	0	0	0	1	SA0469
gi|29165615|ref|NC_002745.2|	542329 +	A	1	1	0	0	0	SA0469
gi|29165615|ref|NC_002745.2|	542330 +	C	0	0	0	0	1	SA0469
gi|29165615|ref|NC_002745.2|	542365 +	C	0	1	0	0	0	SA0469
gi|29165615|ref|NC_002745.2|	542380 +	U	2	0	0	0	0	SA0469
gi|29165615|ref|NC_002745.2|	542385 +	C	1	0	0	0	0	SA0469
gi|29165615|ref|NC_002745.2|	542411 +	A	0	1	0	0	0	SA0469
gi|29165615|ref|NC_002745.2|	542432 +	C	1	0	1	0	0	SA0469
gi|29165615|ref|NC_002745.2|	542435 +	U	0	0	0	0	2	SA0469
gi|29165615|ref|NC_002745.2|	542445 +	G	1	0	0	0	0	SA0469
gi|29165615|ref|NC_002745.2|	542446 +	U	0	0	0	1	0	SA0469
gi|29165615|ref|NC_002745.2|	542447 +	A	0	1	0	0	0	SA0469
gi|29165615|ref|NC_002745.2|	542450 +	A	0	0	1	0	0	SA0469
gi|29165615|ref|NC_002745.2|	542454 +	U	2	0	0	0	0	SA0469
gi|29165615|ref|NC_002745.2|	542464 +	A	0	1	0	0	0	SA0469
gi|29165615|ref|NC_002745.2|	542465 +	C	0	1	0	0	1	SA0469
gi|29165615|ref|NC_002745.2|	542475 +	A	0	0	0	2	0	SA0469
gi|29165615|ref|NC_002745.2|	542476 +	U	0	0	1	0	0	SA0469
gi|29165615|ref|NC_002745.2|	542494 +	C	1	0	0	0	0	SA0469
gi|29165615|ref|NC_002745.2|	542495 +	U	1	0	0	0	0	SA0469
gi|29165615|ref|NC_002745.2|	542499 +	G	0	1	0	0	1	SA0469
gi|29165615|ref|NC_002745.2|	542502 +	A	0	0	1	0	0	SA0469
gi|29165615|ref|NC_002745.2|	542506 +	U	4	1	1	1	0	SA0469
gi|29165615|ref|NC_002745.2|	542507 +	A	1	0	0	0	0	SA0469
gi|29165615|ref|NC_002745.2|	542509 +	G	0	0	0	0	1	SA0469
gi|29165615|ref|NC_002745.2|	542510 +	C	1	0	2	0	0	SA0469
gi|29165615|ref|NC_002745.2|	542511 +	A	1	0	0	0	0	SA0469
gi|29165615|ref|NC_002745.2|	542530 +	A	0	0	0	2	0	SA0469
gi|29165615|ref|NC_002745.2|	542549 +	A	0	1	1	1	2	SA0469
gi|29165615|ref|NC_002745.2|	542550 +	U	1	0	0	0	0	SA0469
gi|29165615|ref|NC_002745.2|	542553 +	A	0	0	0	0	1	SA0469
gi|29165615|ref|NC_002745.2|	542554 +	U	0	0	0	0	1	SA0469
gi|29165615|ref|NC_002745.2|	542564 +	C	0	0	0	0	1	SA0469
gi|29165615|ref|NC_002745.2|	542612 +	G	0	1	0	0	0	SA0469
gi|29165615|ref|NC_002745.2|	542617 +	C	1	0	0	0	0	SA0469
gi|29165615|ref|NC_002745.2|	542633 +	U	0	0	0	1	0	SA0469
gi|29165615|ref|NC_002745.2|	542646 +	C	0	1	0	0	0	SA0469
gi|29165615|ref|NC_002745.2|	542647 +	A	0	0	1	0	0	SA0469
gi|29165615|ref|NC_002745.2|	542661 +	A	0	0	0	0	1	SA0469
gi|29165615|ref|NC_002745.2|	542664 +	C	1	1	0	0	1	SA0469
gi|29165615|ref|NC_002745.2|	542669 +	G	1	0	0	0	0	SA0469
gi|29165615|ref|NC_002745.2|	542681 +	A	0	2	0	0	0	SA0469
gi|29165615|ref|NC_002745.2|	542688 +	C	2	0	0	0	0	SA0469
gi|29165615|ref|NC_002745.2|	542691 +	U	0	1	0	0	0	SA0469
gi|29165615|ref|NC_002745.2|	542692 +	A	0	0	0	0	1	SA0469
gi|29165615|ref|NC_002745.2|	542699 +	G	0	2	0	0	0	SA0469
gi|29165615|ref|NC_002745.2|	542700 +	A	0	1	0	0	0	SA0469
gi|29165615|ref|NC_002745.2|	542707 +	A	0	1	0	0	0	SA0469
gi|29165615|ref|NC_002745.2|	542709 +	U	1	0	0	0	0	SA0469
gi|29165615|ref|NC_002745.2|	542712 +	C	0	0	1	1	1	SA0469
gi|29165615|ref|NC_002745.2|	542717 +	A	0	0	0	0	1	SA0469
gi|29165615|ref|NC_002745.2|	542720 +	G	1	0	0	0	0	SA0469
gi|29165615|ref|NC_002745.2|	542721 +	A	0	2	0	0	0	SA0469
gi|29165615|ref|NC_002745.2|	542723 +	U	0	0	0	1	0	SA0469
gi|29165615|ref|NC_002745.2|	542724 +	A	0	0	0	0	2	SA0469
gi|29165615|ref|NC_002745.2|	542750 +	G	0	0	1	0	0	SA0469
gi|29165615|ref|NC_002745.2|	542757 +	C	0	2	0	0	0	SA0469
gi|29165615|ref|NC_002745.2|	542761 +	A	0	1	0	0	0	SA0469
gi|29165615|ref|NC_002745.2|	542765 +	A	0	0	1	0	1	SA0469
gi|29165615|ref|NC_002745.2|	542776 +	A	0	0	0	0	1	SA0469
gi|29165615|ref|NC_002745.2|	542788 +	U	1	0	0	0	0	SA0469
gi|29165615|ref|NC_002745.2|	542791 +	U	0	0	1	0	0	SA0469
gi|29165615|ref|NC_002745.2|	542810 +	C	0	0	0	0	1	SA0469
gi|29165615|ref|NC_002745.2|	542824 +	A	0	2	0	0	0	SA0469
gi|29165615|ref|NC_002745.2|	542827 +	A	0	1	0	0	0	SA0469
gi|29165615|ref|NC_002745.2|	542831 +	G	0	0	0	1	0	SA0469
gi|29165615|ref|NC_002745.2|	543014 +	A	0	0	0	1	0	-
gi|29165615|ref|NC_002745.2|	543015 +	U	0	1	0	0	0	-
gi|29165615|ref|NC_002745.2|	543016 +	A	1	0	0	0	0	-
gi|29165615|ref|NC_002745.2|	543017 +	G	1	0	1	2	0	-
gi|29165615|ref|NC_002745.2|	543018 +	A	0	1	2	1	2	-
gi|29165615|ref|NC_002745.2|	543019 +	A	0	0	2	0	0	-
gi|29165615|ref|NC_002745.2|	543020 +	A	0	1	1	0	0	-
gi|29165615|ref|NC_002745.2|	543048 +	C	0	0	2	0	0	-
gi|29165615|ref|NC_002745.2|	543055 +	G	0	0	1	0	0	-
gi|29165615|ref|NC_002745.2|	543056 +	A	0	0	2	0	0	-
gi|29165615|ref|NC_002745.2|	543061 +	A	0	1	0	0	1	-
gi|29165615|ref|NC_002745.2|	543064 +	U	0	1	0	0	0	-
gi|29165615|ref|NC_002745.2|	543065 +	C	0	0	1	0	0	-
gi|29165615|ref|NC_002745.2|	543069 +	G	0	0	1	0	0	-

gi|29165615|ref|NC_002745.2|	543070	+	A	0	0	1	0	0	-
gi|29165615|ref|NC_002745.2|	543071	+	A	0	0	0	0	1	-
gi|29165615|ref|NC_002745.2|	543083	+	G	0	0	1	0	0	-
gi|29165615|ref|NC_002745.2|	543090	+	A	0	1	0	0	0	-
gi|29165615|ref|NC_002745.2|	543094	+	U	0	0	0	1	0	-
gi|29165615|ref|NC_002745.2|	543095	+	A	0	0	1	0	0	-
gi|29165615|ref|NC_002745.2|	543096	+	A	0	0	0	0	1	-
gi|29165615|ref|NC_002745.2|	543116	+	U	0	0	0	1	0	-
gi|29165615|ref|NC_002745.2|	543125	+	G	0	1	0	0	0	-
gi|29165615|ref|NC_002745.2|	543142	+	A	26	30	39	25	40	-
gi|29165615|ref|NC_002745.2|	543143	+	A	1	0	8	1	2	-
gi|29165615|ref|NC_002745.2|	543144	+	U	0	0	1	0	0	-
gi|29165615|ref|NC_002745.2|	543146	+	G	0	0	1	0	0	-
gi|29165615|ref|NC_002745.2|	543150	+	C	0	0	0	1	0	-
gi|29165615|ref|NC_002745.2|	543153	+	U	1	0	0	0	0	-
gi|29165615|ref|NC_002745.2|	543155	+	U	0	0	1	0	0	-
gi|29165615|ref|NC_002745.2|	543156	+	A	0	0	3	0	0	-
gi|29165615|ref|NC_002745.2|	543157	+	U	2	0	0	0	1	-
gi|29165615|ref|NC_002745.2|	543158	+	G	6	7	3	1	9	-
gi|29165615|ref|NC_002745.2|	543159	+	A	2	18	9	7	14	-
gi|29165615|ref|NC_002745.2|	543160	+	A	0	0	5	1	0	-
gi|29165615|ref|NC_002745.2|	543161	+	G	0	1	1	1	2	-
gi|29165615|ref|NC_002745.2|	543162	+	U	0	3	1	3	1	-
gi|29165615|ref|NC_002745.2|	543163	+	A	0	12	11	11	9	-
gi|29165615|ref|NC_002745.2|	543164	+	A	0	3	3	0	0	-
gi|29165615|ref|NC_002745.2|	543165	+	A	0	1	1	0	1	-
gi|29165615|ref|NC_002745.2|	543166	+	G	0	0	5	0	1	-
gi|29165615|ref|NC_002745.2|	543167	+	U	0	0	0	0	1	-
gi|29165615|ref|NC_002745.2|	543168	+	A	0	2	8	2	3	-
gi|29165615|ref|NC_002745.2|	543169	+	A	0	1	0	0	0	-
gi|29165615|ref|NC_002745.2|	543170	+	U	2	0	1	2	1	-
gi|29165615|ref|NC_002745.2|	543171	+	U	0	1	0	0	0	-
gi|29165615|ref|NC_002745.2|	543173	+	U	1	4	1	3	0	-
gi|29165615|ref|NC_002745.2|	543174	+	A	0	10	2	4	8	-
gi|29165615|ref|NC_002745.2|	543175	+	A	0	16	8	10	27	-
gi|29165615|ref|NC_002745.2|	543176	+	A	0	8	3	7	8	-
gi|29165615|ref|NC_002745.2|	543177	+	A	0	1	0	1	3	-
gi|29165615|ref|NC_002745.2|	543178	+	U	0	2	1	0	0	-
gi|29165615|ref|NC_002745.2|	543179	+	A	0	2	1	0	3	-
gi|29165615|ref|NC_002745.2|	543180	+	U	0	1	1	0	3	-
gi|29165615|ref|NC_002745.2|	543181	+	A	0	11	6	4	18	-
gi|29165615|ref|NC_002745.2|	543182	+	G	0	3	4	2	4	-
gi|29165615|ref|NC_002745.2|	543183	+	A	1	15	9	10	16	-
gi|29165615|ref|NC_002745.2|	543184	+	G	2	6	1	0	0	-
gi|29165615|ref|NC_002745.2|	543185	+	U	0	1	1	1	2	-
gi|29165615|ref|NC_002745.2|	543186	+	A	0	15	9	2	14	-
gi|29165615|ref|NC_002745.2|	543187	+	A	1	8	2	0	9	-
gi|29165615|ref|NC_002745.2|	543188	+	U	0	2	0	0	0	-
gi|29165615|ref|NC_002745.2|	543189	+	A	0	0	1	0	3	-
gi|29165615|ref|NC_002745.2|	543190	+	G	0	7	5	3	2	-
gi|29165615|ref|NC_002745.2|	543191	+	A	0	2	0	1	1	-
gi|29165615|ref|NC_002745.2|	543192	+	G	0	1	0	0	0	-
gi|29165615|ref|NC_002745.2|	543193	+	G	0	1	2	2	9	-
gi|29165615|ref|NC_002745.2|	543194	+	A	0	7	8	6	11	-
gi|29165615|ref|NC_002745.2|	543195	+	G	0	5	7	3	10	-
gi|29165615|ref|NC_002745.2|	543196	+	A	0	0	1	0	2	-
gi|29165615|ref|NC_002745.2|	543197	+	U	0	0	1	0	0	-
gi|29165615|ref|NC_002745.2|	543199	+	G	0	12	8	2	42	-
gi|29165615|ref|NC_002745.2|	543200	+	A	0	2	4	0	6	-
gi|29165615|ref|NC_002745.2|	543201	+	A	0	3	2	1	5	-
gi|29165615|ref|NC_002745.2|	543202	+	A	0	2	1	0	1	-
gi|29165615|ref|NC_002745.2|	543203	+	U	0	1	0	1	0	-
gi|29165615|ref|NC_002745.2|	543204	+	A	2	7	1	1	2	-
gi|29165615|ref|NC_002745.2|	543206	+	U	0	1	0	0	0	SA0470
gi|29165615|ref|NC_002745.2|	543207	+	G	0	1	1	0	1	SA0470
gi|29165615|ref|NC_002745.2|	543208	+	A	0	6	2	0	3	SA0470
gi|29165615|ref|NC_002745.2|	543209	+	C	0	0	1	1	3	SA0470
gi|29165615|ref|NC_002745.2|	543210	+	A	0	1	1	1	2	SA0470
gi|29165615|ref|NC_002745.2|	543211	+	C	0	0	1	0	1	SA0470
gi|29165615|ref|NC_002745.2|	543212	+	A	0	1	0	0	1	SA0470
gi|29165615|ref|NC_002745.2|	543213	+	C	0	0	1	1	0	SA0470
gi|29165615|ref|NC_002745.2|	543214	+	G	0	5	6	5	1	SA0470
gi|29165615|ref|NC_002745.2|	543215	+	A	1	7	6	7	7	SA0470
gi|29165615|ref|NC_002745.2|	543216	+	U	0	0	0	1	0	SA0470
gi|29165615|ref|NC_002745.2|	543217	+	U	0	0	1	0	0	SA0470
gi|29165615|ref|NC_002745.2|	543218	+	A	0	0	1	0	0	SA0470
gi|29165615|ref|NC_002745.2|	543220	+	A	0	0	0	0	2	SA0470
gi|29165615|ref|NC_002745.2|	543221	+	U	0	0	1	1	0	SA0470
gi|29165615|ref|NC_002745.2|	543223	+	G	0	1	0	0	1	SA0470
gi|29165615|ref|NC_002745.2|	543224	+	U	0	0	1	0	0	SA0470
gi|29165615|ref|NC_002745.2|	543225	+	U	0	1	0	0	1	SA0470

gi|29165615|ref|NC_002745.2|	543226 +	A	0	2	1	0	2	SA0470
gi|29165615|ref|NC_002745.2|	543227 +	A	0	1	0	0	0	SA0470
gi|29165615|ref|NC_002745.2|	543229 +	G	0	0	0	0	1	SA0470
gi|29165615|ref|NC_002745.2|	543230 +	C	0	1	0	0	0	SA0470
gi|29165615|ref|NC_002745.2|	543236 +	C	0	0	1	0	0	SA0470
gi|29165615|ref|NC_002745.2|	543241 +	G	0	0	1	0	0	SA0470
gi|29165615|ref|NC_002745.2|	543245 +	G	1	0	0	0	0	SA0470
gi|29165615|ref|NC_002745.2|	543247 +	G	0	1	1	0	0	SA0470
gi|29165615|ref|NC_002745.2|	543248 +	A	0	1	0	0	0	SA0470
gi|29165615|ref|NC_002745.2|	543249 +	G	0	0	1	0	1	SA0470
gi|29165615|ref|NC_002745.2|	543252 +	U	0	0	1	0	0	SA0470
gi|29165615|ref|NC_002745.2|	543257 +	C	1	0	0	0	0	SA0470
gi|29165615|ref|NC_002745.2|	543263 +	C	0	1	0	0	0	SA0470
gi|29165615|ref|NC_002745.2|	543284 +	U	0	1	1	4	1	SA0470
gi|29165615|ref|NC_002745.2|	543285 +	U	0	0	1	0	0	SA0470
gi|29165615|ref|NC_002745.2|	543286 +	C	0	2	1	0	0	SA0470
gi|29165615|ref|NC_002745.2|	543287 +	A	0	1	0	0	0	SA0470
gi|29165615|ref|NC_002745.2|	543289 +	G	0	0	0	0	1	SA0470
gi|29165615|ref|NC_002745.2|	543290 +	A	0	0	0	0	1	SA0470
gi|29165615|ref|NC_002745.2|	543291 +	A	0	1	0	0	0	SA0470
gi|29165615|ref|NC_002745.2|	543293 +	C	0	0	0	0	1	SA0470
gi|29165615|ref|NC_002745.2|	543295 +	C	0	0	1	0	0	SA0470
gi|29165615|ref|NC_002745.2|	543296 +	A	0	0	0	0	1	SA0470
gi|29165615|ref|NC_002745.2|	543298 +	A	0	1	0	0	0	SA0470
gi|29165615|ref|NC_002745.2|	543303 +	A	0	1	0	0	2	SA0470
gi|29165615|ref|NC_002745.2|	543304 +	C	0	0	0	1	0	SA0470
gi|29165615|ref|NC_002745.2|	543305 +	A	0	1	0	0	0	SA0470
gi|29165615|ref|NC_002745.2|	543308 +	A	0	0	0	1	0	SA0470
gi|29165615|ref|NC_002745.2|	543309 +	U	0	1	1	0	1	SA0470
gi|29165615|ref|NC_002745.2|	543312 +	A	1	0	0	0	0	SA0470
gi|29165615|ref|NC_002745.2|	543326 +	C	1	0	0	0	0	SA0470
gi|29165615|ref|NC_002745.2|	543346 +	A	0	0	0	0	1	SA0470
gi|29165615|ref|NC_002745.2|	543352 +	G	0	0	0	0	1	SA0470
gi|29165615|ref|NC_002745.2|	543361 +	A	0	1	0	0	0	SA0470
gi|29165615|ref|NC_002745.2|	543362 +	U	0	0	1	0	0	SA0470
gi|29165615|ref|NC_002745.2|	543364 +	A	0	0	1	0	0	SA0470
gi|29165615|ref|NC_002745.2|	543371 +	C	2	0	0	0	0	SA0470
gi|29165615|ref|NC_002745.2|	543374 +	U	0	0	1	0	0	SA0470
gi|29165615|ref|NC_002745.2|	543384 +	U	0	1	0	0	0	SA0470
gi|29165615|ref|NC_002745.2|	543386 +	A	0	0	0	1	0	SA0470
gi|29165615|ref|NC_002745.2|	543387 +	U	0	0	0	0	1	SA0470
gi|29165615|ref|NC_002745.2|	543388 +	C	0	0	1	0	0	SA0470
gi|29165615|ref|NC_002745.2|	543389 +	A	0	1	0	0	0	SA0470
gi|29165615|ref|NC_002745.2|	543390 +	A	0	0	0	0	1	SA0470
gi|29165615|ref|NC_002745.2|	543392 +	A	0	0	1	1	0	SA0470
gi|29165615|ref|NC_002745.2|	543393 +	A	0	1	0	0	0	SA0470
gi|29165615|ref|NC_002745.2|	543394 +	U	0	1	0	0	0	SA0470
gi|29165615|ref|NC_002745.2|	543395 +	U	0	0	0	1	0	SA0470
gi|29165615|ref|NC_002745.2|	543397 +	A	0	1	0	0	0	SA0470
gi|29165615|ref|NC_002745.2|	543402 +	C	0	0	1	0	0	SA0470
gi|29165615|ref|NC_002745.2|	543407 +	U	2	0	0	0	0	SA0470
gi|29165615|ref|NC_002745.2|	543408 +	A	0	1	0	0	0	SA0470
gi|29165615|ref|NC_002745.2|	543413 +	G	0	0	1	0	0	SA0470
gi|29165615|ref|NC_002745.2|	543415 +	C	0	3	0	1	0	SA0470
gi|29165615|ref|NC_002745.2|	543421 +	C	0	0	1	0	0	SA0470
gi|29165615|ref|NC_002745.2|	543425 +	U	1	0	0	0	0	SA0470
gi|29165615|ref|NC_002745.2|	543430 +	C	0	1	0	0	0	SA0470
gi|29165615|ref|NC_002745.2|	543432 +	A	0	0	1	0	1	SA0470
gi|29165615|ref|NC_002745.2|	543433 +	A	0	1	1	0	0	SA0470
gi|29165615|ref|NC_002745.2|	543434 +	U	1	0	0	0	1	SA0470
gi|29165615|ref|NC_002745.2|	543435 +	U	0	0	1	0	0	SA0470
gi|29165615|ref|NC_002745.2|	543436 +	A	1	0	1	0	1	SA0470
gi|29165615|ref|NC_002745.2|	543437 +	U	1	0	1	0	0	SA0470
gi|29165615|ref|NC_002745.2|	543438 +	U	0	0	1	0	0	SA0470
gi|29165615|ref|NC_002745.2|	543440 +	C	0	0	1	0	0	SA0470
gi|29165615|ref|NC_002745.2|	543442 +	G	0	2	1	0	1	SA0470
gi|29165615|ref|NC_002745.2|	543447 +	A	3	2	0	0	0	SA0470
gi|29165615|ref|NC_002745.2|	543448 +	A	0	0	0	0	2	SA0470
gi|29165615|ref|NC_002745.2|	543451 +	G	0	1	0	0	1	SA0470
gi|29165615|ref|NC_002745.2|	543454 +	A	0	0	2	0	0	SA0470
gi|29165615|ref|NC_002745.2|	543458 +	G	0	0	2	0	0	SA0470
gi|29165615|ref|NC_002745.2|	543461 +	A	0	0	0	0	1	SA0470
gi|29165615|ref|NC_002745.2|	543480 +	C	0	0	1	0	0	SA0470
gi|29165615|ref|NC_002745.2|	543493 +	C	0	0	1	0	0	SA0470
gi|29165615|ref|NC_002745.2|	543508 +	G	0	0	1	0	0	SA0470
gi|29165615|ref|NC_002745.2|	543511 +	C	0	1	0	0	0	SA0470
gi|29165615|ref|NC_002745.2|	543521 +	U	0	0	1	0	0	SA0470
gi|29165615|ref|NC_002745.2|	543523 +	G	1	0	0	0	0	SA0470
gi|29165615|ref|NC_002745.2|	543524 +	A	0	0	0	1	0	SA0470
gi|29165615|ref|NC_002745.2|	543552 +	A	0	0	0	0	1	SA0470
gi|29165615|ref|NC_002745.2|	543588 +	C	0	1	0	0	0	SA0470

gi|29165615|ref|NC_002745.2|	543641	+	C	0	0	0	1	0	SA0470
gi|29165615|ref|NC_002745.2|	543704	+	C	0	0	0	0	1	SA0470
gi|29165615|ref|NC_002745.2|	543727	+	G	0	0	1	0	0	SA0470
gi|29165615|ref|NC_002745.2|	543786	+	A	0	0	0	1	0	SA0470
gi|29165615|ref|NC_002745.2|	543787	+	A	0	0	0	0	2	SA0470
gi|29165615|ref|NC_002745.2|	543791	+	C	0	0	0	1	1	SA0470
gi|29165615|ref|NC_002745.2|	543792	+	A	0	1	0	0	0	SA0470
gi|29165615|ref|NC_002745.2|	543831	+	A	0	0	0	1	0	SA0470
gi|29165615|ref|NC_002745.2|	543895	+	A	0	0	1	0	0	SA0470
gi|29165615|ref|NC_002745.2|	543898	+	A	0	0	0	0	1	SA0470
gi|29165615|ref|NC_002745.2|	543934	+	A	0	0	0	0	1	SA0470
gi|29165615|ref|NC_002745.2|	543941	+	U	0	0	1	0	0	SA0470
gi|29165615|ref|NC_002745.2|	543946	+	G	0	0	0	1	1	SA0470
gi|29165615|ref|NC_002745.2|	543947	+	C	0	1	0	0	0	SA0470
gi|29165615|ref|NC_002745.2|	543954	+	A	0	1	0	0	0	SA0470
gi|29165615|ref|NC_002745.2|	543977	+	A	0	0	0	0	1	SA0470
gi|29165615|ref|NC_002745.2|	543979	+	A	0	0	0	1	0	SA0470
gi|29165615|ref|NC_002745.2|	543985	+	A	0	0	0	0	1	SA0470
gi|29165615|ref|NC_002745.2|	543988	+	A	0	0	0	0	2	SA0470
gi|29165615|ref|NC_002745.2|	543992	+	A	0	0	0	1	0	SA0470
gi|29165615|ref|NC_002745.2|	543995	+	A	0	0	0	1	0	SA0470
gi|29165615|ref|NC_002745.2|	544034	+	A	0	0	0	1	0	SA0470
gi|29165615|ref|NC_002745.2|	544055	+	A	0	1	0	0	0	SA0470
gi|29165615|ref|NC_002745.2|	544056	+	A	0	0	1	0	0	SA0470
gi|29165615|ref|NC_002745.2|	544135	+	A	0	1	0	0	0	-
gi|29165615|ref|NC_002745.2|	544142	+	G	0	0	0	1	0	-
gi|29165615|ref|NC_002745.2|	544169	+	G	0	0	1	0	0	-
gi|29165615|ref|NC_002745.2|	544207	+	U	0	0	0	0	1	-
gi|29165615|ref|NC_002745.2|	544209	+	C	2	8	4	6	6	-
gi|29165615|ref|NC_002745.2|	544211	+	A	18	32	26	44	28	-
gi|29165615|ref|NC_002745.2|	544212	+	U	0	1	0	1	0	-
gi|29165615|ref|NC_002745.2|	544215	+	C	1	1	0	0	0	-
gi|29165615|ref|NC_002745.2|	544220	+	A	0	1	0	0	0	-
gi|29165615|ref|NC_002745.2|	544221	+	A	0	2	0	2	0	-
gi|29165615|ref|NC_002745.2|	544222	+	A	0	1	0	0	0	-
gi|29165615|ref|NC_002745.2|	544223	+	U	0	1	0	0	0	-
gi|29165615|ref|NC_002745.2|	544224	+	A	0	1	1	2	0	-
gi|29165615|ref|NC_002745.2|	544225	+	A	0	0	0	1	1	-
gi|29165615|ref|NC_002745.2|	544226	+	A	0	1	0	0	0	-
gi|29165615|ref|NC_002745.2|	544227	+	C	0	0	0	0	1	-
gi|29165615|ref|NC_002745.2|	544228	+	U	0	1	0	0	0	-
gi|29165615|ref|NC_002745.2|	544229	+	A	0	0	0	1	0	-
gi|29165615|ref|NC_002745.2|	544230	+	A	0	1	1	0	0	-
gi|29165615|ref|NC_002745.2|	544231	+	G	0	1	0	1	0	-
gi|29165615|ref|NC_002745.2|	544232	+	A	1	13	7	11	3	-
gi|29165615|ref|NC_002745.2|	544233	+	U	0	0	0	0	1	-
gi|29165615|ref|NC_002745.2|	544235	+	U	1	0	1	0	0	-
gi|29165615|ref|NC_002745.2|	544236	+	C	0	1	0	0	0	-
gi|29165615|ref|NC_002745.2|	544237	+	A	0	0	0	1	1	-
gi|29165615|ref|NC_002745.2|	544238	+	A	1	2	2	2	1	-
gi|29165615|ref|NC_002745.2|	544239	+	C	0	1	0	0	3	-
gi|29165615|ref|NC_002745.2|	544240	+	A	0	1	0	0	0	-
gi|29165615|ref|NC_002745.2|	544241	+	A	0	0	0	0	1	-
gi|29165615|ref|NC_002745.2|	544242	+	C	0	1	0	0	1	-
gi|29165615|ref|NC_002745.2|	544244	+	A	0	0	0	1	0	-
gi|29165615|ref|NC_002745.2|	544245	+	A	0	1	0	0	0	-
gi|29165615|ref|NC_002745.2|	544248	+	A	0	3	3	1	1	-
gi|29165615|ref|NC_002745.2|	544249	+	A	0	0	0	1	0	-
gi|29165615|ref|NC_002745.2|	544251	+	A	1	0	0	0	0	-
gi|29165615|ref|NC_002745.2|	544253	+	G	0	0	0	1	0	-
gi|29165615|ref|NC_002745.2|	544254	+	G	0	7	1	3	3	-
gi|29165615|ref|NC_002745.2|	544255	+	A	0	0	0	0	1	-
gi|29165615|ref|NC_002745.2|	544257	+	U	0	0	1	0	0	-
gi|29165615|ref|NC_002745.2|	544259	+	U	1	1	0	0	0	-
gi|29165615|ref|NC_002745.2|	544272	+	A	0	1	0	0	0	SA0471
gi|29165615|ref|NC_002745.2|	544275	+	A	0	1	0	0	0	SA0471
gi|29165615|ref|NC_002745.2|	544281	+	U	0	2	0	1	0	SA0471
gi|29165615|ref|NC_002745.2|	544282	+	A	0	1	0	0	1	SA0471
gi|29165615|ref|NC_002745.2|	544283	+	G	0	0	0	0	1	SA0471
gi|29165615|ref|NC_002745.2|	544284	+	A	0	0	0	0	1	SA0471
gi|29165615|ref|NC_002745.2|	544286	+	A	0	0	1	0	0	SA0471
gi|29165615|ref|NC_002745.2|	544287	+	A	0	1	0	0	0	SA0471
gi|29165615|ref|NC_002745.2|	544295	+	C	0	0	3	3	4	SA0471
gi|29165615|ref|NC_002745.2|	544296	+	A	0	0	1	0	1	SA0471
gi|29165615|ref|NC_002745.2|	544297	+	A	0	1	0	0	1	SA0471
gi|29165615|ref|NC_002745.2|	544298	+	A	0	0	1	0	1	SA0471
gi|29165615|ref|NC_002745.2|	544299	+	U	0	0	0	0	1	SA0471
gi|29165615|ref|NC_002745.2|	544301	+	A	0	0	0	0	1	SA0471
gi|29165615|ref|NC_002745.2|	544303	+	U	0	0	0	0	1	SA0471
gi|29165615|ref|NC_002745.2|	544314	+	C	0	2	0	0	0	SA0471
gi|29165615|ref|NC_002745.2|	544321	+	C	0	0	0	1	0	SA0471

gi|29165615|ref|NC_002745.2|	544322	+	A	1	0	0	0	0	SA0471
gi|29165615|ref|NC_002745.2|	544323	+	A	0	0	0	0	1	SA0471
gi|29165615|ref|NC_002745.2|	544325	+	U	0	1	0	0	0	SA0471
gi|29165615|ref|NC_002745.2|	544330	+	A	0	0	0	2	0	SA0471
gi|29165615|ref|NC_002745.2|	544331	+	A	0	2	1	0	0	SA0471
gi|29165615|ref|NC_002745.2|	544338	+	U	0	3	0	0	0	SA0471
gi|29165615|ref|NC_002745.2|	544339	+	A	0	1	0	1	0	SA0471
gi|29165615|ref|NC_002745.2|	544341	+	A	0	5	2	7	4	SA0471
gi|29165615|ref|NC_002745.2|	544342	+	U	0	0	1	0	0	SA0471
gi|29165615|ref|NC_002745.2|	544343	+	G	0	1	0	0	1	SA0471
gi|29165615|ref|NC_002745.2|	544344	+	A	1	1	4	3	3	SA0471
gi|29165615|ref|NC_002745.2|	544345	+	C	0	0	0	0	2	SA0471
gi|29165615|ref|NC_002745.2|	544347	+	A	0	0	1	0	0	SA0471
gi|29165615|ref|NC_002745.2|	544348	+	U	1	0	0	0	0	SA0471
gi|29165615|ref|NC_002745.2|	544350	+	C	0	0	2	4	1	SA0471
gi|29165615|ref|NC_002745.2|	544351	+	A	0	0	1	0	0	SA0471
gi|29165615|ref|NC_002745.2|	544353	+	C	0	2	0	4	0	SA0471
gi|29165615|ref|NC_002745.2|	544354	+	A	0	1	0	1	0	SA0471
gi|29165615|ref|NC_002745.2|	544356	+	A	0	2	0	0	2	SA0471
gi|29165615|ref|NC_002745.2|	544359	+	U	1	2	1	0	0	SA0471
gi|29165615|ref|NC_002745.2|	544360	+	U	0	0	0	0	1	SA0471
gi|29165615|ref|NC_002745.2|	544361	+	U	0	0	0	1	0	SA0471
gi|29165615|ref|NC_002745.2|	544363	+	U	0	1	0	0	0	SA0471
gi|29165615|ref|NC_002745.2|	544364	+	G	0	0	3	0	1	SA0471
gi|29165615|ref|NC_002745.2|	544366	+	A	0	2	1	0	0	SA0471
gi|29165615|ref|NC_002745.2|	544367	+	A	1	5	4	2	3	SA0471
gi|29165615|ref|NC_002745.2|	544368	+	A	0	1	2	3	3	SA0471
gi|29165615|ref|NC_002745.2|	544369	+	A	0	1	0	0	1	SA0471
gi|29165615|ref|NC_002745.2|	544370	+	U	0	0	0	0	1	SA0471
gi|29165615|ref|NC_002745.2|	544371	+	U	0	0	0	1	0	SA0471
gi|29165615|ref|NC_002745.2|	544372	+	G	0	1	0	0	0	SA0471
gi|29165615|ref|NC_002745.2|	544374	+	A	0	0	0	1	1	SA0471
gi|29165615|ref|NC_002745.2|	544377	+	A	2	4	6	4	1	SA0471
gi|29165615|ref|NC_002745.2|	544378	+	U	1	1	0	0	0	SA0471
gi|29165615|ref|NC_002745.2|	544379	+	C	0	0	0	0	1	SA0471
gi|29165615|ref|NC_002745.2|	544390	+	U	0	0	1	0	0	SA0471
gi|29165615|ref|NC_002745.2|	544393	+	U	1	0	0	0	0	SA0471
gi|29165615|ref|NC_002745.2|	544395	+	C	0	0	0	0	1	SA0471
gi|29165615|ref|NC_002745.2|	544397	+	G	0	0	0	0	1	SA0471
gi|29165615|ref|NC_002745.2|	544398	+	U	1	0	0	0	0	SA0471
gi|29165615|ref|NC_002745.2|	544403	+	G	0	0	1	0	0	SA0471
gi|29165615|ref|NC_002745.2|	544434	+	C	0	0	1	0	0	SA0471
gi|29165615|ref|NC_002745.2|	544435	+	A	1	0	0	0	0	SA0471
gi|29165615|ref|NC_002745.2|	544439	+	C	0	0	0	0	1	SA0471
gi|29165615|ref|NC_002745.2|	544453	+	U	2	0	0	0	0	SA0471
gi|29165615|ref|NC_002745.2|	544471	+	U	0	0	0	0	1	SA0471
gi|29165615|ref|NC_002745.2|	544475	+	G	0	0	1	0	0	SA0471
gi|29165615|ref|NC_002745.2|	544478	+	C	1	0	0	0	0	SA0471
gi|29165615|ref|NC_002745.2|	544483	+	A	0	0	0	0	1	SA0471
gi|29165615|ref|NC_002745.2|	544489	+	U	1	0	0	0	0	SA0471
gi|29165615|ref|NC_002745.2|	544490	+	A	0	0	0	0	1	SA0471
gi|29165615|ref|NC_002745.2|	544498	+	U	0	0	0	0	1	SA0471
gi|29165615|ref|NC_002745.2|	544517	+	U	0	0	1	0	0	SA0471
gi|29165615|ref|NC_002745.2|	544524	+	C	0	0	0	1	0	SA0471
gi|29165615|ref|NC_002745.2|	544532	+	U	0	1	0	0	0	SA0471
gi|29165615|ref|NC_002745.2|	544560	+	C	1	1	0	0	0	SA0471
gi|29165615|ref|NC_002745.2|	544580	+	A	0	0	0	0	1	SA0471
gi|29165615|ref|NC_002745.2|	544584	+	U	0	0	1	0	0	SA0471
gi|29165615|ref|NC_002745.2|	544587	+	U	0	0	1	0	0	SA0471
gi|29165615|ref|NC_002745.2|	544588	+	A	0	0	1	0	0	SA0471
gi|29165615|ref|NC_002745.2|	544591	+	A	0	0	0	1	0	SA0471
gi|29165615|ref|NC_002745.2|	544595	+	U	0	1	0	0	1	SA0471
gi|29165615|ref|NC_002745.2|	544604	+	G	0	0	1	0	0	SA0471
gi|29165615|ref|NC_002745.2|	544611	+	U	0	1	0	0	0	SA0471
gi|29165615|ref|NC_002745.2|	544616	+	A	0	0	0	1	0	SA0471
gi|29165615|ref|NC_002745.2|	544620	+	C	1	0	0	0	0	SA0471
gi|29165615|ref|NC_002745.2|	544628	+	G	1	0	0	0	1	SA0471
gi|29165615|ref|NC_002745.2|	544629	+	A	0	1	0	0	0	SA0471
gi|29165615|ref|NC_002745.2|	544631	+	G	0	1	0	0	0	SA0471
gi|29165615|ref|NC_002745.2|	544635	+	U	0	0	0	0	1	SA0471
gi|29165615|ref|NC_002745.2|	544636	+	G	0	4	1	0	0	SA0471
gi|29165615|ref|NC_002745.2|	544637	+	A	0	0	0	1	1	SA0471
gi|29165615|ref|NC_002745.2|	544644	+	C	0	0	2	0	5	SA0471
gi|29165615|ref|NC_002745.2|	544651	+	A	0	1	0	0	0	SA0471
gi|29165615|ref|NC_002745.2|	544652	+	A	0	2	2	1	3	SA0471
gi|29165615|ref|NC_002745.2|	544656	+	C	0	3	0	2	0	SA0471
gi|29165615|ref|NC_002745.2|	544658	+	A	0	2	0	1	1	SA0471
gi|29165615|ref|NC_002745.2|	544659	+	A	0	0	1	0	0	SA0471
gi|29165615|ref|NC_002745.2|	544660	+	A	0	0	0	0	2	SA0471
gi|29165615|ref|NC_002745.2|	544661	+	G	0	0	0	2	4	SA0471
gi|29165615|ref|NC_002745.2|	544662	+	A	0	2	0	1	4	SA0471

gi|29165615|ref|NC_002745.2|	544665 +	U	0	0	0	0	1	SA0471
gi|29165615|ref|NC_002745.2|	544666 +	A	0	1	0	5	4	SA0471
gi|29165615|ref|NC_002745.2|	544667 +	A	0	0	0	1	0	SA0471
gi|29165615|ref|NC_002745.2|	544668 +	A	0	0	1	1	0	SA0471
gi|29165615|ref|NC_002745.2|	544669 +	A	0	1	0	0	2	SA0471
gi|29165615|ref|NC_002745.2|	544671 +	A	0	1	2	1	3	SA0471
gi|29165615|ref|NC_002745.2|	544672 +	A	0	0	1	0	0	SA0471
gi|29165615|ref|NC_002745.2|	544673 +	G	0	0	0	1	0	SA0471
gi|29165615|ref|NC_002745.2|	544674 +	A	0	1	0	0	3	SA0471
gi|29165615|ref|NC_002745.2|	544676 +	C	0	0	1	0	1	SA0471
gi|29165615|ref|NC_002745.2|	544677 +	A	0	0	1	0	1	SA0471
gi|29165615|ref|NC_002745.2|	544680 +	G	0	1	0	0	1	SA0471
gi|29165615|ref|NC_002745.2|	544681 +	U	0	0	0	0	1	SA0471
gi|29165615|ref|NC_002745.2|	544686 +	U	0	0	0	0	1	SA0471
gi|29165615|ref|NC_002745.2|	544687 +	C	0	0	0	0	1	SA0471
gi|29165615|ref|NC_002745.2|	544688 +	G	0	0	0	0	1	SA0471
gi|29165615|ref|NC_002745.2|	544695 +	A	0	0	0	0	1	SA0471
gi|29165615|ref|NC_002745.2|	544696 +	A	0	1	0	0	0	SA0471
gi|29165615|ref|NC_002745.2|	544697 +	C	0	1	0	0	0	SA0471
gi|29165615|ref|NC_002745.2|	544699 +	A	0	0	0	1	1	SA0471
gi|29165615|ref|NC_002745.2|	544700 +	U	0	0	0	1	0	SA0471
gi|29165615|ref|NC_002745.2|	544701 +	U	0	0	0	0	1	SA0471
gi|29165615|ref|NC_002745.2|	544702 +	U	1	7	5	6	14	SA0471
gi|29165615|ref|NC_002745.2|	544703 +	G	1	4	2	3	2	SA0471
gi|29165615|ref|NC_002745.2|	544704 +	A	0	0	1	0	3	SA0471
gi|29165615|ref|NC_002745.2|	544718 +	C	1	0	0	0	0	SA0471
gi|29165615|ref|NC_002745.2|	544719 +	C	0	1	0	0	0	SA0471
gi|29165615|ref|NC_002745.2|	544721 +	G	0	0	0	0	1	SA0471
gi|29165615|ref|NC_002745.2|	544722 +	A	0	0	0	1	1	SA0471
gi|29165615|ref|NC_002745.2|	544733 +	U	0	0	1	1	0	SA0471
gi|29165615|ref|NC_002745.2|	544736 +	A	0	1	0	0	0	SA0471
gi|29165615|ref|NC_002745.2|	544737 +	C	1	0	0	0	0	SA0471
gi|29165615|ref|NC_002745.2|	544749 +	A	0	1	0	0	0	SA0471
gi|29165615|ref|NC_002745.2|	544750 +	G	0	0	1	1	2	SA0471
gi|29165615|ref|NC_002745.2|	544753 +	A	0	0	0	2	0	SA0471
gi|29165615|ref|NC_002745.2|	544756 +	A	0	0	0	3	0	SA0471
gi|29165615|ref|NC_002745.2|	544762 +	A	0	0	1	0	0	SA0471
gi|29165615|ref|NC_002745.2|	544766 +	G	0	2	0	0	2	SA0471
gi|29165615|ref|NC_002745.2|	544767 +	A	0	1	0	2	0	SA0471
gi|29165615|ref|NC_002745.2|	544772 +	A	0	0	0	2	0	SA0471
gi|29165615|ref|NC_002745.2|	544773 +	A	0	0	0	1	0	SA0471
gi|29165615|ref|NC_002745.2|	544782 +	A	1	1	0	0	0	SA0471
gi|29165615|ref|NC_002745.2|	544785 +	C	0	0	0	1	0	SA0471
gi|29165615|ref|NC_002745.2|	544797 +	G	1	0	0	0	0	SA0471
gi|29165615|ref|NC_002745.2|	544799 +	G	1	0	0	0	0	SA0471
gi|29165615|ref|NC_002745.2|	544802 +	G	0	0	0	0	1	SA0471
gi|29165615|ref|NC_002745.2|	544811 +	G	0	0	0	0	1	SA0471
gi|29165615|ref|NC_002745.2|	544820 +	U	0	2	0	1	0	SA0471
gi|29165615|ref|NC_002745.2|	544821 +	C	1	0	1	0	0	SA0471
gi|29165615|ref|NC_002745.2|	544838 +	C	0	1	0	0	0	SA0471
gi|29165615|ref|NC_002745.2|	544848 +	A	0	0	0	1	0	SA0471
gi|29165615|ref|NC_002745.2|	544850 +	U	0	0	0	0	1	SA0471
gi|29165615|ref|NC_002745.2|	544859 +	A	1	1	0	0	0	SA0471
gi|29165615|ref|NC_002745.2|	544863 +	A	0	1	1	1	0	SA0471
gi|29165615|ref|NC_002745.2|	544866 +	U	0	0	0	0	1	SA0471
gi|29165615|ref|NC_002745.2|	544868 +	G	0	0	0	0	2	SA0471
gi|29165615|ref|NC_002745.2|	544869 +	U	1	0	1	0	0	SA0471
gi|29165615|ref|NC_002745.2|	544872 +	C	0	2	2	2	3	SA0471
gi|29165615|ref|NC_002745.2|	544884 +	A	0	0	0	0	1	SA0471
gi|29165615|ref|NC_002745.2|	544890 +	C	1	0	0	1	0	SA0471
gi|29165615|ref|NC_002745.2|	544895 +	G	0	1	1	0	0	SA0471
gi|29165615|ref|NC_002745.2|	544896 +	U	0	0	1	0	0	SA0471
gi|29165615|ref|NC_002745.2|	544897 +	A	0	0	1	0	0	SA0471
gi|29165615|ref|NC_002745.2|	544898 +	U	0	1	0	0	0	SA0471
gi|29165615|ref|NC_002745.2|	544901 +	A	0	0	1	0	0	SA0471
gi|29165615|ref|NC_002745.2|	544905 +	G	1	0	0	0	0	SA0471
gi|29165615|ref|NC_002745.2|	544915 +	A	0	0	0	1	1	SA0471
gi|29165615|ref|NC_002745.2|	544926 +	A	0	0	0	0	1	SA0471
gi|29165615|ref|NC_002745.2|	544927 +	A	0	3	2	0	1	SA0471
gi|29165615|ref|NC_002745.2|	544930 +	A	0	2	0	0	1	SA0471
gi|29165615|ref|NC_002745.2|	544931 +	C	0	5	2	3	8	SA0471
gi|29165615|ref|NC_002745.2|	544932 +	A	0	0	0	2	2	SA0471
gi|29165615|ref|NC_002745.2|	544933 +	A	2	36	20	17	37	SA0471
gi|29165615|ref|NC_002745.2|	544934 +	G	0	2	0	2	2	SA0471
gi|29165615|ref|NC_002745.2|	544936 +	U	1	0	0	0	0	SA0471
gi|29165615|ref|NC_002745.2|	544951 +	U	0	1	0	0	0	SA0471
gi|29165615|ref|NC_002745.2|	544956 +	C	0	0	0	0	1	SA0471
gi|29165615|ref|NC_002745.2|	544957 +	A	0	1	0	0	1	SA0471
gi|29165615|ref|NC_002745.2|	544967 +	A	0	0	0	0	2	SA0471
gi|29165615|ref|NC_002745.2|	544971 +	C	0	0	0	1	0	SA0471
gi|29165615|ref|NC_002745.2|	544973 +	G	0	8	5	9	14	SA0471

gi|29165615|ref|NC_002745.2|	544974 +	A	4	21	8	15	43	SA0471
gi|29165615|ref|NC_002745.2|	544975 +	A	0	8	2	3	8	SA0471
gi|29165615|ref|NC_002745.2|	544976 +	A	0	6	3	0	8	SA0471
gi|29165615|ref|NC_002745.2|	544977 +	U	0	6	3	1	3	SA0471
gi|29165615|ref|NC_002745.2|	544978 +	C	0	0	0	0	2	SA0471
gi|29165615|ref|NC_002745.2|	544979 +	U	1	0	1	0	2	SA0471
gi|29165615|ref|NC_002745.2|	544980 +	A	0	1	1	2	5	SA0471
gi|29165615|ref|NC_002745.2|	544982 +	G	0	2	0	0	1	SA0471
gi|29165615|ref|NC_002745.2|	544983 +	A	0	7	5	6	8	SA0471
gi|29165615|ref|NC_002745.2|	544984 +	C	0	0	0	1	2	SA0471
gi|29165615|ref|NC_002745.2|	544987 +	U	0	0	0	2	1	SA0471
gi|29165615|ref|NC_002745.2|	544988 +	A	1	7	3	2	10	SA0471
gi|29165615|ref|NC_002745.2|	544989 +	U	0	8	3	6	26	SA0471
gi|29165615|ref|NC_002745.2|	544990 +	U	0	0	1	2	3	SA0471
gi|29165615|ref|NC_002745.2|	544991 +	A	0	27	12	12	32	SA0471
gi|29165615|ref|NC_002745.2|	544992 +	U	0	1	0	0	0	SA0471
gi|29165615|ref|NC_002745.2|	544993 +	U	0	0	1	0	4	SA0471
gi|29165615|ref|NC_002745.2|	544994 +	A	0	11	3	8	18	SA0471
gi|29165615|ref|NC_002745.2|	544995 +	A	0	6	6	5	7	SA0471
gi|29165615|ref|NC_002745.2|	544996 +	A	0	1	0	1	4	SA0471
gi|29165615|ref|NC_002745.2|	544997 +	G	0	1	1	1	6	SA0471
gi|29165615|ref|NC_002745.2|	544998 +	U	0	3	1	2	3	SA0471
gi|29165615|ref|NC_002745.2|	544999 +	A	0	10	6	3	20	SA0471
gi|29165615|ref|NC_002745.2|	545000 +	G	0	3	4	4	4	SA0471
gi|29165615|ref|NC_002745.2|	545001 +	G	1	39	10	24	75	SA0471
gi|29165615|ref|NC_002745.2|	545002 +	A	0	24	4	14	56	SA0471
gi|29165615|ref|NC_002745.2|	545003 +	A	0	12	5	16	16	SA0471
gi|29165615|ref|NC_002745.2|	545004 +	A	0	2	1	2	2	SA0471
gi|29165615|ref|NC_002745.2|	545005 +	U	0	0	0	1	0	SA0471
gi|29165615|ref|NC_002745.2|	545006 +	G	0	1	1	1	1	SA0471
gi|29165615|ref|NC_002745.2|	545007 +	A	0	2	3	2	4	SA0471
gi|29165615|ref|NC_002745.2|	545008 +	U	0	1	0	0	2	SA0471
gi|29165615|ref|NC_002745.2|	545009 +	A	0	2	1	2	7	SA0471
gi|29165615|ref|NC_002745.2|	545010 +	C	0	3	0	1	1	SA0471
gi|29165615|ref|NC_002745.2|	545012 +	G	0	2	2	1	2	SA0471
gi|29165615|ref|NC_002745.2|	545013 +	C	1	7	2	2	6	SA0471
gi|29165615|ref|NC_002745.2|	545014 +	G	0	1	1	2	3	SA0471
gi|29165615|ref|NC_002745.2|	545015 +	A	0	8	2	5	11	SA0471
gi|29165615|ref|NC_002745.2|	545017 +	G	0	0	1	1	0	SA0471
gi|29165615|ref|NC_002745.2|	545018 +	G	0	1	0	1	4	SA0471
gi|29165615|ref|NC_002745.2|	545019 +	A	0	10	2	3	9	SA0471
gi|29165615|ref|NC_002745.2|	545020 +	A	0	2	0	3	5	SA0471
gi|29165615|ref|NC_002745.2|	545021 +	A	0	0	0	2	2	SA0471
gi|29165615|ref|NC_002745.2|	545023 +	G	0	1	0	0	0	SA0471
gi|29165615|ref|NC_002745.2|	545024 +	U	0	0	2	2	0	SA0471
gi|29165615|ref|NC_002745.2|	545025 +	C	1	0	1	0	2	SA0471
gi|29165615|ref|NC_002745.2|	545029 +	U	0	1	0	0	0	SA0471
gi|29165615|ref|NC_002745.2|	545030 +	C	0	0	0	0	2	SA0471
gi|29165615|ref|NC_002745.2|	545031 +	G	0	1	1	0	4	SA0471
gi|29165615|ref|NC_002745.2|	545032 +	A	0	1	1	0	0	SA0471
gi|29165615|ref|NC_002745.2|	545035 +	U	2	0	1	0	0	SA0471
gi|29165615|ref|NC_002745.2|	545037 +	C	0	0	0	0	1	SA0471
gi|29165615|ref|NC_002745.2|	545039 +	A	0	4	0	2	1	SA0471
gi|29165615|ref|NC_002745.2|	545040 +	A	0	0	2	0	2	SA0471
gi|29165615|ref|NC_002745.2|	545041 +	A	0	0	0	3	0	SA0471
gi|29165615|ref|NC_002745.2|	545045 +	G	0	3	0	1	3	SA0471
gi|29165615|ref|NC_002745.2|	545046 +	A	0	0	0	2	0	SA0471
gi|29165615|ref|NC_002745.2|	545048 +	G	0	0	0	0	1	SA0471
gi|29165615|ref|NC_002745.2|	545051 +	A	0	0	0	0	1	SA0471
gi|29165615|ref|NC_002745.2|	545052 +	U	0	0	1	0	0	SA0471
gi|29165615|ref|NC_002745.2|	545059 +	A	0	1	0	0	1	SA0471
gi|29165615|ref|NC_002745.2|	545062 +	U	0	0	0	1	0	SA0471
gi|29165615|ref|NC_002745.2|	545063 +	A	0	0	0	0	1	SA0471
gi|29165615|ref|NC_002745.2|	545079 +	C	0	1	0	0	0	SA0471
gi|29165615|ref|NC_002745.2|	545080 +	G	0	0	0	0	1	SA0471
gi|29165615|ref|NC_002745.2|	545118 +	A	0	1	0	0	0	SA0471
gi|29165615|ref|NC_002745.2|	545126 +	A	0	0	0	0	1	SA0471
gi|29165615|ref|NC_002745.2|	545179 +	A	0	1	0	0	0	SA0471
gi|29165615|ref|NC_002745.2|	545354 +	U	0	0	1	0	0	-
gi|29165615|ref|NC_002745.2|	545356 +	A	0	1	0	0	0	-
gi|29165615|ref|NC_002745.2|	545384 +	A	0	0	1	0	0	-
gi|29165615|ref|NC_002745.2|	545396 +	A	0	0	0	1	0	-
gi|29165615|ref|NC_002745.2|	545419 +	A	0	0	1	1	0	SA0472
gi|29165615|ref|NC_002745.2|	545423 +	C	0	0	0	1	0	SA0472
gi|29165615|ref|NC_002745.2|	545424 +	A	0	2	0	0	0	SA0472
gi|29165615|ref|NC_002745.2|	545425 +	A	0	1	0	0	1	SA0472
gi|29165615|ref|NC_002745.2|	545426 +	A	0	1	0	0	0	SA0472
gi|29165615|ref|NC_002745.2|	545427 +	A	0	0	2	0	0	SA0472
gi|29165615|ref|NC_002745.2|	545498 +	C	0	0	0	1	0	SA0472
gi|29165615|ref|NC_002745.2|	545504 +	A	0	0	0	0	1	SA0472
gi|29165615|ref|NC_002745.2|	545508 +	A	0	0	0	1	0	SA0472

gi|29165615|ref|NC_002745.2|	545526	+	U	0	0	0	1	0	SA0472
gi|29165615|ref|NC_002745.2|	545528	+	A	0	0	0	1	0	SA0472
gi|29165615|ref|NC_002745.2|	545572	+	G	0	1	0	0	0	SA0472
gi|29165615|ref|NC_002745.2|	545598	+	A	0	0	0	0	1	SA0472
gi|29165615|ref|NC_002745.2|	545604	+	G	0	0	1	0	0	SA0472
gi|29165615|ref|NC_002745.2|	545615	+	U	0	0	1	0	0	SA0472
gi|29165615|ref|NC_002745.2|	545659	+	G	1	0	0	0	0	SA0472
gi|29165615|ref|NC_002745.2|	545673	+	A	0	3	0	1	1	SA0472
gi|29165615|ref|NC_002745.2|	545680	+	G	0	2	0	0	0	SA0472
gi|29165615|ref|NC_002745.2|	545698	+	A	0	1	0	0	0	SA0472
gi|29165615|ref|NC_002745.2|	545713	+	A	0	0	0	0	1	SA0472
gi|29165615|ref|NC_002745.2|	545809	+	G	0	0	0	1	0	SA0472
gi|29165615|ref|NC_002745.2|	545810	+	G	0	0	1	0	0	SA0472
gi|29165615|ref|NC_002745.2|	545912	+	A	0	0	1	0	0	SA0472
gi|29165615|ref|NC_002745.2|	545935	+	A	0	0	0	0	1	SA0472
gi|29165615|ref|NC_002745.2|	546018	+	G	0	1	0	0	0	SA0472
gi|29165615|ref|NC_002745.2|	546037	+	A	0	0	0	0	1	SA0472
gi|29165615|ref|NC_002745.2|	546061	+	G	0	0	0	0	1	SA0472
gi|29165615|ref|NC_002745.2|	546073	+	G	0	1	0	0	0	SA0472
gi|29165615|ref|NC_002745.2|	546074	+	A	0	0	1	0	0	SA0472
gi|29165615|ref|NC_002745.2|	546115	+	G	0	3	2	0	2	SA0472
gi|29165615|ref|NC_002745.2|	546132	+	U	0	0	1	0	0	SA0472
gi|29165615|ref|NC_002745.2|	546143	+	A	0	0	1	0	0	SA0472
gi|29165615|ref|NC_002745.2|	546152	+	C	0	1	0	0	0	SA0472
gi|29165615|ref|NC_002745.2|	546154	+	A	0	1	0	0	1	SA0472
gi|29165615|ref|NC_002745.2|	546165	+	A	0	0	0	1	0	SA0472
gi|29165615|ref|NC_002745.2|	546166	+	G	0	1	0	0	0	SA0472
gi|29165615|ref|NC_002745.2|	546167	+	G	0	0	0	0	1	SA0472
gi|29165615|ref|NC_002745.2|	546173	+	A	0	1	0	0	0	SA0472
gi|29165615|ref|NC_002745.2|	546176	+	U	0	1	0	0	0	SA0472
gi|29165615|ref|NC_002745.2|	546179	+	U	0	1	0	0	0	SA0472
gi|29165615|ref|NC_002745.2|	546180	+	A	0	2	0	0	1	SA0472
gi|29165615|ref|NC_002745.2|	546181	+	A	0	1	0	0	0	SA0472
gi|29165615|ref|NC_002745.2|	546182	+	A	0	0	1	1	0	SA0472
gi|29165615|ref|NC_002745.2|	546186	+	G	0	0	0	0	2	SA0472
gi|29165615|ref|NC_002745.2|	546191	+	A	0	1	1	2	3	SA0472
gi|29165615|ref|NC_002745.2|	546192	+	A	0	0	0	0	1	SA0472
gi|29165615|ref|NC_002745.2|	546193	+	A	0	1	0	0	0	SA0472
gi|29165615|ref|NC_002745.2|	546197	+	C	0	2	0	2	0	SA0472_SA0473
gi|29165615|ref|NC_002745.2|	546199	+	A	0	0	0	0	1	SA0472_SA0473
gi|29165615|ref|NC_002745.2|	546200	+	G	0	0	1	0	0	SA0472_SA0473
gi|29165615|ref|NC_002745.2|	546201	+	A	0	1	0	0	0	SA0472_SA0473
gi|29165615|ref|NC_002745.2|	546204	+	C	0	1	0	0	0	SA0472_SA0473
gi|29165615|ref|NC_002745.2|	546208	+	C	0	0	1	0	0	SA0472_SA0473
gi|29165615|ref|NC_002745.2|	546212	+	C	0	0	0	1	0	SA0472_SA0473
gi|29165615|ref|NC_002745.2|	546214	+	U	0	1	1	0	0	SA0472_SA0473
gi|29165615|ref|NC_002745.2|	546215	+	A	0	1	1	0	1	SA0472_SA0473
gi|29165615|ref|NC_002745.2|	546224	+	C	0	0	1	1	1	SA0473
gi|29165615|ref|NC_002745.2|	546226	+	C	0	0	0	0	1	SA0473
gi|29165615|ref|NC_002745.2|	546234	+	G	0	2	0	0	0	SA0473
gi|29165615|ref|NC_002745.2|	546246	+	C	1	0	0	0	1	SA0473
gi|29165615|ref|NC_002745.2|	546263	+	G	0	0	0	1	1	SA0473
gi|29165615|ref|NC_002745.2|	546266	+	A	1	3	0	0	0	SA0473
gi|29165615|ref|NC_002745.2|	546300	+	A	0	0	1	0	0	SA0473
gi|29165615|ref|NC_002745.2|	546303	+	U	0	0	1	0	0	SA0473
gi|29165615|ref|NC_002745.2|	546326	+	A	0	0	0	1	0	SA0473
gi|29165615|ref|NC_002745.2|	546336	+	U	0	0	1	0	0	SA0473
gi|29165615|ref|NC_002745.2|	546366	+	U	0	0	0	0	1	SA0473
gi|29165615|ref|NC_002745.2|	546368	+	G	0	0	1	0	0	SA0473
gi|29165615|ref|NC_002745.2|	546370	+	A	0	0	1	0	0	SA0473
gi|29165615|ref|NC_002745.2|	546414	+	A	0	0	0	0	1	SA0473
gi|29165615|ref|NC_002745.2|	546452	+	C	0	0	0	1	1	SA0473
gi|29165615|ref|NC_002745.2|	546454	+	A	0	0	0	0	1	SA0473
gi|29165615|ref|NC_002745.2|	546458	+	A	0	1	0	0	0	SA0473
gi|29165615|ref|NC_002745.2|	546467	+	A	7	12	4	5	13	SA0473
gi|29165615|ref|NC_002745.2|	546469	+	G	0	1	1	0	0	SA0473
gi|29165615|ref|NC_002745.2|	546471	+	A	0	0	0	1	0	SA0473
gi|29165615|ref|NC_002745.2|	546472	+	A	0	1	0	0	0	SA0473
gi|29165615|ref|NC_002745.2|	546487	+	C	0	0	0	0	1	SA0473
gi|29165615|ref|NC_002745.2|	546504	+	C	0	0	1	0	0	SA0473
gi|29165615|ref|NC_002745.2|	546506	+	A	0	0	0	1	0	SA0473
gi|29165615|ref|NC_002745.2|	546530	+	G	0	0	0	0	1	SA0473
gi|29165615|ref|NC_002745.2|	546539	+	A	0	2	0	0	0	SA0473
gi|29165615|ref|NC_002745.2|	546547	+	G	0	0	1	0	0	SA0473
gi|29165615|ref|NC_002745.2|	546554	+	A	0	0	1	0	0	SA0473
gi|29165615|ref|NC_002745.2|	546575	+	G	0	0	1	0	1	SA0474
gi|29165615|ref|NC_002745.2|	546578	+	U	0	0	1	0	0	SA0474
gi|29165615|ref|NC_002745.2|	546581	+	G	0	2	0	2	1	SA0474
gi|29165615|ref|NC_002745.2|	546597	+	U	0	0	0	0	1	SA0474
gi|29165615|ref|NC_002745.2|	546600	+	A	0	1	0	0	0	SA0474
gi|29165615|ref|NC_002745.2|	546602	+	A	0	1	0	0	0	SA0474

gi|29165615|ref|NC_002745.2|	546620	+	C	0	0	0	0	1	SA0474
gi|29165615|ref|NC_002745.2|	546623	+	U	0	1	0	0	0	SA0474
gi|29165615|ref|NC_002745.2|	546628	+	A	0	0	1	0	0	SA0474
gi|29165615|ref|NC_002745.2|	546638	+	A	0	1	1	2	3	SA0474
gi|29165615|ref|NC_002745.2|	546640	+	U	0	0	0	0	1	SA0474
gi|29165615|ref|NC_002745.2|	546644	+	A	0	1	0	0	0	SA0474
gi|29165615|ref|NC_002745.2|	546652	+	A	0	0	0	0	1	SA0474
gi|29165615|ref|NC_002745.2|	546680	+	A	0	0	0	0	1	SA0474
gi|29165615|ref|NC_002745.2|	546683	+	C	0	0	1	0	0	SA0474
gi|29165615|ref|NC_002745.2|	546686	+	C	0	0	1	0	0	SA0474
gi|29165615|ref|NC_002745.2|	546689	+	C	0	0	1	0	0	SA0474
gi|29165615|ref|NC_002745.2|	546691	+	G	1	0	0	0	0	SA0474
gi|29165615|ref|NC_002745.2|	546712	+	A	0	0	0	0	1	SA0474
gi|29165615|ref|NC_002745.2|	546715	+	U	0	0	1	0	0	SA0474
gi|29165615|ref|NC_002745.2|	546717	+	U	1	0	0	0	0	SA0474
gi|29165615|ref|NC_002745.2|	546721	+	A	0	0	0	0	1	SA0474
gi|29165615|ref|NC_002745.2|	546722	+	A	0	1	1	0	0	SA0474
gi|29165615|ref|NC_002745.2|	546727	+	U	0	1	1	0	1	SA0474
gi|29165615|ref|NC_002745.2|	546745	+	A	0	0	0	0	1	SA0474
gi|29165615|ref|NC_002745.2|	546765	+	G	2	0	0	0	0	SA0474
gi|29165615|ref|NC_002745.2|	546772	+	U	0	0	1	0	0	SA0474
gi|29165615|ref|NC_002745.2|	546781	+	A	0	0	0	0	1	SA0474
gi|29165615|ref|NC_002745.2|	546788	+	A	0	0	0	0	3	SA0474
gi|29165615|ref|NC_002745.2|	546791	+	A	0	0	0	0	1	SA0474
gi|29165615|ref|NC_002745.2|	546795	+	U	0	4	0	0	1	SA0474
gi|29165615|ref|NC_002745.2|	546796	+	U	0	0	0	1	0	SA0474
gi|29165615|ref|NC_002745.2|	546798	+	A	0	1	0	0	1	SA0474
gi|29165615|ref|NC_002745.2|	546804	+	U	0	1	2	0	2	SA0474
gi|29165615|ref|NC_002745.2|	546805	+	A	0	1	0	0	1	SA0474
gi|29165615|ref|NC_002745.2|	546813	+	G	0	0	0	0	1	SA0474
gi|29165615|ref|NC_002745.2|	546815	+	A	0	1	0	0	0	SA0474
gi|29165615|ref|NC_002745.2|	546817	+	C	0	0	0	0	1	SA0474
gi|29165615|ref|NC_002745.2|	546819	+	A	0	2	0	0	0	SA0474
gi|29165615|ref|NC_002745.2|	546831	+	A	0	1	0	0	0	SA0474
gi|29165615|ref|NC_002745.2|	546844	+	G	0	1	0	1	1	SA0474
gi|29165615|ref|NC_002745.2|	546845	+	A	0	2	3	1	4	SA0474
gi|29165615|ref|NC_002745.2|	546846	+	U	1	1	0	0	0	SA0474
gi|29165615|ref|NC_002745.2|	546848	+	U	1	0	0	0	0	SA0474
gi|29165615|ref|NC_002745.2|	546851	+	U	1	0	0	0	0	SA0474
gi|29165615|ref|NC_002745.2|	546852	+	G	1	0	0	0	1	SA0474
gi|29165615|ref|NC_002745.2|	546856	+	U	0	0	0	1	1	SA0474
gi|29165615|ref|NC_002745.2|	546860	+	G	2	0	1	0	0	SA0474
gi|29165615|ref|NC_002745.2|	546862	+	G	0	1	0	0	0	SA0474
gi|29165615|ref|NC_002745.2|	546864	+	A	0	0	0	1	0	SA0474
gi|29165615|ref|NC_002745.2|	546865	+	G	0	0	0	1	0	SA0474
gi|29165615|ref|NC_002745.2|	546866	+	A	0	2	0	0	0	SA0474
gi|29165615|ref|NC_002745.2|	546867	+	A	0	0	0	1	0	SA0474
gi|29165615|ref|NC_002745.2|	546868	+	A	0	0	0	0	1	SA0474
gi|29165615|ref|NC_002745.2|	546871	+	A	0	2	2	1	1	SA0474
gi|29165615|ref|NC_002745.2|	546872	+	U	0	1	1	0	0	SA0474
gi|29165615|ref|NC_002745.2|	546874	+	G	0	0	1	1	0	SA0474
gi|29165615|ref|NC_002745.2|	546875	+	A	0	1	2	0	1	SA0474
gi|29165615|ref|NC_002745.2|	546876	+	U	0	0	1	0	0	SA0474
gi|29165615|ref|NC_002745.2|	546878	+	U	0	1	0	0	0	SA0474
gi|29165615|ref|NC_002745.2|	546879	+	A	0	0	2	0	5	SA0474
gi|29165615|ref|NC_002745.2|	546881	+	C	0	0	1	0	0	SA0474
gi|29165615|ref|NC_002745.2|	546882	+	A	0	1	2	1	3	SA0474
gi|29165615|ref|NC_002745.2|	546883	+	A	0	2	0	1	0	SA0474
gi|29165615|ref|NC_002745.2|	546885	+	A	0	0	2	0	0	SA0474
gi|29165615|ref|NC_002745.2|	546886	+	C	0	0	0	1	0	SA0474
gi|29165615|ref|NC_002745.2|	546887	+	U	0	0	1	0	0	SA0474
gi|29165615|ref|NC_002745.2|	546914	+	A	0	0	0	1	0	SA0474
gi|29165615|ref|NC_002745.2|	546920	+	C	0	0	1	0	0	SA0474
gi|29165615|ref|NC_002745.2|	546938	+	U	0	0	1	0	0	SA0474
gi|29165615|ref|NC_002745.2|	546946	+	A	0	2	0	0	0	SA0474
gi|29165615|ref|NC_002745.2|	546955	+	A	0	0	1	0	0	SA0474
gi|29165615|ref|NC_002745.2|	546962	+	U	0	0	0	1	0	SA0474
gi|29165615|ref|NC_002745.2|	546976	+	A	0	0	1	1	0	SA0474
gi|29165615|ref|NC_002745.2|	546978	+	A	0	1	0	0	0	SA0474
gi|29165615|ref|NC_002745.2|	546988	+	A	0	2	2	2	1	SA0474
gi|29165615|ref|NC_002745.2|	546989	+	A	0	0	1	2	2	SA0474
gi|29165615|ref|NC_002745.2|	546990	+	A	0	1	0	0	0	SA0474
gi|29165615|ref|NC_002745.2|	546992	+	A	0	2	1	0	2	SA0474
gi|29165615|ref|NC_002745.2|	546993	+	U	0	0	1	0	0	SA0474
gi|29165615|ref|NC_002745.2|	546995	+	U	0	6	0	1	0	SA0474
gi|29165615|ref|NC_002745.2|	546996	+	A	0	0	1	0	0	SA0474
gi|29165615|ref|NC_002745.2|	546997	+	G	1	0	0	0	0	SA0474
gi|29165615|ref|NC_002745.2|	546998	+	U	0	0	0	0	1	SA0474
gi|29165615|ref|NC_002745.2|	547004	+	U	0	2	1	0	0	SA0474
gi|29165615|ref|NC_002745.2|	547005	+	C	0	1	0	0	0	SA0474
gi|29165615|ref|NC_002745.2|	547007	+	A	0	0	1	0	1	SA0474

gi|29165615|ref|NC_002745.2|	547016 +	U	0	0	1	0	1	SA0474
gi|29165615|ref|NC_002745.2|	547017 +	A	0	0	1	0	2	SA0474
gi|29165615|ref|NC_002745.2|	547018 +	A	0	1	0	0	1	SA0474
gi|29165615|ref|NC_002745.2|	547019 +	A	0	2	0	0	3	SA0474
gi|29165615|ref|NC_002745.2|	547020 +	G	0	1	0	0	0	SA0474
gi|29165615|ref|NC_002745.2|	547021 +	A	0	0	1	0	0	SA0474
gi|29165615|ref|NC_002745.2|	547022 +	G	0	0	0	1	2	SA0474
gi|29165615|ref|NC_002745.2|	547023 +	A	0	0	1	0	0	SA0474
gi|29165615|ref|NC_002745.2|	547026 +	U	0	0	0	0	1	SA0474
gi|29165615|ref|NC_002745.2|	547028 +	A	0	2	1	0	1	SA0474
gi|29165615|ref|NC_002745.2|	547035 +	U	0	2	1	2	1	-
gi|29165615|ref|NC_002745.2|	547036 +	A	0	6	3	0	3	-
gi|29165615|ref|NC_002745.2|	547037 +	U	0	2	0	0	0	-
gi|29165615|ref|NC_002745.2|	547039 +	G	1	1	2	0	1	-
gi|29165615|ref|NC_002745.2|	547040 +	U	0	6	2	0	1	-
gi|29165615|ref|NC_002745.2|	547041 +	U	0	0	1	0	0	-
gi|29165615|ref|NC_002745.2|	547042 +	G	0	3	1	3	3	-
gi|29165615|ref|NC_002745.2|	547043 +	A	0	3	0	3	5	-
gi|29165615|ref|NC_002745.2|	547044 +	G	0	1	0	0	2	-
gi|29165615|ref|NC_002745.2|	547045 +	A	0	5	0	1	4	-
gi|29165615|ref|NC_002745.2|	547046 +	A	0	0	0	0	1	-
gi|29165615|ref|NC_002745.2|	547054 +	A	0	0	0	0	1	-
gi|29165615|ref|NC_002745.2|	547057 +	U	0	0	0	0	2	-
gi|29165615|ref|NC_002745.2|	547058 +	A	0	1	1	0	0	-
gi|29165615|ref|NC_002745.2|	547062 +	G	0	0	0	0	1	-
gi|29165615|ref|NC_002745.2|	547064 +	A	0	0	1	1	0	-
gi|29165615|ref|NC_002745.2|	547066 +	U	0	1	0	0	0	-
gi|29165615|ref|NC_002745.2|	547068 +	G	0	0	0	1	1	-
gi|29165615|ref|NC_002745.2|	547069 +	A	0	1	0	0	1	-
gi|29165615|ref|NC_002745.2|	547071 +	G	0	0	1	0	0	-
gi|29165615|ref|NC_002745.2|	547083 +	G	0	0	1	0	1	-
gi|29165615|ref|NC_002745.2|	547095 +	C	0	1	0	0	0	-
gi|29165615|ref|NC_002745.2|	547105 +	A	0	1	0	0	0	-
gi|29165615|ref|NC_002745.2|	547117 +	A	0	0	0	1	0	-
gi|29165615|ref|NC_002745.2|	547126 +	C	0	0	0	1	0	-
gi|29165615|ref|NC_002745.2|	547128 +	A	0	1	2	1	0	-
gi|29165615|ref|NC_002745.2|	547134 +	U	0	0	0	3	3	-
gi|29165615|ref|NC_002745.2|	547136 +	U	0	0	0	1	0	-
gi|29165615|ref|NC_002745.2|	547140 +	A	0	1	0	0	0	-
gi|29165615|ref|NC_002745.2|	547142 +	G	0	1	0	0	0	-
gi|29165615|ref|NC_002745.2|	547148 +	G	2	5	1	0	3	-
gi|29165615|ref|NC_002745.2|	547150 +	A	1	0	1	0	0	-
gi|29165615|ref|NC_002745.2|	547152 +	G	0	0	1	0	0	-
gi|29165615|ref|NC_002745.2|	547158 +	G	0	0	0	0	1	-
gi|29165615|ref|NC_002745.2|	547160 +	A	0	0	1	0	0	-
gi|29165615|ref|NC_002745.2|	547164 +	A	0	0	0	1	0	-
gi|29165615|ref|NC_002745.2|	547168 +	G	1	3	0	0	0	-
gi|29165615|ref|NC_002745.2|	547172 +	C	0	0	0	0	1	-
gi|29165615|ref|NC_002745.2|	547176 +	A	0	1	0	0	0	-
gi|29165615|ref|NC_002745.2|	547178 +	A	0	2	0	0	1	-
gi|29165615|ref|NC_002745.2|	547181 +	A	0	2	0	0	0	-
gi|29165615|ref|NC_002745.2|	547195 +	U	0	1	0	0	0	-
gi|29165615|ref|NC_002745.2|	547197 +	G	0	1	0	1	1	-
gi|29165615|ref|NC_002745.2|	547200 +	A	0	0	1	0	1	-
gi|29165615|ref|NC_002745.2|	547201 +	A	0	1	0	0	0	-
gi|29165615|ref|NC_002745.2|	547203 +	U	0	0	0	1	0	-
gi|29165615|ref|NC_002745.2|	547210 +	A	0	2	0	0	0	-
gi|29165615|ref|NC_002745.2|	547287 +	U	1	0	0	0	0	-
gi|29165615|ref|NC_002745.2|	547288 +	A	0	1	0	0	0	-
gi|29165615|ref|NC_002745.2|	547345 +	A	0	0	0	0	1	-
gi|29165615|ref|NC_002745.2|	547363 +	A	0	0	0	1	3	-
gi|29165615|ref|NC_002745.2|	547365 +	A	0	0	0	1	0	-
gi|29165615|ref|NC_002745.2|	547366 +	A	1	5	5	2	3	-
gi|29165615|ref|NC_002745.2|	547367 +	G	3	1	5	2	4	-
gi|29165615|ref|NC_002745.2|	547368 +	U	1	0	0	0	0	-
gi|29165615|ref|NC_002745.2|	547369 +	G	0	5	0	1	2	-
gi|29165615|ref|NC_002745.2|	547370 +	A	0	1	3	0	2	-
gi|29165615|ref|NC_002745.2|	547372 +	A	0	0	0	1	0	-
gi|29165615|ref|NC_002745.2|	547373 +	A	0	0	2	1	1	-
gi|29165615|ref|NC_002745.2|	547374 +	A	0	0	0	0	2	-
gi|29165615|ref|NC_002745.2|	547375 +	A	1	1	2	0	2	-
gi|29165615|ref|NC_002745.2|	547379 +	A	0	0	1	0	0	-
gi|29165615|ref|NC_002745.2|	547384 +	A	2	2	7	3	1	-
gi|29165615|ref|NC_002745.2|	547385 +	U	0	0	0	0	1	-
gi|29165615|ref|NC_002745.2|	547386 +	G	0	1	0	1	0	-
gi|29165615|ref|NC_002745.2|	547387 +	A	0	3	4	2	7	-
gi|29165615|ref|NC_002745.2|	547388 +	A	0	0	0	2	1	-
gi|29165615|ref|NC_002745.2|	547389 +	U	0	1	0	0	0	-
gi|29165615|ref|NC_002745.2|	547390 +	A	0	3	0	0	1	-
gi|29165615|ref|NC_002745.2|	547391 +	U	0	1	0	0	1	-
gi|29165615|ref|NC_002745.2|	547392 +	A	0	0	0	1	2	-

gi|29165615|ref|NC_002745.2|	547393 +	A	0	0	1	0	2	-
gi|29165615|ref|NC_002745.2|	547394 +	G	0	0	1	1	2	-
gi|29165615|ref|NC_002745.2|	547395 +	A	0	5	3	2	7	-
gi|29165615|ref|NC_002745.2|	547396 +	A	0	1	0	1	1	-
gi|29165615|ref|NC_002745.2|	547397 +	A	0	0	0	2	0	-
gi|29165615|ref|NC_002745.2|	547398 +	A	0	2	0	1	2	-
gi|29165615|ref|NC_002745.2|	547399 +	C	1	5	0	6	2	-
gi|29165615|ref|NC_002745.2|	547400 +	A	1	1	0	0	0	-
gi|29165615|ref|NC_002745.2|	547403 +	C	0	0	1	0	1	-
gi|29165615|ref|NC_002745.2|	547404 +	C	0	1	0	1	0	-
gi|29165615|ref|NC_002745.2|	547406 +	C	1	0	1	0	0	-
gi|29165615|ref|NC_002745.2|	547408 +	U	0	0	1	0	0	-
gi|29165615|ref|NC_002745.2|	547410 +	A	0	0	0	1	1	-
gi|29165615|ref|NC_002745.2|	547412 +	A	0	1	0	0	0	-
gi|29165615|ref|NC_002745.2|	547413 +	A	0	0	1	1	2	-
gi|29165615|ref|NC_002745.2|	547416 +	U	0	0	0	1	0	-
gi|29165615|ref|NC_002745.2|	547417 +	U	0	0	0	1	0	-
gi|29165615|ref|NC_002745.2|	547418 +	A	0	0	1	0	1	-
gi|29165615|ref|NC_002745.2|	547419 +	A	1	8	5	8	9	-
gi|29165615|ref|NC_002745.2|	547420 +	G	1	2	4	1	3	-
gi|29165615|ref|NC_002745.2|	547421 +	G	0	1	2	0	2	-
gi|29165615|ref|NC_002745.2|	547422 +	U	0	2	1	0	2	-
gi|29165615|ref|NC_002745.2|	547423 +	G	0	3	1	0	2	-
gi|29165615|ref|NC_002745.2|	547424 +	U	0	4	5	1	1	-
gi|29165615|ref|NC_002745.2|	547425 +	A	0	4	10	4	12	-
gi|29165615|ref|NC_002745.2|	547426 +	A	0	8	5	5	13	-
gi|29165615|ref|NC_002745.2|	547427 +	U	0	2	1	0	0	-
gi|29165615|ref|NC_002745.2|	547429 +	G	1	3	2	3	6	-
gi|29165615|ref|NC_002745.2|	547430 +	U	1	0	4	2	4	-
gi|29165615|ref|NC_002745.2|	547431 +	U	0	2	0	0	0	-
gi|29165615|ref|NC_002745.2|	547432 +	A	1	1	1	2	3	-
gi|29165615|ref|NC_002745.2|	547433 +	A	1	1	1	0	0	-
gi|29165615|ref|NC_002745.2|	547435 +	G	0	0	0	1	0	-
gi|29165615|ref|NC_002745.2|	547437 +	U	0	0	0	0	1	-
gi|29165615|ref|NC_002745.2|	547438 +	A	0	1	1	0	0	-
gi|29165615|ref|NC_002745.2|	547439 +	A	0	2	1	1	2	-
gi|29165615|ref|NC_002745.2|	547440 +	A	0	1	3	1	0	-
gi|29165615|ref|NC_002745.2|	547441 +	G	0	2	0	0	0	-
gi|29165615|ref|NC_002745.2|	547442 +	U	0	3	8	5	7	-
gi|29165615|ref|NC_002745.2|	547443 +	A	0	9	6	9	18	-
gi|29165615|ref|NC_002745.2|	547444 +	U	0	0	2	0	2	-
gi|29165615|ref|NC_002745.2|	547445 +	U	0	0	1	0	0	-
gi|29165615|ref|NC_002745.2|	547446 +	A	0	0	0	0	1	-
gi|29165615|ref|NC_002745.2|	547447 +	G	0	1	0	1	2	-
gi|29165615|ref|NC_002745.2|	547448 +	A	0	1	0	1	1	-
gi|29165615|ref|NC_002745.2|	547449 +	A	0	3	0	1	2	-
gi|29165615|ref|NC_002745.2|	547450 +	A	0	1	0	0	0	-
gi|29165615|ref|NC_002745.2|	547452 +	A	1	0	0	2	1	-
gi|29165615|ref|NC_002745.2|	547453 +	U	0	1	0	0	0	-
gi|29165615|ref|NC_002745.2|	547455 +	G	0	0	0	0	2	-
gi|29165615|ref|NC_002745.2|	547456 +	A	0	1	2	3	4	-
gi|29165615|ref|NC_002745.2|	547457 +	A	0	1	0	0	0	-
gi|29165615|ref|NC_002745.2|	547458 +	A	0	0	0	1	0	-
gi|29165615|ref|NC_002745.2|	547459 +	G	0	1	0	0	2	-
gi|29165615|ref|NC_002745.2|	547461 +	G	0	0	0	3	0	-
gi|29165615|ref|NC_002745.2|	547462 +	A	0	0	0	1	0	-
gi|29165615|ref|NC_002745.2|	547463 +	U	1	0	0	0	0	-
gi|29165615|ref|NC_002745.2|	547464 +	G	0	0	0	0	2	-
gi|29165615|ref|NC_002745.2|	547465 +	U	0	1	3	1	1	-
gi|29165615|ref|NC_002745.2|	547467 +	U	0	1	0	0	0	-
gi|29165615|ref|NC_002745.2|	547469 +	U	0	3	0	3	1	-
gi|29165615|ref|NC_002745.2|	547470 +	U	0	0	0	1	1	-
gi|29165615|ref|NC_002745.2|	547471 +	G	0	0	0	2	0	-
gi|29165615|ref|NC_002745.2|	547472 +	A	0	1	2	8	3	-
gi|29165615|ref|NC_002745.2|	547473 +	G	0	1	3	1	2	-
gi|29165615|ref|NC_002745.2|	547474 +	C	0	0	0	0	3	-
gi|29165615|ref|NC_002745.2|	547475 +	A	0	3	0	0	1	-
gi|29165615|ref|NC_002745.2|	547476 +	A	0	5	3	3	6	-
gi|29165615|ref|NC_002745.2|	547477 +	A	0	1	1	2	3	-
gi|29165615|ref|NC_002745.2|	547478 +	A	0	1	1	3	4	-
gi|29165615|ref|NC_002745.2|	547480 +	U	0	1	0	0	0	-
gi|29165615|ref|NC_002745.2|	547482 +	U	0	1	1	2	0	-
gi|29165615|ref|NC_002745.2|	547483 +	C	0	0	1	0	0	-
gi|29165615|ref|NC_002745.2|	547485 +	G	0	0	0	0	1	-
gi|29165615|ref|NC_002745.2|	547486 +	A	0	8	6	9	5	-
gi|29165615|ref|NC_002745.2|	547487 +	U	0	0	0	1	1	-
gi|29165615|ref|NC_002745.2|	547488 +	A	0	1	3	0	2	-
gi|29165615|ref|NC_002745.2|	547489 +	U	0	0	2	2	1	-
gi|29165615|ref|NC_002745.2|	547490 +	U	0	2	0	1	1	-
gi|29165615|ref|NC_002745.2|	547492 +	U	0	1	2	3	0	-
gi|29165615|ref|NC_002745.2|	547493 +	A	2	13	9	9	15	-

gi|29165615|ref|NC_002745.2|	547494 +	U	0	1	0	0	2	-
gi|29165615|ref|NC_002745.2|	547495 +	A	1	6	5	10	14	-
gi|29165615|ref|NC_002745.2|	547496 +	A	0	6	3	8	5	-
gi|29165615|ref|NC_002745.2|	547497 +	A	1	13	6	7	15	-
gi|29165615|ref|NC_002745.2|	547498 +	A	0	11	6	10	13	-
gi|29165615|ref|NC_002745.2|	547499 +	C	0	6	5	3	6	-
gi|29165615|ref|NC_002745.2|	547500 +	U	0	9	1	3	4	-
gi|29165615|ref|NC_002745.2|	547501 +	U	1	2	1	5	7	-
gi|29165615|ref|NC_002745.2|	547502 +	U	0	20	5	14	16	-
gi|29165615|ref|NC_002745.2|	547503 +	A	6	182	113	119	247	-
gi|29165615|ref|NC_002745.2|	547504 +	A	9	192	85	109	208	-
gi|29165615|ref|NC_002745.2|	547505 +	A	1	43	17	15	28	-
gi|29165615|ref|NC_002745.2|	547506 +	G	0	6	3	2	2	-
gi|29165615|ref|NC_002745.2|	547507 +	G	0	9	12	8	16	-
gi|29165615|ref|NC_002745.2|	547508 +	A	4	63	37	46	55	-
gi|29165615|ref|NC_002745.2|	547509 +	A	0	39	30	20	34	-
gi|29165615|ref|NC_002745.2|	547510 +	A	5	103	62	41	93	-
gi|29165615|ref|NC_002745.2|	547511 +	A	2	33	41	19	28	-
gi|29165615|ref|NC_002745.2|	547512 +	U	0	2	7	2	7	-
gi|29165615|ref|NC_002745.2|	547513 +	G	1	4	1	7	2	-
gi|29165615|ref|NC_002745.2|	547514 +	U	2	32	26	20	33	-
gi|29165615|ref|NC_002745.2|	547515 +	U	1	6	6	2	3	-
gi|29165615|ref|NC_002745.2|	547516 +	U	0	7	7	2	6	-
gi|29165615|ref|NC_002745.2|	547517 +	G	1	25	11	12	10	-
gi|29165615|ref|NC_002745.2|	547518 +	A	3	41	41	35	32	-
gi|29165615|ref|NC_002745.2|	547519 +	G	0	2	2	2	2	-
gi|29165615|ref|NC_002745.2|	547520 +	U	0	5	5	1	3	-
gi|29165615|ref|NC_002745.2|	547521 +	G	0	4	4	0	2	-
gi|29165615|ref|NC_002745.2|	547522 +	U	0	3	2	0	1	-
gi|29165615|ref|NC_002745.2|	547523 +	A	0	0	0	1	0	-
gi|29165615|ref|NC_002745.2|	547524 +	C	0	5	5	3	3	-
gi|29165615|ref|NC_002745.2|	547525 +	C	0	0	2	0	0	-
gi|29165615|ref|NC_002745.2|	547526 +	A	1	1	1	0	0	-
gi|29165615|ref|NC_002745.2|	547531 +	G	0	1	3	0	1	-
gi|29165615|ref|NC_002745.2|	547532 +	A	1	4	1	4	4	-
gi|29165615|ref|NC_002745.2|	547533 +	A	0	2	0	0	1	-
gi|29165615|ref|NC_002745.2|	547535 +	A	0	0	0	1	0	-
gi|29165615|ref|NC_002745.2|	547536 +	C	0	1	1	0	0	-
gi|29165615|ref|NC_002745.2|	547538 +	A	0	1	5	4	1	-
gi|29165615|ref|NC_002745.2|	547539 +	A	0	0	1	0	1	-
gi|29165615|ref|NC_002745.2|	547541 +	G	0	0	0	0	1	-
gi|29165615|ref|NC_002745.2|	547542 +	G	0	2	1	2	0	-
gi|29165615|ref|NC_002745.2|	547543 +	A	1	6	7	4	5	-
gi|29165615|ref|NC_002745.2|	547544 +	U	1	0	0	0	0	-
gi|29165615|ref|NC_002745.2|	547546 +	A	0	7	4	3	3	-
gi|29165615|ref|NC_002745.2|	547547 +	C	0	1	0	1	1	-
gi|29165615|ref|NC_002745.2|	547548 +	A	0	0	4	1	0	-
gi|29165615|ref|NC_002745.2|	547549 +	A	0	2	1	0	1	-
gi|29165615|ref|NC_002745.2|	547550 +	C	0	5	2	1	2	-
gi|29165615|ref|NC_002745.2|	547551 +	A	0	2	1	1	0	-
gi|29165615|ref|NC_002745.2|	547552 +	A	0	2	2	1	1	-
gi|29165615|ref|NC_002745.2|	547553 +	G	0	1	4	0	1	-
gi|29165615|ref|NC_002745.2|	547554 +	U	0	0	0	2	1	-
gi|29165615|ref|NC_002745.2|	547555 +	U	0	0	0	0	1	-
gi|29165615|ref|NC_002745.2|	547556 +	A	0	4	6	4	4	-
gi|29165615|ref|NC_002745.2|	547557 +	A	1	8	8	3	6	-
gi|29165615|ref|NC_002745.2|	547558 +	A	0	0	1	1	1	-
gi|29165615|ref|NC_002745.2|	547559 +	G	0	6	1	1	4	-
gi|29165615|ref|NC_002745.2|	547560 +	G	0	2	0	1	5	-
gi|29165615|ref|NC_002745.2|	547561 +	A	1	1	0	0	3	-
gi|29165615|ref|NC_002745.2|	547562 +	G	0	1	0	1	1	-
gi|29165615|ref|NC_002745.2|	547563 +	A	0	3	1	1	3	-
gi|29165615|ref|NC_002745.2|	547564 +	G	0	0	1	0	2	-
gi|29165615|ref|NC_002745.2|	547565 +	A	1	10	4	4	18	-
gi|29165615|ref|NC_002745.2|	547566 +	A	1	1	2	0	0	-
gi|29165615|ref|NC_002745.2|	547567 +	A	0	1	1	0	0	-
gi|29165615|ref|NC_002745.2|	547568 +	G	0	0	1	0	1	-
gi|29165615|ref|NC_002745.2|	547569 +	U	2	0	2	0	0	-
gi|29165615|ref|NC_002745.2|	547570 +	U	0	0	1	0	1	-
gi|29165615|ref|NC_002745.2|	547571 +	A	0	1	1	0	0	SA0475
gi|29165615|ref|NC_002745.2|	547572 +	U	0	0	1	0	0	SA0475
gi|29165615|ref|NC_002745.2|	547573 +	G	0	1	0	1	0	SA0475
gi|29165615|ref|NC_002745.2|	547574 +	U	0	0	0	1	2	SA0475
gi|29165615|ref|NC_002745.2|	547575 +	C	1	0	2	0	1	SA0475
gi|29165615|ref|NC_002745.2|	547576 +	A	0	2	2	2	2	SA0475
gi|29165615|ref|NC_002745.2|	547577 +	G	0	2	4	2	8	SA0475
gi|29165615|ref|NC_002745.2|	547578 +	A	0	2	0	1	0	SA0475
gi|29165615|ref|NC_002745.2|	547579 +	A	0	1	0	0	1	SA0475
gi|29165615|ref|NC_002745.2|	547580 +	G	0	1	1	0	2	SA0475
gi|29165615|ref|NC_002745.2|	547581 +	A	1	4	2	4	4	SA0475
gi|29165615|ref|NC_002745.2|	547582 +	A	0	2	0	1	0	SA0475

gi|29165615|ref|NC_002745.2|	547583	+	A	0	1	1	0	0	SA0475
gi|29165615|ref|NC_002745.2|	547584	+	U	0	0	1	0	2	SA0475
gi|29165615|ref|NC_002745.2|	547585	+	G	0	0	1	0	0	SA0475
gi|29165615|ref|NC_002745.2|	547586	+	A	0	1	2	1	1	SA0475
gi|29165615|ref|NC_002745.2|	547587	+	A	2	0	0	0	0	SA0475
gi|29165615|ref|NC_002745.2|	547589	+	G	0	0	0	0	1	SA0475
gi|29165615|ref|NC_002745.2|	547590	+	A	1	0	0	0	1	SA0475
gi|29165615|ref|NC_002745.2|	547591	+	C	1	0	0	0	0	SA0475
gi|29165615|ref|NC_002745.2|	547593	+	A	0	4	2	2	0	SA0475
gi|29165615|ref|NC_002745.2|	547594	+	A	0	0	1	0	1	SA0475
gi|29165615|ref|NC_002745.2|	547597	+	G	1	0	0	1	0	SA0475
gi|29165615|ref|NC_002745.2|	547599	+	U	0	2	0	0	0	SA0475
gi|29165615|ref|NC_002745.2|	547602	+	U	3	0	0	0	0	SA0475
gi|29165615|ref|NC_002745.2|	547604	+	C	0	1	1	0	0	SA0475
gi|29165615|ref|NC_002745.2|	547605	+	G	0	0	0	0	2	SA0475
gi|29165615|ref|NC_002745.2|	547612	+	A	0	1	0	1	0	SA0475
gi|29165615|ref|NC_002745.2|	547614	+	A	0	2	2	1	2	SA0475
gi|29165615|ref|NC_002745.2|	547615	+	A	1	27	13	6	24	SA0475
gi|29165615|ref|NC_002745.2|	547616	+	U	0	0	0	0	1	SA0475
gi|29165615|ref|NC_002745.2|	547617	+	U	0	0	1	1	0	SA0475
gi|29165615|ref|NC_002745.2|	547618	+	A	0	2	1	0	1	SA0475
gi|29165615|ref|NC_002745.2|	547619	+	C	0	2	1	4	0	SA0475
gi|29165615|ref|NC_002745.2|	547620	+	A	0	0	0	1	1	SA0475
gi|29165615|ref|NC_002745.2|	547621	+	A	0	5	3	5	5	SA0475
gi|29165615|ref|NC_002745.2|	547622	+	G	0	0	1	2	3	SA0475
gi|29165615|ref|NC_002745.2|	547623	+	A	0	14	4	4	16	SA0475
gi|29165615|ref|NC_002745.2|	547624	+	A	0	6	1	2	6	SA0475
gi|29165615|ref|NC_002745.2|	547625	+	U	0	1	1	0	1	SA0475
gi|29165615|ref|NC_002745.2|	547626	+	U	2	5	1	0	1	SA0475
gi|29165615|ref|NC_002745.2|	547627	+	A	0	0	0	2	2	SA0475
gi|29165615|ref|NC_002745.2|	547628	+	U	0	0	0	0	1	SA0475
gi|29165615|ref|NC_002745.2|	547629	+	A	0	0	1	0	0	SA0475
gi|29165615|ref|NC_002745.2|	547630	+	U	0	0	0	0	1	SA0475
gi|29165615|ref|NC_002745.2|	547632	+	A	2	0	0	1	0	SA0475
gi|29165615|ref|NC_002745.2|	547633	+	U	0	0	1	0	0	SA0475
gi|29165615|ref|NC_002745.2|	547634	+	C	0	1	2	0	0	SA0475
gi|29165615|ref|NC_002745.2|	547635	+	U	0	0	2	0	0	SA0475
gi|29165615|ref|NC_002745.2|	547637	+	G	0	0	1	0	0	SA0475
gi|29165615|ref|NC_002745.2|	547639	+	U	1	0	0	0	0	SA0475
gi|29165615|ref|NC_002745.2|	547640	+	A	0	1	0	0	0	SA0475
gi|29165615|ref|NC_002745.2|	547643	+	G	0	0	1	0	0	SA0475
gi|29165615|ref|NC_002745.2|	547647	+	C	0	0	1	0	0	SA0475
gi|29165615|ref|NC_002745.2|	547648	+	G	0	8	4	1	5	SA0475
gi|29165615|ref|NC_002745.2|	547649	+	U	0	2	2	2	0	SA0475
gi|29165615|ref|NC_002745.2|	547650	+	U	2	0	0	0	0	SA0475
gi|29165615|ref|NC_002745.2|	547651	+	U	1	0	0	0	0	SA0475
gi|29165615|ref|NC_002745.2|	547654	+	U	1	0	0	0	0	SA0475
gi|29165615|ref|NC_002745.2|	547656	+	C	4	1	2	0	0	SA0475
gi|29165615|ref|NC_002745.2|	547657	+	U	0	0	0	0	1	SA0475
gi|29165615|ref|NC_002745.2|	547658	+	A	2	0	0	0	2	SA0475
gi|29165615|ref|NC_002745.2|	547659	+	A	0	0	1	0	0	SA0475
gi|29165615|ref|NC_002745.2|	547660	+	A	0	2	2	0	0	SA0475
gi|29165615|ref|NC_002745.2|	547661	+	U	0	2	2	0	0	SA0475
gi|29165615|ref|NC_002745.2|	547662	+	U	0	0	4	0	0	SA0475
gi|29165615|ref|NC_002745.2|	547663	+	U	0	1	1	0	0	SA0475
gi|29165615|ref|NC_002745.2|	547664	+	G	0	0	1	0	0	SA0475
gi|29165615|ref|NC_002745.2|	547665	+	A	1	9	3	1	8	SA0475
gi|29165615|ref|NC_002745.2|	547666	+	C	1	0	1	0	1	SA0475
gi|29165615|ref|NC_002745.2|	547668	+	G	0	1	0	0	0	SA0475
gi|29165615|ref|NC_002745.2|	547669	+	U	0	0	0	0	1	SA0475
gi|29165615|ref|NC_002745.2|	547670	+	U	0	1	0	0	0	SA0475
gi|29165615|ref|NC_002745.2|	547671	+	C	0	1	0	0	2	SA0475
gi|29165615|ref|NC_002745.2|	547672	+	A	1	0	0	0	0	SA0475
gi|29165615|ref|NC_002745.2|	547673	+	G	0	0	1	0	0	SA0475
gi|29165615|ref|NC_002745.2|	547674	+	G	0	0	2	0	0	SA0475
gi|29165615|ref|NC_002745.2|	547677	+	U	1	0	2	0	0	SA0475
gi|29165615|ref|NC_002745.2|	547678	+	A	3	0	1	0	0	SA0475
gi|29165615|ref|NC_002745.2|	547679	+	U	0	0	0	0	1	SA0475
gi|29165615|ref|NC_002745.2|	547680	+	C	0	2	1	1	0	SA0475
gi|29165615|ref|NC_002745.2|	547681	+	U	1	0	1	0	0	SA0475
gi|29165615|ref|NC_002745.2|	547682	+	A	0	0	0	0	1	SA0475
gi|29165615|ref|NC_002745.2|	547683	+	G	0	0	1	0	0	SA0475
gi|29165615|ref|NC_002745.2|	547685	+	G	0	1	0	0	1	SA0475
gi|29165615|ref|NC_002745.2|	547686	+	A	0	5	2	1	5	SA0475
gi|29165615|ref|NC_002745.2|	547687	+	U	0	0	0	2	0	SA0475
gi|29165615|ref|NC_002745.2|	547688	+	U	1	0	1	0	0	SA0475
gi|29165615|ref|NC_002745.2|	547689	+	U	0	0	1	0	0	SA0475
gi|29165615|ref|NC_002745.2|	547690	+	G	1	1	1	1	1	SA0475
gi|29165615|ref|NC_002745.2|	547691	+	A	0	1	2	3	5	SA0475
gi|29165615|ref|NC_002745.2|	547692	+	A	0	3	3	0	0	SA0475
gi|29165615|ref|NC_002745.2|	547693	+	A	0	2	0	1	1	SA0475

gi|29165615|ref|NC_002745.2|	547694	+	G	0	0	1	1	1	SA0475
gi|29165615|ref|NC_002745.2|	547695	+	A	0	2	2	1	0	SA0475
gi|29165615|ref|NC_002745.2|	547696	+	A	0	1	0	1	1	SA0475
gi|29165615|ref|NC_002745.2|	547697	+	G	0	3	1	0	0	SA0475
gi|29165615|ref|NC_002745.2|	547698	+	A	0	3	3	1	5	SA0475
gi|29165615|ref|NC_002745.2|	547705	+	C	1	0	0	0	0	SA0475
gi|29165615|ref|NC_002745.2|	547706	+	C	0	0	2	0	0	SA0475
gi|29165615|ref|NC_002745.2|	547709	+	U	0	1	2	0	0	SA0475
gi|29165615|ref|NC_002745.2|	547710	+	A	0	0	1	1	1	SA0475
gi|29165615|ref|NC_002745.2|	547712	+	U	1	0	0	0	0	SA0475
gi|29165615|ref|NC_002745.2|	547715	+	A	0	0	0	0	1	SA0475
gi|29165615|ref|NC_002745.2|	547718	+	G	0	1	0	0	0	SA0475
gi|29165615|ref|NC_002745.2|	547722	+	A	0	3	2	1	1	SA0475
gi|29165615|ref|NC_002745.2|	547723	+	A	0	3	2	0	0	SA0475
gi|29165615|ref|NC_002745.2|	547726	+	G	0	1	0	0	0	SA0475
gi|29165615|ref|NC_002745.2|	547727	+	G	0	4	5	3	5	SA0475
gi|29165615|ref|NC_002745.2|	547728	+	U	0	0	0	0	2	SA0475
gi|29165615|ref|NC_002745.2|	547729	+	A	0	4	0	0	1	SA0475
gi|29165615|ref|NC_002745.2|	547730	+	G	0	0	2	0	1	SA0475
gi|29165615|ref|NC_002745.2|	547731	+	A	0	2	1	1	1	SA0475
gi|29165615|ref|NC_002745.2|	547732	+	A	0	0	0	0	3	SA0475
gi|29165615|ref|NC_002745.2|	547733	+	A	0	0	0	1	0	SA0475
gi|29165615|ref|NC_002745.2|	547737	+	A	0	0	0	2	0	SA0475
gi|29165615|ref|NC_002745.2|	547738	+	A	1	0	0	0	0	SA0475
gi|29165615|ref|NC_002745.2|	547743	+	A	0	0	0	0	1	SA0475
gi|29165615|ref|NC_002745.2|	547744	+	U	0	1	0	0	0	SA0475
gi|29165615|ref|NC_002745.2|	547747	+	U	0	0	1	0	0	SA0475
gi|29165615|ref|NC_002745.2|	547748	+	C	0	0	1	0	0	SA0475
gi|29165615|ref|NC_002745.2|	547750	+	U	0	0	1	0	0	SA0475
gi|29165615|ref|NC_002745.2|	547754	+	G	0	0	1	0	0	SA0475
gi|29165615|ref|NC_002745.2|	547755	+	C	0	0	1	0	0	SA0475
gi|29165615|ref|NC_002745.2|	547756	+	U	1	3	0	0	0	SA0475
gi|29165615|ref|NC_002745.2|	547757	+	A	0	0	1	0	0	SA0475
gi|29165615|ref|NC_002745.2|	547758	+	U	0	0	1	0	0	SA0475
gi|29165615|ref|NC_002745.2|	547761	+	C	0	0	1	0	0	SA0475
gi|29165615|ref|NC_002745.2|	547766	+	C	0	0	0	0	1	SA0475
gi|29165615|ref|NC_002745.2|	547768	+	A	0	0	0	0	1	SA0475
gi|29165615|ref|NC_002745.2|	547769	+	U	0	1	0	1	0	SA0475
gi|29165615|ref|NC_002745.2|	547770	+	U	2	0	0	0	0	SA0475
gi|29165615|ref|NC_002745.2|	547771	+	A	0	0	0	0	2	SA0475
gi|29165615|ref|NC_002745.2|	547772	+	A	0	0	2	1	4	SA0475
gi|29165615|ref|NC_002745.2|	547773	+	U	0	0	0	0	1	SA0475
gi|29165615|ref|NC_002745.2|	547774	+	G	0	0	1	0	0	SA0475
gi|29165615|ref|NC_002745.2|	547776	+	C	0	0	1	0	0	SA0475
gi|29165615|ref|NC_002745.2|	547777	+	U	0	0	0	1	0	SA0475
gi|29165615|ref|NC_002745.2|	547779	+	A	0	1	0	0	0	SA0475
gi|29165615|ref|NC_002745.2|	547783	+	U	1	0	0	0	0	SA0475
gi|29165615|ref|NC_002745.2|	547787	+	A	0	1	0	0	0	SA0475
gi|29165615|ref|NC_002745.2|	547789	+	A	0	0	0	0	2	SA0475
gi|29165615|ref|NC_002745.2|	547792	+	U	0	1	0	0	0	SA0475
gi|29165615|ref|NC_002745.2|	547793	+	A	0	1	0	0	0	SA0475
gi|29165615|ref|NC_002745.2|	547801	+	A	0	0	0	0	1	SA0475
gi|29165615|ref|NC_002745.2|	547807	+	A	0	0	0	0	1	SA0475
gi|29165615|ref|NC_002745.2|	547820	+	U	0	1	0	0	0	SA0475
gi|29165615|ref|NC_002745.2|	547821	+	U	0	0	0	0	2	SA0475
gi|29165615|ref|NC_002745.2|	547825	+	U	0	1	0	0	0	SA0475
gi|29165615|ref|NC_002745.2|	547827	+	G	0	0	0	1	0	SA0475
gi|29165615|ref|NC_002745.2|	547838	+	A	0	1	0	0	0	SA0475
gi|29165615|ref|NC_002745.2|	547845	+	U	1	2	0	0	1	SA0475
gi|29165615|ref|NC_002745.2|	547846	+	U	0	0	1	0	0	SA0475
gi|29165615|ref|NC_002745.2|	547849	+	U	2	0	2	0	0	SA0475
gi|29165615|ref|NC_002745.2|	547850	+	A	0	0	0	0	1	SA0475
gi|29165615|ref|NC_002745.2|	547851	+	A	0	0	0	0	1	SA0475
gi|29165615|ref|NC_002745.2|	547856	+	C	0	1	0	1	0	SA0475
gi|29165615|ref|NC_002745.2|	547860	+	U	0	0	1	0	0	SA0475
gi|29165615|ref|NC_002745.2|	547865	+	G	0	0	0	1	0	SA0475
gi|29165615|ref|NC_002745.2|	547869	+	A	1	0	0	1	1	SA0475
gi|29165615|ref|NC_002745.2|	547875	+	U	0	0	0	0	1	SA0475
gi|29165615|ref|NC_002745.2|	547878	+	A	0	0	0	1	0	SA0475
gi|29165615|ref|NC_002745.2|	547881	+	U	2	0	1	0	0	SA0475
gi|29165615|ref|NC_002745.2|	547882	+	A	1	0	0	0	0	SA0475
gi|29165615|ref|NC_002745.2|	547884	+	G	0	0	1	0	0	SA0475
gi|29165615|ref|NC_002745.2|	547886	+	A	0	0	0	1	0	SA0475
gi|29165615|ref|NC_002745.2|	547888	+	A	1	0	2	1	2	SA0475
gi|29165615|ref|NC_002745.2|	547889	+	A	0	1	0	0	1	SA0475
gi|29165615|ref|NC_002745.2|	547890	+	A	0	1	0	0	1	SA0475
gi|29165615|ref|NC_002745.2|	547893	+	C	0	0	1	0	0	SA0475
gi|29165615|ref|NC_002745.2|	547899	+	U	0	2	0	0	0	SA0475
gi|29165615|ref|NC_002745.2|	547903	+	C	0	0	1	0	0	SA0475
gi|29165615|ref|NC_002745.2|	547905	+	A	1	0	0	0	0	SA0475
gi|29165615|ref|NC_002745.2|	547906	+	U	1	0	1	1	0	SA0475

gi|29165615|ref|NC_002745.2|	547908 +	U	5	0	1	0	0	SA0475
gi|29165615|ref|NC_002745.2|	547909 +	C	0	0	1	0	1	SA0475
gi|29165615|ref|NC_002745.2|	547911 +	U	1	0	0	0	0	SA0475
gi|29165615|ref|NC_002745.2|	547915 +	U	0	1	0	0	0	SA0475
gi|29165615|ref|NC_002745.2|	547916 +	G	0	0	0	1	0	SA0475
gi|29165615|ref|NC_002745.2|	547917 +	U	2	0	0	1	0	SA0475
gi|29165615|ref|NC_002745.2|	547919 +	G	0	1	0	1	0	SA0475
gi|29165615|ref|NC_002745.2|	547933 +	C	0	1	0	0	1	SA0475
gi|29165615|ref|NC_002745.2|	547938 +	C	0	0	1	0	0	SA0475
gi|29165615|ref|NC_002745.2|	547940 +	A	0	0	1	1	0	SA0475
gi|29165615|ref|NC_002745.2|	547941 +	A	0	0	0	0	1	SA0475
gi|29165615|ref|NC_002745.2|	547942 +	U	0	1	0	0	0	SA0475
gi|29165615|ref|NC_002745.2|	547943 +	A	0	0	0	1	0	SA0475
gi|29165615|ref|NC_002745.2|	547944 +	C	0	0	1	0	0	SA0475
gi|29165615|ref|NC_002745.2|	547948 +	C	0	0	0	0	1	SA0475
gi|29165615|ref|NC_002745.2|	547951 +	A	0	0	1	0	0	SA0475
gi|29165615|ref|NC_002745.2|	547953 +	U	0	0	2	0	0	SA0475
gi|29165615|ref|NC_002745.2|	547956 +	C	0	0	0	1	0	SA0475
gi|29165615|ref|NC_002745.2|	547961 +	A	0	1	0	0	1	SA0475
gi|29165615|ref|NC_002745.2|	547967 +	A	0	1	0	0	0	SA0475
gi|29165615|ref|NC_002745.2|	547970 +	A	0	0	0	0	1	SA0475
gi|29165615|ref|NC_002745.2|	547972 +	A	0	0	0	0	1	SA0475
gi|29165615|ref|NC_002745.2|	547977 +	C	0	0	0	0	1	SA0475
gi|29165615|ref|NC_002745.2|	547982 +	C	0	0	1	0	1	SA0475
gi|29165615|ref|NC_002745.2|	547983 +	U	0	0	0	0	3	SA0475
gi|29165615|ref|NC_002745.2|	547985 +	A	0	1	0	0	0	SA0475
gi|29165615|ref|NC_002745.2|	547986 +	C	0	1	0	0	0	SA0475
gi|29165615|ref|NC_002745.2|	547988 +	A	0	0	1	0	0	SA0475
gi|29165615|ref|NC_002745.2|	547990 +	A	0	0	1	0	0	SA0475
gi|29165615|ref|NC_002745.2|	547991 +	U	1	0	1	0	0	SA0475
gi|29165615|ref|NC_002745.2|	547992 +	C	2	2	2	0	1	SA0475
gi|29165615|ref|NC_002745.2|	547993 +	A	0	0	1	0	0	SA0475
gi|29165615|ref|NC_002745.2|	547994 +	U	0	0	1	0	0	SA0475
gi|29165615|ref|NC_002745.2|	548006 +	C	0	0	0	0	1	SA0475
gi|29165615|ref|NC_002745.2|	548007 +	C	0	0	0	1	0	SA0475
gi|29165615|ref|NC_002745.2|	548014 +	A	0	1	0	0	1	SA0475
gi|29165615|ref|NC_002745.2|	548030 +	G	0	0	0	0	1	SA0475
gi|29165615|ref|NC_002745.2|	548031 +	A	0	1	0	0	0	SA0475
gi|29165615|ref|NC_002745.2|	548060 +	U	0	0	0	0	1	SA0475
gi|29165615|ref|NC_002745.2|	548106 +	G	0	0	0	1	0	SA0475
gi|29165615|ref|NC_002745.2|	548124 +	A	0	0	0	1	0	SA0475
gi|29165615|ref|NC_002745.2|	548127 +	U	0	0	0	0	1	SA0475
gi|29165615|ref|NC_002745.2|	548141 +	A	0	1	0	0	0	SA0475
gi|29165615|ref|NC_002745.2|	548142 +	A	0	1	0	0	0	SA0475
gi|29165615|ref|NC_002745.2|	548149 +	A	0	0	0	0	1	SA0475
gi|29165615|ref|NC_002745.2|	548168 +	A	0	1	0	0	0	SA0475
gi|29165615|ref|NC_002745.2|	548171 +	C	0	0	1	0	1	SA0475
gi|29165615|ref|NC_002745.2|	548175 +	U	0	0	1	0	0	SA0475
gi|29165615|ref|NC_002745.2|	548179 +	G	0	0	1	0	0	SA0475
gi|29165615|ref|NC_002745.2|	548184 +	A	0	0	0	1	0	SA0475
gi|29165615|ref|NC_002745.2|	548185 +	A	0	0	1	0	0	SA0475
gi|29165615|ref|NC_002745.2|	548188 +	U	0	0	1	0	0	SA0475
gi|29165615|ref|NC_002745.2|	548189 +	G	0	0	1	0	0	SA0475
gi|29165615|ref|NC_002745.2|	548194 +	U	0	0	1	0	0	SA0475
gi|29165615|ref|NC_002745.2|	548195 +	G	0	0	0	0	1	SA0475
gi|29165615|ref|NC_002745.2|	548196 +	G	0	0	0	0	2	SA0475
gi|29165615|ref|NC_002745.2|	548240 +	G	0	1	0	0	0	SA0475
gi|29165615|ref|NC_002745.2|	548246 +	U	0	0	1	0	0	SA0475
gi|29165615|ref|NC_002745.2|	548254 +	G	0	1	0	0	0	SA0475
gi|29165615|ref|NC_002745.2|	548259 +	U	0	0	0	0	1	SA0475
gi|29165615|ref|NC_002745.2|	548262 +	C	1	0	0	0	0	SA0475
gi|29165615|ref|NC_002745.2|	548266 +	U	0	0	1	0	0	SA0475
gi|29165615|ref|NC_002745.2|	548283 +	G	0	0	2	0	1	SA0475
gi|29165615|ref|NC_002745.2|	548285 +	U	0	0	1	0	0	SA0475
gi|29165615|ref|NC_002745.2|	548286 +	U	0	2	0	0	0	SA0475
gi|29165615|ref|NC_002745.2|	548287 +	A	0	0	0	0	1	SA0475
gi|29165615|ref|NC_002745.2|	548288 +	A	0	1	0	2	3	SA0475
gi|29165615|ref|NC_002745.2|	548291 +	G	0	0	0	0	1	SA0475
gi|29165615|ref|NC_002745.2|	548292 +	U	0	0	3	0	0	SA0475
gi|29165615|ref|NC_002745.2|	548298 +	G	0	1	1	1	0	SA0475
gi|29165615|ref|NC_002745.2|	548299 +	A	0	0	0	0	1	SA0475
gi|29165615|ref|NC_002745.2|	548303 +	G	0	1	0	0	0	SA0475
gi|29165615|ref|NC_002745.2|	548304 +	A	0	1	0	0	0	SA0475
gi|29165615|ref|NC_002745.2|	548305 +	A	0	0	0	0	1	SA0475
gi|29165615|ref|NC_002745.2|	548306 +	A	0	0	0	1	0	SA0475
gi|29165615|ref|NC_002745.2|	548310 +	U	0	0	1	0	0	SA0475
gi|29165615|ref|NC_002745.2|	548311 +	A	0	3	2	2	3	SA0475
gi|29165615|ref|NC_002745.2|	548316 +	A	0	0	0	0	1	SA0475
gi|29165615|ref|NC_002745.2|	548318 +	A	0	1	0	0	0	SA0475
gi|29165615|ref|NC_002745.2|	548322 +	G	0	2	0	1	0	SA0475
gi|29165615|ref|NC_002745.2|	548326 +	A	0	1	0	1	1	SA0475

gi|29165615|ref|NC_002745.2|	548328 +	U	0	1	0	0	0	SA0475
gi|29165615|ref|NC_002745.2|	548330 +	U	1	0	0	0	0	SA0475
gi|29165615|ref|NC_002745.2|	548336 +	A	0	4	1	1	1	SA0475
gi|29165615|ref|NC_002745.2|	548339 +	G	0	2	0	0	0	SA0475
gi|29165615|ref|NC_002745.2|	548350 +	A	0	0	1	0	0	SA0475
gi|29165615|ref|NC_002745.2|	548353 +	U	0	1	0	0	0	SA0475
gi|29165615|ref|NC_002745.2|	548356 +	A	0	1	0	1	0	SA0475
gi|29165615|ref|NC_002745.2|	548357 +	C	0	0	0	2	0	SA0475
gi|29165615|ref|NC_002745.2|	548358 +	A	1	0	0	0	0	SA0475
gi|29165615|ref|NC_002745.2|	548367 +	A	0	0	1	0	0	SA0475
gi|29165615|ref|NC_002745.2|	548368 +	A	0	0	0	1	0	SA0475
gi|29165615|ref|NC_002745.2|	548371 +	C	0	0	1	0	0	SA0475
gi|29165615|ref|NC_002745.2|	548373 +	C	0	2	0	0	0	SA0475
gi|29165615|ref|NC_002745.2|	548375 +	A	0	1	0	0	0	SA0475
gi|29165615|ref|NC_002745.2|	548378 +	A	0	0	1	1	0	SA0475
gi|29165615|ref|NC_002745.2|	548380 +	U	0	0	0	0	1	SA0475
gi|29165615|ref|NC_002745.2|	548381 +	G	0	0	0	0	1	SA0475
gi|29165615|ref|NC_002745.2|	548382 +	A	0	0	1	3	1	SA0475
gi|29165615|ref|NC_002745.2|	548383 +	A	0	0	1	1	3	SA0475
gi|29165615|ref|NC_002745.2|	548385 +	U	0	0	1	0	0	SA0475
gi|29165615|ref|NC_002745.2|	548386 +	A	0	2	0	0	0	SA0475
gi|29165615|ref|NC_002745.2|	548388 +	A	0	0	0	1	0	SA0475
gi|29165615|ref|NC_002745.2|	548390 +	G	0	0	0	0	1	SA0475
gi|29165615|ref|NC_002745.2|	548391 +	A	2	0	1	0	1	SA0475
gi|29165615|ref|NC_002745.2|	548393 +	G	0	1	1	0	0	SA0475
gi|29165615|ref|NC_002745.2|	548394 +	C	0	1	0	0	0	SA0475
gi|29165615|ref|NC_002745.2|	548396 +	U	0	1	0	0	0	SA0475
gi|29165615|ref|NC_002745.2|	548399 +	G	0	0	0	1	0	SA0475
gi|29165615|ref|NC_002745.2|	548400 +	C	0	0	0	0	1	SA0475
gi|29165615|ref|NC_002745.2|	548402 +	G	0	0	0	0	1	SA0475
gi|29165615|ref|NC_002745.2|	548403 +	A	0	0	0	1	1	SA0475
gi|29165615|ref|NC_002745.2|	548406 +	A	0	0	1	0	0	SA0475
gi|29165615|ref|NC_002745.2|	548410 +	U	0	0	0	1	0	SA0475
gi|29165615|ref|NC_002745.2|	548412 +	A	0	0	0	0	1	SA0475
gi|29165615|ref|NC_002745.2|	548420 +	G	0	2	0	0	1	SA0475
gi|29165615|ref|NC_002745.2|	548428 +	A	0	0	0	0	1	SA0475
gi|29165615|ref|NC_002745.2|	548433 +	C	0	0	1	0	0	SA0475
gi|29165615|ref|NC_002745.2|	548438 +	G	0	0	0	0	1	SA0475
gi|29165615|ref|NC_002745.2|	548439 +	U	2	0	0	0	0	SA0475
gi|29165615|ref|NC_002745.2|	548441 +	A	0	0	0	0	1	SA0475
gi|29165615|ref|NC_002745.2|	548443 +	A	0	1	0	0	0	SA0475
gi|29165615|ref|NC_002745.2|	548446 +	U	0	0	1	0	0	SA0475
gi|29165615|ref|NC_002745.2|	548447 +	A	0	0	2	0	0	SA0475
gi|29165615|ref|NC_002745.2|	548454 +	A	0	0	0	1	0	SA0475
gi|29165615|ref|NC_002745.2|	548456 +	G	0	0	0	0	1	SA0475
gi|29165615|ref|NC_002745.2|	548457 +	A	0	1	0	1	1	SA0475
gi|29165615|ref|NC_002745.2|	548459 +	G	0	0	0	1	0	SA0475
gi|29165615|ref|NC_002745.2|	548460 +	U	0	0	3	1	0	SA0475
gi|29165615|ref|NC_002745.2|	548461 +	A	0	0	1	0	0	SA0475
gi|29165615|ref|NC_002745.2|	548463 +	U	0	0	0	0	1	SA0475
gi|29165615|ref|NC_002745.2|	548464 +	A	0	1	0	0	0	SA0475
gi|29165615|ref|NC_002745.2|	548465 +	G	0	0	1	0	0	SA0475
gi|29165615|ref|NC_002745.2|	548469 +	C	1	0	0	0	0	SA0475
gi|29165615|ref|NC_002745.2|	548472 +	C	0	0	0	1	0	SA0475
gi|29165615|ref|NC_002745.2|	548473 +	A	0	0	0	1	0	SA0475
gi|29165615|ref|NC_002745.2|	548476 +	A	0	0	0	0	1	SA0475
gi|29165615|ref|NC_002745.2|	548477 +	G	0	0	0	1	0	SA0475
gi|29165615|ref|NC_002745.2|	548478 +	U	0	3	1	0	0	SA0475
gi|29165615|ref|NC_002745.2|	548480 +	C	0	1	0	1	1	SA0475
gi|29165615|ref|NC_002745.2|	548481 +	A	0	0	0	1	0	SA0475
gi|29165615|ref|NC_002745.2|	548482 +	A	0	2	0	0	0	SA0475
gi|29165615|ref|NC_002745.2|	548484 +	A	1	0	0	0	0	SA0475
gi|29165615|ref|NC_002745.2|	548485 +	C	0	1	0	0	0	SA0475
gi|29165615|ref|NC_002745.2|	548487 +	A	0	0	1	1	0	SA0475
gi|29165615|ref|NC_002745.2|	548492 +	G	0	0	1	0	0	SA0475
gi|29165615|ref|NC_002745.2|	548496 +	C	0	1	0	0	0	SA0475
gi|29165615|ref|NC_002745.2|	548497 +	G	1	1	0	0	0	SA0475
gi|29165615|ref|NC_002745.2|	548498 +	A	0	2	0	0	0	SA0475
gi|29165615|ref|NC_002745.2|	548500 +	U	0	0	0	0	1	SA0475
gi|29165615|ref|NC_002745.2|	548501 +	G	0	1	1	0	0	SA0475
gi|29165615|ref|NC_002745.2|	548502 +	A	0	2	0	1	0	SA0475
gi|29165615|ref|NC_002745.2|	548504 +	U	0	0	1	0	1	SA0475
gi|29165615|ref|NC_002745.2|	548505 +	U	0	1	0	0	0	SA0475
gi|29165615|ref|NC_002745.2|	548508 +	A	0	0	0	1	1	SA0475
gi|29165615|ref|NC_002745.2|	548509 +	A	0	1	0	0	0	SA0475
gi|29165615|ref|NC_002745.2|	548510 +	U	0	0	0	0	1	SA0475
gi|29165615|ref|NC_002745.2|	548511 +	C	0	0	0	0	1	SA0475
gi|29165615|ref|NC_002745.2|	548513 +	G	1	0	0	0	0	SA0475
gi|29165615|ref|NC_002745.2|	548514 +	C	0	0	0	0	1	SA0475
gi|29165615|ref|NC_002745.2|	548515 +	U	1	0	0	0	0	SA0475
gi|29165615|ref|NC_002745.2|	548519 +	A	0	0	0	0	1	SA0475

gi|29165615|ref|NC_002745.2|	548523	+	G	0	0	1	0	0	SA0475
gi|29165615|ref|NC_002745.2|	548524	+	U	0	1	0	2	2	SA0475
gi|29165615|ref|NC_002745.2|	548526	+	U	0	2	0	0	0	SA0475
gi|29165615|ref|NC_002745.2|	548527	+	G	0	0	0	0	1	SA0475
gi|29165615|ref|NC_002745.2|	548530	+	U	0	0	0	0	1	SA0475
gi|29165615|ref|NC_002745.2|	548531	+	A	0	0	0	1	0	SA0475
gi|29165615|ref|NC_002745.2|	548532	+	U	1	0	0	0	0	SA0475
gi|29165615|ref|NC_002745.2|	548534	+	G	0	0	1	0	0	SA0475
gi|29165615|ref|NC_002745.2|	548535	+	U	0	0	1	0	0	SA0475
gi|29165615|ref|NC_002745.2|	548538	+	A	0	1	0	0	0	SA0475
gi|29165615|ref|NC_002745.2|	548543	+	G	0	0	0	0	1	SA0475
gi|29165615|ref|NC_002745.2|	548544	+	U	0	0	1	0	0	SA0475
gi|29165615|ref|NC_002745.2|	548546	+	A	0	0	0	1	0	SA0475
gi|29165615|ref|NC_002745.2|	548556	+	C	0	0	1	0	0	SA0475
gi|29165615|ref|NC_002745.2|	548572	+	U	0	1	0	0	0	SA0475
gi|29165615|ref|NC_002745.2|	548573	+	G	0	0	0	0	1	SA0475
gi|29165615|ref|NC_002745.2|	548575	+	A	0	0	0	0	1	SA0475
gi|29165615|ref|NC_002745.2|	548576	+	G	0	0	1	0	0	SA0475
gi|29165615|ref|NC_002745.2|	548582	+	A	0	0	0	0	1	SA0475
gi|29165615|ref|NC_002745.2|	548615	+	C	0	2	0	0	0	SA0475
gi|29165615|ref|NC_002745.2|	548619	+	G	0	0	2	0	0	SA0475
gi|29165615|ref|NC_002745.2|	548624	+	G	0	0	1	0	0	SA0475
gi|29165615|ref|NC_002745.2|	548630	+	A	0	1	0	0	0	SA0475
gi|29165615|ref|NC_002745.2|	548631	+	A	1	0	0	0	1	SA0475
gi|29165615|ref|NC_002745.2|	548633	+	G	0	0	0	0	1	SA0475
gi|29165615|ref|NC_002745.2|	548638	+	A	0	1	0	0	0	SA0475
gi|29165615|ref|NC_002745.2|	548644	+	A	0	2	0	0	1	SA0475
gi|29165615|ref|NC_002745.2|	548645	+	U	0	0	0	0	1	SA0475
gi|29165615|ref|NC_002745.2|	548647	+	U	0	1	0	0	0	SA0475
gi|29165615|ref|NC_002745.2|	548657	+	U	0	0	0	0	1	SA0475
gi|29165615|ref|NC_002745.2|	548661	+	A	0	2	0	0	0	SA0475
gi|29165615|ref|NC_002745.2|	548690	+	A	0	0	0	0	1	SA0475
gi|29165615|ref|NC_002745.2|	548691	+	C	0	0	1	0	0	SA0475
gi|29165615|ref|NC_002745.2|	548693	+	C	0	0	0	0	1	SA0475
gi|29165615|ref|NC_002745.2|	548695	+	U	0	1	0	0	0	SA0475
gi|29165615|ref|NC_002745.2|	548706	+	C	0	0	1	0	0	SA0475
gi|29165615|ref|NC_002745.2|	548708	+	U	0	1	0	0	0	SA0475
gi|29165615|ref|NC_002745.2|	548709	+	U	2	0	0	0	0	SA0475
gi|29165615|ref|NC_002745.2|	548710	+	U	1	0	0	0	0	SA0475
gi|29165615|ref|NC_002745.2|	548714	+	U	1	1	0	0	0	SA0475
gi|29165615|ref|NC_002745.2|	548726	+	A	0	0	1	0	0	SA0475
gi|29165615|ref|NC_002745.2|	548732	+	A	0	1	0	0	0	SA0475
gi|29165615|ref|NC_002745.2|	548736	+	C	0	1	0	0	0	SA0475
gi|29165615|ref|NC_002745.2|	548752	+	A	0	1	0	0	0	SA0475
gi|29165615|ref|NC_002745.2|	548753	+	A	0	0	0	0	1	SA0475
gi|29165615|ref|NC_002745.2|	548754	+	A	0	0	1	0	0	SA0475
gi|29165615|ref|NC_002745.2|	548766	+	C	0	0	0	0	1	SA0475
gi|29165615|ref|NC_002745.2|	548770	+	A	0	0	0	0	1	SA0475
gi|29165615|ref|NC_002745.2|	548787	+	A	0	0	0	1	0	SA0475
gi|29165615|ref|NC_002745.2|	548788	+	A	0	0	0	0	2	SA0475
gi|29165615|ref|NC_002745.2|	548793	+	U	1	0	0	0	0	SA0475
gi|29165615|ref|NC_002745.2|	548802	+	G	0	0	1	0	0	SA0475
gi|29165615|ref|NC_002745.2|	548805	+	G	0	1	0	0	0	SA0475
gi|29165615|ref|NC_002745.2|	548820	+	C	0	0	0	1	0	SA0475
gi|29165615|ref|NC_002745.2|	548826	+	C	0	0	0	0	1	SA0475
gi|29165615|ref|NC_002745.2|	548832	+	U	0	1	1	0	0	SA0475
gi|29165615|ref|NC_002745.2|	548835	+	A	0	1	0	1	0	SA0475
gi|29165615|ref|NC_002745.2|	548841	+	C	0	1	0	1	0	SA0475
gi|29165615|ref|NC_002745.2|	548846	+	G	0	0	0	0	1	SA0475
gi|29165615|ref|NC_002745.2|	548847	+	A	0	1	0	0	0	SA0475
gi|29165615|ref|NC_002745.2|	548851	+	A	0	0	1	0	3	SA0475
gi|29165615|ref|NC_002745.2|	548853	+	A	0	2	1	0	0	SA0475
gi|29165615|ref|NC_002745.2|	548854	+	A	0	0	0	0	1	SA0475
gi|29165615|ref|NC_002745.2|	548856	+	G	0	0	0	0	2	SA0475
gi|29165615|ref|NC_002745.2|	548863	+	U	0	0	0	0	1	SA0475
gi|29165615|ref|NC_002745.2|	548870	+	C	0	0	0	0	1	SA0475
gi|29165615|ref|NC_002745.2|	548872	+	A	0	0	0	1	0	SA0475
gi|29165615|ref|NC_002745.2|	548873	+	C	0	0	0	0	2	SA0475
gi|29165615|ref|NC_002745.2|	548874	+	U	0	1	0	0	0	SA0475
gi|29165615|ref|NC_002745.2|	548876	+	G	0	0	1	0	0	SA0475
gi|29165615|ref|NC_002745.2|	548881	+	A	0	0	1	0	0	SA0475
gi|29165615|ref|NC_002745.2|	548882	+	A	0	0	1	0	0	SA0475
gi|29165615|ref|NC_002745.2|	548885	+	G	0	1	0	0	0	SA0475
gi|29165615|ref|NC_002745.2|	548894	+	A	0	1	0	0	0	SA0475
gi|29165615|ref|NC_002745.2|	548895	+	A	0	0	0	1	0	SA0475
gi|29165615|ref|NC_002745.2|	548896	+	U	0	1	0	0	0	SA0475
gi|29165615|ref|NC_002745.2|	548897	+	G	0	2	0	0	1	SA0475
gi|29165615|ref|NC_002745.2|	548898	+	A	0	0	0	0	1	SA0475
gi|29165615|ref|NC_002745.2|	548899	+	U	0	0	0	1	0	SA0475
gi|29165615|ref|NC_002745.2|	548904	+	C	0	0	0	0	1	SA0475
gi|29165615|ref|NC_002745.2|	548909	+	G	0	0	0	1	0	SA0475

gi|29165615|ref|NC_002745.2|	548910	+	A	0	0	0	1	0	SA0475
gi|29165615|ref|NC_002745.2|	548911	+	A	0	2	0	0	0	SA0475
gi|29165615|ref|NC_002745.2|	548914	+	G	1	0	0	0	0	SA0475
gi|29165615|ref|NC_002745.2|	548915	+	G	0	0	1	0	0	SA0475
gi|29165615|ref|NC_002745.2|	548925	+	A	0	0	0	1	0	SA0475
gi|29165615|ref|NC_002745.2|	548931	+	A	0	2	0	0	0	SA0475
gi|29165615|ref|NC_002745.2|	548932	+	A	0	0	1	0	0	SA0475
gi|29165615|ref|NC_002745.2|	548940	+	A	0	1	1	1	2	SA0475
gi|29165615|ref|NC_002745.2|	548941	+	A	0	1	0	0	1	SA0475
gi|29165615|ref|NC_002745.2|	548942	+	U	0	0	0	0	2	SA0475
gi|29165615|ref|NC_002745.2|	548946	+	G	0	0	0	1	1	SA0475
gi|29165615|ref|NC_002745.2|	548954	+	C	0	0	0	1	0	SA0475
gi|29165615|ref|NC_002745.2|	548956	+	G	0	1	0	0	0	SA0475
gi|29165615|ref|NC_002745.2|	548958	+	C	0	0	0	0	1	SA0475
gi|29165615|ref|NC_002745.2|	549015	+	C	0	0	1	0	0	SA0475
gi|29165615|ref|NC_002745.2|	549056	+	U	0	0	1	0	0	SA0475
gi|29165615|ref|NC_002745.2|	549072	+	U	1	0	0	0	0	-
gi|29165615|ref|NC_002745.2|	549073	+	U	2	0	0	0	0	-
gi|29165615|ref|NC_002745.2|	549156	+	U	1	0	0	0	0	-
gi|29165615|ref|NC_002745.2|	549436	+	A	0	0	0	0	1	-
gi|29165615|ref|NC_002745.2|	549440	+	A	0	1	0	0	0	-
gi|29165615|ref|NC_002745.2|	549442	+	A	0	0	0	0	1	-
gi|29165615|ref|NC_002745.2|	549497	+	G	1	1	0	0	2	-
gi|29165615|ref|NC_002745.2|	549502	+	C	0	1	0	0	0	-
gi|29165615|ref|NC_002745.2|	549519	+	C	1	0	0	0	0	-
gi|29165615|ref|NC_002745.2|	549557	+	A	1	4	4	0	0	-
gi|29165615|ref|NC_002745.2|	549558	+	A	0	5	0	0	1	-
gi|29165615|ref|NC_002745.2|	549560	+	A	0	0	1	0	0	-
gi|29165615|ref|NC_002745.2|	549564	+	A	0	2	0	0	0	-
gi|29165615|ref|NC_002745.2|	549571	+	C	0	0	1	0	0	-
gi|29165615|ref|NC_002745.2|	549601	+	A	0	1	0	0	1	-
gi|29165615|ref|NC_002745.2|	549603	+	G	3	0	1	2	2	-
gi|29165615|ref|NC_002745.2|	549604	+	U	0	0	0	0	1	-
gi|29165615|ref|NC_002745.2|	549605	+	A	8	5	1	3	0	-
gi|29165615|ref|NC_002745.2|	549614	+	U	0	0	0	0	2	-
gi|29165615|ref|NC_002745.2|	549616	+	U	8	9	4	1	3	SArRNA04
gi|29165615|ref|NC_002745.2|	549619	+	G	1	0	0	0	0	SArRNA04
gi|29165615|ref|NC_002745.2|	549731	+	A	3	1	0	1	0	-
gi|29165615|ref|NC_002745.2|	549990	+	U	1	1	1	0	1	-
gi|29165615|ref|NC_002745.2|	549991	+	U	0	0	0	1	2	-
gi|29165615|ref|NC_002745.2|	549998	+	U	0	1	0	0	0	-
gi|29165615|ref|NC_002745.2|	550180	+	G	1	0	0	0	0	SAtRNA10
gi|29165615|ref|NC_002745.2|	550276	+	U	0	0	0	0	1	-
gi|29165615|ref|NC_002745.2|	550370	+	U	0	0	1	0	1	-
gi|29165615|ref|NC_002745.2|	550371	+	U	0	2	1	5	5	-
gi|29165615|ref|NC_002745.2|	550372	+	A	0	0	0	0	1	-
gi|29165615|ref|NC_002745.2|	550469	+	U	0	0	0	0	2	-
gi|29165615|ref|NC_002745.2|	550470	+	U	0	1	0	1	0	-
gi|29165615|ref|NC_002745.2|	550471	+	U	0	0	0	0	1	-
gi|29165615|ref|NC_002745.2|	550475	+	U	0	1	0	0	0	-
gi|29165615|ref|NC_002745.2|	550477	+	U	0	2	0	0	0	-
gi|29165615|ref|NC_002745.2|	550478	+	A	0	0	2	0	1	-
gi|29165615|ref|NC_002745.2|	550479	+	C	1	2	0	1	1	-
gi|29165615|ref|NC_002745.2|	550641	+	U	0	0	0	0	1	SArRNA05
gi|29165615|ref|NC_002745.2|	550642	+	A	1	0	0	0	0	SArRNA05
gi|29165615|ref|NC_002745.2|	550659	+	A	0	0	1	0	0	SArRNA05
gi|29165615|ref|NC_002745.2|	552208	+	U	1	0	0	0	0	-
gi|29165615|ref|NC_002745.2|	552218	+	U	0	1	0	0	0	-
gi|29165615|ref|NC_002745.2|	552440	+	C	1	0	1	2	0	-
gi|29165615|ref|NC_002745.2|	552441	+	A	1	1	1	2	0	-
gi|29165615|ref|NC_002745.2|	552442	+	C	3	3	1	2	1	-
gi|29165615|ref|NC_002745.2|	552443	+	A	0	0	1	4	1	-
gi|29165615|ref|NC_002745.2|	552444	+	A	0	7	2	35	4	-
gi|29165615|ref|NC_002745.2|	552445	+	G	1	1	0	9	0	-
gi|29165615|ref|NC_002745.2|	552446	+	A	0	8	3	42	5	-
gi|29165615|ref|NC_002745.2|	552447	+	U	0	1	0	5	0	-
gi|29165615|ref|NC_002745.2|	552448	+	U	0	2	0	5	4	-
gi|29165615|ref|NC_002745.2|	552449	+	A	4	4	5	54	6	-
gi|29165615|ref|NC_002745.2|	552450	+	A	6	20	6	200	13	-
gi|29165615|ref|NC_002745.2|	552451	+	U	7	5	3	39	3	-
gi|29165615|ref|NC_002745.2|	552452	+	A	3	14	2	113	13	-
gi|29165615|ref|NC_002745.2|	552453	+	A	2	9	9	42	7	-
gi|29165615|ref|NC_002745.2|	552454	+	C	7	3	0	5	2	-
gi|29165615|ref|NC_002745.2|	552455	+	G	2	1	0	8	1	-
gi|29165615|ref|NC_002745.2|	552456	+	C	0	2	0	10	1	-
gi|29165615|ref|NC_002745.2|	552457	+	G	0	0	0	1	0	-
gi|29165615|ref|NC_002745.2|	552458	+	U	0	0	0	1	0	-
gi|29165615|ref|NC_002745.2|	552459	+	U	0	0	0	0	1	-
gi|29165615|ref|NC_002745.2|	552460	+	U	2	0	0	0	0	-
gi|29165615|ref|NC_002745.2|	552461	+	C	0	0	0	5	1	-
gi|29165615|ref|NC_002745.2|	552463	+	U	4	0	0	1	1	-

gi|29165615|ref|NC_002745.2|	552464 +	G	2	2	2	10	1	-
gi|29165615|ref|NC_002745.2|	552465 +	U	5	0	2	2	0	-
gi|29165615|ref|NC_002745.2|	552746 +	G	0	0	0	0	1	SArRNA06
gi|29165615|ref|NC_002745.2|	552750 +	C	0	1	0	0	0	SArRNA06
gi|29165615|ref|NC_002745.2|	552757 +	A	0	0	0	0	1	SArRNA06
gi|29165615|ref|NC_002745.2|	556124 +	U	0	1	0	0	0	-
gi|29165615|ref|NC_002745.2|	556136 +	U	0	0	1	0	0	-
gi|29165615|ref|NC_002745.2|	556384 +	U	0	1	0	0	0	-
gi|29165615|ref|NC_002745.2|	556545 +	A	0	1	0	0	0	-
gi|29165615|ref|NC_002745.2|	556546 +	A	0	1	0	0	0	-
gi|29165615|ref|NC_002745.2|	556585 +	U	0	0	0	1	0	-
gi|29165615|ref|NC_002745.2|	556631 +	A	0	0	0	1	0	-
gi|29165615|ref|NC_002745.2|	557436 +	C	0	0	1	0	0	-
gi|29165615|ref|NC_002745.2|	557442 +	A	0	0	0	0	1	-
gi|29165615|ref|NC_002745.2|	557565 +	A	0	0	0	5	4	-
gi|29165615|ref|NC_002745.2|	557566 +	U	0	0	1	0	0	-
gi|29165615|ref|NC_002745.2|	557567 +	U	0	0	1	0	0	-
gi|29165615|ref|NC_002745.2|	557568 +	U	16	24	27	40	28	-
gi|29165615|ref|NC_002745.2|	557569 +	A	241	401	383	526	350	-
gi|29165615|ref|NC_002745.2|	557570 +	A	7	100	102	70	63	-
gi|29165615|ref|NC_002745.2|	557571 +	U	0	2	0	0	0	-
gi|29165615|ref|NC_002745.2|	557572 +	C	0	6	2	4	5	-
gi|29165615|ref|NC_002745.2|	557573 +	A	0	12	8	5	7	-
gi|29165615|ref|NC_002745.2|	557574 +	A	0	6	3	0	2	-
gi|29165615|ref|NC_002745.2|	557575 +	C	0	0	0	3	0	-
gi|29165615|ref|NC_002745.2|	557576 +	U	1	0	0	0	0	-
gi|29165615|ref|NC_002745.2|	557577 +	U	0	2	2	0	1	-
gi|29165615|ref|NC_002745.2|	557578 +	G	0	9	6	5	4	-
gi|29165615|ref|NC_002745.2|	557579 +	A	1	22	6	10	18	-
gi|29165615|ref|NC_002745.2|	557580 +	A	0	6	2	5	3	-
gi|29165615|ref|NC_002745.2|	557581 +	A	1	0	1	1	0	-
gi|29165615|ref|NC_002745.2|	557585 +	G	0	1	0	0	2	-
gi|29165615|ref|NC_002745.2|	557586 +	G	0	0	3	1	1	-
gi|29165615|ref|NC_002745.2|	557587 +	U	0	0	1	0	0	-
gi|29165615|ref|NC_002745.2|	557589 +	U	0	0	0	0	1	-
gi|29165615|ref|NC_002745.2|	557590 +	U	0	1	0	0	0	-
gi|29165615|ref|NC_002745.2|	557592 +	U	0	1	0	0	1	-
gi|29165615|ref|NC_002745.2|	557593 +	G	0	2	0	2	1	-
gi|29165615|ref|NC_002745.2|	557595 +	A	1	2	0	0	0	-
gi|29165615|ref|NC_002745.2|	557597 +	A	0	1	2	1	0	-
gi|29165615|ref|NC_002745.2|	557598 +	A	0	2	2	0	0	SA0477
gi|29165615|ref|NC_002745.2|	557599 +	U	0	1	0	0	0	SA0477
gi|29165615|ref|NC_002745.2|	557600 +	G	0	1	0	0	1	SA0477
gi|29165615|ref|NC_002745.2|	557601 +	A	0	2	2	2	7	SA0477
gi|29165615|ref|NC_002745.2|	557602 +	G	0	1	0	0	3	SA0477
gi|29165615|ref|NC_002745.2|	557604 +	A	0	0	4	3	6	SA0477
gi|29165615|ref|NC_002745.2|	557605 +	A	1	2	3	5	3	SA0477
gi|29165615|ref|NC_002745.2|	557606 +	A	0	1	2	4	1	SA0477
gi|29165615|ref|NC_002745.2|	557607 +	A	0	5	1	1	5	SA0477
gi|29165615|ref|NC_002745.2|	557608 +	U	0	1	2	0	0	SA0477
gi|29165615|ref|NC_002745.2|	557609 +	U	0	4	2	1	2	SA0477
gi|29165615|ref|NC_002745.2|	557610 +	A	0	1	1	0	0	SA0477
gi|29165615|ref|NC_002745.2|	557611 +	U	0	0	0	0	1	SA0477
gi|29165615|ref|NC_002745.2|	557613 +	G	0	1	1	0	1	SA0477
gi|29165615|ref|NC_002745.2|	557614 +	G	0	9	0	1	1	SA0477
gi|29165615|ref|NC_002745.2|	557615 +	A	0	0	1	1	0	SA0477
gi|29165615|ref|NC_002745.2|	557616 +	U	0	3	1	0	0	SA0477
gi|29165615|ref|NC_002745.2|	557617 +	C	0	1	0	0	0	SA0477
gi|29165615|ref|NC_002745.2|	557618 +	A	0	0	1	0	0	SA0477
gi|29165615|ref|NC_002745.2|	557619 +	G	0	2	1	2	5	SA0477
gi|29165615|ref|NC_002745.2|	557620 +	A	1	2	3	8	1	SA0477
gi|29165615|ref|NC_002745.2|	557621 +	C	0	0	1	2	3	SA0477
gi|29165615|ref|NC_002745.2|	557622 +	A	0	2	0	0	2	SA0477
gi|29165615|ref|NC_002745.2|	557623 +	G	0	1	3	2	1	SA0477
gi|29165615|ref|NC_002745.2|	557624 +	A	0	2	1	0	1	SA0477
gi|29165615|ref|NC_002745.2|	557626 +	U	0	0	0	2	0	SA0477
gi|29165615|ref|NC_002745.2|	557627 +	C	2	1	1	1	2	SA0477
gi|29165615|ref|NC_002745.2|	557628 +	A	0	7	1	1	6	SA0477
gi|29165615|ref|NC_002745.2|	557629 +	A	0	5	2	2	2	SA0477
gi|29165615|ref|NC_002745.2|	557630 +	A	1	1	3	1	5	SA0477
gi|29165615|ref|NC_002745.2|	557631 +	A	0	0	0	0	1	SA0477
gi|29165615|ref|NC_002745.2|	557636 +	U	0	1	0	0	1	SA0477
gi|29165615|ref|NC_002745.2|	557638 +	U	0	1	0	0	0	SA0477
gi|29165615|ref|NC_002745.2|	557639 +	G	0	0	1	0	0	SA0477
gi|29165615|ref|NC_002745.2|	557641 +	C	1	0	0	0	0	SA0477
gi|29165615|ref|NC_002745.2|	557643 +	G	0	0	1	0	1	SA0477
gi|29165615|ref|NC_002745.2|	557644 +	A	0	0	0	0	2	SA0477
gi|29165615|ref|NC_002745.2|	557645 +	A	0	1	0	0	1	SA0477
gi|29165615|ref|NC_002745.2|	557646 +	A	0	0	1	0	0	SA0477
gi|29165615|ref|NC_002745.2|	557649 +	C	0	0	0	1	0	SA0477
gi|29165615|ref|NC_002745.2|	557659 +	G	0	0	0	1	0	SA0477

gi|29165615|ref|NC_002745.2|	557661 +	G	0	1	0	0	0	SA0477
gi|29165615|ref|NC_002745.2|	557662 +	U	0	2	0	0	0	SA0477
gi|29165615|ref|NC_002745.2|	557666 +	U	0	0	0	0	1	SA0477
gi|29165615|ref|NC_002745.2|	557667 +	A	0	0	1	0	0	SA0477
gi|29165615|ref|NC_002745.2|	557673 +	G	0	0	1	0	0	SA0477
gi|29165615|ref|NC_002745.2|	557675 +	C	1	0	0	0	0	SA0477
gi|29165615|ref|NC_002745.2|	557679 +	A	0	0	0	0	2	SA0477
gi|29165615|ref|NC_002745.2|	557680 +	A	0	1	0	0	0	SA0477
gi|29165615|ref|NC_002745.2|	557685 +	G	0	0	0	1	0	SA0477
gi|29165615|ref|NC_002745.2|	557689 +	A	0	0	0	0	1	SA0477
gi|29165615|ref|NC_002745.2|	557694 +	A	0	0	1	0	0	SA0477
gi|29165615|ref|NC_002745.2|	557696 +	A	0	1	0	0	0	SA0477
gi|29165615|ref|NC_002745.2|	557697 +	A	0	0	1	0	0	SA0477
gi|29165615|ref|NC_002745.2|	557701 +	C	0	1	0	1	0	SA0477
gi|29165615|ref|NC_002745.2|	557704 +	A	0	0	2	0	0	SA0477
gi|29165615|ref|NC_002745.2|	557706 +	G	0	0	0	2	1	SA0477
gi|29165615|ref|NC_002745.2|	557710 +	C	0	0	1	0	0	SA0477
gi|29165615|ref|NC_002745.2|	557719 +	U	2	0	0	0	0	SA0477
gi|29165615|ref|NC_002745.2|	557720 +	A	1	0	0	0	0	SA0477
gi|29165615|ref|NC_002745.2|	557722 +	C	0	0	1	0	1	SA0477
gi|29165615|ref|NC_002745.2|	557724 +	G	0	1	0	0	0	SA0477
gi|29165615|ref|NC_002745.2|	557726 +	U	0	0	0	0	1	SA0477
gi|29165615|ref|NC_002745.2|	557730 +	G	0	1	2	0	0	SA0477
gi|29165615|ref|NC_002745.2|	557731 +	C	0	0	0	0	1	SA0477
gi|29165615|ref|NC_002745.2|	557735 +	A	0	1	0	0	0	SA0477
gi|29165615|ref|NC_002745.2|	557736 +	G	0	0	1	0	0	SA0477
gi|29165615|ref|NC_002745.2|	557737 +	A	0	0	1	0	0	SA0477
gi|29165615|ref|NC_002745.2|	557745 +	C	0	1	0	0	0	SA0477
gi|29165615|ref|NC_002745.2|	557749 +	C	2	0	0	0	0	SA0477
gi|29165615|ref|NC_002745.2|	557751 +	G	0	0	2	0	0	SA0477
gi|29165615|ref|NC_002745.2|	557752 +	A	1	0	0	0	1	SA0477
gi|29165615|ref|NC_002745.2|	557753 +	U	0	0	0	0	1	SA0477
gi|29165615|ref|NC_002745.2|	557754 +	A	0	0	0	0	1	SA0477
gi|29165615|ref|NC_002745.2|	557761 +	C	1	0	0	0	0	SA0477
gi|29165615|ref|NC_002745.2|	557764 +	C	3	0	0	0	1	SA0477
gi|29165615|ref|NC_002745.2|	557765 +	U	0	0	1	0	0	SA0477
gi|29165615|ref|NC_002745.2|	557766 +	G	0	0	1	0	0	SA0477
gi|29165615|ref|NC_002745.2|	557770 +	G	0	2	0	0	0	SA0477
gi|29165615|ref|NC_002745.2|	557772 +	G	0	0	0	1	0	SA0477
gi|29165615|ref|NC_002745.2|	557775 +	G	2	0	0	0	0	SA0477
gi|29165615|ref|NC_002745.2|	557777 +	A	0	0	0	1	0	SA0477
gi|29165615|ref|NC_002745.2|	557778 +	C	0	1	1	0	0	SA0477
gi|29165615|ref|NC_002745.2|	557779 +	G	3	0	0	0	0	SA0477
gi|29165615|ref|NC_002745.2|	557780 +	U	1	0	0	0	0	SA0477
gi|29165615|ref|NC_002745.2|	557781 +	A	0	1	0	0	0	SA0477
gi|29165615|ref|NC_002745.2|	557785 +	C	0	0	0	1	1	SA0477
gi|29165615|ref|NC_002745.2|	557789 +	C	0	0	1	0	0	SA0477
gi|29165615|ref|NC_002745.2|	557791 +	C	1	0	0	0	0	SA0477
gi|29165615|ref|NC_002745.2|	557792 +	U	9	10	4	3	8	SA0477
gi|29165615|ref|NC_002745.2|	557793 +	A	0	0	0	1	0	SA0477
gi|29165615|ref|NC_002745.2|	557795 +	A	0	1	0	0	0	SA0477
gi|29165615|ref|NC_002745.2|	557796 +	A	9	55	23	5	42	SA0477
gi|29165615|ref|NC_002745.2|	557798 +	U	0	0	0	0	1	SA0477
gi|29165615|ref|NC_002745.2|	557800 +	U	0	0	1	7	1	SA0477
gi|29165615|ref|NC_002745.2|	557801 +	A	1	7	0	6	6	SA0477
gi|29165615|ref|NC_002745.2|	557802 +	G	0	0	2	2	0	SA0477
gi|29165615|ref|NC_002745.2|	557803 +	A	0	2	2	3	3	SA0477
gi|29165615|ref|NC_002745.2|	557804 +	A	0	0	2	2	0	SA0477
gi|29165615|ref|NC_002745.2|	557805 +	G	0	4	3	5	10	SA0477
gi|29165615|ref|NC_002745.2|	557806 +	A	0	6	2	3	9	SA0477
gi|29165615|ref|NC_002745.2|	557807 +	A	0	2	0	1	3	SA0477
gi|29165615|ref|NC_002745.2|	557808 +	G	0	4	0	2	3	SA0477
gi|29165615|ref|NC_002745.2|	557809 +	U	0	5	0	1	4	SA0477
gi|29165615|ref|NC_002745.2|	557810 +	A	0	4	3	3	2	SA0477
gi|29165615|ref|NC_002745.2|	557811 +	A	0	3	4	1	1	SA0477
gi|29165615|ref|NC_002745.2|	557812 +	U	0	0	0	0	1	SA0477
gi|29165615|ref|NC_002745.2|	557813 +	G	0	2	0	0	2	SA0477
gi|29165615|ref|NC_002745.2|	557814 +	A	0	4	3	2	4	SA0477
gi|29165615|ref|NC_002745.2|	557815 +	A	0	0	1	0	0	SA0477
gi|29165615|ref|NC_002745.2|	557816 +	U	0	0	2	0	0	SA0477
gi|29165615|ref|NC_002745.2|	557817 +	G	0	0	1	0	0	SA0477
gi|29165615|ref|NC_002745.2|	557818 +	C	0	1	1	4	7	SA0477
gi|29165615|ref|NC_002745.2|	557819 +	U	0	0	0	1	0	SA0477
gi|29165615|ref|NC_002745.2|	557821 +	U	0	0	0	0	2	SA0477
gi|29165615|ref|NC_002745.2|	557823 +	U	0	0	0	0	1	SA0477
gi|29165615|ref|NC_002745.2|	557824 +	C	1	1	1	1	0	SA0477
gi|29165615|ref|NC_002745.2|	557826 +	A	0	1	0	0	0	SA0477
gi|29165615|ref|NC_002745.2|	557834 +	C	0	1	0	0	1	SA0477
gi|29165615|ref|NC_002745.2|	557839 +	C	0	0	1	0	0	SA0477
gi|29165615|ref|NC_002745.2|	557841 +	A	0	3	1	0	0	SA0477
gi|29165615|ref|NC_002745.2|	557845 +	C	0	0	1	1	0	SA0477

gi|29165615|ref|NC_002745.2|	557849 +	U	0	0	2	0	0	SA0477
gi|29165615|ref|NC_002745.2|	557854 +	G	2	0	1	2	0	SA0477
gi|29165615|ref|NC_002745.2|	557856 +	C	1	0	0	0	0	SA0477
gi|29165615|ref|NC_002745.2|	557860 +	U	0	0	0	0	1	SA0477
gi|29165615|ref|NC_002745.2|	557862 +	A	0	0	0	0	1	SA0477
gi|29165615|ref|NC_002745.2|	557868 +	G	0	0	1	0	0	SA0477
gi|29165615|ref|NC_002745.2|	557869 +	C	0	1	1	0	0	SA0477
gi|29165615|ref|NC_002745.2|	557871 +	A	0	1	0	0	0	SA0477
gi|29165615|ref|NC_002745.2|	557872 +	G	0	0	0	1	0	SA0477
gi|29165615|ref|NC_002745.2|	557873 +	A	0	0	0	7	2	SA0477
gi|29165615|ref|NC_002745.2|	557874 +	G	2	0	1	1	1	SA0477
gi|29165615|ref|NC_002745.2|	557876 +	A	0	0	0	1	0	SA0477
gi|29165615|ref|NC_002745.2|	557879 +	A	0	1	0	0	1	SA0477
gi|29165615|ref|NC_002745.2|	557880 +	G	0	0	0	0	1	SA0477
gi|29165615|ref|NC_002745.2|	557891 +	U	2	0	0	0	0	SA0477
gi|29165615|ref|NC_002745.2|	557907 +	G	0	0	0	0	1	SA0477
gi|29165615|ref|NC_002745.2|	557910 +	U	0	0	0	1	0	SA0477
gi|29165615|ref|NC_002745.2|	557914 +	A	0	0	1	0	1	SA0477
gi|29165615|ref|NC_002745.2|	557915 +	A	0	0	0	0	1	SA0477
gi|29165615|ref|NC_002745.2|	557917 +	U	1	0	0	0	0	SA0477
gi|29165615|ref|NC_002745.2|	557918 +	G	0	0	0	1	0	SA0477
gi|29165615|ref|NC_002745.2|	557919 +	U	0	0	0	0	1	SA0477
gi|29165615|ref|NC_002745.2|	557923 +	C	0	0	1	0	0	SA0477
gi|29165615|ref|NC_002745.2|	557931 +	G	0	0	0	0	2	SA0477
gi|29165615|ref|NC_002745.2|	557932 +	A	2	0	0	0	0	SA0477
gi|29165615|ref|NC_002745.2|	557934 +	G	1	0	0	0	0	SA0477
gi|29165615|ref|NC_002745.2|	557935 +	A	0	0	1	0	1	SA0477
gi|29165615|ref|NC_002745.2|	557937 +	G	0	0	1	0	2	SA0477
gi|29165615|ref|NC_002745.2|	557938 +	A	0	1	2	5	3	SA0477
gi|29165615|ref|NC_002745.2|	557942 +	U	0	0	1	1	1	SA0477
gi|29165615|ref|NC_002745.2|	557943 +	C	0	2	0	1	2	SA0477
gi|29165615|ref|NC_002745.2|	557945 +	C	0	0	2	0	0	SA0477
gi|29165615|ref|NC_002745.2|	557946 +	U	0	0	0	1	0	SA0477
gi|29165615|ref|NC_002745.2|	557948 +	A	0	2	0	0	1	SA0477
gi|29165615|ref|NC_002745.2|	557949 +	A	0	0	0	0	3	SA0477
gi|29165615|ref|NC_002745.2|	557950 +	G	0	2	1	1	8	SA0477
gi|29165615|ref|NC_002745.2|	557951 +	A	1	4	6	8	19	SA0477
gi|29165615|ref|NC_002745.2|	557952 +	A	0	1	3	6	0	SA0477
gi|29165615|ref|NC_002745.2|	557953 +	A	0	0	1	1	1	SA0477
gi|29165615|ref|NC_002745.2|	557954 +	A	0	13	11	7	12	SA0477
gi|29165615|ref|NC_002745.2|	557955 +	G	0	5	1	2	1	SA0477
gi|29165615|ref|NC_002745.2|	557956 +	A	0	3	6	5	6	SA0477
gi|29165615|ref|NC_002745.2|	557957 +	U	0	1	1	1	2	SA0477
gi|29165615|ref|NC_002745.2|	557958 +	C	0	2	2	0	3	SA0477
gi|29165615|ref|NC_002745.2|	557959 +	A	0	1	1	1	4	SA0477
gi|29165615|ref|NC_002745.2|	557960 +	A	0	0	0	0	2	SA0477
gi|29165615|ref|NC_002745.2|	557961 +	U	0	2	0	0	0	SA0477
gi|29165615|ref|NC_002745.2|	557962 +	U	0	0	0	1	0	SA0477
gi|29165615|ref|NC_002745.2|	557963 +	U	0	0	0	1	0	SA0477
gi|29165615|ref|NC_002745.2|	557964 +	A	0	0	0	3	0	SA0477
gi|29165615|ref|NC_002745.2|	557965 +	C	0	1	6	3	0	SA0477
gi|29165615|ref|NC_002745.2|	557966 +	A	0	0	2	0	0	SA0477
gi|29165615|ref|NC_002745.2|	557967 +	G	0	0	0	4	1	SA0477
gi|29165615|ref|NC_002745.2|	557968 +	U	1	0	0	0	0	SA0477
gi|29165615|ref|NC_002745.2|	557970 +	C	0	0	1	1	0	SA0477
gi|29165615|ref|NC_002745.2|	557971 +	C	0	0	2	0	0	SA0477
gi|29165615|ref|NC_002745.2|	557972 +	A	0	0	0	0	1	SA0477
gi|29165615|ref|NC_002745.2|	557973 +	U	0	0	0	4	0	SA0477
gi|29165615|ref|NC_002745.2|	557974 +	U	0	0	0	3	0	SA0477
gi|29165615|ref|NC_002745.2|	557975 +	U	0	0	1	0	0	SA0477
gi|29165615|ref|NC_002745.2|	557977 +	U	0	1	4	1	1	SA0477
gi|29165615|ref|NC_002745.2|	557978 +	A	0	0	2	0	0	SA0477
gi|29165615|ref|NC_002745.2|	557979 +	U	0	0	1	0	0	SA0477
gi|29165615|ref|NC_002745.2|	557982 +	G	0	1	2	0	1	SA0477
gi|29165615|ref|NC_002745.2|	557983 +	G	0	1	1	1	0	SA0477
gi|29165615|ref|NC_002745.2|	557984 +	A	0	1	0	0	1	SA0477
gi|29165615|ref|NC_002745.2|	557987 +	U	1	0	0	0	0	SA0477
gi|29165615|ref|NC_002745.2|	557989 +	G	0	0	0	0	1	SA0477
gi|29165615|ref|NC_002745.2|	557990 +	U	0	0	1	1	0	SA0477
gi|29165615|ref|NC_002745.2|	557991 +	A	0	1	0	1	1	SA0477
gi|29165615|ref|NC_002745.2|	557996 +	A	0	0	0	0	1	SA0477
gi|29165615|ref|NC_002745.2|	557998 +	G	0	1	0	0	1	SA0477
gi|29165615|ref|NC_002745.2|	558001 +	A	0	0	0	0	2	SA0477
gi|29165615|ref|NC_002745.2|	558005 +	U	1	0	0	0	0	SA0477
gi|29165615|ref|NC_002745.2|	558006 +	G	0	0	2	0	1	SA0477
gi|29165615|ref|NC_002745.2|	558008 +	G	1	0	0	0	0	SA0477
gi|29165615|ref|NC_002745.2|	558012 +	A	0	0	1	0	0	SA0477
gi|29165615|ref|NC_002745.2|	558013 +	G	0	0	0	0	2	SA0477
gi|29165615|ref|NC_002745.2|	558014 +	A	0	2	2	0	0	SA0477
gi|29165615|ref|NC_002745.2|	558015 +	A	0	1	0	0	0	SA0477
gi|29165615|ref|NC_002745.2|	558019 +	G	0	0	0	1	0	SA0477

gi|29165615|ref|NC_002745.2|	558030	+	G	0	0	1	0	0	SA0477
gi|29165615|ref|NC_002745.2|	558047	+	A	0	0	0	0	1	SA0477
gi|29165615|ref|NC_002745.2|	558059	+	U	0	1	0	0	0	SA0477
gi|29165615|ref|NC_002745.2|	558065	+	U	0	1	0	0	0	SA0477
gi|29165615|ref|NC_002745.2|	558068	+	U	1	1	1	0	0	SA0477
gi|29165615|ref|NC_002745.2|	558070	+	U	0	1	1	1	1	SA0477
gi|29165615|ref|NC_002745.2|	558071	+	U	0	0	1	0	0	SA0477
gi|29165615|ref|NC_002745.2|	558075	+	G	0	0	1	0	1	SA0477
gi|29165615|ref|NC_002745.2|	558076	+	A	0	0	2	0	1	SA0477
gi|29165615|ref|NC_002745.2|	558082	+	U	0	1	0	0	0	SA0477
gi|29165615|ref|NC_002745.2|	558083	+	A	0	0	0	0	1	SA0477
gi|29165615|ref|NC_002745.2|	558086	+	A	0	4	1	1	0	SA0477
gi|29165615|ref|NC_002745.2|	558087	+	C	0	2	0	0	0	SA0477
gi|29165615|ref|NC_002745.2|	558088	+	A	0	0	0	0	1	SA0477
gi|29165615|ref|NC_002745.2|	558089	+	U	0	0	1	0	2	SA0477
gi|29165615|ref|NC_002745.2|	558090	+	A	0	0	2	0	0	SA0477
gi|29165615|ref|NC_002745.2|	558092	+	G	0	0	0	1	0	SA0477
gi|29165615|ref|NC_002745.2|	558093	+	A	0	0	0	0	1	SA0477
gi|29165615|ref|NC_002745.2|	558095	+	A	1	1	1	1	0	SA0477
gi|29165615|ref|NC_002745.2|	558096	+	C	0	0	3	0	1	SA0477
gi|29165615|ref|NC_002745.2|	558100	+	U	0	0	1	0	0	SA0477
gi|29165615|ref|NC_002745.2|	558108	+	G	0	3	2	0	0	SA0477
gi|29165615|ref|NC_002745.2|	558109	+	A	0	2	1	0	0	SA0477
gi|29165615|ref|NC_002745.2|	558111	+	G	0	0	1	0	0	SA0477
gi|29165615|ref|NC_002745.2|	558112	+	U	1	0	0	0	0	SA0477
gi|29165615|ref|NC_002745.2|	558116	+	U	0	0	0	0	1	SA0477
gi|29165615|ref|NC_002745.2|	558118	+	G	0	1	0	0	0	SA0477
gi|29165615|ref|NC_002745.2|	558121	+	U	0	0	0	0	1	SA0477
gi|29165615|ref|NC_002745.2|	558122	+	G	0	0	0	0	1	SA0477
gi|29165615|ref|NC_002745.2|	558123	+	A	0	2	0	0	0	SA0477
gi|29165615|ref|NC_002745.2|	558129	+	A	0	1	0	0	0	SA0477
gi|29165615|ref|NC_002745.2|	558135	+	G	0	0	0	1	0	SA0477
gi|29165615|ref|NC_002745.2|	558136	+	A	0	0	1	2	0	SA0477
gi|29165615|ref|NC_002745.2|	558142	+	A	0	1	1	0	0	SA0477
gi|29165615|ref|NC_002745.2|	558144	+	A	0	0	1	3	0	SA0477
gi|29165615|ref|NC_002745.2|	558149	+	G	0	0	0	0	1	SA0477
gi|29165615|ref|NC_002745.2|	558150	+	A	0	0	1	2	1	SA0477
gi|29165615|ref|NC_002745.2|	558152	+	U	0	0	0	1	0	SA0477
gi|29165615|ref|NC_002745.2|	558183	+	A	0	0	0	0	1	SA0477
gi|29165615|ref|NC_002745.2|	558188	+	A	1	0	0	0	1	SA0477
gi|29165615|ref|NC_002745.2|	558189	+	A	0	0	0	0	1	SA0477
gi|29165615|ref|NC_002745.2|	558193	+	A	0	0	1	0	0	SA0477
gi|29165615|ref|NC_002745.2|	558195	+	A	0	0	0	0	1	SA0477
gi|29165615|ref|NC_002745.2|	558196	+	U	0	1	0	0	1	SA0477
gi|29165615|ref|NC_002745.2|	558198	+	A	1	0	1	1	1	SA0477
gi|29165615|ref|NC_002745.2|	558201	+	G	0	0	1	0	0	SA0477
gi|29165615|ref|NC_002745.2|	558202	+	A	0	0	0	0	3	SA0477
gi|29165615|ref|NC_002745.2|	558203	+	C	0	2	1	0	0	SA0477
gi|29165615|ref|NC_002745.2|	558204	+	A	0	0	1	0	0	SA0477
gi|29165615|ref|NC_002745.2|	558208	+	G	0	0	1	0	0	SA0477
gi|29165615|ref|NC_002745.2|	558209	+	U	0	1	0	0	0	SA0477
gi|29165615|ref|NC_002745.2|	558212	+	U	2	0	0	0	0	SA0477
gi|29165615|ref|NC_002745.2|	558214	+	U	0	0	2	2	0	SA0477
gi|29165615|ref|NC_002745.2|	558215	+	A	0	0	0	0	1	SA0477
gi|29165615|ref|NC_002745.2|	558216	+	C	0	0	1	0	0	SA0477
gi|29165615|ref|NC_002745.2|	558225	+	A	0	1	0	0	0	SA0477
gi|29165615|ref|NC_002745.2|	558227	+	C	0	0	0	1	1	SA0477
gi|29165615|ref|NC_002745.2|	558232	+	C	0	0	1	0	0	SA0477
gi|29165615|ref|NC_002745.2|	558234	+	G	0	0	1	0	0	SA0477
gi|29165615|ref|NC_002745.2|	558239	+	U	1	0	0	0	0	SA0477
gi|29165615|ref|NC_002745.2|	558245	+	U	0	0	0	0	1	SA0477
gi|29165615|ref|NC_002745.2|	558247	+	C	0	0	0	1	0	SA0477
gi|29165615|ref|NC_002745.2|	558262	+	C	2	0	0	0	0	SA0477
gi|29165615|ref|NC_002745.2|	558267	+	U	0	0	1	0	0	SA0477
gi|29165615|ref|NC_002745.2|	558274	+	U	0	0	1	0	0	SA0477
gi|29165615|ref|NC_002745.2|	558276	+	G	0	0	1	1	1	SA0477
gi|29165615|ref|NC_002745.2|	558277	+	A	1	0	0	0	0	SA0477
gi|29165615|ref|NC_002745.2|	558298	+	U	0	0	0	0	1	SA0477
gi|29165615|ref|NC_002745.2|	558307	+	C	0	0	0	1	0	SA0477
gi|29165615|ref|NC_002745.2|	558319	+	A	0	0	0	1	0	SA0477
gi|29165615|ref|NC_002745.2|	558330	+	C	0	0	0	1	0	SA0477
gi|29165615|ref|NC_002745.2|	558332	+	A	0	0	2	0	0	SA0477
gi|29165615|ref|NC_002745.2|	558338	+	A	0	2	0	0	1	SA0477
gi|29165615|ref|NC_002745.2|	558346	+	A	0	0	0	0	1	SA0477
gi|29165615|ref|NC_002745.2|	558351	+	A	0	1	0	0	0	SA0477
gi|29165615|ref|NC_002745.2|	558368	+	A	0	0	0	2	0	SA0477
gi|29165615|ref|NC_002745.2|	558373	+	A	0	0	0	1	0	SA0477
gi|29165615|ref|NC_002745.2|	558375	+	C	2	0	0	0	1	SA0477
gi|29165615|ref|NC_002745.2|	558379	+	A	0	0	0	0	1	SA0477
gi|29165615|ref|NC_002745.2|	558381	+	U	0	0	0	0	1	SA0477
gi|29165615|ref|NC_002745.2|	558387	+	C	0	0	0	0	1	SA0477

gi|29165615|ref|NC_002745.2|	558389 +	A	0	0	0	0	1	SA0477
gi|29165615|ref|NC_002745.2|	558391 +	U	0	0	0	1	0	SA0477
gi|29165615|ref|NC_002745.2|	558398 +	A	0	0	0	0	1	SA0477
gi|29165615|ref|NC_002745.2|	558410 +	A	1	0	0	0	0	SA0477
gi|29165615|ref|NC_002745.2|	558412 +	U	0	1	0	0	0	SA0477
gi|29165615|ref|NC_002745.2|	558413 +	U	1	4	0	0	6	SA0477
gi|29165615|ref|NC_002745.2|	558414 +	G	0	0	1	0	0	SA0477
gi|29165615|ref|NC_002745.2|	558415 +	G	0	1	0	0	0	SA0477
gi|29165615|ref|NC_002745.2|	558418 +	C	0	1	0	1	0	SA0477
gi|29165615|ref|NC_002745.2|	558430 +	G	0	0	0	2	0	SA0477
gi|29165615|ref|NC_002745.2|	558436 +	A	0	2	0	0	0	SA0477
gi|29165615|ref|NC_002745.2|	558440 +	C	1	1	0	0	0	SA0477
gi|29165615|ref|NC_002745.2|	558441 +	A	0	1	0	1	2	SA0477
gi|29165615|ref|NC_002745.2|	558442 +	A	1	0	0	0	0	SA0477
gi|29165615|ref|NC_002745.2|	558443 +	U	0	0	0	1	0	SA0477
gi|29165615|ref|NC_002745.2|	558444 +	C	0	0	1	0	0	SA0477
gi|29165615|ref|NC_002745.2|	558446 +	A	0	1	0	1	0	SA0477
gi|29165615|ref|NC_002745.2|	558448 +	U	1	0	0	0	2	SA0477
gi|29165615|ref|NC_002745.2|	558449 +	A	0	1	0	0	1	SA0477
gi|29165615|ref|NC_002745.2|	558451 +	C	0	2	0	0	0	SA0477
gi|29165615|ref|NC_002745.2|	558452 +	A	0	0	0	0	1	SA0477
gi|29165615|ref|NC_002745.2|	558454 +	U	0	0	1	0	0	SA0477
gi|29165615|ref|NC_002745.2|	558455 +	A	0	0	1	2	2	SA0477
gi|29165615|ref|NC_002745.2|	558456 +	G	0	3	0	1	0	SA0477
gi|29165615|ref|NC_002745.2|	558457 +	A	0	0	2	0	0	SA0477
gi|29165615|ref|NC_002745.2|	558458 +	A	0	0	0	0	1	SA0477
gi|29165615|ref|NC_002745.2|	558460 +	A	0	1	0	0	0	SA0477
gi|29165615|ref|NC_002745.2|	558464 +	U	0	0	1	3	3	SA0477
gi|29165615|ref|NC_002745.2|	558465 +	A	1	0	0	1	2	SA0477
gi|29165615|ref|NC_002745.2|	558468 +	C	0	0	0	4	3	SA0477
gi|29165615|ref|NC_002745.2|	558469 +	A	0	0	0	1	0	SA0477
gi|29165615|ref|NC_002745.2|	558470 +	A	0	1	0	1	0	SA0477
gi|29165615|ref|NC_002745.2|	558471 +	G	0	1	2	3	0	SA0477
gi|29165615|ref|NC_002745.2|	558486 +	G	0	1	0	0	0	-
gi|29165615|ref|NC_002745.2|	558489 +	A	0	0	0	0	1	SA0478
gi|29165615|ref|NC_002745.2|	558493 +	A	0	0	1	0	0	SA0478
gi|29165615|ref|NC_002745.2|	558495 +	A	0	0	0	0	1	SA0478
gi|29165615|ref|NC_002745.2|	558533 +	U	0	0	1	0	0	SA0478
gi|29165615|ref|NC_002745.2|	558539 +	A	0	0	0	0	1	SA0478
gi|29165615|ref|NC_002745.2|	558540 +	C	0	0	0	1	0	SA0478
gi|29165615|ref|NC_002745.2|	558542 +	U	0	0	0	1	0	SA0478
gi|29165615|ref|NC_002745.2|	558544 +	U	0	0	0	0	1	SA0478
gi|29165615|ref|NC_002745.2|	558546 +	G	0	1	2	1	0	SA0478
gi|29165615|ref|NC_002745.2|	558566 +	U	0	1	0	0	0	SA0478
gi|29165615|ref|NC_002745.2|	558584 +	U	0	0	0	1	0	SA0478
gi|29165615|ref|NC_002745.2|	558593 +	A	0	1	0	0	0	SA0478
gi|29165615|ref|NC_002745.2|	558594 +	G	0	0	0	1	0	SA0478
gi|29165615|ref|NC_002745.2|	558602 +	C	0	0	1	0	0	SA0478
gi|29165615|ref|NC_002745.2|	558608 +	C	0	0	0	1	0	SA0478
gi|29165615|ref|NC_002745.2|	558613 +	U	1	0	0	0	0	SA0478
gi|29165615|ref|NC_002745.2|	558624 +	G	0	0	0	1	0	SA0478
gi|29165615|ref|NC_002745.2|	558671 +	A	0	0	0	1	0	SA0478
gi|29165615|ref|NC_002745.2|	558744 +	G	0	0	0	0	1	SA0478
gi|29165615|ref|NC_002745.2|	558745 +	U	0	0	0	0	1	SA0478
gi|29165615|ref|NC_002745.2|	558767 +	C	0	0	2	0	0	SA0478
gi|29165615|ref|NC_002745.2|	558775 +	A	0	1	0	0	0	SA0478
gi|29165615|ref|NC_002745.2|	558787 +	A	0	0	0	0	1	SA0478
gi|29165615|ref|NC_002745.2|	558798 +	U	0	0	0	1	0	SA0478
gi|29165615|ref|NC_002745.2|	558826 +	C	0	0	0	0	1	SA0478
gi|29165615|ref|NC_002745.2|	558835 +	A	0	1	0	0	0	SA0478
gi|29165615|ref|NC_002745.2|	558837 +	A	2	0	0	1	0	SA0478
gi|29165615|ref|NC_002745.2|	558838 +	U	0	1	0	0	0	SA0478
gi|29165615|ref|NC_002745.2|	558840 +	A	0	0	0	0	3	SA0478
gi|29165615|ref|NC_002745.2|	558841 +	A	0	0	0	1	0	SA0478
gi|29165615|ref|NC_002745.2|	558846 +	A	0	5	4	3	1	SA0478
gi|29165615|ref|NC_002745.2|	558856 +	A	0	0	0	0	1	SA0478
gi|29165615|ref|NC_002745.2|	558858 +	A	0	0	1	0	1	SA0478
gi|29165615|ref|NC_002745.2|	558882 +	C	0	0	0	1	0	SA0478
gi|29165615|ref|NC_002745.2|	558887 +	U	0	0	0	0	1	SA0478
gi|29165615|ref|NC_002745.2|	558888 +	A	0	0	0	1	0	SA0478
gi|29165615|ref|NC_002745.2|	558889 +	U	0	0	0	1	0	SA0478
gi|29165615|ref|NC_002745.2|	558893 +	A	0	0	0	2	1	SA0478
gi|29165615|ref|NC_002745.2|	558894 +	A	0	2	0	0	0	SA0478
gi|29165615|ref|NC_002745.2|	558895 +	A	0	0	0	1	0	SA0478
gi|29165615|ref|NC_002745.2|	558896 +	A	0	2	0	1	0	SA0478
gi|29165615|ref|NC_002745.2|	558915 +	A	0	0	0	1	0	SA0478
gi|29165615|ref|NC_002745.2|	558927 +	G	0	1	0	0	0	SA0478
gi|29165615|ref|NC_002745.2|	558938 +	A	0	0	1	2	0	SA0478
gi|29165615|ref|NC_002745.2|	559081 +	U	0	0	0	0	1	-
gi|29165615|ref|NC_002745.2|	560345 +	C	0	1	0	0	0	-
gi|29165615|ref|NC_002745.2|	560590 +	U	0	0	0	1	0	-

gi|29165615|ref|NC_002745.2|	560600	+	U	1	1	3	2	2	-
gi|29165615|ref|NC_002745.2|	560601	+	C	2	5	6	3	7	-
gi|29165615|ref|NC_002745.2|	560602	+	A	0	0	0	2	4	-
gi|29165615|ref|NC_002745.2|	560613	+	A	0	0	0	1	0	-
gi|29165615|ref|NC_002745.2|	560684	+	C	0	0	0	0	1	SA0480
gi|29165615|ref|NC_002745.2|	560748	+	A	0	0	1	0	0	SA0480
gi|29165615|ref|NC_002745.2|	560764	+	A	0	1	0	0	0	SA0480
gi|29165615|ref|NC_002745.2|	560780	+	C	0	1	0	0	0	SA0480
gi|29165615|ref|NC_002745.2|	560802	+	C	0	0	0	1	0	SA0480
gi|29165615|ref|NC_002745.2|	560956	+	U	1	0	0	0	1	SA0480
gi|29165615|ref|NC_002745.2|	561061	+	C	0	0	0	0	2	SA0480
gi|29165615|ref|NC_002745.2|	561063	+	U	0	1	0	0	0	SA0480
gi|29165615|ref|NC_002745.2|	561071	+	C	0	0	1	0	0	SA0480
gi|29165615|ref|NC_002745.2|	561077	+	U	0	1	0	0	2	SA0480
gi|29165615|ref|NC_002745.2|	561078	+	A	0	0	2	0	0	SA0480
gi|29165615|ref|NC_002745.2|	561080	+	A	0	0	1	0	0	SA0480
gi|29165615|ref|NC_002745.2|	561083	+	A	0	0	1	1	0	SA0480
gi|29165615|ref|NC_002745.2|	561084	+	U	0	1	0	0	0	SA0480
gi|29165615|ref|NC_002745.2|	561085	+	U	0	0	1	0	0	SA0480
gi|29165615|ref|NC_002745.2|	561086	+	A	1	20	6	4	14	SA0480
gi|29165615|ref|NC_002745.2|	561087	+	C	0	0	1	0	1	SA0480
gi|29165615|ref|NC_002745.2|	561089	+	A	0	2	0	0	0	SA0480
gi|29165615|ref|NC_002745.2|	561095	+	A	0	1	0	0	0	-
gi|29165615|ref|NC_002745.2|	561129	+	A	0	0	0	0	1	SA0481
gi|29165615|ref|NC_002745.2|	561375	+	A	0	0	1	0	0	SA0481
gi|29165615|ref|NC_002745.2|	561397	+	A	1	0	0	0	0	SA0481
gi|29165615|ref|NC_002745.2|	561411	+	U	4	0	0	0	0	SA0481
gi|29165615|ref|NC_002745.2|	561456	+	C	1	0	0	0	0	SA0481
gi|29165615|ref|NC_002745.2|	561529	+	A	0	1	0	0	0	SA0481
gi|29165615|ref|NC_002745.2|	561550	+	A	0	1	0	0	2	SA0481
gi|29165615|ref|NC_002745.2|	561557	+	A	0	0	1	0	0	SA0481
gi|29165615|ref|NC_002745.2|	561563	+	A	0	0	0	0	1	SA0481
gi|29165615|ref|NC_002745.2|	561580	+	C	0	2	2	1	4	SA0481
gi|29165615|ref|NC_002745.2|	561581	+	U	0	0	1	0	0	SA0481
gi|29165615|ref|NC_002745.2|	561595	+	G	0	1	0	0	0	SA0481
gi|29165615|ref|NC_002745.2|	561635	+	C	0	0	0	1	0	SA0481
gi|29165615|ref|NC_002745.2|	561696	+	A	0	0	1	0	0	SA0482
gi|29165615|ref|NC_002745.2|	561697	+	A	0	0	0	1	0	SA0482
gi|29165615|ref|NC_002745.2|	561852	+	A	0	1	0	0	0	SA0482
gi|29165615|ref|NC_002745.2|	561953	+	U	0	1	0	0	0	SA0482
gi|29165615|ref|NC_002745.2|	562208	+	A	0	1	2	0	0	SA0482
gi|29165615|ref|NC_002745.2|	562233	+	A	0	1	0	0	0	SA0482
gi|29165615|ref|NC_002745.2|	562312	+	A	0	1	0	0	0	SA0482
gi|29165615|ref|NC_002745.2|	562433	+	C	0	0	1	0	0	SA0482
gi|29165615|ref|NC_002745.2|	562434	+	A	0	0	1	0	0	SA0482
gi|29165615|ref|NC_002745.2|	562448	+	C	0	1	0	0	0	SA0482
gi|29165615|ref|NC_002745.2|	562537	+	A	0	0	1	0	0	SA0482
gi|29165615|ref|NC_002745.2|	562542	+	A	0	0	0	1	0	SA0482
gi|29165615|ref|NC_002745.2|	562549	+	A	0	1	0	0	0	SA0482
gi|29165615|ref|NC_002745.2|	562562	+	A	0	0	0	0	1	SA0482
gi|29165615|ref|NC_002745.2|	562568	+	A	0	0	0	1	0	SA0482
gi|29165615|ref|NC_002745.2|	562569	+	A	0	2	0	0	0	SA0482
gi|29165615|ref|NC_002745.2|	562570	+	U	0	3	1	2	1	SA0482
gi|29165615|ref|NC_002745.2|	562571	+	G	0	1	0	0	0	SA0482
gi|29165615|ref|NC_002745.2|	562614	+	A	0	0	0	2	0	SA0482
gi|29165615|ref|NC_002745.2|	562617	+	A	0	1	0	0	1	SA0482
gi|29165615|ref|NC_002745.2|	562633	+	A	0	0	1	1	0	SA0482
gi|29165615|ref|NC_002745.2|	562644	+	C	0	0	0	1	1	SA0482
gi|29165615|ref|NC_002745.2|	562646	+	G	0	0	0	1	0	SA0482
gi|29165615|ref|NC_002745.2|	562650	+	U	0	0	0	0	1	SA0482
gi|29165615|ref|NC_002745.2|	562651	+	A	0	0	0	1	0	SA0482
gi|29165615|ref|NC_002745.2|	562654	+	A	0	0	0	0	1	SA0482
gi|29165615|ref|NC_002745.2|	562660	+	A	0	1	0	0	1	SA0482
gi|29165615|ref|NC_002745.2|	562670	+	U	0	0	0	0	1	SA0482
gi|29165615|ref|NC_002745.2|	562689	+	U	0	1	0	0	0	SA0483
gi|29165615|ref|NC_002745.2|	562697	+	U	0	1	0	0	0	SA0483
gi|29165615|ref|NC_002745.2|	562759	+	A	2	0	0	0	0	SA0483
gi|29165615|ref|NC_002745.2|	562763	+	U	0	0	1	0	0	SA0483
gi|29165615|ref|NC_002745.2|	562769	+	U	0	0	2	2	1	SA0483
gi|29165615|ref|NC_002745.2|	562770	+	A	0	0	1	0	0	SA0483
gi|29165615|ref|NC_002745.2|	562774	+	G	0	0	0	0	1	SA0483
gi|29165615|ref|NC_002745.2|	562775	+	A	0	0	1	0	0	SA0483
gi|29165615|ref|NC_002745.2|	562780	+	A	0	1	0	0	0	SA0483
gi|29165615|ref|NC_002745.2|	562787	+	A	0	0	0	0	1	SA0483
gi|29165615|ref|NC_002745.2|	562799	+	A	0	1	0	0	1	SA0483
gi|29165615|ref|NC_002745.2|	562810	+	C	0	0	0	1	0	SA0483
gi|29165615|ref|NC_002745.2|	562818	+	A	1	0	0	0	0	SA0483
gi|29165615|ref|NC_002745.2|	562819	+	U	0	0	0	0	1	SA0483
gi|29165615|ref|NC_002745.2|	562834	+	U	0	0	0	0	1	SA0483
gi|29165615|ref|NC_002745.2|	562837	+	A	0	0	1	1	3	SA0483
gi|29165615|ref|NC_002745.2|	562844	+	U	0	0	0	0	1	SA0483

gi|29165615|ref|NC_002745.2|	562845	+	A	0	0	0	1	0	SA0483
gi|29165615|ref|NC_002745.2|	562853	+	U	0	0	0	1	0	SA0483
gi|29165615|ref|NC_002745.2|	562855	+	A	0	2	2	1	0	SA0483
gi|29165615|ref|NC_002745.2|	562856	+	A	0	1	0	0	0	SA0483
gi|29165615|ref|NC_002745.2|	562864	+	U	0	1	3	1	2	SA0483
gi|29165615|ref|NC_002745.2|	562865	+	A	0	1	1	0	0	SA0483
gi|29165615|ref|NC_002745.2|	562870	+	A	0	0	1	0	0	SA0483
gi|29165615|ref|NC_002745.2|	562873	+	A	0	1	1	0	0	SA0483
gi|29165615|ref|NC_002745.2|	562876	+	U	0	0	0	1	0	SA0483
gi|29165615|ref|NC_002745.2|	562877	+	U	0	0	0	1	0	SA0483
gi|29165615|ref|NC_002745.2|	562879	+	A	0	0	1	2	0	SA0483
gi|29165615|ref|NC_002745.2|	562880	+	A	0	0	0	0	1	SA0483
gi|29165615|ref|NC_002745.2|	562883	+	A	0	0	0	0	1	SA0483
gi|29165615|ref|NC_002745.2|	562889	+	C	0	0	0	0	1	SA0483
gi|29165615|ref|NC_002745.2|	562890	+	G	0	1	0	0	0	SA0483
gi|29165615|ref|NC_002745.2|	562892	+	A	0	0	0	0	1	SA0483
gi|29165615|ref|NC_002745.2|	562893	+	C	0	0	0	0	1	SA0483
gi|29165615|ref|NC_002745.2|	562903	+	A	0	0	1	0	0	SA0483
gi|29165615|ref|NC_002745.2|	562920	+	C	0	0	0	2	0	SA0483
gi|29165615|ref|NC_002745.2|	562936	+	C	0	0	1	0	0	SA0483
gi|29165615|ref|NC_002745.2|	562938	+	A	0	1	0	0	0	SA0483
gi|29165615|ref|NC_002745.2|	562946	+	C	1	0	0	0	0	SA0483
gi|29165615|ref|NC_002745.2|	562947	+	A	1	0	0	0	0	SA0483
gi|29165615|ref|NC_002745.2|	562957	+	C	1	0	0	0	0	SA0483
gi|29165615|ref|NC_002745.2|	562958	+	G	0	1	0	0	0	SA0483
gi|29165615|ref|NC_002745.2|	562976	+	A	0	1	0	0	0	SA0483
gi|29165615|ref|NC_002745.2|	562985	+	C	0	1	0	0	0	SA0483
gi|29165615|ref|NC_002745.2|	563068	+	A	0	1	0	0	0	SA0483
gi|29165615|ref|NC_002745.2|	563117	+	C	1	0	0	0	0	SA0483
gi|29165615|ref|NC_002745.2|	563127	+	A	0	0	0	0	1	SA0483
gi|29165615|ref|NC_002745.2|	563137	+	A	0	1	0	0	0	SA0483
gi|29165615|ref|NC_002745.2|	563142	+	C	0	0	0	1	1	SA0483
gi|29165615|ref|NC_002745.2|	563154	+	U	0	0	0	0	1	SA0483
gi|29165615|ref|NC_002745.2|	563156	+	A	0	0	2	0	0	SA0483
gi|29165615|ref|NC_002745.2|	563159	+	U	0	0	0	1	0	SA0483
gi|29165615|ref|NC_002745.2|	563160	+	A	0	1	0	0	1	SA0483
gi|29165615|ref|NC_002745.2|	563161	+	A	0	0	0	0	1	SA0483
gi|29165615|ref|NC_002745.2|	563162	+	U	0	1	0	0	0	SA0483
gi|29165615|ref|NC_002745.2|	563163	+	A	0	0	0	2	2	SA0483
gi|29165615|ref|NC_002745.2|	563166	+	C	0	1	0	0	0	SA0483
gi|29165615|ref|NC_002745.2|	563168	+	A	0	0	1	0	0	SA0483
gi|29165615|ref|NC_002745.2|	563169	+	A	0	0	0	1	0	SA0483
gi|29165615|ref|NC_002745.2|	563173	+	U	1	0	0	0	0	SA0483
gi|29165615|ref|NC_002745.2|	563176	+	A	0	0	0	0	1	SA0483
gi|29165615|ref|NC_002745.2|	563177	+	U	0	0	0	0	1	SA0483
gi|29165615|ref|NC_002745.2|	563184	+	G	0	0	0	0	1	SA0483
gi|29165615|ref|NC_002745.2|	563200	+	U	0	1	0	0	0	SA0483
gi|29165615|ref|NC_002745.2|	563207	+	C	0	0	0	0	1	SA0483
gi|29165615|ref|NC_002745.2|	563229	+	G	0	0	0	0	1	SA0483
gi|29165615|ref|NC_002745.2|	563248	+	A	0	0	1	0	0	SA0483
gi|29165615|ref|NC_002745.2|	563254	+	C	1	0	0	0	0	SA0483
gi|29165615|ref|NC_002745.2|	563260	+	U	0	0	0	1	0	SA0483
gi|29165615|ref|NC_002745.2|	563265	+	G	0	1	0	0	0	SA0483
gi|29165615|ref|NC_002745.2|	563268	+	G	0	0	0	2	0	SA0483
gi|29165615|ref|NC_002745.2|	563269	+	U	1	0	0	0	0	SA0483
gi|29165615|ref|NC_002745.2|	563279	+	A	0	1	0	0	0	SA0483
gi|29165615|ref|NC_002745.2|	563280	+	C	0	0	0	2	0	SA0483
gi|29165615|ref|NC_002745.2|	563282	+	U	0	0	0	0	1	SA0483
gi|29165615|ref|NC_002745.2|	563285	+	G	0	0	0	0	1	SA0483
gi|29165615|ref|NC_002745.2|	563286	+	A	0	0	0	3	0	SA0483
gi|29165615|ref|NC_002745.2|	563287	+	A	1	0	0	1	0	SA0483
gi|29165615|ref|NC_002745.2|	563290	+	A	2	0	0	1	0	SA0483
gi|29165615|ref|NC_002745.2|	563293	+	A	0	0	0	0	1	SA0483
gi|29165615|ref|NC_002745.2|	563296	+	C	1	0	0	0	0	SA0483
gi|29165615|ref|NC_002745.2|	563300	+	A	0	0	2	0	0	SA0483
gi|29165615|ref|NC_002745.2|	563302	+	U	0	0	1	0	0	SA0483
gi|29165615|ref|NC_002745.2|	563331	+	G	0	0	1	0	0	SA0483
gi|29165615|ref|NC_002745.2|	563332	+	C	6	0	0	0	0	SA0483
gi|29165615|ref|NC_002745.2|	563339	+	U	0	0	0	0	1	SA0483
gi|29165615|ref|NC_002745.2|	563358	+	A	0	0	0	0	1	SA0483
gi|29165615|ref|NC_002745.2|	563366	+	U	0	0	0	0	1	SA0483
gi|29165615|ref|NC_002745.2|	563386	+	U	0	0	0	0	1	SA0483
gi|29165615|ref|NC_002745.2|	563388	+	A	0	0	0	0	1	SA0483
gi|29165615|ref|NC_002745.2|	563404	+	U	0	0	0	0	1	SA0483
gi|29165615|ref|NC_002745.2|	563487	+	A	0	1	0	0	0	SA0483
gi|29165615|ref|NC_002745.2|	563494	+	A	1	0	0	0	0	SA0483
gi|29165615|ref|NC_002745.2|	563524	+	A	1	0	0	0	0	SA0483
gi|29165615|ref|NC_002745.2|	563529	+	C	0	0	0	0	1	SA0483
gi|29165615|ref|NC_002745.2|	563530	+	A	0	1	0	0	0	SA0483
gi|29165615|ref|NC_002745.2|	563540	+	U	1	0	0	0	0	SA0483
gi|29165615|ref|NC_002745.2|	563545	+	C	1	0	0	0	0	SA0483

gi|29165615|ref|NC_002745.2|	563596	+	U	2	0	0	0	0	SA0483
gi|29165615|ref|NC_002745.2|	563653	+	A	0	2	0	0	0	SA0483
gi|29165615|ref|NC_002745.2|	563710	+	C	0	1	0	0	0	SA0483
gi|29165615|ref|NC_002745.2|	563753	+	U	0	0	1	0	0	SA0483
gi|29165615|ref|NC_002745.2|	563779	+	A	0	0	1	0	0	SA0483
gi|29165615|ref|NC_002745.2|	563859	+	A	0	0	0	1	0	SA0483
gi|29165615|ref|NC_002745.2|	563874	+	G	0	0	1	0	0	SA0483
gi|29165615|ref|NC_002745.2|	563877	+	G	0	0	1	0	0	SA0483
gi|29165615|ref|NC_002745.2|	563878	+	C	0	0	1	0	0	SA0483
gi|29165615|ref|NC_002745.2|	563891	+	A	0	0	0	0	1	SA0483
gi|29165615|ref|NC_002745.2|	563897	+	U	1	0	0	0	0	SA0483
gi|29165615|ref|NC_002745.2|	563919	+	A	0	1	0	0	0	SA0483
gi|29165615|ref|NC_002745.2|	563924	+	A	0	0	0	0	1	SA0483
gi|29165615|ref|NC_002745.2|	563925	+	A	0	1	0	0	0	SA0483
gi|29165615|ref|NC_002745.2|	563929	+	A	0	0	0	1	0	SA0483
gi|29165615|ref|NC_002745.2|	563941	+	A	0	1	0	0	0	SA0483
gi|29165615|ref|NC_002745.2|	563952	+	G	0	0	0	0	1	SA0483
gi|29165615|ref|NC_002745.2|	563957	+	A	0	0	0	1	0	SA0483
gi|29165615|ref|NC_002745.2|	563977	+	U	0	0	0	0	1	SA0483
gi|29165615|ref|NC_002745.2|	563980	+	A	1	0	0	0	0	SA0483
gi|29165615|ref|NC_002745.2|	563981	+	U	1	0	0	0	0	SA0483
gi|29165615|ref|NC_002745.2|	564043	+	A	0	0	0	1	0	SA0483
gi|29165615|ref|NC_002745.2|	564094	+	C	0	1	0	0	0	SA0483
gi|29165615|ref|NC_002745.2|	564106	+	A	0	0	0	0	1	SA0483
gi|29165615|ref|NC_002745.2|	564127	+	C	1	0	0	0	0	SA0483
gi|29165615|ref|NC_002745.2|	564155	+	A	0	0	0	1	0	SA0483
gi|29165615|ref|NC_002745.2|	564158	+	C	0	2	0	0	0	SA0483
gi|29165615|ref|NC_002745.2|	564162	+	G	0	0	0	1	0	SA0483
gi|29165615|ref|NC_002745.2|	564168	+	G	1	0	0	0	0	SA0483
gi|29165615|ref|NC_002745.2|	564169	+	A	0	0	0	0	2	SA0483
gi|29165615|ref|NC_002745.2|	564193	+	A	0	0	0	1	0	SA0483
gi|29165615|ref|NC_002745.2|	564197	+	U	0	0	1	0	0	SA0483
gi|29165615|ref|NC_002745.2|	564210	+	A	1	0	0	2	0	SA0483
gi|29165615|ref|NC_002745.2|	564232	+	C	2	0	0	0	0	SA0483
gi|29165615|ref|NC_002745.2|	564238	+	A	1	0	0	0	0	SA0483
gi|29165615|ref|NC_002745.2|	564241	+	C	0	1	0	1	0	SA0483
gi|29165615|ref|NC_002745.2|	564243	+	A	0	1	0	0	0	SA0483
gi|29165615|ref|NC_002745.2|	564252	+	G	0	0	0	1	0	SA0483
gi|29165615|ref|NC_002745.2|	564261	+	C	2	0	0	0	0	SA0483
gi|29165615|ref|NC_002745.2|	564262	+	G	0	0	1	0	0	SA0483
gi|29165615|ref|NC_002745.2|	564269	+	U	2	0	0	0	0	SA0483
gi|29165615|ref|NC_002745.2|	564307	+	U	0	1	0	0	0	SA0483
gi|29165615|ref|NC_002745.2|	564430	+	C	0	0	0	1	0	SA0483
gi|29165615|ref|NC_002745.2|	564457	+	C	0	0	1	0	0	SA0483
gi|29165615|ref|NC_002745.2|	564560	+	U	0	0	0	1	0	SA0483
gi|29165615|ref|NC_002745.2|	564684	+	G	0	0	0	0	1	SA0483
gi|29165615|ref|NC_002745.2|	564889	+	C	0	1	0	0	0	SA0483
gi|29165615|ref|NC_002745.2|	564897	+	A	0	2	0	0	0	SA0483
gi|29165615|ref|NC_002745.2|	564920	+	U	0	1	0	0	0	SA0483
gi|29165615|ref|NC_002745.2|	564922	+	A	0	0	0	0	1	SA0483
gi|29165615|ref|NC_002745.2|	564936	+	A	0	0	0	1	1	SA0483
gi|29165615|ref|NC_002745.2|	564949	+	G	0	0	0	1	0	SA0483
gi|29165615|ref|NC_002745.2|	564955	+	A	0	0	1	0	0	SA0483
gi|29165615|ref|NC_002745.2|	564964	+	A	1	0	0	0	0	SA0483
gi|29165615|ref|NC_002745.2|	565376	+	G	0	0	1	0	0	-
gi|29165615|ref|NC_002745.2|	565385	+	A	0	0	1	0	0	-
gi|29165615|ref|NC_002745.2|	565471	+	A	0	0	1	0	0	-
gi|29165615|ref|NC_002745.2|	565481	+	A	1	2	1	1	0	-
gi|29165615|ref|NC_002745.2|	565483	+	A	0	0	1	0	0	-
gi|29165615|ref|NC_002745.2|	565489	+	C	1	1	3	6	3	-
gi|29165615|ref|NC_002745.2|	565490	+	A	5	3	5	6	0	-
gi|29165615|ref|NC_002745.2|	565491	+	U	1	11	8	5	3	-
gi|29165615|ref|NC_002745.2|	565492	+	A	0	2	5	3	3	-
gi|29165615|ref|NC_002745.2|	565493	+	A	0	0	1	1	0	-
gi|29165615|ref|NC_002745.2|	565494	+	A	0	2	0	0	0	-
gi|29165615|ref|NC_002745.2|	565495	+	A	0	1	1	1	1	-
gi|29165615|ref|NC_002745.2|	565496	+	A	0	0	1	0	0	-
gi|29165615|ref|NC_002745.2|	565500	+	A	0	1	0	0	0	-
gi|29165615|ref|NC_002745.2|	565501	+	A	0	0	1	0	0	-
gi|29165615|ref|NC_002745.2|	565503	+	C	0	0	2	0	1	-
gi|29165615|ref|NC_002745.2|	565505	+	G	0	0	1	1	0	-
gi|29165615|ref|NC_002745.2|	565506	+	G	0	0	2	3	0	-
gi|29165615|ref|NC_002745.2|	565507	+	U	1	0	0	3	2	-
gi|29165615|ref|NC_002745.2|	565508	+	A	0	1	3	2	2	-
gi|29165615|ref|NC_002745.2|	565509	+	A	0	0	1	0	0	-
gi|29165615|ref|NC_002745.2|	565510	+	A	0	0	8	2	0	-
gi|29165615|ref|NC_002745.2|	565512	+	C	0	4	1	0	1	-
gi|29165615|ref|NC_002745.2|	565515	+	U	0	0	3	0	0	-
gi|29165615|ref|NC_002745.2|	565521	+	U	0	0	1	0	1	-
gi|29165615|ref|NC_002745.2|	565523	+	A	0	0	1	0	0	-
gi|29165615|ref|NC_002745.2|	565524	+	G	0	0	1	0	1	-

gi|29165615|ref|NC_002745.2|	565525	+	U	0	1	0	0	0	-
gi|29165615|ref|NC_002745.2|	565526	+	A	1	0	0	0	0	-
gi|29165615|ref|NC_002745.2|	565528	+	U	0	0	0	0	1	-
gi|29165615|ref|NC_002745.2|	565529	+	C	1	1	0	0	0	-
gi|29165615|ref|NC_002745.2|	565530	+	A	1	0	0	0	2	-
gi|29165615|ref|NC_002745.2|	565531	+	U	0	0	0	0	1	-
gi|29165615|ref|NC_002745.2|	565533	+	U	1	0	0	0	0	-
gi|29165615|ref|NC_002745.2|	565534	+	U	0	1	1	0	0	-
gi|29165615|ref|NC_002745.2|	565535	+	A	0	0	1	1	0	-
gi|29165615|ref|NC_002745.2|	565536	+	A	0	0	1	1	0	-
gi|29165615|ref|NC_002745.2|	565537	+	A	0	3	2	1	4	-
gi|29165615|ref|NC_002745.2|	565538	+	A	0	1	0	0	4	-
gi|29165615|ref|NC_002745.2|	565541	+	C	1	2	6	5	2	-
gi|29165615|ref|NC_002745.2|	565542	+	A	0	0	0	0	3	-
gi|29165615|ref|NC_002745.2|	565543	+	A	0	1	0	1	2	-
gi|29165615|ref|NC_002745.2|	565544	+	A	0	3	3	2	4	-
gi|29165615|ref|NC_002745.2|	565545	+	C	1	12	8	4	10	-
gi|29165615|ref|NC_002745.2|	565546	+	A	1	3	2	0	0	-
gi|29165615|ref|NC_002745.2|	565547	+	A	7	23	32	13	21	-
gi|29165615|ref|NC_002745.2|	565548	+	A	0	1	1	1	0	-
gi|29165615|ref|NC_002745.2|	565554	+	C	2	0	0	0	0	-
gi|29165615|ref|NC_002745.2|	565558	+	C	0	6	7	2	10	-
gi|29165615|ref|NC_002745.2|	565559	+	A	0	0	0	0	1	-
gi|29165615|ref|NC_002745.2|	565560	+	U	1	0	1	0	1	-
gi|29165615|ref|NC_002745.2|	565561	+	U	0	1	0	0	0	-
gi|29165615|ref|NC_002745.2|	565564	+	A	0	0	0	0	1	-
gi|29165615|ref|NC_002745.2|	565565	+	U	0	0	1	0	0	-
gi|29165615|ref|NC_002745.2|	565567	+	U	0	0	1	0	0	-
gi|29165615|ref|NC_002745.2|	565568	+	G	1	2	3	0	1	-
gi|29165615|ref|NC_002745.2|	565569	+	U	0	6	12	6	8	-
gi|29165615|ref|NC_002745.2|	565570	+	C	0	3	6	2	2	-
gi|29165615|ref|NC_002745.2|	565571	+	A	0	3	1	0	0	-
gi|29165615|ref|NC_002745.2|	565573	+	A	0	2	1	0	1	-
gi|29165615|ref|NC_002745.2|	565574	+	A	0	1	0	0	1	-
gi|29165615|ref|NC_002745.2|	565577	+	G	0	1	0	0	0	-
gi|29165615|ref|NC_002745.2|	565591	+	A	0	0	0	1	0	-
gi|29165615|ref|NC_002745.2|	565598	+	U	0	0	1	0	0	-
gi|29165615|ref|NC_002745.2|	565599	+	U	0	0	0	1	0	-
gi|29165615|ref|NC_002745.2|	565600	+	U	0	0	0	0	1	-
gi|29165615|ref|NC_002745.2|	565601	+	G	56	46	65	27	30	-
gi|29165615|ref|NC_002745.2|	565602	+	U	0	4	0	0	1	-
gi|29165615|ref|NC_002745.2|	565603	+	U	0	1	3	0	0	-
gi|29165615|ref|NC_002745.2|	565604	+	C	0	0	0	1	0	-
gi|29165615|ref|NC_002745.2|	565610	+	A	0	0	0	0	2	-
gi|29165615|ref|NC_002745.2|	565616	+	G	0	0	0	2	0	-
gi|29165615|ref|NC_002745.2|	565624	+	A	0	0	1	0	1	-
gi|29165615|ref|NC_002745.2|	565637	+	A	0	0	1	0	0	SA0484
gi|29165615|ref|NC_002745.2|	565640	+	A	0	1	0	0	0	SA0484
gi|29165615|ref|NC_002745.2|	565644	+	G	0	0	1	0	0	SA0484
gi|29165615|ref|NC_002745.2|	565645	+	A	0	0	2	1	2	SA0484
gi|29165615|ref|NC_002745.2|	565651	+	G	0	0	0	1	0	SA0484
gi|29165615|ref|NC_002745.2|	565652	+	A	0	0	3	1	0	SA0484
gi|29165615|ref|NC_002745.2|	565654	+	U	0	0	0	0	1	SA0484
gi|29165615|ref|NC_002745.2|	565659	+	G	0	0	1	0	0	SA0484
gi|29165615|ref|NC_002745.2|	565661	+	C	1	0	0	0	0	SA0484
gi|29165615|ref|NC_002745.2|	565667	+	G	0	0	0	0	1	SA0484
gi|29165615|ref|NC_002745.2|	565674	+	A	0	1	0	0	0	SA0484
gi|29165615|ref|NC_002745.2|	565676	+	C	0	1	0	0	0	SA0484
gi|29165615|ref|NC_002745.2|	565681	+	A	0	1	0	0	0	SA0484
gi|29165615|ref|NC_002745.2|	565687	+	A	1	0	0	0	0	SA0484
gi|29165615|ref|NC_002745.2|	565692	+	G	0	1	0	0	0	SA0484
gi|29165615|ref|NC_002745.2|	565722	+	A	0	0	1	0	0	SA0484
gi|29165615|ref|NC_002745.2|	565726	+	G	0	0	1	0	0	SA0484
gi|29165615|ref|NC_002745.2|	565749	+	C	1	0	0	0	0	SA0484
gi|29165615|ref|NC_002745.2|	565763	+	G	0	0	1	0	0	SA0484
gi|29165615|ref|NC_002745.2|	565784	+	C	0	0	0	0	1	SA0484
gi|29165615|ref|NC_002745.2|	565788	+	U	0	0	1	0	0	SA0484
gi|29165615|ref|NC_002745.2|	565797	+	A	0	0	1	0	0	SA0484
gi|29165615|ref|NC_002745.2|	565803	+	A	0	1	0	0	0	SA0484
gi|29165615|ref|NC_002745.2|	565805	+	A	0	0	0	0	1	SA0484
gi|29165615|ref|NC_002745.2|	565810	+	A	0	0	1	0	1	SA0484
gi|29165615|ref|NC_002745.2|	565813	+	A	0	0	1	0	0	SA0484
gi|29165615|ref|NC_002745.2|	565858	+	A	0	0	1	0	0	SA0484
gi|29165615|ref|NC_002745.2|	565898	+	U	1	0	0	0	0	SA0484
gi|29165615|ref|NC_002745.2|	565926	+	U	0	0	1	0	0	SA0484
gi|29165615|ref|NC_002745.2|	565966	+	C	0	0	0	1	0	SA0484
gi|29165615|ref|NC_002745.2|	566001	+	A	0	1	0	0	0	SA0484
gi|29165615|ref|NC_002745.2|	566011	+	C	0	0	0	1	0	SA0484
gi|29165615|ref|NC_002745.2|	566019	+	A	0	1	0	0	0	SA0484
gi|29165615|ref|NC_002745.2|	566021	+	U	0	0	0	0	1	SA0484
gi|29165615|ref|NC_002745.2|	566022	+	A	1	0	0	0	0	SA0484

gi|29165615|ref|NC_002745.2|	566149 +	A	0	2	0	0	0	SA0484
gi|29165615|ref|NC_002745.2|	566150 +	U	0	1	0	0	0	SA0484
gi|29165615|ref|NC_002745.2|	566165 +	U	0	0	1	0	0	SA0484
gi|29165615|ref|NC_002745.2|	566181 +	U	1	0	0	0	0	SA0484
gi|29165615|ref|NC_002745.2|	566191 +	C	0	0	1	0	0	SA0484
gi|29165615|ref|NC_002745.2|	566196 +	U	0	0	1	0	0	SA0484
gi|29165615|ref|NC_002745.2|	566206 +	A	0	0	0	0	1	SA0484
gi|29165615|ref|NC_002745.2|	566224 +	A	0	0	0	0	1	SA0484
gi|29165615|ref|NC_002745.2|	566239 +	A	0	0	0	0	1	SA0484
gi|29165615|ref|NC_002745.2|	566246 +	C	0	0	1	0	0	SA0484
gi|29165615|ref|NC_002745.2|	566274 +	A	0	0	0	0	2	SA0484
gi|29165615|ref|NC_002745.2|	566327 +	U	2	0	0	0	0	SA0484
gi|29165615|ref|NC_002745.2|	566471 +	A	1	0	0	0	0	SA0484
gi|29165615|ref|NC_002745.2|	566510 +	C	0	0	1	0	0	SA0484
gi|29165615|ref|NC_002745.2|	566576 +	A	0	0	0	1	0	SA0484
gi|29165615|ref|NC_002745.2|	566579 +	A	0	1	0	0	0	SA0484
gi|29165615|ref|NC_002745.2|	566582 +	C	1	0	0	0	0	SA0484
gi|29165615|ref|NC_002745.2|	566599 +	G	0	0	1	0	0	SA0484
gi|29165615|ref|NC_002745.2|	566652 +	A	0	1	0	0	0	SA0484
gi|29165615|ref|NC_002745.2|	566654 +	A	0	1	0	0	0	SA0484
gi|29165615|ref|NC_002745.2|	566655 +	U	0	0	0	0	1	SA0484
gi|29165615|ref|NC_002745.2|	566658 +	U	0	0	1	0	0	SA0484
gi|29165615|ref|NC_002745.2|	566690 +	U	1	0	0	0	0	SA0484
gi|29165615|ref|NC_002745.2|	566699 +	G	1	0	0	0	0	SA0484
gi|29165615|ref|NC_002745.2|	566753 +	C	1	0	0	0	0	SA0484
gi|29165615|ref|NC_002745.2|	566766 +	U	0	0	1	0	0	SA0484
gi|29165615|ref|NC_002745.2|	566804 +	U	0	0	0	1	0	SA0484
gi|29165615|ref|NC_002745.2|	566807 +	G	0	0	0	1	0	SA0484
gi|29165615|ref|NC_002745.2|	566819 +	A	0	0	0	1	0	SA0484
gi|29165615|ref|NC_002745.2|	566840 +	U	0	0	1	0	0	SA0484
gi|29165615|ref|NC_002745.2|	566844 +	A	1	0	0	0	0	SA0484
gi|29165615|ref|NC_002745.2|	566846 +	A	0	0	0	0	1	SA0484
gi|29165615|ref|NC_002745.2|	566851 +	G	0	0	1	0	0	SA0484
gi|29165615|ref|NC_002745.2|	566908 +	G	0	1	0	0	0	SA0484
gi|29165615|ref|NC_002745.2|	566916 +	G	0	1	0	0	0	SA0484
gi|29165615|ref|NC_002745.2|	566932 +	A	0	0	1	0	0	SA0484
gi|29165615|ref|NC_002745.2|	566951 +	C	1	0	0	0	0	SA0484
gi|29165615|ref|NC_002745.2|	566957 +	U	0	0	0	2	1	SA0484
gi|29165615|ref|NC_002745.2|	567043 +	A	0	1	0	0	0	SA0485
gi|29165615|ref|NC_002745.2|	567106 +	C	1	0	0	0	0	SA0485
gi|29165615|ref|NC_002745.2|	567126 +	C	0	0	0	0	1	SA0485
gi|29165615|ref|NC_002745.2|	567137 +	A	0	0	0	0	1	SA0485
gi|29165615|ref|NC_002745.2|	567139 +	C	0	1	0	0	0	SA0485
gi|29165615|ref|NC_002745.2|	567141 +	C	0	1	0	0	0	SA0485
gi|29165615|ref|NC_002745.2|	567153 +	C	0	0	0	0	1	SA0485
gi|29165615|ref|NC_002745.2|	567165 +	U	0	0	1	0	0	SA0485
gi|29165615|ref|NC_002745.2|	567206 +	U	1	0	0	0	0	SA0485
gi|29165615|ref|NC_002745.2|	567234 +	U	0	0	1	0	0	SA0485
gi|29165615|ref|NC_002745.2|	567284 +	U	0	0	1	0	0	SA0485
gi|29165615|ref|NC_002745.2|	567285 +	A	0	2	0	0	0	SA0485
gi|29165615|ref|NC_002745.2|	567287 +	U	0	0	0	0	1	SA0485
gi|29165615|ref|NC_002745.2|	567288 +	A	0	0	1	0	0	SA0485
gi|29165615|ref|NC_002745.2|	567291 +	C	0	0	1	0	0	SA0485
gi|29165615|ref|NC_002745.2|	567293 +	G	0	1	0	0	0	SA0485
gi|29165615|ref|NC_002745.2|	567294 +	A	0	1	1	0	1	SA0485
gi|29165615|ref|NC_002745.2|	567298 +	C	0	0	1	0	0	SA0485
gi|29165615|ref|NC_002745.2|	567299 +	U	0	0	1	0	0	SA0485
gi|29165615|ref|NC_002745.2|	567301 +	A	0	0	0	0	1	SA0485
gi|29165615|ref|NC_002745.2|	567304 +	U	0	1	0	0	0	SA0485
gi|29165615|ref|NC_002745.2|	567305 +	C	1	0	0	0	0	SA0485
gi|29165615|ref|NC_002745.2|	567306 +	U	0	0	1	0	0	SA0485
gi|29165615|ref|NC_002745.2|	567334 +	A	0	0	0	0	1	SA0485
gi|29165615|ref|NC_002745.2|	567363 +	U	0	0	1	0	0	SA0485
gi|29165615|ref|NC_002745.2|	567368 +	U	0	7	6	4	4	SA0485
gi|29165615|ref|NC_002745.2|	567369 +	C	0	1	0	2	1	SA0485
gi|29165615|ref|NC_002745.2|	567370 +	A	0	1	1	0	1	SA0485
gi|29165615|ref|NC_002745.2|	567379 +	A	0	2	1	0	1	SA0485
gi|29165615|ref|NC_002745.2|	567386 +	U	0	1	0	0	0	SA0485
gi|29165615|ref|NC_002745.2|	567390 +	U	0	1	0	0	0	SA0485
gi|29165615|ref|NC_002745.2|	567398 +	G	0	0	1	0	0	SA0485
gi|29165615|ref|NC_002745.2|	567451 +	A	0	0	2	0	0	SA0485
gi|29165615|ref|NC_002745.2|	567474 +	A	0	3	0	0	0	SA0485
gi|29165615|ref|NC_002745.2|	567479 +	C	1	0	0	0	0	SA0485
gi|29165615|ref|NC_002745.2|	567483 +	A	0	0	1	0	0	SA0485
gi|29165615|ref|NC_002745.2|	567489 +	U	0	0	0	0	1	SA0485
gi|29165615|ref|NC_002745.2|	567504 +	C	0	0	0	0	1	SA0485
gi|29165615|ref|NC_002745.2|	567509 +	C	0	0	0	1	0	SA0485
gi|29165615|ref|NC_002745.2|	567516 +	A	1	1	3	2	5	SA0485
gi|29165615|ref|NC_002745.2|	567517 +	A	0	0	0	0	1	SA0485
gi|29165615|ref|NC_002745.2|	567539 +	A	0	0	0	0	1	SA0485
gi|29165615|ref|NC_002745.2|	567555 +	U	3	0	0	0	0	SA0485

gi|29165615|ref|NC_002745.2|	567604	+	C	1	0	1	0	0	SA0485
gi|29165615|ref|NC_002745.2|	567606	+	A	0	0	1	0	0	SA0485
gi|29165615|ref|NC_002745.2|	567635	+	A	0	0	0	1	0	SA0485
gi|29165615|ref|NC_002745.2|	567669	+	A	0	1	0	0	0	SA0485
gi|29165615|ref|NC_002745.2|	567691	+	U	0	0	0	0	1	SA0485
gi|29165615|ref|NC_002745.2|	567745	+	C	0	0	0	0	1	SA0485
gi|29165615|ref|NC_002745.2|	567785	+	C	1	0	0	0	0	SA0485
gi|29165615|ref|NC_002745.2|	567854	+	C	0	1	0	0	0	SA0485
gi|29165615|ref|NC_002745.2|	567861	+	A	0	0	0	1	0	SA0485
gi|29165615|ref|NC_002745.2|	568000	+	C	0	1	0	0	0	SA0485
gi|29165615|ref|NC_002745.2|	568066	+	A	0	1	0	0	0	SA0485
gi|29165615|ref|NC_002745.2|	568113	+	A	0	1	0	0	0	-
gi|29165615|ref|NC_002745.2|	568322	+	A	0	1	0	0	0	-
gi|29165615|ref|NC_002745.2|	568325	+	G	0	0	1	0	0	-
gi|29165615|ref|NC_002745.2|	568329	+	A	0	1	0	0	0	-
gi|29165615|ref|NC_002745.2|	568354	+	U	0	1	0	0	0	-
gi|29165615|ref|NC_002745.2|	568432	+	A	0	0	0	1	0	-
gi|29165615|ref|NC_002745.2|	568464	+	A	0	0	0	1	0	-
gi|29165615|ref|NC_002745.2|	568475	+	U	0	0	1	0	1	-
gi|29165615|ref|NC_002745.2|	568476	+	G	0	1	0	0	1	-
gi|29165615|ref|NC_002745.2|	568484	+	U	0	0	1	0	1	-
gi|29165615|ref|NC_002745.2|	568494	+	A	2	1	2	0	1	-
gi|29165615|ref|NC_002745.2|	568495	+	C	0	0	1	0	3	-
gi|29165615|ref|NC_002745.2|	568496	+	A	0	8	7	2	9	-
gi|29165615|ref|NC_002745.2|	568497	+	A	24	36	49	43	39	-
gi|29165615|ref|NC_002745.2|	568498	+	U	0	0	1	1	1	-
gi|29165615|ref|NC_002745.2|	568499	+	G	0	5	6	2	3	-
gi|29165615|ref|NC_002745.2|	568500	+	A	0	3	5	8	6	-
gi|29165615|ref|NC_002745.2|	568501	+	A	1	2	1	0	0	-
gi|29165615|ref|NC_002745.2|	568502	+	A	0	0	2	0	1	-
gi|29165615|ref|NC_002745.2|	568503	+	U	0	0	1	0	0	-
gi|29165615|ref|NC_002745.2|	568504	+	U	1	1	3	0	0	-
gi|29165615|ref|NC_002745.2|	568505	+	A	0	1	0	0	2	-
gi|29165615|ref|NC_002745.2|	568506	+	U	0	3	1	0	0	-
gi|29165615|ref|NC_002745.2|	568507	+	U	1	3	2	1	3	-
gi|29165615|ref|NC_002745.2|	568508	+	U	0	0	3	0	1	-
gi|29165615|ref|NC_002745.2|	568509	+	U	0	2	0	1	1	-
gi|29165615|ref|NC_002745.2|	568510	+	A	0	0	1	0	1	-
gi|29165615|ref|NC_002745.2|	568511	+	A	0	0	1	0	0	-
gi|29165615|ref|NC_002745.2|	568515	+	C	0	0	0	1	2	-
gi|29165615|ref|NC_002745.2|	568521	+	A	1	0	0	0	0	-
gi|29165615|ref|NC_002745.2|	568522	+	A	0	1	0	0	0	-
gi|29165615|ref|NC_002745.2|	568523	+	A	0	0	1	0	0	-
gi|29165615|ref|NC_002745.2|	568524	+	A	0	0	0	0	1	-
gi|29165615|ref|NC_002745.2|	568527	+	C	0	2	0	1	0	-
gi|29165615|ref|NC_002745.2|	568529	+	U	0	0	0	1	0	-
gi|29165615|ref|NC_002745.2|	568532	+	U	0	2	0	0	0	-
gi|29165615|ref|NC_002745.2|	568534	+	U	0	0	2	0	0	-
gi|29165615|ref|NC_002745.2|	568540	+	U	0	1	0	0	0	-
gi|29165615|ref|NC_002745.2|	568541	+	A	0	3	0	1	3	-
gi|29165615|ref|NC_002745.2|	568542	+	A	0	2	2	0	3	-
gi|29165615|ref|NC_002745.2|	568543	+	C	0	0	1	1	0	-
gi|29165615|ref|NC_002745.2|	568544	+	A	0	1	0	0	0	-
gi|29165615|ref|NC_002745.2|	568545	+	A	1	0	0	0	0	-
gi|29165615|ref|NC_002745.2|	568550	+	A	0	0	1	0	0	-
gi|29165615|ref|NC_002745.2|	568553	+	A	0	0	0	0	1	-
gi|29165615|ref|NC_002745.2|	568555	+	U	0	0	0	1	0	-
gi|29165615|ref|NC_002745.2|	568570	+	G	0	1	0	0	0	-
gi|29165615|ref|NC_002745.2|	568571	+	A	0	0	0	0	1	-
gi|29165615|ref|NC_002745.2|	568585	+	C	0	0	1	0	0	-
gi|29165615|ref|NC_002745.2|	568586	+	A	0	1	0	0	0	-
gi|29165615|ref|NC_002745.2|	568592	+	A	0	0	0	0	1	-
gi|29165615|ref|NC_002745.2|	568598	+	U	0	3	2	0	1	-
gi|29165615|ref|NC_002745.2|	568599	+	A	0	4	1	2	1	-
gi|29165615|ref|NC_002745.2|	568600	+	U	0	0	2	1	0	-
gi|29165615|ref|NC_002745.2|	568601	+	A	0	0	1	1	4	-
gi|29165615|ref|NC_002745.2|	568602	+	U	0	0	0	1	0	-
gi|29165615|ref|NC_002745.2|	568604	+	A	0	0	1	2	1	-
gi|29165615|ref|NC_002745.2|	568605	+	A	0	0	1	0	0	-
gi|29165615|ref|NC_002745.2|	568608	+	A	0	1	3	2	2	-
gi|29165615|ref|NC_002745.2|	568609	+	A	0	2	3	2	3	-
gi|29165615|ref|NC_002745.2|	568610	+	C	0	1	3	2	3	-
gi|29165615|ref|NC_002745.2|	568611	+	U	0	0	0	0	1	-
gi|29165615|ref|NC_002745.2|	568612	+	U	0	0	0	1	0	-
gi|29165615|ref|NC_002745.2|	568613	+	G	0	1	0	0	0	-
gi|29165615|ref|NC_002745.2|	568614	+	A	0	5	3	2	4	-
gi|29165615|ref|NC_002745.2|	568615	+	A	0	3	2	3	2	-
gi|29165615|ref|NC_002745.2|	568616	+	A	0	0	2	1	0	-
gi|29165615|ref|NC_002745.2|	568617	+	A	0	3	1	1	2	-
gi|29165615|ref|NC_002745.2|	568621	+	G	0	1	1	0	0	-
gi|29165615|ref|NC_002745.2|	568622	+	A	1	38	23	10	26	-

gi|29165615|ref|NC_002745.2|	568623 +	U	0	5	1	0	0	-
gi|29165615|ref|NC_002745.2|	568624 +	A	0	0	1	0	0	-
gi|29165615|ref|NC_002745.2|	568625 +	U	0	0	0	1	1	-
gi|29165615|ref|NC_002745.2|	568627 +	U	0	1	0	0	2	-
gi|29165615|ref|NC_002745.2|	568628 +	A	0	3	2	2	4	-
gi|29165615|ref|NC_002745.2|	568631 +	U	0	0	2	0	2	-
gi|29165615|ref|NC_002745.2|	568632 +	U	1	0	0	0	0	-
gi|29165615|ref|NC_002745.2|	568633 +	A	2	0	0	0	0	-
gi|29165615|ref|NC_002745.2|	568634 +	G	0	2	1	1	2	-
gi|29165615|ref|NC_002745.2|	568635 +	G	1	1	3	4	5	-
gi|29165615|ref|NC_002745.2|	568636 +	A	1	4	1	1	6	-
gi|29165615|ref|NC_002745.2|	568637 +	G	0	0	1	0	0	-
gi|29165615|ref|NC_002745.2|	568638 +	U	0	0	0	1	1	-
gi|29165615|ref|NC_002745.2|	568639 +	G	0	0	1	1	7	-
gi|29165615|ref|NC_002745.2|	568640 +	A	0	6	6	5	24	-
gi|29165615|ref|NC_002745.2|	568641 +	A	0	3	2	0	1	-
gi|29165615|ref|NC_002745.2|	568642 +	A	0	2	0	1	2	-
gi|29165615|ref|NC_002745.2|	568643 +	A	0	4	2	0	5	-
gi|29165615|ref|NC_002745.2|	568644 +	A	0	0	1	0	0	-
gi|29165615|ref|NC_002745.2|	568645 +	A	0	0	0	0	2	-
gi|29165615|ref|NC_002745.2|	568646 +	A	0	1	1	0	3	SA0486
gi|29165615|ref|NC_002745.2|	568647 +	U	0	3	1	1	3	SA0486
gi|29165615|ref|NC_002745.2|	568648 +	G	0	1	2	1	1	SA0486
gi|29165615|ref|NC_002745.2|	568649 +	A	0	0	1	0	0	SA0486
gi|29165615|ref|NC_002745.2|	568650 +	G	0	0	0	1	0	SA0486
gi|29165615|ref|NC_002745.2|	568651 +	C	0	2	1	1	0	SA0486
gi|29165615|ref|NC_002745.2|	568652 +	G	0	1	0	1	1	SA0486
gi|29165615|ref|NC_002745.2|	568653 +	A	0	1	0	0	0	SA0486
gi|29165615|ref|NC_002745.2|	568655 +	C	0	0	0	0	2	SA0486
gi|29165615|ref|NC_002745.2|	568656 +	G	1	0	0	0	0	SA0486
gi|29165615|ref|NC_002745.2|	568657 +	U	0	1	1	0	0	SA0486
gi|29165615|ref|NC_002745.2|	568658 +	A	0	2	0	0	3	SA0486
gi|29165615|ref|NC_002745.2|	568659 +	U	0	1	0	0	0	SA0486
gi|29165615|ref|NC_002745.2|	568660 +	A	0	0	2	3	1	SA0486
gi|29165615|ref|NC_002745.2|	568661 +	A	0	0	0	2	3	SA0486
gi|29165615|ref|NC_002745.2|	568663 +	A	0	0	2	0	1	SA0486
gi|29165615|ref|NC_002745.2|	568665 +	U	0	1	2	0	1	SA0486
gi|29165615|ref|NC_002745.2|	568666 +	A	0	1	1	0	0	SA0486
gi|29165615|ref|NC_002745.2|	568668 +	G	0	0	1	0	0	SA0486
gi|29165615|ref|NC_002745.2|	568669 +	A	0	2	0	0	1	SA0486
gi|29165615|ref|NC_002745.2|	568670 +	U	0	0	1	0	0	SA0486
gi|29165615|ref|NC_002745.2|	568671 +	A	0	0	0	0	2	SA0486
gi|29165615|ref|NC_002745.2|	568676 +	C	0	0	1	0	0	SA0486
gi|29165615|ref|NC_002745.2|	568677 +	C	2	0	0	0	1	SA0486
gi|29165615|ref|NC_002745.2|	568681 +	U	0	0	0	0	1	SA0486
gi|29165615|ref|NC_002745.2|	568684 +	A	0	1	0	0	0	SA0486
gi|29165615|ref|NC_002745.2|	568690 +	U	1	0	0	0	0	SA0486
gi|29165615|ref|NC_002745.2|	568694 +	C	1	0	0	0	0	SA0486
gi|29165615|ref|NC_002745.2|	568707 +	A	0	0	0	0	1	SA0486
gi|29165615|ref|NC_002745.2|	568722 +	U	0	0	0	1	0	SA0486
gi|29165615|ref|NC_002745.2|	568726 +	C	0	0	0	1	1	SA0486
gi|29165615|ref|NC_002745.2|	568731 +	A	0	0	0	0	1	SA0486
gi|29165615|ref|NC_002745.2|	568740 +	C	1	0	0	0	0	SA0486
gi|29165615|ref|NC_002745.2|	568750 +	C	0	0	0	0	2	SA0486
gi|29165615|ref|NC_002745.2|	568756 +	A	0	0	1	0	1	SA0486
gi|29165615|ref|NC_002745.2|	568758 +	A	0	0	1	0	0	SA0486
gi|29165615|ref|NC_002745.2|	568764 +	U	2	0	0	0	0	SA0486
gi|29165615|ref|NC_002745.2|	568769 +	C	0	0	1	0	0	SA0486
gi|29165615|ref|NC_002745.2|	568771 +	A	0	3	1	0	1	SA0486
gi|29165615|ref|NC_002745.2|	568775 +	G	1	1	0	0	0	SA0486
gi|29165615|ref|NC_002745.2|	568776 +	A	0	0	1	1	0	SA0486
gi|29165615|ref|NC_002745.2|	568781 +	A	0	1	0	0	0	SA0486
gi|29165615|ref|NC_002745.2|	568782 +	C	0	0	0	1	0	SA0486
gi|29165615|ref|NC_002745.2|	568783 +	U	0	0	1	0	0	SA0486
gi|29165615|ref|NC_002745.2|	568787 +	A	0	0	0	0	1	SA0486
gi|29165615|ref|NC_002745.2|	568789 +	A	0	0	0	0	1	SA0486
gi|29165615|ref|NC_002745.2|	568795 +	U	0	4	1	1	2	SA0486
gi|29165615|ref|NC_002745.2|	568796 +	A	0	0	0	0	5	SA0486
gi|29165615|ref|NC_002745.2|	568797 +	A	0	0	0	0	1	SA0486
gi|29165615|ref|NC_002745.2|	568798 +	U	0	0	1	0	0	SA0486
gi|29165615|ref|NC_002745.2|	568799 +	U	0	0	1	0	0	SA0486
gi|29165615|ref|NC_002745.2|	568803 +	A	0	0	1	0	0	SA0486
gi|29165615|ref|NC_002745.2|	568806 +	A	0	0	0	0	1	SA0486
gi|29165615|ref|NC_002745.2|	568809 +	G	0	0	0	0	1	SA0486
gi|29165615|ref|NC_002745.2|	568816 +	A	1	0	0	0	0	SA0486
gi|29165615|ref|NC_002745.2|	568820 +	C	0	0	1	0	0	SA0486
gi|29165615|ref|NC_002745.2|	568857 +	A	3	0	0	0	0	SA0486
gi|29165615|ref|NC_002745.2|	568864 +	U	1	0	0	0	0	SA0486
gi|29165615|ref|NC_002745.2|	568866 +	U	1	0	0	0	0	SA0486
gi|29165615|ref|NC_002745.2|	568871 +	A	0	0	1	0	0	SA0486
gi|29165615|ref|NC_002745.2|	568876 +	C	0	0	1	1	0	SA0486

gi|29165615|ref|NC_002745.2|	568880 +	G	0	1	0	0	0	SA0486
gi|29165615|ref|NC_002745.2|	568882 +	C	1	0	0	0	0	SA0486
gi|29165615|ref|NC_002745.2|	568883 +	U	1	0	0	0	0	SA0486
gi|29165615|ref|NC_002745.2|	568891 +	A	0	0	1	0	0	SA0486
gi|29165615|ref|NC_002745.2|	568894 +	U	0	0	2	0	0	SA0486
gi|29165615|ref|NC_002745.2|	568899 +	A	1	0	0	0	0	SA0486
gi|29165615|ref|NC_002745.2|	568912 +	A	0	0	0	0	1	SA0486
gi|29165615|ref|NC_002745.2|	568915 +	U	0	0	0	0	1	SA0486
gi|29165615|ref|NC_002745.2|	568930 +	A	0	0	0	1	0	SA0486
gi|29165615|ref|NC_002745.2|	568935 +	A	0	0	1	0	0	SA0486
gi|29165615|ref|NC_002745.2|	568942 +	A	0	1	0	0	0	SA0486
gi|29165615|ref|NC_002745.2|	568943 +	C	0	2	0	0	1	SA0486
gi|29165615|ref|NC_002745.2|	568945 +	A	0	0	0	0	1	SA0486
gi|29165615|ref|NC_002745.2|	568947 +	C	0	0	1	0	0	SA0486
gi|29165615|ref|NC_002745.2|	568949 +	G	0	1	1	2	3	SA0486
gi|29165615|ref|NC_002745.2|	568950 +	A	0	2	0	1	3	SA0486
gi|29165615|ref|NC_002745.2|	568952 +	G	0	0	0	0	1	SA0486
gi|29165615|ref|NC_002745.2|	568959 +	C	0	1	1	0	1	SA0486
gi|29165615|ref|NC_002745.2|	568976 +	A	0	4	1	0	2	SA0486
gi|29165615|ref|NC_002745.2|	568978 +	A	0	0	1	0	0	SA0486
gi|29165615|ref|NC_002745.2|	568979 +	G	0	1	0	0	0	SA0486
gi|29165615|ref|NC_002745.2|	568981 +	A	0	1	0	0	0	SA0486
gi|29165615|ref|NC_002745.2|	568982 +	G	0	1	2	0	1	SA0486
gi|29165615|ref|NC_002745.2|	568983 +	A	0	1	3	6	6	SA0486
gi|29165615|ref|NC_002745.2|	568984 +	A	0	4	3	1	4	SA0486
gi|29165615|ref|NC_002745.2|	568985 +	G	0	1	1	0	1	SA0486
gi|29165615|ref|NC_002745.2|	568986 +	A	2	9	3	7	7	SA0486
gi|29165615|ref|NC_002745.2|	568987 +	A	0	1	0	0	1	SA0486
gi|29165615|ref|NC_002745.2|	568988 +	U	0	1	0	0	2	SA0486
gi|29165615|ref|NC_002745.2|	568989 +	U	0	0	1	0	0	SA0486
gi|29165615|ref|NC_002745.2|	568990 +	A	0	0	1	1	0	SA0486
gi|29165615|ref|NC_002745.2|	568991 +	G	0	2	1	2	0	SA0486
gi|29165615|ref|NC_002745.2|	568992 +	A	0	8	2	1	4	SA0486
gi|29165615|ref|NC_002745.2|	568997 +	G	0	2	0	0	2	SA0486
gi|29165615|ref|NC_002745.2|	568998 +	A	0	3	4	2	3	SA0486
gi|29165615|ref|NC_002745.2|	569004 +	A	0	1	0	0	0	SA0486
gi|29165615|ref|NC_002745.2|	569016 +	C	1	0	0	0	0	SA0486
gi|29165615|ref|NC_002745.2|	569029 +	G	1	0	0	0	0	SA0486
gi|29165615|ref|NC_002745.2|	569081 +	U	0	1	0	0	0	SA0486
gi|29165615|ref|NC_002745.2|	569127 +	A	0	0	0	0	1	SA0486
gi|29165615|ref|NC_002745.2|	569166 +	A	0	1	0	0	0	SA0486
gi|29165615|ref|NC_002745.2|	569236 +	U	2	0	0	0	0	SA0486
gi|29165615|ref|NC_002745.2|	569238 +	U	2	0	0	0	0	SA0486
gi|29165615|ref|NC_002745.2|	569244 +	U	0	0	0	1	0	SA0486
gi|29165615|ref|NC_002745.2|	569246 +	G	0	0	0	1	0	SA0486
gi|29165615|ref|NC_002745.2|	569264 +	A	0	0	0	0	1	SA0486
gi|29165615|ref|NC_002745.2|	569268 +	A	1	0	0	0	0	SA0486
gi|29165615|ref|NC_002745.2|	569276 +	G	0	0	1	0	0	SA0486
gi|29165615|ref|NC_002745.2|	569283 +	U	1	0	0	0	0	SA0486
gi|29165615|ref|NC_002745.2|	569287 +	U	0	0	1	0	0	SA0486
gi|29165615|ref|NC_002745.2|	569288 +	G	0	0	1	0	0	SA0486
gi|29165615|ref|NC_002745.2|	569312 +	C	1	0	1	0	1	SA0486
gi|29165615|ref|NC_002745.2|	569314 +	U	0	0	1	0	0	SA0486
gi|29165615|ref|NC_002745.2|	569317 +	A	0	1	0	0	0	SA0486
gi|29165615|ref|NC_002745.2|	569324 +	A	0	1	0	0	0	SA0486
gi|29165615|ref|NC_002745.2|	569326 +	G	0	0	1	0	0	SA0486
gi|29165615|ref|NC_002745.2|	569331 +	A	0	1	0	0	0	SA0486
gi|29165615|ref|NC_002745.2|	569334 +	A	0	0	1	0	0	SA0486
gi|29165615|ref|NC_002745.2|	569337 +	C	0	0	1	0	0	SA0486
gi|29165615|ref|NC_002745.2|	569340 +	U	2	0	0	0	0	SA0486
gi|29165615|ref|NC_002745.2|	569354 +	C	0	0	0	0	1	SA0486
gi|29165615|ref|NC_002745.2|	569366 +	C	0	0	1	0	0	SA0486
gi|29165615|ref|NC_002745.2|	569403 +	U	0	0	1	0	0	SA0486
gi|29165615|ref|NC_002745.2|	569423 +	A	0	0	0	0	2	SA0486
gi|29165615|ref|NC_002745.2|	569491 +	G	0	0	0	0	1	SA0486
gi|29165615|ref|NC_002745.2|	569554 +	C	0	0	0	0	1	SA0486
gi|29165615|ref|NC_002745.2|	569602 +	A	0	0	0	1	0	SA0486
gi|29165615|ref|NC_002745.2|	569603 +	A	0	1	0	0	0	SA0486
gi|29165615|ref|NC_002745.2|	569634 +	A	0	0	0	0	1	SA0486
gi|29165615|ref|NC_002745.2|	569637 +	A	0	1	0	0	0	SA0486
gi|29165615|ref|NC_002745.2|	569667 +	C	0	0	0	1	0	SA0486
gi|29165615|ref|NC_002745.2|	569688 +	C	0	0	1	0	0	SA0486
gi|29165615|ref|NC_002745.2|	569730 +	G	0	0	0	0	1	SA0486
gi|29165615|ref|NC_002745.2|	569754 +	A	1	0	0	0	0	SA0486
gi|29165615|ref|NC_002745.2|	569764 +	A	1	0	0	0	0	SA0486
gi|29165615|ref|NC_002745.2|	569765 +	A	0	0	0	0	1	SA0486
gi|29165615|ref|NC_002745.2|	569787 +	U	0	0	1	0	0	SA0486
gi|29165615|ref|NC_002745.2|	569793 +	U	0	0	0	1	0	SA0486
gi|29165615|ref|NC_002745.2|	569797 +	A	0	0	1	0	0	SA0486
gi|29165615|ref|NC_002745.2|	569815 +	A	0	1	0	0	0	SA0486
gi|29165615|ref|NC_002745.2|	569825 +	C	1	0	0	0	0	SA0486

gi|29165615|ref|NC_002745.2|	569836	+	A	0	0	0	0	2	SA0486
gi|29165615|ref|NC_002745.2|	569842	+	A	0	1	0	0	0	SA0486
gi|29165615|ref|NC_002745.2|	569861	+	C	0	0	0	0	1	SA0486
gi|29165615|ref|NC_002745.2|	569878	+	G	0	0	0	0	2	SA0486
gi|29165615|ref|NC_002745.2|	569879	+	A	1	0	0	0	0	SA0486
gi|29165615|ref|NC_002745.2|	569916	+	U	1	0	0	0	0	SA0486
gi|29165615|ref|NC_002745.2|	569988	+	U	2	0	0	0	0	SA0486
gi|29165615|ref|NC_002745.2|	569989	+	U	0	0	0	1	0	SA0486
gi|29165615|ref|NC_002745.2|	570089	+	C	0	1	0	0	0	SA0486
gi|29165615|ref|NC_002745.2|	570105	+	A	0	0	0	1	0	-
gi|29165615|ref|NC_002745.2|	570116	+	C	1	0	0	0	0	-
gi|29165615|ref|NC_002745.2|	570142	+	A	0	0	1	0	0	-
gi|29165615|ref|NC_002745.2|	570152	+	C	0	0	1	0	0	-
gi|29165615|ref|NC_002745.2|	570166	+	G	1	0	0	0	0	-
gi|29165615|ref|NC_002745.2|	570178	+	U	0	0	0	1	0	-
gi|29165615|ref|NC_002745.2|	570182	+	A	0	0	0	0	1	-
gi|29165615|ref|NC_002745.2|	570185	+	A	0	1	0	0	0	-
gi|29165615|ref|NC_002745.2|	570188	+	U	0	1	0	0	1	-
gi|29165615|ref|NC_002745.2|	570189	+	G	0	1	0	0	0	-
gi|29165615|ref|NC_002745.2|	570190	+	A	0	4	0	2	1	-
gi|29165615|ref|NC_002745.2|	570191	+	A	0	0	0	0	1	-
gi|29165615|ref|NC_002745.2|	570192	+	U	0	0	0	0	1	-
gi|29165615|ref|NC_002745.2|	570193	+	A	0	2	0	5	2	-
gi|29165615|ref|NC_002745.2|	570195	+	A	0	0	0	2	0	-
gi|29165615|ref|NC_002745.2|	570196	+	G	0	0	0	0	1	-
gi|29165615|ref|NC_002745.2|	570197	+	U	0	5	3	8	4	-
gi|29165615|ref|NC_002745.2|	570198	+	A	0	3	1	0	0	-
gi|29165615|ref|NC_002745.2|	570199	+	U	0	0	0	0	1	-
gi|29165615|ref|NC_002745.2|	570202	+	U	0	1	0	1	0	-
gi|29165615|ref|NC_002745.2|	570203	+	A	0	0	1	0	0	-
gi|29165615|ref|NC_002745.2|	570208	+	A	0	0	1	0	0	-
gi|29165615|ref|NC_002745.2|	570209	+	A	0	1	0	0	0	-
gi|29165615|ref|NC_002745.2|	570215	+	G	0	0	0	2	0	-
gi|29165615|ref|NC_002745.2|	570217	+	A	0	0	0	0	1	-
gi|29165615|ref|NC_002745.2|	570222	+	U	0	0	0	1	1	-
gi|29165615|ref|NC_002745.2|	570223	+	A	0	1	0	0	0	-
gi|29165615|ref|NC_002745.2|	570235	+	A	0	0	1	0	1	-
gi|29165615|ref|NC_002745.2|	570236	+	C	4	0	0	0	0	-
gi|29165615|ref|NC_002745.2|	570237	+	G	0	0	2	1	0	-
gi|29165615|ref|NC_002745.2|	570242	+	A	1	0	0	0	1	-
gi|29165615|ref|NC_002745.2|	570281	+	A	0	1	0	0	0	-
gi|29165615|ref|NC_002745.2|	570288	+	C	0	0	0	0	1	-
gi|29165615|ref|NC_002745.2|	570303	+	A	0	0	0	0	2	-
gi|29165615|ref|NC_002745.2|	570304	+	A	0	1	0	0	0	-
gi|29165615|ref|NC_002745.2|	570307	+	A	0	2	1	0	1	-
gi|29165615|ref|NC_002745.2|	570308	+	A	0	3	0	1	6	-
gi|29165615|ref|NC_002745.2|	570309	+	A	0	7	2	2	8	-
gi|29165615|ref|NC_002745.2|	570310	+	U	0	2	1	0	15	-
gi|29165615|ref|NC_002745.2|	570311	+	A	1	9	4	2	14	-
gi|29165615|ref|NC_002745.2|	570312	+	U	0	1	0	1	1	-
gi|29165615|ref|NC_002745.2|	570313	+	A	0	25	0	2	25	-
gi|29165615|ref|NC_002745.2|	570314	+	U	0	2	1	0	2	-
gi|29165615|ref|NC_002745.2|	570315	+	A	0	6	0	1	7	-
gi|29165615|ref|NC_002745.2|	570316	+	A	0	3	0	0	3	-
gi|29165615|ref|NC_002745.2|	570318	+	G	0	1	0	1	1	-
gi|29165615|ref|NC_002745.2|	570319	+	A	3	6	1	3	4	-
gi|29165615|ref|NC_002745.2|	570320	+	G	0	1	0	1	0	-
gi|29165615|ref|NC_002745.2|	570322	+	G	0	0	1	2	3	-
gi|29165615|ref|NC_002745.2|	570323	+	U	0	0	0	1	0	-
gi|29165615|ref|NC_002745.2|	570324	+	G	0	4	2	2	3	-
gi|29165615|ref|NC_002745.2|	570325	+	A	0	6	6	3	5	-
gi|29165615|ref|NC_002745.2|	570326	+	U	0	0	0	1	0	-
gi|29165615|ref|NC_002745.2|	570328	+	A	1	4	2	2	4	-
gi|29165615|ref|NC_002745.2|	570330	+	C	0	2	0	4	1	-
gi|29165615|ref|NC_002745.2|	570331	+	A	0	0	0	1	2	-
gi|29165615|ref|NC_002745.2|	570333	+	U	0	0	0	1	0	-
gi|29165615|ref|NC_002745.2|	570334	+	A	0	15	4	38	23	-
gi|29165615|ref|NC_002745.2|	570335	+	A	1	5	2	4	2	-
gi|29165615|ref|NC_002745.2|	570336	+	G	0	2	2	1	1	-
gi|29165615|ref|NC_002745.2|	570337	+	U	0	1	0	1	0	-
gi|29165615|ref|NC_002745.2|	570339	+	G	0	0	1	2	0	-
gi|29165615|ref|NC_002745.2|	570341	+	C	0	1	0	0	0	-
gi|29165615|ref|NC_002745.2|	570343	+	U	0	0	0	1	0	-
gi|29165615|ref|NC_002745.2|	570354	+	A	0	0	0	1	1	-
gi|29165615|ref|NC_002745.2|	570371	+	G	0	0	0	0	1	-
gi|29165615|ref|NC_002745.2|	570372	+	C	0	0	0	0	1	-
gi|29165615|ref|NC_002745.2|	570373	+	A	0	3	0	2	1	-
gi|29165615|ref|NC_002745.2|	570381	+	U	0	0	0	1	0	-
gi|29165615|ref|NC_002745.2|	570386	+	U	0	0	0	0	1	-
gi|29165615|ref|NC_002745.2|	570393	+	C	0	1	0	0	0	-
gi|29165615|ref|NC_002745.2|	570402	+	G	0	0	0	0	1	-

gi|29165615|ref|NC_002745.2|	570410 +	C	1	0	0	0	0	-
gi|29165615|ref|NC_002745.2|	570424 +	C	0	0	1	0	0	-
gi|29165615|ref|NC_002745.2|	570427 +	U	0	0	1	0	0	-
gi|29165615|ref|NC_002745.2|	570435 +	C	0	0	1	0	0	-
gi|29165615|ref|NC_002745.2|	570436 +	A	0	0	0	0	1	-
gi|29165615|ref|NC_002745.2|	570442 +	U	0	0	0	0	2	-
gi|29165615|ref|NC_002745.2|	570443 +	A	0	0	0	0	1	-
gi|29165615|ref|NC_002745.2|	570445 +	G	0	0	1	0	0	-
gi|29165615|ref|NC_002745.2|	570479 +	G	0	1	0	0	0	-
gi|29165615|ref|NC_002745.2|	570493 +	U	0	1	0	0	0	-
gi|29165615|ref|NC_002745.2|	570497 +	U	0	1	0	0	0	-
gi|29165615|ref|NC_002745.2|	570501 +	U	0	1	0	1	1	-
gi|29165615|ref|NC_002745.2|	570505 +	U	0	0	0	0	1	-
gi|29165615|ref|NC_002745.2|	570510 +	C	0	1	0	0	0	-
gi|29165615|ref|NC_002745.2|	570514 +	G	0	0	0	0	2	-
gi|29165615|ref|NC_002745.2|	570515 +	A	0	3	0	0	0	-
gi|29165615|ref|NC_002745.2|	570517 +	G	0	4	0	0	2	-
gi|29165615|ref|NC_002745.2|	570520 +	G	0	0	1	0	4	-
gi|29165615|ref|NC_002745.2|	570521 +	A	0	1	2	0	1	-
gi|29165615|ref|NC_002745.2|	570525 +	G	0	0	0	0	1	-
gi|29165615|ref|NC_002745.2|	570534 +	A	0	2	0	0	0	SA0487
gi|29165615|ref|NC_002745.2|	570535 +	A	0	0	1	0	1	SA0487
gi|29165615|ref|NC_002745.2|	570536 +	A	0	1	0	0	0	SA0487
gi|29165615|ref|NC_002745.2|	570538 +	A	0	0	0	2	0	SA0487
gi|29165615|ref|NC_002745.2|	570540 +	A	0	1	2	1	3	SA0487
gi|29165615|ref|NC_002745.2|	570546 +	A	0	1	0	0	0	SA0487
gi|29165615|ref|NC_002745.2|	570547 +	G	0	1	0	0	0	SA0487
gi|29165615|ref|NC_002745.2|	570551 +	A	0	0	0	0	1	SA0487
gi|29165615|ref|NC_002745.2|	570558 +	A	0	2	0	0	0	SA0487
gi|29165615|ref|NC_002745.2|	570562 +	G	0	0	1	0	1	SA0487
gi|29165615|ref|NC_002745.2|	570563 +	U	0	1	1	0	0	SA0487
gi|29165615|ref|NC_002745.2|	570566 +	U	1	0	0	0	0	SA0487
gi|29165615|ref|NC_002745.2|	570568 +	G	0	1	0	0	0	SA0487
gi|29165615|ref|NC_002745.2|	570578 +	C	0	0	0	1	0	SA0487
gi|29165615|ref|NC_002745.2|	570581 +	C	0	2	0	0	0	SA0487
gi|29165615|ref|NC_002745.2|	570582 +	G	1	0	0	0	0	SA0487
gi|29165615|ref|NC_002745.2|	570591 +	A	0	0	0	0	1	SA0487
gi|29165615|ref|NC_002745.2|	570593 +	C	0	1	0	0	0	SA0487
gi|29165615|ref|NC_002745.2|	570596 +	U	1	0	0	0	0	SA0487
gi|29165615|ref|NC_002745.2|	570599 +	A	0	0	1	0	1	SA0487
gi|29165615|ref|NC_002745.2|	570602 +	U	0	1	1	0	0	SA0487
gi|29165615|ref|NC_002745.2|	570603 +	C	0	1	0	0	0	SA0487
gi|29165615|ref|NC_002745.2|	570607 +	A	1	0	0	0	0	SA0487
gi|29165615|ref|NC_002745.2|	570612 +	G	0	0	1	0	0	SA0487
gi|29165615|ref|NC_002745.2|	570613 +	U	0	1	0	0	0	SA0487
gi|29165615|ref|NC_002745.2|	570617 +	C	0	1	0	0	0	SA0487
gi|29165615|ref|NC_002745.2|	570620 +	G	0	0	2	0	0	SA0487
gi|29165615|ref|NC_002745.2|	570626 +	A	1	0	0	0	0	SA0487
gi|29165615|ref|NC_002745.2|	570631 +	G	0	0	1	0	0	SA0487
gi|29165615|ref|NC_002745.2|	570632 +	U	0	0	1	0	0	SA0487
gi|29165615|ref|NC_002745.2|	570658 +	U	0	1	0	0	0	SA0487
gi|29165615|ref|NC_002745.2|	570664 +	A	0	1	0	0	0	SA0487
gi|29165615|ref|NC_002745.2|	570671 +	A	0	0	0	0	1	SA0487
gi|29165615|ref|NC_002745.2|	570675 +	A	0	1	0	0	0	SA0487
gi|29165615|ref|NC_002745.2|	570703 +	A	0	0	1	0	0	SA0487
gi|29165615|ref|NC_002745.2|	570708 +	A	0	0	1	0	0	SA0487
gi|29165615|ref|NC_002745.2|	570709 +	A	0	1	0	0	0	SA0487
gi|29165615|ref|NC_002745.2|	570712 +	U	1	0	0	0	0	SA0487
gi|29165615|ref|NC_002745.2|	570719 +	C	2	0	0	0	0	SA0487
gi|29165615|ref|NC_002745.2|	570722 +	G	0	0	1	0	0	SA0487
gi|29165615|ref|NC_002745.2|	570723 +	U	0	1	1	0	0	SA0487
gi|29165615|ref|NC_002745.2|	570724 +	A	0	0	2	0	0	SA0487
gi|29165615|ref|NC_002745.2|	570727 +	G	0	0	1	0	0	SA0487
gi|29165615|ref|NC_002745.2|	570728 +	A	0	0	0	0	1	SA0487
gi|29165615|ref|NC_002745.2|	570729 +	A	0	0	0	1	0	SA0487
gi|29165615|ref|NC_002745.2|	570732 +	C	0	0	0	2	0	SA0487
gi|29165615|ref|NC_002745.2|	570738 +	A	0	0	1	0	0	SA0487
gi|29165615|ref|NC_002745.2|	570744 +	U	0	1	0	0	0	SA0487
gi|29165615|ref|NC_002745.2|	570745 +	A	0	0	0	0	1	SA0487
gi|29165615|ref|NC_002745.2|	570746 +	A	1	2	3	0	0	SA0487
gi|29165615|ref|NC_002745.2|	570747 +	A	0	3	5	1	6	SA0487
gi|29165615|ref|NC_002745.2|	570749 +	U	0	0	0	1	1	SA0487
gi|29165615|ref|NC_002745.2|	570752 +	G	0	0	1	0	0	SA0487
gi|29165615|ref|NC_002745.2|	570755 +	A	0	1	2	0	0	SA0487
gi|29165615|ref|NC_002745.2|	570756 +	G	5	3	7	1	5	SA0487
gi|29165615|ref|NC_002745.2|	570758 +	G	0	2	0	0	0	SA0487
gi|29165615|ref|NC_002745.2|	570764 +	U	0	2	0	0	0	SA0487
gi|29165615|ref|NC_002745.2|	570766 +	A	0	0	0	0	1	SA0487
gi|29165615|ref|NC_002745.2|	570767 +	U	0	0	0	0	1	SA0487
gi|29165615|ref|NC_002745.2|	570770 +	A	0	0	0	1	1	SA0487
gi|29165615|ref|NC_002745.2|	570771 +	U	0	0	1	0	1	SA0487

gi|29165615|ref|NC_002745.2|	570772 +	C	0	0	1	0	0	SA0487
gi|29165615|ref|NC_002745.2|	570775 +	G	0	1	0	0	0	SA0487
gi|29165615|ref|NC_002745.2|	570776 +	G	0	1	1	1	2	SA0487
gi|29165615|ref|NC_002745.2|	570777 +	U	0	1	1	0	0	SA0487
gi|29165615|ref|NC_002745.2|	570783 +	C	0	0	0	0	1	SA0487
gi|29165615|ref|NC_002745.2|	570784 +	G	0	1	0	0	1	SA0487
gi|29165615|ref|NC_002745.2|	570788 +	U	0	0	1	0	0	SA0487
gi|29165615|ref|NC_002745.2|	570789 +	A	0	0	0	0	1	SA0487
gi|29165615|ref|NC_002745.2|	570790 +	A	0	2	0	0	0	SA0487
gi|29165615|ref|NC_002745.2|	570791 +	U	0	2	0	0	1	SA0487
gi|29165615|ref|NC_002745.2|	570794 +	G	0	0	1	0	1	SA0487
gi|29165615|ref|NC_002745.2|	570797 +	A	0	1	1	0	1	SA0487
gi|29165615|ref|NC_002745.2|	570799 +	A	0	1	1	0	2	SA0487
gi|29165615|ref|NC_002745.2|	570800 +	C	0	4	3	0	8	SA0487
gi|29165615|ref|NC_002745.2|	570801 +	A	0	0	1	0	0	SA0487
gi|29165615|ref|NC_002745.2|	570804 +	U	1	0	0	0	0	SA0487
gi|29165615|ref|NC_002745.2|	570806 +	C	0	1	1	0	1	SA0487
gi|29165615|ref|NC_002745.2|	570807 +	A	0	0	1	0	0	SA0487
gi|29165615|ref|NC_002745.2|	570808 +	A	1	0	0	0	1	SA0487
gi|29165615|ref|NC_002745.2|	570812 +	G	0	0	1	0	1	SA0487
gi|29165615|ref|NC_002745.2|	570814 +	G	0	2	0	0	0	SA0487
gi|29165615|ref|NC_002745.2|	570815 +	A	0	1	1	0	0	SA0487
gi|29165615|ref|NC_002745.2|	570817 +	A	0	2	0	0	2	SA0487
gi|29165615|ref|NC_002745.2|	570818 +	A	0	0	0	1	0	SA0487
gi|29165615|ref|NC_002745.2|	570821 +	U	0	0	0	0	1	SA0487
gi|29165615|ref|NC_002745.2|	570824 +	C	0	0	1	0	0	SA0487
gi|29165615|ref|NC_002745.2|	570829 +	U	0	0	1	1	0	SA0487
gi|29165615|ref|NC_002745.2|	570833 +	A	0	1	0	0	0	SA0487
gi|29165615|ref|NC_002745.2|	570843 +	A	0	0	1	0	0	SA0487
gi|29165615|ref|NC_002745.2|	570844 +	C	0	1	0	0	0	SA0487
gi|29165615|ref|NC_002745.2|	570852 +	G	0	0	1	0	0	SA0487
gi|29165615|ref|NC_002745.2|	570853 +	A	0	0	0	0	1	SA0487
gi|29165615|ref|NC_002745.2|	570854 +	C	0	1	0	0	0	SA0487
gi|29165615|ref|NC_002745.2|	570859 +	A	0	0	0	0	1	SA0487
gi|29165615|ref|NC_002745.2|	570862 +	G	0	0	0	0	1	SA0487
gi|29165615|ref|NC_002745.2|	570904 +	A	0	0	1	0	0	SA0487
gi|29165615|ref|NC_002745.2|	570937 +	A	0	1	0	0	1	SA0487
gi|29165615|ref|NC_002745.2|	570938 +	A	0	0	0	0	1	SA0487
gi|29165615|ref|NC_002745.2|	570941 +	U	0	1	0	0	0	SA0487
gi|29165615|ref|NC_002745.2|	570943 +	A	0	0	1	0	0	SA0487
gi|29165615|ref|NC_002745.2|	570944 +	A	0	0	0	0	1	SA0487
gi|29165615|ref|NC_002745.2|	570947 +	C	0	0	0	0	2	SA0487
gi|29165615|ref|NC_002745.2|	570949 +	A	0	0	0	2	0	SA0487
gi|29165615|ref|NC_002745.2|	570982 +	C	0	1	0	0	0	SA0487
gi|29165615|ref|NC_002745.2|	571041 +	U	1	0	0	0	0	SA0487
gi|29165615|ref|NC_002745.2|	571092 +	U	0	1	0	0	0	SA0487
gi|29165615|ref|NC_002745.2|	571098 +	A	0	0	0	0	1	SA0487
gi|29165615|ref|NC_002745.2|	571108 +	C	0	0	0	0	1	SA0487
gi|29165615|ref|NC_002745.2|	571129 +	A	0	0	0	0	1	SA0487
gi|29165615|ref|NC_002745.2|	571139 +	A	0	0	0	0	1	SA0487
gi|29165615|ref|NC_002745.2|	571170 +	A	1	0	0	0	0	SA0487_SA0488
gi|29165615|ref|NC_002745.2|	571212 +	C	0	1	0	0	0	SA0488
gi|29165615|ref|NC_002745.2|	571228 +	G	0	0	2	0	0	SA0488
gi|29165615|ref|NC_002745.2|	571233 +	U	0	0	0	0	1	SA0488
gi|29165615|ref|NC_002745.2|	571276 +	A	0	0	0	0	1	SA0488
gi|29165615|ref|NC_002745.2|	571336 +	A	0	1	0	0	0	SA0488
gi|29165615|ref|NC_002745.2|	571369 +	U	0	0	1	0	0	SA0488
gi|29165615|ref|NC_002745.2|	571372 +	A	0	1	0	0	0	SA0488
gi|29165615|ref|NC_002745.2|	571392 +	A	0	0	1	0	0	SA0488
gi|29165615|ref|NC_002745.2|	571396 +	A	0	0	0	0	1	SA0488
gi|29165615|ref|NC_002745.2|	571401 +	A	0	1	0	0	0	SA0488
gi|29165615|ref|NC_002745.2|	571403 +	U	0	0	1	0	0	SA0488
gi|29165615|ref|NC_002745.2|	571408 +	U	1	0	0	0	0	SA0488
gi|29165615|ref|NC_002745.2|	571410 +	C	1	0	0	0	0	SA0488
gi|29165615|ref|NC_002745.2|	571434 +	C	1	0	0	0	0	SA0488
gi|29165615|ref|NC_002745.2|	571438 +	U	0	0	1	0	0	SA0488
gi|29165615|ref|NC_002745.2|	571439 +	U	1	0	0	0	0	SA0488
gi|29165615|ref|NC_002745.2|	571440 +	U	2	0	0	0	0	SA0488
gi|29165615|ref|NC_002745.2|	571485 +	A	0	1	1	0	2	SA0488
gi|29165615|ref|NC_002745.2|	571487 +	C	0	0	0	0	1	SA0488
gi|29165615|ref|NC_002745.2|	571504 +	U	0	1	0	0	0	SA0488
gi|29165615|ref|NC_002745.2|	571505 +	A	0	0	0	0	1	SA0488
gi|29165615|ref|NC_002745.2|	571529 +	A	0	0	0	0	1	SA0488
gi|29165615|ref|NC_002745.2|	571539 +	U	0	1	0	0	0	SA0488
gi|29165615|ref|NC_002745.2|	571567 +	U	0	0	1	0	0	SA0488
gi|29165615|ref|NC_002745.2|	571594 +	A	0	1	0	0	0	SA0488
gi|29165615|ref|NC_002745.2|	571599 +	A	0	0	1	0	0	SA0488
gi|29165615|ref|NC_002745.2|	571619 +	C	0	0	1	0	0	SA0488
gi|29165615|ref|NC_002745.2|	571620 +	A	1	0	0	0	0	SA0488
gi|29165615|ref|NC_002745.2|	571677 +	A	1	0	0	0	0	SA0488
gi|29165615|ref|NC_002745.2|	571689 +	A	1	0	0	0	0	SA0488

gi|29165615|ref|NC_002745.2|	571706 +	A	0	0	0	0	1	SA0488
gi|29165615|ref|NC_002745.2|	571952 +	A	0	0	0	1	0	SA0488
gi|29165615|ref|NC_002745.2|	571983 +	A	0	0	0	0	1	SA0488
gi|29165615|ref|NC_002745.2|	572115 +	G	1	0	0	0	0	SA0488
gi|29165615|ref|NC_002745.2|	572165 +	C	1	0	0	0	0	SA0488
gi|29165615|ref|NC_002745.2|	572218 +	U	0	0	0	1	0	SA0488
gi|29165615|ref|NC_002745.2|	572241 +	C	0	0	1	0	0	SA0488
gi|29165615|ref|NC_002745.2|	572247 +	C	0	1	0	0	0	SA0488
gi|29165615|ref|NC_002745.2|	572253 +	C	1	0	0	0	0	SA0488
gi|29165615|ref|NC_002745.2|	572254 +	A	1	0	0	0	0	SA0488
gi|29165615|ref|NC_002745.2|	572339 +	G	0	1	0	0	0	SA0488
gi|29165615|ref|NC_002745.2|	572348 +	A	0	0	1	0	0	SA0488
gi|29165615|ref|NC_002745.2|	572351 +	U	0	0	0	0	1	SA0488
gi|29165615|ref|NC_002745.2|	572357 +	G	0	1	0	0	0	SA0488
gi|29165615|ref|NC_002745.2|	572358 +	A	3	0	0	0	0	SA0488
gi|29165615|ref|NC_002745.2|	572361 +	U	1	0	0	0	0	SA0488
gi|29165615|ref|NC_002745.2|	572364 +	U	0	1	1	0	0	SA0488
gi|29165615|ref|NC_002745.2|	572371 +	A	0	0	0	1	0	SA0488
gi|29165615|ref|NC_002745.2|	572380 +	A	0	4	1	0	0	SA0488
gi|29165615|ref|NC_002745.2|	572388 +	A	0	0	1	0	0	SA0488
gi|29165615|ref|NC_002745.2|	572391 +	C	0	1	0	0	0	SA0488
gi|29165615|ref|NC_002745.2|	572394 +	A	0	0	1	0	0	SA0488
gi|29165615|ref|NC_002745.2|	572419 +	A	0	0	1	0	0	SA0488
gi|29165615|ref|NC_002745.2|	572422 +	A	0	1	0	0	0	SA0488
gi|29165615|ref|NC_002745.2|	572424 +	U	0	0	0	0	1	SA0488
gi|29165615|ref|NC_002745.2|	572432 +	G	0	0	2	0	0	SA0488
gi|29165615|ref|NC_002745.2|	572463 +	U	0	0	1	0	0	SA0488
gi|29165615|ref|NC_002745.2|	572472 +	C	0	0	0	0	1	SA0488
gi|29165615|ref|NC_002745.2|	572554 +	A	0	0	1	0	0	SA0488_SA0489
gi|29165615|ref|NC_002745.2|	572572 +	U	0	2	0	0	0	SA0489
gi|29165615|ref|NC_002745.2|	572583 +	A	0	0	0	0	1	SA0489
gi|29165615|ref|NC_002745.2|	572599 +	C	0	0	0	0	1	SA0489
gi|29165615|ref|NC_002745.2|	572693 +	A	0	0	1	0	0	SA0489
gi|29165615|ref|NC_002745.2|	572697 +	U	0	0	1	0	0	SA0489
gi|29165615|ref|NC_002745.2|	572712 +	A	0	0	0	1	0	SA0489
gi|29165615|ref|NC_002745.2|	572741 +	G	1	0	1	0	0	SA0489
gi|29165615|ref|NC_002745.2|	572744 +	G	1	0	0	0	0	SA0489
gi|29165615|ref|NC_002745.2|	572763 +	G	0	0	0	1	0	SA0489
gi|29165615|ref|NC_002745.2|	572769 +	A	0	0	0	0	1	SA0489
gi|29165615|ref|NC_002745.2|	572772 +	G	0	0	1	0	0	SA0489
gi|29165615|ref|NC_002745.2|	572773 +	A	0	0	0	0	1	SA0489
gi|29165615|ref|NC_002745.2|	572782 +	A	0	0	0	0	1	SA0489
gi|29165615|ref|NC_002745.2|	572795 +	C	0	1	0	0	0	SA0489
gi|29165615|ref|NC_002745.2|	572799 +	A	0	0	1	0	0	SA0489
gi|29165615|ref|NC_002745.2|	572822 +	C	0	0	1	0	0	SA0489
gi|29165615|ref|NC_002745.2|	572872 +	G	0	0	0	1	0	SA0489
gi|29165615|ref|NC_002745.2|	572887 +	A	0	0	0	1	0	SA0489
gi|29165615|ref|NC_002745.2|	572903 +	A	0	1	1	0	1	SA0489
gi|29165615|ref|NC_002745.2|	572911 +	C	0	0	0	0	1	SA0489
gi|29165615|ref|NC_002745.2|	572914 +	U	0	0	1	0	0	SA0489
gi|29165615|ref|NC_002745.2|	572922 +	A	0	0	0	1	0	SA0489
gi|29165615|ref|NC_002745.2|	572925 +	A	0	7	5	1	2	SA0489
gi|29165615|ref|NC_002745.2|	572927 +	A	0	1	0	0	0	SA0489
gi|29165615|ref|NC_002745.2|	572928 +	A	0	0	1	0	0	SA0489
gi|29165615|ref|NC_002745.2|	572933 +	A	0	3	0	0	0	SA0489
gi|29165615|ref|NC_002745.2|	572934 +	A	2	2	0	0	1	SA0489
gi|29165615|ref|NC_002745.2|	572935 +	U	0	1	0	0	0	SA0489
gi|29165615|ref|NC_002745.2|	572939 +	A	0	0	0	1	0	SA0489
gi|29165615|ref|NC_002745.2|	572948 +	G	1	0	0	0	0	SA0489
gi|29165615|ref|NC_002745.2|	572951 +	G	0	1	0	0	0	SA0489
gi|29165615|ref|NC_002745.2|	572953 +	G	0	1	0	0	0	-
gi|29165615|ref|NC_002745.2|	572956 +	G	0	1	0	0	0	-
gi|29165615|ref|NC_002745.2|	572960 +	U	0	0	0	0	1	SA0490
gi|29165615|ref|NC_002745.2|	572962 +	G	0	0	0	1	0	SA0490
gi|29165615|ref|NC_002745.2|	572963 +	A	0	0	0	0	1	SA0490
gi|29165615|ref|NC_002745.2|	572969 +	C	0	3	0	0	0	SA0490
gi|29165615|ref|NC_002745.2|	572975 +	U	1	0	0	0	0	SA0490
gi|29165615|ref|NC_002745.2|	573008 +	U	2	0	0	0	0	SA0490
gi|29165615|ref|NC_002745.2|	573109 +	C	0	0	1	0	0	SA0490
gi|29165615|ref|NC_002745.2|	573186 +	U	0	0	0	0	1	SA0490
gi|29165615|ref|NC_002745.2|	573189 +	A	0	1	0	0	0	SA0490
gi|29165615|ref|NC_002745.2|	573216 +	A	0	0	0	0	1	SA0490
gi|29165615|ref|NC_002745.2|	573225 +	A	0	1	0	0	0	SA0490
gi|29165615|ref|NC_002745.2|	573270 +	A	0	0	0	1	0	SA0490
gi|29165615|ref|NC_002745.2|	573503 +	A	0	0	1	0	0	SA0490
gi|29165615|ref|NC_002745.2|	573584 +	A	0	0	0	0	1	SA0490
gi|29165615|ref|NC_002745.2|	573590 +	A	0	0	1	0	0	SA0490
gi|29165615|ref|NC_002745.2|	573619 +	C	0	0	1	0	2	SA0490
gi|29165615|ref|NC_002745.2|	573644 +	U	0	0	1	0	0	SA0490
gi|29165615|ref|NC_002745.2|	573654 +	U	0	0	1	0	0	SA0490
gi|29165615|ref|NC_002745.2|	573685 +	C	1	0	0	0	0	SA0490

gi|29165615|ref|NC_002745.2|	573701	+	U	0	3	0	0	0	SA0490
gi|29165615|ref|NC_002745.2|	573713	+	A	0	0	1	0	1	SA0491
gi|29165615|ref|NC_002745.2|	573717	+	U	0	1	0	0	0	SA0491
gi|29165615|ref|NC_002745.2|	573718	+	A	0	0	1	0	0	SA0491
gi|29165615|ref|NC_002745.2|	573725	+	C	0	1	0	0	0	SA0491
gi|29165615|ref|NC_002745.2|	573731	+	U	1	0	0	0	0	SA0491
gi|29165615|ref|NC_002745.2|	573739	+	A	0	2	0	0	0	SA0491
gi|29165615|ref|NC_002745.2|	573744	+	A	0	0	1	1	0	SA0491
gi|29165615|ref|NC_002745.2|	573746	+	A	0	0	0	0	1	SA0491
gi|29165615|ref|NC_002745.2|	573863	+	U	1	0	0	0	0	SA0491
gi|29165615|ref|NC_002745.2|	573878	+	C	0	0	0	0	1	SA0491
gi|29165615|ref|NC_002745.2|	573961	+	U	0	1	0	0	0	SA0491
gi|29165615|ref|NC_002745.2|	574365	+	A	0	1	0	0	0	SA0492
gi|29165615|ref|NC_002745.2|	574677	+	A	0	2	0	0	0	SA0492
gi|29165615|ref|NC_002745.2|	574700	+	A	0	0	1	0	0	SA0492
gi|29165615|ref|NC_002745.2|	574746	+	A	0	0	0	0	1	SA0492
gi|29165615|ref|NC_002745.2|	574897	+	G	0	1	0	0	0	-
gi|29165615|ref|NC_002745.2|	574920	+	U	0	1	0	0	0	-
gi|29165615|ref|NC_002745.2|	574923	+	U	0	0	0	1	1	-
gi|29165615|ref|NC_002745.2|	574928	+	G	0	0	2	0	0	-
gi|29165615|ref|NC_002745.2|	574956	+	U	0	0	0	0	1	-
gi|29165615|ref|NC_002745.2|	574966	+	G	23	24	24	24	30	-
gi|29165615|ref|NC_002745.2|	574967	+	U	0	1	0	0	1	-
gi|29165615|ref|NC_002745.2|	574968	+	A	0	0	5	3	2	-
gi|29165615|ref|NC_002745.2|	574969	+	U	1	0	0	0	1	-
gi|29165615|ref|NC_002745.2|	574970	+	U	0	2	0	0	0	-
gi|29165615|ref|NC_002745.2|	574971	+	A	6	8	8	7	7	-
gi|29165615|ref|NC_002745.2|	574972	+	U	0	0	0	1	0	-
gi|29165615|ref|NC_002745.2|	574975	+	U	0	1	1	0	0	-
gi|29165615|ref|NC_002745.2|	574976	+	G	0	0	1	1	0	-
gi|29165615|ref|NC_002745.2|	574978	+	A	0	0	0	0	1	-
gi|29165615|ref|NC_002745.2|	574981	+	A	0	1	0	0	0	-
gi|29165615|ref|NC_002745.2|	574982	+	A	0	0	1	0	0	-
gi|29165615|ref|NC_002745.2|	574984	+	G	0	0	1	0	0	-
gi|29165615|ref|NC_002745.2|	574985	+	U	0	0	0	0	1	-
gi|29165615|ref|NC_002745.2|	574990	+	U	0	0	0	1	0	-
gi|29165615|ref|NC_002745.2|	574994	+	G	0	1	0	0	0	-
gi|29165615|ref|NC_002745.2|	574995	+	U	2	0	1	0	0	-
gi|29165615|ref|NC_002745.2|	574996	+	G	0	1	1	0	2	-
gi|29165615|ref|NC_002745.2|	574997	+	A	0	2	1	0	0	-
gi|29165615|ref|NC_002745.2|	574999	+	A	0	0	1	1	1	-
gi|29165615|ref|NC_002745.2|	575000	+	A	0	2	1	1	4	-
gi|29165615|ref|NC_002745.2|	575002	+	A	0	0	1	0	1	-
gi|29165615|ref|NC_002745.2|	575003	+	A	0	1	0	0	0	-
gi|29165615|ref|NC_002745.2|	575004	+	U	0	0	0	0	1	-
gi|29165615|ref|NC_002745.2|	575006	+	C	0	2	1	0	0	-
gi|29165615|ref|NC_002745.2|	575010	+	U	0	0	1	0	0	-
gi|29165615|ref|NC_002745.2|	575011	+	A	0	1	0	0	1	-
gi|29165615|ref|NC_002745.2|	575012	+	A	0	2	2	1	1	-
gi|29165615|ref|NC_002745.2|	575013	+	A	0	0	2	0	0	-
gi|29165615|ref|NC_002745.2|	575018	+	G	0	1	1	1	0	-
gi|29165615|ref|NC_002745.2|	575019	+	A	0	1	4	3	1	-
gi|29165615|ref|NC_002745.2|	575022	+	C	0	1	0	1	0	-
gi|29165615|ref|NC_002745.2|	575031	+	A	0	1	0	0	0	-
gi|29165615|ref|NC_002745.2|	575032	+	U	0	0	0	0	1	-
gi|29165615|ref|NC_002745.2|	575034	+	G	0	0	0	0	1	-
gi|29165615|ref|NC_002745.2|	575035	+	A	0	2	2	1	1	-
gi|29165615|ref|NC_002745.2|	575036	+	A	0	0	0	0	1	-
gi|29165615|ref|NC_002745.2|	575037	+	A	0	1	1	0	4	-
gi|29165615|ref|NC_002745.2|	575051	+	A	1	2	0	0	0	-
gi|29165615|ref|NC_002745.2|	575071	+	A	0	1	0	0	0	-
gi|29165615|ref|NC_002745.2|	575078	+	A	0	0	1	0	0	-
gi|29165615|ref|NC_002745.2|	575079	+	C	0	1	0	0	0	-
gi|29165615|ref|NC_002745.2|	575081	+	U	0	0	1	0	0	-
gi|29165615|ref|NC_002745.2|	575083	+	A	0	0	1	0	1	-
gi|29165615|ref|NC_002745.2|	575084	+	A	0	0	0	1	0	-
gi|29165615|ref|NC_002745.2|	575085	+	A	0	0	0	1	0	-
gi|29165615|ref|NC_002745.2|	575086	+	G	0	0	2	2	2	-
gi|29165615|ref|NC_002745.2|	575088	+	A	0	0	1	0	0	-
gi|29165615|ref|NC_002745.2|	575089	+	A	0	0	1	0	0	-
gi|29165615|ref|NC_002745.2|	575091	+	A	0	1	1	0	0	-
gi|29165615|ref|NC_002745.2|	575094	+	G	0	0	1	0	0	-
gi|29165615|ref|NC_002745.2|	575095	+	U	0	1	0	1	0	-
gi|29165615|ref|NC_002745.2|	575098	+	A	0	1	1	1	1	-
gi|29165615|ref|NC_002745.2|	575099	+	A	0	1	0	2	0	-
gi|29165615|ref|NC_002745.2|	575100	+	A	1	0	0	1	3	-
gi|29165615|ref|NC_002745.2|	575101	+	A	0	0	0	0	1	-
gi|29165615|ref|NC_002745.2|	575102	+	U	0	1	0	0	0	-
gi|29165615|ref|NC_002745.2|	575103	+	G	2	0	0	0	0	-
gi|29165615|ref|NC_002745.2|	575104	+	U	0	0	0	2	0	-
gi|29165615|ref|NC_002745.2|	575105	+	A	0	0	0	0	1	-

gi|29165615|ref|NC_002745.2|	575107 +	C	0	0	0	1	1	-
gi|29165615|ref|NC_002745.2|	575108 +	G	0	3	0	0	1	-
gi|29165615|ref|NC_002745.2|	575109 +	C	0	0	0	3	2	-
gi|29165615|ref|NC_002745.2|	575110 +	G	0	0	0	0	1	-
gi|29165615|ref|NC_002745.2|	575111 +	C	2	17	10	11	26	-
gi|29165615|ref|NC_002745.2|	575112 +	A	1	3	1	3	1	-
gi|29165615|ref|NC_002745.2|	575114 +	A	0	0	2	1	0	-
gi|29165615|ref|NC_002745.2|	575115 +	C	0	0	2	0	0	-
gi|29165615|ref|NC_002745.2|	575116 +	A	0	0	0	0	1	-
gi|29165615|ref|NC_002745.2|	575117 +	A	0	7	4	1	5	-
gi|29165615|ref|NC_002745.2|	575120 +	C	0	0	1	0	1	-
gi|29165615|ref|NC_002745.2|	575121 +	A	0	0	0	0	2	-
gi|29165615|ref|NC_002745.2|	575122 +	U	0	0	1	2	3	-
gi|29165615|ref|NC_002745.2|	575123 +	A	0	6	5	4	8	-
gi|29165615|ref|NC_002745.2|	575124 +	A	0	0	3	1	3	-
gi|29165615|ref|NC_002745.2|	575125 +	A	0	0	1	0	0	-
gi|29165615|ref|NC_002745.2|	575126 +	G	0	3	4	8	2	-
gi|29165615|ref|NC_002745.2|	575127 +	A	0	4	4	2	4	-
gi|29165615|ref|NC_002745.2|	575128 +	A	0	0	1	1	0	-
gi|29165615|ref|NC_002745.2|	575130 +	C	0	0	1	0	0	-
gi|29165615|ref|NC_002745.2|	575131 +	G	0	2	1	5	4	-
gi|29165615|ref|NC_002745.2|	575132 +	A	0	8	7	11	18	-
gi|29165615|ref|NC_002745.2|	575133 +	A	0	6	2	2	3	-
gi|29165615|ref|NC_002745.2|	575134 +	A	1	3	0	1	3	-
gi|29165615|ref|NC_002745.2|	575135 +	U	0	1	1	0	1	-
gi|29165615|ref|NC_002745.2|	575136 +	A	0	0	2	1	0	-
gi|29165615|ref|NC_002745.2|	575137 +	A	0	0	2	1	1	-
gi|29165615|ref|NC_002745.2|	575138 +	A	0	2	0	0	4	-
gi|29165615|ref|NC_002745.2|	575139 +	U	0	1	1	0	2	-
gi|29165615|ref|NC_002745.2|	575140 +	A	0	1	0	0	0	-
gi|29165615|ref|NC_002745.2|	575141 +	C	0	6	4	1	2	-
gi|29165615|ref|NC_002745.2|	575142 +	A	0	2	0	0	1	-
gi|29165615|ref|NC_002745.2|	575143 +	U	1	2	7	7	8	-
gi|29165615|ref|NC_002745.2|	575144 +	U	0	0	0	1	0	-
gi|29165615|ref|NC_002745.2|	575145 +	C	0	5	8	4	7	-
gi|29165615|ref|NC_002745.2|	575146 +	G	1	11	13	3	8	-
gi|29165615|ref|NC_002745.2|	575147 +	A	2	35	20	14	30	-
gi|29165615|ref|NC_002745.2|	575148 +	A	1	6	3	3	5	-
gi|29165615|ref|NC_002745.2|	575149 +	A	0	1	0	0	1	-
gi|29165615|ref|NC_002745.2|	575150 +	U	0	1	0	0	0	-
gi|29165615|ref|NC_002745.2|	575151 +	A	0	0	3	0	0	-
gi|29165615|ref|NC_002745.2|	575152 +	A	0	0	0	1	1	-
gi|29165615|ref|NC_002745.2|	575159 +	G	0	0	2	1	4	-
gi|29165615|ref|NC_002745.2|	575160 +	A	0	4	0	4	5	-
gi|29165615|ref|NC_002745.2|	575161 +	U	0	0	1	0	0	-
gi|29165615|ref|NC_002745.2|	575162 +	A	1	0	0	1	0	-
gi|29165615|ref|NC_002745.2|	575163 +	A	0	1	0	1	2	-
gi|29165615|ref|NC_002745.2|	575164 +	U	0	1	0	0	0	-
gi|29165615|ref|NC_002745.2|	575166 +	U	1	1	2	0	0	-
gi|29165615|ref|NC_002745.2|	575167 +	G	0	0	1	0	0	-
gi|29165615|ref|NC_002745.2|	575168 +	U	0	2	1	0	1	-
gi|29165615|ref|NC_002745.2|	575170 +	C	0	0	0	1	1	-
gi|29165615|ref|NC_002745.2|	575171 +	A	0	0	2	1	1	-
gi|29165615|ref|NC_002745.2|	575172 +	A	1	1	0	0	0	-
gi|29165615|ref|NC_002745.2|	575173 +	A	0	0	1	1	2	-
gi|29165615|ref|NC_002745.2|	575175 +	G	1	4	4	1	4	-
gi|29165615|ref|NC_002745.2|	575176 +	A	4	3	1	0	1	-
gi|29165615|ref|NC_002745.2|	575177 +	U	3	1	1	0	0	-
gi|29165615|ref|NC_002745.2|	575178 +	U	6	0	1	0	2	-
gi|29165615|ref|NC_002745.2|	575179 +	U	7	0	0	0	0	-
gi|29165615|ref|NC_002745.2|	575181 +	G	2	2	0	5	6	-
gi|29165615|ref|NC_002745.2|	575182 +	A	0	2	3	5	2	-
gi|29165615|ref|NC_002745.2|	575183 +	G	1	0	0	0	0	-
gi|29165615|ref|NC_002745.2|	575184 +	G	0	0	0	1	0	-
gi|29165615|ref|NC_002745.2|	575185 +	U	2	0	0	0	0	-
gi|29165615|ref|NC_002745.2|	575186 +	U	1	0	0	0	0	-
gi|29165615|ref|NC_002745.2|	575190 +	C	1	1	1	1	0	-
gi|29165615|ref|NC_002745.2|	575194 +	U	2	0	0	0	0	SA0493
gi|29165615|ref|NC_002745.2|	575196 +	G	0	0	0	0	1	SA0493
gi|29165615|ref|NC_002745.2|	575197 +	C	0	0	0	0	1	SA0493
gi|29165615|ref|NC_002745.2|	575199 +	A	1	1	0	0	0	SA0493
gi|29165615|ref|NC_002745.2|	575200 +	A	0	3	1	2	0	SA0493
gi|29165615|ref|NC_002745.2|	575201 +	A	0	0	1	0	1	SA0493
gi|29165615|ref|NC_002745.2|	575202 +	A	0	4	2	0	2	SA0493
gi|29165615|ref|NC_002745.2|	575203 +	A	0	0	1	1	0	SA0493
gi|29165615|ref|NC_002745.2|	575205 +	G	0	1	2	1	2	SA0493
gi|29165615|ref|NC_002745.2|	575206 +	A	1	2	2	0	1	SA0493
gi|29165615|ref|NC_002745.2|	575210 +	U	3	0	1	0	0	SA0493
gi|29165615|ref|NC_002745.2|	575213 +	C	0	1	2	0	0	SA0493
gi|29165615|ref|NC_002745.2|	575214 +	U	2	0	0	0	0	SA0493
gi|29165615|ref|NC_002745.2|	575218 +	A	0	0	1	0	0	SA0493

gi|29165615|ref|NC_002745.2|	575219 +	A	1	0	1	0	1	SA0493
gi|29165615|ref|NC_002745.2|	575221 +	G	0	1	0	0	0	SA0493
gi|29165615|ref|NC_002745.2|	575222 +	C	1	0	1	0	0	SA0493
gi|29165615|ref|NC_002745.2|	575223 +	G	0	0	0	0	1	SA0493
gi|29165615|ref|NC_002745.2|	575225 +	U	0	0	1	1	0	SA0493
gi|29165615|ref|NC_002745.2|	575226 +	A	0	0	0	1	0	SA0493
gi|29165615|ref|NC_002745.2|	575230 +	C	2	0	0	0	0	SA0493
gi|29165615|ref|NC_002745.2|	575232 +	G	0	0	0	0	1	SA0493
gi|29165615|ref|NC_002745.2|	575233 +	A	2	1	0	0	0	SA0493
gi|29165615|ref|NC_002745.2|	575234 +	A	0	0	1	0	0	SA0493
gi|29165615|ref|NC_002745.2|	575235 +	A	0	0	1	0	0	SA0493
gi|29165615|ref|NC_002745.2|	575236 +	U	1	0	0	0	0	SA0493
gi|29165615|ref|NC_002745.2|	575238 +	G	0	0	1	0	0	SA0493
gi|29165615|ref|NC_002745.2|	575239 +	A	0	2	1	3	2	SA0493
gi|29165615|ref|NC_002745.2|	575240 +	A	0	1	3	0	0	SA0493
gi|29165615|ref|NC_002745.2|	575241 +	A	0	4	11	13	23	SA0493
gi|29165615|ref|NC_002745.2|	575242 +	A	0	4	4	2	5	SA0493
gi|29165615|ref|NC_002745.2|	575243 +	A	0	0	2	1	4	SA0493
gi|29165615|ref|NC_002745.2|	575244 +	A	0	2	0	0	0	SA0493
gi|29165615|ref|NC_002745.2|	575245 +	C	0	0	0	2	0	SA0493
gi|29165615|ref|NC_002745.2|	575246 +	A	0	0	0	1	0	SA0493
gi|29165615|ref|NC_002745.2|	575247 +	A	0	1	1	0	0	SA0493
gi|29165615|ref|NC_002745.2|	575254 +	C	6	0	1	0	1	SA0493
gi|29165615|ref|NC_002745.2|	575255 +	G	1	1	0	1	0	SA0493
gi|29165615|ref|NC_002745.2|	575256 +	A	0	0	1	0	0	SA0493
gi|29165615|ref|NC_002745.2|	575259 +	A	1	1	0	1	0	SA0493
gi|29165615|ref|NC_002745.2|	575268 +	C	0	4	0	0	4	SA0493
gi|29165615|ref|NC_002745.2|	575273 +	U	0	0	1	0	0	SA0493
gi|29165615|ref|NC_002745.2|	575274 +	A	0	0	1	1	3	SA0493
gi|29165615|ref|NC_002745.2|	575275 +	A	1	2	4	1	4	SA0493
gi|29165615|ref|NC_002745.2|	575276 +	A	0	3	0	0	0	SA0493
gi|29165615|ref|NC_002745.2|	575280 +	A	0	0	1	0	0	SA0493
gi|29165615|ref|NC_002745.2|	575281 +	C	0	1	1	0	1	SA0493
gi|29165615|ref|NC_002745.2|	575282 +	U	0	0	0	1	0	SA0493
gi|29165615|ref|NC_002745.2|	575284 +	U	0	1	0	0	2	SA0493
gi|29165615|ref|NC_002745.2|	575285 +	A	0	0	1	0	0	SA0493
gi|29165615|ref|NC_002745.2|	575286 +	A	0	2	0	0	0	SA0493
gi|29165615|ref|NC_002745.2|	575289 +	G	1	0	0	0	1	SA0493
gi|29165615|ref|NC_002745.2|	575290 +	U	0	2	1	0	2	SA0493
gi|29165615|ref|NC_002745.2|	575291 +	A	0	0	0	0	1	SA0493
gi|29165615|ref|NC_002745.2|	575293 +	U	0	0	0	0	1	SA0493
gi|29165615|ref|NC_002745.2|	575300 +	U	1	0	1	0	0	SA0493
gi|29165615|ref|NC_002745.2|	575302 +	U	0	0	1	0	1	SA0493
gi|29165615|ref|NC_002745.2|	575308 +	U	0	0	0	0	1	SA0493
gi|29165615|ref|NC_002745.2|	575310 +	U	0	0	0	0	1	SA0493
gi|29165615|ref|NC_002745.2|	575312 +	C	0	0	0	0	3	SA0493
gi|29165615|ref|NC_002745.2|	575318 +	C	2	0	0	0	0	SA0493
gi|29165615|ref|NC_002745.2|	575319 +	U	0	0	0	1	0	SA0493
gi|29165615|ref|NC_002745.2|	575320 +	U	2	0	0	0	0	SA0493
gi|29165615|ref|NC_002745.2|	575321 +	U	1	0	0	0	0	SA0493
gi|29165615|ref|NC_002745.2|	575322 +	U	0	0	1	0	0	SA0493
gi|29165615|ref|NC_002745.2|	575323 +	U	0	0	0	1	0	SA0493
gi|29165615|ref|NC_002745.2|	575324 +	C	1	1	1	0	1	SA0493
gi|29165615|ref|NC_002745.2|	575325 +	U	0	0	0	1	0	SA0493
gi|29165615|ref|NC_002745.2|	575329 +	C	0	0	0	0	1	SA0493
gi|29165615|ref|NC_002745.2|	575332 +	U	0	0	1	0	0	SA0493
gi|29165615|ref|NC_002745.2|	575333 +	A	0	1	2	0	1	SA0493
gi|29165615|ref|NC_002745.2|	575334 +	G	1	0	0	0	1	SA0493
gi|29165615|ref|NC_002745.2|	575335 +	A	0	3	3	2	4	SA0493
gi|29165615|ref|NC_002745.2|	575336 +	U	2	0	0	0	0	SA0493
gi|29165615|ref|NC_002745.2|	575338 +	U	0	0	1	0	1	SA0493
gi|29165615|ref|NC_002745.2|	575340 +	G	0	3	1	3	2	SA0493
gi|29165615|ref|NC_002745.2|	575341 +	G	2	15	5	3	2	SA0493
gi|29165615|ref|NC_002745.2|	575342 +	A	0	6	6	1	0	SA0493
gi|29165615|ref|NC_002745.2|	575343 +	A	0	1	0	1	1	SA0493
gi|29165615|ref|NC_002745.2|	575345 +	U	0	2	0	0	2	SA0493
gi|29165615|ref|NC_002745.2|	575346 +	A	0	0	0	0	1	SA0493
gi|29165615|ref|NC_002745.2|	575347 +	C	0	0	1	0	0	SA0493
gi|29165615|ref|NC_002745.2|	575348 +	A	0	1	0	0	0	SA0493
gi|29165615|ref|NC_002745.2|	575349 +	G	1	1	3	1	1	SA0493
gi|29165615|ref|NC_002745.2|	575350 +	C	0	4	2	1	1	SA0493
gi|29165615|ref|NC_002745.2|	575351 +	A	0	4	1	1	1	SA0493
gi|29165615|ref|NC_002745.2|	575352 +	U	0	1	0	0	0	SA0493
gi|29165615|ref|NC_002745.2|	575354 +	G	0	1	0	0	0	SA0493
gi|29165615|ref|NC_002745.2|	575355 +	A	0	5	5	4	4	SA0493
gi|29165615|ref|NC_002745.2|	575356 +	A	0	5	5	1	2	SA0493
gi|29165615|ref|NC_002745.2|	575357 +	A	2	4	8	1	3	SA0493
gi|29165615|ref|NC_002745.2|	575358 +	A	2	4	4	1	1	SA0493
gi|29165615|ref|NC_002745.2|	575359 +	A	0	3	0	0	2	SA0493
gi|29165615|ref|NC_002745.2|	575360 +	U	0	3	0	2	3	SA0493
gi|29165615|ref|NC_002745.2|	575361 +	U	0	2	2	15	0	SA0493

gi|29165615|ref|NC_002745.2|	575362	+	U	1	1	1	5	1	SA0493
gi|29165615|ref|NC_002745.2|	575363	+	A	1	1	4	0	0	SA0493
gi|29165615|ref|NC_002745.2|	575364	+	U	0	0	3	0	0	SA0493
gi|29165615|ref|NC_002745.2|	575365	+	U	0	2	0	0	1	SA0493
gi|29165615|ref|NC_002745.2|	575366	+	A	0	1	1	0	0	SA0493
gi|29165615|ref|NC_002745.2|	575369	+	U	0	0	0	1	0	SA0493
gi|29165615|ref|NC_002745.2|	575370	+	G	0	0	1	0	0	SA0493
gi|29165615|ref|NC_002745.2|	575371	+	G	0	2	1	0	3	SA0493
gi|29165615|ref|NC_002745.2|	575372	+	U	1	3	4	0	2	SA0493
gi|29165615|ref|NC_002745.2|	575373	+	U	0	0	0	1	0	SA0493
gi|29165615|ref|NC_002745.2|	575374	+	A	0	0	0	0	1	SA0493
gi|29165615|ref|NC_002745.2|	575375	+	G	0	0	0	0	1	SA0493
gi|29165615|ref|NC_002745.2|	575377	+	G	0	1	0	0	0	-
gi|29165615|ref|NC_002745.2|	575378	+	G	0	0	4	0	2	-
gi|29165615|ref|NC_002745.2|	575379	+	A	0	1	0	0	0	-
gi|29165615|ref|NC_002745.2|	575383	+	A	0	0	0	0	1	-
gi|29165615|ref|NC_002745.2|	575390	+	G	0	1	0	0	0	SA0494
gi|29165615|ref|NC_002745.2|	575392	+	C	1	0	0	0	0	SA0494
gi|29165615|ref|NC_002745.2|	575395	+	A	2	0	0	0	0	SA0494
gi|29165615|ref|NC_002745.2|	575400	+	G	0	0	1	0	0	SA0494
gi|29165615|ref|NC_002745.2|	575405	+	C	0	0	0	1	0	SA0494
gi|29165615|ref|NC_002745.2|	575409	+	A	0	0	0	1	0	SA0494
gi|29165615|ref|NC_002745.2|	575422	+	C	0	1	0	0	0	SA0494
gi|29165615|ref|NC_002745.2|	575447	+	A	1	1	0	0	1	SA0494
gi|29165615|ref|NC_002745.2|	575452	+	A	0	0	0	1	0	SA0494
gi|29165615|ref|NC_002745.2|	575454	+	G	0	0	1	0	0	SA0494
gi|29165615|ref|NC_002745.2|	575458	+	A	0	0	2	0	1	SA0494
gi|29165615|ref|NC_002745.2|	575459	+	A	0	1	0	0	0	SA0494
gi|29165615|ref|NC_002745.2|	575463	+	A	1	1	0	0	0	SA0494
gi|29165615|ref|NC_002745.2|	575464	+	A	0	1	0	0	0	SA0494
gi|29165615|ref|NC_002745.2|	575470	+	A	0	1	0	1	1	SA0494
gi|29165615|ref|NC_002745.2|	575477	+	A	0	0	1	0	0	SA0494
gi|29165615|ref|NC_002745.2|	575478	+	G	0	0	2	0	1	SA0494
gi|29165615|ref|NC_002745.2|	575480	+	A	0	2	0	0	0	SA0494
gi|29165615|ref|NC_002745.2|	575481	+	G	0	0	1	0	0	SA0494
gi|29165615|ref|NC_002745.2|	575495	+	G	0	0	0	1	0	SA0494
gi|29165615|ref|NC_002745.2|	575496	+	A	0	1	1	0	0	SA0494
gi|29165615|ref|NC_002745.2|	575505	+	A	0	0	1	0	0	SA0494
gi|29165615|ref|NC_002745.2|	575514	+	G	1	0	0	0	0	SA0494
gi|29165615|ref|NC_002745.2|	575515	+	U	0	0	0	1	1	SA0494
gi|29165615|ref|NC_002745.2|	575521	+	U	1	0	0	0	0	SA0494
gi|29165615|ref|NC_002745.2|	575524	+	C	1	0	0	0	0	SA0494
gi|29165615|ref|NC_002745.2|	575530	+	A	0	0	1	0	1	SA0494
gi|29165615|ref|NC_002745.2|	575533	+	A	0	0	1	0	0	SA0494
gi|29165615|ref|NC_002745.2|	575534	+	A	1	0	0	0	0	SA0494
gi|29165615|ref|NC_002745.2|	575536	+	A	0	0	1	0	0	SA0494
gi|29165615|ref|NC_002745.2|	575538	+	A	0	0	1	0	0	SA0494
gi|29165615|ref|NC_002745.2|	575540	+	U	1	0	0	0	0	SA0494
gi|29165615|ref|NC_002745.2|	575541	+	C	0	0	1	0	0	SA0494
gi|29165615|ref|NC_002745.2|	575542	+	A	0	0	0	0	1	SA0494
gi|29165615|ref|NC_002745.2|	575543	+	A	1	0	0	1	1	SA0494
gi|29165615|ref|NC_002745.2|	575544	+	G	0	1	0	0	0	SA0494
gi|29165615|ref|NC_002745.2|	575549	+	A	0	2	1	0	0	SA0494
gi|29165615|ref|NC_002745.2|	575551	+	A	1	1	1	0	1	SA0494
gi|29165615|ref|NC_002745.2|	575553	+	G	0	1	0	0	0	SA0494
gi|29165615|ref|NC_002745.2|	575554	+	G	0	0	1	0	0	SA0494
gi|29165615|ref|NC_002745.2|	575555	+	C	0	1	0	0	0	SA0494
gi|29165615|ref|NC_002745.2|	575557	+	A	0	0	0	1	0	SA0494
gi|29165615|ref|NC_002745.2|	575561	+	U	0	0	0	1	1	SA0494
gi|29165615|ref|NC_002745.2|	575562	+	A	0	1	0	0	0	SA0494
gi|29165615|ref|NC_002745.2|	575568	+	A	1	0	0	0	0	SA0494
gi|29165615|ref|NC_002745.2|	575573	+	U	0	1	2	1	1	SA0494
gi|29165615|ref|NC_002745.2|	575574	+	A	0	1	0	1	3	SA0494
gi|29165615|ref|NC_002745.2|	575575	+	A	0	3	0	0	0	SA0494
gi|29165615|ref|NC_002745.2|	575576	+	A	0	1	0	0	1	SA0494
gi|29165615|ref|NC_002745.2|	575577	+	A	0	0	0	1	0	SA0494
gi|29165615|ref|NC_002745.2|	575591	+	A	0	1	0	0	0	SA0494
gi|29165615|ref|NC_002745.2|	575602	+	U	1	0	0	0	0	SA0494
gi|29165615|ref|NC_002745.2|	575603	+	G	0	1	0	0	1	SA0494
gi|29165615|ref|NC_002745.2|	575604	+	G	0	0	0	1	0	SA0494
gi|29165615|ref|NC_002745.2|	575605	+	A	1	0	0	0	0	SA0494
gi|29165615|ref|NC_002745.2|	575610	+	A	0	1	0	0	0	SA0494
gi|29165615|ref|NC_002745.2|	575616	+	A	0	0	3	1	0	SA0494
gi|29165615|ref|NC_002745.2|	575621	+	U	0	0	0	0	1	SA0494
gi|29165615|ref|NC_002745.2|	575626	+	C	1	0	0	0	0	SA0494
gi|29165615|ref|NC_002745.2|	575629	+	G	1	0	0	0	0	SA0494
gi|29165615|ref|NC_002745.2|	575630	+	G	0	0	0	0	1	SA0494
gi|29165615|ref|NC_002745.2|	575632	+	A	0	0	1	0	0	SA0494
gi|29165615|ref|NC_002745.2|	575635	+	U	3	0	0	0	0	SA0494
gi|29165615|ref|NC_002745.2|	575637	+	G	0	0	0	1	0	SA0494
gi|29165615|ref|NC_002745.2|	575638	+	U	0	0	0	2	0	SA0494

gi|29165615|ref|NC_002745.2|	575639	+	A	0	0	0	1	0	SA0494
gi|29165615|ref|NC_002745.2|	575640	+	A	2	1	1	0	0	SA0494
gi|29165615|ref|NC_002745.2|	575641	+	G	1	5	2	2	2	SA0494
gi|29165615|ref|NC_002745.2|	575642	+	A	0	0	2	0	0	SA0494
gi|29165615|ref|NC_002745.2|	575647	+	C	2	2	0	0	0	SA0494
gi|29165615|ref|NC_002745.2|	575650	+	C	1	0	0	0	1	SA0494
gi|29165615|ref|NC_002745.2|	575651	+	A	0	0	0	1	0	SA0494
gi|29165615|ref|NC_002745.2|	575657	+	U	0	0	0	0	1	SA0494
gi|29165615|ref|NC_002745.2|	575659	+	C	1	0	0	0	0	SA0494
gi|29165615|ref|NC_002745.2|	575660	+	U	0	0	1	0	0	SA0494
gi|29165615|ref|NC_002745.2|	575664	+	U	0	0	0	0	1	SA0494
gi|29165615|ref|NC_002745.2|	575665	+	U	1	1	0	0	0	SA0494
gi|29165615|ref|NC_002745.2|	575672	+	U	1	0	1	0	0	SA0494
gi|29165615|ref|NC_002745.2|	575674	+	C	4	0	0	0	0	SA0494
gi|29165615|ref|NC_002745.2|	575678	+	A	0	1	0	0	0	SA0494
gi|29165615|ref|NC_002745.2|	575680	+	G	0	1	0	0	0	SA0494
gi|29165615|ref|NC_002745.2|	575701	+	C	1	0	0	0	0	SA0494
gi|29165615|ref|NC_002745.2|	575705	+	G	0	0	0	1	1	SA0494
gi|29165615|ref|NC_002745.2|	575711	+	A	0	0	0	0	2	SA0494
gi|29165615|ref|NC_002745.2|	575724	+	U	0	0	0	0	1	SA0494
gi|29165615|ref|NC_002745.2|	575728	+	U	0	0	0	1	0	SA0494
gi|29165615|ref|NC_002745.2|	575733	+	A	1	0	0	0	0	SA0494
gi|29165615|ref|NC_002745.2|	575735	+	A	0	0	0	0	1	SA0494
gi|29165615|ref|NC_002745.2|	575736	+	C	0	0	0	0	1	SA0494
gi|29165615|ref|NC_002745.2|	575737	+	A	0	0	0	0	1	SA0494
gi|29165615|ref|NC_002745.2|	575739	+	A	0	1	0	0	0	SA0494
gi|29165615|ref|NC_002745.2|	575749	+	A	0	1	0	0	0	SA0494
gi|29165615|ref|NC_002745.2|	575753	+	A	0	0	1	0	0	SA0494
gi|29165615|ref|NC_002745.2|	575754	+	A	0	1	0	0	0	SA0494
gi|29165615|ref|NC_002745.2|	575762	+	C	0	0	0	0	1	SA0494
gi|29165615|ref|NC_002745.2|	575764	+	A	0	2	1	0	1	SA0494
gi|29165615|ref|NC_002745.2|	575767	+	U	2	0	0	0	0	SA0494
gi|29165615|ref|NC_002745.2|	575769	+	G	0	0	0	0	1	SA0494
gi|29165615|ref|NC_002745.2|	575793	+	C	0	0	0	0	1	SA0494
gi|29165615|ref|NC_002745.2|	575798	+	U	0	1	0	0	0	SA0494
gi|29165615|ref|NC_002745.2|	575799	+	A	0	0	1	0	0	SA0494
gi|29165615|ref|NC_002745.2|	575801	+	A	0	0	1	0	0	SA0494
gi|29165615|ref|NC_002745.2|	575809	+	C	0	2	0	0	0	SA0494
gi|29165615|ref|NC_002745.2|	575817	+	A	0	0	0	0	1	SA0494
gi|29165615|ref|NC_002745.2|	575820	+	C	2	0	0	0	2	SA0494
gi|29165615|ref|NC_002745.2|	575841	+	A	0	0	0	0	1	SA0494
gi|29165615|ref|NC_002745.2|	575844	+	G	0	0	1	0	0	SA0494
gi|29165615|ref|NC_002745.2|	575862	+	C	0	1	0	0	0	SA0494
gi|29165615|ref|NC_002745.2|	575865	+	A	0	0	0	0	1	SA0494
gi|29165615|ref|NC_002745.2|	575869	+	U	0	0	0	0	1	SA0494
gi|29165615|ref|NC_002745.2|	575922	+	A	0	1	1	0	0	SA0494
gi|29165615|ref|NC_002745.2|	575953	+	U	0	0	0	1	0	-
gi|29165615|ref|NC_002745.2|	575960	+	A	0	0	0	1	0	-
gi|29165615|ref|NC_002745.2|	575963	+	C	0	0	1	0	0	-
gi|29165615|ref|NC_002745.2|	575966	+	A	0	2	0	1	0	-
gi|29165615|ref|NC_002745.2|	575967	+	A	0	1	1	0	0	-
gi|29165615|ref|NC_002745.2|	575968	+	G	0	0	0	1	2	-
gi|29165615|ref|NC_002745.2|	575969	+	A	0	0	0	1	0	-
gi|29165615|ref|NC_002745.2|	575972	+	A	0	0	0	0	1	-
gi|29165615|ref|NC_002745.2|	575974	+	A	0	0	0	0	1	-
gi|29165615|ref|NC_002745.2|	575975	+	A	0	0	0	1	0	-
gi|29165615|ref|NC_002745.2|	575978	+	A	0	0	2	4	2	-
gi|29165615|ref|NC_002745.2|	575979	+	G	0	0	0	0	1	-
gi|29165615|ref|NC_002745.2|	575985	+	A	0	1	0	1	0	-
gi|29165615|ref|NC_002745.2|	575990	+	U	0	0	1	1	0	-
gi|29165615|ref|NC_002745.2|	575992	+	A	0	3	1	0	1	-
gi|29165615|ref|NC_002745.2|	575993	+	A	0	3	0	0	0	-
gi|29165615|ref|NC_002745.2|	575994	+	U	0	0	0	0	1	-
gi|29165615|ref|NC_002745.2|	575998	+	A	0	0	2	0	0	-
gi|29165615|ref|NC_002745.2|	575999	+	A	0	1	1	3	0	-
gi|29165615|ref|NC_002745.2|	576000	+	A	0	3	1	1	1	-
gi|29165615|ref|NC_002745.2|	576002	+	G	0	0	0	1	0	-
gi|29165615|ref|NC_002745.2|	576003	+	U	0	0	0	0	1	-
gi|29165615|ref|NC_002745.2|	576004	+	G	0	0	1	0	0	-
gi|29165615|ref|NC_002745.2|	576007	+	A	0	0	0	2	2	-
gi|29165615|ref|NC_002745.2|	576008	+	A	0	0	0	0	1	-
gi|29165615|ref|NC_002745.2|	576009	+	A	0	1	1	0	0	-
gi|29165615|ref|NC_002745.2|	576012	+	U	0	0	0	0	1	-
gi|29165615|ref|NC_002745.2|	576014	+	U	2	1	0	0	0	-
gi|29165615|ref|NC_002745.2|	576015	+	G	2	0	0	0	0	-
gi|29165615|ref|NC_002745.2|	576018	+	G	7	0	1	1	1	-
gi|29165615|ref|NC_002745.2|	576021	+	G	1	0	0	0	0	-
gi|29165615|ref|NC_002745.2|	576042	+	C	1	0	0	0	0	-
gi|29165615|ref|NC_002745.2|	576049	+	A	0	0	1	0	0	-
gi|29165615|ref|NC_002745.2|	576074	+	C	1	0	0	0	0	SAS015
gi|29165615|ref|NC_002745.2|	576075	+	U	1	0	0	0	0	SAS015

gi|29165615|ref|NC_002745.2|	576089	+	A	1	0	0	0	0	SAS015
gi|29165615|ref|NC_002745.2|	576096	+	G	5	0	0	0	1	SAS015
gi|29165615|ref|NC_002745.2|	576097	+	A	0	0	0	1	0	SAS015
gi|29165615|ref|NC_002745.2|	576100	+	U	1	0	0	0	0	SAS015
gi|29165615|ref|NC_002745.2|	576101	+	C	0	0	1	1	1	SAS015
gi|29165615|ref|NC_002745.2|	576102	+	A	0	0	1	0	0	SAS015
gi|29165615|ref|NC_002745.2|	576103	+	A	0	0	1	0	0	SAS015
gi|29165615|ref|NC_002745.2|	576129	+	G	1	0	0	0	0	SA0495
gi|29165615|ref|NC_002745.2|	576130	+	U	0	1	0	0	0	SA0495
gi|29165615|ref|NC_002745.2|	576152	+	A	0	0	1	0	0	SA0495
gi|29165615|ref|NC_002745.2|	576153	+	A	0	0	0	0	1	SA0495
gi|29165615|ref|NC_002745.2|	576161	+	A	0	0	1	0	0	SA0495
gi|29165615|ref|NC_002745.2|	576164	+	U	0	0	0	1	0	SA0495
gi|29165615|ref|NC_002745.2|	576170	+	G	2	0	0	0	0	SA0495
gi|29165615|ref|NC_002745.2|	576178	+	C	0	1	0	0	0	SA0495
gi|29165615|ref|NC_002745.2|	576180	+	C	0	0	0	0	1	SA0495
gi|29165615|ref|NC_002745.2|	576190	+	G	0	1	0	0	0	SA0495
gi|29165615|ref|NC_002745.2|	576198	+	U	0	1	0	0	0	SA0495
gi|29165615|ref|NC_002745.2|	576199	+	U	0	0	0	0	1	SA0495
gi|29165615|ref|NC_002745.2|	576221	+	C	0	1	0	0	0	SA0495
gi|29165615|ref|NC_002745.2|	576226	+	G	0	1	0	0	0	SA0495
gi|29165615|ref|NC_002745.2|	576342	+	C	1	0	0	0	0	SA0495
gi|29165615|ref|NC_002745.2|	576396	+	A	0	0	1	0	0	SA0495
gi|29165615|ref|NC_002745.2|	576402	+	A	2	0	0	0	0	SA0495
gi|29165615|ref|NC_002745.2|	576404	+	A	0	0	0	0	1	SA0495
gi|29165615|ref|NC_002745.2|	576417	+	A	0	0	0	0	2	SA0495
gi|29165615|ref|NC_002745.2|	576420	+	A	0	1	0	0	1	SA0495
gi|29165615|ref|NC_002745.2|	576421	+	A	0	1	1	0	0	SA0495
gi|29165615|ref|NC_002745.2|	576422	+	A	0	0	1	1	1	SA0495
gi|29165615|ref|NC_002745.2|	576424	+	A	0	0	0	1	0	SA0495
gi|29165615|ref|NC_002745.2|	576465	+	A	0	0	0	0	1	SA0495
gi|29165615|ref|NC_002745.2|	576472	+	C	2	0	0	0	0	SA0495
gi|29165615|ref|NC_002745.2|	576480	+	G	1	0	0	0	0	SA0495
gi|29165615|ref|NC_002745.2|	576489	+	A	0	1	0	0	0	SA0495
gi|29165615|ref|NC_002745.2|	576496	+	U	0	1	0	0	0	SA0495
gi|29165615|ref|NC_002745.2|	576498	+	A	0	0	1	0	0	SA0495
gi|29165615|ref|NC_002745.2|	576522	+	G	0	1	0	0	0	SA0495
gi|29165615|ref|NC_002745.2|	576533	+	A	0	1	0	0	0	SA0495
gi|29165615|ref|NC_002745.2|	576534	+	G	0	1	0	0	0	SA0495
gi|29165615|ref|NC_002745.2|	576540	+	U	1	0	0	0	0	-
gi|29165615|ref|NC_002745.2|	576547	+	A	0	1	0	1	0	-
gi|29165615|ref|NC_002745.2|	576554	+	A	0	1	2	0	0	-
gi|29165615|ref|NC_002745.2|	576606	+	G	1	0	0	0	0	-
gi|29165615|ref|NC_002745.2|	576631	+	U	0	0	0	0	1	-
gi|29165615|ref|NC_002745.2|	576656	+	A	0	1	0	0	0	-
gi|29165615|ref|NC_002745.2|	576659	+	U	1	0	0	0	0	-
gi|29165615|ref|NC_002745.2|	576691	+	U	0	0	1	0	0	-
gi|29165615|ref|NC_002745.2|	576700	+	U	1	0	0	0	0	-
gi|29165615|ref|NC_002745.2|	576720	+	C	0	1	0	0	0	-
gi|29165615|ref|NC_002745.2|	576725	+	A	1	1	0	0	0	-
gi|29165615|ref|NC_002745.2|	576731	+	A	0	0	1	0	0	-
gi|29165615|ref|NC_002745.2|	576732	+	A	0	0	1	0	0	-
gi|29165615|ref|NC_002745.2|	576741	+	C	0	0	1	0	0	-
gi|29165615|ref|NC_002745.2|	576747	+	A	0	2	0	0	2	SA0496
gi|29165615|ref|NC_002745.2|	576751	+	C	1	0	1	3	0	SA0496
gi|29165615|ref|NC_002745.2|	576754	+	A	0	0	2	0	0	SA0496
gi|29165615|ref|NC_002745.2|	576755	+	A	0	0	0	1	0	SA0496
gi|29165615|ref|NC_002745.2|	576756	+	A	1	0	0	1	0	SA0496
gi|29165615|ref|NC_002745.2|	576760	+	G	0	0	0	0	1	SA0496
gi|29165615|ref|NC_002745.2|	576762	+	A	0	0	1	0	0	SA0496
gi|29165615|ref|NC_002745.2|	576773	+	A	0	1	0	0	0	SA0496
gi|29165615|ref|NC_002745.2|	576795	+	C	0	0	0	0	1	SA0496
gi|29165615|ref|NC_002745.2|	576809	+	C	0	0	0	0	1	SA0496
gi|29165615|ref|NC_002745.2|	576810	+	A	0	0	0	0	1	SA0496
gi|29165615|ref|NC_002745.2|	576848	+	C	1	0	0	0	0	SA0496
gi|29165615|ref|NC_002745.2|	576853	+	C	1	0	0	0	0	SA0496
gi|29165615|ref|NC_002745.2|	576860	+	U	1	0	0	0	0	SA0496
gi|29165615|ref|NC_002745.2|	576862	+	A	1	0	0	0	0	SA0496
gi|29165615|ref|NC_002745.2|	576865	+	C	5	1	0	0	0	SA0496
gi|29165615|ref|NC_002745.2|	576866	+	U	4	1	0	0	0	SA0496
gi|29165615|ref|NC_002745.2|	576868	+	C	2	0	0	0	0	SA0496
gi|29165615|ref|NC_002745.2|	576871	+	U	0	0	0	1	0	SA0496
gi|29165615|ref|NC_002745.2|	576875	+	A	2	0	0	0	0	SA0496
gi|29165615|ref|NC_002745.2|	576895	+	U	0	0	0	0	1	SA0496
gi|29165615|ref|NC_002745.2|	576896	+	U	0	1	0	0	0	SA0496
gi|29165615|ref|NC_002745.2|	576903	+	C	0	0	0	0	2	SA0496
gi|29165615|ref|NC_002745.2|	576904	+	G	1	0	0	0	0	SA0496
gi|29165615|ref|NC_002745.2|	576905	+	U	1	0	0	0	0	SA0496
gi|29165615|ref|NC_002745.2|	576910	+	A	0	2	0	0	3	SA0496
gi|29165615|ref|NC_002745.2|	576912	+	G	0	0	0	1	0	SA0496
gi|29165615|ref|NC_002745.2|	576916	+	A	0	0	1	0	0	SA0496

gi|29165615|ref|NC_002745.2|	576920	+	A	0	1	1	0	0	SA0496
gi|29165615|ref|NC_002745.2|	576922	+	U	1	0	0	0	0	SA0496
gi|29165615|ref|NC_002745.2|	576924	+	C	1	0	0	0	0	SA0496
gi|29165615|ref|NC_002745.2|	576926	+	U	1	0	0	0	0	SA0496
gi|29165615|ref|NC_002745.2|	576929	+	U	1	0	0	0	0	SA0496
gi|29165615|ref|NC_002745.2|	576934	+	U	0	0	1	0	0	SA0496
gi|29165615|ref|NC_002745.2|	576940	+	U	2	0	0	0	0	SA0496
gi|29165615|ref|NC_002745.2|	576956	+	U	1	0	0	0	0	SA0496
gi|29165615|ref|NC_002745.2|	576961	+	C	0	1	0	0	0	SA0496
gi|29165615|ref|NC_002745.2|	576965	+	A	0	1	0	0	0	SA0496
gi|29165615|ref|NC_002745.2|	576970	+	U	1	0	0	0	0	SA0496
gi|29165615|ref|NC_002745.2|	576971	+	A	0	0	2	0	0	SA0496
gi|29165615|ref|NC_002745.2|	576973	+	U	1	1	0	0	0	SA0496
gi|29165615|ref|NC_002745.2|	576974	+	A	1	0	0	0	0	SA0496
gi|29165615|ref|NC_002745.2|	576976	+	U	0	0	1	0	0	SA0496
gi|29165615|ref|NC_002745.2|	576978	+	U	1	0	0	0	0	SA0496
gi|29165615|ref|NC_002745.2|	576979	+	U	2	0	0	0	0	SA0496
gi|29165615|ref|NC_002745.2|	576996	+	A	1	4	0	0	0	SA0496
gi|29165615|ref|NC_002745.2|	577014	+	G	1	0	0	0	0	SA0496
gi|29165615|ref|NC_002745.2|	577020	+	A	0	0	1	0	0	SA0496
gi|29165615|ref|NC_002745.2|	577061	+	A	0	0	1	0	0	SA0496
gi|29165615|ref|NC_002745.2|	577089	+	G	1	0	0	0	0	SA0496
gi|29165615|ref|NC_002745.2|	577166	+	U	0	1	0	0	0	SA0496
gi|29165615|ref|NC_002745.2|	577170	+	A	0	0	0	0	1	SA0496
gi|29165615|ref|NC_002745.2|	577172	+	U	1	0	0	0	0	SA0496
gi|29165615|ref|NC_002745.2|	577238	+	U	1	0	0	0	0	SA0496
gi|29165615|ref|NC_002745.2|	577282	+	C	1	0	1	0	0	SA0496
gi|29165615|ref|NC_002745.2|	577295	+	A	0	0	1	0	0	SA0496
gi|29165615|ref|NC_002745.2|	577312	+	U	0	1	0	0	0	SA0496
gi|29165615|ref|NC_002745.2|	577345	+	A	0	1	0	0	0	SA0496
gi|29165615|ref|NC_002745.2|	577347	+	C	2	0	0	0	0	SA0496
gi|29165615|ref|NC_002745.2|	577375	+	A	0	0	0	1	0	SA0496
gi|29165615|ref|NC_002745.2|	577384	+	C	0	0	0	1	0	SA0496
gi|29165615|ref|NC_002745.2|	577385	+	U	0	1	0	0	0	SA0496
gi|29165615|ref|NC_002745.2|	577387	+	U	1	0	0	0	0	SA0496
gi|29165615|ref|NC_002745.2|	577396	+	C	0	0	1	0	0	SA0496
gi|29165615|ref|NC_002745.2|	577420	+	A	1	0	0	0	0	SA0496
gi|29165615|ref|NC_002745.2|	577433	+	C	0	1	0	0	0	SA0496
gi|29165615|ref|NC_002745.2|	577449	+	U	0	0	1	0	0	-
gi|29165615|ref|NC_002745.2|	577452	+	A	2	0	6	3	1	-
gi|29165615|ref|NC_002745.2|	577459	+	C	0	0	0	1	0	-
gi|29165615|ref|NC_002745.2|	577474	+	A	0	1	0	0	0	-
gi|29165615|ref|NC_002745.2|	577530	+	A	1	0	1	0	0	-
gi|29165615|ref|NC_002745.2|	577531	+	U	10	44	82	39	31	-
gi|29165615|ref|NC_002745.2|	577532	+	U	5	5	4	0	5	-
gi|29165615|ref|NC_002745.2|	577533	+	G	68	258	336	140	104	-
gi|29165615|ref|NC_002745.2|	577534	+	U	1	6	6	4	0	-
gi|29165615|ref|NC_002745.2|	577535	+	G	0	5	6	1	0	-
gi|29165615|ref|NC_002745.2|	577536	+	A	0	1	0	2	0	-
gi|29165615|ref|NC_002745.2|	577537	+	U	0	1	0	0	1	-
gi|29165615|ref|NC_002745.2|	577538	+	U	0	0	1	0	0	-
gi|29165615|ref|NC_002745.2|	577541	+	U	2	0	1	0	0	-
gi|29165615|ref|NC_002745.2|	577542	+	U	0	1	0	0	0	-
gi|29165615|ref|NC_002745.2|	577543	+	U	0	1	1	1	0	-
gi|29165615|ref|NC_002745.2|	577544	+	A	0	0	1	0	0	-
gi|29165615|ref|NC_002745.2|	577545	+	C	1	0	0	0	0	-
gi|29165615|ref|NC_002745.2|	577550	+	G	0	0	0	0	1	-
gi|29165615|ref|NC_002745.2|	577551	+	A	0	0	0	1	0	-
gi|29165615|ref|NC_002745.2|	577552	+	C	0	1	1	1	0	-
gi|29165615|ref|NC_002745.2|	577555	+	U	0	1	0	0	0	-
gi|29165615|ref|NC_002745.2|	577559	+	A	0	0	1	0	0	-
gi|29165615|ref|NC_002745.2|	577560	+	G	0	0	1	0	0	-
gi|29165615|ref|NC_002745.2|	577564	+	U	0	1	0	0	0	-
gi|29165615|ref|NC_002745.2|	577567	+	A	0	1	0	0	0	-
gi|29165615|ref|NC_002745.2|	577570	+	A	0	1	0	0	0	-
gi|29165615|ref|NC_002745.2|	577571	+	C	0	0	0	1	1	-
gi|29165615|ref|NC_002745.2|	577574	+	A	0	1	1	0	0	-
gi|29165615|ref|NC_002745.2|	577575	+	A	0	0	1	0	0	-
gi|29165615|ref|NC_002745.2|	577576	+	A	0	0	1	1	0	-
gi|29165615|ref|NC_002745.2|	577577	+	A	0	0	0	0	1	-
gi|29165615|ref|NC_002745.2|	577580	+	U	1	0	0	0	0	-
gi|29165615|ref|NC_002745.2|	577582	+	U	0	0	1	0	0	-
gi|29165615|ref|NC_002745.2|	577584	+	C	0	1	0	0	0	-
gi|29165615|ref|NC_002745.2|	577585	+	U	0	0	0	1	0	-
gi|29165615|ref|NC_002745.2|	577593	+	C	0	1	0	0	0	-
gi|29165615|ref|NC_002745.2|	577623	+	A	2	12	14	2	7	-
gi|29165615|ref|NC_002745.2|	577624	+	U	0	0	1	0	0	-
gi|29165615|ref|NC_002745.2|	577627	+	U	0	0	0	0	1	-
gi|29165615|ref|NC_002745.2|	577636	+	U	1	0	0	0	0	-
gi|29165615|ref|NC_002745.2|	577669	+	A	0	2	0	0	1	-
gi|29165615|ref|NC_002745.2|	577670	+	A	0	2	2	2	0	-

gi|29165615|ref|NC_002745.2|	577674 +	U	0	0	0	0	1	-
gi|29165615|ref|NC_002745.2|	577675 +	A	0	5	4	2	1	-
gi|29165615|ref|NC_002745.2|	577676 +	A	0	1	2	0	2	-
gi|29165615|ref|NC_002745.2|	577677 +	A	0	2	1	0	0	-
gi|29165615|ref|NC_002745.2|	577678 +	A	0	0	0	0	1	-
gi|29165615|ref|NC_002745.2|	577679 +	A	0	2	0	0	0	-
gi|29165615|ref|NC_002745.2|	577681 +	A	0	0	0	0	1	-
gi|29165615|ref|NC_002745.2|	577682 +	G	0	1	0	0	0	-
gi|29165615|ref|NC_002745.2|	577683 +	C	1	0	0	0	0	-
gi|29165615|ref|NC_002745.2|	577685 +	C	0	2	1	0	1	-
gi|29165615|ref|NC_002745.2|	577687 +	A	1	0	1	0	1	-
gi|29165615|ref|NC_002745.2|	577688 +	A	0	0	0	3	1	-
gi|29165615|ref|NC_002745.2|	577689 +	A	0	3	3	0	1	-
gi|29165615|ref|NC_002745.2|	577690 +	A	0	7	3	0	1	-
gi|29165615|ref|NC_002745.2|	577691 +	A	0	1	0	0	0	-
gi|29165615|ref|NC_002745.2|	577693 +	U	0	1	1	0	0	-
gi|29165615|ref|NC_002745.2|	577694 +	U	0	1	1	1	0	-
gi|29165615|ref|NC_002745.2|	577695 +	A	0	13	6	2	7	-
gi|29165615|ref|NC_002745.2|	577696 +	A	2	3	5	0	4	-
gi|29165615|ref|NC_002745.2|	577698 +	U	1	0	0	0	0	-
gi|29165615|ref|NC_002745.2|	577700 +	G	2	1	0	0	1	-
gi|29165615|ref|NC_002745.2|	577701 +	A	0	0	0	0	2	-
gi|29165615|ref|NC_002745.2|	577703 +	G	0	1	0	0	0	-
gi|29165615|ref|NC_002745.2|	577704 +	U	0	1	0	0	0	-
gi|29165615|ref|NC_002745.2|	577706 +	U	0	0	3	0	0	-
gi|29165615|ref|NC_002745.2|	577707 +	C	0	0	0	0	1	-
gi|29165615|ref|NC_002745.2|	577710 +	A	0	1	0	0	0	-
gi|29165615|ref|NC_002745.2|	577718 +	C	0	2	1	1	0	SA0497
gi|29165615|ref|NC_002745.2|	577719 +	U	2	0	0	0	0	SA0497
gi|29165615|ref|NC_002745.2|	577723 +	A	0	1	0	0	0	SA0497
gi|29165615|ref|NC_002745.2|	577727 +	A	1	0	0	0	2	SA0497
gi|29165615|ref|NC_002745.2|	577730 +	C	0	1	1	0	2	SA0497
gi|29165615|ref|NC_002745.2|	577732 +	A	0	1	0	0	0	SA0497
gi|29165615|ref|NC_002745.2|	577733 +	A	0	1	0	0	0	SA0497
gi|29165615|ref|NC_002745.2|	577734 +	A	0	3	0	2	4	SA0497
gi|29165615|ref|NC_002745.2|	577741 +	C	0	1	0	0	0	SA0497
gi|29165615|ref|NC_002745.2|	577744 +	G	0	1	0	0	0	SA0497
gi|29165615|ref|NC_002745.2|	577745 +	U	0	1	1	2	0	SA0497
gi|29165615|ref|NC_002745.2|	577747 +	G	0	0	1	0	0	SA0497
gi|29165615|ref|NC_002745.2|	577748 +	A	0	0	0	2	0	SA0497
gi|29165615|ref|NC_002745.2|	577751 +	A	0	0	0	1	0	SA0497
gi|29165615|ref|NC_002745.2|	577753 +	A	0	1	0	0	0	SA0497
gi|29165615|ref|NC_002745.2|	577754 +	U	4	0	0	0	0	SA0497
gi|29165615|ref|NC_002745.2|	577764 +	A	0	0	0	0	1	SA0497
gi|29165615|ref|NC_002745.2|	577783 +	A	0	1	0	0	0	SA0497
gi|29165615|ref|NC_002745.2|	577793 +	U	2	0	0	0	0	SA0497
gi|29165615|ref|NC_002745.2|	577796 +	A	0	1	0	0	0	SA0497
gi|29165615|ref|NC_002745.2|	577797 +	C	1	0	0	0	0	SA0497
gi|29165615|ref|NC_002745.2|	577802 +	G	4	0	0	0	0	SA0497
gi|29165615|ref|NC_002745.2|	577814 +	U	1	0	0	0	0	SA0497
gi|29165615|ref|NC_002745.2|	577822 +	G	0	0	0	0	1	SA0497
gi|29165615|ref|NC_002745.2|	577832 +	U	1	0	0	0	0	SA0497
gi|29165615|ref|NC_002745.2|	577848 +	U	0	0	1	0	0	SA0497
gi|29165615|ref|NC_002745.2|	577852 +	G	0	0	1	0	0	SA0497
gi|29165615|ref|NC_002745.2|	577860 +	U	0	0	1	0	0	SA0497
gi|29165615|ref|NC_002745.2|	577866 +	U	1	0	0	0	0	SA0497
gi|29165615|ref|NC_002745.2|	577871 +	U	0	0	1	0	0	SA0497
gi|29165615|ref|NC_002745.2|	577873 +	U	1	0	0	0	0	SA0497
gi|29165615|ref|NC_002745.2|	577875 +	C	0	0	0	0	1	SA0497
gi|29165615|ref|NC_002745.2|	577884 +	U	2	0	0	0	0	SA0497
gi|29165615|ref|NC_002745.2|	577919 +	A	0	1	0	0	0	SA0497
gi|29165615|ref|NC_002745.2|	577921 +	G	1	0	0	0	0	SA0497
gi|29165615|ref|NC_002745.2|	577928 +	A	1	0	0	0	1	SA0497
gi|29165615|ref|NC_002745.2|	577936 +	U	1	0	0	0	0	SA0497
gi|29165615|ref|NC_002745.2|	577937 +	U	2	0	0	0	0	SA0497
gi|29165615|ref|NC_002745.2|	577938 +	A	0	1	0	0	0	SA0497
gi|29165615|ref|NC_002745.2|	577943 +	G	0	0	1	0	0	SA0497
gi|29165615|ref|NC_002745.2|	577947 +	U	5	0	0	0	0	SA0497
gi|29165615|ref|NC_002745.2|	577949 +	C	1	0	0	0	0	SA0497
gi|29165615|ref|NC_002745.2|	577952 +	C	2	0	0	0	0	SA0497
gi|29165615|ref|NC_002745.2|	577964 +	C	2	1	0	1	2	SA0497
gi|29165615|ref|NC_002745.2|	577966 +	A	0	1	0	0	1	SA0497
gi|29165615|ref|NC_002745.2|	577968 +	U	0	0	1	0	0	SA0497
gi|29165615|ref|NC_002745.2|	577973 +	A	1	0	0	0	0	SA0497
gi|29165615|ref|NC_002745.2|	577979 +	U	4	0	0	0	0	SA0497
gi|29165615|ref|NC_002745.2|	577982 +	C	1	0	0	0	0	SA0497
gi|29165615|ref|NC_002745.2|	577987 +	G	1	0	0	0	0	SA0497
gi|29165615|ref|NC_002745.2|	577988 +	C	1	0	0	0	0	SA0497
gi|29165615|ref|NC_002745.2|	577990 +	A	0	0	0	1	0	SA0497
gi|29165615|ref|NC_002745.2|	577994 +	U	0	1	0	0	0	SA0497
gi|29165615|ref|NC_002745.2|	577995 +	A	0	0	2	0	0	SA0497

gi|29165615|ref|NC_002745.2|	577996	+	A	0	1	0	0	1	SA0497
gi|29165615|ref|NC_002745.2|	577997	+	U	1	0	0	0	0	SA0497
gi|29165615|ref|NC_002745.2|	578009	+	C	0	1	0	1	0	SA0497
gi|29165615|ref|NC_002745.2|	578012	+	A	1	0	0	0	0	SA0497
gi|29165615|ref|NC_002745.2|	578023	+	G	1	0	0	0	0	SA0497
gi|29165615|ref|NC_002745.2|	578024	+	C	1	0	0	0	0	SA0497
gi|29165615|ref|NC_002745.2|	578029	+	G	0	0	0	0	1	SA0497
gi|29165615|ref|NC_002745.2|	578030	+	A	0	1	0	0	0	SA0497
gi|29165615|ref|NC_002745.2|	578042	+	G	1	0	0	0	0	SA0497
gi|29165615|ref|NC_002745.2|	578046	+	U	0	2	0	0	0	SA0497
gi|29165615|ref|NC_002745.2|	578047	+	A	2	0	0	0	0	SA0497
gi|29165615|ref|NC_002745.2|	578048	+	U	2	0	0	1	0	SA0497
gi|29165615|ref|NC_002745.2|	578051	+	A	0	1	0	0	0	SA0497
gi|29165615|ref|NC_002745.2|	578052	+	A	1	0	0	0	2	SA0497
gi|29165615|ref|NC_002745.2|	578055	+	C	0	2	0	0	0	SA0497
gi|29165615|ref|NC_002745.2|	578068	+	G	0	0	0	0	1	SA0497
gi|29165615|ref|NC_002745.2|	578075	+	A	1	0	0	0	0	SA0497
gi|29165615|ref|NC_002745.2|	578085	+	U	1	0	0	0	1	SA0497
gi|29165615|ref|NC_002745.2|	578091	+	U	1	1	0	0	0	SA0497
gi|29165615|ref|NC_002745.2|	578095	+	U	0	1	0	0	0	SA0497
gi|29165615|ref|NC_002745.2|	578096	+	U	0	1	0	0	0	SA0497
gi|29165615|ref|NC_002745.2|	578100	+	U	2	0	0	0	0	SA0497
gi|29165615|ref|NC_002745.2|	578106	+	C	1	0	0	0	0	SA0497
gi|29165615|ref|NC_002745.2|	578111	+	G	3	0	0	0	0	SA0497
gi|29165615|ref|NC_002745.2|	578117	+	U	0	1	0	0	0	SA0497
gi|29165615|ref|NC_002745.2|	578125	+	C	0	0	3	0	0	SA0497
gi|29165615|ref|NC_002745.2|	578131	+	U	0	1	2	0	0	SA0497
gi|29165615|ref|NC_002745.2|	578132	+	C	0	0	1	0	0	SA0497
gi|29165615|ref|NC_002745.2|	578133	+	A	0	0	0	2	0	SA0497
gi|29165615|ref|NC_002745.2|	578135	+	U	1	2	8	2	3	SA0497
gi|29165615|ref|NC_002745.2|	578136	+	A	0	3	28	4	6	SA0497
gi|29165615|ref|NC_002745.2|	578137	+	U	1	0	1	0	0	SA0497
gi|29165615|ref|NC_002745.2|	578139	+	A	0	0	1	0	0	SA0497
gi|29165615|ref|NC_002745.2|	578140	+	C	0	0	1	0	0	SA0497
gi|29165615|ref|NC_002745.2|	578145	+	U	1	0	0	0	0	SA0497
gi|29165615|ref|NC_002745.2|	578147	+	C	2	0	0	0	0	SA0497
gi|29165615|ref|NC_002745.2|	578150	+	U	1	0	2	0	0	SA0497
gi|29165615|ref|NC_002745.2|	578153	+	G	0	0	0	0	1	SA0497
gi|29165615|ref|NC_002745.2|	578157	+	C	0	1	1	0	0	SA0497
gi|29165615|ref|NC_002745.2|	578158	+	U	0	0	1	0	0	SA0497
gi|29165615|ref|NC_002745.2|	578162	+	C	1	3	3	2	0	SA0497
gi|29165615|ref|NC_002745.2|	578163	+	U	1	0	0	0	0	SA0497
gi|29165615|ref|NC_002745.2|	578165	+	A	1	0	0	0	0	SA0497
gi|29165615|ref|NC_002745.2|	578168	+	C	0	2	1	0	0	SA0497
gi|29165615|ref|NC_002745.2|	578170	+	G	0	1	0	0	0	SA0497
gi|29165615|ref|NC_002745.2|	578173	+	A	0	1	1	0	0	SA0497
gi|29165615|ref|NC_002745.2|	578183	+	G	0	1	0	0	0	SA0497
gi|29165615|ref|NC_002745.2|	578184	+	A	0	0	0	1	0	SA0497
gi|29165615|ref|NC_002745.2|	578186	+	A	0	1	0	0	0	SA0497
gi|29165615|ref|NC_002745.2|	578189	+	A	0	0	0	0	1	SA0497
gi|29165615|ref|NC_002745.2|	578193	+	A	0	0	1	0	0	SA0497
gi|29165615|ref|NC_002745.2|	578194	+	G	0	2	0	0	0	SA0497
gi|29165615|ref|NC_002745.2|	578195	+	A	0	1	1	1	0	SA0497
gi|29165615|ref|NC_002745.2|	578202	+	C	1	0	0	0	0	SA0497
gi|29165615|ref|NC_002745.2|	578213	+	U	0	0	0	0	1	-
gi|29165615|ref|NC_002745.2|	578227	+	A	0	2	0	0	0	-
gi|29165615|ref|NC_002745.2|	578228	+	A	0	4	2	3	2	-
gi|29165615|ref|NC_002745.2|	578229	+	U	0	0	0	0	1	-
gi|29165615|ref|NC_002745.2|	578230	+	U	1	0	0	0	0	-
gi|29165615|ref|NC_002745.2|	578231	+	A	0	0	1	0	1	-
gi|29165615|ref|NC_002745.2|	578232	+	A	1	7	6	6	4	-
gi|29165615|ref|NC_002745.2|	578233	+	A	0	0	1	1	1	-
gi|29165615|ref|NC_002745.2|	578234	+	A	0	0	1	0	0	-
gi|29165615|ref|NC_002745.2|	578235	+	A	1	11	9	0	11	-
gi|29165615|ref|NC_002745.2|	578236	+	U	2	0	0	1	0	-
gi|29165615|ref|NC_002745.2|	578237	+	A	0	0	1	0	0	-
gi|29165615|ref|NC_002745.2|	578238	+	A	0	1	5	0	3	-
gi|29165615|ref|NC_002745.2|	578239	+	U	0	0	0	1	1	-
gi|29165615|ref|NC_002745.2|	578243	+	G	1	0	1	0	0	-
gi|29165615|ref|NC_002745.2|	578244	+	G	0	0	4	0	2	-
gi|29165615|ref|NC_002745.2|	578245	+	A	0	5	1	0	2	-
gi|29165615|ref|NC_002745.2|	578246	+	A	0	1	1	0	0	-
gi|29165615|ref|NC_002745.2|	578249	+	A	0	0	0	0	1	-
gi|29165615|ref|NC_002745.2|	578253	+	A	0	1	0	0	0	-
gi|29165615|ref|NC_002745.2|	578254	+	A	1	6	4	0	7	SA0498
gi|29165615|ref|NC_002745.2|	578257	+	G	0	1	0	0	0	SA0498
gi|29165615|ref|NC_002745.2|	578258	+	C	0	1	2	0	0	SA0498
gi|29165615|ref|NC_002745.2|	578263	+	C	0	2	0	0	0	SA0498
gi|29165615|ref|NC_002745.2|	578265	+	U	0	0	1	0	0	SA0498
gi|29165615|ref|NC_002745.2|	578266	+	G	0	0	2	0	1	SA0498
gi|29165615|ref|NC_002745.2|	578267	+	A	0	1	0	0	1	SA0498

gi|29165615|ref|NC_002745.2|	578268	+	A	1	0	0	0	0	SA0498
gi|29165615|ref|NC_002745.2|	578271	+	A	0	0	0	1	0	SA0498
gi|29165615|ref|NC_002745.2|	578272	+	A	0	1	5	0	0	SA0498
gi|29165615|ref|NC_002745.2|	578273	+	U	0	1	0	0	0	SA0498
gi|29165615|ref|NC_002745.2|	578274	+	C	0	0	2	0	0	SA0498
gi|29165615|ref|NC_002745.2|	578275	+	A	0	0	1	0	0	SA0498
gi|29165615|ref|NC_002745.2|	578276	+	U	0	0	1	0	0	SA0498
gi|29165615|ref|NC_002745.2|	578278	+	G	1	1	0	0	1	SA0498
gi|29165615|ref|NC_002745.2|	578282	+	C	1	0	1	1	1	SA0498
gi|29165615|ref|NC_002745.2|	578283	+	G	1	0	2	0	0	SA0498
gi|29165615|ref|NC_002745.2|	578284	+	A	0	1	1	0	1	SA0498
gi|29165615|ref|NC_002745.2|	578287	+	A	0	0	1	0	2	SA0498
gi|29165615|ref|NC_002745.2|	578288	+	A	0	1	0	0	1	SA0498
gi|29165615|ref|NC_002745.2|	578290	+	G	0	0	0	0	2	SA0498
gi|29165615|ref|NC_002745.2|	578291	+	A	1	3	0	0	0	SA0498
gi|29165615|ref|NC_002745.2|	578292	+	A	0	0	0	0	1	SA0498
gi|29165615|ref|NC_002745.2|	578296	+	U	0	2	0	0	0	SA0498
gi|29165615|ref|NC_002745.2|	578297	+	C	3	0	1	0	0	SA0498
gi|29165615|ref|NC_002745.2|	578298	+	A	0	0	0	0	1	SA0498
gi|29165615|ref|NC_002745.2|	578299	+	G	0	1	0	0	0	SA0498
gi|29165615|ref|NC_002745.2|	578300	+	U	0	0	0	1	1	SA0498
gi|29165615|ref|NC_002745.2|	578301	+	A	0	1	0	0	0	SA0498
gi|29165615|ref|NC_002745.2|	578303	+	U	0	0	2	0	1	SA0498
gi|29165615|ref|NC_002745.2|	578304	+	A	0	3	2	1	1	SA0498
gi|29165615|ref|NC_002745.2|	578305	+	G	0	5	1	0	2	SA0498
gi|29165615|ref|NC_002745.2|	578306	+	A	1	6	3	1	2	SA0498
gi|29165615|ref|NC_002745.2|	578307	+	A	0	2	0	0	0	SA0498
gi|29165615|ref|NC_002745.2|	578309	+	U	0	0	2	0	1	SA0498
gi|29165615|ref|NC_002745.2|	578311	+	A	0	0	0	0	1	SA0498
gi|29165615|ref|NC_002745.2|	578313	+	C	0	0	1	0	0	SA0498
gi|29165615|ref|NC_002745.2|	578314	+	G	0	1	0	0	0	SA0498
gi|29165615|ref|NC_002745.2|	578315	+	A	0	0	1	0	1	SA0498
gi|29165615|ref|NC_002745.2|	578316	+	C	0	1	0	0	0	SA0498
gi|29165615|ref|NC_002745.2|	578319	+	A	0	3	0	1	0	SA0498
gi|29165615|ref|NC_002745.2|	578320	+	G	0	1	0	0	0	SA0498
gi|29165615|ref|NC_002745.2|	578322	+	A	0	3	0	0	0	SA0498
gi|29165615|ref|NC_002745.2|	578323	+	A	0	1	4	1	0	SA0498
gi|29165615|ref|NC_002745.2|	578324	+	A	0	0	0	0	1	SA0498
gi|29165615|ref|NC_002745.2|	578326	+	G	0	1	0	0	0	SA0498
gi|29165615|ref|NC_002745.2|	578327	+	C	0	2	1	0	3	SA0498
gi|29165615|ref|NC_002745.2|	578328	+	A	1	5	1	0	0	SA0498
gi|29165615|ref|NC_002745.2|	578329	+	A	0	6	3	1	3	SA0498
gi|29165615|ref|NC_002745.2|	578331	+	U	1	0	0	0	0	SA0498
gi|29165615|ref|NC_002745.2|	578332	+	G	0	0	0	0	2	SA0498
gi|29165615|ref|NC_002745.2|	578333	+	A	1	2	2	0	0	SA0498
gi|29165615|ref|NC_002745.2|	578334	+	A	0	1	1	0	0	SA0498
gi|29165615|ref|NC_002745.2|	578335	+	G	0	1	0	0	5	SA0498
gi|29165615|ref|NC_002745.2|	578336	+	A	0	2	0	0	0	SA0498
gi|29165615|ref|NC_002745.2|	578337	+	A	0	1	0	0	1	SA0498
gi|29165615|ref|NC_002745.2|	578338	+	G	0	7	4	1	6	SA0498
gi|29165615|ref|NC_002745.2|	578339	+	A	0	15	8	1	5	SA0498
gi|29165615|ref|NC_002745.2|	578340	+	A	0	0	1	0	0	SA0498
gi|29165615|ref|NC_002745.2|	578341	+	U	0	1	0	0	0	SA0498
gi|29165615|ref|NC_002745.2|	578342	+	U	2	0	0	0	0	SA0498
gi|29165615|ref|NC_002745.2|	578343	+	U	1	0	0	0	0	SA0498
gi|29165615|ref|NC_002745.2|	578345	+	G	5	0	0	1	0	SA0498
gi|29165615|ref|NC_002745.2|	578348	+	U	8	0	0	0	1	SA0498
gi|29165615|ref|NC_002745.2|	578349	+	A	1	0	0	0	0	SA0498
gi|29165615|ref|NC_002745.2|	578350	+	A	0	0	1	0	0	SA0498
gi|29165615|ref|NC_002745.2|	578351	+	C	2	0	0	0	0	SA0498
gi|29165615|ref|NC_002745.2|	578352	+	U	1	0	0	0	0	SA0498
gi|29165615|ref|NC_002745.2|	578355	+	A	0	0	0	1	0	SA0498
gi|29165615|ref|NC_002745.2|	578356	+	G	1	0	0	0	0	SA0498
gi|29165615|ref|NC_002745.2|	578357	+	C	4	0	0	0	0	SA0498
gi|29165615|ref|NC_002745.2|	578359	+	G	0	0	0	0	1	SA0498
gi|29165615|ref|NC_002745.2|	578360	+	C	1	0	0	0	0	SA0498
gi|29165615|ref|NC_002745.2|	578366	+	U	2	0	0	0	0	SA0498
gi|29165615|ref|NC_002745.2|	578371	+	G	0	4	0	0	0	SA0498
gi|29165615|ref|NC_002745.2|	578372	+	U	3	0	0	0	0	SA0498
gi|29165615|ref|NC_002745.2|	578380	+	G	1	0	0	0	0	SA0498
gi|29165615|ref|NC_002745.2|	578381	+	C	1	0	0	0	0	SA0498
gi|29165615|ref|NC_002745.2|	578383	+	G	1	0	0	0	0	SA0498
gi|29165615|ref|NC_002745.2|	578384	+	C	2	0	0	0	0	SA0498
gi|29165615|ref|NC_002745.2|	578385	+	U	0	0	2	0	0	SA0498
gi|29165615|ref|NC_002745.2|	578387	+	G	6	0	0	0	1	SA0498
gi|29165615|ref|NC_002745.2|	578388	+	U	3	0	0	0	0	SA0498
gi|29165615|ref|NC_002745.2|	578390	+	G	5	1	1	0	1	SA0498
gi|29165615|ref|NC_002745.2|	578391	+	C	0	0	0	1	0	SA0498
gi|29165615|ref|NC_002745.2|	578392	+	G	0	0	0	0	1	SA0498
gi|29165615|ref|NC_002745.2|	578393	+	C	2	0	0	0	0	SA0498
gi|29165615|ref|NC_002745.2|	578395	+	G	1	0	0	0	1	SA0498

gi|29165615|ref|NC_002745.2|	578396	+	A	0	1	0	0	0	SA0498
gi|29165615|ref|NC_002745.2|	578399	+	C	1	0	0	0	0	SA0498
gi|29165615|ref|NC_002745.2|	578405	+	C	0	0	1	0	0	SA0498
gi|29165615|ref|NC_002745.2|	578417	+	A	0	1	0	0	0	SA0498
gi|29165615|ref|NC_002745.2|	578422	+	G	0	0	0	0	1	SA0498
gi|29165615|ref|NC_002745.2|	578423	+	A	0	0	0	0	1	SA0498
gi|29165615|ref|NC_002745.2|	578426	+	U	1	0	0	0	0	SA0498
gi|29165615|ref|NC_002745.2|	578428	+	G	0	1	0	0	0	SA0498
gi|29165615|ref|NC_002745.2|	578435	+	C	1	0	0	0	0	SA0498
gi|29165615|ref|NC_002745.2|	578437	+	U	0	1	0	0	0	SA0498
gi|29165615|ref|NC_002745.2|	578447	+	C	0	0	1	0	0	SA0498
gi|29165615|ref|NC_002745.2|	578453	+	A	0	2	1	0	0	SA0498
gi|29165615|ref|NC_002745.2|	578454	+	A	0	1	0	0	0	SA0498
gi|29165615|ref|NC_002745.2|	578459	+	A	0	1	0	0	0	SA0498
gi|29165615|ref|NC_002745.2|	578462	+	U	0	0	1	0	0	SA0498
gi|29165615|ref|NC_002745.2|	578464	+	G	0	1	1	0	0	SA0498
gi|29165615|ref|NC_002745.2|	578466	+	U	1	0	0	0	0	SA0498
gi|29165615|ref|NC_002745.2|	578469	+	A	0	1	0	0	0	SA0498
gi|29165615|ref|NC_002745.2|	578493	+	A	1	0	0	0	0	SA0498
gi|29165615|ref|NC_002745.2|	578528	+	C	0	0	1	0	0	SA0498
gi|29165615|ref|NC_002745.2|	578537	+	U	0	0	1	0	0	SA0498
gi|29165615|ref|NC_002745.2|	578564	+	A	0	1	0	0	0	SA0498
gi|29165615|ref|NC_002745.2|	578576	+	U	0	0	0	0	1	SA0498
gi|29165615|ref|NC_002745.2|	578636	+	A	0	0	1	0	0	-
gi|29165615|ref|NC_002745.2|	578735	+	A	0	0	1	0	0	-
gi|29165615|ref|NC_002745.2|	578736	+	A	0	1	1	0	1	-
gi|29165615|ref|NC_002745.2|	578737	+	A	0	0	1	0	0	-
gi|29165615|ref|NC_002745.2|	578739	+	A	0	0	0	0	1	-
gi|29165615|ref|NC_002745.2|	578743	+	A	0	1	0	0	1	-
gi|29165615|ref|NC_002745.2|	578746	+	G	0	1	0	0	0	-
gi|29165615|ref|NC_002745.2|	578757	+	A	0	2	0	0	0	-
gi|29165615|ref|NC_002745.2|	578762	+	A	0	1	0	0	0	-
gi|29165615|ref|NC_002745.2|	578765	+	G	0	2	2	0	4	-
gi|29165615|ref|NC_002745.2|	578777	+	A	0	0	1	0	0	-
gi|29165615|ref|NC_002745.2|	578787	+	A	1	0	0	0	0	-
gi|29165615|ref|NC_002745.2|	578795	+	U	0	0	0	1	0	-
gi|29165615|ref|NC_002745.2|	578867	+	A	1	0	0	0	0	SA0499
gi|29165615|ref|NC_002745.2|	578914	+	A	0	1	0	0	0	SA0499
gi|29165615|ref|NC_002745.2|	578915	+	A	0	0	1	0	0	SA0499
gi|29165615|ref|NC_002745.2|	578965	+	C	0	1	0	0	0	SA0499
gi|29165615|ref|NC_002745.2|	578967	+	A	0	0	1	0	0	SA0499
gi|29165615|ref|NC_002745.2|	579011	+	G	1	0	0	0	0	SA0499
gi|29165615|ref|NC_002745.2|	579015	+	A	1	0	0	0	0	SA0499
gi|29165615|ref|NC_002745.2|	579041	+	C	1	0	0	0	0	SA0499
gi|29165615|ref|NC_002745.2|	579087	+	A	1	0	0	0	0	SA0499
gi|29165615|ref|NC_002745.2|	579114	+	A	0	1	0	0	0	SA0499
gi|29165615|ref|NC_002745.2|	579115	+	A	0	1	0	0	0	SA0499
gi|29165615|ref|NC_002745.2|	579116	+	A	0	0	1	0	0	SA0499
gi|29165615|ref|NC_002745.2|	579123	+	U	0	1	0	0	0	SA0499
gi|29165615|ref|NC_002745.2|	579127	+	A	0	0	0	1	0	SA0499
gi|29165615|ref|NC_002745.2|	579138	+	A	0	0	0	0	1	SA0499
gi|29165615|ref|NC_002745.2|	579139	+	A	0	1	0	0	0	SA0499
gi|29165615|ref|NC_002745.2|	579142	+	G	0	0	1	0	0	SA0499
gi|29165615|ref|NC_002745.2|	579172	+	A	0	0	0	0	1	SA0499
gi|29165615|ref|NC_002745.2|	579177	+	U	0	0	1	0	1	SA0499
gi|29165615|ref|NC_002745.2|	579197	+	A	0	0	0	0	2	SA0499
gi|29165615|ref|NC_002745.2|	579212	+	C	0	0	1	0	0	SA0499
gi|29165615|ref|NC_002745.2|	579252	+	A	0	0	1	0	0	SA0499
gi|29165615|ref|NC_002745.2|	579295	+	A	0	2	0	0	2	SA0499
gi|29165615|ref|NC_002745.2|	579296	+	A	0	0	0	0	1	SA0499
gi|29165615|ref|NC_002745.2|	579364	+	A	1	0	0	0	0	SA0499
gi|29165615|ref|NC_002745.2|	579405	+	A	0	0	0	0	1	SA0499
gi|29165615|ref|NC_002745.2|	579416	+	A	0	0	0	1	0	-
gi|29165615|ref|NC_002745.2|	579435	+	U	0	0	0	0	1	-
gi|29165615|ref|NC_002745.2|	579436	+	A	1	4	3	0	1	-
gi|29165615|ref|NC_002745.2|	579437	+	A	11	13	18	12	12	-
gi|29165615|ref|NC_002745.2|	579438	+	U	2	1	0	1	2	-
gi|29165615|ref|NC_002745.2|	579439	+	A	9	26	25	22	29	-
gi|29165615|ref|NC_002745.2|	579440	+	U	0	0	1	0	0	-
gi|29165615|ref|NC_002745.2|	579441	+	A	0	2	2	2	0	-
gi|29165615|ref|NC_002745.2|	579442	+	A	0	2	1	1	0	-
gi|29165615|ref|NC_002745.2|	579444	+	A	0	0	1	0	0	-
gi|29165615|ref|NC_002745.2|	579445	+	A	0	1	2	0	0	-
gi|29165615|ref|NC_002745.2|	579448	+	U	0	1	0	1	0	-
gi|29165615|ref|NC_002745.2|	579449	+	A	1	0	1	0	1	-
gi|29165615|ref|NC_002745.2|	579450	+	U	0	1	0	0	2	-
gi|29165615|ref|NC_002745.2|	579451	+	G	0	1	3	0	1	-
gi|29165615|ref|NC_002745.2|	579452	+	U	0	2	3	0	1	-
gi|29165615|ref|NC_002745.2|	579453	+	U	0	1	3	0	1	-
gi|29165615|ref|NC_002745.2|	579454	+	C	0	0	1	0	1	-
gi|29165615|ref|NC_002745.2|	579455	+	A	0	0	0	0	1	-

gi|29165615|ref|NC_002745.2|	579456	+	A	0	0	1	0	0	-
gi|29165615|ref|NC_002745.2|	579458	+	A	0	3	0	1	0	-
gi|29165615|ref|NC_002745.2|	579470	+	U	0	0	0	0	1	-
gi|29165615|ref|NC_002745.2|	579477	+	A	0	0	1	0	0	-
gi|29165615|ref|NC_002745.2|	579478	+	A	0	0	0	0	1	-
gi|29165615|ref|NC_002745.2|	579483	+	A	0	0	0	1	0	-
gi|29165615|ref|NC_002745.2|	579484	+	U	0	0	0	0	1	-
gi|29165615|ref|NC_002745.2|	579490	+	A	0	3	2	2	0	-
gi|29165615|ref|NC_002745.2|	579496	+	G	0	1	3	0	1	-
gi|29165615|ref|NC_002745.2|	579497	+	A	0	0	1	0	1	-
gi|29165615|ref|NC_002745.2|	579498	+	A	2	0	1	0	0	-
gi|29165615|ref|NC_002745.2|	579500	+	A	0	3	0	0	0	-
gi|29165615|ref|NC_002745.2|	579501	+	A	0	4	3	0	0	-
gi|29165615|ref|NC_002745.2|	579502	+	A	0	1	0	0	0	-
gi|29165615|ref|NC_002745.2|	579503	+	U	0	2	1	0	0	-
gi|29165615|ref|NC_002745.2|	579508	+	A	0	0	1	1	0	-
gi|29165615|ref|NC_002745.2|	579509	+	A	0	1	0	0	0	-
gi|29165615|ref|NC_002745.2|	579513	+	A	0	1	1	1	0	-
gi|29165615|ref|NC_002745.2|	579514	+	A	0	3	3	0	2	-
gi|29165615|ref|NC_002745.2|	579515	+	A	0	11	16	4	4	-
gi|29165615|ref|NC_002745.2|	579516	+	A	0	0	2	1	0	-
gi|29165615|ref|NC_002745.2|	579521	+	G	1	0	0	0	0	-
gi|29165615|ref|NC_002745.2|	579525	+	U	0	0	1	0	0	-
gi|29165615|ref|NC_002745.2|	579526	+	C	0	0	1	0	0	-
gi|29165615|ref|NC_002745.2|	579527	+	A	1	0	1	0	0	-
gi|29165615|ref|NC_002745.2|	579529	+	U	1	1	1	1	0	-
gi|29165615|ref|NC_002745.2|	579531	+	G	0	1	0	0	0	-
gi|29165615|ref|NC_002745.2|	579533	+	G	0	0	0	0	1	-
gi|29165615|ref|NC_002745.2|	579550	+	G	0	1	0	0	0	-
gi|29165615|ref|NC_002745.2|	579552	+	C	0	1	0	0	0	-
gi|29165615|ref|NC_002745.2|	579558	+	A	0	0	0	1	0	-
gi|29165615|ref|NC_002745.2|	579560	+	U	0	1	0	0	0	-
gi|29165615|ref|NC_002745.2|	579561	+	A	0	1	0	0	0	-
gi|29165615|ref|NC_002745.2|	579562	+	A	0	0	0	0	1	-
gi|29165615|ref|NC_002745.2|	579566	+	G	0	0	1	0	0	-
gi|29165615|ref|NC_002745.2|	579567	+	C	0	0	2	1	0	-
gi|29165615|ref|NC_002745.2|	579574	+	U	0	0	0	0	1	-
gi|29165615|ref|NC_002745.2|	579577	+	U	0	0	0	0	1	-
gi|29165615|ref|NC_002745.2|	579593	+	C	0	0	0	0	1	-
gi|29165615|ref|NC_002745.2|	579597	+	U	0	1	2	0	0	-
gi|29165615|ref|NC_002745.2|	579598	+	A	0	0	0	0	1	-
gi|29165615|ref|NC_002745.2|	579613	+	A	0	0	0	1	0	-
gi|29165615|ref|NC_002745.2|	579676	+	A	0	0	1	0	0	SA0500
gi|29165615|ref|NC_002745.2|	579687	+	U	1	0	1	0	0	SA0500
gi|29165615|ref|NC_002745.2|	579690	+	U	0	0	0	0	1	SA0500
gi|29165615|ref|NC_002745.2|	579707	+	A	0	0	1	0	0	SA0500
gi|29165615|ref|NC_002745.2|	579783	+	U	0	1	0	0	0	SA0500
gi|29165615|ref|NC_002745.2|	579786	+	A	0	0	1	0	0	SA0500
gi|29165615|ref|NC_002745.2|	579788	+	G	0	0	2	0	0	SA0500
gi|29165615|ref|NC_002745.2|	579789	+	A	0	0	1	0	0	SA0500
gi|29165615|ref|NC_002745.2|	579795	+	C	0	0	1	1	0	SA0500
gi|29165615|ref|NC_002745.2|	579807	+	C	0	0	2	0	0	SA0500
gi|29165615|ref|NC_002745.2|	579811	+	A	0	0	1	0	0	SA0500
gi|29165615|ref|NC_002745.2|	579822	+	A	0	0	1	0	0	SA0500
gi|29165615|ref|NC_002745.2|	579824	+	U	0	0	0	1	0	SA0500
gi|29165615|ref|NC_002745.2|	579836	+	G	0	0	0	1	0	SA0500
gi|29165615|ref|NC_002745.2|	579841	+	A	0	0	0	0	1	SA0500
gi|29165615|ref|NC_002745.2|	579848	+	G	0	0	0	1	0	SA0500
gi|29165615|ref|NC_002745.2|	579849	+	A	0	1	0	0	0	SA0500
gi|29165615|ref|NC_002745.2|	579853	+	A	0	0	0	0	1	SA0500
gi|29165615|ref|NC_002745.2|	579867	+	A	0	1	0	0	0	SA0500
gi|29165615|ref|NC_002745.2|	579869	+	C	1	0	1	0	0	SA0500
gi|29165615|ref|NC_002745.2|	579870	+	G	1	0	0	0	0	SA0500
gi|29165615|ref|NC_002745.2|	579883	+	U	0	1	0	0	0	SA0500
gi|29165615|ref|NC_002745.2|	579894	+	U	0	0	1	0	0	SA0500
gi|29165615|ref|NC_002745.2|	579904	+	A	0	1	0	0	0	SA0500
gi|29165615|ref|NC_002745.2|	579954	+	U	2	0	0	0	0	SA0500
gi|29165615|ref|NC_002745.2|	579966	+	U	2	0	1	0	0	SA0500
gi|29165615|ref|NC_002745.2|	579974	+	A	0	1	0	0	0	SA0500
gi|29165615|ref|NC_002745.2|	579990	+	C	1	0	0	0	0	SA0500
gi|29165615|ref|NC_002745.2|	580003	+	U	0	0	0	1	1	SA0500
gi|29165615|ref|NC_002745.2|	580043	+	C	1	0	0	0	0	SA0500
gi|29165615|ref|NC_002745.2|	580057	+	C	0	1	0	0	1	SA0500
gi|29165615|ref|NC_002745.2|	580067	+	A	1	8	2	1	4	SA0500
gi|29165615|ref|NC_002745.2|	580068	+	U	0	1	0	0	0	SA0500
gi|29165615|ref|NC_002745.2|	580073	+	A	0	0	0	0	1	SA0500
gi|29165615|ref|NC_002745.2|	580083	+	G	0	0	1	0	0	SA0500
gi|29165615|ref|NC_002745.2|	580090	+	C	0	0	1	0	0	SA0500
gi|29165615|ref|NC_002745.2|	580112	+	A	0	0	0	0	2	SA0500
gi|29165615|ref|NC_002745.2|	580115	+	C	2	0	0	0	0	SA0500
gi|29165615|ref|NC_002745.2|	580123	+	A	1	0	0	0	0	SA0500

gi|29165615|ref|NC_002745.2|	580133 +	U	0	1	0	0	0	SA0500
gi|29165615|ref|NC_002745.2|	580143 +	A	0	2	0	0	0	SA0500
gi|29165615|ref|NC_002745.2|	580152 +	A	0	0	1	0	0	SA0500
gi|29165615|ref|NC_002745.2|	580171 +	U	1	0	0	0	0	SA0500
gi|29165615|ref|NC_002745.2|	580174 +	U	0	1	0	0	0	SA0500
gi|29165615|ref|NC_002745.2|	580190 +	C	0	0	1	0	0	SA0500
gi|29165615|ref|NC_002745.2|	580191 +	C	2	0	0	0	0	SA0500
gi|29165615|ref|NC_002745.2|	580194 +	U	0	2	0	0	0	SA0500
gi|29165615|ref|NC_002745.2|	580195 +	A	0	0	0	0	1	SA0500
gi|29165615|ref|NC_002745.2|	580203 +	U	1	0	0	0	0	SA0500
gi|29165615|ref|NC_002745.2|	580242 +	U	0	1	0	0	0	SA0500
gi|29165615|ref|NC_002745.2|	580263 +	A	0	2	3	1	2	SA0500
gi|29165615|ref|NC_002745.2|	580289 +	G	1	0	0	0	0	SA0500
gi|29165615|ref|NC_002745.2|	580291 +	C	0	0	0	0	1	SA0500
gi|29165615|ref|NC_002745.2|	580325 +	A	0	1	0	0	0	SA0500
gi|29165615|ref|NC_002745.2|	580344 +	C	1	0	0	0	0	SA0500
gi|29165615|ref|NC_002745.2|	580359 +	C	0	0	1	0	0	SA0500
gi|29165615|ref|NC_002745.2|	580368 +	A	0	1	0	0	0	SA0500
gi|29165615|ref|NC_002745.2|	580389 +	C	1	0	0	0	0	SA0500
gi|29165615|ref|NC_002745.2|	580405 +	A	0	0	0	0	1	SA0500
gi|29165615|ref|NC_002745.2|	580412 +	U	0	0	0	0	1	SA0500
gi|29165615|ref|NC_002745.2|	580443 +	C	0	1	0	0	1	SA0500
gi|29165615|ref|NC_002745.2|	580444 +	A	0	0	2	0	0	SA0500
gi|29165615|ref|NC_002745.2|	580445 +	A	0	0	0	0	1	SA0500
gi|29165615|ref|NC_002745.2|	580447 +	C	0	0	0	1	1	SA0500
gi|29165615|ref|NC_002745.2|	580448 +	A	0	2	0	0	0	SA0500
gi|29165615|ref|NC_002745.2|	580450 +	A	0	0	0	1	1	SA0500
gi|29165615|ref|NC_002745.2|	580451 +	A	0	0	1	0	0	SA0500
gi|29165615|ref|NC_002745.2|	580452 +	A	1	2	0	0	1	SA0500
gi|29165615|ref|NC_002745.2|	580453 +	A	0	0	1	0	0	SA0500
gi|29165615|ref|NC_002745.2|	580456 +	A	0	1	0	0	0	SA0500
gi|29165615|ref|NC_002745.2|	580457 +	C	0	4	5	1	0	SA0500
gi|29165615|ref|NC_002745.2|	580458 +	A	0	0	0	0	1	SA0500
gi|29165615|ref|NC_002745.2|	580462 +	A	0	0	1	0	1	SA0500
gi|29165615|ref|NC_002745.2|	580463 +	A	0	0	1	0	0	SA0500
gi|29165615|ref|NC_002745.2|	580464 +	A	0	0	1	0	0	SA0500
gi|29165615|ref|NC_002745.2|	580465 +	A	0	2	0	1	0	SA0500
gi|29165615|ref|NC_002745.2|	580471 +	U	0	1	1	1	2	SA0500
gi|29165615|ref|NC_002745.2|	580472 +	U	0	0	0	0	1	SA0500
gi|29165615|ref|NC_002745.2|	580473 +	U	0	0	1	0	0	SA0500
gi|29165615|ref|NC_002745.2|	580475 +	U	1	0	0	0	0	SA0500
gi|29165615|ref|NC_002745.2|	580477 +	U	0	1	0	0	1	SA0500
gi|29165615|ref|NC_002745.2|	580478 +	A	0	2	1	1	2	SA0500
gi|29165615|ref|NC_002745.2|	580479 +	A	1	3	1	0	3	SA0500
gi|29165615|ref|NC_002745.2|	580480 +	U	0	1	0	1	0	SA0500
gi|29165615|ref|NC_002745.2|	580481 +	C	0	1	0	1	1	SA0500
gi|29165615|ref|NC_002745.2|	580482 +	A	0	1	3	1	1	SA0500
gi|29165615|ref|NC_002745.2|	580483 +	A	0	7	5	2	4	SA0500
gi|29165615|ref|NC_002745.2|	580484 +	A	0	7	2	2	1	SA0500
gi|29165615|ref|NC_002745.2|	580485 +	A	0	6	0	1	1	SA0500
gi|29165615|ref|NC_002745.2|	580486 +	A	0	3	1	0	1	SA0500
gi|29165615|ref|NC_002745.2|	580489 +	A	0	0	0	1	0	SA0500
gi|29165615|ref|NC_002745.2|	580498 +	A	0	0	1	1	0	SA0500
gi|29165615|ref|NC_002745.2|	580505 +	A	0	0	0	0	1	SA0500
gi|29165615|ref|NC_002745.2|	580513 +	A	0	1	0	0	0	SA0500
gi|29165615|ref|NC_002745.2|	580522 +	A	0	0	0	0	1	SA0500
gi|29165615|ref|NC_002745.2|	580546 +	G	1	0	0	0	0	SA0500
gi|29165615|ref|NC_002745.2|	580555 +	U	1	0	0	0	0	SA0500
gi|29165615|ref|NC_002745.2|	580567 +	C	0	0	0	0	1	SA0500
gi|29165615|ref|NC_002745.2|	580580 +	G	0	2	0	0	0	SA0500
gi|29165615|ref|NC_002745.2|	580597 +	A	1	0	0	0	1	SA0500
gi|29165615|ref|NC_002745.2|	580619 +	C	0	0	0	1	0	SA0500
gi|29165615|ref|NC_002745.2|	580629 +	U	1	0	0	0	0	SA0500
gi|29165615|ref|NC_002745.2|	580630 +	U	0	1	0	0	0	SA0500
gi|29165615|ref|NC_002745.2|	580631 +	A	0	0	0	0	1	SA0500
gi|29165615|ref|NC_002745.2|	580640 +	C	0	2	0	0	1	SA0500
gi|29165615|ref|NC_002745.2|	580645 +	A	0	0	0	0	1	SA0500
gi|29165615|ref|NC_002745.2|	580648 +	A	0	3	0	0	1	SA0500
gi|29165615|ref|NC_002745.2|	580649 +	A	0	0	1	0	0	SA0500
gi|29165615|ref|NC_002745.2|	580652 +	C	0	0	1	1	0	SA0500
gi|29165615|ref|NC_002745.2|	580653 +	A	0	0	0	0	1	SA0500
gi|29165615|ref|NC_002745.2|	580656 +	C	0	0	2	1	1	SA0500
gi|29165615|ref|NC_002745.2|	580657 +	A	0	1	0	0	0	SA0500
gi|29165615|ref|NC_002745.2|	580658 +	A	0	0	0	0	1	SA0500
gi|29165615|ref|NC_002745.2|	580659 +	U	0	0	1	0	0	SA0500
gi|29165615|ref|NC_002745.2|	580661 +	A	0	0	4	0	0	SA0500
gi|29165615|ref|NC_002745.2|	580662 +	A	0	2	1	1	0	SA0500
gi|29165615|ref|NC_002745.2|	580665 +	U	0	3	0	0	1	SA0500
gi|29165615|ref|NC_002745.2|	580666 +	A	0	3	0	0	3	SA0500
gi|29165615|ref|NC_002745.2|	580667 +	U	0	1	1	0	0	SA0500
gi|29165615|ref|NC_002745.2|	580668 +	A	0	2	0	0	0	SA0500

gi|29165615|ref|NC_002745.2|	580677	+	A	0	1	0	0	0	SA0500
gi|29165615|ref|NC_002745.2|	580680	+	A	0	5	1	1	0	SA0500
gi|29165615|ref|NC_002745.2|	580683	+	A	0	4	0	0	3	SA0500
gi|29165615|ref|NC_002745.2|	580698	+	C	0	0	1	0	0	SA0500
gi|29165615|ref|NC_002745.2|	580699	+	A	0	1	0	0	0	SA0500
gi|29165615|ref|NC_002745.2|	580704	+	U	0	1	0	0	0	SA0500
gi|29165615|ref|NC_002745.2|	580706	+	A	0	2	0	0	0	SA0500
gi|29165615|ref|NC_002745.2|	580712	+	A	0	0	1	0	0	SA0500
gi|29165615|ref|NC_002745.2|	580716	+	C	0	1	0	0	0	SA0500
gi|29165615|ref|NC_002745.2|	580721	+	C	0	1	0	0	0	SA0500
gi|29165615|ref|NC_002745.2|	580722	+	C	0	0	0	0	1	SA0500
gi|29165615|ref|NC_002745.2|	580759	+	C	0	0	0	0	1	SA0500
gi|29165615|ref|NC_002745.2|	580768	+	A	0	1	0	0	0	SA0500
gi|29165615|ref|NC_002745.2|	580777	+	C	0	0	0	1	0	SA0500
gi|29165615|ref|NC_002745.2|	580809	+	C	0	0	0	1	0	SA0500
gi|29165615|ref|NC_002745.2|	580819	+	U	0	1	0	0	0	SA0500
gi|29165615|ref|NC_002745.2|	580824	+	A	0	1	0	0	0	SA0500
gi|29165615|ref|NC_002745.2|	580862	+	C	0	1	0	0	0	SA0500
gi|29165615|ref|NC_002745.2|	580878	+	G	0	1	0	0	0	SA0500
gi|29165615|ref|NC_002745.2|	580908	+	U	1	0	0	0	0	SA0500
gi|29165615|ref|NC_002745.2|	580922	+	U	0	1	0	0	0	SA0500
gi|29165615|ref|NC_002745.2|	580923	+	C	0	0	1	0	1	SA0500
gi|29165615|ref|NC_002745.2|	580928	+	C	0	0	3	0	0	SA0500
gi|29165615|ref|NC_002745.2|	580930	+	A	0	1	0	0	0	SA0500
gi|29165615|ref|NC_002745.2|	580931	+	G	0	0	1	0	0	SA0500
gi|29165615|ref|NC_002745.2|	580932	+	A	0	2	0	0	0	SA0500
gi|29165615|ref|NC_002745.2|	580933	+	U	0	0	0	0	1	SA0500
gi|29165615|ref|NC_002745.2|	580934	+	A	0	1	0	0	0	SA0500
gi|29165615|ref|NC_002745.2|	580940	+	U	0	1	1	0	1	SA0500
gi|29165615|ref|NC_002745.2|	580952	+	C	0	0	0	0	1	SA0500
gi|29165615|ref|NC_002745.2|	580954	+	A	0	1	0	0	3	SA0500
gi|29165615|ref|NC_002745.2|	580955	+	C	0	0	1	1	0	SA0500
gi|29165615|ref|NC_002745.2|	580957	+	A	0	0	0	0	1	SA0500
gi|29165615|ref|NC_002745.2|	580959	+	U	0	2	2	0	0	SA0500
gi|29165615|ref|NC_002745.2|	580960	+	A	0	1	1	0	3	SA0500
gi|29165615|ref|NC_002745.2|	580961	+	A	0	3	1	0	5	SA0500
gi|29165615|ref|NC_002745.2|	580962	+	U	0	0	1	0	0	SA0500
gi|29165615|ref|NC_002745.2|	580964	+	A	0	2	2	1	6	SA0500
gi|29165615|ref|NC_002745.2|	580965	+	A	0	0	0	0	3	SA0500
gi|29165615|ref|NC_002745.2|	580966	+	U	0	4	0	0	3	SA0500
gi|29165615|ref|NC_002745.2|	580967	+	A	0	2	0	0	1	SA0500
gi|29165615|ref|NC_002745.2|	580972	+	A	0	2	1	0	2	SA0500
gi|29165615|ref|NC_002745.2|	580973	+	C	0	1	0	0	0	SA0500
gi|29165615|ref|NC_002745.2|	580974	+	C	0	0	1	0	0	SA0500
gi|29165615|ref|NC_002745.2|	580983	+	C	1	0	0	0	0	SA0500
gi|29165615|ref|NC_002745.2|	580984	+	A	1	0	0	0	1	SA0500
gi|29165615|ref|NC_002745.2|	580991	+	A	0	0	1	0	0	SA0500
gi|29165615|ref|NC_002745.2|	580996	+	A	0	2	0	0	0	SA0500
gi|29165615|ref|NC_002745.2|	581019	+	C	0	0	1	0	0	SA0500
gi|29165615|ref|NC_002745.2|	581021	+	C	0	1	0	0	0	SA0500
gi|29165615|ref|NC_002745.2|	581023	+	A	0	3	0	0	4	SA0500
gi|29165615|ref|NC_002745.2|	581026	+	C	0	0	1	0	0	SA0500
gi|29165615|ref|NC_002745.2|	581030	+	G	0	0	0	1	0	SA0500
gi|29165615|ref|NC_002745.2|	581037	+	C	1	0	0	0	1	SA0500
gi|29165615|ref|NC_002745.2|	581038	+	A	0	1	0	0	0	SA0500
gi|29165615|ref|NC_002745.2|	581041	+	C	0	1	2	0	0	SA0500
gi|29165615|ref|NC_002745.2|	581042	+	C	1	0	0	0	0	SA0500
gi|29165615|ref|NC_002745.2|	581044	+	A	0	2	0	2	1	SA0500
gi|29165615|ref|NC_002745.2|	581046	+	U	1	1	1	0	0	SA0500
gi|29165615|ref|NC_002745.2|	581049	+	C	1	0	0	0	0	SA0500
gi|29165615|ref|NC_002745.2|	581055	+	U	1	0	0	0	0	SA0500
gi|29165615|ref|NC_002745.2|	581057	+	A	0	1	0	0	0	SA0500
gi|29165615|ref|NC_002745.2|	581062	+	U	0	0	0	0	1	SA0500
gi|29165615|ref|NC_002745.2|	581073	+	U	1	0	0	0	0	SA0500
gi|29165615|ref|NC_002745.2|	581075	+	U	0	0	0	0	1	SA0500
gi|29165615|ref|NC_002745.2|	581079	+	C	0	1	0	0	0	SA0500
gi|29165615|ref|NC_002745.2|	581083	+	A	0	0	0	0	1	SA0500
gi|29165615|ref|NC_002745.2|	581088	+	C	1	0	1	0	0	SA0500
gi|29165615|ref|NC_002745.2|	581095	+	U	0	0	0	1	0	SA0500
gi|29165615|ref|NC_002745.2|	581125	+	A	0	1	0	0	0	SA0500
gi|29165615|ref|NC_002745.2|	581140	+	C	0	0	0	0	1	SA0500
gi|29165615|ref|NC_002745.2|	581142	+	C	0	0	0	0	1	SA0500
gi|29165615|ref|NC_002745.2|	581144	+	C	0	0	1	0	0	SA0500
gi|29165615|ref|NC_002745.2|	581146	+	C	1	0	0	0	0	SA0500
gi|29165615|ref|NC_002745.2|	581148	+	A	1	0	0	0	0	SA0500
gi|29165615|ref|NC_002745.2|	581151	+	G	1	0	0	0	0	SA0500
gi|29165615|ref|NC_002745.2|	581170	+	A	0	1	0	0	0	SA0500
gi|29165615|ref|NC_002745.2|	581173	+	A	1	0	0	0	0	SA0500
gi|29165615|ref|NC_002745.2|	581174	+	C	0	0	1	0	0	SA0500
gi|29165615|ref|NC_002745.2|	581177	+	G	0	0	2	0	1	SA0500
gi|29165615|ref|NC_002745.2|	581178	+	A	0	1	0	0	0	SA0500

gi|29165615|ref|NC_002745.2|	581181 +	G	0	1	0	1	0	SA0500
gi|29165615|ref|NC_002745.2|	581185 +	A	0	0	0	0	1	SA0500
gi|29165615|ref|NC_002745.2|	581186 +	A	0	2	0	0	0	SA0500
gi|29165615|ref|NC_002745.2|	581188 +	C	0	0	1	0	0	SA0500
gi|29165615|ref|NC_002745.2|	581189 +	A	0	0	1	0	0	SA0500
gi|29165615|ref|NC_002745.2|	581193 +	G	0	1	1	0	1	SA0500
gi|29165615|ref|NC_002745.2|	581198 +	A	0	2	0	1	1	SA0500
gi|29165615|ref|NC_002745.2|	581199 +	U	1	0	0	1	0	SA0500
gi|29165615|ref|NC_002745.2|	581201 +	A	0	1	1	0	0	SA0500
gi|29165615|ref|NC_002745.2|	581202 +	A	0	4	1	0	0	SA0500
gi|29165615|ref|NC_002745.2|	581203 +	C	0	0	1	0	0	SA0500
gi|29165615|ref|NC_002745.2|	581205 +	C	0	2	0	0	1	SA0500
gi|29165615|ref|NC_002745.2|	581207 +	U	0	1	0	0	0	SA0500
gi|29165615|ref|NC_002745.2|	581211 +	C	2	6	1	1	2	SA0500
gi|29165615|ref|NC_002745.2|	581213 +	A	0	1	0	0	0	SA0500
gi|29165615|ref|NC_002745.2|	581217 +	A	0	0	0	0	1	SA0500
gi|29165615|ref|NC_002745.2|	581255 +	U	0	0	0	1	0	SA0500
gi|29165615|ref|NC_002745.2|	581283 +	C	0	1	0	0	0	SA0500
gi|29165615|ref|NC_002745.2|	581287 +	C	1	0	1	0	1	SA0500
gi|29165615|ref|NC_002745.2|	581289 +	C	0	1	0	0	0	SA0500
gi|29165615|ref|NC_002745.2|	581295 +	A	0	0	0	0	1	SA0500
gi|29165615|ref|NC_002745.2|	581298 +	U	0	0	0	0	1	SA0500
gi|29165615|ref|NC_002745.2|	581301 +	A	0	12	3	5	7	SA0500
gi|29165615|ref|NC_002745.2|	581302 +	C	0	1	1	2	2	SA0500
gi|29165615|ref|NC_002745.2|	581303 +	U	0	0	0	1	0	SA0500
gi|29165615|ref|NC_002745.2|	581305 +	U	0	1	0	0	1	SA0500
gi|29165615|ref|NC_002745.2|	581306 +	U	0	1	0	0	0	SA0500
gi|29165615|ref|NC_002745.2|	581308 +	A	0	2	0	0	0	SA0500
gi|29165615|ref|NC_002745.2|	581309 +	A	0	2	4	0	2	SA0500
gi|29165615|ref|NC_002745.2|	581310 +	C	0	2	1	0	0	SA0500
gi|29165615|ref|NC_002745.2|	581313 +	C	0	2	0	0	0	SA0500
gi|29165615|ref|NC_002745.2|	581315 +	G	0	0	0	0	1	SA0500
gi|29165615|ref|NC_002745.2|	581316 +	A	0	2	0	2	0	SA0500
gi|29165615|ref|NC_002745.2|	581318 +	G	0	1	0	0	0	SA0500
gi|29165615|ref|NC_002745.2|	581319 +	A	0	1	0	0	0	SA0500
gi|29165615|ref|NC_002745.2|	581322 +	A	0	1	0	0	0	SA0500
gi|29165615|ref|NC_002745.2|	581325 +	A	0	0	0	1	2	SA0500
gi|29165615|ref|NC_002745.2|	581329 +	C	0	2	0	0	0	SA0500
gi|29165615|ref|NC_002745.2|	581331 +	A	0	0	2	0	0	SA0500
gi|29165615|ref|NC_002745.2|	581337 +	U	0	0	0	0	2	SA0500
gi|29165615|ref|NC_002745.2|	581338 +	A	0	0	0	0	1	SA0500
gi|29165615|ref|NC_002745.2|	581342 +	C	0	0	0	1	1	SA0500
gi|29165615|ref|NC_002745.2|	581343 +	A	0	2	0	0	0	SA0500
gi|29165615|ref|NC_002745.2|	581350 +	C	0	1	0	0	0	SA0500
gi|29165615|ref|NC_002745.2|	581353 +	U	0	1	0	0	0	SA0500
gi|29165615|ref|NC_002745.2|	581354 +	A	0	3	1	0	2	SA0500
gi|29165615|ref|NC_002745.2|	581355 +	A	0	6	2	3	3	SA0500
gi|29165615|ref|NC_002745.2|	581356 +	A	0	3	0	1	1	SA0500
gi|29165615|ref|NC_002745.2|	581357 +	U	0	0	1	0	0	SA0500
gi|29165615|ref|NC_002745.2|	581358 +	U	0	0	0	1	0	SA0500
gi|29165615|ref|NC_002745.2|	581359 +	A	0	1	0	0	1	SA0500
gi|29165615|ref|NC_002745.2|	581360 +	G	0	1	2	0	0	SA0500
gi|29165615|ref|NC_002745.2|	581361 +	A	0	0	2	0	0	SA0500
gi|29165615|ref|NC_002745.2|	581363 +	G	1	0	0	0	0	SA0500
gi|29165615|ref|NC_002745.2|	581364 +	A	0	2	0	0	0	SA0500
gi|29165615|ref|NC_002745.2|	581365 +	A	0	1	0	0	0	SA0500
gi|29165615|ref|NC_002745.2|	581374 +	U	2	0	0	0	0	SA0500
gi|29165615|ref|NC_002745.2|	581378 +	A	0	1	0	0	0	SA0500
gi|29165615|ref|NC_002745.2|	581379 +	U	2	1	0	0	0	SA0500
gi|29165615|ref|NC_002745.2|	581381 +	G	0	0	1	0	0	SA0500
gi|29165615|ref|NC_002745.2|	581388 +	A	0	1	1	1	0	SA0500
gi|29165615|ref|NC_002745.2|	581394 +	U	0	1	0	0	0	SA0500
gi|29165615|ref|NC_002745.2|	581395 +	A	1	0	0	0	0	SA0500
gi|29165615|ref|NC_002745.2|	581396 +	U	0	0	0	1	0	SA0500
gi|29165615|ref|NC_002745.2|	581412 +	A	0	1	0	0	0	SA0500
gi|29165615|ref|NC_002745.2|	581415 +	A	0	0	0	0	1	SA0500
gi|29165615|ref|NC_002745.2|	581418 +	C	0	0	0	0	1	SA0500
gi|29165615|ref|NC_002745.2|	581420 +	G	0	1	0	0	1	SA0500
gi|29165615|ref|NC_002745.2|	581422 +	U	0	0	0	0	1	SA0500
gi|29165615|ref|NC_002745.2|	581427 +	C	1	0	0	0	1	SA0500
gi|29165615|ref|NC_002745.2|	581428 +	U	0	2	0	0	0	SA0500
gi|29165615|ref|NC_002745.2|	581429 +	A	0	2	0	0	1	SA0500
gi|29165615|ref|NC_002745.2|	581430 +	A	0	0	0	0	2	SA0500
gi|29165615|ref|NC_002745.2|	581431 +	A	0	0	1	0	0	SA0500
gi|29165615|ref|NC_002745.2|	581447 +	A	1	0	0	0	0	SA0500
gi|29165615|ref|NC_002745.2|	581457 +	C	1	0	0	0	0	SA0500
gi|29165615|ref|NC_002745.2|	581461 +	G	0	0	0	0	1	SA0500
gi|29165615|ref|NC_002745.2|	581475 +	C	2	0	0	0	0	SA0500
gi|29165615|ref|NC_002745.2|	581478 +	C	0	0	1	0	0	SA0500
gi|29165615|ref|NC_002745.2|	581479 +	A	0	1	0	0	0	SA0500
gi|29165615|ref|NC_002745.2|	581485 +	A	0	0	0	0	1	SA0500

gi|29165615|ref|NC_002745.2|	581496 +	C	0	0	1	0	0	SA0500
gi|29165615|ref|NC_002745.2|	581502 +	U	0	0	1	0	0	SA0500
gi|29165615|ref|NC_002745.2|	581508 +	A	0	2	0	1	1	SA0500
gi|29165615|ref|NC_002745.2|	581509 +	C	0	1	0	0	0	SA0500
gi|29165615|ref|NC_002745.2|	581510 +	G	0	0	1	0	0	SA0500
gi|29165615|ref|NC_002745.2|	581511 +	A	0	3	1	0	2	SA0500
gi|29165615|ref|NC_002745.2|	581514 +	A	0	3	0	1	2	SA0500
gi|29165615|ref|NC_002745.2|	581517 +	C	0	1	1	1	0	SA0500
gi|29165615|ref|NC_002745.2|	581518 +	A	0	0	1	0	0	SA0500
gi|29165615|ref|NC_002745.2|	581519 +	A	0	1	0	0	0	SA0500
gi|29165615|ref|NC_002745.2|	581520 +	A	0	0	1	0	0	SA0500
gi|29165615|ref|NC_002745.2|	581521 +	C	2	2	0	0	1	SA0500
gi|29165615|ref|NC_002745.2|	581522 +	C	2	0	2	0	0	SA0500
gi|29165615|ref|NC_002745.2|	581523 +	G	0	1	0	0	0	SA0500
gi|29165615|ref|NC_002745.2|	581526 +	C	0	0	2	0	0	SA0500
gi|29165615|ref|NC_002745.2|	581535 +	G	0	1	0	0	0	SA0500
gi|29165615|ref|NC_002745.2|	581542 +	C	0	2	0	0	0	SA0500
gi|29165615|ref|NC_002745.2|	581543 +	A	0	0	1	0	1	SA0500
gi|29165615|ref|NC_002745.2|	581546 +	C	1	0	0	0	0	SA0500
gi|29165615|ref|NC_002745.2|	581547 +	A	1	0	1	0	0	SA0500
gi|29165615|ref|NC_002745.2|	581550 +	G	0	0	1	0	0	SA0500
gi|29165615|ref|NC_002745.2|	581551 +	U	0	0	0	0	1	SA0500
gi|29165615|ref|NC_002745.2|	581561 +	C	0	2	0	0	0	SA0500
gi|29165615|ref|NC_002745.2|	581567 +	A	1	0	0	0	0	SA0500
gi|29165615|ref|NC_002745.2|	581576 +	G	1	0	0	0	0	SA0500
gi|29165615|ref|NC_002745.2|	581577 +	A	0	1	0	0	0	SA0500
gi|29165615|ref|NC_002745.2|	581585 +	U	0	0	1	0	0	SA0500
gi|29165615|ref|NC_002745.2|	581595 +	C	1	0	2	0	0	SA0500
gi|29165615|ref|NC_002745.2|	581596 +	A	0	1	0	0	0	SA0500
gi|29165615|ref|NC_002745.2|	581599 +	U	0	0	1	0	0	SA0500
gi|29165615|ref|NC_002745.2|	581616 +	C	0	1	0	0	0	SA0500
gi|29165615|ref|NC_002745.2|	581621 +	G	0	0	0	0	1	SA0500
gi|29165615|ref|NC_002745.2|	581622 +	A	0	0	0	0	1	SA0500
gi|29165615|ref|NC_002745.2|	581625 +	C	1	0	0	0	0	SA0500
gi|29165615|ref|NC_002745.2|	581634 +	C	0	0	0	0	1	SA0500
gi|29165615|ref|NC_002745.2|	581640 +	C	2	0	0	0	0	SA0500
gi|29165615|ref|NC_002745.2|	581642 +	G	0	0	1	0	1	SA0500
gi|29165615|ref|NC_002745.2|	581648 +	C	1	0	0	1	0	SA0500
gi|29165615|ref|NC_002745.2|	581661 +	U	1	0	0	0	0	SA0500
gi|29165615|ref|NC_002745.2|	581671 +	U	1	1	0	0	0	SA0500
gi|29165615|ref|NC_002745.2|	581674 +	A	0	2	0	0	0	SA0500
gi|29165615|ref|NC_002745.2|	581682 +	A	0	0	0	1	0	SA0500
gi|29165615|ref|NC_002745.2|	581683 +	A	0	1	0	0	0	SA0500
gi|29165615|ref|NC_002745.2|	581688 +	U	0	0	1	0	0	SA0500
gi|29165615|ref|NC_002745.2|	581696 +	C	0	0	1	0	0	SA0500
gi|29165615|ref|NC_002745.2|	581698 +	U	0	1	0	0	0	SA0500
gi|29165615|ref|NC_002745.2|	581701 +	A	0	1	0	0	0	SA0500
gi|29165615|ref|NC_002745.2|	581702 +	G	0	1	0	0	0	SA0500
gi|29165615|ref|NC_002745.2|	581706 +	A	0	1	0	0	0	SA0500
gi|29165615|ref|NC_002745.2|	581723 +	C	1	0	0	1	1	SA0500
gi|29165615|ref|NC_002745.2|	581727 +	A	0	0	0	3	0	SA0500
gi|29165615|ref|NC_002745.2|	581733 +	A	0	0	0	0	1	SA0500
gi|29165615|ref|NC_002745.2|	581747 +	C	0	0	1	0	0	SA0500
gi|29165615|ref|NC_002745.2|	581749 +	A	0	0	0	0	1	SA0500
gi|29165615|ref|NC_002745.2|	581756 +	A	0	0	0	0	2	SA0500
gi|29165615|ref|NC_002745.2|	581757 +	A	0	2	0	0	2	SA0500
gi|29165615|ref|NC_002745.2|	581758 +	A	2	3	2	0	6	SA0500
gi|29165615|ref|NC_002745.2|	581759 +	U	0	1	0	0	0	SA0500
gi|29165615|ref|NC_002745.2|	581760 +	U	0	1	0	0	0	SA0500
gi|29165615|ref|NC_002745.2|	581761 +	U	0	0	0	1	1	SA0500
gi|29165615|ref|NC_002745.2|	581763 +	A	0	3	2	0	3	SA0500
gi|29165615|ref|NC_002745.2|	581764 +	A	1	1	1	1	1	SA0500
gi|29165615|ref|NC_002745.2|	581765 +	C	0	7	6	1	6	SA0500
gi|29165615|ref|NC_002745.2|	581767 +	U	0	0	0	0	1	SA0500
gi|29165615|ref|NC_002745.2|	581769 +	C	0	0	1	0	0	SA0500
gi|29165615|ref|NC_002745.2|	581771 +	A	0	1	0	0	1	SA0500
gi|29165615|ref|NC_002745.2|	581779 +	U	0	0	1	0	0	SA0500
gi|29165615|ref|NC_002745.2|	581781 +	C	0	0	0	1	2	SA0500
gi|29165615|ref|NC_002745.2|	581785 +	U	1	0	1	0	0	SA0500
gi|29165615|ref|NC_002745.2|	581793 +	A	0	0	0	1	0	SA0500
gi|29165615|ref|NC_002745.2|	581798 +	C	0	1	0	0	0	SA0500
gi|29165615|ref|NC_002745.2|	581799 +	C	1	2	0	1	1	SA0500
gi|29165615|ref|NC_002745.2|	581800 +	A	0	1	0	0	0	SA0500
gi|29165615|ref|NC_002745.2|	581801 +	A	0	1	0	0	0	SA0500
gi|29165615|ref|NC_002745.2|	581802 +	U	1	0	0	0	0	SA0500
gi|29165615|ref|NC_002745.2|	581805 +	U	2	2	0	0	0	SA0500
gi|29165615|ref|NC_002745.2|	581807 +	G	0	1	0	0	0	SA0500
gi|29165615|ref|NC_002745.2|	581808 +	C	0	0	0	1	0	SA0500
gi|29165615|ref|NC_002745.2|	581810 +	G	0	1	0	0	0	SA0500
gi|29165615|ref|NC_002745.2|	581814 +	G	0	1	0	0	0	SA0500
gi|29165615|ref|NC_002745.2|	581817 +	A	0	0	0	1	0	SA0500

gi|29165615|ref|NC_002745.2|	581823 +	U	4	0	0	0	0	SA0500
gi|29165615|ref|NC_002745.2|	581837 +	A	0	2	0	0	1	SA0500
gi|29165615|ref|NC_002745.2|	581841 +	U	2	1	0	0	0	SA0500
gi|29165615|ref|NC_002745.2|	581844 +	C	0	1	0	0	0	SA0500
gi|29165615|ref|NC_002745.2|	581853 +	C	0	2	0	0	1	SA0500
gi|29165615|ref|NC_002745.2|	581854 +	A	0	0	0	0	1	SA0500
gi|29165615|ref|NC_002745.2|	581856 +	C	0	1	1	0	0	SA0500
gi|29165615|ref|NC_002745.2|	581861 +	G	0	1	0	2	2	SA0500
gi|29165615|ref|NC_002745.2|	581862 +	A	0	4	1	1	2	SA0500
gi|29165615|ref|NC_002745.2|	581863 +	A	0	0	1	1	2	SA0500
gi|29165615|ref|NC_002745.2|	581865 +	U	0	2	1	0	0	SA0500
gi|29165615|ref|NC_002745.2|	581866 +	A	0	0	0	1	0	SA0500
gi|29165615|ref|NC_002745.2|	581867 +	G	0	6	1	1	0	SA0500
gi|29165615|ref|NC_002745.2|	581868 +	G	0	2	0	1	1	SA0500
gi|29165615|ref|NC_002745.2|	581869 +	A	0	2	0	0	0	SA0500
gi|29165615|ref|NC_002745.2|	581870 +	G	0	1	0	0	0	SA0500
gi|29165615|ref|NC_002745.2|	581871 +	A	0	2	0	0	0	SA0500
gi|29165615|ref|NC_002745.2|	581872 +	A	0	0	0	0	1	SA0500
gi|29165615|ref|NC_002745.2|	581873 +	A	0	2	1	0	0	SA0500
gi|29165615|ref|NC_002745.2|	581874 +	U	1	0	0	0	0	SA0500
gi|29165615|ref|NC_002745.2|	581878 +	A	0	1	1	0	0	SA0500
gi|29165615|ref|NC_002745.2|	581883 +	G	0	0	0	0	1	SA0500
gi|29165615|ref|NC_002745.2|	581889 +	A	0	1	0	0	0	SA0500
gi|29165615|ref|NC_002745.2|	581890 +	C	1	1	0	0	0	SA0500
gi|29165615|ref|NC_002745.2|	581893 +	A	0	0	0	0	1	SA0500
gi|29165615|ref|NC_002745.2|	581902 +	U	0	1	0	0	0	SA0500
gi|29165615|ref|NC_002745.2|	581907 +	U	1	0	0	0	0	SA0500
gi|29165615|ref|NC_002745.2|	581908 +	G	0	0	1	0	0	SA0500
gi|29165615|ref|NC_002745.2|	581921 +	U	0	0	1	0	0	SA0500
gi|29165615|ref|NC_002745.2|	581923 +	C	0	4	1	0	0	SA0500
gi|29165615|ref|NC_002745.2|	581924 +	A	0	1	0	0	2	SA0500
gi|29165615|ref|NC_002745.2|	581926 +	C	0	0	0	0	2	SA0500
gi|29165615|ref|NC_002745.2|	581927 +	U	0	0	0	0	1	SA0500
gi|29165615|ref|NC_002745.2|	581928 +	A	0	1	1	0	0	SA0500
gi|29165615|ref|NC_002745.2|	581929 +	U	0	0	1	0	0	SA0500
gi|29165615|ref|NC_002745.2|	581940 +	U	0	0	0	0	1	SA0500
gi|29165615|ref|NC_002745.2|	581943 +	U	0	0	0	0	3	SA0500
gi|29165615|ref|NC_002745.2|	581944 +	C	0	0	0	0	1	SA0500
gi|29165615|ref|NC_002745.2|	581945 +	A	0	1	0	0	0	SA0500
gi|29165615|ref|NC_002745.2|	581957 +	C	0	0	1	0	0	SA0500
gi|29165615|ref|NC_002745.2|	581962 +	G	0	0	0	1	0	SA0500
gi|29165615|ref|NC_002745.2|	581963 +	A	0	0	0	1	0	SA0500
gi|29165615|ref|NC_002745.2|	581966 +	G	0	1	0	0	0	SA0500
gi|29165615|ref|NC_002745.2|	581981 +	U	0	1	0	0	0	SA0500
gi|29165615|ref|NC_002745.2|	581983 +	U	1	0	1	0	0	SA0500
gi|29165615|ref|NC_002745.2|	581987 +	C	0	0	1	0	0	SA0500
gi|29165615|ref|NC_002745.2|	581988 +	A	1	0	0	0	0	SA0500
gi|29165615|ref|NC_002745.2|	581994 +	A	0	0	1	0	0	SA0500
gi|29165615|ref|NC_002745.2|	581997 +	A	0	0	1	0	0	SA0500
gi|29165615|ref|NC_002745.2|	582002 +	G	0	0	0	0	1	SA0500
gi|29165615|ref|NC_002745.2|	582007 +	A	2	0	0	0	0	SA0500
gi|29165615|ref|NC_002745.2|	582008 +	G	0	3	0	0	0	SA0500
gi|29165615|ref|NC_002745.2|	582009 +	A	5	5	0	0	0	SA0500
gi|29165615|ref|NC_002745.2|	582021 +	C	0	0	1	0	0	SA0500
gi|29165615|ref|NC_002745.2|	582024 +	A	2	0	0	0	0	SA0500
gi|29165615|ref|NC_002745.2|	582028 +	A	0	0	0	0	1	SA0500
gi|29165615|ref|NC_002745.2|	582030 +	G	1	0	0	0	0	SA0500
gi|29165615|ref|NC_002745.2|	582040 +	A	0	1	0	0	0	SA0500
gi|29165615|ref|NC_002745.2|	582041 +	A	1	4	0	0	7	SA0500
gi|29165615|ref|NC_002745.2|	582042 +	U	0	0	1	0	0	SA0500
gi|29165615|ref|NC_002745.2|	582043 +	C	0	1	0	0	0	SA0500
gi|29165615|ref|NC_002745.2|	582045 +	C	0	0	0	0	1	SA0500
gi|29165615|ref|NC_002745.2|	582047 +	A	0	1	0	0	0	SA0500
gi|29165615|ref|NC_002745.2|	582051 +	A	0	0	1	0	1	SA0500
gi|29165615|ref|NC_002745.2|	582054 +	U	0	1	0	0	0	SA0500
gi|29165615|ref|NC_002745.2|	582056 +	C	0	0	1	0	0	SA0500
gi|29165615|ref|NC_002745.2|	582059 +	A	0	0	0	1	0	SA0500
gi|29165615|ref|NC_002745.2|	582063 +	U	0	3	1	1	0	SA0500
gi|29165615|ref|NC_002745.2|	582064 +	U	0	0	1	0	0	SA0500
gi|29165615|ref|NC_002745.2|	582075 +	C	0	0	0	0	1	SA0500
gi|29165615|ref|NC_002745.2|	582078 +	U	1	0	0	0	0	SA0500
gi|29165615|ref|NC_002745.2|	582080 +	A	0	0	0	0	1	SA0500
gi|29165615|ref|NC_002745.2|	582082 +	G	0	1	0	0	0	SA0500
gi|29165615|ref|NC_002745.2|	582083 +	A	0	1	0	0	3	SA0500
gi|29165615|ref|NC_002745.2|	582094 +	U	0	0	1	0	0	SA0500
gi|29165615|ref|NC_002745.2|	582095 +	C	0	0	0	0	1	SA0500
gi|29165615|ref|NC_002745.2|	582107 +	U	0	2	0	0	0	SA0500
gi|29165615|ref|NC_002745.2|	582125 +	A	0	1	0	0	0	SA0500
gi|29165615|ref|NC_002745.2|	582126 +	A	0	1	0	0	0	SA0500
gi|29165615|ref|NC_002745.2|	582127 +	A	0	2	0	0	0	SA0500
gi|29165615|ref|NC_002745.2|	582135 +	A	0	0	1	0	3	SA0500

gi|29165615|ref|NC_002745.2|	582136 +	U	0	0	0	0	1	SA0500
gi|29165615|ref|NC_002745.2|	582143 +	G	0	1	1	0	0	SA0500
gi|29165615|ref|NC_002745.2|	582144 +	U	0	3	1	0	3	SA0500
gi|29165615|ref|NC_002745.2|	582145 +	U	0	0	0	0	1	SA0500
gi|29165615|ref|NC_002745.2|	582147 +	G	0	1	0	0	0	SA0500
gi|29165615|ref|NC_002745.2|	582149 +	A	0	3	0	0	0	SA0500
gi|29165615|ref|NC_002745.2|	582161 +	A	0	0	1	0	0	SA0500
gi|29165615|ref|NC_002745.2|	582163 +	A	0	0	0	0	1	SA0500
gi|29165615|ref|NC_002745.2|	582169 +	A	0	0	1	0	0	SA0500
gi|29165615|ref|NC_002745.2|	582175 +	G	0	1	0	0	0	SA0500
gi|29165615|ref|NC_002745.2|	582210 +	U	1	0	0	0	0	SA0500
gi|29165615|ref|NC_002745.2|	582218 +	A	0	1	0	0	0	SA0500
gi|29165615|ref|NC_002745.2|	582259 +	C	0	0	0	0	1	SA0500
gi|29165615|ref|NC_002745.2|	582283 +	U	1	0	0	0	0	SA0500
gi|29165615|ref|NC_002745.2|	582297 +	A	0	3	0	0	0	SA0500
gi|29165615|ref|NC_002745.2|	582298 +	U	0	0	0	0	1	SA0500
gi|29165615|ref|NC_002745.2|	582323 +	U	0	0	0	0	1	SA0500
gi|29165615|ref|NC_002745.2|	582328 +	U	1	0	0	0	0	SA0500
gi|29165615|ref|NC_002745.2|	582333 +	U	1	0	0	0	1	SA0500
gi|29165615|ref|NC_002745.2|	582340 +	A	0	1	0	0	0	SA0500
gi|29165615|ref|NC_002745.2|	582345 +	U	1	0	0	0	0	SA0500
gi|29165615|ref|NC_002745.2|	582347 +	C	0	1	0	0	0	SA0500
gi|29165615|ref|NC_002745.2|	582357 +	U	3	0	0	0	0	SA0500
gi|29165615|ref|NC_002745.2|	582358 +	C	0	1	0	0	0	SA0500
gi|29165615|ref|NC_002745.2|	582359 +	G	0	0	1	0	0	SA0500
gi|29165615|ref|NC_002745.2|	582366 +	A	0	0	0	0	2	SA0500
gi|29165615|ref|NC_002745.2|	582374 +	A	0	3	0	0	0	SA0500
gi|29165615|ref|NC_002745.2|	582376 +	U	0	0	0	1	0	SA0500
gi|29165615|ref|NC_002745.2|	582377 +	C	0	1	0	0	0	SA0500
gi|29165615|ref|NC_002745.2|	582378 +	A	1	0	0	0	0	SA0500
gi|29165615|ref|NC_002745.2|	582379 +	U	0	0	0	0	1	SA0500
gi|29165615|ref|NC_002745.2|	582382 +	U	0	0	0	1	0	SA0500
gi|29165615|ref|NC_002745.2|	582409 +	U	0	0	0	0	2	SA0500
gi|29165615|ref|NC_002745.2|	582422 +	A	0	0	1	0	0	SA0500
gi|29165615|ref|NC_002745.2|	582435 +	U	0	1	0	0	0	SA0500
gi|29165615|ref|NC_002745.2|	582473 +	C	1	0	0	0	0	SA0500
gi|29165615|ref|NC_002745.2|	582481 +	U	1	0	0	0	0	SA0500
gi|29165615|ref|NC_002745.2|	582492 +	A	0	1	0	0	0	SA0500
gi|29165615|ref|NC_002745.2|	582495 +	C	0	0	0	0	2	SA0500
gi|29165615|ref|NC_002745.2|	582508 +	A	1	0	0	0	0	SA0500
gi|29165615|ref|NC_002745.2|	582544 +	C	0	0	1	0	0	SA0500
gi|29165615|ref|NC_002745.2|	582545 +	U	0	1	0	0	0	SA0500
gi|29165615|ref|NC_002745.2|	582550 +	U	0	0	0	0	1	SA0500
gi|29165615|ref|NC_002745.2|	582556 +	U	0	1	0	0	0	SA0500
gi|29165615|ref|NC_002745.2|	582564 +	A	0	1	0	0	0	SA0500
gi|29165615|ref|NC_002745.2|	582570 +	G	0	1	0	0	0	SA0500
gi|29165615|ref|NC_002745.2|	582575 +	C	0	1	0	0	0	SA0500
gi|29165615|ref|NC_002745.2|	582584 +	U	0	0	0	0	1	SA0500
gi|29165615|ref|NC_002745.2|	582596 +	G	0	0	1	0	0	SA0500
gi|29165615|ref|NC_002745.2|	582604 +	A	0	0	1	0	0	SA0500
gi|29165615|ref|NC_002745.2|	582610 +	U	0	1	0	0	0	SA0500
gi|29165615|ref|NC_002745.2|	582613 +	C	0	1	0	0	0	SA0500
gi|29165615|ref|NC_002745.2|	582616 +	U	1	0	0	0	0	SA0500
gi|29165615|ref|NC_002745.2|	582629 +	A	0	2	0	0	0	SA0500
gi|29165615|ref|NC_002745.2|	582631 +	U	0	1	0	0	0	SA0500
gi|29165615|ref|NC_002745.2|	582645 +	U	0	0	0	0	1	SA0500
gi|29165615|ref|NC_002745.2|	582681 +	G	3	1	0	0	0	SA0500
gi|29165615|ref|NC_002745.2|	582684 +	C	0	0	0	0	1	SA0500
gi|29165615|ref|NC_002745.2|	582704 +	C	0	1	0	0	0	SA0500
gi|29165615|ref|NC_002745.2|	582712 +	A	0	1	0	0	0	SA0500
gi|29165615|ref|NC_002745.2|	582713 +	G	0	1	0	0	0	SA0500
gi|29165615|ref|NC_002745.2|	582745 +	C	1	0	0	0	0	SA0500
gi|29165615|ref|NC_002745.2|	582764 +	C	0	1	0	0	0	SA0500
gi|29165615|ref|NC_002745.2|	582767 +	G	0	1	0	0	0	SA0500
gi|29165615|ref|NC_002745.2|	582768 +	C	1	0	0	0	0	SA0500
gi|29165615|ref|NC_002745.2|	582791 +	C	0	0	0	0	1	SA0500
gi|29165615|ref|NC_002745.2|	582836 +	C	0	0	1	0	0	SA0500
gi|29165615|ref|NC_002745.2|	582877 +	U	1	0	0	0	0	SA0500
gi|29165615|ref|NC_002745.2|	582891 +	C	1	0	0	0	0	SA0500
gi|29165615|ref|NC_002745.2|	582892 +	A	0	0	0	0	1	SA0500
gi|29165615|ref|NC_002745.2|	582916 +	A	0	2	0	0	1	SA0500
gi|29165615|ref|NC_002745.2|	582919 +	C	1	1	0	0	0	SA0500
gi|29165615|ref|NC_002745.2|	582927 +	C	0	1	0	0	0	SA0500
gi|29165615|ref|NC_002745.2|	582936 +	G	0	0	1	0	0	SA0500
gi|29165615|ref|NC_002745.2|	582937 +	U	0	0	1	0	0	SA0500
gi|29165615|ref|NC_002745.2|	582944 +	A	0	1	0	0	0	SA0500
gi|29165615|ref|NC_002745.2|	582950 +	G	0	1	0	0	0	SA0500
gi|29165615|ref|NC_002745.2|	582954 +	C	0	0	1	0	0	SA0500
gi|29165615|ref|NC_002745.2|	582978 +	C	0	1	1	0	1	SA0500
gi|29165615|ref|NC_002745.2|	582982 +	A	0	0	0	0	1	SA0500
gi|29165615|ref|NC_002745.2|	582983 +	C	0	1	0	0	0	SA0500

gi|29165615|ref|NC_002745.2|	582993 +	C	0	2	0	0	0	SA0500
gi|29165615|ref|NC_002745.2|	582995 +	G	1	0	1	0	1	SA0500
gi|29165615|ref|NC_002745.2|	582996 +	A	0	0	0	0	1	SA0500
gi|29165615|ref|NC_002745.2|	582997 +	A	0	0	1	0	0	SA0500
gi|29165615|ref|NC_002745.2|	582999 +	C	0	0	1	0	0	SA0500
gi|29165615|ref|NC_002745.2|	583000 +	A	0	0	0	0	1	SA0500
gi|29165615|ref|NC_002745.2|	583004 +	C	0	2	0	0	0	SA0500
gi|29165615|ref|NC_002745.2|	583008 +	U	0	0	1	0	0	SA0500
gi|29165615|ref|NC_002745.2|	583011 +	U	0	0	2	0	0	SA0500
gi|29165615|ref|NC_002745.2|	583012 +	G	0	2	0	0	0	SA0500
gi|29165615|ref|NC_002745.2|	583018 +	A	0	3	0	0	0	SA0500
gi|29165615|ref|NC_002745.2|	583021 +	A	0	1	0	0	0	SA0500
gi|29165615|ref|NC_002745.2|	583025 +	C	0	0	1	0	0	SA0500
gi|29165615|ref|NC_002745.2|	583029 +	G	0	0	0	0	1	SA0500
gi|29165615|ref|NC_002745.2|	583033 +	A	0	0	0	0	1	SA0500
gi|29165615|ref|NC_002745.2|	583037 +	U	0	2	0	0	0	SA0500
gi|29165615|ref|NC_002745.2|	583046 +	A	1	1	0	0	0	SA0500
gi|29165615|ref|NC_002745.2|	583057 +	U	0	2	0	0	0	SA0500
gi|29165615|ref|NC_002745.2|	583059 +	A	0	0	0	0	1	SA0500
gi|29165615|ref|NC_002745.2|	583061 +	C	0	3	1	0	1	SA0500
gi|29165615|ref|NC_002745.2|	583062 +	A	0	0	1	0	0	SA0500
gi|29165615|ref|NC_002745.2|	583065 +	A	0	1	0	0	1	SA0500
gi|29165615|ref|NC_002745.2|	583066 +	U	0	0	1	0	0	SA0500
gi|29165615|ref|NC_002745.2|	583069 +	U	0	0	1	0	0	SA0500
gi|29165615|ref|NC_002745.2|	583073 +	A	0	1	1	0	0	SA0500
gi|29165615|ref|NC_002745.2|	583077 +	A	0	1	0	0	0	SA0500
gi|29165615|ref|NC_002745.2|	583078 +	A	0	3	0	2	0	SA0500
gi|29165615|ref|NC_002745.2|	583083 +	C	0	1	0	0	0	SA0500
gi|29165615|ref|NC_002745.2|	583086 +	A	0	0	0	1	0	SA0500
gi|29165615|ref|NC_002745.2|	583089 +	U	0	2	0	1	1	SA0500
gi|29165615|ref|NC_002745.2|	583092 +	A	0	1	0	0	0	SA0500
gi|29165615|ref|NC_002745.2|	583095 +	A	0	1	0	0	0	SA0500
gi|29165615|ref|NC_002745.2|	583099 +	U	0	0	1	0	0	SA0500
gi|29165615|ref|NC_002745.2|	583101 +	A	0	0	0	1	0	SA0500
gi|29165615|ref|NC_002745.2|	583104 +	U	0	0	0	0	1	SA0500
gi|29165615|ref|NC_002745.2|	583105 +	U	0	2	1	0	0	SA0500
gi|29165615|ref|NC_002745.2|	583107 +	U	2	0	1	0	0	SA0500
gi|29165615|ref|NC_002745.2|	583110 +	A	0	0	1	0	0	SA0500
gi|29165615|ref|NC_002745.2|	583111 +	A	0	1	0	0	0	SA0500
gi|29165615|ref|NC_002745.2|	583114 +	C	0	0	1	0	0	SA0500
gi|29165615|ref|NC_002745.2|	583116 +	A	0	1	0	0	1	SA0500
gi|29165615|ref|NC_002745.2|	583119 +	U	0	0	1	1	0	SA0500
gi|29165615|ref|NC_002745.2|	583126 +	A	0	0	1	0	0	SA0500
gi|29165615|ref|NC_002745.2|	583129 +	A	0	1	0	0	0	SA0500
gi|29165615|ref|NC_002745.2|	583134 +	A	0	0	0	1	0	SA0500
gi|29165615|ref|NC_002745.2|	583377 +	U	1	0	0	0	0	SA0501
gi|29165615|ref|NC_002745.2|	583405 +	C	0	0	0	0	1	SA0501
gi|29165615|ref|NC_002745.2|	583408 +	U	0	1	0	0	0	SA0501
gi|29165615|ref|NC_002745.2|	583417 +	G	0	0	0	1	0	SA0501
gi|29165615|ref|NC_002745.2|	583421 +	A	0	0	0	1	0	SA0501
gi|29165615|ref|NC_002745.2|	583427 +	A	0	5	0	0	2	SA0501
gi|29165615|ref|NC_002745.2|	583429 +	C	0	0	0	0	1	SA0501
gi|29165615|ref|NC_002745.2|	583450 +	G	1	0	0	0	0	SA0501
gi|29165615|ref|NC_002745.2|	583471 +	C	0	2	0	0	0	SA0501
gi|29165615|ref|NC_002745.2|	583483 +	A	0	0	1	0	0	SA0501
gi|29165615|ref|NC_002745.2|	583488 +	A	0	0	0	1	0	SA0501
gi|29165615|ref|NC_002745.2|	583496 +	U	0	0	0	1	0	SA0501
gi|29165615|ref|NC_002745.2|	583510 +	U	1	0	0	0	0	SA0501
gi|29165615|ref|NC_002745.2|	583519 +	A	1	0	0	0	0	SA0501
gi|29165615|ref|NC_002745.2|	583547 +	U	0	0	1	0	0	SA0501
gi|29165615|ref|NC_002745.2|	583555 +	C	1	0	0	0	0	SA0501
gi|29165615|ref|NC_002745.2|	583558 +	A	1	0	0	0	0	SA0501
gi|29165615|ref|NC_002745.2|	583559 +	A	0	4	0	1	0	SA0501
gi|29165615|ref|NC_002745.2|	583560 +	U	0	1	0	0	0	SA0501
gi|29165615|ref|NC_002745.2|	583561 +	C	0	0	0	2	0	SA0501
gi|29165615|ref|NC_002745.2|	583562 +	U	3	1	0	0	0	SA0501
gi|29165615|ref|NC_002745.2|	583563 +	A	1	0	0	0	0	SA0501
gi|29165615|ref|NC_002745.2|	583564 +	A	0	0	0	1	0	SA0501
gi|29165615|ref|NC_002745.2|	583565 +	A	0	1	0	1	0	SA0501
gi|29165615|ref|NC_002745.2|	583570 +	G	1	0	1	0	0	SA0501
gi|29165615|ref|NC_002745.2|	583571 +	U	0	0	0	0	1	SA0501
gi|29165615|ref|NC_002745.2|	583572 +	C	0	0	1	0	0	SA0501
gi|29165615|ref|NC_002745.2|	583573 +	G	0	1	1	0	0	SA0501
gi|29165615|ref|NC_002745.2|	583590 +	A	0	0	1	0	0	SA0501
gi|29165615|ref|NC_002745.2|	583594 +	A	0	0	2	0	0	SA0501
gi|29165615|ref|NC_002745.2|	583596 +	C	0	0	0	0	1	SA0501
gi|29165615|ref|NC_002745.2|	583597 +	U	1	0	0	0	0	SA0501
gi|29165615|ref|NC_002745.2|	583605 +	C	0	0	1	0	0	SA0501
gi|29165615|ref|NC_002745.2|	583633 +	G	1	0	0	0	0	SA0501
gi|29165615|ref|NC_002745.2|	583639 +	C	1	1	0	0	0	SA0501
gi|29165615|ref|NC_002745.2|	583663 +	A	0	1	0	0	0	SA0501

gi|29165615|ref|NC_002745.2|	583664 +	C	0	0	1	0	0	SA0501
gi|29165615|ref|NC_002745.2|	583702 +	C	0	0	1	0	0	SA0501
gi|29165615|ref|NC_002745.2|	583714 +	U	1	0	0	0	0	SA0501
gi|29165615|ref|NC_002745.2|	583738 +	U	0	2	0	0	0	SA0501
gi|29165615|ref|NC_002745.2|	583741 +	A	1	0	0	0	0	SA0501
gi|29165615|ref|NC_002745.2|	583746 +	A	0	0	0	1	1	SA0501
gi|29165615|ref|NC_002745.2|	583748 +	A	0	0	0	1	0	SA0501
gi|29165615|ref|NC_002745.2|	583749 +	A	0	0	0	1	0	SA0501
gi|29165615|ref|NC_002745.2|	583751 +	U	0	0	0	0	1	SA0501
gi|29165615|ref|NC_002745.2|	583757 +	A	1	0	0	0	0	SA0501
gi|29165615|ref|NC_002745.2|	583761 +	G	0	0	0	1	0	SA0501
gi|29165615|ref|NC_002745.2|	583766 +	U	0	0	1	0	0	SA0501
gi|29165615|ref|NC_002745.2|	583769 +	A	2	0	0	0	0	SA0501
gi|29165615|ref|NC_002745.2|	583772 +	C	0	0	1	0	0	SA0501
gi|29165615|ref|NC_002745.2|	583790 +	A	0	1	0	0	0	SA0501
gi|29165615|ref|NC_002745.2|	583791 +	U	0	0	1	0	0	SA0501
gi|29165615|ref|NC_002745.2|	583796 +	A	0	1	0	0	0	SA0501
gi|29165615|ref|NC_002745.2|	583805 +	C	2	0	1	0	0	SA0501
gi|29165615|ref|NC_002745.2|	583806 +	G	0	0	0	0	2	SA0501
gi|29165615|ref|NC_002745.2|	583811 +	A	0	0	0	1	2	SA0501
gi|29165615|ref|NC_002745.2|	583815 +	A	0	1	0	0	0	SA0501
gi|29165615|ref|NC_002745.2|	583816 +	U	2	0	0	0	0	SA0501
gi|29165615|ref|NC_002745.2|	583879 +	U	2	0	0	0	0	SA0501
gi|29165615|ref|NC_002745.2|	583892 +	G	1	0	0	0	0	SA0501
gi|29165615|ref|NC_002745.2|	583903 +	C	2	0	0	0	0	SA0501
gi|29165615|ref|NC_002745.2|	583907 +	U	0	0	0	1	0	SA0501
gi|29165615|ref|NC_002745.2|	583910 +	A	0	0	1	0	0	SA0501
gi|29165615|ref|NC_002745.2|	583915 +	U	1	0	0	0	0	SA0501
gi|29165615|ref|NC_002745.2|	583996 +	U	0	0	0	1	0	SA0501
gi|29165615|ref|NC_002745.2|	584034 +	C	0	2	0	0	0	SA0501
gi|29165615|ref|NC_002745.2|	584072 +	C	0	0	1	0	0	SA0501
gi|29165615|ref|NC_002745.2|	584099 +	A	0	0	0	1	0	SA0501
gi|29165615|ref|NC_002745.2|	584108 +	U	0	1	1	0	0	SA0501
gi|29165615|ref|NC_002745.2|	584158 +	A	0	0	0	1	0	SA0501
gi|29165615|ref|NC_002745.2|	584165 +	U	0	4	0	2	0	SA0501
gi|29165615|ref|NC_002745.2|	584171 +	A	0	0	0	0	1	SA0501
gi|29165615|ref|NC_002745.2|	584175 +	G	1	0	0	0	0	SA0501
gi|29165615|ref|NC_002745.2|	584191 +	U	0	0	1	0	0	SA0501
gi|29165615|ref|NC_002745.2|	584194 +	U	0	0	0	1	0	SA0501
gi|29165615|ref|NC_002745.2|	584198 +	U	1	0	0	0	0	SA0501
gi|29165615|ref|NC_002745.2|	584200 +	A	0	1	0	0	0	SA0501
gi|29165615|ref|NC_002745.2|	584221 +	U	0	0	1	0	0	SA0501
gi|29165615|ref|NC_002745.2|	584231 +	A	0	0	0	0	1	SA0501
gi|29165615|ref|NC_002745.2|	584249 +	A	0	3	0	0	1	SA0501
gi|29165615|ref|NC_002745.2|	584258 +	U	2	0	0	0	0	SA0501
gi|29165615|ref|NC_002745.2|	584261 +	U	0	0	0	1	0	SA0501
gi|29165615|ref|NC_002745.2|	584277 +	C	0	0	0	1	0	SA0501
gi|29165615|ref|NC_002745.2|	584286 +	U	2	0	0	0	0	SA0501
gi|29165615|ref|NC_002745.2|	584300 +	A	0	0	1	0	0	SA0501
gi|29165615|ref|NC_002745.2|	584301 +	C	0	0	0	2	1	SA0501
gi|29165615|ref|NC_002745.2|	584304 +	G	0	1	0	0	0	SA0501
gi|29165615|ref|NC_002745.2|	584305 +	G	1	0	0	0	0	SA0501
gi|29165615|ref|NC_002745.2|	584340 +	G	0	0	0	0	1	SA0501
gi|29165615|ref|NC_002745.2|	584377 +	U	0	0	2	0	0	SA0501
gi|29165615|ref|NC_002745.2|	584380 +	C	0	0	0	0	1	SA0501
gi|29165615|ref|NC_002745.2|	584388 +	A	0	2	0	0	0	SA0501
gi|29165615|ref|NC_002745.2|	584398 +	A	0	0	1	0	0	SA0501
gi|29165615|ref|NC_002745.2|	584400 +	C	0	1	0	0	0	SA0501
gi|29165615|ref|NC_002745.2|	584405 +	U	0	0	0	1	0	SA0501
gi|29165615|ref|NC_002745.2|	584409 +	C	2	0	0	0	0	SA0501
gi|29165615|ref|NC_002745.2|	584411 +	A	0	0	0	0	1	SA0501
gi|29165615|ref|NC_002745.2|	584417 +	A	0	0	0	0	1	SA0501
gi|29165615|ref|NC_002745.2|	584422 +	A	0	0	0	0	1	SA0501
gi|29165615|ref|NC_002745.2|	584435 +	A	1	0	0	0	0	SA0501
gi|29165615|ref|NC_002745.2|	584436 +	C	1	0	0	0	0	SA0501
gi|29165615|ref|NC_002745.2|	584438 +	U	0	0	0	0	2	SA0501
gi|29165615|ref|NC_002745.2|	584442 +	A	0	0	1	0	2	SA0501
gi|29165615|ref|NC_002745.2|	584460 +	A	1	0	0	0	0	SA0501
gi|29165615|ref|NC_002745.2|	584467 +	A	0	0	1	0	0	SA0501
gi|29165615|ref|NC_002745.2|	584475 +	A	0	1	0	0	1	SA0501
gi|29165615|ref|NC_002745.2|	584477 +	C	0	1	0	0	0	SA0501
gi|29165615|ref|NC_002745.2|	584530 +	A	1	0	0	0	0	SA0501
gi|29165615|ref|NC_002745.2|	584554 +	C	0	0	0	1	0	SA0501
gi|29165615|ref|NC_002745.2|	584557 +	C	2	0	0	0	0	SA0501
gi|29165615|ref|NC_002745.2|	584561 +	U	0	0	0	0	1	SA0501
gi|29165615|ref|NC_002745.2|	584569 +	U	0	0	1	0	0	SA0501
gi|29165615|ref|NC_002745.2|	584583 +	U	0	1	0	1	0	SA0501
gi|29165615|ref|NC_002745.2|	584596 +	U	0	0	0	0	1	SA0501
gi|29165615|ref|NC_002745.2|	584599 +	U	0	0	0	0	1	SA0501
gi|29165615|ref|NC_002745.2|	584621 +	A	0	0	0	0	1	SA0501
gi|29165615|ref|NC_002745.2|	584645 +	U	1	0	1	0	0	SA0501

gi|29165615|ref|NC_002745.2|	584647	+	A	5	0	0	0	0	SA0501
gi|29165615|ref|NC_002745.2|	584663	+	U	0	1	0	0	0	SA0501
gi|29165615|ref|NC_002745.2|	584687	+	U	1	0	1	1	0	SA0501
gi|29165615|ref|NC_002745.2|	584689	+	U	0	0	0	0	1	SA0501
gi|29165615|ref|NC_002745.2|	584697	+	G	0	0	0	0	1	SA0501
gi|29165615|ref|NC_002745.2|	584740	+	A	0	1	0	0	0	SA0501
gi|29165615|ref|NC_002745.2|	584777	+	A	0	2	0	0	0	SA0501
gi|29165615|ref|NC_002745.2|	584820	+	G	0	1	0	0	0	SA0501
gi|29165615|ref|NC_002745.2|	584839	+	A	0	0	0	0	1	SA0501
gi|29165615|ref|NC_002745.2|	584860	+	A	0	1	2	0	0	SA0501
gi|29165615|ref|NC_002745.2|	584869	+	A	0	0	0	1	0	SA0501
gi|29165615|ref|NC_002745.2|	584872	+	U	0	0	1	0	0	SA0501
gi|29165615|ref|NC_002745.2|	584875	+	U	0	0	0	0	1	SA0501
gi|29165615|ref|NC_002745.2|	584883	+	U	0	1	0	0	0	SA0501
gi|29165615|ref|NC_002745.2|	584939	+	C	0	0	0	1	0	SA0501
gi|29165615|ref|NC_002745.2|	584941	+	A	0	1	0	0	0	SA0501
gi|29165615|ref|NC_002745.2|	584950	+	C	0	0	0	1	0	SA0501
gi|29165615|ref|NC_002745.2|	584954	+	U	0	0	1	0	0	SA0501
gi|29165615|ref|NC_002745.2|	584966	+	A	0	1	0	0	0	SA0501
gi|29165615|ref|NC_002745.2|	584979	+	C	0	0	1	0	0	SA0501
gi|29165615|ref|NC_002745.2|	584980	+	U	8	0	0	0	0	SA0501
gi|29165615|ref|NC_002745.2|	584981	+	U	1	0	0	0	0	SA0501
gi|29165615|ref|NC_002745.2|	584988	+	U	1	0	0	0	0	SA0501
gi|29165615|ref|NC_002745.2|	585000	+	A	0	0	2	0	0	SA0501
gi|29165615|ref|NC_002745.2|	585008	+	C	1	0	1	0	1	SA0501
gi|29165615|ref|NC_002745.2|	585016	+	U	1	0	0	0	0	SA0501
gi|29165615|ref|NC_002745.2|	585021	+	C	0	0	2	0	1	SA0501
gi|29165615|ref|NC_002745.2|	585022	+	C	0	0	0	0	1	SA0501
gi|29165615|ref|NC_002745.2|	585040	+	U	0	0	0	0	3	SA0501
gi|29165615|ref|NC_002745.2|	585066	+	G	0	1	0	0	0	SA0501
gi|29165615|ref|NC_002745.2|	585131	+	A	0	0	0	0	1	SA0501
gi|29165615|ref|NC_002745.2|	585143	+	U	1	0	0	0	0	SA0501
gi|29165615|ref|NC_002745.2|	585173	+	A	1	0	0	0	0	SA0501
gi|29165615|ref|NC_002745.2|	585193	+	C	1	0	0	0	0	SA0501
gi|29165615|ref|NC_002745.2|	585256	+	U	2	0	1	0	0	SA0501
gi|29165615|ref|NC_002745.2|	585268	+	U	0	1	0	0	0	SA0501
gi|29165615|ref|NC_002745.2|	585269	+	C	0	1	0	0	0	SA0501
gi|29165615|ref|NC_002745.2|	585270	+	U	1	0	0	0	0	SA0501
gi|29165615|ref|NC_002745.2|	585271	+	C	1	0	0	0	0	SA0501
gi|29165615|ref|NC_002745.2|	585293	+	A	1	0	0	0	0	SA0501
gi|29165615|ref|NC_002745.2|	585301	+	C	3	0	0	0	0	SA0501
gi|29165615|ref|NC_002745.2|	585305	+	U	0	0	0	1	0	SA0501
gi|29165615|ref|NC_002745.2|	585313	+	U	0	0	1	0	0	SA0501
gi|29165615|ref|NC_002745.2|	585316	+	U	0	0	0	0	1	SA0501
gi|29165615|ref|NC_002745.2|	585318	+	C	0	0	1	0	0	SA0501
gi|29165615|ref|NC_002745.2|	585323	+	U	0	0	1	0	0	SA0501
gi|29165615|ref|NC_002745.2|	585329	+	A	0	0	0	1	0	SA0501
gi|29165615|ref|NC_002745.2|	585341	+	U	0	1	0	0	0	SA0501
gi|29165615|ref|NC_002745.2|	585353	+	A	0	3	0	0	1	SA0501
gi|29165615|ref|NC_002745.2|	585358	+	U	0	0	0	1	0	SA0501
gi|29165615|ref|NC_002745.2|	585371	+	A	0	0	1	0	1	SA0501
gi|29165615|ref|NC_002745.2|	585375	+	C	0	1	2	1	0	SA0501
gi|29165615|ref|NC_002745.2|	585376	+	A	0	1	0	0	1	SA0501
gi|29165615|ref|NC_002745.2|	585377	+	A	0	1	0	0	0	SA0501
gi|29165615|ref|NC_002745.2|	585378	+	U	0	1	0	0	0	SA0501
gi|29165615|ref|NC_002745.2|	585402	+	G	0	0	0	0	1	SA0501
gi|29165615|ref|NC_002745.2|	585403	+	A	0	1	0	0	0	SA0501
gi|29165615|ref|NC_002745.2|	585487	+	C	0	0	1	0	1	SA0501
gi|29165615|ref|NC_002745.2|	585530	+	C	0	0	0	0	1	SA0501
gi|29165615|ref|NC_002745.2|	585554	+	A	1	0	0	0	0	SA0501
gi|29165615|ref|NC_002745.2|	585574	+	C	0	0	1	0	0	SA0501
gi|29165615|ref|NC_002745.2|	585580	+	C	0	1	0	0	0	SA0501
gi|29165615|ref|NC_002745.2|	585603	+	C	0	1	0	1	0	SA0501
gi|29165615|ref|NC_002745.2|	585606	+	A	1	0	0	0	0	SA0501
gi|29165615|ref|NC_002745.2|	585615	+	U	0	0	0	1	0	SA0501
gi|29165615|ref|NC_002745.2|	585657	+	A	0	0	1	0	0	SA0501
gi|29165615|ref|NC_002745.2|	585660	+	C	1	0	0	0	1	SA0501
gi|29165615|ref|NC_002745.2|	585661	+	A	0	0	1	0	0	SA0501
gi|29165615|ref|NC_002745.2|	585664	+	G	1	0	0	0	0	SA0501
gi|29165615|ref|NC_002745.2|	585670	+	G	1	0	0	0	0	SA0501
gi|29165615|ref|NC_002745.2|	585699	+	A	0	1	1	0	1	SA0501
gi|29165615|ref|NC_002745.2|	585700	+	C	0	0	1	0	0	SA0501
gi|29165615|ref|NC_002745.2|	585701	+	A	0	0	0	1	0	SA0501
gi|29165615|ref|NC_002745.2|	585728	+	U	0	0	1	0	0	SA0501
gi|29165615|ref|NC_002745.2|	585729	+	C	2	0	0	0	0	SA0501
gi|29165615|ref|NC_002745.2|	585731	+	U	0	1	0	0	0	SA0501
gi|29165615|ref|NC_002745.2|	585739	+	U	1	1	0	0	1	SA0501
gi|29165615|ref|NC_002745.2|	585747	+	G	0	1	0	0	0	SA0501
gi|29165615|ref|NC_002745.2|	585775	+	G	0	0	0	0	1	SA0501
gi|29165615|ref|NC_002745.2|	585812	+	A	1	0	0	0	0	SA0501
gi|29165615|ref|NC_002745.2|	585844	+	G	2	0	0	0	0	SA0501

gi|29165615|ref|NC_002745.2|	585848 +	U	0	1	0	0	0	SA0501
gi|29165615|ref|NC_002745.2|	585879 +	C	1	0	0	0	0	SA0501
gi|29165615|ref|NC_002745.2|	585887 +	A	0	0	1	0	0	SA0501
gi|29165615|ref|NC_002745.2|	585908 +	U	0	1	0	0	0	SA0501
gi|29165615|ref|NC_002745.2|	585913 +	A	0	0	0	0	1	SA0501
gi|29165615|ref|NC_002745.2|	585933 +	A	0	0	0	0	1	SA0501
gi|29165615|ref|NC_002745.2|	585937 +	C	0	0	1	0	0	SA0501
gi|29165615|ref|NC_002745.2|	585945 +	A	0	0	0	0	1	SA0501
gi|29165615|ref|NC_002745.2|	585997 +	A	1	0	0	0	1	SA0501
gi|29165615|ref|NC_002745.2|	586028 +	C	0	0	0	1	0	SA0501
gi|29165615|ref|NC_002745.2|	586045 +	C	0	1	0	0	0	SA0501
gi|29165615|ref|NC_002745.2|	586081 +	C	0	1	0	0	0	SA0501
gi|29165615|ref|NC_002745.2|	586100 +	C	2	0	0	0	0	SA0501
gi|29165615|ref|NC_002745.2|	586108 +	C	1	2	0	0	1	SA0501
gi|29165615|ref|NC_002745.2|	586109 +	A	0	0	0	1	0	SA0501
gi|29165615|ref|NC_002745.2|	586114 +	C	0	0	0	0	1	SA0501
gi|29165615|ref|NC_002745.2|	586123 +	C	0	0	1	0	0	SA0501
gi|29165615|ref|NC_002745.2|	586137 +	C	0	0	1	0	0	SA0501
gi|29165615|ref|NC_002745.2|	586151 +	A	0	1	0	0	0	SA0501
gi|29165615|ref|NC_002745.2|	586153 +	C	0	0	2	0	1	SA0501
gi|29165615|ref|NC_002745.2|	586167 +	A	0	1	0	0	0	SA0501
gi|29165615|ref|NC_002745.2|	586184 +	U	1	0	0	0	0	SA0501
gi|29165615|ref|NC_002745.2|	586211 +	C	1	0	0	0	0	SA0501
gi|29165615|ref|NC_002745.2|	586240 +	U	1	0	0	0	0	SA0501
gi|29165615|ref|NC_002745.2|	586255 +	A	0	1	0	0	0	SA0501
gi|29165615|ref|NC_002745.2|	586273 +	A	0	1	0	0	0	SA0501
gi|29165615|ref|NC_002745.2|	586286 +	A	0	0	0	0	1	SA0501
gi|29165615|ref|NC_002745.2|	586289 +	A	0	0	0	0	1	SA0501
gi|29165615|ref|NC_002745.2|	586294 +	U	1	0	0	0	0	SA0501
gi|29165615|ref|NC_002745.2|	586298 +	U	1	0	0	0	0	SA0501
gi|29165615|ref|NC_002745.2|	586374 +	C	0	1	0	0	1	SA0501
gi|29165615|ref|NC_002745.2|	586384 +	A	1	0	0	0	0	SA0501
gi|29165615|ref|NC_002745.2|	586402 +	C	1	0	0	0	0	SA0501
gi|29165615|ref|NC_002745.2|	586421 +	C	0	1	0	0	0	SA0501
gi|29165615|ref|NC_002745.2|	586424 +	A	1	0	0	0	0	SA0501
gi|29165615|ref|NC_002745.2|	586436 +	A	0	0	1	0	0	SA0501
gi|29165615|ref|NC_002745.2|	586442 +	C	1	0	0	0	0	SA0501
gi|29165615|ref|NC_002745.2|	586503 +	A	0	1	0	0	0	SA0501
gi|29165615|ref|NC_002745.2|	586519 +	U	0	0	0	1	0	SA0501
gi|29165615|ref|NC_002745.2|	586538 +	A	0	1	0	1	0	SA0501
gi|29165615|ref|NC_002745.2|	586539 +	A	0	1	0	0	0	SA0501
gi|29165615|ref|NC_002745.2|	586588 +	C	1	0	1	0	0	SA0501
gi|29165615|ref|NC_002745.2|	586597 +	U	0	2	0	0	0	SA0501
gi|29165615|ref|NC_002745.2|	586602 +	G	0	0	0	1	0	SA0501
gi|29165615|ref|NC_002745.2|	586636 +	C	0	0	0	2	0	SA0501
gi|29165615|ref|NC_002745.2|	586647 +	C	1	0	0	0	0	SA0501
gi|29165615|ref|NC_002745.2|	586662 +	A	0	1	0	0	0	SA0501
gi|29165615|ref|NC_002745.2|	586688 +	U	0	0	0	0	1	SA0501
gi|29165615|ref|NC_002745.2|	586723 +	C	0	1	0	0	0	SA0501
gi|29165615|ref|NC_002745.2|	586727 +	A	1	0	0	0	0	SA0501
gi|29165615|ref|NC_002745.2|	586733 +	A	0	0	0	0	1	SA0501
gi|29165615|ref|NC_002745.2|	586749 +	A	0	1	0	0	0	SA0501
gi|29165615|ref|NC_002745.2|	586767 +	G	0	0	0	0	1	SA0501
gi|29165615|ref|NC_002745.2|	586795 +	U	0	0	1	0	0	SA0501
gi|29165615|ref|NC_002745.2|	586805 +	A	0	1	0	0	0	SA0501
gi|29165615|ref|NC_002745.2|	586834 +	C	0	0	0	1	0	SA0501
gi|29165615|ref|NC_002745.2|	586916 +	U	0	0	0	1	0	SA0501
gi|29165615|ref|NC_002745.2|	586932 +	C	0	0	0	2	0	-
gi|29165615|ref|NC_002745.2|	586933 +	A	0	0	1	0	0	-
gi|29165615|ref|NC_002745.2|	586936 +	U	0	0	0	0	1	-
gi|29165615|ref|NC_002745.2|	586977 +	U	0	0	0	0	1	-
gi|29165615|ref|NC_002745.2|	586981 +	A	0	0	1	1	0	-
gi|29165615|ref|NC_002745.2|	586982 +	A	0	0	1	0	1	-
gi|29165615|ref|NC_002745.2|	586984 +	U	0	0	1	0	0	-
gi|29165615|ref|NC_002745.2|	586988 +	U	0	1	0	0	1	-
gi|29165615|ref|NC_002745.2|	586989 +	G	0	0	1	0	0	-
gi|29165615|ref|NC_002745.2|	587007 +	U	1	0	0	0	0	-
gi|29165615|ref|NC_002745.2|	587010 +	C	0	0	1	0	0	-
gi|29165615|ref|NC_002745.2|	587026 +	A	0	1	0	0	0	-
gi|29165615|ref|NC_002745.2|	587033 +	A	0	1	1	1	0	-
gi|29165615|ref|NC_002745.2|	587034 +	G	41	11	26	8	6	-
gi|29165615|ref|NC_002745.2|	587035 +	G	52	4	7	4	1	-
gi|29165615|ref|NC_002745.2|	587043 +	A	0	0	0	0	1	-
gi|29165615|ref|NC_002745.2|	587046 +	C	1	0	0	0	0	-
gi|29165615|ref|NC_002745.2|	587056 +	G	2	0	0	0	0	-
gi|29165615|ref|NC_002745.2|	587074 +	A	0	2	0	0	0	SA0502
gi|29165615|ref|NC_002745.2|	587077 +	G	0	1	0	0	0	SA0502
gi|29165615|ref|NC_002745.2|	587078 +	A	0	0	1	0	0	SA0502
gi|29165615|ref|NC_002745.2|	587081 +	A	0	0	1	0	0	SA0502
gi|29165615|ref|NC_002745.2|	587084 +	U	0	0	0	0	1	SA0502
gi|29165615|ref|NC_002745.2|	587168 +	U	0	0	1	0	0	SA0502

gi|29165615|ref|NC_002745.2|	587170	+	A	1	0	0	0	0	SA0502
gi|29165615|ref|NC_002745.2|	587185	+	G	0	0	0	0	1	SA0502
gi|29165615|ref|NC_002745.2|	587189	+	U	0	0	0	1	0	SA0502
gi|29165615|ref|NC_002745.2|	587190	+	A	0	0	1	0	0	SA0502
gi|29165615|ref|NC_002745.2|	587191	+	U	1	0	0	0	0	SA0502
gi|29165615|ref|NC_002745.2|	587192	+	A	0	1	0	1	0	SA0502
gi|29165615|ref|NC_002745.2|	587193	+	U	0	0	1	0	0	SA0502
gi|29165615|ref|NC_002745.2|	587197	+	A	3	0	0	1	0	SA0502
gi|29165615|ref|NC_002745.2|	587198	+	U	0	1	0	0	0	SA0502
gi|29165615|ref|NC_002745.2|	587200	+	A	0	0	1	0	0	SA0502
gi|29165615|ref|NC_002745.2|	587205	+	C	2	0	0	0	0	SA0502
gi|29165615|ref|NC_002745.2|	587220	+	C	1	0	0	1	0	SA0502
gi|29165615|ref|NC_002745.2|	587224	+	C	1	0	0	0	0	SA0502
gi|29165615|ref|NC_002745.2|	587225	+	A	0	1	0	1	1	SA0502
gi|29165615|ref|NC_002745.2|	587226	+	G	0	0	1	0	0	SA0502
gi|29165615|ref|NC_002745.2|	587227	+	A	2	3	8	9	12	SA0502
gi|29165615|ref|NC_002745.2|	587228	+	A	0	1	2	3	3	SA0502
gi|29165615|ref|NC_002745.2|	587231	+	A	2	0	0	0	1	SA0502
gi|29165615|ref|NC_002745.2|	587236	+	C	0	0	1	0	0	SA0502
gi|29165615|ref|NC_002745.2|	587238	+	U	1	0	0	0	0	SA0502
gi|29165615|ref|NC_002745.2|	587243	+	C	1	1	0	0	0	SA0502
gi|29165615|ref|NC_002745.2|	587245	+	U	0	1	0	1	0	SA0502
gi|29165615|ref|NC_002745.2|	587246	+	U	0	2	2	3	3	SA0502
gi|29165615|ref|NC_002745.2|	587247	+	U	0	0	2	0	0	SA0502
gi|29165615|ref|NC_002745.2|	587250	+	C	0	0	1	0	0	SA0502
gi|29165615|ref|NC_002745.2|	587251	+	A	0	0	0	0	1	SA0502
gi|29165615|ref|NC_002745.2|	587252	+	A	0	0	0	1	0	SA0502
gi|29165615|ref|NC_002745.2|	587253	+	A	0	0	1	0	0	SA0502
gi|29165615|ref|NC_002745.2|	587257	+	A	0	0	0	1	0	SA0502
gi|29165615|ref|NC_002745.2|	587258	+	A	0	0	0	0	1	SA0502
gi|29165615|ref|NC_002745.2|	587271	+	U	0	1	0	0	0	SA0502
gi|29165615|ref|NC_002745.2|	587275	+	C	3	2	6	2	2	SA0502
gi|29165615|ref|NC_002745.2|	587276	+	A	0	1	1	0	0	SA0502
gi|29165615|ref|NC_002745.2|	587278	+	G	2	1	0	0	0	SA0502
gi|29165615|ref|NC_002745.2|	587279	+	U	0	0	2	0	0	SA0502
gi|29165615|ref|NC_002745.2|	587280	+	A	1	0	0	0	0	SA0502
gi|29165615|ref|NC_002745.2|	587282	+	G	0	0	0	0	1	SA0502
gi|29165615|ref|NC_002745.2|	587284	+	A	0	0	0	1	0	SA0502
gi|29165615|ref|NC_002745.2|	587293	+	A	1	0	0	0	0	SA0502
gi|29165615|ref|NC_002745.2|	587299	+	A	0	1	0	0	0	SA0502
gi|29165615|ref|NC_002745.2|	587302	+	A	1	0	1	0	0	SA0502
gi|29165615|ref|NC_002745.2|	587303	+	U	1	0	0	0	0	SA0502
gi|29165615|ref|NC_002745.2|	587318	+	A	0	0	1	1	0	SA0502
gi|29165615|ref|NC_002745.2|	587323	+	G	1	0	0	0	0	-
gi|29165615|ref|NC_002745.2|	587339	+	U	1	0	0	0	0	-
gi|29165615|ref|NC_002745.2|	587349	+	A	1	0	0	0	0	-
gi|29165615|ref|NC_002745.2|	587353	+	U	1	0	0	0	0	-
gi|29165615|ref|NC_002745.2|	587354	+	A	0	0	1	0	0	-
gi|29165615|ref|NC_002745.2|	587358	+	A	0	1	0	0	0	-
gi|29165615|ref|NC_002745.2|	587359	+	A	0	0	2	1	1	-
gi|29165615|ref|NC_002745.2|	587360	+	C	0	2	2	3	4	-
gi|29165615|ref|NC_002745.2|	587361	+	C	3	0	2	1	0	-
gi|29165615|ref|NC_002745.2|	587362	+	U	0	1	0	0	0	-
gi|29165615|ref|NC_002745.2|	587363	+	A	0	6	2	0	6	-
gi|29165615|ref|NC_002745.2|	587364	+	A	1	73	51	17	29	-
gi|29165615|ref|NC_002745.2|	587365	+	A	3	15	16	7	8	-
gi|29165615|ref|NC_002745.2|	587366	+	A	2	9	1	0	5	-
gi|29165615|ref|NC_002745.2|	587367	+	A	0	2	1	0	0	-
gi|29165615|ref|NC_002745.2|	587368	+	U	1	1	0	0	1	-
gi|29165615|ref|NC_002745.2|	587369	+	G	0	0	0	0	2	-
gi|29165615|ref|NC_002745.2|	587370	+	A	0	1	0	0	0	-
gi|29165615|ref|NC_002745.2|	587373	+	C	2	1	0	0	1	-
gi|29165615|ref|NC_002745.2|	587374	+	A	1	0	1	0	0	-
gi|29165615|ref|NC_002745.2|	587375	+	C	0	0	1	0	0	-
gi|29165615|ref|NC_002745.2|	587376	+	C	1	0	0	1	0	-
gi|29165615|ref|NC_002745.2|	587379	+	G	1	1	0	0	0	-
gi|29165615|ref|NC_002745.2|	587383	+	U	0	0	1	0	0	-
gi|29165615|ref|NC_002745.2|	587394	+	A	1	0	0	0	1	-
gi|29165615|ref|NC_002745.2|	587395	+	A	0	0	1	0	1	-
gi|29165615|ref|NC_002745.2|	587396	+	A	2	0	0	0	0	-
gi|29165615|ref|NC_002745.2|	587397	+	G	1	0	0	0	0	-
gi|29165615|ref|NC_002745.2|	587398	+	U	2	0	1	0	0	-
gi|29165615|ref|NC_002745.2|	587399	+	G	0	0	1	0	2	-
gi|29165615|ref|NC_002745.2|	587400	+	A	0	2	0	1	1	-
gi|29165615|ref|NC_002745.2|	587412	+	C	0	0	1	0	0	-
gi|29165615|ref|NC_002745.2|	587414	+	U	1	0	0	0	0	-
gi|29165615|ref|NC_002745.2|	587424	+	C	1	0	0	0	0	SA0503
gi|29165615|ref|NC_002745.2|	587440	+	A	0	0	0	0	1	SA0503
gi|29165615|ref|NC_002745.2|	587464	+	A	1	0	0	0	0	SA0503
gi|29165615|ref|NC_002745.2|	587476	+	A	0	0	0	0	1	SA0503
gi|29165615|ref|NC_002745.2|	587517	+	A	0	1	0	0	0	SA0503

gi|29165615|ref|NC_002745.2|	587518 +	A	0	0	0	0	1	SA0503
gi|29165615|ref|NC_002745.2|	587529 +	A	0	0	0	1	0	SA0503
gi|29165615|ref|NC_002745.2|	587541 +	C	0	0	0	1	0	SA0503
gi|29165615|ref|NC_002745.2|	587556 +	U	0	1	0	0	0	SA0503
gi|29165615|ref|NC_002745.2|	587575 +	A	0	0	1	0	0	SA0503
gi|29165615|ref|NC_002745.2|	587585 +	A	0	1	0	0	0	SA0503
gi|29165615|ref|NC_002745.2|	587589 +	A	1	0	0	0	0	SA0503
gi|29165615|ref|NC_002745.2|	587596 +	C	0	0	1	0	0	SA0503
gi|29165615|ref|NC_002745.2|	587604 +	U	2	0	0	0	0	SA0503
gi|29165615|ref|NC_002745.2|	587608 +	U	1	0	0	0	0	SA0503
gi|29165615|ref|NC_002745.2|	587609 +	A	0	1	0	0	0	SA0503
gi|29165615|ref|NC_002745.2|	587616 +	C	1	0	1	0	0	SA0503
gi|29165615|ref|NC_002745.2|	587620 +	U	1	0	0	1	0	SA0503
gi|29165615|ref|NC_002745.2|	587627 +	U	0	2	1	0	0	SA0503
gi|29165615|ref|NC_002745.2|	587631 +	C	0	0	0	1	0	SA0503
gi|29165615|ref|NC_002745.2|	587635 +	C	0	0	1	0	0	SA0503
gi|29165615|ref|NC_002745.2|	587636 +	A	0	0	0	0	1	SA0503
gi|29165615|ref|NC_002745.2|	587639 +	A	0	0	0	1	0	SA0503
gi|29165615|ref|NC_002745.2|	587643 +	A	0	0	1	3	0	SA0503
gi|29165615|ref|NC_002745.2|	587656 +	C	1	0	0	0	0	SA0503
gi|29165615|ref|NC_002745.2|	587658 +	U	1	0	0	0	0	SA0503
gi|29165615|ref|NC_002745.2|	587659 +	C	2	0	0	0	0	SA0503
gi|29165615|ref|NC_002745.2|	587661 +	C	2	0	0	0	0	SA0503
gi|29165615|ref|NC_002745.2|	587665 +	U	2	0	0	0	0	SA0503
gi|29165615|ref|NC_002745.2|	587673 +	A	0	1	0	0	0	SA0503
gi|29165615|ref|NC_002745.2|	587677 +	C	1	0	0	0	0	SA0503
gi|29165615|ref|NC_002745.2|	587680 +	A	1	0	0	0	0	SA0503
gi|29165615|ref|NC_002745.2|	587682 +	A	0	1	0	0	0	SA0503
gi|29165615|ref|NC_002745.2|	587688 +	A	0	0	0	0	1	SA0503
gi|29165615|ref|NC_002745.2|	587691 +	G	0	0	0	0	1	SA0503
gi|29165615|ref|NC_002745.2|	587699 +	C	0	0	0	0	1	SA0503
gi|29165615|ref|NC_002745.2|	587763 +	U	1	0	0	0	0	SA0503
gi|29165615|ref|NC_002745.2|	587782 +	C	0	0	0	0	1	SA0503
gi|29165615|ref|NC_002745.2|	587788 +	U	1	0	0	0	0	SA0503
gi|29165615|ref|NC_002745.2|	587803 +	A	0	0	1	0	0	SA0503
gi|29165615|ref|NC_002745.2|	587811 +	G	1	1	1	0	1	SA0503
gi|29165615|ref|NC_002745.2|	587818 +	G	1	0	0	0	0	SA0503
gi|29165615|ref|NC_002745.2|	587819 +	A	0	1	0	1	0	SA0503
gi|29165615|ref|NC_002745.2|	587847 +	A	0	0	0	1	0	-
gi|29165615|ref|NC_002745.2|	587853 +	A	0	1	0	0	0	-
gi|29165615|ref|NC_002745.2|	587858 +	A	0	2	5	1	0	-
gi|29165615|ref|NC_002745.2|	587859 +	A	0	1	1	1	1	-
gi|29165615|ref|NC_002745.2|	587864 +	A	0	1	0	0	0	-
gi|29165615|ref|NC_002745.2|	587866 +	A	0	1	0	0	0	-
gi|29165615|ref|NC_002745.2|	587867 +	A	0	1	1	0	0	-
gi|29165615|ref|NC_002745.2|	587868 +	U	0	1	0	0	0	-
gi|29165615|ref|NC_002745.2|	587870 +	U	0	1	0	0	0	-
gi|29165615|ref|NC_002745.2|	587873 +	A	0	1	2	0	0	-
gi|29165615|ref|NC_002745.2|	587874 +	A	0	0	0	0	1	-
gi|29165615|ref|NC_002745.2|	587875 +	U	0	0	0	0	1	-
gi|29165615|ref|NC_002745.2|	587876 +	A	0	1	0	0	1	-
gi|29165615|ref|NC_002745.2|	587878 +	A	0	5	2	0	0	-
gi|29165615|ref|NC_002745.2|	587883 +	A	0	0	0	1	0	-
gi|29165615|ref|NC_002745.2|	587891 +	U	1	0	0	0	0	-
gi|29165615|ref|NC_002745.2|	587908 +	A	0	1	0	0	0	SA0504
gi|29165615|ref|NC_002745.2|	587938 +	C	0	1	1	0	0	SA0504
gi|29165615|ref|NC_002745.2|	587939 +	C	0	0	0	0	1	SA0504
gi|29165615|ref|NC_002745.2|	587942 +	A	1	0	0	0	0	SA0504
gi|29165615|ref|NC_002745.2|	587946 +	A	3	1	0	0	4	SA0504
gi|29165615|ref|NC_002745.2|	587947 +	A	0	0	1	0	0	SA0504
gi|29165615|ref|NC_002745.2|	587949 +	U	0	0	1	0	0	SA0504
gi|29165615|ref|NC_002745.2|	587963 +	U	0	0	1	0	0	SA0504
gi|29165615|ref|NC_002745.2|	587966 +	U	0	0	0	1	0	SA0504
gi|29165615|ref|NC_002745.2|	587967 +	A	0	0	0	0	1	SA0504
gi|29165615|ref|NC_002745.2|	587969 +	C	0	1	0	0	0	SA0504
gi|29165615|ref|NC_002745.2|	587971 +	A	0	0	0	0	1	SA0504
gi|29165615|ref|NC_002745.2|	587980 +	A	0	1	0	0	0	SA0504
gi|29165615|ref|NC_002745.2|	587982 +	C	0	0	0	1	0	SA0504
gi|29165615|ref|NC_002745.2|	587983 +	A	0	0	0	0	1	SA0504
gi|29165615|ref|NC_002745.2|	587985 +	A	0	0	0	0	1	SA0504
gi|29165615|ref|NC_002745.2|	587986 +	A	0	2	0	0	0	SA0504
gi|29165615|ref|NC_002745.2|	587988 +	U	2	0	0	0	0	SA0504
gi|29165615|ref|NC_002745.2|	587992 +	U	0	2	0	0	0	SA0504
gi|29165615|ref|NC_002745.2|	587996 +	A	0	0	0	1	0	SA0504
gi|29165615|ref|NC_002745.2|	588001 +	A	0	0	0	1	0	SA0504
gi|29165615|ref|NC_002745.2|	588002 +	A	2	0	0	0	0	SA0504
gi|29165615|ref|NC_002745.2|	588005 +	G	0	0	1	0	0	SA0504
gi|29165615|ref|NC_002745.2|	588011 +	C	1	1	0	0	0	SA0504
gi|29165615|ref|NC_002745.2|	588016 +	C	0	0	0	1	0	SA0504
gi|29165615|ref|NC_002745.2|	588022 +	A	0	0	0	0	1	SA0504
gi|29165615|ref|NC_002745.2|	588038 +	U	1	0	0	0	0	SA0504

gi|29165615|ref|NC_002745.2|	588039 +	C	1	0	0	0	0	SA0504
gi|29165615|ref|NC_002745.2|	588043 +	C	0	1	0	0	0	SA0504
gi|29165615|ref|NC_002745.2|	588044 +	U	0	0	1	0	0	SA0504
gi|29165615|ref|NC_002745.2|	588047 +	U	0	0	0	0	1	SA0504
gi|29165615|ref|NC_002745.2|	588054 +	A	0	0	1	0	0	SA0504
gi|29165615|ref|NC_002745.2|	588055 +	C	0	0	1	0	0	SA0504
gi|29165615|ref|NC_002745.2|	588056 +	G	1	0	0	0	0	SA0504
gi|29165615|ref|NC_002745.2|	588057 +	C	0	0	1	0	0	SA0504
gi|29165615|ref|NC_002745.2|	588071 +	C	1	0	0	0	0	SA0504
gi|29165615|ref|NC_002745.2|	588084 +	C	0	0	1	0	0	SA0504
gi|29165615|ref|NC_002745.2|	588093 +	A	1	0	0	0	0	SA0504
gi|29165615|ref|NC_002745.2|	588094 +	A	0	1	0	0	0	SA0504
gi|29165615|ref|NC_002745.2|	588097 +	A	0	1	0	0	0	SA0504
gi|29165615|ref|NC_002745.2|	588105 +	U	0	1	0	0	0	SA0504
gi|29165615|ref|NC_002745.2|	588109 +	C	0	1	0	0	0	SA0504
gi|29165615|ref|NC_002745.2|	588118 +	G	0	0	1	0	0	SA0504
gi|29165615|ref|NC_002745.2|	588126 +	A	0	0	0	0	1	SA0504
gi|29165615|ref|NC_002745.2|	588128 +	C	2	1	0	0	0	SA0504
gi|29165615|ref|NC_002745.2|	588134 +	G	1	0	0	0	0	SA0504
gi|29165615|ref|NC_002745.2|	588147 +	U	1	0	0	0	0	SA0504
gi|29165615|ref|NC_002745.2|	588157 +	G	0	0	0	0	1	SA0504
gi|29165615|ref|NC_002745.2|	588161 +	C	0	0	0	1	0	SA0504
gi|29165615|ref|NC_002745.2|	588164 +	U	0	0	0	1	0	SA0504
gi|29165615|ref|NC_002745.2|	588177 +	A	0	0	1	1	0	SA0504
gi|29165615|ref|NC_002745.2|	588179 +	A	0	1	0	0	0	SA0504
gi|29165615|ref|NC_002745.2|	588209 +	U	1	0	0	0	0	SA0504
gi|29165615|ref|NC_002745.2|	588230 +	G	0	0	0	1	0	SA0504
gi|29165615|ref|NC_002745.2|	588244 +	A	0	0	0	0	1	SA0504
gi|29165615|ref|NC_002745.2|	588270 +	U	2	0	0	0	0	SA0504
gi|29165615|ref|NC_002745.2|	588287 +	A	0	0	0	0	2	SA0504
gi|29165615|ref|NC_002745.2|	588290 +	C	2	0	0	0	0	SA0504
gi|29165615|ref|NC_002745.2|	588291 +	A	0	1	0	0	0	SA0504
gi|29165615|ref|NC_002745.2|	588319 +	A	0	0	1	0	0	SA0504
gi|29165615|ref|NC_002745.2|	588321 +	U	0	0	0	0	1	SA0504
gi|29165615|ref|NC_002745.2|	588347 +	C	0	0	1	0	0	SA0504
gi|29165615|ref|NC_002745.2|	588350 +	U	2	0	0	0	0	SA0504
gi|29165615|ref|NC_002745.2|	588406 +	U	0	1	0	0	0	-
gi|29165615|ref|NC_002745.2|	588425 +	C	0	1	0	0	0	-
gi|29165615|ref|NC_002745.2|	588433 +	G	1	3	0	0	0	-
gi|29165615|ref|NC_002745.2|	588467 +	U	0	0	1	0	0	-
gi|29165615|ref|NC_002745.2|	588474 +	U	0	1	0	0	0	-
gi|29165615|ref|NC_002745.2|	588496 +	C	0	1	1	0	0	SA0505
gi|29165615|ref|NC_002745.2|	588502 +	A	0	1	0	0	0	SA0505
gi|29165615|ref|NC_002745.2|	588508 +	C	0	2	0	0	0	SA0505
gi|29165615|ref|NC_002745.2|	588509 +	A	1	0	0	0	0	SA0505
gi|29165615|ref|NC_002745.2|	588513 +	G	0	0	1	0	0	SA0505
gi|29165615|ref|NC_002745.2|	588514 +	A	0	0	0	1	0	SA0505
gi|29165615|ref|NC_002745.2|	588519 +	A	0	2	0	0	2	SA0505
gi|29165615|ref|NC_002745.2|	588542 +	U	0	0	0	1	0	SA0505
gi|29165615|ref|NC_002745.2|	588547 +	U	0	1	0	0	0	SA0505
gi|29165615|ref|NC_002745.2|	588551 +	U	0	1	0	0	0	SA0505
gi|29165615|ref|NC_002745.2|	588561 +	A	0	0	0	0	1	SA0505
gi|29165615|ref|NC_002745.2|	588566 +	U	0	1	0	0	0	SA0505
gi|29165615|ref|NC_002745.2|	588591 +	A	2	0	0	0	0	SA0505
gi|29165615|ref|NC_002745.2|	588599 +	U	1	0	0	0	0	SA0505
gi|29165615|ref|NC_002745.2|	588600 +	A	0	1	0	0	0	SA0505
gi|29165615|ref|NC_002745.2|	588631 +	C	1	0	0	0	0	SA0505
gi|29165615|ref|NC_002745.2|	588632 +	U	2	0	0	0	0	SA0505
gi|29165615|ref|NC_002745.2|	588633 +	U	0	0	1	0	0	SA0505
gi|29165615|ref|NC_002745.2|	588643 +	A	0	0	1	0	0	SA0505
gi|29165615|ref|NC_002745.2|	588644 +	C	0	0	0	1	0	SA0505
gi|29165615|ref|NC_002745.2|	588645 +	U	0	0	1	0	0	SA0505
gi|29165615|ref|NC_002745.2|	588661 +	A	1	0	1	0	0	SA0505
gi|29165615|ref|NC_002745.2|	588676 +	C	2	0	0	0	0	SA0505
gi|29165615|ref|NC_002745.2|	588685 +	C	1	0	0	0	0	SA0505
gi|29165615|ref|NC_002745.2|	588688 +	C	2	0	0	0	0	SA0505
gi|29165615|ref|NC_002745.2|	588691 +	C	2	0	0	1	0	SA0505
gi|29165615|ref|NC_002745.2|	588694 +	C	1	0	0	0	0	SA0505
gi|29165615|ref|NC_002745.2|	588714 +	C	2	1	1	0	0	SA0505
gi|29165615|ref|NC_002745.2|	588734 +	U	0	0	1	0	0	SA0505
gi|29165615|ref|NC_002745.2|	588739 +	C	0	2	0	0	0	SA0505
gi|29165615|ref|NC_002745.2|	588754 +	U	0	0	0	0	1	SA0505
gi|29165615|ref|NC_002745.2|	588760 +	U	1	0	0	0	0	SA0505
gi|29165615|ref|NC_002745.2|	588763 +	A	0	0	0	1	0	SA0505
gi|29165615|ref|NC_002745.2|	588797 +	A	1	0	0	0	0	SA0505
gi|29165615|ref|NC_002745.2|	588829 +	A	1	0	0	0	0	SA0505
gi|29165615|ref|NC_002745.2|	588890 +	U	0	0	0	0	1	SA0505
gi|29165615|ref|NC_002745.2|	588899 +	A	0	1	0	0	0	SA0505
gi|29165615|ref|NC_002745.2|	588900 +	A	0	7	0	0	0	SA0505
gi|29165615|ref|NC_002745.2|	588901 +	U	0	1	0	0	0	SA0505
gi|29165615|ref|NC_002745.2|	588905 +	C	0	1	0	0	1	SA0505

gi|29165615|ref|NC_002745.2|	588908	+	A	0	1	0	0	0	SA0505
gi|29165615|ref|NC_002745.2|	588926	+	A	0	0	0	1	0	SA0505
gi|29165615|ref|NC_002745.2|	588932	+	U	1	0	0	0	0	SA0505
gi|29165615|ref|NC_002745.2|	588941	+	A	0	1	0	0	0	SA0505
gi|29165615|ref|NC_002745.2|	588951	+	C	0	0	1	0	0	SA0505
gi|29165615|ref|NC_002745.2|	588955	+	U	0	0	0	0	1	SA0505
gi|29165615|ref|NC_002745.2|	588981	+	U	0	1	0	0	0	SA0505
gi|29165615|ref|NC_002745.2|	588983	+	A	0	1	0	0	0	SA0505
gi|29165615|ref|NC_002745.2|	589014	+	A	0	1	0	0	1	SA0505
gi|29165615|ref|NC_002745.2|	589016	+	C	0	0	1	0	0	SA0505
gi|29165615|ref|NC_002745.2|	589023	+	U	0	0	0	0	1	SA0505
gi|29165615|ref|NC_002745.2|	589080	+	A	0	0	1	0	0	SA0505
gi|29165615|ref|NC_002745.2|	589084	+	A	0	0	1	0	0	SA0505
gi|29165615|ref|NC_002745.2|	589089	+	C	1	0	0	0	0	SA0505
gi|29165615|ref|NC_002745.2|	589090	+	C	1	0	0	0	0	SA0505
gi|29165615|ref|NC_002745.2|	589093	+	A	0	2	0	0	0	SA0505
gi|29165615|ref|NC_002745.2|	589098	+	C	0	1	0	0	0	SA0505
gi|29165615|ref|NC_002745.2|	589100	+	C	1	0	0	0	0	SA0505
gi|29165615|ref|NC_002745.2|	589101	+	U	0	1	0	0	0	SA0505
gi|29165615|ref|NC_002745.2|	589103	+	A	0	0	0	0	1	SA0505
gi|29165615|ref|NC_002745.2|	589105	+	A	1	0	0	0	0	SA0505
gi|29165615|ref|NC_002745.2|	589106	+	U	2	0	0	0	0	SA0505
gi|29165615|ref|NC_002745.2|	589108	+	G	0	2	0	0	0	SA0505
gi|29165615|ref|NC_002745.2|	589109	+	A	1	0	0	0	0	SA0505
gi|29165615|ref|NC_002745.2|	589111	+	C	4	0	0	0	1	SA0505
gi|29165615|ref|NC_002745.2|	589112	+	U	3	0	0	1	2	SA0505
gi|29165615|ref|NC_002745.2|	589113	+	G	0	3	1	0	0	SA0505
gi|29165615|ref|NC_002745.2|	589114	+	A	2	0	0	0	0	SA0505
gi|29165615|ref|NC_002745.2|	589115	+	A	1	0	0	0	1	SA0505
gi|29165615|ref|NC_002745.2|	589117	+	A	0	1	0	0	0	SA0505
gi|29165615|ref|NC_002745.2|	589118	+	A	0	1	0	0	0	SA0505
gi|29165615|ref|NC_002745.2|	589120	+	C	3	0	0	0	0	SA0505
gi|29165615|ref|NC_002745.2|	589126	+	C	0	0	0	1	0	SA0505
gi|29165615|ref|NC_002745.2|	589133	+	A	1	0	0	0	0	SA0505
gi|29165615|ref|NC_002745.2|	589136	+	C	0	0	0	0	1	SA0505
gi|29165615|ref|NC_002745.2|	589141	+	C	0	1	1	0	0	SA0505
gi|29165615|ref|NC_002745.2|	589150	+	A	0	1	0	0	0	SA0505
gi|29165615|ref|NC_002745.2|	589249	+	U	2	0	0	0	0	SA0505
gi|29165615|ref|NC_002745.2|	589306	+	A	1	0	0	0	0	SA0505
gi|29165615|ref|NC_002745.2|	589338	+	G	0	0	1	0	0	SA0505
gi|29165615|ref|NC_002745.2|	589342	+	U	1	0	0	0	0	SA0505
gi|29165615|ref|NC_002745.2|	589346	+	A	1	0	0	0	0	SA0505
gi|29165615|ref|NC_002745.2|	589348	+	C	0	0	0	0	1	SA0505
gi|29165615|ref|NC_002745.2|	589349	+	A	0	0	1	0	0	SA0505
gi|29165615|ref|NC_002745.2|	589351	+	U	0	1	0	0	0	SA0505
gi|29165615|ref|NC_002745.2|	589353	+	A	0	1	0	0	0	SA0505
gi|29165615|ref|NC_002745.2|	589360	+	A	2	0	0	0	0	SA0505
gi|29165615|ref|NC_002745.2|	589373	+	C	0	0	0	0	3	SA0505
gi|29165615|ref|NC_002745.2|	589387	+	U	0	0	0	0	1	SA0505
gi|29165615|ref|NC_002745.2|	589451	+	U	0	1	0	0	0	SA0505
gi|29165615|ref|NC_002745.2|	589461	+	A	0	0	0	1	0	SA0505
gi|29165615|ref|NC_002745.2|	589468	+	C	0	1	1	0	0	SA0505
gi|29165615|ref|NC_002745.2|	589473	+	U	3	0	0	0	0	SA0505
gi|29165615|ref|NC_002745.2|	589486	+	C	0	1	0	0	0	SA0505
gi|29165615|ref|NC_002745.2|	589493	+	A	0	1	0	0	0	SA0505
gi|29165615|ref|NC_002745.2|	589522	+	C	0	1	0	0	0	SA0505
gi|29165615|ref|NC_002745.2|	589523	+	U	2	0	0	0	0	SA0505
gi|29165615|ref|NC_002745.2|	589525	+	C	0	1	0	0	0	SA0505
gi|29165615|ref|NC_002745.2|	589526	+	U	0	0	0	0	1	SA0505
gi|29165615|ref|NC_002745.2|	589527	+	A	0	1	0	0	0	SA0505
gi|29165615|ref|NC_002745.2|	589528	+	A	0	0	1	0	0	SA0505
gi|29165615|ref|NC_002745.2|	589557	+	U	1	0	0	0	0	SA0505
gi|29165615|ref|NC_002745.2|	589561	+	A	0	0	0	1	0	SA0505
gi|29165615|ref|NC_002745.2|	589570	+	C	1	0	0	0	0	SA0505
gi|29165615|ref|NC_002745.2|	589582	+	A	0	0	0	0	2	SA0505
gi|29165615|ref|NC_002745.2|	589585	+	A	0	1	0	0	0	SA0505
gi|29165615|ref|NC_002745.2|	589587	+	A	0	4	2	0	1	SA0505
gi|29165615|ref|NC_002745.2|	589591	+	A	0	1	0	0	0	SA0505
gi|29165615|ref|NC_002745.2|	589592	+	U	0	0	1	0	0	SA0505
gi|29165615|ref|NC_002745.2|	589593	+	A	0	0	1	0	0	SA0505
gi|29165615|ref|NC_002745.2|	589594	+	C	1	0	0	0	0	SA0505
gi|29165615|ref|NC_002745.2|	589595	+	U	3	0	0	0	0	SA0505
gi|29165615|ref|NC_002745.2|	589597	+	U	0	0	2	0	3	SA0505
gi|29165615|ref|NC_002745.2|	589598	+	A	0	0	2	0	1	SA0505
gi|29165615|ref|NC_002745.2|	589601	+	C	2	0	0	0	0	SA0505
gi|29165615|ref|NC_002745.2|	589603	+	C	0	0	0	0	1	SA0505
gi|29165615|ref|NC_002745.2|	589614	+	G	1	0	0	0	0	SA0505
gi|29165615|ref|NC_002745.2|	589621	+	C	0	0	0	0	1	SA0505
gi|29165615|ref|NC_002745.2|	589631	+	U	0	0	0	0	1	SA0505
gi|29165615|ref|NC_002745.2|	589636	+	A	1	0	0	0	0	SA0505
gi|29165615|ref|NC_002745.2|	589639	+	C	0	1	1	0	0	SA0505

gi|29165615|ref|NC_002745.2|	589655	+	U	0	0	1	0	0	SA0505
gi|29165615|ref|NC_002745.2|	589703	+	A	1	0	0	0	0	SA0505
gi|29165615|ref|NC_002745.2|	589708	+	C	0	0	1	0	0	SA0505
gi|29165615|ref|NC_002745.2|	589709	+	A	0	1	0	0	0	SA0505
gi|29165615|ref|NC_002745.2|	589723	+	C	0	1	0	0	0	SA0505
gi|29165615|ref|NC_002745.2|	589734	+	A	0	1	0	0	0	SA0505
gi|29165615|ref|NC_002745.2|	589736	+	A	0	1	0	0	0	SA0505
gi|29165615|ref|NC_002745.2|	589748	+	C	2	0	0	0	0	SA0505
gi|29165615|ref|NC_002745.2|	589779	+	U	0	1	0	0	0	SA0505
gi|29165615|ref|NC_002745.2|	589796	+	A	2	1	0	0	0	SA0505
gi|29165615|ref|NC_002745.2|	589806	+	G	0	0	1	0	0	SA0505
gi|29165615|ref|NC_002745.2|	589807	+	C	1	0	0	0	0	SA0505
gi|29165615|ref|NC_002745.2|	589847	+	U	1	0	0	0	0	SA0505
gi|29165615|ref|NC_002745.2|	589860	+	C	0	0	1	0	0	SA0505
gi|29165615|ref|NC_002745.2|	590004	+	C	0	1	0	0	0	SA0505
gi|29165615|ref|NC_002745.2|	590035	+	C	2	0	0	0	0	SA0505
gi|29165615|ref|NC_002745.2|	590043	+	A	2	0	0	0	0	SA0505
gi|29165615|ref|NC_002745.2|	590065	+	A	0	0	1	0	0	SA0505
gi|29165615|ref|NC_002745.2|	590103	+	A	1	0	0	0	0	SA0505
gi|29165615|ref|NC_002745.2|	590126	+	U	1	0	0	0	0	SA0505
gi|29165615|ref|NC_002745.2|	590134	+	C	1	0	0	0	0	SA0505
gi|29165615|ref|NC_002745.2|	590135	+	U	0	1	1	0	0	SA0505
gi|29165615|ref|NC_002745.2|	590144	+	U	1	0	0	0	0	SA0505
gi|29165615|ref|NC_002745.2|	590152	+	U	1	0	0	0	0	SA0505
gi|29165615|ref|NC_002745.2|	590158	+	G	0	0	1	0	0	SA0505
gi|29165615|ref|NC_002745.2|	590159	+	U	2	0	0	0	0	SA0505
gi|29165615|ref|NC_002745.2|	590164	+	C	0	0	0	0	1	SA0505
gi|29165615|ref|NC_002745.2|	590169	+	A	0	2	0	0	0	SA0505
gi|29165615|ref|NC_002745.2|	590170	+	U	1	0	0	0	0	SA0505
gi|29165615|ref|NC_002745.2|	590172	+	G	0	1	0	0	0	SA0505
gi|29165615|ref|NC_002745.2|	590176	+	U	1	0	0	0	0	SA0505
gi|29165615|ref|NC_002745.2|	590185	+	A	0	0	0	1	0	SA0505
gi|29165615|ref|NC_002745.2|	590186	+	A	0	0	1	0	0	SA0505
gi|29165615|ref|NC_002745.2|	590188	+	U	0	0	0	0	1	SA0505
gi|29165615|ref|NC_002745.2|	590190	+	U	0	1	0	0	0	SA0505
gi|29165615|ref|NC_002745.2|	590192	+	U	2	0	0	0	0	SA0505
gi|29165615|ref|NC_002745.2|	590200	+	C	1	0	0	0	0	SA0505
gi|29165615|ref|NC_002745.2|	590213	+	C	0	0	0	1	0	SA0505
gi|29165615|ref|NC_002745.2|	590221	+	C	0	0	1	0	0	SA0505
gi|29165615|ref|NC_002745.2|	590248	+	C	2	0	0	0	0	SA0505
gi|29165615|ref|NC_002745.2|	590251	+	U	2	0	0	0	0	SA0505
gi|29165615|ref|NC_002745.2|	590253	+	G	0	1	0	0	0	SA0505
gi|29165615|ref|NC_002745.2|	590272	+	A	1	0	0	0	0	SA0505
gi|29165615|ref|NC_002745.2|	590291	+	C	0	0	1	0	0	SA0505
gi|29165615|ref|NC_002745.2|	590305	+	U	1	0	0	0	0	SA0505
gi|29165615|ref|NC_002745.2|	590308	+	A	1	0	0	0	0	SA0505
gi|29165615|ref|NC_002745.2|	590315	+	U	1	0	0	0	0	SA0505
gi|29165615|ref|NC_002745.2|	590322	+	A	0	0	0	0	1	SA0505
gi|29165615|ref|NC_002745.2|	590326	+	C	2	0	0	1	0	SA0505
gi|29165615|ref|NC_002745.2|	590333	+	G	0	0	0	0	2	SA0505
gi|29165615|ref|NC_002745.2|	590338	+	U	1	0	0	0	0	SA0505
gi|29165615|ref|NC_002745.2|	590348	+	C	2	0	0	0	1	SA0505
gi|29165615|ref|NC_002745.2|	590365	+	C	1	0	0	0	0	SA0505
gi|29165615|ref|NC_002745.2|	590370	+	C	0	1	0	0	0	SA0505
gi|29165615|ref|NC_002745.2|	590380	+	U	2	0	0	0	0	SA0505
gi|29165615|ref|NC_002745.2|	590402	+	U	0	1	0	0	0	SA0505
gi|29165615|ref|NC_002745.2|	590410	+	A	0	0	0	0	1	SA0505
gi|29165615|ref|NC_002745.2|	590448	+	G	0	1	0	0	0	SA0505
gi|29165615|ref|NC_002745.2|	590455	+	C	1	0	0	0	0	SA0505
gi|29165615|ref|NC_002745.2|	590472	+	A	0	0	0	0	1	SA0505
gi|29165615|ref|NC_002745.2|	590478	+	C	1	0	0	0	0	SA0505
gi|29165615|ref|NC_002745.2|	590498	+	U	0	1	0	0	0	SA0505
gi|29165615|ref|NC_002745.2|	590509	+	A	1	0	0	0	0	SA0505
gi|29165615|ref|NC_002745.2|	590514	+	U	1	0	0	1	0	SA0505
gi|29165615|ref|NC_002745.2|	590526	+	C	0	0	0	2	0	SA0505
gi|29165615|ref|NC_002745.2|	590535	+	A	0	0	1	0	0	SA0505
gi|29165615|ref|NC_002745.2|	590537	+	C	0	1	0	0	0	SA0505
gi|29165615|ref|NC_002745.2|	590542	+	A	0	1	0	2	0	SA0505
gi|29165615|ref|NC_002745.2|	590545	+	A	0	0	0	1	0	SA0505
gi|29165615|ref|NC_002745.2|	590552	+	C	0	1	0	0	0	SA0505
gi|29165615|ref|NC_002745.2|	590553	+	A	0	0	1	0	0	SA0505
gi|29165615|ref|NC_002745.2|	590578	+	A	0	1	0	1	0	-
gi|29165615|ref|NC_002745.2|	590583	+	U	0	0	0	0	1	-
gi|29165615|ref|NC_002745.2|	590590	+	U	0	0	0	0	1	-
gi|29165615|ref|NC_002745.2|	590608	+	A	0	1	1	0	2	-
gi|29165615|ref|NC_002745.2|	590615	+	G	0	0	0	0	1	-
gi|29165615|ref|NC_002745.2|	590616	+	U	0	0	1	1	0	-
gi|29165615|ref|NC_002745.2|	590617	+	G	4	4	2	4	6	-
gi|29165615|ref|NC_002745.2|	590625	+	A	0	0	1	0	0	-
gi|29165615|ref|NC_002745.2|	590642	+	U	0	0	0	0	1	-
gi|29165615|ref|NC_002745.2|	590643	+	A	0	2	0	0	1	-

gi|29165615|ref|NC_002745.2|	590644	+	U	0	0	0	1	0	-
gi|29165615|ref|NC_002745.2|	590645	+	A	0	0	1	0	0	-
gi|29165615|ref|NC_002745.2|	590648	+	U	0	0	0	0	1	-
gi|29165615|ref|NC_002745.2|	590650	+	G	0	3	2	1	2	-
gi|29165615|ref|NC_002745.2|	590651	+	A	3	7	6	4	4	-
gi|29165615|ref|NC_002745.2|	590652	+	U	0	0	0	1	0	-
gi|29165615|ref|NC_002745.2|	590653	+	U	0	1	0	0	1	-
gi|29165615|ref|NC_002745.2|	590659	+	G	1	1	1	1	1	-
gi|29165615|ref|NC_002745.2|	590668	+	C	0	1	0	1	0	-
gi|29165615|ref|NC_002745.2|	590683	+	G	4	4	2	2	1	-
gi|29165615|ref|NC_002745.2|	590685	+	A	0	0	1	0	0	-
gi|29165615|ref|NC_002745.2|	590688	+	A	0	0	1	0	0	-
gi|29165615|ref|NC_002745.2|	590690	+	U	0	0	1	0	0	-
gi|29165615|ref|NC_002745.2|	590691	+	C	1	0	0	0	0	-
gi|29165615|ref|NC_002745.2|	590698	+	C	0	0	1	1	0	-
gi|29165615|ref|NC_002745.2|	590699	+	A	0	0	0	2	2	-
gi|29165615|ref|NC_002745.2|	590701	+	C	0	1	0	0	0	-
gi|29165615|ref|NC_002745.2|	590702	+	G	0	0	0	0	1	-
gi|29165615|ref|NC_002745.2|	590703	+	C	4	0	3	7	9	-
gi|29165615|ref|NC_002745.2|	590704	+	U	4	0	1	2	1	-
gi|29165615|ref|NC_002745.2|	590706	+	U	4	0	0	3	2	-
gi|29165615|ref|NC_002745.2|	590707	+	A	0	0	0	1	1	-
gi|29165615|ref|NC_002745.2|	590709	+	A	0	1	0	0	0	-
gi|29165615|ref|NC_002745.2|	590715	+	U	5	0	0	2	0	-
gi|29165615|ref|NC_002745.2|	590723	+	U	0	0	1	0	0	-
gi|29165615|ref|NC_002745.2|	590731	+	A	1	0	0	0	0	-
gi|29165615|ref|NC_002745.2|	590733	+	C	0	1	0	1	0	-
gi|29165615|ref|NC_002745.2|	590734	+	U	0	1	0	0	0	-
gi|29165615|ref|NC_002745.2|	590737	+	C	0	0	0	0	1	-
gi|29165615|ref|NC_002745.2|	590741	+	A	0	1	0	0	0	-
gi|29165615|ref|NC_002745.2|	590746	+	U	4	3	0	3	0	-
gi|29165615|ref|NC_002745.2|	590747	+	A	0	1	0	3	1	-
gi|29165615|ref|NC_002745.2|	590748	+	A	0	0	1	0	2	-
gi|29165615|ref|NC_002745.2|	590749	+	A	0	0	1	0	0	-
gi|29165615|ref|NC_002745.2|	590750	+	U	0	0	1	0	0	-
gi|29165615|ref|NC_002745.2|	590751	+	U	0	0	0	1	0	-
gi|29165615|ref|NC_002745.2|	590753	+	A	0	1	0	2	1	-
gi|29165615|ref|NC_002745.2|	590754	+	A	3	25	16	23	17	-
gi|29165615|ref|NC_002745.2|	590755	+	A	0	3	2	0	3	-
gi|29165615|ref|NC_002745.2|	590756	+	U	1	0	0	0	0	-
gi|29165615|ref|NC_002745.2|	590757	+	A	0	0	1	1	2	-
gi|29165615|ref|NC_002745.2|	590758	+	U	0	0	0	1	0	-
gi|29165615|ref|NC_002745.2|	590760	+	A	0	1	2	1	2	-
gi|29165615|ref|NC_002745.2|	590761	+	U	0	0	2	0	0	-
gi|29165615|ref|NC_002745.2|	590764	+	U	0	0	0	1	1	-
gi|29165615|ref|NC_002745.2|	590767	+	A	0	1	0	0	0	-
gi|29165615|ref|NC_002745.2|	590770	+	A	0	1	0	0	0	-
gi|29165615|ref|NC_002745.2|	590771	+	G	0	0	1	0	0	-
gi|29165615|ref|NC_002745.2|	590772	+	A	0	2	0	2	1	-
gi|29165615|ref|NC_002745.2|	590776	+	G	0	0	1	0	0	-
gi|29165615|ref|NC_002745.2|	590778	+	A	0	0	1	0	0	-
gi|29165615|ref|NC_002745.2|	590788	+	U	1	0	0	0	0	-
gi|29165615|ref|NC_002745.2|	590797	+	A	0	2	0	0	0	SA0506
gi|29165615|ref|NC_002745.2|	590800	+	A	1	0	0	0	0	SA0506
gi|29165615|ref|NC_002745.2|	590801	+	A	0	0	0	0	2	SA0506
gi|29165615|ref|NC_002745.2|	590802	+	A	2	0	0	2	0	SA0506
gi|29165615|ref|NC_002745.2|	590803	+	A	0	0	1	0	2	SA0506
gi|29165615|ref|NC_002745.2|	590804	+	A	0	1	0	2	0	SA0506
gi|29165615|ref|NC_002745.2|	590806	+	U	0	0	0	0	1	SA0506
gi|29165615|ref|NC_002745.2|	590807	+	C	0	0	0	2	0	SA0506
gi|29165615|ref|NC_002745.2|	590808	+	G	0	0	0	1	0	SA0506
gi|29165615|ref|NC_002745.2|	590811	+	C	0	0	0	0	1	SA0506
gi|29165615|ref|NC_002745.2|	590812	+	G	1	0	0	0	0	SA0506
gi|29165615|ref|NC_002745.2|	590815	+	C	0	1	1	0	0	SA0506
gi|29165615|ref|NC_002745.2|	590816	+	U	1	0	0	0	0	SA0506
gi|29165615|ref|NC_002745.2|	590817	+	A	0	0	1	0	0	SA0506
gi|29165615|ref|NC_002745.2|	590831	+	U	0	0	0	1	1	SA0506
gi|29165615|ref|NC_002745.2|	590837	+	U	0	0	0	0	1	SA0506
gi|29165615|ref|NC_002745.2|	590855	+	C	0	0	0	0	1	SA0506
gi|29165615|ref|NC_002745.2|	590856	+	C	0	0	0	0	1	SA0506
gi|29165615|ref|NC_002745.2|	590865	+	A	0	1	0	0	1	SA0506
gi|29165615|ref|NC_002745.2|	590867	+	A	1	0	0	0	0	SA0506
gi|29165615|ref|NC_002745.2|	590868	+	A	0	1	1	1	2	SA0506
gi|29165615|ref|NC_002745.2|	590872	+	U	1	0	0	0	0	SA0506
gi|29165615|ref|NC_002745.2|	590883	+	A	0	1	0	0	0	SA0506
gi|29165615|ref|NC_002745.2|	590887	+	C	1	0	0	0	0	SA0506
gi|29165615|ref|NC_002745.2|	590890	+	C	1	0	1	0	0	SA0506
gi|29165615|ref|NC_002745.2|	590893	+	U	3	0	1	0	0	SA0506
gi|29165615|ref|NC_002745.2|	590894	+	A	2	0	0	0	0	SA0506
gi|29165615|ref|NC_002745.2|	590896	+	U	0	0	0	0	1	SA0506
gi|29165615|ref|NC_002745.2|	590897	+	A	1	0	0	0	0	SA0506

gi|29165615|ref|NC_002745.2|	590901	+	A	0	0	0	2	0	SA0506
gi|29165615|ref|NC_002745.2|	590905	+	A	0	2	2	0	0	SA0506
gi|29165615|ref|NC_002745.2|	590912	+	C	1	0	0	0	0	SA0506
gi|29165615|ref|NC_002745.2|	590914	+	C	0	0	0	1	0	SA0506
gi|29165615|ref|NC_002745.2|	590917	+	U	1	0	0	0	0	SA0506
gi|29165615|ref|NC_002745.2|	590920	+	C	1	0	0	0	0	SA0506
gi|29165615|ref|NC_002745.2|	590925	+	U	0	0	1	0	0	SA0506
gi|29165615|ref|NC_002745.2|	590926	+	C	1	0	0	0	0	SA0506
gi|29165615|ref|NC_002745.2|	590927	+	A	0	0	1	0	0	SA0506
gi|29165615|ref|NC_002745.2|	590928	+	U	2	0	0	0	0	SA0506
gi|29165615|ref|NC_002745.2|	590933	+	C	0	0	1	1	0	SA0506
gi|29165615|ref|NC_002745.2|	590937	+	A	1	0	0	0	0	SA0506
gi|29165615|ref|NC_002745.2|	590946	+	G	1	0	1	0	0	SA0506
gi|29165615|ref|NC_002745.2|	590947	+	C	2	0	0	0	0	SA0506
gi|29165615|ref|NC_002745.2|	590948	+	U	0	1	0	0	0	SA0506
gi|29165615|ref|NC_002745.2|	590949	+	C	0	2	1	0	0	SA0506
gi|29165615|ref|NC_002745.2|	590950	+	C	1	0	0	0	0	SA0506
gi|29165615|ref|NC_002745.2|	590951	+	A	0	1	0	0	0	SA0506
gi|29165615|ref|NC_002745.2|	590952	+	G	1	0	0	0	0	SA0506
gi|29165615|ref|NC_002745.2|	590958	+	A	0	2	0	0	0	SA0506
gi|29165615|ref|NC_002745.2|	590962	+	A	1	0	0	0	0	SA0506
gi|29165615|ref|NC_002745.2|	590965	+	G	0	0	0	0	1	SA0506
gi|29165615|ref|NC_002745.2|	590968	+	G	0	0	0	0	1	SA0506
gi|29165615|ref|NC_002745.2|	590969	+	U	1	0	1	0	0	SA0506
gi|29165615|ref|NC_002745.2|	590970	+	A	0	2	0	0	0	SA0506
gi|29165615|ref|NC_002745.2|	590972	+	C	0	1	0	0	0	SA0506
gi|29165615|ref|NC_002745.2|	590973	+	A	1	0	0	0	1	SA0506
gi|29165615|ref|NC_002745.2|	590974	+	C	3	0	0	1	0	SA0506
gi|29165615|ref|NC_002745.2|	590975	+	A	1	0	0	0	0	SA0506
gi|29165615|ref|NC_002745.2|	590977	+	U	0	1	1	0	0	SA0506
gi|29165615|ref|NC_002745.2|	590978	+	C	0	0	0	1	0	SA0506
gi|29165615|ref|NC_002745.2|	590980	+	A	1	3	0	0	0	SA0506
gi|29165615|ref|NC_002745.2|	590981	+	U	2	0	0	0	0	SA0506
gi|29165615|ref|NC_002745.2|	590982	+	A	1	0	0	0	0	SA0506
gi|29165615|ref|NC_002745.2|	590983	+	C	7	0	0	0	0	SA0506
gi|29165615|ref|NC_002745.2|	590985	+	U	0	0	1	0	1	SA0506
gi|29165615|ref|NC_002745.2|	590986	+	C	3	0	0	0	0	SA0506
gi|29165615|ref|NC_002745.2|	590987	+	U	1	0	0	0	0	SA0506
gi|29165615|ref|NC_002745.2|	590988	+	C	0	1	1	0	0	SA0506
gi|29165615|ref|NC_002745.2|	590989	+	A	2	0	0	0	0	SA0506
gi|29165615|ref|NC_002745.2|	590990	+	C	2	0	0	1	0	SA0506
gi|29165615|ref|NC_002745.2|	590992	+	U	1	0	0	0	0	SA0506
gi|29165615|ref|NC_002745.2|	590993	+	U	1	0	0	0	1	SA0506
gi|29165615|ref|NC_002745.2|	590995	+	A	0	1	0	0	0	SA0506
gi|29165615|ref|NC_002745.2|	590996	+	G	1	0	0	0	0	SA0506
gi|29165615|ref|NC_002745.2|	590998	+	A	0	0	1	0	0	SA0506
gi|29165615|ref|NC_002745.2|	591000	+	C	0	0	1	0	0	SA0506
gi|29165615|ref|NC_002745.2|	591002	+	A	0	1	0	0	0	SA0506
gi|29165615|ref|NC_002745.2|	591003	+	A	1	0	0	0	0	SA0506
gi|29165615|ref|NC_002745.2|	591004	+	C	1	0	0	0	0	SA0506
gi|29165615|ref|NC_002745.2|	591006	+	G	0	0	1	0	1	SA0506
gi|29165615|ref|NC_002745.2|	591007	+	A	1	0	0	0	0	SA0506
gi|29165615|ref|NC_002745.2|	591008	+	C	4	0	0	0	0	SA0506
gi|29165615|ref|NC_002745.2|	591011	+	A	0	0	0	0	1	SA0506
gi|29165615|ref|NC_002745.2|	591012	+	C	1	0	0	0	0	SA0506
gi|29165615|ref|NC_002745.2|	591013	+	G	1	0	0	0	0	SA0506
gi|29165615|ref|NC_002745.2|	591014	+	U	3	5	9	2	6	SA0506
gi|29165615|ref|NC_002745.2|	591015	+	C	1	0	1	1	7	SA0506
gi|29165615|ref|NC_002745.2|	591017	+	C	4	0	0	0	1	SA0506
gi|29165615|ref|NC_002745.2|	591018	+	U	0	0	0	0	1	SA0506
gi|29165615|ref|NC_002745.2|	591021	+	G	0	0	1	0	0	SA0506
gi|29165615|ref|NC_002745.2|	591022	+	C	5	0	0	0	0	SA0506
gi|29165615|ref|NC_002745.2|	591023	+	U	11	0	1	0	0	SA0506
gi|29165615|ref|NC_002745.2|	591024	+	C	3	0	0	0	0	SA0506
gi|29165615|ref|NC_002745.2|	591025	+	A	3	0	0	0	0	SA0506
gi|29165615|ref|NC_002745.2|	591028	+	U	2	1	0	0	0	SA0506
gi|29165615|ref|NC_002745.2|	591032	+	C	1	1	0	0	0	SA0506
gi|29165615|ref|NC_002745.2|	591036	+	C	0	2	0	0	0	SA0506
gi|29165615|ref|NC_002745.2|	591038	+	A	2	0	0	0	2	SA0506
gi|29165615|ref|NC_002745.2|	591041	+	A	1	0	0	0	0	SA0506
gi|29165615|ref|NC_002745.2|	591042	+	C	2	0	1	0	1	SA0506
gi|29165615|ref|NC_002745.2|	591043	+	A	2	0	0	0	0	SA0506
gi|29165615|ref|NC_002745.2|	591045	+	G	1	0	2	0	0	SA0506
gi|29165615|ref|NC_002745.2|	591046	+	C	0	0	0	0	1	SA0506
gi|29165615|ref|NC_002745.2|	591047	+	U	1	0	0	0	0	SA0506
gi|29165615|ref|NC_002745.2|	591048	+	G	1	0	0	0	0	SA0506
gi|29165615|ref|NC_002745.2|	591049	+	A	1	0	0	0	0	SA0506
gi|29165615|ref|NC_002745.2|	591050	+	C	1	0	0	0	0	SA0506
gi|29165615|ref|NC_002745.2|	591052	+	A	2	0	0	0	0	SA0506
gi|29165615|ref|NC_002745.2|	591054	+	G	3	0	0	0	0	SA0506
gi|29165615|ref|NC_002745.2|	591055	+	U	2	0	0	0	0	SA0506

gi|29165615|ref|NC_002745.2|	591056 +	U	1	0	0	0	0	SA0506
gi|29165615|ref|NC_002745.2|	591057 +	A	0	2	0	0	1	SA0506
gi|29165615|ref|NC_002745.2|	591058 +	A	1	1	0	0	0	SA0506
gi|29165615|ref|NC_002745.2|	591059 +	A	2	0	0	1	1	SA0506
gi|29165615|ref|NC_002745.2|	591060 +	A	1	0	0	0	1	SA0506
gi|29165615|ref|NC_002745.2|	591061 +	A	2	0	0	0	1	SA0506
gi|29165615|ref|NC_002745.2|	591062 +	C	0	2	2	0	1	SA0506
gi|29165615|ref|NC_002745.2|	591063 +	A	0	0	0	2	1	SA0506
gi|29165615|ref|NC_002745.2|	591064 +	U	1	0	1	0	0	SA0506
gi|29165615|ref|NC_002745.2|	591065 +	G	0	1	0	0	1	SA0506
gi|29165615|ref|NC_002745.2|	591066 +	A	0	0	1	1	0	SA0506
gi|29165615|ref|NC_002745.2|	591067 +	U	2	1	0	0	0	SA0506
gi|29165615|ref|NC_002745.2|	591068 +	C	1	0	0	0	0	SA0506
gi|29165615|ref|NC_002745.2|	591069 +	A	0	1	0	0	0	SA0506
gi|29165615|ref|NC_002745.2|	591070 +	C	1	0	0	0	0	SA0506
gi|29165615|ref|NC_002745.2|	591071 +	U	4	0	0	0	1	SA0506
gi|29165615|ref|NC_002745.2|	591072 +	G	1	0	0	0	0	SA0506
gi|29165615|ref|NC_002745.2|	591073 +	G	1	0	0	0	0	SA0506
gi|29165615|ref|NC_002745.2|	591074 +	U	11	2	0	0	1	SA0506
gi|29165615|ref|NC_002745.2|	591076 +	C	5	0	0	0	0	SA0506
gi|29165615|ref|NC_002745.2|	591079 +	C	2	0	0	0	0	SA0506
gi|29165615|ref|NC_002745.2|	591081 +	C	0	0	1	0	0	SA0506
gi|29165615|ref|NC_002745.2|	591085 +	U	1	0	0	0	0	SA0506
gi|29165615|ref|NC_002745.2|	591089 +	C	0	0	0	1	0	SA0506
gi|29165615|ref|NC_002745.2|	591092 +	C	1	0	0	0	0	SA0506
gi|29165615|ref|NC_002745.2|	591100 +	U	3	1	0	1	1	SA0506
gi|29165615|ref|NC_002745.2|	591103 +	U	4	0	0	0	1	SA0506
gi|29165615|ref|NC_002745.2|	591106 +	U	4	0	1	0	0	SA0506
gi|29165615|ref|NC_002745.2|	591107 +	A	1	0	0	0	0	SA0506
gi|29165615|ref|NC_002745.2|	591109 +	C	3	0	1	0	0	SA0506
gi|29165615|ref|NC_002745.2|	591110 +	U	2	0	0	0	0	SA0506
gi|29165615|ref|NC_002745.2|	591111 +	G	1	0	1	0	0	SA0506
gi|29165615|ref|NC_002745.2|	591112 +	C	2	0	0	0	0	SA0506
gi|29165615|ref|NC_002745.2|	591113 +	U	0	0	1	0	0	SA0506
gi|29165615|ref|NC_002745.2|	591115 +	C	5	0	0	0	0	SA0506
gi|29165615|ref|NC_002745.2|	591117 +	G	1	1	0	0	0	SA0506
gi|29165615|ref|NC_002745.2|	591119 +	C	1	0	0	0	0	SA0506
gi|29165615|ref|NC_002745.2|	591121 +	G	0	0	0	0	1	SA0506
gi|29165615|ref|NC_002745.2|	591122 +	U	0	0	1	0	0	SA0506
gi|29165615|ref|NC_002745.2|	591124 +	C	1	2	0	0	1	SA0506
gi|29165615|ref|NC_002745.2|	591125 +	A	2	0	0	0	0	SA0506
gi|29165615|ref|NC_002745.2|	591126 +	A	0	0	1	0	0	SA0506
gi|29165615|ref|NC_002745.2|	591127 +	U	0	1	0	0	0	SA0506
gi|29165615|ref|NC_002745.2|	591129 +	C	0	2	2	0	0	SA0506
gi|29165615|ref|NC_002745.2|	591130 +	C	1	0	0	0	1	SA0506
gi|29165615|ref|NC_002745.2|	591132 +	C	1	0	0	0	0	SA0506
gi|29165615|ref|NC_002745.2|	591135 +	A	0	1	0	0	0	SA0506
gi|29165615|ref|NC_002745.2|	591136 +	C	0	1	0	0	0	SA0506
gi|29165615|ref|NC_002745.2|	591137 +	U	0	0	1	0	0	SA0506
gi|29165615|ref|NC_002745.2|	591143 +	A	0	0	1	0	0	SA0506
gi|29165615|ref|NC_002745.2|	591146 +	C	0	0	1	1	0	SA0506
gi|29165615|ref|NC_002745.2|	591147 +	A	3	1	0	0	0	SA0506
gi|29165615|ref|NC_002745.2|	591148 +	U	1	3	2	2	2	SA0506
gi|29165615|ref|NC_002745.2|	591150 +	C	0	0	0	1	0	SA0506
gi|29165615|ref|NC_002745.2|	591151 +	U	1	1	0	0	0	SA0506
gi|29165615|ref|NC_002745.2|	591153 +	U	2	0	0	0	0	SA0506
gi|29165615|ref|NC_002745.2|	591154 +	U	3	0	1	0	0	SA0506
gi|29165615|ref|NC_002745.2|	591155 +	A	0	0	0	0	1	SA0506
gi|29165615|ref|NC_002745.2|	591157 +	C	4	2	0	0	0	SA0506
gi|29165615|ref|NC_002745.2|	591158 +	A	0	1	1	0	0	SA0506
gi|29165615|ref|NC_002745.2|	591159 +	C	4	0	0	0	0	SA0506
gi|29165615|ref|NC_002745.2|	591161 +	U	1	0	0	0	0	SA0506
gi|29165615|ref|NC_002745.2|	591163 +	A	0	0	0	0	1	SA0506
gi|29165615|ref|NC_002745.2|	591164 +	C	1	1	0	0	0	SA0506
gi|29165615|ref|NC_002745.2|	591165 +	G	0	2	0	0	0	SA0506
gi|29165615|ref|NC_002745.2|	591166 +	U	2	0	0	0	0	SA0506
gi|29165615|ref|NC_002745.2|	591167 +	U	2	0	0	0	0	SA0506
gi|29165615|ref|NC_002745.2|	591168 +	G	0	0	0	1	0	SA0506
gi|29165615|ref|NC_002745.2|	591170 +	U	1	0	0	0	0	SA0506
gi|29165615|ref|NC_002745.2|	591172 +	U	1	1	1	0	0	SA0506
gi|29165615|ref|NC_002745.2|	591176 +	A	0	0	1	0	1	SA0506
gi|29165615|ref|NC_002745.2|	591178 +	C	0	0	1	0	0	SA0506
gi|29165615|ref|NC_002745.2|	591183 +	G	0	0	0	0	1	SA0506
gi|29165615|ref|NC_002745.2|	591184 +	U	2	0	0	0	0	SA0506
gi|29165615|ref|NC_002745.2|	591187 +	U	3	0	0	0	0	SA0506
gi|29165615|ref|NC_002745.2|	591190 +	U	1	0	0	0	0	SA0506
gi|29165615|ref|NC_002745.2|	591192 +	U	1	0	0	1	1	SA0506
gi|29165615|ref|NC_002745.2|	591193 +	U	0	0	0	0	1	SA0506
gi|29165615|ref|NC_002745.2|	591196 +	A	0	0	0	1	0	SA0506
gi|29165615|ref|NC_002745.2|	591197 +	C	0	0	0	0	2	SA0506
gi|29165615|ref|NC_002745.2|	591204 +	G	1	1	0	0	0	SA0506

gi|29165615|ref|NC_002745.2|	591205	+	A	0	1	1	1	0	SA0506
gi|29165615|ref|NC_002745.2|	591206	+	C	0	0	0	1	0	SA0506
gi|29165615|ref|NC_002745.2|	591210	+	G	1	0	0	0	0	SA0506
gi|29165615|ref|NC_002745.2|	591215	+	C	2	0	1	0	0	SA0506
gi|29165615|ref|NC_002745.2|	591216	+	G	1	0	0	0	0	SA0506
gi|29165615|ref|NC_002745.2|	591220	+	A	0	0	0	0	1	SA0506
gi|29165615|ref|NC_002745.2|	591224	+	A	0	1	0	1	0	SA0506
gi|29165615|ref|NC_002745.2|	591226	+	U	0	0	0	1	3	SA0506
gi|29165615|ref|NC_002745.2|	591227	+	A	0	0	0	0	1	SA0506
gi|29165615|ref|NC_002745.2|	591229	+	U	1	0	0	0	0	SA0506
gi|29165615|ref|NC_002745.2|	591231	+	G	0	0	0	0	2	SA0506
gi|29165615|ref|NC_002745.2|	591232	+	A	0	3	0	1	0	SA0506
gi|29165615|ref|NC_002745.2|	591233	+	A	0	1	0	0	0	SA0506
gi|29165615|ref|NC_002745.2|	591238	+	U	2	0	0	2	0	SA0506
gi|29165615|ref|NC_002745.2|	591255	+	G	0	0	0	0	1	SA0506
gi|29165615|ref|NC_002745.2|	591259	+	U	0	2	0	0	0	SA0506
gi|29165615|ref|NC_002745.2|	591261	+	U	2	0	0	0	0	SA0506
gi|29165615|ref|NC_002745.2|	591268	+	A	2	0	1	0	0	SA0506
gi|29165615|ref|NC_002745.2|	591272	+	U	1	2	0	0	0	SA0506
gi|29165615|ref|NC_002745.2|	591273	+	G	0	0	1	0	0	SA0506
gi|29165615|ref|NC_002745.2|	591274	+	A	0	1	0	0	0	SA0506
gi|29165615|ref|NC_002745.2|	591275	+	C	3	0	0	0	1	SA0506
gi|29165615|ref|NC_002745.2|	591276	+	U	0	1	0	0	0	SA0506
gi|29165615|ref|NC_002745.2|	591277	+	U	2	0	0	0	0	SA0506
gi|29165615|ref|NC_002745.2|	591278	+	C	1	0	0	0	1	SA0506
gi|29165615|ref|NC_002745.2|	591279	+	C	0	0	0	0	2	SA0506
gi|29165615|ref|NC_002745.2|	591280	+	C	1	0	0	0	0	SA0506
gi|29165615|ref|NC_002745.2|	591286	+	A	1	0	1	0	0	SA0506
gi|29165615|ref|NC_002745.2|	591287	+	C	1	0	0	0	1	SA0506
gi|29165615|ref|NC_002745.2|	591295	+	C	2	0	0	0	0	SA0506
gi|29165615|ref|NC_002745.2|	591300	+	A	0	0	0	0	1	SA0506
gi|29165615|ref|NC_002745.2|	591301	+	U	1	0	0	0	0	SA0506
gi|29165615|ref|NC_002745.2|	591302	+	C	1	2	1	0	0	SA0506
gi|29165615|ref|NC_002745.2|	591303	+	G	0	0	1	1	0	SA0506
gi|29165615|ref|NC_002745.2|	591305	+	U	0	0	1	0	0	SA0506
gi|29165615|ref|NC_002745.2|	591308	+	U	2	0	0	0	0	SA0506
gi|29165615|ref|NC_002745.2|	591310	+	C	2	1	0	0	0	SA0506
gi|29165615|ref|NC_002745.2|	591313	+	C	3	0	0	0	0	SA0506
gi|29165615|ref|NC_002745.2|	591314	+	A	2	0	0	0	0	SA0506
gi|29165615|ref|NC_002745.2|	591316	+	U	3	0	0	0	0	SA0506
gi|29165615|ref|NC_002745.2|	591319	+	A	1	0	1	0	1	SA0506
gi|29165615|ref|NC_002745.2|	591320	+	A	0	0	1	0	0	SA0506
gi|29165615|ref|NC_002745.2|	591322	+	C	2	0	0	0	0	SA0506
gi|29165615|ref|NC_002745.2|	591324	+	U	1	1	0	0	0	SA0506
gi|29165615|ref|NC_002745.2|	591328	+	A	0	1	0	0	0	SA0506
gi|29165615|ref|NC_002745.2|	591329	+	A	2	0	0	0	0	SA0506
gi|29165615|ref|NC_002745.2|	591334	+	A	0	0	0	0	1	SA0506
gi|29165615|ref|NC_002745.2|	591336	+	G	1	0	0	0	0	SA0506
gi|29165615|ref|NC_002745.2|	591337	+	C	1	1	0	0	0	SA0506
gi|29165615|ref|NC_002745.2|	591341	+	A	0	2	0	0	0	SA0506
gi|29165615|ref|NC_002745.2|	591342	+	U	1	2	0	3	1	SA0506
gi|29165615|ref|NC_002745.2|	591343	+	A	1	0	0	0	0	SA0506
gi|29165615|ref|NC_002745.2|	591344	+	C	1	0	0	1	0	SA0506
gi|29165615|ref|NC_002745.2|	591345	+	G	0	2	0	1	2	SA0506
gi|29165615|ref|NC_002745.2|	591346	+	A	0	6	2	3	3	SA0506
gi|29165615|ref|NC_002745.2|	591347	+	A	1	2	0	0	0	SA0506
gi|29165615|ref|NC_002745.2|	591348	+	G	0	2	2	2	0	SA0506
gi|29165615|ref|NC_002745.2|	591349	+	A	0	1	1	1	0	SA0506
gi|29165615|ref|NC_002745.2|	591350	+	A	1	1	1	3	2	SA0506
gi|29165615|ref|NC_002745.2|	591351	+	A	0	1	1	0	1	SA0506
gi|29165615|ref|NC_002745.2|	591352	+	A	0	2	2	2	1	SA0506
gi|29165615|ref|NC_002745.2|	591353	+	A	0	1	0	3	1	SA0506
gi|29165615|ref|NC_002745.2|	591354	+	A	0	13	1	0	2	SA0506
gi|29165615|ref|NC_002745.2|	591355	+	U	3	0	0	1	1	SA0506
gi|29165615|ref|NC_002745.2|	591356	+	C	0	0	1	4	0	SA0506
gi|29165615|ref|NC_002745.2|	591357	+	U	2	2	0	0	0	SA0506
gi|29165615|ref|NC_002745.2|	591358	+	U	0	0	0	1	1	SA0506
gi|29165615|ref|NC_002745.2|	591359	+	A	0	0	0	2	0	SA0506
gi|29165615|ref|NC_002745.2|	591360	+	G	1	4	4	2	4	SA0506
gi|29165615|ref|NC_002745.2|	591361	+	A	2	2	4	0	0	SA0506
gi|29165615|ref|NC_002745.2|	591362	+	A	0	0	2	1	0	SA0506
gi|29165615|ref|NC_002745.2|	591363	+	U	0	0	1	0	0	SA0506
gi|29165615|ref|NC_002745.2|	591364	+	U	1	0	1	3	0	SA0506
gi|29165615|ref|NC_002745.2|	591366	+	A	0	2	1	2	0	SA0506
gi|29165615|ref|NC_002745.2|	591367	+	U	1	0	0	0	0	SA0506
gi|29165615|ref|NC_002745.2|	591369	+	G	0	2	0	0	2	SA0506
gi|29165615|ref|NC_002745.2|	591370	+	A	1	1	1	2	1	SA0506
gi|29165615|ref|NC_002745.2|	591371	+	A	0	1	0	0	0	SA0506
gi|29165615|ref|NC_002745.2|	591372	+	G	0	1	0	0	0	SA0506
gi|29165615|ref|NC_002745.2|	591373	+	C	2	1	0	1	1	SA0506
gi|29165615|ref|NC_002745.2|	591374	+	U	0	0	1	1	0	SA0506

gi|29165615|ref|NC_002745.2|	591375 +	G	0	0	0	0	1	SA0506
gi|29165615|ref|NC_002745.2|	591376 +	U	1	0	0	0	0	SA0506
gi|29165615|ref|NC_002745.2|	591378 +	G	0	0	1	0	0	SA0506
gi|29165615|ref|NC_002745.2|	591380 +	U	0	1	0	0	0	SA0506
gi|29165615|ref|NC_002745.2|	591382 +	C	4	0	1	0	0	SA0506
gi|29165615|ref|NC_002745.2|	591383 +	U	0	0	0	1	0	SA0506
gi|29165615|ref|NC_002745.2|	591384 +	U	0	0	2	1	0	SA0506
gi|29165615|ref|NC_002745.2|	591385 +	A	1	0	0	1	0	SA0506
gi|29165615|ref|NC_002745.2|	591386 +	C	0	0	0	1	0	SA0506
gi|29165615|ref|NC_002745.2|	591388 +	U	1	0	0	0	0	SA0506
gi|29165615|ref|NC_002745.2|	591391 +	C	2	0	0	0	0	SA0506
gi|29165615|ref|NC_002745.2|	591392 +	A	1	0	0	1	0	SA0506
gi|29165615|ref|NC_002745.2|	591393 +	A	3	0	0	0	0	SA0506
gi|29165615|ref|NC_002745.2|	591394 +	C	7	0	0	0	0	SA0506
gi|29165615|ref|NC_002745.2|	591395 +	U	0	0	1	0	0	SA0506
gi|29165615|ref|NC_002745.2|	591396 +	C	0	0	0	2	0	SA0506
gi|29165615|ref|NC_002745.2|	591397 +	C	2	1	0	0	1	SA0506
gi|29165615|ref|NC_002745.2|	591399 +	G	6	0	0	1	0	SA0506
gi|29165615|ref|NC_002745.2|	591400 +	A	0	3	0	0	0	SA0506
gi|29165615|ref|NC_002745.2|	591403 +	G	1	0	1	0	0	SA0506
gi|29165615|ref|NC_002745.2|	591406 +	A	2	0	1	2	0	SA0506
gi|29165615|ref|NC_002745.2|	591408 +	U	0	0	1	0	0	SA0506
gi|29165615|ref|NC_002745.2|	591409 +	C	8	0	1	0	1	SA0506
gi|29165615|ref|NC_002745.2|	591410 +	U	3	0	0	0	0	SA0506
gi|29165615|ref|NC_002745.2|	591411 +	G	2	0	0	2	1	SA0506
gi|29165615|ref|NC_002745.2|	591412 +	A	3	0	0	0	1	SA0506
gi|29165615|ref|NC_002745.2|	591413 +	C	2	0	0	2	0	SA0506
gi|29165615|ref|NC_002745.2|	591414 +	A	0	0	4	0	0	SA0506
gi|29165615|ref|NC_002745.2|	591415 +	A	0	1	0	0	0	SA0506
gi|29165615|ref|NC_002745.2|	591416 +	A	0	0	0	0	1	SA0506
gi|29165615|ref|NC_002745.2|	591417 +	C	0	1	1	0	0	SA0506
gi|29165615|ref|NC_002745.2|	591418 +	C	1	0	1	0	1	SA0506
gi|29165615|ref|NC_002745.2|	591419 +	A	1	1	0	0	0	SA0506
gi|29165615|ref|NC_002745.2|	591420 +	U	1	0	0	0	2	SA0506
gi|29165615|ref|NC_002745.2|	591421 +	U	4	0	1	0	0	SA0506
gi|29165615|ref|NC_002745.2|	591422 +	C	3	0	2	0	0	SA0506
gi|29165615|ref|NC_002745.2|	591424 +	U	1	1	0	0	0	SA0506
gi|29165615|ref|NC_002745.2|	591425 +	G	1	0	0	0	0	SA0506
gi|29165615|ref|NC_002745.2|	591426 +	A	0	0	0	0	5	SA0506
gi|29165615|ref|NC_002745.2|	591427 +	U	3	0	1	0	0	SA0506
gi|29165615|ref|NC_002745.2|	591428 +	G	0	1	0	0	0	SA0506
gi|29165615|ref|NC_002745.2|	591430 +	C	1	0	0	0	0	SA0506
gi|29165615|ref|NC_002745.2|	591431 +	A	0	2	0	0	0	SA0506
gi|29165615|ref|NC_002745.2|	591433 +	U	0	0	0	1	0	SA0506
gi|29165615|ref|NC_002745.2|	591434 +	U	1	0	0	0	0	SA0506
gi|29165615|ref|NC_002745.2|	591436 +	A	1	0	0	0	0	SA0506
gi|29165615|ref|NC_002745.2|	591437 +	G	0	0	0	1	0	SA0506
gi|29165615|ref|NC_002745.2|	591441 +	G	0	0	0	0	1	SA0506
gi|29165615|ref|NC_002745.2|	591442 +	U	2	1	0	0	0	SA0506
gi|29165615|ref|NC_002745.2|	591444 +	U	0	0	1	1	0	SA0506
gi|29165615|ref|NC_002745.2|	591445 +	U	3	0	1	1	0	SA0506
gi|29165615|ref|NC_002745.2|	591446 +	C	2	0	0	0	0	SA0506
gi|29165615|ref|NC_002745.2|	591447 +	U	1	0	1	0	1	SA0506
gi|29165615|ref|NC_002745.2|	591448 +	C	3	0	0	0	1	SA0506
gi|29165615|ref|NC_002745.2|	591452 +	C	1	1	0	0	0	SA0506
gi|29165615|ref|NC_002745.2|	591454 +	C	2	0	1	0	1	SA0506
gi|29165615|ref|NC_002745.2|	591455 +	U	1	1	0	0	1	SA0506
gi|29165615|ref|NC_002745.2|	591456 +	G	0	0	1	1	0	SA0506
gi|29165615|ref|NC_002745.2|	591457 +	G	0	1	0	0	1	SA0506
gi|29165615|ref|NC_002745.2|	591458 +	U	2	0	2	0	1	SA0506
gi|29165615|ref|NC_002745.2|	591459 +	C	1	1	3	0	0	SA0506
gi|29165615|ref|NC_002745.2|	591460 +	G	1	0	0	0	0	SA0506
gi|29165615|ref|NC_002745.2|	591463 +	G	2	0	0	0	0	SA0506
gi|29165615|ref|NC_002745.2|	591464 +	U	6	1	0	0	1	SA0506
gi|29165615|ref|NC_002745.2|	591466 +	C	4	0	1	0	0	SA0506
gi|29165615|ref|NC_002745.2|	591467 +	U	1	4	1	0	0	SA0506
gi|29165615|ref|NC_002745.2|	591470 +	U	1	0	0	0	0	SA0506
gi|29165615|ref|NC_002745.2|	591471 +	G	0	0	0	1	0	SA0506
gi|29165615|ref|NC_002745.2|	591472 +	C	3	1	0	1	0	SA0506
gi|29165615|ref|NC_002745.2|	591475 +	C	1	1	0	0	2	SA0506
gi|29165615|ref|NC_002745.2|	591476 +	A	1	1	0	0	0	SA0506
gi|29165615|ref|NC_002745.2|	591479 +	C	0	1	0	0	0	SA0506
gi|29165615|ref|NC_002745.2|	591482 +	U	0	0	1	0	0	SA0506
gi|29165615|ref|NC_002745.2|	591483 +	G	0	1	0	0	0	SA0506
gi|29165615|ref|NC_002745.2|	591484 +	U	1	0	0	0	0	SA0506
gi|29165615|ref|NC_002745.2|	591486 +	G	1	0	0	0	1	SA0506
gi|29165615|ref|NC_002745.2|	591487 +	A	0	2	0	0	0	SA0506
gi|29165615|ref|NC_002745.2|	591488 +	A	1	0	0	0	0	SA0506
gi|29165615|ref|NC_002745.2|	591496 +	A	1	1	0	0	0	SA0506
gi|29165615|ref|NC_002745.2|	591497 +	A	1	0	0	0	0	SA0506
gi|29165615|ref|NC_002745.2|	591500 +	C	1	0	0	0	0	SA0506

gi|29165615|ref|NC_002745.2|	591501	+	A	0	0	1	0	0	SA0506
gi|29165615|ref|NC_002745.2|	591504	+	G	1	0	1	0	0	SA0506
gi|29165615|ref|NC_002745.2|	591505	+	U	3	0	0	0	0	SA0506
gi|29165615|ref|NC_002745.2|	591507	+	G	0	1	0	0	0	SA0506
gi|29165615|ref|NC_002745.2|	591511	+	A	1	0	0	0	0	SA0506
gi|29165615|ref|NC_002745.2|	591512	+	A	0	1	0	2	0	SA0506
gi|29165615|ref|NC_002745.2|	591513	+	G	1	0	2	0	0	SA0506
gi|29165615|ref|NC_002745.2|	591514	+	A	0	0	0	1	0	SA0506
gi|29165615|ref|NC_002745.2|	591515	+	A	1	0	1	0	0	SA0506
gi|29165615|ref|NC_002745.2|	591516	+	G	0	1	0	0	0	SA0506
gi|29165615|ref|NC_002745.2|	591517	+	U	0	1	0	0	0	SA0506
gi|29165615|ref|NC_002745.2|	591519	+	G	0	0	0	1	1	SA0506
gi|29165615|ref|NC_002745.2|	591520	+	A	6	12	4	5	6	SA0506
gi|29165615|ref|NC_002745.2|	591521	+	A	1	0	1	0	0	SA0506
gi|29165615|ref|NC_002745.2|	591522	+	A	0	2	1	1	1	SA0506
gi|29165615|ref|NC_002745.2|	591523	+	U	4	0	1	0	0	SA0506
gi|29165615|ref|NC_002745.2|	591524	+	C	6	0	1	0	0	SA0506
gi|29165615|ref|NC_002745.2|	591525	+	A	0	0	0	0	1	SA0506
gi|29165615|ref|NC_002745.2|	591526	+	U	1	0	0	1	0	SA0506
gi|29165615|ref|NC_002745.2|	591527	+	C	1	0	1	0	0	SA0506
gi|29165615|ref|NC_002745.2|	591532	+	U	1	1	0	0	0	SA0506
gi|29165615|ref|NC_002745.2|	591534	+	C	0	2	1	0	1	SA0506
gi|29165615|ref|NC_002745.2|	591535	+	A	4	0	0	1	0	SA0506
gi|29165615|ref|NC_002745.2|	591538	+	A	2	0	0	0	0	SA0506
gi|29165615|ref|NC_002745.2|	591539	+	C	0	0	0	0	1	SA0506
gi|29165615|ref|NC_002745.2|	591541	+	C	0	1	0	1	0	SA0506
gi|29165615|ref|NC_002745.2|	591542	+	A	0	0	0	0	1	SA0506
gi|29165615|ref|NC_002745.2|	591543	+	U	0	2	0	0	0	SA0506
gi|29165615|ref|NC_002745.2|	591544	+	C	1	0	0	0	0	SA0506
gi|29165615|ref|NC_002745.2|	591546	+	A	0	1	0	0	0	SA0506
gi|29165615|ref|NC_002745.2|	591549	+	A	0	0	1	0	0	SA0506
gi|29165615|ref|NC_002745.2|	591552	+	A	0	3	0	0	0	SA0506
gi|29165615|ref|NC_002745.2|	591553	+	C	1	0	0	0	0	SA0506
gi|29165615|ref|NC_002745.2|	591555	+	G	0	0	0	1	0	SA0506
gi|29165615|ref|NC_002745.2|	591557	+	U	3	0	0	0	0	SA0506
gi|29165615|ref|NC_002745.2|	591558	+	A	0	1	1	0	0	SA0506
gi|29165615|ref|NC_002745.2|	591559	+	C	2	0	0	0	0	SA0506
gi|29165615|ref|NC_002745.2|	591563	+	U	1	0	0	0	0	SA0506
gi|29165615|ref|NC_002745.2|	591565	+	U	1	0	0	1	0	SA0506
gi|29165615|ref|NC_002745.2|	591567	+	G	0	1	0	0	1	SA0506
gi|29165615|ref|NC_002745.2|	591568	+	A	4	0	2	0	0	SA0506
gi|29165615|ref|NC_002745.2|	591569	+	A	3	0	2	0	1	SA0506
gi|29165615|ref|NC_002745.2|	591570	+	A	1	2	0	2	0	SA0506
gi|29165615|ref|NC_002745.2|	591571	+	U	0	0	2	0	1	SA0506
gi|29165615|ref|NC_002745.2|	591572	+	G	0	1	0	0	0	SA0506
gi|29165615|ref|NC_002745.2|	591573	+	U	0	0	0	1	0	SA0506
gi|29165615|ref|NC_002745.2|	591574	+	U	2	0	0	3	1	SA0506
gi|29165615|ref|NC_002745.2|	591575	+	C	1	1	0	3	0	SA0506
gi|29165615|ref|NC_002745.2|	591577	+	G	4	2	0	0	1	SA0506
gi|29165615|ref|NC_002745.2|	591578	+	U	2	2	1	1	2	SA0506
gi|29165615|ref|NC_002745.2|	591579	+	A	3	1	0	1	0	SA0506
gi|29165615|ref|NC_002745.2|	591580	+	A	2	0	0	1	1	SA0506
gi|29165615|ref|NC_002745.2|	591581	+	A	1	8	1	0	1	SA0506
gi|29165615|ref|NC_002745.2|	591582	+	U	0	1	0	0	0	SA0506
gi|29165615|ref|NC_002745.2|	591583	+	U	0	3	1	0	1	SA0506
gi|29165615|ref|NC_002745.2|	591584	+	A	5	0	0	0	0	SA0506
gi|29165615|ref|NC_002745.2|	591585	+	U	0	1	1	2	0	SA0506
gi|29165615|ref|NC_002745.2|	591586	+	U	8	1	1	0	2	SA0506
gi|29165615|ref|NC_002745.2|	591587	+	A	4	0	2	0	0	SA0506
gi|29165615|ref|NC_002745.2|	591588	+	G	2	0	0	0	0	SA0506
gi|29165615|ref|NC_002745.2|	591589	+	A	5	3	1	1	0	SA0506
gi|29165615|ref|NC_002745.2|	591590	+	C	2	0	0	0	0	SA0506
gi|29165615|ref|NC_002745.2|	591591	+	U	3	1	0	1	1	SA0506
gi|29165615|ref|NC_002745.2|	591592	+	A	4	0	1	0	0	SA0506
gi|29165615|ref|NC_002745.2|	591594	+	G	2	3	0	0	0	SA0506
gi|29165615|ref|NC_002745.2|	591595	+	C	5	2	0	0	0	SA0506
gi|29165615|ref|NC_002745.2|	591596	+	U	3	0	0	0	0	SA0506
gi|29165615|ref|NC_002745.2|	591597	+	G	0	1	0	0	3	SA0506
gi|29165615|ref|NC_002745.2|	591598	+	A	0	0	0	1	0	SA0506
gi|29165615|ref|NC_002745.2|	591599	+	A	1	0	0	0	0	SA0506
gi|29165615|ref|NC_002745.2|	591600	+	G	0	1	0	0	0	SA0506
gi|29165615|ref|NC_002745.2|	591602	+	U	1	0	1	0	1	SA0506
gi|29165615|ref|NC_002745.2|	591603	+	G	1	0	0	0	0	SA0506
gi|29165615|ref|NC_002745.2|	591605	+	U	2	1	0	0	1	SA0506
gi|29165615|ref|NC_002745.2|	591606	+	G	1	3	0	0	0	SA0506
gi|29165615|ref|NC_002745.2|	591608	+	C	0	0	2	0	0	SA0506
gi|29165615|ref|NC_002745.2|	591609	+	A	0	0	0	2	0	SA0506
gi|29165615|ref|NC_002745.2|	591610	+	A	1	1	0	0	0	SA0506
gi|29165615|ref|NC_002745.2|	591611	+	C	0	0	0	0	1	SA0506
gi|29165615|ref|NC_002745.2|	591612	+	A	0	1	0	0	0	SA0506
gi|29165615|ref|NC_002745.2|	591617	+	U	0	1	0	0	0	SA0506

gi|29165615|ref|NC_002745.2|	591618 +	G	0	1	2	3	0	SA0506
gi|29165615|ref|NC_002745.2|	591620 +	A	2	0	1	3	0	SA0506
gi|29165615|ref|NC_002745.2|	591621 +	U	1	0	0	1	0	SA0506
gi|29165615|ref|NC_002745.2|	591622 +	U	1	1	2	0	1	SA0506
gi|29165615|ref|NC_002745.2|	591623 +	A	1	0	1	1	0	SA0506
gi|29165615|ref|NC_002745.2|	591624 +	U	0	0	0	0	3	SA0506
gi|29165615|ref|NC_002745.2|	591625 +	U	4	0	1	0	0	SA0506
gi|29165615|ref|NC_002745.2|	591626 +	A	0	0	0	1	0	SA0506
gi|29165615|ref|NC_002745.2|	591627 +	C	1	0	1	0	0	SA0506
gi|29165615|ref|NC_002745.2|	591629 +	U	1	0	0	0	0	SA0506
gi|29165615|ref|NC_002745.2|	591630 +	G	0	0	0	0	1	SA0506
gi|29165615|ref|NC_002745.2|	591631 +	G	4	1	0	0	1	SA0506
gi|29165615|ref|NC_002745.2|	591632 +	U	1	0	0	0	1	SA0506
gi|29165615|ref|NC_002745.2|	591634 +	U	1	0	2	0	0	SA0506
gi|29165615|ref|NC_002745.2|	591635 +	U	1	0	0	0	2	SA0506
gi|29165615|ref|NC_002745.2|	591636 +	G	1	0	0	0	0	SA0506
gi|29165615|ref|NC_002745.2|	591637 +	C	4	0	0	0	0	SA0506
gi|29165615|ref|NC_002745.2|	591639 +	C	0	1	1	0	0	SA0506
gi|29165615|ref|NC_002745.2|	591642 +	G	0	1	1	0	0	SA0506
gi|29165615|ref|NC_002745.2|	591643 +	A	1	0	0	0	0	SA0506
gi|29165615|ref|NC_002745.2|	591645 +	G	1	0	0	0	0	SA0506
gi|29165615|ref|NC_002745.2|	591647 +	C	0	0	0	1	0	SA0506
gi|29165615|ref|NC_002745.2|	591649 +	U	4	0	0	0	1	SA0506
gi|29165615|ref|NC_002745.2|	591651 +	C	1	0	0	0	0	SA0506
gi|29165615|ref|NC_002745.2|	591653 +	A	2	0	0	1	2	SA0506
gi|29165615|ref|NC_002745.2|	591657 +	G	0	0	0	1	0	SA0506
gi|29165615|ref|NC_002745.2|	591658 +	G	1	0	0	0	0	SA0506
gi|29165615|ref|NC_002745.2|	591659 +	U	2	1	1	0	1	SA0506
gi|29165615|ref|NC_002745.2|	591660 +	C	0	2	0	0	1	SA0506
gi|29165615|ref|NC_002745.2|	591663 +	G	0	1	0	0	0	SA0506
gi|29165615|ref|NC_002745.2|	591664 +	U	1	0	0	1	1	SA0506
gi|29165615|ref|NC_002745.2|	591665 +	A	1	0	0	0	0	SA0506
gi|29165615|ref|NC_002745.2|	591676 +	C	1	1	0	0	0	SA0506
gi|29165615|ref|NC_002745.2|	591683 +	A	1	0	0	0	0	SA0506
gi|29165615|ref|NC_002745.2|	591684 +	A	0	2	0	0	0	SA0506
gi|29165615|ref|NC_002745.2|	591688 +	C	0	0	0	1	0	SA0506
gi|29165615|ref|NC_002745.2|	591690 +	C	3	0	0	0	1	SA0506
gi|29165615|ref|NC_002745.2|	591691 +	C	1	0	0	0	0	SA0506
gi|29165615|ref|NC_002745.2|	591693 +	C	0	1	0	0	1	SA0506
gi|29165615|ref|NC_002745.2|	591697 +	C	1	0	0	0	0	SA0506
gi|29165615|ref|NC_002745.2|	591700 +	A	0	0	1	1	0	SA0506
gi|29165615|ref|NC_002745.2|	591712 +	A	1	0	1	0	0	SA0506
gi|29165615|ref|NC_002745.2|	591720 +	G	0	2	0	0	0	SA0506
gi|29165615|ref|NC_002745.2|	591721 +	U	0	0	0	1	0	SA0506
gi|29165615|ref|NC_002745.2|	591727 +	C	0	0	0	1	0	SA0506
gi|29165615|ref|NC_002745.2|	591749 +	C	0	0	0	0	1	SA0506
gi|29165615|ref|NC_002745.2|	591758 +	C	0	1	0	0	0	SA0506
gi|29165615|ref|NC_002745.2|	591759 +	U	0	0	0	0	1	SA0506
gi|29165615|ref|NC_002745.2|	591776 +	A	0	1	0	0	0	SA0506
gi|29165615|ref|NC_002745.2|	591779 +	A	0	1	0	0	3	SA0506
gi|29165615|ref|NC_002745.2|	591780 +	U	0	0	1	0	0	SA0506
gi|29165615|ref|NC_002745.2|	591794 +	U	0	0	0	0	1	SA0506
gi|29165615|ref|NC_002745.2|	591796 +	C	3	0	0	0	0	SA0506
gi|29165615|ref|NC_002745.2|	591799 +	A	3	0	0	0	0	SA0506
gi|29165615|ref|NC_002745.2|	591826 +	A	0	0	0	1	0	SA0506
gi|29165615|ref|NC_002745.2|	591842 +	A	1	0	0	0	0	SA0506
gi|29165615|ref|NC_002745.2|	593099 +	U	0	0	0	0	1	-
gi|29165615|ref|NC_002745.2|	593316 +	A	0	0	0	0	2	-
gi|29165615|ref|NC_002745.2|	593479 +	A	0	1	0	0	0	-
gi|29165615|ref|NC_002745.2|	593565 +	G	0	1	0	1	0	-
gi|29165615|ref|NC_002745.2|	593566 +	G	0	8	3	3	3	-
gi|29165615|ref|NC_002745.2|	593567 +	U	0	16	7	8	9	-
gi|29165615|ref|NC_002745.2|	593568 +	A	32	182	105	70	120	-
gi|29165615|ref|NC_002745.2|	593569 +	U	0	3	1	2	0	-
gi|29165615|ref|NC_002745.2|	593570 +	U	0	3	1	2	3	-
gi|29165615|ref|NC_002745.2|	593571 +	A	0	0	1	2	1	-
gi|29165615|ref|NC_002745.2|	593572 +	C	1	2	0	0	2	-
gi|29165615|ref|NC_002745.2|	593573 +	C	0	8	9	3	11	-
gi|29165615|ref|NC_002745.2|	593574 +	A	0	7	3	2	3	-
gi|29165615|ref|NC_002745.2|	593575 +	A	0	3	1	0	8	-
gi|29165615|ref|NC_002745.2|	593576 +	A	0	5	1	0	2	-
gi|29165615|ref|NC_002745.2|	593577 +	C	0	3	1	0	1	-
gi|29165615|ref|NC_002745.2|	593578 +	A	0	1	1	0	1	-
gi|29165615|ref|NC_002745.2|	593579 +	A	0	8	2	0	4	-
gi|29165615|ref|NC_002745.2|	593580 +	U	0	2	1	0	0	-
gi|29165615|ref|NC_002745.2|	593581 +	A	0	1	1	0	0	-
gi|29165615|ref|NC_002745.2|	593582 +	C	0	2	1	0	1	-
gi|29165615|ref|NC_002745.2|	593583 +	U	0	1	0	0	0	-
gi|29165615|ref|NC_002745.2|	593584 +	U	1	0	1	0	0	-
gi|29165615|ref|NC_002745.2|	593585 +	A	0	0	1	0	0	-
gi|29165615|ref|NC_002745.2|	593589 +	G	0	0	1	1	0	-

gi|29165615|ref|NC_002745.2|	593591	+	G	0	0	1	1	0	-
gi|29165615|ref|NC_002745.2|	593594	+	U	0	1	0	0	0	-
gi|29165615|ref|NC_002745.2|	593621	+	A	0	0	0	0	2	-
gi|29165615|ref|NC_002745.2|	593623	+	U	1	0	0	0	0	-
gi|29165615|ref|NC_002745.2|	593633	+	A	0	0	0	0	1	-
gi|29165615|ref|NC_002745.2|	593635	+	A	0	0	0	1	0	-
gi|29165615|ref|NC_002745.2|	593649	+	A	0	0	1	0	0	SA0508
gi|29165615|ref|NC_002745.2|	593650	+	A	0	0	1	2	2	SA0508
gi|29165615|ref|NC_002745.2|	593651	+	A	0	2	1	1	2	SA0508
gi|29165615|ref|NC_002745.2|	593653	+	G	0	1	0	0	0	SA0508
gi|29165615|ref|NC_002745.2|	593654	+	A	0	4	1	3	4	SA0508
gi|29165615|ref|NC_002745.2|	593655	+	A	0	0	0	1	0	SA0508
gi|29165615|ref|NC_002745.2|	593657	+	A	0	1	0	0	0	SA0508
gi|29165615|ref|NC_002745.2|	593681	+	C	0	1	0	0	0	SA0508
gi|29165615|ref|NC_002745.2|	593683	+	A	0	0	0	1	1	SA0508
gi|29165615|ref|NC_002745.2|	593705	+	A	0	1	1	2	0	SA0508
gi|29165615|ref|NC_002745.2|	593706	+	A	0	0	0	1	0	SA0508
gi|29165615|ref|NC_002745.2|	593707	+	A	0	2	0	0	0	SA0508
gi|29165615|ref|NC_002745.2|	593711	+	A	0	2	2	1	2	SA0508
gi|29165615|ref|NC_002745.2|	593712	+	A	0	1	0	0	1	SA0508
gi|29165615|ref|NC_002745.2|	593713	+	A	0	0	0	0	1	SA0508
gi|29165615|ref|NC_002745.2|	593714	+	U	0	0	0	0	1	SA0508
gi|29165615|ref|NC_002745.2|	593720	+	G	0	0	0	0	1	SA0508
gi|29165615|ref|NC_002745.2|	593721	+	G	0	0	0	0	1	SA0508
gi|29165615|ref|NC_002745.2|	593722	+	A	0	1	0	0	0	SA0508
gi|29165615|ref|NC_002745.2|	593727	+	A	0	0	0	1	0	SA0508
gi|29165615|ref|NC_002745.2|	593728	+	U	0	1	0	1	2	SA0508
gi|29165615|ref|NC_002745.2|	593729	+	A	0	0	1	0	0	SA0508
gi|29165615|ref|NC_002745.2|	593730	+	C	0	0	0	1	0	SA0508
gi|29165615|ref|NC_002745.2|	593736	+	C	1	0	0	0	0	SA0508
gi|29165615|ref|NC_002745.2|	593756	+	U	0	0	0	0	1	SA0508
gi|29165615|ref|NC_002745.2|	593761	+	C	0	1	0	1	0	SA0508
gi|29165615|ref|NC_002745.2|	593765	+	C	0	1	0	0	0	SA0508
gi|29165615|ref|NC_002745.2|	593769	+	A	0	0	0	0	1	SA0508
gi|29165615|ref|NC_002745.2|	593774	+	A	0	0	0	0	2	SA0508
gi|29165615|ref|NC_002745.2|	593776	+	G	0	0	1	1	1	SA0508
gi|29165615|ref|NC_002745.2|	593777	+	A	0	1	0	1	1	SA0508
gi|29165615|ref|NC_002745.2|	593778	+	U	0	0	0	1	1	SA0508
gi|29165615|ref|NC_002745.2|	593780	+	U	0	1	0	0	0	SA0508
gi|29165615|ref|NC_002745.2|	593781	+	G	0	3	1	7	5	SA0508
gi|29165615|ref|NC_002745.2|	593784	+	A	3	0	0	0	0	SA0508
gi|29165615|ref|NC_002745.2|	593796	+	A	0	1	0	0	0	SA0508
gi|29165615|ref|NC_002745.2|	593804	+	U	0	0	0	1	0	SA0508
gi|29165615|ref|NC_002745.2|	593819	+	U	0	0	0	1	0	SA0508
gi|29165615|ref|NC_002745.2|	593823	+	U	0	0	0	0	1	SA0508
gi|29165615|ref|NC_002745.2|	593825	+	C	0	0	1	0	0	SA0508
gi|29165615|ref|NC_002745.2|	593826	+	A	0	0	1	0	0	SA0508
gi|29165615|ref|NC_002745.2|	593840	+	C	0	0	1	0	0	SA0508
gi|29165615|ref|NC_002745.2|	593846	+	A	0	4	0	0	0	SA0508
gi|29165615|ref|NC_002745.2|	593874	+	A	0	1	0	0	0	SA0508
gi|29165615|ref|NC_002745.2|	593875	+	G	0	0	0	0	1	SA0508
gi|29165615|ref|NC_002745.2|	593888	+	C	0	1	0	0	0	SA0508
gi|29165615|ref|NC_002745.2|	593896	+	A	0	2	1	0	2	SA0508
gi|29165615|ref|NC_002745.2|	593897	+	A	0	1	0	0	0	SA0508
gi|29165615|ref|NC_002745.2|	593934	+	U	0	0	0	0	1	SA0508
gi|29165615|ref|NC_002745.2|	593936	+	A	0	0	0	1	0	SA0508
gi|29165615|ref|NC_002745.2|	593945	+	U	1	0	1	0	0	SA0508
gi|29165615|ref|NC_002745.2|	593957	+	C	1	0	0	0	0	SA0508
gi|29165615|ref|NC_002745.2|	593965	+	A	0	1	0	0	0	SA0508
gi|29165615|ref|NC_002745.2|	593989	+	U	0	1	0	0	0	SA0508
gi|29165615|ref|NC_002745.2|	594046	+	A	0	0	0	0	1	SA0508
gi|29165615|ref|NC_002745.2|	594047	+	U	0	0	0	0	1	SA0508
gi|29165615|ref|NC_002745.2|	594059	+	A	0	0	0	0	1	SA0508
gi|29165615|ref|NC_002745.2|	594088	+	A	0	2	0	1	2	SA0508
gi|29165615|ref|NC_002745.2|	594089	+	A	0	1	0	0	0	SA0508
gi|29165615|ref|NC_002745.2|	594090	+	A	0	0	0	0	1	SA0508
gi|29165615|ref|NC_002745.2|	594092	+	C	0	1	1	2	3	SA0508
gi|29165615|ref|NC_002745.2|	594093	+	A	1	0	0	1	2	SA0508
gi|29165615|ref|NC_002745.2|	594094	+	A	0	3	0	1	4	SA0508
gi|29165615|ref|NC_002745.2|	594101	+	C	0	0	1	1	1	SA0508
gi|29165615|ref|NC_002745.2|	594115	+	C	0	0	0	0	1	SA0508
gi|29165615|ref|NC_002745.2|	594128	+	U	1	0	0	0	0	SA0508
gi|29165615|ref|NC_002745.2|	594130	+	A	0	1	0	0	2	SA0508
gi|29165615|ref|NC_002745.2|	594135	+	U	0	1	0	0	0	SA0508
gi|29165615|ref|NC_002745.2|	594154	+	A	0	0	1	0	0	SA0508
gi|29165615|ref|NC_002745.2|	594161	+	A	0	0	0	0	1	SA0508
gi|29165615|ref|NC_002745.2|	594167	+	A	0	0	1	0	0	SA0508
gi|29165615|ref|NC_002745.2|	594172	+	G	0	0	0	0	1	SA0508
gi|29165615|ref|NC_002745.2|	594174	+	U	0	0	0	0	1	SA0508
gi|29165615|ref|NC_002745.2|	594175	+	A	0	0	0	0	1	SA0508
gi|29165615|ref|NC_002745.2|	594177	+	A	0	1	0	0	0	SA0508

gi|29165615|ref|NC_002745.2|	594195	+	A	0	0	2	0	0	SA0508
gi|29165615|ref|NC_002745.2|	594196	+	A	0	3	0	0	2	SA0508
gi|29165615|ref|NC_002745.2|	594200	+	C	0	1	0	0	0	SA0508
gi|29165615|ref|NC_002745.2|	594212	+	G	0	0	0	0	1	SA0508
gi|29165615|ref|NC_002745.2|	594213	+	U	0	0	0	0	2	SA0508
gi|29165615|ref|NC_002745.2|	594214	+	U	0	0	0	0	1	SA0508
gi|29165615|ref|NC_002745.2|	594219	+	A	0	0	0	0	1	SA0508
gi|29165615|ref|NC_002745.2|	594220	+	A	0	0	0	0	1	SA0508
gi|29165615|ref|NC_002745.2|	594221	+	C	0	0	0	0	1	SA0508
gi|29165615|ref|NC_002745.2|	594225	+	U	0	0	1	0	0	SA0508
gi|29165615|ref|NC_002745.2|	594227	+	U	0	0	0	1	3	SA0508
gi|29165615|ref|NC_002745.2|	594231	+	C	0	0	1	0	0	SA0508
gi|29165615|ref|NC_002745.2|	594232	+	G	0	0	0	0	1	SA0508
gi|29165615|ref|NC_002745.2|	594233	+	A	0	1	0	1	0	SA0508
gi|29165615|ref|NC_002745.2|	594234	+	C	0	0	0	1	0	SA0508
gi|29165615|ref|NC_002745.2|	594248	+	G	1	0	0	0	1	SA0508
gi|29165615|ref|NC_002745.2|	594251	+	U	0	1	0	0	0	SA0508
gi|29165615|ref|NC_002745.2|	594257	+	G	0	0	1	0	0	SA0508
gi|29165615|ref|NC_002745.2|	594263	+	G	0	1	0	1	0	SA0508
gi|29165615|ref|NC_002745.2|	594299	+	A	0	0	1	0	0	SA0508
gi|29165615|ref|NC_002745.2|	594301	+	A	0	1	0	0	0	SA0508
gi|29165615|ref|NC_002745.2|	594311	+	A	0	0	0	0	2	SA0508
gi|29165615|ref|NC_002745.2|	594313	+	A	0	0	0	0	1	SA0508
gi|29165615|ref|NC_002745.2|	594314	+	U	0	1	0	0	1	SA0508
gi|29165615|ref|NC_002745.2|	594334	+	A	0	1	0	0	0	SA0508
gi|29165615|ref|NC_002745.2|	594339	+	U	1	0	0	0	0	SA0508
gi|29165615|ref|NC_002745.2|	594341	+	U	2	0	0	0	0	SA0508
gi|29165615|ref|NC_002745.2|	594359	+	C	0	0	0	1	0	SA0508
gi|29165615|ref|NC_002745.2|	594372	+	G	2	0	0	0	0	SA0508
gi|29165615|ref|NC_002745.2|	594377	+	U	2	0	0	0	0	SA0508
gi|29165615|ref|NC_002745.2|	594389	+	A	0	0	0	0	1	SA0508
gi|29165615|ref|NC_002745.2|	594419	+	C	0	0	0	0	2	SA0508
gi|29165615|ref|NC_002745.2|	594424	+	U	0	0	0	0	1	SA0508
gi|29165615|ref|NC_002745.2|	594447	+	A	0	1	0	0	0	SA0508
gi|29165615|ref|NC_002745.2|	594448	+	A	0	1	0	0	2	SA0508
gi|29165615|ref|NC_002745.2|	594494	+	A	0	3	0	0	1	SA0508
gi|29165615|ref|NC_002745.2|	594497	+	A	0	1	0	0	0	SA0508
gi|29165615|ref|NC_002745.2|	594498	+	A	0	2	0	0	0	SA0508
gi|29165615|ref|NC_002745.2|	594517	+	U	2	0	0	0	0	SA0508
gi|29165615|ref|NC_002745.2|	594524	+	A	0	0	1	0	0	SA0508
gi|29165615|ref|NC_002745.2|	594551	+	A	0	0	0	1	0	SA0508
gi|29165615|ref|NC_002745.2|	594568	+	A	0	0	0	0	1	SA0508
gi|29165615|ref|NC_002745.2|	594586	+	A	0	0	1	0	0	SA0508
gi|29165615|ref|NC_002745.2|	594607	+	A	0	1	0	0	0	SA0508
gi|29165615|ref|NC_002745.2|	594612	+	A	0	0	1	0	0	SA0508
gi|29165615|ref|NC_002745.2|	594613	+	G	0	0	1	0	3	SA0508
gi|29165615|ref|NC_002745.2|	594614	+	A	0	5	0	4	3	SA0508
gi|29165615|ref|NC_002745.2|	594615	+	A	0	0	1	0	0	SA0508
gi|29165615|ref|NC_002745.2|	594616	+	U	0	1	0	0	0	SA0508
gi|29165615|ref|NC_002745.2|	594617	+	U	0	0	0	1	0	SA0508
gi|29165615|ref|NC_002745.2|	594620	+	G	0	1	0	0	0	SA0508
gi|29165615|ref|NC_002745.2|	594621	+	U	0	2	2	6	4	SA0508
gi|29165615|ref|NC_002745.2|	594622	+	A	0	0	0	1	0	SA0508
gi|29165615|ref|NC_002745.2|	594624	+	G	0	1	0	0	0	SA0508
gi|29165615|ref|NC_002745.2|	594625	+	C	0	2	0	2	3	SA0508
gi|29165615|ref|NC_002745.2|	594627	+	U	0	0	1	1	0	SA0508
gi|29165615|ref|NC_002745.2|	594630	+	A	0	0	0	0	1	SA0508
gi|29165615|ref|NC_002745.2|	594632	+	A	0	0	0	0	2	SA0508
gi|29165615|ref|NC_002745.2|	594637	+	G	0	0	0	0	1	SA0508
gi|29165615|ref|NC_002745.2|	594638	+	A	0	2	0	0	0	SA0508
gi|29165615|ref|NC_002745.2|	594652	+	A	0	0	0	0	1	SA0508
gi|29165615|ref|NC_002745.2|	594682	+	G	0	0	0	1	0	SA0508
gi|29165615|ref|NC_002745.2|	594701	+	A	0	0	1	0	0	SA0508
gi|29165615|ref|NC_002745.2|	594712	+	G	0	1	0	0	0	SA0508
gi|29165615|ref|NC_002745.2|	594720	+	U	0	2	0	0	0	SA0508
gi|29165615|ref|NC_002745.2|	594729	+	C	0	0	1	0	0	SA0508
gi|29165615|ref|NC_002745.2|	595020	+	A	0	0	1	0	0	-
gi|29165615|ref|NC_002745.2|	595022	+	A	0	0	0	1	0	-
gi|29165615|ref|NC_002745.2|	595023	+	C	0	0	0	0	1	-
gi|29165615|ref|NC_002745.2|	595031	+	A	1	2	0	1	1	-
gi|29165615|ref|NC_002745.2|	595059	+	A	0	0	0	0	1	SA0509
gi|29165615|ref|NC_002745.2|	595069	+	A	0	1	0	0	0	SA0509
gi|29165615|ref|NC_002745.2|	595120	+	C	1	0	0	0	0	SA0509
gi|29165615|ref|NC_002745.2|	595148	+	U	2	0	0	0	0	SA0509
gi|29165615|ref|NC_002745.2|	595150	+	A	0	0	0	0	1	SA0509
gi|29165615|ref|NC_002745.2|	595154	+	U	0	1	0	0	0	SA0509
gi|29165615|ref|NC_002745.2|	595168	+	C	0	0	0	0	1	SA0509
gi|29165615|ref|NC_002745.2|	595178	+	U	0	0	0	0	1	SA0509
gi|29165615|ref|NC_002745.2|	595188	+	C	1	0	0	0	0	SA0509
gi|29165615|ref|NC_002745.2|	595194	+	G	1	0	0	0	0	SA0509
gi|29165615|ref|NC_002745.2|	595196	+	U	1	0	0	0	0	SA0509

gi|29165615|ref|NC_002745.2|	595276	+	U	0	0	0	1	0	SA0509
gi|29165615|ref|NC_002745.2|	595305	+	U	0	1	0	0	0	SA0509
gi|29165615|ref|NC_002745.2|	595330	+	A	1	0	0	0	0	SA0509
gi|29165615|ref|NC_002745.2|	595383	+	G	0	0	0	0	1	SA0509
gi|29165615|ref|NC_002745.2|	595384	+	A	0	0	0	1	0	SA0509
gi|29165615|ref|NC_002745.2|	595399	+	C	0	0	0	0	1	SA0509
gi|29165615|ref|NC_002745.2|	595447	+	A	0	0	0	1	0	SA0509
gi|29165615|ref|NC_002745.2|	595448	+	A	0	0	0	0	1	SA0509
gi|29165615|ref|NC_002745.2|	595460	+	A	0	0	1	0	0	SA0509
gi|29165615|ref|NC_002745.2|	595461	+	A	0	0	0	1	0	SA0509
gi|29165615|ref|NC_002745.2|	595463	+	A	0	2	0	1	1	SA0509
gi|29165615|ref|NC_002745.2|	595473	+	G	0	0	0	1	0	SA0509
gi|29165615|ref|NC_002745.2|	595489	+	A	0	1	1	1	0	SA0509
gi|29165615|ref|NC_002745.2|	595495	+	G	1	0	0	1	0	SA0509
gi|29165615|ref|NC_002745.2|	595507	+	A	0	1	0	0	0	SA0509
gi|29165615|ref|NC_002745.2|	595519	+	U	0	0	1	0	0	SA0509
gi|29165615|ref|NC_002745.2|	595521	+	U	0	1	0	0	0	SA0509
gi|29165615|ref|NC_002745.2|	595535	+	A	0	0	1	0	0	SA0509
gi|29165615|ref|NC_002745.2|	595561	+	A	0	0	0	1	0	SA0509
gi|29165615|ref|NC_002745.2|	595601	+	C	0	1	0	0	0	SA0509
gi|29165615|ref|NC_002745.2|	595680	+	U	0	1	0	0	0	SA0509
gi|29165615|ref|NC_002745.2|	595682	+	A	0	1	0	0	0	SA0509
gi|29165615|ref|NC_002745.2|	595683	+	G	0	0	0	1	0	SA0509
gi|29165615|ref|NC_002745.2|	595693	+	C	1	0	0	1	0	SA0509
gi|29165615|ref|NC_002745.2|	595720	+	A	0	0	0	2	0	SA0509
gi|29165615|ref|NC_002745.2|	595722	+	G	0	0	1	0	0	SA0509
gi|29165615|ref|NC_002745.2|	595753	+	C	2	0	0	0	0	SA0509
gi|29165615|ref|NC_002745.2|	595760	+	C	0	0	0	0	1	SA0509
gi|29165615|ref|NC_002745.2|	595762	+	U	1	0	0	0	0	SA0509
gi|29165615|ref|NC_002745.2|	595763	+	G	0	2	0	2	3	SA0509
gi|29165615|ref|NC_002745.2|	596024	+	C	0	0	1	0	1	-
gi|29165615|ref|NC_002745.2|	596027	+	A	1	0	2	4	5	-
gi|29165615|ref|NC_002745.2|	596028	+	U	0	0	1	0	0	-
gi|29165615|ref|NC_002745.2|	596042	+	U	0	0	0	1	0	-
gi|29165615|ref|NC_002745.2|	596044	+	A	0	0	0	1	0	-
gi|29165615|ref|NC_002745.2|	596060	+	A	0	0	0	0	1	-
gi|29165615|ref|NC_002745.2|	596085	+	G	0	0	0	1	0	-
gi|29165615|ref|NC_002745.2|	596118	+	A	0	0	0	0	1	SA0510
gi|29165615|ref|NC_002745.2|	596220	+	A	0	0	1	1	0	SA0510
gi|29165615|ref|NC_002745.2|	596254	+	A	0	0	1	0	0	SA0510
gi|29165615|ref|NC_002745.2|	596315	+	G	0	0	0	1	0	SA0510
gi|29165615|ref|NC_002745.2|	596391	+	A	0	0	0	0	1	SA0510
gi|29165615|ref|NC_002745.2|	596427	+	A	0	0	1	0	0	SA0510
gi|29165615|ref|NC_002745.2|	596433	+	A	0	0	0	0	1	SA0510
gi|29165615|ref|NC_002745.2|	596517	+	A	0	1	0	1	2	SA0510
gi|29165615|ref|NC_002745.2|	596518	+	U	0	1	0	0	1	SA0510
gi|29165615|ref|NC_002745.2|	596541	+	G	0	0	0	0	1	SA0510
gi|29165615|ref|NC_002745.2|	596546	+	A	0	0	2	0	0	SA0510
gi|29165615|ref|NC_002745.2|	596555	+	A	0	0	0	1	2	SA0510
gi|29165615|ref|NC_002745.2|	596557	+	U	0	0	0	0	1	SA0510
gi|29165615|ref|NC_002745.2|	596590	+	G	0	1	0	0	0	SA0510
gi|29165615|ref|NC_002745.2|	596675	+	G	0	1	0	0	0	SA0510
gi|29165615|ref|NC_002745.2|	596770	+	A	0	0	0	0	1	SA0510
gi|29165615|ref|NC_002745.2|	596771	+	A	0	2	0	0	0	SA0510
gi|29165615|ref|NC_002745.2|	596787	+	A	0	0	0	0	1	SA0510
gi|29165615|ref|NC_002745.2|	596860	+	A	0	0	0	0	1	SA0510
gi|29165615|ref|NC_002745.2|	596948	+	A	0	1	0	0	0	SA0510
gi|29165615|ref|NC_002745.2|	596949	+	C	0	0	0	0	1	SA0510
gi|29165615|ref|NC_002745.2|	596950	+	U	0	2	0	0	0	SA0510
gi|29165615|ref|NC_002745.2|	596956	+	G	0	0	0	0	1	SA0510
gi|29165615|ref|NC_002745.2|	596970	+	C	0	1	0	0	0	SA0510
gi|29165615|ref|NC_002745.2|	596980	+	U	0	0	0	0	1	SA0510
gi|29165615|ref|NC_002745.2|	597164	+	G	0	0	0	0	1	SA0510
gi|29165615|ref|NC_002745.2|	597171	+	U	0	0	0	1	0	SA0510
gi|29165615|ref|NC_002745.2|	597180	+	A	0	0	0	1	0	SA0510
gi|29165615|ref|NC_002745.2|	597182	+	A	0	0	0	0	1	SA0510
gi|29165615|ref|NC_002745.2|	597183	+	U	0	0	0	1	0	SA0510
gi|29165615|ref|NC_002745.2|	597184	+	A	0	0	0	0	1	SA0510
gi|29165615|ref|NC_002745.2|	597203	+	G	0	0	0	0	1	SA0510
gi|29165615|ref|NC_002745.2|	597307	+	U	0	1	0	0	0	SA0510
gi|29165615|ref|NC_002745.2|	597357	+	U	0	1	0	0	0	SA0510
gi|29165615|ref|NC_002745.2|	597452	+	U	0	0	1	0	0	SA0510
gi|29165615|ref|NC_002745.2|	597552	+	A	0	0	0	0	1	SA0510
gi|29165615|ref|NC_002745.2|	597602	+	A	0	0	0	0	1	SA0510
gi|29165615|ref|NC_002745.2|	597865	+	U	0	1	0	0	0	-
gi|29165615|ref|NC_002745.2|	597875	+	A	0	0	0	1	0	-
gi|29165615|ref|NC_002745.2|	597880	+	G	8	70	66	25	61	-
gi|29165615|ref|NC_002745.2|	597881	+	A	1	39	15	12	28	-
gi|29165615|ref|NC_002745.2|	597882	+	A	0	14	12	3	17	-
gi|29165615|ref|NC_002745.2|	597883	+	A	0	5	4	2	6	-
gi|29165615|ref|NC_002745.2|	597884	+	A	0	6	3	1	2	-

gi|29165615|ref|NC_002745.2|	597885 +	U	0	0	1	1	0	-
gi|29165615|ref|NC_002745.2|	597886 +	G	0	3	1	0	0	-
gi|29165615|ref|NC_002745.2|	597887 +	A	1	23	5	22	42	-
gi|29165615|ref|NC_002745.2|	597888 +	A	0	2	2	4	9	-
gi|29165615|ref|NC_002745.2|	597890 +	A	0	3	0	0	3	-
gi|29165615|ref|NC_002745.2|	597891 +	U	0	0	0	0	1	-
gi|29165615|ref|NC_002745.2|	597895 +	G	0	0	0	0	1	-
gi|29165615|ref|NC_002745.2|	597916 +	G	0	0	1	0	0	-
gi|29165615|ref|NC_002745.2|	597920 +	U	0	1	0	0	0	-
gi|29165615|ref|NC_002745.2|	597921 +	A	0	0	0	0	1	-
gi|29165615|ref|NC_002745.2|	597922 +	U	0	3	2	1	1	-
gi|29165615|ref|NC_002745.2|	597923 +	A	0	1	2	2	1	-
gi|29165615|ref|NC_002745.2|	597924 +	G	0	7	2	7	6	-
gi|29165615|ref|NC_002745.2|	597925 +	A	0	3	0	0	2	-
gi|29165615|ref|NC_002745.2|	597926 +	A	0	1	0	1	3	-
gi|29165615|ref|NC_002745.2|	597927 +	G	0	1	1	1	2	-
gi|29165615|ref|NC_002745.2|	597928 +	A	0	0	0	1	0	-
gi|29165615|ref|NC_002745.2|	597929 +	G	0	2	0	0	0	-
gi|29165615|ref|NC_002745.2|	597930 +	A	0	6	1	4	1	-
gi|29165615|ref|NC_002745.2|	597931 +	C	0	3	2	0	2	-
gi|29165615|ref|NC_002745.2|	597932 +	A	0	1	0	0	2	-
gi|29165615|ref|NC_002745.2|	597933 +	A	0	3	1	3	1	-
gi|29165615|ref|NC_002745.2|	597936 +	G	0	4	3	1	7	-
gi|29165615|ref|NC_002745.2|	597938 +	G	0	1	0	0	0	-
gi|29165615|ref|NC_002745.2|	597939 +	A	0	0	0	0	2	-
gi|29165615|ref|NC_002745.2|	597940 +	U	2	0	0	0	0	-
gi|29165615|ref|NC_002745.2|	597944 +	U	0	0	0	0	2	-
gi|29165615|ref|NC_002745.2|	597945 +	C	0	0	0	0	2	-
gi|29165615|ref|NC_002745.2|	597946 +	U	0	0	1	0	0	-
gi|29165615|ref|NC_002745.2|	597947 +	A	0	0	0	1	0	SA0511
gi|29165615|ref|NC_002745.2|	597948 +	U	0	1	0	0	1	SA0511
gi|29165615|ref|NC_002745.2|	597949 +	G	0	2	0	1	5	SA0511
gi|29165615|ref|NC_002745.2|	597950 +	A	0	6	5	8	8	SA0511
gi|29165615|ref|NC_002745.2|	597951 +	A	0	3	0	3	2	SA0511
gi|29165615|ref|NC_002745.2|	597952 +	A	0	3	1	1	2	SA0511
gi|29165615|ref|NC_002745.2|	597953 +	A	0	6	3	17	9	SA0511
gi|29165615|ref|NC_002745.2|	597954 +	A	0	7	1	5	4	SA0511
gi|29165615|ref|NC_002745.2|	597955 +	A	1	5	6	7	5	SA0511
gi|29165615|ref|NC_002745.2|	597956 +	A	1	54	21	51	71	SA0511
gi|29165615|ref|NC_002745.2|	597957 +	U	0	2	3	4	6	SA0511
gi|29165615|ref|NC_002745.2|	597958 +	U	0	2	0	0	2	SA0511
gi|29165615|ref|NC_002745.2|	597959 +	A	1	10	8	16	14	SA0511
gi|29165615|ref|NC_002745.2|	597960 +	U	1	2	1	5	11	SA0511
gi|29165615|ref|NC_002745.2|	597961 +	G	0	3	1	5	4	SA0511
gi|29165615|ref|NC_002745.2|	597962 +	A	0	16	11	14	15	SA0511
gi|29165615|ref|NC_002745.2|	597963 +	U	0	0	0	1	0	SA0511
gi|29165615|ref|NC_002745.2|	597964 +	U	0	0	0	1	2	SA0511
gi|29165615|ref|NC_002745.2|	597966 +	C	1	1	0	1	0	SA0511
gi|29165615|ref|NC_002745.2|	597967 +	U	0	0	0	0	1	SA0511
gi|29165615|ref|NC_002745.2|	597970 +	U	0	0	0	1	0	SA0511
gi|29165615|ref|NC_002745.2|	597971 +	G	0	0	0	1	0	SA0511
gi|29165615|ref|NC_002745.2|	597972 +	C	0	8	1	4	7	SA0511
gi|29165615|ref|NC_002745.2|	597973 +	A	0	1	0	0	2	SA0511
gi|29165615|ref|NC_002745.2|	597974 +	U	1	0	0	0	0	SA0511
gi|29165615|ref|NC_002745.2|	597975 +	U	0	1	0	0	2	SA0511
gi|29165615|ref|NC_002745.2|	597976 +	A	0	1	0	1	1	SA0511
gi|29165615|ref|NC_002745.2|	597977 +	G	0	0	0	0	1	SA0511
gi|29165615|ref|NC_002745.2|	597978 +	G	1	0	0	1	0	SA0511
gi|29165615|ref|NC_002745.2|	597983 +	A	0	0	0	0	1	SA0511
gi|29165615|ref|NC_002745.2|	597984 +	U	1	0	0	0	0	SA0511
gi|29165615|ref|NC_002745.2|	597987 +	G	1	0	0	0	2	SA0511
gi|29165615|ref|NC_002745.2|	597988 +	U	0	1	0	0	0	SA0511
gi|29165615|ref|NC_002745.2|	597989 +	A	0	1	0	0	0	SA0511
gi|29165615|ref|NC_002745.2|	597990 +	C	2	3	0	0	1	SA0511
gi|29165615|ref|NC_002745.2|	597991 +	A	0	1	0	1	1	SA0511
gi|29165615|ref|NC_002745.2|	597993 +	A	1	2	1	5	8	SA0511
gi|29165615|ref|NC_002745.2|	597994 +	A	0	1	2	0	0	SA0511
gi|29165615|ref|NC_002745.2|	597995 +	U	0	0	1	0	0	SA0511
gi|29165615|ref|NC_002745.2|	597996 +	U	0	1	1	0	0	SA0511
gi|29165615|ref|NC_002745.2|	597998 +	G	0	0	0	2	0	SA0511
gi|29165615|ref|NC_002745.2|	597999 +	U	2	1	1	4	0	SA0511
gi|29165615|ref|NC_002745.2|	598002 +	U	2	0	0	0	0	SA0511
gi|29165615|ref|NC_002745.2|	598004 +	A	0	3	0	3	2	SA0511
gi|29165615|ref|NC_002745.2|	598005 +	A	0	0	0	0	1	SA0511
gi|29165615|ref|NC_002745.2|	598009 +	C	0	0	1	1	0	SA0511
gi|29165615|ref|NC_002745.2|	598011 +	G	0	0	0	1	0	SA0511
gi|29165615|ref|NC_002745.2|	598012 +	A	0	0	0	0	1	SA0511
gi|29165615|ref|NC_002745.2|	598014 +	A	0	1	1	1	1	SA0511
gi|29165615|ref|NC_002745.2|	598015 +	A	1	0	2	0	0	SA0511
gi|29165615|ref|NC_002745.2|	598016 +	A	0	3	1	0	1	SA0511
gi|29165615|ref|NC_002745.2|	598017 +	U	0	1	0	0	0	SA0511

gi|29165615|ref|NC_002745.2|	598031	+	A	0	1	0	0	0	SA0511
gi|29165615|ref|NC_002745.2|	598035	+	U	2	0	0	0	0	SA0511
gi|29165615|ref|NC_002745.2|	598037	+	C	0	2	0	0	1	SA0511
gi|29165615|ref|NC_002745.2|	598038	+	U	0	0	1	0	0	SA0511
gi|29165615|ref|NC_002745.2|	598041	+	C	1	0	0	1	0	SA0511
gi|29165615|ref|NC_002745.2|	598042	+	U	0	0	1	0	0	SA0511
gi|29165615|ref|NC_002745.2|	598043	+	A	0	0	1	2	1	SA0511
gi|29165615|ref|NC_002745.2|	598044	+	C	0	0	0	1	1	SA0511
gi|29165615|ref|NC_002745.2|	598045	+	A	0	0	1	1	0	SA0511
gi|29165615|ref|NC_002745.2|	598046	+	G	0	1	0	3	4	SA0511
gi|29165615|ref|NC_002745.2|	598047	+	A	3	0	3	3	0	SA0511
gi|29165615|ref|NC_002745.2|	598048	+	U	0	0	1	0	1	SA0511
gi|29165615|ref|NC_002745.2|	598049	+	A	0	0	1	6	0	SA0511
gi|29165615|ref|NC_002745.2|	598050	+	U	1	1	0	1	1	SA0511
gi|29165615|ref|NC_002745.2|	598051	+	U	2	0	2	0	0	SA0511
gi|29165615|ref|NC_002745.2|	598056	+	A	1	0	0	0	0	SA0511
gi|29165615|ref|NC_002745.2|	598059	+	C	0	0	0	1	0	SA0511
gi|29165615|ref|NC_002745.2|	598065	+	C	0	0	0	1	0	SA0511
gi|29165615|ref|NC_002745.2|	598073	+	C	0	1	0	0	0	SA0511
gi|29165615|ref|NC_002745.2|	598074	+	C	0	0	0	0	1	SA0511
gi|29165615|ref|NC_002745.2|	598079	+	C	0	0	1	0	0	SA0511
gi|29165615|ref|NC_002745.2|	598081	+	A	0	0	0	1	0	SA0511
gi|29165615|ref|NC_002745.2|	598083	+	A	0	1	1	1	1	SA0511
gi|29165615|ref|NC_002745.2|	598087	+	A	0	0	0	0	1	SA0511
gi|29165615|ref|NC_002745.2|	598090	+	A	0	1	0	0	0	SA0511
gi|29165615|ref|NC_002745.2|	598091	+	U	0	0	1	0	0	SA0511
gi|29165615|ref|NC_002745.2|	598095	+	A	0	3	0	0	1	SA0511
gi|29165615|ref|NC_002745.2|	598096	+	A	0	1	0	0	0	SA0511
gi|29165615|ref|NC_002745.2|	598098	+	U	0	0	0	0	1	SA0511
gi|29165615|ref|NC_002745.2|	598099	+	U	0	2	0	0	0	SA0511
gi|29165615|ref|NC_002745.2|	598100	+	U	0	0	0	0	1	SA0511
gi|29165615|ref|NC_002745.2|	598103	+	G	1	5	0	2	4	SA0511
gi|29165615|ref|NC_002745.2|	598104	+	A	0	0	0	1	0	SA0511
gi|29165615|ref|NC_002745.2|	598107	+	U	0	0	0	1	1	SA0511
gi|29165615|ref|NC_002745.2|	598109	+	A	1	2	1	0	0	SA0511
gi|29165615|ref|NC_002745.2|	598110	+	C	0	0	0	0	1	SA0511
gi|29165615|ref|NC_002745.2|	598111	+	A	0	1	0	0	0	SA0511
gi|29165615|ref|NC_002745.2|	598112	+	G	0	1	0	0	0	SA0511
gi|29165615|ref|NC_002745.2|	598113	+	A	0	0	0	0	1	SA0511
gi|29165615|ref|NC_002745.2|	598117	+	U	1	0	0	0	0	SA0511
gi|29165615|ref|NC_002745.2|	598129	+	U	2	0	0	0	0	SA0511
gi|29165615|ref|NC_002745.2|	598133	+	U	0	1	0	0	0	SA0511
gi|29165615|ref|NC_002745.2|	598154	+	G	0	0	0	0	1	SA0511
gi|29165615|ref|NC_002745.2|	598168	+	U	0	1	0	0	0	SA0511
gi|29165615|ref|NC_002745.2|	598203	+	A	0	1	0	0	0	SA0511
gi|29165615|ref|NC_002745.2|	598208	+	A	0	0	0	0	1	SA0511
gi|29165615|ref|NC_002745.2|	598224	+	U	1	0	0	0	0	SA0511
gi|29165615|ref|NC_002745.2|	598248	+	C	0	0	0	0	1	SA0511
gi|29165615|ref|NC_002745.2|	598254	+	A	0	1	0	0	0	SA0511
gi|29165615|ref|NC_002745.2|	598271	+	A	0	0	0	0	1	SA0511
gi|29165615|ref|NC_002745.2|	598335	+	C	0	1	0	0	1	SA0511
gi|29165615|ref|NC_002745.2|	598340	+	C	0	0	0	0	1	SA0511
gi|29165615|ref|NC_002745.2|	598381	+	U	0	0	0	0	2	SA0511
gi|29165615|ref|NC_002745.2|	598413	+	A	0	0	0	1	0	SA0511
gi|29165615|ref|NC_002745.2|	598417	+	C	0	0	0	1	0	SA0511
gi|29165615|ref|NC_002745.2|	598424	+	U	0	1	0	0	0	SA0511
gi|29165615|ref|NC_002745.2|	598428	+	G	0	0	0	1	0	SA0511
gi|29165615|ref|NC_002745.2|	598431	+	U	0	1	0	0	0	SA0511
gi|29165615|ref|NC_002745.2|	598453	+	C	0	1	0	0	0	SA0511
gi|29165615|ref|NC_002745.2|	598454	+	C	0	0	0	0	1	SA0511
gi|29165615|ref|NC_002745.2|	598466	+	U	0	1	0	0	0	SA0511
gi|29165615|ref|NC_002745.2|	598467	+	C	0	1	0	0	0	SA0511
gi|29165615|ref|NC_002745.2|	598469	+	C	0	0	0	0	1	SA0511
gi|29165615|ref|NC_002745.2|	598484	+	G	0	0	0	0	1	SA0511
gi|29165615|ref|NC_002745.2|	598486	+	U	2	0	0	0	0	SA0511
gi|29165615|ref|NC_002745.2|	598489	+	C	0	0	0	0	1	SA0511
gi|29165615|ref|NC_002745.2|	598490	+	G	0	0	0	0	1	SA0511
gi|29165615|ref|NC_002745.2|	598491	+	G	0	0	1	0	0	SA0511
gi|29165615|ref|NC_002745.2|	598492	+	U	0	1	1	0	0	SA0511
gi|29165615|ref|NC_002745.2|	598493	+	A	0	0	0	1	0	SA0511
gi|29165615|ref|NC_002745.2|	598497	+	C	1	0	0	0	0	SA0511
gi|29165615|ref|NC_002745.2|	598499	+	G	0	1	0	0	0	SA0511
gi|29165615|ref|NC_002745.2|	598500	+	A	0	0	1	1	0	SA0511
gi|29165615|ref|NC_002745.2|	598502	+	U	0	0	0	0	1	SA0511
gi|29165615|ref|NC_002745.2|	598504	+	U	0	0	0	1	0	SA0511
gi|29165615|ref|NC_002745.2|	598507	+	U	0	0	0	0	1	SA0511
gi|29165615|ref|NC_002745.2|	598511	+	G	0	0	0	0	1	SA0511
gi|29165615|ref|NC_002745.2|	598512	+	A	0	1	0	0	0	SA0511
gi|29165615|ref|NC_002745.2|	598514	+	A	0	0	0	0	1	SA0511
gi|29165615|ref|NC_002745.2|	598515	+	U	0	1	0	0	0	SA0511
gi|29165615|ref|NC_002745.2|	598517	+	U	0	0	0	1	1	SA0511

gi|29165615|ref|NC_002745.2|	598520 +	U	0	0	0	0	1	SA0511
gi|29165615|ref|NC_002745.2|	598522 +	C	0	0	1	0	0	SA0511
gi|29165615|ref|NC_002745.2|	598525 +	A	0	2	0	0	0	SA0511
gi|29165615|ref|NC_002745.2|	598528 +	A	0	0	0	0	1	SA0511
gi|29165615|ref|NC_002745.2|	598530 +	U	0	0	0	1	0	SA0511
gi|29165615|ref|NC_002745.2|	598532 +	A	1	0	0	0	0	SA0511
gi|29165615|ref|NC_002745.2|	598542 +	A	0	0	1	0	1	SA0511
gi|29165615|ref|NC_002745.2|	598553 +	U	0	0	0	0	1	SA0511
gi|29165615|ref|NC_002745.2|	598556 +	A	0	0	0	0	1	SA0511
gi|29165615|ref|NC_002745.2|	598559 +	G	0	0	2	0	0	SA0511
gi|29165615|ref|NC_002745.2|	598560 +	A	0	0	0	1	0	SA0511
gi|29165615|ref|NC_002745.2|	598563 +	A	0	1	1	1	1	SA0511
gi|29165615|ref|NC_002745.2|	598564 +	A	0	5	0	0	4	SA0511
gi|29165615|ref|NC_002745.2|	598571 +	U	0	1	0	1	3	SA0511
gi|29165615|ref|NC_002745.2|	598572 +	A	0	2	0	1	0	SA0511
gi|29165615|ref|NC_002745.2|	598574 +	A	0	0	1	0	0	SA0511
gi|29165615|ref|NC_002745.2|	598577 +	G	0	1	0	0	0	SA0511
gi|29165615|ref|NC_002745.2|	598583 +	A	0	0	0	1	0	SA0511
gi|29165615|ref|NC_002745.2|	598586 +	U	0	1	0	0	0	SA0511
gi|29165615|ref|NC_002745.2|	598588 +	U	0	0	1	0	0	SA0511
gi|29165615|ref|NC_002745.2|	598600 +	A	0	0	0	1	0	SA0511
gi|29165615|ref|NC_002745.2|	598608 +	C	0	0	0	1	0	SA0511
gi|29165615|ref|NC_002745.2|	598619 +	C	0	0	0	1	0	SA0511
gi|29165615|ref|NC_002745.2|	598625 +	G	0	1	0	0	0	SA0511
gi|29165615|ref|NC_002745.2|	598666 +	U	0	0	0	1	0	SA0511
gi|29165615|ref|NC_002745.2|	598668 +	U	0	1	0	0	0	SA0511
gi|29165615|ref|NC_002745.2|	598669 +	G	0	0	0	1	0	SA0511
gi|29165615|ref|NC_002745.2|	598680 +	G	0	0	0	0	1	SA0511
gi|29165615|ref|NC_002745.2|	598685 +	G	0	2	0	0	0	SA0511
gi|29165615|ref|NC_002745.2|	598700 +	A	0	0	1	0	0	SA0511
gi|29165615|ref|NC_002745.2|	598707 +	C	0	1	0	0	0	SA0511
gi|29165615|ref|NC_002745.2|	598708 +	A	0	0	0	1	1	SA0511
gi|29165615|ref|NC_002745.2|	598716 +	A	0	1	0	1	1	SA0511
gi|29165615|ref|NC_002745.2|	598717 +	A	0	4	0	0	0	SA0511
gi|29165615|ref|NC_002745.2|	598718 +	U	0	0	0	0	1	SA0511
gi|29165615|ref|NC_002745.2|	598719 +	A	0	0	0	0	1	SA0511
gi|29165615|ref|NC_002745.2|	598720 +	C	1	0	0	1	0	SA0511
gi|29165615|ref|NC_002745.2|	598721 +	U	0	0	0	0	1	SA0511
gi|29165615|ref|NC_002745.2|	598722 +	A	0	1	0	0	1	SA0511
gi|29165615|ref|NC_002745.2|	598724 +	C	0	0	1	0	0	SA0511
gi|29165615|ref|NC_002745.2|	598726 +	A	0	0	0	0	1	SA0511
gi|29165615|ref|NC_002745.2|	598728 +	A	0	5	0	0	0	SA0511
gi|29165615|ref|NC_002745.2|	598730 +	U	0	0	0	0	2	SA0511
gi|29165615|ref|NC_002745.2|	598732 +	U	0	0	0	1	2	SA0511
gi|29165615|ref|NC_002745.2|	598734 +	C	0	0	0	1	0	SA0511
gi|29165615|ref|NC_002745.2|	598735 +	A	0	0	0	0	2	SA0511
gi|29165615|ref|NC_002745.2|	598736 +	U	0	1	0	0	0	SA0511
gi|29165615|ref|NC_002745.2|	598737 +	U	0	1	0	0	0	SA0511
gi|29165615|ref|NC_002745.2|	598738 +	A	0	1	2	0	0	SA0511
gi|29165615|ref|NC_002745.2|	598739 +	G	0	0	1	0	3	SA0511
gi|29165615|ref|NC_002745.2|	598740 +	A	1	28	12	13	24	SA0511
gi|29165615|ref|NC_002745.2|	598742 +	U	0	2	0	0	0	SA0511
gi|29165615|ref|NC_002745.2|	598743 +	A	1	0	0	0	0	SA0511
gi|29165615|ref|NC_002745.2|	598744 +	U	0	0	1	0	1	SA0511
gi|29165615|ref|NC_002745.2|	598746 +	A	0	6	5	6	1	SA0511
gi|29165615|ref|NC_002745.2|	598755 +	C	0	0	0	1	0	SA0511
gi|29165615|ref|NC_002745.2|	598757 +	A	0	0	0	0	1	SA0511
gi|29165615|ref|NC_002745.2|	598763 +	C	0	0	1	0	0	SA0511
gi|29165615|ref|NC_002745.2|	598767 +	G	0	1	0	0	0	SA0511
gi|29165615|ref|NC_002745.2|	598769 +	A	0	1	1	1	3	SA0511
gi|29165615|ref|NC_002745.2|	598776 +	A	1	2	0	0	0	SA0511
gi|29165615|ref|NC_002745.2|	598780 +	U	2	1	0	0	0	SA0511
gi|29165615|ref|NC_002745.2|	598797 +	A	0	1	0	0	0	SA0511
gi|29165615|ref|NC_002745.2|	598829 +	G	0	0	0	0	2	SA0511
gi|29165615|ref|NC_002745.2|	598834 +	U	0	0	1	0	0	SA0511
gi|29165615|ref|NC_002745.2|	598890 +	A	0	0	0	0	1	SA0511
gi|29165615|ref|NC_002745.2|	599118 +	A	0	0	1	0	0	-
gi|29165615|ref|NC_002745.2|	599120 +	A	2	2	0	0	0	-
gi|29165615|ref|NC_002745.2|	599122 +	A	0	1	0	0	0	-
gi|29165615|ref|NC_002745.2|	599181 +	C	0	6	1	4	4	-
gi|29165615|ref|NC_002745.2|	599182 +	A	0	2	0	3	2	-
gi|29165615|ref|NC_002745.2|	599183 +	A	0	0	1	0	0	-
gi|29165615|ref|NC_002745.2|	599187 +	A	0	0	0	1	0	-
gi|29165615|ref|NC_002745.2|	599190 +	G	0	0	0	1	0	-
gi|29165615|ref|NC_002745.2|	599191 +	A	0	1	2	0	0	-
gi|29165615|ref|NC_002745.2|	599194 +	G	0	0	0	1	0	-
gi|29165615|ref|NC_002745.2|	599197 +	A	0	0	1	1	1	-
gi|29165615|ref|NC_002745.2|	599198 +	U	0	0	0	0	1	-
gi|29165615|ref|NC_002745.2|	599199 +	A	0	2	0	1	1	-
gi|29165615|ref|NC_002745.2|	599200 +	A	0	0	0	1	0	-
gi|29165615|ref|NC_002745.2|	599203 +	U	0	1	0	2	2	-

gi|29165615|ref|NC_002745.2|	599204	+	A	1	4	5	3	4	-
gi|29165615|ref|NC_002745.2|	599205	+	A	0	4	2	0	3	-
gi|29165615|ref|NC_002745.2|	599207	+	U	0	0	1	3	3	-
gi|29165615|ref|NC_002745.2|	599208	+	U	2	3	1	3	3	-
gi|29165615|ref|NC_002745.2|	599209	+	A	1	5	5	6	14	-
gi|29165615|ref|NC_002745.2|	599210	+	A	0	6	5	5	5	-
gi|29165615|ref|NC_002745.2|	599211	+	A	0	1	0	2	2	-
gi|29165615|ref|NC_002745.2|	599212	+	C	0	1	1	0	2	-
gi|29165615|ref|NC_002745.2|	599213	+	A	0	2	0	1	0	-
gi|29165615|ref|NC_002745.2|	599214	+	A	0	0	1	0	0	-
gi|29165615|ref|NC_002745.2|	599215	+	A	1	1	0	1	0	-
gi|29165615|ref|NC_002745.2|	599216	+	U	0	2	0	0	1	-
gi|29165615|ref|NC_002745.2|	599218	+	C	0	0	1	0	0	-
gi|29165615|ref|NC_002745.2|	599220	+	U	0	0	1	1	0	-
gi|29165615|ref|NC_002745.2|	599221	+	A	0	0	2	0	0	-
gi|29165615|ref|NC_002745.2|	599223	+	A	0	1	0	0	0	-
gi|29165615|ref|NC_002745.2|	599224	+	C	0	0	0	0	1	-
gi|29165615|ref|NC_002745.2|	599225	+	A	0	1	0	1	1	-
gi|29165615|ref|NC_002745.2|	599226	+	A	0	0	1	0	1	-
gi|29165615|ref|NC_002745.2|	599227	+	U	0	0	1	0	1	-
gi|29165615|ref|NC_002745.2|	599228	+	A	0	1	1	0	1	-
gi|29165615|ref|NC_002745.2|	599232	+	U	0	0	0	1	0	-
gi|29165615|ref|NC_002745.2|	599236	+	G	0	0	2	0	1	-
gi|29165615|ref|NC_002745.2|	599237	+	A	0	0	1	0	2	-
gi|29165615|ref|NC_002745.2|	599240	+	C	0	1	0	1	0	-
gi|29165615|ref|NC_002745.2|	599241	+	A	0	1	0	0	1	-
gi|29165615|ref|NC_002745.2|	599254	+	C	0	0	0	1	0	SA0512
gi|29165615|ref|NC_002745.2|	599264	+	A	0	0	0	0	2	SA0512
gi|29165615|ref|NC_002745.2|	599271	+	A	0	1	1	0	0	SA0512
gi|29165615|ref|NC_002745.2|	599273	+	G	0	0	0	1	0	SA0512
gi|29165615|ref|NC_002745.2|	599275	+	C	0	0	0	1	0	SA0512
gi|29165615|ref|NC_002745.2|	599276	+	G	0	0	1	0	0	SA0512
gi|29165615|ref|NC_002745.2|	599279	+	A	0	0	0	0	1	SA0512
gi|29165615|ref|NC_002745.2|	599280	+	A	0	0	0	1	1	SA0512
gi|29165615|ref|NC_002745.2|	599281	+	A	0	0	1	0	0	SA0512
gi|29165615|ref|NC_002745.2|	599286	+	A	0	2	0	2	0	SA0512
gi|29165615|ref|NC_002745.2|	599287	+	A	0	1	0	1	0	SA0512
gi|29165615|ref|NC_002745.2|	599288	+	A	0	2	0	0	1	SA0512
gi|29165615|ref|NC_002745.2|	599292	+	A	0	2	1	0	0	SA0512
gi|29165615|ref|NC_002745.2|	599293	+	A	0	0	0	2	0	SA0512
gi|29165615|ref|NC_002745.2|	599297	+	C	0	0	0	0	1	SA0512
gi|29165615|ref|NC_002745.2|	599299	+	A	0	3	0	2	5	SA0512
gi|29165615|ref|NC_002745.2|	599300	+	A	0	1	0	0	0	SA0512
gi|29165615|ref|NC_002745.2|	599301	+	U	0	1	0	0	0	SA0512
gi|29165615|ref|NC_002745.2|	599303	+	C	0	1	0	0	0	SA0512
gi|29165615|ref|NC_002745.2|	599307	+	U	2	0	0	0	0	SA0512
gi|29165615|ref|NC_002745.2|	599317	+	U	0	0	0	1	0	SA0512
gi|29165615|ref|NC_002745.2|	599325	+	A	0	1	0	0	0	SA0512
gi|29165615|ref|NC_002745.2|	599328	+	U	0	0	0	0	1	SA0512
gi|29165615|ref|NC_002745.2|	599329	+	U	0	0	0	0	1	SA0512
gi|29165615|ref|NC_002745.2|	599353	+	G	0	0	0	0	1	SA0512
gi|29165615|ref|NC_002745.2|	599357	+	A	1	0	0	0	0	SA0512
gi|29165615|ref|NC_002745.2|	599363	+	C	0	0	0	2	0	SA0512
gi|29165615|ref|NC_002745.2|	599365	+	G	0	0	1	0	0	SA0512
gi|29165615|ref|NC_002745.2|	599368	+	A	0	0	0	0	2	SA0512
gi|29165615|ref|NC_002745.2|	599372	+	G	1	0	0	0	0	SA0512
gi|29165615|ref|NC_002745.2|	599376	+	G	0	0	0	0	1	SA0512
gi|29165615|ref|NC_002745.2|	599378	+	A	0	0	0	0	1	SA0512
gi|29165615|ref|NC_002745.2|	599435	+	U	0	0	0	0	1	SA0512
gi|29165615|ref|NC_002745.2|	599442	+	U	0	0	0	0	1	SA0512
gi|29165615|ref|NC_002745.2|	599453	+	U	0	1	0	0	0	SA0512
gi|29165615|ref|NC_002745.2|	599466	+	A	0	0	0	1	0	SA0512
gi|29165615|ref|NC_002745.2|	599468	+	A	0	0	0	1	0	SA0512
gi|29165615|ref|NC_002745.2|	599471	+	C	0	1	0	1	2	SA0512
gi|29165615|ref|NC_002745.2|	599472	+	A	0	0	1	0	0	SA0512
gi|29165615|ref|NC_002745.2|	599473	+	U	0	1	1	0	0	SA0512
gi|29165615|ref|NC_002745.2|	599474	+	A	2	1	0	1	0	SA0512
gi|29165615|ref|NC_002745.2|	599480	+	G	0	0	0	0	1	SA0512
gi|29165615|ref|NC_002745.2|	599487	+	G	0	0	0	1	0	SA0512
gi|29165615|ref|NC_002745.2|	599490	+	A	1	0	0	0	0	SA0512
gi|29165615|ref|NC_002745.2|	599503	+	C	0	0	1	0	0	SA0512
gi|29165615|ref|NC_002745.2|	599513	+	A	0	0	0	2	0	SA0512
gi|29165615|ref|NC_002745.2|	599515	+	A	0	0	0	0	2	SA0512
gi|29165615|ref|NC_002745.2|	599516	+	A	1	2	0	0	1	SA0512
gi|29165615|ref|NC_002745.2|	599517	+	U	1	0	0	0	0	SA0512
gi|29165615|ref|NC_002745.2|	599520	+	U	0	1	0	0	0	SA0512
gi|29165615|ref|NC_002745.2|	599531	+	A	0	1	0	0	0	SA0512
gi|29165615|ref|NC_002745.2|	599535	+	C	1	0	0	0	0	SA0512
gi|29165615|ref|NC_002745.2|	599539	+	U	0	0	1	0	0	SA0512
gi|29165615|ref|NC_002745.2|	599547	+	A	0	1	0	0	0	SA0512
gi|29165615|ref|NC_002745.2|	599597	+	A	0	0	0	0	1	SA0512

gi|29165615|ref|NC_002745.2|	599606	+	A	0	0	0	0	2	SA0512
gi|29165615|ref|NC_002745.2|	599608	+	A	0	1	0	0	1	SA0512
gi|29165615|ref|NC_002745.2|	599609	+	U	0	2	0	0	0	SA0512
gi|29165615|ref|NC_002745.2|	599610	+	A	0	0	1	1	1	SA0512
gi|29165615|ref|NC_002745.2|	599611	+	G	0	0	0	1	0	SA0512
gi|29165615|ref|NC_002745.2|	599612	+	A	0	0	1	0	0	SA0512
gi|29165615|ref|NC_002745.2|	599619	+	U	0	0	0	0	1	SA0512
gi|29165615|ref|NC_002745.2|	599647	+	U	0	1	0	0	0	SA0512
gi|29165615|ref|NC_002745.2|	599669	+	U	0	0	0	1	0	SA0512
gi|29165615|ref|NC_002745.2|	599681	+	G	0	1	0	0	0	SA0512
gi|29165615|ref|NC_002745.2|	599715	+	A	0	1	1	0	1	SA0512
gi|29165615|ref|NC_002745.2|	599718	+	A	1	0	0	0	1	SA0512
gi|29165615|ref|NC_002745.2|	599719	+	U	0	0	1	1	0	SA0512
gi|29165615|ref|NC_002745.2|	599721	+	A	0	0	1	0	1	SA0512
gi|29165615|ref|NC_002745.2|	599722	+	A	0	1	1	1	0	SA0512
gi|29165615|ref|NC_002745.2|	599724	+	U	0	1	0	0	0	SA0512
gi|29165615|ref|NC_002745.2|	599725	+	A	0	1	0	0	0	SA0512
gi|29165615|ref|NC_002745.2|	599728	+	U	0	0	0	0	1	SA0512
gi|29165615|ref|NC_002745.2|	599746	+	U	0	0	0	1	0	SA0512
gi|29165615|ref|NC_002745.2|	599764	+	U	1	0	0	0	0	SA0512
gi|29165615|ref|NC_002745.2|	599768	+	A	1	0	0	0	0	SA0512
gi|29165615|ref|NC_002745.2|	599773	+	A	0	0	1	0	0	SA0512
gi|29165615|ref|NC_002745.2|	599775	+	U	0	0	0	1	0	SA0512
gi|29165615|ref|NC_002745.2|	599777	+	A	0	1	0	1	0	SA0512
gi|29165615|ref|NC_002745.2|	599779	+	A	0	2	0	0	0	SA0512
gi|29165615|ref|NC_002745.2|	599781	+	C	0	1	0	0	0	SA0512
gi|29165615|ref|NC_002745.2|	599785	+	G	0	0	0	0	1	SA0512
gi|29165615|ref|NC_002745.2|	599786	+	U	0	1	0	1	6	SA0512
gi|29165615|ref|NC_002745.2|	599787	+	A	0	3	2	6	10	SA0512
gi|29165615|ref|NC_002745.2|	599788	+	G	1	1	5	1	4	SA0512
gi|29165615|ref|NC_002745.2|	599789	+	A	0	20	10	27	52	SA0512
gi|29165615|ref|NC_002745.2|	599790	+	A	0	4	6	7	17	SA0512
gi|29165615|ref|NC_002745.2|	599791	+	G	0	2	2	4	0	SA0512
gi|29165615|ref|NC_002745.2|	599792	+	A	0	0	1	2	4	SA0512
gi|29165615|ref|NC_002745.2|	599793	+	U	0	0	1	0	0	SA0512
gi|29165615|ref|NC_002745.2|	599795	+	A	0	0	1	1	0	SA0512
gi|29165615|ref|NC_002745.2|	599796	+	A	1	0	1	0	0	SA0512
gi|29165615|ref|NC_002745.2|	599803	+	C	1	0	0	0	0	SA0512
gi|29165615|ref|NC_002745.2|	599840	+	C	1	0	0	0	0	SA0512
gi|29165615|ref|NC_002745.2|	599852	+	C	0	1	0	0	0	SA0512
gi|29165615|ref|NC_002745.2|	599869	+	C	0	0	0	0	1	SA0512
gi|29165615|ref|NC_002745.2|	599875	+	A	0	3	0	0	0	SA0512
gi|29165615|ref|NC_002745.2|	599879	+	C	0	0	1	1	0	SA0512
gi|29165615|ref|NC_002745.2|	599881	+	A	0	1	0	0	0	SA0512
gi|29165615|ref|NC_002745.2|	599886	+	A	0	0	0	1	0	SA0512
gi|29165615|ref|NC_002745.2|	599888	+	U	0	0	1	0	0	SA0512
gi|29165615|ref|NC_002745.2|	599891	+	G	1	2	1	0	1	SA0512
gi|29165615|ref|NC_002745.2|	599901	+	A	0	0	1	2	0	SA0512
gi|29165615|ref|NC_002745.2|	599903	+	U	0	0	1	2	1	SA0512
gi|29165615|ref|NC_002745.2|	599904	+	A	0	0	1	1	0	SA0512
gi|29165615|ref|NC_002745.2|	599905	+	U	1	0	1	0	0	SA0512
gi|29165615|ref|NC_002745.2|	599911	+	C	0	1	0	0	0	SA0512
gi|29165615|ref|NC_002745.2|	599912	+	U	0	0	0	1	0	SA0512
gi|29165615|ref|NC_002745.2|	599915	+	A	0	1	0	0	0	SA0512
gi|29165615|ref|NC_002745.2|	599918	+	G	0	0	0	1	0	SA0512
gi|29165615|ref|NC_002745.2|	599924	+	A	0	0	0	1	0	SA0512
gi|29165615|ref|NC_002745.2|	599925	+	A	0	0	0	1	0	SA0512
gi|29165615|ref|NC_002745.2|	599929	+	A	0	0	0	1	1	SA0512
gi|29165615|ref|NC_002745.2|	599930	+	A	0	1	0	4	1	SA0512
gi|29165615|ref|NC_002745.2|	599936	+	U	1	0	0	0	0	SA0512
gi|29165615|ref|NC_002745.2|	599939	+	A	1	0	0	0	0	SA0512
gi|29165615|ref|NC_002745.2|	599957	+	A	0	1	0	0	0	SA0512
gi|29165615|ref|NC_002745.2|	599971	+	G	0	1	0	0	0	SA0512
gi|29165615|ref|NC_002745.2|	599975	+	A	0	0	1	0	1	SA0512
gi|29165615|ref|NC_002745.2|	599979	+	A	0	1	0	0	1	SA0512
gi|29165615|ref|NC_002745.2|	599984	+	U	0	1	0	3	0	SA0512
gi|29165615|ref|NC_002745.2|	599985	+	A	0	0	0	1	0	SA0512
gi|29165615|ref|NC_002745.2|	599986	+	A	0	1	0	0	0	SA0512
gi|29165615|ref|NC_002745.2|	599990	+	C	0	0	0	1	0	SA0512
gi|29165615|ref|NC_002745.2|	599996	+	A	0	1	0	0	0	SA0512
gi|29165615|ref|NC_002745.2|	599999	+	U	1	0	0	0	0	SA0512
gi|29165615|ref|NC_002745.2|	600011	+	U	1	0	0	0	0	SA0512
gi|29165615|ref|NC_002745.2|	600013	+	U	0	1	0	0	1	SA0512
gi|29165615|ref|NC_002745.2|	600014	+	U	1	0	0	0	0	SA0512
gi|29165615|ref|NC_002745.2|	600025	+	A	0	1	0	0	0	SA0512
gi|29165615|ref|NC_002745.2|	600026	+	C	0	1	0	0	0	SA0512
gi|29165615|ref|NC_002745.2|	600027	+	A	2	0	0	0	0	SA0512
gi|29165615|ref|NC_002745.2|	600031	+	A	0	2	0	0	2	SA0512
gi|29165615|ref|NC_002745.2|	600032	+	A	0	1	0	0	2	SA0512
gi|29165615|ref|NC_002745.2|	600034	+	U	0	0	1	1	1	SA0512
gi|29165615|ref|NC_002745.2|	600035	+	C	0	0	0	0	2	SA0512

gi|29165615|ref|NC_002745.2|	600037 +	A	0	1	1	0	0	SA0512
gi|29165615|ref|NC_002745.2|	600038 +	U	2	7	3	3	3	SA0512
gi|29165615|ref|NC_002745.2|	600039 +	U	0	1	0	3	0	SA0512
gi|29165615|ref|NC_002745.2|	600040 +	A	0	0	3	0	2	SA0512
gi|29165615|ref|NC_002745.2|	600042 +	C	0	0	0	0	1	SA0512
gi|29165615|ref|NC_002745.2|	600044 +	A	0	3	2	0	5	SA0512
gi|29165615|ref|NC_002745.2|	600045 +	A	0	3	2	0	0	SA0512
gi|29165615|ref|NC_002745.2|	600046 +	U	0	0	1	1	0	SA0512
gi|29165615|ref|NC_002745.2|	600049 +	G	0	2	0	0	1	SA0512
gi|29165615|ref|NC_002745.2|	600050 +	C	0	12	1	12	13	SA0512
gi|29165615|ref|NC_002745.2|	600051 +	U	0	1	1	0	1	SA0512
gi|29165615|ref|NC_002745.2|	600052 +	A	0	0	1	0	4	SA0512
gi|29165615|ref|NC_002745.2|	600053 +	A	0	1	1	1	3	SA0512
gi|29165615|ref|NC_002745.2|	600054 +	A	0	1	0	0	1	SA0512
gi|29165615|ref|NC_002745.2|	600055 +	A	0	3	0	0	0	SA0512
gi|29165615|ref|NC_002745.2|	600056 +	A	0	1	0	0	0	SA0512
gi|29165615|ref|NC_002745.2|	600057 +	C	0	1	0	0	0	SA0512
gi|29165615|ref|NC_002745.2|	600060 +	A	0	1	0	0	0	SA0512
gi|29165615|ref|NC_002745.2|	600062 +	G	1	0	1	0	4	SA0512
gi|29165615|ref|NC_002745.2|	600063 +	A	0	0	0	1	0	SA0512
gi|29165615|ref|NC_002745.2|	600065 +	A	0	0	1	0	0	SA0512
gi|29165615|ref|NC_002745.2|	600068 +	A	0	3	0	3	1	SA0512
gi|29165615|ref|NC_002745.2|	600073 +	G	0	0	0	0	1	SA0512
gi|29165615|ref|NC_002745.2|	600074 +	A	0	0	1	0	1	SA0512
gi|29165615|ref|NC_002745.2|	600075 +	A	1	0	0	0	0	SA0512
gi|29165615|ref|NC_002745.2|	600089 +	C	1	0	1	0	0	SA0512
gi|29165615|ref|NC_002745.2|	600091 +	A	0	6	0	0	2	SA0512
gi|29165615|ref|NC_002745.2|	600094 +	G	0	1	0	0	0	SA0512
gi|29165615|ref|NC_002745.2|	600095 +	A	0	1	0	0	1	SA0512
gi|29165615|ref|NC_002745.2|	600097 +	G	0	1	0	0	0	SA0512
gi|29165615|ref|NC_002745.2|	600099 +	A	0	0	0	0	1	SA0512
gi|29165615|ref|NC_002745.2|	600102 +	A	0	0	1	0	0	SA0512
gi|29165615|ref|NC_002745.2|	600103 +	U	0	1	0	0	0	SA0512
gi|29165615|ref|NC_002745.2|	600105 +	C	0	2	0	0	0	SA0512
gi|29165615|ref|NC_002745.2|	600107 +	A	0	0	0	0	1	SA0512
gi|29165615|ref|NC_002745.2|	600108 +	A	0	1	0	0	1	SA0512
gi|29165615|ref|NC_002745.2|	600110 +	C	0	1	1	1	1	SA0512
gi|29165615|ref|NC_002745.2|	600112 +	U	0	1	0	0	1	SA0512
gi|29165615|ref|NC_002745.2|	600113 +	A	0	0	2	0	0	SA0512
gi|29165615|ref|NC_002745.2|	600114 +	U	0	0	0	0	1	SA0512
gi|29165615|ref|NC_002745.2|	600115 +	G	1	0	0	0	0	SA0512
gi|29165615|ref|NC_002745.2|	600116 +	A	0	3	1	5	0	SA0512
gi|29165615|ref|NC_002745.2|	600117 +	U	0	1	0	0	0	SA0512
gi|29165615|ref|NC_002745.2|	600118 +	A	0	0	0	0	3	SA0512
gi|29165615|ref|NC_002745.2|	600119 +	A	0	2	2	0	0	SA0512
gi|29165615|ref|NC_002745.2|	600122 +	G	1	1	0	0	1	SA0512
gi|29165615|ref|NC_002745.2|	600123 +	U	0	1	1	0	3	SA0512
gi|29165615|ref|NC_002745.2|	600124 +	G	0	0	0	1	0	SA0512
gi|29165615|ref|NC_002745.2|	600125 +	A	0	0	0	2	1	SA0512
gi|29165615|ref|NC_002745.2|	600127 +	U	0	0	0	0	3	SA0512
gi|29165615|ref|NC_002745.2|	600129 +	A	0	1	0	0	1	SA0512
gi|29165615|ref|NC_002745.2|	600131 +	C	0	1	0	0	0	SA0512
gi|29165615|ref|NC_002745.2|	600133 +	G	0	2	0	0	0	SA0512
gi|29165615|ref|NC_002745.2|	600134 +	A	0	0	0	0	1	SA0512
gi|29165615|ref|NC_002745.2|	600135 +	A	0	0	1	0	0	SA0512
gi|29165615|ref|NC_002745.2|	600137 +	U	0	3	1	2	0	SA0512
gi|29165615|ref|NC_002745.2|	600138 +	A	0	2	0	1	0	SA0512
gi|29165615|ref|NC_002745.2|	600139 +	U	0	0	1	0	0	SA0512
gi|29165615|ref|NC_002745.2|	600140 +	U	0	0	0	1	0	SA0512
gi|29165615|ref|NC_002745.2|	600142 +	G	0	1	1	0	0	SA0512
gi|29165615|ref|NC_002745.2|	600143 +	G	0	1	0	0	0	SA0512
gi|29165615|ref|NC_002745.2|	600149 +	G	1	0	0	0	0	SA0512
gi|29165615|ref|NC_002745.2|	600214 +	A	0	0	0	0	1	SA0512
gi|29165615|ref|NC_002745.2|	600216 +	U	0	1	0	0	0	SA0512
gi|29165615|ref|NC_002745.2|	600251 +	A	0	0	0	1	0	SA0512
gi|29165615|ref|NC_002745.2|	600276 +	U	0	0	0	1	0	SA0512
gi|29165615|ref|NC_002745.2|	600390 +	G	0	0	0	0	1	-
gi|29165615|ref|NC_002745.2|	600405 +	A	0	1	0	0	0	-
gi|29165615|ref|NC_002745.2|	600464 +	U	0	2	0	0	0	-
gi|29165615|ref|NC_002745.2|	600483 +	A	0	1	0	0	0	-
gi|29165615|ref|NC_002745.2|	600489 +	C	0	0	0	2	0	-
gi|29165615|ref|NC_002745.2|	600547 +	A	0	0	0	1	0	-
gi|29165615|ref|NC_002745.2|	600561 +	A	0	0	0	1	0	-
gi|29165615|ref|NC_002745.2|	600619 +	G	0	0	1	0	0	SA0513
gi|29165615|ref|NC_002745.2|	600620 +	A	0	0	0	1	0	SA0513
gi|29165615|ref|NC_002745.2|	600681 +	A	0	0	0	0	1	SA0513
gi|29165615|ref|NC_002745.2|	600683 +	A	0	0	0	0	1	SA0513
gi|29165615|ref|NC_002745.2|	600686 +	A	0	0	0	1	0	SA0513
gi|29165615|ref|NC_002745.2|	600715 +	G	0	0	0	1	0	SA0513
gi|29165615|ref|NC_002745.2|	600950 +	A	0	1	0	0	0	SA0513
gi|29165615|ref|NC_002745.2|	601019 +	A	0	0	1	0	0	SA0513

gi|29165615|ref|NC_002745.2|	601102 +	A	0	0	0	0	1	SA0513
gi|29165615|ref|NC_002745.2|	601136 +	C	0	0	0	0	1	SA0513
gi|29165615|ref|NC_002745.2|	601353 +	U	0	1	0	0	0	-
gi|29165615|ref|NC_002745.2|	601381 +	U	1	0	0	0	1	-
gi|29165615|ref|NC_002745.2|	602677 +	G	0	0	0	1	1	-
gi|29165615|ref|NC_002745.2|	602886 +	A	0	0	0	0	1	SA0516
gi|29165615|ref|NC_002745.2|	603066 +	C	0	0	0	1	0	SA0516
gi|29165615|ref|NC_002745.2|	603067 +	A	0	0	0	0	1	SA0516
gi|29165615|ref|NC_002745.2|	603072 +	A	0	2	0	0	1	SA0516
gi|29165615|ref|NC_002745.2|	603073 +	A	0	0	0	2	1	SA0516
gi|29165615|ref|NC_002745.2|	603078 +	A	0	0	1	0	0	SA0516
gi|29165615|ref|NC_002745.2|	603079 +	A	0	0	0	0	1	SA0516
gi|29165615|ref|NC_002745.2|	603080 +	U	0	0	1	0	2	SA0516
gi|29165615|ref|NC_002745.2|	603085 +	G	0	0	0	0	1	SA0516
gi|29165615|ref|NC_002745.2|	603125 +	C	0	1	0	0	0	SA0516
gi|29165615|ref|NC_002745.2|	603193 +	A	2	0	0	0	0	-
gi|29165615|ref|NC_002745.2|	603210 +	U	0	0	0	0	1	-
gi|29165615|ref|NC_002745.2|	603214 +	A	0	2	0	0	0	-
gi|29165615|ref|NC_002745.2|	603221 +	U	0	1	4	0	1	-
gi|29165615|ref|NC_002745.2|	603223 +	A	1	4	2	2	6	-
gi|29165615|ref|NC_002745.2|	603224 +	U	0	0	1	0	0	-
gi|29165615|ref|NC_002745.2|	603227 +	G	0	0	0	0	1	-
gi|29165615|ref|NC_002745.2|	603230 +	A	0	1	2	0	0	-
gi|29165615|ref|NC_002745.2|	603235 +	A	0	0	0	1	0	-
gi|29165615|ref|NC_002745.2|	603236 +	A	0	0	2	4	0	-
gi|29165615|ref|NC_002745.2|	603239 +	A	0	1	1	1	0	-
gi|29165615|ref|NC_002745.2|	603240 +	C	0	0	0	0	1	-
gi|29165615|ref|NC_002745.2|	603241 +	A	0	1	4	1	0	-
gi|29165615|ref|NC_002745.2|	603242 +	A	0	0	1	0	1	-
gi|29165615|ref|NC_002745.2|	603245 +	U	0	0	0	0	1	-
gi|29165615|ref|NC_002745.2|	603247 +	A	0	0	0	1	0	-
gi|29165615|ref|NC_002745.2|	603248 +	A	0	0	0	1	0	-
gi|29165615|ref|NC_002745.2|	603251 +	U	0	0	0	1	0	-
gi|29165615|ref|NC_002745.2|	603253 +	A	0	3	0	0	1	-
gi|29165615|ref|NC_002745.2|	603254 +	A	0	1	1	0	1	-
gi|29165615|ref|NC_002745.2|	603258 +	A	0	3	4	0	2	-
gi|29165615|ref|NC_002745.2|	603259 +	A	1	1	0	2	5	-
gi|29165615|ref|NC_002745.2|	603260 +	A	0	1	0	0	0	-
gi|29165615|ref|NC_002745.2|	603263 +	U	0	0	1	0	0	-
gi|29165615|ref|NC_002745.2|	603273 +	G	0	0	0	0	1	-
gi|29165615|ref|NC_002745.2|	603274 +	A	0	0	0	0	1	-
gi|29165615|ref|NC_002745.2|	603278 +	U	0	2	0	0	0	-
gi|29165615|ref|NC_002745.2|	603286 +	U	0	0	0	1	0	-
gi|29165615|ref|NC_002745.2|	603292 +	A	0	2	0	0	0	-
gi|29165615|ref|NC_002745.2|	603294 +	A	0	1	0	0	1	-
gi|29165615|ref|NC_002745.2|	603299 +	G	0	0	1	0	0	-
gi|29165615|ref|NC_002745.2|	603300 +	A	0	1	0	0	1	-
gi|29165615|ref|NC_002745.2|	603301 +	U	0	1	0	0	0	-
gi|29165615|ref|NC_002745.2|	603306 +	A	0	0	1	1	1	-
gi|29165615|ref|NC_002745.2|	603307 +	A	0	3	0	0	0	-
gi|29165615|ref|NC_002745.2|	603312 +	C	0	0	0	1	0	-
gi|29165615|ref|NC_002745.2|	603313 +	G	0	1	0	0	0	-
gi|29165615|ref|NC_002745.2|	603314 +	A	0	0	0	1	0	-
gi|29165615|ref|NC_002745.2|	603315 +	A	0	0	1	0	0	-
gi|29165615|ref|NC_002745.2|	603322 +	U	0	1	1	0	0	-
gi|29165615|ref|NC_002745.2|	603328 +	A	0	1	0	0	0	-
gi|29165615|ref|NC_002745.2|	603351 +	A	0	0	0	0	1	SA0517
gi|29165615|ref|NC_002745.2|	603358 +	U	0	0	1	0	0	SA0517
gi|29165615|ref|NC_002745.2|	603366 +	U	1	0	0	0	0	SA0517
gi|29165615|ref|NC_002745.2|	603471 +	A	0	0	0	0	1	SA0517
gi|29165615|ref|NC_002745.2|	603482 +	C	1	1	0	0	0	SA0517
gi|29165615|ref|NC_002745.2|	603507 +	U	1	0	0	0	0	SA0517
gi|29165615|ref|NC_002745.2|	603508 +	A	0	0	0	2	0	SA0517
gi|29165615|ref|NC_002745.2|	603553 +	C	0	1	1	0	0	SA0517
gi|29165615|ref|NC_002745.2|	603554 +	A	1	0	0	0	0	SA0517
gi|29165615|ref|NC_002745.2|	603566 +	A	0	0	0	0	1	SA0517
gi|29165615|ref|NC_002745.2|	603570 +	C	0	0	1	0	0	SA0517
gi|29165615|ref|NC_002745.2|	603597 +	A	0	1	0	0	0	SA0517
gi|29165615|ref|NC_002745.2|	603609 +	U	0	0	0	1	0	SA0517
gi|29165615|ref|NC_002745.2|	603614 +	A	0	1	0	0	0	SA0517
gi|29165615|ref|NC_002745.2|	603615 +	A	0	1	0	0	0	SA0517
gi|29165615|ref|NC_002745.2|	603616 +	A	0	0	0	1	0	SA0517
gi|29165615|ref|NC_002745.2|	603639 +	U	0	0	0	0	2	SA0517
gi|29165615|ref|NC_002745.2|	603654 +	C	0	0	1	0	0	SA0517
gi|29165615|ref|NC_002745.2|	603656 +	A	0	1	0	0	0	SA0517
gi|29165615|ref|NC_002745.2|	603673 +	A	0	0	0	0	2	SA0517
gi|29165615|ref|NC_002745.2|	603676 +	A	0	0	0	0	1	SA0517
gi|29165615|ref|NC_002745.2|	603679 +	A	0	1	0	1	1	SA0517
gi|29165615|ref|NC_002745.2|	603680 +	G	0	2	0	0	0	SA0517
gi|29165615|ref|NC_002745.2|	603687 +	A	0	0	0	0	1	SA0517
gi|29165615|ref|NC_002745.2|	603693 +	A	0	0	0	1	1	SA0517

gi|29165615|ref|NC_002745.2|	603694 +	C	0	1	0	0	0	SA0517
gi|29165615|ref|NC_002745.2|	603695 +	A	0	2	0	0	0	SA0517
gi|29165615|ref|NC_002745.2|	603696 +	U	0	0	0	0	1	SA0517
gi|29165615|ref|NC_002745.2|	603698 +	G	0	1	0	0	0	SA0517
gi|29165615|ref|NC_002745.2|	603705 +	C	1	0	0	0	0	SA0517
gi|29165615|ref|NC_002745.2|	603707 +	G	0	0	1	0	0	SA0517
gi|29165615|ref|NC_002745.2|	603735 +	A	0	1	0	0	0	SA0517
gi|29165615|ref|NC_002745.2|	603749 +	G	0	1	0	0	0	SA0517
gi|29165615|ref|NC_002745.2|	603751 +	U	1	0	0	0	0	SA0517
gi|29165615|ref|NC_002745.2|	603761 +	C	0	1	0	0	0	SA0517
gi|29165615|ref|NC_002745.2|	603767 +	G	0	0	1	0	0	SA0517
gi|29165615|ref|NC_002745.2|	603768 +	A	0	0	0	0	1	SA0517
gi|29165615|ref|NC_002745.2|	603792 +	A	0	0	1	1	1	SA0517
gi|29165615|ref|NC_002745.2|	603795 +	A	0	0	0	1	0	SA0517
gi|29165615|ref|NC_002745.2|	603835 +	G	0	0	0	1	0	SA0517
gi|29165615|ref|NC_002745.2|	603836 +	A	0	1	1	0	1	SA0517
gi|29165615|ref|NC_002745.2|	603839 +	A	0	0	0	1	0	SA0517
gi|29165615|ref|NC_002745.2|	603851 +	G	0	0	0	0	1	SA0517
gi|29165615|ref|NC_002745.2|	603856 +	A	0	0	0	0	1	SA0517
gi|29165615|ref|NC_002745.2|	603864 +	A	0	1	0	0	0	SA0517
gi|29165615|ref|NC_002745.2|	603869 +	A	0	2	0	1	0	SA0517
gi|29165615|ref|NC_002745.2|	603879 +	C	0	0	0	1	0	SA0517
gi|29165615|ref|NC_002745.2|	603888 +	U	0	0	0	2	0	SA0517
gi|29165615|ref|NC_002745.2|	603889 +	U	2	0	0	0	0	SA0517
gi|29165615|ref|NC_002745.2|	603921 +	C	0	1	0	0	0	SA0517
gi|29165615|ref|NC_002745.2|	603923 +	U	0	1	0	2	0	SA0517
gi|29165615|ref|NC_002745.2|	603924 +	C	0	1	0	0	0	SA0517
gi|29165615|ref|NC_002745.2|	603928 +	G	0	0	1	0	0	SA0517
gi|29165615|ref|NC_002745.2|	603929 +	A	0	0	2	1	3	SA0517
gi|29165615|ref|NC_002745.2|	603930 +	G	0	0	0	0	2	SA0517
gi|29165615|ref|NC_002745.2|	603933 +	G	0	0	0	1	0	SA0517
gi|29165615|ref|NC_002745.2|	603934 +	A	0	0	2	0	2	SA0517
gi|29165615|ref|NC_002745.2|	603935 +	A	0	2	0	0	0	SA0517
gi|29165615|ref|NC_002745.2|	603936 +	A	1	0	0	0	0	SA0517
gi|29165615|ref|NC_002745.2|	603937 +	U	0	0	0	0	3	SA0517
gi|29165615|ref|NC_002745.2|	603938 +	A	0	2	0	0	0	SA0517
gi|29165615|ref|NC_002745.2|	603941 +	G	0	10	9	9	17	SA0517
gi|29165615|ref|NC_002745.2|	603942 +	A	0	4	2	2	14	SA0517
gi|29165615|ref|NC_002745.2|	603943 +	A	0	1	0	1	1	SA0517
gi|29165615|ref|NC_002745.2|	603946 +	A	1	0	0	1	0	SA0517
gi|29165615|ref|NC_002745.2|	603948 +	C	0	0	0	0	1	SA0517
gi|29165615|ref|NC_002745.2|	603950 +	C	0	1	1	1	0	SA0517
gi|29165615|ref|NC_002745.2|	603960 +	C	0	0	0	1	0	SA0517
gi|29165615|ref|NC_002745.2|	603961 +	A	0	1	0	0	0	SA0517
gi|29165615|ref|NC_002745.2|	603962 +	C	0	0	0	2	0	SA0517
gi|29165615|ref|NC_002745.2|	603964 +	A	0	1	0	1	0	SA0517
gi|29165615|ref|NC_002745.2|	603965 +	A	0	0	1	1	0	SA0517
gi|29165615|ref|NC_002745.2|	603975 +	C	0	0	1	1	0	SA0517
gi|29165615|ref|NC_002745.2|	603980 +	G	0	0	0	0	2	SA0517
gi|29165615|ref|NC_002745.2|	603986 +	A	0	5	3	0	3	SA0517
gi|29165615|ref|NC_002745.2|	603993 +	A	0	1	0	2	1	SA0517
gi|29165615|ref|NC_002745.2|	603994 +	A	0	0	0	1	1	SA0517
gi|29165615|ref|NC_002745.2|	603996 +	G	1	0	1	1	0	SA0517
gi|29165615|ref|NC_002745.2|	603997 +	A	0	1	0	0	0	SA0517
gi|29165615|ref|NC_002745.2|	604003 +	G	0	0	1	0	0	SA0517
gi|29165615|ref|NC_002745.2|	604008 +	A	0	0	0	1	0	SA0517
gi|29165615|ref|NC_002745.2|	604009 +	A	0	0	0	3	0	SA0517
gi|29165615|ref|NC_002745.2|	604014 +	A	0	0	0	1	1	SA0517
gi|29165615|ref|NC_002745.2|	604020 +	U	0	0	0	1	0	SA0517
gi|29165615|ref|NC_002745.2|	604039 +	U	0	0	1	0	0	SA0517
gi|29165615|ref|NC_002745.2|	604043 +	A	0	0	0	1	0	SA0517
gi|29165615|ref|NC_002745.2|	604047 +	A	0	0	0	0	1	SA0517
gi|29165615|ref|NC_002745.2|	604053 +	C	0	0	0	1	0	SA0517
gi|29165615|ref|NC_002745.2|	604054 +	A	0	0	0	0	2	SA0517
gi|29165615|ref|NC_002745.2|	604055 +	A	0	1	1	0	0	SA0517
gi|29165615|ref|NC_002745.2|	604060 +	A	0	1	0	2	1	SA0517
gi|29165615|ref|NC_002745.2|	604061 +	G	0	0	0	2	1	SA0517
gi|29165615|ref|NC_002745.2|	604064 +	A	0	0	0	0	2	SA0517
gi|29165615|ref|NC_002745.2|	604070 +	G	0	0	0	0	1	SA0517
gi|29165615|ref|NC_002745.2|	604098 +	C	0	1	0	0	0	SA0517
gi|29165615|ref|NC_002745.2|	604099 +	A	0	0	1	0	0	SA0517
gi|29165615|ref|NC_002745.2|	604102 +	A	0	0	1	0	1	SA0517
gi|29165615|ref|NC_002745.2|	604103 +	G	0	0	0	0	1	SA0517
gi|29165615|ref|NC_002745.2|	604104 +	A	0	0	0	1	1	SA0517
gi|29165615|ref|NC_002745.2|	604105 +	A	1	2	0	0	2	SA0517
gi|29165615|ref|NC_002745.2|	604108 +	U	0	0	1	0	0	SA0517
gi|29165615|ref|NC_002745.2|	604111 +	A	0	0	0	1	2	SA0517
gi|29165615|ref|NC_002745.2|	604115 +	A	0	3	2	0	0	SA0517
gi|29165615|ref|NC_002745.2|	604119 +	C	0	0	0	0	2	SA0517
gi|29165615|ref|NC_002745.2|	604124 +	U	0	1	0	0	0	SA0517
gi|29165615|ref|NC_002745.2|	604133 +	G	0	0	1	0	0	SA0517

gi|29165615|ref|NC_002745.2|	604136 +	A	0	0	0	0	1	SA0517
gi|29165615|ref|NC_002745.2|	604139 +	A	0	2	0	1	1	SA0517
gi|29165615|ref|NC_002745.2|	604143 +	A	0	0	0	1	0	SA0517
gi|29165615|ref|NC_002745.2|	604146 +	A	0	3	0	0	1	SA0517
gi|29165615|ref|NC_002745.2|	604147 +	U	0	1	0	0	0	SA0517
gi|29165615|ref|NC_002745.2|	604158 +	G	0	0	0	2	0	SA0517
gi|29165615|ref|NC_002745.2|	604159 +	A	0	0	0	0	1	SA0517
gi|29165615|ref|NC_002745.2|	604164 +	C	1	0	0	0	0	SA0517
gi|29165615|ref|NC_002745.2|	604168 +	U	0	1	0	0	1	SA0517
gi|29165615|ref|NC_002745.2|	604171 +	G	0	0	1	0	0	SA0517
gi|29165615|ref|NC_002745.2|	604172 +	A	0	0	0	0	2	SA0517
gi|29165615|ref|NC_002745.2|	604179 +	U	1	1	2	1	4	SA0517
gi|29165615|ref|NC_002745.2|	604180 +	A	1	2	4	3	5	SA0517
gi|29165615|ref|NC_002745.2|	604181 +	C	0	0	2	0	0	SA0517
gi|29165615|ref|NC_002745.2|	604182 +	G	0	0	1	0	0	SA0517
gi|29165615|ref|NC_002745.2|	604183 +	U	0	5	3	2	6	SA0517
gi|29165615|ref|NC_002745.2|	604184 +	G	0	1	0	3	1	SA0517
gi|29165615|ref|NC_002745.2|	604185 +	A	1	14	13	5	8	SA0517
gi|29165615|ref|NC_002745.2|	604186 +	A	1	12	9	7	10	SA0517
gi|29165615|ref|NC_002745.2|	604187 +	C	0	0	0	0	1	SA0517
gi|29165615|ref|NC_002745.2|	604188 +	A	0	0	0	0	3	SA0517
gi|29165615|ref|NC_002745.2|	604189 +	A	0	1	1	0	1	SA0517
gi|29165615|ref|NC_002745.2|	604190 +	C	0	0	1	1	0	SA0517
gi|29165615|ref|NC_002745.2|	604191 +	A	0	0	3	0	1	SA0517
gi|29165615|ref|NC_002745.2|	604192 +	A	0	1	7	3	6	SA0517
gi|29165615|ref|NC_002745.2|	604193 +	G	0	0	1	0	0	SA0517
gi|29165615|ref|NC_002745.2|	604194 +	U	1	0	0	0	0	SA0517
gi|29165615|ref|NC_002745.2|	604195 +	U	0	0	1	1	1	SA0517
gi|29165615|ref|NC_002745.2|	604197 +	A	0	4	1	1	2	SA0517
gi|29165615|ref|NC_002745.2|	604198 +	A	0	0	1	0	1	SA0517
gi|29165615|ref|NC_002745.2|	604200 +	A	0	1	2	1	0	-
gi|29165615|ref|NC_002745.2|	604201 +	A	0	0	0	2	2	-
gi|29165615|ref|NC_002745.2|	604202 +	A	0	0	0	0	1	-
gi|29165615|ref|NC_002745.2|	604203 +	A	0	1	0	1	1	-
gi|29165615|ref|NC_002745.2|	604204 +	A	0	2	0	0	0	-
gi|29165615|ref|NC_002745.2|	604205 +	A	0	0	0	1	0	-
gi|29165615|ref|NC_002745.2|	604206 +	A	0	0	1	0	1	-
gi|29165615|ref|NC_002745.2|	604207 +	A	0	0	1	0	0	-
gi|29165615|ref|NC_002745.2|	604209 +	A	1	0	0	0	0	-
gi|29165615|ref|NC_002745.2|	604218 +	A	0	0	0	0	1	-
gi|29165615|ref|NC_002745.2|	604219 +	U	1	0	0	0	0	-
gi|29165615|ref|NC_002745.2|	604231 +	A	0	2	0	0	0	SA0518
gi|29165615|ref|NC_002745.2|	604234 +	U	0	1	0	0	0	SA0518
gi|29165615|ref|NC_002745.2|	604245 +	G	0	2	0	0	0	SA0518
gi|29165615|ref|NC_002745.2|	604269 +	G	0	0	0	0	2	SA0518
gi|29165615|ref|NC_002745.2|	604278 +	C	0	0	0	0	1	SA0518
gi|29165615|ref|NC_002745.2|	604404 +	A	0	1	0	0	0	SA0518
gi|29165615|ref|NC_002745.2|	604420 +	A	0	0	0	1	0	SA0518
gi|29165615|ref|NC_002745.2|	604500 +	C	0	0	1	0	0	SA0518
gi|29165615|ref|NC_002745.2|	604518 +	U	0	1	0	0	0	SA0518
gi|29165615|ref|NC_002745.2|	604532 +	A	0	0	1	0	0	SA0518
gi|29165615|ref|NC_002745.2|	604590 +	C	0	1	0	0	0	SA0518
gi|29165615|ref|NC_002745.2|	604595 +	U	0	1	0	1	0	SA0518
gi|29165615|ref|NC_002745.2|	604604 +	A	0	1	0	0	0	SA0518
gi|29165615|ref|NC_002745.2|	604612 +	A	0	0	1	0	0	SA0518
gi|29165615|ref|NC_002745.2|	604615 +	U	0	0	1	0	0	SA0518
gi|29165615|ref|NC_002745.2|	604617 +	U	0	0	0	0	1	SA0518
gi|29165615|ref|NC_002745.2|	604618 +	A	0	0	1	0	0	SA0518
gi|29165615|ref|NC_002745.2|	604624 +	C	0	0	1	0	0	SA0518
gi|29165615|ref|NC_002745.2|	604748 +	U	0	0	0	1	0	SA0518
gi|29165615|ref|NC_002745.2|	606743 +	A	0	0	0	1	0	SA0519
gi|29165615|ref|NC_002745.2|	608442 +	A	0	0	0	0	1	-
gi|29165615|ref|NC_002745.2|	611045 +	G	0	0	0	1	0	SA0520
gi|29165615|ref|NC_002745.2|	612951 +	A	0	0	0	1	0	-
gi|29165615|ref|NC_002745.2|	613253 +	A	1	0	0	0	0	SA0521
gi|29165615|ref|NC_002745.2|	613265 +	C	0	0	1	0	0	SA0521
gi|29165615|ref|NC_002745.2|	613273 +	A	0	0	0	0	1	SA0521
gi|29165615|ref|NC_002745.2|	613276 +	G	0	0	0	0	1	SA0521
gi|29165615|ref|NC_002745.2|	613331 +	C	0	0	0	1	0	SA0521
gi|29165615|ref|NC_002745.2|	613362 +	A	0	0	1	0	0	SA0521
gi|29165615|ref|NC_002745.2|	613367 +	A	0	0	0	0	3	SA0521
gi|29165615|ref|NC_002745.2|	613387 +	G	0	0	0	0	1	SA0521
gi|29165615|ref|NC_002745.2|	613399 +	G	0	0	1	0	0	SA0521
gi|29165615|ref|NC_002745.2|	613400 +	U	0	0	1	0	0	SA0521
gi|29165615|ref|NC_002745.2|	613671 +	A	0	1	0	0	0	SA0521
gi|29165615|ref|NC_002745.2|	613691 +	A	0	0	0	0	1	SA0521
gi|29165615|ref|NC_002745.2|	613697 +	A	0	0	0	1	0	SA0521
gi|29165615|ref|NC_002745.2|	613817 +	U	0	0	0	0	1	SA0521
gi|29165615|ref|NC_002745.2|	613825 +	U	0	0	0	0	2	SA0521
gi|29165615|ref|NC_002745.2|	613917 +	A	0	0	0	0	1	SA0521
gi|29165615|ref|NC_002745.2|	614115 +	A	0	2	0	0	0	SA0521

gi|29165615|ref|NC_002745.2|	614457	+	A	0	0	0	0	1	SA0521
gi|29165615|ref|NC_002745.2|	614636	+	A	0	0	0	0	2	SA0521
gi|29165615|ref|NC_002745.2|	614726	+	G	0	0	0	1	0	SA0521
gi|29165615|ref|NC_002745.2|	614732	+	C	0	1	0	0	0	SA0521
gi|29165615|ref|NC_002745.2|	614751	+	U	0	1	1	0	0	SA0521
gi|29165615|ref|NC_002745.2|	614755	+	G	0	0	0	0	1	SA0521
gi|29165615|ref|NC_002745.2|	614786	+	C	0	0	0	0	1	SA0521
gi|29165615|ref|NC_002745.2|	614848	+	C	0	0	1	0	0	SA0521
gi|29165615|ref|NC_002745.2|	615089	+	A	0	1	0	0	0	SA0521
gi|29165615|ref|NC_002745.2|	615178	+	G	0	1	0	0	0	SA0521
gi|29165615|ref|NC_002745.2|	615191	+	A	0	0	0	0	1	SA0521
gi|29165615|ref|NC_002745.2|	615228	+	A	0	0	0	0	1	SA0521
gi|29165615|ref|NC_002745.2|	615247	+	A	0	0	1	0	0	SA0521
gi|29165615|ref|NC_002745.2|	615259	+	A	0	0	2	0	0	SA0521
gi|29165615|ref|NC_002745.2|	615266	+	C	0	1	0	0	0	SA0521
gi|29165615|ref|NC_002745.2|	615322	+	G	0	0	0	0	1	SA0521
gi|29165615|ref|NC_002745.2|	615328	+	U	0	0	0	1	0	SA0521
gi|29165615|ref|NC_002745.2|	615364	+	A	1	0	0	1	0	SA0521
gi|29165615|ref|NC_002745.2|	615367	+	A	0	0	0	0	1	SA0521
gi|29165615|ref|NC_002745.2|	615370	+	A	0	0	0	0	1	SA0521
gi|29165615|ref|NC_002745.2|	615371	+	C	0	0	0	1	0	SA0521
gi|29165615|ref|NC_002745.2|	616503	+	A	0	4	0	0	0	-
gi|29165615|ref|NC_002745.2|	616504	+	U	0	1	1	0	0	-
gi|29165615|ref|NC_002745.2|	616505	+	A	1	1	1	2	2	-
gi|29165615|ref|NC_002745.2|	616524	+	U	0	0	1	0	0	-
gi|29165615|ref|NC_002745.2|	616526	+	U	0	1	1	0	0	-
gi|29165615|ref|NC_002745.2|	616835	+	A	0	1	0	0	0	SA0522
gi|29165615|ref|NC_002745.2|	616852	+	G	1	0	0	0	0	SA0522
gi|29165615|ref|NC_002745.2|	616890	+	A	0	0	1	0	0	SA0522
gi|29165615|ref|NC_002745.2|	616904	+	U	0	0	0	1	0	SA0522
gi|29165615|ref|NC_002745.2|	616907	+	A	0	1	0	0	0	SA0522
gi|29165615|ref|NC_002745.2|	616913	+	U	0	1	0	0	0	SA0522
gi|29165615|ref|NC_002745.2|	616925	+	A	1	0	0	0	0	SA0522
gi|29165615|ref|NC_002745.2|	616939	+	A	0	0	0	0	1	SA0522
gi|29165615|ref|NC_002745.2|	617039	+	A	0	0	1	0	0	SA0522
gi|29165615|ref|NC_002745.2|	617396	+	C	0	0	1	0	0	SA0522
gi|29165615|ref|NC_002745.2|	617399	+	A	0	0	1	0	0	SA0522
gi|29165615|ref|NC_002745.2|	617408	+	A	0	0	0	1	0	SA0522
gi|29165615|ref|NC_002745.2|	617419	+	C	0	0	1	0	0	SA0522
gi|29165615|ref|NC_002745.2|	617423	+	U	0	0	0	0	1	SA0522
gi|29165615|ref|NC_002745.2|	617437	+	G	0	0	1	0	0	SA0522
gi|29165615|ref|NC_002745.2|	617507	+	U	1	0	0	0	0	SA0522
gi|29165615|ref|NC_002745.2|	617527	+	A	0	0	1	0	0	SA0522
gi|29165615|ref|NC_002745.2|	617543	+	A	0	0	0	1	0	SA0522
gi|29165615|ref|NC_002745.2|	617574	+	A	0	1	0	0	0	SA0522
gi|29165615|ref|NC_002745.2|	617636	+	U	0	0	0	0	1	SA0522
gi|29165615|ref|NC_002745.2|	617645	+	A	0	3	0	0	0	SA0522
gi|29165615|ref|NC_002745.2|	617650	+	C	0	0	0	0	1	SA0522
gi|29165615|ref|NC_002745.2|	617704	+	A	0	1	0	0	0	SA0522
gi|29165615|ref|NC_002745.2|	620111	+	A	0	1	0	0	0	-
gi|29165615|ref|NC_002745.2|	621722	+	A	0	0	0	0	1	-
gi|29165615|ref|NC_002745.2|	622060	+	G	1	1	1	2	2	-
gi|29165615|ref|NC_002745.2|	622085	+	A	0	0	0	0	1	-
gi|29165615|ref|NC_002745.2|	622091	+	A	0	1	0	0	0	-
gi|29165615|ref|NC_002745.2|	622140	+	G	0	0	0	0	2	SA0527
gi|29165615|ref|NC_002745.2|	622147	+	C	0	1	0	0	0	SA0527
gi|29165615|ref|NC_002745.2|	622273	+	A	0	0	0	0	1	SA0527
gi|29165615|ref|NC_002745.2|	622320	+	A	0	0	0	0	1	SA0527
gi|29165615|ref|NC_002745.2|	622325	+	U	0	1	0	0	0	SA0527
gi|29165615|ref|NC_002745.2|	622326	+	A	0	1	0	0	1	SA0527
gi|29165615|ref|NC_002745.2|	622400	+	U	0	0	0	1	0	SA0527
gi|29165615|ref|NC_002745.2|	622485	+	A	0	0	0	0	1	SA0527
gi|29165615|ref|NC_002745.2|	622486	+	C	0	0	0	0	1	SA0527
gi|29165615|ref|NC_002745.2|	622491	+	U	0	1	0	0	0	SA0527
gi|29165615|ref|NC_002745.2|	622506	+	A	0	2	1	0	1	SA0527
gi|29165615|ref|NC_002745.2|	622520	+	G	0	0	0	0	1	SA0527
gi|29165615|ref|NC_002745.2|	622529	+	G	1	0	0	0	0	SA0527
gi|29165615|ref|NC_002745.2|	622628	+	A	0	0	0	0	1	SA0527
gi|29165615|ref|NC_002745.2|	622634	+	A	0	0	0	0	1	SA0527
gi|29165615|ref|NC_002745.2|	622673	+	C	0	0	0	1	0	SA0527
gi|29165615|ref|NC_002745.2|	622698	+	C	0	0	0	0	2	SA0527
gi|29165615|ref|NC_002745.2|	622700	+	U	0	0	0	1	0	SA0527
gi|29165615|ref|NC_002745.2|	622735	+	A	0	0	1	0	0	SA0527
gi|29165615|ref|NC_002745.2|	622741	+	C	0	0	0	1	0	SA0527
gi|29165615|ref|NC_002745.2|	622749	+	A	0	0	0	1	2	SA0527
gi|29165615|ref|NC_002745.2|	622751	+	A	0	0	1	0	2	SA0527
gi|29165615|ref|NC_002745.2|	622756	+	G	0	1	1	0	0	SA0527
gi|29165615|ref|NC_002745.2|	622757	+	A	0	0	0	0	1	SA0527
gi|29165615|ref|NC_002745.2|	622758	+	A	0	0	0	0	1	SA0527
gi|29165615|ref|NC_002745.2|	622791	+	C	0	0	1	0	0	SA0527
gi|29165615|ref|NC_002745.2|	622792	+	A	0	0	0	1	0	SA0527

gi|29165615|ref|NC_002745.2|	622810	+	A	0	0	1	0	0	SA0527
gi|29165615|ref|NC_002745.2|	622817	+	U	0	0	0	0	1	SA0527
gi|29165615|ref|NC_002745.2|	622820	+	A	1	0	0	0	1	SA0527
gi|29165615|ref|NC_002745.2|	622821	+	C	0	1	0	0	0	SA0527
gi|29165615|ref|NC_002745.2|	622822	+	G	2	0	0	0	1	SA0527
gi|29165615|ref|NC_002745.2|	622823	+	A	29	8	5	5	3	SA0527
gi|29165615|ref|NC_002745.2|	622825	+	G	3	1	0	0	0	SA0527
gi|29165615|ref|NC_002745.2|	622830	+	U	0	0	0	0	1	SA0527
gi|29165615|ref|NC_002745.2|	622888	+	A	0	1	0	0	0	-
gi|29165615|ref|NC_002745.2|	622918	+	A	1	1	1	1	3	-
gi|29165615|ref|NC_002745.2|	622920	+	A	0	1	0	0	0	-
gi|29165615|ref|NC_002745.2|	622928	+	A	0	1	0	0	0	-
gi|29165615|ref|NC_002745.2|	622932	+	C	0	0	0	0	1	-
gi|29165615|ref|NC_002745.2|	622946	+	U	0	0	0	1	0	-
gi|29165615|ref|NC_002745.2|	622948	+	U	0	1	0	0	0	-
gi|29165615|ref|NC_002745.2|	622956	+	A	1	2	1	0	1	SA0528
gi|29165615|ref|NC_002745.2|	622957	+	A	0	0	0	0	1	SA0528
gi|29165615|ref|NC_002745.2|	622961	+	C	0	1	0	0	0	SA0528
gi|29165615|ref|NC_002745.2|	622962	+	A	0	1	0	0	0	SA0528
gi|29165615|ref|NC_002745.2|	622965	+	U	0	0	0	0	2	SA0528
gi|29165615|ref|NC_002745.2|	622974	+	A	0	0	1	0	0	SA0528
gi|29165615|ref|NC_002745.2|	622980	+	U	0	1	0	0	0	SA0528
gi|29165615|ref|NC_002745.2|	622984	+	C	0	0	0	0	1	SA0528
gi|29165615|ref|NC_002745.2|	622985	+	A	0	0	0	0	1	SA0528
gi|29165615|ref|NC_002745.2|	622986	+	A	0	2	4	2	4	SA0528
gi|29165615|ref|NC_002745.2|	622987	+	A	5	1	2	0	1	SA0528
gi|29165615|ref|NC_002745.2|	622988	+	G	0	0	0	2	5	SA0528
gi|29165615|ref|NC_002745.2|	622989	+	A	0	4	1	1	1	SA0528
gi|29165615|ref|NC_002745.2|	622995	+	C	0	0	1	0	0	SA0528
gi|29165615|ref|NC_002745.2|	622998	+	C	1	0	0	0	0	SA0528
gi|29165615|ref|NC_002745.2|	623028	+	U	0	0	0	1	0	SA0528
gi|29165615|ref|NC_002745.2|	623075	+	C	0	0	0	0	1	SA0528
gi|29165615|ref|NC_002745.2|	623085	+	A	0	1	0	0	0	SA0528
gi|29165615|ref|NC_002745.2|	623222	+	U	0	0	0	0	1	SA0528
gi|29165615|ref|NC_002745.2|	623223	+	C	0	0	1	0	0	SA0528
gi|29165615|ref|NC_002745.2|	623225	+	A	0	0	0	1	0	SA0528
gi|29165615|ref|NC_002745.2|	623231	+	G	0	1	0	0	0	SA0528
gi|29165615|ref|NC_002745.2|	623232	+	C	0	0	0	1	0	SA0528
gi|29165615|ref|NC_002745.2|	623233	+	A	0	0	1	0	0	SA0528
gi|29165615|ref|NC_002745.2|	623235	+	C	0	2	3	4	2	SA0528
gi|29165615|ref|NC_002745.2|	623244	+	A	0	1	0	0	0	SA0528
gi|29165615|ref|NC_002745.2|	623247	+	C	1	0	0	0	0	SA0528
gi|29165615|ref|NC_002745.2|	623256	+	A	0	0	0	0	1	SA0528
gi|29165615|ref|NC_002745.2|	623259	+	A	0	0	1	0	0	SA0528
gi|29165615|ref|NC_002745.2|	623260	+	U	0	2	0	0	2	SA0528
gi|29165615|ref|NC_002745.2|	623261	+	A	0	0	1	0	0	SA0528
gi|29165615|ref|NC_002745.2|	623262	+	A	0	1	0	1	4	SA0528
gi|29165615|ref|NC_002745.2|	623263	+	A	0	5	3	4	5	SA0528
gi|29165615|ref|NC_002745.2|	623264	+	C	0	2	1	1	1	SA0528
gi|29165615|ref|NC_002745.2|	623265	+	A	0	0	0	1	0	SA0528
gi|29165615|ref|NC_002745.2|	623266	+	A	0	1	1	0	2	SA0528
gi|29165615|ref|NC_002745.2|	623273	+	G	0	2	0	0	0	SA0528
gi|29165615|ref|NC_002745.2|	623274	+	U	0	3	1	2	1	SA0528
gi|29165615|ref|NC_002745.2|	623276	+	G	0	1	2	0	0	SA0528
gi|29165615|ref|NC_002745.2|	623282	+	A	0	0	0	1	0	SA0528
gi|29165615|ref|NC_002745.2|	623283	+	U	1	0	0	0	0	SA0528
gi|29165615|ref|NC_002745.2|	623284	+	U	1	0	0	0	0	SA0528
gi|29165615|ref|NC_002745.2|	623286	+	C	0	0	1	0	0	SA0528
gi|29165615|ref|NC_002745.2|	623290	+	U	0	0	0	0	1	SA0528
gi|29165615|ref|NC_002745.2|	623293	+	A	0	0	2	1	0	SA0528
gi|29165615|ref|NC_002745.2|	623294	+	G	0	0	0	0	1	SA0528
gi|29165615|ref|NC_002745.2|	623295	+	A	0	1	0	0	0	SA0528
gi|29165615|ref|NC_002745.2|	623299	+	A	0	0	0	1	0	SA0528
gi|29165615|ref|NC_002745.2|	623301	+	A	0	1	0	0	2	SA0528
gi|29165615|ref|NC_002745.2|	623302	+	A	0	0	2	0	1	SA0528
gi|29165615|ref|NC_002745.2|	623303	+	A	0	1	2	0	0	SA0528
gi|29165615|ref|NC_002745.2|	623304	+	A	0	1	0	1	0	SA0528
gi|29165615|ref|NC_002745.2|	623306	+	C	0	3	1	0	1	SA0528
gi|29165615|ref|NC_002745.2|	623309	+	G	0	0	0	0	1	SA0528
gi|29165615|ref|NC_002745.2|	623310	+	C	0	0	0	0	1	SA0528
gi|29165615|ref|NC_002745.2|	623311	+	A	0	0	1	0	0	SA0528
gi|29165615|ref|NC_002745.2|	623312	+	A	0	0	0	0	1	SA0528
gi|29165615|ref|NC_002745.2|	623341	+	U	0	1	0	0	0	SA0528
gi|29165615|ref|NC_002745.2|	623430	+	C	0	0	0	0	1	SA0528
gi|29165615|ref|NC_002745.2|	623442	+	U	0	1	0	0	0	SA0528
gi|29165615|ref|NC_002745.2|	623468	+	A	0	0	0	0	1	SA0528
gi|29165615|ref|NC_002745.2|	623471	+	A	0	0	0	0	1	SA0528
gi|29165615|ref|NC_002745.2|	623473	+	A	0	1	0	0	0	SA0528
gi|29165615|ref|NC_002745.2|	623475	+	A	0	5	2	0	0	SA0528
gi|29165615|ref|NC_002745.2|	623476	+	U	0	0	1	0	0	SA0528
gi|29165615|ref|NC_002745.2|	623477	+	A	0	1	1	0	0	SA0528

gi|29165615|ref|NC_002745.2|	623481 +	U	1	0	0	1	1	SA0528
gi|29165615|ref|NC_002745.2|	623484 +	C	0	0	1	0	0	SA0528
gi|29165615|ref|NC_002745.2|	623561 +	C	1	0	0	0	0	SA0528
gi|29165615|ref|NC_002745.2|	623688 +	C	1	0	0	0	0	SA0529
gi|29165615|ref|NC_002745.2|	623700 +	A	0	0	0	0	1	SA0529
gi|29165615|ref|NC_002745.2|	623776 +	A	0	0	0	0	1	SA0529
gi|29165615|ref|NC_002745.2|	623860 +	A	0	0	0	1	0	SA0529
gi|29165615|ref|NC_002745.2|	623867 +	A	0	0	0	1	0	SA0529
gi|29165615|ref|NC_002745.2|	623887 +	C	0	0	0	0	1	SA0529
gi|29165615|ref|NC_002745.2|	623916 +	A	0	2	0	0	0	SA0529
gi|29165615|ref|NC_002745.2|	623926 +	A	0	0	0	0	1	SA0529
gi|29165615|ref|NC_002745.2|	623928 +	A	0	0	0	1	0	SA0529
gi|29165615|ref|NC_002745.2|	623932 +	A	0	0	0	0	1	SA0529
gi|29165615|ref|NC_002745.2|	623968 +	U	0	0	0	1	0	SA0529
gi|29165615|ref|NC_002745.2|	623973 +	C	0	0	1	0	0	SA0529
gi|29165615|ref|NC_002745.2|	623989 +	A	0	0	0	1	0	SA0529
gi|29165615|ref|NC_002745.2|	624010 +	A	0	0	1	0	0	SA0529
gi|29165615|ref|NC_002745.2|	624038 +	A	0	1	0	0	0	SA0529
gi|29165615|ref|NC_002745.2|	624064 +	U	0	0	0	2	0	SA0529
gi|29165615|ref|NC_002745.2|	624221 +	A	0	0	2	0	0	-
gi|29165615|ref|NC_002745.2|	624222 +	U	0	1	2	1	0	-
gi|29165615|ref|NC_002745.2|	624223 +	A	2	4	5	3	10	-
gi|29165615|ref|NC_002745.2|	624224 +	A	0	0	3	0	0	-
gi|29165615|ref|NC_002745.2|	624250 +	A	0	0	0	1	0	SA0530
gi|29165615|ref|NC_002745.2|	624359 +	C	0	1	0	0	0	SA0530
gi|29165615|ref|NC_002745.2|	624376 +	C	0	1	0	0	0	SA0530
gi|29165615|ref|NC_002745.2|	624394 +	A	0	0	0	1	0	SA0530
gi|29165615|ref|NC_002745.2|	624398 +	A	0	0	0	0	1	SA0530
gi|29165615|ref|NC_002745.2|	624412 +	A	0	0	0	1	0	SA0530
gi|29165615|ref|NC_002745.2|	624476 +	C	0	1	0	0	0	SA0530
gi|29165615|ref|NC_002745.2|	624490 +	G	0	0	0	0	1	SA0530
gi|29165615|ref|NC_002745.2|	624491 +	A	0	0	0	0	3	SA0530
gi|29165615|ref|NC_002745.2|	624492 +	A	0	1	0	0	0	SA0530
gi|29165615|ref|NC_002745.2|	624494 +	A	0	1	0	0	0	SA0530
gi|29165615|ref|NC_002745.2|	624509 +	A	0	0	1	0	0	SA0530
gi|29165615|ref|NC_002745.2|	624683 +	C	0	1	0	0	0	SA0530
gi|29165615|ref|NC_002745.2|	624719 +	U	2	0	0	0	0	SA0530
gi|29165615|ref|NC_002745.2|	624954 +	C	0	0	0	1	0	-
gi|29165615|ref|NC_002745.2|	624955 +	A	0	0	0	0	1	-
gi|29165615|ref|NC_002745.2|	624973 +	A	0	1	0	0	0	-
gi|29165615|ref|NC_002745.2|	624979 +	G	0	0	0	0	1	-
gi|29165615|ref|NC_002745.2|	624980 +	G	0	0	0	1	0	-
gi|29165615|ref|NC_002745.2|	624985 +	U	0	0	0	1	0	-
gi|29165615|ref|NC_002745.2|	624989 +	A	0	1	0	0	0	-
gi|29165615|ref|NC_002745.2|	624993 +	A	0	1	0	0	0	-
gi|29165615|ref|NC_002745.2|	625000 +	U	0	0	1	0	0	-
gi|29165615|ref|NC_002745.2|	625002 +	A	0	0	0	1	1	-
gi|29165615|ref|NC_002745.2|	625003 +	A	0	0	0	0	1	-
gi|29165615|ref|NC_002745.2|	625005 +	A	0	1	1	0	0	-
gi|29165615|ref|NC_002745.2|	625006 +	A	0	1	1	1	0	-
gi|29165615|ref|NC_002745.2|	625011 +	G	0	0	1	0	0	-
gi|29165615|ref|NC_002745.2|	625012 +	U	0	1	1	0	0	-
gi|29165615|ref|NC_002745.2|	625015 +	G	0	1	0	0	0	-
gi|29165615|ref|NC_002745.2|	625017 +	U	0	0	1	0	0	-
gi|29165615|ref|NC_002745.2|	625021 +	A	0	0	1	0	0	-
gi|29165615|ref|NC_002745.2|	625022 +	G	0	0	0	1	0	-
gi|29165615|ref|NC_002745.2|	625372 +	A	4	2	0	1	2	-
gi|29165615|ref|NC_002745.2|	625374 +	A	2	0	0	0	0	-
gi|29165615|ref|NC_002745.2|	625414 +	A	0	1	0	1	0	SA0531
gi|29165615|ref|NC_002745.2|	625415 +	A	0	1	0	0	0	SA0531
gi|29165615|ref|NC_002745.2|	625416 +	U	0	1	0	0	0	SA0531
gi|29165615|ref|NC_002745.2|	625429 +	G	0	0	0	1	0	SA0531
gi|29165615|ref|NC_002745.2|	625620 +	U	1	0	0	0	0	SA0531
gi|29165615|ref|NC_002745.2|	625692 +	A	0	0	0	2	0	SA0531
gi|29165615|ref|NC_002745.2|	625714 +	A	0	0	0	1	0	SA0531
gi|29165615|ref|NC_002745.2|	625739 +	A	0	0	0	0	1	SA0531
gi|29165615|ref|NC_002745.2|	626771 +	G	0	0	0	0	1	SA0531
gi|29165615|ref|NC_002745.2|	627301 +	A	0	0	1	0	0	SA0532
gi|29165615|ref|NC_002745.2|	627560 +	A	0	1	0	0	0	SA0533
gi|29165615|ref|NC_002745.2|	627654 +	A	0	1	0	0	0	SA0533
gi|29165615|ref|NC_002745.2|	627973 +	A	0	0	0	0	1	SA0533
gi|29165615|ref|NC_002745.2|	628340 +	A	0	1	0	0	0	SA0533
gi|29165615|ref|NC_002745.2|	628687 +	A	0	0	1	0	0	SA0533
gi|29165615|ref|NC_002745.2|	628724 +	C	0	0	0	1	0	SA0534
gi|29165615|ref|NC_002745.2|	628725 +	A	0	1	0	1	1	SA0534
gi|29165615|ref|NC_002745.2|	628938 +	A	0	0	0	1	0	SA0534
gi|29165615|ref|NC_002745.2|	629030 +	G	0	1	0	0	0	SA0534
gi|29165615|ref|NC_002745.2|	629465 +	G	0	0	0	1	0	SA0534
gi|29165615|ref|NC_002745.2|	629588 +	A	0	0	0	0	1	SA0534
gi|29165615|ref|NC_002745.2|	629609 +	G	1	0	0	0	0	SA0534
gi|29165615|ref|NC_002745.2|	629610 +	A	0	0	0	1	1	SA0534

gi|29165615|ref|NC_002745.2|	629615 +	A	0	1	0	0	1	SA0534
gi|29165615|ref|NC_002745.2|	629628 +	A	0	0	0	0	1	SA0534
gi|29165615|ref|NC_002745.2|	629690 +	A	0	0	0	1	0	SA0534
gi|29165615|ref|NC_002745.2|	629694 +	A	0	0	0	1	0	SA0534
gi|29165615|ref|NC_002745.2|	629695 +	A	0	0	0	1	0	SA0534
gi|29165615|ref|NC_002745.2|	629696 +	U	0	0	1	0	0	SA0534
gi|29165615|ref|NC_002745.2|	629698 +	A	0	3	0	0	0	SA0534
gi|29165615|ref|NC_002745.2|	629699 +	A	0	0	0	0	1	SA0534
gi|29165615|ref|NC_002745.2|	629700 +	A	0	1	0	0	0	SA0534
gi|29165615|ref|NC_002745.2|	629702 +	A	0	0	1	0	0	SA0534
gi|29165615|ref|NC_002745.2|	629703 +	U	3	0	2	0	1	SA0534
gi|29165615|ref|NC_002745.2|	629704 +	A	2	0	0	0	0	SA0534
gi|29165615|ref|NC_002745.2|	629817 +	G	1	0	0	0	0	SA0534
gi|29165615|ref|NC_002745.2|	630264 +	A	1	0	1	0	1	SA0536
gi|29165615|ref|NC_002745.2|	630273 +	U	0	0	0	1	0	SA0536
gi|29165615|ref|NC_002745.2|	630405 +	A	0	0	1	0	0	SA0536
gi|29165615|ref|NC_002745.2|	630991 +	A	0	0	0	1	0	-
gi|29165615|ref|NC_002745.2|	631602 +	A	0	0	1	0	0	-
gi|29165615|ref|NC_002745.2|	632345 +	G	0	2	5	3	1	-
gi|29165615|ref|NC_002745.2|	632360 +	G	0	1	0	0	0	SA0538
gi|29165615|ref|NC_002745.2|	632398 +	A	0	0	0	2	0	SA0538
gi|29165615|ref|NC_002745.2|	632440 +	C	0	0	0	1	0	SA0538
gi|29165615|ref|NC_002745.2|	632446 +	C	0	0	0	1	0	SA0538
gi|29165615|ref|NC_002745.2|	632448 +	A	0	0	0	1	0	SA0538
gi|29165615|ref|NC_002745.2|	632506 +	A	0	1	0	0	0	SA0538
gi|29165615|ref|NC_002745.2|	632507 +	U	0	0	1	0	0	SA0538
gi|29165615|ref|NC_002745.2|	632518 +	U	0	0	0	0	2	SA0538
gi|29165615|ref|NC_002745.2|	632519 +	U	0	0	1	0	0	SA0538
gi|29165615|ref|NC_002745.2|	632522 +	A	0	0	0	0	1	SA0538
gi|29165615|ref|NC_002745.2|	632557 +	C	0	1	0	0	0	SA0538
gi|29165615|ref|NC_002745.2|	632569 +	C	0	1	0	0	0	SA0538
gi|29165615|ref|NC_002745.2|	632611 +	G	0	0	2	0	0	SA0538
gi|29165615|ref|NC_002745.2|	632616 +	A	0	0	0	0	1	SA0538
gi|29165615|ref|NC_002745.2|	632617 +	U	0	1	0	0	0	SA0538
gi|29165615|ref|NC_002745.2|	632618 +	G	0	0	1	0	0	SA0538
gi|29165615|ref|NC_002745.2|	632629 +	U	0	2	0	0	0	SA0538
gi|29165615|ref|NC_002745.2|	632632 +	C	0	0	0	0	1	SA0538
gi|29165615|ref|NC_002745.2|	632638 +	A	0	6	2	0	1	SA0538
gi|29165615|ref|NC_002745.2|	632664 +	C	0	1	1	0	0	SA0538
gi|29165615|ref|NC_002745.2|	632666 +	U	0	0	0	0	2	SA0538
gi|29165615|ref|NC_002745.2|	632669 +	A	2	1	0	0	0	SA0538
gi|29165615|ref|NC_002745.2|	632670 +	C	0	0	0	0	2	SA0538
gi|29165615|ref|NC_002745.2|	632671 +	A	0	0	0	0	2	SA0538
gi|29165615|ref|NC_002745.2|	632672 +	A	0	1	0	0	1	SA0538
gi|29165615|ref|NC_002745.2|	632676 +	U	0	0	0	0	1	SA0538
gi|29165615|ref|NC_002745.2|	632680 +	C	0	0	0	0	1	SA0538
gi|29165615|ref|NC_002745.2|	632709 +	G	0	0	0	1	0	SA0538
gi|29165615|ref|NC_002745.2|	632715 +	A	0	0	0	0	1	SA0538
gi|29165615|ref|NC_002745.2|	632719 +	U	0	0	1	0	0	SA0538
gi|29165615|ref|NC_002745.2|	632776 +	A	0	2	0	0	0	SA0538
gi|29165615|ref|NC_002745.2|	632808 +	C	0	0	2	0	0	SA0538
gi|29165615|ref|NC_002745.2|	632929 +	U	0	0	0	0	1	SA0538
gi|29165615|ref|NC_002745.2|	632940 +	C	0	1	0	0	0	SA0538
gi|29165615|ref|NC_002745.2|	632944 +	A	0	0	0	0	1	SA0538
gi|29165615|ref|NC_002745.2|	632949 +	A	0	0	0	0	1	SA0538
gi|29165615|ref|NC_002745.2|	632950 +	A	1	0	0	0	0	SA0538
gi|29165615|ref|NC_002745.2|	633000 +	G	0	0	0	1	0	SA0538
gi|29165615|ref|NC_002745.2|	633001 +	A	0	0	0	1	0	SA0538
gi|29165615|ref|NC_002745.2|	633012 +	U	0	1	0	0	0	SA0538
gi|29165615|ref|NC_002745.2|	633036 +	A	0	1	0	0	0	SA0539
gi|29165615|ref|NC_002745.2|	633154 +	C	1	0	0	0	0	SA0539
gi|29165615|ref|NC_002745.2|	633171 +	C	0	1	0	0	0	SA0539
gi|29165615|ref|NC_002745.2|	633200 +	U	0	0	0	1	0	SA0539
gi|29165615|ref|NC_002745.2|	633250 +	C	1	0	0	0	0	SA0539
gi|29165615|ref|NC_002745.2|	633292 +	C	0	1	0	0	0	SA0539
gi|29165615|ref|NC_002745.2|	633307 +	A	0	0	0	0	1	SA0539
gi|29165615|ref|NC_002745.2|	633313 +	C	0	0	1	0	0	SA0539
gi|29165615|ref|NC_002745.2|	633333 +	C	0	1	0	0	0	SA0539
gi|29165615|ref|NC_002745.2|	633339 +	C	1	0	0	0	0	SA0539
gi|29165615|ref|NC_002745.2|	633459 +	C	0	1	1	0	1	-
gi|29165615|ref|NC_002745.2|	633463 +	U	0	0	0	0	1	-
gi|29165615|ref|NC_002745.2|	633483 +	G	0	0	0	0	1	-
gi|29165615|ref|NC_002745.2|	633500 +	A	0	0	1	0	0	-
gi|29165615|ref|NC_002745.2|	633502 +	A	0	0	1	0	0	-
gi|29165615|ref|NC_002745.2|	633503 +	C	1	0	0	0	0	-
gi|29165615|ref|NC_002745.2|	633512 +	A	0	0	2	0	0	-
gi|29165615|ref|NC_002745.2|	633513 +	U	0	0	0	0	1	-
gi|29165615|ref|NC_002745.2|	633522 +	G	0	0	2	0	0	-
gi|29165615|ref|NC_002745.2|	633560 +	C	0	0	0	1	0	SA0540
gi|29165615|ref|NC_002745.2|	633653 +	G	0	1	0	0	0	SA0540
gi|29165615|ref|NC_002745.2|	633674 +	C	0	1	0	0	0	SA0540

gi|29165615|ref|NC_002745.2|	633697	+	U	1	0	0	0	0	SA0540
gi|29165615|ref|NC_002745.2|	633721	+	A	1	0	0	0	0	SA0540
gi|29165615|ref|NC_002745.2|	633726	+	A	0	1	0	1	0	SA0540
gi|29165615|ref|NC_002745.2|	633996	+	C	0	0	1	0	0	-
gi|29165615|ref|NC_002745.2|	633997	+	U	2	0	0	1	4	-
gi|29165615|ref|NC_002745.2|	633998	+	U	0	0	0	1	0	-
gi|29165615|ref|NC_002745.2|	634000	+	G	2	0	0	0	1	-
gi|29165615|ref|NC_002745.2|	634031	+	U	0	0	0	0	1	SA0541
gi|29165615|ref|NC_002745.2|	634035	+	C	0	0	0	0	1	SA0541
gi|29165615|ref|NC_002745.2|	634068	+	U	0	0	1	0	0	SA0541
gi|29165615|ref|NC_002745.2|	634095	+	U	1	0	0	0	0	SA0541
gi|29165615|ref|NC_002745.2|	634152	+	U	0	1	0	0	0	SA0541
gi|29165615|ref|NC_002745.2|	634200	+	A	1	0	0	0	0	SA0541
gi|29165615|ref|NC_002745.2|	634203	+	A	0	2	0	0	0	SA0541
gi|29165615|ref|NC_002745.2|	634213	+	C	0	1	0	0	0	SA0541
gi|29165615|ref|NC_002745.2|	634223	+	U	2	0	0	0	0	SA0541
gi|29165615|ref|NC_002745.2|	634269	+	U	1	0	0	0	0	SA0541
gi|29165615|ref|NC_002745.2|	634292	+	A	1	0	0	0	0	SA0541
gi|29165615|ref|NC_002745.2|	634301	+	U	0	0	1	0	0	SA0541
gi|29165615|ref|NC_002745.2|	634303	+	U	0	1	0	0	0	SA0541
gi|29165615|ref|NC_002745.2|	634316	+	G	0	0	0	1	0	SA0541
gi|29165615|ref|NC_002745.2|	634407	+	A	0	1	0	0	0	SA0541
gi|29165615|ref|NC_002745.2|	634424	+	C	0	0	0	2	0	SA0541
gi|29165615|ref|NC_002745.2|	634425	+	A	4	0	1	0	0	SA0541
gi|29165615|ref|NC_002745.2|	634539	+	U	1	0	0	0	0	SA0541
gi|29165615|ref|NC_002745.2|	634555	+	U	0	1	0	0	0	SA0541
gi|29165615|ref|NC_002745.2|	634556	+	A	0	0	1	0	0	SA0541
gi|29165615|ref|NC_002745.2|	634564	+	U	0	0	1	0	0	SA0541
gi|29165615|ref|NC_002745.2|	634594	+	G	0	0	0	0	1	SA0541
gi|29165615|ref|NC_002745.2|	634595	+	U	3	0	0	0	0	SA0541
gi|29165615|ref|NC_002745.2|	634598	+	U	0	0	0	1	0	SA0541
gi|29165615|ref|NC_002745.2|	634619	+	A	0	0	0	1	0	SA0541
gi|29165615|ref|NC_002745.2|	634626	+	U	0	0	1	0	0	SA0541
gi|29165615|ref|NC_002745.2|	634636	+	C	3	0	0	0	0	SA0541
gi|29165615|ref|NC_002745.2|	634669	+	U	1	0	0	0	0	SA0541
gi|29165615|ref|NC_002745.2|	634758	+	A	0	1	0	0	1	SA0541
gi|29165615|ref|NC_002745.2|	634841	+	U	0	0	0	1	0	SA0541
gi|29165615|ref|NC_002745.2|	634915	+	U	0	0	1	0	0	SA0541
gi|29165615|ref|NC_002745.2|	635071	+	U	0	0	0	1	0	SA0541
gi|29165615|ref|NC_002745.2|	635072	+	A	2	0	0	0	0	SA0541
gi|29165615|ref|NC_002745.2|	635207	+	U	1	0	0	0	0	SA0541
gi|29165615|ref|NC_002745.2|	635432	+	G	2	0	0	0	0	SA0541
gi|29165615|ref|NC_002745.2|	635449	+	A	3	0	0	0	0	SA0541
gi|29165615|ref|NC_002745.2|	635564	+	A	0	1	0	0	0	-
gi|29165615|ref|NC_002745.2|	635566	+	A	0	1	0	0	0	-
gi|29165615|ref|NC_002745.2|	635600	+	U	0	0	0	1	0	-
gi|29165615|ref|NC_002745.2|	635601	+	A	0	0	0	0	1	-
gi|29165615|ref|NC_002745.2|	635603	+	A	0	1	3	1	3	-
gi|29165615|ref|NC_002745.2|	635644	+	A	0	1	0	0	0	-
gi|29165615|ref|NC_002745.2|	635654	+	A	0	0	1	0	0	-
gi|29165615|ref|NC_002745.2|	635664	+	A	0	0	1	0	0	-
gi|29165615|ref|NC_002745.2|	635716	+	A	0	1	0	0	0	-
gi|29165615|ref|NC_002745.2|	635743	+	C	0	1	0	0	0	-
gi|29165615|ref|NC_002745.2|	636443	+	C	0	0	1	0	0	-
gi|29165615|ref|NC_002745.2|	636540	+	U	0	0	0	0	1	-
gi|29165615|ref|NC_002745.2|	636544	+	C	0	0	0	0	1	-
gi|29165615|ref|NC_002745.2|	637120	+	A	0	0	0	1	0	-
gi|29165615|ref|NC_002745.2|	637121	+	U	0	0	1	0	0	-
gi|29165615|ref|NC_002745.2|	637129	+	A	0	0	0	1	0	-
gi|29165615|ref|NC_002745.2|	637258	+	A	1	0	1	0	1	-
gi|29165615|ref|NC_002745.2|	637264	+	C	0	0	2	0	2	-
gi|29165615|ref|NC_002745.2|	637270	+	C	0	0	0	0	1	-
gi|29165615|ref|NC_002745.2|	637272	+	C	0	1	0	0	0	-
gi|29165615|ref|NC_002745.2|	637443	+	C	0	0	0	0	1	-
gi|29165615|ref|NC_002745.2|	638202	+	U	0	0	0	0	1	-
gi|29165615|ref|NC_002745.2|	638250	+	A	0	0	1	0	1	-
gi|29165615|ref|NC_002745.2|	638253	+	A	0	0	0	3	0	-
gi|29165615|ref|NC_002745.2|	638254	+	C	0	0	0	1	0	-
gi|29165615|ref|NC_002745.2|	638261	+	G	0	0	0	1	0	-
gi|29165615|ref|NC_002745.2|	638276	+	G	0	0	0	0	1	-
gi|29165615|ref|NC_002745.2|	638281	+	A	0	1	0	0	0	-
gi|29165615|ref|NC_002745.2|	638302	+	G	22	76	51	26	64	-
gi|29165615|ref|NC_002745.2|	638303	+	U	0	4	1	1	2	-
gi|29165615|ref|NC_002745.2|	638304	+	G	5	15	5	4	8	-
gi|29165615|ref|NC_002745.2|	638305	+	A	1	3	7	7	9	-
gi|29165615|ref|NC_002745.2|	638306	+	A	0	5	6	0	2	-
gi|29165615|ref|NC_002745.2|	638308	+	G	0	1	0	0	0	-
gi|29165615|ref|NC_002745.2|	638309	+	A	0	2	0	0	3	-
gi|29165615|ref|NC_002745.2|	638311	+	U	0	1	0	0	0	-
gi|29165615|ref|NC_002745.2|	638312	+	A	0	2	0	1	2	-
gi|29165615|ref|NC_002745.2|	638313	+	A	0	1	3	0	0	-

gi|29165615|ref|NC_002745.2|	638314 +	U	0	0	0	0	2	-
gi|29165615|ref|NC_002745.2|	638315 +	A	0	1	0	1	2	-
gi|29165615|ref|NC_002745.2|	638316 +	A	0	0	4	0	4	-
gi|29165615|ref|NC_002745.2|	638317 +	G	0	0	2	0	0	-
gi|29165615|ref|NC_002745.2|	638318 +	A	0	7	2	1	2	-
gi|29165615|ref|NC_002745.2|	638319 +	U	0	1	1	1	0	-
gi|29165615|ref|NC_002745.2|	638321 +	U	0	1	0	1	3	-
gi|29165615|ref|NC_002745.2|	638322 +	A	0	1	1	0	2	-
gi|29165615|ref|NC_002745.2|	638323 +	U	0	0	0	0	1	-
gi|29165615|ref|NC_002745.2|	638324 +	A	0	2	0	0	0	-
gi|29165615|ref|NC_002745.2|	638325 +	U	0	1	0	2	0	-
gi|29165615|ref|NC_002745.2|	638327 +	A	0	2	0	0	3	-
gi|29165615|ref|NC_002745.2|	638328 +	C	0	1	3	0	1	-
gi|29165615|ref|NC_002745.2|	638329 +	A	0	2	0	0	0	-
gi|29165615|ref|NC_002745.2|	638331 +	G	0	1	4	0	2	-
gi|29165615|ref|NC_002745.2|	638334 +	G	0	1	1	2	2	-
gi|29165615|ref|NC_002745.2|	638335 +	A	0	1	0	0	6	-
gi|29165615|ref|NC_002745.2|	638336 +	C	0	0	0	1	0	-
gi|29165615|ref|NC_002745.2|	638337 +	A	0	1	1	0	0	-
gi|29165615|ref|NC_002745.2|	638338 +	U	0	2	0	0	2	-
gi|29165615|ref|NC_002745.2|	638339 +	U	0	0	2	1	1	-
gi|29165615|ref|NC_002745.2|	638340 +	A	0	1	0	0	0	SA0545
gi|29165615|ref|NC_002745.2|	638347 +	A	0	4	2	1	6	SA0545
gi|29165615|ref|NC_002745.2|	638350 +	U	0	1	0	2	2	SA0545
gi|29165615|ref|NC_002745.2|	638351 +	A	0	0	0	0	1	SA0545
gi|29165615|ref|NC_002745.2|	638352 +	U	1	0	0	0	0	SA0545
gi|29165615|ref|NC_002745.2|	638354 +	A	0	2	1	1	2	SA0545
gi|29165615|ref|NC_002745.2|	638355 +	A	0	2	0	0	2	SA0545
gi|29165615|ref|NC_002745.2|	638358 +	G	0	1	0	0	0	SA0545
gi|29165615|ref|NC_002745.2|	638359 +	U	0	0	0	0	1	SA0545
gi|29165615|ref|NC_002745.2|	638360 +	A	0	1	0	0	1	SA0545
gi|29165615|ref|NC_002745.2|	638363 +	A	0	1	2	0	3	SA0545
gi|29165615|ref|NC_002745.2|	638364 +	A	0	0	1	1	2	SA0545
gi|29165615|ref|NC_002745.2|	638365 +	A	0	3	1	1	2	SA0545
gi|29165615|ref|NC_002745.2|	638366 +	A	0	0	0	0	2	SA0545
gi|29165615|ref|NC_002745.2|	638371 +	A	0	0	0	0	1	SA0545
gi|29165615|ref|NC_002745.2|	638372 +	A	0	0	1	0	1	SA0545
gi|29165615|ref|NC_002745.2|	638382 +	A	0	1	0	0	0	SA0545
gi|29165615|ref|NC_002745.2|	638384 +	A	0	0	0	0	1	SA0545
gi|29165615|ref|NC_002745.2|	638393 +	A	0	1	0	0	0	SA0545
gi|29165615|ref|NC_002745.2|	638400 +	U	0	0	0	0	1	SA0545
gi|29165615|ref|NC_002745.2|	638401 +	U	1	0	0	0	0	SA0545
gi|29165615|ref|NC_002745.2|	638411 +	A	1	0	0	0	0	SA0545
gi|29165615|ref|NC_002745.2|	638419 +	A	0	0	1	0	0	SA0545
gi|29165615|ref|NC_002745.2|	638422 +	G	0	0	1	0	0	SA0545
gi|29165615|ref|NC_002745.2|	638425 +	U	0	1	1	0	0	SA0545
gi|29165615|ref|NC_002745.2|	638428 +	U	0	0	1	0	0	SA0545
gi|29165615|ref|NC_002745.2|	638431 +	C	0	1	0	0	1	SA0545
gi|29165615|ref|NC_002745.2|	638444 +	A	0	1	0	0	0	SA0545
gi|29165615|ref|NC_002745.2|	638448 +	C	0	2	0	0	0	SA0545
gi|29165615|ref|NC_002745.2|	638452 +	C	0	0	0	0	1	SA0545
gi|29165615|ref|NC_002745.2|	638458 +	A	0	1	0	0	1	SA0545
gi|29165615|ref|NC_002745.2|	638460 +	U	0	0	1	0	0	SA0545
gi|29165615|ref|NC_002745.2|	638476 +	U	1	0	0	0	0	SA0545
gi|29165615|ref|NC_002745.2|	638517 +	C	0	0	1	0	0	SA0545
gi|29165615|ref|NC_002745.2|	638527 +	A	0	0	1	0	0	SA0545
gi|29165615|ref|NC_002745.2|	638534 +	U	0	0	1	0	0	SA0545
gi|29165615|ref|NC_002745.2|	638535 +	A	0	0	1	1	0	SA0545
gi|29165615|ref|NC_002745.2|	638543 +	A	0	0	0	0	1	SA0545
gi|29165615|ref|NC_002745.2|	638545 +	U	0	0	0	0	1	SA0545
gi|29165615|ref|NC_002745.2|	638546 +	A	0	0	0	0	1	SA0545
gi|29165615|ref|NC_002745.2|	638551 +	A	0	1	0	0	0	SA0545
gi|29165615|ref|NC_002745.2|	638554 +	C	0	0	0	0	1	SA0545
gi|29165615|ref|NC_002745.2|	638589 +	U	0	1	0	0	0	SA0545
gi|29165615|ref|NC_002745.2|	638596 +	U	0	0	0	0	1	SA0545
gi|29165615|ref|NC_002745.2|	638598 +	G	0	1	0	0	0	SA0545
gi|29165615|ref|NC_002745.2|	638602 +	G	0	0	0	1	0	SA0545
gi|29165615|ref|NC_002745.2|	638608 +	A	0	0	0	1	0	SA0545
gi|29165615|ref|NC_002745.2|	638613 +	A	0	0	0	0	1	SA0545
gi|29165615|ref|NC_002745.2|	638617 +	C	0	0	0	1	0	SA0545
gi|29165615|ref|NC_002745.2|	638621 +	U	0	1	0	0	0	SA0545
gi|29165615|ref|NC_002745.2|	638632 +	C	0	0	1	1	0	SA0545
gi|29165615|ref|NC_002745.2|	638638 +	A	0	0	1	0	0	SA0545
gi|29165615|ref|NC_002745.2|	638646 +	A	0	1	0	0	0	SA0545
gi|29165615|ref|NC_002745.2|	638648 +	C	0	1	0	0	0	SA0545
gi|29165615|ref|NC_002745.2|	638656 +	A	0	0	0	0	1	SA0545
gi|29165615|ref|NC_002745.2|	638671 +	U	0	0	1	0	0	SA0545
gi|29165615|ref|NC_002745.2|	638692 +	C	0	0	1	0	0	SA0545
gi|29165615|ref|NC_002745.2|	638725 +	C	1	0	0	0	0	SA0545
gi|29165615|ref|NC_002745.2|	638802 +	A	0	0	1	0	0	SA0545
gi|29165615|ref|NC_002745.2|	638803 +	U	0	0	2	0	0	SA0545

gi|29165615|ref|NC_002745.2|	638805 +	A	0	0	1	0	0	SA0545
gi|29165615|ref|NC_002745.2|	638816 +	U	0	0	0	0	1	SA0545
gi|29165615|ref|NC_002745.2|	638818 +	A	0	0	0	1	0	SA0545
gi|29165615|ref|NC_002745.2|	638819 +	A	0	0	0	2	1	SA0545
gi|29165615|ref|NC_002745.2|	638825 +	C	0	1	0	0	0	SA0545
gi|29165615|ref|NC_002745.2|	638878 +	A	0	0	1	0	0	SA0545
gi|29165615|ref|NC_002745.2|	638881 +	U	1	1	0	0	0	SA0545
gi|29165615|ref|NC_002745.2|	638883 +	G	0	0	0	1	1	SA0545
gi|29165615|ref|NC_002745.2|	638885 +	A	1	0	0	0	0	SA0545
gi|29165615|ref|NC_002745.2|	638887 +	U	0	0	0	0	1	SA0545
gi|29165615|ref|NC_002745.2|	638893 +	G	0	0	0	0	1	SA0545
gi|29165615|ref|NC_002745.2|	638897 +	A	0	1	0	0	0	SA0545
gi|29165615|ref|NC_002745.2|	638900 +	A	0	0	0	0	1	SA0545
gi|29165615|ref|NC_002745.2|	638923 +	A	0	1	0	0	0	SA0545
gi|29165615|ref|NC_002745.2|	638925 +	C	0	0	1	0	0	SA0545
gi|29165615|ref|NC_002745.2|	638937 +	A	1	0	0	0	0	SA0545
gi|29165615|ref|NC_002745.2|	638967 +	A	1	0	0	0	0	SA0545
gi|29165615|ref|NC_002745.2|	638969 +	A	0	1	0	0	0	SA0545
gi|29165615|ref|NC_002745.2|	638985 +	A	0	1	0	0	0	SA0545
gi|29165615|ref|NC_002745.2|	638999 +	U	0	0	0	0	1	SA0545
gi|29165615|ref|NC_002745.2|	639025 +	A	0	1	0	0	0	SA0545
gi|29165615|ref|NC_002745.2|	639034 +	A	0	0	0	1	1	SA0545
gi|29165615|ref|NC_002745.2|	639043 +	C	0	1	0	0	0	SA0545
gi|29165615|ref|NC_002745.2|	639056 +	C	0	0	1	0	0	SA0545
gi|29165615|ref|NC_002745.2|	639073 +	C	1	0	0	0	0	SA0545
gi|29165615|ref|NC_002745.2|	639133 +	C	0	0	1	0	0	SA0545
gi|29165615|ref|NC_002745.2|	639155 +	U	1	0	0	0	0	SA0545
gi|29165615|ref|NC_002745.2|	639163 +	C	0	1	0	0	0	SA0545
gi|29165615|ref|NC_002745.2|	639164 +	U	1	0	0	0	0	SA0545
gi|29165615|ref|NC_002745.2|	639223 +	U	0	0	1	0	0	SA0545
gi|29165615|ref|NC_002745.2|	639241 +	C	0	0	0	0	1	SA0545
gi|29165615|ref|NC_002745.2|	639244 +	C	0	1	0	0	0	SA0545
gi|29165615|ref|NC_002745.2|	639261 +	C	0	0	0	0	1	SA0545
gi|29165615|ref|NC_002745.2|	639271 +	C	1	0	0	0	0	SA0545
gi|29165615|ref|NC_002745.2|	639288 +	A	0	0	0	0	1	SA0545
gi|29165615|ref|NC_002745.2|	639290 +	U	0	0	0	0	1	SA0545
gi|29165615|ref|NC_002745.2|	639298 +	U	0	0	0	1	0	SA0545
gi|29165615|ref|NC_002745.2|	639304 +	C	0	1	0	0	0	SA0545
gi|29165615|ref|NC_002745.2|	639309 +	G	0	0	0	0	1	SA0545
gi|29165615|ref|NC_002745.2|	639311 +	G	0	0	0	1	0	SA0545
gi|29165615|ref|NC_002745.2|	639312 +	C	1	0	0	2	0	SA0545
gi|29165615|ref|NC_002745.2|	639313 +	A	0	0	0	1	0	SA0545
gi|29165615|ref|NC_002745.2|	639320 +	A	0	2	0	0	0	SA0545
gi|29165615|ref|NC_002745.2|	639323 +	A	0	0	1	0	1	SA0545
gi|29165615|ref|NC_002745.2|	639326 +	A	0	1	2	0	0	SA0545
gi|29165615|ref|NC_002745.2|	639329 +	A	0	0	0	0	1	SA0546
gi|29165615|ref|NC_002745.2|	639332 +	G	0	0	1	0	0	SA0546
gi|29165615|ref|NC_002745.2|	639336 +	U	0	0	0	1	0	SA0546
gi|29165615|ref|NC_002745.2|	639344 +	A	0	2	0	0	0	SA0546
gi|29165615|ref|NC_002745.2|	639346 +	A	0	0	0	0	1	SA0546
gi|29165615|ref|NC_002745.2|	639348 +	A	0	0	0	1	0	SA0546
gi|29165615|ref|NC_002745.2|	639350 +	U	0	0	1	0	0	SA0546
gi|29165615|ref|NC_002745.2|	639352 +	U	1	0	0	0	0	SA0546
gi|29165615|ref|NC_002745.2|	639353 +	A	0	0	0	1	0	SA0546
gi|29165615|ref|NC_002745.2|	639363 +	A	0	0	0	0	1	SA0546
gi|29165615|ref|NC_002745.2|	639368 +	C	0	0	0	0	1	SA0546
gi|29165615|ref|NC_002745.2|	639370 +	A	0	0	0	0	1	SA0546
gi|29165615|ref|NC_002745.2|	639371 +	U	0	0	1	0	0	SA0546
gi|29165615|ref|NC_002745.2|	639385 +	U	1	0	0	0	0	SA0546
gi|29165615|ref|NC_002745.2|	639387 +	C	2	0	0	0	0	SA0546
gi|29165615|ref|NC_002745.2|	639391 +	A	0	1	0	0	0	SA0546
gi|29165615|ref|NC_002745.2|	639418 +	C	0	0	0	0	1	SA0546
gi|29165615|ref|NC_002745.2|	639420 +	A	1	0	1	0	0	SA0546
gi|29165615|ref|NC_002745.2|	639426 +	C	0	1	0	0	1	SA0546
gi|29165615|ref|NC_002745.2|	639430 +	U	1	0	0	0	0	SA0546
gi|29165615|ref|NC_002745.2|	639434 +	G	0	1	0	0	0	SA0546
gi|29165615|ref|NC_002745.2|	639435 +	A	0	0	1	0	0	SA0546
gi|29165615|ref|NC_002745.2|	639437 +	A	1	0	0	0	0	SA0546
gi|29165615|ref|NC_002745.2|	639451 +	U	0	1	0	0	0	SA0546
gi|29165615|ref|NC_002745.2|	639453 +	U	1	0	0	0	0	SA0546
gi|29165615|ref|NC_002745.2|	639459 +	A	0	1	0	0	1	SA0546
gi|29165615|ref|NC_002745.2|	639460 +	U	0	1	0	0	0	SA0546
gi|29165615|ref|NC_002745.2|	639462 +	A	1	1	1	0	1	SA0546
gi|29165615|ref|NC_002745.2|	639465 +	U	0	0	1	0	0	SA0546
gi|29165615|ref|NC_002745.2|	639486 +	A	0	0	2	0	0	SA0546
gi|29165615|ref|NC_002745.2|	639488 +	C	1	0	1	0	0	SA0546
gi|29165615|ref|NC_002745.2|	639489 +	A	0	1	0	0	0	SA0546
gi|29165615|ref|NC_002745.2|	639490 +	U	0	0	1	0	0	SA0546
gi|29165615|ref|NC_002745.2|	639493 +	U	0	0	1	1	0	SA0546
gi|29165615|ref|NC_002745.2|	639495 +	U	0	0	1	0	0	SA0546
gi|29165615|ref|NC_002745.2|	639505 +	U	0	0	1	0	0	SA0546

gi|29165615|ref|NC_002745.2|	639509 +	C	1	0	1	1	0	SA0546
gi|29165615|ref|NC_002745.2|	639510 +	A	1	0	0	0	0	SA0546
gi|29165615|ref|NC_002745.2|	639515 +	U	0	0	0	0	1	SA0546
gi|29165615|ref|NC_002745.2|	639516 +	C	0	0	0	1	0	SA0546
gi|29165615|ref|NC_002745.2|	639552 +	U	0	0	0	0	2	SA0546
gi|29165615|ref|NC_002745.2|	639553 +	A	1	1	0	0	0	SA0546
gi|29165615|ref|NC_002745.2|	639557 +	A	0	1	0	0	1	SA0546
gi|29165615|ref|NC_002745.2|	639566 +	G	0	0	0	0	1	SA0546
gi|29165615|ref|NC_002745.2|	639571 +	U	0	0	0	0	1	SA0546
gi|29165615|ref|NC_002745.2|	639581 +	G	0	0	0	0	1	SA0546
gi|29165615|ref|NC_002745.2|	639583 +	U	0	2	0	0	0	SA0546
gi|29165615|ref|NC_002745.2|	639588 +	A	0	0	0	0	1	SA0546
gi|29165615|ref|NC_002745.2|	639589 +	U	0	0	0	0	1	SA0546
gi|29165615|ref|NC_002745.2|	639625 +	A	0	0	0	0	1	SA0546
gi|29165615|ref|NC_002745.2|	639630 +	A	0	1	0	0	0	SA0546
gi|29165615|ref|NC_002745.2|	639632 +	A	0	1	0	0	0	SA0546
gi|29165615|ref|NC_002745.2|	639639 +	U	0	2	0	0	0	SA0546
gi|29165615|ref|NC_002745.2|	639654 +	A	0	2	0	0	0	SA0546
gi|29165615|ref|NC_002745.2|	639659 +	G	0	0	1	0	0	SA0546
gi|29165615|ref|NC_002745.2|	639663 +	C	0	0	1	0	0	SA0546
gi|29165615|ref|NC_002745.2|	639668 +	A	0	0	1	0	0	SA0546
gi|29165615|ref|NC_002745.2|	639671 +	G	0	0	0	0	1	SA0546
gi|29165615|ref|NC_002745.2|	639678 +	C	1	0	0	1	0	SA0546
gi|29165615|ref|NC_002745.2|	639735 +	U	1	0	0	0	0	SA0546
gi|29165615|ref|NC_002745.2|	639749 +	A	0	1	0	0	0	SA0546
gi|29165615|ref|NC_002745.2|	639760 +	U	0	0	1	0	0	SA0546
gi|29165615|ref|NC_002745.2|	639764 +	U	1	0	0	0	0	SA0546
gi|29165615|ref|NC_002745.2|	639767 +	C	0	1	0	0	0	SA0546
gi|29165615|ref|NC_002745.2|	639769 +	A	0	1	0	0	0	SA0546
gi|29165615|ref|NC_002745.2|	639775 +	A	0	0	0	0	1	SA0546
gi|29165615|ref|NC_002745.2|	639789 +	U	0	0	0	1	0	SA0546
gi|29165615|ref|NC_002745.2|	639792 +	A	0	0	1	0	0	SA0546
gi|29165615|ref|NC_002745.2|	639796 +	U	0	0	0	1	0	SA0546
gi|29165615|ref|NC_002745.2|	639802 +	A	0	1	0	0	0	SA0546
gi|29165615|ref|NC_002745.2|	639807 +	C	0	0	1	0	0	SA0546
gi|29165615|ref|NC_002745.2|	639816 +	C	0	0	0	0	1	SA0546
gi|29165615|ref|NC_002745.2|	639817 +	G	0	0	0	1	0	SA0546
gi|29165615|ref|NC_002745.2|	639826 +	A	0	0	0	1	0	SA0546
gi|29165615|ref|NC_002745.2|	639857 +	C	0	0	1	0	0	SA0546
gi|29165615|ref|NC_002745.2|	639914 +	C	0	2	0	0	0	SA0546
gi|29165615|ref|NC_002745.2|	639918 +	C	0	0	0	1	0	SA0546
gi|29165615|ref|NC_002745.2|	639929 +	G	0	1	0	0	0	SA0546
gi|29165615|ref|NC_002745.2|	639945 +	A	0	1	0	0	0	SA0546
gi|29165615|ref|NC_002745.2|	639946 +	A	0	0	0	0	2	SA0546
gi|29165615|ref|NC_002745.2|	639950 +	C	0	0	0	1	0	SA0546
gi|29165615|ref|NC_002745.2|	640007 +	A	0	0	0	0	1	SA0546
gi|29165615|ref|NC_002745.2|	640009 +	U	0	0	0	0	1	SA0546
gi|29165615|ref|NC_002745.2|	640023 +	U	1	0	0	0	0	SA0546
gi|29165615|ref|NC_002745.2|	640031 +	C	0	0	1	0	0	SA0546
gi|29165615|ref|NC_002745.2|	640089 +	A	0	0	0	0	1	SA0546
gi|29165615|ref|NC_002745.2|	640101 +	G	0	1	0	0	0	SA0546
gi|29165615|ref|NC_002745.2|	640304 +	A	0	1	0	0	0	-
gi|29165615|ref|NC_002745.2|	640330 +	U	0	1	0	0	0	-
gi|29165615|ref|NC_002745.2|	640346 +	G	0	0	0	0	1	-
gi|29165615|ref|NC_002745.2|	640372 +	G	3	0	0	1	1	-
gi|29165615|ref|NC_002745.2|	640383 +	A	0	0	0	0	1	-
gi|29165615|ref|NC_002745.2|	640467 +	G	0	0	0	0	1	-
gi|29165615|ref|NC_002745.2|	640587 +	C	0	0	1	0	0	-
gi|29165615|ref|NC_002745.2|	640601 +	A	0	0	0	2	1	-
gi|29165615|ref|NC_002745.2|	640603 +	A	0	0	1	0	0	-
gi|29165615|ref|NC_002745.2|	640625 +	A	0	0	0	1	0	-
gi|29165615|ref|NC_002745.2|	640626 +	U	0	0	1	0	0	-
gi|29165615|ref|NC_002745.2|	640630 +	G	0	0	0	1	0	-
gi|29165615|ref|NC_002745.2|	640631 +	U	1	2	3	1	5	-
gi|29165615|ref|NC_002745.2|	640632 +	G	0	0	1	0	0	-
gi|29165615|ref|NC_002745.2|	640633 +	A	0	0	1	0	0	-
gi|29165615|ref|NC_002745.2|	640634 +	G	0	0	1	0	0	-
gi|29165615|ref|NC_002745.2|	640638 +	A	1	0	0	0	0	-
gi|29165615|ref|NC_002745.2|	640645 +	C	0	0	0	1	0	-
gi|29165615|ref|NC_002745.2|	640650 +	A	0	0	0	2	1	-
gi|29165615|ref|NC_002745.2|	640655 +	U	1	0	0	0	0	-
gi|29165615|ref|NC_002745.2|	640656 +	A	0	0	0	1	0	-
gi|29165615|ref|NC_002745.2|	640671 +	G	2	0	0	0	0	-
gi|29165615|ref|NC_002745.2|	640680 +	A	0	0	1	1	2	-
gi|29165615|ref|NC_002745.2|	640684 +	U	0	0	1	0	0	-
gi|29165615|ref|NC_002745.2|	640696 +	C	0	1	0	0	0	-
gi|29165615|ref|NC_002745.2|	640701 +	A	0	0	1	0	0	-
gi|29165615|ref|NC_002745.2|	640702 +	A	0	0	0	1	0	-
gi|29165615|ref|NC_002745.2|	640704 +	U	0	0	0	1	0	-
gi|29165615|ref|NC_002745.2|	640705 +	A	0	0	1	0	0	-
gi|29165615|ref|NC_002745.2|	640706 +	A	1	3	7	3	7	-

gi|29165615|ref|NC_002745.2|	640707 +	A	0	0	1	3	2	-
gi|29165615|ref|NC_002745.2|	640708 +	A	0	0	0	0	1	-
gi|29165615|ref|NC_002745.2|	640709 +	A	0	0	0	0	1	-
gi|29165615|ref|NC_002745.2|	640711 +	A	0	0	0	1	0	-
gi|29165615|ref|NC_002745.2|	640712 +	A	0	2	0	1	2	-
gi|29165615|ref|NC_002745.2|	640716 +	A	0	0	0	1	0	-
gi|29165615|ref|NC_002745.2|	640718 +	C	0	0	1	1	3	-
gi|29165615|ref|NC_002745.2|	640719 +	A	0	1	0	0	0	-
gi|29165615|ref|NC_002745.2|	640721 +	G	0	0	0	0	1	-
gi|29165615|ref|NC_002745.2|	640722 +	U	0	0	1	0	2	-
gi|29165615|ref|NC_002745.2|	640723 +	A	0	0	2	1	0	-
gi|29165615|ref|NC_002745.2|	640727 +	G	1	0	2	1	1	-
gi|29165615|ref|NC_002745.2|	640730 +	A	0	0	0	0	2	-
gi|29165615|ref|NC_002745.2|	640731 +	A	0	0	0	2	0	-
gi|29165615|ref|NC_002745.2|	640734 +	A	0	0	0	1	1	-
gi|29165615|ref|NC_002745.2|	640736 +	A	0	1	0	1	0	-
gi|29165615|ref|NC_002745.2|	640737 +	A	0	2	0	1	0	-
gi|29165615|ref|NC_002745.2|	640738 +	A	0	0	1	0	1	-
gi|29165615|ref|NC_002745.2|	640739 +	A	0	0	0	0	1	-
gi|29165615|ref|NC_002745.2|	640742 +	U	0	0	0	0	1	-
gi|29165615|ref|NC_002745.2|	640744 +	A	0	3	3	2	0	-
gi|29165615|ref|NC_002745.2|	640749 +	G	2	0	0	0	0	-
gi|29165615|ref|NC_002745.2|	640755 +	A	0	1	0	1	1	-
gi|29165615|ref|NC_002745.2|	640756 +	C	0	1	2	0	1	-
gi|29165615|ref|NC_002745.2|	640757 +	A	0	0	0	0	1	-
gi|29165615|ref|NC_002745.2|	640760 +	A	0	0	1	1	0	-
gi|29165615|ref|NC_002745.2|	640761 +	A	1	0	1	1	0	-
gi|29165615|ref|NC_002745.2|	640765 +	G	0	0	0	0	2	-
gi|29165615|ref|NC_002745.2|	640767 +	U	0	0	0	0	1	-
gi|29165615|ref|NC_002745.2|	640768 +	A	0	0	1	0	0	-
gi|29165615|ref|NC_002745.2|	640769 +	U	0	0	1	0	0	-
gi|29165615|ref|NC_002745.2|	640773 +	G	0	1	0	0	0	-
gi|29165615|ref|NC_002745.2|	640780 +	C	0	0	0	1	0	-
gi|29165615|ref|NC_002745.2|	640781 +	A	1	0	0	0	0	-
gi|29165615|ref|NC_002745.2|	640787 +	G	0	1	0	1	0	-
gi|29165615|ref|NC_002745.2|	640796 +	A	0	0	1	3	0	-
gi|29165615|ref|NC_002745.2|	640797 +	A	0	0	0	1	0	-
gi|29165615|ref|NC_002745.2|	640803 +	G	0	0	1	0	0	-
gi|29165615|ref|NC_002745.2|	640810 +	C	0	0	0	0	2	-
gi|29165615|ref|NC_002745.2|	640849 +	A	0	0	0	0	1	SA0547
gi|29165615|ref|NC_002745.2|	640906 +	U	0	0	1	0	0	SA0547
gi|29165615|ref|NC_002745.2|	640918 +	U	0	0	1	0	0	SA0547
gi|29165615|ref|NC_002745.2|	640928 +	U	0	0	0	0	1	SA0547
gi|29165615|ref|NC_002745.2|	640989 +	A	0	0	1	1	0	SA0547
gi|29165615|ref|NC_002745.2|	641004 +	C	1	0	0	0	0	SA0547
gi|29165615|ref|NC_002745.2|	641035 +	C	1	0	0	0	0	SA0547
gi|29165615|ref|NC_002745.2|	641062 +	C	1	0	0	0	0	SA0547
gi|29165615|ref|NC_002745.2|	641064 +	G	0	0	0	1	0	SA0547
gi|29165615|ref|NC_002745.2|	641066 +	C	0	1	0	0	0	SA0547
gi|29165615|ref|NC_002745.2|	641077 +	U	0	0	1	0	0	SA0547
gi|29165615|ref|NC_002745.2|	641078 +	U	1	0	0	0	0	SA0547
gi|29165615|ref|NC_002745.2|	641120 +	A	1	0	0	0	0	SA0547
gi|29165615|ref|NC_002745.2|	641158 +	U	1	0	0	0	0	SA0547
gi|29165615|ref|NC_002745.2|	641175 +	A	0	0	0	0	1	SA0547
gi|29165615|ref|NC_002745.2|	641188 +	C	1	0	0	0	0	SA0547
gi|29165615|ref|NC_002745.2|	641194 +	C	1	0	0	0	0	SA0547
gi|29165615|ref|NC_002745.2|	641197 +	U	1	0	0	0	0	SA0547
gi|29165615|ref|NC_002745.2|	641200 +	U	3	0	0	0	0	SA0547
gi|29165615|ref|NC_002745.2|	641208 +	A	0	0	1	0	0	SA0547
gi|29165615|ref|NC_002745.2|	641219 +	G	1	0	0	0	0	SA0547
gi|29165615|ref|NC_002745.2|	641240 +	A	0	1	0	0	0	SA0547
gi|29165615|ref|NC_002745.2|	641284 +	A	1	0	0	0	0	SA0547
gi|29165615|ref|NC_002745.2|	641305 +	C	1	0	0	0	0	SA0547
gi|29165615|ref|NC_002745.2|	641429 +	C	2	0	0	0	0	SA0547
gi|29165615|ref|NC_002745.2|	641434 +	C	1	0	0	0	0	SA0547
gi|29165615|ref|NC_002745.2|	641499 +	C	1	0	0	0	0	SA0547
gi|29165615|ref|NC_002745.2|	641500 +	A	0	2	0	2	2	SA0547
gi|29165615|ref|NC_002745.2|	641501 +	A	1	4	3	0	1	SA0547
gi|29165615|ref|NC_002745.2|	641511 +	A	0	0	0	0	1	SA0547
gi|29165615|ref|NC_002745.2|	641512 +	U	2	0	0	0	0	SA0547
gi|29165615|ref|NC_002745.2|	641514 +	G	0	0	0	0	1	SA0547
gi|29165615|ref|NC_002745.2|	641515 +	G	0	0	0	1	0	SA0547
gi|29165615|ref|NC_002745.2|	641516 +	U	0	0	0	0	1	SA0547
gi|29165615|ref|NC_002745.2|	641517 +	A	0	1	0	0	0	SA0547
gi|29165615|ref|NC_002745.2|	641521 +	A	0	0	0	1	0	SA0547
gi|29165615|ref|NC_002745.2|	641528 +	U	1	0	0	0	0	SA0547
gi|29165615|ref|NC_002745.2|	641529 +	G	2	0	0	0	0	SA0547
gi|29165615|ref|NC_002745.2|	641532 +	A	0	1	0	0	0	SA0547
gi|29165615|ref|NC_002745.2|	641539 +	G	0	1	2	0	0	SA0547
gi|29165615|ref|NC_002745.2|	641540 +	A	0	1	0	0	0	SA0547
gi|29165615|ref|NC_002745.2|	641541 +	A	0	0	1	0	1	SA0547

gi|29165615|ref|NC_002745.2|	641545 +	U	0	0	0	0	1	SA0547
gi|29165615|ref|NC_002745.2|	641551 +	G	1	0	0	0	0	SA0547
gi|29165615|ref|NC_002745.2|	641552 +	U	6	0	0	0	0	SA0547
gi|29165615|ref|NC_002745.2|	641556 +	G	1	0	0	0	0	SA0547
gi|29165615|ref|NC_002745.2|	641576 +	A	4	0	0	0	0	SA0547
gi|29165615|ref|NC_002745.2|	641580 +	C	1	0	1	0	0	SA0547
gi|29165615|ref|NC_002745.2|	641594 +	A	0	1	0	0	0	SA0547
gi|29165615|ref|NC_002745.2|	641597 +	A	2	0	0	0	0	SA0547
gi|29165615|ref|NC_002745.2|	641599 +	C	0	0	0	1	0	SA0547
gi|29165615|ref|NC_002745.2|	641606 +	A	2	0	0	0	0	SA0547
gi|29165615|ref|NC_002745.2|	641607 +	A	0	0	1	0	0	SA0547
gi|29165615|ref|NC_002745.2|	641608 +	A	0	1	0	0	0	SA0547
gi|29165615|ref|NC_002745.2|	641609 +	U	0	0	2	0	1	SA0547
gi|29165615|ref|NC_002745.2|	641610 +	A	0	0	0	1	0	SA0547
gi|29165615|ref|NC_002745.2|	641615 +	G	0	1	0	1	0	SA0547
gi|29165615|ref|NC_002745.2|	641618 +	A	0	0	0	0	1	SA0547
gi|29165615|ref|NC_002745.2|	641623 +	U	0	0	0	0	1	SA0547
gi|29165615|ref|NC_002745.2|	641649 +	U	1	0	0	0	0	SA0547
gi|29165615|ref|NC_002745.2|	641653 +	U	3	0	1	0	0	SA0547
gi|29165615|ref|NC_002745.2|	641654 +	U	3	0	1	0	1	SA0547
gi|29165615|ref|NC_002745.2|	641661 +	U	1	0	0	0	0	SA0547
gi|29165615|ref|NC_002745.2|	641670 +	U	0	1	0	0	0	SA0547
gi|29165615|ref|NC_002745.2|	641844 +	U	2	0	0	0	0	SA0548
gi|29165615|ref|NC_002745.2|	641873 +	G	0	0	1	0	0	SA0548
gi|29165615|ref|NC_002745.2|	641930 +	G	2	0	0	0	0	SA0548
gi|29165615|ref|NC_002745.2|	641940 +	G	1	0	0	0	0	SA0548
gi|29165615|ref|NC_002745.2|	641953 +	G	0	0	0	1	0	SA0548
gi|29165615|ref|NC_002745.2|	641960 +	A	1	0	0	0	0	SA0548
gi|29165615|ref|NC_002745.2|	641962 +	A	0	0	0	0	1	SA0548
gi|29165615|ref|NC_002745.2|	641966 +	U	1	0	0	0	0	SA0548
gi|29165615|ref|NC_002745.2|	641969 +	U	0	0	1	0	0	SA0548
gi|29165615|ref|NC_002745.2|	641978 +	C	2	0	0	0	0	SA0548
gi|29165615|ref|NC_002745.2|	641987 +	U	2	0	0	0	0	SA0548
gi|29165615|ref|NC_002745.2|	642042 +	A	0	0	0	0	1	SA0548
gi|29165615|ref|NC_002745.2|	642156 +	G	1	0	0	0	0	SA0548
gi|29165615|ref|NC_002745.2|	642263 +	U	1	0	0	0	0	SA0548
gi|29165615|ref|NC_002745.2|	642269 +	U	1	0	0	0	0	SA0548
gi|29165615|ref|NC_002745.2|	642300 +	U	1	0	0	0	0	SA0548
gi|29165615|ref|NC_002745.2|	642360 +	U	1	0	0	0	0	SA0548
gi|29165615|ref|NC_002745.2|	642370 +	C	2	0	0	0	0	SA0548
gi|29165615|ref|NC_002745.2|	642398 +	U	1	0	0	0	0	SA0548
gi|29165615|ref|NC_002745.2|	642400 +	C	1	0	0	0	0	SA0548
gi|29165615|ref|NC_002745.2|	642401 +	G	1	0	0	0	0	SA0548
gi|29165615|ref|NC_002745.2|	642402 +	U	1	0	0	0	0	SA0548
gi|29165615|ref|NC_002745.2|	642406 +	C	1	0	0	0	0	SA0548
gi|29165615|ref|NC_002745.2|	642419 +	U	1	0	0	0	0	SA0548
gi|29165615|ref|NC_002745.2|	642436 +	U	1	0	0	0	0	SA0548
gi|29165615|ref|NC_002745.2|	642443 +	A	0	1	0	0	0	SA0548
gi|29165615|ref|NC_002745.2|	642459 +	U	1	0	0	0	0	SA0548
gi|29165615|ref|NC_002745.2|	642461 +	A	1	0	0	0	0	SA0548
gi|29165615|ref|NC_002745.2|	642468 +	C	2	0	0	0	0	SA0548
gi|29165615|ref|NC_002745.2|	642477 +	C	1	0	0	0	0	SA0548
gi|29165615|ref|NC_002745.2|	642488 +	G	1	0	0	0	0	SA0548
gi|29165615|ref|NC_002745.2|	642500 +	G	1	0	0	0	0	SA0548
gi|29165615|ref|NC_002745.2|	642512 +	A	0	0	0	0	1	SA0548
gi|29165615|ref|NC_002745.2|	642516 +	U	1	0	0	0	0	SA0548
gi|29165615|ref|NC_002745.2|	642519 +	G	1	0	0	0	0	SA0548
gi|29165615|ref|NC_002745.2|	642559 +	A	0	0	0	0	1	SA0548
gi|29165615|ref|NC_002745.2|	642560 +	A	1	0	1	0	0	SA0548
gi|29165615|ref|NC_002745.2|	642564 +	C	1	0	1	1	1	SA0548
gi|29165615|ref|NC_002745.2|	642565 +	A	0	0	0	2	0	SA0548
gi|29165615|ref|NC_002745.2|	642591 +	A	0	0	1	0	0	SA0548
gi|29165615|ref|NC_002745.2|	642603 +	U	0	1	0	0	0	SA0548
gi|29165615|ref|NC_002745.2|	642610 +	C	1	0	0	0	0	SA0548
gi|29165615|ref|NC_002745.2|	642620 +	A	0	0	1	0	0	SA0548
gi|29165615|ref|NC_002745.2|	642624 +	U	1	0	0	0	0	SA0548
gi|29165615|ref|NC_002745.2|	642626 +	A	0	0	1	0	0	SA0548
gi|29165615|ref|NC_002745.2|	642631 +	A	2	0	0	0	0	SA0548
gi|29165615|ref|NC_002745.2|	642632 +	U	3	0	0	0	0	SA0548
gi|29165615|ref|NC_002745.2|	642657 +	G	0	0	1	0	0	SA0548
gi|29165615|ref|NC_002745.2|	642663 +	A	1	0	0	0	0	-
gi|29165615|ref|NC_002745.2|	642671 +	A	1	0	0	0	0	-
gi|29165615|ref|NC_002745.2|	642672 +	A	0	0	1	1	0	-
gi|29165615|ref|NC_002745.2|	642673 +	A	1	0	0	0	1	SA0549
gi|29165615|ref|NC_002745.2|	642675 +	G	1	0	0	0	0	SA0549
gi|29165615|ref|NC_002745.2|	642687 +	A	1	0	0	0	0	SA0549
gi|29165615|ref|NC_002745.2|	642701 +	U	2	0	0	0	0	SA0549
gi|29165615|ref|NC_002745.2|	642722 +	C	1	0	0	0	0	SA0549
gi|29165615|ref|NC_002745.2|	642752 +	U	0	0	0	0	1	SA0549
gi|29165615|ref|NC_002745.2|	642893 +	C	1	0	0	0	0	SA0549
gi|29165615|ref|NC_002745.2|	643076 +	A	0	0	0	0	2	SA0549

gi|29165615|ref|NC_002745.2|	643130 +	U	1	0	0	0	0	SA0549
gi|29165615|ref|NC_002745.2|	643172 +	U	1	0	0	0	0	SA0549
gi|29165615|ref|NC_002745.2|	643186 +	U	1	0	0	0	0	SA0549
gi|29165615|ref|NC_002745.2|	643237 +	C	1	0	0	0	0	SA0549
gi|29165615|ref|NC_002745.2|	643277 +	A	0	0	0	0	1	SA0549
gi|29165615|ref|NC_002745.2|	643356 +	G	0	0	0	0	1	SA0549
gi|29165615|ref|NC_002745.2|	643459 +	C	0	0	0	0	1	SA0549
gi|29165615|ref|NC_002745.2|	643476 +	U	1	0	0	0	0	SA0549
gi|29165615|ref|NC_002745.2|	643485 +	A	1	0	0	0	0	SA0549
gi|29165615|ref|NC_002745.2|	643730 +	A	1	0	0	0	0	SA0549
gi|29165615|ref|NC_002745.2|	643864 +	A	1	0	0	0	1	-
gi|29165615|ref|NC_002745.2|	643865 +	G	2	3	0	1	1	-
gi|29165615|ref|NC_002745.2|	643866 +	U	1	0	0	0	0	-
gi|29165615|ref|NC_002745.2|	643875 +	A	1	1	5	0	8	-
gi|29165615|ref|NC_002745.2|	643877 +	A	0	0	1	0	0	-
gi|29165615|ref|NC_002745.2|	643884 +	A	0	0	0	0	1	-
gi|29165615|ref|NC_002745.2|	643890 +	A	0	0	1	4	1	-
gi|29165615|ref|NC_002745.2|	643891 +	C	1	0	0	1	0	-
gi|29165615|ref|NC_002745.2|	643892 +	G	0	0	0	1	0	-
gi|29165615|ref|NC_002745.2|	643895 +	A	0	0	1	0	0	-
gi|29165615|ref|NC_002745.2|	643898 +	G	0	0	0	1	1	-
gi|29165615|ref|NC_002745.2|	643899 +	A	0	1	1	0	0	-
gi|29165615|ref|NC_002745.2|	643902 +	U	0	0	1	0	0	-
gi|29165615|ref|NC_002745.2|	643904 +	A	0	0	2	0	0	-
gi|29165615|ref|NC_002745.2|	643905 +	U	4	0	1	0	1	-
gi|29165615|ref|NC_002745.2|	643909 +	G	1	0	0	0	0	-
gi|29165615|ref|NC_002745.2|	643910 +	A	0	1	1	1	0	-
gi|29165615|ref|NC_002745.2|	643912 +	A	1	0	0	1	1	-
gi|29165615|ref|NC_002745.2|	643915 +	U	2	0	0	0	0	-
gi|29165615|ref|NC_002745.2|	643932 +	A	0	0	2	0	0	SA0550
gi|29165615|ref|NC_002745.2|	643936 +	C	0	0	1	0	0	SA0550
gi|29165615|ref|NC_002745.2|	643954 +	A	0	0	0	1	0	SA0550
gi|29165615|ref|NC_002745.2|	643984 +	A	0	0	0	2	0	SA0550
gi|29165615|ref|NC_002745.2|	644000 +	A	1	0	0	0	0	SA0550
gi|29165615|ref|NC_002745.2|	644002 +	U	1	0	0	0	0	SA0550
gi|29165615|ref|NC_002745.2|	644005 +	A	0	0	1	0	0	SA0550
gi|29165615|ref|NC_002745.2|	644015 +	U	1	0	0	0	0	SA0550
gi|29165615|ref|NC_002745.2|	644021 +	C	1	0	0	0	0	SA0550
gi|29165615|ref|NC_002745.2|	644040 +	U	0	0	1	0	0	SA0550
gi|29165615|ref|NC_002745.2|	644041 +	A	1	0	0	0	0	SA0550
gi|29165615|ref|NC_002745.2|	644044 +	G	2	0	0	0	0	SA0550
gi|29165615|ref|NC_002745.2|	644045 +	U	2	0	0	0	0	SA0550
gi|29165615|ref|NC_002745.2|	644050 +	C	2	0	2	1	0	SA0550
gi|29165615|ref|NC_002745.2|	644074 +	U	1	0	0	0	0	SA0550
gi|29165615|ref|NC_002745.2|	644075 +	U	1	0	0	0	0	SA0550
gi|29165615|ref|NC_002745.2|	644077 +	U	0	2	0	0	0	SA0550
gi|29165615|ref|NC_002745.2|	644082 +	A	0	0	1	0	0	SA0550
gi|29165615|ref|NC_002745.2|	644090 +	A	0	0	0	0	1	SA0550
gi|29165615|ref|NC_002745.2|	644092 +	U	3	0	0	0	0	SA0550
gi|29165615|ref|NC_002745.2|	644095 +	C	1	0	0	0	0	SA0550
gi|29165615|ref|NC_002745.2|	644104 +	C	0	2	0	0	0	SA0550
gi|29165615|ref|NC_002745.2|	644106 +	G	0	1	0	0	0	SA0550
gi|29165615|ref|NC_002745.2|	644107 +	A	0	0	3	0	0	SA0550
gi|29165615|ref|NC_002745.2|	644110 +	A	0	0	1	0	1	SA0550
gi|29165615|ref|NC_002745.2|	644111 +	A	0	0	0	0	1	SA0550
gi|29165615|ref|NC_002745.2|	644113 +	A	0	0	3	1	3	SA0550
gi|29165615|ref|NC_002745.2|	644114 +	A	0	1	0	0	0	SA0550
gi|29165615|ref|NC_002745.2|	644116 +	U	0	0	0	2	0	SA0550
gi|29165615|ref|NC_002745.2|	644117 +	A	0	0	1	0	1	SA0550
gi|29165615|ref|NC_002745.2|	644118 +	A	0	0	0	1	1	SA0550
gi|29165615|ref|NC_002745.2|	644124 +	A	0	1	0	1	0	SA0550
gi|29165615|ref|NC_002745.2|	644131 +	C	1	0	0	0	0	SA0550
gi|29165615|ref|NC_002745.2|	644135 +	A	0	0	1	1	0	SA0550
gi|29165615|ref|NC_002745.2|	644136 +	C	0	0	1	0	0	SA0550
gi|29165615|ref|NC_002745.2|	644137 +	A	0	0	1	0	0	SA0550
gi|29165615|ref|NC_002745.2|	644141 +	A	1	2	3	1	0	SA0550
gi|29165615|ref|NC_002745.2|	644142 +	A	1	1	2	0	0	SA0550
gi|29165615|ref|NC_002745.2|	644143 +	A	0	1	2	0	3	SA0550
gi|29165615|ref|NC_002745.2|	644144 +	G	1	3	0	1	2	SA0550
gi|29165615|ref|NC_002745.2|	644145 +	A	0	0	2	1	0	SA0550
gi|29165615|ref|NC_002745.2|	644148 +	C	3	0	0	0	0	SA0550
gi|29165615|ref|NC_002745.2|	644149 +	C	0	0	1	0	0	SA0550
gi|29165615|ref|NC_002745.2|	644155 +	A	0	0	1	0	0	SA0550
gi|29165615|ref|NC_002745.2|	644161 +	A	0	0	0	1	0	SA0550
gi|29165615|ref|NC_002745.2|	646530 +	U	0	1	0	0	0	-
gi|29165615|ref|NC_002745.2|	646531 +	A	0	1	0	0	1	-
gi|29165615|ref|NC_002745.2|	646534 +	A	0	1	0	0	0	-
gi|29165615|ref|NC_002745.2|	646535 +	A	1	0	0	0	0	-
gi|29165615|ref|NC_002745.2|	646645 +	A	0	1	0	0	0	-
gi|29165615|ref|NC_002745.2|	646660 +	A	0	1	0	0	0	-
gi|29165615|ref|NC_002745.2|	646667 +	U	0	0	1	0	0	-

gi|29165615|ref|NC_002745.2|	646677 +	A	0	0	0	0	1	-
gi|29165615|ref|NC_002745.2|	646770 +	A	0	1	0	0	0	SA0553
gi|29165615|ref|NC_002745.2|	646802 +	A	1	0	0	0	0	SA0553
gi|29165615|ref|NC_002745.2|	646876 +	C	0	0	0	0	1	SA0553
gi|29165615|ref|NC_002745.2|	647018 +	A	0	0	0	1	0	SA0553
gi|29165615|ref|NC_002745.2|	647132 +	A	0	0	0	0	1	SA0553
gi|29165615|ref|NC_002745.2|	647182 +	G	0	0	1	0	0	SA0553
gi|29165615|ref|NC_002745.2|	647202 +	A	0	0	0	1	0	SA0553
gi|29165615|ref|NC_002745.2|	647308 +	A	0	0	0	0	2	SA0553
gi|29165615|ref|NC_002745.2|	647309 +	A	0	0	0	0	1	SA0553
gi|29165615|ref|NC_002745.2|	647367 +	G	0	0	0	1	0	SA0553
gi|29165615|ref|NC_002745.2|	647379 +	A	0	0	1	0	0	SA0553
gi|29165615|ref|NC_002745.2|	647383 +	A	0	0	1	0	0	SA0553
gi|29165615|ref|NC_002745.2|	647387 +	C	0	0	0	0	1	SA0553
gi|29165615|ref|NC_002745.2|	647422 +	C	0	0	0	0	1	SA0553
gi|29165615|ref|NC_002745.2|	647458 +	U	0	0	0	1	1	SA0553
gi|29165615|ref|NC_002745.2|	647463 +	A	0	0	0	0	2	SA0553
gi|29165615|ref|NC_002745.2|	647468 +	A	0	1	1	0	0	SA0553
gi|29165615|ref|NC_002745.2|	647469 +	C	0	1	0	0	0	SA0553
gi|29165615|ref|NC_002745.2|	647476 +	G	0	0	0	0	1	SA0553
gi|29165615|ref|NC_002745.2|	647477 +	A	0	1	0	0	0	SA0553
gi|29165615|ref|NC_002745.2|	647479 +	A	0	1	0	0	0	SA0553
gi|29165615|ref|NC_002745.2|	647481 +	U	0	1	0	0	0	SA0553
gi|29165615|ref|NC_002745.2|	647482 +	A	1	0	0	0	0	SA0553
gi|29165615|ref|NC_002745.2|	647498 +	G	0	1	0	0	0	SA0553
gi|29165615|ref|NC_002745.2|	647504 +	A	0	0	0	0	1	SA0553
gi|29165615|ref|NC_002745.2|	647525 +	A	1	0	0	0	0	SA0553
gi|29165615|ref|NC_002745.2|	647550 +	A	0	1	0	0	0	SA0553
gi|29165615|ref|NC_002745.2|	647579 +	G	1	0	0	0	0	SA0553
gi|29165615|ref|NC_002745.2|	647673 +	C	1	0	0	0	0	SA0553
gi|29165615|ref|NC_002745.2|	647679 +	A	0	1	0	0	0	SA0553
gi|29165615|ref|NC_002745.2|	647683 +	C	1	0	0	0	0	SA0553
gi|29165615|ref|NC_002745.2|	647688 +	G	0	0	0	0	1	SA0553
gi|29165615|ref|NC_002745.2|	647720 +	G	0	0	0	0	1	SA0553
gi|29165615|ref|NC_002745.2|	647744 +	A	0	0	0	0	1	SA0553
gi|29165615|ref|NC_002745.2|	647756 +	U	0	0	1	0	0	SA0553
gi|29165615|ref|NC_002745.2|	647791 +	G	2	0	0	0	0	SA0553
gi|29165615|ref|NC_002745.2|	647815 +	G	0	0	0	0	1	SA0553
gi|29165615|ref|NC_002745.2|	647842 +	G	0	0	1	0	0	SA0553
gi|29165615|ref|NC_002745.2|	647847 +	U	1	0	0	0	0	SA0553
gi|29165615|ref|NC_002745.2|	647849 +	G	1	0	0	0	0	SA0553
gi|29165615|ref|NC_002745.2|	647850 +	G	1	0	0	0	0	SA0553
gi|29165615|ref|NC_002745.2|	648055 +	A	0	0	0	1	0	SA0553
gi|29165615|ref|NC_002745.2|	648082 +	U	1	0	0	0	0	SA0553
gi|29165615|ref|NC_002745.2|	648094 +	C	0	0	0	1	0	SA0554
gi|29165615|ref|NC_002745.2|	648249 +	G	0	0	0	1	1	SA0554
gi|29165615|ref|NC_002745.2|	648261 +	A	0	0	0	1	0	SA0554
gi|29165615|ref|NC_002745.2|	648263 +	A	0	1	0	0	0	SA0554
gi|29165615|ref|NC_002745.2|	648270 +	U	0	0	1	0	0	SA0554
gi|29165615|ref|NC_002745.2|	648278 +	A	1	0	0	0	0	SA0554
gi|29165615|ref|NC_002745.2|	648283 +	G	0	1	0	0	0	SA0554
gi|29165615|ref|NC_002745.2|	648298 +	A	0	0	0	0	1	SA0554
gi|29165615|ref|NC_002745.2|	648310 +	A	1	0	0	0	0	SA0554
gi|29165615|ref|NC_002745.2|	648314 +	U	1	0	0	0	0	SA0554
gi|29165615|ref|NC_002745.2|	648329 +	U	0	0	0	0	1	SA0554
gi|29165615|ref|NC_002745.2|	648344 +	U	0	0	0	0	1	SA0554
gi|29165615|ref|NC_002745.2|	648356 +	C	0	1	0	0	0	SA0554
gi|29165615|ref|NC_002745.2|	648720 +	A	0	0	0	0	1	SA0554
gi|29165615|ref|NC_002745.2|	648826 +	U	0	1	0	0	0	SA0555
gi|29165615|ref|NC_002745.2|	649282 +	U	0	1	0	0	0	SA0555
gi|29165615|ref|NC_002745.2|	649499 +	U	0	0	0	0	1	-
gi|29165615|ref|NC_002745.2|	649507 +	A	0	0	0	0	1	-
gi|29165615|ref|NC_002745.2|	649595 +	A	0	2	0	0	0	SA0556
gi|29165615|ref|NC_002745.2|	649689 +	G	0	0	0	0	1	SA0556
gi|29165615|ref|NC_002745.2|	649690 +	A	0	2	0	0	0	SA0556
gi|29165615|ref|NC_002745.2|	649696 +	U	1	0	0	0	0	SA0556
gi|29165615|ref|NC_002745.2|	649729 +	A	0	0	0	0	1	SA0556
gi|29165615|ref|NC_002745.2|	649843 +	U	1	0	0	0	0	SA0556
gi|29165615|ref|NC_002745.2|	649945 +	U	1	0	0	0	0	SA0556
gi|29165615|ref|NC_002745.2|	649965 +	U	0	1	0	0	0	SA0556
gi|29165615|ref|NC_002745.2|	649966 +	U	0	0	0	1	0	SA0556
gi|29165615|ref|NC_002745.2|	650328 +	A	0	0	0	0	1	-
gi|29165615|ref|NC_002745.2|	650350 +	A	0	0	0	0	1	-
gi|29165615|ref|NC_002745.2|	650404 +	G	0	1	0	0	0	-
gi|29165615|ref|NC_002745.2|	650421 +	A	0	1	0	0	0	-
gi|29165615|ref|NC_002745.2|	650428 +	A	0	1	0	0	0	-
gi|29165615|ref|NC_002745.2|	650470 +	A	0	0	0	0	1	-
gi|29165615|ref|NC_002745.2|	650494 +	A	0	1	0	0	0	-
gi|29165615|ref|NC_002745.2|	650531 +	U	0	1	0	0	0	-
gi|29165615|ref|NC_002745.2|	650578 +	G	5	6	6	2	11	-
gi|29165615|ref|NC_002745.2|	650579 +	G	0	2	1	4	4	-

gi|29165615|ref|NC_002745.2|	650580	+	A	0	0	0	0	1	-
gi|29165615|ref|NC_002745.2|	650589	+	A	0	0	0	1	0	-
gi|29165615|ref|NC_002745.2|	650590	+	A	0	0	0	0	1	-
gi|29165615|ref|NC_002745.2|	650608	+	U	0	0	1	1	0	-
gi|29165615|ref|NC_002745.2|	650623	+	U	1	0	0	0	0	SA0557
gi|29165615|ref|NC_002745.2|	650685	+	G	1	0	0	0	0	SA0557
gi|29165615|ref|NC_002745.2|	650908	+	A	0	1	0	0	0	SA0557
gi|29165615|ref|NC_002745.2|	650910	+	C	0	0	1	0	0	SA0557
gi|29165615|ref|NC_002745.2|	650942	+	U	0	0	0	1	0	SA0557
gi|29165615|ref|NC_002745.2|	650958	+	C	0	1	0	0	0	SA0557
gi|29165615|ref|NC_002745.2|	651152	+	U	1	0	0	0	0	SA0557
gi|29165615|ref|NC_002745.2|	651154	+	U	1	0	0	0	0	SA0557
gi|29165615|ref|NC_002745.2|	651254	+	A	0	1	0	0	0	SA0557
gi|29165615|ref|NC_002745.2|	651290	+	U	0	0	0	1	0	SA0557
gi|29165615|ref|NC_002745.2|	651410	+	A	0	0	1	0	0	SA0557
gi|29165615|ref|NC_002745.2|	651471	+	A	0	1	1	0	0	SA0557
gi|29165615|ref|NC_002745.2|	651472	+	A	0	2	0	0	1	SA0557
gi|29165615|ref|NC_002745.2|	651477	+	A	0	1	0	0	0	SA0557
gi|29165615|ref|NC_002745.2|	651516	+	U	0	0	0	0	1	SA0557
gi|29165615|ref|NC_002745.2|	651535	+	C	0	1	0	0	0	SA0557
gi|29165615|ref|NC_002745.2|	651542	+	A	0	0	0	0	1	SA0557
gi|29165615|ref|NC_002745.2|	651557	+	U	0	1	0	0	0	-
gi|29165615|ref|NC_002745.2|	651853	+	U	0	0	1	0	0	-
gi|29165615|ref|NC_002745.2|	651941	+	U	0	1	0	0	0	-
gi|29165615|ref|NC_002745.2|	651945	+	G	2	3	4	5	4	-
gi|29165615|ref|NC_002745.2|	651947	+	A	0	0	1	0	1	-
gi|29165615|ref|NC_002745.2|	651951	+	A	0	1	0	0	1	-
gi|29165615|ref|NC_002745.2|	651954	+	A	0	0	0	0	1	-
gi|29165615|ref|NC_002745.2|	651956	+	C	0	1	0	0	0	-
gi|29165615|ref|NC_002745.2|	651961	+	A	0	0	0	0	1	-
gi|29165615|ref|NC_002745.2|	651962	+	U	0	1	0	0	0	-
gi|29165615|ref|NC_002745.2|	651970	+	A	0	0	0	0	1	-
gi|29165615|ref|NC_002745.2|	651971	+	A	0	0	0	0	1	-
gi|29165615|ref|NC_002745.2|	651974	+	A	0	1	0	1	0	-
gi|29165615|ref|NC_002745.2|	651975	+	A	0	0	1	0	1	-
gi|29165615|ref|NC_002745.2|	651976	+	A	0	2	1	0	1	-
gi|29165615|ref|NC_002745.2|	651977	+	A	0	0	1	0	0	-
gi|29165615|ref|NC_002745.2|	651992	+	A	0	2	0	0	0	-
gi|29165615|ref|NC_002745.2|	651993	+	A	0	0	2	1	0	-
gi|29165615|ref|NC_002745.2|	652033	+	A	0	0	1	0	1	SA0558
gi|29165615|ref|NC_002745.2|	652037	+	C	0	1	0	0	0	SA0558
gi|29165615|ref|NC_002745.2|	652043	+	A	0	2	0	0	0	SA0558
gi|29165615|ref|NC_002745.2|	652044	+	A	0	0	0	1	0	SA0558
gi|29165615|ref|NC_002745.2|	652063	+	C	0	1	0	0	0	SA0558
gi|29165615|ref|NC_002745.2|	652080	+	A	0	2	0	0	0	SA0558
gi|29165615|ref|NC_002745.2|	652091	+	U	0	1	0	0	0	SA0558
gi|29165615|ref|NC_002745.2|	652092	+	G	0	1	0	0	0	SA0558
gi|29165615|ref|NC_002745.2|	652098	+	A	0	1	0	0	0	SA0558
gi|29165615|ref|NC_002745.2|	652109	+	A	1	0	0	0	0	SA0558
gi|29165615|ref|NC_002745.2|	652110	+	A	0	1	0	0	0	SA0558
gi|29165615|ref|NC_002745.2|	652160	+	A	0	1	0	0	0	SA0558
gi|29165615|ref|NC_002745.2|	652187	+	U	0	0	0	0	1	SA0558
gi|29165615|ref|NC_002745.2|	652232	+	A	0	0	0	1	0	SA0558
gi|29165615|ref|NC_002745.2|	652325	+	A	0	0	0	0	1	SA0558
gi|29165615|ref|NC_002745.2|	652362	+	U	0	0	0	1	0	SA0558
gi|29165615|ref|NC_002745.2|	652372	+	A	0	0	1	1	0	SA0558
gi|29165615|ref|NC_002745.2|	652381	+	C	0	0	0	1	0	SA0558
gi|29165615|ref|NC_002745.2|	652390	+	A	0	0	0	1	0	SA0558
gi|29165615|ref|NC_002745.2|	652393	+	C	0	0	0	1	0	SA0558
gi|29165615|ref|NC_002745.2|	652394	+	A	0	0	0	1	0	SA0558
gi|29165615|ref|NC_002745.2|	652396	+	A	0	0	0	1	0	SA0558
gi|29165615|ref|NC_002745.2|	652399	+	A	0	3	0	1	1	SA0558
gi|29165615|ref|NC_002745.2|	652400	+	A	0	1	0	0	2	SA0558
gi|29165615|ref|NC_002745.2|	652434	+	U	1	0	0	0	0	SA0558
gi|29165615|ref|NC_002745.2|	652441	+	A	0	0	0	1	0	SA0558
gi|29165615|ref|NC_002745.2|	652584	+	A	0	0	0	1	0	-
gi|29165615|ref|NC_002745.2|	652589	+	A	0	0	1	0	0	-
gi|29165615|ref|NC_002745.2|	652590	+	A	0	0	1	0	0	-
gi|29165615|ref|NC_002745.2|	652632	+	U	0	0	0	0	1	-
gi|29165615|ref|NC_002745.2|	652668	+	A	1	6	3	6	2	-
gi|29165615|ref|NC_002745.2|	652669	+	U	0	0	0	0	1	-
gi|29165615|ref|NC_002745.2|	652670	+	U	2	0	0	0	0	-
gi|29165615|ref|NC_002745.2|	652678	+	G	0	0	1	0	0	-
gi|29165615|ref|NC_002745.2|	652679	+	U	1	0	0	0	0	-
gi|29165615|ref|NC_002745.2|	652681	+	A	0	0	1	0	0	-
gi|29165615|ref|NC_002745.2|	652766	+	G	0	2	2	3	3	SA0559
gi|29165615|ref|NC_002745.2|	652775	+	A	0	1	1	0	1	SA0559
gi|29165615|ref|NC_002745.2|	652776	+	U	0	1	0	0	0	SA0559
gi|29165615|ref|NC_002745.2|	652782	+	A	0	0	0	0	2	SA0559
gi|29165615|ref|NC_002745.2|	652783	+	A	0	1	0	1	1	SA0559
gi|29165615|ref|NC_002745.2|	652784	+	U	0	2	2	0	3	SA0559

gi|29165615|ref|NC_002745.2|	652787	+	A	0	0	0	1	0	SA0559
gi|29165615|ref|NC_002745.2|	652857	+	C	0	0	0	0	1	SA0559
gi|29165615|ref|NC_002745.2|	652937	+	U	0	1	0	0	0	SA0559
gi|29165615|ref|NC_002745.2|	652941	+	U	0	0	0	1	0	SA0559
gi|29165615|ref|NC_002745.2|	652945	+	C	0	0	1	0	0	SA0559
gi|29165615|ref|NC_002745.2|	652957	+	A	0	0	0	1	0	SA0559
gi|29165615|ref|NC_002745.2|	653001	+	G	0	0	1	0	0	SA0559
gi|29165615|ref|NC_002745.2|	653002	+	A	0	0	1	1	0	SA0559
gi|29165615|ref|NC_002745.2|	653003	+	A	0	0	0	0	2	SA0559
gi|29165615|ref|NC_002745.2|	653005	+	G	0	3	2	1	2	SA0559
gi|29165615|ref|NC_002745.2|	653006	+	A	0	5	3	2	1	SA0559
gi|29165615|ref|NC_002745.2|	653020	+	A	0	0	0	2	0	SA0559
gi|29165615|ref|NC_002745.2|	653026	+	A	0	1	0	1	0	SA0559
gi|29165615|ref|NC_002745.2|	653032	+	A	0	0	0	1	0	SA0559
gi|29165615|ref|NC_002745.2|	653051	+	A	0	0	0	0	1	SA0559
gi|29165615|ref|NC_002745.2|	653052	+	G	0	0	1	0	0	SA0559
gi|29165615|ref|NC_002745.2|	653062	+	A	0	0	1	1	0	SA0559
gi|29165615|ref|NC_002745.2|	653064	+	G	0	1	0	0	0	SA0559
gi|29165615|ref|NC_002745.2|	653109	+	C	0	1	0	0	0	SA0559
gi|29165615|ref|NC_002745.2|	653134	+	G	0	1	0	0	0	SA0559
gi|29165615|ref|NC_002745.2|	653151	+	G	0	0	0	1	0	SA0559
gi|29165615|ref|NC_002745.2|	653152	+	C	0	1	0	0	0	SA0559
gi|29165615|ref|NC_002745.2|	653156	+	U	0	0	1	0	1	SA0559
gi|29165615|ref|NC_002745.2|	653157	+	A	0	0	0	0	1	SA0559
gi|29165615|ref|NC_002745.2|	653158	+	U	0	0	0	0	1	SA0559
gi|29165615|ref|NC_002745.2|	653159	+	G	0	0	1	1	0	SA0559
gi|29165615|ref|NC_002745.2|	653166	+	G	0	0	0	0	1	SA0559
gi|29165615|ref|NC_002745.2|	653167	+	C	0	7	0	3	1	SA0559
gi|29165615|ref|NC_002745.2|	653176	+	U	0	0	0	1	0	-
gi|29165615|ref|NC_002745.2|	653177	+	A	0	0	0	0	1	-
gi|29165615|ref|NC_002745.2|	653180	+	U	0	0	0	1	0	-
gi|29165615|ref|NC_002745.2|	653183	+	A	0	1	0	0	0	-
gi|29165615|ref|NC_002745.2|	653184	+	A	0	0	1	0	0	-
gi|29165615|ref|NC_002745.2|	653189	+	A	0	0	1	0	1	-
gi|29165615|ref|NC_002745.2|	653193	+	U	0	1	0	0	0	-
gi|29165615|ref|NC_002745.2|	653197	+	C	0	0	0	1	0	-
gi|29165615|ref|NC_002745.2|	653199	+	A	0	0	1	0	0	-
gi|29165615|ref|NC_002745.2|	653200	+	A	0	2	0	0	1	-
gi|29165615|ref|NC_002745.2|	653245	+	U	0	0	0	0	1	SA0560
gi|29165615|ref|NC_002745.2|	653254	+	A	0	1	1	0	0	SA0560
gi|29165615|ref|NC_002745.2|	653273	+	A	0	0	1	0	0	SA0560
gi|29165615|ref|NC_002745.2|	653281	+	A	0	0	0	0	1	SA0560
gi|29165615|ref|NC_002745.2|	653310	+	G	0	2	0	0	0	SA0560
gi|29165615|ref|NC_002745.2|	653311	+	A	0	1	0	0	0	SA0560
gi|29165615|ref|NC_002745.2|	653336	+	A	0	1	0	0	0	SA0560
gi|29165615|ref|NC_002745.2|	653354	+	A	0	0	1	0	0	SA0560
gi|29165615|ref|NC_002745.2|	653358	+	C	0	2	0	0	0	SA0560
gi|29165615|ref|NC_002745.2|	653376	+	U	0	0	0	0	1	SA0560
gi|29165615|ref|NC_002745.2|	653401	+	G	0	0	1	0	0	SA0560
gi|29165615|ref|NC_002745.2|	653409	+	C	1	0	0	0	0	SA0560
gi|29165615|ref|NC_002745.2|	653410	+	A	1	0	0	0	0	SA0560
gi|29165615|ref|NC_002745.2|	653441	+	A	0	0	1	0	0	SA0560
gi|29165615|ref|NC_002745.2|	653445	+	A	0	1	0	0	0	SA0560
gi|29165615|ref|NC_002745.2|	653465	+	U	1	0	0	0	0	SA0560
gi|29165615|ref|NC_002745.2|	653467	+	A	0	0	0	0	1	SA0560
gi|29165615|ref|NC_002745.2|	653472	+	G	0	0	1	0	0	SA0560
gi|29165615|ref|NC_002745.2|	653494	+	C	0	0	1	0	0	SA0560
gi|29165615|ref|NC_002745.2|	653527	+	U	1	0	0	0	0	SA0560
gi|29165615|ref|NC_002745.2|	653561	+	A	0	0	0	0	1	SA0560
gi|29165615|ref|NC_002745.2|	653567	+	U	0	0	1	0	0	SA0560
gi|29165615|ref|NC_002745.2|	653568	+	A	0	0	0	0	1	SA0560
gi|29165615|ref|NC_002745.2|	653576	+	C	0	0	0	0	1	SA0560
gi|29165615|ref|NC_002745.2|	653592	+	C	0	0	0	0	1	SA0560
gi|29165615|ref|NC_002745.2|	653612	+	U	2	0	1	0	0	SA0560
gi|29165615|ref|NC_002745.2|	653614	+	C	1	0	0	0	1	SA0560
gi|29165615|ref|NC_002745.2|	653620	+	U	0	1	0	0	0	SA0560
gi|29165615|ref|NC_002745.2|	653621	+	G	0	0	0	0	1	SA0560
gi|29165615|ref|NC_002745.2|	653627	+	U	0	0	0	1	0	SA0560
gi|29165615|ref|NC_002745.2|	653668	+	C	0	1	0	0	0	SA0560
gi|29165615|ref|NC_002745.2|	653686	+	C	0	0	0	0	1	SA0560
gi|29165615|ref|NC_002745.2|	653742	+	A	0	0	0	0	1	SA0560
gi|29165615|ref|NC_002745.2|	653766	+	U	0	0	1	0	0	SA0560
gi|29165615|ref|NC_002745.2|	653808	+	A	0	0	0	1	0	SA0560
gi|29165615|ref|NC_002745.2|	653911	+	A	0	1	0	0	1	SA0560
gi|29165615|ref|NC_002745.2|	653912	+	A	0	0	0	0	1	SA0560
gi|29165615|ref|NC_002745.2|	653918	+	C	1	0	0	0	0	SA0560
gi|29165615|ref|NC_002745.2|	653935	+	G	1	0	0	0	0	SA0560
gi|29165615|ref|NC_002745.2|	653936	+	U	0	1	0	0	0	SA0560
gi|29165615|ref|NC_002745.2|	653959	+	A	0	1	0	0	3	SA0560
gi|29165615|ref|NC_002745.2|	653978	+	A	1	0	1	0	1	SA0560
gi|29165615|ref|NC_002745.2|	653982	+	C	0	0	0	1	0	SA0560

gi|29165615|ref|NC_002745.2|	653990	+	U	0	0	0	0	1	SA0560
gi|29165615|ref|NC_002745.2|	654012	+	C	0	1	0	0	0	SA0560
gi|29165615|ref|NC_002745.2|	654022	+	A	1	0	0	0	0	SA0560
gi|29165615|ref|NC_002745.2|	654040	+	A	0	1	0	0	0	SA0560
gi|29165615|ref|NC_002745.2|	654062	+	A	0	1	0	0	0	SA0560
gi|29165615|ref|NC_002745.2|	654063	+	U	0	0	1	0	0	SA0560
gi|29165615|ref|NC_002745.2|	654091	+	C	0	0	1	0	0	SA0560
gi|29165615|ref|NC_002745.2|	654139	+	A	0	1	0	0	1	SA0560
gi|29165615|ref|NC_002745.2|	654174	+	A	0	0	0	1	0	SA0560
gi|29165615|ref|NC_002745.2|	654179	+	U	1	0	0	0	0	SA0560
gi|29165615|ref|NC_002745.2|	654190	+	A	1	0	0	0	0	SA0560
gi|29165615|ref|NC_002745.2|	654196	+	C	0	1	0	0	0	SA0560
gi|29165615|ref|NC_002745.2|	654201	+	A	0	0	1	0	0	SA0560
gi|29165615|ref|NC_002745.2|	654207	+	A	0	2	0	0	0	SA0560
gi|29165615|ref|NC_002745.2|	654209	+	A	0	0	1	0	0	SA0560
gi|29165615|ref|NC_002745.2|	654227	+	G	0	1	0	0	0	SA0560
gi|29165615|ref|NC_002745.2|	654259	+	A	0	0	0	1	0	SA0560
gi|29165615|ref|NC_002745.2|	654265	+	U	0	0	0	0	1	SA0560
gi|29165615|ref|NC_002745.2|	654271	+	A	0	0	0	0	1	SA0560
gi|29165615|ref|NC_002745.2|	654274	+	C	1	0	0	1	0	SA0560
gi|29165615|ref|NC_002745.2|	654285	+	U	0	0	0	0	1	SA0560
gi|29165615|ref|NC_002745.2|	654295	+	A	1	0	0	0	0	SA0560
gi|29165615|ref|NC_002745.2|	654383	+	C	0	0	1	0	0	SA0560
gi|29165615|ref|NC_002745.2|	654387	+	C	0	0	1	0	0	SA0560
gi|29165615|ref|NC_002745.2|	654391	+	G	0	0	0	1	0	SA0560
gi|29165615|ref|NC_002745.2|	654397	+	C	0	0	1	0	0	SA0560
gi|29165615|ref|NC_002745.2|	654444	+	G	0	0	1	0	0	SA0560
gi|29165615|ref|NC_002745.2|	654445	+	U	1	0	0	0	0	SA0560
gi|29165615|ref|NC_002745.2|	654458	+	A	0	0	1	0	0	SA0560
gi|29165615|ref|NC_002745.2|	654465	+	A	0	0	1	0	0	SA0560
gi|29165615|ref|NC_002745.2|	654468	+	A	0	0	0	0	1	SA0560
gi|29165615|ref|NC_002745.2|	654490	+	U	1	0	0	0	0	SA0560
gi|29165615|ref|NC_002745.2|	654503	+	U	0	0	2	0	0	SA0560
gi|29165615|ref|NC_002745.2|	654506	+	U	0	0	0	0	1	SA0560
gi|29165615|ref|NC_002745.2|	654510	+	A	0	0	1	0	0	-
gi|29165615|ref|NC_002745.2|	654518	+	A	0	0	0	2	0	-
gi|29165615|ref|NC_002745.2|	654534	+	A	0	0	1	0	0	-
gi|29165615|ref|NC_002745.2|	654535	+	A	0	0	0	1	1	-
gi|29165615|ref|NC_002745.2|	654543	+	G	0	1	0	0	0	-
gi|29165615|ref|NC_002745.2|	654549	+	U	0	0	0	1	0	-
gi|29165615|ref|NC_002745.2|	654561	+	A	0	0	0	1	0	SA0561
gi|29165615|ref|NC_002745.2|	654565	+	A	0	1	0	0	0	SA0561
gi|29165615|ref|NC_002745.2|	654574	+	A	0	0	0	0	1	SA0561
gi|29165615|ref|NC_002745.2|	654586	+	U	0	0	1	0	0	SA0561
gi|29165615|ref|NC_002745.2|	654601	+	U	0	0	1	2	0	SA0561
gi|29165615|ref|NC_002745.2|	654604	+	A	0	1	0	0	0	SA0561
gi|29165615|ref|NC_002745.2|	654611	+	U	0	0	1	0	0	SA0561
gi|29165615|ref|NC_002745.2|	654619	+	C	0	1	0	0	0	SA0561
gi|29165615|ref|NC_002745.2|	654625	+	U	2	0	0	0	1	SA0561
gi|29165615|ref|NC_002745.2|	654636	+	A	0	1	0	0	0	SA0561
gi|29165615|ref|NC_002745.2|	654643	+	A	0	0	0	0	2	SA0561
gi|29165615|ref|NC_002745.2|	654646	+	A	0	0	0	1	0	SA0561
gi|29165615|ref|NC_002745.2|	654661	+	U	0	0	0	1	0	SA0561
gi|29165615|ref|NC_002745.2|	654662	+	U	2	0	0	0	0	SA0561
gi|29165615|ref|NC_002745.2|	654664	+	U	0	1	0	0	1	SA0561
gi|29165615|ref|NC_002745.2|	654665	+	C	0	0	1	0	0	SA0561
gi|29165615|ref|NC_002745.2|	654666	+	G	0	1	0	0	0	SA0561
gi|29165615|ref|NC_002745.2|	654682	+	A	2	0	0	0	0	SA0561
gi|29165615|ref|NC_002745.2|	654683	+	A	1	0	0	0	0	SA0561
gi|29165615|ref|NC_002745.2|	654685	+	C	0	1	2	0	0	SA0561
gi|29165615|ref|NC_002745.2|	654686	+	A	0	1	0	0	0	SA0561
gi|29165615|ref|NC_002745.2|	654694	+	A	0	0	0	1	0	SA0561
gi|29165615|ref|NC_002745.2|	654700	+	G	1	0	0	0	0	SA0561
gi|29165615|ref|NC_002745.2|	654701	+	A	0	1	0	0	1	SA0561
gi|29165615|ref|NC_002745.2|	654707	+	G	1	0	0	0	0	SA0561
gi|29165615|ref|NC_002745.2|	654709	+	A	1	0	0	0	0	SA0561
gi|29165615|ref|NC_002745.2|	654727	+	A	0	1	0	0	0	SA0561
gi|29165615|ref|NC_002745.2|	654729	+	A	0	1	0	0	0	SA0561
gi|29165615|ref|NC_002745.2|	654730	+	A	0	1	0	0	0	SA0561
gi|29165615|ref|NC_002745.2|	654733	+	G	0	0	0	1	0	SA0561
gi|29165615|ref|NC_002745.2|	654734	+	U	0	1	0	1	0	SA0561
gi|29165615|ref|NC_002745.2|	654744	+	A	0	0	0	1	0	SA0561
gi|29165615|ref|NC_002745.2|	654748	+	C	0	0	0	2	0	SA0561
gi|29165615|ref|NC_002745.2|	654750	+	G	2	1	0	0	0	SA0561
gi|29165615|ref|NC_002745.2|	654751	+	A	2	1	1	2	1	SA0561
gi|29165615|ref|NC_002745.2|	654757	+	C	1	0	0	0	0	SA0561
gi|29165615|ref|NC_002745.2|	654758	+	U	2	1	0	1	0	SA0561
gi|29165615|ref|NC_002745.2|	654760	+	C	0	0	2	1	0	SA0561
gi|29165615|ref|NC_002745.2|	654767	+	A	1	0	0	0	0	SA0561
gi|29165615|ref|NC_002745.2|	654771	+	G	0	0	0	1	0	SA0561
gi|29165615|ref|NC_002745.2|	654775	+	A	0	0	1	0	0	SA0561

gi|29165615|ref|NC_002745.2|	654784	+	A	0	1	0	0	0	SA0561
gi|29165615|ref|NC_002745.2|	654802	+	U	1	0	0	0	0	SA0561
gi|29165615|ref|NC_002745.2|	654810	+	G	0	1	0	0	0	SA0561
gi|29165615|ref|NC_002745.2|	654815	+	U	0	0	0	1	0	SA0561
gi|29165615|ref|NC_002745.2|	654822	+	C	0	1	0	0	0	SA0561
gi|29165615|ref|NC_002745.2|	654823	+	A	0	0	1	0	0	SA0561
gi|29165615|ref|NC_002745.2|	654831	+	U	0	0	0	0	1	SA0561
gi|29165615|ref|NC_002745.2|	654866	+	G	0	0	1	0	0	SA0561
gi|29165615|ref|NC_002745.2|	654871	+	A	0	0	1	0	0	SA0561
gi|29165615|ref|NC_002745.2|	654874	+	C	0	0	0	1	0	SA0561
gi|29165615|ref|NC_002745.2|	654878	+	U	1	0	0	0	0	SA0561
gi|29165615|ref|NC_002745.2|	654897	+	A	0	0	0	0	1	SA0561
gi|29165615|ref|NC_002745.2|	655088	+	C	0	1	0	0	0	-
gi|29165615|ref|NC_002745.2|	655092	+	A	0	0	1	0	0	-
gi|29165615|ref|NC_002745.2|	655095	+	G	0	0	1	0	0	-
gi|29165615|ref|NC_002745.2|	655097	+	U	0	0	1	0	0	-
gi|29165615|ref|NC_002745.2|	655104	+	C	0	0	0	0	1	-
gi|29165615|ref|NC_002745.2|	655121	+	G	0	0	1	0	0	-
gi|29165615|ref|NC_002745.2|	655482	+	U	0	0	0	1	0	-
gi|29165615|ref|NC_002745.2|	655527	+	U	0	1	0	0	0	-
gi|29165615|ref|NC_002745.2|	655530	+	U	0	1	0	0	0	-
gi|29165615|ref|NC_002745.2|	655559	+	G	0	1	0	0	0	SA0562
gi|29165615|ref|NC_002745.2|	655560	+	A	0	0	0	1	0	SA0562
gi|29165615|ref|NC_002745.2|	655655	+	U	0	0	0	0	1	SA0562
gi|29165615|ref|NC_002745.2|	655709	+	U	1	0	0	0	0	SA0562
gi|29165615|ref|NC_002745.2|	655720	+	C	1	0	0	0	0	SA0562
gi|29165615|ref|NC_002745.2|	655782	+	A	0	1	0	0	0	SA0562
gi|29165615|ref|NC_002745.2|	656292	+	C	0	0	0	1	0	SA0562
gi|29165615|ref|NC_002745.2|	656713	+	U	0	1	0	0	0	-
gi|29165615|ref|NC_002745.2|	656808	+	A	0	1	1	1	6	-
gi|29165615|ref|NC_002745.2|	656809	+	U	0	1	0	0	0	-
gi|29165615|ref|NC_002745.2|	656811	+	U	4	35	38	34	36	-
gi|29165615|ref|NC_002745.2|	656812	+	C	0	1	2	0	0	-
gi|29165615|ref|NC_002745.2|	656813	+	U	0	1	2	1	2	-
gi|29165615|ref|NC_002745.2|	656814	+	A	29	47	106	56	50	-
gi|29165615|ref|NC_002745.2|	656815	+	U	0	2	3	1	1	-
gi|29165615|ref|NC_002745.2|	656816	+	U	0	2	2	0	1	-
gi|29165615|ref|NC_002745.2|	656822	+	A	0	1	5	1	0	-
gi|29165615|ref|NC_002745.2|	656823	+	A	0	1	1	1	0	-
gi|29165615|ref|NC_002745.2|	656825	+	A	0	0	4	2	0	-
gi|29165615|ref|NC_002745.2|	656828	+	A	2	0	0	0	0	-
gi|29165615|ref|NC_002745.2|	656833	+	G	0	0	1	0	1	SA0563
gi|29165615|ref|NC_002745.2|	656837	+	A	0	1	0	0	0	SA0563
gi|29165615|ref|NC_002745.2|	656840	+	A	0	0	1	0	0	SA0563
gi|29165615|ref|NC_002745.2|	656851	+	A	0	0	0	0	1	SA0563
gi|29165615|ref|NC_002745.2|	656852	+	A	0	0	0	1	0	SA0563
gi|29165615|ref|NC_002745.2|	656864	+	C	0	1	0	0	2	SA0563
gi|29165615|ref|NC_002745.2|	656865	+	A	0	0	2	0	0	SA0563
gi|29165615|ref|NC_002745.2|	656874	+	A	0	4	0	0	1	SA0563
gi|29165615|ref|NC_002745.2|	656891	+	A	0	0	0	0	1	SA0563
gi|29165615|ref|NC_002745.2|	656910	+	A	0	0	0	0	1	SA0563
gi|29165615|ref|NC_002745.2|	656916	+	C	2	1	1	0	0	SA0563
gi|29165615|ref|NC_002745.2|	656934	+	A	0	0	0	0	1	SA0563
gi|29165615|ref|NC_002745.2|	656939	+	A	2	0	0	0	2	SA0563
gi|29165615|ref|NC_002745.2|	656948	+	A	0	0	2	0	0	SA0563
gi|29165615|ref|NC_002745.2|	656953	+	U	0	1	0	1	0	SA0563
gi|29165615|ref|NC_002745.2|	656954	+	A	0	1	0	0	0	SA0563
gi|29165615|ref|NC_002745.2|	656955	+	U	0	0	0	0	2	SA0563
gi|29165615|ref|NC_002745.2|	656959	+	G	0	0	0	0	2	SA0563
gi|29165615|ref|NC_002745.2|	656960	+	U	0	1	0	0	2	SA0563
gi|29165615|ref|NC_002745.2|	656961	+	A	0	1	0	1	0	SA0563
gi|29165615|ref|NC_002745.2|	656963	+	G	0	0	1	0	1	SA0563
gi|29165615|ref|NC_002745.2|	656964	+	U	1	2	0	1	1	SA0563
gi|29165615|ref|NC_002745.2|	656965	+	A	0	0	0	1	1	SA0563
gi|29165615|ref|NC_002745.2|	656970	+	A	0	0	0	0	1	SA0563
gi|29165615|ref|NC_002745.2|	656971	+	A	0	1	3	0	1	SA0563
gi|29165615|ref|NC_002745.2|	656972	+	A	0	1	0	0	1	SA0563
gi|29165615|ref|NC_002745.2|	656978	+	G	0	4	2	0	2	SA0563
gi|29165615|ref|NC_002745.2|	656982	+	C	0	1	0	0	3	SA0563
gi|29165615|ref|NC_002745.2|	656983	+	A	0	1	0	0	0	SA0563
gi|29165615|ref|NC_002745.2|	656984	+	A	0	0	0	0	1	SA0563
gi|29165615|ref|NC_002745.2|	656985	+	C	0	2	0	0	0	SA0563
gi|29165615|ref|NC_002745.2|	656997	+	C	0	0	0	0	3	SA0563
gi|29165615|ref|NC_002745.2|	656998	+	A	0	0	0	1	0	SA0563
gi|29165615|ref|NC_002745.2|	656999	+	A	0	1	0	0	0	SA0563
gi|29165615|ref|NC_002745.2|	657007	+	G	1	1	1	3	1	SA0563
gi|29165615|ref|NC_002745.2|	657008	+	G	0	0	0	2	0	SA0563
gi|29165615|ref|NC_002745.2|	657009	+	A	0	1	0	0	1	SA0563
gi|29165615|ref|NC_002745.2|	657012	+	A	0	1	0	0	0	SA0563
gi|29165615|ref|NC_002745.2|	657046	+	U	0	1	0	0	0	SA0563
gi|29165615|ref|NC_002745.2|	657053	+	A	0	1	0	0	0	SA0563

gi|29165615|ref|NC_002745.2|	657060	+	A	1	0	0	0	0	SA0563
gi|29165615|ref|NC_002745.2|	657076	+	A	0	0	0	1	0	SA0563
gi|29165615|ref|NC_002745.2|	657080	+	A	0	0	0	1	2	SA0563
gi|29165615|ref|NC_002745.2|	657084	+	C	0	1	0	0	0	SA0563
gi|29165615|ref|NC_002745.2|	657096	+	A	0	1	0	0	0	SA0563
gi|29165615|ref|NC_002745.2|	657120	+	U	1	0	0	0	0	SA0563
gi|29165615|ref|NC_002745.2|	657130	+	A	0	0	0	0	1	SA0563
gi|29165615|ref|NC_002745.2|	657131	+	A	0	0	0	1	0	SA0563
gi|29165615|ref|NC_002745.2|	657139	+	C	0	2	0	0	0	SA0563
gi|29165615|ref|NC_002745.2|	657143	+	U	0	1	0	0	0	SA0563
gi|29165615|ref|NC_002745.2|	657153	+	U	0	3	7	5	5	SA0563
gi|29165615|ref|NC_002745.2|	657154	+	A	1	0	2	0	2	SA0563
gi|29165615|ref|NC_002745.2|	657159	+	G	0	1	0	0	0	SA0563
gi|29165615|ref|NC_002745.2|	657161	+	G	0	0	0	1	0	SA0563
gi|29165615|ref|NC_002745.2|	657165	+	G	0	2	1	0	0	SA0563
gi|29165615|ref|NC_002745.2|	657168	+	G	1	1	0	0	0	SA0563
gi|29165615|ref|NC_002745.2|	657169	+	C	0	6	6	6	3	SA0563
gi|29165615|ref|NC_002745.2|	657170	+	A	0	0	1	0	2	SA0563
gi|29165615|ref|NC_002745.2|	657173	+	C	0	5	1	0	1	SA0563
gi|29165615|ref|NC_002745.2|	657174	+	U	0	0	1	0	0	SA0563
gi|29165615|ref|NC_002745.2|	657176	+	A	0	1	0	0	0	SA0563
gi|29165615|ref|NC_002745.2|	657178	+	G	1	0	0	0	0	SA0563
gi|29165615|ref|NC_002745.2|	657179	+	A	1	1	0	0	0	SA0563
gi|29165615|ref|NC_002745.2|	657188	+	G	0	0	0	0	1	SA0563
gi|29165615|ref|NC_002745.2|	657189	+	A	0	1	1	3	1	SA0563
gi|29165615|ref|NC_002745.2|	657193	+	A	0	2	2	1	0	SA0563
gi|29165615|ref|NC_002745.2|	657195	+	A	0	1	0	1	0	SA0563
gi|29165615|ref|NC_002745.2|	657197	+	C	0	0	1	0	1	SA0563
gi|29165615|ref|NC_002745.2|	657198	+	A	0	1	1	0	0	SA0563
gi|29165615|ref|NC_002745.2|	657199	+	A	0	1	0	1	0	SA0563
gi|29165615|ref|NC_002745.2|	657200	+	G	0	1	1	0	0	SA0563
gi|29165615|ref|NC_002745.2|	657201	+	A	0	3	5	0	1	SA0563
gi|29165615|ref|NC_002745.2|	657202	+	U	0	0	0	1	1	SA0563
gi|29165615|ref|NC_002745.2|	657203	+	A	2	1	0	1	2	SA0563
gi|29165615|ref|NC_002745.2|	657204	+	A	0	0	2	4	3	SA0563
gi|29165615|ref|NC_002745.2|	657206	+	G	0	4	4	1	7	SA0563
gi|29165615|ref|NC_002745.2|	657207	+	A	0	6	6	6	2	SA0563
gi|29165615|ref|NC_002745.2|	657208	+	A	0	4	3	3	3	SA0563
gi|29165615|ref|NC_002745.2|	657209	+	A	0	5	6	5	6	SA0563
gi|29165615|ref|NC_002745.2|	657210	+	A	1	2	1	3	1	SA0563
gi|29165615|ref|NC_002745.2|	657211	+	A	0	0	2	0	3	SA0563
gi|29165615|ref|NC_002745.2|	657224	+	C	2	2	0	0	1	SA0563
gi|29165615|ref|NC_002745.2|	657226	+	A	2	1	1	0	0	SA0563
gi|29165615|ref|NC_002745.2|	657231	+	A	1	4	8	5	5	SA0563
gi|29165615|ref|NC_002745.2|	657232	+	A	0	3	0	0	1	SA0563
gi|29165615|ref|NC_002745.2|	657234	+	U	0	0	1	0	0	SA0563
gi|29165615|ref|NC_002745.2|	657237	+	A	0	1	1	0	3	SA0563
gi|29165615|ref|NC_002745.2|	657239	+	U	0	2	0	0	0	SA0563
gi|29165615|ref|NC_002745.2|	657240	+	A	1	0	0	0	1	SA0563
gi|29165615|ref|NC_002745.2|	657249	+	U	1	0	0	0	0	SA0563
gi|29165615|ref|NC_002745.2|	657253	+	C	0	0	0	1	0	SA0563
gi|29165615|ref|NC_002745.2|	657255	+	A	0	1	0	0	0	SA0563
gi|29165615|ref|NC_002745.2|	657259	+	A	0	3	0	0	0	SA0563_SA0564
gi|29165615|ref|NC_002745.2|	657262	+	A	0	1	0	0	0	SA0564
gi|29165615|ref|NC_002745.2|	657263	+	U	0	0	0	0	1	SA0564
gi|29165615|ref|NC_002745.2|	657269	+	A	0	0	0	1	0	SA0564
gi|29165615|ref|NC_002745.2|	657271	+	C	0	1	0	0	0	SA0564
gi|29165615|ref|NC_002745.2|	657273	+	A	0	1	0	0	0	SA0564
gi|29165615|ref|NC_002745.2|	657275	+	U	0	1	0	0	0	SA0564
gi|29165615|ref|NC_002745.2|	657276	+	G	0	0	1	3	2	SA0564
gi|29165615|ref|NC_002745.2|	657277	+	A	0	0	1	0	1	SA0564
gi|29165615|ref|NC_002745.2|	657278	+	A	0	0	2	0	0	SA0564
gi|29165615|ref|NC_002745.2|	657281	+	A	0	1	1	0	0	SA0564
gi|29165615|ref|NC_002745.2|	657287	+	U	0	1	0	0	0	SA0564
gi|29165615|ref|NC_002745.2|	657290	+	U	0	0	0	1	0	SA0564
gi|29165615|ref|NC_002745.2|	657298	+	A	0	1	0	0	0	SA0564
gi|29165615|ref|NC_002745.2|	657305	+	C	0	0	2	0	2	SA0564
gi|29165615|ref|NC_002745.2|	657310	+	A	0	0	0	0	1	SA0564
gi|29165615|ref|NC_002745.2|	657311	+	U	0	0	1	1	0	SA0564
gi|29165615|ref|NC_002745.2|	657327	+	A	1	0	0	0	0	SA0564
gi|29165615|ref|NC_002745.2|	657350	+	A	0	0	0	1	0	SA0564
gi|29165615|ref|NC_002745.2|	657351	+	A	0	0	1	0	0	SA0564
gi|29165615|ref|NC_002745.2|	657352	+	A	0	0	1	0	0	SA0564
gi|29165615|ref|NC_002745.2|	657366	+	A	1	0	0	0	0	SA0564
gi|29165615|ref|NC_002745.2|	657371	+	C	0	1	0	0	0	SA0564
gi|29165615|ref|NC_002745.2|	657377	+	A	0	0	0	1	0	SA0564
gi|29165615|ref|NC_002745.2|	657392	+	C	2	0	0	0	0	SA0564
gi|29165615|ref|NC_002745.2|	657398	+	U	2	0	0	0	0	SA0564
gi|29165615|ref|NC_002745.2|	657409	+	C	0	1	0	0	0	SA0564
gi|29165615|ref|NC_002745.2|	657422	+	C	0	0	1	0	0	SA0564
gi|29165615|ref|NC_002745.2|	657434	+	C	0	1	0	0	0	SA0564

gi|29165615|ref|NC_002745.2|	657442	+	A	0	1	0	0	0	SA0564
gi|29165615|ref|NC_002745.2|	657467	+	U	2	0	0	0	0	SA0564
gi|29165615|ref|NC_002745.2|	657469	+	G	0	1	0	0	0	SA0564
gi|29165615|ref|NC_002745.2|	657471	+	U	1	0	0	0	0	SA0564
gi|29165615|ref|NC_002745.2|	657512	+	C	1	0	0	0	0	SA0564
gi|29165615|ref|NC_002745.2|	657532	+	U	0	0	1	0	0	SA0564
gi|29165615|ref|NC_002745.2|	657575	+	A	1	0	0	0	0	SA0564
gi|29165615|ref|NC_002745.2|	657578	+	A	0	0	0	0	1	SA0564
gi|29165615|ref|NC_002745.2|	657583	+	G	0	0	0	1	0	SA0564
gi|29165615|ref|NC_002745.2|	657585	+	U	0	1	0	0	1	SA0564
gi|29165615|ref|NC_002745.2|	657601	+	A	0	0	1	0	0	SA0564
gi|29165615|ref|NC_002745.2|	657605	+	A	0	0	0	1	0	SA0564
gi|29165615|ref|NC_002745.2|	657619	+	A	0	1	2	0	0	SA0564
gi|29165615|ref|NC_002745.2|	657656	+	G	0	0	0	1	0	SA0564
gi|29165615|ref|NC_002745.2|	657660	+	U	1	0	0	1	0	SA0564
gi|29165615|ref|NC_002745.2|	657661	+	U	0	0	0	0	1	SA0564
gi|29165615|ref|NC_002745.2|	657663	+	A	0	0	1	0	0	SA0564
gi|29165615|ref|NC_002745.2|	657664	+	C	1	0	0	0	0	SA0564
gi|29165615|ref|NC_002745.2|	657665	+	A	0	0	1	0	1	SA0564
gi|29165615|ref|NC_002745.2|	657672	+	U	2	0	0	0	0	SA0564
gi|29165615|ref|NC_002745.2|	657678	+	U	0	0	0	1	0	SA0564
gi|29165615|ref|NC_002745.2|	657686	+	C	1	0	0	0	0	SA0564
gi|29165615|ref|NC_002745.2|	657711	+	U	0	0	1	0	0	SA0564
gi|29165615|ref|NC_002745.2|	657715	+	U	0	0	0	0	1	SA0564
gi|29165615|ref|NC_002745.2|	657718	+	A	0	0	1	0	0	SA0564
gi|29165615|ref|NC_002745.2|	657732	+	U	0	1	0	0	0	SA0564
gi|29165615|ref|NC_002745.2|	657746	+	A	0	0	0	1	0	SA0564
gi|29165615|ref|NC_002745.2|	657749	+	A	0	0	0	1	0	SA0564
gi|29165615|ref|NC_002745.2|	657752	+	A	3	8	2	1	13	SA0564
gi|29165615|ref|NC_002745.2|	657753	+	U	0	0	1	0	1	SA0564
gi|29165615|ref|NC_002745.2|	657755	+	U	2	1	3	1	0	SA0564
gi|29165615|ref|NC_002745.2|	657756	+	U	0	0	1	0	0	SA0564
gi|29165615|ref|NC_002745.2|	657757	+	A	0	0	0	0	1	SA0564
gi|29165615|ref|NC_002745.2|	657765	+	U	0	1	0	2	0	SA0564
gi|29165615|ref|NC_002745.2|	657768	+	U	0	0	1	0	1	SA0564
gi|29165615|ref|NC_002745.2|	657775	+	A	1	0	0	0	0	SA0564
gi|29165615|ref|NC_002745.2|	657776	+	U	1	1	0	0	0	SA0564
gi|29165615|ref|NC_002745.2|	657788	+	C	1	0	0	0	0	SA0564
gi|29165615|ref|NC_002745.2|	657794	+	C	0	0	2	0	0	SA0564
gi|29165615|ref|NC_002745.2|	657806	+	G	0	0	0	0	1	SA0564
gi|29165615|ref|NC_002745.2|	657830	+	A	0	2	0	0	0	SA0564
gi|29165615|ref|NC_002745.2|	657834	+	U	0	0	0	1	0	SA0564
gi|29165615|ref|NC_002745.2|	657861	+	U	0	1	0	0	0	SA0564
gi|29165615|ref|NC_002745.2|	657868	+	G	0	1	0	0	0	SA0564
gi|29165615|ref|NC_002745.2|	657869	+	A	0	0	1	0	1	SA0564
gi|29165615|ref|NC_002745.2|	657878	+	A	0	1	0	0	0	SA0564
gi|29165615|ref|NC_002745.2|	657884	+	G	0	0	1	0	0	SA0564
gi|29165615|ref|NC_002745.2|	657887	+	A	0	0	2	0	0	SA0564
gi|29165615|ref|NC_002745.2|	657896	+	C	0	0	1	0	0	SA0564
gi|29165615|ref|NC_002745.2|	657917	+	A	0	0	0	0	1	SA0564
gi|29165615|ref|NC_002745.2|	657923	+	A	0	0	0	2	0	SA0564
gi|29165615|ref|NC_002745.2|	657954	+	A	0	0	2	0	2	SA0564
gi|29165615|ref|NC_002745.2|	657957	+	A	0	1	0	0	0	SA0564
gi|29165615|ref|NC_002745.2|	657967	+	G	0	0	1	0	0	SA0564
gi|29165615|ref|NC_002745.2|	657993	+	U	0	0	0	0	1	SA0564
gi|29165615|ref|NC_002745.2|	658009	+	A	0	0	1	0	0	SA0564
gi|29165615|ref|NC_002745.2|	658027	+	U	0	0	0	0	1	SA0564
gi|29165615|ref|NC_002745.2|	658032	+	U	0	1	0	1	0	SA0564
gi|29165615|ref|NC_002745.2|	658039	+	A	0	0	0	1	0	SA0564
gi|29165615|ref|NC_002745.2|	658044	+	U	1	0	0	0	0	SA0564
gi|29165615|ref|NC_002745.2|	658046	+	U	0	0	0	1	0	SA0564
gi|29165615|ref|NC_002745.2|	658047	+	A	0	0	0	1	0	SA0564
gi|29165615|ref|NC_002745.2|	658048	+	U	0	1	0	0	0	SA0564
gi|29165615|ref|NC_002745.2|	658052	+	A	0	0	1	1	1	SA0564
gi|29165615|ref|NC_002745.2|	658054	+	A	0	0	0	1	0	SA0564
gi|29165615|ref|NC_002745.2|	658055	+	A	0	0	1	1	1	SA0564
gi|29165615|ref|NC_002745.2|	658057	+	G	0	0	0	1	0	SA0564
gi|29165615|ref|NC_002745.2|	658058	+	G	0	1	0	0	0	SA0564
gi|29165615|ref|NC_002745.2|	658063	+	A	0	0	1	0	0	SA0564
gi|29165615|ref|NC_002745.2|	658083	+	A	0	1	0	0	0	SA0564
gi|29165615|ref|NC_002745.2|	658084	+	A	0	0	1	0	1	SA0564
gi|29165615|ref|NC_002745.2|	658090	+	G	0	0	1	0	0	SA0564
gi|29165615|ref|NC_002745.2|	658097	+	G	0	0	1	0	0	SA0564
gi|29165615|ref|NC_002745.2|	658103	+	C	0	1	0	1	0	SA0564
gi|29165615|ref|NC_002745.2|	658104	+	G	0	2	0	0	0	SA0564
gi|29165615|ref|NC_002745.2|	658105	+	U	0	1	0	1	0	SA0564
gi|29165615|ref|NC_002745.2|	658106	+	A	1	0	0	0	1	SA0564
gi|29165615|ref|NC_002745.2|	658124	+	C	0	2	0	0	0	SA0564
gi|29165615|ref|NC_002745.2|	658139	+	C	0	1	0	0	0	SA0564
gi|29165615|ref|NC_002745.2|	658142	+	A	0	1	0	0	1	SA0564
gi|29165615|ref|NC_002745.2|	658145	+	U	0	0	1	0	0	SA0564

gi|29165615|ref|NC_002745.2|	658146 +	U	0	0	0	1	0	SA0564
gi|29165615|ref|NC_002745.2|	658151 +	A	0	1	1	1	1	SA0564
gi|29165615|ref|NC_002745.2|	658152 +	C	1	0	0	0	0	SA0564
gi|29165615|ref|NC_002745.2|	658154 +	A	0	3	2	0	2	SA0564
gi|29165615|ref|NC_002745.2|	658155 +	C	0	0	0	0	1	SA0564
gi|29165615|ref|NC_002745.2|	658157 +	A	0	1	0	0	0	SA0564
gi|29165615|ref|NC_002745.2|	658160 +	A	0	1	1	0	0	SA0564
gi|29165615|ref|NC_002745.2|	658164 +	A	0	3	2	1	2	SA0564
gi|29165615|ref|NC_002745.2|	658165 +	G	0	0	0	1	0	SA0564
gi|29165615|ref|NC_002745.2|	658166 +	U	0	0	1	0	0	SA0564
gi|29165615|ref|NC_002745.2|	658170 +	A	0	0	1	0	0	SA0564
gi|29165615|ref|NC_002745.2|	658171 +	A	0	1	2	1	2	SA0564
gi|29165615|ref|NC_002745.2|	658174 +	A	0	1	1	0	1	SA0564
gi|29165615|ref|NC_002745.2|	658175 +	A	0	2	0	2	1	SA0564
gi|29165615|ref|NC_002745.2|	658180 +	G	0	0	0	1	0	SA0564
gi|29165615|ref|NC_002745.2|	658181 +	A	0	0	0	1	0	SA0564
gi|29165615|ref|NC_002745.2|	658184 +	G	0	0	0	0	1	SA0564
gi|29165615|ref|NC_002745.2|	658185 +	U	0	0	1	0	0	SA0564
gi|29165615|ref|NC_002745.2|	658186 +	A	0	1	0	0	0	SA0564
gi|29165615|ref|NC_002745.2|	658187 +	C	0	0	0	1	1	SA0564
gi|29165615|ref|NC_002745.2|	658188 +	A	0	2	0	2	1	SA0564
gi|29165615|ref|NC_002745.2|	658191 +	U	0	0	2	1	0	SA0564
gi|29165615|ref|NC_002745.2|	658192 +	A	1	0	0	0	0	SA0564
gi|29165615|ref|NC_002745.2|	658193 +	C	1	0	0	0	1	SA0564
gi|29165615|ref|NC_002745.2|	658195 +	U	0	0	1	1	0	SA0564
gi|29165615|ref|NC_002745.2|	658197 +	U	0	0	0	0	1	SA0564
gi|29165615|ref|NC_002745.2|	658201 +	U	0	0	1	0	0	SA0564
gi|29165615|ref|NC_002745.2|	658208 +	A	0	0	0	1	0	SA0564
gi|29165615|ref|NC_002745.2|	658209 +	U	0	0	1	0	0	SA0564
gi|29165615|ref|NC_002745.2|	658247 +	A	0	2	0	0	0	SA0564
gi|29165615|ref|NC_002745.2|	658248 +	C	0	1	0	0	0	SA0564
gi|29165615|ref|NC_002745.2|	658249 +	A	0	0	0	0	1	SA0564
gi|29165615|ref|NC_002745.2|	658259 +	A	0	1	1	1	0	SA0564
gi|29165615|ref|NC_002745.2|	658269 +	U	0	0	1	0	0	SA0564
gi|29165615|ref|NC_002745.2|	658274 +	A	0	0	0	1	0	SA0564
gi|29165615|ref|NC_002745.2|	658282 +	G	0	0	0	0	1	SA0564
gi|29165615|ref|NC_002745.2|	658286 +	A	1	0	0	0	0	SA0564
gi|29165615|ref|NC_002745.2|	658289 +	U	0	1	1	0	0	SA0564
gi|29165615|ref|NC_002745.2|	658293 +	U	0	0	1	0	0	SA0564
gi|29165615|ref|NC_002745.2|	658296 +	U	0	0	0	0	1	SA0564
gi|29165615|ref|NC_002745.2|	658299 +	G	0	0	1	0	0	SA0564
gi|29165615|ref|NC_002745.2|	658300 +	A	0	0	0	1	0	SA0564
gi|29165615|ref|NC_002745.2|	658309 +	C	0	0	2	0	0	SA0564
gi|29165615|ref|NC_002745.2|	658315 +	A	0	0	1	0	0	SA0564
gi|29165615|ref|NC_002745.2|	658316 +	C	0	1	0	0	0	SA0564
gi|29165615|ref|NC_002745.2|	658318 +	U	0	0	2	0	1	SA0564
gi|29165615|ref|NC_002745.2|	658319 +	U	4	0	0	0	0	SA0564
gi|29165615|ref|NC_002745.2|	658328 +	A	0	0	0	1	0	SA0564
gi|29165615|ref|NC_002745.2|	658332 +	U	0	0	1	1	0	SA0564
gi|29165615|ref|NC_002745.2|	658338 +	U	0	0	1	1	0	SA0564
gi|29165615|ref|NC_002745.2|	658340 +	U	0	2	0	0	1	SA0564
gi|29165615|ref|NC_002745.2|	658342 +	G	0	0	1	0	0	SA0564
gi|29165615|ref|NC_002745.2|	658343 +	A	0	1	1	0	1	SA0564
gi|29165615|ref|NC_002745.2|	658348 +	C	0	1	0	1	0	SA0564
gi|29165615|ref|NC_002745.2|	658351 +	A	0	2	0	0	1	SA0564
gi|29165615|ref|NC_002745.2|	658354 +	A	0	0	1	0	0	SA0564
gi|29165615|ref|NC_002745.2|	658356 +	G	0	0	1	0	0	SA0564
gi|29165615|ref|NC_002745.2|	658357 +	C	0	3	1	1	5	SA0564
gi|29165615|ref|NC_002745.2|	658360 +	A	0	0	3	0	1	SA0564
gi|29165615|ref|NC_002745.2|	658368 +	U	0	1	0	0	0	SA0564
gi|29165615|ref|NC_002745.2|	658369 +	U	0	0	1	0	0	SA0564
gi|29165615|ref|NC_002745.2|	658370 +	U	0	1	0	1	0	SA0564
gi|29165615|ref|NC_002745.2|	658371 +	A	0	0	0	0	2	SA0564
gi|29165615|ref|NC_002745.2|	658372 +	A	0	1	0	0	0	SA0564
gi|29165615|ref|NC_002745.2|	658375 +	G	0	0	1	0	0	SA0564
gi|29165615|ref|NC_002745.2|	658376 +	A	0	0	0	2	0	SA0564
gi|29165615|ref|NC_002745.2|	658378 +	A	0	3	2	4	0	SA0564
gi|29165615|ref|NC_002745.2|	658379 +	A	0	18	6	2	5	SA0564
gi|29165615|ref|NC_002745.2|	658382 +	G	0	0	2	1	1	SA0564
gi|29165615|ref|NC_002745.2|	658383 +	U	1	0	0	0	0	SA0564
gi|29165615|ref|NC_002745.2|	658384 +	A	0	0	0	2	0	SA0564
gi|29165615|ref|NC_002745.2|	658386 +	A	0	0	1	0	0	SA0564
gi|29165615|ref|NC_002745.2|	658387 +	G	0	0	0	1	1	SA0564
gi|29165615|ref|NC_002745.2|	658388 +	A	1	0	0	3	2	SA0564
gi|29165615|ref|NC_002745.2|	658389 +	A	0	0	1	0	0	SA0564
gi|29165615|ref|NC_002745.2|	658391 +	U	1	1	0	0	0	SA0564
gi|29165615|ref|NC_002745.2|	658393 +	A	0	2	0	2	1	SA0564
gi|29165615|ref|NC_002745.2|	658394 +	A	0	0	0	1	0	SA0564
gi|29165615|ref|NC_002745.2|	658395 +	G	0	0	1	0	0	SA0564
gi|29165615|ref|NC_002745.2|	658396 +	A	0	0	0	1	0	SA0564
gi|29165615|ref|NC_002745.2|	658399 +	A	0	1	0	0	0	SA0564

gi|29165615|ref|NC_002745.2|	658401	+	U	0	0	0	1	1	SA0564
gi|29165615|ref|NC_002745.2|	658402	+	A	0	2	1	0	0	SA0564
gi|29165615|ref|NC_002745.2|	658403	+	A	0	0	0	1	0	SA0564
gi|29165615|ref|NC_002745.2|	658404	+	A	0	0	1	1	0	SA0564
gi|29165615|ref|NC_002745.2|	658405	+	C	0	0	3	0	0	SA0564
gi|29165615|ref|NC_002745.2|	658406	+	G	3	0	0	0	1	SA0564
gi|29165615|ref|NC_002745.2|	658407	+	U	2	1	2	0	0	SA0564
gi|29165615|ref|NC_002745.2|	658408	+	A	0	0	1	0	0	SA0564
gi|29165615|ref|NC_002745.2|	658409	+	C	2	0	0	0	0	SA0564
gi|29165615|ref|NC_002745.2|	658410	+	U	0	0	1	0	0	SA0564
gi|29165615|ref|NC_002745.2|	658412	+	G	0	0	0	1	0	SA0564
gi|29165615|ref|NC_002745.2|	658413	+	U	0	0	1	0	0	SA0564
gi|29165615|ref|NC_002745.2|	658414	+	A	0	2	0	2	0	SA0564
gi|29165615|ref|NC_002745.2|	658418	+	C	0	1	1	1	1	SA0564
gi|29165615|ref|NC_002745.2|	658419	+	G	1	0	0	0	0	SA0564
gi|29165615|ref|NC_002745.2|	658420	+	A	0	2	1	0	0	SA0564
gi|29165615|ref|NC_002745.2|	658421	+	U	0	0	1	0	0	SA0564
gi|29165615|ref|NC_002745.2|	658422	+	U	0	2	0	0	1	SA0564
gi|29165615|ref|NC_002745.2|	658423	+	A	0	4	4	4	7	SA0564
gi|29165615|ref|NC_002745.2|	658424	+	C	0	3	5	2	1	SA0564
gi|29165615|ref|NC_002745.2|	658425	+	A	0	1	0	1	0	SA0564
gi|29165615|ref|NC_002745.2|	658426	+	U	0	3	2	0	0	SA0564
gi|29165615|ref|NC_002745.2|	658427	+	U	0	3	1	4	2	SA0564
gi|29165615|ref|NC_002745.2|	658428	+	A	0	8	9	0	7	SA0564
gi|29165615|ref|NC_002745.2|	658429	+	A	0	10	3	5	7	SA0564
gi|29165615|ref|NC_002745.2|	658430	+	G	0	0	0	0	1	SA0564
gi|29165615|ref|NC_002745.2|	658431	+	A	0	5	1	5	8	SA0564
gi|29165615|ref|NC_002745.2|	658432	+	G	1	12	5	3	4	SA0564
gi|29165615|ref|NC_002745.2|	658433	+	A	0	46	24	20	31	SA0564
gi|29165615|ref|NC_002745.2|	658434	+	A	0	18	13	11	12	SA0564
gi|29165615|ref|NC_002745.2|	658435	+	A	1	33	19	19	32	SA0564
gi|29165615|ref|NC_002745.2|	658436	+	U	0	7	3	0	4	SA0564
gi|29165615|ref|NC_002745.2|	658437	+	U	0	3	2	1	0	SA0564
gi|29165615|ref|NC_002745.2|	658438	+	A	1	2	3	0	2	SA0564
gi|29165615|ref|NC_002745.2|	658439	+	U	3	4	0	1	0	SA0564
gi|29165615|ref|NC_002745.2|	658441	+	G	0	0	0	2	0	SA0564
gi|29165615|ref|NC_002745.2|	658442	+	A	0	0	0	0	2	SA0564
gi|29165615|ref|NC_002745.2|	658443	+	C	1	0	0	0	0	SA0564
gi|29165615|ref|NC_002745.2|	658445	+	A	0	1	4	3	2	SA0564
gi|29165615|ref|NC_002745.2|	658446	+	A	0	0	1	0	2	SA0564
gi|29165615|ref|NC_002745.2|	658448	+	U	0	1	1	0	1	SA0564
gi|29165615|ref|NC_002745.2|	658451	+	G	0	0	0	3	0	SA0564
gi|29165615|ref|NC_002745.2|	658452	+	C	0	0	1	1	0	SA0564
gi|29165615|ref|NC_002745.2|	658453	+	G	0	1	0	0	0	SA0564
gi|29165615|ref|NC_002745.2|	658454	+	U	0	0	0	0	1	SA0564
gi|29165615|ref|NC_002745.2|	658458	+	C	2	0	0	0	0	SA0564
gi|29165615|ref|NC_002745.2|	658460	+	C	1	0	1	0	0	SA0564
gi|29165615|ref|NC_002745.2|	658462	+	G	0	0	0	0	1	SA0564
gi|29165615|ref|NC_002745.2|	658463	+	C	1	0	0	0	0	SA0564
gi|29165615|ref|NC_002745.2|	658467	+	U	7	1	0	0	1	SA0564
gi|29165615|ref|NC_002745.2|	658472	+	U	2	0	0	0	0	SA0564
gi|29165615|ref|NC_002745.2|	658473	+	C	0	0	0	3	0	SA0564
gi|29165615|ref|NC_002745.2|	658475	+	U	0	1	0	0	0	SA0564
gi|29165615|ref|NC_002745.2|	658477	+	A	0	1	1	0	0	SA0564
gi|29165615|ref|NC_002745.2|	658479	+	U	0	0	0	1	0	SA0564
gi|29165615|ref|NC_002745.2|	658480	+	A	0	1	3	0	0	SA0564
gi|29165615|ref|NC_002745.2|	658483	+	C	0	2	0	1	2	SA0564
gi|29165615|ref|NC_002745.2|	658484	+	G	0	0	0	2	0	SA0564
gi|29165615|ref|NC_002745.2|	658485	+	U	0	0	0	0	1	SA0564
gi|29165615|ref|NC_002745.2|	658491	+	U	0	0	0	0	1	SA0564
gi|29165615|ref|NC_002745.2|	658493	+	A	0	1	1	0	1	SA0564
gi|29165615|ref|NC_002745.2|	658505	+	A	0	2	0	1	2	SA0564
gi|29165615|ref|NC_002745.2|	658510	+	G	0	1	0	0	0	SA0564
gi|29165615|ref|NC_002745.2|	658511	+	A	0	1	0	0	2	SA0564
gi|29165615|ref|NC_002745.2|	658516	+	G	0	0	1	0	0	SA0564
gi|29165615|ref|NC_002745.2|	658518	+	A	0	0	0	0	2	SA0564
gi|29165615|ref|NC_002745.2|	658521	+	A	1	0	0	1	0	SA0564
gi|29165615|ref|NC_002745.2|	658523	+	C	0	1	1	0	1	SA0564
gi|29165615|ref|NC_002745.2|	658525	+	A	0	0	0	0	1	SA0564
gi|29165615|ref|NC_002745.2|	658533	+	A	0	2	0	0	0	SA0564
gi|29165615|ref|NC_002745.2|	658534	+	U	0	0	1	0	0	SA0564
gi|29165615|ref|NC_002745.2|	658535	+	C	0	0	1	0	0	SA0564
gi|29165615|ref|NC_002745.2|	658537	+	C	0	1	0	0	0	SA0564
gi|29165615|ref|NC_002745.2|	658541	+	A	0	0	0	1	0	SA0564
gi|29165615|ref|NC_002745.2|	658542	+	C	0	2	1	1	0	SA0564
gi|29165615|ref|NC_002745.2|	658543	+	A	0	1	1	2	0	SA0564
gi|29165615|ref|NC_002745.2|	658545	+	U	0	0	0	0	1	SA0564
gi|29165615|ref|NC_002745.2|	658546	+	C	0	2	0	0	1	SA0564
gi|29165615|ref|NC_002745.2|	658547	+	C	0	0	0	0	2	SA0564
gi|29165615|ref|NC_002745.2|	658548	+	A	0	1	1	0	0	SA0564
gi|29165615|ref|NC_002745.2|	658552	+	U	1	0	0	0	0	SA0564

gi|29165615|ref|NC_002745.2|	658554	+	C	0	0	1	0	0	SA0564
gi|29165615|ref|NC_002745.2|	658565	+	A	0	0	0	0	1	SA0564
gi|29165615|ref|NC_002745.2|	658570	+	C	0	1	1	0	0	SA0564
gi|29165615|ref|NC_002745.2|	658657	+	G	0	0	0	0	1	SA0564
gi|29165615|ref|NC_002745.2|	658661	+	A	0	0	0	1	0	SA0564
gi|29165615|ref|NC_002745.2|	658664	+	C	0	1	0	2	0	SA0564
gi|29165615|ref|NC_002745.2|	658679	+	A	0	0	1	0	0	SA0564
gi|29165615|ref|NC_002745.2|	658680	+	A	0	0	0	1	0	SA0564
gi|29165615|ref|NC_002745.2|	658688	+	C	0	0	1	0	0	SA0564
gi|29165615|ref|NC_002745.2|	658697	+	A	0	2	0	0	0	SA0564
gi|29165615|ref|NC_002745.2|	658699	+	C	0	1	0	0	0	SA0564
gi|29165615|ref|NC_002745.2|	658702	+	A	0	0	1	0	0	SA0564
gi|29165615|ref|NC_002745.2|	658703	+	C	0	0	2	0	0	SA0564
gi|29165615|ref|NC_002745.2|	658704	+	A	0	1	0	0	0	SA0564
gi|29165615|ref|NC_002745.2|	658705	+	A	0	0	0	0	1	SA0564
gi|29165615|ref|NC_002745.2|	658715	+	C	1	0	0	0	0	SA0564
gi|29165615|ref|NC_002745.2|	658716	+	U	2	0	0	0	0	SA0564
gi|29165615|ref|NC_002745.2|	658733	+	C	1	0	0	0	0	SA0564
gi|29165615|ref|NC_002745.2|	658738	+	A	0	0	1	0	0	SA0564
gi|29165615|ref|NC_002745.2|	658739	+	G	0	0	0	0	1	SA0564
gi|29165615|ref|NC_002745.2|	658740	+	A	0	0	0	0	1	SA0564
gi|29165615|ref|NC_002745.2|	658745	+	C	0	0	1	1	0	SA0564
gi|29165615|ref|NC_002745.2|	658747	+	A	0	1	0	0	0	SA0564
gi|29165615|ref|NC_002745.2|	658750	+	U	0	0	1	0	0	SA0564
gi|29165615|ref|NC_002745.2|	658751	+	A	0	0	1	0	0	SA0564
gi|29165615|ref|NC_002745.2|	658754	+	U	0	0	0	1	0	SA0564
gi|29165615|ref|NC_002745.2|	658762	+	U	1	0	0	0	0	SA0564
gi|29165615|ref|NC_002745.2|	658766	+	C	1	0	0	0	0	SA0564
gi|29165615|ref|NC_002745.2|	658769	+	C	1	0	0	0	0	SA0564
gi|29165615|ref|NC_002745.2|	658770	+	U	1	0	0	0	0	SA0564
gi|29165615|ref|NC_002745.2|	658771	+	C	0	0	0	1	0	SA0564
gi|29165615|ref|NC_002745.2|	658772	+	A	0	0	1	0	0	SA0564
gi|29165615|ref|NC_002745.2|	658776	+	C	1	0	0	0	0	SA0564
gi|29165615|ref|NC_002745.2|	658782	+	A	0	0	0	0	1	SA0564
gi|29165615|ref|NC_002745.2|	658784	+	U	0	1	0	2	0	SA0564
gi|29165615|ref|NC_002745.2|	658787	+	A	0	0	2	0	2	SA0564
gi|29165615|ref|NC_002745.2|	658789	+	A	0	2	0	0	0	SA0564
gi|29165615|ref|NC_002745.2|	658800	+	A	0	0	1	0	1	SA0564
gi|29165615|ref|NC_002745.2|	658805	+	U	0	1	0	0	0	SA0564
gi|29165615|ref|NC_002745.2|	658806	+	A	0	1	1	0	1	SA0564
gi|29165615|ref|NC_002745.2|	658809	+	A	0	0	1	0	0	SA0564
gi|29165615|ref|NC_002745.2|	658814	+	A	0	0	0	4	0	SA0564
gi|29165615|ref|NC_002745.2|	658816	+	A	0	1	0	1	0	SA0564
gi|29165615|ref|NC_002745.2|	658820	+	A	0	0	0	0	2	SA0564
gi|29165615|ref|NC_002745.2|	658821	+	A	0	0	0	3	0	SA0564
gi|29165615|ref|NC_002745.2|	658822	+	A	0	0	0	0	3	SA0564
gi|29165615|ref|NC_002745.2|	658823	+	A	0	1	0	0	2	SA0564
gi|29165615|ref|NC_002745.2|	658824	+	A	0	0	0	1	0	SA0564
gi|29165615|ref|NC_002745.2|	658825	+	A	0	0	1	0	0	SA0564
gi|29165615|ref|NC_002745.2|	658828	+	A	0	0	2	0	1	SA0564
gi|29165615|ref|NC_002745.2|	658829	+	A	0	2	0	0	0	SA0564
gi|29165615|ref|NC_002745.2|	658832	+	C	0	1	0	0	0	SA0564
gi|29165615|ref|NC_002745.2|	658836	+	U	0	0	1	0	0	SA0564
gi|29165615|ref|NC_002745.2|	658850	+	A	0	0	0	1	0	SA0564
gi|29165615|ref|NC_002745.2|	658852	+	G	0	1	0	0	0	SA0564
gi|29165615|ref|NC_002745.2|	658865	+	C	0	0	1	0	0	SA0564
gi|29165615|ref|NC_002745.2|	658879	+	U	0	0	0	1	0	SA0564
gi|29165615|ref|NC_002745.2|	658917	+	A	0	2	0	0	0	SA0564
gi|29165615|ref|NC_002745.2|	658919	+	A	0	1	0	0	1	-
gi|29165615|ref|NC_002745.2|	658920	+	A	0	1	0	0	0	-
gi|29165615|ref|NC_002745.2|	658923	+	U	0	2	0	1	1	-
gi|29165615|ref|NC_002745.2|	658984	+	A	0	1	0	0	0	-
gi|29165615|ref|NC_002745.2|	658986	+	G	0	4	0	1	4	-
gi|29165615|ref|NC_002745.2|	658987	+	U	0	11	4	8	10	-
gi|29165615|ref|NC_002745.2|	658988	+	A	0	5	3	3	3	-
gi|29165615|ref|NC_002745.2|	658989	+	U	0	0	0	0	1	-
gi|29165615|ref|NC_002745.2|	658991	+	U	0	1	1	0	0	-
gi|29165615|ref|NC_002745.2|	658992	+	G	0	1	1	1	3	-
gi|29165615|ref|NC_002745.2|	658993	+	A	0	2	0	1	1	-
gi|29165615|ref|NC_002745.2|	658995	+	A	0	0	1	0	0	-
gi|29165615|ref|NC_002745.2|	658996	+	U	0	1	0	0	0	-
gi|29165615|ref|NC_002745.2|	658997	+	A	0	0	1	0	0	-
gi|29165615|ref|NC_002745.2|	658998	+	U	1	0	0	0	0	-
gi|29165615|ref|NC_002745.2|	659018	+	G	0	0	0	1	0	-
gi|29165615|ref|NC_002745.2|	659020	+	A	0	0	2	0	0	-
gi|29165615|ref|NC_002745.2|	659030	+	G	0	0	0	1	0	-
gi|29165615|ref|NC_002745.2|	659422	+	U	1	0	0	0	0	SA0565
gi|29165615|ref|NC_002745.2|	659448	+	A	0	0	1	0	0	SA0565
gi|29165615|ref|NC_002745.2|	659490	+	C	0	0	0	0	1	SA0565
gi|29165615|ref|NC_002745.2|	659652	+	G	0	0	0	1	0	SA0565
gi|29165615|ref|NC_002745.2|	659665	+	A	0	0	1	0	0	SA0565

gi|29165615|ref|NC_002745.2|	659668	+	A	0	1	0	0	0	SA0565
gi|29165615|ref|NC_002745.2|	659863	+	A	0	1	0	0	0	-
gi|29165615|ref|NC_002745.2|	659911	+	G	0	0	0	1	0	-
gi|29165615|ref|NC_002745.2|	659912	+	A	0	0	0	1	2	-
gi|29165615|ref|NC_002745.2|	659913	+	A	1	1	0	0	0	-
gi|29165615|ref|NC_002745.2|	660053	+	U	1	0	0	0	0	-
gi|29165615|ref|NC_002745.2|	660061	+	G	0	0	1	0	0	-
gi|29165615|ref|NC_002745.2|	660062	+	A	0	0	3	0	0	-
gi|29165615|ref|NC_002745.2|	660063	+	G	0	1	1	1	0	-
gi|29165615|ref|NC_002745.2|	660068	+	G	0	0	1	0	0	-
gi|29165615|ref|NC_002745.2|	660069	+	A	0	2	1	1	1	-
gi|29165615|ref|NC_002745.2|	660071	+	A	0	0	1	0	0	-
gi|29165615|ref|NC_002745.2|	660083	+	U	0	1	0	1	0	SA0566
gi|29165615|ref|NC_002745.2|	660086	+	U	0	0	1	0	2	SA0566
gi|29165615|ref|NC_002745.2|	660093	+	C	0	1	0	0	2	SA0566
gi|29165615|ref|NC_002745.2|	660098	+	A	0	0	0	1	1	SA0566
gi|29165615|ref|NC_002745.2|	660104	+	A	0	3	3	3	0	SA0566
gi|29165615|ref|NC_002745.2|	660162	+	A	1	0	0	0	0	SA0566
gi|29165615|ref|NC_002745.2|	660186	+	C	0	1	0	0	0	SA0566
gi|29165615|ref|NC_002745.2|	660218	+	A	0	1	0	0	0	SA0566
gi|29165615|ref|NC_002745.2|	660223	+	U	0	0	1	0	0	SA0566
gi|29165615|ref|NC_002745.2|	660235	+	U	0	0	0	0	1	SA0566
gi|29165615|ref|NC_002745.2|	660295	+	U	0	0	0	0	1	SA0566
gi|29165615|ref|NC_002745.2|	660382	+	U	0	0	0	0	2	SA0566
gi|29165615|ref|NC_002745.2|	660418	+	A	0	1	0	0	0	SA0566
gi|29165615|ref|NC_002745.2|	660444	+	U	0	0	0	0	1	SA0566
gi|29165615|ref|NC_002745.2|	660459	+	A	0	0	0	0	1	SA0566
gi|29165615|ref|NC_002745.2|	660499	+	A	0	0	1	0	0	SA0566
gi|29165615|ref|NC_002745.2|	660506	+	A	0	2	0	0	0	SA0566
gi|29165615|ref|NC_002745.2|	660614	+	U	1	1	0	0	1	SA0566
gi|29165615|ref|NC_002745.2|	660618	+	U	2	0	0	0	0	SA0566
gi|29165615|ref|NC_002745.2|	660736	+	A	0	0	0	1	0	SA0566
gi|29165615|ref|NC_002745.2|	660746	+	A	0	0	1	0	0	SA0566
gi|29165615|ref|NC_002745.2|	660750	+	U	0	0	1	0	0	SA0566
gi|29165615|ref|NC_002745.2|	660752	+	A	0	0	1	0	0	SA0566
gi|29165615|ref|NC_002745.2|	660756	+	A	0	1	1	0	0	SA0566
gi|29165615|ref|NC_002745.2|	660760	+	G	0	1	1	0	0	SA0566
gi|29165615|ref|NC_002745.2|	660775	+	A	0	0	0	0	1	SA0566
gi|29165615|ref|NC_002745.2|	660776	+	A	0	0	0	1	0	SA0566
gi|29165615|ref|NC_002745.2|	660781	+	G	0	0	1	1	0	SA0566
gi|29165615|ref|NC_002745.2|	660782	+	A	0	1	0	0	2	SA0566
gi|29165615|ref|NC_002745.2|	660785	+	G	0	1	3	0	0	SA0566
gi|29165615|ref|NC_002745.2|	660786	+	A	0	1	1	2	0	SA0566
gi|29165615|ref|NC_002745.2|	660787	+	A	0	1	0	0	0	SA0566
gi|29165615|ref|NC_002745.2|	660791	+	A	0	2	0	0	1	SA0566
gi|29165615|ref|NC_002745.2|	660792	+	A	0	1	0	0	0	SA0566
gi|29165615|ref|NC_002745.2|	660793	+	G	0	1	0	0	0	SA0566
gi|29165615|ref|NC_002745.2|	660795	+	C	0	0	1	0	0	SA0566
gi|29165615|ref|NC_002745.2|	660796	+	A	0	0	0	0	1	SA0566
gi|29165615|ref|NC_002745.2|	660804	+	A	0	1	0	1	0	SA0566
gi|29165615|ref|NC_002745.2|	660818	+	A	0	0	1	0	0	SA0566
gi|29165615|ref|NC_002745.2|	660821	+	A	0	0	0	1	0	SA0566
gi|29165615|ref|NC_002745.2|	660822	+	A	0	0	1	0	1	SA0566
gi|29165615|ref|NC_002745.2|	660834	+	G	0	0	0	0	2	SA0566
gi|29165615|ref|NC_002745.2|	660839	+	A	0	2	0	0	0	SA0566
gi|29165615|ref|NC_002745.2|	660841	+	U	0	1	0	1	2	SA0566
gi|29165615|ref|NC_002745.2|	660842	+	A	0	1	1	3	1	SA0566
gi|29165615|ref|NC_002745.2|	660843	+	A	0	6	2	3	1	SA0566
gi|29165615|ref|NC_002745.2|	660844	+	A	0	2	0	3	5	SA0566
gi|29165615|ref|NC_002745.2|	660845	+	A	0	1	0	0	0	SA0566
gi|29165615|ref|NC_002745.2|	660846	+	U	0	0	0	3	0	SA0566
gi|29165615|ref|NC_002745.2|	660847	+	U	0	0	2	1	0	SA0566
gi|29165615|ref|NC_002745.2|	660848	+	A	0	2	1	0	0	SA0566
gi|29165615|ref|NC_002745.2|	660850	+	U	0	0	0	0	1	SA0566
gi|29165615|ref|NC_002745.2|	660852	+	C	0	1	0	0	0	SA0566
gi|29165615|ref|NC_002745.2|	660854	+	G	0	0	0	1	0	SA0566
gi|29165615|ref|NC_002745.2|	660856	+	C	0	1	0	0	1	SA0566
gi|29165615|ref|NC_002745.2|	660857	+	A	0	0	1	0	0	SA0566
gi|29165615|ref|NC_002745.2|	660859	+	G	0	0	0	0	1	SA0566
gi|29165615|ref|NC_002745.2|	660860	+	A	0	0	0	1	1	SA0566
gi|29165615|ref|NC_002745.2|	660864	+	C	0	1	0	0	0	SA0566
gi|29165615|ref|NC_002745.2|	660866	+	C	0	0	0	0	1	SA0566
gi|29165615|ref|NC_002745.2|	660869	+	A	0	1	0	0	1	SA0566
gi|29165615|ref|NC_002745.2|	660872	+	G	0	0	0	1	0	SA0566
gi|29165615|ref|NC_002745.2|	660875	+	G	0	0	0	1	0	SA0566
gi|29165615|ref|NC_002745.2|	660878	+	G	0	0	1	0	0	SA0566
gi|29165615|ref|NC_002745.2|	660882	+	A	0	1	1	0	0	SA0566
gi|29165615|ref|NC_002745.2|	660891	+	A	1	0	0	1	0	SA0566
gi|29165615|ref|NC_002745.2|	660898	+	A	0	0	1	0	0	SA0566
gi|29165615|ref|NC_002745.2|	660962	+	A	0	0	0	1	0	-
gi|29165615|ref|NC_002745.2|	660968	+	C	0	0	0	2	0	-

gi|29165615|ref|NC_002745.2|	661032	+	A	0	0	1	0	0	-
gi|29165615|ref|NC_002745.2|	661040	+	U	0	0	0	1	0	-
gi|29165615|ref|NC_002745.2|	661059	+	U	0	1	0	0	0	-
gi|29165615|ref|NC_002745.2|	661082	+	A	0	12	4	9	5	-
gi|29165615|ref|NC_002745.2|	661083	+	A	0	0	0	1	0	-
gi|29165615|ref|NC_002745.2|	661089	+	G	0	0	0	0	1	-
gi|29165615|ref|NC_002745.2|	661093	+	A	0	2	2	1	2	-
gi|29165615|ref|NC_002745.2|	661094	+	A	0	0	0	0	1	-
gi|29165615|ref|NC_002745.2|	661095	+	A	0	1	1	0	0	-
gi|29165615|ref|NC_002745.2|	661097	+	A	0	0	0	0	1	-
gi|29165615|ref|NC_002745.2|	661098	+	A	0	3	0	0	0	-
gi|29165615|ref|NC_002745.2|	661099	+	A	0	0	0	1	0	-
gi|29165615|ref|NC_002745.2|	661100	+	A	0	0	0	0	1	-
gi|29165615|ref|NC_002745.2|	661112	+	G	0	0	1	0	0	SA0567
gi|29165615|ref|NC_002745.2|	661113	+	A	0	1	0	0	0	SA0567
gi|29165615|ref|NC_002745.2|	661115	+	A	0	0	0	0	1	SA0567
gi|29165615|ref|NC_002745.2|	661120	+	A	0	0	0	0	1	SA0567
gi|29165615|ref|NC_002745.2|	661122	+	A	0	0	0	1	2	SA0567
gi|29165615|ref|NC_002745.2|	661126	+	U	0	0	0	0	2	SA0567
gi|29165615|ref|NC_002745.2|	661127	+	A	0	0	1	1	0	SA0567
gi|29165615|ref|NC_002745.2|	661152	+	A	0	0	0	0	1	SA0567
gi|29165615|ref|NC_002745.2|	661167	+	A	0	0	0	0	1	SA0567
gi|29165615|ref|NC_002745.2|	661204	+	U	0	0	0	1	0	SA0567
gi|29165615|ref|NC_002745.2|	661208	+	U	0	0	0	0	1	SA0567
gi|29165615|ref|NC_002745.2|	661223	+	A	0	1	0	0	0	SA0567
gi|29165615|ref|NC_002745.2|	661224	+	A	0	1	0	0	0	SA0567
gi|29165615|ref|NC_002745.2|	661225	+	U	0	0	0	0	2	SA0567
gi|29165615|ref|NC_002745.2|	661227	+	A	0	0	0	1	0	SA0567
gi|29165615|ref|NC_002745.2|	661228	+	A	0	0	0	2	0	SA0567
gi|29165615|ref|NC_002745.2|	661229	+	U	1	0	0	0	0	SA0567
gi|29165615|ref|NC_002745.2|	661231	+	U	0	1	1	1	2	SA0567
gi|29165615|ref|NC_002745.2|	661232	+	U	0	1	0	0	0	SA0567
gi|29165615|ref|NC_002745.2|	661233	+	A	0	1	0	0	0	SA0567
gi|29165615|ref|NC_002745.2|	661234	+	G	0	0	0	1	0	SA0567
gi|29165615|ref|NC_002745.2|	661242	+	A	0	0	0	1	0	SA0567
gi|29165615|ref|NC_002745.2|	661243	+	G	0	0	0	1	0	SA0567
gi|29165615|ref|NC_002745.2|	661244	+	A	0	1	2	2	2	SA0567
gi|29165615|ref|NC_002745.2|	661245	+	U	0	1	0	0	2	SA0567
gi|29165615|ref|NC_002745.2|	661247	+	A	0	1	0	0	0	SA0567
gi|29165615|ref|NC_002745.2|	661248	+	U	0	0	0	2	0	SA0567
gi|29165615|ref|NC_002745.2|	661250	+	A	0	1	2	0	1	SA0567
gi|29165615|ref|NC_002745.2|	661251	+	G	0	3	1	0	3	SA0567
gi|29165615|ref|NC_002745.2|	661252	+	A	0	1	1	0	4	SA0567
gi|29165615|ref|NC_002745.2|	661253	+	A	0	0	0	1	1	SA0567
gi|29165615|ref|NC_002745.2|	661254	+	G	0	0	1	2	0	SA0567
gi|29165615|ref|NC_002745.2|	661255	+	C	0	4	0	0	1	SA0567
gi|29165615|ref|NC_002745.2|	661257	+	U	0	0	0	0	2	SA0567
gi|29165615|ref|NC_002745.2|	661258	+	U	0	0	1	0	0	SA0567
gi|29165615|ref|NC_002745.2|	661260	+	U	1	2	0	0	0	SA0567
gi|29165615|ref|NC_002745.2|	661262	+	G	0	1	0	0	0	SA0567
gi|29165615|ref|NC_002745.2|	661263	+	A	0	1	0	0	1	SA0567
gi|29165615|ref|NC_002745.2|	661268	+	U	0	0	0	1	2	SA0567
gi|29165615|ref|NC_002745.2|	661269	+	A	0	0	0	3	0	SA0567
gi|29165615|ref|NC_002745.2|	661271	+	G	0	2	0	1	1	SA0567
gi|29165615|ref|NC_002745.2|	661272	+	A	0	15	7	6	19	SA0567
gi|29165615|ref|NC_002745.2|	661273	+	U	0	0	1	0	2	SA0567
gi|29165615|ref|NC_002745.2|	661274	+	A	0	2	0	0	0	SA0567
gi|29165615|ref|NC_002745.2|	661275	+	U	0	5	2	1	4	SA0567
gi|29165615|ref|NC_002745.2|	661276	+	U	0	0	0	0	2	SA0567
gi|29165615|ref|NC_002745.2|	661278	+	A	0	3	1	2	2	SA0567
gi|29165615|ref|NC_002745.2|	661279	+	C	0	0	0	2	0	SA0567
gi|29165615|ref|NC_002745.2|	661280	+	A	0	2	0	0	0	SA0567
gi|29165615|ref|NC_002745.2|	661282	+	U	0	0	0	0	1	SA0567
gi|29165615|ref|NC_002745.2|	661283	+	U	0	0	1	0	0	SA0567
gi|29165615|ref|NC_002745.2|	661285	+	C	0	2	1	0	0	SA0567
gi|29165615|ref|NC_002745.2|	661289	+	U	0	0	1	0	0	SA0567
gi|29165615|ref|NC_002745.2|	661294	+	U	0	0	1	1	0	SA0567
gi|29165615|ref|NC_002745.2|	661299	+	C	0	1	0	0	0	SA0567
gi|29165615|ref|NC_002745.2|	661302	+	A	0	0	0	0	1	SA0567
gi|29165615|ref|NC_002745.2|	661306	+	U	0	3	3	0	1	SA0567
gi|29165615|ref|NC_002745.2|	661308	+	U	0	0	1	0	0	SA0567
gi|29165615|ref|NC_002745.2|	661309	+	U	0	2	0	0	0	SA0567
gi|29165615|ref|NC_002745.2|	661311	+	A	0	0	0	0	1	SA0567
gi|29165615|ref|NC_002745.2|	661318	+	C	0	0	0	1	0	SA0567
gi|29165615|ref|NC_002745.2|	661323	+	G	0	0	2	1	0	SA0567
gi|29165615|ref|NC_002745.2|	661326	+	G	0	0	1	0	0	SA0567
gi|29165615|ref|NC_002745.2|	661331	+	C	0	0	0	1	0	SA0567
gi|29165615|ref|NC_002745.2|	661348	+	C	0	0	1	0	0	SA0567
gi|29165615|ref|NC_002745.2|	661355	+	U	0	0	1	0	0	SA0567
gi|29165615|ref|NC_002745.2|	661503	+	A	0	1	0	0	0	SA0567
gi|29165615|ref|NC_002745.2|	661591	+	C	0	1	0	0	0	SA0567

gi|29165615|ref|NC_002745.2|	661644	+	A	0	0	0	0	1	SA0567
gi|29165615|ref|NC_002745.2|	661691	+	U	0	1	0	0	0	SA0567
gi|29165615|ref|NC_002745.2|	661700	+	A	0	0	0	1	0	SA0567
gi|29165615|ref|NC_002745.2|	661761	+	A	0	1	0	0	2	SA0567
gi|29165615|ref|NC_002745.2|	661889	+	A	1	0	0	0	0	SA0567
gi|29165615|ref|NC_002745.2|	662032	+	U	0	0	0	1	0	SA0567
gi|29165615|ref|NC_002745.2|	662034	+	A	0	0	0	0	1	SA0567
gi|29165615|ref|NC_002745.2|	662044	+	C	0	0	0	0	1	SA0567
gi|29165615|ref|NC_002745.2|	662051	+	G	0	0	0	1	0	SA0567
gi|29165615|ref|NC_002745.2|	662074	+	C	0	0	1	0	0	-
gi|29165615|ref|NC_002745.2|	662076	+	A	0	1	0	0	0	-
gi|29165615|ref|NC_002745.2|	662082	+	A	0	0	0	0	2	-
gi|29165615|ref|NC_002745.2|	662086	+	U	2	0	0	0	0	-
gi|29165615|ref|NC_002745.2|	662087	+	A	10	147	94	55	174	-
gi|29165615|ref|NC_002745.2|	662088	+	A	0	11	5	7	9	-
gi|29165615|ref|NC_002745.2|	662089	+	C	0	1	0	0	2	-
gi|29165615|ref|NC_002745.2|	662090	+	U	0	3	1	1	0	-
gi|29165615|ref|NC_002745.2|	662091	+	A	0	5	1	1	3	-
gi|29165615|ref|NC_002745.2|	662092	+	U	1	1	0	1	3	-
gi|29165615|ref|NC_002745.2|	662093	+	A	1	2	0	0	5	-
gi|29165615|ref|NC_002745.2|	662094	+	A	0	9	5	5	12	-
gi|29165615|ref|NC_002745.2|	662095	+	A	0	6	0	0	2	-
gi|29165615|ref|NC_002745.2|	662096	+	C	0	0	1	3	2	-
gi|29165615|ref|NC_002745.2|	662097	+	A	1	1	5	0	2	-
gi|29165615|ref|NC_002745.2|	662098	+	U	0	12	4	1	6	-
gi|29165615|ref|NC_002745.2|	662099	+	A	0	4	4	1	8	-
gi|29165615|ref|NC_002745.2|	662100	+	A	0	8	2	2	1	-
gi|29165615|ref|NC_002745.2|	662101	+	A	0	1	0	0	1	-
gi|29165615|ref|NC_002745.2|	662102	+	G	0	0	1	0	0	-
gi|29165615|ref|NC_002745.2|	662103	+	A	0	0	0	0	1	-
gi|29165615|ref|NC_002745.2|	662106	+	G	0	0	0	0	1	-
gi|29165615|ref|NC_002745.2|	662107	+	C	0	1	0	0	0	-
gi|29165615|ref|NC_002745.2|	662108	+	A	0	1	0	0	0	-
gi|29165615|ref|NC_002745.2|	662109	+	U	0	0	0	1	0	-
gi|29165615|ref|NC_002745.2|	662110	+	A	0	1	1	0	0	-
gi|29165615|ref|NC_002745.2|	662113	+	C	0	1	0	0	0	-
gi|29165615|ref|NC_002745.2|	662117	+	G	0	1	1	0	0	SA0568
gi|29165615|ref|NC_002745.2|	662118	+	G	0	2	0	2	3	SA0568
gi|29165615|ref|NC_002745.2|	662119	+	A	0	0	1	0	0	SA0568
gi|29165615|ref|NC_002745.2|	662129	+	A	0	0	1	1	0	SA0568
gi|29165615|ref|NC_002745.2|	662130	+	A	0	1	0	0	0	SA0568
gi|29165615|ref|NC_002745.2|	662145	+	U	0	0	0	0	2	SA0568
gi|29165615|ref|NC_002745.2|	662146	+	U	0	1	0	0	0	SA0568
gi|29165615|ref|NC_002745.2|	662148	+	G	0	0	0	1	0	SA0568
gi|29165615|ref|NC_002745.2|	662152	+	U	0	2	0	0	0	SA0568
gi|29165615|ref|NC_002745.2|	662163	+	U	0	0	1	0	0	SA0568
gi|29165615|ref|NC_002745.2|	662170	+	A	0	0	0	1	0	SA0568
gi|29165615|ref|NC_002745.2|	662202	+	G	0	1	0	0	0	SA0568
gi|29165615|ref|NC_002745.2|	662212	+	A	0	1	0	0	1	SA0568
gi|29165615|ref|NC_002745.2|	662410	+	A	0	1	0	0	0	SA0568
gi|29165615|ref|NC_002745.2|	662413	+	A	0	0	1	0	0	SA0568
gi|29165615|ref|NC_002745.2|	662449	+	G	0	1	0	0	0	SA0568
gi|29165615|ref|NC_002745.2|	662450	+	C	0	0	1	0	0	SA0568
gi|29165615|ref|NC_002745.2|	662461	+	G	0	1	0	0	0	SA0568
gi|29165615|ref|NC_002745.2|	662466	+	A	1	0	0	0	0	SA0568
gi|29165615|ref|NC_002745.2|	662471	+	A	0	1	0	0	1	SA0568
gi|29165615|ref|NC_002745.2|	662474	+	U	0	0	1	0	0	SA0568
gi|29165615|ref|NC_002745.2|	662484	+	A	0	1	1	0	0	SA0568
gi|29165615|ref|NC_002745.2|	662485	+	A	0	1	0	1	1	SA0568
gi|29165615|ref|NC_002745.2|	662486	+	G	0	1	1	0	0	SA0568
gi|29165615|ref|NC_002745.2|	662487	+	A	0	0	0	1	0	SA0568
gi|29165615|ref|NC_002745.2|	662493	+	C	0	0	2	0	0	SA0568
gi|29165615|ref|NC_002745.2|	662498	+	U	0	2	0	0	0	SA0568
gi|29165615|ref|NC_002745.2|	662500	+	G	1	0	0	0	0	SA0568
gi|29165615|ref|NC_002745.2|	662501	+	A	0	1	1	2	0	SA0568
gi|29165615|ref|NC_002745.2|	662504	+	A	0	0	1	1	1	SA0568
gi|29165615|ref|NC_002745.2|	662505	+	C	0	2	0	0	2	SA0568
gi|29165615|ref|NC_002745.2|	662523	+	C	0	0	1	0	0	SA0568
gi|29165615|ref|NC_002745.2|	662528	+	U	0	0	0	1	0	SA0568
gi|29165615|ref|NC_002745.2|	662547	+	U	0	0	0	0	1	SA0568
gi|29165615|ref|NC_002745.2|	662548	+	C	0	0	1	0	0	SA0568
gi|29165615|ref|NC_002745.2|	662550	+	G	0	0	0	1	0	SA0568
gi|29165615|ref|NC_002745.2|	662555	+	A	1	0	0	0	0	SA0568
gi|29165615|ref|NC_002745.2|	662583	+	A	0	2	0	0	0	SA0568
gi|29165615|ref|NC_002745.2|	662584	+	U	0	0	1	0	0	SA0568
gi|29165615|ref|NC_002745.2|	662588	+	U	0	0	0	0	1	SA0568
gi|29165615|ref|NC_002745.2|	662589	+	G	0	3	0	0	1	SA0568
gi|29165615|ref|NC_002745.2|	662590	+	A	0	2	1	1	0	SA0568
gi|29165615|ref|NC_002745.2|	662596	+	U	0	0	0	0	1	SA0568
gi|29165615|ref|NC_002745.2|	662599	+	U	0	1	0	0	0	SA0568
gi|29165615|ref|NC_002745.2|	662602	+	A	0	1	1	0	0	SA0568

gi|29165615|ref|NC_002745.2|	662617 +	U	0	0	0	0	1	SA0568
gi|29165615|ref|NC_002745.2|	662621 +	U	0	1	0	0	0	SA0568
gi|29165615|ref|NC_002745.2|	662625 +	C	0	1	1	0	0	SA0568
gi|29165615|ref|NC_002745.2|	662627 +	A	0	0	1	1	0	SA0568
gi|29165615|ref|NC_002745.2|	662629 +	U	0	0	0	1	0	SA0568
gi|29165615|ref|NC_002745.2|	662631 +	A	0	0	1	0	0	SA0568
gi|29165615|ref|NC_002745.2|	662632 +	U	0	0	1	0	0	SA0568
gi|29165615|ref|NC_002745.2|	662634 +	U	0	1	0	1	0	SA0568
gi|29165615|ref|NC_002745.2|	662661 +	G	0	0	0	0	1	SA0568
gi|29165615|ref|NC_002745.2|	662674 +	G	0	0	0	0	1	SA0568
gi|29165615|ref|NC_002745.2|	662703 +	A	0	0	0	1	0	SA0568
gi|29165615|ref|NC_002745.2|	662706 +	C	0	0	1	0	0	SA0568
gi|29165615|ref|NC_002745.2|	662707 +	A	0	0	0	0	1	SA0568
gi|29165615|ref|NC_002745.2|	662710 +	A	1	0	0	0	1	SA0568
gi|29165615|ref|NC_002745.2|	662719 +	A	0	0	0	1	0	SA0568
gi|29165615|ref|NC_002745.2|	662727 +	C	0	1	0	0	0	SA0568
gi|29165615|ref|NC_002745.2|	662757 +	A	0	0	0	1	0	SA0568
gi|29165615|ref|NC_002745.2|	662769 +	G	0	0	1	0	0	SA0568
gi|29165615|ref|NC_002745.2|	662770 +	A	0	0	0	1	0	SA0568
gi|29165615|ref|NC_002745.2|	662800 +	A	0	0	0	2	1	SA0568
gi|29165615|ref|NC_002745.2|	662803 +	A	0	0	0	1	0	SA0568
gi|29165615|ref|NC_002745.2|	662807 +	A	1	0	0	0	0	SA0568
gi|29165615|ref|NC_002745.2|	662810 +	A	0	0	0	0	1	SA0568
gi|29165615|ref|NC_002745.2|	662812 +	A	0	1	0	0	0	SA0568
gi|29165615|ref|NC_002745.2|	662862 +	U	0	1	0	0	0	SA0569
gi|29165615|ref|NC_002745.2|	662900 +	C	1	0	0	0	0	SA0569
gi|29165615|ref|NC_002745.2|	662909 +	A	0	2	0	0	1	SA0569
gi|29165615|ref|NC_002745.2|	662914 +	U	0	0	0	0	1	SA0569
gi|29165615|ref|NC_002745.2|	662952 +	A	0	1	0	0	0	SA0569
gi|29165615|ref|NC_002745.2|	662956 +	U	0	0	1	0	0	SA0569
gi|29165615|ref|NC_002745.2|	663037 +	G	0	0	1	0	0	SA0569
gi|29165615|ref|NC_002745.2|	663044 +	U	0	0	1	0	1	SA0569
gi|29165615|ref|NC_002745.2|	663056 +	A	0	0	1	1	0	SA0569
gi|29165615|ref|NC_002745.2|	663062 +	U	0	0	1	0	1	SA0569
gi|29165615|ref|NC_002745.2|	663064 +	U	0	1	0	0	0	SA0569
gi|29165615|ref|NC_002745.2|	663066 +	C	0	1	0	0	0	SA0569
gi|29165615|ref|NC_002745.2|	663071 +	A	0	0	0	0	1	SA0569
gi|29165615|ref|NC_002745.2|	663109 +	A	0	1	0	0	0	SA0569
gi|29165615|ref|NC_002745.2|	663146 +	C	1	0	0	0	0	SA0569
gi|29165615|ref|NC_002745.2|	663238 +	U	0	0	0	1	0	SA0569
gi|29165615|ref|NC_002745.2|	663251 +	A	0	0	0	1	0	SA0569
gi|29165615|ref|NC_002745.2|	663278 +	A	0	0	0	0	1	SA0569
gi|29165615|ref|NC_002745.2|	663313 +	A	0	0	0	1	0	SA0569
gi|29165615|ref|NC_002745.2|	663329 +	C	0	0	1	0	0	SA0569
gi|29165615|ref|NC_002745.2|	663337 +	A	0	0	1	0	0	SA0569
gi|29165615|ref|NC_002745.2|	663341 +	C	2	0	0	1	0	SA0569
gi|29165615|ref|NC_002745.2|	663342 +	A	0	0	0	0	1	SA0569
gi|29165615|ref|NC_002745.2|	663362 +	C	0	0	0	1	0	SA0569
gi|29165615|ref|NC_002745.2|	663368 +	A	0	0	0	1	0	SA0569
gi|29165615|ref|NC_002745.2|	663382 +	A	0	1	0	0	0	SA0569
gi|29165615|ref|NC_002745.2|	663397 +	C	0	0	0	1	0	SA0569
gi|29165615|ref|NC_002745.2|	663413 +	U	3	0	0	0	0	SA0569
gi|29165615|ref|NC_002745.2|	663419 +	U	2	0	0	0	0	SA0569
gi|29165615|ref|NC_002745.2|	663431 +	U	0	1	0	0	0	SA0569
gi|29165615|ref|NC_002745.2|	663437 +	C	0	0	0	0	1	SA0569
gi|29165615|ref|NC_002745.2|	663447 +	C	0	0	1	0	0	SA0569
gi|29165615|ref|NC_002745.2|	663461 +	C	0	1	0	1	0	SA0569
gi|29165615|ref|NC_002745.2|	663465 +	A	0	0	1	0	0	SA0569
gi|29165615|ref|NC_002745.2|	663470 +	A	0	0	0	1	2	SA0569
gi|29165615|ref|NC_002745.2|	663489 +	U	0	0	0	1	0	SA0569
gi|29165615|ref|NC_002745.2|	663500 +	A	0	0	0	0	1	SA0569
gi|29165615|ref|NC_002745.2|	663503 +	C	0	0	1	0	0	SA0569
gi|29165615|ref|NC_002745.2|	663585 +	U	0	1	0	0	0	SA0569
gi|29165615|ref|NC_002745.2|	663634 +	G	0	0	0	0	1	-
gi|29165615|ref|NC_002745.2|	663644 +	A	0	0	0	1	0	-
gi|29165615|ref|NC_002745.2|	663649 +	U	0	0	0	1	0	-
gi|29165615|ref|NC_002745.2|	663654 +	A	0	0	1	0	0	-
gi|29165615|ref|NC_002745.2|	663662 +	U	0	0	0	1	0	-
gi|29165615|ref|NC_002745.2|	663666 +	A	0	0	0	0	2	-
gi|29165615|ref|NC_002745.2|	663667 +	U	0	0	1	0	1	-
gi|29165615|ref|NC_002745.2|	663668 +	A	0	0	0	1	0	-
gi|29165615|ref|NC_002745.2|	663669 +	A	0	1	0	0	0	-
gi|29165615|ref|NC_002745.2|	663671 +	U	0	2	0	0	0	-
gi|29165615|ref|NC_002745.2|	663676 +	U	0	1	0	0	0	-
gi|29165615|ref|NC_002745.2|	663680 +	A	0	0	0	0	1	-
gi|29165615|ref|NC_002745.2|	663705 +	U	0	1	0	0	0	-
gi|29165615|ref|NC_002745.2|	663717 +	G	16	9	17	16	7	-
gi|29165615|ref|NC_002745.2|	663733 +	A	0	1	0	0	0	-
gi|29165615|ref|NC_002745.2|	663734 +	A	0	0	0	0	1	-
gi|29165615|ref|NC_002745.2|	663745 +	A	0	1	0	0	0	-
gi|29165615|ref|NC_002745.2|	663763 +	C	0	0	0	1	2	SA0570

gi|29165615|ref|NC_002745.2|	663766	+	C	0	0	0	1	2	SA0570
gi|29165615|ref|NC_002745.2|	663769	+	A	1	0	0	0	0	SA0570
gi|29165615|ref|NC_002745.2|	663773	+	C	0	0	1	0	0	SA0570
gi|29165615|ref|NC_002745.2|	663781	+	A	0	1	0	0	0	SA0570
gi|29165615|ref|NC_002745.2|	663784	+	G	1	0	0	0	0	SA0570
gi|29165615|ref|NC_002745.2|	663785	+	C	0	0	0	0	1	SA0570
gi|29165615|ref|NC_002745.2|	663791	+	C	0	1	0	0	0	SA0570
gi|29165615|ref|NC_002745.2|	663806	+	U	0	2	0	0	0	SA0570
gi|29165615|ref|NC_002745.2|	663816	+	G	0	0	0	1	0	SA0570
gi|29165615|ref|NC_002745.2|	663821	+	C	2	0	0	0	0	SA0570
gi|29165615|ref|NC_002745.2|	663846	+	C	0	0	1	0	0	SA0570
gi|29165615|ref|NC_002745.2|	663860	+	U	0	2	3	7	2	SA0570
gi|29165615|ref|NC_002745.2|	663861	+	U	0	4	3	4	2	SA0570
gi|29165615|ref|NC_002745.2|	663862	+	A	0	2	3	2	1	SA0570
gi|29165615|ref|NC_002745.2|	663863	+	A	0	6	9	5	2	SA0570
gi|29165615|ref|NC_002745.2|	663864	+	A	0	12	11	2	7	SA0570
gi|29165615|ref|NC_002745.2|	663865	+	C	0	4	5	4	3	SA0570
gi|29165615|ref|NC_002745.2|	663866	+	A	0	2	2	0	4	SA0570
gi|29165615|ref|NC_002745.2|	663867	+	A	0	0	2	0	1	SA0570
gi|29165615|ref|NC_002745.2|	663868	+	U	0	5	3	2	2	SA0570
gi|29165615|ref|NC_002745.2|	663869	+	U	0	7	4	10	6	SA0570
gi|29165615|ref|NC_002745.2|	663870	+	A	0	4	3	3	5	SA0570
gi|29165615|ref|NC_002745.2|	663871	+	A	0	3	3	3	2	SA0570
gi|29165615|ref|NC_002745.2|	663872	+	U	0	2	1	1	0	SA0570
gi|29165615|ref|NC_002745.2|	663873	+	U	0	3	0	0	0	SA0570
gi|29165615|ref|NC_002745.2|	663874	+	A	0	1	0	1	0	SA0570
gi|29165615|ref|NC_002745.2|	663875	+	A	0	0	0	1	2	SA0570
gi|29165615|ref|NC_002745.2|	663877	+	G	0	0	1	0	0	SA0570
gi|29165615|ref|NC_002745.2|	663878	+	G	1	5	1	1	0	SA0570
gi|29165615|ref|NC_002745.2|	663883	+	C	0	0	1	0	0	SA0570
gi|29165615|ref|NC_002745.2|	663885	+	G	0	0	0	1	0	SA0570
gi|29165615|ref|NC_002745.2|	663886	+	U	0	1	0	0	0	SA0570
gi|29165615|ref|NC_002745.2|	663887	+	C	0	0	1	1	0	SA0570
gi|29165615|ref|NC_002745.2|	663888	+	A	0	1	1	0	0	SA0570
gi|29165615|ref|NC_002745.2|	663889	+	U	0	0	0	0	3	SA0570
gi|29165615|ref|NC_002745.2|	663893	+	A	0	1	0	2	1	SA0570
gi|29165615|ref|NC_002745.2|	663894	+	A	0	0	1	0	0	SA0570
gi|29165615|ref|NC_002745.2|	663895	+	A	0	2	0	0	1	SA0570
gi|29165615|ref|NC_002745.2|	663896	+	A	0	1	1	0	0	SA0570
gi|29165615|ref|NC_002745.2|	663900	+	G	0	0	0	0	1	SA0570
gi|29165615|ref|NC_002745.2|	663901	+	A	0	0	0	1	1	SA0570
gi|29165615|ref|NC_002745.2|	663902	+	A	0	0	0	0	3	SA0570
gi|29165615|ref|NC_002745.2|	663903	+	A	0	0	0	1	0	SA0570
gi|29165615|ref|NC_002745.2|	663904	+	A	0	1	0	0	1	SA0570
gi|29165615|ref|NC_002745.2|	663909	+	C	0	3	0	0	0	SA0570
gi|29165615|ref|NC_002745.2|	663923	+	A	0	0	0	0	1	SA0570
gi|29165615|ref|NC_002745.2|	663929	+	U	0	2	0	0	1	SA0570
gi|29165615|ref|NC_002745.2|	663932	+	C	0	0	0	0	1	SA0570
gi|29165615|ref|NC_002745.2|	663944	+	A	0	0	0	0	1	SA0570
gi|29165615|ref|NC_002745.2|	664057	+	C	0	0	0	0	1	SA0570
gi|29165615|ref|NC_002745.2|	664069	+	A	0	1	0	0	0	SA0570
gi|29165615|ref|NC_002745.2|	664084	+	A	1	0	1	0	0	SA0570
gi|29165615|ref|NC_002745.2|	664103	+	C	0	1	0	0	0	SA0570
gi|29165615|ref|NC_002745.2|	664141	+	G	0	0	0	1	0	SA0570
gi|29165615|ref|NC_002745.2|	664142	+	A	0	1	0	0	0	SA0570
gi|29165615|ref|NC_002745.2|	664175	+	U	0	0	0	1	0	SA0570
gi|29165615|ref|NC_002745.2|	664217	+	A	0	1	0	0	0	SA0570
gi|29165615|ref|NC_002745.2|	664226	+	A	0	0	0	1	0	SA0570
gi|29165615|ref|NC_002745.2|	664230	+	A	0	0	1	1	0	SA0570
gi|29165615|ref|NC_002745.2|	664231	+	A	0	0	0	1	0	SA0570
gi|29165615|ref|NC_002745.2|	664247	+	A	0	0	1	0	0	SA0570
gi|29165615|ref|NC_002745.2|	664253	+	A	0	0	0	0	1	SA0570
gi|29165615|ref|NC_002745.2|	664257	+	A	0	1	0	0	0	SA0570
gi|29165615|ref|NC_002745.2|	664266	+	A	0	3	2	1	1	-
gi|29165615|ref|NC_002745.2|	664267	+	A	1	2	0	1	0	-
gi|29165615|ref|NC_002745.2|	664271	+	A	1	1	0	0	0	-
gi|29165615|ref|NC_002745.2|	664340	+	A	0	2	0	0	1	-
gi|29165615|ref|NC_002745.2|	664356	+	A	0	1	0	0	0	-
gi|29165615|ref|NC_002745.2|	664447	+	U	0	1	0	0	0	SA0571
gi|29165615|ref|NC_002745.2|	664451	+	A	0	1	0	0	0	SA0571
gi|29165615|ref|NC_002745.2|	664709	+	A	0	0	1	0	0	SA0571
gi|29165615|ref|NC_002745.2|	664717	+	C	0	1	0	0	0	SA0571
gi|29165615|ref|NC_002745.2|	664736	+	U	0	0	1	0	0	SA0571
gi|29165615|ref|NC_002745.2|	664755	+	A	0	0	1	0	0	SA0571
gi|29165615|ref|NC_002745.2|	664758	+	A	0	1	1	0	0	SA0571
gi|29165615|ref|NC_002745.2|	664759	+	C	1	0	0	0	0	SA0571
gi|29165615|ref|NC_002745.2|	664760	+	U	0	1	0	0	0	SA0571
gi|29165615|ref|NC_002745.2|	664763	+	U	1	0	0	0	0	SA0571
gi|29165615|ref|NC_002745.2|	664820	+	G	0	1	0	0	0	SA0571
gi|29165615|ref|NC_002745.2|	664873	+	C	0	0	1	0	0	SA0571
gi|29165615|ref|NC_002745.2|	664879	+	A	0	0	0	1	0	SA0571

gi|29165615|ref|NC_002745.2|	664885	+	C	1	0	0	0	0	SA0571
gi|29165615|ref|NC_002745.2|	664908	+	A	0	0	1	0	0	SA0571
gi|29165615|ref|NC_002745.2|	664957	+	C	0	0	0	1	0	SA0571
gi|29165615|ref|NC_002745.2|	665016	+	A	0	1	0	0	0	SA0571
gi|29165615|ref|NC_002745.2|	665047	+	C	1	0	0	0	0	SA0571
gi|29165615|ref|NC_002745.2|	665083	+	U	1	0	0	0	0	SA0571
gi|29165615|ref|NC_002745.2|	665292	+	G	0	0	0	1	0	-
gi|29165615|ref|NC_002745.2|	665409	+	G	0	0	0	0	1	SA0572
gi|29165615|ref|NC_002745.2|	665506	+	A	1	0	0	0	0	SA0572
gi|29165615|ref|NC_002745.2|	665535	+	A	0	1	0	0	0	SA0572
gi|29165615|ref|NC_002745.2|	665578	+	G	0	1	0	0	0	SA0572
gi|29165615|ref|NC_002745.2|	665781	+	G	1	0	0	0	0	SA0572
gi|29165615|ref|NC_002745.2|	667449	+	A	0	0	0	1	0	-
gi|29165615|ref|NC_002745.2|	667673	+	A	0	0	0	1	0	-
gi|29165615|ref|NC_002745.2|	669338	+	G	1	0	0	0	0	-
gi|29165615|ref|NC_002745.2|	670221	+	U	1	0	0	1	0	SA0578
gi|29165615|ref|NC_002745.2|	670326	+	U	0	0	1	0	0	SA0578
gi|29165615|ref|NC_002745.2|	670328	+	A	0	0	0	0	1	SA0578
gi|29165615|ref|NC_002745.2|	670337	+	C	0	0	0	0	1	SA0578
gi|29165615|ref|NC_002745.2|	670398	+	A	0	0	0	1	0	SA0578
gi|29165615|ref|NC_002745.2|	670403	+	U	0	0	0	0	1	SA0578
gi|29165615|ref|NC_002745.2|	670422	+	A	0	2	0	0	0	SA0578
gi|29165615|ref|NC_002745.2|	670442	+	U	1	0	0	0	0	SA0578
gi|29165615|ref|NC_002745.2|	670467	+	C	0	1	0	0	0	SA0578
gi|29165615|ref|NC_002745.2|	670482	+	A	0	0	0	0	1	SA0578
gi|29165615|ref|NC_002745.2|	670532	+	C	0	0	0	1	0	SA0578
gi|29165615|ref|NC_002745.2|	670533	+	A	2	0	0	0	0	SA0578
gi|29165615|ref|NC_002745.2|	670683	+	A	0	0	1	0	0	SA0578
gi|29165615|ref|NC_002745.2|	670728	+	A	0	0	0	0	1	SA0578
gi|29165615|ref|NC_002745.2|	670760	+	A	0	0	0	0	1	SA0578
gi|29165615|ref|NC_002745.2|	670797	+	U	0	0	1	0	0	SA0578
gi|29165615|ref|NC_002745.2|	670975	+	U	1	0	0	0	0	SA0578
[truncated: 653,544 more chars]
